# The rules in co-infection of multiple viruses across diverse lineages in a fungal host

**DOI:** 10.1128/mbio.00262-25

**Published:** 2025-05-20

**Authors:** Jie Duan, Yuduo Yao, Jialing Xu, Anmeng Zhang, Xiaojing Kong, Yang Lin, Jiatao Xie, Jiasen Cheng, Yanping Fu, Tao Chen, Bo Li, Xiao Yu, Xueliang Lyu, Xueqiong Xiao, Amir Sharon, Naomi Kagan Trushina, Ioly Kotta-Loizou, Daohong Jiang

**Affiliations:** 1 State Key Laboratory of Agricultural Microbiology, Huazhong Agricultural University 47895, Wuhan, Hubei, China; 2 Hubei Key Laboratory of Plant Pathology, Huazhong Agricultural University 47895, Wuhan, Hubei, China; 3 Hubei Hongshan Laboratory, Wuhan, Hubei, China; 4 School of Plant Sciences and Food Security, Tel Aviv University 616782, Tel Aviv, Israel; 5 Department of Clinical, Pharmaceutical and Biological Science, School of Medical Sciences, University of Hertfordshire 572625 , Hatfield, United Kingdom; 6 Department of Life Sciences, Faculty of Natural Sciences, Imperial College London, South Kensington Campus 4615, London, United Kingdom; University of California, Berkeley, Berkeley, California, USA

**Keywords:** virus, co-infection, ecology, compatibility, fungi

## Abstract

Viruses, ubiquitous non-cellular organisms, pose significant threats to human health and to the agricultural productivity of both livestock and crops. Emerging evidence indicates that multiple viruses can infect a single host, and viral co-infection can exert a profound influence on host physiology. However, our understanding of the prevalence of co-infection and the compatibility of phylogenetically distant viruses is still limited. In this study, we surveyed 406 field strains of the plant fungal pathogen *Botrytis cinerea* and identified 76 mycoviruses. Strikingly, 404 strains were co-infected with two or more viruses, with some harboring up to 25 viruses simultaneously. We discerned significant preference patterns among viruses in their host. Specifically, we identified “one-to-one” and “two-to-one” rules, wherein one or two viruses could be used to reliably predict the presence or absence of other viruses in the same host, and validated these predicted rules by using five *B. cinerea* strains. Furthermore, through the RNA-sequencing approach, we uncovered *B. cinerea* genes associated with the differences caused by different sets of co-infecting viruses. These are implicated in integral components of membrane, transmembrane transporter activity, autophagy pathways, mitophagy pathway, fatty acid biosynthetic process, sphingolipid metabolism, and glycosphingolipid biosynthesis. Our findings underscore the high prevalence of co-infection by multiple viruses in a fungal host within a population and highlight compatibility dynamics among phylogenetically diverse viruses. These insights contribute to our understanding of viral ecology and hold promise for informing strategies to manage viral diseases effectively.

**IMPORTANCE:**

Viruses, pervasive threats to both humans and agriculture, often infect hosts concurrently, profoundly impacting physiology. Despite this, the prevalence and compatibility of co-infecting viruses remain poorly understood. In the study of 406 *Botrytis cinerea* strains, we discovered a striking phenomenon: 404 out of the 406 strains hosted multiple viruses, some with up to 25 at once. Through rigorous analysis, we unveiled distinct preference patterns among these viruses within hosts, identifying predictive co-infection rules validated by experimentation. Furthermore, we identified genes linked to these dynamics, shedding light on critical cellular processes involved in the regulation of the co-infection rules. These findings highlight the widespread nature of viral co-infection and offer insights crucial for effectively managing viral diseases.

## INTRODUCTION

Viruses, non-cellular symbiotic organisms, proliferate within cellular hosts. To date, over 10,000 viral species have been identified, spanning 3,769 genera in 368 families (1). Novel viruses continue to be unearthed through high-throughput sequencing techniques (2–6). Integral to the global ecosystem, viruses play a significant role in maintaining ecosystems (7–12). Viruses are often associated with diseases of humans, animals, and plants, inflicting significant economic losses and reshaping societal norms (5, 7, 13–15). Their elusive nature, particularly in the past, has altered human existence and societal dynamics, underscoring the importance of comprehensive understanding and mitigation strategies (3).

Emerging evidence has shown that multiple viruses, even phylogenetically distant species, co-infect a single host. For instance, influenza A viruses and coronaviruses can coexist and co-infect humans and various animals (16), co-infection of plant viruses from different families is also common in nature (17), and viruses of various genome types can also co-infect the same fungus (18). Interactions among viruses have been identified, with both positive and negative relationships observed between them (19). While assessing compatibility or incompatibility between two viruses within the same host would be relatively straightforward, understanding how multiple viruses (e.g., three or more viruses) coexist within a host and whether they exhibit compatibility or incompatibility remains an ongoing challenge.

Fungi and fungi-like organisms are prevalently infected by viruses called mycoviruses (viruses that infect fungi) (20–22). Mycoviruses, akin to viruses infecting animals and plants, exhibit substantial diversity, spanning 29 families (https://ictv.global/taxonomy), while numerous novel mycoviruses remain unclassified (21, 23). As integral constituents of the virosphere, the ongoing discovery of mycoviruses significantly enhances our understanding of the diversity, evolution, and ecological roles of viruses. Although the co-occurrence of multiple viruses in a single fungal host has been observed (18, 24, 25), the regulatory principle underlying the co-occurrence remains elusive.

In this study, we collected 406 strains of *Botrytis cinerea*, a widely distributed fungal pathogen with a broad plant host range, and identified 76 mycoviruses in 405 strains. Intriguingly, 405 strains showed infection by one or more viruses, with 37 strains demonstrating co-infection by 20 or more viruses. This unprecedented occurrence provided a unique opportunity to explore the intricate cross-interactions among multiple viruses. Through comprehensive analysis, we discerned the rules underlying the compatibility and incompatibility among viruses in a single fungal host.

## RESULTS

### Diversity of mycoviruses in 406 *B. cinerea* strains isolated in Israel

**Fig 1:**
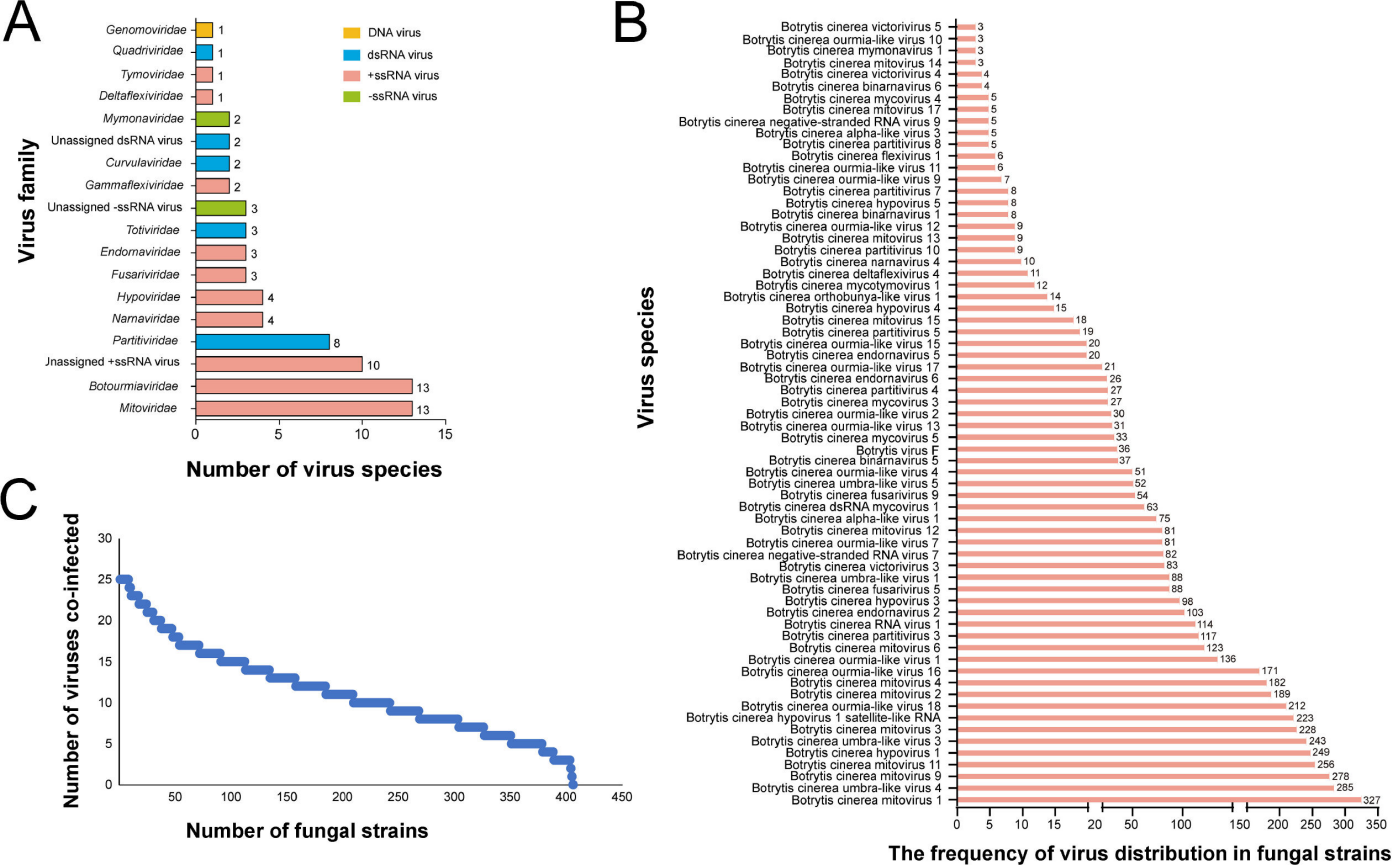
Viruses detected in 406 strains of *Botrytis cinerea* isolated from Israel. (**A**) The virus genome type and number of virus species at the family level (see Table S3 for details). (**B**) Multiple viruses that co-infected the 406 strains; each strain was examined by RT-PCR with specific primers designed based on the genome sequences of 76 viruses. Only one strain was not infected by any viruses; others were co-infected by two or up to 25 viruses. See Table S4 for details. (**C**) Frequency of the 76 viruses in 405 strains. Five viruses in only one strain among the 405 tested strains were not exhibited; these viruses are Botrytis cinerea fusarivirus 4, Botrytis cinerea alpha-like virus 2, Botrytis cinerea poty-like virus 1, Botrytis cinerea mitovirus 16, and Botrytis cinerea ourmia-like virus 19. See Table S4 for details.

All strains were isolated from greenhouse-grown cucumbers and tomatoes, as well as from field-grown strawberries in Israel between January and May of 2018 (Table S1). To further confirm that the isolated strains were indeed *B. cinerea*, we conducted additional molecular identification (Fig. S1). RNA samples were extracted from 406 strains to conduct virome analysis via high-throughput sequencing. This analysis yielded 92 contigs, representing 76 distinct virus species (Table S2). The identified viral genomes encompassed various types, including positive-sense single-stranded RNA (+ssRNA), negative-sense single-stranded RNA (–ssRNA), double-stranded RNA (dsRNA), and single-stranded DNA (ssDNA) (Fig. 1A).

Among the 76 viruses identified, 59 were highly similar to previously reported viruses from *B. cinerea* or other fungi with 90% identity of RNA-dependent RNA polymerases (RdRps). Ten viruses represented novel mycoviruses, displaying less than 70% identity, while seven mycoviruses shared 70% to 90% identity to RdRPs of reported mycoviruses (Table S2).

At the family level, 64 of the 76 mycoviruses (84.21%) were classified into 15 families. However, the remaining 12 viruses spanning six orders (*Elliovirales*, *Ghabrivirales*, *Hepelivirales*, *Martellivirales*, *Patatavirales*, and *Tolivirales*) could not be classified into any existing families (Table S3). At the genus level, 53 mycoviruses were assigned to 18 genera, while 23 mycoviruses were yet to be allocated to a specific genus (Table S3).

Out of the 76 viruses, 54 had +ssRNA genomes, constituting 71.05% of the total viruses, and viruses belonging to *Mitoviridae* and *Botourmiaviridae* accounted for the largest proportion, comprising 50% of the +ssRNA viruses. Additionally, 16 viruses harbored dsRNA genomes, representing 21.05% of the total viruses. Among these, *Partitiviridae* and *Totiriridae* were the predominant groups, encompassing approximately 70% of the total dsRNA viruses. Furthermore, five viruses were characterized as –ssRNA genomes, distributed across *Mymonaviridae* and *Elliovirales*, collectively representing 6.58% of the total viruses. Only one ssDNA virus, belonging to the family *Genomoviridae*, was identified among the tested strains (Fig. 1A).

### The patterns of mycovirus distribution in *B. cinerea* strains

We found that the prevalence of individual viruses varied across the tested strains. Mitovirus, umbra-like virus, botourmiavirus, and hypovirus were notably prevalent within the population of *B. cinerea*. For instance, Botrytis cinerea mitovirus 1 was detected in 327 out of 406 strains. Seventeen viruses were observed in over 100 strains, 28 viruses were detected in over 50 strains, 45 viruses were distributed over 10 strains, and 70 viruses were detected in over two strains. Six viruses, including Botrytis cinerea partitivirus 9, Botrytis cinerea partitivirus 6, Botrytis cinerea negative-stranded RNA virus 6, Botrytis cinerea brome-like virus 1, Botrytis cinerea hypovirulence-associated DNA virus 1, and Botrytis cinerea virga-like virus 1, were detected in a single strain, indicating lower prevalence within the *B. cinerea* population (Fig. 1B).

### Co-occurrence of mycoviruses in *B. cinerea*

The widespread distribution of mycovirus in strains suggested that co-infection of viruses in *B. cinerea* individuals might be very common, thus viruses in individuals were determined by using RT-PCR amplification. Virus-specific primers were designed based on the genome sequence of all 76 viruses and were used to determine their presence among the 406 strains. The findings revealed that out of the 406 strains, only one strain (IBc-111) remained uninfected by any viruses, one strain (IBc-133) harbored just one virus, and one strain (IBc-428) carried two viruses. Remarkably, all other strains were co-infected by three or more viruses. Among the 405 virus-infected strains, 53 strains (13.09%) were co-infected by three to five viruses, 141 strains (34.81%) carried 6 to 10 viruses, 120 strains (29.63%) hosted 11 to 15 viruses, 60 strains (14.81%) were co-infected by 16 to 20 viruses, and 29 strains (7.16%) possessed 21 or more viruses (Fig. 1C; Table S3). These results underscore the prevalence of co-infection by multiple mycoviruses in *B. cinerea*, highlighting its common occurrence in nature.

### “One-to-one” virus co-occurrence rule

**Fig 2:**
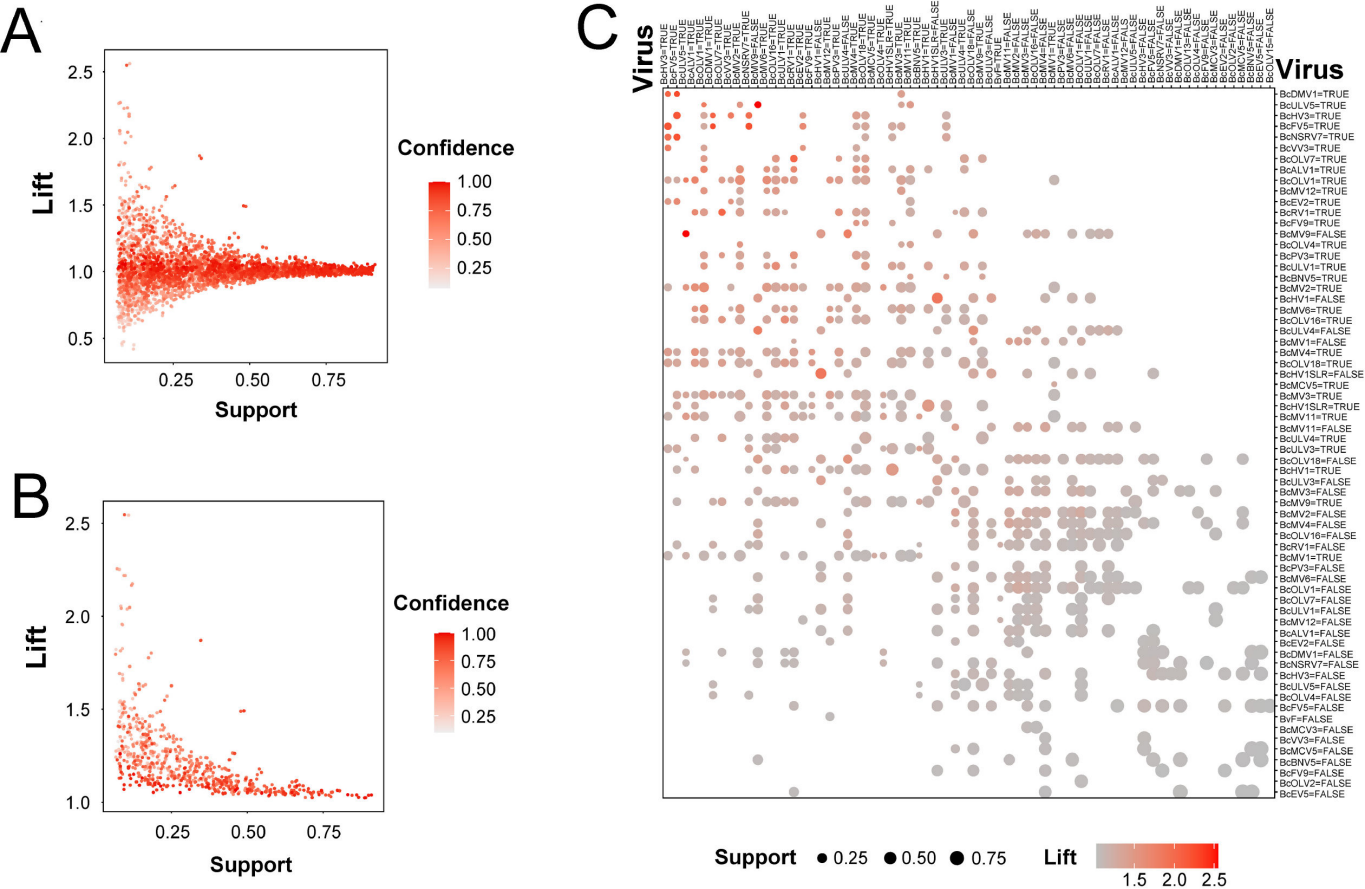
"One-to-one" rule among mycoviruses in *B. cinerea*. (**A**) Candidate rules (4,242) were displayed by using a scatter plot, which uses Support and Lift on the axis. Confidence was shown with the color (red level) of the points. (**B**) A total of 690 significant and non-random rules were displayed by using a scatter plot, which uses Support and Lift on the axis. Confidence was shown with the color (red level) of the points. (**C**) The grouped matrix containing 690 significant and non-random rules was displayed by using a balloon plot, which uses antecedent groups, i.e., RHS as columns and consequents, and LHS as rows. The color of the balloons represents the size of the Lift value, and the size of the balloons shows the size of the Support value. See Table S4 for details of association rules. The association rules were predicted by using the ARM program.

To address the issue that geographical isolation and host population genetic structure may affect the co-occurrence of viruses observed in *B. cinerea* strains, we conducted a principal component analysis (PCA). The results of the PCA indicated that neither the collection sites nor the host plants had an effect on the viruses harbored by *B. cinerea* strains (Fig. S2). Subsequently, we explored the mycovirus co-occurrence patterns. For instance, the presence or absence of virus A in a fungal strain may determine the presence or absence of virus B in the same strain. Initially, we obtained 4,242 pairs of viral interactions (Fig. 2A). Subsequently, after applying a dynamic threshold (Lift = 1.032, Support ≥ 0.073) and false-positive rate (FPR) < 5%, 690 relevant pairs were identified (Fig. 2B). We called the rule governing the presence and absence of virus co-occurrence patterns “one-to-one”: type I, the absence of virus A explains the absence of virus B; type II, the presence of virus A explains the presence of virus B; type III, the presence of virus A explains the absence of virus B; and type IV, the absence of virus A explains the presence of virus B (Fig. 2C; Table S4).

#### Type I

For example, in instances where Botrytis cinerea mitovirus 9 (BcMV9) was absent in a fungal strain, it was improbable for any of the following viruses in that strain: Botrytis cinerea hypovirus 1 (BcHV1), Botrytis cinerea hypovirus 1 satellite-like RNA (BcH1SLR), Botrytis cinerea umbra-like virus 4 (BcULV4), Botrytis cinerea ourmia-like virus 18 (BcOLV18), Botrytis cinerea mitovirus 3 (BcMV3), Botrytis cinerea mitovirus 4 (BcMV4), Botrytis cinerea ourmia-like virus 7 (BcOLV7), Botrytis cinerea umbra-like virus 1 (BcULV1), Botrytis cinerea negative-stranded RNA virus 7 (BcNSRV7), or Botrytis cinerea binarnavirus 5 (BcBNV5).

#### Type II

For example, if Botrytis cinerea hypovirus 3 (BcHV3) was present in a fungal strain, it was highly probable that one of the following viruses was also present in that strain: BcDMV1, Botrytis cinerea fusarivirus 5 (BcFV5), BcNSRV7, Botrytis cinerea victorivirus 3 (BcVV3), Botrytis cinerea ourmia-like virus 1 (BcOLV1), Botrytis cinerea endornavirus 2 (BcEV2), Botrytis cinerea mitovirus 2 (BcMV2), BcMV4, BcOLV7, Botrytis cinerea mitovirus 11 (BcMV11), Botrytis cinerea umbra-like virus 3 (BcULV3), or Botrytis cinerea mitovirus 1 (BcMV1).

#### Type III

For example, if Botrytis cinerea umbra-like virus 5 (BcULV5) was present in a strain, it was improbable for BcMV9 to be present in the same strain. Likewise, if BcDMV1 was present in a strain, BcRV1 was unlikely to appear in that strain. Additionally, if BcOLV7 was present in a strain, BcDMV1 was absent in that strain. Finally, if BcNSRV7 was present in a strain, BcULV1 did not appear in that strain.

#### Type IV

For example, if BcULV5 was absent in a strain, BcMV9 was most likely present in that strain. Overall, the “one-to-one” revealed positive and negative two-way viral interactions within a single fungal host.

### "Two-to-one" virus co-occurrence rule

Building upon the previous PCA analysis, which demonstrated that neither collection sites nor host plants influenced the viruses in *B. cinerea* strains, we further delved into the co-occurrence patterns. We explored whether the co-occurrence patterns of two viruses may determine the presence or absence of other viruses and identified such a rule as “two-to-one.” We initially obtained 102,525 virus interactions (Fig. S3A) and eventually identified 65 “two-to-one” rules (Lift = 1.046, Support ≥ 0.073) and an FPR of less than 5% (Fig. S3B). These “two-to-one” rules contained eight types in total (Table S5), and some of the interactions were displayed according to the ranking of Lift (Fig. S3C).

One type of “two-to-one” rule is exemplified by {Virus A = True, Virus B = True} => {Virus C = True}, indicating that the co-occurrence of virus A and virus B in a strain correlates with a high probability of virus C being present in that strain. For example, when BcMV6 and BcMV11 were present in a strain, that strain was likely infected by Botrytis cinerea alpha-like virus 1 (BcALV1).

Another type of “two-to-one” rule is {Virus A = False, Virus B = False} => {Virus C = True}, meaning that when two viruses are absent in a strain, there is a high probability of other viruses being present in that strain. For example, in instances where neither BcHV1 nor BcULV5 was detected in a strain, BcOLV1 was most likely present in that strain. Similarly, if both BcULV4 and Botrytis cinerea mitovirus 12 (BcMV12) were absent in a strain, BcEV2 was most likely to be detected in that strain. Likewise, if neither BcULV4 nor Botrytis cinerea umbra-like virus 3 (BcULV3) was present in a strain, BcMV1 was most likely to be detected in that strain.

A further category of the “two-to-one” rule is {Virus A = True, Virus B = True} => {Virus C = False}, indicating that when virus A and virus B coexist in a strain, the probability of virus C being present in that strain is considerably low. For example, if BcMV12 and BcMV3 coexisted in a strain, BcEV2 was improbable to exist in that strain. If BcMV3 and BcULV3 were present in a strain, BcULV5 was unlikely to be detected in that strain.

### Experimental validation of the virus co-occurrence rules

**Fig 3:**
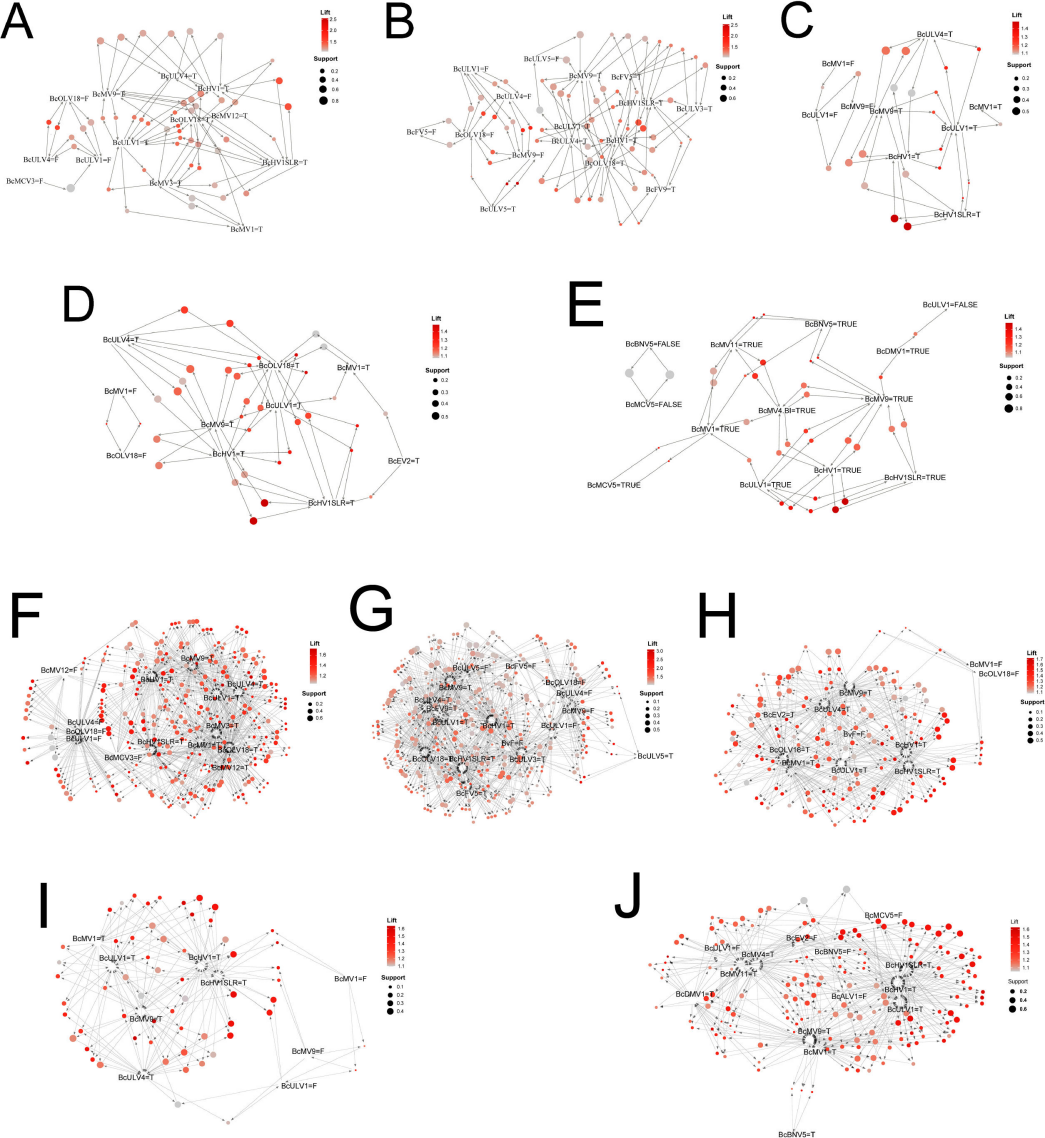
Experimental verification of rules among viruses with *B. cinerea* strains and their offsprings. (A–E) Graph-based visualization with items and rules of “one-to-one” in strains IBc-230, Skr-1, Ca-1, Ca-13, and IBc-352. (F–J) “Two-to-one” rules in strains IBc-230, Skr-1, Ca-1, Ca-13, and IBc-35. The color of the balloons represents the size of the Lift value, and the size of the balloons shows the size of the Support value. The balloons and items were connected by directional arrows; each ball and two arrows together represent the relationship between LHS virus and RHS virus of a rule; each balloon was connected by two arrows; and each arrow connected an item and a balloon. Arrows pointed by the ball represent the RHS virus of the rules, and arrows pointed to the ball represent the LHS virus of the rules. See Table 1 for the number of viruses and number of offsprings used in each tested strain, Figure S2 for virus composition in each strain and its offsprings, and Tables S6 to S10 for rules of viruses in tested strains. “T” = “true,” and “F” = “false.”

**Fig 4:**
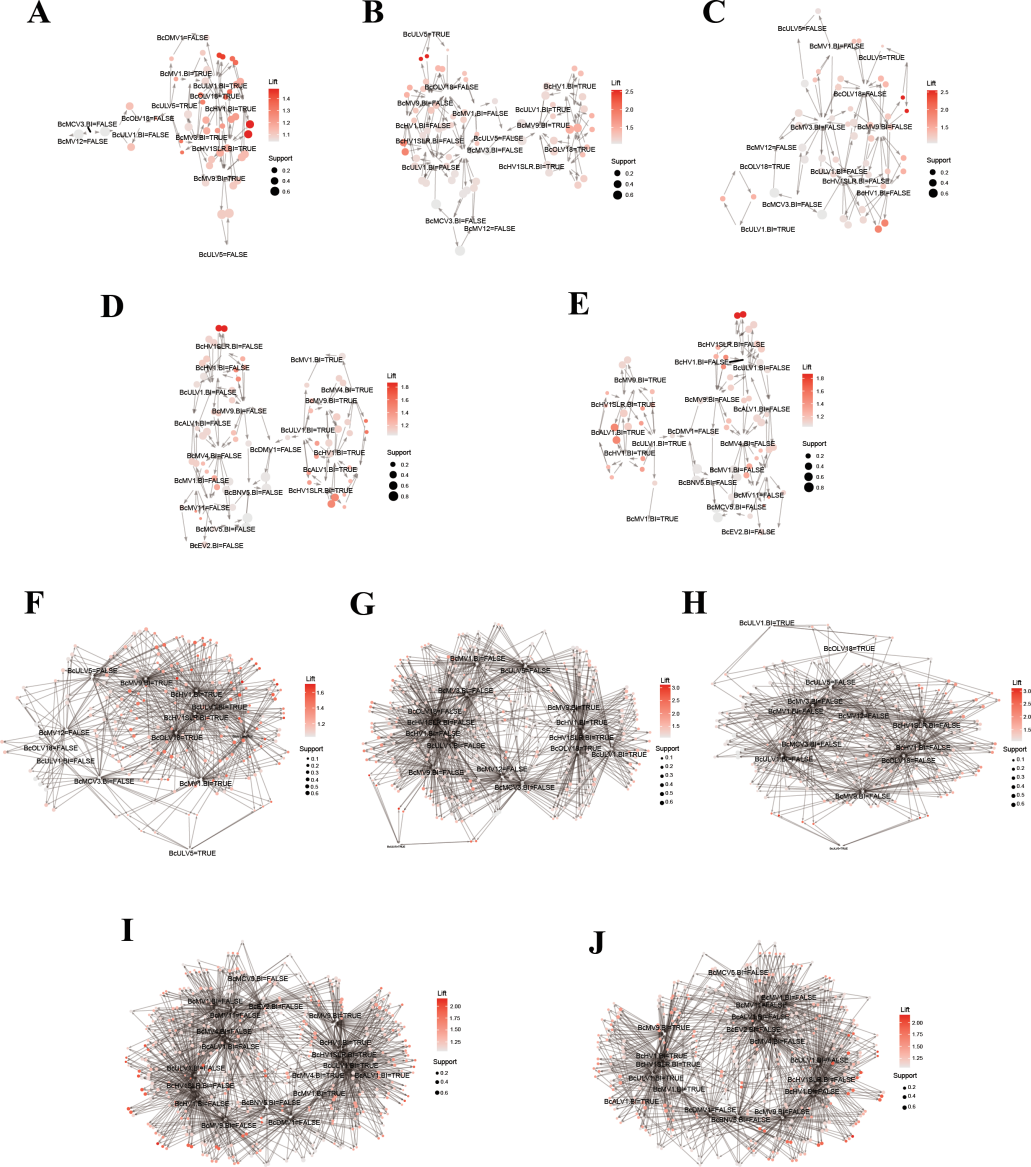
Experimental verification of rules among viruses with *B. cinerea* strains and their transfectants. Graph-based visualization with items and rules of “one-to-one” in transfectants resulting from transfecting strain IBc-230-26-16 with strain IBc-230 (**A**), transfectants resulting from transfecting strain B05.10 with strain IBc-230 (**B**), transfectants resulting from transfecting strain IBc-111 with strain IBc-230 (**C**), transfectants resulting from transfecting strain B05.10 with strain IBc-352 (**D**), and transfectants resulting from transfecting strain IBc-111 with strain IBc-352 (**E**). (F–J) “Two-to-one” rules in transfectants resulting from transfecting strain IBc-230-26-16 with strain IBc-230 (**F**), transfectants resulting from transfecting strain B05.10 with strain IBc-230 (**G**), transfectants resulting from transfecting strain IBc-111 with strain IBc-230 (**H**), transfectants resulting from transfecting strain B05.10 with strain IBc-352 (**I**), and transfectants resulting from transfecting strain IBc-111 with strain IBc-352 (**J**). The color of the balloons represents the size of the Lift value, and the size of the balloons shows the size of the Support value. The balloons and items were connected by directional arrows; each ball and two arrows together represent the relationship between LHS virus and RHS virus of a rule; each balloon was connected by two arrows; and each arrow connected an item and a balloon. Arrows pointed by the ball represent the RHS virus of the rules, and arrows pointed to the ball represent the LHS virus of the rules. See Table 1 for the number of viruses and number of offsprings used in each tested strain, Figure S2 for virus composition in each strain and its offsprings, and Tables S11 and S15 for rules of viruses in tested strains. “T” = “true,” and “F” = “false.”

The above prediction results only indicated the probability of two or three viruses coexisting in an individual in nature. During conidiation, mycoviruses that are co-infected will unevenly enter conidia, some viruses are lost, and some are persisted in offsprings. The disappearance or persistence of specific viruses in offsprings also indicates the likelihood of viral coexistence. By comparing the prediction results of the field strains and their offsprings, we could verify the reliability of the prediction outcomes. Using wild-type strains (Ca-1, Ca-13, IBc-230, IBc-352, and Skr-1) and transfectants derived from IBc-230/IBc-352 total RNA transfection into recipient strains B05.10, IBc-111, and IBc-230-26-16, we validated the predicted virus co-occurrence rules by identifying 35-65 “one-to-one” and 59-406 “two-to-one” interaction cases per strain in their single-spore/protoplast offsprings (Fig. 3 and 4; Table 1; Tables S6 to S15). Eight fungal strains, namely, Ca-1, Ca-13, IBc-230, IBc-352, Skr-1, IBc-230-26-16, B05.10, and IBc-111, exhibited co-infection by 10, 5, 10, 12, 13, 4, 0, and 0 viruses, respectively (Fig. S4 and S5). To assess the accuracy of the predicted virus co-occurrence rules, we isolated single-spore offsprings from fungal strains Ca-1 (*n* = 8), Ca-13 (*n* = 9), IBc-230 (*n* = 10), and Skr-1 (*n* = 16), and generated six protoplast regenerants from the conidium-deficient strain IBc-352. For transfection experiments, we produced three sets of viral transfectants in the IBc-230 background: 19 transfectants transfected with IBc-230-26-16, 11 with B05.10, and 6 with IBc-111. Similarly, in the IBc-352 background, we obtained 13 B05.10-transfected and 6 IBc-111-transfected transfectants. Using RT-PCR (Fig. S4 to S6), we determined the virus compositions in these progenies. The results showed that the “one-to-one” rule had an accuracy rate over 95% (with only two exceptions), and the “two-to-one” rule had an overall accuracy over 94%, although single-spore offsprings of Skr-1 and IBc-111 (transfectants from IBc-111 with IBc-352) had rates of 87.6% and 89.1%, respectively (Table 1).

**TABLE 1:** Examination of the accuracy of predicted compatibility rules of viruses by using *B. cinerea* strains and their offspring, and introduced viruses into strain IBc-230-26-16 and those without viruses^*a*^

Strain	No. of viruses	No. of offspring/virus transfectant	Rules of one-to-one			Rules of two-to-one		
			Predicted	Confirmed	Accuracy (%)	Predicted	Confirmed	Accuracy (%)
Ca-1 (the single-spore offspring of conidia of Ca-1)	10	8	35	35	100	146	146	100
Ca-13 (the single-spore offspring of conidia of Ca-13)	5	9	22	22	100	59	59	100
IBc-230 (the single-spore offspring of conidia of IBc-230)	10	10	50	50	100	261	257	98.5
IBc-352 (the protoplast offspring of IBc-352)	12	7	33	33	100	146	146	100
Skr-1 (the single-spore offspring of conidia of Skr-1)	13	16	63	63	100	340	298	87.6
IBc-230-26-16 (the transfectants resulting from transfecting strain IBc-230-26-16 with strain IBc-230)	4	20	42	41	97.61	189	186	98.4
B05.10 (the transfectants resulting from transfecting strain B05.10 with strain IBc-230)	0							

^*a*^ Viruses in the offspring and transfectants of tested strains were confirmed individually by using RT-PCR.

### Fungal differential expressed genes associated with infection of multiple mycoviruses

**Fig 5:**
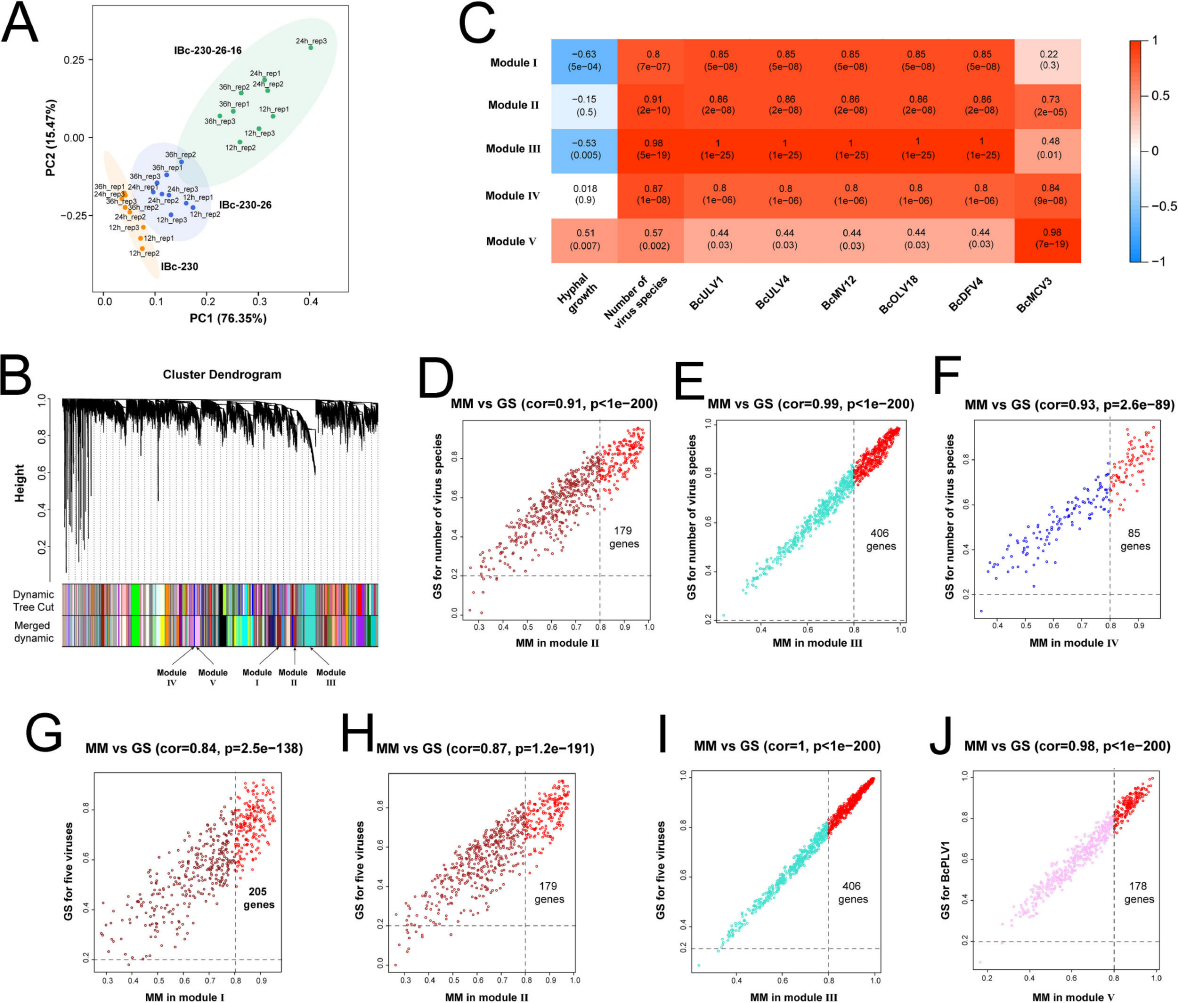
Construction of WGCNA modules of multiple virus co-infection in *B. cinerea* strain IBc-230 and its asexual offsprings. (**A**) PCA plot of all expressed genes after removing the outlier sample. IBc-230 (dark orange, *n* = 8), IBc-230-26 (royal blue, *n* = 9), and IBc-230-26-16 (medium sea green, *n* = 9) samples are plotted along the first two principal component axes (PC1 and PC2). (**B**) Heatmap of module–trait associations. Each column corresponds to a trait, and each row corresponds to an Module Eigengene (ME). The correlation coefficient and corresponding *P* value are marked in their respective rectangles. Red indicates a positive correlation between modules and traits, while blue indicates a negative correlation between modules and traits. (**C**) Clustering dendrograms of 11,553 expressed genes. Each branch in the figure represents one gene, and every color below represents one co-expression module; 58 co-expression modules were constructed and displayed in different colors. (D–F) Scatter plot for correlation between MM and GS of the number of virus species in Module II, Module III, and Module IV, respectively. Red dots represent genes with GS ≥ 0.2 and MM ≥ 0.8 in the modules. (G–I) Scatter plot for correlation between gene MM and GS of five viruses in Module I, Module II, and Module III, and GS of five viruses, respectively. Red dots represent genes with GS ≥ 0.2 and MM ≥ 0.8 in the modules. (**J**) Scatter plot for correlation between gene MM in Module V and GS of BcMCV3. Red dots represent genes with GS ≥ 0.2 and MM ≥ 0.8 in Module V.

We investigated fungal transcriptomes to explore the mechanisms underlying the virus co-occurrence patterns by using strain IBc-230 and its asexual offsprings IBc-230-26 and IBc-230-26-16 (Fig. S5). A total of 36 cDNA libraries were constructed and sequenced using Illumina HiSeq and NovaSeq 6000, generating 722 million clean reads. PCA indicated distinct clustering patterns, with eight samples of IBc-230 segregating from nine samples of IBc-230-26, and similarly from nine samples of IBc-230-26-16. Additionally, nine samples of IBc-230-26 formed a separate cluster from nine samples of IBc-230-26-16 (Fig. 5A).

To elucidate gene expression profiles associated with multiple virus infections in IBc-230, IBc-230-26, and IBc-230-26-16, weighted gene co-expression network analysis (WGCNA) was employed. This analysis aimed at mining expression modules of highly correlated genes and linking these modules to virus infection patterns in host fungal strains. Following data preprocessing, a total of 11,553 genes were included in WGCNA construction with a threshold of β = 10 chosen to ensure a scale-free network (scale-free *R*^2^ = 0.9, slope = −1.87) (Fig. S7).

Initially, 91 modules comprising varying numbers of genes (ranging from 38 to 842) were identified by WGCNA. Following dynamic tree cut and merging the modules with a correlation coefficient > 0.85, 58 modules were ultimately obtained (Fig. 5B). Among these, five modules demonstrating high correlation (cor ≥ 0.85, *P* > 0.05) with the number of co-occurred viruses and the presence of specific virus in a single strain were selected and labeled as Module I, Module II, Module III, Module IV, and Module V.

Module I, Module II, and Module III were associated in the number of virus species in a strain. Module I, Module II, and Module III were associated with the presence of the viruses BcULV1, BcULV4, BcMV12, BcOLV18, and BcDFV4, while Module V was linked to the presence of BcMCV3 (Fig. 5C).

To identify genes linked to viruses within modules, specific criteria were used: gene significance (GS) for the corresponding trait ≥ 0.2 and the module membership (MM) of each gene ≥ 0.8 (Table S16). In Module II, 179 genes associated with co-infection and the presence of five viruses (BcULV1, BcULV4, BcMV12, BcOLV18, and BcDFV4) were identified (Fig. 5D through H), while 406 genes related to the number of virus species co-infection and the presence of these five viruses were identified in Module III (Fig. 5E and I). Module IV encompassed 85 genes associated with co-infection that were identified in Module IV (Fig. 5F), Module I contained 205 genes linked to the presence of these five viruses (Fig. 5G), and Module V comprised 178 genes related to the presence of BcMCV3 (Fig. 5J).

**Fig 6:**
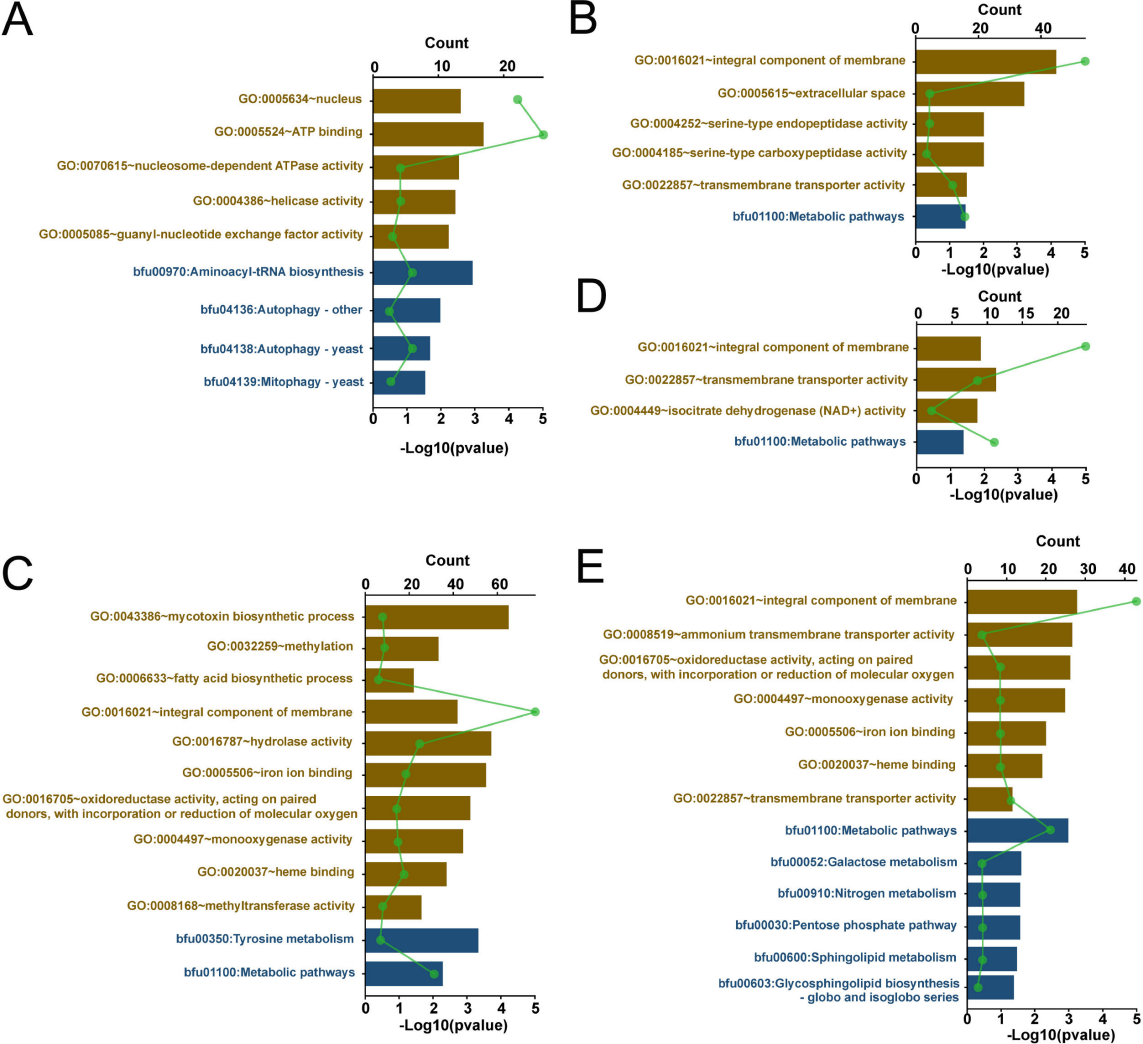
Pathways enriched in the five WGCNA modules by using GO enrichment and KEGG enrichment. (A–E) Double *x*-axis histogram and line chart for the GO and KEGG analyses of genes in Module I, Module II, Module III, Module IV, and Module V. See Table S10 for details. The brown column and blue column represent the enriched GO terms and KEGG pathways, respectively. The height of the histogram represents the normalized *P* value of each enriched pathway in the corresponding module, and the green line chart represents the number of genes in each enriched pathway in the corresponding module.

Furthermore, we performed Gene Ontology (GO) and Kyoto Encyclopedia of Genes and Genomes (KEGG) analyses for genes within the five modules and found the enrichment of the integral component of membrane (GO: 0016021) and metabolic pathways (bfu01100) in all four modules except Module I. Transmembrane transporter activity (GO: 0022857) was enriched in Module II, IV, and V. Autophagy pathways (bfu04136 and bfu04138) and mitophagy pathway (bfu04139) were enriched in Module I, while the fatty acid biosynthetic process (GO: 0006633) was enriched in Module III. Additionally, pathways, such as sphingolipid metabolism (bfu00600) and glycosphingolipid biosynthesis—globo and isoglobo series (bfu00603), were enriched in Module V (Fig. 6; Table S17).

## DISCUSSION

Co-infection by two or three viruses is frequently reported in plants, animals, and humans (26–32), and co-infection by more than three viruses has also been documented only in multiple fungal species and fungus-like species (18, 24, 33–35). Here, we demonstrate that co-infection by a number of viruses is prevalent in the plant pathogenic fungus *B. cinerea*. Out of 406 strains analyzed, only one was devoid of virus infection, two strains were infected by one or two viruses, while the vast majority of strains were co-infected by three or more viruses, with over 86.42% of strains co-infected by six or up to 25 viruses.

Hypovirulence-associated mycoviruses have the potential to control fungal diseases. Nowadays, a single hypovirulence-associated mycovirus has been explored to control fungal diseases (36, 37); future research efforts should focus on exploring hypovirulence induced by multiple co-infecting mycoviruses as a promising strategy for disease management (18, 38). Traditionally, most mycoviruses are thought to be innocuous to their hosts, and the titer of viruses is disregarded. In this study, it was found that among a group of single-spore offsprings with identical genetic background and composition of virus species, some demonstrated debilitation, including slow growth, weak virulence, and poor conidiation, in which the titer (copy) of each virus varied. Notably, the titer (copy) of each virus varied among the symptomatic and asymptomatic offsprings (Fig. S8). This suggests that maintaining equilibrium among viruses within host cells is crucial, as any disruption to this balance may unfavorably impact the host’s development. The potential benefits of co-infection by multiple viruses to their hosts warrant thorough investigation in the future.

The phenomenon of suppression or elimination of virulent strains by mild strains of viruses has been observed initially in animals and subsequently in plants. This concept is being developed for potential use as vaccines to treat virus diseases in humans and animals, or as a form of cross-protection to prevent and treat plant virus diseases (26, 27). While viral interactions in fungi often occur between strains of the same species, exceptional cases of cross-species dependence have been observed, such as the YkV1-YnV1 trans-encapsidation system in *Rosellinia necatrix* (39). Co-infection by two viruses exacerbates disease symptoms compared to infection by a single virus (28, 29, 40), suggesting that there are also interactions between different viruses.

In this study, we have unveiled two sets of virus co-occurrence rules, namely, “One-to-one” and “two-to-one” rules, pertaining to multiple virus infections in *B. cinerea*. Furthermore, we have identified the association rules of “three viruses to one virus” among viruses under the Lift dynamic threshold (Lift = 1.197, Support ≥ 0.073) and FPR < 5% (Fig. S5A and B). Many viruses in co-occurrence rules have distant phylogenetic relationships with each other. These findings suggest that certain viruses, irrespective of phylogenetic relationships, exhibit compatibility or incompatibility. However, whether co-existing viruses have adapted to each other over an extended time period compared to viruses lacking associations is not well known. Individual offspring from strains co-infected with multiple viruses may lose some viruses during reproduction and potentially acquire other viruses horizontally in fields, from other fungi or non-fungal organisms (41, 42). The phenomenon wherein two or multiple viruses collectively determine the co-occurrence of other viruses within a strain has an important implication because this suggests a potential avenue for treating virus disease in humans, animals, and plants by introducing non-pathogenic viruses.

We have unveiled host gene modules associated with specific virus infection using the strain IBc-230 and its two asexual offsprings. We have identified five modules that play a role in the presence or absence of viruses in a co-infected strain. These five modules encompass a multitude of genes, implying that the complex host processes determine virus preference. We further discovered that several pathways, including metabolic pathways, integral component of membrane, transmembrane transporter activity, autophagy pathways, mitophagy pathway, fatty acid biosynthetic process, sphingolipid metabolism, and glycosphingolipid biosynthesis, might be involved in virus-specific host expression. Intracellular membranes are of vital importance for virus replication and survival in host cells, as many viruses form virus factories or viroplasm with host membrane (43–46). Fatty acid, sphingolipid, and glycosphingolipid are integral components of membranes and have been found to play pivotal roles in virus replication (41, 47–52). Furthermore, autophagy pathways are known to be involved in virus replication (52, 53). Genes related to the RNAi pathway and genes associated with the SAGA complex were identified in these modules. Both RNAi pathways and the SAGA complex are involved in antivirus defense mechanisms in fungi and other organisms (54–57). Based on the results of the enrichment analysis in this study and in previous studies, it can be deduced that these five gene modules are associated with viral replication and the interaction between viruses and the fungal antiviral response.

Our results demonstrate that mycoviruses naturally exhibit broad host ranges across fungal species. Previously, mycovirus transmission among phylogenetic distant fungi has been rarely found (40). Among the viruses detected in our study in *B. cinerea*, 30 out of 76 have been previously reported to occur in *Sclerotinia sclerotiorum* and two viruses in *Sclerotinia nivalis*. Given that both *Sclerotinia* and *Botrytis* belong to the family Sclerotiniaceae, it is plausible that these viruses may be transmitted among fungi within this family. Moreover, three viruses, namely, BcMV1, Botrytis cinerea partitivirus 10, and Botrytis cinerea victorivirus 3, were previously identified in *Ophiostoma novo-ulmi*, *Alternaria tenuissima*, and *Penicillium digitatum*, respectively, suggesting a cross-class transmission as these fungi belong to classes different from *B. cinerea*. The possibility of cross-transmission of mycoviruses among different fungi raises questions about potential ecological functions, which warrant careful evaluation in the future (28).

In summary, 76 viruses were identified from 405 field strains of *B. cinerea*, and virus preference patterns in co-infected strains were unveiled. Many host processes may be involved in the host’s preference for certain viruses and virus co-occurrence. Our study highlights the prevalence of multiple virus infections and paves the way to a further understanding of virus co-occurrence and its ecological significance. Such insights would provide new ideas to treat viral diseases of humans, animals, and plants.

## MATERIALS AND METHODS

### Fungal strains and incubation

*B. cinerea* strains were collected from symptomatic plant tissues grown in commercial fields or greenhouses around Israel (Table S1). Fungi were cultured on potato dextrose agar (PDA) supplemented with cephalosporin. Small hyphal plugs from the edge of the colony were transferred to fresh media, and this process was repeated until the appearance of clean-looking cultures. Glycerol stock was prepared from each culture and stored at −80°C, and selected cultures were stored on PDA slants at 4°C. For shipment, conidia or dry mycelia (in case of strains that did not produce conidia) were adsorbed onto sterile filter paper. The strains were recovered by placing on PDA plate under 20°C, and developing hyphae were removed into fresh PDA slants, grown under 20°C, and then stored at 4°C. All strains are listed in Table S1.

### Virome assay

Virome assay was carried out using a method described by Ruiz-Padilla et al. (58). To harvest the mycelia, 406 strains were cultured on cellophane laid on PDA plates for up to 3 d at 20°C. To extract RNA samples conveniently, fresh mycelia of each five strains were mixed equally (1 g for each strain) and ground in liquid nitrogen with a mortar and pestle to a fine powder. The total RNA was extracted using a TRIzol RNA extraction kit (TaKaRa Biotechnology Co. Ltd., Dalian, China). RNA samples were further combined equally into seven groups; after removing rRNA using an Illumina Ribo Zero rRNA Removal Kit, the RNA samples were then sequenced by using the HiSeq 2500 platform (Illumina, San Diego, CA, USA). All library preparation and sequencing were performed by GENEWIZ Inc. (Suzhou, China). Assembled contigs annotated with “virus” or “viral” were retrieved as putative viruses.

The total RNA sample from each of the *B. cinerea* strains was used for cDNA synthesis with EasyScript One-Step gDNA Removal and cDNA Synthesis SuperMix (TransGen Biotech). A dilution of the synthesized cDNA was used as a template in a PCR (2× Hieff PCR Master Mix [With Dye] [YEASEN Biotech]) with specific primers designed based on viral contig sequences.

### Analysis of co-occurrence among mycoviruses in *B. cinerea*

Mycoviruses with occurrence correlation were selected for association rule mining by using the Apriori algorithm (ARM) (59, 60). A series of rules was generated from our data set where occurrence (present or absent) between each virus in 405 strains of *B. cinerea* was calculated. For example, virus A in our data set has two levels, “True” and “False,” representing the presence or absence of virus A in a strain, respectively. ARM would generate two features for virus A, namely, Virus A = True and Virus A = False. The rule came in the form of {Virus A = True/False} => {Virus B = True/False} was called “one-to-one.” If the left-hand side (LHS) contained two viruses while the right-hand side (RHS) had one virus, the rule was called “two-to-one,” such as {Virus A = True/False, Virus B = True/False} => {Virus C = True/False}. If the LHS contained three viruses and the RHS had only one virus, the rule was called “Three to One,” such as {Virus A = True/False, Virus B = True/False, Virus C = True/False} => {Virus D = True/False}.

The algorithm employed three fundamental metrics to quantify the power and significance of the rules. These metrics are Support, Confidence, and Lift. Support means the frequency of the rule occurrence containing both virus A and virus B in the total data set, Support = *P* (Virus A ∩ Virus B). Confidence means the frequency of rule occurrence in the cases of the total data set fulfilling the LHS of the rule; here, Confidence = *P* (Virus A ∩ Virus B)/*P* (Virus A). Lift means a measure of significance; here, Lift = *P* (Virus A ∩ Virus B)/[*P* (Virus A) × *P* (Virus B)]. If the occurrence of virus A and virus B is independent, then the Lift would be equal to 1 theoretically. If the occurrence of virus A and virus B is dependent, then the Lift would be greater than 1, and the value is proportional to the power of the rule. The original data were converted into a sparse matrix. At the species level, almost all strains are co-infected with three or more viruses, except for two strains, which were co-infected by one virus or two viruses, so minimum Support and Confidence values were set as 3/(the total number of virus species identified in this study), and all rules with minimum Support and Confidence below this value would be discarded (61). To ensure the results are statistically significant, the Lift threshold would be controlled by the FPR being less than 5%. Because there was a different Lift threshold for each Support and Confidence value, the dynamic thresholding method was used to determine the threshold (62), and then the Lift threshold was determined as FPR = 5%. Each actual rule would be evaluated according to its corresponding Lift threshold, and eventually, a series of rules was generated. The R language package arulesViz was used to visualize association rules (63).

### Verification of association rules by using strains and their offsprings or transfectants

Eight strains, Ca-1, Ca-13, IBc-230, IBc-352, Skr-1, IBc-230-26-16, B05.10, and IBc-111, were co-infected by 10, 5, 10, 12, 13, 4, 0, and 0 viruses, respectively (Table 1; Tables S6 to S15), and were used to verify the association rules.

Strains Ca-13, Ca-1, IBc-230, and Skr-1 were cultured on PDA under 20°C to produce conidia, and then conidia were collected by using sterilized water. A total of 100 µL conidial suspension (10^2^ spores/mL) was spread on a fresh PDA plate and further incubated for 48 h. The small colonies that appeared were moved into a new PDA plate one by one, and each colony was regarded as a single-spore-isolation offspring.

Because strain IBc-352 could not produce any conidia on PDA, a protoplast regeneration method (62) was used to make asexual clones (regenerants). Protoplasts were serially diluted and spread on a regeneration medium with 50 mg/mL cephalosporin and 100 mg/mL ribavirin. Colonies were picked up and moved onto new PDA plates individually. All clones were used to detect viruses.

Three strains (IBc-230-26-16, B05.10, and IBc-111) were also used to generate protoplasts. The total RNA (1 μg) of IBc-230 was added to 100 µL of protoplasts (about 1 × 10^8^/mL) of strains IBc-230-26-16, B05.10, and IBc-111 for a transfection assay; PEG-mediated transfection was conducted. Transfected protoplasts were mixed with 48°C regeneration medium and incubated for 5 to 10 d at 20°C. The colonies were transferred onto a fresh PDA plate overlaid with cellophane and incubated for 2 d at 20°C. The total RNA of the strains was extracted and used for virus detection with RT-PCR.

To determine viruses in each single-spore-isolation offspring, the total RNA of each culture was extracted and subjected to RT-PCR detection with viral-specific primers. To verify whether the viruses in the conidial offspring are consistent with the previously predicted association rules, the predicted association rules according to the types of viruses that co-infected strains were screened by using the Apriori algorithm.

### Identification of host genes involved in the compatibility of multiple viruses

Strain IBc-230 and two asexual offsprings, IBc-230-26 and IBc-230-26-16, which were co-infected by different species of viruses, were used to explore the potential mechanism for the co-infection of multiple viruses in a single strain.

Mycelia (wet weight 3 g) of strains IBc-230, IBc-230-26, and IBc-230-26-16 were ground with a sterilized mortar and pestle, and the mycelial fragment suspension was placed into a 100 mL potato dextrose broth (PDB) in a 250 mL flask and was incubated for 6 h at 20°C to restore the activity of mycelial fragments. The mycelial fragments were collected through filter paper and washed three times with sterilized distilled water, and then, about 8 g of the mycelial mass was resuspended in sterilized H_2_O as an inoculum. To inoculate tomato leaves, the inoculum was dropped on a sterilized lens paper and stuck on tomato leaves, and then the inoculated plants were kept at 100% relative humidity. Only one leaf at the same age was inoculated on each plant, and three plants were used for each strain. The lens paper with hypha was collected from the leaves at 12, 24, and 36 hours post-inoculation (hpi). The mycelial mass was used to extract the total RNA. After enriching mRNA with A-T base pairing with magnetic beads with Oligo (dT), the RNA samples were used to perform RNA sequencing by using Illumina NovaSeq 6000 (NOVOGENE Technology, Beijing, China), and the analysis of RNA sequence data was carried out by using a method previously described (63). WGCNA R package (version: 1.69) ( 64)was used to construct the gene co-expression network and identify significant modules. The selected modules with significance were used to identify genes involved in the co-infection of multiple viruses. Modules were combined with virus species of co-infection, and the presence of specific viruses in all strains was used to calculate the GS, and the MM was calculated for each gene. Virus-involved genes were screened against the criteria (the absolute value of GS ≥ 0.20; the absolute value of MM ≥ 0.80). To obtain the biological functions and signaling pathways of virus-related genes, the functional annotation of the GO terms and the KEGG was analyzed by DAVID (https://david.ncifcrf.gov/conversion.jsp). The thresholds for identification of the GO functions and the KEGG pathways of virus-related genes were set as *P* value < 0.05.

### Screening of candidate genes

The co-expression network diagrams of genes in the five modules were visualized using Cytoscape (version 3.10.3). The heatmap of candidate genes was generated using the pheatmap package in Rstudio. Total RNA was extracted from three strains, namely, IBc-230, IBc-230-26, and IBc-230-26-16, and was reverse transcribed into cDNA using reverse transcriptase. QRT-PCR amplification of candidate genes was performed with specific primers, and reference genes were set simultaneously. Fluorescent signals were detected by a real-time PCR instrument (Bio-Rad CFX Connect Real-Time PCR Detection System, Bio-Rad Laboratories, Inc., USA). The qRT-PCR reaction mixture contained 10 µL of 2× SYBR Green Master Mix, 0.5 µL of forward primer (10 µM), 0.5 µL of reverse primer (10 µM), 1 µL of cDNA template, and ddH_₂_O to 20 µL, with cycling conditions of 95°C for 10 min (initial denaturation), 40 cycles of 95°C for 15 s (denaturation), and 60°C for 1 min (annealing and extension). The relative expression level of candidate genes in different samples was calculated using the standard curve method or the 2^(−ΔΔCT)^ method.

## SUPPLEMENTAL MATERIAL

Supplemental figures mbio.00262-25-s0001.docx Fig. S1 to S8.

Supplemental tables mbio.00262-25-s0002.docx Tables S1 to S17.

ASM does not own the copyrights to Supplemental Material that may be linked to, or accessed through, an article. The authors have granted ASM a non-exclusive, world-wide license to publish the Supplemental Material files. Please contact the corresponding author directly for reuse.
