# Supplementary material for: The rules in co-infection of multiple viruses across diverse lineages in a fungal host
Source: mBio. 2025 May 20;16(6):e00262-25. doi: 10.1128/mbio.00262-25 (PMC12153359; doi:10.1128/mbio.00262-25)
Supplement: Supplemental figures — Fig. S1 to S8. [file mbio.00262-25-s0001.docx]

## Supplementary information

Supplementary figures


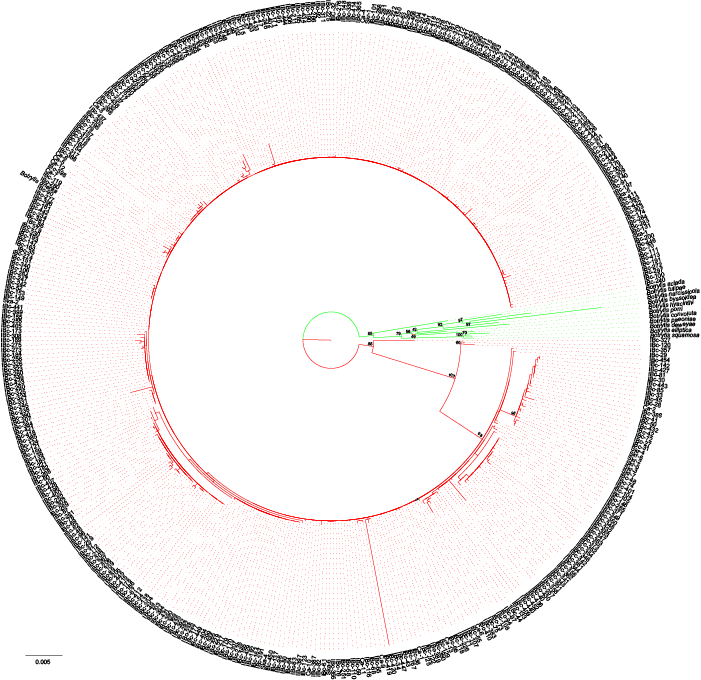


**Fig. S1** **Molecular identification of 406 strains based on multigene phylogenetic analysis.** A maximum-likelihood phylogenetic tree was inferred from the sequences of internal transcribed spacer (ITS), *beta-tubulin*, *elongation factor-1α* (EF-1α), *glyceraldehyde-3-phosphate dehydrogenase* (g3pdh), *heat shock protein 60* (hsp60) and *RNA polymerase II subunit B2* (RPB2) genes. The values above the nodes represent the bootstrap support percentages based on 1000 replicates.


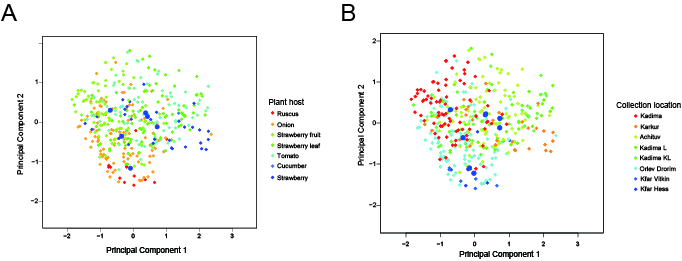


**Fig. S2 The principal component analysis (PCA) of mycoviruses in 406 *B. cinerea* strains, their source plants and collection sites.**

(A). PCA analysis of the relationship between mycoviruses and source plants in 406 *B. cinerea* Strains.

(B). PCA analysis of the relationship between mycoviruses and collection sites in 406 *B. cinerea* Strains.


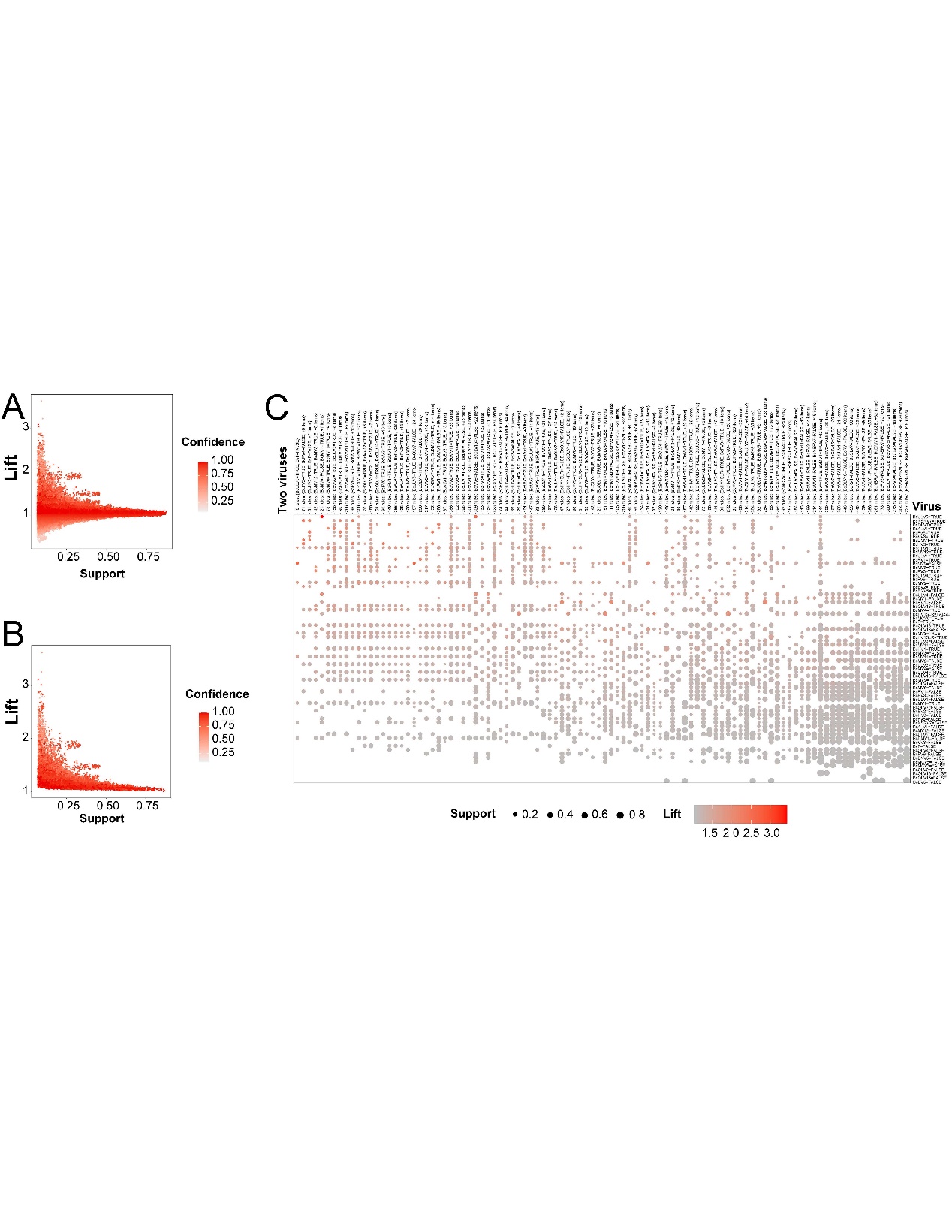


**Fig. S3 “Two-to-One” association rule among viruses in *B. cinerea*.** (A) 102525 candidate association rules were displayed by using a scatter plot, which uses support and lift on the axis. Confidence was shown with the color (red level) of the points. (B) 17765 significant and no random association rules were displayed by using a scatter plot, which uses support and lift on the axis. Confidence was shown with the color (red level) of the points. See Supplementary Table S5 for all association rules and their details. (C) Grouped matrix for partial significant and no random "Two-to-One" association rule among mycoviruses in *B. cinerea*. The grouped matrix containing part of 17765 significant and no random association rules was displayed by using a balloon plot, which uses antecedent groups, i.e. Right Hand Side (RHS) as columns and consequents, i.e., Left Hand Side (LHS) as rows. The color of the balloons represents the size of the Lift value and the size of the balloons shows the size of the Support value. See Supplementary Table S5 for all 17765 rules and their details. The association rules were predicted by using Apriori algorithm (ARM) program.


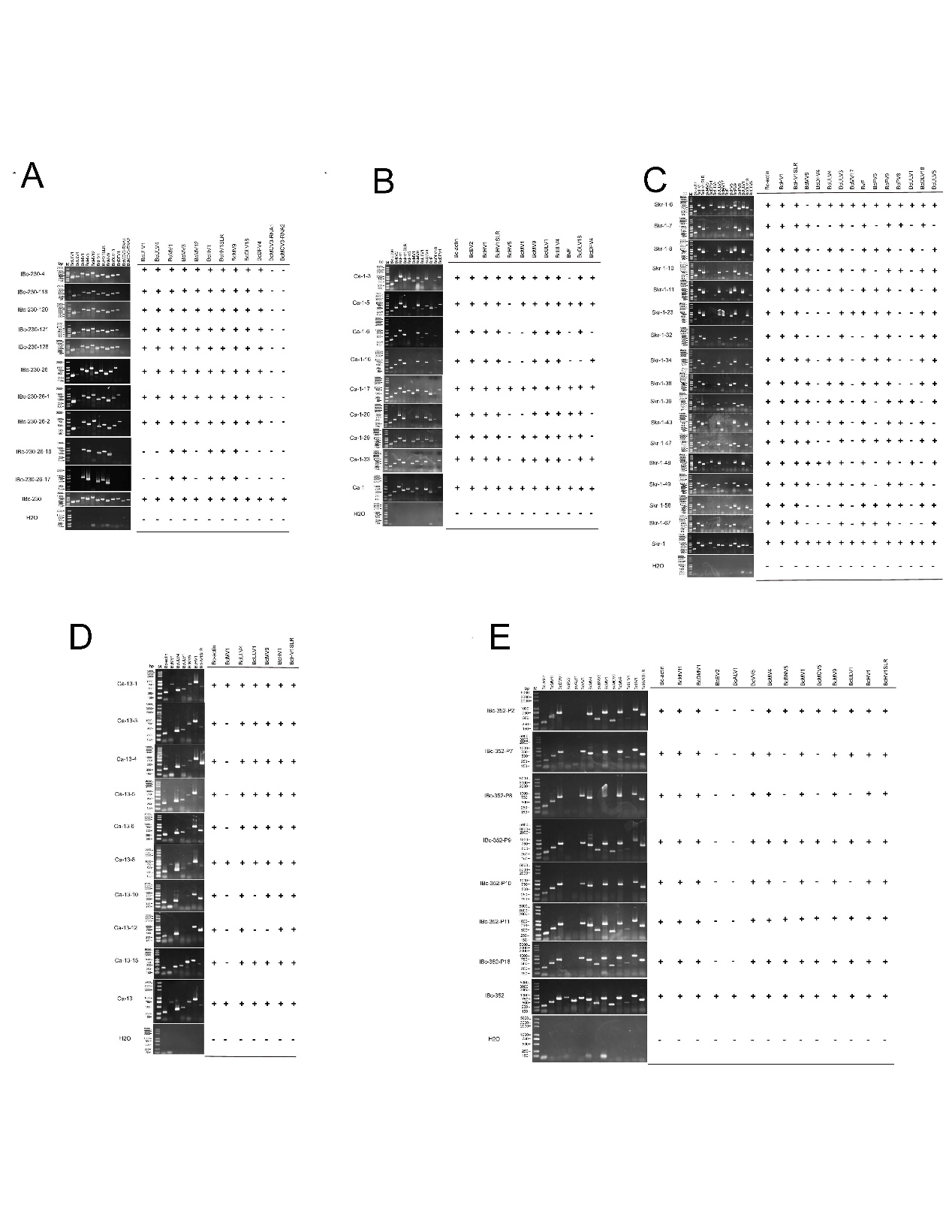


**Fig. S4 RT-PCR Confirmation of the viruses in five strains of *B. cinerea* and their asexual offspring.** (A) Strain IBc-230 and its offspring. Lane 1 to 12: abbreviations of viruses (see Supplementary Table S1 for detail). Lane M: DL2000 DNA molecular weight Marker (Takara Dalian, China). (B) Strain Ca-1 and its offspring. Lane 1 to 12: abbreviations of viruses (see Supplementary Table S1 for detail). (C) Strain Skr-1 and its offspring. Lane 1 to 15: abbreviations of viruses (see Supplementary Table S1 for detail). (D) Strain Skr-1 and its offspring. Lane 1 to 7: abbreviations of viruses (see Supplementary Table S1 for detail). (E) Strain IBc-352 and its offspring. Lane 1 to 14: abbreviations of viruses (see Supplementary Table S1 for detail). Lane M: DL5000 DNA molecular weight Marker (Takara Dalian, China).


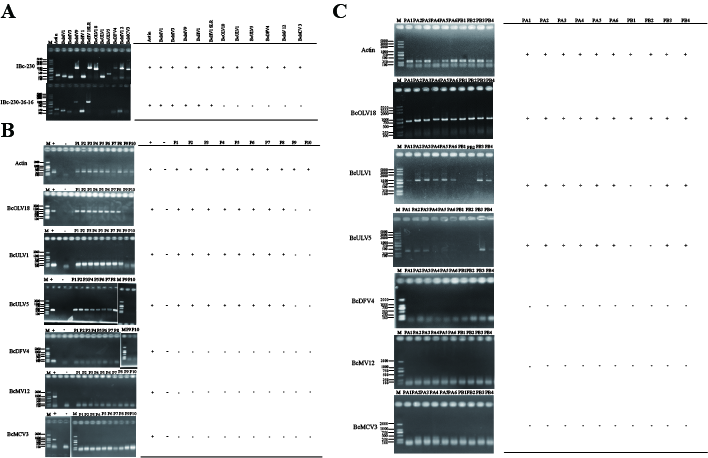


**Fig. S5 RT-PCR confirmation of viruses in transfectants of Strain IBc-230-26-16 following transfection with total RNA of IBc–230.**

(A) Strain IBc-230 and Strain IBc-230-26-16. Lane 1: Actin is the actin gene of *B. cinerea*. Lane 2 to 12: abbreviations of viruses (see Table S1 for detail). Lane M: DL5000 DNA molecular weight Marker (Takara Dalian, China).

(B) Strains P1-P10. The seven electrophoresis gels, from top to bottom, show the distribution of Actin and six viruses (BcOLV18, BcULV1, BcULV5, BcDFV4, BcMV12, and BcMCV3) in ten transfectants. Lane 1: Actin is the actin gene of *B. cinerea*. Lane 3: negative control. Lane 5 to 14: abbreviations of 10 transfectants.

(c) Strains PA1-PA6 and PB1-PB4. The seven electrophoresis gels, from top to bottom, show the distribution of Actin and six viruses (BcOLV18, BcULV1, BcULV5, BcDFV4, BcMV12, and BcMCV3) in ten transfectants. Lane 1 to 10: abbreviations of 10 transfectants.


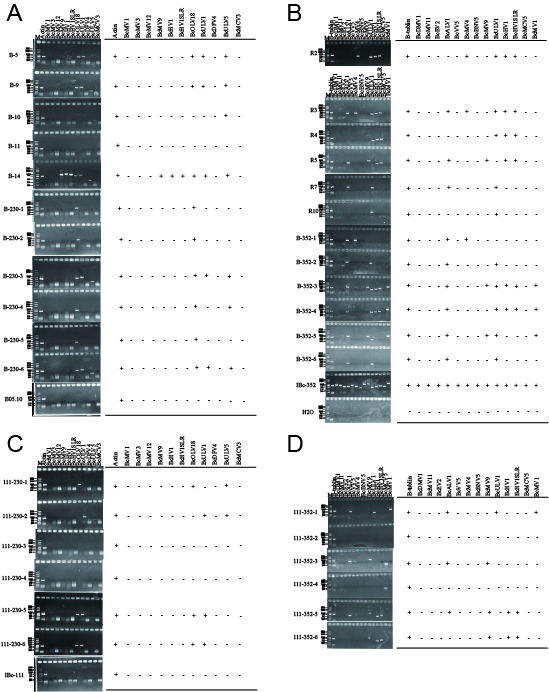


**Fig. S6 RT-PCR confirmation of viruses in transfectants of strains B05.10 and IBc-111 following transfection with total RNA of strains IBc-230 and IBc–352, respectively.**

(A) Transfectants obtained after transfecting strain B05.10 with strain IBc-230. Lane 1: Actin is the actin gene of *B. cinerea*. Lane 2 to 12: abbreviations of viruses (see Table S1 for detail). Lane M: DL5000 DNA molecular weight Marker (Takara Dalian, China).

(B) Transfectants obtained after transfecting strain B05.10 with strain IBc-352. Lane 1: Actin is the actin gene of *B. cinerea*. Lane 2 to 14: abbreviations of viruses (see Table S1 for detail).

(C) Transfectants obtained after transfecting strain IBc-111 with strain IBc-230. Lane 1: Actin is the actin gene of *B. cinerea*. Lane 2 to 12: abbreviations of viruses (see Table S1 for detail).

(D) Transfectants obtained after transfecting strain IBc-111 with strain IBc-352. Lane 1: Actin is the actin gene of *B. cinerea*. Lane 2 to 14: abbreviations of viruses (see Table S1 for detail).


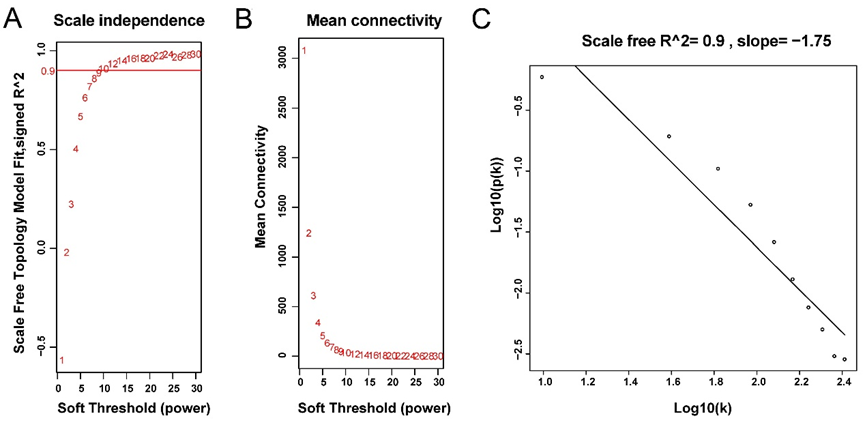


**Fig. S7 Weighting coefficient β selection of gene expression in *B. cinerea* strain IBc-230 and its two asexual offspring.**

(A) Analysis of network topology for various soft-thresholding powers (weighting coefficient, β). The x-axis represents different soft-thresholding powers. The y-axis represents the correlation coefficient between log (k) and log [P(k)]. The red line indicates a correlation coefficient of 0.9. (B) Average network connectivity under different weighting coefficients. (C) Correlation of log (k) and log [P(k)] when β=10.


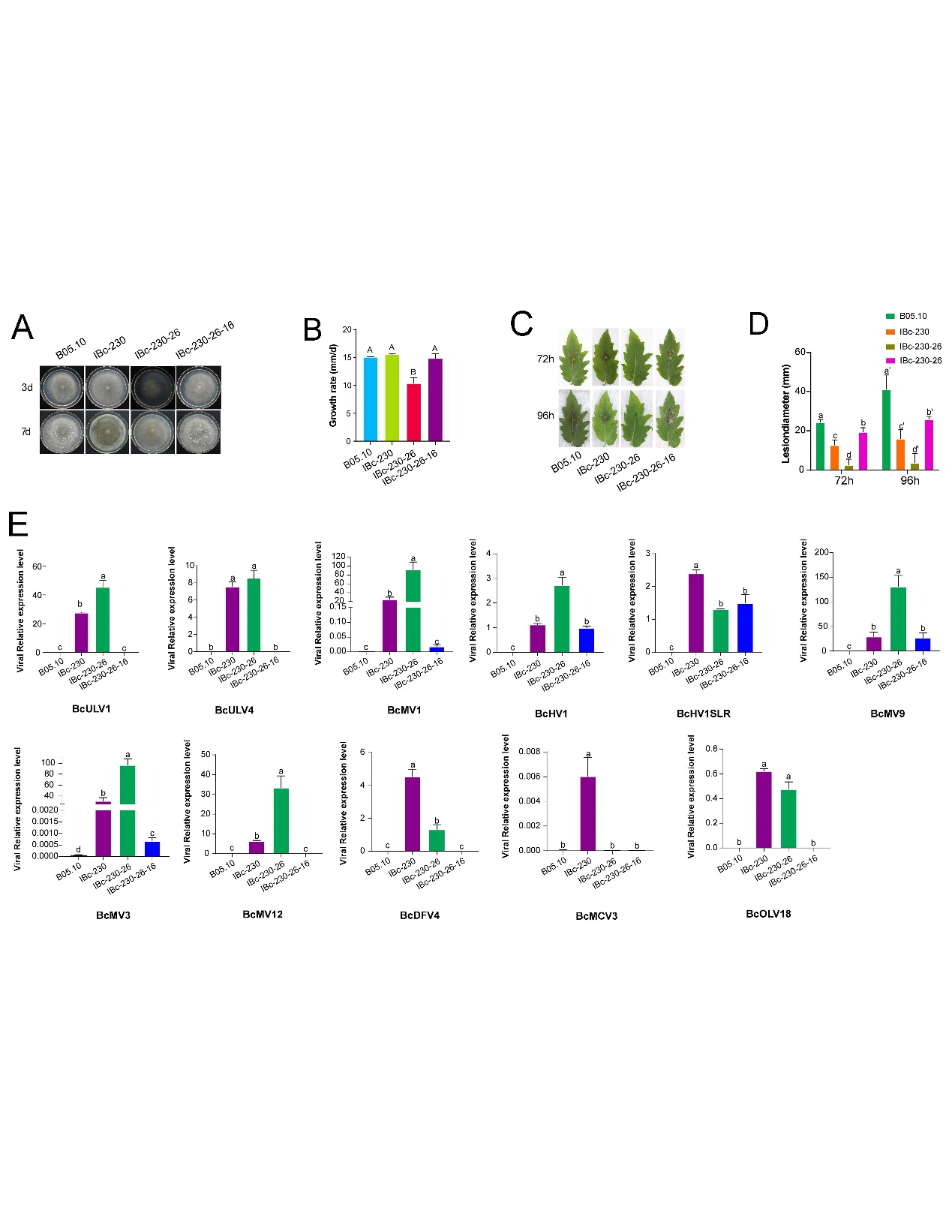


**Fig. S8 The biological characteristics and virus accumulation of *B. cinerea* strain IBc-230 and its asexual offspring.**

(A) Three-day-old and seven-day-old cultures of three strains (IBc-230, IBc-230-26, IBc-230-26-16) (20°C) on potato dextrose agar (PDA). (B) Histogram showing average mycelial growth rates of three strains (IBc-230, IBc-230-26, IBc-230-26-16). Results are means ± standard deviation (SD; n = 3) (p < 0.05). (C) Pathogenicity of three strains (IBc-230, IBc-230-26, IBc-230-26-16) on detached tomato leaves following intact inoculation with mycelia. (D) Histogram showing average lesion diameters of tomato leaves by three strains (IBc-230, IBc-230-26, IBc-230-26-16). (E) Histogram showing viral relative expression level (BcULV1, BcULV4, BcMV1, BcHV1, BcHV1SLR, BcMV9, BcMV3, BcMV12, BcDFV4, BcMCV3 and BcOLV18) in three strains (IBc-230, IBc-230-26, IBc-230-26-16) by qRT-PCR.
