# Supplementary material for: The rules in co-infection of multiple viruses across diverse lineages in a fungal host
Source: mBio. 2025 May 20;16(6):e00262-25. doi: 10.1128/mbio.00262-25 (PMC12153359; doi:10.1128/mbio.00262-25)
Supplement: Supplemental tables — Tables S1 to S17. [file mbio.00262-25-s0002.docx]

Table S1 Collection of *Botrytis cinerea* strains

| **Tube #** | **name** | **source plant** | **place collected** | **date collected** |
| --- | --- | --- | --- | --- |
| 1 | Ska-1 | strawberry | Kadima | 27.3.2018 |
| 2 | Ska-2 | strawberry | Kadima | 27.3.2018 |
| 3 | Ska-3 | strawberry | Kadima | 27.3.2018 |
| 4 | Ska-4 | strawberry | Kadima | 27.3.2018 |
| 5 | Ska-5 | strawberry | Kadima | 27.3.2018 |
| 6 | Ska-6 | strawberry | Kadima | 27.3.2018 |
| 7 | Skr-1 | strawberry | Karkur | 27.3.2018 |
| 8 | Skr-2 | strawberry | Karkur | 27.3.2018 |
| 9 | Skr-3 | strawberry | Karkur | 27.3.2018 |
| 10 | Skr-4 | strawberry | Karkur | 27.3.2018 |
| 11 | Skr-5 | strawberry | Karkur | 27.3.2018 |
| 12 | Skr-6 | strawberry | Karkur | 27.3.2018 |
| 13 | Skr-7 | strawberry | Karkur | 27.3.2018 |
| 14 | Ca-1 | cucumber | Achituv | 27.3.2018 |
| 15 | Ca-2 | cucumber | Achituv | 27.3.2018 |
| 16 | Ca-3 | cucumber | Achituv | 27.3.2018 |
| 17 | Ca-4 | cucumber | Achituv | 27.3.2018 |
| 18 | Ca-5 | cucumber | Achituv | 27.3.2018 |
| 19 | Ca-6 | cucumber | Achituv | 27.3.2018 |
| 20 | Ca-7 | cucumber | Achituv | 27.3.2018 |
| 21 | Ca-8 | cucumber | Achituv | 27.3.2018 |
| 22 | Ca-9 | cucumber | Achituv | 27.3.2018 |
| 23 | Ca-10 | cucumber | Achituv | 27.3.2018 |
| 24 | Ca-11 | cucumber | Achituv | 27.3.2018 |
| 25 | Ca-12 | cucumber | Achituv | 27.3.2018 |
| 26 | Ca-13 | cucumber | Achituv | 27.3.2018 |
| 27 | IBc-27 | cucumber | Achituv | 13.1.2019 |
| 28 | IBc-28 | cucumber | Achituv | 13.1.2019 |
| 29 | IBc-29 | cucumber | Achituv | 13.1.2019 |
| 30 | IBc-30 | cucumber | Achituv | 13.1.2019 |
| 31 | IBc-31 | cucumber | Achituv | 13.1.2019 |
| 32 | IBc-32 | cucumber | Achituv | 13.1.2019 |
| 33 | IBc-33 | cucumber | Achituv | 13.1.2019 |
| 34 | IBc-34 | cucumber | Achituv | 13.1.2019 |
| 35 | IBc-35 | cucumber | Achituv | 13.1.2019 |
| 36 | IBc-36 | cucumber | Achituv | 13.1.2019 |
| 37 | IBc-37 | cucumber | Achituv | 13.1.2019 |

| 38 | IBc-38 | cucumber | Achituv | 13.1.2019 |
| --- | --- | --- | --- | --- |
| 39 | IBc-39 | cucumber | Achituv | 13.1.2019 |
| 40 | IBc-40 | cucumber | Achituv | 13.1.2019 |
| 41 | IBc-41 | cucumber | Achituv | 13.1.2019 |
| 42 | IBc-42 | cucumber | Achituv | 13.1.2019 |
| 43 | IBc-43 | cucumber | Achituv | 13.1.2019 |
| 44 | IBc-44 | cucumber | Achituv | 13.1.2019 |
| 45 | IBc-45 | cucumber | Achituv | 13.1.2019 |
| 46 | IBc-46 | cucumber | Achituv | 13.1.2019 |
| 47 | IBc-47 | cucumber | Achituv | 13.1.2019 |
| 48 | IBc-48 | cucumber | Achituv | 13.1.2019 |
| 49 | IBc-49 | cucumber | Achituv | 13.1.2019 |
| 50 | IBc-50 | cucumber | Achituv | 13.1.2019 |
| 51 | IBc-51 | cucumber | Achituv | 13.1.2019 |
| 52 | IBc-52 | cucumber | Achituv | 13.1.2019 |
| 53 | IBc-53 | cucumber | Achituv | 13.1.2019 |
| 54 | IBc-54 | cucumber | Achituv | 13.1.2019 |
| 55 | IBc-55 | cucumber | Achituv | 13.1.2019 |
| 56 | IBc-56 | cucumber | Achituv | 13.1.2019 |
| 57 | IBc-57 | cucumber | Achituv | 13.1.2019 |
| 58 | IBc-58 | cucumber | Achituv | 13.1.2019 |
| 59 | IBc-59 | cucumber | Achituv | 13.1.2019 |
| 60 | IBc-60 | cucumber | Achituv | 13.1.2019 |
| 61 | IBc-61 | cucumber | Achituv | 13.1.2019 |
| 62 | IBc-62 | cucumber | Achituv | 13.1.2019 |
| 63 | IBc-63 | cucumber | Achituv | 13.1.2019 |
| 64 | IBc-64 | cucumber | Achituv | 13.1.2019 |
| 65 | IBc-65 | cucumber | Achituv | 13.1.2019 |
| 66 | IBc-66 | cucumber | Achituv | 13.1.2019 |
| 67 | IBc-67 | cucumber | Achituv | 13.1.2019 |
| 68 | IBc-68 | cucumber | Achituv | 13.1.2019 |
| 69 | IBc-69 | cucumber | Achituv | 13.1.2019 |
| 70 | IBc-70 | cucumber | Achituv | 13.1.2019 |
| 71 | IBc-71 | cucumber | Achituv | 13.1.2019 |
| 72 | IBc-72 | cucumber | Achituv | 13.1.2019 |
| 73 | IBc-73 | cucumber | Achituv | 13.1.2019 |
| 74 | IBc-74 | cucumber | Achituv | 13.1.2019 |
| 75 | IBc-75 | cucumber | Achituv | 13.1.2019 |
| 76 | IBc-76 | tomato | Achituv | 13.1.2019 |

| 77 | IBc-77 | cucumber | Achituv | 14.1.2019 |
| --- | --- | --- | --- | --- |
| 78 | IBc-78 | cucumber | Achituv | 14.1.2019 |
| 79 | IBc-79 | cucumber | Achituv | 14.1.2019 |
| 80 | IBc-80 | cucumber | Achituv | 14.1.2019 |
| 81 | IBc-81 | cucumber | Achituv | 14.1.2019 |
| 82 | IBc-82 | cucumber | Achituv | 14.1.2019 |
| 83 | IBc-83 | cucumber | Achituv | 14.1.2019 |
| 84 | IBc-84 | cucumber | Achituv | 14.1.2019 |
| 85 | IBc-85 | cucumber | Achituv | 14.1.2019 |
| 87 | IBc-87 | cucumber | Achituv | 14.1.2019 |
| 88 | IBc-88 | cucumber | Achituv | 14.1.2019 |
| 89 | IBc-89 | cucumber | Achituv | 14.1.2019 |
| 90 | IBc-90 | cucumber | Achituv | 14.1.2019 |
| 91 | IBc-91 | cucumber | Achituv | 14.1.2019 |
| 92 | IBc-92 | cucumber | Achituv | 14.1.2019 |
| 93 | IBc-93 | cucumber | Achituv | 14.1.2019 |
| 94 | IBc-94 | cucumber | Achituv | 14.1.2019 |
| 95 | IBc-95 | cucumber | Achituv | 14.1.2019 |
| 96 | IBc-96 | cucumber | Achituv | 14.1.2019 |
| 97 | IBc-97 | cucumber | Achituv | 14.1.2019 |
| 98 | IBc-98 | cucumber | Achituv | 14.1.2019 |
| 99 | IBc-99 | cucumber | Achituv | 14.1.2019 |
| 100 | IBc-100 | cucumber | Achituv | 14.1.2019 |
| 101 | IBc-101 | cucumber | Achituv | 14.1.2019 |
| 102 | IBc-102 | cucumber | Achituv | 14.1.2019 |
| 103 | IBc-103 | cucumber | Achituv | 14.1.2019 |
| 104 | IBc-104 | cucumber | Achituv | 14.1.2019 |
| 105 | IBc-105 | cucumber | Achituv | 14.1.2019 |
| 106 | IBc-106 | cucumber | Achituv | 14.1.2019 |
| 107 | IBc-107 | cucumber | Achituv | 14.1.2019 |
| 108 | IBc-108 | cucumber | Achituv | 14.1.2019 |
| 109 | IBc-109 | cucumber | Achituv | 14.1.2019 |
| 110 | IBc-110 | cucumber | Achituv | 14.1.2019 |
| 111 | IBc-111 | cucumber | Achituv | 14.1.2019 |
| 112 | IBc-112 | cucumber | Achituv | 14.1.2019 |
| 113 | IBc-113 | cucumber | Achituv | 14.1.2019 |
| 115 | IBc-115 | cucumber | Achituv | 14.1.2019 |
| 116 | IBc-116 | cucumber | Achituv | 14.1.2019 |
| 117 | IBc-117 | cucumber | Achituv | 14.1.2019 |

| 118 | IBc-118 | cucumber | Achituv | 14.1.2019 |
| --- | --- | --- | --- | --- |
| 119 | IBc-119 | cucumber | Achituv | 14.1.2019 |
| 120 | IBc-120 | cucumber | Achituv | 14.1.2019 |
| 121 | IBc-121 | cucumber | Achituv | 14.1.2019 |
| 122 | IBc-122 | cucumber | Achituv | 14.1.2019 |
| 123 | IBc-123 | cucumber | Achituv | 14.1.2019 |
| 124 | IBc-124 | cucumber | Achituv | 14.1.2019 |
| 125 | IBc-125 | cucumber | Achituv | 14.1.2019 |
| 126 | IBc-126 | cucumber | Achituv | 14.1.2019 |
| 129 | IBc-129 | strawberry leaf | Kadima L | 14.1.2019 |
| 131 | IBc-131 | strawberry leaf | Kadima L | 14.1.2019 |
| 132 | IBc-132 | strawberry leaf | Kadima L | 14.1.2019 |
| 133 | IBc-133 | strawberry leaf | Kadima L | 14.1.2019 |
| 135 | IBc-135 | strawberry leaf | Kadima L | 14.1.2019 |
| 136 | IBc-136 | strawberry leaf | Kadima L | 14.1.2019 |
| 137 | IBc-137 | strawberry leaf | Kadima L | 14.1.2019 |
| 138 | IBc-13 | strawberry leaf | Kadima L | 14.1.2019 |
| 139 | IBc-139 | strawberry leaf | Kadima L | 14.1.2019 |
| 140 | IBc-140 | strawberry leaf | Kadima L | 14.1.2019 |
| 141 | IBc-141 | strawberry leaf | Kadima L | 14.1.2019 |
| 142 | IBc-142 | strawberry leaf | Kadima L | 14.1.2019 |
| 144 | IBc-144 | strawberry leaf | Kadima L | 14.1.2019 |
| 145 | IBc-145 | strawberry leaf | Kadima L | 14.1.2019 |
| 146 | IBc-146 | strawberry leaf | Kadima L | 14.1.2019 |
| 147 | IBc-147 | strawberry leaf | Kadima L | 14.1.2019 |
| 148 | IBc-148 | strawberry leaf | Kadima L | 14.1.2019 |
| 149 | IBc-149 | strawberry leaf | Kadima L | 14.1.2019 |
| 150 | IBc-150 | strawberry leaf | Kadima L | 14.1.2019 |
| 151 | IBc-151 | strawberry leaf | Kadima L | 14.1.2019 |
| 152 | IBc-152 | strawberry leaf | Kadima L | 14.1.2019 |
| 153 | IBc-153 | strawberry leaf | Kadima L | 14.1.2019 |
| 154 | IBc-154 | strawberry leaf | Kadima L | 14.1.2019 |
| 155 | IBc-155 | strawberry leaf | Kadima L | 14.1.2019 |
| 156 | IBc-156 | strawberry leaf | Kadima L | 14.1.2019 |
| 157 | IBc-157 | strawberry leaf | Kadima L | 14.1.2019 |
| 159 | IBc-159 | strawberry leaf | Kadima L | 14.1.2019 |
| 160 | IBc-160 | strawberry leaf | Kadima L | 14.1.2019 |
| 161 | IBc-161 | strawberry leaf | Kadima L | 14.1.2019 |
| 162 | IBc-162 | strawberry leaf | Kadima L | 14.1.2019 |

| 163 | IBc-163 | strawberry leaf | Kadima L | 14.1.2019 |
| --- | --- | --- | --- | --- |
| 164 | IBc-164 | strawberry leaf | Kadima L | 14.1.2019 |
| 165 | IBc-165 | strawberry leaf | Kadima L | 14.1.2019 |
| 166 | IBc-166 | strawberry leaf | Kadima L | 14.1.2019 |
| 167 | IBc-167 | strawberry leaf | Kadima L | 14.1.2019 |
| 169 | IBc-169 | strawberry leaf | Kadima L | 14.1.2019 |
| 170 | IBc-170 | strawberry leaf | Kadima L | 14.1.2019 |
| 171 | IBc-171 | strawberry leaf | Kadima L | 14.1.2019 |
| 172 | IBc-172 | strawberry leaf | Kadima L | 14.1.2019 |
| 173 | IBc-173 | strawberry leaf | Kadima L | 14.1.2019 |
| 174 | IBc-174 | strawberry leaf | Kadima L | 14.1.2019 |
| 175 | IBc-175 | strawberry leaf | Kadima L | 14.1.2019 |
| 176 | IBc-176 | strawberry leaf | Kadima L | 14.1.2019 |
| 177 | IBc-177 | strawberry fruit | Kadima KL | 14.1.2019 |
| 178 | IBc-178 | strawberry fruit | Kadima KL | 14.1.2019 |
| 179 | IBc-179 | strawberry fruit | Kadima KL | 14.1.2019 |
| 180 | IBc-180 | strawberry fruit | Kadima KL | 14.1.2019 |
| 181 | IBc-181 | strawberry fruit | Kadima KL | 14.1.2019 |
| 183 | IBc-183 | strawberry fruit | Kadima KL | 14.1.2019 |
| 184 | IBc-184 | strawberry fruit | Kadima KL | 14.1.2019 |
| 185 | IBc-185 | strawberry fruit | Kadima KL | 14.1.2019 |
| 188 | IBc-188 | strawberry fruit | Kadima KL | 14.1.2019 |
| 189 | IBc-189 | strawberry fruit | Kadima KL | 14.1.2019 |
| 190 | IBc-190 | strawberry fruit | Kadima KL | 14.1.2019 |
| 191 | IBc-191 | strawberry fruit | Kadima KL | 14.1.2019 |
| 192 | IBc-192 | strawberry fruit | Kadima KL | 14.1.2019 |
| 193 | IBc-193 | strawberry fruit | Kadima KL | 14.1.2019 |
| 194 | IBc-194 | strawberry fruit | Kadima KL | 14.1.2019 |
| 195 | IBc-195 | strawberry fruit | Kadima KL | 14.1.2019 |
| 196 | IBc-196 | strawberry fruit | Kadima KL | 14.1.2019 |
| 197 | IBc-197 | strawberry fruit | Kadima KL | 14.1.2019 |
| 198 | IBc-198 | strawberry fruit | Kadima KL | 14.1.2019 |
| 200 | IBc-200 | strawberry fruit | Kadima KL | 14.1.2019 |
| 202 | IBc-202 | strawberry fruit | Kadima KL | 14.1.2019 |
| 203 | IBc-203 | strawberry fruit | Kadima KL | 14.1.2019 |
| 204 | IBc-204 | strawberry fruit | Kadima KL | 14.1.2019 |
| 205 | IBc-205 | strawberry fruit | Kadima KL | 14.1.2019 |
| 207 | IBc-207 | strawberry fruit | Kadima KL | 14.1.2019 |
| 208 | IBc-208 | strawberry fruit | Kadima KL | 14.1.2019 |

| 210 | IBc-210 | strawberry fruit | Kadima KL | 14.1.2019 |
| --- | --- | --- | --- | --- |
| 211 | IBc-211 | strawberry fruit | Kadima KL | 14.1.2019 |
| 213 | IBc-213 | strawberry fruit | Kadima KL | 14.1.2019 |
| 214 | IBc-214 | strawberry fruit | Kadima KL | 14.1.2019 |
| 216 | IBc-216 | strawberry fruit | Kadima KL | 14.1.2019 |
| 217 | IBc-217 | strawberry fruit | Kadima KL | 14.1.2019 |
| 218 | IBc-218 | strawberry fruit | Kadima KL | 14.1.2019 |
| 219 | IBc-219 | strawberry fruit | Kadima KL | 14.1.2019 |
| 221 | IBc-221 | strawberry fruit | Kadima KL | 14.1.2019 |
| 222 | IBc-222 | strawberry fruit | Kadima KL | 14.1.2019 |
| 223 | IBc-223 | strawberry fruit | Kadima KL | 14.1.2019 |
| 224 | IBc-224 | strawberry fruit | Kadima KL | 14.1.2019 |
| 225 | IBc-225 | strawberry fruit | Kadima KL | 14.1.2019 |
| 226 | IBc-226 | strawberry fruit | Kadima KL | 14.1.2019 |
| 228 | IBc-228 | strawberry leaf | Orlev Drorim | 14.1.2019 |
| 229 | IBc-229 | strawberry leaf | Orlev Drorim | 14.1.2019 |
| 230 | IBc-230 | strawberry leaf | Orlev Drorim | 14.1.2019 |
| 231 | IBc-231 | strawberry leaf | Orlev Drorim | 14.1.2019 |
| 232 | IBc-232 | strawberry leaf | Orlev Drorim | 14.1.2019 |
| 233 | IBc-233 | strawberry leaf | Orlev Drorim | 14.1.2019 |
| 234 | IBc-234 | strawberry leaf | Orlev Drorim | 14.1.2019 |
| 235 | IBc-235 | strawberry leaf | Orlev Drorim | 14.1.2019 |
| 236 | IBc-236 | strawberry leaf | Orlev Drorim | 14.1.2019 |
| 237 | IBc-237 | strawberry leaf | Orlev Drorim | 14.1.2019 |
| 238 | IBc-238 | strawberry leaf | Orlev Drorim | 14.1.2019 |
| 240 | IBc-240 | strawberry leaf | Orlev Drorim | 14.1.2019 |
| 241 | IBc-241 | strawberry leaf | Orlev Drorim | 14.1.2019 |
| 242 | IBc-242 | strawberry leaf | Orlev Drorim | 14.1.2019 |
| 243 | IBc-243 | strawberry leaf | Orlev Drorim | 14.1.2019 |
| 244 | IBc-244 | strawberry leaf | Orlev Drorim | 14.1.2019 |
| 245 | IBc-245 | strawberry leaf | Orlev Drorim | 14.1.2019 |
| 246 | IBc-246 | strawberry leaf | Orlev Drorim | 14.1.2019 |
| 247 | IBc-247 | strawberry leaf | Orlev Drorim | 14.1.2019 |
| 248 | IBc-248 | strawberry leaf | Orlev Drorim | 14.1.2019 |
| 249 | IBc-249 | strawberry leaf | Orlev Drorim | 14.1.2019 |
| 250 | IBc-250 | strawberry leaf | Orlev Drorim | 14.1.2019 |
| 251 | IBc-251 | strawberry leaf | Orlev Drorim | 14.1.2019 |
| 252 | IBc-252 | strawberry leaf | Orlev Drorim | 14.1.2019 |
| 253 | IBc-253 | strawberry fruit | Orlev Drorim | 14.1.2019 |

| 255 | IBc-255 | strawberry fruit | Orlev Drorim | 14.1.2019 |
| --- | --- | --- | --- | --- |
| 256 | IBc-256 | strawberry fruit | Orlev Drorim | 14.1.2019 |
| 257 | IBc-257 | strawberry fruit | Orlev Drorim | 14.1.2019 |
| 258 | IBc-258 | strawberry fruit | Orlev Drorim | 14.1.2019 |
| 259 | IBc-259 | strawberry fruit | Orlev Drorim | 14.1.2019 |
| 260 | IBc-260 | strawberry fruit | Orlev Drorim | 14.1.2019 |
| 261 | IBc-261 | strawberry fruit | Orlev Drorim | 14.1.2019 |
| 262 | IBc-262 | strawberry fruit | Orlev Drorim | 14.1.2019 |
| 263 | IBc-263 | strawberry fruit | Orlev Drorim | 14.1.2019 |
| 264 | IBc-264 | strawberry fruit | Orlev Drorim | 14.1.2019 |
| 265 | IBc-265 | strawberry fruit | Orlev Drorim | 14.1.2019 |
| 266 | IBc-266 | strawberry fruit | Orlev Drorim | 14.1.2019 |
| 267 | IBc-267 | strawberry fruit | Orlev Drorim | 14.1.2019 |
| 268 | IBc-268 | strawberry fruit | Orlev Drorim | 14.1.2019 |
| 269 | IBc-269 | strawberry fruit | Orlev Drorim | 14.1.2019 |
| 271 | IBc-271 | strawberry fruit | Orlev Drorim | 14.1.2019 |
| 272 | IBc-272 | strawberry fruit | Orlev Drorim | 14.1.2019 |
| 273 | IBc-273 | strawberry fruit | Orlev Drorim | 14.1.2019 |
| 274 | IBc-274 | strawberry fruit | Orlev Drorim | 14.1.2019 |
| 275 | IBc-275 | strawberry fruit | Orlev Drorim | 14.1.2019 |
| 276 | IBc-276 | strawberry fruit | Orlev Drorim | 14.1.2019 |
| 277 | IBc-277 | strawberry fruit | Orlev Drorim | 14.1.2019 |
| 278 | IBc-278 | strawberry fruit | Orlev Drorim | 14.1.2019 |
| 279 | IBc-279 | strawberry fruit | Orlev Drorim | 14.1.2019 |
| 280 | IBc-280 | strawberry fruit | Orlev Drorim | 14.1.2019 |
| 281 | IBc-281 | strawberry fruit | Orlev Drorim | 14.1.2019 |
| 282 | IBc-282 | strawberry fruit | Orlev Drorim | 14.1.2019 |
| 283 | IBc-283 | strawberry fruit | Orlev Drorim | 14.1.2019 |
| 284 | IBc-284 | strawberry fruit | Orlev Drorim | 14.1.2019 |
| 285 | IBc-285 | strawberry fruit | Orlev Drorim | 14.1.2019 |
| 286 | IBc-286 | strawberry fruit | Orlev Drorim | 14.1.2019 |
| 287 | IBc-287 | strawberry fruit | Orlev Drorim | 14.1.2019 |
| 288 | IBc-288 | strawberry fruit | Orlev Drorim | 14.1.2019 |
| 290 | IBc-290 | strawberry fruit | Orlev Drorim | 14.1.2019 |
| 291 | IBc-291 | strawberry fruit | Orlev Drorim | 14.1.2019 |
| 292 | IBc-292 | strawberry fruit | Orlev Drorim | 14.1.2019 |
| 293 | IBc-293 | strawberry fruit | Orlev Drorim | 14.1.2019 |
| 294 | IBc-294 | strawberry fruit | Orlev Drorim | 14.1.2019 |
| 295 | IBc-295 | strawberry fruit | Orlev Drorim | 14.1.2019 |

| 296 | IBc-296 | strawberry fruit | Orlev Drorim | 14.1.2019 |
| --- | --- | --- | --- | --- |
| 297 | IBc-297 | strawberry fruit | Orlev Drorim | 14.1.2019 |
| 298 | IBc-298 | strawberry fruit | Orlev Drorim | 14.1.2019 |
| 299 | IBc-299 | strawberry fruit | Orlev Drorim | 14.1.2019 |
| 300 | IBc-300 | strawberry leaf | Orlev Drorim | 14.1.2019 |
| 301 | IBc-301 | strawberry leaf | Orlev Drorim | 14.1.2019 |
| 302 | IBc-302 | strawberry leaf | Orlev Drorim | 14.1.2019 |
| 303 | IBc-303 | strawberry leaf | Orlev Drorim | 14.1.2019 |
| 304 | IBc-304 | strawberry leaf | Orlev Drorim | 14.1.2019 |
| 305 | IBc-305 | strawberry leaf | Orlev Drorim | 14.1.2019 |
| 306 | IBc-306 | strawberry leaf | Orlev Drorim | 14.1.2019 |
| 307 | IBc-307 | strawberry leaf | Orlev Drorim | 14.1.2019 |
| 308 | IBc-308 | strawberry leaf | Orlev Drorim | 14.1.2019 |
| 309 | IBc-309 | strawberry leaf | Orlev Drorim | 14.1.2019 |
| 310 | IBc-310 | strawberry leaf | Orlev Drorim | 14.1.2019 |
| 311 | IBc-311 | strawberry leaf | Orlev Drorim | 14.1.2019 |
| 313 | IBc-313 | strawberry leaf | Orlev Drorim | 14.1.2019 |
| 315 | IBc-315 | strawberry leaf | Orlev Drorim | 14.1.2019 |
| 316 | IBc-316 | strawberry leaf | Orlev Drorim | 14.1.2019 |
| 317 | IBc-317 | strawberry leaf | Orlev Drorim | 14.1.2019 |
| 319 | IBc-319 | strawberry leaf | Orlev Drorim | 14.1.2019 |
| 321 | IBc-321 | strawberry leaf | Orlev Drorim | 14.1.2019 |
| 322 | IBc-322 | strawberry leaf | Orlev Drorim | 14.1.2019 |
| 323 | IBc-323 | strawberry leaf | Orlev Drorim | 14.1.2019 |
| 324 | IBc-324 | strawberry leaf | Orlev Drorim | 14.1.2019 |
| 325 | IBc-325 | strawberry leaf | Orlev Drorim | 14.1.2019 |
| 326 | IBc-326 | strawberry leaf | Orlev Drorim | 14.1.2019 |
| 327 | IBc-327 | Onion |  | 14.1.2019 |
| 331 | IBc-331 | Ruscus | Kfar Vitkin | 13.3.2019 |
| 332 | IBc-332 | Ruscus | Kfar Vitkin | 13.3.2019 |
| 334 | IBc-334 | Ruscus | Kfar Vitkin | 13.3.2019 |
| 338 | IBc-338 | Ruscus | Kfar Vitkin | 13.3.2019 |
| 340 | IBc-340 | Ruscus | Kfar Vitkin | 13.3.2019 |
| 341 | IBc-341 | Ruscus | Kfar Vitkin | 13.3.2019 |
| 342 | IBc-342 | Ruscus | Kfar Vitkin | 13.3.2019 |
| 343 | IBc-343 | Ruscus | Kfar Vitkin | 13.3.2019 |
| 344 | IBc-344 | Ruscus | Kfar Vitkin | 13.3.2019 |
| 346 | IBc-346 | Ruscus | Kfar Vitkin | 13.3.2019 |
| 347 | IBc-347 | Ruscus | Kfar Vitkin | 13.3.2019 |

| 348 | IBc-348 | Ruscus | Kfar Vitkin | 13.3.2019 |
| --- | --- | --- | --- | --- |
| 349 | IBc-349 | Ruscus | Kfar Vitkin | 13.3.2019 |
| 350 | IBc-350 | Ruscus | Kfar Vitkin | 13.3.2019 |
| 351 | IBc-351 | Ruscus | Kfar Vitkin | 13.3.2019 |
| 352 | IBc-352 | Ruscus | Kfar Vitkin | 13.3.2019 |
| 353 | IBc-353 | Ruscus | Kfar Vitkin | 13.3.2019 |
| 354 | IBc-354 | Ruscus | Kfar Vitkin | 13.3.2019 |
| 356 | IBc-356 | Ruscus | Kfar Vitkin | 13.3.2019 |
| 357 | IBc-357 | Ruscus | Kfar Vitkin | 13.3.2019 |
| 358 | IBc-358 | Ruscus | Kfar Vitkin | 13.3.2019 |
| 359 | IBc-359 | Ruscus | Kfar Vitkin | 13.3.2019 |
| 360 | IBc-360 | Ruscus | Kfar Vitkin | 13.3.2019 |
| 361 | IBc-361 | Ruscus | Kfar Vitkin | 13.3.2019 |
| 362 | IBc-362 | Ruscus | Kfar Vitkin | 13.3.2019 |
| 363 | IBc-363 | Ruscus | Kfar Vitkin | 13.3.2019 |
| 365 | IBc-365 | Ruscus | Kfar Vitkin | 13.3.2019 |
| 369 | IBc-369 | Ruscus | Kfar Vitkin | 13.3.2019 |
| 371 | IBc-371 | Ruscus | Kfar Vitkin | 13.3.2019 |
| 373 | IBc-373 | Ruscus | Kfar Vitkin | 13.3.2019 |
| 378 | IBc-378 | Tomato | Kfar Hess | 13.3.2019 |
| 379 | IBc-379 | Tomato | Kfar Hess | 13.3.2019 |
| 380 | IBc-380 | Tomato | Kfar Hess | 13.3.2019 |
| 381 | IBc-381 | Tomato | Kfar Hess | 13.3.2019 |
| 382 | IBc-382 | Tomato | Kfar Hess | 13.3.2019 |
| 383 | IBc-383 | Tomato | Kfar Hess | 13.3.2019 |
| 384 | IBc-384 | Tomato | Kfar Hess | 13.3.2019 |
| 385 | IBc-385 | Tomato | Kfar Hess | 13.3.2019 |
| 386 | IBc-386 | Tomato | Kfar Hess | 13.3.2019 |
| 387 | IBc-387 | Tomato | Kfar Hess | 13.3.2019 |
| 388 | IBc-388 | Tomato | Kfar Hess | 13.3.2019 |
| 389 | IBc-389 | Tomato | Kfar Hess | 13.3.2019 |
| 390 | IBc-390 | Tomato | Kfar Hess | 13.3.2019 |
| 391 | IBc-391 | Tomato | Kfar Hess | 13.3.2019 |
| 392 | IBc-392 | Tomato | Kfar Hess | 13.3.2019 |
| 393 | IBc-393 | Tomato | Kfar Hess | 13.3.2019 |
| 394 | IBc-394 | Tomato | Kfar Hess | 13.3.2019 |
| 395 | IBc-395 | Tomato | Kfar Hess | 13.3.2019 |
| 396 | IBc-396 | Tomato | Kfar Hess | 13.3.2019 |
| 397 | IBc-397 | Tomato | Kfar Hess | 13.3.2019 |

| 398 | IBc-398 | Tomato | Kfar Hess | 13.3.2019 |
| --- | --- | --- | --- | --- |
| 399 | IBc-399 | Tomato | Kfar Hess | 13.3.2019 |
| 400 | IBc-400 | Tomato | Kfar Hess | 13.3.2019 |
| 401 | IBc-401 | Tomato | Kfar Hess | 13.3.2019 |
| 402 | IBc-402 | Tomato | Kfar Hess | 13.3.2019 |
| 403 | IBc-403 | Tomato | Kfar Hess | 13.3.2019 |
| 404 | IBc-404 | Tomato | Kfar Hess | 13.3.2019 |
| 405 | IBc-405 | Tomato | Kfar Hess | 13.3.2019 |
| 406 | IBc-406 | Tomato | Kfar Hess | 13.3.2019 |
| 407 | IBc-407 | Tomato | Kfar Hess | 13.3.2019 |
| 408 | IBc-408 | Tomato | Kfar Hess | 13.3.2019 |
| 409 | IBc-409 | Tomato | Kfar Hess | 13.3.2019 |
| 410 | IBc-410 | Tomato | Kfar Hess | 13.3.2019 |
| 411 | IBc-411 | Tomato | Kfar Hess | 13.3.2019 |
| 412 | IBc-412 | Tomato | Kfar Hess | 13.3.2019 |
| 413 | IBc-413 | Tomato | Kfar Hess | 13.3.2019 |
| 414 | IBc-414 | Tomato | Kfar Hess | 13.3.2019 |
| 415 | IBc-415 | Tomato | Kfar Hess | 13.3.2019 |
| 416 | IBc-416 | Tomato | Kfar Hess | 13.3.2019 |
| 417 | IBc-417 | Tomato | Kfar Hess | 13.3.2019 |
| 418 | IBc-418 | Tomato | Kfar Hess | 13.3.2019 |
| 419 | IBc-419 | Tomato | Kfar Hess | 13.3.2019 |
| 420 | IBc-420 | Tomato | Kfar Hess | 13.3.2019 |
| 421 | IBc-421 | Tomato | Kfar Hess | 13.3.2019 |
| 422 | IBc-422 | Tomato | Kfar Hess | 13.3.2019 |
| 423 | IBc-423 | Tomato | Kfar Hess | 13.3.2019 |
| 424 | IBc-424 | Tomato | Kfar Hess | 13.3.2019 |
| 425 | IBc-425 | Tomato | Kfar Hess | 13.3.2019 |
| 426 | IBc-426 | Tomato | Kfar Hess | 13.3.2019 |
| 427 | IBc-427 | Tomato | Kfar Hess | 13.3.2019 |
| 428 | IBc-428 | Tomato | Kfar Hess | 13.3.2019 |
| 429 | IBc-429 | Tomato | Kfar Hess | 13.3.2019 |
| 430 | IBc-430 | Tomato | Kfar Hess | 13.3.2019 |
| 431 | IBc-431 | Tomato | Kfar Hess | 13.3.2019 |
| 432 | IBc-432 | Tomato | Kfar Hess | 13.3.2019 |
| 433 | IBc-433 | Tomato | Kfar Hess | 13.3.2019 |
| 434 | IBc-434 | Tomato | Kfar Hess | 13.3.2019 |
| 435 | IBc-435 | Tomato | Kfar Hess | 13.3.2019 |
| 436 | IBc-436 | Tomato | Kfar Hess | 13.3.2019 |

| 437 | IBc-437 | Tomato | Kfar Hess | 13.3.2019 |
| --- | --- | --- | --- | --- |
| 438 | IBc-438 | Tomato | Kfar Hess | 13.3.2019 |
| 439 | IBc-439 | Tomato | Kfar Hess | 13.3.2019 |
| 440 | IBc-440 | Tomato | Kfar Hess | 13.3.2019 |
| 441 | IBc-441 | Tomato | Kfar Hess | 13.3.2019 |
| 442 | IBc-442 | Tomato | Kfar Hess | 13.3.2019 |
| 443 | IBc-443 | Tomato | Kfar Hess | 13.3.2019 |
| 444 | IBc-444 | Tomato | Kfar Hess | 13.3.2019 |
| 445 | IBc-445 | Tomato | Kfar Hess | 13.3.2019 |
| 446 | IBc-446 | Tomato | Kfar Hess | 13.3.2019 |
| 447 | IBc-447 | Tomato | Kfar Hess | 13.3.2019 |
| 448 | IBc-448 | Tomato | Kfar Hess | 13.3.2019 |
| 449 | IBc-449 | Tomato | Kfar Hess | 13.3.2019 |
| 450 | IBc-450 | Tomato | Kfar Hess | 13.3.2019 |
| 451 | IBc-451 | Tomato | Kfar Hess | 13.3.2019 |
| 452 | IBc-452 | Tomato | Kfar Hess | 13.3.2019 |
| 453 | IBc-453 | Tomato | Kfar Hess | 13.3.2019 |
| 454 | IBc-454 | Tomato | Kfar Hess | 13.3.2019 |

**Table S2 Viral contigs obtained from 406 strains of *Botrytis cinerea* collected from Israel by using High-throughput sequencing analysis**

| **Contig name** | **GenBase accession numbers** | **Contig length (bp)** | **Virus name (virus strain)** | **Virus abbrev** | **Genome type** | **Best hit** | **Accession (best hit)** | **Identity (%)** | **Query Cover (%)** | **Host(Best hit)** | **Virus length(bp)** | **Taxonomic status** | | **References** |
| --- | --- | --- | --- | --- | --- | --- | --- | --- | --- | --- | --- | --- | --- | --- |
|  |  |  |  |  |  |  |  |  |  |  |  | Family | Genus |  |
| yang3_Contig518 | C_AA045425.1 | 2255 | Botrytis cinerea binarnavirus 5-BcIsrael | BcBNV5-BI | +ssRNA | Sclerotinia sclerotiorum narnavirus 2 | MW442882.1 | 98.87% | 94.00% | *Sclerotinia sclerotiorum* | 2259 | *Narnaviridae* | *Narnavirus* | Jia,J, et al 2021 |
|  |  |  |  |  |  | Botrytis cinerea binarnavirus 5 | MT711187.1 | 95.90% | 94.00% | *Botrytis cinerea* | 2253 | *Narnaviridae* | *Narnavirus* | Ruiz-Padilla,A et al 2021 |
| yang3_Contig462 | C_AA045426.1 | 2373 |  |  | +ssRNA | Botrytis cinerea binarnavirus 5 | MN619799.1 | 98.14% | 93.00% | *Botrytis cinerea* | 2411 | *Narnaviridae* | *Narnavirus* | Ruiz-Padilla,A et al 2021 |
|  |  |  |  |  |  | Sclerotinia sclerotiorum narnavirus 2 | MW442881.1 | 98.41% | 93.00% | *Sclerotinia sclerotiorum* | 2384 | *Narnaviridae* | *Narnavirus* | Jia,J, et al 2021 |
| BC-2_Contig457 | C_AA045495.1 | 2144 | Botrytis cinerea binarnavirus 6 | BcBNV6 | +ssRNA | Erysiphe necator associated narnavirus 30 | MN605443.1 | 62.98% | 83% | *Botrytis cinerea* | 2154 | *Narnaviridae* | *Narnavirus* | Rodriguez-Romero,J et al 2019 |
| BC-1_Contig86 | C_AA045500.1 | 2298 |  |  | +ssRNA | Cryphonectria naterciae splipalmivirus 1 | LC634420.1 | 53.47% | 85% | *Botrytis cinerea* | 2313 | *Narnaviridae* | *Narnavirus* | Sato,Y et al 2022 |
| yang3_Contig450 | C_AA045427.1 | 2469 | Botrytis cinerea narnavirus 4-BcIsrael | BcBNV4-BI | +ssRNA | Botrytis cinerea narnavirus 4 | MN619798.1 | 98.92% | 89% | *Botrytis cinerea* | 2471 | *Narnaviridae* | *Narnavirus* | Ruiz-Padilla,A et al 2021 |
| BC-1_Contig740 | C_AA045428.1 | 2279 | Botrytis cinerea binarnavirus 1-BcIsrael | BcBNV1-BI | +ssRNA | Botrytis cinerea binarnavirus 1 | MT711186.1 | 99.58% | 94% | *Botrytis cinerea* | 2288 | *Narnaviridae* | *Narnavirus* | Ruiz-Padilla,A et al 2021 |
| BC-1_Contig230 | C_AA045429.1 | 2532 |  |  |  | Botrytis cinerea binarnavirus 1 | MN619795.1 | 97.27% | 96% | *Botrytis cinerea* | 2572 | *Narnaviridae* | *Narnavirus* | Ruiz-Padilla,A et al 2021 |
|  |  |  |  |  |  | Erysiphe necator associated narnavirus 12 | MN605425.1 | 92.35% | 96.00% | *Erysiphe necator* | 2540 | *Narnaviridae* | *Narnavirus* | Rodriguez-Romero,J et al 2019 |
| yang1_Contig263 | C_AA045430.1 | 2572 | Botrytis cinerea mitovirus 7 | BcMV11 | +ssRNA | Sclerotinia sclerotiorum mitovirus 3-WX | MT646425.1 | 96.91% | 83.00% | *Sclerotinia sclerotiorum* | 2581 | *Mitoviridae* | *Mitovirus* | Jia,J, et al 2021 |
|  |  |  |  |  |  | Sclerotinia sclerotiorum mitovirus 3 | NC_028475.1 | 96.91% | 82.00% | *Sclerotinia sclerotiorum* | 2617 | *Mitoviridae* | *Mitovirus* | Donaire,L et al 2017 |
|  |  |  |  |  |  | Erysiphe necator associated mitovirus 27 | MN611673.1 | 93.04% | 87.00% | *Erysiphe necator* | 2530 | *Mitoviridae* | *Mitovirus* | Rodriguez-Romero,J et al 2019 |
|  |  |  |  |  |  | Sclerotinia sclerotiorum mitovirus 3 | JX401537.1 | 96.07% | 83.00% | *Sclerotinia sclerotiorum* | 2588 | *Mitoviridae* | *Mitovirus* | Khalifa,M.E 2013 |
|  |  |  |  |  |  | Sclerotinia nivalis mitovirus 2 | KT365896.1 | 95.37% | 83.00% | *Sclerotinia nivalis* | 2583 | *Mitoviridae* | *Mitovirus* | Wu,M et al 2015 |
|  |  |  |  |  |  | Sclerotinia sclerotiorum mitovirus 3 | MN954876.1 | 95.83% | 69.00% | *Sclerotinia sclerotiorum* | 2974 | *Mitoviridae* | *Mitovirus* | Ruiz-Padilla,A et al 2021 |
| BC-3_Contig306 | C_AA045487.1 | 2873 | Botrytis cinerea mitovirus 12 | BcMV12 | +ssRNA | Sclerotinia sclerotiorum mitovirus 4 | MN954877.1 | 96.78% | 68.00% | *Sclerotinia sclerotiorum* | 2737 | *Mitoviridae* | *Mitovirus* | Ruiz-Padilla,A et al 2021 |
|  |  |  |  |  |  | Sclerotinia sclerotiorum mitovirus 4 | JX401538.1 | 96.22% | 68.00% | *Sclerotinia sclerotiorum* | 2744 | *Mitoviridae* | *Mitovirus* | Khalifa,M.E 2013 |
|  |  |  |  |  |  | Sclerotinia sclerotiorum mitovirus 4-A | MF444237.1 | 94.27% | 68.00% | *Sclerotinia sclerotiorum* | 2737 | *Mitoviridae* | *Mitovirus* | Mu,F et al 2018 |
|  |  |  |  |  |  | Sclerotinia sclerotiorum mitovirus 4 | KT962974.1 | 93.84% | 68.00% | *Sclerotinia sclerotiorum* | 2752 | *Mitoviridae* | *Mitovirus* | Ran,H et al 2016 |
| BC-2_Contig422 | C_AA045431.1 | 2711 | Botrytis cinerea mitovirus 4-BcIsrael | BcMV4-BI | +ssRNA | Botrytis cinerea mitovirus 4 | NC_028474.1 | 96.85% | 80.00% | *Botrytis cinerea* | 2768 | *Mitoviridae* | *Mitovirus* | Donaire,L et al 2017 |
|  |  |  |  |  |  | Sclerotinia nivalis mitovirus 1 | KT365895.1 | 95.49% | 80.00% | *Sclerotinia nivalis* | 2720 | *Mitoviridae* | *Mitovirus* | Wu,M et al 2015 |
|  |  |  |  |  |  | Sclerotinia sclerotiorum mitovirus 36 | MT646380.1 | 94.05% | 81.00% | *Sclerotinia sclerotiorum* | 2732 | *Mitoviridae* | *Mitovirus* | Jia,J et al 2021 |
|  |  |  |  |  |  | Botrytis cinerea mitovirus 4 | MN954875.1 | 94.13% | 79.00% | *Botrytis cinerea* | 2709 | *Mitoviridae* | *Mitovirus* | Ruiz-Padilla,A et al 2021 |
| BC-1_Contig244 | C_AA045432.1 | 2406 | Botrytis cinerea mitovirus 3-BcIsrael | BcMV3-BI | +ssRNA | Botrytis cinerea mitovirus 3 | NC_028472.1 | 92.40% | 100.00% | *Botrytis cinerea* | 2922 | *Mitoviridae* | *Mitovirus* | Donaire,L et al 2017 |
|  |  |  |  |  |  | Botrytis cinerea mitovirus 3 | OL602091.1 | 91.53% | 100.00% | *Botrytis cinerea* | 4467 | *Mitoviridae* | *Mitovirus* | Deng,Y et al 2021 |
|  |  |  |  |  |  | Sclerotinia sclerotiorum mitovirus 35 | MT646369.1 | 91.53% | 100.00% | *Sclerotinia sclerotiorum* | 2879 | *Mitoviridae* | *Mitovirus* | Jia,J, et al 2021 |
|  |  |  |  |  |  | Botrytis cinerea mitovirus 3 | MN617166.1 | 91.28% | 100.00% | *Botrytis cinerea* | 2977 | *Mitoviridae* | *Mitovirus* | Ruiz-Padilla,A et al 2021 |
|  |  |  |  |  |  | Botrytis cinerea mitovirus 3 | LN827946.1 | 91.03% | 100.00% | *Botrytis cinerea* | 2930 | *Mitoviridae* | *Mitovirus* | Donaire,L et al 2017 |
| yang3_Contig297 | C_AA045433.1 | 2796 | Botrytis cinerea mitovirus 1-BcIsrael | BcMV1-BI | +ssRNA | Botrytis cinerea mitovirus 1 | LN827944.1 | 95.66% | 79.00% | *Botrytis cinerea* | 2820 | *Mitoviridae* | *Mitovirus* | Donaire,L et al 2017 |
|  |  |  |  |  |  | Botrytis cinerea mitovirus 1 | LN827940.1 | 95.53% | 79.00% | *Botrytis cinerea* | 2873 | *Mitoviridae* | *Mitovirus* | Donaire,L et al 2017 |
|  |  |  |  |  |  | Botrytis cinerea mitovirus 1 | MT119677.1 | 94.72% | 79.00% | *Botrytis cinerea* | 2788 | *Mitoviridae* | *Mitovirus* | Ruiz-Padilla,A et al 2021 |
|  |  |  |  |  |  | Botrytis cinerea mitovirus 1 | NC_011372.2 | 93.77% | 79.00% | *Botrytis cinerea* | 2804 | *Mitoviridae* | *Mitovirus* | Wu,M et al 2010 |
|  |  |  |  |  |  | Ophiostoma mitovirus 3b | AM087550.1 | 93.09% | 79.00% | *Ophiostoma novoulmi* | 2332 | *Mitoviridae* | *Mitovirus* | Doherty,M et al 2006 |
| Unigene_Contig1809 | C_AA045488.1 | 2517 | Botrytis cinerea mitovirus 13 | BcMV13 | +ssRNA | Erysiphe necator associated mitovirus 26 | MN611672.1 | 97.87% | 95.00% | *Erysiphe necator* | 2893 | *Mitoviridae* | *Mitovirus* | Rodriguez-Romero,J et al 2019 |
|  |  |  |  |  |  | Plasmopara viticola lesion associated mitovirus 49 | MN539810.1 | 97.87% | 95.00% | *Plasmopara viticola* | 2874 | *Mitoviridae* | *Mitovirus* | Chiapello,M et al 2020 |
| BC-3_Contig482 | C_AA045489.1 | 2565 | Botrytis cinerea mitovirus 14 | BcMV14 | +ssRNA | Sclerotinia sclerotiorum mitovirus 6 | KJ462513.1 | 88.95% | 82.00% | *Sclerotinia sclerotiorum* | 2586 | *Mitoviridae* | *Mitovirus* | Khalifa,M.E et al 2014 |
| BC-3_Contig1026 | C_AA045490.1 | 2214 | Botrytis cinerea mitovirus 15 | BcMV15 | +ssRNA | Sclerotinia sclerotiorum mitovirus 17 | KP900924.1 | 96.67% | 95.00% | *Sclerotinia sclerotiorum* | 2447 | *Mitoviridae* | *Mitovirus* | Marzano,S.-Y.L et al 2016 |
|  |  |  |  |  |  | Sclerotinia sclerotiorum mitovirus 17-U | MW454897.1 | 95.37% | 95.00% | *Sclerotinia sclerotiorum* | 3021 | *Mitoviridae* | *Mitovirus* | Liang,W et al 2021 |
|  |  |  |  |  |  | Sclerotinia sclerotiorum mitovirus 2 | KJ462508.1 | 92.78% | 95.00% | *Sclerotinia sclerotiorum* | 2438 | *Mitoviridae* | *Mitovirus* | Khalifa,M.E 2014 |
|  |  |  |  |  |  | Sclerotinia sclerotiorum mitovirus 2 | NC_040434.1 | 93.33% | 95.00% | *Sclerotinia sclerotiorum* | 2445 | *Mitoviridae* | *Mitovirus* | Xie,J et al 2012 |
|  |  |  |  |  |  | Sclerotinia sclerotiorum mitovirus 2 | JX401536.1 | 92.96% | 95.00% | *Sclerotinia sclerotiorum* | 2438 | *Mitoviridae* | *Mitovirus* | Khalifa,M.E et al 2013 |
|  |  |  |  |  |  | Sclerotinia sclerotiorum mitovirus 2-A | MF444236.1 | 94.91% | 87.00% | *Sclerotinia sclerotiorum* | 1897 | *Mitoviridae* | *Mitovirus* | Mu,F et al 2018 |
| BC-3_Contig1325 | C_AA045491.1 | 1459 | Botrytis cinerea mitovirus 16 | BcMV16 | +ssRNA | Sclerotinia sclerotiorum mitovirus 1-U | UEP19799.1 | 93.98% | 99.00% | *Sclerotinia sclerotiorum* | 2933 | *Mitoviridae* | *Mitovirus* | Liang,W et al 2021 |
|  |  |  |  |  |  | Sclerotinia sclerotiorum mitovirus 1-A2 | AWY10962.1 | 90.65% | 99.00% | *Sclerotinia sclerotiorum* | 2086 | *Mitoviridae* | *Mitovirus* | Mu,F et al 2018 |
|  |  |  |  |  |  | Sclerotinia sclerotiorum mitovirus 1 | AEX91878.1 | 90.25% | 99.00% | *Sclerotinia sclerotiorum* | 2513 | *Mitoviridae* | *Mitovirus* | Xie,J et al 2012 |
| BC-3_Contig521 | C_AA045492.1 | 2462 | Botrytis cinerea mitovirus 17 | BcMV17 | +ssRNA | Sclerotinia sclerotiorum mitovirus 7-A | MF444241.1 | 97.43% | 85.00% | *Sclerotinia sclerotiorum* | 2602 | *Mitoviridae* | *Mitovirus* | Mu,F et al 2018 |
|  |  |  |  |  |  | Sclerotinia sclerotiorum mitovirus 7-U | MW454892.1 | 92.15% | 85.00% | *Sclerotinia sclerotiorum* | 2842 | *Mitoviridae* | *Mitovirus* | Liang,W et al 2021 |
| BC-3_Contig507 | C_AA045434.1 | 2569 | Botrytis cinerea mitovirus 2-BcIsrael | BcMV2-BI | +ssRNA | Botrytis cinerea mitovirus 2 | NC_028471.1 | 97.18% | 85.00% | *Botrytis cinerea* | 2497 | *Mitoviridae* | *Mitovirus* | Donaire,L et al 2017 |
|  |  |  |  |  |  | Botrytis cinerea mitovirus 2 | LN827945.1 | 96.76% | 85.00% | *Botrytis cinerea* | 2496 | *Mitoviridae* | *Mitovirus* | Donaire,L et al 2017 |
|  |  |  |  |  |  | Botrytis cinerea mitovirus 2 | MN617165.1 | 96.62% | 85.00% | *Botrytis cinerea* | 2502 | *Mitoviridae* | *Mitovirus* | Ruiz-Padilla,A et al 2021 |
| yang3_Contig32 | C_AA045435.1 | 2496 | Botrytis cinerea mitovirus 6-BcIsrael | BcMV6-BI | +ssRNA | Botrytis cinerea mitovirus 6 | MN625252.1 | 96.88% | 81.00% | *Botrytis cinerea* | 2519 | *Mitoviridae* | *Mitovirus* | Ruiz-Padilla,A et al 2021 |
| BC-1_Contig348 | C_AA045436.1 | 2713 | Botrytis cinerea mitovirus 9-BcIsrael | BcMV9-BI | +ssRNA | Botrytis cinerea mitovirus 9 | MT089704.1 | 97.91% | 65.00% | *Botrytis cinerea* | 2720 | *Mitoviridae* | *Mitovirus* | Ruiz-Padilla,A et al 2021 |
|  |  |  |  |  |  | Grapevine associated narnavirus-1 | LN827948.1 | 96.38% | 63.00% | */* | 2735 | *Mitoviridae* | *Mitovirus* | Donaire,L et al 2017 |
|  |  |  |  |  |  | Grapevine associated narnavirus-1 | NC_028473.1 | 93.88% | 63.00% | */* | 2733 | *Mitoviridae* | *Mitovirus* | Donaire,L et al 2017 |
| BC-2_Contig442 | C_AA045437.1 | 2679 | Botrytis cinerea ourmia-like virus 10-BcIsrael | BcOLV10-BI | +ssRNA | Botrytis cinerea ourmia-like virus 10 | MN605476.1 | 93.71% | 54.00% | *Botrytis cinerea* | 2430 | *Botourmiaviridae* | */* | Ruiz-Padilla,A et al 2021 |
| BC-1_Contig397 | C_AA045438.1 | 2894 | Botrytis cinerea ourmia-like virus 13-BcIsrael | BcOLV13-BI | +ssRNA | Botrytis cinerea ourmia-like virus 13 | MN605479.1 | 93.66% | 73.00% | *Botrytis cinerea* | 2877 | *Botourmiaviridae* | *Botoulivirus* | Ruiz-Padilla,A et al 2021 |
| BC-1_Contig398 | C_AA045439.1 | 2837 | Botrytis cinerea ourmia-like virus 11-BcIsrael | BcOLV11-BI | +ssRNA | Botrytis cinerea ourmia-like virus 11 | MN605477.1 | 99.11% | 70.00% | *Botrytis cinerea* | 2850 | *Botourmiaviridae* | *Botoulivirus* | Ruiz-Padilla,A et al 2021 |
| BC-2_Contig504 | C_AA045440.1 | 2439 | Botrytis cinerea ourmia-like virus 15-BcIsrael | BcOLV15-BI | +ssRNA | Sclerotinia sclerotiorum ourmia-like virus 3-U | MW454910.1 | 98.29% | 79.00% | *Sclerotinia sclerotiorum* | 2469 | *Botourmiaviridae* | *Botoulivirus* | Liang,W et al 2021 |
|  |  |  |  |  |  | Botrytis cinerea ourmia-like virus 15 | MN605481.1 | 97.98% | 79.00% | *Botrytis cinerea* | 2428 | *Botourmiaviridae* | *Botoulivirus* | Ruiz-Padilla,A et al 2021 |
|  |  |  |  |  |  | Sclerotinia sclerotiorum ourmia-like virus 3 | MF444276.1 | 98.29% | 79.00% | *Sclerotinia sclerotiorum* | 2442 | *Botourmiaviridae* | *Botoulivirus* | Mu,F et al 2018 |
| BC-1_Contig276 | C_AA045441.1 | 2559 | Botrytis cinerea ourmia-like virus 16-BcIsrael | BcOLV16-BI | +ssRNA | Sclerotinia sclerotiorum ourmia-like virus 12 | MT646377.1 | 98.15% | 76.00% | *Sclerotinia sclerotiorum* | 2498 | *Botourmiaviridae* | *Botoulivirus* | Jia,J et al 2021 |
|  |  |  |  |  |  | Botrytis cinerea ourmia-like virus 16 | MN605482.1 | 96.92% | 76.00% | *Botrytis cinerea* | 2493 | *Botourmiaviridae* | *Botoulivirus* | Ruiz-Padilla,A et al 2021 |
| BC-2_Contig387 | C_AA045442.1 | 2038 | Botrytis cinerea ourmia-like virus 17-BcIsrael | BcOLV17-BI | +ssRNA | Botrytis cinerea ourmia-like virus 17 | MN605483.1 | 97.63% | 62.00% | *Botrytis cinerea* | 2837 | *Botourmiaviridae* | *Botoulivirus* | Ruiz-Padilla,A et al 2021 |
|  |  |  |  |  |  | Sclerotinia sclerotiorum ourmia-like virus 17 | MW452526.1 | 97.87% | 62.00% | *Sclerotinia sclerotiorum* | 2802 | *Botourmiaviridae* | *Botoulivirus* | Mu,F et al 2018 |
|  |  |  |  |  |  | Sclerotinia sclerotiorum ourmia-like virus 13 | MT646375.1 | 97.39% | 62.00% | *Sclerotinia sclerotiorum* | 2831 | *Botourmiaviridae* | *Botoulivirus* | Jia,J et al 2021 |
| BC-2_Contig359 | C_AA045443.1 | 3601 | Botrytis cinerea ourmia-like virus 4-BcIsrael | BcOLV4-BI | +ssRNA | Botrytis cinerea ourmia-like virus 4 | MN605470.1 | 98.50% | 68.00% | *Botrytis cinerea* | 2861 | *Botourmiaviridae* | *Botoulivirus* | Ruiz-Padilla,A et al 2021 |
| BC-2_Contig476 | C_AA045444.1 | 2685 | Botrytis cinerea ourmia-like virus 7-BcIsrael | BcOLV7-BI | +ssRNA | Sclerotinia sclerotiorum ourmia-like virus 19 | MW454911.1 | 98.01% | 68.00% | *Sclerotinia sclerotiorum* | 2697 | *Botourmiaviridae* | *Magoulivirus* | Liang,W et al 2021 |
|  |  |  |  |  |  | Botrytis cinerea ourmia-like virus 7 | MN605473.1 | 98.64% | 50.00% | *Botrytis cinerea* | 2100 | *Botourmiaviridae* | *Magoulivirus* | Ruiz-Padilla,A et al 2021 |
| BC-1_Contig289 | C_AA045445.1 | 3458 | Botrytis cinerea ourmia-like virus 1-BcIsrael | BcOLV1-BI | +ssRNA | Botrytis cinerea ourmia-like virus 1 | MN605467.1 | 92.44% | 80.00% | *Botrytis cinerea* | 5185 | *Botourmiaviridae* | *Penoulivirus* | Ruiz-Padilla,A et al 2021 |
| BC-2_Contig355 | C_AA045446.1 | 2934 | Botrytis cinerea ourmia-like virus 9-BcIsrael | BcOLV9-BI | +ssRNA | Botrytis cinerea ourmia-like virus 9 | MN605475.1 | 96.15% | 74.00% | *Botrytis cinerea* | 2941 | *Botourmiaviridae* | *Penoulivirus* | Ruiz-Padilla,A et al 2021 |
|  |  |  |  |  |  | Sclerotinia sclerotiorum ourmia-like virus 4 | MT706021.1 | 96.27% | 74.00% | *Sclerotinia sclerotiorum* | 2905 | *Botourmiaviridae* | *Penoulivirus* | Mu,F et al 2018 |
|  |  |  |  |  |  | Sclerotinia sclerotiorum ourmia-like virus 4 | MN715322.1 | 94.91% | 73.00% | *Sclerotinia sclerotiorum* | 2892 | *Botourmiaviridae* | *Penoulivirus* | Wang,Q 2019 |
|  |  |  |  |  |  | Botrytis cinerea ourmia-like virus 9 | OL602099.1 | 97.67% | 63.00% | *Botrytis cinerea* | 2190 | *Botourmiaviridae* | *Penoulivirus* | Deng,Y et al 2021 |
| BC-1_Contig930 | C_AA045493.1 | 2712 | Botrytis cinerea ourmia-like virus 18 | BcOLV18 | +ssRNA | Plasmopara viticola lesion associated ourmia-like virus 80 | MN532667.1 | 94.16% | 81.00% | *Plasmopara viticola* | 3000 | *Botourmiaviridae* | */* | Chiapello,M et al 2020 |
|  |  |  |  |  |  | Sclerotinia sclerotiorum ourmia-like virus 15 | MT646399.1 | 93.14% | 81.00% | *Sclerotinia sclerotiorum* | 3210 | *Botourmiaviridae* | */* | Jia,J et al 2021 |
| BC-3_Contig361 | C_AA045447.1 | 2876 | Botrytis cinerea ourmia-like virus 12-BcIsrael | BcOLV12-BI | +ssRNA | Botrytis cinerea ourmia-like virus 12 | MN605478.1 | 96.51% | 71.00% | *Botrytis cinerea* | 2898 | *Botourmiaviridae* | *Botoulivirus* | Ruiz-Padilla,A et al 2021 |
|  |  |  |  |  |  | Sclerotinia sclerotiorum ourmia-like virus 5 | MT706022.1 | 95.78% | 71.00% | *Sclerotinia sclerotiorum* | 2823 | *Botourmiaviridae* | *Botoulivirus* | Mu,F et al 2018 |
|  |  |  |  |  |  | Botrytis cinerea ourmia-like virus 12 | OL602094.1 | 95.49% | 71.00% | *Botrytis cinerea* | 2941 | *Botourmiaviridae* | *Botoulivirus* | Deng,Y et al 2021 |
|  |  |  |  |  |  | Sclerotinia sclerotiorum ourmia-like virus 10 | MT646379.1 | 95.35% | 71.00% | *Sclerotinia sclerotiorum* | 2888 | *Botourmiaviridae* | *Botoulivirus* | Jia,J et al 2021 |
|  |  |  |  |  |  | Sclerotinia minor botoulivirus 1 | MN599068.1 | 95.90% | 71.00% | *Sclerotinia minor* | 2882 | *Botourmiaviridae* | *Botoulivirus* | Liang,N 2020 |
| BC-1_Contig362 | C_AA045471.1 | 3866 | Botrytis cinerea ourmia-like virus 2-BcIsrael | BcOLV2-BI | +ssRNA | Botrytis cinerea ourmia-like virus 2 | MN605468.1 | 92.42% | 100.00% | *Botrytis cinerea* | 3561 | *Botourmiaviridae* | *Penoulivirus* | Ruiz-Padilla,A et al 2021 |
|  |  |  |  |  |  | Sclerotinia sclerotiorum ourmia-like virus 7 | MT646365.1 | 92.40% | 99.00% | *Sclerotinia sclerotiorum* | 3848 | *Botourmiaviridae* | *Penoulivirus* | Jia,J et al 2021 |
| BC-3_Contig366 | C_AA045494.1 | 2860 | Botrytis cinerea ourmia-like virus 19 | BcOLV19 | +ssRNA | Erysiphe necator associated ourmia-like virus 1 | MN552390.1 | 37.64% | 62.00% | *Erysiphe necator* | 3192 | *Botourmiaviridae* | */* | Rodriguez-Romero,J et al 2019 |
| BC-1_Contig693 | C_AA045449.1 | 4029 | Botrytis cinerea hypovirus 1 satellite-like RNA-BI | BcH1SLR-BI | +ssRNA | Sclerotinia sclerotiorum hypovirus 7 satellite-like RNA | MT646409.1 | 98.36% | 48.00% | *Sclerotinia sclerotiorum* | 3904 | *Hypoviridae* | *Betahypovirus* | Jia,J et al 2021 |
|  |  |  |  |  |  | Sclerotinia sclerotiorum hypovirus 7 satellite-like RNA | MW454886.1 | 98.36% | 48.00% | *Sclerotinia sclerotiorum* | 4316 | *Hypoviridae* | *Betahypovirus* | Liang,W et al 2021 |
|  |  |  |  |  |  | Botrytis cinerea hypovirus 1 satellite-like RNA | NC_037663.1 | 98.66% | 48.00% | *Botrytis cinerea* | 4149 | *Hypoviridae* | *Betahypovirus* | Hao,F 2018 |
| BC-1_Contig65 | C_AA045448.1 | 10276 | Botrytis cinerea hypovirus 1-BcIsrael | BcHV1-BI | +ssRNA | Sclerotinia sclerotiorum hypovirus 7 | MW454885.1 | 99.43% | 86.00% | *Sclerotinia sclerotiorum* | 10218 | *Hypoviridae* | *Betahypovirus* | Liang,W et al 2021 |
|  |  |  |  |  |  | Botrytis cinerea hypovirus 1 | NC_037659.1 | 98.48% | 86.00% | *Botrytis cinerea* | 10252 | *Hypoviridae* | *Betahypovirus* | Hao,F 2018 |
|  |  |  |  |  |  | Botrytis cinerea hypovirus 1 | MH347277.1 | 98.55% | 86.00% | *Botrytis cinerea* | 10250 | *Hypoviridae* | *Betahypovirus* | He,G 2018 |
|  |  |  |  |  |  | Botrytis cinerea hypovirus 1 | OL602084.1 | 98.52% | 86.00% | *Botrytis cinerea* | 10061 | *Hypoviridae* | *Betahypovirus* | Deng,Y et al 2021 |
|  |  |  |  |  |  | Sclerotinia sclerotiorum hypovirus 7 | MT646394.1 | 98.58% | 86.00% | *Sclerotinia sclerotiorum* | 10208 | *Hypoviridae* | *Betahypovirus* | Jia,J et al 2021 |
| BC-1_Contig7 | C_AA045472.1 | 14431 | Botrytis cinerea hypovirus 5-BcIsrael | BcHV5-BI | +ssRNA | Botrytis cinerea hypovirus 5 | MT157414.1 | 98.44% | 97.00% | *Botrytis cinerea* | 15353 | *Hypoviridae* | *Gammahypovirus* | Ruiz-Padilla,A et al 2021 |
|  |  |  |  |  |  | Sclerotinia sclerotiorum hypovirus 2-WX | MT646366.1 | 92.89% | 92.00% | *Sclerotinia sclerotiorum* | 15229 | *Hypoviridae* | *Gammahypovirus* | Jia,J et al 2021 |
|  |  |  |  |  |  | Sclerotinia sclerotiorum hypovirus 2 | MH347276.1 | 93.03% | 92.00% | *Sclerotinia sclerotiorum* | 15249 | *Hypoviridae* | *Gammahypovirus* | He,G 2018 |
|  |  |  |  |  |  | Sclerotinia sclerotiorum hypovirus 2 | KJ561218.1 | 92.80% | 92.00% | *Sclerotinia sclerotiorum* | 15239 | *Hypoviridae* | *Gammahypovirus* | Hu,Z 2014 |
|  |  |  |  |  |  | Sclerotinia sclerotiorum hypovirus 2 | MH665657.1 | 96.55% | 87.00% | *Sclerotinia sclerotiorum* | 13535 | *Hypoviridae* | *Gammahypovirus* | Tran,T.T 2018 |
|  |  |  |  |  |  | Sclerotinia sclerotiorum hypovirus 2 | KF898354.2 | 94.91% | 89.00% | *Sclerotinia sclerotiorum* | 14580 | *Hypoviridae* | *Gammahypovirus* | Marzano,S.Y 2015 |
|  |  |  |  |  |  | Sclerotinia sclerotiorum hypovirus 2 | NC_022896.1 | 94.65% | 88.00% | *Sclerotinia sclerotiorum* | 14581 | *Hypoviridae* | *Gammahypovirus* | Khalifa,M.E 2013 |
| BC-1_Contig86 | C_AA045450.1 | 10374 | Botrytis cinerea hypovirus 3-BcIsrael | BcHV3-BI | +ssRNA | Sclerotinia sclerotiorum hypovirus 1-A | MF444220.1 | 91.42% | 85.00% | *Sclerotinia sclerotiorum* | 10205 | *Hypoviridae* | *Betahypovirus* | Mu,F et al 2018 |
|  |  |  |  |  |  | Sclerotinia sclerotiorum hypovirus 1/SX276 | MT829322.1 | 91.28% | 85.00% | *Sclerotinia sclerotiorum* | 10430 | *Hypoviridae* | *Betahypovirus* | Mu,F 2020 |
|  |  |  |  |  |  | Sclerotinia sclerotiorum hypovirus 1 | NC_015939.1 | 91.06% | 85.00% | *Sclerotinia sclerotiorum* | 10438 | *Hypoviridae* | *Betahypovirus* | Xie,J et al 2011 |
|  |  |  |  |  |  | Botrytis cinerea hypovirus 3 | MN617170.1 | 93.75% | 83.00% | *Botrytis cinerea* | 10863 | *Hypoviridae* | *Betahypovirus* | Ruiz-Padilla,A et al 2021 |
| BC-1_Contig6 | MN617171.1 | 16145 | Botrytis cinerea hypovirus 4-BcIsrael | BcHV4-BI | +ssRNA | Botrytis cinerea hypovirus 4 | MN617171.1 | 96.50% | 62.00% | *Botrytis cinerea* | 17631 | *Hypoviridae* | *Gammahypovirus* | Ruiz-Padilla,A et al 2021 |
| BC-1_Contig77 | C_AA045451.1 | 6349 | Botrytis cinerea fusarivirus 5-BcIsrael | BcFV5-BI | +ssRNA | Botrytis cinerea fusarivirus 5 | MN617764.1 | 96.35% | 72.00% | *Botrytis cinerea* | 6313 | *Fusariviridae* | *Alphafusarivirus* | Ruiz-Padilla,A et al 2021 |
|  |  |  |  |  |  | Erysiphe necator associated fusarivirus 3 | MN627479.1 | 92.20% | 73.00% | *Erysiphe necator* | 6282 | *Fusariviridae* | *Alphafusarivirus* | Rodriguez-Romero,J et al 2019 |
| BC-1_Contig29 | C_AA045496.1 | 8461 | Botrytis cinerea fusarivirus 9 | BcFV9 | +ssRNA | Botrytis cinerea fusarivirus 1 | NC_037660.1 | 86.29% | 58.00% | *Botrytis cinerea* | 8448 | *Fusariviridae* | *Betafusarivirus* | Hao,F 2018 |
| BC-1_Contig31 | C_AA045473.1 | 8339 | Botrytis cinerea fusarivirus 4-BcIsrael | BcFV4-BI | +ssRNA | Botrytis cinerea fusarivirus 4 | MN617763.1 | 94.87% | 59.00% | *Botrytis cinerea* | 8349 | *Fusariviridae* | *Betafusarivirus* | Ruiz-Padilla,A et al 2021 |
| BC-2_Contig1096 | C_AA045497.1 | 11097 | Botrytis cinerea endornavirus 5 | BcEV5 | +ssRNA | Botrytis cinerea endornavirus 1 | NC_031752.1 | 67.68% | 98.00% | *Botrytis cinerea* | 11557 | *Endornaviridae* | *Betaendornavirus* | Hao,F 2018 |
| BC-2_Contig1606 | C_AA045452.1 | 12604 | Botrytis cinerea endornavirus 2-BcIsrael | BcEV2-BI | +ssRNA | Sclerotinia sclerotiorum endornavirus 3 | MT706020.1 | 93.15% | 99.00% | *Sclerotinia sclerotiorum* | 12631 | *Endornaviridae* | *Betaendornavirus* | Mu,F et al 2020 |
|  |  |  |  |  |  | Botrytis cinerea endornavirus 2 | MN617758.1 | 98.62% | 99.00% | *Botrytis cinerea* | 13581 | *Endornaviridae* | *Betaendornavirus* | Ruiz-Padilla,A et al 2021 |
|  |  |  |  |  |  | Sclerotinia sclerotiorum endornavirus 9 | MT646421.1 | 94.63% | 99.00% | *Sclerotinia sclerotiorum* | 13562 | *Endornaviridae* | *Betaendornavirus* | Jia,J et al 2021 |
|  |  |  |  |  |  | Botrytis cinerea endornavirus 3 | MN839443.1 | 92.83% | 99.00% | *Botrytis cinerea* | 13582 | *Endornaviridae* | *Betaendornavirus* | Ruiz-Padilla,A et al 2021 |
| BC-2_Contig1904 | C_AA045498.1 | 11541 | Botrytis cinerea endornavirus 6 | BcEV6 | +ssRNA | Sclerotinia sclerotiorum endornavirus 8 | MT646356.1 | 82.66% | 98.00% | *Sclerotinia sclerotiorum* | 11543 | *Endornaviridae* | *Betaendornavirus* | Jia,J et al 2021 |
| BC-2_Contig48 | OM928008.1 | 6872 | Botrytis virus F-BcIsrael | BvF-BI | +ssRNA | Botrytis virus F | MH338171.1 | 94.30% | 82.00% | *Botrytis cinerea* | 6828 | *Gammaflexiviridae* | *Mycoflexivirus* | Svanella-Dumas,L 2018 |
|  |  |  |  |  |  | Sclerotinia sclerotiorum gammaflexivirus 1 | MT646392.1 | 93.83% | 82.00% | *Sclerotinia sclerotiorum* | 6818 | *Gammaflexiviridae* | *Mycoflexivirus* | Jia,J et al 2021 |
|  |  |  |  |  |  | Botrytis virus F | NC_002604.1 | 91.77% | 82.00% | *Botrytis cinerea* | 6827 | *Gammaflexiviridae* | *Mycoflexivirus* | Howitt,R.L 2001 |
|  |  |  |  |  |  | Botrytis virus F | LN827953.1 | 92.25% | 82.00% | *Botrytis cinerea* | 6814 | *Gammaflexiviridae* | *Mycoflexivirus* | Donaire,L et al 2017 |
| BC-2_Contig101 | C_AA045499.1 | 6648 | Botrytis cinerea deltaflexivirus 4 | BcDFV4 | +ssRNA | Sclerotinia sclerotiorum deltaflexivirus 2 | MN954874.1 | 99.28% | 94.00% | *Sclerotinia sclerotiorum* | 6628 | *Deltaflexiviridae* | *Deltaflexivirus* | Ruiz-Padilla,A et al 2021 |
|  |  |  |  |  |  | Sclerotinia sclerotiorum deltaflexivirus 2-WX | MT646426.1 | 96.65% | 97.00% | *Sclerotinia sclerotiorum* | 6651 | *Deltaflexiviridae* | *Deltaflexivirus* | Jia,J et al 2021 |
|  |  |  |  |  |  | Sclerotinia sclerotiorum deltaflexivirus 2 | NC_040649.1 | 92.79% | 96.00% | *Sclerotinia sclerotiorum* | 6735 | *Deltaflexiviridae* | *Deltaflexivirus* | Hamid,M.R 2018 |
|  |  |  |  |  |  | Sclerotinia sclerotiorum deltaflexivirus 2-U | MW454907.1 | 99.29% | 63.00% | *Sclerotinia sclerotiorum* | 4375 | *Deltaflexiviridae* | *Deltaflexivirus* | Liang,W et al 2021 |
|  |  |  |  |  |  | Sclerotinia sclerotiorum tymo-like RNA virus 6 | MF444270.1 | 97.53% | 32.00% | *Sclerotinia sclerotiorum* | 2306 | *Deltaflexiviridae* | *Deltaflexivirus* | Mu,F 2018 |
| BC-2_Contig133 | C_AA045453.1 | 6284 | Botrytis cinerea mycotymovirus 1-BcIsrael | BcMTV1-BI | +ssRNA | Sclerotinia sclerotiorum mycotymovirus 1-WX | MT646427.1 | 98.53% | 97.00% | *Sclerotinia sclerotiorum* | 7320 | *Tymoviridae* | */* | Jia,J et al 2021 |
|  |  |  |  |  |  | Sclerotinia sclerotiorum mycotymovirus 1-U | MW454905.1 | 98.72% | 97.00% | *Sclerotinia sclerotiorum* | 7284 | *Tymoviridae* | */* | Liang,W et al 2021 |
|  |  |  |  |  |  | Botrytis cinerea mycotymovirus 1 | OL602087.1 | 98.58% | 97.00% | *Botrytis cinerea* | 7282 | *Tymoviridae* | */* | Deng,Y et al 2021 |
|  |  |  |  |  |  | Sclerotinia sclerotiorum mycotymovirus 2 | MT706023.1 | 97.79% | 97.00% | *Sclerotinia sclerotiorum* | 7145 | *Tymoviridae* | */* | Mu,F et al 2020 |
|  |  |  |  |  |  | Botrytis cinerea mycotymovirus 1 | MN954873.1 | 97.20% | 97.00% | *Botrytis cinerea* | 7293 | *Tymoviridae* | */* | Ruiz-Padilla,A et al 2021 |
|  |  |  |  |  |  | Sclerotinia sclerotiorum mycotymovirus 1 | MK530705.1 | 95.45% | 93.00% | *Sclerotinia sclerotiorum* | 6414 | *Tymoviridae* | */* | Qian,W.Q 2019 |
| BC-2_Contig569 | C_AA045458.1 | 10491 | Botrytis cinerea flexivirus 1-BcIsrael | BcFV1-BI | +ssRNA | Botrytis cinerea flexivirus 1 | MN625248.1 | 99.51% | 99.00% | *Botrytis cinerea* | 13985 | *Gammaflexiviridae* | *Botryflexivirus* | Ruiz-Padilla,A et al 2021 |
| BC-2_Contig212 | C_AA045501.1 | 4423 | Botrytis cinerea umbra-like virus 4 | BcULV3 | +ssRNA | Sclerotinia sclerotiorum umbra-like virus 3-WX2 | MT646364.1 | 97.69% | 64.00% | *Sclerotinia sclerotiorum* | 3945 | Unassigned | / | Jia,J et al 2021 |
|  |  |  |  |  |  | Sclerotinia sclerotiorum umbra-like virus 3-WX1 | MT646363.1 | 91.61% | 64.00% | *Sclerotinia sclerotiorum* | 4088 | Unassigned | / | Jia,J et al 2021 |
| BC-2_Contig132 | C_AA045454.1 | 3556 | Botrytis cinerea umbra-like virus 1-BcIsrael | BcULV1-BI | +ssRNA | Botrytis cinerea umbra-like virus 1 | MN625251.1 | 98.46% | 43.00% | *Botrytis cinerea* | 3865 | Unassigned | / | Ruiz-Padilla,A et al 2021 |
| BC-2_Contig214 | C_AA045502.1 | 4279 | Botrytis cinerea umbra-like virus 5 | BcULV4 | +ssRNA | Botrytis cinerea umbra-like virus 1 | MN625251.1 | 72.99% | 35.00% | *Botrytis cinerea* | 3865 | Unassigned | / | Ruiz-Padilla,A et al 2021 |
| BC-2_Contig50 | C_AA045503.1 | 1417 | Botrytis cinerea umbra-like virus 6 | BcULV5 | +ssRNA | Botrytis cinerea umbra-like virus 1 | MN625251.1 | 81.54% | 63.00% | *Botrytis cinerea* | 3865 | Unassigned | / | Ruiz-Padilla,A et al 2021 |
| BC-1_Contig50 | C_AA045455.1 | 7992 | Botrytis cinerea alpha-like virus 1-BcIsrael | BcALV1-BI | +ssRNA | Botrytis cinerea alpha-like virus 1 | MN625250.1 | 99.09% | 73.00% | *Botrytis cinerea* | 8008 | Unassigned | */* | Ruiz-Padilla,A et al 2021 |
| BC-1_Contig51 | C_AA045504.1 | 2904 | Botrytis cinerea alpha-like virus 2 | BcALV2 | +ssRNA | Sclerotinia sclerotiorum mycoalphavirus virus 1 | MT706025.1 | 56.82% | 100.00% | *Sclerotinia sclerotiorum* | 7586 | Unassigned | */* | Mu,F et al 2020 |
| BC-1_Contig53 | C_AA045505.1 | 3436 | Botrytis cinerea alpha-like virus 3 | BcALV3 | +ssRNA | Sclerotinia sclerotiorum mycoalphavirus virus 1 | MT706025.1 | 86.52% | 99.00% | *Sclerotinia sclerotiorum* | 7586 | Unassigned | */* | Mu,F et al 2020 |
| BC-3_Contig63 | C_AA045459.1 | 8938 | Botrytis cinerea RNA virus 1-BcIsrael | BcRV1-BI | dsRNA | Botrytis cinerea RNA virus 1 | MN443879.1 | 92.68% | 44.00% | *Botrytis cinerea* | 8905 | Unassigned | */* | Gallo,Y 2018 |
|  |  |  |  |  |  | Botrytis cinerea RNA virus 1 | NC_026139.1 | 90.81% | 44.00% | *Botrytis cinerea* | 8952 | Unassigned | */* | Yu,L 2015 |
|  |  |  |  |  |  | Botrytis cinerea RNA virus 1 | NC_026139.1 | 94.06% | 44.00% | *Botrytis cinerea* | 8952 | Unassigned | */* | Yu,L 2015 |
|  |  |  |  |  |  | Grapevine associated totivirus-2 | GU108594.1 | 94.22% | 44.00% | */* | 6595 | Unassigned | */* | Al Rwahnih,M 2010 |
|  |  |  |  |  |  | Botrytis cinerea RNA virus 1 | MN443879.1 | 93.99% | 44.00% | *Botrytis cinerea* | 8905 | Unassigned | */* | Gallo,Y 2019 |
| BC-3_Contig30 | C_AA045476.1 | 9401 | Botrytis cinerea dsRNA mycovirus 1 | BcDMV1 | dsRNA | Sclerotinia sclerotiorum dsRNA mycovirus-L | LN827952.1 | 94.02% | 42.00% | *Sclerotinia sclerotiorum* | 8937 | Unassigned | */* | Donaire,L et al 2017 |
|  |  |  |  |  |  | Sclerotinia sclerotiorum dsRNA mycovirus-L-WX1 | MT646387.1 | 93.42% | 42.00% | *Sclerotinia sclerotiorum* | 9095 | Unassigned | */* | Jia,J et al 2020 |
|  |  |  |  |  |  | Sclerotinia sclerotiorum dsRNA mycovirus-L-WX2 | MT646415.1 | 92.68% | 42.00% | *Sclerotinia sclerotiorum* | 9097 | Unassigned | */* | Jia,J et al 2021 |
|  |  |  |  |  |  | Sclerotinia sclerotiorum dsRNA mycovirus-L | NC_017915.1 | 90.06% | 42.00% | *Sclerotinia sclerotiorum* | 9124 | Unassigned | */* | Liu,H et al 2012 |
| BC-3_Contig1063 | C_AA045460.1 | 1665 | Botrytis cinerea mycovirus 4-BcIsrael | BcMCV4-BI | dsRNA | Botrytis cinerea mycovirus 4 | MN617034.1 | 98.38% | 100.00% | *Botrytis cinerea* | 3944 | *Quadriviridae* | / | Ruiz-Padilla,A et al 2021 |
|  |  |  |  |  |  | Botrytis cinerea RNA virus 2 | MH347281.1 | 98.38% | 100.00% | *Botrytis cinerea* | 3931 | *Quadriviridae* | / | He,G et al 2018 |
| BC-3_Contig1431 | C_AA045461.1 | 2103 |  |  | dsRNA | Botrytis cinerea mycovirus 4 | MN954885.1 | 96.74% | 100.00% | *Botrytis cinerea* | 4267 | *Quadriviridae* | / | Ruiz-Padilla,A et al 2021 |
|  |  |  |  |  |  | Botrytis cinerea RNA virus 2 | MH347280.1 | 95.18% | 99.00% | *Botrytis cinerea* | 4263 | *Quadriviridae* | / | He,G et al 2018 |
|  |  |  |  |  |  | Botrytis cinerea RNA virus 2 | OL602101.1 | 95.96% | 62.00% | *Botrytis cinerea* | 1836 | *Quadriviridae* | / | Deng,Y et al 2021 |
| BC-3_Contig1298 | C_AA045462.1 | 2178 | Botrytis cinerea mycovirus 5-BcIsrael | BcMCV5-BI | dsRNA | Botrytis cinerea mycovirus 5 | MN617757.1 | 99.26% | 92.00% | *Botrytis cinerea* | 2184 | *Curvulaviridae* | / | Ruiz-Padilla,A et al 2021 |
| BC-3_Contig57 | C_AA045463.1 | 1153 |  |  | dsRNA | Botrytis cinerea mycovirus 5 | MN617755.1 | 100.00% | 82.00% | *Botrytis cinerea* | 1522 | *Curvulaviridae* | / | Ruiz-Padilla,A et al 2021 |
|  |  |  |  |  |  |  | MN617756.1 | 91.86% | 82.00% | *Botrytis cinerea* | 1433 | *Curvulaviridae* | / | Ruiz-Padilla,A et al 2021 |
| BC-3_Contig1405 | C_AA045464.1 | 2026 | Botrytis cinerea mycovirus 3-BcIsrael | BcMCV3-BI | dsRNA | Botrytis cinerea mycovirus 3 | MN617031.1 | 93.25% | 89.00% | *Botrytis cinerea* | 2024 | *Curvulaviridae* | / | Ruiz-Padilla,A et al 2021 |
| BC-3_Contig1406 | C_AA045465.1 | 1618 |  |  |  |  | MN617032.1 | 94.95% | 55.00% | *Botrytis cinerea* | 1780 | *Curvulaviridae* | / | Ruiz-Padilla,A et al 2021 |
| BC-1_Contig1612 | C_AA045477.1 | 1278 | Botrytis cinerea partitivirus 5 | BcPV5 | dsRNA | Aspergillus fumigatus partitivirus 1 | LR746165.1 | 34.35% | 53.00% | *Aspergillus fumigatus* | 1112 | *Partitiviridae* | *Gammapartitivirus* | Kotta-Loizou,I 2020 |
| yang3_Contig1181 | C_AA045478.1 | 1405 | Botrytis cinerea partitivirus 6 | BcPV6 | dsRNA | Ophiostoma partitivirus 1 | NC_038917.1 | 26.27% | 45.00% | *Ophiostoma himal-ulmi* | 1567 | *Partitiviridae* | *Gammapartitivirus* | Crawford,L.J 2006 |
| BC-1_Contig63 | C_AA045479.1 | 1534 | Botrytis cinerea partitivirus 7 | BcPV7 | dsRNA | Plasmopara viticola lesion associated Partitivirus 10 | MN556985.1 | 99.77% | 84.00% | *Plasmopara viticola* | 1478 | *Partitiviridae* | *Gammapartitivirus* | Chiapello,M et al 2020 |
|  |  |  |  |  |  | Erysiphe necator associated partitivirus 8 | MN605502.1 | 99.05% | 82.00% | *Erysiphe necator* | 1523 | *Partitiviridae* | *Gammapartitivirus* | Rodriguez-Romero,J et al 2019 |
| BC-1_Contig1080 | C_AA045466.1 | 1770 | Botrytis cinerea partitivirus 4-BcIsrael | BcPV4-BI | dsRNA | Botryotinia fuckeliana partitivirus 1 | MN954881.1 | 99.07% | 91.00% | *Botryotinia fuckeliana* | 1780 | *Partitiviridae* | *Gammapartitivirus* | Ruiz-Padilla,A et al 2021 |
|  |  |  |  |  |  | Botryotinia fuckeliana partitivirus 1 | NC_010349.1 | 98.89% | 91.00% | *Botryotinia fuckeliana* | 1793 | *Partitiviridae* | *Gammapartitivirus* | De Guido,M.A et al 2008 |
| BC-1_Contig1309 | C_AA045467.1 | 1560 |  |  |  | Botryotinia fuckeliana partitivirus 1 | MN954882.1 | 96.33% | 83.00% | *Botryotinia fuckeliana* | 1597 | *Partitiviridae* | *Gammapartitivirus* | Ruiz-Padilla,A et al 2021 |
|  |  |  |  |  |  | Botrytis cinerea partitivirus 4 | OL602102.1 | 95.87% | 83.00% | *Botrytis cinerea* | 1457 | *Partitiviridae* | *Gammapartitivirus* | Deng,Y et al 2021 |
|  |  |  |  |  |  | Botryotinia fuckeliana partitivirus 1 | NC_010350.1 | 95.18% | 83.00% | *Botryotinia fuckeliana* | 1566 | *Partitiviridae* | *Gammapartitivirus* | De Guido,M.A et al 2008 |
| BC-1_Contig68 | C_AA045480.1 | 1682 | Botrytis cinerea partitivirus 8 | BcPV8 | dsRNA | Plasmopara viticola lesion associated Partitivirus 4 | MN556990.1 | 99.61% | 91.00% | *Plasmopara viticola* | 1677 | *Partitiviridae* | *Gammapartitivirus* | Chiapello,M et al 2020 |
| BC-1_Contig69 | C_AA045481.1 | 1345 |  |  |  |  | MN556994.1 | 99.47% | 83.00% | *Plasmopara viticola* | 1250 | *Partitiviridae* | *Gammapartitivirus* | Chiapello,M et al 2020 |
| yang3_Contig736 | C_AA045482.1 | 1815 | Botrytis cinerea partitivirus 9 | BcPV9 | dsRNA | Colletotrichum eremochloae partitivirus 1 | MK279452.1 | 79.58% | 94.00% | *Colletotrichum eremochloae* | 1815 | *Partitiviridae* | *Epsilonpartitivirus* | Gilbert,K.B et al 2019 |
| yang3_Contig71 | C_AA045483.1 | 1641 |  |  |  |  | MK279453.1 | 74.29% | 90.00% | *Colletotrichum eremochloae* | 1696 | *Partitiviridae* | *Epsilonpartitivirus* | Gilbert,K.B et al 2019 |
| BC-1_Contig12088 |  | 1670 | Botrytis cinerea partitivirus 10 | BcPV10 | dsRNA | Alternaria tenuissima partitivirus 1 | MT648466.1 | 95.15% | 96.00% | *Alternaria tenuissima* | 1785 | *Partitiviridae* | *Gammapartitivirus* | Liang,Z et al 2021 |
| BC-3_Contig1238 | C_AA045468.1 | 1525 | Botrytis cinerea partitivirus 3-BcIsrael | BcPV3-BI | dsRNA | Sclerotinia sclerotiorum partitivirus 2 | MF444213.1 | 99.52% | 82.00% | *Sclerotinia sclerotiorum* | 1547 | *Partitiviridae* | *Gammapartitivirus* | Mu,F et al 2018 |
|  |  |  |  |  |  | Botrytis cinerea partitivirus 3 | MN954883.1 | 92.38% | 82.00% | *Botrytis cinerea* | 1537 | *Partitiviridae* | *Gammapartitivirus* | Ruiz-Padilla,A et al 2021 |
| BC-1_Contig1282 | C_AA045469.1 | 1770 |  |  |  | Sclerotinia sclerotiorum partitivirus 3 | MF444214.1 | 100.00% | 93.00% | *Sclerotinia sclerotiorum* | 1769 | *Partitiviridae* | *Gammapartitivirus* | Mu,F et al 2018 |
|  |  |  |  |  |  | Botrytis cinerea partitivirus 3 | MN954884.1 | 99.63% | 91.00% | *Botrytis cinerea* | 1762 | *Partitiviridae* | *Gammapartitivirus* | Ruiz-Padilla,A et al 2021 |
| BC-1_Contig226 | C_AA045485.1 | 4022 | Botrytis cinerea victorivirus 4 | BcVV4 | dsRNA | Sclerotinia nivalis victorivirus 1 | NC_030392.1 | 78.05% | 52.00% | *Sclerotinia nivalis* | 5162 | *Totiviridae* | *Victorivirus* | Wu,M 2016 |
| BC-1_Contig765 | C_AA045470.1 | 5111 | Botrytis cinerea victorivirus 3-BcIsrael | BcVV3-BI | dsRNA | Botrytis cinerea victorivirus 3-BCS16 | MN839451.1 | 96.65% | 49.00% | *Botrytis cinerea* | 5171 | *Totiviridae* | *Victorivirus* | Ruiz-Padilla,A et al 2021 |
|  |  |  |  |  |  | Botrytis cinerea victorivirus 2-BCI8 | MN839448.1 | 93.18% | 49.00% | *Botrytis cinerea* | 4360 | *Totiviridae* | *Victorivirus* | Ruiz-Padilla,A et al 2021 |
| BC-1_Contig80 | C_AA045486.1 | 5284 | Botrytis cinerea victorivirus 5 | BcVV5 | dsRNA | Fusarium asiaticum victorivirus 2 | MZ042926.1 | 58.11% | 47.00% | *Fusarium asiaticum* | 5296 | *Totiviridae* | *Victorivirus* | Li,W et al 2021 |
| BC-1_Contig81 | C_AA045470.1 | 1265 | Botrytis cinerea victorivirus 3-BcIsrael | BcVV3-BI | dsRNA | Botrytis cinerea victorivirus 1 | MH347278.1 | 94.03% | 83.00% | *Botrytis cinerea* | 5228 | *Totiviridae* | *Victorivirus* | He,G et al 2018 |
|  |  |  |  |  |  | Penicillium digitatum totivirus 2 | MK279494.1 | 92.33% | 83.00% | *Penicillium digitatum* | 5184 | *Totiviridae* | *Victorivirus* | Gilbert,K.B et al 2019 |
|  |  |  |  |  |  | Botrytis cinerea victorivirus 2 | MN617038.1 | 94.01% | 79.00% | *Botrytis cinerea* | 5186 | *Totiviridae* | *Victorivirus* | Ruiz-Padilla,A et al 2021 |
|  |  |  |  |  |  | Botrytis cinerea victorivirus 3-BCS16 | MN839451.1 | 91.32% | 79.00% | *Botrytis cinerea* | 5171 | *Totiviridae* | *Victorivirus* | Ruiz-Padilla,A et al 2021 |
|  |  |  |  |  |  | Botryotinia fuckeliana totivirus 1 | NC_009224.1 | 95.92% | 75.00% | *Botryotinia fuckeliana* | 5261 | *Totiviridae* | *Victorivirus* | De Guido,M.A et al 2007 |
| BC-1_Contig82 | C_AA045475.1 | 7850 | Botrytis cinerea mymonavirus 1-BcIsrael | BcMNV1-BI | -ssRNA | Botrytis cinerea mymonavirus 1 | MH648611.1 | 99.10% | 75.00% | *Botrytis cinerea* | 7863 | *Mymonaviridae* | *Sclerotimonavirus* | Hao,F 2018 |
|  |  |  |  |  |  | Sclerotinia sclerotiorum negative-stranded RNA virus 7 | MF444285.1 | 98.98% | 75.00% | *Sclerotinia sclerotiorum* | 7819 | *Mymonaviridae* | *Sclerotimonavirus* | Mu,F et al 2018 |
| BC-1_Contig87 | C_AA045509.1 | 3816 | Botrytis cinerea brome-like virus 1 | BcBLV1 | +ssRNA | Adelphocoris suturalis virus | BK059735.1 | 25.05% | 31.00% | *parasitic flatworms* | 18887 | */* | */* | Dheilly,N.M et al 2022 |
| BC-1_Contig89 | C_AA045508.1 | 3476 | Botrytis cinerea virga-like virus 1 | BcVLV1 | +ssRNA | Erysiphe necator associated virga-like virus 14 | MN630183.1 | 25.66% | 93.00% | *Erysiphe necator* | 3450 | Related toVirgaviridae | */* | Rodriguez-Romero,J et al 2019 |
| BC-1_Contig88 | C_AA045507.1 | 3236 | Botrytis cinerea poty-like virus 1 | BcPLV1 | +ssRNA | Erysiphe necator associated poty-like virus 3 | MN630178.1 | 31.47% | 78.00% | *Erysiphe necator* | 3148 | Related toPotyviridae | */* | Rodriguez-Romero,J et al 2019 |
| BC-1_Contig83 | C_AA045474.1 | 9956 | Botrytis cinerea negative-stranded RNA virus 7-BcIsrael | BcNSRV7-BI | -ssRNA | Botrytis cinerea negative-stranded RNA virus 7 | MT157412.1 | 99.59% | 58.00% | *Botrytis cinerea* | 10153 | *Mymonaviridae* | *Botrytimonavirus* | Ruiz-Padilla,A et al 2021 |
|  |  |  |  |  |  | Sclerotinia sclerotiorum negative-stranded RNA virus 11 | MT646406.1 | 98.11% | 58.00% | *Sclerotinia sclerotiorum* | 9982 | *Mymonaviridae* | *Botrytimonavirus* | Jia,J et al 2021 |
| BC-1_Contig86 | C_AA045457.1 | 7014 | Botrytis cinerea negative-stranded RNA virus 6-BcIsrael | BcNSRV6-BI | -ssRNA | Botrytis cinerea negative-stranded RNA virus 6 | MN617082.1 | 99.11% | 96.00% | *Botrytis cinerea* | 7071 | *Bunyavirales* | */* | Ruiz-Padilla,A et al 2021 |
| BC-1_Contig85 | C_AA045456.1 | 6445 | Botrytis cinerea orthobunya-like virus 1-BcIsrael | BcOrLV1-BI | -ssRNA | Botrytis cinerea orthobunya-like virus 1 | MT157407.1 | 94.99% | 99.00% | *Botrytis cinerea* | 8027 | *Bunyavirales* | */* | Ruiz-Padilla,A et al 2021 |
|  |  |  |  |  |  | Sclerotinia sclerotiorum bunyavirus 1 | MT646389.1 | 92.36% | 99.00% | *Sclerotinia sclerotiorum* | 8046 | *Bunyavirales* | */* | Jia,J et al 2021 |
|  |  |  |  |  |  | Sclerotinia sclerotiorum bunyavirus 2 | MT646388.1 | 92.55% | 99.00% | *Sclerotinia sclerotiorum* | 8066 | *Bunyavirales* | */* | Jia,J et al 2021 |
| BC-1_Contig84 | C_AA045506.1 | 2620 | Botrytis cinerea negative-stranded RNA virus 13-BcIsrael | BcNSRV9-BI | -ssRNA | Botrytis cinerea negative stranded RNA virus 13 | MT157409.1 | 77.43% | 99.00% | *Botrytis cinerea* | 10287 | *Bunyavirales* | */* | Ruiz-Padilla,A et al 2021 |
| Unigene_Contig26 | MT425545.1 | 1693 | Botrytis cinerea hypovirulence-associated DNA virus 1 | BcHADV1 | ssDNA | Botrytis cinerea ssDNA virus 1 | MN625247.2 | 96.07% | 65.00% | *Botrytis cinerea* | 1694 | *Genomoviridae* | */* | Ruiz-Padilla,A et al 2021 |
| Unigene_Contig27 | MT425546.1 | 1692 |  |  |  | Botrytis cinerea hypovirulence-associated DNA virus 1 | MT425546.1 | 100.00% | 57.00% | *Botrytis cinerea* | 1692 | *Genomoviridae* | */* | Zhang,H et al 2020 |
| Unigene_Contig28 | MT425547.1 | 1675 |  |  |  | Botrytis cinerea hypovirulence-associated DNA virus 1 | MT425547.1 | 100.00% | 40.00% | *Botrytis cinerea* | 1675 | *Genomoviridae* | */* | Zhang,H et al 2020 |
| Unigene_Contig29 | MT425548.1 | 1763 |  |  |  | Botrytis cinerea hypovirulence-associated DNA virus 1 | MT425548.1 | 100.00% | 45.00% | *Botrytis cinerea* | 1763 | *Genomoviridae* | */* | Zhang,H et al 2020 |

| **Strain** | **number** | **Infected virus name (abbreviation)** |
| --- | --- | --- |
| IBc-365 | 25 | BcULV3, BcFV5-BI, BcMV6-BI, BcEV5, BcMV3-BI, BcMV1-BI, BcMV9-BI, BcMV4-BI, BcPV3-BI, BcHV1-BI, BcNSRV7-BI, BcULV4, BcHV3-BI, BcDMV1, BcALV1-BI, BcOLV16-BI, BcMCV5-BI, BcVV3-BI, BcBNV1-BI, BcBNV5-BI, BcMV2-BI,  BcOLV1-BI, BcEV2-BI, BcOLV18, BcMV11, BcMV11 |
| IBc-359 | 25 | BcULV3, BcFV5-BI, BcMV6-BI, BcEV5, BcMV3-BI, BcMV1-BI, BcMV9-BI, BcMV4-BI, BcMV12, BcPV3-BI, BcHV1-BI, BcNSRV7-BI, BcULV4, BcHV3-BI, BcDMV1, BcOLV16-BI, BcMCV5-BI, BcBNV1-BI, BcBNV5-BI, BcMV2-BI, BcULV1-BI,  BcULV5, BcOLV1-BI, BcOLV18, BcMV11, BcMV11 |
| IBc-350 | 25 | BcULV3, BcFV5-BI, BcMV6-BI, BcEV5, BcMV3-BI, BcMV1-BI, BcMV9-BI, BcMV4-BI, BcMV12, BcPV3-BI, BcNSRV7-BI, BcULV4, BcHV3-BI, BcDMV1, BcALV1-BI, BcOLV16-BI, BcMCV5-BI, BcVV3-BI, BcBNV1-BI, BcBNV5-BI, BcMV2-BI,  BcOLV1-BI, BcOLV18, BcMV11, BcALV3, BcALV3 |
| IBc-245 | 25 | BcULV3, BcFV5-BI, BcMV6-BI, BcRV1-BI, BcMV3-BI, BcMV1-BI, BcMV9-BI, BcMV12, BcPV3-BI, BcHV1-BI, BcULV4, BcHV3-BI, BcOLV16-BI, BcMCV5-BI, BcOLV7-BI,  BcOLV4-BI, BcMCV3-BI, BcMCV5-BI, BcMV2-BI, BcULV1-BI,  BcOLV1-BI, BcPV4-BI, BcEV2-BI, BcOLV18, BcMV11, BcMV11 |
| IBc-208 | 25 | BcULV3, BcFV5-BIBcRV1-BI, BcMV3-BI, BcMV1-BI, BcMV9-BI, BcMV4-BI, BcPV3-BI, BcULV4, BcHV3-BI, BcALV1-BI, BcOLV17-BI, BcOLV16-BI, BcOLV7-BI, BcOrLV1-BI, BcOLV4-BI, BcMV2-BI, BcULV1-BI, BcULV5, BcOLV1-BI, BcOLV18, BcMV11, BcHV1SLR-BI, BcOLV2-BI,  BcOLV2-BI |
| IBc-179 | 25 | BcULV3, BcMV6-BI, BcRV1-BI, BcMV3-BI, BcMV1-BI, BcMV9-BI, BcMV4-BI, BcPV3-BI, BcHV1-BI, BcNSRV7-BI, BcULV4, BcOLV17-BI, BcOLV16-BI, BcMCV5-BI, BcOLV7-BI, BcOrLV1-BI, BcOLV4-BI, BcFV9, BcMCV3-BI, BcMV2-BI, BcOLV1-BI, BcOLV13-BI, BcEV2-BI, BcOLV18, BcMV11,  BcMV11 |
| IBc-91 | 25 | BcULV3, BcFV5-BI, BcMV6-BI, BcALV2, BcRV1-BI, BcMV3-BI, BcMV1-BI, BcMV9-BI, BcMV4-BI, BcMV12, BcHV1-BI, BcNSRV7-BI, BcULV4, BcHV3-BI, BcDMV1, BcALV1-BI, BcOLV16-BI, BcOLV7-BI, BcFV9, BcMV2-BI, BcULV1-BI,  BcOLV1-BI, BcOLV18, BcMV11, BcHV1SLR-BI, BcHV1SLR-BI |
| IBc-358 | 24 | BcULV3, BcFV5-BI, BcMV6-BI, BcEV5, BcMV3-BI, BcMV1-BI, BcMV9-BI, BcMV4-BI, BcHV1-BI, BcNSRV7-BI, BcULV4, BcHV3-BI, BcDMV1, BcALV1-BI, BcOLV12-BI, BcOLV16-BI, BcMCV5-BI, BcBNV5-BI, BcMV2-BI, BcULV1-BI, BcOLV1-BI,  BcEV2-BI, BcOLV18, BcMV11, BcHV1SLR-BI, BcHV1SLR-BI |
| IBc-348 | 24 | BcOLV9-BI, BcULV3, BcFV5-BI, BcMV6-BI, BcEV5, BcMV3-BI, |

**Table S3 Viruses in each strain of *Botrytis cinerea***

T

|  |  | BcMV1-BI, BcMV9-BI, BcMV4-BI, BcHV1-BI, BcNSRV7-BI, BcULV4, BcHV3-BI, BcDMV1, BcALV1-BI, BcOLV16-BI, BcMCV5-BI, BcBNV1-BI, BcBNV5-BI, BcMV2-BI, BcOLV1-BI,  BcEV2-BI, BcOLV18, BcMV11, BcHV1SLR-BI, BcHV1SLR-BI |
| --- | --- | --- |
| IBc-341 | 23 | BcOLV9-BI, BcULV3, BcFV5-BI, BcMV6-BI, BcEV5, BcMV3-BI, BcMV1-BI, BcMV9-BI, BcMV4-BI, BcHV1-BI, BcNSRV7-BI, BcULV4, BcHV3-BI, BcDMV1, BcALV1-BI, BcOLV16-BI, BcMCV5-BI, BcVV3-BI, BcBNV5-BI, BcMV2-BI, BcOLV1-BI,  BcOLV18, BcMV11, BcHV1SLR-BI |
| IBc-275 | 23 | BcMV1-BI, BcMV9-BI, BcMV4-BI, BcULV4, BcOLV16-BI, BcOLV7-BI, BcOLV4-BI, BcOLV1-BI, BcOLV18, BcMV11, BcOLV2-BI, BcULV3, BcMV1-BI, BcMV9-BI, BcMV4-BI, BcULV4, BcOLV16-BI, BcOLV7-BI, BcOLV4-BI, BcOLV1-BI,  BcOLV18, BcMV11, BcOLV2-BI |
| IBc-213 | 23 | BcULV3, BcMV6-BI, BcOLV11-BI, BcRV1-BI, BcMV3-BI, BcMV1-BI, BcMV9-BI, BcMV4-BI, BcPV3-BI, BcHV1-BI, BcULV4, BcALV1-BI, BcOLV17-BI, BcOLV16-BI, BcOrLV1-BI, BcOLV4-BI, BcBNV1-BI, BcMV2-BI, BcOLV1-BI, BcOLV13-BI,  BcEV2-BI, BcOLV18, BcMV11, BcHV1SLR-BI |
| IBc-153 | 23 | BcULV3, BcMV6-BI, BcOLV11-BI, BcRV1-BI, BcPV5, BcMV3-BI, BcMV1-BI, BcMV9-BI, BcMV4-BI, BcMV12, BcPV3-BI, BcHV1-BI, BcULV4, BcOLV16-BI, BcOLV7-BI, BcOrLV1-BI, BcMV2-BI, BcULV1-BI, BcULV5, BcOLV1-BI,  BcOLV18, BcMV11, BcHV1SLR-BI, BcOLV2-BI |
| IBc-145 | 23 | BcMV6-BI, BcRV1-BI, BcMV3-BI, BcMV1-BI, BcMV9-BI, BcMV4-BI, BcPV3-BI, BcHV1-BI, BcULV4, BcHV3-BI, BcOLV17-BI, BcOLV16-BI, BcOLV7-BI, BcOrLV1-BI,  BcVV3-BI, BcNSRV9-BI, BcMV2-BI, BcULV1-BI, BcOLV1-BI,  BcOLV13-BI, BcEV2-BI, BcOLV18, BcMV11, BcHV1SLR-BI |
| IBc-142 | 23 | BcULV3, BcMV6-BI, BcRV1-BI, BcMV3-BI, BcMV1-BI, BcMV4-BI, BcMV12, BcHV1-BI, BcFV9, BcULV4, BcOLV17-BI, BcOLV16-BI, BcMCV5-BI, BcOLV4-BI, BcVV3-BI, BcPV3-BI,  BcMV2-BI, BcULV5, BcOLV1-BI, BcOLV13-BI, BcOLV18,  BcMV11, BcHV1SLR-BI, BcOLV2-BI |
| IBc-75 | 23 | BcMV1-BI, BcMV9-BI, BcMV4-BI, BcHV1-BI, BcULV4, BcHV3-BI, BcMCV5-BI, BcOLV7-BI, BcOrLV1-BI, BcVV3-BI, BcOLV18, BcHV1SLR-BI, BcULV3, BcMV1-BI, BcMV9-BI, BcMV4-BI, BcHV1-BI, BcULV4, BcHV3-BI, BcMCV5-BI,  BcOLV7-BI, BcOrLV1-BI, BcVV3-BI, BcOLV18, BcHV1SLR-BI,  BcHV1SLR-BI |
| IBc-362 | 22 | BcULV3, BcFV5-BI, BcMV6-BI, BcEV5, BcMV3-BI, BcMV1-BI, BcMV9-BI, BcMV4-BI, BcHV1-BI, BcULV4, BcHV3-BI, BcDMV1, BcALV1-BI, BcOLV16-BI, BcMCV5-BI, BcOLV4-BI,  BcVV3-BI, BcBNV5-BI, BcMV2-BI, BcOLV1-BI, BcOLV18, |

|  |  | BcMV11, BcHV1SLR-BI |
| --- | --- | --- |
| IBc-346 | 22 | BcULV3, BcFV5-BI, BcMV6-BI, BcEV5, BcMV3-BI, BcMV1-BI, BcMV9-BI, BcMV4-BI, BcHV1-BI, BcULV4, BcHV3-BI, BcDMV1, BcALV1-BI, BcMCV5-BI, BcVV3-BI, BcBNV5-BI, BcMV2-BI, BcOLV1-BI, BcHV4-BI, BcEV2-BI, BcOLV18,  BcMV11 |
| IBc-343 | 22 | BcULV3, BcFV5-BI, BcMV6-BI, BcEV5, BcMV3-BI, BcMV1-BI, BcMV9-BI, BcMV4-BI, BcNSRV7-BI, BcULV4, BcHV3-BI, BcDMV1, BcALV1-BI, BcOLV16-BI, BcMCV5-BI, BcVV3-BI, BcBNV5-BI, BcMV2-BI, BcOLV1-BI, BcVV5, BcOLV18,  BcMV11, BcHV1SLR-BI |
| IBc-309 | 22 | BcRV1-BI, BcPV5, BcMV3-BI, BcMV1-BI, BcMV9-BI, BcMV4-BI, BcPV3-BI, BcULV4, BcHV3-BI, BcALV1-BI, BcOLV16-BI, BcMCV5-BI, BcOLV7-BI, BcOLV4-BI, BcFV9,  BcMCV4-BI, BcNSRV9-BI, BcMV2-BI, BcOLV1-BI, BcEV2-BI,  BcOLV18, BcMV11 |
| IBc-223 | 22 | BcFV5-BI, BcMV6-BI, BcRV1-BI, BcMV3-BI, BcMV1-BI, BcMV9-BI, BcMV4-BI, BcPV3-BI, BcHV1-BI, BcULV4, BcDMV1, BcALV1-BI, BvF-BI, BcFV9, BcBNV5-BI, BcMV2-BI, BcOLV1-BI, BcHV4-BI, BcEV2-BI, BcOLV18, BcMV11,  BcHV1SLR-BI, BcMV15 |
| IBc-152 | 22 | BcMV6-BI, BcRV1-BI, BcMV3-BI, BcMV1-BI, BcMV9-BI, BcMV4-BI, BcMV12, BcPV3-BI, BcHV1-BI, BcULV4, BcHV3-BI, BcOLV17-BI, BcOLV7-BI, BcOrLV1-BI, BcVV3-BI, BcMV2-BI, BcULV1-BI, BcOLV1-BI, BcOLV13-BI, BcOLV18, BcMV11,  BcHV1SLR-BI, BcOLV2-BI |
| IBc-126 | 22 | BcULV3, BcFV5-BI, BcPV5, BcMV3-BI, BcMV1-BI, BcULV5, BcMV4-BI, BcMV12, BcHV1-BI, BcFV9, BcNSRV7-BI, BcULV4, BcHV3-BI, BcOLV16-BI, BcFV1-BI, BcOLV7-BI, BcVV3-BI, BcPV3-BI, BcULV5, BcEV2-BI, BcOLV18, BcMV11,  BcHV1SLR-BI |
| IBc-356 | 21 | BcULV3, BcFV5-BI, BcMV6-BI, BcEV5, BcMV3-BI, BcMV1-BI, BcMV9-BI, BcMV4-BI, BcNSRV7-BI, BcULV4, BcHV3-BI, BcDMV1, BcMCV5-BI, BcVV3-BI, BcBNV5-BI, BcMV2-BI,  BcULV1-BI, BcOLV1-BI, BcEV2-BI, BcOLV18, BcMV11 |
| IBc-331 | 21 | BcULV3, BcFV5-BI, BcMV6-BI, BcMV3-BI, BcMV1-BI, BcMV9-BI, BcMV4-BI, BcHV1-BI, BcNSRV7-BI, BcULV4, BcDMV1, BcALV1-BI, BcFV9, BcVV3-BI, BcBNV5-BI, BcMV2-BI, BcOLV1-BI, BcHV4-BI, BcOLV18, BcMV11,  BcHV1SLR-BI, BcEV6 |
| IBc-207 | 21 | BcULV3, BcFV5-BI, BcMV6-BI, BcRV1-BI, BcMV3-BI, BcMV1-BI, BcMV9-BI, BcMV4-BI, BcPV3-BI, BcHV1-BI, BcULV4, BcALV1-BI, BcOLV16-BI, BcOLV7-BI, BcMCV3-BI,  BcMV2-BI, BcULV1-BI, BcOLV1-BI, BcOLV18, BcMV11, |

|  |  | BcHV1SLR-BI, BcOLV2-BI |
| --- | --- | --- |
| IBc-204 | 21 | BcULV3, BcFV5-BI, BcOLV11-BI, BcRV1-BI, BcMV3-BI, BcMV1-BI, BcMV9-BI, BcMV4-BI, BcPV3-BI, BcHV1-BI, BcHV3-BI, BcALV1-BI, BcOLV16-BI, BcMCV5-BI, BcOrLV1-BI, BcOLV4-BI, BcMV2-BI, BcOLV13-BI, BcEV2-BI, BcOLV18,  BcMV11 |
| IBc-147 | 21 | BcULV3, BcMV6-BI, BcRV1-BI, BcMV3-BI, BcMV1-BI, BcMV9-BI, BcMV4-BI, BcMV12, BcHV1-BI, BcNSRV7-BI, BcULV4, BcOLV17-BI, BcOLV16-BI, BcOrLV1-BI, BcOLV4-BI, BcMV2-BI, BcOLV1-BI, BcOLV13-BI, BcEV2-BI, BcOLV18,  BcMV11, BcHV1SLR-BI |
| IBc-81 | 21 | BcULV3, BcMV6-BI, BcRV1-BI, BcMV3-BI, BcMV1-BI, BcMV9-BI, BcHV1-BI, BcULV4, BcOLV16-BI, BcULV1-BI, BcMV11, BcHV1SLR-BI, BcULV3, BcRV1-BI, BcMV3-BI, BcMV1-BI, BcMV9-BI, BcHV1-BI, BcULV4, BcOLV16-BI,  BcULV1-BI, BcMV11, BcHV1SLR-BI |
| Skr-7 | 20 | BcALV1-BI, BcHV1-BI, BcHV1SLR-BI, BcHV5-BI, BcMV1-BI, BcMV2-BI, BcMV3-BI, BcMV4-BI, BcMV9-BI, BcOLV4-BI, BcOLV7-BI, BcULV4, BvF-BI, BcOLV19, BcOLV18, BcDFV4,  BcMV11, BcOLV7-BI, BcOLV15-BI, BcULV3, BcOLV16-BI |
| Skr-4 | 20 | BcFV9, BcNSRV7-BI, BcHV1-BI, BcMV3-BI, BcULV4, BcBNV5-BI, BcULV3, BcOLV15-BI, BcMV9-BI, BcEV2-BI, BcMV1-BI, BcMV11, BcFV5-BI, BcMV4-BI, BcDFV4, BcOLV13-BI, BcHV5-BI, BcOLV18, BcHV1SLR-BI, BcEV6,  BcHADV1 |
| IBc-316 | 20 | BcULV3, BcMV6-BI, BcRV1-BI, BcMV3-BI, BcMV1-BI, BcMV9-BI, BcMV4-BI, BcMV12, BcPV3-BI, BcHV1-BI, BcULV4, BcOLV16-BI, BcMCV5-BI, BcOLV7-BI, BcMCV3-BI,  BcBNV5-BI, BcULV1-BI, BcOLV1-BI, BcEV2-BI, BcOLV18 |
| IBc-211 | 20 | BcULV3, BcMV6-BI, BcRV1-BI, BcMV3-BI, BcMV1-BI, BcMV9-BI, BcMV4-BI, BcPV3-BI, BcHV1-BI, BcULV4, BcALV1-BI, BcOLV16-BI, BcFV9, BcBNV5-BI, BcMV2-BI, BcOLV1-BI, BcEV2-BI, BcOLV18, BcMV11, BcHV1SLR-BI,  BcOLV2-BI |
| IBc-171 | 20 | BcMV6-BI, BcRV1-BI, BcMV3-BI, BcMV1-BI, BcMV9-BI, BcMV4-BI, BcMV12, BcPV3-BI, BcHV1-BI, BcULV4,  BcALV1-BI, BcOLV16-BI, BcOrLV1-BI, BcMV2-BI, BcULV1-BI, BcOLV1-BI, BcOLV18, BcMV11, BcHV1SLR-BI, BcOLV2-BI,  BcMV15 |
| IBc-155 | 20 | BcULV3, BcMV6-BI, BcRV1-BI, BcMV3-BI, BcMV1-BI, BcMV9-BI, BcMV12, BcHV1-BI, BcNSRV7-BI, BcULV4, BcHV3-BI, BcOLV17-BI, BcOLV16-BI, BcOLV7-BI, BcFV9, BcBNV5-BI, BcMV2-BI, BcOLV1-BI, BcOLV18, BcMV11,  BcHV1SLR-BI |

| IBc-95 | 20 | BcULV3, BcFV5-BI, BcMV1-BI, BcOLV15-BI, BcMV4-BI, BcHV1-BI, BcFV9, BcNSRV7-BI, BcHV3-BI, BcMTV1-BI, BcMNV1-BI, BvF-BI, BcOLV16-BI, BcOLV4-BI, BcBNV1-BI, BcPV3-BI, BcULV1-BI, BcEV2-BI, BcOLV18, BcMV11,  BcHV1SLR-BI |
| --- | --- | --- |
| IBc-361 | 19 | BcULV3, BcMV6-BI, BcEV5, BcMV3-BI, BcMV1-BI, BcMV9-BI, BcMV4-BI, BcHV1-BI, BcFV9, BcNSRV7-BI, BcULV4,  BcHV3-BI, BcDMV1, BcALV1-BI, BcMCV5-BI, BcVV3-BI,  BcBNV5-BI, BcMV2-BI, BcMV11, BcHV1SLR-BI |
| IBc-327 | 19 | BcMV6-BI, BcRV1-BI, BcMV3-BI, BcMV1-BI, BcMV9-BI, BcMV4-BI, BcPV3-BI, BcHV1-BI, BcULV4, BcDMV1,  BcALV1-BI, BvF-BI, BcFV9, BcMV2-BI, BcOLV1-BI, BcHV4-BI,  BcOLV18, BcMV11, BcHV1SLR-BI, BcEV6 |
| IBc-267 | 19 | BcULV3, BcMV6-BI, BcMV3-BI, BcMV1-BI, BcMV9-BI, BcMV12, BcHV1-BI, BcNSRV7-BI, BcULV4, BcHV3-BI, BcDMV1, BcOLV16-BI, BcPV3-BI, BcMV2-BI, BcULV1-BI,  BcOLV13-BI, BcPV4-BI, BcOLV18, BcMV11, BcHV1SLR-BI |
| IBc-236 | 19 | BcULV3, BcMV6-BI, BcRV1-BI, BcMV3-BI, BcMV1-BI, BcMV9-BI, BcMV12, BcPV3-BI, BcHV1-BI, BcULV4,  BcALV1-BI, BcOLV16-BI, BcOLV7-BI, BcMCV3-BI, BcMV2-BI,  BcULV1-BI, BcOLV1-BI, BcOLV18, BcMV11, BcHV1SLR-BI |
| IBc-225 | 19 | BcRV1-BI, BcMV3-BI, BcMV1-BI, BcMV9-BI, BcMV4-BI, BcPV3-BI, BcHV1-BI, BcULV4, BcOLV16-BI, BcOLV7-BI, BcOLV4-BI, BcMV2-BI, BcULV1-BI, BcULV5, BcOLV1-BI,  BcEV2-BI, BcOLV18, BcMV11, BcHV1SLR-BI, BcMV15 |
| IBc-219 | 19 | BcULV3, BcMV6-BI, BcRV1-BI, BcMV3-BI, BcMV9-BI, BcMV4-BI, BcPV3-BI, BcHV1-BI, BcULV4, BcALV1-BI,  BcOLV16-BI, BcOLV7-BI, BcMCV3-BI, BcMV2-BI, BcULV1-BI,  BcOLV1-BI, BcOLV13-BI, BcOLV18, BcMV11, BcHV1SLR-BI |
| IBc-205 | 19 | BcFV5-BI, BcOLV11-BI, BcRV1-BI, BcMV3-BI, BcMV1-BI, BcMV9-BI, BcMV4-BI, BcPV3-BI, BcHV1-BI, BcULV4, BcALV1-BI, BcOLV16-BI, BcOrLV1-BI, BcFV9, BcVV3-BI,  BcMCV3-BI, BcMV2-BI, BcEV2-BI, BcOLV18, BcHV1SLR-BI |
| IBc-180 | 19 | BcULV3, BcMV6-BI, BcRV1-BI, BcMV3-BI, BcMV1-BI, BcMV9-BI, BcMV4-BI, BcMV12, BcHV1-BI, BcNSRV7-BI, BcULV4, BcALV1-BI, BcOLV16-BI, BcOrLV1-BI, BcMV2-BI,  BcOLV1-BI, BcOLV18, BcMV11, BcHV1SLR-BI, BcOLV2-BI |
| IBc-141 | 19 | BcRV1-BI, BcMV1-BI, BcOLV15-BI, BcMV4-BI, BcFV9, BcULV4, BcHV3-BI, BcOLV17-BI, BcFV1-BI, BcMCV5-BI, BcOLV4-BI, BcVV3-BI, BcMV2-BI, BcULV5, BcOLV1-BI,  BcOLV13-BI, BcEV2-BI, BcOLV18, BcMV11 |
| IBc-131 | 19 | BcULV3, BcFV5-BI, BcMV6-BI, BcRV1-BI, BcMV3-BI,  BcMV1-BI, BcMV4-BI, BcMV12, BcHV1-BI, BcULV4, |

|  |  | BcHV3-BI, BcOLV16-BI, BcMCV5-BI, BcOLV7-BI, BcMV2-BI,  BcULV5, BcEV2-BI, BcOLV18, BcMV11, BcHV1SLR-BI |
| --- | --- | --- |
| IBc-353 | 18 | BcULV3, BcFV5-BI, BcMV6-BI, BcEV5, BcMV3-BI, BcMV1-BI, BcMV9-BI, BcMV4-BI, BcHV1-BI, BcNSRV7-BI, BcHV3-BI, BcDMV1, BcMCV5-BI, BcBNV5-BI, BcMV2-BI, BcOLV1-BI,  BcHV4-BI, BcMV11, BcHV1SLR-BI |
| IBc-317 | 18 | BcULV3, BcRV1-BI, BcMV3-BI, BcMV1-BI, BcMV9-BI, BcMV4-BI, BcPV3-BI, BcHV1-BI, BcULV4, BcHV3-BI,  BcOLV17-BI, BcOLV16-BI, BcOLV7-BI, BcVV3-BI, BcULV1-BI,  BcOLV1-BI, BcOLV13-BI, BcOLV18, BcHV1SLR-BI, |
| IBc-248 | 18 | BcULV3, BcMV6-BI, BcMV1-BI, BcMV12, BcMV4-BI, BcMV3-BI, BcOLV16-BI, BcOLV4-BI, BcMV17, BcMCV3-BI, BcMV2-BI, BcULV1-BI, BcULV5, BcOLV1-BI, BcOLV13-BI,  BcMV16, BcHV1SLR-BI, BcOLV2-BI, BcMV15 |
| IBc-151 | 18 | BcULV3, BcMV6-BI, BcRV1-BI, BcMV3-BI, BcMV9-BI, BcMV4-BI, BcPV3-BI, BcHV1-BI, BcULV4, BcOLV17-BI, BcOrLV1-BI, BcFV9, BcMCV3-BI, BcMV2-BI, BcULV1-BI,  BcOLV1-BI, BcOLV18, BcMV11, BcHV1SLR-BI |
| IBc-108 | 18 | BcULV3, BcMV3-BI, BcMV1-BI, BcHV1-BI, BcFV9, BcNSRV7-BI, BcULV4, BcOLV16-BI, BcVV3-BI, BcPV3-BI,  BcMV2-BI, BcULV5, BcVV5, BcPV4-BI, BcHV4-BI, BcEV2-BI,  BcOLV18, BcMV11, BcHV1SLR-BI, |
| IBc-52 | 18 | BcMV2-BI, BvF-BI, BcMV1-BI, BcMV3-BI, BcMV4-BI, BcOLV4-BI, BcALV1-BI, BcMV9-BI, BcPV7, BcHV1-BI,  BcHV3-BI, BcMV11, BcULV3, BcPV3-BI, BcMV3-BI, BcEV2-BI,  BcFV5-BI, BcBNV4-BI, BcHV1SLR-BI |
| Ska-2 | 17 | BcEV6, BcFV9, BcHV5-BI, BcMV1-BI, BcMV2-BI, BcMCV5-BI, BcOLV10-BI, BcOLV9-BI, BcOLV1-BI, BcOLV4-BI, BvF-BI, BcMV9-BI, BcOLV18, BcHV1-BI, BcHV1SLR-BI, BcMTV1-BI,  BcOLV16-BI, BcOLV9-BI |
| IBc-430 | 17 | BcULV3, BcMV3-BI, BcMV1-BI, BcMV4-BI, BcHV1-BI, BcULV4, BcALV1-BI, BcMCV5-BI, BcOLV7-BI, BcOLV4-BI, BcFV9, BcVV3-BI, BcBNV5-BI, BcOLV1-BI, BcHV4-BI,  BcOLV18, BcMV11, BcHV1SLR-BI |
| IBc-405 | 17 | BcULV3, BcMV6-BI, BcMV3-BI, BcMV1-BI, BcULV4, BcMCV4-BI, BcULV5, BcMV11, BcALV3, BcBNV6, BcULV3, BcMV3-BI, BcMV1-BI, BcULV4, BcMCV4-BI, BcULV5,  BcMV11 |
| IBc-354 | 17 | BcULV3, BcMV6-BI, BcEV5, BcMV3-BI, BcMV1-BI, BcMV9-BI, BcMV4-BI, BcFV9, BcNSRV7-BI, BcHV3-BI, BcDMV1, BcVV3-BI, BcPV3-BI, BcBNV5-BI, BcMV2-BI, BcOLV18,  BcMV11 |
| IBc-315 | 17 | BcULV3, BcRV1-BI, BcMV3-BI, BcMV1-BI, BcMV9-BI, |

|  |  | BcMV4-BI, BcHV1-BI, BcULV4, BcHV3-BI, BcOLV16-BI,  BcMCV5-BI, BcOLV7-BI, BcVV3-BI, BcNSRV9-BI, BcULV1-BI, BcOLV18, BcMV11, BcHV1SLR-BI |
| --- | --- | --- |
| IBc-297 | 17 | BcULV3, BcMV6-BI, BcRV1-BI, BcMV3-BI, BcMV1-BI, BcMV9-BI, BcMV12, BcPV3-BI, BcHV1-BI, BcULV4,  BcOLV16-BI, BcMCV3-BI, BcMV2-BI, BcULV1-BI, BcOLV1-BI,  BcPV4-BI, BcMV11, BcHV1SLR-BI |
| IBc-277 | 17 | BcULV3, BcMV3-BI, BcMV1-BI, BcMV9-BI, BcMV12, BcULV4, BcOLV16-BI, BcOLV7-BI, BcOLV4-BI, BcVV3-BI, BcPV3-BI, BcMV2-BI, BcULV1-BI, BcOLV1-BI, BcEV2-BI, BcOLV18,  BcMV11, BcHV1SLR-BI |
| IBc-250 | 17 | BcULV3, BcMV1-BI, BcMV12, BcMV4-BI, BcMV3-BI, BcPV3-BI, BcHV1-BI, BcOLV16-BI, BcOLV4-BI, BcMCV3-BI, BcMV2-BI, BcULV1-BI, BcULV5, BcOLV1-BI, BcOLV13-BI,  BcMV11, BcHV1SLR-BI, BcOLV2-BI |
| IBc-233 | 17 | BcULV3, BcFV5-BI, BcMV3-BI, BcMV1-BI, BcMV9-BI, BcHV1-BI, BcNSRV7-BI, BcULV4, BcHV3-BI, BcVV3-BI, BcMV2-BI, BcOLV1-BI, BcEV2-BI, BcOLV18, BcMV11,  BcHV1SLR-BI, BcOLV2-BI, BcMV15 |
| IBc-197 | 17 | BcULV3, BcMV6-BI, BcRV1-BI, BcMV3-BI, BcMV1-BI, BcMV9-BI, BcMV4-BI, BcPV3-BI, BcHV1-BI, BcULV4, BcOLV16-BI, BcOLV7-BI, BcMV2-BI, BcOLV1-BI, BcOLV18,  BcMV11, BcHV1SLR-BI, BcEV6 |
| IBc-174 | 17 | BcULV3, BcRV1-BI, BcMV3-BI, BcMV1-BI, BcMV9-BI, BcMV4-BI, BcHV1-BI, BcULV4, BcHV3-BI, BcALV1-BI, BcOLV16-BI, BcMCV5-BI, BcMV2-BI, BcULV1-BI, BcULV5,  BcOLV18, BcMV11, BcHV1SLR-BI |
| IBc-172 | 17 | BcMV6-BI, BcRV1-BI, BcMV3-BI, BcMV1-BI, BcMV9-BI, BcMV4-BI, BcPV3-BI, BcHV1-BI, BcULV4, BcHV3-BI, BcOLV16-BI, BcOLV7-BI, BcMV2-BI, BcULV1-BI, BcULV5,  BcOLV18, BcMV11, BcHV1SLR-BI |
| IBc-167 | 17 | BcULV3, BcMV6-BI, BcRV1-BI, BcMV3-BI, BcMV1-BI, BcMV9-BI, BcHV1-BI, BcULV4, BcALV1-BI, BcOLV16-BI, BcOLV7-BI, BcMV2-BI, BcULV1-BI, BcOLV1-BI, BcOLV18,  BcMV11, BcHV1SLR-BI, BcMV15 |
| IBc-138 | 17 | BcRV1-BI, BcMV3-BI, BcMV1-BI, BcOLV15-BI, BcMV4-BI, BcFV9, BcOLV17-BI, BcOLV16-BI, BcFV1-BI, BcMCV5-BI, BcOLV4-BI, BcMV2-BI, BcOLV1-BI, BcOLV13-BI, BcPV4-BI,  BcOLV18, BcMV11 |
| IBc-125 | 17 | BcULV3, BcFV5-BI, BcMV6-BI, BcALV1-BI, BcMV3-BI, BcMV1-BI, BcMV4-BI, BcHV1-BI, BcNSRV7-BI, BcULV4, BvF-BI, BcULV1-BI, BcULV5, BcEV2-BI, BcMV11,  BcHV1SLR-BI, BcALV2, BcALV3 |

| IBc-74 | 17 | BcMV3-BI, BcMV1-BI, BcMV9-BI, BcHV1-BI, BcULV4, BvF-BI, BcOLV18, BcMV11, BcULV3, BcMV3-BI, BcMV1-BI,  BcMV9-BI, BcHV1-BI, BcULV4, BvF-BI, BcOLV18, BcMV11,  BcHV1SLR-BI |
| --- | --- | --- |
| IBc-57 | 17 | BcMV2-BI, BcPV4-BI, BcMV1-BI, BcMV4-BI, BcOLV4-BI, BcMV9-BI, BcHV1-BI, BcMV11, BcMV12, BcNSRV7-BI, BcULV3, BcPV3-BI, BcEV2-BI, BcFV5-BI, BcOLV18,  BcBNV4-BI, BcHV1SLR-BI, BcMV6-BI |
| IBc-30 | 17 | BcULV3, BcFV5-BI, BcMV3-BI, BcMV1-BI, BcMV9-BI, BcPV3-BI, BcHV1-BI, BcULV4, BcHV3-BI, BcDMV1, BcOLV12-BI, BcOLV16-BI, BcMV2-BI, BcEV2-BI, BcOLV18,  BcMV11, BcHV1SLR-BI, BcEV6 |
| Ca-5 | 16 | BcFV9, BcHV1-BI, BcMV3-BI, BcULV4, BcBNV5-BI, BcULV3, BcDMV1, BcOLV15-BI, BcMV9-BI, BcEV2-BI, BcMV1-BI, BcMV11, BcFV5-BI, BcVV3-BI, BcMV4-BI, BcOLV18,  BcHV1SLR-BI |
| Ca-4 | 16 | BcFV9, BcNSRV7-BI, BcHV1-BI, BcMV3-BI, BcULV4, BcBNV5-BI, BcULV3, BcMTV1-BI, BcMV9-BI, BcEV2-BI, BcMV1-BI, BcMV11, BcFV5-BI, BcPV3-BI, BcHV5-BI,  BcOLV18, BcHV1SLR-BI |
| IBc-342 | 16 | BcULV3, BcMV6-BI, BcMV3-BI, BcMV1-BI, BcMV9-BI, BcMV4-BI, BcHV1-BI, BcNSRV7-BI, BcULV4, BcHV3-BI, BcDMV1, BcALV1-BI, BcVV3-BI, BcMV2-BI, BcOLV18,  BcMV11, BcHV1SLR-BI |
| IBc-326 | 16 | BcMV11, BcMV6-BI, BcRV1-BI, BcMV3-BI, BcMV1-BI, BcMV9-BI, BcMV4-BI, BcPV3-BI, BcHV1-BI, BcULV4, BcALV1-BI, BcVV3-BI, BcMV2-BI, BcULV1-BI, BcOLV1-BI,  BcOLV18 |
| IBc-323 | 16 | BcMV6-BI, BcRV1-BI, BcMV3-BI, BcMV1-BI, BcMV9-BI, BcMV4-BI, BcPV3-BI, BcHV1-BI, BcULV4, BcHV3-BI, BcOLV7-BI, BcVV3-BI, BcOLV1-BI, BcOLV18, BcMV11,  BcHV1SLR-BI, BcOLV2-BI |
| IBc-302 | 16 | BcFV5-BI, BcMV6-BI, BcMV3-BI, BcMV1-BI, BcMV9-BI, BcMV12, BcHV1-BI, BcULV4, BcHV3-BI, BcOLV16-BI, BcVV3-BI, BcMV2-BI, BcULV1-BI, BcOLV1-BI, BcEV2-BI,  BcOLV18, BcHV1SLR-BI |
| IBc-268 | 16 | BcMV6-BI, BcPV5, BcMV1-BI, BcMV12, BcMV4-BI, BcMV3-BI, BcPV3-BI, BcHV1-BI, BcULV4, BcOLV4-BI, BcVV3-BI, BcMV2-BI, BcULV5, BcOLV1-BI, BcOLV13-BI, BcMV11,  BcHV1SLR-BI |
| IBc-260 | 16 | BcOLV9-BI, BcULV3, BcPV5, BcMV1-BI, BcMV4-BI, BcHV1-BI, BcHV3-BI, BcALV1-BI, BcOLV16-BI, BcOLV4-BI, BcVV3-BI,  BcMV2-BI, BcULV5, BcOLV1-BI, BcMV11, BcEV6 |

| IBc-257 | 16 | BcOLV9-BI, BcULV3, BcMV6-BI, BcMV1-BI, BcMV12, BcMV4-BI, BcMV3-BI, BcHV1-BI, BcULV4, BcMTV1-BI, BcOLV16-BI, BcOLV4-BI, BcMV2-BI, BcULV5, BcOLV1-BI,  BcMV11, BcHV1SLR-BI |
| --- | --- | --- |
| IBc-246 | 16 | BcULV3, BcMV6-BI, BcMV1-BI, BcMV12, BcMV3-BI, BcPV3-BI, BcHV1-BI, BcOLV16-BI, BcOLV4-BI, BcMCV3-BI, BcMV2-BI, BcULV1-BI, BcULV5, BcOLV1-BI, BcOLV13-BI,  BcPV4-BI, BcHV1SLR-BI |
| IBc-243 | 16 | BcULV3, BcMV6-BI, BcRV1-BI, BcMV3-BI, BcMV9-BI, BcMV4-BI, BcHV1-BI, BcULV4, BcHV3-BI, BcOLV16-BI, BcOLV7-BI, BcMV13, BcMV2-BI, BcOLV1-BI, BcOLV18,  BcMV11, BcHV1SLR-BI |
| IBc-231 | 16 | BcRV1-BI, BcMV3-BI, BcMV1-BI, BcMV9-BI, BcMV4-BI, BcPV3-BI, BcHV1-BI, BcULV4, BcALV1-BI, BcMV2-BI, BcULV5, BcHV4-BI, BcEV2-BI, BcOLV18, BcMV11,  BcHV1SLR-BI, BcMV15 |
| IBc-228 | 16 | BcMV6-BI, BcMV3-BI, BcMV1-BI, BcMV9-BI, BcMV12, BcHV1-BI, BcULV4, BcOLV16-BI, BcVV3-BI, BcMCV3-BI, BcMV2-BI, BcULV1-BI, BcOLV1-BI, BcPV4-BI, BcOLV18,  BcHV1SLR-BI, BcOLV2-BI |
| IBc-203 | 16 | BcMV6-BI, BcRV1-BI, BcMV3-BI, BcMV1-BI, BcMV9-BI, BcHV1-BI, BcULV4, BcALV1-BI, BcOLV16-BI, BcOLV7-BI, BcMV2-BI, BcULV1-BI, BcOLV1-BI, BcOLV18, BcMV11,  BcEV6 |
| IBc-177 | 16 | BcULV3, BcMV1-BI, BcMV9-BI, BcHV1-BI, BcULV4, BcALV1-BI, BcOLV17-BI, BcOLV16-BI, BcOLV7-BI, BcOLV4-BI, BcFV9, BcMV2-BI, BcULV1-BI, BcEV2-BI,  BcOLV18, BcMV11, BcHV1SLR-BI |
| IBc-148 | 16 | BcULV3, BcMV6-BI, BcRV1-BI, BcMV3-BI, BcMV1-BI, BcMV9-BI, BcMV12, BcHV1-BI, BcNSRV7-BI, BcULV4,  BcDMV1, BcOLV17-BI, BcOLV16-BI, BcOLV7-BI, BcOLV13-BI,  BcMV11 |
| IBc-106 | 16 | BcULV3, BcMV3-BI, BcMV1-BI, BcMV4-BI, BcHV1-BI, BcULV4, BcVV3-BI, BcMV11, BcHV1SLR-BI, BcULV3, BcMV3-BI, BcMV1-BI, BcMV4-BI, BcHV1-BI, BcULV4,  BcVV3-BI, BcMV11, BcHV1SLR-BI |
| IBc-67 | 16 | BcFV5-BI, BcRV1-BI, BcMV3-BI, BcMV1-BI, BcMV9-BI, BcMV4-BI, BcNSRV7-BI, BcULV4, BcHV3-BI, BcALV1-BI, BcOLV7-BI, BcMV2-BI, BcULV1-BI, BcOLV1-BI, BcOLV18,  BcMV11 |
| IBc-36 | 16 | BcULV3, BcFV5-BI, BcMV1-BI, BcMV9-BI, BcMV12, BcPV3-BI, BcHV1-BI, BcULV4, BcHV3-BI, BcVLV1, BcPLV1, BcMV2-BI,  BcULV1-BI, BcOLV1-BI, BcOLV18, BcBLV1, BcHV1SLR-BI |

| Ska-6 | 15 | BcFV9, BcNSRV7-BI, BcHV1-BI, BcMV3-BI, BcULV4, BcULV1-BI, BcHV3-BI, BcULV3, BcOLV15-BI, BcMV9-BI, BcEV2-BI, BcMV1-BI, BcFV5-BI, BcMV4-BI, BcOLV13-BI,  BcHV1SLR-BI |
| --- | --- | --- |
| Ca-8 | 15 | BcNSRV7-BI, BcHV1-BI, BcMV3-BI, BcULV4, BcBNV5-BI, BcHV3-BI, BcDMV1, BcOLV15-BI, BcMV9-BI, BcEV2-BI, BcMV1-BI, BcMV11, BcFV5-BI, BcOLV18, BcHV1SLR-BI,  BcOLV2-BI |
| Ca-7 | 15 | BcNSRV7-BI, BcHV1-BI, BcMV3-BI, BcBNV5-BI, BcHV3-BI, BcULV3, BcDMV1, BcOLV15-BI, BcMV9-BI, BcMV1-BI, BcMV11, BcFV5-BI, BvF-BI, BcOLV18, BcHV1SLR-BI,  BcOLV2-BI |
| Ca-11 | 15 | BcFV9, BcPV8, BcNSRV7-BI, BcHV1-BI, BcMV3-BI, BcHV3-BI, BcULV3, BcOLV15-BI, BcMV9-BI, BcMV1-BI, BcFV5-BI,  BcMV12, BcMV4-BI, BcDFV4, BcOLV18, BcHV1SLR-BI |
| IBc-440 | 15 | BcULV3, BcFV5-BI, BcPV5, BcMV3-BI, BcMV1-BI, BcMV9-BI, BcMV4-BI, BcHV1-BI, BcULV4, BcHV3-BI, BcOLV12-BI,  BcOLV7-BI, BcMV2-BI, BcPV4-BI, BcMV11 |
| IBc-332 | 15 | BcULV3, BcMV6-BI, BcEV5, BcMV3-BI, BcMV1-BI, BcMV9-BI, BcMV4-BI, BcHV1-BI, BcHV3-BI, BcDMV1, BcMCV5-BI,  BcFV9, BcBNV5-BI, BcOLV1-BI, BcMV11, BcHV1SLR-BI |
| IBc-304 | 15 | BcULV3, BcRV1-BI, BcMV3-BI, BcMV1-BI, BcMV9-BI, BcMV4-BI, BcPV3-BI, BcHV1-BI, BcULV4, BcHV3-BI,  BcOLV16-BI, BcOLV7-BI, BcOLV1-BI, BcOLV13-BI, BcMV11 |
| IBc-299 | 15 | BcULV3, BcFV5-BI, BcEV5, BcMV3-BI, BcMV1-BI, BcMV4-BI, BcPV3-BI, BcHV1-BI, BcULV4, BcHV3-BI, BcDMV1,  BcNSRV6-BI, BcPLV1, BcMV11, BcMV14, BcHV1SLR-BI |
| IBc-294 | 15 | BcULV3, BcMV6-BI, BcMV3-BI, BcMV1-BI, BcMV9-BI, BcMV12, BcHV1-BI, BcULV4, BcOLV16-BI, BcMCV3-BI, BcMV2-BI, BcULV1-BI, BcOLV1-BI, BcOLV18, BcHV1SLR-BI,  BcOLV2-BI |
| IBc-274 | 15 | BcFV5-BI, BcRV1-BI, BcMV1-BI, BcMV9-BI, BcMV4-BI,  BcPV3-BI, BcHV1-BI, BcULV4, BcALV1-BI, BcOLV16-BI, BcOLV7-BI, BcMV2-BI, BcOLV1-BI, BcOLV18, BcMV11 |
| IBc-261 | 15 | BcULV3, BcMV1-BI, BcMV12, BcMV4-BI, BcMV3-BI, BcPV3-BI, BcHV1-BI, BcVV3-BI, BcOLV16-BI, BcOLV4-BI, BcMV2-BI, BcULV5, BcOLV1-BI, BcOLV13-BI, BcMV11,  BcHV1SLR-BI |
| IBc-251 | 15 | BcULV3, BcMV6-BI, BcMV1-BI, BcMV12, BcMV3-BI, BcHV1-BI, BcULV4, BcOLV16-BI, BcOLV4-BI, BcMCV3-BI, BcMV2-BI, BcULV1-BI, BcOLV1-BI, BcOLV13-BI, BcMV11,  BcHV1SLR-BI |
| IBc-242 | 15 | BcULV3, BcPV5, BcMV3-BI, BcMV1-BI, BcMV9-BI, BcULV4, |

|  |  | BcOLV16-BI, BcOLV7-BI, BcBNV5-BI, BcMV2-BI, BcPV4-BI,  BcEV2-BI, BcOLV18, BcMV11, BcPV6 |
| --- | --- | --- |
| IBc-241 | 15 | BcFV5-BI, BcOLV11-BI, BcMV3-BI, BcMV1-BI, BcMV9-BI, BcMV12, BcHV1-BI, BcULV4, BcALV1-BI, BcOLV16-BI, BcMCV3-BI, BcMV2-BI, BcULV1-BI, BcPV4-BI, BcOLV18,  BcHV1SLR-BI |
| IBc-226 | 15 | BcULV3, BcRV1-BI, BcMV3-BI, BcMV1-BI, BcMV9-BI, BcMV4-BI, BcPV3-BI, BcHV1-BI, BcULV4, BcOLV16-BI, BcOLV7-BI, BcMV2-BI, BcULV1-BI, BcOLV18, BcMV11,  BcHV1SLR-BI |
| IBc-200 | 15 | BcULV3, BcMV6-BI, BcRV1-BI, BcMV3-BI, BcOLV15-BI, BcMV9-BI, BcMV4-BI, BcHV1-BI, BcULV4, BcOLV16-BI, BcMV2-BI, BcOLV1-BI, BcOLV18, BcMV11, BcHV1SLR-BI,  BcOLV2-BI |
| IBc-194 | 15 | BcULV3, BcFV5-BI, BcRV1-BI, BcMV3-BI, BcMV9-BI, BcMV4-BI, BcHV1-BI, BcULV4, BcALV1-BI, BcOLV16-BI, BcFV9, BcMV2-BI, BcULV1-BI, BcOLV18, BcMV11,  BcHV1SLR-BI |
| IBc-166 | 15 | BcULV3, BcMV6-BI, BcRV1-BI, BcMV3-BI, BcMV1-BI, BcMV9-BI, BcMV4-BI, BcHV1-BI, BcULV4, BcALV1-BI, BcOLV16-BI, BcMV2-BI, BcOLV1-BI, BcOLV18, BcMV11,  BcHV1SLR-BI |
| IBc-150 | 15 | BcULV3, BcRV1-BI, BcMV3-BI, BcMV1-BI, BcMV9-BI, BcMV4-BI, BcPV3-BI, BcHV1-BI, BcULV4, BcOLV16-BI, BcOLV7-BI, BcMV2-BI, BcOLV1-BI, BcOLV18, BcMV11,  BcHV1SLR-BI |
| IBc-116 | 15 | BcFV5-BI, BcMV3-BI, BcMV1-BI, BcULV4, BcHV3-BI, BvF-BI, BcFV1-BI, BcOLV4-BI, BcVV3-BI, BcMV2-BI, BcULV1-BI,  BcOLV1-BI, BcHV4-BI, BcEV2-BI, BcMV11 |
| IBc-84 | 15 | BcULV3, BcMV3-BI, BcMV1-BI, BcMV9-BI, BcMV4-BI, BcNSRV7-BI, BcULV4, BcHV3-BI, BcOLV16-BI, BcMV2-BI,  BcULV1-BI, BcOLV1-BI, BcEV2-BI, BcOLV18, BcMV11 |
| IBc-48 | 15 | BcMV2-BI, BvF-BI, BcPV10, BcMV1-BI, BcMV4-BI, BcALV1-BI, BcPV7, BcHV1-BI, BcHV3-BI, BcMV11, BcMV12,  BcULV3, BcEV2-BI, BcOLV18, BcEV6 |
| Skr-6 | 14 | BcNSRV7-BI, BcHV3-BI, BcULV3, BcDMV1, BcMTV1-BI, BcOLV15-BI, BcMV9-BI, BcEV2-BI, BcMV1-BI, BcFV5-BI,  BcOLV2-BI, BcDFV4, BcHV5-BI, BcOLV18 |
| Ca-6 | 14 | BcPV8, BcHV1-BI, BcMV3-BI, BcULV4, BcBNV5-BI,  BcULV1-BI, BcMV9-BI, BcEV2-BI, BcMV11, BcOLV2-BI, BcVV3-BI, BcDFV4, BcMV6-BI, BcHV1SLR-BI, BcOLV10-BI |
| Ca-2 | 14 | BcFV9, BcPV8, BcNSRV7-BI, BcHV1-BI, BcMV3-BI, BcULV4,  BcHV3-BI, BcOLV15-BI, BcMV9-BI, BcMV1-BI, BcFV5-BI, |

|  |  | BcMV4-BI, BvF-BI, BcOLV18, BcHV1SLR-BI |
| --- | --- | --- |
| IBc-449 | 14 | BcULV3, BcFV5-BI, BcPV5, BcMV3-BI, BcMV1-BI, BcMV4-BI,  BcHV1-BI, BcNSRV7-BI, BcULV4, BcHV3-BI, BcOLV12-BI, BcOLV7-BI, BcPV4-BI, BcOLV18, BcHV1SLR-BI |
| IBc-392 | 14 | BcULV3, BcOLV11-BI, BcRV1-BI, BcMV1-BI, BcMV3-BI, BcHV1-BI, BcULV4, BcALV1-BI, BcOLV7-BI, BcMV2-BI,  BcOLV1-BI, BcEV2-BI, BcOLV18, BcMV11, BcHV1SLR-BI |
| IBc-378 | 14 | BcULV3, BcFV5-BI, BcMV1-BI, BcMV9-BI, BcPV3-BI, BcNSRV7-BI, BcULV4, BcHV3-BI, BcMCV5-BI, BcMV2-BI,  BcOLV1-BI, BcEV2-BI, BcOLV18, BcMV11 |
| IBc-349 | 14 | BcULV3, BcMV6-BI, BcMV3-BI, BcMV1-BI, BcMV9-BI, BcMV4-BI, BcPV3-BI, BcHV1-BI, BcNSRV7-BI, BcULV4,  BcDMV1, BcOLV7-BI, BcMV2-BI, BcMV11 |
| IBc-347 | 14 | BcULV3, BcFV5-BI, BcMV6-BI, BcEV5, BcMV3-BI, BcMV1-BI,  BcMV9-BI, BcMV4-BI, BcHV1-BI, BcHV3-BI, BcDMV1, BcFV9, BcMV2-BI, BcMV11, BcHV1SLR-BI |
| IBc-325 | 14 | BcULV3, BcMV3-BI, BcMV1-BI, BcMV9-BI, BcPV3-BI,  BcULV4, BcHV3-BI, BvF-BI, BcOLV7-BI, BcVV3-BI, BcMV2-BI, BcOLV1-BI, BcEV2-BI, BcOLV18 |
| IBc-322 | 14 | BcMV6-BI, BcMV3-BI, BcMV1-BI, BcMV9-BI, BcMV4-BI, BcMV12, BcPV3-BI, BcHV1-BI, BcULV4, BcDMV1, BcFV9,  BcVV3-BI, BcOLV18, BcMV11, BcHV1SLR-BI |
| IBc-319 | 14 | BcULV3, BcRV1-BI, BcMV9-BI, BcMV4-BI, BcHV1-BI, BcULV4, BcHV3-BI, BcOLV16-BI, BcVV3-BI, BcNSRV9-BI,  BcMV2-BI, BcULV1-BI, BcOLV18, BcMV11, BcHV1SLR-BI |
| IBc-305 | 14 | BcMV6-BI, BcRV1-BI, BcMV3-BI, BcMV1-BI, BcMV9-BI, BcMV4-BI, BcPV3-BI, BcHV1-BI, BcULV4, BcMCV3-BI,  BcMV2-BI, BcEV2-BI, BcOLV18, BcMV11, BcHV1SLR-BI |
| IBc-296 | 14 | BcULV3, BcPV5, BcMV3-BI, BcMV1-BI, BcMV9-BI, BcMV4-BI,  BcHV1-BI, BcULV4, BcMTV1-BI, BcOLV16-BI, BcMCV3-BI, BcMV2-BI, BcULV1-BI, BcOLV18, BcHV1SLR-BI |
| IBc-276 | 14 | BcULV3, BcMV3-BI, BcMV1-BI, BcMV9-BI, BcMV4-BI,  BcHV1-BI, BcULV4, BcOLV16-BI, BcMCV3-BI, BcMV2-BI, BcULV1-BI, BcEV2-BI, BcOLV18, BcMV11 |
| IBc-263 | 14 | BcULV3, BcMV1-BI, BcMV12, BcMV4-BI, BcOLV16-BI, BcVV3-BI, BcPV3-BI, BcBNV5-BI, BcMV2-BI, BcULV1-BI,  BcULV5, BcOLV1-BI, BcMV11, BcEV6 |
| IBc-218 | 14 | BcMV6-BI, BcRV1-BI, BcMV1-BI, BcMV9-BI, BcPV3-BI, BcHV1-BI, BcULV4, BcALV1-BI, BcOLV16-BI, BcOLV7-BI,  BcMV2-BI, BcOLV1-BI, BcOLV18, BcMV11, BcHV1SLR-BI |
| IBc-217 | 14 | BcULV3, BcMV6-BI, BcMV3-BI, BcMV1-BI, BcMV9-BI,  BcMV4-BI, BcPV3-BI, BcULV4, BvF-BI, BcVV3-BI, BcMV2-BI, BcVV4, BcHV4-BI, BcEV2-BI, BcHV1SLR-BI |

| IBc-195 | 14 | BcULV3, BcFV5-BI, BcMV6-BI, BcRV1-BI, BcMV9-BI,  BcMV4-BI, BcHV1-BI, BcULV4, BcALV1-BI, BcOLV16-BI, BcBNV5-BI, BcMV2-BI, BcOLV18, BcMV11, BcHV1SLR-BI |
| --- | --- | --- |
| IBc-156 | 14 | BcULV3, BcMV6-BI, BcRV1-BI, BcMV3-BI, BcMV1-BI, BcMV9-BI, BcMV4-BI, BcPV3-BI, BcHV1-BI, BcNSRV7-BI,  BcULV4, BcOLV16-BI, BcMV2-BI, BcMV11, BcHV1SLR-BI |
| IBc-137 | 14 | BcULV3, BcMV6-BI, BcRV1-BI, BcPV5, BcMV3-BI, BcMV1-BI, BcMV4-BI, BcULV4, BcOLV16-BI, BcMV2-BI, BcPV4-BI,  BcEV2-BI, BcOLV18, BcMV11 |
| IBc-87 | 14 | BcULV3, BcRV1-BI, BcMV1-BI, BcMV9-BI, BcMV4-BI,  BcHV1-BI, BcNSRV7-BI, BcULV4, BcHV3-BI, BcALV1-BI, BcMV2-BI, BcOLV1-BI, BcOLV18, BcMV11, BcHV1SLR-BI |
| IBc-83 | 14 | BcFV5-BI, BcRV1-BI, BcMV3-BI, BcMV9-BI, BcMV12, BcULV4,  BcALV1-BI, BcOLV16-BI, BcFV9, BcMV2-BI, BcOLV1-BI, BcOLV18, BcMV11, BcMV15 |
| Skr-5 | 13 | BcNSRV7-BI, BcHV1-BI, BcMV3-BI, BcULV4, BcULV3, BcOLV15-BI, BcMV9-BI, BcEV2-BI, BcMV11, BcFV5-BI,  BcDFV4, BcHV5-BI, BcOLV18, BcHV1SLR-BI |
| SKr-1 | 13 | BcFV9, BcHV1-BI, BcHV1SLR-BI, BcMV9-BI, BcULV1-BI, BcULV4, BcULV5, BvF-BI, BcFV5-BI, BcOLV18, BcPV8,  BcDFV4, BcMV15, BcULV3 |
| IBc-444 | 13 | BcULV3, BcFV5-BI, BcMV3-BI, BcMV1-BI, BcHV1-BI, BcULV4, BcALV1-BI, BcOLV16-BI, BcBNV5-BI, BcULV1-BI, BcOLV1-BI,  BcOLV18, BcMV11 |
| IBc-369 | 13 | BcMV6-BI, BcMV1-BI, BcMV9-BI, BcMV4-BI, BcULV4, BcHV3-BI, BcOLV12-BI, BcVV3-BI, BcMV2-BI, BcULV1-BI,  BcEV2-BI, BcMV11, BcFV4-BI |
| IBc-344 | 13 | BcFV5-BI, BcMV6-BI, BcEV5, BcMV3-BI, BcMV1-BI,  BcMV9-BI, BcMV4-BI, BcDMV1, BcOLV16-BI, BcMV2-BI, BcOLV1-BI, BcEV2-BI, BcMV11 |
| IBc-311 | 13 | BcULV3, BcMV3-BI, BcMV1-BI, BcMV9-BI, BcMV12, BcHV1-BI, BcULV4, BcOLV16-BI, BcOLV7-BI, BcMCV3-BI,  BcULV1-BI, BcOLV18, BcMV11, BcHV1SLR-BI |
| IBc-301 | 13 | BcRV1-BI, BcMV3-BI, BcMV1-BI, BcMV9-BI, BcMV4-BI,  BcPV3-BI, BcULV4, BcHV3-BI, BcOLV7-BI, BcVV3-BI, BcMV2-BI, BcOLV13-BI, BcOLV18 |
| IBc-279 | 13 | BcMV6-BI, BcRV1-BI, BcMV1-BI, BcMV9-BI, BcMV4-BI, BcHV1-BI, BcULV4, BcBNV4-BI, BcOLV4-BI, BcMV2-BI,  BcULV1-BI, BcOLV18, BcMV11, BcHV1SLR-BI |
| IBc-252 | 13 | BcULV3, BcMV6-BI, BcMV1-BI, BcMV12, BcMV3-BI, BcPV3-BI, BcULV4, BcOLV16-BI, BcMCV3-BI, BcMV2-BI,  BcULV5, BcOLV1-BI, BcMV11 |
| IBc-247 | 13 | BcULV3, BcMV6-BI, BcMV1-BI, BcMV12, BcMV3-BI, |

|  |  | BcHV1-BI, BcOLV16-BI, BcMCV3-BI, BcMV2-BI, BcULV1-BI,  BcULV5, BcOLV1-BI, BcMV11, BcHV1SLR-BI |
| --- | --- | --- |
| IBc-244 | 13 | BcRV1-BI, BcMV3-BI, BcMV1-BI, BcMV9-BI, BcPV3-BI,  BcHV1-BI, BcULV4, BvF-BI, BcMV2-BI, BcULV1-BI, BcOLV1-BI, BcOLV18, BcHV1SLR-BI, BcMV15 |
| IBc-222 | 13 | BcULV3, BcRV1-BI, BcMV1-BI, BcMV9-BI, BcMV4-BI,  BcPV3-BI, BcHV1-BI, BcNSRV7-BI, BcULV4, BcDMV1, BcMV2-BI, BcOLV18, BcMV11, BcHV1SLR-BI |
| IBc-198 | 13 | BcFV5-BI, BcMV3-BI, BcMV1-BI, BcMV9-BI, BcMV4-BI, BcHV1-BI, BcULV4, BcALV1-BI, BcOLV16-BI, BcOLV7-BI,  BcMV2-BI, BcOLV18, BcMV11, BcHV1SLR-BI |
| IBc-183 | 13 | BcULV3, BcMV6-BI, BcRV1-BI, BcMV3-BI, BcMV9-BI, BcMV4-BI, BcPV3-BI, BcHV1-BI, BcMV2-BI, BcOLV13-BI,  BcOLV18, BcMV11, BcHV1SLR-BI, BcOLV2-BI |
| IBc-178 | 13 | BcMV9-BI, BcMV4-BI, BcMV12, BcNSRV7-BI, BcULV4, BcHV3-BI, BcDMV1, BcOLV16-BI, BcFV9, BcMV2-BI,  BcULV1-BI, BcOLV1-BI, BcOLV18 |
| IBc-170 | 13 | BcRV1-BI, BcPV5, BcMV9-BI, BcHV1-BI, BcULV4, BcOLV17-BI, BcULV1-BI, BcOLV1-BI, BcOLV13-BI, BcOLV18,  BcHV1SLR-BI, BcEV6, BcOLV2-BI, BcMV15 |
| IBc-160 | 13 | BcULV3, BcMV6-BI, BcMV1-BI, BcMV9-BI, BcPV3-BI, BcNSRV7-BI, BcULV4, BcOLV17-BI, BcOLV16-BI, BcOLV7-BI,  BcVV3-BI, BcOLV18, BcMV11 |
| IBc-96 | 13 | BcULV3, BcFV5-BI, BcMV3-BI, BcMV1-BI, BcMV9-BI, BcMV4-BI, BcULV4, BcHV3-BI, BcOLV4-BI, BcMV2-BI,  BcEV2-BI, BcOLV18, BcMV11, |
| IBc-92 | 13 | BcULV3, BcFV5-BI, BcMV1-BI, BcHV1-BI, BcHV3-BI, BcOLV17-BI, BcFV1-BI, BcVV3-BI, BcPV3-BI, BcMV2-BI,  BcULV5, BcEV2-BI, BcMV11, BcHV1SLR-BI |
| IBc-78 | 13 | BcMV9-BI, BcMV4-BI, BcMV12, BcHV1-BI, BcNSRV7-BI, BcULV4, BcALV1-BI, BcOLV16-BI, BcOLV7-BI, BcFV9,  BcEV2-BI, BcMV11, BcEV6 |
| IBc-69 | 13 | BcULV3, BcFV5-BI, BcRV1-BI, BcMV1-BI, BcMV9-BI, BcMV4-BI, BcMV12, BcHV1-BI, BcNSRV7-BI, BcULV4,  BcALV1-BI, BcOLV18, BcMV11, BcHV1SLR-BI |
| IBc-68 | 13 | BcULV3, BcFV5-BI, BcMV1-BI, BcMV9-BI, BcMV4-BI,  BcHV1-BI, BcULV4, BcOLV16-BI, BcOLV7-BI, BcFV9, BcOLV1-BI, BcOLV18, BcMV11, BcHV1SLR-BI |
| IBc-27 | 13 | BcULV3, BcFV5-BI, BcMV6-BI, BcMV1-BI, BcMV9-BI,  BcHV1-BI, BcULV4, BcHV3-BI, BcMV2-BI, BcOLV1-BI, BcEV2-BI, BcOLV18, BcHV1SLR-BI, BcEV6 |
| Ska-1 | 12 | BcFV9, BcHV1-BI, BcULV4, BcULV3, BcMTV1-BI,  BcOLV15-BI, BcMV9-BI, BcEV2-BI, BcMV1-BI, BcFV5-BI, |

|  |  | BcOLV13-BI, BcOLV18, BcHV1SLR-BI |
| --- | --- | --- |
| Ca-10 | 12 | BcFV9, BcNSRV7-BI, BcHV1-BI, BcMV3-BI, BcBNV5-BI,  BcULV3, BcOLV15-BI, BcMV9-BI, BcMV1-BI, BcFV5-BI, BcDFV4, BcHV5-BI, BcHV1SLR-BI |
| IBc-445 | 12 | BcULV3, BcFV5-BI, BcMV1-BI, BcNSRV7-BI, BcULV4, BcDMV1, BcOLV12-BI, BcOLV16-BI, BcMV17, BcOLV1-BI,  BcHV4-BI, BcOLV18 |
| IBc-397 | 12 | BcMV1-BI, BcMV4-BI, BcMV3-BI, BcULV4, BcMNV1-BI, BcALV1-BI, BcVV3-BI, BcMV2-BI, BcOLV1-BI, BcEV2-BI,  BcOLV18, BcMV11, BcHV1SLR-BI |
| IBc-373 | 12 | BcULV3, BcFV5-BI, BcPV5, BcEV5, BcMV3-BI, BcMV1-BI, BcNSRV7-BI, BcHV3-BI, BcOLV7-BI, BcVV3-BI, BcMV2-BI,  BcOLV1-BI |
| IBc-352 | 12 | BcMV11, BcDMV1, BcEV2-BI, BcALV1-BI, BcVV5, BcMV4-BI,  BcBNV5-BI, BcMV1-BI, BcMCV5-BI, BcMV9-BI, BcULV1-BI, BcHV1-BI, BcHV1SLR-BI |
| IBc-334 | 12 | BcMV3-BI, BcMV1-BI, BcMV9-BI, BcPV3-BI, BcHV1-BI,  BcULV4, BcDMV1, BcULV1-BI, BcULV5, BcOLV1-BI, BcOLV18, BcEV6 |
| IBc-313 | 12 | BcULV3, BcMV3-BI, BcMV1-BI, BcMV9-BI, BcMV4-BI, BcMV12, BcPV3-BI, BcHV1-BI, BcULV4, BcOLV16-BI,  BcMCV3-BI, BcOLV18 |
| IBc-310 | 12 | BcULV3, BcMV3-BI, BcMV1-BI, BcMV9-BI, BcMV4-BI, BcHV1-BI, BcULV4, BcOLV16-BI, BcULV1-BI, BcPV4-BI,  BcOLV18, BcMV11 |
| IBc-307 | 12 | BcULV3, BcMV3-BI, BcMV1-BI, BcMV9-BI, BcMV12, BcHV1-BI, BcULV4, BcOLV16-BI, BcFV9, BcULV1-BI,  BcOLV1-BI, BcHV1SLR-BI, BcOLV2-BI |
| IBc-306 | 12 | BcMV6-BI, BcMV9-BI, BcMV4-BI, BcHV1-BI, BcDFV4,  BcULV4, BcALV1-BI, BcOLV12-BI, BvF-BI, BcVV3-BI, BcMV2-BI, BcEV2-BI, BcHV1SLR-BI |
| IBc-300 | 12 | BcMV6-BI, BcMV3-BI, BcMV1-BI, BcMV9-BI, BcMV4-BI,  BcMV12, BcHV1-BI, BcULV4, BcOLV16-BI, BcMV2-BI, BcOLV18, BcMV15 |
| IBc-273 | 12 | BcMV1-BI, BcMV9-BI, BcMV4-BI, BcMV12, BcULV4, BcMTV1-BI, BcOLV16-BI, BcMV2-BI, BcULV1-BI, BcPV4-BI,  BcOLV18, BcMV11 |
| IBc-271 | 12 | BcMV3-BI, BcMV1-BI, BcMV9-BI, BcMV12, BcHV1-BI, BcOLV16-BI, BcOLV7-BI, BcMCV3-BI, BcMV13, BcMV2-BI,  BcOLV1-BI, BcEV2-BI, BcHV1SLR-BI |
| IBc-265 | 12 | BcMV6-BI, BcPV5, BcMV1-BI, BcMV4-BI, BcMV3-BI,  BcPV3-BI, BcOLV16-BI, BcVV3-BI, BcULV5, BcOLV1-BI, BcPV4-BI, BcMV11 |

| IBc-262 | 12 | BcMV12, BcMV3-BI, BcPV3-BI, BcHV1-BI, BcULV4,  BcALV1-BI, BcOLV16-BI, BcMV2-BI, BcULV5, BcOLV1-BI, BcOLV13-BI, BcMV11, BcHV1SLR-BI |
| --- | --- | --- |
| IBc-258 | 12 | BcULV3, BcMV1-BI, BcMV4-BI, BcMV3-BI, BcHV1-BI, BcHV3-BI, BcOLV16-BI, BcVV3-BI, BcULV5, BcOLV1-BI,  BcOLV13-BI, BcMV11 |
| IBc-256 | 12 | BcULV3, BcMV6-BI, BcMV12, BcMV4-BI, BcMV3-BI, BcPV3-BI, BcOLV16-BI, BcOLV4-BI, BcMCV3-BI, BcMV2-BI,  BcOLV1-BI, BcOLV2-BI |
| IBc-221 | 12 | BcULV3, BcMV6-BI, BcRV1-BI, BcMV9-BI, BcMV4-BI,  BcPV3-BI, BcHV1-BI, BcULV4, BcALV1-BI, BcOLV7-BI, BcOLV1-BI, BcOLV18, BcHV1SLR-BI |
| IBc-202 | 12 | BcULV3, BcMV3-BI, BcMV1-BI, BcMV9-BI, BcULV4,  BcOLV16-BI, BcOLV7-BI, BcOLV4-BI, BcMV2-BI, BcULV1-BI, BcOLV18, BcMV11 |
| IBc-192 | 12 | BcRV1-BI, BcMV1-BI, BcMV9-BI, BcMV4-BI, BcULV4, BcALV1-BI, BcOLV16-BI, BcOLV7-BI, BcFV9, BcMV2-BI,  BcOLV1-BI, BcOLV18 |
| IBc-189 | 12 | BcULV3, BcRV1-BI, BcMV3-BI, BcMV9-BI, BcMV4-BI, BcMV12, BcHV1-BI, BcNSRV7-BI, BcULV4, BcOLV7-BI,  BcMV2-BI, BcOLV18, BcHV1SLR-BI |
| IBc-135 | 12 | BcRV1-BI, BcMV3-BI, BcMV1-BI, BcFV9, BcOLV16-BI, BcMV2-BI, BcULV5, BcOLV1-BI, BcOLV13-BI, BcEV2-BI,  BcMV11, BcOLV2-BI |
| IBc-79 | 12 | BcULV3, BcFV5-BI, BcRV1-BI, BcMV1-BI, BcMV9-BI, BcMV12, BcNSRV7-BI, BcULV4, BcALV1-BI, BcOLV16-BI, BcMV2-BI,  BcMV11, BcHV1SLR-BI |
| IBc-76 | 12 | BcULV3, BcRV1-BI, BcMV1-BI, BcMV9-BI, BcMV4-BI,  BcHV1-BI, BcNSRV7-BI, BcULV4, BcHV3-BI, BcFV9, BcOLV18, BcMV11, BcHV1SLR-BI |
| IBc-70 | 12 | BcULV3, BcRV1-BI, BcMV1-BI, BcMV9-BI, BcMV4-BI, BcHV1-BI, BcULV4, BcHV3-BI, BcOLV16-BI, BcULV1-BI,  BcOLV18, BcMV11, BcHV1SLR-BI |
| IBc-44 | 12 | BcPV4-BI, BcOLV16-BI, BcMV1-BI, BcMV3-BI, BcMV4-BI,  BcMV9-BI, BcHV1-BI, BcULV3, BcMV3-BI, BcFV5-BI, BcOLV18, BcMCV4-BI, BcHV1SLR-BI |
| IBc-42 | 12 | BcMV1-BI, BcMV9-BI, BcMV4-BI, BcPV3-BI, BcHV1-BI, BcNSRV7-BI, BcULV4, BcOLV12-BI, BcMV2-BI, BcULV1-BI,  BcOLV1-BI, BcOLV18 |
| SKr-2 | 11 | BcFV9, BcNSRV7-BI, BcHV1-BI, BcULV4, BcULV3, BcOLV15-BI, BcMV9-BI, BcEV2-BI, BcMV1-BI, BcMV4-BI,  BcHV5-BI, BcHV1SLR-BI |
| IBc-453 | 11 | BcULV3, BcFV5-BI, BcMV3-BI, BcHV1-BI, BcNSRV7-BI, |

|  |  | BcULV4, BcHV3-BI, BcDMV1, BcOLV12-BI, BcOLV1-BI,  BcOLV18, BcHV1SLR-BI |
| --- | --- | --- |
| IBc-420 | 11 | BcULV3, BcMV6-BI, BcPV7, BcMV3-BI, BcMV1-BI, BcPV10,  BcULV5, BcMV11, BcALV3, BcBNV6, BcEV6 |
| IBc-404 | 11 | BcULV3, BcMV6-BI, BcMV3-BI, BcMV1-BI, BcMV9-BI, BcMV4-BI, BcHV1-BI, BcULV4, BcULV1-BI, BcPV4-BI,  BcMV11, BcHV1SLR-BI |
| IBc-398 | 11 | BcRV1-BI, BcMV1-BI, BcHV1-BI, BcULV4, BcALV1-BI, BcPV3-BI, BcMV2-BI, BcOLV1-BI, BcEV2-BI, BcOLV18,  BcMV11, BcHV1SLR-BI |
| IBc-381 | 11 | BcMV6-BI, BcRV1-BI, BcMV1-BI, BcMV9-BI, BcPV3-BI, BcNSRV7-BI, BcULV4, BcOLV16-BI, BcOLV7-BI, BcBNV5-BI,  BcMV2-BI |
| IBc-380 | 11 | BcULV3, BcMV6-BI, BcMV1-BI, BcMV3-BI, BcHV1-BI,  BcPV10, BcMV2-BI, BcULV5, BcOLV18, BcMV11, BcHV1SLR-BI, BcEV6 |
| IBc-291 | 11 | BcULV3, BcMV6-BI, BcMV3-BI, BcMV1-BI, BcMV9-BI,  BcMV4-BI, BcHV1-BI, BcULV4, BcOLV16-BI, BcMV2-BI, BcMV11 |
| IBc-286 | 11 | BcMV3-BI, BcMV1-BI, BcMV9-BI, BcMV4-BI, BcMV12, BcPV3-BI, BcOLV16-BI, BcOLV4-BI, BcMV17, BcOLV18,  BcMV11 |
| IBc-285 | 11 | BcULV3, BcMV3-BI, BcMV1-BI, BcMV9-BI, BcMV4-BI, BcHV1-BI, BcULV4, BcOLV16-BI, BcMV2-BI, BcULV1-BI,  BcOLV18, BcHV1SLR-BI |
| IBc-282 | 11 | BcPV5, BcMV1-BI, BcMV9-BI, BcMV4-BI, BcPV3-BI, BcULV4,  BcVV3-BI, BcMV13, BcULV1-BI, BcOLV18, BcMV11 |
| IBc-278 | 11 | BcMV3-BI, BcMV1-BI, BcMV9-BI, BcMV12, BcOLV16-BI, BcOLV7-BI, BcVV3-BI, BcMV13, BcMV2-BI, BcOLV1-BI,  BcOLV18, BcHV1SLR-BI |
| IBc-272 | 11 | BcULV3, BcMV3-BI, BcMV1-BI, BcMV9-BI, BcMV12, BcHV1-BI, BcULV4, BcOLV16-BI, BcULV1-BI, BcEV2-BI,  BcOLV18, BcHV1SLR-BI |
| IBc-266 | 11 | BcULV3, BcMV3-BI, BcMV1-BI, BcMV9-BI, BcMV4-BI, BcULV4, BcOLV16-BI, BcOLV7-BI, BcOLV18, BcMV11,  BcHV1SLR-BI, BcOLV2-BI |
| IBc-238 | 11 | BcULV3, BcMV6-BI, BcRV1-BI, BcMV9-BI, BcULV4, BcALV1-BI, BcOLV16-BI, BcOLV7-BI, BcULV1-BI, BcPV4-BI,  BcMV11 |
| IBc-191 | 11 | BcMV3-BI, BcMV1-BI, BcMV9-BI, BcMV4-BI, BcPV3-BI, BcHV1-BI, BcULV4, BcALV1-BI, BcFV9, BcULV1-BI,  BcOLV18, BcHV1SLR-BI |
| IBc-190 | 11 | BcULV3, BcFV5-BI, BcMV6-BI, BcMV1-BI, BcMV9-BI, |

|  |  | BcHV1-BI, BcULV4, BcOLV16-BI, BcBNV5-BI, BcMV2-BI,  BcMV11, BcHV1SLR-BI |
| --- | --- | --- |
| IBc-149 | 11 | BcULV3, BcRV1-BI, BcMV1-BI, BcMV9-BI, BcMV4-BI,  BcPV3-BI, BcHV1-BI, BcULV4, BcOLV17-BI, BcMV2-BI, BcMV11 |
| IBc-140 | 11 | BcULV3, BcMV1-BI, BcHV1-BI, BcMCV5-BI, BcVV3-BI,  BcMV2-BI, BcULV1-BI, BcULV5, BcEV2-BI, BcOLV18, BcMV11, BcHV1SLR-BI |
| IBc-122 | 11 | BcULV3, BcMV1-BI, BcHV1-BI, BcFV9, BcULV4, BcHV3-BI, BcOLV4-BI, BcULV5, BcPV4-BI, BcOLV18, BcMV11,  BcHV1SLR-BI |
| IBc-119 | 11 | BcFV5-BI, BcRV1-BI, BcPV5, BcMV3-BI, BcMV1-BI, BcMV12, BcULV4, BcMV2-BI, BcULV5, BcPV4-BI, BcMV11,  BcHV1SLR-BI |
| IBc-117 | 11 | BcMV1-BI, BcHV1-BI, BcHV3-BI, BcOLV16-BI, BcVV3-BI, BcPV3-BI, BcULV5, BcOLV1-BI, BcHV4-BI, BcOLV18,  BcMV11, BcHV1SLR-BI |
| IBc-38 | 11 | BcULV3, BcFV5-BI, BcMV3-BI, BcMV1-BI, BcMV9-BI,  BcULV4, BcDMV1, BcOLV16-BI, BcVV3-BI, BcOLV18, BcMV15 |
| IBc-32 | 11 | BcMV1-BI, BcHV1-BI, BcNSRV7-BI, BcMV2-BI, BcMV11, BcHV1SLR-BI, BcULV3, BcMV1-BI, BcHV1-BI, BcNSRV7-BI,  BcMV2-BI, BcMV11, BcHV1SLR-BI |
| IBc-28 | 11 | BcULV3, BcMV6-BI, BcMV1-BI, BcMV9-BI, BcMV4-BI, BcHV1-BI, BcULV4, BcVV3-BI, BcMV13, BcEV2-BI,  BcHV1SLR-BI, BcEV6 |
| Ska-4 | 10 | BcEV6, BcHV1-BI, BcHV1SLR-BI, BcMV1-BI, BcMV9-BI, BcULV1-BI, BcULV4, BcOLV18, BcMTV1-BI, BcOLV16-BI,  BcULV3 |
| Ska-3 | 10 | BcMV1-BI, BcMV9-BI, BcULV1-BI, BcULV4, BcOLV19, BcOLV18, BcDFV4, BcHV1-BI, BcHV1SLR-BI, BcOLV16-BI,  BcULV3 |
| Ca-1 | 10 | BcEV2, BcHV1, BcHV1SLR, BcHV5, BcMV1, BcMV9, BcULV1,  BcULV4, BvF, BcOLV18, BcDFV4 |
| IBc-450 | 10 | BcFV5-BI, BcMV3-BI, BcMV1-BI, BcNSRV7-BI, BcULV4, BcHV3-BI, BcDMV1, BcOLV1-BI, BcHV4-BI, BcOLV18,  BcHV1SLR-BI |
| IBc-447 | 10 | BcMV6-BI, BcMV1-BI, BcMV9-BI, BcPV3-BI, BcHV1-BI,  BcULV4, BcHV3-BI, BcOLV7-BI, BcOLV18, BcMV11 |
| IBc-436 | 10 | BcMV1-BI, BcMV4-BI, BcPV3-BI, BcULV4, BcHV3-BI,  BcDMV1, BcFV9, BcMV17, BcOLV18, BcMV11 |
| IBc-424 | 10 | BcULV3, BcMV6-BI, BcPV7, BcMV3-BI, BcMV1-BI, BcMV4-BI,  BcMV2-BI, BcULV5, BcOLV1-BI, BcMV11 |

| IBc-422 | 10 | BcMV1-BI, BcMV9-BI, BcPV3-BI, BcNSRV7-BI, BcULV4,  BcHV3-BI, BcVV3-BI, BcMV2-BI, BcEV2-BI, BcOLV18 |
| --- | --- | --- |
| IBc-419 | 10 | BcULV3, BcFV5-BI, BcMV3-BI, BcMV1-BI, BcMV4-BI,  BcMCV4-BI, BcMV2-BI, BcULV5, BcOLV1-BI, BcMV11 |
| IBc-399 | 10 | BcULV3, BcMV6-BI, BcMV1-BI, BcHV1-BI, BcULV4, BcALV1-BI, BcMV2-BI, BcOLV1-BI, BcOLV18, BcMV11,  BcHV1SLR-BI |
| IBc-383 | 10 | BcULV3, BcRV1-BI, BcMV1-BI, BcMV12, BcMV4-BI,  BcALV1-BI, BcVV3-BI, BcMV2-BI, BcEV2-BI, BcMV11 |
| IBc-371 | 10 | BcMV1-BI, BcMV9-BI, BcNSRV7-BI, BcULV4, BcOLV16-BI,  BcMCV5-BI, BcVV3-BI, BcMV2-BI, BcOLV18, BcMV11 |
| IBc-360 | 10 | BcULV3, BcMV6-BI, BcMV1-BI, BcMV9-BI, BcMV4-BI,  BcULV4, BcHV3-BI, BcVV3-BI, BcMV2-BI, BcMV11 |
| IBc-338 | 10 | BcULV3, BcMV1-BI, BcMV9-BI, BcFV9, BcNSRV7-BI, BcULV4,  BcOLV16-BI, BcEV2-BI, BcOLV18, BcMV11 |
| IBc-308 | 10 | BcRV1-BI, BcMV3-BI, BcMV9-BI, BcMV4-BI, BcPV3-BI,  BcHV1-BI, BcULV4, BcNSRV9-BI, BcOLV1-BI, BcOLV18 |
| IBc-281 | 10 | BcULV3, BcMV1-BI, BcMV9-BI, BcULV4, BcOLV16-BI,  BcMV2-BI, BcULV1-BI, BcEV2-BI, BcOLV18, BcMV11 |
| IBc-234 | 10 | BcRV1-BI, BcMV3-BI, BcMV1-BI, BcMV9-BI, BcMV12, BcPV3-BI, BcHV1-BI, BcULV4, BcOLV1-BI, BcOLV18,  BcHV1SLR-BI |
| IBc-232 | 10 | BcRV1-BI, BcMV1-BI, BcMV9-BI, BcMV4-BI, BcHV1-BI, BcULV4, BcOLV16-BI, BcOLV7-BI, BcMV2-BI, BcOLV18,  BcHV1SLR-BI |
| IBc-230 | 10 | BcULV1-BI, BcULV4, BcMV1-BI, BcMV3-BI, BcMV12,  BcHV1-BI, BcHV1SLR-BI, BcMV9-BI, BcOLV18, BcDFV4, BcMCV3-BI |
| IBc-210 | 10 | BcMV3-BI, BcMV1-BI, BcMV9-BI, BcMV12, BcHV1-BI, BcULV4, BcALV1-BI, BcOLV7-BI, BcOLV18, BcMV11,  BcHV1SLR-BI |
| IBc-162 | 10 | BcULV3, BcRV1-BI, BcMV1-BI, BcMV9-BI, BcMV12,  BcHV1-BI, BcULV4, BcOLV7-BI, BcOLV1-BI, BcOLV18 |
| IBc-161 | 10 | BcULV3, BcMV3-BI, BcMV9-BI, BcMV12, BcHV1-BI,  BcNSRV7-BI, BcULV4, BcOLV1-BI, BcOLV18, BcMV11, BcHV1SLR-BI |
| IBc-103 | 10 | BcMV3-BI, BcMV1-BI, BvF-BI, BcOLV4-BI, BcVV3-BI,  BcMV2-BI, BcULV1-BI, BcOLV1-BI, BcOLV13-BI, BcMV11 |
| IBc-101 | 10 | BcULV3, BcMV6-BI, BcMV1-BI, BcHV1-BI, BcNSRV7-BI,  BcHV3-BI, BcVV3-BI, BcULV5, BcEV2-BI, BcMV11, BcHV1SLR-BI |
| IBc-97 | 10 | BcULV3, BcMV1-BI, BcMV4-BI, BcFV9, BcVV3-BI, BcPV3-BI,  BcMV2-BI, BcEV2-BI, BcOLV18, BcMV11 |

| IBc-90 | 10 | BcULV3, BcFV5-BI, BcMV9-BI, BcMV4-BI, BcHV1-BI,  BcNSRV7-BI, BcULV4, BcOLV16-BI, BcMV2-BI, BcMV11, BcHV1SLR-BI |
| --- | --- | --- |
| IBc-80 | 10 | BcULV3, BcMV3-BI, BcMV1-BI, BcMV9-BI, BcHV1-BI, BcULV4, BcOLV4-BI, BcEV2-BI, BcOLV18, BcMV11,  BcHV1SLR-BI |
| IBc-66 | 10 | BcOLV16-BI, BcPV3-BI, BcMV1-BI, BcMV4-BI, BcMV9-BI, BcHV1-BI, BcNSRV7-BI, BcULV3, BcEV2-BI, BcBNV4-BI,  BcHV1SLR-BI |
| IBc-64 | 10 | BvF-BI, BcMV3-BI, BcOLV4-BI, BcHV1-BI, BcHV3-BI,  BcMV11, BcULV3, BcULV4, BcMV3-BI, BcOLV18, BcHV1SLR-BI |
| IBc-51 | 10 | BvF-BI, BcOLV16-BI, BcMV4-BI, BcOLV4-BI, BcALV1-BI,  BcHV1-BI, BcHV3-BI, BcULV3, BcPV3-BI, BcBNV4-BI, BcHV1SLR-BI |
| IBc-45 | 10 | BcMV1-BI, BcMV3-BI, BcOLV4-BI, BcMV9-BI, BcHV1-BI, BcMV11, BcULV3, BcMV3-BI, BcEV2-BI, BcHV1SLR-BI,  BcMV6-BI |
| IBc-37 | 10 | BcULV3, BcMV3-BI, BcMV1-BI, BcMV9-BI, BcMV4-BI,  BcPV3-BI, BcULV4, BcDMV1, BcOLV16-BI, BcMV11 |
| IBc-33 | 10 | BcFV5-BI, BcMV1-BI, BcMV9-BI, BcPV3-BI, BcULV4,  BcDMV1, BcOLV16-BI, BcMV2-BI, BcOLV1-BI, BcOLV18 |
| Skr-3 | 9 | BcHV1-BI, BcULV3, BcOLV15-BI, BcMV9-BI, BcEV2-BI,  BcMV1-BI, BcFV5-BI, BcMV12, BcOLV2-BI, BcHV1SLR-BI |
| Ska-5 | 9 | BcPV8, BcHV1-BI, BcHV3-BI, BcULV3, BcDMV1, BcMTV1-BI,  BcFV5-BI, BcMV12, BcOLV18, BcHV1SLR-BI |
| IBc-452 | 9 | BcMV9-BI, BcHV1-BI, BcULV4, BcDMV1, BvF-BI,  BcOLV16-BI, BcVV3-BI, BcHV1SLR-BI, BcBNV6, BcMV15 |
| IBc-448 | 9 | BcFV5-BI, BcMV3-BI, BcMV1-BI, BcHV1-BI, BcNSRV7-BI,  BcULV4, BcHV3-BI, BcDMV1, BcALV1-BI |
| IBc-439 | 9 | BcMV1-BI, BcMV12, BcPV9, BcULV4, BcHV3-BI, BcDMV1,  BcALV1-BI, BcOLV7-BI, BcBNV5-BI |
| IBc-435 | 9 | BcMV6-BI, BcPV7, BcMV1-BI, BcMV9-BI, BcULV4, BcDMV1,  BcOLV7-BI, BcMV11, BcEV6 |
| IBc-391 | 9 | BcULV3, BcFV5-BI, BcMV1-BI, BcMV9-BI, BcULV4, BcHV3-BI,  BcEV2-BI, BcOLV18, BcMV11 |
| IBc-382 | 9 | BcMV6-BI, BcMV1-BI, BcMV4-BI, BcULV4, BcALV1-BI,  BcMV2-BI, BcOLV1-BI, BcOLV18, BcMV11 |
| IBc-363 | 9 | BcULV3, BcMV6-BI, BcMV3-BI, BcMV9-BI, BcMV4-BI,  BcULV4, BcDMV1, BcOLV16-BI, BcMV11 |
| IBc-303 | 9 | BcMV6-BI, BcMV1-BI, BcMV9-BI, BcPV3-BI, BcHV1-BI,  BcULV4, BcOLV16-BI, BcULV1-BI, BcOLV18 |
| IBc-298 | 9 | BcMV3-BI, BcMV1-BI, BcMV9-BI, BcHV1-BI, BcULV4, |

|  |  | BcOLV16-BI, BcVV3-BI, BcMV11, BcHV1SLR-BI, BcEV6 |
| --- | --- | --- |
| IBc-264 | 9 | BcMV3-BI, BcMV1-BI, BcMV9-BI, BcMV12, BcULV4, BcDMV1,  BcOLV16-BI, BcOLV18, BcMV11 |
| IBc-249 | 9 | BcMV3-BI, BcMV1-BI, BcMV9-BI, BcMV4-BI, BcMV12,  BcULV4, BvF-BI, BcOLV16-BI, BcOLV18 |
| IBc-240 | 9 | BcMV6-BI, BcMV3-BI, BcMV1-BI, BcMV9-BI, BcMV12,  BcNSRV7-BI, BcULV4, BcOLV16-BI, BcOLV18 |
| IBc-229 | 9 | BcULV3, BcMV3-BI, BcMV1-BI, BcMV9-BI, BcHV1-BI,  BcULV4, BcULV1-BI, BcOLV18, BcHV1SLR-BI, BcBNV6 |
| IBc-176 | 9 | BcULV3, BcRV1-BI, BcMV1-BI, BcMV9-BI, BcULV4,  BcHV3-BI, BcOLV16-BI, BcOLV7-BI, BcOLV18 |
| IBc-157 | 9 | BcULV3, BcRV1-BI, BcMV1-BI, BcMV9-BI, BcPV3-BI,  BcHV1-BI, BcULV4, BcOLV16-BI, BcMV11, BcHV1SLR-BI |
| IBc-146 | 9 | BcULV3, BcMV6-BI, BcRV1-BI, BcMV9-BI, BcHV1-BI,  BcULV4, BcBNV5-BI, BcOLV18, BcMV11 |
| IBc-129 | 9 | BcULV3, BcMV6-BI, BcMV1-BI, BcOLV4-BI, BcMV2-BI,  BcULV5, BcOLV1-BI, BcOLV18, BcMV11, BcHV1SLR-BI |
| IBc-120 | 9 | BcULV3, BcMV3-BI, BcMV9-BI, BcHV1-BI, BcULV4, BvF-BI,  BcOLV18, BcMV11, BcHV1SLR-BI, BcFV4-BI |
| IBc-113 | 9 | BcRV1-BI, BcMV1-BI, BcMTV1-BI, BcFV1-BI, BcVV3-BI,  BcULV5, BcOLV1-BI, BcEV2-BI, BcMV11 |
| IBc-73 | 9 | BcULV3, BcMV1-BI, BcMV9-BI, BcMV4-BI, BcHV1-BI,  BcULV4, BcOLV17-BI, BcOLV18, BcMV11, BcHV1SLR-BI |
| IBc-62 | 9 | BcOLV7-BI, BcBNV1-BI, BcMV1-BI, BcMV4-BI, BcMV9-BI,  BcHV3-BI, BcMV11, BcPV3-BI, BcBNV4-BI |
| IBc-54 | 9 | BcMV1-BI, BcMV4-BI, BcMV9-BI, BcHV3-BI, BcMV11,  BcULV3, BcPV3-BI, BcEV2-BI, BcBNV4-BI, BcHV1SLR-BI |
| IBc-47 | 9 | BvF-BI, BcBNV1-BI, BcMV9-BI, BcHV1-BI, BcHV3-BI,  BcMV11, BcEV2-BI, BcBNV4-BI, BcHV1SLR-BI, BcEV6 |
| IBc-41 | 9 | BcULV3, BcMV1-BI, BcMV9-BI, BcMV12, BcHV1-BI, BcULV4,  BcVV3-BI, BcMV13, BcVV4, BcHV1SLR-BI |
| IBc-451 | 8 | BcULV3, BcRV1-BI, BcMV9-BI, BcHV1-BI, BcULV4,  BcOLV16-BI, BcOLV7-BI, BcMV11 |
| IBc-446 | 8 | BcMV6-BI, BcMV3-BI, BcMV1-BI, BcMV9-BI, BcHV1-BI,  BcULV4, BcMV2-BI, BcHV1SLR-BI, BcMV15 |
| IBc-443 | 8 | BcMV1-BI, BcMV9-BI, BcNSRV7-BI, BcULV4, BcDMV1,  BvF-BI, BcOLV16-BI, BcMV11 |
| IBc-434 | 8 | BcULV3, BcRV1-BI, BcMV3-BI, BcMV9-BI, BcULV4, BcDMV1,  BcOLV16-BI, BcVV3-BI |
| IBc-427 | 8 | BcMV3-BI, BcMV1-BI, BcMV4-BI, BcPV10, BcMV2-BI,  BcULV5, BcOLV1-BI, BcMV11 |
| IBc-413 | 8 | BcMV3-BI, BcMV1-BI, BcMV4-BI, BcHV1-BI, BcMV2-BI,  BcOLV1-BI, BcEV2-BI, BcMV11 |

| IBc-409 | 8 | BcMV1-BI, BcMV4-BI, BcULV4, BcMV2-BI, BcULV5,  BcOLV1-BI, BcVV4, BcMV11 |
| --- | --- | --- |
| IBc-385 | 8 | BcMV1-BI, BcMV12, BcMV3-BI, BcALV1-BI, BcMV2-BI,  BcOLV1-BI, BcOLV18, BcMV11 |
| IBc-379 | 8 | BcULV3, BcFV5-BI, BcMV1-BI, BcMV9-BI, BcULV4, BcVV3-BI,  BcOLV1-BI, BcMV11 |
| IBc-351 | 8 | BcFV5-BI, BcEV5, BcMV3-BI, BcMV4-BI, BcNSRV7-BI,  BcDMV1, BcVV3-BI, BcHV4-BI |
| IBc-324 | 8 | BcMV1-BI, BcMV9-BI, BcHV1-BI, BcULV4, BcALV1-BI,  BcOLV16-BI, BcOLV1-BI, BcOLV18, BcHV1SLR-BI |
| IBc-321 | 8 | BcRV1-BI, BcMV9-BI, BcMV4-BI, BcPV3-BI, BcHV1-BI,  BcULV4, BcOLV7-BI, BcVV3-BI |
| IBc-292 | 8 | BcULV3, BcMV1-BI, BcMV9-BI, BcHV1-BI, BcULV4,  BcULV1-BI, BcOLV18, BcHV1SLR-BI, BcEV6 |
| IBc-288 | 8 | BcULV3, BcMV6-BI, BcMV3-BI, BcMV1-BI, BcMV9-BI,  BcMV12, BcHV1-BI, BcMCV3-BI, BcHV1SLR-BI |
| IBc-255 | 8 | BcMV3-BI, BcMV9-BI, BcMV4-BI, BcDMV1, BvF-BI,  BcOLV16-BI, BcOLV18, BcMV11 |
| IBc-253 | 8 | BcULV3, BcMV1-BI, BcMV4-BI, BcPV3-BI, BcOLV16-BI,  BcOLV4-BI, BcMV2-BI, BcMV11 |
| IBc-235 | 8 | BcULV3, BcMV1-BI, BcMV9-BI, BcHV1-BI, BcNSRV7-BI,  BcULV4, BcMV2-BI, BcOLV18, BcHV1SLR-BI |
| IBc-224 | 8 | BcULV3, BcRV1-BI, BcMV3-BI, BcMV9-BI, BcMV4-BI,  BcPV3-BI, BcOLV18, BcMV11 |
| IBc-216 | 8 | BcMV3-BI, BcMV1-BI, BcMV9-BI, BcMV12, BcHV3-BI,  BcOLV4-BI, BcMV2-BI, BcOLV1-BI |
| IBc-196 | 8 | BcFV5-BI, BcMV6-BI, BcMV3-BI, BcMV1-BI, BcOLV15-BI,  BcULV4, BcOLV1-BI, BcOLV18, BcHV1SLR-BI |
| IBc-181 | 8 | BcRV1-BI, BcMV3-BI, BcMV9-BI, BcMV12, BcPV3-BI,  BcHV1-BI, BcULV4, BcMV11 |
| IBc-165 | 8 | BcULV3, BcMV6-BI, BcMV1-BI, BcMV9-BI, BcHV1-BI,  BcULV4, BcOLV16-BI, BcVV4 |
| IBc-144 | 8 | BcRV1-BI, BcMV1-BI, BcMV9-BI, BcMV4-BI, BcULV4,  BcOLV7-BI, BcOLV1-BI, BcMV11 |
| IBc-139 | 8 | BcULV3, BcMV6-BI, BcMV9-BI, BcHV1-BI, BcULV4,  BcOLV16-BI, BcOLV1-BI, BcHV1SLR-BI, BcOLV2-BI |
| IBc-123 | 8 | BcULV3, BcMV3-BI, BcMV1-BI, BcMV12, BcPV3-BI,  BcVV3-BI, BcOLV7-BI, BcFV9 |
| IBc-100 | 8 | BcOLV9-BI, BcULV3, BcMV3-BI, BcMV9-BI, BcHV1-BI,  BcNSRV7-BI, BcULV4, BcDMV1, BcHV1SLR-BI |
| IBc-98 | 8 | BcMV3-BI, BcHV1-BI, BcULV4, BcOLV16-BI, BcULV1-BI,  BcULV5, BcEV2-BI, BcMV11 |
| IBc-72 | 8 | BcULV3, BcRV1-BI, BcMV9-BI, BcHV1-BI, BcULV4, BcHV3-BI, |

|  |  | BcVV3-BI, BcEV2-BI, BcHV1SLR-BI |
| --- | --- | --- |
| IBc-71 | 8 | BcULV3, BcMV3-BI, BcMV1-BI, BcMV9-BI, BcMV4-BI,  BcHV1-BI, BcULV4, BcMV11 |
| IBc-65 | 8 | BcPV4-BI, BvF-BI, BcMV1-BI, BcMV9-BI, BcHV1-BI, BcULV3,  BcEV2-BI, BcHV1SLR-BI, BcMV6-BI |
| IBc-53 | 8 | BvF-BI, BcMV1-BI, BcMV4-BI, BcMV9-BI, BcHV1-BI,  BcEV2-BI, BcFV5-BI, BcBNV4-BI, BcHV1SLR-BI |
| IBc-50 | 8 | BvF-BI, BcMV4-BI, BcMV9-BI, BcHV1-BI, BcNSRV7-BI,  BcULV3, BcEV2-BI, BcFV5-BI, BcHV1SLR-BI |
| IBc-49 | 8 | BcMV1-BI, BcMV4-BI, BcMV9-BI, BcHV1-BI, BcHV3-BI,  BcMV11, BcPV3-BI, BcOLV18, BcHV1SLR-BI |
| IBc-40 | 8 | BcMV1-BI, BcHV1-BI, BcULV4, BcMV2-BI, BcULV1-BI,  BcEV2-BI, BcOLV18, BcMV11, BcHV1SLR-BI |
| IBc-39 | 8 | BcMV6-BI, BcMV1-BI, BcMV9-BI, BcHV1-BI, BcULV4,  BcULV1-BI, BcMV11, BcHV1SLR-BI, BcMV15 |
| Ca-9 | 7 | BcNSRV7-BI, BcHV1-BI, BcBNV5-BI, BcHV3-BI, BcULV3,  BcMV9-BI, BcMV1-BI, BcHV1SLR-BI |
| Ca-12 | 7 | BcFV9, BcMV3-BI, BcDMV1, BcMV9-BI, BcEV2-BI, BcMV1-BI,  BcMV11 |
| IBc-441 | 7 | BcULV3, BcRV1-BI, BcMV3-BI, BcMV9-BI, BcMV4-BI,  BcULV4, BcMV11, BcHV1SLR-BI |
| IBc-425 | 7 | BcULV3, BcRV1-BI, BcMV1-BI, BcMV9-BI, BcPV3-BI,  BcHV1-BI, BcULV4 |
| IBc-418 | 7 | BcULV3, BcMV1-BI, BcHV1-BI, BcPV3-BI, BcEV2-BI,  BcOLV18, BcMV11, BcHV1SLR-BI |
| IBc-295 | 7 | BcMV3-BI, BcMV1-BI, BcMV9-BI, BcMV4-BI, BcHV1-BI,  BcOLV16-BI, BcMV2-BI, BcHV1SLR-BI |
| IBc-293 | 7 | BcULV3, BcRV1-BI, BcMV1-BI, BcMV9-BI, BcULV4,  BcOLV16-BI, BcMV2-BI |
| IBc-287 | 7 | BcMV1-BI, BcMV9-BI, BcMV4-BI, BcPV3-BI, BcOLV16-BI,  BcMV2-BI, BcMV11 |
| IBc-280 | 7 | BcULV3, BcRV1-BI, BcMV1-BI, BcMV9-BI, BcULV4,  BcOLV7-BI, BcVV3-BI |
| IBc-214 | 7 | BcULV3, BcMV6-BI, BcRV1-BI, BcMV3-BI, BcMV1-BI,  BcMV9-BI, BcULV4 |
| IBc-193 | 7 | BcMV1-BI, BcMV9-BI, BcNSRV7-BI, BcULV4, BcALV1-BI,  BcOLV16-BI, BcOLV18 |
| IBc-184 | 7 | BcMV3-BI, BcMV1-BI, BcMV9-BI, BcMV12, BcNSRV7-BI,  BcULV4, BcMV2-BI |
| IBc-173 | 7 | BcULV3, BcMV6-BI, BcMV9-BI, BcHV1-BI, BcNSRV7-BI,  BcULV4, BcOLV16-BI |
| IBc-164 | 7 | BcPV5, BcMV3-BI, BcMV1-BI, BcMV9-BI, BcDMV1,  BcOLV7-BI, BcPV4-BI |

| IBc-163 | 7 | BcPV5, BcMV3-BI, BcMV9-BI, BcULV4, BcOLV7-BI, BcVV3-BI,  BcMV13 |
| --- | --- | --- |
| IBc-99 | 7 | BcRV1-BI, BcMV3-BI, BcMV1-BI, BcULV4, BcHV3-BI,  BcDMV1, BcMV11 |
| IBc-88 | 7 | BcULV3, BcMV3-BI, BcMV1-BI, BcMV9-BI, BcHV1-BI,  BcOLV4-BI, BcMV11, BcHV1SLR-BI |
| IBc-85 | 7 | BcULV3, BcMV3-BI, BcMV1-BI, BcMV9-BI, BcULV4,  BcOLV4-BI, BcMV11, BcHV1SLR-BI |
| IBc-82 | 7 | BcMV1-BI, BcMV9-BI, BcPV3-BI, BcULV4, BcOLV17-BI,  BcMV2-BI, BcEV2-BI |
| IBc-63 | 7 | BvF-BI, BcOLV16-BI, BcMV1-BI, BcMV9-BI, BcHV1-BI,  BcPV3-BI, BcOLV18, BcHV1SLR-BI |
| IBc-59 | 7 | BcPV4-BI, BvF-BI, BcMV1-BI, BcMV9-BI, BcHV1-BI, BcULV3,  BcEV2-BI |
| IBc-34 | 7 | BcULV3, BcMV6-BI, BcULV4, BcHV3-BI, BcALV1-BI,  BcOLV16-BI, BcVV3-BI |
| IBc-29 | 7 | BcULV3, BcPV3-BI, BcHV1-BI, BcNSRV7-BI, BcULV4,  BcHV1SLR-BI, BcEV6, BcMV15 |
| IBc-438 | 6 | BcULV3, BcPV5, BcMV1-BI, BcULV4, BcALV1-BI, BcMV11,  BcHV1SLR-BI |
| IBc-421 | 6 | BcULV3, BcMV1-BI, BcHV1-BI, BcPV3-BI, BcMV2-BI, BcMV11 |
| IBc-411 | 6 | BcMV3-BI, BcMV1-BI, BcMV2-BI, BcULV5, BcOLV1-BI,  BcMV11 |
| IBc-408 | 6 | BcMV3-BI, BcMV1-BI, BcMV2-BI, BcULV5, BcOLV1-BI,  BcMV11 |
| IBc-403 | 6 | BcMV1-BI, BcMV3-BI, BcPV3-BI, BcMV2-BI, BcULV5,  BcOLV18 |
| IBc-401 | 6 | BcULV3, BcRV1-BI, BcMV1-BI, BcMV3-BI, BcPV3-BI,  BcMV2-BI |
| IBc-396 | 6 | BcULV3, BcRV1-BI, BcULV4, BcMV2-BI, BcEV2-BI, BcOLV18 |
| IBc-390 | 6 | BcMV6-BI, BcPV7, BcHV1-BI, BcPV10, BcOLV1-BI, BcMV11,  BcHV1SLR-BI |
| IBc-388 | 6 | BcULV3, BcMV3-BI, BcMV1-BI, BcMV9-BI, BcULV4,  BcOLV16-BI |
| IBc-357 | 6 | BcMV1-BI, BcMV9-BI, BcHV1-BI, BcDMV1, BcMV11, BcEV6 |
| IBc-290 | 6 | BcULV3, BcMV1-BI, BcMV9-BI, BcMV4-BI, BcULV4,  BcOLV16-BI |
| IBc-284 | 6 | BcPV5, BcMV1-BI, BcMV9-BI, BcULV4, BcOLV7-BI, BcPV4-BI |
| IBc-269 | 6 | BcULV3, BcMV6-BI, BcMV3-BI, BcMV9-BI, BcMV12,  BcMV2-BI |
| IBc-159 | 6 | BcULV3, BcMV1-BI, BcMV9-BI, BcMV12, BcHV1-BI, BcULV4 |
| IBc-132 | 6 | BcMV1-BI, BcMV9-BI, BcHV1-BI, BcULV4, BcPV4-BI,  BcOLV18 |

| IBc-118 | 6 | BcHV1-BI, BcFV9, BcNSRV7-BI, BcVV3-BI, BcEV2-BI,  BcMV11, BcHV1SLR-BI |
| --- | --- | --- |
| IBc-115 | 6 | BcULV3, BcHV1-BI, BcVV3-BI, BcHV4-BI, BcEV2-BI, BcMV11,  BcHV1SLR-BI |
| IBc-112 | 6 | BcULV3, BcFV5-BI, BcMV3-BI, BcPV3-BI, BcULV4, BcDMV1 |
| IBc-107 | 6 | BcRV1-BI, BcMV3-BI, BcMV9-BI, BcDMV1, BvF-BI, BcMV11 |
| IBc-93 | 6 | BcFV5-BI, BcMV4-BI, BcHV3-BI, BcOLV16-BI, BcEV2-BI,  BcMV11 |
| IBc-77 | 6 | BcMV6-BI, BcMV1-BI, BcHV1-BI, BcULV4, BcOLV7-BI,  BcOLV4-BI |
| IBc-60 | 6 | BcMV1-BI, BcMV9-BI, BcULV4, BcOLV7-BI, BcOLV18,  BcMV11, BcHV1SLR-BI |
| IBc-43 | 6 | BcPV4-BI, BcHV1-BI, BcMV12, BcULV3, BcOLV18,  BcBNV4-BI, BcHV1SLR-BI |
| IBc-35 | 6 | BcHV1-BI, BcULV4, BcOLV16-BI, BcMV2-BI, BcULV1-BI,  BcHV1SLR-BI, BcEV6 |
| Ca-3 | 5 | BcHV1-BI, BcULV3, BcMV9-BI, BcFV5-BI, BcMV12,  BcHV1SLR-BI |
| Ca-13 | 5 | BcMV1-BI, BcULV4, BcULV1-BI, BcMV9-BI, BcHV1-BI,  BcHV1SLR-BI |
| IBc-454 | 5 | BcMV1-BI, BcMV9-BI, BcHV1-BI, BcULV4, BcDMV1,  BcHV1SLR-BI |
| IBc-433 | 5 | BcULV3, BcMV1-BI, BcMV4-BI, BcULV4, BcOLV16-BI |
| IBc-429 | 5 | BcRV1-BI, BcPV7, BcMV1-BI, BcPV10, BcMV11 |
| IBc-423 | 5 | BcULV3, BcMV1-BI, BcNSRV7-BI, BcULV5, BcMV11,  BcHV1SLR-BI |
| IBc-415 | 5 | BcULV3, BcMV1-BI, BcMV4-BI, BcALV1-BI, BcMV11 |
| IBc-414 | 5 | BcRV1-BI, BcMV3-BI, BcMV1-BI, BcOLV4-BI, BcMV11 |
| IBc-407 | 5 | BcMV3-BI, BcMV1-BI, BcMV2-BI, BcEV2-BI, BcMV11 |
| IBc-402 | 5 | BcRV1-BI, BcMV1-BI, BcHV1-BI, BcULV4, BcMV2-BI,  BcHV1SLR-BI |
| IBc-389 | 5 | BcMV6-BI, BcMV1-BI, BcMV12, BcMV3-BI, BcOLV18 |
| IBc-387 | 5 | BcMV1-BI, BcPV3-BI, BcOLV1-BI, BcEV2-BI, BcMV11 |
| IBc-384 | 5 | BcULV3, BcMV1-BI, BcPV3-BI, BcOLV1-BI, BcOLV18 |
| IBc-283 | 5 | BcFV5-BI, BcMV3-BI, BcMV9-BI, BcOLV16-BI, BcVV3-BI |
| IBc-259 | 5 | BcULV3, BcMV1-BI, BcOLV16-BI, BcOLV1-BI, BcOLV2-BI |
| IBc-237 | 5 | BcULV3, BcMV1-BI, BcMV9-BI, BcHV1-BI, BcMV2-BI |
| IBc-188 | 5 | BcRV1-BI, BcULV4, BcOLV16-BI, BcOLV18, BcMV11 |
| IBc-185 | 5 | BcRV1-BI, BcMV9-BI, BcULV4, BcOLV16-BI, BcMV13 |
| IBc-169 | 5 | BcMV6-BI, BcMV9-BI, BcHV1-BI, BcULV4, BcOLV16-BI,  BcHV1SLR-BI |
| IBc-154 | 5 | BcMV3-BI, BcULV4, BcDMV1, BcVV3-BI, BcOLV18 |

| IBc-136 | 5 | BcRV1-BI, BcMV3-BI, BcFV9, BcEV2-BI, BcMV11 |
| --- | --- | --- |
| IBc-121 | 5 | BcULV3, BcMV1-BI, BcHV1-BI, BcOLV17-BI, BcPV3-BI,  BcHV1SLR-BI |
| IBc-105 | 5 | BcULV3, BcMV3-BI, BcMV1-BI, BcMV9-BI, BcMV11 |
| IBc-104 | 5 | BcULV3, BcMV6-BI, BcMV1-BI, BcHV1-BI, BcFV9 |
| IBc-102 | 5 | BcFV5-BI, BcMV3-BI, BcMV9-BI, BcULV4, BvF-BI |
| IBc-94 | 5 | BcULV3, BcMV1-BI, BcMV9-BI, BvF-BI, BcMV11,  BcHV1SLR-BI |
| IBc-89 | 5 | BcMV9-BI, BcULV4, BcDMV1, BcOLV16-BI, BcMV2-BI |
| IBc-56 | 5 | BcMV1-BI, BcMV9-BI, BcHV1-BI, BcMV11, BcULV3,  BcHV1SLR-BI |
| IBc-432 | 4 | BcMV1-BI, BcPV3-BI, BcULV4, BcOLV4-BI |
| IBc-417 | 4 | BcMV1-BI, BcOLV4-BI, BcALV1-BI, BcMV11 |
| IBc-416 | 4 | BcULV3, BcMV1-BI, BcMV4-BI, BcMV11 |
| IBc-400 | 4 | BcULV3, BcMV9-BI, BcHV1-BI, BcMV2-BI |
| IBc-386 | 4 | BcRV1-BI, BcULV4, BcMNV1-BI, BcMV2-BI |
| IBc-175 | 4 | BcMV9-BI, BcULV4, BcOLV16-BI, BcOLV18 |
| IBc-124 | 4 | BcMV1-BI, BcMV4-BI, BcULV4, BcPV3-BI |
| IBc-61 | 4 | BcMV1-BI, BcMV4-BI, BcMV9-BI, BcEV2-BI |
| IBc-58 | 4 | BcMV1-BI, BcMV9-BI, BcULV4, BcMV11, BcHV1SLR-BI |
| IBc-31 | 4 | BcMV9-BI, BcULV4, BcMV13, BcEV6 |
| IBc-442 | 3 | BcMV12, BcULV4, BcMV14 |
| IBc-437 | 3 | BcMV1-BI, BcULV4, BcHV3-BI |
| IBc-431 | 3 | BcEV5, BcULV4, BcOLV18 |
| IBc-426 | 3 | BcMV1-BI, BcPV10, BcMV11 |
| IBc-412 | 3 | BcMV1-BI, BcULV5, BcMV11 |
| IBc-410 | 3 | BcHV1-BI, BcOLV18, BcMV11, BcHV1SLR-BI |
| IBc-406 | 3 | BcRV1-BI, BcPV7, BcMV11 |
| IBc-395 | 3 | BcRV1-BI, BcMV4-BI, BcEV2-BI |
| IBc-394 | 3 | BcMV4-BI, BcULV4, BcMV2-BI |
| IBc-393 | 3 | BcMV1-BI, BcULV4, BcMV2-BI |
| IBc-340 | 3 | BcULV3, BcPV10, BcMV11, BcHV1SLR-BI |
| IBc-110 | 3 | BcMV9-BI, BcNSRV7-BI, BcMV11 |
| IBc-109 | 3 | BcULV3, BcHV1-BI, BcULV4 |
| IBc-55 | 3 | BcPV3-BI, BcHV1-BI, BcULV3, BcHV1SLR-BI |
| IBc-46 | 3 | BvF-BI, BcMV1-BI, BcEV2-BI, BcHV1SLR-BI |
| IBc-428 | 2 | BcPV10, BcMV11 |
| IBc-133 | 1 | BcMV6-BI |
| IBc-111 | 0 |  |

Table S4 The “One to One” association rules of viruses that infected *B. cinerea*

| **Rules** | **Support** | **Confidence** | **Coverage** | **Lift** | **Count** |
| --- | --- | --- | --- | --- | --- |
| {BcMCV5=TRUE} =>  {BcMV1=TRUE} | 0.076733 | 1 | 0.076733 | 1.266458 | 31 |
| {BcMV1=TRUE} =>  {BcMCV5=TRUE} | 0.076733 | 0.097179 | 0.789604 | 1.266458 | 31 |
| {BvF=TRUE} =>  {BcRV1=FALSE} | 0.076733 | 0.885714 | 0.086634 | 1.22544 | 31 |
| {BvF=TRUE} =>  {BcMV12=FALSE} | 0.081683 | 0.942857 | 0.086634 | 1.179301 | 33 |
| {BvF=TRUE} =>  {BcOLV7=FALSE} | 0.079208 | 0.914286 | 0.086634 | 1.133041 | 32 |
| {BcBNV5=TRUE} =>  {BcULV3=TRUE} | 0.076733 | 0.837838 | 0.091584 | 1.41036 | 31 |
| {BcULV3=TRUE} =>  {BcBNV5=TRUE} | 0.076733 | 0.129167 | 0.594059 | 1.41036 | 31 |
| {BcBNV5=TRUE} =>  {BcMV11=TRUE} | 0.079208 | 0.864865 | 0.091584 | 1.403235 | 32 |
| {BcMV11=TRUE} =>  {BcBNV5=TRUE} | 0.079208 | 0.128514 | 0.616337 | 1.403235 | 32 |
| {BcBNV5=TRUE} =>  {BcMV9=TRUE} | 0.081683 | 0.891892 | 0.091584 | 1.315052 | 33 |
| {BcMV9=TRUE} =>  {BcBNV5=TRUE} | 0.081683 | 0.120438 | 0.678218 | 1.315052 | 33 |
| {BcBNV5=TRUE} =>  {BcRV1=FALSE} | 0.074257 | 0.810811 | 0.091584 | 1.121807 | 30 |
| {BcBNV5=TRUE} =>  {BcMV1=TRUE} | 0.084158 | 0.918919 | 0.091584 | 1.163772 | 34 |
| {BcBNV5=TRUE} =>  {BcOLV4=FALSE} | 0.086634 | 0.945946 | 0.091584 | 1.079554 | 35 |
| {BcBNV5=TRUE} =>  {BcULV5=FALSE} | 0.086634 | 0.945946 | 0.091584 | 1.079554 | 35 |
| {BcOLV4=TRUE} =>  {BcMV2=TRUE} | 0.086634 | 0.7 | 0.123762 | 1.512299 | 35 |
| {BcMV2=TRUE} =>  {BcOLV4=TRUE} | 0.086634 | 0.187166 | 0.462871 | 1.512299 | 35 |
| {BcOLV4=TRUE} =>  {BcHV1SLR=TRUE} | 0.079208 | 0.64 | 0.123762 | 1.180639 | 32 |
| {BcOLV4=TRUE} =>  {BcMV3=TRUE} | 0.086634 | 0.7 | 0.123762 | 1.291324 | 35 |
| {BcMV3=TRUE} =>  {BcOLV4=TRUE} | 0.086634 | 0.159817 | 0.542079 | 1.291324 | 35 |
| {BcOLV4=TRUE} =>  {BcULV3=TRUE} | 0.086634 | 0.7 | 0.123762 | 1.178333 | 35 |

| {BcOLV4=TRUE} =>  {BcMV11=TRUE} | 0.10396 | 0.84 | 0.123762 | 1.362892 | 42 |
| --- | --- | --- | --- | --- | --- |
| {BcMV11=TRUE} =>  {BcOLV4=TRUE} | 0.10396 | 0.168675 | 0.616337 | 1.362892 | 42 |
| {BcOLV4=TRUE} =>  {BcMV1=TRUE} | 0.116337 | 0.94 | 0.123762 | 1.19047 | 47 |
| {BcOLV4=TRUE} =>  {BcNSRV7=FALSE} | 0.113861 | 0.92 | 0.123762 | 1.150712 | 46 |
| {BcOLV4=TRUE} =>  {BcDMV1=FALSE} | 0.121287 | 0.98 | 0.123762 | 1.161056 | 49 |
| {BcULV5=TRUE} =>  {BcMV9=FALSE} | 0.101485 | 0.82 | 0.123762 | 2.548308 | 41 |
| {BcMV9=FALSE} =>  {BcULV5=TRUE} | 0.101485 | 0.315385 | 0.321782 | 2.548308 | 41 |
| {BcULV5=TRUE} =>  {BcOLV1=TRUE} | 0.074257 | 0.6 | 0.123762 | 1.808955 | 30 |
| {BcOLV1=TRUE} =>  {BcULV5=TRUE} | 0.074257 | 0.223881 | 0.331683 | 1.808955 | 30 |
| {BcULV5=TRUE} =>  {BcMV2=TRUE} | 0.086634 | 0.7 | 0.123762 | 1.512299 | 35 |
| {BcMV2=TRUE} =>  {BcULV5=TRUE} | 0.086634 | 0.187166 | 0.462871 | 1.512299 | 35 |
| {BcULV5=TRUE} =>  {BcOLV18=FALSE} | 0.074257 | 0.6 | 0.123762 | 1.243077 | 30 |
| {BcULV5=TRUE} =>  {BcMV3=TRUE} | 0.089109 | 0.72 | 0.123762 | 1.328219 | 36 |
| {BcMV3=TRUE} =>  {BcULV5=TRUE} | 0.089109 | 0.164384 | 0.542079 | 1.328219 | 36 |
| {BcULV5=TRUE} =>  {BcMV11=TRUE} | 0.111386 | 0.9 | 0.123762 | 1.460241 | 45 |
| {BcMV11=TRUE} =>  {BcULV5=TRUE} | 0.111386 | 0.180723 | 0.616337 | 1.460241 | 45 |
| {BcULV5=TRUE} =>  {BcMV1=TRUE} | 0.116337 | 0.94 | 0.123762 | 1.19047 | 47 |
| {BcULV5=TRUE} =>  {BcNSRV7=FALSE} | 0.108911 | 0.88 | 0.123762 | 1.100681 | 44 |
| {BcULV5=TRUE} =>  {BcDMV1=FALSE} | 0.118812 | 0.96 | 0.123762 | 1.137361 | 48 |
| {BcFV9=TRUE} =>  {BcMV4=TRUE} | 0.086634 | 0.660377 | 0.131188 | 1.490461 | 35 |
| {BcMV4=TRUE} =>  {BcFV9=TRUE} | 0.086634 | 0.195531 | 0.443069 | 1.490461 | 35 |
| {BcFV9=TRUE} =>  {BcOLV18=TRUE} | 0.096535 | 0.735849 | 0.131188 | 1.422407 | 39 |

| {BcOLV18=TRUE} =>  {BcFV9=TRUE} | 0.096535 | 0.186603 | 0.517327 | 1.422407 | 39 |
| --- | --- | --- | --- | --- | --- |
| {BcFV9=TRUE} =>  {BcHV1SLR=TRUE} | 0.091584 | 0.698113 | 0.131188 | 1.287844 | 37 |
| {BcHV1SLR=TRUE}  => {BcFV9=TRUE} | 0.091584 | 0.16895 | 0.542079 | 1.287844 | 37 |
| {BcFV9=TRUE} =>  {BcMV3=TRUE} | 0.086634 | 0.660377 | 0.131188 | 1.21823 | 35 |
| {BcFV9=TRUE} =>  {BcHV1=TRUE} | 0.096535 | 0.735849 | 0.131188 | 1.218373 | 39 |
| {BcFV9=TRUE} =>  {BcMV11=TRUE} | 0.094059 | 0.716981 | 0.131188 | 1.163295 | 38 |
| {BcDMV1=TRUE} =>  {BcFV5=TRUE} | 0.076733 | 0.492063 | 0.155941 | 2.259019 | 31 |
| {BcFV5=TRUE} =>  {BcDMV1=TRUE} | 0.076733 | 0.352273 | 0.217822 | 2.259019 | 31 |
| {BcDMV1=TRUE} =>  {BcHV3=TRUE} | 0.076733 | 0.492063 | 0.155941 | 2.049419 | 31 |
| {BcHV3=TRUE} =>  {BcDMV1=TRUE} | 0.076733 | 0.319588 | 0.240099 | 2.049419 | 31 |
| {BcDMV1=TRUE} =>  {BcMV3=TRUE} | 0.118812 | 0.761905 | 0.155941 | 1.405523 | 48 |
| {BcMV3=TRUE} =>  {BcDMV1=TRUE} | 0.118812 | 0.219178 | 0.542079 | 1.405523 | 48 |
| {BcDMV1=TRUE} =>  {BcMV9=TRUE} | 0.126238 | 0.809524 | 0.155941 | 1.193604 | 51 |
| {BcDMV1=TRUE} =>  {BcRV1=FALSE} | 0.136139 | 0.873016 | 0.155941 | 1.207871 | 55 |
| {BcDMV1=TRUE} =>  {BcULV1=FALSE} | 0.136139 | 0.873016 | 0.155941 | 1.112613 | 55 |
| {BcDMV1=TRUE} =>  {BcOLV7=FALSE} | 0.141089 | 0.904762 | 0.155941 | 1.121239 | 57 |
| {BcDMV1=TRUE} =>  {BcOLV4=FALSE} | 0.153465 | 0.984127 | 0.155941 | 1.123128 | 62 |
| {BcDMV1=TRUE} =>  {BcULV5=FALSE} | 0.15099 | 0.968254 | 0.155941 | 1.105013 | 61 |
| {BcALV1=TRUE} =>  {BcRV1=TRUE} | 0.086634 | 0.466667 | 0.185644 | 1.683333 | 35 |
| {BcRV1=TRUE} =>  {BcALV1=TRUE} | 0.086634 | 0.3125 | 0.277228 | 1.683333 | 35 |
| {BcALV1=TRUE} =>  {BcMV6=TRUE} | 0.086634 | 0.466667 | 0.185644 | 1.532791 | 35 |
| {BcMV6=TRUE} =>  {BcALV1=TRUE} | 0.086634 | 0.284553 | 0.304455 | 1.532791 | 35 |

| {BcALV1=TRUE} =>  {BcOLV1=TRUE} | 0.106436 | 0.573333 | 0.185644 | 1.728557 | 43 |
| --- | --- | --- | --- | --- | --- |
| {BcOLV1=TRUE} =>  {BcALV1=TRUE} | 0.106436 | 0.320896 | 0.331683 | 1.728557 | 43 |
| {BcALV1=TRUE} =>  {BcOLV16=TRUE} | 0.106436 | 0.573333 | 0.185644 | 1.37873 | 43 |
| {BcOLV16=TRUE} =>  {BcALV1=TRUE} | 0.106436 | 0.255952 | 0.415842 | 1.37873 | 43 |
| {BcALV1=TRUE} =>  {BcMV4=TRUE} | 0.128713 | 0.693333 | 0.185644 | 1.564842 | 52 |
| {BcMV4=TRUE} =>  {BcALV1=TRUE} | 0.128713 | 0.290503 | 0.443069 | 1.564842 | 52 |
| {BcALV1=TRUE} =>  {BcMV2=TRUE} | 0.138614 | 0.746667 | 0.185644 | 1.613119 | 56 |
| {BcMV2=TRUE} =>  {BcALV1=TRUE} | 0.138614 | 0.299465 | 0.462871 | 1.613119 | 56 |
| {BcALV1=TRUE} =>  {BcOLV18=TRUE} | 0.141089 | 0.76 | 0.185644 | 1.469091 | 57 |
| {BcOLV18=TRUE} =>  {BcALV1=TRUE} | 0.141089 | 0.272727 | 0.517327 | 1.469091 | 57 |
| {BcALV1=TRUE} =>  {BcHV1SLR=TRUE} | 0.126238 | 0.68 | 0.185644 | 1.254429 | 51 |
| {BcHV1SLR=TRUE}  => {BcALV1=TRUE} | 0.126238 | 0.232877 | 0.542079 | 1.254429 | 51 |
| {BcALV1=TRUE} =>  {BcMV3=TRUE} | 0.118812 | 0.64 | 0.185644 | 1.180639 | 48 |
| {BcALV1=TRUE} =>  {BcHV1=TRUE} | 0.138614 | 0.746667 | 0.185644 | 1.236284 | 56 |
| {BcHV1=TRUE} =>  {BcALV1=TRUE} | 0.138614 | 0.229508 | 0.60396 | 1.236284 | 56 |
| {BcALV1=TRUE} =>  {BcMV11=TRUE} | 0.155941 | 0.84 | 0.185644 | 1.362892 | 63 |
| {BcMV11=TRUE} =>  {BcALV1=TRUE} | 0.155941 | 0.253012 | 0.616337 | 1.362892 | 63 |
| {BcALV1=TRUE} =>  {BcULV4=TRUE} | 0.160891 | 0.866667 | 0.185644 | 1.254958 | 65 |
| {BcULV4=TRUE} =>  {BcALV1=TRUE} | 0.160891 | 0.232975 | 0.690594 | 1.254958 | 65 |
| {BcOLV7=TRUE} =>  {BcRV1=TRUE} | 0.108911 | 0.564103 | 0.193069 | 2.034799 | 44 |
| {BcRV1=TRUE} =>  {BcOLV7=TRUE} | 0.108911 | 0.392857 | 0.277228 | 2.034799 | 44 |
| {BcOLV7=TRUE} =>  {BcPV3=TRUE} | 0.081683 | 0.423077 | 0.193069 | 1.460881 | 33 |

| {BcPV3=TRUE} =>  {BcOLV7=TRUE} | 0.081683 | 0.282051 | 0.289604 | 1.460881 | 33 |
| --- | --- | --- | --- | --- | --- |
| {BcOLV7=TRUE} =>  {BcOLV1=TRUE} | 0.094059 | 0.487179 | 0.193069 | 1.46881 | 38 |
| {BcOLV1=TRUE} =>  {BcOLV7=TRUE} | 0.094059 | 0.283582 | 0.331683 | 1.46881 | 38 |
| {BcOLV7=TRUE} =>  {BcOLV16=TRUE} | 0.121287 | 0.628205 | 0.193069 | 1.510684 | 49 |
| {BcOLV16=TRUE} =>  {BcOLV7=TRUE} | 0.121287 | 0.291667 | 0.415842 | 1.510684 | 49 |
| {BcOLV7=TRUE} =>  {BcMV4=TRUE} | 0.106436 | 0.551282 | 0.193069 | 1.244234 | 43 |
| {BcMV4=TRUE} =>  {BcOLV7=TRUE} | 0.106436 | 0.240223 | 0.443069 | 1.244234 | 43 |
| {BcOLV7=TRUE} =>  {BcOLV18=TRUE} | 0.141089 | 0.730769 | 0.193069 | 1.412587 | 57 |
| {BcOLV18=TRUE} =>  {BcOLV7=TRUE} | 0.141089 | 0.272727 | 0.517327 | 1.412587 | 57 |
| {BcOLV7=TRUE} =>  {BcMV3=TRUE} | 0.126238 | 0.653846 | 0.193069 | 1.206182 | 51 |
| {BcOLV7=TRUE} =>  {BcMV9=TRUE} | 0.170792 | 0.884615 | 0.193069 | 1.304323 | 69 |
| {BcMV9=TRUE} =>  {BcOLV7=TRUE} | 0.170792 | 0.251825 | 0.678218 | 1.304323 | 69 |
| {BcOLV7=TRUE} =>  {BcULV4=TRUE} | 0.178218 | 0.923077 | 0.193069 | 1.336642 | 72 |
| {BcULV4=TRUE} =>  {BcOLV7=TRUE} | 0.178218 | 0.258065 | 0.690594 | 1.336642 | 72 |
| {BcOLV7=TRUE} =>  {BcMV1=TRUE} | 0.168317 | 0.871795 | 0.193069 | 1.104091 | 68 |
| {BcOLV7=TRUE} =>  {BcDMV1=FALSE} | 0.178218 | 0.923077 | 0.193069 | 1.093616 | 72 |
| {BcVV3=TRUE} =>  {BcHV3=TRUE} | 0.086634 | 0.4375 | 0.19802 | 1.822165 | 35 |
| {BcHV3=TRUE} =>  {BcVV3=TRUE} | 0.086634 | 0.360825 | 0.240099 | 1.822165 | 35 |
| {BcVV3=TRUE} =>  {BcEV2=TRUE} | 0.076733 | 0.3875 | 0.19802 | 1.534804 | 31 |
| {BcEV2=TRUE} =>  {BcVV3=TRUE} | 0.076733 | 0.303922 | 0.252475 | 1.534804 | 31 |
| {BcVV3=TRUE} =>  {BcMV6=TRUE} | 0.074257 | 0.375 | 0.19802 | 1.231707 | 30 |
| {BcVV3=TRUE} =>  {BcOLV1=TRUE} | 0.089109 | 0.45 | 0.19802 | 1.356716 | 36 |

| {BcOLV1=TRUE} =>  {BcVV3=TRUE} | 0.089109 | 0.268657 | 0.331683 | 1.356716 | 36 |
| --- | --- | --- | --- | --- | --- |
| {BcVV3=TRUE} =>  {BcMV4=TRUE} | 0.111386 | 0.5625 | 0.19802 | 1.269553 | 45 |
| {BcMV4=TRUE} =>  {BcVV3=TRUE} | 0.111386 | 0.251397 | 0.443069 | 1.269553 | 45 |
| {BcVV3=TRUE} =>  {BcMV2=TRUE} | 0.111386 | 0.5625 | 0.19802 | 1.215241 | 45 |
| {BcVV3=TRUE} =>  {BcMV3=TRUE} | 0.126238 | 0.6375 | 0.19802 | 1.176027 | 51 |
| {BcNSRV7=TRUE} =>  {BcFV5=TRUE} | 0.096535 | 0.481481 | 0.200495 | 2.210438 | 39 |
| {BcFV5=TRUE} =>  {BcNSRV7=TRUE} | 0.096535 | 0.443182 | 0.217822 | 2.210438 | 39 |
| {BcNSRV7=TRUE} =>  {BcHV3=TRUE} | 0.094059 | 0.469136 | 0.200495 | 1.953926 | 38 |
| {BcHV3=TRUE} =>  {BcNSRV7=TRUE} | 0.094059 | 0.391753 | 0.240099 | 1.953926 | 38 |
| {BcNSRV7=TRUE} =>  {BcOLV18=TRUE} | 0.128713 | 0.641975 | 0.200495 | 1.240947 | 52 |
| {BcOLV18=TRUE} =>  {BcNSRV7=TRUE} | 0.128713 | 0.248804 | 0.517327 | 1.240947 | 52 |
| {BcNSRV7=TRUE} =>  {BcHV1SLR=TRUE} | 0.133663 | 0.666667 | 0.200495 | 1.229833 | 54 |
| {BcHV1SLR=TRUE}  => {BcNSRV7=TRUE} | 0.133663 | 0.246575 | 0.542079 | 1.229833 | 54 |
| {BcNSRV7=TRUE} =>  {BcULV3=TRUE} | 0.155941 | 0.777778 | 0.200495 | 1.309259 | 63 |
| {BcULV3=TRUE} =>  {BcNSRV7=TRUE} | 0.155941 | 0.2625 | 0.594059 | 1.309259 | 63 |
| {BcNSRV7=TRUE} =>  {BcHV1=TRUE} | 0.138614 | 0.691358 | 0.200495 | 1.144708 | 56 |
| {BcNSRV7=TRUE} =>  {BcMV9=TRUE} | 0.160891 | 0.802469 | 0.200495 | 1.183203 | 65 |
| {BcNSRV7=TRUE} =>  {BcULV4=TRUE} | 0.155941 | 0.777778 | 0.200495 | 1.126245 | 63 |
| {BcNSRV7=TRUE} =>  {BcRV1=FALSE} | 0.163366 | 0.814815 | 0.200495 | 1.127347 | 66 |
| {BcNSRV7=TRUE} =>  {BcULV1=FALSE} | 0.170792 | 0.851852 | 0.200495 | 1.085641 | 69 |
| {BcNSRV7=TRUE} =>  {BcOLV4=FALSE} | 0.190594 | 0.950617 | 0.200495 | 1.084885 | 77 |
| {BcMV12=TRUE} =>  {BcMV6=TRUE} | 0.086634 | 0.432099 | 0.200495 | 1.419251 | 35 |

| {BcMV6=TRUE} =>  {BcMV12=TRUE} | 0.086634 | 0.284553 | 0.304455 | 1.419251 | 35 |
| --- | --- | --- | --- | --- | --- |
| {BcMV12=TRUE} =>  {BcOLV1=TRUE} | 0.101485 | 0.506173 | 0.200495 | 1.526073 | 41 |
| {BcOLV1=TRUE} =>  {BcMV12=TRUE} | 0.101485 | 0.30597 | 0.331683 | 1.526073 | 41 |
| {BcMV12=TRUE} =>  {BcOLV16=TRUE} | 0.121287 | 0.604938 | 0.200495 | 1.454733 | 49 |
| {BcOLV16=TRUE} =>  {BcMV12=TRUE} | 0.121287 | 0.291667 | 0.415842 | 1.454733 | 49 |
| {BcMV12=TRUE} =>  {BcMV2=TRUE} | 0.121287 | 0.604938 | 0.200495 | 1.306925 | 49 |
| {BcMV2=TRUE} =>  {BcMV12=TRUE} | 0.121287 | 0.262032 | 0.462871 | 1.306925 | 49 |
| {BcMV12=TRUE} =>  {BcHV1SLR=TRUE} | 0.128713 | 0.641975 | 0.200495 | 1.184283 | 52 |
| {BcMV12=TRUE} =>  {BcMV3=TRUE} | 0.153465 | 0.765432 | 0.200495 | 1.41203 | 62 |
| {BcMV3=TRUE} =>  {BcMV12=TRUE} | 0.153465 | 0.283105 | 0.542079 | 1.41203 | 62 |
| {BcMV12=TRUE} =>  {BcHV1=TRUE} | 0.138614 | 0.691358 | 0.200495 | 1.144708 | 56 |
| {BcMV12=TRUE} =>  {BcEV2=FALSE} | 0.165842 | 0.82716 | 0.200495 | 1.106533 | 67 |
| {BcMV12=TRUE} =>  {BvF=FALSE} | 0.195545 | 0.975309 | 0.200495 | 1.067818 | 79 |
| {BcMV1=FALSE} =>  {BcRV1=TRUE} | 0.074257 | 0.352941 | 0.210396 | 1.273109 | 30 |
| {BcRV1=TRUE} =>  {BcMV1=FALSE} | 0.074257 | 0.267857 | 0.277228 | 1.273109 | 30 |
| {BcMV1=FALSE} =>  {BcMV11=FALSE} | 0.113861 | 0.541176 | 0.210396 | 1.41055 | 46 |
| {BcMV11=FALSE} =>  {BcMV1=FALSE} | 0.113861 | 0.296774 | 0.383663 | 1.41055 | 46 |
| {BcMV1=FALSE} =>  {BcMV3=FALSE} | 0.126238 | 0.6 | 0.210396 | 1.31027 | 51 |
| {BcMV3=FALSE} =>  {BcMV1=FALSE} | 0.126238 | 0.275676 | 0.457921 | 1.31027 | 51 |
| {BcMV1=FALSE} =>  {BcOLV18=FALSE} | 0.133663 | 0.635294 | 0.210396 | 1.316199 | 54 |
| {BcOLV18=FALSE}  => {BcMV1=FALSE} | 0.133663 | 0.276923 | 0.482673 | 1.316199 | 54 |
| {BcMV1=FALSE} =>  {BcMV2=FALSE} | 0.155941 | 0.741176 | 0.210396 | 1.379886 | 63 |

| {BcMV2=FALSE} =>  {BcMV1=FALSE} | 0.155941 | 0.290323 | 0.537129 | 1.379886 | 63 |
| --- | --- | --- | --- | --- | --- |
| {BcMV1=FALSE} =>  {BcMV4=FALSE} | 0.143564 | 0.682353 | 0.210396 | 1.225203 | 58 |
| {BcMV4=FALSE} =>  {BcMV1=FALSE} | 0.143564 | 0.257778 | 0.556931 | 1.225203 | 58 |
| {BcMV1=FALSE} =>  {BcOLV1=FALSE} | 0.173267 | 0.823529 | 0.210396 | 1.232244 | 70 |
| {BcOLV1=FALSE} =>  {BcMV1=FALSE} | 0.173267 | 0.259259 | 0.668317 | 1.232244 | 70 |
| {BcMV1=FALSE} =>  {BcPV3=FALSE} | 0.175743 | 0.835294 | 0.210396 | 1.175815 | 71 |
| {BcPV3=FALSE} =>  {BcMV1=FALSE} | 0.175743 | 0.247387 | 0.710396 | 1.175815 | 71 |
| {BcMV1=FALSE} =>  {BcEV2=FALSE} | 0.175743 | 0.835294 | 0.210396 | 1.117413 | 71 |
| {BcMV1=FALSE} =>  {BcHV3=FALSE} | 0.183168 | 0.870588 | 0.210396 | 1.14566 | 74 |
| {BcMV1=FALSE} =>  {BcULV1=FALSE} | 0.183168 | 0.870588 | 0.210396 | 1.109519 | 74 |
| {BcMV1=FALSE} =>  {BcOLV7=FALSE} | 0.185644 | 0.882353 | 0.210396 | 1.093468 | 75 |
| {BcMV1=FALSE} =>  {BcOLV4=FALSE} | 0.20297 | 0.964706 | 0.210396 | 1.100964 | 82 |
| {BcMV1=FALSE} =>  {BcULV5=FALSE} | 0.20297 | 0.964706 | 0.210396 | 1.100964 | 82 |
| {BcMV1=FALSE} =>  {BcMCV5=FALSE} | 0.210396 | 1 | 0.210396 | 1.08311 | 85 |
| {BcULV1=TRUE} =>  {BcRV1=TRUE} | 0.076733 | 0.356322 | 0.215347 | 1.285304 | 31 |
| {BcRV1=TRUE} =>  {BcULV1=TRUE} | 0.076733 | 0.276786 | 0.277228 | 1.285304 | 31 |
| {BcULV1=TRUE} =>  {BcMV6=TRUE} | 0.091584 | 0.425287 | 0.215347 | 1.396879 | 37 |
| {BcMV6=TRUE} =>  {BcULV1=TRUE} | 0.091584 | 0.300813 | 0.304455 | 1.396879 | 37 |
| {BcULV1=TRUE} =>  {BcOLV1=TRUE} | 0.106436 | 0.494253 | 0.215347 | 1.490136 | 43 |
| {BcOLV1=TRUE} =>  {BcULV1=TRUE} | 0.106436 | 0.320896 | 0.331683 | 1.490136 | 43 |
| {BcULV1=TRUE} =>  {BcOLV16=TRUE} | 0.14604 | 0.678161 | 0.215347 | 1.630816 | 59 |
| {BcOLV16=TRUE} =>  {BcULV1=TRUE} | 0.14604 | 0.35119 | 0.415842 | 1.630816 | 59 |

| {BcULV1=TRUE} =>  {BcMV2=TRUE} | 0.138614 | 0.643678 | 0.215347 | 1.39062 | 56 |
| --- | --- | --- | --- | --- | --- |
| {BcMV2=TRUE} =>  {BcULV1=TRUE} | 0.138614 | 0.299465 | 0.462871 | 1.39062 | 56 |
| {BcULV1=TRUE} =>  {BcOLV18=TRUE} | 0.160891 | 0.747126 | 0.215347 | 1.444206 | 65 |
| {BcOLV18=TRUE} =>  {BcULV1=TRUE} | 0.160891 | 0.311005 | 0.517327 | 1.444206 | 65 |
| {BcULV1=TRUE} =>  {BcHV1SLR=TRUE} | 0.158416 | 0.735632 | 0.215347 | 1.357057 | 64 |
| {BcHV1SLR=TRUE}  => {BcULV1=TRUE} | 0.158416 | 0.292237 | 0.542079 | 1.357057 | 64 |
| {BcULV1=TRUE} =>  {BcMV3=TRUE} | 0.15099 | 0.701149 | 0.215347 | 1.293445 | 61 |
| {BcMV3=TRUE} =>  {BcULV1=TRUE} | 0.15099 | 0.278539 | 0.542079 | 1.293445 | 61 |
| {BcULV1=TRUE} =>  {BcHV1=TRUE} | 0.175743 | 0.816092 | 0.215347 | 1.351234 | 71 |
| {BcHV1=TRUE} =>  {BcULV1=TRUE} | 0.175743 | 0.290984 | 0.60396 | 1.351234 | 71 |
| {BcULV1=TRUE} =>  {BcMV9=TRUE} | 0.178218 | 0.827586 | 0.215347 | 1.220237 | 72 |
| {BcMV9=TRUE} =>  {BcULV1=TRUE} | 0.178218 | 0.262774 | 0.678218 | 1.220237 | 72 |
| {BcULV1=TRUE} =>  {BcULV4=TRUE} | 0.193069 | 0.896552 | 0.215347 | 1.298233 | 78 |
| {BcULV4=TRUE} =>  {BcULV1=TRUE} | 0.193069 | 0.27957 | 0.690594 | 1.298233 | 78 |
| {BcULV1=TRUE} =>  {BcMV1=TRUE} | 0.188119 | 0.873563 | 0.215347 | 1.106331 | 76 |
| {BcULV1=TRUE} =>  {BcNSRV7=FALSE} | 0.185644 | 0.862069 | 0.215347 | 1.078253 | 75 |
| {BcULV1=TRUE} =>  {BcDMV1=FALSE} | 0.195545 | 0.908046 | 0.215347 | 1.075808 | 79 |
| {BcFV5=TRUE} =>  {BcHV3=TRUE} | 0.113861 | 0.522727 | 0.217822 | 2.177132 | 46 |
| {BcHV3=TRUE} =>  {BcFV5=TRUE} | 0.113861 | 0.474227 | 0.240099 | 2.177132 | 46 |
| {BcFV5=TRUE} =>  {BcEV2=TRUE} | 0.091584 | 0.420455 | 0.217822 | 1.66533 | 37 |
| {BcEV2=TRUE} =>  {BcFV5=TRUE} | 0.091584 | 0.362745 | 0.252475 | 1.66533 | 37 |
| {BcFV5=TRUE} =>  {BcOLV1=TRUE} | 0.091584 | 0.420455 | 0.217822 | 1.267639 | 37 |

| {BcOLV1=TRUE} =>  {BcFV5=TRUE} | 0.091584 | 0.276119 | 0.331683 | 1.267639 | 37 |
| --- | --- | --- | --- | --- | --- |
| {BcFV5=TRUE} =>  {BcMV4=TRUE} | 0.121287 | 0.556818 | 0.217822 | 1.256729 | 49 |
| {BcMV4=TRUE} =>  {BcFV5=TRUE} | 0.121287 | 0.273743 | 0.443069 | 1.256729 | 49 |
| {BcFV5=TRUE} =>  {BcOLV18=TRUE} | 0.148515 | 0.681818 | 0.217822 | 1.317964 | 60 |
| {BcOLV18=TRUE} =>  {BcFV5=TRUE} | 0.148515 | 0.287081 | 0.517327 | 1.317964 | 60 |
| {BcFV5=TRUE} =>  {BcHV1SLR=TRUE} | 0.15099 | 0.693182 | 0.217822 | 1.278746 | 61 |
| {BcHV1SLR=TRUE}  => {BcFV5=TRUE} | 0.15099 | 0.278539 | 0.542079 | 1.278746 | 61 |
| {BcFV5=TRUE} =>  {BcMV3=TRUE} | 0.153465 | 0.704545 | 0.217822 | 1.299709 | 62 |
| {BcMV3=TRUE} =>  {BcFV5=TRUE} | 0.153465 | 0.283105 | 0.542079 | 1.299709 | 62 |
| {BcFV5=TRUE} =>  {BcULV3=TRUE} | 0.163366 | 0.75 | 0.217822 | 1.2625 | 66 |
| {BcULV3=TRUE} =>  {BcFV5=TRUE} | 0.163366 | 0.275 | 0.594059 | 1.2625 | 66 |
| {BcFV5=TRUE} =>  {BcHV1=TRUE} | 0.15099 | 0.693182 | 0.217822 | 1.147727 | 61 |
| {BcFV5=TRUE} =>  {BcMV9=TRUE} | 0.165842 | 0.761364 | 0.217822 | 1.122595 | 67 |
| {BcFV5=TRUE} =>  {BcRV1=FALSE} | 0.178218 | 0.818182 | 0.217822 | 1.132005 | 72 |
| {BcHV3=TRUE} =>  {BcEV2=TRUE} | 0.089109 | 0.371134 | 0.240099 | 1.469982 | 36 |
| {BcEV2=TRUE} =>  {BcHV3=TRUE} | 0.089109 | 0.352941 | 0.252475 | 1.469982 | 36 |
| {BcHV3=TRUE} =>  {BcOLV1=TRUE} | 0.10396 | 0.43299 | 0.240099 | 1.305432 | 42 |
| {BcOLV1=TRUE} =>  {BcHV3=TRUE} | 0.10396 | 0.313433 | 0.331683 | 1.305432 | 42 |
| {BcHV3=TRUE} =>  {BcMV4=TRUE} | 0.15099 | 0.628866 | 0.240099 | 1.41934 | 61 |
| {BcMV4=TRUE} =>  {BcHV3=TRUE} | 0.15099 | 0.340782 | 0.443069 | 1.41934 | 61 |
| {BcHV3=TRUE} =>  {BcMV2=TRUE} | 0.133663 | 0.556701 | 0.240099 | 1.202712 | 54 |
| {BcHV3=TRUE} =>  {BcOLV18=TRUE} | 0.165842 | 0.690722 | 0.240099 | 1.335175 | 67 |

| {BcOLV18=TRUE} =>  {BcHV3=TRUE} | 0.165842 | 0.320574 | 0.517327 | 1.335175 | 67 |
| --- | --- | --- | --- | --- | --- |
| {BcHV3=TRUE} =>  {BcULV3=TRUE} | 0.170792 | 0.71134 | 0.240099 | 1.197423 | 69 |
| {BcULV3=TRUE} =>  {BcHV3=TRUE} | 0.170792 | 0.2875 | 0.594059 | 1.197423 | 69 |
| {BcHV3=TRUE} =>  {BcMV11=TRUE} | 0.173267 | 0.721649 | 0.240099 | 1.170869 | 70 |
| {BcHV3=TRUE} =>  {BcMV1=TRUE} | 0.212871 | 0.886598 | 0.240099 | 1.122839 | 86 |
| {BcEV2=TRUE} =>  {BcMV2=TRUE} | 0.143564 | 0.568627 | 0.252475 | 1.228479 | 58 |
| {BcMV2=TRUE} =>  {BcEV2=TRUE} | 0.143564 | 0.31016 | 0.462871 | 1.228479 | 58 |
| {BcEV2=TRUE} =>  {BcHV1SLR=TRUE} | 0.155941 | 0.617647 | 0.252475 | 1.139404 | 63 |
| {BcEV2=TRUE} =>  {BcMV11=TRUE} | 0.185644 | 0.735294 | 0.252475 | 1.193007 | 75 |
| {BcMV11=TRUE} =>  {BcEV2=TRUE} | 0.185644 | 0.301205 | 0.616337 | 1.193007 | 75 |
| {BcEV2=TRUE} =>  {BcMV1=TRUE} | 0.217822 | 0.862745 | 0.252475 | 1.09263 | 88 |
| {BcEV2=TRUE} =>  {BcMV12=FALSE} | 0.217822 | 0.862745 | 0.252475 | 1.079099 | 88 |
| {BcRV1=TRUE} =>  {BcPV3=TRUE} | 0.126238 | 0.455357 | 0.277228 | 1.572344 | 51 |
| {BcPV3=TRUE} =>  {BcRV1=TRUE} | 0.126238 | 0.435897 | 0.289604 | 1.572344 | 51 |
| {BcRV1=TRUE} =>  {BcMV6=TRUE} | 0.113861 | 0.410714 | 0.277228 | 1.349013 | 46 |
| {BcMV6=TRUE} =>  {BcRV1=TRUE} | 0.113861 | 0.373984 | 0.304455 | 1.349013 | 46 |
| {BcRV1=TRUE} =>  {BcOLV1=TRUE} | 0.133663 | 0.482143 | 0.277228 | 1.453625 | 54 |
| {BcOLV1=TRUE} =>  {BcRV1=TRUE} | 0.133663 | 0.402985 | 0.331683 | 1.453625 | 54 |
| {BcRV1=TRUE} =>  {BcOLV16=TRUE} | 0.15099 | 0.544643 | 0.277228 | 1.309736 | 61 |
| {BcOLV16=TRUE} =>  {BcRV1=TRUE} | 0.15099 | 0.363095 | 0.415842 | 1.309736 | 61 |
| {BcRV1=TRUE} =>  {BcMV4=TRUE} | 0.165842 | 0.598214 | 0.277228 | 1.35016 | 67 |
| {BcMV4=TRUE} =>  {BcRV1=TRUE} | 0.165842 | 0.374302 | 0.443069 | 1.35016 | 67 |

| {BcRV1=TRUE} =>  {BcMV2=TRUE} | 0.178218 | 0.642857 | 0.277228 | 1.388846 | 72 |
| --- | --- | --- | --- | --- | --- |
| {BcMV2=TRUE} =>  {BcRV1=TRUE} | 0.178218 | 0.385027 | 0.462871 | 1.388846 | 72 |
| {BcRV1=TRUE} =>  {BcOLV18=TRUE} | 0.188119 | 0.678571 | 0.277228 | 1.311688 | 76 |
| {BcOLV18=TRUE} =>  {BcRV1=TRUE} | 0.188119 | 0.363636 | 0.517327 | 1.311688 | 76 |
| {BcRV1=TRUE} =>  {BcMV3=TRUE} | 0.175743 | 0.633929 | 0.277228 | 1.169439 | 71 |
| {BcRV1=TRUE} =>  {BcHV1=TRUE} | 0.188119 | 0.678571 | 0.277228 | 1.123536 | 76 |
| {BcRV1=TRUE} =>  {BcMV11=TRUE} | 0.207921 | 0.75 | 0.277228 | 1.216867 | 84 |
| {BcMV11=TRUE} =>  {BcRV1=TRUE} | 0.207921 | 0.337349 | 0.616337 | 1.216867 | 84 |
| {BcRV1=TRUE} =>  {BcMV9=TRUE} | 0.222772 | 0.803571 | 0.277228 | 1.184828 | 90 |
| {BcMV9=TRUE} =>  {BcRV1=TRUE} | 0.222772 | 0.328467 | 0.678218 | 1.184828 | 90 |
| {BcRV1=TRUE} =>  {BcULV4=TRUE} | 0.242574 | 0.875 | 0.277228 | 1.267025 | 98 |
| {BcULV4=TRUE} =>  {BcRV1=TRUE} | 0.242574 | 0.351254 | 0.690594 | 1.267025 | 98 |
| {BcRV1=TRUE} =>  {BcFV5=FALSE} | 0.237624 | 0.857143 | 0.277228 | 1.095841 | 96 |
| {BcRV1=TRUE} =>  {BcNSRV7=FALSE} | 0.240099 | 0.866071 | 0.277228 | 1.08326 | 97 |
| {BcRV1=TRUE} =>  {BcDMV1=FALSE} | 0.257426 | 0.928571 | 0.277228 | 1.100126 | 104 |
| {BcRV1=TRUE} =>  {BcEV5=FALSE} | 0.277228 | 1 | 0.277228 | 1.052083 | 112 |
| {BcPV3=TRUE} =>  {BcMV6=TRUE} | 0.111386 | 0.384615 | 0.289604 | 1.26329 | 45 |
| {BcMV6=TRUE} =>  {BcPV3=TRUE} | 0.111386 | 0.365854 | 0.304455 | 1.26329 | 45 |
| {BcPV3=TRUE} =>  {BcOLV1=TRUE} | 0.136139 | 0.470085 | 0.289604 | 1.417273 | 55 |
| {BcOLV1=TRUE} =>  {BcPV3=TRUE} | 0.136139 | 0.410448 | 0.331683 | 1.417273 | 55 |
| {BcPV3=TRUE} =>  {BcOLV16=TRUE} | 0.143564 | 0.495726 | 0.289604 | 1.192104 | 58 |
| {BcPV3=TRUE} =>  {BcMV4=TRUE} | 0.180693 | 0.623932 | 0.289604 | 1.408203 | 73 |

| {BcMV4=TRUE} =>  {BcPV3=TRUE} | 0.180693 | 0.407821 | 0.443069 | 1.408203 | 73 |
| --- | --- | --- | --- | --- | --- |
| {BcPV3=TRUE} =>  {BcMV2=TRUE} | 0.178218 | 0.615385 | 0.289604 | 1.329494 | 72 |
| {BcMV2=TRUE} =>  {BcPV3=TRUE} | 0.178218 | 0.385027 | 0.462871 | 1.329494 | 72 |
| {BcPV3=TRUE} =>  {BcOLV18=TRUE} | 0.188119 | 0.649573 | 0.289604 | 1.255633 | 76 |
| {BcOLV18=TRUE} =>  {BcPV3=TRUE} | 0.188119 | 0.363636 | 0.517327 | 1.255633 | 76 |
| {BcPV3=TRUE} =>  {BcMV3=TRUE} | 0.180693 | 0.623932 | 0.289604 | 1.150997 | 73 |
| {BcPV3=TRUE} =>  {BcHV1=TRUE} | 0.200495 | 0.692308 | 0.289604 | 1.14628 | 81 |
| {BcPV3=TRUE} =>  {BcMV11=TRUE} | 0.19802 | 0.683761 | 0.289604 | 1.109395 | 80 |
| {BcPV3=TRUE} =>  {BcMV1=TRUE} | 0.25495 | 0.880342 | 0.289604 | 1.114916 | 103 |
| {BcMV6=TRUE} =>  {BcOLV1=TRUE} | 0.163366 | 0.536585 | 0.304455 | 1.617765 | 66 |
| {BcOLV1=TRUE} =>  {BcMV6=TRUE} | 0.163366 | 0.492537 | 0.331683 | 1.617765 | 66 |
| {BcMV6=TRUE} =>  {BcOLV16=TRUE} | 0.165842 | 0.544715 | 0.304455 | 1.309911 | 67 |
| {BcOLV16=TRUE} =>  {BcMV6=TRUE} | 0.165842 | 0.39881 | 0.415842 | 1.309911 | 67 |
| {BcMV6=TRUE} =>  {BcMV4=TRUE} | 0.175743 | 0.577236 | 0.304455 | 1.302811 | 71 |
| {BcMV4=TRUE} =>  {BcMV6=TRUE} | 0.175743 | 0.396648 | 0.443069 | 1.302811 | 71 |
| {BcMV6=TRUE} =>  {BcMV2=TRUE} | 0.207921 | 0.682927 | 0.304455 | 1.475414 | 84 |
| {BcMV2=TRUE} =>  {BcMV6=TRUE} | 0.207921 | 0.449198 | 0.462871 | 1.475414 | 84 |
| {BcMV6=TRUE} =>  {BcHV1SLR=TRUE} | 0.205446 | 0.674797 | 0.304455 | 1.244831 | 83 |
| {BcHV1SLR=TRUE}  => {BcMV6=TRUE} | 0.205446 | 0.378995 | 0.542079 | 1.244831 | 83 |
| {BcMV6=TRUE} =>  {BcMV3=TRUE} | 0.222772 | 0.731707 | 0.304455 | 1.349816 | 90 |
| {BcMV3=TRUE} =>  {BcMV6=TRUE} | 0.222772 | 0.410959 | 0.542079 | 1.349816 | 90 |
| {BcMV6=TRUE} =>  {BcULV3=TRUE} | 0.215347 | 0.707317 | 0.304455 | 1.19065 | 87 |

| {BcULV3=TRUE} =>  {BcMV6=TRUE} | 0.215347 | 0.3625 | 0.594059 | 1.19065 | 87 |
| --- | --- | --- | --- | --- | --- |
| {BcMV6=TRUE} =>  {BcHV1=TRUE} | 0.227723 | 0.747967 | 0.304455 | 1.238438 | 92 |
| {BcHV1=TRUE} =>  {BcMV6=TRUE} | 0.227723 | 0.377049 | 0.60396 | 1.238438 | 92 |
| {BcMV6=TRUE} =>  {BcMV11=TRUE} | 0.227723 | 0.747967 | 0.304455 | 1.21357 | 92 |
| {BcMV11=TRUE} =>  {BcMV6=TRUE} | 0.227723 | 0.369478 | 0.616337 | 1.21357 | 92 |
| {BcMV6=TRUE} =>  {BcMV9=TRUE} | 0.235149 | 0.772358 | 0.304455 | 1.138805 | 95 |
| {BcMV6=TRUE} =>  {BcULV4=TRUE} | 0.242574 | 0.796748 | 0.304455 | 1.153714 | 98 |
| {BcULV4=TRUE} =>  {BcMV6=TRUE} | 0.242574 | 0.351254 | 0.690594 | 1.153714 | 98 |
| {BcULV4=FALSE} =>  {BcMV9=FALSE} | 0.178218 | 0.576 | 0.309406 | 1.790031 | 72 |
| {BcMV9=FALSE} =>  {BcULV4=FALSE} | 0.178218 | 0.553846 | 0.321782 | 1.790031 | 72 |
| {BcULV4=FALSE} =>  {BcHV1=FALSE} | 0.160891 | 0.52 | 0.309406 | 1.313 | 65 |
| {BcHV1=FALSE} =>  {BcULV4=FALSE} | 0.160891 | 0.40625 | 0.39604 | 1.313 | 65 |
| {BcULV4=FALSE} =>  {BcHV1SLR=FALSE} | 0.168317 | 0.544 | 0.309406 | 1.187978 | 68 |
| {BcHV1SLR=FALSE}  => {BcULV4=FALSE} | 0.168317 | 0.367568 | 0.457921 | 1.187978 | 68 |
| {BcULV4=FALSE} =>  {BcMV3=FALSE} | 0.168317 | 0.544 | 0.309406 | 1.187978 | 68 |
| {BcMV3=FALSE} =>  {BcULV4=FALSE} | 0.168317 | 0.367568 | 0.457921 | 1.187978 | 68 |
| {BcULV4=FALSE} =>  {BcOLV18=FALSE} | 0.232673 | 0.752 | 0.309406 | 1.55799 | 94 |
| {BcOLV18=FALSE}  => {BcULV4=FALSE} | 0.232673 | 0.482051 | 0.482673 | 1.55799 | 94 |
| {BcULV4=FALSE} =>  {BcMV2=FALSE} | 0.188119 | 0.608 | 0.309406 | 1.131945 | 76 |
| {BcULV4=FALSE} =>  {BcOLV16=FALSE} | 0.232673 | 0.752 | 0.309406 | 1.287322 | 94 |
| {BcOLV16=FALSE}  => {BcULV4=FALSE} | 0.232673 | 0.398305 | 0.584158 | 1.287322 | 94 |
| {BcULV4=FALSE} =>  {BcMV6=FALSE} | 0.247525 | 0.8 | 0.309406 | 1.150178 | 100 |

| {BcMV6=FALSE} =>  {BcULV4=FALSE} | 0.247525 | 0.355872 | 0.695545 | 1.150178 | 100 |
| --- | --- | --- | --- | --- | --- |
| {BcULV4=FALSE} =>  {BcRV1=FALSE} | 0.274752 | 0.888 | 0.309406 | 1.228603 | 111 |
| {BcRV1=FALSE} =>  {BcULV4=FALSE} | 0.274752 | 0.380137 | 0.722772 | 1.228603 | 111 |
| {BcULV4=FALSE} =>  {BcULV1=FALSE} | 0.287129 | 0.928 | 0.309406 | 1.182688 | 116 |
| {BcULV1=FALSE} =>  {BcULV4=FALSE} | 0.287129 | 0.365931 | 0.784653 | 1.182688 | 116 |
| {BcULV4=FALSE} =>  {BcOLV7=FALSE} | 0.294554 | 0.952 | 0.309406 | 1.179779 | 119 |
| {BcOLV7=FALSE} =>  {BcULV4=FALSE} | 0.294554 | 0.365031 | 0.806931 | 1.179779 | 119 |
| {BcULV4=FALSE} =>  {BcALV1=FALSE} | 0.284653 | 0.92 | 0.309406 | 1.129726 | 115 |
| {BcALV1=FALSE} =>  {BcULV4=FALSE} | 0.284653 | 0.349544 | 0.814356 | 1.129726 | 115 |
| {BcMV9=FALSE} =>  {BcHV1=FALSE} | 0.185644 | 0.576923 | 0.321782 | 1.456731 | 75 |
| {BcHV1=FALSE} =>  {BcMV9=FALSE} | 0.185644 | 0.46875 | 0.39604 | 1.456731 | 75 |
| {BcMV9=FALSE} =>  {BcHV1SLR=FALSE} | 0.188119 | 0.584615 | 0.321782 | 1.276674 | 76 |
| {BcHV1SLR=FALSE}  => {BcMV9=FALSE} | 0.188119 | 0.410811 | 0.457921 | 1.276674 | 76 |
| {BcMV9=FALSE} =>  {BcMV3=FALSE} | 0.175743 | 0.546154 | 0.321782 | 1.192682 | 71 |
| {BcMV3=FALSE} =>  {BcMV9=FALSE} | 0.175743 | 0.383784 | 0.457921 | 1.192682 | 71 |
| {BcMV9=FALSE} =>  {BcOLV18=FALSE} | 0.217822 | 0.676923 | 0.321782 | 1.402446 | 88 |
| {BcOLV18=FALSE}  => {BcMV9=FALSE} | 0.217822 | 0.451282 | 0.482673 | 1.402446 | 88 |
| {BcMV9=FALSE} =>  {BcMV4=FALSE} | 0.215347 | 0.669231 | 0.321782 | 1.201641 | 87 |
| {BcMV4=FALSE} =>  {BcMV9=FALSE} | 0.215347 | 0.386667 | 0.556931 | 1.201641 | 87 |
| {BcMV9=FALSE} =>  {BcOLV16=FALSE} | 0.237624 | 0.738462 | 0.321782 | 1.264146 | 96 |
| {BcOLV16=FALSE}  => {BcMV9=FALSE} | 0.237624 | 0.40678 | 0.584158 | 1.264146 | 96 |
| {BcMV9=FALSE} =>  {BcMV6=FALSE} | 0.252475 | 0.784615 | 0.321782 | 1.128059 | 102 |

| {BcMV6=FALSE} =>  {BcMV9=FALSE} | 0.252475 | 0.362989 | 0.695545 | 1.128059 | 102 |
| --- | --- | --- | --- | --- | --- |
| {BcMV9=FALSE} =>  {BcRV1=FALSE} | 0.267327 | 0.830769 | 0.321782 | 1.14942 | 108 |
| {BcRV1=FALSE} =>  {BcMV9=FALSE} | 0.267327 | 0.369863 | 0.722772 | 1.14942 | 108 |
| {BcMV9=FALSE} =>  {BcULV1=FALSE} | 0.284653 | 0.884615 | 0.321782 | 1.127396 | 115 |
| {BcULV1=FALSE} =>  {BcMV9=FALSE} | 0.284653 | 0.362776 | 0.784653 | 1.127396 | 115 |
| {BcMV9=FALSE} =>  {BcNSRV7=FALSE} | 0.282178 | 0.876923 | 0.321782 | 1.096833 | 114 |
| {BcMV9=FALSE} =>  {BcOLV7=FALSE} | 0.299505 | 0.930769 | 0.321782 | 1.153469 | 121 |
| {BcOLV7=FALSE} =>  {BcMV9=FALSE} | 0.299505 | 0.371166 | 0.806931 | 1.153469 | 121 |
| {BcMV9=FALSE} =>  {BcDMV1=FALSE} | 0.292079 | 0.907692 | 0.321782 | 1.075389 | 118 |
| {BcMV9=FALSE} =>  {BcBNV5=FALSE} | 0.311881 | 0.969231 | 0.321782 | 1.066946 | 126 |
| {BcOLV1=TRUE} =>  {BcOLV16=TRUE} | 0.190594 | 0.574627 | 0.331683 | 1.381841 | 77 |
| {BcOLV16=TRUE} =>  {BcOLV1=TRUE} | 0.190594 | 0.458333 | 0.415842 | 1.381841 | 77 |
| {BcOLV1=TRUE} =>  {BcMV4=TRUE} | 0.185644 | 0.559701 | 0.331683 | 1.263237 | 75 |
| {BcMV4=TRUE} =>  {BcOLV1=TRUE} | 0.185644 | 0.418994 | 0.443069 | 1.263237 | 75 |
| {BcOLV1=TRUE} =>  {BcMV2=TRUE} | 0.25 | 0.753731 | 0.331683 | 1.628382 | 101 |
| {BcMV2=TRUE} =>  {BcOLV1=TRUE} | 0.25 | 0.540107 | 0.462871 | 1.628382 | 101 |
| {BcOLV1=TRUE} =>  {BcOLV18=TRUE} | 0.230198 | 0.69403 | 0.331683 | 1.34157 | 93 |
| {BcOLV18=TRUE} =>  {BcOLV1=TRUE} | 0.230198 | 0.444976 | 0.517327 | 1.34157 | 93 |
| {BcOLV1=TRUE} =>  {BcMV3=TRUE} | 0.24505 | 0.738806 | 0.331683 | 1.362911 | 99 |
| {BcMV3=TRUE} =>  {BcOLV1=TRUE} | 0.24505 | 0.452055 | 0.542079 | 1.362911 | 99 |
| {BcOLV1=TRUE} =>  {BcMV11=TRUE} | 0.242574 | 0.731343 | 0.331683 | 1.186597 | 98 |
| {BcMV11=TRUE} =>  {BcOLV1=TRUE} | 0.242574 | 0.393574 | 0.616337 | 1.186597 | 98 |

| {BcOLV1=TRUE} =>  {BcMV1=TRUE} | 0.294554 | 0.88806 | 0.331683 | 1.12469 | 119 |
| --- | --- | --- | --- | --- | --- |
| {BcMV1=TRUE} =>  {BcOLV1=TRUE} | 0.294554 | 0.373041 | 0.789604 | 1.12469 | 119 |
| {BcMV11=FALSE} =>  {BcULV3=FALSE} | 0.185644 | 0.483871 | 0.383663 | 1.191975 | 75 |
| {BcULV3=FALSE} =>  {BcMV11=FALSE} | 0.185644 | 0.457317 | 0.405941 | 1.191975 | 75 |
| {BcMV11=FALSE} =>  {BcMV3=FALSE} | 0.225248 | 0.587097 | 0.383663 | 1.282092 | 91 |
| {BcMV3=FALSE} =>  {BcMV11=FALSE} | 0.225248 | 0.491892 | 0.457921 | 1.282092 | 91 |
| {BcMV11=FALSE} =>  {BcOLV18=FALSE} | 0.212871 | 0.554839 | 0.383663 | 1.149512 | 86 |
| {BcMV11=FALSE} =>  {BcMV2=FALSE} | 0.262376 | 0.683871 | 0.383663 | 1.273198 | 106 |
| {BcMV2=FALSE} =>  {BcMV11=FALSE} | 0.262376 | 0.488479 | 0.537129 | 1.273198 | 106 |
| {BcMV11=FALSE} =>  {BcMV4=FALSE} | 0.279703 | 0.729032 | 0.383663 | 1.309018 | 113 |
| {BcMV4=FALSE} =>  {BcMV11=FALSE} | 0.279703 | 0.502222 | 0.556931 | 1.309018 | 113 |
| {BcMV11=FALSE} =>  {BcOLV1=FALSE} | 0.294554 | 0.767742 | 0.383663 | 1.148769 | 119 |
| {BcOLV1=FALSE} =>  {BcMV11=FALSE} | 0.294554 | 0.440741 | 0.668317 | 1.148769 | 119 |
| {BcMV11=FALSE} =>  {BcMV6=FALSE} | 0.306931 | 0.8 | 0.383663 | 1.150178 | 124 |
| {BcMV6=FALSE} =>  {BcMV11=FALSE} | 0.306931 | 0.441281 | 0.695545 | 1.150178 | 124 |
| {BcMV11=FALSE} =>  {BcRV1=FALSE} | 0.314356 | 0.819355 | 0.383663 | 1.133628 | 127 |
| {BcRV1=FALSE} =>  {BcMV11=FALSE} | 0.314356 | 0.434932 | 0.722772 | 1.133628 | 127 |
| {BcMV11=FALSE} =>  {BcEV2=FALSE} | 0.316832 | 0.825806 | 0.383663 | 1.104721 | 128 |
| {BcMV11=FALSE} =>  {BcHV3=FALSE} | 0.316832 | 0.825806 | 0.383663 | 1.086729 | 128 |
| {BcMV11=FALSE} =>  {BcALV1=FALSE} | 0.35396 | 0.922581 | 0.383663 | 1.132895 | 143 |
| {BcALV1=FALSE} =>  {BcMV11=FALSE} | 0.35396 | 0.43465 | 0.814356 | 1.132895 | 143 |
| {BcMV11=FALSE} =>  {BcOLV4=FALSE} | 0.363861 | 0.948387 | 0.383663 | 1.08234 | 147 |

| {BcMV11=FALSE} =>  {BcULV5=FALSE} | 0.371287 | 0.967742 | 0.383663 | 1.104429 | 150 |
| --- | --- | --- | --- | --- | --- |
| {BcULV5=FALSE} =>  {BcMV11=FALSE} | 0.371287 | 0.423729 | 0.876238 | 1.104429 | 150 |
| {BcMV11=FALSE} =>  {BcBNV5=FALSE} | 0.371287 | 0.967742 | 0.383663 | 1.065307 | 150 |
| {BcHV1=FALSE} =>  {BcULV3=FALSE} | 0.225248 | 0.56875 | 0.39604 | 1.401067 | 91 |
| {BcULV3=FALSE} =>  {BcHV1=FALSE} | 0.225248 | 0.554878 | 0.405941 | 1.401067 | 91 |
| {BcHV1=FALSE} =>  {BcHV1SLR=FALSE} | 0.339109 | 0.85625 | 0.39604 | 1.869865 | 137 |
| {BcHV1SLR=FALSE}  => {BcHV1=FALSE} | 0.339109 | 0.740541 | 0.457921 | 1.869865 | 137 |
| {BcHV1=FALSE} =>  {BcOLV18=FALSE} | 0.24505 | 0.61875 | 0.39604 | 1.281923 | 99 |
| {BcOLV18=FALSE}  => {BcHV1=FALSE} | 0.24505 | 0.507692 | 0.482673 | 1.281923 | 99 |
| {BcHV1=FALSE} =>  {BcMV4=FALSE} | 0.252475 | 0.6375 | 0.39604 | 1.144667 | 102 |
| {BcMV4=FALSE} =>  {BcHV1=FALSE} | 0.252475 | 0.453333 | 0.556931 | 1.144667 | 102 |
| {BcHV1=FALSE} =>  {BcMV6=FALSE} | 0.319307 | 0.80625 | 0.39604 | 1.159164 | 129 |
| {BcMV6=FALSE} =>  {BcHV1=FALSE} | 0.319307 | 0.459075 | 0.695545 | 1.159164 | 129 |
| {BcHV1=FALSE} =>  {BcPV3=FALSE} | 0.306931 | 0.775 | 0.39604 | 1.090941 | 124 |
| {BcHV1=FALSE} =>  {BcULV1=FALSE} | 0.356436 | 0.9 | 0.39604 | 1.147003 | 144 |
| {BcULV1=FALSE} =>  {BcHV1=FALSE} | 0.356436 | 0.454259 | 0.784653 | 1.147003 | 144 |
| {BcHV1=FALSE} =>  {BcALV1=FALSE} | 0.34901 | 0.88125 | 0.39604 | 1.082143 | 141 |
| {BcULV3=FALSE} =>  {BcHV1SLR=FALSE} | 0.25 | 0.615854 | 0.405941 | 1.344891 | 101 |
| {BcHV1SLR=FALSE}  => {BcULV3=FALSE} | 0.25 | 0.545946 | 0.457921 | 1.344891 | 101 |
| {BcULV3=FALSE} =>  {BcMV4=FALSE} | 0.257426 | 0.634146 | 0.405941 | 1.138645 | 104 |
| {BcMV4=FALSE} =>  {BcULV3=FALSE} | 0.257426 | 0.462222 | 0.556931 | 1.138645 | 104 |
| {BcULV3=FALSE} =>  {BcOLV16=FALSE} | 0.269802 | 0.664634 | 0.405941 | 1.137764 | 109 |

| {BcOLV16=FALSE}  => {BcULV3=FALSE} | 0.269802 | 0.461864 | 0.584158 | 1.137764 | 109 |
| --- | --- | --- | --- | --- | --- |
| {BcULV3=FALSE} =>  {BcMV6=FALSE} | 0.316832 | 0.780488 | 0.405941 | 1.122125 | 128 |
| {BcMV6=FALSE} =>  {BcULV3=FALSE} | 0.316832 | 0.455516 | 0.695545 | 1.122125 | 128 |
| {BcULV3=FALSE} =>  {BcHV3=FALSE} | 0.336634 | 0.829268 | 0.405941 | 1.091285 | 136 |
| {BcULV3=FALSE} =>  {BcFV5=FALSE} | 0.351485 | 0.865854 | 0.405941 | 1.106977 | 142 |
| {BcFV5=FALSE} =>  {BcULV3=FALSE} | 0.351485 | 0.449367 | 0.782178 | 1.106977 | 142 |
| {BcULV3=FALSE} =>  {BcNSRV7=FALSE} | 0.361386 | 0.890244 | 0.405941 | 1.113494 | 146 |
| {BcNSRV7=FALSE}  => {BcULV3=FALSE} | 0.361386 | 0.452012 | 0.799505 | 1.113494 | 146 |
| {BcOLV16=TRUE} =>  {BcMV4=TRUE} | 0.225248 | 0.541667 | 0.415842 | 1.222533 | 91 |
| {BcMV4=TRUE} =>  {BcOLV16=TRUE} | 0.225248 | 0.50838 | 0.443069 | 1.222533 | 91 |
| {BcOLV16=TRUE} =>  {BcMV2=TRUE} | 0.252475 | 0.607143 | 0.415842 | 1.311688 | 102 |
| {BcMV2=TRUE} =>  {BcOLV16=TRUE} | 0.252475 | 0.545455 | 0.462871 | 1.311688 | 102 |
| {BcOLV16=TRUE} =>  {BcOLV18=TRUE} | 0.269802 | 0.64881 | 0.415842 | 1.254158 | 109 |
| {BcOLV18=TRUE} =>  {BcOLV16=TRUE} | 0.269802 | 0.521531 | 0.517327 | 1.254158 | 109 |
| {BcOLV16=TRUE} =>  {BcMV3=TRUE} | 0.267327 | 0.642857 | 0.415842 | 1.18591 | 108 |
| {BcMV3=TRUE} =>  {BcOLV16=TRUE} | 0.267327 | 0.493151 | 0.542079 | 1.18591 | 108 |
| {BcOLV16=TRUE} =>  {BcULV3=TRUE} | 0.279703 | 0.672619 | 0.415842 | 1.132242 | 113 |
| {BcULV3=TRUE} =>  {BcOLV16=TRUE} | 0.279703 | 0.470833 | 0.594059 | 1.132242 | 113 |
| {BcOLV16=TRUE} =>  {BcMV9=TRUE} | 0.331683 | 0.797619 | 0.415842 | 1.176051 | 134 |
| {BcMV9=TRUE} =>  {BcOLV16=TRUE} | 0.331683 | 0.489051 | 0.678218 | 1.176051 | 134 |
| {BcOLV16=TRUE} =>  {BcULV4=TRUE} | 0.339109 | 0.815476 | 0.415842 | 1.180833 | 137 |
| {BcULV4=TRUE} =>  {BcOLV16=TRUE} | 0.339109 | 0.491039 | 0.690594 | 1.180833 | 137 |

| {BcMV4=TRUE} =>  {BcMV2=TRUE} | 0.274752 | 0.620112 | 0.443069 | 1.339707 | 111 |
| --- | --- | --- | --- | --- | --- |
| {BcMV2=TRUE} =>  {BcMV4=TRUE} | 0.274752 | 0.593583 | 0.462871 | 1.339707 | 111 |
| {BcMV4=TRUE} =>  {BcOLV18=TRUE} | 0.277228 | 0.625698 | 0.443069 | 1.209484 | 112 |
| {BcOLV18=TRUE} =>  {BcMV4=TRUE} | 0.277228 | 0.535885 | 0.517327 | 1.209484 | 112 |
| {BcMV4=TRUE} =>  {BcMV3=TRUE} | 0.294554 | 0.664804 | 0.443069 | 1.226397 | 119 |
| {BcMV3=TRUE} =>  {BcMV4=TRUE} | 0.294554 | 0.543379 | 0.542079 | 1.226397 | 119 |
| {BcMV4=TRUE} =>  {BcULV3=TRUE} | 0.294554 | 0.664804 | 0.443069 | 1.119088 | 119 |
| {BcULV3=TRUE} =>  {BcMV4=TRUE} | 0.294554 | 0.495833 | 0.594059 | 1.119088 | 119 |
| {BcMV4=TRUE} =>  {BcHV1=TRUE} | 0.299505 | 0.675978 | 0.443069 | 1.119242 | 121 |
| {BcHV1=TRUE} =>  {BcMV4=TRUE} | 0.299505 | 0.495902 | 0.60396 | 1.119242 | 121 |
| {BcMV4=TRUE} =>  {BcMV11=TRUE} | 0.339109 | 0.765363 | 0.443069 | 1.241794 | 137 |
| {BcMV11=TRUE} =>  {BcMV4=TRUE} | 0.339109 | 0.550201 | 0.616337 | 1.241794 | 137 |
| {BcMV4=TRUE} =>  {BcMV9=TRUE} | 0.336634 | 0.759777 | 0.443069 | 1.120254 | 136 |
| {BcMV9=TRUE} =>  {BcMV4=TRUE} | 0.336634 | 0.49635 | 0.678218 | 1.120254 | 136 |
| {BcMV4=TRUE} =>  {BcMV1=TRUE} | 0.376238 | 0.849162 | 0.443069 | 1.075428 | 152 |
| {BcHV1SLR=FALSE}  => {BcMV3=FALSE} | 0.24505 | 0.535135 | 0.457921 | 1.168619 | 99 |
| {BcMV3=FALSE} =>  {BcHV1SLR=FALSE} | 0.24505 | 0.535135 | 0.457921 | 1.168619 | 99 |
| {BcHV1SLR=FALSE}  =>  {BcOLV18=FALSE} | 0.282178 | 0.616216 | 0.457921 | 1.276674 | 114 |
| {BcOLV18=FALSE}  =>  {BcHV1SLR=FALSE} | 0.282178 | 0.584615 | 0.482673 | 1.276674 | 114 |
| {BcHV1SLR=FALSE}  => {BcMV6=FALSE} | 0.358911 | 0.783784 | 0.457921 | 1.126864 | 145 |
| {BcMV6=FALSE} =>  {BcHV1SLR=FALSE} | 0.358911 | 0.516014 | 0.695545 | 1.126864 | 145 |

| {BcHV1SLR=FALSE}  => {BcFV5=FALSE} | 0.391089 | 0.854054 | 0.457921 | 1.091892 | 158 |
| --- | --- | --- | --- | --- | --- |
| {BcFV5=FALSE} =>  {BcHV1SLR=FALSE} | 0.391089 | 0.5 | 0.782178 | 1.091892 | 158 |
| {BcHV1SLR=FALSE}  => {BcULV1=FALSE} | 0.40099 | 0.875676 | 0.457921 | 1.116003 | 162 |
| {BcULV1=FALSE} =>  {BcHV1SLR=FALSE} | 0.40099 | 0.511041 | 0.784653 | 1.116003 | 162 |
| {BcHV1SLR=FALSE}  =>  {BcNSRV7=FALSE} | 0.391089 | 0.854054 | 0.457921 | 1.068229 | 158 |
| {BcHV1SLR=FALSE}  => {BcALV1=FALSE} | 0.398515 | 0.87027 | 0.457921 | 1.06866 | 161 |
| {BcHV1SLR=FALSE}  => {BcFV9=FALSE} | 0.418317 | 0.913514 | 0.457921 | 1.051451 | 169 |
| {BcMV3=FALSE} =>  {BcOLV18=FALSE} | 0.269802 | 0.589189 | 0.457921 | 1.220679 | 109 |
| {BcOLV18=FALSE}  => {BcMV3=FALSE} | 0.269802 | 0.558974 | 0.482673 | 1.220679 | 109 |
| {BcMV3=FALSE} =>  {BcMV2=FALSE} | 0.309406 | 0.675676 | 0.457921 | 1.25794 | 125 |
| {BcMV2=FALSE} =>  {BcMV3=FALSE} | 0.309406 | 0.576037 | 0.537129 | 1.25794 | 125 |
| {BcMV3=FALSE} =>  {BcMV4=FALSE} | 0.309406 | 0.675676 | 0.457921 | 1.213213 | 125 |
| {BcMV4=FALSE} =>  {BcMV3=FALSE} | 0.309406 | 0.555556 | 0.556931 | 1.213213 | 125 |
| {BcMV3=FALSE} =>  {BcOLV16=FALSE} | 0.309406 | 0.675676 | 0.457921 | 1.156665 | 125 |
| {BcOLV16=FALSE}  => {BcMV3=FALSE} | 0.309406 | 0.529661 | 0.584158 | 1.156665 | 125 |
| {BcMV3=FALSE} =>  {BcOLV1=FALSE} | 0.371287 | 0.810811 | 0.457921 | 1.213213 | 150 |
| {BcOLV1=FALSE} =>  {BcMV3=FALSE} | 0.371287 | 0.555556 | 0.668317 | 1.213213 | 150 |
| {BcMV3=FALSE} =>  {BcMV6=FALSE} | 0.376238 | 0.821622 | 0.457921 | 1.181264 | 152 |
| {BcMV6=FALSE} =>  {BcMV3=FALSE} | 0.376238 | 0.540925 | 0.695545 | 1.181264 | 152 |
| {BcMV3=FALSE} =>  {BcFV5=FALSE} | 0.393564 | 0.859459 | 0.457921 | 1.098803 | 159 |
| {BcFV5=FALSE} =>  {BcMV3=FALSE} | 0.393564 | 0.503165 | 0.782178 | 1.098803 | 159 |
| {BcMV3=FALSE} => | 0.393564 | 0.859459 | 0.457921 | 1.095336 | 159 |

| {BcULV1=FALSE} |  |  |  |  |  |
| --- | --- | --- | --- | --- | --- |
| {BcULV1=FALSE} =>  {BcMV3=FALSE} | 0.393564 | 0.501577 | 0.784653 | 1.095336 | 159 |
| {BcMV3=FALSE} =>  {BcMV12=FALSE} | 0.410891 | 0.897297 | 0.457921 | 1.122316 | 166 |
| {BcMV12=FALSE} =>  {BcMV3=FALSE} | 0.410891 | 0.513932 | 0.799505 | 1.122316 | 166 |
| {BcMV3=FALSE} =>  {BcOLV7=FALSE} | 0.391089 | 0.854054 | 0.457921 | 1.058398 | 158 |
| {BcMV3=FALSE} =>  {BcDMV1=FALSE} | 0.420792 | 0.918919 | 0.457921 | 1.08869 | 170 |
| {BcDMV1=FALSE} =>  {BcMV3=FALSE} | 0.420792 | 0.498534 | 0.844059 | 1.08869 | 170 |
| {BcMV3=FALSE} =>  {BcOLV4=FALSE} | 0.420792 | 0.918919 | 0.457921 | 1.04871 | 170 |
| {BcMV3=FALSE} =>  {BcULV5=FALSE} | 0.423267 | 0.924324 | 0.457921 | 1.054879 | 171 |
| {BcOLV13=FALSE}  => {BcMV3=FALSE} | 0.450495 | 0.486631 | 0.925743 | 1.062697 | 182 |
| {BcMV3=FALSE} =>  {BcMCV3=FALSE} | 0.457921 | 1 | 0.457921 | 1.071618 | 185 |
| {BcMCV3=FALSE} =>  {BcMV3=FALSE} | 0.457921 | 0.490716 | 0.933168 | 1.071618 | 185 |
| {BcMV2=TRUE} =>  {BcOLV18=TRUE} | 0.294554 | 0.636364 | 0.462871 | 1.2301 | 119 |
| {BcOLV18=TRUE} =>  {BcMV2=TRUE} | 0.294554 | 0.569378 | 0.517327 | 1.2301 | 119 |
| {BcMV2=TRUE} =>  {BcMV3=TRUE} | 0.314356 | 0.679144 | 0.462871 | 1.252851 | 127 |
| {BcMV3=TRUE} =>  {BcMV2=TRUE} | 0.314356 | 0.579909 | 0.542079 | 1.252851 | 127 |
| {BcMV2=TRUE} =>  {BcMV11=TRUE} | 0.341584 | 0.737968 | 0.462871 | 1.197346 | 138 |
| {BcMV11=TRUE} =>  {BcMV2=TRUE} | 0.341584 | 0.554217 | 0.616337 | 1.197346 | 138 |
| {BcMV2=TRUE} =>  {BcMV1=TRUE} | 0.408416 | 0.882353 | 0.462871 | 1.117463 | 165 |
| {BcMV1=TRUE} =>  {BcMV2=TRUE} | 0.408416 | 0.517241 | 0.789604 | 1.117463 | 165 |
| {BcOLV18=FALSE}  => {BcMV2=FALSE} | 0.314356 | 0.651282 | 0.482673 | 1.212525 | 127 |
| {BcMV2=FALSE} =>  {BcOLV18=FALSE} | 0.314356 | 0.585253 | 0.537129 | 1.212525 | 127 |

| {BcOLV18=FALSE}  => {BcMV4=FALSE} | 0.316832 | 0.65641 | 0.482673 | 1.178621 | 128 |
| --- | --- | --- | --- | --- | --- |
| {BcMV4=FALSE} =>  {BcOLV18=FALSE} | 0.316832 | 0.568889 | 0.556931 | 1.178621 | 128 |
| {BcOLV18=FALSE}  =>  {BcOLV16=FALSE} | 0.336634 | 0.697436 | 0.482673 | 1.193916 | 136 |
| {BcOLV16=FALSE}  =>  {BcOLV18=FALSE} | 0.336634 | 0.576271 | 0.584158 | 1.193916 | 136 |
| {BcOLV18=FALSE}  => {BcOLV1=FALSE} | 0.381188 | 0.789744 | 0.482673 | 1.18169 | 154 |
| {BcOLV1=FALSE} =>  {BcOLV18=FALSE} | 0.381188 | 0.57037 | 0.668317 | 1.18169 | 154 |
| {BcOLV18=FALSE}  => {BcPV3=FALSE} | 0.381188 | 0.789744 | 0.482673 | 1.111695 | 154 |
| {BcPV3=FALSE} =>  {BcOLV18=FALSE} | 0.381188 | 0.536585 | 0.710396 | 1.111695 | 154 |
| {BcOLV18=FALSE}  => {BcRV1=FALSE} | 0.393564 | 0.815385 | 0.482673 | 1.128135 | 159 |
| {BcRV1=FALSE} =>  {BcOLV18=FALSE} | 0.393564 | 0.544521 | 0.722772 | 1.128135 | 159 |
| {BcOLV18=FALSE}  => {BcHV3=FALSE} | 0.408416 | 0.846154 | 0.482673 | 1.113505 | 165 |
| {BcHV3=FALSE} =>  {BcOLV18=FALSE} | 0.408416 | 0.537459 | 0.759901 | 1.113505 | 165 |
| {BcOLV18=FALSE}  => {BcFV5=FALSE} | 0.413366 | 0.85641 | 0.482673 | 1.094904 | 167 |
| {BcFV5=FALSE} =>  {BcOLV18=FALSE} | 0.413366 | 0.528481 | 0.782178 | 1.094904 | 167 |
| {BcOLV18=FALSE}  => {BcULV1=FALSE} | 0.428218 | 0.887179 | 0.482673 | 1.130664 | 173 |
| {BcULV1=FALSE} =>  {BcOLV18=FALSE} | 0.428218 | 0.545741 | 0.784653 | 1.130664 | 173 |
| {BcOLV18=FALSE}  =>  {BcNSRV7=FALSE} | 0.410891 | 0.851282 | 0.482673 | 1.064761 | 166 |
| {BcOLV18=FALSE}  => {BcOLV7=FALSE} | 0.430693 | 0.892308 | 0.482673 | 1.105805 | 174 |
| {BcOLV7=FALSE} =>  {BcOLV18=FALSE} | 0.430693 | 0.533742 | 0.806931 | 1.105805 | 174 |
| {BcOLV18=FALSE}  => {BcALV1=FALSE} | 0.438119 | 0.907692 | 0.482673 | 1.114613 | 177 |
| {BcALV1=FALSE} => | 0.438119 | 0.537994 | 0.814356 | 1.114613 | 177 |

| {BcOLV18=FALSE} |  |  |  |  |  |
| --- | --- | --- | --- | --- | --- |
| {BcOLV18=FALSE}  => {BcFV9=FALSE} | 0.44802 | 0.928205 | 0.482673 | 1.068361 | 181 |
| {BcFV9=FALSE} =>  {BcOLV18=FALSE} | 0.44802 | 0.51567 | 0.868812 | 1.068361 | 181 |
| {BcOLV18=FALSE}  => {BcMCV5=FALSE} | 0.472772 | 0.979487 | 0.482673 | 1.060892 | 191 |
| {BcMCV5=FALSE} =>  {BcOLV18=FALSE} | 0.472772 | 0.512064 | 0.923267 | 1.060892 | 191 |
| {BcOLV18=TRUE} =>  {BcHV1SLR=TRUE} | 0.341584 | 0.660287 | 0.517327 | 1.218064 | 138 |
| {BcHV1SLR=TRUE}  => {BcOLV18=TRUE} | 0.341584 | 0.630137 | 0.542079 | 1.218064 | 138 |
| {BcOLV18=TRUE} =>  {BcMV3=TRUE} | 0.329208 | 0.636364 | 0.517327 | 1.173931 | 133 |
| {BcMV3=TRUE} =>  {BcOLV18=TRUE} | 0.329208 | 0.607306 | 0.542079 | 1.173931 | 133 |
| {BcOLV18=TRUE} =>  {BcHV1=TRUE} | 0.366337 | 0.708134 | 0.517327 | 1.172484 | 148 |
| {BcHV1=TRUE} =>  {BcOLV18=TRUE} | 0.366337 | 0.606557 | 0.60396 | 1.172484 | 148 |
| {BcOLV18=TRUE} =>  {BcMV11=TRUE} | 0.346535 | 0.669856 | 0.517327 | 1.086835 | 140 |
| {BcOLV18=TRUE} =>  {BcMV9=TRUE} | 0.413366 | 0.799043 | 0.517327 | 1.178151 | 167 |
| {BcMV9=TRUE} =>  {BcOLV18=TRUE} | 0.413366 | 0.609489 | 0.678218 | 1.178151 | 167 |
| {BcOLV18=TRUE} =>  {BcULV4=TRUE} | 0.440594 | 0.851675 | 0.517327 | 1.233249 | 178 |
| {BcULV4=TRUE} =>  {BcOLV18=TRUE} | 0.440594 | 0.637993 | 0.690594 | 1.233249 | 178 |
| {BcOLV18=TRUE} =>  {BcMV1=TRUE} | 0.440594 | 0.851675 | 0.517327 | 1.07861 | 178 |
| {BcMV1=TRUE} =>  {BcOLV18=TRUE} | 0.440594 | 0.557994 | 0.789604 | 1.07861 | 178 |
| {BcMV2=FALSE} =>  {BcMV4=FALSE} | 0.368812 | 0.686636 | 0.537129 | 1.232893 | 149 |
| {BcMV4=FALSE} =>  {BcMV2=FALSE} | 0.368812 | 0.662222 | 0.556931 | 1.232893 | 149 |
| {BcMV2=FALSE} =>  {BcOLV16=FALSE} | 0.373762 | 0.695853 | 0.537129 | 1.191205 | 151 |
| {BcOLV16=FALSE}  => {BcMV2=FALSE} | 0.373762 | 0.639831 | 0.584158 | 1.191205 | 151 |

| {BcMV2=FALSE} =>  {BcOLV1=FALSE} | 0.455446 | 0.847926 | 0.537129 | 1.268749 | 184 |
| --- | --- | --- | --- | --- | --- |
| {BcOLV1=FALSE} =>  {BcMV2=FALSE} | 0.455446 | 0.681481 | 0.668317 | 1.268749 | 184 |
| {BcMV2=FALSE} =>  {BcMV6=FALSE} | 0.440594 | 0.820276 | 0.537129 | 1.17933 | 178 |
| {BcMV6=FALSE} =>  {BcMV2=FALSE} | 0.440594 | 0.633452 | 0.695545 | 1.17933 | 178 |
| {BcMV2=FALSE} =>  {BcPV3=FALSE} | 0.425743 | 0.792627 | 0.537129 | 1.115753 | 172 |
| {BcPV3=FALSE} =>  {BcMV2=FALSE} | 0.425743 | 0.599303 | 0.710396 | 1.115753 | 172 |
| {BcMV2=FALSE} =>  {BcRV1=FALSE} | 0.438119 | 0.815668 | 0.537129 | 1.128527 | 177 |
| {BcRV1=FALSE} =>  {BcMV2=FALSE} | 0.438119 | 0.606164 | 0.722772 | 1.128527 | 177 |
| {BcMV2=FALSE} =>  {BcEV2=FALSE} | 0.428218 | 0.797235 | 0.537129 | 1.0665 | 173 |
| {BcMV2=FALSE} =>  {BcULV1=FALSE} | 0.460396 | 0.857143 | 0.537129 | 1.092384 | 186 |
| {BcULV1=FALSE} =>  {BcMV2=FALSE} | 0.460396 | 0.586751 | 0.784653 | 1.092384 | 186 |
| {BcMV2=FALSE} =>  {BcMV12=FALSE} | 0.457921 | 0.852535 | 0.537129 | 1.066328 | 185 |
| {BcMV12=FALSE} =>  {BcMV2=FALSE} | 0.457921 | 0.572755 | 0.799505 | 1.066328 | 185 |
| {BcMV2=FALSE} =>  {BcALV1=FALSE} | 0.490099 | 0.912442 | 0.537129 | 1.120446 | 198 |
| {BcALV1=FALSE} =>  {BcMV2=FALSE} | 0.490099 | 0.601824 | 0.814356 | 1.120446 | 198 |
| {BcMV2=FALSE} =>  {BcOLV4=FALSE} | 0.5 | 0.930876 | 0.537129 | 1.062355 | 202 |
| {BcOLV4=FALSE} =>  {BcMV2=FALSE} | 0.5 | 0.570621 | 0.876238 | 1.062355 | 202 |
| {BcMV2=FALSE} =>  {BcULV5=FALSE} | 0.5 | 0.930876 | 0.537129 | 1.062355 | 202 |
| {BcULV5=FALSE} =>  {BcMV2=FALSE} | 0.5 | 0.570621 | 0.876238 | 1.062355 | 202 |
| {BcMCV5=FALSE} =>  {BcMV2=FALSE} | 0.522277 | 0.565684 | 0.923267 | 1.053162 | 211 |
| {BcHV1SLR=TRUE}  => {BcMV3=TRUE} | 0.329208 | 0.607306 | 0.542079 | 1.120327 | 133 |
| {BcMV3=TRUE} =>  {BcHV1SLR=TRUE} | 0.329208 | 0.607306 | 0.542079 | 1.120327 | 133 |

| {BcHV1SLR=TRUE}  => {BcULV3=TRUE} | 0.386139 | 0.712329 | 0.542079 | 1.199087 | 156 |
| --- | --- | --- | --- | --- | --- |
| {BcULV3=TRUE} =>  {BcHV1SLR=TRUE} | 0.386139 | 0.65 | 0.594059 | 1.199087 | 156 |
| {BcHV1SLR=TRUE}  => {BcHV1=TRUE} | 0.485149 | 0.894977 | 0.542079 | 1.481847 | 196 |
| {BcHV1=TRUE} =>  {BcHV1SLR=TRUE} | 0.485149 | 0.803279 | 0.60396 | 1.481847 | 196 |
| {BcHV1SLR=TRUE}  => {BcMV9=TRUE} | 0.408416 | 0.753425 | 0.542079 | 1.110889 | 165 |
| {BcMV9=TRUE} =>  {BcHV1SLR=TRUE} | 0.408416 | 0.60219 | 0.678218 | 1.110889 | 165 |
| {BcMV3=TRUE} =>  {BcMV11=TRUE} | 0.383663 | 0.707763 | 0.542079 | 1.148338 | 155 |
| {BcMV11=TRUE} =>  {BcMV3=TRUE} | 0.383663 | 0.62249 | 0.616337 | 1.148338 | 155 |
| {BcMV3=TRUE} =>  {BcMV9=TRUE} | 0.39604 | 0.730594 | 0.542079 | 1.077226 | 160 |
| {BcMV3=TRUE} =>  {BcMV1=TRUE} | 0.457921 | 0.844749 | 0.542079 | 1.069839 | 185 |
| {BcMV1=TRUE} =>  {BcMV3=TRUE} | 0.457921 | 0.579937 | 0.789604 | 1.069839 | 185 |
| {BcMV4=FALSE} =>  {BcOLV16=FALSE} | 0.366337 | 0.657778 | 0.556931 | 1.126026 | 148 |
| {BcOLV16=FALSE}  => {BcMV4=FALSE} | 0.366337 | 0.627119 | 0.584158 | 1.126026 | 148 |
| {BcMV4=FALSE} =>  {BcOLV1=FALSE} | 0.410891 | 0.737778 | 0.556931 | 1.103934 | 166 |
| {BcOLV1=FALSE} =>  {BcMV4=FALSE} | 0.410891 | 0.614815 | 0.668317 | 1.103934 | 166 |
| {BcMV4=FALSE} =>  {BcMV6=FALSE} | 0.428218 | 0.768889 | 0.556931 | 1.105449 | 173 |
| {BcMV6=FALSE} =>  {BcMV4=FALSE} | 0.428218 | 0.615658 | 0.695545 | 1.105449 | 173 |
| {BcMV4=FALSE} =>  {BcPV3=FALSE} | 0.44802 | 0.804444 | 0.556931 | 1.132389 | 181 |
| {BcPV3=FALSE} =>  {BcMV4=FALSE} | 0.44802 | 0.630662 | 0.710396 | 1.132389 | 181 |
| {BcMV4=FALSE} =>  {BcRV1=FALSE} | 0.445545 | 0.8 | 0.556931 | 1.106849 | 180 |
| {BcRV1=FALSE} =>  {BcMV4=FALSE} | 0.445545 | 0.616438 | 0.722772 | 1.106849 | 180 |
| {BcMV4=FALSE} =>  {BcHV3=FALSE} | 0.467822 | 0.84 | 0.556931 | 1.105407 | 189 |

| {BcHV3=FALSE} =>  {BcMV4=FALSE} | 0.467822 | 0.615635 | 0.759901 | 1.105407 | 189 |
| --- | --- | --- | --- | --- | --- |
| {BcMV4=FALSE} =>  {BcFV5=FALSE} | 0.460396 | 0.826667 | 0.556931 | 1.056878 | 186 |
| {BcFV5=FALSE} =>  {BcMV4=FALSE} | 0.460396 | 0.588608 | 0.782178 | 1.056878 | 186 |
| {BcMV4=FALSE} =>  {BcVV3=FALSE} | 0.470297 | 0.844444 | 0.556931 | 1.052949 | 190 |
| {BcMV4=FALSE} =>  {BcALV1=FALSE} | 0.5 | 0.897778 | 0.556931 | 1.102438 | 202 |
| {BcALV1=FALSE} =>  {BcMV4=FALSE} | 0.5 | 0.613982 | 0.814356 | 1.102438 | 202 |
| {BcMV4=FALSE} =>  {BcFV9=FALSE} | 0.512376 | 0.92 | 0.556931 | 1.058917 | 207 |
| {BcFV9=FALSE} =>  {BcMV4=FALSE} | 0.512376 | 0.589744 | 0.868812 | 1.058917 | 207 |
| {BcMV4=FALSE} =>  {BcMCV5=FALSE} | 0.544554 | 0.977778 | 0.556931 | 1.059041 | 220 |
| {BcMCV5=FALSE} =>  {BcMV4=FALSE} | 0.544554 | 0.589812 | 0.923267 | 1.059041 | 220 |
| {BcMV4=FALSE} =>  {BcEV5=FALSE} | 0.55198 | 0.991111 | 0.556931 | 1.042731 | 223 |
| {BcOLV16=FALSE}  => {BcOLV1=FALSE} | 0.443069 | 0.758475 | 0.584158 | 1.134903 | 179 |
| {BcOLV1=FALSE} =>  {BcOLV16=FALSE} | 0.443069 | 0.662963 | 0.668317 | 1.134903 | 179 |
| {BcOLV16=FALSE}  => {BcMV6=FALSE} | 0.445545 | 0.762712 | 0.584158 | 1.096568 | 180 |
| {BcMV6=FALSE} =>  {BcOLV16=FALSE} | 0.445545 | 0.640569 | 0.695545 | 1.096568 | 180 |
| {BcOLV16=FALSE}  => {BcRV1=FALSE} | 0.457921 | 0.783898 | 0.584158 | 1.084572 | 185 |
| {BcRV1=FALSE} =>  {BcOLV16=FALSE} | 0.457921 | 0.633562 | 0.722772 | 1.084572 | 185 |
| {BcOLV16=FALSE}  => {BcULV1=FALSE} | 0.514851 | 0.881356 | 0.584158 | 1.123242 | 208 |
| {BcULV1=FALSE} =>  {BcOLV16=FALSE} | 0.514851 | 0.656151 | 0.784653 | 1.123242 | 208 |
| {BcOLV16=FALSE}  => {BcMV12=FALSE} | 0.50495 | 0.864407 | 0.584158 | 1.081178 | 204 |
| {BcMV12=FALSE} =>  {BcOLV16=FALSE} | 0.50495 | 0.631579 | 0.799505 | 1.081178 | 204 |
| {BcOLV16=FALSE}  => {BcOLV7=FALSE} | 0.512376 | 0.877119 | 0.584158 | 1.086981 | 207 |

| {BcOLV7=FALSE} =>  {BcOLV16=FALSE} | 0.512376 | 0.634969 | 0.806931 | 1.086981 | 207 |
| --- | --- | --- | --- | --- | --- |
| {BcOLV16=FALSE}  => {BcALV1=FALSE} | 0.50495 | 0.864407 | 0.584158 | 1.06146 | 204 |
| {BcALV1=FALSE} =>  {BcOLV16=FALSE} | 0.50495 | 0.620061 | 0.814356 | 1.06146 | 204 |
| {BcOLV16=FALSE}  => {BcMCV3=FALSE} | 0.574257 | 0.983051 | 0.584158 | 1.053455 | 232 |
| {BcMCV3=FALSE} =>  {BcOLV16=FALSE} | 0.574257 | 0.615385 | 0.933168 | 1.053455 | 232 |
| {BcULV3=TRUE} =>  {BcHV1=TRUE} | 0.423267 | 0.7125 | 0.594059 | 1.179713 | 171 |
| {BcHV1=TRUE} =>  {BcULV3=TRUE} | 0.423267 | 0.70082 | 0.60396 | 1.179713 | 171 |
| {BcULV3=TRUE} =>  {BcMV11=TRUE} | 0.39604 | 0.666667 | 0.594059 | 1.08166 | 160 |
| {BcMV11=TRUE} =>  {BcULV3=TRUE} | 0.39604 | 0.64257 | 0.616337 | 1.08166 | 160 |
| {BcHV1=TRUE} =>  {BcMV9=TRUE} | 0.467822 | 0.77459 | 0.60396 | 1.142096 | 189 |
| {BcMV9=TRUE} =>  {BcHV1=TRUE} | 0.467822 | 0.689781 | 0.678218 | 1.142096 | 189 |
| {BcHV1=TRUE} =>  {BcULV4=TRUE} | 0.455446 | 0.754098 | 0.60396 | 1.091956 | 184 |
| {BcULV4=TRUE} =>  {BcHV1=TRUE} | 0.455446 | 0.659498 | 0.690594 | 1.091956 | 184 |
| {BcMV11=TRUE} =>  {BcMV1=TRUE} | 0.519802 | 0.843373 | 0.616337 | 1.068097 | 210 |
| {BcMV1=TRUE} =>  {BcMV11=TRUE} | 0.519802 | 0.658307 | 0.789604 | 1.068097 | 210 |
| {BcOLV1=FALSE} =>  {BcMV6=FALSE} | 0.527228 | 0.788889 | 0.668317 | 1.134203 | 213 |
| {BcMV6=FALSE} =>  {BcOLV1=FALSE} | 0.527228 | 0.758007 | 0.695545 | 1.134203 | 213 |
| {BcOLV1=FALSE} =>  {BcPV3=FALSE} | 0.514851 | 0.77037 | 0.668317 | 1.084424 | 208 |
| {BcPV3=FALSE} =>  {BcOLV1=FALSE} | 0.514851 | 0.724739 | 0.710396 | 1.084424 | 208 |
| {BcOLV1=FALSE} =>  {BcRV1=FALSE} | 0.524752 | 0.785185 | 0.668317 | 1.086352 | 212 |
| {BcRV1=FALSE} =>  {BcOLV1=FALSE} | 0.524752 | 0.726027 | 0.722772 | 1.086352 | 212 |
| {BcOLV1=FALSE} =>  {BcHV3=FALSE} | 0.532178 | 0.796296 | 0.668317 | 1.047895 | 215 |

| {BcOLV1=FALSE} =>  {BcULV1=FALSE} | 0.559406 | 0.837037 | 0.668317 | 1.06676 | 226 |
| --- | --- | --- | --- | --- | --- |
| {BcULV1=FALSE} =>  {BcOLV1=FALSE} | 0.559406 | 0.712934 | 0.784653 | 1.06676 | 226 |
| {BcOLV1=FALSE} =>  {BcMV12=FALSE} | 0.569307 | 0.851852 | 0.668317 | 1.065474 | 230 |
| {BcMV12=FALSE} =>  {BcOLV1=FALSE} | 0.569307 | 0.712074 | 0.799505 | 1.065474 | 230 |
| {BcOLV1=FALSE} =>  {BcOLV7=FALSE} | 0.569307 | 0.851852 | 0.668317 | 1.055669 | 230 |
| {BcOLV7=FALSE} =>  {BcOLV1=FALSE} | 0.569307 | 0.705521 | 0.806931 | 1.055669 | 230 |
| {BcOLV1=FALSE} =>  {BcALV1=FALSE} | 0.589109 | 0.881481 | 0.668317 | 1.082427 | 238 |
| {BcALV1=FALSE} =>  {BcOLV1=FALSE} | 0.589109 | 0.723404 | 0.814356 | 1.082427 | 238 |
| {BcOLV1=FALSE} =>  {BcOLV4=FALSE} | 0.613861 | 0.918519 | 0.668317 | 1.048253 | 248 |
| {BcOLV4=FALSE} =>  {BcOLV1=FALSE} | 0.613861 | 0.700565 | 0.876238 | 1.048253 | 248 |
| {BcOLV1=FALSE} =>  {BcULV5=FALSE} | 0.618812 | 0.925926 | 0.668317 | 1.056706 | 250 |
| {BcULV5=FALSE} =>  {BcOLV1=FALSE} | 0.618812 | 0.706215 | 0.876238 | 1.056706 | 250 |
| {BcMCV5=FALSE} =>  {BcOLV1=FALSE} | 0.64604 | 0.699732 | 0.923267 | 1.047006 | 261 |
| {BcOLV13=FALSE}  => {BcOLV1=FALSE} | 0.648515 | 0.700535 | 0.925743 | 1.048208 | 262 |
| {BcOLV1=FALSE} =>  {BcOLV2=FALSE} | 0.65099 | 0.974074 | 0.668317 | 1.049402 | 263 |
| {BcOLV2=FALSE} =>  {BcOLV1=FALSE} | 0.65099 | 0.701333 | 0.928218 | 1.049402 | 263 |
| {BcMV9=TRUE} =>  {BcULV4=TRUE} | 0.54703 | 0.806569 | 0.678218 | 1.167936 | 221 |
| {BcULV4=TRUE} =>  {BcMV9=TRUE} | 0.54703 | 0.792115 | 0.690594 | 1.167936 | 221 |
| {BcMV9=TRUE} =>  {BcULV5=FALSE} | 0.655941 | 0.967153 | 0.678218 | 1.103757 | 265 |
| {BcULV5=FALSE} =>  {BcMV9=TRUE} | 0.655941 | 0.748588 | 0.876238 | 1.103757 | 265 |
| {BcULV4=TRUE} =>  {BcULV5=FALSE} | 0.631188 | 0.913978 | 0.690594 | 1.043072 | 255 |
| {BcMV6=FALSE} =>  {BcRV1=FALSE} | 0.532178 | 0.765125 | 0.695545 | 1.058597 | 215 |

| {BcRV1=FALSE} =>  {BcMV6=FALSE} | 0.532178 | 0.736301 | 0.722772 | 1.058597 | 215 |
| --- | --- | --- | --- | --- | --- |
| {BcMV6=FALSE} =>  {BcULV1=FALSE} | 0.571782 | 0.822064 | 0.695545 | 1.047678 | 231 |
| {BcULV1=FALSE} =>  {BcMV6=FALSE} | 0.571782 | 0.728707 | 0.784653 | 1.047678 | 231 |
| {BcMV6=FALSE} =>  {BcALV1=FALSE} | 0.596535 | 0.857651 | 0.695545 | 1.053164 | 241 |
| {BcALV1=FALSE} =>  {BcMV6=FALSE} | 0.596535 | 0.732523 | 0.814356 | 1.053164 | 241 |
| {BcMV6=FALSE} =>  {BcBNV5=FALSE} | 0.663366 | 0.953737 | 0.695545 | 1.04989 | 268 |
| {BcBNV5=FALSE} =>  {BcMV6=FALSE} | 0.663366 | 0.730245 | 0.908416 | 1.04989 | 268 |
| {BcEV5=FALSE} =>  {BcMV6=FALSE} | 0.685644 | 0.721354 | 0.950495 | 1.037107 | 277 |
| {BcPV3=FALSE} =>  {BcRV1=FALSE} | 0.559406 | 0.787456 | 0.710396 | 1.089495 | 226 |
| {BcRV1=FALSE} =>  {BcPV3=FALSE} | 0.559406 | 0.773973 | 0.722772 | 1.089495 | 226 |
| {BcRV1=FALSE} =>  {BcOLV7=FALSE} | 0.638614 | 0.883562 | 0.722772 | 1.094966 | 258 |
| {BcOLV7=FALSE} =>  {BcRV1=FALSE} | 0.638614 | 0.791411 | 0.806931 | 1.094966 | 258 |
| {BcRV1=FALSE} =>  {BcALV1=FALSE} | 0.623762 | 0.863014 | 0.722772 | 1.059749 | 252 |
| {BcALV1=FALSE} =>  {BcRV1=FALSE} | 0.623762 | 0.765957 | 0.814356 | 1.059749 | 252 |
| {BcEV2=FALSE} =>  {BcHV3=FALSE} | 0.596535 | 0.798013 | 0.747525 | 1.050154 | 241 |
| {BcHV3=FALSE} =>  {BcEV2=FALSE} | 0.596535 | 0.785016 | 0.759901 | 1.050154 | 241 |
| {BcEV2=FALSE} =>  {BcFV5=FALSE} | 0.621287 | 0.831126 | 0.747525 | 1.062579 | 251 |
| {BcFV5=FALSE} =>  {BcEV2=FALSE} | 0.621287 | 0.794304 | 0.782178 | 1.062579 | 251 |
| {BcEV2=FALSE} =>  {BcFV9=FALSE} | 0.673267 | 0.900662 | 0.747525 | 1.03666 | 272 |
| {BcHV3=FALSE} =>  {BcFV5=FALSE} | 0.655941 | 0.863192 | 0.759901 | 1.103575 | 265 |
| {BcFV5=FALSE} =>  {BcHV3=FALSE} | 0.655941 | 0.838608 | 0.782178 | 1.103575 | 265 |
| {BcHV3=FALSE} =>  {BcNSRV7=FALSE} | 0.653465 | 0.859935 | 0.759901 | 1.075584 | 264 |

| {BcNSRV7=FALSE}  => {BcHV3=FALSE} | 0.653465 | 0.817337 | 0.799505 | 1.075584 | 264 |
| --- | --- | --- | --- | --- | --- |
| {BcHV3=FALSE} =>  {BcVV3=FALSE} | 0.648515 | 0.85342 | 0.759901 | 1.064141 | 262 |
| {BcVV3=FALSE} =>  {BcHV3=FALSE} | 0.648515 | 0.808642 | 0.80198 | 1.064141 | 262 |
| {BcHV3=FALSE} =>  {BcDMV1=FALSE} | 0.680693 | 0.895765 | 0.759901 | 1.061259 | 275 |
| {BcDMV1=FALSE} =>  {BcHV3=FALSE} | 0.680693 | 0.806452 | 0.844059 | 1.061259 | 275 |
| {BcHV3=FALSE} =>  {BcMCV5=FALSE} | 0.737624 | 0.970684 | 0.759901 | 1.051358 | 298 |
| {BcMCV5=FALSE} =>  {BcHV3=FALSE} | 0.737624 | 0.798928 | 0.923267 | 1.051358 | 298 |
| {BcEV5=FALSE} =>  {BcHV3=FALSE} | 0.752475 | 0.791667 | 0.950495 | 1.041802 | 304 |
| {BcFV5=FALSE} =>  {BcNSRV7=FALSE} | 0.678218 | 0.867089 | 0.782178 | 1.084532 | 274 |
| {BcNSRV7=FALSE}  => {BcFV5=FALSE} | 0.678218 | 0.848297 | 0.799505 | 1.084532 | 274 |
| {BcFV5=FALSE} =>  {BcALV1=FALSE} | 0.665842 | 0.851266 | 0.782178 | 1.045323 | 269 |
| {BcALV1=FALSE} =>  {BcFV5=FALSE} | 0.665842 | 0.817629 | 0.814356 | 1.045323 | 269 |
| {BcFV5=FALSE} =>  {BcDMV1=FALSE} | 0.70297 | 0.898734 | 0.782178 | 1.064776 | 284 |
| {BcDMV1=FALSE} =>  {BcFV5=FALSE} | 0.70297 | 0.832845 | 0.844059 | 1.064776 | 284 |
| {BcFV5=FALSE} =>  {BcBNV5=FALSE} | 0.74505 | 0.952532 | 0.782178 | 1.048563 | 301 |
| {BcBNV5=FALSE} =>  {BcFV5=FALSE} | 0.74505 | 0.820163 | 0.908416 | 1.048563 | 301 |
| {BcOLV15.BI=FALSE}  => {BcFV5=FALSE} | 0.769802 | 0.809896 | 0.950495 | 1.035436 | 311 |
| {BcEV5=FALSE} =>  {BcFV5=FALSE} | 0.772277 | 0.8125 | 0.950495 | 1.038766 | 312 |
| {BcMCV3=FALSE} =>  {BcULV1=FALSE} | 0.764851 | 0.819629 | 0.933168 | 1.044574 | 309 |
| {BcMCV3=FALSE} =>  {BcMV12=FALSE} | 0.779703 | 0.835544 | 0.933168 | 1.045076 | 315 |
| {BcNSRV7=FALSE}  => {BcDMV1=FALSE} | 0.715347 | 0.894737 | 0.799505 | 1.06004 | 289 |
| {BcDMV1=FALSE} =>  {BcNSRV7=FALSE} | 0.715347 | 0.847507 | 0.844059 | 1.06004 | 289 |

| {BcNSRV7=FALSE}  => {BcFV9=FALSE} | 0.720297 | 0.900929 | 0.799505 | 1.036966 | 291 |
| --- | --- | --- | --- | --- | --- |
| {BcFV9=FALSE} =>  {BcNSRV7=FALSE} | 0.720297 | 0.82906 | 0.868812 | 1.036966 | 291 |
| {BcBNV5=FALSE} =>  {BcNSRV7=FALSE} | 0.757426 | 0.833787 | 0.908416 | 1.04288 | 306 |
| {BcDMV1=FALSE} =>  {BcBNV5=FALSE} | 0.804455 | 0.953079 | 0.844059 | 1.049166 | 325 |
| {BcBNV5=FALSE} =>  {BcDMV1=FALSE} | 0.804455 | 0.885559 | 0.908416 | 1.049166 | 325 |
| {BcDMV1=FALSE} =>  {BcEV5=FALSE} | 0.839109 | 0.994135 | 0.844059 | 1.045913 | 339 |
| {BcEV5=FALSE} =>  {BcDMV1=FALSE} | 0.839109 | 0.882813 | 0.950495 | 1.045913 | 339 |
| {BcBNV5=FALSE} =>  {BcMCV5=FALSE} | 0.871287 | 0.959128 | 0.908416 | 1.038841 | 352 |
| {BcMCV5=FALSE} =>  {BcBNV5=FALSE} | 0.871287 | 0.9437 | 0.923267 | 1.038841 | 352 |
| {BcBNV5=FALSE} =>  {BcEV5=FALSE} | 0.893564 | 0.983651 | 0.908416 | 1.034883 | 361 |
| {BcEV5=FALSE} =>  {BcBNV5=FALSE} | 0.893564 | 0.940104 | 0.950495 | 1.034883 | 361 |
| {BcMCV5=FALSE} =>  {BcEV5=FALSE} | 0.905941 | 0.981233 | 0.923267 | 1.032339 | 366 |
| {BcEV5=FALSE} =>  {BcMCV5=FALSE} | 0.905941 | 0.953125 | 0.950495 | 1.032339 | 366 |

Table S5 The “Two to One” association rules of viruses that infected *B. cinerea*

| Rules | Supp  ort | Confid  ence | Cove  rage | Lift | Co  unt |
| --- | --- | --- | --- | --- | --- |
| {BcMCV5.BI=TRUE, BvF.BI=FALSE} =>  {BcMV1.BI=TRUE} | 0.07  4257 | 1 | 0.074  257 | 1.26  6458 | 30 |
| {BcMV1.BI=TRUE, BvF.BI=FALSE} =>  {BcMCV5.BI=TRUE} | 0.07  4257 | 0.101  695 | 0.730  198 | 1.32  5314 | 30 |
| {BcMCV5.BI=TRUE, BcOLV2.BI=FALSE} =>  {BcMV1.BI=TRUE} | 0.07  4257 | 1 | 0.074  257 | 1.26  6458 | 30 |
| {BcMV1.BI=TRUE, BcOLV2.BI=FALSE} =>  {BcMCV5.BI=TRUE} | 0.07  4257 | 0.101  351 | 0.732  673 | 1.32  0837 | 30 |
| {BcEV6=FALSE, BcMCV5.BI=TRUE} =>  {BcMV1.BI=TRUE} | 0.07  4257 | 1 | 0.074  257 | 1.26  6458 | 30 |
| {BcEV6=FALSE, BcMV1.BI=TRUE} =>  {BcMCV5.BI=TRUE} | 0.07  4257 | 0.100  334 | 0.740  099 | 1.30  7584 | 30 |
| {BcBNV5.BI=FALSE, BvF.BI=TRUE} =>  {BcRV1.BI=FALSE} | 0.07  4257 | 0.909  091 | 0.081  683 | 1.25  7783 | 30 |
| {BcBNV5.BI=FALSE, BcRV1.BI=FALSE} =>  {BvF.BI=TRUE} | 0.07  4257 | 0.114  504 | 0.648  515 | 1.32  1701 | 30 |
| {BcMCV5.BI=FALSE, BvF.BI=TRUE} =>  {BcRV1.BI=FALSE} | 0.07  4257 | 0.882  353 | 0.084  158 | 1.22  079 | 30 |
| {BcOLV13.BI=FALSE, BvF.BI=TRUE} => | 0.07 | 0.882 | 0.084 | 1.22 | 30 |

| {BcRV1.BI=FALSE} | 4257 | 353 | 158 | 079 |  |
| --- | --- | --- | --- | --- | --- |
| {BcOLV2.BI=FALSE, BvF.BI=TRUE} =>  {BcRV1.BI=FALSE} | 0.07  4257 | 0.882  353 | 0.084  158 | 1.22  079 | 30 |
| {BcMCV3.BI=FALSE, BvF.BI=TRUE} =>  {BcRV1.BI=FALSE} | 0.07  6733 | 0.885  714 | 0.086  634 | 1.22  544 | 31 |
| {BcMCV3.BI=FALSE, BcRV1.BI=FALSE} =>  {BvF.BI=TRUE} | 0.07  6733 | 0.112  727 | 0.680  693 | 1.30  1195 | 31 |
| {BcOLV17.BI=FALSE, BvF.BI=TRUE} =>  {BcRV1.BI=FALSE} | 0.07  6733 | 0.885  714 | 0.086  634 | 1.22  544 | 31 |
| {BcEV5=FALSE, BvF.BI=TRUE} =>  {BcRV1.BI=FALSE} | 0.07  6733 | 0.885  714 | 0.086  634 | 1.22  544 | 31 |
| {BcEV5=FALSE, BcRV1.BI=FALSE} =>  {BvF.BI=TRUE} | 0.07  6733 | 0.113  971 | 0.673  267 | 1.31  5546 | 31 |
| {BcMV12=FALSE, BvF.BI=TRUE} =>  {BcOLV7.BI=FALSE} | 0.07  4257 | 0.909  091 | 0.081  683 | 1.12  6603 | 30 |
| {BcOLV7.BI=FALSE, BvF.BI=TRUE} =>  {BcMV12=FALSE} | 0.07  4257 | 0.937  5 | 0.079  208 | 1.17  2601 | 30 |
| {BcMV12=FALSE, BcOLV7.BI=FALSE} =>  {BvF.BI=TRUE} | 0.07  4257 | 0.113  208 | 0.655  941 | 1.30  6739 | 30 |
| {BcULV5=FALSE, BvF.BI=TRUE} =>  {BcMV12=FALSE} | 0.07  6733 | 0.939  394 | 0.081  683 | 1.17  497 | 31 |
| {BcBNV5.BI=FALSE, BvF.BI=TRUE} => | 0.07 | 0.939 | 0.081 | 1.17 | 31 |

| {BcMV12=FALSE} | 6733 | 394 | 683 | 497 |  |
| --- | --- | --- | --- | --- | --- |
| {BcMCV5.BI=FALSE, BvF.BI=TRUE} =>  {BcMV12=FALSE} | 0.07  9208 | 0.941  176 | 0.084  158 | 1.17  7199 | 32 |
| {BcOLV13.BI=FALSE, BvF.BI=TRUE} =>  {BcMV12=FALSE} | 0.07  9208 | 0.941  176 | 0.084  158 | 1.17  7199 | 32 |
| {BcOLV2.BI=FALSE, BvF.BI=TRUE} =>  {BcMV12=FALSE} | 0.07  9208 | 0.941  176 | 0.084  158 | 1.17  7199 | 32 |
| {BcMCV3.BI=FALSE, BvF.BI=TRUE} =>  {BcMV12=FALSE} | 0.08  1683 | 0.942  857 | 0.086  634 | 1.17  9301 | 33 |
| {BcPV4.BI=FALSE, BvF.BI=TRUE} =>  {BcMV12=FALSE} | 0.07  6733 | 0.939  394 | 0.081  683 | 1.17  497 | 31 |
| {BcEV6=FALSE, BvF.BI=TRUE} =>  {BcMV12=FALSE} | 0.07  4257 | 0.967  742 | 0.076  733 | 1.21  0426 | 30 |
| {BcOLV17.BI=FALSE, BvF.BI=TRUE} =>  {BcMV12=FALSE} | 0.08  1683 | 0.942  857 | 0.086  634 | 1.17  9301 | 33 |
| {BcEV5=FALSE, BvF.BI=TRUE} =>  {BcMV12=FALSE} | 0.08  1683 | 0.942  857 | 0.086  634 | 1.17  9301 | 33 |
| {BcOLV7.BI=FALSE, BvF.BI=TRUE} =>  {BcULV5=FALSE} | 0.07  6733 | 0.968  75 | 0.079  208 | 1.10  5579 | 31 |
| {BcULV5=FALSE, BvF.BI=TRUE} =>  {BcOLV7.BI=FALSE} | 0.07  6733 | 0.939  394 | 0.081  683 | 1.16  4157 | 31 |
| {BcBNV5.BI=FALSE, BvF.BI=TRUE} => | 0.07 | 0.909 | 0.081 | 1.12 | 30 |

| {BcOLV7.BI=FALSE} | 4257 | 091 | 683 | 6603 |  |
| --- | --- | --- | --- | --- | --- |
| {BcMCV5.BI=FALSE, BvF.BI=TRUE} =>  {BcOLV7.BI=FALSE} | 0.07  6733 | 0.911  765 | 0.084  158 | 1.12  9917 | 31 |
| {BcOLV13.BI=FALSE, BvF.BI=TRUE} =>  {BcOLV7.BI=FALSE} | 0.07  6733 | 0.911  765 | 0.084  158 | 1.12  9917 | 31 |
| {BcOLV2.BI=FALSE, BvF.BI=TRUE} =>  {BcOLV7.BI=FALSE} | 0.07  6733 | 0.911  765 | 0.084  158 | 1.12  9917 | 31 |
| {BcMCV3.BI=FALSE, BvF.BI=TRUE} =>  {BcOLV7.BI=FALSE} | 0.07  9208 | 0.914  286 | 0.086  634 | 1.13  3041 | 32 |
| {BcPV4.BI=FALSE, BvF.BI=TRUE} =>  {BcOLV7.BI=FALSE} | 0.07  4257 | 0.909  091 | 0.081  683 | 1.12  6603 | 30 |
| {BcOLV17.BI=FALSE, BvF.BI=TRUE} =>  {BcOLV7.BI=FALSE} | 0.07  9208 | 0.914  286 | 0.086  634 | 1.13  3041 | 32 |
| {BcEV5=FALSE, BvF.BI=TRUE} =>  {BcOLV7.BI=FALSE} | 0.07  9208 | 0.914  286 | 0.086  634 | 1.13  3041 | 32 |
| {BcBNV5.BI=TRUE, BvF.BI=FALSE} =>  {BcULV3=TRUE} | 0.07  4257 | 0.857  143 | 0.086  634 | 1.44  2857 | 30 |
| {BcULV3=TRUE, BvF.BI=FALSE} =>  {BcBNV5.BI=TRUE} | 0.07  4257 | 0.134  529 | 0.551  98 | 1.46  8913 | 30 |
| {BcBNV5.BI=TRUE, BcOLV13.BI=FALSE}  => {BcULV3=TRUE} | 0.07  4257 | 0.833  333 | 0.089  109 | 1.40  2778 | 30 |
| {BcOLV13.BI=FALSE, BcULV3=TRUE} => | 0.07 | 0.136 | 0.544 | 1.48 | 30 |

| {BcBNV5.BI=TRUE} | 4257 | 364 | 554 | 8943 |  |
| --- | --- | --- | --- | --- | --- |
| {BcBNV5.BI=TRUE, BcMCV3.BI=FALSE} =>  {BcULV3=TRUE} | 0.07  4257 | 0.833  333 | 0.089  109 | 1.40  2778 | 30 |
| {BcMCV3.BI=FALSE, BcULV3=TRUE} =>  {BcBNV5.BI=TRUE} | 0.07  4257 | 0.136  986 | 0.542  079 | 1.49  5742 | 30 |
| {BcBNV5.BI=TRUE, BcPV4.BI=FALSE} =>  {BcULV3=TRUE} | 0.07  4257 | 0.833  333 | 0.089  109 | 1.40  2778 | 30 |
| {BcPV4.BI=FALSE, BcULV3=TRUE} =>  {BcBNV5.BI=TRUE} | 0.07  4257 | 0.135  135 | 0.549  505 | 1.47  553 | 30 |
| {BcBNV5.BI=TRUE, BcOLV17.BI=FALSE}  => {BcULV3=TRUE} | 0.07  4257 | 0.833  333 | 0.089  109 | 1.40  2778 | 30 |
| {BcOLV17.BI=FALSE, BcULV3=TRUE} =>  {BcBNV5.BI=TRUE} | 0.07  4257 | 0.133  333 | 0.556  931 | 1.45  5856 | 30 |
| {BcBNV5.BI=TRUE, BcOLV4.BI=FALSE} =>  {BcMV11=TRUE} | 0.07  4257 | 0.857  143 | 0.086  634 | 1.39  0706 | 30 |
| {BcMV11=TRUE, BcOLV4.BI=FALSE} =>  {BcBNV5.BI=TRUE} | 0.07  4257 | 0.144  928 | 0.512  376 | 1.58  2452 | 30 |
| {BcBNV5.BI=TRUE, BcULV5=FALSE} =>  {BcMV11=TRUE} | 0.07  4257 | 0.857  143 | 0.086  634 | 1.39  0706 | 30 |
| {BcMV11=TRUE, BcULV5=FALSE} =>  {BcBNV5.BI=TRUE} | 0.07  4257 | 0.147  059 | 0.504  95 | 1.60  5723 | 30 |
| {BcBNV5.BI=TRUE, BvF.BI=FALSE} => | 0.07 | 0.857 | 0.086 | 1.39 | 30 |

| {BcMV11=TRUE} | 4257 | 143 | 634 | 0706 |  |
| --- | --- | --- | --- | --- | --- |
| {BcMV11=TRUE, BvF.BI=FALSE} =>  {BcBNV5.BI=TRUE} | 0.07  4257 | 0.129  87 | 0.571  782 | 1.41  8041 | 30 |
| {BcBNV5.BI=TRUE, BcOLV13.BI=FALSE}  => {BcMV11=TRUE} | 0.07  6733 | 0.861  111 | 0.089  109 | 1.39  7144 | 31 |
| {BcMV11=TRUE, BcOLV13.BI=FALSE} =>  {BcBNV5.BI=TRUE} | 0.07  6733 | 0.137  168 | 0.559  406 | 1.49  7728 | 31 |
| {BcBNV5.BI=TRUE, BcMCV3.BI=FALSE} =>  {BcMV11=TRUE} | 0.07  9208 | 0.888  889 | 0.089  109 | 1.44  2213 | 32 |
| {BcMCV3.BI=FALSE, BcMV11=TRUE} =>  {BcBNV5.BI=TRUE} | 0.07  9208 | 0.136  17 | 0.581  683 | 1.48  6832 | 32 |
| {BcBNV5.BI=TRUE, BcPV4.BI=FALSE} =>  {BcMV11=TRUE} | 0.07  6733 | 0.861  111 | 0.089  109 | 1.39  7144 | 31 |
| {BcMV11=TRUE, BcPV4.BI=FALSE} =>  {BcBNV5.BI=TRUE} | 0.07  6733 | 0.133  047 | 0.576  733 | 1.45  2732 | 31 |
| {BcBNV5.BI=TRUE, BcOLV17.BI=FALSE}  => {BcMV11=TRUE} | 0.07  6733 | 0.861  111 | 0.089  109 | 1.39  7144 | 31 |
| {BcMV11=TRUE, BcOLV17.BI=FALSE} =>  {BcBNV5.BI=TRUE} | 0.07  6733 | 0.133  621 | 0.574  257 | 1.45  8993 | 31 |
| {BcBNV5.BI=TRUE, BcMV9.BI=TRUE} =>  {BcMV1.BI=TRUE} | 0.07  4257 | 0.909  091 | 0.081  683 | 1.15  1325 | 30 |
| {BcBNV5.BI=TRUE, BcMV1.BI=TRUE} => | 0.07 | 0.882 | 0.084 | 1.30 | 30 |

| {BcMV9.BI=TRUE} | 4257 | 353 | 158 | 0988 |  |
| --- | --- | --- | --- | --- | --- |
| {BcMV1.BI=TRUE, BcMV9.BI=TRUE} =>  {BcBNV5.BI=TRUE} | 0.07  4257 | 0.135  135 | 0.549  505 | 1.47  553 | 30 |
| {BcBNV5.BI=TRUE, BcMV9.BI=TRUE} =>  {BcOLV4.BI=FALSE} | 0.07  9208 | 0.969  697 | 0.081  683 | 1.10  666 | 32 |
| {BcBNV5.BI=TRUE, BcOLV4.BI=FALSE} =>  {BcMV9.BI=TRUE} | 0.07  9208 | 0.914  286 | 0.086  634 | 1.34  8071 | 32 |
| {BcMV9.BI=TRUE, BcOLV4.BI=FALSE} =>  {BcBNV5.BI=TRUE} | 0.07  9208 | 0.128  514 | 0.616  337 | 1.40  3235 | 32 |
| {BcBNV5.BI=TRUE, BcMV9.BI=TRUE} =>  {BcULV5=FALSE} | 0.07  9208 | 0.969  697 | 0.081  683 | 1.10  666 | 32 |
| {BcBNV5.BI=TRUE, BcULV5=FALSE} =>  {BcMV9.BI=TRUE} | 0.07  9208 | 0.914  286 | 0.086  634 | 1.34  8071 | 32 |
| {BcMV9.BI=TRUE, BcULV5=FALSE} =>  {BcBNV5.BI=TRUE} | 0.07  9208 | 0.120  755 | 0.655  941 | 1.31  8511 | 32 |
| {BcBNV5.BI=TRUE, BvF.BI=FALSE} =>  {BcMV9.BI=TRUE} | 0.07  6733 | 0.885  714 | 0.086  634 | 1.30  5944 | 31 |
| {BcMV9.BI=TRUE, BvF.BI=FALSE} =>  {BcBNV5.BI=TRUE} | 0.07  6733 | 0.125  506 | 0.611  386 | 1.37  0391 | 31 |
| {BcBNV5.BI=TRUE, BcOLV13.BI=FALSE}  => {BcMV9.BI=TRUE} | 0.07  9208 | 0.888  889 | 0.089  109 | 1.31  0624 | 32 |
| {BcMV9.BI=TRUE, BcOLV13.BI=FALSE} => | 0.07 | 0.124 | 0.636 | 1.35 | 32 |

| {BcBNV5.BI=TRUE} | 9208 | 514 | 139 | 9554 |  |
| --- | --- | --- | --- | --- | --- |
| {BcBNV5.BI=TRUE, BcMCV3.BI=FALSE} =>  {BcMV9.BI=TRUE} | 0.07  9208 | 0.888  889 | 0.089  109 | 1.31  0624 | 32 |
| {BcMCV3.BI=FALSE, BcMV9.BI=TRUE} =>  {BcBNV5.BI=TRUE} | 0.07  9208 | 0.125  984 | 0.628  713 | 1.37  5612 | 32 |
| {BcBNV5.BI=TRUE, BcPV4.BI=FALSE} =>  {BcMV9.BI=TRUE} | 0.07  9208 | 0.888  889 | 0.089  109 | 1.31  0624 | 32 |
| {BcMV9.BI=TRUE, BcPV4.BI=FALSE} =>  {BcBNV5.BI=TRUE} | 0.07  9208 | 0.125 | 0.633  663 | 1.36  4865 | 32 |
| {BcBNV5.BI=TRUE, BcEV6=FALSE} =>  {BcMV9.BI=TRUE} | 0.07  6733 | 0.911  765 | 0.084  158 | 1.34  4354 | 31 |
| {BcEV6=FALSE, BcMV9.BI=TRUE} =>  {BcBNV5.BI=TRUE} | 0.07  6733 | 0.121  569 | 0.631  188 | 1.32  7398 | 31 |
| {BcBNV5.BI=TRUE, BcOLV17.BI=FALSE}  => {BcMV9.BI=TRUE} | 0.07  9208 | 0.888  889 | 0.089  109 | 1.31  0624 | 32 |
| {BcMV9.BI=TRUE, BcOLV17.BI=FALSE} =>  {BcBNV5.BI=TRUE} | 0.07  9208 | 0.124  031 | 0.638  614 | 1.35  4285 | 32 |
| {BcBNV5.BI=TRUE, BcMCV3.BI=FALSE} =>  {BcRV1.BI=FALSE} | 0.07  4257 | 0.833  333 | 0.089  109 | 1.15  2968 | 30 |
| {BcBNV5.BI=TRUE, BcOLV17.BI=FALSE}  => {BcRV1.BI=FALSE} | 0.07  4257 | 0.833  333 | 0.089  109 | 1.15  2968 | 30 |
| {BcBNV5.BI=TRUE, BcOLV4.BI=FALSE} => | 0.07 | 0.914 | 0.086 | 1.15 | 32 |

| {BcMV1.BI=TRUE} | 9208 | 286 | 634 | 7904 |  |
| --- | --- | --- | --- | --- | --- |
| {BcMV1.BI=TRUE, BcOLV4.BI=FALSE} =>  {BcBNV5.BI=TRUE} | 0.07  9208 | 0.117  647 | 0.673  267 | 1.28  4579 | 32 |
| {BcBNV5.BI=TRUE, BcULV5=FALSE} =>  {BcMV1.BI=TRUE} | 0.07  9208 | 0.914  286 | 0.086  634 | 1.15  7904 | 32 |
| {BcMV1.BI=TRUE, BcULV5=FALSE} =>  {BcBNV5.BI=TRUE} | 0.07  9208 | 0.117  647 | 0.673  267 | 1.28  4579 | 32 |
| {BcBNV5.BI=TRUE, BvF.BI=FALSE} =>  {BcMV1.BI=TRUE} | 0.07  9208 | 0.914  286 | 0.086  634 | 1.15  7904 | 32 |
| {BcBNV5.BI=TRUE, BcOLV13.BI=FALSE}  => {BcMV1.BI=TRUE} | 0.08  1683 | 0.916  667 | 0.089  109 | 1.16  092 | 33 |
| {BcBNV5.BI=TRUE, BcOLV2.BI=FALSE} =>  {BcMV1.BI=TRUE} | 0.07  6733 | 0.939  394 | 0.081  683 | 1.18  9703 | 31 |
| {BcBNV5.BI=TRUE, BcMCV3.BI=FALSE} =>  {BcMV1.BI=TRUE} | 0.08  1683 | 0.916  667 | 0.089  109 | 1.16  092 | 33 |
| {BcBNV5.BI=TRUE, BcPV4.BI=FALSE} =>  {BcMV1.BI=TRUE} | 0.08  1683 | 0.916  667 | 0.089  109 | 1.16  092 | 33 |
| {BcBNV5.BI=TRUE, BcEV6=FALSE} =>  {BcMV1.BI=TRUE} | 0.07  6733 | 0.911  765 | 0.084  158 | 1.15  4711 | 31 |
| {BcBNV5.BI=TRUE, BcOLV17.BI=FALSE}  => {BcMV1.BI=TRUE} | 0.08  1683 | 0.916  667 | 0.089  109 | 1.16  092 | 33 |
| {BcBNV5.BI=TRUE, BcMV12=FALSE} => | 0.07 | 1 | 0.076 | 1.14 | 31 |

| {BcULV5=FALSE} | 6733 |  | 733 | 1243 |  |
| --- | --- | --- | --- | --- | --- |
| {BcBNV5.BI=TRUE, BcEV6=FALSE} =>  {BcULV5=FALSE} | 0.08  1683 | 0.970  588 | 0.084  158 | 1.10  7677 | 33 |
| {BcMV2.BI=TRUE, BcOLV4.BI=TRUE} =>  {BcMV11=TRUE} | 0.07  4257 | 0.857  143 | 0.086  634 | 1.39  0706 | 30 |
| {BcMV11=TRUE, BcOLV4.BI=TRUE} =>  {BcMV2.BI=TRUE} | 0.07  4257 | 0.714  286 | 0.103  96 | 1.54  3163 | 30 |
| {BcMV11=TRUE, BcMV2.BI=TRUE} =>  {BcOLV4.BI=TRUE} | 0.07  4257 | 0.217  391 | 0.341  584 | 1.75  6522 | 30 |
| {BcMV2.BI=TRUE, BcOLV4.BI=TRUE} =>  {BcMV1.BI=TRUE} | 0.08  4158 | 0.971  429 | 0.086  634 | 1.23  0273 | 34 |
| {BcMV1.BI=TRUE, BcOLV4.BI=TRUE} =>  {BcMV2.BI=TRUE} | 0.08  4158 | 0.723  404 | 0.116  337 | 1.56  2863 | 34 |
| {BcMV1.BI=TRUE, BcMV2.BI=TRUE} =>  {BcOLV4.BI=TRUE} | 0.08  4158 | 0.206  061 | 0.408  416 | 1.66  497 | 34 |
| {BcMV2.BI=TRUE, BcOLV4.BI=TRUE} =>  {BcNSRV7.BI=FALSE} | 0.07  9208 | 0.914  286 | 0.086  634 | 1.14  3565 | 32 |
| {BcNSRV7.BI=FALSE, BcOLV4.BI=TRUE}  => {BcMV2.BI=TRUE} | 0.07  9208 | 0.695  652 | 0.113  861 | 1.50  2906 | 32 |
| {BcMV2.BI=TRUE, BcNSRV7.BI=FALSE} =>  {BcOLV4.BI=TRUE} | 0.07  9208 | 0.219  178 | 0.361  386 | 1.77  0959 | 32 |
| {BcMV2.BI=TRUE, BcOLV4.BI=TRUE} => | 0.08 | 0.971 | 0.086 | 1.15 | 34 |

| {BcDMV1=FALSE} | 4158 | 429 | 634 | 0901 |  |
| --- | --- | --- | --- | --- | --- |
| {BcDMV1=FALSE, BcOLV4.BI=TRUE} =>  {BcMV2.BI=TRUE} | 0.08  4158 | 0.693  878 | 0.121  287 | 1.49  9072 | 34 |
| {BcDMV1=FALSE, BcMV2.BI=TRUE} =>  {BcOLV4.BI=TRUE} | 0.08  4158 | 0.212  5 | 0.396  04 | 1.71  7 | 34 |
| {BcBNV5.BI=FALSE, BcOLV4.BI=TRUE} =>  {BcMV2.BI=TRUE} | 0.08  4158 | 0.708  333 | 0.118  812 | 1.53  0303 | 34 |
| {BcBNV5.BI=FALSE, BcMV2.BI=TRUE} =>  {BcOLV4.BI=TRUE} | 0.08  4158 | 0.206  061 | 0.408  416 | 1.66  497 | 34 |
| {BcOLV4.BI=TRUE, BvF.BI=FALSE} =>  {BcMV2.BI=TRUE} | 0.07  4257 | 0.714  286 | 0.103  96 | 1.54  3163 | 30 |
| {BcMV2.BI=TRUE, BvF.BI=FALSE} =>  {BcOLV4.BI=TRUE} | 0.07  4257 | 0.171  429 | 0.433  168 | 1.38  5143 | 30 |
| {BcOLV2.BI=FALSE, BcOLV4.BI=TRUE} =>  {BcMV2.BI=TRUE} | 0.07  4257 | 0.681  818 | 0.108  911 | 1.47  3019 | 30 |
| {BcMV2.BI=TRUE, BcOLV2.BI=FALSE} =>  {BcOLV4.BI=TRUE} | 0.07  4257 | 0.176  471 | 0.420  792 | 1.42  5882 | 30 |
| {BcOLV4.BI=TRUE, BcPV4.BI=FALSE} =>  {BcMV2.BI=TRUE} | 0.07  6733 | 0.688  889 | 0.111  386 | 1.48  8295 | 31 |
| {BcMV2.BI=TRUE, BcPV4.BI=FALSE} =>  {BcOLV4.BI=TRUE} | 0.07  6733 | 0.179  191 | 0.428  218 | 1.44  7861 | 31 |
| {BcEV6=FALSE, BcOLV4.BI=TRUE} => | 0.08 | 0.687 | 0.118 | 1.48 | 33 |

| {BcMV2.BI=TRUE} | 1683 | 5 | 812 | 5294 |  |
| --- | --- | --- | --- | --- | --- |
| {BcEV6=FALSE, BcMV2.BI=TRUE} =>  {BcOLV4.BI=TRUE} | 0.08  1683 | 0.188  571 | 0.433  168 | 1.52  3657 | 33 |
| {BcOLV15.BI=FALSE, BcOLV4.BI=TRUE}  => {BcMV2.BI=TRUE} | 0.07  9208 | 0.695  652 | 0.113  861 | 1.50  2906 | 32 |
| {BcMV2.BI=TRUE, BcOLV15.BI=FALSE} =>  {BcOLV4.BI=TRUE} | 0.07  9208 | 0.174  863 | 0.452  97 | 1.41  2896 | 32 |
| {BcEV5=FALSE, BcOLV4.BI=TRUE} =>  {BcMV2.BI=TRUE} | 0.08  4158 | 0.693  878 | 0.121  287 | 1.49  9072 | 34 |
| {BcEV5=FALSE, BcMV2.BI=TRUE} =>  {BcOLV4.BI=TRUE} | 0.08  4158 | 0.198  83 | 0.423  267 | 1.60  655 | 34 |
| {BcHV1SLR.BI=TRUE, BcOLV4.BI=TRUE}  => {BcMV1.BI=TRUE} | 0.07  4257 | 0.937  5 | 0.079  208 | 1.18  7304 | 30 |
| {BcHV1SLR.BI=TRUE, BcMV1.BI=TRUE} =>  {BcOLV4.BI=TRUE} | 0.07  4257 | 0.168  539 | 0.440  594 | 1.36  1798 | 30 |
| {BcHV1SLR.BI=TRUE, BcOLV4.BI=TRUE}  => {BcDMV1=FALSE} | 0.07  6733 | 0.968  75 | 0.079  208 | 1.14  7727 | 31 |
| {BcDMV1=FALSE, BcHV1SLR.BI=TRUE} =>  {BcOLV4.BI=TRUE} | 0.07  6733 | 0.164  894 | 0.465  347 | 1.33  234 | 31 |
| {BcMV3.BI=TRUE, BcOLV4.BI=TRUE} =>  {BcMV11=TRUE} | 0.07  6733 | 0.885  714 | 0.086  634 | 1.43  7063 | 31 |
| {BcMV11=TRUE, BcOLV4.BI=TRUE} => | 0.07 | 0.738 | 0.103 | 1.36 | 31 |

| {BcMV3.BI=TRUE} | 6733 | 095 | 96 | 16 |  |
| --- | --- | --- | --- | --- | --- |
| {BcMV11=TRUE, BcMV3.BI=TRUE} =>  {BcOLV4.BI=TRUE} | 0.07  6733 | 0.2 | 0.383  663 | 1.61  6 | 31 |
| {BcMV3.BI=TRUE, BcOLV4.BI=TRUE} =>  {BcMV1.BI=TRUE} | 0.08  1683 | 0.942  857 | 0.086  634 | 1.19  4089 | 33 |
| {BcMV1.BI=TRUE, BcOLV4.BI=TRUE} =>  {BcMV3.BI=TRUE} | 0.08  1683 | 0.702  128 | 0.116  337 | 1.29  5249 | 33 |
| {BcMV1.BI=TRUE, BcMV3.BI=TRUE} =>  {BcOLV4.BI=TRUE} | 0.08  1683 | 0.178  378 | 0.457  921 | 1.44  1297 | 33 |
| {BcMV3.BI=TRUE, BcOLV4.BI=TRUE} =>  {BcNSRV7.BI=FALSE} | 0.08  1683 | 0.942  857 | 0.086  634 | 1.17  9301 | 33 |
| {BcNSRV7.BI=FALSE, BcOLV4.BI=TRUE}  => {BcMV3.BI=TRUE} | 0.08  1683 | 0.717  391 | 0.113  861 | 1.32  3407 | 33 |
| {BcMV3.BI=TRUE, BcNSRV7.BI=FALSE} =>  {BcOLV4.BI=TRUE} | 0.08  1683 | 0.192  982 | 0.423  267 | 1.55  9298 | 33 |
| {BcMV3.BI=TRUE, BcOLV4.BI=TRUE} =>  {BcDMV1=FALSE} | 0.08  4158 | 0.971  429 | 0.086  634 | 1.15  0901 | 34 |
| {BcDMV1=FALSE, BcOLV4.BI=TRUE} =>  {BcMV3.BI=TRUE} | 0.08  4158 | 0.693  878 | 0.121  287 | 1.28  003 | 34 |
| {BcDMV1=FALSE, BcMV3.BI=TRUE} =>  {BcOLV4.BI=TRUE} | 0.08  4158 | 0.198  83 | 0.423  267 | 1.60  655 | 34 |
| {BcFV9=FALSE, BcOLV4.BI=TRUE} => | 0.07 | 0.75 | 0.099 | 1.38 | 30 |

| {BcMV3.BI=TRUE} | 4257 |  | 01 | 3562 |  |
| --- | --- | --- | --- | --- | --- |
| {BcFV9=FALSE, BcMV3.BI=TRUE} =>  {BcOLV4.BI=TRUE} | 0.07  4257 | 0.163  043 | 0.455  446 | 1.31  7391 | 30 |
| {BcBNV5.BI=FALSE, BcOLV4.BI=TRUE} =>  {BcMV3.BI=TRUE} | 0.08  1683 | 0.687  5 | 0.118  812 | 1.26  8265 | 33 |
| {BcBNV5.BI=FALSE, BcMV3.BI=TRUE} =>  {BcOLV4.BI=TRUE} | 0.08  1683 | 0.173  684 | 0.470  297 | 1.40  3368 | 33 |
| {BcOLV4.BI=TRUE, BvF.BI=FALSE} =>  {BcMV3.BI=TRUE} | 0.07  4257 | 0.714  286 | 0.103  96 | 1.31  7678 | 30 |
| {BcOLV2.BI=FALSE, BcOLV4.BI=TRUE} =>  {BcMV3.BI=TRUE} | 0.07  4257 | 0.681  818 | 0.108  911 | 1.25  7783 | 30 |
| {BcOLV4.BI=TRUE, BcPV4.BI=FALSE} =>  {BcMV3.BI=TRUE} | 0.07  9208 | 0.711  111 | 0.111  386 | 1.31  1821 | 32 |
| {BcMV3.BI=TRUE, BcPV4.BI=FALSE} =>  {BcOLV4.BI=TRUE} | 0.07  9208 | 0.159  204 | 0.497  525 | 1.28  6368 | 32 |
| {BcEV6=FALSE, BcOLV4.BI=TRUE} =>  {BcMV3.BI=TRUE} | 0.08  6634 | 0.729  167 | 0.118  812 | 1.34  5129 | 35 |
| {BcEV6=FALSE, BcMV3.BI=TRUE} =>  {BcOLV4.BI=TRUE} | 0.08  6634 | 0.167  464 | 0.517  327 | 1.35  311 | 35 |
| {BcOLV15.BI=FALSE, BcOLV4.BI=TRUE}  => {BcMV3.BI=TRUE} | 0.08  1683 | 0.717  391 | 0.113  861 | 1.32  3407 | 33 |
| {BcMV3.BI=TRUE, BcOLV15.BI=FALSE} => | 0.08 | 0.160 | 0.507 | 1.30 | 33 |

| {BcOLV4.BI=TRUE} | 1683 | 976 | 426 | 0683 |  |
| --- | --- | --- | --- | --- | --- |
| {BcEV5=FALSE, BcOLV4.BI=TRUE} =>  {BcMV3.BI=TRUE} | 0.08  4158 | 0.693  878 | 0.121  287 | 1.28  003 | 34 |
| {BcEV5=FALSE, BcMV3.BI=TRUE} =>  {BcOLV4.BI=TRUE} | 0.08  4158 | 0.17 | 0.495  05 | 1.37  36 | 34 |
| {BcOLV4.BI=TRUE, BcULV3=TRUE} =>  {BcMV11=TRUE} | 0.07  6733 | 0.885  714 | 0.086  634 | 1.43  7063 | 31 |
| {BcMV11=TRUE, BcOLV4.BI=TRUE} =>  {BcULV3=TRUE} | 0.07  6733 | 0.738  095 | 0.103  96 | 1.24  246 | 31 |
| {BcMV11=TRUE, BcULV3=TRUE} =>  {BcOLV4.BI=TRUE} | 0.07  6733 | 0.193  75 | 0.396  04 | 1.56  55 | 31 |
| {BcOLV4.BI=TRUE, BcULV3=TRUE} =>  {BcMV1.BI=TRUE} | 0.07  9208 | 0.914  286 | 0.086  634 | 1.15  7904 | 32 |
| {BcMV1.BI=TRUE, BcULV3=TRUE} =>  {BcOLV4.BI=TRUE} | 0.07  9208 | 0.163  265 | 0.485  149 | 1.31  9184 | 32 |
| {BcNSRV7.BI=FALSE, BcULV3=TRUE} =>  {BcOLV4.BI=TRUE} | 0.07  6733 | 0.175  141 | 0.438  119 | 1.41  5141 | 31 |
| {BcOLV4.BI=TRUE, BcULV3=TRUE} =>  {BcDMV1=FALSE} | 0.08  4158 | 0.971  429 | 0.086  634 | 1.15  0901 | 34 |
| {BcDMV1=FALSE, BcULV3=TRUE} =>  {BcOLV4.BI=TRUE} | 0.08  4158 | 0.166  667 | 0.504  95 | 1.34  6667 | 34 |
| {BcOLV4.BI=TRUE, BvF.BI=FALSE} => | 0.07 | 0.714 | 0.103 | 1.20 | 30 |

| {BcULV3=TRUE} | 4257 | 286 | 96 | 2381 |  |
| --- | --- | --- | --- | --- | --- |
| {BcEV6=FALSE, BcOLV4.BI=TRUE} =>  {BcULV3=TRUE} | 0.08  4158 | 0.708  333 | 0.118  812 | 1.19  2361 | 34 |
| {BcOLV15.BI=FALSE, BcOLV4.BI=TRUE}  => {BcULV3=TRUE} | 0.08  1683 | 0.717  391 | 0.113  861 | 1.20  7609 | 33 |
| {BcFV5.BI=FALSE, BcOLV4.BI=TRUE} =>  {BcMV11=TRUE} | 0.08  1683 | 0.804  878 | 0.101  485 | 1.30  5907 | 33 |
| {BcFV5.BI=FALSE, BcMV11=TRUE} =>  {BcOLV4.BI=TRUE} | 0.08  1683 | 0.171  875 | 0.475  248 | 1.38  875 | 33 |
| {BcOLV4.BI=TRUE, BcULV1.BI=FALSE} =>  {BcMV11=TRUE} | 0.07  4257 | 0.833  333 | 0.089  109 | 1.35  2075 | 30 |
| {BcMV11=TRUE, BcOLV4.BI=TRUE} =>  {BcMV1.BI=TRUE} | 0.10  1485 | 0.976  19 | 0.103  96 | 1.23  6304 | 41 |
| {BcMV1.BI=TRUE, BcOLV4.BI=TRUE} =>  {BcMV11=TRUE} | 0.10  1485 | 0.872  34 | 0.116  337 | 1.41  5364 | 41 |
| {BcMV1.BI=TRUE, BcMV11=TRUE} =>  {BcOLV4.BI=TRUE} | 0.10  1485 | 0.195  238 | 0.519  802 | 1.57  7524 | 41 |
| {BcMV12=FALSE, BcOLV4.BI=TRUE} =>  {BcMV11=TRUE} | 0.07  6733 | 0.885  714 | 0.086  634 | 1.43  7063 | 31 |
| {BcMV11=TRUE, BcOLV4.BI=TRUE} =>  {BcNSRV7.BI=FALSE} | 0.09  4059 | 0.904  762 | 0.103  96 | 1.13  1653 | 38 |
| {BcNSRV7.BI=FALSE, BcOLV4.BI=TRUE} | 0.09 | 0.826 | 0.113 | 1.34 | 38 |

| => {BcMV11=TRUE} | 4059 | 087 | 861 | 0318 |  |
| --- | --- | --- | --- | --- | --- |
| {BcMV11=TRUE, BcNSRV7.BI=FALSE} =>  {BcOLV4.BI=TRUE} | 0.09  4059 | 0.193  878 | 0.485  149 | 1.56  6531 | 38 |
| {BcOLV4.BI=TRUE, BcVV3.BI=FALSE} =>  {BcMV11=TRUE} | 0.07  9208 | 0.8 | 0.099  01 | 1.29  7992 | 32 |
| {BcMV11=TRUE, BcVV3.BI=FALSE} =>  {BcOLV4.BI=TRUE} | 0.07  9208 | 0.164  103 | 0.482  673 | 1.32  5949 | 32 |
| {BcOLV4.BI=TRUE, BcOLV7.BI=FALSE} =>  {BcMV11=TRUE} | 0.07  6733 | 0.815  789 | 0.094  059 | 1.32  361 | 31 |
| {BcALV1.BI=FALSE, BcOLV4.BI=TRUE} =>  {BcMV11=TRUE} | 0.07  6733 | 0.815  789 | 0.094  059 | 1.32  361 | 31 |
| {BcALV1.BI=FALSE, BcMV11=TRUE} =>  {BcOLV4.BI=TRUE} | 0.07  6733 | 0.166  667 | 0.460  396 | 1.34  6667 | 31 |
| {BcMV11=TRUE, BcOLV4.BI=TRUE} =>  {BcDMV1=FALSE} | 0.10  1485 | 0.976  19 | 0.103  96 | 1.15  6542 | 41 |
| {BcDMV1=FALSE, BcOLV4.BI=TRUE} =>  {BcMV11=TRUE} | 0.10  1485 | 0.836  735 | 0.121  287 | 1.35  7594 | 41 |
| {BcDMV1=FALSE, BcMV11=TRUE} =>  {BcOLV4.BI=TRUE} | 0.10  1485 | 0.199  029 | 0.509  901 | 1.60  8155 | 41 |
| {BcFV9=FALSE, BcOLV4.BI=TRUE} =>  {BcMV11=TRUE} | 0.08  1683 | 0.825 | 0.099  01 | 1.33  8554 | 33 |
| {BcOLV4.BI=TRUE, BcULV5=FALSE} => | 0.07 | 0.837 | 0.091 | 1.35 | 31 |

| {BcMV11=TRUE} | 6733 | 838 | 584 | 9383 |  |
| --- | --- | --- | --- | --- | --- |
| {BcBNV5.BI=FALSE, BcOLV4.BI=TRUE} =>  {BcMV11=TRUE} | 0.09  901 | 0.833  333 | 0.118  812 | 1.35  2075 | 40 |
| {BcBNV5.BI=FALSE, BcMV11=TRUE} =>  {BcOLV4.BI=TRUE} | 0.09  901 | 0.184  332 | 0.537  129 | 1.48  9401 | 40 |
| {BcOLV4.BI=TRUE, BvF.BI=FALSE} =>  {BcMV11=TRUE} | 0.08  9109 | 0.857  143 | 0.103  96 | 1.39  0706 | 36 |
| {BcMCV5.BI=FALSE, BcOLV4.BI=TRUE} =>  {BcMV11=TRUE} | 0.08  1683 | 0.825 | 0.099  01 | 1.33  8554 | 33 |
| {BcOLV13.BI=FALSE, BcOLV4.BI=TRUE}  => {BcMV11=TRUE} | 0.07  4257 | 0.833  333 | 0.089  109 | 1.35  2075 | 30 |
| {BcOLV2.BI=FALSE, BcOLV4.BI=TRUE} =>  {BcMV11=TRUE} | 0.09  4059 | 0.863  636 | 0.108  911 | 1.40  1241 | 38 |
| {BcMV11=TRUE, BcOLV2.BI=FALSE} =>  {BcOLV4.BI=TRUE} | 0.09  4059 | 0.165  217 | 0.569  307 | 1.33  4957 | 38 |
| {BcMCV3.BI=FALSE, BcOLV4.BI=TRUE} =>  {BcMV11=TRUE} | 0.09  4059 | 0.883  721 | 0.106  436 | 1.43  3828 | 38 |
| {BcMCV3.BI=FALSE, BcMV11=TRUE} =>  {BcOLV4.BI=TRUE} | 0.09  4059 | 0.161  702 | 0.581  683 | 1.30  6553 | 38 |
| {BcOLV4.BI=TRUE, BcPV4.BI=FALSE} =>  {BcMV11=TRUE} | 0.09  4059 | 0.844  444 | 0.111  386 | 1.37  0103 | 38 |
| {BcMV11=TRUE, BcPV4.BI=FALSE} => | 0.09 | 0.163 | 0.576 | 1.31 | 38 |

| {BcOLV4.BI=TRUE} | 4059 | 09 | 733 | 7768 |  |
| --- | --- | --- | --- | --- | --- |
| {BcEV6=FALSE, BcOLV4.BI=TRUE} =>  {BcMV11=TRUE} | 0.10  1485 | 0.854  167 | 0.118  812 | 1.38  5877 | 41 |
| {BcEV6=FALSE, BcMV11=TRUE} =>  {BcOLV4.BI=TRUE} | 0.10  1485 | 0.175  966 | 0.576  733 | 1.42  1803 | 41 |
| {BcOLV17.BI=FALSE, BcOLV4.BI=TRUE}  => {BcMV11=TRUE} | 0.08  4158 | 0.809  524 | 0.103  96 | 1.31  3444 | 34 |
| {BcOLV15.BI=FALSE, BcOLV4.BI=TRUE}  => {BcMV11=TRUE} | 0.09  4059 | 0.826  087 | 0.113  861 | 1.34  0318 | 38 |
| {BcEV5=FALSE, BcOLV4.BI=TRUE} =>  {BcMV11=TRUE} | 0.10  1485 | 0.836  735 | 0.121  287 | 1.35  7594 | 41 |
| {BcEV5=FALSE, BcMV11=TRUE} =>  {BcOLV4.BI=TRUE} | 0.10  1485 | 0.176  724 | 0.574  257 | 1.42  7931 | 41 |
| {BcMV6.BI=FALSE, BcOLV4.BI=TRUE} =>  {BcMV1.BI=TRUE} | 0.07  4257 | 0.937  5 | 0.079  208 | 1.18  7304 | 30 |
| {BcMV6.BI=FALSE, BcOLV4.BI=TRUE} =>  {BcNSRV7.BI=FALSE} | 0.07  6733 | 0.968  75 | 0.079  208 | 1.21  1687 | 31 |
| {BcMV6.BI=FALSE, BcOLV4.BI=TRUE} =>  {BcDMV1=FALSE} | 0.07  9208 | 1 | 0.079  208 | 1.18  4751 | 32 |
| {BcOLV4.BI=TRUE, BcRV1.BI=FALSE} =>  {BcMV1.BI=TRUE} | 0.08  4158 | 0.918  919 | 0.091  584 | 1.16  3772 | 34 |
| {BcOLV4.BI=TRUE, BcRV1.BI=FALSE} => | 0.08 | 0.945 | 0.091 | 1.18 | 35 |

| {BcNSRV7.BI=FALSE} | 6634 | 946 | 584 | 3165 |  |
| --- | --- | --- | --- | --- | --- |
| {BcOLV4.BI=TRUE, BcRV1.BI=FALSE} =>  {BcDMV1=FALSE} | 0.08  9109 | 0.972  973 | 0.091  584 | 1.15  273 | 36 |
| {BcMCV5.BI=FALSE, BcOLV4.BI=TRUE} =>  {BcRV1.BI=FALSE} | 0.08  4158 | 0.85 | 0.099  01 | 1.17  6027 | 34 |
| {BcOLV13.BI=FALSE, BcOLV4.BI=TRUE}  => {BcRV1.BI=FALSE} | 0.07  4257 | 0.833  333 | 0.089  109 | 1.15  2968 | 30 |
| {BcOLV17.BI=FALSE, BcOLV4.BI=TRUE}  => {BcRV1.BI=FALSE} | 0.08  9109 | 0.857  143 | 0.103  96 | 1.18  591 | 36 |
| {BcEV2.BI=FALSE, BcOLV4.BI=TRUE} =>  {BcFV5.BI=FALSE} | 0.07  6733 | 0.939  394 | 0.081  683 | 1.20  0997 | 31 |
| {BcEV2.BI=FALSE, BcOLV4.BI=TRUE} =>  {BcMV1.BI=TRUE} | 0.07  4257 | 0.909  091 | 0.081  683 | 1.15  1325 | 30 |
| {BcEV2.BI=FALSE, BcOLV4.BI=TRUE} =>  {BcNSRV7.BI=FALSE} | 0.08  1683 | 1 | 0.081  683 | 1.25  0774 | 33 |
| {BcEV2.BI=FALSE, BcOLV4.BI=TRUE} =>  {BcDMV1=FALSE} | 0.07  9208 | 0.969  697 | 0.081  683 | 1.14  8849 | 32 |
| {BcHV3.BI=FALSE, BcOLV4.BI=TRUE} =>  {BcFV5.BI=FALSE} | 0.08  4158 | 0.971  429 | 0.086  634 | 1.24  1953 | 34 |
| {BcHV3.BI=FALSE, BcOLV4.BI=TRUE} =>  {BcMV1.BI=TRUE} | 0.08  4158 | 0.971  429 | 0.086  634 | 1.23  0273 | 34 |
| {BcHV3.BI=FALSE, BcOLV4.BI=TRUE} => | 0.07 | 0.914 | 0.086 | 1.14 | 32 |

| {BcNSRV7.BI=FALSE} | 9208 | 286 | 634 | 3565 |  |
| --- | --- | --- | --- | --- | --- |
| {BcHV3.BI=FALSE, BcOLV4.BI=TRUE} =>  {BcDMV1=FALSE} | 0.08  6634 | 1 | 0.086  634 | 1.18  4751 | 35 |
| {BcFV5.BI=FALSE, BcOLV4.BI=TRUE} =>  {BcMV1.BI=TRUE} | 0.09  4059 | 0.926  829 | 0.101  485 | 1.17  379 | 38 |
| {BcFV5.BI=FALSE, BcOLV4.BI=TRUE} =>  {BcNSRV7.BI=FALSE} | 0.09  6535 | 0.951  22 | 0.101  485 | 1.18  9761 | 39 |
| {BcFV5.BI=FALSE, BcOLV4.BI=TRUE} =>  {BcDMV1=FALSE} | 0.10  1485 | 1 | 0.101  485 | 1.18  4751 | 41 |
| {BcOLV4.BI=TRUE, BcULV1.BI=FALSE} =>  {BcMV1.BI=TRUE} | 0.08  1683 | 0.916  667 | 0.089  109 | 1.16  092 | 33 |
| {BcOLV4.BI=TRUE, BcULV1.BI=FALSE} =>  {BcNSRV7.BI=FALSE} | 0.08  1683 | 0.916  667 | 0.089  109 | 1.14  6543 | 33 |
| {BcOLV4.BI=TRUE, BcULV1.BI=FALSE} =>  {BcDMV1=FALSE} | 0.08  6634 | 0.972  222 | 0.089  109 | 1.15  1841 | 35 |
| {BcMV12=FALSE, BcOLV4.BI=TRUE} =>  {BcMV1.BI=TRUE} | 0.08  1683 | 0.942  857 | 0.086  634 | 1.19  4089 | 33 |
| {BcMV1.BI=TRUE, BcOLV4.BI=TRUE} =>  {BcNSRV7.BI=FALSE} | 0.10  6436 | 0.914  894 | 0.116  337 | 1.14  4325 | 43 |
| {BcNSRV7.BI=FALSE, BcOLV4.BI=TRUE}  => {BcMV1.BI=TRUE} | 0.10  6436 | 0.934  783 | 0.113  861 | 1.18  3863 | 43 |
| {BcMV1.BI=TRUE, BcNSRV7.BI=FALSE} => | 0.10 | 0.170 | 0.623 | 1.37 | 43 |

| {BcOLV4.BI=TRUE} | 6436 | 635 | 762 | 873 |  |
| --- | --- | --- | --- | --- | --- |
| {BcOLV4.BI=TRUE, BcVV3.BI=FALSE} =>  {BcMV1.BI=TRUE} | 0.09  1584 | 0.925 | 0.099  01 | 1.17  1473 | 37 |
| {BcOLV4.BI=TRUE, BcOLV7.BI=FALSE} =>  {BcMV1.BI=TRUE} | 0.08  6634 | 0.921  053 | 0.094  059 | 1.16  6474 | 35 |
| {BcALV1.BI=FALSE, BcOLV4.BI=TRUE} =>  {BcMV1.BI=TRUE} | 0.08  9109 | 0.947  368 | 0.094  059 | 1.19  9802 | 36 |
| {BcMV1.BI=TRUE, BcOLV4.BI=TRUE} =>  {BcDMV1=FALSE} | 0.11  3861 | 0.978  723 | 0.116  337 | 1.15  9543 | 46 |
| {BcDMV1=FALSE, BcOLV4.BI=TRUE} =>  {BcMV1.BI=TRUE} | 0.11  3861 | 0.938  776 | 0.121  287 | 1.18  8919 | 46 |
| {BcDMV1=FALSE, BcMV1.BI=TRUE} =>  {BcOLV4.BI=TRUE} | 0.11  3861 | 0.171  004 | 0.665  842 | 1.38  171 | 46 |
| {BcFV9=FALSE, BcOLV4.BI=TRUE} =>  {BcMV1.BI=TRUE} | 0.09  1584 | 0.925 | 0.099  01 | 1.17  1473 | 37 |
| {BcOLV4.BI=TRUE, BcULV5=FALSE} =>  {BcMV1.BI=TRUE} | 0.08  4158 | 0.918  919 | 0.091  584 | 1.16  3772 | 34 |
| {BcBNV5.BI=FALSE, BcOLV4.BI=TRUE} =>  {BcMV1.BI=TRUE} | 0.11  1386 | 0.937  5 | 0.118  812 | 1.18  7304 | 45 |
| {BcBNV5.BI=FALSE, BcMV1.BI=TRUE} =>  {BcOLV4.BI=TRUE} | 0.11  1386 | 0.157  895 | 0.705  446 | 1.27  5789 | 45 |
| {BcOLV4.BI=TRUE, BvF.BI=FALSE} => | 0.10 | 0.976 | 0.103 | 1.23 | 41 |

| {BcMV1.BI=TRUE} | 1485 | 19 | 96 | 6304 |  |
| --- | --- | --- | --- | --- | --- |
| {BcMCV5.BI=FALSE, BcOLV4.BI=TRUE} =>  {BcMV1.BI=TRUE} | 0.09  1584 | 0.925 | 0.099  01 | 1.17  1473 | 37 |
| {BcOLV13.BI=FALSE, BcOLV4.BI=TRUE}  => {BcMV1.BI=TRUE} | 0.08  1683 | 0.916  667 | 0.089  109 | 1.16  092 | 33 |
| {BcOLV2.BI=FALSE, BcOLV4.BI=TRUE} =>  {BcMV1.BI=TRUE} | 0.10  396 | 0.954  545 | 0.108  911 | 1.20  8891 | 42 |
| {BcMCV3.BI=FALSE, BcOLV4.BI=TRUE} =>  {BcMV1.BI=TRUE} | 0.10  1485 | 0.953  488 | 0.106  436 | 1.20  7553 | 41 |
| {BcOLV4.BI=TRUE, BcPV4.BI=FALSE} =>  {BcMV1.BI=TRUE} | 0.10  396 | 0.933  333 | 0.111  386 | 1.18  2027 | 42 |
| {BcEV6=FALSE, BcOLV4.BI=TRUE} =>  {BcMV1.BI=TRUE} | 0.11  1386 | 0.937  5 | 0.118  812 | 1.18  7304 | 45 |
| {BcOLV17.BI=FALSE, BcOLV4.BI=TRUE}  => {BcMV1.BI=TRUE} | 0.09  6535 | 0.928  571 | 0.103  96 | 1.17  5996 | 39 |
| {BcOLV15.BI=FALSE, BcOLV4.BI=TRUE}  => {BcMV1.BI=TRUE} | 0.10  6436 | 0.934  783 | 0.113  861 | 1.18  3863 | 43 |
| {BcEV5=FALSE, BcOLV4.BI=TRUE} =>  {BcMV1.BI=TRUE} | 0.11  3861 | 0.938  776 | 0.121  287 | 1.18  8919 | 46 |
| {BcMV12=FALSE, BcOLV4.BI=TRUE} =>  {BcNSRV7.BI=FALSE} | 0.08  1683 | 0.942  857 | 0.086  634 | 1.17  9301 | 33 |
| {BcMV12=FALSE, BcOLV4.BI=TRUE} => | 0.08 | 0.971 | 0.086 | 1.15 | 34 |

| {BcDMV1=FALSE} | 4158 | 429 | 634 | 0901 |  |
| --- | --- | --- | --- | --- | --- |
| {BcOLV4.BI=TRUE, BcVV3.BI=FALSE} =>  {BcNSRV7.BI=FALSE} | 0.08  9109 | 0.9 | 0.099  01 | 1.12  5697 | 36 |
| {BcOLV4.BI=TRUE, BcOLV7.BI=FALSE} =>  {BcNSRV7.BI=FALSE} | 0.08  6634 | 0.921  053 | 0.094  059 | 1.15  2029 | 35 |
| {BcALV1.BI=FALSE, BcOLV4.BI=TRUE} =>  {BcNSRV7.BI=FALSE} | 0.08  4158 | 0.894  737 | 0.094  059 | 1.11  9114 | 34 |
| {BcNSRV7.BI=FALSE, BcOLV4.BI=TRUE}  => {BcDMV1=FALSE} | 0.11  1386 | 0.978  261 | 0.113  861 | 1.15  8995 | 45 |
| {BcDMV1=FALSE, BcOLV4.BI=TRUE} =>  {BcNSRV7.BI=FALSE} | 0.11  1386 | 0.918  367 | 0.121  287 | 1.14  867 | 45 |
| {BcFV9=FALSE, BcOLV4.BI=TRUE} =>  {BcNSRV7.BI=FALSE} | 0.09  4059 | 0.95 | 0.099  01 | 1.18  8235 | 38 |
| {BcBNV5.BI=FALSE, BcOLV4.BI=TRUE} =>  {BcNSRV7.BI=FALSE} | 0.10  8911 | 0.916  667 | 0.118  812 | 1.14  6543 | 44 |
| {BcOLV4.BI=TRUE, BvF.BI=FALSE} =>  {BcNSRV7.BI=FALSE} | 0.09  6535 | 0.928  571 | 0.103  96 | 1.16  1433 | 39 |
| {BcMCV5.BI=FALSE, BcOLV4.BI=TRUE} =>  {BcNSRV7.BI=FALSE} | 0.09  1584 | 0.925 | 0.099  01 | 1.15  6966 | 37 |
| {BcOLV13.BI=FALSE, BcOLV4.BI=TRUE}  => {BcNSRV7.BI=FALSE} | 0.08  4158 | 0.944  444 | 0.089  109 | 1.18  1287 | 34 |
| {BcOLV2.BI=FALSE, BcOLV4.BI=TRUE} => | 0.09 | 0.909 | 0.108 | 1.13 | 40 |

| {BcNSRV7.BI=FALSE} | 901 | 091 | 911 | 7067 |  |
| --- | --- | --- | --- | --- | --- |
| {BcMCV3.BI=FALSE, BcOLV4.BI=TRUE} =>  {BcNSRV7.BI=FALSE} | 0.09  901 | 0.930  233 | 0.106  436 | 1.16  3511 | 40 |
| {BcOLV4.BI=TRUE, BcPV4.BI=FALSE} =>  {BcNSRV7.BI=FALSE} | 0.10  396 | 0.933  333 | 0.111  386 | 1.16  7389 | 42 |
| {BcEV6=FALSE, BcOLV4.BI=TRUE} =>  {BcNSRV7.BI=FALSE} | 0.10  8911 | 0.916  667 | 0.118  812 | 1.14  6543 | 44 |
| {BcOLV17.BI=FALSE, BcOLV4.BI=TRUE}  => {BcNSRV7.BI=FALSE} | 0.09  901 | 0.952  381 | 0.103  96 | 1.19  1213 | 40 |
| {BcOLV15.BI=FALSE, BcOLV4.BI=TRUE}  => {BcNSRV7.BI=FALSE} | 0.10  6436 | 0.934  783 | 0.113  861 | 1.16  9202 | 43 |
| {BcEV5=FALSE, BcOLV4.BI=TRUE} =>  {BcNSRV7.BI=FALSE} | 0.11  1386 | 0.918  367 | 0.121  287 | 1.14  867 | 45 |
| {BcOLV4.BI=TRUE, BcVV3.BI=FALSE} =>  {BcDMV1=FALSE} | 0.09  901 | 1 | 0.099  01 | 1.18  4751 | 40 |
| {BcOLV4.BI=TRUE, BcVV3.BI=FALSE} =>  {BcBNV5.BI=FALSE} | 0.09  901 | 1 | 0.099  01 | 1.10  0817 | 40 |
| {BcOLV4.BI=TRUE, BcOLV7.BI=FALSE} =>  {BcDMV1=FALSE} | 0.09  1584 | 0.973  684 | 0.094  059 | 1.15  3573 | 37 |
| {BcALV1.BI=FALSE, BcOLV4.BI=TRUE} =>  {BcDMV1=FALSE} | 0.09  4059 | 1 | 0.094  059 | 1.18  4751 | 38 |
| {BcFV9=FALSE, BcOLV4.BI=TRUE} => | 0.09 | 0.975 | 0.099 | 1.15 | 39 |

| {BcDMV1=FALSE} | 6535 |  | 01 | 5132 |  |
| --- | --- | --- | --- | --- | --- |
| {BcOLV4.BI=TRUE, BcULV5=FALSE} =>  {BcDMV1=FALSE} | 0.08  9109 | 0.972  973 | 0.091  584 | 1.15  273 | 36 |
| {BcBNV5.BI=FALSE, BcOLV4.BI=TRUE} =>  {BcDMV1=FALSE} | 0.11  8812 | 1 | 0.118  812 | 1.18  4751 | 48 |
| {BcOLV4.BI=TRUE, BvF.BI=FALSE} =>  {BcDMV1=FALSE} | 0.10  1485 | 0.976  19 | 0.103  96 | 1.15  6542 | 41 |
| {BcMCV5.BI=FALSE, BcOLV4.BI=TRUE} =>  {BcDMV1=FALSE} | 0.09  901 | 1 | 0.099  01 | 1.18  4751 | 40 |
| {BcOLV13.BI=FALSE, BcOLV4.BI=TRUE}  => {BcDMV1=FALSE} | 0.08  6634 | 0.972  222 | 0.089  109 | 1.15  1841 | 35 |
| {BcOLV2.BI=FALSE, BcOLV4.BI=TRUE} =>  {BcDMV1=FALSE} | 0.10  6436 | 0.977  273 | 0.108  911 | 1.15  7825 | 43 |
| {BcMCV3.BI=FALSE, BcOLV4.BI=TRUE} =>  {BcDMV1=FALSE} | 0.10  396 | 0.976  744 | 0.106  436 | 1.15  7198 | 42 |
| {BcOLV4.BI=TRUE, BcPV4.BI=FALSE} =>  {BcDMV1=FALSE} | 0.10  8911 | 0.977  778 | 0.111  386 | 1.15  8423 | 44 |
| {BcEV6=FALSE, BcOLV4.BI=TRUE} =>  {BcDMV1=FALSE} | 0.11  6337 | 0.979  167 | 0.118  812 | 1.16  0068 | 47 |
| {BcOLV17.BI=FALSE, BcOLV4.BI=TRUE}  => {BcDMV1=FALSE} | 0.10  1485 | 0.976  19 | 0.103  96 | 1.15  6542 | 41 |
| {BcOLV15.BI=FALSE, BcOLV4.BI=TRUE} | 0.11 | 0.978 | 0.113 | 1.15 | 45 |

| => {BcDMV1=FALSE} | 1386 | 261 | 861 | 8995 |  |
| --- | --- | --- | --- | --- | --- |
| {BcEV5=FALSE, BcOLV4.BI=TRUE} =>  {BcDMV1=FALSE} | 0.12  1287 | 1 | 0.121  287 | 1.18  4751 | 49 |
| {BcMCV5.BI=FALSE, BcOLV4.BI=TRUE} =>  {BcBNV5.BI=FALSE} | 0.09  901 | 1 | 0.099  01 | 1.10  0817 | 40 |
| {BcMV9.BI=FALSE, BcULV5=TRUE} =>  {BcOLV18=FALSE} | 0.07  4257 | 0.731  707 | 0.101  485 | 1.51  5947 | 30 |
| {BcOLV18=FALSE, BcULV5=TRUE} =>  {BcMV9.BI=FALSE} | 0.07  4257 | 1 | 0.074  257 | 3.10  7692 | 30 |
| {BcMV9.BI=FALSE, BcOLV18=FALSE} =>  {BcULV5=TRUE} | 0.07  4257 | 0.340  909 | 0.217  822 | 2.75  4545 | 30 |
| {BcMV9.BI=FALSE, BcULV5=TRUE} =>  {BcMV11=TRUE} | 0.09  4059 | 0.926  829 | 0.101  485 | 1.50  3771 | 38 |
| {BcMV11=TRUE, BcULV5=TRUE} =>  {BcMV9.BI=FALSE} | 0.09  4059 | 0.844  444 | 0.111  386 | 2.62  4274 | 38 |
| {BcMV11=TRUE, BcMV9.BI=FALSE} =>  {BcULV5=TRUE} | 0.09  4059 | 0.447  059 | 0.210  396 | 3.61  2235 | 38 |
| {BcMV9.BI=FALSE, BcULV5=TRUE} =>  {BcRV1.BI=FALSE} | 0.08  6634 | 0.853  659 | 0.101  485 | 1.18  1089 | 35 |
| {BcRV1.BI=FALSE, BcULV5=TRUE} =>  {BcMV9.BI=FALSE} | 0.08  6634 | 0.921  053 | 0.094  059 | 2.86  2348 | 35 |
| {BcMV9.BI=FALSE, BcRV1.BI=FALSE} => | 0.08 | 0.324 | 0.267 | 2.61 | 35 |

| {BcULV5=TRUE} | 6634 | 074 | 327 | 8519 |  |
| --- | --- | --- | --- | --- | --- |
| {BcEV2.BI=FALSE, BcULV5=TRUE} =>  {BcMV9.BI=FALSE} | 0.07  4257 | 0.810  811 | 0.091  584 | 2.51  9751 | 30 |
| {BcEV2.BI=FALSE, BcMV9.BI=FALSE} =>  {BcULV5=TRUE} | 0.07  4257 | 0.306  122 | 0.242  574 | 2.47  3469 | 30 |
| {BcHV3.BI=FALSE, BcULV5=TRUE} =>  {BcMV9.BI=FALSE} | 0.07  9208 | 0.864  865 | 0.091  584 | 2.68  7734 | 32 |
| {BcHV3.BI=FALSE, BcMV9.BI=FALSE} =>  {BcULV5=TRUE} | 0.07  9208 | 0.310  68 | 0.254  95 | 2.51  0291 | 32 |
| {BcFV5.BI=FALSE, BcULV5=TRUE} =>  {BcMV9.BI=FALSE} | 0.08  6634 | 0.853  659 | 0.101  485 | 2.65  2908 | 35 |
| {BcFV5.BI=FALSE, BcMV9.BI=FALSE} =>  {BcULV5=TRUE} | 0.08  6634 | 0.321  101 | 0.269  802 | 2.59  4495 | 35 |
| {BcULV1.BI=FALSE, BcULV5=TRUE} =>  {BcMV9.BI=FALSE} | 0.08  1683 | 0.970  588 | 0.084  158 | 3.01  629 | 33 |
| {BcMV9.BI=FALSE, BcULV1.BI=FALSE} =>  {BcULV5=TRUE} | 0.08  1683 | 0.286  957 | 0.284  653 | 2.31  8609 | 33 |
| {BcMV9.BI=FALSE, BcULV5=TRUE} =>  {BcMV1.BI=TRUE} | 0.09  6535 | 0.951  22 | 0.101  485 | 1.20  4679 | 39 |
| {BcMV1.BI=TRUE, BcULV5=TRUE} =>  {BcMV9.BI=FALSE} | 0.09  6535 | 0.829  787 | 0.116  337 | 2.57  8723 | 39 |
| {BcMV1.BI=TRUE, BcMV9.BI=FALSE} => | 0.09 | 0.402 | 0.240 | 3.24 | 39 |

| {BcULV5=TRUE} | 6535 | 062 | 099 | 866 |  |
| --- | --- | --- | --- | --- | --- |
| {BcNSRV7.BI=FALSE, BcULV5=TRUE} =>  {BcMV9.BI=FALSE} | 0.08  9109 | 0.818  182 | 0.108  911 | 2.54  2657 | 36 |
| {BcMV9.BI=FALSE, BcNSRV7.BI=FALSE}  => {BcULV5=TRUE} | 0.08  9109 | 0.315  789 | 0.282  178 | 2.55  1579 | 36 |
| {BcMV9.BI=FALSE, BcULV5=TRUE} =>  {BcOLV7.BI=FALSE} | 0.09  6535 | 0.951  22 | 0.101  485 | 1.17  8812 | 39 |
| {BcOLV7.BI=FALSE, BcULV5=TRUE} =>  {BcMV9.BI=FALSE} | 0.09  6535 | 0.906  977 | 0.106  436 | 2.81  8605 | 39 |
| {BcMV9.BI=FALSE, BcOLV7.BI=FALSE} =>  {BcULV5=TRUE} | 0.09  6535 | 0.322  314 | 0.299  505 | 2.60  4298 | 39 |
| {BcMV9.BI=FALSE, BcULV5=TRUE} =>  {BcALV1.BI=FALSE} | 0.09  4059 | 0.926  829 | 0.101  485 | 1.13  8113 | 38 |
| {BcALV1.BI=FALSE, BcULV5=TRUE} =>  {BcMV9.BI=FALSE} | 0.09  4059 | 0.863  636 | 0.108  911 | 2.68  3916 | 38 |
| {BcALV1.BI=FALSE, BcMV9.BI=FALSE} =>  {BcULV5=TRUE} | 0.09  4059 | 0.345  455 | 0.272  277 | 2.79  1273 | 38 |
| {BcMV9.BI=FALSE, BcULV5=TRUE} =>  {BcDMV1=FALSE} | 0.10  1485 | 1 | 0.101  485 | 1.18  4751 | 41 |
| {BcDMV1=FALSE, BcULV5=TRUE} =>  {BcMV9.BI=FALSE} | 0.10  1485 | 0.854  167 | 0.118  812 | 2.65  4487 | 41 |
| {BcDMV1=FALSE, BcMV9.BI=FALSE} => | 0.10 | 0.347 | 0.292 | 2.80 | 41 |

| {BcULV5=TRUE} | 1485 | 458 | 079 | 7458 |  |
| --- | --- | --- | --- | --- | --- |
| {BcFV9=FALSE, BcULV5=TRUE} =>  {BcMV9.BI=FALSE} | 0.08  6634 | 0.813  953 | 0.106  436 | 2.52  9517 | 35 |
| {BcFV9=FALSE, BcMV9.BI=FALSE} =>  {BcULV5=TRUE} | 0.08  6634 | 0.304  348 | 0.284  653 | 2.45  913 | 35 |
| {BcOLV4.BI=FALSE, BcULV5=TRUE} =>  {BcMV9.BI=FALSE} | 0.07  4257 | 0.810  811 | 0.091  584 | 2.51  9751 | 30 |
| {BcMV9.BI=FALSE, BcOLV4.BI=FALSE} =>  {BcULV5=TRUE} | 0.07  4257 | 0.285  714 | 0.259  901 | 2.30  8571 | 30 |
| {BcBNV5.BI=FALSE, BcULV5=TRUE} =>  {BcMV9.BI=FALSE} | 0.09  901 | 0.833  333 | 0.118  812 | 2.58  9744 | 40 |
| {BcBNV5.BI=FALSE, BcMV9.BI=FALSE} =>  {BcULV5=TRUE} | 0.09  901 | 0.317  46 | 0.311  881 | 2.56  5079 | 40 |
| {BcULV5=TRUE, BvF.BI=FALSE} =>  {BcMV9.BI=FALSE} | 0.09  901 | 0.833  333 | 0.118  812 | 2.58  9744 | 40 |
| {BcMV9.BI=FALSE, BvF.BI=FALSE} =>  {BcULV5=TRUE} | 0.09  901 | 0.327  869 | 0.301  98 | 2.64  918 | 40 |
| {BcMCV5.BI=FALSE, BcULV5=TRUE} =>  {BcMV9.BI=FALSE} | 0.09  1584 | 0.840  909 | 0.108  911 | 2.61  3287 | 37 |
| {BcMCV5.BI=FALSE, BcMV9.BI=FALSE} =>  {BcULV5=TRUE} | 0.09  1584 | 0.298  387 | 0.306  931 | 2.41  0968 | 37 |
| {BcOLV13.BI=FALSE, BcULV5=TRUE} => | 0.07 | 0.775 | 0.099 | 2.40 | 31 |

| {BcMV9.BI=FALSE} | 6733 |  | 01 | 8462 |  |
| --- | --- | --- | --- | --- | --- |
| {BcMV9.BI=FALSE, BcOLV13.BI=FALSE} =>  {BcULV5=TRUE} | 0.07  6733 | 0.264  957 | 0.289  604 | 2.14  0855 | 31 |
| {BcOLV2.BI=FALSE, BcULV5=TRUE} =>  {BcMV9.BI=FALSE} | 0.09  1584 | 0.840  909 | 0.108  911 | 2.61  3287 | 37 |
| {BcMV9.BI=FALSE, BcOLV2.BI=FALSE} =>  {BcULV5=TRUE} | 0.09  1584 | 0.298  387 | 0.306  931 | 2.41  0968 | 37 |
| {BcMCV3.BI=FALSE, BcULV5=TRUE} =>  {BcMV9.BI=FALSE} | 0.08  9109 | 0.8 | 0.111  386 | 2.48  6154 | 36 |
| {BcMCV3.BI=FALSE, BcMV9.BI=FALSE} =>  {BcULV5=TRUE} | 0.08  9109 | 0.292  683 | 0.304  455 | 2.36  4878 | 36 |
| {BcPV4.BI=FALSE, BcULV5=TRUE} =>  {BcMV9.BI=FALSE} | 0.08  9109 | 0.8 | 0.111  386 | 2.48  6154 | 36 |
| {BcMV9.BI=FALSE, BcPV4.BI=FALSE} =>  {BcULV5=TRUE} | 0.08  9109 | 0.297  521 | 0.299  505 | 2.40  3967 | 36 |
| {BcEV6=FALSE, BcULV5=TRUE} =>  {BcMV9.BI=FALSE} | 0.09  1584 | 0.822  222 | 0.111  386 | 2.55  5214 | 37 |
| {BcEV6=FALSE, BcMV9.BI=FALSE} =>  {BcULV5=TRUE} | 0.09  1584 | 0.300  813 | 0.304  455 | 2.43  0569 | 37 |
| {BcOLV17.BI=FALSE, BcULV5=TRUE} =>  {BcMV9.BI=FALSE} | 0.09  4059 | 0.826  087 | 0.113  861 | 2.56  7224 | 38 |
| {BcMV9.BI=FALSE, BcOLV17.BI=FALSE} => | 0.09 | 0.304 | 0.309 | 2.45 | 38 |

| {BcULV5=TRUE} | 4059 |  | 406 | 632 |  |
| --- | --- | --- | --- | --- | --- |
| {BcOLV15.BI=FALSE, BcULV5=TRUE} =>  {BcMV9.BI=FALSE} | 0.09  6535 | 0.812  5 | 0.118  812 | 2.52  5 | 39 |
| {BcMV9.BI=FALSE, BcOLV15.BI=FALSE} =>  {BcULV5=TRUE} | 0.09  6535 | 0.312 | 0.309  406 | 2.52  096 | 39 |
| {BcEV5=FALSE, BcULV5=TRUE} =>  {BcMV9.BI=FALSE} | 0.10  1485 | 0.836  735 | 0.121  287 | 2.60  0314 | 41 |
| {BcEV5=FALSE, BcMV9.BI=FALSE} =>  {BcULV5=TRUE} | 0.10  1485 | 0.325  397 | 0.311  881 | 2.62  9206 | 41 |
| {BcULV5=TRUE, BvF.BI=FALSE} =>  {BcOLV1.BI.MOVA.=TRUE} | 0.07  4257 | 0.625 | 0.118  812 | 1.88  4328 | 30 |
| {BcOLV1.BI.MOVA.=TRUE, BvF.BI=FALSE}  => {BcULV5=TRUE} | 0.07  4257 | 0.236  22 | 0.314  356 | 1.90  8661 | 30 |
| {BcMV2.BI=TRUE, BcULV5=TRUE} =>  {BcMV11=TRUE} | 0.07  9208 | 0.914  286 | 0.086  634 | 1.48  3419 | 32 |
| {BcMV11=TRUE, BcULV5=TRUE} =>  {BcMV2.BI=TRUE} | 0.07  9208 | 0.711  111 | 0.111  386 | 1.53  6304 | 32 |
| {BcMV11=TRUE, BcMV2.BI=TRUE} =>  {BcULV5=TRUE} | 0.07  9208 | 0.231  884 | 0.341  584 | 1.87  3623 | 32 |
| {BcMV2.BI=TRUE, BcULV5=TRUE} =>  {BcMV1.BI=TRUE} | 0.08  4158 | 0.971  429 | 0.086  634 | 1.23  0273 | 34 |
| {BcMV1.BI=TRUE, BcULV5=TRUE} => | 0.08 | 0.723 | 0.116 | 1.56 | 34 |

| {BcMV2.BI=TRUE} | 4158 | 404 | 337 | 2863 |  |
| --- | --- | --- | --- | --- | --- |
| {BcMV1.BI=TRUE, BcMV2.BI=TRUE} =>  {BcULV5=TRUE} | 0.08  4158 | 0.206  061 | 0.408  416 | 1.66  497 | 34 |
| {BcMV2.BI=TRUE, BcULV5=TRUE} =>  {BcNSRV7.BI=FALSE} | 0.08  1683 | 0.942  857 | 0.086  634 | 1.17  9301 | 33 |
| {BcNSRV7.BI=FALSE, BcULV5=TRUE} =>  {BcMV2.BI=TRUE} | 0.08  1683 | 0.75 | 0.108  911 | 1.62  0321 | 33 |
| {BcMV2.BI=TRUE, BcNSRV7.BI=FALSE} =>  {BcULV5=TRUE} | 0.08  1683 | 0.226  027 | 0.361  386 | 1.82  6301 | 33 |
| {BcOLV7.BI=FALSE, BcULV5=TRUE} =>  {BcMV2.BI=TRUE} | 0.07  4257 | 0.697  674 | 0.106  436 | 1.50  7275 | 30 |
| {BcMV2.BI=TRUE, BcOLV7.BI=FALSE} =>  {BcULV5=TRUE} | 0.07  4257 | 0.206  897 | 0.358  911 | 1.67  1724 | 30 |
| {BcALV1.BI=FALSE, BcULV5=TRUE} =>  {BcMV2.BI=TRUE} | 0.07  4257 | 0.681  818 | 0.108  911 | 1.47  3019 | 30 |
| {BcALV1.BI=FALSE, BcMV2.BI=TRUE} =>  {BcULV5=TRUE} | 0.07  4257 | 0.229  008 | 0.324  257 | 1.85  0382 | 30 |
| {BcMV2.BI=TRUE, BcULV5=TRUE} =>  {BcDMV1=FALSE} | 0.08  4158 | 0.971  429 | 0.086  634 | 1.15  0901 | 34 |
| {BcDMV1=FALSE, BcULV5=TRUE} =>  {BcMV2.BI=TRUE} | 0.08  4158 | 0.708  333 | 0.118  812 | 1.53  0303 | 34 |
| {BcDMV1=FALSE, BcMV2.BI=TRUE} => | 0.08 | 0.212 | 0.396 | 1.71 | 34 |

| {BcULV5=TRUE} | 4158 | 5 | 04 | 7 |  |
| --- | --- | --- | --- | --- | --- |
| {BcFV9=FALSE, BcULV5=TRUE} =>  {BcMV2.BI=TRUE} | 0.07  6733 | 0.720  93 | 0.106  436 | 1.55  7518 | 31 |
| {BcFV9=FALSE, BcMV2.BI=TRUE} =>  {BcULV5=TRUE} | 0.07  6733 | 0.191  358 | 0.400  99 | 1.54  6173 | 31 |
| {BcBNV5.BI=FALSE, BcULV5=TRUE} =>  {BcMV2.BI=TRUE} | 0.08  1683 | 0.687  5 | 0.118  812 | 1.48  5294 | 33 |
| {BcBNV5.BI=FALSE, BcMV2.BI=TRUE} =>  {BcULV5=TRUE} | 0.08  1683 | 0.2 | 0.408  416 | 1.61  6 | 33 |
| {BcULV5=TRUE, BvF.BI=FALSE} =>  {BcMV2.BI=TRUE} | 0.08  6634 | 0.729  167 | 0.118  812 | 1.57  5312 | 35 |
| {BcMV2.BI=TRUE, BvF.BI=FALSE} =>  {BcULV5=TRUE} | 0.08  6634 | 0.2 | 0.433  168 | 1.61  6 | 35 |
| {BcMCV3.BI=FALSE, BcULV5=TRUE} =>  {BcMV2.BI=TRUE} | 0.07  4257 | 0.666  667 | 0.111  386 | 1.44  0285 | 30 |
| {BcMCV3.BI=FALSE, BcMV2.BI=TRUE} =>  {BcULV5=TRUE} | 0.07  4257 | 0.181  818 | 0.408  416 | 1.46  9091 | 30 |
| {BcPV4.BI=FALSE, BcULV5=TRUE} =>  {BcMV2.BI=TRUE} | 0.07  9208 | 0.711  111 | 0.111  386 | 1.53  6304 | 32 |
| {BcMV2.BI=TRUE, BcPV4.BI=FALSE} =>  {BcULV5=TRUE} | 0.07  9208 | 0.184  971 | 0.428  218 | 1.49  4566 | 32 |
| {BcEV6=FALSE, BcULV5=TRUE} => | 0.07 | 0.711 | 0.111 | 1.53 | 32 |

| {BcMV2.BI=TRUE} | 9208 | 111 | 386 | 6304 |  |
| --- | --- | --- | --- | --- | --- |
| {BcEV6=FALSE, BcMV2.BI=TRUE} =>  {BcULV5=TRUE} | 0.07  9208 | 0.182  857 | 0.433  168 | 1.47  7486 | 32 |
| {BcOLV17.BI=FALSE, BcULV5=TRUE} =>  {BcMV2.BI=TRUE} | 0.07  6733 | 0.673  913 | 0.113  861 | 1.45  594 | 31 |
| {BcMV2.BI=TRUE, BcOLV17.BI=FALSE} =>  {BcULV5=TRUE} | 0.07  6733 | 0.180  233 | 0.425  743 | 1.45  6279 | 31 |
| {BcOLV15.BI=FALSE, BcULV5=TRUE} =>  {BcMV2.BI=TRUE} | 0.08  4158 | 0.708  333 | 0.118  812 | 1.53  0303 | 34 |
| {BcMV2.BI=TRUE, BcOLV15.BI=FALSE} =>  {BcULV5=TRUE} | 0.08  4158 | 0.185  792 | 0.452  97 | 1.50  1202 | 34 |
| {BcEV5=FALSE, BcULV5=TRUE} =>  {BcMV2.BI=TRUE} | 0.08  4158 | 0.693  878 | 0.121  287 | 1.49  9072 | 34 |
| {BcEV5=FALSE, BcMV2.BI=TRUE} =>  {BcULV5=TRUE} | 0.08  4158 | 0.198  83 | 0.423  267 | 1.60  655 | 34 |
| {BcOLV18=FALSE, BcULV5=TRUE} =>  {BcOLV7.BI=FALSE} | 0.07  4257 | 1 | 0.074  257 | 1.23  9264 | 30 |
| {BcOLV7.BI=FALSE, BcULV5=TRUE} =>  {BcOLV18=FALSE} | 0.07  4257 | 0.697  674 | 0.106  436 | 1.44  5438 | 30 |
| {BcOLV18=FALSE, BcOLV7.BI=FALSE} =>  {BcULV5=TRUE} | 0.07  4257 | 0.172  414 | 0.430  693 | 1.39  3103 | 30 |
| {BcOLV18=FALSE, BcULV5=TRUE} => | 0.07 | 1 | 0.074 | 1.18 | 30 |

| {BcDMV1=FALSE} | 4257 |  | 257 | 4751 |  |
| --- | --- | --- | --- | --- | --- |
| {BcDMV1=FALSE, BcULV5=TRUE} =>  {BcOLV18=FALSE} | 0.07  4257 | 0.625 | 0.118  812 | 1.29  4872 | 30 |
| {BcDMV1=FALSE, BcOLV18=FALSE} =>  {BcULV5=TRUE} | 0.07  4257 | 0.178  571 | 0.415  842 | 1.44  2857 | 30 |
| {BcMCV5.BI=FALSE, BcULV5=TRUE} =>  {BcOLV18=FALSE} | 0.07  4257 | 0.681  818 | 0.108  911 | 1.41  2587 | 30 |
| {BcOLV15.BI=FALSE, BcULV5=TRUE} =>  {BcOLV18=FALSE} | 0.07  4257 | 0.625 | 0.118  812 | 1.29  4872 | 30 |
| {BcEV5=FALSE, BcULV5=TRUE} =>  {BcOLV18=FALSE} | 0.07  4257 | 0.612  245 | 0.121  287 | 1.26  8446 | 30 |
| {BcEV5=FALSE, BcOLV18=FALSE} =>  {BcULV5=TRUE} | 0.07  4257 | 0.160  428 | 0.462  871 | 1.29  6257 | 30 |
| {BcMV3.BI=TRUE, BcULV5=TRUE} =>  {BcMV11=TRUE} | 0.07  9208 | 0.888  889 | 0.089  109 | 1.44  2213 | 32 |
| {BcMV11=TRUE, BcULV5=TRUE} =>  {BcMV3.BI=TRUE} | 0.07  9208 | 0.711  111 | 0.111  386 | 1.31  1821 | 32 |
| {BcMV11=TRUE, BcMV3.BI=TRUE} =>  {BcULV5=TRUE} | 0.07  9208 | 0.206  452 | 0.383  663 | 1.66  8129 | 32 |
| {BcMV3.BI=TRUE, BcULV5=TRUE} =>  {BcMV1.BI=TRUE} | 0.08  4158 | 0.944  444 | 0.089  109 | 1.19  6099 | 34 |
| {BcMV1.BI=TRUE, BcULV5=TRUE} => | 0.08 | 0.723 | 0.116 | 1.33 | 34 |

| {BcMV3.BI=TRUE} | 4158 | 404 | 337 | 4499 |  |
| --- | --- | --- | --- | --- | --- |
| {BcMV1.BI=TRUE, BcMV3.BI=TRUE} =>  {BcULV5=TRUE} | 0.08  4158 | 0.183  784 | 0.457  921 | 1.48  4973 | 34 |
| {BcNSRV7.BI=FALSE, BcULV5=TRUE} =>  {BcMV3.BI=TRUE} | 0.07  9208 | 0.727  273 | 0.108  911 | 1.34  1636 | 32 |
| {BcMV3.BI=TRUE, BcNSRV7.BI=FALSE} =>  {BcULV5=TRUE} | 0.07  9208 | 0.187  135 | 0.423  267 | 1.51  2047 | 32 |
| {BcOLV7.BI=FALSE, BcULV5=TRUE} =>  {BcMV3.BI=TRUE} | 0.07  4257 | 0.697  674 | 0.106  436 | 1.28  7034 | 30 |
| {BcMV3.BI=TRUE, BcOLV7.BI=FALSE} =>  {BcULV5=TRUE} | 0.07  4257 | 0.178  571 | 0.415  842 | 1.44  2857 | 30 |
| {BcALV1.BI=FALSE, BcULV5=TRUE} =>  {BcMV3.BI=TRUE} | 0.07  6733 | 0.704  545 | 0.108  911 | 1.29  9709 | 31 |
| {BcALV1.BI=FALSE, BcMV3.BI=TRUE} =>  {BcULV5=TRUE} | 0.07  6733 | 0.181  287 | 0.423  267 | 1.46  4795 | 31 |
| {BcMV3.BI=TRUE, BcULV5=TRUE} =>  {BcDMV1=FALSE} | 0.08  4158 | 0.944  444 | 0.089  109 | 1.11  8931 | 34 |
| {BcDMV1=FALSE, BcULV5=TRUE} =>  {BcMV3.BI=TRUE} | 0.08  4158 | 0.708  333 | 0.118  812 | 1.30  6697 | 34 |
| {BcDMV1=FALSE, BcMV3.BI=TRUE} =>  {BcULV5=TRUE} | 0.08  4158 | 0.198  83 | 0.423  267 | 1.60  655 | 34 |
| {BcFV9=FALSE, BcULV5=TRUE} => | 0.07 | 0.744 | 0.106 | 1.37 | 32 |

| {BcMV3.BI=TRUE} | 9208 | 186 | 436 | 2836 |  |
| --- | --- | --- | --- | --- | --- |
| {BcFV9=FALSE, BcMV3.BI=TRUE} =>  {BcULV5=TRUE} | 0.07  9208 | 0.173  913 | 0.455  446 | 1.40  5217 | 32 |
| {BcBNV5.BI=FALSE, BcULV5=TRUE} =>  {BcMV3.BI=TRUE} | 0.08  6634 | 0.729  167 | 0.118  812 | 1.34  5129 | 35 |
| {BcBNV5.BI=FALSE, BcMV3.BI=TRUE} =>  {BcULV5=TRUE} | 0.08  6634 | 0.184  211 | 0.470  297 | 1.48  8421 | 35 |
| {BcULV5=TRUE, BvF.BI=FALSE} =>  {BcMV3.BI=TRUE} | 0.08  6634 | 0.729  167 | 0.118  812 | 1.34  5129 | 35 |
| {BcMV3.BI=TRUE, BvF.BI=FALSE} =>  {BcULV5=TRUE} | 0.08  6634 | 0.175 | 0.495  05 | 1.41  4 | 35 |
| {BcMCV5.BI=FALSE, BcULV5=TRUE} =>  {BcMV3.BI=TRUE} | 0.07  9208 | 0.727  273 | 0.108  911 | 1.34  1636 | 32 |
| {BcMCV5.BI=FALSE, BcMV3.BI=TRUE} =>  {BcULV5=TRUE} | 0.07  9208 | 0.164  103 | 0.482  673 | 1.32  5949 | 32 |
| {BcOLV2.BI=FALSE, BcULV5=TRUE} =>  {BcMV3.BI=TRUE} | 0.07  4257 | 0.681  818 | 0.108  911 | 1.25  7783 | 30 |
| {BcMCV3.BI=FALSE, BcULV5=TRUE} =>  {BcMV3.BI=TRUE} | 0.07  6733 | 0.688  889 | 0.111  386 | 1.27  0827 | 31 |
| {BcMCV3.BI=FALSE, BcMV3.BI=TRUE} =>  {BcULV5=TRUE} | 0.07  6733 | 0.161  458 | 0.475  248 | 1.30  4583 | 31 |
| {BcPV4.BI=FALSE, BcULV5=TRUE} => | 0.07 | 0.711 | 0.111 | 1.31 | 32 |

| {BcMV3.BI=TRUE} | 9208 | 111 | 386 | 1821 |  |
| --- | --- | --- | --- | --- | --- |
| {BcMV3.BI=TRUE, BcPV4.BI=FALSE} =>  {BcULV5=TRUE} | 0.07  9208 | 0.159  204 | 0.497  525 | 1.28  6368 | 32 |
| {BcEV6=FALSE, BcULV5=TRUE} =>  {BcMV3.BI=TRUE} | 0.08  1683 | 0.733  333 | 0.111  386 | 1.35  2816 | 33 |
| {BcOLV17.BI=FALSE, BcULV5=TRUE} =>  {BcMV3.BI=TRUE} | 0.08  4158 | 0.739  13 | 0.113  861 | 1.36  351 | 34 |
| {BcMV3.BI=TRUE, BcOLV17.BI=FALSE} =>  {BcULV5=TRUE} | 0.08  4158 | 0.164  251 | 0.512  376 | 1.32  715 | 34 |
| {BcOLV15.BI=FALSE, BcULV5=TRUE} =>  {BcMV3.BI=TRUE} | 0.08  6634 | 0.729  167 | 0.118  812 | 1.34  5129 | 35 |
| {BcMV3.BI=TRUE, BcOLV15.BI=FALSE} =>  {BcULV5=TRUE} | 0.08  6634 | 0.170  732 | 0.507  426 | 1.37  9512 | 35 |
| {BcEV5=FALSE, BcULV5=TRUE} =>  {BcMV3.BI=TRUE} | 0.08  6634 | 0.714  286 | 0.121  287 | 1.31  7678 | 35 |
| {BcEV5=FALSE, BcMV3.BI=TRUE} =>  {BcULV5=TRUE} | 0.08  6634 | 0.175 | 0.495  05 | 1.41  4 | 35 |
| {BcULV3=TRUE, BcULV5=TRUE} =>  {BcMV1.BI=TRUE} | 0.07  4257 | 0.967  742 | 0.076  733 | 1.22  5604 | 30 |
| {BcULV3=TRUE, BcULV5=TRUE} =>  {BcDMV1=FALSE} | 0.07  4257 | 0.967  742 | 0.076  733 | 1.14  6533 | 30 |
| {BcRV1.BI=FALSE, BcULV5=TRUE} => | 0.08 | 0.868 | 0.094 | 1.40 | 33 |

| {BcMV11=TRUE} | 1683 | 421 | 059 | 9004 |  |
| --- | --- | --- | --- | --- | --- |
| {BcMV11=TRUE, BcRV1.BI=FALSE} =>  {BcULV5=TRUE} | 0.08  1683 | 0.2 | 0.408  416 | 1.61  6 | 33 |
| {BcEV2.BI=FALSE, BcULV5=TRUE} =>  {BcMV11=TRUE} | 0.07  9208 | 0.864  865 | 0.091  584 | 1.40  3235 | 32 |
| {BcEV2.BI=FALSE, BcMV11=TRUE} =>  {BcULV5=TRUE} | 0.07  9208 | 0.183  908 | 0.430  693 | 1.48  5977 | 32 |
| {BcHV3.BI=FALSE, BcULV5=TRUE} =>  {BcMV11=TRUE} | 0.07  9208 | 0.864  865 | 0.091  584 | 1.40  3235 | 32 |
| {BcHV3.BI=FALSE, BcMV11=TRUE} =>  {BcULV5=TRUE} | 0.07  9208 | 0.178  771 | 0.443  069 | 1.44  4469 | 32 |
| {BcFV5.BI=FALSE, BcULV5=TRUE} =>  {BcMV11=TRUE} | 0.09  1584 | 0.902  439 | 0.101  485 | 1.46  4198 | 37 |
| {BcFV5.BI=FALSE, BcMV11=TRUE} =>  {BcULV5=TRUE} | 0.09  1584 | 0.192  708 | 0.475  248 | 1.55  7083 | 37 |
| {BcULV1.BI=FALSE, BcULV5=TRUE} =>  {BcMV11=TRUE} | 0.08  1683 | 0.970  588 | 0.084  158 | 1.57  477 | 33 |
| {BcMV11=TRUE, BcULV1.BI=FALSE} =>  {BcULV5=TRUE} | 0.08  1683 | 0.172  775 | 0.472  772 | 1.39  6021 | 33 |
| {BcMV11=TRUE, BcULV5=TRUE} =>  {BcMV1.BI=TRUE} | 0.10  6436 | 0.955  556 | 0.111  386 | 1.21  0171 | 43 |
| {BcMV1.BI=TRUE, BcULV5=TRUE} => | 0.10 | 0.914 | 0.116 | 1.48 | 43 |

| {BcMV11=TRUE} | 6436 | 894 | 337 | 4406 |  |
| --- | --- | --- | --- | --- | --- |
| {BcMV1.BI=TRUE, BcMV11=TRUE} =>  {BcULV5=TRUE} | 0.10  6436 | 0.204  762 | 0.519  802 | 1.65  4476 | 43 |
| {BcMV12=FALSE, BcULV5=TRUE} =>  {BcMV11=TRUE} | 0.07  6733 | 0.911  765 | 0.084  158 | 1.47  9329 | 31 |
| {BcNSRV7.BI=FALSE, BcULV5=TRUE} =>  {BcMV11=TRUE} | 0.09  6535 | 0.886  364 | 0.108  911 | 1.43  8116 | 39 |
| {BcMV11=TRUE, BcNSRV7.BI=FALSE} =>  {BcULV5=TRUE} | 0.09  6535 | 0.198  98 | 0.485  149 | 1.60  7755 | 39 |
| {BcOLV7.BI=FALSE, BcULV5=TRUE} =>  {BcMV11=TRUE} | 0.09  6535 | 0.906  977 | 0.106  436 | 1.47  1561 | 39 |
| {BcMV11=TRUE, BcOLV7.BI=FALSE} =>  {BcULV5=TRUE} | 0.09  6535 | 0.198  98 | 0.485  149 | 1.60  7755 | 39 |
| {BcALV1.BI=FALSE, BcULV5=TRUE} =>  {BcMV11=TRUE} | 0.09  6535 | 0.886  364 | 0.108  911 | 1.43  8116 | 39 |
| {BcALV1.BI=FALSE, BcMV11=TRUE} =>  {BcULV5=TRUE} | 0.09  6535 | 0.209  677 | 0.460  396 | 1.69  4194 | 39 |
| {BcMV11=TRUE, BcULV5=TRUE} =>  {BcDMV1=FALSE} | 0.10  8911 | 0.977  778 | 0.111  386 | 1.15  8423 | 44 |
| {BcDMV1=FALSE, BcULV5=TRUE} =>  {BcMV11=TRUE} | 0.10  8911 | 0.916  667 | 0.118  812 | 1.48  7282 | 44 |
| {BcDMV1=FALSE, BcMV11=TRUE} => | 0.10 | 0.213 | 0.509 | 1.72 | 44 |

| {BcULV5=TRUE} | 8911 | 592 | 901 | 5825 |  |
| --- | --- | --- | --- | --- | --- |
| {BcFV9=FALSE, BcULV5=TRUE} =>  {BcMV11=TRUE} | 0.09  6535 | 0.906  977 | 0.106  436 | 1.47  1561 | 39 |
| {BcFV9=FALSE, BcMV11=TRUE} =>  {BcULV5=TRUE} | 0.09  6535 | 0.184  834 | 0.522  277 | 1.49  346 | 39 |
| {BcOLV4.BI=FALSE, BcULV5=TRUE} =>  {BcMV11=TRUE} | 0.08  4158 | 0.918  919 | 0.091  584 | 1.49  0937 | 34 |
| {BcMV11=TRUE, BcOLV4.BI=FALSE} =>  {BcULV5=TRUE} | 0.08  4158 | 0.164  251 | 0.512  376 | 1.32  715 | 34 |
| {BcBNV5.BI=FALSE, BcULV5=TRUE} =>  {BcMV11=TRUE} | 0.10  6436 | 0.895  833 | 0.118  812 | 1.45  3481 | 43 |
| {BcBNV5.BI=FALSE, BcMV11=TRUE} =>  {BcULV5=TRUE} | 0.10  6436 | 0.198  157 | 0.537  129 | 1.60  1106 | 43 |
| {BcULV5=TRUE, BvF.BI=FALSE} =>  {BcMV11=TRUE} | 0.10  8911 | 0.916  667 | 0.118  812 | 1.48  7282 | 44 |
| {BcMV11=TRUE, BvF.BI=FALSE} =>  {BcULV5=TRUE} | 0.10  8911 | 0.190  476 | 0.571  782 | 1.53  9048 | 44 |
| {BcMCV5.BI=FALSE, BcULV5=TRUE} =>  {BcMV11=TRUE} | 0.09  6535 | 0.886  364 | 0.108  911 | 1.43  8116 | 39 |
| {BcMCV5.BI=FALSE, BcMV11=TRUE} =>  {BcULV5=TRUE} | 0.09  6535 | 0.176  471 | 0.547  03 | 1.42  5882 | 39 |
| {BcOLV13.BI=FALSE, BcULV5=TRUE} => | 0.09 | 0.925 | 0.099 | 1.50 | 37 |

| {BcMV11=TRUE} | 1584 |  | 01 | 0803 |  |
| --- | --- | --- | --- | --- | --- |
| {BcMV11=TRUE, BcOLV13.BI=FALSE} =>  {BcULV5=TRUE} | 0.09  1584 | 0.163  717 | 0.559  406 | 1.32  2832 | 37 |
| {BcOLV2.BI=FALSE, BcULV5=TRUE} =>  {BcMV11=TRUE} | 0.09  901 | 0.909  091 | 0.108  911 | 1.47  4991 | 40 |
| {BcMV11=TRUE, BcOLV2.BI=FALSE} =>  {BcULV5=TRUE} | 0.09  901 | 0.173  913 | 0.569  307 | 1.40  5217 | 40 |
| {BcMCV3.BI=FALSE, BcULV5=TRUE} =>  {BcMV11=TRUE} | 0.10  396 | 0.933  333 | 0.111  386 | 1.51  4324 | 42 |
| {BcMCV3.BI=FALSE, BcMV11=TRUE} =>  {BcULV5=TRUE} | 0.10  396 | 0.178  723 | 0.581  683 | 1.44  4085 | 42 |
| {BcPV4.BI=FALSE, BcULV5=TRUE} =>  {BcMV11=TRUE} | 0.10  1485 | 0.911  111 | 0.111  386 | 1.47  8269 | 41 |
| {BcMV11=TRUE, BcPV4.BI=FALSE} =>  {BcULV5=TRUE} | 0.10  1485 | 0.175  966 | 0.576  733 | 1.42  1803 | 41 |
| {BcEV6=FALSE, BcULV5=TRUE} =>  {BcMV11=TRUE} | 0.10  1485 | 0.911  111 | 0.111  386 | 1.47  8269 | 41 |
| {BcEV6=FALSE, BcMV11=TRUE} =>  {BcULV5=TRUE} | 0.10  1485 | 0.175  966 | 0.576  733 | 1.42  1803 | 41 |
| {BcOLV17.BI=FALSE, BcULV5=TRUE} =>  {BcMV11=TRUE} | 0.10  1485 | 0.891  304 | 0.113  861 | 1.44  6132 | 41 |
| {BcMV11=TRUE, BcOLV17.BI=FALSE} => | 0.10 | 0.176 | 0.574 | 1.42 | 41 |

| {BcULV5=TRUE} | 1485 | 724 | 257 | 7931 |  |
| --- | --- | --- | --- | --- | --- |
| {BcOLV15.BI=FALSE, BcULV5=TRUE} =>  {BcMV11=TRUE} | 0.10  6436 | 0.895  833 | 0.118  812 | 1.45  3481 | 43 |
| {BcMV11=TRUE, BcOLV15.BI=FALSE} =>  {BcULV5=TRUE} | 0.10  6436 | 0.180  672 | 0.589  109 | 1.45  9832 | 43 |
| {BcEV5=FALSE, BcULV5=TRUE} =>  {BcMV11=TRUE} | 0.10  8911 | 0.897  959 | 0.121  287 | 1.45  693 | 44 |
| {BcEV5=FALSE, BcMV11=TRUE} =>  {BcULV5=TRUE} | 0.10  8911 | 0.189  655 | 0.574  257 | 1.53  2414 | 44 |
| {BcEV2.BI=FALSE, BcULV5=TRUE} =>  {BcRV1.BI=FALSE} | 0.07  6733 | 0.837  838 | 0.091  584 | 1.15  92 | 31 |
| {BcRV1.BI=FALSE, BcULV5=TRUE} =>  {BcMV1.BI=TRUE} | 0.08  6634 | 0.921  053 | 0.094  059 | 1.16  6474 | 35 |
| {BcRV1.BI=FALSE, BcULV5=TRUE} =>  {BcOLV7.BI=FALSE} | 0.08  9109 | 0.947  368 | 0.094  059 | 1.17  4039 | 36 |
| {BcOLV7.BI=FALSE, BcULV5=TRUE} =>  {BcRV1.BI=FALSE} | 0.08  9109 | 0.837  209 | 0.106  436 | 1.15  8331 | 36 |
| {BcRV1.BI=FALSE, BcULV5=TRUE} =>  {BcALV1.BI=FALSE} | 0.08  6634 | 0.921  053 | 0.094  059 | 1.13  1019 | 35 |
| {BcRV1.BI=FALSE, BcULV5=TRUE} =>  {BcDMV1=FALSE} | 0.08  9109 | 0.947  368 | 0.094  059 | 1.12  2395 | 36 |
| {BcMCV5.BI=FALSE, BcULV5=TRUE} => | 0.08 | 0.818 | 0.108 | 1.13 | 36 |

| {BcRV1.BI=FALSE} | 9109 | 182 | 911 | 2005 |  |
| --- | --- | --- | --- | --- | --- |
| {BcOLV2.BI=FALSE, BcULV5=TRUE} =>  {BcRV1.BI=FALSE} | 0.08  9109 | 0.818  182 | 0.108  911 | 1.13  2005 | 36 |
| {BcEV2.BI=FALSE, BcULV5=TRUE} =>  {BcMV1.BI=TRUE} | 0.08  6634 | 0.945  946 | 0.091  584 | 1.19  8001 | 35 |
| {BcEV2.BI=FALSE, BcULV5=TRUE} =>  {BcNSRV7.BI=FALSE} | 0.08  6634 | 0.945  946 | 0.091  584 | 1.18  3165 | 35 |
| {BcEV2.BI=FALSE, BcULV5=TRUE} =>  {BcDMV1=FALSE} | 0.08  6634 | 0.945  946 | 0.091  584 | 1.12  071 | 35 |
| {BcHV3.BI=FALSE, BcULV5=TRUE} =>  {BcFV5.BI=FALSE} | 0.08  1683 | 0.891  892 | 0.091  584 | 1.14  0267 | 33 |
| {BcHV3.BI=FALSE, BcULV5=TRUE} =>  {BcMV1.BI=TRUE} | 0.08  4158 | 0.918  919 | 0.091  584 | 1.16  3772 | 34 |
| {BcHV3.BI=FALSE, BcULV5=TRUE} =>  {BcNSRV7.BI=FALSE} | 0.08  4158 | 0.918  919 | 0.091  584 | 1.14  936 | 34 |
| {BcHV3.BI=FALSE, BcULV5=TRUE} =>  {BcOLV7.BI=FALSE} | 0.08  4158 | 0.918  919 | 0.091  584 | 1.13  8783 | 34 |
| {BcHV3.BI=FALSE, BcULV5=TRUE} =>  {BcALV1.BI=FALSE} | 0.08  4158 | 0.918  919 | 0.091  584 | 1.12  8399 | 34 |
| {BcHV3.BI=FALSE, BcULV5=TRUE} =>  {BcDMV1=FALSE} | 0.08  9109 | 0.972  973 | 0.091  584 | 1.15  273 | 36 |
| {BcFV5.BI=FALSE, BcULV5=TRUE} => | 0.09 | 0.951 | 0.101 | 1.20 | 39 |

| {BcMV1.BI=TRUE} | 6535 | 22 | 485 | 4679 |  |
| --- | --- | --- | --- | --- | --- |
| {BcFV5.BI=FALSE, BcULV5=TRUE} =>  {BcNSRV7.BI=FALSE} | 0.09  4059 | 0.926  829 | 0.101  485 | 1.15  9254 | 38 |
| {BcFV5.BI=FALSE, BcULV5=TRUE} =>  {BcOLV7.BI=FALSE} | 0.09  4059 | 0.926  829 | 0.101  485 | 1.14  8586 | 38 |
| {BcOLV7.BI=FALSE, BcULV5=TRUE} =>  {BcFV5.BI=FALSE} | 0.09  4059 | 0.883  721 | 0.106  436 | 1.12  982 | 38 |
| {BcFV5.BI=FALSE, BcULV5=TRUE} =>  {BcDMV1=FALSE} | 0.09  901 | 0.975  61 | 0.101  485 | 1.15  5854 | 40 |
| {BcULV1.BI=FALSE, BcULV5=TRUE} =>  {BcMV1.BI=TRUE} | 0.08  1683 | 0.970  588 | 0.084  158 | 1.22  9209 | 33 |
| {BcULV1.BI=FALSE, BcULV5=TRUE} =>  {BcOLV7.BI=FALSE} | 0.07  9208 | 0.941  176 | 0.084  158 | 1.16  6366 | 32 |
| {BcULV1.BI=FALSE, BcULV5=TRUE} =>  {BcALV1.BI=FALSE} | 0.07  6733 | 0.911  765 | 0.084  158 | 1.11  9614 | 31 |
| {BcULV1.BI=FALSE, BcULV5=TRUE} =>  {BcDMV1=FALSE} | 0.08  4158 | 1 | 0.084  158 | 1.18  4751 | 34 |
| {BcMV12=FALSE, BcULV5=TRUE} =>  {BcMV1.BI=TRUE} | 0.07  9208 | 0.941  176 | 0.084  158 | 1.19  196 | 32 |
| {BcNSRV7.BI=FALSE, BcULV5=TRUE} =>  {BcMV1.BI=TRUE} | 0.10  1485 | 0.931  818 | 0.108  911 | 1.18  0108 | 41 |
| {BcMV1.BI=TRUE, BcNSRV7.BI=FALSE} => | 0.10 | 0.162 | 0.623 | 1.31 | 41 |

| {BcULV5=TRUE} | 1485 | 698 | 762 | 4603 |  |
| --- | --- | --- | --- | --- | --- |
| {BcULV5=TRUE, BcVV3.BI=FALSE} =>  {BcMV1.BI=TRUE} | 0.07  6733 | 0.911  765 | 0.084  158 | 1.15  4711 | 31 |
| {BcOLV7.BI=FALSE, BcULV5=TRUE} =>  {BcMV1.BI=TRUE} | 0.10  1485 | 0.953  488 | 0.106  436 | 1.20  7553 | 41 |
| {BcMV1.BI=TRUE, BcOLV7.BI=FALSE} =>  {BcULV5=TRUE} | 0.10  1485 | 0.163  347 | 0.621  287 | 1.31  9841 | 41 |
| {BcALV1.BI=FALSE, BcULV5=TRUE} =>  {BcMV1.BI=TRUE} | 0.10  396 | 0.954  545 | 0.108  911 | 1.20  8891 | 42 |
| {BcALV1.BI=FALSE, BcMV1.BI=TRUE} =>  {BcULV5=TRUE} | 0.10  396 | 0.164  706 | 0.631  188 | 1.33  0824 | 42 |
| {BcMV1.BI=TRUE, BcULV5=TRUE} =>  {BcDMV1=FALSE} | 0.11  1386 | 0.957  447 | 0.116  337 | 1.13  4336 | 45 |
| {BcDMV1=FALSE, BcULV5=TRUE} =>  {BcMV1.BI=TRUE} | 0.11  1386 | 0.937  5 | 0.118  812 | 1.18  7304 | 45 |
| {BcDMV1=FALSE, BcMV1.BI=TRUE} =>  {BcULV5=TRUE} | 0.11  1386 | 0.167  286 | 0.665  842 | 1.35  1673 | 45 |
| {BcFV9=FALSE, BcULV5=TRUE} =>  {BcMV1.BI=TRUE} | 0.10  1485 | 0.953  488 | 0.106  436 | 1.20  7553 | 41 |
| {BcOLV4.BI=FALSE, BcULV5=TRUE} =>  {BcMV1.BI=TRUE} | 0.08  4158 | 0.918  919 | 0.091  584 | 1.16  3772 | 34 |
| {BcBNV5.BI=FALSE, BcULV5=TRUE} => | 0.11 | 0.937 | 0.118 | 1.18 | 45 |

| {BcMV1.BI=TRUE} | 1386 | 5 | 812 | 7304 |  |
| --- | --- | --- | --- | --- | --- |
| {BcBNV5.BI=FALSE, BcMV1.BI=TRUE} =>  {BcULV5=TRUE} | 0.11  1386 | 0.157  895 | 0.705  446 | 1.27  5789 | 45 |
| {BcULV5=TRUE, BvF.BI=FALSE} =>  {BcMV1.BI=TRUE} | 0.11  3861 | 0.958  333 | 0.118  812 | 1.21  3689 | 46 |
| {BcMCV5.BI=FALSE, BcULV5=TRUE} =>  {BcMV1.BI=TRUE} | 0.10  1485 | 0.931  818 | 0.108  911 | 1.18  0108 | 41 |
| {BcOLV13.BI=FALSE, BcULV5=TRUE} =>  {BcMV1.BI=TRUE} | 0.09  4059 | 0.95 | 0.099  01 | 1.20  3135 | 38 |
| {BcOLV2.BI=FALSE, BcULV5=TRUE} =>  {BcMV1.BI=TRUE} | 0.10  1485 | 0.931  818 | 0.108  911 | 1.18  0108 | 41 |
| {BcMCV3.BI=FALSE, BcULV5=TRUE} =>  {BcMV1.BI=TRUE} | 0.10  396 | 0.933  333 | 0.111  386 | 1.18  2027 | 42 |
| {BcPV4.BI=FALSE, BcULV5=TRUE} =>  {BcMV1.BI=TRUE} | 0.10  396 | 0.933  333 | 0.111  386 | 1.18  2027 | 42 |
| {BcEV6=FALSE, BcULV5=TRUE} =>  {BcMV1.BI=TRUE} | 0.10  396 | 0.933  333 | 0.111  386 | 1.18  2027 | 42 |
| {BcOLV17.BI=FALSE, BcULV5=TRUE} =>  {BcMV1.BI=TRUE} | 0.10  6436 | 0.934  783 | 0.113  861 | 1.18  3863 | 43 |
| {BcOLV15.BI=FALSE, BcULV5=TRUE} =>  {BcMV1.BI=TRUE} | 0.11  1386 | 0.937  5 | 0.118  812 | 1.18  7304 | 45 |
| {BcEV5=FALSE, BcULV5=TRUE} => | 0.11 | 0.938 | 0.121 | 1.18 | 46 |

| {BcMV1.BI=TRUE} | 3861 | 776 | 287 | 8919 |  |
| --- | --- | --- | --- | --- | --- |
| {BcMV12=FALSE, BcULV5=TRUE} =>  {BcDMV1=FALSE} | 0.08  1683 | 0.970  588 | 0.084  158 | 1.14  9905 | 33 |
| {BcULV5=TRUE, BcVV3.BI=FALSE} =>  {BcNSRV7.BI=FALSE} | 0.07  6733 | 0.911  765 | 0.084  158 | 1.14  0412 | 31 |
| {BcNSRV7.BI=FALSE, BcULV5=TRUE} =>  {BcDMV1=FALSE} | 0.10  6436 | 0.977  273 | 0.108  911 | 1.15  7825 | 43 |
| {BcDMV1=FALSE, BcULV5=TRUE} =>  {BcNSRV7.BI=FALSE} | 0.10  6436 | 0.895  833 | 0.118  812 | 1.12  0485 | 43 |
| {BcFV9=FALSE, BcULV5=TRUE} =>  {BcNSRV7.BI=FALSE} | 0.09  6535 | 0.906  977 | 0.106  436 | 1.13  4423 | 39 |
| {BcBNV5.BI=FALSE, BcULV5=TRUE} =>  {BcNSRV7.BI=FALSE} | 0.10  6436 | 0.895  833 | 0.118  812 | 1.12  0485 | 43 |
| {BcULV5=TRUE, BvF.BI=FALSE} =>  {BcNSRV7.BI=FALSE} | 0.10  6436 | 0.895  833 | 0.118  812 | 1.12  0485 | 43 |
| {BcOLV15.BI=FALSE, BcULV5=TRUE} =>  {BcNSRV7.BI=FALSE} | 0.10  6436 | 0.895  833 | 0.118  812 | 1.12  0485 | 43 |
| {BcEV5=FALSE, BcULV5=TRUE} =>  {BcNSRV7.BI=FALSE} | 0.10  8911 | 0.897  959 | 0.121  287 | 1.12  3144 | 44 |
| {BcULV5=TRUE, BcVV3.BI=FALSE} =>  {BcDMV1=FALSE} | 0.07  9208 | 0.941  176 | 0.084  158 | 1.11  506 | 32 |
| {BcOLV7.BI=FALSE, BcULV5=TRUE} => | 0.10 | 0.953 | 0.106 | 1.12 | 41 |

| {BcDMV1=FALSE} | 1485 | 488 | 436 | 9646 |  |
| --- | --- | --- | --- | --- | --- |
| {BcALV1.BI=FALSE, BcULV5=TRUE} =>  {BcDMV1=FALSE} | 0.10  396 | 0.954  545 | 0.108  911 | 1.13  0898 | 42 |
| {BcFV9=FALSE, BcULV5=TRUE} =>  {BcDMV1=FALSE} | 0.10  1485 | 0.953  488 | 0.106  436 | 1.12  9646 | 41 |
| {BcOLV4.BI=FALSE, BcULV5=TRUE} =>  {BcDMV1=FALSE} | 0.08  6634 | 0.945  946 | 0.091  584 | 1.12  071 | 35 |
| {BcBNV5.BI=FALSE, BcULV5=TRUE} =>  {BcDMV1=FALSE} | 0.11  6337 | 0.979  167 | 0.118  812 | 1.16  0068 | 47 |
| {BcULV5=TRUE, BvF.BI=FALSE} =>  {BcDMV1=FALSE} | 0.11  3861 | 0.958  333 | 0.118  812 | 1.13  5386 | 46 |
| {BcMCV5.BI=FALSE, BcULV5=TRUE} =>  {BcDMV1=FALSE} | 0.10  6436 | 0.977  273 | 0.108  911 | 1.15  7825 | 43 |
| {BcOLV13.BI=FALSE, BcULV5=TRUE} =>  {BcDMV1=FALSE} | 0.09  4059 | 0.95 | 0.099  01 | 1.12  5513 | 38 |
| {BcOLV2.BI=FALSE, BcULV5=TRUE} =>  {BcDMV1=FALSE} | 0.10  396 | 0.954  545 | 0.108  911 | 1.13  0898 | 42 |
| {BcMCV3.BI=FALSE, BcULV5=TRUE} =>  {BcDMV1=FALSE} | 0.10  6436 | 0.955  556 | 0.111  386 | 1.13  2095 | 43 |
| {BcPV4.BI=FALSE, BcULV5=TRUE} =>  {BcDMV1=FALSE} | 0.10  6436 | 0.955  556 | 0.111  386 | 1.13  2095 | 43 |
| {BcEV6=FALSE, BcULV5=TRUE} => | 0.10 | 0.977 | 0.111 | 1.15 | 44 |

| {BcDMV1=FALSE} | 8911 | 778 | 386 | 8423 |  |
| --- | --- | --- | --- | --- | --- |
| {BcOLV17.BI=FALSE, BcULV5=TRUE} =>  {BcDMV1=FALSE} | 0.10  8911 | 0.956  522 | 0.113  861 | 1.13  324 | 44 |
| {BcOLV15.BI=FALSE, BcULV5=TRUE} =>  {BcDMV1=FALSE} | 0.11  3861 | 0.958  333 | 0.118  812 | 1.13  5386 | 46 |
| {BcEV5=FALSE, BcULV5=TRUE} =>  {BcDMV1=FALSE} | 0.11  8812 | 0.979  592 | 0.121  287 | 1.16  0572 | 48 |
| {BcFV9=TRUE, BcMV1.BI=TRUE} =>  {BcMV4.BI=TRUE} | 0.07  6733 | 0.688  889 | 0.111  386 | 1.55  4811 | 31 |
| {BcMV1.BI=TRUE, BcMV4.BI=TRUE} =>  {BcFV9=TRUE} | 0.07  6733 | 0.203  947 | 0.376  238 | 1.55  4618 | 31 |
| {BcFV9=TRUE, BcULV5=FALSE} =>  {BcMV4.BI=TRUE} | 0.07  9208 | 0.695  652 | 0.113  861 | 1.57  0075 | 32 |
| {BcMV4.BI=TRUE, BcULV5=FALSE} =>  {BcFV9=TRUE} | 0.07  9208 | 0.207  792 | 0.381  188 | 1.58  3926 | 32 |
| {BcFV9=TRUE, BvF.BI=FALSE} =>  {BcMV4.BI=TRUE} | 0.07  6733 | 0.659  574 | 0.116  337 | 1.48  8649 | 31 |
| {BcMV4.BI=TRUE, BvF.BI=FALSE} =>  {BcFV9=TRUE} | 0.07  6733 | 0.189  024 | 0.405  941 | 1.44  0865 | 31 |
| {BcFV9=TRUE, BcOLV2.BI=FALSE} =>  {BcMV4.BI=TRUE} | 0.08  1683 | 0.673  469 | 0.121  287 | 1.52  0009 | 33 |
| {BcMV4.BI=TRUE, BcOLV2.BI=FALSE} => | 0.08 | 0.202 | 0.403 | 1.54 | 33 |

| {BcFV9=TRUE} | 1683 | 454 | 465 | 3234 |  |
| --- | --- | --- | --- | --- | --- |
| {BcFV9=TRUE, BcMCV3.BI=FALSE} =>  {BcMV4.BI=TRUE} | 0.07  9208 | 0.64 | 0.123  762 | 1.44  4469 | 32 |
| {BcMCV3.BI=FALSE, BcMV4.BI=TRUE} =>  {BcFV9=TRUE} | 0.07  9208 | 0.192  771 | 0.410  891 | 1.46  9425 | 32 |
| {BcFV9=TRUE, BcPV4.BI=FALSE} =>  {BcMV4.BI=TRUE} | 0.08  4158 | 0.68 | 0.123  762 | 1.53  4749 | 34 |
| {BcMV4.BI=TRUE, BcPV4.BI=FALSE} =>  {BcFV9=TRUE} | 0.08  4158 | 0.201  183 | 0.418  317 | 1.53  3549 | 34 |
| {BcEV6=FALSE, BcFV9=TRUE} =>  {BcMV4.BI=TRUE} | 0.07  6733 | 0.645  833 | 0.118  812 | 1.45  7635 | 31 |
| {BcEV6=FALSE, BcMV4.BI=TRUE} =>  {BcFV9=TRUE} | 0.07  6733 | 0.182  353 | 0.420  792 | 1.39  0011 | 31 |
| {BcFV9=TRUE, BcOLV17.BI=FALSE} =>  {BcMV4.BI=TRUE} | 0.07  4257 | 0.652  174 | 0.113  861 | 1.47  1946 | 30 |
| {BcMV4.BI=TRUE, BcOLV17.BI=FALSE} =>  {BcFV9=TRUE} | 0.07  4257 | 0.180  723 | 0.410  891 | 1.37  7586 | 30 |
| {BcEV5=FALSE, BcFV9=TRUE} =>  {BcMV4.BI=TRUE} | 0.07  6733 | 0.632  653 | 0.121  287 | 1.42  7887 | 31 |
| {BcEV5=FALSE, BcMV4.BI=TRUE} =>  {BcFV9=TRUE} | 0.07  6733 | 0.192  547 | 0.398  515 | 1.46  7714 | 31 |
| {BcFV9=TRUE, BcOLV18=TRUE} => | 0.07 | 0.769 | 0.096 | 1.24 | 30 |

| {BcMV11=TRUE} | 4257 | 231 | 535 | 8069 |  |
| --- | --- | --- | --- | --- | --- |
| {BcFV9=TRUE, BcMV11=TRUE} =>  {BcOLV18=TRUE} | 0.07  4257 | 0.789  474 | 0.094  059 | 1.52  6064 | 30 |
| {BcMV11=TRUE, BcOLV18=TRUE} =>  {BcFV9=TRUE} | 0.07  4257 | 0.214  286 | 0.346  535 | 1.63  3423 | 30 |
| {BcFV9=TRUE, BcOLV18=TRUE} =>  {BcULV4=TRUE} | 0.08  1683 | 0.846  154 | 0.096  535 | 1.22  5255 | 33 |
| {BcFV9=TRUE, BcULV4=TRUE} =>  {BcOLV18=TRUE} | 0.08  1683 | 0.868  421 | 0.094  059 | 1.67  867 | 33 |
| {BcOLV18=TRUE, BcULV4=TRUE} =>  {BcFV9=TRUE} | 0.08  1683 | 0.185  393 | 0.440  594 | 1.41  3186 | 33 |
| {BcFV9=TRUE, BcULV1.BI=FALSE} =>  {BcOLV18=TRUE} | 0.07  6733 | 0.720  93 | 0.106  436 | 1.39  3568 | 31 |
| {BcOLV18=TRUE, BcULV1.BI=FALSE} =>  {BcFV9=TRUE} | 0.07  6733 | 0.215  278 | 0.356  436 | 1.64  0985 | 31 |
| {BcFV9=TRUE, BcMV1.BI=TRUE} =>  {BcOLV18=TRUE} | 0.08  4158 | 0.755  556 | 0.111  386 | 1.46  05 | 34 |
| {BcMV1.BI=TRUE, BcOLV18=TRUE} =>  {BcFV9=TRUE} | 0.08  4158 | 0.191  011 | 0.440  594 | 1.45  601 | 34 |
| {BcFV9=TRUE, BcMV12=FALSE} =>  {BcOLV18=TRUE} | 0.07  6733 | 0.738  095 | 0.103  96 | 1.42  6749 | 31 |
| {BcMV12=FALSE, BcOLV18=TRUE} => | 0.07 | 0.193 | 0.396 | 1.47 | 31 |

| {BcFV9=TRUE} | 6733 | 75 | 04 | 6887 |  |
| --- | --- | --- | --- | --- | --- |
| {BcDMV1=FALSE, BcFV9=TRUE} =>  {BcOLV18=TRUE} | 0.07  4257 | 0.75 | 0.099  01 | 1.44  9761 | 30 |
| {BcDMV1=FALSE, BcOLV18=TRUE} =>  {BcFV9=TRUE} | 0.07  4257 | 0.173  41 | 0.428  218 | 1.32  1845 | 30 |
| {BcFV9=TRUE, BcULV5=FALSE} =>  {BcOLV18=TRUE} | 0.08  1683 | 0.717  391 | 0.113  861 | 1.38  6728 | 33 |
| {BcOLV18=TRUE, BcULV5=FALSE} =>  {BcFV9=TRUE} | 0.08  1683 | 0.174  603 | 0.467  822 | 1.33  0937 | 33 |
| {BcBNV5.BI=FALSE, BcFV9=TRUE} =>  {BcOLV18=TRUE} | 0.07  4257 | 0.731  707 | 0.101  485 | 1.41  4401 | 30 |
| {BcFV9=TRUE, BvF.BI=FALSE} =>  {BcOLV18=TRUE} | 0.08  1683 | 0.702  128 | 0.116  337 | 1.35  7223 | 33 |
| {BcOLV18=TRUE, BvF.BI=FALSE} =>  {BcFV9=TRUE} | 0.08  1683 | 0.171  875 | 0.475  248 | 1.31  0142 | 33 |
| {BcFV9=TRUE, BcMCV5.BI=FALSE} =>  {BcOLV18=TRUE} | 0.07  9208 | 0.727  273 | 0.108  911 | 1.40  5829 | 32 |
| {BcMCV5.BI=FALSE, BcOLV18=TRUE} =>  {BcFV9=TRUE} | 0.07  9208 | 0.175  824 | 0.450  495 | 1.34  0245 | 32 |
| {BcFV9=TRUE, BcOLV13.BI=FALSE} =>  {BcOLV18=TRUE} | 0.08  1683 | 0.733  333 | 0.111  386 | 1.41  7544 | 33 |
| {BcOLV13.BI=FALSE, BcOLV18=TRUE} => | 0.08 | 0.171 | 0.475 | 1.31 | 33 |

| {BcFV9=TRUE} | 1683 | 875 | 248 | 0142 |  |
| --- | --- | --- | --- | --- | --- |
| {BcFV9=TRUE, BcOLV2.BI=FALSE} =>  {BcOLV18=TRUE} | 0.09  1584 | 0.755  102 | 0.121  287 | 1.45  9623 | 37 |
| {BcOLV18=TRUE, BcOLV2.BI=FALSE} =>  {BcFV9=TRUE} | 0.09  1584 | 0.195  767 | 0.467  822 | 1.49  2263 | 37 |
| {BcFV9=TRUE, BcMCV3.BI=FALSE} =>  {BcOLV18=TRUE} | 0.08  9109 | 0.72 | 0.123  762 | 1.39  177 | 36 |
| {BcMCV3.BI=FALSE, BcOLV18=TRUE} =>  {BcFV9=TRUE} | 0.08  9109 | 0.187  5 | 0.475  248 | 1.42  9245 | 36 |
| {BcFV9=TRUE, BcPV4.BI=FALSE} =>  {BcOLV18=TRUE} | 0.08  9109 | 0.72 | 0.123  762 | 1.39  177 | 36 |
| {BcOLV18=TRUE, BcPV4.BI=FALSE} =>  {BcFV9=TRUE} | 0.08  9109 | 0.186  528 | 0.477  723 | 1.42  184 | 36 |
| {BcEV6=FALSE, BcFV9=TRUE} =>  {BcOLV18=TRUE} | 0.08  6634 | 0.729  167 | 0.118  812 | 1.40  949 | 35 |
| {BcEV6=FALSE, BcOLV18=TRUE} =>  {BcFV9=TRUE} | 0.08  6634 | 0.179  487 | 0.482  673 | 1.36  8166 | 35 |
| {BcFV9=TRUE, BcOLV17.BI=FALSE} =>  {BcOLV18=TRUE} | 0.07  9208 | 0.695  652 | 0.113  861 | 1.34  4706 | 32 |
| {BcFV9=TRUE, BcOLV15.BI=FALSE} =>  {BcOLV18=TRUE} | 0.07  4257 | 0.731  707 | 0.101  485 | 1.41  4401 | 30 |
| {BcEV5=FALSE, BcFV9=TRUE} => | 0.09 | 0.775 | 0.121 | 1.49 | 38 |

| {BcOLV18=TRUE} | 4059 | 51 | 287 | 9072 |  |
| --- | --- | --- | --- | --- | --- |
| {BcEV5=FALSE, BcOLV18=TRUE} =>  {BcFV9=TRUE} | 0.09  4059 | 0.192  893 | 0.487  624 | 1.47  0357 | 38 |
| {BcFV9=TRUE, BcHV1SLR.BI=TRUE} =>  {BcHV1.BI=TRUE} | 0.09  1584 | 1 | 0.091  584 | 1.65  5738 | 37 |
| {BcFV9=TRUE, BcHV1.BI=TRUE} =>  {BcHV1SLR.BI=TRUE} | 0.09  1584 | 0.948  718 | 0.096  535 | 1.75  0146 | 37 |
| {BcHV1.BI=TRUE, BcHV1SLR.BI=TRUE} =>  {BcFV9=TRUE} | 0.09  1584 | 0.188  776 | 0.485  149 | 1.43  8968 | 37 |
| {BcFV9=TRUE, BcHV1SLR.BI=TRUE} =>  {BcMV9.BI=TRUE} | 0.07  4257 | 0.810  811 | 0.091  584 | 1.19  5502 | 30 |
| {BcFV9=TRUE, BcMV9.BI=TRUE} =>  {BcHV1SLR.BI=TRUE} | 0.07  4257 | 0.789  474 | 0.094  059 | 1.45  6381 | 30 |
| {BcHV1SLR.BI=TRUE, BcMV9.BI=TRUE} =>  {BcFV9=TRUE} | 0.07  4257 | 0.181  818 | 0.408  416 | 1.38  5935 | 30 |
| {BcFV9=TRUE, BcHV1SLR.BI=TRUE} =>  {BcULV4=TRUE} | 0.07  4257 | 0.810  811 | 0.091  584 | 1.17  4077 | 30 |
| {BcFV9=TRUE, BcULV4=TRUE} =>  {BcHV1SLR.BI=TRUE} | 0.07  4257 | 0.789  474 | 0.094  059 | 1.45  6381 | 30 |
| {BcHV1SLR.BI=TRUE, BcULV4=TRUE} =>  {BcFV9=TRUE} | 0.07  4257 | 0.185  185 | 0.400  99 | 1.41  16 | 30 |
| {BcFV9=TRUE, BcHV1SLR.BI=TRUE} => | 0.08 | 0.891 | 0.091 | 1.12 | 33 |

| {BcMV1.BI=TRUE} | 1683 | 892 | 584 | 9543 |  |
| --- | --- | --- | --- | --- | --- |
| {BcFV9=TRUE, BcMV1.BI=TRUE} =>  {BcHV1SLR.BI=TRUE} | 0.08  1683 | 0.733  333 | 0.111  386 | 1.35  2816 | 33 |
| {BcHV1SLR.BI=TRUE, BcMV1.BI=TRUE} =>  {BcFV9=TRUE} | 0.08  1683 | 0.185  393 | 0.440  594 | 1.41  3186 | 33 |
| {BcFV9=TRUE, BcMV12=FALSE} =>  {BcHV1SLR.BI=TRUE} | 0.07  4257 | 0.714  286 | 0.103  96 | 1.31  7678 | 30 |
| {BcHV1SLR.BI=TRUE, BcMV12=FALSE} =>  {BcFV9=TRUE} | 0.07  4257 | 0.179  641 | 0.413  366 | 1.36  9337 | 30 |
| {BcFV9=TRUE, BcOLV4.BI=FALSE} =>  {BcHV1SLR.BI=TRUE} | 0.07  4257 | 0.697  674 | 0.106  436 | 1.28  7034 | 30 |
| {BcFV9=TRUE, BcULV5=FALSE} =>  {BcHV1SLR.BI=TRUE} | 0.07  9208 | 0.695  652 | 0.113  861 | 1.28  3304 | 32 |
| {BcHV1SLR.BI=TRUE, BcULV5=FALSE} =>  {BcFV9=TRUE} | 0.07  9208 | 0.169  312 | 0.467  822 | 1.29  0606 | 32 |
| {BcFV9=TRUE, BcMCV5.BI=FALSE} =>  {BcHV1SLR.BI=TRUE} | 0.07  6733 | 0.704  545 | 0.108  911 | 1.29  9709 | 31 |
| {BcFV9=TRUE, BcOLV13.BI=FALSE} =>  {BcHV1SLR.BI=TRUE} | 0.07  9208 | 0.711  111 | 0.111  386 | 1.31  1821 | 32 |
| {BcFV9=TRUE, BcOLV2.BI=FALSE} =>  {BcHV1SLR.BI=TRUE} | 0.08  4158 | 0.693  878 | 0.121  287 | 1.28  003 | 34 |
| {BcHV1SLR.BI=TRUE, BcOLV2.BI=FALSE} | 0.08 | 0.174 | 0.482 | 1.32 | 34 |

| => {BcFV9=TRUE} | 4158 | 359 | 673 | 9076 |  |
| --- | --- | --- | --- | --- | --- |
| {BcFV9=TRUE, BcMCV3.BI=FALSE} =>  {BcHV1SLR.BI=TRUE} | 0.08  4158 | 0.68 | 0.123  762 | 1.25  4429 | 34 |
| {BcHV1SLR.BI=TRUE, BcMCV3.BI=FALSE}  => {BcFV9=TRUE} | 0.08  4158 | 0.172  589 | 0.487  624 | 1.31  5583 | 34 |
| {BcFV9=TRUE, BcPV4.BI=FALSE} =>  {BcHV1SLR.BI=TRUE} | 0.08  6634 | 0.7 | 0.123  762 | 1.29  1324 | 35 |
| {BcHV1SLR.BI=TRUE, BcPV4.BI=FALSE}  => {BcFV9=TRUE} | 0.08  6634 | 0.171  569 | 0.504  95 | 1.30  7806 | 35 |
| {BcEV6=FALSE, BcFV9=TRUE} =>  {BcHV1SLR.BI=TRUE} | 0.08  1683 | 0.687  5 | 0.118  812 | 1.26  8265 | 33 |
| {BcFV9=TRUE, BcOLV17.BI=FALSE} =>  {BcHV1SLR.BI=TRUE} | 0.07  9208 | 0.695  652 | 0.113  861 | 1.28  3304 | 32 |
| {BcEV5=FALSE, BcFV9=TRUE} =>  {BcHV1SLR.BI=TRUE} | 0.08  4158 | 0.693  878 | 0.121  287 | 1.28  003 | 34 |
| {BcFV9=TRUE, BcMV1.BI=TRUE} =>  {BcMV3.BI=TRUE} | 0.07  6733 | 0.688  889 | 0.111  386 | 1.27  0827 | 31 |
| {BcFV9=TRUE, BcOLV4.BI=FALSE} =>  {BcMV3.BI=TRUE} | 0.07  4257 | 0.697  674 | 0.106  436 | 1.28  7034 | 30 |
| {BcFV9=TRUE, BcULV5=FALSE} =>  {BcMV3.BI=TRUE} | 0.07  6733 | 0.673  913 | 0.113  861 | 1.24  32 | 31 |
| {BcMV3.BI=TRUE, BcULV5=FALSE} => | 0.07 | 0.169 | 0.452 | 1.29 | 31 |

| {BcFV9=TRUE} | 6733 | 399 | 97 | 1267 |  |
| --- | --- | --- | --- | --- | --- |
| {BcFV9=TRUE, BvF.BI=FALSE} =>  {BcMV3.BI=TRUE} | 0.07  9208 | 0.680  851 | 0.116  337 | 1.25  5999 | 32 |
| {BcEV6=FALSE, BcFV9=TRUE} =>  {BcMV3.BI=TRUE} | 0.07  9208 | 0.666  667 | 0.118  812 | 1.22  9833 | 32 |
| {BcFV9=TRUE, BcULV3=TRUE} =>  {BcHV1.BI=TRUE} | 0.07  4257 | 0.882  353 | 0.084  158 | 1.46  0945 | 30 |
| {BcFV9=TRUE, BcHV1.BI=TRUE} =>  {BcULV3=TRUE} | 0.07  4257 | 0.769  231 | 0.096  535 | 1.29  4872 | 30 |
| {BcHV1.BI=TRUE, BcULV3=TRUE} =>  {BcFV9=TRUE} | 0.07  4257 | 0.175  439 | 0.423  267 | 1.33  7306 | 30 |
| {BcFV9=TRUE, BcULV3=TRUE} =>  {BcMV1.BI=TRUE} | 0.07  6733 | 0.911  765 | 0.084  158 | 1.15  4711 | 31 |
| {BcFV9=TRUE, BcHV1.BI=TRUE} =>  {BcMV9.BI=TRUE} | 0.07  6733 | 0.794  872 | 0.096  535 | 1.17  2001 | 31 |
| {BcFV9=TRUE, BcMV9.BI=TRUE} =>  {BcHV1.BI=TRUE} | 0.07  6733 | 0.815  789 | 0.094  059 | 1.35  0733 | 31 |
| {BcFV9=TRUE, BcHV1.BI=TRUE} =>  {BcULV4=TRUE} | 0.07  6733 | 0.794  872 | 0.096  535 | 1.15  0997 | 31 |
| {BcFV9=TRUE, BcULV4=TRUE} =>  {BcHV1.BI=TRUE} | 0.07  6733 | 0.815  789 | 0.094  059 | 1.35  0733 | 31 |
| {BcHV1.BI=TRUE, BcULV4=TRUE} => | 0.07 | 0.168 | 0.455 | 1.28 | 31 |

| {BcFV9=TRUE} | 6733 | 478 | 446 | 4249 |  |
| --- | --- | --- | --- | --- | --- |
| {BcHV1.BI=TRUE, BcULV1.BI=FALSE} =>  {BcFV9=TRUE} | 0.07  4257 | 0.173  41 | 0.428  218 | 1.32  1845 | 30 |
| {BcFV9=TRUE, BcMV1.BI=TRUE} =>  {BcHV1.BI=TRUE} | 0.08  4158 | 0.755  556 | 0.111  386 | 1.25  1002 | 34 |
| {BcHV1.BI=TRUE, BcMV1.BI=TRUE} =>  {BcFV9=TRUE} | 0.08  4158 | 0.174  359 | 0.482  673 | 1.32  9076 | 34 |
| {BcFV9=TRUE, BcMV12=FALSE} =>  {BcHV1.BI=TRUE} | 0.07  6733 | 0.738  095 | 0.103  96 | 1.22  2092 | 31 |
| {BcFV9=TRUE, BcOLV7.BI=FALSE} =>  {BcHV1.BI=TRUE} | 0.07  4257 | 0.731  707 | 0.101  485 | 1.21  1515 | 30 |
| {BcDMV1=FALSE, BcFV9=TRUE} =>  {BcHV1.BI=TRUE} | 0.07  4257 | 0.75 | 0.099  01 | 1.24  1803 | 30 |
| {BcFV9=TRUE, BcOLV4.BI=FALSE} =>  {BcHV1.BI=TRUE} | 0.07  9208 | 0.744  186 | 0.106  436 | 1.23  2177 | 32 |
| {BcFV9=TRUE, BcULV5=FALSE} =>  {BcHV1.BI=TRUE} | 0.08  4158 | 0.739  13 | 0.113  861 | 1.22  3806 | 34 |
| {BcFV9=TRUE, BcMCV5.BI=FALSE} =>  {BcHV1.BI=TRUE} | 0.08  1683 | 0.75 | 0.108  911 | 1.24  1803 | 33 |
| {BcFV9=TRUE, BcOLV13.BI=FALSE} =>  {BcHV1.BI=TRUE} | 0.08  4158 | 0.755  556 | 0.111  386 | 1.25  1002 | 34 |
| {BcFV9=TRUE, BcOLV2.BI=FALSE} => | 0.08 | 0.734 | 0.121 | 1.21 | 36 |

| {BcHV1.BI=TRUE} | 9109 | 694 | 287 | 646 |  |
| --- | --- | --- | --- | --- | --- |
| {BcFV9=TRUE, BcMCV3.BI=FALSE} =>  {BcHV1.BI=TRUE} | 0.08  9109 | 0.72 | 0.123  762 | 1.19  2131 | 36 |
| {BcFV9=TRUE, BcPV4.BI=FALSE} =>  {BcHV1.BI=TRUE} | 0.09  1584 | 0.74 | 0.123  762 | 1.22  5246 | 37 |
| {BcFV9=TRUE, BcOLV17.BI=FALSE} =>  {BcHV1.BI=TRUE} | 0.08  4158 | 0.739  13 | 0.113  861 | 1.22  3806 | 34 |
| {BcEV5=FALSE, BcFV9=TRUE} =>  {BcHV1.BI=TRUE} | 0.08  9109 | 0.734  694 | 0.121  287 | 1.21  646 | 36 |
| {BcFV9=TRUE, BcULV1.BI=FALSE} =>  {BcMV11=TRUE} | 0.08  1683 | 0.767  442 | 0.106  436 | 1.24  5167 | 33 |
| {BcMV11=TRUE, BcULV1.BI=FALSE} =>  {BcFV9=TRUE} | 0.08  1683 | 0.172  775 | 0.472  772 | 1.31  7001 | 33 |
| {BcFV9=TRUE, BcMV12=FALSE} =>  {BcMV11=TRUE} | 0.07  6733 | 0.738  095 | 0.103  96 | 1.19  7552 | 31 |
| {BcFV9=TRUE, BvF.BI=FALSE} =>  {BcMV11=TRUE} | 0.08  6634 | 0.744  681 | 0.116  337 | 1.20  8237 | 35 |
| {BcFV9=TRUE, BcOLV15.BI=FALSE} =>  {BcMV11=TRUE} | 0.07  9208 | 0.780  488 | 0.101  485 | 1.26  6334 | 32 |
| {BcFV9=TRUE, BcMV9.BI=TRUE} =>  {BcULV4=TRUE} | 0.07  6733 | 0.815  789 | 0.094  059 | 1.18  1287 | 31 |
| {BcFV9=TRUE, BcULV4=TRUE} => | 0.07 | 0.815 | 0.094 | 1.20 | 31 |

| {BcMV9.BI=TRUE} | 6733 | 789 | 059 | 2843 |  |
| --- | --- | --- | --- | --- | --- |
| {BcFV9=TRUE, BcVV3.BI=FALSE} =>  {BcMV9.BI=TRUE} | 0.07  9208 | 0.842  105 | 0.094  059 | 1.24  1644 | 32 |
| {BcFV9=TRUE, BcOLV4.BI=FALSE} =>  {BcMV9.BI=TRUE} | 0.08  4158 | 0.790  698 | 0.106  436 | 1.16  5846 | 34 |
| {BcFV9=TRUE, BcMV9.BI=TRUE} =>  {BcULV5=FALSE} | 0.09  1584 | 0.973  684 | 0.094  059 | 1.11  121 | 37 |
| {BcFV9=TRUE, BcULV5=FALSE} =>  {BcMV9.BI=TRUE} | 0.09  1584 | 0.804  348 | 0.113  861 | 1.18  5973 | 37 |
| {BcDMV1=FALSE, BcFV9=TRUE} =>  {BcMV6.BI=FALSE} | 0.08  4158 | 0.85 | 0.099  01 | 1.22  2064 | 34 |
| {BcBNV5.BI=FALSE, BcFV9=TRUE} =>  {BcMV6.BI=FALSE} | 0.08  1683 | 0.804  878 | 0.101  485 | 1.15  7191 | 33 |
| {BcFV9=TRUE, BcULV1.BI=FALSE} =>  {BcMV1.BI=TRUE} | 0.09  6535 | 0.906  977 | 0.106  436 | 1.14  8648 | 39 |
| {BcDMV1=TRUE, BcFV5.BI=TRUE} =>  {BcOLV7.BI=FALSE} | 0.07  4257 | 0.967  742 | 0.076  733 | 1.19  9288 | 30 |
| {BcDMV1=TRUE, BcOLV7.BI=FALSE} =>  {BcFV5.BI=TRUE} | 0.07  4257 | 0.526  316 | 0.141  089 | 2.41  6268 | 30 |
| {BcFV5.BI=TRUE, BcOLV7.BI=FALSE} =>  {BcDMV1=TRUE} | 0.07  4257 | 0.405  405 | 0.183  168 | 2.59  9743 | 30 |
| {BcDMV1=TRUE, BcOLV4.BI=FALSE} => | 0.07 | 0.483 | 0.153 | 2.22 | 30 |

| {BcFV5.BI=TRUE} | 4257 | 871 | 465 | 1408 |  |
| --- | --- | --- | --- | --- | --- |
| {BcFV5.BI=TRUE, BcOLV4.BI=FALSE} =>  {BcDMV1=TRUE} | 0.07  4257 | 0.379  747 | 0.195  545 | 2.43  5202 | 30 |
| {BcDMV1=TRUE, BcULV5=FALSE} =>  {BcFV5.BI=TRUE} | 0.07  4257 | 0.491  803 | 0.150  99 | 2.25  7824 | 30 |
| {BcFV5.BI=TRUE, BcULV5=FALSE} =>  {BcDMV1=TRUE} | 0.07  4257 | 0.379  747 | 0.195  545 | 2.43  5202 | 30 |
| {BcDMV1=TRUE, BcOLV13.BI=FALSE} =>  {BcFV5.BI=TRUE} | 0.07  6733 | 0.508  197 | 0.150  99 | 2.33  3085 | 31 |
| {BcFV5.BI=TRUE, BcOLV13.BI=FALSE} =>  {BcDMV1=TRUE} | 0.07  6733 | 0.369  048 | 0.207  921 | 2.36  6591 | 31 |
| {BcDMV1=TRUE, BcMCV3.BI=FALSE} =>  {BcFV5.BI=TRUE} | 0.07  6733 | 0.492  063 | 0.155  941 | 2.25  9019 | 31 |
| {BcFV5.BI=TRUE, BcMCV3.BI=FALSE} =>  {BcDMV1=TRUE} | 0.07  6733 | 0.369  048 | 0.207  921 | 2.36  6591 | 31 |
| {BcDMV1=TRUE, BcPV4.BI=FALSE} =>  {BcFV5.BI=TRUE} | 0.07  6733 | 0.508  197 | 0.150  99 | 2.33  3085 | 31 |
| {BcFV5.BI=TRUE, BcPV4.BI=FALSE} =>  {BcDMV1=TRUE} | 0.07  6733 | 0.382  716 | 0.200  495 | 2.45  4243 | 31 |
| {BcDMV1=TRUE, BcOLV17.BI=FALSE} =>  {BcFV5.BI=TRUE} | 0.07  6733 | 0.5 | 0.153  465 | 2.29  5455 | 31 |
| {BcFV5.BI=TRUE, BcOLV17.BI=FALSE} => | 0.07 | 0.360 | 0.212 | 2.31 | 31 |

| {BcDMV1=TRUE} | 6733 | 465 | 871 | 1554 |  |
| --- | --- | --- | --- | --- | --- |
| {BcDMV1=TRUE, BcOLV4.BI=FALSE} =>  {BcHV3.BI=TRUE} | 0.07  4257 | 0.483  871 | 0.153  465 | 2.01  5298 | 30 |
| {BcHV3.BI=TRUE, BcOLV4.BI=FALSE} =>  {BcDMV1=TRUE} | 0.07  4257 | 0.365  854 | 0.202  97 | 2.34  6109 | 30 |
| {BcDMV1=TRUE, BcULV5=FALSE} =>  {BcHV3.BI=TRUE} | 0.07  4257 | 0.491  803 | 0.150  99 | 2.04  8335 | 30 |
| {BcHV3.BI=TRUE, BcULV5=FALSE} =>  {BcDMV1=TRUE} | 0.07  4257 | 0.357  143 | 0.207  921 | 2.29  0249 | 30 |
| {BcDMV1=TRUE, BvF.BI=FALSE} =>  {BcHV3.BI=TRUE} | 0.07  4257 | 0.535  714 | 0.138  614 | 2.23  1222 | 30 |
| {BcHV3.BI=TRUE, BvF.BI=FALSE} =>  {BcDMV1=TRUE} | 0.07  4257 | 0.344  828 | 0.215  347 | 2.21  1275 | 30 |
| {BcDMV1=TRUE, BcOLV13.BI=FALSE} =>  {BcHV3.BI=TRUE} | 0.07  4257 | 0.491  803 | 0.150  99 | 2.04  8335 | 30 |
| {BcHV3.BI=TRUE, BcOLV13.BI=FALSE} =>  {BcDMV1=TRUE} | 0.07  4257 | 0.344  828 | 0.215  347 | 2.21  1275 | 30 |
| {BcDMV1=TRUE, BcMCV3.BI=FALSE} =>  {BcHV3.BI=TRUE} | 0.07  6733 | 0.492  063 | 0.155  941 | 2.04  9419 | 31 |
| {BcHV3.BI=TRUE, BcMCV3.BI=FALSE} =>  {BcDMV1=TRUE} | 0.07  6733 | 0.322  917 | 0.237  624 | 2.07  0767 | 31 |
| {BcDMV1=TRUE, BcPV4.BI=FALSE} => | 0.07 | 0.491 | 0.150 | 2.04 | 30 |

| {BcHV3.BI=TRUE} | 4257 | 803 | 99 | 8335 |  |
| --- | --- | --- | --- | --- | --- |
| {BcHV3.BI=TRUE, BcPV4.BI=FALSE} =>  {BcDMV1=TRUE} | 0.07  4257 | 0.326  087 | 0.227  723 | 2.09  1097 | 30 |
| {BcDMV1=TRUE, BcEV6=FALSE} =>  {BcHV3.BI=TRUE} | 0.07  4257 | 0.526  316 | 0.141  089 | 2.19  2078 | 30 |
| {BcEV6=FALSE, BcHV3.BI=TRUE} =>  {BcDMV1=TRUE} | 0.07  4257 | 0.326  087 | 0.227  723 | 2.09  1097 | 30 |
| {BcDMV1=TRUE, BcOLV17.BI=FALSE} =>  {BcHV3.BI=TRUE} | 0.07  6733 | 0.5 | 0.153  465 | 2.08  2474 | 31 |
| {BcHV3.BI=TRUE, BcOLV17.BI=FALSE} =>  {BcDMV1=TRUE} | 0.07  6733 | 0.344  444 | 0.222  772 | 2.20  8818 | 31 |
| {BcDMV1=TRUE, BcMV4.BI=TRUE} =>  {BcMV11=TRUE} | 0.07  6733 | 0.939  394 | 0.081  683 | 1.52  4157 | 31 |
| {BcDMV1=TRUE, BcMV11=TRUE} =>  {BcMV4.BI=TRUE} | 0.07  6733 | 0.720  93 | 0.106  436 | 1.62  7127 | 31 |
| {BcMV11=TRUE, BcMV4.BI=TRUE} =>  {BcDMV1=TRUE} | 0.07  6733 | 0.226  277 | 0.339  109 | 1.45  1049 | 31 |
| {BcDMV1=TRUE, BcMV4.BI=TRUE} =>  {BcMV9.BI=TRUE} | 0.07  4257 | 0.909  091 | 0.081  683 | 1.34  0411 | 30 |
| {BcDMV1=TRUE, BcMV9.BI=TRUE} =>  {BcMV4.BI=TRUE} | 0.07  4257 | 0.588  235 | 0.126  238 | 1.32  7637 | 30 |
| {BcMV4.BI=TRUE, BcMV9.BI=TRUE} => | 0.07 | 0.220 | 0.336 | 1.41 | 30 |

| {BcDMV1=TRUE} | 4257 | 588 | 634 | 4566 |  |
| --- | --- | --- | --- | --- | --- |
| {BcDMV1=TRUE, BcMV4.BI=TRUE} =>  {BcOLV7.BI=FALSE} | 0.07  6733 | 0.939  394 | 0.081  683 | 1.16  4157 | 31 |
| {BcMV4.BI=TRUE, BcOLV7.BI=FALSE} =>  {BcDMV1=TRUE} | 0.07  6733 | 0.227  941 | 0.336  634 | 1.46  1718 | 31 |
| {BcDMV1=TRUE, BcMV4.BI=TRUE} =>  {BcOLV4.BI=FALSE} | 0.07  9208 | 0.969  697 | 0.081  683 | 1.10  666 | 32 |
| {BcMV4.BI=TRUE, BcOLV4.BI=FALSE} =>  {BcDMV1=TRUE} | 0.07  9208 | 0.213  333 | 0.371  287 | 1.36  8042 | 32 |
| {BcDMV1=TRUE, BcMV4.BI=TRUE} =>  {BcULV5=FALSE} | 0.07  9208 | 0.969  697 | 0.081  683 | 1.10  666 | 32 |
| {BcMV4.BI=TRUE, BcULV5=FALSE} =>  {BcDMV1=TRUE} | 0.07  9208 | 0.207  792 | 0.381  188 | 1.33  2509 | 32 |
| {BcMV4.BI=TRUE, BcOLV13.BI=FALSE} =>  {BcDMV1=TRUE} | 0.08  1683 | 0.208  861 | 0.391  089 | 1.33  9361 | 33 |
| {BcMV4.BI=TRUE, BcOLV2.BI=FALSE} =>  {BcDMV1=TRUE} | 0.08  1683 | 0.202  454 | 0.403  465 | 1.29  8276 | 33 |
| {BcDMV1=TRUE, BcHV1SLR.BI=FALSE} =>  {BcOLV4.BI=FALSE} | 0.07  9208 | 1 | 0.079  208 | 1.14  1243 | 32 |
| {BcDMV1=TRUE, BcHV1SLR.BI=FALSE} =>  {BcULV5=FALSE} | 0.07  6733 | 0.968  75 | 0.079  208 | 1.10  5579 | 31 |
| {BcDMV1=TRUE, BcOLV18=TRUE} => | 0.07 | 0.833 | 0.089 | 1.22 | 30 |

| {BcMV9.BI=TRUE} | | 4257 | 333 | 109 | 871 |  |
| --- | --- | --- | --- | --- | --- | --- |
| {BcDMV1=TRUE, | BcOLV18=TRUE} => | 0.07 | 0.861 | 0.089 | 1.24 |  |
|  | |  |  |  |  | 31 |
| {BcULV4=TRUE} | | 6733 | 111 | 109 | 6914 |  |
| {BcDMV1=TRUE, | BcULV4=TRUE} => | 0.07 | 0.645 | 0.118 | 1.24 |  |
|  | |  |  |  |  | 31 |
| {BcOLV18=TRUE} | | 6733 | 833 | 812 | 8405 |  |
| {BcDMV1=TRUE, | BcOLV18=TRUE} => | 0.07 | 0.888 | 0.089 | 1.22 |  |
|  | |  |  |  |  | 32 |
| {BcRV1.BI=FALSE} | | 9208 | 889 | 109 | 9833 |  |
| {BcOLV18=TRUE, | BcRV1.BI=FALSE} => | 0.07 | 0.240 | 0.329 | 1.54 |  |
|  | |  |  |  |  | 32 |
| {BcDMV1=TRUE} | | 9208 | 602 | 208 | 2905 |  |
| {BcDMV1=TRUE, | BcOLV18=TRUE} => | 0.08 | 0.972 | 0.089 | 1.20 |  |
|  | |  |  |  |  | 35 |
| {BcOLV7.BI=FALSE} | | 6634 | 222 | 109 | 484 |  |
| {BcOLV18=TRUE, | BcOLV7.BI=FALSE} => | 0.08 | 0.230 | 0.376 | 1.47 |  |
|  | |  |  |  |  | 35 |
| {BcDMV1=TRUE} | | 6634 | 263 | 238 | 6608 |  |
| {BcDMV1=TRUE, | BcOLV18=TRUE} => | 0.08 | 0.972 | 0.089 | 1.10 |  |
|  | |  |  |  |  | 35 |
| {BcOLV4.BI=FALSE} | | 6634 | 222 | 109 | 9542 |  |
| {BcDMV1=TRUE, | BcMV2.BI=FALSE} => | 0.07 | 0.861 | 0.089 | 1.28 |  |
|  | |  |  |  |  | 31 |
| {BcOLV1.BI.MOVA.=FALSE} | | 6733 | 111 | 109 | 8477 |  |
| {BcDMV1=TRUE, | |  |  |  |  |  |
|  | | 0.07 |  | 0.099 | 1.44 |  |
| BcOLV1.BI.MOVA.=FALSE} => | |  | 0.775 |  |  | 31 |
|  | | 6733 |  | 01 | 2857 |  |
| {BcMV2.BI=FALSE} | |  |  |  |  |  |
| {BcDMV1=TRUE, | BcMV2.BI=FALSE} => | 0.07 | 0.861 | 0.089 | 1.23 |  |
|  | |  |  |  |  | 31 |
| {BcMV6.BI=FALSE} | | 6733 | 111 | 109 | 8039 |  |

| {BcDMV1=TRUE, BcMV6.BI=FALSE} =>  {BcMV2.BI=FALSE} | 0.07  6733 | 0.861  111 | 0.089  109 | 1.60  3175 | 31 |
| --- | --- | --- | --- | --- | --- |
| {BcDMV1=TRUE, BcMV2.BI=FALSE} =>  {BcPV3.BI=FALSE} | 0.07  4257 | 0.833  333 | 0.089  109 | 1.17  3055 | 30 |
| {BcDMV1=TRUE, BcMV2.BI=FALSE} =>  {BcRV1.BI=FALSE} | 0.07  9208 | 0.888  889 | 0.089  109 | 1.22  9833 | 32 |
| {BcDMV1=TRUE, BcMV2.BI=FALSE} =>  {BcEV2.BI=FALSE} | 0.07  6733 | 0.861  111 | 0.089  109 | 1.15  195 | 31 |
| {BcDMV1=TRUE, BcMV2.BI=FALSE} =>  {BcULV1.BI=FALSE} | 0.08  4158 | 0.944  444 | 0.089  109 | 1.20  3645 | 34 |
| {BcDMV1=TRUE, BcMV2.BI=FALSE} =>  {BcALV1.BI=FALSE} | 0.08  1683 | 0.916  667 | 0.089  109 | 1.12  5633 | 33 |
| {BcALV1.BI=FALSE, BcDMV1=TRUE} =>  {BcMV2.BI=FALSE} | 0.08  1683 | 0.717  391 | 0.113  861 | 1.33  5604 | 33 |
| {BcDMV1=TRUE, BcMV2.BI=FALSE} =>  {BcOLV4.BI=FALSE} | 0.08  9109 | 1 | 0.089  109 | 1.14  1243 | 36 |
| {BcDMV1=TRUE, BcMV2.BI=FALSE} =>  {BcULV5=FALSE} | 0.08  6634 | 0.972  222 | 0.089  109 | 1.10  9542 | 35 |
| {BcBNV5.BI=FALSE, BcDMV1=TRUE} =>  {BcMV2.BI=FALSE} | 0.07  4257 | 0.714  286 | 0.103  96 | 1.32  9822 | 30 |
| {BcDMV1=TRUE, BcMCV5.BI=FALSE} =>  {BcMV2.BI=FALSE} | 0.08  4158 | 0.693  878 | 0.121  287 | 1.29  1827 | 34 |

| {BcDMV1=TRUE, BcEV5=FALSE} =>  {BcMV2.BI=FALSE} | 0.08  1683 | 0.733  333 | 0.111  386 | 1.36  5284 | 33 |
| --- | --- | --- | --- | --- | --- |
| {BcDMV1=TRUE, BcHV1SLR.BI=TRUE} =>  {BcOLV7.BI=FALSE} | 0.07  4257 | 0.967  742 | 0.076  733 | 1.19  9288 | 30 |
| {BcDMV1=TRUE, BcULV3=TRUE} =>  {BcMV3.BI=TRUE} | 0.07  9208 | 0.888  889 | 0.089  109 | 1.63  9777 | 32 |
| {BcMV3.BI=TRUE, BcULV3=TRUE} =>  {BcDMV1=TRUE} | 0.07  9208 | 0.228  571 | 0.346  535 | 1.46  576 | 32 |
| {BcDMV1=TRUE, BcMV3.BI=TRUE} =>  {BcMV11=TRUE} | 0.09  1584 | 0.770  833 | 0.118  812 | 1.25  0669 | 37 |
| {BcDMV1=TRUE, BcMV11=TRUE} =>  {BcMV3.BI=TRUE} | 0.09  1584 | 0.860  465 | 0.106  436 | 1.58  7342 | 37 |
| {BcMV11=TRUE, BcMV3.BI=TRUE} =>  {BcDMV1=TRUE} | 0.09  1584 | 0.238  71 | 0.383  663 | 1.53  0773 | 37 |
| {BcDMV1=TRUE, BcMV3.BI=TRUE} =>  {BcMV9.BI=TRUE} | 0.09  901 | 0.833  333 | 0.118  812 | 1.22  871 | 40 |
| {BcDMV1=TRUE, BcMV9.BI=TRUE} =>  {BcMV3.BI=TRUE} | 0.09  901 | 0.784  314 | 0.126  238 | 1.44  6862 | 40 |
| {BcMV3.BI=TRUE, BcMV9.BI=TRUE} =>  {BcDMV1=TRUE} | 0.09  901 | 0.25 | 0.396  04 | 1.60  3175 | 40 |
| {BcDMV1=TRUE, BcULV4=TRUE} =>  {BcMV3.BI=TRUE} | 0.09  1584 | 0.770  833 | 0.118  812 | 1.42  1994 | 37 |

| {BcMV3.BI=TRUE, BcULV4=TRUE} =>  {BcDMV1=TRUE} | 0.09  1584 | 0.228  395 | 0.400  99 | 1.46  4629 | 37 |
| --- | --- | --- | --- | --- | --- |
| {BcDMV1=TRUE, BcPV3.BI=FALSE} =>  {BcMV3.BI=TRUE} | 0.08  4158 | 0.739  13 | 0.113  861 | 1.36  351 | 34 |
| {BcMV3.BI=TRUE, BcPV3.BI=FALSE} =>  {BcDMV1=TRUE} | 0.08  4158 | 0.232  877 | 0.361  386 | 1.49  3368 | 34 |
| {BcDMV1=TRUE, BcMV3.BI=TRUE} =>  {BcRV1.BI=FALSE} | 0.10  1485 | 0.854  167 | 0.118  812 | 1.18  1792 | 41 |
| {BcDMV1=TRUE, BcRV1.BI=FALSE} =>  {BcMV3.BI=TRUE} | 0.10  1485 | 0.745  455 | 0.136  139 | 1.37  5176 | 41 |
| {BcMV3.BI=TRUE, BcRV1.BI=FALSE} =>  {BcDMV1=TRUE} | 0.10  1485 | 0.277  027 | 0.366  337 | 1.77  6491 | 41 |
| {BcDMV1=TRUE, BcEV2.BI=FALSE} =>  {BcMV3.BI=TRUE} | 0.09  1584 | 0.74 | 0.123  762 | 1.36  5114 | 37 |
| {BcEV2.BI=FALSE, BcMV3.BI=TRUE} =>  {BcDMV1=TRUE} | 0.09  1584 | 0.226  994 | 0.403  465 | 1.45  5643 | 37 |
| {BcDMV1=TRUE, BcULV1.BI=FALSE} =>  {BcMV3.BI=TRUE} | 0.10  396 | 0.763  636 | 0.136  139 | 1.40  8717 | 42 |
| {BcMV3.BI=TRUE, BcULV1.BI=FALSE} =>  {BcDMV1=TRUE} | 0.10  396 | 0.265  823 | 0.391  089 | 1.70  4641 | 42 |
| {BcDMV1=TRUE, BcMV1.BI=TRUE} =>  {BcMV3.BI=TRUE} | 0.09  6535 | 0.78 | 0.123  762 | 1.43  8904 | 39 |

| {BcMV1.BI=TRUE, BcMV3.BI=TRUE} =>  {BcDMV1=TRUE} | 0.09  6535 | 0.210  811 | 0.457  921 | 1.35  1866 | 39 |
| --- | --- | --- | --- | --- | --- |
| {BcDMV1=TRUE, BcMV12=FALSE} =>  {BcMV3.BI=TRUE} | 0.10  1485 | 0.773  585 | 0.131  188 | 1.42  707 | 41 |
| {BcMV12=FALSE, BcMV3.BI=TRUE} =>  {BcDMV1=TRUE} | 0.10  1485 | 0.261  146 | 0.388  614 | 1.67  4654 | 41 |
| {BcDMV1=TRUE, BcVV3.BI=FALSE} =>  {BcMV3.BI=TRUE} | 0.07  6733 | 0.688  889 | 0.111  386 | 1.27  0827 | 31 |
| {BcDMV1=TRUE, BcMV3.BI=TRUE} =>  {BcOLV7.BI=FALSE} | 0.10  8911 | 0.916  667 | 0.118  812 | 1.13  5992 | 44 |
| {BcDMV1=TRUE, BcOLV7.BI=FALSE} =>  {BcMV3.BI=TRUE} | 0.10  8911 | 0.771  93 | 0.141  089 | 1.42  4017 | 44 |
| {BcMV3.BI=TRUE, BcOLV7.BI=FALSE} =>  {BcDMV1=TRUE} | 0.10  8911 | 0.261  905 | 0.415  842 | 1.67  9516 | 44 |
| {BcALV1.BI=FALSE, BcDMV1=TRUE} =>  {BcMV3.BI=TRUE} | 0.08  1683 | 0.717  391 | 0.113  861 | 1.32  3407 | 33 |
| {BcDMV1=TRUE, BcFV9=FALSE} =>  {BcMV3.BI=TRUE} | 0.09  1584 | 0.74 | 0.123  762 | 1.36  5114 | 37 |
| {BcDMV1=TRUE, BcMV3.BI=TRUE} =>  {BcOLV4.BI=FALSE} | 0.11  6337 | 0.979  167 | 0.118  812 | 1.11  7467 | 47 |
| {BcDMV1=TRUE, BcOLV4.BI=FALSE} =>  {BcMV3.BI=TRUE} | 0.11  6337 | 0.758  065 | 0.153  465 | 1.39  8439 | 47 |

| {BcMV3.BI=TRUE, BcOLV4.BI=FALSE} =>  {BcDMV1=TRUE} | 0.11  6337 | 0.255  435 | 0.455  446 | 1.63  8026 | 47 |
| --- | --- | --- | --- | --- | --- |
| {BcDMV1=TRUE, BcULV5=FALSE} =>  {BcMV3.BI=TRUE} | 0.11  3861 | 0.754  098 | 0.150  99 | 1.39  1122 | 46 |
| {BcMV3.BI=TRUE, BcULV5=FALSE} =>  {BcDMV1=TRUE} | 0.11  3861 | 0.251  366 | 0.452  97 | 1.61  1935 | 46 |
| {BcDMV1=TRUE, BvF.BI=FALSE} =>  {BcMV3.BI=TRUE} | 0.10  6436 | 0.767  857 | 0.138  614 | 1.41  6504 | 43 |
| {BcMV3.BI=TRUE, BvF.BI=FALSE} =>  {BcDMV1=TRUE} | 0.10  6436 | 0.215 | 0.495  05 | 1.37  873 | 43 |
| {BcDMV1=TRUE, BcMCV5.BI=FALSE} =>  {BcMV3.BI=TRUE} | 0.08  6634 | 0.714  286 | 0.121  287 | 1.31  7678 | 35 |
| {BcDMV1=TRUE, BcOLV13.BI=FALSE} =>  {BcMV3.BI=TRUE} | 0.11  3861 | 0.754  098 | 0.150  99 | 1.39  1122 | 46 |
| {BcMV3.BI=TRUE, BcOLV13.BI=FALSE} =>  {BcDMV1=TRUE} | 0.11  3861 | 0.239  583 | 0.475  248 | 1.53  6376 | 46 |
| {BcDMV1=TRUE, BcOLV2.BI=FALSE} =>  {BcMV3.BI=TRUE} | 0.11  3861 | 0.766  667 | 0.148  515 | 1.41  4307 | 46 |
| {BcMV3.BI=TRUE, BcOLV2.BI=FALSE} =>  {BcDMV1=TRUE} | 0.11  3861 | 0.234  694 | 0.485  149 | 1.50  5021 | 46 |
| {BcDMV1=TRUE, BcMCV3.BI=FALSE} =>  {BcMV3.BI=TRUE} | 0.11  8812 | 0.761  905 | 0.155  941 | 1.40  5523 | 48 |

| {BcMCV3.BI=FALSE, BcMV3.BI=TRUE} =>  {BcDMV1=TRUE} | 0.11  8812 | 0.25 | 0.475  248 | 1.60  3175 | 48 |
| --- | --- | --- | --- | --- | --- |
| {BcDMV1=TRUE, BcPV4.BI=FALSE} =>  {BcMV3.BI=TRUE} | 0.11  3861 | 0.754  098 | 0.150  99 | 1.39  1122 | 46 |
| {BcMV3.BI=TRUE, BcPV4.BI=FALSE} =>  {BcDMV1=TRUE} | 0.11  3861 | 0.228  856 | 0.497  525 | 1.46  7583 | 46 |
| {BcDMV1=TRUE, BcEV6=FALSE} =>  {BcMV3.BI=TRUE} | 0.10  8911 | 0.771  93 | 0.141  089 | 1.42  4017 | 44 |
| {BcEV6=FALSE, BcMV3.BI=TRUE} =>  {BcDMV1=TRUE} | 0.10  8911 | 0.210  526 | 0.517  327 | 1.35  0042 | 44 |
| {BcDMV1=TRUE, BcOLV17.BI=FALSE} =>  {BcMV3.BI=TRUE} | 0.11  6337 | 0.758  065 | 0.153  465 | 1.39  8439 | 47 |
| {BcMV3.BI=TRUE, BcOLV17.BI=FALSE} =>  {BcDMV1=TRUE} | 0.11  6337 | 0.227  053 | 0.512  376 | 1.45  6023 | 47 |
| {BcDMV1=TRUE, BcOLV15.BI=FALSE} =>  {BcMV3.BI=TRUE} | 0.11  1386 | 0.762  712 | 0.146  04 | 1.40  7012 | 45 |
| {BcMV3.BI=TRUE, BcOLV15.BI=FALSE} =>  {BcDMV1=TRUE} | 0.11  1386 | 0.219  512 | 0.507  426 | 1.40  7666 | 45 |
| {BcDMV1=TRUE, BcEV5=FALSE} =>  {BcMV3.BI=TRUE} | 0.07  4257 | 0.666  667 | 0.111  386 | 1.22  9833 | 30 |
| {BcDMV1=TRUE, BcMV4.BI=FALSE} =>  {BcOLV4.BI=FALSE} | 0.07  4257 | 1 | 0.074  257 | 1.14  1243 | 30 |

| {BcDMV1=TRUE, BcOLV16.BI=FALSE} =>  {BcRV1.BI=FALSE} | 0.08  1683 | 0.868  421 | 0.094  059 | 1.20  1514 | 33 |
| --- | --- | --- | --- | --- | --- |
| {BcDMV1=TRUE, BcOLV16.BI=FALSE} =>  {BcULV1.BI=FALSE} | 0.08  6634 | 0.921  053 | 0.094  059 | 1.17  3834 | 35 |
| {BcDMV1=TRUE, BcOLV16.BI=FALSE} =>  {BcMV12=FALSE} | 0.08  6634 | 0.921  053 | 0.094  059 | 1.15  2029 | 35 |
| {BcDMV1=TRUE, BcOLV16.BI=FALSE} =>  {BcOLV4.BI=FALSE} | 0.09  4059 | 1 | 0.094  059 | 1.14  1243 | 38 |
| {BcDMV1=TRUE, BcOLV16.BI=FALSE} =>  {BcULV5=FALSE} | 0.09  1584 | 0.973  684 | 0.094  059 | 1.11  121 | 37 |
| {BcDMV1=TRUE, BcULV3=TRUE} =>  {BcMV9.BI=TRUE} | 0.07  6733 | 0.861  111 | 0.089  109 | 1.26  9667 | 31 |
| {BcDMV1=TRUE, BcULV3=TRUE} =>  {BcRV1.BI=FALSE} | 0.07  9208 | 0.888  889 | 0.089  109 | 1.22  9833 | 32 |
| {BcDMV1=TRUE, BcULV3=TRUE} =>  {BcOLV7.BI=FALSE} | 0.08  1683 | 0.916  667 | 0.089  109 | 1.13  5992 | 33 |
| {BcDMV1=TRUE, BcULV3=TRUE} =>  {BcOLV4.BI=FALSE} | 0.08  6634 | 0.972  222 | 0.089  109 | 1.10  9542 | 35 |
| {BcDMV1=TRUE, BcULV3=TRUE} =>  {BcULV5=FALSE} | 0.08  6634 | 0.972  222 | 0.089  109 | 1.10  9542 | 35 |
| {BcDMV1=TRUE, BcHV1.BI=TRUE} =>  {BcMV9.BI=TRUE} | 0.07  6733 | 0.885  714 | 0.086  634 | 1.30  5944 | 31 |

| {BcDMV1=TRUE, BcHV1.BI=TRUE} =>  {BcRV1.BI=FALSE} | 0.07  4257 | 0.857  143 | 0.086  634 | 1.18  591 | 30 |
| --- | --- | --- | --- | --- | --- |
| {BcDMV1=TRUE, BcHV1.BI=TRUE} =>  {BcOLV7.BI=FALSE} | 0.07  9208 | 0.914  286 | 0.086  634 | 1.13  3041 | 32 |
| {BcDMV1=TRUE, BcHV1.BI=TRUE} =>  {BcOLV4.BI=FALSE} | 0.08  4158 | 0.971  429 | 0.086  634 | 1.10  8636 | 34 |
| {BcDMV1=TRUE, BcMV11=TRUE} =>  {BcMV9.BI=TRUE} | 0.09  901 | 0.930  233 | 0.106  436 | 1.37  1584 | 40 |
| {BcDMV1=TRUE, BcMV9.BI=TRUE} =>  {BcMV11=TRUE} | 0.09  901 | 0.784  314 | 0.126  238 | 1.27  2541 | 40 |
| {BcMV11=TRUE, BcMV9.BI=TRUE} =>  {BcDMV1=TRUE} | 0.09  901 | 0.243  902 | 0.405  941 | 1.56  4073 | 40 |
| {BcDMV1=TRUE, BcMV11=TRUE} =>  {BcRV1.BI=FALSE} | 0.08  9109 | 0.837  209 | 0.106  436 | 1.15  8331 | 36 |
| {BcMV11=TRUE, BcRV1.BI=FALSE} =>  {BcDMV1=TRUE} | 0.08  9109 | 0.218  182 | 0.408  416 | 1.39  9134 | 36 |
| {BcDMV1=TRUE, BcMV11=TRUE} =>  {BcMV1.BI=TRUE} | 0.09  901 | 0.930  233 | 0.106  436 | 1.17  81 | 40 |
| {BcDMV1=TRUE, BcMV1.BI=TRUE} =>  {BcMV11=TRUE} | 0.09  901 | 0.8 | 0.123  762 | 1.29  7992 | 40 |
| {BcDMV1=TRUE, BcMV11=TRUE} =>  {BcOLV7.BI=FALSE} | 0.09  6535 | 0.906  977 | 0.106  436 | 1.12  3983 | 39 |

| {BcDMV1=TRUE, BcMV11=TRUE} =>  {BcOLV4.BI=FALSE} | 0.10  396 | 0.976  744 | 0.106  436 | 1.11  4702 | 42 |
| --- | --- | --- | --- | --- | --- |
| {BcMV11=TRUE, BcOLV4.BI=FALSE} =>  {BcDMV1=TRUE} | 0.10  396 | 0.202  899 | 0.512  376 | 1.30  1127 | 42 |
| {BcDMV1=TRUE, BcMV11=TRUE} =>  {BcULV5=FALSE} | 0.10  396 | 0.976  744 | 0.106  436 | 1.11  4702 | 42 |
| {BcMV11=TRUE, BcULV5=FALSE} =>  {BcDMV1=TRUE} | 0.10  396 | 0.205  882 | 0.504  95 | 1.32  0261 | 42 |
| {BcDMV1=TRUE, BcMV6.BI=FALSE} =>  {BcOLV1.BI.MOVA.=FALSE} | 0.07  4257 | 0.833  333 | 0.089  109 | 1.24  6914 | 30 |
| {BcDMV1=TRUE, BcOLV1.BI.MOVA.=FALSE} =>  {BcRV1.BI=FALSE} | 0.08  6634 | 0.875 | 0.099  01 | 1.21  0616 | 35 |
| {BcDMV1=TRUE, BcOLV1.BI.MOVA.=FALSE} =>  {BcEV2.BI=FALSE} | 0.08  4158 | 0.85 | 0.099  01 | 1.13  7086 | 34 |
| {BcDMV1=TRUE, BcOLV1.BI.MOVA.=FALSE} =>  {BcULV1.BI=FALSE} | 0.09  4059 | 0.95 | 0.099  01 | 1.21  0726 | 38 |
| {BcDMV1=TRUE, BcOLV1.BI.MOVA.=FALSE} =>  {BcOLV4.BI=FALSE} | 0.09  901 | 1 | 0.099  01 | 1.14  1243 | 40 |

| {BcDMV1=TRUE, BcOLV1.BI.MOVA.=FALSE} =>  {BcULV5=FALSE} | 0.09  901 | 1 | 0.099  01 | 1.14  1243 | 40 |
| --- | --- | --- | --- | --- | --- |
| {BcBNV5.BI=FALSE, BcDMV1=TRUE} =>  {BcOLV1.BI.MOVA.=FALSE} | 0.08  1683 | 0.785  714 | 0.103  96 | 1.17  5661 | 33 |
| {BcDMV1=TRUE, BcMCV5.BI=FALSE} =>  {BcOLV1.BI.MOVA.=FALSE} | 0.09  4059 | 0.775  51 | 0.121  287 | 1.16  0393 | 38 |
| {BcDMV1=TRUE, BcEV5=FALSE} =>  {BcOLV1.BI.MOVA.=FALSE} | 0.08  6634 | 0.777  778 | 0.111  386 | 1.16  3786 | 35 |
| {BcDMV1=TRUE, BcULV4=TRUE} =>  {BcMV9.BI=TRUE} | 0.09  4059 | 0.791  667 | 0.118  812 | 1.16  7275 | 38 |
| {BcDMV1=TRUE, BcPV3.BI=FALSE} =>  {BcMV9.BI=TRUE} | 0.09  1584 | 0.804  348 | 0.113  861 | 1.18  5973 | 37 |
| {BcDMV1=TRUE, BcMV9.BI=TRUE} =>  {BcRV1.BI=FALSE} | 0.10  8911 | 0.862  745 | 0.126  238 | 1.19  3661 | 44 |
| {BcDMV1=TRUE, BcRV1.BI=FALSE} =>  {BcMV9.BI=TRUE} | 0.10  8911 | 0.8 | 0.136  139 | 1.17  9562 | 44 |
| {BcMV9.BI=TRUE, BcRV1.BI=FALSE} =>  {BcDMV1=TRUE} | 0.10  8911 | 0.239  13 | 0.455  446 | 1.53  3471 | 44 |
| {BcDMV1=TRUE, BcULV1.BI=FALSE} =>  {BcMV9.BI=TRUE} | 0.10  6436 | 0.781  818 | 0.136  139 | 1.15  2754 | 43 |
| {BcMV9.BI=TRUE, BcULV1.BI=FALSE} => | 0.10 | 0.212 | 0.5 | 1.36 | 43 |

| {BcDMV1=TRUE} | 6436 | 871 |  | 5079 |  |
| --- | --- | --- | --- | --- | --- |
| {BcDMV1=TRUE, BcMV1.BI=TRUE} =>  {BcMV9.BI=TRUE} | 0.10  6436 | 0.86 | 0.123  762 | 1.26  8029 | 43 |
| {BcDMV1=TRUE, BcMV12=FALSE} =>  {BcMV9.BI=TRUE} | 0.10  6436 | 0.811  321 | 0.131  188 | 1.19  6254 | 43 |
| {BcDMV1=TRUE, BcMV9.BI=TRUE} =>  {BcOLV7.BI=FALSE} | 0.11  3861 | 0.901  961 | 0.126  238 | 1.11  7767 | 46 |
| {BcDMV1=TRUE, BcOLV7.BI=FALSE} =>  {BcMV9.BI=TRUE} | 0.11  3861 | 0.807  018 | 0.141  089 | 1.18  9909 | 46 |
| {BcMV9.BI=TRUE, BcOLV7.BI=FALSE} =>  {BcDMV1=TRUE} | 0.11  3861 | 0.224  39 | 0.507  426 | 1.43  8947 | 46 |
| {BcALV1.BI=FALSE, BcDMV1=TRUE} =>  {BcMV9.BI=TRUE} | 0.08  9109 | 0.782  609 | 0.113  861 | 1.15  3919 | 36 |
| {BcDMV1=TRUE, BcMV9.BI=TRUE} =>  {BcOLV4.BI=FALSE} | 0.12  3762 | 0.980  392 | 0.126  238 | 1.11  8866 | 50 |
| {BcDMV1=TRUE, BcOLV4.BI=FALSE} =>  {BcMV9.BI=TRUE} | 0.12  3762 | 0.806  452 | 0.153  465 | 1.18  9075 | 50 |
| {BcMV9.BI=TRUE, BcOLV4.BI=FALSE} =>  {BcDMV1=TRUE} | 0.12  3762 | 0.200  803 | 0.616  337 | 1.28  769 | 50 |
| {BcDMV1=TRUE, BcMV9.BI=TRUE} =>  {BcULV5=FALSE} | 0.12  1287 | 0.960  784 | 0.126  238 | 1.09  6488 | 49 |
| {BcDMV1=TRUE, BcULV5=FALSE} => | 0.12 | 0.803 | 0.150 | 1.18 | 49 |

| {BcMV9.BI=TRUE} | 1287 | 279 | 99 | 4396 |  |
| --- | --- | --- | --- | --- | --- |
| {BcDMV1=TRUE, BvF.BI=FALSE} =>  {BcMV9.BI=TRUE} | 0.10  8911 | 0.785  714 | 0.138  614 | 1.15  8498 | 44 |
| {BcDMV1=TRUE, BcOLV13.BI=FALSE} =>  {BcMV9.BI=TRUE} | 0.12  1287 | 0.803  279 | 0.150  99 | 1.18  4396 | 49 |
| {BcDMV1=TRUE, BcOLV2.BI=FALSE} =>  {BcMV9.BI=TRUE} | 0.11  8812 | 0.8 | 0.148  515 | 1.17  9562 | 48 |
| {BcDMV1=TRUE, BcMCV3.BI=FALSE} =>  {BcMV9.BI=TRUE} | 0.12  6238 | 0.809  524 | 0.155  941 | 1.19  3604 | 51 |
| {BcMCV3.BI=FALSE, BcMV9.BI=TRUE} =>  {BcDMV1=TRUE} | 0.12  6238 | 0.200  787 | 0.628  713 | 1.28  7589 | 51 |
| {BcDMV1=TRUE, BcPV4.BI=FALSE} =>  {BcMV9.BI=TRUE} | 0.12  1287 | 0.803  279 | 0.150  99 | 1.18  4396 | 49 |
| {BcDMV1=TRUE, BcEV6=FALSE} =>  {BcMV9.BI=TRUE} | 0.11  1386 | 0.789  474 | 0.141  089 | 1.16  4041 | 45 |
| {BcDMV1=TRUE, BcOLV17.BI=FALSE} =>  {BcMV9.BI=TRUE} | 0.12  3762 | 0.806  452 | 0.153  465 | 1.18  9075 | 50 |
| {BcDMV1=TRUE, BcOLV15.BI=FALSE} =>  {BcMV9.BI=TRUE} | 0.11  6337 | 0.796  61 | 0.146  04 | 1.17  4564 | 47 |
| {BcDMV1=TRUE, BcULV4=TRUE} =>  {BcRV1.BI=FALSE} | 0.10  1485 | 0.854  167 | 0.118  812 | 1.18  1792 | 41 |
| {BcRV1.BI=FALSE, BcULV4=TRUE} => | 0.10 | 0.226 | 0.448 | 1.45 | 41 |

| {BcDMV1=TRUE} | 1485 | 519 | 02 | 26 |  |
| --- | --- | --- | --- | --- | --- |
| {BcULV1.BI=FALSE, BcULV4=TRUE} =>  {BcDMV1=TRUE} | 0.10  1485 | 0.203  98 | 0.497  525 | 1.30  8063 | 41 |
| {BcDMV1=TRUE, BcULV4=TRUE} =>  {BcOLV7.BI=FALSE} | 0.10  6436 | 0.895  833 | 0.118  812 | 1.11  0174 | 43 |
| {BcOLV7.BI=FALSE, BcULV4=TRUE} =>  {BcDMV1=TRUE} | 0.10  6436 | 0.207  729 | 0.512  376 | 1.33  2106 | 43 |
| {BcDMV1=TRUE, BcULV4=TRUE} =>  {BcOLV4.BI=FALSE} | 0.11  6337 | 0.979  167 | 0.118  812 | 1.11  7467 | 47 |
| {BcDMV1=TRUE, BcEV5=FALSE} =>  {BcULV4=TRUE} | 0.08  9109 | 0.8 | 0.111  386 | 1.15  8423 | 36 |
| {BcDMV1=TRUE, BcMV6.BI=FALSE} =>  {BcRV1.BI=FALSE} | 0.07  9208 | 0.888  889 | 0.089  109 | 1.22  9833 | 32 |
| {BcDMV1=TRUE, BcMV6.BI=FALSE} =>  {BcULV1.BI=FALSE} | 0.08  1683 | 0.916  667 | 0.089  109 | 1.16  8244 | 33 |
| {BcDMV1=TRUE, BcMV6.BI=FALSE} =>  {BcOLV7.BI=FALSE} | 0.08  4158 | 0.944  444 | 0.089  109 | 1.17  0416 | 34 |
| {BcDMV1=TRUE, BcMV6.BI=FALSE} =>  {BcALV1.BI=FALSE} | 0.08  1683 | 0.916  667 | 0.089  109 | 1.12  5633 | 33 |
| {BcDMV1=TRUE, BcMV6.BI=FALSE} =>  {BcOLV4.BI=FALSE} | 0.08  9109 | 1 | 0.089  109 | 1.14  1243 | 36 |
| {BcDMV1=TRUE, BcMV6.BI=FALSE} => | 0.08 | 0.972 | 0.089 | 1.10 | 35 |

| {BcULV5=FALSE} | 6634 | 222 | 109 | 9542 |  |
| --- | --- | --- | --- | --- | --- |
| {BcDMV1=TRUE, BcPV3.BI=FALSE} =>  {BcRV1.BI=FALSE} | 0.10  1485 | 0.891  304 | 0.113  861 | 1.23  3175 | 41 |
| {BcDMV1=TRUE, BcPV3.BI=FALSE} =>  {BcULV1.BI=FALSE} | 0.10  1485 | 0.891  304 | 0.113  861 | 1.13  5921 | 41 |
| {BcDMV1=TRUE, BcPV3.BI=FALSE} =>  {BcOLV4.BI=FALSE} | 0.11  1386 | 0.978  261 | 0.113  861 | 1.11  6433 | 45 |
| {BcDMV1=TRUE, BcPV3.BI=FALSE} =>  {BcULV5=FALSE} | 0.11  3861 | 1 | 0.113  861 | 1.14  1243 | 46 |
| {BcDMV1=TRUE, BcEV2.BI=FALSE} =>  {BcRV1.BI=FALSE} | 0.10  6436 | 0.86 | 0.123  762 | 1.18  9863 | 43 |
| {BcEV2.BI=FALSE, BcRV1.BI=FALSE} =>  {BcDMV1=TRUE} | 0.10  6436 | 0.199  074 | 0.534  653 | 1.27  6602 | 43 |
| {BcDMV1=TRUE, BcULV1.BI=FALSE} =>  {BcRV1.BI=FALSE} | 0.11  8812 | 0.872  727 | 0.136  139 | 1.20  7472 | 48 |
| {BcRV1.BI=FALSE, BcULV1.BI=FALSE} =>  {BcDMV1=TRUE} | 0.11  8812 | 0.203  39 | 0.584  158 | 1.30  4278 | 48 |
| {BcDMV1=TRUE, BcMV1.BI=TRUE} =>  {BcRV1.BI=FALSE} | 0.10  8911 | 0.88 | 0.123  762 | 1.21  7534 | 44 |
| {BcDMV1=TRUE, BcMV12=FALSE} =>  {BcRV1.BI=FALSE} | 0.11  6337 | 0.886  792 | 0.131  188 | 1.22  6932 | 47 |
| {BcMV12=FALSE, BcRV1.BI=FALSE} => | 0.11 | 0.200 | 0.579 | 1.28 | 47 |

| {BcDMV1=TRUE} | 6337 | 855 | 208 | 8021 |  |
| --- | --- | --- | --- | --- | --- |
| {BcDMV1=TRUE, BcVV3.BI=FALSE} =>  {BcRV1.BI=FALSE} | 0.09  4059 | 0.844  444 | 0.111  386 | 1.16  8341 | 38 |
| {BcDMV1=TRUE, BcRV1.BI=FALSE} =>  {BcOLV7.BI=FALSE} | 0.12  6238 | 0.927  273 | 0.136  139 | 1.14  9136 | 51 |
| {BcDMV1=TRUE, BcOLV7.BI=FALSE} =>  {BcRV1.BI=FALSE} | 0.12  6238 | 0.894  737 | 0.141  089 | 1.23  7924 | 51 |
| {BcOLV7.BI=FALSE, BcRV1.BI=FALSE} =>  {BcDMV1=TRUE} | 0.12  6238 | 0.197  674 | 0.638  614 | 1.26  7626 | 51 |
| {BcALV1.BI=FALSE, BcDMV1=TRUE} =>  {BcRV1.BI=FALSE} | 0.10  1485 | 0.891  304 | 0.113  861 | 1.23  3175 | 41 |
| {BcDMV1=TRUE, BcFV9=FALSE} =>  {BcRV1.BI=FALSE} | 0.11  1386 | 0.9 | 0.123  762 | 1.24  5205 | 45 |
| {BcDMV1=TRUE, BcRV1.BI=FALSE} =>  {BcOLV4.BI=FALSE} | 0.13  3663 | 0.981  818 | 0.136  139 | 1.12  0493 | 54 |
| {BcDMV1=TRUE, BcOLV4.BI=FALSE} =>  {BcRV1.BI=FALSE} | 0.13  3663 | 0.870  968 | 0.153  465 | 1.20  5038 | 54 |
| {BcOLV4.BI=FALSE, BcRV1.BI=FALSE} =>  {BcDMV1=TRUE} | 0.13  3663 | 0.211  765 | 0.631  188 | 1.35  7983 | 54 |
| {BcDMV1=TRUE, BcRV1.BI=FALSE} =>  {BcULV5=FALSE} | 0.13  1188 | 0.963  636 | 0.136  139 | 1.09  9743 | 53 |
| {BcDMV1=TRUE, BcULV5=FALSE} => | 0.13 | 0.868 | 0.150 | 1.20 | 53 |

| {BcRV1.BI=FALSE} | 1188 | 852 | 99 | 2111 |  |
| --- | --- | --- | --- | --- | --- |
| {BcRV1.BI=FALSE, BcULV5=FALSE} =>  {BcDMV1=TRUE} | 0.13  1188 | 0.208  661 | 0.628  713 | 1.33  8083 | 53 |
| {BcBNV5.BI=FALSE, BcDMV1=TRUE} =>  {BcRV1.BI=FALSE} | 0.08  6634 | 0.833  333 | 0.103  96 | 1.15  2968 | 35 |
| {BcDMV1=TRUE, BvF.BI=FALSE} =>  {BcRV1.BI=FALSE} | 0.12  6238 | 0.910  714 | 0.138  614 | 1.26  0029 | 51 |
| {BcRV1.BI=FALSE, BvF.BI=FALSE} =>  {BcDMV1=TRUE} | 0.12  6238 | 0.195  402 | 0.646  04 | 1.25  3056 | 51 |
| {BcDMV1=TRUE, BcMCV5.BI=FALSE} =>  {BcRV1.BI=FALSE} | 0.10  1485 | 0.836  735 | 0.121  287 | 1.15  7674 | 41 |
| {BcDMV1=TRUE, BcOLV13.BI=FALSE} =>  {BcRV1.BI=FALSE} | 0.13  3663 | 0.885  246 | 0.150  99 | 1.22  4792 | 54 |
| {BcDMV1=TRUE, BcOLV2.BI=FALSE} =>  {BcRV1.BI=FALSE} | 0.12  8713 | 0.866  667 | 0.148  515 | 1.19  9087 | 52 |
| {BcDMV1=TRUE, BcMCV3.BI=FALSE} =>  {BcRV1.BI=FALSE} | 0.13  6139 | 0.873  016 | 0.155  941 | 1.20  7871 | 55 |
| {BcMCV3.BI=FALSE, BcRV1.BI=FALSE} =>  {BcDMV1=TRUE} | 0.13  6139 | 0.2 | 0.680  693 | 1.28  254 | 55 |
| {BcDMV1=TRUE, BcPV4.BI=FALSE} =>  {BcRV1.BI=FALSE} | 0.13  1188 | 0.868  852 | 0.150  99 | 1.20  2111 | 53 |
| {BcPV4.BI=FALSE, BcRV1.BI=FALSE} => | 0.13 | 0.195 | 0.670 | 1.25 | 53 |

| {BcDMV1=TRUE} | 1188 | 572 | 792 | 4144 |  |
| --- | --- | --- | --- | --- | --- |
| {BcDMV1=TRUE, BcEV6=FALSE} =>  {BcRV1.BI=FALSE} | 0.12  3762 | 0.877  193 | 0.141  089 | 1.21  3651 | 50 |
| {BcDMV1=TRUE, BcOLV17.BI=FALSE} =>  {BcRV1.BI=FALSE} | 0.13  6139 | 0.887  097 | 0.153  465 | 1.22  7353 | 55 |
| {BcOLV17.BI=FALSE, BcRV1.BI=FALSE} =>  {BcDMV1=TRUE} | 0.13  6139 | 0.192  308 | 0.707  921 | 1.23  3211 | 55 |
| {BcDMV1=TRUE, BcOLV15.BI=FALSE} =>  {BcRV1.BI=FALSE} | 0.12  6238 | 0.864  407 | 0.146  04 | 1.19  596 | 51 |
| {BcDMV1=TRUE, BcEV5=FALSE} =>  {BcRV1.BI=FALSE} | 0.09  1584 | 0.822  222 | 0.111  386 | 1.13  7595 | 37 |
| {BcDMV1=TRUE, BcFV5.BI=FALSE} =>  {BcEV2.BI=FALSE} | 0.07  4257 | 0.937  5 | 0.079  208 | 1.25  4139 | 30 |
| {BcDMV1=TRUE, BcEV2.BI=FALSE} =>  {BcULV1.BI=FALSE} | 0.11  1386 | 0.9 | 0.123  762 | 1.14  7003 | 45 |
| {BcALV1.BI=FALSE, BcDMV1=TRUE} =>  {BcEV2.BI=FALSE} | 0.09  6535 | 0.847  826 | 0.113  861 | 1.13  4178 | 39 |
| {BcDMV1=TRUE, BcEV2.BI=FALSE} =>  {BcOLV4.BI=FALSE} | 0.12  1287 | 0.98 | 0.123  762 | 1.11  8418 | 49 |
| {BcDMV1=TRUE, BcEV2.BI=FALSE} =>  {BcULV5=FALSE} | 0.11  8812 | 0.96 | 0.123  762 | 1.09  5593 | 48 |
| {BcBNV5.BI=FALSE, BcDMV1=TRUE} => | 0.09 | 0.904 | 0.103 | 1.21 | 38 |

| {BcEV2.BI=FALSE} | 4059 | 762 | 96 | 0344 |  |
| --- | --- | --- | --- | --- | --- |
| {BcDMV1=TRUE, BcMCV5.BI=FALSE} =>  {BcEV2.BI=FALSE} | 0.10  396 | 0.857  143 | 0.121  287 | 1.14  6641 | 42 |
| {BcDMV1=TRUE, BcEV5=FALSE} =>  {BcEV2.BI=FALSE} | 0.09  4059 | 0.844  444 | 0.111  386 | 1.12  9654 | 38 |
| {BcDMV1=TRUE, BcHV3.BI=FALSE} =>  {BcULV1.BI=FALSE} | 0.07  4257 | 0.937  5 | 0.079  208 | 1.19  4795 | 30 |
| {BcDMV1=TRUE, BcHV3.BI=FALSE} =>  {BcOLV4.BI=FALSE} | 0.07  9208 | 1 | 0.079  208 | 1.14  1243 | 32 |
| {BcDMV1=TRUE, BcHV3.BI=FALSE} =>  {BcULV5=FALSE} | 0.07  6733 | 0.968  75 | 0.079  208 | 1.10  5579 | 31 |
| {BcDMV1=TRUE, BcFV5.BI=FALSE} =>  {BcOLV4.BI=FALSE} | 0.07  9208 | 1 | 0.079  208 | 1.14  1243 | 32 |
| {BcDMV1=TRUE, BcFV5.BI=FALSE} =>  {BcULV5=FALSE} | 0.07  6733 | 0.968  75 | 0.079  208 | 1.10  5579 | 31 |
| {BcDMV1=TRUE, BcULV1.BI=FALSE} =>  {BcMV12=FALSE} | 0.12  1287 | 0.890  909 | 0.136  139 | 1.11  4326 | 49 |
| {BcDMV1=TRUE, BcMV12=FALSE} =>  {BcULV1.BI=FALSE} | 0.12  1287 | 0.924  528 | 0.131  188 | 1.17  8263 | 49 |
| {BcDMV1=TRUE, BcNSRV7.BI=FALSE} =>  {BcULV1.BI=FALSE} | 0.07  9208 | 0.941  176 | 0.084  158 | 1.19  948 | 32 |
| {BcDMV1=TRUE, BcULV1.BI=FALSE} => | 0.12 | 0.909 | 0.136 | 1.12 | 50 |

| {BcOLV7.BI=FALSE} | 3762 | 091 | 139 | 6603 |  |
| --- | --- | --- | --- | --- | --- |
| {BcDMV1=TRUE, BcOLV7.BI=FALSE} =>  {BcULV1.BI=FALSE} | 0.12  3762 | 0.877  193 | 0.141  089 | 1.11  7937 | 50 |
| {BcALV1.BI=FALSE, BcDMV1=TRUE} =>  {BcULV1.BI=FALSE} | 0.10  1485 | 0.891  304 | 0.113  861 | 1.13  5921 | 41 |
| {BcDMV1=TRUE, BcFV9=FALSE} =>  {BcULV1.BI=FALSE} | 0.10  8911 | 0.88 | 0.123  762 | 1.12  1514 | 44 |
| {BcDMV1=TRUE, BcULV1.BI=FALSE} =>  {BcOLV4.BI=FALSE} | 0.13  3663 | 0.981  818 | 0.136  139 | 1.12  0493 | 54 |
| {BcDMV1=TRUE, BcULV1.BI=FALSE} =>  {BcULV5=FALSE} | 0.13  6139 | 1 | 0.136  139 | 1.14  1243 | 55 |
| {BcDMV1=TRUE, BcULV5=FALSE} =>  {BcULV1.BI=FALSE} | 0.13  6139 | 0.901  639 | 0.150  99 | 1.14  9092 | 55 |
| {BcULV1.BI=FALSE, BcULV5=FALSE} =>  {BcDMV1=TRUE} | 0.13  6139 | 0.194  346 | 0.700  495 | 1.24  6284 | 55 |
| {BcBNV5.BI=FALSE, BcDMV1=TRUE} =>  {BcULV1.BI=FALSE} | 0.09  4059 | 0.904  762 | 0.103  96 | 1.15  3072 | 38 |
| {BcDMV1=TRUE, BcMCV5.BI=FALSE} =>  {BcULV1.BI=FALSE} | 0.11  1386 | 0.918  367 | 0.121  287 | 1.17  0411 | 45 |
| {BcDMV1=TRUE, BcOLV13.BI=FALSE} =>  {BcULV1.BI=FALSE} | 0.13  3663 | 0.885  246 | 0.150  99 | 1.12  82 | 54 |
| {BcDMV1=TRUE, BcPV4.BI=FALSE} => | 0.13 | 0.885 | 0.150 | 1.12 | 54 |

| {BcULV1.BI=FALSE} | 3663 | 246 | 99 | 82 |  |
| --- | --- | --- | --- | --- | --- |
| {BcDMV1=TRUE, BcEV6=FALSE} =>  {BcULV1.BI=FALSE} | 0.12  3762 | 0.877  193 | 0.141  089 | 1.11  7937 | 50 |
| {BcDMV1=TRUE, BcEV5=FALSE} =>  {BcULV1.BI=FALSE} | 0.09  901 | 0.888  889 | 0.111  386 | 1.13  2843 | 40 |
| {BcDMV1=TRUE, BcMV1.BI=TRUE} =>  {BcOLV4.BI=FALSE} | 0.12  1287 | 0.98 | 0.123  762 | 1.11  8418 | 49 |
| {BcDMV1=TRUE, BcMV1.BI=TRUE} =>  {BcULV5=FALSE} | 0.11  8812 | 0.96 | 0.123  762 | 1.09  5593 | 48 |
| {BcDMV1=TRUE, BcMV12=FALSE} =>  {BcOLV7.BI=FALSE} | 0.12  3762 | 0.943  396 | 0.131  188 | 1.16  9117 | 50 |
| {BcDMV1=TRUE, BcMV12=FALSE} =>  {BcOLV4.BI=FALSE} | 0.12  8713 | 0.981  132 | 0.131  188 | 1.11  971 | 52 |
| {BcDMV1=TRUE, BcMV12=FALSE} =>  {BcULV5=FALSE} | 0.12  8713 | 0.981  132 | 0.131  188 | 1.11  971 | 52 |
| {BcDMV1=TRUE, BcNSRV7.BI=FALSE} =>  {BcOLV7.BI=FALSE} | 0.07  6733 | 0.911  765 | 0.084  158 | 1.12  9917 | 31 |
| {BcDMV1=TRUE, BcNSRV7.BI=FALSE} =>  {BcOLV4.BI=FALSE} | 0.08  1683 | 0.970  588 | 0.084  158 | 1.10  7677 | 33 |
| {BcDMV1=TRUE, BcNSRV7.BI=FALSE} =>  {BcULV5=FALSE} | 0.08  1683 | 0.970  588 | 0.084  158 | 1.10  7677 | 33 |
| {BcDMV1=TRUE, BcVV3.BI=FALSE} => | 0.11 | 1 | 0.111 | 1.14 | 45 |

| {BcOLV4.BI=FALSE} | 1386 |  | 386 | 1243 |  |
| --- | --- | --- | --- | --- | --- |
| {BcALV1.BI=FALSE, BcDMV1=TRUE} =>  {BcOLV7.BI=FALSE} | 0.10  396 | 0.913  043 | 0.113  861 | 1.13  1502 | 42 |
| {BcDMV1=TRUE, BcFV9=FALSE} =>  {BcOLV7.BI=FALSE} | 0.11  1386 | 0.9 | 0.123  762 | 1.11  5337 | 45 |
| {BcDMV1=TRUE, BcOLV7.BI=FALSE} =>  {BcOLV4.BI=FALSE} | 0.13  8614 | 0.982  456 | 0.141  089 | 1.12  1221 | 56 |
| {BcDMV1=TRUE, BcOLV4.BI=FALSE} =>  {BcOLV7.BI=FALSE} | 0.13  8614 | 0.903  226 | 0.153  465 | 1.11  9335 | 56 |
| {BcOLV4.BI=FALSE, BcOLV7.BI=FALSE} =>  {BcDMV1=TRUE} | 0.13  8614 | 0.194  444 | 0.712  871 | 1.24  6914 | 56 |
| {BcDMV1=TRUE, BcOLV7.BI=FALSE} =>  {BcULV5=FALSE} | 0.13  6139 | 0.964  912 | 0.141  089 | 1.10  1199 | 55 |
| {BcDMV1=TRUE, BcULV5=FALSE} =>  {BcOLV7.BI=FALSE} | 0.13  6139 | 0.901  639 | 0.150  99 | 1.11  7369 | 55 |
| {BcOLV7.BI=FALSE, BcULV5=FALSE} =>  {BcDMV1=TRUE} | 0.13  6139 | 0.194  346 | 0.700  495 | 1.24  6284 | 55 |
| {BcDMV1=TRUE, BvF.BI=FALSE} =>  {BcOLV7.BI=FALSE} | 0.12  3762 | 0.892  857 | 0.138  614 | 1.10  6486 | 50 |
| {BcDMV1=TRUE, BcOLV13.BI=FALSE} =>  {BcOLV7.BI=FALSE} | 0.13  8614 | 0.918  033 | 0.150  99 | 1.13  7685 | 56 |
| {BcDMV1=TRUE, BcOLV2.BI=FALSE} => | 0.13 | 0.9 | 0.148 | 1.11 | 54 |

| {BcOLV7.BI=FALSE} | 3663 |  | 515 | 5337 |  |
| --- | --- | --- | --- | --- | --- |
| {BcDMV1=TRUE, BcMCV3.BI=FALSE} =>  {BcOLV7.BI=FALSE} | 0.14  1089 | 0.904  762 | 0.155  941 | 1.12  1239 | 57 |
| {BcDMV1=TRUE, BcPV4.BI=FALSE} =>  {BcOLV7.BI=FALSE} | 0.13  8614 | 0.918  033 | 0.150  99 | 1.13  7685 | 56 |
| {BcDMV1=TRUE, BcEV6=FALSE} =>  {BcOLV7.BI=FALSE} | 0.12  8713 | 0.912  281 | 0.141  089 | 1.13  0556 | 52 |
| {BcDMV1=TRUE, BcOLV17.BI=FALSE} =>  {BcOLV7.BI=FALSE} | 0.14  1089 | 0.919  355 | 0.153  465 | 1.13  9323 | 57 |
| {BcDMV1=TRUE, BcOLV15.BI=FALSE} =>  {BcOLV7.BI=FALSE} | 0.13  1188 | 0.898  305 | 0.146  04 | 1.11  3237 | 53 |
| {BcALV1.BI=FALSE, BcDMV1=TRUE} =>  {BcOLV4.BI=FALSE} | 0.11  3861 | 1 | 0.113  861 | 1.14  1243 | 46 |
| {BcBNV5.BI=FALSE, BcDMV1=TRUE} =>  {BcALV1.BI=FALSE} | 0.09  4059 | 0.904  762 | 0.103  96 | 1.11  1015 | 38 |
| {BcDMV1=TRUE, BcFV9=FALSE} =>  {BcOLV4.BI=FALSE} | 0.12  1287 | 0.98 | 0.123  762 | 1.11  8418 | 49 |
| {BcDMV1=TRUE, BcFV9=FALSE} =>  {BcULV5=FALSE} | 0.11  8812 | 0.96 | 0.123  762 | 1.09  5593 | 48 |
| {BcDMV1=TRUE, BcOLV4.BI=FALSE} =>  {BcULV5=FALSE} | 0.14  8515 | 0.967  742 | 0.153  465 | 1.10  4429 | 60 |
| {BcDMV1=TRUE, BcULV5=FALSE} => | 0.14 | 0.983 | 0.150 | 1.12 | 60 |

| {BcOLV4.BI=FALSE} | 8515 | 607 | 99 | 2534 |  |
| --- | --- | --- | --- | --- | --- |
| {BcBNV5.BI=FALSE, BcDMV1=TRUE} =>  {BcOLV4.BI=FALSE} | 0.10  396 | 1 | 0.103  96 | 1.14  1243 | 42 |
| {BcDMV1=TRUE, BvF.BI=FALSE} =>  {BcOLV4.BI=FALSE} | 0.13  6139 | 0.982  143 | 0.138  614 | 1.12  0864 | 55 |
| {BcDMV1=TRUE, BcMCV5.BI=FALSE} =>  {BcOLV4.BI=FALSE} | 0.12  1287 | 1 | 0.121  287 | 1.14  1243 | 49 |
| {BcDMV1=TRUE, BcOLV13.BI=FALSE} =>  {BcOLV4.BI=FALSE} | 0.14  8515 | 0.983  607 | 0.150  99 | 1.12  2534 | 60 |
| {BcDMV1=TRUE, BcOLV2.BI=FALSE} =>  {BcOLV4.BI=FALSE} | 0.14  604 | 0.983  333 | 0.148  515 | 1.12  2222 | 59 |
| {BcDMV1=TRUE, BcMCV3.BI=FALSE} =>  {BcOLV4.BI=FALSE} | 0.15  3465 | 0.984  127 | 0.155  941 | 1.12  3128 | 62 |
| {BcDMV1=TRUE, BcPV4.BI=FALSE} =>  {BcOLV4.BI=FALSE} | 0.14  8515 | 0.983  607 | 0.150  99 | 1.12  2534 | 60 |
| {BcDMV1=TRUE, BcEV6=FALSE} =>  {BcOLV4.BI=FALSE} | 0.13  8614 | 0.982  456 | 0.141  089 | 1.12  1221 | 56 |
| {BcDMV1=TRUE, BcOLV17.BI=FALSE} =>  {BcOLV4.BI=FALSE} | 0.15  099 | 0.983  871 | 0.153  465 | 1.12  2836 | 61 |
| {BcDMV1=TRUE, BcOLV15.BI=FALSE} =>  {BcOLV4.BI=FALSE} | 0.14  3564 | 0.983  051 | 0.146  04 | 1.12  19 | 58 |
| {BcDMV1=TRUE, BcEV5=FALSE} => | 0.11 | 1 | 0.111 | 1.14 | 45 |

| {BcOLV4.BI=FALSE} | 1386 |  | 386 | 1243 |  |
| --- | --- | --- | --- | --- | --- |
| {BcBNV5.BI=FALSE, BcDMV1=TRUE} =>  {BcULV5=FALSE} | 0.10  1485 | 0.976  19 | 0.103  96 | 1.11  407 | 41 |
| {BcDMV1=TRUE, BvF.BI=FALSE} =>  {BcULV5=FALSE} | 0.13  3663 | 0.964  286 | 0.138  614 | 1.10  0484 | 54 |
| {BcDMV1=TRUE, BcMCV5.BI=FALSE} =>  {BcULV5=FALSE} | 0.11  8812 | 0.979  592 | 0.121  287 | 1.11  7952 | 48 |
| {BcDMV1=TRUE, BcOLV13.BI=FALSE} =>  {BcULV5=FALSE} | 0.14  604 | 0.967  213 | 0.150  99 | 1.10  3825 | 59 |
| {BcDMV1=TRUE, BcOLV2.BI=FALSE} =>  {BcULV5=FALSE} | 0.14  3564 | 0.966  667 | 0.148  515 | 1.10  3202 | 58 |
| {BcDMV1=TRUE, BcMCV3.BI=FALSE} =>  {BcULV5=FALSE} | 0.15  099 | 0.968  254 | 0.155  941 | 1.10  5013 | 61 |
| {BcDMV1=TRUE, BcPV4.BI=FALSE} =>  {BcULV5=FALSE} | 0.14  604 | 0.967  213 | 0.150  99 | 1.10  3825 | 59 |
| {BcDMV1=TRUE, BcEV6=FALSE} =>  {BcULV5=FALSE} | 0.13  8614 | 0.982  456 | 0.141  089 | 1.12  1221 | 56 |
| {BcDMV1=TRUE, BcOLV17.BI=FALSE} =>  {BcULV5=FALSE} | 0.14  8515 | 0.967  742 | 0.153  465 | 1.10  4429 | 60 |
| {BcDMV1=TRUE, BcOLV15.BI=FALSE} =>  {BcULV5=FALSE} | 0.14  1089 | 0.966  102 | 0.146  04 | 1.10  2557 | 57 |
| {BcDMV1=TRUE, BcEV5=FALSE} => | 0.10 | 0.977 | 0.111 | 1.11 | 44 |

| {BcULV5=FALSE} | 8911 | 778 | 386 | 5882 |  |
| --- | --- | --- | --- | --- | --- |
| {BcALV1.BI=TRUE, BcRV1.BI=TRUE} =>  {BcMV2.BI=TRUE} | 0.07  9208 | 0.914  286 | 0.086  634 | 1.97  5248 | 32 |
| {BcALV1.BI=TRUE, BcMV2.BI=TRUE} =>  {BcRV1.BI=TRUE} | 0.07  9208 | 0.571  429 | 0.138  614 | 2.06  1224 | 32 |
| {BcMV2.BI=TRUE, BcRV1.BI=TRUE} =>  {BcALV1.BI=TRUE} | 0.07  9208 | 0.444  444 | 0.178  218 | 2.39  4074 | 32 |
| {BcALV1.BI=TRUE, BcRV1.BI=TRUE} =>  {BcOLV18=TRUE} | 0.07  9208 | 0.914  286 | 0.086  634 | 1.76  7327 | 32 |
| {BcALV1.BI=TRUE, BcOLV18=TRUE} =>  {BcRV1.BI=TRUE} | 0.07  9208 | 0.561  404 | 0.141  089 | 2.02  5063 | 32 |
| {BcOLV18=TRUE, BcRV1.BI=TRUE} =>  {BcALV1.BI=TRUE} | 0.07  9208 | 0.421  053 | 0.188  119 | 2.26  807 | 32 |
| {BcALV1.BI=TRUE, BcRV1.BI=TRUE} =>  {BcMV11=TRUE} | 0.07  9208 | 0.914  286 | 0.086  634 | 1.48  3419 | 32 |
| {BcALV1.BI=TRUE, BcMV11=TRUE} =>  {BcRV1.BI=TRUE} | 0.07  9208 | 0.507  937 | 0.155  941 | 1.83  22 | 32 |
| {BcMV11=TRUE, BcRV1.BI=TRUE} =>  {BcALV1.BI=TRUE} | 0.07  9208 | 0.380  952 | 0.207  921 | 2.05  2063 | 32 |
| {BcALV1.BI=TRUE, BcRV1.BI=TRUE} =>  {BcMV9.BI=TRUE} | 0.07  9208 | 0.914  286 | 0.086  634 | 1.34  8071 | 32 |
| {BcALV1.BI=TRUE, BcMV9.BI=TRUE} => | 0.07 | 0.581 | 0.136 | 2.09 | 32 |

| {BcRV1.BI=TRUE} | 9208 | 818 | 139 | 8701 |  |
| --- | --- | --- | --- | --- | --- |
| {BcMV9.BI=TRUE, BcRV1.BI=TRUE} =>  {BcALV1.BI=TRUE} | 0.07  9208 | 0.355  556 | 0.222  772 | 1.91  5259 | 32 |
| {BcALV1.BI=TRUE, BcRV1.BI=TRUE} =>  {BcULV4=TRUE} | 0.08  1683 | 0.942  857 | 0.086  634 | 1.36  5284 | 33 |
| {BcALV1.BI=TRUE, BcULV4=TRUE} =>  {BcRV1.BI=TRUE} | 0.08  1683 | 0.507  692 | 0.160  891 | 1.83  1319 | 33 |
| {BcRV1.BI=TRUE, BcULV4=TRUE} =>  {BcALV1.BI=TRUE} | 0.08  1683 | 0.336  735 | 0.242  574 | 1.81  3878 | 33 |
| {BcALV1.BI=TRUE, BcRV1.BI=TRUE} =>  {BcVV3.BI=FALSE} | 0.07  9208 | 0.914  286 | 0.086  634 | 1.14  0035 | 32 |
| {BcALV1.BI=TRUE, BcVV3.BI=FALSE} =>  {BcRV1.BI=TRUE} | 0.07  9208 | 0.551  724 | 0.143  564 | 1.99  0148 | 32 |
| {BcRV1.BI=TRUE, BcVV3.BI=FALSE} =>  {BcALV1.BI=TRUE} | 0.07  9208 | 0.340  426 | 0.232  673 | 1.83  3759 | 32 |
| {BcALV1.BI=TRUE, BcDMV1=FALSE} =>  {BcRV1.BI=TRUE} | 0.07  9208 | 0.551  724 | 0.143  564 | 1.99  0148 | 32 |
| {BcDMV1=FALSE, BcRV1.BI=TRUE} =>  {BcALV1.BI=TRUE} | 0.07  9208 | 0.307  692 | 0.257  426 | 1.65  7436 | 32 |
| {BcALV1.BI=TRUE, BcOLV4.BI=FALSE} =>  {BcRV1.BI=TRUE} | 0.07  6733 | 0.492  063 | 0.155  941 | 1.77  4943 | 31 |
| {BcOLV4.BI=FALSE, BcRV1.BI=TRUE} => | 0.07 | 0.313 | 0.245 | 1.68 | 31 |

| {BcALV1.BI=TRUE} | 6733 | 131 | 05 | 6734 |  |
| --- | --- | --- | --- | --- | --- |
| {BcALV1.BI=TRUE, BcULV5=FALSE} =>  {BcRV1.BI=TRUE} | 0.07  9208 | 0.463  768 | 0.170  792 | 1.67  2878 | 32 |
| {BcRV1.BI=TRUE, BcULV5=FALSE} =>  {BcALV1.BI=TRUE} | 0.07  9208 | 0.32 | 0.247  525 | 1.72  3733 | 32 |
| {BcALV1.BI=TRUE, BcBNV5.BI=FALSE} =>  {BcRV1.BI=TRUE} | 0.07  9208 | 0.551  724 | 0.143  564 | 1.99  0148 | 32 |
| {BcBNV5.BI=FALSE, BcRV1.BI=TRUE} =>  {BcALV1.BI=TRUE} | 0.07  9208 | 0.304  762 | 0.259  901 | 1.64  1651 | 32 |
| {BcALV1.BI=TRUE, BvF.BI=FALSE} =>  {BcRV1.BI=TRUE} | 0.08  1683 | 0.492  537 | 0.165  842 | 1.77  6652 | 33 |
| {BcRV1.BI=TRUE, BvF.BI=FALSE} =>  {BcALV1.BI=TRUE} | 0.08  1683 | 0.305  556 | 0.267  327 | 1.64  5926 | 33 |
| {BcALV1.BI=TRUE, BcMCV5.BI=FALSE} =>  {BcRV1.BI=TRUE} | 0.07  9208 | 0.524  59 | 0.150  99 | 1.89  2272 | 32 |
| {BcMCV5.BI=FALSE, BcRV1.BI=TRUE} =>  {BcALV1.BI=TRUE} | 0.07  9208 | 0.316  832 | 0.25 | 1.70  6667 | 32 |
| {BcALV1.BI=TRUE, BcOLV13.BI=FALSE} =>  {BcRV1.BI=TRUE} | 0.07  9208 | 0.450  704 | 0.175  743 | 1.62  5755 | 32 |
| {BcOLV13.BI=FALSE, BcRV1.BI=TRUE} =>  {BcALV1.BI=TRUE} | 0.07  9208 | 0.336  842 | 0.235  149 | 1.81  4456 | 32 |
| {BcALV1.BI=TRUE, BcOLV2.BI=FALSE} => | 0.07 | 0.428 | 0.173 | 1.54 | 30 |

| {BcRV1.BI=TRUE} | 4257 | 571 | 267 | 5918 |  |
| --- | --- | --- | --- | --- | --- |
| {BcOLV2.BI=FALSE, BcRV1.BI=TRUE} =>  {BcALV1.BI=TRUE} | 0.07  4257 | 0.303  03 | 0.245  05 | 1.63  2323 | 30 |
| {BcALV1.BI=TRUE, BcMCV3.BI=FALSE} =>  {BcRV1.BI=TRUE} | 0.07  6733 | 0.442  857 | 0.173  267 | 1.59  7449 | 31 |
| {BcMCV3.BI=FALSE, BcRV1.BI=TRUE} =>  {BcALV1.BI=TRUE} | 0.07  6733 | 0.303  922 | 0.252  475 | 1.63  7124 | 31 |
| {BcALV1.BI=TRUE, BcPV4.BI=FALSE} =>  {BcRV1.BI=TRUE} | 0.08  4158 | 0.465  753 | 0.180  693 | 1.68  0039 | 34 |
| {BcPV4.BI=FALSE, BcRV1.BI=TRUE} =>  {BcALV1.BI=TRUE} | 0.08  4158 | 0.320  755 | 0.262  376 | 1.72  7799 | 34 |
| {BcALV1.BI=TRUE, BcEV6=FALSE} =>  {BcRV1.BI=TRUE} | 0.08  1683 | 0.478  261 | 0.170  792 | 1.72  5155 | 33 |
| {BcEV6=FALSE, BcRV1.BI=TRUE} =>  {BcALV1.BI=TRUE} | 0.08  1683 | 0.305  556 | 0.267  327 | 1.64  5926 | 33 |
| {BcALV1.BI=TRUE, BcOLV17.BI=FALSE} =>  {BcRV1.BI=TRUE} | 0.08  1683 | 0.458  333 | 0.178  218 | 1.65  3274 | 33 |
| {BcOLV17.BI=FALSE, BcRV1.BI=TRUE} =>  {BcALV1.BI=TRUE} | 0.08  1683 | 0.340  206 | 0.240  099 | 1.83  2577 | 33 |
| {BcALV1.BI=TRUE, BcOLV15.BI=FALSE} =>  {BcRV1.BI=TRUE} | 0.08  6634 | 0.472  973 | 0.183  168 | 1.70  6081 | 35 |
| {BcOLV15.BI=FALSE, BcRV1.BI=TRUE} => | 0.08 | 0.321 | 0.269 | 1.72 | 35 |

| {BcALV1.BI=TRUE} | 6634 | 101 | 802 | 9664 |  |
| --- | --- | --- | --- | --- | --- |
| {BcALV1.BI=TRUE, BcEV5=FALSE} =>  {BcRV1.BI=TRUE} | 0.08  6634 | 0.530  303 | 0.163  366 | 1.91  2879 | 35 |
| {BcEV5=FALSE, BcRV1.BI=TRUE} =>  {BcALV1.BI=TRUE} | 0.08  6634 | 0.312  5 | 0.277  228 | 1.68  3333 | 35 |
| {BcALV1.BI=TRUE, BcMV6.BI=TRUE} =>  {BcMV2.BI=TRUE} | 0.07  6733 | 0.885  714 | 0.086  634 | 1.91  3522 | 31 |
| {BcALV1.BI=TRUE, BcMV2.BI=TRUE} =>  {BcMV6.BI=TRUE} | 0.07  6733 | 0.553  571 | 0.138  614 | 1.81  8235 | 31 |
| {BcMV2.BI=TRUE, BcMV6.BI=TRUE} =>  {BcALV1.BI=TRUE} | 0.07  6733 | 0.369  048 | 0.207  921 | 1.98  7937 | 31 |
| {BcALV1.BI=TRUE, BcMV6.BI=TRUE} =>  {BcOLV18=TRUE} | 0.07  4257 | 0.857  143 | 0.086  634 | 1.65  6869 | 30 |
| {BcALV1.BI=TRUE, BcOLV18=TRUE} =>  {BcMV6.BI=TRUE} | 0.07  4257 | 0.526  316 | 0.141  089 | 1.72  8712 | 30 |
| {BcMV6.BI=TRUE, BcOLV18=TRUE} =>  {BcALV1.BI=TRUE} | 0.07  4257 | 0.428  571 | 0.173  267 | 2.30  8571 | 30 |
| {BcALV1.BI=TRUE, BcMV6.BI=TRUE} =>  {BcMV11=TRUE} | 0.07  9208 | 0.914  286 | 0.086  634 | 1.48  3419 | 32 |
| {BcALV1.BI=TRUE, BcMV11=TRUE} =>  {BcMV6.BI=TRUE} | 0.07  9208 | 0.507  937 | 0.155  941 | 1.66  8344 | 32 |
| {BcMV11=TRUE, BcMV6.BI=TRUE} => | 0.07 | 0.347 | 0.227 | 1.87 | 32 |

| {BcALV1.BI=TRUE} | 9208 | 826 | 723 | 3623 |  |
| --- | --- | --- | --- | --- | --- |
| {BcALV1.BI=TRUE, BcMV6.BI=TRUE} =>  {BcMV9.BI=TRUE} | 0.07  6733 | 0.885  714 | 0.086  634 | 1.30  5944 | 31 |
| {BcALV1.BI=TRUE, BcMV9.BI=TRUE} =>  {BcMV6.BI=TRUE} | 0.07  6733 | 0.563  636 | 0.136  139 | 1.85  1293 | 31 |
| {BcMV6.BI=TRUE, BcMV9.BI=TRUE} =>  {BcALV1.BI=TRUE} | 0.07  6733 | 0.326  316 | 0.235  149 | 1.75  7754 | 31 |
| {BcALV1.BI=TRUE, BcMV6.BI=TRUE} =>  {BcULV4=TRUE} | 0.08  6634 | 1 | 0.086  634 | 1.44  8029 | 35 |
| {BcALV1.BI=TRUE, BcULV4=TRUE} =>  {BcMV6.BI=TRUE} | 0.08  6634 | 0.538  462 | 0.160  891 | 1.76  8605 | 35 |
| {BcMV6.BI=TRUE, BcULV4=TRUE} =>  {BcALV1.BI=TRUE} | 0.08  6634 | 0.357  143 | 0.242  574 | 1.92  381 | 35 |
| {BcALV1.BI=TRUE, BcMV12=FALSE} =>  {BcMV6.BI=TRUE} | 0.07  4257 | 0.508  475 | 0.146  04 | 1.67  0112 | 30 |
| {BcMV12=FALSE, BcMV6.BI=TRUE} =>  {BcALV1.BI=TRUE} | 0.07  4257 | 0.340  909 | 0.217  822 | 1.83  6364 | 30 |
| {BcALV1.BI=TRUE, BcOLV4.BI=FALSE} =>  {BcMV6.BI=TRUE} | 0.07  9208 | 0.507  937 | 0.155  941 | 1.66  8344 | 32 |
| {BcMV6.BI=TRUE, BcOLV4.BI=FALSE} =>  {BcALV1.BI=TRUE} | 0.07  9208 | 0.304  762 | 0.259  901 | 1.64  1651 | 32 |
| {BcALV1.BI=TRUE, BcULV5=FALSE} => | 0.08 | 0.478 | 0.170 | 1.57 | 33 |

| {BcMV6.BI=TRUE} | 1683 | 261 | 792 | 0873 |  |
| --- | --- | --- | --- | --- | --- |
| {BcMV6.BI=TRUE, BcULV5=FALSE} =>  {BcALV1.BI=TRUE} | 0.08  1683 | 0.320  388 | 0.254  95 | 1.72  5825 | 33 |
| {BcALV1.BI=TRUE, BvF.BI=FALSE} =>  {BcMV6.BI=TRUE} | 0.07  6733 | 0.462  687 | 0.165  842 | 1.51  9718 | 31 |
| {BcMV6.BI=TRUE, BvF.BI=FALSE} =>  {BcALV1.BI=TRUE} | 0.07  6733 | 0.264  957 | 0.289  604 | 1.42  7236 | 31 |
| {BcALV1.BI=TRUE, BcOLV13.BI=FALSE} =>  {BcMV6.BI=TRUE} | 0.08  1683 | 0.464  789 | 0.175  743 | 1.52  6623 | 33 |
| {BcMV6.BI=TRUE, BcOLV13.BI=FALSE} =>  {BcALV1.BI=TRUE} | 0.08  1683 | 0.302  752 | 0.269  802 | 1.63  0826 | 33 |
| {BcALV1.BI=TRUE, BcOLV2.BI=FALSE} =>  {BcMV6.BI=TRUE} | 0.07  4257 | 0.428  571 | 0.173  267 | 1.40  7666 | 30 |
| {BcMV6.BI=TRUE, BcOLV2.BI=FALSE} =>  {BcALV1.BI=TRUE} | 0.07  4257 | 0.283  019 | 0.262  376 | 1.52  4528 | 30 |
| {BcALV1.BI=TRUE, BcMCV3.BI=FALSE} =>  {BcMV6.BI=TRUE} | 0.07  9208 | 0.457  143 | 0.173  267 | 1.50  151 | 32 |
| {BcMCV3.BI=FALSE, BcMV6.BI=TRUE} =>  {BcALV1.BI=TRUE} | 0.07  9208 | 0.304  762 | 0.259  901 | 1.64  1651 | 32 |
| {BcALV1.BI=TRUE, BcPV4.BI=FALSE} =>  {BcMV6.BI=TRUE} | 0.08  4158 | 0.465  753 | 0.180  693 | 1.52  9792 | 34 |
| {BcMV6.BI=TRUE, BcPV4.BI=FALSE} => | 0.08 | 0.303 | 0.277 | 1.63 | 34 |

| {BcALV1.BI=TRUE} | 4158 | 571 | 228 | 5238 |  |
| --- | --- | --- | --- | --- | --- |
| {BcALV1.BI=TRUE, BcEV6=FALSE} =>  {BcMV6.BI=TRUE} | 0.07  9208 | 0.463  768 | 0.170  792 | 1.52  3271 | 32 |
| {BcEV6=FALSE, BcMV6.BI=TRUE} =>  {BcALV1.BI=TRUE} | 0.07  9208 | 0.280  702 | 0.282  178 | 1.51  2047 | 32 |
| {BcALV1.BI=TRUE, BcOLV17.BI=FALSE} =>  {BcMV6.BI=TRUE} | 0.08  1683 | 0.458  333 | 0.178  218 | 1.50  542 | 33 |
| {BcMV6.BI=TRUE, BcOLV17.BI=FALSE} =>  {BcALV1.BI=TRUE} | 0.08  1683 | 0.294  643 | 0.277  228 | 1.58  7143 | 33 |
| {BcALV1.BI=TRUE, BcOLV15.BI=FALSE} =>  {BcMV6.BI=TRUE} | 0.08  6634 | 0.472  973 | 0.183  168 | 1.55  3505 | 35 |
| {BcMV6.BI=TRUE, BcOLV15.BI=FALSE} =>  {BcALV1.BI=TRUE} | 0.08  6634 | 0.289  256 | 0.299  505 | 1.55  8127 | 35 |
| {BcALV1.BI=TRUE, BcOLV1.BI.MOVA.=TRUE} =>  {BcMV4.BI=TRUE} | 0.07  6733 | 0.720  93 | 0.106  436 | 1.62  7127 | 31 |
| {BcALV1.BI=TRUE, BcMV4.BI=TRUE} =>  {BcOLV1.BI.MOVA.=TRUE} | 0.07  6733 | 0.596  154 | 0.128  713 | 1.79  7359 | 31 |
| {BcMV4.BI=TRUE, BcOLV1.BI.MOVA.=TRUE} =>  {BcALV1.BI=TRUE} | 0.07  6733 | 0.413  333 | 0.185  644 | 2.22  6489 | 31 |
| {BcALV1.BI=TRUE, | 0.09 | 0.906 | 0.106 | 1.95 | 39 |

| BcOLV1.BI.MOVA.=TRUE} =>  {BcMV2.BI=TRUE} | 6535 | 977 | 436 | 9458 |  |
| --- | --- | --- | --- | --- | --- |
| {BcALV1.BI=TRUE, BcMV2.BI=TRUE} => | 0.09 | 0.696 | 0.138 | 2.09 |  |
|  |  |  |  |  | 39 |
| {BcOLV1.BI.MOVA.=TRUE} | 6535 | 429 | 614 | 968 |  |
| {BcMV2.BI=TRUE, |  |  |  |  |  |
|  | 0.09 | 0.386 |  |  |  |
| BcOLV1.BI.MOVA.=TRUE} => |  |  | 0.25 | 2.08 | 39 |
|  | 6535 | 139 |  |  |  |
| {BcALV1.BI=TRUE} |  |  |  |  |  |
| {BcALV1.BI=TRUE, |  |  |  |  |  |
|  | 0.10 | 0.953 | 0.106 | 1.84 |  |
| BcOLV1.BI.MOVA.=TRUE} => |  |  |  |  | 41 |
|  | 1485 | 488 | 436 | 3107 |  |
| {BcOLV18=TRUE} |  |  |  |  |  |
| {BcALV1.BI=TRUE, BcOLV18=TRUE} => | 0.10 | 0.719 | 0.141 | 2.16 |  |
|  |  |  |  |  | 41 |
| {BcOLV1.BI.MOVA.=TRUE} | 1485 | 298 | 089 | 8631 |  |
| {BcOLV1.BI.MOVA.=TRUE, | 0.10 | 0.440 | 0.230 | 2.37 |  |
|  |  |  |  |  | 41 |
| BcOLV18=TRUE} => {BcALV1.BI=TRUE} | 1485 | 86 | 198 | 4767 |  |
| {BcALV1.BI=TRUE, |  |  |  |  |  |
|  | 0.07 | 0.697 | 0.106 | 1.28 |  |
| BcOLV1.BI.MOVA.=TRUE} => |  |  |  |  | 30 |
|  | 4257 | 674 | 436 | 7034 |  |
| {BcHV1SLR.BI=TRUE} |  |  |  |  |  |
| {BcALV1.BI=TRUE, BcHV1SLR.BI=TRUE} | 0.07 | 0.588 | 0.126 | 1.77 |  |
|  |  |  |  |  | 30 |
| => {BcOLV1.BI.MOVA.=TRUE} | 4257 | 235 | 238 | 3486 |  |
| {BcHV1SLR.BI=TRUE, |  |  |  |  |  |
|  | 0.07 | 0.370 | 0.200 | 1.99 |  |
| BcOLV1.BI.MOVA.=TRUE} => |  |  |  |  | 30 |
|  | 4257 | 37 | 495 | 5062 |  |
| {BcALV1.BI=TRUE} |  |  |  |  |  |

| {BcALV1.BI=TRUE, BcOLV1.BI.MOVA.=TRUE} =>  {BcMV3.BI=TRUE} | 0.08  1683 | 0.767  442 | 0.106  436 | 1.41  5737 | 33 |
| --- | --- | --- | --- | --- | --- |
| {BcALV1.BI=TRUE, BcMV3.BI=TRUE} =>  {BcOLV1.BI.MOVA.=TRUE} | 0.08  1683 | 0.687  5 | 0.118  812 | 2.07  2761 | 33 |
| {BcMV3.BI=TRUE, BcOLV1.BI.MOVA.=TRUE} =>  {BcALV1.BI=TRUE} | 0.08  1683 | 0.333  333 | 0.245  05 | 1.79  5556 | 33 |
| {BcALV1.BI=TRUE, BcOLV1.BI.MOVA.=TRUE} =>  {BcHV1.BI=TRUE} | 0.08  1683 | 0.767  442 | 0.106  436 | 1.27  0682 | 33 |
| {BcALV1.BI=TRUE, BcHV1.BI=TRUE} =>  {BcOLV1.BI.MOVA.=TRUE} | 0.08  1683 | 0.589  286 | 0.138  614 | 1.77  6652 | 33 |
| {BcHV1.BI=TRUE, BcOLV1.BI.MOVA.=TRUE} =>  {BcALV1.BI=TRUE} | 0.08  1683 | 0.383  721 | 0.212  871 | 2.06  6977 | 33 |
| {BcALV1.BI=TRUE, BcOLV1.BI.MOVA.=TRUE} =>  {BcMV11=TRUE} | 0.09  901 | 0.930  233 | 0.106  436 | 1.50  9293 | 40 |
| {BcALV1.BI=TRUE, BcMV11=TRUE} =>  {BcOLV1.BI.MOVA.=TRUE} | 0.09  901 | 0.634  921 | 0.155  941 | 1.91  4238 | 40 |
| {BcMV11=TRUE, BcOLV1.BI.MOVA.=TRUE} | 0.09 | 0.408 | 0.242 | 2.19 | 40 |

| => {BcALV1.BI=TRUE} | 901 | 163 | 574 | 8639 |  |
| --- | --- | --- | --- | --- | --- |
| {BcALV1.BI=TRUE, BcMV9.BI=TRUE} =>  {BcOLV1.BI.MOVA.=TRUE} | 0.08  1683 | 0.6 | 0.136  139 | 1.80  8955 | 33 |
| {BcMV9.BI=TRUE, |  |  |  |  |  |
|  | 0.08 | 0.383 | 0.212 | 2.06 |  |
| BcOLV1.BI.MOVA.=TRUE} => |  |  |  |  | 33 |
|  | 1683 | 721 | 871 | 6977 |  |
| {BcALV1.BI=TRUE} |  |  |  |  |  |
| {BcALV1.BI=TRUE, |  |  |  |  |  |
|  | 0.10 | 0.953 | 0.106 | 1.38 |  |
| BcOLV1.BI.MOVA.=TRUE} => |  |  |  |  | 41 |
|  | 1485 | 488 | 436 | 0679 |  |
| {BcULV4=TRUE} |  |  |  |  |  |
| {BcALV1.BI=TRUE, BcULV4=TRUE} => | 0.10 | 0.630 | 0.160 | 1.90 |  |
|  |  |  |  |  | 41 |
| {BcOLV1.BI.MOVA.=TRUE} | 1485 | 769 | 891 | 1722 |  |
| {BcOLV1.BI.MOVA.=TRUE, BcULV4=TRUE} | 0.10 | 0.414 | 0.245 | 2.23 |  |
|  |  |  |  |  | 41 |
| => {BcALV1.BI=TRUE} | 1485 | 141 | 05 | 0842 |  |
| {BcALV1.BI=TRUE, BcEV2.BI=FALSE} => | 0.07 | 0.603 | 0.131 | 1.82 |  |
|  |  |  |  |  | 32 |
| {BcOLV1.BI.MOVA.=TRUE} | 9208 | 774 | 188 | 0332 |  |
| {BcEV2.BI=FALSE, |  |  |  |  |  |
|  | 0.07 | 0.316 |  | 1.70 |  |
| BcOLV1.BI.MOVA.=TRUE} => |  |  | 0.25 |  | 32 |
|  | 9208 | 832 |  | 6667 |  |
| {BcALV1.BI=TRUE} |  |  |  |  |  |
| {BcALV1.BI=TRUE, BcULV1.BI=FALSE} => | 0.07 | 0.563 | 0.136 | 1.69 |  |
|  |  |  |  |  | 31 |
| {BcOLV1.BI.MOVA.=TRUE} | 6733 | 636 | 139 | 9322 |  |
| {BcOLV1.BI.MOVA.=TRUE, | 0.07 | 0.340 | 0.225 | 1.83 |  |
|  |  |  |  |  | 31 |
| BcULV1.BI=FALSE} => {BcALV1.BI=TRUE} | 6733 | 659 | 248 | 5018 |  |

| {BcALV1.BI=TRUE, BcOLV1.BI.MOVA.=TRUE} =>  {BcMV1.BI=TRUE} | 0.09  6535 | 0.906  977 | 0.106  436 | 1.14  8648 | 39 |
| --- | --- | --- | --- | --- | --- |
| {BcALV1.BI=TRUE, BcMV1.BI=TRUE} =>  {BcOLV1.BI.MOVA.=TRUE} | 0.09  6535 | 0.609  375 | 0.158  416 | 1.83  722 | 39 |
| {BcMV1.BI=TRUE, BcOLV1.BI.MOVA.=TRUE} =>  {BcALV1.BI=TRUE} | 0.09  6535 | 0.327  731 | 0.294  554 | 1.76  5378 | 39 |
| {BcALV1.BI=TRUE, BcMV12=FALSE} =>  {BcOLV1.BI.MOVA.=TRUE} | 0.08  6634 | 0.593  22 | 0.146  04 | 1.78  8515 | 35 |
| {BcMV12=FALSE, BcOLV1.BI.MOVA.=TRUE} =>  {BcALV1.BI=TRUE} | 0.08  6634 | 0.376  344 | 0.230  198 | 2.02  724 | 35 |
| {BcALV1.BI=TRUE, BcNSRV7.BI=FALSE}  => {BcOLV1.BI.MOVA.=TRUE} | 0.07  9208 | 0.571  429 | 0.138  614 | 1.72  2814 | 32 |
| {BcNSRV7.BI=FALSE, BcOLV1.BI.MOVA.=TRUE} =>  {BcALV1.BI=TRUE} | 0.07  9208 | 0.299  065 | 0.264  851 | 1.61  0966 | 32 |
| {BcALV1.BI=TRUE, BcVV3.BI=FALSE} =>  {BcOLV1.BI.MOVA.=TRUE} | 0.07  9208 | 0.551  724 | 0.143  564 | 1.66  3407 | 32 |
| {BcOLV1.BI.MOVA.=TRUE,  BcVV3.BI=FALSE} => {BcALV1.BI=TRUE} | 0.07  9208 | 0.326  531 | 0.242  574 | 1.75  8912 | 32 |

| {BcALV1.BI=TRUE, BcDMV1=FALSE} =>  {BcOLV1.BI.MOVA.=TRUE} | 0.07  6733 | 0.534  483 | 0.143  564 | 1.61  1426 | 31 |
| --- | --- | --- | --- | --- | --- |
| {BcDMV1=FALSE, BcOLV1.BI.MOVA.=TRUE} =>  {BcALV1.BI=TRUE} | 0.07  6733 | 0.279  279 | 0.274  752 | 1.50  4384 | 31 |
| {BcALV1.BI=TRUE, BcFV9=FALSE} =>  {BcOLV1.BI.MOVA.=TRUE} | 0.08  4158 | 0.566  667 | 0.148  515 | 1.70  8458 | 34 |
| {BcFV9=FALSE, BcOLV1.BI.MOVA.=TRUE}  => {BcALV1.BI=TRUE} | 0.08  4158 | 0.300  885 | 0.279  703 | 1.62  0767 | 34 |
| {BcALV1.BI=TRUE, BcOLV4.BI=FALSE} =>  {BcOLV1.BI.MOVA.=TRUE} | 0.09  1584 | 0.587  302 | 0.155  941 | 1.77  067 | 37 |
| {BcOLV1.BI.MOVA.=TRUE,  BcOLV4.BI=FALSE} => {BcALV1.BI=TRUE} | 0.09  1584 | 0.349  057 | 0.262  376 | 1.88  0252 | 37 |
| {BcALV1.BI=TRUE, BcULV5=FALSE} =>  {BcOLV1.BI.MOVA.=TRUE} | 0.09  901 | 0.579  71 | 0.170  792 | 1.74  7783 | 40 |
| {BcOLV1.BI.MOVA.=TRUE,  BcULV5=FALSE} => {BcALV1.BI=TRUE} | 0.09  901 | 0.384  615 | 0.257  426 | 2.07  1795 | 40 |
| {BcALV1.BI=TRUE, BcBNV5.BI=FALSE} =>  {BcOLV1.BI.MOVA.=TRUE} | 0.07  4257 | 0.517  241 | 0.143  564 | 1.55  9444 | 30 |
| {BcBNV5.BI=FALSE, BcOLV1.BI.MOVA.=TRUE} =>  {BcALV1.BI=TRUE} | 0.07  4257 | 0.263  158 | 0.282  178 | 1.41  7544 | 30 |

| {BcALV1.BI=TRUE, BvF.BI=FALSE} =>  {BcOLV1.BI.MOVA.=TRUE} | 0.10  1485 | 0.611  94 | 0.165  842 | 1.84  4954 | 41 |
| --- | --- | --- | --- | --- | --- |
| {BcOLV1.BI.MOVA.=TRUE, BvF.BI=FALSE}  => {BcALV1.BI=TRUE} | 0.10  1485 | 0.322  835 | 0.314  356 | 1.73  9003 | 41 |
| {BcALV1.BI=TRUE, BcMCV5.BI=FALSE} =>  {BcOLV1.BI.MOVA.=TRUE} | 0.08  1683 | 0.540  984 | 0.150  99 | 1.63  1025 | 33 |
| {BcMCV5.BI=FALSE, BcOLV1.BI.MOVA.=TRUE} =>  {BcALV1.BI=TRUE} | 0.08  1683 | 0.294  643 | 0.277  228 | 1.58  7143 | 33 |
| {BcALV1.BI=TRUE, BcOLV13.BI=FALSE} =>  {BcOLV1.BI.MOVA.=TRUE} | 0.09  901 | 0.563  38 | 0.175  743 | 1.69  855 | 40 |
| {BcOLV1.BI.MOVA.=TRUE,  BcOLV13.BI=FALSE} => {BcALV1.BI=TRUE} | 0.09  901 | 0.357  143 | 0.277  228 | 1.92  381 | 40 |
| {BcALV1.BI=TRUE, BcOLV2.BI=FALSE} =>  {BcOLV1.BI.MOVA.=TRUE} | 0.09  4059 | 0.542  857 | 0.173  267 | 1.63  6674 | 38 |
| {BcOLV1.BI.MOVA.=TRUE,  BcOLV2.BI=FALSE} => {BcALV1.BI=TRUE} | 0.09  4059 | 0.339  286 | 0.277  228 | 1.82  7619 | 38 |
| {BcALV1.BI=TRUE, BcMCV3.BI=FALSE} =>  {BcOLV1.BI.MOVA.=TRUE} | 0.09  901 | 0.571  429 | 0.173  267 | 1.72  2814 | 40 |
| {BcMCV3.BI=FALSE, BcOLV1.BI.MOVA.=TRUE} =>  {BcALV1.BI=TRUE} | 0.09  901 | 0.344  828 | 0.287  129 | 1.85  7471 | 40 |

| {BcALV1.BI=TRUE, BcPV4.BI=FALSE} =>  {BcOLV1.BI.MOVA.=TRUE} | 0.10  6436 | 0.589  041 | 0.180  693 | 1.77  5915 | 43 |
| --- | --- | --- | --- | --- | --- |
| {BcOLV1.BI.MOVA.=TRUE,  BcPV4.BI=FALSE} => {BcALV1.BI=TRUE} | 0.10  6436 | 0.335  938 | 0.316  832 | 1.80  9583 | 43 |
| {BcALV1.BI=TRUE, BcEV6=FALSE} =>  {BcOLV1.BI.MOVA.=TRUE} | 0.09  6535 | 0.565  217 | 0.170  792 | 1.70  4088 | 39 |
| {BcEV6=FALSE, BcOLV1.BI.MOVA.=TRUE}  => {BcALV1.BI=TRUE} | 0.09  6535 | 0.314  516 | 0.306  931 | 1.69  4194 | 39 |
| {BcALV1.BI=TRUE, BcOLV17.BI=FALSE} =>  {BcOLV1.BI.MOVA.=TRUE} | 0.10  1485 | 0.569  444 | 0.178  218 | 1.71  6833 | 41 |
| {BcOLV1.BI.MOVA.=TRUE,  BcOLV17.BI=FALSE} => {BcALV1.BI=TRUE} | 0.10  1485 | 0.338  843 | 0.299  505 | 1.82  5234 | 41 |
| {BcALV1.BI=TRUE, BcOLV15.BI=FALSE} =>  {BcOLV1.BI.MOVA.=TRUE} | 0.10  6436 | 0.581  081 | 0.183  168 | 1.75  1916 | 43 |
| {BcOLV1.BI.MOVA.=TRUE,  BcOLV15.BI=FALSE} => {BcALV1.BI=TRUE} | 0.10  6436 | 0.330  769 | 0.321  782 | 1.78  1744 | 43 |
| {BcALV1.BI=TRUE, BcEV5=FALSE} =>  {BcOLV1.BI.MOVA.=TRUE} | 0.08  6634 | 0.530  303 | 0.163  366 | 1.59  8824 | 35 |
| {BcEV5=FALSE, BcOLV1.BI.MOVA.=TRUE}  => {BcALV1.BI=TRUE} | 0.08  6634 | 0.291  667 | 0.297  03 | 1.57  1111 | 35 |
| {BcALV1.BI=TRUE, BcOLV16.BI=TRUE} =>  {BcMV2.BI=TRUE} | 0.08  9109 | 0.837  209 | 0.106  436 | 1.80  873 | 36 |

| {BcALV1.BI=TRUE, BcMV2.BI=TRUE} =>  {BcOLV16.BI=TRUE} | 0.08  9109 | 0.642  857 | 0.138  614 | 1.54  5918 | 36 |
| --- | --- | --- | --- | --- | --- |
| {BcMV2.BI=TRUE, BcOLV16.BI=TRUE} =>  {BcALV1.BI=TRUE} | 0.08  9109 | 0.352  941 | 0.252  475 | 1.90  1176 | 36 |
| {BcALV1.BI=TRUE, BcOLV16.BI=TRUE} =>  {BcOLV18=TRUE} | 0.08  9109 | 0.837  209 | 0.106  436 | 1.61  8338 | 36 |
| {BcALV1.BI=TRUE, BcOLV18=TRUE} =>  {BcOLV16.BI=TRUE} | 0.08  9109 | 0.631  579 | 0.141  089 | 1.51  8797 | 36 |
| {BcOLV16.BI=TRUE, BcOLV18=TRUE} =>  {BcALV1.BI=TRUE} | 0.08  9109 | 0.330  275 | 0.269  802 | 1.77  9083 | 36 |
| {BcALV1.BI=TRUE, BcOLV16.BI=TRUE} =>  {BcHV1SLR.BI=TRUE} | 0.07  4257 | 0.697  674 | 0.106  436 | 1.28  7034 | 30 |
| {BcALV1.BI=TRUE, BcHV1SLR.BI=TRUE}  => {BcOLV16.BI=TRUE} | 0.07  4257 | 0.588  235 | 0.126  238 | 1.41  4566 | 30 |
| {BcHV1SLR.BI=TRUE, BcOLV16.BI=TRUE}  => {BcALV1.BI=TRUE} | 0.07  4257 | 0.312  5 | 0.237  624 | 1.68  3333 | 30 |
| {BcALV1.BI=TRUE, BcOLV16.BI=TRUE} =>  {BcMV3.BI=TRUE} | 0.07  4257 | 0.697  674 | 0.106  436 | 1.28  7034 | 30 |
| {BcALV1.BI=TRUE, BcMV3.BI=TRUE} =>  {BcOLV16.BI=TRUE} | 0.07  4257 | 0.625 | 0.118  812 | 1.50  2976 | 30 |
| {BcMV3.BI=TRUE, BcOLV16.BI=TRUE} =>  {BcALV1.BI=TRUE} | 0.07  4257 | 0.277  778 | 0.267  327 | 1.49  6296 | 30 |

| {BcALV1.BI=TRUE, BcOLV16.BI=TRUE} =>  {BcHV1.BI=TRUE} | 0.08  1683 | 0.767  442 | 0.106  436 | 1.27  0682 | 33 |
| --- | --- | --- | --- | --- | --- |
| {BcALV1.BI=TRUE, BcHV1.BI=TRUE} =>  {BcOLV16.BI=TRUE} | 0.08  1683 | 0.589  286 | 0.138  614 | 1.41  7092 | 33 |
| {BcHV1.BI=TRUE, BcOLV16.BI=TRUE} =>  {BcALV1.BI=TRUE} | 0.08  1683 | 0.302  752 | 0.269  802 | 1.63  0826 | 33 |
| {BcALV1.BI=TRUE, BcOLV16.BI=TRUE} =>  {BcMV11=TRUE} | 0.08  9109 | 0.837  209 | 0.106  436 | 1.35  8364 | 36 |
| {BcALV1.BI=TRUE, BcMV11=TRUE} =>  {BcOLV16.BI=TRUE} | 0.08  9109 | 0.571  429 | 0.155  941 | 1.37  415 | 36 |
| {BcMV11=TRUE, BcOLV16.BI=TRUE} =>  {BcALV1.BI=TRUE} | 0.08  9109 | 0.324  324 | 0.274  752 | 1.74  7027 | 36 |
| {BcALV1.BI=TRUE, BcOLV16.BI=TRUE} =>  {BcMV9.BI=TRUE} | 0.09  4059 | 0.883  721 | 0.106  436 | 1.30  3005 | 38 |
| {BcALV1.BI=TRUE, BcMV9.BI=TRUE} =>  {BcOLV16.BI=TRUE} | 0.09  4059 | 0.690  909 | 0.136  139 | 1.66  1472 | 38 |
| {BcMV9.BI=TRUE, BcOLV16.BI=TRUE} =>  {BcALV1.BI=TRUE} | 0.09  4059 | 0.283  582 | 0.331  683 | 1.52  7562 | 38 |
| {BcALV1.BI=TRUE, BcOLV16.BI=TRUE} =>  {BcULV4=TRUE} | 0.09  901 | 0.930  233 | 0.106  436 | 1.34  7003 | 40 |
| {BcALV1.BI=TRUE, BcULV4=TRUE} =>  {BcOLV16.BI=TRUE} | 0.09  901 | 0.615  385 | 0.160  891 | 1.47  9853 | 40 |

| {BcOLV16.BI=TRUE, BcULV4=TRUE} =>  {BcALV1.BI=TRUE} | 0.09  901 | 0.291  971 | 0.339  109 | 1.57  2749 | 40 |
| --- | --- | --- | --- | --- | --- |
| {BcALV1.BI=TRUE, BcEV2.BI=FALSE} =>  {BcOLV16.BI=TRUE} | 0.08  1683 | 0.622  642 | 0.131  188 | 1.49  7305 | 33 |
| {BcEV2.BI=FALSE, BcOLV16.BI=TRUE} =>  {BcALV1.BI=TRUE} | 0.08  1683 | 0.25 | 0.326  733 | 1.34  6667 | 33 |
| {BcALV1.BI=TRUE, BcMV1.BI=TRUE} =>  {BcOLV16.BI=TRUE} | 0.08  4158 | 0.531  25 | 0.158  416 | 1.27  753 | 34 |
| {BcMV1.BI=TRUE, BcOLV16.BI=TRUE} =>  {BcALV1.BI=TRUE} | 0.08  4158 | 0.25 | 0.336  634 | 1.34  6667 | 34 |
| {BcALV1.BI=TRUE, BcMV12=FALSE} =>  {BcOLV16.BI=TRUE} | 0.08  1683 | 0.559  322 | 0.146  04 | 1.34  5036 | 33 |
| {BcMV12=FALSE, BcOLV16.BI=TRUE} =>  {BcALV1.BI=TRUE} | 0.08  1683 | 0.277  311 | 0.294  554 | 1.49  3782 | 33 |
| {BcALV1.BI=TRUE, BcNSRV7.BI=FALSE}  => {BcOLV16.BI=TRUE} | 0.07  9208 | 0.571  429 | 0.138  614 | 1.37  415 | 32 |
| {BcALV1.BI=TRUE, BcVV3.BI=FALSE} =>  {BcOLV16.BI=TRUE} | 0.08  6634 | 0.603  448 | 0.143  564 | 1.45  1149 | 35 |
| {BcOLV16.BI=TRUE, BcVV3.BI=FALSE} =>  {BcALV1.BI=TRUE} | 0.08  6634 | 0.257  353 | 0.336  634 | 1.38  6275 | 35 |
| {BcALV1.BI=TRUE, BcDMV1=FALSE} =>  {BcOLV16.BI=TRUE} | 0.08  6634 | 0.603  448 | 0.143  564 | 1.45  1149 | 35 |

| {BcDMV1=FALSE, BcOLV16.BI=TRUE} =>  {BcALV1.BI=TRUE} | 0.08  6634 | 0.244  755 | 0.353  96 | 1.31  8415 | 35 |
| --- | --- | --- | --- | --- | --- |
| {BcALV1.BI=TRUE, BcFV9=FALSE} =>  {BcOLV16.BI=TRUE} | 0.08  4158 | 0.566  667 | 0.148  515 | 1.36  2698 | 34 |
| {BcALV1.BI=TRUE, BcOLV4.BI=FALSE} =>  {BcOLV16.BI=TRUE} | 0.08  4158 | 0.539  683 | 0.155  941 | 1.29  7808 | 34 |
| {BcOLV16.BI=TRUE, BcOLV4.BI=FALSE}  => {BcALV1.BI=TRUE} | 0.08  4158 | 0.244  604 | 0.344  059 | 1.31  7602 | 34 |
| {BcALV1.BI=TRUE, BcULV5=FALSE} =>  {BcOLV16.BI=TRUE} | 0.09  6535 | 0.565  217 | 0.170  792 | 1.35  9213 | 39 |
| {BcOLV16.BI=TRUE, BcULV5=FALSE} =>  {BcALV1.BI=TRUE} | 0.09  6535 | 0.274  648 | 0.351  485 | 1.47  9437 | 39 |
| {BcALV1.BI=TRUE, BcBNV5.BI=FALSE} =>  {BcOLV16.BI=TRUE} | 0.08  1683 | 0.568  966 | 0.143  564 | 1.36  8227 | 33 |
| {BcALV1.BI=TRUE, BvF.BI=FALSE} =>  {BcOLV16.BI=TRUE} | 0.10  1485 | 0.611  94 | 0.165  842 | 1.47  1571 | 41 |
| {BcOLV16.BI=TRUE, BvF.BI=FALSE} =>  {BcALV1.BI=TRUE} | 0.10  1485 | 0.259  494 | 0.391  089 | 1.39  7806 | 41 |
| {BcALV1.BI=TRUE, BcMCV5.BI=FALSE} =>  {BcOLV16.BI=TRUE} | 0.08  1683 | 0.540  984 | 0.150  99 | 1.30  0937 | 33 |
| {BcALV1.BI=TRUE, BcOLV13.BI=FALSE} =>  {BcOLV16.BI=TRUE} | 0.09  6535 | 0.549  296 | 0.175  743 | 1.32  0926 | 39 |

| {BcOLV13.BI=FALSE, BcOLV16.BI=TRUE}  => {BcALV1.BI=TRUE} | 0.09  6535 | 0.263  514 | 0.366  337 | 1.41  9459 | 39 |
| --- | --- | --- | --- | --- | --- |
| {BcALV1.BI=TRUE, BcOLV2.BI=FALSE} =>  {BcOLV16.BI=TRUE} | 0.09  4059 | 0.542  857 | 0.173  267 | 1.30  5442 | 38 |
| {BcOLV16.BI=TRUE, BcOLV2.BI=FALSE}  => {BcALV1.BI=TRUE} | 0.09  4059 | 0.255  034 | 0.368  812 | 1.37  3781 | 38 |
| {BcALV1.BI=TRUE, BcMCV3.BI=FALSE} =>  {BcOLV16.BI=TRUE} | 0.09  4059 | 0.542  857 | 0.173  267 | 1.30  5442 | 38 |
| {BcMCV3.BI=FALSE, BcOLV16.BI=TRUE}  => {BcALV1.BI=TRUE} | 0.09  4059 | 0.262  069 | 0.358  911 | 1.41  1678 | 38 |
| {BcALV1.BI=TRUE, BcPV4.BI=FALSE} =>  {BcOLV16.BI=TRUE} | 0.10  1485 | 0.561  644 | 0.180  693 | 1.35  062 | 41 |
| {BcOLV16.BI=TRUE, BcPV4.BI=FALSE} =>  {BcALV1.BI=TRUE} | 0.10  1485 | 0.267  974 | 0.378  713 | 1.44  3486 | 41 |
| {BcALV1.BI=TRUE, BcEV6=FALSE} =>  {BcOLV16.BI=TRUE} | 0.09  901 | 0.579  71 | 0.170  792 | 1.39  4065 | 40 |
| {BcEV6=FALSE, BcOLV16.BI=TRUE} =>  {BcALV1.BI=TRUE} | 0.09  901 | 0.253  165 | 0.391  089 | 1.36  3713 | 40 |
| {BcALV1.BI=TRUE, BcOLV17.BI=FALSE} =>  {BcOLV16.BI=TRUE} | 0.09  901 | 0.555  556 | 0.178  218 | 1.33  5979 | 40 |
| {BcOLV16.BI=TRUE, BcOLV17.BI=FALSE}  => {BcALV1.BI=TRUE} | 0.09  901 | 0.256  41 | 0.386  139 | 1.38  1197 | 40 |

| {BcALV1.BI=TRUE, BcOLV15.BI=FALSE} =>  {BcOLV16.BI=TRUE} | 0.10  396 | 0.567  568 | 0.183  168 | 1.36  4865 | 42 |
| --- | --- | --- | --- | --- | --- |
| {BcOLV15.BI=FALSE, BcOLV16.BI=TRUE}  => {BcALV1.BI=TRUE} | 0.10  396 | 0.257  669 | 0.403  465 | 1.38  7975 | 42 |
| {BcALV1.BI=TRUE, BcEV5=FALSE} =>  {BcOLV16.BI=TRUE} | 0.08  9109 | 0.545  455 | 0.163  366 | 1.31  1688 | 36 |
| {BcALV1.BI=TRUE, BcMV4.BI=TRUE} =>  {BcMV2.BI=TRUE} | 0.10  6436 | 0.826  923 | 0.128  713 | 1.78  6508 | 43 |
| {BcALV1.BI=TRUE, BcMV2.BI=TRUE} =>  {BcMV4.BI=TRUE} | 0.10  6436 | 0.767  857 | 0.138  614 | 1.73  3041 | 43 |
| {BcMV2.BI=TRUE, BcMV4.BI=TRUE} =>  {BcALV1.BI=TRUE} | 0.10  6436 | 0.387  387 | 0.274  752 | 2.08  6727 | 43 |
| {BcALV1.BI=TRUE, BcMV4.BI=TRUE} =>  {BcOLV18=TRUE} | 0.10  396 | 0.807  692 | 0.128  713 | 1.56  1281 | 42 |
| {BcALV1.BI=TRUE, BcOLV18=TRUE} =>  {BcMV4.BI=TRUE} | 0.10  396 | 0.736  842 | 0.141  089 | 1.66  304 | 42 |
| {BcMV4.BI=TRUE, BcOLV18=TRUE} =>  {BcALV1.BI=TRUE} | 0.10  396 | 0.375 | 0.277  228 | 2.02 | 42 |
| {BcALV1.BI=TRUE, BcMV4.BI=TRUE} =>  {BcHV1SLR.BI=TRUE} | 0.09  4059 | 0.730  769 | 0.128  713 | 1.34  8086 | 38 |
| {BcALV1.BI=TRUE, BcHV1SLR.BI=TRUE}  => {BcMV4.BI=TRUE} | 0.09  4059 | 0.745  098 | 0.126  238 | 1.68  1674 | 38 |

| {BcHV1SLR.BI=TRUE, BcMV4.BI=TRUE} =>  {BcALV1.BI=TRUE} | 0.09  4059 | 0.358  491 | 0.262  376 | 1.93  1069 | 38 |
| --- | --- | --- | --- | --- | --- |
| {BcALV1.BI=TRUE, BcMV4.BI=TRUE} =>  {BcMV3.BI=TRUE} | 0.09  1584 | 0.711  538 | 0.128  713 | 1.31  261 | 37 |
| {BcALV1.BI=TRUE, BcMV3.BI=TRUE} =>  {BcMV4.BI=TRUE} | 0.09  1584 | 0.770  833 | 0.118  812 | 1.73  9758 | 37 |
| {BcMV3.BI=TRUE, BcMV4.BI=TRUE} =>  {BcALV1.BI=TRUE} | 0.09  1584 | 0.310  924 | 0.294  554 | 1.67  4846 | 37 |
| {BcALV1.BI=TRUE, BcULV3=TRUE} =>  {BcMV4.BI=TRUE} | 0.08  6634 | 0.777  778 | 0.111  386 | 1.75  5431 | 35 |
| {BcMV4.BI=TRUE, BcULV3=TRUE} =>  {BcALV1.BI=TRUE} | 0.08  6634 | 0.294  118 | 0.294  554 | 1.58  4314 | 35 |
| {BcALV1.BI=TRUE, BcMV4.BI=TRUE} =>  {BcHV1.BI=TRUE} | 0.10  396 | 0.807  692 | 0.128  713 | 1.33  7327 | 42 |
| {BcALV1.BI=TRUE, BcHV1.BI=TRUE} =>  {BcMV4.BI=TRUE} | 0.10  396 | 0.75 | 0.138  614 | 1.69  2737 | 42 |
| {BcHV1.BI=TRUE, BcMV4.BI=TRUE} =>  {BcALV1.BI=TRUE} | 0.10  396 | 0.347  107 | 0.299  505 | 1.86  9752 | 42 |
| {BcALV1.BI=TRUE, BcMV4.BI=TRUE} =>  {BcMV11=TRUE} | 0.11  3861 | 0.884  615 | 0.128  713 | 1.43  528 | 46 |
| {BcALV1.BI=TRUE, BcMV11=TRUE} =>  {BcMV4.BI=TRUE} | 0.11  3861 | 0.730  159 | 0.155  941 | 1.64  7956 | 46 |

| {BcMV11=TRUE, BcMV4.BI=TRUE} =>  {BcALV1.BI=TRUE} | 0.11  3861 | 0.335  766 | 0.339  109 | 1.80  8662 | 46 |
| --- | --- | --- | --- | --- | --- |
| {BcALV1.BI=TRUE, BcMV4.BI=TRUE} =>  {BcMV9.BI=TRUE} | 0.10  6436 | 0.826  923 | 0.128  713 | 1.21  9259 | 43 |
| {BcALV1.BI=TRUE, BcMV9.BI=TRUE} =>  {BcMV4.BI=TRUE} | 0.10  6436 | 0.781  818 | 0.136  139 | 1.76  4551 | 43 |
| {BcMV4.BI=TRUE, BcMV9.BI=TRUE} =>  {BcALV1.BI=TRUE} | 0.10  6436 | 0.316  176 | 0.336  634 | 1.70  3137 | 43 |
| {BcALV1.BI=TRUE, BcMV4.BI=TRUE} =>  {BcULV4=TRUE} | 0.10  8911 | 0.846  154 | 0.128  713 | 1.22  5255 | 44 |
| {BcALV1.BI=TRUE, BcULV4=TRUE} =>  {BcMV4.BI=TRUE} | 0.10  8911 | 0.676  923 | 0.160  891 | 1.52  7804 | 44 |
| {BcMV4.BI=TRUE, BcULV4=TRUE} =>  {BcALV1.BI=TRUE} | 0.10  8911 | 0.335  878 | 0.324  257 | 1.80  9262 | 44 |
| {BcALV1.BI=TRUE, BcPV3.BI=FALSE} =>  {BcMV4.BI=TRUE} | 0.07  9208 | 0.627  451 | 0.126  238 | 1.41  6146 | 32 |
| {BcMV4.BI=TRUE, BcPV3.BI=FALSE} =>  {BcALV1.BI=TRUE} | 0.07  9208 | 0.301  887 | 0.262  376 | 1.62  6164 | 32 |
| {BcALV1.BI=TRUE, BcEV2.BI=FALSE} =>  {BcMV4.BI=TRUE} | 0.08  1683 | 0.622  642 | 0.131  188 | 1.40  5291 | 33 |
| {BcEV2.BI=FALSE, BcMV4.BI=TRUE} =>  {BcALV1.BI=TRUE} | 0.08  1683 | 0.259  843 | 0.314  356 | 1.39  9685 | 33 |

| {BcALV1.BI=TRUE, BcHV3.BI=FALSE} =>  {BcMV4.BI=TRUE} | 0.07  6733 | 0.607  843 | 0.126  238 | 1.37  1892 | 31 |
| --- | --- | --- | --- | --- | --- |
| {BcHV3.BI=FALSE, BcMV4.BI=TRUE} =>  {BcALV1.BI=TRUE} | 0.07  6733 | 0.262  712 | 0.292  079 | 1.41  5141 | 31 |
| {BcALV1.BI=TRUE, BcULV1.BI=FALSE} =>  {BcMV4.BI=TRUE} | 0.09  6535 | 0.709  091 | 0.136  139 | 1.60  0406 | 39 |
| {BcMV4.BI=TRUE, BcULV1.BI=FALSE} =>  {BcALV1.BI=TRUE} | 0.09  6535 | 0.288  889 | 0.334  158 | 1.55  6148 | 39 |
| {BcALV1.BI=TRUE, BcMV1.BI=TRUE} =>  {BcMV4.BI=TRUE} | 0.11  1386 | 0.703  125 | 0.158  416 | 1.58  6941 | 45 |
| {BcMV1.BI=TRUE, BcMV4.BI=TRUE} =>  {BcALV1.BI=TRUE} | 0.11  1386 | 0.296  053 | 0.376  238 | 1.59  4737 | 45 |
| {BcALV1.BI=TRUE, BcMV12=FALSE} =>  {BcMV4.BI=TRUE} | 0.10  8911 | 0.745  763 | 0.146  04 | 1.68  3174 | 44 |
| {BcMV12=FALSE, BcMV4.BI=TRUE} =>  {BcALV1.BI=TRUE} | 0.10  8911 | 0.301  37 | 0.361  386 | 1.62  3379 | 44 |
| {BcALV1.BI=TRUE, BcNSRV7.BI=FALSE}  => {BcMV4.BI=TRUE} | 0.08  9109 | 0.642  857 | 0.138  614 | 1.45  0918 | 36 |
| {BcMV4.BI=TRUE, BcNSRV7.BI=FALSE} =>  {BcALV1.BI=TRUE} | 0.08  9109 | 0.264  706 | 0.336  634 | 1.42  5882 | 36 |
| {BcALV1.BI=TRUE, BcVV3.BI=FALSE} =>  {BcMV4.BI=TRUE} | 0.08  9109 | 0.620  69 | 0.143  564 | 1.40  0886 | 36 |

| {BcMV4.BI=TRUE, BcVV3.BI=FALSE} =>  {BcALV1.BI=TRUE} | 0.08  9109 | 0.268  657 | 0.331  683 | 1.44  7164 | 36 |
| --- | --- | --- | --- | --- | --- |
| {BcALV1.BI=TRUE, BcOLV7.BI=FALSE} =>  {BcMV4.BI=TRUE} | 0.09  6535 | 0.735  849 | 0.131  188 | 1.66  0799 | 39 |
| {BcMV4.BI=TRUE, BcOLV7.BI=FALSE} =>  {BcALV1.BI=TRUE} | 0.09  6535 | 0.286  765 | 0.336  634 | 1.54  4706 | 39 |
| {BcALV1.BI=TRUE, BcDMV1=FALSE} =>  {BcMV4.BI=TRUE} | 0.09  1584 | 0.637  931 | 0.143  564 | 1.43  98 | 37 |
| {BcDMV1=FALSE, BcMV4.BI=TRUE} =>  {BcALV1.BI=TRUE} | 0.09  1584 | 0.253  425 | 0.361  386 | 1.36  5114 | 37 |
| {BcALV1.BI=TRUE, BcFV9=FALSE} =>  {BcMV4.BI=TRUE} | 0.09  6535 | 0.65 | 0.148  515 | 1.46  7039 | 39 |
| {BcFV9=FALSE, BcMV4.BI=TRUE} =>  {BcALV1.BI=TRUE} | 0.09  6535 | 0.270  833 | 0.356  436 | 1.45  8889 | 39 |
| {BcALV1.BI=TRUE, BcOLV4.BI=FALSE} =>  {BcMV4.BI=TRUE} | 0.10  396 | 0.666  667 | 0.155  941 | 1.50  4655 | 42 |
| {BcMV4.BI=TRUE, BcOLV4.BI=FALSE} =>  {BcALV1.BI=TRUE} | 0.10  396 | 0.28 | 0.371  287 | 1.50  8267 | 42 |
| {BcALV1.BI=TRUE, BcULV5=FALSE} =>  {BcMV4.BI=TRUE} | 0.11  6337 | 0.681  159 | 0.170  792 | 1.53  7365 | 47 |
| {BcMV4.BI=TRUE, BcULV5=FALSE} =>  {BcALV1.BI=TRUE} | 0.11  6337 | 0.305  195 | 0.381  188 | 1.64  3983 | 47 |

| {BcALV1.BI=TRUE, BcBNV5.BI=FALSE} =>  {BcMV4.BI=TRUE} | 0.09  1584 | 0.637  931 | 0.143  564 | 1.43  98 | 37 |
| --- | --- | --- | --- | --- | --- |
| {BcALV1.BI=TRUE, BvF.BI=FALSE} =>  {BcMV4.BI=TRUE} | 0.10  8911 | 0.656  716 | 0.165  842 | 1.48  2198 | 44 |
| {BcMV4.BI=TRUE, BvF.BI=FALSE} =>  {BcALV1.BI=TRUE} | 0.10  8911 | 0.268  293 | 0.405  941 | 1.44  5203 | 44 |
| {BcALV1.BI=TRUE, BcMCV5.BI=FALSE} =>  {BcMV4.BI=TRUE} | 0.09  4059 | 0.622  951 | 0.150  99 | 1.40  599 | 38 |
| {BcMCV5.BI=FALSE, BcMV4.BI=TRUE} =>  {BcALV1.BI=TRUE} | 0.09  4059 | 0.248  366 | 0.378  713 | 1.33  7865 | 38 |
| {BcALV1.BI=TRUE, BcOLV13.BI=FALSE} =>  {BcMV4.BI=TRUE} | 0.12  1287 | 0.690  141 | 0.175  743 | 1.55  7636 | 49 |
| {BcMV4.BI=TRUE, BcOLV13.BI=FALSE} =>  {BcALV1.BI=TRUE} | 0.12  1287 | 0.310  127 | 0.391  089 | 1.67  0549 | 49 |
| {BcALV1.BI=TRUE, BcOLV2.BI=FALSE} =>  {BcMV4.BI=TRUE} | 0.11  6337 | 0.671  429 | 0.173  267 | 1.51  5403 | 47 |
| {BcMV4.BI=TRUE, BcOLV2.BI=FALSE} =>  {BcALV1.BI=TRUE} | 0.11  6337 | 0.288  344 | 0.403  465 | 1.55  3211 | 47 |
| {BcALV1.BI=TRUE, BcMCV3.BI=FALSE} =>  {BcMV4.BI=TRUE} | 0.12  1287 | 0.7 | 0.173  267 | 1.57  9888 | 49 |
| {BcMCV3.BI=FALSE, BcMV4.BI=TRUE} =>  {BcALV1.BI=TRUE} | 0.12  1287 | 0.295  181 | 0.410  891 | 1.59  004 | 49 |

| {BcALV1.BI=TRUE, BcPV4.BI=FALSE} =>  {BcMV4.BI=TRUE} | 0.12  8713 | 0.712  329 | 0.180  693 | 1.60  7714 | 52 |
| --- | --- | --- | --- | --- | --- |
| {BcMV4.BI=TRUE, BcPV4.BI=FALSE} =>  {BcALV1.BI=TRUE} | 0.12  8713 | 0.307  692 | 0.418  317 | 1.65  7436 | 52 |
| {BcALV1.BI=TRUE, BcEV6=FALSE} =>  {BcMV4.BI=TRUE} | 0.11  6337 | 0.681  159 | 0.170  792 | 1.53  7365 | 47 |
| {BcEV6=FALSE, BcMV4.BI=TRUE} =>  {BcALV1.BI=TRUE} | 0.11  6337 | 0.276  471 | 0.420  792 | 1.48  9255 | 47 |
| {BcALV1.BI=TRUE, BcOLV17.BI=FALSE} =>  {BcMV4.BI=TRUE} | 0.12  3762 | 0.694  444 | 0.178  218 | 1.56  7349 | 50 |
| {BcMV4.BI=TRUE, BcOLV17.BI=FALSE} =>  {BcALV1.BI=TRUE} | 0.12  3762 | 0.301  205 | 0.410  891 | 1.62  249 | 50 |
| {BcALV1.BI=TRUE, BcOLV15.BI=FALSE} =>  {BcMV4.BI=TRUE} | 0.12  6238 | 0.689  189 | 0.183  168 | 1.55  5488 | 51 |
| {BcMV4.BI=TRUE, BcOLV15.BI=FALSE} =>  {BcALV1.BI=TRUE} | 0.12  6238 | 0.305  389 | 0.413  366 | 1.64  503 | 51 |
| {BcALV1.BI=TRUE, BcEV5=FALSE} =>  {BcMV4.BI=TRUE} | 0.10  6436 | 0.651  515 | 0.163  366 | 1.47  0459 | 43 |
| {BcEV5=FALSE, BcMV4.BI=TRUE} =>  {BcALV1.BI=TRUE} | 0.10  6436 | 0.267  081 | 0.398  515 | 1.43  8675 | 43 |
| {BcALV1.BI=TRUE, BcMV2.BI=TRUE} =>  {BcOLV18=TRUE} | 0.12  1287 | 0.875 | 0.138  614 | 1.69  1388 | 49 |

| {BcALV1.BI=TRUE, BcOLV18=TRUE} =>  {BcMV2.BI=TRUE} | 0.12  1287 | 0.859  649 | 0.141  089 | 1.85  721 | 49 |
| --- | --- | --- | --- | --- | --- |
| {BcMV2.BI=TRUE, BcOLV18=TRUE} =>  {BcALV1.BI=TRUE} | 0.12  1287 | 0.411  765 | 0.294  554 | 2.21  8039 | 49 |
| {BcALV1.BI=TRUE, BcMV2.BI=TRUE} =>  {BcHV1SLR.BI=TRUE} | 0.10  1485 | 0.732  143 | 0.138  614 | 1.35  062 | 41 |
| {BcALV1.BI=TRUE, BcHV1SLR.BI=TRUE}  => {BcMV2.BI=TRUE} | 0.10  1485 | 0.803  922 | 0.126  238 | 1.73  6815 | 41 |
| {BcHV1SLR.BI=TRUE, BcMV2.BI=TRUE} =>  {BcALV1.BI=TRUE} | 0.10  1485 | 0.372  727 | 0.272  277 | 2.00  7758 | 41 |
| {BcALV1.BI=TRUE, BcMV2.BI=TRUE} =>  {BcMV3.BI=TRUE} | 0.10  396 | 0.75 | 0.138  614 | 1.38  3562 | 42 |
| {BcALV1.BI=TRUE, BcMV3.BI=TRUE} =>  {BcMV2.BI=TRUE} | 0.10  396 | 0.875 | 0.118  812 | 1.89  0374 | 42 |
| {BcMV2.BI=TRUE, BcMV3.BI=TRUE} =>  {BcALV1.BI=TRUE} | 0.10  396 | 0.330  709 | 0.314  356 | 1.78  1417 | 42 |
| {BcALV1.BI=TRUE, BcULV3=TRUE} =>  {BcMV2.BI=TRUE} | 0.08  6634 | 0.777  778 | 0.111  386 | 1.68  0333 | 35 |
| {BcMV2.BI=TRUE, BcULV3=TRUE} =>  {BcALV1.BI=TRUE} | 0.08  6634 | 0.304  348 | 0.284  653 | 1.63  942 | 35 |
| {BcALV1.BI=TRUE, BcMV2.BI=TRUE} =>  {BcHV1.BI=TRUE} | 0.10  8911 | 0.785  714 | 0.138  614 | 1.30  0937 | 44 |

| {BcALV1.BI=TRUE, BcHV1.BI=TRUE} =>  {BcMV2.BI=TRUE} | 0.10  8911 | 0.785  714 | 0.138  614 | 1.69  7479 | 44 |
| --- | --- | --- | --- | --- | --- |
| {BcHV1.BI=TRUE, BcMV2.BI=TRUE} =>  {BcALV1.BI=TRUE} | 0.10  8911 | 0.372  881 | 0.292  079 | 2.00  8588 | 44 |
| {BcALV1.BI=TRUE, BcMV2.BI=TRUE} =>  {BcMV11=TRUE} | 0.12  8713 | 0.928  571 | 0.138  614 | 1.50  6598 | 52 |
| {BcALV1.BI=TRUE, BcMV11=TRUE} =>  {BcMV2.BI=TRUE} | 0.12  8713 | 0.825  397 | 0.155  941 | 1.78  321 | 52 |
| {BcMV11=TRUE, BcMV2.BI=TRUE} =>  {BcALV1.BI=TRUE} | 0.12  8713 | 0.376  812 | 0.341  584 | 2.02  9758 | 52 |
| {BcALV1.BI=TRUE, BcMV2.BI=TRUE} =>  {BcMV9.BI=TRUE} | 0.11  3861 | 0.821  429 | 0.138  614 | 1.21  1157 | 46 |
| {BcALV1.BI=TRUE, BcMV9.BI=TRUE} =>  {BcMV2.BI=TRUE} | 0.11  3861 | 0.836  364 | 0.136  139 | 1.80  6903 | 46 |
| {BcMV2.BI=TRUE, BcMV9.BI=TRUE} =>  {BcALV1.BI=TRUE} | 0.11  3861 | 0.353  846 | 0.321  782 | 1.90  6051 | 46 |
| {BcALV1.BI=TRUE, BcMV2.BI=TRUE} =>  {BcULV4=TRUE} | 0.12  3762 | 0.892  857 | 0.138  614 | 1.29  2883 | 50 |
| {BcALV1.BI=TRUE, BcULV4=TRUE} =>  {BcMV2.BI=TRUE} | 0.12  3762 | 0.769  231 | 0.160  891 | 1.66  1868 | 50 |
| {BcMV2.BI=TRUE, BcULV4=TRUE} =>  {BcALV1.BI=TRUE} | 0.12  3762 | 0.362  319 | 0.341  584 | 1.95  1691 | 50 |

| {BcALV1.BI=TRUE, BcPV3.BI=FALSE} =>  {BcMV2.BI=TRUE} | 0.08  6634 | 0.686  275 | 0.126  238 | 1.48  2647 | 35 |
| --- | --- | --- | --- | --- | --- |
| {BcMV2.BI=TRUE, BcPV3.BI=FALSE} =>  {BcALV1.BI=TRUE} | 0.08  6634 | 0.304  348 | 0.284  653 | 1.63  942 | 35 |
| {BcALV1.BI=TRUE, BcEV2.BI=FALSE} =>  {BcMV2.BI=TRUE} | 0.09  1584 | 0.698  113 | 0.131  188 | 1.50  8223 | 37 |
| {BcEV2.BI=FALSE, BcMV2.BI=TRUE} =>  {BcALV1.BI=TRUE} | 0.09  1584 | 0.286  822 | 0.319  307 | 1.54  5013 | 37 |
| {BcALV1.BI=TRUE, BcHV3.BI=FALSE} =>  {BcMV2.BI=TRUE} | 0.08  9109 | 0.705  882 | 0.126  238 | 1.52  5008 | 36 |
| {BcHV3.BI=FALSE, BcMV2.BI=TRUE} =>  {BcALV1.BI=TRUE} | 0.08  9109 | 0.270  677 | 0.329  208 | 1.45  8045 | 36 |
| {BcALV1.BI=TRUE, BcFV5.BI=FALSE} =>  {BcMV2.BI=TRUE} | 0.07  9208 | 0.680  851 | 0.116  337 | 1.47  093 | 32 |
| {BcALV1.BI=TRUE, BcULV1.BI=FALSE} =>  {BcMV2.BI=TRUE} | 0.10  1485 | 0.745  455 | 0.136  139 | 1.61  0501 | 41 |
| {BcMV2.BI=TRUE, BcULV1.BI=FALSE} =>  {BcALV1.BI=TRUE} | 0.10  1485 | 0.312  977 | 0.324  257 | 1.68  5903 | 41 |
| {BcALV1.BI=TRUE, BcMV2.BI=TRUE} =>  {BcMV1.BI=TRUE} | 0.12  3762 | 0.892  857 | 0.138  614 | 1.13  0766 | 50 |
| {BcALV1.BI=TRUE, BcMV1.BI=TRUE} =>  {BcMV2.BI=TRUE} | 0.12  3762 | 0.781  25 | 0.158  416 | 1.68  7834 | 50 |

| {BcMV1.BI=TRUE, BcMV2.BI=TRUE} =>  {BcALV1.BI=TRUE} | 0.12  3762 | 0.303  03 | 0.408  416 | 1.63  2323 | 50 |
| --- | --- | --- | --- | --- | --- |
| {BcALV1.BI=TRUE, BcMV12=FALSE} =>  {BcMV2.BI=TRUE} | 0.10  8911 | 0.745  763 | 0.146  04 | 1.61  1167 | 44 |
| {BcMV12=FALSE, BcMV2.BI=TRUE} =>  {BcALV1.BI=TRUE} | 0.10  8911 | 0.318  841 | 0.341  584 | 1.71  7488 | 44 |
| {BcALV1.BI=TRUE, BcNSRV7.BI=FALSE}  => {BcMV2.BI=TRUE} | 0.10  396 | 0.75 | 0.138  614 | 1.62  0321 | 42 |
| {BcMV2.BI=TRUE, BcNSRV7.BI=FALSE} =>  {BcALV1.BI=TRUE} | 0.10  396 | 0.287  671 | 0.361  386 | 1.54  9589 | 42 |
| {BcALV1.BI=TRUE, BcVV3.BI=FALSE} =>  {BcMV2.BI=TRUE} | 0.10  1485 | 0.706  897 | 0.143  564 | 1.52  7199 | 41 |
| {BcMV2.BI=TRUE, BcVV3.BI=FALSE} =>  {BcALV1.BI=TRUE} | 0.10  1485 | 0.288  732 | 0.351  485 | 1.55  5305 | 41 |
| {BcALV1.BI=TRUE, BcOLV7.BI=FALSE} =>  {BcMV2.BI=TRUE} | 0.09  901 | 0.754  717 | 0.131  188 | 1.63  0512 | 40 |
| {BcMV2.BI=TRUE, BcOLV7.BI=FALSE} =>  {BcALV1.BI=TRUE} | 0.09  901 | 0.275  862 | 0.358  911 | 1.48  5977 | 40 |
| {BcALV1.BI=TRUE, BcDMV1=FALSE} =>  {BcMV2.BI=TRUE} | 0.10  396 | 0.724  138 | 0.143  564 | 1.56  4448 | 42 |
| {BcDMV1=FALSE, BcMV2.BI=TRUE} =>  {BcALV1.BI=TRUE} | 0.10  396 | 0.262  5 | 0.396  04 | 1.41  4 | 42 |

| {BcALV1.BI=TRUE, BcFV9=FALSE} =>  {BcMV2.BI=TRUE} | 0.10  8911 | 0.733  333 | 0.148  515 | 1.58  4314 | 44 |
| --- | --- | --- | --- | --- | --- |
| {BcFV9=FALSE, BcMV2.BI=TRUE} =>  {BcALV1.BI=TRUE} | 0.10  8911 | 0.271  605 | 0.400  99 | 1.46  3045 | 44 |
| {BcALV1.BI=TRUE, BcOLV4.BI=FALSE} =>  {BcMV2.BI=TRUE} | 0.11  6337 | 0.746  032 | 0.155  941 | 1.61  1748 | 47 |
| {BcMV2.BI=TRUE, BcOLV4.BI=FALSE} =>  {BcALV1.BI=TRUE} | 0.11  6337 | 0.309  211 | 0.376  238 | 1.66  5614 | 47 |
| {BcALV1.BI=TRUE, BcULV5=FALSE} =>  {BcMV2.BI=TRUE} | 0.12  6238 | 0.739  13 | 0.170  792 | 1.59  6838 | 51 |
| {BcMV2.BI=TRUE, BcULV5=FALSE} =>  {BcALV1.BI=TRUE} | 0.12  6238 | 0.335  526 | 0.376  238 | 1.80  7368 | 51 |
| {BcALV1.BI=TRUE, BcBNV5.BI=FALSE} =>  {BcMV2.BI=TRUE} | 0.10  6436 | 0.741  379 | 0.143  564 | 1.60  1696 | 43 |
| {BcBNV5.BI=FALSE, BcMV2.BI=TRUE} =>  {BcALV1.BI=TRUE} | 0.10  6436 | 0.260  606 | 0.408  416 | 1.40  3798 | 43 |
| {BcALV1.BI=TRUE, BvF.BI=FALSE} =>  {BcMV2.BI=TRUE} | 0.12  3762 | 0.746  269 | 0.165  842 | 1.61  226 | 50 |
| {BcMV2.BI=TRUE, BvF.BI=FALSE} =>  {BcALV1.BI=TRUE} | 0.12  3762 | 0.285  714 | 0.433  168 | 1.53  9048 | 50 |
| {BcALV1.BI=TRUE, BcMCV5.BI=FALSE} =>  {BcMV2.BI=TRUE} | 0.10  8911 | 0.721  311 | 0.150  99 | 1.55  8341 | 44 |

| {BcMCV5.BI=FALSE, BcMV2.BI=TRUE} =>  {BcALV1.BI=TRUE} | 0.10  8911 | 0.271  605 | 0.400  99 | 1.46  3045 | 44 |
| --- | --- | --- | --- | --- | --- |
| {BcALV1.BI=TRUE, BcOLV13.BI=FALSE} =>  {BcMV2.BI=TRUE} | 0.12  8713 | 0.732  394 | 0.175  743 | 1.58  2285 | 52 |
| {BcMV2.BI=TRUE, BcOLV13.BI=FALSE} =>  {BcALV1.BI=TRUE} | 0.12  8713 | 0.315  152 | 0.408  416 | 1.69  7616 | 52 |
| {BcALV1.BI=TRUE, BcOLV2.BI=FALSE} =>  {BcMV2.BI=TRUE} | 0.12  6238 | 0.728  571 | 0.173  267 | 1.57  4026 | 51 |
| {BcMV2.BI=TRUE, BcOLV2.BI=FALSE} =>  {BcALV1.BI=TRUE} | 0.12  6238 | 0.3 | 0.420  792 | 1.61  6 | 51 |
| {BcALV1.BI=TRUE, BcMCV3.BI=FALSE} =>  {BcMV2.BI=TRUE} | 0.12  6238 | 0.728  571 | 0.173  267 | 1.57  4026 | 51 |
| {BcMCV3.BI=FALSE, BcMV2.BI=TRUE} =>  {BcALV1.BI=TRUE} | 0.12  6238 | 0.309  091 | 0.408  416 | 1.66  497 | 51 |
| {BcALV1.BI=TRUE, BcPV4.BI=FALSE} =>  {BcMV2.BI=TRUE} | 0.13  6139 | 0.753  425 | 0.180  693 | 1.62  772 | 55 |
| {BcMV2.BI=TRUE, BcPV4.BI=FALSE} =>  {BcALV1.BI=TRUE} | 0.13  6139 | 0.317  919 | 0.428  218 | 1.71  2524 | 55 |
| {BcALV1.BI=TRUE, BcEV6=FALSE} =>  {BcMV2.BI=TRUE} | 0.12  6238 | 0.739  13 | 0.170  792 | 1.59  6838 | 51 |
| {BcEV6=FALSE, BcMV2.BI=TRUE} =>  {BcALV1.BI=TRUE} | 0.12  6238 | 0.291  429 | 0.433  168 | 1.56  9829 | 51 |

| {BcALV1.BI=TRUE, BcOLV17.BI=FALSE} =>  {BcMV2.BI=TRUE} | 0.13  1188 | 0.736  111 | 0.178  218 | 1.59  0315 | 53 |
| --- | --- | --- | --- | --- | --- |
| {BcMV2.BI=TRUE, BcOLV17.BI=FALSE} =>  {BcALV1.BI=TRUE} | 0.13  1188 | 0.308  14 | 0.425  743 | 1.65  9845 | 53 |
| {BcALV1.BI=TRUE, BcOLV15.BI=FALSE} =>  {BcMV2.BI=TRUE} | 0.13  6139 | 0.743  243 | 0.183  168 | 1.60  5723 | 55 |
| {BcMV2.BI=TRUE, BcOLV15.BI=FALSE} =>  {BcALV1.BI=TRUE} | 0.13  6139 | 0.300  546 | 0.452  97 | 1.61  8944 | 55 |
| {BcALV1.BI=TRUE, BcEV5=FALSE} =>  {BcMV2.BI=TRUE} | 0.11  6337 | 0.712  121 | 0.163  366 | 1.53  8486 | 47 |
| {BcEV5=FALSE, BcMV2.BI=TRUE} =>  {BcALV1.BI=TRUE} | 0.11  6337 | 0.274  854 | 0.423  267 | 1.48  0546 | 47 |
| {BcALV1.BI=TRUE, BcOLV18=TRUE} =>  {BcHV1SLR.BI=TRUE} | 0.10  396 | 0.736  842 | 0.141  089 | 1.35  9289 | 42 |
| {BcALV1.BI=TRUE, BcHV1SLR.BI=TRUE}  => {BcOLV18=TRUE} | 0.10  396 | 0.823  529 | 0.126  238 | 1.59  1894 | 42 |
| {BcHV1SLR.BI=TRUE, BcOLV18=TRUE} =>  {BcALV1.BI=TRUE} | 0.10  396 | 0.304  348 | 0.341  584 | 1.63  942 | 42 |
| {BcALV1.BI=TRUE, BcOLV18=TRUE} =>  {BcMV3.BI=TRUE} | 0.10  6436 | 0.754  386 | 0.141  089 | 1.39  1653 | 43 |
| {BcALV1.BI=TRUE, BcMV3.BI=TRUE} =>  {BcOLV18=TRUE} | 0.10  6436 | 0.895  833 | 0.118  812 | 1.73  1659 | 43 |

| {BcMV3.BI=TRUE, BcOLV18=TRUE} =>  {BcALV1.BI=TRUE} | 0.10  6436 | 0.323  308 | 0.329  208 | 1.74  1554 | 43 |
| --- | --- | --- | --- | --- | --- |
| {BcALV1.BI=TRUE, BcULV3=TRUE} =>  {BcOLV18=TRUE} | 0.08  4158 | 0.755  556 | 0.111  386 | 1.46  05 | 34 |
| {BcOLV18=TRUE, BcULV3=TRUE} =>  {BcALV1.BI=TRUE} | 0.08  4158 | 0.259  542 | 0.324  257 | 1.39  8066 | 34 |
| {BcALV1.BI=TRUE, BcOLV18=TRUE} =>  {BcHV1.BI=TRUE} | 0.11  3861 | 0.807  018 | 0.141  089 | 1.33  6209 | 46 |
| {BcALV1.BI=TRUE, BcHV1.BI=TRUE} =>  {BcOLV18=TRUE} | 0.11  3861 | 0.821  429 | 0.138  614 | 1.58  7833 | 46 |
| {BcHV1.BI=TRUE, BcOLV18=TRUE} =>  {BcALV1.BI=TRUE} | 0.11  3861 | 0.310  811 | 0.366  337 | 1.67  4234 | 46 |
| {BcALV1.BI=TRUE, BcOLV18=TRUE} =>  {BcMV11=TRUE} | 0.12  3762 | 0.877  193 | 0.141  089 | 1.42  3237 | 50 |
| {BcALV1.BI=TRUE, BcMV11=TRUE} =>  {BcOLV18=TRUE} | 0.12  3762 | 0.793  651 | 0.155  941 | 1.53  4138 | 50 |
| {BcMV11=TRUE, BcOLV18=TRUE} =>  {BcALV1.BI=TRUE} | 0.12  3762 | 0.357  143 | 0.346  535 | 1.92  381 | 50 |
| {BcALV1.BI=TRUE, BcOLV18=TRUE} =>  {BcMV9.BI=TRUE} | 0.11  8812 | 0.842  105 | 0.141  089 | 1.24  1644 | 48 |
| {BcALV1.BI=TRUE, BcMV9.BI=TRUE} =>  {BcOLV18=TRUE} | 0.11  8812 | 0.872  727 | 0.136  139 | 1.68  6994 | 48 |

| {BcMV9.BI=TRUE, BcOLV18=TRUE} =>  {BcALV1.BI=TRUE} | 0.11  8812 | 0.287  425 | 0.413  366 | 1.54  8263 | 48 |
| --- | --- | --- | --- | --- | --- |
| {BcALV1.BI=TRUE, BcOLV18=TRUE} =>  {BcULV4=TRUE} | 0.13  3663 | 0.947  368 | 0.141  089 | 1.37  1817 | 54 |
| {BcALV1.BI=TRUE, BcULV4=TRUE} =>  {BcOLV18=TRUE} | 0.13  3663 | 0.830  769 | 0.160  891 | 1.60  5889 | 54 |
| {BcOLV18=TRUE, BcULV4=TRUE} =>  {BcALV1.BI=TRUE} | 0.13  3663 | 0.303  371 | 0.440  594 | 1.63  4157 | 54 |
| {BcALV1.BI=TRUE, BcPV3.BI=FALSE} =>  {BcOLV18=TRUE} | 0.08  9109 | 0.705  882 | 0.126  238 | 1.36  4481 | 36 |
| {BcOLV18=TRUE, BcPV3.BI=FALSE} =>  {BcALV1.BI=TRUE} | 0.08  9109 | 0.270  677 | 0.329  208 | 1.45  8045 | 36 |
| {BcALV1.BI=TRUE, BcEV2.BI=FALSE} =>  {BcOLV18=TRUE} | 0.10  1485 | 0.773  585 | 0.131  188 | 1.49  5351 | 41 |
| {BcEV2.BI=FALSE, BcOLV18=TRUE} =>  {BcALV1.BI=TRUE} | 0.10  1485 | 0.275  168 | 0.368  812 | 1.48  2237 | 41 |
| {BcALV1.BI=TRUE, BcHV3.BI=FALSE} =>  {BcOLV18=TRUE} | 0.09  901 | 0.784  314 | 0.126  238 | 1.51  609 | 40 |
| {BcHV3.BI=FALSE, BcOLV18=TRUE} =>  {BcALV1.BI=TRUE} | 0.09  901 | 0.281  69 | 0.351  485 | 1.51  7371 | 40 |
| {BcALV1.BI=TRUE, BcFV5.BI=FALSE} =>  {BcOLV18=TRUE} | 0.08  1683 | 0.702  128 | 0.116  337 | 1.35  7223 | 33 |

| {BcALV1.BI=TRUE, BcULV1.BI=FALSE} =>  {BcOLV18=TRUE} | 0.09  901 | 0.727  273 | 0.136  139 | 1.40  5829 | 40 |
| --- | --- | --- | --- | --- | --- |
| {BcOLV18=TRUE, BcULV1.BI=FALSE} =>  {BcALV1.BI=TRUE} | 0.09  901 | 0.277  778 | 0.356  436 | 1.49  6296 | 40 |
| {BcALV1.BI=TRUE, BcOLV18=TRUE} =>  {BcMV1.BI=TRUE} | 0.12  8713 | 0.912  281 | 0.141  089 | 1.15  5365 | 52 |
| {BcALV1.BI=TRUE, BcMV1.BI=TRUE} =>  {BcOLV18=TRUE} | 0.12  8713 | 0.812  5 | 0.158  416 | 1.57  0574 | 52 |
| {BcMV1.BI=TRUE, BcOLV18=TRUE} =>  {BcALV1.BI=TRUE} | 0.12  8713 | 0.292  135 | 0.440  594 | 1.57  3633 | 52 |
| {BcALV1.BI=TRUE, BcMV12=FALSE} =>  {BcOLV18=TRUE} | 0.11  3861 | 0.779  661 | 0.146  04 | 1.50  7096 | 46 |
| {BcMV12=FALSE, BcOLV18=TRUE} =>  {BcALV1.BI=TRUE} | 0.11  3861 | 0.287  5 | 0.396  04 | 1.54  8667 | 46 |
| {BcALV1.BI=TRUE, BcNSRV7.BI=FALSE}  => {BcOLV18=TRUE} | 0.10  6436 | 0.767  857 | 0.138  614 | 1.48  4279 | 43 |
| {BcNSRV7.BI=FALSE, BcOLV18=TRUE} =>  {BcALV1.BI=TRUE} | 0.10  6436 | 0.273  885 | 0.388  614 | 1.47  5329 | 43 |
| {BcALV1.BI=TRUE, BcVV3.BI=FALSE} =>  {BcOLV18=TRUE} | 0.11  1386 | 0.775  862 | 0.143  564 | 1.49  9753 | 45 |
| {BcOLV18=TRUE, BcVV3.BI=FALSE} =>  {BcALV1.BI=TRUE} | 0.11  1386 | 0.271  084 | 0.410  891 | 1.46  0241 | 45 |

| {BcALV1.BI=TRUE, BcOLV7.BI=FALSE} =>  {BcOLV18=TRUE} | 0.09  4059 | 0.716  981 | 0.131  188 | 1.38  5935 | 38 |
| --- | --- | --- | --- | --- | --- |
| {BcOLV18=TRUE, BcOLV7.BI=FALSE} =>  {BcALV1.BI=TRUE} | 0.09  4059 | 0.25 | 0.376  238 | 1.34  6667 | 38 |
| {BcALV1.BI=TRUE, BcDMV1=FALSE} =>  {BcOLV18=TRUE} | 0.10  8911 | 0.758  621 | 0.143  564 | 1.46  6425 | 44 |
| {BcDMV1=FALSE, BcOLV18=TRUE} =>  {BcALV1.BI=TRUE} | 0.10  8911 | 0.254  335 | 0.428  218 | 1.37  0019 | 44 |
| {BcALV1.BI=TRUE, BcFV9=FALSE} =>  {BcOLV18=TRUE} | 0.10  8911 | 0.733  333 | 0.148  515 | 1.41  7544 | 44 |
| {BcFV9=FALSE, BcOLV18=TRUE} =>  {BcALV1.BI=TRUE} | 0.10  8911 | 0.258  824 | 0.420  792 | 1.39  4196 | 44 |
| {BcALV1.BI=TRUE, BcOLV4.BI=FALSE} =>  {BcOLV18=TRUE} | 0.12  1287 | 0.777  778 | 0.155  941 | 1.50  3456 | 49 |
| {BcOLV18=TRUE, BcOLV4.BI=FALSE} =>  {BcALV1.BI=TRUE} | 0.12  1287 | 0.270  718 | 0.448  02 | 1.45  8269 | 49 |
| {BcALV1.BI=TRUE, BcULV5=FALSE} =>  {BcOLV18=TRUE} | 0.13  3663 | 0.782  609 | 0.170  792 | 1.51  2794 | 54 |
| {BcOLV18=TRUE, BcULV5=FALSE} =>  {BcALV1.BI=TRUE} | 0.13  3663 | 0.285  714 | 0.467  822 | 1.53  9048 | 54 |
| {BcALV1.BI=TRUE, BcBNV5.BI=FALSE} =>  {BcOLV18=TRUE} | 0.10  6436 | 0.741  379 | 0.143  564 | 1.43  3097 | 43 |

| {BcALV1.BI=TRUE, BvF.BI=FALSE} =>  {BcOLV18=TRUE} | 0.13  1188 | 0.791  045 | 0.165  842 | 1.52  9101 | 53 |
| --- | --- | --- | --- | --- | --- |
| {BcOLV18=TRUE, BvF.BI=FALSE} =>  {BcALV1.BI=TRUE} | 0.13  1188 | 0.276  042 | 0.475  248 | 1.48  6944 | 53 |
| {BcALV1.BI=TRUE, BcMCV5.BI=FALSE} =>  {BcOLV18=TRUE} | 0.11  1386 | 0.737  705 | 0.150  99 | 1.42  5994 | 45 |
| {BcMCV5.BI=FALSE, BcOLV18=TRUE} =>  {BcALV1.BI=TRUE} | 0.11  1386 | 0.247  253 | 0.450  495 | 1.33  1868 | 45 |
| {BcALV1.BI=TRUE, BcOLV13.BI=FALSE} =>  {BcOLV18=TRUE} | 0.13  3663 | 0.760  563 | 0.175  743 | 1.47  018 | 54 |
| {BcOLV13.BI=FALSE, BcOLV18=TRUE} =>  {BcALV1.BI=TRUE} | 0.13  3663 | 0.281  25 | 0.475  248 | 1.51  5 | 54 |
| {BcALV1.BI=TRUE, BcOLV2.BI=FALSE} =>  {BcOLV18=TRUE} | 0.12  8713 | 0.742  857 | 0.173  267 | 1.43  5954 | 52 |
| {BcOLV18=TRUE, BcOLV2.BI=FALSE} =>  {BcALV1.BI=TRUE} | 0.12  8713 | 0.275  132 | 0.467  822 | 1.48  2046 | 52 |
| {BcALV1.BI=TRUE, BcMCV3.BI=FALSE} =>  {BcOLV18=TRUE} | 0.12  8713 | 0.742  857 | 0.173  267 | 1.43  5954 | 52 |
| {BcMCV3.BI=FALSE, BcOLV18=TRUE} =>  {BcALV1.BI=TRUE} | 0.12  8713 | 0.270  833 | 0.475  248 | 1.45  8889 | 52 |
| {BcALV1.BI=TRUE, BcPV4.BI=FALSE} =>  {BcOLV18=TRUE} | 0.13  8614 | 0.767  123 | 0.180  693 | 1.48  286 | 56 |

| {BcOLV18=TRUE, BcPV4.BI=FALSE} =>  {BcALV1.BI=TRUE} | 0.13  8614 | 0.290  155 | 0.477  723 | 1.56  2971 | 56 |
| --- | --- | --- | --- | --- | --- |
| {BcALV1.BI=TRUE, BcEV6=FALSE} =>  {BcOLV18=TRUE} | 0.13  1188 | 0.768  116 | 0.170  792 | 1.48  4779 | 53 |
| {BcEV6=FALSE, BcOLV18=TRUE} =>  {BcALV1.BI=TRUE} | 0.13  1188 | 0.271  795 | 0.482  673 | 1.46  4068 | 53 |
| {BcALV1.BI=TRUE, BcOLV17.BI=FALSE} =>  {BcOLV18=TRUE} | 0.13  3663 | 0.75 | 0.178  218 | 1.44  9761 | 54 |
| {BcOLV17.BI=FALSE, BcOLV18=TRUE} =>  {BcALV1.BI=TRUE} | 0.13  3663 | 0.279  793 | 0.477  723 | 1.50  715 | 54 |
| {BcALV1.BI=TRUE, BcOLV15.BI=FALSE} =>  {BcOLV18=TRUE} | 0.13  8614 | 0.756  757 | 0.183  168 | 1.46  2822 | 56 |
| {BcOLV15.BI=FALSE, BcOLV18=TRUE} =>  {BcALV1.BI=TRUE} | 0.13  8614 | 0.290  155 | 0.477  723 | 1.56  2971 | 56 |
| {BcALV1.BI=TRUE, BcEV5=FALSE} =>  {BcOLV18=TRUE} | 0.12  1287 | 0.742  424 | 0.163  366 | 1.43  5117 | 49 |
| {BcEV5=FALSE, BcOLV18=TRUE} =>  {BcALV1.BI=TRUE} | 0.12  1287 | 0.248  731 | 0.487  624 | 1.33  9831 | 49 |
| {BcALV1.BI=TRUE, BcHV1SLR.BI=TRUE}  => {BcMV3.BI=TRUE} | 0.09  1584 | 0.725  49 | 0.126  238 | 1.33  8347 | 37 |
| {BcALV1.BI=TRUE, BcMV3.BI=TRUE} =>  {BcHV1SLR.BI=TRUE} | 0.09  1584 | 0.770  833 | 0.118  812 | 1.42  1994 | 37 |

| {BcHV1SLR.BI=TRUE, BcMV3.BI=TRUE} =>  {BcALV1.BI=TRUE} | 0.09  1584 | 0.278  195 | 0.329  208 | 1.49  8546 | 37 |
| --- | --- | --- | --- | --- | --- |
| {BcALV1.BI=TRUE, BcULV3=TRUE} =>  {BcHV1SLR.BI=TRUE} | 0.08  6634 | 0.777  778 | 0.111  386 | 1.43  4805 | 35 |
| {BcALV1.BI=TRUE, BcHV1SLR.BI=TRUE}  => {BcHV1.BI=TRUE} | 0.11  3861 | 0.901  961 | 0.126  238 | 1.49  341 | 46 |
| {BcALV1.BI=TRUE, BcHV1.BI=TRUE} =>  {BcHV1SLR.BI=TRUE} | 0.11  3861 | 0.821  429 | 0.138  614 | 1.51  5329 | 46 |
| {BcALV1.BI=TRUE, BcHV1SLR.BI=TRUE}  => {BcMV11=TRUE} | 0.10  8911 | 0.862  745 | 0.126  238 | 1.39  9795 | 44 |
| {BcALV1.BI=TRUE, BcMV11=TRUE} =>  {BcHV1SLR.BI=TRUE} | 0.10  8911 | 0.698  413 | 0.155  941 | 1.28  8396 | 44 |
| {BcHV1SLR.BI=TRUE, BcMV11=TRUE} =>  {BcALV1.BI=TRUE} | 0.10  8911 | 0.305  556 | 0.356  436 | 1.64  5926 | 44 |
| {BcALV1.BI=TRUE, BcHV1SLR.BI=TRUE}  => {BcMV9.BI=TRUE} | 0.10  396 | 0.823  529 | 0.126  238 | 1.21  4255 | 42 |
| {BcALV1.BI=TRUE, BcMV9.BI=TRUE} =>  {BcHV1SLR.BI=TRUE} | 0.10  396 | 0.763  636 | 0.136  139 | 1.40  8717 | 42 |
| {BcHV1SLR.BI=TRUE, BcMV9.BI=TRUE} =>  {BcALV1.BI=TRUE} | 0.10  396 | 0.254  545 | 0.408  416 | 1.37  1152 | 42 |
| {BcALV1.BI=TRUE, BcHV1SLR.BI=TRUE}  => {BcULV4=TRUE} | 0.11  8812 | 0.941  176 | 0.126  238 | 1.36  2851 | 48 |

| {BcALV1.BI=TRUE, BcULV4=TRUE} =>  {BcHV1SLR.BI=TRUE} | 0.11  8812 | 0.738  462 | 0.160  891 | 1.36  2276 | 48 |
| --- | --- | --- | --- | --- | --- |
| {BcHV1SLR.BI=TRUE, BcULV4=TRUE} =>  {BcALV1.BI=TRUE} | 0.11  8812 | 0.296  296 | 0.400  99 | 1.59  6049 | 48 |
| {BcALV1.BI=TRUE, BcHV3.BI=FALSE} =>  {BcHV1SLR.BI=TRUE} | 0.09  1584 | 0.725  49 | 0.126  238 | 1.33  8347 | 37 |
| {BcALV1.BI=TRUE, BcMV1.BI=TRUE} =>  {BcHV1SLR.BI=TRUE} | 0.10  8911 | 0.687  5 | 0.158  416 | 1.26  8265 | 44 |
| {BcHV1SLR.BI=TRUE, BcMV1.BI=TRUE} =>  {BcALV1.BI=TRUE} | 0.10  8911 | 0.247  191 | 0.440  594 | 1.33  1536 | 44 |
| {BcALV1.BI=TRUE, BcMV12=FALSE} =>  {BcHV1SLR.BI=TRUE} | 0.10  396 | 0.711  864 | 0.146  04 | 1.31  3211 | 42 |
| {BcHV1SLR.BI=TRUE, BcMV12=FALSE} =>  {BcALV1.BI=TRUE} | 0.10  396 | 0.251  497 | 0.413  366 | 1.35  4731 | 42 |
| {BcALV1.BI=TRUE, BcVV3.BI=FALSE} =>  {BcHV1SLR.BI=TRUE} | 0.09  901 | 0.689  655 | 0.143  564 | 1.27  2241 | 40 |
| {BcALV1.BI=TRUE, BcOLV7.BI=FALSE} =>  {BcHV1SLR.BI=TRUE} | 0.09  1584 | 0.698  113 | 0.131  188 | 1.28  7844 | 37 |
| {BcALV1.BI=TRUE, BcFV9=FALSE} =>  {BcHV1SLR.BI=TRUE} | 0.09  901 | 0.666  667 | 0.148  515 | 1.22  9833 | 40 |
| {BcALV1.BI=TRUE, BcOLV4.BI=FALSE} =>  {BcHV1SLR.BI=TRUE} | 0.10  6436 | 0.682  54 | 0.155  941 | 1.25  9114 | 43 |

| {BcALV1.BI=TRUE, BcULV5=FALSE} =>  {BcHV1SLR.BI=TRUE} | 0.11  3861 | 0.666  667 | 0.170  792 | 1.22  9833 | 46 |
| --- | --- | --- | --- | --- | --- |
| {BcHV1SLR.BI=TRUE, BcULV5=FALSE} =>  {BcALV1.BI=TRUE} | 0.11  3861 | 0.243  386 | 0.467  822 | 1.31  1041 | 46 |
| {BcALV1.BI=TRUE, BvF.BI=FALSE} =>  {BcHV1SLR.BI=TRUE} | 0.10  8911 | 0.656  716 | 0.165  842 | 1.21  1477 | 44 |
| {BcALV1.BI=TRUE, BcMCV5.BI=FALSE} =>  {BcHV1SLR.BI=TRUE} | 0.10  1485 | 0.672  131 | 0.150  99 | 1.23  9913 | 41 |
| {BcALV1.BI=TRUE, BcOLV13.BI=FALSE} =>  {BcHV1SLR.BI=TRUE} | 0.11  8812 | 0.676  056 | 0.175  743 | 1.24  7154 | 48 |
| {BcHV1SLR.BI=TRUE, BcOLV13.BI=FALSE}  => {BcALV1.BI=TRUE} | 0.11  8812 | 0.242  424 | 0.490  099 | 1.30  5859 | 48 |
| {BcALV1.BI=TRUE, BcOLV2.BI=FALSE} =>  {BcHV1SLR.BI=TRUE} | 0.11  3861 | 0.657  143 | 0.173  267 | 1.21  2264 | 46 |
| {BcALV1.BI=TRUE, BcMCV3.BI=FALSE} =>  {BcHV1SLR.BI=TRUE} | 0.11  3861 | 0.657  143 | 0.173  267 | 1.21  2264 | 46 |
| {BcALV1.BI=TRUE, BcPV4.BI=FALSE} =>  {BcHV1SLR.BI=TRUE} | 0.12  3762 | 0.684  932 | 0.180  693 | 1.26  3527 | 50 |
| {BcHV1SLR.BI=TRUE, BcPV4.BI=FALSE}  => {BcALV1.BI=TRUE} | 0.12  3762 | 0.245  098 | 0.504  95 | 1.32  0261 | 50 |
| {BcALV1.BI=TRUE, BcEV6=FALSE} =>  {BcHV1SLR.BI=TRUE} | 0.12  1287 | 0.710  145 | 0.170  792 | 1.31  0039 | 49 |

| {BcEV6=FALSE, BcHV1SLR.BI=TRUE} =>  {BcALV1.BI=TRUE} | 0.12  1287 | 0.241  379 | 0.502  475 | 1.30  023 | 49 |
| --- | --- | --- | --- | --- | --- |
| {BcALV1.BI=TRUE, BcOLV17.BI=FALSE} =>  {BcHV1SLR.BI=TRUE} | 0.11  8812 | 0.666  667 | 0.178  218 | 1.22  9833 | 48 |
| {BcALV1.BI=TRUE, BcOLV15.BI=FALSE} =>  {BcHV1SLR.BI=TRUE} | 0.12  3762 | 0.675  676 | 0.183  168 | 1.24  6452 | 50 |
| {BcHV1SLR.BI=TRUE, BcOLV15.BI=FALSE}  => {BcALV1.BI=TRUE} | 0.12  3762 | 0.247  525 | 0.5 | 1.33  3333 | 50 |
| {BcALV1.BI=TRUE, BcEV5=FALSE} =>  {BcHV1SLR.BI=TRUE} | 0.10  8911 | 0.666  667 | 0.163  366 | 1.22  9833 | 44 |
| {BcALV1.BI=TRUE, BcULV3=TRUE} =>  {BcMV3.BI=TRUE} | 0.07  4257 | 0.666  667 | 0.111  386 | 1.22  9833 | 30 |
| {BcALV1.BI=TRUE, BcMV3.BI=TRUE} =>  {BcHV1.BI=TRUE} | 0.09  901 | 0.833  333 | 0.118  812 | 1.37  9781 | 40 |
| {BcALV1.BI=TRUE, BcHV1.BI=TRUE} =>  {BcMV3.BI=TRUE} | 0.09  901 | 0.714  286 | 0.138  614 | 1.31  7678 | 40 |
| {BcHV1.BI=TRUE, BcMV3.BI=TRUE} =>  {BcALV1.BI=TRUE} | 0.09  901 | 0.279  72 | 0.353  96 | 1.50  676 | 40 |
| {BcALV1.BI=TRUE, BcMV3.BI=TRUE} =>  {BcMV11=TRUE} | 0.10  8911 | 0.916  667 | 0.118  812 | 1.48  7282 | 44 |
| {BcALV1.BI=TRUE, BcMV11=TRUE} =>  {BcMV3.BI=TRUE} | 0.10  8911 | 0.698  413 | 0.155  941 | 1.28  8396 | 44 |

| {BcMV11=TRUE, BcMV3.BI=TRUE} =>  {BcALV1.BI=TRUE} | 0.10  8911 | 0.283  871 | 0.383  663 | 1.52  9118 | 44 |
| --- | --- | --- | --- | --- | --- |
| {BcALV1.BI=TRUE, BcMV3.BI=TRUE} =>  {BcMV9.BI=TRUE} | 0.09  901 | 0.833  333 | 0.118  812 | 1.22  871 | 40 |
| {BcALV1.BI=TRUE, BcMV9.BI=TRUE} =>  {BcMV3.BI=TRUE} | 0.09  901 | 0.727  273 | 0.136  139 | 1.34  1636 | 40 |
| {BcMV3.BI=TRUE, BcMV9.BI=TRUE} =>  {BcALV1.BI=TRUE} | 0.09  901 | 0.25 | 0.396  04 | 1.34  6667 | 40 |
| {BcALV1.BI=TRUE, BcMV3.BI=TRUE} =>  {BcULV4=TRUE} | 0.11  1386 | 0.937  5 | 0.118  812 | 1.35  7527 | 45 |
| {BcALV1.BI=TRUE, BcULV4=TRUE} =>  {BcMV3.BI=TRUE} | 0.11  1386 | 0.692  308 | 0.160  891 | 1.27  7134 | 45 |
| {BcMV3.BI=TRUE, BcULV4=TRUE} =>  {BcALV1.BI=TRUE} | 0.11  1386 | 0.277  778 | 0.400  99 | 1.49  6296 | 45 |
| {BcALV1.BI=TRUE, BcMV3.BI=TRUE} =>  {BcMV1.BI=TRUE} | 0.10  8911 | 0.916  667 | 0.118  812 | 1.16  092 | 44 |
| {BcALV1.BI=TRUE, BcMV1.BI=TRUE} =>  {BcMV3.BI=TRUE} | 0.10  8911 | 0.687  5 | 0.158  416 | 1.26  8265 | 44 |
| {BcMV1.BI=TRUE, BcMV3.BI=TRUE} =>  {BcALV1.BI=TRUE} | 0.10  8911 | 0.237  838 | 0.457  921 | 1.28  1153 | 44 |
| {BcMV12=FALSE, BcMV3.BI=TRUE} =>  {BcALV1.BI=TRUE} | 0.09  4059 | 0.242  038 | 0.388  614 | 1.30  3779 | 38 |

| {BcALV1.BI=TRUE, BcOLV16.BI=FALSE} =>  {BcMV1.BI=TRUE} | 0.07  4257 | 0.937  5 | 0.079  208 | 1.18  7304 | 30 |
| --- | --- | --- | --- | --- | --- |
| {BcALV1.BI=TRUE, BcULV3=TRUE} =>  {BcHV1.BI=TRUE} | 0.08  9109 | 0.8 | 0.111  386 | 1.32  459 | 36 |
| {BcALV1.BI=TRUE, BcULV3=TRUE} =>  {BcMV11=TRUE} | 0.10  396 | 0.933  333 | 0.111  386 | 1.51  4324 | 42 |
| {BcMV11=TRUE, BcULV3=TRUE} =>  {BcALV1.BI=TRUE} | 0.10  396 | 0.262  5 | 0.396  04 | 1.41  4 | 42 |
| {BcALV1.BI=TRUE, BcULV3=TRUE} =>  {BcULV4=TRUE} | 0.09  4059 | 0.844  444 | 0.111  386 | 1.22  278 | 38 |
| {BcALV1.BI=TRUE, BcHV1.BI=TRUE} =>  {BcMV11=TRUE} | 0.11  8812 | 0.857  143 | 0.138  614 | 1.39  0706 | 48 |
| {BcALV1.BI=TRUE, BcMV11=TRUE} =>  {BcHV1.BI=TRUE} | 0.11  8812 | 0.761  905 | 0.155  941 | 1.26  1514 | 48 |
| {BcHV1.BI=TRUE, BcMV11=TRUE} =>  {BcALV1.BI=TRUE} | 0.11  8812 | 0.311  688 | 0.381  188 | 1.67  8961 | 48 |
| {BcALV1.BI=TRUE, BcHV1.BI=TRUE} =>  {BcMV9.BI=TRUE} | 0.11  1386 | 0.803  571 | 0.138  614 | 1.18  4828 | 45 |
| {BcALV1.BI=TRUE, BcMV9.BI=TRUE} =>  {BcHV1.BI=TRUE} | 0.11  1386 | 0.818  182 | 0.136  139 | 1.35  4694 | 45 |
| {BcHV1.BI=TRUE, BcMV9.BI=TRUE} =>  {BcALV1.BI=TRUE} | 0.11  1386 | 0.238  095 | 0.467  822 | 1.28  254 | 45 |

| {BcALV1.BI=TRUE, BcHV1.BI=TRUE} =>  {BcULV4=TRUE} | 0.12  3762 | 0.892  857 | 0.138  614 | 1.29  2883 | 50 |
| --- | --- | --- | --- | --- | --- |
| {BcALV1.BI=TRUE, BcULV4=TRUE} =>  {BcHV1.BI=TRUE} | 0.12  3762 | 0.769  231 | 0.160  891 | 1.27  3644 | 50 |
| {BcHV1.BI=TRUE, BcULV4=TRUE} =>  {BcALV1.BI=TRUE} | 0.12  3762 | 0.271  739 | 0.455  446 | 1.46  3768 | 50 |
| {BcALV1.BI=TRUE, BcHV3.BI=FALSE} =>  {BcHV1.BI=TRUE} | 0.09  6535 | 0.764  706 | 0.126  238 | 1.26  6152 | 39 |
| {BcALV1.BI=TRUE, BcFV5.BI=FALSE} =>  {BcHV1.BI=TRUE} | 0.08  4158 | 0.723  404 | 0.116  337 | 1.19  7768 | 34 |
| {BcALV1.BI=TRUE, BcULV1.BI=FALSE} =>  {BcHV1.BI=TRUE} | 0.09  6535 | 0.709  091 | 0.136  139 | 1.17  4069 | 39 |
| {BcALV1.BI=TRUE, BcMV1.BI=TRUE} =>  {BcHV1.BI=TRUE} | 0.11  8812 | 0.75 | 0.158  416 | 1.24  1803 | 48 |
| {BcHV1.BI=TRUE, BcMV1.BI=TRUE} =>  {BcALV1.BI=TRUE} | 0.11  8812 | 0.246  154 | 0.482  673 | 1.32  5949 | 48 |
| {BcALV1.BI=TRUE, BcMV12=FALSE} =>  {BcHV1.BI=TRUE} | 0.11  3861 | 0.779  661 | 0.146  04 | 1.29  0914 | 46 |
| {BcHV1.BI=TRUE, BcMV12=FALSE} =>  {BcALV1.BI=TRUE} | 0.11  3861 | 0.244  681 | 0.465  347 | 1.31  8014 | 46 |
| {BcALV1.BI=TRUE, BcNSRV7.BI=FALSE}  => {BcHV1.BI=TRUE} | 0.10  396 | 0.75 | 0.138  614 | 1.24  1803 | 42 |

| {BcALV1.BI=TRUE, BcVV3.BI=FALSE} =>  {BcHV1.BI=TRUE} | 0.10  8911 | 0.758  621 | 0.143  564 | 1.25  6077 | 44 |
| --- | --- | --- | --- | --- | --- |
| {BcALV1.BI=TRUE, BcOLV7.BI=FALSE} =>  {BcHV1.BI=TRUE} | 0.09  901 | 0.754  717 | 0.131  188 | 1.24  9613 | 40 |
| {BcALV1.BI=TRUE, BcDMV1=FALSE} =>  {BcHV1.BI=TRUE} | 0.10  396 | 0.724  138 | 0.143  564 | 1.19  8982 | 42 |
| {BcALV1.BI=TRUE, BcFV9=FALSE} =>  {BcHV1.BI=TRUE} | 0.10  8911 | 0.733  333 | 0.148  515 | 1.21  4208 | 44 |
| {BcALV1.BI=TRUE, BcOLV4.BI=FALSE} =>  {BcHV1.BI=TRUE} | 0.11  6337 | 0.746  032 | 0.155  941 | 1.23  5233 | 47 |
| {BcALV1.BI=TRUE, BcULV5=FALSE} =>  {BcHV1.BI=TRUE} | 0.12  6238 | 0.739  13 | 0.170  792 | 1.22  3806 | 51 |
| {BcHV1.BI=TRUE, BcULV5=FALSE} =>  {BcALV1.BI=TRUE} | 0.12  6238 | 0.237  209 | 0.532  178 | 1.27  7767 | 51 |
| {BcALV1.BI=TRUE, BcBNV5.BI=FALSE} =>  {BcHV1.BI=TRUE} | 0.10  396 | 0.724  138 | 0.143  564 | 1.19  8982 | 42 |
| {BcALV1.BI=TRUE, BvF.BI=FALSE} =>  {BcHV1.BI=TRUE} | 0.11  8812 | 0.716  418 | 0.165  842 | 1.18  62 | 48 |
| {BcALV1.BI=TRUE, BcMCV5.BI=FALSE} =>  {BcHV1.BI=TRUE} | 0.11  1386 | 0.737  705 | 0.150  99 | 1.22  1446 | 45 |
| {BcALV1.BI=TRUE, BcOLV13.BI=FALSE} =>  {BcHV1.BI=TRUE} | 0.12  8713 | 0.732  394 | 0.175  743 | 1.21  2653 | 52 |

| {BcHV1.BI=TRUE, BcOLV13.BI=FALSE} =>  {BcALV1.BI=TRUE} | 0.12  8713 | 0.236  364 | 0.544  554 | 1.27  3212 | 52 |
| --- | --- | --- | --- | --- | --- |
| {BcALV1.BI=TRUE, BcOLV2.BI=FALSE} =>  {BcHV1.BI=TRUE} | 0.12  8713 | 0.742  857 | 0.173  267 | 1.22  9977 | 52 |
| {BcHV1.BI=TRUE, BcOLV2.BI=FALSE} =>  {BcALV1.BI=TRUE} | 0.12  8713 | 0.233  184 | 0.551  98 | 1.25  6084 | 52 |
| {BcALV1.BI=TRUE, BcMCV3.BI=FALSE} =>  {BcHV1.BI=TRUE} | 0.12  6238 | 0.728  571 | 0.173  267 | 1.20  6323 | 51 |
| {BcHV1.BI=TRUE, BcMCV3.BI=FALSE} =>  {BcALV1.BI=TRUE} | 0.12  6238 | 0.231  818 | 0.544  554 | 1.24  8727 | 51 |
| {BcALV1.BI=TRUE, BcPV4.BI=FALSE} =>  {BcHV1.BI=TRUE} | 0.13  6139 | 0.753  425 | 0.180  693 | 1.24  7474 | 55 |
| {BcHV1.BI=TRUE, BcPV4.BI=FALSE} =>  {BcALV1.BI=TRUE} | 0.13  6139 | 0.243  363 | 0.559  406 | 1.31  0914 | 55 |
| {BcALV1.BI=TRUE, BcEV6=FALSE} =>  {BcHV1.BI=TRUE} | 0.12  3762 | 0.724  638 | 0.170  792 | 1.19  981 | 50 |
| {BcALV1.BI=TRUE, BcOLV17.BI=FALSE} =>  {BcHV1.BI=TRUE} | 0.13  3663 | 0.75 | 0.178  218 | 1.24  1803 | 54 |
| {BcHV1.BI=TRUE, BcOLV17.BI=FALSE} =>  {BcALV1.BI=TRUE} | 0.13  3663 | 0.236  842 | 0.564  356 | 1.27  5789 | 54 |
| {BcALV1.BI=TRUE, BcOLV15.BI=FALSE} =>  {BcHV1.BI=TRUE} | 0.13  6139 | 0.743  243 | 0.183  168 | 1.23  0616 | 55 |

| {BcHV1.BI=TRUE, BcOLV15.BI=FALSE} =>  {BcALV1.BI=TRUE} | 0.13  6139 | 0.241  228 | 0.564  356 | 1.29  9415 | 55 |
| --- | --- | --- | --- | --- | --- |
| {BcALV1.BI=TRUE, BcEV5=FALSE} =>  {BcHV1.BI=TRUE} | 0.12  1287 | 0.742  424 | 0.163  366 | 1.22  926 | 49 |
| {BcALV1.BI=TRUE, BcMV9.BI=TRUE} =>  {BcMV11=TRUE} | 0.11  6337 | 0.854  545 | 0.136  139 | 1.38  6491 | 47 |
| {BcMV11=TRUE, BcMV9.BI=TRUE} =>  {BcALV1.BI=TRUE} | 0.11  6337 | 0.286  585 | 0.405  941 | 1.54  374 | 47 |
| {BcALV1.BI=TRUE, BcMV11=TRUE} =>  {BcULV4=TRUE} | 0.13  3663 | 0.857  143 | 0.155  941 | 1.24  1167 | 54 |
| {BcALV1.BI=TRUE, BcULV4=TRUE} =>  {BcMV11=TRUE} | 0.13  3663 | 0.830  769 | 0.160  891 | 1.34  7915 | 54 |
| {BcMV11=TRUE, BcULV4=TRUE} =>  {BcALV1.BI=TRUE} | 0.13  3663 | 0.319  527 | 0.418  317 | 1.72  1183 | 54 |
| {BcALV1.BI=TRUE, BcMV6.BI=FALSE} =>  {BcMV11=TRUE} | 0.07  6733 | 0.775 | 0.099  01 | 1.25  743 | 31 |
| {BcALV1.BI=TRUE, BcPV3.BI=FALSE} =>  {BcMV11=TRUE} | 0.10  6436 | 0.843  137 | 0.126  238 | 1.36  7982 | 43 |
| {BcMV11=TRUE, BcPV3.BI=FALSE} =>  {BcALV1.BI=TRUE} | 0.10  6436 | 0.254  438 | 0.418  317 | 1.37  0572 | 43 |
| {BcALV1.BI=TRUE, BcRV1.BI=FALSE} =>  {BcMV11=TRUE} | 0.07  6733 | 0.775 | 0.099  01 | 1.25  743 | 31 |

| {BcALV1.BI=TRUE, BcEV2.BI=FALSE} =>  {BcMV11=TRUE} | 0.10  6436 | 0.811  321 | 0.131  188 | 1.31  636 | 43 |
| --- | --- | --- | --- | --- | --- |
| {BcEV2.BI=FALSE, BcMV11=TRUE} =>  {BcALV1.BI=TRUE} | 0.10  6436 | 0.247  126 | 0.430  693 | 1.33  1188 | 43 |
| {BcALV1.BI=TRUE, BcHV3.BI=FALSE} =>  {BcMV11=TRUE} | 0.10  6436 | 0.843  137 | 0.126  238 | 1.36  7982 | 43 |
| {BcHV3.BI=FALSE, BcMV11=TRUE} =>  {BcALV1.BI=TRUE} | 0.10  6436 | 0.240  223 | 0.443  069 | 1.29  4004 | 43 |
| {BcALV1.BI=TRUE, BcFV5.BI=FALSE} =>  {BcMV11=TRUE} | 0.09  4059 | 0.808  511 | 0.116  337 | 1.31  18 | 38 |
| {BcALV1.BI=TRUE, BcULV1.BI=FALSE} =>  {BcMV11=TRUE} | 0.11  1386 | 0.818  182 | 0.136  139 | 1.32  7492 | 45 |
| {BcALV1.BI=TRUE, BcMV11=TRUE} =>  {BcMV1.BI=TRUE} | 0.13  8614 | 0.888  889 | 0.155  941 | 1.12  574 | 56 |
| {BcALV1.BI=TRUE, BcMV1.BI=TRUE} =>  {BcMV11=TRUE} | 0.13  8614 | 0.875 | 0.158  416 | 1.41  9679 | 56 |
| {BcMV1.BI=TRUE, BcMV11=TRUE} =>  {BcALV1.BI=TRUE} | 0.13  8614 | 0.266  667 | 0.519  802 | 1.43  6444 | 56 |
| {BcALV1.BI=TRUE, BcMV12=FALSE} =>  {BcMV11=TRUE} | 0.12  1287 | 0.830  508 | 0.146  04 | 1.34  7492 | 49 |
| {BcMV11=TRUE, BcMV12=FALSE} =>  {BcALV1.BI=TRUE} | 0.12  1287 | 0.239  024 | 0.507  426 | 1.28  7545 | 49 |

| {BcALV1.BI=TRUE, BcNSRV7.BI=FALSE}  => {BcMV11=TRUE} | 0.11  3861 | 0.821  429 | 0.138  614 | 1.33  276 | 46 |
| --- | --- | --- | --- | --- | --- |
| {BcALV1.BI=TRUE, BcVV3.BI=FALSE} =>  {BcMV11=TRUE} | 0.12  1287 | 0.844  828 | 0.143  564 | 1.37  0724 | 49 |
| {BcMV11=TRUE, BcVV3.BI=FALSE} =>  {BcALV1.BI=TRUE} | 0.12  1287 | 0.251  282 | 0.482  673 | 1.35  3573 | 49 |
| {BcALV1.BI=TRUE, BcOLV7.BI=FALSE} =>  {BcMV11=TRUE} | 0.10  8911 | 0.830  189 | 0.131  188 | 1.34  6973 | 44 |
| {BcALV1.BI=TRUE, BcDMV1=FALSE} =>  {BcMV11=TRUE} | 0.11  8812 | 0.827  586 | 0.143  564 | 1.34  275 | 48 |
| {BcALV1.BI=TRUE, BcFV9=FALSE} =>  {BcMV11=TRUE} | 0.12  6238 | 0.85 | 0.148  515 | 1.37  9116 | 51 |
| {BcFV9=FALSE, BcMV11=TRUE} =>  {BcALV1.BI=TRUE} | 0.12  6238 | 0.241  706 | 0.522  277 | 1.30  1991 | 51 |
| {BcALV1.BI=TRUE, BcOLV4.BI=FALSE} =>  {BcMV11=TRUE} | 0.12  8713 | 0.825  397 | 0.155  941 | 1.33  9198 | 52 |
| {BcMV11=TRUE, BcOLV4.BI=FALSE} =>  {BcALV1.BI=TRUE} | 0.12  8713 | 0.251  208 | 0.512  376 | 1.35  3172 | 52 |
| {BcALV1.BI=TRUE, BcULV5=FALSE} =>  {BcMV11=TRUE} | 0.14  1089 | 0.826  087 | 0.170  792 | 1.34  0318 | 57 |
| {BcMV11=TRUE, BcULV5=FALSE} =>  {BcALV1.BI=TRUE} | 0.14  1089 | 0.279  412 | 0.504  95 | 1.50  5098 | 57 |

| {BcALV1.BI=TRUE, BcBNV5.BI=FALSE} =>  {BcMV11=TRUE} | 0.11  6337 | 0.810  345 | 0.143  564 | 1.31  4776 | 47 |
| --- | --- | --- | --- | --- | --- |
| {BcALV1.BI=TRUE, BvF.BI=FALSE} =>  {BcMV11=TRUE} | 0.14  1089 | 0.850  746 | 0.165  842 | 1.38  0327 | 57 |
| {BcMV11=TRUE, BvF.BI=FALSE} =>  {BcALV1.BI=TRUE} | 0.14  1089 | 0.246  753 | 0.571  782 | 1.32  9177 | 57 |
| {BcALV1.BI=TRUE, BcMCV5.BI=FALSE} =>  {BcMV11=TRUE} | 0.12  1287 | 0.803  279 | 0.150  99 | 1.30  3312 | 49 |
| {BcALV1.BI=TRUE, BcOLV13.BI=FALSE} =>  {BcMV11=TRUE} | 0.14  604 | 0.830  986 | 0.175  743 | 1.34  8266 | 59 |
| {BcMV11=TRUE, BcOLV13.BI=FALSE} =>  {BcALV1.BI=TRUE} | 0.14  604 | 0.261  062 | 0.559  406 | 1.40  6254 | 59 |
| {BcALV1.BI=TRUE, BcOLV2.BI=FALSE} =>  {BcMV11=TRUE} | 0.14  3564 | 0.828  571 | 0.173  267 | 1.34  4349 | 58 |
| {BcMV11=TRUE, BcOLV2.BI=FALSE} =>  {BcALV1.BI=TRUE} | 0.14  3564 | 0.252  174 | 0.569  307 | 1.35  8377 | 58 |
| {BcALV1.BI=TRUE, BcMCV3.BI=FALSE} =>  {BcMV11=TRUE} | 0.14  8515 | 0.857  143 | 0.173  267 | 1.39  0706 | 60 |
| {BcMCV3.BI=FALSE, BcMV11=TRUE} =>  {BcALV1.BI=TRUE} | 0.14  8515 | 0.255  319 | 0.581  683 | 1.37  5319 | 60 |
| {BcALV1.BI=TRUE, BcPV4.BI=FALSE} =>  {BcMV11=TRUE} | 0.15  3465 | 0.849  315 | 0.180  693 | 1.37  8005 | 62 |

| {BcMV11=TRUE, BcPV4.BI=FALSE} =>  {BcALV1.BI=TRUE} | 0.15  3465 | 0.266  094 | 0.576  733 | 1.43  3362 | 62 |
| --- | --- | --- | --- | --- | --- |
| {BcALV1.BI=TRUE, BcEV6=FALSE} =>  {BcMV11=TRUE} | 0.14  1089 | 0.826  087 | 0.170  792 | 1.34  0318 | 57 |
| {BcEV6=FALSE, BcMV11=TRUE} =>  {BcALV1.BI=TRUE} | 0.14  1089 | 0.244  635 | 0.576  733 | 1.31  7768 | 57 |
| {BcALV1.BI=TRUE, BcOLV17.BI=FALSE} =>  {BcMV11=TRUE} | 0.14  8515 | 0.833  333 | 0.178  218 | 1.35  2075 | 60 |
| {BcMV11=TRUE, BcOLV17.BI=FALSE} =>  {BcALV1.BI=TRUE} | 0.14  8515 | 0.258  621 | 0.574  257 | 1.39  3103 | 60 |
| {BcALV1.BI=TRUE, BcOLV15.BI=FALSE} =>  {BcMV11=TRUE} | 0.15  3465 | 0.837  838 | 0.183  168 | 1.35  9383 | 62 |
| {BcMV11=TRUE, BcOLV15.BI=FALSE} =>  {BcALV1.BI=TRUE} | 0.15  3465 | 0.260  504 | 0.589  109 | 1.40  3249 | 62 |
| {BcALV1.BI=TRUE, BcEV5=FALSE} =>  {BcMV11=TRUE} | 0.13  3663 | 0.818  182 | 0.163  366 | 1.32  7492 | 54 |
| {BcEV5=FALSE, BcMV11=TRUE} =>  {BcALV1.BI=TRUE} | 0.13  3663 | 0.232  759 | 0.574  257 | 1.25  3793 | 54 |
| {BcALV1.BI=TRUE, BcMV9.BI=TRUE} =>  {BcULV4=TRUE} | 0.12  8713 | 0.945  455 | 0.136  139 | 1.36  9045 | 52 |
| {BcALV1.BI=TRUE, BcULV4=TRUE} =>  {BcMV9.BI=TRUE} | 0.12  8713 | 0.8 | 0.160  891 | 1.17  9562 | 52 |

| {BcMV9.BI=TRUE, BcULV4=TRUE} =>  {BcALV1.BI=TRUE} | 0.12  8713 | 0.235  294 | 0.547  03 | 1.26  7451 | 52 |
| --- | --- | --- | --- | --- | --- |
| {BcALV1.BI=TRUE, BcPV3.BI=FALSE} =>  {BcULV4=TRUE} | 0.10  8911 | 0.862  745 | 0.126  238 | 1.24  928 | 44 |
| {BcALV1.BI=TRUE, BcRV1.BI=FALSE} =>  {BcULV4=TRUE} | 0.07  9208 | 0.8 | 0.099  01 | 1.15  8423 | 32 |
| {BcALV1.BI=TRUE, BcEV2.BI=FALSE} =>  {BcULV4=TRUE} | 0.11  8812 | 0.905  66 | 0.131  188 | 1.31  1422 | 48 |
| {BcALV1.BI=TRUE, BcHV3.BI=FALSE} =>  {BcULV4=TRUE} | 0.11  3861 | 0.901  961 | 0.126  238 | 1.30  6065 | 46 |
| {BcALV1.BI=TRUE, BcFV5.BI=FALSE} =>  {BcULV4=TRUE} | 0.09  6535 | 0.829  787 | 0.116  337 | 1.20  1556 | 39 |
| {BcALV1.BI=TRUE, BcULV1.BI=FALSE} =>  {BcULV4=TRUE} | 0.11  3861 | 0.836  364 | 0.136  139 | 1.21  1079 | 46 |
| {BcALV1.BI=TRUE, BcMV1.BI=TRUE} =>  {BcULV4=TRUE} | 0.13  6139 | 0.859  375 | 0.158  416 | 1.24  44 | 55 |
| {BcMV1.BI=TRUE, BcULV4=TRUE} =>  {BcALV1.BI=TRUE} | 0.13  6139 | 0.247  748 | 0.549  505 | 1.33  4535 | 55 |
| {BcALV1.BI=TRUE, BcMV12=FALSE} =>  {BcULV4=TRUE} | 0.12  8713 | 0.881  356 | 0.146  04 | 1.27  6229 | 52 |
| {BcMV12=FALSE, BcULV4=TRUE} =>  {BcALV1.BI=TRUE} | 0.12  8713 | 0.234  234 | 0.549  505 | 1.26  1742 | 52 |

| {BcALV1.BI=TRUE, BcNSRV7.BI=FALSE}  => {BcULV4=TRUE} | 0.11  3861 | 0.821  429 | 0.138  614 | 1.18  9452 | 46 |
| --- | --- | --- | --- | --- | --- |
| {BcALV1.BI=TRUE, BcVV3.BI=FALSE} =>  {BcULV4=TRUE} | 0.12  3762 | 0.862  069 | 0.143  564 | 1.24  8301 | 50 |
| {BcALV1.BI=TRUE, BcOLV7.BI=FALSE} =>  {BcULV4=TRUE} | 0.10  6436 | 0.811  321 | 0.131  188 | 1.17  4816 | 43 |
| {BcALV1.BI=TRUE, BcDMV1=FALSE} =>  {BcULV4=TRUE} | 0.12  1287 | 0.844  828 | 0.143  564 | 1.22  3335 | 49 |
| {BcALV1.BI=TRUE, BcFV9=FALSE} =>  {BcULV4=TRUE} | 0.12  3762 | 0.833  333 | 0.148  515 | 1.20  6691 | 50 |
| {BcALV1.BI=TRUE, BcOLV4.BI=FALSE} =>  {BcULV4=TRUE} | 0.14  3564 | 0.920  635 | 0.155  941 | 1.33  3106 | 58 |
| {BcOLV4.BI=FALSE, BcULV4=TRUE} =>  {BcALV1.BI=TRUE} | 0.14  3564 | 0.231  076 | 0.621  287 | 1.24  4728 | 58 |
| {BcALV1.BI=TRUE, BcULV5=FALSE} =>  {BcULV4=TRUE} | 0.14  8515 | 0.869  565 | 0.170  792 | 1.25  9155 | 60 |
| {BcULV4=TRUE, BcULV5=FALSE} =>  {BcALV1.BI=TRUE} | 0.14  8515 | 0.235  294 | 0.631  188 | 1.26  7451 | 60 |
| {BcALV1.BI=TRUE, BcBNV5.BI=FALSE} =>  {BcULV4=TRUE} | 0.12  1287 | 0.844  828 | 0.143  564 | 1.22  3335 | 49 |
| {BcALV1.BI=TRUE, BvF.BI=FALSE} =>  {BcULV4=TRUE} | 0.14  8515 | 0.895  522 | 0.165  842 | 1.29  6742 | 60 |

| {BcULV4=TRUE, BvF.BI=FALSE} =>  {BcALV1.BI=TRUE} | 0.14  8515 | 0.229  885 | 0.646  04 | 1.23  8314 | 60 |
| --- | --- | --- | --- | --- | --- |
| {BcALV1.BI=TRUE, BcMCV5.BI=FALSE} =>  {BcULV4=TRUE} | 0.13  1188 | 0.868  852 | 0.150  99 | 1.25  8123 | 53 |
| {BcALV1.BI=TRUE, BcOLV13.BI=FALSE} =>  {BcULV4=TRUE} | 0.15  3465 | 0.873  239 | 0.175  743 | 1.26  4476 | 62 |
| {BcOLV13.BI=FALSE, BcULV4=TRUE} =>  {BcALV1.BI=TRUE} | 0.15  3465 | 0.239  382 | 0.641  089 | 1.28  9472 | 62 |
| {BcALV1.BI=TRUE, BcOLV2.BI=FALSE} =>  {BcULV4=TRUE} | 0.14  8515 | 0.857  143 | 0.173  267 | 1.24  1167 | 60 |
| {BcOLV2.BI=FALSE, BcULV4=TRUE} =>  {BcALV1.BI=TRUE} | 0.14  8515 | 0.231  66 | 0.641  089 | 1.24  7876 | 60 |
| {BcALV1.BI=TRUE, BcMCV3.BI=FALSE} =>  {BcULV4=TRUE} | 0.14  8515 | 0.857  143 | 0.173  267 | 1.24  1167 | 60 |
| {BcMCV3.BI=FALSE, BcULV4=TRUE} =>  {BcALV1.BI=TRUE} | 0.14  8515 | 0.231  66 | 0.641  089 | 1.24  7876 | 60 |
| {BcALV1.BI=TRUE, BcPV4.BI=FALSE} =>  {BcULV4=TRUE} | 0.15  5941 | 0.863  014 | 0.180  693 | 1.24  9669 | 63 |
| {BcPV4.BI=FALSE, BcULV4=TRUE} =>  {BcALV1.BI=TRUE} | 0.15  5941 | 0.241  379 | 0.646  04 | 1.30  023 | 63 |
| {BcALV1.BI=TRUE, BcEV6=FALSE} =>  {BcULV4=TRUE} | 0.15  099 | 0.884  058 | 0.170  792 | 1.28  0141 | 61 |

| {BcEV6=FALSE, BcULV4=TRUE} =>  {BcALV1.BI=TRUE} | 0.15  099 | 0.233  716 | 0.646  04 | 1.25  8953 | 61 |
| --- | --- | --- | --- | --- | --- |
| {BcALV1.BI=TRUE, BcOLV17.BI=FALSE} =>  {BcULV4=TRUE} | 0.15  3465 | 0.861  111 | 0.178  218 | 1.24  6914 | 62 |
| {BcOLV17.BI=FALSE, BcULV4=TRUE} =>  {BcALV1.BI=TRUE} | 0.15  3465 | 0.237  548 | 0.646  04 | 1.27  9591 | 62 |
| {BcALV1.BI=TRUE, BcOLV15.BI=FALSE} =>  {BcULV4=TRUE} | 0.15  8416 | 0.864  865 | 0.183  168 | 1.25  2349 | 64 |
| {BcOLV15.BI=FALSE, BcULV4=TRUE} =>  {BcALV1.BI=TRUE} | 0.15  8416 | 0.240  602 | 0.658  416 | 1.29  604 | 64 |
| {BcALV1.BI=TRUE, BcEV5=FALSE} =>  {BcULV4=TRUE} | 0.13  8614 | 0.848  485 | 0.163  366 | 1.22  863 | 56 |
| {BcALV1.BI=TRUE, BcMV6.BI=FALSE} =>  {BcDMV1=FALSE} | 0.09  1584 | 0.925 | 0.099  01 | 1.09  5894 | 37 |
| {BcALV1.BI=TRUE, BcRV1.BI=FALSE} =>  {BcPV3.BI=FALSE} | 0.08  4158 | 0.85 | 0.099  01 | 1.19  6516 | 34 |
| {BcALV1.BI=TRUE, BcHV3.BI=FALSE} =>  {BcDMV1=FALSE} | 0.11  6337 | 0.921  569 | 0.126  238 | 1.09  1829 | 47 |
| {BcALV1.BI=TRUE, BcOLV13.BI=FALSE} =>  {BcMV1.BI=TRUE} | 0.15  3465 | 0.873  239 | 0.175  743 | 1.10  5921 | 62 |
| {BcALV1.BI=TRUE, BcBNV5.BI=FALSE} =>  {BcDMV1=FALSE} | 0.13  3663 | 0.931  034 | 0.143  564 | 1.10  3044 | 54 |

| {BcOLV7.BI=TRUE, BcRV1.BI=TRUE} =>  {BcOLV1.BI.MOVA.=TRUE} | 0.07  4257 | 0.681  818 | 0.108  911 | 2.05  5631 | 30 |
| --- | --- | --- | --- | --- | --- |
| {BcOLV1.BI.MOVA.=TRUE,  BcOLV7.BI=TRUE} => {BcRV1.BI=TRUE} | 0.07  4257 | 0.789  474 | 0.094  059 | 2.84  7744 | 30 |
| {BcOLV1.BI.MOVA.=TRUE,  BcRV1.BI=TRUE} => {BcOLV7.BI=TRUE} | 0.07  4257 | 0.555  556 | 0.133  663 | 2.87  7493 | 30 |
| {BcOLV7.BI=TRUE, BcRV1.BI=TRUE} =>  {BcOLV16.BI=TRUE} | 0.08  1683 | 0.75 | 0.108  911 | 1.80  3571 | 33 |
| {BcOLV16.BI=TRUE, BcOLV7.BI=TRUE} =>  {BcRV1.BI=TRUE} | 0.08  1683 | 0.673  469 | 0.121  287 | 2.42  93 | 33 |
| {BcOLV16.BI=TRUE, BcRV1.BI=TRUE} =>  {BcOLV7.BI=TRUE} | 0.08  1683 | 0.540  984 | 0.150  99 | 2.80  2018 | 33 |
| {BcOLV7.BI=TRUE, BcRV1.BI=TRUE} =>  {BcMV4.BI=TRUE} | 0.07  4257 | 0.681  818 | 0.108  911 | 1.53  8852 | 30 |
| {BcMV4.BI=TRUE, BcOLV7.BI=TRUE} =>  {BcRV1.BI=TRUE} | 0.07  4257 | 0.697  674 | 0.106  436 | 2.51  6611 | 30 |
| {BcMV4.BI=TRUE, BcRV1.BI=TRUE} =>  {BcOLV7.BI=TRUE} | 0.07  4257 | 0.447  761 | 0.165  842 | 2.31  9173 | 30 |
| {BcOLV7.BI=TRUE, BcRV1.BI=TRUE} =>  {BcMV2.BI=TRUE} | 0.07  4257 | 0.681  818 | 0.108  911 | 1.47  3019 | 30 |
| {BcMV2.BI=TRUE, BcOLV7.BI=TRUE} =>  {BcRV1.BI=TRUE} | 0.07  4257 | 0.714  286 | 0.103  96 | 2.57  6531 | 30 |

| {BcMV2.BI=TRUE, BcRV1.BI=TRUE} =>  {BcOLV7.BI=TRUE} | 0.07  4257 | 0.416  667 | 0.178  218 | 2.15  812 | 30 |
| --- | --- | --- | --- | --- | --- |
| {BcOLV7.BI=TRUE, BcRV1.BI=TRUE} =>  {BcOLV18=TRUE} | 0.08  9109 | 0.818  182 | 0.108  911 | 1.58  1557 | 36 |
| {BcOLV18=TRUE, BcOLV7.BI=TRUE} =>  {BcRV1.BI=TRUE} | 0.08  9109 | 0.631  579 | 0.141  089 | 2.27  8195 | 36 |
| {BcOLV18=TRUE, BcRV1.BI=TRUE} =>  {BcOLV7.BI=TRUE} | 0.08  9109 | 0.473  684 | 0.188  119 | 2.45  3441 | 36 |
| {BcOLV7.BI=TRUE, BcRV1.BI=TRUE} =>  {BcMV3.BI=TRUE} | 0.07  6733 | 0.704  545 | 0.108  911 | 1.29  9709 | 31 |
| {BcMV3.BI=TRUE, BcOLV7.BI=TRUE} =>  {BcRV1.BI=TRUE} | 0.07  6733 | 0.607  843 | 0.126  238 | 2.19  2577 | 31 |
| {BcMV3.BI=TRUE, BcRV1.BI=TRUE} =>  {BcOLV7.BI=TRUE} | 0.07  6733 | 0.436  62 | 0.175  743 | 2.26  1466 | 31 |
| {BcOLV7.BI=TRUE, BcRV1.BI=TRUE} =>  {BcHV1.BI=TRUE} | 0.08  4158 | 0.772  727 | 0.108  911 | 1.27  9434 | 34 |
| {BcHV1.BI=TRUE, BcOLV7.BI=TRUE} =>  {BcRV1.BI=TRUE} | 0.08  4158 | 0.666  667 | 0.126  238 | 2.40  4762 | 34 |
| {BcHV1.BI=TRUE, BcRV1.BI=TRUE} =>  {BcOLV7.BI=TRUE} | 0.08  4158 | 0.447  368 | 0.188  119 | 2.31  7139 | 34 |
| {BcOLV7.BI=TRUE, BcRV1.BI=TRUE} =>  {BcMV11=TRUE} | 0.07  9208 | 0.727  273 | 0.108  911 | 1.17  9993 | 32 |

| {BcMV11=TRUE, BcOLV7.BI=TRUE} =>  {BcRV1.BI=TRUE} | 0.07  9208 | 0.603  774 | 0.131  188 | 2.17  7898 | 32 |
| --- | --- | --- | --- | --- | --- |
| {BcMV11=TRUE, BcRV1.BI=TRUE} =>  {BcOLV7.BI=TRUE} | 0.07  9208 | 0.380  952 | 0.207  921 | 1.97  3138 | 32 |
| {BcOLV7.BI=TRUE, BcRV1.BI=TRUE} =>  {BcMV9.BI=TRUE} | 0.10  396 | 0.954  545 | 0.108  911 | 1.40  7432 | 42 |
| {BcMV9.BI=TRUE, BcOLV7.BI=TRUE} =>  {BcRV1.BI=TRUE} | 0.10  396 | 0.608  696 | 0.170  792 | 2.19  5652 | 42 |
| {BcMV9.BI=TRUE, BcRV1.BI=TRUE} =>  {BcOLV7.BI=TRUE} | 0.10  396 | 0.466  667 | 0.222  772 | 2.41  7094 | 42 |
| {BcOLV7.BI=TRUE, BcRV1.BI=TRUE} =>  {BcULV4=TRUE} | 0.10  8911 | 1 | 0.108  911 | 1.44  8029 | 44 |
| {BcOLV7.BI=TRUE, BcULV4=TRUE} =>  {BcRV1.BI=TRUE} | 0.10  8911 | 0.611  111 | 0.178  218 | 2.20  4365 | 44 |
| {BcRV1.BI=TRUE, BcULV4=TRUE} =>  {BcOLV7.BI=TRUE} | 0.10  8911 | 0.448  98 | 0.242  574 | 2.32  5484 | 44 |
| {BcEV2.BI=FALSE, BcOLV7.BI=TRUE} =>  {BcRV1.BI=TRUE} | 0.08  9109 | 0.571  429 | 0.155  941 | 2.06  1224 | 36 |
| {BcEV2.BI=FALSE, BcRV1.BI=TRUE} =>  {BcOLV7.BI=TRUE} | 0.08  9109 | 0.418  605 | 0.212  871 | 2.16  8157 | 36 |
| {BcFV5.BI=FALSE, BcOLV7.BI=TRUE} =>  {BcRV1.BI=TRUE} | 0.09  1584 | 0.578  125 | 0.158  416 | 2.08  5379 | 37 |

| {BcFV5.BI=FALSE, BcRV1.BI=TRUE} =>  {BcOLV7.BI=TRUE} | 0.09  1584 | 0.385  417 | 0.237  624 | 1.99  6261 | 37 |
| --- | --- | --- | --- | --- | --- |
| {BcMV1.BI=TRUE, BcOLV7.BI=TRUE} =>  {BcRV1.BI=TRUE} | 0.09  1584 | 0.544  118 | 0.168  317 | 1.96  271 | 37 |
| {BcMV1.BI=TRUE, BcRV1.BI=TRUE} =>  {BcOLV7.BI=TRUE} | 0.09  1584 | 0.451  22 | 0.202  97 | 2.33  7086 | 37 |
| {BcMV12=FALSE, BcOLV7.BI=TRUE} =>  {BcRV1.BI=TRUE} | 0.08  1683 | 0.568  966 | 0.143  564 | 2.05  234 | 33 |
| {BcMV12=FALSE, BcRV1.BI=TRUE} =>  {BcOLV7.BI=TRUE} | 0.08  1683 | 0.370  787 | 0.220  297 | 1.92  0484 | 33 |
| {BcNSRV7.BI=FALSE, BcOLV7.BI=TRUE}  => {BcRV1.BI=TRUE} | 0.09  1584 | 0.569  231 | 0.160  891 | 2.05  3297 | 37 |
| {BcNSRV7.BI=FALSE, BcRV1.BI=TRUE} =>  {BcOLV7.BI=TRUE} | 0.09  1584 | 0.381  443 | 0.240  099 | 1.97  5681 | 37 |
| {BcOLV7.BI=TRUE, BcVV3.BI=FALSE} =>  {BcRV1.BI=TRUE} | 0.08  9109 | 0.6 | 0.148  515 | 2.16  4286 | 36 |
| {BcRV1.BI=TRUE, BcVV3.BI=FALSE} =>  {BcOLV7.BI=TRUE} | 0.08  9109 | 0.382  979 | 0.232  673 | 1.98  3633 | 36 |
| {BcOLV7.BI=TRUE, BcRV1.BI=TRUE} =>  {BcDMV1=FALSE} | 0.10  396 | 0.954  545 | 0.108  911 | 1.13  0898 | 42 |
| {BcDMV1=FALSE, BcOLV7.BI=TRUE} =>  {BcRV1.BI=TRUE} | 0.10  396 | 0.583  333 | 0.178  218 | 2.10  4167 | 42 |

| {BcDMV1=FALSE, BcRV1.BI=TRUE} =>  {BcOLV7.BI=TRUE} | 0.10  396 | 0.403  846 | 0.257  426 | 2.09  1716 | 42 |
| --- | --- | --- | --- | --- | --- |
| {BcFV9=FALSE, BcOLV7.BI=TRUE} =>  {BcRV1.BI=TRUE} | 0.09  6535 | 0.590  909 | 0.163  366 | 2.13  1494 | 39 |
| {BcFV9=FALSE, BcRV1.BI=TRUE} =>  {BcOLV7.BI=TRUE} | 0.09  6535 | 0.414  894 | 0.232  673 | 2.14  8936 | 39 |
| {BcOLV4.BI=FALSE, BcOLV7.BI=TRUE} =>  {BcRV1.BI=TRUE} | 0.09  6535 | 0.590  909 | 0.163  366 | 2.13  1494 | 39 |
| {BcOLV4.BI=FALSE, BcRV1.BI=TRUE} =>  {BcOLV7.BI=TRUE} | 0.09  6535 | 0.393  939 | 0.245  05 | 2.04  0404 | 39 |
| {BcOLV7.BI=TRUE, BcULV5=FALSE} =>  {BcRV1.BI=TRUE} | 0.09  6535 | 0.549  296 | 0.175  743 | 1.98  1388 | 39 |
| {BcRV1.BI=TRUE, BcULV5=FALSE} =>  {BcOLV7.BI=TRUE} | 0.09  6535 | 0.39 | 0.247  525 | 2.02 | 39 |
| {BcBNV5.BI=FALSE, BcOLV7.BI=TRUE} =>  {BcRV1.BI=TRUE} | 0.10  1485 | 0.569  444 | 0.178  218 | 2.05  4067 | 41 |
| {BcBNV5.BI=FALSE, BcRV1.BI=TRUE} =>  {BcOLV7.BI=TRUE} | 0.10  1485 | 0.390  476 | 0.259  901 | 2.02  2466 | 41 |
| {BcOLV7.BI=TRUE, BcRV1.BI=TRUE} =>  {BvF.BI=FALSE} | 0.10  8911 | 1 | 0.108  911 | 1.09  4851 | 44 |
| {BcOLV7.BI=TRUE, BvF.BI=FALSE} =>  {BcRV1.BI=TRUE} | 0.10  8911 | 0.586  667 | 0.185  644 | 2.11  619 | 44 |

| {BcRV1.BI=TRUE, BvF.BI=FALSE} =>  {BcOLV7.BI=TRUE} | 0.10  8911 | 0.407  407 | 0.267  327 | 2.11  0161 | 44 |
| --- | --- | --- | --- | --- | --- |
| {BcMCV5.BI=FALSE, BcOLV7.BI=TRUE} =>  {BcRV1.BI=TRUE} | 0.09  4059 | 0.542  857 | 0.173  267 | 1.95  8163 | 38 |
| {BcMCV5.BI=FALSE, BcRV1.BI=TRUE} =>  {BcOLV7.BI=TRUE} | 0.09  4059 | 0.376  238 | 0.25 | 1.94  8718 | 38 |
| {BcOLV13.BI=FALSE, BcOLV7.BI=TRUE}  => {BcRV1.BI=TRUE} | 0.08  9109 | 0.514  286 | 0.173  267 | 1.85  5102 | 36 |
| {BcOLV13.BI=FALSE, BcRV1.BI=TRUE} =>  {BcOLV7.BI=TRUE} | 0.08  9109 | 0.378  947 | 0.235  149 | 1.96  2753 | 36 |
| {BcOLV2.BI=FALSE, BcOLV7.BI=TRUE} =>  {BcRV1.BI=TRUE} | 0.09  6535 | 0.549  296 | 0.175  743 | 1.98  1388 | 39 |
| {BcOLV2.BI=FALSE, BcRV1.BI=TRUE} =>  {BcOLV7.BI=TRUE} | 0.09  6535 | 0.393  939 | 0.245  05 | 2.04  0404 | 39 |
| {BcMCV3.BI=FALSE, BcOLV7.BI=TRUE} =>  {BcRV1.BI=TRUE} | 0.09  4059 | 0.542  857 | 0.173  267 | 1.95  8163 | 38 |
| {BcMCV3.BI=FALSE, BcRV1.BI=TRUE} =>  {BcOLV7.BI=TRUE} | 0.09  4059 | 0.372  549 | 0.252  475 | 1.92  9613 | 38 |
| {BcOLV7.BI=TRUE, BcPV4.BI=FALSE} =>  {BcRV1.BI=TRUE} | 0.10  396 | 0.591  549 | 0.175  743 | 2.13  3803 | 42 |
| {BcPV4.BI=FALSE, BcRV1.BI=TRUE} =>  {BcOLV7.BI=TRUE} | 0.10  396 | 0.396  226 | 0.262  376 | 2.05  225 | 42 |

| {BcEV6=FALSE, BcOLV7.BI=TRUE} =>  {BcRV1.BI=TRUE} | 0.10  396 | 0.567  568 | 0.183  168 | 2.04  7297 | 42 |
| --- | --- | --- | --- | --- | --- |
| {BcEV6=FALSE, BcRV1.BI=TRUE} =>  {BcOLV7.BI=TRUE} | 0.10  396 | 0.388  889 | 0.267  327 | 2.01  4245 | 42 |
| {BcOLV17.BI=FALSE, BcOLV7.BI=TRUE}  => {BcRV1.BI=TRUE} | 0.09  1584 | 0.536  232 | 0.170  792 | 1.93  4265 | 37 |
| {BcOLV17.BI=FALSE, BcRV1.BI=TRUE} =>  {BcOLV7.BI=TRUE} | 0.09  1584 | 0.381  443 | 0.240  099 | 1.97  5681 | 37 |
| {BcOLV15.BI=FALSE, BcOLV7.BI=TRUE}  => {BcRV1.BI=TRUE} | 0.10  8911 | 0.578  947 | 0.188  119 | 2.08  8346 | 44 |
| {BcOLV15.BI=FALSE, BcRV1.BI=TRUE} =>  {BcOLV7.BI=TRUE} | 0.10  8911 | 0.403  67 | 0.269  802 | 2.09  0802 | 44 |
| {BcEV5=FALSE, BcOLV7.BI=TRUE} =>  {BcRV1.BI=TRUE} | 0.10  8911 | 0.571  429 | 0.190  594 | 2.06  1224 | 44 |
| {BcEV5=FALSE, BcRV1.BI=TRUE} =>  {BcOLV7.BI=TRUE} | 0.10  8911 | 0.392  857 | 0.277  228 | 2.03  4799 | 44 |
| {BcOLV7.BI=TRUE, BcPV3.BI=TRUE} =>  {BcMV9.BI=TRUE} | 0.07  6733 | 0.939  394 | 0.081  683 | 1.38  5092 | 31 |
| {BcMV9.BI=TRUE, BcOLV7.BI=TRUE} =>  {BcPV3.BI=TRUE} | 0.07  6733 | 0.449  275 | 0.170  792 | 1.55  1344 | 31 |
| {BcMV9.BI=TRUE, BcPV3.BI=TRUE} =>  {BcOLV7.BI=TRUE} | 0.07  6733 | 0.373  494 | 0.205  446 | 1.93  4507 | 31 |

| {BcOLV7.BI=TRUE, BcPV3.BI=TRUE} =>  {BcULV4=TRUE} | 0.07  6733 | 0.939  394 | 0.081  683 | 1.36  0269 | 31 |
| --- | --- | --- | --- | --- | --- |
| {BcOLV7.BI=TRUE, BcULV4=TRUE} =>  {BcPV3.BI=TRUE} | 0.07  6733 | 0.430  556 | 0.178  218 | 1.48  6705 | 31 |
| {BcPV3.BI=TRUE, BcULV4=TRUE} =>  {BcOLV7.BI=TRUE} | 0.07  6733 | 0.373  494 | 0.205  446 | 1.93  4507 | 31 |
| {BcOLV7.BI=TRUE, BcPV3.BI=TRUE} =>  {BcMV1.BI=TRUE} | 0.07  4257 | 0.909  091 | 0.081  683 | 1.15  1325 | 30 |
| {BcMV1.BI=TRUE, BcOLV7.BI=TRUE} =>  {BcPV3.BI=TRUE} | 0.07  4257 | 0.441  176 | 0.168  317 | 1.52  3379 | 30 |
| {BcMV1.BI=TRUE, BcPV3.BI=TRUE} =>  {BcOLV7.BI=TRUE} | 0.07  4257 | 0.291  262 | 0.254  95 | 1.50  8588 | 30 |
| {BcOLV7.BI=TRUE, BcPV3.BI=TRUE} =>  {BcDMV1=FALSE} | 0.07  9208 | 0.969  697 | 0.081  683 | 1.14  8849 | 32 |
| {BcDMV1=FALSE, BcOLV7.BI=TRUE} =>  {BcPV3.BI=TRUE} | 0.07  9208 | 0.444  444 | 0.178  218 | 1.53  4663 | 32 |
| {BcDMV1=FALSE, BcPV3.BI=TRUE} =>  {BcOLV7.BI=TRUE} | 0.07  9208 | 0.32 | 0.247  525 | 1.65  7436 | 32 |
| {BcBNV5.BI=FALSE, BcOLV7.BI=TRUE} =>  {BcPV3.BI=TRUE} | 0.07  6733 | 0.430  556 | 0.178  218 | 1.48  6705 | 31 |
| {BcBNV5.BI=FALSE, BcPV3.BI=TRUE} =>  {BcOLV7.BI=TRUE} | 0.07  6733 | 0.289  72 | 0.264  851 | 1.50  0599 | 31 |

| {BcOLV7.BI=TRUE, BvF.BI=FALSE} =>  {BcPV3.BI=TRUE} | 0.07  9208 | 0.426  667 | 0.185  644 | 1.47  3276 | 32 |
| --- | --- | --- | --- | --- | --- |
| {BcPV3.BI=TRUE, BvF.BI=FALSE} =>  {BcOLV7.BI=TRUE} | 0.07  9208 | 0.296  296 | 0.267  327 | 1.53  4663 | 32 |
| {BcOLV7.BI=TRUE, BcPV4.BI=FALSE} =>  {BcPV3.BI=TRUE} | 0.07  9208 | 0.450  704 | 0.175  743 | 1.55  6278 | 32 |
| {BcPV3.BI=TRUE, BcPV4.BI=FALSE} =>  {BcOLV7.BI=TRUE} | 0.07  9208 | 0.290  909 | 0.272  277 | 1.50  676 | 32 |
| {BcEV6=FALSE, BcOLV7.BI=TRUE} =>  {BcPV3.BI=TRUE} | 0.07  9208 | 0.432  432 | 0.183  168 | 1.49  3185 | 32 |
| {BcEV6=FALSE, BcPV3.BI=TRUE} =>  {BcOLV7.BI=TRUE} | 0.07  9208 | 0.288  288 | 0.274  752 | 1.49  3185 | 32 |
| {BcOLV15.BI=FALSE, BcOLV7.BI=TRUE}  => {BcPV3.BI=TRUE} | 0.07  9208 | 0.421  053 | 0.188  119 | 1.45  3891 | 32 |
| {BcOLV15.BI=FALSE, BcPV3.BI=TRUE} =>  {BcOLV7.BI=TRUE} | 0.07  9208 | 0.278  261 | 0.284  653 | 1.44  1249 | 32 |
| {BcEV5=FALSE, BcOLV7.BI=TRUE} =>  {BcPV3.BI=TRUE} | 0.08  1683 | 0.428  571 | 0.190  594 | 1.47  9853 | 33 |
| {BcEV5=FALSE, BcPV3.BI=TRUE} =>  {BcOLV7.BI=TRUE} | 0.08  1683 | 0.294  643 | 0.277  228 | 1.52  6099 | 33 |
| {BcOLV1.BI.MOVA.=TRUE,  BcOLV7.BI=TRUE} => {BcOLV18=TRUE} | 0.08  4158 | 0.894  737 | 0.094  059 | 1.72  9539 | 34 |

| {BcOLV18=TRUE, BcOLV7.BI=TRUE} =>  {BcOLV1.BI.MOVA.=TRUE} | 0.08  4158 | 0.596  491 | 0.141  089 | 1.79  8377 | 34 |
| --- | --- | --- | --- | --- | --- |
| {BcOLV1.BI.MOVA.=TRUE,  BcOLV18=TRUE} => {BcOLV7.BI=TRUE} | 0.08  4158 | 0.365  591 | 0.230  198 | 1.89  3576 | 34 |
| {BcOLV1.BI.MOVA.=TRUE,  BcOLV7.BI=TRUE} => {BcMV3.BI=TRUE} | 0.07  4257 | 0.789  474 | 0.094  059 | 1.45  6381 | 30 |
| {BcMV3.BI=TRUE, BcOLV7.BI=TRUE} =>  {BcOLV1.BI.MOVA.=TRUE} | 0.07  4257 | 0.588  235 | 0.126  238 | 1.77  3486 | 30 |
| {BcMV3.BI=TRUE, BcOLV1.BI.MOVA.=TRUE} =>  {BcOLV7.BI=TRUE} | 0.07  4257 | 0.303  03 | 0.245  05 | 1.56  9542 | 30 |
| {BcOLV1.BI.MOVA.=TRUE,  BcOLV7.BI=TRUE} => {BcMV9.BI=TRUE} | 0.08  6634 | 0.921  053 | 0.094  059 | 1.35  8048 | 35 |
| {BcMV9.BI=TRUE, BcOLV7.BI=TRUE} =>  {BcOLV1.BI.MOVA.=TRUE} | 0.08  6634 | 0.507  246 | 0.170  792 | 1.52  931 | 35 |
| {BcMV9.BI=TRUE, BcOLV1.BI.MOVA.=TRUE} =>  {BcOLV7.BI=TRUE} | 0.08  6634 | 0.406  977 | 0.212  871 | 2.10  7931 | 35 |
| {BcOLV1.BI.MOVA.=TRUE,  BcOLV7.BI=TRUE} => {BcULV4=TRUE} | 0.08  6634 | 0.921  053 | 0.094  059 | 1.33  3711 | 35 |
| {BcOLV7.BI=TRUE, BcULV4=TRUE} =>  {BcOLV1.BI.MOVA.=TRUE} | 0.08  6634 | 0.486  111 | 0.178  218 | 1.46  5589 | 35 |

| {BcOLV1.BI.MOVA.=TRUE, BcULV4=TRUE}  => {BcOLV7.BI=TRUE} | 0.08  6634 | 0.353  535 | 0.245  05 | 1.83  1132 | 35 |
| --- | --- | --- | --- | --- | --- |
| {BcFV5.BI=FALSE, BcOLV7.BI=TRUE} =>  {BcOLV1.BI.MOVA.=TRUE} | 0.07  4257 | 0.468  75 | 0.158  416 | 1.41  3246 | 30 |
| {BcFV5.BI=FALSE, BcOLV1.BI.MOVA.=TRUE} =>  {BcOLV7.BI=TRUE} | 0.07  4257 | 0.309  278 | 0.240  099 | 1.60  1903 | 30 |
| {BcOLV1.BI.MOVA.=TRUE,  BcOLV7.BI=TRUE} => {BcMV1.BI=TRUE} | 0.08  6634 | 0.921  053 | 0.094  059 | 1.16  6474 | 35 |
| {BcMV1.BI=TRUE, BcOLV7.BI=TRUE} =>  {BcOLV1.BI.MOVA.=TRUE} | 0.08  6634 | 0.514  706 | 0.168  317 | 1.55  18 | 35 |
| {BcMV1.BI=TRUE, BcOLV1.BI.MOVA.=TRUE} =>  {BcOLV7.BI=TRUE} | 0.08  6634 | 0.294  118 | 0.294  554 | 1.52  3379 | 35 |
| {BcNSRV7.BI=FALSE, BcOLV7.BI=TRUE}  => {BcOLV1.BI.MOVA.=TRUE} | 0.08  1683 | 0.507  692 | 0.160  891 | 1.53  0654 | 33 |
| {BcNSRV7.BI=FALSE, BcOLV1.BI.MOVA.=TRUE} =>  {BcOLV7.BI=TRUE} | 0.08  1683 | 0.308  411 | 0.264  851 | 1.59  7412 | 33 |
| {BcOLV1.BI.MOVA.=TRUE,  BcOLV7.BI=TRUE} => {BcDMV1=FALSE} | 0.09  1584 | 0.973  684 | 0.094  059 | 1.15  3573 | 37 |
| {BcDMV1=FALSE, BcOLV7.BI=TRUE} => | 0.09 | 0.513 | 0.178 | 1.54 | 37 |

| {BcOLV1.BI.MOVA.=TRUE} | 1584 | 889 | 218 | 9337 |  |
| --- | --- | --- | --- | --- | --- |
| {BcDMV1=FALSE, |  |  |  |  |  |
|  | 0.09 | 0.333 | 0.274 | 1.72 |  |
| BcOLV1.BI.MOVA.=TRUE} => |  |  |  |  | 37 |
|  | 1584 | 333 | 752 | 6496 |  |
| {BcOLV7.BI=TRUE} |  |  |  |  |  |
| {BcFV9=FALSE, BcOLV7.BI=TRUE} => | 0.07 | 0.469 | 0.163 | 1.41 |  |
|  |  |  |  |  | 31 |
| {BcOLV1.BI.MOVA.=TRUE} | 6733 | 697 | 366 | 6101 |  |
| {BcFV9=FALSE, BcOLV1.BI.MOVA.=TRUE} | 0.07 | 0.274 | 0.279 | 1.42 |  |
|  |  |  |  |  | 31 |
| => {BcOLV7.BI=TRUE} | 6733 | 336 | 703 | 0921 |  |
| {BcOLV4.BI=FALSE, BcOLV7.BI=TRUE} => | 0.07 | 0.454 | 0.163 | 1.37 |  |
|  |  |  |  |  | 30 |
| {BcOLV1.BI.MOVA.=TRUE} | 4257 | 545 | 366 | 0421 |  |
| {BcOLV1.BI.MOVA.=TRUE, | 0.07 | 0.283 | 0.262 | 1.46 |  |
|  |  |  |  |  | 30 |
| BcOLV4.BI=FALSE} => {BcOLV7.BI=TRUE} | 4257 | 019 | 376 | 5893 |  |
| {BcOLV7.BI=TRUE, BcULV5=FALSE} => | 0.08 | 0.492 | 0.175 | 1.48 |  |
|  |  |  |  |  | 35 |
| {BcOLV1.BI.MOVA.=TRUE} | 6634 | 958 | 743 | 6231 |  |
| {BcOLV1.BI.MOVA.=TRUE, | 0.08 | 0.336 | 0.257 | 1.74 |  |
|  |  |  |  |  | 35 |
| BcULV5=FALSE} => {BcOLV7.BI=TRUE} | 6634 | 538 | 426 | 3097 |  |
| {BcBNV5.BI=FALSE, BcOLV7.BI=TRUE} => | 0.08 | 0.486 | 0.178 | 1.46 |  |
|  |  |  |  |  | 35 |
| {BcOLV1.BI.MOVA.=TRUE} | 6634 | 111 | 218 | 5589 |  |
| {BcBNV5.BI=FALSE, |  |  |  |  |  |
|  | 0.08 | 0.307 | 0.282 | 1.59 |  |
| BcOLV1.BI.MOVA.=TRUE} => |  |  |  |  | 35 |
|  | 6634 | 018 | 178 | 0193 |  |
| {BcOLV7.BI=TRUE} |  |  |  |  |  |
| {BcOLV7.BI=TRUE, BvF.BI=FALSE} => | 0.09 | 0.493 | 0.185 | 1.48 | 37 |

| {BcOLV1.BI.MOVA.=TRUE} | 1584 | 333 | 644 | 7363 |  |
| --- | --- | --- | --- | --- | --- |
| {BcOLV1.BI.MOVA.=TRUE, BvF.BI=FALSE} | 0.09 | 0.291 | 0.314 | 1.50 |  |
|  |  |  |  |  | 37 |
| => {BcOLV7.BI=TRUE} | 1584 | 339 | 356 | 8984 |  |
| {BcMCV5.BI=FALSE, BcOLV7.BI=TRUE} => | 0.08 | 0.471 | 0.173 | 1.42 |  |
|  |  |  |  |  | 33 |
| {BcOLV1.BI.MOVA.=TRUE} | 1683 | 429 | 267 | 1322 |  |
| {BcMCV5.BI=FALSE, |  |  |  |  |  |
|  | 0.08 | 0.294 | 0.277 | 1.52 |  |
| BcOLV1.BI.MOVA.=TRUE} => |  |  |  |  | 33 |
|  | 1683 | 643 | 228 | 6099 |  |
| {BcOLV7.BI=TRUE} |  |  |  |  |  |
| {BcOLV13.BI=FALSE, BcOLV7.BI=TRUE} | 0.07 | 0.457 | 0.173 | 1.37 |  |
|  |  |  |  |  | 32 |
| => {BcOLV1.BI.MOVA.=TRUE} | 9208 | 143 | 267 | 8252 |  |
| {BcOLV1.BI.MOVA.=TRUE, | 0.07 | 0.285 | 0.277 | 1.47 |  |
|  |  |  |  |  | 32 |
| BcOLV13.BI=FALSE} => {BcOLV7.BI=TRUE} | 9208 | 714 | 228 | 9853 |  |
| {BcOLV2.BI=FALSE, BcOLV7.BI=TRUE} => | 0.07 | 0.450 | 0.175 | 1.35 |  |
|  |  |  |  |  | 32 |
| {BcOLV1.BI.MOVA.=TRUE} | 9208 | 704 | 743 | 884 |  |
| {BcOLV1.BI.MOVA.=TRUE, | 0.07 | 0.285 | 0.277 | 1.47 |  |
|  |  |  |  |  | 32 |
| BcOLV2.BI=FALSE} => {BcOLV7.BI=TRUE} | 9208 | 714 | 228 | 9853 |  |
| {BcMCV3.BI=FALSE, BcOLV7.BI=TRUE} => | 0.07 | 0.442 | 0.173 | 1.33 |  |
|  |  |  |  |  | 31 |
| {BcOLV1.BI.MOVA.=TRUE} | 6733 | 857 | 267 | 5181 |  |
| {BcMCV3.BI=FALSE, |  |  |  |  |  |
|  | 0.07 | 0.267 | 0.287 | 1.38 |  |
| BcOLV1.BI.MOVA.=TRUE} => |  |  |  |  | 31 |
|  | 6733 | 241 | 129 | 4173 |  |
| {BcOLV7.BI=TRUE} |  |  |  |  |  |
| {BcOLV7.BI=TRUE, BcPV4.BI=FALSE} => | 0.09 | 0.521 | 0.175 | 1.57 | 37 |

| {BcOLV1.BI.MOVA.=TRUE} | 1584 | 127 | 743 | 1158 |  |
| --- | --- | --- | --- | --- | --- |
| {BcOLV1.BI.MOVA.=TRUE,  BcPV4.BI=FALSE} => {BcOLV7.BI=TRUE} | 0.09  1584 | 0.289  063 | 0.316  832 | 1.49  7196 | 37 |
| {BcEV6=FALSE, BcOLV7.BI=TRUE} =>  {BcOLV1.BI.MOVA.=TRUE} | 0.08  9109 | 0.486  486 | 0.183  168 | 1.46  672 | 36 |
| {BcEV6=FALSE, BcOLV1.BI.MOVA.=TRUE}  => {BcOLV7.BI=TRUE} | 0.08  9109 | 0.290  323 | 0.306  931 | 1.50  3722 | 36 |
| {BcOLV17.BI=FALSE, BcOLV7.BI=TRUE}  => {BcOLV1.BI.MOVA.=TRUE} | 0.07  9208 | 0.463  768 | 0.170  792 | 1.39  8226 | 32 |
| {BcOLV1.BI.MOVA.=TRUE,  BcOLV17.BI=FALSE} => {BcOLV7.BI=TRUE} | 0.07  9208 | 0.264  463 | 0.299  505 | 1.36  9782 | 32 |
| {BcOLV15.BI=FALSE, BcOLV7.BI=TRUE}  => {BcOLV1.BI.MOVA.=TRUE} | 0.09  4059 | 0.5 | 0.188  119 | 1.50  7463 | 38 |
| {BcOLV1.BI.MOVA.=TRUE,  BcOLV15.BI=FALSE} => {BcOLV7.BI=TRUE} | 0.09  4059 | 0.292  308 | 0.321  782 | 1.51  4004 | 38 |
| {BcEV5=FALSE, BcOLV7.BI=TRUE} =>  {BcOLV1.BI.MOVA.=TRUE} | 0.09  1584 | 0.480  519 | 0.190  594 | 1.44  873 | 37 |
| {BcEV5=FALSE, BcOLV1.BI.MOVA.=TRUE}  => {BcOLV7.BI=TRUE} | 0.09  1584 | 0.308  333 | 0.297  03 | 1.59  7009 | 37 |
| {BcOLV16.BI=TRUE, BcOLV7.BI=TRUE} =>  {BcMV2.BI=TRUE} | 0.08  1683 | 0.673  469 | 0.121  287 | 1.45  4982 | 33 |
| {BcMV2.BI=TRUE, BcOLV7.BI=TRUE} => | 0.08 | 0.785 | 0.103 | 1.88 | 33 |

| {BcOLV16.BI=TRUE} | 1683 | 714 | 96 | 9456 |  |
| --- | --- | --- | --- | --- | --- |
| {BcMV2.BI=TRUE, BcOLV16.BI=TRUE} =>  {BcOLV7.BI=TRUE} | 0.08  1683 | 0.323  529 | 0.252  475 | 1.67  5716 | 33 |
| {BcOLV16.BI=TRUE, BcOLV7.BI=TRUE} =>  {BcOLV18=TRUE} | 0.10  396 | 0.857  143 | 0.121  287 | 1.65  6869 | 42 |
| {BcOLV18=TRUE, BcOLV7.BI=TRUE} =>  {BcOLV16.BI=TRUE} | 0.10  396 | 0.736  842 | 0.141  089 | 1.77  193 | 42 |
| {BcOLV16.BI=TRUE, BcOLV18=TRUE} =>  {BcOLV7.BI=TRUE} | 0.10  396 | 0.385  321 | 0.269  802 | 1.99  5766 | 42 |
| {BcOLV16.BI=TRUE, BcOLV7.BI=TRUE} =>  {BcHV1SLR.BI=TRUE} | 0.08  1683 | 0.673  469 | 0.121  287 | 1.24  2382 | 33 |
| {BcHV1SLR.BI=TRUE, BcOLV7.BI=TRUE}  => {BcOLV16.BI=TRUE} | 0.08  1683 | 0.767  442 | 0.106  436 | 1.84  5515 | 33 |
| {BcHV1SLR.BI=TRUE, BcOLV16.BI=TRUE}  => {BcOLV7.BI=TRUE} | 0.08  1683 | 0.343  75 | 0.237  624 | 1.78  0449 | 33 |
| {BcOLV16.BI=TRUE, BcOLV7.BI=TRUE} =>  {BcMV3.BI=TRUE} | 0.08  6634 | 0.714  286 | 0.121  287 | 1.31  7678 | 35 |
| {BcMV3.BI=TRUE, BcOLV7.BI=TRUE} =>  {BcOLV16.BI=TRUE} | 0.08  6634 | 0.686  275 | 0.126  238 | 1.65  0327 | 35 |
| {BcMV3.BI=TRUE, BcOLV16.BI=TRUE} =>  {BcOLV7.BI=TRUE} | 0.08  6634 | 0.324  074 | 0.267  327 | 1.67  8538 | 35 |
| {BcOLV16.BI=TRUE, BcOLV7.BI=TRUE} => | 0.08 | 0.714 | 0.121 | 1.20 | 35 |

| {BcULV3=TRUE} | 6634 | 286 | 287 | 2381 |  |
| --- | --- | --- | --- | --- | --- |
| {BcOLV7.BI=TRUE, BcULV3=TRUE} =>  {BcOLV16.BI=TRUE} | 0.08  6634 | 0.729  167 | 0.118  812 | 1.75  3472 | 35 |
| {BcOLV16.BI=TRUE, BcULV3=TRUE} =>  {BcOLV7.BI=TRUE} | 0.08  6634 | 0.309  735 | 0.279  703 | 1.60  4266 | 35 |
| {BcOLV16.BI=TRUE, BcOLV7.BI=TRUE} =>  {BcHV1.BI=TRUE} | 0.08  9109 | 0.734  694 | 0.121  287 | 1.21  646 | 36 |
| {BcHV1.BI=TRUE, BcOLV7.BI=TRUE} =>  {BcOLV16.BI=TRUE} | 0.08  9109 | 0.705  882 | 0.126  238 | 1.69  7479 | 36 |
| {BcHV1.BI=TRUE, BcOLV16.BI=TRUE} =>  {BcOLV7.BI=TRUE} | 0.08  9109 | 0.330  275 | 0.269  802 | 1.71  0656 | 36 |
| {BcOLV16.BI=TRUE, BcOLV7.BI=TRUE} =>  {BcMV11=TRUE} | 0.09  901 | 0.816  327 | 0.121  287 | 1.32  4482 | 40 |
| {BcMV11=TRUE, BcOLV7.BI=TRUE} =>  {BcOLV16.BI=TRUE} | 0.09  901 | 0.754  717 | 0.131  188 | 1.81  4915 | 40 |
| {BcMV11=TRUE, BcOLV16.BI=TRUE} =>  {BcOLV7.BI=TRUE} | 0.09  901 | 0.360  36 | 0.274  752 | 1.86  6482 | 40 |
| {BcOLV16.BI=TRUE, BcOLV7.BI=TRUE} =>  {BcMV9.BI=TRUE} | 0.11  6337 | 0.959  184 | 0.121  287 | 1.41  4271 | 47 |
| {BcMV9.BI=TRUE, BcOLV7.BI=TRUE} =>  {BcOLV16.BI=TRUE} | 0.11  6337 | 0.681  159 | 0.170  792 | 1.63  8026 | 47 |
| {BcMV9.BI=TRUE, BcOLV16.BI=TRUE} => | 0.11 | 0.350 | 0.331 | 1.81 | 47 |

| {BcOLV7.BI=TRUE} | 6337 | 746 | 683 | 6686 |  |
| --- | --- | --- | --- | --- | --- |
| {BcOLV16.BI=TRUE, BcOLV7.BI=TRUE} =>  {BcULV4=TRUE} | 0.11  6337 | 0.959  184 | 0.121  287 | 1.38  8925 | 47 |
| {BcOLV7.BI=TRUE, BcULV4=TRUE} =>  {BcOLV16.BI=TRUE} | 0.11  6337 | 0.652  778 | 0.178  218 | 1.56  9775 | 47 |
| {BcOLV16.BI=TRUE, BcULV4=TRUE} =>  {BcOLV7.BI=TRUE} | 0.11  6337 | 0.343  066 | 0.339  109 | 1.77  6904 | 47 |
| {BcEV2.BI=FALSE, BcOLV7.BI=TRUE} =>  {BcOLV16.BI=TRUE} | 0.08  9109 | 0.571  429 | 0.155  941 | 1.37  415 | 36 |
| {BcEV2.BI=FALSE, BcOLV16.BI=TRUE} =>  {BcOLV7.BI=TRUE} | 0.08  9109 | 0.272  727 | 0.326  733 | 1.41  2587 | 36 |
| {BcHV3.BI=FALSE, BcOLV7.BI=TRUE} =>  {BcOLV16.BI=TRUE} | 0.08  6634 | 0.673  077 | 0.128  713 | 1.61  859 | 35 |
| {BcHV3.BI=FALSE, BcOLV16.BI=TRUE} =>  {BcOLV7.BI=TRUE} | 0.08  6634 | 0.269  231 | 0.321  782 | 1.39  4477 | 35 |
| {BcFV5.BI=FALSE, BcOLV7.BI=TRUE} =>  {BcOLV16.BI=TRUE} | 0.09  6535 | 0.609  375 | 0.158  416 | 1.46  5402 | 39 |
| {BcFV5.BI=FALSE, BcOLV16.BI=TRUE} =>  {BcOLV7.BI=TRUE} | 0.09  6535 | 0.3 | 0.321  782 | 1.55  3846 | 39 |
| {BcMV1.BI=TRUE, BcOLV7.BI=TRUE} =>  {BcOLV16.BI=TRUE} | 0.10  6436 | 0.632  353 | 0.168  317 | 1.52  0658 | 43 |
| {BcMV1.BI=TRUE, BcOLV16.BI=TRUE} => | 0.10 | 0.316 | 0.336 | 1.63 | 43 |

| {BcOLV7.BI=TRUE} | 6436 | 176 | 634 | 7632 |  |
| --- | --- | --- | --- | --- | --- |
| {BcMV12=FALSE, BcOLV7.BI=TRUE} =>  {BcOLV16.BI=TRUE} | 0.08  6634 | 0.603  448 | 0.143  564 | 1.45  1149 | 35 |
| {BcMV12=FALSE, BcOLV16.BI=TRUE} =>  {BcOLV7.BI=TRUE} | 0.08  6634 | 0.294  118 | 0.294  554 | 1.52  3379 | 35 |
| {BcNSRV7.BI=FALSE, BcOLV7.BI=TRUE}  => {BcOLV16.BI=TRUE} | 0.10  1485 | 0.630  769 | 0.160  891 | 1.51  685 | 41 |
| {BcNSRV7.BI=FALSE, BcOLV16.BI=TRUE}  => {BcOLV7.BI=TRUE} | 0.10  1485 | 0.303  704 | 0.334  158 | 1.57  3029 | 41 |
| {BcOLV7.BI=TRUE, BcVV3.BI=FALSE} =>  {BcOLV16.BI=TRUE} | 0.10  396 | 0.7 | 0.148  515 | 1.68  3333 | 42 |
| {BcOLV16.BI=TRUE, BcVV3.BI=FALSE} =>  {BcOLV7.BI=TRUE} | 0.10  396 | 0.308  824 | 0.336  634 | 1.59  9548 | 42 |
| {BcALV1.BI=FALSE, BcOLV7.BI=TRUE} =>  {BcOLV16.BI=TRUE} | 0.08  1683 | 0.589  286 | 0.138  614 | 1.41  7092 | 33 |
| {BcALV1.BI=FALSE, BcOLV16.BI=TRUE} =>  {BcOLV7.BI=TRUE} | 0.08  1683 | 0.264 | 0.309  406 | 1.36  7385 | 33 |
| {BcOLV16.BI=TRUE, BcOLV7.BI=TRUE} =>  {BcDMV1=FALSE} | 0.11  6337 | 0.959  184 | 0.121  287 | 1.13  6394 | 47 |
| {BcDMV1=FALSE, BcOLV7.BI=TRUE} =>  {BcOLV16.BI=TRUE} | 0.11  6337 | 0.652  778 | 0.178  218 | 1.56  9775 | 47 |
| {BcDMV1=FALSE, BcOLV16.BI=TRUE} => | 0.11 | 0.328 | 0.353 | 1.70 | 47 |

| {BcOLV7.BI=TRUE} | 6337 | 671 | 96 | 2349 |  |
| --- | --- | --- | --- | --- | --- |
| {BcFV9=FALSE, BcOLV7.BI=TRUE} =>  {BcOLV16.BI=TRUE} | 0.09  6535 | 0.590  909 | 0.163  366 | 1.42  0996 | 39 |
| {BcFV9=FALSE, BcOLV16.BI=TRUE} =>  {BcOLV7.BI=TRUE} | 0.09  6535 | 0.268  966 | 0.358  911 | 1.39  3103 | 39 |
| {BcOLV4.BI=FALSE, BcOLV7.BI=TRUE} =>  {BcOLV16.BI=TRUE} | 0.09  6535 | 0.590  909 | 0.163  366 | 1.42  0996 | 39 |
| {BcOLV16.BI=TRUE, BcOLV4.BI=FALSE}  => {BcOLV7.BI=TRUE} | 0.09  6535 | 0.280  576 | 0.344  059 | 1.45  3237 | 39 |
| {BcOLV7.BI=TRUE, BcULV5=FALSE} =>  {BcOLV16.BI=TRUE} | 0.10  396 | 0.591  549 | 0.175  743 | 1.42  2535 | 42 |
| {BcOLV16.BI=TRUE, BcULV5=FALSE} =>  {BcOLV7.BI=TRUE} | 0.10  396 | 0.295  775 | 0.351  485 | 1.53  1961 | 42 |
| {BcBNV5.BI=FALSE, BcOLV7.BI=TRUE} =>  {BcOLV16.BI=TRUE} | 0.11  1386 | 0.625 | 0.178  218 | 1.50  2976 | 45 |
| {BcBNV5.BI=FALSE, BcOLV16.BI=TRUE}  => {BcOLV7.BI=TRUE} | 0.11  1386 | 0.298  013 | 0.373  762 | 1.54  3556 | 45 |
| {BcOLV7.BI=TRUE, BvF.BI=FALSE} =>  {BcOLV16.BI=TRUE} | 0.11  6337 | 0.626  667 | 0.185  644 | 1.50  6984 | 47 |
| {BcOLV16.BI=TRUE, BvF.BI=FALSE} =>  {BcOLV7.BI=TRUE} | 0.11  6337 | 0.297  468 | 0.391  089 | 1.54  0734 | 47 |
| {BcMCV5.BI=FALSE, BcOLV7.BI=TRUE} => | 0.10 | 0.614 | 0.173 | 1.47 | 43 |

| {BcOLV16.BI=TRUE} | 6436 | 286 | 267 | 7211 |  |
| --- | --- | --- | --- | --- | --- |
| {BcMCV5.BI=FALSE, BcOLV16.BI=TRUE}  => {BcOLV7.BI=TRUE} | 0.10  6436 | 0.290  541 | 0.366  337 | 1.50  4851 | 43 |
| {BcOLV13.BI=FALSE, BcOLV7.BI=TRUE}  => {BcOLV16.BI=TRUE} | 0.10  6436 | 0.614  286 | 0.173  267 | 1.47  7211 | 43 |
| {BcOLV13.BI=FALSE, BcOLV16.BI=TRUE}  => {BcOLV7.BI=TRUE} | 0.10  6436 | 0.290  541 | 0.366  337 | 1.50  4851 | 43 |
| {BcOLV2.BI=FALSE, BcOLV7.BI=TRUE} =>  {BcOLV16.BI=TRUE} | 0.10  8911 | 0.619  718 | 0.175  743 | 1.49  0275 | 44 |
| {BcOLV16.BI=TRUE, BcOLV2.BI=FALSE}  => {BcOLV7.BI=TRUE} | 0.10  8911 | 0.295  302 | 0.368  812 | 1.52  9513 | 44 |
| {BcMCV3.BI=FALSE, BcOLV7.BI=TRUE} =>  {BcOLV16.BI=TRUE} | 0.10  1485 | 0.585  714 | 0.173  267 | 1.40  8503 | 41 |
| {BcMCV3.BI=FALSE, BcOLV16.BI=TRUE}  => {BcOLV7.BI=TRUE} | 0.10  1485 | 0.282  759 | 0.358  911 | 1.46  4545 | 41 |
| {BcOLV7.BI=TRUE, BcPV4.BI=FALSE} =>  {BcOLV16.BI=TRUE} | 0.11  3861 | 0.647  887 | 0.175  743 | 1.55  8015 | 46 |
| {BcOLV16.BI=TRUE, BcPV4.BI=FALSE} =>  {BcOLV7.BI=TRUE} | 0.11  3861 | 0.300  654 | 0.378  713 | 1.55  7231 | 46 |
| {BcEV6=FALSE, BcOLV7.BI=TRUE} =>  {BcOLV16.BI=TRUE} | 0.11  3861 | 0.621  622 | 0.183  168 | 1.49  4852 | 46 |
| {BcEV6=FALSE, BcOLV16.BI=TRUE} => | 0.11 | 0.291 | 0.391 | 1.50 | 46 |

| {BcOLV7.BI=TRUE} | 3861 | 139 | 089 | 7952 |  |
| --- | --- | --- | --- | --- | --- |
| {BcOLV17.BI=FALSE, BcOLV7.BI=TRUE}  => {BcOLV16.BI=TRUE} | 0.10  1485 | 0.594  203 | 0.170  792 | 1.42  8916 | 41 |
| {BcOLV16.BI=TRUE, BcOLV17.BI=FALSE}  => {BcOLV7.BI=TRUE} | 0.10  1485 | 0.262  821 | 0.386  139 | 1.36  1275 | 41 |
| {BcOLV15.BI=FALSE, BcOLV7.BI=TRUE}  => {BcOLV16.BI=TRUE} | 0.11  6337 | 0.618  421 | 0.188  119 | 1.48  7155 | 47 |
| {BcOLV15.BI=FALSE, BcOLV16.BI=TRUE}  => {BcOLV7.BI=TRUE} | 0.11  6337 | 0.288  344 | 0.403  465 | 1.49  3472 | 47 |
| {BcEV5=FALSE, BcOLV7.BI=TRUE} =>  {BcOLV16.BI=TRUE} | 0.12  1287 | 0.636  364 | 0.190  594 | 1.53  0303 | 49 |
| {BcEV5=FALSE, BcOLV16.BI=TRUE} =>  {BcOLV7.BI=TRUE} | 0.12  1287 | 0.308  176 | 0.393  564 | 1.59  6194 | 49 |
| {BcMV4.BI=TRUE, BcOLV7.BI=TRUE} =>  {BcOLV18=TRUE} | 0.08  9109 | 0.837  209 | 0.106  436 | 1.61  8338 | 36 |
| {BcOLV18=TRUE, BcOLV7.BI=TRUE} =>  {BcMV4.BI=TRUE} | 0.08  9109 | 0.631  579 | 0.141  089 | 1.42  5463 | 36 |
| {BcMV4.BI=TRUE, BcOLV18=TRUE} =>  {BcOLV7.BI=TRUE} | 0.08  9109 | 0.321  429 | 0.277  228 | 1.66  4835 | 36 |
| {BcMV4.BI=TRUE, BcOLV7.BI=TRUE} =>  {BcMV3.BI=TRUE} | 0.07  9208 | 0.744  186 | 0.106  436 | 1.37  2836 | 32 |
| {BcMV3.BI=TRUE, BcOLV7.BI=TRUE} => | 0.07 | 0.627 | 0.126 | 1.41 | 32 |

| {BcMV4.BI=TRUE} | 9208 | 451 | 238 | 6146 |  |
| --- | --- | --- | --- | --- | --- |
| {BcMV3.BI=TRUE, BcMV4.BI=TRUE} =>  {BcOLV7.BI=TRUE} | 0.07  9208 | 0.268  908 | 0.294  554 | 1.39  2803 | 32 |
| {BcMV4.BI=TRUE, BcOLV7.BI=TRUE} =>  {BcHV1.BI=TRUE} | 0.08  4158 | 0.790  698 | 0.106  436 | 1.30  9188 | 34 |
| {BcHV1.BI=TRUE, BcOLV7.BI=TRUE} =>  {BcMV4.BI=TRUE} | 0.08  4158 | 0.666  667 | 0.126  238 | 1.50  4655 | 34 |
| {BcHV1.BI=TRUE, BcMV4.BI=TRUE} =>  {BcOLV7.BI=TRUE} | 0.08  4158 | 0.280  992 | 0.299  505 | 1.45  5393 | 34 |
| {BcMV4.BI=TRUE, BcOLV7.BI=TRUE} =>  {BcMV11=TRUE} | 0.08  1683 | 0.767  442 | 0.106  436 | 1.24  5167 | 33 |
| {BcMV11=TRUE, BcOLV7.BI=TRUE} =>  {BcMV4.BI=TRUE} | 0.08  1683 | 0.622  642 | 0.131  188 | 1.40  5291 | 33 |
| {BcMV4.BI=TRUE, BcOLV7.BI=TRUE} =>  {BcMV9.BI=TRUE} | 0.09  6535 | 0.906  977 | 0.106  436 | 1.33  7294 | 39 |
| {BcMV9.BI=TRUE, BcOLV7.BI=TRUE} =>  {BcMV4.BI=TRUE} | 0.09  6535 | 0.565  217 | 0.170  792 | 1.27  5686 | 39 |
| {BcMV4.BI=TRUE, BcMV9.BI=TRUE} =>  {BcOLV7.BI=TRUE} | 0.09  6535 | 0.286  765 | 0.336  634 | 1.48  5294 | 39 |
| {BcMV4.BI=TRUE, BcOLV7.BI=TRUE} =>  {BcULV4=TRUE} | 0.10  396 | 0.976  744 | 0.106  436 | 1.41  4354 | 42 |
| {BcOLV7.BI=TRUE, BcULV4=TRUE} => | 0.10 | 0.583 | 0.178 | 1.31 | 42 |

| {BcMV4.BI=TRUE} | 396 | 333 | 218 | 6574 |  |
| --- | --- | --- | --- | --- | --- |
| {BcMV4.BI=TRUE, BcULV4=TRUE} =>  {BcOLV7.BI=TRUE} | 0.10  396 | 0.320  611 | 0.324  257 | 1.66  0599 | 42 |
| {BcEV2.BI=FALSE, BcOLV7.BI=TRUE} =>  {BcMV4.BI=TRUE} | 0.08  6634 | 0.555  556 | 0.155  941 | 1.25  388 | 35 |
| {BcEV2.BI=FALSE, BcMV4.BI=TRUE} =>  {BcOLV7.BI=TRUE} | 0.08  6634 | 0.275  591 | 0.314  356 | 1.42  7418 | 35 |
| {BcMV12=FALSE, BcOLV7.BI=TRUE} =>  {BcMV4.BI=TRUE} | 0.08  6634 | 0.603  448 | 0.143  564 | 1.36  1973 | 35 |
| {BcMV4.BI=TRUE, BcNSRV7.BI=FALSE} =>  {BcOLV7.BI=TRUE} | 0.08  6634 | 0.257  353 | 0.336  634 | 1.33  2956 | 35 |
| {BcMV4.BI=TRUE, BcOLV7.BI=TRUE} =>  {BcDMV1=FALSE} | 0.10  1485 | 0.953  488 | 0.106  436 | 1.12  9646 | 41 |
| {BcDMV1=FALSE, BcOLV7.BI=TRUE} =>  {BcMV4.BI=TRUE} | 0.10  1485 | 0.569  444 | 0.178  218 | 1.28  5227 | 41 |
| {BcDMV1=FALSE, BcMV4.BI=TRUE} =>  {BcOLV7.BI=TRUE} | 0.10  1485 | 0.280  822 | 0.361  386 | 1.45  4514 | 41 |
| {BcBNV5.BI=FALSE, BcOLV7.BI=TRUE} =>  {BcMV4.BI=TRUE} | 0.10  1485 | 0.569  444 | 0.178  218 | 1.28  5227 | 41 |
| {BcBNV5.BI=FALSE, BcMV4.BI=TRUE} =>  {BcOLV7.BI=TRUE} | 0.10  1485 | 0.264  516 | 0.383  663 | 1.37  0058 | 41 |
| {BcOLV7.BI=TRUE, BvF.BI=FALSE} => | 0.10 | 0.56 | 0.185 | 1.26 | 42 |

| {BcMV4.BI=TRUE} | 396 |  | 644 | 3911 |  |
| --- | --- | --- | --- | --- | --- |
| {BcMV4.BI=TRUE, BvF.BI=FALSE} =>  {BcOLV7.BI=TRUE} | 0.10  396 | 0.256  098 | 0.405  941 | 1.32  6454 | 42 |
| {BcMCV3.BI=FALSE, BcOLV7.BI=TRUE} =>  {BcMV4.BI=TRUE} | 0.09  6535 | 0.557  143 | 0.173  267 | 1.25  7462 | 39 |
| {BcOLV7.BI=TRUE, BcPV4.BI=FALSE} =>  {BcMV4.BI=TRUE} | 0.10  1485 | 0.577  465 | 0.175  743 | 1.30  3328 | 41 |
| {BcEV6=FALSE, BcOLV7.BI=TRUE} =>  {BcMV4.BI=TRUE} | 0.10  1485 | 0.554  054 | 0.183  168 | 1.25  0491 | 41 |
| {BcEV5=FALSE, BcOLV7.BI=TRUE} =>  {BcMV4.BI=TRUE} | 0.10  6436 | 0.558  442 | 0.190  594 | 1.26  0393 | 43 |
| {BcEV5=FALSE, BcMV4.BI=TRUE} =>  {BcOLV7.BI=TRUE} | 0.10  6436 | 0.267  081 | 0.398  515 | 1.38  3341 | 43 |
| {BcHV1SLR.BI=FALSE, BcOLV7.BI=TRUE}  => {BcMV9.BI=TRUE} | 0.07  6733 | 0.885  714 | 0.086  634 | 1.30  5944 | 31 |
| {BcHV1SLR.BI=FALSE, BcMV9.BI=TRUE}  => {BcOLV7.BI=TRUE} | 0.07  6733 | 0.284  404 | 0.269  802 | 1.47  3065 | 31 |
| {BcHV1SLR.BI=FALSE, BcOLV7.BI=TRUE}  => {BcULV4=TRUE} | 0.07  6733 | 0.885  714 | 0.086  634 | 1.28  254 | 31 |
| {BcHV1SLR.BI=FALSE, BcULV4=TRUE} =>  {BcOLV7.BI=TRUE} | 0.07  6733 | 0.264  957 | 0.289  604 | 1.37  2343 | 31 |
| {BcHV1SLR.BI=FALSE, BcOLV7.BI=TRUE} | 0.07 | 0.857 | 0.086 | 1.14 | 30 |

| => {BcEV2.BI=FALSE} | 4257 | 143 | 634 | 6641 |  |
| --- | --- | --- | --- | --- | --- |
| {BcHV1SLR.BI=FALSE, BcOLV7.BI=TRUE}  => {BcFV5.BI=FALSE} | 0.07  6733 | 0.885  714 | 0.086  634 | 1.13  2369 | 31 |
| {BcHV1SLR.BI=FALSE, BcOLV7.BI=TRUE}  => {BcULV5=FALSE} | 0.08  6634 | 1 | 0.086  634 | 1.14  1243 | 35 |
| {BcMV2.BI=TRUE, BcOLV7.BI=TRUE} =>  {BcOLV18=TRUE} | 0.09  1584 | 0.880  952 | 0.103  96 | 1.70  2894 | 37 |
| {BcOLV18=TRUE, BcOLV7.BI=TRUE} =>  {BcMV2.BI=TRUE} | 0.09  1584 | 0.649  123 | 0.141  089 | 1.40  2383 | 37 |
| {BcMV2.BI=TRUE, BcOLV18=TRUE} =>  {BcOLV7.BI=TRUE} | 0.09  1584 | 0.310  924 | 0.294  554 | 1.61  0429 | 37 |
| {BcMV2.BI=TRUE, BcOLV7.BI=TRUE} =>  {BcMV3.BI=TRUE} | 0.08  9109 | 0.857  143 | 0.103  96 | 1.58  1213 | 36 |
| {BcMV3.BI=TRUE, BcOLV7.BI=TRUE} =>  {BcMV2.BI=TRUE} | 0.08  9109 | 0.705  882 | 0.126  238 | 1.52  5008 | 36 |
| {BcMV2.BI=TRUE, BcMV3.BI=TRUE} =>  {BcOLV7.BI=TRUE} | 0.08  9109 | 0.283  465 | 0.314  356 | 1.46  8201 | 36 |
| {BcHV1.BI=TRUE, BcOLV7.BI=TRUE} =>  {BcMV2.BI=TRUE} | 0.07  4257 | 0.588  235 | 0.126  238 | 1.27  084 | 30 |
| {BcMV2.BI=TRUE, BcOLV7.BI=TRUE} =>  {BcMV11=TRUE} | 0.08  1683 | 0.785  714 | 0.103  96 | 1.27  4814 | 33 |
| {BcMV11=TRUE, BcOLV7.BI=TRUE} => | 0.08 | 0.622 | 0.131 | 1.34 | 33 |

| {BcMV2.BI=TRUE} | 1683 | 642 | 188 | 5172 |  |
| --- | --- | --- | --- | --- | --- |
| {BcMV2.BI=TRUE, BcOLV7.BI=TRUE} =>  {BcMV9.BI=TRUE} | 0.09  6535 | 0.928  571 | 0.103  96 | 1.36  9135 | 39 |
| {BcMV2.BI=TRUE, BcMV9.BI=TRUE} =>  {BcOLV7.BI=TRUE} | 0.09  6535 | 0.3 | 0.321  782 | 1.55  3846 | 39 |
| {BcMV2.BI=TRUE, BcOLV7.BI=TRUE} =>  {BcULV4=TRUE} | 0.09  6535 | 0.928  571 | 0.103  96 | 1.34  4598 | 39 |
| {BcMV2.BI=TRUE, BcULV4=TRUE} =>  {BcOLV7.BI=TRUE} | 0.09  6535 | 0.282  609 | 0.341  584 | 1.46  3768 | 39 |
| {BcMV2.BI=TRUE, BcOLV7.BI=TRUE} =>  {BcMV1.BI=TRUE} | 0.09  6535 | 0.928  571 | 0.103  96 | 1.17  5996 | 39 |
| {BcOLV7.BI=TRUE, BcVV3.BI=FALSE} =>  {BcMV2.BI=TRUE} | 0.08  6634 | 0.583  333 | 0.148  515 | 1.26  025 | 35 |
| {BcMV2.BI=TRUE, BcOLV7.BI=TRUE} =>  {BcDMV1=FALSE} | 0.09  901 | 0.952  381 | 0.103  96 | 1.12  8334 | 40 |
| {BcOLV18=TRUE, BcOLV7.BI=TRUE} =>  {BcHV1SLR.BI=TRUE} | 0.10  396 | 0.736  842 | 0.141  089 | 1.35  9289 | 42 |
| {BcHV1SLR.BI=TRUE, BcOLV7.BI=TRUE}  => {BcOLV18=TRUE} | 0.10  396 | 0.976  744 | 0.106  436 | 1.88  8061 | 42 |
| {BcHV1SLR.BI=TRUE, BcOLV18=TRUE} =>  {BcOLV7.BI=TRUE} | 0.10  396 | 0.304  348 | 0.341  584 | 1.57  6366 | 42 |
| {BcOLV18=TRUE, BcOLV7.BI=TRUE} => | 0.10 | 0.736 | 0.141 | 1.35 | 42 |

| {BcMV3.BI=TRUE} | 396 | 842 | 089 | 9289 |  |
| --- | --- | --- | --- | --- | --- |
| {BcMV3.BI=TRUE, BcOLV7.BI=TRUE} =>  {BcOLV18=TRUE} | 0.10  396 | 0.823  529 | 0.126  238 | 1.59  1894 | 42 |
| {BcMV3.BI=TRUE, BcOLV18=TRUE} =>  {BcOLV7.BI=TRUE} | 0.10  396 | 0.315  789 | 0.329  208 | 1.63  5628 | 42 |
| {BcOLV7.BI=TRUE, BcULV3=TRUE} =>  {BcOLV18=TRUE} | 0.09  6535 | 0.812  5 | 0.118  812 | 1.57  0574 | 39 |
| {BcOLV18=TRUE, BcULV3=TRUE} =>  {BcOLV7.BI=TRUE} | 0.09  6535 | 0.297  71 | 0.324  257 | 1.54  1985 | 39 |
| {BcOLV18=TRUE, BcOLV7.BI=TRUE} =>  {BcHV1.BI=TRUE} | 0.10  396 | 0.736  842 | 0.141  089 | 1.22  0017 | 42 |
| {BcHV1.BI=TRUE, BcOLV7.BI=TRUE} =>  {BcOLV18=TRUE} | 0.10  396 | 0.823  529 | 0.126  238 | 1.59  1894 | 42 |
| {BcHV1.BI=TRUE, BcOLV18=TRUE} =>  {BcOLV7.BI=TRUE} | 0.10  396 | 0.283  784 | 0.366  337 | 1.46  9854 | 42 |
| {BcOLV18=TRUE, BcOLV7.BI=TRUE} =>  {BcMV11=TRUE} | 0.10  6436 | 0.754  386 | 0.141  089 | 1.22  3984 | 43 |
| {BcMV11=TRUE, BcOLV7.BI=TRUE} =>  {BcOLV18=TRUE} | 0.10  6436 | 0.811  321 | 0.131  188 | 1.56  8295 | 43 |
| {BcMV11=TRUE, BcOLV18=TRUE} =>  {BcOLV7.BI=TRUE} | 0.10  6436 | 0.307  143 | 0.346  535 | 1.59  0842 | 43 |
| {BcOLV18=TRUE, BcOLV7.BI=TRUE} => | 0.12 | 0.912 | 0.141 | 1.34 | 52 |

| {BcMV9.BI=TRUE} | 8713 | 281 | 089 | 5115 |  |
| --- | --- | --- | --- | --- | --- |
| {BcMV9.BI=TRUE, BcOLV7.BI=TRUE} =>  {BcOLV18=TRUE} | 0.12  8713 | 0.753  623 | 0.170  792 | 1.45  6764 | 52 |
| {BcMV9.BI=TRUE, BcOLV18=TRUE} =>  {BcOLV7.BI=TRUE} | 0.12  8713 | 0.311  377 | 0.413  366 | 1.61  2774 | 52 |
| {BcOLV18=TRUE, BcOLV7.BI=TRUE} =>  {BcULV4=TRUE} | 0.13  8614 | 0.982  456 | 0.141  089 | 1.42  2625 | 56 |
| {BcOLV7.BI=TRUE, BcULV4=TRUE} =>  {BcOLV18=TRUE} | 0.13  8614 | 0.777  778 | 0.178  218 | 1.50  3456 | 56 |
| {BcOLV18=TRUE, BcULV4=TRUE} =>  {BcOLV7.BI=TRUE} | 0.13  8614 | 0.314  607 | 0.440  594 | 1.62  9502 | 56 |
| {BcMV6.BI=FALSE, BcOLV7.BI=TRUE} =>  {BcOLV18=TRUE} | 0.08  4158 | 0.693  878 | 0.121  287 | 1.34  1275 | 34 |
| {BcOLV7.BI=TRUE, BcPV3.BI=FALSE} =>  {BcOLV18=TRUE} | 0.07  4257 | 0.666  667 | 0.111  386 | 1.28  8676 | 30 |
| {BcEV2.BI=FALSE, BcOLV7.BI=TRUE} =>  {BcOLV18=TRUE} | 0.10  8911 | 0.698  413 | 0.155  941 | 1.35  0042 | 44 |
| {BcEV2.BI=FALSE, BcOLV18=TRUE} =>  {BcOLV7.BI=TRUE} | 0.10  8911 | 0.295  302 | 0.368  812 | 1.52  9513 | 44 |
| {BcHV3.BI=FALSE, BcOLV7.BI=TRUE} =>  {BcOLV18=TRUE} | 0.08  9109 | 0.692  308 | 0.128  713 | 1.33  8241 | 36 |
| {BcHV3.BI=FALSE, BcOLV18=TRUE} => | 0.08 | 0.253 | 0.351 | 1.31 | 36 |

| {BcOLV7.BI=TRUE} | 9109 | 521 | 485 | 3109 |  |
| --- | --- | --- | --- | --- | --- |
| {BcFV5.BI=FALSE, BcOLV7.BI=TRUE} =>  {BcOLV18=TRUE} | 0.11  1386 | 0.703  125 | 0.158  416 | 1.35  9151 | 45 |
| {BcFV5.BI=FALSE, BcOLV18=TRUE} =>  {BcOLV7.BI=TRUE} | 0.11  1386 | 0.302  013 | 0.368  812 | 1.56  4275 | 45 |
| {BcOLV18=TRUE, BcOLV7.BI=TRUE} =>  {BcMV1.BI=TRUE} | 0.12  8713 | 0.912  281 | 0.141  089 | 1.15  5365 | 52 |
| {BcMV1.BI=TRUE, BcOLV7.BI=TRUE} =>  {BcOLV18=TRUE} | 0.12  8713 | 0.764  706 | 0.168  317 | 1.47  8187 | 52 |
| {BcMV1.BI=TRUE, BcOLV18=TRUE} =>  {BcOLV7.BI=TRUE} | 0.12  8713 | 0.292  135 | 0.440  594 | 1.51  3109 | 52 |
| {BcMV12=FALSE, BcOLV7.BI=TRUE} =>  {BcOLV18=TRUE} | 0.10  396 | 0.724  138 | 0.143  564 | 1.39  9769 | 42 |
| {BcMV12=FALSE, BcOLV18=TRUE} =>  {BcOLV7.BI=TRUE} | 0.10  396 | 0.262  5 | 0.396  04 | 1.35  9615 | 42 |
| {BcNSRV7.BI=FALSE, BcOLV7.BI=TRUE}  => {BcOLV18=TRUE} | 0.12  1287 | 0.753  846 | 0.160  891 | 1.45  7195 | 49 |
| {BcNSRV7.BI=FALSE, BcOLV18=TRUE} =>  {BcOLV7.BI=TRUE} | 0.12  1287 | 0.312  102 | 0.388  614 | 1.61  6528 | 49 |
| {BcOLV7.BI=TRUE, BcVV3.BI=FALSE} =>  {BcOLV18=TRUE} | 0.10  8911 | 0.733  333 | 0.148  515 | 1.41  7544 | 44 |
| {BcOLV18=TRUE, BcVV3.BI=FALSE} => | 0.10 | 0.265 | 0.410 | 1.37 | 44 |

| {BcOLV7.BI=TRUE} | 8911 | 06 | 891 | 2876 |  |
| --- | --- | --- | --- | --- | --- |
| {BcALV1.BI=FALSE, BcOLV7.BI=TRUE} =>  {BcOLV18=TRUE} | 0.09  4059 | 0.678  571 | 0.138  614 | 1.31  1688 | 38 |
| {BcOLV18=TRUE, BcOLV7.BI=TRUE} =>  {BcDMV1=FALSE} | 0.13  8614 | 0.982  456 | 0.141  089 | 1.16  3966 | 56 |
| {BcDMV1=FALSE, BcOLV7.BI=TRUE} =>  {BcOLV18=TRUE} | 0.13  8614 | 0.777  778 | 0.178  218 | 1.50  3456 | 56 |
| {BcDMV1=FALSE, BcOLV18=TRUE} =>  {BcOLV7.BI=TRUE} | 0.13  8614 | 0.323  699 | 0.428  218 | 1.67  6597 | 56 |
| {BcFV9=FALSE, BcOLV7.BI=TRUE} =>  {BcOLV18=TRUE} | 0.11  6337 | 0.712  121 | 0.163  366 | 1.37  6541 | 47 |
| {BcFV9=FALSE, BcOLV18=TRUE} =>  {BcOLV7.BI=TRUE} | 0.11  6337 | 0.276  471 | 0.420  792 | 1.43  1976 | 47 |
| {BcOLV4.BI=FALSE, BcOLV7.BI=TRUE} =>  {BcOLV18=TRUE} | 0.11  3861 | 0.696  97 | 0.163  366 | 1.34  7252 | 46 |
| {BcOLV18=TRUE, BcOLV4.BI=FALSE} =>  {BcOLV7.BI=TRUE} | 0.11  3861 | 0.254  144 | 0.448  02 | 1.31  6334 | 46 |
| {BcOLV7.BI=TRUE, BcULV5=FALSE} =>  {BcOLV18=TRUE} | 0.12  3762 | 0.704  225 | 0.175  743 | 1.36  1278 | 50 |
| {BcOLV18=TRUE, BcULV5=FALSE} =>  {BcOLV7.BI=TRUE} | 0.12  3762 | 0.264  55 | 0.467  822 | 1.37  0235 | 50 |
| {BcBNV5.BI=FALSE, BcOLV7.BI=TRUE} => | 0.13 | 0.736 | 0.178 | 1.42 | 53 |

| {BcOLV18=TRUE} | 1188 | 111 | 218 | 2913 |  |
| --- | --- | --- | --- | --- | --- |
| {BcBNV5.BI=FALSE, BcOLV18=TRUE} =>  {BcOLV7.BI=TRUE} | 0.13  1188 | 0.289  617 | 0.452  97 | 1.50  007 | 53 |
| {BcOLV7.BI=TRUE, BvF.BI=FALSE} =>  {BcOLV18=TRUE} | 0.13  3663 | 0.72 | 0.185  644 | 1.39  177 | 54 |
| {BcOLV18=TRUE, BvF.BI=FALSE} =>  {BcOLV7.BI=TRUE} | 0.13  3663 | 0.281  25 | 0.475  248 | 1.45  6731 | 54 |
| {BcMCV5.BI=FALSE, BcOLV7.BI=TRUE} =>  {BcOLV18=TRUE} | 0.12  1287 | 0.7 | 0.173  267 | 1.35  311 | 49 |
| {BcMCV5.BI=FALSE, BcOLV18=TRUE} =>  {BcOLV7.BI=TRUE} | 0.12  1287 | 0.269  231 | 0.450  495 | 1.39  4477 | 49 |
| {BcOLV13.BI=FALSE, BcOLV7.BI=TRUE}  => {BcOLV18=TRUE} | 0.12  6238 | 0.728  571 | 0.173  267 | 1.40  8339 | 51 |
| {BcOLV13.BI=FALSE, BcOLV18=TRUE} =>  {BcOLV7.BI=TRUE} | 0.12  6238 | 0.265  625 | 0.475  248 | 1.37  5801 | 51 |
| {BcOLV2.BI=FALSE, BcOLV7.BI=TRUE} =>  {BcOLV18=TRUE} | 0.12  3762 | 0.704  225 | 0.175  743 | 1.36  1278 | 50 |
| {BcOLV18=TRUE, BcOLV2.BI=FALSE} =>  {BcOLV7.BI=TRUE} | 0.12  3762 | 0.264  55 | 0.467  822 | 1.37  0235 | 50 |
| {BcMCV3.BI=FALSE, BcOLV7.BI=TRUE} =>  {BcOLV18=TRUE} | 0.12  3762 | 0.714  286 | 0.173  267 | 1.38  0725 | 50 |
| {BcMCV3.BI=FALSE, BcOLV18=TRUE} => | 0.12 | 0.260 | 0.475 | 1.34 | 50 |

| {BcOLV7.BI=TRUE} | 3762 | 417 | 248 | 8825 |  |
| --- | --- | --- | --- | --- | --- |
| {BcOLV7.BI=TRUE, BcPV4.BI=FALSE} =>  {BcOLV18=TRUE} | 0.13  3663 | 0.760  563 | 0.175  743 | 1.47  018 | 54 |
| {BcOLV18=TRUE, BcPV4.BI=FALSE} =>  {BcOLV7.BI=TRUE} | 0.13  3663 | 0.279  793 | 0.477  723 | 1.44  9183 | 54 |
| {BcEV6=FALSE, BcOLV7.BI=TRUE} =>  {BcOLV18=TRUE} | 0.13  6139 | 0.743  243 | 0.183  168 | 1.43  67 | 55 |
| {BcEV6=FALSE, BcOLV18=TRUE} =>  {BcOLV7.BI=TRUE} | 0.13  6139 | 0.282  051 | 0.482  673 | 1.46  0881 | 55 |
| {BcOLV17.BI=FALSE, BcOLV7.BI=TRUE}  => {BcOLV18=TRUE} | 0.12  1287 | 0.710  145 | 0.170  792 | 1.37  272 | 49 |
| {BcOLV17.BI=FALSE, BcOLV18=TRUE} =>  {BcOLV7.BI=TRUE} | 0.12  1287 | 0.253  886 | 0.477  723 | 1.31  4999 | 49 |
| {BcOLV15.BI=FALSE, BcOLV7.BI=TRUE}  => {BcOLV18=TRUE} | 0.13  6139 | 0.723  684 | 0.188  119 | 1.39  8892 | 55 |
| {BcOLV15.BI=FALSE, BcOLV18=TRUE} =>  {BcOLV7.BI=TRUE} | 0.13  6139 | 0.284  974 | 0.477  723 | 1.47  602 | 55 |
| {BcEV5=FALSE, BcOLV7.BI=TRUE} =>  {BcOLV18=TRUE} | 0.14  1089 | 0.740  26 | 0.190  594 | 1.43  0933 | 57 |
| {BcEV5=FALSE, BcOLV18=TRUE} =>  {BcOLV7.BI=TRUE} | 0.14  1089 | 0.289  34 | 0.487  624 | 1.49  8633 | 57 |
| {BcMV2.BI=FALSE, BcOLV7.BI=TRUE} => | 0.07 | 0.833 | 0.089 | 1.22 | 30 |

| {BcMV9.BI=TRUE} | 4257 | 333 | 109 | 871 |  |
| --- | --- | --- | --- | --- | --- |
| {BcMV2.BI=FALSE, BcOLV7.BI=TRUE} =>  {BcULV4=TRUE} | 0.08  1683 | 0.916  667 | 0.089  109 | 1.32  736 | 33 |
| {BcMV2.BI=FALSE, BcOLV7.BI=TRUE} =>  {BcEV2.BI=FALSE} | 0.08  1683 | 0.916  667 | 0.089  109 | 1.22  6269 | 33 |
| {BcMV2.BI=FALSE, BcOLV7.BI=TRUE} =>  {BcFV5.BI=FALSE} | 0.07  9208 | 0.888  889 | 0.089  109 | 1.13  6428 | 32 |
| {BcHV1SLR.BI=TRUE, BcOLV7.BI=TRUE}  => {BcMV3.BI=TRUE} | 0.08  6634 | 0.813  953 | 0.106  436 | 1.50  154 | 35 |
| {BcMV3.BI=TRUE, BcOLV7.BI=TRUE} =>  {BcHV1SLR.BI=TRUE} | 0.08  6634 | 0.686  275 | 0.126  238 | 1.26  6004 | 35 |
| {BcHV1SLR.BI=TRUE, BcMV3.BI=TRUE} =>  {BcOLV7.BI=TRUE} | 0.08  6634 | 0.263  158 | 0.329  208 | 1.36  3023 | 35 |
| {BcHV1SLR.BI=TRUE, BcOLV7.BI=TRUE}  => {BcULV3=TRUE} | 0.07  6733 | 0.720  93 | 0.106  436 | 1.21  3566 | 31 |
| {BcHV1SLR.BI=TRUE, BcOLV7.BI=TRUE}  => {BcHV1.BI=TRUE} | 0.09  4059 | 0.883  721 | 0.106  436 | 1.46  321 | 38 |
| {BcHV1.BI=TRUE, BcOLV7.BI=TRUE} =>  {BcHV1SLR.BI=TRUE} | 0.09  4059 | 0.745  098 | 0.126  238 | 1.37  4519 | 38 |
| {BcHV1SLR.BI=TRUE, BcOLV7.BI=TRUE}  => {BcMV11=TRUE} | 0.08  4158 | 0.790  698 | 0.106  436 | 1.28  2899 | 34 |
| {BcHV1SLR.BI=TRUE, BcOLV7.BI=TRUE} | 0.09 | 0.883 | 0.106 | 1.30 | 38 |

| => {BcMV9.BI=TRUE} | 4059 | 721 | 436 | 3005 |  |
| --- | --- | --- | --- | --- | --- |
| {BcHV1SLR.BI=TRUE, BcOLV7.BI=TRUE}  => {BcULV4=TRUE} | 0.10  1485 | 0.953  488 | 0.106  436 | 1.38  0679 | 41 |
| {BcHV1SLR.BI=TRUE, BcULV4=TRUE} =>  {BcOLV7.BI=TRUE} | 0.10  1485 | 0.253  086 | 0.400  99 | 1.31  0858 | 41 |
| {BcHV1SLR.BI=TRUE, BcOLV7.BI=TRUE}  => {BcDMV1=FALSE} | 0.10  396 | 0.976  744 | 0.106  436 | 1.15  7198 | 42 |
| {BcMV3.BI=TRUE, BcOLV7.BI=TRUE} =>  {BcULV3=TRUE} | 0.08  9109 | 0.705  882 | 0.126  238 | 1.18  8235 | 36 |
| {BcOLV7.BI=TRUE, BcULV3=TRUE} =>  {BcMV3.BI=TRUE} | 0.08  9109 | 0.75 | 0.118  812 | 1.38  3562 | 36 |
| {BcMV3.BI=TRUE, BcULV3=TRUE} =>  {BcOLV7.BI=TRUE} | 0.08  9109 | 0.257  143 | 0.346  535 | 1.33  1868 | 36 |
| {BcMV3.BI=TRUE, BcOLV7.BI=TRUE} =>  {BcHV1.BI=TRUE} | 0.09  1584 | 0.725  49 | 0.126  238 | 1.20  1221 | 37 |
| {BcHV1.BI=TRUE, BcOLV7.BI=TRUE} =>  {BcMV3.BI=TRUE} | 0.09  1584 | 0.725  49 | 0.126  238 | 1.33  8347 | 37 |
| {BcHV1.BI=TRUE, BcMV3.BI=TRUE} =>  {BcOLV7.BI=TRUE} | 0.09  1584 | 0.258  741 | 0.353  96 | 1.34  0147 | 37 |
| {BcMV3.BI=TRUE, BcOLV7.BI=TRUE} =>  {BcMV11=TRUE} | 0.09  6535 | 0.764  706 | 0.126  238 | 1.24  0728 | 39 |
| {BcMV11=TRUE, BcOLV7.BI=TRUE} => | 0.09 | 0.735 | 0.131 | 1.35 | 39 |

| {BcMV3.BI=TRUE} | 6535 | 849 | 188 | 7457 |  |
| --- | --- | --- | --- | --- | --- |
| {BcMV11=TRUE, BcMV3.BI=TRUE} =>  {BcOLV7.BI=TRUE} | 0.09  6535 | 0.251  613 | 0.383  663 | 1.30  3226 | 39 |
| {BcMV3.BI=TRUE, BcOLV7.BI=TRUE} =>  {BcMV9.BI=TRUE} | 0.10  8911 | 0.862  745 | 0.126  238 | 1.27  2077 | 44 |
| {BcMV3.BI=TRUE, BcMV9.BI=TRUE} =>  {BcOLV7.BI=TRUE} | 0.10  8911 | 0.275 | 0.396  04 | 1.42  4359 | 44 |
| {BcMV3.BI=TRUE, BcOLV7.BI=TRUE} =>  {BcULV4=TRUE} | 0.11  3861 | 0.901  961 | 0.126  238 | 1.30  6065 | 46 |
| {BcMV3.BI=TRUE, BcULV4=TRUE} =>  {BcOLV7.BI=TRUE} | 0.11  3861 | 0.283  951 | 0.400  99 | 1.47  0719 | 46 |
| {BcFV5.BI=FALSE, BcMV3.BI=TRUE} =>  {BcOLV7.BI=TRUE} | 0.09  901 | 0.254  777 | 0.388  614 | 1.31  9615 | 40 |
| {BcMV3.BI=TRUE, BcOLV7.BI=TRUE} =>  {BcMV1.BI=TRUE} | 0.11  6337 | 0.921  569 | 0.126  238 | 1.16  7128 | 47 |
| {BcMV1.BI=TRUE, BcOLV7.BI=TRUE} =>  {BcMV3.BI=TRUE} | 0.11  6337 | 0.691  176 | 0.168  317 | 1.27  5047 | 47 |
| {BcMV1.BI=TRUE, BcMV3.BI=TRUE} =>  {BcOLV7.BI=TRUE} | 0.11  6337 | 0.254  054 | 0.457  921 | 1.31  587 | 47 |
| {BcMV3.BI=TRUE, BcOLV7.BI=TRUE} =>  {BcDMV1=FALSE} | 0.11  6337 | 0.921  569 | 0.126  238 | 1.09  1829 | 47 |
| {BcDMV1=FALSE, BcOLV7.BI=TRUE} => | 0.11 | 0.652 | 0.178 | 1.20 | 47 |

| {BcMV3.BI=TRUE} | 6337 | 778 | 218 | 4211 |  |
| --- | --- | --- | --- | --- | --- |
| {BcDMV1=FALSE, BcMV3.BI=TRUE} =>  {BcOLV7.BI=TRUE} | 0.11  6337 | 0.274  854 | 0.423  267 | 1.42  3602 | 47 |
| {BcFV9=FALSE, BcOLV7.BI=TRUE} =>  {BcMV3.BI=TRUE} | 0.10  8911 | 0.666  667 | 0.163  366 | 1.22  9833 | 44 |
| {BcMV3.BI=TRUE, BcULV5=FALSE} =>  {BcOLV7.BI=TRUE} | 0.11  1386 | 0.245  902 | 0.452  97 | 1.27  3644 | 45 |
| {BcBNV5.BI=FALSE, BcOLV7.BI=TRUE} =>  {BcMV3.BI=TRUE} | 0.11  6337 | 0.652  778 | 0.178  218 | 1.20  4211 | 47 |
| {BcBNV5.BI=FALSE, BcMV3.BI=TRUE} =>  {BcOLV7.BI=TRUE} | 0.11  6337 | 0.247  368 | 0.470  297 | 1.28  1242 | 47 |
| {BcOLV7.BI=TRUE, BvF.BI=FALSE} =>  {BcMV3.BI=TRUE} | 0.12  1287 | 0.653  333 | 0.185  644 | 1.20  5236 | 49 |
| {BcEV6=FALSE, BcOLV7.BI=TRUE} =>  {BcMV3.BI=TRUE} | 0.12  1287 | 0.662  162 | 0.183  168 | 1.22  1523 | 49 |
| {BcEV5=FALSE, BcOLV7.BI=TRUE} =>  {BcMV3.BI=TRUE} | 0.12  3762 | 0.649  351 | 0.190  594 | 1.19  7889 | 50 |
| {BcEV5=FALSE, BcMV3.BI=TRUE} =>  {BcOLV7.BI=TRUE} | 0.12  3762 | 0.25 | 0.495  05 | 1.29  4872 | 50 |
| {BcMV4.BI=FALSE, BcOLV7.BI=TRUE} =>  {BcMV9.BI=TRUE} | 0.07  4257 | 0.857  143 | 0.086  634 | 1.26  3816 | 30 |
| {BcMV4.BI=FALSE, BcOLV7.BI=TRUE} => | 0.07 | 0.857 | 0.086 | 1.24 | 30 |

| {BcULV4=TRUE} | 4257 | 143 | 634 | 1167 |  |
| --- | --- | --- | --- | --- | --- |
| {BcMV4.BI=FALSE, BcOLV7.BI=TRUE} =>  {BcFV5.BI=FALSE} | 0.07  9208 | 0.914  286 | 0.086  634 | 1.16  8897 | 32 |
| {BcMV4.BI=FALSE, BcOLV7.BI=TRUE} =>  {BcULV5=FALSE} | 0.08  4158 | 0.971  429 | 0.086  634 | 1.10  8636 | 34 |
| {BcOLV7.BI=TRUE, BcULV3=TRUE} =>  {BcHV1.BI=TRUE} | 0.08  6634 | 0.729  167 | 0.118  812 | 1.20  7309 | 35 |
| {BcOLV7.BI=TRUE, BcULV3=TRUE} =>  {BcMV11=TRUE} | 0.08  6634 | 0.729  167 | 0.118  812 | 1.18  3066 | 35 |
| {BcOLV7.BI=TRUE, BcULV3=TRUE} =>  {BcMV9.BI=TRUE} | 0.10  1485 | 0.854  167 | 0.118  812 | 1.25  9428 | 41 |
| {BcOLV7.BI=TRUE, BcULV3=TRUE} =>  {BcULV4=TRUE} | 0.11  3861 | 0.958  333 | 0.118  812 | 1.38  7694 | 46 |
| {BcULV3=TRUE, BcULV4=TRUE} =>  {BcOLV7.BI=TRUE} | 0.11  3861 | 0.273  81 | 0.415  842 | 1.41  8193 | 46 |
| {BcOLV7.BI=TRUE, BcULV3=TRUE} =>  {BcDMV1=FALSE} | 0.11  1386 | 0.937  5 | 0.118  812 | 1.11  0704 | 45 |
| {BcHV1.BI=TRUE, BcOLV7.BI=TRUE} =>  {BcMV11=TRUE} | 0.09  6535 | 0.764  706 | 0.126  238 | 1.24  0728 | 39 |
| {BcMV11=TRUE, BcOLV7.BI=TRUE} =>  {BcHV1.BI=TRUE} | 0.09  6535 | 0.735  849 | 0.131  188 | 1.21  8373 | 39 |
| {BcHV1.BI=TRUE, BcMV11=TRUE} => | 0.09 | 0.253 | 0.381 | 1.31 | 39 |

| {BcOLV7.BI=TRUE} | 6535 | 247 | 188 | 1688 |  |
| --- | --- | --- | --- | --- | --- |
| {BcHV1.BI=TRUE, BcOLV7.BI=TRUE} =>  {BcMV9.BI=TRUE} | 0.11  1386 | 0.882  353 | 0.126  238 | 1.30  0988 | 45 |
| {BcHV1.BI=TRUE, BcOLV7.BI=TRUE} =>  {BcULV4=TRUE} | 0.12  3762 | 0.980  392 | 0.126  238 | 1.41  9636 | 50 |
| {BcHV1.BI=TRUE, BcULV4=TRUE} =>  {BcOLV7.BI=TRUE} | 0.12  3762 | 0.271  739 | 0.455  446 | 1.40  7469 | 50 |
| {BcHV1.BI=TRUE, BcOLV7.BI=TRUE} =>  {BcDMV1=FALSE} | 0.11  8812 | 0.941  176 | 0.126  238 | 1.11  506 | 48 |
| {BcMV11=TRUE, BcOLV7.BI=TRUE} =>  {BcMV9.BI=TRUE} | 0.12  1287 | 0.924  528 | 0.131  188 | 1.36  3173 | 49 |
| {BcMV11=TRUE, BcMV9.BI=TRUE} =>  {BcOLV7.BI=TRUE} | 0.12  1287 | 0.298  78 | 0.405  941 | 1.54  753 | 49 |
| {BcMV11=TRUE, BcOLV7.BI=TRUE} =>  {BcULV4=TRUE} | 0.12  8713 | 0.981  132 | 0.131  188 | 1.42  0707 | 52 |
| {BcOLV7.BI=TRUE, BcULV4=TRUE} =>  {BcMV11=TRUE} | 0.12  8713 | 0.722  222 | 0.178  218 | 1.17  1798 | 52 |
| {BcMV11=TRUE, BcULV4=TRUE} =>  {BcOLV7.BI=TRUE} | 0.12  8713 | 0.307  692 | 0.418  317 | 1.59  3688 | 52 |
| {BcMV11=TRUE, BcOLV7.BI=TRUE} =>  {BcMV1.BI=TRUE} | 0.11  8812 | 0.905  66 | 0.131  188 | 1.14  6981 | 48 |
| {BcOLV7.BI=TRUE, BcVV3.BI=FALSE} => | 0.11 | 0.75 | 0.148 | 1.21 | 45 |

| {BcMV11=TRUE} | 1386 |  | 515 | 6867 |  |
| --- | --- | --- | --- | --- | --- |
| {BcMV11=TRUE, BcOLV7.BI=TRUE} =>  {BcDMV1=FALSE} | 0.12  1287 | 0.924  528 | 0.131  188 | 1.09  5336 | 49 |
| {BcOLV1.BI.MOVA.=FALSE,  BcOLV7.BI=TRUE} => {BcMV9.BI=TRUE} | 0.08  4158 | 0.85 | 0.099  01 | 1.25  3285 | 34 |
| {BcOLV1.BI.MOVA.=FALSE,  BcOLV7.BI=TRUE} => {BcULV4=TRUE} | 0.09  1584 | 0.925 | 0.099  01 | 1.33  9427 | 37 |
| {BcOLV1.BI.MOVA.=FALSE,  BcOLV7.BI=TRUE} => {BcEV2.BI=FALSE} | 0.08  6634 | 0.875 | 0.099  01 | 1.17  053 | 35 |
| {BcMV9.BI=TRUE, BcOLV7.BI=TRUE} =>  {BcULV4=TRUE} | 0.16  0891 | 0.942  029 | 0.170  792 | 1.36  4085 | 65 |
| {BcOLV7.BI=TRUE, BcULV4=TRUE} =>  {BcMV9.BI=TRUE} | 0.16  0891 | 0.902  778 | 0.178  218 | 1.33  1103 | 65 |
| {BcMV9.BI=TRUE, BcULV4=TRUE} =>  {BcOLV7.BI=TRUE} | 0.16  0891 | 0.294  118 | 0.547  03 | 1.52  3379 | 65 |
| {BcMV6.BI=FALSE, BcOLV7.BI=TRUE} =>  {BcMV9.BI=TRUE} | 0.10  396 | 0.857  143 | 0.121  287 | 1.26  3816 | 42 |
| {BcOLV7.BI=TRUE, BcPV3.BI=FALSE} =>  {BcMV9.BI=TRUE} | 0.09  4059 | 0.844  444 | 0.111  386 | 1.24  5093 | 38 |
| {BcEV2.BI=FALSE, BcOLV7.BI=TRUE} =>  {BcMV9.BI=TRUE} | 0.14  1089 | 0.904  762 | 0.155  941 | 1.33  4029 | 57 |
| {BcEV2.BI=FALSE, BcMV9.BI=TRUE} => | 0.14 | 0.279 | 0.504 | 1.44 | 57 |

| {BcOLV7.BI=TRUE} | 1089 | 412 | 95 | 721 |  |
| --- | --- | --- | --- | --- | --- |
| {BcHV3.BI=FALSE, BcOLV7.BI=TRUE} =>  {BcMV9.BI=TRUE} | 0.11  8812 | 0.923  077 | 0.128  713 | 1.36  1033 | 48 |
| {BcFV5.BI=FALSE, BcOLV7.BI=TRUE} =>  {BcMV9.BI=TRUE} | 0.14  604 | 0.921  875 | 0.158  416 | 1.35  9261 | 59 |
| {BcFV5.BI=FALSE, BcMV9.BI=TRUE} =>  {BcOLV7.BI=TRUE} | 0.14  604 | 0.285  024 | 0.512  376 | 1.47  6279 | 59 |
| {BcOLV7.BI=TRUE, BcULV1.BI=FALSE} =>  {BcMV9.BI=TRUE} | 0.11  1386 | 0.833  333 | 0.133  663 | 1.22  871 | 45 |
| {BcMV1.BI=TRUE, BcOLV7.BI=TRUE} =>  {BcMV9.BI=TRUE} | 0.14  604 | 0.867  647 | 0.168  317 | 1.27  9304 | 59 |
| {BcMV1.BI=TRUE, BcMV9.BI=TRUE} =>  {BcOLV7.BI=TRUE} | 0.14  604 | 0.265  766 | 0.549  505 | 1.37  653 | 59 |
| {BcMV12=FALSE, BcOLV7.BI=TRUE} =>  {BcMV9.BI=TRUE} | 0.13  1188 | 0.913  793 | 0.143  564 | 1.34  7345 | 53 |
| {BcMV12=FALSE, BcMV9.BI=TRUE} =>  {BcOLV7.BI=TRUE} | 0.13  1188 | 0.243  119 | 0.539  604 | 1.25  9233 | 53 |
| {BcNSRV7.BI=FALSE, BcOLV7.BI=TRUE}  => {BcMV9.BI=TRUE} | 0.14  604 | 0.907  692 | 0.160  891 | 1.33  8349 | 59 |
| {BcMV9.BI=TRUE, BcNSRV7.BI=FALSE} =>  {BcOLV7.BI=TRUE} | 0.14  604 | 0.282  297 | 0.517  327 | 1.46  2152 | 59 |
| {BcOLV7.BI=TRUE, BcVV3.BI=FALSE} => | 0.13 | 0.916 | 0.148 | 1.35 | 55 |

| {BcMV9.BI=TRUE} | 6139 | 667 | 515 | 1582 |  |
| --- | --- | --- | --- | --- | --- |
| {BcMV9.BI=TRUE, BcVV3.BI=FALSE} =>  {BcOLV7.BI=TRUE} | 0.13  6139 | 0.245  536 | 0.554  455 | 1.27  1749 | 55 |
| {BcALV1.BI=FALSE, BcOLV7.BI=TRUE} =>  {BcMV9.BI=TRUE} | 0.12  3762 | 0.892  857 | 0.138  614 | 1.31  6475 | 50 |
| {BcMV9.BI=TRUE, BcOLV7.BI=TRUE} =>  {BcDMV1=FALSE} | 0.15  8416 | 0.927  536 | 0.170  792 | 1.09  8899 | 64 |
| {BcDMV1=FALSE, BcOLV7.BI=TRUE} =>  {BcMV9.BI=TRUE} | 0.15  8416 | 0.888  889 | 0.178  218 | 1.31  0624 | 64 |
| {BcDMV1=FALSE, BcMV9.BI=TRUE} =>  {BcOLV7.BI=TRUE} | 0.15  8416 | 0.286  996 | 0.551  98 | 1.48  649 | 64 |
| {BcFV9=FALSE, BcOLV7.BI=TRUE} =>  {BcMV9.BI=TRUE} | 0.14  8515 | 0.909  091 | 0.163  366 | 1.34  0411 | 60 |
| {BcFV9=FALSE, BcMV9.BI=TRUE} =>  {BcOLV7.BI=TRUE} | 0.14  8515 | 0.254  237 | 0.584  158 | 1.31  6819 | 60 |
| {BcOLV4.BI=FALSE, BcOLV7.BI=TRUE} =>  {BcMV9.BI=TRUE} | 0.14  604 | 0.893  939 | 0.163  366 | 1.31  8071 | 59 |
| {BcMV9.BI=TRUE, BcOLV4.BI=FALSE} =>  {BcOLV7.BI=TRUE} | 0.14  604 | 0.236  948 | 0.616  337 | 1.22  7268 | 59 |
| {BcOLV7.BI=TRUE, BcULV5=FALSE} =>  {BcMV9.BI=TRUE} | 0.15  8416 | 0.901  408 | 0.175  743 | 1.32  9084 | 64 |
| {BcMV9.BI=TRUE, BcULV5=FALSE} => | 0.15 | 0.241 | 0.655 | 1.25 | 64 |

| {BcOLV7.BI=TRUE} | 8416 | 509 | 941 | 0895 |  |
| --- | --- | --- | --- | --- | --- |
| {BcBNV5.BI=FALSE, BcOLV7.BI=TRUE} =>  {BcMV9.BI=TRUE} | 0.16  0891 | 0.902  778 | 0.178  218 | 1.33  1103 | 65 |
| {BcBNV5.BI=FALSE, BcMV9.BI=TRUE} =>  {BcOLV7.BI=TRUE} | 0.16  0891 | 0.269  71 | 0.596  535 | 1.39  6957 | 65 |
| {BcOLV7.BI=TRUE, BvF.BI=FALSE} =>  {BcMV9.BI=TRUE} | 0.16  3366 | 0.88 | 0.185  644 | 1.29  7518 | 66 |
| {BcMV9.BI=TRUE, BvF.BI=FALSE} =>  {BcOLV7.BI=TRUE} | 0.16  3366 | 0.267  206 | 0.611  386 | 1.38  3993 | 66 |
| {BcMCV5.BI=FALSE, BcOLV7.BI=TRUE} =>  {BcMV9.BI=TRUE} | 0.15  5941 | 0.9 | 0.173  267 | 1.32  7007 | 63 |
| {BcMCV5.BI=FALSE, BcMV9.BI=TRUE} =>  {BcOLV7.BI=TRUE} | 0.15  5941 | 0.253  012 | 0.616  337 | 1.31  0473 | 63 |
| {BcOLV13.BI=FALSE, BcOLV7.BI=TRUE}  => {BcMV9.BI=TRUE} | 0.15  099 | 0.871  429 | 0.173  267 | 1.28  488 | 61 |
| {BcMV9.BI=TRUE, BcOLV13.BI=FALSE} =>  {BcOLV7.BI=TRUE} | 0.15  099 | 0.237  354 | 0.636  139 | 1.22  9372 | 61 |
| {BcOLV2.BI=FALSE, BcOLV7.BI=TRUE} =>  {BcMV9.BI=TRUE} | 0.15  3465 | 0.873  239 | 0.175  743 | 1.28  755 | 62 |
| {BcMV9.BI=TRUE, BcOLV2.BI=FALSE} =>  {BcOLV7.BI=TRUE} | 0.15  3465 | 0.247  012 | 0.621  287 | 1.27  9395 | 62 |
| {BcMCV3.BI=FALSE, BcOLV7.BI=TRUE} => | 0.15 | 0.871 | 0.173 | 1.28 | 61 |

| {BcMV9.BI=TRUE} | 099 | 429 | 267 | 488 |  |
| --- | --- | --- | --- | --- | --- |
| {BcMCV3.BI=FALSE, BcMV9.BI=TRUE} =>  {BcOLV7.BI=TRUE} | 0.15  099 | 0.240  157 | 0.628  713 | 1.24  3893 | 61 |
| {BcOLV7.BI=TRUE, BcPV4.BI=FALSE} =>  {BcMV9.BI=TRUE} | 0.15  5941 | 0.887  324 | 0.175  743 | 1.30  8317 | 63 |
| {BcMV9.BI=TRUE, BcPV4.BI=FALSE} =>  {BcOLV7.BI=TRUE} | 0.15  5941 | 0.246  094 | 0.633  663 | 1.27  4639 | 63 |
| {BcEV6=FALSE, BcOLV7.BI=TRUE} =>  {BcMV9.BI=TRUE} | 0.16  0891 | 0.878  378 | 0.183  168 | 1.29  5127 | 65 |
| {BcEV6=FALSE, BcMV9.BI=TRUE} =>  {BcOLV7.BI=TRUE} | 0.16  0891 | 0.254  902 | 0.631  188 | 1.32  0261 | 65 |
| {BcOLV17.BI=FALSE, BcOLV7.BI=TRUE}  => {BcMV9.BI=TRUE} | 0.14  8515 | 0.869  565 | 0.170  792 | 1.28  2133 | 60 |
| {BcOLV15.BI=FALSE, BcOLV7.BI=TRUE}  => {BcMV9.BI=TRUE} | 0.16  8317 | 0.894  737 | 0.188  119 | 1.31  9247 | 68 |
| {BcMV9.BI=TRUE, BcOLV15.BI=FALSE} =>  {BcOLV7.BI=TRUE} | 0.16  8317 | 0.262  548 | 0.641  089 | 1.35  9865 | 68 |
| {BcEV5=FALSE, BcOLV7.BI=TRUE} =>  {BcMV9.BI=TRUE} | 0.17  0792 | 0.896  104 | 0.190  594 | 1.32  1263 | 69 |
| {BcEV5=FALSE, BcMV9.BI=TRUE} =>  {BcOLV7.BI=TRUE} | 0.17  0792 | 0.267  442 | 0.638  614 | 1.38  5212 | 69 |
| {BcMV6.BI=FALSE, BcOLV7.BI=TRUE} => | 0.10 | 0.877 | 0.121 | 1.27 | 43 |

| {BcULV4=TRUE} | 6436 | 551 | 287 | 0719 |  |
| --- | --- | --- | --- | --- | --- |
| {BcOLV7.BI=TRUE, BcPV3.BI=FALSE} =>  {BcULV4=TRUE} | 0.10  1485 | 0.911  111 | 0.111  386 | 1.31  9315 | 41 |
| {BcEV2.BI=FALSE, BcOLV7.BI=TRUE} =>  {BcULV4=TRUE} | 0.14  3564 | 0.920  635 | 0.155  941 | 1.33  3106 | 58 |
| {BcEV2.BI=FALSE, BcULV4=TRUE} =>  {BcOLV7.BI=TRUE} | 0.14  3564 | 0.271  028 | 0.529  703 | 1.40  3786 | 58 |
| {BcHV3.BI=FALSE, BcOLV7.BI=TRUE} =>  {BcULV4=TRUE} | 0.11  8812 | 0.923  077 | 0.128  713 | 1.33  6642 | 48 |
| {BcFV5.BI=FALSE, BcOLV7.BI=TRUE} =>  {BcULV4=TRUE} | 0.14  604 | 0.921  875 | 0.158  416 | 1.33  4901 | 59 |
| {BcFV5.BI=FALSE, BcULV4=TRUE} =>  {BcOLV7.BI=TRUE} | 0.14  604 | 0.275  701 | 0.529  703 | 1.42  7989 | 59 |
| {BcOLV7.BI=TRUE, BcULV1.BI=FALSE} =>  {BcULV4=TRUE} | 0.11  8812 | 0.888  889 | 0.133  663 | 1.28  7137 | 48 |
| {BcMV1.BI=TRUE, BcOLV7.BI=TRUE} =>  {BcULV4=TRUE} | 0.15  3465 | 0.911  765 | 0.168  317 | 1.32  0261 | 62 |
| {BcMV1.BI=TRUE, BcULV4=TRUE} =>  {BcOLV7.BI=TRUE} | 0.15  3465 | 0.279  279 | 0.549  505 | 1.44  6523 | 62 |
| {BcMV12=FALSE, BcOLV7.BI=TRUE} =>  {BcULV4=TRUE} | 0.13  6139 | 0.948  276 | 0.143  564 | 1.37  3131 | 55 |
| {BcMV12=FALSE, BcULV4=TRUE} => | 0.13 | 0.247 | 0.549 | 1.28 | 55 |

| {BcOLV7.BI=TRUE} | 6139 | 748 | 505 | 3206 |  |
| --- | --- | --- | --- | --- | --- |
| {BcNSRV7.BI=FALSE, BcOLV7.BI=TRUE}  => {BcULV4=TRUE} | 0.14  8515 | 0.923  077 | 0.160  891 | 1.33  6642 | 60 |
| {BcNSRV7.BI=FALSE, BcULV4=TRUE} =>  {BcOLV7.BI=TRUE} | 0.14  8515 | 0.277  778 | 0.534  653 | 1.43  8746 | 60 |
| {BcOLV7.BI=TRUE, BcVV3.BI=FALSE} =>  {BcULV4=TRUE} | 0.14  1089 | 0.95 | 0.148  515 | 1.37  5627 | 57 |
| {BcULV4=TRUE, BcVV3.BI=FALSE} =>  {BcOLV7.BI=TRUE} | 0.14  1089 | 0.257  919 | 0.547  03 | 1.33  5886 | 57 |
| {BcALV1.BI=FALSE, BcOLV7.BI=TRUE} =>  {BcULV4=TRUE} | 0.12  3762 | 0.892  857 | 0.138  614 | 1.29  2883 | 50 |
| {BcOLV7.BI=TRUE, BcULV4=TRUE} =>  {BcDMV1=FALSE} | 0.16  5842 | 0.930  556 | 0.178  218 | 1.10  2476 | 67 |
| {BcDMV1=FALSE, BcOLV7.BI=TRUE} =>  {BcULV4=TRUE} | 0.16  5842 | 0.930  556 | 0.178  218 | 1.34  7471 | 67 |
| {BcDMV1=FALSE, BcULV4=TRUE} =>  {BcOLV7.BI=TRUE} | 0.16  5842 | 0.290  043 | 0.571  782 | 1.50  2276 | 67 |
| {BcFV9=FALSE, BcOLV7.BI=TRUE} =>  {BcULV4=TRUE} | 0.15  099 | 0.924  242 | 0.163  366 | 1.33  833 | 61 |
| {BcFV9=FALSE, BcULV4=TRUE} =>  {BcOLV7.BI=TRUE} | 0.15  099 | 0.253  112 | 0.596  535 | 1.31  0991 | 61 |
| {BcOLV4.BI=FALSE, BcOLV7.BI=TRUE} => | 0.14 | 0.909 | 0.163 | 1.31 | 60 |

| {BcULV4=TRUE} | 8515 | 091 | 366 | 639 |  |
| --- | --- | --- | --- | --- | --- |
| {BcOLV4.BI=FALSE, BcULV4=TRUE} =>  {BcOLV7.BI=TRUE} | 0.14  8515 | 0.239  044 | 0.621  287 | 1.23  8124 | 60 |
| {BcOLV7.BI=TRUE, BcULV5=FALSE} =>  {BcULV4=TRUE} | 0.16  0891 | 0.915  493 | 0.175  743 | 1.32  566 | 65 |
| {BcULV4=TRUE, BcULV5=FALSE} =>  {BcOLV7.BI=TRUE} | 0.16  0891 | 0.254  902 | 0.631  188 | 1.32  0261 | 65 |
| {BcBNV5.BI=FALSE, BcOLV7.BI=TRUE} =>  {BcULV4=TRUE} | 0.16  3366 | 0.916  667 | 0.178  218 | 1.32  736 | 66 |
| {BcBNV5.BI=FALSE, BcULV4=TRUE} =>  {BcOLV7.BI=TRUE} | 0.16  3366 | 0.264 | 0.618  812 | 1.36  7385 | 66 |
| {BcOLV7.BI=TRUE, BvF.BI=FALSE} =>  {BcULV4=TRUE} | 0.17  0792 | 0.92 | 0.185  644 | 1.33  2186 | 69 |
| {BcULV4=TRUE, BvF.BI=FALSE} =>  {BcOLV7.BI=TRUE} | 0.17  0792 | 0.264  368 | 0.646  04 | 1.36  929 | 69 |
| {BcMCV5.BI=FALSE, BcOLV7.BI=TRUE} =>  {BcULV4=TRUE} | 0.15  8416 | 0.914  286 | 0.173  267 | 1.32  3912 | 64 |
| {BcMCV5.BI=FALSE, BcULV4=TRUE} =>  {BcOLV7.BI=TRUE} | 0.15  8416 | 0.250  98 | 0.631  188 | 1.29  995 | 64 |
| {BcOLV13.BI=FALSE, BcOLV7.BI=TRUE}  => {BcULV4=TRUE} | 0.15  8416 | 0.914  286 | 0.173  267 | 1.32  3912 | 64 |
| {BcOLV13.BI=FALSE, BcULV4=TRUE} => | 0.15 | 0.247 | 0.641 | 1.27 | 64 |

| {BcOLV7.BI=TRUE} | 8416 | 104 | 089 | 9873 |  |
| --- | --- | --- | --- | --- | --- |
| {BcOLV2.BI=FALSE, BcOLV7.BI=TRUE} =>  {BcULV4=TRUE} | 0.16  0891 | 0.915  493 | 0.175  743 | 1.32  566 | 65 |
| {BcOLV2.BI=FALSE, BcULV4=TRUE} =>  {BcOLV7.BI=TRUE} | 0.16  0891 | 0.250  965 | 0.641  089 | 1.29  9871 | 65 |
| {BcMCV3.BI=FALSE, BcOLV7.BI=TRUE} =>  {BcULV4=TRUE} | 0.16  0891 | 0.928  571 | 0.173  267 | 1.34  4598 | 65 |
| {BcMCV3.BI=FALSE, BcULV4=TRUE} =>  {BcOLV7.BI=TRUE} | 0.16  0891 | 0.250  965 | 0.641  089 | 1.29  9871 | 65 |
| {BcOLV7.BI=TRUE, BcPV4.BI=FALSE} =>  {BcULV4=TRUE} | 0.16  3366 | 0.929  577 | 0.175  743 | 1.34  6055 | 66 |
| {BcPV4.BI=FALSE, BcULV4=TRUE} =>  {BcOLV7.BI=TRUE} | 0.16  3366 | 0.252  874 | 0.646  04 | 1.30  9755 | 66 |
| {BcEV6=FALSE, BcOLV7.BI=TRUE} =>  {BcULV4=TRUE} | 0.16  8317 | 0.918  919 | 0.183  168 | 1.33  0621 | 68 |
| {BcEV6=FALSE, BcULV4=TRUE} =>  {BcOLV7.BI=TRUE} | 0.16  8317 | 0.260  536 | 0.646  04 | 1.34  9445 | 68 |
| {BcOLV17.BI=FALSE, BcOLV7.BI=TRUE}  => {BcULV4=TRUE} | 0.15  5941 | 0.913  043 | 0.170  792 | 1.32  2113 | 63 |
| {BcOLV17.BI=FALSE, BcULV4=TRUE} =>  {BcOLV7.BI=TRUE} | 0.15  5941 | 0.241  379 | 0.646  04 | 1.25  0221 | 63 |
| {BcOLV15.BI=FALSE, BcOLV7.BI=TRUE} | 0.17 | 0.921 | 0.188 | 1.33 | 70 |

| => {BcULV4=TRUE} | 3267 | 053 | 119 | 3711 |  |
| --- | --- | --- | --- | --- | --- |
| {BcOLV15.BI=FALSE, BcULV4=TRUE} =>  {BcOLV7.BI=TRUE} | 0.17  3267 | 0.263  158 | 0.658  416 | 1.36  3023 | 70 |
| {BcEV5=FALSE, BcOLV7.BI=TRUE} =>  {BcULV4=TRUE} | 0.17  8218 | 0.935  065 | 0.190  594 | 1.35  4001 | 72 |
| {BcEV5=FALSE, BcULV4=TRUE} =>  {BcOLV7.BI=TRUE} | 0.17  8218 | 0.270  677 | 0.658  416 | 1.40  1966 | 72 |
| {BcMV6.BI=FALSE, BcOLV7.BI=TRUE} =>  {BcDMV1=FALSE} | 0.11  6337 | 0.959  184 | 0.121  287 | 1.13  6394 | 47 |
| {BcOLV7.BI=TRUE, BcPV3.BI=FALSE} =>  {BcEV2.BI=FALSE} | 0.09  6535 | 0.866  667 | 0.111  386 | 1.15  9382 | 39 |
| {BcOLV7.BI=TRUE, BcRV1.BI=FALSE} =>  {BcMV1.BI=TRUE} | 0.07  6733 | 0.911  765 | 0.084  158 | 1.15  4711 | 31 |
| {BcMV12=FALSE, BcOLV7.BI=TRUE} =>  {BcEV2.BI=FALSE} | 0.12  3762 | 0.862  069 | 0.143  564 | 1.15  3231 | 50 |
| {BcFV9=FALSE, BcOLV7.BI=TRUE} =>  {BcEV2.BI=FALSE} | 0.13  8614 | 0.848  485 | 0.163  366 | 1.13  5059 | 56 |
| {BcOLV4.BI=FALSE, BcOLV7.BI=TRUE} =>  {BcEV2.BI=FALSE} | 0.14  1089 | 0.863  636 | 0.163  366 | 1.15  5328 | 57 |
| {BcMCV5.BI=FALSE, BcOLV7.BI=TRUE} =>  {BcEV2.BI=FALSE} | 0.14  8515 | 0.857  143 | 0.173  267 | 1.14  6641 | 60 |
| {BcMCV3.BI=FALSE, BcOLV7.BI=TRUE} => | 0.14 | 0.842 | 0.173 | 1.12 | 59 |

| {BcEV2.BI=FALSE} | 604 | 857 | 267 | 7531 |  |
| --- | --- | --- | --- | --- | --- |
| {BcHV3.BI=FALSE, BcOLV7.BI=TRUE} =>  {BcFV5.BI=FALSE} | 0.11  6337 | 0.903  846 | 0.128  713 | 1.15  555 | 47 |
| {BcHV3.BI=FALSE, BcOLV7.BI=TRUE} =>  {BcDMV1=FALSE} | 0.11  8812 | 0.923  077 | 0.128  713 | 1.09  3616 | 48 |
| {BcFV5.BI=FALSE, BcOLV7.BI=TRUE} =>  {BcDMV1=FALSE} | 0.14  604 | 0.921  875 | 0.158  416 | 1.09  2192 | 59 |
| {BcFV5.BI=FALSE, BcOLV7.BI=TRUE} =>  {BcULV5=FALSE} | 0.15  099 | 0.953  125 | 0.158  416 | 1.08  7747 | 61 |
| {BcOLV7.BI=TRUE, BcULV1.BI=FALSE} =>  {BcULV5=FALSE} | 0.12  8713 | 0.962  963 | 0.133  663 | 1.09  8975 | 52 |
| {BcALV1.BI=FALSE, BcOLV7.BI=TRUE} =>  {BcMV1.BI=TRUE} | 0.12  3762 | 0.892  857 | 0.138  614 | 1.13  0766 | 50 |
| {BcMV1.BI=TRUE, BcOLV7.BI=TRUE} =>  {BcDMV1=FALSE} | 0.15  3465 | 0.911  765 | 0.168  317 | 1.08  0214 | 62 |
| {BcOLV7.BI=TRUE, BcULV5=FALSE} =>  {BcMV1.BI=TRUE} | 0.15  3465 | 0.873  239 | 0.175  743 | 1.10  5921 | 62 |
| {BcOLV7.BI=TRUE, BvF.BI=FALSE} =>  {BcMV1.BI=TRUE} | 0.16  3366 | 0.88 | 0.185  644 | 1.11  4483 | 66 |
| {BcOLV7.BI=TRUE, BcPV4.BI=FALSE} =>  {BcMV1.BI=TRUE} | 0.15  3465 | 0.873  239 | 0.175  743 | 1.10  5921 | 62 |
| {BcEV6=FALSE, BcOLV7.BI=TRUE} => | 0.16 | 0.878 | 0.183 | 1.11 | 65 |

| {BcMV1.BI=TRUE} | 0891 | 378 | 168 | 2429 |  |
| --- | --- | --- | --- | --- | --- |
| {BcMV12=FALSE, BcOLV7.BI=TRUE} =>  {BcDMV1=FALSE} | 0.13  6139 | 0.948  276 | 0.143  564 | 1.12  3471 | 55 |
| {BcNSRV7.BI=FALSE, BcOLV7.BI=TRUE}  => {BcDMV1=FALSE} | 0.15  3465 | 0.953  846 | 0.160  891 | 1.13  007 | 62 |
| {BcALV1.BI=FALSE, BcOLV7.BI=TRUE} =>  {BcDMV1=FALSE} | 0.12  8713 | 0.928  571 | 0.138  614 | 1.10  0126 | 52 |
| {BcFV9=FALSE, BcOLV7.BI=TRUE} =>  {BcDMV1=FALSE} | 0.15  099 | 0.924  242 | 0.163  366 | 1.09  4997 | 61 |
| {BcOLV7.BI=TRUE, BcULV5=FALSE} =>  {BcDMV1=FALSE} | 0.16  0891 | 0.915  493 | 0.175  743 | 1.08  4631 | 65 |
| {BcBNV5.BI=FALSE, BcOLV7.BI=TRUE} =>  {BcDMV1=FALSE} | 0.16  5842 | 0.930  556 | 0.178  218 | 1.10  2476 | 67 |
| {BcOLV7.BI=TRUE, BvF.BI=FALSE} =>  {BcDMV1=FALSE} | 0.17  0792 | 0.92 | 0.185  644 | 1.08  9971 | 69 |
| {BcMCV5.BI=FALSE, BcOLV7.BI=TRUE} =>  {BcDMV1=FALSE} | 0.15  8416 | 0.914  286 | 0.173  267 | 1.08  3201 | 64 |
| {BcOLV13.BI=FALSE, BcOLV7.BI=TRUE}  => {BcDMV1=FALSE} | 0.16  0891 | 0.928  571 | 0.173  267 | 1.10  0126 | 65 |
| {BcOLV2.BI=FALSE, BcOLV7.BI=TRUE} =>  {BcDMV1=FALSE} | 0.16  0891 | 0.915  493 | 0.175  743 | 1.08  4631 | 65 |
| {BcMCV3.BI=FALSE, BcOLV7.BI=TRUE} => | 0.15 | 0.914 | 0.173 | 1.08 | 64 |

| {BcDMV1=FALSE} | 8416 | 286 | 267 | 3201 |  |
| --- | --- | --- | --- | --- | --- |
| {BcOLV7.BI=TRUE, BcPV4.BI=FALSE} =>  {BcDMV1=FALSE} | 0.16  3366 | 0.929  577 | 0.175  743 | 1.10  1318 | 66 |
| {BcEV6=FALSE, BcOLV7.BI=TRUE} =>  {BcDMV1=FALSE} | 0.17  0792 | 0.932  432 | 0.183  168 | 1.10  47 | 69 |
| {BcOLV17.BI=FALSE, BcOLV7.BI=TRUE}  => {BcDMV1=FALSE} | 0.15  8416 | 0.927  536 | 0.170  792 | 1.09  8899 | 64 |
| {BcOLV15.BI=FALSE, BcOLV7.BI=TRUE}  => {BcDMV1=FALSE} | 0.17  3267 | 0.921  053 | 0.188  119 | 1.09  1218 | 70 |
| {BcEV5=FALSE, BcOLV7.BI=TRUE} =>  {BcDMV1=FALSE} | 0.17  5743 | 0.922  078 | 0.190  594 | 1.09  2432 | 71 |
| {BcHV3.BI=TRUE, BcVV3.BI=TRUE} =>  {BcMV1.BI=TRUE} | 0.07  9208 | 0.914  286 | 0.086  634 | 1.15  7904 | 32 |
| {BcMV1.BI=TRUE, BcVV3.BI=TRUE} =>  {BcHV3.BI=TRUE} | 0.07  9208 | 0.484  848 | 0.163  366 | 2.01  9369 | 32 |
| {BcHV3.BI=TRUE, BcMV1.BI=TRUE} =>  {BcVV3.BI=TRUE} | 0.07  9208 | 0.372  093 | 0.212  871 | 1.87  907 | 32 |
| {BcMV12=FALSE, BcVV3.BI=TRUE} =>  {BcHV3.BI=TRUE} | 0.07  6733 | 0.476  923 | 0.160  891 | 1.98  636 | 31 |
| {BcHV3.BI=TRUE, BcMV12=FALSE} =>  {BcVV3.BI=TRUE} | 0.07  6733 | 0.387  5 | 0.198  02 | 1.95  6875 | 31 |
| {BcFV9=FALSE, BcVV3.BI=TRUE} => | 0.07 | 0.476 | 0.160 | 1.98 | 31 |

| {BcHV3.BI=TRUE} | 6733 | 923 | 891 | 636 |  |
| --- | --- | --- | --- | --- | --- |
| {BcFV9=FALSE, BcHV3.BI=TRUE} =>  {BcVV3.BI=TRUE} | 0.07  6733 | 0.387  5 | 0.198  02 | 1.95  6875 | 31 |
| {BcOLV4.BI=FALSE, BcVV3.BI=TRUE} =>  {BcHV3.BI=TRUE} | 0.07  6733 | 0.442  857 | 0.173  267 | 1.84  4477 | 31 |
| {BcHV3.BI=TRUE, BcOLV4.BI=FALSE} =>  {BcVV3.BI=TRUE} | 0.07  6733 | 0.378  049 | 0.202  97 | 1.90  9146 | 31 |
| {BcVV3.BI=TRUE, BvF.BI=FALSE} =>  {BcHV3.BI=TRUE} | 0.08  1683 | 0.445  946 | 0.183  168 | 1.85  7342 | 33 |
| {BcHV3.BI=TRUE, BvF.BI=FALSE} =>  {BcVV3.BI=TRUE} | 0.08  1683 | 0.379  31 | 0.215  347 | 1.91  5517 | 33 |
| {BcOLV2.BI=FALSE, BcVV3.BI=TRUE} =>  {BcHV3.BI=TRUE} | 0.07  9208 | 0.438  356 | 0.180  693 | 1.82  5731 | 32 |
| {BcHV3.BI=TRUE, BcOLV2.BI=FALSE} =>  {BcVV3.BI=TRUE} | 0.07  9208 | 0.355  556 | 0.222  772 | 1.79  5556 | 32 |
| {BcMCV3.BI=FALSE, BcVV3.BI=TRUE} =>  {BcHV3.BI=TRUE} | 0.08  6634 | 0.448  718 | 0.193  069 | 1.86  8887 | 35 |
| {BcHV3.BI=TRUE, BcMCV3.BI=FALSE} =>  {BcVV3.BI=TRUE} | 0.08  6634 | 0.364  583 | 0.237  624 | 1.84  1146 | 35 |
| {BcPV4.BI=FALSE, BcVV3.BI=TRUE} =>  {BcHV3.BI=TRUE} | 0.08  6634 | 0.454  545 | 0.190  594 | 1.89  3158 | 35 |
| {BcHV3.BI=TRUE, BcPV4.BI=FALSE} => | 0.08 | 0.380 | 0.227 | 1.92 | 35 |

| {BcVV3.BI=TRUE} | 6634 | 435 | 723 | 1196 |  |
| --- | --- | --- | --- | --- | --- |
| {BcEV6=FALSE, BcVV3.BI=TRUE} =>  {BcHV3.BI=TRUE} | 0.08  4158 | 0.453  333 | 0.185  644 | 1.88  811 | 34 |
| {BcEV6=FALSE, BcHV3.BI=TRUE} =>  {BcVV3.BI=TRUE} | 0.08  4158 | 0.369  565 | 0.227  723 | 1.86  6304 | 34 |
| {BcOLV17.BI=FALSE, BcVV3.BI=TRUE} =>  {BcHV3.BI=TRUE} | 0.07  4257 | 0.410  959 | 0.180  693 | 1.71  1623 | 30 |
| {BcHV3.BI=TRUE, BcOLV17.BI=FALSE} =>  {BcVV3.BI=TRUE} | 0.07  4257 | 0.333  333 | 0.222  772 | 1.68  3333 | 30 |
| {BcOLV15.BI=FALSE, BcVV3.BI=TRUE} =>  {BcHV3.BI=TRUE} | 0.08  1683 | 0.428  571 | 0.190  594 | 1.78  4978 | 33 |
| {BcHV3.BI=TRUE, BcOLV15.BI=FALSE} =>  {BcVV3.BI=TRUE} | 0.08  1683 | 0.375 | 0.217  822 | 1.89  375 | 33 |
| {BcMCV3.BI=FALSE, BcVV3.BI=TRUE} =>  {BcEV2.BI=TRUE} | 0.07  4257 | 0.384  615 | 0.193  069 | 1.52  3379 | 30 |
| {BcEV2.BI=TRUE, BcMCV3.BI=FALSE} =>  {BcVV3.BI=TRUE} | 0.07  4257 | 0.315  789 | 0.235  149 | 1.59  4737 | 30 |
| {BcPV4.BI=FALSE, BcVV3.BI=TRUE} =>  {BcEV2.BI=TRUE} | 0.07  4257 | 0.389  61 | 0.190  594 | 1.54  3163 | 30 |
| {BcEV2.BI=TRUE, BcPV4.BI=FALSE} =>  {BcVV3.BI=TRUE} | 0.07  4257 | 0.315  789 | 0.235  149 | 1.59  4737 | 30 |
| {BcEV6=FALSE, BcVV3.BI=TRUE} => | 0.07 | 0.4 | 0.185 | 1.58 | 30 |

| {BcEV2.BI=TRUE} | 4257 |  | 644 | 4314 |  |
| --- | --- | --- | --- | --- | --- |
| {BcEV2.BI=TRUE, BcEV6=FALSE} => | 0.07 | 0.315 | 0.235 | 1.59 |  |
|  |  |  |  |  | 30 |
| {BcVV3.BI=TRUE} | 4257 | 789 | 149 | 4737 |  |
| {BcOLV1.BI.MOVA.=TRUE, | 0.07 | 0.833 | 0.089 | 1.53 |  |
|  |  |  |  |  | 30 |
| BcVV3.BI=TRUE} => {BcMV3.BI=TRUE} | 4257 | 333 | 109 | 7291 |  |
| {BcMV3.BI=TRUE, BcVV3.BI=TRUE} => | 0.07 | 0.588 | 0.126 | 1.77 |  |
|  |  |  |  |  | 30 |
| {BcOLV1.BI.MOVA.=TRUE} | 4257 | 235 | 238 | 3486 |  |
| {BcMV3.BI=TRUE, |  |  |  |  |  |
|  | 0.07 | 0.303 | 0.245 | 1.53 |  |
| BcOLV1.BI.MOVA.=TRUE} => |  |  |  |  | 30 |
|  | 4257 | 03 | 05 | 0303 |  |
| {BcVV3.BI=TRUE} |  |  |  |  |  |
| {BcOLV1.BI.MOVA.=TRUE, | 0.07 | 0.833 | 0.089 | 1.35 |  |
|  |  |  |  |  | 30 |
| BcVV3.BI=TRUE} => {BcMV11=TRUE} | 4257 | 333 | 109 | 2075 |  |
| {BcMV11=TRUE, BcVV3.BI=TRUE} => | 0.07 | 0.555 | 0.133 | 1.67 |  |
|  |  |  |  |  | 30 |
| {BcOLV1.BI.MOVA.=TRUE} | 4257 | 556 | 663 | 4959 |  |
| {BcMV11=TRUE, BcOLV1.BI.MOVA.=TRUE} | 0.07 | 0.306 | 0.242 | 1.54 |  |
|  |  |  |  |  | 30 |
| => {BcVV3.BI=TRUE} | 4257 | 122 | 574 | 5918 |  |
| {BcOLV1.BI.MOVA.=TRUE, | 0.08 |  | 0.089 | 1.26 |  |
|  |  | 1 |  |  | 36 |
| BcVV3.BI=TRUE} => {BcMV1.BI=TRUE} | 9109 |  | 109 | 6458 |  |
| {BcMV1.BI=TRUE, BcVV3.BI=TRUE} => | 0.08 | 0.545 | 0.163 | 1.64 |  |
|  |  |  |  |  | 36 |
| {BcOLV1.BI.MOVA.=TRUE} | 9109 | 455 | 366 | 4505 |  |
| {BcMV1.BI=TRUE, | 0.08 | 0.302 | 0.294 | 1.52 |  |
|  |  |  |  |  | 36 |
| BcOLV1.BI.MOVA.=TRUE} => | 9109 | 521 | 554 | 7731 |  |

| {BcVV3.BI=TRUE} |  |  |  |  |  |
| --- | --- | --- | --- | --- | --- |
| {BcFV9=FALSE, BcVV3.BI=TRUE} => | 0.07 | 0.476 | 0.160 | 1.43 |  |
|  |  |  |  |  | 31 |
| {BcOLV1.BI.MOVA.=TRUE} | 6733 | 923 | 891 | 7887 |  |
| {BcFV9=FALSE, BcOLV1.BI.MOVA.=TRUE} | 0.07 | 0.274 | 0.279 | 1.38 |  |
|  |  |  |  |  | 31 |
| => {BcVV3.BI=TRUE} | 6733 | 336 | 703 | 5398 |  |
| {BcVV3.BI=TRUE, BvF.BI=FALSE} => | 0.08 | 0.445 | 0.183 | 1.34 |  |
|  |  |  |  |  | 33 |
| {BcOLV1.BI.MOVA.=TRUE} | 1683 | 946 | 168 | 4494 |  |
| {BcOLV1.BI.MOVA.=TRUE, | 0.07 | 0.267 | 0.277 | 1.35 |  |
|  |  |  |  |  | 30 |
| BcOLV2.BI=FALSE} => {BcVV3.BI=TRUE} | 4257 | 857 | 228 | 2679 |  |
| {BcMCV3.BI=FALSE, BcVV3.BI=TRUE} => | 0.08 | 0.448 | 0.193 | 1.35 |  |
|  |  |  |  |  | 35 |
| {BcOLV1.BI.MOVA.=TRUE} | 6634 | 718 | 069 | 2851 |  |
| {BcMCV3.BI=FALSE, |  |  |  |  |  |
|  | 0.08 | 0.301 | 0.287 | 1.52 |  |
| BcOLV1.BI.MOVA.=TRUE} => |  |  |  |  | 35 |
|  | 6634 | 724 | 129 | 3707 |  |
| {BcVV3.BI=TRUE} |  |  |  |  |  |
| {BcPV4.BI=FALSE, BcVV3.BI=TRUE} => | 0.08 | 0.441 | 0.190 | 1.33 |  |
|  |  |  |  |  | 34 |
| {BcOLV1.BI.MOVA.=TRUE} | 4158 | 558 | 594 | 1266 |  |
| {BcOLV1.BI.MOVA.=TRUE, | 0.08 | 0.265 | 0.316 | 1.34 |  |
|  |  |  |  |  | 34 |
| BcPV4.BI=FALSE} => {BcVV3.BI=TRUE} | 4158 | 625 | 832 | 1406 |  |
| {BcEV6=FALSE, BcVV3.BI=TRUE} => | 0.08 |  | 0.185 | 1.32 |  |
|  |  | 0.44 |  |  | 33 |
| {BcOLV1.BI.MOVA.=TRUE} | 1683 |  | 644 | 6567 |  |
| {BcEV6=FALSE, BcOLV1.BI.MOVA.=TRUE} | 0.08 | 0.266 | 0.306 | 1.34 |  |
|  |  |  |  |  | 33 |
| => {BcVV3.BI=TRUE} | 1683 | 129 | 931 | 3952 |  |

| {BcOLV15.BI=FALSE, BcVV3.BI=TRUE} =>  {BcOLV1.BI.MOVA.=TRUE} | 0.08  6634 | 0.454  545 | 0.190  594 | 1.37  0421 | 35 |
| --- | --- | --- | --- | --- | --- |
| {BcOLV1.BI.MOVA.=TRUE,  BcOLV15.BI=FALSE} => {BcVV3.BI=TRUE} | 0.08  6634 | 0.269  231 | 0.321  782 | 1.35  9615 | 35 |
| {BcHV1.BI=FALSE, BcVV3.BI=TRUE} =>  {BcHV1SLR.BI=FALSE} | 0.07  6733 | 0.861  111 | 0.089  109 | 1.88  048 | 31 |
| {BcHV1SLR.BI=FALSE, BcVV3.BI=TRUE}  => {BcHV1.BI=FALSE} | 0.07  6733 | 0.861  111 | 0.089  109 | 2.17  4306 | 31 |
| {BcMV4.BI=TRUE, BcVV3.BI=TRUE} =>  {BcMV2.BI=TRUE} | 0.07  4257 | 0.666  667 | 0.111  386 | 1.44  0285 | 30 |
| {BcMV2.BI=TRUE, BcVV3.BI=TRUE} =>  {BcMV4.BI=TRUE} | 0.07  4257 | 0.666  667 | 0.111  386 | 1.50  4655 | 30 |
| {BcMV2.BI=TRUE, BcMV4.BI=TRUE} =>  {BcVV3.BI=TRUE} | 0.07  4257 | 0.270  27 | 0.274  752 | 1.36  4865 | 30 |
| {BcMV4.BI=TRUE, BcVV3.BI=TRUE} =>  {BcMV3.BI=TRUE} | 0.07  9208 | 0.711  111 | 0.111  386 | 1.31  1821 | 32 |
| {BcMV3.BI=TRUE, BcVV3.BI=TRUE} =>  {BcMV4.BI=TRUE} | 0.07  9208 | 0.627  451 | 0.126  238 | 1.41  6146 | 32 |
| {BcMV3.BI=TRUE, BcMV4.BI=TRUE} =>  {BcVV3.BI=TRUE} | 0.07  9208 | 0.268  908 | 0.294  554 | 1.35  7983 | 32 |
| {BcMV4.BI=TRUE, BcVV3.BI=TRUE} =>  {BcMV11=TRUE} | 0.08  9109 | 0.8 | 0.111  386 | 1.29  7992 | 36 |

| {BcMV11=TRUE, BcVV3.BI=TRUE} =>  {BcMV4.BI=TRUE} | 0.08  9109 | 0.666  667 | 0.133  663 | 1.50  4655 | 36 |
| --- | --- | --- | --- | --- | --- |
| {BcMV11=TRUE, BcMV4.BI=TRUE} =>  {BcVV3.BI=TRUE} | 0.08  9109 | 0.262  774 | 0.339  109 | 1.32  7007 | 36 |
| {BcMV9.BI=TRUE, BcVV3.BI=TRUE} =>  {BcMV4.BI=TRUE} | 0.07  4257 | 0.6 | 0.123  762 | 1.35  419 | 30 |
| {BcMV4.BI=TRUE, BcVV3.BI=TRUE} =>  {BcULV4=TRUE} | 0.08  9109 | 0.8 | 0.111  386 | 1.15  8423 | 36 |
| {BcULV4=TRUE, BcVV3.BI=TRUE} =>  {BcMV4.BI=TRUE} | 0.08  9109 | 0.620  69 | 0.143  564 | 1.40  0886 | 36 |
| {BcMV4.BI=TRUE, BcULV4=TRUE} =>  {BcVV3.BI=TRUE} | 0.08  9109 | 0.274  809 | 0.324  257 | 1.38  7786 | 36 |
| {BcMV4.BI=TRUE, BcRV1.BI=FALSE} =>  {BcVV3.BI=TRUE} | 0.07  9208 | 0.285  714 | 0.277  228 | 1.44  2857 | 32 |
| {BcEV2.BI=FALSE, BcVV3.BI=TRUE} =>  {BcMV4.BI=TRUE} | 0.07  4257 | 0.612  245 | 0.121  287 | 1.38  1826 | 30 |
| {BcULV1.BI=FALSE, BcVV3.BI=TRUE} =>  {BcMV4.BI=TRUE} | 0.08  6634 | 0.555  556 | 0.155  941 | 1.25  388 | 35 |
| {BcMV4.BI=TRUE, BcULV1.BI=FALSE} =>  {BcVV3.BI=TRUE} | 0.08  6634 | 0.259  259 | 0.334  158 | 1.30  9259 | 35 |
| {BcMV4.BI=TRUE, BcVV3.BI=TRUE} =>  {BcMV1.BI=TRUE} | 0.10  1485 | 0.911  111 | 0.111  386 | 1.15  3884 | 41 |

| {BcMV1.BI=TRUE, BcVV3.BI=TRUE} =>  {BcMV4.BI=TRUE} | 0.10  1485 | 0.621  212 | 0.163  366 | 1.40  2065 | 41 |
| --- | --- | --- | --- | --- | --- |
| {BcMV1.BI=TRUE, BcMV4.BI=TRUE} =>  {BcVV3.BI=TRUE} | 0.10  1485 | 0.269  737 | 0.376  238 | 1.36  2171 | 41 |
| {BcMV12=FALSE, BcVV3.BI=TRUE} =>  {BcMV4.BI=TRUE} | 0.08  9109 | 0.553  846 | 0.160  891 | 1.25  0021 | 36 |
| {BcNSRV7.BI=FALSE, BcVV3.BI=TRUE} =>  {BcMV4.BI=TRUE} | 0.08  4158 | 0.557  377 | 0.150  99 | 1.25  7991 | 34 |
| {BcOLV7.BI=FALSE, BcVV3.BI=TRUE} =>  {BcMV4.BI=TRUE} | 0.08  6634 | 0.564  516 | 0.153  465 | 1.27  4103 | 35 |
| {BcMV4.BI=TRUE, BcOLV7.BI=FALSE} =>  {BcVV3.BI=TRUE} | 0.08  6634 | 0.257  353 | 0.336  634 | 1.29  9632 | 35 |
| {BcULV5=FALSE, BcVV3.BI=TRUE} =>  {BcMV4.BI=TRUE} | 0.08  9109 | 0.562  5 | 0.158  416 | 1.26  9553 | 36 |
| {BcVV3.BI=TRUE, BvF.BI=FALSE} =>  {BcMV4.BI=TRUE} | 0.10  6436 | 0.581  081 | 0.183  168 | 1.31  149 | 43 |
| {BcMV4.BI=TRUE, BvF.BI=FALSE} =>  {BcVV3.BI=TRUE} | 0.10  6436 | 0.262  195 | 0.405  941 | 1.32  4085 | 43 |
| {BcOLV2.BI=FALSE, BcVV3.BI=TRUE} =>  {BcMV4.BI=TRUE} | 0.10  396 | 0.575  342 | 0.180  693 | 1.29  8538 | 42 |
| {BcMV4.BI=TRUE, BcOLV2.BI=FALSE} =>  {BcVV3.BI=TRUE} | 0.10  396 | 0.257  669 | 0.403  465 | 1.30  1227 | 42 |

| {BcMCV3.BI=FALSE, BcVV3.BI=TRUE} =>  {BcMV4.BI=TRUE} | 0.10  8911 | 0.564  103 | 0.193  069 | 1.27  317 | 44 |
| --- | --- | --- | --- | --- | --- |
| {BcMCV3.BI=FALSE, BcMV4.BI=TRUE} =>  {BcVV3.BI=TRUE} | 0.10  8911 | 0.265  06 | 0.410  891 | 1.33  8554 | 44 |
| {BcPV4.BI=FALSE, BcVV3.BI=TRUE} =>  {BcMV4.BI=TRUE} | 0.10  8911 | 0.571  429 | 0.190  594 | 1.28  9705 | 44 |
| {BcMV4.BI=TRUE, BcPV4.BI=FALSE} =>  {BcVV3.BI=TRUE} | 0.10  8911 | 0.260  355 | 0.418  317 | 1.31  4793 | 44 |
| {BcMV2.BI=TRUE, BcVV3.BI=TRUE} =>  {BcOLV18=TRUE} | 0.07  4257 | 0.666  667 | 0.111  386 | 1.28  8676 | 30 |
| {BcOLV18=TRUE, BcVV3.BI=TRUE} =>  {BcMV2.BI=TRUE} | 0.07  4257 | 0.697  674 | 0.106  436 | 1.50  7275 | 30 |
| {BcMV2.BI=TRUE, BcVV3.BI=TRUE} =>  {BcMV3.BI=TRUE} | 0.07  9208 | 0.711  111 | 0.111  386 | 1.31  1821 | 32 |
| {BcMV3.BI=TRUE, BcVV3.BI=TRUE} =>  {BcMV2.BI=TRUE} | 0.07  9208 | 0.627  451 | 0.126  238 | 1.35  5563 | 32 |
| {BcMV2.BI=TRUE, BcVV3.BI=TRUE} =>  {BcMV11=TRUE} | 0.08  6634 | 0.777  778 | 0.111  386 | 1.26  1937 | 35 |
| {BcMV11=TRUE, BcVV3.BI=TRUE} =>  {BcMV2.BI=TRUE} | 0.08  6634 | 0.648  148 | 0.133  663 | 1.40  0277 | 35 |
| {BcMV2.BI=TRUE, BcRV1.BI=FALSE} =>  {BcVV3.BI=TRUE} | 0.08  6634 | 0.304  348 | 0.284  653 | 1.53  6957 | 35 |

| {BcMV2.BI=TRUE, BcVV3.BI=TRUE} =>  {BcMV1.BI=TRUE} | 0.10  6436 | 0.955  556 | 0.111  386 | 1.21  0171 | 43 |
| --- | --- | --- | --- | --- | --- |
| {BcMV1.BI=TRUE, BcVV3.BI=TRUE} =>  {BcMV2.BI=TRUE} | 0.10  6436 | 0.651  515 | 0.163  366 | 1.40  7551 | 43 |
| {BcMV1.BI=TRUE, BcMV2.BI=TRUE} =>  {BcVV3.BI=TRUE} | 0.10  6436 | 0.260  606 | 0.408  416 | 1.31  6061 | 43 |
| {BcOLV7.BI=FALSE, BcVV3.BI=TRUE} =>  {BcMV2.BI=TRUE} | 0.09  4059 | 0.612  903 | 0.153  465 | 1.32  4133 | 38 |
| {BcMV2.BI=TRUE, BcOLV7.BI=FALSE} =>  {BcVV3.BI=TRUE} | 0.09  4059 | 0.262  069 | 0.358  911 | 1.32  3448 | 38 |
| {BcMCV3.BI=FALSE, BcMV2.BI=TRUE} =>  {BcVV3.BI=TRUE} | 0.10  6436 | 0.260  606 | 0.408  416 | 1.31  6061 | 43 |
| {BcOLV18=TRUE, BcVV3.BI=TRUE} =>  {BcMV3.BI=TRUE} | 0.08  1683 | 0.767  442 | 0.106  436 | 1.41  5737 | 33 |
| {BcMV3.BI=TRUE, BcVV3.BI=TRUE} =>  {BcOLV18=TRUE} | 0.08  1683 | 0.647  059 | 0.126  238 | 1.25  0774 | 33 |
| {BcOLV18=TRUE, BcVV3.BI=TRUE} =>  {BcMV11=TRUE} | 0.07  9208 | 0.744  186 | 0.106  436 | 1.20  7434 | 32 |
| {BcMV9.BI=TRUE, BcVV3.BI=TRUE} =>  {BcOLV18=TRUE} | 0.08  1683 | 0.66 | 0.123  762 | 1.27  5789 | 33 |
| {BcOLV18=TRUE, BcVV3.BI=TRUE} =>  {BcULV4=TRUE} | 0.09  4059 | 0.883  721 | 0.106  436 | 1.27  9653 | 38 |

| {BcULV4=TRUE, BcVV3.BI=TRUE} =>  {BcOLV18=TRUE} | 0.09  4059 | 0.655  172 | 0.143  564 | 1.26  6458 | 38 |
| --- | --- | --- | --- | --- | --- |
| {BcOLV18=TRUE, BcVV3.BI=TRUE} =>  {BcMV1.BI=TRUE} | 0.10  1485 | 0.953  488 | 0.106  436 | 1.20  7553 | 41 |
| {BcMV2.BI=FALSE, BcVV3.BI=TRUE} =>  {BcULV1.BI=FALSE} | 0.07  6733 | 0.885  714 | 0.086  634 | 1.12  8797 | 31 |
| {BcMV2.BI=FALSE, BcVV3.BI=TRUE} =>  {BcALV1.BI=FALSE} | 0.08  1683 | 0.942  857 | 0.086  634 | 1.15  7794 | 33 |
| {BcMV2.BI=FALSE, BcVV3.BI=TRUE} =>  {BcOLV4.BI=FALSE} | 0.08  4158 | 0.971  429 | 0.086  634 | 1.10  8636 | 34 |
| {BcHV1SLR.BI=TRUE, BcVV3.BI=TRUE} =>  {BcMV3.BI=TRUE} | 0.07  6733 | 0.704  545 | 0.108  911 | 1.29  9709 | 31 |
| {BcHV1SLR.BI=TRUE, BcVV3.BI=TRUE} =>  {BcHV1.BI=TRUE} | 0.09  6535 | 0.886  364 | 0.108  911 | 1.46  7586 | 39 |
| {BcHV1.BI=TRUE, BcVV3.BI=TRUE} =>  {BcHV1SLR.BI=TRUE} | 0.09  6535 | 0.886  364 | 0.108  911 | 1.63  5118 | 39 |
| {BcHV1SLR.BI=TRUE, BcVV3.BI=TRUE} =>  {BcMV11=TRUE} | 0.07  9208 | 0.727  273 | 0.108  911 | 1.17  9993 | 32 |
| {BcHV1SLR.BI=TRUE, BcVV3.BI=TRUE} =>  {BcULV4=TRUE} | 0.08  9109 | 0.818  182 | 0.108  911 | 1.18  4751 | 36 |
| {BcMV9.BI=TRUE, BcVV3.BI=TRUE} =>  {BcMV3.BI=TRUE} | 0.08  4158 | 0.68 | 0.123  762 | 1.25  4429 | 34 |

| {BcULV4=TRUE, BcVV3.BI=TRUE} =>  {BcMV3.BI=TRUE} | 0.09  901 | 0.689  655 | 0.143  564 | 1.27  2241 | 40 |
| --- | --- | --- | --- | --- | --- |
| {BcMV3.BI=TRUE, BcRV1.BI=FALSE} =>  {BcVV3.BI=TRUE} | 0.09  901 | 0.270  27 | 0.366  337 | 1.36  4865 | 40 |
| {BcEV2.BI=FALSE, BcVV3.BI=TRUE} =>  {BcMV3.BI=TRUE} | 0.08  4158 | 0.693  878 | 0.121  287 | 1.28  003 | 34 |
| {BcMV3.BI=TRUE, BcVV3.BI=TRUE} =>  {BcMV1.BI=TRUE} | 0.11  1386 | 0.882  353 | 0.126  238 | 1.11  7463 | 45 |
| {BcMV1.BI=TRUE, BcVV3.BI=TRUE} =>  {BcMV3.BI=TRUE} | 0.11  1386 | 0.681  818 | 0.163  366 | 1.25  7783 | 45 |
| {BcULV5=FALSE, BcVV3.BI=TRUE} =>  {BcMV3.BI=TRUE} | 0.10  6436 | 0.671  875 | 0.158  416 | 1.23  9441 | 43 |
| {BcMCV3.BI=FALSE, BcMV3.BI=TRUE} =>  {BcVV3.BI=TRUE} | 0.12  1287 | 0.255  208 | 0.475  248 | 1.28  8802 | 49 |
| {BcEV6=FALSE, BcVV3.BI=TRUE} =>  {BcMV3.BI=TRUE} | 0.12  1287 | 0.653  333 | 0.185  644 | 1.20  5236 | 49 |
| {BcMV4.BI=FALSE, BcVV3.BI=TRUE} =>  {BcRV1.BI=FALSE} | 0.07  4257 | 0.857  143 | 0.086  634 | 1.18  591 | 30 |
| {BcMV4.BI=FALSE, BcVV3.BI=TRUE} =>  {BcALV1.BI=FALSE} | 0.08  4158 | 0.971  429 | 0.086  634 | 1.19  2879 | 34 |
| {BcOLV1.BI.MOVA.=FALSE,  BcVV3.BI=TRUE} => {BcOLV16.BI=FALSE} | 0.07  6733 | 0.704  545 | 0.108  911 | 1.20  6086 | 31 |

| {BcULV3=TRUE, BcVV3.BI=TRUE} =>  {BcMV11=TRUE} | 0.08  4158 | 0.723  404 | 0.116  337 | 1.17  3716 | 34 |
| --- | --- | --- | --- | --- | --- |
| {BcULV3=TRUE, BcVV3.BI=TRUE} =>  {BcRV1.BI=FALSE} | 0.09  6535 | 0.829  787 | 0.116  337 | 1.14  8062 | 39 |
| {BcULV3=TRUE, BcVV3.BI=TRUE} =>  {BcMV1.BI=TRUE} | 0.10  396 | 0.893  617 | 0.116  337 | 1.13  1728 | 42 |
| {BcHV1.BI=TRUE, BcVV3.BI=TRUE} =>  {BcMV11=TRUE} | 0.08  1683 | 0.75 | 0.108  911 | 1.21  6867 | 33 |
| {BcHV1.BI=TRUE, BcVV3.BI=TRUE} =>  {BcULV4=TRUE} | 0.08  6634 | 0.795  455 | 0.108  911 | 1.15  1841 | 35 |
| {BcMV11=TRUE, BcRV1.BI=FALSE} =>  {BcVV3.BI=TRUE} | 0.10  6436 | 0.260  606 | 0.408  416 | 1.31  6061 | 43 |
| {BcMV11=TRUE, BcVV3.BI=TRUE} =>  {BcMV1.BI=TRUE} | 0.12  3762 | 0.925  926 | 0.133  663 | 1.17  2646 | 50 |
| {BcMV1.BI=TRUE, BcVV3.BI=TRUE} =>  {BcMV11=TRUE} | 0.12  3762 | 0.757  576 | 0.163  366 | 1.22  9159 | 50 |
| {BcOLV7.BI=FALSE, BcVV3.BI=TRUE} =>  {BcMV11=TRUE} | 0.11  3861 | 0.741  935 | 0.153  465 | 1.20  3783 | 46 |
| {BcOLV1.BI.MOVA.=FALSE,  BcVV3.BI=TRUE} => {BcOLV4.BI=FALSE} | 0.10  8911 | 1 | 0.108  911 | 1.14  1243 | 44 |
| {BcMV9.BI=TRUE, BcVV3.BI=TRUE} =>  {BcULV4=TRUE} | 0.11  6337 | 0.94 | 0.123  762 | 1.36  1147 | 47 |

| {BcULV4=TRUE, BcVV3.BI=TRUE} =>  {BcMV9.BI=TRUE} | 0.11  6337 | 0.810  345 | 0.143  564 | 1.19  4815 | 47 |
| --- | --- | --- | --- | --- | --- |
| {BcMV9.BI=TRUE, BcVV3.BI=TRUE} =>  {BcOLV4.BI=FALSE} | 0.11  8812 | 0.96 | 0.123  762 | 1.09  5593 | 48 |
| {BcMV9.BI=TRUE, BcVV3.BI=TRUE} =>  {BcULV5=FALSE} | 0.12  3762 | 1 | 0.123  762 | 1.14  1243 | 50 |
| {BcULV5=FALSE, BcVV3.BI=TRUE} =>  {BcMV9.BI=TRUE} | 0.12  3762 | 0.781  25 | 0.158  416 | 1.15  1916 | 50 |
| {BcULV5=FALSE, BcVV3.BI=TRUE} =>  {BcULV4=TRUE} | 0.13  1188 | 0.828  125 | 0.158  416 | 1.19  9149 | 53 |
| {BcPV3.BI=FALSE, BcVV3.BI=TRUE} =>  {BcRV1.BI=FALSE} | 0.10  396 | 0.823  529 | 0.126  238 | 1.13  9404 | 42 |
| {BcPV3.BI=FALSE, BcVV3.BI=TRUE} =>  {BcMV12=FALSE} | 0.11  3861 | 0.901  961 | 0.126  238 | 1.12  8149 | 46 |
| {BcOLV7.BI=FALSE, BcVV3.BI=TRUE} =>  {BcRV1.BI=FALSE} | 0.12  8713 | 0.838  71 | 0.153  465 | 1.16  0407 | 52 |
| {BcOLV13.BI=FALSE, BcVV3.BI=TRUE} =>  {BcRV1.BI=FALSE} | 0.14  3564 | 0.840  58 | 0.170  792 | 1.16  2994 | 58 |
| {BcOLV17.BI=FALSE, BcVV3.BI=TRUE} =>  {BcRV1.BI=FALSE} | 0.14  8515 | 0.821  918 | 0.180  693 | 1.13  7174 | 60 |
| {BcFV5.BI=TRUE, BcNSRV7.BI=TRUE} =>  {BcHV1SLR.BI=TRUE} | 0.07  4257 | 0.769  231 | 0.096  535 | 1.41  9038 | 30 |

| {BcHV1SLR.BI=TRUE, BcNSRV7.BI=TRUE}  => {BcFV5.BI=TRUE} | 0.07  4257 | 0.555  556 | 0.133  663 | 2.55  0505 | 30 |
| --- | --- | --- | --- | --- | --- |
| {BcFV5.BI=TRUE, BcHV1SLR.BI=TRUE} =>  {BcNSRV7.BI=TRUE} | 0.07  4257 | 0.491  803 | 0.150  99 | 2.45  2945 | 30 |
| {BcFV5.BI=TRUE, BcNSRV7.BI=TRUE} =>  {BcMV3.BI=TRUE} | 0.07  4257 | 0.769  231 | 0.096  535 | 1.41  9038 | 30 |
| {BcMV3.BI=TRUE, BcNSRV7.BI=TRUE} =>  {BcFV5.BI=TRUE} | 0.07  4257 | 0.625 | 0.118  812 | 2.86  9318 | 30 |
| {BcFV5.BI=TRUE, BcMV3.BI=TRUE} =>  {BcNSRV7.BI=TRUE} | 0.07  4257 | 0.483  871 | 0.153  465 | 2.41  3381 | 30 |
| {BcFV5.BI=TRUE, BcNSRV7.BI=TRUE} =>  {BcULV3=TRUE} | 0.08  1683 | 0.846  154 | 0.096  535 | 1.42  4359 | 33 |
| {BcNSRV7.BI=TRUE, BcULV3=TRUE} =>  {BcFV5.BI=TRUE} | 0.08  1683 | 0.523  81 | 0.155  941 | 2.40  4762 | 33 |
| {BcFV5.BI=TRUE, BcULV3=TRUE} =>  {BcNSRV7.BI=TRUE} | 0.08  1683 | 0.5 | 0.163  366 | 2.49  3827 | 33 |
| {BcNSRV7.BI=TRUE, BcPV3.BI=FALSE} =>  {BcFV5.BI=TRUE} | 0.07  6733 | 0.516  667 | 0.148  515 | 2.37  197 | 31 |
| {BcFV5.BI=TRUE, BcPV3.BI=FALSE} =>  {BcNSRV7.BI=TRUE} | 0.07  6733 | 0.469  697 | 0.163  366 | 2.34  2686 | 31 |
| {BcFV5.BI=TRUE, BcNSRV7.BI=TRUE} =>  {BcRV1.BI=FALSE} | 0.08  6634 | 0.897  436 | 0.096  535 | 1.24  1658 | 35 |

| {BcNSRV7.BI=TRUE, BcRV1.BI=FALSE} =>  {BcFV5.BI=TRUE} | 0.08  6634 | 0.530  303 | 0.163  366 | 2.43  4573 | 35 |
| --- | --- | --- | --- | --- | --- |
| {BcFV5.BI=TRUE, BcRV1.BI=FALSE} =>  {BcNSRV7.BI=TRUE} | 0.08  6634 | 0.486  111 | 0.178  218 | 2.42  4554 | 35 |
| {BcNSRV7.BI=TRUE, BcULV1.BI=FALSE}  => {BcFV5.BI=TRUE} | 0.07  6733 | 0.449  275 | 0.170  792 | 2.06  2582 | 31 |
| {BcFV5.BI=TRUE, BcULV1.BI=FALSE} =>  {BcNSRV7.BI=TRUE} | 0.07  6733 | 0.442  857 | 0.173  267 | 2.20  8818 | 31 |
| {BcMV1.BI=TRUE, BcNSRV7.BI=TRUE} =>  {BcFV5.BI=TRUE} | 0.08  4158 | 0.507  463 | 0.165  842 | 2.32  9715 | 34 |
| {BcFV5.BI=TRUE, BcMV1.BI=TRUE} =>  {BcNSRV7.BI=TRUE} | 0.08  4158 | 0.465  753 | 0.180  693 | 2.32  3017 | 34 |
| {BcMV12=FALSE, BcNSRV7.BI=TRUE} =>  {BcFV5.BI=TRUE} | 0.07  6733 | 0.5 | 0.153  465 | 2.29  5455 | 31 |
| {BcFV5.BI=TRUE, BcMV12=FALSE} =>  {BcNSRV7.BI=TRUE} | 0.07  6733 | 0.442  857 | 0.173  267 | 2.20  8818 | 31 |
| {BcNSRV7.BI=TRUE, BcOLV7.BI=FALSE}  => {BcFV5.BI=TRUE} | 0.08  4158 | 0.5 | 0.168  317 | 2.29  5455 | 34 |
| {BcFV5.BI=TRUE, BcOLV7.BI=FALSE} =>  {BcNSRV7.BI=TRUE} | 0.08  4158 | 0.459  459 | 0.183  168 | 2.29  1625 | 34 |
| {BcNSRV7.BI=TRUE, BcOLV4.BI=FALSE}  => {BcFV5.BI=TRUE} | 0.09  1584 | 0.480  519 | 0.190  594 | 2.20  6021 | 37 |

| {BcFV5.BI=TRUE, BcOLV4.BI=FALSE} =>  {BcNSRV7.BI=TRUE} | 0.09  1584 | 0.468  354 | 0.195  545 | 2.33  599 | 37 |
| --- | --- | --- | --- | --- | --- |
| {BcNSRV7.BI=TRUE, BcULV5=FALSE} =>  {BcFV5.BI=TRUE} | 0.08  9109 | 0.48 | 0.185  644 | 2.20  3636 | 36 |
| {BcFV5.BI=TRUE, BcULV5=FALSE} =>  {BcNSRV7.BI=TRUE} | 0.08  9109 | 0.455  696 | 0.195  545 | 2.27  2855 | 36 |
| {BcNSRV7.BI=TRUE, BvF.BI=FALSE} =>  {BcFV5.BI=TRUE} | 0.08  4158 | 0.453  333 | 0.185  644 | 2.08  1212 | 34 |
| {BcFV5.BI=TRUE, BvF.BI=FALSE} =>  {BcNSRV7.BI=TRUE} | 0.08  4158 | 0.441  558 | 0.190  594 | 2.20  2341 | 34 |
| {BcNSRV7.BI=TRUE, BcOLV13.BI=FALSE}  => {BcFV5.BI=TRUE} | 0.09  1584 | 0.493  333 | 0.185  644 | 2.26  4848 | 37 |
| {BcFV5.BI=TRUE, BcOLV13.BI=FALSE} =>  {BcNSRV7.BI=TRUE} | 0.09  1584 | 0.440  476 | 0.207  921 | 2.19  6943 | 37 |
| {BcNSRV7.BI=TRUE, BcOLV2.BI=FALSE}  => {BcFV5.BI=TRUE} | 0.08  6634 | 0.460  526 | 0.188  119 | 2.11  4234 | 35 |
| {BcFV5.BI=TRUE, BcOLV2.BI=FALSE} =>  {BcNSRV7.BI=TRUE} | 0.08  6634 | 0.432  099 | 0.200  495 | 2.15  5159 | 35 |
| {BcMCV3.BI=FALSE, BcNSRV7.BI=TRUE}  => {BcFV5.BI=TRUE} | 0.09  6535 | 0.487  5 | 0.198  02 | 2.23  8068 | 39 |
| {BcFV5.BI=TRUE, BcMCV3.BI=FALSE} =>  {BcNSRV7.BI=TRUE} | 0.09  6535 | 0.464  286 | 0.207  921 | 2.31  5697 | 39 |

| {BcNSRV7.BI=TRUE, BcPV4.BI=FALSE} =>  {BcFV5.BI=TRUE} | 0.09  1584 | 0.480  519 | 0.190  594 | 2.20  6021 | 37 |
| --- | --- | --- | --- | --- | --- |
| {BcFV5.BI=TRUE, BcPV4.BI=FALSE} =>  {BcNSRV7.BI=TRUE} | 0.09  1584 | 0.456  79 | 0.200  495 | 2.27  8311 | 37 |
| {BcEV6=FALSE, BcNSRV7.BI=TRUE} =>  {BcFV5.BI=TRUE} | 0.09  1584 | 0.480  519 | 0.190  594 | 2.20  6021 | 37 |
| {BcEV6=FALSE, BcFV5.BI=TRUE} =>  {BcNSRV7.BI=TRUE} | 0.09  1584 | 0.440  476 | 0.207  921 | 2.19  6943 | 37 |
| {BcNSRV7.BI=TRUE, BcOLV17.BI=FALSE}  => {BcFV5.BI=TRUE} | 0.09  6535 | 0.513  158 | 0.188  119 | 2.35  5861 | 39 |
| {BcFV5.BI=TRUE, BcOLV17.BI=FALSE} =>  {BcNSRV7.BI=TRUE} | 0.09  6535 | 0.453  488 | 0.212  871 | 2.26  1843 | 39 |
| {BcHV3.BI=TRUE, BcNSRV7.BI=TRUE} =>  {BcOLV18=TRUE} | 0.07  6733 | 0.815  789 | 0.094  059 | 1.57  6933 | 31 |
| {BcNSRV7.BI=TRUE, BcOLV18=TRUE} =>  {BcHV3.BI=TRUE} | 0.07  6733 | 0.596  154 | 0.128  713 | 2.48  295 | 31 |
| {BcHV3.BI=TRUE, BcOLV18=TRUE} =>  {BcNSRV7.BI=TRUE} | 0.07  6733 | 0.462  687 | 0.165  842 | 2.30  7721 | 31 |
| {BcHV3.BI=TRUE, BcNSRV7.BI=TRUE} =>  {BcULV3=TRUE} | 0.07  6733 | 0.815  789 | 0.094  059 | 1.37  3246 | 31 |
| {BcNSRV7.BI=TRUE, BcULV3=TRUE} =>  {BcHV3.BI=TRUE} | 0.07  6733 | 0.492  063 | 0.155  941 | 2.04  9419 | 31 |

| {BcHV3.BI=TRUE, BcULV3=TRUE} =>  {BcNSRV7.BI=TRUE} | 0.07  6733 | 0.449  275 | 0.170  792 | 2.24  083 | 31 |
| --- | --- | --- | --- | --- | --- |
| {BcHV3.BI=TRUE, BcNSRV7.BI=TRUE} =>  {BcMV9.BI=TRUE} | 0.07  4257 | 0.789  474 | 0.094  059 | 1.16  4041 | 30 |
| {BcMV9.BI=TRUE, BcNSRV7.BI=TRUE} =>  {BcHV3.BI=TRUE} | 0.07  4257 | 0.461  538 | 0.160  891 | 1.92  2284 | 30 |
| {BcHV3.BI=TRUE, BcMV9.BI=TRUE} =>  {BcNSRV7.BI=TRUE} | 0.07  4257 | 0.428  571 | 0.173  267 | 2.13  7566 | 30 |
| {BcHV3.BI=TRUE, BcNSRV7.BI=TRUE} =>  {BcRV1.BI=FALSE} | 0.08  1683 | 0.868  421 | 0.094  059 | 1.20  1514 | 33 |
| {BcNSRV7.BI=TRUE, BcRV1.BI=FALSE} =>  {BcHV3.BI=TRUE} | 0.08  1683 | 0.5 | 0.163  366 | 2.08  2474 | 33 |
| {BcHV3.BI=TRUE, BcRV1.BI=FALSE} =>  {BcNSRV7.BI=TRUE} | 0.08  1683 | 0.464  789 | 0.175  743 | 2.31  8206 | 33 |
| {BcHV3.BI=TRUE, BcNSRV7.BI=TRUE} =>  {BcMV1.BI=TRUE} | 0.08  9109 | 0.947  368 | 0.094  059 | 1.19  9802 | 36 |
| {BcMV1.BI=TRUE, BcNSRV7.BI=TRUE} =>  {BcHV3.BI=TRUE} | 0.08  9109 | 0.537  313 | 0.165  842 | 2.23  7883 | 36 |
| {BcHV3.BI=TRUE, BcMV1.BI=TRUE} =>  {BcNSRV7.BI=TRUE} | 0.08  9109 | 0.418  605 | 0.212  871 | 2.08  7855 | 36 |
| {BcMV12=FALSE, BcNSRV7.BI=TRUE} =>  {BcHV3.BI=TRUE} | 0.07  4257 | 0.483  871 | 0.153  465 | 2.01  5298 | 30 |

| {BcHV3.BI=TRUE, BcMV12=FALSE} =>  {BcNSRV7.BI=TRUE} | 0.07  4257 | 0.375 | 0.198  02 | 1.87  037 | 30 |
| --- | --- | --- | --- | --- | --- |
| {BcNSRV7.BI=TRUE, BcOLV7.BI=FALSE}  => {BcHV3.BI=TRUE} | 0.07  9208 | 0.470  588 | 0.168  317 | 1.95  9976 | 32 |
| {BcHV3.BI=TRUE, BcOLV7.BI=FALSE} =>  {BcNSRV7.BI=TRUE} | 0.07  9208 | 0.450  704 | 0.175  743 | 2.24  7957 | 32 |
| {BcHV3.BI=TRUE, BcNSRV7.BI=TRUE} =>  {BcOLV4.BI=FALSE} | 0.09  1584 | 0.973  684 | 0.094  059 | 1.11  121 | 37 |
| {BcNSRV7.BI=TRUE, BcOLV4.BI=FALSE}  => {BcHV3.BI=TRUE} | 0.09  1584 | 0.480  519 | 0.190  594 | 2.00  1339 | 37 |
| {BcHV3.BI=TRUE, BcOLV4.BI=FALSE} =>  {BcNSRV7.BI=TRUE} | 0.09  1584 | 0.451  22 | 0.202  97 | 2.25  0527 | 37 |
| {BcNSRV7.BI=TRUE, BcULV5=FALSE} =>  {BcHV3.BI=TRUE} | 0.08  6634 | 0.466  667 | 0.185  644 | 1.94  3643 | 35 |
| {BcHV3.BI=TRUE, BcULV5=FALSE} =>  {BcNSRV7.BI=TRUE} | 0.08  6634 | 0.416  667 | 0.207  921 | 2.07  8189 | 35 |
| {BcNSRV7.BI=TRUE, BvF.BI=FALSE} =>  {BcHV3.BI=TRUE} | 0.08  6634 | 0.466  667 | 0.185  644 | 1.94  3643 | 35 |
| {BcHV3.BI=TRUE, BvF.BI=FALSE} =>  {BcNSRV7.BI=TRUE} | 0.08  6634 | 0.402  299 | 0.215  347 | 2.00  6528 | 35 |
| {BcNSRV7.BI=TRUE, BcOLV13.BI=FALSE}  => {BcHV3.BI=TRUE} | 0.08  9109 | 0.48 | 0.185  644 | 1.99  9175 | 36 |

| {BcHV3.BI=TRUE, BcOLV13.BI=FALSE} =>  {BcNSRV7.BI=TRUE} | 0.08  9109 | 0.413  793 | 0.215  347 | 2.06  3857 | 36 |
| --- | --- | --- | --- | --- | --- |
| {BcNSRV7.BI=TRUE, BcOLV2.BI=FALSE}  => {BcHV3.BI=TRUE} | 0.08  4158 | 0.447  368 | 0.188  119 | 1.86  3266 | 34 |
| {BcHV3.BI=TRUE, BcOLV2.BI=FALSE} =>  {BcNSRV7.BI=TRUE} | 0.08  4158 | 0.377  778 | 0.222  772 | 1.88  4225 | 34 |
| {BcMCV3.BI=FALSE, BcNSRV7.BI=TRUE}  => {BcHV3.BI=TRUE} | 0.09  4059 | 0.475 | 0.198  02 | 1.97  8351 | 38 |
| {BcHV3.BI=TRUE, BcMCV3.BI=FALSE} =>  {BcNSRV7.BI=TRUE} | 0.09  4059 | 0.395  833 | 0.237  624 | 1.97  428 | 38 |
| {BcNSRV7.BI=TRUE, BcPV4.BI=FALSE} =>  {BcHV3.BI=TRUE} | 0.08  9109 | 0.467  532 | 0.190  594 | 1.94  7249 | 36 |
| {BcHV3.BI=TRUE, BcPV4.BI=FALSE} =>  {BcNSRV7.BI=TRUE} | 0.08  9109 | 0.391  304 | 0.227  723 | 1.95  1691 | 36 |
| {BcEV6=FALSE, BcNSRV7.BI=TRUE} =>  {BcHV3.BI=TRUE} | 0.09  4059 | 0.493  506 | 0.190  594 | 2.05  5429 | 38 |
| {BcEV6=FALSE, BcHV3.BI=TRUE} =>  {BcNSRV7.BI=TRUE} | 0.09  4059 | 0.413  043 | 0.227  723 | 2.06  0118 | 38 |
| {BcNSRV7.BI=TRUE, BcOLV17.BI=FALSE}  => {BcHV3.BI=TRUE} | 0.09  1584 | 0.486  842 | 0.188  119 | 2.02  7672 | 37 |
| {BcHV3.BI=TRUE, BcOLV17.BI=FALSE} =>  {BcNSRV7.BI=TRUE} | 0.09  1584 | 0.411  111 | 0.222  772 | 2.05  048 | 37 |

| {BcNSRV7.BI=TRUE, BcOLV15.BI=FALSE}  => {BcHV3.BI=TRUE} | 0.07  4257 | 0.434  783 | 0.170  792 | 1.81  0847 | 30 |
| --- | --- | --- | --- | --- | --- |
| {BcHV3.BI=TRUE, BcOLV15.BI=FALSE} =>  {BcNSRV7.BI=TRUE} | 0.07  4257 | 0.340  909 | 0.217  822 | 1.70  0337 | 30 |
| {BcNSRV7.BI=TRUE, BcOLV16.BI=TRUE}  => {BcULV4=TRUE} | 0.07  6733 | 0.939  394 | 0.081  683 | 1.36  0269 | 31 |
| {BcMV4.BI=TRUE, BcNSRV7.BI=TRUE} =>  {BcOLV18=TRUE} | 0.07  6733 | 0.720  93 | 0.106  436 | 1.39  3568 | 31 |
| {BcNSRV7.BI=TRUE, BcOLV18=TRUE} =>  {BcMV4.BI=TRUE} | 0.07  6733 | 0.596  154 | 0.128  713 | 1.34  5509 | 31 |
| {BcMV4.BI=TRUE, BcOLV18=TRUE} =>  {BcNSRV7.BI=TRUE} | 0.07  6733 | 0.276  786 | 0.277  228 | 1.38  0511 | 31 |
| {BcMV4.BI=TRUE, BcNSRV7.BI=TRUE} =>  {BcHV1SLR.BI=TRUE} | 0.08  1683 | 0.767  442 | 0.106  436 | 1.41  5737 | 33 |
| {BcHV1SLR.BI=TRUE, BcNSRV7.BI=TRUE}  => {BcMV4.BI=TRUE} | 0.08  1683 | 0.611  111 | 0.133  663 | 1.37  9268 | 33 |
| {BcHV1SLR.BI=TRUE, BcMV4.BI=TRUE} =>  {BcNSRV7.BI=TRUE} | 0.08  1683 | 0.311  321 | 0.262  376 | 1.55  276 | 33 |
| {BcMV4.BI=TRUE, BcNSRV7.BI=TRUE} =>  {BcMV3.BI=TRUE} | 0.07  4257 | 0.697  674 | 0.106  436 | 1.28  7034 | 30 |
| {BcMV3.BI=TRUE, BcNSRV7.BI=TRUE} =>  {BcMV4.BI=TRUE} | 0.07  4257 | 0.625 | 0.118  812 | 1.41  0615 | 30 |

| {BcMV4.BI=TRUE, BcNSRV7.BI=TRUE} =>  {BcULV3=TRUE} | 0.09  1584 | 0.860  465 | 0.106  436 | 1.44  845 | 37 |
| --- | --- | --- | --- | --- | --- |
| {BcNSRV7.BI=TRUE, BcULV3=TRUE} =>  {BcMV4.BI=TRUE} | 0.09  1584 | 0.587  302 | 0.155  941 | 1.32  553 | 37 |
| {BcMV4.BI=TRUE, BcULV3=TRUE} =>  {BcNSRV7.BI=TRUE} | 0.09  1584 | 0.310  924 | 0.294  554 | 1.55  0783 | 37 |
| {BcMV4.BI=TRUE, BcNSRV7.BI=TRUE} =>  {BcHV1.BI=TRUE} | 0.08  6634 | 0.813  953 | 0.106  436 | 1.34  7693 | 35 |
| {BcHV1.BI=TRUE, BcNSRV7.BI=TRUE} =>  {BcMV4.BI=TRUE} | 0.08  6634 | 0.625 | 0.138  614 | 1.41  0615 | 35 |
| {BcHV1.BI=TRUE, BcMV4.BI=TRUE} =>  {BcNSRV7.BI=TRUE} | 0.08  6634 | 0.289  256 | 0.299  505 | 1.44  271 | 35 |
| {BcMV4.BI=TRUE, BcNSRV7.BI=TRUE} =>  {BcMV11=TRUE} | 0.07  9208 | 0.744  186 | 0.106  436 | 1.20  7434 | 32 |
| {BcMV11=TRUE, BcNSRV7.BI=TRUE} =>  {BcMV4.BI=TRUE} | 0.07  9208 | 0.603  774 | 0.131  188 | 1.36  2707 | 32 |
| {BcMV4.BI=TRUE, BcNSRV7.BI=TRUE} =>  {BcMV9.BI=TRUE} | 0.09  4059 | 0.883  721 | 0.106  436 | 1.30  3005 | 38 |
| {BcMV9.BI=TRUE, BcNSRV7.BI=TRUE} =>  {BcMV4.BI=TRUE} | 0.09  4059 | 0.584  615 | 0.160  891 | 1.31  9467 | 38 |
| {BcMV4.BI=TRUE, BcMV9.BI=TRUE} =>  {BcNSRV7.BI=TRUE} | 0.09  4059 | 0.279  412 | 0.336  634 | 1.39  3609 | 38 |

| {BcMV4.BI=TRUE, BcNSRV7.BI=TRUE} =>  {BcULV4=TRUE} | 0.08  6634 | 0.813  953 | 0.106  436 | 1.17  8628 | 35 |
| --- | --- | --- | --- | --- | --- |
| {BcNSRV7.BI=TRUE, BcULV4=TRUE} =>  {BcMV4.BI=TRUE} | 0.08  6634 | 0.555  556 | 0.155  941 | 1.25  388 | 35 |
| {BcMV4.BI=TRUE, BcULV4=TRUE} =>  {BcNSRV7.BI=TRUE} | 0.08  6634 | 0.267  176 | 0.324  257 | 1.33  2579 | 35 |
| {BcMV4.BI=TRUE, BcPV3.BI=FALSE} =>  {BcNSRV7.BI=TRUE} | 0.07  4257 | 0.283  019 | 0.262  376 | 1.41  16 | 30 |
| {BcMV4.BI=TRUE, BcRV1.BI=FALSE} =>  {BcNSRV7.BI=TRUE} | 0.07  9208 | 0.285  714 | 0.277  228 | 1.42  5044 | 32 |
| {BcMV1.BI=TRUE, BcNSRV7.BI=TRUE} =>  {BcMV4.BI=TRUE} | 0.09  1584 | 0.552  239 | 0.165  842 | 1.24  6394 | 37 |
| {BcNSRV7.BI=TRUE, BcOLV2.BI=FALSE}  => {BcMV4.BI=TRUE} | 0.10  396 | 0.552  632 | 0.188  119 | 1.24  728 | 42 |
| {BcMV4.BI=TRUE, BcOLV2.BI=FALSE} =>  {BcNSRV7.BI=TRUE} | 0.10  396 | 0.257  669 | 0.403  465 | 1.28  5162 | 42 |
| {BcMV3.BI=FALSE, BcNSRV7.BI=TRUE} =>  {BcULV1.BI=FALSE} | 0.07  4257 | 0.909  091 | 0.081  683 | 1.15  8589 | 30 |
| {BcMV3.BI=FALSE, BcNSRV7.BI=TRUE} =>  {BcOLV7.BI=FALSE} | 0.07  4257 | 0.909  091 | 0.081  683 | 1.12  6603 | 30 |
| {BcMV2.BI=TRUE, BcNSRV7.BI=TRUE} =>  {BcOLV18=TRUE} | 0.07  6733 | 0.756  098 | 0.101  485 | 1.46  1547 | 31 |

| {BcNSRV7.BI=TRUE, BcOLV18=TRUE} =>  {BcMV2.BI=TRUE} | 0.07  6733 | 0.596  154 | 0.128  713 | 1.28  7947 | 31 |
| --- | --- | --- | --- | --- | --- |
| {BcMV2.BI=TRUE, BcNSRV7.BI=TRUE} =>  {BcULV3=TRUE} | 0.08  4158 | 0.829  268 | 0.101  485 | 1.39  5935 | 34 |
| {BcMV2.BI=TRUE, BcULV3=TRUE} =>  {BcNSRV7.BI=TRUE} | 0.08  4158 | 0.295  652 | 0.284  653 | 1.47  4611 | 34 |
| {BcMV2.BI=TRUE, BcNSRV7.BI=TRUE} =>  {BcMV11=TRUE} | 0.08  1683 | 0.804  878 | 0.101  485 | 1.30  5907 | 33 |
| {BcMV11=TRUE, BcNSRV7.BI=TRUE} =>  {BcMV2.BI=TRUE} | 0.08  1683 | 0.622  642 | 0.131  188 | 1.34  5172 | 33 |
| {BcMV2.BI=TRUE, BcNSRV7.BI=TRUE} =>  {BcMV9.BI=TRUE} | 0.09  4059 | 0.926  829 | 0.101  485 | 1.36  6566 | 38 |
| {BcMV9.BI=TRUE, BcNSRV7.BI=TRUE} =>  {BcMV2.BI=TRUE} | 0.09  4059 | 0.584  615 | 0.160  891 | 1.26  3019 | 38 |
| {BcMV2.BI=TRUE, BcMV9.BI=TRUE} =>  {BcNSRV7.BI=TRUE} | 0.09  4059 | 0.292  308 | 0.321  782 | 1.45  793 | 38 |
| {BcMV2.BI=TRUE, BcNSRV7.BI=TRUE} =>  {BcULV4=TRUE} | 0.08  9109 | 0.878  049 | 0.101  485 | 1.27  144 | 36 |
| {BcMV2.BI=TRUE, BcULV4=TRUE} =>  {BcNSRV7.BI=TRUE} | 0.08  9109 | 0.260  87 | 0.341  584 | 1.30  1127 | 36 |
| {BcMV2.BI=TRUE, BcNSRV7.BI=TRUE} =>  {BcMV1.BI=TRUE} | 0.09  4059 | 0.926  829 | 0.101  485 | 1.17  379 | 38 |

| {BcNSRV7.BI=TRUE, BcOLV15.BI=FALSE}  => {BcMV2.BI=TRUE} | 0.10  1485 | 0.594  203 | 0.170  792 | 1.28  3732 | 41 |
| --- | --- | --- | --- | --- | --- |
| {BcNSRV7.BI=TRUE, BcOLV18=TRUE} =>  {BcHV1SLR.BI=TRUE} | 0.08  9109 | 0.692  308 | 0.128  713 | 1.27  7134 | 36 |
| {BcHV1SLR.BI=TRUE, BcNSRV7.BI=TRUE}  => {BcOLV18=TRUE} | 0.08  9109 | 0.666  667 | 0.133  663 | 1.28  8676 | 36 |
| {BcHV1SLR.BI=TRUE, BcOLV18=TRUE} =>  {BcNSRV7.BI=TRUE} | 0.08  9109 | 0.260  87 | 0.341  584 | 1.30  1127 | 36 |
| {BcNSRV7.BI=TRUE, BcOLV18=TRUE} =>  {BcMV3.BI=TRUE} | 0.08  6634 | 0.673  077 | 0.128  713 | 1.24  1658 | 35 |
| {BcMV3.BI=TRUE, BcNSRV7.BI=TRUE} =>  {BcOLV18=TRUE} | 0.08  6634 | 0.729  167 | 0.118  812 | 1.40  949 | 35 |
| {BcMV3.BI=TRUE, BcOLV18=TRUE} =>  {BcNSRV7.BI=TRUE} | 0.08  6634 | 0.263  158 | 0.329  208 | 1.31  2541 | 35 |
| {BcNSRV7.BI=TRUE, BcOLV18=TRUE} =>  {BcULV3=TRUE} | 0.10  396 | 0.807  692 | 0.128  713 | 1.35  9615 | 42 |
| {BcNSRV7.BI=TRUE, BcULV3=TRUE} =>  {BcOLV18=TRUE} | 0.10  396 | 0.666  667 | 0.155  941 | 1.28  8676 | 42 |
| {BcOLV18=TRUE, BcULV3=TRUE} =>  {BcNSRV7.BI=TRUE} | 0.10  396 | 0.320  611 | 0.324  257 | 1.59  9095 | 42 |
| {BcNSRV7.BI=TRUE, BcOLV18=TRUE} =>  {BcMV11=TRUE} | 0.09  4059 | 0.730  769 | 0.128  713 | 1.18  5666 | 38 |

| {BcMV11=TRUE, BcNSRV7.BI=TRUE} =>  {BcOLV18=TRUE} | 0.09  4059 | 0.716  981 | 0.131  188 | 1.38  5935 | 38 |
| --- | --- | --- | --- | --- | --- |
| {BcMV11=TRUE, BcOLV18=TRUE} =>  {BcNSRV7.BI=TRUE} | 0.09  4059 | 0.271  429 | 0.346  535 | 1.35  3792 | 38 |
| {BcNSRV7.BI=TRUE, BcOLV18=TRUE} =>  {BcMV9.BI=TRUE} | 0.11  1386 | 0.865  385 | 0.128  713 | 1.27  5969 | 45 |
| {BcMV9.BI=TRUE, BcNSRV7.BI=TRUE} =>  {BcOLV18=TRUE} | 0.11  1386 | 0.692  308 | 0.160  891 | 1.33  8241 | 45 |
| {BcMV9.BI=TRUE, BcOLV18=TRUE} =>  {BcNSRV7.BI=TRUE} | 0.11  1386 | 0.269  461 | 0.413  366 | 1.34  3979 | 45 |
| {BcNSRV7.BI=TRUE, BcOLV18=TRUE} =>  {BcULV4=TRUE} | 0.11  3861 | 0.884  615 | 0.128  713 | 1.28  0948 | 46 |
| {BcNSRV7.BI=TRUE, BcULV4=TRUE} =>  {BcOLV18=TRUE} | 0.11  3861 | 0.730  159 | 0.155  941 | 1.41  1407 | 46 |
| {BcOLV18=TRUE, BcULV4=TRUE} =>  {BcNSRV7.BI=TRUE} | 0.11  3861 | 0.258  427 | 0.440  594 | 1.28  8944 | 46 |
| {BcOLV18=TRUE, BcPV3.BI=FALSE} =>  {BcNSRV7.BI=TRUE} | 0.08  9109 | 0.270  677 | 0.329  208 | 1.35  0042 | 36 |
| {BcOLV18=TRUE, BcRV1.BI=FALSE} =>  {BcNSRV7.BI=TRUE} | 0.10  1485 | 0.308  271 | 0.329  208 | 1.53  7548 | 41 |
| {BcOLV18=TRUE, BcULV1.BI=FALSE} =>  {BcNSRV7.BI=TRUE} | 0.10  396 | 0.291  667 | 0.356  436 | 1.45  4733 | 42 |

| {BcNSRV7.BI=TRUE, BcOLV18=TRUE} =>  {BcMV1.BI=TRUE} | 0.11  6337 | 0.903  846 | 0.128  713 | 1.14  4683 | 47 |
| --- | --- | --- | --- | --- | --- |
| {BcMV1.BI=TRUE, BcNSRV7.BI=TRUE} =>  {BcOLV18=TRUE} | 0.11  6337 | 0.701  493 | 0.165  842 | 1.35  5995 | 47 |
| {BcMV1.BI=TRUE, BcOLV18=TRUE} =>  {BcNSRV7.BI=TRUE} | 0.11  6337 | 0.264  045 | 0.440  594 | 1.31  6965 | 47 |
| {BcNSRV7.BI=TRUE, BcOLV7.BI=FALSE}  => {BcOLV18=TRUE} | 0.10  8911 | 0.647  059 | 0.168  317 | 1.25  0774 | 44 |
| {BcOLV18=TRUE, BcOLV7.BI=FALSE} =>  {BcNSRV7.BI=TRUE} | 0.10  8911 | 0.289  474 | 0.376  238 | 1.44  3795 | 44 |
| {BcOLV18=TRUE, BcOLV4.BI=FALSE} =>  {BcNSRV7.BI=TRUE} | 0.11  8812 | 0.265  193 | 0.448  02 | 1.32  2693 | 48 |
| {BcNSRV7.BI=TRUE, BcULV5=FALSE} =>  {BcOLV18=TRUE} | 0.12  1287 | 0.653  333 | 0.185  644 | 1.26  2903 | 49 |
| {BcOLV18=TRUE, BcULV5=FALSE} =>  {BcNSRV7.BI=TRUE} | 0.12  1287 | 0.259  259 | 0.467  822 | 1.29  3096 | 49 |
| {BcNSRV7.BI=TRUE, BvF.BI=FALSE} =>  {BcOLV18=TRUE} | 0.12  1287 | 0.653  333 | 0.185  644 | 1.26  2903 | 49 |
| {BcOLV18=TRUE, BvF.BI=FALSE} =>  {BcNSRV7.BI=TRUE} | 0.12  1287 | 0.255  208 | 0.475  248 | 1.27  2891 | 49 |
| {BcNSRV7.BI=TRUE, BcOLV13.BI=FALSE}  => {BcOLV18=TRUE} | 0.11  8812 | 0.64 | 0.185  644 | 1.23  7129 | 48 |

| {BcMCV3.BI=FALSE, BcNSRV7.BI=TRUE}  => {BcOLV18=TRUE} | 0.12  6238 | 0.637  5 | 0.198  02 | 1.23  2297 | 51 |
| --- | --- | --- | --- | --- | --- |
| {BcMCV3.BI=FALSE, BcOLV18=TRUE} =>  {BcNSRV7.BI=TRUE} | 0.12  6238 | 0.265  625 | 0.475  248 | 1.32  4846 | 51 |
| {BcEV6=FALSE, BcNSRV7.BI=TRUE} =>  {BcOLV18=TRUE} | 0.12  3762 | 0.649  351 | 0.190  594 | 1.25  5204 | 50 |
| {BcEV6=FALSE, BcOLV18=TRUE} =>  {BcNSRV7.BI=TRUE} | 0.12  3762 | 0.256  41 | 0.482  673 | 1.27  8886 | 50 |
| {BcNSRV7.BI=TRUE, BcOLV17.BI=FALSE}  => {BcOLV18=TRUE} | 0.11  8812 | 0.631  579 | 0.188  119 | 1.22  0851 | 48 |
| {BcEV5=FALSE, BcNSRV7.BI=TRUE} =>  {BcOLV18=TRUE} | 0.10  6436 | 0.632  353 | 0.168  317 | 1.22  2347 | 43 |
| {BcMV2.BI=FALSE, BcNSRV7.BI=TRUE} =>  {BcOLV1.BI.MOVA.=FALSE} | 0.08  9109 | 0.9 | 0.099  01 | 1.34  6667 | 36 |
| {BcNSRV7.BI=TRUE, BcOLV1.BI.MOVA.=FALSE} =>  {BcMV2.BI=FALSE} | 0.08  9109 | 0.666  667 | 0.133  663 | 1.24  1167 | 36 |
| {BcMV2.BI=FALSE, BcNSRV7.BI=TRUE} =>  {BcMV6.BI=FALSE} | 0.08  4158 | 0.85 | 0.099  01 | 1.22  2064 | 34 |
| {BcMV2.BI=FALSE, BcNSRV7.BI=TRUE} =>  {BcPV3.BI=FALSE} | 0.08  4158 | 0.85 | 0.099  01 | 1.19  6516 | 34 |
| {BcMV2.BI=FALSE, BcNSRV7.BI=TRUE} => | 0.09 | 0.925 | 0.099 | 1.27 | 37 |

| {BcRV1.BI=FALSE} | 1584 |  | 01 | 9795 |  |
| --- | --- | --- | --- | --- | --- |
| {BcMV2.BI=FALSE, BcNSRV7.BI=TRUE} =>  {BcULV1.BI=FALSE} | 0.09  1584 | 0.925 | 0.099  01 | 1.17  8864 | 37 |
| {BcMV2.BI=FALSE, BcNSRV7.BI=TRUE} =>  {BcOLV4.BI=FALSE} | 0.09  6535 | 0.975 | 0.099  01 | 1.11  2712 | 39 |
| {BcHV1SLR.BI=TRUE, BcNSRV7.BI=TRUE}  => {BcMV3.BI=TRUE} | 0.08  9109 | 0.666  667 | 0.133  663 | 1.22  9833 | 36 |
| {BcMV3.BI=TRUE, BcNSRV7.BI=TRUE} =>  {BcHV1SLR.BI=TRUE} | 0.08  9109 | 0.75 | 0.118  812 | 1.38  3562 | 36 |
| {BcHV1SLR.BI=TRUE, BcMV3.BI=TRUE} =>  {BcNSRV7.BI=TRUE} | 0.08  9109 | 0.270  677 | 0.329  208 | 1.35  0042 | 36 |
| {BcNSRV7.BI=TRUE, BcOLV16.BI=FALSE}  => {BcHV1SLR.BI=TRUE} | 0.08  6634 | 0.729  167 | 0.118  812 | 1.34  5129 | 35 |
| {BcHV1SLR.BI=TRUE, BcOLV16.BI=FALSE}  => {BcNSRV7.BI=TRUE} | 0.08  6634 | 0.284  553 | 0.304  455 | 1.41  9251 | 35 |
| {BcHV1SLR.BI=TRUE, BcNSRV7.BI=TRUE}  => {BcULV3=TRUE} | 0.12  3762 | 0.925  926 | 0.133  663 | 1.55  8642 | 50 |
| {BcNSRV7.BI=TRUE, BcULV3=TRUE} =>  {BcHV1SLR.BI=TRUE} | 0.12  3762 | 0.793  651 | 0.155  941 | 1.46  4086 | 50 |
| {BcHV1SLR.BI=TRUE, BcULV3=TRUE} =>  {BcNSRV7.BI=TRUE} | 0.12  3762 | 0.320  513 | 0.386  139 | 1.59  8607 | 50 |
| {BcHV1SLR.BI=TRUE, BcNSRV7.BI=TRUE} | 0.12 | 0.925 | 0.133 | 1.53 | 50 |

| => {BcHV1.BI=TRUE} | 3762 | 926 | 663 | 309 |  |
| --- | --- | --- | --- | --- | --- |
| {BcHV1.BI=TRUE, BcNSRV7.BI=TRUE} => | 0.12 | 0.892 | 0.138 | 1.64 |  |
|  |  |  |  |  | 50 |
| {BcHV1SLR.BI=TRUE} | 3762 | 857 | 614 | 7097 |  |
| {BcHV1.BI=TRUE, BcHV1SLR.BI=TRUE} => | 0.12 | 0.255 | 0.485 | 1.27 |  |
|  |  |  |  |  | 50 |
| {BcNSRV7.BI=TRUE} | 3762 | 102 | 149 | 2361 |  |
| {BcMV11=TRUE, BcNSRV7.BI=TRUE} => | 0.09 | 0.735 | 0.131 | 1.35 |  |
|  |  |  |  |  | 39 |
| {BcHV1SLR.BI=TRUE} | 6535 | 849 | 188 | 7457 |  |
| {BcHV1SLR.BI=TRUE, BcMV11=TRUE} => | 0.09 | 0.270 | 0.356 | 1.35 |  |
|  |  |  |  |  | 39 |
| {BcNSRV7.BI=TRUE} | 6535 | 833 | 436 | 0823 |  |
| {BcNSRV7.BI=TRUE, |  |  |  |  |  |
|  | 0.08 | 0.666 | 0.133 | 1.22 |  |
| BcOLV1.BI.MOVA.=FALSE} => |  |  |  |  | 36 |
|  | 9109 | 667 | 663 | 9833 |  |
| {BcHV1SLR.BI=TRUE} |  |  |  |  |  |
| {BcHV1SLR.BI=TRUE, |  |  |  |  |  |
|  | 0.08 | 0.260 | 0.341 | 1.30 |  |
| BcOLV1.BI.MOVA.=FALSE} => |  |  |  |  | 36 |
|  | 9109 | 87 | 584 | 1127 |  |
| {BcNSRV7.BI=TRUE} |  |  |  |  |  |
| {BcNSRV7.BI=TRUE, BcULV4=TRUE} => | 0.10 | 0.650 | 0.155 | 1.20 |  |
|  |  |  |  |  | 41 |
| {BcHV1SLR.BI=TRUE} | 1485 | 794 | 941 | 0551 |  |
| {BcNSRV7.BI=TRUE, BcPV3.BI=FALSE} => | 0.10 | 0.683 | 0.148 | 1.26 |  |
|  |  |  |  |  | 41 |
| {BcHV1SLR.BI=TRUE} | 1485 | 333 | 515 | 0578 |  |
| {BcHV1SLR.BI=TRUE, BcPV3.BI=FALSE} | 0.10 | 0.271 | 0.373 | 1.35 |  |
|  |  |  |  |  | 41 |
| => {BcNSRV7.BI=TRUE} | 1485 | 523 | 762 | 4264 |  |
| {BcHV1SLR.BI=TRUE, BcRV1.BI=FALSE} | 0.10 | 0.272 | 0.381 | 1.36 | 42 |

| => {BcNSRV7.BI=TRUE} | 396 | 727 | 188 | 0269 |  |
| --- | --- | --- | --- | --- | --- |
| {BcNSRV7.BI=TRUE, BcULV1.BI=FALSE}  => {BcHV1SLR.BI=TRUE} | 0.11  6337 | 0.681  159 | 0.170  792 | 1.25  6568 | 47 |
| {BcHV1SLR.BI=TRUE, BcULV1.BI=FALSE}  => {BcNSRV7.BI=TRUE} | 0.11  6337 | 0.303  226 | 0.383  663 | 1.51  2386 | 47 |
| {BcMV1.BI=TRUE, BcNSRV7.BI=TRUE} =>  {BcHV1SLR.BI=TRUE} | 0.11  1386 | 0.671  642 | 0.165  842 | 1.23  901 | 45 |
| {BcMV12=FALSE, BcNSRV7.BI=TRUE} =>  {BcHV1SLR.BI=TRUE} | 0.10  1485 | 0.661  29 | 0.153  465 | 1.21  9915 | 41 |
| {BcNSRV7.BI=TRUE, BcVV3.BI=FALSE} =>  {BcHV1SLR.BI=TRUE} | 0.10  6436 | 0.693  548 | 0.153  465 | 1.27  9423 | 43 |
| {BcNSRV7.BI=TRUE, BcOLV7.BI=FALSE}  => {BcHV1SLR.BI=TRUE} | 0.11  8812 | 0.705  882 | 0.168  317 | 1.30  2176 | 48 |
| {BcHV1SLR.BI=TRUE, BcOLV7.BI=FALSE}  => {BcNSRV7.BI=TRUE} | 0.11  8812 | 0.272  727 | 0.435  644 | 1.36  0269 | 48 |
| {BcDMV1=FALSE, BcNSRV7.BI=TRUE} =>  {BcHV1SLR.BI=TRUE} | 0.08  9109 | 0.692  308 | 0.128  713 | 1.27  7134 | 36 |
| {BcNSRV7.BI=TRUE, BcOLV4.BI=FALSE}  => {BcHV1SLR.BI=TRUE} | 0.12  3762 | 0.649  351 | 0.190  594 | 1.19  7889 | 50 |
| {BcHV1SLR.BI=TRUE, BcOLV4.BI=FALSE}  => {BcNSRV7.BI=TRUE} | 0.12  3762 | 0.267  38 | 0.462  871 | 1.33  3597 | 50 |
| {BcNSRV7.BI=TRUE, BvF.BI=FALSE} => | 0.12 | 0.653 | 0.185 | 1.20 | 49 |

| {BcHV1SLR.BI=TRUE} | 1287 | 333 | 644 | 5236 |  |
| --- | --- | --- | --- | --- | --- |
| {BcMCV5.BI=FALSE, BcNSRV7.BI=TRUE}  => {BcHV1SLR.BI=TRUE} | 0.11  1386 | 0.661  765 | 0.168  317 | 1.22  079 | 45 |
| {BcNSRV7.BI=TRUE, BcOLV13.BI=FALSE}  => {BcHV1SLR.BI=TRUE} | 0.12  1287 | 0.653  333 | 0.185  644 | 1.20  5236 | 49 |
| {BcNSRV7.BI=TRUE, BcOLV2.BI=FALSE}  => {BcHV1SLR.BI=TRUE} | 0.12  3762 | 0.657  895 | 0.188  119 | 1.21  3651 | 50 |
| {BcHV1SLR.BI=TRUE, BcOLV2.BI=FALSE}  => {BcNSRV7.BI=TRUE} | 0.12  3762 | 0.256  41 | 0.482  673 | 1.27  8886 | 50 |
| {BcMCV3.BI=FALSE, BcNSRV7.BI=TRUE}  => {BcHV1SLR.BI=TRUE} | 0.13  1188 | 0.662  5 | 0.198  02 | 1.22  2146 | 53 |
| {BcHV1SLR.BI=TRUE, BcMCV3.BI=FALSE}  => {BcNSRV7.BI=TRUE} | 0.13  1188 | 0.269  036 | 0.487  624 | 1.34  1856 | 53 |
| {BcNSRV7.BI=TRUE, BcPV4.BI=FALSE} =>  {BcHV1SLR.BI=TRUE} | 0.12  3762 | 0.649  351 | 0.190  594 | 1.19  7889 | 50 |
| {BcEV6=FALSE, BcNSRV7.BI=TRUE} =>  {BcHV1SLR.BI=TRUE} | 0.12  6238 | 0.662  338 | 0.190  594 | 1.22  1847 | 51 |
| {BcEV6=FALSE, BcHV1SLR.BI=TRUE} =>  {BcNSRV7.BI=TRUE} | 0.12  6238 | 0.251  232 | 0.502  475 | 1.25  3056 | 51 |
| {BcNSRV7.BI=TRUE, BcOLV17.BI=FALSE}  => {BcHV1SLR.BI=TRUE} | 0.12  6238 | 0.671  053 | 0.188  119 | 1.23  7924 | 51 |
| {BcHV1SLR.BI=TRUE, BcOLV17.BI=FALSE} | 0.12 | 0.25 | 0.504 | 1.24 | 51 |

| => {BcNSRV7.BI=TRUE} | 6238 |  | 95 | 6914 |  |
| --- | --- | --- | --- | --- | --- |
| {BcEV5=FALSE, BcNSRV7.BI=TRUE} =>  {BcHV1SLR.BI=TRUE} | 0.11  3861 | 0.676  471 | 0.168  317 | 1.24  7918 | 46 |
| {BcMV3.BI=TRUE, BcNSRV7.BI=TRUE} =>  {BcULV3=TRUE} | 0.09  901 | 0.833  333 | 0.118  812 | 1.40  2778 | 40 |
| {BcMV3.BI=TRUE, BcULV3=TRUE} =>  {BcNSRV7.BI=TRUE} | 0.09  901 | 0.285  714 | 0.346  535 | 1.42  5044 | 40 |
| {BcMV3.BI=TRUE, BcNSRV7.BI=TRUE} =>  {BcHV1.BI=TRUE} | 0.09  1584 | 0.770  833 | 0.118  812 | 1.27  6298 | 37 |
| {BcMV3.BI=TRUE, BcNSRV7.BI=TRUE} =>  {BcMV9.BI=TRUE} | 0.09  6535 | 0.812  5 | 0.118  812 | 1.19  7993 | 39 |
| {BcMV3.BI=TRUE, BcNSRV7.BI=TRUE} =>  {BcULV4=TRUE} | 0.10  1485 | 0.854  167 | 0.118  812 | 1.23  6858 | 41 |
| {BcNSRV7.BI=TRUE, BcULV4=TRUE} =>  {BcMV3.BI=TRUE} | 0.10  1485 | 0.650  794 | 0.155  941 | 1.20  0551 | 41 |
| {BcMV3.BI=TRUE, BcRV1.BI=FALSE} =>  {BcNSRV7.BI=TRUE} | 0.09  6535 | 0.263  514 | 0.366  337 | 1.31  4314 | 39 |
| {BcMV4.BI=FALSE, BcNSRV7.BI=TRUE} =>  {BcOLV1.BI.MOVA.=FALSE} | 0.07  4257 | 0.789  474 | 0.094  059 | 1.18  1287 | 30 |
| {BcMV4.BI=FALSE, BcNSRV7.BI=TRUE} =>  {BcRV1.BI=FALSE} | 0.08  4158 | 0.894  737 | 0.094  059 | 1.23  7924 | 34 |
| {BcMV4.BI=FALSE, BcNSRV7.BI=TRUE} => | 0.09 | 0.973 | 0.094 | 1.24 | 37 |

| {BcULV1.BI=FALSE} | 1584 | 684 | 059 | 091 |  |
| --- | --- | --- | --- | --- | --- |
| {BcMV4.BI=FALSE, BcNSRV7.BI=TRUE} =>  {BcALV1.BI=FALSE} | 0.08  6634 | 0.921  053 | 0.094  059 | 1.13  1019 | 35 |
| {BcMV4.BI=FALSE, BcNSRV7.BI=TRUE} =>  {BcOLV4.BI=FALSE} | 0.09  4059 | 1 | 0.094  059 | 1.14  1243 | 38 |
| {BcNSRV7.BI=TRUE, BcOLV16.BI=FALSE}  => {BcULV3=TRUE} | 0.09  1584 | 0.770  833 | 0.118  812 | 1.29  7569 | 37 |
| {BcOLV16.BI=FALSE, BcULV3=TRUE} =>  {BcNSRV7.BI=TRUE} | 0.09  1584 | 0.291  339 | 0.314  356 | 1.45  3096 | 37 |
| {BcNSRV7.BI=TRUE, BcOLV16.BI=FALSE}  => {BcHV1.BI=TRUE} | 0.08  9109 | 0.75 | 0.118  812 | 1.24  1803 | 36 |
| {BcHV1.BI=TRUE, BcOLV16.BI=FALSE} =>  {BcNSRV7.BI=TRUE} | 0.08  9109 | 0.266  667 | 0.334  158 | 1.33  0041 | 36 |
| {BcMV6.BI=FALSE, BcNSRV7.BI=TRUE} =>  {BcOLV16.BI=FALSE} | 0.09  4059 | 0.730  769 | 0.128  713 | 1.25  0978 | 38 |
| {BcNSRV7.BI=TRUE, BcOLV16.BI=FALSE}  => {BcPV3.BI=FALSE} | 0.09  6535 | 0.812  5 | 0.118  812 | 1.14  3728 | 39 |
| {BcNSRV7.BI=TRUE, BcOLV16.BI=FALSE}  => {BcRV1.BI=FALSE} | 0.10  396 | 0.875 | 0.118  812 | 1.21  0616 | 42 |
| {BcNSRV7.BI=TRUE, BcOLV16.BI=FALSE}  => {BcULV1.BI=FALSE} | 0.10  6436 | 0.895  833 | 0.118  812 | 1.14  1693 | 43 |
| {BcNSRV7.BI=TRUE, BcOLV16.BI=FALSE} | 0.10 | 0.895 | 0.118 | 1.11 | 43 |

| => {BcOLV7.BI=FALSE} | 6436 | 833 | 812 | 0174 |  |
| --- | --- | --- | --- | --- | --- |
| {BcNSRV7.BI=TRUE, BcOLV16.BI=FALSE} | 0.11 | 0.979 | 0.118 | 1.11 |  |
|  |  |  |  |  | 47 |
| => {BcOLV4.BI=FALSE} | 6337 | 167 | 812 | 7467 |  |
| {BcNSRV7.BI=TRUE, BcULV3=TRUE} => | 0.12 | 0.793 | 0.155 | 1.31 |  |
|  |  |  |  |  | 50 |
| {BcHV1.BI=TRUE} | 3762 | 651 | 941 | 4078 |  |
| {BcHV1.BI=TRUE, BcNSRV7.BI=TRUE} => | 0.12 | 0.892 | 0.138 | 1.50 |  |
|  |  |  |  |  | 50 |
| {BcULV3=TRUE} | 3762 | 857 | 614 | 2976 |  |
| {BcHV1.BI=TRUE, BcULV3=TRUE} => | 0.12 | 0.292 | 0.423 | 1.45 |  |
|  |  |  |  |  | 50 |
| {BcNSRV7.BI=TRUE} | 3762 | 398 | 267 | 8378 |  |
| {BcNSRV7.BI=TRUE, BcULV3=TRUE} => | 0.11 | 0.730 | 0.155 | 1.18 |  |
|  |  |  |  |  | 46 |
| {BcMV11=TRUE} | 3861 | 159 | 941 | 4675 |  |
| {BcMV11=TRUE, BcNSRV7.BI=TRUE} => | 0.11 | 0.867 | 0.131 | 1.46 |  |
|  |  |  |  |  | 46 |
| {BcULV3=TRUE} | 3861 | 925 | 188 | 1006 |  |
| {BcMV11=TRUE, BcULV3=TRUE} => | 0.11 | 0.287 | 0.396 | 1.43 |  |
|  |  |  |  |  | 46 |
| {BcNSRV7.BI=TRUE} | 3861 | 5 | 04 | 3951 |  |
| {BcNSRV7.BI=TRUE, |  |  |  |  |  |
|  | 0.09 | 0.740 | 0.133 | 1.24 |  |
| BcOLV1.BI.MOVA.=FALSE} => |  |  |  |  | 40 |
|  | 901 | 741 | 663 | 6914 |  |
| {BcULV3=TRUE} |  |  |  |  |  |
| {BcNSRV7.BI=TRUE, BcULV3=TRUE} => | 0.12 | 0.809 | 0.155 | 1.19 |  |
|  |  |  |  |  | 51 |
| {BcMV9.BI=TRUE} | 6238 | 524 | 941 | 3604 |  |
| {BcMV9.BI=TRUE, BcNSRV7.BI=TRUE} => | 0.12 | 0.784 | 0.160 | 1.32 |  |
|  |  |  |  |  | 51 |
| {BcULV3=TRUE} | 6238 | 615 | 891 | 0769 |  |

| {BcMV9.BI=TRUE, BcULV3=TRUE} =>  {BcNSRV7.BI=TRUE} | 0.12  6238 | 0.298  246 | 0.423  267 | 1.48  7546 | 51 |
| --- | --- | --- | --- | --- | --- |
| {BcNSRV7.BI=TRUE, BcULV4=TRUE} =>  {BcULV3=TRUE} | 0.11  8812 | 0.761  905 | 0.155  941 | 1.28  254 | 48 |
| {BcULV3=TRUE, BcULV4=TRUE} =>  {BcNSRV7.BI=TRUE} | 0.11  8812 | 0.285  714 | 0.415  842 | 1.42  5044 | 48 |
| {BcNSRV7.BI=TRUE, BcPV3.BI=FALSE} =>  {BcULV3=TRUE} | 0.11  1386 | 0.75 | 0.148  515 | 1.26  25 | 45 |
| {BcPV3.BI=FALSE, BcULV3=TRUE} =>  {BcNSRV7.BI=TRUE} | 0.11  1386 | 0.264  706 | 0.420  792 | 1.32  0261 | 45 |
| {BcNSRV7.BI=TRUE, BcRV1.BI=FALSE} =>  {BcULV3=TRUE} | 0.12  3762 | 0.757  576 | 0.163  366 | 1.27  5253 | 50 |
| {BcRV1.BI=FALSE, BcULV3=TRUE} =>  {BcNSRV7.BI=TRUE} | 0.12  3762 | 0.285  714 | 0.433  168 | 1.42  5044 | 50 |
| {BcEV2.BI=FALSE, BcNSRV7.BI=TRUE} =>  {BcULV3=TRUE} | 0.09  6535 | 0.735  849 | 0.131  188 | 1.23  8679 | 39 |
| {BcHV3.BI=FALSE, BcNSRV7.BI=TRUE} =>  {BcULV3=TRUE} | 0.07  9208 | 0.744  186 | 0.106  436 | 1.25  2713 | 32 |
| {BcFV5.BI=FALSE, BcNSRV7.BI=TRUE} =>  {BcULV3=TRUE} | 0.07  4257 | 0.714  286 | 0.103  96 | 1.20  2381 | 30 |
| {BcNSRV7.BI=TRUE, BcULV1.BI=FALSE}  => {BcULV3=TRUE} | 0.13  3663 | 0.782  609 | 0.170  792 | 1.31  7391 | 54 |

| {BcULV1.BI=FALSE, BcULV3=TRUE} =>  {BcNSRV7.BI=TRUE} | 0.13  3663 | 0.291  892 | 0.457  921 | 1.45  5856 | 54 |
| --- | --- | --- | --- | --- | --- |
| {BcMV1.BI=TRUE, BcNSRV7.BI=TRUE} =>  {BcULV3=TRUE} | 0.13  3663 | 0.805  97 | 0.165  842 | 1.35  6716 | 54 |
| {BcMV1.BI=TRUE, BcULV3=TRUE} =>  {BcNSRV7.BI=TRUE} | 0.13  3663 | 0.275  51 | 0.485  149 | 1.37  415 | 54 |
| {BcMV12=FALSE, BcNSRV7.BI=TRUE} =>  {BcULV3=TRUE} | 0.11  8812 | 0.774  194 | 0.153  465 | 1.30  3226 | 48 |
| {BcNSRV7.BI=TRUE, BcVV3.BI=FALSE} =>  {BcULV3=TRUE} | 0.11  8812 | 0.774  194 | 0.153  465 | 1.30  3226 | 48 |
| {BcNSRV7.BI=TRUE, BcOLV7.BI=FALSE}  => {BcULV3=TRUE} | 0.13  1188 | 0.779  412 | 0.168  317 | 1.31  201 | 53 |
| {BcOLV7.BI=FALSE, BcULV3=TRUE} =>  {BcNSRV7.BI=TRUE} | 0.13  1188 | 0.276  042 | 0.475  248 | 1.37  68 | 53 |
| {BcALV1.BI=FALSE, BcNSRV7.BI=TRUE}  => {BcULV3=TRUE} | 0.11  8812 | 0.774  194 | 0.153  465 | 1.30  3226 | 48 |
| {BcDMV1=FALSE, BcNSRV7.BI=TRUE} =>  {BcULV3=TRUE} | 0.09  901 | 0.769  231 | 0.128  713 | 1.29  4872 | 40 |
| {BcFV9=FALSE, BcNSRV7.BI=TRUE} =>  {BcULV3=TRUE} | 0.11  3861 | 0.766  667 | 0.148  515 | 1.29  0556 | 46 |
| {BcNSRV7.BI=TRUE, BcOLV4.BI=FALSE}  => {BcULV3=TRUE} | 0.14  604 | 0.766  234 | 0.190  594 | 1.28  9827 | 59 |

| {BcOLV4.BI=FALSE, BcULV3=TRUE} =>  {BcNSRV7.BI=TRUE} | 0.14  604 | 0.287  805 | 0.507  426 | 1.43  5471 | 59 |
| --- | --- | --- | --- | --- | --- |
| {BcNSRV7.BI=TRUE, BcULV5=FALSE} =>  {BcULV3=TRUE} | 0.14  1089 | 0.76 | 0.185  644 | 1.27  9333 | 57 |
| {BcULV3=TRUE, BcULV5=FALSE} =>  {BcNSRV7.BI=TRUE} | 0.14  1089 | 0.272  727 | 0.517  327 | 1.36  0269 | 57 |
| {BcBNV5.BI=FALSE, BcNSRV7.BI=TRUE}  => {BcULV3=TRUE} | 0.11  1386 | 0.737  705 | 0.150  99 | 1.24  1803 | 45 |
| {BcNSRV7.BI=TRUE, BvF.BI=FALSE} =>  {BcULV3=TRUE} | 0.14  604 | 0.786  667 | 0.185  644 | 1.32  4222 | 59 |
| {BcULV3=TRUE, BvF.BI=FALSE} =>  {BcNSRV7.BI=TRUE} | 0.14  604 | 0.264  574 | 0.551  98 | 1.31  9604 | 59 |
| {BcMCV5.BI=FALSE, BcNSRV7.BI=TRUE}  => {BcULV3=TRUE} | 0.12  6238 | 0.75 | 0.168  317 | 1.26  25 | 51 |
| {BcNSRV7.BI=TRUE, BcOLV13.BI=FALSE}  => {BcULV3=TRUE} | 0.14  1089 | 0.76 | 0.185  644 | 1.27  9333 | 57 |
| {BcOLV13.BI=FALSE, BcULV3=TRUE} =>  {BcNSRV7.BI=TRUE} | 0.14  1089 | 0.259  091 | 0.544  554 | 1.29  2256 | 57 |
| {BcNSRV7.BI=TRUE, BcOLV2.BI=FALSE}  => {BcULV3=TRUE} | 0.14  604 | 0.776  316 | 0.188  119 | 1.30  6798 | 59 |
| {BcOLV2.BI=FALSE, BcULV3=TRUE} =>  {BcNSRV7.BI=TRUE} | 0.14  604 | 0.269  406 | 0.542  079 | 1.34  3706 | 59 |

| {BcMCV3.BI=FALSE, BcNSRV7.BI=TRUE}  => {BcULV3=TRUE} | 0.15  3465 | 0.775 | 0.198  02 | 1.30  4583 | 62 |
| --- | --- | --- | --- | --- | --- |
| {BcMCV3.BI=FALSE, BcULV3=TRUE} =>  {BcNSRV7.BI=TRUE} | 0.15  3465 | 0.283  105 | 0.542  079 | 1.41  203 | 62 |
| {BcNSRV7.BI=TRUE, BcPV4.BI=FALSE} =>  {BcULV3=TRUE} | 0.14  604 | 0.766  234 | 0.190  594 | 1.28  9827 | 59 |
| {BcPV4.BI=FALSE, BcULV3=TRUE} =>  {BcNSRV7.BI=TRUE} | 0.14  604 | 0.265  766 | 0.549  505 | 1.32  5548 | 59 |
| {BcEV6=FALSE, BcNSRV7.BI=TRUE} =>  {BcULV3=TRUE} | 0.14  8515 | 0.779  221 | 0.190  594 | 1.31  1688 | 60 |
| {BcEV6=FALSE, BcULV3=TRUE} =>  {BcNSRV7.BI=TRUE} | 0.14  8515 | 0.265  487 | 0.559  406 | 1.32  4156 | 60 |
| {BcNSRV7.BI=TRUE, BcOLV17.BI=FALSE}  => {BcULV3=TRUE} | 0.14  3564 | 0.763  158 | 0.188  119 | 1.28  4649 | 58 |
| {BcOLV17.BI=FALSE, BcULV3=TRUE} =>  {BcNSRV7.BI=TRUE} | 0.14  3564 | 0.257  778 | 0.556  931 | 1.28  5706 | 58 |
| {BcNSRV7.BI=TRUE, BcOLV15.BI=FALSE}  => {BcULV3=TRUE} | 0.13  1188 | 0.768  116 | 0.170  792 | 1.29  2995 | 53 |
| {BcEV5=FALSE, BcNSRV7.BI=TRUE} =>  {BcULV3=TRUE} | 0.12  6238 | 0.75 | 0.168  317 | 1.26  25 | 51 |
| {BcMV11=TRUE, BcNSRV7.BI=TRUE} =>  {BcHV1.BI=TRUE} | 0.09  6535 | 0.735  849 | 0.131  188 | 1.21  8373 | 39 |

| {BcNSRV7.BI=TRUE, BcOLV1.BI.MOVA.=FALSE} =>  {BcHV1.BI=TRUE} | 0.09  6535 | 0.722  222 | 0.133  663 | 1.19  5811 | 39 |
| --- | --- | --- | --- | --- | --- |
| {BcHV1.BI=TRUE, BcNSRV7.BI=TRUE} =>  {BcMV9.BI=TRUE} | 0.11  1386 | 0.803  571 | 0.138  614 | 1.18  4828 | 45 |
| {BcHV1.BI=TRUE, BcRV1.BI=FALSE} =>  {BcNSRV7.BI=TRUE} | 0.10  8911 | 0.261  905 | 0.415  842 | 1.30  629 | 44 |
| {BcHV1.BI=TRUE, BcULV1.BI=FALSE} =>  {BcNSRV7.BI=TRUE} | 0.11  8812 | 0.277  457 | 0.428  218 | 1.38  3858 | 48 |
| {BcNSRV7.BI=TRUE, BcVV3.BI=FALSE} =>  {BcHV1.BI=TRUE} | 0.11  3861 | 0.741  935 | 0.153  465 | 1.22  8451 | 46 |
| {BcDMV1=FALSE, BcNSRV7.BI=TRUE} =>  {BcHV1.BI=TRUE} | 0.09  1584 | 0.711  538 | 0.128  713 | 1.17  8121 | 37 |
| {BcHV1.BI=TRUE, BcMCV3.BI=FALSE} =>  {BcNSRV7.BI=TRUE} | 0.13  6139 | 0.25 | 0.544  554 | 1.24  6914 | 55 |
| {BcEV5=FALSE, BcNSRV7.BI=TRUE} =>  {BcHV1.BI=TRUE} | 0.12  1287 | 0.720  588 | 0.168  317 | 1.19  3105 | 49 |
| {BcMV11=TRUE, BcNSRV7.BI=TRUE} =>  {BcMV9.BI=TRUE} | 0.11  1386 | 0.849  057 | 0.131  188 | 1.25  1894 | 45 |
| {BcMV11=TRUE, BcMV9.BI=TRUE} =>  {BcNSRV7.BI=TRUE} | 0.11  1386 | 0.274  39 | 0.405  941 | 1.36  8564 | 45 |
| {BcMV11=TRUE, BcNSRV7.BI=TRUE} => | 0.10 | 0.811 | 0.131 | 1.17 | 43 |

| {BcULV4=TRUE} | 6436 | 321 | 188 | 4816 |  |
| --- | --- | --- | --- | --- | --- |
| {BcMV11=TRUE, BcNSRV7.BI=TRUE} =>  {BcMV1.BI=TRUE} | 0.11  6337 | 0.886  792 | 0.131  188 | 1.12  3085 | 47 |
| {BcMV6.BI=FALSE, BcNSRV7.BI=TRUE} =>  {BcOLV1.BI.MOVA.=FALSE} | 0.09  901 | 0.769  231 | 0.128  713 | 1.15  0997 | 40 |
| {BcNSRV7.BI=TRUE, |  |  |  |  |  |
|  | 0.11 | 0.851 | 0.133 | 1.17 |  |
| BcOLV1.BI.MOVA.=FALSE} => |  |  |  |  | 46 |
|  | 3861 | 852 | 663 | 859 |  |
| {BcRV1.BI=FALSE} |  |  |  |  |  |
| {BcHV3.BI=FALSE, BcNSRV7.BI=TRUE} => | 0.08 | 0.837 | 0.106 | 1.25 |  |
|  |  |  |  |  | 36 |
| {BcOLV1.BI.MOVA.=FALSE} | 9109 | 209 | 436 | 2713 |  |
| {BcFV5.BI=FALSE, BcNSRV7.BI=TRUE} => | 0.08 | 0.785 | 0.103 | 1.17 |  |
|  |  |  |  |  | 33 |
| {BcOLV1.BI.MOVA.=FALSE} | 1683 | 714 | 96 | 5661 |  |
| {BcNSRV7.BI=TRUE, |  |  |  |  |  |
|  | 0.12 | 0.925 | 0.133 | 1.18 |  |
| BcOLV1.BI.MOVA.=FALSE} => |  |  |  |  | 50 |
|  | 3762 | 926 | 663 | 0044 |  |
| {BcULV1.BI=FALSE} |  |  |  |  |  |
| {BcDMV1=FALSE, BcNSRV7.BI=TRUE} => | 0.09 | 0.769 | 0.128 | 1.15 |  |
|  |  |  |  |  | 40 |
| {BcOLV1.BI.MOVA.=FALSE} | 901 | 231 | 713 | 0997 |  |
| {BcNSRV7.BI=TRUE, |  |  |  |  |  |
|  | 0.12 | 0.962 | 0.133 | 1.09 |  |
| BcOLV1.BI.MOVA.=FALSE} => |  |  |  |  | 52 |
|  | 8713 | 963 | 663 | 8975 |  |
| {BcOLV4.BI=FALSE} |  |  |  |  |  |
| {BcMCV5.BI=FALSE, BcNSRV7.BI=TRUE} | 0.12 | 0.764 | 0.168 | 1.14 |  |
|  |  |  |  |  | 52 |
| => {BcOLV1.BI.MOVA.=FALSE} | 8713 | 706 | 317 | 4227 |  |

| {BcMV9.BI=TRUE, BcNSRV7.BI=TRUE} =>  {BcULV4=TRUE} | 0.13  3663 | 0.830  769 | 0.160  891 | 1.20  2978 | 54 |
| --- | --- | --- | --- | --- | --- |
| {BcNSRV7.BI=TRUE, BcULV4=TRUE} =>  {BcMV9.BI=TRUE} | 0.13  3663 | 0.857  143 | 0.155  941 | 1.26  3816 | 54 |
| {BcNSRV7.BI=TRUE, BcPV3.BI=FALSE} =>  {BcMV9.BI=TRUE} | 0.11  8812 | 0.8 | 0.148  515 | 1.17  9562 | 48 |
| {BcMV9.BI=TRUE, BcRV1.BI=FALSE} =>  {BcNSRV7.BI=TRUE} | 0.12  3762 | 0.271  739 | 0.455  446 | 1.35  5341 | 50 |
| {BcEV2.BI=FALSE, BcNSRV7.BI=TRUE} =>  {BcMV9.BI=TRUE} | 0.10  6436 | 0.811  321 | 0.131  188 | 1.19  6254 | 43 |
| {BcHV3.BI=FALSE, BcNSRV7.BI=TRUE} =>  {BcMV9.BI=TRUE} | 0.08  6634 | 0.813  953 | 0.106  436 | 1.20  0136 | 35 |
| {BcFV5.BI=FALSE, BcNSRV7.BI=TRUE} =>  {BcMV9.BI=TRUE} | 0.08  9109 | 0.857  143 | 0.103  96 | 1.26  3816 | 36 |
| {BcNSRV7.BI=TRUE, BcULV1.BI=FALSE}  => {BcMV9.BI=TRUE} | 0.13  6139 | 0.797  101 | 0.170  792 | 1.17  5288 | 55 |
| {BcMV9.BI=TRUE, BcULV1.BI=FALSE} =>  {BcNSRV7.BI=TRUE} | 0.13  6139 | 0.272  277 | 0.5 | 1.35  8025 | 55 |
| {BcMV1.BI=TRUE, BcNSRV7.BI=TRUE} =>  {BcMV9.BI=TRUE} | 0.13  6139 | 0.820  896 | 0.165  842 | 1.21  0372 | 55 |
| {BcNSRV7.BI=TRUE, BcVV3.BI=FALSE} =>  {BcMV9.BI=TRUE} | 0.12  8713 | 0.838  71 | 0.153  465 | 1.23  6638 | 52 |

| {BcNSRV7.BI=TRUE, BcOLV7.BI=FALSE}  => {BcMV9.BI=TRUE} | 0.13  6139 | 0.808  824 | 0.168  317 | 1.19  2572 | 55 |
| --- | --- | --- | --- | --- | --- |
| {BcMV9.BI=TRUE, BcOLV7.BI=FALSE} =>  {BcNSRV7.BI=TRUE} | 0.13  6139 | 0.268  293 | 0.507  426 | 1.33  8151 | 55 |
| {BcDMV1=FALSE, BcNSRV7.BI=TRUE} =>  {BcMV9.BI=TRUE} | 0.10  1485 | 0.788  462 | 0.128  713 | 1.16  2549 | 41 |
| {BcFV9=FALSE, BcNSRV7.BI=TRUE} =>  {BcMV9.BI=TRUE} | 0.11  8812 | 0.8 | 0.148  515 | 1.17  9562 | 48 |
| {BcMV9.BI=TRUE, BcNSRV7.BI=TRUE} =>  {BcOLV4.BI=FALSE} | 0.15  3465 | 0.953  846 | 0.160  891 | 1.08  857 | 62 |
| {BcNSRV7.BI=TRUE, BcOLV4.BI=FALSE}  => {BcMV9.BI=TRUE} | 0.15  3465 | 0.805  195 | 0.190  594 | 1.18  7222 | 62 |
| {BcMV9.BI=TRUE, BcOLV4.BI=FALSE} =>  {BcNSRV7.BI=TRUE} | 0.15  3465 | 0.248  996 | 0.616  337 | 1.24  1906 | 62 |
| {BcMV9.BI=TRUE, BcNSRV7.BI=TRUE} =>  {BcULV5=FALSE} | 0.15  8416 | 0.984  615 | 0.160  891 | 1.12  3685 | 64 |
| {BcNSRV7.BI=TRUE, BcULV5=FALSE} =>  {BcMV9.BI=TRUE} | 0.15  8416 | 0.853  333 | 0.185  644 | 1.25  82 | 64 |
| {BcNSRV7.BI=TRUE, BvF.BI=FALSE} =>  {BcMV9.BI=TRUE} | 0.15  099 | 0.813  333 | 0.185  644 | 1.19  9221 | 61 |
| {BcMV9.BI=TRUE, BvF.BI=FALSE} =>  {BcNSRV7.BI=TRUE} | 0.15  099 | 0.246  964 | 0.611  386 | 1.23  1769 | 61 |

| {BcNSRV7.BI=TRUE, BcOLV13.BI=FALSE}  => {BcMV9.BI=TRUE} | 0.14  604 | 0.786  667 | 0.185  644 | 1.15  9903 | 59 |
| --- | --- | --- | --- | --- | --- |
| {BcNSRV7.BI=TRUE, BcOLV2.BI=FALSE}  => {BcMV9.BI=TRUE} | 0.14  8515 | 0.789  474 | 0.188  119 | 1.16  4041 | 60 |
| {BcMCV3.BI=FALSE, BcNSRV7.BI=TRUE}  => {BcMV9.BI=TRUE} | 0.15  8416 | 0.8 | 0.198  02 | 1.17  9562 | 64 |
| {BcMCV3.BI=FALSE, BcMV9.BI=TRUE} =>  {BcNSRV7.BI=TRUE} | 0.15  8416 | 0.251  969 | 0.628  713 | 1.25  6732 | 64 |
| {BcNSRV7.BI=TRUE, BcPV4.BI=FALSE} =>  {BcMV9.BI=TRUE} | 0.15  5941 | 0.818  182 | 0.190  594 | 1.20  637 | 63 |
| {BcMV9.BI=TRUE, BcPV4.BI=FALSE} =>  {BcNSRV7.BI=TRUE} | 0.15  5941 | 0.246  094 | 0.633  663 | 1.22  7431 | 63 |
| {BcEV6=FALSE, BcNSRV7.BI=TRUE} =>  {BcMV9.BI=TRUE} | 0.15  3465 | 0.805  195 | 0.190  594 | 1.18  7222 | 62 |
| {BcNSRV7.BI=TRUE, BcOLV17.BI=FALSE}  => {BcMV9.BI=TRUE} | 0.14  8515 | 0.789  474 | 0.188  119 | 1.16  4041 | 60 |
| {BcNSRV7.BI=TRUE, BcOLV15.BI=FALSE}  => {BcMV9.BI=TRUE} | 0.13  6139 | 0.797  101 | 0.170  792 | 1.17  5288 | 55 |
| {BcEV5=FALSE, BcNSRV7.BI=TRUE} =>  {BcMV9.BI=TRUE} | 0.13  3663 | 0.794  118 | 0.168  317 | 1.17  0889 | 54 |
| {BcRV1.BI=FALSE, BcULV4=TRUE} =>  {BcNSRV7.BI=TRUE} | 0.11  8812 | 0.265  193 | 0.448  02 | 1.32  2693 | 48 |

| {BcFV5.BI=FALSE, BcNSRV7.BI=TRUE} =>  {BcULV4=TRUE} | 0.08  4158 | 0.809  524 | 0.103  96 | 1.17  2214 | 34 |
| --- | --- | --- | --- | --- | --- |
| {BcULV1.BI=FALSE, BcULV4=TRUE} =>  {BcNSRV7.BI=TRUE} | 0.12  8713 | 0.258  706 | 0.497  525 | 1.29  0338 | 52 |
| {BcMV1.BI=TRUE, BcNSRV7.BI=TRUE} =>  {BcULV4=TRUE} | 0.13  1188 | 0.791  045 | 0.165  842 | 1.14  5456 | 53 |
| {BcNSRV7.BI=TRUE, BcVV3.BI=FALSE} =>  {BcULV4=TRUE} | 0.12  1287 | 0.790  323 | 0.153  465 | 1.14  441 | 49 |
| {BcNSRV7.BI=TRUE, BcULV4=TRUE} =>  {BcOLV4.BI=FALSE} | 0.15  099 | 0.968  254 | 0.155  941 | 1.10  5013 | 61 |
| {BcNSRV7.BI=TRUE, BcOLV4.BI=FALSE}  => {BcULV4=TRUE} | 0.15  099 | 0.792  208 | 0.190  594 | 1.14  714 | 61 |
| {BcNSRV7.BI=TRUE, BcULV5=FALSE} =>  {BcULV4=TRUE} | 0.14  604 | 0.786  667 | 0.185  644 | 1.13  9116 | 59 |
| {BcBNV5.BI=FALSE, BcNSRV7.BI=TRUE}  => {BcULV4=TRUE} | 0.11  8812 | 0.786  885 | 0.150  99 | 1.13  9432 | 48 |
| {BcNSRV7.BI=TRUE, BvF.BI=FALSE} =>  {BcULV4=TRUE} | 0.14  8515 | 0.8 | 0.185  644 | 1.15  8423 | 60 |
| {BcNSRV7.BI=TRUE, BcOLV2.BI=FALSE}  => {BcULV4=TRUE} | 0.14  8515 | 0.789  474 | 0.188  119 | 1.14  3181 | 60 |
| {BcNSRV7.BI=TRUE, BcOLV15.BI=FALSE}  => {BcULV4=TRUE} | 0.13  8614 | 0.811  594 | 0.170  792 | 1.17  5212 | 56 |

| {BcEV5=FALSE, BcNSRV7.BI=TRUE} =>  {BcULV4=TRUE} | 0.13  3663 | 0.794  118 | 0.168  317 | 1.14  9905 | 54 |
| --- | --- | --- | --- | --- | --- |
| {BcMV6.BI=FALSE, BcNSRV7.BI=TRUE} =>  {BcRV1.BI=FALSE} | 0.11  1386 | 0.865  385 | 0.128  713 | 1.19  7313 | 45 |
| {BcMV6.BI=FALSE, BcNSRV7.BI=TRUE} =>  {BcULV1.BI=FALSE} | 0.11  3861 | 0.884  615 | 0.128  713 | 1.12  7396 | 46 |
| {BcMV6.BI=FALSE, BcNSRV7.BI=TRUE} =>  {BcOLV4.BI=FALSE} | 0.12  6238 | 0.980  769 | 0.128  713 | 1.11  9296 | 51 |
| {BcNSRV7.BI=TRUE, BcPV3.BI=FALSE} =>  {BcRV1.BI=FALSE} | 0.12  1287 | 0.816  667 | 0.148  515 | 1.12  9909 | 49 |
| {BcNSRV7.BI=TRUE, BcPV3.BI=FALSE} =>  {BcOLV4.BI=FALSE} | 0.14  604 | 0.983  333 | 0.148  515 | 1.12  2222 | 59 |
| {BcNSRV7.BI=TRUE, BcPV3.BI=FALSE} =>  {BcULV5=FALSE} | 0.14  1089 | 0.95 | 0.148  515 | 1.08  4181 | 57 |
| {BcMV12=FALSE, BcNSRV7.BI=TRUE} =>  {BcRV1.BI=FALSE} | 0.13  6139 | 0.887  097 | 0.153  465 | 1.22  7353 | 55 |
| {BcNSRV7.BI=TRUE, BcRV1.BI=FALSE} =>  {BcOLV7.BI=FALSE} | 0.14  8515 | 0.909  091 | 0.163  366 | 1.12  6603 | 60 |
| {BcNSRV7.BI=TRUE, BcOLV7.BI=FALSE}  => {BcRV1.BI=FALSE} | 0.14  8515 | 0.882  353 | 0.168  317 | 1.22  079 | 60 |
| {BcALV1.BI=FALSE, BcNSRV7.BI=TRUE}  => {BcRV1.BI=FALSE} | 0.13  1188 | 0.854  839 | 0.153  465 | 1.18  2722 | 53 |

| {BcFV9=FALSE, BcNSRV7.BI=TRUE} =>  {BcRV1.BI=FALSE} | 0.12  1287 | 0.816  667 | 0.148  515 | 1.12  9909 | 49 |
| --- | --- | --- | --- | --- | --- |
| {BcNSRV7.BI=TRUE, BcRV1.BI=FALSE} =>  {BcOLV4.BI=FALSE} | 0.15  8416 | 0.969  697 | 0.163  366 | 1.10  666 | 64 |
| {BcNSRV7.BI=TRUE, BcOLV4.BI=FALSE}  => {BcRV1.BI=FALSE} | 0.15  8416 | 0.831  169 | 0.190  594 | 1.14  9973 | 64 |
| {BcOLV4.BI=FALSE, BcRV1.BI=FALSE} =>  {BcNSRV7.BI=TRUE} | 0.15  8416 | 0.250  98 | 0.631  188 | 1.25  1803 | 64 |
| {BcNSRV7.BI=TRUE, BcOLV13.BI=FALSE}  => {BcRV1.BI=FALSE} | 0.15  5941 | 0.84 | 0.185  644 | 1.16  2192 | 63 |
| {BcNSRV7.BI=TRUE, BcOLV2.BI=FALSE}  => {BcRV1.BI=FALSE} | 0.15  3465 | 0.815  789 | 0.188  119 | 1.12  8695 | 62 |
| {BcMCV3.BI=FALSE, BcNSRV7.BI=TRUE}  => {BcRV1.BI=FALSE} | 0.16  3366 | 0.825 | 0.198  02 | 1.14  1438 | 66 |
| {BcNSRV7.BI=TRUE, BcOLV17.BI=FALSE}  => {BcRV1.BI=FALSE} | 0.16  0891 | 0.855  263 | 0.188  119 | 1.18  3309 | 65 |
| {BcEV2.BI=FALSE, BcNSRV7.BI=TRUE} =>  {BcULV1.BI=FALSE} | 0.11  6337 | 0.886  792 | 0.131  188 | 1.13  0171 | 47 |
| {BcEV2.BI=FALSE, BcNSRV7.BI=TRUE} =>  {BcOLV4.BI=FALSE} | 0.13  1188 | 1 | 0.131  188 | 1.14  1243 | 53 |
| {BcEV2.BI=FALSE, BcNSRV7.BI=TRUE} =>  {BcULV5=FALSE} | 0.12  6238 | 0.962  264 | 0.131  188 | 1.09  8177 | 51 |

| {BcHV3.BI=FALSE, BcNSRV7.BI=TRUE} =>  {BcULV1.BI=FALSE} | 0.10  1485 | 0.953  488 | 0.106  436 | 1.21  5171 | 41 |
| --- | --- | --- | --- | --- | --- |
| {BcFV5.BI=FALSE, BcNSRV7.BI=TRUE} =>  {BcULV1.BI=FALSE} | 0.09  4059 | 0.904  762 | 0.103  96 | 1.15  3072 | 38 |
| {BcDMV1=FALSE, BcNSRV7.BI=TRUE} =>  {BcULV1.BI=FALSE} | 0.11  3861 | 0.884  615 | 0.128  713 | 1.12  7396 | 46 |
| {BcNSRV7.BI=TRUE, BcULV1.BI=FALSE}  => {BcOLV4.BI=FALSE} | 0.16  3366 | 0.956  522 | 0.170  792 | 1.09  1624 | 66 |
| {BcMV12=FALSE, BcNSRV7.BI=TRUE} =>  {BcOLV4.BI=FALSE} | 0.14  8515 | 0.967  742 | 0.153  465 | 1.10  4429 | 60 |
| {BcNSRV7.BI=TRUE, BcVV3.BI=FALSE} =>  {BcULV5=FALSE} | 0.14  604 | 0.951  613 | 0.153  465 | 1.08  6022 | 59 |
| {BcNSRV7.BI=TRUE, BcOLV7.BI=FALSE}  => {BcOLV4.BI=FALSE} | 0.16  0891 | 0.955  882 | 0.168  317 | 1.09  0894 | 65 |
| {BcFV9=FALSE, BcNSRV7.BI=TRUE} =>  {BcOLV4.BI=FALSE} | 0.14  3564 | 0.966  667 | 0.148  515 | 1.10  3202 | 58 |
| {BcNSRV7.BI=TRUE, BcULV5=FALSE} =>  {BcOLV4.BI=FALSE} | 0.17  5743 | 0.946  667 | 0.185  644 | 1.08  0377 | 71 |
| {BcNSRV7.BI=TRUE, BvF.BI=FALSE} =>  {BcOLV4.BI=FALSE} | 0.17  8218 | 0.96 | 0.185  644 | 1.09  5593 | 72 |
| {BcMCV5.BI=FALSE, BcNSRV7.BI=TRUE}  => {BcOLV4.BI=FALSE} | 0.16  0891 | 0.955  882 | 0.168  317 | 1.09  0894 | 65 |

| {BcNSRV7.BI=TRUE, BcOLV13.BI=FALSE}  => {BcOLV4.BI=FALSE} | 0.18  0693 | 0.973  333 | 0.185  644 | 1.11  081 | 73 |
| --- | --- | --- | --- | --- | --- |
| {BcNSRV7.BI=TRUE, BcOLV2.BI=FALSE}  => {BcOLV4.BI=FALSE} | 0.17  8218 | 0.947  368 | 0.188  119 | 1.08  1178 | 72 |
| {BcMCV3.BI=FALSE, BcNSRV7.BI=TRUE}  => {BcOLV4.BI=FALSE} | 0.19  0594 | 0.962  5 | 0.198  02 | 1.09  8446 | 77 |
| {BcNSRV7.BI=TRUE, BcPV4.BI=FALSE} =>  {BcOLV4.BI=FALSE} | 0.18  3168 | 0.961  039 | 0.190  594 | 1.09  6779 | 74 |
| {BcEV6=FALSE, BcNSRV7.BI=TRUE} =>  {BcOLV4.BI=FALSE} | 0.18  0693 | 0.948  052 | 0.190  594 | 1.08  1958 | 73 |
| {BcNSRV7.BI=TRUE, BcOLV17.BI=FALSE}  => {BcOLV4.BI=FALSE} | 0.18  3168 | 0.973  684 | 0.188  119 | 1.11  121 | 74 |
| {BcNSRV7.BI=TRUE, BcOLV15.BI=FALSE}  => {BcOLV4.BI=FALSE} | 0.16  3366 | 0.956  522 | 0.170  792 | 1.09  1624 | 66 |
| {BcMV12=TRUE, BcMV6.BI=TRUE} =>  {BcMV3.BI=TRUE} | 0.08  4158 | 0.971  429 | 0.086  634 | 1.79  2042 | 34 |
| {BcMV12=TRUE, BcMV3.BI=TRUE} =>  {BcMV6.BI=TRUE} | 0.08  4158 | 0.548  387 | 0.153  465 | 1.80  1206 | 34 |
| {BcMV3.BI=TRUE, BcMV6.BI=TRUE} =>  {BcMV12=TRUE} | 0.08  4158 | 0.377  778 | 0.222  772 | 1.88  4225 | 34 |
| {BcMV12=TRUE, BcMV6.BI=TRUE} =>  {BcMV1.BI=TRUE} | 0.08  1683 | 0.942  857 | 0.086  634 | 1.19  4089 | 33 |

| {BcMV1.BI=TRUE, BcMV12=TRUE} =>  {BcMV6.BI=TRUE} | 0.08  1683 | 0.485  294 | 0.168  317 | 1.59  3974 | 33 |
| --- | --- | --- | --- | --- | --- |
| {BcMV1.BI=TRUE, BcMV6.BI=TRUE} =>  {BcMV12=TRUE} | 0.08  1683 | 0.320  388 | 0.254  95 | 1.59  7986 | 33 |
| {BcALV1.BI=FALSE, BcMV12=TRUE} =>  {BcMV6.BI=TRUE} | 0.07  4257 | 0.461  538 | 0.160  891 | 1.51  5947 | 30 |
| {BcALV1.BI=FALSE, BcMV6.BI=TRUE} =>  {BcMV12=TRUE} | 0.07  4257 | 0.340  909 | 0.217  822 | 1.70  0337 | 30 |
| {BcFV9=FALSE, BcMV12=TRUE} =>  {BcMV6.BI=TRUE} | 0.07  6733 | 0.442  857 | 0.173  267 | 1.45  4588 | 31 |
| {BcFV9=FALSE, BcMV6.BI=TRUE} =>  {BcMV12=TRUE} | 0.07  6733 | 0.287  037 | 0.267  327 | 1.43  1642 | 31 |
| {BcBNV5.BI=FALSE, BcMV12=TRUE} =>  {BcMV6.BI=TRUE} | 0.07  6733 | 0.413  333 | 0.185  644 | 1.35  7615 | 31 |
| {BcBNV5.BI=FALSE, BcMV6.BI=TRUE} =>  {BcMV12=TRUE} | 0.07  6733 | 0.313  131 | 0.245  05 | 1.56  1791 | 31 |
| {BcMV12=TRUE, BvF.BI=FALSE} =>  {BcMV6.BI=TRUE} | 0.08  6634 | 0.443  038 | 0.195  545 | 1.45  5182 | 35 |
| {BcMV6.BI=TRUE, BvF.BI=FALSE} =>  {BcMV12=TRUE} | 0.08  6634 | 0.299  145 | 0.289  604 | 1.49  2033 | 35 |
| {BcEV6=FALSE, BcMV12=TRUE} =>  {BcMV6.BI=TRUE} | 0.08  6634 | 0.448  718 | 0.193  069 | 1.47  3838 | 35 |

| {BcEV6=FALSE, BcMV6.BI=TRUE} =>  {BcMV12=TRUE} | 0.08  6634 | 0.307  018 | 0.282  178 | 1.53  1297 | 35 |
| --- | --- | --- | --- | --- | --- |
| {BcMV6.BI=TRUE, BcOLV17.BI=FALSE} =>  {BcMV12=TRUE} | 0.07  4257 | 0.267  857 | 0.277  228 | 1.33  5979 | 30 |
| {BcMV12=TRUE, BcOLV15.BI=FALSE} =>  {BcMV6.BI=TRUE} | 0.08  6634 | 0.448  718 | 0.193  069 | 1.47  3838 | 35 |
| {BcMV6.BI=TRUE, BcOLV15.BI=FALSE} =>  {BcMV12=TRUE} | 0.08  6634 | 0.289  256 | 0.299  505 | 1.44  271 | 35 |
| {BcEV5=FALSE, BcMV12=TRUE} =>  {BcMV6.BI=TRUE} | 0.08  1683 | 0.417  722 | 0.195  545 | 1.37  2028 | 33 |
| {BcEV5=FALSE, BcMV6.BI=TRUE} =>  {BcMV12=TRUE} | 0.08  1683 | 0.308  411 | 0.264  851 | 1.53  8249 | 33 |
| {BcMV12=TRUE, BcOLV1.BI.MOVA.=TRUE}  => {BcOLV16.BI=TRUE} | 0.08  1683 | 0.804  878 | 0.101  485 | 1.93  554 | 33 |
| {BcMV12=TRUE, BcOLV16.BI=TRUE} =>  {BcOLV1.BI.MOVA.=TRUE} | 0.08  1683 | 0.673  469 | 0.121  287 | 2.03  046 | 33 |
| {BcOLV1.BI.MOVA.=TRUE,  BcOLV16.BI=TRUE} => {BcMV12=TRUE} | 0.08  1683 | 0.428  571 | 0.190  594 | 2.13  7566 | 33 |
| {BcMV12=TRUE, BcOLV1.BI.MOVA.=TRUE}  => {BcMV2.BI=TRUE} | 0.08  9109 | 0.878  049 | 0.101  485 | 1.89  6961 | 36 |
| {BcMV12=TRUE, BcMV2.BI=TRUE} =>  {BcOLV1.BI.MOVA.=TRUE} | 0.08  9109 | 0.734  694 | 0.121  287 | 2.21  5047 | 36 |

| {BcMV2.BI=TRUE, BcOLV1.BI.MOVA.=TRUE} =>  {BcMV12=TRUE} | 0.08  9109 | 0.356  436 | 0.25 | 1.77  7778 | 36 |
| --- | --- | --- | --- | --- | --- |
| {BcMV12=TRUE, BcOLV1.BI.MOVA.=TRUE}  => {BcHV1SLR.BI=TRUE} | 0.07  6733 | 0.756  098 | 0.101  485 | 1.39  481 | 31 |
| {BcHV1SLR.BI=TRUE, BcMV12=TRUE} =>  {BcOLV1.BI.MOVA.=TRUE} | 0.07  6733 | 0.596  154 | 0.128  713 | 1.79  7359 | 31 |
| {BcHV1SLR.BI=TRUE, BcOLV1.BI.MOVA.=TRUE} =>  {BcMV12=TRUE} | 0.07  6733 | 0.382  716 | 0.200  495 | 1.90  8855 | 31 |
| {BcMV12=TRUE, BcOLV1.BI.MOVA.=TRUE}  => {BcMV3.BI=TRUE} | 0.09  1584 | 0.902  439 | 0.101  485 | 1.66  4773 | 37 |
| {BcMV12=TRUE, BcMV3.BI=TRUE} =>  {BcOLV1.BI.MOVA.=TRUE} | 0.09  1584 | 0.596  774 | 0.153  465 | 1.79  923 | 37 |
| {BcMV3.BI=TRUE, BcOLV1.BI.MOVA.=TRUE} =>  {BcMV12=TRUE} | 0.09  1584 | 0.373  737 | 0.245  05 | 1.86  4073 | 37 |
| {BcMV12=TRUE, BcOLV1.BI.MOVA.=TRUE}  => {BcHV1.BI=TRUE} | 0.07  4257 | 0.731  707 | 0.101  485 | 1.21  1515 | 30 |
| {BcHV1.BI=TRUE, BcMV12=TRUE} =>  {BcOLV1.BI.MOVA.=TRUE} | 0.07  4257 | 0.535  714 | 0.138  614 | 1.61  5139 | 30 |
| {BcHV1.BI=TRUE, | 0.07 | 0.348 | 0.212 | 1.73 | 30 |

| BcOLV1.BI.MOVA.=TRUE} =>  {BcMV12=TRUE} | 4257 | 837 | 871 | 9879 |  |
| --- | --- | --- | --- | --- | --- |
| {BcMV12=TRUE, BcULV4=TRUE} => | 0.07 | 0.526 | 0.141 | 1.58 |  |
|  |  |  |  |  | 30 |
| {BcOLV1.BI.MOVA.=TRUE} | 4257 | 316 | 089 | 6803 |  |
| {BcOLV1.BI.MOVA.=TRUE, BcULV4=TRUE} | 0.07 | 0.303 | 0.245 | 1.51 |  |
|  |  |  |  |  | 30 |
| => {BcMV12=TRUE} | 4257 | 03 | 05 | 141 |  |
| {BcMV12=TRUE, BcOLV1.BI.MOVA.=TRUE} | 0.08 | 0.853 | 0.101 | 1.14 |  |
|  |  |  |  |  | 35 |
| => {BcEV2.BI=FALSE} | 6634 | 659 | 485 | 198 |  |
| {BcEV2.BI=FALSE, BcMV12=TRUE} => | 0.08 | 0.522 | 0.165 | 1.57 |  |
|  |  |  |  |  | 35 |
| {BcOLV1.BI.MOVA.=TRUE} | 6634 | 388 | 842 | 4961 |  |
| {BcEV2.BI=FALSE, |  |  |  |  |  |
|  | 0.08 | 0.346 |  | 1.72 |  |
| BcOLV1.BI.MOVA.=TRUE} => |  |  | 0.25 |  | 35 |
|  | 6634 | 535 |  | 8395 |  |
| {BcMV12=TRUE} |  |  |  |  |  |
| {BcHV3.BI=FALSE, BcMV12=TRUE} => | 0.07 | 0.484 | 0.158 | 1.46 |  |
|  |  |  |  |  | 31 |
| {BcOLV1.BI.MOVA.=TRUE} | 6733 | 375 | 416 | 0354 |  |
| {BcHV3.BI=FALSE, |  |  |  |  |  |
|  | 0.07 | 0.336 | 0.227 | 1.68 |  |
| BcOLV1.BI.MOVA.=TRUE} => |  |  |  |  | 31 |
|  | 6733 | 957 | 723 | 0623 |  |
| {BcMV12=TRUE} |  |  |  |  |  |
| {BcFV5.BI=FALSE, BcMV12=TRUE} => | 0.08 | 0.539 | 0.155 | 1.62 |  |
|  |  |  |  |  | 34 |
| {BcOLV1.BI.MOVA.=TRUE} | 4158 | 683 | 941 | 7103 |  |
| {BcFV5.BI=FALSE, | 0.08 | 0.350 | 0.240 | 1.74 |  |
|  |  |  |  |  | 34 |
| BcOLV1.BI.MOVA.=TRUE} => | 4158 | 515 | 099 | 825 |  |

| {BcMV12=TRUE} |  |  |  |  |  |
| --- | --- | --- | --- | --- | --- |
| {BcMV1.BI=TRUE, BcMV12=TRUE} => | 0.08 | 0.529 | 0.168 | 1.59 |  |
|  |  |  |  |  | 36 |
| {BcOLV1.BI.MOVA.=TRUE} | 9109 | 412 | 317 | 6137 |  |
| {BcMV1.BI=TRUE, |  |  |  |  |  |
|  | 0.08 | 0.302 | 0.294 | 1.50 |  |
| BcOLV1.BI.MOVA.=TRUE} => |  |  |  |  | 36 |
|  | 9109 | 521 | 554 | 887 |  |
| {BcMV12=TRUE} |  |  |  |  |  |
| {BcMV12=TRUE, BcNSRV7.BI=FALSE} => | 0.08 | 0.532 | 0.153 | 1.60 |  |
|  |  |  |  |  | 33 |
| {BcOLV1.BI.MOVA.=TRUE} | 1683 | 258 | 465 | 4718 |  |
| {BcNSRV7.BI=FALSE, |  |  |  |  |  |
|  | 0.08 | 0.308 | 0.264 | 1.53 |  |
| BcOLV1.BI.MOVA.=TRUE} => |  |  |  |  | 33 |
|  | 1683 | 411 | 851 | 8249 |  |
| {BcMV12=TRUE} |  |  |  |  |  |
| {BcMV12=TRUE, BcVV3.BI=FALSE} => | 0.07 | 0.469 | 0.163 | 1.41 |  |
|  |  |  |  |  | 31 |
| {BcOLV1.BI.MOVA.=TRUE} | 6733 | 697 | 366 | 6101 |  |
| {BcOLV1.BI.MOVA.=TRUE, | 0.07 | 0.316 | 0.242 | 1.57 |  |
|  |  |  |  |  | 31 |
| BcVV3.BI=FALSE} => {BcMV12=TRUE} | 6733 | 327 | 574 | 7727 |  |
| {BcMV12=TRUE, BcOLV7.BI=FALSE} => | 0.07 | 0.491 | 0.150 | 1.48 |  |
|  |  |  |  |  | 30 |
| {BcOLV1.BI.MOVA.=TRUE} | 4257 | 803 | 99 | 275 |  |
| {BcOLV1.BI.MOVA.=TRUE, | 0.07 | 0.312 | 0.237 | 1.55 |  |
|  |  |  |  |  | 30 |
| BcOLV7.BI=FALSE} => {BcMV12=TRUE} | 4257 | 5 | 624 | 8642 |  |
| {BcALV1.BI=FALSE, BcMV12=TRUE} => | 0.08 | 0.507 | 0.160 | 1.53 |  |
|  |  |  |  |  | 33 |
| {BcOLV1.BI.MOVA.=TRUE} | 1683 | 692 | 891 | 0654 |  |
| {BcALV1.BI=FALSE, | 0.08 | 0.362 | 0.225 | 1.80 | 33 |

| BcOLV1.BI.MOVA.=TRUE} =>  {BcMV12=TRUE} | 1683 | 637 | 248 | 871 |  |
| --- | --- | --- | --- | --- | --- |
| {BcDMV1=FALSE, BcMV12=TRUE} => | 0.09 | 0.521 | 0.175 | 1.57 |  |
|  |  |  |  |  | 37 |
| {BcOLV1.BI.MOVA.=TRUE} | 1584 | 127 | 743 | 1158 |  |
| {BcDMV1=FALSE, |  |  |  |  |  |
|  | 0.09 | 0.333 | 0.274 | 1.66 |  |
| BcOLV1.BI.MOVA.=TRUE} => |  |  |  |  | 37 |
|  | 1584 | 333 | 752 | 2551 |  |
| {BcMV12=TRUE} |  |  |  |  |  |
| {BcFV9=FALSE, BcMV12=TRUE} => | 0.08 |  | 0.173 | 1.50 |  |
|  |  | 0.5 |  |  | 35 |
| {BcOLV1.BI.MOVA.=TRUE} | 6634 |  | 267 | 7463 |  |
| {BcFV9=FALSE, BcOLV1.BI.MOVA.=TRUE} | 0.08 | 0.309 | 0.279 | 1.54 |  |
|  |  |  |  |  | 35 |
| => {BcMV12=TRUE} | 6634 | 735 | 703 | 4849 |  |
| {BcBNV5.BI=FALSE, BcMV12=TRUE} => | 0.08 |  | 0.185 | 1.44 |  |
|  |  | 0.48 |  |  | 36 |
| {BcOLV1.BI.MOVA.=TRUE} | 9109 |  | 644 | 7164 |  |
| {BcBNV5.BI=FALSE, |  |  |  |  |  |
|  | 0.08 | 0.315 | 0.282 | 1.57 |  |
| BcOLV1.BI.MOVA.=TRUE} => |  |  |  |  | 36 |
|  | 9109 | 789 | 178 | 5049 |  |
| {BcMV12=TRUE} |  |  |  |  |  |
| {BcMV12=TRUE, BvF.BI=FALSE} => | 0.10 | 0.518 | 0.195 | 1.56 |  |
|  |  |  |  |  | 41 |
| {BcOLV1.BI.MOVA.=TRUE} | 1485 | 987 | 545 | 4708 |  |
| {BcOLV1.BI.MOVA.=TRUE, BvF.BI=FALSE} | 0.10 | 0.322 | 0.314 | 1.61 |  |
|  |  |  |  |  | 41 |
| => {BcMV12=TRUE} | 1485 | 835 | 356 | 0188 |  |
| {BcMCV5.BI=FALSE, BcMV12=TRUE} => | 0.08 |  | 0.185 | 1.44 |  |
|  |  | 0.48 |  |  | 36 |
| {BcOLV1.BI.MOVA.=TRUE} | 9109 |  | 644 | 7164 |  |

| {BcMCV5.BI=FALSE, BcOLV1.BI.MOVA.=TRUE} =>  {BcMV12=TRUE} | 0.08  9109 | 0.321  429 | 0.277  228 | 1.60  3175 | 36 |
| --- | --- | --- | --- | --- | --- |
| {BcMV12=TRUE, BcOLV13.BI=FALSE} =>  {BcOLV1.BI.MOVA.=TRUE} | 0.07  6733 | 0.449  275 | 0.170  792 | 1.35  4532 | 31 |
| {BcOLV1.BI.MOVA.=TRUE,  BcOLV13.BI=FALSE} => {BcMV12=TRUE} | 0.07  6733 | 0.276  786 | 0.277  228 | 1.38  0511 | 31 |
| {BcMV12=TRUE, BcOLV2.BI=FALSE} =>  {BcOLV1.BI.MOVA.=TRUE} | 0.07  4257 | 0.434  783 | 0.170  792 | 1.31  0837 | 30 |
| {BcOLV1.BI.MOVA.=TRUE,  BcOLV2.BI=FALSE} => {BcMV12=TRUE} | 0.07  4257 | 0.267  857 | 0.277  228 | 1.33  5979 | 30 |
| {BcMV12=TRUE, BcPV4.BI=FALSE} =>  {BcOLV1.BI.MOVA.=TRUE} | 0.09  1584 | 0.521  127 | 0.175  743 | 1.57  1158 | 37 |
| {BcOLV1.BI.MOVA.=TRUE,  BcPV4.BI=FALSE} => {BcMV12=TRUE} | 0.09  1584 | 0.289  063 | 0.316  832 | 1.44  1744 | 37 |
| {BcEV6=FALSE, BcMV12=TRUE} =>  {BcOLV1.BI.MOVA.=TRUE} | 0.09  901 | 0.512  821 | 0.193  069 | 1.54  6116 | 40 |
| {BcEV6=FALSE, BcOLV1.BI.MOVA.=TRUE}  => {BcMV12=TRUE} | 0.09  901 | 0.322  581 | 0.306  931 | 1.60  8921 | 40 |
| {BcMV12=TRUE, BcOLV17.BI=FALSE} =>  {BcOLV1.BI.MOVA.=TRUE} | 0.09  1584 | 0.486  842 | 0.188  119 | 1.46  7793 | 37 |
| {BcOLV1.BI.MOVA.=TRUE, | 0.09 | 0.305 | 0.299 | 1.52 | 37 |

| BcOLV17.BI=FALSE} => {BcMV12=TRUE} | 1584 | 785 | 505 | 515 |  |
| --- | --- | --- | --- | --- | --- |
| {BcMV12=TRUE, BcOLV15.BI=FALSE} =>  {BcOLV1.BI.MOVA.=TRUE} | 0.10  1485 | 0.525  641 | 0.193  069 | 1.58  4768 | 41 |
| {BcOLV1.BI.MOVA.=TRUE,  BcOLV15.BI=FALSE} => {BcMV12=TRUE} | 0.10  1485 | 0.315  385 | 0.321  782 | 1.57  3029 | 41 |
| {BcEV5=FALSE, BcMV12=TRUE} =>  {BcOLV1.BI.MOVA.=TRUE} | 0.09  6535 | 0.493  671 | 0.195  545 | 1.48  8381 | 39 |
| {BcEV5=FALSE, BcOLV1.BI.MOVA.=TRUE}  => {BcMV12=TRUE} | 0.09  6535 | 0.325 | 0.297  03 | 1.62  0988 | 39 |
| {BcMV12=TRUE, BcPV3.BI=FALSE} =>  {BcMV11=FALSE} | 0.07  4257 | 0.576  923 | 0.128  713 | 1.50  3722 | 30 |
| {BcMV11=FALSE, BcMV12=TRUE} =>  {BcRV1.BI=FALSE} | 0.08  1683 | 0.891  892 | 0.091  584 | 1.23  3987 | 33 |
| {BcMV12=TRUE, BcRV1.BI=FALSE} =>  {BcMV11=FALSE} | 0.08  1683 | 0.568  966 | 0.143  564 | 1.48  2981 | 33 |
| {BcMV11=FALSE, BcMV12=TRUE} =>  {BcEV2.BI=FALSE} | 0.07  9208 | 0.864  865 | 0.091  584 | 1.15  6972 | 32 |
| {BcMV12=TRUE, BcNSRV7.BI=FALSE} =>  {BcMV11=FALSE} | 0.07  9208 | 0.516  129 | 0.153  465 | 1.34  5265 | 32 |
| {BcMV12=TRUE, BcOLV7.BI=FALSE} =>  {BcMV11=FALSE} | 0.07  4257 | 0.491  803 | 0.150  99 | 1.28  1861 | 30 |
| {BcMV11=FALSE, BcMV12=TRUE} => | 0.08 | 0.945 | 0.091 | 1.16 | 35 |

| {BcALV1.BI=FALSE} | 6634 | 946 | 584 | 1587 |  |
| --- | --- | --- | --- | --- | --- |
| {BcALV1.BI=FALSE, BcMV12=TRUE} =>  {BcMV11=FALSE} | 0.08  6634 | 0.538  462 | 0.160  891 | 1.40  3474 | 35 |
| {BcMV12=TRUE, BcOLV4.BI=FALSE} =>  {BcMV11=FALSE} | 0.08  1683 | 0.5 | 0.163  366 | 1.30  3226 | 33 |
| {BcMV12=TRUE, BcULV5=FALSE} =>  {BcMV11=FALSE} | 0.08  6634 | 0.538  462 | 0.160  891 | 1.40  3474 | 35 |
| {BcMV11=FALSE, BvF.BI=FALSE} =>  {BcMV12=TRUE} | 0.08  9109 | 0.260  87 | 0.341  584 | 1.30  1127 | 36 |
| {BcMV12=TRUE, BcOLV13.BI=FALSE} =>  {BcMV11=FALSE} | 0.08  6634 | 0.507  246 | 0.170  792 | 1.32  2113 | 35 |
| {BcMV12=TRUE, BcOLV16.BI=TRUE} =>  {BcMV2.BI=TRUE} | 0.09  1584 | 0.755  102 | 0.121  287 | 1.63  1343 | 37 |
| {BcMV12=TRUE, BcMV2.BI=TRUE} =>  {BcOLV16.BI=TRUE} | 0.09  1584 | 0.755  102 | 0.121  287 | 1.81  5841 | 37 |
| {BcMV2.BI=TRUE, BcOLV16.BI=TRUE} =>  {BcMV12=TRUE} | 0.09  1584 | 0.362  745 | 0.252  475 | 1.80  9247 | 37 |
| {BcMV12=TRUE, BcOLV16.BI=TRUE} =>  {BcOLV18=TRUE} | 0.07  9208 | 0.653  061 | 0.121  287 | 1.26  2377 | 32 |
| {BcMV12=TRUE, BcOLV18=TRUE} =>  {BcOLV16.BI=TRUE} | 0.07  9208 | 0.653  061 | 0.121  287 | 1.57  0457 | 32 |
| {BcOLV16.BI=TRUE, BcOLV18=TRUE} => | 0.07 | 0.293 | 0.269 | 1.46 | 32 |

| {BcMV12=TRUE} | 9208 | 578 | 802 | 4265 |  |
| --- | --- | --- | --- | --- | --- |
| {BcMV12=TRUE, BcOLV16.BI=TRUE} =>  {BcHV1SLR.BI=TRUE} | 0.08  1683 | 0.673  469 | 0.121  287 | 1.24  2382 | 33 |
| {BcHV1SLR.BI=TRUE, BcMV12=TRUE} =>  {BcOLV16.BI=TRUE} | 0.08  1683 | 0.634  615 | 0.128  713 | 1.52  6099 | 33 |
| {BcHV1SLR.BI=TRUE, BcOLV16.BI=TRUE}  => {BcMV12=TRUE} | 0.08  1683 | 0.343  75 | 0.237  624 | 1.71  4506 | 33 |
| {BcMV12=TRUE, BcOLV16.BI=TRUE} =>  {BcMV3.BI=TRUE} | 0.10  8911 | 0.897  959 | 0.121  287 | 1.65  6509 | 44 |
| {BcMV12=TRUE, BcMV3.BI=TRUE} =>  {BcOLV16.BI=TRUE} | 0.10  8911 | 0.709  677 | 0.153  465 | 1.70  6605 | 44 |
| {BcMV3.BI=TRUE, BcOLV16.BI=TRUE} =>  {BcMV12=TRUE} | 0.10  8911 | 0.407  407 | 0.267  327 | 2.03  2007 | 44 |
| {BcMV12=TRUE, BcULV3=TRUE} =>  {BcOLV16.BI=TRUE} | 0.08  1683 | 0.647  059 | 0.126  238 | 1.55  6022 | 33 |
| {BcOLV16.BI=TRUE, BcULV3=TRUE} =>  {BcMV12=TRUE} | 0.08  1683 | 0.292  035 | 0.279  703 | 1.45  6572 | 33 |
| {BcHV1.BI=TRUE, BcMV12=TRUE} =>  {BcOLV16.BI=TRUE} | 0.08  4158 | 0.607  143 | 0.138  614 | 1.46  0034 | 34 |
| {BcHV1.BI=TRUE, BcOLV16.BI=TRUE} =>  {BcMV12=TRUE} | 0.08  4158 | 0.311  927 | 0.269  802 | 1.55  5782 | 34 |
| {BcMV11=TRUE, BcMV12=TRUE} => | 0.07 | 0.727 | 0.108 | 1.74 | 32 |

| {BcOLV16.BI=TRUE} | 9208 | 273 | 911 | 8918 |  |
| --- | --- | --- | --- | --- | --- |
| {BcMV11=TRUE, BcOLV16.BI=TRUE} =>  {BcMV12=TRUE} | 0.07  9208 | 0.288  288 | 0.274  752 | 1.43  7882 | 32 |
| {BcMV12=TRUE, BcMV9.BI=TRUE} =>  {BcOLV16.BI=TRUE} | 0.08  6634 | 0.625 | 0.138  614 | 1.50  2976 | 35 |
| {BcMV9.BI=TRUE, BcOLV16.BI=TRUE} =>  {BcMV12=TRUE} | 0.08  6634 | 0.261  194 | 0.331  683 | 1.30  2746 | 35 |
| {BcMV12=TRUE, BcOLV16.BI=TRUE} =>  {BcULV4=TRUE} | 0.09  6535 | 0.795  918 | 0.121  287 | 1.15  2513 | 39 |
| {BcMV12=TRUE, BcULV4=TRUE} =>  {BcOLV16.BI=TRUE} | 0.09  6535 | 0.684  211 | 0.141  089 | 1.64  5363 | 39 |
| {BcOLV16.BI=TRUE, BcULV4=TRUE} =>  {BcMV12=TRUE} | 0.09  6535 | 0.284  672 | 0.339  109 | 1.41  9843 | 39 |
| {BcMV12=TRUE, BcRV1.BI=FALSE} =>  {BcOLV16.BI=TRUE} | 0.08  4158 | 0.586  207 | 0.143  564 | 1.40  9688 | 34 |
| {BcOLV16.BI=TRUE, BcRV1.BI=FALSE} =>  {BcMV12=TRUE} | 0.08  4158 | 0.317  757 | 0.264  851 | 1.58  4862 | 34 |
| {BcEV2.BI=FALSE, BcMV12=TRUE} =>  {BcOLV16.BI=TRUE} | 0.09  6535 | 0.582  09 | 0.165  842 | 1.39  9787 | 39 |
| {BcEV2.BI=FALSE, BcOLV16.BI=TRUE} =>  {BcMV12=TRUE} | 0.09  6535 | 0.295  455 | 0.326  733 | 1.47  3625 | 39 |
| {BcHV3.BI=FALSE, BcMV12=TRUE} => | 0.09 | 0.609 | 0.158 | 1.46 | 39 |

| {BcOLV16.BI=TRUE} | 6535 | 375 | 416 | 5402 |  |
| --- | --- | --- | --- | --- | --- |
| {BcHV3.BI=FALSE, BcOLV16.BI=TRUE} =>  {BcMV12=TRUE} | 0.09  6535 | 0.3 | 0.321  782 | 1.49  6296 | 39 |
| {BcFV5.BI=FALSE, BcMV12=TRUE} =>  {BcOLV16.BI=TRUE} | 0.09  6535 | 0.619  048 | 0.155  941 | 1.48  8662 | 39 |
| {BcFV5.BI=FALSE, BcOLV16.BI=TRUE} =>  {BcMV12=TRUE} | 0.09  6535 | 0.3 | 0.321  782 | 1.49  6296 | 39 |
| {BcMV12=TRUE, BcOLV16.BI=TRUE} =>  {BcMV1.BI=TRUE} | 0.10  8911 | 0.897  959 | 0.121  287 | 1.13  7227 | 44 |
| {BcMV1.BI=TRUE, BcMV12=TRUE} =>  {BcOLV16.BI=TRUE} | 0.10  8911 | 0.647  059 | 0.168  317 | 1.55  6022 | 44 |
| {BcMV1.BI=TRUE, BcOLV16.BI=TRUE} =>  {BcMV12=TRUE} | 0.10  8911 | 0.323  529 | 0.336  634 | 1.61  3653 | 44 |
| {BcMV12=TRUE, BcNSRV7.BI=FALSE} =>  {BcOLV16.BI=TRUE} | 0.08  9109 | 0.580  645 | 0.153  465 | 1.39  6313 | 36 |
| {BcNSRV7.BI=FALSE, BcOLV16.BI=TRUE}  => {BcMV12=TRUE} | 0.08  9109 | 0.266  667 | 0.334  158 | 1.33  0041 | 36 |
| {BcMV12=TRUE, BcVV3.BI=FALSE} =>  {BcOLV16.BI=TRUE} | 0.09  901 | 0.606  061 | 0.163  366 | 1.45  7431 | 40 |
| {BcOLV16.BI=TRUE, BcVV3.BI=FALSE} =>  {BcMV12=TRUE} | 0.09  901 | 0.294  118 | 0.336  634 | 1.46  6957 | 40 |
| {BcMV12=TRUE, BcOLV7.BI=FALSE} => | 0.08 | 0.573 | 0.150 | 1.37 | 35 |

| {BcOLV16.BI=TRUE} | 6634 | 77 | 99 | 9781 |  |
| --- | --- | --- | --- | --- | --- |
| {BcOLV16.BI=TRUE, BcOLV7.BI=FALSE}  => {BcMV12=TRUE} | 0.08  6634 | 0.294  118 | 0.294  554 | 1.46  6957 | 35 |
| {BcALV1.BI=FALSE, BcMV12=TRUE} =>  {BcOLV16.BI=TRUE} | 0.09  6535 | 0.6 | 0.160  891 | 1.44  2857 | 39 |
| {BcALV1.BI=FALSE, BcOLV16.BI=TRUE} =>  {BcMV12=TRUE} | 0.09  6535 | 0.312 | 0.309  406 | 1.55  6148 | 39 |
| {BcDMV1=FALSE, BcMV12=TRUE} =>  {BcOLV16.BI=TRUE} | 0.10  396 | 0.591  549 | 0.175  743 | 1.42  2535 | 42 |
| {BcDMV1=FALSE, BcOLV16.BI=TRUE} =>  {BcMV12=TRUE} | 0.10  396 | 0.293  706 | 0.353  96 | 1.46  4905 | 42 |
| {BcFV9=FALSE, BcMV12=TRUE} =>  {BcOLV16.BI=TRUE} | 0.10  1485 | 0.585  714 | 0.173  267 | 1.40  8503 | 41 |
| {BcFV9=FALSE, BcOLV16.BI=TRUE} =>  {BcMV12=TRUE} | 0.10  1485 | 0.282  759 | 0.358  911 | 1.41  0302 | 41 |
| {BcMV12=TRUE, BcOLV4.BI=FALSE} =>  {BcOLV16.BI=TRUE} | 0.09  1584 | 0.560  606 | 0.163  366 | 1.34  8124 | 37 |
| {BcOLV16.BI=TRUE, BcOLV4.BI=FALSE}  => {BcMV12=TRUE} | 0.09  1584 | 0.266  187 | 0.344  059 | 1.32  7649 | 37 |
| {BcMV12=TRUE, BcULV5=FALSE} =>  {BcOLV16.BI=TRUE} | 0.08  6634 | 0.538  462 | 0.160  891 | 1.29  4872 | 35 |
| {BcBNV5.BI=FALSE, BcMV12=TRUE} => | 0.10 | 0.586 | 0.185 | 1.41 | 44 |

| {BcOLV16.BI=TRUE} | 8911 | 667 | 644 | 0794 |  |
| --- | --- | --- | --- | --- | --- |
| {BcBNV5.BI=FALSE, BcOLV16.BI=TRUE}  => {BcMV12=TRUE} | 0.10  8911 | 0.291  391 | 0.373  762 | 1.45  3356 | 44 |
| {BcMV12=TRUE, BvF.BI=FALSE} =>  {BcOLV16.BI=TRUE} | 0.11  8812 | 0.607  595 | 0.195  545 | 1.46  1121 | 48 |
| {BcOLV16.BI=TRUE, BvF.BI=FALSE} =>  {BcMV12=TRUE} | 0.11  8812 | 0.303  797 | 0.391  089 | 1.51  5237 | 48 |
| {BcMCV5.BI=FALSE, BcMV12=TRUE} =>  {BcOLV16.BI=TRUE} | 0.10  6436 | 0.573  333 | 0.185  644 | 1.37  873 | 43 |
| {BcMCV5.BI=FALSE, BcOLV16.BI=TRUE}  => {BcMV12=TRUE} | 0.10  6436 | 0.290  541 | 0.366  337 | 1.44  9116 | 43 |
| {BcMV12=TRUE, BcOLV13.BI=FALSE} =>  {BcOLV16.BI=TRUE} | 0.09  6535 | 0.565  217 | 0.170  792 | 1.35  9213 | 39 |
| {BcOLV13.BI=FALSE, BcOLV16.BI=TRUE}  => {BcMV12=TRUE} | 0.09  6535 | 0.263  514 | 0.366  337 | 1.31  4314 | 39 |
| {BcMV12=TRUE, BcOLV2.BI=FALSE} =>  {BcOLV16.BI=TRUE} | 0.09  6535 | 0.565  217 | 0.170  792 | 1.35  9213 | 39 |
| {BcOLV16.BI=TRUE, BcOLV2.BI=FALSE}  => {BcMV12=TRUE} | 0.09  6535 | 0.261  745 | 0.368  812 | 1.30  5493 | 39 |
| {BcMV12=TRUE, BcPV4.BI=FALSE} =>  {BcOLV16.BI=TRUE} | 0.10  396 | 0.591  549 | 0.175  743 | 1.42  2535 | 42 |
| {BcOLV16.BI=TRUE, BcPV4.BI=FALSE} => | 0.10 | 0.274 | 0.378 | 1.36 | 42 |

| {BcMV12=TRUE} | 396 | 51 | 713 | 916 |  |
| --- | --- | --- | --- | --- | --- |
| {BcEV6=FALSE, BcMV12=TRUE} =>  {BcOLV16.BI=TRUE} | 0.11  6337 | 0.602  564 | 0.193  069 | 1.44  9023 | 47 |
| {BcEV6=FALSE, BcOLV16.BI=TRUE} =>  {BcMV12=TRUE} | 0.11  6337 | 0.297  468 | 0.391  089 | 1.48  3669 | 47 |
| {BcMV12=TRUE, BcOLV17.BI=FALSE} =>  {BcOLV16.BI=TRUE} | 0.11  1386 | 0.592  105 | 0.188  119 | 1.42  3872 | 45 |
| {BcOLV16.BI=TRUE, BcOLV17.BI=FALSE}  => {BcMV12=TRUE} | 0.11  1386 | 0.288  462 | 0.386  139 | 1.43  8746 | 45 |
| {BcMV12=TRUE, BcOLV15.BI=FALSE} =>  {BcOLV16.BI=TRUE} | 0.11  8812 | 0.615  385 | 0.193  069 | 1.47  9853 | 48 |
| {BcOLV15.BI=FALSE, BcOLV16.BI=TRUE}  => {BcMV12=TRUE} | 0.11  8812 | 0.294  479 | 0.403  465 | 1.46  8757 | 48 |
| {BcEV5=FALSE, BcMV12=TRUE} =>  {BcOLV16.BI=TRUE} | 0.11  6337 | 0.594  937 | 0.195  545 | 1.43  0681 | 47 |
| {BcEV5=FALSE, BcOLV16.BI=TRUE} =>  {BcMV12=TRUE} | 0.11  6337 | 0.295  597 | 0.393  564 | 1.47  4338 | 47 |
| {BcMV12=TRUE, BcMV2.BI=TRUE} =>  {BcHV1SLR.BI=TRUE} | 0.08  6634 | 0.714  286 | 0.121  287 | 1.31  7678 | 35 |
| {BcHV1SLR.BI=TRUE, BcMV12=TRUE} =>  {BcMV2.BI=TRUE} | 0.08  6634 | 0.673  077 | 0.128  713 | 1.45  4134 | 35 |
| {BcHV1SLR.BI=TRUE, BcMV2.BI=TRUE} => | 0.08 | 0.318 | 0.272 | 1.58 | 35 |

| {BcMV12=TRUE} | 6634 | 182 | 277 | 6981 |  |
| --- | --- | --- | --- | --- | --- |
| {BcMV12=TRUE, BcMV2.BI=TRUE} =>  {BcMV3.BI=TRUE} | 0.10  1485 | 0.836  735 | 0.121  287 | 1.54  3565 | 41 |
| {BcMV12=TRUE, BcMV3.BI=TRUE} =>  {BcMV2.BI=TRUE} | 0.10  1485 | 0.661  29 | 0.153  465 | 1.42  867 | 41 |
| {BcMV2.BI=TRUE, BcMV3.BI=TRUE} =>  {BcMV12=TRUE} | 0.10  1485 | 0.322  835 | 0.314  356 | 1.61  0188 | 41 |
| {BcMV12=TRUE, BcULV3=TRUE} =>  {BcMV2.BI=TRUE} | 0.07  9208 | 0.627  451 | 0.126  238 | 1.35  5563 | 32 |
| {BcMV2.BI=TRUE, BcULV3=TRUE} =>  {BcMV12=TRUE} | 0.07  9208 | 0.278  261 | 0.284  653 | 1.38  7869 | 32 |
| {BcHV1.BI=TRUE, BcMV2.BI=TRUE} =>  {BcMV12=TRUE} | 0.07  9208 | 0.271  186 | 0.292  079 | 1.35  2584 | 32 |
| {BcMV11=TRUE, BcMV12=TRUE} =>  {BcMV2.BI=TRUE} | 0.08  1683 | 0.75 | 0.108  911 | 1.62  0321 | 33 |
| {BcMV12=TRUE, BcULV4=TRUE} =>  {BcMV2.BI=TRUE} | 0.08  4158 | 0.596  491 | 0.141  089 | 1.28  8676 | 34 |
| {BcMV2.BI=TRUE, BcRV1.BI=FALSE} =>  {BcMV12=TRUE} | 0.07  9208 | 0.278  261 | 0.284  653 | 1.38  7869 | 32 |
| {BcEV2.BI=FALSE, BcMV12=TRUE} =>  {BcMV2.BI=TRUE} | 0.09  901 | 0.597  015 | 0.165  842 | 1.28  9808 | 40 |
| {BcEV2.BI=FALSE, BcMV2.BI=TRUE} => | 0.09 | 0.310 | 0.319 | 1.54 | 40 |

| {BcMV12=TRUE} | 901 | 078 | 307 | 6559 |  |
| --- | --- | --- | --- | --- | --- |
| {BcHV3.BI=FALSE, BcMV2.BI=TRUE} =>  {BcMV12=TRUE} | 0.08  9109 | 0.270  677 | 0.329  208 | 1.35  0042 | 36 |
| {BcFV5.BI=FALSE, BcMV12=TRUE} =>  {BcMV2.BI=TRUE} | 0.09  1584 | 0.587  302 | 0.155  941 | 1.26  8823 | 37 |
| {BcFV5.BI=FALSE, BcMV2.BI=TRUE} =>  {BcMV12=TRUE} | 0.09  1584 | 0.266  187 | 0.344  059 | 1.32  7649 | 37 |
| {BcMV1.BI=TRUE, BcMV12=TRUE} =>  {BcMV2.BI=TRUE} | 0.10  6436 | 0.632  353 | 0.168  317 | 1.36  6153 | 43 |
| {BcMV1.BI=TRUE, BcMV2.BI=TRUE} =>  {BcMV12=TRUE} | 0.10  6436 | 0.260  606 | 0.408  416 | 1.29  9813 | 43 |
| {BcMV12=TRUE, BcNSRV7.BI=FALSE} =>  {BcMV2.BI=TRUE} | 0.09  1584 | 0.596  774 | 0.153  465 | 1.28  9288 | 37 |
| {BcMV2.BI=TRUE, BcVV3.BI=FALSE} =>  {BcMV12=TRUE} | 0.09  4059 | 0.267  606 | 0.351  485 | 1.33  4724 | 38 |
| {BcMV12=TRUE, BcOLV7.BI=FALSE} =>  {BcMV2.BI=TRUE} | 0.09  4059 | 0.622  951 | 0.150  99 | 1.34  584 | 38 |
| {BcMV2.BI=TRUE, BcOLV7.BI=FALSE} =>  {BcMV12=TRUE} | 0.09  4059 | 0.262  069 | 0.358  911 | 1.30  7109 | 38 |
| {BcALV1.BI=FALSE, BcMV2.BI=TRUE} =>  {BcMV12=TRUE} | 0.09  1584 | 0.282  443 | 0.324  257 | 1.40  8727 | 37 |
| {BcDMV1=FALSE, BcMV12=TRUE} => | 0.10 | 0.619 | 0.175 | 1.33 | 44 |

| {BcMV2.BI=TRUE} | 8911 | 718 | 743 | 8857 |  |
| --- | --- | --- | --- | --- | --- |
| {BcDMV1=FALSE, BcMV2.BI=TRUE} =>  {BcMV12=TRUE} | 0.10  8911 | 0.275 | 0.396  04 | 1.37  1605 | 44 |
| {BcFV9=FALSE, BcMV12=TRUE} =>  {BcMV2.BI=TRUE} | 0.10  8911 | 0.628  571 | 0.173  267 | 1.35  7983 | 44 |
| {BcFV9=FALSE, BcMV2.BI=TRUE} =>  {BcMV12=TRUE} | 0.10  8911 | 0.271  605 | 0.400  99 | 1.35  4672 | 44 |
| {BcBNV5.BI=FALSE, BcMV12=TRUE} =>  {BcMV2.BI=TRUE} | 0.11  1386 | 0.6 | 0.185  644 | 1.29  6257 | 45 |
| {BcBNV5.BI=FALSE, BcMV2.BI=TRUE} =>  {BcMV12=TRUE} | 0.11  1386 | 0.272  727 | 0.408  416 | 1.36  0269 | 45 |
| {BcMV12=TRUE, BvF.BI=FALSE} =>  {BcMV2.BI=TRUE} | 0.11  8812 | 0.607  595 | 0.195  545 | 1.31  2665 | 48 |
| {BcMV2.BI=TRUE, BvF.BI=FALSE} =>  {BcMV12=TRUE} | 0.11  8812 | 0.274  286 | 0.433  168 | 1.36  8042 | 48 |
| {BcMCV5.BI=FALSE, BcMV12=TRUE} =>  {BcMV2.BI=TRUE} | 0.10  8911 | 0.586  667 | 0.185  644 | 1.26  7451 | 44 |
| {BcMCV5.BI=FALSE, BcMV2.BI=TRUE} =>  {BcMV12=TRUE} | 0.10  8911 | 0.271  605 | 0.400  99 | 1.35  4672 | 44 |
| {BcEV6=FALSE, BcMV12=TRUE} =>  {BcMV2.BI=TRUE} | 0.11  6337 | 0.602  564 | 0.193  069 | 1.30  1796 | 47 |
| {BcEV6=FALSE, BcMV2.BI=TRUE} => | 0.11 | 0.268 | 0.433 | 1.33 | 47 |

| {BcMV12=TRUE} | 6337 | 571 | 168 | 9541 |  |
| --- | --- | --- | --- | --- | --- |
| {BcMV12=TRUE, BcOLV17.BI=FALSE} =>  {BcMV2.BI=TRUE} | 0.11  1386 | 0.592  105 | 0.188  119 | 1.27  9201 | 45 |
| {BcMV2.BI=TRUE, BcOLV17.BI=FALSE} =>  {BcMV12=TRUE} | 0.11  1386 | 0.261  628 | 0.425  743 | 1.30  491 | 45 |
| {BcMV12=TRUE, BcOLV15.BI=FALSE} =>  {BcMV2.BI=TRUE} | 0.12  1287 | 0.628  205 | 0.193  069 | 1.35  7192 | 49 |
| {BcMV2.BI=TRUE, BcOLV15.BI=FALSE} =>  {BcMV12=TRUE} | 0.12  1287 | 0.267  76 | 0.452  97 | 1.33  5492 | 49 |
| {BcEV5=FALSE, BcMV12=TRUE} =>  {BcMV2.BI=TRUE} | 0.11  6337 | 0.594  937 | 0.195  545 | 1.28  5318 | 47 |
| {BcEV5=FALSE, BcMV2.BI=TRUE} =>  {BcMV12=TRUE} | 0.11  6337 | 0.274  854 | 0.423  267 | 1.37  0876 | 47 |
| {BcMV12=TRUE, BcOLV18=FALSE} =>  {BcHV3.BI=FALSE} | 0.07  4257 | 0.937  5 | 0.079  208 | 1.23  3713 | 30 |
| {BcMV12=TRUE, BcOLV18=FALSE} =>  {BcDMV1=FALSE} | 0.07  4257 | 0.937  5 | 0.079  208 | 1.11  0704 | 30 |
| {BcMV12=TRUE, BcOLV18=TRUE} =>  {BcHV1SLR.BI=TRUE} | 0.08  4158 | 0.693  878 | 0.121  287 | 1.28  003 | 34 |
| {BcHV1SLR.BI=TRUE, BcMV12=TRUE} =>  {BcOLV18=TRUE} | 0.08  4158 | 0.653  846 | 0.128  713 | 1.26  3894 | 34 |
| {BcMV12=TRUE, BcOLV18=TRUE} => | 0.09 | 0.816 | 0.121 | 1.50 | 40 |

| {BcMV3.BI=TRUE} | 901 | 327 | 287 | 5917 |  |
| --- | --- | --- | --- | --- | --- |
| {BcMV12=TRUE, BcMV3.BI=TRUE} =>  {BcOLV18=TRUE} | 0.09  901 | 0.645  161 | 0.153  465 | 1.24  7106 | 40 |
| {BcMV3.BI=TRUE, BcOLV18=TRUE} =>  {BcMV12=TRUE} | 0.09  901 | 0.300  752 | 0.329  208 | 1.50  0046 | 40 |
| {BcMV12=TRUE, BcOLV18=TRUE} =>  {BcHV1.BI=TRUE} | 0.09  1584 | 0.755  102 | 0.121  287 | 1.25  0251 | 37 |
| {BcHV1.BI=TRUE, BcMV12=TRUE} =>  {BcOLV18=TRUE} | 0.09  1584 | 0.660  714 | 0.138  614 | 1.27  717 | 37 |
| {BcMV12=TRUE, BcOLV18=TRUE} =>  {BcMV9.BI=TRUE} | 0.10  1485 | 0.836  735 | 0.121  287 | 1.23  3726 | 41 |
| {BcMV12=TRUE, BcMV9.BI=TRUE} =>  {BcOLV18=TRUE} | 0.10  1485 | 0.732  143 | 0.138  614 | 1.41  5243 | 41 |
| {BcMV12=TRUE, BcOLV18=TRUE} =>  {BcULV4=TRUE} | 0.09  901 | 0.816  327 | 0.121  287 | 1.18  2064 | 40 |
| {BcMV12=TRUE, BcULV4=TRUE} =>  {BcOLV18=TRUE} | 0.09  901 | 0.701  754 | 0.141  089 | 1.35  6501 | 40 |
| {BcEV2.BI=FALSE, BcOLV18=TRUE} =>  {BcMV12=TRUE} | 0.09  6535 | 0.261  745 | 0.368  812 | 1.30  5493 | 39 |
| {BcMV1.BI=TRUE, BcMV12=TRUE} =>  {BcOLV18=TRUE} | 0.10  6436 | 0.632  353 | 0.168  317 | 1.22  2347 | 43 |
| {BcMV12=TRUE, BcOLV4.BI=FALSE} => | 0.10 | 0.651 | 0.163 | 1.25 | 43 |

| {BcOLV18=TRUE} | 6436 | 515 | 366 | 9388 |  |
| --- | --- | --- | --- | --- | --- |
| {BcMV12=TRUE, BcULV5=FALSE} =>  {BcOLV18=TRUE} | 0.10  8911 | 0.676  923 | 0.160  891 | 1.30  8502 | 44 |
| {BcMV12=TRUE, BcOLV13.BI=FALSE} =>  {BcOLV18=TRUE} | 0.11  1386 | 0.652  174 | 0.170  792 | 1.26  0662 | 45 |
| {BcMCV3.BI=FALSE, BcMV12=TRUE} =>  {BcOLV18=TRUE} | 0.09  901 | 0.645  161 | 0.153  465 | 1.24  7106 | 40 |
| {BcMV12=TRUE, BcMV2.BI=FALSE} =>  {BcOLV4.BI=FALSE} | 0.07  6733 | 0.968  75 | 0.079  208 | 1.10  5579 | 31 |
| {BcMV12=TRUE, BcMV2.BI=FALSE} =>  {BcULV5=FALSE} | 0.07  6733 | 0.968  75 | 0.079  208 | 1.10  5579 | 31 |
| {BcHV1SLR.BI=TRUE, BcMV12=TRUE} =>  {BcMV3.BI=TRUE} | 0.10  6436 | 0.826  923 | 0.128  713 | 1.52  5465 | 43 |
| {BcMV12=TRUE, BcMV3.BI=TRUE} =>  {BcHV1SLR.BI=TRUE} | 0.10  6436 | 0.693  548 | 0.153  465 | 1.27  9423 | 43 |
| {BcHV1SLR.BI=TRUE, BcMV3.BI=TRUE} =>  {BcMV12=TRUE} | 0.10  6436 | 0.323  308 | 0.329  208 | 1.61  255 | 43 |
| {BcMV12=TRUE, BcMV4.BI=FALSE} =>  {BcHV1SLR.BI=TRUE} | 0.07  9208 | 0.666  667 | 0.118  812 | 1.22  9833 | 32 |
| {BcHV1SLR.BI=TRUE, BcMV4.BI=FALSE}  => {BcMV12=TRUE} | 0.07  9208 | 0.283  186 | 0.279  703 | 1.41  2433 | 32 |
| {BcHV1SLR.BI=TRUE, BcMV12=TRUE} => | 0.09 | 0.730 | 0.128 | 1.23 | 38 |

| {BcULV3=TRUE} | 4059 | 769 | 713 | 0128 |  |
| --- | --- | --- | --- | --- | --- |
| {BcMV12=TRUE, BcULV3=TRUE} =>  {BcHV1SLR.BI=TRUE} | 0.09  4059 | 0.745  098 | 0.126  238 | 1.37  4519 | 38 |
| {BcHV1SLR.BI=TRUE, BcMV12=TRUE} =>  {BcHV1.BI=TRUE} | 0.11  6337 | 0.903  846 | 0.128  713 | 1.49  6532 | 47 |
| {BcHV1.BI=TRUE, BcMV12=TRUE} =>  {BcHV1SLR.BI=TRUE} | 0.11  6337 | 0.839  286 | 0.138  614 | 1.54  8271 | 47 |
| {BcMV11=TRUE, BcMV12=TRUE} =>  {BcHV1SLR.BI=TRUE} | 0.07  6733 | 0.704  545 | 0.108  911 | 1.29  9709 | 31 |
| {BcMV12=TRUE, BcULV4=TRUE} =>  {BcHV1SLR.BI=TRUE} | 0.09  4059 | 0.666  667 | 0.141  089 | 1.22  9833 | 38 |
| {BcEV2.BI=FALSE, BcHV1SLR.BI=TRUE}  => {BcMV12=TRUE} | 0.10  396 | 0.269  231 | 0.386  139 | 1.34  283 | 42 |
| {BcHV1SLR.BI=TRUE, BcMV12=TRUE} =>  {BcMV1.BI=TRUE} | 0.11  3861 | 0.884  615 | 0.128  713 | 1.12  0328 | 46 |
| {BcMV1.BI=TRUE, BcMV12=TRUE} =>  {BcHV1SLR.BI=TRUE} | 0.11  3861 | 0.676  471 | 0.168  317 | 1.24  7918 | 46 |
| {BcHV1SLR.BI=TRUE, BcMV1.BI=TRUE} =>  {BcMV12=TRUE} | 0.11  3861 | 0.258  427 | 0.440  594 | 1.28  8944 | 46 |
| {BcALV1.BI=FALSE, BcMV12=TRUE} =>  {BcHV1SLR.BI=TRUE} | 0.10  6436 | 0.661  538 | 0.160  891 | 1.22  0372 | 43 |
| {BcALV1.BI=FALSE, BcHV1SLR.BI=TRUE} | 0.10 | 0.255 | 0.415 | 1.27 | 43 |

| => {BcMV12=TRUE} | 6436 | 952 | 842 | 6602 |  |
| --- | --- | --- | --- | --- | --- |
| {BcDMV1=FALSE, BcMV12=TRUE} =>  {BcHV1SLR.BI=TRUE} | 0.11  6337 | 0.661  972 | 0.175  743 | 1.22  1172 | 47 |
| {BcBNV5.BI=FALSE, BcMV12=TRUE} =>  {BcHV1SLR.BI=TRUE} | 0.12  3762 | 0.666  667 | 0.185  644 | 1.22  9833 | 50 |
| {BcBNV5.BI=FALSE, BcHV1SLR.BI=TRUE}  => {BcMV12=TRUE} | 0.12  3762 | 0.259  067 | 0.477  723 | 1.29  2138 | 50 |
| {BcHV1SLR.BI=TRUE, BcMV12=TRUE} =>  {BvF.BI=FALSE} | 0.12  8713 | 1 | 0.128  713 | 1.09  4851 | 52 |
| {BcMV12=TRUE, BvF.BI=FALSE} =>  {BcHV1SLR.BI=TRUE} | 0.12  8713 | 0.658  228 | 0.195  545 | 1.21  4265 | 52 |
| {BcHV1SLR.BI=TRUE, BvF.BI=FALSE} =>  {BcMV12=TRUE} | 0.12  8713 | 0.268  041 | 0.480  198 | 1.33  6897 | 52 |
| {BcEV6=FALSE, BcMV12=TRUE} =>  {BcHV1SLR.BI=TRUE} | 0.12  8713 | 0.666  667 | 0.193  069 | 1.22  9833 | 52 |
| {BcEV6=FALSE, BcHV1SLR.BI=TRUE} =>  {BcMV12=TRUE} | 0.12  8713 | 0.256  158 | 0.502  475 | 1.27  7626 | 52 |
| {BcMV12=TRUE, BcMV4.BI=FALSE} =>  {BcMV3.BI=TRUE} | 0.09  1584 | 0.770  833 | 0.118  812 | 1.42  1994 | 37 |
| {BcMV3.BI=TRUE, BcMV4.BI=FALSE} =>  {BcMV12=TRUE} | 0.09  1584 | 0.37 | 0.247  525 | 1.84  5432 | 37 |
| {BcMV12=TRUE, BcULV3=TRUE} => | 0.09 | 0.725 | 0.126 | 1.33 | 37 |

| {BcMV3.BI=TRUE} | 1584 | 49 | 238 | 8347 |  |
| --- | --- | --- | --- | --- | --- |
| {BcMV3.BI=TRUE, BcULV3=TRUE} =>  {BcMV12=TRUE} | 0.09  1584 | 0.264  286 | 0.346  535 | 1.31  8166 | 37 |
| {BcMV12=TRUE, BcMV3.BI=TRUE} =>  {BcHV1.BI=TRUE} | 0.10  8911 | 0.709  677 | 0.153  465 | 1.17  504 | 44 |
| {BcHV1.BI=TRUE, BcMV12=TRUE} =>  {BcMV3.BI=TRUE} | 0.10  8911 | 0.785  714 | 0.138  614 | 1.44  9446 | 44 |
| {BcHV1.BI=TRUE, BcMV3.BI=TRUE} =>  {BcMV12=TRUE} | 0.10  8911 | 0.307  692 | 0.353  96 | 1.53  4663 | 44 |
| {BcMV11=TRUE, BcMV12=TRUE} =>  {BcMV3.BI=TRUE} | 0.08  9109 | 0.818  182 | 0.108  911 | 1.50  934 | 36 |
| {BcMV12=TRUE, BcMV9.BI=TRUE} =>  {BcMV3.BI=TRUE} | 0.10  8911 | 0.785  714 | 0.138  614 | 1.44  9446 | 44 |
| {BcMV3.BI=TRUE, BcMV9.BI=TRUE} =>  {BcMV12=TRUE} | 0.10  8911 | 0.275 | 0.396  04 | 1.37  1605 | 44 |
| {BcMV12=TRUE, BcULV4=TRUE} =>  {BcMV3.BI=TRUE} | 0.11  3861 | 0.807  018 | 0.141  089 | 1.48  8745 | 46 |
| {BcMV3.BI=TRUE, BcULV4=TRUE} =>  {BcMV12=TRUE} | 0.11  3861 | 0.283  951 | 0.400  99 | 1.41  6248 | 46 |
| {BcMV12=TRUE, BcPV3.BI=FALSE} =>  {BcMV3.BI=TRUE} | 0.08  9109 | 0.692  308 | 0.128  713 | 1.27  7134 | 36 |
| {BcMV12=TRUE, BcRV1.BI=FALSE} => | 0.10 | 0.741 | 0.143 | 1.36 | 43 |

| {BcMV3.BI=TRUE} | 6436 | 379 | 564 | 7659 |  |
| --- | --- | --- | --- | --- | --- |
| {BcMV3.BI=TRUE, BcRV1.BI=FALSE} =>  {BcMV12=TRUE} | 0.10  6436 | 0.290  541 | 0.366  337 | 1.44  9116 | 43 |
| {BcMV12=TRUE, BcMV3.BI=TRUE} =>  {BcEV2.BI=FALSE} | 0.13  1188 | 0.854  839 | 0.153  465 | 1.14  3559 | 53 |
| {BcEV2.BI=FALSE, BcMV12=TRUE} =>  {BcMV3.BI=TRUE} | 0.13  1188 | 0.791  045 | 0.165  842 | 1.45  9279 | 53 |
| {BcEV2.BI=FALSE, BcMV3.BI=TRUE} =>  {BcMV12=TRUE} | 0.13  1188 | 0.325  153 | 0.403  465 | 1.62  1753 | 53 |
| {BcHV3.BI=FALSE, BcMV12=TRUE} =>  {BcMV3.BI=TRUE} | 0.12  3762 | 0.781  25 | 0.158  416 | 1.44  121 | 50 |
| {BcHV3.BI=FALSE, BcMV3.BI=TRUE} =>  {BcMV12=TRUE} | 0.12  3762 | 0.314  465 | 0.393  564 | 1.56  8445 | 50 |
| {BcFV5.BI=FALSE, BcMV12=TRUE} =>  {BcMV3.BI=TRUE} | 0.12  6238 | 0.809  524 | 0.155  941 | 1.49  3368 | 51 |
| {BcFV5.BI=FALSE, BcMV3.BI=TRUE} =>  {BcMV12=TRUE} | 0.12  6238 | 0.324  841 | 0.388  614 | 1.62  0193 | 51 |
| {BcMV12=TRUE, BcULV1.BI=FALSE} =>  {BcMV3.BI=TRUE} | 0.09  4059 | 0.716  981 | 0.131  188 | 1.32  265 | 38 |
| {BcMV12=TRUE, BcMV3.BI=TRUE} =>  {BcMV1.BI=TRUE} | 0.13  6139 | 0.887  097 | 0.153  465 | 1.12  3471 | 55 |
| {BcMV1.BI=TRUE, BcMV12=TRUE} => | 0.13 | 0.808 | 0.168 | 1.49 | 55 |

| {BcMV3.BI=TRUE} | 6139 | 824 | 317 | 2076 |  |
| --- | --- | --- | --- | --- | --- |
| {BcMV1.BI=TRUE, BcMV3.BI=TRUE} =>  {BcMV12=TRUE} | 0.13  6139 | 0.297  297 | 0.457  921 | 1.48  2816 | 55 |
| {BcMV12=TRUE, BcNSRV7.BI=FALSE} =>  {BcMV3.BI=TRUE} | 0.11  8812 | 0.774  194 | 0.153  465 | 1.42  8193 | 48 |
| {BcMV3.BI=TRUE, BcNSRV7.BI=FALSE} =>  {BcMV12=TRUE} | 0.11  8812 | 0.280  702 | 0.423  267 | 1.40  0043 | 48 |
| {BcMV12=TRUE, BcVV3.BI=FALSE} =>  {BcMV3.BI=TRUE} | 0.12  3762 | 0.757  576 | 0.163  366 | 1.39  7537 | 50 |
| {BcMV3.BI=TRUE, BcVV3.BI=FALSE} =>  {BcMV12=TRUE} | 0.12  3762 | 0.297  619 | 0.415  842 | 1.48  4421 | 50 |
| {BcMV12=TRUE, BcOLV7.BI=FALSE} =>  {BcMV3.BI=TRUE} | 0.11  1386 | 0.737  705 | 0.150  99 | 1.36  088 | 45 |
| {BcMV3.BI=TRUE, BcOLV7.BI=FALSE} =>  {BcMV12=TRUE} | 0.11  1386 | 0.267  857 | 0.415  842 | 1.33  5979 | 45 |
| {BcALV1.BI=FALSE, BcMV12=TRUE} =>  {BcMV3.BI=TRUE} | 0.12  8713 | 0.8 | 0.160  891 | 1.47  5799 | 52 |
| {BcALV1.BI=FALSE, BcMV3.BI=TRUE} =>  {BcMV12=TRUE} | 0.12  8713 | 0.304  094 | 0.423  267 | 1.51  6714 | 52 |
| {BcDMV1=FALSE, BcMV12=TRUE} =>  {BcMV3.BI=TRUE} | 0.13  6139 | 0.774  648 | 0.175  743 | 1.42  9031 | 55 |
| {BcDMV1=FALSE, BcMV3.BI=TRUE} => | 0.13 | 0.321 | 0.423 | 1.60 | 55 |

| {BcMV12=TRUE} | 6139 | 637 | 267 | 4216 |  |
| --- | --- | --- | --- | --- | --- |
| {BcFV9=FALSE, BcMV12=TRUE} =>  {BcMV3.BI=TRUE} | 0.13  1188 | 0.757  143 | 0.173  267 | 1.39  6738 | 53 |
| {BcFV9=FALSE, BcMV3.BI=TRUE} =>  {BcMV12=TRUE} | 0.13  1188 | 0.288  043 | 0.455  446 | 1.43  6661 | 53 |
| {BcMV12=TRUE, BcOLV4.BI=FALSE} =>  {BcMV3.BI=TRUE} | 0.11  8812 | 0.727  273 | 0.163  366 | 1.34  1636 | 48 |
| {BcMV3.BI=TRUE, BcOLV4.BI=FALSE} =>  {BcMV12=TRUE} | 0.11  8812 | 0.260  87 | 0.455  446 | 1.30  1127 | 48 |
| {BcMV12=TRUE, BcULV5=FALSE} =>  {BcMV3.BI=TRUE} | 0.11  6337 | 0.723  077 | 0.160  891 | 1.33  3895 | 47 |
| {BcMV3.BI=TRUE, BcULV5=FALSE} =>  {BcMV12=TRUE} | 0.11  6337 | 0.256  831 | 0.452  97 | 1.28  0982 | 47 |
| {BcBNV5.BI=FALSE, BcMV12=TRUE} =>  {BcMV3.BI=TRUE} | 0.14  3564 | 0.773  333 | 0.185  644 | 1.42  6606 | 58 |
| {BcBNV5.BI=FALSE, BcMV3.BI=TRUE} =>  {BcMV12=TRUE} | 0.14  3564 | 0.305  263 | 0.470  297 | 1.52  2547 | 58 |
| {BcMV12=TRUE, BvF.BI=FALSE} =>  {BcMV3.BI=TRUE} | 0.15  099 | 0.772  152 | 0.195  545 | 1.42  4426 | 61 |
| {BcMV3.BI=TRUE, BvF.BI=FALSE} =>  {BcMV12=TRUE} | 0.15  099 | 0.305 | 0.495  05 | 1.52  1235 | 61 |
| {BcMCV5.BI=FALSE, BcMV12=TRUE} => | 0.13 | 0.746 | 0.185 | 1.37 | 56 |

| {BcMV3.BI=TRUE} | 8614 | 667 | 644 | 7412 |  |
| --- | --- | --- | --- | --- | --- |
| {BcMCV5.BI=FALSE, BcMV3.BI=TRUE} =>  {BcMV12=TRUE} | 0.13  8614 | 0.287  179 | 0.482  673 | 1.43  2352 | 56 |
| {BcMV12=TRUE, BcOLV13.BI=FALSE} =>  {BcMV3.BI=TRUE} | 0.12  3762 | 0.724  638 | 0.170  792 | 1.33  6775 | 50 |
| {BcMV3.BI=TRUE, BcOLV13.BI=FALSE} =>  {BcMV12=TRUE} | 0.12  3762 | 0.260  417 | 0.475  248 | 1.29  8868 | 50 |
| {BcMV12=TRUE, BcOLV2.BI=FALSE} =>  {BcMV3.BI=TRUE} | 0.12  6238 | 0.739  13 | 0.170  792 | 1.36  351 | 51 |
| {BcMV3.BI=TRUE, BcOLV2.BI=FALSE} =>  {BcMV12=TRUE} | 0.12  6238 | 0.260  204 | 0.485  149 | 1.29  7808 | 51 |
| {BcMCV3.BI=FALSE, BcMV12=TRUE} =>  {BcMV3.BI=TRUE} | 0.10  6436 | 0.693  548 | 0.153  465 | 1.27  9423 | 43 |
| {BcMV12=TRUE, BcPV4.BI=FALSE} =>  {BcMV3.BI=TRUE} | 0.13  6139 | 0.774  648 | 0.175  743 | 1.42  9031 | 55 |
| {BcMV3.BI=TRUE, BcPV4.BI=FALSE} =>  {BcMV12=TRUE} | 0.13  6139 | 0.273  632 | 0.497  525 | 1.36  4781 | 55 |
| {BcEV6=FALSE, BcMV12=TRUE} =>  {BcMV3.BI=TRUE} | 0.15  3465 | 0.794  872 | 0.193  069 | 1.46  6339 | 62 |
| {BcEV6=FALSE, BcMV3.BI=TRUE} =>  {BcMV12=TRUE} | 0.15  3465 | 0.296  651 | 0.517  327 | 1.47  9591 | 62 |
| {BcMV12=TRUE, BcOLV17.BI=FALSE} => | 0.14 | 0.75 | 0.188 | 1.38 | 57 |

| {BcMV3.BI=TRUE} | 1089 |  | 119 | 3562 |  |
| --- | --- | --- | --- | --- | --- |
| {BcMV3.BI=TRUE, BcOLV17.BI=FALSE} =>  {BcMV12=TRUE} | 0.14  1089 | 0.275  362 | 0.512  376 | 1.37  3412 | 57 |
| {BcMV12=TRUE, BcOLV15.BI=FALSE} =>  {BcMV3.BI=TRUE} | 0.14  8515 | 0.769  231 | 0.193  069 | 1.41  9038 | 60 |
| {BcMV3.BI=TRUE, BcOLV15.BI=FALSE} =>  {BcMV12=TRUE} | 0.14  8515 | 0.292  683 | 0.507  426 | 1.45  9801 | 60 |
| {BcEV5=FALSE, BcMV12=TRUE} =>  {BcMV3.BI=TRUE} | 0.14  8515 | 0.759  494 | 0.195  545 | 1.40  1075 | 60 |
| {BcEV5=FALSE, BcMV3.BI=TRUE} =>  {BcMV12=TRUE} | 0.14  8515 | 0.3 | 0.495  05 | 1.49  6296 | 60 |
| {BcMV12=TRUE, BcPV3.BI=FALSE} =>  {BcMV4.BI=FALSE} | 0.08  9109 | 0.692  308 | 0.128  713 | 1.24  3077 | 36 |
| {BcMV12=TRUE, BcMV4.BI=FALSE} =>  {BcEV2.BI=FALSE} | 0.10  396 | 0.875 | 0.118  812 | 1.17  053 | 42 |
| {BcMV12=TRUE, BcMV4.BI=FALSE} =>  {BvF.BI=FALSE} | 0.11  8812 | 1 | 0.118  812 | 1.09  4851 | 48 |
| {BcMV12=TRUE, BcULV3=TRUE} =>  {BcHV1.BI=TRUE} | 0.10  1485 | 0.803  922 | 0.126  238 | 1.33  1083 | 41 |
| {BcHV1.BI=TRUE, BcMV12=TRUE} =>  {BcULV3=TRUE} | 0.10  1485 | 0.732  143 | 0.138  614 | 1.23  244 | 41 |
| {BcMV11=TRUE, BcMV12=TRUE} => | 0.07 | 0.727 | 0.108 | 1.20 | 32 |

| {BcHV1.BI=TRUE} | 9208 | 273 | 911 | 4173 |  |
| --- | --- | --- | --- | --- | --- |
| {BcMV12=TRUE, BcMV9.BI=TRUE} =>  {BcHV1.BI=TRUE} | 0.10  396 | 0.75 | 0.138  614 | 1.24  1803 | 42 |
| {BcMV12=TRUE, BcULV4=TRUE} =>  {BcHV1.BI=TRUE} | 0.10  6436 | 0.754  386 | 0.141  089 | 1.24  9065 | 43 |
| {BcMV12=TRUE, BcVV3.BI=FALSE} =>  {BcHV1.BI=TRUE} | 0.11  6337 | 0.712  121 | 0.163  366 | 1.17  9086 | 47 |
| {BcBNV5.BI=FALSE, BcMV12=TRUE} =>  {BcHV1.BI=TRUE} | 0.13  1188 | 0.706  667 | 0.185  644 | 1.17  0055 | 53 |
| {BcHV1.BI=TRUE, BvF.BI=FALSE} =>  {BcMV12=TRUE} | 0.13  6139 | 0.25 | 0.544  554 | 1.24  6914 | 55 |
| {BcMV11=TRUE, BcMV12=TRUE} =>  {BcULV4=TRUE} | 0.08  6634 | 0.795  455 | 0.108  911 | 1.15  1841 | 35 |
| {BcMV11=TRUE, BcMV12=TRUE} =>  {BcMV1.BI=TRUE} | 0.09  6535 | 0.886  364 | 0.108  911 | 1.12  2542 | 39 |
| {BcMV12=TRUE, BcMV9.BI=TRUE} =>  {BcULV4=TRUE} | 0.11  3861 | 0.821  429 | 0.138  614 | 1.18  9452 | 46 |
| {BcMV12=TRUE, BcULV4=TRUE} =>  {BcMV9.BI=TRUE} | 0.11  3861 | 0.807  018 | 0.141  089 | 1.18  9909 | 46 |
| {BcMV12=TRUE, BcMV9.BI=TRUE} =>  {BcULV5=FALSE} | 0.13  3663 | 0.964  286 | 0.138  614 | 1.10  0484 | 54 |
| {BcMV12=TRUE, BcULV5=FALSE} => | 0.13 | 0.830 | 0.160 | 1.22 | 54 |

| {BcMV9.BI=TRUE} | 3663 | 769 | 891 | 493 |  |
| --- | --- | --- | --- | --- | --- |
| {BcMV12=TRUE, BcULV4=TRUE} =>  {BcEV2.BI=FALSE} | 0.11  8812 | 0.842  105 | 0.141  089 | 1.12  6525 | 48 |
| {BcMV12=TRUE, BcPV3.BI=FALSE} =>  {BcVV3.BI=FALSE} | 0.11  6337 | 0.903  846 | 0.128  713 | 1.12  7018 | 47 |
| {BcMV12=TRUE, BcRV1.BI=FALSE} =>  {BcEV2.BI=FALSE} | 0.12  1287 | 0.844  828 | 0.143  564 | 1.13  0167 | 49 |
| {BcHV3.BI=FALSE, BcMV12=TRUE} =>  {BcEV2.BI=FALSE} | 0.13  6139 | 0.859  375 | 0.158  416 | 1.14  9627 | 55 |
| {BcFV5.BI=FALSE, BcMV12=TRUE} =>  {BcEV2.BI=FALSE} | 0.13  6139 | 0.873  016 | 0.155  941 | 1.16  7876 | 55 |
| {BcMV12=TRUE, BcNSRV7.BI=FALSE} =>  {BcEV2.BI=FALSE} | 0.12  8713 | 0.838  71 | 0.153  465 | 1.12  1982 | 52 |
| {BcMV12=TRUE, BcVV3.BI=FALSE} =>  {BcEV2.BI=FALSE} | 0.13  8614 | 0.848  485 | 0.163  366 | 1.13  5059 | 56 |
| {BcMV12=TRUE, BcOLV7.BI=FALSE} =>  {BcEV2.BI=FALSE} | 0.13  3663 | 0.885  246 | 0.150  99 | 1.18  4236 | 54 |
| {BcMV12=TRUE, BcOLV4.BI=FALSE} =>  {BcEV2.BI=FALSE} | 0.13  8614 | 0.848  485 | 0.163  366 | 1.13  5059 | 56 |
| {BcEV2.BI=FALSE, BcMV12=TRUE} =>  {BvF.BI=FALSE} | 0.16  3366 | 0.985  075 | 0.165  842 | 1.07  851 | 66 |
| {BcMV12=TRUE, BvF.BI=FALSE} => | 0.16 | 0.835 | 0.195 | 1.11 | 66 |

| {BcEV2.BI=FALSE} | 3366 | 443 | 545 | 7613 |  |
| --- | --- | --- | --- | --- | --- |
| {BcMCV5.BI=FALSE, BcMV12=TRUE} =>  {BcEV2.BI=FALSE} | 0.15  8416 | 0.853  333 | 0.185  644 | 1.14  1545 | 64 |
| {BcEV6=FALSE, BcMV12=TRUE} =>  {BcEV2.BI=FALSE} | 0.16  3366 | 0.846  154 | 0.193  069 | 1.13  1941 | 66 |
| {BcMV12=TRUE, BcOLV15.BI=FALSE} =>  {BcEV2.BI=FALSE} | 0.16  3366 | 0.846  154 | 0.193  069 | 1.13  1941 | 66 |
| {BcHV3.BI=FALSE, BcMV12=TRUE} =>  {BcFV5.BI=FALSE} | 0.13  8614 | 0.875 | 0.158  416 | 1.11  8671 | 56 |
| {BcFV5.BI=FALSE, BcMV12=TRUE} =>  {BcHV3.BI=FALSE} | 0.13  8614 | 0.888  889 | 0.155  941 | 1.16  9743 | 56 |
| {BcMV12=TRUE, BcNSRV7.BI=FALSE} =>  {BcHV3.BI=FALSE} | 0.13  1188 | 0.854  839 | 0.153  465 | 1.12  4934 | 53 |
| {BcHV3.BI=FALSE, BcMV12=TRUE} =>  {BcDMV1=FALSE} | 0.15  099 | 0.953  125 | 0.158  416 | 1.12  9216 | 61 |
| {BcDMV1=FALSE, BcMV12=TRUE} =>  {BcHV3.BI=FALSE} | 0.15  099 | 0.859  155 | 0.175  743 | 1.13  0614 | 61 |
| {BcMV12=TRUE, BcNSRV7.BI=FALSE} =>  {BcDMV1=FALSE} | 0.14  3564 | 0.935  484 | 0.153  465 | 1.10  8315 | 58 |
| {BcALV1.BI=FALSE, BcMV12=TRUE} =>  {BvF.BI=FALSE} | 0.15  8416 | 0.984  615 | 0.160  891 | 1.07  8007 | 64 |
| {BcBNV5.BI=FALSE, BcMV12=TRUE} => | 0.16 | 0.906 | 0.185 | 1.07 | 68 |

| {BcDMV1=FALSE} | 8317 | 667 | 644 | 4174 |  |
| --- | --- | --- | --- | --- | --- |
| {BcMV1.BI=FALSE, BcRV1.BI=TRUE} =>  {BcOLV4.BI=FALSE} | 0.07  4257 | 1 | 0.074  257 | 1.14  1243 | 30 |
| {BcMV1.BI=FALSE, BcOLV4.BI=FALSE} =>  {BcRV1.BI=TRUE} | 0.07  4257 | 0.365  854 | 0.202  97 | 1.31  9686 | 30 |
| {BcOLV4.BI=FALSE, BcRV1.BI=TRUE} =>  {BcMV1.BI=FALSE} | 0.07  4257 | 0.303  03 | 0.245  05 | 1.44  0285 | 30 |
| {BcMV1.BI=FALSE, BcRV1.BI=TRUE} =>  {BcULV5=FALSE} | 0.07  4257 | 1 | 0.074  257 | 1.14  1243 | 30 |
| {BcMV1.BI=FALSE, BcULV5=FALSE} =>  {BcRV1.BI=TRUE} | 0.07  4257 | 0.365  854 | 0.202  97 | 1.31  9686 | 30 |
| {BcRV1.BI=TRUE, BcULV5=FALSE} =>  {BcMV1.BI=FALSE} | 0.07  4257 | 0.3 | 0.247  525 | 1.42  5882 | 30 |
| {BcMCV5.BI=FALSE, BcRV1.BI=TRUE} =>  {BcMV1.BI=FALSE} | 0.07  4257 | 0.297  03 | 0.25 | 1.41  1765 | 30 |
| {BcEV5=FALSE, BcMV1.BI=FALSE} =>  {BcRV1.BI=TRUE} | 0.07  4257 | 0.361  446 | 0.205  446 | 1.30  3787 | 30 |
| {BcMV1.BI=FALSE, BcMV9.BI=FALSE} =>  {BcULV1.BI=FALSE} | 0.07  6733 | 0.939  394 | 0.081  683 | 1.19  7209 | 31 |
| {BcMV1.BI=FALSE, BcULV1.BI=FALSE} =>  {BcMV9.BI=FALSE} | 0.07  6733 | 0.418  919 | 0.183  168 | 1.30  1871 | 31 |
| {BcMV1.BI=FALSE, BcMV9.BI=FALSE} => | 0.08 | 1 | 0.081 | 1.23 | 33 |

| {BcOLV7.BI=FALSE} | 1683 |  | 683 | 9264 |  |
| --- | --- | --- | --- | --- | --- |
| {BcMV1.BI=FALSE, BcOLV7.BI=FALSE} =>  {BcMV9.BI=FALSE} | 0.08  1683 | 0.44 | 0.185  644 | 1.36  7385 | 33 |
| {BcMV1.BI=FALSE, BcMV9.BI=FALSE} =>  {BcALV1.BI=FALSE} | 0.07  4257 | 0.909  091 | 0.081  683 | 1.11  633 | 30 |
| {BcMV9.BI=FALSE, BcOLV4.BI=FALSE} =>  {BcMV1.BI=FALSE} | 0.07  4257 | 0.285  714 | 0.259  901 | 1.35  7983 | 30 |
| {BcMV9.BI=FALSE, BcULV5=FALSE} =>  {BcMV1.BI=FALSE} | 0.07  6733 | 0.348  315 | 0.220  297 | 1.65  5519 | 31 |
| {BcMV1.BI=FALSE, BvF.BI=FALSE} =>  {BcMV9.BI=FALSE} | 0.07  6733 | 0.418  919 | 0.183  168 | 1.30  1871 | 31 |
| {BcMV1.BI=FALSE, BcMV11=FALSE} =>  {BcMV3.BI=FALSE} | 0.08  1683 | 0.717  391 | 0.113  861 | 1.56  6627 | 33 |
| {BcMV1.BI=FALSE, BcMV3.BI=FALSE} =>  {BcMV11=FALSE} | 0.08  1683 | 0.647  059 | 0.126  238 | 1.68  6528 | 33 |
| {BcMV11=FALSE, BcMV3.BI=FALSE} =>  {BcMV1.BI=FALSE} | 0.08  1683 | 0.362  637 | 0.225  248 | 1.72  3594 | 33 |
| {BcMV1.BI=FALSE, BcMV11=FALSE} =>  {BcOLV18=FALSE} | 0.08  1683 | 0.717  391 | 0.113  861 | 1.48  6288 | 33 |
| {BcMV1.BI=FALSE, BcOLV18=FALSE} =>  {BcMV11=FALSE} | 0.08  1683 | 0.611  111 | 0.133  663 | 1.59  2832 | 33 |
| {BcMV11=FALSE, BcOLV18=FALSE} => | 0.08 | 0.383 | 0.212 | 1.82 | 33 |

| {BcMV1.BI=FALSE} | 1683 | 721 | 871 | 3803 |  |
| --- | --- | --- | --- | --- | --- |
| {BcMV1.BI=FALSE, BcMV11=FALSE} => | 0.08 | 0.760 | 0.113 | 1.41 |  |
|  |  |  |  |  | 35 |
| {BcMV2.BI=FALSE} | 6634 | 87 | 861 | 655 |  |
| {BcMV1.BI=FALSE, BcMV2.BI=FALSE} => | 0.08 | 0.555 | 0.155 | 1.44 |  |
|  |  |  |  |  | 35 |
| {BcMV11=FALSE} | 6634 | 556 | 941 | 8029 |  |
| {BcMV11=FALSE, BcMV2.BI=FALSE} => | 0.08 | 0.330 | 0.262 | 1.56 |  |
|  |  |  |  |  | 35 |
| {BcMV1.BI=FALSE} | 6634 | 189 | 376 | 9367 |  |
| {BcMV1.BI=FALSE, BcMV11=FALSE} => | 0.08 | 0.739 | 0.113 | 1.32 |  |
|  |  |  |  |  | 34 |
| {BcMV4.BI=FALSE} | 4158 | 13 | 861 | 715 |  |
| {BcMV1.BI=FALSE, BcMV4.BI=FALSE} => | 0.08 | 0.586 | 0.143 | 1.52 |  |
|  |  |  |  |  | 34 |
| {BcMV11=FALSE} | 4158 | 207 | 564 | 792 |  |
| {BcMV11=FALSE, BcMV4.BI=FALSE} => | 0.08 | 0.300 | 0.279 | 1.43 |  |
|  |  |  |  |  | 34 |
| {BcMV1.BI=FALSE} | 4158 | 885 | 703 | 0088 |  |
| {BcMV1.BI=FALSE, BcOLV16.BI=FALSE} => | 0.07 | 0.584 | 0.131 | 1.52 |  |
|  |  |  |  |  | 31 |
| {BcMV11=FALSE} | 6733 | 906 | 188 | 4528 |  |
| {BcMV11=FALSE, BcOLV16.BI=FALSE} => | 0.07 | 0.316 | 0.242 | 1.50 |  |
|  |  |  |  |  | 31 |
| {BcMV1.BI=FALSE} | 6733 | 327 | 574 | 3481 |  |
| {BcMV1.BI=FALSE, BcMV11=FALSE} => | 0.09 | 0.847 | 0.113 | 1.26 |  |
|  |  |  |  |  | 39 |
| {BcOLV1.BI.MOVA.=FALSE} | 6535 | 826 | 861 | 8599 |  |
| {BcMV1.BI=FALSE, |  |  |  |  |  |
|  | 0.09 | 0.557 | 0.173 | 1.45 |  |
| BcOLV1.BI.MOVA.=FALSE} => |  |  |  |  | 39 |
|  | 6535 | 143 | 267 | 2166 |  |
| {BcMV11=FALSE} |  |  |  |  |  |

| {BcMV11=FALSE, BcOLV1.BI.MOVA.=FALSE} =>  {BcMV1.BI=FALSE} | 0.09  6535 | 0.327  731 | 0.294  554 | 1.55  7687 | 39 |
| --- | --- | --- | --- | --- | --- |
| {BcMV1.BI=FALSE, BcULV4=TRUE} =>  {BcMV11=FALSE} | 0.08  1683 | 0.578  947 | 0.141  089 | 1.50  8998 | 33 |
| {BcMV11=FALSE, BcULV4=TRUE} =>  {BcMV1.BI=FALSE} | 0.08  1683 | 0.3 | 0.272  277 | 1.42  5882 | 33 |
| {BcMV1.BI=FALSE, BcMV11=FALSE} =>  {BcMV6.BI=FALSE} | 0.09  1584 | 0.804  348 | 0.113  861 | 1.15  6429 | 37 |
| {BcMV1.BI=FALSE, BcMV6.BI=FALSE} =>  {BcMV11=FALSE} | 0.09  1584 | 0.569  231 | 0.160  891 | 1.48  3672 | 37 |
| {BcMV11=FALSE, BcMV6.BI=FALSE} =>  {BcMV1.BI=FALSE} | 0.09  1584 | 0.298  387 | 0.306  931 | 1.41  8216 | 37 |
| {BcMV1.BI=FALSE, BcMV11=FALSE} =>  {BcPV3.BI=FALSE} | 0.09  4059 | 0.826  087 | 0.113  861 | 1.16  2854 | 38 |
| {BcMV1.BI=FALSE, BcPV3.BI=FALSE} =>  {BcMV11=FALSE} | 0.09  4059 | 0.535  211 | 0.175  743 | 1.39  5002 | 38 |
| {BcMV11=FALSE, BcPV3.BI=FALSE} =>  {BcMV1.BI=FALSE} | 0.09  4059 | 0.322  034 | 0.292  079 | 1.53  0608 | 38 |
| {BcMV1.BI=FALSE, BcRV1.BI=FALSE} =>  {BcMV11=FALSE} | 0.08  6634 | 0.636  364 | 0.136  139 | 1.65  8651 | 35 |
| {BcMV11=FALSE, BcRV1.BI=FALSE} => | 0.08 | 0.275 | 0.314 | 1.30 | 35 |

| {BcMV1.BI=FALSE} | 6634 | 591 | 356 | 9866 |  |
| --- | --- | --- | --- | --- | --- |
| {BcMV1.BI=FALSE, BcMV11=FALSE} =>  {BcEV2.BI=FALSE} | 0.10  1485 | 0.891  304 | 0.113  861 | 1.19  2341 | 41 |
| {BcEV2.BI=FALSE, BcMV1.BI=FALSE} =>  {BcMV11=FALSE} | 0.10  1485 | 0.577  465 | 0.175  743 | 1.50  5134 | 41 |
| {BcEV2.BI=FALSE, BcMV11=FALSE} =>  {BcMV1.BI=FALSE} | 0.10  1485 | 0.320  313 | 0.316  832 | 1.52  2426 | 41 |
| {BcMV1.BI=FALSE, BcMV11=FALSE} =>  {BcHV3.BI=FALSE} | 0.09  901 | 0.869  565 | 0.113  861 | 1.14  4314 | 40 |
| {BcHV3.BI=FALSE, BcMV1.BI=FALSE} =>  {BcMV11=FALSE} | 0.09  901 | 0.540  541 | 0.183  168 | 1.40  8893 | 40 |
| {BcHV3.BI=FALSE, BcMV11=FALSE} =>  {BcMV1.BI=FALSE} | 0.09  901 | 0.312  5 | 0.316  832 | 1.48  5294 | 40 |
| {BcFV5.BI=FALSE, BcMV1.BI=FALSE} =>  {BcMV11=FALSE} | 0.09  1584 | 0.528  571 | 0.173  267 | 1.37  7696 | 37 |
| {BcFV5.BI=FALSE, BcMV11=FALSE} =>  {BcMV1.BI=FALSE} | 0.09  1584 | 0.298  387 | 0.306  931 | 1.41  8216 | 37 |
| {BcMV1.BI=FALSE, BcMV11=FALSE} =>  {BcULV1.BI=FALSE} | 0.10  396 | 0.913  043 | 0.113  861 | 1.16  3626 | 42 |
| {BcMV1.BI=FALSE, BcULV1.BI=FALSE} =>  {BcMV11=FALSE} | 0.10  396 | 0.567  568 | 0.183  168 | 1.47  9337 | 42 |
| {BcMV11=FALSE, BcULV1.BI=FALSE} => | 0.10 | 0.333 | 0.311 | 1.58 | 42 |

| {BcMV1.BI=FALSE} | 396 | 333 | 881 | 4314 |  |
| --- | --- | --- | --- | --- | --- |
| {BcMV1.BI=FALSE, BcMV12=FALSE} =>  {BcMV11=FALSE} | 0.09  4059 | 0.527  778 | 0.178  218 | 1.37  5627 | 38 |
| {BcMV11=FALSE, BcMV12=FALSE} =>  {BcMV1.BI=FALSE} | 0.09  4059 | 0.322  034 | 0.292  079 | 1.53  0608 | 38 |
| {BcMV1.BI=FALSE, BcNSRV7.BI=FALSE}  => {BcMV11=FALSE} | 0.09  4059 | 0.535  211 | 0.175  743 | 1.39  5002 | 38 |
| {BcMV11=FALSE, BcNSRV7.BI=FALSE} =>  {BcMV1.BI=FALSE} | 0.09  4059 | 0.299  213 | 0.314  356 | 1.42  214 | 38 |
| {BcMV1.BI=FALSE, BcVV3.BI=FALSE} =>  {BcMV11=FALSE} | 0.08  9109 | 0.507  042 | 0.175  743 | 1.32  1581 | 36 |
| {BcMV11=FALSE, BcVV3.BI=FALSE} =>  {BcMV1.BI=FALSE} | 0.08  9109 | 0.279  07 | 0.319  307 | 1.32  6402 | 36 |
| {BcMV1.BI=FALSE, BcOLV7.BI=FALSE} =>  {BcMV11=FALSE} | 0.10  1485 | 0.546  667 | 0.185  644 | 1.42  486 | 41 |
| {BcMV11=FALSE, BcOLV7.BI=FALSE} =>  {BcMV1.BI=FALSE} | 0.10  1485 | 0.315  385 | 0.321  782 | 1.49  9005 | 41 |
| {BcMV1.BI=FALSE, BcMV11=FALSE} =>  {BcALV1.BI=FALSE} | 0.10  396 | 0.913  043 | 0.113  861 | 1.12  1184 | 42 |
| {BcALV1.BI=FALSE, BcMV1.BI=FALSE} =>  {BcMV11=FALSE} | 0.10  396 | 0.567  568 | 0.183  168 | 1.47  9337 | 42 |
| {BcALV1.BI=FALSE, BcMV11=FALSE} => | 0.10 | 0.293 | 0.353 | 1.39 | 42 |

| {BcMV1.BI=FALSE} | 396 | 706 | 96 | 5969 |  |
| --- | --- | --- | --- | --- | --- |
| {BcDMV1=FALSE, BcMV1.BI=FALSE} =>  {BcMV11=FALSE} | 0.08  9109 | 0.5 | 0.178  218 | 1.30  3226 | 36 |
| {BcMV1.BI=FALSE, BcMV11=FALSE} =>  {BcFV9=FALSE} | 0.10  8911 | 0.956  522 | 0.113  861 | 1.10  0954 | 44 |
| {BcFV9=FALSE, BcMV1.BI=FALSE} =>  {BcMV11=FALSE} | 0.10  8911 | 0.571  429 | 0.190  594 | 1.48  9401 | 44 |
| {BcFV9=FALSE, BcMV11=FALSE} =>  {BcMV1.BI=FALSE} | 0.10  8911 | 0.314  286 | 0.346  535 | 1.49  3782 | 44 |
| {BcMV1.BI=FALSE, BcOLV4.BI=FALSE} =>  {BcMV11=FALSE} | 0.10  8911 | 0.536  585 | 0.202  97 | 1.39  8584 | 44 |
| {BcMV11=FALSE, BcOLV4.BI=FALSE} =>  {BcMV1.BI=FALSE} | 0.10  8911 | 0.299  32 | 0.363  861 | 1.42  2649 | 44 |
| {BcMV1.BI=FALSE, BcMV11=FALSE} =>  {BcULV5=FALSE} | 0.11  1386 | 0.978  261 | 0.113  861 | 1.11  6433 | 45 |
| {BcMV1.BI=FALSE, BcULV5=FALSE} =>  {BcMV11=FALSE} | 0.11  1386 | 0.548  78 | 0.202  97 | 1.43  037 | 45 |
| {BcMV11=FALSE, BcULV5=FALSE} =>  {BcMV1.BI=FALSE} | 0.11  1386 | 0.3 | 0.371  287 | 1.42  5882 | 45 |
| {BcMV1.BI=FALSE, BcMV11=FALSE} =>  {BcBNV5.BI=FALSE} | 0.11  3861 | 1 | 0.113  861 | 1.10  0817 | 46 |
| {BcBNV5.BI=FALSE, BcMV1.BI=FALSE} => | 0.11 | 0.560 | 0.202 | 1.46 | 46 |

| {BcMV11=FALSE} | 3861 | 976 | 97 | 2156 |  |
| --- | --- | --- | --- | --- | --- |
| {BcBNV5.BI=FALSE, BcMV11=FALSE} =>  {BcMV1.BI=FALSE} | 0.11  3861 | 0.306  667 | 0.371  287 | 1.45  7569 | 46 |
| {BcMV1.BI=FALSE, BvF.BI=FALSE} =>  {BcMV11=FALSE} | 0.09  901 | 0.540  541 | 0.183  168 | 1.40  8893 | 40 |
| {BcMV11=FALSE, BvF.BI=FALSE} =>  {BcMV1.BI=FALSE} | 0.09  901 | 0.289  855 | 0.341  584 | 1.37  7664 | 40 |
| {BcMCV5.BI=FALSE, BcMV1.BI=FALSE} =>  {BcMV11=FALSE} | 0.11  3861 | 0.541  176 | 0.210  396 | 1.41  055 | 46 |
| {BcMCV5.BI=FALSE, BcMV11=FALSE} =>  {BcMV1.BI=FALSE} | 0.11  3861 | 0.302  632 | 0.376  238 | 1.43  839 | 46 |
| {BcMV1.BI=FALSE, BcOLV13.BI=FALSE} =>  {BcMV11=FALSE} | 0.11  1386 | 0.555  556 | 0.200  495 | 1.44  8029 | 45 |
| {BcMV11=FALSE, BcOLV13.BI=FALSE} =>  {BcMV1.BI=FALSE} | 0.11  1386 | 0.304  054 | 0.366  337 | 1.44  5151 | 45 |
| {BcMV1.BI=FALSE, BcOLV2.BI=FALSE} =>  {BcMV11=FALSE} | 0.10  6436 | 0.544  304 | 0.195  545 | 1.41  8702 | 43 |
| {BcMV11=FALSE, BcOLV2.BI=FALSE} =>  {BcMV1.BI=FALSE} | 0.10  6436 | 0.296  552 | 0.358  911 | 1.40  9493 | 43 |
| {BcMCV3.BI=FALSE, BcMV1.BI=FALSE} =>  {BcMV11=FALSE} | 0.11  1386 | 0.548  78 | 0.202  97 | 1.43  037 | 45 |
| {BcMCV3.BI=FALSE, BcMV11=FALSE} => | 0.11 | 0.316 | 0.351 | 1.50 | 45 |

| {BcMV1.BI=FALSE} | 1386 | 901 | 485 | 6214 |  |
| --- | --- | --- | --- | --- | --- |
| {BcMV1.BI=FALSE, BcPV4.BI=FALSE} =>  {BcMV11=FALSE} | 0.11  1386 | 0.542  169 | 0.205  446 | 1.41  3136 | 45 |
| {BcMV11=FALSE, BcPV4.BI=FALSE} =>  {BcMV1.BI=FALSE} | 0.11  1386 | 0.312  5 | 0.356  436 | 1.48  5294 | 45 |
| {BcEV6=FALSE, BcMV1.BI=FALSE} =>  {BcMV11=FALSE} | 0.10  396 | 0.531  646 | 0.195  545 | 1.38  5708 | 42 |
| {BcEV6=FALSE, BcMV11=FALSE} =>  {BcMV1.BI=FALSE} | 0.10  396 | 0.289  655 | 0.358  911 | 1.37  6714 | 42 |
| {BcMV1.BI=FALSE, BcOLV17.BI=FALSE} =>  {BcMV11=FALSE} | 0.11  1386 | 0.542  169 | 0.205  446 | 1.41  3136 | 45 |
| {BcMV11=FALSE, BcOLV17.BI=FALSE} =>  {BcMV1.BI=FALSE} | 0.11  1386 | 0.298  013 | 0.373  762 | 1.41  6439 | 45 |
| {BcMV1.BI=FALSE, BcOLV15.BI=FALSE} =>  {BcMV11=FALSE} | 0.11  3861 | 0.554  217 | 0.205  446 | 1.44  4539 | 46 |
| {BcMV11=FALSE, BcOLV15.BI=FALSE} =>  {BcMV1.BI=FALSE} | 0.11  3861 | 0.315  068 | 0.361  386 | 1.49  7502 | 46 |
| {BcEV5=FALSE, BcMV1.BI=FALSE} =>  {BcMV11=FALSE} | 0.10  8911 | 0.530  12 | 0.205  446 | 1.38  1733 | 44 |
| {BcEV5=FALSE, BcMV11=FALSE} =>  {BcMV1.BI=FALSE} | 0.10  8911 | 0.289  474 | 0.376  238 | 1.37  5851 | 44 |
| {BcHV1.BI=FALSE, BcMV1.BI=FALSE} => | 0.08 | 0.944 | 0.089 | 2.06 | 34 |

| {BcHV1SLR.BI=FALSE} | 4158 | 444 | 109 | 2462 |  |
| --- | --- | --- | --- | --- | --- |
| {BcHV1SLR.BI=FALSE, BcMV1.BI=FALSE}  => {BcHV1.BI=FALSE} | 0.08  4158 | 0.772  727 | 0.108  911 | 1.95  1136 | 34 |
| {BcHV1.BI=FALSE, BcMV1.BI=FALSE} =>  {BcOLV1.BI.MOVA.=FALSE} | 0.08  1683 | 0.916  667 | 0.089  109 | 1.37  1605 | 33 |
| {BcHV1.BI=FALSE, BcOLV1.BI.MOVA.=FALSE} =>  {BcMV1.BI=FALSE} | 0.08  1683 | 0.294  643 | 0.277  228 | 1.40  042 | 33 |
| {BcHV1.BI=FALSE, BcMV1.BI=FALSE} => | 0.07 | 0.833 | 0.089 | 1.19 |  |
|  |  |  |  |  | 30 |
| {BcMV6.BI=FALSE} | 4257 | 333 | 109 | 8102 |  |
| {BcHV1.BI=FALSE, BcMV1.BI=FALSE} => | 0.08 | 0.916 | 0.089 | 1.29 |  |
|  |  |  |  |  | 33 |
| {BcPV3.BI=FALSE} | 1683 | 667 | 109 | 036 |  |
| {BcHV1.BI=FALSE, BcMV1.BI=FALSE} => | 0.07 | 0.888 | 0.089 | 1.18 |  |
|  |  |  |  |  | 32 |
| {BcEV2.BI=FALSE} | 9208 | 889 | 109 | 911 |  |
| {BcHV1.BI=FALSE, BcMV1.BI=FALSE} => | 0.08 | 0.916 | 0.089 | 1.20 |  |
|  |  |  |  |  | 33 |
| {BcHV3.BI=FALSE} | 1683 | 667 | 109 | 6298 |  |
| {BcHV1.BI=FALSE, BcMV1.BI=FALSE} => | 0.08 | 0.944 | 0.089 | 1.20 |  |
|  |  |  |  |  | 34 |
| {BcULV1.BI=FALSE} | 4158 | 444 | 109 | 3645 |  |
| {BcHV1.BI=FALSE, BcMV1.BI=FALSE} => | 0.08 | 0.916 | 0.089 | 1.14 |  |
|  |  |  |  |  | 33 |
| {BcNSRV7.BI=FALSE} | 1683 | 667 | 109 | 6543 |  |
| {BcHV1.BI=FALSE, BcMV1.BI=FALSE} => | 0.08 | 0.944 | 0.089 | 1.17 |  |
|  |  |  |  |  | 34 |
| {BcOLV7.BI=FALSE} | 4158 | 444 | 109 | 0416 |  |

| {BcHV1.BI=FALSE, BcMV1.BI=FALSE} =>  {BcALV1.BI=FALSE} | 0.08  1683 | 0.916  667 | 0.089  109 | 1.12  5633 | 33 |
| --- | --- | --- | --- | --- | --- |
| {BcHV1.BI=FALSE, BcMV1.BI=FALSE} =>  {BcOLV4.BI=FALSE} | 0.08  6634 | 0.972  222 | 0.089  109 | 1.10  9542 | 35 |
| {BcHV1.BI=FALSE, BcMV1.BI=FALSE} =>  {BcULV5=FALSE} | 0.08  9109 | 1 | 0.089  109 | 1.14  1243 | 36 |
| {BcMV1.BI=FALSE, BcULV3=FALSE} =>  {BcHV1SLR.BI=FALSE} | 0.07  4257 | 0.731  707 | 0.101  485 | 1.59  7891 | 30 |
| {BcHV1SLR.BI=FALSE, BcMV1.BI=FALSE}  => {BcULV3=FALSE} | 0.07  4257 | 0.681  818 | 0.108  911 | 1.67  9601 | 30 |
| {BcHV1SLR.BI=FALSE, BcULV3=FALSE} =>  {BcMV1.BI=FALSE} | 0.07  4257 | 0.297  03 | 0.25 | 1.41  1765 | 30 |
| {BcMV1.BI=FALSE, BcULV3=FALSE} =>  {BcOLV18=FALSE} | 0.07  6733 | 0.756  098 | 0.101  485 | 1.56  6479 | 31 |
| {BcMV1.BI=FALSE, BcOLV18=FALSE} =>  {BcULV3=FALSE} | 0.07  6733 | 0.574  074 | 0.133  663 | 1.41  4182 | 31 |
| {BcOLV18=FALSE, BcULV3=FALSE} =>  {BcMV1.BI=FALSE} | 0.07  6733 | 0.360  465 | 0.212  871 | 1.71  3269 | 31 |
| {BcMV1.BI=FALSE, BcULV3=FALSE} =>  {BcMV2.BI=FALSE} | 0.08  1683 | 0.804  878 | 0.101  485 | 1.49  8483 | 33 |
| {BcMV1.BI=FALSE, BcMV2.BI=FALSE} =>  {BcULV3=FALSE} | 0.08  1683 | 0.523  81 | 0.155  941 | 1.29  036 | 33 |

| {BcMV2.BI=FALSE, BcULV3=FALSE} =>  {BcMV1.BI=FALSE} | 0.08  1683 | 0.358  696 | 0.227  723 | 1.70  4859 | 33 |
| --- | --- | --- | --- | --- | --- |
| {BcMV1.BI=FALSE, BcULV3=FALSE} =>  {BcMV4.BI=FALSE} | 0.07  6733 | 0.756  098 | 0.101  485 | 1.35  7615 | 31 |
| {BcMV1.BI=FALSE, BcMV4.BI=FALSE} =>  {BcULV3=FALSE} | 0.07  6733 | 0.534  483 | 0.143  564 | 1.31  6653 | 31 |
| {BcMV4.BI=FALSE, BcULV3=FALSE} =>  {BcMV1.BI=FALSE} | 0.07  6733 | 0.298  077 | 0.257  426 | 1.41  6742 | 31 |
| {BcMV1.BI=FALSE, BcULV3=FALSE} =>  {BcOLV1.BI.MOVA.=FALSE} | 0.08  6634 | 0.853  659 | 0.101  485 | 1.27  7326 | 35 |
| {BcOLV1.BI.MOVA.=FALSE,  BcULV3=FALSE} => {BcMV1.BI=FALSE} | 0.08  6634 | 0.321  101 | 0.269  802 | 1.52  6174 | 35 |
| {BcMV1.BI=FALSE, BcULV3=FALSE} =>  {BcMV6.BI=FALSE} | 0.08  9109 | 0.878  049 | 0.101  485 | 1.26  239 | 36 |
| {BcMV1.BI=FALSE, BcMV6.BI=FALSE} =>  {BcULV3=FALSE} | 0.08  9109 | 0.553  846 | 0.160  891 | 1.36  4353 | 36 |
| {BcMV6.BI=FALSE, BcULV3=FALSE} =>  {BcMV1.BI=FALSE} | 0.08  9109 | 0.281  25 | 0.316  832 | 1.33  6765 | 36 |
| {BcMV1.BI=FALSE, BcULV3=FALSE} =>  {BcPV3.BI=FALSE} | 0.09  1584 | 0.902  439 | 0.101  485 | 1.27  0332 | 37 |
| {BcMV1.BI=FALSE, BcPV3.BI=FALSE} =>  {BcULV3=FALSE} | 0.09  1584 | 0.521  127 | 0.175  743 | 1.28  3751 | 37 |

| {BcPV3.BI=FALSE, BcULV3=FALSE} =>  {BcMV1.BI=FALSE} | 0.09  1584 | 0.316  239 | 0.289  604 | 1.50  3067 | 37 |
| --- | --- | --- | --- | --- | --- |
| {BcMV1.BI=FALSE, BcULV3=FALSE} =>  {BcHV3.BI=FALSE} | 0.09  4059 | 0.926  829 | 0.101  485 | 1.21  9671 | 38 |
| {BcHV3.BI=FALSE, BcMV1.BI=FALSE} =>  {BcULV3=FALSE} | 0.09  4059 | 0.513  514 | 0.183  168 | 1.26  4997 | 38 |
| {BcHV3.BI=FALSE, BcULV3=FALSE} =>  {BcMV1.BI=FALSE} | 0.09  4059 | 0.279  412 | 0.336  634 | 1.32  8028 | 38 |
| {BcMV1.BI=FALSE, BcULV3=FALSE} =>  {BcOLV7.BI=FALSE} | 0.09  4059 | 0.926  829 | 0.101  485 | 1.14  8586 | 38 |
| {BcMV1.BI=FALSE, BcOLV7.BI=FALSE} =>  {BcULV3=FALSE} | 0.09  4059 | 0.506  667 | 0.185  644 | 1.24  813 | 38 |
| {BcOLV7.BI=FALSE, BcULV3=FALSE} =>  {BcMV1.BI=FALSE} | 0.09  4059 | 0.283  582 | 0.331  683 | 1.34  7849 | 38 |
| {BcALV1.BI=FALSE, BcULV3=FALSE} =>  {BcMV1.BI=FALSE} | 0.09  1584 | 0.276  119 | 0.331  683 | 1.31  2379 | 37 |
| {BcMV1.BI=FALSE, BcULV3=FALSE} =>  {BcOLV4.BI=FALSE} | 0.10  1485 | 1 | 0.101  485 | 1.14  1243 | 41 |
| {BcOLV4.BI=FALSE, BcULV3=FALSE} =>  {BcMV1.BI=FALSE} | 0.10  1485 | 0.275  168 | 0.368  812 | 1.30  7856 | 41 |
| {BcHV1SLR.BI=FALSE, BcMV1.BI=FALSE}  => {BcOLV18=FALSE} | 0.08  1683 | 0.75 | 0.108  911 | 1.55  3846 | 33 |

| {BcMV1.BI=FALSE, BcOLV18=FALSE} =>  {BcHV1SLR.BI=FALSE} | 0.08  1683 | 0.611  111 | 0.133  663 | 1.33  4535 | 33 |
| --- | --- | --- | --- | --- | --- |
| {BcHV1SLR.BI=FALSE, BcOLV18=FALSE}  => {BcMV1.BI=FALSE} | 0.08  1683 | 0.289  474 | 0.282  178 | 1.37  5851 | 33 |
| {BcHV1SLR.BI=FALSE, BcMV1.BI=FALSE}  => {BcMV2.BI=FALSE} | 0.08  6634 | 0.795  455 | 0.108  911 | 1.48  0938 | 35 |
| {BcHV1SLR.BI=FALSE, BcMV2.BI=FALSE}  => {BcMV1.BI=FALSE} | 0.08  6634 | 0.324  074 | 0.267  327 | 1.54  0305 | 35 |
| {BcHV1SLR.BI=FALSE, BcMV1.BI=FALSE}  => {BcMV4.BI=FALSE} | 0.07  9208 | 0.727  273 | 0.108  911 | 1.30  5859 | 32 |
| {BcHV1SLR.BI=FALSE, BcMV4.BI=FALSE}  => {BcMV1.BI=FALSE} | 0.07  9208 | 0.285  714 | 0.277  228 | 1.35  7983 | 32 |
| {BcHV1SLR.BI=FALSE, BcMV1.BI=FALSE}  => {BcOLV1.BI.MOVA.=FALSE} | 0.09  901 | 0.909  091 | 0.108  911 | 1.36  0269 | 40 |
| {BcMV1.BI=FALSE, BcOLV1.BI.MOVA.=FALSE} =>  {BcHV1SLR.BI=FALSE} | 0.09  901 | 0.571  429 | 0.173  267 | 1.24  7876 | 40 |
| {BcHV1SLR.BI=FALSE, BcOLV1.BI.MOVA.=FALSE} =>  {BcMV1.BI=FALSE} | 0.09  901 | 0.303  03 | 0.326  733 | 1.44  0285 | 40 |
| {BcHV1SLR.BI=FALSE, BcMV1.BI=FALSE}  => {BcMV6.BI=FALSE} | 0.08  9109 | 0.818  182 | 0.108  911 | 1.17  6318 | 36 |

| {BcHV1SLR.BI=FALSE, BcMV1.BI=FALSE}  => {BcPV3.BI=FALSE} | 0.09  4059 | 0.863  636 | 0.108  911 | 1.21  5711 | 38 |
| --- | --- | --- | --- | --- | --- |
| {BcHV1SLR.BI=FALSE, BcPV3.BI=FALSE}  => {BcMV1.BI=FALSE} | 0.09  4059 | 0.279  412 | 0.336  634 | 1.32  8028 | 38 |
| {BcHV1SLR.BI=FALSE, BcMV1.BI=FALSE}  => {BcEV2.BI=FALSE} | 0.09  4059 | 0.863  636 | 0.108  911 | 1.15  5328 | 38 |
| {BcHV1SLR.BI=FALSE, BcMV1.BI=FALSE}  => {BcHV3.BI=FALSE} | 0.10  1485 | 0.931  818 | 0.108  911 | 1.22  6236 | 41 |
| {BcHV1SLR.BI=FALSE, BcHV3.BI=FALSE}  => {BcMV1.BI=FALSE} | 0.10  1485 | 0.277  027 | 0.366  337 | 1.31  6693 | 41 |
| {BcHV1SLR.BI=FALSE, BcMV1.BI=FALSE}  => {BcULV1.BI=FALSE} | 0.10  1485 | 0.931  818 | 0.108  911 | 1.18  7554 | 41 |
| {BcHV1SLR.BI=FALSE, BcMV1.BI=FALSE}  => {BcALV1.BI=FALSE} | 0.09  901 | 0.909  091 | 0.108  911 | 1.11  633 | 40 |
| {BcHV1SLR.BI=FALSE, BcMV1.BI=FALSE}  => {BcOLV4.BI=FALSE} | 0.10  6436 | 0.977  273 | 0.108  911 | 1.11  5306 | 43 |
| {BcHV1SLR.BI=FALSE, BcMV1.BI=FALSE}  => {BcULV5=FALSE} | 0.10  6436 | 0.977  273 | 0.108  911 | 1.11  5306 | 43 |
| {BcMV1.BI=FALSE, BcMV3.BI=FALSE} =>  {BcOLV18=FALSE} | 0.09  1584 | 0.725  49 | 0.126  238 | 1.50  3067 | 37 |
| {BcMV1.BI=FALSE, BcOLV18=FALSE} =>  {BcMV3.BI=FALSE} | 0.09  1584 | 0.685  185 | 0.133  663 | 1.49  6296 | 37 |

| {BcMV3.BI=FALSE, BcOLV18=FALSE} =>  {BcMV1.BI=FALSE} | 0.09  1584 | 0.339  45 | 0.269  802 | 1.61  3384 | 37 |
| --- | --- | --- | --- | --- | --- |
| {BcMV1.BI=FALSE, BcMV3.BI=FALSE} =>  {BcMV2.BI=FALSE} | 0.09  901 | 0.784  314 | 0.126  238 | 1.46  0197 | 40 |
| {BcMV1.BI=FALSE, BcMV2.BI=FALSE} =>  {BcMV3.BI=FALSE} | 0.09  901 | 0.634  921 | 0.155  941 | 1.38  6529 | 40 |
| {BcMV2.BI=FALSE, BcMV3.BI=FALSE} =>  {BcMV1.BI=FALSE} | 0.09  901 | 0.32 | 0.309  406 | 1.52  0941 | 40 |
| {BcMV1.BI=FALSE, BcMV3.BI=FALSE} =>  {BcMV4.BI=FALSE} | 0.09  4059 | 0.745  098 | 0.126  238 | 1.33  7865 | 38 |
| {BcMV1.BI=FALSE, BcMV4.BI=FALSE} =>  {BcMV3.BI=FALSE} | 0.09  4059 | 0.655  172 | 0.143  564 | 1.43  0755 | 38 |
| {BcMV3.BI=FALSE, BcMV4.BI=FALSE} =>  {BcMV1.BI=FALSE} | 0.09  4059 | 0.304 | 0.309  406 | 1.44  4894 | 38 |
| {BcMV1.BI=FALSE, BcOLV16.BI=FALSE} =>  {BcMV3.BI=FALSE} | 0.07  6733 | 0.584  906 | 0.131  188 | 1.27  7307 | 31 |
| {BcHV1.BI=TRUE, BcMV1.BI=FALSE} =>  {BcMV3.BI=FALSE} | 0.07  6733 | 0.632  653 | 0.121  287 | 1.38  1577 | 31 |
| {BcHV1.BI=TRUE, BcMV3.BI=FALSE} =>  {BcMV1.BI=FALSE} | 0.07  6733 | 0.306  931 | 0.25 | 1.45  8824 | 31 |
| {BcMV1.BI=FALSE, BcMV3.BI=FALSE} =>  {BcOLV1.BI.MOVA.=FALSE} | 0.11  3861 | 0.901  961 | 0.126  238 | 1.34  9601 | 46 |

| {BcMV1.BI=FALSE, BcOLV1.BI.MOVA.=FALSE} =>  {BcMV3.BI=FALSE} | 0.11  3861 | 0.657  143 | 0.173  267 | 1.43  5058 | 46 |
| --- | --- | --- | --- | --- | --- |
| {BcMV3.BI=FALSE, BcOLV1.BI.MOVA.=FALSE} =>  {BcMV1.BI=FALSE} | 0.11  3861 | 0.306  667 | 0.371  287 | 1.45  7569 | 46 |
| {BcMV1.BI=FALSE, BcMV6.BI=FALSE} =>  {BcMV3.BI=FALSE} | 0.09  901 | 0.615  385 | 0.160  891 | 1.34  3867 | 40 |
| {BcMV1.BI=FALSE, BcMV3.BI=FALSE} =>  {BcPV3.BI=FALSE} | 0.11  3861 | 0.901  961 | 0.126  238 | 1.26  9659 | 46 |
| {BcMV1.BI=FALSE, BcPV3.BI=FALSE} =>  {BcMV3.BI=FALSE} | 0.11  3861 | 0.647  887 | 0.175  743 | 1.41  4846 | 46 |
| {BcMV3.BI=FALSE, BcPV3.BI=FALSE} =>  {BcMV1.BI=FALSE} | 0.11  3861 | 0.326  241 | 0.349  01 | 1.55  0605 | 46 |
| {BcMV1.BI=FALSE, BcRV1.BI=FALSE} =>  {BcMV3.BI=FALSE} | 0.08  9109 | 0.654  545 | 0.136  139 | 1.42  9386 | 36 |
| {BcEV2.BI=FALSE, BcMV1.BI=FALSE} =>  {BcMV3.BI=FALSE} | 0.10  1485 | 0.577  465 | 0.175  743 | 1.26  1058 | 41 |
| {BcEV2.BI=FALSE, BcMV3.BI=FALSE} =>  {BcMV1.BI=FALSE} | 0.10  1485 | 0.294  964 | 0.344  059 | 1.40  1947 | 41 |
| {BcHV3.BI=FALSE, BcMV1.BI=FALSE} =>  {BcMV3.BI=FALSE} | 0.10  6436 | 0.581  081 | 0.183  168 | 1.26  8955 | 43 |

| {BcHV3.BI=FALSE, BcMV3.BI=FALSE} =>  {BcMV1.BI=FALSE} | 0.10  6436 | 0.290  541 | 0.366  337 | 1.38  0922 | 43 |
| --- | --- | --- | --- | --- | --- |
| {BcFV5.BI=FALSE, BcMV1.BI=FALSE} =>  {BcMV3.BI=FALSE} | 0.10  8911 | 0.628  571 | 0.173  267 | 1.37  2664 | 44 |
| {BcFV5.BI=FALSE, BcMV3.BI=FALSE} =>  {BcMV1.BI=FALSE} | 0.10  8911 | 0.276  73 | 0.393  564 | 1.31  5279 | 44 |
| {BcMV1.BI=FALSE, BcMV3.BI=FALSE} =>  {BcULV1.BI=FALSE} | 0.11  1386 | 0.882  353 | 0.126  238 | 1.12  4513 | 45 |
| {BcMV1.BI=FALSE, BcULV1.BI=FALSE} =>  {BcMV3.BI=FALSE} | 0.11  1386 | 0.608  108 | 0.183  168 | 1.32  7977 | 45 |
| {BcMV3.BI=FALSE, BcULV1.BI=FALSE} =>  {BcMV1.BI=FALSE} | 0.11  1386 | 0.283  019 | 0.393  564 | 1.34  5172 | 45 |
| {BcMV1.BI=FALSE, BcMV12=FALSE} =>  {BcMV3.BI=FALSE} | 0.11  1386 | 0.625 | 0.178  218 | 1.36  4865 | 45 |
| {BcMV12=FALSE, BcMV3.BI=FALSE} =>  {BcMV1.BI=FALSE} | 0.11  1386 | 0.271  084 | 0.410  891 | 1.28  8448 | 45 |
| {BcMV1.BI=FALSE, BcNSRV7.BI=FALSE}  => {BcMV3.BI=FALSE} | 0.10  6436 | 0.605  634 | 0.175  743 | 1.32  2573 | 43 |
| {BcMV3.BI=FALSE, BcNSRV7.BI=FALSE}  => {BcMV1.BI=FALSE} | 0.10  6436 | 0.282  895 | 0.376  238 | 1.34  4582 | 43 |
| {BcMV1.BI=FALSE, BcVV3.BI=FALSE} =>  {BcMV3.BI=FALSE} | 0.10  6436 | 0.605  634 | 0.175  743 | 1.32  2573 | 43 |

| {BcMV3.BI=FALSE, BcVV3.BI=FALSE} =>  {BcMV1.BI=FALSE} | 0.10  6436 | 0.275  641 | 0.386  139 | 1.31  0106 | 43 |
| --- | --- | --- | --- | --- | --- |
| {BcMV1.BI=FALSE, BcOLV7.BI=FALSE} =>  {BcMV3.BI=FALSE} | 0.11  1386 | 0.6 | 0.185  644 | 1.31  027 | 45 |
| {BcMV3.BI=FALSE, BcOLV7.BI=FALSE} =>  {BcMV1.BI=FALSE} | 0.11  1386 | 0.284  81 | 0.391  089 | 1.35  3686 | 45 |
| {BcALV1.BI=FALSE, BcMV1.BI=FALSE} =>  {BcMV3.BI=FALSE} | 0.10  8911 | 0.594  595 | 0.183  168 | 1.29  8466 | 44 |
| {BcALV1.BI=FALSE, BcMV3.BI=FALSE} =>  {BcMV1.BI=FALSE} | 0.10  8911 | 0.278  481 | 0.391  089 | 1.32  3604 | 44 |
| {BcMV1.BI=FALSE, BcMV3.BI=FALSE} =>  {BcDMV1=FALSE} | 0.11  6337 | 0.921  569 | 0.126  238 | 1.09  1829 | 47 |
| {BcDMV1=FALSE, BcMV1.BI=FALSE} =>  {BcMV3.BI=FALSE} | 0.11  6337 | 0.652  778 | 0.178  218 | 1.42  5526 | 47 |
| {BcDMV1=FALSE, BcMV3.BI=FALSE} =>  {BcMV1.BI=FALSE} | 0.11  6337 | 0.276  471 | 0.420  792 | 1.31  4048 | 47 |
| {BcFV9=FALSE, BcMV1.BI=FALSE} =>  {BcMV3.BI=FALSE} | 0.11  6337 | 0.610  39 | 0.190  594 | 1.33  2959 | 47 |
| {BcFV9=FALSE, BcMV3.BI=FALSE} =>  {BcMV1.BI=FALSE} | 0.11  6337 | 0.281  437 | 0.413  366 | 1.33  7654 | 47 |
| {BcMV1.BI=FALSE, BcMV3.BI=FALSE} =>  {BcOLV4.BI=FALSE} | 0.12  3762 | 0.980  392 | 0.126  238 | 1.11  8866 | 50 |

| {BcMV1.BI=FALSE, BcOLV4.BI=FALSE} =>  {BcMV3.BI=FALSE} | 0.12  3762 | 0.609  756 | 0.202  97 | 1.33  1575 | 50 |
| --- | --- | --- | --- | --- | --- |
| {BcMV3.BI=FALSE, BcOLV4.BI=FALSE} =>  {BcMV1.BI=FALSE} | 0.12  3762 | 0.294  118 | 0.420  792 | 1.39  7924 | 50 |
| {BcMV1.BI=FALSE, BcMV3.BI=FALSE} =>  {BcULV5=FALSE} | 0.12  3762 | 0.980  392 | 0.126  238 | 1.11  8866 | 50 |
| {BcMV1.BI=FALSE, BcULV5=FALSE} =>  {BcMV3.BI=FALSE} | 0.12  3762 | 0.609  756 | 0.202  97 | 1.33  1575 | 50 |
| {BcMV3.BI=FALSE, BcULV5=FALSE} =>  {BcMV1.BI=FALSE} | 0.12  3762 | 0.292  398 | 0.423  267 | 1.38  9749 | 50 |
| {BcBNV5.BI=FALSE, BcMV1.BI=FALSE} =>  {BcMV3.BI=FALSE} | 0.12  1287 | 0.597  561 | 0.202  97 | 1.30  4944 | 49 |
| {BcBNV5.BI=FALSE, BcMV3.BI=FALSE} =>  {BcMV1.BI=FALSE} | 0.12  1287 | 0.276  836 | 0.438  119 | 1.31  5786 | 49 |
| {BcMV1.BI=FALSE, BvF.BI=FALSE} =>  {BcMV3.BI=FALSE} | 0.11  1386 | 0.608  108 | 0.183  168 | 1.32  7977 | 45 |
| {BcMV1.BI=FALSE, BcMV3.BI=FALSE} =>  {BcMCV5.BI=FALSE} | 0.12  6238 | 1 | 0.126  238 | 1.08  311 | 51 |
| {BcMCV5.BI=FALSE, BcMV1.BI=FALSE} =>  {BcMV3.BI=FALSE} | 0.12  6238 | 0.6 | 0.210  396 | 1.31  027 | 51 |
| {BcMCV5.BI=FALSE, BcMV3.BI=FALSE} =>  {BcMV1.BI=FALSE} | 0.12  6238 | 0.286  517 | 0.440  594 | 1.36  1798 | 51 |

| {BcMV1.BI=FALSE, BcOLV13.BI=FALSE} =>  {BcMV3.BI=FALSE} | 0.12  3762 | 0.617  284 | 0.200  495 | 1.34  8015 | 50 |
| --- | --- | --- | --- | --- | --- |
| {BcMV3.BI=FALSE, BcOLV13.BI=FALSE} =>  {BcMV1.BI=FALSE} | 0.12  3762 | 0.274  725 | 0.450  495 | 1.30  5753 | 50 |
| {BcMV1.BI=FALSE, BcOLV2.BI=FALSE} =>  {BcMV3.BI=FALSE} | 0.12  1287 | 0.620  253 | 0.195  545 | 1.35  4499 | 49 |
| {BcMV3.BI=FALSE, BcOLV2.BI=FALSE} =>  {BcMV1.BI=FALSE} | 0.12  1287 | 0.273  743 | 0.443  069 | 1.30  1084 | 49 |
| {BcMCV3.BI=FALSE, BcMV1.BI=FALSE} =>  {BcMV3.BI=FALSE} | 0.12  6238 | 0.621  951 | 0.202  97 | 1.35  8207 | 51 |
| {BcMCV3.BI=FALSE, BcMV3.BI=FALSE} =>  {BcMV1.BI=FALSE} | 0.12  6238 | 0.275  676 | 0.457  921 | 1.31  027 | 51 |
| {BcMV1.BI=FALSE, BcPV4.BI=FALSE} =>  {BcMV3.BI=FALSE} | 0.12  1287 | 0.590  361 | 0.205  446 | 1.28  9222 | 49 |
| {BcMV3.BI=FALSE, BcPV4.BI=FALSE} =>  {BcMV1.BI=FALSE} | 0.12  1287 | 0.278  409 | 0.435  644 | 1.32  3262 | 49 |
| {BcEV6=FALSE, BcMV1.BI=FALSE} =>  {BcMV3.BI=FALSE} | 0.11  1386 | 0.569  62 | 0.195  545 | 1.24  3927 | 45 |
| {BcMV1.BI=FALSE, BcOLV17.BI=FALSE} =>  {BcMV3.BI=FALSE} | 0.12  3762 | 0.602  41 | 0.205  446 | 1.31  5532 | 50 |
| {BcMV3.BI=FALSE, BcOLV17.BI=FALSE} =>  {BcMV1.BI=FALSE} | 0.12  3762 | 0.284  091 | 0.435  644 | 1.35  0267 | 50 |

| {BcMV1.BI=FALSE, BcOLV15.BI=FALSE} =>  {BcMV3.BI=FALSE} | 0.12  6238 | 0.614  458 | 0.205  446 | 1.34  1843 | 51 |
| --- | --- | --- | --- | --- | --- |
| {BcMV3.BI=FALSE, BcOLV15.BI=FALSE} =>  {BcMV1.BI=FALSE} | 0.12  6238 | 0.284  916 | 0.443  069 | 1.35  419 | 51 |
| {BcEV5=FALSE, BcMV1.BI=FALSE} =>  {BcMV3.BI=FALSE} | 0.12  3762 | 0.602  41 | 0.205  446 | 1.31  5532 | 50 |
| {BcEV5=FALSE, BcMV3.BI=FALSE} =>  {BcMV1.BI=FALSE} | 0.12  3762 | 0.271  739 | 0.455  446 | 1.29  156 | 50 |
| {BcMV1.BI=FALSE, BcOLV18=FALSE} =>  {BcMV2.BI=FALSE} | 0.10  8911 | 0.814  815 | 0.133  663 | 1.51  6982 | 44 |
| {BcMV1.BI=FALSE, BcMV2.BI=FALSE} =>  {BcOLV18=FALSE} | 0.10  8911 | 0.698  413 | 0.155  941 | 1.44  6968 | 44 |
| {BcMV2.BI=FALSE, BcOLV18=FALSE} =>  {BcMV1.BI=FALSE} | 0.10  8911 | 0.346  457 | 0.314  356 | 1.64  6688 | 44 |
| {BcMV1.BI=FALSE, BcOLV18=FALSE} =>  {BcMV4.BI=FALSE} | 0.10  1485 | 0.759  259 | 0.133  663 | 1.36  3292 | 41 |
| {BcMV1.BI=FALSE, BcMV4.BI=FALSE} =>  {BcOLV18=FALSE} | 0.10  1485 | 0.706  897 | 0.143  564 | 1.46  4545 | 41 |
| {BcMV4.BI=FALSE, BcOLV18=FALSE} =>  {BcMV1.BI=FALSE} | 0.10  1485 | 0.320  313 | 0.316  832 | 1.52  2426 | 41 |
| {BcMV1.BI=FALSE, BcOLV16.BI=FALSE} =>  {BcOLV18=FALSE} | 0.08  4158 | 0.641  509 | 0.131  188 | 1.32  9076 | 34 |

| {BcMV1.BI=FALSE, BcOLV18=FALSE} =>  {BcOLV1.BI.MOVA.=FALSE} | 0.12  3762 | 0.925  926 | 0.133  663 | 1.38  546 | 50 |
| --- | --- | --- | --- | --- | --- |
| {BcMV1.BI=FALSE, BcOLV1.BI.MOVA.=FALSE} =>  {BcOLV18=FALSE} | 0.12  3762 | 0.714  286 | 0.173  267 | 1.47  9853 | 50 |
| {BcOLV1.BI.MOVA.=FALSE,  BcOLV18=FALSE} => {BcMV1.BI=FALSE} | 0.12  3762 | 0.324  675 | 0.381  188 | 1.54  3163 | 50 |
| {BcMV9.BI=TRUE, BcOLV18=FALSE} =>  {BcMV1.BI=FALSE} | 0.07  4257 | 0.280  374 | 0.264  851 | 1.33  26 | 30 |
| {BcOLV18=FALSE, BcULV4=TRUE} =>  {BcMV1.BI=FALSE} | 0.07  9208 | 0.316  832 | 0.25 | 1.50  5882 | 32 |
| {BcMV1.BI=FALSE, BcMV6.BI=FALSE} =>  {BcOLV18=FALSE} | 0.10  396 | 0.646  154 | 0.160  891 | 1.33  8698 | 42 |
| {BcMV6.BI=FALSE, BcOLV18=FALSE} =>  {BcMV1.BI=FALSE} | 0.10  396 | 0.295  775 | 0.351  485 | 1.40  58 | 42 |
| {BcMV1.BI=FALSE, BcOLV18=FALSE} =>  {BcPV3.BI=FALSE} | 0.11  3861 | 0.851  852 | 0.133  663 | 1.19  9122 | 46 |
| {BcMV1.BI=FALSE, BcPV3.BI=FALSE} =>  {BcOLV18=FALSE} | 0.11  3861 | 0.647  887 | 0.175  743 | 1.34  229 | 46 |
| {BcOLV18=FALSE, BcPV3.BI=FALSE} =>  {BcMV1.BI=FALSE} | 0.11  3861 | 0.298  701 | 0.381  188 | 1.41  971 | 46 |
| {BcMV1.BI=FALSE, BcRV1.BI=FALSE} => | 0.10 | 0.745 | 0.136 | 1.54 | 41 |

| {BcOLV18=FALSE} | 1485 | 455 | 139 | 4429 |  |
| --- | --- | --- | --- | --- | --- |
| {BcEV2.BI=FALSE, BcMV1.BI=FALSE} =>  {BcOLV18=FALSE} | 0.10  396 | 0.591  549 | 0.175  743 | 1.22  5569 | 42 |
| {BcEV2.BI=FALSE, BcOLV18=FALSE} =>  {BcMV1.BI=FALSE} | 0.10  396 | 0.274  51 | 0.378  713 | 1.30  4729 | 42 |
| {BcMV1.BI=FALSE, BcOLV18=FALSE} =>  {BcHV3.BI=FALSE} | 0.12  1287 | 0.907  407 | 0.133  663 | 1.19  4113 | 49 |
| {BcHV3.BI=FALSE, BcMV1.BI=FALSE} =>  {BcOLV18=FALSE} | 0.12  1287 | 0.662  162 | 0.183  168 | 1.37  1864 | 49 |
| {BcHV3.BI=FALSE, BcOLV18=FALSE} =>  {BcMV1.BI=FALSE} | 0.12  1287 | 0.296  97 | 0.408  416 | 1.41  148 | 49 |
| {BcFV5.BI=FALSE, BcMV1.BI=FALSE} =>  {BcOLV18=FALSE} | 0.11  3861 | 0.657  143 | 0.173  267 | 1.36  1465 | 46 |
| {BcFV5.BI=FALSE, BcOLV18=FALSE} =>  {BcMV1.BI=FALSE} | 0.11  3861 | 0.275  449 | 0.413  366 | 1.30  9193 | 46 |
| {BcMV1.BI=FALSE, BcOLV18=FALSE} =>  {BcULV1.BI=FALSE} | 0.12  3762 | 0.925  926 | 0.133  663 | 1.18  0044 | 50 |
| {BcMV1.BI=FALSE, BcULV1.BI=FALSE} =>  {BcOLV18=FALSE} | 0.12  3762 | 0.675  676 | 0.183  168 | 1.39  9861 | 50 |
| {BcOLV18=FALSE, BcULV1.BI=FALSE} =>  {BcMV1.BI=FALSE} | 0.12  3762 | 0.289  017 | 0.428  218 | 1.37  3682 | 50 |
| {BcMV1.BI=FALSE, BcMV12=FALSE} => | 0.11 | 0.652 | 0.178 | 1.35 | 47 |

| {BcOLV18=FALSE} | 6337 | 778 | 218 | 2422 |  |
| --- | --- | --- | --- | --- | --- |
| {BcMV12=FALSE, BcOLV18=FALSE} =>  {BcMV1.BI=FALSE} | 0.11  6337 | 0.288  344 | 0.403  465 | 1.37  048 | 47 |
| {BcMV1.BI=FALSE, BcNSRV7.BI=FALSE}  => {BcOLV18=FALSE} | 0.11  1386 | 0.633  803 | 0.175  743 | 1.31  3109 | 45 |
| {BcNSRV7.BI=FALSE, BcOLV18=FALSE}  => {BcMV1.BI=FALSE} | 0.11  1386 | 0.271  084 | 0.410  891 | 1.28  8448 | 45 |
| {BcMV1.BI=FALSE, BcVV3.BI=FALSE} =>  {BcOLV18=FALSE} | 0.10  396 | 0.591  549 | 0.175  743 | 1.22  5569 | 42 |
| {BcMV1.BI=FALSE, BcOLV18=FALSE} =>  {BcOLV7.BI=FALSE} | 0.12  1287 | 0.907  407 | 0.133  663 | 1.12  4517 | 49 |
| {BcMV1.BI=FALSE, BcOLV7.BI=FALSE} =>  {BcOLV18=FALSE} | 0.12  1287 | 0.653  333 | 0.185  644 | 1.35  3573 | 49 |
| {BcOLV18=FALSE, BcOLV7.BI=FALSE} =>  {BcMV1.BI=FALSE} | 0.12  1287 | 0.281  609 | 0.430  693 | 1.33  8472 | 49 |
| {BcALV1.BI=FALSE, BcMV1.BI=FALSE} =>  {BcOLV18=FALSE} | 0.11  8812 | 0.648  649 | 0.183  168 | 1.34  3867 | 48 |
| {BcALV1.BI=FALSE, BcOLV18=FALSE} =>  {BcMV1.BI=FALSE} | 0.11  8812 | 0.271  186 | 0.438  119 | 1.28  8933 | 48 |
| {BcDMV1=FALSE, BcMV1.BI=FALSE} =>  {BcOLV18=FALSE} | 0.11  3861 | 0.638  889 | 0.178  218 | 1.32  3647 | 46 |
| {BcDMV1=FALSE, BcOLV18=FALSE} => | 0.11 | 0.273 | 0.415 | 1.30 | 46 |

| {BcMV1.BI=FALSE} | 3861 | 81 | 842 | 1401 |  |
| --- | --- | --- | --- | --- | --- |
| {BcMV1.BI=FALSE, BcOLV18=FALSE} =>  {BcFV9=FALSE} | 0.12  6238 | 0.944  444 | 0.133  663 | 1.08  7053 | 51 |
| {BcFV9=FALSE, BcMV1.BI=FALSE} =>  {BcOLV18=FALSE} | 0.12  6238 | 0.662  338 | 0.190  594 | 1.37  2228 | 51 |
| {BcFV9=FALSE, BcOLV18=FALSE} =>  {BcMV1.BI=FALSE} | 0.12  6238 | 0.281  768 | 0.448  02 | 1.33  9227 | 51 |
| {BcMV1.BI=FALSE, BcOLV18=FALSE} =>  {BcOLV4.BI=FALSE} | 0.12  8713 | 0.962  963 | 0.133  663 | 1.09  8975 | 52 |
| {BcMV1.BI=FALSE, BcOLV4.BI=FALSE} =>  {BcOLV18=FALSE} | 0.12  8713 | 0.634  146 | 0.202  97 | 1.31  3821 | 52 |
| {BcOLV18=FALSE, BcOLV4.BI=FALSE} =>  {BcMV1.BI=FALSE} | 0.12  8713 | 0.300  578 | 0.428  218 | 1.42  863 | 52 |
| {BcMV1.BI=FALSE, BcOLV18=FALSE} =>  {BcULV5=FALSE} | 0.12  8713 | 0.962  963 | 0.133  663 | 1.09  8975 | 52 |
| {BcMV1.BI=FALSE, BcULV5=FALSE} =>  {BcOLV18=FALSE} | 0.12  8713 | 0.634  146 | 0.202  97 | 1.31  3821 | 52 |
| {BcOLV18=FALSE, BcULV5=FALSE} =>  {BcMV1.BI=FALSE} | 0.12  8713 | 0.315  152 | 0.408  416 | 1.49  7897 | 52 |
| {BcMV1.BI=FALSE, BcOLV18=FALSE} =>  {BcBNV5.BI=FALSE} | 0.13  1188 | 0.981  481 | 0.133  663 | 1.08  0432 | 53 |
| {BcBNV5.BI=FALSE, BcMV1.BI=FALSE} => | 0.13 | 0.646 | 0.202 | 1.33 | 53 |

| {BcOLV18=FALSE} | 1188 | 341 | 97 | 9087 |  |
| --- | --- | --- | --- | --- | --- |
| {BcBNV5.BI=FALSE, BcOLV18=FALSE} =>  {BcMV1.BI=FALSE} | 0.13  1188 | 0.288  043 | 0.455  446 | 1.36  9054 | 53 |
| {BcMV1.BI=FALSE, BvF.BI=FALSE} =>  {BcOLV18=FALSE} | 0.11  6337 | 0.635  135 | 0.183  168 | 1.31  587 | 47 |
| {BcMV1.BI=FALSE, BcOLV18=FALSE} =>  {BcMCV5.BI=FALSE} | 0.13  3663 | 1 | 0.133  663 | 1.08  311 | 54 |
| {BcMCV5.BI=FALSE, BcMV1.BI=FALSE} =>  {BcOLV18=FALSE} | 0.13  3663 | 0.635  294 | 0.210  396 | 1.31  6199 | 54 |
| {BcMCV5.BI=FALSE, BcOLV18=FALSE} =>  {BcMV1.BI=FALSE} | 0.13  3663 | 0.282  723 | 0.472  772 | 1.34  3763 | 54 |
| {BcMV1.BI=FALSE, BcOLV13.BI=FALSE} =>  {BcOLV18=FALSE} | 0.13  1188 | 0.654  321 | 0.200  495 | 1.35  5619 | 53 |
| {BcOLV13.BI=FALSE, BcOLV18=FALSE} =>  {BcMV1.BI=FALSE} | 0.13  1188 | 0.291  209 | 0.450  495 | 1.38  4098 | 53 |
| {BcMV1.BI=FALSE, BcOLV2.BI=FALSE} =>  {BcOLV18=FALSE} | 0.12  6238 | 0.645  57 | 0.195  545 | 1.33  7488 | 51 |
| {BcOLV18=FALSE, BcOLV2.BI=FALSE} =>  {BcMV1.BI=FALSE} | 0.12  6238 | 0.274  194 | 0.460  396 | 1.30  3226 | 51 |
| {BcMCV3.BI=FALSE, BcMV1.BI=FALSE} =>  {BcOLV18=FALSE} | 0.13  1188 | 0.646  341 | 0.202  97 | 1.33  9087 | 53 |
| {BcMCV3.BI=FALSE, BcOLV18=FALSE} => | 0.13 | 0.286 | 0.457 | 1.36 | 53 |

| {BcMV1.BI=FALSE} | 1188 | 486 | 921 | 1653 |  |
| --- | --- | --- | --- | --- | --- |
| {BcMV1.BI=FALSE, BcPV4.BI=FALSE} =>  {BcOLV18=FALSE} | 0.13  1188 | 0.638  554 | 0.205  446 | 1.32  2953 | 53 |
| {BcOLV18=FALSE, BcPV4.BI=FALSE} =>  {BcMV1.BI=FALSE} | 0.13  1188 | 0.288  043 | 0.455  446 | 1.36  9054 | 53 |
| {BcEV6=FALSE, BcMV1.BI=FALSE} =>  {BcOLV18=FALSE} | 0.12  1287 | 0.620  253 | 0.195  545 | 1.28  5037 | 49 |
| {BcEV6=FALSE, BcOLV18=FALSE} =>  {BcMV1.BI=FALSE} | 0.12  1287 | 0.267  76 | 0.452  97 | 1.27  2645 | 49 |
| {BcMV1.BI=FALSE, BcOLV17.BI=FALSE} =>  {BcOLV18=FALSE} | 0.13  3663 | 0.650  602 | 0.205  446 | 1.34  7915 | 54 |
| {BcOLV17.BI=FALSE, BcOLV18=FALSE} =>  {BcMV1.BI=FALSE} | 0.13  3663 | 0.284  211 | 0.470  297 | 1.35  0836 | 54 |
| {BcMV1.BI=FALSE, BcOLV15.BI=FALSE} =>  {BcOLV18=FALSE} | 0.13  3663 | 0.650  602 | 0.205  446 | 1.34  7915 | 54 |
| {BcOLV15.BI=FALSE, BcOLV18=FALSE} =>  {BcMV1.BI=FALSE} | 0.13  3663 | 0.282  723 | 0.472  772 | 1.34  3763 | 54 |
| {BcEV5=FALSE, BcMV1.BI=FALSE} =>  {BcOLV18=FALSE} | 0.13  1188 | 0.638  554 | 0.205  446 | 1.32  2953 | 53 |
| {BcEV5=FALSE, BcOLV18=FALSE} =>  {BcMV1.BI=FALSE} | 0.13  1188 | 0.283  422 | 0.462  871 | 1.34  709 | 53 |
| {BcMV1.BI=FALSE, BcMV2.BI=FALSE} => | 0.12 | 0.793 | 0.155 | 1.42 | 50 |

| {BcMV4.BI=FALSE} | 3762 | 651 | 941 | 5044 |  |
| --- | --- | --- | --- | --- | --- |
| {BcMV1.BI=FALSE, BcMV4.BI=FALSE} =>  {BcMV2.BI=FALSE} | 0.12  3762 | 0.862  069 | 0.143  564 | 1.60  4958 | 50 |
| {BcMV2.BI=FALSE, BcMV4.BI=FALSE} =>  {BcMV1.BI=FALSE} | 0.12  3762 | 0.335  57 | 0.368  812 | 1.59  4947 | 50 |
| {BcMV1.BI=FALSE, BcMV2.BI=FALSE} =>  {BcOLV16.BI=FALSE} | 0.10  8911 | 0.698  413 | 0.155  941 | 1.19  5588 | 44 |
| {BcMV1.BI=FALSE, BcOLV16.BI=FALSE} =>  {BcMV2.BI=FALSE} | 0.10  8911 | 0.830  189 | 0.131  188 | 1.54  5605 | 44 |
| {BcMV2.BI=FALSE, BcOLV16.BI=FALSE} =>  {BcMV1.BI=FALSE} | 0.10  8911 | 0.291  391 | 0.373  762 | 1.38  4963 | 44 |
| {BcMV1.BI=FALSE, BcULV3=TRUE} =>  {BcMV2.BI=FALSE} | 0.07  4257 | 0.681  818 | 0.108  911 | 1.26  9376 | 30 |
| {BcHV1.BI=TRUE, BcMV1.BI=FALSE} =>  {BcMV2.BI=FALSE} | 0.08  6634 | 0.714  286 | 0.121  287 | 1.32  9822 | 35 |
| {BcHV1.BI=TRUE, BcMV2.BI=FALSE} =>  {BcMV1.BI=FALSE} | 0.08  6634 | 0.277  778 | 0.311  881 | 1.32  0261 | 35 |
| {BcMV1.BI=FALSE, BcMV2.BI=FALSE} =>  {BcOLV1.BI.MOVA.=FALSE} | 0.13  8614 | 0.888  889 | 0.155  941 | 1.33  0041 | 56 |
| {BcMV1.BI=FALSE, BcOLV1.BI.MOVA.=FALSE} =>  {BcMV2.BI=FALSE} | 0.13  8614 | 0.8 | 0.173  267 | 1.48  9401 | 56 |

| {BcMV2.BI=FALSE, BcOLV1.BI.MOVA.=FALSE} =>  {BcMV1.BI=FALSE} | 0.13  8614 | 0.304  348 | 0.455  446 | 1.44  6547 | 56 |
| --- | --- | --- | --- | --- | --- |
| {BcMV1.BI=FALSE, BcMV9.BI=TRUE} =>  {BcMV2.BI=FALSE} | 0.08  9109 | 0.692  308 | 0.128  713 | 1.28  8905 | 36 |
| {BcMV1.BI=FALSE, BcULV4=TRUE} =>  {BcMV2.BI=FALSE} | 0.09  6535 | 0.684  211 | 0.141  089 | 1.27  383 | 39 |
| {BcMV2.BI=FALSE, BcULV4=TRUE} =>  {BcMV1.BI=FALSE} | 0.09  6535 | 0.276  596 | 0.349  01 | 1.31  4643 | 39 |
| {BcMV1.BI=FALSE, BcMV2.BI=FALSE} =>  {BcMV6.BI=FALSE} | 0.12  8713 | 0.825  397 | 0.155  941 | 1.18  6692 | 52 |
| {BcMV1.BI=FALSE, BcMV6.BI=FALSE} =>  {BcMV2.BI=FALSE} | 0.12  8713 | 0.8 | 0.160  891 | 1.48  9401 | 52 |
| {BcMV2.BI=FALSE, BcMV6.BI=FALSE} =>  {BcMV1.BI=FALSE} | 0.12  8713 | 0.292  135 | 0.440  594 | 1.38  85 | 52 |
| {BcMV1.BI=FALSE, BcMV2.BI=FALSE} =>  {BcPV3.BI=FALSE} | 0.13  3663 | 0.857  143 | 0.155  941 | 1.20  657 | 54 |
| {BcMV1.BI=FALSE, BcPV3.BI=FALSE} =>  {BcMV2.BI=FALSE} | 0.13  3663 | 0.760  563 | 0.175  743 | 1.41  598 | 54 |
| {BcMV2.BI=FALSE, BcPV3.BI=FALSE} =>  {BcMV1.BI=FALSE} | 0.13  3663 | 0.313  953 | 0.425  743 | 1.49  2202 | 54 |
| {BcMV1.BI=FALSE, BcRV1.BI=FALSE} => | 0.11 | 0.818 | 0.136 | 1.52 | 45 |

| {BcMV2.BI=FALSE} | 1386 | 182 | 139 | 3251 |  |
| --- | --- | --- | --- | --- | --- |
| {BcEV2.BI=FALSE, BcMV1.BI=FALSE} =>  {BcMV2.BI=FALSE} | 0.12  6238 | 0.718  31 | 0.175  743 | 1.33  7314 | 51 |
| {BcEV2.BI=FALSE, BcMV2.BI=FALSE} =>  {BcMV1.BI=FALSE} | 0.12  6238 | 0.294  798 | 0.428  218 | 1.40  1156 | 51 |
| {BcMV1.BI=FALSE, BcMV2.BI=FALSE} =>  {BcHV3.BI=FALSE} | 0.13  6139 | 0.873  016 | 0.155  941 | 1.14  8855 | 55 |
| {BcHV3.BI=FALSE, BcMV1.BI=FALSE} =>  {BcMV2.BI=FALSE} | 0.13  6139 | 0.743  243 | 0.183  168 | 1.38  3734 | 55 |
| {BcHV3.BI=FALSE, BcMV2.BI=FALSE} =>  {BcMV1.BI=FALSE} | 0.13  6139 | 0.316  092 | 0.430  693 | 1.50  2366 | 55 |
| {BcFV5.BI=FALSE, BcMV1.BI=FALSE} =>  {BcMV2.BI=FALSE} | 0.12  8713 | 0.742  857 | 0.173  267 | 1.38  3015 | 52 |
| {BcFV5.BI=FALSE, BcMV2.BI=FALSE} =>  {BcMV1.BI=FALSE} | 0.12  8713 | 0.293  785 | 0.438  119 | 1.39  6344 | 52 |
| {BcMV1.BI=FALSE, BcMV2.BI=FALSE} =>  {BcULV1.BI=FALSE} | 0.14  3564 | 0.920  635 | 0.155  941 | 1.17  3301 | 58 |
| {BcMV1.BI=FALSE, BcULV1.BI=FALSE} =>  {BcMV2.BI=FALSE} | 0.14  3564 | 0.783  784 | 0.183  168 | 1.45  921 | 58 |
| {BcMV2.BI=FALSE, BcULV1.BI=FALSE} =>  {BcMV1.BI=FALSE} | 0.14  3564 | 0.311  828 | 0.460  396 | 1.48  21 | 58 |
| {BcMV1.BI=FALSE, BcMV2.BI=FALSE} => | 0.13 | 0.888 | 0.155 | 1.11 | 56 |

| {BcMV12=FALSE} | 8614 | 889 | 941 | 1799 |  |
| --- | --- | --- | --- | --- | --- |
| {BcMV1.BI=FALSE, BcMV12=FALSE} =>  {BcMV2.BI=FALSE} | 0.13  8614 | 0.777  778 | 0.178  218 | 1.44  8029 | 56 |
| {BcMV12=FALSE, BcMV2.BI=FALSE} =>  {BcMV1.BI=FALSE} | 0.13  8614 | 0.302  703 | 0.457  921 | 1.43  8728 | 56 |
| {BcMV1.BI=FALSE, BcNSRV7.BI=FALSE}  => {BcMV2.BI=FALSE} | 0.12  8713 | 0.732  394 | 0.175  743 | 1.36  3536 | 52 |
| {BcMV2.BI=FALSE, BcNSRV7.BI=FALSE}  => {BcMV1.BI=FALSE} | 0.12  8713 | 0.293  785 | 0.438  119 | 1.39  6344 | 52 |
| {BcMV1.BI=FALSE, BcVV3.BI=FALSE} =>  {BcMV2.BI=FALSE} | 0.12  6238 | 0.718  31 | 0.175  743 | 1.33  7314 | 51 |
| {BcMV2.BI=FALSE, BcVV3.BI=FALSE} =>  {BcMV1.BI=FALSE} | 0.12  6238 | 0.280  22 | 0.450  495 | 1.33  1868 | 51 |
| {BcMV1.BI=FALSE, BcOLV7.BI=FALSE} =>  {BcMV2.BI=FALSE} | 0.13  8614 | 0.746  667 | 0.185  644 | 1.39  0108 | 56 |
| {BcMV2.BI=FALSE, BcOLV7.BI=FALSE} =>  {BcMV1.BI=FALSE} | 0.13  8614 | 0.309  392 | 0.448  02 | 1.47  0523 | 56 |
| {BcMV1.BI=FALSE, BcMV2.BI=FALSE} =>  {BcALV1.BI=FALSE} | 0.14  3564 | 0.920  635 | 0.155  941 | 1.13  0506 | 58 |
| {BcALV1.BI=FALSE, BcMV1.BI=FALSE} =>  {BcMV2.BI=FALSE} | 0.14  3564 | 0.783  784 | 0.183  168 | 1.45  921 | 58 |
| {BcALV1.BI=FALSE, BcMV2.BI=FALSE} => | 0.14 | 0.292 | 0.490 | 1.39 | 58 |

| {BcMV1.BI=FALSE} | 3564 | 929 | 099 | 2276 |  |
| --- | --- | --- | --- | --- | --- |
| {BcDMV1=FALSE, BcMV1.BI=FALSE} =>  {BcMV2.BI=FALSE} | 0.12  8713 | 0.722  222 | 0.178  218 | 1.34  4598 | 52 |
| {BcDMV1=FALSE, BcMV2.BI=FALSE} =>  {BcMV1.BI=FALSE} | 0.12  8713 | 0.287  293 | 0.448  02 | 1.36  5486 | 52 |
| {BcMV1.BI=FALSE, BcMV2.BI=FALSE} =>  {BcFV9=FALSE} | 0.14  604 | 0.936  508 | 0.155  941 | 1.07  7918 | 59 |
| {BcFV9=FALSE, BcMV1.BI=FALSE} =>  {BcMV2.BI=FALSE} | 0.14  604 | 0.766  234 | 0.190  594 | 1.42  6537 | 59 |
| {BcFV9=FALSE, BcMV2.BI=FALSE} =>  {BcMV1.BI=FALSE} | 0.14  604 | 0.312  169 | 0.467  822 | 1.48  3722 | 59 |
| {BcMV1.BI=FALSE, BcMV2.BI=FALSE} =>  {BcOLV4.BI=FALSE} | 0.15  099 | 0.968  254 | 0.155  941 | 1.10  5013 | 61 |
| {BcMV1.BI=FALSE, BcOLV4.BI=FALSE} =>  {BcMV2.BI=FALSE} | 0.15  099 | 0.743  902 | 0.202  97 | 1.38  4961 | 61 |
| {BcMV2.BI=FALSE, BcOLV4.BI=FALSE} =>  {BcMV1.BI=FALSE} | 0.15  099 | 0.301  98 | 0.5 | 1.43  5294 | 61 |
| {BcMV1.BI=FALSE, BcMV2.BI=FALSE} =>  {BcULV5=FALSE} | 0.15  099 | 0.968  254 | 0.155  941 | 1.10  5013 | 61 |
| {BcMV1.BI=FALSE, BcULV5=FALSE} =>  {BcMV2.BI=FALSE} | 0.15  099 | 0.743  902 | 0.202  97 | 1.38  4961 | 61 |
| {BcMV2.BI=FALSE, BcULV5=FALSE} => | 0.15 | 0.301 | 0.5 | 1.43 | 61 |

| {BcMV1.BI=FALSE} | 099 | 98 |  | 5294 |  |
| --- | --- | --- | --- | --- | --- |
| {BcBNV5.BI=FALSE, BcMV1.BI=FALSE} =>  {BcMV2.BI=FALSE} | 0.15  099 | 0.743  902 | 0.202  97 | 1.38  4961 | 61 |
| {BcBNV5.BI=FALSE, BcMV2.BI=FALSE} =>  {BcMV1.BI=FALSE} | 0.15  099 | 0.301  98 | 0.5 | 1.43  5294 | 61 |
| {BcMV1.BI=FALSE, BvF.BI=FALSE} =>  {BcMV2.BI=FALSE} | 0.13  1188 | 0.716  216 | 0.183  168 | 1.33  3416 | 53 |
| {BcMV2.BI=FALSE, BvF.BI=FALSE} =>  {BcMV1.BI=FALSE} | 0.13  1188 | 0.273  196 | 0.480  198 | 1.29  8484 | 53 |
| {BcMV1.BI=FALSE, BcMV2.BI=FALSE} =>  {BcMCV5.BI=FALSE} | 0.15  5941 | 1 | 0.155  941 | 1.08  311 | 63 |
| {BcMCV5.BI=FALSE, BcMV1.BI=FALSE} =>  {BcMV2.BI=FALSE} | 0.15  5941 | 0.741  176 | 0.210  396 | 1.37  9886 | 63 |
| {BcMCV5.BI=FALSE, BcMV2.BI=FALSE} =>  {BcMV1.BI=FALSE} | 0.15  5941 | 0.298  578 | 0.522  277 | 1.41  9125 | 63 |
| {BcMV1.BI=FALSE, BcOLV13.BI=FALSE} =>  {BcMV2.BI=FALSE} | 0.15  3465 | 0.765  432 | 0.200  495 | 1.42  5044 | 62 |
| {BcMV2.BI=FALSE, BcOLV13.BI=FALSE} =>  {BcMV1.BI=FALSE} | 0.15  3465 | 0.296  651 | 0.517  327 | 1.40  9963 | 62 |
| {BcMV1.BI=FALSE, BcOLV2.BI=FALSE} =>  {BcMV2.BI=FALSE} | 0.14  8515 | 0.759  494 | 0.195  545 | 1.41  3988 | 60 |
| {BcMV2.BI=FALSE, BcOLV2.BI=FALSE} => | 0.14 | 0.292 | 0.507 | 1.39 | 60 |

| {BcMV1.BI=FALSE} | 8515 | 683 | 426 | 1105 |  |
| --- | --- | --- | --- | --- | --- |
| {BcMCV3.BI=FALSE, BcMV1.BI=FALSE} =>  {BcMV2.BI=FALSE} | 0.15  5941 | 0.768  293 | 0.202  97 | 1.43  037 | 63 |
| {BcMCV3.BI=FALSE, BcMV2.BI=FALSE} =>  {BcMV1.BI=FALSE} | 0.15  5941 | 0.297  17 | 0.524  752 | 1.41  2431 | 63 |
| {BcMV1.BI=FALSE, BcPV4.BI=FALSE} =>  {BcMV2.BI=FALSE} | 0.15  099 | 0.734  94 | 0.205  446 | 1.36  8275 | 61 |
| {BcMV2.BI=FALSE, BcPV4.BI=FALSE} =>  {BcMV1.BI=FALSE} | 0.15  099 | 0.299  02 | 0.504  95 | 1.42  1223 | 61 |
| {BcEV6=FALSE, BcMV1.BI=FALSE} =>  {BcMV2.BI=FALSE} | 0.14  3564 | 0.734  177 | 0.195  545 | 1.36  6855 | 58 |
| {BcEV6=FALSE, BcMV2.BI=FALSE} =>  {BcMV1.BI=FALSE} | 0.14  3564 | 0.285  714 | 0.502  475 | 1.35  7983 | 58 |
| {BcMV1.BI=FALSE, BcOLV17.BI=FALSE} =>  {BcMV2.BI=FALSE} | 0.15  3465 | 0.746  988 | 0.205  446 | 1.39  0706 | 62 |
| {BcMV2.BI=FALSE, BcOLV17.BI=FALSE} =>  {BcMV1.BI=FALSE} | 0.15  3465 | 0.293  839 | 0.522  277 | 1.39  6599 | 62 |
| {BcMV1.BI=FALSE, BcOLV15.BI=FALSE} =>  {BcMV2.BI=FALSE} | 0.15  3465 | 0.746  988 | 0.205  446 | 1.39  0706 | 62 |
| {BcMV2.BI=FALSE, BcOLV15.BI=FALSE} =>  {BcMV1.BI=FALSE} | 0.15  3465 | 0.308  458 | 0.497  525 | 1.46  6081 | 62 |
| {BcEV5=FALSE, BcMV1.BI=FALSE} => | 0.15 | 0.734 | 0.205 | 1.36 | 61 |

| {BcMV2.BI=FALSE} | 099 | 94 | 446 | 8275 |  |
| --- | --- | --- | --- | --- | --- |
| {BcEV5=FALSE, BcMV2.BI=FALSE} =>  {BcMV1.BI=FALSE} | 0.15  099 | 0.286  385 | 0.527  228 | 1.36  1171 | 61 |
| {BcHV1SLR.BI=TRUE, BcMV1.BI=FALSE}  => {BcULV3=TRUE} | 0.07  4257 | 0.731  707 | 0.101  485 | 1.23  1707 | 30 |
| {BcMV1.BI=FALSE, BcULV3=TRUE} =>  {BcHV1SLR.BI=TRUE} | 0.07  4257 | 0.681  818 | 0.108  911 | 1.25  7783 | 30 |
| {BcHV1SLR.BI=TRUE, BcMV1.BI=FALSE}  => {BcHV1.BI=TRUE} | 0.09  6535 | 0.951  22 | 0.101  485 | 1.57  497 | 39 |
| {BcHV1.BI=TRUE, BcMV1.BI=FALSE} =>  {BcHV1SLR.BI=TRUE} | 0.09  6535 | 0.795  918 | 0.121  287 | 1.46  827 | 39 |
| {BcMV1.BI=FALSE, BcMV3.BI=TRUE} =>  {BcEV2.BI=FALSE} | 0.07  4257 | 0.882  353 | 0.084  158 | 1.18  0366 | 30 |
| {BcMV1.BI=FALSE, BcMV3.BI=TRUE} =>  {BcHV3.BI=FALSE} | 0.07  6733 | 0.911  765 | 0.084  158 | 1.19  9847 | 31 |
| {BcMV1.BI=FALSE, BcOLV16.BI=FALSE} =>  {BcMV4.BI=FALSE} | 0.09  901 | 0.754  717 | 0.131  188 | 1.35  5136 | 40 |
| {BcMV1.BI=FALSE, BcMV4.BI=FALSE} =>  {BcOLV1.BI.MOVA.=FALSE} | 0.12  6238 | 0.879  31 | 0.143  564 | 1.31  5709 | 51 |
| {BcMV1.BI=FALSE, BcOLV1.BI.MOVA.=FALSE} =>  {BcMV4.BI=FALSE} | 0.12  6238 | 0.728  571 | 0.173  267 | 1.30  819 | 51 |

| {BcMV4.BI=FALSE, BcOLV1.BI.MOVA.=FALSE} =>  {BcMV1.BI=FALSE} | 0.12  6238 | 0.307  229 | 0.410  891 | 1.46  0241 | 51 |
| --- | --- | --- | --- | --- | --- |
| {BcMV1.BI=FALSE, BcULV4=TRUE} =>  {BcMV4.BI=FALSE} | 0.09  6535 | 0.684  211 | 0.141  089 | 1.22  8538 | 39 |
| {BcMV1.BI=FALSE, BcMV4.BI=FALSE} =>  {BcMV6.BI=FALSE} | 0.11  8812 | 0.827  586 | 0.143  564 | 1.18  9839 | 48 |
| {BcMV1.BI=FALSE, BcMV6.BI=FALSE} =>  {BcMV4.BI=FALSE} | 0.11  8812 | 0.738  462 | 0.160  891 | 1.32  5949 | 48 |
| {BcMV4.BI=FALSE, BcMV6.BI=FALSE} =>  {BcMV1.BI=FALSE} | 0.11  8812 | 0.277  457 | 0.428  218 | 1.31  8735 | 48 |
| {BcMV1.BI=FALSE, BcMV4.BI=FALSE} =>  {BcPV3.BI=FALSE} | 0.13  1188 | 0.913  793 | 0.143  564 | 1.28  6315 | 53 |
| {BcMV1.BI=FALSE, BcPV3.BI=FALSE} =>  {BcMV4.BI=FALSE} | 0.13  1188 | 0.746  479 | 0.175  743 | 1.34  0344 | 53 |
| {BcMV4.BI=FALSE, BcPV3.BI=FALSE} =>  {BcMV1.BI=FALSE} | 0.13  1188 | 0.292  818 | 0.448  02 | 1.39  1745 | 53 |
| {BcMV1.BI=FALSE, BcRV1.BI=FALSE} =>  {BcMV4.BI=FALSE} | 0.10  6436 | 0.781  818 | 0.136  139 | 1.40  3798 | 43 |
| {BcMV1.BI=FALSE, BcMV4.BI=FALSE} =>  {BcEV2.BI=FALSE} | 0.12  1287 | 0.844  828 | 0.143  564 | 1.13  0167 | 49 |
| {BcEV2.BI=FALSE, BcMV1.BI=FALSE} => | 0.12 | 0.690 | 0.175 | 1.23 | 49 |

| {BcMV4.BI=FALSE} | 1287 | 141 | 743 | 9186 |  |
| --- | --- | --- | --- | --- | --- |
| {BcEV2.BI=FALSE, BcMV4.BI=FALSE} =>  {BcMV1.BI=FALSE} | 0.12  1287 | 0.28 | 0.433  168 | 1.33  0824 | 49 |
| {BcMV1.BI=FALSE, BcMV4.BI=FALSE} =>  {BcHV3.BI=FALSE} | 0.12  8713 | 0.896  552 | 0.143  564 | 1.17  9827 | 52 |
| {BcHV3.BI=FALSE, BcMV1.BI=FALSE} =>  {BcMV4.BI=FALSE} | 0.12  8713 | 0.702  703 | 0.183  168 | 1.26  1742 | 52 |
| {BcHV3.BI=FALSE, BcMV4.BI=FALSE} =>  {BcMV1.BI=FALSE} | 0.12  8713 | 0.275  132 | 0.467  822 | 1.30  7688 | 52 |
| {BcFV5.BI=FALSE, BcMV1.BI=FALSE} =>  {BcMV4.BI=FALSE} | 0.12  1287 | 0.7 | 0.173  267 | 1.25  6889 | 49 |
| {BcMV1.BI=FALSE, BcMV4.BI=FALSE} =>  {BcULV1.BI=FALSE} | 0.12  8713 | 0.896  552 | 0.143  564 | 1.14  2609 | 52 |
| {BcMV1.BI=FALSE, BcULV1.BI=FALSE} =>  {BcMV4.BI=FALSE} | 0.12  8713 | 0.702  703 | 0.183  168 | 1.26  1742 | 52 |
| {BcMV4.BI=FALSE, BcULV1.BI=FALSE} =>  {BcMV1.BI=FALSE} | 0.12  8713 | 0.285  714 | 0.450  495 | 1.35  7983 | 52 |
| {BcMV1.BI=FALSE, BcMV12=FALSE} =>  {BcMV4.BI=FALSE} | 0.12  1287 | 0.680  556 | 0.178  218 | 1.22  1975 | 49 |
| {BcMV12=FALSE, BcMV4.BI=FALSE} =>  {BcMV1.BI=FALSE} | 0.12  1287 | 0.276  836 | 0.438  119 | 1.31  5786 | 49 |
| {BcMV1.BI=FALSE, BcNSRV7.BI=FALSE} | 0.12 | 0.704 | 0.175 | 1.26 | 50 |

| => {BcMV4.BI=FALSE} | 3762 | 225 | 743 | 4476 |  |
| --- | --- | --- | --- | --- | --- |
| {BcMV4.BI=FALSE, BcNSRV7.BI=FALSE}  => {BcMV1.BI=FALSE} | 0.12  3762 | 0.267  38 | 0.462  871 | 1.27  084 | 50 |
| {BcMV1.BI=FALSE, BcVV3.BI=FALSE} =>  {BcMV4.BI=FALSE} | 0.11  8812 | 0.676  056 | 0.175  743 | 1.21  3897 | 48 |
| {BcMV1.BI=FALSE, BcMV4.BI=FALSE} =>  {BcOLV7.BI=FALSE} | 0.13  3663 | 0.931  034 | 0.143  564 | 1.15  3797 | 54 |
| {BcMV1.BI=FALSE, BcOLV7.BI=FALSE} =>  {BcMV4.BI=FALSE} | 0.13  3663 | 0.72 | 0.185  644 | 1.29  28 | 54 |
| {BcMV4.BI=FALSE, BcOLV7.BI=FALSE} =>  {BcMV1.BI=FALSE} | 0.13  3663 | 0.284  211 | 0.470  297 | 1.35  0836 | 54 |
| {BcMV1.BI=FALSE, BcMV4.BI=FALSE} =>  {BcALV1.BI=FALSE} | 0.13  3663 | 0.931  034 | 0.143  564 | 1.14  3276 | 54 |
| {BcALV1.BI=FALSE, BcMV1.BI=FALSE} =>  {BcMV4.BI=FALSE} | 0.13  3663 | 0.729  73 | 0.183  168 | 1.31  027 | 54 |
| {BcALV1.BI=FALSE, BcMV4.BI=FALSE} =>  {BcMV1.BI=FALSE} | 0.13  3663 | 0.267  327 | 0.5 | 1.27  0588 | 54 |
| {BcDMV1=FALSE, BcMV1.BI=FALSE} =>  {BcMV4.BI=FALSE} | 0.12  1287 | 0.680  556 | 0.178  218 | 1.22  1975 | 49 |
| {BcFV9=FALSE, BcMV1.BI=FALSE} =>  {BcMV4.BI=FALSE} | 0.13  3663 | 0.701  299 | 0.190  594 | 1.25  9221 | 54 |
| {BcMV1.BI=FALSE, BcMV4.BI=FALSE} => | 0.14 | 0.982 | 0.143 | 1.12 | 57 |

| {BcOLV4.BI=FALSE} | 1089 | 759 | 564 | 1566 |  |
| --- | --- | --- | --- | --- | --- |
| {BcMV1.BI=FALSE, BcOLV4.BI=FALSE} =>  {BcMV4.BI=FALSE} | 0.14  1089 | 0.695  122 | 0.202  97 | 1.24  813 | 57 |
| {BcMV4.BI=FALSE, BcOLV4.BI=FALSE} =>  {BcMV1.BI=FALSE} | 0.14  1089 | 0.279  412 | 0.504  95 | 1.32  8028 | 57 |
| {BcMV1.BI=FALSE, BcMV4.BI=FALSE} =>  {BcULV5=FALSE} | 0.13  6139 | 0.948  276 | 0.143  564 | 1.08  2213 | 55 |
| {BcMV1.BI=FALSE, BcULV5=FALSE} =>  {BcMV4.BI=FALSE} | 0.13  6139 | 0.670  732 | 0.202  97 | 1.20  4336 | 55 |
| {BcMV4.BI=FALSE, BcULV5=FALSE} =>  {BcMV1.BI=FALSE} | 0.13  6139 | 0.275 | 0.495  05 | 1.30  7059 | 55 |
| {BcBNV5.BI=FALSE, BcMV1.BI=FALSE} =>  {BcMV4.BI=FALSE} | 0.13  8614 | 0.682  927 | 0.202  97 | 1.22  6233 | 56 |
| {BcBNV5.BI=FALSE, BcMV4.BI=FALSE} =>  {BcMV1.BI=FALSE} | 0.13  8614 | 0.264  151 | 0.524  752 | 1.25  5494 | 56 |
| {BcMV1.BI=FALSE, BvF.BI=FALSE} =>  {BcMV4.BI=FALSE} | 0.12  6238 | 0.689  189 | 0.183  168 | 1.23  7477 | 51 |
| {BcMV1.BI=FALSE, BcMV4.BI=FALSE} =>  {BcMCV5.BI=FALSE} | 0.14  3564 | 1 | 0.143  564 | 1.08  311 | 58 |
| {BcMCV5.BI=FALSE, BcMV1.BI=FALSE} =>  {BcMV4.BI=FALSE} | 0.14  3564 | 0.682  353 | 0.210  396 | 1.22  5203 | 58 |
| {BcMCV5.BI=FALSE, BcMV4.BI=FALSE} => | 0.14 | 0.263 | 0.544 | 1.25 | 58 |

| {BcMV1.BI=FALSE} | 3564 | 636 | 554 | 3048 |  |
| --- | --- | --- | --- | --- | --- |
| {BcMV1.BI=FALSE, BcOLV13.BI=FALSE} =>  {BcMV4.BI=FALSE} | 0.13  8614 | 0.691  358 | 0.200  495 | 1.24  1372 | 56 |
| {BcMV4.BI=FALSE, BcOLV13.BI=FALSE} =>  {BcMV1.BI=FALSE} | 0.13  8614 | 0.259  259 | 0.534  653 | 1.23  2244 | 56 |
| {BcMV1.BI=FALSE, BcOLV2.BI=FALSE} =>  {BcMV4.BI=FALSE} | 0.13  6139 | 0.696  203 | 0.195  545 | 1.25  007 | 55 |
| {BcMCV3.BI=FALSE, BcMV1.BI=FALSE} =>  {BcMV4.BI=FALSE} | 0.14  3564 | 0.707  317 | 0.202  97 | 1.27  0027 | 58 |
| {BcMCV3.BI=FALSE, BcMV4.BI=FALSE} =>  {BcMV1.BI=FALSE} | 0.14  3564 | 0.274  882 | 0.522  277 | 1.30  6496 | 58 |
| {BcMV1.BI=FALSE, BcPV4.BI=FALSE} =>  {BcMV4.BI=FALSE} | 0.13  8614 | 0.674  699 | 0.205  446 | 1.21  1459 | 56 |
| {BcMV4.BI=FALSE, BcPV4.BI=FALSE} =>  {BcMV1.BI=FALSE} | 0.13  8614 | 0.269  231 | 0.514  851 | 1.27  9638 | 56 |
| {BcEV6=FALSE, BcMV1.BI=FALSE} =>  {BcMV4.BI=FALSE} | 0.13  1188 | 0.670  886 | 0.195  545 | 1.20  4613 | 53 |
| {BcMV1.BI=FALSE, BcOLV17.BI=FALSE} =>  {BcMV4.BI=FALSE} | 0.14  1089 | 0.686  747 | 0.205  446 | 1.23  3092 | 57 |
| {BcMV4.BI=FALSE, BcOLV17.BI=FALSE} =>  {BcMV1.BI=FALSE} | 0.14  1089 | 0.262  673 | 0.537  129 | 1.24  8468 | 57 |
| {BcMV1.BI=FALSE, BcOLV15.BI=FALSE} => | 0.14 | 0.686 | 0.205 | 1.23 | 57 |

| {BcMV4.BI=FALSE} | 1089 | 747 | 446 | 3092 |  |
| --- | --- | --- | --- | --- | --- |
| {BcMV4.BI=FALSE, BcOLV15.BI=FALSE} =>  {BcMV1.BI=FALSE} | 0.14  1089 | 0.262  673 | 0.537  129 | 1.24  8468 | 57 |
| {BcEV5=FALSE, BcMV1.BI=FALSE} =>  {BcMV4.BI=FALSE} | 0.14  1089 | 0.686  747 | 0.205  446 | 1.23  3092 | 57 |
| {BcMV1.BI=FALSE, BcOLV16.BI=FALSE} =>  {BcOLV1.BI.MOVA.=FALSE} | 0.11  3861 | 0.867  925 | 0.131  188 | 1.29  8672 | 46 |
| {BcMV1.BI=FALSE, BcOLV16.BI=FALSE} =>  {BcMV6.BI=FALSE} | 0.10  8911 | 0.830  189 | 0.131  188 | 1.19  3581 | 44 |
| {BcMV1.BI=FALSE, BcOLV16.BI=FALSE} =>  {BcPV3.BI=FALSE} | 0.10  6436 | 0.811  321 | 0.131  188 | 1.14  2068 | 43 |
| {BcMV1.BI=FALSE, BcOLV16.BI=FALSE} =>  {BcHV3.BI=FALSE} | 0.11  8812 | 0.905  66 | 0.131  188 | 1.19  1814 | 48 |
| {BcHV3.BI=FALSE, BcOLV16.BI=FALSE} =>  {BcMV1.BI=FALSE} | 0.11  8812 | 0.271  186 | 0.438  119 | 1.28  8933 | 48 |
| {BcMV1.BI=FALSE, BcOLV16.BI=FALSE} =>  {BcULV1.BI=FALSE} | 0.12  3762 | 0.943  396 | 0.131  188 | 1.20  2309 | 50 |
| {BcMV1.BI=FALSE, BcOLV16.BI=FALSE} =>  {BcOLV7.BI=FALSE} | 0.12  1287 | 0.924  528 | 0.131  188 | 1.14  5734 | 49 |
| {BcMV1.BI=FALSE, BcOLV16.BI=FALSE} =>  {BcALV1.BI=FALSE} | 0.12  6238 | 0.962  264 | 0.131  188 | 1.18  1625 | 51 |
| {BcALV1.BI=FALSE, BcMV1.BI=FALSE} => | 0.12 | 0.689 | 0.183 | 1.17 | 51 |

| {BcOLV16.BI=FALSE} | 6238 | 189 | 168 | 9798 |  |
| --- | --- | --- | --- | --- | --- |
| {BcMV1.BI=FALSE, BcOLV16.BI=FALSE} =>  {BcFV9=FALSE} | 0.12  3762 | 0.943  396 | 0.131  188 | 1.08  5846 | 50 |
| {BcMV1.BI=FALSE, BcOLV16.BI=FALSE} =>  {BcOLV4.BI=FALSE} | 0.12  8713 | 0.981  132 | 0.131  188 | 1.11  971 | 52 |
| {BcMV1.BI=FALSE, BcOLV16.BI=FALSE} =>  {BcULV5=FALSE} | 0.13  1188 | 1 | 0.131  188 | 1.14  1243 | 53 |
| {BcMV1.BI=FALSE, BcOLV16.BI=FALSE} =>  {BcMCV5.BI=FALSE} | 0.13  1188 | 1 | 0.131  188 | 1.08  311 | 53 |
| {BcMV1.BI=FALSE, BcULV3=TRUE} =>  {BcHV1.BI=TRUE} | 0.08  1683 | 0.75 | 0.108  911 | 1.24  1803 | 33 |
| {BcMV1.BI=FALSE, BcULV3=TRUE} =>  {BcOLV1.BI.MOVA.=FALSE} | 0.08  6634 | 0.795  455 | 0.108  911 | 1.19  0236 | 35 |
| {BcMV1.BI=FALSE, BcULV3=TRUE} =>  {BcEV2.BI=FALSE} | 0.09  6535 | 0.886  364 | 0.108  911 | 1.18  5731 | 39 |
| {BcMV1.BI=FALSE, BcULV3=TRUE} =>  {BcULV5=FALSE} | 0.10  6436 | 0.977  273 | 0.108  911 | 1.11  5306 | 43 |
| {BcHV1.BI=TRUE, BcMV1.BI=FALSE} =>  {BcOLV4.BI=FALSE} | 0.11  6337 | 0.959  184 | 0.121  287 | 1.09  4662 | 47 |
| {BcHV1.BI=TRUE, BcMV1.BI=FALSE} =>  {BcMCV5.BI=FALSE} | 0.12  1287 | 1 | 0.121  287 | 1.08  311 | 49 |
| {BcMV1.BI=FALSE, BcMV11=TRUE} => | 0.07 | 0.794 | 0.096 | 1.18 | 31 |

| {BcOLV1.BI.MOVA.=FALSE} | | 6733 | 872 | 535 | 9364 |  |
| --- | --- | --- | --- | --- | --- | --- |
| {BcMV1.BI=FALSE, | BcMV11=TRUE} => | 0.08 | 0.846 | 0.096 | 1.19 |  |
|  | |  |  |  |  | 33 |
| {BcPV3.BI=FALSE} | | 1683 | 154 | 535 | 1102 |  |
| {BcMV1.BI=FALSE, | BcMV11=TRUE} => | 0.08 | 0.871 | 0.096 | 1.14 |  |
|  | |  |  |  |  | 34 |
| {BcHV3.BI=FALSE} | | 4158 | 795 | 535 | 7248 |  |
| {BcMV1.BI=FALSE, | BcMV11=TRUE} => | 0.08 | 0.897 | 0.096 | 1.11 |  |
|  | |  |  |  |  | 35 |
| {BcVV3.BI=FALSE} | | 6634 | 436 | 535 | 9025 |  |
| {BcMV1.BI=FALSE, | BcMV11=TRUE} => | 0.09 | 0.974 | 0.096 | 1.11 |  |
|  | |  |  |  |  | 38 |
| {BcOLV4.BI=FALSE} | | 4059 | 359 | 535 | 198 |  |
| {BcMV1.BI=FALSE, | BcMV9.BI=TRUE} => | 0.10 | 0.788 | 0.128 | 1.17 |  |
|  | |  |  |  |  | 41 |
| {BcOLV1.BI.MOVA.=FALSE} | | 1485 | 462 | 713 | 9772 |  |
| {BcMV1.BI=FALSE, | BcULV4=TRUE} => | 0.10 | 0.771 | 0.141 | 1.15 |  |
|  | |  |  |  |  | 44 |
| {BcOLV1.BI.MOVA.=FALSE} | | 8911 | 93 | 089 | 5036 |  |
| {BcMV1.BI=FALSE, | |  |  |  |  |  |
|  | | 0.14 | 0.828 | 0.173 | 1.19 |  |
| BcOLV1.BI.MOVA.=FALSE} => | |  |  |  |  | 58 |
|  | | 3564 | 571 | 267 | 1256 |  |
| {BcMV6.BI=FALSE} | |  |  |  |  |  |
| {BcMV1.BI=FALSE, | BcMV6.BI=FALSE} => | 0.14 | 0.892 | 0.160 | 1.33 |  |
|  | |  |  |  |  | 58 |
| {BcOLV1.BI.MOVA.=FALSE} | | 3564 | 308 | 891 | 5157 |  |
| {BcMV6.BI=FALSE, | |  |  |  |  |  |
|  | | 0.14 | 0.272 | 0.527 | 1.29 |  |
| BcOLV1.BI.MOVA.=FALSE} => | |  |  |  |  | 58 |
|  | | 3564 | 3 | 228 | 4228 |  |
| {BcMV1.BI=FALSE} | |  |  |  |  |  |
| {BcMV1.BI=FALSE, | | 0.15 | 0.885 | 0.173 | 1.24 | 62 |

| BcOLV1.BI.MOVA.=FALSE} =>  {BcPV3.BI=FALSE} | 3465 | 714 | 267 | 6789 |  |
| --- | --- | --- | --- | --- | --- |
| {BcMV1.BI=FALSE, BcPV3.BI=FALSE} => | 0.15 | 0.873 | 0.175 | 1.30 |  |
|  |  |  |  |  | 62 |
| {BcOLV1.BI.MOVA.=FALSE} | 3465 | 239 | 743 | 6625 |  |
| {BcOLV1.BI.MOVA.=FALSE, | 0.15 | 0.298 | 0.514 | 1.41 |  |
|  |  |  |  |  | 62 |
| BcPV3.BI=FALSE} => {BcMV1.BI=FALSE} | 3465 | 077 | 851 | 6742 |  |
| {BcMV1.BI=FALSE, BcRV1.BI=FALSE} => | 0.11 | 0.872 | 0.136 | 1.30 |  |
|  |  |  |  |  | 48 |
| {BcOLV1.BI.MOVA.=FALSE} | 8812 | 727 | 139 | 5859 |  |
| {BcEV2.BI=FALSE, BcMV1.BI=FALSE} => | 0.13 | 0.788 | 0.175 | 1.18 |  |
|  |  |  |  |  | 56 |
| {BcOLV1.BI.MOVA.=FALSE} | 8614 | 732 | 743 | 0177 |  |
| {BcEV2.BI=FALSE, |  |  |  |  |  |
|  | 0.13 | 0.278 | 0.497 | 1.32 |  |
| BcOLV1.BI.MOVA.=FALSE} => |  |  |  |  | 56 |
|  | 8614 | 607 | 525 | 4203 |  |
| {BcMV1.BI=FALSE} |  |  |  |  |  |
| {BcMV1.BI=FALSE, |  |  |  |  |  |
|  | 0.15 | 0.885 | 0.173 | 1.16 |  |
| BcOLV1.BI.MOVA.=FALSE} => |  |  |  |  | 62 |
|  | 3465 | 714 | 267 | 5565 |  |
| {BcHV3.BI=FALSE} |  |  |  |  |  |
| {BcHV3.BI=FALSE, BcMV1.BI=FALSE} => | 0.15 | 0.837 | 0.183 | 1.25 |  |
|  |  |  |  |  | 62 |
| {BcOLV1.BI.MOVA.=FALSE} | 3465 | 838 | 168 | 3654 |  |
| {BcHV3.BI=FALSE, |  |  |  |  |  |
|  | 0.15 | 0.288 | 0.532 | 1.37 |  |
| BcOLV1.BI.MOVA.=FALSE} => |  |  |  |  | 62 |
|  | 3465 | 372 | 178 | 0616 |  |
| {BcMV1.BI=FALSE} |  |  |  |  |  |
| {BcFV5.BI=FALSE, BcMV1.BI=FALSE} => | 0.14 | 0.814 | 0.173 | 1.21 | 57 |

| {BcOLV1.BI.MOVA.=FALSE} | 1089 | 286 | 267 | 8413 |  |
| --- | --- | --- | --- | --- | --- |
| {BcFV5.BI=FALSE, BcOLV1.BI.MOVA.=FALSE} =>  {BcMV1.BI=FALSE} | 0.14  1089 | 0.260  274 | 0.542  079 | 1.23  7067 | 57 |
| {BcMV1.BI=FALSE, BcOLV1.BI.MOVA.=FALSE} =>  {BcULV1.BI=FALSE} | 0.15  5941 | 0.9 | 0.173  267 | 1.14  7003 | 63 |
| {BcMV1.BI=FALSE, BcULV1.BI=FALSE} => | 0.15 | 0.851 | 0.183 | 1.27 |  |
|  |  |  |  |  | 63 |
| {BcOLV1.BI.MOVA.=FALSE} | 5941 | 351 | 168 | 3874 |  |
| {BcOLV1.BI.MOVA.=FALSE, | 0.15 | 0.278 | 0.559 | 1.32 |  |
|  |  |  |  |  | 63 |
| BcULV1.BI=FALSE} => {BcMV1.BI=FALSE} | 5941 | 761 | 406 | 4935 |  |
| {BcMV1.BI=FALSE, |  |  |  |  |  |
|  | 0.15 | 0.885 | 0.173 | 1.10 |  |
| BcOLV1.BI.MOVA.=FALSE} => |  |  |  |  | 62 |
|  | 3465 | 714 | 267 | 7828 |  |
| {BcMV12=FALSE} |  |  |  |  |  |
| {BcMV1.BI=FALSE, BcMV12=FALSE} => | 0.15 | 0.861 | 0.178 | 1.28 |  |
|  |  |  |  |  | 62 |
| {BcOLV1.BI.MOVA.=FALSE} | 3465 | 111 | 218 | 8477 |  |
| {BcMV12=FALSE, |  |  |  |  |  |
|  | 0.15 | 0.269 | 0.569 | 1.28 |  |
| BcOLV1.BI.MOVA.=FALSE} => |  |  |  |  | 62 |
|  | 3465 | 565 | 307 | 1228 |  |
| {BcMV1.BI=FALSE} |  |  |  |  |  |
| {BcMV1.BI=FALSE, BcNSRV7.BI=FALSE} | 0.14 | 0.830 | 0.175 | 1.24 |  |
|  |  |  |  |  | 59 |
| => {BcOLV1.BI.MOVA.=FALSE} | 604 | 986 | 743 | 3401 |  |
| {BcNSRV7.BI=FALSE, | 0.14 | 0.273 | 0.534 | 1.29 | 59 |

| BcOLV1.BI.MOVA.=FALSE} =>  {BcMV1.BI=FALSE} | 604 | 148 | 653 | 8257 |  |
| --- | --- | --- | --- | --- | --- |
| {BcMV1.BI=FALSE, BcVV3.BI=FALSE} => | 0.13 | 0.788 | 0.175 | 1.18 |  |
|  |  |  |  |  | 56 |
| {BcOLV1.BI.MOVA.=FALSE} | 8614 | 732 | 743 | 0177 |  |
| {BcMV1.BI=FALSE, |  |  |  |  |  |
|  | 0.15 |  | 0.173 | 1.11 |  |
| BcOLV1.BI.MOVA.=FALSE} => |  | 0.9 |  |  | 63 |
|  | 5941 |  | 267 | 5337 |  |
| {BcOLV7.BI=FALSE} |  |  |  |  |  |
| {BcMV1.BI=FALSE, BcOLV7.BI=FALSE} => | 0.15 |  | 0.185 | 1.25 |  |
|  |  | 0.84 |  |  | 63 |
| {BcOLV1.BI.MOVA.=FALSE} | 5941 |  | 644 | 6889 |  |
| {BcOLV1.BI.MOVA.=FALSE, | 0.15 | 0.273 | 0.569 | 1.30 |  |
|  |  |  |  |  | 63 |
| BcOLV7.BI=FALSE} => {BcMV1.BI=FALSE} | 5941 | 913 | 307 | 1893 |  |
| {BcMV1.BI=FALSE, |  |  |  |  |  |
|  | 0.15 |  | 0.173 | 1.10 |  |
| BcOLV1.BI.MOVA.=FALSE} => |  | 0.9 |  |  | 63 |
|  | 5941 |  | 267 | 5167 |  |
| {BcALV1.BI=FALSE} |  |  |  |  |  |
| {BcALV1.BI=FALSE, BcMV1.BI=FALSE} => | 0.15 | 0.851 | 0.183 | 1.27 |  |
|  |  |  |  |  | 63 |
| {BcOLV1.BI.MOVA.=FALSE} | 5941 | 351 | 168 | 3874 |  |
| {BcALV1.BI=FALSE, |  |  |  |  |  |
|  | 0.15 | 0.264 | 0.589 | 1.25 |  |
| BcOLV1.BI.MOVA.=FALSE} => |  |  |  |  | 63 |
|  | 5941 | 706 | 109 | 8131 |  |
| {BcMV1.BI=FALSE} |  |  |  |  |  |
| {BcDMV1=FALSE, BcMV1.BI=FALSE} => | 0.14 | 0.819 | 0.178 | 1.22 |  |
|  |  |  |  |  | 59 |
| {BcOLV1.BI.MOVA.=FALSE} | 604 | 444 | 218 | 6132 |  |
| {BcFV9=FALSE, BcMV1.BI=FALSE} => | 0.16 | 0.844 | 0.190 | 1.26 | 65 |

| {BcOLV1.BI.MOVA.=FALSE} | 0891 | 156 | 594 | 3107 |  |
| --- | --- | --- | --- | --- | --- |
| {BcFV9=FALSE, BcOLV1.BI.MOVA.=FALSE} =>  {BcMV1.BI=FALSE} | 0.16  0891 | 0.273  109 | 0.589  109 | 1.29  8072 | 65 |
| {BcMV1.BI=FALSE, BcOLV1.BI.MOVA.=FALSE} =>  {BcOLV4.BI=FALSE} | 0.16  8317 | 0.971  429 | 0.173  267 | 1.10  8636 | 68 |
| {BcMV1.BI=FALSE, BcOLV4.BI=FALSE} => | 0.16 | 0.829 | 0.202 | 1.24 |  |
|  |  |  |  |  | 68 |
| {BcOLV1.BI.MOVA.=FALSE} | 8317 | 268 | 97 | 0831 |  |
| {BcOLV1.BI.MOVA.=FALSE, | 0.16 | 0.274 | 0.613 | 1.30 |  |
|  |  |  |  |  | 68 |
| BcOLV4.BI=FALSE} => {BcMV1.BI=FALSE} | 8317 | 194 | 861 | 3226 |  |
| {BcMV1.BI=FALSE, |  |  |  |  |  |
|  | 0.16 | 0.971 | 0.173 | 1.10 |  |
| BcOLV1.BI.MOVA.=FALSE} => |  |  |  |  | 68 |
|  | 8317 | 429 | 267 | 8636 |  |
| {BcULV5=FALSE} |  |  |  |  |  |
| {BcMV1.BI=FALSE, BcULV5=FALSE} => | 0.16 | 0.829 | 0.202 | 1.24 |  |
|  |  |  |  |  | 68 |
| {BcOLV1.BI.MOVA.=FALSE} | 8317 | 268 | 97 | 0831 |  |
| {BcOLV1.BI.MOVA.=FALSE, | 0.16 |  | 0.618 | 1.29 |  |
|  |  | 0.272 |  |  | 68 |
| BcULV5=FALSE} => {BcMV1.BI=FALSE} | 8317 |  | 812 | 28 |  |
| {BcBNV5.BI=FALSE, BcMV1.BI=FALSE} => | 0.16 | 0.817 | 0.202 | 1.22 |  |
|  |  |  |  |  | 67 |
| {BcOLV1.BI.MOVA.=FALSE} | 5842 | 073 | 97 | 2584 |  |
| {BcBNV5.BI=FALSE, | 0.16 | 0.264 | 0.626 | 1.25 |  |
|  |  |  |  |  | 67 |
| BcOLV1.BI.MOVA.=FALSE} => | 5842 | 822 | 238 | 8684 |  |

| {BcMV1.BI=FALSE} |  |  |  |  |  |
| --- | --- | --- | --- | --- | --- |
| {BcMV1.BI=FALSE, BvF.BI=FALSE} => | 0.14 | 0.797 | 0.183 | 1.19 |  |
|  |  |  |  |  | 59 |
| {BcOLV1.BI.MOVA.=FALSE} | 604 | 297 | 168 | 2993 |  |
| {BcMV1.BI=FALSE, |  |  |  |  |  |
|  | 0.17 |  | 0.173 | 1.08 |  |
| BcOLV1.BI.MOVA.=FALSE} => |  | 1 |  |  | 70 |
|  | 3267 |  | 267 | 311 |  |
| {BcMCV5.BI=FALSE} |  |  |  |  |  |
| {BcMCV5.BI=FALSE, BcMV1.BI=FALSE} => | 0.17 | 0.823 | 0.210 | 1.23 |  |
|  |  |  |  |  | 70 |
| {BcOLV1.BI.MOVA.=FALSE} | 3267 | 529 | 396 | 2244 |  |
| {BcMCV5.BI=FALSE, |  |  |  |  |  |
|  | 0.17 | 0.268 | 0.646 | 1.27 |  |
| BcOLV1.BI.MOVA.=FALSE} => |  |  |  |  | 70 |
|  | 3267 | 199 | 04 | 4735 |  |
| {BcMV1.BI=FALSE} |  |  |  |  |  |
| {BcMV1.BI=FALSE, BcOLV13.BI=FALSE} => | 0.17 | 0.851 | 0.200 | 1.27 |  |
|  |  |  |  |  | 69 |
| {BcOLV1.BI.MOVA.=FALSE} | 0792 | 852 | 495 | 4623 |  |
| {BcOLV1.BI.MOVA.=FALSE, | 0.17 | 0.263 | 0.648 | 1.25 |  |
|  |  |  |  |  | 69 |
| BcOLV13.BI=FALSE} => {BcMV1.BI=FALSE} | 0792 | 359 | 515 | 1729 |  |
| {BcMV1.BI=FALSE, BcOLV2.BI=FALSE} => | 0.16 | 0.860 | 0.195 | 1.28 |  |
|  |  |  |  |  | 68 |
| {BcOLV1.BI.MOVA.=FALSE} | 8317 | 759 | 545 | 7951 |  |
| {BcOLV1.BI.MOVA.=FALSE, | 0.16 | 0.258 | 0.650 | 1.22 |  |
|  |  |  |  |  | 68 |
| BcOLV2.BI=FALSE} => {BcMV1.BI=FALSE} | 8317 | 555 | 99 | 8897 |  |
| {BcMCV3.BI=FALSE, BcMV1.BI=FALSE} => | 0.17 | 0.853 | 0.202 | 1.27 |  |
|  |  |  |  |  | 70 |
| {BcOLV1.BI.MOVA.=FALSE} | 3267 | 659 | 97 | 7326 |  |
| {BcMCV3.BI=FALSE, | 0.17 | 0.268 | 0.646 | 1.27 | 70 |

| BcOLV1.BI.MOVA.=FALSE} =>  {BcMV1.BI=FALSE} | 3267 | 199 | 04 | 4735 |  |
| --- | --- | --- | --- | --- | --- |
| {BcMV1.BI=FALSE, BcPV4.BI=FALSE} => | 0.16 | 0.819 | 0.205 | 1.22 |  |
|  |  |  |  |  | 68 |
| {BcOLV1.BI.MOVA.=FALSE} | 8317 | 277 | 446 | 5881 |  |
| {BcOLV1.BI.MOVA.=FALSE, | 0.16 | 0.273 | 0.616 | 1.29 |  |
|  |  |  |  |  | 68 |
| BcPV4.BI=FALSE} => {BcMV1.BI=FALSE} | 8317 | 092 | 337 | 7992 |  |
| {BcEV6=FALSE, BcMV1.BI=FALSE} => | 0.16 | 0.822 | 0.195 | 1.23 |  |
|  |  |  |  |  | 65 |
| {BcOLV1.BI.MOVA.=FALSE} | 0891 | 785 | 545 | 113 |  |
| {BcMV1.BI=FALSE, BcOLV17.BI=FALSE} => | 0.17 | 0.843 | 0.205 | 1.26 |  |
|  |  |  |  |  | 70 |
| {BcOLV1.BI.MOVA.=FALSE} | 3267 | 373 | 446 | 1937 |  |
| {BcOLV1.BI.MOVA.=FALSE, | 0.17 | 0.267 | 0.648 | 1.26 |  |
|  |  |  |  |  | 70 |
| BcOLV17.BI=FALSE} => {BcMV1.BI=FALSE} | 3267 | 176 | 515 | 987 |  |
| {BcMV1.BI=FALSE, BcOLV15.BI=FALSE} => | 0.17 | 0.831 | 0.205 | 1.24 |  |
|  |  |  |  |  | 69 |
| {BcOLV1.BI.MOVA.=FALSE} | 0792 | 325 | 446 | 3909 |  |
| {BcOLV1.BI.MOVA.=FALSE, | 0.17 | 0.271 | 0.628 | 1.29 |  |
|  |  |  |  |  | 69 |
| BcOLV15.BI=FALSE} => {BcMV1.BI=FALSE} | 0792 | 654 | 713 | 1153 |  |
| {BcEV5=FALSE, BcMV1.BI=FALSE} => | 0.16 | 0.819 | 0.205 | 1.22 |  |
|  |  |  |  |  | 68 |
| {BcOLV1.BI.MOVA.=FALSE} | 8317 | 277 | 446 | 5881 |  |
| {BcEV5=FALSE, |  |  |  |  |  |
|  | 0.16 | 0.257 | 0.653 | 1.22 |  |
| BcOLV1.BI.MOVA.=FALSE} => |  |  |  |  | 68 |
|  | 8317 | 576 | 465 | 4242 |  |
| {BcMV1.BI=FALSE} |  |  |  |  |  |
| {BcMV1.BI=FALSE, BcMV9.BI=TRUE} => | 0.10 | 0.846 | 0.128 | 1.19 | 44 |

| {BcPV3.BI=FALSE} | 8911 | 154 | 713 | 1102 |  |
| --- | --- | --- | --- | --- | --- |
| {BcMV1.BI=FALSE, BcMV9.BI=TRUE} =>  {BcEV2.BI=FALSE} | 0.11  1386 | 0.865  385 | 0.128  713 | 1.15  7667 | 45 |
| {BcMV1.BI=FALSE, BcMV9.BI=TRUE} =>  {BcHV3.BI=FALSE} | 0.11  6337 | 0.903  846 | 0.128  713 | 1.18  9426 | 47 |
| {BcMV1.BI=FALSE, BcMV9.BI=TRUE} =>  {BcOLV4.BI=FALSE} | 0.12  8713 | 1 | 0.128  713 | 1.14  1243 | 52 |
| {BcMV1.BI=FALSE, BcMV9.BI=TRUE} =>  {BcULV5=FALSE} | 0.12  6238 | 0.980  769 | 0.128  713 | 1.11  9296 | 51 |
| {BcMV1.BI=FALSE, BcMV9.BI=TRUE} =>  {BcMCV5.BI=FALSE} | 0.12  8713 | 1 | 0.128  713 | 1.08  311 | 52 |
| {BcMV1.BI=FALSE, BcULV4=TRUE} =>  {BcPV3.BI=FALSE} | 0.11  8812 | 0.842  105 | 0.141  089 | 1.18  5403 | 48 |
| {BcMV1.BI=FALSE, BcULV4=TRUE} =>  {BcEV2.BI=FALSE} | 0.12  3762 | 0.877  193 | 0.141  089 | 1.17  3463 | 50 |
| {BcMV1.BI=FALSE, BcULV4=TRUE} =>  {BcHV3.BI=FALSE} | 0.12  3762 | 0.877  193 | 0.141  089 | 1.15  4352 | 50 |
| {BcMV1.BI=FALSE, BcULV4=TRUE} =>  {BcOLV4.BI=FALSE} | 0.13  8614 | 0.982  456 | 0.141  089 | 1.12  1221 | 56 |
| {BcMV1.BI=FALSE, BcULV4=TRUE} =>  {BcMCV5.BI=FALSE} | 0.14  1089 | 1 | 0.141  089 | 1.08  311 | 57 |
| {BcMV1.BI=FALSE, BcMV6.BI=FALSE} => | 0.13 | 0.861 | 0.160 | 1.21 | 56 |

| {BcPV3.BI=FALSE} | 8614 | 538 | 891 | 2758 |  |
| --- | --- | --- | --- | --- | --- |
| {BcMV6.BI=FALSE, BcPV3.BI=FALSE} =>  {BcMV1.BI=FALSE} | 0.13  8614 | 0.267  943 | 0.517  327 | 1.27  3515 | 56 |
| {BcMV1.BI=FALSE, BcRV1.BI=FALSE} =>  {BcMV6.BI=FALSE} | 0.10  8911 | 0.8 | 0.136  139 | 1.15  0178 | 44 |
| {BcMV1.BI=FALSE, BcMV6.BI=FALSE} =>  {BcHV3.BI=FALSE} | 0.13  8614 | 0.861  538 | 0.160  891 | 1.13  3751 | 56 |
| {BcMV1.BI=FALSE, BcMV6.BI=FALSE} =>  {BcULV1.BI=FALSE} | 0.14  3564 | 0.892  308 | 0.160  891 | 1.13  72 | 58 |
| {BcMV1.BI=FALSE, BcMV6.BI=FALSE} =>  {BcOLV7.BI=FALSE} | 0.14  604 | 0.907  692 | 0.160  891 | 1.12  487 | 59 |
| {BcMV1.BI=FALSE, BcMV6.BI=FALSE} =>  {BcALV1.BI=FALSE} | 0.14  8515 | 0.923  077 | 0.160  891 | 1.13  3505 | 60 |
| {BcALV1.BI=FALSE, BcMV1.BI=FALSE} =>  {BcMV6.BI=FALSE} | 0.14  8515 | 0.810  811 | 0.183  168 | 1.16  5721 | 60 |
| {BcMV1.BI=FALSE, BcMV6.BI=FALSE} =>  {BcOLV4.BI=FALSE} | 0.15  5941 | 0.969  231 | 0.160  891 | 1.10  6128 | 63 |
| {BcMV1.BI=FALSE, BcMV6.BI=FALSE} =>  {BcULV5=FALSE} | 0.15  3465 | 0.953  846 | 0.160  891 | 1.08  857 | 62 |
| {BcMV1.BI=FALSE, BcMV6.BI=FALSE} =>  {BcBNV5.BI=FALSE} | 0.16  0891 | 1 | 0.160  891 | 1.10  0817 | 65 |
| {BcBNV5.BI=FALSE, BcMV1.BI=FALSE} => | 0.16 | 0.792 | 0.202 | 1.13 | 65 |

| {BcMV6.BI=FALSE} | 0891 | 683 | 97 | 9658 |  |
| --- | --- | --- | --- | --- | --- |
| {BcMV1.BI=FALSE, BcMV6.BI=FALSE} =>  {BcMCV5.BI=FALSE} | 0.16  0891 | 1 | 0.160  891 | 1.08  311 | 65 |
| {BcMV1.BI=FALSE, BcOLV2.BI=FALSE} =>  {BcMV6.BI=FALSE} | 0.15  8416 | 0.810  127 | 0.195  545 | 1.16  4737 | 64 |
| {BcMCV3.BI=FALSE, BcMV1.BI=FALSE} =>  {BcMV6.BI=FALSE} | 0.16  0891 | 0.792  683 | 0.202  97 | 1.13  9658 | 65 |
| {BcMV1.BI=FALSE, BcRV1.BI=FALSE} =>  {BcPV3.BI=FALSE} | 0.12  1287 | 0.890  909 | 0.136  139 | 1.25  4102 | 49 |
| {BcEV2.BI=FALSE, BcMV1.BI=FALSE} =>  {BcPV3.BI=FALSE} | 0.14  1089 | 0.802  817 | 0.175  743 | 1.13  0098 | 57 |
| {BcEV2.BI=FALSE, BcPV3.BI=FALSE} =>  {BcMV1.BI=FALSE} | 0.14  1089 | 0.260  274 | 0.542  079 | 1.23  7067 | 57 |
| {BcMV1.BI=FALSE, BcPV3.BI=FALSE} =>  {BcHV3.BI=FALSE} | 0.15  099 | 0.859  155 | 0.175  743 | 1.13  0614 | 61 |
| {BcHV3.BI=FALSE, BcMV1.BI=FALSE} =>  {BcPV3.BI=FALSE} | 0.15  099 | 0.824  324 | 0.183  168 | 1.16  0373 | 61 |
| {BcHV3.BI=FALSE, BcPV3.BI=FALSE} =>  {BcMV1.BI=FALSE} | 0.15  099 | 0.273  543 | 0.551  98 | 1.30  0132 | 61 |
| {BcFV5.BI=FALSE, BcMV1.BI=FALSE} =>  {BcPV3.BI=FALSE} | 0.14  1089 | 0.814  286 | 0.173  267 | 1.14  6242 | 57 |
| {BcFV5.BI=FALSE, BcPV3.BI=FALSE} => | 0.14 | 0.257 | 0.547 | 1.22 | 57 |

| {BcMV1.BI=FALSE} | 1089 | 919 | 03 | 5872 |  |
| --- | --- | --- | --- | --- | --- |
| {BcMV1.BI=FALSE, BcPV3.BI=FALSE} =>  {BcULV1.BI=FALSE} | 0.15  3465 | 0.873  239 | 0.175  743 | 1.11  2898 | 62 |
| {BcMV1.BI=FALSE, BcULV1.BI=FALSE} =>  {BcPV3.BI=FALSE} | 0.15  3465 | 0.837  838 | 0.183  168 | 1.17  9395 | 62 |
| {BcPV3.BI=FALSE, BcULV1.BI=FALSE} =>  {BcMV1.BI=FALSE} | 0.15  3465 | 0.268  398 | 0.571  782 | 1.27  5681 | 62 |
| {BcMV1.BI=FALSE, BcMV12=FALSE} =>  {BcPV3.BI=FALSE} | 0.15  099 | 0.847  222 | 0.178  218 | 1.19  2605 | 61 |
| {BcMV12=FALSE, BcPV3.BI=FALSE} =>  {BcMV1.BI=FALSE} | 0.15  099 | 0.259  574 | 0.581  683 | 1.23  3742 | 61 |
| {BcMV1.BI=FALSE, BcNSRV7.BI=FALSE}  => {BcPV3.BI=FALSE} | 0.14  3564 | 0.816  901 | 0.175  743 | 1.14  9924 | 58 |
| {BcMV1.BI=FALSE, BcVV3.BI=FALSE} =>  {BcPV3.BI=FALSE} | 0.14  3564 | 0.816  901 | 0.175  743 | 1.14  9924 | 58 |
| {BcMV1.BI=FALSE, BcPV3.BI=FALSE} =>  {BcOLV7.BI=FALSE} | 0.15  8416 | 0.901  408 | 0.175  743 | 1.11  7083 | 64 |
| {BcMV1.BI=FALSE, BcOLV7.BI=FALSE} =>  {BcPV3.BI=FALSE} | 0.15  8416 | 0.853  333 | 0.185  644 | 1.20  1208 | 64 |
| {BcOLV7.BI=FALSE, BcPV3.BI=FALSE} =>  {BcMV1.BI=FALSE} | 0.15  8416 | 0.264  463 | 0.599  01 | 1.25  6976 | 64 |
| {BcMV1.BI=FALSE, BcPV3.BI=FALSE} => | 0.15 | 0.901 | 0.175 | 1.10 | 64 |

| {BcALV1.BI=FALSE} | 8416 | 408 | 743 | 6897 |  |
| --- | --- | --- | --- | --- | --- |
| {BcALV1.BI=FALSE, BcMV1.BI=FALSE} =>  {BcPV3.BI=FALSE} | 0.15  8416 | 0.864  865 | 0.183  168 | 1.21  744 | 64 |
| {BcALV1.BI=FALSE, BcPV3.BI=FALSE} =>  {BcMV1.BI=FALSE} | 0.15  8416 | 0.271  186 | 0.584  158 | 1.28  8933 | 64 |
| {BcDMV1=FALSE, BcMV1.BI=FALSE} =>  {BcPV3.BI=FALSE} | 0.14  604 | 0.819  444 | 0.178  218 | 1.15  3504 | 59 |
| {BcFV9=FALSE, BcMV1.BI=FALSE} =>  {BcPV3.BI=FALSE} | 0.15  8416 | 0.831  169 | 0.190  594 | 1.17  0008 | 64 |
| {BcMV1.BI=FALSE, BcPV3.BI=FALSE} =>  {BcOLV4.BI=FALSE} | 0.17  3267 | 0.985  915 | 0.175  743 | 1.12  5169 | 70 |
| {BcMV1.BI=FALSE, BcOLV4.BI=FALSE} =>  {BcPV3.BI=FALSE} | 0.17  3267 | 0.853  659 | 0.202  97 | 1.20  1666 | 70 |
| {BcOLV4.BI=FALSE, BcPV3.BI=FALSE} =>  {BcMV1.BI=FALSE} | 0.17  3267 | 0.271  318 | 0.638  614 | 1.28  9558 | 70 |
| {BcMV1.BI=FALSE, BcPV3.BI=FALSE} =>  {BcULV5=FALSE} | 0.17  0792 | 0.971  831 | 0.175  743 | 1.10  9095 | 69 |
| {BcMV1.BI=FALSE, BcULV5=FALSE} =>  {BcPV3.BI=FALSE} | 0.17  0792 | 0.841  463 | 0.202  97 | 1.18  4499 | 69 |
| {BcPV3.BI=FALSE, BcULV5=FALSE} =>  {BcMV1.BI=FALSE} | 0.17  0792 | 0.267  442 | 0.638  614 | 1.27  1135 | 69 |
| {BcBNV5.BI=FALSE, BcMV1.BI=FALSE} => | 0.16 | 0.829 | 0.202 | 1.16 | 68 |

| {BcPV3.BI=FALSE} | 8317 | 268 | 97 | 7332 |  |
| --- | --- | --- | --- | --- | --- |
| {BcBNV5.BI=FALSE, BcPV3.BI=FALSE} =>  {BcMV1.BI=FALSE} | 0.16  8317 | 0.261  538 | 0.643  564 | 1.24  3077 | 68 |
| {BcMV1.BI=FALSE, BvF.BI=FALSE} =>  {BcPV3.BI=FALSE} | 0.15  099 | 0.824  324 | 0.183  168 | 1.16  0373 | 61 |
| {BcMV1.BI=FALSE, BcPV3.BI=FALSE} =>  {BcMCV5.BI=FALSE} | 0.17  5743 | 1 | 0.175  743 | 1.08  311 | 71 |
| {BcMCV5.BI=FALSE, BcMV1.BI=FALSE} =>  {BcPV3.BI=FALSE} | 0.17  5743 | 0.835  294 | 0.210  396 | 1.17  5815 | 71 |
| {BcMCV5.BI=FALSE, BcPV3.BI=FALSE} =>  {BcMV1.BI=FALSE} | 0.17  5743 | 0.266  917 | 0.658  416 | 1.26  8642 | 71 |
| {BcMV1.BI=FALSE, BcOLV13.BI=FALSE} =>  {BcPV3.BI=FALSE} | 0.17  3267 | 0.864  198 | 0.200  495 | 1.21  6501 | 70 |
| {BcOLV13.BI=FALSE, BcPV3.BI=FALSE} =>  {BcMV1.BI=FALSE} | 0.17  3267 | 0.255  474 | 0.678  218 | 1.21  4255 | 70 |
| {BcMV1.BI=FALSE, BcOLV2.BI=FALSE} =>  {BcPV3.BI=FALSE} | 0.16  5842 | 0.848  101 | 0.195  545 | 1.19  3843 | 67 |
| {BcMCV3.BI=FALSE, BcMV1.BI=FALSE} =>  {BcPV3.BI=FALSE} | 0.17  5743 | 0.865  854 | 0.202  97 | 1.21  8832 | 71 |
| {BcMCV3.BI=FALSE, BcPV3.BI=FALSE} =>  {BcMV1.BI=FALSE} | 0.17  5743 | 0.258  182 | 0.680  693 | 1.22  7123 | 71 |
| {BcMV1.BI=FALSE, BcPV4.BI=FALSE} => | 0.17 | 0.831 | 0.205 | 1.17 | 69 |

| {BcPV3.BI=FALSE} | 0792 | 325 | 446 | 0228 |  |
| --- | --- | --- | --- | --- | --- |
| {BcPV3.BI=FALSE, BcPV4.BI=FALSE} =>  {BcMV1.BI=FALSE} | 0.17  0792 | 0.258  427 | 0.660  891 | 1.22  8288 | 69 |
| {BcEV6=FALSE, BcMV1.BI=FALSE} =>  {BcPV3.BI=FALSE} | 0.16  3366 | 0.835  443 | 0.195  545 | 1.17  6024 | 66 |
| {BcMV1.BI=FALSE, BcOLV17.BI=FALSE} =>  {BcPV3.BI=FALSE} | 0.17  3267 | 0.843  373 | 0.205  446 | 1.18  7188 | 70 |
| {BcMV1.BI=FALSE, BcOLV15.BI=FALSE} =>  {BcPV3.BI=FALSE} | 0.17  0792 | 0.831  325 | 0.205  446 | 1.17  0228 | 69 |
| {BcOLV15.BI=FALSE, BcPV3.BI=FALSE} =>  {BcMV1.BI=FALSE} | 0.17  0792 | 0.256  506 | 0.665  842 | 1.21  9156 | 69 |
| {BcEV5=FALSE, BcMV1.BI=FALSE} =>  {BcPV3.BI=FALSE} | 0.17  0792 | 0.831  325 | 0.205  446 | 1.17  0228 | 69 |
| {BcEV5=FALSE, BcPV3.BI=FALSE} =>  {BcMV1.BI=FALSE} | 0.17  0792 | 0.253  676 | 0.673  267 | 1.20  5709 | 69 |
| {BcMV1.BI=FALSE, BcRV1.BI=FALSE} =>  {BcHV3.BI=FALSE} | 0.11  6337 | 0.854  545 | 0.136  139 | 1.12  4548 | 47 |
| {BcMV1.BI=FALSE, BcRV1.BI=FALSE} =>  {BcULV1.BI=FALSE} | 0.12  3762 | 0.909  091 | 0.136  139 | 1.15  8589 | 50 |
| {BcMV1.BI=FALSE, BcRV1.BI=FALSE} =>  {BcOLV7.BI=FALSE} | 0.12  8713 | 0.945  455 | 0.136  139 | 1.17  1668 | 52 |
| {BcMV1.BI=FALSE, BcRV1.BI=FALSE} => | 0.12 | 0.909 | 0.136 | 1.11 | 50 |

| {BcALV1.BI=FALSE} | 3762 | 091 | 139 | 633 |  |
| --- | --- | --- | --- | --- | --- |
| {BcMV1.BI=FALSE, BcRV1.BI=FALSE} =>  {BcBNV5.BI=FALSE} | 0.13  3663 | 0.981  818 | 0.136  139 | 1.08  0803 | 54 |
| {BcMV1.BI=FALSE, BcRV1.BI=FALSE} =>  {BcMCV5.BI=FALSE} | 0.13  6139 | 1 | 0.136  139 | 1.08  311 | 55 |
| {BcEV2.BI=FALSE, BcMV1.BI=FALSE} =>  {BcHV3.BI=FALSE} | 0.15  5941 | 0.887  324 | 0.175  743 | 1.16  7684 | 63 |
| {BcHV3.BI=FALSE, BcMV1.BI=FALSE} =>  {BcEV2.BI=FALSE} | 0.15  5941 | 0.851  351 | 0.183  168 | 1.13  8894 | 63 |
| {BcEV2.BI=FALSE, BcHV3.BI=FALSE} =>  {BcMV1.BI=FALSE} | 0.15  5941 | 0.261  411 | 0.596  535 | 1.24  247 | 63 |
| {BcFV5.BI=FALSE, BcMV1.BI=FALSE} =>  {BcEV2.BI=FALSE} | 0.14  604 | 0.842  857 | 0.173  267 | 1.12  7531 | 59 |
| {BcEV2.BI=FALSE, BcMV1.BI=FALSE} =>  {BcULV1.BI=FALSE} | 0.15  3465 | 0.873  239 | 0.175  743 | 1.11  2898 | 62 |
| {BcMV1.BI=FALSE, BcULV1.BI=FALSE} =>  {BcEV2.BI=FALSE} | 0.15  3465 | 0.837  838 | 0.183  168 | 1.12  0816 | 62 |
| {BcEV2.BI=FALSE, BcULV1.BI=FALSE} =>  {BcMV1.BI=FALSE} | 0.15  3465 | 0.260  504 | 0.589  109 | 1.23  8161 | 62 |
| {BcMV1.BI=FALSE, BcNSRV7.BI=FALSE}  => {BcEV2.BI=FALSE} | 0.15  099 | 0.859  155 | 0.175  743 | 1.14  9333 | 61 |
| {BcMV1.BI=FALSE, BcVV3.BI=FALSE} => | 0.15 | 0.873 | 0.175 | 1.16 | 62 |

| {BcEV2.BI=FALSE} | 3465 | 239 | 743 | 8175 |  |
| --- | --- | --- | --- | --- | --- |
| {BcEV2.BI=FALSE, BcOLV7.BI=FALSE} =>  {BcMV1.BI=FALSE} | 0.15  3465 | 0.259  414 | 0.591  584 | 1.23  2981 | 62 |
| {BcALV1.BI=FALSE, BcMV1.BI=FALSE} =>  {BcEV2.BI=FALSE} | 0.15  3465 | 0.837  838 | 0.183  168 | 1.12  0816 | 62 |
| {BcFV9=FALSE, BcMV1.BI=FALSE} =>  {BcEV2.BI=FALSE} | 0.16  3366 | 0.857  143 | 0.190  594 | 1.14  6641 | 66 |
| {BcEV2.BI=FALSE, BcMV1.BI=FALSE} =>  {BcOLV4.BI=FALSE} | 0.16  8317 | 0.957  746 | 0.175  743 | 1.09  3021 | 68 |
| {BcEV2.BI=FALSE, BcOLV4.BI=FALSE} =>  {BcMV1.BI=FALSE} | 0.16  8317 | 0.252  788 | 0.665  842 | 1.20  1487 | 68 |
| {BcEV2.BI=FALSE, BcMV1.BI=FALSE} =>  {BcULV5=FALSE} | 0.17  0792 | 0.971  831 | 0.175  743 | 1.10  9095 | 69 |
| {BcMV1.BI=FALSE, BcULV5=FALSE} =>  {BcEV2.BI=FALSE} | 0.17  0792 | 0.841  463 | 0.202  97 | 1.12  5666 | 69 |
| {BcEV2.BI=FALSE, BcULV5=FALSE} =>  {BcMV1.BI=FALSE} | 0.17  0792 | 0.260  377 | 0.655  941 | 1.23  7558 | 69 |
| {BcBNV5.BI=FALSE, BcMV1.BI=FALSE} =>  {BcEV2.BI=FALSE} | 0.17  0792 | 0.841  463 | 0.202  97 | 1.12  5666 | 69 |
| {BcMV1.BI=FALSE, BvF.BI=FALSE} =>  {BcEV2.BI=FALSE} | 0.15  5941 | 0.851  351 | 0.183  168 | 1.13  8894 | 63 |
| {BcEV2.BI=FALSE, BcMV1.BI=FALSE} => | 0.17 | 1 | 0.175 | 1.08 | 71 |

| {BcMCV5.BI=FALSE} | 5743 |  | 743 | 311 |  |
| --- | --- | --- | --- | --- | --- |
| {BcMCV5.BI=FALSE, BcMV1.BI=FALSE} =>  {BcEV2.BI=FALSE} | 0.17  5743 | 0.835  294 | 0.210  396 | 1.11  7413 | 71 |
| {BcMV1.BI=FALSE, BcOLV2.BI=FALSE} =>  {BcEV2.BI=FALSE} | 0.16  3366 | 0.835  443 | 0.195  545 | 1.11  7613 | 66 |
| {BcEV6=FALSE, BcMV1.BI=FALSE} =>  {BcEV2.BI=FALSE} | 0.16  5842 | 0.848  101 | 0.195  545 | 1.13  4546 | 67 |
| {BcMV1.BI=FALSE, BcOLV15.BI=FALSE} =>  {BcEV2.BI=FALSE} | 0.17  3267 | 0.843  373 | 0.205  446 | 1.12  8221 | 70 |
| {BcFV5.BI=FALSE, BcMV1.BI=FALSE} =>  {BcHV3.BI=FALSE} | 0.15  3465 | 0.885  714 | 0.173  267 | 1.16  5565 | 62 |
| {BcHV3.BI=FALSE, BcMV1.BI=FALSE} =>  {BcULV1.BI=FALSE} | 0.16  0891 | 0.878  378 | 0.183  168 | 1.11  9448 | 65 |
| {BcMV1.BI=FALSE, BcULV1.BI=FALSE} =>  {BcHV3.BI=FALSE} | 0.16  0891 | 0.878  378 | 0.183  168 | 1.15  5912 | 65 |
| {BcHV3.BI=FALSE, BcULV1.BI=FALSE} =>  {BcMV1.BI=FALSE} | 0.16  0891 | 0.266  393 | 0.603  96 | 1.26  6152 | 65 |
| {BcMV1.BI=FALSE, BcMV12=FALSE} =>  {BcHV3.BI=FALSE} | 0.15  5941 | 0.875 | 0.178  218 | 1.15  1466 | 63 |
| {BcHV3.BI=FALSE, BcMV12=FALSE} =>  {BcMV1.BI=FALSE} | 0.15  5941 | 0.259  259 | 0.601  485 | 1.23  2244 | 63 |
| {BcMV1.BI=FALSE, BcNSRV7.BI=FALSE} | 0.15 | 0.873 | 0.175 | 1.14 | 62 |

| => {BcHV3.BI=FALSE} | 3465 | 239 | 743 | 9149 |  |
| --- | --- | --- | --- | --- | --- |
| {BcMV1.BI=FALSE, BcVV3.BI=FALSE} =>  {BcHV3.BI=FALSE} | 0.15  5941 | 0.887  324 | 0.175  743 | 1.16  7684 | 63 |
| {BcMV1.BI=FALSE, BcOLV7.BI=FALSE} =>  {BcHV3.BI=FALSE} | 0.16  0891 | 0.866  667 | 0.185  644 | 1.14  0499 | 65 |
| {BcALV1.BI=FALSE, BcMV1.BI=FALSE} =>  {BcHV3.BI=FALSE} | 0.16  0891 | 0.878  378 | 0.183  168 | 1.15  5912 | 65 |
| {BcDMV1=FALSE, BcMV1.BI=FALSE} =>  {BcHV3.BI=FALSE} | 0.15  8416 | 0.888  889 | 0.178  218 | 1.16  9743 | 64 |
| {BcFV9=FALSE, BcMV1.BI=FALSE} =>  {BcHV3.BI=FALSE} | 0.16  5842 | 0.870  13 | 0.190  594 | 1.14  5057 | 67 |
| {BcHV3.BI=FALSE, BcMV1.BI=FALSE} =>  {BcOLV4.BI=FALSE} | 0.18  0693 | 0.986  486 | 0.183  168 | 1.12  5821 | 73 |
| {BcMV1.BI=FALSE, BcOLV4.BI=FALSE} =>  {BcHV3.BI=FALSE} | 0.18  0693 | 0.890  244 | 0.202  97 | 1.17  1526 | 73 |
| {BcHV3.BI=FALSE, BcOLV4.BI=FALSE} =>  {BcMV1.BI=FALSE} | 0.18  0693 | 0.268  382 | 0.673  267 | 1.27  5606 | 73 |
| {BcHV3.BI=FALSE, BcMV1.BI=FALSE} =>  {BcULV5=FALSE} | 0.17  5743 | 0.959  459 | 0.183  168 | 1.09  4976 | 71 |
| {BcMV1.BI=FALSE, BcULV5=FALSE} =>  {BcHV3.BI=FALSE} | 0.17  5743 | 0.865  854 | 0.202  97 | 1.13  943 | 71 |
| {BcHV3.BI=FALSE, BcULV5=FALSE} => | 0.17 | 0.262 | 0.668 | 1.24 | 71 |

| {BcMV1.BI=FALSE} | 5743 | 963 | 317 | 9847 |  |
| --- | --- | --- | --- | --- | --- |
| {BcBNV5.BI=FALSE, BcMV1.BI=FALSE} =>  {BcHV3.BI=FALSE} | 0.17  5743 | 0.865  854 | 0.202  97 | 1.13  943 | 71 |
| {BcMV1.BI=FALSE, BvF.BI=FALSE} =>  {BcHV3.BI=FALSE} | 0.16  3366 | 0.891  892 | 0.183  168 | 1.17  3695 | 66 |
| {BcHV3.BI=FALSE, BcMV1.BI=FALSE} =>  {BcMCV5.BI=FALSE} | 0.18  3168 | 1 | 0.183  168 | 1.08  311 | 74 |
| {BcMCV5.BI=FALSE, BcMV1.BI=FALSE} =>  {BcHV3.BI=FALSE} | 0.18  3168 | 0.870  588 | 0.210  396 | 1.14  566 | 74 |
| {BcMV1.BI=FALSE, BcOLV13.BI=FALSE} =>  {BcHV3.BI=FALSE} | 0.17  3267 | 0.864  198 | 0.200  495 | 1.13  725 | 70 |
| {BcMV1.BI=FALSE, BcOLV2.BI=FALSE} =>  {BcHV3.BI=FALSE} | 0.16  8317 | 0.860  759 | 0.195  545 | 1.13  2726 | 68 |
| {BcMCV3.BI=FALSE, BcMV1.BI=FALSE} =>  {BcHV3.BI=FALSE} | 0.17  5743 | 0.865  854 | 0.202  97 | 1.13  943 | 71 |
| {BcHV3.BI=FALSE, BcMCV3.BI=FALSE} =>  {BcMV1.BI=FALSE} | 0.17  5743 | 0.252  669 | 0.695  545 | 1.20  0921 | 71 |
| {BcMV1.BI=FALSE, BcPV4.BI=FALSE} =>  {BcHV3.BI=FALSE} | 0.17  8218 | 0.867  47 | 0.205  446 | 1.14  1556 | 72 |
| {BcHV3.BI=FALSE, BcPV4.BI=FALSE} =>  {BcMV1.BI=FALSE} | 0.17  8218 | 0.252  632 | 0.705  446 | 1.20  0743 | 72 |
| {BcEV6=FALSE, BcMV1.BI=FALSE} => | 0.17 | 0.873 | 0.195 | 1.14 | 69 |

| {BcHV3.BI=FALSE} | 0792 | 418 | 545 | 9384 |  |
| --- | --- | --- | --- | --- | --- |
| {BcMV1.BI=FALSE, BcOLV17.BI=FALSE} =>  {BcHV3.BI=FALSE} | 0.17  8218 | 0.867  47 | 0.205  446 | 1.14  1556 | 72 |
| {BcMV1.BI=FALSE, BcOLV15.BI=FALSE} =>  {BcHV3.BI=FALSE} | 0.17  8218 | 0.867  47 | 0.205  446 | 1.14  1556 | 72 |
| {BcEV5=FALSE, BcMV1.BI=FALSE} =>  {BcHV3.BI=FALSE} | 0.17  8218 | 0.867  47 | 0.205  446 | 1.14  1556 | 72 |
| {BcFV5.BI=FALSE, BcMV1.BI=FALSE} =>  {BcULV1.BI=FALSE} | 0.15  099 | 0.871  429 | 0.173  267 | 1.11  059 | 61 |
| {BcFV5.BI=FALSE, BcMV1.BI=FALSE} =>  {BcOLV4.BI=FALSE} | 0.16  5842 | 0.957  143 | 0.173  267 | 1.09  2333 | 67 |
| {BcFV5.BI=FALSE, BcMV1.BI=FALSE} =>  {BcULV5=FALSE} | 0.16  8317 | 0.971  429 | 0.173  267 | 1.10  8636 | 68 |
| {BcFV5.BI=FALSE, BcMV1.BI=FALSE} =>  {BcMCV5.BI=FALSE} | 0.17  3267 | 1 | 0.173  267 | 1.08  311 | 70 |
| {BcMV1.BI=FALSE, BcVV3.BI=FALSE} =>  {BcULV1.BI=FALSE} | 0.15  3465 | 0.873  239 | 0.175  743 | 1.11  2898 | 62 |
| {BcMV1.BI=FALSE, BcULV1.BI=FALSE} =>  {BcOLV7.BI=FALSE} | 0.16  5842 | 0.905  405 | 0.183  168 | 1.12  2036 | 67 |
| {BcMV1.BI=FALSE, BcOLV7.BI=FALSE} =>  {BcULV1.BI=FALSE} | 0.16  5842 | 0.893  333 | 0.185  644 | 1.13  8507 | 67 |
| {BcOLV7.BI=FALSE, BcULV1.BI=FALSE} => | 0.16 | 0.254 | 0.650 | 1.21 | 67 |

| {BcMV1.BI=FALSE} | 5842 | 753 | 99 | 0825 |  |
| --- | --- | --- | --- | --- | --- |
| {BcMV1.BI=FALSE, BcULV1.BI=FALSE} =>  {BcALV1.BI=FALSE} | 0.16  3366 | 0.891  892 | 0.183  168 | 1.09  5211 | 66 |
| {BcALV1.BI=FALSE, BcMV1.BI=FALSE} =>  {BcULV1.BI=FALSE} | 0.16  3366 | 0.891  892 | 0.183  168 | 1.13  667 | 66 |
| {BcALV1.BI=FALSE, BcULV1.BI=FALSE} =>  {BcMV1.BI=FALSE} | 0.16  3366 | 0.251  908 | 0.648  515 | 1.19  7306 | 66 |
| {BcMV1.BI=FALSE, BcULV1.BI=FALSE} =>  {BcFV9=FALSE} | 0.17  3267 | 0.945  946 | 0.183  168 | 1.08  8781 | 70 |
| {BcFV9=FALSE, BcMV1.BI=FALSE} =>  {BcULV1.BI=FALSE} | 0.17  3267 | 0.909  091 | 0.190  594 | 1.15  8589 | 70 |
| {BcFV9=FALSE, BcULV1.BI=FALSE} =>  {BcMV1.BI=FALSE} | 0.17  3267 | 0.255  474 | 0.678  218 | 1.21  4255 | 70 |
| {BcMV1.BI=FALSE, BcULV1.BI=FALSE} =>  {BcOLV4.BI=FALSE} | 0.17  5743 | 0.959  459 | 0.183  168 | 1.09  4976 | 71 |
| {BcOLV4.BI=FALSE, BcULV1.BI=FALSE} =>  {BcMV1.BI=FALSE} | 0.17  5743 | 0.252  669 | 0.695  545 | 1.20  0921 | 71 |
| {BcMV1.BI=FALSE, BcULV1.BI=FALSE} =>  {BcULV5=FALSE} | 0.18  0693 | 0.986  486 | 0.183  168 | 1.12  5821 | 73 |
| {BcMV1.BI=FALSE, BcULV5=FALSE} =>  {BcULV1.BI=FALSE} | 0.18  0693 | 0.890  244 | 0.202  97 | 1.13  457 | 73 |
| {BcULV1.BI=FALSE, BcULV5=FALSE} => | 0.18 | 0.257 | 0.700 | 1.22 | 73 |

| {BcMV1.BI=FALSE} | 0693 | 951 | 495 | 6024 |  |
| --- | --- | --- | --- | --- | --- |
| {BcBNV5.BI=FALSE, BcMV1.BI=FALSE} =>  {BcULV1.BI=FALSE} | 0.17  8218 | 0.878  049 | 0.202  97 | 1.11  9027 | 72 |
| {BcMV1.BI=FALSE, BcULV1.BI=FALSE} =>  {BcMCV5.BI=FALSE} | 0.18  3168 | 1 | 0.183  168 | 1.08  311 | 74 |
| {BcMCV5.BI=FALSE, BcMV1.BI=FALSE} =>  {BcULV1.BI=FALSE} | 0.18  3168 | 0.870  588 | 0.210  396 | 1.10  9519 | 74 |
| {BcMV1.BI=FALSE, BcOLV13.BI=FALSE} =>  {BcULV1.BI=FALSE} | 0.17  8218 | 0.888  889 | 0.200  495 | 1.13  2843 | 72 |
| {BcMV1.BI=FALSE, BcOLV2.BI=FALSE} =>  {BcULV1.BI=FALSE} | 0.17  3267 | 0.886  076 | 0.195  545 | 1.12  9258 | 70 |
| {BcMCV3.BI=FALSE, BcMV1.BI=FALSE} =>  {BcULV1.BI=FALSE} | 0.18  0693 | 0.890  244 | 0.202  97 | 1.13  457 | 73 |
| {BcMV1.BI=FALSE, BcPV4.BI=FALSE} =>  {BcULV1.BI=FALSE} | 0.18  0693 | 0.879  518 | 0.205  446 | 1.12  09 | 73 |
| {BcEV6=FALSE, BcMV1.BI=FALSE} =>  {BcULV1.BI=FALSE} | 0.17  3267 | 0.886  076 | 0.195  545 | 1.12  9258 | 70 |
| {BcMV1.BI=FALSE, BcOLV17.BI=FALSE} =>  {BcULV1.BI=FALSE} | 0.18  3168 | 0.891  566 | 0.205  446 | 1.13  6255 | 74 |
| {BcMV1.BI=FALSE, BcOLV15.BI=FALSE} =>  {BcULV1.BI=FALSE} | 0.17  8218 | 0.867  47 | 0.205  446 | 1.10  5545 | 72 |
| {BcEV5=FALSE, BcMV1.BI=FALSE} => | 0.17 | 0.867 | 0.205 | 1.10 | 72 |

| {BcULV1.BI=FALSE} | 8218 | 47 | 446 | 5545 |  |
| --- | --- | --- | --- | --- | --- |
| {BcMV1.BI=FALSE, BcMV12=FALSE} =>  {BcOLV4.BI=FALSE} | 0.17  3267 | 0.972  222 | 0.178  218 | 1.10  9542 | 70 |
| {BcMV1.BI=FALSE, BcMV12=FALSE} =>  {BcULV5=FALSE} | 0.17  3267 | 0.972  222 | 0.178  218 | 1.10  9542 | 70 |
| {BcMV1.BI=FALSE, BcMV12=FALSE} =>  {BcMCV5.BI=FALSE} | 0.17  8218 | 1 | 0.178  218 | 1.08  311 | 72 |
| {BcMV1.BI=FALSE, BcNSRV7.BI=FALSE}  => {BcOLV4.BI=FALSE} | 0.16  8317 | 0.957  746 | 0.175  743 | 1.09  3021 | 68 |
| {BcMV1.BI=FALSE, BcNSRV7.BI=FALSE}  => {BcULV5=FALSE} | 0.16  8317 | 0.957  746 | 0.175  743 | 1.09  3021 | 68 |
| {BcMV1.BI=FALSE, BcNSRV7.BI=FALSE}  => {BcMCV5.BI=FALSE} | 0.17  5743 | 1 | 0.175  743 | 1.08  311 | 71 |
| {BcMV1.BI=FALSE, BcVV3.BI=FALSE} =>  {BcOLV4.BI=FALSE} | 0.16  8317 | 0.957  746 | 0.175  743 | 1.09  3021 | 68 |
| {BcMV1.BI=FALSE, BcVV3.BI=FALSE} =>  {BcULV5=FALSE} | 0.16  8317 | 0.957  746 | 0.175  743 | 1.09  3021 | 68 |
| {BcMV1.BI=FALSE, BcVV3.BI=FALSE} =>  {BcMCV5.BI=FALSE} | 0.17  5743 | 1 | 0.175  743 | 1.08  311 | 71 |
| {BcMV1.BI=FALSE, BcOLV7.BI=FALSE} =>  {BcALV1.BI=FALSE} | 0.16  8317 | 0.906  667 | 0.185  644 | 1.11  3354 | 68 |
| {BcALV1.BI=FALSE, BcMV1.BI=FALSE} => | 0.16 | 0.918 | 0.183 | 1.13 | 68 |

| {BcOLV7.BI=FALSE} | 8317 | 919 | 168 | 8783 |  |
| --- | --- | --- | --- | --- | --- |
| {BcFV9=FALSE, BcMV1.BI=FALSE} =>  {BcOLV7.BI=FALSE} | 0.17  0792 | 0.896  104 | 0.190  594 | 1.11  0509 | 69 |
| {BcMV1.BI=FALSE, BcOLV7.BI=FALSE} =>  {BcOLV4.BI=FALSE} | 0.17  8218 | 0.96 | 0.185  644 | 1.09  5593 | 72 |
| {BcMV1.BI=FALSE, BcOLV7.BI=FALSE} =>  {BcULV5=FALSE} | 0.18  0693 | 0.973  333 | 0.185  644 | 1.11  081 | 73 |
| {BcMV1.BI=FALSE, BcULV5=FALSE} =>  {BcOLV7.BI=FALSE} | 0.18  0693 | 0.890  244 | 0.202  97 | 1.10  3247 | 73 |
| {BcOLV7.BI=FALSE, BcULV5=FALSE} =>  {BcMV1.BI=FALSE} | 0.18  0693 | 0.257  951 | 0.700  495 | 1.22  6024 | 73 |
| {BcMV1.BI=FALSE, BcOLV7.BI=FALSE} =>  {BcMCV5.BI=FALSE} | 0.18  5644 | 1 | 0.185  644 | 1.08  311 | 75 |
| {BcMV1.BI=FALSE, BcOLV13.BI=FALSE} =>  {BcOLV7.BI=FALSE} | 0.17  8218 | 0.888  889 | 0.200  495 | 1.10  1568 | 72 |
| {BcMCV3.BI=FALSE, BcMV1.BI=FALSE} =>  {BcOLV7.BI=FALSE} | 0.18  0693 | 0.890  244 | 0.202  97 | 1.10  3247 | 73 |
| {BcMV1.BI=FALSE, BcPV4.BI=FALSE} =>  {BcOLV7.BI=FALSE} | 0.18  3168 | 0.891  566 | 0.205  446 | 1.10  4886 | 74 |
| {BcEV6=FALSE, BcMV1.BI=FALSE} =>  {BcOLV7.BI=FALSE} | 0.17  3267 | 0.886  076 | 0.195  545 | 1.09  8082 | 70 |
| {BcALV1.BI=FALSE, BcMV1.BI=FALSE} => | 0.17 | 0.932 | 0.183 | 1.07 | 69 |

| {BcFV9=FALSE} | 0792 | 432 | 168 | 3227 |  |
| --- | --- | --- | --- | --- | --- |
| {BcFV9=FALSE, BcMV1.BI=FALSE} =>  {BcALV1.BI=FALSE} | 0.17  0792 | 0.896  104 | 0.190  594 | 1.10  0383 | 69 |
| {BcALV1.BI=FALSE, BcMV1.BI=FALSE} =>  {BcOLV4.BI=FALSE} | 0.17  8218 | 0.972  973 | 0.183  168 | 1.11  0399 | 72 |
| {BcALV1.BI=FALSE, BcMV1.BI=FALSE} =>  {BcULV5=FALSE} | 0.17  8218 | 0.972  973 | 0.183  168 | 1.11  0399 | 72 |
| {BcALV1.BI=FALSE, BcULV5=FALSE} =>  {BcMV1.BI=FALSE} | 0.17  8218 | 0.252  632 | 0.705  446 | 1.20  0743 | 72 |
| {BcALV1.BI=FALSE, BcMV1.BI=FALSE} =>  {BcMCV5.BI=FALSE} | 0.18  3168 | 1 | 0.183  168 | 1.08  311 | 74 |
| {BcDMV1=FALSE, BcMV1.BI=FALSE} =>  {BcOLV4.BI=FALSE} | 0.17  0792 | 0.958  333 | 0.178  218 | 1.09  3691 | 69 |
| {BcDMV1=FALSE, BcMV1.BI=FALSE} =>  {BcULV5=FALSE} | 0.17  0792 | 0.958  333 | 0.178  218 | 1.09  3691 | 69 |
| {BcDMV1=FALSE, BcMV1.BI=FALSE} =>  {BcMCV5.BI=FALSE} | 0.17  8218 | 1 | 0.178  218 | 1.08  311 | 72 |
| {BcFV9=FALSE, BcMV1.BI=FALSE} =>  {BcOLV4.BI=FALSE} | 0.18  3168 | 0.961  039 | 0.190  594 | 1.09  6779 | 74 |
| {BcFV9=FALSE, BcMV1.BI=FALSE} =>  {BcULV5=FALSE} | 0.18  5644 | 0.974  026 | 0.190  594 | 1.11  16 | 75 |
| {BcFV9=FALSE, BcMV1.BI=FALSE} => | 0.19 | 1 | 0.190 | 1.08 | 77 |

| {BcMCV5.BI=FALSE} | 0594 |  | 594 | 311 |  |
| --- | --- | --- | --- | --- | --- |
| {BcMV1.BI=FALSE, BcOLV4.BI=FALSE} =>  {BcULV5=FALSE} | 0.19  5545 | 0.963  415 | 0.202  97 | 1.09  949 | 79 |
| {BcMV1.BI=FALSE, BcULV5=FALSE} =>  {BcOLV4.BI=FALSE} | 0.19  5545 | 0.963  415 | 0.202  97 | 1.09  949 | 79 |
| {BcOLV4.BI=FALSE, BcULV5=FALSE} =>  {BcMV1.BI=FALSE} | 0.19  5545 | 0.249  211 | 0.784  653 | 1.18  4487 | 79 |
| {BcBNV5.BI=FALSE, BcMV1.BI=FALSE} =>  {BcOLV4.BI=FALSE} | 0.19  5545 | 0.963  415 | 0.202  97 | 1.09  949 | 79 |
| {BcBNV5.BI=FALSE, BcOLV4.BI=FALSE} =>  {BcMV1.BI=FALSE} | 0.19  5545 | 0.247  649 | 0.789  604 | 1.17  7061 | 79 |
| {BcMV1.BI=FALSE, BvF.BI=FALSE} =>  {BcOLV4.BI=FALSE} | 0.18  0693 | 0.986  486 | 0.183  168 | 1.12  5821 | 73 |
| {BcMV1.BI=FALSE, BcOLV4.BI=FALSE} =>  {BcMCV5.BI=FALSE} | 0.20  297 | 1 | 0.202  97 | 1.08  311 | 82 |
| {BcMCV5.BI=FALSE, BcMV1.BI=FALSE} =>  {BcOLV4.BI=FALSE} | 0.20  297 | 0.964  706 | 0.210  396 | 1.10  0964 | 82 |
| {BcMV1.BI=FALSE, BcOLV13.BI=FALSE} =>  {BcOLV4.BI=FALSE} | 0.19  3069 | 0.962  963 | 0.200  495 | 1.09  8975 | 78 |
| {BcMV1.BI=FALSE, BcOLV2.BI=FALSE} =>  {BcOLV4.BI=FALSE} | 0.19  0594 | 0.974  684 | 0.195  545 | 1.11  2351 | 77 |
| {BcMCV3.BI=FALSE, BcMV1.BI=FALSE} => | 0.19 | 0.975 | 0.202 | 1.11 | 80 |

| {BcOLV4.BI=FALSE} | 802 | 61 | 97 | 3408 |  |
| --- | --- | --- | --- | --- | --- |
| {BcMV1.BI=FALSE, BcPV4.BI=FALSE} =>  {BcOLV4.BI=FALSE} | 0.19  802 | 0.963  855 | 0.205  446 | 1.09  9993 | 80 |
| {BcEV6=FALSE, BcMV1.BI=FALSE} =>  {BcOLV4.BI=FALSE} | 0.18  8119 | 0.962  025 | 0.195  545 | 1.09  7905 | 76 |
| {BcMV1.BI=FALSE, BcOLV17.BI=FALSE} =>  {BcOLV4.BI=FALSE} | 0.19  802 | 0.963  855 | 0.205  446 | 1.09  9993 | 80 |
| {BcMV1.BI=FALSE, BcOLV15.BI=FALSE} =>  {BcOLV4.BI=FALSE} | 0.19  802 | 0.963  855 | 0.205  446 | 1.09  9993 | 80 |
| {BcEV5=FALSE, BcMV1.BI=FALSE} =>  {BcOLV4.BI=FALSE} | 0.19  802 | 0.963  855 | 0.205  446 | 1.09  9993 | 80 |
| {BcBNV5.BI=FALSE, BcMV1.BI=FALSE} =>  {BcULV5=FALSE} | 0.19  5545 | 0.963  415 | 0.202  97 | 1.09  949 | 79 |
| {BcBNV5.BI=FALSE, BcULV5=FALSE} =>  {BcMV1.BI=FALSE} | 0.19  5545 | 0.247  649 | 0.789  604 | 1.17  7061 | 79 |
| {BcMV1.BI=FALSE, BvF.BI=FALSE} =>  {BcULV5=FALSE} | 0.17  8218 | 0.972  973 | 0.183  168 | 1.11  0399 | 72 |
| {BcMV1.BI=FALSE, BcULV5=FALSE} =>  {BcMCV5.BI=FALSE} | 0.20  297 | 1 | 0.202  97 | 1.08  311 | 82 |
| {BcMCV5.BI=FALSE, BcMV1.BI=FALSE} =>  {BcULV5=FALSE} | 0.20  297 | 0.964  706 | 0.210  396 | 1.10  0964 | 82 |
| {BcMCV5.BI=FALSE, BcULV5=FALSE} => | 0.20 | 0.249 | 0.814 | 1.18 | 82 |

| {BcMV1.BI=FALSE} | 297 | 24 | 356 | 4624 |  |
| --- | --- | --- | --- | --- | --- |
| {BcMV1.BI=FALSE, BcOLV13.BI=FALSE} =>  {BcULV5=FALSE} | 0.19  5545 | 0.975  309 | 0.200  495 | 1.11  3064 | 79 |
| {BcMV1.BI=FALSE, BcOLV2.BI=FALSE} =>  {BcULV5=FALSE} | 0.18  8119 | 0.962  025 | 0.195  545 | 1.09  7905 | 76 |
| {BcMCV3.BI=FALSE, BcMV1.BI=FALSE} =>  {BcULV5=FALSE} | 0.19  5545 | 0.963  415 | 0.202  97 | 1.09  949 | 79 |
| {BcMV1.BI=FALSE, BcPV4.BI=FALSE} =>  {BcULV5=FALSE} | 0.19  802 | 0.963  855 | 0.205  446 | 1.09  9993 | 80 |
| {BcEV6=FALSE, BcMV1.BI=FALSE} =>  {BcULV5=FALSE} | 0.18  8119 | 0.962  025 | 0.195  545 | 1.09  7905 | 76 |
| {BcMV1.BI=FALSE, BcOLV17.BI=FALSE} =>  {BcULV5=FALSE} | 0.19  802 | 0.963  855 | 0.205  446 | 1.09  9993 | 80 |
| {BcMV1.BI=FALSE, BcOLV15.BI=FALSE} =>  {BcULV5=FALSE} | 0.19  802 | 0.963  855 | 0.205  446 | 1.09  9993 | 80 |
| {BcEV5=FALSE, BcMV1.BI=FALSE} =>  {BcULV5=FALSE} | 0.19  802 | 0.963  855 | 0.205  446 | 1.09  9993 | 80 |
| {BcBNV5.BI=FALSE, BcMV1.BI=FALSE} =>  {BcMCV5.BI=FALSE} | 0.20  297 | 1 | 0.202  97 | 1.08  311 | 82 |
| {BcMV1.BI=FALSE, BvF.BI=FALSE} =>  {BcMCV5.BI=FALSE} | 0.18  3168 | 1 | 0.183  168 | 1.08  311 | 74 |
| {BcMV1.BI=FALSE, BcOLV13.BI=FALSE} => | 0.20 | 1 | 0.200 | 1.08 | 81 |

| {BcMCV5.BI=FALSE} | 0495 |  | 495 | 311 |  |
| --- | --- | --- | --- | --- | --- |
| {BcMV1.BI=FALSE, BcOLV2.BI=FALSE} =>  {BcMCV5.BI=FALSE} | 0.19  5545 | 1 | 0.195  545 | 1.08  311 | 79 |
| {BcMCV3.BI=FALSE, BcMV1.BI=FALSE} =>  {BcMCV5.BI=FALSE} | 0.20  297 | 1 | 0.202  97 | 1.08  311 | 82 |
| {BcMV1.BI=FALSE, BcPV4.BI=FALSE} =>  {BcMCV5.BI=FALSE} | 0.20  5446 | 1 | 0.205  446 | 1.08  311 | 83 |
| {BcEV6=FALSE, BcMV1.BI=FALSE} =>  {BcMCV5.BI=FALSE} | 0.19  5545 | 1 | 0.195  545 | 1.08  311 | 79 |
| {BcMV1.BI=FALSE, BcOLV17.BI=FALSE} =>  {BcMCV5.BI=FALSE} | 0.20  5446 | 1 | 0.205  446 | 1.08  311 | 83 |
| {BcMV1.BI=FALSE, BcOLV15.BI=FALSE} =>  {BcMCV5.BI=FALSE} | 0.20  5446 | 1 | 0.205  446 | 1.08  311 | 83 |
| {BcEV5=FALSE, BcMV1.BI=FALSE} =>  {BcMCV5.BI=FALSE} | 0.20  5446 | 1 | 0.205  446 | 1.08  311 | 83 |
| {BcRV1.BI=TRUE, BcULV1.BI=TRUE} =>  {BcMV9.BI=TRUE} | 0.07  6733 | 1 | 0.076  733 | 1.47  4453 | 31 |
| {BcMV9.BI=TRUE, BcULV1.BI=TRUE} =>  {BcRV1.BI=TRUE} | 0.07  6733 | 0.430  556 | 0.178  218 | 1.55  3075 | 31 |
| {BcMV9.BI=TRUE, BcRV1.BI=TRUE} =>  {BcULV1.BI=TRUE} | 0.07  6733 | 0.344  444 | 0.222  772 | 1.59  9489 | 31 |
| {BcRV1.BI=TRUE, BcULV1.BI=TRUE} => | 0.07 | 1 | 0.076 | 1.44 | 31 |

| {BcULV4=TRUE} | 6733 |  | 733 | 8029 |  |
| --- | --- | --- | --- | --- | --- |
| {BcULV1.BI=TRUE, BcULV4=TRUE} =>  {BcRV1.BI=TRUE} | 0.07  6733 | 0.397  436 | 0.193  069 | 1.43  3608 | 31 |
| {BcRV1.BI=TRUE, BcULV4=TRUE} =>  {BcULV1.BI=TRUE} | 0.07  6733 | 0.316  327 | 0.242  574 | 1.46  8919 | 31 |
| {BcRV1.BI=TRUE, BcULV1.BI=TRUE} =>  {BcDMV1=FALSE} | 0.07  4257 | 0.967  742 | 0.076  733 | 1.14  6533 | 30 |
| {BcDMV1=FALSE, BcULV1.BI=TRUE} =>  {BcRV1.BI=TRUE} | 0.07  4257 | 0.379  747 | 0.195  545 | 1.36  9801 | 30 |
| {BcDMV1=FALSE, BcRV1.BI=TRUE} =>  {BcULV1.BI=TRUE} | 0.07  4257 | 0.288  462 | 0.257  426 | 1.33  9523 | 30 |
| {BcBNV5.BI=FALSE, BcULV1.BI=TRUE} =>  {BcRV1.BI=TRUE} | 0.07  4257 | 0.379  747 | 0.195  545 | 1.36  9801 | 30 |
| {BcBNV5.BI=FALSE, BcRV1.BI=TRUE} =>  {BcULV1.BI=TRUE} | 0.07  4257 | 0.285  714 | 0.259  901 | 1.32  6765 | 30 |
| {BcULV1.BI=TRUE, BvF.BI=FALSE} =>  {BcRV1.BI=TRUE} | 0.07  4257 | 0.370  37 | 0.200  495 | 1.33  5979 | 30 |
| {BcOLV15.BI=FALSE, BcULV1.BI=TRUE} =>  {BcRV1.BI=TRUE} | 0.07  6733 | 0.364  706 | 0.210  396 | 1.31  5546 | 31 |
| {BcEV5=FALSE, BcULV1.BI=TRUE} =>  {BcRV1.BI=TRUE} | 0.07  6733 | 0.369  048 | 0.207  921 | 1.33  1207 | 31 |
| {BcMV6.BI=TRUE, BcULV1.BI=TRUE} => | 0.07 | 0.810 | 0.091 | 1.49 | 30 |

| {BcHV1SLR.BI=TRUE} | 4257 | 811 | 584 | 5742 |  |
| --- | --- | --- | --- | --- | --- |
| {BcHV1SLR.BI=TRUE, BcULV1.BI=TRUE}  => {BcMV6.BI=TRUE} | 0.07  4257 | 0.468  75 | 0.158  416 | 1.53  9634 | 30 |
| {BcHV1SLR.BI=TRUE, BcMV6.BI=TRUE} =>  {BcULV1.BI=TRUE} | 0.07  4257 | 0.361  446 | 0.205  446 | 1.67  8438 | 30 |
| {BcMV6.BI=TRUE, BcULV1.BI=TRUE} =>  {BcMV3.BI=TRUE} | 0.07  9208 | 0.864  865 | 0.091  584 | 1.59  5458 | 32 |
| {BcMV3.BI=TRUE, BcULV1.BI=TRUE} =>  {BcMV6.BI=TRUE} | 0.07  9208 | 0.524  59 | 0.150  99 | 1.72  3044 | 32 |
| {BcMV3.BI=TRUE, BcMV6.BI=TRUE} =>  {BcULV1.BI=TRUE} | 0.07  9208 | 0.355  556 | 0.222  772 | 1.65  1086 | 32 |
| {BcMV6.BI=TRUE, BcULV1.BI=TRUE} =>  {BcHV1.BI=TRUE} | 0.07  9208 | 0.864  865 | 0.091  584 | 1.43  1989 | 32 |
| {BcHV1.BI=TRUE, BcULV1.BI=TRUE} =>  {BcMV6.BI=TRUE} | 0.07  9208 | 0.450  704 | 0.175  743 | 1.48  0362 | 32 |
| {BcHV1.BI=TRUE, BcMV6.BI=TRUE} =>  {BcULV1.BI=TRUE} | 0.07  9208 | 0.347  826 | 0.227  723 | 1.61  5192 | 32 |
| {BcMV6.BI=TRUE, BcULV1.BI=TRUE} =>  {BcMV11=TRUE} | 0.07  4257 | 0.810  811 | 0.091  584 | 1.31  5532 | 30 |
| {BcMV11=TRUE, BcULV1.BI=TRUE} =>  {BcMV6.BI=TRUE} | 0.07  4257 | 0.517  241 | 0.143  564 | 1.69  8907 | 30 |
| {BcMV11=TRUE, BcMV6.BI=TRUE} => | 0.07 | 0.326 | 0.227 | 1.51 | 30 |

| {BcULV1.BI=TRUE} | 4257 | 087 | 723 | 4243 |  |
| --- | --- | --- | --- | --- | --- |
| {BcMV6.BI=TRUE, BcULV1.BI=TRUE} =>  {BcMV9.BI=TRUE} | 0.07  9208 | 0.864  865 | 0.091  584 | 1.27  5202 | 32 |
| {BcMV9.BI=TRUE, BcULV1.BI=TRUE} =>  {BcMV6.BI=TRUE} | 0.07  9208 | 0.444  444 | 0.178  218 | 1.45  9801 | 32 |
| {BcMV6.BI=TRUE, BcMV9.BI=TRUE} =>  {BcULV1.BI=TRUE} | 0.07  9208 | 0.336  842 | 0.235  149 | 1.56  4186 | 32 |
| {BcMV6.BI=TRUE, BcULV1.BI=TRUE} =>  {BcULV4=TRUE} | 0.08  4158 | 0.918  919 | 0.091  584 | 1.33  0621 | 34 |
| {BcULV1.BI=TRUE, BcULV4=TRUE} =>  {BcMV6.BI=TRUE} | 0.08  4158 | 0.435  897 | 0.193  069 | 1.43  1728 | 34 |
| {BcMV6.BI=TRUE, BcULV4=TRUE} =>  {BcULV1.BI=TRUE} | 0.08  4158 | 0.346  939 | 0.242  574 | 1.61  1072 | 34 |
| {BcMV6.BI=TRUE, BcULV1.BI=TRUE} =>  {BcMV1.BI=TRUE} | 0.08  1683 | 0.891  892 | 0.091  584 | 1.12  9543 | 33 |
| {BcMV1.BI=TRUE, BcULV1.BI=TRUE} =>  {BcMV6.BI=TRUE} | 0.08  1683 | 0.434  211 | 0.188  119 | 1.42  6187 | 33 |
| {BcMV1.BI=TRUE, BcMV6.BI=TRUE} =>  {BcULV1.BI=TRUE} | 0.08  1683 | 0.320  388 | 0.254  95 | 1.48  778 | 33 |
| {BcNSRV7.BI=FALSE, BcULV1.BI=TRUE}  => {BcMV6.BI=TRUE} | 0.07  6733 | 0.413  333 | 0.185  644 | 1.35  7615 | 31 |
| {BcMV6.BI=TRUE, BcNSRV7.BI=FALSE} => | 0.07 | 0.329 | 0.232 | 1.53 | 31 |

| {BcULV1.BI=TRUE} | 6733 | 787 | 673 | 1426 |  |
| --- | --- | --- | --- | --- | --- |
| {BcDMV1=FALSE, BcULV1.BI=TRUE} =>  {BcMV6.BI=TRUE} | 0.07  9208 | 0.405  063 | 0.195  545 | 1.33  0452 | 32 |
| {BcDMV1=FALSE, BcMV6.BI=TRUE} =>  {BcULV1.BI=TRUE} | 0.07  9208 | 0.333  333 | 0.237  624 | 1.54  7893 | 32 |
| {BcFV9=FALSE, BcULV1.BI=TRUE} =>  {BcMV6.BI=TRUE} | 0.08  6634 | 0.454  545 | 0.190  594 | 1.49  2979 | 35 |
| {BcFV9=FALSE, BcMV6.BI=TRUE} =>  {BcULV1.BI=TRUE} | 0.08  6634 | 0.324  074 | 0.267  327 | 1.50  4896 | 35 |
| {BcOLV4.BI=FALSE, BcULV1.BI=TRUE} =>  {BcMV6.BI=TRUE} | 0.07  6733 | 0.424  658 | 0.180  693 | 1.39  481 | 31 |
| {BcMV6.BI=TRUE, BcOLV4.BI=FALSE} =>  {BcULV1.BI=TRUE} | 0.07  6733 | 0.295  238 | 0.259  901 | 1.37  0991 | 31 |
| {BcBNV5.BI=FALSE, BcULV1.BI=TRUE} =>  {BcMV6.BI=TRUE} | 0.07  9208 | 0.405  063 | 0.195  545 | 1.33  0452 | 32 |
| {BcBNV5.BI=FALSE, BcMV6.BI=TRUE} =>  {BcULV1.BI=TRUE} | 0.07  9208 | 0.323  232 | 0.245  05 | 1.50  0987 | 32 |
| {BcULV1.BI=TRUE, BvF.BI=FALSE} =>  {BcMV6.BI=TRUE} | 0.08  9109 | 0.444  444 | 0.200  495 | 1.45  9801 | 36 |
| {BcMV6.BI=TRUE, BvF.BI=FALSE} =>  {BcULV1.BI=TRUE} | 0.08  9109 | 0.307  692 | 0.289  604 | 1.42  8824 | 36 |
| {BcMCV5.BI=FALSE, BcULV1.BI=TRUE} => | 0.07 | 0.410 | 0.193 | 1.34 | 32 |

| {BcMV6.BI=TRUE} | 9208 | 256 | 069 | 7509 |  |
| --- | --- | --- | --- | --- | --- |
| {BcMCV5.BI=FALSE, BcMV6.BI=TRUE} =>  {BcULV1.BI=TRUE} | 0.07  9208 | 0.304  762 | 0.259  901 | 1.41  5216 | 32 |
| {BcOLV13.BI=FALSE, BcULV1.BI=TRUE} =>  {BcMV6.BI=TRUE} | 0.07  4257 | 0.4 | 0.185  644 | 1.31  3821 | 30 |
| {BcEV6=FALSE, BcULV1.BI=TRUE} =>  {BcMV6.BI=TRUE} | 0.08  9109 | 0.45 | 0.198  02 | 1.47  8049 | 36 |
| {BcEV6=FALSE, BcMV6.BI=TRUE} =>  {BcULV1.BI=TRUE} | 0.08  9109 | 0.315  789 | 0.282  178 | 1.46  6425 | 36 |
| {BcOLV17.BI=FALSE, BcULV1.BI=TRUE} =>  {BcMV6.BI=TRUE} | 0.08  1683 | 0.412  5 | 0.198  02 | 1.35  4878 | 33 |
| {BcMV6.BI=TRUE, BcOLV17.BI=FALSE} =>  {BcULV1.BI=TRUE} | 0.08  1683 | 0.294  643 | 0.277  228 | 1.36  8227 | 33 |
| {BcOLV15.BI=FALSE, BcULV1.BI=TRUE} =>  {BcMV6.BI=TRUE} | 0.09  1584 | 0.435  294 | 0.210  396 | 1.42  9747 | 37 |
| {BcMV6.BI=TRUE, BcOLV15.BI=FALSE} =>  {BcULV1.BI=TRUE} | 0.09  1584 | 0.305  785 | 0.299  505 | 1.41  9968 | 37 |
| {BcEV5=FALSE, BcULV1.BI=TRUE} =>  {BcMV6.BI=TRUE} | 0.08  4158 | 0.404  762 | 0.207  921 | 1.32  9462 | 34 |
| {BcEV5=FALSE, BcMV6.BI=TRUE} =>  {BcULV1.BI=TRUE} | 0.08  4158 | 0.317  757 | 0.264  851 | 1.47  5561 | 34 |
| {BcOLV1.BI.MOVA.=TRUE, | 0.07 | 0.720 | 0.106 | 1.73 | 31 |

| BcULV1.BI=TRUE} => {BcOLV16.BI=TRUE} | 6733 | 93 | 436 | 3666 |  |
| --- | --- | --- | --- | --- | --- |
| {BcOLV16.BI=TRUE, BcULV1.BI=TRUE} => | 0.07 | 0.525 | 0.146 | 1.58 |  |
|  |  |  |  |  | 31 |
| {BcOLV1.BI.MOVA.=TRUE} | 6733 | 424 | 04 | 4113 |  |
| {BcOLV1.BI.MOVA.=TRUE, | 0.07 | 0.402 | 0.190 | 1.86 |  |
|  |  |  |  |  | 31 |
| BcOLV16.BI=TRUE} => {BcULV1.BI=TRUE} | 6733 | 597 | 594 | 9533 |  |
| {BcOLV1.BI.MOVA.=TRUE, | 0.09 | 0.860 | 0.106 | 1.85 |  |
|  |  |  |  |  | 37 |
| BcULV1.BI=TRUE} => {BcMV2.BI=TRUE} | 1584 | 465 | 436 | 8973 |  |
| {BcMV2.BI=TRUE, BcULV1.BI=TRUE} => | 0.09 | 0.660 | 0.138 | 1.99 |  |
|  |  |  |  |  | 37 |
| {BcOLV1.BI.MOVA.=TRUE} | 1584 | 714 | 614 | 2004 |  |
| {BcMV2.BI=TRUE, |  |  |  |  |  |
|  | 0.09 | 0.366 |  | 1.70 |  |
| BcOLV1.BI.MOVA.=TRUE} => |  |  | 0.25 |  | 37 |
|  | 1584 | 337 |  | 1149 |  |
| {BcULV1.BI=TRUE} |  |  |  |  |  |
| {BcOLV1.BI.MOVA.=TRUE, | 0.08 | 0.767 | 0.106 | 1.48 |  |
|  |  |  |  |  | 33 |
| BcULV1.BI=TRUE} => {BcOLV18=TRUE} | 1683 | 442 | 436 | 3476 |  |
| {BcOLV18=TRUE, BcULV1.BI=TRUE} => | 0.08 | 0.507 | 0.160 | 1.53 |  |
|  |  |  |  |  | 33 |
| {BcOLV1.BI.MOVA.=TRUE} | 1683 | 692 | 891 | 0654 |  |
| {BcOLV1.BI.MOVA.=TRUE, | 0.08 | 0.354 | 0.230 | 1.64 |  |
|  |  |  |  |  | 33 |
| BcOLV18=TRUE} => {BcULV1.BI=TRUE} | 1683 | 839 | 198 | 7757 |  |
| {BcOLV1.BI.MOVA.=TRUE, | 0.07 | 0.697 | 0.106 | 1.28 |  |
|  |  |  |  |  | 30 |
| BcULV1.BI=TRUE} => {BcHV1SLR.BI=TRUE} | 4257 | 674 | 436 | 7034 |  |
| {BcHV1SLR.BI=TRUE, BcULV1.BI=TRUE} | 0.07 | 0.468 | 0.158 | 1.41 |  |
|  |  |  |  |  | 30 |
| => {BcOLV1.BI.MOVA.=TRUE} | 4257 | 75 | 416 | 3246 |  |

| {BcHV1SLR.BI=TRUE, BcOLV1.BI.MOVA.=TRUE} =>  {BcULV1.BI=TRUE} | 0.07  4257 | 0.370  37 | 0.200  495 | 1.71  9881 | 30 |
| --- | --- | --- | --- | --- | --- |
| {BcOLV1.BI.MOVA.=TRUE,  BcULV1.BI=TRUE} => {BcMV3.BI=TRUE} | 0.09  4059 | 0.883  721 | 0.106  436 | 1.63  0243 | 38 |
| {BcMV3.BI=TRUE, BcULV1.BI=TRUE} =>  {BcOLV1.BI.MOVA.=TRUE} | 0.09  4059 | 0.622  951 | 0.150  99 | 1.87  815 | 38 |
| {BcMV3.BI=TRUE, BcOLV1.BI.MOVA.=TRUE} =>  {BcULV1.BI=TRUE} | 0.09  4059 | 0.383  838 | 0.245  05 | 1.78  2422 | 38 |
| {BcOLV1.BI.MOVA.=TRUE,  BcULV1.BI=TRUE} => {BcHV1.BI=TRUE} | 0.08  1683 | 0.767  442 | 0.106  436 | 1.27  0682 | 33 |
| {BcHV1.BI=TRUE, BcULV1.BI=TRUE} =>  {BcOLV1.BI.MOVA.=TRUE} | 0.08  1683 | 0.464  789 | 0.175  743 | 1.40  1303 | 33 |
| {BcHV1.BI=TRUE, BcOLV1.BI.MOVA.=TRUE} =>  {BcULV1.BI=TRUE} | 0.08  1683 | 0.383  721 | 0.212  871 | 1.78  1877 | 33 |
| {BcOLV1.BI.MOVA.=TRUE,  BcULV1.BI=TRUE} => {BcMV9.BI=TRUE} | 0.08  4158 | 0.790  698 | 0.106  436 | 1.16  5846 | 34 |
| {BcMV9.BI=TRUE, BcULV1.BI=TRUE} =>  {BcOLV1.BI.MOVA.=TRUE} | 0.08  4158 | 0.472  222 | 0.178  218 | 1.42  3715 | 34 |
| {BcMV9.BI=TRUE, | 0.08 | 0.395 | 0.212 | 1.83 | 34 |

| BcOLV1.BI.MOVA.=TRUE} =>  {BcULV1.BI=TRUE} | 4158 | 349 | 871 | 5873 |  |
| --- | --- | --- | --- | --- | --- |
| {BcOLV1.BI.MOVA.=TRUE, | 0.09 | 0.860 | 0.106 | 1.24 |  |
|  |  |  |  |  | 37 |
| BcULV1.BI=TRUE} => {BcULV4=TRUE} | 1584 | 465 | 436 | 5978 |  |
| {BcULV1.BI=TRUE, BcULV4=TRUE} => | 0.09 | 0.474 | 0.193 | 1.43 |  |
|  |  |  |  |  | 37 |
| {BcOLV1.BI.MOVA.=TRUE} | 1584 | 359 | 069 | 0157 |  |
| {BcOLV1.BI.MOVA.=TRUE, BcULV4=TRUE} | 0.09 | 0.373 | 0.245 | 1.73 |  |
|  |  |  |  |  | 37 |
| => {BcULV1.BI=TRUE} | 1584 | 737 | 05 | 5516 |  |
| {BcEV2.BI=FALSE, BcULV1.BI=TRUE} => | 0.08 | 0.515 | 0.158 | 1.55 |  |
|  |  |  |  |  | 33 |
| {BcOLV1.BI.MOVA.=TRUE} | 1683 | 625 | 416 | 4571 |  |
| {BcEV2.BI=FALSE, |  |  |  |  |  |
|  | 0.08 | 0.326 |  | 1.51 |  |
| BcOLV1.BI.MOVA.=TRUE} => |  |  | 0.25 |  | 33 |
|  | 1683 | 733 |  | 7241 |  |
| {BcULV1.BI=TRUE} |  |  |  |  |  |
| {BcFV5.BI=FALSE, BcULV1.BI=TRUE} => | 0.07 | 0.449 | 0.170 | 1.35 |  |
|  |  |  |  |  | 31 |
| {BcOLV1.BI.MOVA.=TRUE} | 6733 | 275 | 792 | 4532 |  |
| {BcFV5.BI=FALSE, |  |  |  |  |  |
|  | 0.07 | 0.319 | 0.240 | 1.48 |  |
| BcOLV1.BI.MOVA.=TRUE} => |  |  |  |  | 31 |
|  | 6733 | 588 | 099 | 4062 |  |
| {BcULV1.BI=TRUE} |  |  |  |  |  |
| {BcOLV1.BI.MOVA.=TRUE, | 0.09 | 0.906 | 0.106 | 1.14 |  |
|  |  |  |  |  | 39 |
| BcULV1.BI=TRUE} => {BcMV1.BI=TRUE} | 6535 | 977 | 436 | 8648 |  |
| {BcMV1.BI=TRUE, BcULV1.BI=TRUE} => | 0.09 | 0.513 | 0.188 | 1.54 |  |
|  |  |  |  |  | 39 |
| {BcOLV1.BI.MOVA.=TRUE} | 6535 | 158 | 119 | 7133 |  |

| {BcMV1.BI=TRUE, BcOLV1.BI.MOVA.=TRUE} =>  {BcULV1.BI=TRUE} | 0.09  6535 | 0.327  731 | 0.294  554 | 1.52  1878 | 39 |
| --- | --- | --- | --- | --- | --- |
| {BcNSRV7.BI=FALSE, BcULV1.BI=TRUE}  => {BcOLV1.BI.MOVA.=TRUE} | 0.08  6634 | 0.466  667 | 0.185  644 | 1.40  6965 | 35 |
| {BcNSRV7.BI=FALSE, BcOLV1.BI.MOVA.=TRUE} =>  {BcULV1.BI=TRUE} | 0.08  6634 | 0.327  103 | 0.264  851 | 1.51  896 | 35 |
| {BcULV1.BI=TRUE, BcVV3.BI=FALSE} =>  {BcOLV1.BI.MOVA.=TRUE} | 0.07  9208 | 0.457  143 | 0.173  267 | 1.37  8252 | 32 |
| {BcOLV1.BI.MOVA.=TRUE,  BcVV3.BI=FALSE} => {BcULV1.BI=TRUE} | 0.07  9208 | 0.326  531 | 0.242  574 | 1.51  6303 | 32 |
| {BcALV1.BI=FALSE, BcULV1.BI=TRUE} =>  {BcOLV1.BI.MOVA.=TRUE} | 0.07  6733 | 0.462  687 | 0.165  842 | 1.39  4965 | 31 |
| {BcALV1.BI=FALSE, BcOLV1.BI.MOVA.=TRUE} =>  {BcULV1.BI=TRUE} | 0.07  6733 | 0.340  659 | 0.225  248 | 1.58  1912 | 31 |
| {BcDMV1=FALSE, BcULV1.BI=TRUE} =>  {BcOLV1.BI.MOVA.=TRUE} | 0.09  1584 | 0.468  354 | 0.195  545 | 1.41  2054 | 37 |
| {BcDMV1=FALSE, BcOLV1.BI.MOVA.=TRUE} =>  {BcULV1.BI=TRUE} | 0.09  1584 | 0.333  333 | 0.274  752 | 1.54  7893 | 37 |

| {BcFV9=FALSE, BcULV1.BI=TRUE} =>  {BcOLV1.BI.MOVA.=TRUE} | 0.09  6535 | 0.506  494 | 0.190  594 | 1.52  704 | 39 |
| --- | --- | --- | --- | --- | --- |
| {BcFV9=FALSE, BcOLV1.BI.MOVA.=TRUE}  => {BcULV1.BI=TRUE} | 0.09  6535 | 0.345  133 | 0.279  703 | 1.60  2685 | 39 |
| {BcOLV4.BI=FALSE, BcULV1.BI=TRUE} =>  {BcOLV1.BI.MOVA.=TRUE} | 0.08  1683 | 0.452  055 | 0.180  693 | 1.36  2911 | 33 |
| {BcOLV1.BI.MOVA.=TRUE,  BcOLV4.BI=FALSE} => {BcULV1.BI=TRUE} | 0.08  1683 | 0.311  321 | 0.262  376 | 1.44  5673 | 33 |
| {BcULV1.BI=TRUE, BcULV5=FALSE} =>  {BcOLV1.BI.MOVA.=TRUE} | 0.08  1683 | 0.464  789 | 0.175  743 | 1.40  1303 | 33 |
| {BcOLV1.BI.MOVA.=TRUE,  BcULV5=FALSE} => {BcULV1.BI=TRUE} | 0.08  1683 | 0.317  308 | 0.257  426 | 1.47  3475 | 33 |
| {BcBNV5.BI=FALSE, BcULV1.BI=TRUE} =>  {BcOLV1.BI.MOVA.=TRUE} | 0.09  1584 | 0.468  354 | 0.195  545 | 1.41  2054 | 37 |
| {BcBNV5.BI=FALSE, BcOLV1.BI.MOVA.=TRUE} =>  {BcULV1.BI=TRUE} | 0.09  1584 | 0.324  561 | 0.282  178 | 1.50  7159 | 37 |
| {BcULV1.BI=TRUE, BvF.BI=FALSE} =>  {BcOLV1.BI.MOVA.=TRUE} | 0.09  901 | 0.493  827 | 0.200  495 | 1.48  8852 | 40 |
| {BcOLV1.BI.MOVA.=TRUE, BvF.BI=FALSE}  => {BcULV1.BI=TRUE} | 0.09  901 | 0.314  961 | 0.314  356 | 1.46  2576 | 40 |
| {BcMCV5.BI=FALSE, BcULV1.BI=TRUE} => | 0.09 | 0.487 | 0.193 | 1.46 | 38 |

| {BcOLV1.BI.MOVA.=TRUE} | 4059 | 179 | 069 | 881 |  |
| --- | --- | --- | --- | --- | --- |
| {BcMCV5.BI=FALSE, BcOLV1.BI.MOVA.=TRUE} =>  {BcULV1.BI=TRUE} | 0.09  4059 | 0.339  286 | 0.277  228 | 1.57  5534 | 38 |
| {BcOLV13.BI=FALSE, BcULV1.BI=TRUE} =>  {BcOLV1.BI.MOVA.=TRUE} | 0.08  1683 | 0.44 | 0.185  644 | 1.32  6567 | 33 |
| {BcOLV1.BI.MOVA.=TRUE, | 0.08 | 0.294 | 0.277 | 1.36 |  |
|  |  |  |  |  | 33 |
| BcOLV13.BI=FALSE} => {BcULV1.BI=TRUE} | 1683 | 643 | 228 | 8227 |  |
| {BcOLV1.BI.MOVA.=TRUE, | 0.07 | 0.285 | 0.277 | 1.32 |  |
|  |  |  |  |  | 32 |
| BcOLV2.BI=FALSE} => {BcULV1.BI=TRUE} | 9208 | 714 | 228 | 6765 |  |
| {BcPV4.BI=FALSE, BcULV1.BI=TRUE} => | 0.09 | 0.506 | 0.190 | 1.52 |  |
|  |  |  |  |  | 39 |
| {BcOLV1.BI.MOVA.=TRUE} | 6535 | 494 | 594 | 704 |  |
| {BcOLV1.BI.MOVA.=TRUE, | 0.09 | 0.304 | 0.316 | 1.41 |  |
|  |  |  |  |  | 39 |
| BcPV4.BI=FALSE} => {BcULV1.BI=TRUE} | 6535 | 688 | 832 | 4871 |  |
| {BcEV6=FALSE, BcULV1.BI=TRUE} => | 0.09 | 0.487 | 0.198 | 1.46 |  |
|  |  |  |  |  | 39 |
| {BcOLV1.BI.MOVA.=TRUE} | 6535 | 5 | 02 | 9776 |  |
| {BcEV6=FALSE, BcOLV1.BI.MOVA.=TRUE} | 0.09 | 0.314 | 0.306 | 1.46 |  |
|  |  |  |  |  | 39 |
| => {BcULV1.BI=TRUE} | 6535 | 516 | 931 | 0512 |  |
| {BcOLV17.BI=FALSE, BcULV1.BI=TRUE} => | 0.09 | 0.462 | 0.198 | 1.39 |  |
|  |  |  |  |  | 37 |
| {BcOLV1.BI.MOVA.=TRUE} | 1584 | 5 | 02 | 4403 |  |
| {BcOLV1.BI.MOVA.=TRUE, | 0.09 | 0.305 | 0.299 | 1.41 |  |
|  |  |  |  |  | 37 |
| BcOLV17.BI=FALSE} => {BcULV1.BI=TRUE} | 1584 | 785 | 505 | 9968 |  |

| {BcOLV15.BI=FALSE, BcULV1.BI=TRUE} =>  {BcOLV1.BI.MOVA.=TRUE} | 0.10  6436 | 0.505  882 | 0.210  396 | 1.52  5198 | 43 |
| --- | --- | --- | --- | --- | --- |
| {BcOLV1.BI.MOVA.=TRUE,  BcOLV15.BI=FALSE} => {BcULV1.BI=TRUE} | 0.10  6436 | 0.330  769 | 0.321  782 | 1.53  5986 | 43 |
| {BcEV5=FALSE, BcULV1.BI=TRUE} =>  {BcOLV1.BI.MOVA.=TRUE} | 0.09  901 | 0.476  19 | 0.207  921 | 1.43  5679 | 40 |
| {BcEV5=FALSE, BcOLV1.BI.MOVA.=TRUE}  => {BcULV1.BI=TRUE} | 0.09  901 | 0.333  333 | 0.297  03 | 1.54  7893 | 40 |
| {BcULV1.BI=TRUE, BcULV3=FALSE} =>  {BcULV4=TRUE} | 0.07  4257 | 0.937  5 | 0.079  208 | 1.35  7527 | 30 |
| {BcMV4.BI=TRUE, BcULV1.BI=TRUE} =>  {BcOLV16.BI=TRUE} | 0.07  4257 | 0.681  818 | 0.108  911 | 1.63  961 | 30 |
| {BcMV4.BI=TRUE, BcOLV16.BI=TRUE} =>  {BcULV1.BI=TRUE} | 0.07  4257 | 0.329  67 | 0.225  248 | 1.53  0883 | 30 |
| {BcOLV16.BI=TRUE, BcULV1.BI=TRUE} =>  {BcMV2.BI=TRUE} | 0.10  396 | 0.711  864 | 0.146  04 | 1.53  7932 | 42 |
| {BcMV2.BI=TRUE, BcULV1.BI=TRUE} =>  {BcOLV16.BI=TRUE} | 0.10  396 | 0.75 | 0.138  614 | 1.80  3571 | 42 |
| {BcMV2.BI=TRUE, BcOLV16.BI=TRUE} =>  {BcULV1.BI=TRUE} | 0.10  396 | 0.411  765 | 0.252  475 | 1.91  2103 | 42 |
| {BcOLV16.BI=TRUE, BcULV1.BI=TRUE} =>  {BcOLV18=TRUE} | 0.11  6337 | 0.796  61 | 0.146  04 | 1.53  9859 | 47 |

| {BcOLV18=TRUE, BcULV1.BI=TRUE} =>  {BcOLV16.BI=TRUE} | 0.11  6337 | 0.723  077 | 0.160  891 | 1.73  8828 | 47 |
| --- | --- | --- | --- | --- | --- |
| {BcOLV16.BI=TRUE, BcOLV18=TRUE} =>  {BcULV1.BI=TRUE} | 0.11  6337 | 0.431  193 | 0.269  802 | 2.00  232 | 47 |
| {BcOLV16.BI=TRUE, BcULV1.BI=TRUE} =>  {BcHV1SLR.BI=TRUE} | 0.11  1386 | 0.762  712 | 0.146  04 | 1.40  7012 | 45 |
| {BcHV1SLR.BI=TRUE, BcULV1.BI=TRUE}  => {BcOLV16.BI=TRUE} | 0.11  1386 | 0.703  125 | 0.158  416 | 1.69  0848 | 45 |
| {BcHV1SLR.BI=TRUE, BcOLV16.BI=TRUE}  => {BcULV1.BI=TRUE} | 0.11  1386 | 0.468  75 | 0.237  624 | 2.17  6724 | 45 |
| {BcOLV16.BI=TRUE, BcULV1.BI=TRUE} =>  {BcMV3.BI=TRUE} | 0.11  1386 | 0.762  712 | 0.146  04 | 1.40  7012 | 45 |
| {BcMV3.BI=TRUE, BcULV1.BI=TRUE} =>  {BcOLV16.BI=TRUE} | 0.11  1386 | 0.737  705 | 0.150  99 | 1.77  4005 | 45 |
| {BcMV3.BI=TRUE, BcOLV16.BI=TRUE} =>  {BcULV1.BI=TRUE} | 0.11  1386 | 0.416  667 | 0.267  327 | 1.93  4866 | 45 |
| {BcOLV16.BI=TRUE, BcULV1.BI=TRUE} =>  {BcULV3=TRUE} | 0.11  3861 | 0.779  661 | 0.146  04 | 1.31  2429 | 46 |
| {BcULV1.BI=TRUE, BcULV3=TRUE} =>  {BcOLV16.BI=TRUE} | 0.11  3861 | 0.836  364 | 0.136  139 | 2.01  1255 | 46 |
| {BcOLV16.BI=TRUE, BcULV3=TRUE} =>  {BcULV1.BI=TRUE} | 0.11  3861 | 0.407  08 | 0.279  703 | 1.89  0347 | 46 |

| {BcOLV16.BI=TRUE, BcULV1.BI=TRUE} =>  {BcHV1.BI=TRUE} | 0.12  1287 | 0.830  508 | 0.146  04 | 1.37  5104 | 49 |
| --- | --- | --- | --- | --- | --- |
| {BcHV1.BI=TRUE, BcULV1.BI=TRUE} =>  {BcOLV16.BI=TRUE} | 0.12  1287 | 0.690  141 | 0.175  743 | 1.65  9624 | 49 |
| {BcHV1.BI=TRUE, BcOLV16.BI=TRUE} =>  {BcULV1.BI=TRUE} | 0.12  1287 | 0.449  541 | 0.269  802 | 2.08  7525 | 49 |
| {BcMV11=TRUE, BcULV1.BI=TRUE} =>  {BcOLV16.BI=TRUE} | 0.10  1485 | 0.706  897 | 0.143  564 | 1.69  9918 | 41 |
| {BcMV11=TRUE, BcOLV16.BI=TRUE} =>  {BcULV1.BI=TRUE} | 0.10  1485 | 0.369  369 | 0.274  752 | 1.71  5232 | 41 |
| {BcOLV16.BI=TRUE, BcULV1.BI=TRUE} =>  {BcMV9.BI=TRUE} | 0.12  1287 | 0.830  508 | 0.146  04 | 1.22  4545 | 49 |
| {BcMV9.BI=TRUE, BcULV1.BI=TRUE} =>  {BcOLV16.BI=TRUE} | 0.12  1287 | 0.680  556 | 0.178  218 | 1.63  6574 | 49 |
| {BcMV9.BI=TRUE, BcOLV16.BI=TRUE} =>  {BcULV1.BI=TRUE} | 0.12  1287 | 0.365  672 | 0.331  683 | 1.69  8061 | 49 |
| {BcOLV16.BI=TRUE, BcULV1.BI=TRUE} =>  {BcULV4=TRUE} | 0.13  1188 | 0.898  305 | 0.146  04 | 1.30  0772 | 53 |
| {BcULV1.BI=TRUE, BcULV4=TRUE} =>  {BcOLV16.BI=TRUE} | 0.13  1188 | 0.679  487 | 0.193  069 | 1.63  4005 | 53 |
| {BcOLV16.BI=TRUE, BcULV4=TRUE} =>  {BcULV1.BI=TRUE} | 0.13  1188 | 0.386  861 | 0.339  109 | 1.79  6459 | 53 |

| {BcMV6.BI=FALSE, BcULV1.BI=TRUE} =>  {BcOLV16.BI=TRUE} | 0.07  9208 | 0.64 | 0.123  762 | 1.53  9048 | 32 |
| --- | --- | --- | --- | --- | --- |
| {BcMV6.BI=FALSE, BcOLV16.BI=TRUE} =>  {BcULV1.BI=TRUE} | 0.07  9208 | 0.316  832 | 0.25 | 1.47  1264 | 32 |
| {BcPV3.BI=FALSE, BcULV1.BI=TRUE} =>  {BcOLV16.BI=TRUE} | 0.09  1584 | 0.660  714 | 0.138  614 | 1.58  8861 | 37 |
| {BcOLV16.BI=TRUE, BcPV3.BI=FALSE} =>  {BcULV1.BI=TRUE} | 0.09  1584 | 0.336  364 | 0.272  277 | 1.56  1964 | 37 |
| {BcRV1.BI=FALSE, BcULV1.BI=TRUE} =>  {BcOLV16.BI=TRUE} | 0.08  6634 | 0.625 | 0.138  614 | 1.50  2976 | 35 |
| {BcOLV16.BI=TRUE, BcRV1.BI=FALSE} =>  {BcULV1.BI=TRUE} | 0.08  6634 | 0.327  103 | 0.264  851 | 1.51  896 | 35 |
| {BcEV2.BI=FALSE, BcULV1.BI=TRUE} =>  {BcOLV16.BI=TRUE} | 0.11  1386 | 0.703  125 | 0.158  416 | 1.69  0848 | 45 |
| {BcEV2.BI=FALSE, BcOLV16.BI=TRUE} =>  {BcULV1.BI=TRUE} | 0.11  1386 | 0.340  909 | 0.326  733 | 1.58  3072 | 45 |
| {BcHV3.BI=FALSE, BcULV1.BI=TRUE} =>  {BcOLV16.BI=TRUE} | 0.10  396 | 0.666  667 | 0.155  941 | 1.60  3175 | 42 |
| {BcHV3.BI=FALSE, BcOLV16.BI=TRUE} =>  {BcULV1.BI=TRUE} | 0.10  396 | 0.323  077 | 0.321  782 | 1.50  0265 | 42 |
| {BcFV5.BI=FALSE, BcULV1.BI=TRUE} =>  {BcOLV16.BI=TRUE} | 0.11  6337 | 0.681  159 | 0.170  792 | 1.63  8026 | 47 |

| {BcFV5.BI=FALSE, BcOLV16.BI=TRUE} =>  {BcULV1.BI=TRUE} | 0.11  6337 | 0.361  538 | 0.321  782 | 1.67  8868 | 47 |
| --- | --- | --- | --- | --- | --- |
| {BcMV1.BI=TRUE, BcULV1.BI=TRUE} =>  {BcOLV16.BI=TRUE} | 0.12  6238 | 0.671  053 | 0.188  119 | 1.61  3722 | 51 |
| {BcMV1.BI=TRUE, BcOLV16.BI=TRUE} =>  {BcULV1.BI=TRUE} | 0.12  6238 | 0.375 | 0.336  634 | 1.74  1379 | 51 |
| {BcMV12=FALSE, BcULV1.BI=TRUE} =>  {BcOLV16.BI=TRUE} | 0.08  4158 | 0.576  271 | 0.146  04 | 1.38  5795 | 34 |
| {BcMV12=FALSE, BcOLV16.BI=TRUE} =>  {BcULV1.BI=TRUE} | 0.08  4158 | 0.285  714 | 0.294  554 | 1.32  6765 | 34 |
| {BcNSRV7.BI=FALSE, BcULV1.BI=TRUE}  => {BcOLV16.BI=TRUE} | 0.12  8713 | 0.693  333 | 0.185  644 | 1.66  7302 | 52 |
| {BcNSRV7.BI=FALSE, BcOLV16.BI=TRUE}  => {BcULV1.BI=TRUE} | 0.12  8713 | 0.385  185 | 0.334  158 | 1.78  8676 | 52 |
| {BcULV1.BI=TRUE, BcVV3.BI=FALSE} =>  {BcOLV16.BI=TRUE} | 0.12  6238 | 0.728  571 | 0.173  267 | 1.75  2041 | 51 |
| {BcOLV16.BI=TRUE, BcVV3.BI=FALSE} =>  {BcULV1.BI=TRUE} | 0.12  6238 | 0.375 | 0.336  634 | 1.74  1379 | 51 |
| {BcOLV7.BI=FALSE, BcULV1.BI=TRUE} =>  {BcOLV16.BI=TRUE} | 0.09  1584 | 0.587  302 | 0.155  941 | 1.41  232 | 37 |
| {BcOLV16.BI=TRUE, BcOLV7.BI=FALSE}  => {BcULV1.BI=TRUE} | 0.09  1584 | 0.310  924 | 0.294  554 | 1.44  3833 | 37 |

| {BcALV1.BI=FALSE, BcULV1.BI=TRUE} =>  {BcOLV16.BI=TRUE} | 0.10  8911 | 0.656  716 | 0.165  842 | 1.57  9247 | 44 |
| --- | --- | --- | --- | --- | --- |
| {BcALV1.BI=FALSE, BcOLV16.BI=TRUE} =>  {BcULV1.BI=TRUE} | 0.10  8911 | 0.352 | 0.309  406 | 1.63  4575 | 44 |
| {BcOLV16.BI=TRUE, BcULV1.BI=TRUE} =>  {BcDMV1=FALSE} | 0.13  3663 | 0.915  254 | 0.146  04 | 1.08  4348 | 54 |
| {BcDMV1=FALSE, BcULV1.BI=TRUE} =>  {BcOLV16.BI=TRUE} | 0.13  3663 | 0.683  544 | 0.195  545 | 1.64  3761 | 54 |
| {BcDMV1=FALSE, BcOLV16.BI=TRUE} =>  {BcULV1.BI=TRUE} | 0.13  3663 | 0.377  622 | 0.353  96 | 1.75  3557 | 54 |
| {BcFV9=FALSE, BcULV1.BI=TRUE} =>  {BcOLV16.BI=TRUE} | 0.12  8713 | 0.675  325 | 0.190  594 | 1.62  3995 | 52 |
| {BcFV9=FALSE, BcOLV16.BI=TRUE} =>  {BcULV1.BI=TRUE} | 0.12  8713 | 0.358  621 | 0.358  911 | 1.66  5319 | 52 |
| {BcOLV4.BI=FALSE, BcULV1.BI=TRUE} =>  {BcOLV16.BI=TRUE} | 0.11  8812 | 0.657  534 | 0.180  693 | 1.58  1213 | 48 |
| {BcOLV16.BI=TRUE, BcOLV4.BI=FALSE}  => {BcULV1.BI=TRUE} | 0.11  8812 | 0.345  324 | 0.344  059 | 1.60  3572 | 48 |
| {BcULV1.BI=TRUE, BcULV5=FALSE} =>  {BcOLV16.BI=TRUE} | 0.11  3861 | 0.647  887 | 0.175  743 | 1.55  8015 | 46 |
| {BcOLV16.BI=TRUE, BcULV5=FALSE} =>  {BcULV1.BI=TRUE} | 0.11  3861 | 0.323  944 | 0.351  485 | 1.50  429 | 46 |

| {BcBNV5.BI=FALSE, BcULV1.BI=TRUE} =>  {BcOLV16.BI=TRUE} | 0.13  3663 | 0.683  544 | 0.195  545 | 1.64  3761 | 54 |
| --- | --- | --- | --- | --- | --- |
| {BcBNV5.BI=FALSE, BcOLV16.BI=TRUE}  => {BcULV1.BI=TRUE} | 0.13  3663 | 0.357  616 | 0.373  762 | 1.66  0653 | 54 |
| {BcULV1.BI=TRUE, BvF.BI=FALSE} =>  {BcOLV16.BI=TRUE} | 0.14  1089 | 0.703  704 | 0.200  495 | 1.69  224 | 57 |
| {BcOLV16.BI=TRUE, BvF.BI=FALSE} =>  {BcULV1.BI=TRUE} | 0.14  1089 | 0.360  759 | 0.391  089 | 1.67  5251 | 57 |
| {BcMCV5.BI=FALSE, BcULV1.BI=TRUE} =>  {BcOLV16.BI=TRUE} | 0.13  1188 | 0.679  487 | 0.193  069 | 1.63  4005 | 53 |
| {BcMCV5.BI=FALSE, BcOLV16.BI=TRUE}  => {BcULV1.BI=TRUE} | 0.13  1188 | 0.358  108 | 0.366  337 | 1.66  2939 | 53 |
| {BcOLV13.BI=FALSE, BcULV1.BI=TRUE} =>  {BcOLV16.BI=TRUE} | 0.12  6238 | 0.68 | 0.185  644 | 1.63  5238 | 51 |
| {BcOLV13.BI=FALSE, BcOLV16.BI=TRUE}  => {BcULV1.BI=TRUE} | 0.12  6238 | 0.344  595 | 0.366  337 | 1.60  0186 | 51 |
| {BcOLV2.BI=FALSE, BcULV1.BI=TRUE} =>  {BcOLV16.BI=TRUE} | 0.12  3762 | 0.666  667 | 0.185  644 | 1.60  3175 | 50 |
| {BcOLV16.BI=TRUE, BcOLV2.BI=FALSE}  => {BcULV1.BI=TRUE} | 0.12  3762 | 0.335  57 | 0.368  812 | 1.55  8281 | 50 |
| {BcMCV3.BI=FALSE, BcULV1.BI=TRUE} =>  {BcOLV16.BI=TRUE} | 0.10  396 | 0.617  647 | 0.168  317 | 1.48  5294 | 42 |

| {BcMCV3.BI=FALSE, BcOLV16.BI=TRUE}  => {BcULV1.BI=TRUE} | 0.10  396 | 0.289  655 | 0.358  911 | 1.34  5065 | 42 |
| --- | --- | --- | --- | --- | --- |
| {BcPV4.BI=FALSE, BcULV1.BI=TRUE} =>  {BcOLV16.BI=TRUE} | 0.12  3762 | 0.649  351 | 0.190  594 | 1.56  1534 | 50 |
| {BcOLV16.BI=TRUE, BcPV4.BI=FALSE} =>  {BcULV1.BI=TRUE} | 0.12  3762 | 0.326  797 | 0.378  713 | 1.51  7542 | 50 |
| {BcEV6=FALSE, BcULV1.BI=TRUE} =>  {BcOLV16.BI=TRUE} | 0.13  6139 | 0.687  5 | 0.198  02 | 1.65  3274 | 55 |
| {BcEV6=FALSE, BcOLV16.BI=TRUE} =>  {BcULV1.BI=TRUE} | 0.13  6139 | 0.348  101 | 0.391  089 | 1.61  647 | 55 |
| {BcOLV17.BI=FALSE, BcULV1.BI=TRUE} =>  {BcOLV16.BI=TRUE} | 0.13  6139 | 0.687  5 | 0.198  02 | 1.65  3274 | 55 |
| {BcOLV16.BI=TRUE, BcOLV17.BI=FALSE}  => {BcULV1.BI=TRUE} | 0.13  6139 | 0.352  564 | 0.386  139 | 1.63  7194 | 55 |
| {BcOLV15.BI=FALSE, BcULV1.BI=TRUE} =>  {BcOLV16.BI=TRUE} | 0.14  3564 | 0.682  353 | 0.210  396 | 1.64  0896 | 58 |
| {BcOLV15.BI=FALSE, BcOLV16.BI=TRUE}  => {BcULV1.BI=TRUE} | 0.14  3564 | 0.355  828 | 0.403  465 | 1.65  2352 | 58 |
| {BcEV5=FALSE, BcULV1.BI=TRUE} =>  {BcOLV16.BI=TRUE} | 0.14  1089 | 0.678  571 | 0.207  921 | 1.63  1803 | 57 |
| {BcEV5=FALSE, BcOLV16.BI=TRUE} =>  {BcULV1.BI=TRUE} | 0.14  1089 | 0.358  491 | 0.393  564 | 1.66  4715 | 57 |

| {BcMV4.BI=TRUE, BcULV1.BI=TRUE} =>  {BcMV2.BI=TRUE} | 0.07  9208 | 0.727  273 | 0.108  911 | 1.57  122 | 32 |
| --- | --- | --- | --- | --- | --- |
| {BcMV2.BI=TRUE, BcULV1.BI=TRUE} =>  {BcMV4.BI=TRUE} | 0.07  9208 | 0.571  429 | 0.138  614 | 1.28  9705 | 32 |
| {BcMV2.BI=TRUE, BcMV4.BI=TRUE} =>  {BcULV1.BI=TRUE} | 0.07  9208 | 0.288  288 | 0.274  752 | 1.33  8718 | 32 |
| {BcMV4.BI=TRUE, BcULV1.BI=TRUE} =>  {BcOLV18=TRUE} | 0.08  9109 | 0.818  182 | 0.108  911 | 1.58  1557 | 36 |
| {BcOLV18=TRUE, BcULV1.BI=TRUE} =>  {BcMV4.BI=TRUE} | 0.08  9109 | 0.553  846 | 0.160  891 | 1.25  0021 | 36 |
| {BcMV4.BI=TRUE, BcOLV18=TRUE} =>  {BcULV1.BI=TRUE} | 0.08  9109 | 0.321  429 | 0.277  228 | 1.49  2611 | 36 |
| {BcMV4.BI=TRUE, BcULV1.BI=TRUE} =>  {BcHV1SLR.BI=TRUE} | 0.07  6733 | 0.704  545 | 0.108  911 | 1.29  9709 | 31 |
| {BcHV1SLR.BI=TRUE, BcMV4.BI=TRUE} =>  {BcULV1.BI=TRUE} | 0.07  6733 | 0.292  453 | 0.262  376 | 1.35  8057 | 31 |
| {BcMV4.BI=TRUE, BcULV1.BI=TRUE} =>  {BcMV3.BI=TRUE} | 0.08  1683 | 0.75 | 0.108  911 | 1.38  3562 | 33 |
| {BcMV4.BI=TRUE, BcULV1.BI=TRUE} =>  {BcHV1.BI=TRUE} | 0.08  4158 | 0.772  727 | 0.108  911 | 1.27  9434 | 34 |
| {BcHV1.BI=TRUE, BcMV4.BI=TRUE} =>  {BcULV1.BI=TRUE} | 0.08  4158 | 0.280  992 | 0.299  505 | 1.30  4835 | 34 |

| {BcMV4.BI=TRUE, BcULV1.BI=TRUE} =>  {BcMV11=TRUE} | 0.08  6634 | 0.795  455 | 0.108  911 | 1.29  0617 | 35 |
| --- | --- | --- | --- | --- | --- |
| {BcMV11=TRUE, BcULV1.BI=TRUE} =>  {BcMV4.BI=TRUE} | 0.08  6634 | 0.603  448 | 0.143  564 | 1.36  1973 | 35 |
| {BcMV4.BI=TRUE, BcULV1.BI=TRUE} =>  {BcMV9.BI=TRUE} | 0.09  6535 | 0.886  364 | 0.108  911 | 1.30  6901 | 39 |
| {BcMV4.BI=TRUE, BcMV9.BI=TRUE} =>  {BcULV1.BI=TRUE} | 0.09  6535 | 0.286  765 | 0.336  634 | 1.33  1643 | 39 |
| {BcMV4.BI=TRUE, BcULV1.BI=TRUE} =>  {BcULV4=TRUE} | 0.09  6535 | 0.886  364 | 0.108  911 | 1.28  348 | 39 |
| {BcMV4.BI=TRUE, BcULV4=TRUE} =>  {BcULV1.BI=TRUE} | 0.09  6535 | 0.297  71 | 0.324  257 | 1.38  2469 | 39 |
| {BcMV4.BI=TRUE, BcULV1.BI=TRUE} =>  {BcMV1.BI=TRUE} | 0.09  6535 | 0.886  364 | 0.108  911 | 1.12  2542 | 39 |
| {BcMV12=FALSE, BcULV1.BI=TRUE} =>  {BcMV4.BI=TRUE} | 0.08  1683 | 0.559  322 | 0.146  04 | 1.26  238 | 33 |
| {BcMV2.BI=TRUE, BcULV1.BI=TRUE} =>  {BcOLV18=TRUE} | 0.11  1386 | 0.803  571 | 0.138  614 | 1.55  3315 | 45 |
| {BcOLV18=TRUE, BcULV1.BI=TRUE} =>  {BcMV2.BI=TRUE} | 0.11  1386 | 0.692  308 | 0.160  891 | 1.49  5681 | 45 |
| {BcMV2.BI=TRUE, BcOLV18=TRUE} =>  {BcULV1.BI=TRUE} | 0.11  1386 | 0.378  151 | 0.294  554 | 1.75  6013 | 45 |

| {BcMV2.BI=TRUE, BcULV1.BI=TRUE} =>  {BcHV1SLR.BI=TRUE} | 0.10  1485 | 0.732  143 | 0.138  614 | 1.35  062 | 41 |
| --- | --- | --- | --- | --- | --- |
| {BcHV1SLR.BI=TRUE, BcULV1.BI=TRUE}  => {BcMV2.BI=TRUE} | 0.10  1485 | 0.640  625 | 0.158  416 | 1.38  4024 | 41 |
| {BcHV1SLR.BI=TRUE, BcMV2.BI=TRUE} =>  {BcULV1.BI=TRUE} | 0.10  1485 | 0.372  727 | 0.272  277 | 1.73  0825 | 41 |
| {BcMV2.BI=TRUE, BcULV1.BI=TRUE} =>  {BcMV3.BI=TRUE} | 0.10  6436 | 0.767  857 | 0.138  614 | 1.41  6504 | 43 |
| {BcMV3.BI=TRUE, BcULV1.BI=TRUE} =>  {BcMV2.BI=TRUE} | 0.10  6436 | 0.704  918 | 0.150  99 | 1.52  2925 | 43 |
| {BcMV2.BI=TRUE, BcMV3.BI=TRUE} =>  {BcULV1.BI=TRUE} | 0.10  6436 | 0.338  583 | 0.314  356 | 1.57  2269 | 43 |
| {BcULV1.BI=TRUE, BcULV3=TRUE} =>  {BcMV2.BI=TRUE} | 0.08  6634 | 0.636  364 | 0.136  139 | 1.37  4818 | 35 |
| {BcMV2.BI=TRUE, BcULV3=TRUE} =>  {BcULV1.BI=TRUE} | 0.08  6634 | 0.304  348 | 0.284  653 | 1.41  3293 | 35 |
| {BcMV2.BI=TRUE, BcULV1.BI=TRUE} =>  {BcHV1.BI=TRUE} | 0.10  396 | 0.75 | 0.138  614 | 1.24  1803 | 42 |
| {BcHV1.BI=TRUE, BcULV1.BI=TRUE} =>  {BcMV2.BI=TRUE} | 0.10  396 | 0.591  549 | 0.175  743 | 1.27  8 | 42 |
| {BcHV1.BI=TRUE, BcMV2.BI=TRUE} =>  {BcULV1.BI=TRUE} | 0.10  396 | 0.355  932 | 0.292  079 | 1.65  2835 | 42 |

| {BcMV2.BI=TRUE, BcULV1.BI=TRUE} =>  {BcMV11=TRUE} | 0.10  6436 | 0.767  857 | 0.138  614 | 1.24  5841 | 43 |
| --- | --- | --- | --- | --- | --- |
| {BcMV11=TRUE, BcULV1.BI=TRUE} =>  {BcMV2.BI=TRUE} | 0.10  6436 | 0.741  379 | 0.143  564 | 1.60  1696 | 43 |
| {BcMV11=TRUE, BcMV2.BI=TRUE} =>  {BcULV1.BI=TRUE} | 0.10  6436 | 0.311  594 | 0.341  584 | 1.44  6943 | 43 |
| {BcMV2.BI=TRUE, BcULV1.BI=TRUE} =>  {BcMV9.BI=TRUE} | 0.11  1386 | 0.803  571 | 0.138  614 | 1.18  4828 | 45 |
| {BcMV9.BI=TRUE, BcULV1.BI=TRUE} =>  {BcMV2.BI=TRUE} | 0.11  1386 | 0.625 | 0.178  218 | 1.35  0267 | 45 |
| {BcMV2.BI=TRUE, BcMV9.BI=TRUE} =>  {BcULV1.BI=TRUE} | 0.11  1386 | 0.346  154 | 0.321  782 | 1.60  7427 | 45 |
| {BcMV2.BI=TRUE, BcULV1.BI=TRUE} =>  {BcULV4=TRUE} | 0.12  1287 | 0.875 | 0.138  614 | 1.26  7025 | 49 |
| {BcULV1.BI=TRUE, BcULV4=TRUE} =>  {BcMV2.BI=TRUE} | 0.12  1287 | 0.628  205 | 0.193  069 | 1.35  7192 | 49 |
| {BcMV2.BI=TRUE, BcULV4=TRUE} =>  {BcULV1.BI=TRUE} | 0.12  1287 | 0.355  072 | 0.341  584 | 1.64  8842 | 49 |
| {BcEV2.BI=FALSE, BcULV1.BI=TRUE} =>  {BcMV2.BI=TRUE} | 0.10  1485 | 0.640  625 | 0.158  416 | 1.38  4024 | 41 |
| {BcEV2.BI=FALSE, BcMV2.BI=TRUE} =>  {BcULV1.BI=TRUE} | 0.10  1485 | 0.317  829 | 0.319  307 | 1.47  5898 | 41 |

| {BcHV3.BI=FALSE, BcULV1.BI=TRUE} =>  {BcMV2.BI=TRUE} | 0.09  1584 | 0.587  302 | 0.155  941 | 1.26  8823 | 37 |
| --- | --- | --- | --- | --- | --- |
| {BcFV5.BI=FALSE, BcULV1.BI=TRUE} =>  {BcMV2.BI=TRUE} | 0.10  6436 | 0.623  188 | 0.170  792 | 1.34  6354 | 43 |
| {BcFV5.BI=FALSE, BcMV2.BI=TRUE} =>  {BcULV1.BI=TRUE} | 0.10  6436 | 0.309  353 | 0.344  059 | 1.43  6534 | 43 |
| {BcMV2.BI=TRUE, BcULV1.BI=TRUE} =>  {BcMV1.BI=TRUE} | 0.12  3762 | 0.892  857 | 0.138  614 | 1.13  0766 | 50 |
| {BcMV1.BI=TRUE, BcULV1.BI=TRUE} =>  {BcMV2.BI=TRUE} | 0.12  3762 | 0.657  895 | 0.188  119 | 1.42  1334 | 50 |
| {BcMV1.BI=TRUE, BcMV2.BI=TRUE} =>  {BcULV1.BI=TRUE} | 0.12  3762 | 0.303  03 | 0.408  416 | 1.40  7175 | 50 |
| {BcNSRV7.BI=FALSE, BcULV1.BI=TRUE}  => {BcMV2.BI=TRUE} | 0.11  6337 | 0.626  667 | 0.185  644 | 1.35  3868 | 47 |
| {BcMV2.BI=TRUE, BcNSRV7.BI=FALSE} =>  {BcULV1.BI=TRUE} | 0.11  6337 | 0.321  918 | 0.361  386 | 1.49  4883 | 47 |
| {BcULV1.BI=TRUE, BcVV3.BI=FALSE} =>  {BcMV2.BI=TRUE} | 0.10  6436 | 0.614  286 | 0.173  267 | 1.32  712 | 43 |
| {BcMV2.BI=TRUE, BcVV3.BI=FALSE} =>  {BcULV1.BI=TRUE} | 0.10  6436 | 0.302  817 | 0.351  485 | 1.40  6184 | 43 |
| {BcOLV7.BI=FALSE, BcULV1.BI=TRUE} =>  {BcMV2.BI=TRUE} | 0.09  4059 | 0.603  175 | 0.155  941 | 1.30  3115 | 38 |

| {BcALV1.BI=FALSE, BcULV1.BI=TRUE} =>  {BcMV2.BI=TRUE} | 0.10  1485 | 0.611  94 | 0.165  842 | 1.32  2053 | 41 |
| --- | --- | --- | --- | --- | --- |
| {BcALV1.BI=FALSE, BcMV2.BI=TRUE} =>  {BcULV1.BI=TRUE} | 0.10  1485 | 0.312  977 | 0.324  257 | 1.45  3365 | 41 |
| {BcDMV1=FALSE, BcULV1.BI=TRUE} =>  {BcMV2.BI=TRUE} | 0.12  3762 | 0.632  911 | 0.195  545 | 1.36  7359 | 50 |
| {BcDMV1=FALSE, BcMV2.BI=TRUE} =>  {BcULV1.BI=TRUE} | 0.12  3762 | 0.312  5 | 0.396  04 | 1.45  1149 | 50 |
| {BcFV9=FALSE, BcULV1.BI=TRUE} =>  {BcMV2.BI=TRUE} | 0.12  6238 | 0.662  338 | 0.190  594 | 1.43  0933 | 51 |
| {BcFV9=FALSE, BcMV2.BI=TRUE} =>  {BcULV1.BI=TRUE} | 0.12  6238 | 0.314  815 | 0.400  99 | 1.46  1899 | 51 |
| {BcOLV4.BI=FALSE, BcULV1.BI=TRUE} =>  {BcMV2.BI=TRUE} | 0.10  6436 | 0.589  041 | 0.180  693 | 1.27  2581 | 43 |
| {BcMV2.BI=TRUE, BcOLV4.BI=FALSE} =>  {BcULV1.BI=TRUE} | 0.10  6436 | 0.282  895 | 0.376  238 | 1.31  3672 | 43 |
| {BcULV1.BI=TRUE, BcULV5=FALSE} =>  {BcMV2.BI=TRUE} | 0.10  8911 | 0.619  718 | 0.175  743 | 1.33  8857 | 44 |
| {BcMV2.BI=TRUE, BcULV5=FALSE} =>  {BcULV1.BI=TRUE} | 0.10  8911 | 0.289  474 | 0.376  238 | 1.34  4223 | 44 |
| {BcBNV5.BI=FALSE, BcULV1.BI=TRUE} =>  {BcMV2.BI=TRUE} | 0.12  8713 | 0.658  228 | 0.195  545 | 1.42  2054 | 52 |

| {BcBNV5.BI=FALSE, BcMV2.BI=TRUE} =>  {BcULV1.BI=TRUE} | 0.12  8713 | 0.315  152 | 0.408  416 | 1.46  3462 | 52 |
| --- | --- | --- | --- | --- | --- |
| {BcULV1.BI=TRUE, BvF.BI=FALSE} =>  {BcMV2.BI=TRUE} | 0.13  1188 | 0.654  321 | 0.200  495 | 1.41  3613 | 53 |
| {BcMV2.BI=TRUE, BvF.BI=FALSE} =>  {BcULV1.BI=TRUE} | 0.13  1188 | 0.302  857 | 0.433  168 | 1.40  6371 | 53 |
| {BcMCV5.BI=FALSE, BcULV1.BI=TRUE} =>  {BcMV2.BI=TRUE} | 0.12  3762 | 0.641  026 | 0.193  069 | 1.38  489 | 50 |
| {BcMCV5.BI=FALSE, BcMV2.BI=TRUE} =>  {BcULV1.BI=TRUE} | 0.12  3762 | 0.308  642 | 0.400  99 | 1.43  3234 | 50 |
| {BcOLV13.BI=FALSE, BcULV1.BI=TRUE} =>  {BcMV2.BI=TRUE} | 0.11  6337 | 0.626  667 | 0.185  644 | 1.35  3868 | 47 |
| {BcMV2.BI=TRUE, BcOLV13.BI=FALSE} =>  {BcULV1.BI=TRUE} | 0.11  6337 | 0.284  848 | 0.408  416 | 1.32  2745 | 47 |
| {BcOLV2.BI=FALSE, BcULV1.BI=TRUE} =>  {BcMV2.BI=TRUE} | 0.11  6337 | 0.626  667 | 0.185  644 | 1.35  3868 | 47 |
| {BcMV2.BI=TRUE, BcOLV2.BI=FALSE} =>  {BcULV1.BI=TRUE} | 0.11  6337 | 0.276  471 | 0.420  792 | 1.28  384 | 47 |
| {BcMCV3.BI=FALSE, BcULV1.BI=TRUE} =>  {BcMV2.BI=TRUE} | 0.09  901 | 0.588  235 | 0.168  317 | 1.27  084 | 40 |
| {BcPV4.BI=FALSE, BcULV1.BI=TRUE} =>  {BcMV2.BI=TRUE} | 0.12  1287 | 0.636  364 | 0.190  594 | 1.37  4818 | 49 |

| {BcMV2.BI=TRUE, BcPV4.BI=FALSE} =>  {BcULV1.BI=TRUE} | 0.12  1287 | 0.283  237 | 0.428  218 | 1.31  5261 | 49 |
| --- | --- | --- | --- | --- | --- |
| {BcEV6=FALSE, BcULV1.BI=TRUE} =>  {BcMV2.BI=TRUE} | 0.13  1188 | 0.662  5 | 0.198  02 | 1.43  1283 | 53 |
| {BcEV6=FALSE, BcMV2.BI=TRUE} =>  {BcULV1.BI=TRUE} | 0.13  1188 | 0.302  857 | 0.433  168 | 1.40  6371 | 53 |
| {BcOLV17.BI=FALSE, BcULV1.BI=TRUE} =>  {BcMV2.BI=TRUE} | 0.12  6238 | 0.637  5 | 0.198  02 | 1.37  7273 | 51 |
| {BcMV2.BI=TRUE, BcOLV17.BI=FALSE} =>  {BcULV1.BI=TRUE} | 0.12  6238 | 0.296  512 | 0.425  743 | 1.37  6905 | 51 |
| {BcOLV15.BI=FALSE, BcULV1.BI=TRUE} =>  {BcMV2.BI=TRUE} | 0.13  8614 | 0.658  824 | 0.210  396 | 1.42  3341 | 56 |
| {BcMV2.BI=TRUE, BcOLV15.BI=FALSE} =>  {BcULV1.BI=TRUE} | 0.13  8614 | 0.306  011 | 0.452  97 | 1.42  1016 | 56 |
| {BcEV5=FALSE, BcULV1.BI=TRUE} =>  {BcMV2.BI=TRUE} | 0.13  1188 | 0.630  952 | 0.207  921 | 1.36  3127 | 53 |
| {BcEV5=FALSE, BcMV2.BI=TRUE} =>  {BcULV1.BI=TRUE} | 0.13  1188 | 0.309  942 | 0.423  267 | 1.43  9269 | 53 |
| {BcOLV18=TRUE, BcULV1.BI=TRUE} =>  {BcHV1SLR.BI=TRUE} | 0.11  8812 | 0.738  462 | 0.160  891 | 1.36  2276 | 48 |
| {BcHV1SLR.BI=TRUE, BcULV1.BI=TRUE}  => {BcOLV18=TRUE} | 0.11  8812 | 0.75 | 0.158  416 | 1.44  9761 | 48 |

| {BcHV1SLR.BI=TRUE, BcOLV18=TRUE} =>  {BcULV1.BI=TRUE} | 0.11  8812 | 0.347  826 | 0.341  584 | 1.61  5192 | 48 |
| --- | --- | --- | --- | --- | --- |
| {BcOLV18=TRUE, BcULV1.BI=TRUE} =>  {BcMV3.BI=TRUE} | 0.11  3861 | 0.707  692 | 0.160  891 | 1.30  5515 | 46 |
| {BcMV3.BI=TRUE, BcULV1.BI=TRUE} =>  {BcOLV18=TRUE} | 0.11  3861 | 0.754  098 | 0.150  99 | 1.45  7683 | 46 |
| {BcMV3.BI=TRUE, BcOLV18=TRUE} =>  {BcULV1.BI=TRUE} | 0.11  3861 | 0.345  865 | 0.329  208 | 1.60  6084 | 46 |
| {BcULV1.BI=TRUE, BcULV3=TRUE} =>  {BcOLV18=TRUE} | 0.10  396 | 0.763  636 | 0.136  139 | 1.47  612 | 42 |
| {BcOLV18=TRUE, BcULV3=TRUE} =>  {BcULV1.BI=TRUE} | 0.10  396 | 0.320  611 | 0.324  257 | 1.48  8813 | 42 |
| {BcOLV18=TRUE, BcULV1.BI=TRUE} =>  {BcHV1.BI=TRUE} | 0.13  6139 | 0.846  154 | 0.160  891 | 1.40  1009 | 55 |
| {BcHV1.BI=TRUE, BcULV1.BI=TRUE} =>  {BcOLV18=TRUE} | 0.13  6139 | 0.774  648 | 0.175  743 | 1.49  7405 | 55 |
| {BcHV1.BI=TRUE, BcOLV18=TRUE} =>  {BcULV1.BI=TRUE} | 0.13  6139 | 0.371  622 | 0.366  337 | 1.72  5691 | 55 |
| {BcMV11=TRUE, BcULV1.BI=TRUE} =>  {BcOLV18=TRUE} | 0.10  396 | 0.724  138 | 0.143  564 | 1.39  9769 | 42 |
| {BcMV11=TRUE, BcOLV18=TRUE} =>  {BcULV1.BI=TRUE} | 0.10  396 | 0.3 | 0.346  535 | 1.39  3103 | 42 |

| {BcOLV1.BI.MOVA.=FALSE,  BcULV1.BI=TRUE} => {BcOLV18=TRUE} | 0.07  9208 | 0.727  273 | 0.108  911 | 1.40  5829 | 32 |
| --- | --- | --- | --- | --- | --- |
| {BcOLV18=TRUE, BcULV1.BI=TRUE} =>  {BcMV9.BI=TRUE} | 0.15  099 | 0.938  462 | 0.160  891 | 1.38  3717 | 61 |
| {BcMV9.BI=TRUE, BcULV1.BI=TRUE} =>  {BcOLV18=TRUE} | 0.15  099 | 0.847  222 | 0.178  218 | 1.63  7693 | 61 |
| {BcMV9.BI=TRUE, BcOLV18=TRUE} =>  {BcULV1.BI=TRUE} | 0.15  099 | 0.365  269 | 0.413  366 | 1.69  6194 | 61 |
| {BcOLV18=TRUE, BcULV1.BI=TRUE} =>  {BcULV4=TRUE} | 0.15  5941 | 0.969  231 | 0.160  891 | 1.40  3474 | 63 |
| {BcULV1.BI=TRUE, BcULV4=TRUE} =>  {BcOLV18=TRUE} | 0.15  5941 | 0.807  692 | 0.193  069 | 1.56  1281 | 63 |
| {BcOLV18=TRUE, BcULV4=TRUE} =>  {BcULV1.BI=TRUE} | 0.15  5941 | 0.353  933 | 0.440  594 | 1.64  3549 | 63 |
| {BcMV6.BI=FALSE, BcULV1.BI=TRUE} =>  {BcOLV18=TRUE} | 0.09  901 | 0.8 | 0.123  762 | 1.54  6411 | 40 |
| {BcMV6.BI=FALSE, BcOLV18=TRUE} =>  {BcULV1.BI=TRUE} | 0.09  901 | 0.287  77 | 0.344  059 | 1.33  631 | 40 |
| {BcPV3.BI=FALSE, BcULV1.BI=TRUE} =>  {BcOLV18=TRUE} | 0.09  4059 | 0.678  571 | 0.138  614 | 1.31  1688 | 38 |
| {BcOLV18=TRUE, BcPV3.BI=FALSE} =>  {BcULV1.BI=TRUE} | 0.09  4059 | 0.285  714 | 0.329  208 | 1.32  6765 | 38 |

| {BcRV1.BI=FALSE, BcULV1.BI=TRUE} =>  {BcOLV18=TRUE} | 0.09  1584 | 0.660  714 | 0.138  614 | 1.27  717 | 37 |
| --- | --- | --- | --- | --- | --- |
| {BcEV2.BI=FALSE, BcULV1.BI=TRUE} =>  {BcOLV18=TRUE} | 0.12  1287 | 0.765  625 | 0.158  416 | 1.47  9964 | 49 |
| {BcEV2.BI=FALSE, BcOLV18=TRUE} =>  {BcULV1.BI=TRUE} | 0.12  1287 | 0.328  859 | 0.368  812 | 1.52  7116 | 49 |
| {BcHV3.BI=FALSE, BcULV1.BI=TRUE} =>  {BcOLV18=TRUE} | 0.10  8911 | 0.698  413 | 0.155  941 | 1.35  0042 | 44 |
| {BcHV3.BI=FALSE, BcOLV18=TRUE} =>  {BcULV1.BI=TRUE} | 0.10  8911 | 0.309  859 | 0.351  485 | 1.43  8886 | 44 |
| {BcFV5.BI=FALSE, BcULV1.BI=TRUE} =>  {BcOLV18=TRUE} | 0.12  3762 | 0.724  638 | 0.170  792 | 1.40  0735 | 50 |
| {BcFV5.BI=FALSE, BcOLV18=TRUE} =>  {BcULV1.BI=TRUE} | 0.12  3762 | 0.335  57 | 0.368  812 | 1.55  8281 | 50 |
| {BcOLV18=TRUE, BcULV1.BI=TRUE} =>  {BcMV1.BI=TRUE} | 0.14  3564 | 0.892  308 | 0.160  891 | 1.13  007 | 58 |
| {BcMV1.BI=TRUE, BcULV1.BI=TRUE} =>  {BcOLV18=TRUE} | 0.14  3564 | 0.763  158 | 0.188  119 | 1.47  5195 | 58 |
| {BcMV1.BI=TRUE, BcOLV18=TRUE} =>  {BcULV1.BI=TRUE} | 0.14  3564 | 0.325  843 | 0.440  594 | 1.51  3109 | 58 |
| {BcMV12=FALSE, BcULV1.BI=TRUE} =>  {BcOLV18=TRUE} | 0.11  1386 | 0.762  712 | 0.146  04 | 1.47  4333 | 45 |

| {BcMV12=FALSE, BcOLV18=TRUE} =>  {BcULV1.BI=TRUE} | 0.11  1386 | 0.281  25 | 0.396  04 | 1.30  6034 | 45 |
| --- | --- | --- | --- | --- | --- |
| {BcNSRV7.BI=FALSE, BcULV1.BI=TRUE}  => {BcOLV18=TRUE} | 0.13  6139 | 0.733  333 | 0.185  644 | 1.41  7544 | 55 |
| {BcNSRV7.BI=FALSE, BcOLV18=TRUE} =>  {BcULV1.BI=TRUE} | 0.13  6139 | 0.350  318 | 0.388  614 | 1.62  6766 | 55 |
| {BcULV1.BI=TRUE, BcVV3.BI=FALSE} =>  {BcOLV18=TRUE} | 0.13  1188 | 0.757  143 | 0.173  267 | 1.46  3568 | 53 |
| {BcOLV18=TRUE, BcVV3.BI=FALSE} =>  {BcULV1.BI=TRUE} | 0.13  1188 | 0.319  277 | 0.410  891 | 1.48  262 | 53 |
| {BcOLV7.BI=FALSE, BcULV1.BI=TRUE} =>  {BcOLV18=TRUE} | 0.10  396 | 0.666  667 | 0.155  941 | 1.28  8676 | 42 |
| {BcOLV18=TRUE, BcOLV7.BI=FALSE} =>  {BcULV1.BI=TRUE} | 0.10  396 | 0.276  316 | 0.376  238 | 1.28  3122 | 42 |
| {BcALV1.BI=FALSE, BcULV1.BI=TRUE} =>  {BcOLV18=TRUE} | 0.11  8812 | 0.716  418 | 0.165  842 | 1.38  4846 | 48 |
| {BcALV1.BI=FALSE, BcOLV18=TRUE} =>  {BcULV1.BI=TRUE} | 0.11  8812 | 0.315  789 | 0.376  238 | 1.46  6425 | 48 |
| {BcDMV1=FALSE, BcULV1.BI=TRUE} =>  {BcOLV18=TRUE} | 0.14  3564 | 0.734  177 | 0.195  545 | 1.41  9175 | 58 |
| {BcDMV1=FALSE, BcOLV18=TRUE} =>  {BcULV1.BI=TRUE} | 0.14  3564 | 0.335  26 | 0.428  218 | 1.55  684 | 58 |

| {BcFV9=FALSE, BcULV1.BI=TRUE} =>  {BcOLV18=TRUE} | 0.14  1089 | 0.740  26 | 0.190  594 | 1.43  0933 | 57 |
| --- | --- | --- | --- | --- | --- |
| {BcFV9=FALSE, BcOLV18=TRUE} =>  {BcULV1.BI=TRUE} | 0.14  1089 | 0.335  294 | 0.420  792 | 1.55  6998 | 57 |
| {BcOLV4.BI=FALSE, BcULV1.BI=TRUE} =>  {BcOLV18=TRUE} | 0.14  1089 | 0.780  822 | 0.180  693 | 1.50  934 | 57 |
| {BcOLV18=TRUE, BcOLV4.BI=FALSE} =>  {BcULV1.BI=TRUE} | 0.14  1089 | 0.314  917 | 0.448  02 | 1.46  2374 | 57 |
| {BcULV1.BI=TRUE, BcULV5=FALSE} =>  {BcOLV18=TRUE} | 0.13  8614 | 0.788  732 | 0.175  743 | 1.52  4631 | 56 |
| {BcOLV18=TRUE, BcULV5=FALSE} =>  {BcULV1.BI=TRUE} | 0.13  8614 | 0.296  296 | 0.467  822 | 1.37  5905 | 56 |
| {BcBNV5.BI=FALSE, BcULV1.BI=TRUE} =>  {BcOLV18=TRUE} | 0.14  8515 | 0.759  494 | 0.195  545 | 1.46  8112 | 60 |
| {BcBNV5.BI=FALSE, BcOLV18=TRUE} =>  {BcULV1.BI=TRUE} | 0.14  8515 | 0.327  869 | 0.452  97 | 1.52  2517 | 60 |
| {BcULV1.BI=TRUE, BvF.BI=FALSE} =>  {BcOLV18=TRUE} | 0.15  3465 | 0.765  432 | 0.200  495 | 1.47  9591 | 62 |
| {BcOLV18=TRUE, BvF.BI=FALSE} =>  {BcULV1.BI=TRUE} | 0.15  3465 | 0.322  917 | 0.475  248 | 1.49  9521 | 62 |
| {BcMCV5.BI=FALSE, BcULV1.BI=TRUE} =>  {BcOLV18=TRUE} | 0.14  1089 | 0.730  769 | 0.193  069 | 1.41  2587 | 57 |

| {BcMCV5.BI=FALSE, BcOLV18=TRUE} =>  {BcULV1.BI=TRUE} | 0.14  1089 | 0.313  187 | 0.450  495 | 1.45  4339 | 57 |
| --- | --- | --- | --- | --- | --- |
| {BcOLV13.BI=FALSE, BcULV1.BI=TRUE} =>  {BcOLV18=TRUE} | 0.14  604 | 0.786  667 | 0.185  644 | 1.52  0638 | 59 |
| {BcOLV13.BI=FALSE, BcOLV18=TRUE} =>  {BcULV1.BI=TRUE} | 0.14  604 | 0.307  292 | 0.475  248 | 1.42  6964 | 59 |
| {BcOLV2.BI=FALSE, BcULV1.BI=TRUE} =>  {BcOLV18=TRUE} | 0.14  1089 | 0.76 | 0.185  644 | 1.46  9091 | 57 |
| {BcOLV18=TRUE, BcOLV2.BI=FALSE} =>  {BcULV1.BI=TRUE} | 0.14  1089 | 0.301  587 | 0.467  822 | 1.40  0474 | 57 |
| {BcMCV3.BI=FALSE, BcULV1.BI=TRUE} =>  {BcOLV18=TRUE} | 0.12  8713 | 0.764  706 | 0.168  317 | 1.47  8187 | 52 |
| {BcMCV3.BI=FALSE, BcOLV18=TRUE} =>  {BcULV1.BI=TRUE} | 0.12  8713 | 0.270  833 | 0.475  248 | 1.25  7663 | 52 |
| {BcPV4.BI=FALSE, BcULV1.BI=TRUE} =>  {BcOLV18=TRUE} | 0.14  604 | 0.766  234 | 0.190  594 | 1.48  1141 | 59 |
| {BcOLV18=TRUE, BcPV4.BI=FALSE} =>  {BcULV1.BI=TRUE} | 0.14  604 | 0.305  699 | 0.477  723 | 1.41  957 | 59 |
| {BcEV6=FALSE, BcULV1.BI=TRUE} =>  {BcOLV18=TRUE} | 0.14  8515 | 0.75 | 0.198  02 | 1.44  9761 | 60 |
| {BcEV6=FALSE, BcOLV18=TRUE} =>  {BcULV1.BI=TRUE} | 0.14  8515 | 0.307  692 | 0.482  673 | 1.42  8824 | 60 |

| {BcOLV17.BI=FALSE, BcULV1.BI=TRUE} =>  {BcOLV18=TRUE} | 0.14  3564 | 0.725 | 0.198  02 | 1.40  1435 | 58 |
| --- | --- | --- | --- | --- | --- |
| {BcOLV17.BI=FALSE, BcOLV18=TRUE} =>  {BcULV1.BI=TRUE} | 0.14  3564 | 0.300  518 | 0.477  723 | 1.39  5509 | 58 |
| {BcOLV15.BI=FALSE, BcULV1.BI=TRUE} =>  {BcOLV18=TRUE} | 0.15  8416 | 0.752  941 | 0.210  396 | 1.45  5446 | 64 |
| {BcOLV15.BI=FALSE, BcOLV18=TRUE} =>  {BcULV1.BI=TRUE} | 0.15  8416 | 0.331  606 | 0.477  723 | 1.53  9873 | 64 |
| {BcEV5=FALSE, BcULV1.BI=TRUE} =>  {BcOLV18=TRUE} | 0.15  3465 | 0.738  095 | 0.207  921 | 1.42  6749 | 62 |
| {BcEV5=FALSE, BcOLV18=TRUE} =>  {BcULV1.BI=TRUE} | 0.15  3465 | 0.314  721 | 0.487  624 | 1.46  1462 | 62 |
| {BcHV1SLR.BI=TRUE, BcULV1.BI=TRUE}  => {BcMV3.BI=TRUE} | 0.11  6337 | 0.734  375 | 0.158  416 | 1.35  4737 | 47 |
| {BcMV3.BI=TRUE, BcULV1.BI=TRUE} =>  {BcHV1SLR.BI=TRUE} | 0.11  6337 | 0.770  492 | 0.150  99 | 1.42  1364 | 47 |
| {BcHV1SLR.BI=TRUE, BcMV3.BI=TRUE} =>  {BcULV1.BI=TRUE} | 0.11  6337 | 0.353  383 | 0.329  208 | 1.64  0999 | 47 |
| {BcMV4.BI=FALSE, BcULV1.BI=TRUE} =>  {BcHV1SLR.BI=TRUE} | 0.08  1683 | 0.767  442 | 0.106  436 | 1.41  5737 | 33 |
| {BcHV1SLR.BI=TRUE, BcMV4.BI=FALSE}  => {BcULV1.BI=TRUE} | 0.08  1683 | 0.292  035 | 0.279  703 | 1.35  6118 | 33 |

| {BcHV1SLR.BI=TRUE, BcULV1.BI=TRUE}  => {BcULV3=TRUE} | 0.11  1386 | 0.703  125 | 0.158  416 | 1.18  3594 | 45 |
| --- | --- | --- | --- | --- | --- |
| {BcULV1.BI=TRUE, BcULV3=TRUE} =>  {BcHV1SLR.BI=TRUE} | 0.11  1386 | 0.818  182 | 0.136  139 | 1.50  934 | 45 |
| {BcHV1SLR.BI=TRUE, BcULV3=TRUE} =>  {BcULV1.BI=TRUE} | 0.11  1386 | 0.288  462 | 0.386  139 | 1.33  9523 | 45 |
| {BcHV1SLR.BI=TRUE, BcULV1.BI=TRUE}  => {BcHV1.BI=TRUE} | 0.15  099 | 0.953  125 | 0.158  416 | 1.57  8125 | 61 |
| {BcHV1.BI=TRUE, BcULV1.BI=TRUE} =>  {BcHV1SLR.BI=TRUE} | 0.15  099 | 0.859  155 | 0.175  743 | 1.58  4925 | 61 |
| {BcHV1.BI=TRUE, BcHV1SLR.BI=TRUE} =>  {BcULV1.BI=TRUE} | 0.15  099 | 0.311  224 | 0.485  149 | 1.44  5226 | 61 |
| {BcMV11=TRUE, BcULV1.BI=TRUE} =>  {BcHV1SLR.BI=TRUE} | 0.09  901 | 0.689  655 | 0.143  564 | 1.27  2241 | 40 |
| {BcOLV1.BI.MOVA.=FALSE,  BcULV1.BI=TRUE} => {BcHV1SLR.BI=TRUE} | 0.08  4158 | 0.772  727 | 0.108  911 | 1.42  5488 | 34 |
| {BcHV1SLR.BI=TRUE, BcULV1.BI=TRUE}  => {BcMV9.BI=TRUE} | 0.13  3663 | 0.843  75 | 0.158  416 | 1.24  4069 | 54 |
| {BcMV9.BI=TRUE, BcULV1.BI=TRUE} =>  {BcHV1SLR.BI=TRUE} | 0.13  3663 | 0.75 | 0.178  218 | 1.38  3562 | 54 |
| {BcHV1SLR.BI=TRUE, BcMV9.BI=TRUE} =>  {BcULV1.BI=TRUE} | 0.13  3663 | 0.327  273 | 0.408  416 | 1.51  9749 | 54 |

| {BcHV1SLR.BI=TRUE, BcULV1.BI=TRUE}  => {BcULV4=TRUE} | 0.14  1089 | 0.890  625 | 0.158  416 | 1.28  9651 | 57 |
| --- | --- | --- | --- | --- | --- |
| {BcULV1.BI=TRUE, BcULV4=TRUE} =>  {BcHV1SLR.BI=TRUE} | 0.14  1089 | 0.730  769 | 0.193  069 | 1.34  8086 | 57 |
| {BcHV1SLR.BI=TRUE, BcULV4=TRUE} =>  {BcULV1.BI=TRUE} | 0.14  1089 | 0.351  852 | 0.400  99 | 1.63  3887 | 57 |
| {BcMV6.BI=FALSE, BcULV1.BI=TRUE} =>  {BcHV1SLR.BI=TRUE} | 0.08  4158 | 0.68 | 0.123  762 | 1.25  4429 | 34 |
| {BcPV3.BI=FALSE, BcULV1.BI=TRUE} =>  {BcHV1SLR.BI=TRUE} | 0.09  901 | 0.714  286 | 0.138  614 | 1.31  7678 | 40 |
| {BcRV1.BI=FALSE, BcULV1.BI=TRUE} =>  {BcHV1SLR.BI=TRUE} | 0.09  4059 | 0.678  571 | 0.138  614 | 1.25  1794 | 38 |
| {BcEV2.BI=FALSE, BcULV1.BI=TRUE} =>  {BcHV1SLR.BI=TRUE} | 0.12  1287 | 0.765  625 | 0.158  416 | 1.41  2386 | 49 |
| {BcEV2.BI=FALSE, BcHV1SLR.BI=TRUE}  => {BcULV1.BI=TRUE} | 0.12  1287 | 0.314  103 | 0.386  139 | 1.45  8591 | 49 |
| {BcHV3.BI=FALSE, BcULV1.BI=TRUE} =>  {BcHV1SLR.BI=TRUE} | 0.11  3861 | 0.730  159 | 0.155  941 | 1.34  6959 | 46 |
| {BcHV1SLR.BI=TRUE, BcHV3.BI=FALSE}  => {BcULV1.BI=TRUE} | 0.11  3861 | 0.289  308 | 0.393  564 | 1.34  3454 | 46 |
| {BcFV5.BI=FALSE, BcULV1.BI=TRUE} =>  {BcHV1SLR.BI=TRUE} | 0.12  3762 | 0.724  638 | 0.170  792 | 1.33  6775 | 50 |

| {BcFV5.BI=FALSE, BcHV1SLR.BI=TRUE}  => {BcULV1.BI=TRUE} | 0.12  3762 | 0.316  456 | 0.391  089 | 1.46  9518 | 50 |
| --- | --- | --- | --- | --- | --- |
| {BcMV1.BI=TRUE, BcULV1.BI=TRUE} =>  {BcHV1SLR.BI=TRUE} | 0.13  8614 | 0.736  842 | 0.188  119 | 1.35  9289 | 56 |
| {BcHV1SLR.BI=TRUE, BcMV1.BI=TRUE} =>  {BcULV1.BI=TRUE} | 0.13  8614 | 0.314  607 | 0.440  594 | 1.46  0932 | 56 |
| {BcMV12=FALSE, BcULV1.BI=TRUE} =>  {BcHV1SLR.BI=TRUE} | 0.09  901 | 0.677  966 | 0.146  04 | 1.25  0677 | 40 |
| {BcHV1SLR.BI=TRUE, BcULV1.BI=TRUE}  => {BcNSRV7.BI=FALSE} | 0.14  1089 | 0.890  625 | 0.158  416 | 1.11  3971 | 57 |
| {BcNSRV7.BI=FALSE, BcULV1.BI=TRUE}  => {BcHV1SLR.BI=TRUE} | 0.14  1089 | 0.76 | 0.185  644 | 1.40  2009 | 57 |
| {BcHV1SLR.BI=TRUE,  BcNSRV7.BI=FALSE} => {BcULV1.BI=TRUE} | 0.14  1089 | 0.345  455 | 0.408  416 | 1.60  418 | 57 |
| {BcULV1.BI=TRUE, BcVV3.BI=FALSE} =>  {BcHV1SLR.BI=TRUE} | 0.13  3663 | 0.771  429 | 0.173  267 | 1.42  3092 | 54 |
| {BcHV1SLR.BI=TRUE, BcVV3.BI=FALSE}  => {BcULV1.BI=TRUE} | 0.13  3663 | 0.308  571 | 0.433  168 | 1.43  2906 | 54 |
| {BcOLV7.BI=FALSE, BcULV1.BI=TRUE} =>  {BcHV1SLR.BI=TRUE} | 0.11  1386 | 0.714  286 | 0.155  941 | 1.31  7678 | 45 |
| {BcALV1.BI=FALSE, BcULV1.BI=TRUE} =>  {BcHV1SLR.BI=TRUE} | 0.12  1287 | 0.731  343 | 0.165  842 | 1.34  9145 | 49 |

| {BcALV1.BI=FALSE, BcHV1SLR.BI=TRUE}  => {BcULV1.BI=TRUE} | 0.12  1287 | 0.291  667 | 0.415  842 | 1.35  4406 | 49 |
| --- | --- | --- | --- | --- | --- |
| {BcHV1SLR.BI=TRUE, BcULV1.BI=TRUE}  => {BcDMV1=FALSE} | 0.14  604 | 0.921  875 | 0.158  416 | 1.09  2192 | 59 |
| {BcDMV1=FALSE, BcULV1.BI=TRUE} =>  {BcHV1SLR.BI=TRUE} | 0.14  604 | 0.746  835 | 0.195  545 | 1.37  7724 | 59 |
| {BcDMV1=FALSE, BcHV1SLR.BI=TRUE} =>  {BcULV1.BI=TRUE} | 0.14  604 | 0.313  83 | 0.465  347 | 1.45  7325 | 59 |
| {BcFV9=FALSE, BcULV1.BI=TRUE} =>  {BcHV1SLR.BI=TRUE} | 0.13  6139 | 0.714  286 | 0.190  594 | 1.31  7678 | 55 |
| {BcFV9=FALSE, BcHV1SLR.BI=TRUE} =>  {BcULV1.BI=TRUE} | 0.13  6139 | 0.302  198 | 0.450  495 | 1.40  3309 | 55 |
| {BcOLV4.BI=FALSE, BcULV1.BI=TRUE} =>  {BcHV1SLR.BI=TRUE} | 0.13  1188 | 0.726  027 | 0.180  693 | 1.33  9338 | 53 |
| {BcHV1SLR.BI=TRUE, BcOLV4.BI=FALSE}  => {BcULV1.BI=TRUE} | 0.13  1188 | 0.283  422 | 0.462  871 | 1.31  6123 | 53 |
| {BcULV1.BI=TRUE, BcULV5=FALSE} =>  {BcHV1SLR.BI=TRUE} | 0.12  6238 | 0.718  31 | 0.175  743 | 1.32  5101 | 51 |
| {BcHV1SLR.BI=TRUE, BcULV5=FALSE} =>  {BcULV1.BI=TRUE} | 0.12  6238 | 0.269  841 | 0.467  822 | 1.25  3056 | 51 |
| {BcBNV5.BI=FALSE, BcULV1.BI=TRUE} =>  {BcHV1SLR.BI=TRUE} | 0.14  8515 | 0.759  494 | 0.195  545 | 1.40  1075 | 60 |

| {BcBNV5.BI=FALSE, BcHV1SLR.BI=TRUE}  => {BcULV1.BI=TRUE} | 0.14  8515 | 0.310  881 | 0.477  723 | 1.44  3631 | 60 |
| --- | --- | --- | --- | --- | --- |
| {BcULV1.BI=TRUE, BvF.BI=FALSE} =>  {BcHV1SLR.BI=TRUE} | 0.14  8515 | 0.740  741 | 0.200  495 | 1.36  6481 | 60 |
| {BcHV1SLR.BI=TRUE, BvF.BI=FALSE} =>  {BcULV1.BI=TRUE} | 0.14  8515 | 0.309  278 | 0.480  198 | 1.43  6189 | 60 |
| {BcMCV5.BI=FALSE, BcULV1.BI=TRUE} =>  {BcHV1SLR.BI=TRUE} | 0.14  1089 | 0.730  769 | 0.193  069 | 1.34  8086 | 57 |
| {BcHV1SLR.BI=TRUE, BcMCV5.BI=FALSE}  => {BcULV1.BI=TRUE} | 0.14  1089 | 0.287  879 | 0.490  099 | 1.33  6816 | 57 |
| {BcOLV13.BI=FALSE, BcULV1.BI=TRUE} =>  {BcHV1SLR.BI=TRUE} | 0.13  1188 | 0.706  667 | 0.185  644 | 1.30  3623 | 53 |
| {BcOLV2.BI=FALSE, BcULV1.BI=TRUE} =>  {BcHV1SLR.BI=TRUE} | 0.12  8713 | 0.693  333 | 0.185  644 | 1.27  9026 | 52 |
| {BcMCV3.BI=FALSE, BcULV1.BI=TRUE} =>  {BcHV1SLR.BI=TRUE} | 0.11  6337 | 0.691  176 | 0.168  317 | 1.27  5047 | 47 |
| {BcPV4.BI=FALSE, BcULV1.BI=TRUE} =>  {BcHV1SLR.BI=TRUE} | 0.14  1089 | 0.740  26 | 0.190  594 | 1.36  5593 | 57 |
| {BcHV1SLR.BI=TRUE, BcPV4.BI=FALSE}  => {BcULV1.BI=TRUE} | 0.14  1089 | 0.279  412 | 0.504  95 | 1.29  7498 | 57 |
| {BcEV6=FALSE, BcULV1.BI=TRUE} =>  {BcHV1SLR.BI=TRUE} | 0.14  8515 | 0.75 | 0.198  02 | 1.38  3562 | 60 |

| {BcEV6=FALSE, BcHV1SLR.BI=TRUE} =>  {BcULV1.BI=TRUE} | 0.14  8515 | 0.295  567 | 0.502  475 | 1.37  2516 | 60 |
| --- | --- | --- | --- | --- | --- |
| {BcOLV17.BI=FALSE, BcULV1.BI=TRUE} =>  {BcHV1SLR.BI=TRUE} | 0.14  1089 | 0.712  5 | 0.198  02 | 1.31  4384 | 57 |
| {BcHV1SLR.BI=TRUE, BcOLV17.BI=FALSE}  => {BcULV1.BI=TRUE} | 0.14  1089 | 0.279  412 | 0.504  95 | 1.29  7498 | 57 |
| {BcOLV15.BI=FALSE, BcULV1.BI=TRUE} =>  {BcHV1SLR.BI=TRUE} | 0.15  3465 | 0.729  412 | 0.210  396 | 1.34  5582 | 62 |
| {BcHV1SLR.BI=TRUE, BcOLV15.BI=FALSE}  => {BcULV1.BI=TRUE} | 0.15  3465 | 0.306  931 | 0.5 | 1.42  5287 | 62 |
| {BcEV5=FALSE, BcULV1.BI=TRUE} =>  {BcHV1SLR.BI=TRUE} | 0.15  3465 | 0.738  095 | 0.207  921 | 1.36  16 | 62 |
| {BcEV5=FALSE, BcHV1SLR.BI=TRUE} =>  {BcULV1.BI=TRUE} | 0.15  3465 | 0.299  517 | 0.512  376 | 1.39  086 | 62 |
| {BcULV1.BI=TRUE, BcULV3=TRUE} =>  {BcMV3.BI=TRUE} | 0.10  396 | 0.763  636 | 0.136  139 | 1.40  8717 | 42 |
| {BcMV3.BI=TRUE, BcULV3=TRUE} =>  {BcULV1.BI=TRUE} | 0.10  396 | 0.3 | 0.346  535 | 1.39  3103 | 42 |
| {BcMV3.BI=TRUE, BcULV1.BI=TRUE} =>  {BcHV1.BI=TRUE} | 0.12  8713 | 0.852  459 | 0.150  99 | 1.41  1449 | 52 |
| {BcHV1.BI=TRUE, BcULV1.BI=TRUE} =>  {BcMV3.BI=TRUE} | 0.12  8713 | 0.732  394 | 0.175  743 | 1.35  1084 | 52 |

| {BcHV1.BI=TRUE, BcMV3.BI=TRUE} =>  {BcULV1.BI=TRUE} | 0.12  8713 | 0.363  636 | 0.353  96 | 1.68  861 | 52 |
| --- | --- | --- | --- | --- | --- |
| {BcMV11=TRUE, BcULV1.BI=TRUE} =>  {BcMV3.BI=TRUE} | 0.10  6436 | 0.741  379 | 0.143  564 | 1.36  7659 | 43 |
| {BcMV11=TRUE, BcMV3.BI=TRUE} =>  {BcULV1.BI=TRUE} | 0.10  6436 | 0.277  419 | 0.383  663 | 1.28  8246 | 43 |
| {BcMV3.BI=TRUE, BcULV1.BI=TRUE} =>  {BcMV9.BI=TRUE} | 0.12  6238 | 0.836  066 | 0.150  99 | 1.23  2739 | 51 |
| {BcMV9.BI=TRUE, BcULV1.BI=TRUE} =>  {BcMV3.BI=TRUE} | 0.12  6238 | 0.708  333 | 0.178  218 | 1.30  6697 | 51 |
| {BcMV3.BI=TRUE, BcMV9.BI=TRUE} =>  {BcULV1.BI=TRUE} | 0.12  6238 | 0.318  75 | 0.396  04 | 1.48  0172 | 51 |
| {BcMV3.BI=TRUE, BcULV1.BI=TRUE} =>  {BcULV4=TRUE} | 0.13  8614 | 0.918  033 | 0.150  99 | 1.32  9338 | 56 |
| {BcULV1.BI=TRUE, BcULV4=TRUE} =>  {BcMV3.BI=TRUE} | 0.13  8614 | 0.717  949 | 0.193  069 | 1.32  4435 | 56 |
| {BcMV3.BI=TRUE, BcULV4=TRUE} =>  {BcULV1.BI=TRUE} | 0.13  8614 | 0.345  679 | 0.400  99 | 1.60  5222 | 56 |
| {BcEV2.BI=FALSE, BcULV1.BI=TRUE} =>  {BcMV3.BI=TRUE} | 0.11  1386 | 0.703  125 | 0.158  416 | 1.29  7089 | 45 |
| {BcEV2.BI=FALSE, BcMV3.BI=TRUE} =>  {BcULV1.BI=TRUE} | 0.11  1386 | 0.276  074 | 0.403  465 | 1.28  1997 | 45 |

| {BcHV3.BI=FALSE, BcULV1.BI=TRUE} =>  {BcMV3.BI=TRUE} | 0.10  6436 | 0.682  54 | 0.155  941 | 1.25  9114 | 43 |
| --- | --- | --- | --- | --- | --- |
| {BcFV5.BI=FALSE, BcULV1.BI=TRUE} =>  {BcMV3.BI=TRUE} | 0.11  3861 | 0.666  667 | 0.170  792 | 1.22  9833 | 46 |
| {BcFV5.BI=FALSE, BcMV3.BI=TRUE} =>  {BcULV1.BI=TRUE} | 0.11  3861 | 0.292  994 | 0.388  614 | 1.36  0568 | 46 |
| {BcMV3.BI=TRUE, BcULV1.BI=TRUE} =>  {BcMV1.BI=TRUE} | 0.13  8614 | 0.918  033 | 0.150  99 | 1.16  265 | 56 |
| {BcMV1.BI=TRUE, BcULV1.BI=TRUE} =>  {BcMV3.BI=TRUE} | 0.13  8614 | 0.736  842 | 0.188  119 | 1.35  9289 | 56 |
| {BcMV1.BI=TRUE, BcMV3.BI=TRUE} =>  {BcULV1.BI=TRUE} | 0.13  8614 | 0.302  703 | 0.457  921 | 1.40  5654 | 56 |
| {BcNSRV7.BI=FALSE, BcULV1.BI=TRUE}  => {BcMV3.BI=TRUE} | 0.12  8713 | 0.693  333 | 0.185  644 | 1.27  9026 | 52 |
| {BcMV3.BI=TRUE, BcNSRV7.BI=FALSE} =>  {BcULV1.BI=TRUE} | 0.12  8713 | 0.304  094 | 0.423  267 | 1.41  2113 | 52 |
| {BcULV1.BI=TRUE, BcVV3.BI=FALSE} =>  {BcMV3.BI=TRUE} | 0.12  1287 | 0.7 | 0.173  267 | 1.29  1324 | 49 |
| {BcMV3.BI=TRUE, BcVV3.BI=FALSE} =>  {BcULV1.BI=TRUE} | 0.12  1287 | 0.291  667 | 0.415  842 | 1.35  4406 | 49 |
| {BcALV1.BI=FALSE, BcULV1.BI=TRUE} =>  {BcMV3.BI=TRUE} | 0.10  8911 | 0.656  716 | 0.165  842 | 1.21  1477 | 44 |

| {BcDMV1=FALSE, BcULV1.BI=TRUE} =>  {BcMV3.BI=TRUE} | 0.13  6139 | 0.696  203 | 0.195  545 | 1.28  4319 | 55 |
| --- | --- | --- | --- | --- | --- |
| {BcDMV1=FALSE, BcMV3.BI=TRUE} =>  {BcULV1.BI=TRUE} | 0.13  6139 | 0.321  637 | 0.423  267 | 1.49  3581 | 55 |
| {BcFV9=FALSE, BcULV1.BI=TRUE} =>  {BcMV3.BI=TRUE} | 0.13  6139 | 0.714  286 | 0.190  594 | 1.31  7678 | 55 |
| {BcFV9=FALSE, BcMV3.BI=TRUE} =>  {BcULV1.BI=TRUE} | 0.13  6139 | 0.298  913 | 0.455  446 | 1.38  8056 | 55 |
| {BcOLV4.BI=FALSE, BcULV1.BI=TRUE} =>  {BcMV3.BI=TRUE} | 0.12  3762 | 0.684  932 | 0.180  693 | 1.26  3527 | 50 |
| {BcMV3.BI=TRUE, BcOLV4.BI=FALSE} =>  {BcULV1.BI=TRUE} | 0.12  3762 | 0.271  739 | 0.455  446 | 1.26  1869 | 50 |
| {BcULV1.BI=TRUE, BcULV5=FALSE} =>  {BcMV3.BI=TRUE} | 0.11  8812 | 0.676  056 | 0.175  743 | 1.24  7154 | 48 |
| {BcBNV5.BI=FALSE, BcULV1.BI=TRUE} =>  {BcMV3.BI=TRUE} | 0.13  6139 | 0.696  203 | 0.195  545 | 1.28  4319 | 55 |
| {BcBNV5.BI=FALSE, BcMV3.BI=TRUE} =>  {BcULV1.BI=TRUE} | 0.13  6139 | 0.289  474 | 0.470  297 | 1.34  4223 | 55 |
| {BcULV1.BI=TRUE, BvF.BI=FALSE} =>  {BcMV3.BI=TRUE} | 0.14  1089 | 0.703  704 | 0.200  495 | 1.29  8157 | 57 |
| {BcMV3.BI=TRUE, BvF.BI=FALSE} =>  {BcULV1.BI=TRUE} | 0.14  1089 | 0.285 | 0.495  05 | 1.32  3448 | 57 |

| {BcMCV5.BI=FALSE, BcULV1.BI=TRUE} =>  {BcMV3.BI=TRUE} | 0.13  3663 | 0.692  308 | 0.193  069 | 1.27  7134 | 54 |
| --- | --- | --- | --- | --- | --- |
| {BcMCV5.BI=FALSE, BcMV3.BI=TRUE} =>  {BcULV1.BI=TRUE} | 0.13  3663 | 0.276  923 | 0.482  673 | 1.28  5942 | 54 |
| {BcOLV13.BI=FALSE, BcULV1.BI=TRUE} =>  {BcMV3.BI=TRUE} | 0.12  3762 | 0.666  667 | 0.185  644 | 1.22  9833 | 50 |
| {BcOLV2.BI=FALSE, BcULV1.BI=TRUE} =>  {BcMV3.BI=TRUE} | 0.12  3762 | 0.666  667 | 0.185  644 | 1.22  9833 | 50 |
| {BcPV4.BI=FALSE, BcULV1.BI=TRUE} =>  {BcMV3.BI=TRUE} | 0.13  1188 | 0.688  312 | 0.190  594 | 1.26  9762 | 53 |
| {BcEV6=FALSE, BcULV1.BI=TRUE} =>  {BcMV3.BI=TRUE} | 0.14  604 | 0.737  5 | 0.198  02 | 1.36  0502 | 59 |
| {BcEV6=FALSE, BcMV3.BI=TRUE} =>  {BcULV1.BI=TRUE} | 0.14  604 | 0.282  297 | 0.517  327 | 1.31  0895 | 59 |
| {BcOLV17.BI=FALSE, BcULV1.BI=TRUE} =>  {BcMV3.BI=TRUE} | 0.13  8614 | 0.7 | 0.198  02 | 1.29  1324 | 56 |
| {BcMV3.BI=TRUE, BcOLV17.BI=FALSE} =>  {BcULV1.BI=TRUE} | 0.13  8614 | 0.270  531 | 0.512  376 | 1.25  6261 | 56 |
| {BcOLV15.BI=FALSE, BcULV1.BI=TRUE} =>  {BcMV3.BI=TRUE} | 0.14  8515 | 0.705  882 | 0.210  396 | 1.30  2176 | 60 |
| {BcMV3.BI=TRUE, BcOLV15.BI=FALSE} =>  {BcULV1.BI=TRUE} | 0.14  8515 | 0.292  683 | 0.507  426 | 1.35  9125 | 60 |

| {BcEV5=FALSE, BcULV1.BI=TRUE} =>  {BcMV3.BI=TRUE} | 0.14  3564 | 0.690  476 | 0.207  921 | 1.27  3755 | 58 |
| --- | --- | --- | --- | --- | --- |
| {BcEV5=FALSE, BcMV3.BI=TRUE} =>  {BcULV1.BI=TRUE} | 0.14  3564 | 0.29 | 0.495  05 | 1.34  6667 | 58 |
| {BcMV4.BI=FALSE, BcULV1.BI=TRUE} =>  {BcHV1.BI=TRUE} | 0.09  1584 | 0.860  465 | 0.106  436 | 1.42  4705 | 37 |
| {BcHV1.BI=TRUE, BcMV4.BI=FALSE} =>  {BcULV1.BI=TRUE} | 0.09  1584 | 0.300  813 | 0.304  455 | 1.39  6879 | 37 |
| {BcMV4.BI=FALSE, BcULV1.BI=TRUE} =>  {BcULV4=TRUE} | 0.09  6535 | 0.906  977 | 0.106  436 | 1.31  3328 | 39 |
| {BcMV4.BI=FALSE, BcULV1.BI=TRUE} =>  {BcHV3.BI=FALSE} | 0.09  4059 | 0.883  721 | 0.106  436 | 1.16  2942 | 38 |
| {BcMV4.BI=FALSE, BcULV1.BI=TRUE} =>  {BcNSRV7.BI=FALSE} | 0.10  396 | 0.976  744 | 0.106  436 | 1.22  1686 | 42 |
| {BcMV4.BI=FALSE, BcULV1.BI=TRUE} =>  {BcDMV1=FALSE} | 0.10  1485 | 0.953  488 | 0.106  436 | 1.12  9646 | 41 |
| {BcULV1.BI=TRUE, BcULV3=TRUE} =>  {BcHV1.BI=TRUE} | 0.11  3861 | 0.836  364 | 0.136  139 | 1.38  4799 | 46 |
| {BcULV1.BI=TRUE, BcULV3=TRUE} =>  {BcMV9.BI=TRUE} | 0.11  1386 | 0.818  182 | 0.136  139 | 1.20  637 | 45 |
| {BcULV1.BI=TRUE, BcULV3=TRUE} =>  {BcULV4=TRUE} | 0.11  8812 | 0.872  727 | 0.136  139 | 1.26  3734 | 48 |

| {BcULV3=TRUE, BcULV4=TRUE} =>  {BcULV1.BI=TRUE} | 0.11  8812 | 0.285  714 | 0.415  842 | 1.32  6765 | 48 |
| --- | --- | --- | --- | --- | --- |
| {BcULV1.BI=TRUE, BcULV3=TRUE} =>  {BcMV1.BI=TRUE} | 0.12  1287 | 0.890  909 | 0.136  139 | 1.12  8299 | 49 |
| {BcMV11=TRUE, BcULV1.BI=TRUE} =>  {BcHV1.BI=TRUE} | 0.10  8911 | 0.758  621 | 0.143  564 | 1.25  6077 | 44 |
| {BcHV1.BI=TRUE, BcMV11=TRUE} =>  {BcULV1.BI=TRUE} | 0.10  8911 | 0.285  714 | 0.381  188 | 1.32  6765 | 44 |
| {BcOLV1.BI.MOVA.=FALSE,  BcULV1.BI=TRUE} => {BcHV1.BI=TRUE} | 0.09  4059 | 0.863  636 | 0.108  911 | 1.42  9955 | 38 |
| {BcHV1.BI=TRUE, BcULV1.BI=TRUE} =>  {BcMV9.BI=TRUE} | 0.14  8515 | 0.845  07 | 0.175  743 | 1.24  6016 | 60 |
| {BcMV9.BI=TRUE, BcULV1.BI=TRUE} =>  {BcHV1.BI=TRUE} | 0.14  8515 | 0.833  333 | 0.178  218 | 1.37  9781 | 60 |
| {BcHV1.BI=TRUE, BcMV9.BI=TRUE} =>  {BcULV1.BI=TRUE} | 0.14  8515 | 0.317  46 | 0.467  822 | 1.47  4184 | 60 |
| {BcHV1.BI=TRUE, BcULV1.BI=TRUE} =>  {BcULV4=TRUE} | 0.16  0891 | 0.915  493 | 0.175  743 | 1.32  566 | 65 |
| {BcULV1.BI=TRUE, BcULV4=TRUE} =>  {BcHV1.BI=TRUE} | 0.16  0891 | 0.833  333 | 0.193  069 | 1.37  9781 | 65 |
| {BcHV1.BI=TRUE, BcULV4=TRUE} =>  {BcULV1.BI=TRUE} | 0.16  0891 | 0.353  261 | 0.455  446 | 1.64  043 | 65 |

| {BcMV6.BI=FALSE, BcULV1.BI=TRUE} =>  {BcHV1.BI=TRUE} | 0.09  6535 | 0.78 | 0.123  762 | 1.29  1475 | 39 |
| --- | --- | --- | --- | --- | --- |
| {BcPV3.BI=FALSE, BcULV1.BI=TRUE} =>  {BcHV1.BI=TRUE} | 0.10  8911 | 0.785  714 | 0.138  614 | 1.30  0937 | 44 |
| {BcRV1.BI=FALSE, BcULV1.BI=TRUE} =>  {BcHV1.BI=TRUE} | 0.10  6436 | 0.767  857 | 0.138  614 | 1.27  137 | 43 |
| {BcEV2.BI=FALSE, BcULV1.BI=TRUE} =>  {BcHV1.BI=TRUE} | 0.13  3663 | 0.843  75 | 0.158  416 | 1.39  7029 | 54 |
| {BcEV2.BI=FALSE, BcHV1.BI=TRUE} =>  {BcULV1.BI=TRUE} | 0.13  3663 | 0.305  085 | 0.438  119 | 1.41  6715 | 54 |
| {BcHV3.BI=FALSE, BcULV1.BI=TRUE} =>  {BcHV1.BI=TRUE} | 0.13  3663 | 0.857  143 | 0.155  941 | 1.41  9204 | 54 |
| {BcHV1.BI=TRUE, BcHV3.BI=FALSE} =>  {BcULV1.BI=TRUE} | 0.13  3663 | 0.301  676 | 0.443  069 | 1.40  0886 | 54 |
| {BcFV5.BI=FALSE, BcULV1.BI=TRUE} =>  {BcHV1.BI=TRUE} | 0.14  1089 | 0.826  087 | 0.170  792 | 1.36  7783 | 57 |
| {BcFV5.BI=FALSE, BcHV1.BI=TRUE} =>  {BcULV1.BI=TRUE} | 0.14  1089 | 0.311  475 | 0.452  97 | 1.44  6392 | 57 |
| {BcHV1.BI=TRUE, BcULV1.BI=TRUE} =>  {BcMV1.BI=TRUE} | 0.15  3465 | 0.873  239 | 0.175  743 | 1.10  5921 | 62 |
| {BcMV1.BI=TRUE, BcULV1.BI=TRUE} =>  {BcHV1.BI=TRUE} | 0.15  3465 | 0.815  789 | 0.188  119 | 1.35  0733 | 62 |

| {BcHV1.BI=TRUE, BcMV1.BI=TRUE} =>  {BcULV1.BI=TRUE} | 0.15  3465 | 0.317  949 | 0.482  673 | 1.47  6452 | 62 |
| --- | --- | --- | --- | --- | --- |
| {BcMV12=FALSE, BcULV1.BI=TRUE} =>  {BcHV1.BI=TRUE} | 0.11  8812 | 0.813  559 | 0.146  04 | 1.34  7041 | 48 |
| {BcHV1.BI=TRUE, BcULV1.BI=TRUE} =>  {BcNSRV7.BI=FALSE} | 0.15  5941 | 0.887  324 | 0.175  743 | 1.10  9842 | 63 |
| {BcNSRV7.BI=FALSE, BcULV1.BI=TRUE}  => {BcHV1.BI=TRUE} | 0.15  5941 | 0.84 | 0.185  644 | 1.39  082 | 63 |
| {BcHV1.BI=TRUE, BcNSRV7.BI=FALSE} =>  {BcULV1.BI=TRUE} | 0.15  5941 | 0.335  106 | 0.465  347 | 1.55  6126 | 63 |
| {BcULV1.BI=TRUE, BcVV3.BI=FALSE} =>  {BcHV1.BI=TRUE} | 0.15  099 | 0.871  429 | 0.173  267 | 1.44  2857 | 61 |
| {BcHV1.BI=TRUE, BcVV3.BI=FALSE} =>  {BcULV1.BI=TRUE} | 0.15  099 | 0.305 | 0.495  05 | 1.41  6322 | 61 |
| {BcOLV7.BI=FALSE, BcULV1.BI=TRUE} =>  {BcHV1.BI=TRUE} | 0.12  8713 | 0.825  397 | 0.155  941 | 1.36  6641 | 52 |
| {BcHV1.BI=TRUE, BcOLV7.BI=FALSE} =>  {BcULV1.BI=TRUE} | 0.12  8713 | 0.269  43 | 0.477  723 | 1.25  1146 | 52 |
| {BcALV1.BI=FALSE, BcULV1.BI=TRUE} =>  {BcHV1.BI=TRUE} | 0.13  3663 | 0.805  97 | 0.165  842 | 1.33  4475 | 54 |
| {BcALV1.BI=FALSE, BcHV1.BI=TRUE} =>  {BcULV1.BI=TRUE} | 0.13  3663 | 0.287  234 | 0.465  347 | 1.33  3822 | 54 |

| {BcHV1.BI=TRUE, BcULV1.BI=TRUE} =>  {BcDMV1=FALSE} | 0.16  0891 | 0.915  493 | 0.175  743 | 1.08  4631 | 65 |
| --- | --- | --- | --- | --- | --- |
| {BcDMV1=FALSE, BcULV1.BI=TRUE} =>  {BcHV1.BI=TRUE} | 0.16  0891 | 0.822  785 | 0.195  545 | 1.36  2316 | 65 |
| {BcDMV1=FALSE, BcHV1.BI=TRUE} =>  {BcULV1.BI=TRUE} | 0.16  0891 | 0.311  005 | 0.517  327 | 1.44  4206 | 65 |
| {BcFV9=FALSE, BcULV1.BI=TRUE} =>  {BcHV1.BI=TRUE} | 0.15  3465 | 0.805  195 | 0.190  594 | 1.33  3191 | 62 |
| {BcFV9=FALSE, BcHV1.BI=TRUE} =>  {BcULV1.BI=TRUE} | 0.15  3465 | 0.302  439 | 0.507  426 | 1.40  4429 | 62 |
| {BcOLV4.BI=FALSE, BcULV1.BI=TRUE} =>  {BcHV1.BI=TRUE} | 0.15  5941 | 0.863  014 | 0.180  693 | 1.42  8924 | 63 |
| {BcHV1.BI=TRUE, BcOLV4.BI=FALSE} =>  {BcULV1.BI=TRUE} | 0.15  5941 | 0.294  393 | 0.529  703 | 1.36  7064 | 63 |
| {BcULV1.BI=TRUE, BcULV5=FALSE} =>  {BcHV1.BI=TRUE} | 0.14  3564 | 0.816  901 | 0.175  743 | 1.35  2574 | 58 |
| {BcHV1.BI=TRUE, BcULV5=FALSE} =>  {BcULV1.BI=TRUE} | 0.14  3564 | 0.269  767 | 0.532  178 | 1.25  2713 | 58 |
| {BcBNV5.BI=FALSE, BcULV1.BI=TRUE} =>  {BcHV1.BI=TRUE} | 0.16  0891 | 0.822  785 | 0.195  545 | 1.36  2316 | 65 |
| {BcBNV5.BI=FALSE, BcHV1.BI=TRUE} =>  {BcULV1.BI=TRUE} | 0.16  0891 | 0.302  326 | 0.532  178 | 1.40  3903 | 65 |

| {BcULV1.BI=TRUE, BvF.BI=FALSE} =>  {BcHV1.BI=TRUE} | 0.16  5842 | 0.827  16 | 0.200  495 | 1.36  9561 | 67 |
| --- | --- | --- | --- | --- | --- |
| {BcHV1.BI=TRUE, BvF.BI=FALSE} =>  {BcULV1.BI=TRUE} | 0.16  5842 | 0.304  545 | 0.544  554 | 1.41  4211 | 67 |
| {BcMCV5.BI=FALSE, BcULV1.BI=TRUE} =>  {BcHV1.BI=TRUE} | 0.15  5941 | 0.807  692 | 0.193  069 | 1.33  7327 | 63 |
| {BcHV1.BI=TRUE, BcMCV5.BI=FALSE} =>  {BcULV1.BI=TRUE} | 0.15  5941 | 0.285  068 | 0.547  03 | 1.32  3763 | 63 |
| {BcOLV13.BI=FALSE, BcULV1.BI=TRUE} =>  {BcHV1.BI=TRUE} | 0.15  099 | 0.813  333 | 0.185  644 | 1.34  6667 | 61 |
| {BcHV1.BI=TRUE, BcOLV13.BI=FALSE} =>  {BcULV1.BI=TRUE} | 0.15  099 | 0.277  273 | 0.544  554 | 1.28  7565 | 61 |
| {BcOLV2.BI=FALSE, BcULV1.BI=TRUE} =>  {BcHV1.BI=TRUE} | 0.15  099 | 0.813  333 | 0.185  644 | 1.34  6667 | 61 |
| {BcHV1.BI=TRUE, BcOLV2.BI=FALSE} =>  {BcULV1.BI=TRUE} | 0.15  099 | 0.273  543 | 0.551  98 | 1.27  0244 | 61 |
| {BcMCV3.BI=FALSE, BcULV1.BI=TRUE} =>  {BcHV1.BI=TRUE} | 0.13  1188 | 0.779  412 | 0.168  317 | 1.29  0501 | 53 |
| {BcPV4.BI=FALSE, BcULV1.BI=TRUE} =>  {BcHV1.BI=TRUE} | 0.15  5941 | 0.818  182 | 0.190  594 | 1.35  4694 | 63 |
| {BcHV1.BI=TRUE, BcPV4.BI=FALSE} =>  {BcULV1.BI=TRUE} | 0.15  5941 | 0.278  761 | 0.559  406 | 1.29  4477 | 63 |

| {BcEV6=FALSE, BcULV1.BI=TRUE} =>  {BcHV1.BI=TRUE} | 0.16  0891 | 0.812  5 | 0.198  02 | 1.34  5287 | 65 |
| --- | --- | --- | --- | --- | --- |
| {BcEV6=FALSE, BcHV1.BI=TRUE} =>  {BcULV1.BI=TRUE} | 0.16  0891 | 0.292  793 | 0.549  505 | 1.35  9635 | 65 |
| {BcOLV17.BI=FALSE, BcULV1.BI=TRUE} =>  {BcHV1.BI=TRUE} | 0.16  0891 | 0.812  5 | 0.198  02 | 1.34  5287 | 65 |
| {BcHV1.BI=TRUE, BcOLV17.BI=FALSE} =>  {BcULV1.BI=TRUE} | 0.16  0891 | 0.285  088 | 0.564  356 | 1.32  3856 | 65 |
| {BcOLV15.BI=FALSE, BcULV1.BI=TRUE} =>  {BcHV1.BI=TRUE} | 0.17  0792 | 0.811  765 | 0.210  396 | 1.34  4069 | 69 |
| {BcHV1.BI=TRUE, BcOLV15.BI=FALSE} =>  {BcULV1.BI=TRUE} | 0.17  0792 | 0.302  632 | 0.564  356 | 1.40  5324 | 69 |
| {BcEV5=FALSE, BcULV1.BI=TRUE} =>  {BcHV1.BI=TRUE} | 0.17  0792 | 0.821  429 | 0.207  921 | 1.36  007 | 69 |
| {BcEV5=FALSE, BcHV1.BI=TRUE} =>  {BcULV1.BI=TRUE} | 0.17  0792 | 0.297  414 | 0.574  257 | 1.38  1094 | 69 |
| {BcMV11=TRUE, BcULV1.BI=TRUE} =>  {BcMV9.BI=TRUE} | 0.11  3861 | 0.793  103 | 0.143  564 | 1.16  9393 | 46 |
| {BcMV11=TRUE, BcMV9.BI=TRUE} =>  {BcULV1.BI=TRUE} | 0.11  3861 | 0.280  488 | 0.405  941 | 1.30  2495 | 46 |
| {BcMV11=TRUE, BcULV1.BI=TRUE} =>  {BcULV4=TRUE} | 0.12  6238 | 0.879  31 | 0.143  564 | 1.27  3267 | 51 |

| {BcMV11=TRUE, BcULV4=TRUE} =>  {BcULV1.BI=TRUE} | 0.12  6238 | 0.301  775 | 0.418  317 | 1.40  1347 | 51 |
| --- | --- | --- | --- | --- | --- |
| {BcOLV1.BI.MOVA.=FALSE,  BcULV1.BI=TRUE} => {BcMV9.BI=TRUE} | 0.09  4059 | 0.863  636 | 0.108  911 | 1.27  3391 | 38 |
| {BcOLV1.BI.MOVA.=FALSE,  BcULV1.BI=TRUE} => {BcULV4=TRUE} | 0.10  1485 | 0.931  818 | 0.108  911 | 1.34  9299 | 41 |
| {BcOLV1.BI.MOVA.=FALSE,  BcULV1.BI=TRUE} => {BcPV3.BI=FALSE} | 0.09  1584 | 0.840  909 | 0.108  911 | 1.18  3719 | 37 |
| {BcOLV1.BI.MOVA.=FALSE,  BcULV1.BI=TRUE} => {BcNSRV7.BI=FALSE} | 0.09  901 | 0.909  091 | 0.108  911 | 1.13  7067 | 40 |
| {BcOLV1.BI.MOVA.=FALSE,  BcULV1.BI=TRUE} => {BcDMV1=FALSE} | 0.10  396 | 0.954  545 | 0.108  911 | 1.13  0898 | 42 |
| {BcMV9.BI=TRUE, BcULV1.BI=TRUE} =>  {BcULV4=TRUE} | 0.17  5743 | 0.986  111 | 0.178  218 | 1.42  7917 | 71 |
| {BcULV1.BI=TRUE, BcULV4=TRUE} =>  {BcMV9.BI=TRUE} | 0.17  5743 | 0.910  256 | 0.193  069 | 1.34  213 | 71 |
| {BcMV9.BI=TRUE, BcULV4=TRUE} =>  {BcULV1.BI=TRUE} | 0.17  5743 | 0.321  267 | 0.547  03 | 1.49  186 | 71 |
| {BcMV6.BI=FALSE, BcULV1.BI=TRUE} =>  {BcMV9.BI=TRUE} | 0.09  901 | 0.8 | 0.123  762 | 1.17  9562 | 40 |
| {BcPV3.BI=FALSE, BcULV1.BI=TRUE} =>  {BcMV9.BI=TRUE} | 0.11  1386 | 0.803  571 | 0.138  614 | 1.18  4828 | 45 |

| {BcEV2.BI=FALSE, BcULV1.BI=TRUE} =>  {BcMV9.BI=TRUE} | 0.13  6139 | 0.859  375 | 0.158  416 | 1.26  7108 | 55 |
| --- | --- | --- | --- | --- | --- |
| {BcEV2.BI=FALSE, BcMV9.BI=TRUE} =>  {BcULV1.BI=TRUE} | 0.13  6139 | 0.269  608 | 0.504  95 | 1.25  1972 | 55 |
| {BcHV3.BI=FALSE, BcULV1.BI=TRUE} =>  {BcMV9.BI=TRUE} | 0.12  3762 | 0.793  651 | 0.155  941 | 1.17  02 | 50 |
| {BcFV5.BI=FALSE, BcULV1.BI=TRUE} =>  {BcMV9.BI=TRUE} | 0.14  3564 | 0.840  58 | 0.170  792 | 1.23  9395 | 58 |
| {BcFV5.BI=FALSE, BcMV9.BI=TRUE} =>  {BcULV1.BI=TRUE} | 0.14  3564 | 0.280  193 | 0.512  376 | 1.30  1127 | 58 |
| {BcMV9.BI=TRUE, BcULV1.BI=TRUE} =>  {BcMV1.BI=TRUE} | 0.15  5941 | 0.875 | 0.178  218 | 1.10  815 | 63 |
| {BcMV1.BI=TRUE, BcULV1.BI=TRUE} =>  {BcMV9.BI=TRUE} | 0.15  5941 | 0.828  947 | 0.188  119 | 1.22  2244 | 63 |
| {BcMV1.BI=TRUE, BcMV9.BI=TRUE} =>  {BcULV1.BI=TRUE} | 0.15  5941 | 0.283  784 | 0.549  505 | 1.31  7801 | 63 |
| {BcMV12=FALSE, BcULV1.BI=TRUE} =>  {BcMV9.BI=TRUE} | 0.12  3762 | 0.847  458 | 0.146  04 | 1.24  9536 | 50 |
| {BcNSRV7.BI=FALSE, BcULV1.BI=TRUE}  => {BcMV9.BI=TRUE} | 0.15  3465 | 0.826  667 | 0.185  644 | 1.21  8881 | 62 |
| {BcMV9.BI=TRUE, BcNSRV7.BI=FALSE} =>  {BcULV1.BI=TRUE} | 0.15  3465 | 0.296  651 | 0.517  327 | 1.37  755 | 62 |

| {BcULV1.BI=TRUE, BcVV3.BI=FALSE} =>  {BcMV9.BI=TRUE} | 0.14  604 | 0.842  857 | 0.173  267 | 1.24  2753 | 59 |
| --- | --- | --- | --- | --- | --- |
| {BcMV9.BI=TRUE, BcVV3.BI=FALSE} =>  {BcULV1.BI=TRUE} | 0.14  604 | 0.263  393 | 0.554  455 | 1.22  3112 | 59 |
| {BcALV1.BI=FALSE, BcULV1.BI=TRUE} =>  {BcMV9.BI=TRUE} | 0.13  3663 | 0.805  97 | 0.165  842 | 1.18  8365 | 54 |
| {BcDMV1=FALSE, BcULV1.BI=TRUE} =>  {BcMV9.BI=TRUE} | 0.15  8416 | 0.810  127 | 0.195  545 | 1.19  4493 | 64 |
| {BcDMV1=FALSE, BcMV9.BI=TRUE} =>  {BcULV1.BI=TRUE} | 0.15  8416 | 0.286  996 | 0.551  98 | 1.33  2715 | 64 |
| {BcFV9=FALSE, BcULV1.BI=TRUE} =>  {BcMV9.BI=TRUE} | 0.15  5941 | 0.818  182 | 0.190  594 | 1.20  637 | 63 |
| {BcFV9=FALSE, BcMV9.BI=TRUE} =>  {BcULV1.BI=TRUE} | 0.15  5941 | 0.266  949 | 0.584  158 | 1.23  9626 | 63 |
| {BcOLV4.BI=FALSE, BcULV1.BI=TRUE} =>  {BcMV9.BI=TRUE} | 0.16  0891 | 0.890  411 | 0.180  693 | 1.31  2869 | 65 |
| {BcULV1.BI=TRUE, BcULV5=FALSE} =>  {BcMV9.BI=TRUE} | 0.15  8416 | 0.901  408 | 0.175  743 | 1.32  9084 | 64 |
| {BcBNV5.BI=FALSE, BcULV1.BI=TRUE} =>  {BcMV9.BI=TRUE} | 0.16  3366 | 0.835  443 | 0.195  545 | 1.23  1821 | 66 |
| {BcBNV5.BI=FALSE, BcMV9.BI=TRUE} =>  {BcULV1.BI=TRUE} | 0.16  3366 | 0.273  859 | 0.596  535 | 1.27  1713 | 66 |

| {BcULV1.BI=TRUE, BvF.BI=FALSE} =>  {BcMV9.BI=TRUE} | 0.17  3267 | 0.864  198 | 0.200  495 | 1.27  4218 | 70 |
| --- | --- | --- | --- | --- | --- |
| {BcMV9.BI=TRUE, BvF.BI=FALSE} =>  {BcULV1.BI=TRUE} | 0.17  3267 | 0.283  401 | 0.611  386 | 1.31  6022 | 70 |
| {BcMCV5.BI=FALSE, BcULV1.BI=TRUE} =>  {BcMV9.BI=TRUE} | 0.15  8416 | 0.820  513 | 0.193  069 | 1.20  9807 | 64 |
| {BcOLV13.BI=FALSE, BcULV1.BI=TRUE} =>  {BcMV9.BI=TRUE} | 0.16  0891 | 0.866  667 | 0.185  644 | 1.27  7859 | 65 |
| {BcOLV2.BI=FALSE, BcULV1.BI=TRUE} =>  {BcMV9.BI=TRUE} | 0.15  3465 | 0.826  667 | 0.185  644 | 1.21  8881 | 62 |
| {BcMCV3.BI=FALSE, BcULV1.BI=TRUE} =>  {BcMV9.BI=TRUE} | 0.14  3564 | 0.852  941 | 0.168  317 | 1.25  7621 | 58 |
| {BcPV4.BI=FALSE, BcULV1.BI=TRUE} =>  {BcMV9.BI=TRUE} | 0.15  5941 | 0.818  182 | 0.190  594 | 1.20  637 | 63 |
| {BcEV6=FALSE, BcULV1.BI=TRUE} =>  {BcMV9.BI=TRUE} | 0.16  5842 | 0.837  5 | 0.198  02 | 1.23  4854 | 67 |
| {BcEV6=FALSE, BcMV9.BI=TRUE} =>  {BcULV1.BI=TRUE} | 0.16  5842 | 0.262  745 | 0.631  188 | 1.22  0104 | 67 |
| {BcOLV17.BI=FALSE, BcULV1.BI=TRUE} =>  {BcMV9.BI=TRUE} | 0.16  0891 | 0.812  5 | 0.198  02 | 1.19  7993 | 65 |
| {BcOLV15.BI=FALSE, BcULV1.BI=TRUE} =>  {BcMV9.BI=TRUE} | 0.17  5743 | 0.835  294 | 0.210  396 | 1.23  1602 | 71 |

| {BcMV9.BI=TRUE, BcOLV15.BI=FALSE} =>  {BcULV1.BI=TRUE} | 0.17  5743 | 0.274  131 | 0.641  089 | 1.27  2977 | 71 |
| --- | --- | --- | --- | --- | --- |
| {BcEV5=FALSE, BcULV1.BI=TRUE} =>  {BcMV9.BI=TRUE} | 0.17  0792 | 0.821  429 | 0.207  921 | 1.21  1157 | 69 |
| {BcEV5=FALSE, BcMV9.BI=TRUE} =>  {BcULV1.BI=TRUE} | 0.17  0792 | 0.267  442 | 0.638  614 | 1.24  1914 | 69 |
| {BcMV6.BI=FALSE, BcULV1.BI=TRUE} =>  {BcULV4=TRUE} | 0.10  8911 | 0.88 | 0.123  762 | 1.27  4265 | 44 |
| {BcPV3.BI=FALSE, BcULV1.BI=TRUE} =>  {BcULV4=TRUE} | 0.12  6238 | 0.910  714 | 0.138  614 | 1.31  874 | 51 |
| {BcRV1.BI=FALSE, BcULV1.BI=TRUE} =>  {BcULV4=TRUE} | 0.11  6337 | 0.839  286 | 0.138  614 | 1.21  531 | 47 |
| {BcEV2.BI=FALSE, BcULV1.BI=TRUE} =>  {BcULV4=TRUE} | 0.14  3564 | 0.906  25 | 0.158  416 | 1.31  2276 | 58 |
| {BcEV2.BI=FALSE, BcULV4=TRUE} =>  {BcULV1.BI=TRUE} | 0.14  3564 | 0.271  028 | 0.529  703 | 1.25  8567 | 58 |
| {BcHV3.BI=FALSE, BcULV1.BI=TRUE} =>  {BcULV4=TRUE} | 0.13  6139 | 0.873  016 | 0.155  941 | 1.26  4152 | 55 |
| {BcFV5.BI=FALSE, BcULV1.BI=TRUE} =>  {BcULV4=TRUE} | 0.15  099 | 0.884  058 | 0.170  792 | 1.28  0141 | 61 |
| {BcFV5.BI=FALSE, BcULV4=TRUE} =>  {BcULV1.BI=TRUE} | 0.15  099 | 0.285  047 | 0.529  703 | 1.32  3665 | 61 |

| {BcMV1.BI=TRUE, BcULV1.BI=TRUE} =>  {BcULV4=TRUE} | 0.16  5842 | 0.881  579 | 0.188  119 | 1.27  6552 | 67 |
| --- | --- | --- | --- | --- | --- |
| {BcMV1.BI=TRUE, BcULV4=TRUE} =>  {BcULV1.BI=TRUE} | 0.16  5842 | 0.301  802 | 0.549  505 | 1.40  147 | 67 |
| {BcMV12=FALSE, BcULV1.BI=TRUE} =>  {BcULV4=TRUE} | 0.13  6139 | 0.932  203 | 0.146  04 | 1.34  9857 | 55 |
| {BcNSRV7.BI=FALSE, BcULV1.BI=TRUE}  => {BcULV4=TRUE} | 0.16  5842 | 0.893  333 | 0.185  644 | 1.29  3572 | 67 |
| {BcNSRV7.BI=FALSE, BcULV4=TRUE} =>  {BcULV1.BI=TRUE} | 0.16  5842 | 0.310  185 | 0.534  653 | 1.44  04 | 67 |
| {BcULV1.BI=TRUE, BcVV3.BI=FALSE} =>  {BcULV4=TRUE} | 0.15  8416 | 0.914  286 | 0.173  267 | 1.32  3912 | 64 |
| {BcULV4=TRUE, BcVV3.BI=FALSE} =>  {BcULV1.BI=TRUE} | 0.15  8416 | 0.289  593 | 0.547  03 | 1.34  4776 | 64 |
| {BcOLV7.BI=FALSE, BcULV1.BI=TRUE} =>  {BcULV4=TRUE} | 0.13  3663 | 0.857  143 | 0.155  941 | 1.24  1167 | 54 |
| {BcALV1.BI=FALSE, BcULV1.BI=TRUE} =>  {BcULV4=TRUE} | 0.14  604 | 0.880  597 | 0.165  842 | 1.27  513 | 59 |
| {BcALV1.BI=FALSE, BcULV4=TRUE} =>  {BcULV1.BI=TRUE} | 0.14  604 | 0.275  701 | 0.529  703 | 1.28  0266 | 59 |
| {BcULV1.BI=TRUE, BcULV4=TRUE} =>  {BcDMV1=FALSE} | 0.17  5743 | 0.910  256 | 0.193  069 | 1.07  8427 | 71 |

| {BcDMV1=FALSE, BcULV1.BI=TRUE} =>  {BcULV4=TRUE} | 0.17  5743 | 0.898  734 | 0.195  545 | 1.30  1393 | 71 |
| --- | --- | --- | --- | --- | --- |
| {BcDMV1=FALSE, BcULV4=TRUE} =>  {BcULV1.BI=TRUE} | 0.17  5743 | 0.307  359 | 0.571  782 | 1.42  7278 | 71 |
| {BcFV9=FALSE, BcULV1.BI=TRUE} =>  {BcULV4=TRUE} | 0.17  0792 | 0.896  104 | 0.190  594 | 1.29  7584 | 69 |
| {BcFV9=FALSE, BcULV4=TRUE} =>  {BcULV1.BI=TRUE} | 0.17  0792 | 0.286  307 | 0.596  535 | 1.32  9518 | 69 |
| {BcOLV4.BI=FALSE, BcULV1.BI=TRUE} =>  {BcULV4=TRUE} | 0.17  0792 | 0.945  205 | 0.180  693 | 1.36  8685 | 69 |
| {BcOLV4.BI=FALSE, BcULV4=TRUE} =>  {BcULV1.BI=TRUE} | 0.17  0792 | 0.274  9 | 0.621  287 | 1.27  6549 | 69 |
| {BcULV1.BI=TRUE, BcULV5=FALSE} =>  {BcULV4=TRUE} | 0.16  8317 | 0.957  746 | 0.175  743 | 1.38  6844 | 68 |
| {BcULV4=TRUE, BcULV5=FALSE} =>  {BcULV1.BI=TRUE} | 0.16  8317 | 0.266  667 | 0.631  188 | 1.23  8314 | 68 |
| {BcBNV5.BI=FALSE, BcULV1.BI=TRUE} =>  {BcULV4=TRUE} | 0.17  8218 | 0.911  392 | 0.195  545 | 1.31  9722 | 72 |
| {BcBNV5.BI=FALSE, BcULV4=TRUE} =>  {BcULV1.BI=TRUE} | 0.17  8218 | 0.288 | 0.618  812 | 1.33  7379 | 72 |
| {BcULV1.BI=TRUE, BvF.BI=FALSE} =>  {BcULV4=TRUE} | 0.18  3168 | 0.913  58 | 0.200  495 | 1.32  289 | 74 |

| {BcULV4=TRUE, BvF.BI=FALSE} =>  {BcULV1.BI=TRUE} | 0.18  3168 | 0.283  525 | 0.646  04 | 1.31  6598 | 74 |
| --- | --- | --- | --- | --- | --- |
| {BcMCV5.BI=FALSE, BcULV1.BI=TRUE} =>  {BcULV4=TRUE} | 0.17  5743 | 0.910  256 | 0.193  069 | 1.31  8077 | 71 |
| {BcMCV5.BI=FALSE, BcULV4=TRUE} =>  {BcULV1.BI=TRUE} | 0.17  5743 | 0.278  431 | 0.631  188 | 1.29  2946 | 71 |
| {BcOLV13.BI=FALSE, BcULV1.BI=TRUE} =>  {BcULV4=TRUE} | 0.17  3267 | 0.933  333 | 0.185  644 | 1.35  1493 | 70 |
| {BcOLV13.BI=FALSE, BcULV4=TRUE} =>  {BcULV1.BI=TRUE} | 0.17  3267 | 0.270  27 | 0.641  089 | 1.25  5048 | 70 |
| {BcOLV2.BI=FALSE, BcULV1.BI=TRUE} =>  {BcULV4=TRUE} | 0.16  8317 | 0.906  667 | 0.185  644 | 1.31  2879 | 68 |
| {BcOLV2.BI=FALSE, BcULV4=TRUE} =>  {BcULV1.BI=TRUE} | 0.16  8317 | 0.262  548 | 0.641  089 | 1.21  919 | 68 |
| {BcMCV3.BI=FALSE, BcULV1.BI=TRUE} =>  {BcULV4=TRUE} | 0.15  5941 | 0.926  471 | 0.168  317 | 1.34  1556 | 63 |
| {BcPV4.BI=FALSE, BcULV1.BI=TRUE} =>  {BcULV4=TRUE} | 0.17  0792 | 0.896  104 | 0.190  594 | 1.29  7584 | 69 |
| {BcPV4.BI=FALSE, BcULV4=TRUE} =>  {BcULV1.BI=TRUE} | 0.17  0792 | 0.264  368 | 0.646  04 | 1.22  7639 | 69 |
| {BcEV6=FALSE, BcULV1.BI=TRUE} =>  {BcULV4=TRUE} | 0.17  8218 | 0.9 | 0.198  02 | 1.30  3226 | 72 |

| {BcEV6=FALSE, BcULV4=TRUE} =>  {BcULV1.BI=TRUE} | 0.17  8218 | 0.275  862 | 0.646  04 | 1.28  1015 | 72 |
| --- | --- | --- | --- | --- | --- |
| {BcOLV17.BI=FALSE, BcULV1.BI=TRUE} =>  {BcULV4=TRUE} | 0.17  5743 | 0.887  5 | 0.198  02 | 1.28  5125 | 71 |
| {BcOLV17.BI=FALSE, BcULV4=TRUE} =>  {BcULV1.BI=TRUE} | 0.17  5743 | 0.272  031 | 0.646  04 | 1.26  3223 | 71 |
| {BcOLV15.BI=FALSE, BcULV1.BI=TRUE} =>  {BcULV4=TRUE} | 0.19  0594 | 0.905  882 | 0.210  396 | 1.31  1744 | 77 |
| {BcOLV15.BI=FALSE, BcULV4=TRUE} =>  {BcULV1.BI=TRUE} | 0.19  0594 | 0.289  474 | 0.658  416 | 1.34  4223 | 77 |
| {BcEV5=FALSE, BcULV1.BI=TRUE} =>  {BcULV4=TRUE} | 0.18  5644 | 0.892  857 | 0.207  921 | 1.29  2883 | 75 |
| {BcEV5=FALSE, BcULV4=TRUE} =>  {BcULV1.BI=TRUE} | 0.18  5644 | 0.281  955 | 0.658  416 | 1.30  9308 | 75 |
| {BcMV6.BI=FALSE, BcULV1.BI=TRUE} =>  {BcDMV1=FALSE} | 0.11  6337 | 0.94 | 0.123  762 | 1.11  3666 | 47 |
| {BcRV1.BI=FALSE, BcULV1.BI=TRUE} =>  {BcMV1.BI=TRUE} | 0.12  6238 | 0.910  714 | 0.138  614 | 1.15  3381 | 51 |
| {BcRV1.BI=FALSE, BcULV1.BI=TRUE} =>  {BcOLV7.BI=FALSE} | 0.12  6238 | 0.910  714 | 0.138  614 | 1.12  8615 | 51 |
| {BcEV2.BI=FALSE, BcULV1.BI=TRUE} =>  {BcNSRV7.BI=FALSE} | 0.14  3564 | 0.906  25 | 0.158  416 | 1.13  3514 | 58 |

| {BcEV2.BI=FALSE, BcULV1.BI=TRUE} =>  {BcDMV1=FALSE} | 0.14  604 | 0.921  875 | 0.158  416 | 1.09  2192 | 59 |
| --- | --- | --- | --- | --- | --- |
| {BcHV3.BI=FALSE, BcULV1.BI=TRUE} =>  {BcFV5.BI=FALSE} | 0.14  1089 | 0.904  762 | 0.155  941 | 1.15  6721 | 57 |
| {BcHV3.BI=FALSE, BcULV1.BI=TRUE} =>  {BcNSRV7.BI=FALSE} | 0.15  099 | 0.968  254 | 0.155  941 | 1.21  1067 | 61 |
| {BcHV3.BI=FALSE, BcULV1.BI=TRUE} =>  {BcDMV1=FALSE} | 0.15  099 | 0.968  254 | 0.155  941 | 1.14  714 | 61 |
| {BcFV5.BI=FALSE, BcULV1.BI=TRUE} =>  {BcNSRV7.BI=FALSE} | 0.16  0891 | 0.942  029 | 0.170  792 | 1.17  8265 | 65 |
| {BcNSRV7.BI=FALSE, BcULV1.BI=TRUE}  => {BcFV5.BI=FALSE} | 0.16  0891 | 0.866  667 | 0.185  644 | 1.10  8017 | 65 |
| {BcFV5.BI=FALSE, BcULV1.BI=TRUE} =>  {BcDMV1=FALSE} | 0.16  0891 | 0.942  029 | 0.170  792 | 1.11  607 | 65 |
| {BcALV1.BI=FALSE, BcULV1.BI=TRUE} =>  {BcMV1.BI=TRUE} | 0.14  604 | 0.880  597 | 0.165  842 | 1.11  5239 | 59 |
| {BcMV1.BI=TRUE, BcULV1.BI=TRUE} =>  {BcDMV1=FALSE} | 0.17  0792 | 0.907  895 | 0.188  119 | 1.07  5629 | 69 |
| {BcDMV1=FALSE, BcULV1.BI=TRUE} =>  {BcMV1.BI=TRUE} | 0.17  0792 | 0.873  418 | 0.195  545 | 1.10  6147 | 69 |
| {BcFV9=FALSE, BcULV1.BI=TRUE} =>  {BcMV1.BI=TRUE} | 0.17  3267 | 0.909  091 | 0.190  594 | 1.15  1325 | 70 |

| {BcULV1.BI=TRUE, BcULV5=FALSE} =>  {BcMV1.BI=TRUE} | 0.15  3465 | 0.873  239 | 0.175  743 | 1.10  5921 | 62 |
| --- | --- | --- | --- | --- | --- |
| {BcBNV5.BI=FALSE, BcULV1.BI=TRUE} =>  {BcMV1.BI=TRUE} | 0.17  0792 | 0.873  418 | 0.195  545 | 1.10  6147 | 69 |
| {BcULV1.BI=TRUE, BvF.BI=FALSE} =>  {BcMV1.BI=TRUE} | 0.17  5743 | 0.876  543 | 0.200  495 | 1.11  0105 | 71 |
| {BcOLV13.BI=FALSE, BcULV1.BI=TRUE} =>  {BcMV1.BI=TRUE} | 0.16  3366 | 0.88 | 0.185  644 | 1.11  4483 | 66 |
| {BcOLV2.BI=FALSE, BcULV1.BI=TRUE} =>  {BcMV1.BI=TRUE} | 0.16  3366 | 0.88 | 0.185  644 | 1.11  4483 | 66 |
| {BcEV6=FALSE, BcULV1.BI=TRUE} =>  {BcMV1.BI=TRUE} | 0.17  5743 | 0.887  5 | 0.198  02 | 1.12  3981 | 71 |
| {BcOLV17.BI=FALSE, BcULV1.BI=TRUE} =>  {BcMV1.BI=TRUE} | 0.17  5743 | 0.887  5 | 0.198  02 | 1.12  3981 | 71 |
| {BcMV12=FALSE, BcULV1.BI=TRUE} =>  {BcDMV1=FALSE} | 0.13  6139 | 0.932  203 | 0.146  04 | 1.10  4429 | 55 |
| {BcNSRV7.BI=FALSE, BcULV1.BI=TRUE}  => {BcDMV1=FALSE} | 0.18  0693 | 0.973  333 | 0.185  644 | 1.15  3157 | 73 |
| {BcDMV1=FALSE, BcULV1.BI=TRUE} =>  {BcNSRV7.BI=FALSE} | 0.18  0693 | 0.924  051 | 0.195  545 | 1.15  5779 | 73 |
| {BcFV9=FALSE, BcULV1.BI=TRUE} =>  {BcNSRV7.BI=FALSE} | 0.17  0792 | 0.896  104 | 0.190  594 | 1.12  0823 | 69 |

| {BcBNV5.BI=FALSE, BcULV1.BI=TRUE} =>  {BcNSRV7.BI=FALSE} | 0.17  3267 | 0.886  076 | 0.195  545 | 1.10  8281 | 70 |
| --- | --- | --- | --- | --- | --- |
| {BcMCV5.BI=FALSE, BcULV1.BI=TRUE} =>  {BcNSRV7.BI=FALSE} | 0.17  0792 | 0.884  615 | 0.193  069 | 1.10  6454 | 69 |
| {BcOLV15.BI=FALSE, BcULV1.BI=TRUE} =>  {BcNSRV7.BI=FALSE} | 0.18  5644 | 0.882  353 | 0.210  396 | 1.10  3624 | 75 |
| {BcEV5=FALSE, BcULV1.BI=TRUE} =>  {BcNSRV7.BI=FALSE} | 0.18  5644 | 0.892  857 | 0.207  921 | 1.11  6762 | 75 |
| {BcALV1.BI=FALSE, BcULV1.BI=TRUE} =>  {BcDMV1=FALSE} | 0.15  3465 | 0.925  373 | 0.165  842 | 1.09  6336 | 62 |
| {BcFV9=FALSE, BcULV1.BI=TRUE} =>  {BcDMV1=FALSE} | 0.17  5743 | 0.922  078 | 0.190  594 | 1.09  2432 | 71 |
| {BcULV1.BI=TRUE, BcULV5=FALSE} =>  {BcDMV1=FALSE} | 0.16  0891 | 0.915  493 | 0.175  743 | 1.08  4631 | 65 |
| {BcBNV5.BI=FALSE, BcULV1.BI=TRUE} =>  {BcDMV1=FALSE} | 0.18  5644 | 0.949  367 | 0.195  545 | 1.12  4763 | 75 |
| {BcMCV5.BI=FALSE, BcULV1.BI=TRUE} =>  {BcDMV1=FALSE} | 0.18  3168 | 0.948  718 | 0.193  069 | 1.12  3994 | 74 |
| {BcOLV13.BI=FALSE, BcULV1.BI=TRUE} =>  {BcDMV1=FALSE} | 0.16  8317 | 0.906  667 | 0.185  644 | 1.07  4174 | 68 |
| {BcPV4.BI=FALSE, BcULV1.BI=TRUE} =>  {BcDMV1=FALSE} | 0.17  3267 | 0.909  091 | 0.190  594 | 1.07  7046 | 70 |

| {BcEV6=FALSE, BcULV1.BI=TRUE} =>  {BcDMV1=FALSE} | 0.18  0693 | 0.912  5 | 0.198  02 | 1.08  1085 | 73 |
| --- | --- | --- | --- | --- | --- |
| {BcOLV15.BI=FALSE, BcULV1.BI=TRUE} =>  {BcDMV1=FALSE} | 0.19  0594 | 0.905  882 | 0.210  396 | 1.07  3245 | 77 |
| {BcEV5=FALSE, BcULV1.BI=TRUE} =>  {BcDMV1=FALSE} | 0.19  5545 | 0.940  476 | 0.207  921 | 1.11  423 | 79 |
| {BcFV5.BI=TRUE, BcHV3.BI=TRUE} =>  {BcMV2.BI=TRUE} | 0.07  4257 | 0.652  174 | 0.113  861 | 1.40  8975 | 30 |
| {BcFV5.BI=TRUE, BcMV2.BI=TRUE} =>  {BcHV3.BI=TRUE} | 0.07  4257 | 0.625 | 0.118  812 | 2.60  3093 | 30 |
| {BcHV3.BI=TRUE, BcMV2.BI=TRUE} =>  {BcFV5.BI=TRUE} | 0.07  4257 | 0.555  556 | 0.133  663 | 2.55  0505 | 30 |
| {BcFV5.BI=TRUE, BcHV3.BI=TRUE} =>  {BcOLV18=TRUE} | 0.08  6634 | 0.760  87 | 0.113  861 | 1.47  0772 | 35 |
| {BcFV5.BI=TRUE, BcOLV18=TRUE} =>  {BcHV3.BI=TRUE} | 0.08  6634 | 0.583  333 | 0.148  515 | 2.42  9553 | 35 |
| {BcHV3.BI=TRUE, BcOLV18=TRUE} =>  {BcFV5.BI=TRUE} | 0.08  6634 | 0.522  388 | 0.165  842 | 2.39  8236 | 35 |
| {BcFV5.BI=TRUE, BcHV3.BI=TRUE} =>  {BcHV1SLR.BI=TRUE} | 0.07  9208 | 0.695  652 | 0.113  861 | 1.28  3304 | 32 |
| {BcFV5.BI=TRUE, BcHV1SLR.BI=TRUE} =>  {BcHV3.BI=TRUE} | 0.07  9208 | 0.524  59 | 0.150  99 | 2.18  4891 | 32 |

| {BcHV1SLR.BI=TRUE, BcHV3.BI=TRUE} =>  {BcFV5.BI=TRUE} | 0.07  9208 | 0.533  333 | 0.148  515 | 2.44  8485 | 32 |
| --- | --- | --- | --- | --- | --- |
| {BcFV5.BI=TRUE, BcHV3.BI=TRUE} =>  {BcMV3.BI=TRUE} | 0.09  1584 | 0.804  348 | 0.113  861 | 1.48  382 | 37 |
| {BcFV5.BI=TRUE, BcMV3.BI=TRUE} =>  {BcHV3.BI=TRUE} | 0.09  1584 | 0.596  774 | 0.153  465 | 2.48  5534 | 37 |
| {BcHV3.BI=TRUE, BcMV3.BI=TRUE} =>  {BcFV5.BI=TRUE} | 0.09  1584 | 0.616  667 | 0.148  515 | 2.83  1061 | 37 |
| {BcFV5.BI=TRUE, BcHV3.BI=TRUE} =>  {BcULV3=TRUE} | 0.09  4059 | 0.826  087 | 0.113  861 | 1.39  058 | 38 |
| {BcFV5.BI=TRUE, BcULV3=TRUE} =>  {BcHV3.BI=TRUE} | 0.09  4059 | 0.575  758 | 0.163  366 | 2.39  8001 | 38 |
| {BcHV3.BI=TRUE, BcULV3=TRUE} =>  {BcFV5.BI=TRUE} | 0.09  4059 | 0.550  725 | 0.170  792 | 2.52  8327 | 38 |
| {BcFV5.BI=TRUE, BcHV3.BI=TRUE} =>  {BcHV1.BI=TRUE} | 0.08  1683 | 0.717  391 | 0.113  861 | 1.18  7812 | 33 |
| {BcFV5.BI=TRUE, BcHV1.BI=TRUE} =>  {BcHV3.BI=TRUE} | 0.08  1683 | 0.540  984 | 0.150  99 | 2.25  3169 | 33 |
| {BcHV1.BI=TRUE, BcHV3.BI=TRUE} =>  {BcFV5.BI=TRUE} | 0.08  1683 | 0.507  692 | 0.160  891 | 2.33  0769 | 33 |
| {BcFV5.BI=TRUE, BcMV11=TRUE} =>  {BcHV3.BI=TRUE} | 0.08  1683 | 0.578  947 | 0.141  089 | 2.41  1286 | 33 |

| {BcHV3.BI=TRUE, BcMV11=TRUE} =>  {BcFV5.BI=TRUE} | 0.08  1683 | 0.471  429 | 0.173  267 | 2.16  4286 | 33 |
| --- | --- | --- | --- | --- | --- |
| {BcFV5.BI=TRUE, BcMV9.BI=TRUE} =>  {BcHV3.BI=TRUE} | 0.08  1683 | 0.492  537 | 0.165  842 | 2.05  1393 | 33 |
| {BcHV3.BI=TRUE, BcMV9.BI=TRUE} =>  {BcFV5.BI=TRUE} | 0.08  1683 | 0.471  429 | 0.173  267 | 2.16  4286 | 33 |
| {BcFV5.BI=TRUE, BcULV4=TRUE} =>  {BcHV3.BI=TRUE} | 0.08  4158 | 0.523  077 | 0.160  891 | 2.17  8588 | 34 |
| {BcHV3.BI=TRUE, BcULV4=TRUE} =>  {BcFV5.BI=TRUE} | 0.08  4158 | 0.478  873 | 0.175  743 | 2.19  8464 | 34 |
| {BcFV5.BI=TRUE, BcPV3.BI=FALSE} =>  {BcHV3.BI=TRUE} | 0.07  9208 | 0.484  848 | 0.163  366 | 2.01  9369 | 32 |
| {BcHV3.BI=TRUE, BcPV3.BI=FALSE} =>  {BcFV5.BI=TRUE} | 0.07  9208 | 0.5 | 0.158  416 | 2.29  5455 | 32 |
| {BcFV5.BI=TRUE, BcHV3.BI=TRUE} =>  {BcRV1.BI=FALSE} | 0.09  901 | 0.869  565 | 0.113  861 | 1.20  3097 | 40 |
| {BcFV5.BI=TRUE, BcRV1.BI=FALSE} =>  {BcHV3.BI=TRUE} | 0.09  901 | 0.555  556 | 0.178  218 | 2.31  386 | 40 |
| {BcHV3.BI=TRUE, BcRV1.BI=FALSE} =>  {BcFV5.BI=TRUE} | 0.09  901 | 0.563  38 | 0.175  743 | 2.58  6428 | 40 |
| {BcFV5.BI=TRUE, BcULV1.BI=FALSE} =>  {BcHV3.BI=TRUE} | 0.08  4158 | 0.485  714 | 0.173  267 | 2.02  2975 | 34 |

| {BcHV3.BI=TRUE, BcULV1.BI=FALSE} =>  {BcFV5.BI=TRUE} | 0.08  4158 | 0.465  753 | 0.180  693 | 2.13  8232 | 34 |
| --- | --- | --- | --- | --- | --- |
| {BcFV5.BI=TRUE, BcHV3.BI=TRUE} =>  {BcMV1.BI=TRUE} | 0.10  6436 | 0.934  783 | 0.113  861 | 1.18  3863 | 43 |
| {BcFV5.BI=TRUE, BcMV1.BI=TRUE} =>  {BcHV3.BI=TRUE} | 0.10  6436 | 0.589  041 | 0.180  693 | 2.45  3326 | 43 |
| {BcHV3.BI=TRUE, BcMV1.BI=TRUE} =>  {BcFV5.BI=TRUE} | 0.10  6436 | 0.5 | 0.212  871 | 2.29  5455 | 43 |
| {BcFV5.BI=TRUE, BcMV12=FALSE} =>  {BcHV3.BI=TRUE} | 0.08  9109 | 0.514  286 | 0.173  267 | 2.14  1973 | 36 |
| {BcHV3.BI=TRUE, BcMV12=FALSE} =>  {BcFV5.BI=TRUE} | 0.08  9109 | 0.45 | 0.198  02 | 2.06  5909 | 36 |
| {BcFV5.BI=TRUE, BcVV3.BI=FALSE} =>  {BcHV3.BI=TRUE} | 0.08  1683 | 0.485  294 | 0.168  317 | 2.02  1225 | 33 |
| {BcHV3.BI=TRUE, BcVV3.BI=FALSE} =>  {BcFV5.BI=TRUE} | 0.08  1683 | 0.532  258 | 0.153  465 | 2.44  3548 | 33 |
| {BcFV5.BI=TRUE, BcOLV7.BI=FALSE} =>  {BcHV3.BI=TRUE} | 0.09  1584 | 0.5 | 0.183  168 | 2.08  2474 | 37 |
| {BcHV3.BI=TRUE, BcOLV7.BI=FALSE} =>  {BcFV5.BI=TRUE} | 0.09  1584 | 0.521  127 | 0.175  743 | 2.39  2446 | 37 |
| {BcALV1.BI=FALSE, BcFV5.BI=TRUE} =>  {BcHV3.BI=TRUE} | 0.07  9208 | 0.533  333 | 0.148  515 | 2.22  1306 | 32 |

| {BcALV1.BI=FALSE, BcHV3.BI=TRUE} =>  {BcFV5.BI=TRUE} | 0.07  9208 | 0.438  356 | 0.180  693 | 2.01  2453 | 32 |
| --- | --- | --- | --- | --- | --- |
| {BcFV5.BI=TRUE, BcFV9=FALSE} =>  {BcHV3.BI=TRUE} | 0.09  6535 | 0.565  217 | 0.170  792 | 2.35  4101 | 39 |
| {BcFV9=FALSE, BcHV3.BI=TRUE} =>  {BcFV5.BI=TRUE} | 0.09  6535 | 0.487  5 | 0.198  02 | 2.23  8068 | 39 |
| {BcFV5.BI=TRUE, BcOLV4.BI=FALSE} =>  {BcHV3.BI=TRUE} | 0.09  4059 | 0.481  013 | 0.195  545 | 2.00  3393 | 38 |
| {BcHV3.BI=TRUE, BcOLV4.BI=FALSE} =>  {BcFV5.BI=TRUE} | 0.09  4059 | 0.463  415 | 0.202  97 | 2.12  7494 | 38 |
| {BcFV5.BI=TRUE, BcULV5=FALSE} =>  {BcHV3.BI=TRUE} | 0.10  1485 | 0.518  987 | 0.195  545 | 2.16  1556 | 41 |
| {BcHV3.BI=TRUE, BcULV5=FALSE} =>  {BcFV5.BI=TRUE} | 0.10  1485 | 0.488  095 | 0.207  921 | 2.24  0801 | 41 |
| {BcBNV5.BI=FALSE, BcFV5.BI=TRUE} =>  {BcHV3.BI=TRUE} | 0.08  1683 | 0.5 | 0.163  366 | 2.08  2474 | 33 |
| {BcBNV5.BI=FALSE, BcHV3.BI=TRUE} =>  {BcFV5.BI=TRUE} | 0.08  1683 | 0.423  077 | 0.193  069 | 1.94  2308 | 33 |
| {BcFV5.BI=TRUE, BvF.BI=FALSE} =>  {BcHV3.BI=TRUE} | 0.10  1485 | 0.532  468 | 0.190  594 | 2.21  77 | 41 |
| {BcHV3.BI=TRUE, BvF.BI=FALSE} =>  {BcFV5.BI=TRUE} | 0.10  1485 | 0.471  264 | 0.215  347 | 2.16  3532 | 41 |

| {BcFV5.BI=TRUE, BcMCV5.BI=FALSE} =>  {BcHV3.BI=TRUE} | 0.07  6733 | 0.424  658 | 0.180  693 | 1.76  8677 | 31 |
| --- | --- | --- | --- | --- | --- |
| {BcHV3.BI=TRUE, BcMCV5.BI=FALSE} =>  {BcFV5.BI=TRUE} | 0.07  6733 | 0.413  333 | 0.185  644 | 1.89  7576 | 31 |
| {BcFV5.BI=TRUE, BcOLV13.BI=FALSE} =>  {BcHV3.BI=TRUE} | 0.10  8911 | 0.523  81 | 0.207  921 | 2.18  164 | 44 |
| {BcHV3.BI=TRUE, BcOLV13.BI=FALSE} =>  {BcFV5.BI=TRUE} | 0.10  8911 | 0.505  747 | 0.215  347 | 2.32  1839 | 44 |
| {BcFV5.BI=TRUE, BcOLV2.BI=FALSE} =>  {BcHV3.BI=TRUE} | 0.10  1485 | 0.506  173 | 0.200  495 | 2.10  8184 | 41 |
| {BcHV3.BI=TRUE, BcOLV2.BI=FALSE} =>  {BcFV5.BI=TRUE} | 0.10  1485 | 0.455  556 | 0.222  772 | 2.09  1414 | 41 |
| {BcFV5.BI=TRUE, BcMCV3.BI=FALSE} =>  {BcHV3.BI=TRUE} | 0.11  1386 | 0.535  714 | 0.207  921 | 2.23  1222 | 45 |
| {BcHV3.BI=TRUE, BcMCV3.BI=FALSE} =>  {BcFV5.BI=TRUE} | 0.11  1386 | 0.468  75 | 0.237  624 | 2.15  1989 | 45 |
| {BcFV5.BI=TRUE, BcPV4.BI=FALSE} =>  {BcHV3.BI=TRUE} | 0.10  6436 | 0.530  864 | 0.200  495 | 2.21  1022 | 43 |
| {BcHV3.BI=TRUE, BcPV4.BI=FALSE} =>  {BcFV5.BI=TRUE} | 0.10  6436 | 0.467  391 | 0.227  723 | 2.14  5751 | 43 |
| {BcEV6=FALSE, BcFV5.BI=TRUE} =>  {BcHV3.BI=TRUE} | 0.10  8911 | 0.523  81 | 0.207  921 | 2.18  164 | 44 |

| {BcEV6=FALSE, BcHV3.BI=TRUE} =>  {BcFV5.BI=TRUE} | 0.10  8911 | 0.478  261 | 0.227  723 | 2.19  5652 | 44 |
| --- | --- | --- | --- | --- | --- |
| {BcFV5.BI=TRUE, BcOLV17.BI=FALSE} =>  {BcHV3.BI=TRUE} | 0.10  8911 | 0.511  628 | 0.212  871 | 2.13  0904 | 44 |
| {BcHV3.BI=TRUE, BcOLV17.BI=FALSE} =>  {BcFV5.BI=TRUE} | 0.10  8911 | 0.488  889 | 0.222  772 | 2.24  4444 | 44 |
| {BcFV5.BI=TRUE, BcOLV15.BI=FALSE} =>  {BcHV3.BI=TRUE} | 0.09  4059 | 0.520  548 | 0.180  693 | 2.16  8055 | 38 |
| {BcHV3.BI=TRUE, BcOLV15.BI=FALSE} =>  {BcFV5.BI=TRUE} | 0.09  4059 | 0.431  818 | 0.217  822 | 1.98  2438 | 38 |
| {BcEV5=FALSE, BcFV5.BI=TRUE} =>  {BcHV3.BI=TRUE} | 0.07  9208 | 0.444  444 | 0.178  218 | 1.85  1088 | 32 |
| {BcEV5=FALSE, BcHV3.BI=TRUE} =>  {BcFV5.BI=TRUE} | 0.07  9208 | 0.4 | 0.198  02 | 1.83  6364 | 32 |
| {BcEV2.BI=TRUE, BcFV5.BI=TRUE} =>  {BcMV9.BI=TRUE} | 0.07  4257 | 0.810  811 | 0.091  584 | 1.19  5502 | 30 |
| {BcFV5.BI=TRUE, BcMV9.BI=TRUE} =>  {BcEV2.BI=TRUE} | 0.07  4257 | 0.447  761 | 0.165  842 | 1.77  3486 | 30 |
| {BcEV2.BI=TRUE, BcMV9.BI=TRUE} =>  {BcFV5.BI=TRUE} | 0.07  4257 | 0.428  571 | 0.173  267 | 1.96  7532 | 30 |
| {BcEV2.BI=TRUE, BcFV5.BI=TRUE} =>  {BcRV1.BI=FALSE} | 0.07  9208 | 0.864  865 | 0.091  584 | 1.19  6594 | 32 |

| {BcFV5.BI=TRUE, BcRV1.BI=FALSE} =>  {BcEV2.BI=TRUE} | 0.07  9208 | 0.444  444 | 0.178  218 | 1.76  0349 | 32 |
| --- | --- | --- | --- | --- | --- |
| {BcEV2.BI=TRUE, BcRV1.BI=FALSE} =>  {BcFV5.BI=TRUE} | 0.07  9208 | 0.421  053 | 0.188  119 | 1.93  3014 | 32 |
| {BcEV2.BI=TRUE, BcFV5.BI=TRUE} =>  {BcMV1.BI=TRUE} | 0.08  4158 | 0.918  919 | 0.091  584 | 1.16  3772 | 34 |
| {BcFV5.BI=TRUE, BcMV1.BI=TRUE} =>  {BcEV2.BI=TRUE} | 0.08  4158 | 0.465  753 | 0.180  693 | 1.84  4749 | 34 |
| {BcEV2.BI=TRUE, BcMV1.BI=TRUE} =>  {BcFV5.BI=TRUE} | 0.08  4158 | 0.386  364 | 0.217  822 | 1.77  376 | 34 |
| {BcFV5.BI=TRUE, BcMV12=FALSE} =>  {BcEV2.BI=TRUE} | 0.07  6733 | 0.442  857 | 0.173  267 | 1.75  4062 | 31 |
| {BcEV2.BI=TRUE, BcMV12=FALSE} =>  {BcFV5.BI=TRUE} | 0.07  6733 | 0.352  273 | 0.217  822 | 1.61  7252 | 31 |
| {BcEV2.BI=TRUE, BcFV5.BI=TRUE} =>  {BcOLV7.BI=FALSE} | 0.08  4158 | 0.918  919 | 0.091  584 | 1.13  8783 | 34 |
| {BcFV5.BI=TRUE, BcOLV7.BI=FALSE} =>  {BcEV2.BI=TRUE} | 0.08  4158 | 0.459  459 | 0.183  168 | 1.81  982 | 34 |
| {BcEV2.BI=TRUE, BcOLV7.BI=FALSE} =>  {BcFV5.BI=TRUE} | 0.08  4158 | 0.390  805 | 0.215  347 | 1.79  4148 | 34 |
| {BcFV5.BI=TRUE, BcOLV4.BI=FALSE} =>  {BcEV2.BI=TRUE} | 0.07  4257 | 0.379  747 | 0.195  545 | 1.50  4095 | 30 |

| {BcEV2.BI=TRUE, BcOLV4.BI=FALSE} =>  {BcFV5.BI=TRUE} | 0.07  4257 | 0.352  941 | 0.210  396 | 1.62  0321 | 30 |
| --- | --- | --- | --- | --- | --- |
| {BcFV5.BI=TRUE, BcULV5=FALSE} =>  {BcEV2.BI=TRUE} | 0.08  1683 | 0.417  722 | 0.195  545 | 1.65  4505 | 33 |
| {BcEV2.BI=TRUE, BcULV5=FALSE} =>  {BcFV5.BI=TRUE} | 0.08  1683 | 0.370  787 | 0.220  297 | 1.70  2247 | 33 |
| {BcFV5.BI=TRUE, BvF.BI=FALSE} =>  {BcEV2.BI=TRUE} | 0.07  4257 | 0.389  61 | 0.190  594 | 1.54  3163 | 30 |
| {BcEV2.BI=TRUE, BvF.BI=FALSE} =>  {BcFV5.BI=TRUE} | 0.07  4257 | 0.344  828 | 0.215  347 | 1.58  3072 | 30 |
| {BcFV5.BI=TRUE, BcOLV13.BI=FALSE} =>  {BcEV2.BI=TRUE} | 0.08  1683 | 0.392  857 | 0.207  921 | 1.55  6022 | 33 |
| {BcEV2.BI=TRUE, BcOLV13.BI=FALSE} =>  {BcFV5.BI=TRUE} | 0.08  1683 | 0.358  696 | 0.227  723 | 1.64  6739 | 33 |
| {BcFV5.BI=TRUE, BcOLV2.BI=FALSE} =>  {BcEV2.BI=TRUE} | 0.08  1683 | 0.407  407 | 0.200  495 | 1.61  3653 | 33 |
| {BcEV2.BI=TRUE, BcOLV2.BI=FALSE} =>  {BcFV5.BI=TRUE} | 0.08  1683 | 0.347  368 | 0.235  149 | 1.59  4737 | 33 |
| {BcFV5.BI=TRUE, BcMCV3.BI=FALSE} =>  {BcEV2.BI=TRUE} | 0.08  6634 | 0.416  667 | 0.207  921 | 1.65  0327 | 35 |
| {BcEV2.BI=TRUE, BcMCV3.BI=FALSE} =>  {BcFV5.BI=TRUE} | 0.08  6634 | 0.368  421 | 0.235  149 | 1.69  1388 | 35 |

| {BcFV5.BI=TRUE, BcPV4.BI=FALSE} =>  {BcEV2.BI=TRUE} | 0.08  6634 | 0.432  099 | 0.200  495 | 1.71  145 | 35 |
| --- | --- | --- | --- | --- | --- |
| {BcEV2.BI=TRUE, BcPV4.BI=FALSE} =>  {BcFV5.BI=TRUE} | 0.08  6634 | 0.368  421 | 0.235  149 | 1.69  1388 | 35 |
| {BcEV6=FALSE, BcFV5.BI=TRUE} =>  {BcEV2.BI=TRUE} | 0.08  4158 | 0.404  762 | 0.207  921 | 1.60  3175 | 34 |
| {BcEV2.BI=TRUE, BcEV6=FALSE} =>  {BcFV5.BI=TRUE} | 0.08  4158 | 0.357  895 | 0.235  149 | 1.64  3062 | 34 |
| {BcFV5.BI=TRUE, BcOLV17.BI=FALSE} =>  {BcEV2.BI=TRUE} | 0.08  9109 | 0.418  605 | 0.212  871 | 1.65  8003 | 36 |
| {BcEV2.BI=TRUE, BcOLV17.BI=FALSE} =>  {BcFV5.BI=TRUE} | 0.08  9109 | 0.382  979 | 0.232  673 | 1.75  8221 | 36 |
| {BcEV5=FALSE, BcFV5.BI=TRUE} =>  {BcEV2.BI=TRUE} | 0.07  6733 | 0.430  556 | 0.178  218 | 1.70  5338 | 31 |
| {BcEV2.BI=TRUE, BcEV5=FALSE} =>  {BcFV5.BI=TRUE} | 0.07  6733 | 0.322  917 | 0.237  624 | 1.48  2481 | 31 |
| {BcFV5.BI=TRUE, BcOLV1.BI.MOVA.=TRUE} =>  {BcMV2.BI=TRUE} | 0.07  4257 | 0.810  811 | 0.091  584 | 1.75  1698 | 30 |
| {BcFV5.BI=TRUE, BcMV2.BI=TRUE} =>  {BcOLV1.BI.MOVA.=TRUE} | 0.07  4257 | 0.625 | 0.118  812 | 1.88  4328 | 30 |
| {BcMV2.BI=TRUE, | 0.07 | 0.297 | 0.25 | 1.36 | 30 |

| BcOLV1.BI.MOVA.=TRUE} =>  {BcFV5.BI=TRUE} | 4257 | 03 |  | 3636 |  |
| --- | --- | --- | --- | --- | --- |
| {BcFV5.BI=TRUE, |  |  |  |  |  |
|  | 0.07 | 0.837 | 0.091 | 1.61 |  |
| BcOLV1.BI.MOVA.=TRUE} => |  |  |  |  | 31 |
|  | 6733 | 838 | 584 | 9553 |  |
| {BcOLV18=TRUE} |  |  |  |  |  |
| {BcFV5.BI=TRUE, BcOLV18=TRUE} => | 0.07 | 0.516 | 0.148 | 1.55 |  |
|  |  |  |  |  | 31 |
| {BcOLV1.BI.MOVA.=TRUE} | 6733 | 667 | 515 | 7711 |  |
| {BcOLV1.BI.MOVA.=TRUE, | 0.07 | 0.333 | 0.230 | 1.53 |  |
|  |  |  |  |  | 31 |
| BcOLV18=TRUE} => {BcFV5.BI=TRUE} | 6733 | 333 | 198 | 0303 |  |
| {BcFV5.BI=TRUE, |  |  |  |  |  |
|  | 0.08 | 0.891 | 0.091 | 1.29 |  |
| BcOLV1.BI.MOVA.=TRUE} => |  |  |  |  | 33 |
|  | 1683 | 892 | 584 | 1485 |  |
| {BcULV4=TRUE} |  |  |  |  |  |
| {BcFV5.BI=TRUE, BcULV4=TRUE} => | 0.08 | 0.507 | 0.160 | 1.53 |  |
|  |  |  |  |  | 33 |
| {BcOLV1.BI.MOVA.=TRUE} | 1683 | 692 | 891 | 0654 |  |
| {BcOLV1.BI.MOVA.=TRUE, BcULV4=TRUE} | 0.08 | 0.333 | 0.245 | 1.53 |  |
|  |  |  |  |  | 33 |
| => {BcFV5.BI=TRUE} | 1683 | 333 | 05 | 0303 |  |
| {BcFV5.BI=TRUE, |  |  |  |  |  |
|  | 0.08 | 0.945 | 0.091 | 1.19 |  |
| BcOLV1.BI.MOVA.=TRUE} => |  |  |  |  | 35 |
|  | 6634 | 946 | 584 | 8001 |  |
| {BcMV1.BI=TRUE} |  |  |  |  |  |
| {BcFV5.BI=TRUE, BcMV1.BI=TRUE} => | 0.08 | 0.479 | 0.180 | 1.44 |  |
|  |  |  |  |  | 35 |
| {BcOLV1.BI.MOVA.=TRUE} | 6634 | 452 | 693 | 5512 |  |
| {BcMV1.BI=TRUE, | 0.08 | 0.294 | 0.294 | 1.35 | 35 |

| BcOLV1.BI.MOVA.=TRUE} =>  {BcFV5.BI=TRUE} | 6634 | 118 | 554 | 0267 |  |
| --- | --- | --- | --- | --- | --- |
| {BcMV12=FALSE, |  |  |  |  |  |
|  | 0.07 | 0.322 | 0.230 | 1.48 |  |
| BcOLV1.BI.MOVA.=TRUE} => |  |  |  |  | 30 |
|  | 4257 | 581 | 198 | 0938 |  |
| {BcFV5.BI=TRUE} |  |  |  |  |  |
| {BcFV5.BI=TRUE, BcFV9=FALSE} => | 0.07 | 0.463 | 0.170 | 1.39 |  |
|  |  |  |  |  | 32 |
| {BcOLV1.BI.MOVA.=TRUE} | 9208 | 768 | 792 | 8226 |  |
| {BcOLV1.BI.MOVA.=TRUE, | 0.08 | 0.311 | 0.262 | 1.42 |  |
|  |  |  |  |  | 33 |
| BcOLV4.BI=FALSE} => {BcFV5.BI=TRUE} | 1683 | 321 | 376 | 9245 |  |
| {BcFV5.BI=TRUE, BcULV5=FALSE} => | 0.08 | 0.430 | 0.195 | 1.29 |  |
|  |  |  |  |  | 34 |
| {BcOLV1.BI.MOVA.=TRUE} | 4158 | 38 | 545 | 7563 |  |
| {BcOLV1.BI.MOVA.=TRUE, | 0.08 | 0.326 | 0.257 | 1.50 |  |
|  |  |  |  |  | 34 |
| BcULV5=FALSE} => {BcFV5.BI=TRUE} | 4158 | 923 | 426 | 0874 |  |
| {BcFV5.BI=TRUE, BvF.BI=FALSE} => | 0.08 | 0.454 | 0.190 | 1.37 |  |
|  |  |  |  |  | 35 |
| {BcOLV1.BI.MOVA.=TRUE} | 6634 | 545 | 594 | 0421 |  |
| {BcFV5.BI=TRUE, BcOLV13.BI=FALSE} => | 0.09 | 0.440 | 0.207 | 1.32 |  |
|  |  |  |  |  | 37 |
| {BcOLV1.BI.MOVA.=TRUE} | 1584 | 476 | 921 | 8003 |  |
| {BcOLV1.BI.MOVA.=TRUE, | 0.09 | 0.330 | 0.277 | 1.51 |  |
|  |  |  |  |  | 37 |
| BcOLV13.BI=FALSE} => {BcFV5.BI=TRUE} | 1584 | 357 | 228 | 664 |  |
| {BcOLV1.BI.MOVA.=TRUE, | 0.08 | 0.303 | 0.277 | 1.39 |  |
|  |  |  |  |  | 34 |
| BcOLV2.BI=FALSE} => {BcFV5.BI=TRUE} | 4158 | 571 | 228 | 3669 |  |
| {BcMCV3.BI=FALSE, | 0.08 | 0.301 | 0.287 | 1.38 | 35 |

| BcOLV1.BI.MOVA.=TRUE} =>  {BcFV5.BI=TRUE} | 6634 | 724 | 129 | 5188 |  |
| --- | --- | --- | --- | --- | --- |
| {BcFV5.BI=TRUE, BcPV4.BI=FALSE} =>  {BcOLV1.BI.MOVA.=TRUE} | 0.08  9109 | 0.444  444 | 0.200  495 | 1.33  9967 | 36 |
| {BcOLV1.BI.MOVA.=TRUE,  BcOLV17.BI=FALSE} => {BcFV5.BI=TRUE} | 0.08  9109 | 0.297  521 | 0.299  505 | 1.36  589 | 36 |
| {BcFV5.BI=TRUE, BcOLV15.BI=FALSE} =>  {BcOLV1.BI.MOVA.=TRUE} | 0.08  9109 | 0.493  151 | 0.180  693 | 1.48  6813 | 36 |
| {BcFV5.BI=TRUE, BcMV11=FALSE} =>  {BcRV1.BI=FALSE} | 0.07  4257 | 0.967  742 | 0.076  733 | 1.33  8931 | 30 |
| {BcFV5.BI=TRUE, BcMV11=FALSE} =>  {BcOLV4.BI=FALSE} | 0.07  6733 | 1 | 0.076  733 | 1.14  1243 | 31 |
| {BcFV5.BI=TRUE, BcOLV16.BI=TRUE} =>  {BcOLV18=TRUE} | 0.07  9208 | 0.842  105 | 0.094  059 | 1.62  7802 | 32 |
| {BcFV5.BI=TRUE, BcOLV18=TRUE} =>  {BcOLV16.BI=TRUE} | 0.07  9208 | 0.533  333 | 0.148  515 | 1.28  254 | 32 |
| {BcOLV16.BI=TRUE, BcOLV18=TRUE} =>  {BcFV5.BI=TRUE} | 0.07  9208 | 0.293  578 | 0.269  802 | 1.34  779 | 32 |
| {BcFV5.BI=TRUE, BcOLV16.BI=TRUE} =>  {BcMV9.BI=TRUE} | 0.07  9208 | 0.842  105 | 0.094  059 | 1.24  1644 | 32 |
| {BcFV5.BI=TRUE, BcOLV16.BI=TRUE} =>  {BcULV4=TRUE} | 0.07  9208 | 0.842  105 | 0.094  059 | 1.21  9393 | 32 |

| {BcFV5.BI=TRUE, BcMV4.BI=TRUE} =>  {BcMV2.BI=TRUE} | 0.07  9208 | 0.653  061 | 0.121  287 | 1.41  0892 | 32 |
| --- | --- | --- | --- | --- | --- |
| {BcFV5.BI=TRUE, BcMV2.BI=TRUE} =>  {BcMV4.BI=TRUE} | 0.07  9208 | 0.666  667 | 0.118  812 | 1.50  4655 | 32 |
| {BcMV2.BI=TRUE, BcMV4.BI=TRUE} =>  {BcFV5.BI=TRUE} | 0.07  9208 | 0.288  288 | 0.274  752 | 1.32  3505 | 32 |
| {BcFV5.BI=TRUE, BcMV4.BI=TRUE} =>  {BcOLV18=TRUE} | 0.08  6634 | 0.714  286 | 0.121  287 | 1.38  0725 | 35 |
| {BcFV5.BI=TRUE, BcOLV18=TRUE} =>  {BcMV4.BI=TRUE} | 0.08  6634 | 0.583  333 | 0.148  515 | 1.31  6574 | 35 |
| {BcMV4.BI=TRUE, BcOLV18=TRUE} =>  {BcFV5.BI=TRUE} | 0.08  6634 | 0.312  5 | 0.277  228 | 1.43  4659 | 35 |
| {BcFV5.BI=TRUE, BcMV4.BI=TRUE} =>  {BcHV1SLR.BI=TRUE} | 0.09  1584 | 0.755  102 | 0.121  287 | 1.39  2974 | 37 |
| {BcFV5.BI=TRUE, BcHV1SLR.BI=TRUE} =>  {BcMV4.BI=TRUE} | 0.09  1584 | 0.606  557 | 0.150  99 | 1.36  899 | 37 |
| {BcHV1SLR.BI=TRUE, BcMV4.BI=TRUE} =>  {BcFV5.BI=TRUE} | 0.09  1584 | 0.349  057 | 0.262  376 | 1.60  2487 | 37 |
| {BcFV5.BI=TRUE, BcMV4.BI=TRUE} =>  {BcMV3.BI=TRUE} | 0.09  6535 | 0.795  918 | 0.121  287 | 1.46  827 | 39 |
| {BcFV5.BI=TRUE, BcMV3.BI=TRUE} =>  {BcMV4.BI=TRUE} | 0.09  6535 | 0.629  032 | 0.153  465 | 1.41  9715 | 39 |

| {BcMV3.BI=TRUE, BcMV4.BI=TRUE} =>  {BcFV5.BI=TRUE} | 0.09  6535 | 0.327  731 | 0.294  554 | 1.50  4584 | 39 |
| --- | --- | --- | --- | --- | --- |
| {BcFV5.BI=TRUE, BcMV4.BI=TRUE} =>  {BcULV3=TRUE} | 0.09  6535 | 0.795  918 | 0.121  287 | 1.33  9796 | 39 |
| {BcFV5.BI=TRUE, BcULV3=TRUE} =>  {BcMV4.BI=TRUE} | 0.09  6535 | 0.590  909 | 0.163  366 | 1.33  3672 | 39 |
| {BcMV4.BI=TRUE, BcULV3=TRUE} =>  {BcFV5.BI=TRUE} | 0.09  6535 | 0.327  731 | 0.294  554 | 1.50  4584 | 39 |
| {BcFV5.BI=TRUE, BcMV4.BI=TRUE} =>  {BcHV1.BI=TRUE} | 0.09  6535 | 0.795  918 | 0.121  287 | 1.31  7832 | 39 |
| {BcFV5.BI=TRUE, BcHV1.BI=TRUE} =>  {BcMV4.BI=TRUE} | 0.09  6535 | 0.639  344 | 0.150  99 | 1.44  2989 | 39 |
| {BcHV1.BI=TRUE, BcMV4.BI=TRUE} =>  {BcFV5.BI=TRUE} | 0.09  6535 | 0.322  314 | 0.299  505 | 1.47  9715 | 39 |
| {BcFV5.BI=TRUE, BcMV4.BI=TRUE} =>  {BcMV11=TRUE} | 0.09  901 | 0.816  327 | 0.121  287 | 1.32  4482 | 40 |
| {BcFV5.BI=TRUE, BcMV11=TRUE} =>  {BcMV4.BI=TRUE} | 0.09  901 | 0.701  754 | 0.141  089 | 1.58  3848 | 40 |
| {BcMV11=TRUE, BcMV4.BI=TRUE} =>  {BcFV5.BI=TRUE} | 0.09  901 | 0.291  971 | 0.339  109 | 1.34  0411 | 40 |
| {BcFV5.BI=TRUE, BcMV4.BI=TRUE} =>  {BcMV9.BI=TRUE} | 0.09  901 | 0.816  327 | 0.121  287 | 1.20  3635 | 40 |

| {BcFV5.BI=TRUE, BcMV9.BI=TRUE} =>  {BcMV4.BI=TRUE} | 0.09  901 | 0.597  015 | 0.165  842 | 1.34  7453 | 40 |
| --- | --- | --- | --- | --- | --- |
| {BcMV4.BI=TRUE, BcMV9.BI=TRUE} =>  {BcFV5.BI=TRUE} | 0.09  901 | 0.294  118 | 0.336  634 | 1.35  0267 | 40 |
| {BcMV4.BI=TRUE, BcPV3.BI=FALSE} =>  {BcFV5.BI=TRUE} | 0.08  6634 | 0.330  189 | 0.262  376 | 1.51  5866 | 35 |
| {BcMV4.BI=TRUE, BcRV1.BI=FALSE} =>  {BcFV5.BI=TRUE} | 0.09  1584 | 0.330  357 | 0.277  228 | 1.51  664 | 37 |
| {BcFV5.BI=TRUE, BcMV1.BI=TRUE} =>  {BcMV4.BI=TRUE} | 0.10  6436 | 0.589  041 | 0.180  693 | 1.32  9456 | 43 |
| {BcMV1.BI=TRUE, BcMV4.BI=TRUE} =>  {BcFV5.BI=TRUE} | 0.10  6436 | 0.282  895 | 0.376  238 | 1.29  8744 | 43 |
| {BcFV5.BI=TRUE, BcMV12=FALSE} =>  {BcMV4.BI=TRUE} | 0.10  1485 | 0.585  714 | 0.173  267 | 1.32  1947 | 41 |
| {BcMV12=FALSE, BcMV4.BI=TRUE} =>  {BcFV5.BI=TRUE} | 0.10  1485 | 0.280  822 | 0.361  386 | 1.28  9228 | 41 |
| {BcMV4.BI=TRUE, BcOLV4.BI=FALSE} =>  {BcFV5.BI=TRUE} | 0.10  396 | 0.28 | 0.371  287 | 1.28  5455 | 42 |
| {BcMV4.BI=TRUE, BcULV5=FALSE} =>  {BcFV5.BI=TRUE} | 0.10  6436 | 0.279  221 | 0.381  188 | 1.28  1877 | 43 |
| {BcMV4.BI=TRUE, BcOLV13.BI=FALSE} =>  {BcFV5.BI=TRUE} | 0.11  3861 | 0.291  139 | 0.391  089 | 1.33  6594 | 46 |

| {BcFV5.BI=TRUE, BcOLV2.BI=FALSE} =>  {BcMV4.BI=TRUE} | 0.11  6337 | 0.580  247 | 0.200  495 | 1.30  9608 | 47 |
| --- | --- | --- | --- | --- | --- |
| {BcMV4.BI=TRUE, BcOLV2.BI=FALSE} =>  {BcFV5.BI=TRUE} | 0.11  6337 | 0.288  344 | 0.403  465 | 1.32  3759 | 47 |
| {BcFV5.BI=TRUE, BcMCV3.BI=FALSE} =>  {BcMV4.BI=TRUE} | 0.11  6337 | 0.559  524 | 0.207  921 | 1.26  2836 | 47 |
| {BcMCV3.BI=FALSE, BcMV4.BI=TRUE} =>  {BcFV5.BI=TRUE} | 0.11  6337 | 0.283  133 | 0.410  891 | 1.29  9836 | 47 |
| {BcFV5.BI=TRUE, BcPV4.BI=FALSE} =>  {BcMV4.BI=TRUE} | 0.11  1386 | 0.555  556 | 0.200  495 | 1.25  388 | 45 |
| {BcEV6=FALSE, BcFV5.BI=TRUE} =>  {BcMV4.BI=TRUE} | 0.11  6337 | 0.559  524 | 0.207  921 | 1.26  2836 | 47 |
| {BcFV5.BI=TRUE, BcOLV17.BI=FALSE} =>  {BcMV4.BI=TRUE} | 0.11  8812 | 0.558  14 | 0.212  871 | 1.25  9712 | 48 |
| {BcMV4.BI=TRUE, BcOLV17.BI=FALSE} =>  {BcFV5.BI=TRUE} | 0.11  8812 | 0.289  157 | 0.410  891 | 1.32  7492 | 48 |
| {BcFV5.BI=TRUE, BcOLV15.BI=FALSE} =>  {BcMV4.BI=TRUE} | 0.10  396 | 0.575  342 | 0.180  693 | 1.29  8538 | 42 |
| {BcFV5.BI=TRUE, BcMV2.BI=TRUE} =>  {BcOLV18=TRUE} | 0.08  6634 | 0.729  167 | 0.118  812 | 1.40  949 | 35 |
| {BcFV5.BI=TRUE, BcOLV18=TRUE} =>  {BcMV2.BI=TRUE} | 0.08  6634 | 0.583  333 | 0.148  515 | 1.26  025 | 35 |

| {BcMV2.BI=TRUE, BcOLV18=TRUE} =>  {BcFV5.BI=TRUE} | 0.08  6634 | 0.294  118 | 0.294  554 | 1.35  0267 | 35 |
| --- | --- | --- | --- | --- | --- |
| {BcFV5.BI=TRUE, BcMV2.BI=TRUE} =>  {BcHV1SLR.BI=TRUE} | 0.08  1683 | 0.687  5 | 0.118  812 | 1.26  8265 | 33 |
| {BcHV1SLR.BI=TRUE, BcMV2.BI=TRUE} =>  {BcFV5.BI=TRUE} | 0.08  1683 | 0.3 | 0.272  277 | 1.37  7273 | 33 |
| {BcFV5.BI=TRUE, BcMV2.BI=TRUE} =>  {BcMV3.BI=TRUE} | 0.09  1584 | 0.770  833 | 0.118  812 | 1.42  1994 | 37 |
| {BcFV5.BI=TRUE, BcMV3.BI=TRUE} =>  {BcMV2.BI=TRUE} | 0.09  1584 | 0.596  774 | 0.153  465 | 1.28  9288 | 37 |
| {BcMV2.BI=TRUE, BcMV3.BI=TRUE} =>  {BcFV5.BI=TRUE} | 0.09  1584 | 0.291  339 | 0.314  356 | 1.33  7509 | 37 |
| {BcFV5.BI=TRUE, BcMV2.BI=TRUE} =>  {BcULV3=TRUE} | 0.08  9109 | 0.75 | 0.118  812 | 1.26  25 | 36 |
| {BcMV2.BI=TRUE, BcULV3=TRUE} =>  {BcFV5.BI=TRUE} | 0.08  9109 | 0.313  043 | 0.284  653 | 1.43  7154 | 36 |
| {BcFV5.BI=TRUE, BcMV2.BI=TRUE} =>  {BcMV11=TRUE} | 0.10  1485 | 0.854  167 | 0.118  812 | 1.38  5877 | 41 |
| {BcFV5.BI=TRUE, BcMV11=TRUE} =>  {BcMV2.BI=TRUE} | 0.10  1485 | 0.719  298 | 0.141  089 | 1.55  3992 | 41 |
| {BcMV11=TRUE, BcMV2.BI=TRUE} =>  {BcFV5.BI=TRUE} | 0.10  1485 | 0.297  101 | 0.341  584 | 1.36  3966 | 41 |

| {BcFV5.BI=TRUE, BcMV2.BI=TRUE} =>  {BcMV9.BI=TRUE} | 0.10  396 | 0.875 | 0.118  812 | 1.29  0146 | 42 |
| --- | --- | --- | --- | --- | --- |
| {BcFV5.BI=TRUE, BcMV9.BI=TRUE} =>  {BcMV2.BI=TRUE} | 0.10  396 | 0.626  866 | 0.165  842 | 1.35  4298 | 42 |
| {BcMV2.BI=TRUE, BcMV9.BI=TRUE} =>  {BcFV5.BI=TRUE} | 0.10  396 | 0.323  077 | 0.321  782 | 1.48  3217 | 42 |
| {BcFV5.BI=TRUE, BcMV2.BI=TRUE} =>  {BcULV4=TRUE} | 0.09  6535 | 0.812  5 | 0.118  812 | 1.17  6523 | 39 |
| {BcFV5.BI=TRUE, BcULV4=TRUE} =>  {BcMV2.BI=TRUE} | 0.09  6535 | 0.6 | 0.160  891 | 1.29  6257 | 39 |
| {BcMV2.BI=TRUE, BcULV4=TRUE} =>  {BcFV5.BI=TRUE} | 0.09  6535 | 0.282  609 | 0.341  584 | 1.29  7431 | 39 |
| {BcMV2.BI=TRUE, BcRV1.BI=FALSE} =>  {BcFV5.BI=TRUE} | 0.08  1683 | 0.286  957 | 0.284  653 | 1.31  7391 | 33 |
| {BcFV5.BI=TRUE, BcMV2.BI=TRUE} =>  {BcMV1.BI=TRUE} | 0.10  8911 | 0.916  667 | 0.118  812 | 1.16  092 | 44 |
| {BcFV5.BI=TRUE, BcMV1.BI=TRUE} =>  {BcMV2.BI=TRUE} | 0.10  8911 | 0.602  74 | 0.180  693 | 1.30  2176 | 44 |
| {BcFV5.BI=TRUE, BcNSRV7.BI=FALSE} =>  {BcMV2.BI=TRUE} | 0.07  4257 | 0.612  245 | 0.121  287 | 1.32  2711 | 30 |
| {BcFV5.BI=TRUE, BcFV9=FALSE} =>  {BcMV2.BI=TRUE} | 0.10  1485 | 0.594  203 | 0.170  792 | 1.28  3732 | 41 |

| {BcFV5.BI=TRUE, BvF.BI=FALSE} =>  {BcMV2.BI=TRUE} | 0.11  1386 | 0.584  416 | 0.190  594 | 1.26  2588 | 45 |
| --- | --- | --- | --- | --- | --- |
| {BcMV2.BI=TRUE, BcOLV13.BI=FALSE} =>  {BcFV5.BI=TRUE} | 0.11  6337 | 0.284  848 | 0.408  416 | 1.30  7713 | 47 |
| {BcFV5.BI=TRUE, BcOLV15.BI=FALSE} =>  {BcMV2.BI=TRUE} | 0.11  8812 | 0.657  534 | 0.180  693 | 1.42  0555 | 48 |
| {BcFV5.BI=TRUE, BcOLV18=TRUE} =>  {BcHV1SLR.BI=TRUE} | 0.11  1386 | 0.75 | 0.148  515 | 1.38  3562 | 45 |
| {BcFV5.BI=TRUE, BcHV1SLR.BI=TRUE} =>  {BcOLV18=TRUE} | 0.11  1386 | 0.737  705 | 0.150  99 | 1.42  5994 | 45 |
| {BcHV1SLR.BI=TRUE, BcOLV18=TRUE} =>  {BcFV5.BI=TRUE} | 0.11  1386 | 0.326  087 | 0.341  584 | 1.49  7036 | 45 |
| {BcFV5.BI=TRUE, BcOLV18=TRUE} =>  {BcMV3.BI=TRUE} | 0.10  8911 | 0.733  333 | 0.148  515 | 1.35  2816 | 44 |
| {BcFV5.BI=TRUE, BcMV3.BI=TRUE} =>  {BcOLV18=TRUE} | 0.10  8911 | 0.709  677 | 0.153  465 | 1.37  1817 | 44 |
| {BcMV3.BI=TRUE, BcOLV18=TRUE} =>  {BcFV5.BI=TRUE} | 0.10  8911 | 0.330  827 | 0.329  208 | 1.51  8797 | 44 |
| {BcFV5.BI=TRUE, BcOLV18=TRUE} =>  {BcULV3=TRUE} | 0.11  6337 | 0.783  333 | 0.148  515 | 1.31  8611 | 47 |
| {BcFV5.BI=TRUE, BcULV3=TRUE} =>  {BcOLV18=TRUE} | 0.11  6337 | 0.712  121 | 0.163  366 | 1.37  6541 | 47 |

| {BcOLV18=TRUE, BcULV3=TRUE} =>  {BcFV5.BI=TRUE} | 0.11  6337 | 0.358  779 | 0.324  257 | 1.64  712 | 47 |
| --- | --- | --- | --- | --- | --- |
| {BcFV5.BI=TRUE, BcOLV18=TRUE} =>  {BcHV1.BI=TRUE} | 0.11  1386 | 0.75 | 0.148  515 | 1.24  1803 | 45 |
| {BcFV5.BI=TRUE, BcHV1.BI=TRUE} =>  {BcOLV18=TRUE} | 0.11  1386 | 0.737  705 | 0.150  99 | 1.42  5994 | 45 |
| {BcHV1.BI=TRUE, BcOLV18=TRUE} =>  {BcFV5.BI=TRUE} | 0.11  1386 | 0.304  054 | 0.366  337 | 1.39  5885 | 45 |
| {BcFV5.BI=TRUE, BcMV11=TRUE} =>  {BcOLV18=TRUE} | 0.10  1485 | 0.719  298 | 0.141  089 | 1.39  0414 | 41 |
| {BcMV11=TRUE, BcOLV18=TRUE} =>  {BcFV5.BI=TRUE} | 0.10  1485 | 0.292  857 | 0.346  535 | 1.34  4481 | 41 |
| {BcFV5.BI=TRUE, BcOLV18=TRUE} =>  {BcMV9.BI=TRUE} | 0.12  3762 | 0.833  333 | 0.148  515 | 1.22  871 | 50 |
| {BcFV5.BI=TRUE, BcMV9.BI=TRUE} =>  {BcOLV18=TRUE} | 0.12  3762 | 0.746  269 | 0.165  842 | 1.44  2548 | 50 |
| {BcMV9.BI=TRUE, BcOLV18=TRUE} =>  {BcFV5.BI=TRUE} | 0.12  3762 | 0.299  401 | 0.413  366 | 1.37  4524 | 50 |
| {BcFV5.BI=TRUE, BcOLV18=TRUE} =>  {BcULV4=TRUE} | 0.12  8713 | 0.866  667 | 0.148  515 | 1.25  4958 | 52 |
| {BcFV5.BI=TRUE, BcULV4=TRUE} =>  {BcOLV18=TRUE} | 0.12  8713 | 0.8 | 0.160  891 | 1.54  6411 | 52 |

| {BcOLV18=TRUE, BcULV4=TRUE} =>  {BcFV5.BI=TRUE} | 0.12  8713 | 0.292  135 | 0.440  594 | 1.34  1164 | 52 |
| --- | --- | --- | --- | --- | --- |
| {BcFV5.BI=TRUE, BcPV3.BI=FALSE} =>  {BcOLV18=TRUE} | 0.10  396 | 0.636  364 | 0.163  366 | 1.23  01 | 42 |
| {BcOLV18=TRUE, BcPV3.BI=FALSE} =>  {BcFV5.BI=TRUE} | 0.10  396 | 0.315  789 | 0.329  208 | 1.44  9761 | 42 |
| {BcFV5.BI=TRUE, BcRV1.BI=FALSE} =>  {BcOLV18=TRUE} | 0.11  3861 | 0.638  889 | 0.178  218 | 1.23  4981 | 46 |
| {BcOLV18=TRUE, BcRV1.BI=FALSE} =>  {BcFV5.BI=TRUE} | 0.11  3861 | 0.345  865 | 0.329  208 | 1.58  7833 | 46 |
| {BcEV2.BI=FALSE, BcFV5.BI=TRUE} =>  {BcOLV18=TRUE} | 0.08  1683 | 0.647  059 | 0.126  238 | 1.25  0774 | 33 |
| {BcFV5.BI=TRUE, BcULV1.BI=FALSE} =>  {BcOLV18=TRUE} | 0.11  1386 | 0.642  857 | 0.173  267 | 1.24  2652 | 45 |
| {BcOLV18=TRUE, BcULV1.BI=FALSE} =>  {BcFV5.BI=TRUE} | 0.11  1386 | 0.312  5 | 0.356  436 | 1.43  4659 | 45 |
| {BcFV5.BI=TRUE, BcOLV18=TRUE} =>  {BcMV1.BI=TRUE} | 0.13  1188 | 0.883  333 | 0.148  515 | 1.11  8704 | 53 |
| {BcFV5.BI=TRUE, BcMV1.BI=TRUE} =>  {BcOLV18=TRUE} | 0.13  1188 | 0.726  027 | 0.180  693 | 1.40  3421 | 53 |
| {BcMV1.BI=TRUE, BcOLV18=TRUE} =>  {BcFV5.BI=TRUE} | 0.13  1188 | 0.297  753 | 0.440  594 | 1.36  6956 | 53 |

| {BcFV5.BI=TRUE, BcMV12=FALSE} =>  {BcOLV18=TRUE} | 0.11  3861 | 0.657  143 | 0.173  267 | 1.27  0267 | 46 |
| --- | --- | --- | --- | --- | --- |
| {BcMV12=FALSE, BcOLV18=TRUE} =>  {BcFV5.BI=TRUE} | 0.11  3861 | 0.287  5 | 0.396  04 | 1.31  9886 | 46 |
| {BcFV5.BI=TRUE, BcNSRV7.BI=FALSE} =>  {BcOLV18=TRUE} | 0.07  6733 | 0.632  653 | 0.121  287 | 1.22  2927 | 31 |
| {BcFV5.BI=TRUE, BcVV3.BI=FALSE} =>  {BcOLV18=TRUE} | 0.11  3861 | 0.676  471 | 0.168  317 | 1.30  7627 | 46 |
| {BcOLV18=TRUE, BcVV3.BI=FALSE} =>  {BcFV5.BI=TRUE} | 0.11  3861 | 0.277  108 | 0.410  891 | 1.27  218 | 46 |
| {BcFV5.BI=TRUE, BcOLV7.BI=FALSE} =>  {BcOLV18=TRUE} | 0.11  8812 | 0.648  649 | 0.183  168 | 1.25  3847 | 48 |
| {BcOLV18=TRUE, BcOLV7.BI=FALSE} =>  {BcFV5.BI=TRUE} | 0.11  8812 | 0.315  789 | 0.376  238 | 1.44  9761 | 48 |
| {BcFV5.BI=TRUE, BcFV9=FALSE} =>  {BcOLV18=TRUE} | 0.10  8911 | 0.637  681 | 0.170  792 | 1.23  2647 | 44 |
| {BcFV5.BI=TRUE, BcOLV4.BI=FALSE} =>  {BcOLV18=TRUE} | 0.13  1188 | 0.670  886 | 0.195  545 | 1.29  6832 | 53 |
| {BcOLV18=TRUE, BcOLV4.BI=FALSE} =>  {BcFV5.BI=TRUE} | 0.13  1188 | 0.292  818 | 0.448  02 | 1.34  4299 | 53 |
| {BcFV5.BI=TRUE, BcULV5=FALSE} =>  {BcOLV18=TRUE} | 0.13  6139 | 0.696  203 | 0.195  545 | 1.34  5769 | 55 |

| {BcOLV18=TRUE, BcULV5=FALSE} =>  {BcFV5.BI=TRUE} | 0.13  6139 | 0.291  005 | 0.467  822 | 1.33  5979 | 55 |
| --- | --- | --- | --- | --- | --- |
| {BcFV5.BI=TRUE, BvF.BI=FALSE} =>  {BcOLV18=TRUE} | 0.13  6139 | 0.714  286 | 0.190  594 | 1.38  0725 | 55 |
| {BcOLV18=TRUE, BvF.BI=FALSE} =>  {BcFV5.BI=TRUE} | 0.13  6139 | 0.286  458 | 0.475  248 | 1.31  5104 | 55 |
| {BcFV5.BI=TRUE, BcMCV5.BI=FALSE} =>  {BcOLV18=TRUE} | 0.11  3861 | 0.630  137 | 0.180  693 | 1.21  8064 | 46 |
| {BcFV5.BI=TRUE, BcOLV13.BI=FALSE} =>  {BcOLV18=TRUE} | 0.14  1089 | 0.678  571 | 0.207  921 | 1.31  1688 | 57 |
| {BcOLV13.BI=FALSE, BcOLV18=TRUE} =>  {BcFV5.BI=TRUE} | 0.14  1089 | 0.296  875 | 0.475  248 | 1.36  2926 | 57 |
| {BcFV5.BI=TRUE, BcOLV2.BI=FALSE} =>  {BcOLV18=TRUE} | 0.13  3663 | 0.666  667 | 0.200  495 | 1.28  8676 | 54 |
| {BcOLV18=TRUE, BcOLV2.BI=FALSE} =>  {BcFV5.BI=TRUE} | 0.13  3663 | 0.285  714 | 0.467  822 | 1.31  1688 | 54 |
| {BcFV5.BI=TRUE, BcMCV3.BI=FALSE} =>  {BcOLV18=TRUE} | 0.13  8614 | 0.666  667 | 0.207  921 | 1.28  8676 | 56 |
| {BcMCV3.BI=FALSE, BcOLV18=TRUE} =>  {BcFV5.BI=TRUE} | 0.13  8614 | 0.291  667 | 0.475  248 | 1.33  9015 | 56 |
| {BcFV5.BI=TRUE, BcPV4.BI=FALSE} =>  {BcOLV18=TRUE} | 0.13  6139 | 0.679  012 | 0.200  495 | 1.31  2541 | 55 |

| {BcOLV18=TRUE, BcPV4.BI=FALSE} =>  {BcFV5.BI=TRUE} | 0.13  6139 | 0.284  974 | 0.477  723 | 1.30  829 | 55 |
| --- | --- | --- | --- | --- | --- |
| {BcEV6=FALSE, BcFV5.BI=TRUE} =>  {BcOLV18=TRUE} | 0.13  8614 | 0.666  667 | 0.207  921 | 1.28  8676 | 56 |
| {BcEV6=FALSE, BcOLV18=TRUE} =>  {BcFV5.BI=TRUE} | 0.13  8614 | 0.287  179 | 0.482  673 | 1.31  8415 | 56 |
| {BcFV5.BI=TRUE, BcOLV17.BI=FALSE} =>  {BcOLV18=TRUE} | 0.14  604 | 0.686  047 | 0.212  871 | 1.32  6138 | 59 |
| {BcOLV17.BI=FALSE, BcOLV18=TRUE} =>  {BcFV5.BI=TRUE} | 0.14  604 | 0.305  699 | 0.477  723 | 1.40  3439 | 59 |
| {BcFV5.BI=TRUE, BcOLV15.BI=FALSE} =>  {BcOLV18=TRUE} | 0.11  8812 | 0.657  534 | 0.180  693 | 1.27  1023 | 48 |
| {BcEV5=FALSE, BcFV5.BI=TRUE} =>  {BcOLV18=TRUE} | 0.12  3762 | 0.694  444 | 0.178  218 | 1.34  2371 | 50 |
| {BcFV5.BI=TRUE, BcMV2.BI=FALSE} =>  {BcOLV16.BI=FALSE} | 0.07  4257 | 0.75 | 0.099  01 | 1.28  3898 | 30 |
| {BcFV5.BI=TRUE, BcMV2.BI=FALSE} =>  {BcULV3=TRUE} | 0.07  4257 | 0.75 | 0.099  01 | 1.26  25 | 30 |
| {BcFV5.BI=TRUE, BcMV2.BI=FALSE} =>  {BcOLV1.BI.MOVA.=FALSE} | 0.08  1683 | 0.825 | 0.099  01 | 1.23  4444 | 33 |
| {BcFV5.BI=TRUE, BcMV2.BI=FALSE} =>  {BcMV6.BI=FALSE} | 0.09  4059 | 0.95 | 0.099  01 | 1.36  5836 | 38 |

| {BcFV5.BI=TRUE, BcMV2.BI=FALSE} =>  {BcPV3.BI=FALSE} | 0.08  6634 | 0.875 | 0.099  01 | 1.23  1707 | 35 |
| --- | --- | --- | --- | --- | --- |
| {BcFV5.BI=TRUE, BcMV2.BI=FALSE} =>  {BcRV1.BI=FALSE} | 0.09  6535 | 0.975 | 0.099  01 | 1.34  8973 | 39 |
| {BcFV5.BI=TRUE, BcMV2.BI=FALSE} =>  {BcOLV4.BI=FALSE} | 0.09  6535 | 0.975 | 0.099  01 | 1.11  2712 | 39 |
| {BcFV5.BI=TRUE, BcHV1SLR.BI=TRUE} =>  {BcMV3.BI=TRUE} | 0.10  6436 | 0.704  918 | 0.150  99 | 1.30  0397 | 43 |
| {BcFV5.BI=TRUE, BcMV3.BI=TRUE} =>  {BcHV1SLR.BI=TRUE} | 0.10  6436 | 0.693  548 | 0.153  465 | 1.27  9423 | 43 |
| {BcHV1SLR.BI=TRUE, BcMV3.BI=TRUE} =>  {BcFV5.BI=TRUE} | 0.10  6436 | 0.323  308 | 0.329  208 | 1.48  4279 | 43 |
| {BcFV5.BI=TRUE, BcOLV16.BI=FALSE} =>  {BcHV1SLR.BI=TRUE} | 0.08  4158 | 0.68 | 0.123  762 | 1.25  4429 | 34 |
| {BcFV5.BI=TRUE, BcHV1SLR.BI=TRUE} =>  {BcULV3=TRUE} | 0.12  3762 | 0.819  672 | 0.150  99 | 1.37  9781 | 50 |
| {BcFV5.BI=TRUE, BcULV3=TRUE} =>  {BcHV1SLR.BI=TRUE} | 0.12  3762 | 0.757  576 | 0.163  366 | 1.39  7537 | 50 |
| {BcHV1SLR.BI=TRUE, BcULV3=TRUE} =>  {BcFV5.BI=TRUE} | 0.12  3762 | 0.320  513 | 0.386  139 | 1.47  1445 | 50 |
| {BcFV5.BI=TRUE, BcHV1SLR.BI=TRUE} =>  {BcHV1.BI=TRUE} | 0.13  6139 | 0.901  639 | 0.150  99 | 1.49  2878 | 55 |

| {BcFV5.BI=TRUE, BcHV1.BI=TRUE} =>  {BcHV1SLR.BI=TRUE} | 0.13  6139 | 0.901  639 | 0.150  99 | 1.66  3298 | 55 |
| --- | --- | --- | --- | --- | --- |
| {BcHV1.BI=TRUE, BcHV1SLR.BI=TRUE} =>  {BcFV5.BI=TRUE} | 0.13  6139 | 0.280  612 | 0.485  149 | 1.28  8265 | 55 |
| {BcFV5.BI=TRUE, BcMV11=TRUE} =>  {BcHV1SLR.BI=TRUE} | 0.09  901 | 0.701  754 | 0.141  089 | 1.29  4561 | 40 |
| {BcFV5.BI=TRUE, BcOLV1.BI.MOVA.=FALSE} =>  {BcHV1SLR.BI=TRUE} | 0.09  6535 | 0.764  706 | 0.126  238 | 1.41  069 | 39 |
| {BcHV1SLR.BI=TRUE, BcOLV1.BI.MOVA.=FALSE} =>  {BcFV5.BI=TRUE} | 0.09  6535 | 0.282  609 | 0.341  584 | 1.29  7431 | 39 |
| {BcFV5.BI=TRUE, BcHV1SLR.BI=TRUE} =>  {BcMV9.BI=TRUE} | 0.12  1287 | 0.803  279 | 0.150  99 | 1.18  4396 | 49 |
| {BcFV5.BI=TRUE, BcMV9.BI=TRUE} =>  {BcHV1SLR.BI=TRUE} | 0.12  1287 | 0.731  343 | 0.165  842 | 1.34  9145 | 49 |
| {BcHV1SLR.BI=TRUE, BcMV9.BI=TRUE} =>  {BcFV5.BI=TRUE} | 0.12  1287 | 0.296  97 | 0.408  416 | 1.36  3361 | 49 |
| {BcFV5.BI=TRUE, BcULV4=TRUE} =>  {BcHV1SLR.BI=TRUE} | 0.11  3861 | 0.707  692 | 0.160  891 | 1.30  5515 | 46 |
| {BcHV1SLR.BI=TRUE, BcULV4=TRUE} =>  {BcFV5.BI=TRUE} | 0.11  3861 | 0.283  951 | 0.400  99 | 1.30  3591 | 46 |

| {BcFV5.BI=TRUE, BcPV3.BI=FALSE} =>  {BcHV1SLR.BI=TRUE} | 0.11  1386 | 0.681  818 | 0.163  366 | 1.25  7783 | 45 |
| --- | --- | --- | --- | --- | --- |
| {BcHV1SLR.BI=TRUE, BcPV3.BI=FALSE}  => {BcFV5.BI=TRUE} | 0.11  1386 | 0.298  013 | 0.373  762 | 1.36  8152 | 45 |
| {BcFV5.BI=TRUE, BcRV1.BI=FALSE} =>  {BcHV1SLR.BI=TRUE} | 0.12  1287 | 0.680  556 | 0.178  218 | 1.25  5454 | 49 |
| {BcHV1SLR.BI=TRUE, BcRV1.BI=FALSE}  => {BcFV5.BI=TRUE} | 0.12  1287 | 0.318  182 | 0.381  188 | 1.46  0744 | 49 |
| {BcEV2.BI=FALSE, BcFV5.BI=TRUE} =>  {BcHV1SLR.BI=TRUE} | 0.08  4158 | 0.666  667 | 0.126  238 | 1.22  9833 | 34 |
| {BcFV5.BI=TRUE, BcULV1.BI=FALSE} =>  {BcHV1SLR.BI=TRUE} | 0.11  6337 | 0.671  429 | 0.173  267 | 1.23  8617 | 47 |
| {BcHV1SLR.BI=TRUE, BcULV1.BI=FALSE}  => {BcFV5.BI=TRUE} | 0.11  6337 | 0.303  226 | 0.383  663 | 1.39  2082 | 47 |
| {BcFV5.BI=TRUE, BcMV1.BI=TRUE} =>  {BcHV1SLR.BI=TRUE} | 0.12  8713 | 0.712  329 | 0.180  693 | 1.31  4068 | 52 |
| {BcHV1SLR.BI=TRUE, BcMV1.BI=TRUE} =>  {BcFV5.BI=TRUE} | 0.12  8713 | 0.292  135 | 0.440  594 | 1.34  1164 | 52 |
| {BcFV5.BI=TRUE, BcVV3.BI=FALSE} =>  {BcHV1SLR.BI=TRUE} | 0.12  3762 | 0.735  294 | 0.168  317 | 1.35  6433 | 50 |
| {BcHV1SLR.BI=TRUE, BcVV3.BI=FALSE}  => {BcFV5.BI=TRUE} | 0.12  3762 | 0.285  714 | 0.433  168 | 1.31  1688 | 50 |

| {BcFV5.BI=TRUE, BcOLV7.BI=FALSE} =>  {BcHV1SLR.BI=TRUE} | 0.12  6238 | 0.689  189 | 0.183  168 | 1.27  1381 | 51 |
| --- | --- | --- | --- | --- | --- |
| {BcHV1SLR.BI=TRUE, BcOLV7.BI=FALSE}  => {BcFV5.BI=TRUE} | 0.12  6238 | 0.289  773 | 0.435  644 | 1.33  032 | 51 |
| {BcALV1.BI=FALSE, BcFV5.BI=TRUE} =>  {BcHV1SLR.BI=TRUE} | 0.10  1485 | 0.683  333 | 0.148  515 | 1.26  0578 | 41 |
| {BcDMV1=FALSE, BcFV5.BI=TRUE} =>  {BcHV1SLR.BI=TRUE} | 0.10  1485 | 0.719  298 | 0.141  089 | 1.32  6925 | 41 |
| {BcFV5.BI=TRUE, BcOLV4.BI=FALSE} =>  {BcHV1SLR.BI=TRUE} | 0.13  6139 | 0.696  203 | 0.195  545 | 1.28  4319 | 55 |
| {BcHV1SLR.BI=TRUE, BcOLV4.BI=FALSE}  => {BcFV5.BI=TRUE} | 0.13  6139 | 0.294  118 | 0.462  871 | 1.35  0267 | 55 |
| {BcFV5.BI=TRUE, BcULV5=FALSE} =>  {BcHV1SLR.BI=TRUE} | 0.13  1188 | 0.670  886 | 0.195  545 | 1.23  7616 | 53 |
| {BcHV1SLR.BI=TRUE, BcULV5=FALSE} =>  {BcFV5.BI=TRUE} | 0.13  1188 | 0.280  423 | 0.467  822 | 1.28  7398 | 53 |
| {BcFV5.BI=TRUE, BvF.BI=FALSE} =>  {BcHV1SLR.BI=TRUE} | 0.12  8713 | 0.675  325 | 0.190  594 | 1.24  5804 | 52 |
| {BcFV5.BI=TRUE, BcMCV5.BI=FALSE} =>  {BcHV1SLR.BI=TRUE} | 0.12  6238 | 0.698  63 | 0.180  693 | 1.28  8797 | 51 |
| {BcFV5.BI=TRUE, BcOLV13.BI=FALSE} =>  {BcHV1SLR.BI=TRUE} | 0.14  3564 | 0.690  476 | 0.207  921 | 1.27  3755 | 58 |

| {BcHV1SLR.BI=TRUE, BcOLV13.BI=FALSE}  => {BcFV5.BI=TRUE} | 0.14  3564 | 0.292  929 | 0.490  099 | 1.34  4812 | 58 |
| --- | --- | --- | --- | --- | --- |
| {BcFV5.BI=TRUE, BcOLV2.BI=FALSE} =>  {BcHV1SLR.BI=TRUE} | 0.13  6139 | 0.679  012 | 0.200  495 | 1.25  2607 | 55 |
| {BcHV1SLR.BI=TRUE, BcOLV2.BI=FALSE}  => {BcFV5.BI=TRUE} | 0.13  6139 | 0.282  051 | 0.482  673 | 1.29  4872 | 55 |
| {BcFV5.BI=TRUE, BcMCV3.BI=FALSE} =>  {BcHV1SLR.BI=TRUE} | 0.14  1089 | 0.678  571 | 0.207  921 | 1.25  1794 | 57 |
| {BcHV1SLR.BI=TRUE, BcMCV3.BI=FALSE}  => {BcFV5.BI=TRUE} | 0.14  1089 | 0.289  34 | 0.487  624 | 1.32  8334 | 57 |
| {BcFV5.BI=TRUE, BcPV4.BI=FALSE} =>  {BcHV1SLR.BI=TRUE} | 0.13  6139 | 0.679  012 | 0.200  495 | 1.25  2607 | 55 |
| {BcEV6=FALSE, BcFV5.BI=TRUE} =>  {BcHV1SLR.BI=TRUE} | 0.14  1089 | 0.678  571 | 0.207  921 | 1.25  1794 | 57 |
| {BcEV6=FALSE, BcHV1SLR.BI=TRUE} =>  {BcFV5.BI=TRUE} | 0.14  1089 | 0.280  788 | 0.502  475 | 1.28  9073 | 57 |
| {BcFV5.BI=TRUE, BcOLV17.BI=FALSE} =>  {BcHV1SLR.BI=TRUE} | 0.14  604 | 0.686  047 | 0.212  871 | 1.26  5584 | 59 |
| {BcHV1SLR.BI=TRUE, BcOLV17.BI=FALSE}  => {BcFV5.BI=TRUE} | 0.14  604 | 0.289  216 | 0.504  95 | 1.32  7763 | 59 |
| {BcEV5=FALSE, BcFV5.BI=TRUE} =>  {BcHV1SLR.BI=TRUE} | 0.12  6238 | 0.708  333 | 0.178  218 | 1.30  6697 | 51 |

| {BcFV5.BI=TRUE, BcOLV16.BI=FALSE} =>  {BcMV3.BI=TRUE} | 0.08  6634 | 0.7 | 0.123  762 | 1.29  1324 | 35 |
| --- | --- | --- | --- | --- | --- |
| {BcMV3.BI=TRUE, BcOLV16.BI=FALSE} =>  {BcFV5.BI=TRUE} | 0.08  6634 | 0.315  315 | 0.274  752 | 1.44  7584 | 35 |
| {BcFV5.BI=TRUE, BcMV3.BI=TRUE} =>  {BcULV3=TRUE} | 0.10  8911 | 0.709  677 | 0.153  465 | 1.19  4624 | 44 |
| {BcFV5.BI=TRUE, BcULV3=TRUE} =>  {BcMV3.BI=TRUE} | 0.10  8911 | 0.666  667 | 0.163  366 | 1.22  9833 | 44 |
| {BcMV3.BI=TRUE, BcULV3=TRUE} =>  {BcFV5.BI=TRUE} | 0.10  8911 | 0.314  286 | 0.346  535 | 1.44  2857 | 44 |
| {BcFV5.BI=TRUE, BcHV1.BI=TRUE} =>  {BcMV3.BI=TRUE} | 0.10  6436 | 0.704  918 | 0.150  99 | 1.30  0397 | 43 |
| {BcHV1.BI=TRUE, BcMV3.BI=TRUE} =>  {BcFV5.BI=TRUE} | 0.10  6436 | 0.300  699 | 0.353  96 | 1.38  0483 | 43 |
| {BcFV5.BI=TRUE, BcMV11=TRUE} =>  {BcMV3.BI=TRUE} | 0.10  6436 | 0.754  386 | 0.141  089 | 1.39  1653 | 43 |
| {BcMV11=TRUE, BcMV3.BI=TRUE} =>  {BcFV5.BI=TRUE} | 0.10  6436 | 0.277  419 | 0.383  663 | 1.27  3607 | 43 |
| {BcFV5.BI=TRUE, BcMV9.BI=TRUE} =>  {BcMV3.BI=TRUE} | 0.11  3861 | 0.686  567 | 0.165  842 | 1.26  6544 | 46 |
| {BcMV3.BI=TRUE, BcMV9.BI=TRUE} =>  {BcFV5.BI=TRUE} | 0.11  3861 | 0.287  5 | 0.396  04 | 1.31  9886 | 46 |

| {BcFV5.BI=TRUE, BcMV3.BI=TRUE} =>  {BcULV4=TRUE} | 0.12  1287 | 0.790  323 | 0.153  465 | 1.14  441 | 49 |
| --- | --- | --- | --- | --- | --- |
| {BcFV5.BI=TRUE, BcULV4=TRUE} =>  {BcMV3.BI=TRUE} | 0.12  1287 | 0.753  846 | 0.160  891 | 1.39  0657 | 49 |
| {BcMV3.BI=TRUE, BcULV4=TRUE} =>  {BcFV5.BI=TRUE} | 0.12  1287 | 0.302  469 | 0.400  99 | 1.38  8608 | 49 |
| {BcMV3.BI=TRUE, BcMV6.BI=FALSE} =>  {BcFV5.BI=TRUE} | 0.09  6535 | 0.302  326 | 0.319  307 | 1.38  7949 | 39 |
| {BcFV5.BI=TRUE, BcPV3.BI=FALSE} =>  {BcMV3.BI=TRUE} | 0.11  6337 | 0.712  121 | 0.163  366 | 1.31  3685 | 47 |
| {BcMV3.BI=TRUE, BcPV3.BI=FALSE} =>  {BcFV5.BI=TRUE} | 0.11  6337 | 0.321  918 | 0.361  386 | 1.47  7895 | 47 |
| {BcFV5.BI=TRUE, BcRV1.BI=FALSE} =>  {BcMV3.BI=TRUE} | 0.12  3762 | 0.694  444 | 0.178  218 | 1.28  1076 | 50 |
| {BcMV3.BI=TRUE, BcRV1.BI=FALSE} =>  {BcFV5.BI=TRUE} | 0.12  3762 | 0.337  838 | 0.366  337 | 1.55  0983 | 50 |
| {BcEV2.BI=FALSE, BcFV5.BI=TRUE} =>  {BcMV3.BI=TRUE} | 0.09  1584 | 0.725  49 | 0.126  238 | 1.33  8347 | 37 |
| {BcFV5.BI=TRUE, BcULV1.BI=FALSE} =>  {BcMV3.BI=TRUE} | 0.11  6337 | 0.671  429 | 0.173  267 | 1.23  8617 | 47 |
| {BcMV3.BI=TRUE, BcULV1.BI=FALSE} =>  {BcFV5.BI=TRUE} | 0.11  6337 | 0.297  468 | 0.391  089 | 1.36  565 | 47 |

| {BcFV5.BI=TRUE, BcMV1.BI=TRUE} =>  {BcMV3.BI=TRUE} | 0.13  3663 | 0.739  726 | 0.180  693 | 1.36  4609 | 54 |
| --- | --- | --- | --- | --- | --- |
| {BcMV1.BI=TRUE, BcMV3.BI=TRUE} =>  {BcFV5.BI=TRUE} | 0.13  3663 | 0.291  892 | 0.457  921 | 1.34  0049 | 54 |
| {BcFV5.BI=TRUE, BcMV12=FALSE} =>  {BcMV3.BI=TRUE} | 0.12  6238 | 0.728  571 | 0.173  267 | 1.34  4031 | 51 |
| {BcMV12=FALSE, BcMV3.BI=TRUE} =>  {BcFV5.BI=TRUE} | 0.12  6238 | 0.324  841 | 0.388  614 | 1.49  1314 | 51 |
| {BcFV5.BI=TRUE, BcOLV7.BI=FALSE} =>  {BcMV3.BI=TRUE} | 0.12  6238 | 0.689  189 | 0.183  168 | 1.27  1381 | 51 |
| {BcMV3.BI=TRUE, BcOLV7.BI=FALSE} =>  {BcFV5.BI=TRUE} | 0.12  6238 | 0.303  571 | 0.415  842 | 1.39  3669 | 51 |
| {BcFV5.BI=TRUE, BcFV9=FALSE} =>  {BcMV3.BI=TRUE} | 0.11  6337 | 0.681  159 | 0.170  792 | 1.25  6568 | 47 |
| {BcFV5.BI=TRUE, BcOLV4.BI=FALSE} =>  {BcMV3.BI=TRUE} | 0.13  6139 | 0.696  203 | 0.195  545 | 1.28  4319 | 55 |
| {BcMV3.BI=TRUE, BcOLV4.BI=FALSE} =>  {BcFV5.BI=TRUE} | 0.13  6139 | 0.298  913 | 0.455  446 | 1.37  2283 | 55 |
| {BcFV5.BI=TRUE, BcULV5=FALSE} =>  {BcMV3.BI=TRUE} | 0.13  6139 | 0.696  203 | 0.195  545 | 1.28  4319 | 55 |
| {BcMV3.BI=TRUE, BcULV5=FALSE} =>  {BcFV5.BI=TRUE} | 0.13  6139 | 0.300  546 | 0.452  97 | 1.37  9781 | 55 |

| {BcFV5.BI=TRUE, BvF.BI=FALSE} =>  {BcMV3.BI=TRUE} | 0.13  6139 | 0.714  286 | 0.190  594 | 1.31  7678 | 55 |
| --- | --- | --- | --- | --- | --- |
| {BcMV3.BI=TRUE, BvF.BI=FALSE} =>  {BcFV5.BI=TRUE} | 0.13  6139 | 0.275 | 0.495  05 | 1.26  25 | 55 |
| {BcFV5.BI=TRUE, BcMCV5.BI=FALSE} =>  {BcMV3.BI=TRUE} | 0.11  8812 | 0.657  534 | 0.180  693 | 1.21  2986 | 48 |
| {BcFV5.BI=TRUE, BcOLV13.BI=FALSE} =>  {BcMV3.BI=TRUE} | 0.14  604 | 0.702  381 | 0.207  921 | 1.29  5716 | 59 |
| {BcMV3.BI=TRUE, BcOLV13.BI=FALSE} =>  {BcFV5.BI=TRUE} | 0.14  604 | 0.307  292 | 0.475  248 | 1.41  0748 | 59 |
| {BcFV5.BI=TRUE, BcOLV2.BI=FALSE} =>  {BcMV3.BI=TRUE} | 0.14  1089 | 0.703  704 | 0.200  495 | 1.29  8157 | 57 |
| {BcMV3.BI=TRUE, BcOLV2.BI=FALSE} =>  {BcFV5.BI=TRUE} | 0.14  1089 | 0.290  816 | 0.485  149 | 1.33  5111 | 57 |
| {BcFV5.BI=TRUE, BcMCV3.BI=FALSE} =>  {BcMV3.BI=TRUE} | 0.14  3564 | 0.690  476 | 0.207  921 | 1.27  3755 | 58 |
| {BcMCV3.BI=FALSE, BcMV3.BI=TRUE} =>  {BcFV5.BI=TRUE} | 0.14  3564 | 0.302  083 | 0.475  248 | 1.38  6837 | 58 |
| {BcFV5.BI=TRUE, BcPV4.BI=FALSE} =>  {BcMV3.BI=TRUE} | 0.13  8614 | 0.691  358 | 0.200  495 | 1.27  5382 | 56 |
| {BcMV3.BI=TRUE, BcPV4.BI=FALSE} =>  {BcFV5.BI=TRUE} | 0.13  8614 | 0.278  607 | 0.497  525 | 1.27  9059 | 56 |

| {BcEV6=FALSE, BcFV5.BI=TRUE} =>  {BcMV3.BI=TRUE} | 0.14  604 | 0.702  381 | 0.207  921 | 1.29  5716 | 59 |
| --- | --- | --- | --- | --- | --- |
| {BcEV6=FALSE, BcMV3.BI=TRUE} =>  {BcFV5.BI=TRUE} | 0.14  604 | 0.282  297 | 0.517  327 | 1.29  5998 | 59 |
| {BcFV5.BI=TRUE, BcOLV17.BI=FALSE} =>  {BcMV3.BI=TRUE} | 0.15  099 | 0.709  302 | 0.212  871 | 1.30  8485 | 61 |
| {BcMV3.BI=TRUE, BcOLV17.BI=FALSE} =>  {BcFV5.BI=TRUE} | 0.15  099 | 0.294  686 | 0.512  376 | 1.35  2877 | 61 |
| {BcFV5.BI=TRUE, BcOLV15.BI=FALSE} =>  {BcMV3.BI=TRUE} | 0.12  6238 | 0.698  63 | 0.180  693 | 1.28  8797 | 51 |
| {BcFV5.BI=TRUE, BcMV4.BI=FALSE} =>  {BcMV6.BI=FALSE} | 0.08  4158 | 0.871  795 | 0.096  535 | 1.25  3399 | 34 |
| {BcFV5.BI=TRUE, BcMV4.BI=FALSE} =>  {BcRV1.BI=FALSE} | 0.08  6634 | 0.897  436 | 0.096  535 | 1.24  1658 | 35 |
| {BcFV5.BI=TRUE, BcMV4.BI=FALSE} =>  {BcOLV7.BI=FALSE} | 0.08  9109 | 0.923  077 | 0.096  535 | 1.14  3936 | 36 |
| {BcFV5.BI=TRUE, BcOLV16.BI=FALSE} =>  {BcULV3=TRUE} | 0.09  4059 | 0.76 | 0.123  762 | 1.27  9333 | 38 |
| {BcOLV16.BI=FALSE, BcULV3=TRUE} =>  {BcFV5.BI=TRUE} | 0.09  4059 | 0.299  213 | 0.314  356 | 1.37  3658 | 38 |
| {BcFV5.BI=TRUE, BcOLV16.BI=FALSE} =>  {BcPV3.BI=FALSE} | 0.10  1485 | 0.82 | 0.123  762 | 1.15  4286 | 41 |

| {BcFV5.BI=TRUE, BcOLV16.BI=FALSE} =>  {BcRV1.BI=FALSE} | 0.11  3861 | 0.92 | 0.123  762 | 1.27  2877 | 46 |
| --- | --- | --- | --- | --- | --- |
| {BcFV5.BI=TRUE, BcOLV16.BI=FALSE} =>  {BcULV1.BI=FALSE} | 0.10  8911 | 0.88 | 0.123  762 | 1.12  1514 | 44 |
| {BcFV5.BI=TRUE, BcOLV16.BI=FALSE} =>  {BcOLV7.BI=FALSE} | 0.11  3861 | 0.92 | 0.123  762 | 1.14  0123 | 46 |
| {BcALV1.BI=FALSE, BcFV5.BI=TRUE} =>  {BcOLV16.BI=FALSE} | 0.10  396 | 0.7 | 0.148  515 | 1.19  8305 | 42 |
| {BcFV5.BI=TRUE, BcULV3=TRUE} =>  {BcHV1.BI=TRUE} | 0.12  6238 | 0.772  727 | 0.163  366 | 1.27  9434 | 51 |
| {BcFV5.BI=TRUE, BcHV1.BI=TRUE} =>  {BcULV3=TRUE} | 0.12  6238 | 0.836  066 | 0.150  99 | 1.40  7377 | 51 |
| {BcHV1.BI=TRUE, BcULV3=TRUE} =>  {BcFV5.BI=TRUE} | 0.12  6238 | 0.298  246 | 0.423  267 | 1.36  9219 | 51 |
| {BcFV5.BI=TRUE, BcMV11=TRUE} =>  {BcULV3=TRUE} | 0.11  6337 | 0.824  561 | 0.141  089 | 1.38  8012 | 47 |
| {BcMV11=TRUE, BcULV3=TRUE} =>  {BcFV5.BI=TRUE} | 0.11  6337 | 0.293  75 | 0.396  04 | 1.34  858 | 47 |
| {BcFV5.BI=TRUE, BcOLV1.BI.MOVA.=FALSE} =>  {BcULV3=TRUE} | 0.09  6535 | 0.764  706 | 0.126  238 | 1.28  7255 | 39 |
| {BcFV5.BI=TRUE, BcULV3=TRUE} => | 0.12 | 0.787 | 0.163 | 1.16 | 52 |

| {BcMV9.BI=TRUE} | 8713 | 879 | 366 | 169 |  |
| --- | --- | --- | --- | --- | --- |
| {BcFV5.BI=TRUE, BcMV9.BI=TRUE} =>  {BcULV3=TRUE} | 0.12  8713 | 0.776  119 | 0.165  842 | 1.30  6468 | 52 |
| {BcMV9.BI=TRUE, BcULV3=TRUE} =>  {BcFV5.BI=TRUE} | 0.12  8713 | 0.304  094 | 0.423  267 | 1.39  6066 | 52 |
| {BcFV5.BI=TRUE, BcULV4=TRUE} =>  {BcULV3=TRUE} | 0.11  8812 | 0.738  462 | 0.160  891 | 1.24  3077 | 48 |
| {BcULV3=TRUE, BcULV4=TRUE} =>  {BcFV5.BI=TRUE} | 0.11  8812 | 0.285  714 | 0.415  842 | 1.31  1688 | 48 |
| {BcFV5.BI=TRUE, BcMV6.BI=FALSE} =>  {BcULV3=TRUE} | 0.10  6436 | 0.704  918 | 0.150  99 | 1.18  6612 | 43 |
| {BcMV6.BI=FALSE, BcULV3=TRUE} =>  {BcFV5.BI=TRUE} | 0.10  6436 | 0.281  046 | 0.378  713 | 1.29  0255 | 43 |
| {BcFV5.BI=TRUE, BcPV3.BI=FALSE} =>  {BcULV3=TRUE} | 0.11  8812 | 0.727  273 | 0.163  366 | 1.22  4242 | 48 |
| {BcPV3.BI=FALSE, BcULV3=TRUE} =>  {BcFV5.BI=TRUE} | 0.11  8812 | 0.282  353 | 0.420  792 | 1.29  6257 | 48 |
| {BcFV5.BI=TRUE, BcULV3=TRUE} =>  {BcRV1.BI=FALSE} | 0.13  8614 | 0.848  485 | 0.163  366 | 1.17  3931 | 56 |
| {BcFV5.BI=TRUE, BcRV1.BI=FALSE} =>  {BcULV3=TRUE} | 0.13  8614 | 0.777  778 | 0.178  218 | 1.30  9259 | 56 |
| {BcRV1.BI=FALSE, BcULV3=TRUE} => | 0.13 | 0.32 | 0.433 | 1.46 | 56 |

| {BcFV5.BI=TRUE} | 8614 |  | 168 | 9091 |  |
| --- | --- | --- | --- | --- | --- |
| {BcEV2.BI=FALSE, BcFV5.BI=TRUE} =>  {BcULV3=TRUE} | 0.09  1584 | 0.725  49 | 0.126  238 | 1.22  1242 | 37 |
| {BcFV5.BI=TRUE, BcULV1.BI=FALSE} =>  {BcULV3=TRUE} | 0.12  8713 | 0.742  857 | 0.173  267 | 1.25  0476 | 52 |
| {BcULV1.BI=FALSE, BcULV3=TRUE} =>  {BcFV5.BI=TRUE} | 0.12  8713 | 0.281  081 | 0.457  921 | 1.29  0418 | 52 |
| {BcFV5.BI=TRUE, BcMV1.BI=TRUE} =>  {BcULV3=TRUE} | 0.13  8614 | 0.767  123 | 0.180  693 | 1.29  1324 | 56 |
| {BcMV1.BI=TRUE, BcULV3=TRUE} =>  {BcFV5.BI=TRUE} | 0.13  8614 | 0.285  714 | 0.485  149 | 1.31  1688 | 56 |
| {BcFV5.BI=TRUE, BcMV12=FALSE} =>  {BcULV3=TRUE} | 0.12  8713 | 0.742  857 | 0.173  267 | 1.25  0476 | 52 |
| {BcMV12=FALSE, BcULV3=TRUE} =>  {BcFV5.BI=TRUE} | 0.12  8713 | 0.275  132 | 0.467  822 | 1.26  3107 | 52 |
| {BcFV5.BI=TRUE, BcVV3.BI=FALSE} =>  {BcULV3=TRUE} | 0.12  6238 | 0.75 | 0.168  317 | 1.26  25 | 51 |
| {BcFV5.BI=TRUE, BcOLV7.BI=FALSE} =>  {BcULV3=TRUE} | 0.13  6139 | 0.743  243 | 0.183  168 | 1.25  1126 | 55 |
| {BcOLV7.BI=FALSE, BcULV3=TRUE} =>  {BcFV5.BI=TRUE} | 0.13  6139 | 0.286  458 | 0.475  248 | 1.31  5104 | 55 |
| {BcALV1.BI=FALSE, BcFV5.BI=TRUE} => | 0.11 | 0.766 | 0.148 | 1.29 | 46 |

| {BcULV3=TRUE} | 3861 | 667 | 515 | 0556 |  |
| --- | --- | --- | --- | --- | --- |
| {BcDMV1=FALSE, BcFV5.BI=TRUE} =>  {BcULV3=TRUE} | 0.10  396 | 0.736  842 | 0.141  089 | 1.24  0351 | 42 |
| {BcFV5.BI=TRUE, BcFV9=FALSE} =>  {BcULV3=TRUE} | 0.12  6238 | 0.739  13 | 0.170  792 | 1.24  4203 | 51 |
| {BcFV5.BI=TRUE, BcOLV4.BI=FALSE} =>  {BcULV3=TRUE} | 0.14  3564 | 0.734  177 | 0.195  545 | 1.23  5865 | 58 |
| {BcOLV4.BI=FALSE, BcULV3=TRUE} =>  {BcFV5.BI=TRUE} | 0.14  3564 | 0.282  927 | 0.507  426 | 1.29  8891 | 58 |
| {BcFV5.BI=TRUE, BcULV5=FALSE} =>  {BcULV3=TRUE} | 0.14  3564 | 0.734  177 | 0.195  545 | 1.23  5865 | 58 |
| {BcULV3=TRUE, BcULV5=FALSE} =>  {BcFV5.BI=TRUE} | 0.14  3564 | 0.277  512 | 0.517  327 | 1.27  4032 | 58 |
| {BcFV5.BI=TRUE, BvF.BI=FALSE} =>  {BcULV3=TRUE} | 0.14  8515 | 0.779  221 | 0.190  594 | 1.31  1688 | 60 |
| {BcULV3=TRUE, BvF.BI=FALSE} =>  {BcFV5.BI=TRUE} | 0.14  8515 | 0.269  058 | 0.551  98 | 1.23  5222 | 60 |
| {BcFV5.BI=TRUE, BcMCV5.BI=FALSE} =>  {BcULV3=TRUE} | 0.12  6238 | 0.698  63 | 0.180  693 | 1.17  6027 | 51 |
| {BcFV5.BI=TRUE, BcOLV13.BI=FALSE} =>  {BcULV3=TRUE} | 0.15  3465 | 0.738  095 | 0.207  921 | 1.24  246 | 62 |
| {BcOLV13.BI=FALSE, BcULV3=TRUE} => | 0.15 | 0.281 | 0.544 | 1.29 | 62 |

| {BcFV5.BI=TRUE} | 3465 | 818 | 554 | 3802 |  |
| --- | --- | --- | --- | --- | --- |
| {BcFV5.BI=TRUE, BcOLV2.BI=FALSE} =>  {BcULV3=TRUE} | 0.14  8515 | 0.740  741 | 0.200  495 | 1.24  6914 | 60 |
| {BcOLV2.BI=FALSE, BcULV3=TRUE} =>  {BcFV5.BI=TRUE} | 0.14  8515 | 0.273  973 | 0.542  079 | 1.25  7783 | 60 |
| {BcFV5.BI=TRUE, BcMCV3.BI=FALSE} =>  {BcULV3=TRUE} | 0.15  8416 | 0.761  905 | 0.207  921 | 1.28  254 | 64 |
| {BcMCV3.BI=FALSE, BcULV3=TRUE} =>  {BcFV5.BI=TRUE} | 0.15  8416 | 0.292  237 | 0.542  079 | 1.34  1636 | 64 |
| {BcFV5.BI=TRUE, BcPV4.BI=FALSE} =>  {BcULV3=TRUE} | 0.15  099 | 0.753  086 | 0.200  495 | 1.26  7695 | 61 |
| {BcPV4.BI=FALSE, BcULV3=TRUE} =>  {BcFV5.BI=TRUE} | 0.15  099 | 0.274  775 | 0.549  505 | 1.26  1466 | 61 |
| {BcEV6=FALSE, BcFV5.BI=TRUE} =>  {BcULV3=TRUE} | 0.15  3465 | 0.738  095 | 0.207  921 | 1.24  246 | 62 |
| {BcEV6=FALSE, BcULV3=TRUE} =>  {BcFV5.BI=TRUE} | 0.15  3465 | 0.274  336 | 0.559  406 | 1.25  9453 | 62 |
| {BcFV5.BI=TRUE, BcOLV17.BI=FALSE} =>  {BcULV3=TRUE} | 0.15  8416 | 0.744  186 | 0.212  871 | 1.25  2713 | 64 |
| {BcOLV17.BI=FALSE, BcULV3=TRUE} =>  {BcFV5.BI=TRUE} | 0.15  8416 | 0.284  444 | 0.556  931 | 1.30  5859 | 64 |
| {BcFV5.BI=TRUE, BcOLV15.BI=FALSE} => | 0.13 | 0.739 | 0.180 | 1.24 | 54 |

| {BcULV3=TRUE} | 3663 | 726 | 693 | 5205 |  |
| --- | --- | --- | --- | --- | --- |
| {BcEV5=FALSE, BcFV5.BI=TRUE} => | 0.12 | 0.722 | 0.178 | 1.21 |  |
|  |  |  |  |  | 52 |
| {BcULV3=TRUE} | 8713 | 222 | 218 | 5741 |  |
| {BcFV5.BI=TRUE, BcMV11=TRUE} => | 0.10 | 0.719 | 0.141 | 1.19 |  |
|  |  |  |  |  | 41 |
| {BcHV1.BI=TRUE} | 1485 | 298 | 089 | 0969 |  |
| {BcFV5.BI=TRUE, |  |  |  |  |  |
|  | 0.09 | 0.784 | 0.126 | 1.29 |  |
| BcOLV1.BI.MOVA.=FALSE} => |  |  |  |  | 40 |
|  | 901 | 314 | 238 | 8618 |  |
| {BcHV1.BI=TRUE} |  |  |  |  |  |
| {BcFV5.BI=TRUE, BcHV1.BI=TRUE} => | 0.12 | 0.819 | 0.150 | 1.20 |  |
|  |  |  |  |  | 50 |
| {BcMV9.BI=TRUE} | 3762 | 672 | 99 | 8568 |  |
| {BcFV5.BI=TRUE, BcMV9.BI=TRUE} => | 0.12 | 0.746 | 0.165 | 1.23 |  |
|  |  |  |  |  | 50 |
| {BcHV1.BI=TRUE} | 3762 | 269 | 842 | 5625 |  |
| {BcFV5.BI=TRUE, BcHV1.BI=TRUE} => | 0.12 | 0.819 | 0.150 | 1.13 |  |
|  |  |  |  |  | 50 |
| {BcRV1.BI=FALSE} | 3762 | 672 | 99 | 4067 |  |
| {BcHV1.BI=TRUE, BcRV1.BI=FALSE} => | 0.12 | 0.297 | 0.415 | 1.36 |  |
|  |  |  |  |  | 50 |
| {BcFV5.BI=TRUE} | 3762 | 619 | 842 | 6342 |  |
| {BcFV5.BI=TRUE, BcMV1.BI=TRUE} => | 0.12 | 0.712 | 0.180 | 1.17 |  |
|  |  |  |  |  | 52 |
| {BcHV1.BI=TRUE} | 8713 | 329 | 693 | 943 |  |
| {BcFV5.BI=TRUE, BcVV3.BI=FALSE} => | 0.12 | 0.735 | 0.168 | 1.21 |  |
|  |  |  |  |  | 50 |
| {BcHV1.BI=TRUE} | 3762 | 294 | 317 | 7454 |  |
| {BcDMV1=FALSE, BcFV5.BI=TRUE} => | 0.10 | 0.719 | 0.141 | 1.19 |  |
|  |  |  |  |  | 41 |
| {BcHV1.BI=TRUE} | 1485 | 298 | 089 | 0969 |  |

| {BcEV5=FALSE, BcFV5.BI=TRUE} =>  {BcHV1.BI=TRUE} | 0.12  6238 | 0.708  333 | 0.178  218 | 1.17  2814 | 51 |
| --- | --- | --- | --- | --- | --- |
| {BcFV5.BI=TRUE, BcMV11=TRUE} =>  {BcMV9.BI=TRUE} | 0.11  3861 | 0.807  018 | 0.141  089 | 1.18  9909 | 46 |
| {BcMV11=TRUE, BcMV9.BI=TRUE} =>  {BcFV5.BI=TRUE} | 0.11  3861 | 0.280  488 | 0.405  941 | 1.28  7694 | 46 |
| {BcFV5.BI=TRUE, BcMV11=TRUE} =>  {BcULV4=TRUE} | 0.11  3861 | 0.807  018 | 0.141  089 | 1.16  8585 | 46 |
| {BcFV5.BI=TRUE, BcMV11=TRUE} =>  {BcMV1.BI=TRUE} | 0.12  6238 | 0.894  737 | 0.141  089 | 1.13  3146 | 51 |
| {BcFV5.BI=TRUE, BcOLV1.BI.MOVA.=FALSE} =>  {BcMV6.BI=FALSE} | 0.11  1386 | 0.882  353 | 0.126  238 | 1.26  8579 | 45 |
| {BcFV5.BI=TRUE, BcOLV1.BI.MOVA.=FALSE} =>  {BcRV1.BI=FALSE} | 0.10  6436 | 0.843  137 | 0.126  238 | 1.16  6532 | 43 |
| {BcFV5.BI=TRUE, BcOLV1.BI.MOVA.=FALSE} =>  {BcULV1.BI=FALSE} | 0.11  1386 | 0.882  353 | 0.126  238 | 1.12  4513 | 45 |
| {BcFV5.BI=TRUE, BcULV4=TRUE} =>  {BcMV9.BI=TRUE} | 0.12  6238 | 0.784  615 | 0.160  891 | 1.15  6878 | 51 |
| {BcMV9.BI=TRUE, BcRV1.BI=FALSE} => | 0.13 | 0.288 | 0.455 | 1.32 | 53 |

| {BcFV5.BI=TRUE} | 1188 | 043 | 446 | 2381 |  |
| --- | --- | --- | --- | --- | --- |
| {BcFV5.BI=TRUE, BcHV3.BI=FALSE} =>  {BcMV9.BI=TRUE} | 0.08  4158 | 0.809  524 | 0.103  96 | 1.19  3604 | 34 |
| {BcFV5.BI=TRUE, BcMV1.BI=TRUE} =>  {BcMV9.BI=TRUE} | 0.14  1089 | 0.780  822 | 0.180  693 | 1.15  1285 | 57 |
| {BcMV9.BI=TRUE, BcOLV7.BI=FALSE} =>  {BcFV5.BI=TRUE} | 0.14  1089 | 0.278  049 | 0.507  426 | 1.27  6497 | 57 |
| {BcFV5.BI=TRUE, BcMV9.BI=TRUE} =>  {BcULV5=FALSE} | 0.15  8416 | 0.955  224 | 0.165  842 | 1.09  0143 | 64 |
| {BcFV5.BI=TRUE, BcULV5=FALSE} =>  {BcMV9.BI=TRUE} | 0.15  8416 | 0.810  127 | 0.195  545 | 1.19  4493 | 64 |
| {BcFV5.BI=TRUE, BcOLV17.BI=FALSE} =>  {BcMV9.BI=TRUE} | 0.16  3366 | 0.767  442 | 0.212  871 | 1.13  1557 | 66 |
| {BcRV1.BI=FALSE, BcULV4=TRUE} =>  {BcFV5.BI=TRUE} | 0.12  3762 | 0.276  243 | 0.448  02 | 1.26  8207 | 50 |
| {BcFV5.BI=TRUE, BcMV6.BI=FALSE} =>  {BcRV1.BI=FALSE} | 0.12  8713 | 0.852  459 | 0.150  99 | 1.17  943 | 52 |
| {BcALV1.BI=FALSE, BcFV5.BI=TRUE} =>  {BcMV6.BI=FALSE} | 0.11  8812 | 0.8 | 0.148  515 | 1.15  0178 | 48 |
| {BcDMV1=FALSE, BcFV5.BI=TRUE} =>  {BcMV6.BI=FALSE} | 0.11  3861 | 0.807  018 | 0.141  089 | 1.16  0267 | 46 |
| {BcBNV5.BI=FALSE, BcFV5.BI=TRUE} => | 0.13 | 0.818 | 0.163 | 1.17 | 54 |

| {BcMV6.BI=FALSE} | 3663 | 182 | 366 | 6318 |  |
| --- | --- | --- | --- | --- | --- |
| {BcFV5.BI=TRUE, BcMCV5.BI=FALSE} =>  {BcMV6.BI=FALSE} | 0.14  604 | 0.808  219 | 0.180  693 | 1.16  1995 | 59 |
| {BcEV5=FALSE, BcFV5.BI=TRUE} =>  {BcMV6.BI=FALSE} | 0.14  3564 | 0.805  556 | 0.178  218 | 1.15  8165 | 58 |
| {BcFV5.BI=TRUE, BcPV3.BI=FALSE} =>  {BcRV1.BI=FALSE} | 0.14  1089 | 0.863  636 | 0.163  366 | 1.19  4894 | 57 |
| {BcEV2.BI=FALSE, BcFV5.BI=TRUE} =>  {BcPV3.BI=FALSE} | 0.10  396 | 0.823  529 | 0.126  238 | 1.15  9254 | 42 |
| {BcFV5.BI=TRUE, BcPV3.BI=FALSE} =>  {BcOLV4.BI=FALSE} | 0.15  5941 | 0.954  545 | 0.163  366 | 1.08  9368 | 63 |
| {BcFV5.BI=TRUE, BcULV1.BI=FALSE} =>  {BcRV1.BI=FALSE} | 0.14  8515 | 0.857  143 | 0.173  267 | 1.18  591 | 60 |
| {BcFV5.BI=TRUE, BcMV1.BI=TRUE} =>  {BcRV1.BI=FALSE} | 0.14  8515 | 0.821  918 | 0.180  693 | 1.13  7174 | 60 |
| {BcFV5.BI=TRUE, BcMV12=FALSE} =>  {BcRV1.BI=FALSE} | 0.15  099 | 0.871  429 | 0.173  267 | 1.20  5675 | 61 |
| {BcFV5.BI=TRUE, BcRV1.BI=FALSE} =>  {BcOLV7.BI=FALSE} | 0.16  0891 | 0.902  778 | 0.178  218 | 1.11  878 | 65 |
| {BcFV5.BI=TRUE, BcOLV7.BI=FALSE} =>  {BcRV1.BI=FALSE} | 0.16  0891 | 0.878  378 | 0.183  168 | 1.21  5291 | 65 |
| {BcALV1.BI=FALSE, BcFV5.BI=TRUE} => | 0.14 | 0.95 | 0.148 | 1.31 | 57 |

| {BcRV1.BI=FALSE} | 1089 |  | 515 | 4384 |  |
| --- | --- | --- | --- | --- | --- |
| {BcFV5.BI=TRUE, BcFV9=FALSE} =>  {BcRV1.BI=FALSE} | 0.14  3564 | 0.840  58 | 0.170  792 | 1.16  2994 | 58 |
| {BcFV5.BI=TRUE, BcOLV4.BI=FALSE} =>  {BcRV1.BI=FALSE} | 0.16  3366 | 0.835  443 | 0.195  545 | 1.15  5887 | 66 |
| {BcFV5.BI=TRUE, BcULV5=FALSE} =>  {BcRV1.BI=FALSE} | 0.16  3366 | 0.835  443 | 0.195  545 | 1.15  5887 | 66 |
| {BcFV5.BI=TRUE, BcMCV5.BI=FALSE} =>  {BcRV1.BI=FALSE} | 0.14  8515 | 0.821  918 | 0.180  693 | 1.13  7174 | 60 |
| {BcFV5.BI=TRUE, BcOLV13.BI=FALSE} =>  {BcRV1.BI=FALSE} | 0.17  0792 | 0.821  429 | 0.207  921 | 1.13  6497 | 69 |
| {BcFV5.BI=TRUE, BcOLV2.BI=FALSE} =>  {BcRV1.BI=FALSE} | 0.16  5842 | 0.827  16 | 0.200  495 | 1.14  4428 | 67 |
| {BcFV5.BI=TRUE, BcMCV3.BI=FALSE} =>  {BcRV1.BI=FALSE} | 0.17  5743 | 0.845  238 | 0.207  921 | 1.16  9439 | 71 |
| {BcFV5.BI=TRUE, BcPV4.BI=FALSE} =>  {BcRV1.BI=FALSE} | 0.16  5842 | 0.827  16 | 0.200  495 | 1.14  4428 | 67 |
| {BcEV6=FALSE, BcFV5.BI=TRUE} =>  {BcRV1.BI=FALSE} | 0.16  8317 | 0.809  524 | 0.207  921 | 1.12  0026 | 68 |
| {BcFV5.BI=TRUE, BcOLV17.BI=FALSE} =>  {BcRV1.BI=FALSE} | 0.17  5743 | 0.825  581 | 0.212  871 | 1.14  2243 | 71 |
| {BcEV2.BI=FALSE, BcFV5.BI=TRUE} => | 0.12 | 0.960 | 0.126 | 1.09 | 49 |

| {BcOLV4.BI=FALSE} | 1287 | 784 | 238 | 6488 |  |
| --- | --- | --- | --- | --- | --- |
| {BcFV5.BI=TRUE, BcHV3.BI=FALSE} =>  {BcOLV4.BI=FALSE} | 0.10  1485 | 0.976  19 | 0.103  96 | 1.11  407 | 41 |
| {BcEV2.BI=TRUE, BcHV3.BI=TRUE} =>  {BcMV1.BI=TRUE} | 0.08  1683 | 0.916  667 | 0.089  109 | 1.16  092 | 33 |
| {BcHV3.BI=TRUE, BcMV1.BI=TRUE} =>  {BcEV2.BI=TRUE} | 0.08  1683 | 0.383  721 | 0.212  871 | 1.51  9836 | 33 |
| {BcEV2.BI=TRUE, BcMV1.BI=TRUE} =>  {BcHV3.BI=TRUE} | 0.08  1683 | 0.375 | 0.217  822 | 1.56  1856 | 33 |
| {BcHV3.BI=TRUE, BcMV12=FALSE} =>  {BcEV2.BI=TRUE} | 0.07  6733 | 0.387  5 | 0.198  02 | 1.53  4804 | 31 |
| {BcEV2.BI=TRUE, BcMV12=FALSE} =>  {BcHV3.BI=TRUE} | 0.07  6733 | 0.352  273 | 0.217  822 | 1.46  7198 | 31 |
| {BcHV3.BI=TRUE, BcOLV7.BI=FALSE} =>  {BcEV2.BI=TRUE} | 0.07  4257 | 0.422  535 | 0.175  743 | 1.67  3571 | 30 |
| {BcEV2.BI=TRUE, BcOLV7.BI=FALSE} =>  {BcHV3.BI=TRUE} | 0.07  4257 | 0.344  828 | 0.215  347 | 1.43  6189 | 30 |
| {BcFV9=FALSE, BcHV3.BI=TRUE} =>  {BcEV2.BI=TRUE} | 0.07  6733 | 0.387  5 | 0.198  02 | 1.53  4804 | 31 |
| {BcEV2.BI=TRUE, BcFV9=FALSE} =>  {BcHV3.BI=TRUE} | 0.07  6733 | 0.392  405 | 0.195  545 | 1.63  4347 | 31 |
| {BcHV3.BI=TRUE, BcULV5=FALSE} => | 0.07 | 0.369 | 0.207 | 1.46 | 31 |

| {BcEV2.BI=TRUE} | 6733 | 048 | 921 | 1718 |  |
| --- | --- | --- | --- | --- | --- |
| {BcEV2.BI=TRUE, BcULV5=FALSE} =>  {BcHV3.BI=TRUE} | 0.07  6733 | 0.348  315 | 0.220  297 | 1.45  0712 | 31 |
| {BcBNV5.BI=FALSE, BcHV3.BI=TRUE} =>  {BcEV2.BI=TRUE} | 0.07  4257 | 0.384  615 | 0.193  069 | 1.52  3379 | 30 |
| {BcBNV5.BI=FALSE, BcEV2.BI=TRUE} =>  {BcHV3.BI=TRUE} | 0.07  4257 | 0.344  828 | 0.215  347 | 1.43  6189 | 30 |
| {BcHV3.BI=TRUE, BvF.BI=FALSE} =>  {BcEV2.BI=TRUE} | 0.07  4257 | 0.344  828 | 0.215  347 | 1.36  5788 | 30 |
| {BcEV2.BI=TRUE, BvF.BI=FALSE} =>  {BcHV3.BI=TRUE} | 0.07  4257 | 0.344  828 | 0.215  347 | 1.43  6189 | 30 |
| {BcHV3.BI=TRUE, BcOLV13.BI=FALSE} =>  {BcEV2.BI=TRUE} | 0.07  9208 | 0.367  816 | 0.215  347 | 1.45  684 | 32 |
| {BcEV2.BI=TRUE, BcOLV13.BI=FALSE} =>  {BcHV3.BI=TRUE} | 0.07  9208 | 0.347  826 | 0.227  723 | 1.44  8678 | 32 |
| {BcHV3.BI=TRUE, BcOLV2.BI=FALSE} =>  {BcEV2.BI=TRUE} | 0.08  1683 | 0.366  667 | 0.222  772 | 1.45  2288 | 33 |
| {BcEV2.BI=TRUE, BcOLV2.BI=FALSE} =>  {BcHV3.BI=TRUE} | 0.08  1683 | 0.347  368 | 0.235  149 | 1.44  6772 | 33 |
| {BcHV3.BI=TRUE, BcMCV3.BI=FALSE} =>  {BcEV2.BI=TRUE} | 0.08  6634 | 0.364  583 | 0.237  624 | 1.44  4036 | 35 |
| {BcEV2.BI=TRUE, BcMCV3.BI=FALSE} => | 0.08 | 0.368 | 0.235 | 1.53 | 35 |

| {BcHV3.BI=TRUE} | 6634 | 421 | 149 | 4455 |  |
| --- | --- | --- | --- | --- | --- |
| {BcHV3.BI=TRUE, BcPV4.BI=FALSE} =>  {BcEV2.BI=TRUE} | 0.08  6634 | 0.380  435 | 0.227  723 | 1.50  682 | 35 |
| {BcEV2.BI=TRUE, BcPV4.BI=FALSE} =>  {BcHV3.BI=TRUE} | 0.08  6634 | 0.368  421 | 0.235  149 | 1.53  4455 | 35 |
| {BcEV6=FALSE, BcHV3.BI=TRUE} =>  {BcEV2.BI=TRUE} | 0.07  9208 | 0.347  826 | 0.227  723 | 1.37  7664 | 32 |
| {BcEV2.BI=TRUE, BcEV6=FALSE} =>  {BcHV3.BI=TRUE} | 0.07  9208 | 0.336  842 | 0.235  149 | 1.40  293 | 32 |
| {BcHV3.BI=TRUE, BcOLV17.BI=FALSE} =>  {BcEV2.BI=TRUE} | 0.08  1683 | 0.366  667 | 0.222  772 | 1.45  2288 | 33 |
| {BcEV2.BI=TRUE, BcOLV17.BI=FALSE} =>  {BcHV3.BI=TRUE} | 0.08  1683 | 0.351  064 | 0.232  673 | 1.46  2163 | 33 |
| {BcHV3.BI=TRUE, BcOLV15.BI=FALSE} =>  {BcEV2.BI=TRUE} | 0.07  4257 | 0.340  909 | 0.217  822 | 1.35  0267 | 30 |
| {BcEV2.BI=TRUE, BcOLV15.BI=FALSE} =>  {BcHV3.BI=TRUE} | 0.07  4257 | 0.333  333 | 0.222  772 | 1.38  8316 | 30 |
| {BcEV5=FALSE, BcHV3.BI=TRUE} =>  {BcEV2.BI=TRUE} | 0.07  6733 | 0.387  5 | 0.198  02 | 1.53  4804 | 31 |
| {BcEV2.BI=TRUE, BcEV5=FALSE} =>  {BcHV3.BI=TRUE} | 0.07  6733 | 0.322  917 | 0.237  624 | 1.34  4931 | 31 |
| {BcHV3.BI=TRUE, BcPV3.BI=TRUE} => | 0.07 | 0.969 | 0.081 | 1.22 | 32 |

| {BcMV1.BI=TRUE} | 9208 | 697 | 683 | 808 |  |
| --- | --- | --- | --- | --- | --- |
| {BcMCV3.BI=FALSE, BcPV3.BI=TRUE} =>  {BcHV3.BI=TRUE} | 0.07  9208 | 0.313  725 | 0.252  475 | 1.30  665 | 32 |
| {BcHV3.BI=TRUE, BcMV6.BI=TRUE} =>  {BcMV11=TRUE} | 0.07  6733 | 0.911  765 | 0.084  158 | 1.47  9329 | 31 |
| {BcHV3.BI=TRUE, BcMV11=TRUE} =>  {BcMV6.BI=TRUE} | 0.07  6733 | 0.442  857 | 0.173  267 | 1.45  4588 | 31 |
| {BcMV11=TRUE, BcMV6.BI=TRUE} =>  {BcHV3.BI=TRUE} | 0.07  6733 | 0.336  957 | 0.227  723 | 1.40  3407 | 31 |
| {BcHV3.BI=TRUE, BcMV6.BI=TRUE} =>  {BcMV9.BI=TRUE} | 0.07  6733 | 0.911  765 | 0.084  158 | 1.34  4354 | 31 |
| {BcHV3.BI=TRUE, BcMV9.BI=TRUE} =>  {BcMV6.BI=TRUE} | 0.07  6733 | 0.442  857 | 0.173  267 | 1.45  4588 | 31 |
| {BcMV6.BI=TRUE, BcMV9.BI=TRUE} =>  {BcHV3.BI=TRUE} | 0.07  6733 | 0.326  316 | 0.235  149 | 1.35  9088 | 31 |
| {BcHV3.BI=TRUE, BcMV6.BI=TRUE} =>  {BcMV1.BI=TRUE} | 0.07  9208 | 0.941  176 | 0.084  158 | 1.19  196 | 32 |
| {BcMCV3.BI=FALSE, BcMV6.BI=TRUE} =>  {BcHV3.BI=TRUE} | 0.08  1683 | 0.314  286 | 0.259  901 | 1.30  8984 | 33 |
| {BcHV3.BI=TRUE, BcOLV1.BI.MOVA.=TRUE}  => {BcMV2.BI=TRUE} | 0.08  4158 | 0.809  524 | 0.103  96 | 1.74  8918 | 34 |
| {BcHV3.BI=TRUE, BcMV2.BI=TRUE} => | 0.08 | 0.629 | 0.133 | 1.89 | 34 |

| {BcOLV1.BI.MOVA.=TRUE} | 4158 | 63 | 663 | 8286 |  |
| --- | --- | --- | --- | --- | --- |
| {BcMV2.BI=TRUE, BcOLV1.BI.MOVA.=TRUE}  => {BcHV3.BI=TRUE} | 0.08  4158 | 0.336  634 | 0.25 | 1.40  2062 | 34 |
| {BcHV3.BI=TRUE, BcOLV1.BI.MOVA.=TRUE}  => {BcOLV18=TRUE} | 0.08  4158 | 0.809  524 | 0.103  96 | 1.56  4821 | 34 |
| {BcHV3.BI=TRUE, BcOLV18=TRUE} =>  {BcOLV1.BI.MOVA.=TRUE} | 0.08  4158 | 0.507  463 | 0.165  842 | 1.52  9962 | 34 |
| {BcOLV1.BI.MOVA.=TRUE, BcOLV18=TRUE}  => {BcHV3.BI=TRUE} | 0.08  4158 | 0.365  591 | 0.230  198 | 1.52  2669 | 34 |
| {BcHV3.BI=TRUE, BcOLV1.BI.MOVA.=TRUE}  => {BcMV3.BI=TRUE} | 0.08  4158 | 0.809  524 | 0.103  96 | 1.49  3368 | 34 |
| {BcHV3.BI=TRUE, BcMV3.BI=TRUE} =>  {BcOLV1.BI.MOVA.=TRUE} | 0.08  4158 | 0.566  667 | 0.148  515 | 1.70  8458 | 34 |
| {BcMV3.BI=TRUE, BcOLV1.BI.MOVA.=TRUE}  => {BcHV3.BI=TRUE} | 0.08  4158 | 0.343  434 | 0.245  05 | 1.43  0386 | 34 |
| {BcHV3.BI=TRUE, BcOLV1.BI.MOVA.=TRUE}  => {BcULV3=TRUE} | 0.07  4257 | 0.714  286 | 0.103  96 | 1.20  2381 | 30 |
| {BcHV3.BI=TRUE, BcULV3=TRUE} =>  {BcOLV1.BI.MOVA.=TRUE} | 0.07  4257 | 0.434  783 | 0.170  792 | 1.31  0837 | 30 |
| {BcOLV1.BI.MOVA.=TRUE, BcULV3=TRUE}  => {BcHV3.BI=TRUE} | 0.07  4257 | 0.379  747 | 0.195  545 | 1.58  1626 | 30 |
| {BcHV3.BI=TRUE, BcOLV1.BI.MOVA.=TRUE} | 0.07 | 0.761 | 0.103 | 1.23 | 32 |

| => {BcMV11=TRUE} | 9208 | 905 | 96 | 6183 |  |
| --- | --- | --- | --- | --- | --- |
| {BcHV3.BI=TRUE, BcMV11=TRUE} =>  {BcOLV1.BI.MOVA.=TRUE} | 0.07  9208 | 0.457  143 | 0.173  267 | 1.37  8252 | 32 |
| {BcMV11=TRUE, BcOLV1.BI.MOVA.=TRUE}  => {BcHV3.BI=TRUE} | 0.07  9208 | 0.326  531 | 0.242  574 | 1.35  9983 | 32 |
| {BcHV3.BI=TRUE, BcOLV1.BI.MOVA.=TRUE}  => {BcMV9.BI=TRUE} | 0.08  4158 | 0.809  524 | 0.103  96 | 1.19  3604 | 34 |
| {BcHV3.BI=TRUE, BcMV9.BI=TRUE} =>  {BcOLV1.BI.MOVA.=TRUE} | 0.08  4158 | 0.485  714 | 0.173  267 | 1.46  4392 | 34 |
| {BcMV9.BI=TRUE, BcOLV1.BI.MOVA.=TRUE}  => {BcHV3.BI=TRUE} | 0.08  4158 | 0.395  349 | 0.212  871 | 1.64  6608 | 34 |
| {BcHV3.BI=TRUE, BcOLV1.BI.MOVA.=TRUE}  => {BcULV4=TRUE} | 0.08  6634 | 0.833  333 | 0.103  96 | 1.20  6691 | 35 |
| {BcHV3.BI=TRUE, BcULV4=TRUE} =>  {BcOLV1.BI.MOVA.=TRUE} | 0.08  6634 | 0.492  958 | 0.175  743 | 1.48  6231 | 35 |
| {BcOLV1.BI.MOVA.=TRUE, BcULV4=TRUE}  => {BcHV3.BI=TRUE} | 0.08  6634 | 0.353  535 | 0.245  05 | 1.47  2457 | 35 |
| {BcHV3.BI=TRUE, BcOLV1.BI.MOVA.=TRUE}  => {BcMV1.BI=TRUE} | 0.09  6535 | 0.928  571 | 0.103  96 | 1.17  5996 | 39 |
| {BcHV3.BI=TRUE, BcMV1.BI=TRUE} =>  {BcOLV1.BI.MOVA.=TRUE} | 0.09  6535 | 0.453  488 | 0.212  871 | 1.36  7234 | 39 |
| {BcMV1.BI=TRUE, BcOLV1.BI.MOVA.=TRUE} | 0.09 | 0.327 | 0.294 | 1.36 | 39 |

| => {BcHV3.BI=TRUE} | 6535 | 731 | 554 | 4983 |  |
| --- | --- | --- | --- | --- | --- |
| {BcMV12=FALSE, BcOLV1.BI.MOVA.=TRUE}  => {BcHV3.BI=TRUE} | 0.07  9208 | 0.344  086 | 0.230  198 | 1.43  3101 | 32 |
| {BcFV9=FALSE, BcHV3.BI=TRUE} =>  {BcOLV1.BI.MOVA.=TRUE} | 0.08  9109 | 0.45 | 0.198  02 | 1.35  6716 | 36 |
| {BcFV9=FALSE, BcOLV1.BI.MOVA.=TRUE}  => {BcHV3.BI=TRUE} | 0.08  9109 | 0.318  584 | 0.279  703 | 1.32  6886 | 36 |
| {BcOLV1.BI.MOVA.=TRUE,  BcOLV4.BI=FALSE} => {BcHV3.BI=TRUE} | 0.08  4158 | 0.320  755 | 0.262  376 | 1.33  5927 | 34 |
| {BcHV3.BI=TRUE, BcULV5=FALSE} =>  {BcOLV1.BI.MOVA.=TRUE} | 0.08  9109 | 0.428  571 | 0.207  921 | 1.29  2111 | 36 |
| {BcOLV1.BI.MOVA.=TRUE, BcULV5=FALSE}  => {BcHV3.BI=TRUE} | 0.08  9109 | 0.346  154 | 0.257  426 | 1.44  1713 | 36 |
| {BcHV3.BI=TRUE, BvF.BI=FALSE} =>  {BcOLV1.BI.MOVA.=TRUE} | 0.09  901 | 0.459  77 | 0.215  347 | 1.38  6173 | 40 |
| {BcOLV1.BI.MOVA.=TRUE, BvF.BI=FALSE}  => {BcHV3.BI=TRUE} | 0.09  901 | 0.314  961 | 0.314  356 | 1.31  1795 | 40 |
| {BcOLV1.BI.MOVA.=TRUE,  BcOLV13.BI=FALSE} => {BcHV3.BI=TRUE} | 0.08  9109 | 0.321  429 | 0.277  228 | 1.33  8733 | 36 |
| {BcHV3.BI=TRUE, BcOLV2.BI=FALSE} =>  {BcOLV1.BI.MOVA.=TRUE} | 0.09  4059 | 0.422  222 | 0.222  772 | 1.27  2968 | 38 |
| {BcOLV1.BI.MOVA.=TRUE, | 0.09 | 0.339 | 0.277 | 1.41 | 38 |

| BcOLV2.BI=FALSE} => {BcHV3.BI=TRUE} | 4059 | 286 | 228 | 3108 |  |
| --- | --- | --- | --- | --- | --- |
| {BcHV3.BI=TRUE, BcMCV3.BI=FALSE} => | 0.10 | 0.427 | 0.237 | 1.28 |  |
|  |  |  |  |  | 41 |
| {BcOLV1.BI.MOVA.=TRUE} | 1485 | 083 | 624 | 7624 |  |
| {BcMCV3.BI=FALSE, |  |  |  |  |  |
|  | 0.10 | 0.353 | 0.287 | 1.47 |  |
| BcOLV1.BI.MOVA.=TRUE} => |  |  |  |  | 41 |
|  | 1485 | 448 | 129 | 2094 |  |
| {BcHV3.BI=TRUE} |  |  |  |  |  |
| {BcHV3.BI=TRUE, BcPV4.BI=FALSE} => | 0.10 | 0.445 | 0.227 | 1.34 |  |
|  |  |  |  |  | 41 |
| {BcOLV1.BI.MOVA.=TRUE} | 1485 | 652 | 723 | 3608 |  |
| {BcOLV1.BI.MOVA.=TRUE, | 0.10 | 0.320 | 0.316 | 1.33 |  |
|  |  |  |  |  | 41 |
| BcPV4.BI=FALSE} => {BcHV3.BI=TRUE} | 1485 | 313 | 832 | 4085 |  |
| {BcEV6=FALSE, BcHV3.BI=TRUE} => | 0.09 | 0.434 | 0.227 | 1.31 |  |
|  |  |  |  |  | 40 |
| {BcOLV1.BI.MOVA.=TRUE} | 901 | 783 | 723 | 0837 |  |
| {BcEV6=FALSE, BcOLV1.BI.MOVA.=TRUE} | 0.09 | 0.322 | 0.306 | 1.34 |  |
|  |  |  |  |  | 40 |
| => {BcHV3.BI=TRUE} | 901 | 581 | 931 | 3532 |  |
| {BcHV3.BI=TRUE, BcOLV15.BI=FALSE} => | 0.10 | 0.465 | 0.217 | 1.40 |  |
|  |  |  |  |  | 41 |
| {BcOLV1.BI.MOVA.=TRUE} | 1485 | 909 | 822 | 4681 |  |
| {BcOLV1.BI.MOVA.=TRUE, | 0.10 | 0.315 | 0.321 | 1.31 |  |
|  |  |  |  |  | 41 |
| BcOLV15.BI=FALSE} => {BcHV3.BI=TRUE} | 1485 | 385 | 782 | 3561 |  |
| {BcHV3.BI=TRUE, BcOLV16.BI=TRUE} => | 0.07 | 0.789 | 0.094 | 1.78 |  |
|  |  |  |  |  | 30 |
| {BcMV4.BI=TRUE} | 4257 | 474 | 059 | 1829 |  |
| {BcMV4.BI=TRUE, BcOLV16.BI=TRUE} => | 0.07 | 0.329 | 0.225 | 1.37 |  |
|  |  |  |  |  | 30 |
| {BcHV3.BI=TRUE} | 4257 | 67 | 248 | 306 |  |

| {BcHV3.BI=TRUE, BcOLV16.BI=TRUE} =>  {BcOLV18=TRUE} | 0.07  9208 | 0.842  105 | 0.094  059 | 1.62  7802 | 32 |
| --- | --- | --- | --- | --- | --- |
| {BcHV3.BI=TRUE, BcOLV16.BI=TRUE} =>  {BcULV3=TRUE} | 0.07  6733 | 0.815  789 | 0.094  059 | 1.37  3246 | 31 |
| {BcHV3.BI=TRUE, BcOLV16.BI=TRUE} =>  {BcMV11=TRUE} | 0.07  9208 | 0.842  105 | 0.094  059 | 1.36  6307 | 32 |
| {BcHV3.BI=TRUE, BcOLV16.BI=TRUE} =>  {BcULV4=TRUE} | 0.07  6733 | 0.815  789 | 0.094  059 | 1.18  1287 | 31 |
| {BcHV3.BI=TRUE, BcMV4.BI=TRUE} =>  {BcMV2.BI=TRUE} | 0.09  6535 | 0.639  344 | 0.150  99 | 1.38  1257 | 39 |
| {BcHV3.BI=TRUE, BcMV2.BI=TRUE} =>  {BcMV4.BI=TRUE} | 0.09  6535 | 0.722  222 | 0.133  663 | 1.63  0043 | 39 |
| {BcMV2.BI=TRUE, BcMV4.BI=TRUE} =>  {BcHV3.BI=TRUE} | 0.09  6535 | 0.351  351 | 0.274  752 | 1.46  336 | 39 |
| {BcHV3.BI=TRUE, BcMV4.BI=TRUE} =>  {BcOLV18=TRUE} | 0.10  8911 | 0.721  311 | 0.150  99 | 1.39  4305 | 44 |
| {BcHV3.BI=TRUE, BcOLV18=TRUE} =>  {BcMV4.BI=TRUE} | 0.10  8911 | 0.656  716 | 0.165  842 | 1.48  2198 | 44 |
| {BcMV4.BI=TRUE, BcOLV18=TRUE} =>  {BcHV3.BI=TRUE} | 0.10  8911 | 0.392  857 | 0.277  228 | 1.63  623 | 44 |
| {BcHV1SLR.BI=TRUE, BcHV3.BI=TRUE} =>  {BcMV4.BI=TRUE} | 0.09  6535 | 0.65 | 0.148  515 | 1.46  7039 | 39 |

| {BcHV1SLR.BI=TRUE, BcMV4.BI=TRUE} =>  {BcHV3.BI=TRUE} | 0.09  6535 | 0.367  925 | 0.262  376 | 1.53  2387 | 39 |
| --- | --- | --- | --- | --- | --- |
| {BcHV3.BI=TRUE, BcMV4.BI=TRUE} =>  {BcMV3.BI=TRUE} | 0.10  6436 | 0.704  918 | 0.150  99 | 1.30  0397 | 43 |
| {BcHV3.BI=TRUE, BcMV3.BI=TRUE} =>  {BcMV4.BI=TRUE} | 0.10  6436 | 0.716  667 | 0.148  515 | 1.61  7505 | 43 |
| {BcMV3.BI=TRUE, BcMV4.BI=TRUE} =>  {BcHV3.BI=TRUE} | 0.10  6436 | 0.361  345 | 0.294  554 | 1.50  4981 | 43 |
| {BcMV4.BI=TRUE, BcOLV16.BI=FALSE} =>  {BcHV3.BI=TRUE} | 0.07  6733 | 0.352  273 | 0.217  822 | 1.46  7198 | 31 |
| {BcHV3.BI=TRUE, BcMV4.BI=TRUE} =>  {BcULV3=TRUE} | 0.11  3861 | 0.754  098 | 0.150  99 | 1.26  9399 | 46 |
| {BcHV3.BI=TRUE, BcULV3=TRUE} =>  {BcMV4.BI=TRUE} | 0.11  3861 | 0.666  667 | 0.170  792 | 1.50  4655 | 46 |
| {BcMV4.BI=TRUE, BcULV3=TRUE} =>  {BcHV3.BI=TRUE} | 0.11  3861 | 0.386  555 | 0.294  554 | 1.60  998 | 46 |
| {BcHV1.BI=TRUE, BcHV3.BI=TRUE} =>  {BcMV4.BI=TRUE} | 0.10  6436 | 0.661  538 | 0.160  891 | 1.49  3081 | 43 |
| {BcHV1.BI=TRUE, BcMV4.BI=TRUE} =>  {BcHV3.BI=TRUE} | 0.10  6436 | 0.355  372 | 0.299  505 | 1.48  0106 | 43 |
| {BcHV3.BI=TRUE, BcMV4.BI=TRUE} =>  {BcMV11=TRUE} | 0.12  8713 | 0.852  459 | 0.150  99 | 1.38  3106 | 52 |

| {BcHV3.BI=TRUE, BcMV11=TRUE} =>  {BcMV4.BI=TRUE} | 0.12  8713 | 0.742  857 | 0.173  267 | 1.67  6616 | 52 |
| --- | --- | --- | --- | --- | --- |
| {BcMV11=TRUE, BcMV4.BI=TRUE} =>  {BcHV3.BI=TRUE} | 0.12  8713 | 0.379  562 | 0.339  109 | 1.58  0856 | 52 |
| {BcHV3.BI=TRUE, BcOLV1.BI.MOVA.=FALSE} =>  {BcMV4.BI=TRUE} | 0.08  1683 | 0.6 | 0.136  139 | 1.35  419 | 33 |
| {BcMV4.BI=TRUE, BcOLV1.BI.MOVA.=FALSE} =>  {BcHV3.BI=TRUE} | 0.08  1683 | 0.317  308 | 0.257  426 | 1.32  157 | 33 |
| {BcHV3.BI=TRUE, BcMV4.BI=TRUE} =>  {BcMV9.BI=TRUE} | 0.12  1287 | 0.803  279 | 0.150  99 | 1.18  4396 | 49 |
| {BcHV3.BI=TRUE, BcMV9.BI=TRUE} =>  {BcMV4.BI=TRUE} | 0.12  1287 | 0.7 | 0.173  267 | 1.57  9888 | 49 |
| {BcMV4.BI=TRUE, BcMV9.BI=TRUE} =>  {BcHV3.BI=TRUE} | 0.12  1287 | 0.360  294 | 0.336  634 | 1.50  0606 | 49 |
| {BcHV3.BI=TRUE, BcULV4=TRUE} =>  {BcMV4.BI=TRUE} | 0.11  1386 | 0.633  803 | 0.175  743 | 1.43  0482 | 45 |
| {BcMV4.BI=TRUE, BcULV4=TRUE} =>  {BcHV3.BI=TRUE} | 0.11  1386 | 0.343  511 | 0.324  257 | 1.43  0707 | 45 |
| {BcHV3.BI=TRUE, BcMV6.BI=FALSE} =>  {BcMV4.BI=TRUE} | 0.08  6634 | 0.555  556 | 0.155  941 | 1.25  388 | 35 |

| {BcMV4.BI=TRUE, BcMV6.BI=FALSE} =>  {BcHV3.BI=TRUE} | 0.08  6634 | 0.324  074 | 0.267  327 | 1.34  9752 | 35 |
| --- | --- | --- | --- | --- | --- |
| {BcHV3.BI=TRUE, BcPV3.BI=FALSE} =>  {BcMV4.BI=TRUE} | 0.09  4059 | 0.593  75 | 0.158  416 | 1.34  0084 | 38 |
| {BcMV4.BI=TRUE, BcPV3.BI=FALSE} =>  {BcHV3.BI=TRUE} | 0.09  4059 | 0.358  491 | 0.262  376 | 1.49  3095 | 38 |
| {BcHV3.BI=TRUE, BcRV1.BI=FALSE} =>  {BcMV4.BI=TRUE} | 0.09  901 | 0.563  38 | 0.175  743 | 1.27  154 | 40 |
| {BcMV4.BI=TRUE, BcRV1.BI=FALSE} =>  {BcHV3.BI=TRUE} | 0.09  901 | 0.357  143 | 0.277  228 | 1.48  7482 | 40 |
| {BcEV2.BI=FALSE, BcHV3.BI=TRUE} =>  {BcMV4.BI=TRUE} | 0.10  1485 | 0.672  131 | 0.150  99 | 1.51  6989 | 41 |
| {BcEV2.BI=FALSE, BcMV4.BI=TRUE} =>  {BcHV3.BI=TRUE} | 0.10  1485 | 0.322  835 | 0.314  356 | 1.34  459 | 41 |
| {BcFV5.BI=FALSE, BcHV3.BI=TRUE} =>  {BcMV4.BI=TRUE} | 0.08  1683 | 0.647  059 | 0.126  238 | 1.46  0401 | 33 |
| {BcHV3.BI=TRUE, BcULV1.BI=FALSE} =>  {BcMV4.BI=TRUE} | 0.10  396 | 0.575  342 | 0.180  693 | 1.29  8538 | 42 |
| {BcMV4.BI=TRUE, BcULV1.BI=FALSE} =>  {BcHV3.BI=TRUE} | 0.10  396 | 0.311  111 | 0.334  158 | 1.29  5762 | 42 |
| {BcHV3.BI=TRUE, BcMV4.BI=TRUE} =>  {BcMV1.BI=TRUE} | 0.13  8614 | 0.918  033 | 0.150  99 | 1.16  265 | 56 |

| {BcHV3.BI=TRUE, BcMV1.BI=TRUE} =>  {BcMV4.BI=TRUE} | 0.13  8614 | 0.651  163 | 0.212  871 | 1.46  9664 | 56 |
| --- | --- | --- | --- | --- | --- |
| {BcMV1.BI=TRUE, BcMV4.BI=TRUE} =>  {BcHV3.BI=TRUE} | 0.13  8614 | 0.368  421 | 0.376  238 | 1.53  4455 | 56 |
| {BcHV3.BI=TRUE, BcMV12=FALSE} =>  {BcMV4.BI=TRUE} | 0.12  8713 | 0.65 | 0.198  02 | 1.46  7039 | 52 |
| {BcMV12=FALSE, BcMV4.BI=TRUE} =>  {BcHV3.BI=TRUE} | 0.12  8713 | 0.356  164 | 0.361  386 | 1.48  3406 | 52 |
| {BcHV3.BI=TRUE, BcNSRV7.BI=FALSE} =>  {BcMV4.BI=TRUE} | 0.09  1584 | 0.627  119 | 0.146  04 | 1.41  5396 | 37 |
| {BcHV3.BI=TRUE, BcVV3.BI=FALSE} =>  {BcMV4.BI=TRUE} | 0.09  1584 | 0.596  774 | 0.153  465 | 1.34  6909 | 37 |
| {BcHV3.BI=TRUE, BcOLV7.BI=FALSE} =>  {BcMV4.BI=TRUE} | 0.10  396 | 0.591  549 | 0.175  743 | 1.33  5117 | 42 |
| {BcMV4.BI=TRUE, BcOLV7.BI=FALSE} =>  {BcHV3.BI=TRUE} | 0.10  396 | 0.308  824 | 0.336  634 | 1.28  6234 | 42 |
| {BcALV1.BI=FALSE, BcMV4.BI=TRUE} =>  {BcHV3.BI=TRUE} | 0.09  901 | 0.314  961 | 0.314  356 | 1.31  1795 | 40 |
| {BcDMV1=FALSE, BcHV3.BI=TRUE} =>  {BcMV4.BI=TRUE} | 0.10  1485 | 0.621  212 | 0.163  366 | 1.40  2065 | 41 |
| {BcFV9=FALSE, BcHV3.BI=TRUE} =>  {BcMV4.BI=TRUE} | 0.11  3861 | 0.575 | 0.198  02 | 1.29  7765 | 46 |

| {BcFV9=FALSE, BcMV4.BI=TRUE} =>  {BcHV3.BI=TRUE} | 0.11  3861 | 0.319  444 | 0.356  436 | 1.33  047 | 46 |
| --- | --- | --- | --- | --- | --- |
| {BcHV3.BI=TRUE, BcOLV4.BI=FALSE} =>  {BcMV4.BI=TRUE} | 0.12  6238 | 0.621  951 | 0.202  97 | 1.40  3733 | 51 |
| {BcMV4.BI=TRUE, BcOLV4.BI=FALSE} =>  {BcHV3.BI=TRUE} | 0.12  6238 | 0.34 | 0.371  287 | 1.41  6082 | 51 |
| {BcHV3.BI=TRUE, BcULV5=FALSE} =>  {BcMV4.BI=TRUE} | 0.12  8713 | 0.619  048 | 0.207  921 | 1.39  718 | 52 |
| {BcMV4.BI=TRUE, BcULV5=FALSE} =>  {BcHV3.BI=TRUE} | 0.12  8713 | 0.337  662 | 0.381  188 | 1.40  6346 | 52 |
| {BcBNV5.BI=FALSE, BcHV3.BI=TRUE} =>  {BcMV4.BI=TRUE} | 0.11  6337 | 0.602  564 | 0.193  069 | 1.35  9977 | 47 |
| {BcBNV5.BI=FALSE, BcMV4.BI=TRUE} =>  {BcHV3.BI=TRUE} | 0.11  6337 | 0.303  226 | 0.383  663 | 1.26  292 | 47 |
| {BcHV3.BI=TRUE, BvF.BI=FALSE} =>  {BcMV4.BI=TRUE} | 0.13  8614 | 0.643  678 | 0.215  347 | 1.45  2771 | 56 |
| {BcMV4.BI=TRUE, BvF.BI=FALSE} =>  {BcHV3.BI=TRUE} | 0.13  8614 | 0.341  463 | 0.405  941 | 1.42  2178 | 56 |
| {BcHV3.BI=TRUE, BcOLV13.BI=FALSE} =>  {BcMV4.BI=TRUE} | 0.12  8713 | 0.597  701 | 0.215  347 | 1.34  9001 | 52 |
| {BcMV4.BI=TRUE, BcOLV13.BI=FALSE} =>  {BcHV3.BI=TRUE} | 0.12  8713 | 0.329  114 | 0.391  089 | 1.37  0743 | 52 |

| {BcHV3.BI=TRUE, BcOLV2.BI=FALSE} =>  {BcMV4.BI=TRUE} | 0.14  3564 | 0.644  444 | 0.222  772 | 1.45  45 | 58 |
| --- | --- | --- | --- | --- | --- |
| {BcMV4.BI=TRUE, BcOLV2.BI=FALSE} =>  {BcHV3.BI=TRUE} | 0.14  3564 | 0.355  828 | 0.403  465 | 1.48  2006 | 58 |
| {BcHV3.BI=TRUE, BcMCV3.BI=FALSE} =>  {BcMV4.BI=TRUE} | 0.15  099 | 0.635  417 | 0.237  624 | 1.43  4125 | 61 |
| {BcMCV3.BI=FALSE, BcMV4.BI=TRUE} =>  {BcHV3.BI=TRUE} | 0.15  099 | 0.367  47 | 0.410  891 | 1.53  0493 | 61 |
| {BcHV3.BI=TRUE, BcPV4.BI=FALSE} =>  {BcMV4.BI=TRUE} | 0.14  604 | 0.641  304 | 0.227  723 | 1.44  7413 | 59 |
| {BcMV4.BI=TRUE, BcPV4.BI=FALSE} =>  {BcHV3.BI=TRUE} | 0.14  604 | 0.349  112 | 0.418  317 | 1.45  4035 | 59 |
| {BcEV6=FALSE, BcHV3.BI=TRUE} =>  {BcMV4.BI=TRUE} | 0.14  604 | 0.641  304 | 0.227  723 | 1.44  7413 | 59 |
| {BcEV6=FALSE, BcMV4.BI=TRUE} =>  {BcHV3.BI=TRUE} | 0.14  604 | 0.347  059 | 0.420  792 | 1.44  5482 | 59 |
| {BcHV3.BI=TRUE, BcOLV17.BI=FALSE} =>  {BcMV4.BI=TRUE} | 0.13  8614 | 0.622  222 | 0.222  772 | 1.40  4345 | 56 |
| {BcMV4.BI=TRUE, BcOLV17.BI=FALSE} =>  {BcHV3.BI=TRUE} | 0.13  8614 | 0.337  349 | 0.410  891 | 1.40  5043 | 56 |
| {BcHV3.BI=TRUE, BcOLV15.BI=FALSE} =>  {BcMV4.BI=TRUE} | 0.13  6139 | 0.625 | 0.217  822 | 1.41  0615 | 55 |

| {BcMV4.BI=TRUE, BcOLV15.BI=FALSE} =>  {BcHV3.BI=TRUE} | 0.13  6139 | 0.329  341 | 0.413  366 | 1.37  169 | 55 |
| --- | --- | --- | --- | --- | --- |
| {BcEV5=FALSE, BcHV3.BI=TRUE} =>  {BcMV4.BI=TRUE} | 0.11  1386 | 0.562  5 | 0.198  02 | 1.26  9553 | 45 |
| {BcHV1SLR.BI=FALSE, BcHV3.BI=TRUE} =>  {BcMV1.BI=TRUE} | 0.08  4158 | 0.918  919 | 0.091  584 | 1.16  3772 | 34 |
| {BcHV3.BI=TRUE, BcMV3.BI=FALSE} =>  {BcMV6.BI=FALSE} | 0.07  6733 | 0.837  838 | 0.091  584 | 1.20  4578 | 31 |
| {BcHV3.BI=TRUE, BcMV2.BI=TRUE} =>  {BcOLV18=TRUE} | 0.10  396 | 0.777  778 | 0.133  663 | 1.50  3456 | 42 |
| {BcHV3.BI=TRUE, BcOLV18=TRUE} =>  {BcMV2.BI=TRUE} | 0.10  396 | 0.626  866 | 0.165  842 | 1.35  4298 | 42 |
| {BcMV2.BI=TRUE, BcOLV18=TRUE} =>  {BcHV3.BI=TRUE} | 0.10  396 | 0.352  941 | 0.294  554 | 1.46  9982 | 42 |
| {BcHV3.BI=TRUE, BcMV2.BI=TRUE} =>  {BcMV3.BI=TRUE} | 0.10  1485 | 0.759  259 | 0.133  663 | 1.40  0643 | 41 |
| {BcHV3.BI=TRUE, BcMV3.BI=TRUE} =>  {BcMV2.BI=TRUE} | 0.10  1485 | 0.683  333 | 0.148  515 | 1.47  6292 | 41 |
| {BcMV2.BI=TRUE, BcMV3.BI=TRUE} =>  {BcHV3.BI=TRUE} | 0.10  1485 | 0.322  835 | 0.314  356 | 1.34  459 | 41 |
| {BcHV3.BI=TRUE, BcMV2.BI=TRUE} =>  {BcULV3=TRUE} | 0.10  1485 | 0.759  259 | 0.133  663 | 1.27  8086 | 41 |

| {BcHV3.BI=TRUE, BcULV3=TRUE} =>  {BcMV2.BI=TRUE} | 0.10  1485 | 0.594  203 | 0.170  792 | 1.28  3732 | 41 |
| --- | --- | --- | --- | --- | --- |
| {BcMV2.BI=TRUE, BcULV3=TRUE} =>  {BcHV3.BI=TRUE} | 0.10  1485 | 0.356  522 | 0.284  653 | 1.48  4895 | 41 |
| {BcHV3.BI=TRUE, BcMV2.BI=TRUE} =>  {BcMV11=TRUE} | 0.11  1386 | 0.833  333 | 0.133  663 | 1.35  2075 | 45 |
| {BcHV3.BI=TRUE, BcMV11=TRUE} =>  {BcMV2.BI=TRUE} | 0.11  1386 | 0.642  857 | 0.173  267 | 1.38  8846 | 45 |
| {BcMV11=TRUE, BcMV2.BI=TRUE} =>  {BcHV3.BI=TRUE} | 0.11  1386 | 0.326  087 | 0.341  584 | 1.35  8135 | 45 |
| {BcHV3.BI=TRUE, BcMV2.BI=TRUE} =>  {BcMV9.BI=TRUE} | 0.11  6337 | 0.870  37 | 0.133  663 | 1.28  332 | 47 |
| {BcHV3.BI=TRUE, BcMV9.BI=TRUE} =>  {BcMV2.BI=TRUE} | 0.11  6337 | 0.671  429 | 0.173  267 | 1.45  0573 | 47 |
| {BcMV2.BI=TRUE, BcMV9.BI=TRUE} =>  {BcHV3.BI=TRUE} | 0.11  6337 | 0.361  538 | 0.321  782 | 1.50  5789 | 47 |
| {BcHV3.BI=TRUE, BcMV2.BI=TRUE} =>  {BcULV4=TRUE} | 0.10  8911 | 0.814  815 | 0.133  663 | 1.17  9875 | 44 |
| {BcHV3.BI=TRUE, BcULV4=TRUE} =>  {BcMV2.BI=TRUE} | 0.10  8911 | 0.619  718 | 0.175  743 | 1.33  8857 | 44 |
| {BcMV2.BI=TRUE, BcULV4=TRUE} =>  {BcHV3.BI=TRUE} | 0.10  8911 | 0.318  841 | 0.341  584 | 1.32  7955 | 44 |

| {BcMV2.BI=TRUE, BcRV1.BI=FALSE} =>  {BcHV3.BI=TRUE} | 0.09  1584 | 0.321  739 | 0.284  653 | 1.34  0027 | 37 |
| --- | --- | --- | --- | --- | --- |
| {BcHV3.BI=TRUE, BcMV2.BI=TRUE} =>  {BcMV1.BI=TRUE} | 0.12  6238 | 0.944  444 | 0.133  663 | 1.19  6099 | 51 |
| {BcHV3.BI=TRUE, BcMV1.BI=TRUE} =>  {BcMV2.BI=TRUE} | 0.12  6238 | 0.593  023 | 0.212  871 | 1.28  1184 | 51 |
| {BcMV1.BI=TRUE, BcMV2.BI=TRUE} =>  {BcHV3.BI=TRUE} | 0.12  6238 | 0.309  091 | 0.408  416 | 1.28  7348 | 51 |
| {BcFV9=FALSE, BcHV3.BI=TRUE} =>  {BcMV2.BI=TRUE} | 0.11  3861 | 0.575 | 0.198  02 | 1.24  2246 | 46 |
| {BcHV3.BI=TRUE, BvF.BI=FALSE} =>  {BcMV2.BI=TRUE} | 0.12  3762 | 0.574  713 | 0.215  347 | 1.24  1625 | 50 |
| {BcHV3.BI=TRUE, BcOLV2.BI=FALSE} =>  {BcMV2.BI=TRUE} | 0.12  6238 | 0.566  667 | 0.222  772 | 1.22  4242 | 51 |
| {BcMV2.BI=TRUE, BcOLV2.BI=FALSE} =>  {BcHV3.BI=TRUE} | 0.12  6238 | 0.3 | 0.420  792 | 1.24  9485 | 51 |
| {BcMCV3.BI=FALSE, BcMV2.BI=TRUE} =>  {BcHV3.BI=TRUE} | 0.13  1188 | 0.321  212 | 0.408  416 | 1.33  7832 | 53 |
| {BcHV3.BI=TRUE, BcOLV15.BI=FALSE} =>  {BcMV2.BI=TRUE} | 0.13  1188 | 0.602  273 | 0.217  822 | 1.30  1167 | 53 |
| {BcHV3.BI=TRUE, BcOLV18=TRUE} =>  {BcHV1SLR.BI=TRUE} | 0.11  3861 | 0.686  567 | 0.165  842 | 1.26  6544 | 46 |

| {BcHV1SLR.BI=TRUE, BcHV3.BI=TRUE} =>  {BcOLV18=TRUE} | 0.11  3861 | 0.766  667 | 0.148  515 | 1.48  1978 | 46 |
| --- | --- | --- | --- | --- | --- |
| {BcHV1SLR.BI=TRUE, BcOLV18=TRUE} =>  {BcHV3.BI=TRUE} | 0.11  3861 | 0.333  333 | 0.341  584 | 1.38  8316 | 46 |
| {BcHV3.BI=TRUE, BcOLV18=TRUE} =>  {BcMV3.BI=TRUE} | 0.11  1386 | 0.671  642 | 0.165  842 | 1.23  901 | 45 |
| {BcHV3.BI=TRUE, BcMV3.BI=TRUE} =>  {BcOLV18=TRUE} | 0.11  1386 | 0.75 | 0.148  515 | 1.44  9761 | 45 |
| {BcMV3.BI=TRUE, BcOLV18=TRUE} =>  {BcHV3.BI=TRUE} | 0.11  1386 | 0.338  346 | 0.329  208 | 1.40  9193 | 45 |
| {BcOLV16.BI=FALSE, BcOLV18=TRUE} =>  {BcHV3.BI=TRUE} | 0.08  6634 | 0.35 | 0.247  525 | 1.45  7732 | 35 |
| {BcHV3.BI=TRUE, BcOLV18=TRUE} =>  {BcULV3=TRUE} | 0.12  1287 | 0.731  343 | 0.165  842 | 1.23  1095 | 49 |
| {BcHV3.BI=TRUE, BcULV3=TRUE} =>  {BcOLV18=TRUE} | 0.12  1287 | 0.710  145 | 0.170  792 | 1.37  272 | 49 |
| {BcOLV18=TRUE, BcULV3=TRUE} =>  {BcHV3.BI=TRUE} | 0.12  1287 | 0.374  046 | 0.324  257 | 1.55  7881 | 49 |
| {BcHV1.BI=TRUE, BcHV3.BI=TRUE} =>  {BcOLV18=TRUE} | 0.11  6337 | 0.723  077 | 0.160  891 | 1.39  7718 | 47 |
| {BcHV1.BI=TRUE, BcOLV18=TRUE} =>  {BcHV3.BI=TRUE} | 0.11  6337 | 0.317  568 | 0.366  337 | 1.32  2653 | 47 |

| {BcHV3.BI=TRUE, BcOLV18=TRUE} =>  {BcMV11=TRUE} | 0.12  3762 | 0.746  269 | 0.165  842 | 1.21  0813 | 50 |
| --- | --- | --- | --- | --- | --- |
| {BcHV3.BI=TRUE, BcMV11=TRUE} =>  {BcOLV18=TRUE} | 0.12  3762 | 0.714  286 | 0.173  267 | 1.38  0725 | 50 |
| {BcMV11=TRUE, BcOLV18=TRUE} =>  {BcHV3.BI=TRUE} | 0.12  3762 | 0.357  143 | 0.346  535 | 1.48  7482 | 50 |
| {BcHV3.BI=TRUE, BcOLV18=TRUE} =>  {BcMV9.BI=TRUE} | 0.13  3663 | 0.805  97 | 0.165  842 | 1.18  8365 | 54 |
| {BcHV3.BI=TRUE, BcMV9.BI=TRUE} =>  {BcOLV18=TRUE} | 0.13  3663 | 0.771  429 | 0.173  267 | 1.49  1183 | 54 |
| {BcMV9.BI=TRUE, BcOLV18=TRUE} =>  {BcHV3.BI=TRUE} | 0.13  3663 | 0.323  353 | 0.413  366 | 1.34  675 | 54 |
| {BcHV3.BI=TRUE, BcOLV18=TRUE} =>  {BcULV4=TRUE} | 0.14  1089 | 0.850  746 | 0.165  842 | 1.23  1905 | 57 |
| {BcHV3.BI=TRUE, BcULV4=TRUE} =>  {BcOLV18=TRUE} | 0.14  1089 | 0.802  817 | 0.175  743 | 1.55  1857 | 57 |
| {BcOLV18=TRUE, BcULV4=TRUE} =>  {BcHV3.BI=TRUE} | 0.14  1089 | 0.320  225 | 0.440  594 | 1.33  3719 | 57 |
| {BcHV3.BI=TRUE, BcMV6.BI=FALSE} =>  {BcOLV18=TRUE} | 0.10  1485 | 0.650  794 | 0.155  941 | 1.25  7993 | 41 |
| {BcHV3.BI=TRUE, BcPV3.BI=FALSE} =>  {BcOLV18=TRUE} | 0.10  1485 | 0.640  625 | 0.158  416 | 1.23  8337 | 41 |

| {BcOLV18=TRUE, BcPV3.BI=FALSE} =>  {BcHV3.BI=TRUE} | 0.10  1485 | 0.308  271 | 0.329  208 | 1.28  3931 | 41 |
| --- | --- | --- | --- | --- | --- |
| {BcOLV18=TRUE, BcRV1.BI=FALSE} =>  {BcHV3.BI=TRUE} | 0.10  8911 | 0.330  827 | 0.329  208 | 1.37  7878 | 44 |
| {BcEV2.BI=FALSE, BcHV3.BI=TRUE} =>  {BcOLV18=TRUE} | 0.10  1485 | 0.672  131 | 0.150  99 | 1.29  9239 | 41 |
| {BcHV3.BI=TRUE, BcULV1.BI=FALSE} =>  {BcOLV18=TRUE} | 0.11  3861 | 0.630  137 | 0.180  693 | 1.21  8064 | 46 |
| {BcOLV18=TRUE, BcULV1.BI=FALSE} =>  {BcHV3.BI=TRUE} | 0.11  3861 | 0.319  444 | 0.356  436 | 1.33  047 | 46 |
| {BcHV3.BI=TRUE, BcOLV18=TRUE} =>  {BcMV1.BI=TRUE} | 0.15  099 | 0.910  448 | 0.165  842 | 1.15  3044 | 61 |
| {BcHV3.BI=TRUE, BcMV1.BI=TRUE} =>  {BcOLV18=TRUE} | 0.15  099 | 0.709  302 | 0.212  871 | 1.37  1092 | 61 |
| {BcMV1.BI=TRUE, BcOLV18=TRUE} =>  {BcHV3.BI=TRUE} | 0.15  099 | 0.342  697 | 0.440  594 | 1.42  7314 | 61 |
| {BcHV3.BI=TRUE, BcMV12=FALSE} =>  {BcOLV18=TRUE} | 0.12  8713 | 0.65 | 0.198  02 | 1.25  6459 | 52 |
| {BcMV12=FALSE, BcOLV18=TRUE} =>  {BcHV3.BI=TRUE} | 0.12  8713 | 0.325 | 0.396  04 | 1.35  3608 | 52 |
| {BcHV3.BI=TRUE, BcVV3.BI=FALSE} =>  {BcOLV18=TRUE} | 0.10  6436 | 0.693  548 | 0.153  465 | 1.34  0639 | 43 |

| {BcHV3.BI=TRUE, BcOLV7.BI=FALSE} =>  {BcOLV18=TRUE} | 0.11  3861 | 0.647  887 | 0.175  743 | 1.25  2375 | 46 |
| --- | --- | --- | --- | --- | --- |
| {BcALV1.BI=FALSE, BcHV3.BI=TRUE} =>  {BcOLV18=TRUE} | 0.12  3762 | 0.684  932 | 0.180  693 | 1.32  3982 | 50 |
| {BcALV1.BI=FALSE, BcOLV18=TRUE} =>  {BcHV3.BI=TRUE} | 0.12  3762 | 0.328  947 | 0.376  238 | 1.37  0049 | 50 |
| {BcDMV1=FALSE, BcHV3.BI=TRUE} =>  {BcOLV18=TRUE} | 0.10  8911 | 0.666  667 | 0.163  366 | 1.28  8676 | 44 |
| {BcFV9=FALSE, BcHV3.BI=TRUE} =>  {BcOLV18=TRUE} | 0.13  3663 | 0.675 | 0.198  02 | 1.30  4785 | 54 |
| {BcFV9=FALSE, BcOLV18=TRUE} =>  {BcHV3.BI=TRUE} | 0.13  3663 | 0.317  647 | 0.420  792 | 1.32  2984 | 54 |
| {BcHV3.BI=TRUE, BcOLV4.BI=FALSE} =>  {BcOLV18=TRUE} | 0.14  1089 | 0.695  122 | 0.202  97 | 1.34  3681 | 57 |
| {BcOLV18=TRUE, BcOLV4.BI=FALSE} =>  {BcHV3.BI=TRUE} | 0.14  1089 | 0.314  917 | 0.448  02 | 1.31  1614 | 57 |
| {BcHV3.BI=TRUE, BcULV5=FALSE} =>  {BcOLV18=TRUE} | 0.14  3564 | 0.690  476 | 0.207  921 | 1.33  47 | 58 |
| {BcOLV18=TRUE, BcULV5=FALSE} =>  {BcHV3.BI=TRUE} | 0.14  3564 | 0.306  878 | 0.467  822 | 1.27  8132 | 58 |
| {BcBNV5.BI=FALSE, BcHV3.BI=TRUE} =>  {BcOLV18=TRUE} | 0.13  1188 | 0.679  487 | 0.193  069 | 1.31  3458 | 53 |

| {BcHV3.BI=TRUE, BvF.BI=FALSE} =>  {BcOLV18=TRUE} | 0.15  099 | 0.701  149 | 0.215  347 | 1.35  5332 | 61 |
| --- | --- | --- | --- | --- | --- |
| {BcOLV18=TRUE, BvF.BI=FALSE} =>  {BcHV3.BI=TRUE} | 0.15  099 | 0.317  708 | 0.475  248 | 1.32  3239 | 61 |
| {BcHV3.BI=TRUE, BcMCV5.BI=FALSE} =>  {BcOLV18=TRUE} | 0.11  8812 | 0.64 | 0.185  644 | 1.23  7129 | 48 |
| {BcHV3.BI=TRUE, BcOLV13.BI=FALSE} =>  {BcOLV18=TRUE} | 0.14  8515 | 0.689  655 | 0.215  347 | 1.33  3113 | 60 |
| {BcOLV13.BI=FALSE, BcOLV18=TRUE} =>  {BcHV3.BI=TRUE} | 0.14  8515 | 0.312  5 | 0.475  248 | 1.30  1546 | 60 |
| {BcHV3.BI=TRUE, BcOLV2.BI=FALSE} =>  {BcOLV18=TRUE} | 0.14  8515 | 0.666  667 | 0.222  772 | 1.28  8676 | 60 |
| {BcOLV18=TRUE, BcOLV2.BI=FALSE} =>  {BcHV3.BI=TRUE} | 0.14  8515 | 0.317  46 | 0.467  822 | 1.32  2206 | 60 |
| {BcHV3.BI=TRUE, BcMCV3.BI=FALSE} =>  {BcOLV18=TRUE} | 0.16  3366 | 0.687  5 | 0.237  624 | 1.32  8947 | 66 |
| {BcMCV3.BI=FALSE, BcOLV18=TRUE} =>  {BcHV3.BI=TRUE} | 0.16  3366 | 0.343  75 | 0.475  248 | 1.43  1701 | 66 |
| {BcHV3.BI=TRUE, BcPV4.BI=FALSE} =>  {BcOLV18=TRUE} | 0.15  5941 | 0.684  783 | 0.227  723 | 1.32  3695 | 63 |
| {BcOLV18=TRUE, BcPV4.BI=FALSE} =>  {BcHV3.BI=TRUE} | 0.15  5941 | 0.326  425 | 0.477  723 | 1.35  9543 | 63 |

| {BcEV6=FALSE, BcHV3.BI=TRUE} =>  {BcOLV18=TRUE} | 0.15  8416 | 0.695  652 | 0.227  723 | 1.34  4706 | 64 |
| --- | --- | --- | --- | --- | --- |
| {BcEV6=FALSE, BcOLV18=TRUE} =>  {BcHV3.BI=TRUE} | 0.15  8416 | 0.328  205 | 0.482  673 | 1.36  6957 | 64 |
| {BcHV3.BI=TRUE, BcOLV17.BI=FALSE} =>  {BcOLV18=TRUE} | 0.15  099 | 0.677  778 | 0.222  772 | 1.31  0154 | 61 |
| {BcOLV17.BI=FALSE, BcOLV18=TRUE} =>  {BcHV3.BI=TRUE} | 0.15  099 | 0.316  062 | 0.477  723 | 1.31  6383 | 61 |
| {BcHV3.BI=TRUE, BcOLV15.BI=FALSE} =>  {BcOLV18=TRUE} | 0.14  604 | 0.670  455 | 0.217  822 | 1.29  5998 | 59 |
| {BcOLV15.BI=FALSE, BcOLV18=TRUE} =>  {BcHV3.BI=TRUE} | 0.14  604 | 0.305  699 | 0.477  723 | 1.27  3223 | 59 |
| {BcEV5=FALSE, BcHV3.BI=TRUE} =>  {BcOLV18=TRUE} | 0.13  8614 | 0.7 | 0.198  02 | 1.35  311 | 56 |
| {BcHV3.BI=TRUE, BcMV2.BI=FALSE} =>  {BcOLV16.BI=FALSE} | 0.07  6733 | 0.720  93 | 0.106  436 | 1.23  4135 | 31 |
| {BcHV3.BI=TRUE, BcMV2.BI=FALSE} =>  {BcHV1.BI=TRUE} | 0.07  6733 | 0.720  93 | 0.106  436 | 1.19  3671 | 31 |
| {BcHV3.BI=TRUE, BcMV2.BI=FALSE} =>  {BcOLV1.BI.MOVA.=FALSE} | 0.08  6634 | 0.813  953 | 0.106  436 | 1.21  7916 | 35 |
| {BcHV3.BI=TRUE, BcMV2.BI=FALSE} =>  {BcMV6.BI=FALSE} | 0.09  4059 | 0.883  721 | 0.106  436 | 1.27  0545 | 38 |

| {BcHV3.BI=TRUE, BcMV2.BI=FALSE} =>  {BcMV12=FALSE} | 0.09  6535 | 0.906  977 | 0.106  436 | 1.13  4423 | 39 |
| --- | --- | --- | --- | --- | --- |
| {BcHV3.BI=TRUE, BcMV2.BI=FALSE} =>  {BcALV1.BI=FALSE} | 0.09  6535 | 0.906  977 | 0.106  436 | 1.11  3734 | 39 |
| {BcHV1SLR.BI=TRUE, BcHV3.BI=TRUE} =>  {BcMV3.BI=TRUE} | 0.10  1485 | 0.683  333 | 0.148  515 | 1.26  0578 | 41 |
| {BcHV3.BI=TRUE, BcMV3.BI=TRUE} =>  {BcHV1SLR.BI=TRUE} | 0.10  1485 | 0.683  333 | 0.148  515 | 1.26  0578 | 41 |
| {BcHV1SLR.BI=TRUE, BcMV3.BI=TRUE} =>  {BcHV3.BI=TRUE} | 0.10  1485 | 0.308  271 | 0.329  208 | 1.28  3931 | 41 |
| {BcHV1SLR.BI=TRUE, BcHV3.BI=TRUE} =>  {BcULV3=TRUE} | 0.12  1287 | 0.816  667 | 0.148  515 | 1.37  4722 | 49 |
| {BcHV3.BI=TRUE, BcULV3=TRUE} =>  {BcHV1SLR.BI=TRUE} | 0.12  1287 | 0.710  145 | 0.170  792 | 1.31  0039 | 49 |
| {BcHV1SLR.BI=TRUE, BcULV3=TRUE} =>  {BcHV3.BI=TRUE} | 0.12  1287 | 0.314  103 | 0.386  139 | 1.30  8221 | 49 |
| {BcHV1SLR.BI=TRUE, BcHV3.BI=TRUE} =>  {BcHV1.BI=TRUE} | 0.13  8614 | 0.933  333 | 0.148  515 | 1.54  5355 | 56 |
| {BcHV1.BI=TRUE, BcHV3.BI=TRUE} =>  {BcHV1SLR.BI=TRUE} | 0.13  8614 | 0.861  538 | 0.160  891 | 1.58  9322 | 56 |
| {BcHV1SLR.BI=TRUE, BcHV3.BI=TRUE} =>  {BcMV11=TRUE} | 0.11  1386 | 0.75 | 0.148  515 | 1.21  6867 | 45 |

| {BcHV1SLR.BI=TRUE, BcMV11=TRUE} =>  {BcHV3.BI=TRUE} | 0.11  1386 | 0.312  5 | 0.356  436 | 1.30  1546 | 45 |
| --- | --- | --- | --- | --- | --- |
| {BcHV3.BI=TRUE, BcMV9.BI=TRUE} =>  {BcHV1SLR.BI=TRUE} | 0.11  3861 | 0.657  143 | 0.173  267 | 1.21  2264 | 46 |
| {BcEV2.BI=FALSE, BcHV3.BI=TRUE} =>  {BcHV1SLR.BI=TRUE} | 0.09  901 | 0.655  738 | 0.150  99 | 1.20  9671 | 40 |
| {BcHV1SLR.BI=TRUE, BcMCV3.BI=FALSE}  => {BcHV3.BI=TRUE} | 0.14  604 | 0.299  492 | 0.487  624 | 1.24  737 | 59 |
| {BcHV3.BI=TRUE, BcMV3.BI=TRUE} =>  {BcULV3=TRUE} | 0.11  1386 | 0.75 | 0.148  515 | 1.26  25 | 45 |
| {BcMV3.BI=TRUE, BcULV3=TRUE} =>  {BcHV3.BI=TRUE} | 0.11  1386 | 0.321  429 | 0.346  535 | 1.33  8733 | 45 |
| {BcHV3.BI=TRUE, BcMV3.BI=TRUE} =>  {BcHV1.BI=TRUE} | 0.10  8911 | 0.733  333 | 0.148  515 | 1.21  4208 | 44 |
| {BcHV1.BI=TRUE, BcHV3.BI=TRUE} =>  {BcMV3.BI=TRUE} | 0.10  8911 | 0.676  923 | 0.160  891 | 1.24  8753 | 44 |
| {BcHV1.BI=TRUE, BcMV3.BI=TRUE} =>  {BcHV3.BI=TRUE} | 0.10  8911 | 0.307  692 | 0.353  96 | 1.28  1523 | 44 |
| {BcHV3.BI=TRUE, BcMV3.BI=TRUE} =>  {BcMV11=TRUE} | 0.11  6337 | 0.783  333 | 0.148  515 | 1.27  095 | 47 |
| {BcHV3.BI=TRUE, BcMV11=TRUE} =>  {BcMV3.BI=TRUE} | 0.11  6337 | 0.671  429 | 0.173  267 | 1.23  8617 | 47 |

| {BcMV11=TRUE, BcMV3.BI=TRUE} =>  {BcHV3.BI=TRUE} | 0.11  6337 | 0.303  226 | 0.383  663 | 1.26  292 | 47 |
| --- | --- | --- | --- | --- | --- |
| {BcHV3.BI=TRUE, BcMV3.BI=TRUE} =>  {BcMV9.BI=TRUE} | 0.11  8812 | 0.8 | 0.148  515 | 1.17  9562 | 48 |
| {BcHV3.BI=TRUE, BcMV9.BI=TRUE} =>  {BcMV3.BI=TRUE} | 0.11  8812 | 0.685  714 | 0.173  267 | 1.26  4971 | 48 |
| {BcMV3.BI=TRUE, BcMV9.BI=TRUE} =>  {BcHV3.BI=TRUE} | 0.11  8812 | 0.3 | 0.396  04 | 1.24  9485 | 48 |
| {BcHV3.BI=TRUE, BcMV3.BI=TRUE} =>  {BcULV4=TRUE} | 0.12  1287 | 0.816  667 | 0.148  515 | 1.18  2557 | 49 |
| {BcHV3.BI=TRUE, BcULV4=TRUE} =>  {BcMV3.BI=TRUE} | 0.12  1287 | 0.690  141 | 0.175  743 | 1.27  3137 | 49 |
| {BcMV3.BI=TRUE, BcULV4=TRUE} =>  {BcHV3.BI=TRUE} | 0.12  1287 | 0.302  469 | 0.400  99 | 1.25  9768 | 49 |
| {BcHV3.BI=TRUE, BcMV3.BI=TRUE} =>  {BcMV1.BI=TRUE} | 0.14  1089 | 0.95 | 0.148  515 | 1.20  3135 | 57 |
| {BcHV3.BI=TRUE, BcMV1.BI=TRUE} =>  {BcMV3.BI=TRUE} | 0.14  1089 | 0.662  791 | 0.212  871 | 1.22  2682 | 57 |
| {BcMV1.BI=TRUE, BcMV3.BI=TRUE} =>  {BcHV3.BI=TRUE} | 0.14  1089 | 0.308  108 | 0.457  921 | 1.28  3254 | 57 |
| {BcMV12=FALSE, BcMV3.BI=TRUE} =>  {BcHV3.BI=TRUE} | 0.11  8812 | 0.305  732 | 0.388  614 | 1.27  336 | 48 |

| {BcMCV3.BI=FALSE, BcMV3.BI=TRUE} =>  {BcHV3.BI=TRUE} | 0.14  604 | 0.307  292 | 0.475  248 | 1.27  9854 | 59 |
| --- | --- | --- | --- | --- | --- |
| {BcHV3.BI=TRUE, BcMV4.BI=FALSE} =>  {BcRV1.BI=FALSE} | 0.07  6733 | 0.861  111 | 0.089  109 | 1.19  14 | 31 |
| {BcHV3.BI=TRUE, BcMV4.BI=FALSE} =>  {BcALV1.BI=FALSE} | 0.08  1683 | 0.916  667 | 0.089  109 | 1.12  5633 | 33 |
| {BcHV3.BI=TRUE, BcOLV1.BI.MOVA.=FALSE} =>  {BcOLV16.BI=FALSE} | 0.09  901 | 0.727  273 | 0.136  139 | 1.24  4992 | 40 |
| {BcHV3.BI=TRUE, BcMV6.BI=FALSE} =>  {BcOLV16.BI=FALSE} | 0.10  8911 | 0.698  413 | 0.155  941 | 1.19  5588 | 44 |
| {BcHV3.BI=TRUE, BcOLV16.BI=FALSE} =>  {BcRV1.BI=FALSE} | 0.12  3762 | 0.847  458 | 0.146  04 | 1.17  251 | 50 |
| {BcHV3.BI=TRUE, BcRV1.BI=FALSE} =>  {BcOLV16.BI=FALSE} | 0.12  3762 | 0.704  225 | 0.175  743 | 1.20  5538 | 50 |
| {BcHV3.BI=TRUE, BcOLV16.BI=FALSE} =>  {BcULV1.BI=FALSE} | 0.12  8713 | 0.881  356 | 0.146  04 | 1.12  3242 | 52 |
| {BcHV3.BI=TRUE, BcULV1.BI=FALSE} =>  {BcOLV16.BI=FALSE} | 0.12  8713 | 0.712  329 | 0.180  693 | 1.21  941 | 52 |
| {BcHV3.BI=TRUE, BcOLV16.BI=FALSE} =>  {BcMV1.BI=TRUE} | 0.13  3663 | 0.915  254 | 0.146  04 | 1.15  9131 | 54 |
| {BcHV3.BI=TRUE, BcULV3=TRUE} => | 0.13 | 0.768 | 0.170 | 1.27 | 53 |

| {BcHV1.BI=TRUE} | 1188 | 116 | 792 | 1799 |  |
| --- | --- | --- | --- | --- | --- |
| {BcHV1.BI=TRUE, BcHV3.BI=TRUE} => | 0.13 | 0.815 | 0.160 | 1.37 |  |
|  |  |  |  |  | 53 |
| {BcULV3=TRUE} | 1188 | 385 | 891 | 2564 |  |
| {BcHV1.BI=TRUE, BcULV3=TRUE} => | 0.13 | 0.309 | 0.423 | 1.29 |  |
|  |  |  |  |  | 53 |
| {BcHV3.BI=TRUE} | 1188 | 942 | 267 | 089 |  |
| {BcHV3.BI=TRUE, BcULV3=TRUE} => | 0.12 | 0.753 | 0.170 | 1.22 |  |
|  |  |  |  |  | 52 |
| {BcMV11=TRUE} | 8713 | 623 | 792 | 2746 |  |
| {BcHV3.BI=TRUE, BcMV11=TRUE} => | 0.12 | 0.742 | 0.173 | 1.25 |  |
|  |  |  |  |  | 52 |
| {BcULV3=TRUE} | 8713 | 857 | 267 | 0476 |  |
| {BcMV11=TRUE, BcULV3=TRUE} => | 0.12 |  | 0.396 | 1.35 |  |
|  |  | 0.325 |  |  | 52 |
| {BcHV3.BI=TRUE} | 8713 |  | 04 | 3608 |  |
| {BcHV3.BI=TRUE, |  |  |  |  |  |
|  | 0.09 | 0.709 | 0.136 | 1.19 |  |
| BcOLV1.BI.MOVA.=FALSE} => |  |  |  |  | 39 |
|  | 6535 | 091 | 139 | 3636 |  |
| {BcULV3=TRUE} |  |  |  |  |  |
| {BcHV3.BI=TRUE, BcMV9.BI=TRUE} => | 0.12 | 0.742 | 0.173 | 1.25 |  |
|  |  |  |  |  | 52 |
| {BcULV3=TRUE} | 8713 | 857 | 267 | 0476 |  |
| {BcMV9.BI=TRUE, BcULV3=TRUE} => | 0.12 | 0.304 | 0.423 | 1.26 |  |
|  |  |  |  |  | 52 |
| {BcHV3.BI=TRUE} | 8713 | 094 | 267 | 6534 |  |
| {BcHV3.BI=TRUE, BcPV3.BI=FALSE} => | 0.11 |  | 0.158 | 1.26 |  |
|  |  | 0.75 |  |  | 48 |
| {BcULV3=TRUE} | 8812 |  | 416 | 25 |  |
| {BcHV3.BI=TRUE, BcRV1.BI=FALSE} => | 0.12 | 0.732 | 0.175 | 1.23 |  |
|  |  |  |  |  | 52 |
| {BcULV3=TRUE} | 8713 | 394 | 743 | 2864 |  |

| {BcEV2.BI=FALSE, BcHV3.BI=TRUE} =>  {BcULV3=TRUE} | 0.10  6436 | 0.704  918 | 0.150  99 | 1.18  6612 | 43 |
| --- | --- | --- | --- | --- | --- |
| {BcHV3.BI=TRUE, BcULV1.BI=FALSE} =>  {BcULV3=TRUE} | 0.13  1188 | 0.726  027 | 0.180  693 | 1.22  2146 | 53 |
| {BcHV3.BI=TRUE, BcULV3=TRUE} =>  {BcMV1.BI=TRUE} | 0.15  099 | 0.884  058 | 0.170  792 | 1.11  9622 | 61 |
| {BcHV3.BI=TRUE, BcMV1.BI=TRUE} =>  {BcULV3=TRUE} | 0.15  099 | 0.709  302 | 0.212  871 | 1.19  3992 | 61 |
| {BcMV1.BI=TRUE, BcULV3=TRUE} =>  {BcHV3.BI=TRUE} | 0.15  099 | 0.311  224 | 0.485  149 | 1.29  6234 | 61 |
| {BcHV3.BI=TRUE, BcMV12=FALSE} =>  {BcULV3=TRUE} | 0.14  1089 | 0.712  5 | 0.198  02 | 1.19  9375 | 57 |
| {BcMV12=FALSE, BcULV3=TRUE} =>  {BcHV3.BI=TRUE} | 0.14  1089 | 0.301  587 | 0.467  822 | 1.25  6096 | 57 |
| {BcHV3.BI=TRUE, BcVV3.BI=FALSE} =>  {BcULV3=TRUE} | 0.10  8911 | 0.709  677 | 0.153  465 | 1.19  4624 | 44 |
| {BcHV3.BI=TRUE, BcOLV7.BI=FALSE} =>  {BcULV3=TRUE} | 0.13  1188 | 0.746  479 | 0.175  743 | 1.25  6573 | 53 |
| {BcFV9=FALSE, BcHV3.BI=TRUE} =>  {BcULV3=TRUE} | 0.14  1089 | 0.712  5 | 0.198  02 | 1.19  9375 | 57 |
| {BcHV3.BI=TRUE, BcOLV4.BI=FALSE} =>  {BcULV3=TRUE} | 0.14  3564 | 0.707  317 | 0.202  97 | 1.19  065 | 58 |

| {BcHV3.BI=TRUE, BcULV5=FALSE} =>  {BcULV3=TRUE} | 0.14  604 | 0.702  381 | 0.207  921 | 1.18  2341 | 59 |
| --- | --- | --- | --- | --- | --- |
| {BcHV3.BI=TRUE, BvF.BI=FALSE} =>  {BcULV3=TRUE} | 0.15  3465 | 0.712  644 | 0.215  347 | 1.19  9617 | 62 |
| {BcHV3.BI=TRUE, BcOLV13.BI=FALSE} =>  {BcULV3=TRUE} | 0.15  5941 | 0.724  138 | 0.215  347 | 1.21  8966 | 63 |
| {BcHV3.BI=TRUE, BcOLV2.BI=FALSE} =>  {BcULV3=TRUE} | 0.16  0891 | 0.722  222 | 0.222  772 | 1.21  5741 | 65 |
| {BcOLV2.BI=FALSE, BcULV3=TRUE} =>  {BcHV3.BI=TRUE} | 0.16  0891 | 0.296  804 | 0.542  079 | 1.23  6172 | 65 |
| {BcHV3.BI=TRUE, BcMCV3.BI=FALSE} =>  {BcULV3=TRUE} | 0.16  8317 | 0.708  333 | 0.237  624 | 1.19  2361 | 68 |
| {BcMCV3.BI=FALSE, BcULV3=TRUE} =>  {BcHV3.BI=TRUE} | 0.16  8317 | 0.310  502 | 0.542  079 | 1.29  3226 | 68 |
| {BcHV3.BI=TRUE, BcPV4.BI=FALSE} =>  {BcULV3=TRUE} | 0.15  8416 | 0.695  652 | 0.227  723 | 1.17  1014 | 64 |
| {BcEV6=FALSE, BcHV3.BI=TRUE} =>  {BcULV3=TRUE} | 0.16  0891 | 0.706  522 | 0.227  723 | 1.18  9312 | 65 |
| {BcHV3.BI=TRUE, BcOLV17.BI=FALSE} =>  {BcULV3=TRUE} | 0.16  0891 | 0.722  222 | 0.222  772 | 1.21  5741 | 65 |
| {BcHV3.BI=TRUE, BcOLV15.BI=FALSE} =>  {BcULV3=TRUE} | 0.15  5941 | 0.715  909 | 0.217  822 | 1.20  5114 | 63 |

| {BcHV1.BI=TRUE, BcHV3.BI=TRUE} =>  {BcMV11=TRUE} | 0.12  3762 | 0.769  231 | 0.160  891 | 1.24  8069 | 50 |
| --- | --- | --- | --- | --- | --- |
| {BcHV3.BI=TRUE, BcMV11=TRUE} =>  {BcHV1.BI=TRUE} | 0.12  3762 | 0.714  286 | 0.173  267 | 1.18  267 | 50 |
| {BcHV1.BI=TRUE, BcMV11=TRUE} =>  {BcHV3.BI=TRUE} | 0.12  3762 | 0.324  675 | 0.381  188 | 1.35  2256 | 50 |
| {BcHV3.BI=TRUE, BcMV9.BI=TRUE} =>  {BcMV11=TRUE} | 0.13  1188 | 0.757  143 | 0.173  267 | 1.22  8457 | 53 |
| {BcMV11=TRUE, BcMV9.BI=TRUE} =>  {BcHV3.BI=TRUE} | 0.13  1188 | 0.323  171 | 0.405  941 | 1.34  5989 | 53 |
| {BcHV3.BI=TRUE, BcULV4=TRUE} =>  {BcMV11=TRUE} | 0.12  6238 | 0.718  31 | 0.175  743 | 1.16  5451 | 51 |
| {BcMV11=TRUE, BcULV4=TRUE} =>  {BcHV3.BI=TRUE} | 0.12  6238 | 0.301  775 | 0.418  317 | 1.25  6878 | 51 |
| {BcFV5.BI=FALSE, BcHV3.BI=TRUE} =>  {BcMV11=TRUE} | 0.09  1584 | 0.725  49 | 0.126  238 | 1.17  7101 | 37 |
| {BcHV3.BI=TRUE, BcMV11=TRUE} =>  {BcMV1.BI=TRUE} | 0.16  0891 | 0.928  571 | 0.173  267 | 1.17  5996 | 65 |
| {BcHV3.BI=TRUE, BcMV1.BI=TRUE} =>  {BcMV11=TRUE} | 0.16  0891 | 0.755  814 | 0.212  871 | 1.22  6301 | 65 |
| {BcMV1.BI=TRUE, BcMV11=TRUE} =>  {BcHV3.BI=TRUE} | 0.16  0891 | 0.309  524 | 0.519  802 | 1.28  9151 | 65 |

| {BcHV3.BI=TRUE, BcMV12=FALSE} =>  {BcMV11=TRUE} | 0.14  8515 | 0.75 | 0.198  02 | 1.21  6867 | 60 |
| --- | --- | --- | --- | --- | --- |
| {BcHV3.BI=TRUE, BcNSRV7.BI=FALSE} =>  {BcMV11=TRUE} | 0.10  8911 | 0.745  763 | 0.146  04 | 1.20  9993 | 44 |
| {BcHV3.BI=TRUE, BcOLV7.BI=FALSE} =>  {BcMV11=TRUE} | 0.12  8713 | 0.732  394 | 0.175  743 | 1.18  8303 | 52 |
| {BcFV9=FALSE, BcHV3.BI=TRUE} =>  {BcMV11=TRUE} | 0.14  1089 | 0.712  5 | 0.198  02 | 1.15  6024 | 57 |
| {BcHV3.BI=TRUE, BvF.BI=FALSE} =>  {BcMV11=TRUE} | 0.15  5941 | 0.724  138 | 0.215  347 | 1.17  4907 | 63 |
| {BcHV3.BI=TRUE, BcOLV13.BI=FALSE} =>  {BcMV11=TRUE} | 0.15  5941 | 0.724  138 | 0.215  347 | 1.17  4907 | 63 |
| {BcHV3.BI=TRUE, BcOLV2.BI=FALSE} =>  {BcMV11=TRUE} | 0.15  8416 | 0.711  111 | 0.222  772 | 1.15  3771 | 64 |
| {BcHV3.BI=TRUE, BcMCV3.BI=FALSE} =>  {BcMV11=TRUE} | 0.17  0792 | 0.718  75 | 0.237  624 | 1.16  6165 | 69 |
| {BcMCV3.BI=FALSE, BcMV11=TRUE} =>  {BcHV3.BI=TRUE} | 0.17  0792 | 0.293  617 | 0.581  683 | 1.22  29 | 69 |
| {BcHV3.BI=TRUE, BcPV4.BI=FALSE} =>  {BcMV11=TRUE} | 0.16  3366 | 0.717  391 | 0.227  723 | 1.16  396 | 66 |
| {BcEV6=FALSE, BcHV3.BI=TRUE} =>  {BcMV11=TRUE} | 0.16  3366 | 0.717  391 | 0.227  723 | 1.16  396 | 66 |

| {BcHV3.BI=TRUE, BcOLV17.BI=FALSE} =>  {BcMV11=TRUE} | 0.15  8416 | 0.711  111 | 0.222  772 | 1.15  3771 | 64 |
| --- | --- | --- | --- | --- | --- |
| {BcHV3.BI=TRUE, BcOLV15.BI=FALSE} =>  {BcMV11=TRUE} | 0.16  0891 | 0.738  636 | 0.217  822 | 1.19  843 | 65 |
| {BcHV3.BI=TRUE, BcULV4=TRUE} =>  {BcMV9.BI=TRUE} | 0.13  6139 | 0.774  648 | 0.175  743 | 1.14  2182 | 55 |
| {BcHV3.BI=TRUE, BcMV9.BI=TRUE} =>  {BcMV1.BI=TRUE} | 0.16  0891 | 0.928  571 | 0.173  267 | 1.17  5996 | 65 |
| {BcHV3.BI=TRUE, BcULV5=FALSE} =>  {BcMV9.BI=TRUE} | 0.16  3366 | 0.785  714 | 0.207  921 | 1.15  8498 | 66 |
| {BcHV3.BI=TRUE, BcULV4=TRUE} =>  {BcMV1.BI=TRUE} | 0.15  8416 | 0.901  408 | 0.175  743 | 1.14  1596 | 64 |
| {BcHV3.BI=TRUE, BcRV1.BI=FALSE} =>  {BcMV1.BI=TRUE} | 0.15  5941 | 0.887  324 | 0.175  743 | 1.12  3758 | 63 |
| {BcHV3.BI=TRUE, BcOLV7.BI=FALSE} =>  {BcRV1.BI=FALSE} | 0.15  3465 | 0.873  239 | 0.175  743 | 1.20  8181 | 62 |
| {BcHV3.BI=TRUE, BcULV1.BI=FALSE} =>  {BcMV1.BI=TRUE} | 0.15  8416 | 0.876  712 | 0.180  693 | 1.11  0319 | 64 |
| {BcHV3.BI=TRUE, BcMV12=FALSE} =>  {BcMV1.BI=TRUE} | 0.17  5743 | 0.887  5 | 0.198  02 | 1.12  3981 | 71 |
| {BcALV1.BI=FALSE, BcHV3.BI=TRUE} =>  {BcMV1.BI=TRUE} | 0.15  8416 | 0.876  712 | 0.180  693 | 1.11  0319 | 64 |

| {BcFV9=FALSE, BcHV3.BI=TRUE} =>  {BcMV1.BI=TRUE} | 0.17  3267 | 0.875 | 0.198  02 | 1.10  815 | 70 |
| --- | --- | --- | --- | --- | --- |
| {BcHV3.BI=TRUE, BcOLV4.BI=FALSE} =>  {BcMV1.BI=TRUE} | 0.18  0693 | 0.890  244 | 0.202  97 | 1.12  7456 | 73 |
| {BcHV3.BI=TRUE, BvF.BI=FALSE} =>  {BcMV1.BI=TRUE} | 0.19  5545 | 0.908  046 | 0.215  347 | 1.15  0002 | 79 |
| {BcHV3.BI=TRUE, BcOLV13.BI=FALSE} =>  {BcMV1.BI=TRUE} | 0.18  8119 | 0.873  563 | 0.215  347 | 1.10  6331 | 76 |
| {BcHV3.BI=TRUE, BcOLV2.BI=FALSE} =>  {BcMV1.BI=TRUE} | 0.19  5545 | 0.877  778 | 0.222  772 | 1.11  1668 | 79 |
| {BcHV3.BI=TRUE, BcMCV3.BI=FALSE} =>  {BcMV1.BI=TRUE} | 0.21  0396 | 0.885  417 | 0.237  624 | 1.12  1343 | 85 |
| {BcMCV3.BI=FALSE, BcMV1.BI=TRUE} =>  {BcHV3.BI=TRUE} | 0.21  0396 | 0.288  136 | 0.730  198 | 1.20  007 | 85 |
| {BcHV3.BI=TRUE, BcPV4.BI=FALSE} =>  {BcMV1.BI=TRUE} | 0.20  0495 | 0.880  435 | 0.227  723 | 1.11  5033 | 81 |
| {BcEV6=FALSE, BcHV3.BI=TRUE} =>  {BcMV1.BI=TRUE} | 0.20  297 | 0.891  304 | 0.227  723 | 1.12  8799 | 82 |
| {BcHV3.BI=TRUE, BcOLV17.BI=FALSE} =>  {BcMV1.BI=TRUE} | 0.19  5545 | 0.877  778 | 0.222  772 | 1.11  1668 | 79 |
| {BcHV3.BI=TRUE, BcOLV15.BI=FALSE} =>  {BcMV1.BI=TRUE} | 0.19  0594 | 0.875 | 0.217  822 | 1.10  815 | 77 |

| {BcEV2.BI=TRUE, BcPV3.BI=TRUE} =>  {BcMV1.BI=TRUE} | 0.08  4158 | 1 | 0.084  158 | 1.26  6458 | 34 |
| --- | --- | --- | --- | --- | --- |
| {BcEV2.BI=TRUE, BcMV1.BI=TRUE} =>  {BcPV3.BI=TRUE} | 0.08  4158 | 0.386  364 | 0.217  822 | 1.33  411 | 34 |
| {BcMV1.BI=TRUE, BcPV3.BI=TRUE} =>  {BcEV2.BI=TRUE} | 0.08  4158 | 0.330  097 | 0.254  95 | 1.30  7443 | 34 |
| {BcEV2.BI=TRUE, BcULV4=FALSE} =>  {BcOLV16.BI=FALSE} | 0.07  4257 | 0.810  811 | 0.091  584 | 1.38  7998 | 30 |
| {BcEV2.BI=TRUE, BcOLV16.BI=FALSE} =>  {BcULV4=FALSE} | 0.07  4257 | 0.454  545 | 0.163  366 | 1.46  9091 | 30 |
| {BcEV2.BI=TRUE, BcULV4=FALSE} =>  {BcOLV1.BI.MOVA.=FALSE} | 0.07  6733 | 0.837  838 | 0.091  584 | 1.25  3654 | 31 |
| {BcEV2.BI=TRUE, BcOLV1.BI.MOVA.=FALSE} =>  {BcULV4=FALSE} | 0.07  6733 | 0.449  275 | 0.170  792 | 1.45  2058 | 31 |
| {BcOLV1.BI.MOVA.=FALSE, BcULV4=FALSE}  => {BcEV2.BI=TRUE} | 0.07  6733 | 0.344  444 | 0.222  772 | 1.36  427 | 31 |
| {BcEV2.BI=TRUE, BcULV4=FALSE} =>  {BcMV6.BI=FALSE} | 0.07  9208 | 0.864  865 | 0.091  584 | 1.24  3436 | 32 |
| {BcEV2.BI=TRUE, BcMV6.BI=FALSE} =>  {BcULV4=FALSE} | 0.07  9208 | 0.438  356 | 0.180  693 | 1.41  6767 | 32 |
| {BcEV2.BI=TRUE, BcULV4=FALSE} => | 0.07 | 0.837 | 0.091 | 1.15 | 31 |

| {BcRV1.BI=FALSE} | 6733 | 838 | 584 | 92 |  |
| --- | --- | --- | --- | --- | --- |
| {BcEV2.BI=TRUE, BcRV1.BI=FALSE} =>  {BcULV4=FALSE} | 0.07  6733 | 0.407  895 | 0.188  119 | 1.31  8316 | 31 |
| {BcEV2.BI=TRUE, BcULV4=FALSE} =>  {BcULV1.BI=FALSE} | 0.08  4158 | 0.918  919 | 0.091  584 | 1.17  1114 | 34 |
| {BcEV2.BI=TRUE, BcULV1.BI=FALSE} =>  {BcULV4=FALSE} | 0.08  4158 | 0.430  38 | 0.195  545 | 1.39  0987 | 34 |
| {BcEV2.BI=TRUE, BcNSRV7.BI=FALSE} =>  {BcULV4=FALSE} | 0.07  4257 | 0.405  405 | 0.183  168 | 1.31  027 | 30 |
| {BcEV2.BI=TRUE, BcULV4=FALSE} =>  {BcOLV7.BI=FALSE} | 0.08  9109 | 0.972  973 | 0.091  584 | 1.20  577 | 36 |
| {BcEV2.BI=TRUE, BcOLV7.BI=FALSE} =>  {BcULV4=FALSE} | 0.08  9109 | 0.413  793 | 0.215  347 | 1.33  7379 | 36 |
| {BcBNV5.BI=FALSE, BcEV2.BI=TRUE} =>  {BcULV4=FALSE} | 0.08  9109 | 0.413  793 | 0.215  347 | 1.33  7379 | 36 |
| {BcEV2.BI=TRUE, BcMV9.BI=FALSE} =>  {BcDMV1=FALSE} | 0.07  9208 | 1 | 0.079  208 | 1.18  4751 | 32 |
| {BcEV2.BI=TRUE, BcOLV1.BI.MOVA.=TRUE}  => {BcMV2.BI=TRUE} | 0.07  4257 | 0.909  091 | 0.081  683 | 1.96  4025 | 30 |
| {BcEV2.BI=TRUE, BcMV2.BI=TRUE} =>  {BcOLV1.BI.MOVA.=TRUE} | 0.07  4257 | 0.517  241 | 0.143  564 | 1.55  9444 | 30 |
| {BcEV2.BI=TRUE, BcOLV1.BI.MOVA.=TRUE} | 0.08 | 1 | 0.081 | 1.26 | 33 |

| => {BcMV1.BI=TRUE} | 1683 |  | 683 | 6458 |  |
| --- | --- | --- | --- | --- | --- |
| {BcEV2.BI=TRUE, BcHV1.BI=FALSE} =>  {BcHV1SLR.BI=FALSE} | 0.07  4257 | 0.857  143 | 0.086  634 | 1.87  1815 | 30 |
| {BcEV2.BI=TRUE, BcHV1SLR.BI=FALSE} =>  {BcHV1.BI=FALSE} | 0.07  4257 | 0.769  231 | 0.096  535 | 1.94  2308 | 30 |
| {BcEV2.BI=TRUE, BcHV1.BI=FALSE} =>  {BcMV6.BI=FALSE} | 0.07  4257 | 0.857  143 | 0.086  634 | 1.23  2334 | 30 |
| {BcEV2.BI=TRUE, BcHV1.BI=FALSE} =>  {BcMV12=FALSE} | 0.08  1683 | 0.942  857 | 0.086  634 | 1.17  9301 | 33 |
| {BcEV2.BI=TRUE, BcHV1.BI=FALSE} =>  {BcALV1.BI=FALSE} | 0.07  9208 | 0.914  286 | 0.086  634 | 1.12  271 | 32 |
| {BcEV2.BI=TRUE, BcULV3=FALSE} =>  {BcMV12=FALSE} | 0.08  6634 | 0.921  053 | 0.094  059 | 1.15  2029 | 35 |
| {BcEV2.BI=TRUE, BcULV3=FALSE} =>  {BcNSRV7.BI=FALSE} | 0.08  4158 | 0.894  737 | 0.094  059 | 1.11  9114 | 34 |
| {BcEV2.BI=TRUE, BcOLV16.BI=TRUE} =>  {BcMV11=TRUE} | 0.07  4257 | 0.833  333 | 0.089  109 | 1.35  2075 | 30 |
| {BcEV2.BI=TRUE, BcOLV16.BI=TRUE} =>  {BcMV1.BI=TRUE} | 0.08  1683 | 0.916  667 | 0.089  109 | 1.16  092 | 33 |
| {BcEV2.BI=TRUE, BcMV4.BI=TRUE} =>  {BcMV2.BI=TRUE} | 0.08  4158 | 0.653  846 | 0.128  713 | 1.41  2587 | 34 |
| {BcEV2.BI=TRUE, BcMV2.BI=TRUE} => | 0.08 | 0.586 | 0.143 | 1.32 | 34 |

| {BcMV4.BI=TRUE} | 4158 | 207 | 564 | 3059 |  |
| --- | --- | --- | --- | --- | --- |
| {BcEV2.BI=TRUE, BcMV3.BI=TRUE} =>  {BcMV4.BI=TRUE} | 0.08  1683 | 0.589  286 | 0.138  614 | 1.33  0008 | 33 |
| {BcEV2.BI=TRUE, BcMV4.BI=TRUE} =>  {BcMV11=TRUE} | 0.09  901 | 0.769  231 | 0.128  713 | 1.24  8069 | 40 |
| {BcEV2.BI=TRUE, BcMV9.BI=TRUE} =>  {BcMV4.BI=TRUE} | 0.09  6535 | 0.557  143 | 0.173  267 | 1.25  7462 | 39 |
| {BcEV2.BI=TRUE, BcMV4.BI=TRUE} =>  {BcMV1.BI=TRUE} | 0.11  6337 | 0.903  846 | 0.128  713 | 1.14  4683 | 47 |
| {BcMV4.BI=TRUE, BcOLV7.BI=FALSE} =>  {BcEV2.BI=TRUE} | 0.10  8911 | 0.323  529 | 0.336  634 | 1.28  143 | 44 |
| {BcEV2.BI=TRUE, BcHV1SLR.BI=FALSE} =>  {BcMV11=TRUE} | 0.07  4257 | 0.769  231 | 0.096  535 | 1.24  8069 | 30 |
| {BcEV2.BI=TRUE, BcHV1SLR.BI=FALSE} =>  {BcMV6.BI=FALSE} | 0.08  1683 | 0.846  154 | 0.096  535 | 1.21  6534 | 33 |
| {BcEV2.BI=TRUE, BcHV1SLR.BI=FALSE} =>  {BcMV12=FALSE} | 0.08  6634 | 0.897  436 | 0.096  535 | 1.12  2489 | 35 |
| {BcEV2.BI=TRUE, BcMV3.BI=FALSE} =>  {BcOLV16.BI=FALSE} | 0.09  6535 | 0.847  826 | 0.113  861 | 1.45  1363 | 39 |
| {BcEV2.BI=TRUE, BcOLV16.BI=FALSE} =>  {BcMV3.BI=FALSE} | 0.09  6535 | 0.590  909 | 0.163  366 | 1.29  0418 | 39 |
| {BcEV2.BI=TRUE, BcMV3.BI=FALSE} => | 0.09 | 0.869 | 0.113 | 1.30 | 40 |

| {BcOLV1.BI.MOVA.=FALSE} | 901 | 565 | 861 | 1127 |  |
| --- | --- | --- | --- | --- | --- |
| {BcEV2.BI=TRUE, BcOLV1.BI.MOVA.=FALSE} =>  {BcMV3.BI=FALSE} | 0.09  901 | 0.579  71 | 0.170  792 | 1.26  5962 | 40 |
| {BcEV2.BI=TRUE, BcMV3.BI=FALSE} => | 0.09 | 0.847 | 0.113 | 1.21 |  |
|  |  |  |  |  | 39 |
| {BcMV6.BI=FALSE} | 6535 | 826 | 861 | 8939 |  |
| {BcEV2.BI=TRUE, BcMV3.BI=FALSE} => | 0.09 | 0.847 | 0.113 | 1.17 |  |
|  |  |  |  |  | 39 |
| {BcRV1.BI=FALSE} | 6535 | 826 | 861 | 302 |  |
| {BcEV2.BI=TRUE, BcMV3.BI=FALSE} => | 0.10 | 0.956 | 0.113 | 1.18 |  |
|  |  |  |  |  | 44 |
| {BcOLV7.BI=FALSE} | 8911 | 522 | 861 | 5383 |  |
| {BcEV2.BI=TRUE, BcMV3.BI=FALSE} => | 0.10 | 0.956 | 0.113 | 1.13 |  |
|  |  |  |  |  | 44 |
| {BcDMV1=FALSE} | 8911 | 522 | 861 | 324 |  |
| {BcEV2.BI=TRUE, BcMV2.BI=TRUE} => | 0.11 | 0.775 | 0.143 | 1.49 |  |
|  |  |  |  |  | 45 |
| {BcOLV18=TRUE} | 1386 | 862 | 564 | 9753 |  |
| {BcEV2.BI=TRUE, BcOLV18=TRUE} => | 0.11 |  | 0.148 | 1.62 |  |
|  |  | 0.75 |  |  | 45 |
| {BcMV2.BI=TRUE} | 1386 |  | 515 | 0321 |  |
| {BcMV2.BI=TRUE, BcOLV18=TRUE} => | 0.11 | 0.378 | 0.294 | 1.49 |  |
|  |  |  |  |  | 45 |
| {BcEV2.BI=TRUE} | 1386 | 151 | 554 | 7776 |  |
| {BcEV2.BI=TRUE, BcMV2.BI=TRUE} => | 0.09 | 0.689 | 0.143 | 1.27 |  |
|  |  |  |  |  | 40 |
| {BcMV3.BI=TRUE} | 901 | 655 | 564 | 2241 |  |
| {BcEV2.BI=TRUE, BcMV3.BI=TRUE} => | 0.09 | 0.714 | 0.138 | 1.54 |  |
|  |  |  |  |  | 40 |
| {BcMV2.BI=TRUE} | 901 | 286 | 614 | 3163 |  |

| {BcMV2.BI=TRUE, BcOLV16.BI=FALSE} =>  {BcEV2.BI=TRUE} | 0.07  6733 | 0.364  706 | 0.210  396 | 1.44  4521 | 31 |
| --- | --- | --- | --- | --- | --- |
| {BcEV2.BI=TRUE, BcMV2.BI=TRUE} =>  {BcMV11=TRUE} | 0.11  8812 | 0.827  586 | 0.143  564 | 1.34  275 | 48 |
| {BcEV2.BI=TRUE, BcMV11=TRUE} =>  {BcMV2.BI=TRUE} | 0.11  8812 | 0.64 | 0.185  644 | 1.38  2674 | 48 |
| {BcMV11=TRUE, BcMV2.BI=TRUE} =>  {BcEV2.BI=TRUE} | 0.11  8812 | 0.347  826 | 0.341  584 | 1.37  7664 | 48 |
| {BcEV2.BI=TRUE, BcULV4=TRUE} =>  {BcMV2.BI=TRUE} | 0.11  1386 | 0.692  308 | 0.160  891 | 1.49  5681 | 45 |
| {BcMV2.BI=TRUE, BcULV4=TRUE} =>  {BcEV2.BI=TRUE} | 0.11  1386 | 0.326  087 | 0.341  584 | 1.29  156 | 45 |
| {BcMV2.BI=TRUE, BcMV6.BI=FALSE} =>  {BcEV2.BI=TRUE} | 0.08  9109 | 0.349  515 | 0.254  95 | 1.38  4352 | 36 |
| {BcMV2.BI=TRUE, BcRV1.BI=FALSE} =>  {BcEV2.BI=TRUE} | 0.09  1584 | 0.321  739 | 0.284  653 | 1.27  4339 | 37 |
| {BcMV2.BI=TRUE, BcULV1.BI=FALSE} =>  {BcEV2.BI=TRUE} | 0.10  6436 | 0.328  244 | 0.324  257 | 1.30  0105 | 43 |
| {BcEV2.BI=TRUE, BcMV2.BI=TRUE} =>  {BcMV1.BI=TRUE} | 0.13  8614 | 0.965  517 | 0.143  564 | 1.22  2787 | 56 |
| {BcEV2.BI=TRUE, BcMV1.BI=TRUE} =>  {BcMV2.BI=TRUE} | 0.13  8614 | 0.636  364 | 0.217  822 | 1.37  4818 | 56 |

| {BcMV1.BI=TRUE, BcMV2.BI=TRUE} =>  {BcEV2.BI=TRUE} | 0.13  8614 | 0.339  394 | 0.408  416 | 1.34  4266 | 56 |
| --- | --- | --- | --- | --- | --- |
| {BcMV12=FALSE, BcMV2.BI=TRUE} =>  {BcEV2.BI=TRUE} | 0.12  1287 | 0.355  072 | 0.341  584 | 1.40  6365 | 49 |
| {BcEV2.BI=TRUE, BcNSRV7.BI=FALSE} =>  {BcMV2.BI=TRUE} | 0.11  3861 | 0.621  622 | 0.183  168 | 1.34  2969 | 46 |
| {BcEV2.BI=TRUE, BcFV9=FALSE} =>  {BcMV2.BI=TRUE} | 0.11  8812 | 0.607  595 | 0.195  545 | 1.31  2665 | 48 |
| {BcMV2.BI=TRUE, BcULV5=FALSE} =>  {BcEV2.BI=TRUE} | 0.12  3762 | 0.328  947 | 0.376  238 | 1.30  289 | 50 |
| {BcBNV5.BI=FALSE, BcEV2.BI=TRUE} =>  {BcMV2.BI=TRUE} | 0.12  3762 | 0.574  713 | 0.215  347 | 1.24  1625 | 50 |
| {BcEV2.BI=TRUE, BvF.BI=FALSE} =>  {BcMV2.BI=TRUE} | 0.12  6238 | 0.586  207 | 0.215  347 | 1.26  6458 | 51 |
| {BcEV2.BI=TRUE, BcOLV2.BI=FALSE} =>  {BcMV2.BI=TRUE} | 0.13  6139 | 0.578  947 | 0.235  149 | 1.25  0774 | 55 |
| {BcMV2.BI=TRUE, BcOLV2.BI=FALSE} =>  {BcEV2.BI=TRUE} | 0.13  6139 | 0.323  529 | 0.420  792 | 1.28  143 | 55 |
| {BcMCV3.BI=FALSE, BcMV2.BI=TRUE} =>  {BcEV2.BI=TRUE} | 0.12  8713 | 0.315  152 | 0.408  416 | 1.24  8247 | 52 |
| {BcEV2.BI=TRUE, BcEV6=FALSE} =>  {BcMV2.BI=TRUE} | 0.13  6139 | 0.578  947 | 0.235  149 | 1.25  0774 | 55 |

| {BcEV6=FALSE, BcMV2.BI=TRUE} =>  {BcEV2.BI=TRUE} | 0.13  6139 | 0.314  286 | 0.433  168 | 1.24  4818 | 55 |
| --- | --- | --- | --- | --- | --- |
| {BcEV2.BI=TRUE, BcOLV15.BI=FALSE} =>  {BcMV2.BI=TRUE} | 0.14  1089 | 0.633  333 | 0.222  772 | 1.36  8271 | 57 |
| {BcMV2.BI=TRUE, BcOLV15.BI=FALSE} =>  {BcEV2.BI=TRUE} | 0.14  1089 | 0.311  475 | 0.452  97 | 1.23  3687 | 57 |
| {BcEV2.BI=TRUE, BcOLV18=FALSE} =>  {BcOLV16.BI=FALSE} | 0.08  6634 | 0.833  333 | 0.103  96 | 1.42  6554 | 35 |
| {BcEV2.BI=TRUE, BcOLV18=FALSE} =>  {BcOLV1.BI.MOVA.=FALSE} | 0.08  6634 | 0.833  333 | 0.103  96 | 1.24  6914 | 35 |
| {BcEV2.BI=TRUE, BcOLV18=FALSE} =>  {BcPV3.BI=FALSE} | 0.08  6634 | 0.833  333 | 0.103  96 | 1.17  3055 | 35 |
| {BcEV2.BI=TRUE, BcOLV18=FALSE} =>  {BcRV1.BI=FALSE} | 0.08  9109 | 0.857  143 | 0.103  96 | 1.18  591 | 36 |
| {BcEV2.BI=TRUE, BcOLV18=FALSE} =>  {BcMV12=FALSE} | 0.09  4059 | 0.904  762 | 0.103  96 | 1.13  1653 | 38 |
| {BcEV2.BI=TRUE, BcOLV18=FALSE} =>  {BcOLV7.BI=FALSE} | 0.09  901 | 0.952  381 | 0.103  96 | 1.18  0251 | 40 |
| {BcEV2.BI=TRUE, BcOLV18=FALSE} =>  {BcDMV1=FALSE} | 0.09  6535 | 0.928  571 | 0.103  96 | 1.10  0126 | 39 |
| {BcEV2.BI=TRUE, BcOLV18=TRUE} =>  {BcMV3.BI=TRUE} | 0.10  1485 | 0.683  333 | 0.148  515 | 1.26  0578 | 41 |

| {BcEV2.BI=TRUE, BcMV3.BI=TRUE} =>  {BcOLV18=TRUE} | 0.10  1485 | 0.732  143 | 0.138  614 | 1.41  5243 | 41 |
| --- | --- | --- | --- | --- | --- |
| {BcEV2.BI=TRUE, BcOLV18=TRUE} =>  {BcULV3=TRUE} | 0.11  3861 | 0.766  667 | 0.148  515 | 1.29  0556 | 46 |
| {BcEV2.BI=TRUE, BcULV3=TRUE} =>  {BcOLV18=TRUE} | 0.11  3861 | 0.718  75 | 0.158  416 | 1.38  9354 | 46 |
| {BcOLV18=TRUE, BcULV3=TRUE} =>  {BcEV2.BI=TRUE} | 0.11  3861 | 0.351  145 | 0.324  257 | 1.39  081 | 46 |
| {BcEV2.BI=TRUE, BcOLV18=TRUE} =>  {BcMV11=TRUE} | 0.12  3762 | 0.833  333 | 0.148  515 | 1.35  2075 | 50 |
| {BcEV2.BI=TRUE, BcMV11=TRUE} =>  {BcOLV18=TRUE} | 0.12  3762 | 0.666  667 | 0.185  644 | 1.28  8676 | 50 |
| {BcMV11=TRUE, BcOLV18=TRUE} =>  {BcEV2.BI=TRUE} | 0.12  3762 | 0.357  143 | 0.346  535 | 1.41  4566 | 50 |
| {BcEV2.BI=TRUE, BcMV9.BI=TRUE} =>  {BcOLV18=TRUE} | 0.11  1386 | 0.642  857 | 0.173  267 | 1.24  2652 | 45 |
| {BcEV2.BI=TRUE, BcOLV18=TRUE} =>  {BcULV4=TRUE} | 0.12  8713 | 0.866  667 | 0.148  515 | 1.25  4958 | 52 |
| {BcEV2.BI=TRUE, BcULV4=TRUE} =>  {BcOLV18=TRUE} | 0.12  8713 | 0.8 | 0.160  891 | 1.54  6411 | 52 |
| {BcEV2.BI=TRUE, BcOLV18=TRUE} =>  {BcMV1.BI=TRUE} | 0.14  3564 | 0.966  667 | 0.148  515 | 1.22  4242 | 58 |

| {BcEV2.BI=TRUE, BcMV1.BI=TRUE} =>  {BcOLV18=TRUE} | 0.14  3564 | 0.659  091 | 0.217  822 | 1.27  4032 | 58 |
| --- | --- | --- | --- | --- | --- |
| {BcMV1.BI=TRUE, BcOLV18=TRUE} =>  {BcEV2.BI=TRUE} | 0.14  3564 | 0.325  843 | 0.440  594 | 1.29  0593 | 58 |
| {BcEV2.BI=TRUE, BvF.BI=FALSE} =>  {BcOLV18=TRUE} | 0.13  8614 | 0.643  678 | 0.215  347 | 1.24  4239 | 56 |
| {BcEV2.BI=TRUE, BcMV2.BI=FALSE} =>  {BcHV1SLR.BI=TRUE} | 0.07  4257 | 0.681  818 | 0.108  911 | 1.25  7783 | 30 |
| {BcEV2.BI=TRUE, BcMV2.BI=FALSE} =>  {BcOLV16.BI=FALSE} | 0.08  6634 | 0.795  455 | 0.108  911 | 1.36  171 | 35 |
| {BcEV2.BI=TRUE, BcMV2.BI=FALSE} =>  {BcHV1.BI=TRUE} | 0.07  9208 | 0.727  273 | 0.108  911 | 1.20  4173 | 32 |
| {BcEV2.BI=TRUE, BcMV2.BI=FALSE} =>  {BcOLV1.BI.MOVA.=FALSE} | 0.10  1485 | 0.931  818 | 0.108  911 | 1.39  4276 | 41 |
| {BcEV2.BI=TRUE, BcMV2.BI=FALSE} =>  {BcMV6.BI=FALSE} | 0.09  1584 | 0.840  909 | 0.108  911 | 1.20  8994 | 37 |
| {BcEV2.BI=TRUE, BcMV2.BI=FALSE} =>  {BcPV3.BI=FALSE} | 0.08  9109 | 0.818  182 | 0.108  911 | 1.15  1726 | 36 |
| {BcEV2.BI=TRUE, BcMV2.BI=FALSE} =>  {BcRV1.BI=FALSE} | 0.09  6535 | 0.886  364 | 0.108  911 | 1.22  6339 | 39 |
| {BcEV2.BI=TRUE, BcMV2.BI=FALSE} =>  {BcOLV7.BI=FALSE} | 0.10  1485 | 0.931  818 | 0.108  911 | 1.15  4769 | 41 |

| {BcEV2.BI=TRUE, BcMV2.BI=FALSE} =>  {BcALV1.BI=FALSE} | 0.10  1485 | 0.931  818 | 0.108  911 | 1.14  4239 | 41 |
| --- | --- | --- | --- | --- | --- |
| {BcHV1SLR.BI=TRUE, BcOLV16.BI=FALSE}  => {BcEV2.BI=TRUE} | 0.10  1485 | 0.333  333 | 0.304  455 | 1.32  0261 | 41 |
| {BcEV2.BI=TRUE, BcULV3=TRUE} =>  {BcHV1SLR.BI=TRUE} | 0.10  8911 | 0.687  5 | 0.158  416 | 1.26  8265 | 44 |
| {BcEV2.BI=TRUE, BcHV1SLR.BI=TRUE} =>  {BcHV1.BI=TRUE} | 0.14  3564 | 0.920  635 | 0.155  941 | 1.52  433 | 58 |
| {BcEV2.BI=TRUE, BcHV1.BI=TRUE} =>  {BcHV1SLR.BI=TRUE} | 0.14  3564 | 0.865  672 | 0.165  842 | 1.59  6947 | 58 |
| {BcEV2.BI=TRUE, BcMV9.BI=TRUE} =>  {BcHV1SLR.BI=TRUE} | 0.11  6337 | 0.671  429 | 0.173  267 | 1.23  8617 | 47 |
| {BcEV2.BI=TRUE, BcULV4=TRUE} =>  {BcHV1SLR.BI=TRUE} | 0.10  6436 | 0.661  538 | 0.160  891 | 1.22  0372 | 43 |
| {BcHV1SLR.BI=TRUE, BcMV1.BI=TRUE} =>  {BcEV2.BI=TRUE} | 0.13  6139 | 0.308  989 | 0.440  594 | 1.22  3838 | 55 |
| {BcHV1SLR.BI=TRUE, BcMV12=FALSE} =>  {BcEV2.BI=TRUE} | 0.13  1188 | 0.317  365 | 0.413  366 | 1.25  7015 | 53 |
| {BcEV2.BI=TRUE, BcMV3.BI=TRUE} =>  {BcHV1.BI=TRUE} | 0.09  901 | 0.714  286 | 0.138  614 | 1.18  267 | 40 |
| {BcEV2.BI=TRUE, BcMV3.BI=TRUE} =>  {BcMV11=TRUE} | 0.11  8812 | 0.857  143 | 0.138  614 | 1.39  0706 | 48 |

| {BcEV2.BI=TRUE, BcMV3.BI=TRUE} =>  {BcULV4=TRUE} | 0.11  3861 | 0.821  429 | 0.138  614 | 1.18  9452 | 46 |
| --- | --- | --- | --- | --- | --- |
| {BcEV2.BI=TRUE, BcULV4=TRUE} =>  {BcMV3.BI=TRUE} | 0.11  3861 | 0.707  692 | 0.160  891 | 1.30  5515 | 46 |
| {BcEV2.BI=TRUE, BcMV3.BI=TRUE} =>  {BcMV1.BI=TRUE} | 0.12  8713 | 0.928  571 | 0.138  614 | 1.17  5996 | 52 |
| {BcEV2.BI=TRUE, BcMV4.BI=FALSE} =>  {BcOLV16.BI=FALSE} | 0.09  1584 | 0.74 | 0.123  762 | 1.26  678 | 37 |
| {BcEV2.BI=TRUE, BcMV4.BI=FALSE} =>  {BcMV6.BI=FALSE} | 0.10  6436 | 0.86 | 0.123  762 | 1.23  6441 | 43 |
| {BcEV2.BI=TRUE, BcMV4.BI=FALSE} =>  {BcRV1.BI=FALSE} | 0.10  396 | 0.84 | 0.123  762 | 1.16  2192 | 42 |
| {BcEV2.BI=TRUE, BcMV4.BI=FALSE} =>  {BcALV1.BI=FALSE} | 0.11  6337 | 0.94 | 0.123  762 | 1.15  4286 | 47 |
| {BcEV2.BI=TRUE, BcMV4.BI=FALSE} =>  {BcDMV1=FALSE} | 0.11  3861 | 0.92 | 0.123  762 | 1.08  9971 | 46 |
| {BcMV11=TRUE, BcOLV16.BI=FALSE} =>  {BcEV2.BI=TRUE} | 0.11  1386 | 0.326  087 | 0.341  584 | 1.29  156 | 45 |
| {BcEV2.BI=TRUE, BcOLV16.BI=FALSE} =>  {BcOLV1.BI.MOVA.=FALSE} | 0.12  6238 | 0.772  727 | 0.163  366 | 1.15  6229 | 51 |
| {BcEV2.BI=TRUE,  BcOLV1.BI.MOVA.=FALSE} => | 0.12  6238 | 0.739  13 | 0.170  792 | 1.26  5291 | 51 |

| {BcOLV16.BI=FALSE} |  |  |  |  |  |
| --- | --- | --- | --- | --- | --- |
| {BcEV2.BI=TRUE, BcMV6.BI=FALSE} =>  {BcOLV16.BI=FALSE} | 0.12  6238 | 0.698  63 | 0.180  693 | 1.19  596 | 51 |
| {BcEV2.BI=TRUE, BcPV3.BI=FALSE} =>  {BcOLV16.BI=FALSE} | 0.12  1287 | 0.720  588 | 0.168  317 | 1.23  3549 | 49 |
| {BcEV2.BI=TRUE, BcOLV16.BI=FALSE} =>  {BcRV1.BI=FALSE} | 0.13  3663 | 0.818  182 | 0.163  366 | 1.13  2005 | 54 |
| {BcEV2.BI=TRUE, BcRV1.BI=FALSE} =>  {BcOLV16.BI=FALSE} | 0.13  3663 | 0.710  526 | 0.188  119 | 1.21  6325 | 54 |
| {BcEV2.BI=TRUE, BcULV1.BI=FALSE} =>  {BcOLV16.BI=FALSE} | 0.14  1089 | 0.721  519 | 0.195  545 | 1.23  5143 | 57 |
| {BcEV2.BI=TRUE, BcOLV16.BI=FALSE} =>  {BcMV12=FALSE} | 0.15  3465 | 0.939  394 | 0.163  366 | 1.17  497 | 62 |
| {BcEV2.BI=TRUE, BcMV12=FALSE} =>  {BcOLV16.BI=FALSE} | 0.15  3465 | 0.704  545 | 0.217  822 | 1.20  6086 | 62 |
| {BcEV2.BI=TRUE, BcOLV16.BI=FALSE} =>  {BcOLV7.BI=FALSE} | 0.15  8416 | 0.969  697 | 0.163  366 | 1.20  171 | 64 |
| {BcEV2.BI=TRUE, BcOLV7.BI=FALSE} =>  {BcOLV16.BI=FALSE} | 0.15  8416 | 0.735  632 | 0.215  347 | 1.25  9303 | 64 |
| {BcOLV16.BI=FALSE, BcOLV7.BI=FALSE} =>  {BcEV2.BI=TRUE} | 0.15  8416 | 0.309  179 | 0.512  376 | 1.22  459 | 64 |
| {BcEV2.BI=TRUE, BcOLV4.BI=FALSE} => | 0.14 | 0.694 | 0.210 | 1.18 | 59 |

| {BcOLV16.BI=FALSE} | 604 | 118 | 396 | 8235 |  |
| --- | --- | --- | --- | --- | --- |
| {BcEV2.BI=TRUE, BcMCV5.BI=FALSE} =>  {BcOLV16.BI=FALSE} | 0.14  8515 | 0.689  655 | 0.215  347 | 1.18  0596 | 60 |
| {BcEV2.BI=TRUE, BcMCV3.BI=FALSE} =>  {BcOLV16.BI=FALSE} | 0.16  0891 | 0.684  211 | 0.235  149 | 1.17  1276 | 65 |
| {BcEV2.BI=TRUE, BcULV3=TRUE} =>  {BcHV1.BI=TRUE} | 0.11  6337 | 0.734  375 | 0.158  416 | 1.21  5932 | 47 |
| {BcEV2.BI=TRUE, BcHV1.BI=TRUE} =>  {BcULV3=TRUE} | 0.11  6337 | 0.701  493 | 0.165  842 | 1.18  0846 | 47 |
| {BcEV2.BI=TRUE, BcULV3=TRUE} =>  {BcMV11=TRUE} | 0.11  6337 | 0.734  375 | 0.158  416 | 1.19  1516 | 47 |
| {BcEV2.BI=TRUE, BcULV3=TRUE} =>  {BcMV1.BI=TRUE} | 0.14  604 | 0.921  875 | 0.158  416 | 1.16  7516 | 59 |
| {BcEV2.BI=TRUE, BcHV1.BI=TRUE} =>  {BcMV11=TRUE} | 0.12  1287 | 0.731  343 | 0.165  842 | 1.18  6597 | 49 |
| {BcHV1.BI=TRUE, BcMV11=TRUE} =>  {BcEV2.BI=TRUE} | 0.12  1287 | 0.318  182 | 0.381  188 | 1.26  025 | 49 |
| {BcEV2.BI=TRUE, BcMV9.BI=TRUE} =>  {BcHV1.BI=TRUE} | 0.12  3762 | 0.714  286 | 0.173  267 | 1.18  267 | 50 |
| {BcEV2.BI=TRUE, BcULV4=TRUE} =>  {BcMV11=TRUE} | 0.12  1287 | 0.753  846 | 0.160  891 | 1.22  3108 | 49 |
| {BcEV2.BI=TRUE, BcMV6.BI=FALSE} => | 0.13 | 0.726 | 0.180 | 1.17 | 53 |

| {BcMV11=TRUE} | 1188 | 027 | 693 | 7972 |  |
| --- | --- | --- | --- | --- | --- |
| {BcMV11=TRUE, BcMV6.BI=FALSE} =>  {BcEV2.BI=TRUE} | 0.13  1188 | 0.337  58 | 0.388  614 | 1.33  708 | 53 |
| {BcMV11=TRUE, BcRV1.BI=FALSE} =>  {BcEV2.BI=TRUE} | 0.13  3663 | 0.327  273 | 0.408  416 | 1.29  6257 | 54 |
| {BcEV2.BI=TRUE, BcFV5.BI=FALSE} =>  {BcMV11=TRUE} | 0.11  6337 | 0.723  077 | 0.160  891 | 1.17  3185 | 47 |
| {BcEV2.BI=TRUE, BcMV11=TRUE} =>  {BcMV1.BI=TRUE} | 0.16  3366 | 0.88 | 0.185  644 | 1.11  4483 | 66 |
| {BcEV2.BI=TRUE, BcMV1.BI=TRUE} =>  {BcMV11=TRUE} | 0.16  3366 | 0.75 | 0.217  822 | 1.21  6867 | 66 |
| {BcMV1.BI=TRUE, BcMV11=TRUE} =>  {BcEV2.BI=TRUE} | 0.16  3366 | 0.314  286 | 0.519  802 | 1.24  4818 | 66 |
| {BcEV2.BI=TRUE, BcMV12=FALSE} =>  {BcMV11=TRUE} | 0.16  3366 | 0.75 | 0.217  822 | 1.21  6867 | 66 |
| {BcMV11=TRUE, BcMV12=FALSE} =>  {BcEV2.BI=TRUE} | 0.16  3366 | 0.321  951 | 0.507  426 | 1.27  5179 | 66 |
| {BcEV2.BI=TRUE, BcNSRV7.BI=FALSE} =>  {BcMV11=TRUE} | 0.13  1188 | 0.716  216 | 0.183  168 | 1.16  2054 | 53 |
| {BcEV2.BI=TRUE, BcVV3.BI=FALSE} =>  {BcMV11=TRUE} | 0.12  8713 | 0.732  394 | 0.175  743 | 1.18  8303 | 52 |
| {BcEV2.BI=TRUE, BcOLV7.BI=FALSE} => | 0.15 | 0.724 | 0.215 | 1.17 | 63 |

| {BcMV11=TRUE} | 5941 | 138 | 347 | 4907 |  |
| --- | --- | --- | --- | --- | --- |
| {BcMV11=TRUE, BcOLV7.BI=FALSE} =>  {BcEV2.BI=TRUE} | 0.15  5941 | 0.321  429 | 0.485  149 | 1.27  3109 | 63 |
| {BcEV2.BI=TRUE, BvF.BI=FALSE} =>  {BcMV11=TRUE} | 0.16  8317 | 0.781  609 | 0.215  347 | 1.26  8153 | 68 |
| {BcEV2.BI=TRUE, BcOLV13.BI=FALSE} =>  {BcMV11=TRUE} | 0.16  5842 | 0.728  261 | 0.227  723 | 1.18  1596 | 67 |
| {BcEV2.BI=TRUE, BcOLV2.BI=FALSE} =>  {BcMV11=TRUE} | 0.17  3267 | 0.736  842 | 0.235  149 | 1.19  5519 | 70 |
| {BcMV11=TRUE, BcOLV2.BI=FALSE} =>  {BcEV2.BI=TRUE} | 0.17  3267 | 0.304  348 | 0.569  307 | 1.20  5456 | 70 |
| {BcEV2.BI=TRUE, BcMCV3.BI=FALSE} =>  {BcMV11=TRUE} | 0.17  5743 | 0.747  368 | 0.235  149 | 1.21  2598 | 71 |
| {BcEV2.BI=TRUE, BcPV4.BI=FALSE} =>  {BcMV11=TRUE} | 0.17  3267 | 0.736  842 | 0.235  149 | 1.19  5519 | 70 |
| {BcEV2.BI=TRUE, BcEV6=FALSE} =>  {BcMV11=TRUE} | 0.17  3267 | 0.736  842 | 0.235  149 | 1.19  5519 | 70 |
| {BcEV2.BI=TRUE, BcOLV17.BI=FALSE} =>  {BcMV11=TRUE} | 0.16  8317 | 0.723  404 | 0.232  673 | 1.17  3716 | 68 |
| {BcEV2.BI=TRUE, BcOLV15.BI=FALSE} =>  {BcMV11=TRUE} | 0.16  8317 | 0.755  556 | 0.222  772 | 1.22  5881 | 68 |
| {BcEV2.BI=TRUE, BcEV5=FALSE} => | 0.17 | 0.718 | 0.237 | 1.16 | 69 |

| {BcMV11=TRUE} | 0792 | 75 | 624 | 6165 |  |
| --- | --- | --- | --- | --- | --- |
| {BcEV2.BI=TRUE, BcOLV1.BI.MOVA.=FALSE} =>  {BcMV6.BI=FALSE} | 0.13  8614 | 0.811  594 | 0.170  792 | 1.16  6847 | 56 |
| {BcEV2.BI=TRUE, BcMV6.BI=FALSE} =>  {BcOLV1.BI.MOVA.=FALSE} | 0.13  8614 | 0.767  123 | 0.180  693 | 1.14  7844 | 56 |
| {BcEV2.BI=TRUE, BcOLV1.BI.MOVA.=FALSE} =>  {BcRV1.BI=FALSE} | 0.14  3564 | 0.840  58 | 0.170  792 | 1.16  2994 | 58 |
| {BcEV2.BI=TRUE, BcRV1.BI=FALSE} => | 0.14 | 0.763 | 0.188 | 1.14 |  |
|  |  |  |  |  | 58 |
| {BcOLV1.BI.MOVA.=FALSE} | 3564 | 158 | 119 | 191 |  |
| {BcEV2.BI=TRUE, |  |  |  |  |  |
|  | 0.15 | 0.884 | 0.170 | 1.10 |  |
| BcOLV1.BI.MOVA.=FALSE} => |  |  |  |  | 61 |
|  | 099 | 058 | 792 | 5757 |  |
| {BcMV12=FALSE} |  |  |  |  |  |
| {BcEV2.BI=TRUE, |  |  |  |  |  |
|  | 0.15 | 0.927 | 0.170 | 1.14 |  |
| BcOLV1.BI.MOVA.=FALSE} => |  |  |  |  | 64 |
|  | 8416 | 536 | 792 | 9462 |  |
| {BcOLV7.BI=FALSE} |  |  |  |  |  |
| {BcEV2.BI=TRUE, |  |  |  |  |  |
|  | 0.15 | 0.913 | 0.170 | 1.08 |  |
| BcOLV1.BI.MOVA.=FALSE} => |  |  |  |  | 63 |
|  | 5941 | 043 | 792 | 1729 |  |
| {BcDMV1=FALSE} |  |  |  |  |  |
| {BcEV2.BI=TRUE, BcULV4=TRUE} => | 0.12 |  | 0.160 | 1.17 |  |
|  |  | 0.8 |  |  | 52 |
| {BcMV9.BI=TRUE} | 8713 |  | 891 | 9562 |  |

| {BcEV2.BI=TRUE, BcMV9.BI=TRUE} =>  {BcMV1.BI=TRUE} | 0.15  5941 | 0.9 | 0.173  267 | 1.13  9812 | 63 |
| --- | --- | --- | --- | --- | --- |
| {BcEV2.BI=TRUE, BcMV9.BI=TRUE} =>  {BcULV5=FALSE} | 0.16  8317 | 0.971  429 | 0.173  267 | 1.10  8636 | 68 |
| {BcEV2.BI=TRUE, BcULV4=TRUE} =>  {BcMV1.BI=TRUE} | 0.14  3564 | 0.892  308 | 0.160  891 | 1.13  007 | 58 |
| {BcEV2.BI=TRUE, BcMV6.BI=FALSE} =>  {BcMV12=FALSE} | 0.16  0891 | 0.890  411 | 0.180  693 | 1.11  3703 | 65 |
| {BcEV2.BI=TRUE, BcMV6.BI=FALSE} =>  {BcDMV1=FALSE} | 0.16  5842 | 0.917  808 | 0.180  693 | 1.08  7374 | 67 |
| {BcEV2.BI=TRUE, BcPV3.BI=FALSE} =>  {BcRV1.BI=FALSE} | 0.13  8614 | 0.823  529 | 0.168  317 | 1.13  9404 | 56 |
| {BcEV2.BI=TRUE, BcPV3.BI=FALSE} =>  {BcOLV7.BI=FALSE} | 0.15  3465 | 0.911  765 | 0.168  317 | 1.12  9917 | 62 |
| {BcEV2.BI=TRUE, BcRV1.BI=FALSE} =>  {BcOLV7.BI=FALSE} | 0.17  0792 | 0.907  895 | 0.188  119 | 1.12  5121 | 69 |
| {BcEV2.BI=TRUE, BcHV3.BI=FALSE} =>  {BcDMV1=FALSE} | 0.15  099 | 0.924  242 | 0.163  366 | 1.09  4997 | 61 |
| {BcEV2.BI=TRUE, BcFV5.BI=FALSE} =>  {BcDMV1=FALSE} | 0.15  5941 | 0.969  231 | 0.160  891 | 1.14  8297 | 63 |
| {BcEV2.BI=TRUE, BcULV1.BI=FALSE} =>  {BcMV12=FALSE} | 0.17  3267 | 0.886  076 | 0.195  545 | 1.10  8281 | 70 |

| {BcEV2.BI=TRUE, BcULV1.BI=FALSE} =>  {BcOLV7.BI=FALSE} | 0.17  3267 | 0.886  076 | 0.195  545 | 1.09  8082 | 70 |
| --- | --- | --- | --- | --- | --- |
| {BcEV2.BI=TRUE, BcVV3.BI=FALSE} =>  {BcMV1.BI=TRUE} | 0.15  3465 | 0.873  239 | 0.175  743 | 1.10  5921 | 62 |
| {BcEV2.BI=TRUE, BvF.BI=FALSE} =>  {BcMV1.BI=TRUE} | 0.18  8119 | 0.873  563 | 0.215  347 | 1.10  6331 | 76 |
| {BcEV2.BI=TRUE, BcEV6=FALSE} =>  {BcMV1.BI=TRUE} | 0.20  5446 | 0.873  684 | 0.235  149 | 1.10  6484 | 83 |
| {BcEV2.BI=TRUE, BcMV12=FALSE} =>  {BcOLV7.BI=FALSE} | 0.19  802 | 0.909  091 | 0.217  822 | 1.12  6603 | 80 |
| {BcEV2.BI=TRUE, BcOLV7.BI=FALSE} =>  {BcMV12=FALSE} | 0.19  802 | 0.919  54 | 0.215  347 | 1.15  0137 | 80 |
| {BcMV12=FALSE, BcOLV7.BI=FALSE} =>  {BcEV2.BI=TRUE} | 0.19  802 | 0.301  887 | 0.655  941 | 1.19  5708 | 80 |
| {BcEV2.BI=TRUE, BcOLV4.BI=FALSE} =>  {BcMV12=FALSE} | 0.18  5644 | 0.882  353 | 0.210  396 | 1.10  3624 | 75 |
| {BcEV2.BI=TRUE, BcMCV3.BI=FALSE} =>  {BcMV12=FALSE} | 0.20  7921 | 0.884  211 | 0.235  149 | 1.10  5948 | 84 |
| {BcEV2.BI=TRUE, BcOLV4.BI=FALSE} =>  {BcOLV7.BI=FALSE} | 0.18  8119 | 0.894  118 | 0.210  396 | 1.10  8048 | 76 |
| {BcEV2.BI=TRUE, BcMCV5.BI=FALSE} =>  {BcOLV7.BI=FALSE} | 0.19  0594 | 0.885  057 | 0.215  347 | 1.09  682 | 77 |

| {BcALV1.BI=FALSE, BcEV2.BI=TRUE} =>  {BcDMV1=FALSE} | 0.18  0693 | 0.912  5 | 0.198  02 | 1.08  1085 | 73 |
| --- | --- | --- | --- | --- | --- |
| {BcBNV5.BI=FALSE, BcEV2.BI=TRUE} =>  {BcDMV1=FALSE} | 0.20  5446 | 0.954  023 | 0.215  347 | 1.13  0279 | 83 |
| {BcEV2.BI=TRUE, BcMCV5.BI=FALSE} =>  {BcDMV1=FALSE} | 0.19  802 | 0.919  54 | 0.215  347 | 1.08  9426 | 80 |
| {BcEV2.BI=TRUE, BcEV5=FALSE} =>  {BcDMV1=FALSE} | 0.22  0297 | 0.927  083 | 0.237  624 | 1.09  8363 | 89 |
| {BcPV3.BI=TRUE, BcRV1.BI=TRUE} =>  {BcOLV1.BI.MOVA.=TRUE} | 0.08  1683 | 0.647  059 | 0.126  238 | 1.95  0834 | 33 |
| {BcOLV1.BI.MOVA.=TRUE, BcRV1.BI=TRUE}  => {BcPV3.BI=TRUE} | 0.08  1683 | 0.611  111 | 0.133  663 | 2.11  0161 | 33 |
| {BcOLV1.BI.MOVA.=TRUE, BcPV3.BI=TRUE}  => {BcRV1.BI=TRUE} | 0.08  1683 | 0.6 | 0.136  139 | 2.16  4286 | 33 |
| {BcPV3.BI=TRUE, BcRV1.BI=TRUE} =>  {BcMV4.BI=TRUE} | 0.09  6535 | 0.764  706 | 0.126  238 | 1.72  5928 | 39 |
| {BcMV4.BI=TRUE, BcRV1.BI=TRUE} =>  {BcPV3.BI=TRUE} | 0.09  6535 | 0.582  09 | 0.165  842 | 2.00  995 | 39 |
| {BcMV4.BI=TRUE, BcPV3.BI=TRUE} =>  {BcRV1.BI=TRUE} | 0.09  6535 | 0.534  247 | 0.180  693 | 1.92  7104 | 39 |
| {BcPV3.BI=TRUE, BcRV1.BI=TRUE} =>  {BcMV2.BI=TRUE} | 0.09  6535 | 0.764  706 | 0.126  238 | 1.65  2092 | 39 |

| {BcMV2.BI=TRUE, BcRV1.BI=TRUE} =>  {BcPV3.BI=TRUE} | 0.09  6535 | 0.541  667 | 0.178  218 | 1.87  037 | 39 |
| --- | --- | --- | --- | --- | --- |
| {BcMV2.BI=TRUE, BcPV3.BI=TRUE} =>  {BcRV1.BI=TRUE} | 0.09  6535 | 0.541  667 | 0.178  218 | 1.95  3869 | 39 |
| {BcPV3.BI=TRUE, BcRV1.BI=TRUE} =>  {BcOLV18=TRUE} | 0.10  1485 | 0.803  922 | 0.126  238 | 1.55  3992 | 41 |
| {BcOLV18=TRUE, BcRV1.BI=TRUE} =>  {BcPV3.BI=TRUE} | 0.10  1485 | 0.539  474 | 0.188  119 | 1.86  2798 | 41 |
| {BcOLV18=TRUE, BcPV3.BI=TRUE} =>  {BcRV1.BI=TRUE} | 0.10  1485 | 0.539  474 | 0.188  119 | 1.94  5959 | 41 |
| {BcPV3.BI=TRUE, BcRV1.BI=TRUE} =>  {BcHV1SLR.BI=TRUE} | 0.08  9109 | 0.705  882 | 0.126  238 | 1.30  2176 | 36 |
| {BcHV1SLR.BI=TRUE, BcRV1.BI=TRUE} =>  {BcPV3.BI=TRUE} | 0.08  9109 | 0.553  846 | 0.160  891 | 1.91  2426 | 36 |
| {BcHV1SLR.BI=TRUE, BcPV3.BI=TRUE} =>  {BcRV1.BI=TRUE} | 0.08  9109 | 0.529  412 | 0.168  317 | 1.90  9664 | 36 |
| {BcPV3.BI=TRUE, BcRV1.BI=TRUE} =>  {BcMV3.BI=TRUE} | 0.10  1485 | 0.803  922 | 0.126  238 | 1.48  3033 | 41 |
| {BcMV3.BI=TRUE, BcRV1.BI=TRUE} =>  {BcPV3.BI=TRUE} | 0.10  1485 | 0.577  465 | 0.175  743 | 1.99  3981 | 41 |
| {BcMV3.BI=TRUE, BcPV3.BI=TRUE} =>  {BcRV1.BI=TRUE} | 0.10  1485 | 0.561  644 | 0.180  693 | 2.02  593 | 41 |

| {BcPV3.BI=TRUE, BcRV1.BI=TRUE} =>  {BcHV1.BI=TRUE} | 0.11  1386 | 0.882  353 | 0.126  238 | 1.46  0945 | 45 |
| --- | --- | --- | --- | --- | --- |
| {BcHV1.BI=TRUE, BcRV1.BI=TRUE} =>  {BcPV3.BI=TRUE} | 0.11  1386 | 0.592  105 | 0.188  119 | 2.04  4534 | 45 |
| {BcHV1.BI=TRUE, BcPV3.BI=TRUE} =>  {BcRV1.BI=TRUE} | 0.11  1386 | 0.555  556 | 0.200  495 | 2.00  3968 | 45 |
| {BcPV3.BI=TRUE, BcRV1.BI=TRUE} =>  {BcMV11=TRUE} | 0.09  6535 | 0.764  706 | 0.126  238 | 1.24  0728 | 39 |
| {BcMV11=TRUE, BcRV1.BI=TRUE} =>  {BcPV3.BI=TRUE} | 0.09  6535 | 0.464  286 | 0.207  921 | 1.60  3175 | 39 |
| {BcMV11=TRUE, BcPV3.BI=TRUE} =>  {BcRV1.BI=TRUE} | 0.09  6535 | 0.487  5 | 0.198  02 | 1.75  8482 | 39 |
| {BcPV3.BI=TRUE, BcRV1.BI=TRUE} =>  {BcMV9.BI=TRUE} | 0.11  8812 | 0.941  176 | 0.126  238 | 1.38  772 | 48 |
| {BcMV9.BI=TRUE, BcRV1.BI=TRUE} =>  {BcPV3.BI=TRUE} | 0.11  8812 | 0.533  333 | 0.222  772 | 1.84  1595 | 48 |
| {BcMV9.BI=TRUE, BcPV3.BI=TRUE} =>  {BcRV1.BI=TRUE} | 0.11  8812 | 0.578  313 | 0.205  446 | 2.08  6059 | 48 |
| {BcPV3.BI=TRUE, BcRV1.BI=TRUE} =>  {BcULV4=TRUE} | 0.11  6337 | 0.921  569 | 0.126  238 | 1.33  4458 | 47 |
| {BcRV1.BI=TRUE, BcULV4=TRUE} =>  {BcPV3.BI=TRUE} | 0.11  6337 | 0.479  592 | 0.242  574 | 1.65  6027 | 47 |

| {BcPV3.BI=TRUE, BcULV4=TRUE} =>  {BcRV1.BI=TRUE} | 0.11  6337 | 0.566  265 | 0.205  446 | 2.04  2599 | 47 |
| --- | --- | --- | --- | --- | --- |
| {BcEV2.BI=FALSE, BcRV1.BI=TRUE} =>  {BcPV3.BI=TRUE} | 0.09  1584 | 0.430  233 | 0.212  871 | 1.48  5589 | 37 |
| {BcEV2.BI=FALSE, BcPV3.BI=TRUE} =>  {BcRV1.BI=TRUE} | 0.09  1584 | 0.445  783 | 0.205  446 | 1.60  8003 | 37 |
| {BcHV3.BI=FALSE, BcRV1.BI=TRUE} =>  {BcPV3.BI=TRUE} | 0.09  901 | 0.465  116 | 0.212  871 | 1.60  6043 | 40 |
| {BcHV3.BI=FALSE, BcPV3.BI=TRUE} =>  {BcRV1.BI=TRUE} | 0.09  901 | 0.476  19 | 0.207  921 | 1.71  7687 | 40 |
| {BcFV5.BI=FALSE, BcRV1.BI=TRUE} =>  {BcPV3.BI=TRUE} | 0.10  8911 | 0.458  333 | 0.237  624 | 1.58  2621 | 44 |
| {BcFV5.BI=FALSE, BcPV3.BI=TRUE} =>  {BcRV1.BI=TRUE} | 0.10  8911 | 0.463  158 | 0.235  149 | 1.67  0677 | 44 |
| {BcRV1.BI=TRUE, BcULV1.BI=FALSE} =>  {BcPV3.BI=TRUE} | 0.08  1683 | 0.407  407 | 0.200  495 | 1.40  6774 | 33 |
| {BcPV3.BI=TRUE, BcULV1.BI=FALSE} =>  {BcRV1.BI=TRUE} | 0.08  1683 | 0.383  721 | 0.212  871 | 1.38  4136 | 33 |
| {BcMV1.BI=TRUE, BcRV1.BI=TRUE} =>  {BcPV3.BI=TRUE} | 0.10  6436 | 0.524  39 | 0.202  97 | 1.81  0715 | 43 |
| {BcMV1.BI=TRUE, BcPV3.BI=TRUE} =>  {BcRV1.BI=TRUE} | 0.10  6436 | 0.417  476 | 0.254  95 | 1.50  5895 | 43 |

| {BcMV12=FALSE, BcRV1.BI=TRUE} =>  {BcPV3.BI=TRUE} | 0.10  1485 | 0.460  674 | 0.220  297 | 1.59  0704 | 41 |
| --- | --- | --- | --- | --- | --- |
| {BcMV12=FALSE, BcPV3.BI=TRUE} =>  {BcRV1.BI=TRUE} | 0.10  1485 | 0.465  909 | 0.217  822 | 1.68  0601 | 41 |
| {BcPV3.BI=TRUE, BcRV1.BI=TRUE} =>  {BcNSRV7.BI=FALSE} | 0.11  6337 | 0.921  569 | 0.126  238 | 1.15  2674 | 47 |
| {BcNSRV7.BI=FALSE, BcRV1.BI=TRUE} =>  {BcPV3.BI=TRUE} | 0.11  6337 | 0.484  536 | 0.240  099 | 1.67  3099 | 47 |
| {BcNSRV7.BI=FALSE, BcPV3.BI=TRUE} =>  {BcRV1.BI=TRUE} | 0.11  6337 | 0.489  583 | 0.237  624 | 1.76  5997 | 47 |
| {BcRV1.BI=TRUE, BcVV3.BI=FALSE} =>  {BcPV3.BI=TRUE} | 0.10  396 | 0.446  809 | 0.232  673 | 1.54  2826 | 42 |
| {BcPV3.BI=TRUE, BcVV3.BI=FALSE} =>  {BcRV1.BI=TRUE} | 0.10  396 | 0.477  273 | 0.217  822 | 1.72  1591 | 42 |
| {BcALV1.BI=FALSE, BcRV1.BI=TRUE} =>  {BcPV3.BI=TRUE} | 0.08  1683 | 0.428  571 | 0.190  594 | 1.47  9853 | 33 |
| {BcPV3.BI=TRUE, BcRV1.BI=TRUE} =>  {BcDMV1=FALSE} | 0.11  8812 | 0.941  176 | 0.126  238 | 1.11  506 | 48 |
| {BcDMV1=FALSE, BcRV1.BI=TRUE} =>  {BcPV3.BI=TRUE} | 0.11  8812 | 0.461  538 | 0.257  426 | 1.59  3688 | 48 |
| {BcDMV1=FALSE, BcPV3.BI=TRUE} =>  {BcRV1.BI=TRUE} | 0.11  8812 | 0.48 | 0.247  525 | 1.73  1429 | 48 |

| {BcFV9=FALSE, BcRV1.BI=TRUE} =>  {BcPV3.BI=TRUE} | 0.10  6436 | 0.457  447 | 0.232  673 | 1.57  956 | 43 |
| --- | --- | --- | --- | --- | --- |
| {BcFV9=FALSE, BcPV3.BI=TRUE} =>  {BcRV1.BI=TRUE} | 0.10  6436 | 0.434  343 | 0.245  05 | 1.56  6739 | 43 |
| {BcOLV4.BI=FALSE, BcRV1.BI=TRUE} =>  {BcPV3.BI=TRUE} | 0.10  6436 | 0.434  343 | 0.245  05 | 1.49  9784 | 43 |
| {BcOLV4.BI=FALSE, BcPV3.BI=TRUE} =>  {BcRV1.BI=TRUE} | 0.10  6436 | 0.447  917 | 0.237  624 | 1.61  5699 | 43 |
| {BcRV1.BI=TRUE, BcULV5=FALSE} =>  {BcPV3.BI=TRUE} | 0.11  1386 | 0.45 | 0.247  525 | 1.55  3846 | 45 |
| {BcPV3.BI=TRUE, BcULV5=FALSE} =>  {BcRV1.BI=TRUE} | 0.11  1386 | 0.468  75 | 0.237  624 | 1.69  0848 | 45 |
| {BcBNV5.BI=FALSE, BcRV1.BI=TRUE} =>  {BcPV3.BI=TRUE} | 0.11  6337 | 0.447  619 | 0.259  901 | 1.54  5625 | 47 |
| {BcBNV5.BI=FALSE, BcPV3.BI=TRUE} =>  {BcRV1.BI=TRUE} | 0.11  6337 | 0.439  252 | 0.264  851 | 1.58  4446 | 47 |
| {BcRV1.BI=TRUE, BvF.BI=FALSE} =>  {BcPV3.BI=TRUE} | 0.11  8812 | 0.444  444 | 0.267  327 | 1.53  4663 | 48 |
| {BcPV3.BI=TRUE, BvF.BI=FALSE} =>  {BcRV1.BI=TRUE} | 0.11  8812 | 0.444  444 | 0.267  327 | 1.60  3175 | 48 |
| {BcMCV5.BI=FALSE, BcRV1.BI=TRUE} =>  {BcPV3.BI=TRUE} | 0.11  1386 | 0.445  545 | 0.25 | 1.53  8462 | 45 |

| {BcMCV5.BI=FALSE, BcPV3.BI=TRUE} =>  {BcRV1.BI=TRUE} | 0.11  1386 | 0.420  561 | 0.264  851 | 1.51  7023 | 45 |
| --- | --- | --- | --- | --- | --- |
| {BcOLV13.BI=FALSE, BcRV1.BI=TRUE} =>  {BcPV3.BI=TRUE} | 0.09  901 | 0.421  053 | 0.235  149 | 1.45  3891 | 40 |
| {BcOLV13.BI=FALSE, BcPV3.BI=TRUE} =>  {BcRV1.BI=TRUE} | 0.09  901 | 0.4 | 0.247  525 | 1.44  2857 | 40 |
| {BcOLV2.BI=FALSE, BcRV1.BI=TRUE} =>  {BcPV3.BI=TRUE} | 0.10  396 | 0.424  242 | 0.245  05 | 1.46  4905 | 42 |
| {BcOLV2.BI=FALSE, BcPV3.BI=TRUE} =>  {BcRV1.BI=TRUE} | 0.10  396 | 0.396  226 | 0.262  376 | 1.42  9245 | 42 |
| {BcMCV3.BI=FALSE, BcRV1.BI=TRUE} =>  {BcPV3.BI=TRUE} | 0.10  1485 | 0.401  961 | 0.252  475 | 1.38  7967 | 41 |
| {BcMCV3.BI=FALSE, BcPV3.BI=TRUE} =>  {BcRV1.BI=TRUE} | 0.10  1485 | 0.401  961 | 0.252  475 | 1.44  993 | 41 |
| {BcPV4.BI=FALSE, BcRV1.BI=TRUE} =>  {BcPV3.BI=TRUE} | 0.12  1287 | 0.462  264 | 0.262  376 | 1.59  6194 | 49 |
| {BcPV3.BI=TRUE, BcPV4.BI=FALSE} =>  {BcRV1.BI=TRUE} | 0.12  1287 | 0.445  455 | 0.272  277 | 1.60  6818 | 49 |
| {BcEV6=FALSE, BcRV1.BI=TRUE} =>  {BcPV3.BI=TRUE} | 0.12  1287 | 0.453  704 | 0.267  327 | 1.56  6635 | 49 |
| {BcEV6=FALSE, BcPV3.BI=TRUE} =>  {BcRV1.BI=TRUE} | 0.12  1287 | 0.441  441 | 0.274  752 | 1.59  2342 | 49 |

| {BcOLV17.BI=FALSE, BcRV1.BI=TRUE} =>  {BcPV3.BI=TRUE} | 0.10  396 | 0.432  99 | 0.240  099 | 1.49  511 | 42 |
| --- | --- | --- | --- | --- | --- |
| {BcOLV17.BI=FALSE, BcPV3.BI=TRUE} =>  {BcRV1.BI=TRUE} | 0.10  396 | 0.403  846 | 0.257  426 | 1.45  6731 | 42 |
| {BcOLV15.BI=FALSE, BcRV1.BI=TRUE} =>  {BcPV3.BI=TRUE} | 0.12  6238 | 0.467  89 | 0.269  802 | 1.61  562 | 51 |
| {BcOLV15.BI=FALSE, BcPV3.BI=TRUE} =>  {BcRV1.BI=TRUE} | 0.12  6238 | 0.443  478 | 0.284  653 | 1.59  9689 | 51 |
| {BcEV5=FALSE, BcRV1.BI=TRUE} =>  {BcPV3.BI=TRUE} | 0.12  6238 | 0.455  357 | 0.277  228 | 1.57  2344 | 51 |
| {BcEV5=FALSE, BcPV3.BI=TRUE} =>  {BcRV1.BI=TRUE} | 0.12  6238 | 0.455  357 | 0.277  228 | 1.64  2538 | 51 |
| {BcMV6.BI=TRUE, BcRV1.BI=TRUE} =>  {BcOLV1.BI.MOVA.=TRUE} | 0.07  9208 | 0.695  652 | 0.113  861 | 2.09  7339 | 32 |
| {BcOLV1.BI.MOVA.=TRUE, BcRV1.BI=TRUE}  => {BcMV6.BI=TRUE} | 0.07  9208 | 0.592  593 | 0.133  663 | 1.94  6402 | 32 |
| {BcMV6.BI=TRUE, BcOLV1.BI.MOVA.=TRUE}  => {BcRV1.BI=TRUE} | 0.07  9208 | 0.484  848 | 0.163  366 | 1.74  8918 | 32 |
| {BcMV6.BI=TRUE, BcRV1.BI=TRUE} =>  {BcOLV16.BI=TRUE} | 0.08  4158 | 0.739  13 | 0.113  861 | 1.77  7433 | 34 |
| {BcOLV16.BI=TRUE, BcRV1.BI=TRUE} =>  {BcMV6.BI=TRUE} | 0.08  4158 | 0.557  377 | 0.150  99 | 1.83  0734 | 34 |

| {BcMV6.BI=TRUE, BcOLV16.BI=TRUE} =>  {BcRV1.BI=TRUE} | 0.08  4158 | 0.507  463 | 0.165  842 | 1.83  049 | 34 |
| --- | --- | --- | --- | --- | --- |
| {BcMV6.BI=TRUE, BcRV1.BI=TRUE} =>  {BcMV4.BI=TRUE} | 0.08  1683 | 0.717  391 | 0.113  861 | 1.61  914 | 33 |
| {BcMV4.BI=TRUE, BcRV1.BI=TRUE} =>  {BcMV6.BI=TRUE} | 0.08  1683 | 0.492  537 | 0.165  842 | 1.61  7765 | 33 |
| {BcMV4.BI=TRUE, BcMV6.BI=TRUE} =>  {BcRV1.BI=TRUE} | 0.08  1683 | 0.464  789 | 0.175  743 | 1.67  6559 | 33 |
| {BcMV6.BI=TRUE, BcRV1.BI=TRUE} =>  {BcMV2.BI=TRUE} | 0.09  4059 | 0.826  087 | 0.113  861 | 1.78  4701 | 38 |
| {BcMV2.BI=TRUE, BcRV1.BI=TRUE} =>  {BcMV6.BI=TRUE} | 0.09  4059 | 0.527  778 | 0.178  218 | 1.73  3514 | 38 |
| {BcMV2.BI=TRUE, BcMV6.BI=TRUE} =>  {BcRV1.BI=TRUE} | 0.09  4059 | 0.452  381 | 0.207  921 | 1.63  1803 | 38 |
| {BcMV6.BI=TRUE, BcRV1.BI=TRUE} =>  {BcOLV18=TRUE} | 0.09  6535 | 0.847  826 | 0.113  861 | 1.63  886 | 39 |
| {BcOLV18=TRUE, BcRV1.BI=TRUE} =>  {BcMV6.BI=TRUE} | 0.09  6535 | 0.513  158 | 0.188  119 | 1.68  5494 | 39 |
| {BcMV6.BI=TRUE, BcOLV18=TRUE} =>  {BcRV1.BI=TRUE} | 0.09  6535 | 0.557  143 | 0.173  267 | 2.00  9694 | 39 |
| {BcMV6.BI=TRUE, BcRV1.BI=TRUE} =>  {BcHV1SLR.BI=TRUE} | 0.09  1584 | 0.804  348 | 0.113  861 | 1.48  382 | 37 |

| {BcHV1SLR.BI=TRUE, BcRV1.BI=TRUE} =>  {BcMV6.BI=TRUE} | 0.09  1584 | 0.569  231 | 0.160  891 | 1.86  9669 | 37 |
| --- | --- | --- | --- | --- | --- |
| {BcHV1SLR.BI=TRUE, BcMV6.BI=TRUE} =>  {BcRV1.BI=TRUE} | 0.09  1584 | 0.445  783 | 0.205  446 | 1.60  8003 | 37 |
| {BcMV6.BI=TRUE, BcRV1.BI=TRUE} =>  {BcMV3.BI=TRUE} | 0.09  6535 | 0.847  826 | 0.113  861 | 1.56  4026 | 39 |
| {BcMV3.BI=TRUE, BcRV1.BI=TRUE} =>  {BcMV6.BI=TRUE} | 0.09  6535 | 0.549  296 | 0.175  743 | 1.80  4191 | 39 |
| {BcMV3.BI=TRUE, BcMV6.BI=TRUE} =>  {BcRV1.BI=TRUE} | 0.09  6535 | 0.433  333 | 0.222  772 | 1.56  3095 | 39 |
| {BcMV6.BI=TRUE, BcRV1.BI=TRUE} =>  {BcULV3=TRUE} | 0.08  1683 | 0.717  391 | 0.113  861 | 1.20  7609 | 33 |
| {BcRV1.BI=TRUE, BcULV3=TRUE} =>  {BcMV6.BI=TRUE} | 0.08  1683 | 0.507  692 | 0.160  891 | 1.66  7542 | 33 |
| {BcMV6.BI=TRUE, BcULV3=TRUE} =>  {BcRV1.BI=TRUE} | 0.08  1683 | 0.379  31 | 0.215  347 | 1.36  8227 | 33 |
| {BcMV6.BI=TRUE, BcRV1.BI=TRUE} =>  {BcHV1.BI=TRUE} | 0.10  1485 | 0.891  304 | 0.113  861 | 1.47  5766 | 41 |
| {BcHV1.BI=TRUE, BcRV1.BI=TRUE} =>  {BcMV6.BI=TRUE} | 0.10  1485 | 0.539  474 | 0.188  119 | 1.77  193 | 41 |
| {BcHV1.BI=TRUE, BcMV6.BI=TRUE} =>  {BcRV1.BI=TRUE} | 0.10  1485 | 0.445  652 | 0.227  723 | 1.60  7531 | 41 |

| {BcMV6.BI=TRUE, BcRV1.BI=TRUE} =>  {BcMV11=TRUE} | 0.10  396 | 0.913  043 | 0.113  861 | 1.48  1404 | 42 |
| --- | --- | --- | --- | --- | --- |
| {BcMV11=TRUE, BcRV1.BI=TRUE} =>  {BcMV6.BI=TRUE} | 0.10  396 | 0.5 | 0.207  921 | 1.64  2276 | 42 |
| {BcMV11=TRUE, BcMV6.BI=TRUE} =>  {BcRV1.BI=TRUE} | 0.10  396 | 0.456  522 | 0.227  723 | 1.64  6739 | 42 |
| {BcMV6.BI=TRUE, BcRV1.BI=TRUE} =>  {BcMV9.BI=TRUE} | 0.10  6436 | 0.934  783 | 0.113  861 | 1.37  8293 | 43 |
| {BcMV9.BI=TRUE, BcRV1.BI=TRUE} =>  {BcMV6.BI=TRUE} | 0.10  6436 | 0.477  778 | 0.222  772 | 1.56  9286 | 43 |
| {BcMV6.BI=TRUE, BcMV9.BI=TRUE} =>  {BcRV1.BI=TRUE} | 0.10  6436 | 0.452  632 | 0.235  149 | 1.63  2707 | 43 |
| {BcMV6.BI=TRUE, BcRV1.BI=TRUE} =>  {BcULV4=TRUE} | 0.11  1386 | 0.978  261 | 0.113  861 | 1.41  655 | 45 |
| {BcRV1.BI=TRUE, BcULV4=TRUE} =>  {BcMV6.BI=TRUE} | 0.11  1386 | 0.459  184 | 0.242  574 | 1.50  8213 | 45 |
| {BcMV6.BI=TRUE, BcULV4=TRUE} =>  {BcRV1.BI=TRUE} | 0.11  1386 | 0.459  184 | 0.242  574 | 1.65  6341 | 45 |
| {BcEV2.BI=FALSE, BcRV1.BI=TRUE} =>  {BcMV6.BI=TRUE} | 0.08  6634 | 0.406  977 | 0.212  871 | 1.33  6737 | 35 |
| {BcEV2.BI=FALSE, BcMV6.BI=TRUE} =>  {BcRV1.BI=TRUE} | 0.08  6634 | 0.372  34 | 0.232  673 | 1.34  3085 | 35 |

| {BcHV3.BI=FALSE, BcRV1.BI=TRUE} =>  {BcMV6.BI=TRUE} | 0.08  9109 | 0.418  605 | 0.212  871 | 1.37  4929 | 36 |
| --- | --- | --- | --- | --- | --- |
| {BcHV3.BI=FALSE, BcMV6.BI=TRUE} =>  {BcRV1.BI=TRUE} | 0.08  9109 | 0.404  494 | 0.220  297 | 1.45  9069 | 36 |
| {BcFV5.BI=FALSE, BcRV1.BI=TRUE} =>  {BcMV6.BI=TRUE} | 0.09  6535 | 0.406  25 | 0.237  624 | 1.33  435 | 39 |
| {BcFV5.BI=FALSE, BcMV6.BI=TRUE} =>  {BcRV1.BI=TRUE} | 0.09  6535 | 0.406  25 | 0.237  624 | 1.46  5402 | 39 |
| {BcMV1.BI=TRUE, BcRV1.BI=TRUE} =>  {BcMV6.BI=TRUE} | 0.09  1584 | 0.451  22 | 0.202  97 | 1.48  2054 | 37 |
| {BcMV1.BI=TRUE, BcMV6.BI=TRUE} =>  {BcRV1.BI=TRUE} | 0.09  1584 | 0.359  223 | 0.254  95 | 1.29  577 | 37 |
| {BcMV12=FALSE, BcMV6.BI=TRUE} =>  {BcRV1.BI=TRUE} | 0.07  9208 | 0.363  636 | 0.217  822 | 1.31  1688 | 32 |
| {BcNSRV7.BI=FALSE, BcRV1.BI=TRUE} =>  {BcMV6.BI=TRUE} | 0.09  4059 | 0.391  753 | 0.240  099 | 1.28  6732 | 38 |
| {BcMV6.BI=TRUE, BcNSRV7.BI=FALSE} =>  {BcRV1.BI=TRUE} | 0.09  4059 | 0.404  255 | 0.232  673 | 1.45  8207 | 38 |
| {BcRV1.BI=TRUE, BcVV3.BI=FALSE} =>  {BcMV6.BI=TRUE} | 0.10  1485 | 0.436  17 | 0.232  673 | 1.43  2624 | 41 |
| {BcMV6.BI=TRUE, BcVV3.BI=FALSE} =>  {BcRV1.BI=TRUE} | 0.10  1485 | 0.440  86 | 0.230  198 | 1.59  0246 | 41 |

| {BcDMV1=FALSE, BcRV1.BI=TRUE} =>  {BcMV6.BI=TRUE} | 0.10  396 | 0.403  846 | 0.257  426 | 1.32  6454 | 42 |
| --- | --- | --- | --- | --- | --- |
| {BcDMV1=FALSE, BcMV6.BI=TRUE} =>  {BcRV1.BI=TRUE} | 0.10  396 | 0.437  5 | 0.237  624 | 1.57  8125 | 42 |
| {BcFV9=FALSE, BcRV1.BI=TRUE} =>  {BcMV6.BI=TRUE} | 0.09  4059 | 0.404  255 | 0.232  673 | 1.32  7798 | 38 |
| {BcOLV4.BI=FALSE, BcRV1.BI=TRUE} =>  {BcMV6.BI=TRUE} | 0.09  6535 | 0.393  939 | 0.245  05 | 1.29  3915 | 39 |
| {BcMV6.BI=TRUE, BcOLV4.BI=FALSE} =>  {BcRV1.BI=TRUE} | 0.09  6535 | 0.371  429 | 0.259  901 | 1.33  9796 | 39 |
| {BcRV1.BI=TRUE, BcULV5=FALSE} =>  {BcMV6.BI=TRUE} | 0.10  1485 | 0.41 | 0.247  525 | 1.34  6667 | 41 |
| {BcMV6.BI=TRUE, BcULV5=FALSE} =>  {BcRV1.BI=TRUE} | 0.10  1485 | 0.398  058 | 0.254  95 | 1.43  5853 | 41 |
| {BcBNV5.BI=FALSE, BcMV6.BI=TRUE} =>  {BcRV1.BI=TRUE} | 0.09  6535 | 0.393  939 | 0.245  05 | 1.42  0996 | 39 |
| {BcRV1.BI=TRUE, BvF.BI=FALSE} =>  {BcMV6.BI=TRUE} | 0.10  8911 | 0.407  407 | 0.267  327 | 1.33  8151 | 44 |
| {BcMV6.BI=TRUE, BvF.BI=FALSE} =>  {BcRV1.BI=TRUE} | 0.10  8911 | 0.376  068 | 0.289  604 | 1.35  6532 | 44 |
| {BcMCV5.BI=FALSE, BcRV1.BI=TRUE} =>  {BcMV6.BI=TRUE} | 0.10  1485 | 0.405  941 | 0.25 | 1.33  3333 | 41 |

| {BcMCV5.BI=FALSE, BcMV6.BI=TRUE} =>  {BcRV1.BI=TRUE} | 0.10  1485 | 0.390  476 | 0.259  901 | 1.40  8503 | 41 |
| --- | --- | --- | --- | --- | --- |
| {BcOLV13.BI=FALSE, BcRV1.BI=TRUE} =>  {BcMV6.BI=TRUE} | 0.09  1584 | 0.389  474 | 0.235  149 | 1.27  9247 | 37 |
| {BcPV4.BI=FALSE, BcRV1.BI=TRUE} =>  {BcMV6.BI=TRUE} | 0.10  396 | 0.396  226 | 0.262  376 | 1.30  1427 | 42 |
| {BcMV6.BI=TRUE, BcPV4.BI=FALSE} =>  {BcRV1.BI=TRUE} | 0.10  396 | 0.375 | 0.277  228 | 1.35  2679 | 42 |
| {BcEV6=FALSE, BcRV1.BI=TRUE} =>  {BcMV6.BI=TRUE} | 0.10  6436 | 0.398  148 | 0.267  327 | 1.30  7739 | 43 |
| {BcEV6=FALSE, BcMV6.BI=TRUE} =>  {BcRV1.BI=TRUE} | 0.10  6436 | 0.377  193 | 0.282  178 | 1.36  0589 | 43 |
| {BcOLV15.BI=FALSE, BcRV1.BI=TRUE} =>  {BcMV6.BI=TRUE} | 0.11  1386 | 0.412  844 | 0.269  802 | 1.35  6008 | 45 |
| {BcMV6.BI=TRUE, BcOLV15.BI=FALSE} =>  {BcRV1.BI=TRUE} | 0.11  1386 | 0.371  901 | 0.299  505 | 1.34  1499 | 45 |
| {BcEV5=FALSE, BcRV1.BI=TRUE} =>  {BcMV6.BI=TRUE} | 0.11  3861 | 0.410  714 | 0.277  228 | 1.34  9013 | 46 |
| {BcEV5=FALSE, BcMV6.BI=TRUE} =>  {BcRV1.BI=TRUE} | 0.11  3861 | 0.429  907 | 0.264  851 | 1.55  0734 | 46 |
| {BcOLV1.BI.MOVA.=TRUE, BcRV1.BI=TRUE}  => {BcOLV16.BI=TRUE} | 0.08  6634 | 0.648  148 | 0.133  663 | 1.55  8642 | 35 |

| {BcOLV16.BI=TRUE, BcRV1.BI=TRUE} =>  {BcOLV1.BI.MOVA.=TRUE} | 0.08  6634 | 0.573  77 | 0.150  99 | 1.72  9875 | 35 |
| --- | --- | --- | --- | --- | --- |
| {BcOLV1.BI.MOVA.=TRUE,  BcOLV16.BI=TRUE} => {BcRV1.BI=TRUE} | 0.08  6634 | 0.454  545 | 0.190  594 | 1.63  961 | 35 |
| {BcOLV1.BI.MOVA.=TRUE, BcRV1.BI=TRUE}  => {BcMV4.BI=TRUE} | 0.09  4059 | 0.703  704 | 0.133  663 | 1.58  8247 | 38 |
| {BcMV4.BI=TRUE, BcRV1.BI=TRUE} =>  {BcOLV1.BI.MOVA.=TRUE} | 0.09  4059 | 0.567  164 | 0.165  842 | 1.70  9958 | 38 |
| {BcMV4.BI=TRUE, BcOLV1.BI.MOVA.=TRUE}  => {BcRV1.BI=TRUE} | 0.09  4059 | 0.506  667 | 0.185  644 | 1.82  7619 | 38 |
| {BcOLV1.BI.MOVA.=TRUE, BcRV1.BI=TRUE}  => {BcMV2.BI=TRUE} | 0.10  6436 | 0.796  296 | 0.133  663 | 1.72  0341 | 43 |
| {BcMV2.BI=TRUE, BcRV1.BI=TRUE} =>  {BcOLV1.BI.MOVA.=TRUE} | 0.10  6436 | 0.597  222 | 0.178  218 | 1.80  058 | 43 |
| {BcMV2.BI=TRUE, BcOLV1.BI.MOVA.=TRUE}  => {BcRV1.BI=TRUE} | 0.10  6436 | 0.425  743 | 0.25 | 1.53  5714 | 43 |
| {BcOLV1.BI.MOVA.=TRUE, BcRV1.BI=TRUE}  => {BcOLV18=TRUE} | 0.12  1287 | 0.907  407 | 0.133  663 | 1.75  4032 | 49 |
| {BcOLV18=TRUE, BcRV1.BI=TRUE} =>  {BcOLV1.BI.MOVA.=TRUE} | 0.12  1287 | 0.644  737 | 0.188  119 | 1.94  3833 | 49 |
| {BcOLV1.BI.MOVA.=TRUE, BcOLV18=TRUE}  => {BcRV1.BI=TRUE} | 0.12  1287 | 0.526  882 | 0.230  198 | 1.90  0538 | 49 |

| {BcOLV1.BI.MOVA.=TRUE, BcRV1.BI=TRUE}  => {BcHV1SLR.BI=TRUE} | 0.09  4059 | 0.703  704 | 0.133  663 | 1.29  8157 | 38 |
| --- | --- | --- | --- | --- | --- |
| {BcHV1SLR.BI=TRUE, BcRV1.BI=TRUE} =>  {BcOLV1.BI.MOVA.=TRUE} | 0.09  4059 | 0.584  615 | 0.160  891 | 1.76  2572 | 38 |
| {BcHV1SLR.BI=TRUE, BcOLV1.BI.MOVA.=TRUE} =>  {BcRV1.BI=TRUE} | 0.09  4059 | 0.469  136 | 0.200  495 | 1.69  224 | 38 |
| {BcOLV1.BI.MOVA.=TRUE, BcRV1.BI=TRUE}  => {BcMV3.BI=TRUE} | 0.10  6436 | 0.796  296 | 0.133  663 | 1.46  8967 | 43 |
| {BcMV3.BI=TRUE, BcRV1.BI=TRUE} =>  {BcOLV1.BI.MOVA.=TRUE} | 0.10  6436 | 0.605  634 | 0.175  743 | 1.82  5941 | 43 |
| {BcMV3.BI=TRUE, BcOLV1.BI.MOVA.=TRUE}  => {BcRV1.BI=TRUE} | 0.10  6436 | 0.434  343 | 0.245  05 | 1.56  6739 | 43 |
| {BcOLV1.BI.MOVA.=TRUE, BcRV1.BI=TRUE}  => {BcHV1.BI=TRUE} | 0.10  8911 | 0.814  815 | 0.133  663 | 1.34  912 | 44 |
| {BcHV1.BI=TRUE, BcRV1.BI=TRUE} =>  {BcOLV1.BI.MOVA.=TRUE} | 0.10  8911 | 0.578  947 | 0.188  119 | 1.74  5483 | 44 |
| {BcHV1.BI=TRUE, BcOLV1.BI.MOVA.=TRUE}  => {BcRV1.BI=TRUE} | 0.10  8911 | 0.511  628 | 0.212  871 | 1.84  5515 | 44 |
| {BcOLV1.BI.MOVA.=TRUE, BcRV1.BI=TRUE}  => {BcMV11=TRUE} | 0.11  1386 | 0.833  333 | 0.133  663 | 1.35  2075 | 45 |
| {BcMV11=TRUE, BcRV1.BI=TRUE} => | 0.11 | 0.535 | 0.207 | 1.61 | 45 |

| {BcOLV1.BI.MOVA.=TRUE} | 1386 | 714 | 921 | 5139 |  |
| --- | --- | --- | --- | --- | --- |
| {BcMV11=TRUE, BcOLV1.BI.MOVA.=TRUE} | 0.11 | 0.459 | 0.242 | 1.65 |  |
|  |  |  |  |  | 45 |
| => {BcRV1.BI=TRUE} | 1386 | 184 | 574 | 6341 |  |
| {BcOLV1.BI.MOVA.=TRUE, BcRV1.BI=TRUE} | 0.11 | 0.870 | 0.133 | 1.28 |  |
|  |  |  |  |  | 47 |
| => {BcMV9.BI=TRUE} | 6337 | 37 | 663 | 332 |  |
| {BcMV9.BI=TRUE, BcRV1.BI=TRUE} => | 0.11 | 0.522 | 0.222 | 1.57 |  |
|  |  |  |  |  | 47 |
| {BcOLV1.BI.MOVA.=TRUE} | 6337 | 222 | 772 | 4461 |  |
| {BcMV9.BI=TRUE, BcOLV1.BI.MOVA.=TRUE} | 0.11 | 0.546 | 0.212 | 1.97 |  |
|  |  |  |  |  | 47 |
| => {BcRV1.BI=TRUE} | 6337 | 512 | 871 | 1346 |  |
| {BcOLV1.BI.MOVA.=TRUE, BcRV1.BI=TRUE} | 0.12 | 0.944 | 0.133 | 1.36 |  |
|  |  |  |  |  | 51 |
| => {BcULV4=TRUE} | 6238 | 444 | 663 | 7583 |  |
| {BcRV1.BI=TRUE, BcULV4=TRUE} => | 0.12 | 0.520 | 0.242 | 1.56 |  |
|  |  |  |  |  | 51 |
| {BcOLV1.BI.MOVA.=TRUE} | 6238 | 408 | 574 | 8992 |  |
| {BcOLV1.BI.MOVA.=TRUE, BcULV4=TRUE} | 0.12 | 0.515 | 0.245 | 1.85 |  |
|  |  |  |  |  | 51 |
| => {BcRV1.BI=TRUE} | 6238 | 152 | 05 | 8225 |  |
| {BcEV2.BI=FALSE, BcRV1.BI=TRUE} => | 0.09 | 0.453 | 0.212 | 1.36 |  |
|  |  |  |  |  | 39 |
| {BcOLV1.BI.MOVA.=TRUE} | 6535 | 488 | 871 | 7234 |  |
| {BcEV2.BI=FALSE, |  |  |  |  |  |
|  | 0.09 | 0.386 |  | 1.39 |  |
| BcOLV1.BI.MOVA.=TRUE} => |  |  | 0.25 |  | 39 |
|  | 6535 | 139 |  | 2857 |  |
| {BcRV1.BI=TRUE} |  |  |  |  |  |
| {BcHV3.BI=FALSE, BcRV1.BI=TRUE} => | 0.09 | 0.465 | 0.212 | 1.40 |  |
|  |  |  |  |  | 40 |
| {BcOLV1.BI.MOVA.=TRUE} | 901 | 116 | 871 | 2291 |  |

| {BcHV3.BI=FALSE, BcOLV1.BI.MOVA.=TRUE} =>  {BcRV1.BI=TRUE} | 0.09  901 | 0.434  783 | 0.227  723 | 1.56  8323 | 40 |
| --- | --- | --- | --- | --- | --- |
| {BcFV5.BI=FALSE, BcRV1.BI=TRUE} =>  {BcOLV1.BI.MOVA.=TRUE} | 0.11  3861 | 0.479  167 | 0.237  624 | 1.44  4652 | 46 |
| {BcFV5.BI=FALSE, BcOLV1.BI.MOVA.=TRUE} =>  {BcRV1.BI=TRUE} | 0.11  3861 | 0.474  227 | 0.240  099 | 1.71  0604 | 46 |
| {BcOLV1.BI.MOVA.=TRUE,  BcULV1.BI=FALSE} => {BcRV1.BI=TRUE} | 0.08  1683 | 0.362  637 | 0.225  248 | 1.30  8085 | 33 |
| {BcMV1.BI=TRUE, BcRV1.BI=TRUE} =>  {BcOLV1.BI.MOVA.=TRUE} | 0.11  3861 | 0.560  976 | 0.202  97 | 1.69  13 | 46 |
| {BcMV1.BI=TRUE, BcOLV1.BI.MOVA.=TRUE}  => {BcRV1.BI=TRUE} | 0.11  3861 | 0.386  555 | 0.294  554 | 1.39  4358 | 46 |
| {BcMV12=FALSE, BcRV1.BI=TRUE} =>  {BcOLV1.BI.MOVA.=TRUE} | 0.09  6535 | 0.438  202 | 0.220  297 | 1.32  1147 | 39 |
| {BcMV12=FALSE, BcOLV1.BI.MOVA.=TRUE}  => {BcRV1.BI=TRUE} | 0.09  6535 | 0.419  355 | 0.230  198 | 1.51  2673 | 39 |
| {BcNSRV7.BI=FALSE, BcRV1.BI=TRUE} =>  {BcOLV1.BI.MOVA.=TRUE} | 0.11  6337 | 0.484  536 | 0.240  099 | 1.46  084 | 47 |
| {BcNSRV7.BI=FALSE,  BcOLV1.BI.MOVA.=TRUE} => | 0.11  6337 | 0.439  252 | 0.264  851 | 1.58  4446 | 47 |

| {BcRV1.BI=TRUE} |  |  |  |  |  |
| --- | --- | --- | --- | --- | --- |
| {BcRV1.BI=TRUE, BcVV3.BI=FALSE} =>  {BcOLV1.BI.MOVA.=TRUE} | 0.11  1386 | 0.478  723 | 0.232  673 | 1.44  3315 | 45 |
| {BcOLV1.BI.MOVA.=TRUE,  BcVV3.BI=FALSE} => {BcRV1.BI=TRUE} | 0.11  1386 | 0.459  184 | 0.242  574 | 1.65  6341 | 45 |
| {BcOLV1.BI.MOVA.=TRUE, BcRV1.BI=TRUE}  => {BcDMV1=FALSE} | 0.12  6238 | 0.944  444 | 0.133  663 | 1.11  8931 | 51 |
| {BcDMV1=FALSE, BcRV1.BI=TRUE} =>  {BcOLV1.BI.MOVA.=TRUE} | 0.12  6238 | 0.490  385 | 0.257  426 | 1.47  8473 | 51 |
| {BcDMV1=FALSE, BcOLV1.BI.MOVA.=TRUE}  => {BcRV1.BI=TRUE} | 0.12  6238 | 0.459  459 | 0.274  752 | 1.65  7336 | 51 |
| {BcFV9=FALSE, BcRV1.BI=TRUE} =>  {BcOLV1.BI.MOVA.=TRUE} | 0.09  901 | 0.425  532 | 0.232  673 | 1.28  2947 | 40 |
| {BcFV9=FALSE, BcOLV1.BI.MOVA.=TRUE}  => {BcRV1.BI=TRUE} | 0.09  901 | 0.353  982 | 0.279  703 | 1.27  6865 | 40 |
| {BcOLV4.BI=FALSE, BcRV1.BI=TRUE} =>  {BcOLV1.BI.MOVA.=TRUE} | 0.10  8911 | 0.444  444 | 0.245  05 | 1.33  9967 | 44 |
| {BcOLV1.BI.MOVA.=TRUE,  BcOLV4.BI=FALSE} => {BcRV1.BI=TRUE} | 0.10  8911 | 0.415  094 | 0.262  376 | 1.49  7305 | 44 |
| {BcRV1.BI=TRUE, BcULV5=FALSE} =>  {BcOLV1.BI.MOVA.=TRUE} | 0.11  6337 | 0.47 | 0.247  525 | 1.41  7015 | 47 |
| {BcOLV1.BI.MOVA.=TRUE, BcULV5=FALSE} | 0.11 | 0.451 | 0.257 | 1.63 | 47 |

| => {BcRV1.BI=TRUE} | 6337 | 923 | 426 | 0151 |  |
| --- | --- | --- | --- | --- | --- |
| {BcBNV5.BI=FALSE, BcRV1.BI=TRUE} =>  {BcOLV1.BI.MOVA.=TRUE} | 0.12  3762 | 0.476  19 | 0.259  901 | 1.43  5679 | 50 |
| {BcBNV5.BI=FALSE, |  |  |  |  |  |
|  | 0.12 | 0.438 | 0.282 | 1.58 |  |
| BcOLV1.BI.MOVA.=TRUE} => |  |  |  |  | 50 |
|  | 3762 | 596 | 178 | 208 |  |
| {BcRV1.BI=TRUE} |  |  |  |  |  |
| {BcRV1.BI=TRUE, BvF.BI=FALSE} => | 0.12 | 0.472 | 0.267 | 1.42 |  |
|  |  |  |  |  | 51 |
| {BcOLV1.BI.MOVA.=TRUE} | 6238 | 222 | 327 | 3715 |  |
| {BcOLV1.BI.MOVA.=TRUE, BvF.BI=FALSE} | 0.12 | 0.401 | 0.314 | 1.44 |  |
|  |  |  |  |  | 51 |
| => {BcRV1.BI=TRUE} | 6238 | 575 | 356 | 8538 |  |
| {BcMCV5.BI=FALSE, BcRV1.BI=TRUE} => | 0.11 | 0.465 |  | 1.40 |  |
|  |  |  | 0.25 |  | 47 |
| {BcOLV1.BI.MOVA.=TRUE} | 6337 | 347 |  | 2985 |  |
| {BcMCV5.BI=FALSE, |  |  |  |  |  |
|  | 0.11 | 0.419 | 0.277 | 1.51 |  |
| BcOLV1.BI.MOVA.=TRUE} => |  |  |  |  | 47 |
|  | 6337 | 643 | 228 | 3712 |  |
| {BcRV1.BI=TRUE} |  |  |  |  |  |
| {BcOLV13.BI=FALSE, BcRV1.BI=TRUE} => | 0.10 | 0.431 | 0.235 | 1.30 |  |
|  |  |  |  |  | 41 |
| {BcOLV1.BI.MOVA.=TRUE} | 1485 | 579 | 149 | 1178 |  |
| {BcOLV1.BI.MOVA.=TRUE, | 0.10 | 0.366 | 0.277 | 1.32 |  |
|  |  |  |  |  | 41 |
| BcOLV13.BI=FALSE} => {BcRV1.BI=TRUE} | 1485 | 071 | 228 | 0472 |  |
| {BcOLV2.BI=FALSE, BcRV1.BI=TRUE} => | 0.10 | 0.424 | 0.245 | 1.27 |  |
|  |  |  |  |  | 42 |
| {BcOLV1.BI.MOVA.=TRUE} | 396 | 242 | 05 | 9059 |  |
| {BcOLV1.BI.MOVA.=TRUE, | 0.10 | 0.375 | 0.277 | 1.35 | 42 |

| BcOLV2.BI=FALSE} => {BcRV1.BI=TRUE} | 396 |  | 228 | 2679 |  |
| --- | --- | --- | --- | --- | --- |
| {BcMCV3.BI=FALSE, BcRV1.BI=TRUE} => | 0.11 | 0.450 | 0.252 | 1.35 |  |
|  |  |  |  |  | 46 |
| {BcOLV1.BI.MOVA.=TRUE} | 3861 | 98 | 475 | 9672 |  |
| {BcMCV3.BI=FALSE, |  |  |  |  |  |
|  | 0.11 | 0.396 | 0.287 | 1.43 |  |
| BcOLV1.BI.MOVA.=TRUE} => |  |  |  |  | 46 |
|  | 3861 | 552 | 129 | 0419 |  |
| {BcRV1.BI=TRUE} |  |  |  |  |  |
| {BcPV4.BI=FALSE, BcRV1.BI=TRUE} => | 0.12 | 0.481 | 0.262 | 1.45 |  |
|  |  |  |  |  | 51 |
| {BcOLV1.BI.MOVA.=TRUE} | 6238 | 132 | 376 | 0577 |  |
| {BcOLV1.BI.MOVA.=TRUE, | 0.12 | 0.398 | 0.316 | 1.43 |  |
|  |  |  |  |  | 51 |
| BcPV4.BI=FALSE} => {BcRV1.BI=TRUE} | 6238 | 438 | 832 | 7221 |  |
| {BcEV6=FALSE, BcRV1.BI=TRUE} => | 0.12 | 0.462 | 0.267 | 1.39 |  |
|  |  |  |  |  | 50 |
| {BcOLV1.BI.MOVA.=TRUE} | 3762 | 963 | 327 | 5799 |  |
| {BcEV6=FALSE, BcOLV1.BI.MOVA.=TRUE} | 0.12 | 0.403 | 0.306 | 1.45 |  |
|  |  |  |  |  | 50 |
| => {BcRV1.BI=TRUE} | 3762 | 226 | 931 | 4493 |  |
| {BcOLV17.BI=FALSE, BcRV1.BI=TRUE} => | 0.10 | 0.422 | 0.240 | 1.27 |  |
|  |  |  |  |  | 41 |
| {BcOLV1.BI.MOVA.=TRUE} | 1485 | 68 | 099 | 435 |  |
| {BcOLV15.BI=FALSE, BcRV1.BI=TRUE} => | 0.12 | 0.467 | 0.269 | 1.41 |  |
|  |  |  |  |  | 51 |
| {BcOLV1.BI.MOVA.=TRUE} | 6238 | 89 | 802 | 0653 |  |
| {BcOLV1.BI.MOVA.=TRUE, | 0.12 | 0.392 | 0.321 | 1.41 |  |
|  |  |  |  |  | 51 |
| BcOLV15.BI=FALSE} => {BcRV1.BI=TRUE} | 6238 | 308 | 782 | 511 |  |
| {BcEV5=FALSE, BcRV1.BI=TRUE} => | 0.13 | 0.482 | 0.277 | 1.45 |  |
|  |  |  |  |  | 54 |
| {BcOLV1.BI.MOVA.=TRUE} | 3663 | 143 | 228 | 3625 |  |

| {BcEV5=FALSE, BcOLV1.BI.MOVA.=TRUE}  => {BcRV1.BI=TRUE} | 0.13  3663 | 0.45 | 0.297  03 | 1.62  3214 | 54 |
| --- | --- | --- | --- | --- | --- |
| {BcHV1.BI=FALSE, BcRV1.BI=TRUE} =>  {BcHV1SLR.BI=FALSE} | 0.07  9208 | 0.888  889 | 0.089  109 | 1.94  1141 | 32 |
| {BcHV1SLR.BI=FALSE, BcRV1.BI=TRUE} =>  {BcHV1.BI=FALSE} | 0.07  9208 | 0.680  851 | 0.116  337 | 1.71  9149 | 32 |
| {BcHV1.BI=FALSE, BcRV1.BI=TRUE} =>  {BcMV6.BI=FALSE} | 0.07  6733 | 0.861  111 | 0.089  109 | 1.23  8039 | 31 |
| {BcHV1.BI=FALSE, BcRV1.BI=TRUE} =>  {BcPV3.BI=FALSE} | 0.07  4257 | 0.833  333 | 0.089  109 | 1.17  3055 | 30 |
| {BcHV1.BI=FALSE, BcRV1.BI=TRUE} =>  {BcULV1.BI=FALSE} | 0.08  1683 | 0.916  667 | 0.089  109 | 1.16  8244 | 33 |
| {BcHV1.BI=FALSE, BcRV1.BI=TRUE} =>  {BcNSRV7.BI=FALSE} | 0.08  1683 | 0.916  667 | 0.089  109 | 1.14  6543 | 33 |
| {BcRV1.BI=TRUE, BcULV3=FALSE} =>  {BcMV2.BI=TRUE} | 0.07  4257 | 0.638  298 | 0.116  337 | 1.37  8996 | 30 |
| {BcMV2.BI=TRUE, BcULV3=FALSE} =>  {BcRV1.BI=TRUE} | 0.07  4257 | 0.416  667 | 0.178  218 | 1.50  2976 | 30 |
| {BcRV1.BI=TRUE, BcULV3=FALSE} =>  {BcOLV18=TRUE} | 0.07  4257 | 0.638  298 | 0.116  337 | 1.23  3839 | 30 |
| {BcOLV18=TRUE, BcULV3=FALSE} =>  {BcRV1.BI=TRUE} | 0.07  4257 | 0.384  615 | 0.193  069 | 1.38  7363 | 30 |

| {BcOLV16.BI=FALSE, BcRV1.BI=TRUE} =>  {BcULV3=FALSE} | 0.07  4257 | 0.588  235 | 0.126  238 | 1.44  9067 | 30 |
| --- | --- | --- | --- | --- | --- |
| {BcMV11=TRUE, BcULV3=FALSE} =>  {BcRV1.BI=TRUE} | 0.08  1683 | 0.370  787 | 0.220  297 | 1.33  748 | 33 |
| {BcRV1.BI=TRUE, BcULV3=FALSE} =>  {BcULV4=TRUE} | 0.09  4059 | 0.808  511 | 0.116  337 | 1.17  0747 | 38 |
| {BcRV1.BI=TRUE, BcULV3=FALSE} =>  {BcNSRV7.BI=FALSE} | 0.11  1386 | 0.957  447 | 0.116  337 | 1.19  755 | 45 |
| {BcOLV16.BI=TRUE, BcRV1.BI=TRUE} =>  {BcMV4.BI=TRUE} | 0.09  901 | 0.655  738 | 0.150  99 | 1.47  9989 | 40 |
| {BcMV4.BI=TRUE, BcRV1.BI=TRUE} =>  {BcOLV16.BI=TRUE} | 0.09  901 | 0.597  015 | 0.165  842 | 1.43  5679 | 40 |
| {BcMV4.BI=TRUE, BcOLV16.BI=TRUE} =>  {BcRV1.BI=TRUE} | 0.09  901 | 0.439  56 | 0.225  248 | 1.58  5557 | 40 |
| {BcOLV16.BI=TRUE, BcRV1.BI=TRUE} =>  {BcMV2.BI=TRUE} | 0.11  6337 | 0.770  492 | 0.150  99 | 1.66  4592 | 47 |
| {BcMV2.BI=TRUE, BcRV1.BI=TRUE} =>  {BcOLV16.BI=TRUE} | 0.11  6337 | 0.652  778 | 0.178  218 | 1.56  9775 | 47 |
| {BcMV2.BI=TRUE, BcOLV16.BI=TRUE} =>  {BcRV1.BI=TRUE} | 0.11  6337 | 0.460  784 | 0.252  475 | 1.66  2115 | 47 |
| {BcOLV16.BI=TRUE, BcRV1.BI=TRUE} =>  {BcOLV18=TRUE} | 0.11  6337 | 0.770  492 | 0.150  99 | 1.48  9372 | 47 |

| {BcOLV18=TRUE, BcRV1.BI=TRUE} =>  {BcOLV16.BI=TRUE} | 0.11  6337 | 0.618  421 | 0.188  119 | 1.48  7155 | 47 |
| --- | --- | --- | --- | --- | --- |
| {BcOLV16.BI=TRUE, BcOLV18=TRUE} =>  {BcRV1.BI=TRUE} | 0.11  6337 | 0.431  193 | 0.269  802 | 1.55  5374 | 47 |
| {BcOLV16.BI=TRUE, BcRV1.BI=TRUE} =>  {BcHV1SLR.BI=TRUE} | 0.10  1485 | 0.672  131 | 0.150  99 | 1.23  9913 | 41 |
| {BcHV1SLR.BI=TRUE, BcRV1.BI=TRUE} =>  {BcOLV16.BI=TRUE} | 0.10  1485 | 0.630  769 | 0.160  891 | 1.51  685 | 41 |
| {BcHV1SLR.BI=TRUE, BcOLV16.BI=TRUE}  => {BcRV1.BI=TRUE} | 0.10  1485 | 0.427  083 | 0.237  624 | 1.54  0551 | 41 |
| {BcOLV16.BI=TRUE, BcRV1.BI=TRUE} =>  {BcMV3.BI=TRUE} | 0.11  1386 | 0.737  705 | 0.150  99 | 1.36  088 | 45 |
| {BcMV3.BI=TRUE, BcRV1.BI=TRUE} =>  {BcOLV16.BI=TRUE} | 0.11  1386 | 0.633  803 | 0.175  743 | 1.52  4145 | 45 |
| {BcMV3.BI=TRUE, BcOLV16.BI=TRUE} =>  {BcRV1.BI=TRUE} | 0.11  1386 | 0.416  667 | 0.267  327 | 1.50  2976 | 45 |
| {BcOLV16.BI=TRUE, BcRV1.BI=TRUE} =>  {BcULV3=TRUE} | 0.10  8911 | 0.721  311 | 0.150  99 | 1.21  4208 | 44 |
| {BcRV1.BI=TRUE, BcULV3=TRUE} =>  {BcOLV16.BI=TRUE} | 0.10  8911 | 0.676  923 | 0.160  891 | 1.62  7839 | 44 |
| {BcOLV16.BI=TRUE, BcULV3=TRUE} =>  {BcRV1.BI=TRUE} | 0.10  8911 | 0.389  381 | 0.279  703 | 1.40  4551 | 44 |

| {BcOLV16.BI=TRUE, BcRV1.BI=TRUE} =>  {BcHV1.BI=TRUE} | 0.11  3861 | 0.754  098 | 0.150  99 | 1.24  8589 | 46 |
| --- | --- | --- | --- | --- | --- |
| {BcHV1.BI=TRUE, BcRV1.BI=TRUE} =>  {BcOLV16.BI=TRUE} | 0.11  3861 | 0.605  263 | 0.188  119 | 1.45  5514 | 46 |
| {BcHV1.BI=TRUE, BcOLV16.BI=TRUE} =>  {BcRV1.BI=TRUE} | 0.11  3861 | 0.422  018 | 0.269  802 | 1.52  228 | 46 |
| {BcOLV16.BI=TRUE, BcRV1.BI=TRUE} =>  {BcMV11=TRUE} | 0.12  6238 | 0.836  066 | 0.150  99 | 1.35  6508 | 51 |
| {BcMV11=TRUE, BcRV1.BI=TRUE} =>  {BcOLV16.BI=TRUE} | 0.12  6238 | 0.607  143 | 0.207  921 | 1.46  0034 | 51 |
| {BcMV11=TRUE, BcOLV16.BI=TRUE} =>  {BcRV1.BI=TRUE} | 0.12  6238 | 0.459  459 | 0.274  752 | 1.65  7336 | 51 |
| {BcOLV16.BI=TRUE, BcRV1.BI=TRUE} =>  {BcMV9.BI=TRUE} | 0.13  6139 | 0.901  639 | 0.150  99 | 1.32  9424 | 55 |
| {BcMV9.BI=TRUE, BcRV1.BI=TRUE} =>  {BcOLV16.BI=TRUE} | 0.13  6139 | 0.611  111 | 0.222  772 | 1.46  9577 | 55 |
| {BcMV9.BI=TRUE, BcOLV16.BI=TRUE} =>  {BcRV1.BI=TRUE} | 0.13  6139 | 0.410  448 | 0.331  683 | 1.48  0544 | 55 |
| {BcOLV16.BI=TRUE, BcRV1.BI=TRUE} =>  {BcULV4=TRUE} | 0.14  3564 | 0.950  82 | 0.150  99 | 1.37  6814 | 58 |
| {BcRV1.BI=TRUE, BcULV4=TRUE} =>  {BcOLV16.BI=TRUE} | 0.14  3564 | 0.591  837 | 0.242  574 | 1.42  3226 | 58 |

| {BcOLV16.BI=TRUE, BcULV4=TRUE} =>  {BcRV1.BI=TRUE} | 0.14  3564 | 0.423  358 | 0.339  109 | 1.52  7112 | 58 |
| --- | --- | --- | --- | --- | --- |
| {BcEV2.BI=FALSE, BcRV1.BI=TRUE} =>  {BcOLV16.BI=TRUE} | 0.11  6337 | 0.546  512 | 0.212  871 | 1.31  423 | 47 |
| {BcEV2.BI=FALSE, BcOLV16.BI=TRUE} =>  {BcRV1.BI=TRUE} | 0.11  6337 | 0.356  061 | 0.326  733 | 1.28  4361 | 47 |
| {BcFV5.BI=FALSE, BcOLV16.BI=TRUE} =>  {BcRV1.BI=TRUE} | 0.12  1287 | 0.376  923 | 0.321  782 | 1.35  9615 | 49 |
| {BcMV1.BI=TRUE, BcRV1.BI=TRUE} =>  {BcOLV16.BI=TRUE} | 0.12  1287 | 0.597  561 | 0.202  97 | 1.43  6992 | 49 |
| {BcMV1.BI=TRUE, BcOLV16.BI=TRUE} =>  {BcRV1.BI=TRUE} | 0.12  1287 | 0.360  294 | 0.336  634 | 1.29  9632 | 49 |
| {BcMV12=FALSE, BcOLV16.BI=TRUE} =>  {BcRV1.BI=TRUE} | 0.11  3861 | 0.386  555 | 0.294  554 | 1.39  4358 | 46 |
| {BcNSRV7.BI=FALSE, BcRV1.BI=TRUE} =>  {BcOLV16.BI=TRUE} | 0.12  8713 | 0.536  082 | 0.240  099 | 1.28  9151 | 52 |
| {BcNSRV7.BI=FALSE, BcOLV16.BI=TRUE}  => {BcRV1.BI=TRUE} | 0.12  8713 | 0.385  185 | 0.334  158 | 1.38  9418 | 52 |
| {BcRV1.BI=TRUE, BcVV3.BI=FALSE} =>  {BcOLV16.BI=TRUE} | 0.13  1188 | 0.563  83 | 0.232  673 | 1.35  5876 | 53 |
| {BcOLV16.BI=TRUE, BcVV3.BI=FALSE} =>  {BcRV1.BI=TRUE} | 0.13  1188 | 0.389  706 | 0.336  634 | 1.40  5725 | 53 |

| {BcOLV16.BI=TRUE, BcRV1.BI=TRUE} =>  {BcDMV1=FALSE} | 0.14  3564 | 0.950  82 | 0.150  99 | 1.12  6484 | 58 |
| --- | --- | --- | --- | --- | --- |
| {BcDMV1=FALSE, BcRV1.BI=TRUE} =>  {BcOLV16.BI=TRUE} | 0.14  3564 | 0.557  692 | 0.257  426 | 1.34  1117 | 58 |
| {BcDMV1=FALSE, BcOLV16.BI=TRUE} =>  {BcRV1.BI=TRUE} | 0.14  3564 | 0.405  594 | 0.353  96 | 1.46  3037 | 58 |
| {BcFV9=FALSE, BcRV1.BI=TRUE} =>  {BcOLV16.BI=TRUE} | 0.12  1287 | 0.521  277 | 0.232  673 | 1.25  3546 | 49 |
| {BcOLV4.BI=FALSE, BcRV1.BI=TRUE} =>  {BcOLV16.BI=TRUE} | 0.12  6238 | 0.515  152 | 0.245  05 | 1.23  8817 | 51 |
| {BcOLV16.BI=TRUE, BcOLV4.BI=FALSE} =>  {BcRV1.BI=TRUE} | 0.12  6238 | 0.366  906 | 0.344  059 | 1.32  3484 | 51 |
| {BcRV1.BI=TRUE, BcULV5=FALSE} =>  {BcOLV16.BI=TRUE} | 0.13  1188 | 0.53 | 0.247  525 | 1.27  4524 | 53 |
| {BcOLV16.BI=TRUE, BcULV5=FALSE} =>  {BcRV1.BI=TRUE} | 0.13  1188 | 0.373  239 | 0.351  485 | 1.34  6328 | 53 |
| {BcBNV5.BI=FALSE, BcRV1.BI=TRUE} =>  {BcOLV16.BI=TRUE} | 0.13  8614 | 0.533  333 | 0.259  901 | 1.28  254 | 56 |
| {BcBNV5.BI=FALSE, BcOLV16.BI=TRUE} =>  {BcRV1.BI=TRUE} | 0.13  8614 | 0.370  861 | 0.373  762 | 1.33  7748 | 56 |
| {BcOLV16.BI=TRUE, BcRV1.BI=TRUE} =>  {BvF.BI=FALSE} | 0.15  099 | 1 | 0.150  99 | 1.09  4851 | 61 |

| {BcRV1.BI=TRUE, BvF.BI=FALSE} =>  {BcOLV16.BI=TRUE} | 0.15  099 | 0.564  815 | 0.267  327 | 1.35  8245 | 61 |
| --- | --- | --- | --- | --- | --- |
| {BcOLV16.BI=TRUE, BvF.BI=FALSE} =>  {BcRV1.BI=TRUE} | 0.15  099 | 0.386  076 | 0.391  089 | 1.39  2631 | 61 |
| {BcOLV2.BI=FALSE, BcRV1.BI=TRUE} =>  {BcOLV16.BI=TRUE} | 0.12  8713 | 0.525  253 | 0.245  05 | 1.26  3107 | 52 |
| {BcOLV16.BI=TRUE, BcOLV2.BI=FALSE} =>  {BcRV1.BI=TRUE} | 0.12  8713 | 0.348  993 | 0.368  812 | 1.25  8869 | 52 |
| {BcMCV3.BI=FALSE, BcRV1.BI=TRUE} =>  {BcOLV16.BI=TRUE} | 0.13  1188 | 0.519  608 | 0.252  475 | 1.24  9533 | 53 |
| {BcMCV3.BI=FALSE, BcOLV16.BI=TRUE} =>  {BcRV1.BI=TRUE} | 0.13  1188 | 0.365  517 | 0.358  911 | 1.31  8473 | 53 |
| {BcPV4.BI=FALSE, BcRV1.BI=TRUE} =>  {BcOLV16.BI=TRUE} | 0.13  8614 | 0.528  302 | 0.262  376 | 1.27  044 | 56 |
| {BcOLV16.BI=TRUE, BcPV4.BI=FALSE} =>  {BcRV1.BI=TRUE} | 0.13  8614 | 0.366  013 | 0.378  713 | 1.32  0261 | 56 |
| {BcEV6=FALSE, BcRV1.BI=TRUE} =>  {BcOLV16.BI=TRUE} | 0.14  604 | 0.546  296 | 0.267  327 | 1.31  3713 | 59 |
| {BcEV6=FALSE, BcOLV16.BI=TRUE} =>  {BcRV1.BI=TRUE} | 0.14  604 | 0.373  418 | 0.391  089 | 1.34  6971 | 59 |
| {BcOLV17.BI=FALSE, BcRV1.BI=TRUE} =>  {BcOLV16.BI=TRUE} | 0.12  6238 | 0.525  773 | 0.240  099 | 1.26  4359 | 51 |

| {BcOLV15.BI=FALSE, BcRV1.BI=TRUE} =>  {BcOLV16.BI=TRUE} | 0.14  604 | 0.541  284 | 0.269  802 | 1.30  166 | 59 |
| --- | --- | --- | --- | --- | --- |
| {BcOLV15.BI=FALSE, BcOLV16.BI=TRUE} =>  {BcRV1.BI=TRUE} | 0.14  604 | 0.361  963 | 0.403  465 | 1.30  5653 | 59 |
| {BcEV5=FALSE, BcRV1.BI=TRUE} =>  {BcOLV16.BI=TRUE} | 0.15  099 | 0.544  643 | 0.277  228 | 1.30  9736 | 61 |
| {BcEV5=FALSE, BcOLV16.BI=TRUE} =>  {BcRV1.BI=TRUE} | 0.15  099 | 0.383  648 | 0.393  564 | 1.38  3872 | 61 |
| {BcMV4.BI=TRUE, BcRV1.BI=TRUE} =>  {BcMV2.BI=TRUE} | 0.12  8713 | 0.776  119 | 0.165  842 | 1.67  675 | 52 |
| {BcMV2.BI=TRUE, BcRV1.BI=TRUE} =>  {BcMV4.BI=TRUE} | 0.12  8713 | 0.722  222 | 0.178  218 | 1.63  0043 | 52 |
| {BcMV2.BI=TRUE, BcMV4.BI=TRUE} =>  {BcRV1.BI=TRUE} | 0.12  8713 | 0.468  468 | 0.274  752 | 1.68  9833 | 52 |
| {BcMV4.BI=TRUE, BcRV1.BI=TRUE} =>  {BcOLV18=TRUE} | 0.14  604 | 0.880  597 | 0.165  842 | 1.70  2207 | 59 |
| {BcOLV18=TRUE, BcRV1.BI=TRUE} =>  {BcMV4.BI=TRUE} | 0.14  604 | 0.776  316 | 0.188  119 | 1.75  2132 | 59 |
| {BcMV4.BI=TRUE, BcOLV18=TRUE} =>  {BcRV1.BI=TRUE} | 0.14  604 | 0.526  786 | 0.277  228 | 1.90  0191 | 59 |
| {BcMV4.BI=TRUE, BcRV1.BI=TRUE} =>  {BcHV1SLR.BI=TRUE} | 0.11  8812 | 0.716  418 | 0.165  842 | 1.32  1611 | 48 |

| {BcHV1SLR.BI=TRUE, BcRV1.BI=TRUE} =>  {BcMV4.BI=TRUE} | 0.11  8812 | 0.738  462 | 0.160  891 | 1.66  6695 | 48 |
| --- | --- | --- | --- | --- | --- |
| {BcHV1SLR.BI=TRUE, BcMV4.BI=TRUE} =>  {BcRV1.BI=TRUE} | 0.11  8812 | 0.452  83 | 0.262  376 | 1.63  3423 | 48 |
| {BcMV4.BI=TRUE, BcRV1.BI=TRUE} =>  {BcMV3.BI=TRUE} | 0.12  1287 | 0.731  343 | 0.165  842 | 1.34  9145 | 49 |
| {BcMV3.BI=TRUE, BcRV1.BI=TRUE} =>  {BcMV4.BI=TRUE} | 0.12  1287 | 0.690  141 | 0.175  743 | 1.55  7636 | 49 |
| {BcMV3.BI=TRUE, BcMV4.BI=TRUE} =>  {BcRV1.BI=TRUE} | 0.12  1287 | 0.411  765 | 0.294  554 | 1.48  5294 | 49 |
| {BcRV1.BI=TRUE, BcULV3=TRUE} =>  {BcMV4.BI=TRUE} | 0.10  396 | 0.646  154 | 0.160  891 | 1.45  8358 | 42 |
| {BcMV4.BI=TRUE, BcULV3=TRUE} =>  {BcRV1.BI=TRUE} | 0.10  396 | 0.352  941 | 0.294  554 | 1.27  3109 | 42 |
| {BcMV4.BI=TRUE, BcRV1.BI=TRUE} =>  {BcHV1.BI=TRUE} | 0.13  3663 | 0.805  97 | 0.165  842 | 1.33  4475 | 54 |
| {BcHV1.BI=TRUE, BcRV1.BI=TRUE} =>  {BcMV4.BI=TRUE} | 0.13  3663 | 0.710  526 | 0.188  119 | 1.60  3646 | 54 |
| {BcHV1.BI=TRUE, BcMV4.BI=TRUE} =>  {BcRV1.BI=TRUE} | 0.13  3663 | 0.446  281 | 0.299  505 | 1.60  9799 | 54 |
| {BcMV4.BI=TRUE, BcRV1.BI=TRUE} =>  {BcMV11=TRUE} | 0.13  8614 | 0.835  821 | 0.165  842 | 1.35  6111 | 56 |

| {BcMV11=TRUE, BcRV1.BI=TRUE} =>  {BcMV4.BI=TRUE} | 0.13  8614 | 0.666  667 | 0.207  921 | 1.50  4655 | 56 |
| --- | --- | --- | --- | --- | --- |
| {BcMV11=TRUE, BcMV4.BI=TRUE} =>  {BcRV1.BI=TRUE} | 0.13  8614 | 0.408  759 | 0.339  109 | 1.47  4453 | 56 |
| {BcMV4.BI=TRUE, BcRV1.BI=TRUE} =>  {BcMV9.BI=TRUE} | 0.14  8515 | 0.895  522 | 0.165  842 | 1.32  0405 | 60 |
| {BcMV9.BI=TRUE, BcRV1.BI=TRUE} =>  {BcMV4.BI=TRUE} | 0.14  8515 | 0.666  667 | 0.222  772 | 1.50  4655 | 60 |
| {BcMV4.BI=TRUE, BcMV9.BI=TRUE} =>  {BcRV1.BI=TRUE} | 0.14  8515 | 0.441  176 | 0.336  634 | 1.59  1387 | 60 |
| {BcMV4.BI=TRUE, BcRV1.BI=TRUE} =>  {BcULV4=TRUE} | 0.15  099 | 0.910  448 | 0.165  842 | 1.31  8354 | 61 |
| {BcRV1.BI=TRUE, BcULV4=TRUE} =>  {BcMV4.BI=TRUE} | 0.15  099 | 0.622  449 | 0.242  574 | 1.40  4857 | 61 |
| {BcMV4.BI=TRUE, BcULV4=TRUE} =>  {BcRV1.BI=TRUE} | 0.15  099 | 0.465  649 | 0.324  257 | 1.67  9662 | 61 |
| {BcEV2.BI=FALSE, BcRV1.BI=TRUE} =>  {BcMV4.BI=TRUE} | 0.12  1287 | 0.569  767 | 0.212  871 | 1.28  5956 | 49 |
| {BcEV2.BI=FALSE, BcMV4.BI=TRUE} =>  {BcRV1.BI=TRUE} | 0.12  1287 | 0.385  827 | 0.314  356 | 1.39  1732 | 49 |
| {BcHV3.BI=FALSE, BcMV4.BI=TRUE} =>  {BcRV1.BI=TRUE} | 0.11  3861 | 0.389  831 | 0.292  079 | 1.40  6174 | 46 |

| {BcFV5.BI=FALSE, BcRV1.BI=TRUE} =>  {BcMV4.BI=TRUE} | 0.13  6139 | 0.572  917 | 0.237  624 | 1.29  3063 | 55 |
| --- | --- | --- | --- | --- | --- |
| {BcFV5.BI=FALSE, BcMV4.BI=TRUE} =>  {BcRV1.BI=TRUE} | 0.13  6139 | 0.423  077 | 0.321  782 | 1.52  6099 | 55 |
| {BcRV1.BI=TRUE, BcULV1.BI=FALSE} =>  {BcMV4.BI=TRUE} | 0.11  1386 | 0.555  556 | 0.200  495 | 1.25  388 | 45 |
| {BcMV1.BI=TRUE, BcRV1.BI=TRUE} =>  {BcMV4.BI=TRUE} | 0.12  8713 | 0.634  146 | 0.202  97 | 1.43  1258 | 52 |
| {BcMV12=FALSE, BcRV1.BI=TRUE} =>  {BcMV4.BI=TRUE} | 0.13  6139 | 0.617  978 | 0.220  297 | 1.39  4765 | 55 |
| {BcMV12=FALSE, BcMV4.BI=TRUE} =>  {BcRV1.BI=TRUE} | 0.13  6139 | 0.376  712 | 0.361  386 | 1.35  8855 | 55 |
| {BcNSRV7.BI=FALSE, BcRV1.BI=TRUE} =>  {BcMV4.BI=TRUE} | 0.13  8614 | 0.577  32 | 0.240  099 | 1.30  3001 | 56 |
| {BcMV4.BI=TRUE, BcNSRV7.BI=FALSE} =>  {BcRV1.BI=TRUE} | 0.13  8614 | 0.411  765 | 0.336  634 | 1.48  5294 | 56 |
| {BcRV1.BI=TRUE, BcVV3.BI=FALSE} =>  {BcMV4.BI=TRUE} | 0.13  3663 | 0.574  468 | 0.232  673 | 1.29  6565 | 54 |
| {BcMV4.BI=TRUE, BcVV3.BI=FALSE} =>  {BcRV1.BI=TRUE} | 0.13  3663 | 0.402  985 | 0.331  683 | 1.45  3625 | 54 |
| {BcMV4.BI=TRUE, BcRV1.BI=TRUE} =>  {BcDMV1=FALSE} | 0.15  5941 | 0.940  299 | 0.165  842 | 1.11  4019 | 63 |

| {BcDMV1=FALSE, BcRV1.BI=TRUE} =>  {BcMV4.BI=TRUE} | 0.15  5941 | 0.605  769 | 0.257  426 | 1.36  7211 | 63 |
| --- | --- | --- | --- | --- | --- |
| {BcDMV1=FALSE, BcMV4.BI=TRUE} =>  {BcRV1.BI=TRUE} | 0.15  5941 | 0.431  507 | 0.361  386 | 1.55  6507 | 63 |
| {BcFV9=FALSE, BcRV1.BI=TRUE} =>  {BcMV4.BI=TRUE} | 0.13  1188 | 0.563  83 | 0.232  673 | 1.27  2554 | 53 |
| {BcFV9=FALSE, BcMV4.BI=TRUE} =>  {BcRV1.BI=TRUE} | 0.13  1188 | 0.368  056 | 0.356  436 | 1.32  7629 | 53 |
| {BcOLV4.BI=FALSE, BcRV1.BI=TRUE} =>  {BcMV4.BI=TRUE} | 0.13  8614 | 0.565  657 | 0.245  05 | 1.27  6677 | 56 |
| {BcMV4.BI=TRUE, BcOLV4.BI=FALSE} =>  {BcRV1.BI=TRUE} | 0.13  8614 | 0.373  333 | 0.371  287 | 1.34  6667 | 56 |
| {BcRV1.BI=TRUE, BcULV5=FALSE} =>  {BcMV4.BI=TRUE} | 0.14  3564 | 0.58 | 0.247  525 | 1.30  905 | 58 |
| {BcMV4.BI=TRUE, BcULV5=FALSE} =>  {BcRV1.BI=TRUE} | 0.14  3564 | 0.376  623 | 0.381  188 | 1.35  8534 | 58 |
| {BcBNV5.BI=FALSE, BcRV1.BI=TRUE} =>  {BcMV4.BI=TRUE} | 0.15  5941 | 0.6 | 0.259  901 | 1.35  419 | 63 |
| {BcBNV5.BI=FALSE, BcMV4.BI=TRUE} =>  {BcRV1.BI=TRUE} | 0.15  5941 | 0.406  452 | 0.383  663 | 1.46  6129 | 63 |
| {BcRV1.BI=TRUE, BvF.BI=FALSE} =>  {BcMV4.BI=TRUE} | 0.16  0891 | 0.601  852 | 0.267  327 | 1.35  837 | 65 |

| {BcMV4.BI=TRUE, BvF.BI=FALSE} =>  {BcRV1.BI=TRUE} | 0.16  0891 | 0.396  341 | 0.405  941 | 1.42  966 | 65 |
| --- | --- | --- | --- | --- | --- |
| {BcMCV5.BI=FALSE, BcRV1.BI=TRUE} =>  {BcMV4.BI=TRUE} | 0.14  1089 | 0.564  356 | 0.25 | 1.27  3743 | 57 |
| {BcMCV5.BI=FALSE, BcMV4.BI=TRUE} =>  {BcRV1.BI=TRUE} | 0.14  1089 | 0.372  549 | 0.378  713 | 1.34  3838 | 57 |
| {BcOLV13.BI=FALSE, BcRV1.BI=TRUE} =>  {BcMV4.BI=TRUE} | 0.13  1188 | 0.557  895 | 0.235  149 | 1.25  9159 | 53 |
| {BcOLV2.BI=FALSE, BcRV1.BI=TRUE} =>  {BcMV4.BI=TRUE} | 0.13  8614 | 0.565  657 | 0.245  05 | 1.27  6677 | 56 |
| {BcMV4.BI=TRUE, BcOLV2.BI=FALSE} =>  {BcRV1.BI=TRUE} | 0.13  8614 | 0.343  558 | 0.403  465 | 1.23  9264 | 56 |
| {BcMCV3.BI=FALSE, BcRV1.BI=TRUE} =>  {BcMV4.BI=TRUE} | 0.14  8515 | 0.588  235 | 0.252  475 | 1.32  7637 | 60 |
| {BcMCV3.BI=FALSE, BcMV4.BI=TRUE} =>  {BcRV1.BI=TRUE} | 0.14  8515 | 0.361  446 | 0.410  891 | 1.30  3787 | 60 |
| {BcPV4.BI=FALSE, BcRV1.BI=TRUE} =>  {BcMV4.BI=TRUE} | 0.16  0891 | 0.613  208 | 0.262  376 | 1.38  3999 | 65 |
| {BcMV4.BI=TRUE, BcPV4.BI=FALSE} =>  {BcRV1.BI=TRUE} | 0.16  0891 | 0.384  615 | 0.418  317 | 1.38  7363 | 65 |
| {BcEV6=FALSE, BcRV1.BI=TRUE} =>  {BcMV4.BI=TRUE} | 0.16  0891 | 0.601  852 | 0.267  327 | 1.35  837 | 65 |

| {BcEV6=FALSE, BcMV4.BI=TRUE} =>  {BcRV1.BI=TRUE} | 0.16  0891 | 0.382  353 | 0.420  792 | 1.37  9202 | 65 |
| --- | --- | --- | --- | --- | --- |
| {BcOLV17.BI=FALSE, BcRV1.BI=TRUE} =>  {BcMV4.BI=TRUE} | 0.13  6139 | 0.567  01 | 0.240  099 | 1.27  9733 | 55 |
| {BcOLV15.BI=FALSE, BcRV1.BI=TRUE} =>  {BcMV4.BI=TRUE} | 0.15  8416 | 0.587  156 | 0.269  802 | 1.32  5201 | 64 |
| {BcMV4.BI=TRUE, BcOLV15.BI=FALSE} =>  {BcRV1.BI=TRUE} | 0.15  8416 | 0.383  234 | 0.413  366 | 1.38  2378 | 64 |
| {BcEV5=FALSE, BcRV1.BI=TRUE} =>  {BcMV4.BI=TRUE} | 0.16  5842 | 0.598  214 | 0.277  228 | 1.35  016 | 67 |
| {BcEV5=FALSE, BcMV4.BI=TRUE} =>  {BcRV1.BI=TRUE} | 0.16  5842 | 0.416  149 | 0.398  515 | 1.50  1109 | 67 |
| {BcHV1SLR.BI=FALSE, BcRV1.BI=TRUE} =>  {BcMV6.BI=FALSE} | 0.09  4059 | 0.808  511 | 0.116  337 | 1.16  2414 | 38 |
| {BcMV6.BI=FALSE, BcRV1.BI=TRUE} =>  {BcHV1SLR.BI=FALSE} | 0.09  4059 | 0.575  758 | 0.163  366 | 1.25  733 | 38 |
| {BcHV1SLR.BI=FALSE, BcRV1.BI=TRUE} =>  {BcFV5.BI=FALSE} | 0.10  6436 | 0.914  894 | 0.116  337 | 1.16  9674 | 43 |
| {BcHV1SLR.BI=FALSE, BcRV1.BI=TRUE} =>  {BcULV1.BI=FALSE} | 0.10  396 | 0.893  617 | 0.116  337 | 1.13  8868 | 42 |
| {BcHV1SLR.BI=FALSE, BcRV1.BI=TRUE} =>  {BcNSRV7.BI=FALSE} | 0.10  8911 | 0.936  17 | 0.116  337 | 1.17  0937 | 44 |

| {BcMV3.BI=FALSE, BcRV1.BI=TRUE} =>  {BcULV4=TRUE} | 0.08  9109 | 0.878  049 | 0.101  485 | 1.27  144 | 36 |
| --- | --- | --- | --- | --- | --- |
| {BcMV3.BI=FALSE, BcRV1.BI=TRUE} =>  {BcMV6.BI=FALSE} | 0.08  4158 | 0.829  268 | 0.101  485 | 1.19  2258 | 34 |
| {BcMV3.BI=FALSE, BcRV1.BI=TRUE} =>  {BcFV5.BI=FALSE} | 0.09  1584 | 0.902  439 | 0.101  485 | 1.15  3751 | 37 |
| {BcMV3.BI=FALSE, BcRV1.BI=TRUE} =>  {BcMV12=FALSE} | 0.09  1584 | 0.902  439 | 0.101  485 | 1.12  8747 | 37 |
| {BcMV3.BI=FALSE, BcRV1.BI=TRUE} =>  {BcDMV1=FALSE} | 0.09  901 | 0.975  61 | 0.101  485 | 1.15  5854 | 40 |
| {BcMV2.BI=TRUE, BcRV1.BI=TRUE} =>  {BcOLV18=TRUE} | 0.14  8515 | 0.833  333 | 0.178  218 | 1.61  0845 | 60 |
| {BcOLV18=TRUE, BcRV1.BI=TRUE} =>  {BcMV2.BI=TRUE} | 0.14  8515 | 0.789  474 | 0.188  119 | 1.70  5601 | 60 |
| {BcMV2.BI=TRUE, BcOLV18=TRUE} =>  {BcRV1.BI=TRUE} | 0.14  8515 | 0.504  202 | 0.294  554 | 1.81  8727 | 60 |
| {BcMV2.BI=TRUE, BcRV1.BI=TRUE} =>  {BcHV1SLR.BI=TRUE} | 0.12  8713 | 0.722  222 | 0.178  218 | 1.33  2319 | 52 |
| {BcHV1SLR.BI=TRUE, BcRV1.BI=TRUE} =>  {BcMV2.BI=TRUE} | 0.12  8713 | 0.8 | 0.160  891 | 1.72  8342 | 52 |
| {BcHV1SLR.BI=TRUE, BcMV2.BI=TRUE} =>  {BcRV1.BI=TRUE} | 0.12  8713 | 0.472  727 | 0.272  277 | 1.70  5195 | 52 |

| {BcMV2.BI=TRUE, BcRV1.BI=TRUE} =>  {BcMV3.BI=TRUE} | 0.13  1188 | 0.736  111 | 0.178  218 | 1.35  794 | 53 |
| --- | --- | --- | --- | --- | --- |
| {BcMV3.BI=TRUE, BcRV1.BI=TRUE} =>  {BcMV2.BI=TRUE} | 0.13  1188 | 0.746  479 | 0.175  743 | 1.61  2714 | 53 |
| {BcMV2.BI=TRUE, BcMV3.BI=TRUE} =>  {BcRV1.BI=TRUE} | 0.13  1188 | 0.417  323 | 0.314  356 | 1.50  5343 | 53 |
| {BcRV1.BI=TRUE, BcULV3=TRUE} =>  {BcMV2.BI=TRUE} | 0.10  396 | 0.646  154 | 0.160  891 | 1.39  5969 | 42 |
| {BcMV2.BI=TRUE, BcULV3=TRUE} =>  {BcRV1.BI=TRUE} | 0.10  396 | 0.365  217 | 0.284  653 | 1.31  7391 | 42 |
| {BcMV2.BI=TRUE, BcRV1.BI=TRUE} =>  {BcHV1.BI=TRUE} | 0.13  3663 | 0.75 | 0.178  218 | 1.24  1803 | 54 |
| {BcHV1.BI=TRUE, BcRV1.BI=TRUE} =>  {BcMV2.BI=TRUE} | 0.13  3663 | 0.710  526 | 0.188  119 | 1.53  5041 | 54 |
| {BcHV1.BI=TRUE, BcMV2.BI=TRUE} =>  {BcRV1.BI=TRUE} | 0.13  3663 | 0.457  627 | 0.292  079 | 1.65  0726 | 54 |
| {BcMV2.BI=TRUE, BcRV1.BI=TRUE} =>  {BcMV11=TRUE} | 0.14  8515 | 0.833  333 | 0.178  218 | 1.35  2075 | 60 |
| {BcMV11=TRUE, BcRV1.BI=TRUE} =>  {BcMV2.BI=TRUE} | 0.14  8515 | 0.714  286 | 0.207  921 | 1.54  3163 | 60 |
| {BcMV11=TRUE, BcMV2.BI=TRUE} =>  {BcRV1.BI=TRUE} | 0.14  8515 | 0.434  783 | 0.341  584 | 1.56  8323 | 60 |

| {BcMV2.BI=TRUE, BcRV1.BI=TRUE} =>  {BcMV9.BI=TRUE} | 0.14  3564 | 0.805  556 | 0.178  218 | 1.18  7753 | 58 |
| --- | --- | --- | --- | --- | --- |
| {BcMV9.BI=TRUE, BcRV1.BI=TRUE} =>  {BcMV2.BI=TRUE} | 0.14  3564 | 0.644  444 | 0.222  772 | 1.39  2276 | 58 |
| {BcMV2.BI=TRUE, BcMV9.BI=TRUE} =>  {BcRV1.BI=TRUE} | 0.14  3564 | 0.446  154 | 0.321  782 | 1.60  9341 | 58 |
| {BcMV2.BI=TRUE, BcRV1.BI=TRUE} =>  {BcULV4=TRUE} | 0.16  3366 | 0.916  667 | 0.178  218 | 1.32  736 | 66 |
| {BcRV1.BI=TRUE, BcULV4=TRUE} =>  {BcMV2.BI=TRUE} | 0.16  3366 | 0.673  469 | 0.242  574 | 1.45  4982 | 66 |
| {BcMV2.BI=TRUE, BcULV4=TRUE} =>  {BcRV1.BI=TRUE} | 0.16  3366 | 0.478  261 | 0.341  584 | 1.72  5155 | 66 |
| {BcEV2.BI=FALSE, BcRV1.BI=TRUE} =>  {BcMV2.BI=TRUE} | 0.12  6238 | 0.593  023 | 0.212  871 | 1.28  1184 | 51 |
| {BcEV2.BI=FALSE, BcMV2.BI=TRUE} =>  {BcRV1.BI=TRUE} | 0.12  6238 | 0.395  349 | 0.319  307 | 1.42  608 | 51 |
| {BcHV3.BI=FALSE, BcRV1.BI=TRUE} =>  {BcMV2.BI=TRUE} | 0.13  6139 | 0.639  535 | 0.212  871 | 1.38  1669 | 55 |
| {BcHV3.BI=FALSE, BcMV2.BI=TRUE} =>  {BcRV1.BI=TRUE} | 0.13  6139 | 0.413  534 | 0.329  208 | 1.49  1676 | 55 |
| {BcFV5.BI=FALSE, BcRV1.BI=TRUE} =>  {BcMV2.BI=TRUE} | 0.14  1089 | 0.593  75 | 0.237  624 | 1.28  2754 | 57 |

| {BcFV5.BI=FALSE, BcMV2.BI=TRUE} =>  {BcRV1.BI=TRUE} | 0.14  1089 | 0.410  072 | 0.344  059 | 1.47  9188 | 57 |
| --- | --- | --- | --- | --- | --- |
| {BcRV1.BI=TRUE, BcULV1.BI=FALSE} =>  {BcMV2.BI=TRUE} | 0.11  8812 | 0.592  593 | 0.200  495 | 1.28  0254 | 48 |
| {BcMV2.BI=TRUE, BcULV1.BI=FALSE} =>  {BcRV1.BI=TRUE} | 0.11  8812 | 0.366  412 | 0.324  257 | 1.32  1701 | 48 |
| {BcMV1.BI=TRUE, BcRV1.BI=TRUE} =>  {BcMV2.BI=TRUE} | 0.14  8515 | 0.731  707 | 0.202  97 | 1.58  0801 | 60 |
| {BcMV1.BI=TRUE, BcMV2.BI=TRUE} =>  {BcRV1.BI=TRUE} | 0.14  8515 | 0.363  636 | 0.408  416 | 1.31  1688 | 60 |
| {BcMV12=FALSE, BcRV1.BI=TRUE} =>  {BcMV2.BI=TRUE} | 0.13  6139 | 0.617  978 | 0.220  297 | 1.33  5096 | 55 |
| {BcMV12=FALSE, BcMV2.BI=TRUE} =>  {BcRV1.BI=TRUE} | 0.13  6139 | 0.398  551 | 0.341  584 | 1.43  7629 | 55 |
| {BcNSRV7.BI=FALSE, BcRV1.BI=TRUE} =>  {BcMV2.BI=TRUE} | 0.14  8515 | 0.618  557 | 0.240  099 | 1.33  6347 | 60 |
| {BcMV2.BI=TRUE, BcNSRV7.BI=FALSE} =>  {BcRV1.BI=TRUE} | 0.14  8515 | 0.410  959 | 0.361  386 | 1.48  2387 | 60 |
| {BcRV1.BI=TRUE, BcVV3.BI=FALSE} =>  {BcMV2.BI=TRUE} | 0.15  3465 | 0.659  574 | 0.232  673 | 1.42  4963 | 62 |
| {BcMV2.BI=TRUE, BcVV3.BI=FALSE} =>  {BcRV1.BI=TRUE} | 0.15  3465 | 0.436  62 | 0.351  485 | 1.57  495 | 62 |

| {BcOLV7.BI=FALSE, BcRV1.BI=TRUE} =>  {BcMV2.BI=TRUE} | 0.10  396 | 0.617  647 | 0.168  317 | 1.33  4382 | 42 |
| --- | --- | --- | --- | --- | --- |
| {BcMV2.BI=TRUE, BcRV1.BI=TRUE} =>  {BcDMV1=FALSE} | 0.16  8317 | 0.944  444 | 0.178  218 | 1.11  8931 | 68 |
| {BcDMV1=FALSE, BcRV1.BI=TRUE} =>  {BcMV2.BI=TRUE} | 0.16  8317 | 0.653  846 | 0.257  426 | 1.41  2587 | 68 |
| {BcDMV1=FALSE, BcMV2.BI=TRUE} =>  {BcRV1.BI=TRUE} | 0.16  8317 | 0.425 | 0.396  04 | 1.53  3036 | 68 |
| {BcFV9=FALSE, BcRV1.BI=TRUE} =>  {BcMV2.BI=TRUE} | 0.13  8614 | 0.595  745 | 0.232  673 | 1.28  7063 | 56 |
| {BcFV9=FALSE, BcMV2.BI=TRUE} =>  {BcRV1.BI=TRUE} | 0.13  8614 | 0.345  679 | 0.400  99 | 1.24  6914 | 56 |
| {BcOLV4.BI=FALSE, BcRV1.BI=TRUE} =>  {BcMV2.BI=TRUE} | 0.14  8515 | 0.606  061 | 0.245  05 | 1.30  935 | 60 |
| {BcMV2.BI=TRUE, BcOLV4.BI=FALSE} =>  {BcRV1.BI=TRUE} | 0.14  8515 | 0.394  737 | 0.376  238 | 1.42  3872 | 60 |
| {BcRV1.BI=TRUE, BcULV5=FALSE} =>  {BcMV2.BI=TRUE} | 0.15  099 | 0.61 | 0.247  525 | 1.31  7861 | 61 |
| {BcMV2.BI=TRUE, BcULV5=FALSE} =>  {BcRV1.BI=TRUE} | 0.15  099 | 0.401  316 | 0.376  238 | 1.44  7603 | 61 |
| {BcBNV5.BI=FALSE, BcRV1.BI=TRUE} =>  {BcMV2.BI=TRUE} | 0.16  5842 | 0.638  095 | 0.259  901 | 1.37  8559 | 67 |

| {BcBNV5.BI=FALSE, BcMV2.BI=TRUE} =>  {BcRV1.BI=TRUE} | 0.16  5842 | 0.406  061 | 0.408  416 | 1.46  4719 | 67 |
| --- | --- | --- | --- | --- | --- |
| {BcRV1.BI=TRUE, BvF.BI=FALSE} =>  {BcMV2.BI=TRUE} | 0.17  0792 | 0.638  889 | 0.267  327 | 1.38  0273 | 69 |
| {BcMV2.BI=TRUE, BvF.BI=FALSE} =>  {BcRV1.BI=TRUE} | 0.17  0792 | 0.394  286 | 0.433  168 | 1.42  2245 | 69 |
| {BcMCV5.BI=FALSE, BcRV1.BI=TRUE} =>  {BcMV2.BI=TRUE} | 0.15  5941 | 0.623  762 | 0.25 | 1.34  7594 | 63 |
| {BcMCV5.BI=FALSE, BcMV2.BI=TRUE} =>  {BcRV1.BI=TRUE} | 0.15  5941 | 0.388  889 | 0.400  99 | 1.40  2778 | 63 |
| {BcOLV13.BI=FALSE, BcRV1.BI=TRUE} =>  {BcMV2.BI=TRUE} | 0.14  604 | 0.621  053 | 0.235  149 | 1.34  1739 | 59 |
| {BcMV2.BI=TRUE, BcOLV13.BI=FALSE} =>  {BcRV1.BI=TRUE} | 0.14  604 | 0.357  576 | 0.408  416 | 1.28  9827 | 59 |
| {BcOLV2.BI=FALSE, BcRV1.BI=TRUE} =>  {BcMV2.BI=TRUE} | 0.15  099 | 0.616  162 | 0.245  05 | 1.33  1173 | 61 |
| {BcMV2.BI=TRUE, BcOLV2.BI=FALSE} =>  {BcRV1.BI=TRUE} | 0.15  099 | 0.358  824 | 0.420  792 | 1.29  4328 | 61 |
| {BcMCV3.BI=FALSE, BcRV1.BI=TRUE} =>  {BcMV2.BI=TRUE} | 0.15  5941 | 0.617  647 | 0.252  475 | 1.33  4382 | 63 |
| {BcMCV3.BI=FALSE, BcMV2.BI=TRUE} =>  {BcRV1.BI=TRUE} | 0.15  5941 | 0.381  818 | 0.408  416 | 1.37  7273 | 63 |

| {BcPV4.BI=FALSE, BcRV1.BI=TRUE} =>  {BcMV2.BI=TRUE} | 0.16  5842 | 0.632  075 | 0.262  376 | 1.36  5553 | 67 |
| --- | --- | --- | --- | --- | --- |
| {BcMV2.BI=TRUE, BcPV4.BI=FALSE} =>  {BcRV1.BI=TRUE} | 0.16  5842 | 0.387  283 | 0.428  218 | 1.39  6986 | 67 |
| {BcEV6=FALSE, BcRV1.BI=TRUE} =>  {BcMV2.BI=TRUE} | 0.17  0792 | 0.638  889 | 0.267  327 | 1.38  0273 | 69 |
| {BcEV6=FALSE, BcMV2.BI=TRUE} =>  {BcRV1.BI=TRUE} | 0.17  0792 | 0.394  286 | 0.433  168 | 1.42  2245 | 69 |
| {BcOLV17.BI=FALSE, BcRV1.BI=TRUE} =>  {BcMV2.BI=TRUE} | 0.14  8515 | 0.618  557 | 0.240  099 | 1.33  6347 | 60 |
| {BcMV2.BI=TRUE, BcOLV17.BI=FALSE} =>  {BcRV1.BI=TRUE} | 0.14  8515 | 0.348  837 | 0.425  743 | 1.25  8306 | 60 |
| {BcOLV15.BI=FALSE, BcRV1.BI=TRUE} =>  {BcMV2.BI=TRUE} | 0.17  0792 | 0.633  028 | 0.269  802 | 1.36  761 | 69 |
| {BcMV2.BI=TRUE, BcOLV15.BI=FALSE} =>  {BcRV1.BI=TRUE} | 0.17  0792 | 0.377  049 | 0.452  97 | 1.36  007 | 69 |
| {BcEV5=FALSE, BcRV1.BI=TRUE} =>  {BcMV2.BI=TRUE} | 0.17  8218 | 0.642  857 | 0.277  228 | 1.38  8846 | 72 |
| {BcEV5=FALSE, BcMV2.BI=TRUE} =>  {BcRV1.BI=TRUE} | 0.17  8218 | 0.421  053 | 0.423  267 | 1.51  8797 | 72 |
| {BcOLV18=FALSE, BcRV1.BI=TRUE} =>  {BcOLV1.BI.MOVA.=FALSE} | 0.07  6733 | 0.861  111 | 0.089  109 | 1.28  8477 | 31 |

| {BcOLV18=FALSE, BcRV1.BI=TRUE} =>  {BcHV3.BI=FALSE} | 0.08  1683 | 0.916  667 | 0.089  109 | 1.20  6298 | 33 |
| --- | --- | --- | --- | --- | --- |
| {BcOLV18=FALSE, BcRV1.BI=TRUE} =>  {BcFV5.BI=FALSE} | 0.08  4158 | 0.944  444 | 0.089  109 | 1.20  7454 | 34 |
| {BcOLV18=FALSE, BcRV1.BI=TRUE} =>  {BcULV1.BI=FALSE} | 0.08  1683 | 0.916  667 | 0.089  109 | 1.16  8244 | 33 |
| {BcOLV18=FALSE, BcRV1.BI=TRUE} =>  {BcALV1.BI=FALSE} | 0.08  1683 | 0.916  667 | 0.089  109 | 1.12  5633 | 33 |
| {BcOLV18=FALSE, BcRV1.BI=TRUE} =>  {BcOLV4.BI=FALSE} | 0.08  6634 | 0.972  222 | 0.089  109 | 1.10  9542 | 35 |
| {BcOLV18=TRUE, BcRV1.BI=TRUE} =>  {BcHV1SLR.BI=TRUE} | 0.13  8614 | 0.736  842 | 0.188  119 | 1.35  9289 | 56 |
| {BcHV1SLR.BI=TRUE, BcRV1.BI=TRUE} =>  {BcOLV18=TRUE} | 0.13  8614 | 0.861  538 | 0.160  891 | 1.66  5366 | 56 |
| {BcHV1SLR.BI=TRUE, BcOLV18=TRUE} =>  {BcRV1.BI=TRUE} | 0.13  8614 | 0.405  797 | 0.341  584 | 1.46  3768 | 56 |
| {BcOLV18=TRUE, BcRV1.BI=TRUE} =>  {BcMV3.BI=TRUE} | 0.13  6139 | 0.723  684 | 0.188  119 | 1.33  5016 | 55 |
| {BcMV3.BI=TRUE, BcRV1.BI=TRUE} =>  {BcOLV18=TRUE} | 0.13  6139 | 0.774  648 | 0.175  743 | 1.49  7405 | 55 |
| {BcMV3.BI=TRUE, BcOLV18=TRUE} =>  {BcRV1.BI=TRUE} | 0.13  6139 | 0.413  534 | 0.329  208 | 1.49  1676 | 55 |

| {BcRV1.BI=TRUE, BcULV3=TRUE} =>  {BcOLV18=TRUE} | 0.11  3861 | 0.707  692 | 0.160  891 | 1.36  7979 | 46 |
| --- | --- | --- | --- | --- | --- |
| {BcOLV18=TRUE, BcULV3=TRUE} =>  {BcRV1.BI=TRUE} | 0.11  3861 | 0.351  145 | 0.324  257 | 1.26  663 | 46 |
| {BcOLV18=TRUE, BcRV1.BI=TRUE} =>  {BcHV1.BI=TRUE} | 0.15  5941 | 0.828  947 | 0.188  119 | 1.37  2519 | 63 |
| {BcHV1.BI=TRUE, BcRV1.BI=TRUE} =>  {BcOLV18=TRUE} | 0.15  5941 | 0.828  947 | 0.188  119 | 1.60  2367 | 63 |
| {BcHV1.BI=TRUE, BcOLV18=TRUE} =>  {BcRV1.BI=TRUE} | 0.15  5941 | 0.425  676 | 0.366  337 | 1.53  5473 | 63 |
| {BcOLV18=TRUE, BcRV1.BI=TRUE} =>  {BcMV11=TRUE} | 0.15  099 | 0.802  632 | 0.188  119 | 1.30  2262 | 61 |
| {BcMV11=TRUE, BcRV1.BI=TRUE} =>  {BcOLV18=TRUE} | 0.15  099 | 0.726  19 | 0.207  921 | 1.40  3737 | 61 |
| {BcMV11=TRUE, BcOLV18=TRUE} =>  {BcRV1.BI=TRUE} | 0.15  099 | 0.435  714 | 0.346  535 | 1.57  1684 | 61 |
| {BcOLV18=TRUE, BcRV1.BI=TRUE} =>  {BcMV9.BI=TRUE} | 0.16  5842 | 0.881  579 | 0.188  119 | 1.29  9846 | 67 |
| {BcMV9.BI=TRUE, BcRV1.BI=TRUE} =>  {BcOLV18=TRUE} | 0.16  5842 | 0.744  444 | 0.222  772 | 1.43  9022 | 67 |
| {BcMV9.BI=TRUE, BcOLV18=TRUE} =>  {BcRV1.BI=TRUE} | 0.16  5842 | 0.401  198 | 0.413  366 | 1.44  7177 | 67 |

| {BcOLV18=TRUE, BcRV1.BI=TRUE} =>  {BcULV4=TRUE} | 0.17  8218 | 0.947  368 | 0.188  119 | 1.37  1817 | 72 |
| --- | --- | --- | --- | --- | --- |
| {BcRV1.BI=TRUE, BcULV4=TRUE} =>  {BcOLV18=TRUE} | 0.17  8218 | 0.734  694 | 0.242  574 | 1.42  0174 | 72 |
| {BcOLV18=TRUE, BcULV4=TRUE} =>  {BcRV1.BI=TRUE} | 0.17  8218 | 0.404  494 | 0.440  594 | 1.45  9069 | 72 |
| {BcEV2.BI=FALSE, BcRV1.BI=TRUE} =>  {BcOLV18=TRUE} | 0.13  8614 | 0.651  163 | 0.212  871 | 1.25  8707 | 56 |
| {BcEV2.BI=FALSE, BcOLV18=TRUE} =>  {BcRV1.BI=TRUE} | 0.13  8614 | 0.375  839 | 0.368  812 | 1.35  5705 | 56 |
| {BcHV3.BI=FALSE, BcOLV18=TRUE} =>  {BcRV1.BI=TRUE} | 0.13  1188 | 0.373  239 | 0.351  485 | 1.34  6328 | 53 |
| {BcFV5.BI=FALSE, BcRV1.BI=TRUE} =>  {BcOLV18=TRUE} | 0.15  3465 | 0.645  833 | 0.237  624 | 1.24  8405 | 62 |
| {BcFV5.BI=FALSE, BcOLV18=TRUE} =>  {BcRV1.BI=TRUE} | 0.15  3465 | 0.416  107 | 0.368  812 | 1.50  0959 | 62 |
| {BcMV1.BI=TRUE, BcRV1.BI=TRUE} =>  {BcOLV18=TRUE} | 0.14  604 | 0.719  512 | 0.202  97 | 1.39  0827 | 59 |
| {BcMV12=FALSE, BcRV1.BI=TRUE} =>  {BcOLV18=TRUE} | 0.14  604 | 0.662  921 | 0.220  297 | 1.28  1436 | 59 |
| {BcMV12=FALSE, BcOLV18=TRUE} =>  {BcRV1.BI=TRUE} | 0.14  604 | 0.368  75 | 0.396  04 | 1.33  0134 | 59 |

| {BcNSRV7.BI=FALSE, BcRV1.BI=TRUE} =>  {BcOLV18=TRUE} | 0.16  0891 | 0.670  103 | 0.240  099 | 1.29  5319 | 65 |
| --- | --- | --- | --- | --- | --- |
| {BcNSRV7.BI=FALSE, BcOLV18=TRUE} =>  {BcRV1.BI=TRUE} | 0.16  0891 | 0.414  013 | 0.388  614 | 1.49  3403 | 65 |
| {BcRV1.BI=TRUE, BcVV3.BI=FALSE} =>  {BcOLV18=TRUE} | 0.16  0891 | 0.691  489 | 0.232  673 | 1.33  6659 | 65 |
| {BcOLV18=TRUE, BcVV3.BI=FALSE} =>  {BcRV1.BI=TRUE} | 0.16  0891 | 0.391  566 | 0.410  891 | 1.41  2435 | 65 |
| {BcOLV18=TRUE, BcRV1.BI=TRUE} =>  {BcDMV1=FALSE} | 0.17  8218 | 0.947  368 | 0.188  119 | 1.12  2395 | 72 |
| {BcDMV1=FALSE, BcRV1.BI=TRUE} =>  {BcOLV18=TRUE} | 0.17  8218 | 0.692  308 | 0.257  426 | 1.33  8241 | 72 |
| {BcDMV1=FALSE, BcOLV18=TRUE} =>  {BcRV1.BI=TRUE} | 0.17  8218 | 0.416  185 | 0.428  218 | 1.50  1239 | 72 |
| {BcFV9=FALSE, BcRV1.BI=TRUE} =>  {BcOLV18=TRUE} | 0.14  8515 | 0.638  298 | 0.232  673 | 1.23  3839 | 60 |
| {BcFV9=FALSE, BcOLV18=TRUE} =>  {BcRV1.BI=TRUE} | 0.14  8515 | 0.352  941 | 0.420  792 | 1.27  3109 | 60 |
| {BcOLV4.BI=FALSE, BcRV1.BI=TRUE} =>  {BcOLV18=TRUE} | 0.15  8416 | 0.646  465 | 0.245  05 | 1.24  9625 | 64 |
| {BcOLV18=TRUE, BcOLV4.BI=FALSE} =>  {BcRV1.BI=TRUE} | 0.15  8416 | 0.353  591 | 0.448  02 | 1.27  5454 | 64 |

| {BcRV1.BI=TRUE, BcULV5=FALSE} =>  {BcOLV18=TRUE} | 0.16  5842 | 0.67 | 0.247  525 | 1.29  512 | 67 |
| --- | --- | --- | --- | --- | --- |
| {BcOLV18=TRUE, BcULV5=FALSE} =>  {BcRV1.BI=TRUE} | 0.16  5842 | 0.354  497 | 0.467  822 | 1.27  8723 | 67 |
| {BcBNV5.BI=FALSE, BcRV1.BI=TRUE} =>  {BcOLV18=TRUE} | 0.17  3267 | 0.666  667 | 0.259  901 | 1.28  8676 | 70 |
| {BcBNV5.BI=FALSE, BcOLV18=TRUE} =>  {BcRV1.BI=TRUE} | 0.17  3267 | 0.382  514 | 0.452  97 | 1.37  9781 | 70 |
| {BcRV1.BI=TRUE, BvF.BI=FALSE} =>  {BcOLV18=TRUE} | 0.18  0693 | 0.675  926 | 0.267  327 | 1.30  6575 | 73 |
| {BcOLV18=TRUE, BvF.BI=FALSE} =>  {BcRV1.BI=TRUE} | 0.18  0693 | 0.380  208 | 0.475  248 | 1.37  1466 | 73 |
| {BcMCV5.BI=FALSE, BcRV1.BI=TRUE} =>  {BcOLV18=TRUE} | 0.16  0891 | 0.643  564 | 0.25 | 1.24  4019 | 65 |
| {BcMCV5.BI=FALSE, BcOLV18=TRUE} =>  {BcRV1.BI=TRUE} | 0.16  0891 | 0.357  143 | 0.450  495 | 1.28  8265 | 65 |
| {BcOLV13.BI=FALSE, BcRV1.BI=TRUE} =>  {BcOLV18=TRUE} | 0.15  3465 | 0.652  632 | 0.235  149 | 1.26  1546 | 62 |
| {BcOLV2.BI=FALSE, BcRV1.BI=TRUE} =>  {BcOLV18=TRUE} | 0.15  8416 | 0.646  465 | 0.245  05 | 1.24  9625 | 64 |
| {BcOLV18=TRUE, BcOLV2.BI=FALSE} =>  {BcRV1.BI=TRUE} | 0.15  8416 | 0.338  624 | 0.467  822 | 1.22  1466 | 64 |

| {BcMCV3.BI=FALSE, BcRV1.BI=TRUE} =>  {BcOLV18=TRUE} | 0.16  5842 | 0.656  863 | 0.252  475 | 1.26  9725 | 67 |
| --- | --- | --- | --- | --- | --- |
| {BcMCV3.BI=FALSE, BcOLV18=TRUE} =>  {BcRV1.BI=TRUE} | 0.16  5842 | 0.348  958 | 0.475  248 | 1.25  8743 | 67 |
| {BcPV4.BI=FALSE, BcRV1.BI=TRUE} =>  {BcOLV18=TRUE} | 0.18  0693 | 0.688  679 | 0.262  376 | 1.33  1227 | 73 |
| {BcOLV18=TRUE, BcPV4.BI=FALSE} =>  {BcRV1.BI=TRUE} | 0.18  0693 | 0.378  238 | 0.477  723 | 1.36  436 | 73 |
| {BcEV6=FALSE, BcRV1.BI=TRUE} =>  {BcOLV18=TRUE} | 0.17  8218 | 0.666  667 | 0.267  327 | 1.28  8676 | 72 |
| {BcEV6=FALSE, BcOLV18=TRUE} =>  {BcRV1.BI=TRUE} | 0.17  8218 | 0.369  231 | 0.482  673 | 1.33  1868 | 72 |
| {BcOLV17.BI=FALSE, BcRV1.BI=TRUE} =>  {BcOLV18=TRUE} | 0.15  5941 | 0.649  485 | 0.240  099 | 1.25  5463 | 63 |
| {BcOLV15.BI=FALSE, BcRV1.BI=TRUE} =>  {BcOLV18=TRUE} | 0.18  0693 | 0.669  725 | 0.269  802 | 1.29  4588 | 73 |
| {BcOLV15.BI=FALSE, BcOLV18=TRUE} =>  {BcRV1.BI=TRUE} | 0.18  0693 | 0.378  238 | 0.477  723 | 1.36  436 | 73 |
| {BcEV5=FALSE, BcRV1.BI=TRUE} =>  {BcOLV18=TRUE} | 0.18  8119 | 0.678  571 | 0.277  228 | 1.31  1688 | 76 |
| {BcEV5=FALSE, BcOLV18=TRUE} =>  {BcRV1.BI=TRUE} | 0.18  8119 | 0.385  787 | 0.487  624 | 1.39  1588 | 76 |

| {BcMV2.BI=FALSE, BcRV1.BI=TRUE} =>  {BcMV9.BI=TRUE} | 0.07  9208 | 0.8 | 0.099  01 | 1.17  9562 | 32 |
| --- | --- | --- | --- | --- | --- |
| {BcMV2.BI=FALSE, BcRV1.BI=TRUE} =>  {BcULV4=TRUE} | 0.07  9208 | 0.8 | 0.099  01 | 1.15  8423 | 32 |
| {BcMV2.BI=FALSE, BcRV1.BI=TRUE} =>  {BcEV2.BI=FALSE} | 0.08  6634 | 0.875 | 0.099  01 | 1.17  053 | 35 |
| {BcMV2.BI=FALSE, BcRV1.BI=TRUE} =>  {BcFV5.BI=FALSE} | 0.09  6535 | 0.975 | 0.099  01 | 1.24  6519 | 39 |
| {BcMV2.BI=FALSE, BcRV1.BI=TRUE} =>  {BcNSRV7.BI=FALSE} | 0.09  1584 | 0.925 | 0.099  01 | 1.15  6966 | 37 |
| {BcMV2.BI=FALSE, BcRV1.BI=TRUE} =>  {BcALV1.BI=FALSE} | 0.09  1584 | 0.925 | 0.099  01 | 1.13  5866 | 37 |
| {BcMV2.BI=FALSE, BcRV1.BI=TRUE} =>  {BcOLV4.BI=FALSE} | 0.09  6535 | 0.975 | 0.099  01 | 1.11  2712 | 39 |
| {BcMV2.BI=FALSE, BcRV1.BI=TRUE} =>  {BcULV5=FALSE} | 0.09  6535 | 0.975 | 0.099  01 | 1.11  2712 | 39 |
| {BcHV1SLR.BI=TRUE, BcRV1.BI=TRUE} =>  {BcMV3.BI=TRUE} | 0.11  8812 | 0.738  462 | 0.160  891 | 1.36  2276 | 48 |
| {BcMV3.BI=TRUE, BcRV1.BI=TRUE} =>  {BcHV1SLR.BI=TRUE} | 0.11  8812 | 0.676  056 | 0.175  743 | 1.24  7154 | 48 |
| {BcHV1SLR.BI=TRUE, BcMV3.BI=TRUE} =>  {BcRV1.BI=TRUE} | 0.11  8812 | 0.360  902 | 0.329  208 | 1.30  1826 | 48 |

| {BcRV1.BI=TRUE, BcULV3=TRUE} =>  {BcHV1SLR.BI=TRUE} | 0.11  1386 | 0.692  308 | 0.160  891 | 1.27  7134 | 45 |
| --- | --- | --- | --- | --- | --- |
| {BcHV1SLR.BI=TRUE, BcRV1.BI=TRUE} =>  {BcHV1.BI=TRUE} | 0.15  099 | 0.938  462 | 0.160  891 | 1.55  3846 | 61 |
| {BcHV1.BI=TRUE, BcRV1.BI=TRUE} =>  {BcHV1SLR.BI=TRUE} | 0.15  099 | 0.802  632 | 0.188  119 | 1.48  0654 | 61 |
| {BcHV1SLR.BI=TRUE, BcRV1.BI=TRUE} =>  {BcMV11=TRUE} | 0.13  6139 | 0.846  154 | 0.160  891 | 1.37  2876 | 55 |
| {BcMV11=TRUE, BcRV1.BI=TRUE} =>  {BcHV1SLR.BI=TRUE} | 0.13  6139 | 0.654  762 | 0.207  921 | 1.20  7871 | 55 |
| {BcHV1SLR.BI=TRUE, BcMV11=TRUE} =>  {BcRV1.BI=TRUE} | 0.13  6139 | 0.381  944 | 0.356  436 | 1.37  7728 | 55 |
| {BcHV1SLR.BI=TRUE, BcRV1.BI=TRUE} =>  {BcMV9.BI=TRUE} | 0.14  604 | 0.907  692 | 0.160  891 | 1.33  8349 | 59 |
| {BcMV9.BI=TRUE, BcRV1.BI=TRUE} =>  {BcHV1SLR.BI=TRUE} | 0.14  604 | 0.655  556 | 0.222  772 | 1.20  9335 | 59 |
| {BcHV1SLR.BI=TRUE, BcMV9.BI=TRUE} =>  {BcRV1.BI=TRUE} | 0.14  604 | 0.357  576 | 0.408  416 | 1.28  9827 | 59 |
| {BcHV1SLR.BI=TRUE, BcRV1.BI=TRUE} =>  {BcULV4=TRUE} | 0.15  8416 | 0.984  615 | 0.160  891 | 1.42  5751 | 64 |
| {BcRV1.BI=TRUE, BcULV4=TRUE} =>  {BcHV1SLR.BI=TRUE} | 0.15  8416 | 0.653  061 | 0.242  574 | 1.20  4734 | 64 |

| {BcHV1SLR.BI=TRUE, BcULV4=TRUE} =>  {BcRV1.BI=TRUE} | 0.15  8416 | 0.395  062 | 0.400  99 | 1.42  5044 | 64 |
| --- | --- | --- | --- | --- | --- |
| {BcHV1SLR.BI=TRUE, BcRV1.BI=TRUE} =>  {BcDMV1=FALSE} | 0.15  099 | 0.938  462 | 0.160  891 | 1.11  1843 | 61 |
| {BcRV1.BI=TRUE, BcULV3=TRUE} =>  {BcMV3.BI=TRUE} | 0.10  6436 | 0.661  538 | 0.160  891 | 1.22  0372 | 43 |
| {BcMV3.BI=TRUE, BcRV1.BI=TRUE} =>  {BcHV1.BI=TRUE} | 0.13  1188 | 0.746  479 | 0.175  743 | 1.23  5973 | 53 |
| {BcHV1.BI=TRUE, BcRV1.BI=TRUE} =>  {BcMV3.BI=TRUE} | 0.13  1188 | 0.697  368 | 0.188  119 | 1.28  647 | 53 |
| {BcHV1.BI=TRUE, BcMV3.BI=TRUE} =>  {BcRV1.BI=TRUE} | 0.13  1188 | 0.370  629 | 0.353  96 | 1.33  6913 | 53 |
| {BcMV3.BI=TRUE, BcRV1.BI=TRUE} =>  {BcMV11=TRUE} | 0.14  8515 | 0.845  07 | 0.175  743 | 1.37  1118 | 60 |
| {BcMV11=TRUE, BcRV1.BI=TRUE} =>  {BcMV3.BI=TRUE} | 0.14  8515 | 0.714  286 | 0.207  921 | 1.31  7678 | 60 |
| {BcMV11=TRUE, BcMV3.BI=TRUE} =>  {BcRV1.BI=TRUE} | 0.14  8515 | 0.387  097 | 0.383  663 | 1.39  6313 | 60 |
| {BcMV3.BI=TRUE, BcRV1.BI=TRUE} =>  {BcMV9.BI=TRUE} | 0.14  8515 | 0.845  07 | 0.175  743 | 1.24  6016 | 60 |
| {BcMV9.BI=TRUE, BcRV1.BI=TRUE} =>  {BcMV3.BI=TRUE} | 0.14  8515 | 0.666  667 | 0.222  772 | 1.22  9833 | 60 |

| {BcMV3.BI=TRUE, BcMV9.BI=TRUE} =>  {BcRV1.BI=TRUE} | 0.14  8515 | 0.375 | 0.396  04 | 1.35  2679 | 60 |
| --- | --- | --- | --- | --- | --- |
| {BcMV3.BI=TRUE, BcRV1.BI=TRUE} =>  {BcULV4=TRUE} | 0.15  3465 | 0.873  239 | 0.175  743 | 1.26  4476 | 62 |
| {BcMV3.BI=TRUE, BcULV4=TRUE} =>  {BcRV1.BI=TRUE} | 0.15  3465 | 0.382  716 | 0.400  99 | 1.38  0511 | 62 |
| {BcFV5.BI=FALSE, BcMV3.BI=TRUE} =>  {BcRV1.BI=TRUE} | 0.14  604 | 0.375  796 | 0.388  614 | 1.35  5551 | 59 |
| {BcMV1.BI=TRUE, BcRV1.BI=TRUE} =>  {BcMV3.BI=TRUE} | 0.13  8614 | 0.682  927 | 0.202  97 | 1.25  9828 | 56 |
| {BcMV3.BI=TRUE, BcNSRV7.BI=FALSE} =>  {BcRV1.BI=TRUE} | 0.15  3465 | 0.362  573 | 0.423  267 | 1.30  7853 | 62 |
| {BcMV3.BI=TRUE, BcVV3.BI=FALSE} =>  {BcRV1.BI=TRUE} | 0.14  8515 | 0.357  143 | 0.415  842 | 1.28  8265 | 60 |
| {BcDMV1=FALSE, BcMV3.BI=TRUE} =>  {BcRV1.BI=TRUE} | 0.15  8416 | 0.374  269 | 0.423  267 | 1.35  0042 | 64 |
| {BcBNV5.BI=FALSE, BcRV1.BI=TRUE} =>  {BcMV3.BI=TRUE} | 0.16  5842 | 0.638  095 | 0.259  901 | 1.17  7125 | 67 |
| {BcBNV5.BI=FALSE, BcMV3.BI=TRUE} =>  {BcRV1.BI=TRUE} | 0.16  5842 | 0.352  632 | 0.470  297 | 1.27  1992 | 67 |
| {BcMV3.BI=TRUE, BvF.BI=FALSE} =>  {BcRV1.BI=TRUE} | 0.16  5842 | 0.335 | 0.495  05 | 1.20  8393 | 67 |

| {BcMV3.BI=TRUE, BcOLV15.BI=FALSE} =>  {BcRV1.BI=TRUE} | 0.17  0792 | 0.336  585 | 0.507  426 | 1.21  4111 | 69 |
| --- | --- | --- | --- | --- | --- |
| {BcEV5=FALSE, BcMV3.BI=TRUE} =>  {BcRV1.BI=TRUE} | 0.17  5743 | 0.355 | 0.495  05 | 1.28  0536 | 71 |
| {BcMV4.BI=FALSE, BcRV1.BI=TRUE} =>  {BcULV4=TRUE} | 0.09  1584 | 0.822  222 | 0.111  386 | 1.19  0601 | 37 |
| {BcMV4.BI=FALSE, BcRV1.BI=TRUE} =>  {BcHV3.BI=FALSE} | 0.09  901 | 0.888  889 | 0.111  386 | 1.16  9743 | 40 |
| {BcMV4.BI=FALSE, BcRV1.BI=TRUE} =>  {BcFV5.BI=FALSE} | 0.10  1485 | 0.911  111 | 0.111  386 | 1.16  4838 | 41 |
| {BcMV4.BI=FALSE, BcRV1.BI=TRUE} =>  {BcNSRV7.BI=FALSE} | 0.10  1485 | 0.911  111 | 0.111  386 | 1.13  9594 | 41 |
| {BcOLV16.BI=FALSE, BcRV1.BI=TRUE} =>  {BcFV5.BI=FALSE} | 0.11  6337 | 0.921  569 | 0.126  238 | 1.17  8208 | 47 |
| {BcRV1.BI=TRUE, BcULV3=TRUE} =>  {BcHV1.BI=TRUE} | 0.12  6238 | 0.784  615 | 0.160  891 | 1.29  9117 | 51 |
| {BcRV1.BI=TRUE, BcULV3=TRUE} =>  {BcMV11=TRUE} | 0.12  6238 | 0.784  615 | 0.160  891 | 1.27  3031 | 51 |
| {BcRV1.BI=TRUE, BcULV3=TRUE} =>  {BcMV9.BI=TRUE} | 0.14  3564 | 0.892  308 | 0.160  891 | 1.31  5665 | 58 |
| {BcMV9.BI=TRUE, BcULV3=TRUE} =>  {BcRV1.BI=TRUE} | 0.14  3564 | 0.339  181 | 0.423  267 | 1.22  3475 | 58 |

| {BcRV1.BI=TRUE, BcULV3=TRUE} =>  {BcULV4=TRUE} | 0.14  8515 | 0.923  077 | 0.160  891 | 1.33  6642 | 60 |
| --- | --- | --- | --- | --- | --- |
| {BcULV3=TRUE, BcULV4=TRUE} =>  {BcRV1.BI=TRUE} | 0.14  8515 | 0.357  143 | 0.415  842 | 1.28  8265 | 60 |
| {BcRV1.BI=TRUE, BcULV3=TRUE} =>  {BcDMV1=FALSE} | 0.15  099 | 0.938  462 | 0.160  891 | 1.11  1843 | 61 |
| {BcRV1.BI=TRUE, BcULV3=TRUE} =>  {BvF.BI=FALSE} | 0.16  0891 | 1 | 0.160  891 | 1.09  4851 | 65 |
| {BcHV1.BI=TRUE, BcRV1.BI=TRUE} =>  {BcMV11=TRUE} | 0.15  099 | 0.802  632 | 0.188  119 | 1.30  2262 | 61 |
| {BcMV11=TRUE, BcRV1.BI=TRUE} =>  {BcHV1.BI=TRUE} | 0.15  099 | 0.726  19 | 0.207  921 | 1.20  2381 | 61 |
| {BcHV1.BI=TRUE, BcMV11=TRUE} =>  {BcRV1.BI=TRUE} | 0.15  099 | 0.396  104 | 0.381  188 | 1.42  8803 | 61 |
| {BcHV1.BI=TRUE, BcRV1.BI=TRUE} =>  {BcMV9.BI=TRUE} | 0.17  5743 | 0.934  211 | 0.188  119 | 1.37  7449 | 71 |
| {BcMV9.BI=TRUE, BcRV1.BI=TRUE} =>  {BcHV1.BI=TRUE} | 0.17  5743 | 0.788  889 | 0.222  772 | 1.30  6193 | 71 |
| {BcHV1.BI=TRUE, BcMV9.BI=TRUE} =>  {BcRV1.BI=TRUE} | 0.17  5743 | 0.375  661 | 0.467  822 | 1.35  5064 | 71 |
| {BcHV1.BI=TRUE, BcRV1.BI=TRUE} =>  {BcULV4=TRUE} | 0.18  3168 | 0.973  684 | 0.188  119 | 1.40  9923 | 74 |

| {BcRV1.BI=TRUE, BcULV4=TRUE} =>  {BcHV1.BI=TRUE} | 0.18  3168 | 0.755  102 | 0.242  574 | 1.25  0251 | 74 |
| --- | --- | --- | --- | --- | --- |
| {BcHV1.BI=TRUE, BcULV4=TRUE} =>  {BcRV1.BI=TRUE} | 0.18  3168 | 0.402  174 | 0.455  446 | 1.45  0699 | 74 |
| {BcFV5.BI=FALSE, BcHV1.BI=TRUE} =>  {BcRV1.BI=TRUE} | 0.16  0891 | 0.355  191 | 0.452  97 | 1.28  1226 | 65 |
| {BcMV1.BI=TRUE, BcRV1.BI=TRUE} =>  {BcHV1.BI=TRUE} | 0.14  604 | 0.719  512 | 0.202  97 | 1.19  1323 | 59 |
| {BcHV1.BI=TRUE, BcNSRV7.BI=FALSE} =>  {BcRV1.BI=TRUE} | 0.15  8416 | 0.340  426 | 0.465  347 | 1.22  7964 | 64 |
| {BcHV1.BI=TRUE, BcRV1.BI=TRUE} =>  {BcDMV1=FALSE} | 0.17  5743 | 0.934  211 | 0.188  119 | 1.10  6807 | 71 |
| {BcDMV1=FALSE, BcHV1.BI=TRUE} =>  {BcRV1.BI=TRUE} | 0.17  5743 | 0.339  713 | 0.517  327 | 1.22  5393 | 71 |
| {BcPV4.BI=FALSE, BcRV1.BI=TRUE} =>  {BcHV1.BI=TRUE} | 0.18  3168 | 0.698  113 | 0.262  376 | 1.15  5892 | 74 |
| {BcMV11=TRUE, BcRV1.BI=TRUE} =>  {BcMV9.BI=TRUE} | 0.16  5842 | 0.797  619 | 0.207  921 | 1.17  6051 | 67 |
| {BcMV9.BI=TRUE, BcRV1.BI=TRUE} =>  {BcMV11=TRUE} | 0.16  5842 | 0.744  444 | 0.222  772 | 1.20  7854 | 67 |
| {BcMV11=TRUE, BcMV9.BI=TRUE} =>  {BcRV1.BI=TRUE} | 0.16  5842 | 0.408  537 | 0.405  941 | 1.47  365 | 67 |

| {BcMV11=TRUE, BcRV1.BI=TRUE} =>  {BcULV4=TRUE} | 0.17  8218 | 0.857  143 | 0.207  921 | 1.24  1167 | 72 |
| --- | --- | --- | --- | --- | --- |
| {BcRV1.BI=TRUE, BcULV4=TRUE} =>  {BcMV11=TRUE} | 0.17  8218 | 0.734  694 | 0.242  574 | 1.19  2033 | 72 |
| {BcMV11=TRUE, BcULV4=TRUE} =>  {BcRV1.BI=TRUE} | 0.17  8218 | 0.426  036 | 0.418  317 | 1.53  6771 | 72 |
| {BcPV3.BI=FALSE, BcRV1.BI=TRUE} =>  {BcMV11=TRUE} | 0.11  1386 | 0.737  705 | 0.150  99 | 1.19  6919 | 45 |
| {BcEV2.BI=FALSE, BcRV1.BI=TRUE} =>  {BcMV11=TRUE} | 0.15  5941 | 0.732  558 | 0.212  871 | 1.18  8568 | 63 |
| {BcEV2.BI=FALSE, BcMV11=TRUE} =>  {BcRV1.BI=TRUE} | 0.15  5941 | 0.362  069 | 0.430  693 | 1.30  6034 | 63 |
| {BcHV3.BI=FALSE, BcRV1.BI=TRUE} =>  {BcMV11=TRUE} | 0.15  3465 | 0.720  93 | 0.212  871 | 1.16  9702 | 62 |
| {BcHV3.BI=FALSE, BcMV11=TRUE} =>  {BcRV1.BI=TRUE} | 0.15  3465 | 0.346  369 | 0.443  069 | 1.24  9401 | 62 |
| {BcFV5.BI=FALSE, BcRV1.BI=TRUE} =>  {BcMV11=TRUE} | 0.17  0792 | 0.718  75 | 0.237  624 | 1.16  6165 | 69 |
| {BcFV5.BI=FALSE, BcMV11=TRUE} =>  {BcRV1.BI=TRUE} | 0.17  0792 | 0.359  375 | 0.475  248 | 1.29  6317 | 69 |
| {BcMV1.BI=TRUE, BcRV1.BI=TRUE} =>  {BcMV11=TRUE} | 0.16  0891 | 0.792  683 | 0.202  97 | 1.28  612 | 65 |

| {BcMV12=FALSE, BcRV1.BI=TRUE} =>  {BcMV11=TRUE} | 0.16  0891 | 0.730  337 | 0.220  297 | 1.18  4965 | 65 |
| --- | --- | --- | --- | --- | --- |
| {BcNSRV7.BI=FALSE, BcRV1.BI=TRUE} =>  {BcMV11=TRUE} | 0.17  5743 | 0.731  959 | 0.240  099 | 1.18  7596 | 71 |
| {BcMV11=TRUE, BcNSRV7.BI=FALSE} =>  {BcRV1.BI=TRUE} | 0.17  5743 | 0.362  245 | 0.485  149 | 1.30  6669 | 71 |
| {BcRV1.BI=TRUE, BcVV3.BI=FALSE} =>  {BcMV11=TRUE} | 0.18  0693 | 0.776  596 | 0.232  673 | 1.26  0019 | 73 |
| {BcMV11=TRUE, BcVV3.BI=FALSE} =>  {BcRV1.BI=TRUE} | 0.18  0693 | 0.374  359 | 0.482  673 | 1.35  0366 | 73 |
| {BcOLV7.BI=FALSE, BcRV1.BI=TRUE} =>  {BcMV11=TRUE} | 0.12  8713 | 0.764  706 | 0.168  317 | 1.24  0728 | 52 |
| {BcMV11=TRUE, BcRV1.BI=TRUE} =>  {BcDMV1=FALSE} | 0.19  0594 | 0.916  667 | 0.207  921 | 1.08  6022 | 77 |
| {BcDMV1=FALSE, BcRV1.BI=TRUE} =>  {BcMV11=TRUE} | 0.19  0594 | 0.740  385 | 0.257  426 | 1.20  1267 | 77 |
| {BcDMV1=FALSE, BcMV11=TRUE} =>  {BcRV1.BI=TRUE} | 0.19  0594 | 0.373  786 | 0.509  901 | 1.34  8301 | 77 |
| {BcFV9=FALSE, BcRV1.BI=TRUE} =>  {BcMV11=TRUE} | 0.16  8317 | 0.723  404 | 0.232  673 | 1.17  3716 | 68 |
| {BcOLV4.BI=FALSE, BcRV1.BI=TRUE} =>  {BcMV11=TRUE} | 0.17  5743 | 0.717  172 | 0.245  05 | 1.16  3604 | 71 |

| {BcMV11=TRUE, BcOLV4.BI=FALSE} =>  {BcRV1.BI=TRUE} | 0.17  5743 | 0.342  995 | 0.512  376 | 1.23  7233 | 71 |
| --- | --- | --- | --- | --- | --- |
| {BcRV1.BI=TRUE, BcULV5=FALSE} =>  {BcMV11=TRUE} | 0.17  8218 | 0.72 | 0.247  525 | 1.16  8193 | 72 |
| {BcMV11=TRUE, BcULV5=FALSE} =>  {BcRV1.BI=TRUE} | 0.17  8218 | 0.352  941 | 0.504  95 | 1.27  3109 | 72 |
| {BcBNV5.BI=FALSE, BcRV1.BI=TRUE} =>  {BcMV11=TRUE} | 0.19  5545 | 0.752  381 | 0.259  901 | 1.22  0731 | 79 |
| {BcBNV5.BI=FALSE, BcMV11=TRUE} =>  {BcRV1.BI=TRUE} | 0.19  5545 | 0.364  055 | 0.537  129 | 1.31  3199 | 79 |
| {BcRV1.BI=TRUE, BvF.BI=FALSE} =>  {BcMV11=TRUE} | 0.20  0495 | 0.75 | 0.267  327 | 1.21  6867 | 81 |
| {BcMV11=TRUE, BvF.BI=FALSE} =>  {BcRV1.BI=TRUE} | 0.20  0495 | 0.350  649 | 0.571  782 | 1.26  4842 | 81 |
| {BcMCV5.BI=FALSE, BcRV1.BI=TRUE} =>  {BcMV11=TRUE} | 0.18  3168 | 0.732  673 | 0.25 | 1.18  8755 | 74 |
| {BcMCV5.BI=FALSE, BcMV11=TRUE} =>  {BcRV1.BI=TRUE} | 0.18  3168 | 0.334  842 | 0.547  03 | 1.20  7822 | 74 |
| {BcOLV13.BI=FALSE, BcRV1.BI=TRUE} =>  {BcMV11=TRUE} | 0.17  3267 | 0.736  842 | 0.235  149 | 1.19  5519 | 70 |
| {BcOLV2.BI=FALSE, BcRV1.BI=TRUE} =>  {BcMV11=TRUE} | 0.17  8218 | 0.727  273 | 0.245  05 | 1.17  9993 | 72 |

| {BcMCV3.BI=FALSE, BcRV1.BI=TRUE} =>  {BcMV11=TRUE} | 0.18  8119 | 0.745  098 | 0.252  475 | 1.20  8914 | 76 |
| --- | --- | --- | --- | --- | --- |
| {BcPV4.BI=FALSE, BcRV1.BI=TRUE} =>  {BcMV11=TRUE} | 0.19  3069 | 0.735  849 | 0.262  376 | 1.19  3908 | 78 |
| {BcMV11=TRUE, BcPV4.BI=FALSE} =>  {BcRV1.BI=TRUE} | 0.19  3069 | 0.334  764 | 0.576  733 | 1.20  7541 | 78 |
| {BcEV6=FALSE, BcRV1.BI=TRUE} =>  {BcMV11=TRUE} | 0.20  0495 | 0.75 | 0.267  327 | 1.21  6867 | 81 |
| {BcEV6=FALSE, BcMV11=TRUE} =>  {BcRV1.BI=TRUE} | 0.20  0495 | 0.347  639 | 0.576  733 | 1.25  3985 | 81 |
| {BcOLV17.BI=FALSE, BcRV1.BI=TRUE} =>  {BcMV11=TRUE} | 0.17  5743 | 0.731  959 | 0.240  099 | 1.18  7596 | 71 |
| {BcOLV15.BI=FALSE, BcRV1.BI=TRUE} =>  {BcMV11=TRUE} | 0.20  0495 | 0.743  119 | 0.269  802 | 1.20  5704 | 81 |
| {BcMV11=TRUE, BcOLV15.BI=FALSE} =>  {BcRV1.BI=TRUE} | 0.20  0495 | 0.340  336 | 0.589  109 | 1.22  7641 | 81 |
| {BcEV5=FALSE, BcRV1.BI=TRUE} =>  {BcMV11=TRUE} | 0.20  7921 | 0.75 | 0.277  228 | 1.21  6867 | 84 |
| {BcEV5=FALSE, BcMV11=TRUE} =>  {BcRV1.BI=TRUE} | 0.20  7921 | 0.362  069 | 0.574  257 | 1.30  6034 | 84 |
| {BcOLV1.BI.MOVA.=FALSE,  BcRV1.BI=TRUE} => {BcULV4=TRUE} | 0.11  6337 | 0.810  345 | 0.143  564 | 1.17  3403 | 47 |

| {BcOLV1.BI.MOVA.=FALSE,  BcRV1.BI=TRUE} => {BcOLV4.BI=FALSE} | 0.13  6139 | 0.948  276 | 0.143  564 | 1.08  2213 | 55 |
| --- | --- | --- | --- | --- | --- |
| {BcMV9.BI=TRUE, BcRV1.BI=TRUE} =>  {BcULV4=TRUE} | 0.21  2871 | 0.955  556 | 0.222  772 | 1.38  3672 | 86 |
| {BcRV1.BI=TRUE, BcULV4=TRUE} =>  {BcMV9.BI=TRUE} | 0.21  2871 | 0.877  551 | 0.242  574 | 1.29  3907 | 86 |
| {BcMV9.BI=TRUE, BcULV4=TRUE} =>  {BcRV1.BI=TRUE} | 0.21  2871 | 0.389  14 | 0.547  03 | 1.40  3685 | 86 |
| {BcMV9.BI=TRUE, BcRV1.BI=TRUE} =>  {BcEV2.BI=FALSE} | 0.18  5644 | 0.833  333 | 0.222  772 | 1.11  479 | 75 |
| {BcEV2.BI=FALSE, BcRV1.BI=TRUE} =>  {BcMV9.BI=TRUE} | 0.18  5644 | 0.872  093 | 0.212  871 | 1.28  586 | 75 |
| {BcEV2.BI=FALSE, BcMV9.BI=TRUE} =>  {BcRV1.BI=TRUE} | 0.18  5644 | 0.367  647 | 0.504  95 | 1.32  6155 | 75 |
| {BcHV3.BI=FALSE, BcRV1.BI=TRUE} =>  {BcMV9.BI=TRUE} | 0.16  5842 | 0.779  07 | 0.212  871 | 1.14  8701 | 67 |
| {BcFV5.BI=FALSE, BcRV1.BI=TRUE} =>  {BcMV9.BI=TRUE} | 0.18  8119 | 0.791  667 | 0.237  624 | 1.16  7275 | 76 |
| {BcFV5.BI=FALSE, BcMV9.BI=TRUE} =>  {BcRV1.BI=TRUE} | 0.18  8119 | 0.367  15 | 0.512  376 | 1.32  4362 | 76 |
| {BcMV1.BI=TRUE, BcRV1.BI=TRUE} =>  {BcMV9.BI=TRUE} | 0.16  3366 | 0.804  878 | 0.202  97 | 1.18  6754 | 66 |

| {BcMV12=FALSE, BcRV1.BI=TRUE} =>  {BcMV9.BI=TRUE} | 0.17  5743 | 0.797  753 | 0.220  297 | 1.17  6249 | 71 |
| --- | --- | --- | --- | --- | --- |
| {BcNSRV7.BI=FALSE, BcRV1.BI=TRUE} =>  {BcMV9.BI=TRUE} | 0.18  5644 | 0.773  196 | 0.240  099 | 1.14  0041 | 75 |
| {BcMV9.BI=TRUE, BcNSRV7.BI=FALSE} =>  {BcRV1.BI=TRUE} | 0.18  5644 | 0.358  852 | 0.517  327 | 1.29  4429 | 75 |
| {BcRV1.BI=TRUE, BcVV3.BI=FALSE} =>  {BcMV9.BI=TRUE} | 0.19  0594 | 0.819  149 | 0.232  673 | 1.20  7796 | 77 |
| {BcMV9.BI=TRUE, BcVV3.BI=FALSE} =>  {BcRV1.BI=TRUE} | 0.19  0594 | 0.343  75 | 0.554  455 | 1.23  9955 | 77 |
| {BcMV9.BI=TRUE, BcRV1.BI=TRUE} =>  {BcDMV1=FALSE} | 0.20  5446 | 0.922  222 | 0.222  772 | 1.09  2603 | 83 |
| {BcDMV1=FALSE, BcRV1.BI=TRUE} =>  {BcMV9.BI=TRUE} | 0.20  5446 | 0.798  077 | 0.257  426 | 1.17  6727 | 83 |
| {BcDMV1=FALSE, BcMV9.BI=TRUE} =>  {BcRV1.BI=TRUE} | 0.20  5446 | 0.372  197 | 0.551  98 | 1.34  2569 | 83 |
| {BcFV9=FALSE, BcRV1.BI=TRUE} =>  {BcMV9.BI=TRUE} | 0.19  0594 | 0.819  149 | 0.232  673 | 1.20  7796 | 77 |
| {BcOLV4.BI=FALSE, BcRV1.BI=TRUE} =>  {BcMV9.BI=TRUE} | 0.20  0495 | 0.818  182 | 0.245  05 | 1.20  637 | 81 |
| {BcRV1.BI=TRUE, BcULV5=FALSE} =>  {BcMV9.BI=TRUE} | 0.20  7921 | 0.84 | 0.247  525 | 1.23  854 | 84 |

| {BcBNV5.BI=FALSE, BcRV1.BI=TRUE} =>  {BcMV9.BI=TRUE} | 0.20  5446 | 0.790  476 | 0.259  901 | 1.16  552 | 83 |
| --- | --- | --- | --- | --- | --- |
| {BcBNV5.BI=FALSE, BcMV9.BI=TRUE} =>  {BcRV1.BI=TRUE} | 0.20  5446 | 0.344  398 | 0.596  535 | 1.24  2294 | 83 |
| {BcRV1.BI=TRUE, BvF.BI=FALSE} =>  {BcMV9.BI=TRUE} | 0.21  2871 | 0.796  296 | 0.267  327 | 1.17  4101 | 86 |
| {BcMV9.BI=TRUE, BvF.BI=FALSE} =>  {BcRV1.BI=TRUE} | 0.21  2871 | 0.348  178 | 0.611  386 | 1.25  5928 | 86 |
| {BcMCV5.BI=FALSE, BcRV1.BI=TRUE} =>  {BcMV9.BI=TRUE} | 0.20  5446 | 0.821  782 | 0.25 | 1.21  1679 | 83 |
| {BcMCV5.BI=FALSE, BcMV9.BI=TRUE} =>  {BcRV1.BI=TRUE} | 0.20  5446 | 0.333  333 | 0.616  337 | 1.20  2381 | 83 |
| {BcOLV13.BI=FALSE, BcRV1.BI=TRUE} =>  {BcMV9.BI=TRUE} | 0.19  0594 | 0.810  526 | 0.235  149 | 1.19  5083 | 77 |
| {BcOLV2.BI=FALSE, BcRV1.BI=TRUE} =>  {BcMV9.BI=TRUE} | 0.19  5545 | 0.797  98 | 0.245  05 | 1.17  6583 | 79 |
| {BcMCV3.BI=FALSE, BcRV1.BI=TRUE} =>  {BcMV9.BI=TRUE} | 0.19  802 | 0.784  314 | 0.252  475 | 1.15  6433 | 80 |
| {BcPV4.BI=FALSE, BcRV1.BI=TRUE} =>  {BcMV9.BI=TRUE} | 0.21  5347 | 0.820  755 | 0.262  376 | 1.21  0164 | 87 |
| {BcMV9.BI=TRUE, BcPV4.BI=FALSE} =>  {BcRV1.BI=TRUE} | 0.21  5347 | 0.339  844 | 0.633  663 | 1.22  5865 | 87 |

| {BcEV6=FALSE, BcRV1.BI=TRUE} =>  {BcMV9.BI=TRUE} | 0.21  2871 | 0.796  296 | 0.267  327 | 1.17  4101 | 86 |
| --- | --- | --- | --- | --- | --- |
| {BcEV6=FALSE, BcMV9.BI=TRUE} =>  {BcRV1.BI=TRUE} | 0.21  2871 | 0.337  255 | 0.631  188 | 1.21  6527 | 86 |
| {BcOLV17.BI=FALSE, BcRV1.BI=TRUE} =>  {BcMV9.BI=TRUE} | 0.19  3069 | 0.804  124 | 0.240  099 | 1.18  5642 | 78 |
| {BcOLV15.BI=FALSE, BcRV1.BI=TRUE} =>  {BcMV9.BI=TRUE} | 0.22  0297 | 0.816  514 | 0.269  802 | 1.20  3911 | 89 |
| {BcMV9.BI=TRUE, BcOLV15.BI=FALSE} =>  {BcRV1.BI=TRUE} | 0.22  0297 | 0.343  629 | 0.641  089 | 1.23  952 | 89 |
| {BcEV5=FALSE, BcRV1.BI=TRUE} =>  {BcMV9.BI=TRUE} | 0.22  2772 | 0.803  571 | 0.277  228 | 1.18  4828 | 90 |
| {BcEV5=FALSE, BcMV9.BI=TRUE} =>  {BcRV1.BI=TRUE} | 0.22  2772 | 0.348  837 | 0.638  614 | 1.25  8306 | 90 |
| {BcMV6.BI=FALSE, BcRV1.BI=TRUE} =>  {BcULV4=TRUE} | 0.13  1188 | 0.803  03 | 0.163  366 | 1.16  2811 | 53 |
| {BcPV3.BI=FALSE, BcRV1.BI=TRUE} =>  {BcULV4=TRUE} | 0.12  6238 | 0.836  066 | 0.150  99 | 1.21  0647 | 51 |
| {BcEV2.BI=FALSE, BcRV1.BI=TRUE} =>  {BcULV4=TRUE} | 0.19  3069 | 0.906  977 | 0.212  871 | 1.31  3328 | 78 |
| {BcEV2.BI=FALSE, BcULV4=TRUE} =>  {BcRV1.BI=TRUE} | 0.19  3069 | 0.364  486 | 0.529  703 | 1.31  4753 | 78 |

| {BcHV3.BI=FALSE, BcRV1.BI=TRUE} =>  {BcULV4=TRUE} | 0.18  0693 | 0.848  837 | 0.212  871 | 1.22  9141 | 73 |
| --- | --- | --- | --- | --- | --- |
| {BcHV3.BI=FALSE, BcULV4=TRUE} =>  {BcRV1.BI=TRUE} | 0.18  0693 | 0.350  962 | 0.514  851 | 1.26  5968 | 73 |
| {BcFV5.BI=FALSE, BcRV1.BI=TRUE} =>  {BcULV4=TRUE} | 0.20  5446 | 0.864  583 | 0.237  624 | 1.25  1941 | 83 |
| {BcFV5.BI=FALSE, BcULV4=TRUE} =>  {BcRV1.BI=TRUE} | 0.20  5446 | 0.387  85 | 0.529  703 | 1.39  9032 | 83 |
| {BcRV1.BI=TRUE, BcULV1.BI=FALSE} =>  {BcULV4=TRUE} | 0.16  5842 | 0.827  16 | 0.200  495 | 1.19  7752 | 67 |
| {BcULV1.BI=FALSE, BcULV4=TRUE} =>  {BcRV1.BI=TRUE} | 0.16  5842 | 0.333  333 | 0.497  525 | 1.20  2381 | 67 |
| {BcMV1.BI=TRUE, BcRV1.BI=TRUE} =>  {BcULV4=TRUE} | 0.18  3168 | 0.902  439 | 0.202  97 | 1.30  6758 | 74 |
| {BcMV1.BI=TRUE, BcULV4=TRUE} =>  {BcRV1.BI=TRUE} | 0.18  3168 | 0.333  333 | 0.549  505 | 1.20  2381 | 74 |
| {BcMV12=FALSE, BcRV1.BI=TRUE} =>  {BcULV4=TRUE} | 0.18  8119 | 0.853  933 | 0.220  297 | 1.23  6519 | 76 |
| {BcMV12=FALSE, BcULV4=TRUE} =>  {BcRV1.BI=TRUE} | 0.18  8119 | 0.342  342 | 0.549  505 | 1.23  4878 | 76 |
| {BcNSRV7.BI=FALSE, BcRV1.BI=TRUE} =>  {BcULV4=TRUE} | 0.20  5446 | 0.855  67 | 0.240  099 | 1.23  9035 | 83 |

| {BcNSRV7.BI=FALSE, BcULV4=TRUE} =>  {BcRV1.BI=TRUE} | 0.20  5446 | 0.384  259 | 0.534  653 | 1.38  6078 | 83 |
| --- | --- | --- | --- | --- | --- |
| {BcRV1.BI=TRUE, BcVV3.BI=FALSE} =>  {BcULV4=TRUE} | 0.20  5446 | 0.882  979 | 0.232  673 | 1.27  8579 | 83 |
| {BcULV4=TRUE, BcVV3.BI=FALSE} =>  {BcRV1.BI=TRUE} | 0.20  5446 | 0.375  566 | 0.547  03 | 1.35  4719 | 83 |
| {BcOLV7.BI=FALSE, BcRV1.BI=TRUE} =>  {BcULV4=TRUE} | 0.13  3663 | 0.794  118 | 0.168  317 | 1.14  9905 | 54 |
| {BcALV1.BI=FALSE, BcRV1.BI=TRUE} =>  {BcULV4=TRUE} | 0.16  0891 | 0.844  156 | 0.190  594 | 1.22  2362 | 65 |
| {BcRV1.BI=TRUE, BcULV4=TRUE} =>  {BcDMV1=FALSE} | 0.22  5248 | 0.928  571 | 0.242  574 | 1.10  0126 | 91 |
| {BcDMV1=FALSE, BcRV1.BI=TRUE} =>  {BcULV4=TRUE} | 0.22  5248 | 0.875 | 0.257  426 | 1.26  7025 | 91 |
| {BcDMV1=FALSE, BcULV4=TRUE} =>  {BcRV1.BI=TRUE} | 0.22  5248 | 0.393  939 | 0.571  782 | 1.42  0996 | 91 |
| {BcFV9=FALSE, BcRV1.BI=TRUE} =>  {BcULV4=TRUE} | 0.20  5446 | 0.882  979 | 0.232  673 | 1.27  8579 | 83 |
| {BcFV9=FALSE, BcULV4=TRUE} =>  {BcRV1.BI=TRUE} | 0.20  5446 | 0.344  398 | 0.596  535 | 1.24  2294 | 83 |
| {BcOLV4.BI=FALSE, BcRV1.BI=TRUE} =>  {BcULV4=TRUE} | 0.21  7822 | 0.888  889 | 0.245  05 | 1.28  7137 | 88 |

| {BcOLV4.BI=FALSE, BcULV4=TRUE} =>  {BcRV1.BI=TRUE} | 0.21  7822 | 0.350  598 | 0.621  287 | 1.26  4656 | 88 |
| --- | --- | --- | --- | --- | --- |
| {BcRV1.BI=TRUE, BcULV5=FALSE} =>  {BcULV4=TRUE} | 0.21  7822 | 0.88 | 0.247  525 | 1.27  4265 | 88 |
| {BcULV4=TRUE, BcULV5=FALSE} =>  {BcRV1.BI=TRUE} | 0.21  7822 | 0.345  098 | 0.631  188 | 1.24  4818 | 88 |
| {BcBNV5.BI=FALSE, BcRV1.BI=TRUE} =>  {BcULV4=TRUE} | 0.22  5248 | 0.866  667 | 0.259  901 | 1.25  4958 | 91 |
| {BcBNV5.BI=FALSE, BcULV4=TRUE} =>  {BcRV1.BI=TRUE} | 0.22  5248 | 0.364 | 0.618  812 | 1.31  3 | 91 |
| {BcRV1.BI=TRUE, BvF.BI=FALSE} =>  {BcULV4=TRUE} | 0.23  5149 | 0.879  63 | 0.267  327 | 1.27  3729 | 95 |
| {BcULV4=TRUE, BvF.BI=FALSE} =>  {BcRV1.BI=TRUE} | 0.23  5149 | 0.363  985 | 0.646  04 | 1.31  2945 | 95 |
| {BcMCV5.BI=FALSE, BcRV1.BI=TRUE} =>  {BcULV4=TRUE} | 0.22  0297 | 0.881  188 | 0.25 | 1.27  5986 | 89 |
| {BcMCV5.BI=FALSE, BcULV4=TRUE} =>  {BcRV1.BI=TRUE} | 0.22  0297 | 0.349  02 | 0.631  188 | 1.25  8964 | 89 |
| {BcOLV13.BI=FALSE, BcRV1.BI=TRUE} =>  {BcULV4=TRUE} | 0.21  0396 | 0.894  737 | 0.235  149 | 1.29  5605 | 85 |
| {BcOLV2.BI=FALSE, BcRV1.BI=TRUE} =>  {BcULV4=TRUE} | 0.21  5347 | 0.878  788 | 0.245  05 | 1.27  251 | 87 |

| {BcOLV2.BI=FALSE, BcULV4=TRUE} =>  {BcRV1.BI=TRUE} | 0.21  5347 | 0.335  907 | 0.641  089 | 1.21  1666 | 87 |
| --- | --- | --- | --- | --- | --- |
| {BcMCV3.BI=FALSE, BcRV1.BI=TRUE} =>  {BcULV4=TRUE} | 0.21  7822 | 0.862  745 | 0.252  475 | 1.24  928 | 88 |
| {BcMCV3.BI=FALSE, BcULV4=TRUE} =>  {BcRV1.BI=TRUE} | 0.21  7822 | 0.339  768 | 0.641  089 | 1.22  5593 | 88 |
| {BcPV4.BI=FALSE, BcRV1.BI=TRUE} =>  {BcULV4=TRUE} | 0.23  0198 | 0.877  358 | 0.262  376 | 1.27  044 | 93 |
| {BcPV4.BI=FALSE, BcULV4=TRUE} =>  {BcRV1.BI=TRUE} | 0.23  0198 | 0.356  322 | 0.646  04 | 1.28  5304 | 93 |
| {BcEV6=FALSE, BcRV1.BI=TRUE} =>  {BcULV4=TRUE} | 0.23  2673 | 0.870  37 | 0.267  327 | 1.26  0321 | 94 |
| {BcEV6=FALSE, BcULV4=TRUE} =>  {BcRV1.BI=TRUE} | 0.23  2673 | 0.360  153 | 0.646  04 | 1.29  9124 | 94 |
| {BcOLV17.BI=FALSE, BcRV1.BI=TRUE} =>  {BcULV4=TRUE} | 0.20  7921 | 0.865  979 | 0.240  099 | 1.25  3963 | 84 |
| {BcOLV15.BI=FALSE, BcRV1.BI=TRUE} =>  {BcULV4=TRUE} | 0.23  7624 | 0.880  734 | 0.269  802 | 1.27  5328 | 96 |
| {BcOLV15.BI=FALSE, BcULV4=TRUE} =>  {BcRV1.BI=TRUE} | 0.23  7624 | 0.360  902 | 0.658  416 | 1.30  1826 | 96 |
| {BcEV5=FALSE, BcRV1.BI=TRUE} =>  {BcULV4=TRUE} | 0.24  2574 | 0.875 | 0.277  228 | 1.26  7025 | 98 |

| {BcEV5=FALSE, BcULV4=TRUE} =>  {BcRV1.BI=TRUE} | 0.24  2574 | 0.368  421 | 0.658  416 | 1.32  8947 | 98 |
| --- | --- | --- | --- | --- | --- |
| {BcMV6.BI=FALSE, BcRV1.BI=TRUE} =>  {BcNSRV7.BI=FALSE} | 0.14  604 | 0.893  939 | 0.163  366 | 1.11  8116 | 59 |
| {BcMV6.BI=FALSE, BcRV1.BI=TRUE} =>  {BcDMV1=FALSE} | 0.15  3465 | 0.939  394 | 0.163  366 | 1.11  2948 | 62 |
| {BcMV6.BI=FALSE, BcRV1.BI=TRUE} =>  {BcBNV5.BI=FALSE} | 0.16  3366 | 1 | 0.163  366 | 1.10  0817 | 66 |
| {BcPV3.BI=FALSE, BcRV1.BI=TRUE} =>  {BcDMV1=FALSE} | 0.13  8614 | 0.918  033 | 0.150  99 | 1.08  764 | 56 |
| {BcEV2.BI=FALSE, BcRV1.BI=TRUE} =>  {BcFV5.BI=FALSE} | 0.18  5644 | 0.872  093 | 0.212  871 | 1.11  4954 | 75 |
| {BcEV2.BI=FALSE, BcRV1.BI=TRUE} =>  {BcDMV1=FALSE} | 0.19  5545 | 0.918  605 | 0.212  871 | 1.08  8318 | 79 |
| {BcEV2.BI=FALSE, BcRV1.BI=TRUE} =>  {BcOLV4.BI=FALSE} | 0.20  0495 | 0.941  86 | 0.212  871 | 1.07  4892 | 81 |
| {BcHV3.BI=FALSE, BcRV1.BI=TRUE} =>  {BcFV5.BI=FALSE} | 0.18  8119 | 0.883  721 | 0.212  871 | 1.12  982 | 76 |
| {BcHV3.BI=FALSE, BcRV1.BI=TRUE} =>  {BcNSRV7.BI=FALSE} | 0.18  8119 | 0.883  721 | 0.212  871 | 1.10  5335 | 76 |
| {BcHV3.BI=FALSE, BcRV1.BI=TRUE} =>  {BcVV3.BI=FALSE} | 0.19  0594 | 0.895  349 | 0.212  871 | 1.11  6423 | 77 |

| {BcOLV7.BI=FALSE, BcRV1.BI=TRUE} =>  {BcHV3.BI=FALSE} | 0.14  604 | 0.867  647 | 0.168  317 | 1.14  179 | 59 |
| --- | --- | --- | --- | --- | --- |
| {BcHV3.BI=FALSE, BcRV1.BI=TRUE} =>  {BcDMV1=FALSE} | 0.19  802 | 0.930  233 | 0.212  871 | 1.10  2094 | 80 |
| {BcRV1.BI=TRUE, BcULV1.BI=FALSE} =>  {BcFV5.BI=FALSE} | 0.17  5743 | 0.876  543 | 0.200  495 | 1.12  0644 | 71 |
| {BcMV12=FALSE, BcRV1.BI=TRUE} =>  {BcFV5.BI=FALSE} | 0.19  802 | 0.898  876 | 0.220  297 | 1.14  9196 | 80 |
| {BcFV5.BI=FALSE, BcRV1.BI=TRUE} =>  {BcNSRV7.BI=FALSE} | 0.21  0396 | 0.885  417 | 0.237  624 | 1.10  7456 | 85 |
| {BcNSRV7.BI=FALSE, BcRV1.BI=TRUE} =>  {BcFV5.BI=FALSE} | 0.21  0396 | 0.876  289 | 0.240  099 | 1.12  0318 | 85 |
| {BcALV1.BI=FALSE, BcRV1.BI=TRUE} =>  {BcFV5.BI=FALSE} | 0.18  3168 | 0.961  039 | 0.190  594 | 1.22  867 | 74 |
| {BcFV5.BI=FALSE, BcRV1.BI=TRUE} =>  {BcDMV1=FALSE} | 0.22  2772 | 0.937  5 | 0.237  624 | 1.11  0704 | 90 |
| {BcDMV1=FALSE, BcRV1.BI=TRUE} =>  {BcFV5.BI=FALSE} | 0.22  2772 | 0.865  385 | 0.257  426 | 1.10  6378 | 90 |
| {BcFV9=FALSE, BcRV1.BI=TRUE} =>  {BcFV5.BI=FALSE} | 0.20  5446 | 0.882  979 | 0.232  673 | 1.12  8872 | 83 |
| {BcOLV4.BI=FALSE, BcRV1.BI=TRUE} =>  {BcFV5.BI=FALSE} | 0.21  2871 | 0.868  687 | 0.245  05 | 1.11  06 | 86 |

| {BcRV1.BI=TRUE, BcULV5=FALSE} =>  {BcFV5.BI=FALSE} | 0.21  5347 | 0.87 | 0.247  525 | 1.11  2278 | 87 |
| --- | --- | --- | --- | --- | --- |
| {BcBNV5.BI=FALSE, BcRV1.BI=TRUE} =>  {BcFV5.BI=FALSE} | 0.22  5248 | 0.866  667 | 0.259  901 | 1.10  8017 | 91 |
| {BcMCV5.BI=FALSE, BcRV1.BI=TRUE} =>  {BcFV5.BI=FALSE} | 0.21  7822 | 0.871  287 | 0.25 | 1.11  3924 | 88 |
| {BcMCV3.BI=FALSE, BcRV1.BI=TRUE} =>  {BcFV5.BI=FALSE} | 0.22  0297 | 0.872  549 | 0.252  475 | 1.11  5537 | 89 |
| {BcPV4.BI=FALSE, BcRV1.BI=TRUE} =>  {BcFV5.BI=FALSE} | 0.22  7723 | 0.867  925 | 0.262  376 | 1.10  9625 | 92 |
| {BcRV1.BI=TRUE, BcULV1.BI=FALSE} =>  {BcDMV1=FALSE} | 0.18  3168 | 0.913  58 | 0.200  495 | 1.08  2365 | 74 |
| {BcMV1.BI=TRUE, BcRV1.BI=TRUE} =>  {BcDMV1=FALSE} | 0.18  8119 | 0.926  829 | 0.202  97 | 1.09  8062 | 76 |
| {BcMV12=FALSE, BcRV1.BI=TRUE} =>  {BcNSRV7.BI=FALSE} | 0.20  297 | 0.921  348 | 0.220  297 | 1.15  2399 | 82 |
| {BcMV12=FALSE, BcRV1.BI=TRUE} =>  {BcDMV1=FALSE} | 0.20  5446 | 0.932  584 | 0.220  297 | 1.10  488 | 83 |
| {BcNSRV7.BI=FALSE, BcRV1.BI=TRUE} =>  {BcDMV1=FALSE} | 0.22  7723 | 0.948  454 | 0.240  099 | 1.12  3681 | 92 |
| {BcDMV1=FALSE, BcRV1.BI=TRUE} =>  {BcNSRV7.BI=FALSE} | 0.22  7723 | 0.884  615 | 0.257  426 | 1.10  6454 | 92 |

| {BcFV9=FALSE, BcRV1.BI=TRUE} =>  {BcNSRV7.BI=FALSE} | 0.20  5446 | 0.882  979 | 0.232  673 | 1.10  4407 | 83 |
| --- | --- | --- | --- | --- | --- |
| {BcOLV17.BI=FALSE, BcRV1.BI=TRUE} =>  {BcNSRV7.BI=FALSE} | 0.21  2871 | 0.886  598 | 0.240  099 | 1.10  8934 | 86 |
| {BcRV1.BI=TRUE, BcVV3.BI=FALSE} =>  {BcDMV1=FALSE} | 0.21  5347 | 0.925  532 | 0.232  673 | 1.09  6525 | 87 |
| {BcOLV13.BI=FALSE, BcRV1.BI=TRUE} =>  {BcVV3.BI=FALSE} | 0.20  7921 | 0.884  211 | 0.235  149 | 1.10  2534 | 84 |
| {BcOLV7.BI=FALSE, BcRV1.BI=TRUE} =>  {BcDMV1=FALSE} | 0.15  3465 | 0.911  765 | 0.168  317 | 1.08  0214 | 62 |
| {BcALV1.BI=FALSE, BcRV1.BI=TRUE} =>  {BcDMV1=FALSE} | 0.17  8218 | 0.935  065 | 0.190  594 | 1.10  7819 | 72 |
| {BcFV9=FALSE, BcRV1.BI=TRUE} =>  {BcDMV1=FALSE} | 0.22  0297 | 0.946  809 | 0.232  673 | 1.12  1732 | 89 |
| {BcOLV4.BI=FALSE, BcRV1.BI=TRUE} =>  {BcDMV1=FALSE} | 0.22  5248 | 0.919  192 | 0.245  05 | 1.08  9013 | 91 |
| {BcRV1.BI=TRUE, BcULV5=FALSE} =>  {BcDMV1=FALSE} | 0.22  7723 | 0.92 | 0.247  525 | 1.08  9971 | 92 |
| {BcBNV5.BI=FALSE, BcRV1.BI=TRUE} =>  {BcDMV1=FALSE} | 0.24  2574 | 0.933  333 | 0.259  901 | 1.10  5767 | 98 |
| {BcDMV1=FALSE, BcRV1.BI=TRUE} =>  {BvF.BI=FALSE} | 0.25  495 | 0.990  385 | 0.257  426 | 1.08  4324 | 10  3 |

| {BcRV1.BI=TRUE, BvF.BI=FALSE} =>  {BcDMV1=FALSE} | 0.25  495 | 0.953  704 | 0.267  327 | 1.12  9901 | 10  3 |
| --- | --- | --- | --- | --- | --- |
| {BcDMV1=FALSE, BvF.BI=FALSE} =>  {BcRV1.BI=TRUE} | 0.25  495 | 0.329  073 | 0.774  752 | 1.18  7015 | 10  3 |
| {BcMCV5.BI=FALSE, BcRV1.BI=TRUE} =>  {BcDMV1=FALSE} | 0.23  0198 | 0.920  792 | 0.25 | 1.09  0909 | 93 |
| {BcOLV13.BI=FALSE, BcRV1.BI=TRUE} =>  {BcDMV1=FALSE} | 0.21  7822 | 0.926  316 | 0.235  149 | 1.09  7453 | 88 |
| {BcOLV2.BI=FALSE, BcRV1.BI=TRUE} =>  {BcDMV1=FALSE} | 0.22  5248 | 0.919  192 | 0.245  05 | 1.08  9013 | 91 |
| {BcMCV3.BI=FALSE, BcRV1.BI=TRUE} =>  {BcDMV1=FALSE} | 0.23  2673 | 0.921  569 | 0.252  475 | 1.09  1829 | 94 |
| {BcPV4.BI=FALSE, BcRV1.BI=TRUE} =>  {BcDMV1=FALSE} | 0.24  2574 | 0.924  528 | 0.262  376 | 1.09  5336 | 98 |
| {BcEV6=FALSE, BcRV1.BI=TRUE} =>  {BcDMV1=FALSE} | 0.25 | 0.935  185 | 0.267  327 | 1.10  7961 | 10  1 |
| {BcOLV17.BI=FALSE, BcRV1.BI=TRUE} =>  {BcDMV1=FALSE} | 0.22  2772 | 0.927  835 | 0.240  099 | 1.09  9253 | 90 |
| {BcOLV15.BI=FALSE, BcRV1.BI=TRUE} =>  {BcDMV1=FALSE} | 0.25 | 0.926  606 | 0.269  802 | 1.09  7797 | 10  1 |
| {BcEV5=FALSE, BcRV1.BI=TRUE} =>  {BcDMV1=FALSE} | 0.25  7426 | 0.928  571 | 0.277  228 | 1.10  0126 | 10  4 |

| {BcMCV5.BI=FALSE, BcRV1.BI=TRUE} =>  {BcOLV4.BI=FALSE} | 0.23  5149 | 0.940  594 | 0.25 | 1.07  3446 | 95 |
| --- | --- | --- | --- | --- | --- |
| {BcOLV13.BI=FALSE, BcRV1.BI=TRUE} =>  {BcOLV4.BI=FALSE} | 0.22  0297 | 0.936  842 | 0.235  149 | 1.06  9164 | 89 |
| {BcOLV17.BI=FALSE, BcRV1.BI=TRUE} =>  {BcOLV4.BI=FALSE} | 0.22  5248 | 0.938  144 | 0.240  099 | 1.07  0651 | 91 |
| {BcMV6.BI=TRUE, BcPV3.BI=TRUE} =>  {BcOLV1.BI.MOVA.=TRUE} | 0.07  6733 | 0.688  889 | 0.111  386 | 2.07  6949 | 31 |
| {BcOLV1.BI.MOVA.=TRUE, BcPV3.BI=TRUE}  => {BcMV6.BI=TRUE} | 0.07  6733 | 0.563  636 | 0.136  139 | 1.85  1293 | 31 |
| {BcMV6.BI=TRUE, BcOLV1.BI.MOVA.=TRUE}  => {BcPV3.BI=TRUE} | 0.07  6733 | 0.469  697 | 0.163  366 | 1.62  186 | 31 |
| {BcMV6.BI=TRUE, BcPV3.BI=TRUE} =>  {BcMV4.BI=TRUE} | 0.08  4158 | 0.755  556 | 0.111  386 | 1.70  5276 | 34 |
| {BcMV4.BI=TRUE, BcPV3.BI=TRUE} =>  {BcMV6.BI=TRUE} | 0.08  4158 | 0.465  753 | 0.180  693 | 1.52  9792 | 34 |
| {BcMV4.BI=TRUE, BcMV6.BI=TRUE} =>  {BcPV3.BI=TRUE} | 0.08  4158 | 0.478  873 | 0.175  743 | 1.65  3545 | 34 |
| {BcMV6.BI=TRUE, BcPV3.BI=TRUE} =>  {BcMV2.BI=TRUE} | 0.09  1584 | 0.822  222 | 0.111  386 | 1.77  6352 | 37 |
| {BcMV2.BI=TRUE, BcPV3.BI=TRUE} =>  {BcMV6.BI=TRUE} | 0.09  1584 | 0.513  889 | 0.178  218 | 1.68  7895 | 37 |

| {BcMV2.BI=TRUE, BcMV6.BI=TRUE} =>  {BcPV3.BI=TRUE} | 0.09  1584 | 0.440  476 | 0.207  921 | 1.52  0961 | 37 |
| --- | --- | --- | --- | --- | --- |
| {BcMV6.BI=TRUE, BcPV3.BI=TRUE} =>  {BcOLV18=TRUE} | 0.08  6634 | 0.777  778 | 0.111  386 | 1.50  3456 | 35 |
| {BcOLV18=TRUE, BcPV3.BI=TRUE} =>  {BcMV6.BI=TRUE} | 0.08  6634 | 0.460  526 | 0.188  119 | 1.51  2623 | 35 |
| {BcMV6.BI=TRUE, BcOLV18=TRUE} =>  {BcPV3.BI=TRUE} | 0.08  6634 | 0.5 | 0.173  267 | 1.72  6496 | 35 |
| {BcMV6.BI=TRUE, BcPV3.BI=TRUE} =>  {BcHV1SLR.BI=TRUE} | 0.08  1683 | 0.733  333 | 0.111  386 | 1.35  2816 | 33 |
| {BcHV1SLR.BI=TRUE, BcPV3.BI=TRUE} =>  {BcMV6.BI=TRUE} | 0.08  1683 | 0.485  294 | 0.168  317 | 1.59  3974 | 33 |
| {BcHV1SLR.BI=TRUE, BcMV6.BI=TRUE} =>  {BcPV3.BI=TRUE} | 0.08  1683 | 0.397  59 | 0.205  446 | 1.37  2876 | 33 |
| {BcMV6.BI=TRUE, BcPV3.BI=TRUE} =>  {BcMV3.BI=TRUE} | 0.09  4059 | 0.844  444 | 0.111  386 | 1.55  7788 | 38 |
| {BcMV3.BI=TRUE, BcPV3.BI=TRUE} =>  {BcMV6.BI=TRUE} | 0.09  4059 | 0.520  548 | 0.180  693 | 1.70  9767 | 38 |
| {BcMV3.BI=TRUE, BcMV6.BI=TRUE} =>  {BcPV3.BI=TRUE} | 0.09  4059 | 0.422  222 | 0.222  772 | 1.45  793 | 38 |
| {BcMV6.BI=TRUE, BcPV3.BI=TRUE} =>  {BcHV1.BI=TRUE} | 0.08  9109 | 0.8 | 0.111  386 | 1.32  459 | 36 |

| {BcHV1.BI=TRUE, BcPV3.BI=TRUE} =>  {BcMV6.BI=TRUE} | 0.08  9109 | 0.444  444 | 0.200  495 | 1.45  9801 | 36 |
| --- | --- | --- | --- | --- | --- |
| {BcHV1.BI=TRUE, BcMV6.BI=TRUE} =>  {BcPV3.BI=TRUE} | 0.08  9109 | 0.391  304 | 0.227  723 | 1.35  1171 | 36 |
| {BcMV6.BI=TRUE, BcPV3.BI=TRUE} =>  {BcMV11=TRUE} | 0.09  4059 | 0.844  444 | 0.111  386 | 1.37  0103 | 38 |
| {BcMV11=TRUE, BcPV3.BI=TRUE} =>  {BcMV6.BI=TRUE} | 0.09  4059 | 0.475 | 0.198  02 | 1.56  0163 | 38 |
| {BcMV11=TRUE, BcMV6.BI=TRUE} =>  {BcPV3.BI=TRUE} | 0.09  4059 | 0.413  043 | 0.227  723 | 1.42  6236 | 38 |
| {BcMV6.BI=TRUE, BcPV3.BI=TRUE} =>  {BcMV9.BI=TRUE} | 0.09  6535 | 0.866  667 | 0.111  386 | 1.27  7859 | 39 |
| {BcMV9.BI=TRUE, BcPV3.BI=TRUE} =>  {BcMV6.BI=TRUE} | 0.09  6535 | 0.469  88 | 0.205  446 | 1.54  3344 | 39 |
| {BcMV6.BI=TRUE, BcMV9.BI=TRUE} =>  {BcPV3.BI=TRUE} | 0.09  6535 | 0.410  526 | 0.235  149 | 1.41  7544 | 39 |
| {BcMV6.BI=TRUE, BcPV3.BI=TRUE} =>  {BcULV4=TRUE} | 0.09  6535 | 0.866  667 | 0.111  386 | 1.25  4958 | 39 |
| {BcPV3.BI=TRUE, BcULV4=TRUE} =>  {BcMV6.BI=TRUE} | 0.09  6535 | 0.469  88 | 0.205  446 | 1.54  3344 | 39 |
| {BcMV6.BI=TRUE, BcULV4=TRUE} =>  {BcPV3.BI=TRUE} | 0.09  6535 | 0.397  959 | 0.242  574 | 1.37  415 | 39 |

| {BcEV2.BI=FALSE, BcPV3.BI=TRUE} =>  {BcMV6.BI=TRUE} | 0.08  4158 | 0.409  639 | 0.205  446 | 1.34  5479 | 34 |
| --- | --- | --- | --- | --- | --- |
| {BcFV5.BI=FALSE, BcPV3.BI=TRUE} =>  {BcMV6.BI=TRUE} | 0.09  1584 | 0.389  474 | 0.235  149 | 1.27  9247 | 37 |
| {BcFV5.BI=FALSE, BcMV6.BI=TRUE} =>  {BcPV3.BI=TRUE} | 0.09  1584 | 0.385  417 | 0.237  624 | 1.33  084 | 37 |
| {BcMV6.BI=TRUE, BcPV3.BI=TRUE} =>  {BcMV1.BI=TRUE} | 0.09  901 | 0.888  889 | 0.111  386 | 1.12  574 | 40 |
| {BcMV1.BI=TRUE, BcPV3.BI=TRUE} =>  {BcMV6.BI=TRUE} | 0.09  901 | 0.388  35 | 0.254  95 | 1.27  5555 | 40 |
| {BcMV1.BI=TRUE, BcMV6.BI=TRUE} =>  {BcPV3.BI=TRUE} | 0.09  901 | 0.388  35 | 0.254  95 | 1.34  0968 | 40 |
| {BcDMV1=FALSE, BcMV6.BI=TRUE} =>  {BcPV3.BI=TRUE} | 0.08  9109 | 0.375 | 0.237  624 | 1.29  4872 | 36 |
| {BcBNV5.BI=FALSE, BcMV6.BI=TRUE} =>  {BcPV3.BI=TRUE} | 0.09  1584 | 0.373  737 | 0.245  05 | 1.29  0512 | 37 |
| {BcPV3.BI=TRUE, BvF.BI=FALSE} =>  {BcMV6.BI=TRUE} | 0.10  396 | 0.388  889 | 0.267  327 | 1.27  7326 | 42 |
| {BcEV6=FALSE, BcPV3.BI=TRUE} =>  {BcMV6.BI=TRUE} | 0.10  6436 | 0.387  387 | 0.274  752 | 1.27  2394 | 43 |
| {BcEV6=FALSE, BcMV6.BI=TRUE} =>  {BcPV3.BI=TRUE} | 0.10  6436 | 0.377  193 | 0.282  178 | 1.30  2444 | 43 |

| {BcOLV15.BI=FALSE, BcPV3.BI=TRUE} =>  {BcMV6.BI=TRUE} | 0.11  1386 | 0.391  304 | 0.284  653 | 1.28  526 | 45 |
| --- | --- | --- | --- | --- | --- |
| {BcMV6.BI=TRUE, BcOLV15.BI=FALSE} =>  {BcPV3.BI=TRUE} | 0.11  1386 | 0.371  901 | 0.299  505 | 1.28  417 | 45 |
| {BcEV5=FALSE, BcMV6.BI=TRUE} =>  {BcPV3.BI=TRUE} | 0.10  1485 | 0.383  178 | 0.264  851 | 1.32  3109 | 41 |
| {BcPV3.BI=TRUE, BcULV4=FALSE} =>  {BcRV1.BI=FALSE} | 0.07  4257 | 0.882  353 | 0.084  158 | 1.22  079 | 30 |
| {BcPV3.BI=TRUE, BcRV1.BI=FALSE} =>  {BcULV4=FALSE} | 0.07  4257 | 0.454  545 | 0.163  366 | 1.46  9091 | 30 |
| {BcPV3.BI=TRUE, BcULV4=FALSE} =>  {BcOLV7.BI=FALSE} | 0.07  9208 | 0.941  176 | 0.084  158 | 1.16  6366 | 32 |
| {BcPV3.BI=TRUE, BcULV4=FALSE} =>  {BcALV1.BI=FALSE} | 0.07  6733 | 0.911  765 | 0.084  158 | 1.11  9614 | 31 |
| {BcPV3.BI=TRUE, BcULV4=FALSE} =>  {BcDMV1=FALSE} | 0.08  1683 | 0.970  588 | 0.084  158 | 1.14  9905 | 33 |
| {BcMV9.BI=FALSE, BcPV3.BI=TRUE} =>  {BcRV1.BI=FALSE} | 0.07  6733 | 0.911  765 | 0.084  158 | 1.26  1483 | 31 |
| {BcPV3.BI=TRUE, BcRV1.BI=FALSE} =>  {BcMV9.BI=FALSE} | 0.07  6733 | 0.469  697 | 0.163  366 | 1.45  9674 | 31 |
| {BcMV9.BI=FALSE, BcPV3.BI=TRUE} =>  {BcOLV7.BI=FALSE} | 0.07  9208 | 0.941  176 | 0.084  158 | 1.16  6366 | 32 |

| {BcMV9.BI=FALSE, BcPV3.BI=TRUE} =>  {BcALV1.BI=FALSE} | 0.07  6733 | 0.911  765 | 0.084  158 | 1.11  9614 | 31 |
| --- | --- | --- | --- | --- | --- |
| {BcOLV1.BI.MOVA.=TRUE, BcPV3.BI=TRUE}  => {BcOLV16.BI=TRUE} | 0.08  9109 | 0.654  545 | 0.136  139 | 1.57  4026 | 36 |
| {BcOLV16.BI=TRUE, BcPV3.BI=TRUE} =>  {BcOLV1.BI.MOVA.=TRUE} | 0.08  9109 | 0.620  69 | 0.143  564 | 1.87  1333 | 36 |
| {BcOLV1.BI.MOVA.=TRUE,  BcOLV16.BI=TRUE} => {BcPV3.BI=TRUE} | 0.08  9109 | 0.467  532 | 0.190  594 | 1.61  4386 | 36 |
| {BcOLV1.BI.MOVA.=TRUE, BcPV3.BI=TRUE}  => {BcMV4.BI=TRUE} | 0.08  9109 | 0.654  545 | 0.136  139 | 1.47  7298 | 36 |
| {BcMV4.BI=TRUE, BcPV3.BI=TRUE} =>  {BcOLV1.BI.MOVA.=TRUE} | 0.08  9109 | 0.493  151 | 0.180  693 | 1.48  6813 | 36 |
| {BcMV4.BI=TRUE, BcOLV1.BI.MOVA.=TRUE}  => {BcPV3.BI=TRUE} | 0.08  9109 | 0.48 | 0.185  644 | 1.65  7436 | 36 |
| {BcOLV1.BI.MOVA.=TRUE, BcPV3.BI=TRUE}  => {BcMV2.BI=TRUE} | 0.10  6436 | 0.781  818 | 0.136  139 | 1.68  9062 | 43 |
| {BcMV2.BI=TRUE, BcPV3.BI=TRUE} =>  {BcOLV1.BI.MOVA.=TRUE} | 0.10  6436 | 0.597  222 | 0.178  218 | 1.80  058 | 43 |
| {BcMV2.BI=TRUE, BcOLV1.BI.MOVA.=TRUE}  => {BcPV3.BI=TRUE} | 0.10  6436 | 0.425  743 | 0.25 | 1.47  0085 | 43 |
| {BcOLV1.BI.MOVA.=TRUE, BcPV3.BI=TRUE}  => {BcOLV18=TRUE} | 0.10  6436 | 0.781  818 | 0.136  139 | 1.51  1266 | 43 |

| {BcOLV18=TRUE, BcPV3.BI=TRUE} =>  {BcOLV1.BI.MOVA.=TRUE} | 0.10  6436 | 0.565  789 | 0.188  119 | 1.70  5813 | 43 |
| --- | --- | --- | --- | --- | --- |
| {BcOLV1.BI.MOVA.=TRUE, BcOLV18=TRUE}  => {BcPV3.BI=TRUE} | 0.10  6436 | 0.462  366 | 0.230  198 | 1.59  6544 | 43 |
| {BcOLV1.BI.MOVA.=TRUE, BcPV3.BI=TRUE}  => {BcHV1SLR.BI=TRUE} | 0.09  1584 | 0.672  727 | 0.136  139 | 1.24  1013 | 37 |
| {BcHV1SLR.BI=TRUE, BcPV3.BI=TRUE} =>  {BcOLV1.BI.MOVA.=TRUE} | 0.09  1584 | 0.544  118 | 0.168  317 | 1.64  0474 | 37 |
| {BcHV1SLR.BI=TRUE, BcOLV1.BI.MOVA.=TRUE} =>  {BcPV3.BI=TRUE} | 0.09  1584 | 0.456  79 | 0.200  495 | 1.57  7292 | 37 |
| {BcOLV1.BI.MOVA.=TRUE, BcPV3.BI=TRUE}  => {BcMV3.BI=TRUE} | 0.10  6436 | 0.781  818 | 0.136  139 | 1.44  2258 | 43 |
| {BcMV3.BI=TRUE, BcPV3.BI=TRUE} =>  {BcOLV1.BI.MOVA.=TRUE} | 0.10  6436 | 0.589  041 | 0.180  693 | 1.77  5915 | 43 |
| {BcMV3.BI=TRUE, BcOLV1.BI.MOVA.=TRUE}  => {BcPV3.BI=TRUE} | 0.10  6436 | 0.434  343 | 0.245  05 | 1.49  9784 | 43 |
| {BcPV3.BI=TRUE, BcULV3=TRUE} =>  {BcOLV1.BI.MOVA.=TRUE} | 0.07  9208 | 0.457  143 | 0.173  267 | 1.37  8252 | 32 |
| {BcOLV1.BI.MOVA.=TRUE, BcULV3=TRUE}  => {BcPV3.BI=TRUE} | 0.07  9208 | 0.405  063 | 0.195  545 | 1.39  868 | 32 |
| {BcOLV1.BI.MOVA.=TRUE, BcPV3.BI=TRUE} | 0.10 | 0.763 | 0.136 | 1.26 | 42 |

| => {BcHV1.BI=TRUE} | 396 | 636 | 139 | 4382 |  |
| --- | --- | --- | --- | --- | --- |
| {BcHV1.BI=TRUE, BcPV3.BI=TRUE} =>  {BcOLV1.BI.MOVA.=TRUE} | 0.10  396 | 0.518  519 | 0.200  495 | 1.56  3295 | 42 |
| {BcHV1.BI=TRUE, BcOLV1.BI.MOVA.=TRUE}  => {BcPV3.BI=TRUE} | 0.10  396 | 0.488  372 | 0.212  871 | 1.68  6345 | 42 |
| {BcOLV1.BI.MOVA.=TRUE, BcPV3.BI=TRUE}  => {BcMV11=TRUE} | 0.10  1485 | 0.745  455 | 0.136  139 | 1.20  9493 | 41 |
| {BcMV11=TRUE, BcPV3.BI=TRUE} =>  {BcOLV1.BI.MOVA.=TRUE} | 0.10  1485 | 0.512  5 | 0.198  02 | 1.54  5149 | 41 |
| {BcMV11=TRUE, BcOLV1.BI.MOVA.=TRUE}  => {BcPV3.BI=TRUE} | 0.10  1485 | 0.418  367 | 0.242  574 | 1.44  4619 | 41 |
| {BcMV9.BI=TRUE, BcPV3.BI=TRUE} =>  {BcOLV1.BI.MOVA.=TRUE} | 0.10  1485 | 0.493  976 | 0.205  446 | 1.48  93 | 41 |
| {BcMV9.BI=TRUE, BcOLV1.BI.MOVA.=TRUE}  => {BcPV3.BI=TRUE} | 0.10  1485 | 0.476  744 | 0.212  871 | 1.64  6194 | 41 |
| {BcOLV1.BI.MOVA.=TRUE, BcPV3.BI=TRUE}  => {BcULV4=TRUE} | 0.11  3861 | 0.836  364 | 0.136  139 | 1.21  1079 | 46 |
| {BcPV3.BI=TRUE, BcULV4=TRUE} =>  {BcOLV1.BI.MOVA.=TRUE} | 0.11  3861 | 0.554  217 | 0.205  446 | 1.67  0922 | 46 |
| {BcOLV1.BI.MOVA.=TRUE, BcULV4=TRUE}  => {BcPV3.BI=TRUE} | 0.11  3861 | 0.464  646 | 0.245  05 | 1.60  442 | 46 |
| {BcEV2.BI=FALSE, BcPV3.BI=TRUE} => | 0.09 | 0.481 | 0.205 | 1.45 | 40 |

| {BcOLV1.BI.MOVA.=TRUE} | 901 | 928 | 446 | 2976 |  |
| --- | --- | --- | --- | --- | --- |
| {BcEV2.BI=FALSE, BcOLV1.BI.MOVA.=TRUE} =>  {BcPV3.BI=TRUE} | 0.09  901 | 0.396  04 | 0.25 | 1.36  7521 | 40 |
| {BcHV3.BI=FALSE, BcPV3.BI=TRUE} =>  {BcOLV1.BI.MOVA.=TRUE} | 0.09  901 | 0.476  19 | 0.207  921 | 1.43  5679 | 40 |
| {BcHV3.BI=FALSE, BcOLV1.BI.MOVA.=TRUE} =>  {BcPV3.BI=TRUE} | 0.09  901 | 0.434  783 | 0.227  723 | 1.50  1301 | 40 |
| {BcFV5.BI=FALSE, BcPV3.BI=TRUE} =>  {BcOLV1.BI.MOVA.=TRUE} | 0.10  8911 | 0.463  158 | 0.235  149 | 1.39  6386 | 44 |
| {BcFV5.BI=FALSE, BcOLV1.BI.MOVA.=TRUE} =>  {BcPV3.BI=TRUE} | 0.10  8911 | 0.453  608 | 0.240  099 | 1.56  6305 | 44 |
| {BcOLV1.BI.MOVA.=TRUE, BcPV3.BI=TRUE}  => {BcMV1.BI=TRUE} | 0.12  1287 | 0.890  909 | 0.136  139 | 1.12  8299 | 49 |
| {BcMV1.BI=TRUE, BcPV3.BI=TRUE} =>  {BcOLV1.BI.MOVA.=TRUE} | 0.12  1287 | 0.475  728 | 0.254  95 | 1.43  4285 | 49 |
| {BcMV1.BI=TRUE, BcOLV1.BI.MOVA.=TRUE}  => {BcPV3.BI=TRUE} | 0.12  1287 | 0.411  765 | 0.294  554 | 1.42  182 | 49 |
| {BcOLV1.BI.MOVA.=TRUE, BcPV3.BI=TRUE}  => {BcNSRV7.BI=FALSE} | 0.12  1287 | 0.890  909 | 0.136  139 | 1.11  4326 | 49 |

| {BcNSRV7.BI=FALSE, BcPV3.BI=TRUE} =>  {BcOLV1.BI.MOVA.=TRUE} | 0.12  1287 | 0.510  417 | 0.237  624 | 1.53  8868 | 49 |
| --- | --- | --- | --- | --- | --- |
| {BcNSRV7.BI=FALSE, BcOLV1.BI.MOVA.=TRUE} =>  {BcPV3.BI=TRUE} | 0.12  1287 | 0.457  944 | 0.264  851 | 1.58  1276 | 49 |
| {BcPV3.BI=TRUE, BcVV3.BI=FALSE} =>  {BcOLV1.BI.MOVA.=TRUE} | 0.09  901 | 0.454  545 | 0.217  822 | 1.37  0421 | 40 |
| {BcOLV1.BI.MOVA.=TRUE,  BcVV3.BI=FALSE} => {BcPV3.BI=TRUE} | 0.09  901 | 0.408  163 | 0.242  574 | 1.40  9384 | 40 |
| {BcALV1.BI=FALSE, BcOLV1.BI.MOVA.=TRUE} =>  {BcPV3.BI=TRUE} | 0.09  1584 | 0.406  593 | 0.225  248 | 1.40  3964 | 37 |
| {BcDMV1=FALSE, BcPV3.BI=TRUE} =>  {BcOLV1.BI.MOVA.=TRUE} | 0.11  8812 | 0.48 | 0.247  525 | 1.44  7164 | 48 |
| {BcDMV1=FALSE, BcOLV1.BI.MOVA.=TRUE}  => {BcPV3.BI=TRUE} | 0.11  8812 | 0.432  432 | 0.274  752 | 1.49  3185 | 48 |
| {BcFV9=FALSE, BcPV3.BI=TRUE} =>  {BcOLV1.BI.MOVA.=TRUE} | 0.11  8812 | 0.484  848 | 0.245  05 | 1.46  1782 | 48 |
| {BcFV9=FALSE, BcOLV1.BI.MOVA.=TRUE}  => {BcPV3.BI=TRUE} | 0.11  8812 | 0.424  779 | 0.279  703 | 1.46  6757 | 48 |
| {BcOLV4.BI=FALSE, BcPV3.BI=TRUE} =>  {BcOLV1.BI.MOVA.=TRUE} | 0.10  396 | 0.437  5 | 0.237  624 | 1.31  903 | 42 |

| {BcOLV1.BI.MOVA.=TRUE,  BcOLV4.BI=FALSE} => {BcPV3.BI=TRUE} | 0.10  396 | 0.396  226 | 0.262  376 | 1.36  8166 | 42 |
| --- | --- | --- | --- | --- | --- |
| {BcOLV1.BI.MOVA.=TRUE, BcULV5=FALSE}  => {BcPV3.BI=TRUE} | 0.09  901 | 0.384  615 | 0.257  426 | 1.32  8074 | 40 |
| {BcBNV5.BI=FALSE, BcPV3.BI=TRUE} =>  {BcOLV1.BI.MOVA.=TRUE} | 0.11  8812 | 0.448  598 | 0.264  851 | 1.35  249 | 48 |
| {BcBNV5.BI=FALSE, BcOLV1.BI.MOVA.=TRUE} =>  {BcPV3.BI=TRUE} | 0.11  8812 | 0.421  053 | 0.282  178 | 1.45  3891 | 48 |
| {BcPV3.BI=TRUE, BvF.BI=FALSE} =>  {BcOLV1.BI.MOVA.=TRUE} | 0.12  6238 | 0.472  222 | 0.267  327 | 1.42  3715 | 51 |
| {BcOLV1.BI.MOVA.=TRUE, BvF.BI=FALSE}  => {BcPV3.BI=TRUE} | 0.12  6238 | 0.401  575 | 0.314  356 | 1.38  6634 | 51 |
| {BcMCV5.BI=FALSE, BcPV3.BI=TRUE} =>  {BcOLV1.BI.MOVA.=TRUE} | 0.11  3861 | 0.429  907 | 0.264  851 | 1.29  6136 | 46 |
| {BcMCV5.BI=FALSE, BcOLV1.BI.MOVA.=TRUE} =>  {BcPV3.BI=TRUE} | 0.11  3861 | 0.410  714 | 0.277  228 | 1.41  8193 | 46 |
| {BcOLV1.BI.MOVA.=TRUE,  BcOLV13.BI=FALSE} => {BcPV3.BI=TRUE} | 0.10  396 | 0.375 | 0.277  228 | 1.29  4872 | 42 |
| {BcOLV2.BI=FALSE, BcPV3.BI=TRUE} =>  {BcOLV1.BI.MOVA.=TRUE} | 0.11  1386 | 0.424  528 | 0.262  376 | 1.27  9921 | 45 |

| {BcOLV1.BI.MOVA.=TRUE,  BcOLV2.BI=FALSE} => {BcPV3.BI=TRUE} | 0.11  1386 | 0.401  786 | 0.277  228 | 1.38  7363 | 45 |
| --- | --- | --- | --- | --- | --- |
| {BcMCV3.BI=FALSE, BcPV3.BI=TRUE} =>  {BcOLV1.BI.MOVA.=TRUE} | 0.10  6436 | 0.421  569 | 0.252  475 | 1.27  0998 | 43 |
| {BcMCV3.BI=FALSE, BcOLV1.BI.MOVA.=TRUE} =>  {BcPV3.BI=TRUE} | 0.10  6436 | 0.370  69 | 0.287  129 | 1.27  9988 | 43 |
| {BcPV3.BI=TRUE, BcPV4.BI=FALSE} =>  {BcOLV1.BI.MOVA.=TRUE} | 0.12  6238 | 0.463  636 | 0.272  277 | 1.39  7829 | 51 |
| {BcOLV1.BI.MOVA.=TRUE,  BcPV4.BI=FALSE} => {BcPV3.BI=TRUE} | 0.12  6238 | 0.398  438 | 0.316  832 | 1.37  5801 | 51 |
| {BcEV6=FALSE, BcPV3.BI=TRUE} =>  {BcOLV1.BI.MOVA.=TRUE} | 0.12  6238 | 0.459  459 | 0.274  752 | 1.38  5236 | 51 |
| {BcEV6=FALSE, BcOLV1.BI.MOVA.=TRUE}  => {BcPV3.BI=TRUE} | 0.12  6238 | 0.411  29 | 0.306  931 | 1.42  0182 | 51 |
| {BcOLV17.BI=FALSE, BcPV3.BI=TRUE} =>  {BcOLV1.BI.MOVA.=TRUE} | 0.11  6337 | 0.451  923 | 0.257  426 | 1.36  2514 | 47 |
| {BcOLV1.BI.MOVA.=TRUE,  BcOLV17.BI=FALSE} => {BcPV3.BI=TRUE} | 0.11  6337 | 0.388  43 | 0.299  505 | 1.34  1245 | 47 |
| {BcOLV15.BI=FALSE, BcPV3.BI=TRUE} =>  {BcOLV1.BI.MOVA.=TRUE} | 0.13  6139 | 0.478  261 | 0.284  653 | 1.44  1921 | 55 |
| {BcOLV1.BI.MOVA.=TRUE, | 0.13 | 0.423 | 0.321 | 1.46 | 55 |

| BcOLV15.BI=FALSE} => {BcPV3.BI=TRUE} | 6139 | 077 | 782 | 0881 |  |
| --- | --- | --- | --- | --- | --- |
| {BcEV5=FALSE, BcPV3.BI=TRUE} =>  {BcOLV1.BI.MOVA.=TRUE} | 0.12  8713 | 0.464  286 | 0.277  228 | 1.39  9787 | 52 |
| {BcEV5=FALSE, BcOLV1.BI.MOVA.=TRUE}  => {BcPV3.BI=TRUE} | 0.12  8713 | 0.433  333 | 0.297  03 | 1.49  6296 | 52 |
| {BcMV11=FALSE, BcPV3.BI=TRUE} =>  {BcMV6.BI=FALSE} | 0.07  4257 | 0.810  811 | 0.091  584 | 1.16  5721 | 30 |
| {BcMV11=FALSE, BcPV3.BI=TRUE} =>  {BcFV5.BI=FALSE} | 0.08  1683 | 0.891  892 | 0.091  584 | 1.14  0267 | 33 |
| {BcHV1.BI=FALSE, BcPV3.BI=TRUE} =>  {BcHV1SLR.BI=FALSE} | 0.07  9208 | 0.888  889 | 0.089  109 | 1.94  1141 | 32 |
| {BcHV1SLR.BI=FALSE, BcPV3.BI=TRUE} =>  {BcHV1.BI=FALSE} | 0.07  9208 | 0.653  061 | 0.121  287 | 1.64  898 | 32 |
| {BcHV1.BI=FALSE, BcPV3.BI=TRUE} =>  {BcRV1.BI=FALSE} | 0.07  4257 | 0.833  333 | 0.089  109 | 1.15  2968 | 30 |
| {BcHV1.BI=FALSE, BcPV3.BI=TRUE} =>  {BcULV1.BI=FALSE} | 0.07  9208 | 0.888  889 | 0.089  109 | 1.13  2843 | 32 |
| {BcHV1.BI=FALSE, BcPV3.BI=TRUE} =>  {BcMV1.BI=TRUE} | 0.08  1683 | 0.916  667 | 0.089  109 | 1.16  092 | 33 |
| {BcHV1.BI=FALSE, BcPV3.BI=TRUE} =>  {BcALV1.BI=FALSE} | 0.08  1683 | 0.916  667 | 0.089  109 | 1.12  5633 | 33 |
| {BcPV3.BI=TRUE, BcULV3=FALSE} => | 0.08 | 0.744 | 0.116 | 1.43 | 35 |

| {BcOLV18=TRUE} | 6634 | 681 | 337 | 9479 |  |
| --- | --- | --- | --- | --- | --- |
| {BcOLV18=TRUE, BcULV3=FALSE} =>  {BcPV3.BI=TRUE} | 0.08  6634 | 0.448  718 | 0.193  069 | 1.54  9419 | 35 |
| {BcHV1.BI=TRUE, BcULV3=FALSE} =>  {BcPV3.BI=TRUE} | 0.07  6733 | 0.424  658 | 0.180  693 | 1.46  6339 | 31 |
| {BcPV3.BI=TRUE, BcULV3=FALSE} =>  {BcMV9.BI=TRUE} | 0.09  1584 | 0.787  234 | 0.116  337 | 1.16  0739 | 37 |
| {BcPV3.BI=TRUE, BcULV3=FALSE} =>  {BcULV4=TRUE} | 0.09  4059 | 0.808  511 | 0.116  337 | 1.17  0747 | 38 |
| {BcPV3.BI=TRUE, BcULV3=FALSE} =>  {BcFV5.BI=FALSE} | 0.10  6436 | 0.914  894 | 0.116  337 | 1.16  9674 | 43 |
| {BcPV3.BI=TRUE, BcULV3=FALSE} =>  {BcMV1.BI=TRUE} | 0.10  6436 | 0.914  894 | 0.116  337 | 1.15  8674 | 43 |
| {BcPV3.BI=TRUE, BcULV3=FALSE} =>  {BcNSRV7.BI=FALSE} | 0.10  8911 | 0.936  17 | 0.116  337 | 1.17  0937 | 44 |
| {BcOLV16.BI=TRUE, BcPV3.BI=TRUE} =>  {BcMV4.BI=TRUE} | 0.09  901 | 0.689  655 | 0.143  564 | 1.55  654 | 40 |
| {BcMV4.BI=TRUE, BcPV3.BI=TRUE} =>  {BcOLV16.BI=TRUE} | 0.09  901 | 0.547  945 | 0.180  693 | 1.31  7678 | 40 |
| {BcMV4.BI=TRUE, BcOLV16.BI=TRUE} =>  {BcPV3.BI=TRUE} | 0.09  901 | 0.439  56 | 0.225  248 | 1.51  7798 | 40 |
| {BcOLV16.BI=TRUE, BcPV3.BI=TRUE} => | 0.10 | 0.724 | 0.143 | 1.56 | 42 |

| {BcMV2.BI=TRUE} | 396 | 138 | 564 | 4448 |  |
| --- | --- | --- | --- | --- | --- |
| {BcMV2.BI=TRUE, BcPV3.BI=TRUE} =>  {BcOLV16.BI=TRUE} | 0.10  396 | 0.583  333 | 0.178  218 | 1.40  2778 | 42 |
| {BcMV2.BI=TRUE, BcOLV16.BI=TRUE} =>  {BcPV3.BI=TRUE} | 0.10  396 | 0.411  765 | 0.252  475 | 1.42  182 | 42 |
| {BcOLV16.BI=TRUE, BcPV3.BI=TRUE} =>  {BcOLV18=TRUE} | 0.09  901 | 0.689  655 | 0.143  564 | 1.33  3113 | 40 |
| {BcOLV18=TRUE, BcPV3.BI=TRUE} =>  {BcOLV16.BI=TRUE} | 0.09  901 | 0.526  316 | 0.188  119 | 1.26  5664 | 40 |
| {BcOLV16.BI=TRUE, BcPV3.BI=TRUE} =>  {BcHV1SLR.BI=TRUE} | 0.09  6535 | 0.672  414 | 0.143  564 | 1.24  0435 | 39 |
| {BcHV1SLR.BI=TRUE, BcPV3.BI=TRUE} =>  {BcOLV16.BI=TRUE} | 0.09  6535 | 0.573  529 | 0.168  317 | 1.37  9202 | 39 |
| {BcHV1SLR.BI=TRUE, BcOLV16.BI=TRUE}  => {BcPV3.BI=TRUE} | 0.09  6535 | 0.406  25 | 0.237  624 | 1.40  2778 | 39 |
| {BcOLV16.BI=TRUE, BcPV3.BI=TRUE} =>  {BcMV3.BI=TRUE} | 0.10  6436 | 0.741  379 | 0.143  564 | 1.36  7659 | 43 |
| {BcMV3.BI=TRUE, BcPV3.BI=TRUE} =>  {BcOLV16.BI=TRUE} | 0.10  6436 | 0.589  041 | 0.180  693 | 1.41  6504 | 43 |
| {BcMV3.BI=TRUE, BcOLV16.BI=TRUE} =>  {BcPV3.BI=TRUE} | 0.10  6436 | 0.398  148 | 0.267  327 | 1.37  4802 | 43 |
| {BcOLV16.BI=TRUE, BcPV3.BI=TRUE} => | 0.10 | 0.706 | 0.143 | 1.18 | 41 |

| {BcULV3=TRUE} | 1485 | 897 | 564 | 9943 |  |
| --- | --- | --- | --- | --- | --- |
| {BcPV3.BI=TRUE, BcULV3=TRUE} =>  {BcOLV16.BI=TRUE} | 0.10  1485 | 0.585  714 | 0.173  267 | 1.40  8503 | 41 |
| {BcOLV16.BI=TRUE, BcPV3.BI=TRUE} =>  {BcHV1.BI=TRUE} | 0.10  6436 | 0.741  379 | 0.143  564 | 1.22  753 | 43 |
| {BcHV1.BI=TRUE, BcPV3.BI=TRUE} =>  {BcOLV16.BI=TRUE} | 0.10  6436 | 0.530  864 | 0.200  495 | 1.27  6602 | 43 |
| {BcHV1.BI=TRUE, BcOLV16.BI=TRUE} =>  {BcPV3.BI=TRUE} | 0.10  6436 | 0.394  495 | 0.269  802 | 1.36  2189 | 43 |
| {BcOLV16.BI=TRUE, BcPV3.BI=TRUE} =>  {BcMV11=TRUE} | 0.11  3861 | 0.793  103 | 0.143  564 | 1.28  6802 | 46 |
| {BcMV11=TRUE, BcPV3.BI=TRUE} =>  {BcOLV16.BI=TRUE} | 0.11  3861 | 0.575 | 0.198  02 | 1.38  2738 | 46 |
| {BcMV11=TRUE, BcOLV16.BI=TRUE} =>  {BcPV3.BI=TRUE} | 0.11  3861 | 0.414  414 | 0.274  752 | 1.43  0969 | 46 |
| {BcOLV16.BI=TRUE, BcPV3.BI=TRUE} =>  {BcMV1.BI=TRUE} | 0.13  3663 | 0.931  034 | 0.143  564 | 1.17  9116 | 54 |
| {BcMV1.BI=TRUE, BcPV3.BI=TRUE} =>  {BcOLV16.BI=TRUE} | 0.13  3663 | 0.524  272 | 0.254  95 | 1.26  0749 | 54 |
| {BcMV1.BI=TRUE, BcOLV16.BI=TRUE} =>  {BcPV3.BI=TRUE} | 0.13  3663 | 0.397  059 | 0.336  634 | 1.37  1041 | 54 |
| {BcDMV1=FALSE, BcPV3.BI=TRUE} => | 0.12 | 0.51 | 0.247 | 1.22 | 51 |

| {BcOLV16.BI=TRUE} | 6238 |  | 525 | 6429 |  |
| --- | --- | --- | --- | --- | --- |
| {BcPV3.BI=TRUE, BvF.BI=FALSE} =>  {BcOLV16.BI=TRUE} | 0.13  6139 | 0.509  259 | 0.267  327 | 1.22  4647 | 55 |
| {BcMV4.BI=TRUE, BcPV3.BI=TRUE} =>  {BcMV2.BI=TRUE} | 0.11  8812 | 0.657  534 | 0.180  693 | 1.42  0555 | 48 |
| {BcMV2.BI=TRUE, BcPV3.BI=TRUE} =>  {BcMV4.BI=TRUE} | 0.11  8812 | 0.666  667 | 0.178  218 | 1.50  4655 | 48 |
| {BcMV2.BI=TRUE, BcMV4.BI=TRUE} =>  {BcPV3.BI=TRUE} | 0.11  8812 | 0.432  432 | 0.274  752 | 1.49  3185 | 48 |
| {BcMV4.BI=TRUE, BcPV3.BI=TRUE} =>  {BcOLV18=TRUE} | 0.12  6238 | 0.698  63 | 0.180  693 | 1.35  0462 | 51 |
| {BcOLV18=TRUE, BcPV3.BI=TRUE} =>  {BcMV4.BI=TRUE} | 0.12  6238 | 0.671  053 | 0.188  119 | 1.51  4555 | 51 |
| {BcMV4.BI=TRUE, BcOLV18=TRUE} =>  {BcPV3.BI=TRUE} | 0.12  6238 | 0.455  357 | 0.277  228 | 1.57  2344 | 51 |
| {BcHV1SLR.BI=TRUE, BcPV3.BI=TRUE} =>  {BcMV4.BI=TRUE} | 0.11  1386 | 0.661  765 | 0.168  317 | 1.49  3592 | 45 |
| {BcHV1SLR.BI=TRUE, BcMV4.BI=TRUE} =>  {BcPV3.BI=TRUE} | 0.11  1386 | 0.424  528 | 0.262  376 | 1.46  5893 | 45 |
| {BcMV4.BI=TRUE, BcPV3.BI=TRUE} =>  {BcMV3.BI=TRUE} | 0.13  1188 | 0.726  027 | 0.180  693 | 1.33  9338 | 53 |
| {BcMV3.BI=TRUE, BcPV3.BI=TRUE} => | 0.13 | 0.726 | 0.180 | 1.63 | 53 |

| {BcMV4.BI=TRUE} | 1188 | 027 | 693 | 8632 |  |
| --- | --- | --- | --- | --- | --- |
| {BcMV3.BI=TRUE, BcMV4.BI=TRUE} =>  {BcPV3.BI=TRUE} | 0.13  1188 | 0.445  378 | 0.294  554 | 1.53  7887 | 53 |
| {BcOLV16.BI=FALSE, BcPV3.BI=TRUE} =>  {BcMV4.BI=TRUE} | 0.08  1683 | 0.559  322 | 0.146  04 | 1.26  238 | 33 |
| {BcPV3.BI=TRUE, BcULV3=TRUE} =>  {BcMV4.BI=TRUE} | 0.10  8911 | 0.628  571 | 0.173  267 | 1.41  8675 | 44 |
| {BcMV4.BI=TRUE, BcULV3=TRUE} =>  {BcPV3.BI=TRUE} | 0.10  8911 | 0.369  748 | 0.294  554 | 1.27  6736 | 44 |
| {BcMV4.BI=TRUE, BcPV3.BI=TRUE} =>  {BcHV1.BI=TRUE} | 0.13  1188 | 0.726  027 | 0.180  693 | 1.20  2111 | 53 |
| {BcHV1.BI=TRUE, BcPV3.BI=TRUE} =>  {BcMV4.BI=TRUE} | 0.13  1188 | 0.654  321 | 0.200  495 | 1.47  6792 | 53 |
| {BcHV1.BI=TRUE, BcMV4.BI=TRUE} =>  {BcPV3.BI=TRUE} | 0.13  1188 | 0.438  017 | 0.299  505 | 1.51  2467 | 53 |
| {BcMV4.BI=TRUE, BcPV3.BI=TRUE} =>  {BcMV11=TRUE} | 0.14  3564 | 0.794  521 | 0.180  693 | 1.28  9102 | 58 |
| {BcMV11=TRUE, BcPV3.BI=TRUE} =>  {BcMV4.BI=TRUE} | 0.14  3564 | 0.725 | 0.198  02 | 1.63  6313 | 58 |
| {BcMV11=TRUE, BcMV4.BI=TRUE} =>  {BcPV3.BI=TRUE} | 0.14  3564 | 0.423  358 | 0.339  109 | 1.46  185 | 58 |
| {BcOLV1.BI.MOVA.=FALSE, | 0.09 | 0.596 | 0.153 | 1.34 | 37 |

| BcPV3.BI=TRUE} => {BcMV4.BI=TRUE} | 1584 | 774 | 465 | 6909 |  |
| --- | --- | --- | --- | --- | --- |
| {BcMV4.BI=TRUE, BcPV3.BI=TRUE} =>  {BcMV9.BI=TRUE} | 0.14  3564 | 0.794  521 | 0.180  693 | 1.17  1483 | 58 |
| {BcMV9.BI=TRUE, BcPV3.BI=TRUE} =>  {BcMV4.BI=TRUE} | 0.14  3564 | 0.698  795 | 0.205  446 | 1.57  7169 | 58 |
| {BcMV4.BI=TRUE, BcMV9.BI=TRUE} =>  {BcPV3.BI=TRUE} | 0.14  3564 | 0.426  471 | 0.336  634 | 1.47  2599 | 58 |
| {BcPV3.BI=TRUE, BcULV4=TRUE} =>  {BcMV4.BI=TRUE} | 0.12  8713 | 0.626  506 | 0.205  446 | 1.41  4014 | 52 |
| {BcMV4.BI=TRUE, BcULV4=TRUE} =>  {BcPV3.BI=TRUE} | 0.12  8713 | 0.396  947 | 0.324  257 | 1.37  0653 | 52 |
| {BcEV2.BI=FALSE, BcPV3.BI=TRUE} =>  {BcMV4.BI=TRUE} | 0.12  8713 | 0.626  506 | 0.205  446 | 1.41  4014 | 52 |
| {BcEV2.BI=FALSE, BcMV4.BI=TRUE} =>  {BcPV3.BI=TRUE} | 0.12  8713 | 0.409  449 | 0.314  356 | 1.41  3823 | 52 |
| {BcHV3.BI=FALSE, BcPV3.BI=TRUE} =>  {BcMV4.BI=TRUE} | 0.12  3762 | 0.595  238 | 0.207  921 | 1.34  3442 | 50 |
| {BcHV3.BI=FALSE, BcMV4.BI=TRUE} =>  {BcPV3.BI=TRUE} | 0.12  3762 | 0.423  729 | 0.292  079 | 1.46  3132 | 50 |
| {BcFV5.BI=FALSE, BcPV3.BI=TRUE} =>  {BcMV4.BI=TRUE} | 0.14  604 | 0.621  053 | 0.235  149 | 1.40  1705 | 59 |
| {BcFV5.BI=FALSE, BcMV4.BI=TRUE} => | 0.14 | 0.453 | 0.321 | 1.56 | 59 |

| {BcPV3.BI=TRUE} | 604 | 846 | 782 | 7127 |  |
| --- | --- | --- | --- | --- | --- |
| {BcPV3.BI=TRUE, BcULV1.BI=FALSE} =>  {BcMV4.BI=TRUE} | 0.12  8713 | 0.604  651 | 0.212  871 | 1.36  4688 | 52 |
| {BcMV4.BI=TRUE, BcULV1.BI=FALSE} =>  {BcPV3.BI=TRUE} | 0.12  8713 | 0.385  185 | 0.334  158 | 1.33  0041 | 52 |
| {BcMV4.BI=TRUE, BcPV3.BI=TRUE} =>  {BcMV1.BI=TRUE} | 0.15  8416 | 0.876  712 | 0.180  693 | 1.11  0319 | 64 |
| {BcMV1.BI=TRUE, BcPV3.BI=TRUE} =>  {BcMV4.BI=TRUE} | 0.15  8416 | 0.621  359 | 0.254  95 | 1.40  2397 | 64 |
| {BcMV1.BI=TRUE, BcMV4.BI=TRUE} =>  {BcPV3.BI=TRUE} | 0.15  8416 | 0.421  053 | 0.376  238 | 1.45  3891 | 64 |
| {BcMV12=FALSE, BcPV3.BI=TRUE} =>  {BcMV4.BI=TRUE} | 0.13  8614 | 0.636  364 | 0.217  822 | 1.43  6262 | 56 |
| {BcMV12=FALSE, BcMV4.BI=TRUE} =>  {BcPV3.BI=TRUE} | 0.13  8614 | 0.383  562 | 0.361  386 | 1.32  4435 | 56 |
| {BcNSRV7.BI=FALSE, BcPV3.BI=TRUE} =>  {BcMV4.BI=TRUE} | 0.14  8515 | 0.625 | 0.237  624 | 1.41  0615 | 60 |
| {BcMV4.BI=TRUE, BcNSRV7.BI=FALSE} =>  {BcPV3.BI=TRUE} | 0.14  8515 | 0.441  176 | 0.336  634 | 1.52  3379 | 60 |
| {BcPV3.BI=TRUE, BcVV3.BI=FALSE} =>  {BcMV4.BI=TRUE} | 0.12  8713 | 0.590  909 | 0.217  822 | 1.33  3672 | 52 |
| {BcMV4.BI=TRUE, BcVV3.BI=FALSE} => | 0.12 | 0.388 | 0.331 | 1.33 | 52 |

| {BcPV3.BI=TRUE} | 8713 | 06 | 683 | 9967 |  |
| --- | --- | --- | --- | --- | --- |
| {BcOLV7.BI=FALSE, BcPV3.BI=TRUE} =>  {BcMV4.BI=TRUE} | 0.12  1287 | 0.583  333 | 0.207  921 | 1.31  6574 | 49 |
| {BcALV1.BI=FALSE, BcPV3.BI=TRUE} =>  {BcMV4.BI=TRUE} | 0.13  1188 | 0.569  892 | 0.230  198 | 1.28  6238 | 53 |
| {BcALV1.BI=FALSE, BcMV4.BI=TRUE} =>  {BcPV3.BI=TRUE} | 0.13  1188 | 0.417  323 | 0.314  356 | 1.44  1012 | 53 |
| {BcDMV1=FALSE, BcPV3.BI=TRUE} =>  {BcMV4.BI=TRUE} | 0.15  099 | 0.61 | 0.247  525 | 1.37  676 | 61 |
| {BcDMV1=FALSE, BcMV4.BI=TRUE} =>  {BcPV3.BI=TRUE} | 0.15  099 | 0.417  808 | 0.361  386 | 1.44  2688 | 61 |
| {BcFV9=FALSE, BcPV3.BI=TRUE} =>  {BcMV4.BI=TRUE} | 0.14  3564 | 0.585  859 | 0.245  05 | 1.32  2273 | 58 |
| {BcFV9=FALSE, BcMV4.BI=TRUE} =>  {BcPV3.BI=TRUE} | 0.14  3564 | 0.402  778 | 0.356  436 | 1.39  0788 | 58 |
| {BcOLV4.BI=FALSE, BcPV3.BI=TRUE} =>  {BcMV4.BI=TRUE} | 0.13  8614 | 0.583  333 | 0.237  624 | 1.31  6574 | 56 |
| {BcMV4.BI=TRUE, BcOLV4.BI=FALSE} =>  {BcPV3.BI=TRUE} | 0.13  8614 | 0.373  333 | 0.371  287 | 1.28  9117 | 56 |
| {BcPV3.BI=TRUE, BcULV5=FALSE} =>  {BcMV4.BI=TRUE} | 0.14  8515 | 0.625 | 0.237  624 | 1.41  0615 | 60 |
| {BcMV4.BI=TRUE, BcULV5=FALSE} => | 0.14 | 0.389 | 0.381 | 1.34 | 60 |

| {BcPV3.BI=TRUE} | 8515 | 61 | 188 | 5321 |  |
| --- | --- | --- | --- | --- | --- |
| {BcBNV5.BI=FALSE, BcPV3.BI=TRUE} =>  {BcMV4.BI=TRUE} | 0.16  0891 | 0.607  477 | 0.264  851 | 1.37  1065 | 65 |
| {BcBNV5.BI=FALSE, BcMV4.BI=TRUE} =>  {BcPV3.BI=TRUE} | 0.16  0891 | 0.419  355 | 0.383  663 | 1.44  8029 | 65 |
| {BcPV3.BI=TRUE, BvF.BI=FALSE} =>  {BcMV4.BI=TRUE} | 0.16  5842 | 0.620  37 | 0.267  327 | 1.40  0166 | 67 |
| {BcMV4.BI=TRUE, BvF.BI=FALSE} =>  {BcPV3.BI=TRUE} | 0.16  5842 | 0.408  537 | 0.405  941 | 1.41  0673 | 67 |
| {BcMCV5.BI=FALSE, BcPV3.BI=TRUE} =>  {BcMV4.BI=TRUE} | 0.16  0891 | 0.607  477 | 0.264  851 | 1.37  1065 | 65 |
| {BcMCV5.BI=FALSE, BcMV4.BI=TRUE} =>  {BcPV3.BI=TRUE} | 0.16  0891 | 0.424  837 | 0.378  713 | 1.46  6957 | 65 |
| {BcOLV13.BI=FALSE, BcPV3.BI=TRUE} =>  {BcMV4.BI=TRUE} | 0.14  604 | 0.59 | 0.247  525 | 1.33  162 | 59 |
| {BcMV4.BI=TRUE, BcOLV13.BI=FALSE} =>  {BcPV3.BI=TRUE} | 0.14  604 | 0.373  418 | 0.391  089 | 1.28  9408 | 59 |
| {BcOLV2.BI=FALSE, BcPV3.BI=TRUE} =>  {BcMV4.BI=TRUE} | 0.15  3465 | 0.584  906 | 0.262  376 | 1.32  0122 | 62 |
| {BcMV4.BI=TRUE, BcOLV2.BI=FALSE} =>  {BcPV3.BI=TRUE} | 0.15  3465 | 0.380  368 | 0.403  465 | 1.31  3408 | 62 |
| {BcMCV3.BI=FALSE, BcPV3.BI=TRUE} => | 0.15 | 0.617 | 0.252 | 1.39 | 63 |

| {BcMV4.BI=TRUE} | 5941 | 647 | 475 | 4019 |  |
| --- | --- | --- | --- | --- | --- |
| {BcMCV3.BI=FALSE, BcMV4.BI=TRUE} =>  {BcPV3.BI=TRUE} | 0.15  5941 | 0.379  518 | 0.410  891 | 1.31  0473 | 63 |
| {BcPV3.BI=TRUE, BcPV4.BI=FALSE} =>  {BcMV4.BI=TRUE} | 0.17  5743 | 0.645  455 | 0.272  277 | 1.45  678 | 71 |
| {BcMV4.BI=TRUE, BcPV4.BI=FALSE} =>  {BcPV3.BI=TRUE} | 0.17  5743 | 0.420  118 | 0.418  317 | 1.45  0665 | 71 |
| {BcEV6=FALSE, BcPV3.BI=TRUE} =>  {BcMV4.BI=TRUE} | 0.17  3267 | 0.630  631 | 0.274  752 | 1.42  3323 | 70 |
| {BcEV6=FALSE, BcMV4.BI=TRUE} =>  {BcPV3.BI=TRUE} | 0.17  3267 | 0.411  765 | 0.420  792 | 1.42  182 | 70 |
| {BcOLV17.BI=FALSE, BcPV3.BI=TRUE} =>  {BcMV4.BI=TRUE} | 0.15  8416 | 0.615  385 | 0.257  426 | 1.38  8913 | 64 |
| {BcMV4.BI=TRUE, BcOLV17.BI=FALSE} =>  {BcPV3.BI=TRUE} | 0.15  8416 | 0.385  542 | 0.410  891 | 1.33  1274 | 64 |
| {BcOLV15.BI=FALSE, BcPV3.BI=TRUE} =>  {BcMV4.BI=TRUE} | 0.17  5743 | 0.617  391 | 0.284  653 | 1.39  3442 | 71 |
| {BcMV4.BI=TRUE, BcOLV15.BI=FALSE} =>  {BcPV3.BI=TRUE} | 0.17  5743 | 0.425  15 | 0.413  366 | 1.46  8038 | 71 |
| {BcEV5=FALSE, BcPV3.BI=TRUE} =>  {BcMV4.BI=TRUE} | 0.16  8317 | 0.607  143 | 0.277  228 | 1.37  0311 | 68 |
| {BcEV5=FALSE, BcMV4.BI=TRUE} => | 0.16 | 0.422 | 0.398 | 1.45 | 68 |

| {BcPV3.BI=TRUE} | 8317 | 36 | 515 | 8406 |  |
| --- | --- | --- | --- | --- | --- |
| {BcHV1SLR.BI=FALSE, BcPV3.BI=TRUE} =>  {BcFV5.BI=FALSE} | 0.10  6436 | 0.877  551 | 0.121  287 | 1.12  1932 | 43 |
| {BcMV3.BI=FALSE, BcPV3.BI=TRUE} =>  {BcMV6.BI=FALSE} | 0.09  1584 | 0.840  909 | 0.108  911 | 1.20  8994 | 37 |
| {BcMV3.BI=FALSE, BcPV3.BI=TRUE} =>  {BcMV1.BI=TRUE} | 0.09  6535 | 0.886  364 | 0.108  911 | 1.12  2542 | 39 |
| {BcMV3.BI=FALSE, BcPV3.BI=TRUE} =>  {BcMV12=FALSE} | 0.10  1485 | 0.931  818 | 0.108  911 | 1.16  5494 | 41 |
| {BcMV3.BI=FALSE, BcPV3.BI=TRUE} =>  {BcDMV1=FALSE} | 0.10  1485 | 0.931  818 | 0.108  911 | 1.10  3972 | 41 |
| {BcMV2.BI=TRUE, BcPV3.BI=TRUE} =>  {BcOLV18=TRUE} | 0.12  6238 | 0.708  333 | 0.178  218 | 1.36  9219 | 51 |
| {BcOLV18=TRUE, BcPV3.BI=TRUE} =>  {BcMV2.BI=TRUE} | 0.12  6238 | 0.671  053 | 0.188  119 | 1.44  9761 | 51 |
| {BcMV2.BI=TRUE, BcOLV18=TRUE} =>  {BcPV3.BI=TRUE} | 0.12  6238 | 0.428  571 | 0.294  554 | 1.47  9853 | 51 |
| {BcMV2.BI=TRUE, BcPV3.BI=TRUE} =>  {BcHV1SLR.BI=TRUE} | 0.11  6337 | 0.652  778 | 0.178  218 | 1.20  4211 | 47 |
| {BcHV1SLR.BI=TRUE, BcPV3.BI=TRUE} =>  {BcMV2.BI=TRUE} | 0.11  6337 | 0.691  176 | 0.168  317 | 1.49  3237 | 47 |
| {BcHV1SLR.BI=TRUE, BcMV2.BI=TRUE} => | 0.11 | 0.427 | 0.272 | 1.47 | 47 |

| {BcPV3.BI=TRUE} | 6337 | 273 | 277 | 5369 |  |
| --- | --- | --- | --- | --- | --- |
| {BcMV2.BI=TRUE, BcPV3.BI=TRUE} =>  {BcMV3.BI=TRUE} | 0.13  1188 | 0.736  111 | 0.178  218 | 1.35  794 | 53 |
| {BcMV3.BI=TRUE, BcPV3.BI=TRUE} =>  {BcMV2.BI=TRUE} | 0.13  1188 | 0.726  027 | 0.180  693 | 1.56  853 | 53 |
| {BcMV2.BI=TRUE, BcMV3.BI=TRUE} =>  {BcPV3.BI=TRUE} | 0.13  1188 | 0.417  323 | 0.314  356 | 1.44  1012 | 53 |
| {BcPV3.BI=TRUE, BcULV3=TRUE} =>  {BcMV2.BI=TRUE} | 0.11  3861 | 0.657  143 | 0.173  267 | 1.41  971 | 46 |
| {BcMV2.BI=TRUE, BcULV3=TRUE} =>  {BcPV3.BI=TRUE} | 0.11  3861 | 0.4 | 0.284  653 | 1.38  1197 | 46 |
| {BcMV2.BI=TRUE, BcPV3.BI=TRUE} =>  {BcHV1.BI=TRUE} | 0.12  6238 | 0.708  333 | 0.178  218 | 1.17  2814 | 51 |
| {BcHV1.BI=TRUE, BcPV3.BI=TRUE} =>  {BcMV2.BI=TRUE} | 0.12  6238 | 0.629  63 | 0.200  495 | 1.36  0269 | 51 |
| {BcHV1.BI=TRUE, BcMV2.BI=TRUE} =>  {BcPV3.BI=TRUE} | 0.12  6238 | 0.432  203 | 0.292  079 | 1.49  2395 | 51 |
| {BcMV2.BI=TRUE, BcPV3.BI=TRUE} =>  {BcMV11=TRUE} | 0.14  1089 | 0.791  667 | 0.178  218 | 1.28  4471 | 57 |
| {BcMV11=TRUE, BcPV3.BI=TRUE} =>  {BcMV2.BI=TRUE} | 0.14  1089 | 0.712  5 | 0.198  02 | 1.53  9305 | 57 |
| {BcMV11=TRUE, BcMV2.BI=TRUE} => | 0.14 | 0.413 | 0.341 | 1.42 | 57 |

| {BcPV3.BI=TRUE} | 1089 | 043 | 584 | 6236 |  |
| --- | --- | --- | --- | --- | --- |
| {BcMV9.BI=TRUE, BcPV3.BI=TRUE} =>  {BcMV2.BI=TRUE} | 0.13  6139 | 0.662  651 | 0.205  446 | 1.43  1609 | 55 |
| {BcMV2.BI=TRUE, BcMV9.BI=TRUE} =>  {BcPV3.BI=TRUE} | 0.13  6139 | 0.423  077 | 0.321  782 | 1.46  0881 | 55 |
| {BcPV3.BI=TRUE, BcULV4=TRUE} =>  {BcMV2.BI=TRUE} | 0.13  6139 | 0.662  651 | 0.205  446 | 1.43  1609 | 55 |
| {BcMV2.BI=TRUE, BcULV4=TRUE} =>  {BcPV3.BI=TRUE} | 0.13  6139 | 0.398  551 | 0.341  584 | 1.37  6192 | 55 |
| {BcHV3.BI=FALSE, BcPV3.BI=TRUE} =>  {BcMV2.BI=TRUE} | 0.12  8713 | 0.619  048 | 0.207  921 | 1.33  7408 | 52 |
| {BcHV3.BI=FALSE, BcMV2.BI=TRUE} =>  {BcPV3.BI=TRUE} | 0.12  8713 | 0.390  977 | 0.329  208 | 1.35  0042 | 52 |
| {BcFV5.BI=FALSE, BcPV3.BI=TRUE} =>  {BcMV2.BI=TRUE} | 0.13  6139 | 0.578  947 | 0.235  149 | 1.25  0774 | 55 |
| {BcFV5.BI=FALSE, BcMV2.BI=TRUE} =>  {BcPV3.BI=TRUE} | 0.13  6139 | 0.395  683 | 0.344  059 | 1.36  6292 | 55 |
| {BcMV2.BI=TRUE, BcULV1.BI=FALSE} =>  {BcPV3.BI=TRUE} | 0.11  8812 | 0.366  412 | 0.324  257 | 1.26  5218 | 48 |
| {BcMV2.BI=TRUE, BcPV3.BI=TRUE} =>  {BcMV1.BI=TRUE} | 0.16  5842 | 0.930  556 | 0.178  218 | 1.17  8509 | 67 |
| {BcMV1.BI=TRUE, BcPV3.BI=TRUE} => | 0.16 | 0.650 | 0.254 | 1.40 | 67 |

| {BcMV2.BI=TRUE} | 5842 | 485 | 95 | 5327 |  |
| --- | --- | --- | --- | --- | --- |
| {BcMV1.BI=TRUE, BcMV2.BI=TRUE} =>  {BcPV3.BI=TRUE} | 0.16  5842 | 0.406  061 | 0.408  416 | 1.40  2124 | 67 |
| {BcMV12=FALSE, BcPV3.BI=TRUE} =>  {BcMV2.BI=TRUE} | 0.12  6238 | 0.579  545 | 0.217  822 | 1.25  2066 | 51 |
| {BcMV12=FALSE, BcMV2.BI=TRUE} =>  {BcPV3.BI=TRUE} | 0.12  6238 | 0.369  565 | 0.341  584 | 1.27  6106 | 51 |
| {BcNSRV7.BI=FALSE, BcPV3.BI=TRUE} =>  {BcMV2.BI=TRUE} | 0.14  1089 | 0.593  75 | 0.237  624 | 1.28  2754 | 57 |
| {BcMV2.BI=TRUE, BcNSRV7.BI=FALSE} =>  {BcPV3.BI=TRUE} | 0.14  1089 | 0.390  411 | 0.361  386 | 1.34  8086 | 57 |
| {BcPV3.BI=TRUE, BcVV3.BI=FALSE} =>  {BcMV2.BI=TRUE} | 0.13  1188 | 0.602  273 | 0.217  822 | 1.30  1167 | 53 |
| {BcMV2.BI=TRUE, BcVV3.BI=FALSE} =>  {BcPV3.BI=TRUE} | 0.13  1188 | 0.373  239 | 0.351  485 | 1.28  8793 | 53 |
| {BcOLV7.BI=FALSE, BcPV3.BI=TRUE} =>  {BcMV2.BI=TRUE} | 0.12  3762 | 0.595  238 | 0.207  921 | 1.28  5969 | 50 |
| {BcALV1.BI=FALSE, BcMV2.BI=TRUE} =>  {BcPV3.BI=TRUE} | 0.12  6238 | 0.389  313 | 0.324  257 | 1.34  4294 | 51 |
| {BcDMV1=FALSE, BcPV3.BI=TRUE} =>  {BcMV2.BI=TRUE} | 0.15  099 | 0.61 | 0.247  525 | 1.31  7861 | 61 |
| {BcDMV1=FALSE, BcMV2.BI=TRUE} => | 0.15 | 0.381 | 0.396 | 1.31 | 61 |

| {BcPV3.BI=TRUE} | 099 | 25 | 04 | 6453 |  |
| --- | --- | --- | --- | --- | --- |
| {BcFV9=FALSE, BcPV3.BI=TRUE} =>  {BcMV2.BI=TRUE} | 0.15  099 | 0.616  162 | 0.245  05 | 1.33  1173 | 61 |
| {BcFV9=FALSE, BcMV2.BI=TRUE} =>  {BcPV3.BI=TRUE} | 0.15  099 | 0.376  543 | 0.400  99 | 1.30  02 | 61 |
| {BcOLV4.BI=FALSE, BcPV3.BI=TRUE} =>  {BcMV2.BI=TRUE} | 0.13  6139 | 0.572  917 | 0.237  624 | 1.23  7745 | 55 |
| {BcMV2.BI=TRUE, BcOLV4.BI=FALSE} =>  {BcPV3.BI=TRUE} | 0.13  6139 | 0.361  842 | 0.376  238 | 1.24  9438 | 55 |
| {BcPV3.BI=TRUE, BcULV5=FALSE} =>  {BcMV2.BI=TRUE} | 0.13  6139 | 0.572  917 | 0.237  624 | 1.23  7745 | 55 |
| {BcMV2.BI=TRUE, BcULV5=FALSE} =>  {BcPV3.BI=TRUE} | 0.13  6139 | 0.361  842 | 0.376  238 | 1.24  9438 | 55 |
| {BcBNV5.BI=FALSE, BcPV3.BI=TRUE} =>  {BcMV2.BI=TRUE} | 0.15  8416 | 0.598  131 | 0.264  851 | 1.29  2219 | 64 |
| {BcBNV5.BI=FALSE, BcMV2.BI=TRUE} =>  {BcPV3.BI=TRUE} | 0.15  8416 | 0.387  879 | 0.408  416 | 1.33  9342 | 64 |
| {BcPV3.BI=TRUE, BvF.BI=FALSE} =>  {BcMV2.BI=TRUE} | 0.16  3366 | 0.611  111 | 0.267  327 | 1.32  0261 | 66 |
| {BcMV2.BI=TRUE, BvF.BI=FALSE} =>  {BcPV3.BI=TRUE} | 0.16  3366 | 0.377  143 | 0.433  168 | 1.30  2271 | 66 |
| {BcMCV5.BI=FALSE, BcPV3.BI=TRUE} => | 0.15 | 0.588 | 0.264 | 1.27 | 63 |

| {BcMV2.BI=TRUE} | 5941 | 785 | 851 | 2028 |  |
| --- | --- | --- | --- | --- | --- |
| {BcMCV5.BI=FALSE, BcMV2.BI=TRUE} =>  {BcPV3.BI=TRUE} | 0.15  5941 | 0.388  889 | 0.400  99 | 1.34  283 | 63 |
| {BcOLV13.BI=FALSE, BcPV3.BI=TRUE} =>  {BcMV2.BI=TRUE} | 0.14  1089 | 0.57 | 0.247  525 | 1.23  1444 | 57 |
| {BcOLV2.BI=FALSE, BcPV3.BI=TRUE} =>  {BcMV2.BI=TRUE} | 0.15  3465 | 0.584  906 | 0.262  376 | 1.26  3646 | 62 |
| {BcMV2.BI=TRUE, BcOLV2.BI=FALSE} =>  {BcPV3.BI=TRUE} | 0.15  3465 | 0.364  706 | 0.420  792 | 1.25  9326 | 62 |
| {BcMCV3.BI=FALSE, BcPV3.BI=TRUE} =>  {BcMV2.BI=TRUE} | 0.14  604 | 0.578  431 | 0.252  475 | 1.24  9659 | 59 |
| {BcMCV3.BI=FALSE, BcMV2.BI=TRUE} =>  {BcPV3.BI=TRUE} | 0.14  604 | 0.357  576 | 0.408  416 | 1.23  4706 | 59 |
| {BcPV3.BI=TRUE, BcPV4.BI=FALSE} =>  {BcMV2.BI=TRUE} | 0.16  3366 | 0.6 | 0.272  277 | 1.29  6257 | 66 |
| {BcMV2.BI=TRUE, BcPV4.BI=FALSE} =>  {BcPV3.BI=TRUE} | 0.16  3366 | 0.381  503 | 0.428  218 | 1.31  7326 | 66 |
| {BcEV6=FALSE, BcPV3.BI=TRUE} =>  {BcMV2.BI=TRUE} | 0.16  8317 | 0.612  613 | 0.274  752 | 1.32  3505 | 68 |
| {BcEV6=FALSE, BcMV2.BI=TRUE} =>  {BcPV3.BI=TRUE} | 0.16  8317 | 0.388  571 | 0.433  168 | 1.34  1734 | 68 |
| {BcOLV17.BI=FALSE, BcPV3.BI=TRUE} => | 0.15 | 0.596 | 0.257 | 1.28 | 62 |

| {BcMV2.BI=TRUE} | 3465 | 154 | 426 | 7947 |  |
| --- | --- | --- | --- | --- | --- |
| {BcMV2.BI=TRUE, BcOLV17.BI=FALSE} =>  {BcPV3.BI=TRUE} | 0.15  3465 | 0.360  465 | 0.425  743 | 1.24  4683 | 62 |
| {BcOLV15.BI=FALSE, BcPV3.BI=TRUE} =>  {BcMV2.BI=TRUE} | 0.17  8218 | 0.626  087 | 0.284  653 | 1.35  2616 | 72 |
| {BcMV2.BI=TRUE, BcOLV15.BI=FALSE} =>  {BcPV3.BI=TRUE} | 0.17  8218 | 0.393  443 | 0.452  97 | 1.35  8554 | 72 |
| {BcEV5=FALSE, BcPV3.BI=TRUE} =>  {BcMV2.BI=TRUE} | 0.16  8317 | 0.607  143 | 0.277  228 | 1.31  1688 | 68 |
| {BcEV5=FALSE, BcMV2.BI=TRUE} =>  {BcPV3.BI=TRUE} | 0.16  8317 | 0.397  661 | 0.423  267 | 1.37  3119 | 68 |
| {BcOLV18=FALSE, BcPV3.BI=TRUE} =>  {BcFV5.BI=FALSE} | 0.09  1584 | 0.902  439 | 0.101  485 | 1.15  3751 | 37 |
| {BcOLV18=FALSE, BcPV3.BI=TRUE} =>  {BcULV1.BI=FALSE} | 0.09  1584 | 0.902  439 | 0.101  485 | 1.15  0112 | 37 |
| {BcOLV18=FALSE, BcPV3.BI=TRUE} =>  {BcALV1.BI=FALSE} | 0.09  4059 | 0.926  829 | 0.101  485 | 1.13  8113 | 38 |
| {BcOLV18=FALSE, BcPV3.BI=TRUE} =>  {BcFV9=FALSE} | 0.09  901 | 0.975  61 | 0.101  485 | 1.12  2924 | 40 |
| {BcOLV18=TRUE, BcPV3.BI=TRUE} =>  {BcHV1SLR.BI=TRUE} | 0.12  3762 | 0.657  895 | 0.188  119 | 1.21  3651 | 50 |
| {BcHV1SLR.BI=TRUE, BcPV3.BI=TRUE} => | 0.12 | 0.735 | 0.168 | 1.42 | 50 |

| {BcOLV18=TRUE} | 3762 | 294 | 317 | 1334 |  |
| --- | --- | --- | --- | --- | --- |
| {BcHV1SLR.BI=TRUE, BcOLV18=TRUE} =>  {BcPV3.BI=TRUE} | 0.12  3762 | 0.362  319 | 0.341  584 | 1.25  1084 | 50 |
| {BcOLV18=TRUE, BcPV3.BI=TRUE} =>  {BcMV3.BI=TRUE} | 0.13  1188 | 0.697  368 | 0.188  119 | 1.28  647 | 53 |
| {BcMV3.BI=TRUE, BcPV3.BI=TRUE} =>  {BcOLV18=TRUE} | 0.13  1188 | 0.726  027 | 0.180  693 | 1.40  3421 | 53 |
| {BcMV3.BI=TRUE, BcOLV18=TRUE} =>  {BcPV3.BI=TRUE} | 0.13  1188 | 0.398  496 | 0.329  208 | 1.37  6004 | 53 |
| {BcOLV18=TRUE, BcPV3.BI=TRUE} =>  {BcHV1.BI=TRUE} | 0.14  3564 | 0.763  158 | 0.188  119 | 1.26  3589 | 58 |
| {BcHV1.BI=TRUE, BcPV3.BI=TRUE} =>  {BcOLV18=TRUE} | 0.14  3564 | 0.716  049 | 0.200  495 | 1.38  4134 | 58 |
| {BcHV1.BI=TRUE, BcOLV18=TRUE} =>  {BcPV3.BI=TRUE} | 0.14  3564 | 0.391  892 | 0.366  337 | 1.35  3199 | 58 |
| {BcOLV18=TRUE, BcPV3.BI=TRUE} =>  {BcMV11=TRUE} | 0.13  8614 | 0.736  842 | 0.188  119 | 1.19  5519 | 56 |
| {BcMV11=TRUE, BcPV3.BI=TRUE} =>  {BcOLV18=TRUE} | 0.13  8614 | 0.7 | 0.198  02 | 1.35  311 | 56 |
| {BcMV11=TRUE, BcOLV18=TRUE} =>  {BcPV3.BI=TRUE} | 0.13  8614 | 0.4 | 0.346  535 | 1.38  1197 | 56 |
| {BcOLV18=TRUE, BcPV3.BI=TRUE} => | 0.16 | 0.855 | 0.188 | 1.26 | 65 |

| {BcMV9.BI=TRUE} | 0891 | 263 | 119 | 1045 |  |
| --- | --- | --- | --- | --- | --- |
| {BcMV9.BI=TRUE, BcPV3.BI=TRUE} =>  {BcOLV18=TRUE} | 0.16  0891 | 0.783  133 | 0.205  446 | 1.51  3806 | 65 |
| {BcMV9.BI=TRUE, BcOLV18=TRUE} =>  {BcPV3.BI=TRUE} | 0.16  0891 | 0.389  222 | 0.413  366 | 1.34  3979 | 65 |
| {BcOLV18=TRUE, BcPV3.BI=TRUE} =>  {BcULV4=TRUE} | 0.15  3465 | 0.815  789 | 0.188  119 | 1.18  1287 | 62 |
| {BcPV3.BI=TRUE, BcULV4=TRUE} =>  {BcOLV18=TRUE} | 0.15  3465 | 0.746  988 | 0.205  446 | 1.44  3938 | 62 |
| {BcFV5.BI=FALSE, BcOLV18=TRUE} =>  {BcPV3.BI=TRUE} | 0.14  3564 | 0.389  262 | 0.368  812 | 1.34  4117 | 58 |
| {BcOLV18=TRUE, BcPV3.BI=TRUE} =>  {BcMV1.BI=TRUE} | 0.17  3267 | 0.921  053 | 0.188  119 | 1.16  6474 | 70 |
| {BcMV1.BI=TRUE, BcPV3.BI=TRUE} =>  {BcOLV18=TRUE} | 0.17  3267 | 0.679  612 | 0.254  95 | 1.31  3699 | 70 |
| {BcMV1.BI=TRUE, BcOLV18=TRUE} =>  {BcPV3.BI=TRUE} | 0.17  3267 | 0.393  258 | 0.440  594 | 1.35  7918 | 70 |
| {BcMV12=FALSE, BcPV3.BI=TRUE} =>  {BcOLV18=TRUE} | 0.14  3564 | 0.659  091 | 0.217  822 | 1.27  4032 | 58 |
| {BcMV12=FALSE, BcOLV18=TRUE} =>  {BcPV3.BI=TRUE} | 0.14  3564 | 0.362  5 | 0.396  04 | 1.25  1709 | 58 |
| {BcNSRV7.BI=FALSE, BcPV3.BI=TRUE} => | 0.14 | 0.625 | 0.237 | 1.20 | 60 |

| {BcOLV18=TRUE} | 8515 |  | 624 | 8134 |  |
| --- | --- | --- | --- | --- | --- |
| {BcNSRV7.BI=FALSE, BcOLV18=TRUE} =>  {BcPV3.BI=TRUE} | 0.14  8515 | 0.382  166 | 0.388  614 | 1.31  9615 | 60 |
| {BcPV3.BI=TRUE, BcVV3.BI=FALSE} =>  {BcOLV18=TRUE} | 0.13  6139 | 0.625 | 0.217  822 | 1.20  8134 | 55 |
| {BcALV1.BI=FALSE, BcOLV18=TRUE} =>  {BcPV3.BI=TRUE} | 0.13  6139 | 0.361  842 | 0.376  238 | 1.24  9438 | 55 |
| {BcDMV1=FALSE, BcPV3.BI=TRUE} =>  {BcOLV18=TRUE} | 0.15  5941 | 0.63 | 0.247  525 | 1.21  7799 | 63 |
| {BcDMV1=FALSE, BcOLV18=TRUE} =>  {BcPV3.BI=TRUE} | 0.15  5941 | 0.364  162 | 0.428  218 | 1.25  7448 | 63 |
| {BcOLV4.BI=FALSE, BcPV3.BI=TRUE} =>  {BcOLV18=TRUE} | 0.15  8416 | 0.666  667 | 0.237  624 | 1.28  8676 | 64 |
| {BcPV3.BI=TRUE, BcULV5=FALSE} =>  {BcOLV18=TRUE} | 0.15  8416 | 0.666  667 | 0.237  624 | 1.28  8676 | 64 |
| {BcBNV5.BI=FALSE, BcPV3.BI=TRUE} =>  {BcOLV18=TRUE} | 0.16  8317 | 0.635  514 | 0.264  851 | 1.22  8458 | 68 |
| {BcBNV5.BI=FALSE, BcOLV18=TRUE} =>  {BcPV3.BI=TRUE} | 0.16  8317 | 0.371  585 | 0.452  97 | 1.28  3079 | 68 |
| {BcPV3.BI=TRUE, BvF.BI=FALSE} =>  {BcOLV18=TRUE} | 0.17  3267 | 0.648  148 | 0.267  327 | 1.25  288 | 70 |
| {BcOLV18=TRUE, BvF.BI=FALSE} => | 0.17 | 0.364 | 0.475 | 1.25 | 70 |

| {BcPV3.BI=TRUE} | 3267 | 583 | 248 | 8903 |  |
| --- | --- | --- | --- | --- | --- |
| {BcMCV5.BI=FALSE, BcPV3.BI=TRUE} =>  {BcOLV18=TRUE} | 0.16  3366 | 0.616  822 | 0.264  851 | 1.19  2327 | 66 |
| {BcMCV5.BI=FALSE, BcOLV18=TRUE} =>  {BcPV3.BI=TRUE} | 0.16  3366 | 0.362  637 | 0.450  495 | 1.25  2184 | 66 |
| {BcOLV13.BI=FALSE, BcPV3.BI=TRUE} =>  {BcOLV18=TRUE} | 0.16  0891 | 0.65 | 0.247  525 | 1.25  6459 | 65 |
| {BcOLV2.BI=FALSE, BcPV3.BI=TRUE} =>  {BcOLV18=TRUE} | 0.16  5842 | 0.632  075 | 0.262  376 | 1.22  1811 | 67 |
| {BcOLV18=TRUE, BcOLV2.BI=FALSE} =>  {BcPV3.BI=TRUE} | 0.16  5842 | 0.354  497 | 0.467  822 | 1.22  4076 | 67 |
| {BcMCV3.BI=FALSE, BcPV3.BI=TRUE} =>  {BcOLV18=TRUE} | 0.16  3366 | 0.647  059 | 0.252  475 | 1.25  0774 | 66 |
| {BcPV3.BI=TRUE, BcPV4.BI=FALSE} =>  {BcOLV18=TRUE} | 0.17  8218 | 0.654  545 | 0.272  277 | 1.26  5246 | 72 |
| {BcOLV18=TRUE, BcPV4.BI=FALSE} =>  {BcPV3.BI=TRUE} | 0.17  8218 | 0.373  057 | 0.477  723 | 1.28  8163 | 72 |
| {BcEV6=FALSE, BcPV3.BI=TRUE} =>  {BcOLV18=TRUE} | 0.17  8218 | 0.648  649 | 0.274  752 | 1.25  3847 | 72 |
| {BcEV6=FALSE, BcOLV18=TRUE} =>  {BcPV3.BI=TRUE} | 0.17  8218 | 0.369  231 | 0.482  673 | 1.27  4951 | 72 |
| {BcOLV17.BI=FALSE, BcPV3.BI=TRUE} => | 0.16 | 0.644 | 0.257 | 1.24 | 67 |

| {BcOLV18=TRUE} | 5842 | 231 | 426 | 5307 |  |
| --- | --- | --- | --- | --- | --- |
| {BcOLV17.BI=FALSE, BcOLV18=TRUE} =>  {BcPV3.BI=TRUE} | 0.16  5842 | 0.347  15 | 0.477  723 | 1.19  8707 | 67 |
| {BcOLV15.BI=FALSE, BcPV3.BI=TRUE} =>  {BcOLV18=TRUE} | 0.18  3168 | 0.643  478 | 0.284  653 | 1.24  3853 | 74 |
| {BcOLV15.BI=FALSE, BcOLV18=TRUE} =>  {BcPV3.BI=TRUE} | 0.18  3168 | 0.383  42 | 0.477  723 | 1.32  3945 | 74 |
| {BcEV5=FALSE, BcPV3.BI=TRUE} =>  {BcOLV18=TRUE} | 0.17  8218 | 0.642  857 | 0.277  228 | 1.24  2652 | 72 |
| {BcEV5=FALSE, BcOLV18=TRUE} =>  {BcPV3.BI=TRUE} | 0.17  8218 | 0.365  482 | 0.487  624 | 1.26  2007 | 72 |
| {BcMV2.BI=FALSE, BcPV3.BI=TRUE} =>  {BcMV6.BI=FALSE} | 0.09  1584 | 0.822  222 | 0.111  386 | 1.18  2127 | 37 |
| {BcMV2.BI=FALSE, BcPV3.BI=TRUE} =>  {BcFV5.BI=FALSE} | 0.09  901 | 0.888  889 | 0.111  386 | 1.13  6428 | 40 |
| {BcMV2.BI=FALSE, BcPV3.BI=TRUE} =>  {BcALV1.BI=FALSE} | 0.10  396 | 0.933  333 | 0.111  386 | 1.14  6099 | 42 |
| {BcHV1SLR.BI=TRUE, BcPV3.BI=TRUE} =>  {BcMV3.BI=TRUE} | 0.12  1287 | 0.720  588 | 0.168  317 | 1.32  9304 | 49 |
| {BcMV3.BI=TRUE, BcPV3.BI=TRUE} =>  {BcHV1SLR.BI=TRUE} | 0.12  1287 | 0.671  233 | 0.180  693 | 1.23  8256 | 49 |
| {BcHV1SLR.BI=TRUE, BcMV3.BI=TRUE} => | 0.12 | 0.368 | 0.329 | 1.27 | 49 |

| {BcPV3.BI=TRUE} | 1287 | 421 | 208 | 2155 |  |
| --- | --- | --- | --- | --- | --- |
| {BcPV3.BI=TRUE, BcULV3=TRUE} =>  {BcHV1SLR.BI=TRUE} | 0.11  3861 | 0.657  143 | 0.173  267 | 1.21  2264 | 46 |
| {BcHV1SLR.BI=TRUE, BcPV3.BI=TRUE} =>  {BcHV1.BI=TRUE} | 0.15  8416 | 0.941  176 | 0.168  317 | 1.55  8341 | 64 |
| {BcHV1.BI=TRUE, BcPV3.BI=TRUE} =>  {BcHV1SLR.BI=TRUE} | 0.15  8416 | 0.790  123 | 0.200  495 | 1.45  7579 | 64 |
| {BcHV1SLR.BI=TRUE, BcPV3.BI=TRUE} =>  {BcMV11=TRUE} | 0.13  1188 | 0.779  412 | 0.168  317 | 1.26  4588 | 53 |
| {BcMV11=TRUE, BcPV3.BI=TRUE} =>  {BcHV1SLR.BI=TRUE} | 0.13  1188 | 0.662  5 | 0.198  02 | 1.22  2146 | 53 |
| {BcHV1SLR.BI=TRUE, BcMV11=TRUE} =>  {BcPV3.BI=TRUE} | 0.13  1188 | 0.368  056 | 0.356  436 | 1.27  0893 | 53 |
| {BcHV1SLR.BI=TRUE, BcPV3.BI=TRUE} =>  {BcMV1.BI=TRUE} | 0.14  8515 | 0.882  353 | 0.168  317 | 1.11  7463 | 60 |
| {BcPV3.BI=TRUE, BcULV3=TRUE} =>  {BcMV3.BI=TRUE} | 0.11  6337 | 0.671  429 | 0.173  267 | 1.23  8617 | 47 |
| {BcMV3.BI=TRUE, BcPV3.BI=TRUE} =>  {BcHV1.BI=TRUE} | 0.13  6139 | 0.753  425 | 0.180  693 | 1.24  7474 | 55 |
| {BcHV1.BI=TRUE, BcPV3.BI=TRUE} =>  {BcMV3.BI=TRUE} | 0.13  6139 | 0.679  012 | 0.200  495 | 1.25  2607 | 55 |
| {BcHV1.BI=TRUE, BcMV3.BI=TRUE} => | 0.13 | 0.384 | 0.353 | 1.32 | 55 |

| {BcPV3.BI=TRUE} | 6139 | 615 | 96 | 8074 |  |
| --- | --- | --- | --- | --- | --- |
| {BcMV3.BI=TRUE, BcPV3.BI=TRUE} =>  {BcMV11=TRUE} | 0.13  6139 | 0.753  425 | 0.180  693 | 1.22  2424 | 55 |
| {BcMV11=TRUE, BcPV3.BI=TRUE} =>  {BcMV3.BI=TRUE} | 0.13  6139 | 0.687  5 | 0.198  02 | 1.26  8265 | 55 |
| {BcMV11=TRUE, BcMV3.BI=TRUE} =>  {BcPV3.BI=TRUE} | 0.13  6139 | 0.354  839 | 0.383  663 | 1.22  5255 | 55 |
| {BcMV3.BI=TRUE, BcPV3.BI=TRUE} =>  {BcMV9.BI=TRUE} | 0.14  1089 | 0.780  822 | 0.180  693 | 1.15  1285 | 57 |
| {BcMV9.BI=TRUE, BcPV3.BI=TRUE} =>  {BcMV3.BI=TRUE} | 0.14  1089 | 0.686  747 | 0.205  446 | 1.26  6876 | 57 |
| {BcMV3.BI=TRUE, BcMV9.BI=TRUE} =>  {BcPV3.BI=TRUE} | 0.14  1089 | 0.356  25 | 0.396  04 | 1.23  0128 | 57 |
| {BcMV3.BI=TRUE, BcPV3.BI=TRUE} =>  {BcULV4=TRUE} | 0.14  604 | 0.808  219 | 0.180  693 | 1.17  0325 | 59 |
| {BcPV3.BI=TRUE, BcULV4=TRUE} =>  {BcMV3.BI=TRUE} | 0.14  604 | 0.710  843 | 0.205  446 | 1.31  1328 | 59 |
| {BcMV3.BI=TRUE, BcULV4=TRUE} =>  {BcPV3.BI=TRUE} | 0.14  604 | 0.364  198 | 0.400  99 | 1.25  7571 | 59 |
| {BcFV5.BI=FALSE, BcMV3.BI=TRUE} =>  {BcPV3.BI=TRUE} | 0.14  3564 | 0.369  427 | 0.388  614 | 1.27  5627 | 58 |
| {BcMV3.BI=TRUE, BcPV3.BI=TRUE} => | 0.15 | 0.876 | 0.180 | 1.11 | 64 |

| {BcMV1.BI=TRUE} | 8416 | 712 | 693 | 0319 |  |
| --- | --- | --- | --- | --- | --- |
| {BcNSRV7.BI=FALSE, BcPV3.BI=TRUE} =>  {BcMV3.BI=TRUE} | 0.15  3465 | 0.645  833 | 0.237  624 | 1.19  14 | 62 |
| {BcMV3.BI=TRUE, BcNSRV7.BI=FALSE} =>  {BcPV3.BI=TRUE} | 0.15  3465 | 0.362  573 | 0.423  267 | 1.25  1962 | 62 |
| {BcMV3.BI=TRUE, BcOLV15.BI=FALSE} =>  {BcPV3.BI=TRUE} | 0.17  8218 | 0.351  22 | 0.507  426 | 1.21  2758 | 72 |
| {BcMV4.BI=FALSE, BcPV3.BI=TRUE} =>  {BcMV1.BI=TRUE} | 0.09  6535 | 0.886  364 | 0.108  911 | 1.12  2542 | 39 |
| {BcMV4.BI=FALSE, BcPV3.BI=TRUE} =>  {BcALV1.BI=FALSE} | 0.09  901 | 0.909  091 | 0.108  911 | 1.11  633 | 40 |
| {BcPV3.BI=TRUE, BcULV3=TRUE} =>  {BcHV1.BI=TRUE} | 0.12  3762 | 0.714  286 | 0.173  267 | 1.18  267 | 50 |
| {BcPV3.BI=TRUE, BcULV3=TRUE} =>  {BcMV11=TRUE} | 0.12  6238 | 0.728  571 | 0.173  267 | 1.18  21 | 51 |
| {BcHV1.BI=TRUE, BcPV3.BI=TRUE} =>  {BcMV11=TRUE} | 0.14  604 | 0.728  395 | 0.200  495 | 1.18  1814 | 59 |
| {BcMV11=TRUE, BcPV3.BI=TRUE} =>  {BcHV1.BI=TRUE} | 0.14  604 | 0.737  5 | 0.198  02 | 1.22  1107 | 59 |
| {BcHV1.BI=TRUE, BcMV11=TRUE} =>  {BcPV3.BI=TRUE} | 0.14  604 | 0.383  117 | 0.381  188 | 1.32  2899 | 59 |
| {BcMV9.BI=TRUE, BcPV3.BI=TRUE} => | 0.15 | 0.746 | 0.205 | 1.23 | 62 |

| {BcHV1.BI=TRUE} | 3465 | 988 | 446 | 6816 |  |
| --- | --- | --- | --- | --- | --- |
| {BcPV3.BI=TRUE, BcULV4=TRUE} =>  {BcHV1.BI=TRUE} | 0.15  5941 | 0.759  036 | 0.205  446 | 1.25  6765 | 63 |
| {BcHV3.BI=FALSE, BcPV3.BI=TRUE} =>  {BcHV1.BI=TRUE} | 0.14  604 | 0.702  381 | 0.207  921 | 1.16  2959 | 59 |
| {BcHV1.BI=TRUE, BcMV1.BI=TRUE} =>  {BcPV3.BI=TRUE} | 0.17  3267 | 0.358  974 | 0.482  673 | 1.23  9535 | 70 |
| {BcHV1.BI=TRUE, BcNSRV7.BI=FALSE} =>  {BcPV3.BI=TRUE} | 0.16  3366 | 0.351  064 | 0.465  347 | 1.21  222 | 66 |
| {BcPV3.BI=TRUE, BcVV3.BI=FALSE} =>  {BcHV1.BI=TRUE} | 0.16  0891 | 0.738  636 | 0.217  822 | 1.22  2988 | 65 |
| {BcDMV1=FALSE, BcPV3.BI=TRUE} =>  {BcHV1.BI=TRUE} | 0.17  3267 | 0.7 | 0.247  525 | 1.15  9016 | 70 |
| {BcOLV4.BI=FALSE, BcPV3.BI=TRUE} =>  {BcHV1.BI=TRUE} | 0.16  5842 | 0.697  917 | 0.237  624 | 1.15  5567 | 67 |
| {BcBNV5.BI=FALSE, BcPV3.BI=TRUE} =>  {BcHV1.BI=TRUE} | 0.18  5644 | 0.700  935 | 0.264  851 | 1.16  0564 | 75 |
| {BcBNV5.BI=FALSE, BcHV1.BI=TRUE} =>  {BcPV3.BI=TRUE} | 0.18  5644 | 0.348  837 | 0.532  178 | 1.20  4532 | 75 |
| {BcHV1.BI=TRUE, BcOLV15.BI=FALSE} =>  {BcPV3.BI=TRUE} | 0.19  5545 | 0.346  491 | 0.564  356 | 1.19  6431 | 79 |
| {BcEV5=FALSE, BcPV3.BI=TRUE} => | 0.19 | 0.696 | 0.277 | 1.15 | 78 |

| {BcHV1.BI=TRUE} | 3069 | 429 | 228 | 3103 |  |
| --- | --- | --- | --- | --- | --- |
| {BcMV9.BI=TRUE, BcPV3.BI=TRUE} =>  {BcMV11=TRUE} | 0.14  604 | 0.710  843 | 0.205  446 | 1.15  3336 | 59 |
| {BcMV11=TRUE, BcMV9.BI=TRUE} =>  {BcPV3.BI=TRUE} | 0.14  604 | 0.359  756 | 0.405  941 | 1.24  2235 | 59 |
| {BcMV11=TRUE, BcPV3.BI=TRUE} =>  {BcMV1.BI=TRUE} | 0.18  3168 | 0.925 | 0.198  02 | 1.17  1473 | 74 |
| {BcMV1.BI=TRUE, BcPV3.BI=TRUE} =>  {BcMV11=TRUE} | 0.18  3168 | 0.718  447 | 0.254  95 | 1.16  5672 | 74 |
| {BcMV1.BI=TRUE, BcMV11=TRUE} =>  {BcPV3.BI=TRUE} | 0.18  3168 | 0.352  381 | 0.519  802 | 1.21  6768 | 74 |
| {BcPV3.BI=TRUE, BvF.BI=FALSE} =>  {BcMV11=TRUE} | 0.18  8119 | 0.703  704 | 0.267  327 | 1.14  1752 | 76 |
| {BcOLV1.BI.MOVA.=FALSE,  BcPV3.BI=TRUE} => {BcULV1.BI=FALSE} | 0.13  6139 | 0.887  097 | 0.153  465 | 1.13  0559 | 55 |
| {BcOLV1.BI.MOVA.=FALSE,  BcPV3.BI=TRUE} => {BcALV1.BI=FALSE} | 0.13  8614 | 0.903  226 | 0.153  465 | 1.10  9128 | 56 |
| {BcMV9.BI=TRUE, BcPV3.BI=TRUE} =>  {BcULV4=TRUE} | 0.17  3267 | 0.843  373 | 0.205  446 | 1.22  1229 | 70 |
| {BcPV3.BI=TRUE, BcULV4=TRUE} =>  {BcMV9.BI=TRUE} | 0.17  3267 | 0.843  373 | 0.205  446 | 1.24  3514 | 70 |
| {BcMV9.BI=TRUE, BcPV3.BI=TRUE} => | 0.18 | 0.903 | 0.205 | 1.14 | 75 |

| {BcMV1.BI=TRUE} | 5644 | 614 | 446 | 4389 |  |
| --- | --- | --- | --- | --- | --- |
| {BcPV3.BI=TRUE, BcULV5=FALSE} =>  {BcMV9.BI=TRUE} | 0.18  8119 | 0.791  667 | 0.237  624 | 1.16  7275 | 76 |
| {BcPV3.BI=TRUE, BcULV4=TRUE} =>  {BcMV1.BI=TRUE} | 0.18  3168 | 0.891  566 | 0.205  446 | 1.12  9131 | 74 |
| {BcMV6.BI=FALSE, BcPV3.BI=TRUE} =>  {BcMV1.BI=TRUE} | 0.15  5941 | 0.875 | 0.178  218 | 1.10  815 | 63 |
| {BcPV3.BI=TRUE, BcRV1.BI=FALSE} =>  {BcMV1.BI=TRUE} | 0.14  8515 | 0.909  091 | 0.163  366 | 1.15  1325 | 60 |
| {BcPV3.BI=TRUE, BcRV1.BI=FALSE} =>  {BcALV1.BI=FALSE} | 0.14  8515 | 0.909  091 | 0.163  366 | 1.11  633 | 60 |
| {BcEV2.BI=FALSE, BcPV3.BI=TRUE} =>  {BcFV5.BI=FALSE} | 0.18  3168 | 0.891  566 | 0.205  446 | 1.13  9851 | 74 |
| {BcHV3.BI=FALSE, BcPV3.BI=TRUE} =>  {BcFV5.BI=FALSE} | 0.18  8119 | 0.904  762 | 0.207  921 | 1.15  6721 | 76 |
| {BcFV5.BI=FALSE, BcPV3.BI=TRUE} =>  {BcDMV1=FALSE} | 0.21  2871 | 0.905  263 | 0.235  149 | 1.07  2511 | 86 |
| {BcMV12=FALSE, BcPV3.BI=TRUE} =>  {BcMV1.BI=TRUE} | 0.19  0594 | 0.875 | 0.217  822 | 1.10  815 | 77 |
| {BcALV1.BI=FALSE, BcPV3.BI=TRUE} =>  {BcMV1.BI=TRUE} | 0.20  5446 | 0.892  473 | 0.230  198 | 1.13  0279 | 83 |
| {BcOLV4.BI=FALSE, BcPV3.BI=TRUE} => | 0.20 | 0.875 | 0.237 | 1.10 | 84 |

| {BcMV1.BI=TRUE} | 7921 |  | 624 | 815 |  |
| --- | --- | --- | --- | --- | --- |
| {BcPV3.BI=TRUE, BvF.BI=FALSE} =>  {BcMV1.BI=TRUE} | 0.23  5149 | 0.879  63 | 0.267  327 | 1.11  4014 | 95 |
| {BcOLV13.BI=FALSE, BcPV3.BI=TRUE} =>  {BcMV1.BI=TRUE} | 0.22  0297 | 0.89 | 0.247  525 | 1.12  7147 | 89 |
| {BcOLV2.BI=FALSE, BcPV3.BI=TRUE} =>  {BcMV1.BI=TRUE} | 0.23  2673 | 0.886  792 | 0.262  376 | 1.12  3085 | 94 |
| {BcMCV3.BI=FALSE, BcPV3.BI=TRUE} =>  {BcMV1.BI=TRUE} | 0.22  5248 | 0.892  157 | 0.252  475 | 1.12  9879 | 91 |
| {BcPV3.BI=TRUE, BcPV4.BI=FALSE} =>  {BcMV1.BI=TRUE} | 0.23  7624 | 0.872  727 | 0.272  277 | 1.10  5272 | 96 |
| {BcEV6=FALSE, BcPV3.BI=TRUE} =>  {BcMV1.BI=TRUE} | 0.24  2574 | 0.882  883 | 0.274  752 | 1.11  8134 | 98 |
| {BcOLV17.BI=FALSE, BcPV3.BI=TRUE} =>  {BcMV1.BI=TRUE} | 0.22  5248 | 0.875 | 0.257  426 | 1.10  815 | 91 |
| {BcOLV15.BI=FALSE, BcPV3.BI=TRUE} =>  {BcMV1.BI=TRUE} | 0.25 | 0.878  261 | 0.284  653 | 1.11  228 | 10  1 |
| {BcEV5=FALSE, BcPV3.BI=TRUE} =>  {BcMV1.BI=TRUE} | 0.24  2574 | 0.875 | 0.277  228 | 1.10  815 | 98 |
| {BcMV6.BI=TRUE, BcOLV1.BI.MOVA.=TRUE}  => {BcOLV16.BI=TRUE} | 0.11  3861 | 0.696  97 | 0.163  366 | 1.67  6046 | 46 |
| {BcMV6.BI=TRUE, BcOLV16.BI=TRUE} => | 0.11 | 0.686 | 0.165 | 2.06 | 46 |

| {BcOLV1.BI.MOVA.=TRUE} | 3861 | 567 | 842 | 9949 |  |
| --- | --- | --- | --- | --- | --- |
| {BcOLV1.BI.MOVA.=TRUE,  BcOLV16.BI=TRUE} => {BcMV6.BI=TRUE} | 0.11  3861 | 0.597  403 | 0.190  594 | 1.96  22 | 46 |
| {BcMV6.BI=TRUE, BcOLV1.BI.MOVA.=TRUE}  => {BcMV4.BI=TRUE} | 0.11  3861 | 0.696  97 | 0.163  366 | 1.57  3049 | 46 |
| {BcMV4.BI=TRUE, BcMV6.BI=TRUE} =>  {BcOLV1.BI.MOVA.=TRUE} | 0.11  3861 | 0.647  887 | 0.175  743 | 1.95  3332 | 46 |
| {BcMV4.BI=TRUE, BcOLV1.BI.MOVA.=TRUE}  => {BcMV6.BI=TRUE} | 0.11  3861 | 0.613  333 | 0.185  644 | 2.01  4526 | 46 |
| {BcMV6.BI=TRUE, BcOLV1.BI.MOVA.=TRUE}  => {BcMV2.BI=TRUE} | 0.14  3564 | 0.878  788 | 0.163  366 | 1.89  8558 | 58 |
| {BcMV2.BI=TRUE, BcMV6.BI=TRUE} =>  {BcOLV1.BI.MOVA.=TRUE} | 0.14  3564 | 0.690  476 | 0.207  921 | 2.08  1734 | 58 |
| {BcMV2.BI=TRUE, BcOLV1.BI.MOVA.=TRUE}  => {BcMV6.BI=TRUE} | 0.14  3564 | 0.574  257 | 0.25 | 1.88  6179 | 58 |
| {BcMV6.BI=TRUE, BcOLV1.BI.MOVA.=TRUE}  => {BcOLV18=TRUE} | 0.12  3762 | 0.757  576 | 0.163  366 | 1.46  4405 | 50 |
| {BcMV6.BI=TRUE, BcOLV18=TRUE} =>  {BcOLV1.BI.MOVA.=TRUE} | 0.12  3762 | 0.714  286 | 0.173  267 | 2.15  3518 | 50 |
| {BcOLV1.BI.MOVA.=TRUE, BcOLV18=TRUE}  => {BcMV6.BI=TRUE} | 0.12  3762 | 0.537  634 | 0.230  198 | 1.76  5889 | 50 |
| {BcMV6.BI=TRUE, BcOLV1.BI.MOVA.=TRUE} | 0.13 | 0.818 | 0.163 | 1.50 | 54 |

| => {BcHV1SLR.BI=TRUE} | 3663 | 182 | 366 | 934 |  |
| --- | --- | --- | --- | --- | --- |
| {BcHV1SLR.BI=TRUE, BcMV6.BI=TRUE} =>  {BcOLV1.BI.MOVA.=TRUE} | 0.13  3663 | 0.650  602 | 0.205  446 | 1.96  1518 | 54 |
| {BcHV1SLR.BI=TRUE, BcOLV1.BI.MOVA.=TRUE} =>  {BcMV6.BI=TRUE} | 0.13  3663 | 0.666  667 | 0.200  495 | 2.18  9702 | 54 |
| {BcMV6.BI=TRUE, BcOLV1.BI.MOVA.=TRUE}  => {BcMV3.BI=TRUE} | 0.14  3564 | 0.878  788 | 0.163  366 | 1.62  1143 | 58 |
| {BcMV3.BI=TRUE, BcMV6.BI=TRUE} =>  {BcOLV1.BI.MOVA.=TRUE} | 0.14  3564 | 0.644  444 | 0.222  772 | 1.94  2952 | 58 |
| {BcMV3.BI=TRUE, BcOLV1.BI.MOVA.=TRUE}  => {BcMV6.BI=TRUE} | 0.14  3564 | 0.585  859 | 0.245  05 | 1.92  4283 | 58 |
| {BcMV6.BI=TRUE, BcOLV1.BI.MOVA.=TRUE}  => {BcULV3=TRUE} | 0.12  1287 | 0.742  424 | 0.163  366 | 1.24  9747 | 49 |
| {BcMV6.BI=TRUE, BcULV3=TRUE} =>  {BcOLV1.BI.MOVA.=TRUE} | 0.12  1287 | 0.563  218 | 0.215  347 | 1.69  8061 | 49 |
| {BcOLV1.BI.MOVA.=TRUE, BcULV3=TRUE}  => {BcMV6.BI=TRUE} | 0.12  1287 | 0.620  253 | 0.195  545 | 2.03  7254 | 49 |
| {BcMV6.BI=TRUE, BcOLV1.BI.MOVA.=TRUE}  => {BcHV1.BI=TRUE} | 0.13  1188 | 0.803  03 | 0.163  366 | 1.32  9608 | 53 |
| {BcHV1.BI=TRUE, BcMV6.BI=TRUE} =>  {BcOLV1.BI.MOVA.=TRUE} | 0.13  1188 | 0.576  087 | 0.227  723 | 1.73  6859 | 53 |

| {BcHV1.BI=TRUE, BcOLV1.BI.MOVA.=TRUE}  => {BcMV6.BI=TRUE} | 0.13  1188 | 0.616  279 | 0.212  871 | 2.02  4201 | 53 |
| --- | --- | --- | --- | --- | --- |
| {BcMV6.BI=TRUE, BcOLV1.BI.MOVA.=TRUE}  => {BcMV11=TRUE} | 0.13  6139 | 0.833  333 | 0.163  366 | 1.35  2075 | 55 |
| {BcMV11=TRUE, BcMV6.BI=TRUE} =>  {BcOLV1.BI.MOVA.=TRUE} | 0.13  6139 | 0.597  826 | 0.227  723 | 1.80  2401 | 55 |
| {BcMV11=TRUE, BcOLV1.BI.MOVA.=TRUE}  => {BcMV6.BI=TRUE} | 0.13  6139 | 0.561  224 | 0.242  574 | 1.84  3371 | 55 |
| {BcMV6.BI=TRUE, BcMV9.BI=TRUE} =>  {BcOLV1.BI.MOVA.=TRUE} | 0.12  3762 | 0.526  316 | 0.235  149 | 1.58  6803 | 50 |
| {BcMV9.BI=TRUE, BcOLV1.BI.MOVA.=TRUE}  => {BcMV6.BI=TRUE} | 0.12  3762 | 0.581  395 | 0.212  871 | 1.90  9624 | 50 |
| {BcMV6.BI=TRUE, BcOLV1.BI.MOVA.=TRUE}  => {BcULV4=TRUE} | 0.13  6139 | 0.833  333 | 0.163  366 | 1.20  6691 | 55 |
| {BcMV6.BI=TRUE, BcULV4=TRUE} =>  {BcOLV1.BI.MOVA.=TRUE} | 0.13  6139 | 0.561  224 | 0.242  574 | 1.69  205 | 55 |
| {BcOLV1.BI.MOVA.=TRUE, BcULV4=TRUE}  => {BcMV6.BI=TRUE} | 0.13  6139 | 0.555  556 | 0.245  05 | 1.82  4752 | 55 |
| {BcMV6.BI=TRUE, BcPV3.BI=FALSE} =>  {BcOLV1.BI.MOVA.=TRUE} | 0.08  6634 | 0.448  718 | 0.193  069 | 1.35  2851 | 35 |
| {BcOLV1.BI.MOVA.=TRUE,  BcPV3.BI=FALSE} => {BcMV6.BI=TRUE} | 0.08  6634 | 0.443  038 | 0.195  545 | 1.45  5182 | 35 |

| {BcMV6.BI=TRUE, BcRV1.BI=FALSE} =>  {BcOLV1.BI.MOVA.=TRUE} | 0.08  4158 | 0.441  558 | 0.190  594 | 1.33  1266 | 34 |
| --- | --- | --- | --- | --- | --- |
| {BcOLV1.BI.MOVA.=TRUE,  BcRV1.BI=FALSE} => {BcMV6.BI=TRUE} | 0.08  4158 | 0.425 | 0.198  02 | 1.39  5935 | 34 |
| {BcEV2.BI=FALSE, BcMV6.BI=TRUE} =>  {BcOLV1.BI.MOVA.=TRUE} | 0.12  3762 | 0.531  915 | 0.232  673 | 1.60  3684 | 50 |
| {BcEV2.BI=FALSE, BcOLV1.BI.MOVA.=TRUE} =>  {BcMV6.BI=TRUE} | 0.12  3762 | 0.495  05 | 0.25 | 1.62  6016 | 50 |
| {BcHV3.BI=FALSE, BcMV6.BI=TRUE} =>  {BcOLV1.BI.MOVA.=TRUE} | 0.10  8911 | 0.494  382 | 0.220  297 | 1.49  0525 | 44 |
| {BcHV3.BI=FALSE, BcOLV1.BI.MOVA.=TRUE} =>  {BcMV6.BI=TRUE} | 0.10  8911 | 0.478  261 | 0.227  723 | 1.57  0873 | 44 |
| {BcFV5.BI=FALSE, BcMV6.BI=TRUE} =>  {BcOLV1.BI.MOVA.=TRUE} | 0.11  1386 | 0.468  75 | 0.237  624 | 1.41  3246 | 45 |
| {BcFV5.BI=FALSE, BcOLV1.BI.MOVA.=TRUE} =>  {BcMV6.BI=TRUE} | 0.11  1386 | 0.463  918 | 0.240  099 | 1.52  3762 | 45 |
| {BcMV6.BI=TRUE, BcULV1.BI=FALSE} =>  {BcOLV1.BI.MOVA.=TRUE} | 0.09  901 | 0.465  116 | 0.212  871 | 1.40  2291 | 40 |
| {BcOLV1.BI.MOVA.=TRUE, | 0.09 | 0.439 | 0.225 | 1.44 | 40 |

| BcULV1.BI=FALSE} => {BcMV6.BI=TRUE} | 901 | 56 | 248 | 3759 |  |
| --- | --- | --- | --- | --- | --- |
| {BcMV1.BI=TRUE, BcMV6.BI=TRUE} => | 0.14 | 0.563 | 0.254 | 1.69 |  |
|  |  |  |  |  | 58 |
| {BcOLV1.BI.MOVA.=TRUE} | 3564 | 107 | 95 | 7725 |  |
| {BcMV1.BI=TRUE, BcOLV1.BI.MOVA.=TRUE} | 0.14 | 0.487 | 0.294 | 1.60 |  |
|  |  |  |  |  | 58 |
| => {BcMV6.BI=TRUE} | 3564 | 395 | 554 | 0874 |  |
| {BcMV12=FALSE, BcMV6.BI=TRUE} => | 0.10 | 0.465 | 0.217 | 1.40 |  |
|  |  |  |  |  | 41 |
| {BcOLV1.BI.MOVA.=TRUE} | 1485 | 909 | 822 | 4681 |  |
| {BcMV12=FALSE, BcOLV1.BI.MOVA.=TRUE} | 0.10 | 0.440 | 0.230 | 1.44 |  |
|  |  |  |  |  | 41 |
| => {BcMV6.BI=TRUE} | 1485 | 86 | 198 | 8029 |  |
| {BcMV6.BI=TRUE, BcNSRV7.BI=FALSE} => | 0.12 | 0.542 | 0.232 | 1.63 |  |
|  |  |  |  |  | 51 |
| {BcOLV1.BI.MOVA.=TRUE} | 6238 | 553 | 673 | 5757 |  |
| {BcNSRV7.BI=FALSE, |  |  |  |  |  |
|  | 0.12 | 0.476 | 0.264 | 1.56 |  |
| BcOLV1.BI.MOVA.=TRUE} => |  |  |  |  | 51 |
|  | 6238 | 636 | 851 | 5535 |  |
| {BcMV6.BI=TRUE} |  |  |  |  |  |
| {BcMV6.BI=TRUE, BcVV3.BI=FALSE} => | 0.12 | 0.526 | 0.230 | 1.58 |  |
|  |  |  |  |  | 49 |
| {BcOLV1.BI.MOVA.=TRUE} | 1287 | 882 | 198 | 8509 |  |
| {BcOLV1.BI.MOVA.=TRUE, | 0.12 |  | 0.242 | 1.64 |  |
|  |  | 0.5 |  |  | 49 |
| BcVV3.BI=FALSE} => {BcMV6.BI=TRUE} | 1287 |  | 574 | 2276 |  |
| {BcMV6.BI=TRUE, BcOLV7.BI=FALSE} => | 0.11 |  | 0.232 | 1.50 |  |
|  |  | 0.5 |  |  | 47 |
| {BcOLV1.BI.MOVA.=TRUE} | 6337 |  | 673 | 7463 |  |
| {BcOLV1.BI.MOVA.=TRUE, | 0.11 | 0.489 | 0.237 | 1.60 |  |
|  |  |  |  |  | 47 |
| BcOLV7.BI=FALSE} => {BcMV6.BI=TRUE} | 6337 | 583 | 624 | 8062 |  |

| {BcALV1.BI=FALSE, BcMV6.BI=TRUE} =>  {BcOLV1.BI.MOVA.=TRUE} | 0.09  4059 | 0.431  818 | 0.217  822 | 1.30  19 | 38 |
| --- | --- | --- | --- | --- | --- |
| {BcALV1.BI=FALSE, BcOLV1.BI.MOVA.=TRUE} =>  {BcMV6.BI=TRUE} | 0.09  4059 | 0.417  582 | 0.225  248 | 1.37  1572 | 38 |
| {BcDMV1=FALSE, BcMV6.BI=TRUE} =>  {BcOLV1.BI.MOVA.=TRUE} | 0.12  1287 | 0.510  417 | 0.237  624 | 1.53  8868 | 49 |
| {BcDMV1=FALSE, BcOLV1.BI.MOVA.=TRUE}  => {BcMV6.BI=TRUE} | 0.12  1287 | 0.441  441 | 0.274  752 | 1.44  9938 | 49 |
| {BcFV9=FALSE, BcMV6.BI=TRUE} =>  {BcOLV1.BI.MOVA.=TRUE} | 0.13  8614 | 0.518  519 | 0.267  327 | 1.56  3295 | 56 |
| {BcFV9=FALSE, BcOLV1.BI.MOVA.=TRUE}  => {BcMV6.BI=TRUE} | 0.13  8614 | 0.495  575 | 0.279  703 | 1.62  7743 | 56 |
| {BcMV6.BI=TRUE, BcOLV4.BI=FALSE} =>  {BcOLV1.BI.MOVA.=TRUE} | 0.12  8713 | 0.495  238 | 0.259  901 | 1.49  3106 | 52 |
| {BcOLV1.BI.MOVA.=TRUE,  BcOLV4.BI=FALSE} => {BcMV6.BI=TRUE} | 0.12  8713 | 0.490  566 | 0.262  376 | 1.61  129 | 52 |
| {BcMV6.BI=TRUE, BcULV5=FALSE} =>  {BcOLV1.BI.MOVA.=TRUE} | 0.13  1188 | 0.514  563 | 0.254  95 | 1.55  1369 | 53 |
| {BcOLV1.BI.MOVA.=TRUE, BcULV5=FALSE}  => {BcMV6.BI=TRUE} | 0.13  1188 | 0.509  615 | 0.257  426 | 1.67  3859 | 53 |
| {BcBNV5.BI=FALSE, BcMV6.BI=TRUE} => | 0.12 | 0.494 | 0.245 | 1.49 | 49 |

| {BcOLV1.BI.MOVA.=TRUE} | 1287 | 949 | 05 | 2236 |  |
| --- | --- | --- | --- | --- | --- |
| {BcBNV5.BI=FALSE, |  |  |  |  |  |
|  | 0.12 | 0.429 | 0.282 | 1.41 |  |
| BcOLV1.BI.MOVA.=TRUE} => |  |  |  |  | 49 |
|  | 1287 | 825 | 178 | 1781 |  |
| {BcMV6.BI=TRUE} |  |  |  |  |  |
| {BcMV6.BI=TRUE, BvF.BI=FALSE} => | 0.15 | 0.547 | 0.289 | 1.64 |  |
|  |  |  |  |  | 64 |
| {BcOLV1.BI.MOVA.=TRUE} | 8416 | 009 | 604 | 919 |  |
| {BcOLV1.BI.MOVA.=TRUE, BvF.BI=FALSE} | 0.15 | 0.503 | 0.314 | 1.65 |  |
|  |  |  |  |  | 64 |
| => {BcMV6.BI=TRUE} | 8416 | 937 | 356 | 5208 |  |
| {BcMCV5.BI=FALSE, BcMV6.BI=TRUE} => | 0.12 | 0.476 | 0.259 | 1.43 |  |
|  |  |  |  |  | 50 |
| {BcOLV1.BI.MOVA.=TRUE} | 3762 | 19 | 901 | 5679 |  |
| {BcMCV5.BI=FALSE, |  |  |  |  |  |
|  | 0.12 | 0.446 | 0.277 | 1.46 |  |
| BcOLV1.BI.MOVA.=TRUE} => |  |  |  |  | 50 |
|  | 3762 | 429 | 228 | 6318 |  |
| {BcMV6.BI=TRUE} |  |  |  |  |  |
| {BcMV6.BI=TRUE, BcOLV13.BI=FALSE} => | 0.13 | 0.504 | 0.269 | 1.52 |  |
|  |  |  |  |  | 55 |
| {BcOLV1.BI.MOVA.=TRUE} | 6139 | 587 | 802 | 1293 |  |
| {BcOLV1.BI.MOVA.=TRUE, | 0.13 | 0.491 | 0.277 | 1.61 |  |
|  |  |  |  |  | 55 |
| BcOLV13.BI=FALSE} => {BcMV6.BI=TRUE} | 6139 | 071 | 228 | 295 |  |
| {BcMV6.BI=TRUE, BcOLV2.BI=FALSE} => | 0.12 | 0.481 | 0.262 | 1.45 |  |
|  |  |  |  |  | 51 |
| {BcOLV1.BI.MOVA.=TRUE} | 6238 | 132 | 376 | 0577 |  |
| {BcOLV1.BI.MOVA.=TRUE, | 0.12 | 0.455 | 0.277 | 1.49 |  |
|  |  |  |  |  | 51 |
| BcOLV2.BI=FALSE} => {BcMV6.BI=TRUE} | 6238 | 357 | 228 | 5645 |  |
| {BcMCV3.BI=FALSE, BcMV6.BI=TRUE} => | 0.12 | 0.476 | 0.259 | 1.43 | 50 |

| {BcOLV1.BI.MOVA.=TRUE} | 3762 | 19 | 901 | 5679 |  |
| --- | --- | --- | --- | --- | --- |
| {BcMCV3.BI=FALSE, |  |  |  |  |  |
|  | 0.12 | 0.431 | 0.287 | 1.41 |  |
| BcOLV1.BI.MOVA.=TRUE} => |  |  |  |  | 50 |
|  | 3762 | 034 | 129 | 5756 |  |
| {BcMV6.BI=TRUE} |  |  |  |  |  |
| {BcMV6.BI=TRUE, BcPV4.BI=FALSE} => | 0.15 | 0.544 | 0.277 | 1.64 |  |
|  |  |  |  |  | 61 |
| {BcOLV1.BI.MOVA.=TRUE} | 099 | 643 | 228 | 2058 |  |
| {BcOLV1.BI.MOVA.=TRUE, | 0.15 | 0.476 | 0.316 | 1.56 |  |
|  |  |  |  |  | 61 |
| BcPV4.BI=FALSE} => {BcMV6.BI=TRUE} | 099 | 563 | 832 | 5295 |  |
| {BcEV6=FALSE, BcMV6.BI=TRUE} => | 0.15 | 0.535 | 0.282 | 1.61 |  |
|  |  |  |  |  | 61 |
| {BcOLV1.BI.MOVA.=TRUE} | 099 | 088 | 178 | 325 |  |
| {BcEV6=FALSE, BcOLV1.BI.MOVA.=TRUE} | 0.15 | 0.491 | 0.306 | 1.61 |  |
|  |  |  |  |  | 61 |
| => {BcMV6.BI=TRUE} | 099 | 935 | 931 | 5788 |  |
| {BcMV6.BI=TRUE, BcOLV17.BI=FALSE} => | 0.14 | 0.508 | 0.277 | 1.53 |  |
|  |  |  |  |  | 57 |
| {BcOLV1.BI.MOVA.=TRUE} | 1089 | 929 | 228 | 4382 |  |
| {BcOLV1.BI.MOVA.=TRUE, | 0.14 | 0.471 | 0.299 | 1.54 |  |
|  |  |  |  |  | 57 |
| BcOLV17.BI=FALSE} => {BcMV6.BI=TRUE} | 1089 | 074 | 505 | 7269 |  |
| {BcMV6.BI=TRUE, BcOLV15.BI=FALSE} => | 0.15 | 0.528 | 0.299 | 1.59 |  |
|  |  |  |  |  | 64 |
| {BcOLV1.BI.MOVA.=TRUE} | 8416 | 926 | 505 | 4671 |  |
| {BcOLV1.BI.MOVA.=TRUE, | 0.15 | 0.492 | 0.321 | 1.61 |  |
|  |  |  |  |  | 64 |
| BcOLV15.BI=FALSE} => {BcMV6.BI=TRUE} | 8416 | 308 | 782 | 7011 |  |
| {BcEV5=FALSE, BcMV6.BI=TRUE} => | 0.13 | 0.495 | 0.264 | 1.49 |  |
|  |  |  |  |  | 53 |
| {BcOLV1.BI.MOVA.=TRUE} | 1188 | 327 | 851 | 3374 |  |

| {BcEV5=FALSE, BcOLV1.BI.MOVA.=TRUE}  => {BcMV6.BI=TRUE} | 0.13  1188 | 0.441  667 | 0.297  03 | 1.45  0678 | 53 |
| --- | --- | --- | --- | --- | --- |
| {BcMV11=FALSE, BcMV6.BI=TRUE} =>  {BcDMV1=FALSE} | 0.07  6733 | 1 | 0.076  733 | 1.18  4751 | 31 |
| {BcMV11=FALSE, BcMV6.BI=TRUE} =>  {BcFV9=FALSE} | 0.07  4257 | 0.967  742 | 0.076  733 | 1.11  3868 | 30 |
| {BcHV1.BI=FALSE, BcMV6.BI=TRUE} =>  {BcFV9=FALSE} | 0.07  4257 | 0.967  742 | 0.076  733 | 1.11  3868 | 30 |
| {BcMV6.BI=TRUE, BcULV3=FALSE} =>  {BcULV4=TRUE} | 0.07  6733 | 0.861  111 | 0.089  109 | 1.24  6914 | 31 |
| {BcMV6.BI=TRUE, BcULV3=FALSE} =>  {BcFV5.BI=FALSE} | 0.07  9208 | 0.888  889 | 0.089  109 | 1.13  6428 | 32 |
| {BcMV6.BI=TRUE, BcULV3=FALSE} =>  {BcNSRV7.BI=FALSE} | 0.08  4158 | 0.944  444 | 0.089  109 | 1.18  1287 | 34 |
| {BcMV6.BI=TRUE, BcOLV16.BI=TRUE} =>  {BcMV4.BI=TRUE} | 0.09  6535 | 0.582  09 | 0.165  842 | 1.31  3766 | 39 |
| {BcMV4.BI=TRUE, BcMV6.BI=TRUE} =>  {BcOLV16.BI=TRUE} | 0.09  6535 | 0.549  296 | 0.175  743 | 1.32  0926 | 39 |
| {BcMV4.BI=TRUE, BcOLV16.BI=TRUE} =>  {BcMV6.BI=TRUE} | 0.09  6535 | 0.428  571 | 0.225  248 | 1.40  7666 | 39 |
| {BcMV6.BI=TRUE, BcOLV16.BI=TRUE} =>  {BcMV2.BI=TRUE} | 0.13  1188 | 0.791  045 | 0.165  842 | 1.70  8995 | 53 |

| {BcMV2.BI=TRUE, BcMV6.BI=TRUE} =>  {BcOLV16.BI=TRUE} | 0.13  1188 | 0.630  952 | 0.207  921 | 1.51  729 | 53 |
| --- | --- | --- | --- | --- | --- |
| {BcMV2.BI=TRUE, BcOLV16.BI=TRUE} =>  {BcMV6.BI=TRUE} | 0.13  1188 | 0.519  608 | 0.252  475 | 1.70  6679 | 53 |
| {BcMV6.BI=TRUE, BcOLV16.BI=TRUE} =>  {BcOLV18=TRUE} | 0.10  8911 | 0.656  716 | 0.165  842 | 1.26  9442 | 44 |
| {BcMV6.BI=TRUE, BcOLV18=TRUE} =>  {BcOLV16.BI=TRUE} | 0.10  8911 | 0.628  571 | 0.173  267 | 1.51  1565 | 44 |
| {BcOLV16.BI=TRUE, BcOLV18=TRUE} =>  {BcMV6.BI=TRUE} | 0.10  8911 | 0.403  67 | 0.269  802 | 1.32  5875 | 44 |
| {BcMV6.BI=TRUE, BcOLV16.BI=TRUE} =>  {BcHV1SLR.BI=TRUE} | 0.11  6337 | 0.701  493 | 0.165  842 | 1.29  4078 | 47 |
| {BcHV1SLR.BI=TRUE, BcMV6.BI=TRUE} =>  {BcOLV16.BI=TRUE} | 0.11  6337 | 0.566  265 | 0.205  446 | 1.36  1733 | 47 |
| {BcHV1SLR.BI=TRUE, BcOLV16.BI=TRUE}  => {BcMV6.BI=TRUE} | 0.11  6337 | 0.489  583 | 0.237  624 | 1.60  8062 | 47 |
| {BcMV6.BI=TRUE, BcOLV16.BI=TRUE} =>  {BcMV3.BI=TRUE} | 0.13  6139 | 0.820  896 | 0.165  842 | 1.51  4346 | 55 |
| {BcMV3.BI=TRUE, BcMV6.BI=TRUE} =>  {BcOLV16.BI=TRUE} | 0.13  6139 | 0.611  111 | 0.222  772 | 1.46  9577 | 55 |
| {BcMV3.BI=TRUE, BcOLV16.BI=TRUE} =>  {BcMV6.BI=TRUE} | 0.13  6139 | 0.509  259 | 0.267  327 | 1.67  2689 | 55 |

| {BcMV6.BI=TRUE, BcOLV16.BI=TRUE} =>  {BcULV3=TRUE} | 0.13  1188 | 0.791  045 | 0.165  842 | 1.33  1592 | 53 |
| --- | --- | --- | --- | --- | --- |
| {BcMV6.BI=TRUE, BcULV3=TRUE} =>  {BcOLV16.BI=TRUE} | 0.13  1188 | 0.609  195 | 0.215  347 | 1.46  497 | 53 |
| {BcOLV16.BI=TRUE, BcULV3=TRUE} =>  {BcMV6.BI=TRUE} | 0.13  1188 | 0.469  027 | 0.279  703 | 1.54  0542 | 53 |
| {BcMV6.BI=TRUE, BcOLV16.BI=TRUE} =>  {BcHV1.BI=TRUE} | 0.12  8713 | 0.776  119 | 0.165  842 | 1.28  505 | 52 |
| {BcHV1.BI=TRUE, BcMV6.BI=TRUE} =>  {BcOLV16.BI=TRUE} | 0.12  8713 | 0.565  217 | 0.227  723 | 1.35  9213 | 52 |
| {BcHV1.BI=TRUE, BcOLV16.BI=TRUE} =>  {BcMV6.BI=TRUE} | 0.12  8713 | 0.477  064 | 0.269  802 | 1.56  6943 | 52 |
| {BcMV6.BI=TRUE, BcOLV16.BI=TRUE} =>  {BcMV11=TRUE} | 0.12  6238 | 0.761  194 | 0.165  842 | 1.23  503 | 51 |
| {BcMV11=TRUE, BcMV6.BI=TRUE} =>  {BcOLV16.BI=TRUE} | 0.12  6238 | 0.554  348 | 0.227  723 | 1.33  3075 | 51 |
| {BcMV11=TRUE, BcOLV16.BI=TRUE} =>  {BcMV6.BI=TRUE} | 0.12  6238 | 0.459  459 | 0.274  752 | 1.50  9119 | 51 |
| {BcMV6.BI=TRUE, BcOLV16.BI=TRUE} =>  {BcMV9.BI=TRUE} | 0.13  6139 | 0.820  896 | 0.165  842 | 1.21  0372 | 55 |
| {BcMV6.BI=TRUE, BcMV9.BI=TRUE} =>  {BcOLV16.BI=TRUE} | 0.13  6139 | 0.578  947 | 0.235  149 | 1.39  2231 | 55 |

| {BcMV9.BI=TRUE, BcOLV16.BI=TRUE} =>  {BcMV6.BI=TRUE} | 0.13  6139 | 0.410  448 | 0.331  683 | 1.34  8137 | 55 |
| --- | --- | --- | --- | --- | --- |
| {BcMV6.BI=TRUE, BcOLV16.BI=TRUE} =>  {BcULV4=TRUE} | 0.15  099 | 0.910  448 | 0.165  842 | 1.31  8354 | 61 |
| {BcMV6.BI=TRUE, BcULV4=TRUE} =>  {BcOLV16.BI=TRUE} | 0.15  099 | 0.622  449 | 0.242  574 | 1.49  6842 | 61 |
| {BcOLV16.BI=TRUE, BcULV4=TRUE} =>  {BcMV6.BI=TRUE} | 0.15  099 | 0.445  255 | 0.339  109 | 1.46  2465 | 61 |
| {BcEV2.BI=FALSE, BcMV6.BI=TRUE} =>  {BcOLV16.BI=TRUE} | 0.13  1188 | 0.563  83 | 0.232  673 | 1.35  5876 | 53 |
| {BcEV2.BI=FALSE, BcOLV16.BI=TRUE} =>  {BcMV6.BI=TRUE} | 0.13  1188 | 0.401  515 | 0.326  733 | 1.31  8798 | 53 |
| {BcHV3.BI=FALSE, BcMV6.BI=TRUE} =>  {BcOLV16.BI=TRUE} | 0.11  8812 | 0.539  326 | 0.220  297 | 1.29  695 | 48 |
| {BcFV5.BI=FALSE, BcMV6.BI=TRUE} =>  {BcOLV16.BI=TRUE} | 0.12  3762 | 0.520  833 | 0.237  624 | 1.25  248 | 50 |
| {BcFV5.BI=FALSE, BcOLV16.BI=TRUE} =>  {BcMV6.BI=TRUE} | 0.12  3762 | 0.384  615 | 0.321  782 | 1.26  329 | 50 |
| {BcMV1.BI=TRUE, BcMV6.BI=TRUE} =>  {BcOLV16.BI=TRUE} | 0.13  8614 | 0.543  689 | 0.254  95 | 1.30  7443 | 56 |
| {BcMV1.BI=TRUE, BcOLV16.BI=TRUE} =>  {BcMV6.BI=TRUE} | 0.13  8614 | 0.411  765 | 0.336  634 | 1.35  2463 | 56 |

| {BcMV6.BI=TRUE, BcVV3.BI=FALSE} =>  {BcOLV16.BI=TRUE} | 0.13  6139 | 0.591  398 | 0.230  198 | 1.42  2171 | 55 |
| --- | --- | --- | --- | --- | --- |
| {BcOLV16.BI=TRUE, BcVV3.BI=FALSE} =>  {BcMV6.BI=TRUE} | 0.13  6139 | 0.404  412 | 0.336  634 | 1.32  8312 | 55 |
| {BcDMV1=FALSE, BcMV6.BI=TRUE} =>  {BcOLV16.BI=TRUE} | 0.13  3663 | 0.562  5 | 0.237  624 | 1.35  2679 | 54 |
| {BcFV9=FALSE, BcMV6.BI=TRUE} =>  {BcOLV16.BI=TRUE} | 0.15  3465 | 0.574  074 | 0.267  327 | 1.38  0511 | 62 |
| {BcFV9=FALSE, BcOLV16.BI=TRUE} =>  {BcMV6.BI=TRUE} | 0.15  3465 | 0.427  586 | 0.358  911 | 1.40  4429 | 62 |
| {BcMV6.BI=TRUE, BcOLV4.BI=FALSE} =>  {BcOLV16.BI=TRUE} | 0.13  6139 | 0.523  81 | 0.259  901 | 1.25  9637 | 55 |
| {BcOLV16.BI=TRUE, BcOLV4.BI=FALSE} =>  {BcMV6.BI=TRUE} | 0.13  6139 | 0.395  683 | 0.344  059 | 1.29  9643 | 55 |
| {BcMV6.BI=TRUE, BcULV5=FALSE} =>  {BcOLV16.BI=TRUE} | 0.13  6139 | 0.533  981 | 0.254  95 | 1.28  4096 | 55 |
| {BcOLV16.BI=TRUE, BcULV5=FALSE} =>  {BcMV6.BI=TRUE} | 0.13  6139 | 0.387  324 | 0.351  485 | 1.27  2186 | 55 |
| {BcBNV5.BI=FALSE, BcMV6.BI=TRUE} =>  {BcOLV16.BI=TRUE} | 0.13  1188 | 0.535  354 | 0.245  05 | 1.28  7398 | 53 |
| {BcMV6.BI=TRUE, BcOLV16.BI=TRUE} =>  {BvF.BI=FALSE} | 0.16  5842 | 1 | 0.165  842 | 1.09  4851 | 67 |

| {BcMV6.BI=TRUE, BvF.BI=FALSE} =>  {BcOLV16.BI=TRUE} | 0.16  5842 | 0.572  65 | 0.289  604 | 1.37  7086 | 67 |
| --- | --- | --- | --- | --- | --- |
| {BcOLV16.BI=TRUE, BvF.BI=FALSE} =>  {BcMV6.BI=TRUE} | 0.16  5842 | 0.424  051 | 0.391  089 | 1.39  2817 | 67 |
| {BcMCV5.BI=FALSE, BcMV6.BI=TRUE} =>  {BcOLV16.BI=TRUE} | 0.13  3663 | 0.514  286 | 0.259  901 | 1.23  6735 | 54 |
| {BcMV6.BI=TRUE, BcOLV13.BI=FALSE} =>  {BcOLV16.BI=TRUE} | 0.13  8614 | 0.513  761 | 0.269  802 | 1.23  5474 | 56 |
| {BcOLV13.BI=FALSE, BcOLV16.BI=TRUE} =>  {BcMV6.BI=TRUE} | 0.13  8614 | 0.378  378 | 0.366  337 | 1.24  2804 | 56 |
| {BcMV6.BI=TRUE, BcOLV2.BI=FALSE} =>  {BcOLV16.BI=TRUE} | 0.13  3663 | 0.509  434 | 0.262  376 | 1.22  5067 | 54 |
| {BcMV6.BI=TRUE, BcPV4.BI=FALSE} =>  {BcOLV16.BI=TRUE} | 0.14  604 | 0.526  786 | 0.277  228 | 1.26  6794 | 59 |
| {BcOLV16.BI=TRUE, BcPV4.BI=FALSE} =>  {BcMV6.BI=TRUE} | 0.14  604 | 0.385  621 | 0.378  713 | 1.26  6592 | 59 |
| {BcEV6=FALSE, BcMV6.BI=TRUE} =>  {BcOLV16.BI=TRUE} | 0.16  0891 | 0.570  175 | 0.282  178 | 1.37  1136 | 65 |
| {BcEV6=FALSE, BcOLV16.BI=TRUE} =>  {BcMV6.BI=TRUE} | 0.16  0891 | 0.411  392 | 0.391  089 | 1.35  124 | 65 |
| {BcMV6.BI=TRUE, BcOLV17.BI=FALSE} =>  {BcOLV16.BI=TRUE} | 0.14  3564 | 0.517  857 | 0.277  228 | 1.24  5323 | 58 |

| {BcMV6.BI=TRUE, BcOLV15.BI=FALSE} =>  {BcOLV16.BI=TRUE} | 0.16  3366 | 0.545  455 | 0.299  505 | 1.31  1688 | 66 |
| --- | --- | --- | --- | --- | --- |
| {BcOLV15.BI=FALSE, BcOLV16.BI=TRUE} =>  {BcMV6.BI=TRUE} | 0.16  3366 | 0.404  908 | 0.403  465 | 1.32  9942 | 66 |
| {BcEV5=FALSE, BcMV6.BI=TRUE} =>  {BcOLV16.BI=TRUE} | 0.14  3564 | 0.542  056 | 0.264  851 | 1.30  3516 | 58 |
| {BcMV4.BI=TRUE, BcMV6.BI=TRUE} =>  {BcMV2.BI=TRUE} | 0.15  099 | 0.859  155 | 0.175  743 | 1.85  6142 | 61 |
| {BcMV2.BI=TRUE, BcMV6.BI=TRUE} =>  {BcMV4.BI=TRUE} | 0.15  099 | 0.726  19 | 0.207  921 | 1.63  9 | 61 |
| {BcMV2.BI=TRUE, BcMV4.BI=TRUE} =>  {BcMV6.BI=TRUE} | 0.15  099 | 0.549  55 | 0.274  752 | 1.80  5025 | 61 |
| {BcMV4.BI=TRUE, BcMV6.BI=TRUE} =>  {BcOLV18=TRUE} | 0.12  1287 | 0.690  141 | 0.175  743 | 1.33  4052 | 49 |
| {BcMV6.BI=TRUE, BcOLV18=TRUE} =>  {BcMV4.BI=TRUE} | 0.12  1287 | 0.7 | 0.173  267 | 1.57  9888 | 49 |
| {BcMV4.BI=TRUE, BcOLV18=TRUE} =>  {BcMV6.BI=TRUE} | 0.12  1287 | 0.437  5 | 0.277  228 | 1.43  6992 | 49 |
| {BcMV4.BI=TRUE, BcMV6.BI=TRUE} =>  {BcHV1SLR.BI=TRUE} | 0.13  1188 | 0.746  479 | 0.175  743 | 1.37  7066 | 53 |
| {BcHV1SLR.BI=TRUE, BcMV6.BI=TRUE} =>  {BcMV4.BI=TRUE} | 0.13  1188 | 0.638  554 | 0.205  446 | 1.44  1206 | 53 |

| {BcHV1SLR.BI=TRUE, BcMV4.BI=TRUE} =>  {BcMV6.BI=TRUE} | 0.13  1188 | 0.5 | 0.262  376 | 1.64  2276 | 53 |
| --- | --- | --- | --- | --- | --- |
| {BcMV4.BI=TRUE, BcMV6.BI=TRUE} =>  {BcMV3.BI=TRUE} | 0.15  3465 | 0.873  239 | 0.175  743 | 1.61  0907 | 62 |
| {BcMV3.BI=TRUE, BcMV6.BI=TRUE} =>  {BcMV4.BI=TRUE} | 0.15  3465 | 0.688  889 | 0.222  772 | 1.55  4811 | 62 |
| {BcMV3.BI=TRUE, BcMV4.BI=TRUE} =>  {BcMV6.BI=TRUE} | 0.15  3465 | 0.521  008 | 0.294  554 | 1.71  128 | 62 |
| {BcMV6.BI=TRUE, BcOLV16.BI=FALSE} =>  {BcMV4.BI=TRUE} | 0.07  9208 | 0.571  429 | 0.138  614 | 1.28  9705 | 32 |
| {BcMV4.BI=TRUE, BcMV6.BI=TRUE} =>  {BcULV3=TRUE} | 0.13  1188 | 0.746  479 | 0.175  743 | 1.25  6573 | 53 |
| {BcMV6.BI=TRUE, BcULV3=TRUE} =>  {BcMV4.BI=TRUE} | 0.13  1188 | 0.609  195 | 0.215  347 | 1.37  4944 | 53 |
| {BcMV4.BI=TRUE, BcULV3=TRUE} =>  {BcMV6.BI=TRUE} | 0.13  1188 | 0.445  378 | 0.294  554 | 1.46  2868 | 53 |
| {BcMV4.BI=TRUE, BcMV6.BI=TRUE} =>  {BcHV1.BI=TRUE} | 0.13  6139 | 0.774  648 | 0.175  743 | 1.28  2614 | 55 |
| {BcHV1.BI=TRUE, BcMV6.BI=TRUE} =>  {BcMV4.BI=TRUE} | 0.13  6139 | 0.597  826 | 0.227  723 | 1.34  9283 | 55 |
| {BcHV1.BI=TRUE, BcMV4.BI=TRUE} =>  {BcMV6.BI=TRUE} | 0.13  6139 | 0.454  545 | 0.299  505 | 1.49  2979 | 55 |

| {BcMV4.BI=TRUE, BcMV6.BI=TRUE} =>  {BcMV11=TRUE} | 0.15  5941 | 0.887  324 | 0.175  743 | 1.43  9674 | 63 |
| --- | --- | --- | --- | --- | --- |
| {BcMV11=TRUE, BcMV6.BI=TRUE} =>  {BcMV4.BI=TRUE} | 0.15  5941 | 0.684  783 | 0.227  723 | 1.54  5543 | 63 |
| {BcMV11=TRUE, BcMV4.BI=TRUE} =>  {BcMV6.BI=TRUE} | 0.15  5941 | 0.459  854 | 0.339  109 | 1.51  0415 | 63 |
| {BcMV4.BI=TRUE, BcMV6.BI=TRUE} =>  {BcMV9.BI=TRUE} | 0.14  8515 | 0.845  07 | 0.175  743 | 1.24  6016 | 60 |
| {BcMV6.BI=TRUE, BcMV9.BI=TRUE} =>  {BcMV4.BI=TRUE} | 0.14  8515 | 0.631  579 | 0.235  149 | 1.42  5463 | 60 |
| {BcMV4.BI=TRUE, BcMV9.BI=TRUE} =>  {BcMV6.BI=TRUE} | 0.14  8515 | 0.441  176 | 0.336  634 | 1.44  9067 | 60 |
| {BcMV4.BI=TRUE, BcMV6.BI=TRUE} =>  {BcULV4=TRUE} | 0.14  8515 | 0.845  07 | 0.175  743 | 1.22  3686 | 60 |
| {BcMV6.BI=TRUE, BcULV4=TRUE} =>  {BcMV4.BI=TRUE} | 0.14  8515 | 0.612  245 | 0.242  574 | 1.38  1826 | 60 |
| {BcMV4.BI=TRUE, BcULV4=TRUE} =>  {BcMV6.BI=TRUE} | 0.14  8515 | 0.458  015 | 0.324  257 | 1.50  4375 | 60 |
| {BcEV2.BI=FALSE, BcMV4.BI=TRUE} =>  {BcMV6.BI=TRUE} | 0.12  1287 | 0.385  827 | 0.314  356 | 1.26  7268 | 49 |
| {BcMV6.BI=TRUE, BcULV1.BI=FALSE} =>  {BcMV4.BI=TRUE} | 0.12  6238 | 0.593  023 | 0.212  871 | 1.33  8444 | 51 |

| {BcMV1.BI=TRUE, BcMV6.BI=TRUE} =>  {BcMV4.BI=TRUE} | 0.15  099 | 0.592  233 | 0.254  95 | 1.33  666 | 61 |
| --- | --- | --- | --- | --- | --- |
| {BcMV1.BI=TRUE, BcMV4.BI=TRUE} =>  {BcMV6.BI=TRUE} | 0.15  099 | 0.401  316 | 0.376  238 | 1.31  8143 | 61 |
| {BcMV12=FALSE, BcMV6.BI=TRUE} =>  {BcMV4.BI=TRUE} | 0.13  1188 | 0.602  273 | 0.217  822 | 1.35  9319 | 53 |
| {BcMV6.BI=TRUE, BcOLV7.BI=FALSE} =>  {BcMV4.BI=TRUE} | 0.13  6139 | 0.585  106 | 0.232  673 | 1.32  0575 | 55 |
| {BcMV4.BI=TRUE, BcOLV7.BI=FALSE} =>  {BcMV6.BI=TRUE} | 0.13  6139 | 0.404  412 | 0.336  634 | 1.32  8312 | 55 |
| {BcFV9=FALSE, BcMV4.BI=TRUE} =>  {BcMV6.BI=TRUE} | 0.14  3564 | 0.402  778 | 0.356  436 | 1.32  2945 | 58 |
| {BcMV6.BI=TRUE, BcOLV4.BI=FALSE} =>  {BcMV4.BI=TRUE} | 0.14  604 | 0.561  905 | 0.259  901 | 1.26  821 | 59 |
| {BcMV4.BI=TRUE, BcOLV4.BI=FALSE} =>  {BcMV6.BI=TRUE} | 0.14  604 | 0.393  333 | 0.371  287 | 1.29  1924 | 59 |
| {BcMV6.BI=TRUE, BcULV5=FALSE} =>  {BcMV4.BI=TRUE} | 0.14  604 | 0.572  816 | 0.254  95 | 1.29  2835 | 59 |
| {BcMV4.BI=TRUE, BcULV5=FALSE} =>  {BcMV6.BI=TRUE} | 0.14  604 | 0.383  117 | 0.381  188 | 1.25  8368 | 59 |
| {BcMV6.BI=TRUE, BvF.BI=FALSE} =>  {BcMV4.BI=TRUE} | 0.16  3366 | 0.564  103 | 0.289  604 | 1.27  317 | 66 |

| {BcMV4.BI=TRUE, BvF.BI=FALSE} =>  {BcMV6.BI=TRUE} | 0.16  3366 | 0.402  439 | 0.405  941 | 1.32  1832 | 66 |
| --- | --- | --- | --- | --- | --- |
| {BcMV6.BI=TRUE, BcOLV13.BI=FALSE} =>  {BcMV4.BI=TRUE} | 0.15  099 | 0.559  633 | 0.269  802 | 1.26  3082 | 61 |
| {BcMV4.BI=TRUE, BcOLV13.BI=FALSE} =>  {BcMV6.BI=TRUE} | 0.15  099 | 0.386  076 | 0.391  089 | 1.26  8087 | 61 |
| {BcMV6.BI=TRUE, BcOLV2.BI=FALSE} =>  {BcMV4.BI=TRUE} | 0.14  3564 | 0.547  17 | 0.262  376 | 1.23  4953 | 58 |
| {BcMCV3.BI=FALSE, BcMV6.BI=TRUE} =>  {BcMV4.BI=TRUE} | 0.15  5941 | 0.6 | 0.259  901 | 1.35  419 | 63 |
| {BcMCV3.BI=FALSE, BcMV4.BI=TRUE} =>  {BcMV6.BI=TRUE} | 0.15  5941 | 0.379  518 | 0.410  891 | 1.24  6547 | 63 |
| {BcMV6.BI=TRUE, BcPV4.BI=FALSE} =>  {BcMV4.BI=TRUE} | 0.16  5842 | 0.598  214 | 0.277  228 | 1.35  016 | 67 |
| {BcMV4.BI=TRUE, BcPV4.BI=FALSE} =>  {BcMV6.BI=TRUE} | 0.16  5842 | 0.396  45 | 0.418  317 | 1.30  216 | 67 |
| {BcEV6=FALSE, BcMV6.BI=TRUE} =>  {BcMV4.BI=TRUE} | 0.16  5842 | 0.587  719 | 0.282  178 | 1.32  6473 | 67 |
| {BcEV6=FALSE, BcMV4.BI=TRUE} =>  {BcMV6.BI=TRUE} | 0.16  5842 | 0.394  118 | 0.420  792 | 1.29  45 | 67 |
| {BcMV6.BI=TRUE, BcOLV17.BI=FALSE} =>  {BcMV4.BI=TRUE} | 0.15  5941 | 0.562  5 | 0.277  228 | 1.26  9553 | 63 |

| {BcMV4.BI=TRUE, BcOLV17.BI=FALSE} =>  {BcMV6.BI=TRUE} | 0.15  5941 | 0.379  518 | 0.410  891 | 1.24  6547 | 63 |
| --- | --- | --- | --- | --- | --- |
| {BcMV6.BI=TRUE, BcOLV15.BI=FALSE} =>  {BcMV4.BI=TRUE} | 0.17  3267 | 0.578  512 | 0.299  505 | 1.30  5693 | 70 |
| {BcMV4.BI=TRUE, BcOLV15.BI=FALSE} =>  {BcMV6.BI=TRUE} | 0.17  3267 | 0.419  162 | 0.413  366 | 1.37  6759 | 70 |
| {BcHV1SLR.BI=FALSE, BcMV6.BI=TRUE} =>  {BcEV2.BI=FALSE} | 0.08  4158 | 0.85 | 0.099  01 | 1.13  7086 | 34 |
| {BcHV1SLR.BI=FALSE, BcMV6.BI=TRUE} =>  {BcFV5.BI=FALSE} | 0.08  9109 | 0.9 | 0.099  01 | 1.15  0633 | 36 |
| {BcMV3.BI=FALSE, BcMV6.BI=TRUE} =>  {BcMV12=FALSE} | 0.07  9208 | 0.969  697 | 0.081  683 | 1.21  2872 | 32 |
| {BcMV3.BI=FALSE, BcMV6.BI=TRUE} =>  {BcDMV1=FALSE} | 0.07  9208 | 0.969  697 | 0.081  683 | 1.14  8849 | 32 |
| {BcMV3.BI=FALSE, BcMV6.BI=TRUE} =>  {BcFV9=FALSE} | 0.07  9208 | 0.969  697 | 0.081  683 | 1.11  6118 | 32 |
| {BcMV2.BI=TRUE, BcMV6.BI=TRUE} =>  {BcOLV18=TRUE} | 0.14  604 | 0.702  381 | 0.207  921 | 1.35  7712 | 59 |
| {BcMV6.BI=TRUE, BcOLV18=TRUE} =>  {BcMV2.BI=TRUE} | 0.14  604 | 0.842  857 | 0.173  267 | 1.82  0932 | 59 |
| {BcMV2.BI=TRUE, BcOLV18=TRUE} =>  {BcMV6.BI=TRUE} | 0.14  604 | 0.495  798 | 0.294  554 | 1.62  8476 | 59 |

| {BcMV2.BI=TRUE, BcMV6.BI=TRUE} =>  {BcHV1SLR.BI=TRUE} | 0.16  0891 | 0.773  81 | 0.207  921 | 1.42  7484 | 65 |
| --- | --- | --- | --- | --- | --- |
| {BcHV1SLR.BI=TRUE, BcMV6.BI=TRUE} =>  {BcMV2.BI=TRUE} | 0.16  0891 | 0.783  133 | 0.205  446 | 1.69  1901 | 65 |
| {BcHV1SLR.BI=TRUE, BcMV2.BI=TRUE} =>  {BcMV6.BI=TRUE} | 0.16  0891 | 0.590  909 | 0.272  277 | 1.94  0872 | 65 |
| {BcMV2.BI=TRUE, BcMV6.BI=TRUE} =>  {BcMV3.BI=TRUE} | 0.17  5743 | 0.845  238 | 0.207  921 | 1.55  9252 | 71 |
| {BcMV3.BI=TRUE, BcMV6.BI=TRUE} =>  {BcMV2.BI=TRUE} | 0.17  5743 | 0.788  889 | 0.222  772 | 1.70  4337 | 71 |
| {BcMV2.BI=TRUE, BcMV3.BI=TRUE} =>  {BcMV6.BI=TRUE} | 0.17  5743 | 0.559  055 | 0.314  356 | 1.83  6246 | 71 |
| {BcMV2.BI=TRUE, BcMV6.BI=TRUE} =>  {BcULV3=TRUE} | 0.15  5941 | 0.75 | 0.207  921 | 1.26  25 | 63 |
| {BcMV6.BI=TRUE, BcULV3=TRUE} =>  {BcMV2.BI=TRUE} | 0.15  5941 | 0.724  138 | 0.215  347 | 1.56  4448 | 63 |
| {BcMV2.BI=TRUE, BcULV3=TRUE} =>  {BcMV6.BI=TRUE} | 0.15  5941 | 0.547  826 | 0.284  653 | 1.79  9364 | 63 |
| {BcMV2.BI=TRUE, BcMV6.BI=TRUE} =>  {BcHV1.BI=TRUE} | 0.16  3366 | 0.785  714 | 0.207  921 | 1.30  0937 | 66 |
| {BcHV1.BI=TRUE, BcMV6.BI=TRUE} =>  {BcMV2.BI=TRUE} | 0.16  3366 | 0.717  391 | 0.227  723 | 1.54  9872 | 66 |

| {BcHV1.BI=TRUE, BcMV2.BI=TRUE} =>  {BcMV6.BI=TRUE} | 0.16  3366 | 0.559  322 | 0.292  079 | 1.83  7123 | 66 |
| --- | --- | --- | --- | --- | --- |
| {BcMV2.BI=TRUE, BcMV6.BI=TRUE} =>  {BcMV11=TRUE} | 0.17  5743 | 0.845  238 | 0.207  921 | 1.37  139 | 71 |
| {BcMV11=TRUE, BcMV6.BI=TRUE} =>  {BcMV2.BI=TRUE} | 0.17  5743 | 0.771  739 | 0.227  723 | 1.66  7287 | 71 |
| {BcMV11=TRUE, BcMV2.BI=TRUE} =>  {BcMV6.BI=TRUE} | 0.17  5743 | 0.514  493 | 0.341  584 | 1.68  9879 | 71 |
| {BcMV2.BI=TRUE, BcMV6.BI=TRUE} =>  {BcMV9.BI=TRUE} | 0.16  8317 | 0.809  524 | 0.207  921 | 1.19  3604 | 68 |
| {BcMV6.BI=TRUE, BcMV9.BI=TRUE} =>  {BcMV2.BI=TRUE} | 0.16  8317 | 0.715  789 | 0.235  149 | 1.54  6411 | 68 |
| {BcMV2.BI=TRUE, BcMV9.BI=TRUE} =>  {BcMV6.BI=TRUE} | 0.16  8317 | 0.523  077 | 0.321  782 | 1.71  8074 | 68 |
| {BcMV2.BI=TRUE, BcMV6.BI=TRUE} =>  {BcULV4=TRUE} | 0.17  3267 | 0.833  333 | 0.207  921 | 1.20  6691 | 70 |
| {BcMV6.BI=TRUE, BcULV4=TRUE} =>  {BcMV2.BI=TRUE} | 0.17  3267 | 0.714  286 | 0.242  574 | 1.54  3163 | 70 |
| {BcMV2.BI=TRUE, BcULV4=TRUE} =>  {BcMV6.BI=TRUE} | 0.17  3267 | 0.507  246 | 0.341  584 | 1.66  6078 | 70 |
| {BcMV6.BI=TRUE, BcPV3.BI=FALSE} =>  {BcMV2.BI=TRUE} | 0.11  6337 | 0.602  564 | 0.193  069 | 1.30  1796 | 47 |

| {BcMV2.BI=TRUE, BcPV3.BI=FALSE} =>  {BcMV6.BI=TRUE} | 0.11  6337 | 0.408  696 | 0.284  653 | 1.34  2382 | 47 |
| --- | --- | --- | --- | --- | --- |
| {BcMV6.BI=TRUE, BcRV1.BI=FALSE} =>  {BcMV2.BI=TRUE} | 0.11  3861 | 0.597  403 | 0.190  594 | 1.29  0645 | 46 |
| {BcMV2.BI=TRUE, BcRV1.BI=FALSE} =>  {BcMV6.BI=TRUE} | 0.11  3861 | 0.4 | 0.284  653 | 1.31  3821 | 46 |
| {BcEV2.BI=FALSE, BcMV6.BI=TRUE} =>  {BcMV2.BI=TRUE} | 0.15  3465 | 0.659  574 | 0.232  673 | 1.42  4963 | 62 |
| {BcEV2.BI=FALSE, BcMV2.BI=TRUE} =>  {BcMV6.BI=TRUE} | 0.15  3465 | 0.480  62 | 0.319  307 | 1.57  8622 | 62 |
| {BcHV3.BI=FALSE, BcMV6.BI=TRUE} =>  {BcMV2.BI=TRUE} | 0.13  6139 | 0.617  978 | 0.220  297 | 1.33  5096 | 55 |
| {BcHV3.BI=FALSE, BcMV2.BI=TRUE} =>  {BcMV6.BI=TRUE} | 0.13  6139 | 0.413  534 | 0.329  208 | 1.35  8274 | 55 |
| {BcFV5.BI=FALSE, BcMV6.BI=TRUE} =>  {BcMV2.BI=TRUE} | 0.14  604 | 0.614  583 | 0.237  624 | 1.32  7763 | 59 |
| {BcFV5.BI=FALSE, BcMV2.BI=TRUE} =>  {BcMV6.BI=TRUE} | 0.14  604 | 0.424  46 | 0.344  059 | 1.39  4163 | 59 |
| {BcMV6.BI=TRUE, BcULV1.BI=FALSE} =>  {BcMV2.BI=TRUE} | 0.13  6139 | 0.639  535 | 0.212  871 | 1.38  1669 | 55 |
| {BcMV2.BI=TRUE, BcULV1.BI=FALSE} =>  {BcMV6.BI=TRUE} | 0.13  6139 | 0.419  847 | 0.324  257 | 1.37  9011 | 55 |

| {BcMV2.BI=TRUE, BcMV6.BI=TRUE} =>  {BcMV1.BI=TRUE} | 0.18  5644 | 0.892  857 | 0.207  921 | 1.13  0766 | 75 |
| --- | --- | --- | --- | --- | --- |
| {BcMV1.BI=TRUE, BcMV6.BI=TRUE} =>  {BcMV2.BI=TRUE} | 0.18  5644 | 0.728  155 | 0.254  95 | 1.57  3127 | 75 |
| {BcMV1.BI=TRUE, BcMV2.BI=TRUE} =>  {BcMV6.BI=TRUE} | 0.18  5644 | 0.454  545 | 0.408  416 | 1.49  2979 | 75 |
| {BcMV12=FALSE, BcMV6.BI=TRUE} =>  {BcMV2.BI=TRUE} | 0.13  6139 | 0.625 | 0.217  822 | 1.35  0267 | 55 |
| {BcMV12=FALSE, BcMV2.BI=TRUE} =>  {BcMV6.BI=TRUE} | 0.13  6139 | 0.398  551 | 0.341  584 | 1.30  9061 | 55 |
| {BcMV6.BI=TRUE, BcNSRV7.BI=FALSE} =>  {BcMV2.BI=TRUE} | 0.15  099 | 0.648  936 | 0.232  673 | 1.40  198 | 61 |
| {BcMV2.BI=TRUE, BcNSRV7.BI=FALSE} =>  {BcMV6.BI=TRUE} | 0.15  099 | 0.417  808 | 0.361  386 | 1.37  2313 | 61 |
| {BcMV6.BI=TRUE, BcVV3.BI=FALSE} =>  {BcMV2.BI=TRUE} | 0.15  3465 | 0.666  667 | 0.230  198 | 1.44  0285 | 62 |
| {BcMV2.BI=TRUE, BcVV3.BI=FALSE} =>  {BcMV6.BI=TRUE} | 0.15  3465 | 0.436  62 | 0.351  485 | 1.43  4101 | 62 |
| {BcMV6.BI=TRUE, BcOLV7.BI=FALSE} =>  {BcMV2.BI=TRUE} | 0.15  8416 | 0.680  851 | 0.232  673 | 1.47  093 | 64 |
| {BcMV2.BI=TRUE, BcOLV7.BI=FALSE} =>  {BcMV6.BI=TRUE} | 0.15  8416 | 0.441  379 | 0.358  911 | 1.44  9734 | 64 |

| {BcALV1.BI=FALSE, BcMV6.BI=TRUE} =>  {BcMV2.BI=TRUE} | 0.13  1188 | 0.602  273 | 0.217  822 | 1.30  1167 | 53 |
| --- | --- | --- | --- | --- | --- |
| {BcALV1.BI=FALSE, BcMV2.BI=TRUE} =>  {BcMV6.BI=TRUE} | 0.13  1188 | 0.404  58 | 0.324  257 | 1.32  8865 | 53 |
| {BcDMV1=FALSE, BcMV6.BI=TRUE} =>  {BcMV2.BI=TRUE} | 0.15  3465 | 0.645  833 | 0.237  624 | 1.39  5276 | 62 |
| {BcDMV1=FALSE, BcMV2.BI=TRUE} =>  {BcMV6.BI=TRUE} | 0.15  3465 | 0.387  5 | 0.396  04 | 1.27  2764 | 62 |
| {BcFV9=FALSE, BcMV6.BI=TRUE} =>  {BcMV2.BI=TRUE} | 0.17  8218 | 0.666  667 | 0.267  327 | 1.44  0285 | 72 |
| {BcFV9=FALSE, BcMV2.BI=TRUE} =>  {BcMV6.BI=TRUE} | 0.17  8218 | 0.444  444 | 0.400  99 | 1.45  9801 | 72 |
| {BcMV6.BI=TRUE, BcOLV4.BI=FALSE} =>  {BcMV2.BI=TRUE} | 0.16  8317 | 0.647  619 | 0.259  901 | 1.39  9134 | 68 |
| {BcMV2.BI=TRUE, BcOLV4.BI=FALSE} =>  {BcMV6.BI=TRUE} | 0.16  8317 | 0.447  368 | 0.376  238 | 1.46  9405 | 68 |
| {BcMV6.BI=TRUE, BcULV5=FALSE} =>  {BcMV2.BI=TRUE} | 0.17  0792 | 0.669  903 | 0.254  95 | 1.44  7277 | 69 |
| {BcMV2.BI=TRUE, BcULV5=FALSE} =>  {BcMV6.BI=TRUE} | 0.17  0792 | 0.453  947 | 0.376  238 | 1.49  1014 | 69 |
| {BcBNV5.BI=FALSE, BcMV6.BI=TRUE} =>  {BcMV2.BI=TRUE} | 0.15  8416 | 0.646  465 | 0.245  05 | 1.39  664 | 64 |

| {BcBNV5.BI=FALSE, BcMV2.BI=TRUE} =>  {BcMV6.BI=TRUE} | 0.15  8416 | 0.387  879 | 0.408  416 | 1.27  4008 | 64 |
| --- | --- | --- | --- | --- | --- |
| {BcMV6.BI=TRUE, BvF.BI=FALSE} =>  {BcMV2.BI=TRUE} | 0.19  802 | 0.683  761 | 0.289  604 | 1.47  7216 | 80 |
| {BcMV2.BI=TRUE, BvF.BI=FALSE} =>  {BcMV6.BI=TRUE} | 0.19  802 | 0.457  143 | 0.433  168 | 1.50  151 | 80 |
| {BcMCV5.BI=FALSE, BcMV6.BI=TRUE} =>  {BcMV2.BI=TRUE} | 0.16  8317 | 0.647  619 | 0.259  901 | 1.39  9134 | 68 |
| {BcMCV5.BI=FALSE, BcMV2.BI=TRUE} =>  {BcMV6.BI=TRUE} | 0.16  8317 | 0.419  753 | 0.400  99 | 1.37  8701 | 68 |
| {BcMV6.BI=TRUE, BcOLV13.BI=FALSE} =>  {BcMV2.BI=TRUE} | 0.17  5743 | 0.651  376 | 0.269  802 | 1.40  7251 | 71 |
| {BcMV2.BI=TRUE, BcOLV13.BI=FALSE} =>  {BcMV6.BI=TRUE} | 0.17  5743 | 0.430  303 | 0.408  416 | 1.41  3353 | 71 |
| {BcMV6.BI=TRUE, BcOLV2.BI=FALSE} =>  {BcMV2.BI=TRUE} | 0.17  3267 | 0.660  377 | 0.262  376 | 1.42  6698 | 70 |
| {BcMV2.BI=TRUE, BcOLV2.BI=FALSE} =>  {BcMV6.BI=TRUE} | 0.17  3267 | 0.411  765 | 0.420  792 | 1.35  2463 | 70 |
| {BcMCV3.BI=FALSE, BcMV6.BI=TRUE} =>  {BcMV2.BI=TRUE} | 0.16  8317 | 0.647  619 | 0.259  901 | 1.39  9134 | 68 |
| {BcMCV3.BI=FALSE, BcMV2.BI=TRUE} =>  {BcMV6.BI=TRUE} | 0.16  8317 | 0.412  121 | 0.408  416 | 1.35  3634 | 68 |

| {BcMV6.BI=TRUE, BcPV4.BI=FALSE} =>  {BcMV2.BI=TRUE} | 0.19  0594 | 0.687  5 | 0.277  228 | 1.48  5294 | 77 |
| --- | --- | --- | --- | --- | --- |
| {BcMV2.BI=TRUE, BcPV4.BI=FALSE} =>  {BcMV6.BI=TRUE} | 0.19  0594 | 0.445  087 | 0.428  218 | 1.46  1911 | 77 |
| {BcEV6=FALSE, BcMV6.BI=TRUE} =>  {BcMV2.BI=TRUE} | 0.19  3069 | 0.684  211 | 0.282  178 | 1.47  8187 | 78 |
| {BcEV6=FALSE, BcMV2.BI=TRUE} =>  {BcMV6.BI=TRUE} | 0.19  3069 | 0.445  714 | 0.433  168 | 1.46  3972 | 78 |
| {BcMV6.BI=TRUE, BcOLV17.BI=FALSE} =>  {BcMV2.BI=TRUE} | 0.18  5644 | 0.669  643 | 0.277  228 | 1.44  6715 | 75 |
| {BcMV2.BI=TRUE, BcOLV17.BI=FALSE} =>  {BcMV6.BI=TRUE} | 0.18  5644 | 0.436  047 | 0.425  743 | 1.43  2218 | 75 |
| {BcMV6.BI=TRUE, BcOLV15.BI=FALSE} =>  {BcMV2.BI=TRUE} | 0.20  5446 | 0.685  95 | 0.299  505 | 1.48  1946 | 83 |
| {BcMV2.BI=TRUE, BcOLV15.BI=FALSE} =>  {BcMV6.BI=TRUE} | 0.20  5446 | 0.453  552 | 0.452  97 | 1.48  9715 | 83 |
| {BcEV5=FALSE, BcMV6.BI=TRUE} =>  {BcMV2.BI=TRUE} | 0.17  0792 | 0.644  86 | 0.264  851 | 1.39  3173 | 69 |
| {BcEV5=FALSE, BcMV2.BI=TRUE} =>  {BcMV6.BI=TRUE} | 0.17  0792 | 0.403  509 | 0.423  267 | 1.32  5346 | 69 |
| {BcMV6.BI=TRUE, BcOLV18=FALSE} =>  {BcULV3=TRUE} | 0.09  6535 | 0.735  849 | 0.131  188 | 1.23  8679 | 39 |

| {BcMV6.BI=TRUE, BcOLV1.BI.MOVA.=FALSE} =>  {BcOLV18=FALSE} | 0.09  1584 | 0.649  123 | 0.141  089 | 1.34  4849 | 37 |
| --- | --- | --- | --- | --- | --- |
| {BcMV6.BI=TRUE, BcOLV18=FALSE} =>  {BcPV3.BI=FALSE} | 0.10  6436 | 0.811  321 | 0.131  188 | 1.14  2068 | 43 |
| {BcMV6.BI=TRUE, BcOLV18=FALSE} =>  {BcRV1.BI=FALSE} | 0.11  3861 | 0.867  925 | 0.131  188 | 1.20  0827 | 46 |
| {BcMV6.BI=TRUE, BcRV1.BI=FALSE} =>  {BcOLV18=FALSE} | 0.11  3861 | 0.597  403 | 0.190  594 | 1.23  7696 | 46 |
| {BcMV6.BI=TRUE, BcOLV18=FALSE} =>  {BcFV5.BI=FALSE} | 0.11  8812 | 0.905  66 | 0.131  188 | 1.15  787 | 48 |
| {BcMV6.BI=TRUE, BcOLV18=FALSE} =>  {BcALV1.BI=FALSE} | 0.11  8812 | 0.905  66 | 0.131  188 | 1.11  2118 | 48 |
| {BcMV6.BI=TRUE, BcOLV18=TRUE} =>  {BcHV1SLR.BI=TRUE} | 0.13  3663 | 0.771  429 | 0.173  267 | 1.42  3092 | 54 |
| {BcHV1SLR.BI=TRUE, BcMV6.BI=TRUE} =>  {BcOLV18=TRUE} | 0.13  3663 | 0.650  602 | 0.205  446 | 1.25  7624 | 54 |
| {BcHV1SLR.BI=TRUE, BcOLV18=TRUE} =>  {BcMV6.BI=TRUE} | 0.13  3663 | 0.391  304 | 0.341  584 | 1.28  526 | 54 |
| {BcMV6.BI=TRUE, BcOLV18=TRUE} =>  {BcMV3.BI=TRUE} | 0.14  1089 | 0.814  286 | 0.173  267 | 1.50  2153 | 57 |
| {BcMV3.BI=TRUE, BcMV6.BI=TRUE} => | 0.14 | 0.633 | 0.222 | 1.22 | 57 |

| {BcOLV18=TRUE} | 1089 | 333 | 772 | 4242 |  |
| --- | --- | --- | --- | --- | --- |
| {BcMV3.BI=TRUE, BcOLV18=TRUE} =>  {BcMV6.BI=TRUE} | 0.14  1089 | 0.428  571 | 0.329  208 | 1.40  7666 | 57 |
| {BcMV6.BI=TRUE, BcOLV18=TRUE} =>  {BcHV1.BI=TRUE} | 0.14  3564 | 0.828  571 | 0.173  267 | 1.37  1897 | 58 |
| {BcHV1.BI=TRUE, BcMV6.BI=TRUE} =>  {BcOLV18=TRUE} | 0.14  3564 | 0.630  435 | 0.227  723 | 1.21  8639 | 58 |
| {BcHV1.BI=TRUE, BcOLV18=TRUE} =>  {BcMV6.BI=TRUE} | 0.14  3564 | 0.391  892 | 0.366  337 | 1.28  719 | 58 |
| {BcMV6.BI=TRUE, BcOLV18=TRUE} =>  {BcMV11=TRUE} | 0.14  604 | 0.842  857 | 0.173  267 | 1.36  7527 | 59 |
| {BcMV11=TRUE, BcMV6.BI=TRUE} =>  {BcOLV18=TRUE} | 0.14  604 | 0.641  304 | 0.227  723 | 1.23  9651 | 59 |
| {BcMV11=TRUE, BcOLV18=TRUE} =>  {BcMV6.BI=TRUE} | 0.14  604 | 0.421  429 | 0.346  535 | 1.38  4204 | 59 |
| {BcMV6.BI=TRUE, BcOLV18=TRUE} =>  {BcMV9.BI=TRUE} | 0.15  099 | 0.871  429 | 0.173  267 | 1.28  488 | 61 |
| {BcMV6.BI=TRUE, BcMV9.BI=TRUE} =>  {BcOLV18=TRUE} | 0.15  099 | 0.642  105 | 0.235  149 | 1.24  1199 | 61 |
| {BcMV6.BI=TRUE, BcOLV18=TRUE} =>  {BcULV4=TRUE} | 0.15  8416 | 0.914  286 | 0.173  267 | 1.32  3912 | 64 |
| {BcMV6.BI=TRUE, BcULV4=TRUE} => | 0.15 | 0.653 | 0.242 | 1.26 | 64 |

| {BcOLV18=TRUE} | 8416 | 061 | 574 | 2377 |  |
| --- | --- | --- | --- | --- | --- |
| {BcMV6.BI=TRUE, BcOLV18=TRUE} =>  {BcMV1.BI=TRUE} | 0.15  3465 | 0.885  714 | 0.173  267 | 1.12  172 | 62 |
| {BcMV2.BI=FALSE, BcMV6.BI=TRUE} =>  {BcOLV1.BI.MOVA.=FALSE} | 0.07  6733 | 0.794  872 | 0.096  535 | 1.18  9364 | 31 |
| {BcMV2.BI=FALSE, BcMV6.BI=TRUE} =>  {BcHV3.BI=FALSE} | 0.08  4158 | 0.871  795 | 0.096  535 | 1.14  7248 | 34 |
| {BcMV2.BI=FALSE, BcMV6.BI=TRUE} =>  {BcFV5.BI=FALSE} | 0.09  1584 | 0.948  718 | 0.096  535 | 1.21  2918 | 37 |
| {BcHV1SLR.BI=TRUE, BcMV6.BI=TRUE} =>  {BcMV3.BI=TRUE} | 0.16  3366 | 0.795  181 | 0.205  446 | 1.46  6909 | 66 |
| {BcMV3.BI=TRUE, BcMV6.BI=TRUE} =>  {BcHV1SLR.BI=TRUE} | 0.16  3366 | 0.733  333 | 0.222  772 | 1.35  2816 | 66 |
| {BcHV1SLR.BI=TRUE, BcMV3.BI=TRUE} =>  {BcMV6.BI=TRUE} | 0.16  3366 | 0.496  241 | 0.329  208 | 1.62  9928 | 66 |
| {BcHV1SLR.BI=TRUE, BcMV6.BI=TRUE} =>  {BcULV3=TRUE} | 0.15  3465 | 0.746  988 | 0.205  446 | 1.25  743 | 62 |
| {BcMV6.BI=TRUE, BcULV3=TRUE} =>  {BcHV1SLR.BI=TRUE} | 0.15  3465 | 0.712  644 | 0.215  347 | 1.31  4649 | 62 |
| {BcHV1SLR.BI=TRUE, BcULV3=TRUE} =>  {BcMV6.BI=TRUE} | 0.15  3465 | 0.397  436 | 0.386  139 | 1.30  5399 | 62 |
| {BcHV1SLR.BI=TRUE, BcMV6.BI=TRUE} => | 0.19 | 0.927 | 0.205 | 1.53 | 77 |

| {BcHV1.BI=TRUE} | 0594 | 711 | 446 | 6046 |  |
| --- | --- | --- | --- | --- | --- |
| {BcHV1.BI=TRUE, BcMV6.BI=TRUE} =>  {BcHV1SLR.BI=TRUE} | 0.19  0594 | 0.836  957 | 0.227  723 | 1.54  3975 | 77 |
| {BcHV1.BI=TRUE, BcHV1SLR.BI=TRUE} =>  {BcMV6.BI=TRUE} | 0.19  0594 | 0.392  857 | 0.485  149 | 1.29  036 | 77 |
| {BcHV1SLR.BI=TRUE, BcMV6.BI=TRUE} =>  {BcMV11=TRUE} | 0.16  5842 | 0.807  229 | 0.205  446 | 1.30  9721 | 67 |
| {BcMV11=TRUE, BcMV6.BI=TRUE} =>  {BcHV1SLR.BI=TRUE} | 0.16  5842 | 0.728  261 | 0.227  723 | 1.34  3458 | 67 |
| {BcHV1SLR.BI=TRUE, BcMV11=TRUE} =>  {BcMV6.BI=TRUE} | 0.16  5842 | 0.465  278 | 0.356  436 | 1.52  8229 | 67 |
| {BcHV1SLR.BI=TRUE, BcMV6.BI=TRUE} =>  {BcMV9.BI=TRUE} | 0.16  8317 | 0.819  277 | 0.205  446 | 1.20  7985 | 68 |
| {BcMV6.BI=TRUE, BcMV9.BI=TRUE} =>  {BcHV1SLR.BI=TRUE} | 0.16  8317 | 0.715  789 | 0.235  149 | 1.32  0452 | 68 |
| {BcHV1SLR.BI=TRUE, BcMV9.BI=TRUE} =>  {BcMV6.BI=TRUE} | 0.16  8317 | 0.412  121 | 0.408  416 | 1.35  3634 | 68 |
| {BcHV1SLR.BI=TRUE, BcMV6.BI=TRUE} =>  {BcULV4=TRUE} | 0.16  8317 | 0.819  277 | 0.205  446 | 1.18  6337 | 68 |
| {BcMV6.BI=TRUE, BcULV4=TRUE} =>  {BcHV1SLR.BI=TRUE} | 0.16  8317 | 0.693  878 | 0.242  574 | 1.28  003 | 68 |
| {BcHV1SLR.BI=TRUE, BcULV4=TRUE} => | 0.16 | 0.419 | 0.400 | 1.37 | 68 |

| {BcMV6.BI=TRUE} | 8317 | 753 | 99 | 8701 |  |
| --- | --- | --- | --- | --- | --- |
| {BcEV2.BI=FALSE, BcHV1SLR.BI=TRUE} =>  {BcMV6.BI=TRUE} | 0.14  8515 | 0.384  615 | 0.386  139 | 1.26  329 | 60 |
| {BcFV5.BI=FALSE, BcHV1SLR.BI=TRUE} =>  {BcMV6.BI=TRUE} | 0.14  8515 | 0.379  747 | 0.391  089 | 1.24  7299 | 60 |
| {BcMV1.BI=TRUE, BcMV6.BI=TRUE} =>  {BcHV1SLR.BI=TRUE} | 0.17  5743 | 0.689  32 | 0.254  95 | 1.27  1623 | 71 |
| {BcHV1SLR.BI=TRUE, BcMV1.BI=TRUE} =>  {BcMV6.BI=TRUE} | 0.17  5743 | 0.398  876 | 0.440  594 | 1.31  0131 | 71 |
| {BcMV12=FALSE, BcMV6.BI=TRUE} =>  {BcHV1SLR.BI=TRUE} | 0.14  1089 | 0.647  727 | 0.217  822 | 1.19  4894 | 57 |
| {BcMV6.BI=TRUE, BcNSRV7.BI=FALSE} =>  {BcHV1SLR.BI=TRUE} | 0.15  5941 | 0.670  213 | 0.232  673 | 1.23  6374 | 63 |
| {BcHV1SLR.BI=TRUE, BcNSRV7.BI=FALSE}  => {BcMV6.BI=TRUE} | 0.15  5941 | 0.381  818 | 0.408  416 | 1.25  4102 | 63 |
| {BcMV6.BI=TRUE, BcVV3.BI=FALSE} =>  {BcHV1SLR.BI=TRUE} | 0.15  5941 | 0.677  419 | 0.230  198 | 1.24  9669 | 63 |
| {BcMV6.BI=TRUE, BcOLV7.BI=FALSE} =>  {BcHV1SLR.BI=TRUE} | 0.15  8416 | 0.680  851 | 0.232  673 | 1.25  5999 | 64 |
| {BcDMV1=FALSE, BcMV6.BI=TRUE} =>  {BcHV1SLR.BI=TRUE} | 0.16  0891 | 0.677  083 | 0.237  624 | 1.24  9049 | 65 |
| {BcFV9=FALSE, BcMV6.BI=TRUE} => | 0.17 | 0.648 | 0.267 | 1.19 | 70 |

| {BcHV1SLR.BI=TRUE} | 3267 | 148 | 327 | 5671 |  |
| --- | --- | --- | --- | --- | --- |
| {BcFV9=FALSE, BcHV1SLR.BI=TRUE} =>  {BcMV6.BI=TRUE} | 0.17  3267 | 0.384  615 | 0.450  495 | 1.26  329 | 70 |
| {BcMV6.BI=TRUE, BcOLV4.BI=FALSE} =>  {BcHV1SLR.BI=TRUE} | 0.16  5842 | 0.638  095 | 0.259  901 | 1.17  7125 | 67 |
| {BcMV6.BI=TRUE, BcULV5=FALSE} =>  {BcHV1SLR.BI=TRUE} | 0.16  8317 | 0.660  194 | 0.254  95 | 1.21  7892 | 68 |
| {BcBNV5.BI=FALSE, BcMV6.BI=TRUE} =>  {BcHV1SLR.BI=TRUE} | 0.16  3366 | 0.666  667 | 0.245  05 | 1.22  9833 | 66 |
| {BcMV6.BI=TRUE, BvF.BI=FALSE} =>  {BcHV1SLR.BI=TRUE} | 0.19  0594 | 0.658  12 | 0.289  604 | 1.21  4065 | 77 |
| {BcHV1SLR.BI=TRUE, BvF.BI=FALSE} =>  {BcMV6.BI=TRUE} | 0.19  0594 | 0.396  907 | 0.480  198 | 1.30  3663 | 77 |
| {BcMCV5.BI=FALSE, BcMV6.BI=TRUE} =>  {BcHV1SLR.BI=TRUE} | 0.17  0792 | 0.657  143 | 0.259  901 | 1.21  2264 | 69 |
| {BcMV6.BI=TRUE, BcOLV13.BI=FALSE} =>  {BcHV1SLR.BI=TRUE} | 0.17  3267 | 0.642  202 | 0.269  802 | 1.18  4701 | 70 |
| {BcMCV3.BI=FALSE, BcMV6.BI=TRUE} =>  {BcHV1SLR.BI=TRUE} | 0.16  8317 | 0.647  619 | 0.259  901 | 1.19  4694 | 68 |
| {BcMV6.BI=TRUE, BcPV4.BI=FALSE} =>  {BcHV1SLR.BI=TRUE} | 0.18  5644 | 0.669  643 | 0.277  228 | 1.23  5323 | 75 |
| {BcHV1SLR.BI=TRUE, BcPV4.BI=FALSE} => | 0.18 | 0.367 | 0.504 | 1.20 | 75 |

| {BcMV6.BI=TRUE} | 5644 | 647 | 95 | 7556 |  |
| --- | --- | --- | --- | --- | --- |
| {BcEV6=FALSE, BcMV6.BI=TRUE} =>  {BcHV1SLR.BI=TRUE} | 0.19  0594 | 0.675  439 | 0.282  178 | 1.24  6015 | 77 |
| {BcEV6=FALSE, BcHV1SLR.BI=TRUE} =>  {BcMV6.BI=TRUE} | 0.19  0594 | 0.379  31 | 0.502  475 | 1.24  5865 | 77 |
| {BcMV6.BI=TRUE, BcOLV17.BI=FALSE} =>  {BcHV1SLR.BI=TRUE} | 0.18  3168 | 0.660  714 | 0.277  228 | 1.21  8852 | 74 |
| {BcMV6.BI=TRUE, BcOLV15.BI=FALSE} =>  {BcHV1SLR.BI=TRUE} | 0.20  0495 | 0.669  421 | 0.299  505 | 1.23  4915 | 81 |
| {BcHV1SLR.BI=TRUE, BcOLV15.BI=FALSE}  => {BcMV6.BI=TRUE} | 0.20  0495 | 0.400  99 | 0.5 | 1.31  7073 | 81 |
| {BcEV5=FALSE, BcMV6.BI=TRUE} =>  {BcHV1SLR.BI=TRUE} | 0.17  8218 | 0.672  897 | 0.264  851 | 1.24  1326 | 72 |
| {BcMV3.BI=TRUE, BcMV6.BI=TRUE} =>  {BcULV3=TRUE} | 0.16  8317 | 0.755  556 | 0.222  772 | 1.27  1852 | 68 |
| {BcMV6.BI=TRUE, BcULV3=TRUE} =>  {BcMV3.BI=TRUE} | 0.16  8317 | 0.781  609 | 0.215  347 | 1.44  1873 | 68 |
| {BcMV3.BI=TRUE, BcULV3=TRUE} =>  {BcMV6.BI=TRUE} | 0.16  8317 | 0.485  714 | 0.346  535 | 1.59  5354 | 68 |
| {BcMV3.BI=TRUE, BcMV6.BI=TRUE} =>  {BcHV1.BI=TRUE} | 0.17  0792 | 0.766  667 | 0.222  772 | 1.26  9399 | 69 |
| {BcHV1.BI=TRUE, BcMV6.BI=TRUE} => | 0.17 | 0.75 | 0.227 | 1.38 | 69 |

| {BcMV3.BI=TRUE} | 0792 |  | 723 | 3562 |  |
| --- | --- | --- | --- | --- | --- |
| {BcHV1.BI=TRUE, BcMV3.BI=TRUE} =>  {BcMV6.BI=TRUE} | 0.17  0792 | 0.482  517 | 0.353  96 | 1.58  4854 | 69 |
| {BcMV3.BI=TRUE, BcMV6.BI=TRUE} =>  {BcMV11=TRUE} | 0.18  3168 | 0.822  222 | 0.222  772 | 1.33  4047 | 74 |
| {BcMV11=TRUE, BcMV6.BI=TRUE} =>  {BcMV3.BI=TRUE} | 0.18  3168 | 0.804  348 | 0.227  723 | 1.48  382 | 74 |
| {BcMV11=TRUE, BcMV3.BI=TRUE} =>  {BcMV6.BI=TRUE} | 0.18  3168 | 0.477  419 | 0.383  663 | 1.56  8109 | 74 |
| {BcMV3.BI=TRUE, BcMV6.BI=TRUE} =>  {BcMV9.BI=TRUE} | 0.17  5743 | 0.788  889 | 0.222  772 | 1.16  3179 | 71 |
| {BcMV6.BI=TRUE, BcMV9.BI=TRUE} =>  {BcMV3.BI=TRUE} | 0.17  5743 | 0.747  368 | 0.235  149 | 1.37  8707 | 71 |
| {BcMV3.BI=TRUE, BcMV9.BI=TRUE} =>  {BcMV6.BI=TRUE} | 0.17  5743 | 0.443  75 | 0.396  04 | 1.45  752 | 71 |
| {BcMV3.BI=TRUE, BcMV6.BI=TRUE} =>  {BcULV4=TRUE} | 0.17  8218 | 0.8 | 0.222  772 | 1.15  8423 | 72 |
| {BcMV6.BI=TRUE, BcULV4=TRUE} =>  {BcMV3.BI=TRUE} | 0.17  8218 | 0.734  694 | 0.242  574 | 1.35  5326 | 72 |
| {BcMV3.BI=TRUE, BcULV4=TRUE} =>  {BcMV6.BI=TRUE} | 0.17  8218 | 0.444  444 | 0.400  99 | 1.45  9801 | 72 |
| {BcMV6.BI=TRUE, BcPV3.BI=FALSE} => | 0.12 | 0.666 | 0.193 | 1.22 | 52 |

| {BcMV3.BI=TRUE} | 8713 | 667 | 069 | 9833 |  |
| --- | --- | --- | --- | --- | --- |
| {BcMV6.BI=TRUE, BcRV1.BI=FALSE} =>  {BcMV3.BI=TRUE} | 0.12  6238 | 0.662  338 | 0.190  594 | 1.22  1847 | 51 |
| {BcEV2.BI=FALSE, BcMV6.BI=TRUE} =>  {BcMV3.BI=TRUE} | 0.16  8317 | 0.723  404 | 0.232  673 | 1.33  4499 | 68 |
| {BcEV2.BI=FALSE, BcMV3.BI=TRUE} =>  {BcMV6.BI=TRUE} | 0.16  8317 | 0.417  178 | 0.403  465 | 1.37  0243 | 68 |
| {BcHV3.BI=FALSE, BcMV6.BI=TRUE} =>  {BcMV3.BI=TRUE} | 0.15  3465 | 0.696  629 | 0.220  297 | 1.28  5106 | 62 |
| {BcHV3.BI=FALSE, BcMV3.BI=TRUE} =>  {BcMV6.BI=TRUE} | 0.15  3465 | 0.389  937 | 0.393  564 | 1.28  0769 | 62 |
| {BcFV5.BI=FALSE, BcMV6.BI=TRUE} =>  {BcMV3.BI=TRUE} | 0.16  5842 | 0.697  917 | 0.237  624 | 1.28  7481 | 67 |
| {BcFV5.BI=FALSE, BcMV3.BI=TRUE} =>  {BcMV6.BI=TRUE} | 0.16  5842 | 0.426  752 | 0.388  614 | 1.40  1688 | 67 |
| {BcMV6.BI=TRUE, BcULV1.BI=FALSE} =>  {BcMV3.BI=TRUE} | 0.14  3564 | 0.674  419 | 0.212  871 | 1.24  4133 | 58 |
| {BcMV3.BI=TRUE, BcMV6.BI=TRUE} =>  {BcMV1.BI=TRUE} | 0.20  0495 | 0.9 | 0.222  772 | 1.13  9812 | 81 |
| {BcMV1.BI=TRUE, BcMV6.BI=TRUE} =>  {BcMV3.BI=TRUE} | 0.20  0495 | 0.786  408 | 0.254  95 | 1.45  0725 | 81 |
| {BcMV1.BI=TRUE, BcMV3.BI=TRUE} => | 0.20 | 0.437 | 0.457 | 1.43 | 81 |

| {BcMV6.BI=TRUE} | 0495 | 838 | 921 | 8102 |  |
| --- | --- | --- | --- | --- | --- |
| {BcMV6.BI=TRUE, BcNSRV7.BI=FALSE} =>  {BcMV3.BI=TRUE} | 0.16  3366 | 0.702  128 | 0.232  673 | 1.29  5249 | 66 |
| {BcMV3.BI=TRUE, BcNSRV7.BI=FALSE} =>  {BcMV6.BI=TRUE} | 0.16  3366 | 0.385  965 | 0.423  267 | 1.26  7722 | 66 |
| {BcMV6.BI=TRUE, BcVV3.BI=FALSE} =>  {BcMV3.BI=TRUE} | 0.16  5842 | 0.720  43 | 0.230  198 | 1.32  9013 | 67 |
| {BcMV3.BI=TRUE, BcVV3.BI=FALSE} =>  {BcMV6.BI=TRUE} | 0.16  5842 | 0.398  81 | 0.415  842 | 1.30  9911 | 67 |
| {BcMV6.BI=TRUE, BcOLV7.BI=FALSE} =>  {BcMV3.BI=TRUE} | 0.17  0792 | 0.734  043 | 0.232  673 | 1.35  4124 | 69 |
| {BcMV3.BI=TRUE, BcOLV7.BI=FALSE} =>  {BcMV6.BI=TRUE} | 0.17  0792 | 0.410  714 | 0.415  842 | 1.34  9013 | 69 |
| {BcALV1.BI=FALSE, BcMV6.BI=TRUE} =>  {BcMV3.BI=TRUE} | 0.15  5941 | 0.715  909 | 0.217  822 | 1.32  0672 | 63 |
| {BcDMV1=FALSE, BcMV6.BI=TRUE} =>  {BcMV3.BI=TRUE} | 0.15  8416 | 0.666  667 | 0.237  624 | 1.22  9833 | 64 |
| {BcDMV1=FALSE, BcMV3.BI=TRUE} =>  {BcMV6.BI=TRUE} | 0.15  8416 | 0.374  269 | 0.423  267 | 1.22  9306 | 64 |
| {BcFV9=FALSE, BcMV6.BI=TRUE} =>  {BcMV3.BI=TRUE} | 0.18  8119 | 0.703  704 | 0.267  327 | 1.29  8157 | 76 |
| {BcFV9=FALSE, BcMV3.BI=TRUE} => | 0.18 | 0.413 | 0.455 | 1.35 | 76 |

| {BcMV6.BI=TRUE} | 8119 | 043 | 446 | 6663 |  |
| --- | --- | --- | --- | --- | --- |
| {BcMV6.BI=TRUE, BcOLV4.BI=FALSE} =>  {BcMV3.BI=TRUE} | 0.18  8119 | 0.723  81 | 0.259  901 | 1.33  5247 | 76 |
| {BcMV3.BI=TRUE, BcOLV4.BI=FALSE} =>  {BcMV6.BI=TRUE} | 0.18  8119 | 0.413  043 | 0.455  446 | 1.35  6663 | 76 |
| {BcMV6.BI=TRUE, BcULV5=FALSE} =>  {BcMV3.BI=TRUE} | 0.17  8218 | 0.699  029 | 0.254  95 | 1.28  9533 | 72 |
| {BcMV3.BI=TRUE, BcULV5=FALSE} =>  {BcMV6.BI=TRUE} | 0.17  8218 | 0.393  443 | 0.452  97 | 1.29  2283 | 72 |
| {BcBNV5.BI=FALSE, BcMV6.BI=TRUE} =>  {BcMV3.BI=TRUE} | 0.17  3267 | 0.707  071 | 0.245  05 | 1.30  4368 | 70 |
| {BcBNV5.BI=FALSE, BcMV3.BI=TRUE} =>  {BcMV6.BI=TRUE} | 0.17  3267 | 0.368  421 | 0.470  297 | 1.21  0098 | 70 |
| {BcMV6.BI=TRUE, BvF.BI=FALSE} =>  {BcMV3.BI=TRUE} | 0.21  2871 | 0.735  043 | 0.289  604 | 1.35  5969 | 86 |
| {BcMV3.BI=TRUE, BvF.BI=FALSE} =>  {BcMV6.BI=TRUE} | 0.21  2871 | 0.43 | 0.495  05 | 1.41  2358 | 86 |
| {BcMCV5.BI=FALSE, BcMV6.BI=TRUE} =>  {BcMV3.BI=TRUE} | 0.17  8218 | 0.685  714 | 0.259  901 | 1.26  4971 | 72 |
| {BcMCV5.BI=FALSE, BcMV3.BI=TRUE} =>  {BcMV6.BI=TRUE} | 0.17  8218 | 0.369  231 | 0.482  673 | 1.21  2758 | 72 |
| {BcMV6.BI=TRUE, BcOLV13.BI=FALSE} => | 0.18 | 0.697 | 0.269 | 1.28 | 76 |

| {BcMV3.BI=TRUE} | 8119 | 248 | 802 | 6247 |  |
| --- | --- | --- | --- | --- | --- |
| {BcMV3.BI=TRUE, BcOLV13.BI=FALSE} =>  {BcMV6.BI=TRUE} | 0.18  8119 | 0.395  833 | 0.475  248 | 1.30  0136 | 76 |
| {BcMV6.BI=TRUE, BcOLV2.BI=FALSE} =>  {BcMV3.BI=TRUE} | 0.18  3168 | 0.698  113 | 0.262  376 | 1.28  7844 | 74 |
| {BcMV3.BI=TRUE, BcOLV2.BI=FALSE} =>  {BcMV6.BI=TRUE} | 0.18  3168 | 0.377  551 | 0.485  149 | 1.24  0086 | 74 |
| {BcMCV3.BI=FALSE, BcMV6.BI=TRUE} =>  {BcMV3.BI=TRUE} | 0.17  8218 | 0.685  714 | 0.259  901 | 1.26  4971 | 72 |
| {BcMCV3.BI=FALSE, BcMV3.BI=TRUE} =>  {BcMV6.BI=TRUE} | 0.17  8218 | 0.375 | 0.475  248 | 1.23  1707 | 72 |
| {BcMV6.BI=TRUE, BcPV4.BI=FALSE} =>  {BcMV3.BI=TRUE} | 0.20  297 | 0.732  143 | 0.277  228 | 1.35  062 | 82 |
| {BcMV3.BI=TRUE, BcPV4.BI=FALSE} =>  {BcMV6.BI=TRUE} | 0.20  297 | 0.407  96 | 0.497  525 | 1.33  9967 | 82 |
| {BcEV6=FALSE, BcMV6.BI=TRUE} =>  {BcMV3.BI=TRUE} | 0.20  7921 | 0.736  842 | 0.282  178 | 1.35  9289 | 84 |
| {BcEV6=FALSE, BcMV3.BI=TRUE} =>  {BcMV6.BI=TRUE} | 0.20  7921 | 0.401  914 | 0.517  327 | 1.32  0107 | 84 |
| {BcMV6.BI=TRUE, BcOLV17.BI=FALSE} =>  {BcMV3.BI=TRUE} | 0.19  802 | 0.714  286 | 0.277  228 | 1.31  7678 | 80 |
| {BcMV3.BI=TRUE, BcOLV17.BI=FALSE} => | 0.19 | 0.386 | 0.512 | 1.26 | 80 |

| {BcMV6.BI=TRUE} | 802 | 473 | 376 | 9392 |  |
| --- | --- | --- | --- | --- | --- |
| {BcMV6.BI=TRUE, BcOLV15.BI=FALSE} =>  {BcMV3.BI=TRUE} | 0.21  7822 | 0.727  273 | 0.299  505 | 1.34  1636 | 88 |
| {BcMV3.BI=TRUE, BcOLV15.BI=FALSE} =>  {BcMV6.BI=TRUE} | 0.21  7822 | 0.429  268 | 0.507  426 | 1.40  9954 | 88 |
| {BcEV5=FALSE, BcMV6.BI=TRUE} =>  {BcMV3.BI=TRUE} | 0.18  3168 | 0.691  589 | 0.264  851 | 1.27  5808 | 74 |
| {BcEV5=FALSE, BcMV3.BI=TRUE} =>  {BcMV6.BI=TRUE} | 0.18  3168 | 0.37 | 0.495  05 | 1.21  5285 | 74 |
| {BcMV4.BI=FALSE, BcMV6.BI=TRUE} =>  {BcHV1.BI=TRUE} | 0.09  1584 | 0.711  538 | 0.128  713 | 1.17  8121 | 37 |
| {BcMV4.BI=FALSE, BcMV6.BI=TRUE} =>  {BcEV2.BI=FALSE} | 0.11  1386 | 0.865  385 | 0.128  713 | 1.15  7667 | 45 |
| {BcMV4.BI=FALSE, BcMV6.BI=TRUE} =>  {BcFV5.BI=FALSE} | 0.11  6337 | 0.903  846 | 0.128  713 | 1.15  555 | 47 |
| {BcMV4.BI=FALSE, BcMV6.BI=TRUE} =>  {BcDMV1=FALSE} | 0.12  1287 | 0.942  308 | 0.128  713 | 1.11  64 | 49 |
| {BcMV4.BI=FALSE, BcMV6.BI=TRUE} =>  {BcFV9=FALSE} | 0.12  3762 | 0.961  538 | 0.128  713 | 1.10  6728 | 50 |
| {BcMV6.BI=TRUE, BcOLV16.BI=FALSE} =>  {BcHV1.BI=TRUE} | 0.09  901 | 0.714  286 | 0.138  614 | 1.18  267 | 40 |
| {BcMV6.BI=TRUE, BcOLV16.BI=FALSE} => | 0.10 | 0.732 | 0.138 | 1.18 | 41 |

| {BcMV11=TRUE} | 1485 | 143 | 614 | 7894 |  |
| --- | --- | --- | --- | --- | --- |
| {BcMV6.BI=TRUE, BcULV3=TRUE} =>  {BcHV1.BI=TRUE} | 0.16  3366 | 0.758  621 | 0.215  347 | 1.25  6077 | 66 |
| {BcHV1.BI=TRUE, BcMV6.BI=TRUE} =>  {BcULV3=TRUE} | 0.16  3366 | 0.717  391 | 0.227  723 | 1.20  7609 | 66 |
| {BcHV1.BI=TRUE, BcULV3=TRUE} =>  {BcMV6.BI=TRUE} | 0.16  3366 | 0.385  965 | 0.423  267 | 1.26  7722 | 66 |
| {BcMV6.BI=TRUE, BcULV3=TRUE} =>  {BcMV11=TRUE} | 0.17  0792 | 0.793  103 | 0.215  347 | 1.28  6802 | 69 |
| {BcMV11=TRUE, BcMV6.BI=TRUE} =>  {BcULV3=TRUE} | 0.17  0792 | 0.75 | 0.227  723 | 1.26  25 | 69 |
| {BcMV11=TRUE, BcULV3=TRUE} =>  {BcMV6.BI=TRUE} | 0.17  0792 | 0.431  25 | 0.396  04 | 1.41  6463 | 69 |
| {BcMV6.BI=TRUE, BcULV3=TRUE} =>  {BcMV9.BI=TRUE} | 0.16  5842 | 0.770  115 | 0.215  347 | 1.13  5498 | 67 |
| {BcMV6.BI=TRUE, BcMV9.BI=TRUE} =>  {BcULV3=TRUE} | 0.16  5842 | 0.705  263 | 0.235  149 | 1.18  7193 | 67 |
| {BcMV9.BI=TRUE, BcULV3=TRUE} =>  {BcMV6.BI=TRUE} | 0.16  5842 | 0.391  813 | 0.423  267 | 1.28  693 | 67 |
| {BcMV6.BI=TRUE, BcULV4=TRUE} =>  {BcULV3=TRUE} | 0.16  5842 | 0.683  673 | 0.242  574 | 1.15  085 | 67 |
| {BcULV3=TRUE, BcULV4=TRUE} => | 0.16 | 0.398 | 0.415 | 1.30 | 67 |

| {BcMV6.BI=TRUE} | 5842 | 81 | 842 | 9911 |  |
| --- | --- | --- | --- | --- | --- |
| {BcMV6.BI=TRUE, BcPV3.BI=FALSE} =>  {BcULV3=TRUE} | 0.14  3564 | 0.743  59 | 0.193  069 | 1.25  1709 | 58 |
| {BcMV6.BI=TRUE, BcRV1.BI=FALSE} =>  {BcULV3=TRUE} | 0.13  3663 | 0.701  299 | 0.190  594 | 1.18  0519 | 54 |
| {BcEV2.BI=FALSE, BcMV6.BI=TRUE} =>  {BcULV3=TRUE} | 0.16  3366 | 0.702  128 | 0.232  673 | 1.18  1915 | 66 |
| {BcEV2.BI=FALSE, BcULV3=TRUE} =>  {BcMV6.BI=TRUE} | 0.16  3366 | 0.375 | 0.435  644 | 1.23  1707 | 66 |
| {BcMV6.BI=TRUE, BcULV1.BI=FALSE} =>  {BcULV3=TRUE} | 0.15  5941 | 0.732  558 | 0.212  871 | 1.23  314 | 63 |
| {BcMV1.BI=TRUE, BcMV6.BI=TRUE} =>  {BcULV3=TRUE} | 0.17  8218 | 0.699  029 | 0.254  95 | 1.17  6699 | 72 |
| {BcMV1.BI=TRUE, BcULV3=TRUE} =>  {BcMV6.BI=TRUE} | 0.17  8218 | 0.367  347 | 0.485  149 | 1.20  657 | 72 |
| {BcMV12=FALSE, BcMV6.BI=TRUE} =>  {BcULV3=TRUE} | 0.15  099 | 0.693  182 | 0.217  822 | 1.16  6856 | 61 |
| {BcMV6.BI=TRUE, BcVV3.BI=FALSE} =>  {BcULV3=TRUE} | 0.17  0792 | 0.741  935 | 0.230  198 | 1.24  8925 | 69 |
| {BcMV6.BI=TRUE, BcOLV7.BI=FALSE} =>  {BcULV3=TRUE} | 0.16  8317 | 0.723  404 | 0.232  673 | 1.21  773 | 68 |
| {BcFV9=FALSE, BcMV6.BI=TRUE} => | 0.18 | 0.694 | 0.267 | 1.16 | 75 |

| {BcULV3=TRUE} | 5644 | 444 | 327 | 8981 |  |
| --- | --- | --- | --- | --- | --- |
| {BcMV6.BI=TRUE, BcOLV4.BI=FALSE} =>  {BcULV3=TRUE} | 0.17  8218 | 0.685  714 | 0.259  901 | 1.15  4286 | 72 |
| {BcMV6.BI=TRUE, BvF.BI=FALSE} =>  {BcULV3=TRUE} | 0.20  7921 | 0.717  949 | 0.289  604 | 1.20  8547 | 84 |
| {BcULV3=TRUE, BvF.BI=FALSE} =>  {BcMV6.BI=TRUE} | 0.20  7921 | 0.376  682 | 0.551  98 | 1.23  7231 | 84 |
| {BcMV6.BI=TRUE, BcOLV13.BI=FALSE} =>  {BcULV3=TRUE} | 0.18  8119 | 0.697  248 | 0.269  802 | 1.17  37 | 76 |
| {BcMV6.BI=TRUE, BcOLV2.BI=FALSE} =>  {BcULV3=TRUE} | 0.18  5644 | 0.707  547 | 0.262  376 | 1.19  1038 | 75 |
| {BcMV6.BI=TRUE, BcPV4.BI=FALSE} =>  {BcULV3=TRUE} | 0.19  3069 | 0.696  429 | 0.277  228 | 1.17  2321 | 78 |
| {BcEV6=FALSE, BcMV6.BI=TRUE} =>  {BcULV3=TRUE} | 0.20  0495 | 0.710  526 | 0.282  178 | 1.19  6053 | 81 |
| {BcMV6.BI=TRUE, BcOLV17.BI=FALSE} =>  {BcULV3=TRUE} | 0.19  3069 | 0.696  429 | 0.277  228 | 1.17  2321 | 78 |
| {BcMV6.BI=TRUE, BcOLV15.BI=FALSE} =>  {BcULV3=TRUE} | 0.21  2871 | 0.710  744 | 0.299  505 | 1.19  6419 | 86 |
| {BcOLV15.BI=FALSE, BcULV3=TRUE} =>  {BcMV6.BI=TRUE} | 0.21  2871 | 0.382  222 | 0.556  931 | 1.25  5429 | 86 |
| {BcHV1.BI=TRUE, BcMV6.BI=TRUE} => | 0.17 | 0.782 | 0.227 | 1.26 | 72 |

| {BcMV11=TRUE} | 8218 | 609 | 723 | 9775 |  |
| --- | --- | --- | --- | --- | --- |
| {BcMV11=TRUE, BcMV6.BI=TRUE} =>  {BcHV1.BI=TRUE} | 0.17  8218 | 0.782  609 | 0.227  723 | 1.29  5795 | 72 |
| {BcHV1.BI=TRUE, BcMV11=TRUE} =>  {BcMV6.BI=TRUE} | 0.17  8218 | 0.467  532 | 0.381  188 | 1.53  5635 | 72 |
| {BcHV1.BI=TRUE, BcMV6.BI=TRUE} =>  {BcMV9.BI=TRUE} | 0.19  3069 | 0.847  826 | 0.227  723 | 1.25  0079 | 78 |
| {BcMV6.BI=TRUE, BcMV9.BI=TRUE} =>  {BcHV1.BI=TRUE} | 0.19  3069 | 0.821  053 | 0.235  149 | 1.35  9448 | 78 |
| {BcHV1.BI=TRUE, BcMV9.BI=TRUE} =>  {BcMV6.BI=TRUE} | 0.19  3069 | 0.412  698 | 0.467  822 | 1.35  553 | 78 |
| {BcHV1.BI=TRUE, BcMV6.BI=TRUE} =>  {BcULV4=TRUE} | 0.19  3069 | 0.847  826 | 0.227  723 | 1.22  7676 | 78 |
| {BcMV6.BI=TRUE, BcULV4=TRUE} =>  {BcHV1.BI=TRUE} | 0.19  3069 | 0.795  918 | 0.242  574 | 1.31  7832 | 78 |
| {BcHV1.BI=TRUE, BcULV4=TRUE} =>  {BcMV6.BI=TRUE} | 0.19  3069 | 0.423  913 | 0.455  446 | 1.39  2365 | 78 |
| {BcMV6.BI=TRUE, BcPV3.BI=FALSE} =>  {BcHV1.BI=TRUE} | 0.13  8614 | 0.717  949 | 0.193  069 | 1.18  8735 | 56 |
| {BcEV2.BI=FALSE, BcMV6.BI=TRUE} =>  {BcHV1.BI=TRUE} | 0.16  8317 | 0.723  404 | 0.232  673 | 1.19  7768 | 68 |
| {BcEV2.BI=FALSE, BcHV1.BI=TRUE} => | 0.16 | 0.384 | 0.438 | 1.26 | 68 |

| {BcMV6.BI=TRUE} | 8317 | 181 | 119 | 1862 |  |
| --- | --- | --- | --- | --- | --- |
| {BcHV3.BI=FALSE, BcMV6.BI=TRUE} =>  {BcHV1.BI=TRUE} | 0.16  3366 | 0.741  573 | 0.220  297 | 1.22  785 | 66 |
| {BcHV1.BI=TRUE, BcHV3.BI=FALSE} =>  {BcMV6.BI=TRUE} | 0.16  3366 | 0.368  715 | 0.443  069 | 1.21  1064 | 66 |
| {BcFV5.BI=FALSE, BcMV6.BI=TRUE} =>  {BcHV1.BI=TRUE} | 0.17  5743 | 0.739  583 | 0.237  624 | 1.22  4556 | 71 |
| {BcFV5.BI=FALSE, BcHV1.BI=TRUE} =>  {BcMV6.BI=TRUE} | 0.17  5743 | 0.387  978 | 0.452  97 | 1.27  4335 | 71 |
| {BcMV1.BI=TRUE, BcMV6.BI=TRUE} =>  {BcHV1.BI=TRUE} | 0.19  3069 | 0.757  282 | 0.254  95 | 1.25  386 | 78 |
| {BcHV1.BI=TRUE, BcMV1.BI=TRUE} =>  {BcMV6.BI=TRUE} | 0.19  3069 | 0.4 | 0.482  673 | 1.31  3821 | 78 |
| {BcMV12=FALSE, BcMV6.BI=TRUE} =>  {BcHV1.BI=TRUE} | 0.15  8416 | 0.727  273 | 0.217  822 | 1.20  4173 | 64 |
| {BcMV6.BI=TRUE, BcNSRV7.BI=FALSE} =>  {BcHV1.BI=TRUE} | 0.17  3267 | 0.744  681 | 0.232  673 | 1.23  2996 | 70 |
| {BcHV1.BI=TRUE, BcNSRV7.BI=FALSE} =>  {BcMV6.BI=TRUE} | 0.17  3267 | 0.372  34 | 0.465  347 | 1.22  2972 | 70 |
| {BcMV6.BI=TRUE, BcVV3.BI=FALSE} =>  {BcHV1.BI=TRUE} | 0.17  8218 | 0.774  194 | 0.230  198 | 1.28  1861 | 72 |
| {BcMV6.BI=TRUE, BcOLV7.BI=FALSE} => | 0.16 | 0.723 | 0.232 | 1.19 | 68 |

| {BcHV1.BI=TRUE} | 8317 | 404 | 673 | 7768 |  |
| --- | --- | --- | --- | --- | --- |
| {BcALV1.BI=FALSE, BcMV6.BI=TRUE} =>  {BcHV1.BI=TRUE} | 0.15  5941 | 0.715  909 | 0.217  822 | 1.18  5358 | 63 |
| {BcDMV1=FALSE, BcMV6.BI=TRUE} =>  {BcHV1.BI=TRUE} | 0.17  8218 | 0.75 | 0.237  624 | 1.24  1803 | 72 |
| {BcFV9=FALSE, BcMV6.BI=TRUE} =>  {BcHV1.BI=TRUE} | 0.19  3069 | 0.722  222 | 0.267  327 | 1.19  5811 | 78 |
| {BcFV9=FALSE, BcHV1.BI=TRUE} =>  {BcMV6.BI=TRUE} | 0.19  3069 | 0.380  488 | 0.507  426 | 1.24  9732 | 78 |
| {BcMV6.BI=TRUE, BcOLV4.BI=FALSE} =>  {BcHV1.BI=TRUE} | 0.19  3069 | 0.742  857 | 0.259  901 | 1.22  9977 | 78 |
| {BcMV6.BI=TRUE, BcULV5=FALSE} =>  {BcHV1.BI=TRUE} | 0.19  802 | 0.776  699 | 0.254  95 | 1.28  601 | 80 |
| {BcHV1.BI=TRUE, BcULV5=FALSE} =>  {BcMV6.BI=TRUE} | 0.19  802 | 0.372  093 | 0.532  178 | 1.22  2159 | 80 |
| {BcBNV5.BI=FALSE, BcMV6.BI=TRUE} =>  {BcHV1.BI=TRUE} | 0.18  0693 | 0.737  374 | 0.245  05 | 1.22  0898 | 73 |
| {BcMV6.BI=TRUE, BvF.BI=FALSE} =>  {BcHV1.BI=TRUE} | 0.21  5347 | 0.743  59 | 0.289  604 | 1.23  119 | 87 |
| {BcHV1.BI=TRUE, BvF.BI=FALSE} =>  {BcMV6.BI=TRUE} | 0.21  5347 | 0.395  455 | 0.544  554 | 1.29  8891 | 87 |
| {BcMCV5.BI=FALSE, BcMV6.BI=TRUE} => | 0.19 | 0.733 | 0.259 | 1.21 | 77 |

| {BcHV1.BI=TRUE} | 0594 | 333 | 901 | 4208 |  |
| --- | --- | --- | --- | --- | --- |
| {BcMV6.BI=TRUE, BcOLV13.BI=FALSE} =>  {BcHV1.BI=TRUE} | 0.19  5545 | 0.724  771 | 0.269  802 | 1.20  003 | 79 |
| {BcMV6.BI=TRUE, BcOLV2.BI=FALSE} =>  {BcHV1.BI=TRUE} | 0.19  3069 | 0.735  849 | 0.262  376 | 1.21  8373 | 78 |
| {BcMCV3.BI=FALSE, BcMV6.BI=TRUE} =>  {BcHV1.BI=TRUE} | 0.19  0594 | 0.733  333 | 0.259  901 | 1.21  4208 | 77 |
| {BcMV6.BI=TRUE, BcPV4.BI=FALSE} =>  {BcHV1.BI=TRUE} | 0.20  7921 | 0.75 | 0.277  228 | 1.24  1803 | 84 |
| {BcHV1.BI=TRUE, BcPV4.BI=FALSE} =>  {BcMV6.BI=TRUE} | 0.20  7921 | 0.371  681 | 0.559  406 | 1.22  0807 | 84 |
| {BcEV6=FALSE, BcMV6.BI=TRUE} =>  {BcHV1.BI=TRUE} | 0.21  0396 | 0.745  614 | 0.282  178 | 1.23  4541 | 85 |
| {BcEV6=FALSE, BcHV1.BI=TRUE} =>  {BcMV6.BI=TRUE} | 0.21  0396 | 0.382  883 | 0.549  505 | 1.25  7599 | 85 |
| {BcMV6.BI=TRUE, BcOLV17.BI=FALSE} =>  {BcHV1.BI=TRUE} | 0.20  5446 | 0.741  071 | 0.277  228 | 1.22  702 | 83 |
| {BcHV1.BI=TRUE, BcOLV17.BI=FALSE} =>  {BcMV6.BI=TRUE} | 0.20  5446 | 0.364  035 | 0.564  356 | 1.19  5692 | 83 |
| {BcMV6.BI=TRUE, BcOLV15.BI=FALSE} =>  {BcHV1.BI=TRUE} | 0.22  5248 | 0.752  066 | 0.299  505 | 1.24  5224 | 91 |
| {BcHV1.BI=TRUE, BcOLV15.BI=FALSE} => | 0.22 | 0.399 | 0.564 | 1.31 | 91 |

| {BcMV6.BI=TRUE} | 5248 | 123 | 356 | 094 |  |
| --- | --- | --- | --- | --- | --- |
| {BcEV5=FALSE, BcMV6.BI=TRUE} =>  {BcHV1.BI=TRUE} | 0.20  0495 | 0.757  009 | 0.264  851 | 1.25  3409 | 81 |
| {BcMV11=TRUE, BcMV6.BI=TRUE} =>  {BcMV9.BI=TRUE} | 0.18  0693 | 0.793  478 | 0.227  723 | 1.16  9946 | 73 |
| {BcMV6.BI=TRUE, BcMV9.BI=TRUE} =>  {BcMV11=TRUE} | 0.18  0693 | 0.768  421 | 0.235  149 | 1.24  6755 | 73 |
| {BcMV11=TRUE, BcMV9.BI=TRUE} =>  {BcMV6.BI=TRUE} | 0.18  0693 | 0.445  122 | 0.405  941 | 1.46  2027 | 73 |
| {BcMV11=TRUE, BcMV6.BI=TRUE} =>  {BcULV4=TRUE} | 0.18  8119 | 0.826  087 | 0.227  723 | 1.19  6198 | 76 |
| {BcMV6.BI=TRUE, BcULV4=TRUE} =>  {BcMV11=TRUE} | 0.18  8119 | 0.775  51 | 0.242  574 | 1.25  8258 | 76 |
| {BcMV11=TRUE, BcULV4=TRUE} =>  {BcMV6.BI=TRUE} | 0.18  8119 | 0.449  704 | 0.418  317 | 1.47  7077 | 76 |
| {BcEV2.BI=FALSE, BcMV6.BI=TRUE} =>  {BcMV11=TRUE} | 0.17  3267 | 0.744  681 | 0.232  673 | 1.20  8237 | 70 |
| {BcEV2.BI=FALSE, BcMV11=TRUE} =>  {BcMV6.BI=TRUE} | 0.17  3267 | 0.402  299 | 0.430  693 | 1.32  1372 | 70 |
| {BcFV5.BI=FALSE, BcMV6.BI=TRUE} =>  {BcMV11=TRUE} | 0.16  8317 | 0.708  333 | 0.237  624 | 1.14  9264 | 68 |
| {BcMV6.BI=TRUE, BcULV1.BI=FALSE} => | 0.15 | 0.720 | 0.212 | 1.16 | 62 |

| {BcMV11=TRUE} | 3465 | 93 | 871 | 9702 |  |
| --- | --- | --- | --- | --- | --- |
| {BcMV11=TRUE, BcMV6.BI=TRUE} =>  {BcMV1.BI=TRUE} | 0.20  0495 | 0.880  435 | 0.227  723 | 1.11  5033 | 81 |
| {BcMV1.BI=TRUE, BcMV6.BI=TRUE} =>  {BcMV11=TRUE} | 0.20  0495 | 0.786  408 | 0.254  95 | 1.27  5939 | 81 |
| {BcMV1.BI=TRUE, BcMV11=TRUE} =>  {BcMV6.BI=TRUE} | 0.20  0495 | 0.385  714 | 0.519  802 | 1.26  6899 | 81 |
| {BcMV12=FALSE, BcMV6.BI=TRUE} =>  {BcMV11=TRUE} | 0.17  0792 | 0.784  091 | 0.217  822 | 1.27  218 | 69 |
| {BcMV6.BI=TRUE, BcVV3.BI=FALSE} =>  {BcMV11=TRUE} | 0.16  8317 | 0.731  183 | 0.230  198 | 1.18  6337 | 68 |
| {BcMV6.BI=TRUE, BcOLV7.BI=FALSE} =>  {BcMV11=TRUE} | 0.16  5842 | 0.712  766 | 0.232  673 | 1.15  6456 | 67 |
| {BcFV9=FALSE, BcMV6.BI=TRUE} =>  {BcMV11=TRUE} | 0.19  3069 | 0.722  222 | 0.267  327 | 1.17  1798 | 78 |
| {BcFV9=FALSE, BcMV11=TRUE} =>  {BcMV6.BI=TRUE} | 0.19  3069 | 0.369  668 | 0.522  277 | 1.21  4195 | 78 |
| {BcMV6.BI=TRUE, BcOLV4.BI=FALSE} =>  {BcMV11=TRUE} | 0.19  3069 | 0.742  857 | 0.259  901 | 1.20  5278 | 78 |
| {BcMV11=TRUE, BcOLV4.BI=FALSE} =>  {BcMV6.BI=TRUE} | 0.19  3069 | 0.376  812 | 0.512  376 | 1.23  7658 | 78 |
| {BcMV6.BI=TRUE, BcULV5=FALSE} => | 0.18 | 0.718 | 0.254 | 1.16 | 74 |

| {BcMV11=TRUE} | 3168 | 447 | 95 | 5672 |  |
| --- | --- | --- | --- | --- | --- |
| {BcBNV5.BI=FALSE, BcMV6.BI=TRUE} =>  {BcMV11=TRUE} | 0.17  3267 | 0.707  071 | 0.245  05 | 1.14  7215 | 70 |
| {BcMV6.BI=TRUE, BvF.BI=FALSE} =>  {BcMV11=TRUE} | 0.22  0297 | 0.760  684 | 0.289  604 | 1.23  4202 | 89 |
| {BcMV11=TRUE, BvF.BI=FALSE} =>  {BcMV6.BI=TRUE} | 0.22  0297 | 0.385  281 | 0.571  782 | 1.26  5477 | 89 |
| {BcMCV5.BI=FALSE, BcMV6.BI=TRUE} =>  {BcMV11=TRUE} | 0.18  5644 | 0.714  286 | 0.259  901 | 1.15  8921 | 75 |
| {BcMV6.BI=TRUE, BcOLV13.BI=FALSE} =>  {BcMV11=TRUE} | 0.19  802 | 0.733  945 | 0.269  802 | 1.19  0818 | 80 |
| {BcMV6.BI=TRUE, BcOLV2.BI=FALSE} =>  {BcMV11=TRUE} | 0.19  802 | 0.754  717 | 0.262  376 | 1.22  4521 | 80 |
| {BcMCV3.BI=FALSE, BcMV6.BI=TRUE} =>  {BcMV11=TRUE} | 0.20  0495 | 0.771  429 | 0.259  901 | 1.25  1635 | 81 |
| {BcMV6.BI=TRUE, BcPV4.BI=FALSE} =>  {BcMV11=TRUE} | 0.20  7921 | 0.75 | 0.277  228 | 1.21  6867 | 84 |
| {BcEV6=FALSE, BcMV6.BI=TRUE} =>  {BcMV11=TRUE} | 0.21  0396 | 0.745  614 | 0.282  178 | 1.20  9751 | 85 |
| {BcEV6=FALSE, BcMV11=TRUE} =>  {BcMV6.BI=TRUE} | 0.21  0396 | 0.364  807 | 0.576  733 | 1.19  8227 | 85 |
| {BcMV6.BI=TRUE, BcOLV17.BI=FALSE} => | 0.20 | 0.723 | 0.277 | 1.17 | 81 |

| {BcMV11=TRUE} | 0495 | 214 | 228 | 3408 |  |
| --- | --- | --- | --- | --- | --- |
| {BcMV6.BI=TRUE, BcOLV15.BI=FALSE} =>  {BcMV11=TRUE} | 0.22  5248 | 0.752  066 | 0.299  505 | 1.22  022 | 91 |
| {BcMV11=TRUE, BcOLV15.BI=FALSE} =>  {BcMV6.BI=TRUE} | 0.22  5248 | 0.382  353 | 0.589  109 | 1.25  5858 | 91 |
| {BcEV5=FALSE, BcMV6.BI=TRUE} =>  {BcMV11=TRUE} | 0.18  8119 | 0.710  28 | 0.264  851 | 1.15  2423 | 76 |
| {BcMV6.BI=TRUE, BcOLV1.BI.MOVA.=FALSE} =>  {BcMV9.BI=TRUE} | 0.11  1386 | 0.789  474 | 0.141  089 | 1.16  4041 | 45 |
| {BcMV6.BI=TRUE, BcOLV1.BI.MOVA.=FALSE} =>  {BcFV5.BI=FALSE} | 0.12  6238 | 0.894  737 | 0.141  089 | 1.14  3904 | 51 |
| {BcMV6.BI=TRUE, BcMV9.BI=TRUE} =>  {BcULV4=TRUE} | 0.20  7921 | 0.884  211 | 0.235  149 | 1.28  0362 | 84 |
| {BcMV6.BI=TRUE, BcULV4=TRUE} =>  {BcMV9.BI=TRUE} | 0.20  7921 | 0.857  143 | 0.242  574 | 1.26  3816 | 84 |
| {BcMV9.BI=TRUE, BcULV4=TRUE} =>  {BcMV6.BI=TRUE} | 0.20  7921 | 0.380  09 | 0.547  03 | 1.24  8427 | 84 |
| {BcMV1.BI=TRUE, BcMV6.BI=TRUE} =>  {BcMV9.BI=TRUE} | 0.19  5545 | 0.766  99 | 0.254  95 | 1.13  0891 | 79 |
| {BcMV12=FALSE, BcMV6.BI=TRUE} => | 0.17 | 0.806 | 0.217 | 1.18 | 71 |

| {BcMV9.BI=TRUE} | 5743 | 818 | 822 | 9615 |  |
| --- | --- | --- | --- | --- | --- |
| {BcMV6.BI=TRUE, BcOLV4.BI=FALSE} =>  {BcMV9.BI=TRUE} | 0.21  2871 | 0.819  048 | 0.259  901 | 1.20  7647 | 86 |
| {BcMV6.BI=TRUE, BcMV9.BI=TRUE} =>  {BcULV5=FALSE} | 0.22  5248 | 0.957  895 | 0.235  149 | 1.09  3191 | 91 |
| {BcMV6.BI=TRUE, BcULV5=FALSE} =>  {BcMV9.BI=TRUE} | 0.22  5248 | 0.883  495 | 0.254  95 | 1.30  2672 | 91 |
| {BcMV6.BI=TRUE, BvF.BI=FALSE} =>  {BcMV9.BI=TRUE} | 0.22  2772 | 0.769  231 | 0.289  604 | 1.13  4194 | 90 |
| {BcMV9.BI=TRUE, BvF.BI=FALSE} =>  {BcMV6.BI=TRUE} | 0.22  2772 | 0.364  372 | 0.611  386 | 1.19  6801 | 90 |
| {BcMV6.BI=TRUE, BcOLV13.BI=FALSE} =>  {BcMV9.BI=TRUE} | 0.21  2871 | 0.788  991 | 0.269  802 | 1.16  333 | 86 |
| {BcMCV3.BI=FALSE, BcMV6.BI=TRUE} =>  {BcMV9.BI=TRUE} | 0.20  5446 | 0.790  476 | 0.259  901 | 1.16  552 | 83 |
| {BcMV6.BI=TRUE, BcPV4.BI=FALSE} =>  {BcMV9.BI=TRUE} | 0.21  5347 | 0.776  786 | 0.277  228 | 1.14  5334 | 87 |
| {BcEV6=FALSE, BcMV6.BI=TRUE} =>  {BcMV9.BI=TRUE} | 0.21  7822 | 0.771  93 | 0.282  178 | 1.13  8174 | 88 |
| {BcMV6.BI=TRUE, BcOLV15.BI=FALSE} =>  {BcMV9.BI=TRUE} | 0.23  2673 | 0.776  86 | 0.299  505 | 1.14  5442 | 94 |
| {BcMV9.BI=TRUE, BcOLV15.BI=FALSE} => | 0.23 | 0.362 | 0.641 | 1.19 | 94 |

| {BcMV6.BI=TRUE} | 2673 | 934 | 089 | 2077 |  |
| --- | --- | --- | --- | --- | --- |
| {BcEV2.BI=FALSE, BcMV6.BI=TRUE} =>  {BcULV4=TRUE} | 0.18  3168 | 0.787  234 | 0.232  673 | 1.13  9937 | 74 |
| {BcFV5.BI=FALSE, BcMV6.BI=TRUE} =>  {BcULV4=TRUE} | 0.18  5644 | 0.781  25 | 0.237  624 | 1.13  1272 | 75 |
| {BcMV1.BI=TRUE, BcMV6.BI=TRUE} =>  {BcULV4=TRUE} | 0.20  5446 | 0.805  825 | 0.254  95 | 1.16  6858 | 83 |
| {BcMV1.BI=TRUE, BcULV4=TRUE} =>  {BcMV6.BI=TRUE} | 0.20  5446 | 0.373  874 | 0.549  505 | 1.22  8008 | 83 |
| {BcMV12=FALSE, BcMV6.BI=TRUE} =>  {BcULV4=TRUE} | 0.17  5743 | 0.806  818 | 0.217  822 | 1.16  8296 | 71 |
| {BcDMV1=FALSE, BcMV6.BI=TRUE} =>  {BcULV4=TRUE} | 0.18  8119 | 0.791  667 | 0.237  624 | 1.14  6356 | 76 |
| {BcFV9=FALSE, BcMV6.BI=TRUE} =>  {BcULV4=TRUE} | 0.21  5347 | 0.805  556 | 0.267  327 | 1.16  6468 | 87 |
| {BcFV9=FALSE, BcULV4=TRUE} =>  {BcMV6.BI=TRUE} | 0.21  5347 | 0.360  996 | 0.596  535 | 1.18  571 | 87 |
| {BcMV6.BI=TRUE, BcOLV4.BI=FALSE} =>  {BcULV4=TRUE} | 0.21  2871 | 0.819  048 | 0.259  901 | 1.18  6004 | 86 |
| {BcMV6.BI=TRUE, BcULV5=FALSE} =>  {BcULV4=TRUE} | 0.21  5347 | 0.844  66 | 0.254  95 | 1.22  3092 | 87 |
| {BcMV6.BI=TRUE, BvF.BI=FALSE} => | 0.23 | 0.794 | 0.289 | 1.15 | 93 |

| {BcULV4=TRUE} | 0198 | 872 | 604 | 0997 |  |
| --- | --- | --- | --- | --- | --- |
| {BcMCV5.BI=FALSE, BcMV6.BI=TRUE} =>  {BcULV4=TRUE} | 0.20  297 | 0.780  952 | 0.259  901 | 1.13  0841 | 82 |
| {BcMV6.BI=TRUE, BcOLV13.BI=FALSE} =>  {BcULV4=TRUE} | 0.21  5347 | 0.798  165 | 0.269  802 | 1.15  5766 | 87 |
| {BcMV6.BI=TRUE, BcOLV2.BI=FALSE} =>  {BcULV4=TRUE} | 0.20  7921 | 0.792  453 | 0.262  376 | 1.14  7494 | 84 |
| {BcMCV3.BI=FALSE, BcMV6.BI=TRUE} =>  {BcULV4=TRUE} | 0.21  0396 | 0.809  524 | 0.259  901 | 1.17  2214 | 85 |
| {BcMV6.BI=TRUE, BcPV4.BI=FALSE} =>  {BcULV4=TRUE} | 0.22  5248 | 0.812  5 | 0.277  228 | 1.17  6523 | 91 |
| {BcEV6=FALSE, BcMV6.BI=TRUE} =>  {BcULV4=TRUE} | 0.22  5248 | 0.798  246 | 0.282  178 | 1.15  5883 | 91 |
| {BcMV6.BI=TRUE, BcOLV15.BI=FALSE} =>  {BcULV4=TRUE} | 0.23  7624 | 0.793  388 | 0.299  505 | 1.14  8849 | 96 |
| {BcOLV15.BI=FALSE, BcULV4=TRUE} =>  {BcMV6.BI=TRUE} | 0.23  7624 | 0.360  902 | 0.658  416 | 1.18  5403 | 96 |
| {BcEV5=FALSE, BcMV6.BI=TRUE} =>  {BcULV4=TRUE} | 0.21  5347 | 0.813  084 | 0.264  851 | 1.17  7369 | 87 |
| {BcMV6.BI=TRUE, BcRV1.BI=FALSE} =>  {BcOLV7.BI=FALSE} | 0.17  8218 | 0.935  065 | 0.190  594 | 1.15  8792 | 72 |
| {BcFV5.BI=FALSE, BcMV6.BI=TRUE} => | 0.19 | 0.833 | 0.237 | 1.11 | 80 |

| {BcEV2.BI=FALSE} | 802 | 333 | 624 | 479 |  |
| --- | --- | --- | --- | --- | --- |
| {BcEV2.BI=FALSE, BcMV6.BI=TRUE} =>  {BvF.BI=FALSE} | 0.23  0198 | 0.989  362 | 0.232  673 | 1.08  3204 | 93 |
| {BcHV3.BI=FALSE, BcMV6.BI=TRUE} =>  {BcFV5.BI=FALSE} | 0.19  802 | 0.898  876 | 0.220  297 | 1.14  9196 | 80 |
| {BcDMV1=FALSE, BcMV6.BI=TRUE} =>  {BcFV5.BI=FALSE} | 0.21  0396 | 0.885  417 | 0.237  624 | 1.13  1988 | 85 |
| {BcBNV5.BI=FALSE, BcMV6.BI=TRUE} =>  {BcFV5.BI=FALSE} | 0.21  5347 | 0.878  788 | 0.245  05 | 1.12  3514 | 87 |
| {BcMCV5.BI=FALSE, BcMV6.BI=TRUE} =>  {BcFV5.BI=FALSE} | 0.22  5248 | 0.866  667 | 0.259  901 | 1.10  8017 | 91 |
| {BcEV5=FALSE, BcMV6.BI=TRUE} =>  {BcFV5.BI=FALSE} | 0.23  0198 | 0.869  159 | 0.264  851 | 1.11  1203 | 93 |
| {BcDMV1=FALSE, BcMV6.BI=TRUE} =>  {BcFV9=FALSE} | 0.22  2772 | 0.937  5 | 0.237  624 | 1.07  906 | 90 |
| {BcMV9.BI=FALSE, BcULV4=FALSE} =>  {BcHV1.BI=FALSE} | 0.11  1386 | 0.625 | 0.178  218 | 1.57  8125 | 45 |
| {BcHV1.BI=FALSE, BcULV4=FALSE} =>  {BcMV9.BI=FALSE} | 0.11  1386 | 0.692  308 | 0.160  891 | 2.15  1479 | 45 |
| {BcHV1.BI=FALSE, BcMV9.BI=FALSE} =>  {BcULV4=FALSE} | 0.11  1386 | 0.6 | 0.185  644 | 1.93  92 | 45 |
| {BcULV3=FALSE, BcULV4=FALSE} => | 0.07 | 0.584 | 0.131 | 1.81 | 31 |

| {BcMV9.BI=FALSE} | 6733 | 906 | 188 | 7707 |  |
| --- | --- | --- | --- | --- | --- |
| {BcMV9.BI=FALSE, BcULV3=FALSE} =>  {BcULV4=FALSE} | 0.07  6733 | 0.508  197 | 0.150  99 | 1.64  2492 | 31 |
| {BcMV9.BI=FALSE, BcULV4=FALSE} =>  {BcHV1SLR.BI=FALSE} | 0.11  3861 | 0.638  889 | 0.178  218 | 1.39  5195 | 46 |
| {BcHV1SLR.BI=FALSE, BcULV4=FALSE} =>  {BcMV9.BI=FALSE} | 0.11  3861 | 0.676  471 | 0.168  317 | 2.10  2262 | 46 |
| {BcHV1SLR.BI=FALSE, BcMV9.BI=FALSE}  => {BcULV4=FALSE} | 0.11  3861 | 0.605  263 | 0.188  119 | 1.95  6211 | 46 |
| {BcMV9.BI=FALSE, BcULV4=FALSE} =>  {BcMV3.BI=FALSE} | 0.10  6436 | 0.597  222 | 0.178  218 | 1.30  4204 | 43 |
| {BcMV3.BI=FALSE, BcULV4=FALSE} =>  {BcMV9.BI=FALSE} | 0.10  6436 | 0.632  353 | 0.168  317 | 1.96  5158 | 43 |
| {BcMV3.BI=FALSE, BcMV9.BI=FALSE} =>  {BcULV4=FALSE} | 0.10  6436 | 0.605  634 | 0.175  743 | 1.95  7408 | 43 |
| {BcMV2.BI=TRUE, BcULV4=FALSE} =>  {BcMV9.BI=FALSE} | 0.07  9208 | 0.653  061 | 0.121  287 | 2.02  9513 | 32 |
| {BcMV2.BI=TRUE, BcMV9.BI=FALSE} =>  {BcULV4=FALSE} | 0.07  9208 | 0.561  404 | 0.141  089 | 1.81  4456 | 32 |
| {BcMV9.BI=FALSE, BcULV4=FALSE} =>  {BcOLV18=FALSE} | 0.13  8614 | 0.777  778 | 0.178  218 | 1.61  1396 | 56 |
| {BcOLV18=FALSE, BcULV4=FALSE} => | 0.13 | 0.595 | 0.232 | 1.85 | 56 |

| {BcMV9.BI=FALSE} | 8614 | 745 | 673 | 1391 |  |
| --- | --- | --- | --- | --- | --- |
| {BcMV9.BI=FALSE, BcOLV18=FALSE} =>  {BcULV4=FALSE} | 0.13  8614 | 0.636  364 | 0.217  822 | 2.05  6727 | 56 |
| {BcMV2.BI=FALSE, BcULV4=FALSE} =>  {BcMV9.BI=FALSE} | 0.09  901 | 0.526  316 | 0.188  119 | 1.63  5628 | 40 |
| {BcMV2.BI=FALSE, BcMV9.BI=FALSE} =>  {BcULV4=FALSE} | 0.09  901 | 0.547  945 | 0.180  693 | 1.77  0959 | 40 |
| {BcMV4.BI=FALSE, BcULV4=FALSE} =>  {BcMV9.BI=FALSE} | 0.11  8812 | 0.623  377 | 0.190  594 | 1.93  7263 | 48 |
| {BcMV4.BI=FALSE, BcMV9.BI=FALSE} =>  {BcULV4=FALSE} | 0.11  8812 | 0.551  724 | 0.215  347 | 1.78  3172 | 48 |
| {BcMV9.BI=FALSE, BcULV4=FALSE} =>  {BcOLV16.BI=FALSE} | 0.13  3663 | 0.75 | 0.178  218 | 1.28  3898 | 54 |
| {BcOLV16.BI=FALSE, BcULV4=FALSE} =>  {BcMV9.BI=FALSE} | 0.13  3663 | 0.574  468 | 0.232  673 | 1.78  527 | 54 |
| {BcMV9.BI=FALSE, BcOLV16.BI=FALSE} =>  {BcULV4=FALSE} | 0.13  3663 | 0.562  5 | 0.237  624 | 1.81  8 | 54 |
| {BcULV3=TRUE, BcULV4=FALSE} =>  {BcMV9.BI=FALSE} | 0.10  1485 | 0.569  444 | 0.178  218 | 1.76  9658 | 41 |
| {BcMV9.BI=FALSE, BcULV3=TRUE} =>  {BcULV4=FALSE} | 0.10  1485 | 0.594  203 | 0.170  792 | 1.92  0464 | 41 |
| {BcMV9.BI=FALSE, BcULV4=FALSE} => | 0.12 | 0.722 | 0.178 | 1.17 | 52 |

| {BcMV11=TRUE} | 8713 | 222 | 218 | 1798 |  |
| --- | --- | --- | --- | --- | --- |
| {BcMV11=TRUE, BcULV4=FALSE} => | 0.12 |  | 0.198 |  |  |
|  |  | 0.65 |  | 2.02 | 52 |
| {BcMV9.BI=FALSE} | 8713 |  | 02 |  |  |
| {BcMV11=TRUE, BcMV9.BI=FALSE} => | 0.12 | 0.611 | 0.210 | 1.97 |  |
|  |  |  |  |  | 52 |
| {BcULV4=FALSE} | 8713 | 765 | 396 | 7224 |  |
| {BcOLV1.BI.MOVA.=FALSE, BcULV4=FALSE} | 0.10 | 0.488 | 0.222 | 1.51 |  |
|  |  |  |  |  | 44 |
| => {BcMV9.BI=FALSE} | 8911 | 889 | 772 | 9316 |  |
| {BcMV9.BI=FALSE, |  |  |  |  |  |
|  | 0.10 | 0.536 | 0.202 | 1.73 |  |
| BcOLV1.BI.MOVA.=FALSE} => |  |  |  |  | 44 |
|  | 8911 | 585 | 97 | 4244 |  |
| {BcULV4=FALSE} |  |  |  |  |  |
| {BcMV9.BI=FALSE, BcULV4=FALSE} => | 0.14 | 0.805 | 0.178 | 1.15 |  |
|  |  |  |  |  | 58 |
| {BcMV6.BI=FALSE} | 3564 | 556 | 218 | 8165 |  |
| {BcMV6.BI=FALSE, BcULV4=FALSE} => | 0.14 |  | 0.247 | 1.80 |  |
|  |  | 0.58 |  |  | 58 |
| {BcMV9.BI=FALSE} | 3564 |  | 525 | 2462 |  |
| {BcMV6.BI=FALSE, BcMV9.BI=FALSE} => | 0.14 | 0.568 | 0.252 | 1.83 |  |
|  |  |  |  |  | 58 |
| {BcULV4=FALSE} | 3564 | 627 | 475 | 7804 |  |
| {BcPV3.BI=FALSE, BcULV4=FALSE} => | 0.12 | 0.560 | 0.225 | 1.74 |  |
|  |  |  |  |  | 51 |
| {BcMV9.BI=FALSE} | 6238 | 44 | 248 | 1674 |  |
| {BcMV9.BI=FALSE, BcPV3.BI=FALSE} => | 0.12 | 0.531 | 0.237 | 1.71 |  |
|  |  |  |  |  | 51 |
| {BcULV4=FALSE} | 6238 | 25 | 624 | 7 |  |
| {BcMV9.BI=FALSE, BcULV4=FALSE} => | 0.15 | 0.861 | 0.178 | 1.19 |  |
|  |  |  |  |  | 62 |
| {BcRV1.BI=FALSE} | 3465 | 111 | 218 | 14 |  |

| {BcRV1.BI=FALSE, BcULV4=FALSE} =>  {BcMV9.BI=FALSE} | 0.15  3465 | 0.558  559 | 0.274  752 | 1.73  5828 | 62 |
| --- | --- | --- | --- | --- | --- |
| {BcMV9.BI=FALSE, BcRV1.BI=FALSE} =>  {BcULV4=FALSE} | 0.15  3465 | 0.574  074 | 0.267  327 | 1.85  5407 | 62 |
| {BcEV2.BI=FALSE, BcULV4=FALSE} =>  {BcMV9.BI=FALSE} | 0.13  1188 | 0.602  273 | 0.217  822 | 1.87  1678 | 53 |
| {BcEV2.BI=FALSE, BcMV9.BI=FALSE} =>  {BcULV4=FALSE} | 0.13  1188 | 0.540  816 | 0.242  574 | 1.74  7918 | 53 |
| {BcHV3.BI=FALSE, BcULV4=FALSE} =>  {BcMV9.BI=FALSE} | 0.15  099 | 0.616  162 | 0.245  05 | 1.91  4841 | 61 |
| {BcHV3.BI=FALSE, BcMV9.BI=FALSE} =>  {BcULV4=FALSE} | 0.15  099 | 0.592  233 | 0.254  95 | 1.91  4097 | 61 |
| {BcMV9.BI=FALSE, BcULV4=FALSE} =>  {BcFV5.BI=FALSE} | 0.16  0891 | 0.902  778 | 0.178  218 | 1.15  4184 | 65 |
| {BcFV5.BI=FALSE, BcULV4=FALSE} =>  {BcMV9.BI=FALSE} | 0.16  0891 | 0.637  255 | 0.252  475 | 1.98  0392 | 65 |
| {BcFV5.BI=FALSE, BcMV9.BI=FALSE} =>  {BcULV4=FALSE} | 0.16  0891 | 0.596  33 | 0.269  802 | 1.92  7339 | 65 |
| {BcMV9.BI=FALSE, BcULV4=FALSE} =>  {BcULV1.BI=FALSE} | 0.15  8416 | 0.888  889 | 0.178  218 | 1.13  2843 | 64 |
| {BcULV1.BI=FALSE, BcULV4=FALSE} =>  {BcMV9.BI=FALSE} | 0.15  8416 | 0.551  724 | 0.287  129 | 1.71  4589 | 64 |

| {BcMV9.BI=FALSE, BcULV1.BI=FALSE} =>  {BcULV4=FALSE} | 0.15  8416 | 0.556  522 | 0.284  653 | 1.79  8678 | 64 |
| --- | --- | --- | --- | --- | --- |
| {BcMV1.BI=TRUE, BcULV4=FALSE} =>  {BcMV9.BI=FALSE} | 0.13  6139 | 0.567  01 | 0.240  099 | 1.76  2094 | 55 |
| {BcMV1.BI=TRUE, BcMV9.BI=FALSE} =>  {BcULV4=FALSE} | 0.13  6139 | 0.567  01 | 0.240  099 | 1.83  2577 | 55 |
| {BcMV12=FALSE, BcULV4=FALSE} =>  {BcMV9.BI=FALSE} | 0.14  3564 | 0.574  257 | 0.25 | 1.78  4615 | 58 |
| {BcMV12=FALSE, BcMV9.BI=FALSE} =>  {BcULV4=FALSE} | 0.14  3564 | 0.552  381 | 0.259  901 | 1.78  5295 | 58 |
| {BcMV9.BI=FALSE, BcULV4=FALSE} =>  {BcNSRV7.BI=FALSE} | 0.16  0891 | 0.902  778 | 0.178  218 | 1.12  9171 | 65 |
| {BcNSRV7.BI=FALSE, BcULV4=FALSE} =>  {BcMV9.BI=FALSE} | 0.16  0891 | 0.607  477 | 0.264  851 | 1.88  785 | 65 |
| {BcMV9.BI=FALSE, BcNSRV7.BI=FALSE} =>  {BcULV4=FALSE} | 0.16  0891 | 0.570  175 | 0.282  178 | 1.84  2807 | 65 |
| {BcULV4=FALSE, BcVV3.BI=FALSE} =>  {BcMV9.BI=FALSE} | 0.13  1188 | 0.514  563 | 0.254  95 | 1.59  9104 | 53 |
| {BcMV9.BI=FALSE, BcVV3.BI=FALSE} =>  {BcULV4=FALSE} | 0.13  1188 | 0.53 | 0.247  525 | 1.71  296 | 53 |
| {BcMV9.BI=FALSE, BcULV4=FALSE} =>  {BcOLV7.BI=FALSE} | 0.17  3267 | 0.972  222 | 0.178  218 | 1.20  484 | 70 |

| {BcOLV7.BI=FALSE, BcULV4=FALSE} =>  {BcMV9.BI=FALSE} | 0.17  3267 | 0.588  235 | 0.294  554 | 1.82  8054 | 70 |
| --- | --- | --- | --- | --- | --- |
| {BcMV9.BI=FALSE, BcOLV7.BI=FALSE} =>  {BcULV4=FALSE} | 0.17  3267 | 0.578  512 | 0.299  505 | 1.86  9752 | 70 |
| {BcMV9.BI=FALSE, BcULV4=FALSE} =>  {BcALV1.BI=FALSE} | 0.16  0891 | 0.902  778 | 0.178  218 | 1.10  8578 | 65 |
| {BcALV1.BI=FALSE, BcULV4=FALSE} =>  {BcMV9.BI=FALSE} | 0.16  0891 | 0.565  217 | 0.284  653 | 1.75  6522 | 65 |
| {BcALV1.BI=FALSE, BcMV9.BI=FALSE} =>  {BcULV4=FALSE} | 0.16  0891 | 0.590  909 | 0.272  277 | 1.90  9818 | 65 |
| {BcMV9.BI=FALSE, BcULV4=FALSE} =>  {BcDMV1=FALSE} | 0.17  3267 | 0.972  222 | 0.178  218 | 1.15  1841 | 70 |
| {BcDMV1=FALSE, BcULV4=FALSE} =>  {BcMV9.BI=FALSE} | 0.17  3267 | 0.636  364 | 0.272  277 | 1.97  7622 | 70 |
| {BcDMV1=FALSE, BcMV9.BI=FALSE} =>  {BcULV4=FALSE} | 0.17  3267 | 0.593  22 | 0.292  079 | 1.91  7288 | 70 |
| {BcFV9=FALSE, BcULV4=FALSE} =>  {BcMV9.BI=FALSE} | 0.15  8416 | 0.581  818 | 0.272  277 | 1.80  8112 | 64 |
| {BcFV9=FALSE, BcMV9.BI=FALSE} =>  {BcULV4=FALSE} | 0.15  8416 | 0.556  522 | 0.284  653 | 1.79  8678 | 64 |
| {BcOLV4.BI=FALSE, BcULV4=FALSE} =>  {BcMV9.BI=FALSE} | 0.14  3564 | 0.563  107 | 0.254  95 | 1.74  9963 | 58 |

| {BcMV9.BI=FALSE, BcOLV4.BI=FALSE} =>  {BcULV4=FALSE} | 0.14  3564 | 0.552  381 | 0.259  901 | 1.78  5295 | 58 |
| --- | --- | --- | --- | --- | --- |
| {BcULV4=FALSE, BcULV5=FALSE} =>  {BcMV9.BI=FALSE} | 0.11  3861 | 0.464  646 | 0.245  05 | 1.44  3978 | 46 |
| {BcMV9.BI=FALSE, BcULV5=FALSE} =>  {BcULV4=FALSE} | 0.11  3861 | 0.516  854 | 0.220  297 | 1.67  0472 | 46 |
| {BcMV9.BI=FALSE, BcULV4=FALSE} =>  {BcBNV5.BI=FALSE} | 0.17  5743 | 0.986  111 | 0.178  218 | 1.08  5528 | 71 |
| {BcBNV5.BI=FALSE, BcULV4=FALSE} =>  {BcMV9.BI=FALSE} | 0.17  5743 | 0.606  838 | 0.289  604 | 1.88  5865 | 71 |
| {BcBNV5.BI=FALSE, BcMV9.BI=FALSE} =>  {BcULV4=FALSE} | 0.17  5743 | 0.563  492 | 0.311  881 | 1.82  1206 | 71 |
| {BcULV4=FALSE, BvF.BI=FALSE} =>  {BcMV9.BI=FALSE} | 0.16  5842 | 0.620  37 | 0.267  327 | 1.92  792 | 67 |
| {BcMV9.BI=FALSE, BvF.BI=FALSE} =>  {BcULV4=FALSE} | 0.16  5842 | 0.549  18 | 0.301  98 | 1.77  4951 | 67 |
| {BcMCV5.BI=FALSE, BcULV4=FALSE} =>  {BcMV9.BI=FALSE} | 0.17  3267 | 0.593  22 | 0.292  079 | 1.84  3546 | 70 |
| {BcMCV5.BI=FALSE, BcMV9.BI=FALSE} =>  {BcULV4=FALSE} | 0.17  3267 | 0.564  516 | 0.306  931 | 1.82  4516 | 70 |
| {BcOLV13.BI=FALSE, BcULV4=FALSE} =>  {BcMV9.BI=FALSE} | 0.15  8416 | 0.556  522 | 0.284  653 | 1.72  9498 | 64 |

| {BcMV9.BI=FALSE, BcOLV13.BI=FALSE} =>  {BcULV4=FALSE} | 0.15  8416 | 0.547  009 | 0.289  604 | 1.76  7932 | 64 |
| --- | --- | --- | --- | --- | --- |
| {BcOLV2.BI=FALSE, BcULV4=FALSE} =>  {BcMV9.BI=FALSE} | 0.16  5842 | 0.577  586 | 0.287  129 | 1.79  496 | 67 |
| {BcMV9.BI=FALSE, BcOLV2.BI=FALSE} =>  {BcULV4=FALSE} | 0.16  5842 | 0.540  323 | 0.306  931 | 1.74  6323 | 67 |
| {BcMCV3.BI=FALSE, BcULV4=FALSE} =>  {BcMV9.BI=FALSE} | 0.16  5842 | 0.567  797 | 0.292  079 | 1.76  4537 | 67 |
| {BcMCV3.BI=FALSE, BcMV9.BI=FALSE} =>  {BcULV4=FALSE} | 0.16  5842 | 0.544  715 | 0.304  455 | 1.76  052 | 67 |
| {BcPV4.BI=FALSE, BcULV4=FALSE} =>  {BcMV9.BI=FALSE} | 0.16  8317 | 0.586  207 | 0.287  129 | 1.82  1751 | 68 |
| {BcMV9.BI=FALSE, BcPV4.BI=FALSE} =>  {BcULV4=FALSE} | 0.16  8317 | 0.561  983 | 0.299  505 | 1.81  6331 | 68 |
| {BcEV6=FALSE, BcULV4=FALSE} =>  {BcMV9.BI=FALSE} | 0.16  5842 | 0.572  65 | 0.289  604 | 1.77  9619 | 67 |
| {BcEV6=FALSE, BcMV9.BI=FALSE} =>  {BcULV4=FALSE} | 0.16  5842 | 0.544  715 | 0.304  455 | 1.76  052 | 67 |
| {BcOLV17.BI=FALSE, BcULV4=FALSE} =>  {BcMV9.BI=FALSE} | 0.17  0792 | 0.565  574 | 0.301  98 | 1.75  7629 | 69 |
| {BcMV9.BI=FALSE, BcOLV17.BI=FALSE} =>  {BcULV4=FALSE} | 0.17  0792 | 0.552 | 0.309  406 | 1.78  4064 | 69 |

| {BcOLV15.BI=FALSE, BcULV4=FALSE} =>  {BcMV9.BI=FALSE} | 0.17  3267 | 0.593  22 | 0.292  079 | 1.84  3546 | 70 |
| --- | --- | --- | --- | --- | --- |
| {BcMV9.BI=FALSE, BcOLV15.BI=FALSE} =>  {BcULV4=FALSE} | 0.17  3267 | 0.56 | 0.309  406 | 1.80  992 | 70 |
| {BcEV5=FALSE, BcULV4=FALSE} =>  {BcMV9.BI=FALSE} | 0.17  3267 | 0.593  22 | 0.292  079 | 1.84  3546 | 70 |
| {BcEV5=FALSE, BcMV9.BI=FALSE} =>  {BcULV4=FALSE} | 0.17  3267 | 0.555  556 | 0.311  881 | 1.79  5556 | 70 |
| {BcOLV1.BI.MOVA.=TRUE, BcULV4=FALSE}  => {BcRV1.BI=FALSE} | 0.07  9208 | 0.914  286 | 0.086  634 | 1.26  4971 | 32 |
| {BcOLV1.BI.MOVA.=TRUE, BcULV4=FALSE}  => {BcFV5.BI=FALSE} | 0.07  6733 | 0.885  714 | 0.086  634 | 1.13  2369 | 31 |
| {BcOLV1.BI.MOVA.=TRUE, BcULV4=FALSE}  => {BcMV1.BI=TRUE} | 0.08  1683 | 0.942  857 | 0.086  634 | 1.19  4089 | 33 |
| {BcOLV1.BI.MOVA.=TRUE, BcULV4=FALSE}  => {BcNSRV7.BI=FALSE} | 0.08  1683 | 0.942  857 | 0.086  634 | 1.17  9301 | 33 |
| {BcOLV1.BI.MOVA.=TRUE, BcULV4=FALSE}  => {BcOLV7.BI=FALSE} | 0.07  9208 | 0.914  286 | 0.086  634 | 1.13  3041 | 32 |
| {BcOLV1.BI.MOVA.=TRUE, BcULV4=FALSE}  => {BcALV1.BI=FALSE} | 0.08  1683 | 0.942  857 | 0.086  634 | 1.15  7794 | 33 |
| {BcMV11=FALSE, BcULV4=FALSE} =>  {BcOLV18=FALSE} | 0.08  4158 | 0.755  556 | 0.111  386 | 1.56  5356 | 34 |

| {BcMV11=FALSE, BcULV4=FALSE} =>  {BcMV2.BI=FALSE} | 0.07  6733 | 0.688  889 | 0.111  386 | 1.28  254 | 31 |
| --- | --- | --- | --- | --- | --- |
| {BcMV11=FALSE, BcULV4=FALSE} =>  {BcMV4.BI=FALSE} | 0.08  1683 | 0.733  333 | 0.111  386 | 1.31  6741 | 33 |
| {BcMV11=FALSE, BcULV4=FALSE} =>  {BcOLV16.BI=FALSE} | 0.07  9208 | 0.711  111 | 0.111  386 | 1.21  7326 | 32 |
| {BcMV11=FALSE, BcULV4=FALSE} =>  {BcOLV1.BI.MOVA.=FALSE} | 0.08  6634 | 0.777  778 | 0.111  386 | 1.16  3786 | 35 |
| {BcMV11=FALSE, BcULV4=FALSE} =>  {BcRV1.BI=FALSE} | 0.10  6436 | 0.955  556 | 0.111  386 | 1.32  207 | 43 |
| {BcMV11=FALSE, BcULV4=FALSE} =>  {BcULV1.BI=FALSE} | 0.10  6436 | 0.955  556 | 0.111  386 | 1.21  7806 | 43 |
| {BcMV11=FALSE, BcULV4=FALSE} =>  {BcALV1.BI=FALSE} | 0.10  8911 | 0.977  778 | 0.111  386 | 1.20  0675 | 44 |
| {BcHV1.BI=FALSE, BcULV4=FALSE} =>  {BcULV3=FALSE} | 0.09  6535 | 0.6 | 0.160  891 | 1.47  8049 | 39 |
| {BcULV3=FALSE, BcULV4=FALSE} =>  {BcHV1.BI=FALSE} | 0.09  6535 | 0.735  849 | 0.131  188 | 1.85  8019 | 39 |
| {BcHV1.BI=FALSE, BcULV3=FALSE} =>  {BcULV4=FALSE} | 0.09  6535 | 0.428  571 | 0.225  248 | 1.38  5143 | 39 |
| {BcHV1.BI=FALSE, BcULV4=FALSE} =>  {BcHV1SLR.BI=FALSE} | 0.14  1089 | 0.876  923 | 0.160  891 | 1.91  501 | 57 |

| {BcHV1SLR.BI=FALSE, BcULV4=FALSE} =>  {BcHV1.BI=FALSE} | 0.14  1089 | 0.838  235 | 0.168  317 | 2.11  6544 | 57 |
| --- | --- | --- | --- | --- | --- |
| {BcHV1.BI=FALSE, BcHV1SLR.BI=FALSE} =>  {BcULV4=FALSE} | 0.14  1089 | 0.416  058 | 0.339  109 | 1.34  4701 | 57 |
| {BcHV1.BI=FALSE, BcULV4=FALSE} =>  {BcOLV18=FALSE} | 0.12  8713 | 0.8 | 0.160  891 | 1.65  7436 | 52 |
| {BcOLV18=FALSE, BcULV4=FALSE} =>  {BcHV1.BI=FALSE} | 0.12  8713 | 0.553  191 | 0.232  673 | 1.39  6809 | 52 |
| {BcHV1.BI=FALSE, BcOLV18=FALSE} =>  {BcULV4=FALSE} | 0.12  8713 | 0.525  253 | 0.245  05 | 1.69  7616 | 52 |
| {BcMV2.BI=FALSE, BcULV4=FALSE} =>  {BcHV1.BI=FALSE} | 0.09  6535 | 0.513  158 | 0.188  119 | 1.29  5724 | 39 |
| {BcHV1.BI=FALSE, BcMV2.BI=FALSE} =>  {BcULV4=FALSE} | 0.09  6535 | 0.428  571 | 0.225  248 | 1.38  5143 | 39 |
| {BcMV3.BI=TRUE, BcULV4=FALSE} =>  {BcHV1.BI=FALSE} | 0.08  6634 | 0.614  035 | 0.141  089 | 1.55  0439 | 35 |
| {BcHV1.BI=FALSE, BcMV3.BI=TRUE} =>  {BcULV4=FALSE} | 0.08  6634 | 0.460  526 | 0.188  119 | 1.48  8421 | 35 |
| {BcMV4.BI=FALSE, BcULV4=FALSE} =>  {BcHV1.BI=FALSE} | 0.09  901 | 0.519  481 | 0.190  594 | 1.31  1688 | 40 |
| {BcHV1.BI=FALSE, BcULV4=FALSE} =>  {BcOLV16.BI=FALSE} | 0.12  3762 | 0.769  231 | 0.160  891 | 1.31  6819 | 50 |

| {BcOLV16.BI=FALSE, BcULV4=FALSE} =>  {BcHV1.BI=FALSE} | 0.12  3762 | 0.531  915 | 0.232  673 | 1.34  3085 | 50 |
| --- | --- | --- | --- | --- | --- |
| {BcHV1.BI=FALSE, BcOLV16.BI=FALSE} =>  {BcULV4=FALSE} | 0.12  3762 | 0.495  05 | 0.25 | 1.6 | 50 |
| {BcMV11=TRUE, BcULV4=FALSE} =>  {BcHV1.BI=FALSE} | 0.11  1386 | 0.562  5 | 0.198  02 | 1.42  0313 | 45 |
| {BcHV1.BI=FALSE, BcMV11=TRUE} =>  {BcULV4=FALSE} | 0.11  1386 | 0.473  684 | 0.235  149 | 1.53  0947 | 45 |
| {BcHV1.BI=FALSE, BcULV4=FALSE} =>  {BcMV6.BI=FALSE} | 0.13  3663 | 0.830  769 | 0.160  891 | 1.19  4416 | 54 |
| {BcMV6.BI=FALSE, BcULV4=FALSE} =>  {BcHV1.BI=FALSE} | 0.13  3663 | 0.54 | 0.247  525 | 1.36  35 | 54 |
| {BcHV1.BI=FALSE, BcMV6.BI=FALSE} =>  {BcULV4=FALSE} | 0.13  3663 | 0.418  605 | 0.319  307 | 1.35  293 | 54 |
| {BcPV3.BI=FALSE, BcULV4=FALSE} =>  {BcHV1.BI=FALSE} | 0.12  1287 | 0.538  462 | 0.225  248 | 1.35  9615 | 49 |
| {BcHV1.BI=FALSE, BcPV3.BI=FALSE} =>  {BcULV4=FALSE} | 0.12  1287 | 0.395  161 | 0.306  931 | 1.27  7161 | 49 |
| {BcHV1.BI=FALSE, BcULV4=FALSE} =>  {BcRV1.BI=FALSE} | 0.13  1188 | 0.815  385 | 0.160  891 | 1.12  8135 | 53 |
| {BcHV1.BI=FALSE, BcRV1.BI=FALSE} =>  {BcULV4=FALSE} | 0.13  1188 | 0.427  419 | 0.306  931 | 1.38  1419 | 53 |

| {BcEV2.BI=FALSE, BcULV4=FALSE} =>  {BcHV1.BI=FALSE} | 0.12  3762 | 0.568  182 | 0.217  822 | 1.43  4659 | 50 |
| --- | --- | --- | --- | --- | --- |
| {BcEV2.BI=FALSE, BcHV1.BI=FALSE} =>  {BcULV4=FALSE} | 0.12  3762 | 0.4 | 0.309  406 | 1.29  28 | 50 |
| {BcHV1.BI=FALSE, BcULV4=FALSE} =>  {BcHV3.BI=FALSE} | 0.14  3564 | 0.892  308 | 0.160  891 | 1.17  4242 | 58 |
| {BcHV3.BI=FALSE, BcULV4=FALSE} =>  {BcHV1.BI=FALSE} | 0.14  3564 | 0.585  859 | 0.245  05 | 1.47  9293 | 58 |
| {BcHV1.BI=FALSE, BcHV3.BI=FALSE} =>  {BcULV4=FALSE} | 0.14  3564 | 0.453  125 | 0.316  832 | 1.46  45 | 58 |
| {BcHV1.BI=FALSE, BcULV4=FALSE} =>  {BcFV5.BI=FALSE} | 0.14  3564 | 0.892  308 | 0.160  891 | 1.14  0798 | 58 |
| {BcFV5.BI=FALSE, BcULV4=FALSE} =>  {BcHV1.BI=FALSE} | 0.14  3564 | 0.568  627 | 0.252  475 | 1.43  5784 | 58 |
| {BcFV5.BI=FALSE, BcHV1.BI=FALSE} =>  {BcULV4=FALSE} | 0.14  3564 | 0.436  09 | 0.329  208 | 1.40  9444 | 58 |
| {BcHV1.BI=FALSE, BcULV4=FALSE} =>  {BcULV1.BI=FALSE} | 0.15  3465 | 0.953  846 | 0.160  891 | 1.21  5627 | 62 |
| {BcULV1.BI=FALSE, BcULV4=FALSE} =>  {BcHV1.BI=FALSE} | 0.15  3465 | 0.534  483 | 0.287  129 | 1.34  9569 | 62 |
| {BcHV1.BI=FALSE, BcULV1.BI=FALSE} =>  {BcULV4=FALSE} | 0.15  3465 | 0.430  556 | 0.356  436 | 1.39  1556 | 62 |

| {BcMV1.BI=TRUE, BcULV4=FALSE} =>  {BcHV1.BI=FALSE} | 0.12  3762 | 0.515  464 | 0.240  099 | 1.30  1546 | 50 |
| --- | --- | --- | --- | --- | --- |
| {BcHV1.BI=FALSE, BcMV1.BI=TRUE} =>  {BcULV4=FALSE} | 0.12  3762 | 0.403  226 | 0.306  931 | 1.30  3226 | 50 |
| {BcMV12=FALSE, BcULV4=FALSE} =>  {BcHV1.BI=FALSE} | 0.13  3663 | 0.534  653 | 0.25 | 1.35 | 54 |
| {BcHV1.BI=FALSE, BcMV12=FALSE} =>  {BcULV4=FALSE} | 0.13  3663 | 0.4 | 0.334  158 | 1.29  28 | 54 |
| {BcHV1.BI=FALSE, BcULV4=FALSE} =>  {BcNSRV7.BI=FALSE} | 0.14  604 | 0.907  692 | 0.160  891 | 1.13  5318 | 59 |
| {BcNSRV7.BI=FALSE, BcULV4=FALSE} =>  {BcHV1.BI=FALSE} | 0.14  604 | 0.551  402 | 0.264  851 | 1.39  229 | 59 |
| {BcHV1.BI=FALSE, BcNSRV7.BI=FALSE} =>  {BcULV4=FALSE} | 0.14  604 | 0.437  037 | 0.334  158 | 1.41  2504 | 59 |
| {BcULV4=FALSE, BcVV3.BI=FALSE} =>  {BcHV1.BI=FALSE} | 0.12  8713 | 0.504  854 | 0.254  95 | 1.27  4757 | 52 |
| {BcHV1.BI=FALSE, BcVV3.BI=FALSE} =>  {BcULV4=FALSE} | 0.12  8713 | 0.419  355 | 0.306  931 | 1.35  5355 | 52 |
| {BcHV1.BI=FALSE, BcULV4=FALSE} =>  {BcOLV7.BI=FALSE} | 0.14  8515 | 0.923  077 | 0.160  891 | 1.14  3936 | 60 |
| {BcOLV7.BI=FALSE, BcULV4=FALSE} =>  {BcHV1.BI=FALSE} | 0.14  8515 | 0.504  202 | 0.294  554 | 1.27  3109 | 60 |

| {BcHV1.BI=FALSE, BcOLV7.BI=FALSE} =>  {BcULV4=FALSE} | 0.14  8515 | 0.451  128 | 0.329  208 | 1.45  8045 | 60 |
| --- | --- | --- | --- | --- | --- |
| {BcHV1.BI=FALSE, BcULV4=FALSE} =>  {BcALV1.BI=FALSE} | 0.15  099 | 0.938  462 | 0.160  891 | 1.15  2397 | 61 |
| {BcALV1.BI=FALSE, BcULV4=FALSE} =>  {BcHV1.BI=FALSE} | 0.15  099 | 0.530  435 | 0.284  653 | 1.33  9348 | 61 |
| {BcALV1.BI=FALSE, BcHV1.BI=FALSE} =>  {BcULV4=FALSE} | 0.15  099 | 0.432  624 | 0.349  01 | 1.39  8241 | 61 |
| {BcDMV1=FALSE, BcULV4=FALSE} =>  {BcHV1.BI=FALSE} | 0.14  1089 | 0.518  182 | 0.272  277 | 1.30  8409 | 57 |
| {BcDMV1=FALSE, BcHV1.BI=FALSE} =>  {BcULV4=FALSE} | 0.14  1089 | 0.431  818 | 0.326  733 | 1.39  5636 | 57 |
| {BcFV9=FALSE, BcULV4=FALSE} =>  {BcHV1.BI=FALSE} | 0.14  3564 | 0.527  273 | 0.272  277 | 1.33  1364 | 58 |
| {BcFV9=FALSE, BcHV1.BI=FALSE} =>  {BcULV4=FALSE} | 0.14  3564 | 0.397  26 | 0.361  386 | 1.28  3945 | 58 |
| {BcOLV4.BI=FALSE, BcULV4=FALSE} =>  {BcHV1.BI=FALSE} | 0.13  6139 | 0.533  981 | 0.254  95 | 1.34  8301 | 55 |
| {BcHV1.BI=FALSE, BcOLV4.BI=FALSE} =>  {BcULV4=FALSE} | 0.13  6139 | 0.392  857 | 0.346  535 | 1.26  9714 | 55 |
| {BcULV4=FALSE, BcULV5=FALSE} =>  {BcHV1.BI=FALSE} | 0.12  3762 | 0.505  051 | 0.245  05 | 1.27  5253 | 50 |

| {BcBNV5.BI=FALSE, BcULV4=FALSE} =>  {BcHV1.BI=FALSE} | 0.15  5941 | 0.538  462 | 0.289  604 | 1.35  9615 | 63 |
| --- | --- | --- | --- | --- | --- |
| {BcBNV5.BI=FALSE, BcHV1.BI=FALSE} =>  {BcULV4=FALSE} | 0.15  5941 | 0.414  474 | 0.376  238 | 1.33  9579 | 63 |
| {BcULV4=FALSE, BvF.BI=FALSE} =>  {BcHV1.BI=FALSE} | 0.14  8515 | 0.555  556 | 0.267  327 | 1.40  2778 | 60 |
| {BcHV1.BI=FALSE, BvF.BI=FALSE} =>  {BcULV4=FALSE} | 0.14  8515 | 0.402  685 | 0.368  812 | 1.30  1477 | 60 |
| {BcMCV5.BI=FALSE, BcULV4=FALSE} =>  {BcHV1.BI=FALSE} | 0.15  8416 | 0.542  373 | 0.292  079 | 1.36  9492 | 64 |
| {BcHV1.BI=FALSE, BcMCV5.BI=FALSE} =>  {BcULV4=FALSE} | 0.15  8416 | 0.421  053 | 0.376  238 | 1.36  0842 | 64 |
| {BcOLV13.BI=FALSE, BcULV4=FALSE} =>  {BcHV1.BI=FALSE} | 0.15  099 | 0.530  435 | 0.284  653 | 1.33  9348 | 61 |
| {BcHV1.BI=FALSE, BcOLV13.BI=FALSE} =>  {BcULV4=FALSE} | 0.15  099 | 0.396  104 | 0.381  188 | 1.28  0208 | 61 |
| {BcOLV2.BI=FALSE, BcULV4=FALSE} =>  {BcHV1.BI=FALSE} | 0.14  8515 | 0.517  241 | 0.287  129 | 1.30  6034 | 60 |
| {BcHV1.BI=FALSE, BcOLV2.BI=FALSE} =>  {BcULV4=FALSE} | 0.14  8515 | 0.394  737 | 0.376  238 | 1.27  5789 | 60 |
| {BcMCV3.BI=FALSE, BcULV4=FALSE} =>  {BcHV1.BI=FALSE} | 0.15  5941 | 0.533  898 | 0.292  079 | 1.34  8093 | 63 |

| {BcHV1.BI=FALSE, BcMCV3.BI=FALSE} =>  {BcULV4=FALSE} | 0.15  5941 | 0.401  274 | 0.388  614 | 1.29  6917 | 63 |
| --- | --- | --- | --- | --- | --- |
| {BcPV4.BI=FALSE, BcULV4=FALSE} =>  {BcHV1.BI=FALSE} | 0.15  3465 | 0.534  483 | 0.287  129 | 1.34  9569 | 62 |
| {BcHV1.BI=FALSE, BcPV4.BI=FALSE} =>  {BcULV4=FALSE} | 0.15  3465 | 0.410  596 | 0.373  762 | 1.32  7046 | 62 |
| {BcEV6=FALSE, BcULV4=FALSE} =>  {BcHV1.BI=FALSE} | 0.15  5941 | 0.538  462 | 0.289  604 | 1.35  9615 | 63 |
| {BcEV6=FALSE, BcHV1.BI=FALSE} =>  {BcULV4=FALSE} | 0.15  5941 | 0.403  846 | 0.386  139 | 1.30  5231 | 63 |
| {BcOLV17.BI=FALSE, BcULV4=FALSE} =>  {BcHV1.BI=FALSE} | 0.15  8416 | 0.524  59 | 0.301  98 | 1.32  459 | 64 |
| {BcHV1.BI=FALSE, BcOLV17.BI=FALSE} =>  {BcULV4=FALSE} | 0.15  8416 | 0.412  903 | 0.383  663 | 1.33  4503 | 64 |
| {BcOLV15.BI=FALSE, BcULV4=FALSE} =>  {BcHV1.BI=FALSE} | 0.15  5941 | 0.533  898 | 0.292  079 | 1.34  8093 | 63 |
| {BcHV1.BI=FALSE, BcOLV15.BI=FALSE} =>  {BcULV4=FALSE} | 0.15  5941 | 0.403  846 | 0.386  139 | 1.30  5231 | 63 |
| {BcEV5=FALSE, BcULV4=FALSE} =>  {BcHV1.BI=FALSE} | 0.15  099 | 0.516  949 | 0.292  079 | 1.30  5297 | 61 |
| {BcEV5=FALSE, BcHV1.BI=FALSE} =>  {BcULV4=FALSE} | 0.15  099 | 0.401  316 | 0.376  238 | 1.29  7053 | 61 |

| {BcULV3=FALSE, BcULV4=FALSE} =>  {BcHV1SLR.BI=FALSE} | 0.09  6535 | 0.735  849 | 0.131  188 | 1.60  6935 | 39 |
| --- | --- | --- | --- | --- | --- |
| {BcHV1SLR.BI=FALSE, BcULV4=FALSE} =>  {BcULV3=FALSE} | 0.09  6535 | 0.573  529 | 0.168  317 | 1.41  2841 | 39 |
| {BcULV3=FALSE, BcULV4=FALSE} =>  {BcOLV18=FALSE} | 0.10  1485 | 0.773  585 | 0.131  188 | 1.60  2709 | 41 |
| {BcOLV18=FALSE, BcULV3=FALSE} =>  {BcULV4=FALSE} | 0.10  1485 | 0.476  744 | 0.212  871 | 1.54  0837 | 41 |
| {BcULV3=FALSE, BcULV4=FALSE} =>  {BcMV2.BI=FALSE} | 0.08  9109 | 0.679  245 | 0.131  188 | 1.26  4586 | 36 |
| {BcULV3=FALSE, BcULV4=FALSE} =>  {BcMV4.BI=FALSE} | 0.08  9109 | 0.679  245 | 0.131  188 | 1.21  9623 | 36 |
| {BcULV3=FALSE, BcULV4=FALSE} =>  {BcOLV16.BI=FALSE} | 0.09  4059 | 0.716  981 | 0.131  188 | 1.22  7374 | 38 |
| {BcMV11=TRUE, BcULV3=FALSE} =>  {BcULV4=FALSE} | 0.09  1584 | 0.415  73 | 0.220  297 | 1.34  364 | 37 |
| {BcULV3=FALSE, BcULV4=FALSE} =>  {BcMV6.BI=FALSE} | 0.11  8812 | 0.905  66 | 0.131  188 | 1.30  2088 | 48 |
| {BcULV3=FALSE, BcULV4=FALSE} =>  {BcPV3.BI=FALSE} | 0.10  8911 | 0.830  189 | 0.131  188 | 1.16  8628 | 44 |
| {BcULV3=FALSE, BcULV4=FALSE} =>  {BcRV1.BI=FALSE} | 0.10  8911 | 0.830  189 | 0.131  188 | 1.14  8617 | 44 |

| {BcULV3=FALSE, BcULV4=FALSE} =>  {BcHV3.BI=FALSE} | 0.11  6337 | 0.886  792 | 0.131  188 | 1.16  6984 | 47 |
| --- | --- | --- | --- | --- | --- |
| {BcULV3=FALSE, BcULV4=FALSE} =>  {BcFV5.BI=FALSE} | 0.11  8812 | 0.905  66 | 0.131  188 | 1.15  787 | 48 |
| {BcULV3=FALSE, BcULV4=FALSE} =>  {BcULV1.BI=FALSE} | 0.12  6238 | 0.962  264 | 0.131  188 | 1.22  6356 | 51 |
| {BcULV1.BI=FALSE, BcULV3=FALSE} =>  {BcULV4=FALSE} | 0.12  6238 | 0.386  364 | 0.326  733 | 1.24  8727 | 51 |
| {BcULV3=FALSE, BcULV4=FALSE} =>  {BcNSRV7.BI=FALSE} | 0.12  3762 | 0.943  396 | 0.131  188 | 1.17  9975 | 50 |
| {BcULV3=FALSE, BcULV4=FALSE} =>  {BcOLV7.BI=FALSE} | 0.12  1287 | 0.924  528 | 0.131  188 | 1.14  5734 | 49 |
| {BcULV3=FALSE, BcULV4=FALSE} =>  {BcALV1.BI=FALSE} | 0.12  3762 | 0.943  396 | 0.131  188 | 1.15  8456 | 50 |
| {BcULV3=FALSE, BcULV4=FALSE} =>  {BcBNV5.BI=FALSE} | 0.12  8713 | 0.981  132 | 0.131  188 | 1.08  0047 | 52 |
| {BcMV4.BI=TRUE, BcULV4=FALSE} =>  {BcOLV18=FALSE} | 0.08  4158 | 0.708  333 | 0.118  812 | 1.46  7521 | 34 |
| {BcMV4.BI=TRUE, BcOLV18=FALSE} =>  {BcULV4=FALSE} | 0.08  4158 | 0.507  463 | 0.165  842 | 1.64  0119 | 34 |
| {BcMV4.BI=TRUE, BcULV4=FALSE} =>  {BcMV11=TRUE} | 0.08  9109 | 0.75 | 0.118  812 | 1.21  6867 | 36 |

| {BcMV4.BI=TRUE, BcULV4=FALSE} =>  {BcRV1.BI=FALSE} | 0.10  396 | 0.875 | 0.118  812 | 1.21  0616 | 42 |
| --- | --- | --- | --- | --- | --- |
| {BcMV4.BI=TRUE, BcULV4=FALSE} =>  {BcULV1.BI=FALSE} | 0.10  6436 | 0.895  833 | 0.118  812 | 1.14  1693 | 43 |
| {BcMV4.BI=TRUE, BcULV4=FALSE} =>  {BcOLV7.BI=FALSE} | 0.11  6337 | 0.979  167 | 0.118  812 | 1.21  3446 | 47 |
| {BcMV2.BI=TRUE, BcULV4=FALSE} =>  {BcHV1SLR.BI=FALSE} | 0.07  4257 | 0.612  245 | 0.121  287 | 1.33  701 | 30 |
| {BcHV1SLR.BI=FALSE, BcULV4=FALSE} =>  {BcOLV18=FALSE} | 0.13  6139 | 0.808  824 | 0.168  317 | 1.67  5716 | 55 |
| {BcOLV18=FALSE, BcULV4=FALSE} =>  {BcHV1SLR.BI=FALSE} | 0.13  6139 | 0.585  106 | 0.232  673 | 1.27  7746 | 55 |
| {BcHV1SLR.BI=FALSE, BcOLV18=FALSE} =>  {BcULV4=FALSE} | 0.13  6139 | 0.482  456 | 0.282  178 | 1.55  9298 | 55 |
| {BcMV3.BI=TRUE, BcULV4=FALSE} =>  {BcHV1SLR.BI=FALSE} | 0.08  9109 | 0.631  579 | 0.141  089 | 1.37  9232 | 36 |
| {BcHV1SLR.BI=FALSE, BcMV3.BI=TRUE} =>  {BcULV4=FALSE} | 0.08  9109 | 0.418  605 | 0.212  871 | 1.35  293 | 36 |
| {BcHV1SLR.BI=FALSE, BcULV4=FALSE} =>  {BcOLV16.BI=FALSE} | 0.12  8713 | 0.764  706 | 0.168  317 | 1.30  9073 | 52 |
| {BcHV1SLR.BI=FALSE, BcOLV16.BI=FALSE}  => {BcULV4=FALSE} | 0.12  8713 | 0.460  177 | 0.279  703 | 1.48  7292 | 52 |

| {BcMV11=TRUE, BcULV4=FALSE} =>  {BcHV1SLR.BI=FALSE} | 0.11  6337 | 0.587  5 | 0.198  02 | 1.28  2973 | 47 |
| --- | --- | --- | --- | --- | --- |
| {BcHV1SLR.BI=FALSE, BcMV11=TRUE} =>  {BcULV4=FALSE} | 0.11  6337 | 0.447  619 | 0.259  901 | 1.44  6705 | 47 |
| {BcHV1SLR.BI=FALSE, BcULV4=FALSE} =>  {BcMV6.BI=FALSE} | 0.14  3564 | 0.852  941 | 0.168  317 | 1.22  6293 | 58 |
| {BcMV6.BI=FALSE, BcULV4=FALSE} =>  {BcHV1SLR.BI=FALSE} | 0.14  3564 | 0.58 | 0.247  525 | 1.26  6595 | 58 |
| {BcHV1SLR.BI=FALSE, BcMV6.BI=FALSE}  => {BcULV4=FALSE} | 0.14  3564 | 0.4 | 0.358  911 | 1.29  28 | 58 |
| {BcPV3.BI=FALSE, BcULV4=FALSE} =>  {BcHV1SLR.BI=FALSE} | 0.12  6238 | 0.560  44 | 0.225  248 | 1.22  3879 | 51 |
| {BcHV1SLR.BI=FALSE, BcRV1.BI=FALSE} =>  {BcULV4=FALSE} | 0.13  6139 | 0.398  551 | 0.341  584 | 1.28  8116 | 55 |
| {BcEV2.BI=FALSE, BcULV4=FALSE} =>  {BcHV1SLR.BI=FALSE} | 0.12  6238 | 0.579  545 | 0.217  822 | 1.26  5602 | 51 |
| {BcHV1SLR.BI=FALSE, BcULV4=FALSE} =>  {BcHV3.BI=FALSE} | 0.14  3564 | 0.852  941 | 0.168  317 | 1.12  2437 | 58 |
| {BcHV3.BI=FALSE, BcULV4=FALSE} =>  {BcHV1SLR.BI=FALSE} | 0.14  3564 | 0.585  859 | 0.245  05 | 1.27  9388 | 58 |
| {BcHV1SLR.BI=FALSE, BcHV3.BI=FALSE} =>  {BcULV4=FALSE} | 0.14  3564 | 0.391  892 | 0.366  337 | 1.26  6595 | 58 |

| {BcHV1SLR.BI=FALSE, BcULV4=FALSE} =>  {BcFV5.BI=FALSE} | 0.14  8515 | 0.882  353 | 0.168  317 | 1.12  8071 | 60 |
| --- | --- | --- | --- | --- | --- |
| {BcFV5.BI=FALSE, BcULV4=FALSE} =>  {BcHV1SLR.BI=FALSE} | 0.14  8515 | 0.588  235 | 0.252  475 | 1.28  4579 | 60 |
| {BcFV5.BI=FALSE, BcHV1SLR.BI=FALSE} =>  {BcULV4=FALSE} | 0.14  8515 | 0.379  747 | 0.391  089 | 1.22  7342 | 60 |
| {BcHV1SLR.BI=FALSE, BcULV4=FALSE} =>  {BcULV1.BI=FALSE} | 0.16  3366 | 0.970  588 | 0.168  317 | 1.23  6964 | 66 |
| {BcULV1.BI=FALSE, BcULV4=FALSE} =>  {BcHV1SLR.BI=FALSE} | 0.16  3366 | 0.568  966 | 0.287  129 | 1.24  2498 | 66 |
| {BcHV1SLR.BI=FALSE, BcULV1.BI=FALSE}  => {BcULV4=FALSE} | 0.16  3366 | 0.407  407 | 0.400  99 | 1.31  6741 | 66 |
| {BcMV12=FALSE, BcULV4=FALSE} =>  {BcHV1SLR.BI=FALSE} | 0.14  3564 | 0.574  257 | 0.25 | 1.25  4054 | 58 |
| {BcHV1SLR.BI=FALSE, BcULV4=FALSE} =>  {BcNSRV7.BI=FALSE} | 0.15  5941 | 0.926  471 | 0.168  317 | 1.15  8805 | 63 |
| {BcNSRV7.BI=FALSE, BcULV4=FALSE} =>  {BcHV1SLR.BI=FALSE} | 0.15  5941 | 0.588  785 | 0.264  851 | 1.28  5779 | 63 |
| {BcHV1SLR.BI=FALSE, BcNSRV7.BI=FALSE}  => {BcULV4=FALSE} | 0.15  5941 | 0.398  734 | 0.391  089 | 1.28  8709 | 63 |
| {BcHV1SLR.BI=FALSE, BcULV4=FALSE} =>  {BcOLV7.BI=FALSE} | 0.15  8416 | 0.941  176 | 0.168  317 | 1.16  6366 | 64 |

| {BcHV1SLR.BI=FALSE, BcOLV7.BI=FALSE}  => {BcULV4=FALSE} | 0.15  8416 | 0.426  667 | 0.371  287 | 1.37  8987 | 64 |
| --- | --- | --- | --- | --- | --- |
| {BcHV1SLR.BI=FALSE, BcULV4=FALSE} =>  {BcALV1.BI=FALSE} | 0.15  099 | 0.897  059 | 0.168  317 | 1.10  1556 | 61 |
| {BcALV1.BI=FALSE, BcHV1SLR.BI=FALSE}  => {BcULV4=FALSE} | 0.15  099 | 0.378  882 | 0.398  515 | 1.22  4547 | 61 |
| {BcDMV1=FALSE, BcHV1SLR.BI=FALSE} =>  {BcULV4=FALSE} | 0.14  604 | 0.385  621 | 0.378  713 | 1.24  6327 | 59 |
| {BcOLV4.BI=FALSE, BcULV4=FALSE} =>  {BcHV1SLR.BI=FALSE} | 0.14  3564 | 0.563  107 | 0.254  95 | 1.22  9703 | 58 |
| {BcBNV5.BI=FALSE, BcULV4=FALSE} =>  {BcHV1SLR.BI=FALSE} | 0.16  3366 | 0.564  103 | 0.289  604 | 1.23  1878 | 66 |
| {BcBNV5.BI=FALSE, BcHV1SLR.BI=FALSE}  => {BcULV4=FALSE} | 0.16  3366 | 0.379  31 | 0.430  693 | 1.22  5931 | 66 |
| {BcULV4=FALSE, BvF.BI=FALSE} =>  {BcHV1SLR.BI=FALSE} | 0.15  5941 | 0.583  333 | 0.267  327 | 1.27  3874 | 63 |
| {BcMCV5.BI=FALSE, BcULV4=FALSE} =>  {BcHV1SLR.BI=FALSE} | 0.16  3366 | 0.559  322 | 0.292  079 | 1.22  1438 | 66 |
| {BcHV1SLR.BI=FALSE, BcMCV5.BI=FALSE}  => {BcULV4=FALSE} | 0.16  3366 | 0.377  143 | 0.433  168 | 1.21  8926 | 66 |
| {BcMCV3.BI=FALSE, BcULV4=FALSE} =>  {BcHV1SLR.BI=FALSE} | 0.16  5842 | 0.567  797 | 0.292  079 | 1.23  9945 | 67 |

| {BcHV1SLR.BI=FALSE, BcMCV3.BI=FALSE}  => {BcULV4=FALSE} | 0.16  5842 | 0.372  222 | 0.445  545 | 1.20  3022 | 67 |
| --- | --- | --- | --- | --- | --- |
| {BcOLV17.BI=FALSE, BcULV4=FALSE} =>  {BcHV1SLR.BI=FALSE} | 0.16  5842 | 0.549  18 | 0.301  98 | 1.19  9291 | 67 |
| {BcHV1SLR.BI=FALSE, BcOLV17.BI=FALSE}  => {BcULV4=FALSE} | 0.16  5842 | 0.374  302 | 0.443  069 | 1.20  9743 | 67 |
| {BcOLV15.BI=FALSE, BcULV4=FALSE} =>  {BcHV1SLR.BI=FALSE} | 0.16  3366 | 0.559  322 | 0.292  079 | 1.22  1438 | 66 |
| {BcMV3.BI=FALSE, BcULV4=FALSE} =>  {BcOLV18=FALSE} | 0.12  8713 | 0.764  706 | 0.168  317 | 1.58  4314 | 52 |
| {BcMV3.BI=FALSE, BcOLV18=FALSE} =>  {BcULV4=FALSE} | 0.12  8713 | 0.477  064 | 0.269  802 | 1.54  1872 | 52 |
| {BcMV3.BI=FALSE, BcULV4=FALSE} =>  {BcMV2.BI=FALSE} | 0.12  8713 | 0.764  706 | 0.168  317 | 1.42  3692 | 52 |
| {BcMV2.BI=FALSE, BcULV4=FALSE} =>  {BcMV3.BI=FALSE} | 0.12  8713 | 0.684  211 | 0.188  119 | 1.49  4168 | 52 |
| {BcMV2.BI=FALSE, BcMV3.BI=FALSE} =>  {BcULV4=FALSE} | 0.12  8713 | 0.416 | 0.309  406 | 1.34  4512 | 52 |
| {BcHV1SLR.BI=TRUE, BcULV4=FALSE} =>  {BcMV3.BI=FALSE} | 0.08  9109 | 0.631  579 | 0.141  089 | 1.37  9232 | 36 |
| {BcHV1SLR.BI=TRUE, BcMV3.BI=FALSE} =>  {BcULV4=FALSE} | 0.08  9109 | 0.418  605 | 0.212  871 | 1.35  293 | 36 |

| {BcMV3.BI=FALSE, BcULV4=FALSE} =>  {BcMV4.BI=FALSE} | 0.11  3861 | 0.676  471 | 0.168  317 | 1.21  4641 | 46 |
| --- | --- | --- | --- | --- | --- |
| {BcMV4.BI=FALSE, BcULV4=FALSE} =>  {BcMV3.BI=FALSE} | 0.11  3861 | 0.597  403 | 0.190  594 | 1.30  4598 | 46 |
| {BcMV3.BI=FALSE, BcULV4=FALSE} =>  {BcOLV16.BI=FALSE} | 0.13  8614 | 0.823  529 | 0.168  317 | 1.40  9771 | 56 |
| {BcOLV16.BI=FALSE, BcULV4=FALSE} =>  {BcMV3.BI=FALSE} | 0.13  8614 | 0.595  745 | 0.232  673 | 1.30  0978 | 56 |
| {BcMV3.BI=FALSE, BcOLV16.BI=FALSE} =>  {BcULV4=FALSE} | 0.13  8614 | 0.448 | 0.309  406 | 1.44  7936 | 56 |
| {BcMV3.BI=FALSE, BcULV3=TRUE} =>  {BcULV4=FALSE} | 0.10  1485 | 0.41 | 0.247  525 | 1.32  512 | 41 |
| {BcHV1.BI=TRUE, BcULV4=FALSE} =>  {BcMV3.BI=FALSE} | 0.09  4059 | 0.633  333 | 0.148  515 | 1.38  3063 | 38 |
| {BcMV11=TRUE, BcMV3.BI=FALSE} =>  {BcULV4=FALSE} | 0.10  6436 | 0.457  447 | 0.232  673 | 1.47  8468 | 43 |
| {BcMV3.BI=FALSE, BcULV4=FALSE} =>  {BcOLV1.BI.MOVA.=FALSE} | 0.14  3564 | 0.852  941 | 0.168  317 | 1.27  6253 | 58 |
| {BcOLV1.BI.MOVA.=FALSE, BcULV4=FALSE}  => {BcMV3.BI=FALSE} | 0.14  3564 | 0.644  444 | 0.222  772 | 1.40  7327 | 58 |
| {BcMV3.BI=FALSE,  BcOLV1.BI.MOVA.=FALSE} => | 0.14  3564 | 0.386  667 | 0.371  287 | 1.24  9707 | 58 |

| {BcULV4=FALSE} |  |  |  |  |  |
| --- | --- | --- | --- | --- | --- |
| {BcMV3.BI=FALSE, BcULV4=FALSE} =>  {BcMV6.BI=FALSE} | 0.15  099 | 0.897  059 | 0.168  317 | 1.28  9722 | 61 |
| {BcMV6.BI=FALSE, BcULV4=FALSE} =>  {BcMV3.BI=FALSE} | 0.15  099 | 0.61 | 0.247  525 | 1.33  2108 | 61 |
| {BcMV3.BI=FALSE, BcMV6.BI=FALSE} =>  {BcULV4=FALSE} | 0.15  099 | 0.401  316 | 0.376  238 | 1.29  7053 | 61 |
| {BcMV3.BI=FALSE, BcULV4=FALSE} =>  {BcRV1.BI=FALSE} | 0.15  5941 | 0.926  471 | 0.168  317 | 1.28  1829 | 63 |
| {BcRV1.BI=FALSE, BcULV4=FALSE} =>  {BcMV3.BI=FALSE} | 0.15  5941 | 0.567  568 | 0.274  752 | 1.23  9445 | 63 |
| {BcMV3.BI=FALSE, BcRV1.BI=FALSE} =>  {BcULV4=FALSE} | 0.15  5941 | 0.437  5 | 0.356  436 | 1.41  4 | 63 |
| {BcFV5.BI=FALSE, BcULV4=FALSE} =>  {BcMV3.BI=FALSE} | 0.14  3564 | 0.568  627 | 0.252  475 | 1.24  1759 | 58 |
| {BcMV3.BI=FALSE, BcULV4=FALSE} =>  {BcULV1.BI=FALSE} | 0.15  8416 | 0.941  176 | 0.168  317 | 1.19  948 | 64 |
| {BcMV3.BI=FALSE, BcULV1.BI=FALSE} =>  {BcULV4=FALSE} | 0.15  8416 | 0.402  516 | 0.393  564 | 1.30  0931 | 64 |
| {BcMV12=FALSE, BcULV4=FALSE} =>  {BcMV3.BI=FALSE} | 0.14  8515 | 0.594  059 | 0.25 | 1.29  7297 | 60 |
| {BcMV3.BI=FALSE, BcULV4=FALSE} => | 0.16 | 0.985 | 0.168 | 1.22 | 67 |

| {BcOLV7.BI=FALSE} | 5842 | 294 | 317 | 1039 |  |
| --- | --- | --- | --- | --- | --- |
| {BcOLV7.BI=FALSE, BcULV4=FALSE} =>  {BcMV3.BI=FALSE} | 0.16  5842 | 0.563  025 | 0.294  554 | 1.22  9525 | 67 |
| {BcMV3.BI=FALSE, BcOLV7.BI=FALSE} =>  {BcULV4=FALSE} | 0.16  5842 | 0.424  051 | 0.391  089 | 1.37  0532 | 67 |
| {BcMV3.BI=FALSE, BcULV4=FALSE} =>  {BcALV1.BI=FALSE} | 0.15  099 | 0.897  059 | 0.168  317 | 1.10  1556 | 61 |
| {BcALV1.BI=FALSE, BcMV3.BI=FALSE} =>  {BcULV4=FALSE} | 0.15  099 | 0.386  076 | 0.391  089 | 1.24  7797 | 61 |
| {BcMV3.BI=FALSE, BcULV4=FALSE} =>  {BcDMV1=FALSE} | 0.15  8416 | 0.941  176 | 0.168  317 | 1.11  506 | 64 |
| {BcDMV1=FALSE, BcULV4=FALSE} =>  {BcMV3.BI=FALSE} | 0.15  8416 | 0.581  818 | 0.272  277 | 1.27  0565 | 64 |
| {BcFV9=FALSE, BcULV4=FALSE} =>  {BcMV3.BI=FALSE} | 0.15  5941 | 0.572  727 | 0.272  277 | 1.25  0713 | 63 |
| {BcOLV4.BI=FALSE, BcULV4=FALSE} =>  {BcMV3.BI=FALSE} | 0.14  8515 | 0.582  524 | 0.254  95 | 1.27  2107 | 60 |
| {BcULV4=FALSE, BcULV5=FALSE} =>  {BcMV3.BI=FALSE} | 0.14  3564 | 0.585  859 | 0.245  05 | 1.27  9388 | 58 |
| {BcBNV5.BI=FALSE, BcULV4=FALSE} =>  {BcMV3.BI=FALSE} | 0.16  0891 | 0.555  556 | 0.289  604 | 1.21  3213 | 65 |
| {BcOLV13.BI=FALSE, BcULV4=FALSE} => | 0.16 | 0.591 | 0.284 | 1.29 | 68 |

| {BcMV3.BI=FALSE} | 8317 | 304 | 653 | 1281 |  |
| --- | --- | --- | --- | --- | --- |
| {BcMV3.BI=FALSE, BcOLV13.BI=FALSE} =>  {BcULV4=FALSE} | 0.16  8317 | 0.373  626 | 0.450  495 | 1.20  756 | 68 |
| {BcOLV2.BI=FALSE, BcULV4=FALSE} =>  {BcMV3.BI=FALSE} | 0.16  0891 | 0.560  345 | 0.287  129 | 1.22  3672 | 65 |
| {BcMCV3.BI=FALSE, BcULV4=FALSE} =>  {BcMV3.BI=FALSE} | 0.16  8317 | 0.576  271 | 0.292  079 | 1.25  8452 | 68 |
| {BcMV3.BI=FALSE, BcOLV17.BI=FALSE} =>  {BcULV4=FALSE} | 0.16  3366 | 0.375 | 0.435  644 | 1.21  2 | 66 |
| {BcEV5=FALSE, BcULV4=FALSE} =>  {BcMV3.BI=FALSE} | 0.16  8317 | 0.576  271 | 0.292  079 | 1.25  8452 | 68 |
| {BcMV2.BI=TRUE, BcULV4=FALSE} =>  {BcOLV18=FALSE} | 0.08  6634 | 0.714  286 | 0.121  287 | 1.47  9853 | 35 |
| {BcMV2.BI=TRUE, BcOLV18=FALSE} =>  {BcULV4=FALSE} | 0.08  6634 | 0.514  706 | 0.168  317 | 1.66  3529 | 35 |
| {BcMV2.BI=TRUE, BcULV4=FALSE} =>  {BcMV3.BI=TRUE} | 0.08  1683 | 0.673  469 | 0.121  287 | 1.24  2382 | 33 |
| {BcMV3.BI=TRUE, BcULV4=FALSE} =>  {BcMV2.BI=TRUE} | 0.08  1683 | 0.578  947 | 0.141  089 | 1.25  0774 | 33 |
| {BcMV2.BI=TRUE, BcULV4=FALSE} =>  {BcRV1.BI=FALSE} | 0.10  6436 | 0.877  551 | 0.121  287 | 1.21  4146 | 43 |
| {BcMV2.BI=TRUE, BcULV4=FALSE} => | 0.11 | 0.918 | 0.121 | 1.16 | 45 |

| {BcMV1.BI=TRUE} | 1386 | 367 | 287 | 3073 |  |
| --- | --- | --- | --- | --- | --- |
| {BcMV2.BI=TRUE, BcULV4=FALSE} =>  {BcNSRV7.BI=FALSE} | 0.10  8911 | 0.897  959 | 0.121  287 | 1.12  3144 | 44 |
| {BcMV2.BI=TRUE, BcULV4=FALSE} =>  {BcOLV7.BI=FALSE} | 0.11  3861 | 0.938  776 | 0.121  287 | 1.16  3391 | 46 |
| {BcMV2.BI=FALSE, BcULV4=FALSE} =>  {BcOLV18=FALSE} | 0.14  604 | 0.776  316 | 0.188  119 | 1.60  8367 | 59 |
| {BcMV2.BI=FALSE, BcOLV18=FALSE} =>  {BcULV4=FALSE} | 0.14  604 | 0.464  567 | 0.314  356 | 1.50  148 | 59 |
| {BcHV1SLR.BI=TRUE, BcULV4=FALSE} =>  {BcOLV18=FALSE} | 0.09  6535 | 0.684  211 | 0.141  089 | 1.41  7544 | 39 |
| {BcHV1SLR.BI=TRUE, BcOLV18=FALSE} =>  {BcULV4=FALSE} | 0.09  6535 | 0.481  481 | 0.200  495 | 1.55  6148 | 39 |
| {BcMV3.BI=TRUE, BcULV4=FALSE} =>  {BcOLV18=FALSE} | 0.10  396 | 0.736  842 | 0.141  089 | 1.52  6586 | 42 |
| {BcMV3.BI=TRUE, BcOLV18=FALSE} =>  {BcULV4=FALSE} | 0.10  396 | 0.488  372 | 0.212  871 | 1.57  8419 | 42 |
| {BcMV4.BI=FALSE, BcULV4=FALSE} =>  {BcOLV18=FALSE} | 0.14  8515 | 0.779  221 | 0.190  594 | 1.61  4386 | 60 |
| {BcMV4.BI=FALSE, BcOLV18=FALSE} =>  {BcULV4=FALSE} | 0.14  8515 | 0.468  75 | 0.316  832 | 1.51  5 | 60 |
| {BcOLV18=FALSE, BcULV4=FALSE} => | 0.18 | 0.776 | 0.232 | 1.32 | 73 |

| {BcOLV16.BI=FALSE} | 0693 | 596 | 673 | 9427 |  |
| --- | --- | --- | --- | --- | --- |
| {BcOLV16.BI=FALSE, BcULV4=FALSE} =>  {BcOLV18=FALSE} | 0.18  0693 | 0.776  596 | 0.232  673 | 1.60  8947 | 73 |
| {BcOLV16.BI=FALSE, BcOLV18=FALSE} =>  {BcULV4=FALSE} | 0.18  0693 | 0.536  765 | 0.336  634 | 1.73  4824 | 73 |
| {BcULV3=TRUE, BcULV4=FALSE} =>  {BcOLV18=FALSE} | 0.13  1188 | 0.736  111 | 0.178  218 | 1.52  5071 | 53 |
| {BcOLV18=FALSE, BcULV3=TRUE} =>  {BcULV4=FALSE} | 0.13  1188 | 0.486  239 | 0.269  802 | 1.57  1523 | 53 |
| {BcHV1.BI=TRUE, BcULV4=FALSE} =>  {BcOLV18=FALSE} | 0.10  396 | 0.7 | 0.148  515 | 1.45  0256 | 42 |
| {BcHV1.BI=TRUE, BcOLV18=FALSE} =>  {BcULV4=FALSE} | 0.10  396 | 0.437  5 | 0.237  624 | 1.41  4 | 42 |
| {BcMV11=TRUE, BcULV4=FALSE} =>  {BcOLV18=FALSE} | 0.14  8515 | 0.75 | 0.198  02 | 1.55  3846 | 60 |
| {BcMV11=TRUE, BcOLV18=FALSE} =>  {BcULV4=FALSE} | 0.14  8515 | 0.550  459 | 0.269  802 | 1.77  9083 | 60 |
| {BcOLV1.BI.MOVA.=FALSE, BcULV4=FALSE}  => {BcOLV18=FALSE} | 0.16  3366 | 0.733  333 | 0.222  772 | 1.51  9316 | 66 |
| {BcOLV1.BI.MOVA.=FALSE,  BcOLV18=FALSE} => {BcULV4=FALSE} | 0.16  3366 | 0.428  571 | 0.381  188 | 1.38  5143 | 66 |
| {BcMV9.BI=TRUE, BcULV4=FALSE} => | 0.09 | 0.716 | 0.131 | 1.48 | 38 |

| {BcOLV18=FALSE} | 4059 | 981 | 188 | 5438 |  |
| --- | --- | --- | --- | --- | --- |
| {BcOLV18=FALSE, BcULV4=FALSE} =>  {BcMV6.BI=FALSE} | 0.18  5644 | 0.797  872 | 0.232  673 | 1.14  7119 | 75 |
| {BcMV6.BI=FALSE, BcULV4=FALSE} =>  {BcOLV18=FALSE} | 0.18  5644 | 0.75 | 0.247  525 | 1.55  3846 | 75 |
| {BcMV6.BI=FALSE, BcOLV18=FALSE} =>  {BcULV4=FALSE} | 0.18  5644 | 0.528  169 | 0.351  485 | 1.70  7042 | 75 |
| {BcPV3.BI=FALSE, BcULV4=FALSE} =>  {BcOLV18=FALSE} | 0.18  3168 | 0.813  187 | 0.225  248 | 1.68  4756 | 74 |
| {BcOLV18=FALSE, BcPV3.BI=FALSE} =>  {BcULV4=FALSE} | 0.18  3168 | 0.480  519 | 0.381  188 | 1.55  3039 | 74 |
| {BcOLV18=FALSE, BcULV4=FALSE} =>  {BcRV1.BI=FALSE} | 0.20  7921 | 0.893  617 | 0.232  673 | 1.23  6374 | 84 |
| {BcRV1.BI=FALSE, BcULV4=FALSE} =>  {BcOLV18=FALSE} | 0.20  7921 | 0.756  757 | 0.274  752 | 1.56  7845 | 84 |
| {BcOLV18=FALSE, BcRV1.BI=FALSE} =>  {BcULV4=FALSE} | 0.20  7921 | 0.528  302 | 0.393  564 | 1.70  7472 | 84 |
| {BcEV2.BI=FALSE, BcULV4=FALSE} =>  {BcOLV18=FALSE} | 0.16  0891 | 0.738  636 | 0.217  822 | 1.53  0303 | 65 |
| {BcEV2.BI=FALSE, BcOLV18=FALSE} =>  {BcULV4=FALSE} | 0.16  0891 | 0.424  837 | 0.378  713 | 1.37  3072 | 65 |
| {BcHV3.BI=FALSE, BcULV4=FALSE} => | 0.19 | 0.787 | 0.245 | 1.63 | 78 |

| {BcOLV18=FALSE} | 3069 | 879 | 05 | 2323 |  |
| --- | --- | --- | --- | --- | --- |
| {BcHV3.BI=FALSE, BcOLV18=FALSE} =>  {BcULV4=FALSE} | 0.19  3069 | 0.472  727 | 0.408  416 | 1.52  7855 | 78 |
| {BcFV5.BI=FALSE, BcULV4=FALSE} =>  {BcOLV18=FALSE} | 0.19  5545 | 0.774  51 | 0.252  475 | 1.60  4625 | 79 |
| {BcFV5.BI=FALSE, BcOLV18=FALSE} =>  {BcULV4=FALSE} | 0.19  5545 | 0.473  054 | 0.413  366 | 1.52  891 | 79 |
| {BcOLV18=FALSE, BcULV4=FALSE} =>  {BcULV1.BI=FALSE} | 0.21  5347 | 0.925  532 | 0.232  673 | 1.17  9542 | 87 |
| {BcULV1.BI=FALSE, BcULV4=FALSE} =>  {BcOLV18=FALSE} | 0.21  5347 | 0.75 | 0.287  129 | 1.55  3846 | 87 |
| {BcOLV18=FALSE, BcULV1.BI=FALSE} =>  {BcULV4=FALSE} | 0.21  5347 | 0.502  89 | 0.428  218 | 1.62  5341 | 87 |
| {BcMV1.BI=TRUE, BcULV4=FALSE} =>  {BcOLV18=FALSE} | 0.17  8218 | 0.742  268 | 0.240  099 | 1.53  7827 | 72 |
| {BcMV1.BI=TRUE, BcOLV18=FALSE} =>  {BcULV4=FALSE} | 0.17  8218 | 0.510  638 | 0.349  01 | 1.65  0383 | 72 |
| {BcMV12=FALSE, BcULV4=FALSE} =>  {BcOLV18=FALSE} | 0.19  5545 | 0.782  178 | 0.25 | 1.62  0513 | 79 |
| {BcMV12=FALSE, BcOLV18=FALSE} =>  {BcULV4=FALSE} | 0.19  5545 | 0.484  663 | 0.403  465 | 1.56  6429 | 79 |
| {BcNSRV7.BI=FALSE, BcULV4=FALSE} => | 0.20 | 0.766 | 0.264 | 1.58 | 82 |

| {BcOLV18=FALSE} | 297 | 355 | 851 | 7731 |  |
| --- | --- | --- | --- | --- | --- |
| {BcNSRV7.BI=FALSE, BcOLV18=FALSE} =>  {BcULV4=FALSE} | 0.20  297 | 0.493  976 | 0.410  891 | 1.59  653 | 82 |
| {BcULV4=FALSE, BcVV3.BI=FALSE} =>  {BcOLV18=FALSE} | 0.19  0594 | 0.747  573 | 0.254  95 | 1.54  8818 | 77 |
| {BcOLV18=FALSE, BcVV3.BI=FALSE} =>  {BcULV4=FALSE} | 0.19  0594 | 0.487  342 | 0.391  089 | 1.57  5089 | 77 |
| {BcOLV18=FALSE, BcULV4=FALSE} =>  {BcOLV7.BI=FALSE} | 0.22  0297 | 0.946  809 | 0.232  673 | 1.17  3346 | 89 |
| {BcOLV7.BI=FALSE, BcULV4=FALSE} =>  {BcOLV18=FALSE} | 0.22  0297 | 0.747  899 | 0.294  554 | 1.54  9494 | 89 |
| {BcOLV18=FALSE, BcOLV7.BI=FALSE} =>  {BcULV4=FALSE} | 0.22  0297 | 0.511  494 | 0.430  693 | 1.65  3149 | 89 |
| {BcOLV18=FALSE, BcULV4=FALSE} =>  {BcALV1.BI=FALSE} | 0.21  5347 | 0.925  532 | 0.232  673 | 1.13  6519 | 87 |
| {BcALV1.BI=FALSE, BcULV4=FALSE} =>  {BcOLV18=FALSE} | 0.21  5347 | 0.756  522 | 0.284  653 | 1.56  7358 | 87 |
| {BcALV1.BI=FALSE, BcOLV18=FALSE} =>  {BcULV4=FALSE} | 0.21  5347 | 0.491  525 | 0.438  119 | 1.58  861 | 87 |
| {BcDMV1=FALSE, BcULV4=FALSE} =>  {BcOLV18=FALSE} | 0.20  7921 | 0.763  636 | 0.272  277 | 1.58  2098 | 84 |
| {BcDMV1=FALSE, BcOLV18=FALSE} => | 0.20 | 0.5 | 0.415 | 1.61 | 84 |

| {BcULV4=FALSE} | 7921 |  | 842 | 6 |  |
| --- | --- | --- | --- | --- | --- |
| {BcFV9=FALSE, BcULV4=FALSE} =>  {BcOLV18=FALSE} | 0.21  0396 | 0.772  727 | 0.272  277 | 1.60  0932 | 85 |
| {BcFV9=FALSE, BcOLV18=FALSE} =>  {BcULV4=FALSE} | 0.21  0396 | 0.469  613 | 0.448  02 | 1.51  779 | 85 |
| {BcOLV4.BI=FALSE, BcULV4=FALSE} =>  {BcOLV18=FALSE} | 0.19  5545 | 0.766  99 | 0.254  95 | 1.58  9047 | 79 |
| {BcOLV18=FALSE, BcOLV4.BI=FALSE} =>  {BcULV4=FALSE} | 0.19  5545 | 0.456  647 | 0.428  218 | 1.47  5884 | 79 |
| {BcULV4=FALSE, BcULV5=FALSE} =>  {BcOLV18=FALSE} | 0.18  0693 | 0.737  374 | 0.245  05 | 1.52  7687 | 73 |
| {BcOLV18=FALSE, BcULV5=FALSE} =>  {BcULV4=FALSE} | 0.18  0693 | 0.442  424 | 0.408  416 | 1.42  9915 | 73 |
| {BcBNV5.BI=FALSE, BcULV4=FALSE} =>  {BcOLV18=FALSE} | 0.21  7822 | 0.752  137 | 0.289  604 | 1.55  8273 | 88 |
| {BcBNV5.BI=FALSE, BcOLV18=FALSE} =>  {BcULV4=FALSE} | 0.21  7822 | 0.478  261 | 0.455  446 | 1.54  5739 | 88 |
| {BcULV4=FALSE, BvF.BI=FALSE} =>  {BcOLV18=FALSE} | 0.20  5446 | 0.768  519 | 0.267  327 | 1.59  2213 | 83 |
| {BcOLV18=FALSE, BvF.BI=FALSE} =>  {BcULV4=FALSE} | 0.20  5446 | 0.468  927 | 0.438  119 | 1.51  5571 | 83 |
| {BcMCV5.BI=FALSE, BcULV4=FALSE} => | 0.22 | 0.771 | 0.292 | 1.59 | 91 |

| {BcOLV18=FALSE} | 5248 | 186 | 079 | 774 |  |
| --- | --- | --- | --- | --- | --- |
| {BcMCV5.BI=FALSE, BcOLV18=FALSE} =>  {BcULV4=FALSE} | 0.22  5248 | 0.476  44 | 0.472  772 | 1.53  9853 | 91 |
| {BcOLV13.BI=FALSE, BcULV4=FALSE} =>  {BcOLV18=FALSE} | 0.21  5347 | 0.756  522 | 0.284  653 | 1.56  7358 | 87 |
| {BcOLV13.BI=FALSE, BcOLV18=FALSE} =>  {BcULV4=FALSE} | 0.21  5347 | 0.478  022 | 0.450  495 | 1.54  4967 | 87 |
| {BcOLV2.BI=FALSE, BcULV4=FALSE} =>  {BcOLV18=FALSE} | 0.21  7822 | 0.758  621 | 0.287  129 | 1.57  1706 | 88 |
| {BcOLV18=FALSE, BcOLV2.BI=FALSE} =>  {BcULV4=FALSE} | 0.21  7822 | 0.473  118 | 0.460  396 | 1.52  9118 | 88 |
| {BcMCV3.BI=FALSE, BcULV4=FALSE} =>  {BcOLV18=FALSE} | 0.21  5347 | 0.737  288 | 0.292  079 | 1.52  751 | 87 |
| {BcMCV3.BI=FALSE, BcOLV18=FALSE} =>  {BcULV4=FALSE} | 0.21  5347 | 0.470  27 | 0.457  921 | 1.51  9914 | 87 |
| {BcPV4.BI=FALSE, BcULV4=FALSE} =>  {BcOLV18=FALSE} | 0.22  0297 | 0.767  241 | 0.287  129 | 1.58  9567 | 89 |
| {BcOLV18=FALSE, BcPV4.BI=FALSE} =>  {BcULV4=FALSE} | 0.22  0297 | 0.483  696 | 0.455  446 | 1.56  3304 | 89 |
| {BcEV6=FALSE, BcULV4=FALSE} =>  {BcOLV18=FALSE} | 0.22  0297 | 0.760  684 | 0.289  604 | 1.57  5981 | 89 |
| {BcEV6=FALSE, BcOLV18=FALSE} => | 0.22 | 0.486 | 0.452 | 1.57 | 89 |

| {BcULV4=FALSE} | 0297 | 339 | 97 | 1847 |  |
| --- | --- | --- | --- | --- | --- |
| {BcOLV17.BI=FALSE, BcULV4=FALSE} =>  {BcOLV18=FALSE} | 0.22  7723 | 0.754  098 | 0.301  98 | 1.56  2337 | 92 |
| {BcOLV17.BI=FALSE, BcOLV18=FALSE} =>  {BcULV4=FALSE} | 0.22  7723 | 0.484  211 | 0.470  297 | 1.56  4968 | 92 |
| {BcOLV15.BI=FALSE, BcULV4=FALSE} =>  {BcOLV18=FALSE} | 0.22  7723 | 0.779  661 | 0.292  079 | 1.61  5298 | 92 |
| {BcOLV15.BI=FALSE, BcOLV18=FALSE} =>  {BcULV4=FALSE} | 0.22  7723 | 0.481  675 | 0.472  772 | 1.55  6775 | 92 |
| {BcEV5=FALSE, BcULV4=FALSE} =>  {BcOLV18=FALSE} | 0.21  7822 | 0.745  763 | 0.292  079 | 1.54  5067 | 88 |
| {BcEV5=FALSE, BcOLV18=FALSE} =>  {BcULV4=FALSE} | 0.21  7822 | 0.470  588 | 0.462  871 | 1.52  0941 | 88 |
| {BcOLV18=TRUE, BcULV4=FALSE} =>  {BcOLV7.BI=FALSE} | 0.07  4257 | 0.967  742 | 0.076  733 | 1.19  9288 | 30 |
| {BcHV1SLR.BI=TRUE, BcULV4=FALSE} =>  {BcMV2.BI=FALSE} | 0.09  4059 | 0.666  667 | 0.141  089 | 1.24  1167 | 38 |
| {BcMV2.BI=FALSE, BcULV4=FALSE} =>  {BcMV4.BI=FALSE} | 0.13  1188 | 0.697  368 | 0.188  119 | 1.25  2164 | 53 |
| {BcMV4.BI=FALSE, BcULV4=FALSE} =>  {BcMV2.BI=FALSE} | 0.13  1188 | 0.688  312 | 0.190  594 | 1.28  1465 | 53 |
| {BcMV2.BI=FALSE, BcULV4=FALSE} => | 0.15 | 0.828 | 0.188 | 1.41 | 63 |

| {BcOLV16.BI=FALSE} | 5941 | 947 | 119 | 9045 |  |
| --- | --- | --- | --- | --- | --- |
| {BcOLV16.BI=FALSE, BcULV4=FALSE} =>  {BcMV2.BI=FALSE} | 0.15  5941 | 0.670  213 | 0.232  673 | 1.24  7769 | 63 |
| {BcMV2.BI=FALSE, BcOLV16.BI=FALSE} =>  {BcULV4=FALSE} | 0.15  5941 | 0.417  219 | 0.373  762 | 1.34  845 | 63 |
| {BcMV11=TRUE, BcMV2.BI=FALSE} =>  {BcULV4=FALSE} | 0.11  1386 | 0.405  405 | 0.274  752 | 1.31  027 | 45 |
| {BcMV2.BI=FALSE, BcULV4=FALSE} =>  {BcOLV1.BI.MOVA.=FALSE} | 0.16  5842 | 0.881  579 | 0.188  119 | 1.31  9103 | 67 |
| {BcOLV1.BI.MOVA.=FALSE, BcULV4=FALSE}  => {BcMV2.BI=FALSE} | 0.16  5842 | 0.744  444 | 0.222  772 | 1.38  597 | 67 |
| {BcMV9.BI=TRUE, BcULV4=FALSE} =>  {BcMV2.BI=FALSE} | 0.08  9109 | 0.679  245 | 0.131  188 | 1.26  4586 | 36 |
| {BcMV2.BI=FALSE, BcULV4=FALSE} =>  {BcMV6.BI=FALSE} | 0.16  0891 | 0.855  263 | 0.188  119 | 1.22  9631 | 65 |
| {BcMV6.BI=FALSE, BcULV4=FALSE} =>  {BcMV2.BI=FALSE} | 0.16  0891 | 0.65 | 0.247  525 | 1.21  0138 | 65 |
| {BcPV3.BI=FALSE, BcULV4=FALSE} =>  {BcMV2.BI=FALSE} | 0.14  604 | 0.648  352 | 0.225  248 | 1.20  7069 | 59 |
| {BcMV2.BI=FALSE, BcULV4=FALSE} =>  {BcRV1.BI=FALSE} | 0.16  8317 | 0.894  737 | 0.188  119 | 1.23  7924 | 68 |
| {BcMV2.BI=FALSE, BcRV1.BI=FALSE} => | 0.16 | 0.384 | 0.438 | 1.24 | 68 |

| {BcULV4=FALSE} | 8317 | 181 | 119 | 1672 |  |
| --- | --- | --- | --- | --- | --- |
| {BcMV2.BI=FALSE, BcULV4=FALSE} =>  {BcULV1.BI=FALSE} | 0.18  3168 | 0.973  684 | 0.188  119 | 1.24  091 | 74 |
| {BcULV1.BI=FALSE, BcULV4=FALSE} =>  {BcMV2.BI=FALSE} | 0.18  3168 | 0.637  931 | 0.287  129 | 1.18  7669 | 74 |
| {BcMV2.BI=FALSE, BcULV1.BI=FALSE} =>  {BcULV4=FALSE} | 0.18  3168 | 0.397  849 | 0.460  396 | 1.28  5849 | 74 |
| {BcMV12=FALSE, BcULV4=FALSE} =>  {BcMV2.BI=FALSE} | 0.16  5842 | 0.663  366 | 0.25 | 1.23  5023 | 67 |
| {BcULV4=FALSE, BcVV3.BI=FALSE} =>  {BcMV2.BI=FALSE} | 0.16  3366 | 0.640  777 | 0.254  95 | 1.19  2967 | 66 |
| {BcMV2.BI=FALSE, BcULV4=FALSE} =>  {BcOLV7.BI=FALSE} | 0.18  0693 | 0.960  526 | 0.188  119 | 1.19  0345 | 73 |
| {BcMV2.BI=FALSE, BcOLV7.BI=FALSE} =>  {BcULV4=FALSE} | 0.18  0693 | 0.403  315 | 0.448  02 | 1.30  3514 | 73 |
| {BcMV2.BI=FALSE, BcULV4=FALSE} =>  {BcALV1.BI=FALSE} | 0.17  8218 | 0.947  368 | 0.188  119 | 1.16  3334 | 72 |
| {BcOLV4.BI=FALSE, BcULV4=FALSE} =>  {BcMV2.BI=FALSE} | 0.17  0792 | 0.669  903 | 0.254  95 | 1.24  7193 | 69 |
| {BcULV4=FALSE, BcULV5=FALSE} =>  {BcMV2.BI=FALSE} | 0.16  8317 | 0.686  869 | 0.245  05 | 1.27  8779 | 68 |
| {BcOLV13.BI=FALSE, BcULV4=FALSE} => | 0.18 | 0.652 | 0.284 | 1.21 | 75 |

| {BcMV2.BI=FALSE} | 5644 | 174 | 653 | 4186 |  |
| --- | --- | --- | --- | --- | --- |
| {BcMCV3.BI=FALSE, BcULV4=FALSE} =>  {BcMV2.BI=FALSE} | 0.18  5644 | 0.635  593 | 0.292  079 | 1.18  3316 | 75 |
| {BcHV1SLR.BI=TRUE, BcULV4=FALSE} =>  {BcOLV16.BI=FALSE} | 0.10  396 | 0.736  842 | 0.141  089 | 1.26  1374 | 42 |
| {BcHV1SLR.BI=TRUE, BcULV4=FALSE} =>  {BcULV3=TRUE} | 0.10  6436 | 0.754  386 | 0.141  089 | 1.26  9883 | 43 |
| {BcHV1SLR.BI=TRUE, BcULV4=FALSE} =>  {BcHV1.BI=TRUE} | 0.12  1287 | 0.859  649 | 0.141  089 | 1.42  3353 | 49 |
| {BcHV1.BI=TRUE, BcULV4=FALSE} =>  {BcHV1SLR.BI=TRUE} | 0.12  1287 | 0.816  667 | 0.148  515 | 1.50  6545 | 49 |
| {BcHV1SLR.BI=TRUE, BcULV4=FALSE} =>  {BcOLV1.BI.MOVA.=FALSE} | 0.10  8911 | 0.771  93 | 0.141  089 | 1.15  5036 | 44 |
| {BcHV1SLR.BI=TRUE, BcULV4=FALSE} =>  {BcRV1.BI=FALSE} | 0.13  8614 | 0.982  456 | 0.141  089 | 1.35  9289 | 56 |
| {BcHV1SLR.BI=TRUE, BcULV4=FALSE} =>  {BcULV1.BI=FALSE} | 0.12  3762 | 0.877  193 | 0.141  089 | 1.11  7937 | 50 |
| {BcHV1SLR.BI=TRUE, BcULV4=FALSE} =>  {BcOLV7.BI=FALSE} | 0.13  6139 | 0.964  912 | 0.141  089 | 1.19  5781 | 55 |
| {BcHV1SLR.BI=TRUE, BcULV4=FALSE} =>  {BcALV1.BI=FALSE} | 0.13  3663 | 0.947  368 | 0.141  089 | 1.16  3334 | 54 |
| {BcMV3.BI=TRUE, BcULV4=FALSE} => | 0.11 | 0.842 | 0.141 | 1.16 | 48 |

| {BcRV1.BI=FALSE} | 8812 | 105 | 089 | 5105 |  |
| --- | --- | --- | --- | --- | --- |
| {BcMV3.BI=TRUE, BcULV4=FALSE} =>  {BcULV1.BI=FALSE} | 0.12  8713 | 0.912  281 | 0.141  089 | 1.16  2654 | 52 |
| {BcMV3.BI=TRUE, BcULV4=FALSE} =>  {BcOLV7.BI=FALSE} | 0.12  8713 | 0.912  281 | 0.141  089 | 1.13  0556 | 52 |
| {BcMV3.BI=TRUE, BcULV4=FALSE} =>  {BcALV1.BI=FALSE} | 0.13  3663 | 0.947  368 | 0.141  089 | 1.16  3334 | 54 |
| {BcMV4.BI=FALSE, BcULV4=FALSE} =>  {BcOLV16.BI=FALSE} | 0.16  5842 | 0.870  13 | 0.190  594 | 1.48  9544 | 67 |
| {BcOLV16.BI=FALSE, BcULV4=FALSE} =>  {BcMV4.BI=FALSE} | 0.16  5842 | 0.712  766 | 0.232  673 | 1.27  9811 | 67 |
| {BcMV4.BI=FALSE, BcOLV16.BI=FALSE} =>  {BcULV4=FALSE} | 0.16  5842 | 0.452  703 | 0.366  337 | 1.46  3135 | 67 |
| {BcMV4.BI=FALSE, BcULV4=FALSE} =>  {BcMV6.BI=FALSE} | 0.15  5941 | 0.818  182 | 0.190  594 | 1.17  6318 | 63 |
| {BcMV4.BI=FALSE, BcULV4=FALSE} =>  {BcPV3.BI=FALSE} | 0.15  8416 | 0.831  169 | 0.190  594 | 1.17  0008 | 64 |
| {BcPV3.BI=FALSE, BcULV4=FALSE} =>  {BcMV4.BI=FALSE} | 0.15  8416 | 0.703  297 | 0.225  248 | 1.26  2808 | 64 |
| {BcMV4.BI=FALSE, BcULV4=FALSE} =>  {BcRV1.BI=FALSE} | 0.17  0792 | 0.896  104 | 0.190  594 | 1.23  9815 | 69 |
| {BcMV4.BI=FALSE, BcRV1.BI=FALSE} => | 0.17 | 0.383 | 0.445 | 1.23 | 69 |

| {BcULV4=FALSE} | 0792 | 333 | 545 | 8933 |  |
| --- | --- | --- | --- | --- | --- |
| {BcMV4.BI=FALSE, BcULV4=FALSE} =>  {BcHV3.BI=FALSE} | 0.16  5842 | 0.870  13 | 0.190  594 | 1.14  5057 | 67 |
| {BcHV3.BI=FALSE, BcULV4=FALSE} =>  {BcMV4.BI=FALSE} | 0.16  5842 | 0.676  768 | 0.245  05 | 1.21  5174 | 67 |
| {BcMV4.BI=FALSE, BcULV4=FALSE} =>  {BcFV5.BI=FALSE} | 0.16  8317 | 0.883  117 | 0.190  594 | 1.12  9048 | 68 |
| {BcFV5.BI=FALSE, BcULV4=FALSE} =>  {BcMV4.BI=FALSE} | 0.16  8317 | 0.666  667 | 0.252  475 | 1.19  7037 | 68 |
| {BcMV4.BI=FALSE, BcULV4=FALSE} =>  {BcULV1.BI=FALSE} | 0.18  0693 | 0.948  052 | 0.190  594 | 1.20  8243 | 73 |
| {BcMV4.BI=FALSE, BcULV1.BI=FALSE} =>  {BcULV4=FALSE} | 0.18  0693 | 0.401  099 | 0.450  495 | 1.29  6352 | 73 |
| {BcMV4.BI=FALSE, BcULV4=FALSE} =>  {BcOLV7.BI=FALSE} | 0.17  8218 | 0.935  065 | 0.190  594 | 1.15  8792 | 72 |
| {BcMV4.BI=FALSE, BcOLV7.BI=FALSE} =>  {BcULV4=FALSE} | 0.17  8218 | 0.378  947 | 0.470  297 | 1.22  4758 | 72 |
| {BcMV4.BI=FALSE, BcULV4=FALSE} =>  {BcALV1.BI=FALSE} | 0.18  5644 | 0.974  026 | 0.190  594 | 1.19  6068 | 75 |
| {BcALV1.BI=FALSE, BcULV4=FALSE} =>  {BcMV4.BI=FALSE} | 0.18  5644 | 0.652  174 | 0.284  653 | 1.17  1014 | 75 |
| {BcALV1.BI=FALSE, BcMV4.BI=FALSE} => | 0.18 | 0.371 | 0.5 | 1.2 | 75 |

| {BcULV4=FALSE} | 5644 | 287 |  |  |  |
| --- | --- | --- | --- | --- | --- |
| {BcMV4.BI=FALSE, BcULV4=FALSE} =>  {BcDMV1=FALSE} | 0.17  3267 | 0.909  091 | 0.190  594 | 1.07  7046 | 70 |
| {BcOLV4.BI=FALSE, BcULV4=FALSE} =>  {BcMV4.BI=FALSE} | 0.16  8317 | 0.660  194 | 0.254  95 | 1.18  5415 | 68 |
| {BcULV3=TRUE, BcULV4=FALSE} =>  {BcOLV16.BI=FALSE} | 0.13  8614 | 0.777  778 | 0.178  218 | 1.33  145 | 56 |
| {BcOLV16.BI=FALSE, BcULV3=TRUE} =>  {BcULV4=FALSE} | 0.13  8614 | 0.440  945 | 0.314  356 | 1.42  5134 | 56 |
| {BcHV1.BI=TRUE, BcULV4=FALSE} =>  {BcOLV16.BI=FALSE} | 0.10  8911 | 0.733  333 | 0.148  515 | 1.25  5367 | 44 |
| {BcMV11=TRUE, BcULV4=FALSE} =>  {BcOLV16.BI=FALSE} | 0.15  3465 | 0.775 | 0.198  02 | 1.32  6695 | 62 |
| {BcMV11=TRUE, BcOLV16.BI=FALSE} =>  {BcULV4=FALSE} | 0.15  3465 | 0.449  275 | 0.341  584 | 1.45  2058 | 62 |
| {BcOLV16.BI=FALSE, BcULV4=FALSE} =>  {BcOLV1.BI.MOVA.=FALSE} | 0.19  0594 | 0.819  149 | 0.232  673 | 1.22  569 | 77 |
| {BcOLV1.BI.MOVA.=FALSE, BcULV4=FALSE}  => {BcOLV16.BI=FALSE} | 0.19  0594 | 0.855  556 | 0.222  772 | 1.46  4595 | 77 |
| {BcOLV1.BI.MOVA.=FALSE,  BcOLV16.BI=FALSE} => {BcULV4=FALSE} | 0.19  0594 | 0.430  168 | 0.443  069 | 1.39  0302 | 77 |
| {BcMV9.BI=TRUE, BcULV4=FALSE} => | 0.09 | 0.754 | 0.131 | 1.29 | 40 |

| {BcOLV16.BI=FALSE} | 901 | 717 | 188 | 1973 |  |
| --- | --- | --- | --- | --- | --- |
| {BcOLV16.BI=FALSE, BcULV4=FALSE} =>  {BcMV6.BI=FALSE} | 0.18  5644 | 0.797  872 | 0.232  673 | 1.14  7119 | 75 |
| {BcMV6.BI=FALSE, BcULV4=FALSE} =>  {BcOLV16.BI=FALSE} | 0.18  5644 | 0.75 | 0.247  525 | 1.28  3898 | 75 |
| {BcMV6.BI=FALSE, BcOLV16.BI=FALSE} =>  {BcULV4=FALSE} | 0.18  5644 | 0.416  667 | 0.445  545 | 1.34  6667 | 75 |
| {BcOLV16.BI=FALSE, BcULV4=FALSE} =>  {BcPV3.BI=FALSE} | 0.18  5644 | 0.797  872 | 0.232  673 | 1.12  3137 | 75 |
| {BcPV3.BI=FALSE, BcULV4=FALSE} =>  {BcOLV16.BI=FALSE} | 0.18  5644 | 0.824  176 | 0.225  248 | 1.41  0877 | 75 |
| {BcOLV16.BI=FALSE, BcPV3.BI=FALSE} =>  {BcULV4=FALSE} | 0.18  5644 | 0.423  729 | 0.438  119 | 1.36  9492 | 75 |
| {BcOLV16.BI=FALSE, BcULV4=FALSE} =>  {BcRV1.BI=FALSE} | 0.20  5446 | 0.882  979 | 0.232  673 | 1.22  1655 | 83 |
| {BcRV1.BI=FALSE, BcULV4=FALSE} =>  {BcOLV16.BI=FALSE} | 0.20  5446 | 0.747  748 | 0.274  752 | 1.28  0043 | 83 |
| {BcOLV16.BI=FALSE, BcRV1.BI=FALSE} =>  {BcULV4=FALSE} | 0.20  5446 | 0.448  649 | 0.457  921 | 1.45  0032 | 83 |
| {BcEV2.BI=FALSE, BcULV4=FALSE} =>  {BcOLV16.BI=FALSE} | 0.15  8416 | 0.727  273 | 0.217  822 | 1.24  4992 | 64 |
| {BcHV3.BI=FALSE, BcULV4=FALSE} => | 0.18 | 0.757 | 0.245 | 1.29 | 75 |

| {BcOLV16.BI=FALSE} | 5644 | 576 | 05 | 6867 |  |
| --- | --- | --- | --- | --- | --- |
| {BcHV3.BI=FALSE, BcOLV16.BI=FALSE} =>  {BcULV4=FALSE} | 0.18  5644 | 0.423  729 | 0.438  119 | 1.36  9492 | 75 |
| {BcFV5.BI=FALSE, BcULV4=FALSE} =>  {BcOLV16.BI=FALSE} | 0.19  0594 | 0.754  902 | 0.252  475 | 1.29  229 | 77 |
| {BcFV5.BI=FALSE, BcOLV16.BI=FALSE} =>  {BcULV4=FALSE} | 0.19  0594 | 0.413  978 | 0.460  396 | 1.33  7978 | 77 |
| {BcOLV16.BI=FALSE, BcULV4=FALSE} =>  {BcULV1.BI=FALSE} | 0.22  5248 | 0.968  085 | 0.232  673 | 1.23  3774 | 91 |
| {BcULV1.BI=FALSE, BcULV4=FALSE} =>  {BcOLV16.BI=FALSE} | 0.22  5248 | 0.784  483 | 0.287  129 | 1.34  2928 | 91 |
| {BcOLV16.BI=FALSE, BcULV1.BI=FALSE} =>  {BcULV4=FALSE} | 0.22  5248 | 0.437  5 | 0.514  851 | 1.41  4 | 91 |
| {BcMV1.BI=TRUE, BcULV4=FALSE} =>  {BcOLV16.BI=FALSE} | 0.17  5743 | 0.731  959 | 0.240  099 | 1.25  3014 | 71 |
| {BcMV1.BI=TRUE, BcOLV16.BI=FALSE} =>  {BcULV4=FALSE} | 0.17  5743 | 0.387  978 | 0.452  97 | 1.25  3945 | 71 |
| {BcMV12=FALSE, BcULV4=FALSE} =>  {BcOLV16.BI=FALSE} | 0.19  802 | 0.792  079 | 0.25 | 1.35  5932 | 80 |
| {BcMV12=FALSE, BcOLV16.BI=FALSE} =>  {BcULV4=FALSE} | 0.19  802 | 0.392  157 | 0.504  95 | 1.26  7451 | 80 |
| {BcNSRV7.BI=FALSE, BcULV4=FALSE} => | 0.19 | 0.728 | 0.264 | 1.24 | 78 |

| {BcOLV16.BI=FALSE} | 3069 | 972 | 851 | 7901 |  |
| --- | --- | --- | --- | --- | --- |
| {BcNSRV7.BI=FALSE, BcOLV16.BI=FALSE}  => {BcULV4=FALSE} | 0.19  3069 | 0.414  894 | 0.465  347 | 1.34  0936 | 78 |
| {BcULV4=FALSE, BcVV3.BI=FALSE} =>  {BcOLV16.BI=FALSE} | 0.20  0495 | 0.786  408 | 0.254  95 | 1.34  6223 | 81 |
| {BcOLV16.BI=FALSE, BcVV3.BI=FALSE} =>  {BcULV4=FALSE} | 0.20  0495 | 0.430  851 | 0.465  347 | 1.39  2511 | 81 |
| {BcOLV16.BI=FALSE, BcULV4=FALSE} =>  {BcOLV7.BI=FALSE} | 0.22  2772 | 0.957  447 | 0.232  673 | 1.18  6529 | 90 |
| {BcOLV7.BI=FALSE, BcULV4=FALSE} =>  {BcOLV16.BI=FALSE} | 0.22  2772 | 0.756  303 | 0.294  554 | 1.29  4687 | 90 |
| {BcOLV16.BI=FALSE, BcOLV7.BI=FALSE} =>  {BcULV4=FALSE} | 0.22  2772 | 0.434  783 | 0.512  376 | 1.40  5217 | 90 |
| {BcOLV16.BI=FALSE, BcULV4=FALSE} =>  {BcALV1.BI=FALSE} | 0.21  5347 | 0.925  532 | 0.232  673 | 1.13  6519 | 87 |
| {BcALV1.BI=FALSE, BcULV4=FALSE} =>  {BcOLV16.BI=FALSE} | 0.21  5347 | 0.756  522 | 0.284  653 | 1.29  5063 | 87 |
| {BcALV1.BI=FALSE, BcOLV16.BI=FALSE} =>  {BcULV4=FALSE} | 0.21  5347 | 0.426  471 | 0.504  95 | 1.37  8353 | 87 |
| {BcDMV1=FALSE, BcULV4=FALSE} =>  {BcOLV16.BI=FALSE} | 0.20  0495 | 0.736  364 | 0.272  277 | 1.26  0555 | 81 |
| {BcDMV1=FALSE, BcOLV16.BI=FALSE} => | 0.20 | 0.409 | 0.490 | 1.32 | 81 |

| {BcULV4=FALSE} | 0495 | 091 | 099 | 2182 |  |
| --- | --- | --- | --- | --- | --- |
| {BcFV9=FALSE, BcULV4=FALSE} =>  {BcOLV16.BI=FALSE} | 0.20  5446 | 0.754  545 | 0.272  277 | 1.29  168 | 83 |
| {BcFV9=FALSE, BcOLV16.BI=FALSE} =>  {BcULV4=FALSE} | 0.20  5446 | 0.402  913 | 0.509  901 | 1.30  2214 | 83 |
| {BcOLV4.BI=FALSE, BcULV4=FALSE} =>  {BcOLV16.BI=FALSE} | 0.21  0396 | 0.825  243 | 0.254  95 | 1.41  2704 | 85 |
| {BcOLV16.BI=FALSE, BcOLV4.BI=FALSE} =>  {BcULV4=FALSE} | 0.21  0396 | 0.395  349 | 0.532  178 | 1.27  7767 | 85 |
| {BcULV4=FALSE, BcULV5=FALSE} =>  {BcOLV16.BI=FALSE} | 0.19  5545 | 0.797  98 | 0.245  05 | 1.36  6033 | 79 |
| {BcOLV16.BI=FALSE, BcULV5=FALSE} =>  {BcULV4=FALSE} | 0.19  5545 | 0.372  642 | 0.524  752 | 1.20  4377 | 79 |
| {BcBNV5.BI=FALSE, BcULV4=FALSE} =>  {BcOLV16.BI=FALSE} | 0.21  5347 | 0.743  59 | 0.289  604 | 1.27  2925 | 87 |
| {BcBNV5.BI=FALSE, BcOLV16.BI=FALSE} =>  {BcULV4=FALSE} | 0.21  5347 | 0.402  778 | 0.534  653 | 1.30  1778 | 87 |
| {BcULV4=FALSE, BvF.BI=FALSE} =>  {BcOLV16.BI=FALSE} | 0.20  297 | 0.759  259 | 0.267  327 | 1.29  9749 | 82 |
| {BcOLV16.BI=FALSE, BvF.BI=FALSE} =>  {BcULV4=FALSE} | 0.20  297 | 0.388  626 | 0.522  277 | 1.25  6038 | 82 |
| {BcMCV5.BI=FALSE, BcULV4=FALSE} => | 0.22 | 0.762 | 0.292 | 1.30 | 90 |

| {BcOLV16.BI=FALSE} | 2772 | 712 | 079 | 5659 |  |
| --- | --- | --- | --- | --- | --- |
| {BcMCV5.BI=FALSE, BcOLV16.BI=FALSE} =>  {BcULV4=FALSE} | 0.22  2772 | 0.4 | 0.556  931 | 1.29  28 | 90 |
| {BcOLV13.BI=FALSE, BcULV4=FALSE} =>  {BcOLV16.BI=FALSE} | 0.22  7723 | 0.8 | 0.284  653 | 1.36  9492 | 92 |
| {BcOLV13.BI=FALSE, BcOLV16.BI=FALSE}  => {BcULV4=FALSE} | 0.22  7723 | 0.407  08 | 0.559  406 | 1.31  5681 | 92 |
| {BcOLV2.BI=FALSE, BcULV4=FALSE} =>  {BcOLV16.BI=FALSE} | 0.22  2772 | 0.775  862 | 0.287  129 | 1.32  8171 | 90 |
| {BcOLV16.BI=FALSE, BcOLV2.BI=FALSE} =>  {BcULV4=FALSE} | 0.22  2772 | 0.398  23 | 0.559  406 | 1.28  708 | 90 |
| {BcMCV3.BI=FALSE, BcULV4=FALSE} =>  {BcOLV16.BI=FALSE} | 0.23  0198 | 0.788  136 | 0.292  079 | 1.34  9181 | 93 |
| {BcMCV3.BI=FALSE, BcOLV16.BI=FALSE} =>  {BcULV4=FALSE} | 0.23  0198 | 0.400  862 | 0.574  257 | 1.29  5586 | 93 |
| {BcPV4.BI=FALSE, BcULV4=FALSE} =>  {BcOLV16.BI=FALSE} | 0.22  0297 | 0.767  241 | 0.287  129 | 1.31  3413 | 89 |
| {BcOLV16.BI=FALSE, BcPV4.BI=FALSE} =>  {BcULV4=FALSE} | 0.22  0297 | 0.397  321 | 0.554  455 | 1.28  4143 | 89 |
| {BcEV6=FALSE, BcULV4=FALSE} =>  {BcOLV16.BI=FALSE} | 0.22  0297 | 0.760  684 | 0.289  604 | 1.30  2187 | 89 |
| {BcEV6=FALSE, BcOLV16.BI=FALSE} => | 0.22 | 0.404 | 0.544 | 1.30 | 89 |

| {BcULV4=FALSE} | 0297 | 545 | 554 | 7491 |  |
| --- | --- | --- | --- | --- | --- |
| {BcOLV17.BI=FALSE, BcULV4=FALSE} =>  {BcOLV16.BI=FALSE} | 0.22  7723 | 0.754  098 | 0.301  98 | 1.29  0914 | 92 |
| {BcOLV16.BI=FALSE, BcOLV17.BI=FALSE}  => {BcULV4=FALSE} | 0.22  7723 | 0.405  286 | 0.561  881 | 1.30  9885 | 92 |
| {BcOLV15.BI=FALSE, BcULV4=FALSE} =>  {BcOLV16.BI=FALSE} | 0.22  0297 | 0.754  237 | 0.292  079 | 1.29  1152 | 89 |
| {BcOLV15.BI=FALSE, BcOLV16.BI=FALSE}  => {BcULV4=FALSE} | 0.22  0297 | 0.402  715 | 0.547  03 | 1.30  1575 | 89 |
| {BcEV5=FALSE, BcULV4=FALSE} =>  {BcOLV16.BI=FALSE} | 0.21  7822 | 0.745  763 | 0.292  079 | 1.27  6645 | 88 |
| {BcEV5=FALSE, BcOLV16.BI=FALSE} =>  {BcULV4=FALSE} | 0.21  7822 | 0.391  111 | 0.556  931 | 1.26  4071 | 88 |
| {BcHV1.BI=TRUE, BcULV4=FALSE} =>  {BcULV3=TRUE} | 0.11  3861 | 0.766  667 | 0.148  515 | 1.29  0556 | 46 |
| {BcULV3=TRUE, BcULV4=FALSE} =>  {BcOLV1.BI.MOVA.=FALSE} | 0.13  6139 | 0.763  889 | 0.178  218 | 1.14  3004 | 55 |
| {BcULV3=TRUE, BcULV4=FALSE} =>  {BcRV1.BI=FALSE} | 0.16  5842 | 0.930  556 | 0.178  218 | 1.28  7481 | 67 |
| {BcRV1.BI=FALSE, BcULV3=TRUE} =>  {BcULV4=FALSE} | 0.16  5842 | 0.382  857 | 0.433  168 | 1.23  7394 | 67 |
| {BcULV3=TRUE, BcULV4=FALSE} => | 0.16 | 0.902 | 0.178 | 1.15 | 65 |

| {BcULV1.BI=FALSE} | 0891 | 778 | 218 | 0543 |  |
| --- | --- | --- | --- | --- | --- |
| {BcULV3=TRUE, BcULV4=FALSE} =>  {BcOLV7.BI=FALSE} | 0.17  3267 | 0.972  222 | 0.178  218 | 1.20  484 | 70 |
| {BcULV3=TRUE, BcULV4=FALSE} =>  {BcALV1.BI=FALSE} | 0.16  0891 | 0.902  778 | 0.178  218 | 1.10  8578 | 65 |
| {BcHV1.BI=TRUE, BcULV4=FALSE} =>  {BcOLV1.BI.MOVA.=FALSE} | 0.11  6337 | 0.783  333 | 0.148  515 | 1.17  2099 | 47 |
| {BcHV1.BI=TRUE, BcULV4=FALSE} =>  {BcRV1.BI=FALSE} | 0.14  3564 | 0.966  667 | 0.148  515 | 1.33  7443 | 58 |
| {BcHV1.BI=TRUE, BcULV4=FALSE} =>  {BcULV1.BI=FALSE} | 0.13  3663 | 0.9 | 0.148  515 | 1.14  7003 | 54 |
| {BcHV1.BI=TRUE, BcULV4=FALSE} =>  {BcOLV7.BI=FALSE} | 0.14  604 | 0.983  333 | 0.148  515 | 1.21  8609 | 59 |
| {BcHV1.BI=TRUE, BcULV4=FALSE} =>  {BcALV1.BI=FALSE} | 0.13  3663 | 0.9 | 0.148  515 | 1.10  5167 | 54 |
| {BcMV11=TRUE, BcULV4=FALSE} =>  {BcMV6.BI=FALSE} | 0.15  8416 | 0.8 | 0.198  02 | 1.15  0178 | 64 |
| {BcMV11=TRUE, BcMV6.BI=FALSE} =>  {BcULV4=FALSE} | 0.15  8416 | 0.407  643 | 0.388  614 | 1.31  7503 | 64 |
| {BcMV11=TRUE, BcULV4=FALSE} =>  {BcRV1.BI=FALSE} | 0.16  8317 | 0.85 | 0.198  02 | 1.17  6027 | 68 |
| {BcMV11=TRUE, BcRV1.BI=FALSE} => | 0.16 | 0.412 | 0.408 | 1.33 | 68 |

| {BcULV4=FALSE} | 8317 | 121 | 416 | 1976 |  |
| --- | --- | --- | --- | --- | --- |
| {BcMV11=TRUE, BcULV4=FALSE} =>  {BcULV1.BI=FALSE} | 0.18  0693 | 0.912  5 | 0.198  02 | 1.16  2934 | 73 |
| {BcMV11=TRUE, BcULV1.BI=FALSE} =>  {BcULV4=FALSE} | 0.18  0693 | 0.382  199 | 0.472  772 | 1.23  5267 | 73 |
| {BcMV11=TRUE, BcULV4=FALSE} =>  {BcMV12=FALSE} | 0.17  5743 | 0.887  5 | 0.198  02 | 1.11  0062 | 71 |
| {BcMV12=FALSE, BcULV4=FALSE} =>  {BcMV11=TRUE} | 0.17  5743 | 0.702  97 | 0.25 | 1.14  0562 | 71 |
| {BcMV11=TRUE, BcULV4=FALSE} =>  {BcOLV7.BI=FALSE} | 0.19  5545 | 0.987  5 | 0.198  02 | 1.22  3773 | 79 |
| {BcMV11=TRUE, BcOLV7.BI=FALSE} =>  {BcULV4=FALSE} | 0.19  5545 | 0.403  061 | 0.485  149 | 1.30  2694 | 79 |
| {BcALV1.BI=FALSE, BcMV11=TRUE} =>  {BcULV4=FALSE} | 0.17  5743 | 0.381  72 | 0.460  396 | 1.23  372 | 71 |
| {BcMV9.BI=TRUE, BcULV4=FALSE} =>  {BcOLV1.BI.MOVA.=FALSE} | 0.11  3861 | 0.867  925 | 0.131  188 | 1.29  8672 | 46 |
| {BcOLV1.BI.MOVA.=FALSE, BcULV4=FALSE}  => {BcMV6.BI=FALSE} | 0.18  8119 | 0.844  444 | 0.222  772 | 1.21  4077 | 76 |
| {BcMV6.BI=FALSE, BcULV4=FALSE} =>  {BcOLV1.BI.MOVA.=FALSE} | 0.18  8119 | 0.76 | 0.247  525 | 1.13  7185 | 76 |
| {BcOLV1.BI.MOVA.=FALSE, BcULV4=FALSE} | 0.19 | 0.877 | 0.222 | 1.21 | 79 |

| => {BcRV1.BI=FALSE} | 5545 | 778 | 772 | 446 |  |
| --- | --- | --- | --- | --- | --- |
| {BcOLV1.BI.MOVA.=FALSE,  BcRV1.BI=FALSE} => {BcULV4=FALSE} | 0.19  5545 | 0.372  642 | 0.524  752 | 1.20  4377 | 79 |
| {BcOLV1.BI.MOVA.=FALSE, BcULV4=FALSE}  => {BcULV1.BI=FALSE} | 0.21  5347 | 0.966  667 | 0.222  772 | 1.23  1966 | 87 |
| {BcOLV1.BI.MOVA.=FALSE,  BcULV1.BI=FALSE} => {BcULV4=FALSE} | 0.21  5347 | 0.384  956 | 0.559  406 | 1.24  4177 | 87 |
| {BcMV12=FALSE, BcULV4=FALSE} =>  {BcOLV1.BI.MOVA.=FALSE} | 0.19  0594 | 0.762  376 | 0.25 | 1.14  0741 | 77 |
| {BcULV4=FALSE, BcVV3.BI=FALSE} =>  {BcOLV1.BI.MOVA.=FALSE} | 0.19  5545 | 0.766  99 | 0.254  95 | 1.14  7645 | 79 |
| {BcOLV1.BI.MOVA.=FALSE, BcULV4=FALSE}  => {BcOLV7.BI=FALSE} | 0.21  5347 | 0.966  667 | 0.222  772 | 1.19  7955 | 87 |
| {BcOLV1.BI.MOVA.=FALSE,  BcOLV7.BI=FALSE} => {BcULV4=FALSE} | 0.21  5347 | 0.378  261 | 0.569  307 | 1.22  2539 | 87 |
| {BcOLV1.BI.MOVA.=FALSE, BcULV4=FALSE}  => {BcALV1.BI=FALSE} | 0.20  297 | 0.911  111 | 0.222  772 | 1.11  8811 | 82 |
| {BcOLV4.BI=FALSE, BcULV4=FALSE} =>  {BcOLV1.BI.MOVA.=FALSE} | 0.19  5545 | 0.766  99 | 0.254  95 | 1.14  7645 | 79 |
| {BcULV4=FALSE, BcULV5=FALSE} =>  {BcOLV1.BI.MOVA.=FALSE} | 0.20  297 | 0.828  283 | 0.245  05 | 1.23  9357 | 82 |
| {BcOLV13.BI=FALSE, BcULV4=FALSE} => | 0.21 | 0.765 | 0.284 | 1.14 | 88 |

| {BcOLV1.BI.MOVA.=FALSE} | 7822 | 217 | 653 | 4992 |  |
| --- | --- | --- | --- | --- | --- |
| {BcMCV3.BI=FALSE, BcULV4=FALSE} =>  {BcOLV1.BI.MOVA.=FALSE} | 0.22  0297 | 0.754  237 | 0.292  079 | 1.12  8562 | 89 |
| {BcMV9.BI=TRUE, BcULV4=FALSE} =>  {BcRV1.BI=FALSE} | 0.12  1287 | 0.924  528 | 0.131  188 | 1.27  9142 | 49 |
| {BcMV9.BI=TRUE, BcULV4=FALSE} =>  {BcULV1.BI=FALSE} | 0.12  8713 | 0.981  132 | 0.131  188 | 1.25  0402 | 52 |
| {BcMV9.BI=TRUE, BcULV4=FALSE} =>  {BcVV3.BI=FALSE} | 0.12  3762 | 0.943  396 | 0.131  188 | 1.17  6334 | 50 |
| {BcMV9.BI=TRUE, BcULV4=FALSE} =>  {BcOLV7.BI=FALSE} | 0.12  1287 | 0.924  528 | 0.131  188 | 1.14  5734 | 49 |
| {BcMV9.BI=TRUE, BcULV4=FALSE} =>  {BcALV1.BI=FALSE} | 0.12  3762 | 0.943  396 | 0.131  188 | 1.15  8456 | 50 |
| {BcMV9.BI=TRUE, BcULV4=FALSE} =>  {BcULV5=FALSE} | 0.13  1188 | 1 | 0.131  188 | 1.14  1243 | 53 |
| {BcPV3.BI=FALSE, BcULV4=FALSE} =>  {BcMV6.BI=FALSE} | 0.17  8218 | 0.791  209 | 0.225  248 | 1.13  7539 | 72 |
| {BcMV6.BI=FALSE, BcULV4=FALSE} =>  {BcRV1.BI=FALSE} | 0.21  5347 | 0.87 | 0.247  525 | 1.20  3699 | 87 |
| {BcMV6.BI=FALSE, BcRV1.BI=FALSE} =>  {BcULV4=FALSE} | 0.21  5347 | 0.404  651 | 0.532  178 | 1.30  7833 | 87 |
| {BcHV3.BI=FALSE, BcULV4=FALSE} => | 0.19 | 0.797 | 0.245 | 1.14 | 79 |

| {BcMV6.BI=FALSE} | 5545 | 98 | 05 | 7273 |  |
| --- | --- | --- | --- | --- | --- |
| {BcFV5.BI=FALSE, BcULV4=FALSE} =>  {BcMV6.BI=FALSE} | 0.20  0495 | 0.794  118 | 0.252  475 | 1.14  1721 | 81 |
| {BcFV5.BI=FALSE, BcMV6.BI=FALSE} =>  {BcULV4=FALSE} | 0.20  0495 | 0.368  182 | 0.544  554 | 1.18  9964 | 81 |
| {BcMV6.BI=FALSE, BcULV4=FALSE} =>  {BcULV1.BI=FALSE} | 0.23  2673 | 0.94 | 0.247  525 | 1.19  7981 | 94 |
| {BcULV1.BI=FALSE, BcULV4=FALSE} =>  {BcMV6.BI=FALSE} | 0.23  2673 | 0.810  345 | 0.287  129 | 1.16  5051 | 94 |
| {BcMV6.BI=FALSE, BcULV1.BI=FALSE} =>  {BcULV4=FALSE} | 0.23  2673 | 0.406  926 | 0.571  782 | 1.31  5186 | 94 |
| {BcMV1.BI=TRUE, BcULV4=FALSE} =>  {BcMV6.BI=FALSE} | 0.19  0594 | 0.793  814 | 0.240  099 | 1.14  1285 | 77 |
| {BcMV12=FALSE, BcULV4=FALSE} =>  {BcMV6.BI=FALSE} | 0.20  7921 | 0.831  683 | 0.25 | 1.19  573 | 84 |
| {BcNSRV7.BI=FALSE, BcULV4=FALSE} =>  {BcMV6.BI=FALSE} | 0.21  2871 | 0.803  738 | 0.264  851 | 1.15  5553 | 86 |
| {BcMV6.BI=FALSE, BcNSRV7.BI=FALSE} =>  {BcULV4=FALSE} | 0.21  2871 | 0.375  546 | 0.566  832 | 1.21  3764 | 86 |
| {BcULV4=FALSE, BcVV3.BI=FALSE} =>  {BcMV6.BI=FALSE} | 0.20  0495 | 0.786  408 | 0.254  95 | 1.13  0636 | 81 |
| {BcMV6.BI=FALSE, BcULV4=FALSE} => | 0.23 | 0.94 | 0.247 | 1.16 | 94 |

| {BcOLV7.BI=FALSE} | 2673 |  | 525 | 4908 |  |
| --- | --- | --- | --- | --- | --- |
| {BcOLV7.BI=FALSE, BcULV4=FALSE} =>  {BcMV6.BI=FALSE} | 0.23  2673 | 0.789  916 | 0.294  554 | 1.13  568 | 94 |
| {BcMV6.BI=FALSE, BcOLV7.BI=FALSE} =>  {BcULV4=FALSE} | 0.23  2673 | 0.405  172 | 0.574  257 | 1.30  9517 | 94 |
| {BcMV6.BI=FALSE, BcULV4=FALSE} =>  {BcALV1.BI=FALSE} | 0.22  2772 | 0.9 | 0.247  525 | 1.10  5167 | 90 |
| {BcALV1.BI=FALSE, BcULV4=FALSE} =>  {BcMV6.BI=FALSE} | 0.22  2772 | 0.782  609 | 0.284  653 | 1.12  5174 | 90 |
| {BcALV1.BI=FALSE, BcMV6.BI=FALSE} =>  {BcULV4=FALSE} | 0.22  2772 | 0.373  444 | 0.596  535 | 1.20  6971 | 90 |
| {BcDMV1=FALSE, BcULV4=FALSE} =>  {BcMV6.BI=FALSE} | 0.22  2772 | 0.818  182 | 0.272  277 | 1.17  6318 | 90 |
| {BcDMV1=FALSE, BcMV6.BI=FALSE} =>  {BcULV4=FALSE} | 0.22  2772 | 0.367  347 | 0.606  436 | 1.18  7265 | 90 |
| {BcFV9=FALSE, BcULV4=FALSE} =>  {BcMV6.BI=FALSE} | 0.22  0297 | 0.809  091 | 0.272  277 | 1.16  3248 | 89 |
| {BcFV9=FALSE, BcMV6.BI=FALSE} =>  {BcULV4=FALSE} | 0.22  0297 | 0.366  255 | 0.601  485 | 1.18  3737 | 89 |
| {BcOLV4.BI=FALSE, BcULV4=FALSE} =>  {BcMV6.BI=FALSE} | 0.20  7921 | 0.815  534 | 0.254  95 | 1.17  2511 | 84 |
| {BcULV4=FALSE, BcULV5=FALSE} => | 0.20 | 0.838 | 0.245 | 1.20 | 83 |

| {BcMV6.BI=FALSE} | 5446 | 384 | 05 | 5363 |  |
| --- | --- | --- | --- | --- | --- |
| {BcBNV5.BI=FALSE, BcULV4=FALSE} =>  {BcMV6.BI=FALSE} | 0.23  5149 | 0.811  966 | 0.289  604 | 1.16  7381 | 95 |
| {BcMCV5.BI=FALSE, BcULV4=FALSE} =>  {BcMV6.BI=FALSE} | 0.23  5149 | 0.805  085 | 0.292  079 | 1.15  7488 | 95 |
| {BcOLV13.BI=FALSE, BcULV4=FALSE} =>  {BcMV6.BI=FALSE} | 0.23  0198 | 0.808  696 | 0.284  653 | 1.16  268 | 93 |
| {BcOLV2.BI=FALSE, BcULV4=FALSE} =>  {BcMV6.BI=FALSE} | 0.23  2673 | 0.810  345 | 0.287  129 | 1.16  5051 | 94 |
| {BcMCV3.BI=FALSE, BcULV4=FALSE} =>  {BcMV6.BI=FALSE} | 0.24  2574 | 0.830  508 | 0.292  079 | 1.19  4041 | 98 |
| {BcMCV3.BI=FALSE, BcMV6.BI=FALSE} =>  {BcULV4=FALSE} | 0.24  2574 | 0.360  294 | 0.673  267 | 1.16  4471 | 98 |
| {BcPV4.BI=FALSE, BcULV4=FALSE} =>  {BcMV6.BI=FALSE} | 0.23  5149 | 0.818  966 | 0.287  129 | 1.17  7445 | 95 |
| {BcEV6=FALSE, BcULV4=FALSE} =>  {BcMV6.BI=FALSE} | 0.23  2673 | 0.803  419 | 0.289  604 | 1.15  5093 | 94 |
| {BcOLV17.BI=FALSE, BcULV4=FALSE} =>  {BcMV6.BI=FALSE} | 0.24  0099 | 0.795  082 | 0.301  98 | 1.14  3107 | 97 |
| {BcOLV15.BI=FALSE, BcULV4=FALSE} =>  {BcMV6.BI=FALSE} | 0.23  0198 | 0.788  136 | 0.292  079 | 1.13  312 | 93 |
| {BcEV5=FALSE, BcULV4=FALSE} => | 0.24 | 0.830 | 0.292 | 1.19 | 98 |

| {BcMV6.BI=FALSE} | 2574 | 508 | 079 | 4041 |  |
| --- | --- | --- | --- | --- | --- |
| {BcPV3.BI=FALSE, BcULV4=FALSE} =>  {BcRV1.BI=FALSE} | 0.20  0495 | 0.890  11 | 0.225  248 | 1.23  1522 | 81 |
| {BcPV3.BI=FALSE, BcULV4=FALSE} =>  {BcULV1.BI=FALSE} | 0.21  2871 | 0.945  055 | 0.225  248 | 1.20  4423 | 86 |
| {BcPV3.BI=FALSE, BcULV1.BI=FALSE} =>  {BcULV4=FALSE} | 0.21  2871 | 0.372  294 | 0.571  782 | 1.20  3255 | 86 |
| {BcPV3.BI=FALSE, BcULV4=FALSE} =>  {BcOLV7.BI=FALSE} | 0.21  5347 | 0.956  044 | 0.225  248 | 1.18  4791 | 87 |
| {BcPV3.BI=FALSE, BcULV4=FALSE} =>  {BcALV1.BI=FALSE} | 0.20  7921 | 0.923  077 | 0.225  248 | 1.13  3505 | 84 |
| {BcEV2.BI=FALSE, BcULV4=FALSE} =>  {BcRV1.BI=FALSE} | 0.19  802 | 0.909  091 | 0.217  822 | 1.25  7783 | 80 |
| {BcEV2.BI=FALSE, BcRV1.BI=FALSE} =>  {BcULV4=FALSE} | 0.19  802 | 0.370  37 | 0.534  653 | 1.19  7037 | 80 |
| {BcHV3.BI=FALSE, BcULV4=FALSE} =>  {BcRV1.BI=FALSE} | 0.21  2871 | 0.868  687 | 0.245  05 | 1.20  1882 | 86 |
| {BcHV3.BI=FALSE, BcRV1.BI=FALSE} =>  {BcULV4=FALSE} | 0.21  2871 | 0.389  14 | 0.547  03 | 1.25  7701 | 86 |
| {BcFV5.BI=FALSE, BcULV4=FALSE} =>  {BcRV1.BI=FALSE} | 0.22  0297 | 0.872  549 | 0.252  475 | 1.20  7225 | 89 |
| {BcFV5.BI=FALSE, BcRV1.BI=FALSE} => | 0.22 | 0.404 | 0.544 | 1.30 | 89 |

| {BcULV4=FALSE} | 0297 | 545 | 554 | 7491 |  |
| --- | --- | --- | --- | --- | --- |
| {BcRV1.BI=FALSE, BcULV4=FALSE} =>  {BcULV1.BI=FALSE} | 0.25  2475 | 0.918  919 | 0.274  752 | 1.17  1114 | 10  2 |
| {BcULV1.BI=FALSE, BcULV4=FALSE} =>  {BcRV1.BI=FALSE} | 0.25  2475 | 0.879  31 | 0.287  129 | 1.21  658 | 10  2 |
| {BcRV1.BI=FALSE, BcULV1.BI=FALSE} =>  {BcULV4=FALSE} | 0.25  2475 | 0.432  203 | 0.584  158 | 1.39  6881 | 10  2 |
| {BcMV1.BI=TRUE, BcULV4=FALSE} =>  {BcRV1.BI=FALSE} | 0.22  0297 | 0.917  526 | 0.240  099 | 1.26  9453 | 89 |
| {BcMV1.BI=TRUE, BcRV1.BI=FALSE} =>  {BcULV4=FALSE} | 0.22  0297 | 0.375  527 | 0.586  634 | 1.21  3705 | 89 |
| {BcMV12=FALSE, BcULV4=FALSE} =>  {BcRV1.BI=FALSE} | 0.21  7822 | 0.871  287 | 0.25 | 1.20  5479 | 88 |
| {BcMV12=FALSE, BcRV1.BI=FALSE} =>  {BcULV4=FALSE} | 0.21  7822 | 0.376  068 | 0.579  208 | 1.21  5453 | 88 |
| {BcNSRV7.BI=FALSE, BcULV4=FALSE} =>  {BcRV1.BI=FALSE} | 0.23  0198 | 0.869  159 | 0.264  851 | 1.20  2535 | 93 |
| {BcNSRV7.BI=FALSE, BcRV1.BI=FALSE} =>  {BcULV4=FALSE} | 0.23  0198 | 0.411  504 | 0.559  406 | 1.32  9982 | 93 |
| {BcULV4=FALSE, BcVV3.BI=FALSE} =>  {BcRV1.BI=FALSE} | 0.22  7723 | 0.893  204 | 0.254  95 | 1.23  5803 | 92 |
| {BcRV1.BI=FALSE, BcVV3.BI=FALSE} => | 0.22 | 0.4 | 0.569 | 1.29 | 92 |

| {BcULV4=FALSE} | 7723 |  | 307 | 28 |  |
| --- | --- | --- | --- | --- | --- |
| {BcRV1.BI=FALSE, BcULV4=FALSE} =>  {BcOLV7.BI=FALSE} | 0.25  9901 | 0.945  946 | 0.274  752 | 1.17  2277 | 10  5 |
| {BcOLV7.BI=FALSE, BcULV4=FALSE} =>  {BcRV1.BI=FALSE} | 0.25  9901 | 0.882  353 | 0.294  554 | 1.22  079 | 10  5 |
| {BcOLV7.BI=FALSE, BcRV1.BI=FALSE} =>  {BcULV4=FALSE} | 0.25  9901 | 0.406  977 | 0.638  614 | 1.31  5349 | 10  5 |
| {BcRV1.BI=FALSE, BcULV4=FALSE} =>  {BcALV1.BI=FALSE} | 0.25  495 | 0.927  928 | 0.274  752 | 1.13  9462 | 10  3 |
| {BcALV1.BI=FALSE, BcULV4=FALSE} =>  {BcRV1.BI=FALSE} | 0.25  495 | 0.895  652 | 0.284  653 | 1.23  919 | 10  3 |
| {BcALV1.BI=FALSE, BcRV1.BI=FALSE} =>  {BcULV4=FALSE} | 0.25  495 | 0.408  73 | 0.623  762 | 1.32  1016 | 10  3 |
| {BcDMV1=FALSE, BcULV4=FALSE} =>  {BcRV1.BI=FALSE} | 0.24  0099 | 0.881  818 | 0.272  277 | 1.22  005 | 97 |
| {BcDMV1=FALSE, BcRV1.BI=FALSE} =>  {BcULV4=FALSE} | 0.24  0099 | 0.409  283 | 0.586  634 | 1.32  2802 | 97 |
| {BcFV9=FALSE, BcULV4=FALSE} =>  {BcRV1.BI=FALSE} | 0.24  505 | 0.9 | 0.272  277 | 1.24  5205 | 99 |
| {BcFV9=FALSE, BcRV1.BI=FALSE} =>  {BcULV4=FALSE} | 0.24  505 | 0.385  214 | 0.636  139 | 1.24  5012 | 99 |
| {BcOLV4.BI=FALSE, BcULV4=FALSE} => | 0.22 | 0.893 | 0.254 | 1.23 | 92 |

| {BcRV1.BI=FALSE} | 7723 | 204 | 95 | 5803 |  |
| --- | --- | --- | --- | --- | --- |
| {BcULV4=FALSE, BcULV5=FALSE} =>  {BcRV1.BI=FALSE} | 0.21  5347 | 0.878  788 | 0.245  05 | 1.21  5857 | 87 |
| {BcBNV5.BI=FALSE, BcULV4=FALSE} =>  {BcRV1.BI=FALSE} | 0.25  495 | 0.880  342 | 0.289  604 | 1.21  8007 | 10  3 |
| {BcBNV5.BI=FALSE, BcRV1.BI=FALSE} =>  {BcULV4=FALSE} | 0.25  495 | 0.393  13 | 0.648  515 | 1.27  0595 | 10  3 |
| {BcULV4=FALSE, BvF.BI=FALSE} =>  {BcRV1.BI=FALSE} | 0.23  5149 | 0.879  63 | 0.267  327 | 1.21  7022 | 95 |
| {BcRV1.BI=FALSE, BvF.BI=FALSE} =>  {BcULV4=FALSE} | 0.23  5149 | 0.363  985 | 0.646  04 | 1.17  6398 | 95 |
| {BcMCV5.BI=FALSE, BcULV4=FALSE} =>  {BcRV1.BI=FALSE} | 0.26  2376 | 0.898  305 | 0.292  079 | 1.24  286 | 10  6 |
| {BcMCV5.BI=FALSE, BcRV1.BI=FALSE} =>  {BcULV4=FALSE} | 0.26  2376 | 0.389  706 | 0.673  267 | 1.25  9529 | 10  6 |
| {BcOLV13.BI=FALSE, BcULV4=FALSE} =>  {BcRV1.BI=FALSE} | 0.25  9901 | 0.913  043 | 0.284  653 | 1.26  3252 | 10  5 |
| {BcOLV13.BI=FALSE, BcRV1.BI=FALSE} =>  {BcULV4=FALSE} | 0.25  9901 | 0.376  344 | 0.690  594 | 1.21  6344 | 10  5 |
| {BcOLV2.BI=FALSE, BcULV4=FALSE} =>  {BcRV1.BI=FALSE} | 0.25  7426 | 0.896  552 | 0.287  129 | 1.24  0435 | 10  4 |
| {BcOLV2.BI=FALSE, BcRV1.BI=FALSE} => | 0.25 | 0.376 | 0.683 | 1.21 | 10 |

| {BcULV4=FALSE} | 7426 | 812 | 168 | 7855 | 4 |
| --- | --- | --- | --- | --- | --- |
| {BcMCV3.BI=FALSE, BcULV4=FALSE} =>  {BcRV1.BI=FALSE} | 0.25  7426 | 0.881  356 | 0.292  079 | 1.21  941 | 10  4 |
| {BcMCV3.BI=FALSE, BcRV1.BI=FALSE} =>  {BcULV4=FALSE} | 0.25  7426 | 0.378  182 | 0.680  693 | 1.22  2284 | 10  4 |
| {BcPV4.BI=FALSE, BcULV4=FALSE} =>  {BcRV1.BI=FALSE} | 0.25  495 | 0.887  931 | 0.287  129 | 1.22  8507 | 10  3 |
| {BcPV4.BI=FALSE, BcRV1.BI=FALSE} =>  {BcULV4=FALSE} | 0.25  495 | 0.380  074 | 0.670  792 | 1.22  8399 | 10  3 |
| {BcEV6=FALSE, BcULV4=FALSE} =>  {BcRV1.BI=FALSE} | 0.25  495 | 0.880  342 | 0.289  604 | 1.21  8007 | 10  3 |
| {BcEV6=FALSE, BcRV1.BI=FALSE} =>  {BcULV4=FALSE} | 0.25  495 | 0.381  481 | 0.668  317 | 1.23  2948 | 10  3 |
| {BcOLV17.BI=FALSE, BcULV4=FALSE} =>  {BcRV1.BI=FALSE} | 0.26  9802 | 0.893  443 | 0.301  98 | 1.23  6133 | 10  9 |
| {BcOLV17.BI=FALSE, BcRV1.BI=FALSE} =>  {BcULV4=FALSE} | 0.26  9802 | 0.381  119 | 0.707  921 | 1.23  1776 | 10  9 |
| {BcOLV15.BI=FALSE, BcULV4=FALSE} =>  {BcRV1.BI=FALSE} | 0.25  9901 | 0.889  831 | 0.292  079 | 1.23  1135 | 10  5 |
| {BcOLV15.BI=FALSE, BcRV1.BI=FALSE} =>  {BcULV4=FALSE} | 0.25  9901 | 0.381  818 | 0.680  693 | 1.23  4036 | 10  5 |
| {BcEV5=FALSE, BcULV4=FALSE} => | 0.25 | 0.881 | 0.292 | 1.21 | 10 |

| {BcRV1.BI=FALSE} | 7426 | 356 | 079 | 941 | 4 |
| --- | --- | --- | --- | --- | --- |
| {BcEV5=FALSE, BcRV1.BI=FALSE} =>  {BcULV4=FALSE} | 0.25  7426 | 0.382  353 | 0.673  267 | 1.23  5765 | 10  4 |
| {BcEV2.BI=FALSE, BcULV4=FALSE} =>  {BcULV1.BI=FALSE} | 0.20  297 | 0.931  818 | 0.217  822 | 1.18  7554 | 82 |
| {BcEV2.BI=FALSE, BcULV4=FALSE} =>  {BcOLV7.BI=FALSE} | 0.20  5446 | 0.943  182 | 0.217  822 | 1.16  8851 | 83 |
| {BcEV2.BI=FALSE, BcULV4=FALSE} =>  {BcALV1.BI=FALSE} | 0.20  5446 | 0.943  182 | 0.217  822 | 1.15  8193 | 83 |
| {BcHV3.BI=FALSE, BcULV4=FALSE} =>  {BcFV5.BI=FALSE} | 0.21  7822 | 0.888  889 | 0.245  05 | 1.13  6428 | 88 |
| {BcFV5.BI=FALSE, BcULV4=FALSE} =>  {BcHV3.BI=FALSE} | 0.21  7822 | 0.862  745 | 0.252  475 | 1.13  5339 | 88 |
| {BcHV3.BI=FALSE, BcULV4=FALSE} =>  {BcULV1.BI=FALSE} | 0.22  5248 | 0.919  192 | 0.245  05 | 1.17  1462 | 91 |
| {BcHV3.BI=FALSE, BcULV1.BI=FALSE} =>  {BcULV4=FALSE} | 0.22  5248 | 0.372  951 | 0.603  96 | 1.20  5377 | 91 |
| {BcHV3.BI=FALSE, BcULV4=FALSE} =>  {BcNSRV7.BI=FALSE} | 0.22  2772 | 0.909  091 | 0.245  05 | 1.13  7067 | 90 |
| {BcNSRV7.BI=FALSE, BcULV4=FALSE} =>  {BcHV3.BI=FALSE} | 0.22  2772 | 0.841  121 | 0.264  851 | 1.10  6883 | 90 |
| {BcHV3.BI=FALSE, BcULV4=FALSE} => | 0.23 | 0.959 | 0.245 | 1.18 | 95 |

| {BcOLV7.BI=FALSE} | 5149 | 596 | 05 | 9193 |  |
| --- | --- | --- | --- | --- | --- |
| {BcHV3.BI=FALSE, BcOLV7.BI=FALSE} =>  {BcULV4=FALSE} | 0.23  5149 | 0.372  549 | 0.631  188 | 1.20  4078 | 95 |
| {BcHV3.BI=FALSE, BcULV4=FALSE} =>  {BcALV1.BI=FALSE} | 0.23  2673 | 0.949  495 | 0.245  05 | 1.16  5945 | 94 |
| {BcALV1.BI=FALSE, BcHV3.BI=FALSE} =>  {BcULV4=FALSE} | 0.23  2673 | 0.367  188 | 0.633  663 | 1.18  675 | 94 |
| {BcHV3.BI=FALSE, BcULV4=FALSE} =>  {BcDMV1=FALSE} | 0.22  5248 | 0.919  192 | 0.245  05 | 1.08  9013 | 91 |
| {BcFV5.BI=FALSE, BcULV4=FALSE} =>  {BcULV1.BI=FALSE} | 0.23  2673 | 0.921  569 | 0.252  475 | 1.17  4491 | 94 |
| {BcFV5.BI=FALSE, BcULV1.BI=FALSE} =>  {BcULV4=FALSE} | 0.23  2673 | 0.380  567 | 0.611  386 | 1.22  9992 | 94 |
| {BcFV5.BI=FALSE, BcULV4=FALSE} =>  {BcNSRV7.BI=FALSE} | 0.23  2673 | 0.921  569 | 0.252  475 | 1.15  2674 | 94 |
| {BcNSRV7.BI=FALSE, BcULV4=FALSE} =>  {BcFV5.BI=FALSE} | 0.23  2673 | 0.878  505 | 0.264  851 | 1.12  3152 | 94 |
| {BcFV5.BI=FALSE, BcULV4=FALSE} =>  {BcOLV7.BI=FALSE} | 0.24  0099 | 0.950  98 | 0.252  475 | 1.17  8516 | 97 |
| {BcFV5.BI=FALSE, BcOLV7.BI=FALSE} =>  {BcULV4=FALSE} | 0.24  0099 | 0.384  921 | 0.623  762 | 1.24  4063 | 97 |
| {BcFV5.BI=FALSE, BcULV4=FALSE} => | 0.23 | 0.921 | 0.252 | 1.13 | 94 |

| {BcALV1.BI=FALSE} | 2673 | 569 | 475 | 1653 |  |
| --- | --- | --- | --- | --- | --- |
| {BcFV5.BI=FALSE, BcULV4=FALSE} =>  {BcDMV1=FALSE} | 0.23  2673 | 0.921  569 | 0.252  475 | 1.09  1829 | 94 |
| {BcOLV15.BI=FALSE, BcULV4=FALSE} =>  {BcFV5.BI=FALSE} | 0.25 | 0.855  932 | 0.292  079 | 1.09  4293 | 10  1 |
| {BcMV1.BI=TRUE, BcULV4=FALSE} =>  {BcULV1.BI=FALSE} | 0.21  7822 | 0.907  216 | 0.240  099 | 1.15  62 | 88 |
| {BcMV12=FALSE, BcULV4=FALSE} =>  {BcULV1.BI=FALSE} | 0.24  0099 | 0.960  396 | 0.25 | 1.22  3975 | 97 |
| {BcMV12=FALSE, BcULV1.BI=FALSE} =>  {BcULV4=FALSE} | 0.24  0099 | 0.367  424 | 0.653  465 | 1.18  7515 | 97 |
| {BcNSRV7.BI=FALSE, BcULV4=FALSE} =>  {BcULV1.BI=FALSE} | 0.24  505 | 0.925  234 | 0.264  851 | 1.17  9162 | 99 |
| {BcNSRV7.BI=FALSE, BcULV1.BI=FALSE} =>  {BcULV4=FALSE} | 0.24  505 | 0.399  194 | 0.613  861 | 1.29  0194 | 99 |
| {BcULV4=FALSE, BcVV3.BI=FALSE} =>  {BcULV1.BI=FALSE} | 0.24  0099 | 0.941  748 | 0.254  95 | 1.20  0208 | 97 |
| {BcULV1.BI=FALSE, BcVV3.BI=FALSE} =>  {BcULV4=FALSE} | 0.24  0099 | 0.381  89 | 0.628  713 | 1.23  4268 | 97 |
| {BcULV1.BI=FALSE, BcULV4=FALSE} =>  {BcOLV7.BI=FALSE} | 0.27  2277 | 0.948  276 | 0.287  129 | 1.17  5164 | 11  0 |
| {BcOLV7.BI=FALSE, BcULV4=FALSE} => | 0.27 | 0.924 | 0.294 | 1.17 | 11 |

| {BcULV1.BI=FALSE} | 2277 | 37 | 554 | 8061 | 0 |
| --- | --- | --- | --- | --- | --- |
| {BcOLV7.BI=FALSE, BcULV1.BI=FALSE} =>  {BcULV4=FALSE} | 0.27  2277 | 0.418  251 | 0.650  99 | 1.35  1787 | 11  0 |
| {BcULV1.BI=FALSE, BcULV4=FALSE} =>  {BcALV1.BI=FALSE} | 0.26  4851 | 0.922  414 | 0.287  129 | 1.13  269 | 10  7 |
| {BcALV1.BI=FALSE, BcULV4=FALSE} =>  {BcULV1.BI=FALSE} | 0.26  4851 | 0.930  435 | 0.284  653 | 1.18  5791 | 10  7 |
| {BcALV1.BI=FALSE, BcULV1.BI=FALSE} =>  {BcULV4=FALSE} | 0.26  4851 | 0.408  397 | 0.648  515 | 1.31  9939 | 10  7 |
| {BcDMV1=FALSE, BcULV4=FALSE} =>  {BcULV1.BI=FALSE} | 0.25  2475 | 0.927  273 | 0.272  277 | 1.18  1761 | 10  2 |
| {BcDMV1=FALSE, BcULV1.BI=FALSE} =>  {BcULV4=FALSE} | 0.25  2475 | 0.389  313 | 0.648  515 | 1.25  826 | 10  2 |
| {BcFV9=FALSE, BcULV4=FALSE} =>  {BcULV1.BI=FALSE} | 0.25  2475 | 0.927  273 | 0.272  277 | 1.18  1761 | 10  2 |
| {BcFV9=FALSE, BcULV1.BI=FALSE} =>  {BcULV4=FALSE} | 0.25  2475 | 0.372  263 | 0.678  218 | 1.20  3153 | 10  2 |
| {BcOLV4.BI=FALSE, BcULV4=FALSE} =>  {BcULV1.BI=FALSE} | 0.24  505 | 0.961  165 | 0.254  95 | 1.22  4955 | 99 |
| {BcULV4=FALSE, BcULV5=FALSE} =>  {BcULV1.BI=FALSE} | 0.23  7624 | 0.969  697 | 0.245  05 | 1.23  5828 | 96 |
| {BcBNV5.BI=FALSE, BcULV4=FALSE} => | 0.27 | 0.940 | 0.289 | 1.19 | 11 |

| {BcULV1.BI=FALSE} | 2277 | 171 | 604 | 8199 | 0 |
| --- | --- | --- | --- | --- | --- |
| {BcBNV5.BI=FALSE, BcULV1.BI=FALSE} =>  {BcULV4=FALSE} | 0.27  2277 | 0.381  944 | 0.712  871 | 1.23  4444 | 11  0 |
| {BcULV4=FALSE, BvF.BI=FALSE} =>  {BcULV1.BI=FALSE} | 0.25 | 0.935  185 | 0.267  327 | 1.19  1845 | 10  1 |
| {BcMCV5.BI=FALSE, BcULV4=FALSE} =>  {BcULV1.BI=FALSE} | 0.27  4752 | 0.940  678 | 0.292  079 | 1.19  8845 | 11  1 |
| {BcMCV5.BI=FALSE, BcULV1.BI=FALSE} =>  {BcULV4=FALSE} | 0.27  4752 | 0.376  271 | 0.730  198 | 1.21  6108 | 11  1 |
| {BcOLV13.BI=FALSE, BcULV4=FALSE} =>  {BcULV1.BI=FALSE} | 0.27  2277 | 0.956  522 | 0.284  653 | 1.21  9037 | 11  0 |
| {BcOLV13.BI=FALSE, BcULV1.BI=FALSE} =>  {BcULV4=FALSE} | 0.27  2277 | 0.367  893 | 0.740  099 | 1.18  903 | 11  0 |
| {BcOLV2.BI=FALSE, BcULV4=FALSE} =>  {BcULV1.BI=FALSE} | 0.26  9802 | 0.939  655 | 0.287  129 | 1.19  7542 | 10  9 |
| {BcOLV2.BI=FALSE, BcULV1.BI=FALSE} =>  {BcULV4=FALSE} | 0.26  9802 | 0.363  333 | 0.742  574 | 1.17  4293 | 10  9 |
| {BcMCV3.BI=FALSE, BcULV4=FALSE} =>  {BcULV1.BI=FALSE} | 0.27  9703 | 0.957  627 | 0.292  079 | 1.22  0446 | 11  3 |
| {BcMCV3.BI=FALSE, BcULV1.BI=FALSE} =>  {BcULV4=FALSE} | 0.27  9703 | 0.365  696 | 0.764  851 | 1.18  1929 | 11  3 |
| {BcPV4.BI=FALSE, BcULV4=FALSE} => | 0.26 | 0.931 | 0.287 | 1.18 | 10 |

| {BcULV1.BI=FALSE} | 7327 | 034 | 129 | 6555 | 8 |
| --- | --- | --- | --- | --- | --- |
| {BcPV4.BI=FALSE, BcULV1.BI=FALSE} =>  {BcULV4=FALSE} | 0.26  7327 | 0.36 | 0.742  574 | 1.16  352 | 10  8 |
| {BcEV6=FALSE, BcULV4=FALSE} =>  {BcULV1.BI=FALSE} | 0.26  9802 | 0.931  624 | 0.289  604 | 1.18  7306 | 10  9 |
| {BcEV6=FALSE, BcULV1.BI=FALSE} =>  {BcULV4=FALSE} | 0.26  9802 | 0.365  772 | 0.737  624 | 1.18  2174 | 10  9 |
| {BcOLV17.BI=FALSE, BcULV4=FALSE} =>  {BcULV1.BI=FALSE} | 0.27  9703 | 0.926  23 | 0.301  98 | 1.18  0431 | 11  3 |
| {BcOLV17.BI=FALSE, BcULV1.BI=FALSE} =>  {BcULV4=FALSE} | 0.27  9703 | 0.372  937 | 0.75 | 1.20  5333 | 11  3 |
| {BcOLV15.BI=FALSE, BcULV4=FALSE} =>  {BcULV1.BI=FALSE} | 0.27  2277 | 0.932  203 | 0.292  079 | 1.18  8045 | 11  0 |
| {BcOLV15.BI=FALSE, BcULV1.BI=FALSE} =>  {BcULV4=FALSE} | 0.27  2277 | 0.367  893 | 0.740  099 | 1.18  903 | 11  0 |
| {BcEV5=FALSE, BcULV4=FALSE} =>  {BcULV1.BI=FALSE} | 0.26  9802 | 0.923  729 | 0.292  079 | 1.17  7244 | 10  9 |
| {BcEV5=FALSE, BcULV1.BI=FALSE} =>  {BcULV4=FALSE} | 0.26  9802 | 0.363  333 | 0.742  574 | 1.17  4293 | 10  9 |
| {BcMV1.BI=TRUE, BcULV4=FALSE} =>  {BcOLV7.BI=FALSE} | 0.22  5248 | 0.938  144 | 0.240  099 | 1.16  2608 | 91 |
| {BcMV1.BI=TRUE, BcULV4=FALSE} => | 0.21 | 0.907 | 0.240 | 1.11 | 88 |

| {BcALV1.BI=FALSE} | 7822 | 216 | 099 | 4029 |  |
| --- | --- | --- | --- | --- | --- |
| {BcMV12=FALSE, BcULV4=FALSE} =>  {BcOLV7.BI=FALSE} | 0.24  2574 | 0.970  297 | 0.25 | 1.20  2454 | 98 |
| {BcMV12=FALSE, BcOLV7.BI=FALSE} =>  {BcULV4=FALSE} | 0.24  2574 | 0.369  811 | 0.655  941 | 1.19  523 | 98 |
| {BcMV12=FALSE, BcULV4=FALSE} =>  {BcALV1.BI=FALSE} | 0.23  2673 | 0.930  693 | 0.25 | 1.14  2857 | 94 |
| {BcNSRV7.BI=FALSE, BcULV4=FALSE} =>  {BcOLV7.BI=FALSE} | 0.25  2475 | 0.953  271 | 0.264  851 | 1.18  1354 | 10  2 |
| {BcNSRV7.BI=FALSE, BcOLV7.BI=FALSE} =>  {BcULV4=FALSE} | 0.25  2475 | 0.395  349 | 0.638  614 | 1.27  7767 | 10  2 |
| {BcNSRV7.BI=FALSE, BcULV4=FALSE} =>  {BcALV1.BI=FALSE} | 0.24  0099 | 0.906  542 | 0.264  851 | 1.11  3201 | 97 |
| {BcALV1.BI=FALSE, BcNSRV7.BI=FALSE} =>  {BcULV4=FALSE} | 0.24  0099 | 0.363  296 | 0.660  891 | 1.17  4172 | 97 |
| {BcNSRV7.BI=FALSE, BcULV4=FALSE} =>  {BcDMV1=FALSE} | 0.24  0099 | 0.906  542 | 0.264  851 | 1.07  4026 | 97 |
| {BcDMV1=FALSE, BcULV4=FALSE} =>  {BcNSRV7.BI=FALSE} | 0.24  0099 | 0.881  818 | 0.272  277 | 1.10  2955 | 97 |
| {BcFV9=FALSE, BcULV4=FALSE} =>  {BcNSRV7.BI=FALSE} | 0.24  0099 | 0.881  818 | 0.272  277 | 1.10  2955 | 97 |
| {BcBNV5.BI=FALSE, BcULV4=FALSE} => | 0.25 | 0.888 | 0.289 | 1.11 | 10 |

| {BcNSRV7.BI=FALSE} | 7426 | 889 | 604 | 1799 | 4 |
| --- | --- | --- | --- | --- | --- |
| {BcOLV15.BI=FALSE, BcULV4=FALSE} =>  {BcNSRV7.BI=FALSE} | 0.25  9901 | 0.889  831 | 0.292  079 | 1.11  2977 | 10  5 |
| {BcEV5=FALSE, BcULV4=FALSE} =>  {BcNSRV7.BI=FALSE} | 0.25  7426 | 0.881  356 | 0.292  079 | 1.10  2377 | 10  4 |
| {BcULV4=FALSE, BcVV3.BI=FALSE} =>  {BcOLV7.BI=FALSE} | 0.24  7525 | 0.970  874 | 0.254  95 | 1.20  3169 | 10  0 |
| {BcOLV7.BI=FALSE, BcVV3.BI=FALSE} =>  {BcULV4=FALSE} | 0.24  7525 | 0.378  788 | 0.653  465 | 1.22  4242 | 10  0 |
| {BcULV4=FALSE, BcVV3.BI=FALSE} =>  {BcALV1.BI=FALSE} | 0.23  5149 | 0.922  33 | 0.254  95 | 1.13  2588 | 95 |
| {BcULV4=FALSE, BcULV5=FALSE} =>  {BcVV3.BI=FALSE} | 0.21  7822 | 0.888  889 | 0.245  05 | 1.10  8368 | 88 |
| {BcOLV7.BI=FALSE, BcULV4=FALSE} =>  {BcALV1.BI=FALSE} | 0.26  9802 | 0.915  966 | 0.294  554 | 1.12  4773 | 10  9 |
| {BcALV1.BI=FALSE, BcULV4=FALSE} =>  {BcOLV7.BI=FALSE} | 0.26  9802 | 0.947  826 | 0.284  653 | 1.17  4607 | 10  9 |
| {BcALV1.BI=FALSE, BcOLV7.BI=FALSE} =>  {BcULV4=FALSE} | 0.26  9802 | 0.399  267 | 0.675  743 | 1.29  0432 | 10  9 |
| {BcDMV1=FALSE, BcULV4=FALSE} =>  {BcOLV7.BI=FALSE} | 0.25  9901 | 0.954  545 | 0.272  277 | 1.18  2934 | 10  5 |
| {BcDMV1=FALSE, BcOLV7.BI=FALSE} => | 0.25 | 0.390 | 0.665 | 1.26 | 10 |

| {BcULV4=FALSE} | 9901 | 335 | 842 | 1561 | 5 |
| --- | --- | --- | --- | --- | --- |
| {BcFV9=FALSE, BcULV4=FALSE} =>  {BcOLV7.BI=FALSE} | 0.25  9901 | 0.954  545 | 0.272  277 | 1.18  2934 | 10  5 |
| {BcFV9=FALSE, BcOLV7.BI=FALSE} =>  {BcULV4=FALSE} | 0.25  9901 | 0.368  421 | 0.705  446 | 1.19  0737 | 10  5 |
| {BcOLV4.BI=FALSE, BcULV4=FALSE} =>  {BcOLV7.BI=FALSE} | 0.24  0099 | 0.941  748 | 0.254  95 | 1.16  7074 | 97 |
| {BcULV4=FALSE, BcULV5=FALSE} =>  {BcOLV7.BI=FALSE} | 0.23  0198 | 0.939  394 | 0.245  05 | 1.16  4157 | 93 |
| {BcBNV5.BI=FALSE, BcULV4=FALSE} =>  {BcOLV7.BI=FALSE} | 0.27  4752 | 0.948  718 | 0.289  604 | 1.17  5712 | 11  1 |
| {BcBNV5.BI=FALSE, BcOLV7.BI=FALSE} =>  {BcULV4=FALSE} | 0.27  4752 | 0.376  271 | 0.730  198 | 1.21  6108 | 11  1 |
| {BcULV4=FALSE, BvF.BI=FALSE} =>  {BcOLV7.BI=FALSE} | 0.25  2475 | 0.944  444 | 0.267  327 | 1.17  0416 | 10  2 |
| {BcMCV5.BI=FALSE, BcULV4=FALSE} =>  {BcOLV7.BI=FALSE} | 0.27  7228 | 0.949  153 | 0.292  079 | 1.17  625 | 11  2 |
| {BcMCV5.BI=FALSE, BcOLV7.BI=FALSE} =>  {BcULV4=FALSE} | 0.27  7228 | 0.369  637 | 0.75 | 1.19  4667 | 11  2 |
| {BcOLV13.BI=FALSE, BcULV4=FALSE} =>  {BcOLV7.BI=FALSE} | 0.26  9802 | 0.947  826 | 0.284  653 | 1.17  4607 | 10  9 |
| {BcOLV13.BI=FALSE, BcOLV7.BI=FALSE} => | 0.26 | 0.358 | 0.752 | 1.15 | 10 |

| {BcULV4=FALSE} | 9802 | 553 | 475 | 8842 | 9 |
| --- | --- | --- | --- | --- | --- |
| {BcOLV2.BI=FALSE, BcULV4=FALSE} =>  {BcOLV7.BI=FALSE} | 0.27  2277 | 0.948  276 | 0.287  129 | 1.17  5164 | 11  0 |
| {BcOLV2.BI=FALSE, BcOLV7.BI=FALSE} =>  {BcULV4=FALSE} | 0.27  2277 | 0.361  842 | 0.752  475 | 1.16  9474 | 11  0 |
| {BcMCV3.BI=FALSE, BcULV4=FALSE} =>  {BcOLV7.BI=FALSE} | 0.27  9703 | 0.957  627 | 0.292  079 | 1.18  6753 | 11  3 |
| {BcMCV3.BI=FALSE, BcOLV7.BI=FALSE} =>  {BcULV4=FALSE} | 0.27  9703 | 0.368  078 | 0.759  901 | 1.18  9629 | 11  3 |
| {BcPV4.BI=FALSE, BcULV4=FALSE} =>  {BcOLV7.BI=FALSE} | 0.27  4752 | 0.956  897 | 0.287  129 | 1.18  5847 | 11  1 |
| {BcOLV7.BI=FALSE, BcPV4.BI=FALSE} =>  {BcULV4=FALSE} | 0.27  4752 | 0.362  745 | 0.757  426 | 1.17  2392 | 11  1 |
| {BcEV6=FALSE, BcULV4=FALSE} =>  {BcOLV7.BI=FALSE} | 0.27  4752 | 0.948  718 | 0.289  604 | 1.17  5712 | 11  1 |
| {BcEV6=FALSE, BcOLV7.BI=FALSE} =>  {BcULV4=FALSE} | 0.27  4752 | 0.365  132 | 0.752  475 | 1.18  0105 | 11  1 |
| {BcOLV17.BI=FALSE, BcULV4=FALSE} =>  {BcOLV7.BI=FALSE} | 0.28  7129 | 0.950  82 | 0.301  98 | 1.17  8316 | 11  6 |
| {BcOLV17.BI=FALSE, BcOLV7.BI=FALSE} =>  {BcULV4=FALSE} | 0.28  7129 | 0.369  427 | 0.777  228 | 1.19  3987 | 11  6 |
| {BcOLV15.BI=FALSE, BcULV4=FALSE} => | 0.27 | 0.949 | 0.292 | 1.17 | 11 |

| {BcOLV7.BI=FALSE} | 7228 | 153 | 079 | 625 | 2 |
| --- | --- | --- | --- | --- | --- |
| {BcOLV15.BI=FALSE, BcOLV7.BI=FALSE} =>  {BcULV4=FALSE} | 0.27  7228 | 0.363  636 | 0.762  376 | 1.17  5273 | 11  2 |
| {BcEV5=FALSE, BcULV4=FALSE} =>  {BcOLV7.BI=FALSE} | 0.27  9703 | 0.957  627 | 0.292  079 | 1.18  6753 | 11  3 |
| {BcEV5=FALSE, BcOLV7.BI=FALSE} =>  {BcULV4=FALSE} | 0.27  9703 | 0.368  078 | 0.759  901 | 1.18  9629 | 11  3 |
| {BcDMV1=FALSE, BcULV4=FALSE} =>  {BcALV1.BI=FALSE} | 0.25 | 0.918  182 | 0.272  277 | 1.12  7494 | 10  1 |
| {BcFV9=FALSE, BcULV4=FALSE} =>  {BcALV1.BI=FALSE} | 0.24  7525 | 0.909  091 | 0.272  277 | 1.11  633 | 10  0 |
| {BcOLV4.BI=FALSE, BcULV4=FALSE} =>  {BcALV1.BI=FALSE} | 0.24  2574 | 0.951  456 | 0.254  95 | 1.16  8354 | 98 |
| {BcULV4=FALSE, BcULV5=FALSE} =>  {BcALV1.BI=FALSE} | 0.22  2772 | 0.909  091 | 0.245  05 | 1.11  633 | 90 |
| {BcBNV5.BI=FALSE, BcULV4=FALSE} =>  {BcALV1.BI=FALSE} | 0.26  7327 | 0.923  077 | 0.289  604 | 1.13  3505 | 10  8 |
| {BcULV4=FALSE, BvF.BI=FALSE} =>  {BcALV1.BI=FALSE} | 0.25 | 0.935  185 | 0.267  327 | 1.14  8373 | 10  1 |
| {BcMCV5.BI=FALSE, BcULV4=FALSE} =>  {BcALV1.BI=FALSE} | 0.27  2277 | 0.932  203 | 0.292  079 | 1.14  4712 | 11  0 |
| {BcOLV13.BI=FALSE, BcULV4=FALSE} => | 0.26 | 0.921 | 0.284 | 1.13 | 10 |

| {BcALV1.BI=FALSE} | 2376 | 739 | 653 | 1862 | 6 |
| --- | --- | --- | --- | --- | --- |
| {BcOLV2.BI=FALSE, BcULV4=FALSE} =>  {BcALV1.BI=FALSE} | 0.26  2376 | 0.913  793 | 0.287  129 | 1.12  2105 | 10  6 |
| {BcMCV3.BI=FALSE, BcULV4=FALSE} =>  {BcALV1.BI=FALSE} | 0.26  7327 | 0.915  254 | 0.292  079 | 1.12  3899 | 10  8 |
| {BcPV4.BI=FALSE, BcULV4=FALSE} =>  {BcALV1.BI=FALSE} | 0.26  2376 | 0.913  793 | 0.287  129 | 1.12  2105 | 10  6 |
| {BcEV6=FALSE, BcULV4=FALSE} =>  {BcALV1.BI=FALSE} | 0.26  9802 | 0.931  624 | 0.289  604 | 1.14  4 | 10  9 |
| {BcOLV17.BI=FALSE, BcULV4=FALSE} =>  {BcALV1.BI=FALSE} | 0.27  7228 | 0.918  033 | 0.301  98 | 1.12  7311 | 11  2 |
| {BcALV1.BI=FALSE, BcOLV17.BI=FALSE} =>  {BcULV4=FALSE} | 0.27  7228 | 0.360  129 | 0.769  802 | 1.16  3936 | 11  2 |
| {BcOLV15.BI=FALSE, BcULV4=FALSE} =>  {BcALV1.BI=FALSE} | 0.26  7327 | 0.915  254 | 0.292  079 | 1.12  3899 | 10  8 |
| {BcEV5=FALSE, BcULV4=FALSE} =>  {BcALV1.BI=FALSE} | 0.26  7327 | 0.915  254 | 0.292  079 | 1.12  3899 | 10  8 |
| {BcBNV5.BI=FALSE, BcULV4=FALSE} =>  {BcDMV1=FALSE} | 0.26  4851 | 0.914  53 | 0.289  604 | 1.08  349 | 10  7 |
| {BcEV5=FALSE, BcULV4=FALSE} =>  {BcDMV1=FALSE} | 0.26  9802 | 0.923  729 | 0.292  079 | 1.09  4388 | 10  9 |
| {BcMV9.BI=FALSE, | 0.08 | 0.708 | 0.118 | 1.53 | 34 |

| BcOLV1.BI.MOVA.=TRUE} =>  {BcMV2.BI=TRUE} | 4158 | 333 | 812 | 0303 |  |
| --- | --- | --- | --- | --- | --- |
| {BcMV2.BI=TRUE, BcMV9.BI=FALSE} => | 0.08 | 0.596 | 0.141 | 1.79 |  |
|  |  |  |  |  | 34 |
| {BcOLV1.BI.MOVA.=TRUE} | 4158 | 491 | 089 | 8377 |  |
| {BcMV9.BI=FALSE, |  |  |  |  |  |
|  | 0.07 |  | 0.118 | 1.29 |  |
| BcOLV1.BI.MOVA.=TRUE} => |  | 0.625 |  |  | 30 |
|  | 4257 |  | 812 | 4872 |  |
| {BcOLV18=FALSE} |  |  |  |  |  |
| {BcOLV1.BI.MOVA.=TRUE, | 0.07 | 0.731 | 0.101 | 2.27 |  |
|  |  |  |  |  | 30 |
| BcOLV18=FALSE} => {BcMV9.BI=FALSE} | 4257 | 707 | 485 | 3921 |  |
| {BcMV9.BI=FALSE, |  |  |  |  |  |
|  | 0.08 | 0.687 | 0.118 | 1.26 |  |
| BcOLV1.BI.MOVA.=TRUE} => |  |  |  |  | 33 |
|  | 1683 | 5 | 812 | 8265 |  |
| {BcMV3.BI=TRUE} |  |  |  |  |  |
| {BcMV3.BI=TRUE, BcMV9.BI=FALSE} => | 0.08 | 0.559 | 0.146 | 1.68 |  |
|  |  |  |  |  | 33 |
| {BcOLV1.BI.MOVA.=TRUE} | 1683 | 322 | 04 | 6314 |  |
| {BcMV9.BI=FALSE, |  |  |  |  |  |
|  | 0.09 | 0.791 | 0.118 | 1.28 |  |
| BcOLV1.BI.MOVA.=TRUE} => |  |  |  |  | 38 |
|  | 4059 | 667 | 812 | 4471 |  |
| {BcMV11=TRUE} |  |  |  |  |  |
| {BcMV11=TRUE, BcMV9.BI=FALSE} => | 0.09 | 0.447 | 0.210 | 1.34 |  |
|  |  |  |  |  | 38 |
| {BcOLV1.BI.MOVA.=TRUE} | 4059 | 059 | 396 | 7849 |  |
| {BcMV6.BI=FALSE, |  |  |  |  |  |
|  | 0.07 | 0.470 | 0.168 | 1.46 |  |
| BcOLV1.BI.MOVA.=TRUE} => |  |  |  |  | 32 |
|  | 9208 | 588 | 317 | 2443 |  |
| {BcMV9.BI=FALSE} |  |  |  |  |  |

| {BcOLV1.BI.MOVA.=TRUE,  BcPV3.BI=FALSE} => {BcMV9.BI=FALSE} | 0.08  4158 | 0.430  38 | 0.195  545 | 1.33  7488 | 34 |
| --- | --- | --- | --- | --- | --- |
| {BcMV9.BI=FALSE, BcOLV1.BI.MOVA.=TRUE} =>  {BcRV1.BI=FALSE} | 0.10  1485 | 0.854  167 | 0.118  812 | 1.18  1792 | 41 |
| {BcOLV1.BI.MOVA.=TRUE,  BcRV1.BI=FALSE} => {BcMV9.BI=FALSE} | 0.10  1485 | 0.512  5 | 0.198  02 | 1.59  2692 | 41 |
| {BcHV3.BI=FALSE, BcOLV1.BI.MOVA.=TRUE} =>  {BcMV9.BI=FALSE} | 0.09  901 | 0.434  783 | 0.227  723 | 1.35  1171 | 40 |
| {BcFV5.BI=FALSE, BcOLV1.BI.MOVA.=TRUE} =>  {BcMV9.BI=FALSE} | 0.09  901 | 0.412  371 | 0.240  099 | 1.28  1523 | 40 |
| {BcOLV1.BI.MOVA.=TRUE,  BcULV1.BI=FALSE} => {BcMV9.BI=FALSE} | 0.09  6535 | 0.428  571 | 0.225  248 | 1.33  1868 | 39 |
| {BcMV9.BI=FALSE, BcOLV1.BI.MOVA.=TRUE} =>  {BcMV1.BI=TRUE} | 0.10  8911 | 0.916  667 | 0.118  812 | 1.16  092 | 44 |
| {BcMV1.BI=TRUE, BcMV9.BI=FALSE} =>  {BcOLV1.BI.MOVA.=TRUE} | 0.10  8911 | 0.453  608 | 0.240  099 | 1.36  7595 | 44 |
| {BcMV9.BI=FALSE,  BcOLV1.BI.MOVA.=TRUE} => | 0.10  8911 | 0.916  667 | 0.118  812 | 1.14  6543 | 44 |

| {BcNSRV7.BI=FALSE} |  |  |  |  |  |
| --- | --- | --- | --- | --- | --- |
| {BcNSRV7.BI=FALSE, |  |  |  |  |  |
|  | 0.10 | 0.411 | 0.264 | 1.27 |  |
| BcOLV1.BI.MOVA.=TRUE} => |  |  |  |  | 44 |
|  | 8911 | 215 | 851 | 793 |  |
| {BcMV9.BI=FALSE} |  |  |  |  |  |
| {BcMV9.BI=FALSE, |  |  |  |  |  |
|  | 0.11 | 0.937 | 0.118 | 1.16 |  |
| BcOLV1.BI.MOVA.=TRUE} => |  |  |  |  | 45 |
|  | 1386 | 5 | 812 | 181 |  |
| {BcOLV7.BI=FALSE} |  |  |  |  |  |
| {BcOLV1.BI.MOVA.=TRUE, | 0.11 | 0.468 | 0.237 | 1.45 |  |
|  |  |  |  |  | 45 |
| BcOLV7.BI=FALSE} => {BcMV9.BI=FALSE} | 1386 | 75 | 624 | 6731 |  |
| {BcALV1.BI=FALSE, |  |  |  |  |  |
|  | 0.09 | 0.417 | 0.225 | 1.29 |  |
| BcOLV1.BI.MOVA.=TRUE} => |  |  |  |  | 38 |
|  | 4059 | 582 | 248 | 7718 |  |
| {BcMV9.BI=FALSE} |  |  |  |  |  |
| {BcMV9.BI=FALSE, |  |  |  |  |  |
|  | 0.11 | 0.937 | 0.118 | 1.11 |  |
| BcOLV1.BI.MOVA.=TRUE} => |  |  |  |  | 45 |
|  | 1386 | 5 | 812 | 0704 |  |
| {BcDMV1=FALSE} |  |  |  |  |  |
| {BcDMV1=FALSE, BcOLV1.BI.MOVA.=TRUE} | 0.11 | 0.405 | 0.274 | 1.25 |  |
|  |  |  |  |  | 45 |
| => {BcMV9.BI=FALSE} | 1386 | 405 | 752 | 9875 |  |
| {BcMV11=FALSE, BcMV9.BI=FALSE} => | 0.07 | 0.666 | 0.111 | 1.68 |  |
|  |  |  |  |  | 30 |
| {BcHV1.BI=FALSE} | 4257 | 667 | 386 | 3333 |  |
| {BcHV1.BI=FALSE, BcMV11=FALSE} => | 0.07 | 0.461 | 0.160 | 1.43 |  |
|  |  |  |  |  | 30 |
| {BcMV9.BI=FALSE} | 4257 | 538 | 891 | 432 |  |
| {BcMV11=FALSE, BcMV9.BI=FALSE} => | 0.07 | 0.666 | 0.111 | 1.45 | 30 |

| {BcHV1SLR.BI=FALSE} | 4257 | 667 | 386 | 5856 |  |
| --- | --- | --- | --- | --- | --- |
| {BcMV11=FALSE, BcMV9.BI=FALSE} =>  {BcOLV18=FALSE} | 0.07  9208 | 0.711  111 | 0.111  386 | 1.47  3276 | 32 |
| {BcMV11=FALSE, BcMV9.BI=FALSE} =>  {BcMV2.BI=FALSE} | 0.08  1683 | 0.733  333 | 0.111  386 | 1.36  5284 | 33 |
| {BcMV11=FALSE, BcMV9.BI=FALSE} =>  {BcMV4.BI=FALSE} | 0.08  9109 | 0.8 | 0.111  386 | 1.43  6444 | 36 |
| {BcMV11=FALSE, BcMV9.BI=FALSE} =>  {BcOLV16.BI=FALSE} | 0.08  9109 | 0.8 | 0.111  386 | 1.36  9492 | 36 |
| {BcMV11=FALSE, BcMV9.BI=FALSE} =>  {BcOLV1.BI.MOVA.=FALSE} | 0.08  6634 | 0.777  778 | 0.111  386 | 1.16  3786 | 35 |
| {BcMV11=FALSE, BcMV9.BI=FALSE} =>  {BcRV1.BI=FALSE} | 0.09  901 | 0.888  889 | 0.111  386 | 1.22  9833 | 40 |
| {BcMV11=FALSE, BcMV9.BI=FALSE} =>  {BcEV2.BI=FALSE} | 0.10  396 | 0.933  333 | 0.111  386 | 1.24  8565 | 42 |
| {BcMV11=FALSE, BcMV9.BI=FALSE} =>  {BcULV1.BI=FALSE} | 0.10  396 | 0.933  333 | 0.111  386 | 1.18  9485 | 42 |
| {BcMV11=FALSE, BcMV9.BI=FALSE} =>  {BcALV1.BI=FALSE} | 0.10  1485 | 0.911  111 | 0.111  386 | 1.11  8811 | 41 |
| {BcMV11=FALSE, BcMV9.BI=FALSE} =>  {BcFV9=FALSE} | 0.10  6436 | 0.955  556 | 0.111  386 | 1.09  9842 | 43 |
| {BcHV1.BI=FALSE, BcMV9.BI=FALSE} => | 0.11 | 0.626 | 0.185 | 1.54 | 47 |

| {BcULV3=FALSE} | 6337 | 667 | 644 | 374 |  |
| --- | --- | --- | --- | --- | --- |
| {BcMV9.BI=FALSE, BcULV3=FALSE} =>  {BcHV1.BI=FALSE} | 0.11  6337 | 0.770  492 | 0.150  99 | 1.94  5492 | 47 |
| {BcHV1.BI=FALSE, BcULV3=FALSE} =>  {BcMV9.BI=FALSE} | 0.11  6337 | 0.516  484 | 0.225  248 | 1.60  5072 | 47 |
| {BcHV1.BI=FALSE, BcMV9.BI=FALSE} =>  {BcHV1SLR.BI=FALSE} | 0.16  0891 | 0.866  667 | 0.185  644 | 1.89  2613 | 65 |
| {BcHV1SLR.BI=FALSE, BcMV9.BI=FALSE}  => {BcHV1.BI=FALSE} | 0.16  0891 | 0.855  263 | 0.188  119 | 2.15  9539 | 65 |
| {BcHV1.BI=FALSE, BcHV1SLR.BI=FALSE} =>  {BcMV9.BI=FALSE} | 0.16  0891 | 0.474  453 | 0.339  109 | 1.47  4453 | 65 |
| {BcMV3.BI=FALSE, BcMV9.BI=FALSE} =>  {BcHV1.BI=FALSE} | 0.10  396 | 0.591  549 | 0.175  743 | 1.49  3662 | 42 |
| {BcHV1.BI=FALSE, BcMV3.BI=FALSE} =>  {BcMV9.BI=FALSE} | 0.10  396 | 0.5 | 0.207  921 | 1.55  3846 | 42 |
| {BcMV2.BI=TRUE, BcMV9.BI=FALSE} =>  {BcHV1.BI=FALSE} | 0.07  9208 | 0.561  404 | 0.141  089 | 1.41  7544 | 32 |
| {BcHV1.BI=FALSE, BcMV2.BI=TRUE} =>  {BcMV9.BI=FALSE} | 0.07  9208 | 0.463  768 | 0.170  792 | 1.44  1249 | 32 |
| {BcHV1.BI=FALSE, BcMV9.BI=FALSE} =>  {BcOLV18=FALSE} | 0.13  8614 | 0.746  667 | 0.185  644 | 1.54  694 | 56 |
| {BcMV9.BI=FALSE, BcOLV18=FALSE} => | 0.13 | 0.636 | 0.217 | 1.60 | 56 |

| {BcHV1.BI=FALSE} | 8614 | 364 | 822 | 6818 |  |
| --- | --- | --- | --- | --- | --- |
| {BcHV1.BI=FALSE, BcOLV18=FALSE} =>  {BcMV9.BI=FALSE} | 0.13  8614 | 0.565  657 | 0.245  05 | 1.75  7887 | 56 |
| {BcMV2.BI=FALSE, BcMV9.BI=FALSE} =>  {BcHV1.BI=FALSE} | 0.10  6436 | 0.589  041 | 0.180  693 | 1.48  7329 | 43 |
| {BcHV1.BI=FALSE, BcMV2.BI=FALSE} =>  {BcMV9.BI=FALSE} | 0.10  6436 | 0.472  527 | 0.225  248 | 1.46  847 | 43 |
| {BcMV3.BI=TRUE, BcMV9.BI=FALSE} =>  {BcHV1.BI=FALSE} | 0.08  1683 | 0.559  322 | 0.146  04 | 1.41  2288 | 33 |
| {BcHV1.BI=FALSE, BcMV3.BI=TRUE} =>  {BcMV9.BI=FALSE} | 0.08  1683 | 0.434  211 | 0.188  119 | 1.34  9393 | 33 |
| {BcHV1.BI=FALSE, BcMV9.BI=FALSE} =>  {BcMV4.BI=FALSE} | 0.12  3762 | 0.666  667 | 0.185  644 | 1.19  7037 | 50 |
| {BcMV4.BI=FALSE, BcMV9.BI=FALSE} =>  {BcHV1.BI=FALSE} | 0.12  3762 | 0.574  713 | 0.215  347 | 1.45  1149 | 50 |
| {BcHV1.BI=FALSE, BcMV4.BI=FALSE} =>  {BcMV9.BI=FALSE} | 0.12  3762 | 0.490  196 | 0.252  475 | 1.52  3379 | 50 |
| {BcHV1.BI=FALSE, BcMV9.BI=FALSE} =>  {BcOLV16.BI=FALSE} | 0.14  8515 | 0.8 | 0.185  644 | 1.36  9492 | 60 |
| {BcMV9.BI=FALSE, BcOLV16.BI=FALSE} =>  {BcHV1.BI=FALSE} | 0.14  8515 | 0.625 | 0.237  624 | 1.57  8125 | 60 |
| {BcHV1.BI=FALSE, BcOLV16.BI=FALSE} => | 0.14 | 0.594 | 0.25 | 1.84 | 60 |

| {BcMV9.BI=FALSE} | 8515 | 059 |  | 6154 |  |
| --- | --- | --- | --- | --- | --- |
| {BcMV11=TRUE, BcMV9.BI=FALSE} =>  {BcHV1.BI=FALSE} | 0.11  1386 | 0.529  412 | 0.210  396 | 1.33  6765 | 45 |
| {BcHV1.BI=FALSE, BcMV11=TRUE} =>  {BcMV9.BI=FALSE} | 0.11  1386 | 0.473  684 | 0.235  149 | 1.47  2065 | 45 |
| {BcMV9.BI=FALSE, BcOLV1.BI.MOVA.=FALSE} =>  {BcHV1.BI=FALSE} | 0.11  6337 | 0.573  171 | 0.202  97 | 1.44  7256 | 47 |
| {BcHV1.BI=FALSE, |  |  |  |  |  |
|  | 0.11 | 0.419 | 0.277 | 1.30 |  |
| BcOLV1.BI.MOVA.=FALSE} => |  |  |  |  | 47 |
|  | 6337 | 643 | 228 | 4121 |  |
| {BcMV9.BI=FALSE} |  |  |  |  |  |
| {BcMV9.BI=FALSE, BcULV4=TRUE} => | 0.07 | 0.517 | 0.143 | 1.30 |  |
|  |  |  |  |  | 30 |
| {BcHV1.BI=FALSE} | 4257 | 241 | 564 | 6034 |  |
| {BcHV1.BI=FALSE, BcMV9.BI=FALSE} => | 0.15 | 0.813 | 0.185 | 1.16 |  |
|  |  |  |  |  | 61 |
| {BcMV6.BI=FALSE} | 099 | 333 | 644 | 9348 |  |
| {BcMV6.BI=FALSE, BcMV9.BI=FALSE} => | 0.15 | 0.598 | 0.252 | 1.51 |  |
|  |  |  |  |  | 61 |
| {BcHV1.BI=FALSE} | 099 | 039 | 475 | 0049 |  |
| {BcHV1.BI=FALSE, BcMV6.BI=FALSE} => | 0.15 | 0.472 | 0.319 | 1.46 |  |
|  |  |  |  |  | 61 |
| {BcMV9.BI=FALSE} | 099 | 868 | 307 | 9529 |  |
| {BcMV9.BI=FALSE, BcPV3.BI=FALSE} => | 0.14 |  | 0.237 | 1.57 |  |
|  |  | 0.625 |  |  | 60 |
| {BcHV1.BI=FALSE} | 8515 |  | 624 | 8125 |  |
| {BcHV1.BI=FALSE, BcPV3.BI=FALSE} => | 0.14 | 0.483 | 0.306 | 1.50 | 60 |

| {BcMV9.BI=FALSE} | 8515 | 871 | 931 | 3722 |  |
| --- | --- | --- | --- | --- | --- |
| {BcMV9.BI=FALSE, BcRV1.BI=FALSE} =>  {BcHV1.BI=FALSE} | 0.14  3564 | 0.537  037 | 0.267  327 | 1.35  6019 | 58 |
| {BcHV1.BI=FALSE, BcRV1.BI=FALSE} =>  {BcMV9.BI=FALSE} | 0.14  3564 | 0.467  742 | 0.306  931 | 1.45  3598 | 58 |
| {BcEV2.BI=FALSE, BcMV9.BI=FALSE} =>  {BcHV1.BI=FALSE} | 0.14  8515 | 0.612  245 | 0.242  574 | 1.54  5918 | 60 |
| {BcEV2.BI=FALSE, BcHV1.BI=FALSE} =>  {BcMV9.BI=FALSE} | 0.14  8515 | 0.48 | 0.309  406 | 1.49  1692 | 60 |
| {BcHV1.BI=FALSE, BcMV9.BI=FALSE} =>  {BcHV3.BI=FALSE} | 0.16  0891 | 0.866  667 | 0.185  644 | 1.14  0499 | 65 |
| {BcHV3.BI=FALSE, BcMV9.BI=FALSE} =>  {BcHV1.BI=FALSE} | 0.16  0891 | 0.631  068 | 0.254  95 | 1.59  3447 | 65 |
| {BcHV1.BI=FALSE, BcHV3.BI=FALSE} =>  {BcMV9.BI=FALSE} | 0.16  0891 | 0.507  813 | 0.316  832 | 1.57  8125 | 65 |
| {BcHV1.BI=FALSE, BcMV9.BI=FALSE} =>  {BcFV5.BI=FALSE} | 0.16  0891 | 0.866  667 | 0.185  644 | 1.10  8017 | 65 |
| {BcFV5.BI=FALSE, BcMV9.BI=FALSE} =>  {BcHV1.BI=FALSE} | 0.16  0891 | 0.596  33 | 0.269  802 | 1.50  5734 | 65 |
| {BcFV5.BI=FALSE, BcHV1.BI=FALSE} =>  {BcMV9.BI=FALSE} | 0.16  0891 | 0.488  722 | 0.329  208 | 1.51  8797 | 65 |
| {BcHV1.BI=FALSE, BcMV9.BI=FALSE} => | 0.17 | 0.946 | 0.185 | 1.20 | 71 |

| {BcULV1.BI=FALSE} | 5743 | 667 | 644 | 6477 |  |
| --- | --- | --- | --- | --- | --- |
| {BcMV9.BI=FALSE, BcULV1.BI=FALSE} =>  {BcHV1.BI=FALSE} | 0.17  5743 | 0.617  391 | 0.284  653 | 1.55  8913 | 71 |
| {BcHV1.BI=FALSE, BcULV1.BI=FALSE} =>  {BcMV9.BI=FALSE} | 0.17  5743 | 0.493  056 | 0.356  436 | 1.53  2265 | 71 |
| {BcMV1.BI=TRUE, BcMV9.BI=FALSE} =>  {BcHV1.BI=FALSE} | 0.14  1089 | 0.587  629 | 0.240  099 | 1.48  3763 | 57 |
| {BcHV1.BI=FALSE, BcMV1.BI=TRUE} =>  {BcMV9.BI=FALSE} | 0.14  1089 | 0.459  677 | 0.306  931 | 1.42  8536 | 57 |
| {BcMV12=FALSE, BcMV9.BI=FALSE} =>  {BcHV1.BI=FALSE} | 0.15  8416 | 0.609  524 | 0.259  901 | 1.53  9048 | 64 |
| {BcHV1.BI=FALSE, BcMV12=FALSE} =>  {BcMV9.BI=FALSE} | 0.15  8416 | 0.474  074 | 0.334  158 | 1.47  3276 | 64 |
| {BcHV1.BI=FALSE, BcMV9.BI=FALSE} =>  {BcNSRV7.BI=FALSE} | 0.17  3267 | 0.933  333 | 0.185  644 | 1.16  7389 | 70 |
| {BcMV9.BI=FALSE, BcNSRV7.BI=FALSE} =>  {BcHV1.BI=FALSE} | 0.17  3267 | 0.614  035 | 0.282  178 | 1.55  0439 | 70 |
| {BcHV1.BI=FALSE, BcNSRV7.BI=FALSE} =>  {BcMV9.BI=FALSE} | 0.17  3267 | 0.518  519 | 0.334  158 | 1.61  1396 | 70 |
| {BcMV9.BI=FALSE, BcVV3.BI=FALSE} =>  {BcHV1.BI=FALSE} | 0.14  8515 | 0.6 | 0.247  525 | 1.51  5 | 60 |
| {BcHV1.BI=FALSE, BcVV3.BI=FALSE} => | 0.14 | 0.483 | 0.306 | 1.50 | 60 |

| {BcMV9.BI=FALSE} | 8515 | 871 | 931 | 3722 |  |
| --- | --- | --- | --- | --- | --- |
| {BcHV1.BI=FALSE, BcMV9.BI=FALSE} =>  {BcOLV7.BI=FALSE} | 0.17  8218 | 0.96 | 0.185  644 | 1.18  9693 | 72 |
| {BcMV9.BI=FALSE, BcOLV7.BI=FALSE} =>  {BcHV1.BI=FALSE} | 0.17  8218 | 0.595  041 | 0.299  505 | 1.50  2479 | 72 |
| {BcHV1.BI=FALSE, BcOLV7.BI=FALSE} =>  {BcMV9.BI=FALSE} | 0.17  8218 | 0.541  353 | 0.329  208 | 1.68  236 | 72 |
| {BcALV1.BI=FALSE, BcMV9.BI=FALSE} =>  {BcHV1.BI=FALSE} | 0.16  3366 | 0.6 | 0.272  277 | 1.51  5 | 66 |
| {BcALV1.BI=FALSE, BcHV1.BI=FALSE} =>  {BcMV9.BI=FALSE} | 0.16  3366 | 0.468  085 | 0.349  01 | 1.45  4664 | 66 |
| {BcDMV1=FALSE, BcMV9.BI=FALSE} =>  {BcHV1.BI=FALSE} | 0.16  5842 | 0.567  797 | 0.292  079 | 1.43  3686 | 67 |
| {BcDMV1=FALSE, BcHV1.BI=FALSE} =>  {BcMV9.BI=FALSE} | 0.16  5842 | 0.507  576 | 0.326  733 | 1.57  7389 | 67 |
| {BcFV9=FALSE, BcMV9.BI=FALSE} =>  {BcHV1.BI=FALSE} | 0.16  8317 | 0.591  304 | 0.284  653 | 1.49  3043 | 68 |
| {BcFV9=FALSE, BcHV1.BI=FALSE} =>  {BcMV9.BI=FALSE} | 0.16  8317 | 0.465  753 | 0.361  386 | 1.44  7418 | 68 |
| {BcMV9.BI=FALSE, BcOLV4.BI=FALSE} =>  {BcHV1.BI=FALSE} | 0.15  8416 | 0.609  524 | 0.259  901 | 1.53  9048 | 64 |
| {BcHV1.BI=FALSE, BcOLV4.BI=FALSE} => | 0.15 | 0.457 | 0.346 | 1.42 | 64 |

| {BcMV9.BI=FALSE} | 8416 | 143 | 535 | 0659 |  |
| --- | --- | --- | --- | --- | --- |
| {BcMV9.BI=FALSE, BcULV5=FALSE} =>  {BcHV1.BI=FALSE} | 0.13  6139 | 0.617  978 | 0.220  297 | 1.56  0393 | 55 |
| {BcHV1.BI=FALSE, BcULV5=FALSE} =>  {BcMV9.BI=FALSE} | 0.13  6139 | 0.395  683 | 0.344  059 | 1.22  9662 | 55 |
| {BcBNV5.BI=FALSE, BcMV9.BI=FALSE} =>  {BcHV1.BI=FALSE} | 0.18  0693 | 0.579  365 | 0.311  881 | 1.46  2897 | 73 |
| {BcBNV5.BI=FALSE, BcHV1.BI=FALSE} =>  {BcMV9.BI=FALSE} | 0.18  0693 | 0.480  263 | 0.376  238 | 1.49  251 | 73 |
| {BcMV9.BI=FALSE, BvF.BI=FALSE} =>  {BcHV1.BI=FALSE} | 0.17  8218 | 0.590  164 | 0.301  98 | 1.49  0164 | 72 |
| {BcHV1.BI=FALSE, BvF.BI=FALSE} =>  {BcMV9.BI=FALSE} | 0.17  8218 | 0.483  221 | 0.368  812 | 1.50  1704 | 72 |
| {BcMCV5.BI=FALSE, BcMV9.BI=FALSE} =>  {BcHV1.BI=FALSE} | 0.18  0693 | 0.588  71 | 0.306  931 | 1.48  6492 | 73 |
| {BcHV1.BI=FALSE, BcMCV5.BI=FALSE} =>  {BcMV9.BI=FALSE} | 0.18  0693 | 0.480  263 | 0.376  238 | 1.49  251 | 73 |
| {BcMV9.BI=FALSE, BcOLV13.BI=FALSE} =>  {BcHV1.BI=FALSE} | 0.17  3267 | 0.598  291 | 0.289  604 | 1.51  0684 | 70 |
| {BcHV1.BI=FALSE, BcOLV13.BI=FALSE} =>  {BcMV9.BI=FALSE} | 0.17  3267 | 0.454  545 | 0.381  188 | 1.41  2587 | 70 |
| {BcMV9.BI=FALSE, BcOLV2.BI=FALSE} => | 0.17 | 0.572 | 0.306 | 1.44 | 71 |

| {BcHV1.BI=FALSE} | 5743 | 581 | 931 | 5766 |  |
| --- | --- | --- | --- | --- | --- |
| {BcHV1.BI=FALSE, BcOLV2.BI=FALSE} =>  {BcMV9.BI=FALSE} | 0.17  5743 | 0.467  105 | 0.376  238 | 1.45  1619 | 71 |
| {BcMCV3.BI=FALSE, BcMV9.BI=FALSE} =>  {BcHV1.BI=FALSE} | 0.17  8218 | 0.585  366 | 0.304  455 | 1.47  8049 | 72 |
| {BcHV1.BI=FALSE, BcMCV3.BI=FALSE} =>  {BcMV9.BI=FALSE} | 0.17  8218 | 0.458  599 | 0.388  614 | 1.42  5184 | 72 |
| {BcMV9.BI=FALSE, BcPV4.BI=FALSE} =>  {BcHV1.BI=FALSE} | 0.17  5743 | 0.586  777 | 0.299  505 | 1.48  1612 | 71 |
| {BcHV1.BI=FALSE, BcPV4.BI=FALSE} =>  {BcMV9.BI=FALSE} | 0.17  5743 | 0.470  199 | 0.373  762 | 1.46  1233 | 71 |
| {BcEV6=FALSE, BcMV9.BI=FALSE} =>  {BcHV1.BI=FALSE} | 0.18  0693 | 0.593  496 | 0.304  455 | 1.49  8577 | 73 |
| {BcEV6=FALSE, BcHV1.BI=FALSE} =>  {BcMV9.BI=FALSE} | 0.18  0693 | 0.467  949 | 0.386  139 | 1.45  4241 | 73 |
| {BcMV9.BI=FALSE, BcOLV17.BI=FALSE} =>  {BcHV1.BI=FALSE} | 0.18  0693 | 0.584 | 0.309  406 | 1.47  46 | 73 |
| {BcHV1.BI=FALSE, BcOLV17.BI=FALSE} =>  {BcMV9.BI=FALSE} | 0.18  0693 | 0.470  968 | 0.383  663 | 1.46  3623 | 73 |
| {BcMV9.BI=FALSE, BcOLV15.BI=FALSE} =>  {BcHV1.BI=FALSE} | 0.17  8218 | 0.576 | 0.309  406 | 1.45  44 | 72 |
| {BcHV1.BI=FALSE, BcOLV15.BI=FALSE} => | 0.17 | 0.461 | 0.386 | 1.43 | 72 |

| {BcMV9.BI=FALSE} | 8218 | 538 | 139 | 432 |  |
| --- | --- | --- | --- | --- | --- |
| {BcEV5=FALSE, BcMV9.BI=FALSE} =>  {BcHV1.BI=FALSE} | 0.17  8218 | 0.571  429 | 0.311  881 | 1.44  2857 | 72 |
| {BcEV5=FALSE, BcHV1.BI=FALSE} =>  {BcMV9.BI=FALSE} | 0.17  8218 | 0.473  684 | 0.376  238 | 1.47  2065 | 72 |
| {BcMV9.BI=FALSE, BcULV3=FALSE} =>  {BcHV1SLR.BI=FALSE} | 0.11  3861 | 0.754  098 | 0.150  99 | 1.64  6788 | 46 |
| {BcHV1SLR.BI=FALSE, BcMV9.BI=FALSE}  => {BcULV3=FALSE} | 0.11  3861 | 0.605  263 | 0.188  119 | 1.49  1014 | 46 |
| {BcHV1SLR.BI=FALSE, BcULV3=FALSE} =>  {BcMV9.BI=FALSE} | 0.11  3861 | 0.455  446 | 0.25 | 1.41  5385 | 46 |
| {BcMV9.BI=FALSE, BcULV3=FALSE} =>  {BcMV3.BI=FALSE} | 0.08  6634 | 0.573  77 | 0.150  99 | 1.25  2991 | 35 |
| {BcMV3.BI=FALSE, BcULV3=FALSE} =>  {BcMV9.BI=FALSE} | 0.08  6634 | 0.411  765 | 0.210  396 | 1.27  9638 | 35 |
| {BcMV9.BI=FALSE, BcULV3=FALSE} =>  {BcOLV18=FALSE} | 0.10  8911 | 0.721  311 | 0.150  99 | 1.49  4409 | 44 |
| {BcOLV18=FALSE, BcULV3=FALSE} =>  {BcMV9.BI=FALSE} | 0.10  8911 | 0.511  628 | 0.212  871 | 1.58  9982 | 44 |
| {BcMV9.BI=FALSE, BcULV3=FALSE} =>  {BcMV4.BI=FALSE} | 0.11  3861 | 0.754  098 | 0.150  99 | 1.35  4026 | 46 |
| {BcMV4.BI=FALSE, BcMV9.BI=FALSE} => | 0.11 | 0.528 | 0.215 | 1.30 | 46 |

| {BcULV3=FALSE} | 3861 | 736 | 347 | 2495 |  |
| --- | --- | --- | --- | --- | --- |
| {BcMV4.BI=FALSE, BcULV3=FALSE} =>  {BcMV9.BI=FALSE} | 0.11  3861 | 0.442  308 | 0.257  426 | 1.37  4556 | 46 |
| {BcMV9.BI=FALSE, BcULV3=FALSE} =>  {BcOLV16.BI=FALSE} | 0.12  8713 | 0.852  459 | 0.150  99 | 1.45  9294 | 52 |
| {BcMV9.BI=FALSE, BcOLV16.BI=FALSE} =>  {BcULV3=FALSE} | 0.12  8713 | 0.541  667 | 0.237  624 | 1.33  435 | 52 |
| {BcOLV16.BI=FALSE, BcULV3=FALSE} =>  {BcMV9.BI=FALSE} | 0.12  8713 | 0.477  064 | 0.269  802 | 1.48  2569 | 52 |
| {BcMV11=TRUE, BcULV3=FALSE} =>  {BcMV9.BI=FALSE} | 0.09  6535 | 0.438  202 | 0.220  297 | 1.36  1798 | 39 |
| {BcMV9.BI=FALSE, BcULV4=TRUE} =>  {BcULV3=FALSE} | 0.07  4257 | 0.517  241 | 0.143  564 | 1.27  418 | 30 |
| {BcMV9.BI=FALSE, BcULV3=FALSE} =>  {BcMV6.BI=FALSE} | 0.13  1188 | 0.868  852 | 0.150  99 | 1.24  9169 | 53 |
| {BcMV6.BI=FALSE, BcMV9.BI=FALSE} =>  {BcULV3=FALSE} | 0.13  1188 | 0.519  608 | 0.252  475 | 1.28  001 | 53 |
| {BcMV6.BI=FALSE, BcULV3=FALSE} =>  {BcMV9.BI=FALSE} | 0.13  1188 | 0.414  063 | 0.316  832 | 1.28  6779 | 53 |
| {BcMV9.BI=FALSE, BcULV3=FALSE} =>  {BcPV3.BI=FALSE} | 0.12  6238 | 0.836  066 | 0.150  99 | 1.17  6901 | 51 |
| {BcMV9.BI=FALSE, BcPV3.BI=FALSE} => | 0.12 | 0.531 | 0.237 | 1.30 | 51 |

| {BcULV3=FALSE} | 6238 | 25 | 624 | 8689 |  |
| --- | --- | --- | --- | --- | --- |
| {BcPV3.BI=FALSE, BcULV3=FALSE} =>  {BcMV9.BI=FALSE} | 0.12  6238 | 0.435  897 | 0.289  604 | 1.35  4635 | 51 |
| {BcMV9.BI=FALSE, BcULV3=FALSE} =>  {BcFV5.BI=FALSE} | 0.13  3663 | 0.885  246 | 0.150  99 | 1.13  177 | 54 |
| {BcMV9.BI=FALSE, BcULV3=FALSE} =>  {BcULV1.BI=FALSE} | 0.13  8614 | 0.918  033 | 0.150  99 | 1.16  9985 | 56 |
| {BcULV1.BI=FALSE, BcULV3=FALSE} =>  {BcMV9.BI=FALSE} | 0.13  8614 | 0.424  242 | 0.326  733 | 1.31  8415 | 56 |
| {BcMV9.BI=FALSE, BcULV3=FALSE} =>  {BcMV12=FALSE} | 0.13  3663 | 0.885  246 | 0.150  99 | 1.10  7243 | 54 |
| {BcMV12=FALSE, BcMV9.BI=FALSE} =>  {BcULV3=FALSE} | 0.13  3663 | 0.514  286 | 0.259  901 | 1.26  6899 | 54 |
| {BcMV12=FALSE, BcULV3=FALSE} =>  {BcMV9.BI=FALSE} | 0.13  3663 | 0.402  985 | 0.331  683 | 1.25  2354 | 54 |
| {BcMV9.BI=FALSE, BcULV3=FALSE} =>  {BcNSRV7.BI=FALSE} | 0.14  1089 | 0.934  426 | 0.150  99 | 1.16  8756 | 57 |
| {BcMV9.BI=FALSE, BcNSRV7.BI=FALSE} =>  {BcULV3=FALSE} | 0.14  1089 | 0.5 | 0.282  178 | 1.23  1707 | 57 |
| {BcMV9.BI=FALSE, BcULV3=FALSE} =>  {BcOLV7.BI=FALSE} | 0.14  604 | 0.967  213 | 0.150  99 | 1.19  8632 | 59 |
| {BcOLV7.BI=FALSE, BcULV3=FALSE} => | 0.14 | 0.440 | 0.331 | 1.36 | 59 |

| {BcMV9.BI=FALSE} | 604 | 299 | 683 | 8312 |  |
| --- | --- | --- | --- | --- | --- |
| {BcMV9.BI=FALSE, BcULV5=FALSE} =>  {BcULV3=FALSE} | 0.11  3861 | 0.516  854 | 0.220  297 | 1.27  3226 | 46 |
| {BcMV9.BI=FALSE, BcULV3=FALSE} =>  {BcBNV5.BI=FALSE} | 0.14  8515 | 0.983  607 | 0.150  99 | 1.08  2771 | 60 |
| {BcULV3=FALSE, BvF.BI=FALSE} =>  {BcMV9.BI=FALSE} | 0.14  3564 | 0.397  26 | 0.361  386 | 1.23  4563 | 58 |
| {BcMCV3.BI=FALSE, BcMV9.BI=FALSE} =>  {BcULV3=FALSE} | 0.15  099 | 0.495  935 | 0.304  455 | 1.22  1693 | 61 |
| {BcEV6=FALSE, BcULV3=FALSE} =>  {BcMV9.BI=FALSE} | 0.14  8515 | 0.394  737 | 0.376  238 | 1.22  6721 | 60 |
| {BcMV9.BI=FALSE, BcOLV16.BI=TRUE} =>  {BcOLV7.BI=FALSE} | 0.07  9208 | 0.941  176 | 0.084  158 | 1.16  6366 | 32 |
| {BcMV9.BI=FALSE, BcOLV16.BI=TRUE} =>  {BcDMV1=FALSE} | 0.08  1683 | 0.970  588 | 0.084  158 | 1.14  9905 | 33 |
| {BcMV4.BI=TRUE, BcMV9.BI=FALSE} =>  {BcMV11=TRUE} | 0.08  4158 | 0.790  698 | 0.106  436 | 1.28  2899 | 34 |
| {BcMV4.BI=TRUE, BcMV9.BI=FALSE} =>  {BcRV1.BI=FALSE} | 0.08  9109 | 0.837  209 | 0.106  436 | 1.15  8331 | 36 |
| {BcMV4.BI=TRUE, BcMV9.BI=FALSE} =>  {BcOLV7.BI=FALSE} | 0.09  6535 | 0.906  977 | 0.106  436 | 1.12  3983 | 39 |
| {BcMV4.BI=TRUE, BcMV9.BI=FALSE} => | 0.09 | 0.930 | 0.106 | 1.10 | 40 |

| {BcDMV1=FALSE} | 901 | 233 | 436 | 2094 |  |
| --- | --- | --- | --- | --- | --- |
| {BcMV3.BI=FALSE, BcMV9.BI=FALSE} =>  {BcHV1SLR.BI=FALSE} | 0.10  6436 | 0.605  634 | 0.175  743 | 1.32  2573 | 43 |
| {BcHV1SLR.BI=FALSE, BcMV3.BI=FALSE}  => {BcMV9.BI=FALSE} | 0.10  6436 | 0.434  343 | 0.245  05 | 1.34  9806 | 43 |
| {BcHV1SLR.BI=FALSE, BcMV9.BI=FALSE}  => {BcOLV18=FALSE} | 0.14  604 | 0.776  316 | 0.188  119 | 1.60  8367 | 59 |
| {BcMV9.BI=FALSE, BcOLV18=FALSE} =>  {BcHV1SLR.BI=FALSE} | 0.14  604 | 0.670  455 | 0.217  822 | 1.46  4128 | 59 |
| {BcHV1SLR.BI=FALSE, BcOLV18=FALSE} =>  {BcMV9.BI=FALSE} | 0.14  604 | 0.517  544 | 0.282  178 | 1.60  8367 | 59 |
| {BcMV2.BI=FALSE, BcMV9.BI=FALSE} =>  {BcHV1SLR.BI=FALSE} | 0.10  8911 | 0.602  74 | 0.180  693 | 1.31  6253 | 44 |
| {BcHV1SLR.BI=FALSE, BcMV2.BI=FALSE}  => {BcMV9.BI=FALSE} | 0.10  8911 | 0.407  407 | 0.267  327 | 1.26  6097 | 44 |
| {BcMV4.BI=FALSE, BcMV9.BI=FALSE} =>  {BcHV1SLR.BI=FALSE} | 0.12  1287 | 0.563  218 | 0.215  347 | 1.22  9947 | 49 |
| {BcHV1SLR.BI=FALSE, BcMV4.BI=FALSE}  => {BcMV9.BI=FALSE} | 0.12  1287 | 0.437  5 | 0.277  228 | 1.35  9615 | 49 |
| {BcHV1SLR.BI=FALSE, BcMV9.BI=FALSE}  => {BcOLV16.BI=FALSE} | 0.14  3564 | 0.763  158 | 0.188  119 | 1.30  6423 | 58 |
| {BcMV9.BI=FALSE, BcOLV16.BI=FALSE} => | 0.14 | 0.604 | 0.237 | 1.31 | 58 |

| {BcHV1SLR.BI=FALSE} | 3564 | 167 | 624 | 9369 |  |
| --- | --- | --- | --- | --- | --- |
| {BcHV1SLR.BI=FALSE, BcOLV16.BI=FALSE}  => {BcMV9.BI=FALSE} | 0.14  3564 | 0.513  274 | 0.279  703 | 1.59  5099 | 58 |
| {BcHV1SLR.BI=FALSE, BcMV11=TRUE} =>  {BcMV9.BI=FALSE} | 0.11  3861 | 0.438  095 | 0.259  901 | 1.36  1465 | 46 |
| {BcMV9.BI=FALSE, BcOLV1.BI.MOVA.=FALSE} =>  {BcHV1SLR.BI=FALSE} | 0.12  1287 | 0.597  561 | 0.202  97 | 1.30  4944 | 49 |
| {BcHV1SLR.BI=FALSE, BcMV9.BI=FALSE}  => {BcMV6.BI=FALSE} | 0.15  5941 | 0.828  947 | 0.188  119 | 1.19  1796 | 63 |
| {BcMV6.BI=FALSE, BcMV9.BI=FALSE} =>  {BcHV1SLR.BI=FALSE} | 0.15  5941 | 0.617  647 | 0.252  475 | 1.34  8808 | 63 |
| {BcHV1SLR.BI=FALSE, BcMV6.BI=FALSE}  => {BcMV9.BI=FALSE} | 0.15  5941 | 0.434  483 | 0.358  911 | 1.35  0239 | 63 |
| {BcMV9.BI=FALSE, BcPV3.BI=FALSE} =>  {BcHV1SLR.BI=FALSE} | 0.14  8515 | 0.625 | 0.237  624 | 1.36  4865 | 60 |
| {BcHV1SLR.BI=FALSE, BcPV3.BI=FALSE} =>  {BcMV9.BI=FALSE} | 0.14  8515 | 0.441  176 | 0.336  634 | 1.37  1041 | 60 |
| {BcMV9.BI=FALSE, BcRV1.BI=FALSE} =>  {BcHV1SLR.BI=FALSE} | 0.14  8515 | 0.555  556 | 0.267  327 | 1.21  3213 | 60 |
| {BcHV1SLR.BI=FALSE, BcRV1.BI=FALSE} =>  {BcMV9.BI=FALSE} | 0.14  8515 | 0.434  783 | 0.341  584 | 1.35  1171 | 60 |

| {BcEV2.BI=FALSE, BcMV9.BI=FALSE} =>  {BcHV1SLR.BI=FALSE} | 0.14  8515 | 0.612  245 | 0.242  574 | 1.33  701 | 60 |
| --- | --- | --- | --- | --- | --- |
| {BcEV2.BI=FALSE, BcHV1SLR.BI=FALSE} =>  {BcMV9.BI=FALSE} | 0.14  8515 | 0.410  959 | 0.361  386 | 1.27  7134 | 60 |
| {BcHV3.BI=FALSE, BcMV9.BI=FALSE} =>  {BcHV1SLR.BI=FALSE} | 0.15  5941 | 0.611  65 | 0.254  95 | 1.33  5712 | 63 |
| {BcHV1SLR.BI=FALSE, BcHV3.BI=FALSE} =>  {BcMV9.BI=FALSE} | 0.15  5941 | 0.425  676 | 0.366  337 | 1.32  2869 | 63 |
| {BcHV1SLR.BI=FALSE, BcMV9.BI=FALSE}  => {BcFV5.BI=FALSE} | 0.16  5842 | 0.881  579 | 0.188  119 | 1.12  7082 | 67 |
| {BcFV5.BI=FALSE, BcMV9.BI=FALSE} =>  {BcHV1SLR.BI=FALSE} | 0.16  5842 | 0.614  679 | 0.269  802 | 1.34  2326 | 67 |
| {BcFV5.BI=FALSE, BcHV1SLR.BI=FALSE} =>  {BcMV9.BI=FALSE} | 0.16  5842 | 0.424  051 | 0.391  089 | 1.31  7819 | 67 |
| {BcHV1SLR.BI=FALSE, BcMV9.BI=FALSE}  => {BcULV1.BI=FALSE} | 0.17  5743 | 0.934  211 | 0.188  119 | 1.19  0603 | 71 |
| {BcMV9.BI=FALSE, BcULV1.BI=FALSE} =>  {BcHV1SLR.BI=FALSE} | 0.17  5743 | 0.617  391 | 0.284  653 | 1.34  8249 | 71 |
| {BcHV1SLR.BI=FALSE, BcULV1.BI=FALSE}  => {BcMV9.BI=FALSE} | 0.17  5743 | 0.438  272 | 0.400  99 | 1.36  2013 | 71 |
| {BcMV1.BI=TRUE, BcMV9.BI=FALSE} =>  {BcHV1SLR.BI=FALSE} | 0.14  1089 | 0.587  629 | 0.240  099 | 1.28  3254 | 57 |

| {BcHV1SLR.BI=FALSE, BcMV1.BI=TRUE} =>  {BcMV9.BI=FALSE} | 0.14  1089 | 0.404  255 | 0.349  01 | 1.25  6301 | 57 |
| --- | --- | --- | --- | --- | --- |
| {BcMV12=FALSE, BcMV9.BI=FALSE} =>  {BcHV1SLR.BI=FALSE} | 0.16  3366 | 0.628  571 | 0.259  901 | 1.37  2664 | 66 |
| {BcHV1SLR.BI=FALSE, BcMV12=FALSE} =>  {BcMV9.BI=FALSE} | 0.16  3366 | 0.423  077 | 0.386  139 | 1.31  4793 | 66 |
| {BcHV1SLR.BI=FALSE, BcMV9.BI=FALSE}  => {BcNSRV7.BI=FALSE} | 0.17  8218 | 0.947  368 | 0.188  119 | 1.18  4944 | 72 |
| {BcMV9.BI=FALSE, BcNSRV7.BI=FALSE} =>  {BcHV1SLR.BI=FALSE} | 0.17  8218 | 0.631  579 | 0.282  178 | 1.37  9232 | 72 |
| {BcHV1SLR.BI=FALSE, BcNSRV7.BI=FALSE}  => {BcMV9.BI=FALSE} | 0.17  8218 | 0.455  696 | 0.391  089 | 1.41  6164 | 72 |
| {BcMV9.BI=FALSE, BcVV3.BI=FALSE} =>  {BcHV1SLR.BI=FALSE} | 0.14  8515 | 0.6 | 0.247  525 | 1.31  027 | 60 |
| {BcHV1SLR.BI=FALSE, BcVV3.BI=FALSE} =>  {BcMV9.BI=FALSE} | 0.14  8515 | 0.402  685 | 0.368  812 | 1.25  142 | 60 |
| {BcHV1SLR.BI=FALSE, BcMV9.BI=FALSE}  => {BcOLV7.BI=FALSE} | 0.17  8218 | 0.947  368 | 0.188  119 | 1.17  4039 | 72 |
| {BcMV9.BI=FALSE, BcOLV7.BI=FALSE} =>  {BcHV1SLR.BI=FALSE} | 0.17  8218 | 0.595  041 | 0.299  505 | 1.29  9442 | 72 |
| {BcHV1SLR.BI=FALSE, BcOLV7.BI=FALSE}  => {BcMV9.BI=FALSE} | 0.17  8218 | 0.48 | 0.371  287 | 1.49  1692 | 72 |

| {BcALV1.BI=FALSE, BcMV9.BI=FALSE} =>  {BcHV1SLR.BI=FALSE} | 0.16  0891 | 0.590  909 | 0.272  277 | 1.29  0418 | 65 |
| --- | --- | --- | --- | --- | --- |
| {BcALV1.BI=FALSE, BcHV1SLR.BI=FALSE}  => {BcMV9.BI=FALSE} | 0.16  0891 | 0.403  727 | 0.398  515 | 1.25  4658 | 65 |
| {BcDMV1=FALSE, BcMV9.BI=FALSE} =>  {BcHV1SLR.BI=FALSE} | 0.16  8317 | 0.576  271 | 0.292  079 | 1.25  8452 | 68 |
| {BcDMV1=FALSE, BcHV1SLR.BI=FALSE} =>  {BcMV9.BI=FALSE} | 0.16  8317 | 0.444  444 | 0.378  713 | 1.38  1197 | 68 |
| {BcFV9=FALSE, BcMV9.BI=FALSE} =>  {BcHV1SLR.BI=FALSE} | 0.16  8317 | 0.591  304 | 0.284  653 | 1.29  1281 | 68 |
| {BcFV9=FALSE, BcHV1SLR.BI=FALSE} =>  {BcMV9.BI=FALSE} | 0.16  8317 | 0.402  367 | 0.418  317 | 1.25  0432 | 68 |
| {BcMV9.BI=FALSE, BcOLV4.BI=FALSE} =>  {BcHV1SLR.BI=FALSE} | 0.16  0891 | 0.619  048 | 0.259  901 | 1.35  1866 | 65 |
| {BcMV9.BI=FALSE, BcULV5=FALSE} =>  {BcHV1SLR.BI=FALSE} | 0.14  1089 | 0.640  449 | 0.220  297 | 1.39  8603 | 57 |
| {BcBNV5.BI=FALSE, BcMV9.BI=FALSE} =>  {BcHV1SLR.BI=FALSE} | 0.18  0693 | 0.579  365 | 0.311  881 | 1.26  5208 | 73 |
| {BcBNV5.BI=FALSE, BcHV1SLR.BI=FALSE}  => {BcMV9.BI=FALSE} | 0.18  0693 | 0.419  54 | 0.430  693 | 1.30  3802 | 73 |
| {BcMV9.BI=FALSE, BvF.BI=FALSE} =>  {BcHV1SLR.BI=FALSE} | 0.18  0693 | 0.598  361 | 0.301  98 | 1.30  669 | 73 |

| {BcHV1SLR.BI=FALSE, BvF.BI=FALSE} =>  {BcMV9.BI=FALSE} | 0.18  0693 | 0.417  143 | 0.433  168 | 1.29  6352 | 73 |
| --- | --- | --- | --- | --- | --- |
| {BcMCV5.BI=FALSE, BcMV9.BI=FALSE} =>  {BcHV1SLR.BI=FALSE} | 0.18  3168 | 0.596  774 | 0.306  931 | 1.30  3226 | 74 |
| {BcHV1SLR.BI=FALSE, BcMCV5.BI=FALSE}  => {BcMV9.BI=FALSE} | 0.18  3168 | 0.422  857 | 0.433  168 | 1.31  411 | 74 |
| {BcMV9.BI=FALSE, BcOLV13.BI=FALSE} =>  {BcHV1SLR.BI=FALSE} | 0.17  5743 | 0.606  838 | 0.289  604 | 1.32  5202 | 71 |
| {BcHV1SLR.BI=FALSE, BcOLV13.BI=FALSE}  => {BcMV9.BI=FALSE} | 0.17  5743 | 0.403  409 | 0.435  644 | 1.25  3671 | 71 |
| {BcMV9.BI=FALSE, BcOLV2.BI=FALSE} =>  {BcHV1SLR.BI=FALSE} | 0.18  0693 | 0.588  71 | 0.306  931 | 1.28  5615 | 73 |
| {BcHV1SLR.BI=FALSE, BcOLV2.BI=FALSE}  => {BcMV9.BI=FALSE} | 0.18  0693 | 0.405  556 | 0.445  545 | 1.26  0342 | 73 |
| {BcMCV3.BI=FALSE, BcMV9.BI=FALSE} =>  {BcHV1SLR.BI=FALSE} | 0.18  3168 | 0.601  626 | 0.304  455 | 1.31  3821 | 74 |
| {BcHV1SLR.BI=FALSE, BcMCV3.BI=FALSE}  => {BcMV9.BI=FALSE} | 0.18  3168 | 0.411  111 | 0.445  545 | 1.27  7607 | 74 |
| {BcMV9.BI=FALSE, BcPV4.BI=FALSE} =>  {BcHV1SLR.BI=FALSE} | 0.18  0693 | 0.603  306 | 0.299  505 | 1.31  7489 | 73 |
| {BcHV1SLR.BI=FALSE, BcPV4.BI=FALSE} =>  {BcMV9.BI=FALSE} | 0.18  0693 | 0.421  965 | 0.428  218 | 1.31  1338 | 73 |

| {BcEV6=FALSE, BcMV9.BI=FALSE} =>  {BcHV1SLR.BI=FALSE} | 0.17  8218 | 0.585  366 | 0.304  455 | 1.27  8312 | 72 |
| --- | --- | --- | --- | --- | --- |
| {BcEV6=FALSE, BcHV1SLR.BI=FALSE} =>  {BcMV9.BI=FALSE} | 0.17  8218 | 0.411  429 | 0.433  168 | 1.27  8593 | 72 |
| {BcMV9.BI=FALSE, BcOLV17.BI=FALSE} =>  {BcHV1SLR.BI=FALSE} | 0.18  3168 | 0.592 | 0.309  406 | 1.29  28 | 74 |
| {BcHV1SLR.BI=FALSE, BcOLV17.BI=FALSE}  => {BcMV9.BI=FALSE} | 0.18  3168 | 0.413  408 | 0.443  069 | 1.28  4744 | 74 |
| {BcMV9.BI=FALSE, BcOLV15.BI=FALSE} =>  {BcHV1SLR.BI=FALSE} | 0.18  3168 | 0.592 | 0.309  406 | 1.29  28 | 74 |
| {BcHV1SLR.BI=FALSE, BcOLV15.BI=FALSE}  => {BcMV9.BI=FALSE} | 0.18  3168 | 0.406  593 | 0.450  495 | 1.26  3567 | 74 |
| {BcEV5=FALSE, BcMV9.BI=FALSE} =>  {BcHV1SLR.BI=FALSE} | 0.18  0693 | 0.579  365 | 0.311  881 | 1.26  5208 | 73 |
| {BcEV5=FALSE, BcHV1SLR.BI=FALSE} =>  {BcMV9.BI=FALSE} | 0.18  0693 | 0.412  429 | 0.438  119 | 1.28  1704 | 73 |
| {BcMV3.BI=FALSE, BcMV9.BI=FALSE} =>  {BcOLV18=FALSE} | 0.12  1287 | 0.690  141 | 0.175  743 | 1.42  983 | 49 |
| {BcMV3.BI=FALSE, BcOLV18=FALSE} =>  {BcMV9.BI=FALSE} | 0.12  1287 | 0.449  541 | 0.269  802 | 1.39  7036 | 49 |
| {BcMV3.BI=FALSE, BcMV9.BI=FALSE} =>  {BcMV2.BI=FALSE} | 0.11  8812 | 0.676  056 | 0.175  743 | 1.25  8649 | 48 |

| {BcMV2.BI=FALSE, BcMV9.BI=FALSE} =>  {BcMV3.BI=FALSE} | 0.11  8812 | 0.657  534 | 0.180  693 | 1.43  5913 | 48 |
| --- | --- | --- | --- | --- | --- |
| {BcMV3.BI=FALSE, BcMV9.BI=FALSE} =>  {BcMV4.BI=FALSE} | 0.12  8713 | 0.732  394 | 0.175  743 | 1.31  5055 | 52 |
| {BcMV4.BI=FALSE, BcMV9.BI=FALSE} =>  {BcMV3.BI=FALSE} | 0.12  8713 | 0.597  701 | 0.215  347 | 1.30  525 | 52 |
| {BcMV3.BI=FALSE, BcMV4.BI=FALSE} =>  {BcMV9.BI=FALSE} | 0.12  8713 | 0.416 | 0.309  406 | 1.29  28 | 52 |
| {BcMV3.BI=FALSE, BcMV9.BI=FALSE} =>  {BcOLV16.BI=FALSE} | 0.14  3564 | 0.816  901 | 0.175  743 | 1.39  8424 | 58 |
| {BcMV9.BI=FALSE, BcOLV16.BI=FALSE} =>  {BcMV3.BI=FALSE} | 0.14  3564 | 0.604  167 | 0.237  624 | 1.31  9369 | 58 |
| {BcMV3.BI=FALSE, BcOLV16.BI=FALSE} =>  {BcMV9.BI=FALSE} | 0.14  3564 | 0.464 | 0.309  406 | 1.44  1969 | 58 |
| {BcMV11=TRUE, BcMV3.BI=FALSE} =>  {BcMV9.BI=FALSE} | 0.10  396 | 0.446  809 | 0.232  673 | 1.38  8543 | 42 |
| {BcMV3.BI=FALSE, BcMV9.BI=FALSE} =>  {BcOLV1.BI.MOVA.=FALSE} | 0.13  8614 | 0.788  732 | 0.175  743 | 1.18  0177 | 56 |
| {BcMV9.BI=FALSE, BcOLV1.BI.MOVA.=FALSE} =>  {BcMV3.BI=FALSE} | 0.13  8614 | 0.682  927 | 0.202  97 | 1.49  1365 | 56 |
| {BcMV3.BI=FALSE, BcMV9.BI=FALSE} => | 0.15 | 0.873 | 0.175 | 1.25 | 62 |

| {BcMV6.BI=FALSE} | 3465 | 239 | 743 | 5476 |  |
| --- | --- | --- | --- | --- | --- |
| {BcMV6.BI=FALSE, BcMV9.BI=FALSE} =>  {BcMV3.BI=FALSE} | 0.15  3465 | 0.607  843 | 0.252  475 | 1.32  7398 | 62 |
| {BcMV3.BI=FALSE, BcMV6.BI=FALSE} =>  {BcMV9.BI=FALSE} | 0.15  3465 | 0.407  895 | 0.376  238 | 1.26  7611 | 62 |
| {BcMV3.BI=FALSE, BcMV9.BI=FALSE} =>  {BcRV1.BI=FALSE} | 0.14  8515 | 0.845  07 | 0.175  743 | 1.16  9207 | 60 |
| {BcMV9.BI=FALSE, BcRV1.BI=FALSE} =>  {BcMV3.BI=FALSE} | 0.14  8515 | 0.555  556 | 0.267  327 | 1.21  3213 | 60 |
| {BcMV3.BI=FALSE, BcRV1.BI=FALSE} =>  {BcMV9.BI=FALSE} | 0.14  8515 | 0.416  667 | 0.356  436 | 1.29  4872 | 60 |
| {BcMV3.BI=FALSE, BcMV9.BI=FALSE} =>  {BcFV5.BI=FALSE} | 0.16  3366 | 0.929  577 | 0.175  743 | 1.18  8447 | 66 |
| {BcFV5.BI=FALSE, BcMV9.BI=FALSE} =>  {BcMV3.BI=FALSE} | 0.16  3366 | 0.605  505 | 0.269  802 | 1.32  2291 | 66 |
| {BcFV5.BI=FALSE, BcMV3.BI=FALSE} =>  {BcMV9.BI=FALSE} | 0.16  3366 | 0.415  094 | 0.393  564 | 1.28  9985 | 66 |
| {BcMV3.BI=FALSE, BcMV9.BI=FALSE} =>  {BcULV1.BI=FALSE} | 0.16  3366 | 0.929  577 | 0.175  743 | 1.18  4698 | 66 |
| {BcMV9.BI=FALSE, BcULV1.BI=FALSE} =>  {BcMV3.BI=FALSE} | 0.16  3366 | 0.573  913 | 0.284  653 | 1.25  3302 | 66 |
| {BcMV3.BI=FALSE, BcULV1.BI=FALSE} => | 0.16 | 0.415 | 0.393 | 1.28 | 66 |

| {BcMV9.BI=FALSE} | 3366 | 094 | 564 | 9985 |  |
| --- | --- | --- | --- | --- | --- |
| {BcMV3.BI=FALSE, BcMV9.BI=FALSE} =>  {BcMV12=FALSE} | 0.15  8416 | 0.901  408 | 0.175  743 | 1.12  7458 | 64 |
| {BcMV12=FALSE, BcMV9.BI=FALSE} =>  {BcMV3.BI=FALSE} | 0.15  8416 | 0.609  524 | 0.259  901 | 1.33  1068 | 64 |
| {BcMV3.BI=FALSE, BcMV9.BI=FALSE} =>  {BcNSRV7.BI=FALSE} | 0.15  8416 | 0.901  408 | 0.175  743 | 1.12  7458 | 64 |
| {BcMV9.BI=FALSE, BcNSRV7.BI=FALSE} =>  {BcMV3.BI=FALSE} | 0.15  8416 | 0.561  404 | 0.282  178 | 1.22  5984 | 64 |
| {BcMV3.BI=FALSE, BcNSRV7.BI=FALSE} =>  {BcMV9.BI=FALSE} | 0.15  8416 | 0.421  053 | 0.376  238 | 1.30  8502 | 64 |
| {BcMV9.BI=FALSE, BcVV3.BI=FALSE} =>  {BcMV3.BI=FALSE} | 0.14  3564 | 0.58 | 0.247  525 | 1.26  6595 | 58 |
| {BcMV3.BI=FALSE, BcMV9.BI=FALSE} =>  {BcOLV7.BI=FALSE} | 0.17  0792 | 0.971  831 | 0.175  743 | 1.20  4355 | 69 |
| {BcMV9.BI=FALSE, BcOLV7.BI=FALSE} =>  {BcMV3.BI=FALSE} | 0.17  0792 | 0.570  248 | 0.299  505 | 1.24  5298 | 69 |
| {BcMV3.BI=FALSE, BcOLV7.BI=FALSE} =>  {BcMV9.BI=FALSE} | 0.17  0792 | 0.436  709 | 0.391  089 | 1.35  7157 | 69 |
| {BcMV3.BI=FALSE, BcMV9.BI=FALSE} =>  {BcDMV1=FALSE} | 0.16  5842 | 0.943  662 | 0.175  743 | 1.11  8004 | 67 |
| {BcDMV1=FALSE, BcMV9.BI=FALSE} => | 0.16 | 0.567 | 0.292 | 1.23 | 67 |

| {BcMV3.BI=FALSE} | 5842 | 797 | 079 | 9945 |  |
| --- | --- | --- | --- | --- | --- |
| {BcDMV1=FALSE, BcMV3.BI=FALSE} =>  {BcMV9.BI=FALSE} | 0.16  5842 | 0.394  118 | 0.420  792 | 1.22  4796 | 67 |
| {BcFV9=FALSE, BcMV9.BI=FALSE} =>  {BcMV3.BI=FALSE} | 0.15  8416 | 0.556  522 | 0.284  653 | 1.21  5323 | 64 |
| {BcMV9.BI=FALSE, BcOLV4.BI=FALSE} =>  {BcMV3.BI=FALSE} | 0.15  099 | 0.580  952 | 0.259  901 | 1.26  8674 | 61 |
| {BcMV9.BI=FALSE, BcULV5=FALSE} =>  {BcMV3.BI=FALSE} | 0.14  3564 | 0.651  685 | 0.220  297 | 1.42  314 | 58 |
| {BcBNV5.BI=FALSE, BcMV3.BI=FALSE} =>  {BcMV9.BI=FALSE} | 0.17  0792 | 0.389  831 | 0.438  119 | 1.21  1473 | 69 |
| {BcMV9.BI=FALSE, BvF.BI=FALSE} =>  {BcMV3.BI=FALSE} | 0.16  5842 | 0.549  18 | 0.301  98 | 1.19  9291 | 67 |
| {BcMV3.BI=FALSE, BvF.BI=FALSE} =>  {BcMV9.BI=FALSE} | 0.16  5842 | 0.396  45 | 0.418  317 | 1.23  2044 | 67 |
| {BcMCV5.BI=FALSE, BcMV9.BI=FALSE} =>  {BcMV3.BI=FALSE} | 0.17  0792 | 0.556  452 | 0.306  931 | 1.21  517 | 69 |
| {BcMCV5.BI=FALSE, BcMV3.BI=FALSE} =>  {BcMV9.BI=FALSE} | 0.17  0792 | 0.387  64 | 0.440  594 | 1.20  4667 | 69 |
| {BcMV9.BI=FALSE, BcOLV13.BI=FALSE} =>  {BcMV3.BI=FALSE} | 0.17  3267 | 0.598  291 | 0.289  604 | 1.30  6537 | 70 |
| {BcMV9.BI=FALSE, BcOLV2.BI=FALSE} => | 0.17 | 0.564 | 0.306 | 1.23 | 70 |

| {BcMV3.BI=FALSE} | 3267 | 516 | 931 | 2781 |  |
| --- | --- | --- | --- | --- | --- |
| {BcMV3.BI=FALSE, BcOLV2.BI=FALSE} =>  {BcMV9.BI=FALSE} | 0.17  3267 | 0.391  061 | 0.443  069 | 1.21  5299 | 70 |
| {BcMCV3.BI=FALSE, BcMV9.BI=FALSE} =>  {BcMV3.BI=FALSE} | 0.17  5743 | 0.577  236 | 0.304  455 | 1.26  0558 | 71 |
| {BcMV9.BI=FALSE, BcPV4.BI=FALSE} =>  {BcMV3.BI=FALSE} | 0.17  0792 | 0.570  248 | 0.299  505 | 1.24  5298 | 69 |
| {BcMV3.BI=FALSE, BcPV4.BI=FALSE} =>  {BcMV9.BI=FALSE} | 0.17  0792 | 0.392  045 | 0.435  644 | 1.21  8357 | 69 |
| {BcEV6=FALSE, BcMV3.BI=FALSE} =>  {BcMV9.BI=FALSE} | 0.16  3366 | 0.390  533 | 0.418  317 | 1.21  3655 | 66 |
| {BcMV3.BI=FALSE, BcOLV17.BI=FALSE} =>  {BcMV9.BI=FALSE} | 0.16  8317 | 0.386  364 | 0.435  644 | 1.20  0699 | 68 |
| {BcMV9.BI=FALSE, BcOLV15.BI=FALSE} =>  {BcMV3.BI=FALSE} | 0.17  0792 | 0.552 | 0.309  406 | 1.20  5449 | 69 |
| {BcMV3.BI=FALSE, BcOLV15.BI=FALSE} =>  {BcMV9.BI=FALSE} | 0.17  0792 | 0.385  475 | 0.443  069 | 1.19  7937 | 69 |
| {BcEV5=FALSE, BcMV9.BI=FALSE} =>  {BcMV3.BI=FALSE} | 0.17  3267 | 0.555  556 | 0.311  881 | 1.21  3213 | 70 |
| {BcMV2.BI=TRUE, BcMV9.BI=FALSE} =>  {BcOLV18=FALSE} | 0.09  1584 | 0.649  123 | 0.141  089 | 1.34  4849 | 37 |
| {BcMV2.BI=TRUE, BcOLV18=FALSE} => | 0.09 | 0.544 | 0.168 | 1.69 | 37 |

| {BcMV9.BI=FALSE} | 1584 | 118 | 317 | 095 |  |
| --- | --- | --- | --- | --- | --- |
| {BcMV2.BI=TRUE, BcMV4.BI=FALSE} =>  {BcMV9.BI=FALSE} | 0.07  9208 | 0.421  053 | 0.188  119 | 1.30  8502 | 32 |
| {BcMV2.BI=TRUE, BcOLV16.BI=FALSE} =>  {BcMV9.BI=FALSE} | 0.09  1584 | 0.435  294 | 0.210  396 | 1.35  276 | 37 |
| {BcMV2.BI=TRUE, BcMV9.BI=FALSE} =>  {BcMV11=TRUE} | 0.11  1386 | 0.789  474 | 0.141  089 | 1.28  0913 | 45 |
| {BcMV2.BI=TRUE, BcMV9.BI=FALSE} =>  {BcHV3.BI=FALSE} | 0.12  3762 | 0.877  193 | 0.141  089 | 1.15  4352 | 50 |
| {BcMV2.BI=TRUE, BcMV9.BI=FALSE} =>  {BcFV5.BI=FALSE} | 0.12  6238 | 0.894  737 | 0.141  089 | 1.14  3904 | 51 |
| {BcMV2.BI=TRUE, BcMV9.BI=FALSE} =>  {BcMV1.BI=TRUE} | 0.12  6238 | 0.894  737 | 0.141  089 | 1.13  3146 | 51 |
| {BcMV2.BI=TRUE, BcMV9.BI=FALSE} =>  {BcNSRV7.BI=FALSE} | 0.13  3663 | 0.947  368 | 0.141  089 | 1.18  4944 | 54 |
| {BcMV2.BI=TRUE, BcMV9.BI=FALSE} =>  {BcOLV7.BI=FALSE} | 0.13  3663 | 0.947  368 | 0.141  089 | 1.17  4039 | 54 |
| {BcMV2.BI=TRUE, BcMV9.BI=FALSE} =>  {BcDMV1=FALSE} | 0.14  1089 | 1 | 0.141  089 | 1.18  4751 | 57 |
| {BcMV2.BI=TRUE, BcMV9.BI=FALSE} =>  {BcBNV5.BI=FALSE} | 0.13  8614 | 0.982  456 | 0.141  089 | 1.08  1505 | 56 |
| {BcMV2.BI=FALSE, BcMV9.BI=FALSE} => | 0.12 | 0.698 | 0.180 | 1.44 | 51 |

| {BcOLV18=FALSE} | 6238 | 63 | 693 | 7418 |  |
| --- | --- | --- | --- | --- | --- |
| {BcMV2.BI=FALSE, BcOLV18=FALSE} =>  {BcMV9.BI=FALSE} | 0.12  6238 | 0.401  575 | 0.314  356 | 1.24  7971 | 51 |
| {BcMV3.BI=TRUE, BcMV9.BI=FALSE} =>  {BcOLV18=FALSE} | 0.09  6535 | 0.661  017 | 0.146  04 | 1.36  9492 | 39 |
| {BcMV3.BI=TRUE, BcOLV18=FALSE} =>  {BcMV9.BI=FALSE} | 0.09  6535 | 0.453  488 | 0.212  871 | 1.40  9302 | 39 |
| {BcMV9.BI=FALSE, BcOLV18=FALSE} =>  {BcMV4.BI=FALSE} | 0.14  604 | 0.670  455 | 0.217  822 | 1.20  3838 | 59 |
| {BcMV4.BI=FALSE, BcMV9.BI=FALSE} =>  {BcOLV18=FALSE} | 0.14  604 | 0.678  161 | 0.215  347 | 1.40  501 | 59 |
| {BcMV4.BI=FALSE, BcOLV18=FALSE} =>  {BcMV9.BI=FALSE} | 0.14  604 | 0.460  938 | 0.316  832 | 1.43  2452 | 59 |
| {BcMV9.BI=FALSE, BcOLV18=FALSE} =>  {BcOLV16.BI=FALSE} | 0.16  0891 | 0.738  636 | 0.217  822 | 1.26  4445 | 65 |
| {BcMV9.BI=FALSE, BcOLV16.BI=FALSE} =>  {BcOLV18=FALSE} | 0.16  0891 | 0.677  083 | 0.237  624 | 1.40  2778 | 65 |
| {BcOLV16.BI=FALSE, BcOLV18=FALSE} =>  {BcMV9.BI=FALSE} | 0.16  0891 | 0.477  941 | 0.336  634 | 1.48  5294 | 65 |
| {BcMV9.BI=FALSE, BcULV3=TRUE} =>  {BcOLV18=FALSE} | 0.10  8911 | 0.637  681 | 0.170  792 | 1.32  1145 | 44 |
| {BcOLV18=FALSE, BcULV3=TRUE} => | 0.10 | 0.403 | 0.269 | 1.25 | 44 |

| {BcMV9.BI=FALSE} | 8911 | 67 | 802 | 4481 |  |
| --- | --- | --- | --- | --- | --- |
| {BcMV11=TRUE, BcMV9.BI=FALSE} => | 0.13 | 0.658 | 0.210 | 1.36 |  |
|  |  |  |  |  | 56 |
| {BcOLV18=FALSE} | 8614 | 824 | 396 | 4947 |  |
| {BcMV11=TRUE, BcOLV18=FALSE} => | 0.13 | 0.513 | 0.269 | 1.59 |  |
|  |  |  |  |  | 56 |
| {BcMV9.BI=FALSE} | 8614 | 761 | 802 | 6613 |  |
| {BcMV9.BI=FALSE, |  |  |  |  |  |
|  | 0.14 | 0.707 | 0.202 | 1.46 |  |
| BcOLV1.BI.MOVA.=FALSE} => |  |  |  |  | 58 |
|  | 3564 | 317 | 97 | 5416 |  |
| {BcOLV18=FALSE} |  |  |  |  |  |
| {BcMV6.BI=FALSE, BcMV9.BI=FALSE} => | 0.17 | 0.676 | 0.252 | 1.40 |  |
|  |  |  |  |  | 69 |
| {BcOLV18=FALSE} | 0792 | 471 | 475 | 1508 |  |
| {BcMV6.BI=FALSE, BcOLV18=FALSE} => | 0.17 | 0.485 | 0.351 | 1.51 |  |
|  |  |  |  |  | 69 |
| {BcMV9.BI=FALSE} | 0792 | 915 | 485 | 0076 |  |
| {BcMV9.BI=FALSE, BcPV3.BI=FALSE} => | 0.16 | 0.677 | 0.237 | 1.40 |  |
|  |  |  |  |  | 65 |
| {BcOLV18=FALSE} | 0891 | 083 | 624 | 2778 |  |
| {BcOLV18=FALSE, BcPV3.BI=FALSE} => | 0.16 | 0.422 | 0.381 | 1.31 |  |
|  |  |  |  |  | 65 |
| {BcMV9.BI=FALSE} | 0891 | 078 | 188 | 1688 |  |
| {BcMV9.BI=FALSE, BcOLV18=FALSE} => | 0.18 | 0.852 | 0.217 | 1.17 |  |
|  |  |  |  |  | 75 |
| {BcRV1.BI=FALSE} | 5644 | 273 | 822 | 9172 |  |
| {BcMV9.BI=FALSE, BcRV1.BI=FALSE} => | 0.18 | 0.694 | 0.267 | 1.43 |  |
|  |  |  |  |  | 75 |
| {BcOLV18=FALSE} | 5644 | 444 | 327 | 8746 |  |
| {BcOLV18=FALSE, BcRV1.BI=FALSE} => | 0.18 | 0.471 | 0.393 | 1.46 |  |
|  |  |  |  |  | 75 |
| {BcMV9.BI=FALSE} | 5644 | 698 | 564 | 5893 |  |

| {BcEV2.BI=FALSE, BcMV9.BI=FALSE} =>  {BcOLV18=FALSE} | 0.17  5743 | 0.724  49 | 0.242  574 | 1.50  0994 | 71 |
| --- | --- | --- | --- | --- | --- |
| {BcEV2.BI=FALSE, BcOLV18=FALSE} =>  {BcMV9.BI=FALSE} | 0.17  5743 | 0.464  052 | 0.378  713 | 1.44  2132 | 71 |
| {BcMV9.BI=FALSE, BcOLV18=FALSE} =>  {BcHV3.BI=FALSE} | 0.18  3168 | 0.840  909 | 0.217  822 | 1.10  6603 | 74 |
| {BcHV3.BI=FALSE, BcMV9.BI=FALSE} =>  {BcOLV18=FALSE} | 0.18  3168 | 0.718  447 | 0.254  95 | 1.48  8474 | 74 |
| {BcHV3.BI=FALSE, BcOLV18=FALSE} =>  {BcMV9.BI=FALSE} | 0.18  3168 | 0.448  485 | 0.408  416 | 1.39  3753 | 74 |
| {BcMV9.BI=FALSE, BcOLV18=FALSE} =>  {BcFV5.BI=FALSE} | 0.19  0594 | 0.875 | 0.217  822 | 1.11  8671 | 77 |
| {BcFV5.BI=FALSE, BcMV9.BI=FALSE} =>  {BcOLV18=FALSE} | 0.19  0594 | 0.706  422 | 0.269  802 | 1.46  3562 | 77 |
| {BcFV5.BI=FALSE, BcOLV18=FALSE} =>  {BcMV9.BI=FALSE} | 0.19  0594 | 0.461  078 | 0.413  366 | 1.43  2888 | 77 |
| {BcMV9.BI=FALSE, BcOLV18=FALSE} =>  {BcULV1.BI=FALSE} | 0.19  0594 | 0.875 | 0.217  822 | 1.11  5142 | 77 |
| {BcMV9.BI=FALSE, BcULV1.BI=FALSE} =>  {BcOLV18=FALSE} | 0.19  0594 | 0.669  565 | 0.284  653 | 1.38  7202 | 77 |
| {BcOLV18=FALSE, BcULV1.BI=FALSE} =>  {BcMV9.BI=FALSE} | 0.19  0594 | 0.445  087 | 0.428  218 | 1.38  3193 | 77 |

| {BcMV1.BI=TRUE, BcMV9.BI=FALSE} =>  {BcOLV18=FALSE} | 0.15  8416 | 0.659  794 | 0.240  099 | 1.36  6957 | 64 |
| --- | --- | --- | --- | --- | --- |
| {BcMV1.BI=TRUE, BcOLV18=FALSE} =>  {BcMV9.BI=FALSE} | 0.15  8416 | 0.453  901 | 0.349  01 | 1.41  0584 | 64 |
| {BcMV12=FALSE, BcMV9.BI=FALSE} =>  {BcOLV18=FALSE} | 0.17  5743 | 0.676  19 | 0.259  901 | 1.40  0928 | 71 |
| {BcMV12=FALSE, BcOLV18=FALSE} =>  {BcMV9.BI=FALSE} | 0.17  5743 | 0.435  583 | 0.403  465 | 1.35  3657 | 71 |
| {BcMV9.BI=FALSE, BcOLV18=FALSE} =>  {BcNSRV7.BI=FALSE} | 0.19  5545 | 0.897  727 | 0.217  822 | 1.12  2854 | 79 |
| {BcMV9.BI=FALSE, BcNSRV7.BI=FALSE} =>  {BcOLV18=FALSE} | 0.19  5545 | 0.692  982 | 0.282  178 | 1.43  5717 | 79 |
| {BcNSRV7.BI=FALSE, BcOLV18=FALSE} =>  {BcMV9.BI=FALSE} | 0.19  5545 | 0.475  904 | 0.410  891 | 1.47  8962 | 79 |
| {BcMV9.BI=FALSE, BcVV3.BI=FALSE} =>  {BcOLV18=FALSE} | 0.16  8317 | 0.68 | 0.247  525 | 1.40  8821 | 68 |
| {BcOLV18=FALSE, BcVV3.BI=FALSE} =>  {BcMV9.BI=FALSE} | 0.16  8317 | 0.430  38 | 0.391  089 | 1.33  7488 | 68 |
| {BcMV9.BI=FALSE, BcOLV18=FALSE} =>  {BcOLV7.BI=FALSE} | 0.20  7921 | 0.954  545 | 0.217  822 | 1.18  2934 | 84 |
| {BcMV9.BI=FALSE, BcOLV7.BI=FALSE} =>  {BcOLV18=FALSE} | 0.20  7921 | 0.694  215 | 0.299  505 | 1.43  8271 | 84 |

| {BcOLV18=FALSE, BcOLV7.BI=FALSE} =>  {BcMV9.BI=FALSE} | 0.20  7921 | 0.482  759 | 0.430  693 | 1.50  0265 | 84 |
| --- | --- | --- | --- | --- | --- |
| {BcALV1.BI=FALSE, BcMV9.BI=FALSE} =>  {BcOLV18=FALSE} | 0.19  0594 | 0.7 | 0.272  277 | 1.45  0256 | 77 |
| {BcALV1.BI=FALSE, BcOLV18=FALSE} =>  {BcMV9.BI=FALSE} | 0.19  0594 | 0.435  028 | 0.438  119 | 1.35  1934 | 77 |
| {BcMV9.BI=FALSE, BcOLV18=FALSE} =>  {BcDMV1=FALSE} | 0.20  297 | 0.931  818 | 0.217  822 | 1.10  3972 | 82 |
| {BcDMV1=FALSE, BcMV9.BI=FALSE} =>  {BcOLV18=FALSE} | 0.20  297 | 0.694  915 | 0.292  079 | 1.43  9722 | 82 |
| {BcDMV1=FALSE, BcOLV18=FALSE} =>  {BcMV9.BI=FALSE} | 0.20  297 | 0.488  095 | 0.415  842 | 1.51  685 | 82 |
| {BcMV9.BI=FALSE, BcOLV18=FALSE} =>  {BcFV9=FALSE} | 0.20  5446 | 0.943  182 | 0.217  822 | 1.08  56 | 83 |
| {BcFV9=FALSE, BcMV9.BI=FALSE} =>  {BcOLV18=FALSE} | 0.20  5446 | 0.721  739 | 0.284  653 | 1.49  5295 | 83 |
| {BcFV9=FALSE, BcOLV18=FALSE} =>  {BcMV9.BI=FALSE} | 0.20  5446 | 0.458  564 | 0.448  02 | 1.42  5074 | 83 |
| {BcMV9.BI=FALSE, BcOLV4.BI=FALSE} =>  {BcOLV18=FALSE} | 0.17  5743 | 0.676  19 | 0.259  901 | 1.40  0928 | 71 |
| {BcOLV18=FALSE, BcOLV4.BI=FALSE} =>  {BcMV9.BI=FALSE} | 0.17  5743 | 0.410  405 | 0.428  218 | 1.27  5411 | 71 |

| {BcMV9.BI=FALSE, BcULV5=FALSE} =>  {BcOLV18=FALSE} | 0.14  3564 | 0.651  685 | 0.220  297 | 1.35  0158 | 58 |
| --- | --- | --- | --- | --- | --- |
| {BcMV9.BI=FALSE, BcOLV18=FALSE} =>  {BcBNV5.BI=FALSE} | 0.21  2871 | 0.977  273 | 0.217  822 | 1.07  5799 | 86 |
| {BcBNV5.BI=FALSE, BcMV9.BI=FALSE} =>  {BcOLV18=FALSE} | 0.21  2871 | 0.682  54 | 0.311  881 | 1.41  4082 | 86 |
| {BcBNV5.BI=FALSE, BcOLV18=FALSE} =>  {BcMV9.BI=FALSE} | 0.21  2871 | 0.467  391 | 0.455  446 | 1.45  2508 | 86 |
| {BcMV9.BI=FALSE, BvF.BI=FALSE} =>  {BcOLV18=FALSE} | 0.20  5446 | 0.680  328 | 0.301  98 | 1.40  95 | 83 |
| {BcOLV18=FALSE, BvF.BI=FALSE} =>  {BcMV9.BI=FALSE} | 0.20  5446 | 0.468  927 | 0.438  119 | 1.45  7279 | 83 |
| {BcMV9.BI=FALSE, BcOLV18=FALSE} =>  {BcMCV5.BI=FALSE} | 0.21  7822 | 1 | 0.217  822 | 1.08  311 | 88 |
| {BcMCV5.BI=FALSE, BcMV9.BI=FALSE} =>  {BcOLV18=FALSE} | 0.21  7822 | 0.709  677 | 0.306  931 | 1.47  0306 | 88 |
| {BcMCV5.BI=FALSE, BcOLV18=FALSE} =>  {BcMV9.BI=FALSE} | 0.21  7822 | 0.460  733 | 0.472  772 | 1.43  1816 | 88 |
| {BcMV9.BI=FALSE, BcOLV13.BI=FALSE} =>  {BcOLV18=FALSE} | 0.19  3069 | 0.666  667 | 0.289  604 | 1.38  1197 | 78 |
| {BcOLV13.BI=FALSE, BcOLV18=FALSE} =>  {BcMV9.BI=FALSE} | 0.19  3069 | 0.428  571 | 0.450  495 | 1.33  1868 | 78 |

| {BcMV9.BI=FALSE, BcOLV2.BI=FALSE} =>  {BcOLV18=FALSE} | 0.20  5446 | 0.669  355 | 0.306  931 | 1.38  6766 | 83 |
| --- | --- | --- | --- | --- | --- |
| {BcOLV18=FALSE, BcOLV2.BI=FALSE} =>  {BcMV9.BI=FALSE} | 0.20  5446 | 0.446  237 | 0.460  396 | 1.38  6766 | 83 |
| {BcMCV3.BI=FALSE, BcMV9.BI=FALSE} =>  {BcOLV18=FALSE} | 0.20  0495 | 0.658  537 | 0.304  455 | 1.36  4353 | 81 |
| {BcMCV3.BI=FALSE, BcOLV18=FALSE} =>  {BcMV9.BI=FALSE} | 0.20  0495 | 0.437  838 | 0.457  921 | 1.36  0665 | 81 |
| {BcMV9.BI=FALSE, BcPV4.BI=FALSE} =>  {BcOLV18=FALSE} | 0.21  0396 | 0.702  479 | 0.299  505 | 1.45  5393 | 85 |
| {BcOLV18=FALSE, BcPV4.BI=FALSE} =>  {BcMV9.BI=FALSE} | 0.21  0396 | 0.461  957 | 0.455  446 | 1.43  5619 | 85 |
| {BcEV6=FALSE, BcMV9.BI=FALSE} =>  {BcOLV18=FALSE} | 0.20  5446 | 0.674  797 | 0.304  455 | 1.39  804 | 83 |
| {BcEV6=FALSE, BcOLV18=FALSE} =>  {BcMV9.BI=FALSE} | 0.20  5446 | 0.453  552 | 0.452  97 | 1.40  95 | 83 |
| {BcMV9.BI=FALSE, BcOLV17.BI=FALSE} =>  {BcOLV18=FALSE} | 0.21  2871 | 0.688 | 0.309  406 | 1.42  5395 | 86 |
| {BcOLV17.BI=FALSE, BcOLV18=FALSE} =>  {BcMV9.BI=FALSE} | 0.21  2871 | 0.452  632 | 0.470  297 | 1.40  664 | 86 |
| {BcMV9.BI=FALSE, BcOLV15.BI=FALSE} =>  {BcOLV18=FALSE} | 0.21  7822 | 0.704 | 0.309  406 | 1.45  8544 | 88 |

| {BcOLV15.BI=FALSE, BcOLV18=FALSE} =>  {BcMV9.BI=FALSE} | 0.21  7822 | 0.460  733 | 0.472  772 | 1.43  1816 | 88 |
| --- | --- | --- | --- | --- | --- |
| {BcEV5=FALSE, BcMV9.BI=FALSE} =>  {BcOLV18=FALSE} | 0.21  0396 | 0.674  603 | 0.311  881 | 1.39  7639 | 85 |
| {BcEV5=FALSE, BcOLV18=FALSE} =>  {BcMV9.BI=FALSE} | 0.21  0396 | 0.454  545 | 0.462  871 | 1.41  2587 | 85 |
| {BcMV9.BI=FALSE, BcOLV18=TRUE} =>  {BcOLV16.BI=FALSE} | 0.07  6733 | 0.738  095 | 0.103  96 | 1.26  3519 | 31 |
| {BcMV9.BI=FALSE, BcOLV18=TRUE} =>  {BcULV1.BI=FALSE} | 0.09  4059 | 0.904  762 | 0.103  96 | 1.15  3072 | 38 |
| {BcMV2.BI=FALSE, BcMV9.BI=FALSE} =>  {BcMV4.BI=FALSE} | 0.13  6139 | 0.753  425 | 0.180  693 | 1.35  2816 | 55 |
| {BcMV2.BI=FALSE, BcMV9.BI=FALSE} =>  {BcOLV16.BI=FALSE} | 0.14  604 | 0.808  219 | 0.180  693 | 1.38  3562 | 59 |
| {BcMV2.BI=FALSE, BcMV9.BI=FALSE} =>  {BcOLV1.BI.MOVA.=FALSE} | 0.14  604 | 0.808  219 | 0.180  693 | 1.20  9335 | 59 |
| {BcMV9.BI=FALSE, BcOLV1.BI.MOVA.=FALSE} =>  {BcMV2.BI=FALSE} | 0.14  604 | 0.719  512 | 0.202  97 | 1.33  9553 | 59 |
| {BcMV2.BI=FALSE, BcMV9.BI=FALSE} =>  {BcMV6.BI=FALSE} | 0.15  099 | 0.835  616 | 0.180  693 | 1.20  1384 | 61 |
| {BcMV2.BI=FALSE, BcMV9.BI=FALSE} => | 0.16 | 0.890 | 0.180 | 1.23 | 65 |

| {BcRV1.BI=FALSE} | 0891 | 411 | 693 | 1938 |  |
| --- | --- | --- | --- | --- | --- |
| {BcMV2.BI=FALSE, BcMV9.BI=FALSE} =>  {BcULV1.BI=FALSE} | 0.17  0792 | 0.945  205 | 0.180  693 | 1.20  4615 | 69 |
| {BcMV2.BI=FALSE, BcMV9.BI=FALSE} =>  {BcMV12=FALSE} | 0.16  3366 | 0.904  11 | 0.180  693 | 1.13  0837 | 66 |
| {BcMV2.BI=FALSE, BcMV9.BI=FALSE} =>  {BcOLV7.BI=FALSE} | 0.16  5842 | 0.917  808 | 0.180  693 | 1.13  7407 | 67 |
| {BcMV9.BI=FALSE, BcULV5=FALSE} =>  {BcMV2.BI=FALSE} | 0.14  8515 | 0.674  157 | 0.220  297 | 1.25  5113 | 60 |
| {BcHV1SLR.BI=TRUE, BcMV9.BI=FALSE} =>  {BcMV4.BI=FALSE} | 0.09  4059 | 0.703  704 | 0.133  663 | 1.26  3539 | 38 |
| {BcHV1SLR.BI=TRUE, BcMV9.BI=FALSE} =>  {BcOLV16.BI=FALSE} | 0.09  4059 | 0.703  704 | 0.133  663 | 1.20  4645 | 38 |
| {BcHV1SLR.BI=TRUE, BcMV9.BI=FALSE} =>  {BcULV3=TRUE} | 0.09  6535 | 0.722  222 | 0.133  663 | 1.21  5741 | 39 |
| {BcHV1SLR.BI=TRUE, BcMV9.BI=FALSE} =>  {BcHV1.BI=TRUE} | 0.10  8911 | 0.814  815 | 0.133  663 | 1.34  912 | 44 |
| {BcHV1.BI=TRUE, BcMV9.BI=FALSE} =>  {BcHV1SLR.BI=TRUE} | 0.10  8911 | 0.8 | 0.136  139 | 1.47  5799 | 44 |
| {BcHV1SLR.BI=TRUE, BcMV9.BI=FALSE} =>  {BcRV1.BI=FALSE} | 0.11  8812 | 0.888  889 | 0.133  663 | 1.22  9833 | 48 |
| {BcHV1SLR.BI=TRUE, BcMV9.BI=FALSE} => | 0.12 | 0.907 | 0.133 | 1.12 | 49 |

| {BcOLV7.BI=FALSE} | 1287 | 407 | 663 | 4517 |  |
| --- | --- | --- | --- | --- | --- |
| {BcHV1SLR.BI=TRUE, BcMV9.BI=FALSE} =>  {BcDMV1=FALSE} | 0.12  3762 | 0.925  926 | 0.133  663 | 1.09  6991 | 50 |
| {BcHV1SLR.BI=TRUE, BcMV9.BI=FALSE} =>  {BcBNV5.BI=FALSE} | 0.13  1188 | 0.981  481 | 0.133  663 | 1.08  0432 | 53 |
| {BcMV3.BI=TRUE, BcMV9.BI=FALSE} =>  {BcMV11=TRUE} | 0.10  6436 | 0.728  814 | 0.146  04 | 1.18  2493 | 43 |
| {BcMV4.BI=FALSE, BcMV9.BI=FALSE} =>  {BcOLV16.BI=FALSE} | 0.17  8218 | 0.827  586 | 0.215  347 | 1.41  6715 | 72 |
| {BcMV9.BI=FALSE, BcOLV16.BI=FALSE} =>  {BcMV4.BI=FALSE} | 0.17  8218 | 0.75 | 0.237  624 | 1.34  6667 | 72 |
| {BcMV4.BI=FALSE, BcOLV16.BI=FALSE} =>  {BcMV9.BI=FALSE} | 0.17  8218 | 0.486  486 | 0.366  337 | 1.51  185 | 72 |
| {BcHV1.BI=TRUE, BcMV9.BI=FALSE} =>  {BcMV4.BI=FALSE} | 0.09  1584 | 0.672  727 | 0.136  139 | 1.20  7919 | 37 |
| {BcMV11=TRUE, BcMV4.BI=FALSE} =>  {BcMV9.BI=FALSE} | 0.12  6238 | 0.455  357 | 0.277  228 | 1.41  511 | 51 |
| {BcMV9.BI=FALSE, BcOLV1.BI.MOVA.=FALSE} =>  {BcMV4.BI=FALSE} | 0.14  8515 | 0.731  707 | 0.202  97 | 1.31  3821 | 60 |
| {BcMV9.BI=FALSE, BcULV4=TRUE} =>  {BcMV4.BI=FALSE} | 0.09  6535 | 0.672  414 | 0.143  564 | 1.20  7356 | 39 |

| {BcMV4.BI=FALSE, BcMV9.BI=FALSE} =>  {BcMV6.BI=FALSE} | 0.17  3267 | 0.804  598 | 0.215  347 | 1.15  6788 | 70 |
| --- | --- | --- | --- | --- | --- |
| {BcMV6.BI=FALSE, BcMV9.BI=FALSE} =>  {BcMV4.BI=FALSE} | 0.17  3267 | 0.686  275 | 0.252  475 | 1.23  2244 | 70 |
| {BcMV4.BI=FALSE, BcMV6.BI=FALSE} =>  {BcMV9.BI=FALSE} | 0.17  3267 | 0.404  624 | 0.428  218 | 1.25  7448 | 70 |
| {BcMV9.BI=FALSE, BcPV3.BI=FALSE} =>  {BcMV4.BI=FALSE} | 0.16  8317 | 0.708  333 | 0.237  624 | 1.27  1852 | 68 |
| {BcMV4.BI=FALSE, BcMV9.BI=FALSE} =>  {BcRV1.BI=FALSE} | 0.17  8218 | 0.827  586 | 0.215  347 | 1.14  5017 | 72 |
| {BcMV9.BI=FALSE, BcRV1.BI=FALSE} =>  {BcMV4.BI=FALSE} | 0.17  8218 | 0.666  667 | 0.267  327 | 1.19  7037 | 72 |
| {BcMV4.BI=FALSE, BcRV1.BI=FALSE} =>  {BcMV9.BI=FALSE} | 0.17  8218 | 0.4 | 0.445  545 | 1.24  3077 | 72 |
| {BcEV2.BI=FALSE, BcMV9.BI=FALSE} =>  {BcMV4.BI=FALSE} | 0.16  8317 | 0.693  878 | 0.242  574 | 1.24  5896 | 68 |
| {BcEV2.BI=FALSE, BcMV4.BI=FALSE} =>  {BcMV9.BI=FALSE} | 0.16  8317 | 0.388  571 | 0.433  168 | 1.20  756 | 68 |
| {BcHV3.BI=FALSE, BcMV9.BI=FALSE} =>  {BcMV4.BI=FALSE} | 0.17  8218 | 0.699  029 | 0.254  95 | 1.25  5146 | 72 |
| {BcFV5.BI=FALSE, BcMV9.BI=FALSE} =>  {BcMV4.BI=FALSE} | 0.18  5644 | 0.688  073 | 0.269  802 | 1.23  5474 | 75 |

| {BcFV5.BI=FALSE, BcMV4.BI=FALSE} =>  {BcMV9.BI=FALSE} | 0.18  5644 | 0.403  226 | 0.460  396 | 1.25  3102 | 75 |
| --- | --- | --- | --- | --- | --- |
| {BcMV4.BI=FALSE, BcMV9.BI=FALSE} =>  {BcULV1.BI=FALSE} | 0.19  0594 | 0.885  057 | 0.215  347 | 1.12  796 | 77 |
| {BcMV9.BI=FALSE, BcULV1.BI=FALSE} =>  {BcMV4.BI=FALSE} | 0.19  0594 | 0.669  565 | 0.284  653 | 1.20  2242 | 77 |
| {BcMV4.BI=FALSE, BcULV1.BI=FALSE} =>  {BcMV9.BI=FALSE} | 0.19  0594 | 0.423  077 | 0.450  495 | 1.31  4793 | 77 |
| {BcMV12=FALSE, BcMV9.BI=FALSE} =>  {BcMV4.BI=FALSE} | 0.18  3168 | 0.704  762 | 0.259  901 | 1.26  5439 | 74 |
| {BcMV12=FALSE, BcMV4.BI=FALSE} =>  {BcMV9.BI=FALSE} | 0.18  3168 | 0.418  079 | 0.438  119 | 1.29  9261 | 74 |
| {BcMV9.BI=FALSE, BcNSRV7.BI=FALSE} =>  {BcMV4.BI=FALSE} | 0.18  8119 | 0.666  667 | 0.282  178 | 1.19  7037 | 76 |
| {BcMV4.BI=FALSE, BcNSRV7.BI=FALSE} =>  {BcMV9.BI=FALSE} | 0.18  8119 | 0.406  417 | 0.462  871 | 1.26  3019 | 76 |
| {BcMV9.BI=FALSE, BcVV3.BI=FALSE} =>  {BcMV4.BI=FALSE} | 0.17  8218 | 0.72 | 0.247  525 | 1.29  28 | 72 |
| {BcMV4.BI=FALSE, BcMV9.BI=FALSE} =>  {BcOLV7.BI=FALSE} | 0.20  297 | 0.942  529 | 0.215  347 | 1.16  8042 | 82 |
| {BcMV9.BI=FALSE, BcOLV7.BI=FALSE} =>  {BcMV4.BI=FALSE} | 0.20  297 | 0.677  686 | 0.299  505 | 1.21  6823 | 82 |

| {BcMV4.BI=FALSE, BcOLV7.BI=FALSE} =>  {BcMV9.BI=FALSE} | 0.20  297 | 0.431  579 | 0.470  297 | 1.34  1215 | 82 |
| --- | --- | --- | --- | --- | --- |
| {BcALV1.BI=FALSE, BcMV9.BI=FALSE} =>  {BcMV4.BI=FALSE} | 0.18  8119 | 0.690  909 | 0.272  277 | 1.24  0566 | 76 |
| {BcDMV1=FALSE, BcMV9.BI=FALSE} =>  {BcMV4.BI=FALSE} | 0.19  3069 | 0.661  017 | 0.292  079 | 1.18  6893 | 78 |
| {BcDMV1=FALSE, BcMV4.BI=FALSE} =>  {BcMV9.BI=FALSE} | 0.19  3069 | 0.4 | 0.482  673 | 1.24  3077 | 78 |
| {BcFV9=FALSE, BcMV9.BI=FALSE} =>  {BcMV4.BI=FALSE} | 0.19  802 | 0.695  652 | 0.284  653 | 1.24  9082 | 80 |
| {BcFV9=FALSE, BcMV4.BI=FALSE} =>  {BcMV9.BI=FALSE} | 0.19  802 | 0.386  473 | 0.512  376 | 1.20  1041 | 80 |
| {BcMV9.BI=FALSE, BcOLV4.BI=FALSE} =>  {BcMV4.BI=FALSE} | 0.18  8119 | 0.723  81 | 0.259  901 | 1.29  964 | 76 |
| {BcMV9.BI=FALSE, BcULV5=FALSE} =>  {BcMV4.BI=FALSE} | 0.15  8416 | 0.719  101 | 0.220  297 | 1.29  1186 | 64 |
| {BcMV4.BI=FALSE, BcMV9.BI=FALSE} =>  {BcBNV5.BI=FALSE} | 0.21  0396 | 0.977  011 | 0.215  347 | 1.07  5511 | 85 |
| {BcBNV5.BI=FALSE, BcMV9.BI=FALSE} =>  {BcMV4.BI=FALSE} | 0.21  0396 | 0.674  603 | 0.311  881 | 1.21  1287 | 85 |
| {BcBNV5.BI=FALSE, BcMV4.BI=FALSE} =>  {BcMV9.BI=FALSE} | 0.21  0396 | 0.400  943 | 0.524  752 | 1.24  6009 | 85 |

| {BcMV9.BI=FALSE, BvF.BI=FALSE} =>  {BcMV4.BI=FALSE} | 0.20  5446 | 0.680  328 | 0.301  98 | 1.22  1566 | 83 |
| --- | --- | --- | --- | --- | --- |
| {BcMV4.BI=FALSE, BvF.BI=FALSE} =>  {BcMV9.BI=FALSE} | 0.20  5446 | 0.404  878 | 0.507  426 | 1.25  8236 | 83 |
| {BcMCV5.BI=FALSE, BcMV9.BI=FALSE} =>  {BcMV4.BI=FALSE} | 0.21  2871 | 0.693  548 | 0.306  931 | 1.24  5305 | 86 |
| {BcMCV5.BI=FALSE, BcMV4.BI=FALSE} =>  {BcMV9.BI=FALSE} | 0.21  2871 | 0.390  909 | 0.544  554 | 1.21  4825 | 86 |
| {BcMV9.BI=FALSE, BcOLV13.BI=FALSE} =>  {BcMV4.BI=FALSE} | 0.20  297 | 0.700  855 | 0.289  604 | 1.25  8424 | 82 |
| {BcMV9.BI=FALSE, BcOLV2.BI=FALSE} =>  {BcMV4.BI=FALSE} | 0.21  0396 | 0.685  484 | 0.306  931 | 1.23  0824 | 85 |
| {BcMV4.BI=FALSE, BcOLV2.BI=FALSE} =>  {BcMV9.BI=FALSE} | 0.21  0396 | 0.400  943 | 0.524  752 | 1.24  6009 | 85 |
| {BcMCV3.BI=FALSE, BcMV9.BI=FALSE} =>  {BcMV4.BI=FALSE} | 0.20  5446 | 0.674  797 | 0.304  455 | 1.21  1635 | 83 |
| {BcMCV3.BI=FALSE, BcMV4.BI=FALSE} =>  {BcMV9.BI=FALSE} | 0.20  5446 | 0.393  365 | 0.522  277 | 1.22  2457 | 83 |
| {BcMV9.BI=FALSE, BcPV4.BI=FALSE} =>  {BcMV4.BI=FALSE} | 0.20  297 | 0.677  686 | 0.299  505 | 1.21  6823 | 82 |
| {BcMV4.BI=FALSE, BcPV4.BI=FALSE} =>  {BcMV9.BI=FALSE} | 0.20  297 | 0.394  231 | 0.514  851 | 1.22  5148 | 82 |

| {BcEV6=FALSE, BcMV9.BI=FALSE} =>  {BcMV4.BI=FALSE} | 0.20  5446 | 0.674  797 | 0.304  455 | 1.21  1635 | 83 |
| --- | --- | --- | --- | --- | --- |
| {BcEV6=FALSE, BcMV4.BI=FALSE} =>  {BcMV9.BI=FALSE} | 0.20  5446 | 0.399  038 | 0.514  851 | 1.24  0089 | 83 |
| {BcMV9.BI=FALSE, BcOLV17.BI=FALSE} =>  {BcMV4.BI=FALSE} | 0.21  0396 | 0.68 | 0.309  406 | 1.22  0978 | 85 |
| {BcMV4.BI=FALSE, BcOLV17.BI=FALSE} =>  {BcMV9.BI=FALSE} | 0.21  0396 | 0.391  705 | 0.537  129 | 1.21  7299 | 85 |
| {BcMV9.BI=FALSE, BcOLV15.BI=FALSE} =>  {BcMV4.BI=FALSE} | 0.21  2871 | 0.688 | 0.309  406 | 1.23  5342 | 86 |
| {BcMV4.BI=FALSE, BcOLV15.BI=FALSE} =>  {BcMV9.BI=FALSE} | 0.21  2871 | 0.396  313 | 0.537  129 | 1.23  162 | 86 |
| {BcEV5=FALSE, BcMV9.BI=FALSE} =>  {BcMV4.BI=FALSE} | 0.21  0396 | 0.674  603 | 0.311  881 | 1.21  1287 | 85 |
| {BcMV11=TRUE, BcMV9.BI=FALSE} =>  {BcOLV16.BI=FALSE} | 0.14  8515 | 0.705  882 | 0.210  396 | 1.20  8375 | 60 |
| {BcMV11=TRUE, BcOLV16.BI=FALSE} =>  {BcMV9.BI=FALSE} | 0.14  8515 | 0.434  783 | 0.341  584 | 1.35  1171 | 60 |
| {BcMV9.BI=FALSE, BcOLV1.BI.MOVA.=FALSE} =>  {BcOLV16.BI=FALSE} | 0.17  0792 | 0.841  463 | 0.202  97 | 1.44  0471 | 69 |
| {BcOLV1.BI.MOVA.=FALSE, | 0.17 | 0.385 | 0.443 | 1.19 | 69 |

| BcOLV16.BI=FALSE} => {BcMV9.BI=FALSE} | 0792 | 475 | 069 | 7937 |  |
| --- | --- | --- | --- | --- | --- |
| {BcMV9.BI=FALSE, BcULV4=TRUE} =>  {BcOLV16.BI=FALSE} | 0.10  396 | 0.724  138 | 0.143  564 | 1.23  9626 | 42 |
| {BcMV9.BI=FALSE, BcOLV16.BI=FALSE} =>  {BcMV6.BI=FALSE} | 0.19  802 | 0.833  333 | 0.237  624 | 1.19  8102 | 80 |
| {BcMV6.BI=FALSE, BcMV9.BI=FALSE} =>  {BcOLV16.BI=FALSE} | 0.19  802 | 0.784  314 | 0.252  475 | 1.34  2639 | 80 |
| {BcMV6.BI=FALSE, BcOLV16.BI=FALSE} =>  {BcMV9.BI=FALSE} | 0.19  802 | 0.444  444 | 0.445  545 | 1.38  1197 | 80 |
| {BcMV9.BI=FALSE, BcOLV16.BI=FALSE} =>  {BcPV3.BI=FALSE} | 0.19  0594 | 0.802  083 | 0.237  624 | 1.12  9065 | 77 |
| {BcMV9.BI=FALSE, BcPV3.BI=FALSE} =>  {BcOLV16.BI=FALSE} | 0.19  0594 | 0.802  083 | 0.237  624 | 1.37  3058 | 77 |
| {BcOLV16.BI=FALSE, BcPV3.BI=FALSE} =>  {BcMV9.BI=FALSE} | 0.19  0594 | 0.435  028 | 0.438  119 | 1.35  1934 | 77 |
| {BcMV9.BI=FALSE, BcOLV16.BI=FALSE} =>  {BcRV1.BI=FALSE} | 0.19  802 | 0.833  333 | 0.237  624 | 1.15  2968 | 80 |
| {BcMV9.BI=FALSE, BcRV1.BI=FALSE} =>  {BcOLV16.BI=FALSE} | 0.19  802 | 0.740  741 | 0.267  327 | 1.26  8048 | 80 |
| {BcOLV16.BI=FALSE, BcRV1.BI=FALSE} =>  {BcMV9.BI=FALSE} | 0.19  802 | 0.432  432 | 0.457  921 | 1.34  3867 | 80 |
| {BcEV2.BI=FALSE, BcMV9.BI=FALSE} => | 0.17 | 0.734 | 0.242 | 1.25 | 72 |

| {BcOLV16.BI=FALSE} | 8218 | 694 | 574 | 7696 |  |
| --- | --- | --- | --- | --- | --- |
| {BcEV2.BI=FALSE, BcOLV16.BI=FALSE} =>  {BcMV9.BI=FALSE} | 0.17  8218 | 0.423  529 | 0.420  792 | 1.31  6199 | 72 |
| {BcHV3.BI=FALSE, BcMV9.BI=FALSE} =>  {BcOLV16.BI=FALSE} | 0.19  3069 | 0.757  282 | 0.254  95 | 1.29  6363 | 78 |
| {BcHV3.BI=FALSE, BcOLV16.BI=FALSE} =>  {BcMV9.BI=FALSE} | 0.19  3069 | 0.440  678 | 0.438  119 | 1.36  9492 | 78 |
| {BcFV5.BI=FALSE, BcMV9.BI=FALSE} =>  {BcOLV16.BI=FALSE} | 0.20  0495 | 0.743  119 | 0.269  802 | 1.27  2119 | 81 |
| {BcFV5.BI=FALSE, BcOLV16.BI=FALSE} =>  {BcMV9.BI=FALSE} | 0.20  0495 | 0.435  484 | 0.460  396 | 1.35  335 | 81 |
| {BcMV9.BI=FALSE, BcOLV16.BI=FALSE} =>  {BcULV1.BI=FALSE} | 0.22  5248 | 0.947  917 | 0.237  624 | 1.20  807 | 91 |
| {BcMV9.BI=FALSE, BcULV1.BI=FALSE} =>  {BcOLV16.BI=FALSE} | 0.22  5248 | 0.791  304 | 0.284  653 | 1.35  4606 | 91 |
| {BcOLV16.BI=FALSE, BcULV1.BI=FALSE} =>  {BcMV9.BI=FALSE} | 0.22  5248 | 0.437  5 | 0.514  851 | 1.35  9615 | 91 |
| {BcMV1.BI=TRUE, BcMV9.BI=FALSE} =>  {BcOLV16.BI=FALSE} | 0.17  5743 | 0.731  959 | 0.240  099 | 1.25  3014 | 71 |
| {BcMV1.BI=TRUE, BcOLV16.BI=FALSE} =>  {BcMV9.BI=FALSE} | 0.17  5743 | 0.387  978 | 0.452  97 | 1.20  5717 | 71 |
| {BcMV9.BI=FALSE, BcOLV16.BI=FALSE} => | 0.21 | 0.885 | 0.237 | 1.10 | 85 |

| {BcMV12=FALSE} | 0396 | 417 | 624 | 7456 |  |
| --- | --- | --- | --- | --- | --- |
| {BcMV12=FALSE, BcMV9.BI=FALSE} =>  {BcOLV16.BI=FALSE} | 0.21  0396 | 0.809  524 | 0.259  901 | 1.38  5795 | 85 |
| {BcMV12=FALSE, BcOLV16.BI=FALSE} =>  {BcMV9.BI=FALSE} | 0.21  0396 | 0.416  667 | 0.504  95 | 1.29  4872 | 85 |
| {BcMV9.BI=FALSE, BcNSRV7.BI=FALSE} =>  {BcOLV16.BI=FALSE} | 0.20  7921 | 0.736  842 | 0.282  178 | 1.26  1374 | 84 |
| {BcNSRV7.BI=FALSE, BcOLV16.BI=FALSE}  => {BcMV9.BI=FALSE} | 0.20  7921 | 0.446  809 | 0.465  347 | 1.38  8543 | 84 |
| {BcMV9.BI=FALSE, BcVV3.BI=FALSE} =>  {BcOLV16.BI=FALSE} | 0.19  0594 | 0.77 | 0.247  525 | 1.31  8136 | 77 |
| {BcOLV16.BI=FALSE, BcVV3.BI=FALSE} =>  {BcMV9.BI=FALSE} | 0.19  0594 | 0.409  574 | 0.465  347 | 1.27  2831 | 77 |
| {BcMV9.BI=FALSE, BcOLV16.BI=FALSE} =>  {BcOLV7.BI=FALSE} | 0.22  0297 | 0.927  083 | 0.237  624 | 1.14  8901 | 89 |
| {BcMV9.BI=FALSE, BcOLV7.BI=FALSE} =>  {BcOLV16.BI=FALSE} | 0.22  0297 | 0.735  537 | 0.299  505 | 1.25  914 | 89 |
| {BcOLV16.BI=FALSE, BcOLV7.BI=FALSE} =>  {BcMV9.BI=FALSE} | 0.22  0297 | 0.429  952 | 0.512  376 | 1.33  6158 | 89 |
| {BcALV1.BI=FALSE, BcMV9.BI=FALSE} =>  {BcOLV16.BI=FALSE} | 0.20  0495 | 0.736  364 | 0.272  277 | 1.26  0555 | 81 |
| {BcALV1.BI=FALSE, BcOLV16.BI=FALSE} => | 0.20 | 0.397 | 0.504 | 1.23 | 81 |

| {BcMV9.BI=FALSE} | 0495 | 059 | 95 | 3937 |  |
| --- | --- | --- | --- | --- | --- |
| {BcDMV1=FALSE, BcMV9.BI=FALSE} =>  {BcOLV16.BI=FALSE} | 0.21  0396 | 0.720  339 | 0.292  079 | 1.23  3123 | 85 |
| {BcDMV1=FALSE, BcOLV16.BI=FALSE} =>  {BcMV9.BI=FALSE} | 0.21  0396 | 0.429  293 | 0.490  099 | 1.33  411 | 85 |
| {BcFV9=FALSE, BcMV9.BI=FALSE} =>  {BcOLV16.BI=FALSE} | 0.21  5347 | 0.756  522 | 0.284  653 | 1.29  5063 | 87 |
| {BcFV9=FALSE, BcOLV16.BI=FALSE} =>  {BcMV9.BI=FALSE} | 0.21  5347 | 0.422  33 | 0.509  901 | 1.31  2472 | 87 |
| {BcMV9.BI=FALSE, BcOLV4.BI=FALSE} =>  {BcOLV16.BI=FALSE} | 0.20  7921 | 0.8 | 0.259  901 | 1.36  9492 | 84 |
| {BcOLV16.BI=FALSE, BcOLV4.BI=FALSE} =>  {BcMV9.BI=FALSE} | 0.20  7921 | 0.390  698 | 0.532  178 | 1.21  4168 | 84 |
| {BcMV9.BI=FALSE, BcULV5=FALSE} =>  {BcOLV16.BI=FALSE} | 0.18  3168 | 0.831  461 | 0.220  297 | 1.42  3348 | 74 |
| {BcMV9.BI=FALSE, BcOLV16.BI=FALSE} =>  {BcBNV5.BI=FALSE} | 0.23  2673 | 0.979  167 | 0.237  624 | 1.07  7884 | 94 |
| {BcBNV5.BI=FALSE, BcMV9.BI=FALSE} =>  {BcOLV16.BI=FALSE} | 0.23  2673 | 0.746  032 | 0.311  881 | 1.27  7105 | 94 |
| {BcBNV5.BI=FALSE, BcOLV16.BI=FALSE} =>  {BcMV9.BI=FALSE} | 0.23  2673 | 0.435  185 | 0.534  653 | 1.35  2422 | 94 |
| {BcMV9.BI=FALSE, BvF.BI=FALSE} => | 0.22 | 0.737 | 0.301 | 1.26 | 90 |

| {BcOLV16.BI=FALSE} | 2772 | 705 | 98 | 2851 |  |
| --- | --- | --- | --- | --- | --- |
| {BcOLV16.BI=FALSE, BvF.BI=FALSE} =>  {BcMV9.BI=FALSE} | 0.22  2772 | 0.426  54 | 0.522  277 | 1.32  5556 | 90 |
| {BcMCV5.BI=FALSE, BcMV9.BI=FALSE} =>  {BcOLV16.BI=FALSE} | 0.23  0198 | 0.75 | 0.306  931 | 1.28  3898 | 93 |
| {BcMCV5.BI=FALSE, BcOLV16.BI=FALSE} =>  {BcMV9.BI=FALSE} | 0.23  0198 | 0.413  333 | 0.556  931 | 1.28  4513 | 93 |
| {BcMV9.BI=FALSE, BcOLV13.BI=FALSE} =>  {BcOLV16.BI=FALSE} | 0.23  0198 | 0.794  872 | 0.289  604 | 1.36  0713 | 93 |
| {BcOLV13.BI=FALSE, BcOLV16.BI=FALSE}  => {BcMV9.BI=FALSE} | 0.23  0198 | 0.411  504 | 0.559  406 | 1.27  8829 | 93 |
| {BcMV9.BI=FALSE, BcOLV2.BI=FALSE} =>  {BcOLV16.BI=FALSE} | 0.23  7624 | 0.774  194 | 0.306  931 | 1.32  5314 | 96 |
| {BcOLV16.BI=FALSE, BcOLV2.BI=FALSE} =>  {BcMV9.BI=FALSE} | 0.23  7624 | 0.424  779 | 0.559  406 | 1.32  0082 | 96 |
| {BcMCV3.BI=FALSE, BcMV9.BI=FALSE} =>  {BcOLV16.BI=FALSE} | 0.23  7624 | 0.780  488 | 0.304  455 | 1.33  6089 | 96 |
| {BcMCV3.BI=FALSE, BcOLV16.BI=FALSE} =>  {BcMV9.BI=FALSE} | 0.23  7624 | 0.413  793 | 0.574  257 | 1.28  5942 | 96 |
| {BcMV9.BI=FALSE, BcPV4.BI=FALSE} =>  {BcOLV16.BI=FALSE} | 0.22  7723 | 0.760  331 | 0.299  505 | 1.30  1583 | 92 |
| {BcOLV16.BI=FALSE, BcPV4.BI=FALSE} => | 0.22 | 0.410 | 0.554 | 1.27 | 92 |

| {BcMV9.BI=FALSE} | 7723 | 714 | 455 | 6374 |  |
| --- | --- | --- | --- | --- | --- |
| {BcEV6=FALSE, BcMV9.BI=FALSE} =>  {BcOLV16.BI=FALSE} | 0.22  7723 | 0.747  967 | 0.304  455 | 1.28  0419 | 92 |
| {BcEV6=FALSE, BcOLV16.BI=FALSE} =>  {BcMV9.BI=FALSE} | 0.22  7723 | 0.418  182 | 0.544  554 | 1.29  958 | 92 |
| {BcMV9.BI=FALSE, BcOLV17.BI=FALSE} =>  {BcOLV16.BI=FALSE} | 0.23  0198 | 0.744 | 0.309  406 | 1.27  3627 | 93 |
| {BcOLV16.BI=FALSE, BcOLV17.BI=FALSE}  => {BcMV9.BI=FALSE} | 0.23  0198 | 0.409  692 | 0.561  881 | 1.27  3196 | 93 |
| {BcMV9.BI=FALSE, BcOLV15.BI=FALSE} =>  {BcOLV16.BI=FALSE} | 0.23  2673 | 0.752 | 0.309  406 | 1.28  7322 | 94 |
| {BcOLV15.BI=FALSE, BcOLV16.BI=FALSE}  => {BcMV9.BI=FALSE} | 0.23  2673 | 0.425  339 | 0.547  03 | 1.32  1824 | 94 |
| {BcEV5=FALSE, BcMV9.BI=FALSE} =>  {BcOLV16.BI=FALSE} | 0.22  7723 | 0.730  159 | 0.311  881 | 1.24  9933 | 92 |
| {BcEV5=FALSE, BcOLV16.BI=FALSE} =>  {BcMV9.BI=FALSE} | 0.22  7723 | 0.408  889 | 0.556  931 | 1.27  0701 | 92 |
| {BcHV1.BI=TRUE, BcMV9.BI=FALSE} =>  {BcULV3=TRUE} | 0.10  1485 | 0.745  455 | 0.136  139 | 1.25  4848 | 41 |
| {BcMV9.BI=FALSE, BcULV3=TRUE} =>  {BcRV1.BI=FALSE} | 0.15  3465 | 0.898  551 | 0.170  792 | 1.24  32 | 62 |
| {BcMV9.BI=FALSE, BcULV3=TRUE} => | 0.15 | 0.898 | 0.170 | 1.11 | 62 |

| {BcOLV7.BI=FALSE} | 3465 | 551 | 792 | 3541 |  |
| --- | --- | --- | --- | --- | --- |
| {BcMV9.BI=FALSE, BcULV3=TRUE} =>  {BcDMV1=FALSE} | 0.15  8416 | 0.927  536 | 0.170  792 | 1.09  8899 | 64 |
| {BcHV1.BI=TRUE, BcMV9.BI=FALSE} =>  {BcMV11=TRUE} | 0.09  901 | 0.727  273 | 0.136  139 | 1.17  9993 | 40 |
| {BcHV1.BI=TRUE, BcMV9.BI=FALSE} =>  {BcRV1.BI=FALSE} | 0.12  3762 | 0.909  091 | 0.136  139 | 1.25  7783 | 50 |
| {BcHV1.BI=TRUE, BcMV9.BI=FALSE} =>  {BcDMV1=FALSE} | 0.12  6238 | 0.927  273 | 0.136  139 | 1.09  8587 | 51 |
| {BcMV11=TRUE, BcMV6.BI=FALSE} =>  {BcMV9.BI=FALSE} | 0.16  3366 | 0.420  382 | 0.388  614 | 1.30  6418 | 66 |
| {BcMV11=TRUE, BcRV1.BI=FALSE} =>  {BcMV9.BI=FALSE} | 0.16  8317 | 0.412  121 | 0.408  416 | 1.28  0746 | 68 |
| {BcMV11=TRUE, BcMV9.BI=FALSE} =>  {BcFV5.BI=FALSE} | 0.18  3168 | 0.870  588 | 0.210  396 | 1.11  3031 | 74 |
| {BcFV5.BI=FALSE, BcMV11=TRUE} =>  {BcMV9.BI=FALSE} | 0.18  3168 | 0.385  417 | 0.475  248 | 1.19  7756 | 74 |
| {BcMV1.BI=TRUE, BcMV9.BI=FALSE} =>  {BcMV11=TRUE} | 0.17  8218 | 0.742  268 | 0.240  099 | 1.20  4322 | 72 |
| {BcMV11=TRUE, BcMV9.BI=FALSE} =>  {BcNSRV7.BI=FALSE} | 0.19  0594 | 0.905  882 | 0.210  396 | 1.13  3054 | 77 |
| {BcMV11=TRUE, BcNSRV7.BI=FALSE} => | 0.19 | 0.392 | 0.485 | 1.22 | 77 |

| {BcMV9.BI=FALSE} | 0594 | 857 | 149 | 0879 |  |
| --- | --- | --- | --- | --- | --- |
| {BcMV11=TRUE, BcMV9.BI=FALSE} => | 0.20 | 0.952 | 0.210 | 1.18 |  |
|  |  |  |  |  | 81 |
| {BcOLV7.BI=FALSE} | 0495 | 941 | 396 | 0946 |  |
| {BcMV11=TRUE, BcOLV7.BI=FALSE} => | 0.20 | 0.413 | 0.485 | 1.28 |  |
|  |  |  |  |  | 81 |
| {BcMV9.BI=FALSE} | 0495 | 265 | 149 | 4301 |  |
| {BcMV11=TRUE, BcMV9.BI=FALSE} => | 0.20 | 0.964 | 0.210 | 1.14 |  |
|  |  |  |  |  | 82 |
| {BcDMV1=FALSE} | 297 | 706 | 396 | 2936 |  |
| {BcDMV1=FALSE, BcMV11=TRUE} => | 0.20 | 0.398 | 0.509 | 1.23 |  |
|  |  |  |  |  | 82 |
| {BcMV9.BI=FALSE} | 297 | 058 | 901 | 7043 |  |
| {BcMV9.BI=FALSE, |  |  |  |  |  |
|  | 0.17 | 0.853 | 0.202 | 1.22 |  |
| BcOLV1.BI.MOVA.=FALSE} => |  |  |  |  | 70 |
|  | 3267 | 659 | 97 | 7324 |  |
| {BcMV6.BI=FALSE} |  |  |  |  |  |
| {BcMV9.BI=FALSE, |  |  |  |  |  |
|  | 0.16 | 0.817 | 0.202 | 1.13 |  |
| BcOLV1.BI.MOVA.=FALSE} => |  |  |  |  | 67 |
|  | 5842 | 073 | 97 | 0471 |  |
| {BcRV1.BI=FALSE} |  |  |  |  |  |
| {BcMV9.BI=FALSE, |  |  |  |  |  |
|  | 0.18 | 0.926 | 0.202 | 1.18 |  |
| BcOLV1.BI.MOVA.=FALSE} => |  |  |  |  | 76 |
|  | 8119 | 829 | 97 | 1196 |  |
| {BcULV1.BI=FALSE} |  |  |  |  |  |
| {BcMV9.BI=FALSE, |  |  |  |  |  |
|  | 0.18 | 0.926 | 0.202 | 1.14 |  |
| BcOLV1.BI.MOVA.=FALSE} => |  |  |  |  | 76 |
|  | 8119 | 829 | 97 | 8586 |  |
| {BcOLV7.BI=FALSE} |  |  |  |  |  |
| {BcMV9.BI=FALSE, | 0.20 | 0.987 | 0.202 | 1.08 | 81 |

| BcOLV1.BI.MOVA.=FALSE} =>  {BcBNV5.BI=FALSE} | 0495 | 805 | 97 | 7393 |  |
| --- | --- | --- | --- | --- | --- |
| {BcMV9.BI=FALSE, BcULV4=TRUE} =>  {BcULV1.BI=FALSE} | 0.12  6238 | 0.879  31 | 0.143  564 | 1.12  0635 | 51 |
| {BcMV6.BI=FALSE, BcMV9.BI=FALSE} =>  {BcRV1.BI=FALSE} | 0.20  5446 | 0.813  725 | 0.252  475 | 1.12  5839 | 83 |
| {BcMV6.BI=FALSE, BcRV1.BI=FALSE} =>  {BcMV9.BI=FALSE} | 0.20  5446 | 0.386  047 | 0.532  178 | 1.19  9714 | 83 |
| {BcFV5.BI=FALSE, BcMV6.BI=FALSE} =>  {BcMV9.BI=FALSE} | 0.20  7921 | 0.381  818 | 0.544  554 | 1.18  6573 | 84 |
| {BcMV6.BI=FALSE, BcMV9.BI=FALSE} =>  {BcULV1.BI=FALSE} | 0.22  7723 | 0.901  961 | 0.252  475 | 1.14  9502 | 92 |
| {BcMV9.BI=FALSE, BcULV1.BI=FALSE} =>  {BcMV6.BI=FALSE} | 0.22  7723 | 0.8 | 0.284  653 | 1.15  0178 | 92 |
| {BcMV6.BI=FALSE, BcULV1.BI=FALSE} =>  {BcMV9.BI=FALSE} | 0.22  7723 | 0.398  268 | 0.571  782 | 1.23  7696 | 92 |
| {BcMV12=FALSE, BcMV9.BI=FALSE} =>  {BcMV6.BI=FALSE} | 0.21  7822 | 0.838  095 | 0.259  901 | 1.20  4948 | 88 |
| {BcMV6.BI=FALSE, BcNSRV7.BI=FALSE} =>  {BcMV9.BI=FALSE} | 0.21  7822 | 0.384  279 | 0.566  832 | 1.19  4222 | 88 |
| {BcMV6.BI=FALSE, BcMV9.BI=FALSE} =>  {BcOLV7.BI=FALSE} | 0.23  5149 | 0.931  373 | 0.252  475 | 1.15  4216 | 95 |

| {BcMV9.BI=FALSE, BcOLV7.BI=FALSE} =>  {BcMV6.BI=FALSE} | 0.23  5149 | 0.785  124 | 0.299  505 | 1.12  879 | 95 |
| --- | --- | --- | --- | --- | --- |
| {BcMV6.BI=FALSE, BcOLV7.BI=FALSE} =>  {BcMV9.BI=FALSE} | 0.23  5149 | 0.409  483 | 0.574  257 | 1.27  2546 | 95 |
| {BcMV9.BI=FALSE, BcOLV4.BI=FALSE} =>  {BcMV6.BI=FALSE} | 0.21  2871 | 0.819  048 | 0.259  901 | 1.17  7563 | 86 |
| {BcMV9.BI=FALSE, BcULV5=FALSE} =>  {BcMV6.BI=FALSE} | 0.19  0594 | 0.865  169 | 0.220  297 | 1.24  3872 | 77 |
| {BcBNV5.BI=FALSE, BcMV9.BI=FALSE} =>  {BcMV6.BI=FALSE} | 0.24  2574 | 0.777  778 | 0.311  881 | 1.11  8229 | 98 |
| {BcMV9.BI=FALSE, BvF.BI=FALSE} =>  {BcMV6.BI=FALSE} | 0.23  5149 | 0.778  689 | 0.301  98 | 1.11  9538 | 95 |
| {BcMV6.BI=FALSE, BvF.BI=FALSE} =>  {BcMV9.BI=FALSE} | 0.23  5149 | 0.376  984 | 0.623  762 | 1.17  1551 | 95 |
| {BcMCV5.BI=FALSE, BcMV9.BI=FALSE} =>  {BcMV6.BI=FALSE} | 0.24  2574 | 0.790  323 | 0.306  931 | 1.13  6264 | 98 |
| {BcMV9.BI=FALSE, BcOLV13.BI=FALSE} =>  {BcMV6.BI=FALSE} | 0.23  2673 | 0.803  419 | 0.289  604 | 1.15  5093 | 94 |
| {BcMV9.BI=FALSE, BcOLV2.BI=FALSE} =>  {BcMV6.BI=FALSE} | 0.24  505 | 0.798  387 | 0.306  931 | 1.14  7859 | 99 |
| {BcMCV3.BI=FALSE, BcMV9.BI=FALSE} =>  {BcMV6.BI=FALSE} | 0.25 | 0.821  138 | 0.304  455 | 1.18  0569 | 10  1 |

| {BcMV9.BI=FALSE, BcPV4.BI=FALSE} =>  {BcMV6.BI=FALSE} | 0.23  7624 | 0.793  388 | 0.299  505 | 1.14  0672 | 96 |
| --- | --- | --- | --- | --- | --- |
| {BcEV6=FALSE, BcMV9.BI=FALSE} =>  {BcMV6.BI=FALSE} | 0.24  0099 | 0.788  618 | 0.304  455 | 1.13  3814 | 97 |
| {BcMV9.BI=FALSE, BcOLV17.BI=FALSE} =>  {BcMV6.BI=FALSE} | 0.24  2574 | 0.784 | 0.309  406 | 1.12  7174 | 98 |
| {BcMV9.BI=FALSE, BcOLV15.BI=FALSE} =>  {BcMV6.BI=FALSE} | 0.24  2574 | 0.784 | 0.309  406 | 1.12  7174 | 98 |
| {BcEV5=FALSE, BcMV9.BI=FALSE} =>  {BcMV6.BI=FALSE} | 0.24  2574 | 0.777  778 | 0.311  881 | 1.11  8229 | 98 |
| {BcMV9.BI=FALSE, BcPV3.BI=FALSE} =>  {BcULV1.BI=FALSE} | 0.21  0396 | 0.885  417 | 0.237  624 | 1.12  8417 | 85 |
| {BcMV9.BI=FALSE, BcPV3.BI=FALSE} =>  {BcOLV7.BI=FALSE} | 0.22  0297 | 0.927  083 | 0.237  624 | 1.14  8901 | 89 |
| {BcMV9.BI=FALSE, BcPV3.BI=FALSE} =>  {BcDMV1=FALSE} | 0.21  5347 | 0.906  25 | 0.237  624 | 1.07  368 | 87 |
| {BcEV2.BI=FALSE, BcMV9.BI=FALSE} =>  {BcRV1.BI=FALSE} | 0.21  5347 | 0.887  755 | 0.242  574 | 1.22  8264 | 87 |
| {BcEV2.BI=FALSE, BcRV1.BI=FALSE} =>  {BcMV9.BI=FALSE} | 0.21  5347 | 0.402  778 | 0.534  653 | 1.25  1709 | 87 |
| {BcHV3.BI=FALSE, BcMV9.BI=FALSE} =>  {BcRV1.BI=FALSE} | 0.20  7921 | 0.815  534 | 0.254  95 | 1.12  8342 | 84 |

| {BcFV5.BI=FALSE, BcMV9.BI=FALSE} =>  {BcRV1.BI=FALSE} | 0.22  0297 | 0.816  514 | 0.269  802 | 1.12  9697 | 89 |
| --- | --- | --- | --- | --- | --- |
| {BcFV5.BI=FALSE, BcRV1.BI=FALSE} =>  {BcMV9.BI=FALSE} | 0.22  0297 | 0.404  545 | 0.544  554 | 1.25  7203 | 89 |
| {BcMV9.BI=FALSE, BcULV1.BI=FALSE} =>  {BcRV1.BI=FALSE} | 0.23  0198 | 0.808  696 | 0.284  653 | 1.11  888 | 93 |
| {BcRV1.BI=FALSE, BcULV1.BI=FALSE} =>  {BcMV9.BI=FALSE} | 0.23  0198 | 0.394  068 | 0.584  158 | 1.22  4641 | 93 |
| {BcMV1.BI=TRUE, BcMV9.BI=FALSE} =>  {BcRV1.BI=FALSE} | 0.20  0495 | 0.835  052 | 0.240  099 | 1.15  5345 | 81 |
| {BcMV12=FALSE, BcMV9.BI=FALSE} =>  {BcRV1.BI=FALSE} | 0.21  5347 | 0.828  571 | 0.259  901 | 1.14  638 | 87 |
| {BcMV9.BI=FALSE, BcNSRV7.BI=FALSE} =>  {BcRV1.BI=FALSE} | 0.22  7723 | 0.807  018 | 0.282  178 | 1.11  6559 | 92 |
| {BcNSRV7.BI=FALSE, BcRV1.BI=FALSE} =>  {BcMV9.BI=FALSE} | 0.22  7723 | 0.407  08 | 0.559  406 | 1.26  5078 | 92 |
| {BcMV9.BI=FALSE, BcVV3.BI=FALSE} =>  {BcRV1.BI=FALSE} | 0.20  5446 | 0.83 | 0.247  525 | 1.14  8356 | 83 |
| {BcMV9.BI=FALSE, BcRV1.BI=FALSE} =>  {BcOLV7.BI=FALSE} | 0.25 | 0.935  185 | 0.267  327 | 1.15  8941 | 10  1 |
| {BcMV9.BI=FALSE, BcOLV7.BI=FALSE} =>  {BcRV1.BI=FALSE} | 0.25 | 0.834  711 | 0.299  505 | 1.15  4874 | 10  1 |

| {BcOLV7.BI=FALSE, BcRV1.BI=FALSE} =>  {BcMV9.BI=FALSE} | 0.25 | 0.391  473 | 0.638  614 | 1.21  6577 | 10  1 |
| --- | --- | --- | --- | --- | --- |
| {BcALV1.BI=FALSE, BcMV9.BI=FALSE} =>  {BcRV1.BI=FALSE} | 0.22  5248 | 0.827  273 | 0.272  277 | 1.14  4583 | 91 |
| {BcDMV1=FALSE, BcMV9.BI=FALSE} =>  {BcRV1.BI=FALSE} | 0.24  0099 | 0.822  034 | 0.292  079 | 1.13  7335 | 97 |
| {BcDMV1=FALSE, BcRV1.BI=FALSE} =>  {BcMV9.BI=FALSE} | 0.24  0099 | 0.409  283 | 0.586  634 | 1.27  1925 | 97 |
| {BcFV9=FALSE, BcMV9.BI=FALSE} =>  {BcRV1.BI=FALSE} | 0.24  2574 | 0.852  174 | 0.284  653 | 1.17  9035 | 98 |
| {BcFV9=FALSE, BcRV1.BI=FALSE} =>  {BcMV9.BI=FALSE} | 0.24  2574 | 0.381  323 | 0.636  139 | 1.18  5034 | 98 |
| {BcMV9.BI=FALSE, BcOLV4.BI=FALSE} =>  {BcRV1.BI=FALSE} | 0.21  5347 | 0.828  571 | 0.259  901 | 1.14  638 | 87 |
| {BcMV9.BI=FALSE, BcULV5=FALSE} =>  {BcRV1.BI=FALSE} | 0.18  0693 | 0.820  225 | 0.220  297 | 1.13  4831 | 73 |
| {BcBNV5.BI=FALSE, BcMV9.BI=FALSE} =>  {BcRV1.BI=FALSE} | 0.25  7426 | 0.825  397 | 0.311  881 | 1.14  1987 | 10  4 |
| {BcBNV5.BI=FALSE, BcRV1.BI=FALSE} =>  {BcMV9.BI=FALSE} | 0.25  7426 | 0.396  947 | 0.648  515 | 1.23  3588 | 10  4 |
| {BcMV9.BI=FALSE, BvF.BI=FALSE} =>  {BcRV1.BI=FALSE} | 0.24  7525 | 0.819  672 | 0.301  98 | 1.13  4067 | 10  0 |

| {BcRV1.BI=FALSE, BvF.BI=FALSE} =>  {BcMV9.BI=FALSE} | 0.24  7525 | 0.383  142 | 0.646  04 | 1.19  0687 | 10  0 |
| --- | --- | --- | --- | --- | --- |
| {BcMCV5.BI=FALSE, BcMV9.BI=FALSE} =>  {BcRV1.BI=FALSE} | 0.26  2376 | 0.854  839 | 0.306  931 | 1.18  2722 | 10  6 |
| {BcMCV5.BI=FALSE, BcRV1.BI=FALSE} =>  {BcMV9.BI=FALSE} | 0.26  2376 | 0.389  706 | 0.673  267 | 1.21  1086 | 10  6 |
| {BcMV9.BI=FALSE, BcOLV13.BI=FALSE} =>  {BcRV1.BI=FALSE} | 0.24  505 | 0.846  154 | 0.289  604 | 1.17  0706 | 99 |
| {BcMV9.BI=FALSE, BcOLV2.BI=FALSE} =>  {BcRV1.BI=FALSE} | 0.25  7426 | 0.838  71 | 0.306  931 | 1.16  0407 | 10  4 |
| {BcOLV2.BI=FALSE, BcRV1.BI=FALSE} =>  {BcMV9.BI=FALSE} | 0.25  7426 | 0.376  812 | 0.683  168 | 1.17  1014 | 10  4 |
| {BcMCV3.BI=FALSE, BcMV9.BI=FALSE} =>  {BcRV1.BI=FALSE} | 0.25 | 0.821  138 | 0.304  455 | 1.13  6095 | 10  1 |
| {BcMV9.BI=FALSE, BcPV4.BI=FALSE} =>  {BcRV1.BI=FALSE} | 0.25  2475 | 0.842  975 | 0.299  505 | 1.16  6308 | 10  2 |
| {BcPV4.BI=FALSE, BcRV1.BI=FALSE} =>  {BcMV9.BI=FALSE} | 0.25  2475 | 0.376  384 | 0.670  792 | 1.16  9685 | 10  2 |
| {BcEV6=FALSE, BcMV9.BI=FALSE} =>  {BcRV1.BI=FALSE} | 0.25 | 0.821  138 | 0.304  455 | 1.13  6095 | 10  1 |
| {BcEV6=FALSE, BcRV1.BI=FALSE} =>  {BcMV9.BI=FALSE} | 0.25 | 0.374  074 | 0.668  317 | 1.16  2507 | 10  1 |

| {BcMV9.BI=FALSE, BcOLV17.BI=FALSE} =>  {BcRV1.BI=FALSE} | 0.26  2376 | 0.848 | 0.309  406 | 1.17  326 | 10  6 |
| --- | --- | --- | --- | --- | --- |
| {BcOLV17.BI=FALSE, BcRV1.BI=FALSE} =>  {BcMV9.BI=FALSE} | 0.26  2376 | 0.370  629 | 0.707  921 | 1.15  1802 | 10  6 |
| {BcMV9.BI=FALSE, BcOLV15.BI=FALSE} =>  {BcRV1.BI=FALSE} | 0.25  9901 | 0.84 | 0.309  406 | 1.16  2192 | 10  5 |
| {BcOLV15.BI=FALSE, BcRV1.BI=FALSE} =>  {BcMV9.BI=FALSE} | 0.25  9901 | 0.381  818 | 0.680  693 | 1.18  6573 | 10  5 |
| {BcEV5=FALSE, BcMV9.BI=FALSE} =>  {BcRV1.BI=FALSE} | 0.25  7426 | 0.825  397 | 0.311  881 | 1.14  1987 | 10  4 |
| {BcEV5=FALSE, BcRV1.BI=FALSE} =>  {BcMV9.BI=FALSE} | 0.25  7426 | 0.382  353 | 0.673  267 | 1.18  8235 | 10  4 |
| {BcEV2.BI=FALSE, BcMV9.BI=FALSE} =>  {BcULV1.BI=FALSE} | 0.22  0297 | 0.908  163 | 0.242  574 | 1.15  7407 | 89 |
| {BcEV2.BI=FALSE, BcMV9.BI=FALSE} =>  {BcNSRV7.BI=FALSE} | 0.21  7822 | 0.897  959 | 0.242  574 | 1.12  3144 | 88 |
| {BcEV2.BI=FALSE, BcMV9.BI=FALSE} =>  {BcOLV7.BI=FALSE} | 0.22  7723 | 0.938  776 | 0.242  574 | 1.16  3391 | 92 |
| {BcEV2.BI=FALSE, BcOLV7.BI=FALSE} =>  {BcMV9.BI=FALSE} | 0.22  7723 | 0.384  937 | 0.591  584 | 1.19  6266 | 92 |
| {BcEV2.BI=FALSE, BcMV9.BI=FALSE} =>  {BcFV9=FALSE} | 0.22  5248 | 0.928  571 | 0.242  574 | 1.06  8783 | 91 |

| {BcHV3.BI=FALSE, BcMV9.BI=FALSE} =>  {BcFV5.BI=FALSE} | 0.23  5149 | 0.922  33 | 0.254  95 | 1.17  9182 | 95 |
| --- | --- | --- | --- | --- | --- |
| {BcFV5.BI=FALSE, BcMV9.BI=FALSE} =>  {BcHV3.BI=FALSE} | 0.23  5149 | 0.871  56 | 0.269  802 | 1.14  6938 | 95 |
| {BcHV3.BI=FALSE, BcMV9.BI=FALSE} =>  {BcULV1.BI=FALSE} | 0.22  2772 | 0.873  786 | 0.254  95 | 1.11  3595 | 90 |
| {BcHV3.BI=FALSE, BcMV9.BI=FALSE} =>  {BcNSRV7.BI=FALSE} | 0.23  5149 | 0.922  33 | 0.254  95 | 1.15  3626 | 95 |
| {BcHV3.BI=FALSE, BcMV9.BI=FALSE} =>  {BcOLV7.BI=FALSE} | 0.24  505 | 0.961  165 | 0.254  95 | 1.19  1137 | 99 |
| {BcHV3.BI=FALSE, BcOLV7.BI=FALSE} =>  {BcMV9.BI=FALSE} | 0.24  505 | 0.388  235 | 0.631  188 | 1.20  6516 | 99 |
| {BcHV3.BI=FALSE, BcMV9.BI=FALSE} =>  {BcDMV1=FALSE} | 0.24  505 | 0.961  165 | 0.254  95 | 1.13  8741 | 99 |
| {BcFV5.BI=FALSE, BcMV9.BI=FALSE} =>  {BcULV1.BI=FALSE} | 0.24  2574 | 0.899  083 | 0.269  802 | 1.14  5834 | 98 |
| {BcFV5.BI=FALSE, BcULV1.BI=FALSE} =>  {BcMV9.BI=FALSE} | 0.24  2574 | 0.396  761 | 0.611  386 | 1.23  3012 | 98 |
| {BcFV5.BI=FALSE, BcMV9.BI=FALSE} =>  {BcNSRV7.BI=FALSE} | 0.25  495 | 0.944  954 | 0.269  802 | 1.18  1924 | 10  3 |
| {BcMV9.BI=FALSE, BcNSRV7.BI=FALSE} =>  {BcFV5.BI=FALSE} | 0.25  495 | 0.903  509 | 0.282  178 | 1.15  5119 | 10  3 |

| {BcFV5.BI=FALSE, BcNSRV7.BI=FALSE} =>  {BcMV9.BI=FALSE} | 0.25  495 | 0.375  912 | 0.678  218 | 1.16  822 | 10  3 |
| --- | --- | --- | --- | --- | --- |
| {BcFV5.BI=FALSE, BcMV9.BI=FALSE} =>  {BcOLV7.BI=FALSE} | 0.25  7426 | 0.954  128 | 0.269  802 | 1.18  2417 | 10  4 |
| {BcMV9.BI=FALSE, BcOLV7.BI=FALSE} =>  {BcFV5.BI=FALSE} | 0.25  7426 | 0.859  504 | 0.299  505 | 1.09  886 | 10  4 |
| {BcFV5.BI=FALSE, BcOLV7.BI=FALSE} =>  {BcMV9.BI=FALSE} | 0.25  7426 | 0.412  698 | 0.623  762 | 1.28  254 | 10  4 |
| {BcFV5.BI=FALSE, BcMV9.BI=FALSE} =>  {BcDMV1=FALSE} | 0.25  9901 | 0.963  303 | 0.269  802 | 1.14  1274 | 10  5 |
| {BcDMV1=FALSE, BcMV9.BI=FALSE} =>  {BcFV5.BI=FALSE} | 0.25  9901 | 0.889  831 | 0.292  079 | 1.13  7631 | 10  5 |
| {BcMV9.BI=FALSE, BcOLV15.BI=FALSE} =>  {BcFV5.BI=FALSE} | 0.26  4851 | 0.856 | 0.309  406 | 1.09  438 | 10  7 |
| {BcEV5=FALSE, BcMV9.BI=FALSE} =>  {BcFV5.BI=FALSE} | 0.26  7327 | 0.857  143 | 0.311  881 | 1.09  5841 | 10  8 |
| {BcMV12=FALSE, BcMV9.BI=FALSE} =>  {BcULV1.BI=FALSE} | 0.23  7624 | 0.914  286 | 0.259  901 | 1.16  521 | 96 |
| {BcMV9.BI=FALSE, BcULV1.BI=FALSE} =>  {BcNSRV7.BI=FALSE} | 0.25 | 0.878  261 | 0.284  653 | 1.09  8506 | 10  1 |
| {BcMV9.BI=FALSE, BcNSRV7.BI=FALSE} =>  {BcULV1.BI=FALSE} | 0.25 | 0.885  965 | 0.282  178 | 1.12  9116 | 10  1 |

| {BcNSRV7.BI=FALSE, BcULV1.BI=FALSE} =>  {BcMV9.BI=FALSE} | 0.25 | 0.407  258 | 0.613  861 | 1.26  5633 | 10  1 |
| --- | --- | --- | --- | --- | --- |
| {BcMV9.BI=FALSE, BcVV3.BI=FALSE} =>  {BcULV1.BI=FALSE} | 0.22  0297 | 0.89 | 0.247  525 | 1.13  4259 | 89 |
| {BcMV9.BI=FALSE, BcULV1.BI=FALSE} =>  {BcOLV7.BI=FALSE} | 0.26  2376 | 0.921  739 | 0.284  653 | 1.14  2278 | 10  6 |
| {BcMV9.BI=FALSE, BcOLV7.BI=FALSE} =>  {BcULV1.BI=FALSE} | 0.26  2376 | 0.876  033 | 0.299  505 | 1.11  6459 | 10  6 |
| {BcOLV7.BI=FALSE, BcULV1.BI=FALSE} =>  {BcMV9.BI=FALSE} | 0.26  2376 | 0.403  042 | 0.650  99 | 1.25  253 | 10  6 |
| {BcALV1.BI=FALSE, BcMV9.BI=FALSE} =>  {BcULV1.BI=FALSE} | 0.24  0099 | 0.881  818 | 0.272  277 | 1.12  3831 | 97 |
| {BcDMV1=FALSE, BcMV9.BI=FALSE} =>  {BcULV1.BI=FALSE} | 0.25  495 | 0.872  881 | 0.292  079 | 1.11  2442 | 10  3 |
| {BcDMV1=FALSE, BcULV1.BI=FALSE} =>  {BcMV9.BI=FALSE} | 0.25  495 | 0.393  13 | 0.648  515 | 1.22  1726 | 10  3 |
| {BcFV9=FALSE, BcMV9.BI=FALSE} =>  {BcULV1.BI=FALSE} | 0.25 | 0.878  261 | 0.284  653 | 1.11  9298 | 10  1 |
| {BcMV9.BI=FALSE, BcOLV4.BI=FALSE} =>  {BcULV1.BI=FALSE} | 0.24  0099 | 0.923  81 | 0.259  901 | 1.17  7347 | 97 |
| {BcMV9.BI=FALSE, BcULV5=FALSE} =>  {BcULV1.BI=FALSE} | 0.20  297 | 0.921  348 | 0.220  297 | 1.17  421 | 82 |

| {BcMV9.BI=FALSE, BcULV1.BI=FALSE} =>  {BcBNV5.BI=FALSE} | 0.27  9703 | 0.982  609 | 0.284  653 | 1.08  1673 | 11  3 |
| --- | --- | --- | --- | --- | --- |
| {BcBNV5.BI=FALSE, BcMV9.BI=FALSE} =>  {BcULV1.BI=FALSE} | 0.27  9703 | 0.896  825 | 0.311  881 | 1.14  2957 | 11  3 |
| {BcBNV5.BI=FALSE, BcULV1.BI=FALSE} =>  {BcMV9.BI=FALSE} | 0.27  9703 | 0.392  361 | 0.712  871 | 1.21  9338 | 11  3 |
| {BcMV9.BI=FALSE, BvF.BI=FALSE} =>  {BcULV1.BI=FALSE} | 0.27  4752 | 0.909  836 | 0.301  98 | 1.15  9539 | 11  1 |
| {BcULV1.BI=FALSE, BvF.BI=FALSE} =>  {BcMV9.BI=FALSE} | 0.27  4752 | 0.385  417 | 0.712  871 | 1.19  7756 | 11  1 |
| {BcMCV5.BI=FALSE, BcMV9.BI=FALSE} =>  {BcULV1.BI=FALSE} | 0.27  2277 | 0.887  097 | 0.306  931 | 1.13  0559 | 11  0 |
| {BcMCV5.BI=FALSE, BcULV1.BI=FALSE} =>  {BcMV9.BI=FALSE} | 0.27  2277 | 0.372  881 | 0.730  198 | 1.15  8801 | 11  0 |
| {BcMV9.BI=FALSE, BcOLV13.BI=FALSE} =>  {BcULV1.BI=FALSE} | 0.26  4851 | 0.914  53 | 0.289  604 | 1.16  5521 | 10  7 |
| {BcMV9.BI=FALSE, BcOLV2.BI=FALSE} =>  {BcULV1.BI=FALSE} | 0.27  4752 | 0.895  161 | 0.306  931 | 1.14  0836 | 11  1 |
| {BcOLV2.BI=FALSE, BcULV1.BI=FALSE} =>  {BcMV9.BI=FALSE} | 0.27  4752 | 0.37 | 0.742  574 | 1.14  9846 | 11  1 |
| {BcMCV3.BI=FALSE, BcMV9.BI=FALSE} =>  {BcULV1.BI=FALSE} | 0.27  9703 | 0.918  699 | 0.304  455 | 1.17  0834 | 11  3 |

| {BcMV9.BI=FALSE, BcPV4.BI=FALSE} =>  {BcULV1.BI=FALSE} | 0.26  4851 | 0.884  298 | 0.299  505 | 1.12  6991 | 10  7 |
| --- | --- | --- | --- | --- | --- |
| {BcEV6=FALSE, BcMV9.BI=FALSE} =>  {BcULV1.BI=FALSE} | 0.27  2277 | 0.894  309 | 0.304  455 | 1.13  975 | 11  0 |
| {BcMV9.BI=FALSE, BcOLV17.BI=FALSE} =>  {BcULV1.BI=FALSE} | 0.27  2277 | 0.88 | 0.309  406 | 1.12  1514 | 11  0 |
| {BcMV9.BI=FALSE, BcOLV15.BI=FALSE} =>  {BcULV1.BI=FALSE} | 0.27  4752 | 0.888 | 0.309  406 | 1.13  171 | 11  1 |
| {BcOLV15.BI=FALSE, BcULV1.BI=FALSE} =>  {BcMV9.BI=FALSE} | 0.27  4752 | 0.371  237 | 0.740  099 | 1.15  3692 | 11  1 |
| {BcEV5=FALSE, BcMV9.BI=FALSE} =>  {BcULV1.BI=FALSE} | 0.27  4752 | 0.880  952 | 0.311  881 | 1.12  2728 | 11  1 |
| {BcEV5=FALSE, BcULV1.BI=FALSE} =>  {BcMV9.BI=FALSE} | 0.27  4752 | 0.37 | 0.742  574 | 1.14  9846 | 11  1 |
| {BcMV1.BI=TRUE, BcMV9.BI=FALSE} =>  {BcOLV7.BI=FALSE} | 0.21  7822 | 0.907  216 | 0.240  099 | 1.12  4281 | 88 |
| {BcMV1.BI=TRUE, BcMV9.BI=FALSE} =>  {BcDMV1=FALSE} | 0.22  2772 | 0.927  835 | 0.240  099 | 1.09  9253 | 90 |
| {BcMV12=FALSE, BcMV9.BI=FALSE} =>  {BcOLV7.BI=FALSE} | 0.24  7525 | 0.952  381 | 0.259  901 | 1.18  0251 | 10  0 |
| {BcMV12=FALSE, BcOLV7.BI=FALSE} =>  {BcMV9.BI=FALSE} | 0.24  7525 | 0.377  358 | 0.655  941 | 1.17  2714 | 10  0 |

| {BcMV12=FALSE, BcMV9.BI=FALSE} =>  {BcBNV5.BI=FALSE} | 0.25  495 | 0.980  952 | 0.259  901 | 1.07  9849 | 10  3 |
| --- | --- | --- | --- | --- | --- |
| {BcMV9.BI=FALSE, BcVV3.BI=FALSE} =>  {BcNSRV7.BI=FALSE} | 0.22  2772 | 0.9 | 0.247  525 | 1.12  5697 | 90 |
| {BcMV9.BI=FALSE, BcNSRV7.BI=FALSE} =>  {BcOLV7.BI=FALSE} | 0.26  7327 | 0.947  368 | 0.282  178 | 1.17  4039 | 10  8 |
| {BcMV9.BI=FALSE, BcOLV7.BI=FALSE} =>  {BcNSRV7.BI=FALSE} | 0.26  7327 | 0.892  562 | 0.299  505 | 1.11  6393 | 10  8 |
| {BcNSRV7.BI=FALSE, BcOLV7.BI=FALSE} =>  {BcMV9.BI=FALSE} | 0.26  7327 | 0.418  605 | 0.638  614 | 1.30  0894 | 10  8 |
| {BcMV9.BI=FALSE, BcNSRV7.BI=FALSE} =>  {BcDMV1=FALSE} | 0.26  4851 | 0.938  596 | 0.282  178 | 1.11  2003 | 10  7 |
| {BcDMV1=FALSE, BcMV9.BI=FALSE} =>  {BcNSRV7.BI=FALSE} | 0.26  4851 | 0.906  78 | 0.292  079 | 1.13  4176 | 10  7 |
| {BcDMV1=FALSE, BcNSRV7.BI=FALSE} =>  {BcMV9.BI=FALSE} | 0.26  4851 | 0.370  242 | 0.715  347 | 1.15  0599 | 10  7 |
| {BcFV9=FALSE, BcMV9.BI=FALSE} =>  {BcNSRV7.BI=FALSE} | 0.25  495 | 0.895  652 | 0.284  653 | 1.12  0258 | 10  3 |
| {BcBNV5.BI=FALSE, BcMV9.BI=FALSE} =>  {BcNSRV7.BI=FALSE} | 0.27  2277 | 0.873  016 | 0.311  881 | 1.09  1946 | 11  0 |
| {BcMV9.BI=FALSE, BvF.BI=FALSE} =>  {BcNSRV7.BI=FALSE} | 0.26  7327 | 0.885  246 | 0.301  98 | 1.10  7243 | 10  8 |

| {BcMV9.BI=FALSE, BcPV4.BI=FALSE} =>  {BcNSRV7.BI=FALSE} | 0.26  4851 | 0.884  298 | 0.299  505 | 1.10  6056 | 10  7 |
| --- | --- | --- | --- | --- | --- |
| {BcEV6=FALSE, BcMV9.BI=FALSE} =>  {BcNSRV7.BI=FALSE} | 0.26  7327 | 0.878  049 | 0.304  455 | 1.09  8241 | 10  8 |
| {BcMV9.BI=FALSE, BcOLV15.BI=FALSE} =>  {BcNSRV7.BI=FALSE} | 0.27  4752 | 0.888 | 0.309  406 | 1.11  0687 | 11  1 |
| {BcEV5=FALSE, BcMV9.BI=FALSE} =>  {BcNSRV7.BI=FALSE} | 0.27  7228 | 0.888  889 | 0.311  881 | 1.11  1799 | 11  2 |
| {BcMV9.BI=FALSE, BcVV3.BI=FALSE} =>  {BcOLV7.BI=FALSE} | 0.23  5149 | 0.95 | 0.247  525 | 1.17  7301 | 95 |
| {BcMV9.BI=FALSE, BcVV3.BI=FALSE} =>  {BcFV9=FALSE} | 0.23  2673 | 0.94 | 0.247  525 | 1.08  1937 | 94 |
| {BcMV9.BI=FALSE, BcVV3.BI=FALSE} =>  {BcBNV5.BI=FALSE} | 0.24  2574 | 0.98 | 0.247  525 | 1.07  8801 | 98 |
| {BcALV1.BI=FALSE, BcMV9.BI=FALSE} =>  {BcOLV7.BI=FALSE} | 0.25  7426 | 0.945  455 | 0.272  277 | 1.17  1668 | 10  4 |
| {BcALV1.BI=FALSE, BcOLV7.BI=FALSE} =>  {BcMV9.BI=FALSE} | 0.25  7426 | 0.380  952 | 0.675  743 | 1.18  3883 | 10  4 |
| {BcMV9.BI=FALSE, BcOLV7.BI=FALSE} =>  {BcDMV1=FALSE} | 0.27  2277 | 0.909  091 | 0.299  505 | 1.07  7046 | 11  0 |
| {BcDMV1=FALSE, BcMV9.BI=FALSE} =>  {BcOLV7.BI=FALSE} | 0.27  2277 | 0.932  203 | 0.292  079 | 1.15  5246 | 11  0 |

| {BcDMV1=FALSE, BcOLV7.BI=FALSE} =>  {BcMV9.BI=FALSE} | 0.27  2277 | 0.408  922 | 0.665  842 | 1.27  0804 | 11  0 |
| --- | --- | --- | --- | --- | --- |
| {BcFV9=FALSE, BcMV9.BI=FALSE} =>  {BcOLV7.BI=FALSE} | 0.26  9802 | 0.947  826 | 0.284  653 | 1.17  4607 | 10  9 |
| {BcFV9=FALSE, BcOLV7.BI=FALSE} =>  {BcMV9.BI=FALSE} | 0.26  9802 | 0.382  456 | 0.705  446 | 1.18  8556 | 10  9 |
| {BcMV9.BI=FALSE, BcOLV4.BI=FALSE} =>  {BcOLV7.BI=FALSE} | 0.24  2574 | 0.933  333 | 0.259  901 | 1.15  6646 | 98 |
| {BcMV9.BI=FALSE, BcULV5=FALSE} =>  {BcOLV7.BI=FALSE} | 0.20  297 | 0.921  348 | 0.220  297 | 1.14  1794 | 82 |
| {BcMV9.BI=FALSE, BcOLV7.BI=FALSE} =>  {BcBNV5.BI=FALSE} | 0.29  4554 | 0.983  471 | 0.299  505 | 1.08  2622 | 11  9 |
| {BcBNV5.BI=FALSE, BcMV9.BI=FALSE} =>  {BcOLV7.BI=FALSE} | 0.29  4554 | 0.944  444 | 0.311  881 | 1.17  0416 | 11  9 |
| {BcBNV5.BI=FALSE, BcOLV7.BI=FALSE} =>  {BcMV9.BI=FALSE} | 0.29  4554 | 0.403  39 | 0.730  198 | 1.25  3611 | 11  9 |
| {BcMV9.BI=FALSE, BvF.BI=FALSE} =>  {BcOLV7.BI=FALSE} | 0.27  9703 | 0.926  23 | 0.301  98 | 1.14  7843 | 11  3 |
| {BcOLV7.BI=FALSE, BvF.BI=FALSE} =>  {BcMV9.BI=FALSE} | 0.27  9703 | 0.384  354 | 0.727  723 | 1.19  4453 | 11  3 |
| {BcMCV5.BI=FALSE, BcMV9.BI=FALSE} =>  {BcOLV7.BI=FALSE} | 0.28  9604 | 0.943  548 | 0.306  931 | 1.16  9305 | 11  7 |

| {BcMCV5.BI=FALSE, BcOLV7.BI=FALSE} =>  {BcMV9.BI=FALSE} | 0.28  9604 | 0.386  139 | 0.75 | 1.2 | 11  7 |
| --- | --- | --- | --- | --- | --- |
| {BcMV9.BI=FALSE, BcOLV13.BI=FALSE} =>  {BcOLV7.BI=FALSE} | 0.26  7327 | 0.923  077 | 0.289  604 | 1.14  3936 | 10  8 |
| {BcMV9.BI=FALSE, BcOLV2.BI=FALSE} =>  {BcOLV7.BI=FALSE} | 0.28  4653 | 0.927  419 | 0.306  931 | 1.14  9317 | 11  5 |
| {BcOLV2.BI=FALSE, BcOLV7.BI=FALSE} =>  {BcMV9.BI=FALSE} | 0.28  4653 | 0.378  289 | 0.752  475 | 1.17  5607 | 11  5 |
| {BcMCV3.BI=FALSE, BcMV9.BI=FALSE} =>  {BcOLV7.BI=FALSE} | 0.28  2178 | 0.926  829 | 0.304  455 | 1.14  8586 | 11  4 |
| {BcMCV3.BI=FALSE, BcOLV7.BI=FALSE} =>  {BcMV9.BI=FALSE} | 0.28  2178 | 0.371  336 | 0.759  901 | 1.15  3996 | 11  4 |
| {BcMV9.BI=FALSE, BcPV4.BI=FALSE} =>  {BcOLV7.BI=FALSE} | 0.27  9703 | 0.933  884 | 0.299  505 | 1.15  7329 | 11  3 |
| {BcOLV7.BI=FALSE, BcPV4.BI=FALSE} =>  {BcMV9.BI=FALSE} | 0.27  9703 | 0.369  281 | 0.757  426 | 1.14  7612 | 11  3 |
| {BcEV6=FALSE, BcMV9.BI=FALSE} =>  {BcOLV7.BI=FALSE} | 0.28  2178 | 0.926  829 | 0.304  455 | 1.14  8586 | 11  4 |
| {BcEV6=FALSE, BcOLV7.BI=FALSE} =>  {BcMV9.BI=FALSE} | 0.28  2178 | 0.375 | 0.752  475 | 1.16  5385 | 11  4 |
| {BcMV9.BI=FALSE, BcOLV17.BI=FALSE} =>  {BcOLV7.BI=FALSE} | 0.28  7129 | 0.928 | 0.309  406 | 1.15  0037 | 11  6 |

| {BcOLV17.BI=FALSE, BcOLV7.BI=FALSE} =>  {BcMV9.BI=FALSE} | 0.28  7129 | 0.369  427 | 0.777  228 | 1.14  8065 | 11  6 |
| --- | --- | --- | --- | --- | --- |
| {BcMV9.BI=FALSE, BcOLV15.BI=FALSE} =>  {BcOLV7.BI=FALSE} | 0.28  9604 | 0.936 | 0.309  406 | 1.15  9951 | 11  7 |
| {BcOLV15.BI=FALSE, BcOLV7.BI=FALSE} =>  {BcMV9.BI=FALSE} | 0.28  9604 | 0.379  87 | 0.762  376 | 1.18  0519 | 11  7 |
| {BcEV5=FALSE, BcMV9.BI=FALSE} =>  {BcOLV7.BI=FALSE} | 0.29  2079 | 0.936  508 | 0.311  881 | 1.16  058 | 11  8 |
| {BcEV5=FALSE, BcOLV7.BI=FALSE} =>  {BcMV9.BI=FALSE} | 0.29  2079 | 0.384  365 | 0.759  901 | 1.19  4488 | 11  8 |
| {BcALV1.BI=FALSE, BcMV9.BI=FALSE} =>  {BcDMV1=FALSE} | 0.24  7525 | 0.909  091 | 0.272  277 | 1.07  7046 | 10  0 |
| {BcALV1.BI=FALSE, BcMV9.BI=FALSE} =>  {BcBNV5.BI=FALSE} | 0.26  9802 | 0.990  909 | 0.272  277 | 1.09  081 | 10  9 |
| {BcFV9=FALSE, BcMV9.BI=FALSE} =>  {BcDMV1=FALSE} | 0.25  7426 | 0.904  348 | 0.284  653 | 1.07  1427 | 10  4 |
| {BcDMV1=FALSE, BcMV9.BI=FALSE} =>  {BcBNV5.BI=FALSE} | 0.28  4653 | 0.974  576 | 0.292  079 | 1.07  2831 | 11  5 |
| {BcBNV5.BI=FALSE, BcMV9.BI=FALSE} =>  {BcDMV1=FALSE} | 0.28  4653 | 0.912  698 | 0.311  881 | 1.08  132 | 11  5 |
| {BcMV9.BI=FALSE, BvF.BI=FALSE} =>  {BcDMV1=FALSE} | 0.27  2277 | 0.901  639 | 0.301  98 | 1.06  8218 | 11  0 |

| {BcMCV5.BI=FALSE, BcMV9.BI=FALSE} =>  {BcDMV1=FALSE} | 0.27  7228 | 0.903  226 | 0.306  931 | 1.07  0097 | 11  2 |
| --- | --- | --- | --- | --- | --- |
| {BcMV9.BI=FALSE, BcOLV2.BI=FALSE} =>  {BcDMV1=FALSE} | 0.27  7228 | 0.903  226 | 0.306  931 | 1.07  0097 | 11  2 |
| {BcMCV3.BI=FALSE, BcMV9.BI=FALSE} =>  {BcDMV1=FALSE} | 0.27  4752 | 0.902  439 | 0.304  455 | 1.06  9165 | 11  1 |
| {BcMV9.BI=FALSE, BcPV4.BI=FALSE} =>  {BcDMV1=FALSE} | 0.26  9802 | 0.900  826 | 0.299  505 | 1.06  7255 | 10  9 |
| {BcEV6=FALSE, BcMV9.BI=FALSE} =>  {BcDMV1=FALSE} | 0.27  4752 | 0.902  439 | 0.304  455 | 1.06  9165 | 11  1 |
| {BcMV9.BI=FALSE, BcOLV17.BI=FALSE} =>  {BcDMV1=FALSE} | 0.27  9703 | 0.904 | 0.309  406 | 1.07  1015 | 11  3 |
| {BcMV9.BI=FALSE, BcOLV15.BI=FALSE} =>  {BcDMV1=FALSE} | 0.27  9703 | 0.904 | 0.309  406 | 1.07  1015 | 11  3 |
| {BcEV5=FALSE, BcMV9.BI=FALSE} =>  {BcDMV1=FALSE} | 0.28  7129 | 0.920  635 | 0.311  881 | 1.09  0723 | 11  6 |
| {BcFV9=FALSE, BcMV9.BI=FALSE} =>  {BcBNV5.BI=FALSE} | 0.27  7228 | 0.973  913 | 0.284  653 | 1.07  21 | 11  2 |
| {BcMCV5.BI=FALSE, BcMV9.BI=FALSE} =>  {BcBNV5.BI=FALSE} | 0.29  9505 | 0.975  806 | 0.306  931 | 1.07  4185 | 12  1 |
| {BcEV6=FALSE, BcMV9.BI=FALSE} =>  {BcBNV5.BI=FALSE} | 0.29  703 | 0.975  61 | 0.304  455 | 1.07  3968 | 12  0 |

| {BcMV11=FALSE, BcOLV1.BI.MOVA.=TRUE}  => {BcEV2.BI=FALSE} | 0.07  6733 | 0.861  111 | 0.089  109 | 1.15  195 | 31 |
| --- | --- | --- | --- | --- | --- |
| {BcMV11=FALSE, BcOLV1.BI.MOVA.=TRUE}  => {BcALV1.BI=FALSE} | 0.08  1683 | 0.916  667 | 0.089  109 | 1.12  5633 | 33 |
| {BcHV1.BI=FALSE, BcOLV1.BI.MOVA.=TRUE} =>  {BcHV1SLR.BI=FALSE} | 0.09  6535 | 0.812  5 | 0.118  812 | 1.77  4324 | 39 |
| {BcHV1SLR.BI=FALSE, BcOLV1.BI.MOVA.=TRUE} =>  {BcHV1.BI=FALSE} | 0.09  6535 | 0.735  849 | 0.131  188 | 1.85  8019 | 39 |
| {BcHV1.BI=FALSE, BcOLV1.BI.MOVA.=TRUE} =>  {BcMV2.BI=TRUE} | 0.09  1584 | 0.770  833 | 0.118  812 | 1.66  533 | 37 |
| {BcHV1.BI=FALSE, BcMV2.BI=TRUE} =>  {BcOLV1.BI.MOVA.=TRUE} | 0.09  1584 | 0.536  232 | 0.170  792 | 1.61  6699 | 37 |
| {BcMV6.BI=FALSE, BcOLV1.BI.MOVA.=TRUE} =>  {BcHV1.BI=FALSE} | 0.08  6634 | 0.514  706 | 0.168  317 | 1.29  9632 | 35 |
| {BcHV1.BI=FALSE, BcOLV1.BI.MOVA.=TRUE} =>  {BcMV1.BI=TRUE} | 0.11  1386 | 0.937  5 | 0.118  812 | 1.18  7304 | 45 |
| {BcHV1SLR.BI=FALSE, | 0.07 | 0.566 | 0.131 | 1.39 | 30 |

| BcOLV1.BI.MOVA.=TRUE} =>  {BcULV3=FALSE} | 4257 | 038 | 188 | 4386 |  |
| --- | --- | --- | --- | --- | --- |
| {BcOLV1.BI.MOVA.=TRUE, BcULV3=FALSE} | 0.10 | 0.745 | 0.136 | 1.61 |  |
|  |  |  |  |  | 41 |
| => {BcMV2.BI=TRUE} | 1485 | 455 | 139 | 0501 |  |
| {BcMV2.BI=TRUE, BcULV3=FALSE} => | 0.10 | 0.569 | 0.178 | 1.71 |  |
|  |  |  |  |  | 41 |
| {BcOLV1.BI.MOVA.=TRUE} | 1485 | 444 | 218 | 6833 |  |
| {BcOLV1.BI.MOVA.=TRUE, BcULV3=FALSE} | 0.09 | 0.672 | 0.136 | 1.30 |  |
|  |  |  |  |  | 37 |
| => {BcOLV18=TRUE} | 1584 | 727 | 139 | 0391 |  |
| {BcOLV18=TRUE, BcULV3=FALSE} => | 0.09 | 0.474 | 0.193 | 1.43 |  |
|  |  |  |  |  | 37 |
| {BcOLV1.BI.MOVA.=TRUE} | 1584 | 359 | 069 | 0157 |  |
| {BcOLV1.BI.MOVA.=TRUE, BcULV3=FALSE} | 0.09 | 0.672 | 0.136 | 1.24 |  |
|  |  |  |  |  | 37 |
| => {BcMV3.BI=TRUE} | 1584 | 727 | 139 | 1013 |  |
| {BcMV3.BI=TRUE, BcULV3=FALSE} => | 0.09 | 0.468 | 0.195 | 1.41 |  |
|  |  |  |  |  | 37 |
| {BcOLV1.BI.MOVA.=TRUE} | 1584 | 354 | 545 | 2054 |  |
| {BcOLV1.BI.MOVA.=TRUE, | 0.07 | 0.561 | 0.141 | 1.38 |  |
|  |  |  |  |  | 32 |
| BcOLV16.BI=FALSE} => {BcULV3=FALSE} | 9208 | 404 | 089 | 297 |  |
| {BcMV6.BI=FALSE, |  |  |  |  |  |
|  | 0.09 | 0.558 | 0.168 | 1.37 |  |
| BcOLV1.BI.MOVA.=TRUE} => |  |  |  |  | 38 |
|  | 4059 | 824 | 317 | 6614 |  |
| {BcULV3=FALSE} |  |  |  |  |  |
| {BcOLV1.BI.MOVA.=TRUE, BcULV3=FALSE} | 0.12 | 0.890 | 0.136 | 1.12 |  |
|  |  |  |  |  | 49 |
| => {BcMV1.BI=TRUE} | 1287 | 909 | 139 | 8299 |  |
| {BcOLV1.BI.MOVA.=TRUE, BcULV3=FALSE} | 0.12 | 0.927 | 0.136 | 1.15 | 51 |

| => {BcNSRV7.BI=FALSE} | 6238 | 273 | 139 | 9809 |  |
| --- | --- | --- | --- | --- | --- |
| {BcOLV1.BI.MOVA.=TRUE, BcULV3=FALSE}  => {BcBNV5.BI=FALSE} | 0.13  3663 | 0.981  818 | 0.136  139 | 1.08  0803 | 54 |
| {BcOLV1.BI.MOVA.=TRUE,  BcOLV16.BI=TRUE} => {BcMV4.BI=TRUE} | 0.11  8812 | 0.623  377 | 0.190  594 | 1.40  6951 | 48 |
| {BcMV4.BI=TRUE, BcOLV1.BI.MOVA.=TRUE}  => {BcOLV16.BI=TRUE} | 0.11  8812 | 0.64 | 0.185  644 | 1.53  9048 | 48 |
| {BcMV4.BI=TRUE, BcOLV16.BI=TRUE} =>  {BcOLV1.BI.MOVA.=TRUE} | 0.11  8812 | 0.527  473 | 0.225  248 | 1.59  029 | 48 |
| {BcOLV1.BI.MOVA.=TRUE,  BcOLV16.BI=TRUE} => {BcMV2.BI=TRUE} | 0.15  5941 | 0.818  182 | 0.190  594 | 1.76  7623 | 63 |
| {BcMV2.BI=TRUE, BcOLV1.BI.MOVA.=TRUE}  => {BcOLV16.BI=TRUE} | 0.15  5941 | 0.623  762 | 0.25 | 1.5 | 63 |
| {BcMV2.BI=TRUE, BcOLV16.BI=TRUE} =>  {BcOLV1.BI.MOVA.=TRUE} | 0.15  5941 | 0.617  647 | 0.252  475 | 1.86  216 | 63 |
| {BcOLV1.BI.MOVA.=TRUE,  BcOLV16.BI=TRUE} => {BcOLV18=TRUE} | 0.13  6139 | 0.714  286 | 0.190  594 | 1.38  0725 | 55 |
| {BcOLV1.BI.MOVA.=TRUE, BcOLV18=TRUE}  => {BcOLV16.BI=TRUE} | 0.13  6139 | 0.591  398 | 0.230  198 | 1.42  2171 | 55 |
| {BcOLV16.BI=TRUE, BcOLV18=TRUE} =>  {BcOLV1.BI.MOVA.=TRUE} | 0.13  6139 | 0.504  587 | 0.269  802 | 1.52  1293 | 55 |
| {BcOLV1.BI.MOVA.=TRUE, | 0.13 | 0.688 | 0.190 | 1.26 | 53 |

| BcOLV16.BI=TRUE} =>  {BcHV1SLR.BI=TRUE} | 1188 | 312 | 594 | 9762 |  |
| --- | --- | --- | --- | --- | --- |
| {BcHV1SLR.BI=TRUE, BcOLV1.BI.MOVA.=TRUE} =>  {BcOLV16.BI=TRUE} | 0.13  1188 | 0.654  321 | 0.200  495 | 1.57  3486 | 53 |
| {BcHV1SLR.BI=TRUE, BcOLV16.BI=TRUE}  => {BcOLV1.BI.MOVA.=TRUE} | 0.13  1188 | 0.552  083 | 0.237  624 | 1.66  449 | 53 |
| {BcOLV1.BI.MOVA.=TRUE,  BcOLV16.BI=TRUE} => {BcMV3.BI=TRUE} | 0.15  3465 | 0.805  195 | 0.190  594 | 1.48  5382 | 62 |
| {BcMV3.BI=TRUE, BcOLV1.BI.MOVA.=TRUE}  => {BcOLV16.BI=TRUE} | 0.15  3465 | 0.626  263 | 0.245  05 | 1.50  6013 | 62 |
| {BcMV3.BI=TRUE, BcOLV16.BI=TRUE} =>  {BcOLV1.BI.MOVA.=TRUE} | 0.15  3465 | 0.574  074 | 0.267  327 | 1.73  079 | 62 |
| {BcOLV1.BI.MOVA.=TRUE,  BcOLV16.BI=TRUE} => {BcULV3=TRUE} | 0.13  3663 | 0.701  299 | 0.190  594 | 1.18  0519 | 54 |
| {BcOLV1.BI.MOVA.=TRUE, BcULV3=TRUE}  => {BcOLV16.BI=TRUE} | 0.13  3663 | 0.683  544 | 0.195  545 | 1.64  3761 | 54 |
| {BcOLV16.BI=TRUE, BcULV3=TRUE} =>  {BcOLV1.BI.MOVA.=TRUE} | 0.13  3663 | 0.477  876 | 0.279  703 | 1.44  0761 | 54 |
| {BcOLV1.BI.MOVA.=TRUE,  BcOLV16.BI=TRUE} => {BcHV1.BI=TRUE} | 0.13  6139 | 0.714  286 | 0.190  594 | 1.18  267 | 55 |
| {BcHV1.BI=TRUE, BcOLV1.BI.MOVA.=TRUE} | 0.13 | 0.639 | 0.212 | 1.53 | 55 |

| => {BcOLV16.BI=TRUE} | 6139 | 535 | 871 | 7929 |  |
| --- | --- | --- | --- | --- | --- |
| {BcHV1.BI=TRUE, BcOLV16.BI=TRUE} =>  {BcOLV1.BI.MOVA.=TRUE} | 0.13  6139 | 0.504  587 | 0.269  802 | 1.52  1293 | 55 |
| {BcOLV1.BI.MOVA.=TRUE,  BcOLV16.BI=TRUE} => {BcMV11=TRUE} | 0.14  3564 | 0.753  247 | 0.190  594 | 1.22  2135 | 58 |
| {BcMV11=TRUE, BcOLV1.BI.MOVA.=TRUE}  => {BcOLV16.BI=TRUE} | 0.14  3564 | 0.591  837 | 0.242  574 | 1.42  3226 | 58 |
| {BcMV11=TRUE, BcOLV16.BI=TRUE} =>  {BcOLV1.BI.MOVA.=TRUE} | 0.14  3564 | 0.522  523 | 0.274  752 | 1.57  5366 | 58 |
| {BcMV9.BI=TRUE, BcOLV1.BI.MOVA.=TRUE}  => {BcOLV16.BI=TRUE} | 0.13  8614 | 0.651  163 | 0.212  871 | 1.56  5891 | 56 |
| {BcMV9.BI=TRUE, BcOLV16.BI=TRUE} =>  {BcOLV1.BI.MOVA.=TRUE} | 0.13  8614 | 0.417  91 | 0.331  683 | 1.25  9969 | 56 |
| {BcOLV1.BI.MOVA.=TRUE, BcULV4=TRUE}  => {BcOLV16.BI=TRUE} | 0.14  604 | 0.595  96 | 0.245  05 | 1.43  3141 | 59 |
| {BcOLV16.BI=TRUE, BcULV4=TRUE} =>  {BcOLV1.BI.MOVA.=TRUE} | 0.14  604 | 0.430  657 | 0.339  109 | 1.29  8399 | 59 |
| {BcOLV1.BI.MOVA.=TRUE,  BcPV3.BI=FALSE} => {BcOLV16.BI=TRUE} | 0.10  1485 | 0.518  987 | 0.195  545 | 1.24  8041 | 41 |
| {BcOLV1.BI.MOVA.=TRUE,  BcRV1.BI=FALSE} => {BcOLV16.BI=TRUE} | 0.10  396 | 0.525 | 0.198  02 | 1.26  25 | 42 |
| {BcEV2.BI=FALSE, | 0.14 | 0.584 | 0.25 | 1.40 | 59 |

| BcOLV1.BI.MOVA.=TRUE} =>  {BcOLV16.BI=TRUE} | 604 | 158 |  | 4762 |  |
| --- | --- | --- | --- | --- | --- |
| {BcEV2.BI=FALSE, BcOLV16.BI=TRUE} =>  {BcOLV1.BI.MOVA.=TRUE} | 0.14  604 | 0.446  97 | 0.326  733 | 1.34  758 | 59 |
| {BcHV3.BI=FALSE, BcOLV1.BI.MOVA.=TRUE} =>  {BcOLV16.BI=TRUE} | 0.13  3663 | 0.586  957 | 0.227  723 | 1.41  1491 | 54 |
| {BcHV3.BI=FALSE, BcOLV16.BI=TRUE} =>  {BcOLV1.BI.MOVA.=TRUE} | 0.13  3663 | 0.415  385 | 0.321  782 | 1.25  2354 | 54 |
| {BcFV5.BI=FALSE, BcOLV1.BI.MOVA.=TRUE} =>  {BcOLV16.BI=TRUE} | 0.14  1089 | 0.587  629 | 0.240  099 | 1.41  3108 | 57 |
| {BcFV5.BI=FALSE, BcOLV16.BI=TRUE} =>  {BcOLV1.BI.MOVA.=TRUE} | 0.14  1089 | 0.438  462 | 0.321  782 | 1.32  1929 | 57 |
| {BcOLV16.BI=TRUE, BcULV1.BI=FALSE} =>  {BcOLV1.BI.MOVA.=TRUE} | 0.11  3861 | 0.422  018 | 0.269  802 | 1.27  2354 | 46 |
| {BcOLV1.BI.MOVA.=TRUE,  BcOLV16.BI=TRUE} => {BcMV1.BI=TRUE} | 0.17  0792 | 0.896  104 | 0.190  594 | 1.13  4878 | 69 |
| {BcMV1.BI=TRUE, BcOLV1.BI.MOVA.=TRUE}  => {BcOLV16.BI=TRUE} | 0.17  0792 | 0.579  832 | 0.294  554 | 1.39  4358 | 69 |
| {BcMV1.BI=TRUE, BcOLV16.BI=TRUE} =>  {BcOLV1.BI.MOVA.=TRUE} | 0.17  0792 | 0.507  353 | 0.336  634 | 1.52  9631 | 69 |

| {BcNSRV7.BI=FALSE, BcOLV1.BI.MOVA.=TRUE} =>  {BcOLV16.BI=TRUE} | 0.15  3465 | 0.579  439 | 0.264  851 | 1.39  3413 | 62 |
| --- | --- | --- | --- | --- | --- |
| {BcNSRV7.BI=FALSE, BcOLV16.BI=TRUE}  => {BcOLV1.BI.MOVA.=TRUE} | 0.15  3465 | 0.459  259 | 0.334  158 | 1.38  4632 | 62 |
| {BcOLV1.BI.MOVA.=TRUE,  BcVV3.BI=FALSE} => {BcOLV16.BI=TRUE} | 0.14  3564 | 0.591  837 | 0.242  574 | 1.42  3226 | 58 |
| {BcOLV16.BI=TRUE, BcVV3.BI=FALSE} =>  {BcOLV1.BI.MOVA.=TRUE} | 0.14  3564 | 0.426  471 | 0.336  634 | 1.28  5777 | 58 |
| {BcALV1.BI=FALSE, BcOLV1.BI.MOVA.=TRUE} =>  {BcOLV16.BI=TRUE} | 0.12  1287 | 0.538  462 | 0.225  248 | 1.29  4872 | 49 |
| {BcDMV1=FALSE, BcOLV1.BI.MOVA.=TRUE}  => {BcOLV16.BI=TRUE} | 0.15  8416 | 0.576  577 | 0.274  752 | 1.38  6529 | 64 |
| {BcDMV1=FALSE, BcOLV16.BI=TRUE} =>  {BcOLV1.BI.MOVA.=TRUE} | 0.15  8416 | 0.447  552 | 0.353  96 | 1.34  9337 | 64 |
| {BcFV9=FALSE, BcOLV1.BI.MOVA.=TRUE}  => {BcOLV16.BI=TRUE} | 0.15  5941 | 0.557  522 | 0.279  703 | 1.34  0708 | 63 |
| {BcFV9=FALSE, BcOLV16.BI=TRUE} =>  {BcOLV1.BI.MOVA.=TRUE} | 0.15  5941 | 0.434  483 | 0.358  911 | 1.30  9933 | 63 |
| {BcOLV1.BI.MOVA.=TRUE,  BcOLV4.BI=FALSE} => {BcOLV16.BI=TRUE} | 0.13  8614 | 0.528  302 | 0.262  376 | 1.27  044 | 56 |

| {BcOLV1.BI.MOVA.=TRUE, BcULV5=FALSE}  => {BcOLV16.BI=TRUE} | 0.14  3564 | 0.557  692 | 0.257  426 | 1.34  1117 | 58 |
| --- | --- | --- | --- | --- | --- |
| {BcOLV16.BI=TRUE, BcULV5=FALSE} =>  {BcOLV1.BI.MOVA.=TRUE} | 0.14  3564 | 0.408  451 | 0.351  485 | 1.23  1448 | 58 |
| {BcBNV5.BI=FALSE, BcOLV1.BI.MOVA.=TRUE} =>  {BcOLV16.BI=TRUE} | 0.15  8416 | 0.561  404 | 0.282  178 | 1.35  0042 | 64 |
| {BcBNV5.BI=FALSE, BcOLV16.BI=TRUE} =>  {BcOLV1.BI.MOVA.=TRUE} | 0.15  8416 | 0.423  841 | 0.373  762 | 1.27  7849 | 64 |
| {BcOLV1.BI.MOVA.=TRUE, BvF.BI=FALSE}  => {BcOLV16.BI=TRUE} | 0.18  8119 | 0.598  425 | 0.314  356 | 1.43  907 | 76 |
| {BcOLV16.BI=TRUE, BvF.BI=FALSE} =>  {BcOLV1.BI.MOVA.=TRUE} | 0.18  8119 | 0.481  013 | 0.391  089 | 1.45  0217 | 76 |
| {BcMCV5.BI=FALSE, BcOLV1.BI.MOVA.=TRUE} =>  {BcOLV16.BI=TRUE} | 0.15  3465 | 0.553  571 | 0.277  228 | 1.33  1207 | 62 |
| {BcMCV5.BI=FALSE, BcOLV16.BI=TRUE} =>  {BcOLV1.BI.MOVA.=TRUE} | 0.15  3465 | 0.418  919 | 0.366  337 | 1.26  3009 | 62 |
| {BcOLV1.BI.MOVA.=TRUE,  BcOLV13.BI=FALSE} => {BcOLV16.BI=TRUE} | 0.14  8515 | 0.535  714 | 0.277  228 | 1.28  8265 | 60 |
| {BcOLV13.BI=FALSE, BcOLV16.BI=TRUE} =>  {BcOLV1.BI.MOVA.=TRUE} | 0.14  8515 | 0.405  405 | 0.366  337 | 1.22  2267 | 60 |

| {BcOLV1.BI.MOVA.=TRUE,  BcOLV2.BI=FALSE} => {BcOLV16.BI=TRUE} | 0.14  604 | 0.526  786 | 0.277  228 | 1.26  6794 | 59 |
| --- | --- | --- | --- | --- | --- |
| {BcMCV3.BI=FALSE, BcOLV1.BI.MOVA.=TRUE} =>  {BcOLV16.BI=TRUE} | 0.14  8515 | 0.517  241 | 0.287  129 | 1.24  3842 | 60 |
| {BcMCV3.BI=FALSE, BcOLV16.BI=TRUE} =>  {BcOLV1.BI.MOVA.=TRUE} | 0.14  8515 | 0.413  793 | 0.358  911 | 1.24  7555 | 60 |
| {BcOLV1.BI.MOVA.=TRUE,  BcPV4.BI=FALSE} => {BcOLV16.BI=TRUE} | 0.17  5743 | 0.554  688 | 0.316  832 | 1.33  3891 | 71 |
| {BcOLV16.BI=TRUE, BcPV4.BI=FALSE} =>  {BcOLV1.BI.MOVA.=TRUE} | 0.17  5743 | 0.464  052 | 0.378  713 | 1.39  9083 | 71 |
| {BcEV6=FALSE, BcOLV1.BI.MOVA.=TRUE}  => {BcOLV16.BI=TRUE} | 0.17  8218 | 0.580  645 | 0.306  931 | 1.39  6313 | 72 |
| {BcEV6=FALSE, BcOLV16.BI=TRUE} =>  {BcOLV1.BI.MOVA.=TRUE} | 0.17  8218 | 0.455  696 | 0.391  089 | 1.37  389 | 72 |
| {BcOLV1.BI.MOVA.=TRUE,  BcOLV17.BI=FALSE} => {BcOLV16.BI=TRUE} | 0.16  8317 | 0.561  983 | 0.299  505 | 1.35  1436 | 68 |
| {BcOLV16.BI=TRUE, BcOLV17.BI=FALSE} =>  {BcOLV1.BI.MOVA.=TRUE} | 0.16  8317 | 0.435  897 | 0.386  139 | 1.31  4198 | 68 |
| {BcOLV1.BI.MOVA.=TRUE,  BcOLV15.BI=FALSE} => {BcOLV16.BI=TRUE} | 0.18  5644 | 0.576  923 | 0.321  782 | 1.38  7363 | 75 |
| {BcOLV15.BI=FALSE, BcOLV16.BI=TRUE} => | 0.18 | 0.460 | 0.403 | 1.38 | 75 |

| {BcOLV1.BI.MOVA.=TRUE} | 5644 | 123 | 465 | 7236 |  |
| --- | --- | --- | --- | --- | --- |
| {BcEV5=FALSE, BcOLV1.BI.MOVA.=TRUE}  => {BcOLV16.BI=TRUE} | 0.16  8317 | 0.566  667 | 0.297  03 | 1.36  2698 | 68 |
| {BcEV5=FALSE, BcOLV16.BI=TRUE} =>  {BcOLV1.BI.MOVA.=TRUE} | 0.16  8317 | 0.427  673 | 0.393  564 | 1.28  9402 | 68 |
| {BcHV1SLR.BI=FALSE, BcOLV1.BI.MOVA.=TRUE} =>  {BcMV4.BI=TRUE} | 0.07  4257 | 0.566  038 | 0.131  188 | 1.27  7538 | 30 |
| {BcMV4.BI=TRUE, BcOLV1.BI.MOVA.=TRUE} | 0.15 | 0.826 | 0.185 | 1.78 |  |
|  |  |  |  |  | 62 |
| => {BcMV2.BI=TRUE} | 3465 | 667 | 644 | 5954 |  |
| {BcMV2.BI=TRUE, BcOLV1.BI.MOVA.=TRUE} | 0.15 | 0.613 |  | 1.38 |  |
|  |  |  | 0.25 |  | 62 |
| => {BcMV4.BI=TRUE} | 3465 | 861 |  | 5475 |  |
| {BcMV2.BI=TRUE, BcMV4.BI=TRUE} => | 0.15 | 0.558 | 0.274 | 1.68 |  |
|  |  |  |  |  | 62 |
| {BcOLV1.BI.MOVA.=TRUE} | 3465 | 559 | 752 | 4012 |  |
| {BcMV4.BI=TRUE, BcOLV1.BI.MOVA.=TRUE} | 0.13 | 0.733 | 0.185 | 1.41 |  |
|  |  |  |  |  | 55 |
| => {BcOLV18=TRUE} | 6139 | 333 | 644 | 7544 |  |
| {BcOLV1.BI.MOVA.=TRUE, BcOLV18=TRUE} | 0.13 | 0.591 | 0.230 | 1.33 |  |
|  |  |  |  |  | 55 |
| => {BcMV4.BI=TRUE} | 6139 | 398 | 198 | 4775 |  |
| {BcMV4.BI=TRUE, BcOLV18=TRUE} => | 0.13 | 0.491 | 0.277 | 1.48 |  |
|  |  |  |  |  | 55 |
| {BcOLV1.BI.MOVA.=TRUE} | 6139 | 071 | 228 | 0544 |  |
| {BcHV1SLR.BI=TRUE, | 0.11 | 0.555 | 0.200 | 1.25 |  |
|  |  |  |  |  | 45 |
| BcOLV1.BI.MOVA.=TRUE} => | 1386 | 556 | 495 | 388 |  |

| {BcMV4.BI=TRUE} |  |  |  |  |  |
| --- | --- | --- | --- | --- | --- |
| {BcHV1SLR.BI=TRUE, BcMV4.BI=TRUE} =>  {BcOLV1.BI.MOVA.=TRUE} | 0.11  1386 | 0.424  528 | 0.262  376 | 1.27  9921 | 45 |
| {BcMV4.BI=TRUE, BcOLV1.BI.MOVA.=TRUE}  => {BcMV3.BI=TRUE} | 0.15  099 | 0.813  333 | 0.185  644 | 1.50  0396 | 61 |
| {BcMV3.BI=TRUE, BcOLV1.BI.MOVA.=TRUE}  => {BcMV4.BI=TRUE} | 0.15  099 | 0.616  162 | 0.245  05 | 1.39  0666 | 61 |
| {BcMV3.BI=TRUE, BcMV4.BI=TRUE} =>  {BcOLV1.BI.MOVA.=TRUE} | 0.15  099 | 0.512  605 | 0.294  554 | 1.54  5466 | 61 |
| {BcOLV1.BI.MOVA.=TRUE, BcULV3=TRUE}  => {BcMV4.BI=TRUE} | 0.12  1287 | 0.620  253 | 0.195  545 | 1.39  9901 | 49 |
| {BcHV1.BI=TRUE, BcOLV1.BI.MOVA.=TRUE}  => {BcMV4.BI=TRUE} | 0.12  6238 | 0.593  023 | 0.212  871 | 1.33  8444 | 51 |
| {BcHV1.BI=TRUE, BcMV4.BI=TRUE} =>  {BcOLV1.BI.MOVA.=TRUE} | 0.12  6238 | 0.421  488 | 0.299  505 | 1.27  0754 | 51 |
| {BcMV4.BI=TRUE, BcOLV1.BI.MOVA.=TRUE}  => {BcMV11=TRUE} | 0.16  3366 | 0.88 | 0.185  644 | 1.42  7791 | 66 |
| {BcMV11=TRUE, BcOLV1.BI.MOVA.=TRUE}  => {BcMV4.BI=TRUE} | 0.16  3366 | 0.673  469 | 0.242  574 | 1.52  0009 | 66 |
| {BcMV11=TRUE, BcMV4.BI=TRUE} =>  {BcOLV1.BI.MOVA.=TRUE} | 0.16  3366 | 0.481  752 | 0.339  109 | 1.45  2446 | 66 |
| {BcMV9.BI=TRUE, BcOLV1.BI.MOVA.=TRUE} | 0.13 | 0.627 | 0.212 | 1.41 | 54 |

| => {BcMV4.BI=TRUE} | 3663 | 907 | 871 | 7176 |  |
| --- | --- | --- | --- | --- | --- |
| {BcMV4.BI=TRUE, BcOLV1.BI.MOVA.=TRUE}  => {BcULV4=TRUE} | 0.14  604 | 0.786  667 | 0.185  644 | 1.13  9116 | 59 |
| {BcOLV1.BI.MOVA.=TRUE, BcULV4=TRUE}  => {BcMV4.BI=TRUE} | 0.14  604 | 0.595  96 | 0.245  05 | 1.34  5071 | 59 |
| {BcMV4.BI=TRUE, BcULV4=TRUE} =>  {BcOLV1.BI.MOVA.=TRUE} | 0.14  604 | 0.450  382 | 0.324  257 | 1.35  7867 | 59 |
| {BcEV2.BI=FALSE, BcOLV1.BI.MOVA.=TRUE} =>  {BcMV4.BI=TRUE} | 0.13  8614 | 0.554  455 | 0.25 | 1.25  1397 | 56 |
| {BcEV2.BI=FALSE, BcMV4.BI=TRUE} =>  {BcOLV1.BI.MOVA.=TRUE} | 0.13  8614 | 0.440  945 | 0.314  356 | 1.32  9416 | 56 |
| {BcFV5.BI=FALSE, BcOLV1.BI.MOVA.=TRUE} =>  {BcMV4.BI=TRUE} | 0.13  3663 | 0.556  701 | 0.240  099 | 1.25  6465 | 54 |
| {BcFV5.BI=FALSE, BcMV4.BI=TRUE} =>  {BcOLV1.BI.MOVA.=TRUE} | 0.13  3663 | 0.415  385 | 0.321  782 | 1.25  2354 | 54 |
| {BcOLV1.BI.MOVA.=TRUE,  BcULV1.BI=FALSE} => {BcMV4.BI=TRUE} | 0.12  8713 | 0.571  429 | 0.225  248 | 1.28  9705 | 52 |
| {BcMV4.BI=TRUE, BcOLV1.BI.MOVA.=TRUE}  => {BcMV1.BI=TRUE} | 0.16  5842 | 0.893  333 | 0.185  644 | 1.13  1369 | 67 |
| {BcMV1.BI=TRUE, BcOLV1.BI.MOVA.=TRUE} | 0.16 | 0.563 | 0.294 | 1.27 | 67 |

| => {BcMV4.BI=TRUE} | 5842 | 025 | 554 | 0738 |  |
| --- | --- | --- | --- | --- | --- |
| {BcMV1.BI=TRUE, BcMV4.BI=TRUE} =>  {BcOLV1.BI.MOVA.=TRUE} | 0.16  5842 | 0.440  789 | 0.376  238 | 1.32  8947 | 67 |
| {BcMV12=FALSE, BcOLV1.BI.MOVA.=TRUE}  => {BcMV4.BI=TRUE} | 0.14  1089 | 0.612  903 | 0.230  198 | 1.38  3312 | 57 |
| {BcMV4.BI=TRUE, BcNSRV7.BI=FALSE} =>  {BcOLV1.BI.MOVA.=TRUE} | 0.13  8614 | 0.411  765 | 0.336  634 | 1.24  144 | 56 |
| {BcFV9=FALSE, BcMV4.BI=TRUE} => | 0.14 | 0.409 | 0.356 | 1.23 |  |
|  |  |  |  |  | 59 |
| {BcOLV1.BI.MOVA.=TRUE} | 604 | 722 | 436 | 5282 |  |
| {BcOLV1.BI.MOVA.=TRUE, BvF.BI=FALSE} | 0.18 | 0.574 | 0.314 | 1.29 |  |
|  |  |  |  |  | 73 |
| => {BcMV4.BI=TRUE} | 0693 | 803 | 356 | 7321 |  |
| {BcMV4.BI=TRUE, BvF.BI=FALSE} => | 0.18 | 0.445 | 0.405 | 1.34 |  |
|  |  |  |  |  | 73 |
| {BcOLV1.BI.MOVA.=TRUE} | 0693 | 122 | 941 | 2009 |  |
| {BcOLV1.BI.MOVA.=TRUE, | 0.15 | 0.544 | 0.277 | 1.22 |  |
|  |  |  |  |  | 61 |
| BcOLV2.BI=FALSE} => {BcMV4.BI=TRUE} | 099 | 643 | 228 | 925 |  |
| {BcMCV3.BI=FALSE, |  |  |  |  |  |
|  | 0.16 | 0.577 | 0.287 | 1.30 |  |
| BcOLV1.BI.MOVA.=TRUE} => |  |  |  |  | 67 |
|  | 5842 | 586 | 129 | 3602 |  |
| {BcMV4.BI=TRUE} |  |  |  |  |  |
| {BcMCV3.BI=FALSE, BcMV4.BI=TRUE} => | 0.16 | 0.403 | 0.410 | 1.21 |  |
|  |  |  |  |  | 67 |
| {BcOLV1.BI.MOVA.=TRUE} | 5842 | 614 | 891 | 6867 |  |
| {BcOLV1.BI.MOVA.=TRUE, | 0.18 | 0.570 | 0.316 | 1.28 |  |
|  |  |  |  |  | 73 |
| BcPV4.BI=FALSE} => {BcMV4.BI=TRUE} | 0693 | 313 | 832 | 7186 |  |

| {BcMV4.BI=TRUE, BcPV4.BI=FALSE} =>  {BcOLV1.BI.MOVA.=TRUE} | 0.18  0693 | 0.431  953 | 0.418  317 | 1.30  2305 | 73 |
| --- | --- | --- | --- | --- | --- |
| {BcEV6=FALSE, BcOLV1.BI.MOVA.=TRUE}  => {BcMV4.BI=TRUE} | 0.17  3267 | 0.564  516 | 0.306  931 | 1.27  4103 | 70 |
| {BcEV6=FALSE, BcMV4.BI=TRUE} =>  {BcOLV1.BI.MOVA.=TRUE} | 0.17  3267 | 0.411  765 | 0.420  792 | 1.24  144 | 70 |
| {BcOLV1.BI.MOVA.=TRUE,  BcOLV15.BI=FALSE} => {BcMV4.BI=TRUE} | 0.17  8218 | 0.553  846 | 0.321  782 | 1.25  0021 | 72 |
| {BcMV4.BI=TRUE, BcOLV15.BI=FALSE} =>  {BcOLV1.BI.MOVA.=TRUE} | 0.17  8218 | 0.431  138 | 0.413  366 | 1.29  9848 | 72 |
| {BcHV1SLR.BI=FALSE, BcOLV1.BI.MOVA.=TRUE} =>  {BcMV2.BI=TRUE} | 0.09  1584 | 0.698  113 | 0.131  188 | 1.50  8223 | 37 |
| {BcHV1SLR.BI=FALSE, BcMV2.BI=TRUE} =>  {BcOLV1.BI.MOVA.=TRUE} | 0.09  1584 | 0.480  519 | 0.190  594 | 1.44  873 | 37 |
| {BcMV6.BI=FALSE, BcOLV1.BI.MOVA.=TRUE} =>  {BcHV1SLR.BI=FALSE} | 0.10  1485 | 0.602  941 | 0.168  317 | 1.31  6693 | 41 |
| {BcHV1SLR.BI=FALSE, BcOLV1.BI.MOVA.=TRUE} =>  {BcMV1.BI=TRUE} | 0.12  1287 | 0.924  528 | 0.131  188 | 1.17  0876 | 49 |
| {BcMV2.BI=TRUE, BcOLV1.BI.MOVA.=TRUE} | 0.17 | 0.712 | 0.25 | 1.37 | 72 |

| => {BcOLV18=TRUE} | 8218 | 871 |  | 799 |  |
| --- | --- | --- | --- | --- | --- |
| {BcOLV1.BI.MOVA.=TRUE, BcOLV18=TRUE} | 0.17 | 0.774 | 0.230 | 1.67 |  |
|  |  |  |  |  | 72 |
| => {BcMV2.BI=TRUE} | 8218 | 194 | 198 | 2589 |  |
| {BcMV2.BI=TRUE, BcOLV18=TRUE} => | 0.17 | 0.605 | 0.294 | 1.82 |  |
|  |  |  |  |  | 72 |
| {BcOLV1.BI.MOVA.=TRUE} | 8218 | 042 | 554 | 4157 |  |
| {BcHV1SLR.BI=TRUE, |  |  |  |  |  |
|  | 0.15 | 0.790 | 0.200 | 1.70 |  |
| BcOLV1.BI.MOVA.=TRUE} => |  |  |  |  | 64 |
|  | 8416 | 123 | 495 | 7005 |  |
| {BcMV2.BI=TRUE} |  |  |  |  |  |
| {BcHV1SLR.BI=TRUE, BcMV2.BI=TRUE} => | 0.15 | 0.581 | 0.272 | 1.75 |  |
|  |  |  |  |  | 64 |
| {BcOLV1.BI.MOVA.=TRUE} | 8416 | 818 | 277 | 4138 |  |
| {BcMV2.BI=TRUE, BcOLV1.BI.MOVA.=TRUE} | 0.20 | 0.811 |  | 1.49 |  |
|  |  |  | 0.25 |  | 82 |
| => {BcMV3.BI=TRUE} | 297 | 881 |  | 7717 |  |
| {BcMV3.BI=TRUE, BcOLV1.BI.MOVA.=TRUE} | 0.20 | 0.828 | 0.245 | 1.78 |  |
|  |  |  |  |  | 82 |
| => {BcMV2.BI=TRUE} | 297 | 283 | 05 | 9445 |  |
| {BcMV2.BI=TRUE, BcMV3.BI=TRUE} => | 0.20 | 0.645 | 0.314 | 1.94 |  |
|  |  |  |  |  | 82 |
| {BcOLV1.BI.MOVA.=TRUE} | 297 | 669 | 356 | 6645 |  |
| {BcMV4.BI=FALSE, |  |  |  |  |  |
|  | 0.09 | 0.661 | 0.146 | 1.42 |  |
| BcOLV1.BI.MOVA.=TRUE} => |  |  |  |  | 39 |
|  | 6535 | 017 | 04 | 8079 |  |
| {BcMV2.BI=TRUE} |  |  |  |  |  |
| {BcMV2.BI=TRUE, BcMV4.BI=FALSE} => | 0.09 | 0.513 | 0.188 | 1.54 |  |
|  |  |  |  |  | 39 |
| {BcOLV1.BI.MOVA.=TRUE} | 6535 | 158 | 119 | 7133 |  |
| {BcOLV1.BI.MOVA.=TRUE, | 0.09 | 0.666 | 0.141 | 1.44 | 38 |

| BcOLV16.BI=FALSE} => {BcMV2.BI=TRUE} | 4059 | 667 | 089 | 0285 |  |
| --- | --- | --- | --- | --- | --- |
| {BcMV2.BI=TRUE, BcOLV16.BI=FALSE} =>  {BcOLV1.BI.MOVA.=TRUE} | 0.09  4059 | 0.447  059 | 0.210  396 | 1.34  7849 | 38 |
| {BcOLV1.BI.MOVA.=TRUE, BcULV3=TRUE}  => {BcMV2.BI=TRUE} | 0.14  8515 | 0.759  494 | 0.195  545 | 1.64  0831 | 60 |
| {BcMV2.BI=TRUE, BcULV3=TRUE} =>  {BcOLV1.BI.MOVA.=TRUE} | 0.14  8515 | 0.521  739 | 0.284  653 | 1.57  3005 | 60 |
| {BcHV1.BI=TRUE, BcOLV1.BI.MOVA.=TRUE}  => {BcMV2.BI=TRUE} | 0.15  8416 | 0.744  186 | 0.212  871 | 1.60  776 | 64 |
| {BcHV1.BI=TRUE, BcMV2.BI=TRUE} =>  {BcOLV1.BI.MOVA.=TRUE} | 0.15  8416 | 0.542  373 | 0.292  079 | 1.63  5214 | 64 |
| {BcMV2.BI=TRUE, BcOLV1.BI.MOVA.=TRUE}  => {BcMV11=TRUE} | 0.20  297 | 0.811  881 | 0.25 | 1.31  7269 | 82 |
| {BcMV11=TRUE, BcOLV1.BI.MOVA.=TRUE}  => {BcMV2.BI=TRUE} | 0.20  297 | 0.836  735 | 0.242  574 | 1.80  7705 | 82 |
| {BcMV11=TRUE, BcMV2.BI=TRUE} =>  {BcOLV1.BI.MOVA.=TRUE} | 0.20  297 | 0.594  203 | 0.341  584 | 1.79  1477 | 82 |
| {BcMV9.BI=TRUE, BcOLV1.BI.MOVA.=TRUE}  => {BcMV2.BI=TRUE} | 0.16  5842 | 0.779  07 | 0.212  871 | 1.68  3124 | 67 |
| {BcMV2.BI=TRUE, BcMV9.BI=TRUE} =>  {BcOLV1.BI.MOVA.=TRUE} | 0.16  5842 | 0.515  385 | 0.321  782 | 1.55  3846 | 67 |
| {BcOLV1.BI.MOVA.=TRUE, BcULV4=TRUE} | 0.18 | 0.757 | 0.245 | 1.63 | 75 |

| => {BcMV2.BI=TRUE} | 5644 | 576 | 05 | 6688 |  |
| --- | --- | --- | --- | --- | --- |
| {BcMV2.BI=TRUE, BcULV4=TRUE} => | 0.18 | 0.543 | 0.341 | 1.63 |  |
|  |  |  |  |  | 75 |
| {BcOLV1.BI.MOVA.=TRUE} | 5644 | 478 | 584 | 8546 |  |
| {BcMV6.BI=FALSE, |  |  |  |  |  |
|  | 0.10 | 0.632 | 0.168 | 1.36 |  |
| BcOLV1.BI.MOVA.=TRUE} => |  |  |  |  | 43 |
|  | 6436 | 353 | 317 | 6153 |  |
| {BcMV2.BI=TRUE} |  |  |  |  |  |
| {BcMV2.BI=TRUE, BcMV6.BI=FALSE} => | 0.10 | 0.417 | 0.254 | 1.25 |  |
|  |  |  |  |  | 43 |
| {BcOLV1.BI.MOVA.=TRUE} | 6436 | 476 | 95 | 8658 |  |
| {BcOLV1.BI.MOVA.=TRUE, | 0.14 | 0.734 | 0.195 | 1.58 |  |
|  |  |  |  |  | 58 |
| BcPV3.BI=FALSE} => {BcMV2.BI=TRUE} | 3564 | 177 | 545 | 6137 |  |
| {BcMV2.BI=TRUE, BcPV3.BI=FALSE} => | 0.14 | 0.504 | 0.284 | 1.52 |  |
|  |  |  |  |  | 58 |
| {BcOLV1.BI.MOVA.=TRUE} | 3564 | 348 | 653 | 0571 |  |
| {BcOLV1.BI.MOVA.=TRUE, | 0.14 |  | 0.198 | 1.56 |  |
|  |  | 0.725 |  |  | 58 |
| BcRV1.BI=FALSE} => {BcMV2.BI=TRUE} | 3564 |  | 02 | 631 |  |
| {BcMV2.BI=TRUE, BcRV1.BI=FALSE} => | 0.14 | 0.504 | 0.284 | 1.52 |  |
|  |  |  |  |  | 58 |
| {BcOLV1.BI.MOVA.=TRUE} | 3564 | 348 | 653 | 0571 |  |
| {BcEV2.BI=FALSE, |  |  |  |  |  |
|  | 0.17 | 0.702 |  | 1.51 |  |
| BcOLV1.BI.MOVA.=TRUE} => |  |  | 0.25 |  | 71 |
|  | 5743 | 97 |  | 8717 |  |
| {BcMV2.BI=TRUE} |  |  |  |  |  |
| {BcEV2.BI=FALSE, BcMV2.BI=TRUE} => | 0.17 | 0.550 | 0.319 | 1.65 |  |
|  |  |  |  |  | 71 |
| {BcOLV1.BI.MOVA.=TRUE} | 5743 | 388 | 307 | 9378 |  |
| {BcHV3.BI=FALSE, | 0.16 | 0.728 | 0.227 | 1.57 | 67 |

| BcOLV1.BI.MOVA.=TRUE} =>  {BcMV2.BI=TRUE} | 5842 | 261 | 723 | 3355 |  |
| --- | --- | --- | --- | --- | --- |
| {BcHV3.BI=FALSE, BcMV2.BI=TRUE} => | 0.16 | 0.503 | 0.329 | 1.51 |  |
|  |  |  |  |  | 67 |
| {BcOLV1.BI.MOVA.=TRUE} | 5842 | 759 | 208 | 8797 |  |
| {BcFV5.BI=FALSE, |  |  |  |  |  |
|  | 0.17 | 0.731 | 0.240 | 1.58 |  |
| BcOLV1.BI.MOVA.=TRUE} => |  |  |  |  | 71 |
|  | 5743 | 959 | 099 | 1344 |  |
| {BcMV2.BI=TRUE} |  |  |  |  |  |
| {BcFV5.BI=FALSE, BcMV2.BI=TRUE} => | 0.17 | 0.510 | 0.344 | 1.53 |  |
|  |  |  |  |  | 71 |
| {BcOLV1.BI.MOVA.=TRUE} | 5743 | 791 | 059 | 9998 |  |
| {BcOLV1.BI.MOVA.=TRUE, | 0.15 | 0.703 | 0.225 | 1.51 |  |
|  |  |  |  |  | 64 |
| BcULV1.BI=FALSE} => {BcMV2.BI=TRUE} | 8416 | 297 | 248 | 9422 |  |
| {BcMV2.BI=TRUE, BcULV1.BI=FALSE} => | 0.15 | 0.488 | 0.324 | 1.47 |  |
|  |  |  |  |  | 64 |
| {BcOLV1.BI.MOVA.=TRUE} | 8416 | 55 | 257 | 2941 |  |
| {BcMV2.BI=TRUE, BcOLV1.BI.MOVA.=TRUE} | 0.23 | 0.920 |  | 1.16 |  |
|  |  |  | 0.25 |  | 93 |
| => {BcMV1.BI=TRUE} | 0198 | 792 |  | 6144 |  |
| {BcMV1.BI=TRUE, BcOLV1.BI.MOVA.=TRUE} | 0.23 | 0.781 | 0.294 | 1.68 |  |
|  |  |  |  |  | 93 |
| => {BcMV2.BI=TRUE} | 0198 | 513 | 554 | 8402 |  |
| {BcMV1.BI=TRUE, BcMV2.BI=TRUE} => | 0.23 | 0.563 | 0.408 | 1.69 |  |
|  |  |  |  |  | 93 |
| {BcOLV1.BI.MOVA.=TRUE} | 0198 | 636 | 416 | 9322 |  |
| {BcMV12=FALSE, BcOLV1.BI.MOVA.=TRUE} | 0.16 | 0.698 | 0.230 | 1.50 |  |
|  |  |  |  |  | 65 |
| => {BcMV2.BI=TRUE} | 0891 | 925 | 198 | 9976 |  |
| {BcMV12=FALSE, BcMV2.BI=TRUE} => | 0.16 | 0.471 | 0.341 | 1.42 | 65 |

| {BcOLV1.BI.MOVA.=TRUE} | 0891 | 014 | 584 | 0074 |  |
| --- | --- | --- | --- | --- | --- |
| {BcNSRV7.BI=FALSE, |  |  |  |  |  |
|  | 0.19 | 0.728 | 0.264 | 1.57 |  |
| BcOLV1.BI.MOVA.=TRUE} => |  |  |  |  | 78 |
|  | 3069 | 972 | 851 | 4891 |  |
| {BcMV2.BI=TRUE} |  |  |  |  |  |
| {BcMV2.BI=TRUE, BcNSRV7.BI=FALSE} => | 0.19 | 0.534 | 0.361 | 1.61 |  |
|  |  |  |  |  | 78 |
| {BcOLV1.BI.MOVA.=TRUE} | 3069 | 247 | 386 | 0714 |  |
| {BcOLV1.BI.MOVA.=TRUE, | 0.18 | 0.744 | 0.242 | 1.60 |  |
|  |  |  |  |  | 73 |
| BcVV3.BI=FALSE} => {BcMV2.BI=TRUE} | 0693 | 898 | 574 | 9298 |  |
| {BcMV2.BI=TRUE, BcVV3.BI=FALSE} => | 0.18 | 0.514 | 0.351 | 1.54 |  |
|  |  |  |  |  | 73 |
| {BcOLV1.BI.MOVA.=TRUE} | 0693 | 085 | 485 | 9926 |  |
| {BcOLV1.BI.MOVA.=TRUE, | 0.18 | 0.760 | 0.237 | 1.64 |  |
|  |  |  |  |  | 73 |
| BcOLV7.BI=FALSE} => {BcMV2.BI=TRUE} | 0693 | 417 | 624 | 2825 |  |
| {BcMV2.BI=TRUE, BcOLV7.BI=FALSE} => | 0.18 | 0.503 | 0.358 | 1.51 |  |
|  |  |  |  |  | 73 |
| {BcOLV1.BI.MOVA.=TRUE} | 0693 | 448 | 911 | 7859 |  |
| {BcALV1.BI=FALSE, |  |  |  |  |  |
|  | 0.15 | 0.681 | 0.225 | 1.47 |  |
| BcOLV1.BI.MOVA.=TRUE} => |  |  |  |  | 62 |
|  | 3465 | 319 | 248 | 194 |  |
| {BcMV2.BI=TRUE} |  |  |  |  |  |
| {BcALV1.BI=FALSE, BcMV2.BI=TRUE} => | 0.15 | 0.473 | 0.324 | 1.42 |  |
|  |  |  |  |  | 62 |
| {BcOLV1.BI.MOVA.=TRUE} | 3465 | 282 | 257 | 6911 |  |
| {BcDMV1=FALSE, BcOLV1.BI.MOVA.=TRUE} | 0.20 | 0.747 | 0.274 | 1.61 |  |
|  |  |  |  |  | 83 |
| => {BcMV2.BI=TRUE} | 5446 | 748 | 752 | 5455 |  |
| {BcDMV1=FALSE, BcMV2.BI=TRUE} => | 0.20 | 0.518 | 0.396 | 1.56 | 83 |

| {BcOLV1.BI.MOVA.=TRUE} | 5446 | 75 | 04 | 3993 |  |
| --- | --- | --- | --- | --- | --- |
| {BcFV9=FALSE, BcOLV1.BI.MOVA.=TRUE} | 0.20 | 0.743 | 0.279 | 1.60 |  |
|  |  |  |  |  | 84 |
| => {BcMV2.BI=TRUE} | 7921 | 363 | 703 | 5982 |  |
| {BcFV9=FALSE, BcMV2.BI=TRUE} => | 0.20 | 0.518 | 0.400 | 1.56 |  |
|  |  |  |  |  | 84 |
| {BcOLV1.BI.MOVA.=TRUE} | 7921 | 519 | 99 | 3295 |  |
| {BcOLV1.BI.MOVA.=TRUE, | 0.18 | 0.707 | 0.262 | 1.52 |  |
|  |  |  |  |  | 75 |
| BcOLV4.BI=FALSE} => {BcMV2.BI=TRUE} | 5644 | 547 | 376 | 8605 |  |
| {BcMV2.BI=TRUE, BcOLV4.BI=FALSE} => | 0.18 | 0.493 | 0.376 | 1.48 |  |
|  |  |  |  |  | 75 |
| {BcOLV1.BI.MOVA.=TRUE} | 5644 | 421 | 238 | 7628 |  |
| {BcOLV1.BI.MOVA.=TRUE, BcULV5=FALSE} | 0.18 | 0.730 | 0.257 | 1.57 |  |
|  |  |  |  |  | 76 |
| => {BcMV2.BI=TRUE} | 8119 | 769 | 426 | 8774 |  |
| {BcMV2.BI=TRUE, BcULV5=FALSE} => | 0.18 |  | 0.376 | 1.50 |  |
|  |  | 0.5 |  |  | 76 |
| {BcOLV1.BI.MOVA.=TRUE} | 8119 |  | 238 | 7463 |  |
| {BcBNV5.BI=FALSE, |  |  |  |  |  |
|  | 0.21 | 0.745 | 0.282 | 1.61 |  |
| BcOLV1.BI.MOVA.=TRUE} => |  |  |  |  | 85 |
|  | 0396 | 614 | 178 | 0845 |  |
| {BcMV2.BI=TRUE} |  |  |  |  |  |
| {BcBNV5.BI=FALSE, BcMV2.BI=TRUE} => | 0.21 | 0.515 | 0.408 | 1.55 |  |
|  |  |  |  |  | 85 |
| {BcOLV1.BI.MOVA.=TRUE} | 0396 | 152 | 416 | 3143 |  |
| {BcOLV1.BI.MOVA.=TRUE, BvF.BI=FALSE} | 0.23 | 0.740 | 0.314 | 1.59 |  |
|  |  |  |  |  | 94 |
| => {BcMV2.BI=TRUE} | 2673 | 157 | 356 | 9057 |  |
| {BcMV2.BI=TRUE, BvF.BI=FALSE} => | 0.23 | 0.537 | 0.433 | 1.61 |  |
|  |  |  |  |  | 94 |
| {BcOLV1.BI.MOVA.=TRUE} | 2673 | 143 | 168 | 9446 |  |

| {BcMCV5.BI=FALSE, BcOLV1.BI.MOVA.=TRUE} =>  {BcMV2.BI=TRUE} | 0.20  297 | 0.732  143 | 0.277  228 | 1.58  1742 | 82 |
| --- | --- | --- | --- | --- | --- |
| {BcMCV5.BI=FALSE, BcMV2.BI=TRUE} =>  {BcOLV1.BI.MOVA.=TRUE} | 0.20  297 | 0.506  173 | 0.400  99 | 1.52  6073 | 82 |
| {BcOLV1.BI.MOVA.=TRUE,  BcOLV13.BI=FALSE} => {BcMV2.BI=TRUE} | 0.20  5446 | 0.741  071 | 0.277  228 | 1.60  1031 | 83 |
| {BcMV2.BI=TRUE, BcOLV13.BI=FALSE} =>  {BcOLV1.BI.MOVA.=TRUE} | 0.20  5446 | 0.503  03 | 0.408  416 | 1.51  6599 | 83 |
| {BcOLV1.BI.MOVA.=TRUE,  BcOLV2.BI=FALSE} => {BcMV2.BI=TRUE} | 0.21  0396 | 0.758  929 | 0.277  228 | 1.63  961 | 85 |
| {BcMV2.BI=TRUE, BcOLV2.BI=FALSE} =>  {BcOLV1.BI.MOVA.=TRUE} | 0.21  0396 | 0.5 | 0.420  792 | 1.50  7463 | 85 |
| {BcMCV3.BI=FALSE, BcOLV1.BI.MOVA.=TRUE} =>  {BcMV2.BI=TRUE} | 0.20  7921 | 0.724  138 | 0.287  129 | 1.56  4448 | 84 |
| {BcMCV3.BI=FALSE, BcMV2.BI=TRUE} =>  {BcOLV1.BI.MOVA.=TRUE} | 0.20  7921 | 0.509  091 | 0.408  416 | 1.53  4871 | 84 |
| {BcOLV1.BI.MOVA.=TRUE,  BcPV4.BI=FALSE} => {BcMV2.BI=TRUE} | 0.23  7624 | 0.75 | 0.316  832 | 1.62  0321 | 96 |
| {BcMV2.BI=TRUE, BcPV4.BI=FALSE} =>  {BcOLV1.BI.MOVA.=TRUE} | 0.23  7624 | 0.554  913 | 0.428  218 | 1.67  3022 | 96 |

| {BcEV6=FALSE, BcOLV1.BI.MOVA.=TRUE}  => {BcMV2.BI=TRUE} | 0.23  0198 | 0.75 | 0.306  931 | 1.62  0321 | 93 |
| --- | --- | --- | --- | --- | --- |
| {BcEV6=FALSE, BcMV2.BI=TRUE} =>  {BcOLV1.BI.MOVA.=TRUE} | 0.23  0198 | 0.531  429 | 0.433  168 | 1.60  2217 | 93 |
| {BcOLV1.BI.MOVA.=TRUE,  BcOLV17.BI=FALSE} => {BcMV2.BI=TRUE} | 0.22  2772 | 0.743  802 | 0.299  505 | 1.60  693 | 90 |
| {BcMV2.BI=TRUE, BcOLV17.BI=FALSE} =>  {BcOLV1.BI.MOVA.=TRUE} | 0.22  2772 | 0.523  256 | 0.425  743 | 1.57  7577 | 90 |
| {BcOLV1.BI.MOVA.=TRUE,  BcOLV15.BI=FALSE} => {BcMV2.BI=TRUE} | 0.24  2574 | 0.753  846 | 0.321  782 | 1.62  863 | 98 |
| {BcMV2.BI=TRUE, BcOLV15.BI=FALSE} =>  {BcOLV1.BI.MOVA.=TRUE} | 0.24  2574 | 0.535  519 | 0.452  97 | 1.61  455 | 98 |
| {BcEV5=FALSE, BcOLV1.BI.MOVA.=TRUE}  => {BcMV2.BI=TRUE} | 0.21  7822 | 0.733  333 | 0.297  03 | 1.58  4314 | 88 |
| {BcEV5=FALSE, BcMV2.BI=TRUE} =>  {BcOLV1.BI.MOVA.=TRUE} | 0.21  7822 | 0.514  62 | 0.423  267 | 1.55  1541 | 88 |
| {BcOLV1.BI.MOVA.=TRUE,  BcOLV18=FALSE} => {BcMV3.BI=TRUE} | 0.07  6733 | 0.756  098 | 0.101  485 | 1.39  481 | 31 |
| {BcOLV1.BI.MOVA.=TRUE,  BcOLV18=FALSE} => {BcMV11=TRUE} | 0.07  9208 | 0.780  488 | 0.101  485 | 1.26  6334 | 32 |
| {BcOLV1.BI.MOVA.=TRUE,  BcOLV18=FALSE} => {BcRV1.BI=FALSE} | 0.08  9109 | 0.878  049 | 0.101  485 | 1.21  4835 | 36 |

| {BcOLV1.BI.MOVA.=TRUE,  BcOLV18=FALSE} => {BcMV1.BI=TRUE} | 0.09  1584 | 0.902  439 | 0.101  485 | 1.14  2901 | 37 |
| --- | --- | --- | --- | --- | --- |
| {BcOLV1.BI.MOVA.=TRUE,  BcOLV18=FALSE} => {BcNSRV7.BI=FALSE} | 0.09  6535 | 0.951  22 | 0.101  485 | 1.18  9761 | 39 |
| {BcOLV1.BI.MOVA.=TRUE,  BcOLV18=FALSE} => {BcOLV7.BI=FALSE} | 0.09  1584 | 0.902  439 | 0.101  485 | 1.11  836 | 37 |
| {BcOLV1.BI.MOVA.=TRUE,  BcOLV18=FALSE} => {BcALV1.BI=FALSE} | 0.09  6535 | 0.951  22 | 0.101  485 | 1.16  8063 | 39 |
| {BcOLV1.BI.MOVA.=TRUE,  BcOLV18=FALSE} => {BcDMV1=FALSE} | 0.09  4059 | 0.926  829 | 0.101  485 | 1.09  8062 | 38 |
| {BcOLV1.BI.MOVA.=TRUE, BcOLV18=TRUE}  => {BcHV1SLR.BI=TRUE} | 0.16  0891 | 0.698  925 | 0.230  198 | 1.28  9341 | 65 |
| {BcHV1SLR.BI=TRUE, BcOLV1.BI.MOVA.=TRUE} =>  {BcOLV18=TRUE} | 0.16  0891 | 0.802  469 | 0.200  495 | 1.55  1184 | 65 |
| {BcHV1SLR.BI=TRUE, BcOLV18=TRUE} =>  {BcOLV1.BI.MOVA.=TRUE} | 0.16  0891 | 0.471  014 | 0.341  584 | 1.42  0074 | 65 |
| {BcOLV1.BI.MOVA.=TRUE, BcOLV18=TRUE}  => {BcMV3.BI=TRUE} | 0.16  8317 | 0.731  183 | 0.230  198 | 1.34  8849 | 68 |
| {BcMV3.BI=TRUE, BcOLV1.BI.MOVA.=TRUE}  => {BcOLV18=TRUE} | 0.16  8317 | 0.686  869 | 0.245  05 | 1.32  7727 | 68 |
| {BcMV3.BI=TRUE, BcOLV18=TRUE} => | 0.16 | 0.511 | 0.329 | 1.54 | 68 |

| {BcOLV1.BI.MOVA.=TRUE} | 8317 | 278 | 208 | 1466 |  |
| --- | --- | --- | --- | --- | --- |
| {BcMV4.BI=FALSE, |  |  |  |  |  |
|  | 0.09 | 0.644 | 0.146 | 1.24 |  |
| BcOLV1.BI.MOVA.=TRUE} => |  |  |  |  | 38 |
|  | 4059 | 068 | 04 | 4992 |  |
| {BcOLV18=TRUE} |  |  |  |  |  |
| {BcOLV1.BI.MOVA.=TRUE, | 0.09 | 0.666 | 0.141 | 1.28 |  |
|  |  |  |  |  | 38 |
| BcOLV16.BI=FALSE} => {BcOLV18=TRUE} | 4059 | 667 | 089 | 8676 |  |
| {BcOLV1.BI.MOVA.=TRUE, BcULV3=TRUE} | 0.13 | 0.708 | 0.195 | 1.37 |  |
|  |  |  |  |  | 56 |
| => {BcOLV18=TRUE} | 8614 | 861 | 545 | 0238 |  |
| {BcOLV18=TRUE, BcULV3=TRUE} => | 0.13 | 0.427 | 0.324 | 1.28 |  |
|  |  |  |  |  | 56 |
| {BcOLV1.BI.MOVA.=TRUE} | 8614 | 481 | 257 | 8823 |  |
| {BcOLV1.BI.MOVA.=TRUE, BcOLV18=TRUE} | 0.16 | 0.720 | 0.230 | 1.19 |  |
|  |  |  |  |  | 67 |
| => {BcHV1.BI=TRUE} | 5842 | 43 | 198 | 2843 |  |
| {BcHV1.BI=TRUE, BcOLV1.BI.MOVA.=TRUE} | 0.16 | 0.779 | 0.212 | 1.50 |  |
|  |  |  |  |  | 67 |
| => {BcOLV18=TRUE} | 5842 | 07 | 871 | 5953 |  |
| {BcHV1.BI=TRUE, BcOLV18=TRUE} => | 0.16 | 0.452 | 0.366 | 1.36 |  |
|  |  |  |  |  | 67 |
| {BcOLV1.BI.MOVA.=TRUE} | 5842 | 703 | 337 | 4865 |  |
| {BcOLV1.BI.MOVA.=TRUE, BcOLV18=TRUE} | 0.16 | 0.709 | 0.230 | 1.15 |  |
|  |  |  |  |  | 66 |
| => {BcMV11=TRUE} | 3366 | 677 | 198 | 1444 |  |
| {BcMV11=TRUE, BcOLV1.BI.MOVA.=TRUE} | 0.16 | 0.673 | 0.242 | 1.30 |  |
|  |  |  |  |  | 66 |
| => {BcOLV18=TRUE} | 3366 | 469 | 574 | 1826 |  |
| {BcMV11=TRUE, BcOLV18=TRUE} => | 0.16 | 0.471 | 0.346 | 1.42 |  |
|  |  |  |  |  | 66 |
| {BcOLV1.BI.MOVA.=TRUE} | 3366 | 429 | 535 | 1322 |  |

| {BcOLV1.BI.MOVA.=TRUE, BcOLV18=TRUE}  => {BcMV9.BI=TRUE} | 0.18  5644 | 0.806  452 | 0.230  198 | 1.18  9075 | 75 |
| --- | --- | --- | --- | --- | --- |
| {BcMV9.BI=TRUE, BcOLV1.BI.MOVA.=TRUE}  => {BcOLV18=TRUE} | 0.18  5644 | 0.872  093 | 0.212  871 | 1.68  5768 | 75 |
| {BcMV9.BI=TRUE, BcOLV18=TRUE} =>  {BcOLV1.BI.MOVA.=TRUE} | 0.18  5644 | 0.449  102 | 0.413  366 | 1.35  4008 | 75 |
| {BcOLV1.BI.MOVA.=TRUE, BcOLV18=TRUE}  => {BcULV4=TRUE} | 0.21  2871 | 0.924  731 | 0.230  198 | 1.33  9037 | 86 |
| {BcOLV1.BI.MOVA.=TRUE, BcULV4=TRUE}  => {BcOLV18=TRUE} | 0.21  2871 | 0.868  687 | 0.245  05 | 1.67  9184 | 86 |
| {BcOLV18=TRUE, BcULV4=TRUE} =>  {BcOLV1.BI.MOVA.=TRUE} | 0.21  2871 | 0.483  146 | 0.440  594 | 1.45  6649 | 86 |
| {BcMV6.BI=FALSE, BcOLV1.BI.MOVA.=TRUE} =>  {BcOLV18=TRUE} | 0.10  6436 | 0.632  353 | 0.168  317 | 1.22  2347 | 43 |
| {BcOLV1.BI.MOVA.=TRUE,  BcPV3.BI=FALSE} => {BcOLV18=TRUE} | 0.12  3762 | 0.632  911 | 0.195  545 | 1.22  3427 | 50 |
| {BcEV2.BI=FALSE, BcOLV1.BI.MOVA.=TRUE} =>  {BcOLV18=TRUE} | 0.16  5842 | 0.663  366 | 0.25 | 1.28  2297 | 67 |
| {BcEV2.BI=FALSE, BcOLV18=TRUE} =>  {BcOLV1.BI.MOVA.=TRUE} | 0.16  5842 | 0.449  664 | 0.368  812 | 1.35  5705 | 67 |

| {BcHV3.BI=FALSE, BcOLV1.BI.MOVA.=TRUE} =>  {BcOLV18=TRUE} | 0.14  604 | 0.641  304 | 0.227  723 | 1.23  9651 | 59 |
| --- | --- | --- | --- | --- | --- |
| {BcHV3.BI=FALSE, BcOLV18=TRUE} =>  {BcOLV1.BI.MOVA.=TRUE} | 0.14  604 | 0.415  493 | 0.351  485 | 1.25  268 | 59 |
| {BcFV5.BI=FALSE, BcOLV1.BI.MOVA.=TRUE} =>  {BcOLV18=TRUE} | 0.15  3465 | 0.639  175 | 0.240  099 | 1.23  5535 | 62 |
| {BcFV5.BI=FALSE, BcOLV18=TRUE} =>  {BcOLV1.BI.MOVA.=TRUE} | 0.15  3465 | 0.416  107 | 0.368  812 | 1.25  4533 | 62 |
| {BcOLV1.BI.MOVA.=TRUE,  BcULV1.BI=FALSE} => {BcOLV18=TRUE} | 0.14  8515 | 0.659  341 | 0.225  248 | 1.27  4515 | 60 |
| {BcOLV18=TRUE, BcULV1.BI=FALSE} =>  {BcOLV1.BI.MOVA.=TRUE} | 0.14  8515 | 0.416  667 | 0.356  436 | 1.25  6219 | 60 |
| {BcOLV1.BI.MOVA.=TRUE, BcOLV18=TRUE}  => {BcMV1.BI=TRUE} | 0.20  297 | 0.881  72 | 0.230  198 | 1.11  6662 | 82 |
| {BcMV1.BI=TRUE, BcOLV1.BI.MOVA.=TRUE}  => {BcOLV18=TRUE} | 0.20  297 | 0.689  076 | 0.294  554 | 1.33  1993 | 82 |
| {BcMV1.BI=TRUE, BcOLV18=TRUE} =>  {BcOLV1.BI.MOVA.=TRUE} | 0.20  297 | 0.460  674 | 0.440  594 | 1.38  8898 | 82 |
| {BcMV12=FALSE, BcOLV1.BI.MOVA.=TRUE}  => {BcOLV18=TRUE} | 0.16  8317 | 0.731  183 | 0.230  198 | 1.41  3387 | 68 |

| {BcMV12=FALSE, BcOLV18=TRUE} =>  {BcOLV1.BI.MOVA.=TRUE} | 0.16  8317 | 0.425 | 0.396  04 | 1.28  1343 | 68 |
| --- | --- | --- | --- | --- | --- |
| {BcNSRV7.BI=FALSE, BcOLV1.BI.MOVA.=TRUE} =>  {BcOLV18=TRUE} | 0.16  8317 | 0.635  514 | 0.264  851 | 1.22  8458 | 68 |
| {BcNSRV7.BI=FALSE, BcOLV18=TRUE} =>  {BcOLV1.BI.MOVA.=TRUE} | 0.16  8317 | 0.433  121 | 0.388  614 | 1.30  5828 | 68 |
| {BcOLV1.BI.MOVA.=TRUE,  BcVV3.BI=FALSE} => {BcOLV18=TRUE} | 0.17  0792 | 0.704  082 | 0.242  574 | 1.36  1 | 69 |
| {BcOLV18=TRUE, BcVV3.BI=FALSE} =>  {BcOLV1.BI.MOVA.=TRUE} | 0.17  0792 | 0.415  663 | 0.410  891 | 1.25  3192 | 69 |
| {BcOLV1.BI.MOVA.=TRUE,  BcOLV7.BI=FALSE} => {BcOLV18=TRUE} | 0.14  604 | 0.614  583 | 0.237  624 | 1.18  7998 | 59 |
| {BcDMV1=FALSE, BcOLV1.BI.MOVA.=TRUE}  => {BcOLV18=TRUE} | 0.18  0693 | 0.657  658 | 0.274  752 | 1.27  1262 | 73 |
| {BcDMV1=FALSE, BcOLV18=TRUE} =>  {BcOLV1.BI.MOVA.=TRUE} | 0.18  0693 | 0.421  965 | 0.428  218 | 1.27  2194 | 73 |
| {BcFV9=FALSE, BcOLV1.BI.MOVA.=TRUE}  => {BcOLV18=TRUE} | 0.18  5644 | 0.663  717 | 0.279  703 | 1.28  2974 | 75 |
| {BcFV9=FALSE, BcOLV18=TRUE} =>  {BcOLV1.BI.MOVA.=TRUE} | 0.18  5644 | 0.441  176 | 0.420  792 | 1.33  0114 | 75 |
| {BcOLV1.BI.MOVA.=TRUE, | 0.19 | 0.726 | 0.262 | 1.40 | 77 |

| BcOLV4.BI=FALSE} => {BcOLV18=TRUE} | 0594 | 415 | 376 | 4171 |  |
| --- | --- | --- | --- | --- | --- |
| {BcOLV18=TRUE, BcOLV4.BI=FALSE} => | 0.19 | 0.425 | 0.448 | 1.28 |  |
|  |  |  |  |  | 77 |
| {BcOLV1.BI.MOVA.=TRUE} | 0594 | 414 | 02 | 2593 |  |
| {BcOLV1.BI.MOVA.=TRUE, BcULV5=FALSE} | 0.20 | 0.807 | 0.257 | 1.56 |  |
|  |  |  |  |  | 84 |
| => {BcOLV18=TRUE} | 7921 | 692 | 426 | 1281 |  |
| {BcOLV18=TRUE, BcULV5=FALSE} => | 0.20 | 0.444 | 0.467 | 1.33 |  |
|  |  |  |  |  | 84 |
| {BcOLV1.BI.MOVA.=TRUE} | 7921 | 444 | 822 | 9967 |  |
| {BcBNV5.BI=FALSE, |  |  |  |  |  |
|  | 0.18 | 0.666 | 0.282 | 1.28 |  |
| BcOLV1.BI.MOVA.=TRUE} => |  |  |  |  | 76 |
|  | 8119 | 667 | 178 | 8676 |  |
| {BcOLV18=TRUE} |  |  |  |  |  |
| {BcBNV5.BI=FALSE, BcOLV18=TRUE} => | 0.18 | 0.415 | 0.452 | 1.25 |  |
|  |  |  |  |  | 76 |
| {BcOLV1.BI.MOVA.=TRUE} | 8119 | 301 | 97 | 21 |  |
| {BcOLV1.BI.MOVA.=TRUE, BvF.BI=FALSE} | 0.21 | 0.692 | 0.314 | 1.33 |  |
|  |  |  |  |  | 88 |
| => {BcOLV18=TRUE} | 7822 | 913 | 356 | 9412 |  |
| {BcOLV18=TRUE, BvF.BI=FALSE} => | 0.21 | 0.458 | 0.475 | 1.38 |  |
|  |  |  |  |  | 88 |
| {BcOLV1.BI.MOVA.=TRUE} | 7822 | 333 | 248 | 1841 |  |
| {BcMCV5.BI=FALSE, |  |  |  |  |  |
|  | 0.18 | 0.651 | 0.277 | 1.25 |  |
| BcOLV1.BI.MOVA.=TRUE} => |  |  |  |  | 73 |
|  | 0693 | 786 | 228 | 9911 |  |
| {BcOLV18=TRUE} |  |  |  |  |  |
| {BcMCV5.BI=FALSE, BcOLV18=TRUE} => | 0.18 | 0.401 | 0.450 | 1.20 |  |
|  |  |  |  |  | 73 |
| {BcOLV1.BI.MOVA.=TRUE} | 0693 | 099 | 495 | 9283 |  |
| {BcOLV1.BI.MOVA.=TRUE, | 0.20 | 0.732 | 0.277 | 1.41 | 82 |

| BcOLV13.BI=FALSE} => {BcOLV18=TRUE} | 297 | 143 | 228 | 5243 |  |
| --- | --- | --- | --- | --- | --- |
| {BcOLV13.BI=FALSE, BcOLV18=TRUE} => | 0.20 | 0.427 | 0.475 | 1.28 |  |
|  |  |  |  |  | 82 |
| {BcOLV1.BI.MOVA.=TRUE} | 297 | 083 | 248 | 7624 |  |
| {BcOLV1.BI.MOVA.=TRUE, | 0.19 | 0.696 | 0.277 | 1.34 |  |
|  |  |  |  |  | 78 |
| BcOLV2.BI=FALSE} => {BcOLV18=TRUE} | 3069 | 429 | 228 | 6206 |  |
| {BcOLV18=TRUE, BcOLV2.BI=FALSE} => | 0.19 | 0.412 | 0.467 | 1.24 |  |
|  |  |  |  |  | 78 |
| {BcOLV1.BI.MOVA.=TRUE} | 3069 | 698 | 822 | 4255 |  |
| {BcMCV3.BI=FALSE, |  |  |  |  |  |
|  | 0.20 | 0.724 | 0.287 | 1.39 |  |
| BcOLV1.BI.MOVA.=TRUE} => |  |  |  |  | 84 |
|  | 7921 | 138 | 129 | 9769 |  |
| {BcOLV18=TRUE} |  |  |  |  |  |
| {BcMCV3.BI=FALSE, BcOLV18=TRUE} => | 0.20 | 0.437 | 0.475 | 1.31 |  |
|  |  |  |  |  | 84 |
| {BcOLV1.BI.MOVA.=TRUE} | 7921 | 5 | 248 | 903 |  |
| {BcOLV1.BI.MOVA.=TRUE, | 0.22 | 0.703 | 0.316 | 1.35 |  |
|  |  |  |  |  | 90 |
| BcPV4.BI=FALSE} => {BcOLV18=TRUE} | 2772 | 125 | 832 | 9151 |  |
| {BcOLV18=TRUE, BcPV4.BI=FALSE} => | 0.22 | 0.466 | 0.477 | 1.40 |  |
|  |  |  |  |  | 90 |
| {BcOLV1.BI.MOVA.=TRUE} | 2772 | 321 | 723 | 5924 |  |
| {BcEV6=FALSE, BcOLV1.BI.MOVA.=TRUE} | 0.21 | 0.685 | 0.306 | 1.32 |  |
|  |  |  |  |  | 85 |
| => {BcOLV18=TRUE} | 0396 | 484 | 931 | 505 |  |
| {BcEV6=FALSE, BcOLV18=TRUE} => | 0.21 | 0.435 | 0.482 | 1.31 |  |
|  |  |  |  |  | 85 |
| {BcOLV1.BI.MOVA.=TRUE} | 0396 | 897 | 673 | 4198 |  |
| {BcOLV1.BI.MOVA.=TRUE, | 0.19 | 0.661 | 0.299 | 1.27 |  |
|  |  |  |  |  | 80 |
| BcOLV17.BI=FALSE} => {BcOLV18=TRUE} | 802 | 157 | 505 | 8026 |  |

| {BcOLV17.BI=FALSE, BcOLV18=TRUE} =>  {BcOLV1.BI.MOVA.=TRUE} | 0.19  802 | 0.414  508 | 0.477  723 | 1.24  971 | 80 |
| --- | --- | --- | --- | --- | --- |
| {BcOLV1.BI.MOVA.=TRUE,  BcOLV15.BI=FALSE} => {BcOLV18=TRUE} | 0.22  0297 | 0.684  615 | 0.321  782 | 1.32  3371 | 89 |
| {BcOLV15.BI=FALSE, BcOLV18=TRUE} =>  {BcOLV1.BI.MOVA.=TRUE} | 0.22  0297 | 0.461  14 | 0.477  723 | 1.39  0302 | 89 |
| {BcEV5=FALSE, BcOLV1.BI.MOVA.=TRUE}  => {BcOLV18=TRUE} | 0.20  5446 | 0.691  667 | 0.297  03 | 1.33  7002 | 83 |
| {BcEV5=FALSE, BcOLV18=TRUE} =>  {BcOLV1.BI.MOVA.=TRUE} | 0.20  5446 | 0.421  32 | 0.487  624 | 1.27  0248 | 83 |
| {BcMV2.BI=FALSE, BcOLV1.BI.MOVA.=TRUE} =>  {BcEV2.BI=FALSE} | 0.07  4257 | 0.909  091 | 0.081  683 | 1.21  6135 | 30 |
| {BcHV1SLR.BI=TRUE, BcOLV1.BI.MOVA.=TRUE} =>  {BcMV3.BI=TRUE} | 0.16  3366 | 0.814  815 | 0.200  495 | 1.50  3129 | 66 |
| {BcMV3.BI=TRUE, BcOLV1.BI.MOVA.=TRUE}  => {BcHV1SLR.BI=TRUE} | 0.16  3366 | 0.666  667 | 0.245  05 | 1.22  9833 | 66 |
| {BcHV1SLR.BI=TRUE, BcMV3.BI=TRUE} =>  {BcOLV1.BI.MOVA.=TRUE} | 0.16  3366 | 0.496  241 | 0.329  208 | 1.49  6128 | 66 |
| {BcHV1SLR.BI=TRUE,  BcOLV1.BI.MOVA.=TRUE} => | 0.13  8614 | 0.691  358 | 0.200  495 | 1.16  3786 | 56 |

| {BcULV3=TRUE} |  |  |  |  |  |
| --- | --- | --- | --- | --- | --- |
| {BcOLV1.BI.MOVA.=TRUE, BcULV3=TRUE} | 0.13 | 0.708 | 0.195 | 1.30 |  |
|  |  |  |  |  | 56 |
| => {BcHV1SLR.BI=TRUE} | 8614 | 861 | 545 | 767 |  |
| {BcHV1SLR.BI=TRUE, |  |  |  |  |  |
|  | 0.17 | 0.888 | 0.200 | 1.47 |  |
| BcOLV1.BI.MOVA.=TRUE} => |  |  |  |  | 72 |
|  | 8218 | 889 | 495 | 1767 |  |
| {BcHV1.BI=TRUE} |  |  |  |  |  |
| {BcHV1.BI=TRUE, BcOLV1.BI.MOVA.=TRUE} | 0.17 | 0.837 | 0.212 | 1.54 |  |
|  |  |  |  |  | 72 |
| => {BcHV1SLR.BI=TRUE} | 8218 | 209 | 871 | 4441 |  |
| {BcHV1SLR.BI=TRUE, |  |  |  |  |  |
|  | 0.14 | 0.740 | 0.200 | 1.20 |  |
| BcOLV1.BI.MOVA.=TRUE} => |  |  |  |  | 60 |
|  | 8515 | 741 | 495 | 1844 |  |
| {BcMV11=TRUE} |  |  |  |  |  |
| {BcHV1SLR.BI=TRUE, BcMV11=TRUE} => | 0.14 | 0.416 | 0.356 | 1.25 |  |
|  |  |  |  |  | 60 |
| {BcOLV1.BI.MOVA.=TRUE} | 8515 | 667 | 436 | 6219 |  |
| {BcMV9.BI=TRUE, BcOLV1.BI.MOVA.=TRUE} | 0.14 | 0.697 | 0.212 | 1.28 |  |
|  |  |  |  |  | 60 |
| => {BcHV1SLR.BI=TRUE} | 8515 | 674 | 871 | 7034 |  |
| {BcHV1SLR.BI=TRUE, |  |  |  |  |  |
|  | 0.16 | 0.839 | 0.200 | 1.21 |  |
| BcOLV1.BI.MOVA.=TRUE} => |  |  |  |  | 68 |
|  | 8317 | 506 | 495 | 5629 |  |
| {BcULV4=TRUE} |  |  |  |  |  |
| {BcOLV1.BI.MOVA.=TRUE, BcULV4=TRUE} | 0.16 | 0.686 | 0.245 | 1.26 |  |
|  |  |  |  |  | 68 |
| => {BcHV1SLR.BI=TRUE} | 8317 | 869 | 05 | 71 |  |
| {BcHV1SLR.BI=TRUE, BcULV4=TRUE} => | 0.16 | 0.419 | 0.400 | 1.26 |  |
|  |  |  |  |  | 68 |
| {BcOLV1.BI.MOVA.=TRUE} | 8317 | 753 | 99 | 5524 |  |

| {BcOLV1.BI.MOVA.=TRUE, BcULV3=TRUE}  => {BcMV3.BI=TRUE} | 0.15  3465 | 0.784  81 | 0.195  545 | 1.44  7778 | 62 |
| --- | --- | --- | --- | --- | --- |
| {BcMV3.BI=TRUE, BcULV3=TRUE} =>  {BcOLV1.BI.MOVA.=TRUE} | 0.15  3465 | 0.442  857 | 0.346  535 | 1.33  5181 | 62 |
| {BcHV1.BI=TRUE, BcOLV1.BI.MOVA.=TRUE}  => {BcMV3.BI=TRUE} | 0.16  8317 | 0.790  698 | 0.212  871 | 1.45  8639 | 68 |
| {BcHV1.BI=TRUE, BcMV3.BI=TRUE} =>  {BcOLV1.BI.MOVA.=TRUE} | 0.16  8317 | 0.475  524 | 0.353  96 | 1.43  3671 | 68 |
| {BcMV3.BI=TRUE, BcOLV1.BI.MOVA.=TRUE}  => {BcMV11=TRUE} | 0.19  3069 | 0.787  879 | 0.245  05 | 1.27  8325 | 78 |
| {BcMV11=TRUE, BcOLV1.BI.MOVA.=TRUE}  => {BcMV3.BI=TRUE} | 0.19  3069 | 0.795  918 | 0.242  574 | 1.46  827 | 78 |
| {BcMV11=TRUE, BcMV3.BI=TRUE} =>  {BcOLV1.BI.MOVA.=TRUE} | 0.19  3069 | 0.503  226 | 0.383  663 | 1.51  7188 | 78 |
| {BcMV9.BI=TRUE, BcOLV1.BI.MOVA.=TRUE}  => {BcMV3.BI=TRUE} | 0.16  3366 | 0.767  442 | 0.212  871 | 1.41  5737 | 66 |
| {BcMV3.BI=TRUE, BcMV9.BI=TRUE} =>  {BcOLV1.BI.MOVA.=TRUE} | 0.16  3366 | 0.412  5 | 0.396  04 | 1.24  3657 | 66 |
| {BcOLV1.BI.MOVA.=TRUE, BcULV4=TRUE}  => {BcMV3.BI=TRUE} | 0.18  3168 | 0.747  475 | 0.245  05 | 1.37  8903 | 74 |
| {BcMV3.BI=TRUE, BcULV4=TRUE} =>  {BcOLV1.BI.MOVA.=TRUE} | 0.18  3168 | 0.456  79 | 0.400  99 | 1.37  7188 | 74 |

| {BcOLV1.BI.MOVA.=TRUE,  BcPV3.BI=FALSE} => {BcMV3.BI=TRUE} | 0.13  8614 | 0.708  861 | 0.195  545 | 1.30  767 | 56 |
| --- | --- | --- | --- | --- | --- |
| {BcOLV1.BI.MOVA.=TRUE,  BcRV1.BI=FALSE} => {BcMV3.BI=TRUE} | 0.13  8614 | 0.7 | 0.198  02 | 1.29  1324 | 56 |
| {BcEV2.BI=FALSE, BcOLV1.BI.MOVA.=TRUE} =>  {BcMV3.BI=TRUE} | 0.17  8218 | 0.712  871 | 0.25 | 1.31  5068 | 72 |
| {BcEV2.BI=FALSE, BcMV3.BI=TRUE} =>  {BcOLV1.BI.MOVA.=TRUE} | 0.17  8218 | 0.441  718 | 0.403  465 | 1.33  1746 | 72 |
| {BcHV3.BI=FALSE, BcOLV1.BI.MOVA.=TRUE} =>  {BcMV3.BI=TRUE} | 0.16  0891 | 0.706  522 | 0.227  723 | 1.30  3355 | 65 |
| {BcHV3.BI=FALSE, BcMV3.BI=TRUE} =>  {BcOLV1.BI.MOVA.=TRUE} | 0.16  0891 | 0.408  805 | 0.393  564 | 1.23  2517 | 65 |
| {BcFV5.BI=FALSE, BcOLV1.BI.MOVA.=TRUE} =>  {BcMV3.BI=TRUE} | 0.17  3267 | 0.721  649 | 0.240  099 | 1.33  1262 | 70 |
| {BcFV5.BI=FALSE, BcMV3.BI=TRUE} =>  {BcOLV1.BI.MOVA.=TRUE} | 0.17  3267 | 0.445  86 | 0.388  614 | 1.34  4234 | 70 |
| {BcOLV1.BI.MOVA.=TRUE,  BcULV1.BI=FALSE} => {BcMV3.BI=TRUE} | 0.15  099 | 0.670  33 | 0.225  248 | 1.23  659 | 61 |
| {BcMV3.BI=TRUE, BcOLV1.BI.MOVA.=TRUE} | 0.22 | 0.898 | 0.245 | 1.13 | 89 |

| => {BcMV1.BI=TRUE} | 0297 | 99 | 05 | 8533 |  |
| --- | --- | --- | --- | --- | --- |
| {BcMV1.BI=TRUE, BcOLV1.BI.MOVA.=TRUE} | 0.22 | 0.747 | 0.294 | 1.37 |  |
|  |  |  |  |  | 89 |
| => {BcMV3.BI=TRUE} | 0297 | 899 | 554 | 9686 |  |
| {BcMV1.BI=TRUE, BcMV3.BI=TRUE} => | 0.22 | 0.481 | 0.457 | 1.45 |  |
|  |  |  |  |  | 89 |
| {BcOLV1.BI.MOVA.=TRUE} | 0297 | 081 | 921 | 0424 |  |
| {BcMV12=FALSE, BcOLV1.BI.MOVA.=TRUE} | 0.15 | 0.666 | 0.230 | 1.22 |  |
|  |  |  |  |  | 62 |
| => {BcMV3.BI=TRUE} | 3465 | 667 | 198 | 9833 |  |
| {BcNSRV7.BI=FALSE, |  |  |  |  |  |
|  | 0.19 | 0.719 | 0.264 | 1.32 |  |
| BcOLV1.BI.MOVA.=TRUE} => |  |  |  |  | 77 |
|  | 0594 | 626 | 851 | 753 |  |
| {BcMV3.BI=TRUE} |  |  |  |  |  |
| {BcMV3.BI=TRUE, BcNSRV7.BI=FALSE} => | 0.19 | 0.450 | 0.423 | 1.35 |  |
|  |  |  |  |  | 77 |
| {BcOLV1.BI.MOVA.=TRUE} | 0594 | 292 | 267 | 7598 |  |
| {BcOLV1.BI.MOVA.=TRUE, | 0.17 | 0.704 | 0.242 | 1.29 |  |
|  |  |  |  |  | 69 |
| BcVV3.BI=FALSE} => {BcMV3.BI=TRUE} | 0792 | 082 | 574 | 8854 |  |
| {BcMV3.BI=TRUE, BcVV3.BI=FALSE} => | 0.17 | 0.410 | 0.415 | 1.23 |  |
|  |  |  |  |  | 69 |
| {BcOLV1.BI.MOVA.=TRUE} | 0792 | 714 | 842 | 8273 |  |
| {BcOLV1.BI.MOVA.=TRUE, | 0.17 | 0.718 | 0.237 | 1.32 |  |
|  |  |  |  |  | 69 |
| BcOLV7.BI=FALSE} => {BcMV3.BI=TRUE} | 0792 | 75 | 624 | 5913 |  |
| {BcMV3.BI=TRUE, BcOLV7.BI=FALSE} => | 0.17 | 0.410 | 0.415 | 1.23 |  |
|  |  |  |  |  | 69 |
| {BcOLV1.BI.MOVA.=TRUE} | 0792 | 714 | 842 | 8273 |  |
| {BcALV1.BI=FALSE, | 0.16 | 0.725 | 0.225 | 1.33 |  |
|  |  |  |  |  | 66 |
| BcOLV1.BI.MOVA.=TRUE} => | 3366 | 275 | 248 | 795 |  |

| {BcMV3.BI=TRUE} |  |  |  |  |  |
| --- | --- | --- | --- | --- | --- |
| {BcDMV1=FALSE, BcOLV1.BI.MOVA.=TRUE} | 0.19 | 0.711 | 0.274 | 1.31 |  |
|  |  |  |  |  | 79 |
| => {BcMV3.BI=TRUE} | 5545 | 712 | 752 | 2929 |  |
| {BcDMV1=FALSE, BcMV3.BI=TRUE} => | 0.19 | 0.461 | 0.423 | 1.39 |  |
|  |  |  |  |  | 79 |
| {BcOLV1.BI.MOVA.=TRUE} | 5545 | 988 | 267 | 286 |  |
| {BcFV9=FALSE, BcOLV1.BI.MOVA.=TRUE} | 0.20 | 0.734 | 0.279 | 1.35 |  |
|  |  |  |  |  | 83 |
| => {BcMV3.BI=TRUE} | 5446 | 513 | 703 | 4993 |  |
| {BcFV9=FALSE, BcMV3.BI=TRUE} => | 0.20 | 0.451 | 0.455 | 1.35 |  |
|  |  |  |  |  | 83 |
| {BcOLV1.BI.MOVA.=TRUE} | 5446 | 087 | 446 | 9994 |  |
| {BcOLV1.BI.MOVA.=TRUE, | 0.18 | 0.716 | 0.262 | 1.32 |  |
|  |  |  |  |  | 76 |
| BcOLV4.BI=FALSE} => {BcMV3.BI=TRUE} | 8119 | 981 | 376 | 265 |  |
| {BcMV3.BI=TRUE, BcOLV4.BI=FALSE} => | 0.18 | 0.413 | 0.455 | 1.24 |  |
|  |  |  |  |  | 76 |
| {BcOLV1.BI.MOVA.=TRUE} | 8119 | 043 | 446 | 5295 |  |
| {BcOLV1.BI.MOVA.=TRUE, BcULV5=FALSE} | 0.18 | 0.730 | 0.257 | 1.34 |  |
|  |  |  |  |  | 76 |
| => {BcMV3.BI=TRUE} | 8119 | 769 | 426 | 8086 |  |
| {BcMV3.BI=TRUE, BcULV5=FALSE} => | 0.18 | 0.415 | 0.452 | 1.25 |  |
|  |  |  |  |  | 76 |
| {BcOLV1.BI.MOVA.=TRUE} | 8119 | 301 | 97 | 21 |  |
| {BcBNV5.BI=FALSE, |  |  |  |  |  |
|  | 0.19 | 0.701 | 0.282 | 1.29 |  |
| BcOLV1.BI.MOVA.=TRUE} => |  |  |  |  | 80 |
|  | 802 | 754 | 178 | 4561 |  |
| {BcMV3.BI=TRUE} |  |  |  |  |  |
| {BcBNV5.BI=FALSE, BcMV3.BI=TRUE} => | 0.19 | 0.421 | 0.470 | 1.26 |  |
|  |  |  |  |  | 80 |
| {BcOLV1.BI.MOVA.=TRUE} | 802 | 053 | 297 | 9442 |  |

| {BcOLV1.BI.MOVA.=TRUE, BvF.BI=FALSE}  => {BcMV3.BI=TRUE} | 0.23  0198 | 0.732  283 | 0.314  356 | 1.35  0879 | 93 |
| --- | --- | --- | --- | --- | --- |
| {BcMV3.BI=TRUE, BvF.BI=FALSE} =>  {BcOLV1.BI.MOVA.=TRUE} | 0.23  0198 | 0.465 | 0.495  05 | 1.40  194 | 93 |
| {BcMCV5.BI=FALSE, BcOLV1.BI.MOVA.=TRUE} =>  {BcMV3.BI=TRUE} | 0.19  802 | 0.714  286 | 0.277  228 | 1.31  7678 | 80 |
| {BcMCV5.BI=FALSE, BcMV3.BI=TRUE} =>  {BcOLV1.BI.MOVA.=TRUE} | 0.19  802 | 0.410  256 | 0.482  673 | 1.23  6892 | 80 |
| {BcOLV1.BI.MOVA.=TRUE,  BcOLV13.BI=FALSE} => {BcMV3.BI=TRUE} | 0.19  5545 | 0.705  357 | 0.277  228 | 1.30  1207 | 79 |
| {BcMV3.BI=TRUE, BcOLV13.BI=FALSE} =>  {BcOLV1.BI.MOVA.=TRUE} | 0.19  5545 | 0.411  458 | 0.475  248 | 1.24  0516 | 79 |
| {BcOLV1.BI.MOVA.=TRUE,  BcOLV2.BI=FALSE} => {BcMV3.BI=TRUE} | 0.20  0495 | 0.723  214 | 0.277  228 | 1.33  4149 | 81 |
| {BcMV3.BI=TRUE, BcOLV2.BI=FALSE} =>  {BcOLV1.BI.MOVA.=TRUE} | 0.20  0495 | 0.413  265 | 0.485  149 | 1.24  5964 | 81 |
| {BcMCV3.BI=FALSE, BcOLV1.BI.MOVA.=TRUE} =>  {BcMV3.BI=TRUE} | 0.20  0495 | 0.698  276 | 0.287  129 | 1.28  8144 | 81 |
| {BcMCV3.BI=FALSE, BcMV3.BI=TRUE} =>  {BcOLV1.BI.MOVA.=TRUE} | 0.20  0495 | 0.421  875 | 0.475  248 | 1.27  1922 | 81 |

| {BcOLV1.BI.MOVA.=TRUE,  BcPV4.BI=FALSE} => {BcMV3.BI=TRUE} | 0.23  0198 | 0.726  563 | 0.316  832 | 1.34  0325 | 93 |
| --- | --- | --- | --- | --- | --- |
| {BcMV3.BI=TRUE, BcPV4.BI=FALSE} =>  {BcOLV1.BI.MOVA.=TRUE} | 0.23  0198 | 0.462  687 | 0.497  525 | 1.39  4965 | 93 |
| {BcEV6=FALSE, BcOLV1.BI.MOVA.=TRUE}  => {BcMV3.BI=TRUE} | 0.23  2673 | 0.758  065 | 0.306  931 | 1.39  8439 | 94 |
| {BcEV6=FALSE, BcMV3.BI=TRUE} =>  {BcOLV1.BI.MOVA.=TRUE} | 0.23  2673 | 0.449  761 | 0.517  327 | 1.35  5995 | 94 |
| {BcOLV1.BI.MOVA.=TRUE,  BcOLV17.BI=FALSE} => {BcMV3.BI=TRUE} | 0.21  7822 | 0.727  273 | 0.299  505 | 1.34  1636 | 88 |
| {BcMV3.BI=TRUE, BcOLV17.BI=FALSE} =>  {BcOLV1.BI.MOVA.=TRUE} | 0.21  7822 | 0.425  121 | 0.512  376 | 1.28  1707 | 88 |
| {BcOLV1.BI.MOVA.=TRUE,  BcOLV15.BI=FALSE} => {BcMV3.BI=TRUE} | 0.23  7624 | 0.738  462 | 0.321  782 | 1.36  2276 | 96 |
| {BcMV3.BI=TRUE, BcOLV15.BI=FALSE} =>  {BcOLV1.BI.MOVA.=TRUE} | 0.23  7624 | 0.468  293 | 0.507  426 | 1.41  1867 | 96 |
| {BcEV5=FALSE, BcOLV1.BI.MOVA.=TRUE}  => {BcMV3.BI=TRUE} | 0.21  0396 | 0.708  333 | 0.297  03 | 1.30  6697 | 85 |
| {BcEV5=FALSE, BcMV3.BI=TRUE} =>  {BcOLV1.BI.MOVA.=TRUE} | 0.21  0396 | 0.425 | 0.495  05 | 1.28  1343 | 85 |
| {BcMV4.BI=FALSE,  BcOLV1.BI.MOVA.=TRUE} => | 0.13  3663 | 0.915  254 | 0.146  04 | 1.08  4348 | 54 |

| {BcDMV1=FALSE} |  |  |  |  |  |
| --- | --- | --- | --- | --- | --- |
| {BcOLV1.BI.MOVA.=TRUE, BcULV3=TRUE}  => {BcHV1.BI=TRUE} | 0.14  3564 | 0.734  177 | 0.195  545 | 1.21  5605 | 58 |
| {BcOLV1.BI.MOVA.=TRUE, BcULV3=TRUE}  => {BcMV11=TRUE} | 0.15  099 | 0.772  152 | 0.195  545 | 1.25  2809 | 61 |
| {BcOLV1.BI.MOVA.=TRUE, BcULV3=TRUE}  => {BcULV4=TRUE} | 0.15  3465 | 0.784  81 | 0.195  545 | 1.13  6428 | 62 |
| {BcOLV1.BI.MOVA.=TRUE, BcULV3=TRUE}  => {BcMV1.BI=TRUE} | 0.17  3267 | 0.886  076 | 0.195  545 | 1.12  2178 | 70 |
| {BcHV1.BI=TRUE, BcOLV1.BI.MOVA.=TRUE}  => {BcMV11=TRUE} | 0.15  8416 | 0.744  186 | 0.212  871 | 1.20  7434 | 64 |
| {BcHV1.BI=TRUE, BcMV11=TRUE} =>  {BcOLV1.BI.MOVA.=TRUE} | 0.15  8416 | 0.415  584 | 0.381  188 | 1.25  2956 | 64 |
| {BcHV1.BI=TRUE, BcOLV1.BI.MOVA.=TRUE}  => {BcMV9.BI=TRUE} | 0.16  3366 | 0.767  442 | 0.212  871 | 1.13  1557 | 66 |
| {BcMV9.BI=TRUE, BcOLV1.BI.MOVA.=TRUE}  => {BcHV1.BI=TRUE} | 0.16  3366 | 0.767  442 | 0.212  871 | 1.27  0682 | 66 |
| {BcHV1.BI=TRUE, BcOLV1.BI.MOVA.=TRUE}  => {BcULV4=TRUE} | 0.18  0693 | 0.848  837 | 0.212  871 | 1.22  9141 | 73 |
| {BcOLV1.BI.MOVA.=TRUE, BcULV4=TRUE}  => {BcHV1.BI=TRUE} | 0.18  0693 | 0.737  374 | 0.245  05 | 1.22  0898 | 73 |
| {BcOLV1.BI.MOVA.=TRUE, BcULV4=TRUE} | 0.18 | 0.737 | 0.245 | 1.19 | 73 |

| => {BcMV11=TRUE} | 0693 | 374 | 05 | 6381 |  |
| --- | --- | --- | --- | --- | --- |
| {BcMV11=TRUE, BcULV4=TRUE} =>  {BcOLV1.BI.MOVA.=TRUE} | 0.18  0693 | 0.431  953 | 0.418  317 | 1.30  2305 | 73 |
| {BcOLV1.BI.MOVA.=TRUE,  BcPV3.BI=FALSE} => {BcMV11=TRUE} | 0.14  1089 | 0.721  519 | 0.195  545 | 1.17  0657 | 57 |
| {BcEV2.BI=FALSE, BcMV11=TRUE} =>  {BcOLV1.BI.MOVA.=TRUE} | 0.17  3267 | 0.402  299 | 0.430  693 | 1.21  2901 | 70 |
| {BcHV3.BI=FALSE, BcOLV1.BI.MOVA.=TRUE} =>  {BcMV11=TRUE} | 0.16  3366 | 0.717  391 | 0.227  723 | 1.16  396 | 66 |
| {BcFV5.BI=FALSE, BcOLV1.BI.MOVA.=TRUE} =>  {BcMV11=TRUE} | 0.17  3267 | 0.721  649 | 0.240  099 | 1.17  0869 | 70 |
| {BcOLV1.BI.MOVA.=TRUE,  BcULV1.BI=FALSE} => {BcMV11=TRUE} | 0.17  0792 | 0.758  242 | 0.225  248 | 1.23  024 | 69 |
| {BcMV11=TRUE, BcOLV1.BI.MOVA.=TRUE}  => {BcMV1.BI=TRUE} | 0.22  2772 | 0.918  367 | 0.242  574 | 1.16  3073 | 90 |
| {BcMV1.BI=TRUE, BcOLV1.BI.MOVA.=TRUE}  => {BcMV11=TRUE} | 0.22  2772 | 0.756  303 | 0.294  554 | 1.22  7093 | 90 |
| {BcMV1.BI=TRUE, BcMV11=TRUE} =>  {BcOLV1.BI.MOVA.=TRUE} | 0.22  2772 | 0.428  571 | 0.519  802 | 1.29  2111 | 90 |
| {BcMV12=FALSE, BcOLV1.BI.MOVA.=TRUE} | 0.17 | 0.774 | 0.230 | 1.25 | 72 |

| => {BcMV11=TRUE} | 8218 | 194 | 198 | 6121 |  |
| --- | --- | --- | --- | --- | --- |
| {BcNSRV7.BI=FALSE, BcOLV1.BI.MOVA.=TRUE} =>  {BcMV11=TRUE} | 0.19  0594 | 0.719  626 | 0.264  851 | 1.16  7586 | 77 |
| {BcOLV1.BI.MOVA.=TRUE,  BcOLV7.BI=FALSE} => {BcMV11=TRUE} | 0.17  0792 | 0.718  75 | 0.237  624 | 1.16  6165 | 69 |
| {BcDMV1=FALSE, BcOLV1.BI.MOVA.=TRUE}  => {BcMV11=TRUE} | 0.20  0495 | 0.729  73 | 0.274  752 | 1.18  3979 | 81 |
| {BcDMV1=FALSE, BcMV11=TRUE} =>  {BcOLV1.BI.MOVA.=TRUE} | 0.20  0495 | 0.393  204 | 0.509  901 | 1.18  548 | 81 |
| {BcFV9=FALSE, BcOLV1.BI.MOVA.=TRUE}  => {BcMV11=TRUE} | 0.20  0495 | 0.716  814 | 0.279  703 | 1.16  3024 | 81 |
| {BcOLV1.BI.MOVA.=TRUE,  BcOLV4.BI=FALSE} => {BcMV11=TRUE} | 0.18  5644 | 0.707  547 | 0.262  376 | 1.14  7988 | 75 |
| {BcOLV1.BI.MOVA.=TRUE, BvF.BI=FALSE}  => {BcMV11=TRUE} | 0.23  2673 | 0.740  157 | 0.314  356 | 1.20  0898 | 94 |
| {BcMV11=TRUE, BvF.BI=FALSE} =>  {BcOLV1.BI.MOVA.=TRUE} | 0.23  2673 | 0.406  926 | 0.571  782 | 1.22  6853 | 94 |
| {BcOLV1.BI.MOVA.=TRUE,  BcOLV13.BI=FALSE} => {BcMV11=TRUE} | 0.19  802 | 0.714  286 | 0.277  228 | 1.15  8921 | 80 |
| {BcOLV1.BI.MOVA.=TRUE,  BcOLV2.BI=FALSE} => {BcMV11=TRUE} | 0.20  7921 | 0.75 | 0.277  228 | 1.21  6867 | 84 |

| {BcMCV3.BI=FALSE, BcOLV1.BI.MOVA.=TRUE} =>  {BcMV11=TRUE} | 0.21  5347 | 0.75 | 0.287  129 | 1.21  6867 | 87 |
| --- | --- | --- | --- | --- | --- |
| {BcOLV1.BI.MOVA.=TRUE,  BcPV4.BI=FALSE} => {BcMV11=TRUE} | 0.23  2673 | 0.734  375 | 0.316  832 | 1.19  1516 | 94 |
| {BcMV11=TRUE, BcPV4.BI=FALSE} =>  {BcOLV1.BI.MOVA.=TRUE} | 0.23  2673 | 0.403  433 | 0.576  733 | 1.21  6322 | 94 |
| {BcEV6=FALSE, BcOLV1.BI.MOVA.=TRUE}  => {BcMV11=TRUE} | 0.22  7723 | 0.741  935 | 0.306  931 | 1.20  3783 | 92 |
| {BcEV6=FALSE, BcMV11=TRUE} =>  {BcOLV1.BI.MOVA.=TRUE} | 0.22  7723 | 0.394  85 | 0.576  733 | 1.19  0443 | 92 |
| {BcOLV1.BI.MOVA.=TRUE,  BcOLV17.BI=FALSE} => {BcMV11=TRUE} | 0.21  5347 | 0.719  008 | 0.299  505 | 1.16  6584 | 87 |
| {BcOLV1.BI.MOVA.=TRUE,  BcOLV15.BI=FALSE} => {BcMV11=TRUE} | 0.23  5149 | 0.730  769 | 0.321  782 | 1.18  5666 | 95 |
| {BcMV11=TRUE, BcOLV15.BI=FALSE} =>  {BcOLV1.BI.MOVA.=TRUE} | 0.23  5149 | 0.399  16 | 0.589  109 | 1.20  3437 | 95 |
| {BcEV5=FALSE, BcOLV1.BI.MOVA.=TRUE}  => {BcMV11=TRUE} | 0.21  0396 | 0.708  333 | 0.297  03 | 1.14  9264 | 85 |
| {BcMV9.BI=TRUE, BcOLV1.BI.MOVA.=TRUE}  => {BcULV4=TRUE} | 0.19  5545 | 0.918  605 | 0.212  871 | 1.33  0166 | 79 |
| {BcOLV1.BI.MOVA.=TRUE, BcULV4=TRUE} | 0.19 | 0.797 | 0.245 | 1.17 | 79 |

| => {BcMV9.BI=TRUE} | 5545 | 98 | 05 | 6583 |  |
| --- | --- | --- | --- | --- | --- |
| {BcMV9.BI=TRUE, BcOLV1.BI.MOVA.=TRUE} | 0.20 | 0.941 | 0.212 | 1.07 |  |
|  |  |  |  |  | 81 |
| => {BcULV5=FALSE} | 0495 | 86 | 871 | 4892 |  |
| {BcOLV1.BI.MOVA.=TRUE, BcULV5=FALSE} | 0.20 | 0.778 | 0.257 | 1.14 |  |
|  |  |  |  |  | 81 |
| => {BcMV9.BI=TRUE} | 0495 | 846 | 426 | 8372 |  |
| {BcOLV1.BI.MOVA.=TRUE, BcULV5=FALSE} | 0.21 | 0.836 | 0.257 | 1.21 |  |
|  |  |  |  |  | 87 |
| => {BcULV4=TRUE} | 5347 | 538 | 426 | 1332 |  |
| {BcMV6.BI=FALSE, |  |  |  |  |  |
|  | 0.15 | 0.897 | 0.168 | 1.13 |  |
| BcOLV1.BI.MOVA.=TRUE} => |  |  |  |  | 61 |
|  | 099 | 059 | 317 | 6087 |  |
| {BcMV1.BI=TRUE} |  |  |  |  |  |
| {BcMV6.BI=FALSE, |  |  |  |  |  |
|  | 0.15 | 0.911 | 0.168 | 1.08 |  |
| BcOLV1.BI.MOVA.=TRUE} => |  |  |  |  | 62 |
|  | 3465 | 765 | 317 | 0214 |  |
| {BcDMV1=FALSE} |  |  |  |  |  |
| {BcOLV1.BI.MOVA.=TRUE, | 0.17 | 0.886 | 0.195 | 1.12 |  |
|  |  |  |  |  | 70 |
| BcPV3.BI=FALSE} => {BcMV1.BI=TRUE} | 3267 | 076 | 545 | 2178 |  |
| {BcOLV1.BI.MOVA.=TRUE, | 0.18 | 0.912 | 0.198 | 1.15 |  |
|  |  |  |  |  | 73 |
| BcRV1.BI=FALSE} => {BcMV1.BI=TRUE} | 0693 | 5 | 02 | 5643 |  |
| {BcOLV1.BI.MOVA.=TRUE, | 0.17 |  | 0.198 | 1.11 |  |
|  |  | 0.9 |  |  | 72 |
| BcRV1.BI=FALSE} => {BcOLV7.BI=FALSE} | 8218 |  | 02 | 5337 |  |
| {BcHV3.BI=FALSE, |  |  |  |  |  |
|  | 0.21 | 0.923 | 0.227 | 1.15 |  |
| BcOLV1.BI.MOVA.=TRUE} => |  |  |  |  | 85 |
|  | 0396 | 913 | 723 | 5606 |  |
| {BcNSRV7.BI=FALSE} |  |  |  |  |  |

| {BcHV3.BI=FALSE, BcOLV1.BI.MOVA.=TRUE} =>  {BcDMV1=FALSE} | 0.21  0396 | 0.923  913 | 0.227  723 | 1.09  4607 | 85 |
| --- | --- | --- | --- | --- | --- |
| {BcFV5.BI=FALSE, BcOLV1.BI.MOVA.=TRUE} =>  {BcNSRV7.BI=FALSE} | 0.21  7822 | 0.907  216 | 0.240  099 | 1.13  4723 | 88 |
| {BcFV5.BI=FALSE, BcOLV1.BI.MOVA.=TRUE} =>  {BcDMV1=FALSE} | 0.23  0198 | 0.958  763 | 0.240  099 | 1.13  5895 | 93 |
| {BcOLV1.BI.MOVA.=TRUE,  BcULV1.BI=FALSE} => {BcMV1.BI=TRUE} | 0.19  802 | 0.879  121 | 0.225  248 | 1.11  3369 | 80 |
| {BcMV12=FALSE, BcOLV1.BI.MOVA.=TRUE}  => {BcMV1.BI=TRUE} | 0.20  5446 | 0.892  473 | 0.230  198 | 1.13  0279 | 83 |
| {BcNSRV7.BI=FALSE, BcOLV1.BI.MOVA.=TRUE} =>  {BcMV1.BI=TRUE} | 0.23  5149 | 0.887  85 | 0.264  851 | 1.12  4425 | 95 |
| {BcOLV1.BI.MOVA.=TRUE,  BcOLV7.BI=FALSE} => {BcMV1.BI=TRUE} | 0.20  7921 | 0.875 | 0.237  624 | 1.10  815 | 84 |
| {BcALV1.BI=FALSE, BcOLV1.BI.MOVA.=TRUE} =>  {BcMV1.BI=TRUE} | 0.19  802 | 0.879  121 | 0.225  248 | 1.11  3369 | 80 |
| {BcDMV1=FALSE, BcOLV1.BI.MOVA.=TRUE} | 0.24 | 0.882 | 0.274 | 1.11 | 98 |

| => {BcMV1.BI=TRUE} | 2574 | 883 | 752 | 8134 |  |
| --- | --- | --- | --- | --- | --- |
| {BcFV9=FALSE, BcOLV1.BI.MOVA.=TRUE}  => {BcMV1.BI=TRUE} | 0.25 | 0.893  805 | 0.279  703 | 1.13  1967 | 10  1 |
| {BcBNV5.BI=FALSE, BcOLV1.BI.MOVA.=TRUE} =>  {BcMV1.BI=TRUE} | 0.24  505 | 0.868  421 | 0.282  178 | 1.09  9819 | 99 |
| {BcOLV1.BI.MOVA.=TRUE, BvF.BI=FALSE} | 0.27 | 0.881 | 0.314 | 1.11 | 11 |
| => {BcMV1.BI=TRUE} | 7228 | 89 | 356 | 6876 | 2 |
| {BcMV1.BI=TRUE, BvF.BI=FALSE} => | 0.27 | 0.379 | 0.730 | 1.14 | 11 |
| {BcOLV1.BI.MOVA.=TRUE} | 7228 | 661 | 198 | 465 | 2 |
| {BcMCV5.BI=FALSE, |  |  |  |  |  |
|  | 0.24 | 0.866 | 0.277 | 1.09 |  |
| BcOLV1.BI.MOVA.=TRUE} => |  |  |  |  | 97 |
|  | 0099 | 071 | 228 | 6843 |  |
| {BcMV1.BI=TRUE} |  |  |  |  |  |
| {BcOLV1.BI.MOVA.=TRUE, | 0.24 | 0.892 | 0.277 | 1.13 | 10 |
| BcOLV13.BI=FALSE} => {BcMV1.BI=TRUE} | 7525 | 857 | 228 | 0766 | 0 |
| {BcOLV1.BI.MOVA.=TRUE, |  | 0.901 | 0.277 | 1.14 | 10 |
|  | 0.25 |  |  |  |  |
| BcOLV2.BI=FALSE} => {BcMV1.BI=TRUE} |  | 786 | 228 | 2073 | 1 |
| {BcMCV3.BI=FALSE, |  |  |  |  |  |
|  | 0.25 | 0.896 | 0.287 | 1.13 | 10 |
| BcOLV1.BI.MOVA.=TRUE} => |  |  |  |  |  |
|  | 7426 | 552 | 129 | 5445 | 4 |
| {BcMV1.BI=TRUE} |  |  |  |  |  |
| {BcOLV1.BI.MOVA.=TRUE, | 0.27 | 0.882 | 0.316 | 1.11 | 11 |
| BcPV4.BI=FALSE} => {BcMV1.BI=TRUE} | 9703 | 813 | 832 | 8045 | 3 |

| {BcMV1.BI=TRUE, BcPV4.BI=FALSE} =>  {BcOLV1.BI.MOVA.=TRUE} | 0.27  9703 | 0.384  354 | 0.727  723 | 1.15  8798 | 11  3 |
| --- | --- | --- | --- | --- | --- |
| {BcEV6=FALSE, BcOLV1.BI.MOVA.=TRUE}  => {BcMV1.BI=TRUE} | 0.27  2277 | 0.887  097 | 0.306  931 | 1.12  3471 | 11  0 |
| {BcOLV1.BI.MOVA.=TRUE,  BcOLV17.BI=FALSE} => {BcMV1.BI=TRUE} | 0.26  7327 | 0.892  562 | 0.299  505 | 1.13  0392 | 10  8 |
| {BcOLV1.BI.MOVA.=TRUE,  BcOLV15.BI=FALSE} => {BcMV1.BI=TRUE} | 0.28  7129 | 0.892  308 | 0.321  782 | 1.13  007 | 11  6 |
| {BcMV1.BI=TRUE, BcOLV15.BI=FALSE} =>  {BcOLV1.BI.MOVA.=TRUE} | 0.28  7129 | 0.385  382 | 0.745  05 | 1.16  1898 | 11  6 |
| {BcEV5=FALSE, BcOLV1.BI.MOVA.=TRUE}  => {BcMV1.BI=TRUE} | 0.25  9901 | 0.875 | 0.297  03 | 1.10  815 | 10  5 |
| {BcNSRV7.BI=FALSE, BcOLV1.BI.MOVA.=TRUE} =>  {BcDMV1=FALSE} | 0.24  505 | 0.925  234 | 0.264  851 | 1.09  6171 | 99 |
| {BcDMV1=FALSE, BcOLV1.BI.MOVA.=TRUE}  => {BcNSRV7.BI=FALSE} | 0.24  505 | 0.891  892 | 0.274  752 | 1.11  5555 | 99 |
| {BcBNV5.BI=FALSE, BcOLV1.BI.MOVA.=TRUE} =>  {BcDMV1=FALSE} | 0.25  9901 | 0.921  053 | 0.282  178 | 1.09  1218 | 10  5 |
| {BcMCV5.BI=FALSE,  BcOLV1.BI.MOVA.=TRUE} => | 0.25 | 0.901  786 | 0.277  228 | 1.06  8391 | 10  1 |

| {BcDMV1=FALSE} |  |  |  |  |  |
| --- | --- | --- | --- | --- | --- |
| {BcEV5=FALSE, BcOLV1.BI.MOVA.=TRUE}  => {BcDMV1=FALSE} | 0.27  2277 | 0.916  667 | 0.297  03 | 1.08  6022 | 11  0 |
| {BcHV1.BI=FALSE, BcMV11=FALSE} =>  {BcULV3=FALSE} | 0.10  1485 | 0.630  769 | 0.160  891 | 1.55  3846 | 41 |
| {BcMV11=FALSE, BcULV3=FALSE} =>  {BcHV1.BI=FALSE} | 0.10  1485 | 0.546  667 | 0.185  644 | 1.38  0333 | 41 |
| {BcHV1.BI=FALSE, BcMV11=FALSE} =>  {BcHV1SLR.BI=FALSE} | 0.14  604 | 0.907  692 | 0.160  891 | 1.98  2204 | 59 |
| {BcHV1SLR.BI=FALSE, BcMV11=FALSE} =>  {BcHV1.BI=FALSE} | 0.14  604 | 0.737  5 | 0.198  02 | 1.86  2188 | 59 |
| {BcHV1.BI=FALSE, BcMV11=FALSE} =>  {BcOLV18=FALSE} | 0.10  396 | 0.646  154 | 0.160  891 | 1.33  8698 | 42 |
| {BcHV1.BI=FALSE, BcMV11=FALSE} =>  {BcMV4.BI=FALSE} | 0.12  6238 | 0.784  615 | 0.160  891 | 1.40  8821 | 51 |
| {BcHV1.BI=FALSE, BcMV4.BI=FALSE} =>  {BcMV11=FALSE} | 0.12  6238 | 0.5 | 0.252  475 | 1.30  3226 | 51 |
| {BcHV1.BI=FALSE, BcMV11=FALSE} =>  {BcOLV1.BI.MOVA.=FALSE} | 0.12  6238 | 0.784  615 | 0.160  891 | 1.17  4017 | 51 |
| {BcHV1.BI=FALSE, BcMV11=FALSE} =>  {BcMV6.BI=FALSE} | 0.13  3663 | 0.830  769 | 0.160  891 | 1.19  4416 | 54 |
| {BcHV1.BI=FALSE, BcMV11=FALSE} => | 0.13 | 0.861 | 0.160 | 1.15 | 56 |

| {BcEV2.BI=FALSE} | 8614 | 538 | 891 | 2522 |  |
| --- | --- | --- | --- | --- | --- |
| {BcHV1.BI=FALSE, BcMV11=FALSE} =>  {BcULV1.BI=FALSE} | 0.15  5941 | 0.969  231 | 0.160  891 | 1.23  5234 | 63 |
| {BcMV11=FALSE, BcULV1.BI=FALSE} =>  {BcHV1.BI=FALSE} | 0.15  5941 | 0.5 | 0.311  881 | 1.26  25 | 63 |
| {BcHV1.BI=FALSE, BcMV11=FALSE} =>  {BcALV1.BI=FALSE} | 0.15  099 | 0.938  462 | 0.160  891 | 1.15  2397 | 61 |
| {BcHV1.BI=FALSE, BcMV11=FALSE} =>  {BcFV9=FALSE} | 0.15  3465 | 0.953  846 | 0.160  891 | 1.09  7874 | 62 |
| {BcHV1.BI=FALSE, BcMV11=FALSE} =>  {BcULV5=FALSE} | 0.15  5941 | 0.969  231 | 0.160  891 | 1.10  6128 | 63 |
| {BcHV1.BI=FALSE, BcMV11=FALSE} =>  {BcMCV5.BI=FALSE} | 0.16  0891 | 1 | 0.160  891 | 1.08  311 | 65 |
| {BcMV11=FALSE, BcULV3=FALSE} =>  {BcHV1SLR.BI=FALSE} | 0.11  3861 | 0.613  333 | 0.185  644 | 1.33  9387 | 46 |
| {BcHV1SLR.BI=FALSE, BcMV11=FALSE} =>  {BcULV3=FALSE} | 0.11  3861 | 0.575 | 0.198  02 | 1.41  6463 | 46 |
| {BcMV11=FALSE, BcULV3=FALSE} =>  {BcMV3.BI=FALSE} | 0.10  6436 | 0.573  333 | 0.185  644 | 1.25  2036 | 43 |
| {BcMV3.BI=FALSE, BcULV3=FALSE} =>  {BcMV11=FALSE} | 0.10  6436 | 0.505  882 | 0.210  396 | 1.31  8558 | 43 |
| {BcMV11=FALSE, BcMV2.BI=TRUE} => | 0.07 | 0.612 | 0.121 | 1.50 | 30 |

| {BcULV3=FALSE} | 4257 | 245 | 287 | 8213 |  |
| --- | --- | --- | --- | --- | --- |
| {BcMV11=FALSE, BcOLV18=TRUE} =>  {BcULV3=FALSE} | 0.08  9109 | 0.521  739 | 0.170  792 | 1.28  526 | 36 |
| {BcMV2.BI=FALSE, BcULV3=FALSE} =>  {BcMV11=FALSE} | 0.11  1386 | 0.489  13 | 0.227  723 | 1.27  4895 | 45 |
| {BcMV11=FALSE, BcULV3=FALSE} =>  {BcMV4.BI=FALSE} | 0.13  6139 | 0.733  333 | 0.185  644 | 1.31  6741 | 55 |
| {BcMV4.BI=FALSE, BcULV3=FALSE} =>  {BcMV11=FALSE} | 0.13  6139 | 0.528  846 | 0.257  426 | 1.37  8412 | 55 |
| {BcOLV1.BI.MOVA.=FALSE, BcULV3=FALSE}  => {BcMV11=FALSE} | 0.14  1089 | 0.522  936 | 0.269  802 | 1.36  3007 | 57 |
| {BcMV9.BI=TRUE, BcULV3=FALSE} =>  {BcMV11=FALSE} | 0.13  1188 | 0.514  563 | 0.254  95 | 1.34  1184 | 53 |
| {BcMV11=FALSE, BcULV3=FALSE} =>  {BcULV4=TRUE} | 0.14  604 | 0.786  667 | 0.185  644 | 1.13  9116 | 59 |
| {BcMV11=FALSE, BcULV4=TRUE} =>  {BcULV3=FALSE} | 0.14  604 | 0.536  364 | 0.272  277 | 1.32  1286 | 59 |
| {BcULV3=FALSE, BcULV4=TRUE} =>  {BcMV11=FALSE} | 0.14  604 | 0.531  532 | 0.274  752 | 1.38  5411 | 59 |
| {BcMV11=FALSE, BcULV3=FALSE} =>  {BcMV6.BI=FALSE} | 0.15  3465 | 0.826  667 | 0.185  644 | 1.18  8517 | 62 |
| {BcMV11=FALSE, BcMV6.BI=FALSE} => | 0.15 | 0.5 | 0.306 | 1.23 | 62 |

| {BcULV3=FALSE} | 3465 |  | 931 | 1707 |  |
| --- | --- | --- | --- | --- | --- |
| {BcMV6.BI=FALSE, BcULV3=FALSE} =>  {BcMV11=FALSE} | 0.15  3465 | 0.484  375 | 0.316  832 | 1.26  25 | 62 |
| {BcPV3.BI=FALSE, BcULV3=FALSE} =>  {BcMV11=FALSE} | 0.14  1089 | 0.487  179 | 0.289  604 | 1.26  981 | 57 |
| {BcRV1.BI=FALSE, BcULV3=FALSE} =>  {BcMV11=FALSE} | 0.15  099 | 0.521  368 | 0.289  604 | 1.35  8919 | 61 |
| {BcMV11=FALSE, BcULV3=FALSE} =>  {BcEV2.BI=FALSE} | 0.16  0891 | 0.866  667 | 0.185  644 | 1.15  9382 | 65 |
| {BcEV2.BI=FALSE, BcMV11=FALSE} =>  {BcULV3=FALSE} | 0.16  0891 | 0.507  813 | 0.316  832 | 1.25  0953 | 65 |
| {BcEV2.BI=FALSE, BcULV3=FALSE} =>  {BcMV11=FALSE} | 0.16  0891 | 0.515  873 | 0.311  881 | 1.34  4598 | 65 |
| {BcMV11=FALSE, BcULV3=FALSE} =>  {BcHV3.BI=FALSE} | 0.16  0891 | 0.866  667 | 0.185  644 | 1.14  0499 | 65 |
| {BcHV3.BI=FALSE, BcMV11=FALSE} =>  {BcULV3=FALSE} | 0.16  0891 | 0.507  813 | 0.316  832 | 1.25  0953 | 65 |
| {BcHV3.BI=FALSE, BcULV3=FALSE} =>  {BcMV11=FALSE} | 0.16  0891 | 0.477  941 | 0.336  634 | 1.24  5731 | 65 |
| {BcFV5.BI=FALSE, BcMV11=FALSE} =>  {BcULV3=FALSE} | 0.15  5941 | 0.508  065 | 0.306  931 | 1.25  1574 | 63 |
| {BcMV11=FALSE, BcULV1.BI=FALSE} => | 0.15 | 0.492 | 0.311 | 1.21 | 62 |

| {BcULV3=FALSE} | 3465 | 063 | 881 | 2156 |  |
| --- | --- | --- | --- | --- | --- |
| {BcULV1.BI=FALSE, BcULV3=FALSE} =>  {BcMV11=FALSE} | 0.15  3465 | 0.469  697 | 0.326  733 | 1.22  4242 | 62 |
| {BcMV11=FALSE, BcMV12=FALSE} =>  {BcULV3=FALSE} | 0.14  604 | 0.5 | 0.292  079 | 1.23  1707 | 59 |
| {BcMV11=FALSE, BcNSRV7.BI=FALSE} =>  {BcULV3=FALSE} | 0.15  8416 | 0.503  937 | 0.314  356 | 1.24  1406 | 64 |
| {BcULV3=FALSE, BcVV3.BI=FALSE} =>  {BcMV11=FALSE} | 0.15  3465 | 0.473  282 | 0.324  257 | 1.23  3588 | 62 |
| {BcOLV7.BI=FALSE, BcULV3=FALSE} =>  {BcMV11=FALSE} | 0.15  5941 | 0.470  149 | 0.331  683 | 1.22  5421 | 63 |
| {BcALV1.BI=FALSE, BcULV3=FALSE} =>  {BcMV11=FALSE} | 0.16  3366 | 0.492  537 | 0.331  683 | 1.28  3775 | 66 |
| {BcDMV1=FALSE, BcULV3=FALSE} =>  {BcMV11=FALSE} | 0.15  5941 | 0.459  854 | 0.339  109 | 1.19  8587 | 63 |
| {BcFV9=FALSE, BcMV11=FALSE} =>  {BcULV3=FALSE} | 0.17  0792 | 0.492  857 | 0.346  535 | 1.21  4111 | 69 |
| {BcFV9=FALSE, BcULV3=FALSE} =>  {BcMV11=FALSE} | 0.17  0792 | 0.475  862 | 0.358  911 | 1.24  0311 | 69 |
| {BcMV11=FALSE, BcULV3=FALSE} =>  {BcOLV4.BI=FALSE} | 0.17  5743 | 0.946  667 | 0.185  644 | 1.08  0377 | 71 |
| {BcOLV4.BI=FALSE, BcULV3=FALSE} => | 0.17 | 0.476 | 0.368 | 1.24 | 71 |

| {BcMV11=FALSE} | 5743 | 51 | 812 | 2 |  |
| --- | --- | --- | --- | --- | --- |
| {BcMV11=FALSE, BcULV3=FALSE} =>  {BcULV5=FALSE} | 0.18  0693 | 0.973  333 | 0.185  644 | 1.11  081 | 73 |
| {BcMV11=FALSE, BcULV5=FALSE} =>  {BcULV3=FALSE} | 0.18  0693 | 0.486  667 | 0.371  287 | 1.19  8862 | 73 |
| {BcULV3=FALSE, BcULV5=FALSE} =>  {BcMV11=FALSE} | 0.18  0693 | 0.503  448 | 0.358  911 | 1.31  2214 | 73 |
| {BcBNV5.BI=FALSE, BcMV11=FALSE} =>  {BcULV3=FALSE} | 0.18  0693 | 0.486  667 | 0.371  287 | 1.19  8862 | 73 |
| {BcBNV5.BI=FALSE, BcULV3=FALSE} =>  {BcMV11=FALSE} | 0.18  0693 | 0.462  025 | 0.391  089 | 1.20  4247 | 73 |
| {BcMCV5.BI=FALSE, BcMV11=FALSE} =>  {BcULV3=FALSE} | 0.18  3168 | 0.486  842 | 0.376  238 | 1.19  9294 | 74 |
| {BcMCV5.BI=FALSE, BcULV3=FALSE} =>  {BcMV11=FALSE} | 0.18  3168 | 0.468  354 | 0.391  089 | 1.22  0743 | 74 |
| {BcMV11=FALSE, BcOLV13.BI=FALSE} =>  {BcULV3=FALSE} | 0.18  0693 | 0.493  243 | 0.366  337 | 1.21  5063 | 73 |
| {BcOLV13.BI=FALSE, BcULV3=FALSE} =>  {BcMV11=FALSE} | 0.18  0693 | 0.474  026 | 0.381  188 | 1.23  5526 | 73 |
| {BcMV11=FALSE, BcOLV2.BI=FALSE} =>  {BcULV3=FALSE} | 0.18  0693 | 0.503  448 | 0.358  911 | 1.24  0202 | 73 |
| {BcOLV2.BI=FALSE, BcULV3=FALSE} => | 0.18 | 0.467 | 0.386 | 1.21 | 73 |

| {BcMV11=FALSE} | 0693 | 949 | 139 | 9686 |  |
| --- | --- | --- | --- | --- | --- |
| {BcMCV3.BI=FALSE, BcMV11=FALSE} =>  {BcULV3=FALSE} | 0.17  3267 | 0.492  958 | 0.351  485 | 1.21  4359 | 70 |
| {BcMV11=FALSE, BcPV4.BI=FALSE} =>  {BcULV3=FALSE} | 0.17  3267 | 0.486  111 | 0.356  436 | 1.19  7493 | 70 |
| {BcEV6=FALSE, BcULV3=FALSE} =>  {BcMV11=FALSE} | 0.17  3267 | 0.460  526 | 0.376  238 | 1.20  034 | 70 |
| {BcOLV17.BI=FALSE, BcULV3=FALSE} =>  {BcMV11=FALSE} | 0.18  0693 | 0.462  025 | 0.391  089 | 1.20  4247 | 73 |
| {BcMV11=FALSE, BcOLV15.BI=FALSE} =>  {BcULV3=FALSE} | 0.18  0693 | 0.5 | 0.361  386 | 1.23  1707 | 73 |
| {BcMV3.BI=FALSE, BcOLV16.BI=TRUE} =>  {BcMV11=FALSE} | 0.07  6733 | 0.516  667 | 0.148  515 | 1.34  6667 | 31 |
| {BcMV2.BI=FALSE, BcOLV16.BI=TRUE} =>  {BcMV11=FALSE} | 0.08  4158 | 0.515  152 | 0.163  366 | 1.34  2717 | 34 |
| {BcMV4.BI=FALSE, BcOLV16.BI=TRUE} =>  {BcMV11=FALSE} | 0.09  4059 | 0.493  506 | 0.190  594 | 1.28  6301 | 38 |
| {BcMV11=FALSE, BcOLV16.BI=TRUE} =>  {BcMV9.BI=TRUE} | 0.11  8812 | 0.842  105 | 0.141  089 | 1.24  1644 | 48 |
| {BcMV11=FALSE, BcOLV16.BI=TRUE} =>  {BcRV1.BI=FALSE} | 0.11  6337 | 0.824  561 | 0.141  089 | 1.14  0832 | 47 |
| {BcMV11=FALSE, BcOLV16.BI=TRUE} => | 0.12 | 0.894 | 0.141 | 1.19 | 51 |

| {BcEV2.BI=FALSE} | 6238 | 737 | 089 | 6933 |  |
| --- | --- | --- | --- | --- | --- |
| {BcMV11=FALSE, BcOLV16.BI=TRUE} =>  {BcHV3.BI=FALSE} | 0.12  6238 | 0.894  737 | 0.141  089 | 1.17  7439 | 51 |
| {BcMV11=FALSE, BcMV4.BI=TRUE} =>  {BcOLV1.BI.MOVA.=FALSE} | 0.08  1683 | 0.785  714 | 0.103  96 | 1.17  5661 | 33 |
| {BcMV11=FALSE, BcMV4.BI=TRUE} =>  {BcMV9.BI=TRUE} | 0.08  1683 | 0.785  714 | 0.103  96 | 1.15  8498 | 33 |
| {BcMV11=FALSE, BcMV4.BI=TRUE} =>  {BcMV6.BI=FALSE} | 0.08  4158 | 0.809  524 | 0.103  96 | 1.16  3871 | 34 |
| {BcMV11=FALSE, BcMV4.BI=TRUE} =>  {BcDMV1=FALSE} | 0.09  901 | 0.952  381 | 0.103  96 | 1.12  8334 | 40 |
| {BcMV11=FALSE, BcMV4.BI=TRUE} =>  {BcULV5=FALSE} | 0.10  1485 | 0.976  19 | 0.103  96 | 1.11  407 | 41 |
| {BcHV1SLR.BI=FALSE, BcMV11=FALSE} =>  {BcMV3.BI=FALSE} | 0.12  3762 | 0.625 | 0.198  02 | 1.36  4865 | 50 |
| {BcHV1SLR.BI=FALSE, BcMV3.BI=FALSE}  => {BcMV11=FALSE} | 0.12  3762 | 0.505  051 | 0.245  05 | 1.31  639 | 50 |
| {BcHV1SLR.BI=FALSE, BcMV11=FALSE} =>  {BcOLV18=FALSE} | 0.12  6238 | 0.637  5 | 0.198  02 | 1.32  0769 | 51 |
| {BcMV11=FALSE, BcOLV18=FALSE} =>  {BcHV1SLR.BI=FALSE} | 0.12  6238 | 0.593  023 | 0.212  871 | 1.29  5035 | 51 |
| {BcHV1SLR.BI=FALSE, BcMV11=FALSE} => | 0.13 | 0.687 | 0.198 | 1.27 | 55 |

| {BcMV2.BI=FALSE} | 6139 | 5 | 02 | 9954 |  |
| --- | --- | --- | --- | --- | --- |
| {BcHV1SLR.BI=FALSE, BcMV2.BI=FALSE} | 0.13 | 0.509 | 0.267 | 1.32 |  |
|  |  |  |  |  | 55 |
| => {BcMV11=FALSE} | 6139 | 259 | 327 | 736 |  |
| {BcHV1SLR.BI=FALSE, BcMV11=FALSE} => | 0.15 |  | 0.198 | 1.39 |  |
|  |  | 0.775 |  |  | 62 |
| {BcMV4.BI=FALSE} | 3465 |  | 02 | 1556 |  |
| {BcHV1SLR.BI=FALSE, BcMV4.BI=FALSE} | 0.15 | 0.553 | 0.277 | 1.44 |  |
|  |  |  |  |  | 62 |
| => {BcMV11=FALSE} | 3465 | 571 | 228 | 2857 |  |
| {BcHV1SLR.BI=FALSE, BcMV11=FALSE} => | 0.16 | 0.812 | 0.198 | 1.21 |  |
|  |  |  |  |  | 65 |
| {BcOLV1.BI.MOVA.=FALSE} | 0891 | 5 | 02 | 5741 |  |
| {BcHV1SLR.BI=FALSE, |  |  |  |  |  |
|  | 0.16 | 0.492 | 0.326 | 1.28 |  |
| BcOLV1.BI.MOVA.=FALSE} => |  |  |  |  | 65 |
|  | 0891 | 424 | 733 | 348 |  |
| {BcMV11=FALSE} |  |  |  |  |  |
| {BcHV1SLR.BI=FALSE, BcULV4=TRUE} => | 0.14 | 0.504 | 0.289 | 1.31 |  |
|  |  |  |  |  | 59 |
| {BcMV11=FALSE} | 604 | 274 | 604 | 4364 |  |
| {BcHV1SLR.BI=FALSE, BcMV11=FALSE} => | 0.16 | 0.812 | 0.198 | 1.16 |  |
|  |  |  |  |  | 65 |
| {BcMV6.BI=FALSE} | 0891 | 5 | 02 | 8149 |  |
| {BcHV1SLR.BI=FALSE, BcMV11=FALSE} => | 0.17 | 0.887 | 0.198 | 1.18 |  |
|  |  |  |  |  | 71 |
| {BcEV2.BI=FALSE} | 5743 | 5 | 02 | 7252 |  |
| {BcEV2.BI=FALSE, BcMV11=FALSE} => | 0.17 | 0.554 | 0.316 | 1.21 |  |
|  |  |  |  |  | 71 |
| {BcHV1SLR.BI=FALSE} | 5743 | 688 | 832 | 1318 |  |
| {BcEV2.BI=FALSE, BcHV1SLR.BI=FALSE} => | 0.17 | 0.486 | 0.361 | 1.26 |  |
|  |  |  |  |  | 71 |
| {BcMV11=FALSE} | 5743 | 301 | 386 | 7521 |  |

| {BcHV1SLR.BI=FALSE, BcMV11=FALSE} =>  {BcHV3.BI=FALSE} | 0.16  8317 | 0.85 | 0.198  02 | 1.11  8567 | 68 |
| --- | --- | --- | --- | --- | --- |
| {BcHV1SLR.BI=FALSE, BcMV11=FALSE} =>  {BcFV5.BI=FALSE} | 0.17  3267 | 0.875 | 0.198  02 | 1.11  8671 | 70 |
| {BcFV5.BI=FALSE, BcMV11=FALSE} =>  {BcHV1SLR.BI=FALSE} | 0.17  3267 | 0.564  516 | 0.306  931 | 1.23  2781 | 70 |
| {BcHV1SLR.BI=FALSE, BcMV11=FALSE} =>  {BcULV1.BI=FALSE} | 0.18  5644 | 0.937  5 | 0.198  02 | 1.19  4795 | 75 |
| {BcMV11=FALSE, BcULV1.BI=FALSE} =>  {BcHV1SLR.BI=FALSE} | 0.18  5644 | 0.595  238 | 0.311  881 | 1.29  9871 | 75 |
| {BcHV1SLR.BI=FALSE, BcULV1.BI=FALSE}  => {BcMV11=FALSE} | 0.18  5644 | 0.462  963 | 0.400  99 | 1.20  6691 | 75 |
| {BcHV1SLR.BI=FALSE, BcMV11=FALSE} =>  {BcALV1.BI=FALSE} | 0.18  5644 | 0.937  5 | 0.198  02 | 1.15  1216 | 75 |
| {BcALV1.BI=FALSE, BcHV1SLR.BI=FALSE}  => {BcMV11=FALSE} | 0.18  5644 | 0.465  839 | 0.398  515 | 1.21  4186 | 75 |
| {BcHV1SLR.BI=FALSE, BcMV11=FALSE} =>  {BcFV9=FALSE} | 0.18  8119 | 0.95 | 0.198  02 | 1.09  3447 | 76 |
| {BcFV9=FALSE, BcMV11=FALSE} =>  {BcHV1SLR.BI=FALSE} | 0.18  8119 | 0.542  857 | 0.346  535 | 1.18  5483 | 76 |
| {BcHV1SLR.BI=FALSE, BcMV11=FALSE} =>  {BcOLV4.BI=FALSE} | 0.18  8119 | 0.95 | 0.198  02 | 1.08  4181 | 76 |

| {BcHV1SLR.BI=FALSE, BcMV11=FALSE} =>  {BcULV5=FALSE} | 0.19  3069 | 0.975 | 0.198  02 | 1.11  2712 | 78 |
| --- | --- | --- | --- | --- | --- |
| {BcHV1SLR.BI=FALSE, BcULV5=FALSE} =>  {BcMV11=FALSE} | 0.19  3069 | 0.472  727 | 0.408  416 | 1.23  2141 | 78 |
| {BcMV11=FALSE, BvF.BI=FALSE} =>  {BcHV1SLR.BI=FALSE} | 0.18  8119 | 0.550  725 | 0.341  584 | 1.20  2664 | 76 |
| {BcMV11=FALSE, BcMV3.BI=FALSE} =>  {BcOLV18=FALSE} | 0.14  3564 | 0.637  363 | 0.225  248 | 1.32  0485 | 58 |
| {BcMV11=FALSE, BcOLV18=FALSE} =>  {BcMV3.BI=FALSE} | 0.14  3564 | 0.674  419 | 0.212  871 | 1.47  2784 | 58 |
| {BcMV3.BI=FALSE, BcOLV18=FALSE} =>  {BcMV11=FALSE} | 0.14  3564 | 0.532  11 | 0.269  802 | 1.38  6919 | 58 |
| {BcMV11=FALSE, BcMV3.BI=FALSE} =>  {BcMV2.BI=FALSE} | 0.16  8317 | 0.747  253 | 0.225  248 | 1.39  1199 | 68 |
| {BcMV11=FALSE, BcMV2.BI=FALSE} =>  {BcMV3.BI=FALSE} | 0.16  8317 | 0.641  509 | 0.262  376 | 1.40  0918 | 68 |
| {BcMV2.BI=FALSE, BcMV3.BI=FALSE} =>  {BcMV11=FALSE} | 0.16  8317 | 0.544 | 0.309  406 | 1.41  791 | 68 |
| {BcMV11=FALSE, BcMV3.BI=FALSE} =>  {BcMV4.BI=FALSE} | 0.17  5743 | 0.780  22 | 0.225  248 | 1.40  0928 | 71 |
| {BcMV11=FALSE, BcMV4.BI=FALSE} =>  {BcMV3.BI=FALSE} | 0.17  5743 | 0.628  319 | 0.279  703 | 1.37  2112 | 71 |

| {BcMV3.BI=FALSE, BcMV4.BI=FALSE} =>  {BcMV11=FALSE} | 0.17  5743 | 0.568 | 0.309  406 | 1.48  0465 | 71 |
| --- | --- | --- | --- | --- | --- |
| {BcMV11=FALSE, BcOLV16.BI=FALSE} =>  {BcMV3.BI=FALSE} | 0.14  8515 | 0.612  245 | 0.242  574 | 1.33  701 | 60 |
| {BcMV3.BI=FALSE, BcOLV16.BI=FALSE} =>  {BcMV11=FALSE} | 0.14  8515 | 0.48 | 0.309  406 | 1.25  1097 | 60 |
| {BcMV11=FALSE, BcULV3=TRUE} =>  {BcMV3.BI=FALSE} | 0.11  8812 | 0.6 | 0.198  02 | 1.31  027 | 48 |
| {BcMV3.BI=FALSE, BcULV3=TRUE} =>  {BcMV11=FALSE} | 0.11  8812 | 0.48 | 0.247  525 | 1.25  1097 | 48 |
| {BcHV1.BI=TRUE, BcMV11=FALSE} =>  {BcMV3.BI=FALSE} | 0.13  6139 | 0.611  111 | 0.222  772 | 1.33  4535 | 55 |
| {BcHV1.BI=TRUE, BcMV3.BI=FALSE} =>  {BcMV11=FALSE} | 0.13  6139 | 0.544  554 | 0.25 | 1.41  9355 | 55 |
| {BcMV11=FALSE, BcMV3.BI=FALSE} =>  {BcOLV1.BI.MOVA.=FALSE} | 0.18  8119 | 0.835  165 | 0.225  248 | 1.24  9654 | 76 |
| {BcMV11=FALSE, BcOLV1.BI.MOVA.=FALSE} =>  {BcMV3.BI=FALSE} | 0.18  8119 | 0.638  655 | 0.294  554 | 1.39  4685 | 76 |
| {BcMV3.BI=FALSE, BcOLV1.BI.MOVA.=FALSE} =>  {BcMV11=FALSE} | 0.18  8119 | 0.506  667 | 0.371  287 | 1.32  0602 | 76 |

| {BcMV11=FALSE, BcMV9.BI=TRUE} =>  {BcMV3.BI=FALSE} | 0.15  3465 | 0.563  636 | 0.272  277 | 1.23  086 | 62 |
| --- | --- | --- | --- | --- | --- |
| {BcMV3.BI=FALSE, BcMV9.BI=TRUE} =>  {BcMV11=FALSE} | 0.15  3465 | 0.543  86 | 0.282  178 | 1.41  7544 | 62 |
| {BcMV11=FALSE, BcULV4=TRUE} =>  {BcMV3.BI=FALSE} | 0.16  3366 | 0.6 | 0.272  277 | 1.31  027 | 66 |
| {BcMV3.BI=FALSE, BcULV4=TRUE} =>  {BcMV11=FALSE} | 0.16  3366 | 0.564  103 | 0.289  604 | 1.47  0306 | 66 |
| {BcMV11=FALSE, BcMV3.BI=FALSE} =>  {BcMV6.BI=FALSE} | 0.18  8119 | 0.835  165 | 0.225  248 | 1.20  0735 | 76 |
| {BcMV11=FALSE, BcMV6.BI=FALSE} =>  {BcMV3.BI=FALSE} | 0.18  8119 | 0.612  903 | 0.306  931 | 1.33  8448 | 76 |
| {BcMV3.BI=FALSE, BcMV6.BI=FALSE} =>  {BcMV11=FALSE} | 0.18  8119 | 0.5 | 0.376  238 | 1.30  3226 | 76 |
| {BcMV11=FALSE, BcPV3.BI=FALSE} =>  {BcMV3.BI=FALSE} | 0.17  8218 | 0.610  169 | 0.292  079 | 1.33  2478 | 72 |
| {BcMV3.BI=FALSE, BcPV3.BI=FALSE} =>  {BcMV11=FALSE} | 0.17  8218 | 0.510  638 | 0.349  01 | 1.33  0954 | 72 |
| {BcMV11=FALSE, BcMV3.BI=FALSE} =>  {BcRV1.BI=FALSE} | 0.18  3168 | 0.813  187 | 0.225  248 | 1.12  5094 | 74 |
| {BcMV11=FALSE, BcRV1.BI=FALSE} =>  {BcMV3.BI=FALSE} | 0.18  3168 | 0.582  677 | 0.314  356 | 1.27  2441 | 74 |

| {BcMV3.BI=FALSE, BcRV1.BI=FALSE} =>  {BcMV11=FALSE} | 0.18  3168 | 0.513  889 | 0.356  436 | 1.33  9427 | 74 |
| --- | --- | --- | --- | --- | --- |
| {BcEV2.BI=FALSE, BcMV11=FALSE} =>  {BcMV3.BI=FALSE} | 0.17  8218 | 0.562  5 | 0.316  832 | 1.22  8378 | 72 |
| {BcEV2.BI=FALSE, BcMV3.BI=FALSE} =>  {BcMV11=FALSE} | 0.17  8218 | 0.517  986 | 0.344  059 | 1.35  0104 | 72 |
| {BcMV11=FALSE, BcMV3.BI=FALSE} =>  {BcHV3.BI=FALSE} | 0.19  0594 | 0.846  154 | 0.225  248 | 1.11  3505 | 77 |
| {BcHV3.BI=FALSE, BcMV11=FALSE} =>  {BcMV3.BI=FALSE} | 0.19  0594 | 0.601  563 | 0.316  832 | 1.31  3682 | 77 |
| {BcHV3.BI=FALSE, BcMV3.BI=FALSE} =>  {BcMV11=FALSE} | 0.19  0594 | 0.520  27 | 0.366  337 | 1.35  6059 | 77 |
| {BcMV11=FALSE, BcMV3.BI=FALSE} =>  {BcFV5.BI=FALSE} | 0.19  5545 | 0.868  132 | 0.225  248 | 1.10  989 | 79 |
| {BcFV5.BI=FALSE, BcMV11=FALSE} =>  {BcMV3.BI=FALSE} | 0.19  5545 | 0.637  097 | 0.306  931 | 1.39  1282 | 79 |
| {BcFV5.BI=FALSE, BcMV3.BI=FALSE} =>  {BcMV11=FALSE} | 0.19  5545 | 0.496  855 | 0.393  564 | 1.29  5029 | 79 |
| {BcMV11=FALSE, BcMV3.BI=FALSE} =>  {BcULV1.BI=FALSE} | 0.19  802 | 0.879  121 | 0.225  248 | 1.12  0394 | 80 |
| {BcMV11=FALSE, BcULV1.BI=FALSE} =>  {BcMV3.BI=FALSE} | 0.19  802 | 0.634  921 | 0.311  881 | 1.38  6529 | 80 |

| {BcMV3.BI=FALSE, BcULV1.BI=FALSE} =>  {BcMV11=FALSE} | 0.19  802 | 0.503  145 | 0.393  564 | 1.31  1422 | 80 |
| --- | --- | --- | --- | --- | --- |
| {BcMV11=FALSE, BcMV12=FALSE} =>  {BcMV3.BI=FALSE} | 0.19  802 | 0.677  966 | 0.292  079 | 1.48  0531 | 80 |
| {BcMV12=FALSE, BcMV3.BI=FALSE} =>  {BcMV11=FALSE} | 0.19  802 | 0.481  928 | 0.410  891 | 1.25  6121 | 80 |
| {BcMV11=FALSE, BcNSRV7.BI=FALSE} =>  {BcMV3.BI=FALSE} | 0.19  0594 | 0.606  299 | 0.314  356 | 1.32  4026 | 77 |
| {BcMV3.BI=FALSE, BcNSRV7.BI=FALSE} =>  {BcMV11=FALSE} | 0.19  0594 | 0.506  579 | 0.376  238 | 1.32  0374 | 77 |
| {BcMV11=FALSE, BcMV3.BI=FALSE} =>  {BcVV3.BI=FALSE} | 0.20  0495 | 0.890  11 | 0.225  248 | 1.10  989 | 81 |
| {BcMV11=FALSE, BcVV3.BI=FALSE} =>  {BcMV3.BI=FALSE} | 0.20  0495 | 0.627  907 | 0.319  307 | 1.37  1213 | 81 |
| {BcMV3.BI=FALSE, BcVV3.BI=FALSE} =>  {BcMV11=FALSE} | 0.20  0495 | 0.519  231 | 0.386  139 | 1.35  335 | 81 |
| {BcMV11=FALSE, BcOLV7.BI=FALSE} =>  {BcMV3.BI=FALSE} | 0.19  3069 | 0.6 | 0.321  782 | 1.31  027 | 78 |
| {BcMV3.BI=FALSE, BcOLV7.BI=FALSE} =>  {BcMV11=FALSE} | 0.19  3069 | 0.493  671 | 0.391  089 | 1.28  6729 | 78 |
| {BcMV11=FALSE, BcMV3.BI=FALSE} =>  {BcALV1.BI=FALSE} | 0.20  5446 | 0.912  088 | 0.225  248 | 1.12  0011 | 83 |

| {BcALV1.BI=FALSE, BcMV11=FALSE} =>  {BcMV3.BI=FALSE} | 0.20  5446 | 0.580  42 | 0.353  96 | 1.26  7511 | 83 |
| --- | --- | --- | --- | --- | --- |
| {BcALV1.BI=FALSE, BcMV3.BI=FALSE} =>  {BcMV11=FALSE} | 0.20  5446 | 0.525  316 | 0.391  089 | 1.36  9212 | 83 |
| {BcDMV1=FALSE, BcMV11=FALSE} =>  {BcMV3.BI=FALSE} | 0.20  297 | 0.607  407 | 0.334  158 | 1.32  6446 | 82 |
| {BcDMV1=FALSE, BcMV3.BI=FALSE} =>  {BcMV11=FALSE} | 0.20  297 | 0.482  353 | 0.420  792 | 1.25  723 | 82 |
| {BcFV9=FALSE, BcMV11=FALSE} =>  {BcMV3.BI=FALSE} | 0.20  7921 | 0.6 | 0.346  535 | 1.31  027 | 84 |
| {BcFV9=FALSE, BcMV3.BI=FALSE} =>  {BcMV11=FALSE} | 0.20  7921 | 0.502  994 | 0.413  366 | 1.31  103 | 84 |
| {BcMV11=FALSE, BcMV3.BI=FALSE} =>  {BcOLV4.BI=FALSE} | 0.21  5347 | 0.956  044 | 0.225  248 | 1.09  1078 | 87 |
| {BcMV11=FALSE, BcOLV4.BI=FALSE} =>  {BcMV3.BI=FALSE} | 0.21  5347 | 0.591  837 | 0.363  861 | 1.29  2443 | 87 |
| {BcMV3.BI=FALSE, BcOLV4.BI=FALSE} =>  {BcMV11=FALSE} | 0.21  5347 | 0.511  765 | 0.420  792 | 1.33  389 | 87 |
| {BcMV11=FALSE, BcMV3.BI=FALSE} =>  {BcULV5=FALSE} | 0.22  2772 | 0.989  011 | 0.225  248 | 1.12  8702 | 90 |
| {BcMV11=FALSE, BcULV5=FALSE} =>  {BcMV3.BI=FALSE} | 0.22  2772 | 0.6 | 0.371  287 | 1.31  027 | 90 |

| {BcMV3.BI=FALSE, BcULV5=FALSE} =>  {BcMV11=FALSE} | 0.22  2772 | 0.526  316 | 0.423  267 | 1.37  1817 | 90 |
| --- | --- | --- | --- | --- | --- |
| {BcBNV5.BI=FALSE, BcMV11=FALSE} =>  {BcMV3.BI=FALSE} | 0.21  7822 | 0.586  667 | 0.371  287 | 1.28  1153 | 88 |
| {BcBNV5.BI=FALSE, BcMV3.BI=FALSE} =>  {BcMV11=FALSE} | 0.21  7822 | 0.497  175 | 0.438  119 | 1.29  5863 | 88 |
| {BcMV11=FALSE, BvF.BI=FALSE} =>  {BcMV3.BI=FALSE} | 0.19  802 | 0.579  71 | 0.341  584 | 1.26  5962 | 80 |
| {BcMV3.BI=FALSE, BvF.BI=FALSE} =>  {BcMV11=FALSE} | 0.19  802 | 0.473  373 | 0.418  317 | 1.23  3823 | 80 |
| {BcMCV5.BI=FALSE, BcMV11=FALSE} =>  {BcMV3.BI=FALSE} | 0.22  0297 | 0.585  526 | 0.376  238 | 1.27  8663 | 89 |
| {BcMCV5.BI=FALSE, BcMV3.BI=FALSE} =>  {BcMV11=FALSE} | 0.22  0297 | 0.5 | 0.440  594 | 1.30  3226 | 89 |
| {BcMV11=FALSE, BcOLV13.BI=FALSE} =>  {BcMV3.BI=FALSE} | 0.22  0297 | 0.601  351 | 0.366  337 | 1.31  3221 | 89 |
| {BcMV3.BI=FALSE, BcOLV13.BI=FALSE} =>  {BcMV11=FALSE} | 0.22  0297 | 0.489  011 | 0.450  495 | 1.27  4583 | 89 |
| {BcMV11=FALSE, BcOLV2.BI=FALSE} =>  {BcMV3.BI=FALSE} | 0.21  2871 | 0.593  103 | 0.358  911 | 1.29  521 | 86 |
| {BcMV3.BI=FALSE, BcOLV2.BI=FALSE} =>  {BcMV11=FALSE} | 0.21  2871 | 0.480  447 | 0.443  069 | 1.25  2262 | 86 |

| {BcMCV3.BI=FALSE, BcMV11=FALSE} =>  {BcMV3.BI=FALSE} | 0.22  5248 | 0.640  845 | 0.351  485 | 1.39  9467 | 91 |
| --- | --- | --- | --- | --- | --- |
| {BcMCV3.BI=FALSE, BcMV3.BI=FALSE} =>  {BcMV11=FALSE} | 0.22  5248 | 0.491  892 | 0.457  921 | 1.28  2092 | 91 |
| {BcMV11=FALSE, BcPV4.BI=FALSE} =>  {BcMV3.BI=FALSE} | 0.21  2871 | 0.597  222 | 0.356  436 | 1.30  4204 | 86 |
| {BcMV3.BI=FALSE, BcPV4.BI=FALSE} =>  {BcMV11=FALSE} | 0.21  2871 | 0.488  636 | 0.435  644 | 1.27  3607 | 86 |
| {BcEV6=FALSE, BcMV11=FALSE} =>  {BcMV3.BI=FALSE} | 0.20  297 | 0.565  517 | 0.358  911 | 1.23  4967 | 82 |
| {BcEV6=FALSE, BcMV3.BI=FALSE} =>  {BcMV11=FALSE} | 0.20  297 | 0.485  207 | 0.418  317 | 1.26  4669 | 82 |
| {BcMV11=FALSE, BcOLV17.BI=FALSE} =>  {BcMV3.BI=FALSE} | 0.21  7822 | 0.582  781 | 0.373  762 | 1.27  2669 | 88 |
| {BcMV3.BI=FALSE, BcOLV17.BI=FALSE} =>  {BcMV11=FALSE} | 0.21  7822 | 0.5 | 0.435  644 | 1.30  3226 | 88 |
| {BcMV11=FALSE, BcOLV15.BI=FALSE} =>  {BcMV3.BI=FALSE} | 0.21  5347 | 0.595  89 | 0.361  386 | 1.30  1296 | 87 |
| {BcMV3.BI=FALSE, BcOLV15.BI=FALSE} =>  {BcMV11=FALSE} | 0.21  5347 | 0.486  034 | 0.443  069 | 1.26  6823 | 87 |
| {BcEV5=FALSE, BcMV11=FALSE} =>  {BcMV3.BI=FALSE} | 0.22  2772 | 0.592  105 | 0.376  238 | 1.29  303 | 90 |

| {BcEV5=FALSE, BcMV3.BI=FALSE} =>  {BcMV11=FALSE} | 0.22  2772 | 0.489  13 | 0.455  446 | 1.27  4895 | 90 |
| --- | --- | --- | --- | --- | --- |
| {BcMV11=FALSE, BcMV2.BI=TRUE} =>  {BcALV1.BI=FALSE} | 0.11  1386 | 0.918  367 | 0.121  287 | 1.12  7722 | 45 |
| {BcMV11=FALSE, BcMV2.BI=TRUE} =>  {BcDMV1=FALSE} | 0.11  3861 | 0.938  776 | 0.121  287 | 1.11  2215 | 46 |
| {BcMV11=FALSE, BcOLV18=FALSE} =>  {BcMV2.BI=FALSE} | 0.15  3465 | 0.720  93 | 0.212  871 | 1.34  2193 | 62 |
| {BcMV11=FALSE, BcMV2.BI=FALSE} =>  {BcOLV18=FALSE} | 0.15  3465 | 0.584  906 | 0.262  376 | 1.21  1805 | 62 |
| {BcMV2.BI=FALSE, BcOLV18=FALSE} =>  {BcMV11=FALSE} | 0.15  3465 | 0.488  189 | 0.314  356 | 1.27  2441 | 62 |
| {BcMV11=FALSE, BcOLV18=FALSE} =>  {BcMV4.BI=FALSE} | 0.16  3366 | 0.767  442 | 0.212  871 | 1.37  7984 | 66 |
| {BcMV11=FALSE, BcMV4.BI=FALSE} =>  {BcOLV18=FALSE} | 0.16  3366 | 0.584  071 | 0.279  703 | 1.21  0075 | 66 |
| {BcMV4.BI=FALSE, BcOLV18=FALSE} =>  {BcMV11=FALSE} | 0.16  3366 | 0.515  625 | 0.316  832 | 1.34  3952 | 66 |
| {BcMV11=FALSE, BcOLV18=FALSE} =>  {BcOLV16.BI=FALSE} | 0.15  099 | 0.709  302 | 0.212  871 | 1.21  4229 | 61 |
| {BcMV11=FALSE, BcOLV16.BI=FALSE} =>  {BcOLV18=FALSE} | 0.15  099 | 0.622  449 | 0.242  574 | 1.28  9587 | 61 |

| {BcMV11=FALSE, BcOLV18=FALSE} =>  {BcOLV1.BI.MOVA.=FALSE} | 0.19  0594 | 0.895  349 | 0.212  871 | 1.33  9707 | 77 |
| --- | --- | --- | --- | --- | --- |
| {BcMV11=FALSE, BcOLV1.BI.MOVA.=FALSE} =>  {BcOLV18=FALSE} | 0.19  0594 | 0.647  059 | 0.294  554 | 1.34  0573 | 77 |
| {BcOLV1.BI.MOVA.=FALSE,  BcOLV18=FALSE} => {BcMV11=FALSE} | 0.19  0594 | 0.5 | 0.381  188 | 1.30  3226 | 77 |
| {BcMV9.BI=TRUE, BcOLV18=FALSE} =>  {BcMV11=FALSE} | 0.13  3663 | 0.504  673 | 0.264  851 | 1.31  5405 | 54 |
| {BcOLV18=FALSE, BcULV4=TRUE} =>  {BcMV11=FALSE} | 0.12  8713 | 0.514  851 | 0.25 | 1.34  1935 | 52 |
| {BcMV6.BI=FALSE, BcOLV18=FALSE} =>  {BcMV11=FALSE} | 0.16  3366 | 0.464  789 | 0.351  485 | 1.21  1449 | 66 |
| {BcMV11=FALSE, BcOLV18=FALSE} =>  {BcPV3.BI=FALSE} | 0.17  0792 | 0.802  326 | 0.212  871 | 1.12  9406 | 69 |
| {BcMV11=FALSE, BcPV3.BI=FALSE} =>  {BcOLV18=FALSE} | 0.17  0792 | 0.584  746 | 0.292  079 | 1.21  1473 | 69 |
| {BcMV11=FALSE, BcOLV18=FALSE} =>  {BcRV1.BI=FALSE} | 0.18  0693 | 0.848  837 | 0.212  871 | 1.17  4419 | 73 |
| {BcMV11=FALSE, BcRV1.BI=FALSE} =>  {BcOLV18=FALSE} | 0.18  0693 | 0.574  803 | 0.314  356 | 1.19  0874 | 73 |
| {BcMV11=FALSE, BcOLV18=FALSE} => | 0.18 | 0.883 | 0.212 | 1.16 | 76 |

| {BcHV3.BI=FALSE} | 8119 | 721 | 871 | 2942 |  |
| --- | --- | --- | --- | --- | --- |
| {BcHV3.BI=FALSE, BcMV11=FALSE} =>  {BcOLV18=FALSE} | 0.18  8119 | 0.593  75 | 0.316  832 | 1.23  0128 | 76 |
| {BcHV3.BI=FALSE, BcOLV18=FALSE} =>  {BcMV11=FALSE} | 0.18  8119 | 0.460  606 | 0.408  416 | 1.20  0547 | 76 |
| {BcFV5.BI=FALSE, BcMV11=FALSE} =>  {BcOLV18=FALSE} | 0.18  3168 | 0.596  774 | 0.306  931 | 1.23  6394 | 74 |
| {BcMV11=FALSE, BcOLV18=FALSE} =>  {BcULV1.BI=FALSE} | 0.19  802 | 0.930  233 | 0.212  871 | 1.18  5533 | 80 |
| {BcMV11=FALSE, BcULV1.BI=FALSE} =>  {BcOLV18=FALSE} | 0.19  802 | 0.634  921 | 0.311  881 | 1.31  5425 | 80 |
| {BcOLV18=FALSE, BcULV1.BI=FALSE} =>  {BcMV11=FALSE} | 0.19  802 | 0.462  428 | 0.428  218 | 1.20  5296 | 80 |
| {BcMV11=FALSE, BcMV12=FALSE} =>  {BcOLV18=FALSE} | 0.17  3267 | 0.593  22 | 0.292  079 | 1.22  9031 | 70 |
| {BcMV11=FALSE, BcOLV7.BI=FALSE} =>  {BcOLV18=FALSE} | 0.18  5644 | 0.576  923 | 0.321  782 | 1.19  5266 | 75 |
| {BcMV11=FALSE, BcOLV18=FALSE} =>  {BcALV1.BI=FALSE} | 0.20  0495 | 0.941  86 | 0.212  871 | 1.15  657 | 81 |
| {BcALV1.BI=FALSE, BcMV11=FALSE} =>  {BcOLV18=FALSE} | 0.20  0495 | 0.566  434 | 0.353  96 | 1.17  3534 | 81 |
| {BcALV1.BI=FALSE, BcOLV18=FALSE} => | 0.20 | 0.457 | 0.438 | 1.19 | 81 |

| {BcMV11=FALSE} | 0495 | 627 | 119 | 2783 |  |
| --- | --- | --- | --- | --- | --- |
| {BcMV11=FALSE, BcOLV18=FALSE} =>  {BcFV9=FALSE} | 0.19  802 | 0.930  233 | 0.212  871 | 1.07  0695 | 80 |
| {BcFV9=FALSE, BcMV11=FALSE} =>  {BcOLV18=FALSE} | 0.19  802 | 0.571  429 | 0.346  535 | 1.18  3883 | 80 |
| {BcOLV18=FALSE, BcOLV4.BI=FALSE} =>  {BcMV11=FALSE} | 0.19  5545 | 0.456  647 | 0.428  218 | 1.19  0229 | 79 |
| {BcMV11=FALSE, BcOLV18=FALSE} =>  {BcULV5=FALSE} | 0.20  7921 | 0.976  744 | 0.212  871 | 1.11  4702 | 84 |
| {BcOLV18=FALSE, BcULV5=FALSE} =>  {BcMV11=FALSE} | 0.20  7921 | 0.509  091 | 0.408  416 | 1.32  6921 | 84 |
| {BcMV11=FALSE, BcOLV18=FALSE} =>  {BcMCV5.BI=FALSE} | 0.21  2871 | 1 | 0.212  871 | 1.08  311 | 86 |
| {BcOLV13.BI=FALSE, BcOLV18=FALSE} =>  {BcMV11=FALSE} | 0.20  5446 | 0.456  044 | 0.450  495 | 1.18  8657 | 83 |
| {BcMCV3.BI=FALSE, BcMV11=FALSE} =>  {BcOLV18=FALSE} | 0.20  0495 | 0.570  423 | 0.351  485 | 1.18  1798 | 81 |
| {BcMV2.BI=FALSE, BcOLV18=TRUE} =>  {BcMV11=FALSE} | 0.10  8911 | 0.488  889 | 0.222  772 | 1.27  4265 | 44 |
| {BcMV11=FALSE, BcOLV18=TRUE} =>  {BcMV4.BI=FALSE} | 0.11  6337 | 0.681  159 | 0.170  792 | 1.22  306 | 47 |
| {BcMV4.BI=FALSE, BcOLV18=TRUE} => | 0.11 | 0.484 | 0.240 | 1.26 | 47 |

| {BcMV11=FALSE} | 6337 | 536 | 099 | 292 |  |
| --- | --- | --- | --- | --- | --- |
| {BcMV11=FALSE, BcOLV18=TRUE} =>  {BcMV9.BI=TRUE} | 0.13  8614 | 0.811  594 | 0.170  792 | 1.19  6657 | 56 |
| {BcMV11=FALSE, BcOLV18=TRUE} =>  {BcULV4=TRUE} | 0.14  3564 | 0.840  58 | 0.170  792 | 1.21  7184 | 58 |
| {BcMV11=FALSE, BcOLV18=TRUE} =>  {BcMV6.BI=FALSE} | 0.14  3564 | 0.840  58 | 0.170  792 | 1.20  852 | 58 |
| {BcMV11=FALSE, BcOLV18=TRUE} =>  {BcEV2.BI=FALSE} | 0.14  604 | 0.855  072 | 0.170  792 | 1.14  3872 | 59 |
| {BcMV11=FALSE, BcOLV18=TRUE} =>  {BcALV1.BI=FALSE} | 0.15  3465 | 0.898  551 | 0.170  792 | 1.10  3388 | 62 |
| {BcMV11=FALSE, BcOLV18=TRUE} =>  {BcOLV4.BI=FALSE} | 0.16  8317 | 0.985  507 | 0.170  792 | 1.12  4703 | 68 |
| {BcMV11=FALSE, BcOLV18=TRUE} =>  {BcULV5=FALSE} | 0.16  3366 | 0.956  522 | 0.170  792 | 1.09  1624 | 66 |
| {BcMV11=FALSE, BcOLV18=TRUE} =>  {BcBNV5.BI=FALSE} | 0.16  8317 | 0.985  507 | 0.170  792 | 1.08  4864 | 68 |
| {BcHV1SLR.BI=TRUE, BcMV11=FALSE} =>  {BcMV2.BI=FALSE} | 0.12  6238 | 0.68 | 0.185  644 | 1.26  5991 | 51 |
| {BcMV11=FALSE, BcMV2.BI=FALSE} =>  {BcMV4.BI=FALSE} | 0.19  802 | 0.754  717 | 0.262  376 | 1.35  5136 | 80 |
| {BcMV11=FALSE, BcMV4.BI=FALSE} => | 0.19 | 0.707 | 0.279 | 1.31 | 80 |

| {BcMV2.BI=FALSE} | 802 | 965 | 703 | 8054 |  |
| --- | --- | --- | --- | --- | --- |
| {BcMV2.BI=FALSE, BcMV4.BI=FALSE} => | 0.19 | 0.536 | 0.368 | 1.39 |  |
|  |  |  |  |  | 80 |
| {BcMV11=FALSE} | 802 | 913 | 812 | 9437 |  |
| {BcMV11=FALSE, BcMV2.BI=FALSE} => | 0.17 | 0.679 | 0.262 | 1.16 |  |
|  |  |  |  |  | 72 |
| {BcOLV16.BI=FALSE} | 8218 | 245 | 376 | 2776 |  |
| {BcMV11=FALSE, BcOLV16.BI=FALSE} => | 0.17 | 0.734 | 0.242 | 1.36 |  |
|  |  |  |  |  | 72 |
| {BcMV2.BI=FALSE} | 8218 | 694 | 574 | 7817 |  |
| {BcMV2.BI=FALSE, BcOLV16.BI=FALSE} => | 0.17 | 0.476 | 0.373 | 1.24 |  |
|  |  |  |  |  | 72 |
| {BcMV11=FALSE} | 8218 | 821 | 762 | 2811 |  |
| {BcMV11=FALSE, BcULV3=TRUE} => | 0.15 | 0.762 | 0.198 | 1.41 |  |
|  |  |  |  |  | 61 |
| {BcMV2.BI=FALSE} | 099 | 5 | 02 | 9585 |  |
| {BcMV2.BI=FALSE, BcULV3=TRUE} => | 0.15 |  | 0.309 | 1.27 |  |
|  |  | 0.488 |  |  | 61 |
| {BcMV11=FALSE} | 099 |  | 406 | 1948 |  |
| {BcHV1.BI=TRUE, BcMV11=FALSE} => | 0.16 | 0.722 | 0.222 | 1.34 |  |
|  |  |  |  |  | 65 |
| {BcMV2.BI=FALSE} | 0891 | 222 | 772 | 4598 |  |
| {BcHV1.BI=TRUE, BcMV2.BI=FALSE} => | 0.16 | 0.515 | 0.311 | 1.34 |  |
|  |  |  |  |  | 65 |
| {BcMV11=FALSE} | 0891 | 873 | 881 | 4598 |  |
| {BcMV11=FALSE, BcMV2.BI=FALSE} => | 0.22 | 0.839 | 0.262 | 1.25 |  |
|  |  |  |  |  | 89 |
| {BcOLV1.BI.MOVA.=FALSE} | 0297 | 623 | 376 | 6324 |  |
| {BcMV11=FALSE, |  |  |  |  |  |
|  | 0.22 | 0.747 | 0.294 | 1.39 |  |
| BcOLV1.BI.MOVA.=FALSE} => |  |  |  |  | 89 |
|  | 0297 | 899 | 554 | 2402 |  |
| {BcMV2.BI=FALSE} |  |  |  |  |  |

| {BcMV2.BI=FALSE, BcOLV1.BI.MOVA.=FALSE} =>  {BcMV11=FALSE} | 0.22  0297 | 0.483  696 | 0.455  446 | 1.26  0729 | 89 |
| --- | --- | --- | --- | --- | --- |
| {BcMV11=FALSE, BcMV9.BI=TRUE} =>  {BcMV2.BI=FALSE} | 0.18  0693 | 0.663  636 | 0.272  277 | 1.23  5526 | 73 |
| {BcMV2.BI=FALSE, BcMV9.BI=TRUE} =>  {BcMV11=FALSE} | 0.18  0693 | 0.506  944 | 0.356  436 | 1.32  1326 | 73 |
| {BcMV11=FALSE, BcULV4=TRUE} =>  {BcMV2.BI=FALSE} | 0.18  5644 | 0.681  818 | 0.272  277 | 1.26  9376 | 75 |
| {BcMV2.BI=FALSE, BcULV4=TRUE} =>  {BcMV11=FALSE} | 0.18  5644 | 0.531  915 | 0.349  01 | 1.38  641 | 75 |
| {BcMV11=FALSE, BcMV2.BI=FALSE} =>  {BcMV6.BI=FALSE} | 0.21  7822 | 0.830  189 | 0.262  376 | 1.19  3581 | 88 |
| {BcMV11=FALSE, BcMV6.BI=FALSE} =>  {BcMV2.BI=FALSE} | 0.21  7822 | 0.709  677 | 0.306  931 | 1.32  1243 | 88 |
| {BcMV2.BI=FALSE, BcMV6.BI=FALSE} =>  {BcMV11=FALSE} | 0.21  7822 | 0.494  382 | 0.440  594 | 1.28  8583 | 88 |
| {BcMV11=FALSE, BcPV3.BI=FALSE} =>  {BcMV2.BI=FALSE} | 0.20  7921 | 0.711  864 | 0.292  079 | 1.32  5314 | 84 |
| {BcMV2.BI=FALSE, BcPV3.BI=FALSE} =>  {BcMV11=FALSE} | 0.20  7921 | 0.488  372 | 0.425  743 | 1.27  2918 | 84 |
| {BcMV11=FALSE, BcMV2.BI=FALSE} => | 0.22 | 0.849 | 0.262 | 1.17 | 90 |

| {BcRV1.BI=FALSE} | 2772 | 057 | 376 | 4722 |  |
| --- | --- | --- | --- | --- | --- |
| {BcMV11=FALSE, BcRV1.BI=FALSE} =>  {BcMV2.BI=FALSE} | 0.22  2772 | 0.708  661 | 0.314  356 | 1.31  9351 | 90 |
| {BcMV2.BI=FALSE, BcRV1.BI=FALSE} =>  {BcMV11=FALSE} | 0.22  2772 | 0.508  475 | 0.438  119 | 1.32  5314 | 90 |
| {BcMV11=FALSE, BcMV2.BI=FALSE} =>  {BcEV2.BI=FALSE} | 0.22  0297 | 0.839  623 | 0.262  376 | 1.12  3204 | 89 |
| {BcEV2.BI=FALSE, BcMV11=FALSE} =>  {BcMV2.BI=FALSE} | 0.22  0297 | 0.695  313 | 0.316  832 | 1.29  4499 | 89 |
| {BcEV2.BI=FALSE, BcMV2.BI=FALSE} =>  {BcMV11=FALSE} | 0.22  0297 | 0.514  451 | 0.428  218 | 1.34  0891 | 89 |
| {BcHV3.BI=FALSE, BcMV11=FALSE} =>  {BcMV2.BI=FALSE} | 0.21  7822 | 0.687  5 | 0.316  832 | 1.27  9954 | 88 |
| {BcHV3.BI=FALSE, BcMV2.BI=FALSE} =>  {BcMV11=FALSE} | 0.21  7822 | 0.505  747 | 0.430  693 | 1.31  8205 | 88 |
| {BcFV5.BI=FALSE, BcMV11=FALSE} =>  {BcMV2.BI=FALSE} | 0.20  297 | 0.661  29 | 0.306  931 | 1.23  1158 | 82 |
| {BcFV5.BI=FALSE, BcMV2.BI=FALSE} =>  {BcMV11=FALSE} | 0.20  297 | 0.463  277 | 0.438  119 | 1.20  7509 | 82 |
| {BcMV11=FALSE, BcULV1.BI=FALSE} =>  {BcMV2.BI=FALSE} | 0.22  2772 | 0.714  286 | 0.311  881 | 1.32  9822 | 90 |
| {BcMV2.BI=FALSE, BcULV1.BI=FALSE} => | 0.22 | 0.483 | 0.460 | 1.26 | 90 |

| {BcMV11=FALSE} | 2772 | 871 | 396 | 1186 |  |
| --- | --- | --- | --- | --- | --- |
| {BcMV1.BI=TRUE, BcMV11=FALSE} =>  {BcMV2.BI=FALSE} | 0.17  5743 | 0.651  376 | 0.269  802 | 1.21  27 | 71 |
| {BcMV1.BI=TRUE, BcMV2.BI=FALSE} =>  {BcMV11=FALSE} | 0.17  5743 | 0.461  039 | 0.381  188 | 1.20  1676 | 71 |
| {BcMV11=FALSE, BcMV12=FALSE} =>  {BcMV2.BI=FALSE} | 0.21  0396 | 0.720  339 | 0.292  079 | 1.34  1092 | 85 |
| {BcMV12=FALSE, BcMV2.BI=FALSE} =>  {BcMV11=FALSE} | 0.21  0396 | 0.459  459 | 0.457  921 | 1.19  7559 | 85 |
| {BcMV11=FALSE, BcNSRV7.BI=FALSE} =>  {BcMV2.BI=FALSE} | 0.21  2871 | 0.677  165 | 0.314  356 | 1.26  0713 | 86 |
| {BcMV2.BI=FALSE, BcNSRV7.BI=FALSE} =>  {BcMV11=FALSE} | 0.21  2871 | 0.485  876 | 0.438  119 | 1.26  6412 | 86 |
| {BcMV11=FALSE, BcVV3.BI=FALSE} =>  {BcMV2.BI=FALSE} | 0.22  2772 | 0.697  674 | 0.319  307 | 1.29  8896 | 90 |
| {BcMV2.BI=FALSE, BcVV3.BI=FALSE} =>  {BcMV11=FALSE} | 0.22  2772 | 0.494  505 | 0.450  495 | 1.28  8905 | 90 |
| {BcMV11=FALSE, BcOLV7.BI=FALSE} =>  {BcMV2.BI=FALSE} | 0.22  2772 | 0.692  308 | 0.321  782 | 1.28  8905 | 90 |
| {BcMV2.BI=FALSE, BcOLV7.BI=FALSE} =>  {BcMV11=FALSE} | 0.22  2772 | 0.497  238 | 0.448  02 | 1.29  6026 | 90 |
| {BcMV11=FALSE, BcMV2.BI=FALSE} => | 0.24 | 0.924 | 0.262 | 1.13 | 98 |

| {BcALV1.BI=FALSE} | 2574 | 528 | 376 | 5287 |  |
| --- | --- | --- | --- | --- | --- |
| {BcALV1.BI=FALSE, BcMV11=FALSE} =>  {BcMV2.BI=FALSE} | 0.24  2574 | 0.685  315 | 0.353  96 | 1.27  5885 | 98 |
| {BcALV1.BI=FALSE, BcMV2.BI=FALSE} =>  {BcMV11=FALSE} | 0.24  2574 | 0.494  949 | 0.490  099 | 1.29  0062 | 98 |
| {BcDMV1=FALSE, BcMV11=FALSE} =>  {BcMV2.BI=FALSE} | 0.22  0297 | 0.659  259 | 0.334  158 | 1.22  7377 | 89 |
| {BcDMV1=FALSE, BcMV2.BI=FALSE} =>  {BcMV11=FALSE} | 0.22  0297 | 0.491  713 | 0.448  02 | 1.28  1625 | 89 |
| {BcFV9=FALSE, BcMV11=FALSE} =>  {BcMV2.BI=FALSE} | 0.23  5149 | 0.678  571 | 0.346  535 | 1.26  3331 | 95 |
| {BcFV9=FALSE, BcMV2.BI=FALSE} =>  {BcMV11=FALSE} | 0.23  5149 | 0.502  646 | 0.467  822 | 1.31  0121 | 95 |
| {BcMV11=FALSE, BcMV2.BI=FALSE} =>  {BcOLV4.BI=FALSE} | 0.25  495 | 0.971  698 | 0.262  376 | 1.10  8944 | 10  3 |
| {BcMV11=FALSE, BcOLV4.BI=FALSE} =>  {BcMV2.BI=FALSE} | 0.25  495 | 0.700  68 | 0.363  861 | 1.30  4492 | 10  3 |
| {BcMV2.BI=FALSE, BcOLV4.BI=FALSE} =>  {BcMV11=FALSE} | 0.25  495 | 0.509  901 | 0.5 | 1.32  9032 | 10  3 |
| {BcMV11=FALSE, BcMV2.BI=FALSE} =>  {BcULV5=FALSE} | 0.25  7426 | 0.981  132 | 0.262  376 | 1.11  971 | 10  4 |
| {BcMV11=FALSE, BcULV5=FALSE} => | 0.25 | 0.693 | 0.371 | 1.29 | 10 |

| {BcMV2.BI=FALSE} | 7426 | 333 | 287 | 0814 | 4 |
| --- | --- | --- | --- | --- | --- |
| {BcMV2.BI=FALSE, BcULV5=FALSE} =>  {BcMV11=FALSE} | 0.25  7426 | 0.514  851 | 0.5 | 1.34  1935 | 10  4 |
| {BcBNV5.BI=FALSE, BcMV11=FALSE} =>  {BcMV2.BI=FALSE} | 0.25  2475 | 0.68 | 0.371  287 | 1.26  5991 | 10  2 |
| {BcBNV5.BI=FALSE, BcMV2.BI=FALSE} =>  {BcMV11=FALSE} | 0.25  2475 | 0.504  95 | 0.5 | 1.31  6129 | 10  2 |
| {BcMV11=FALSE, BvF.BI=FALSE} =>  {BcMV2.BI=FALSE} | 0.23  2673 | 0.681  159 | 0.341  584 | 1.26  8149 | 94 |
| {BcMV2.BI=FALSE, BvF.BI=FALSE} =>  {BcMV11=FALSE} | 0.23  2673 | 0.484  536 | 0.480  198 | 1.26  292 | 94 |
| {BcMCV5.BI=FALSE, BcMV11=FALSE} =>  {BcMV2.BI=FALSE} | 0.25  7426 | 0.684  211 | 0.376  238 | 1.27  383 | 10  4 |
| {BcMCV5.BI=FALSE, BcMV2.BI=FALSE} =>  {BcMV11=FALSE} | 0.25  7426 | 0.492  891 | 0.522  277 | 1.28  4697 | 10  4 |
| {BcMV11=FALSE, BcOLV13.BI=FALSE} =>  {BcMV2.BI=FALSE} | 0.25  2475 | 0.689  189 | 0.366  337 | 1.28  3099 | 10  2 |
| {BcMV2.BI=FALSE, BcOLV13.BI=FALSE} =>  {BcMV11=FALSE} | 0.25  2475 | 0.488  038 | 0.517  327 | 1.27  2048 | 10  2 |
| {BcMV11=FALSE, BcOLV2.BI=FALSE} =>  {BcMV2.BI=FALSE} | 0.24  7525 | 0.689  655 | 0.358  911 | 1.28  3966 | 10  0 |
| {BcMV2.BI=FALSE, BcOLV2.BI=FALSE} => | 0.24 | 0.487 | 0.507 | 1.27 | 10 |

| {BcMV11=FALSE} | 7525 | 805 | 426 | 144 | 0 |
| --- | --- | --- | --- | --- | --- |
| {BcMCV3.BI=FALSE, BcMV11=FALSE} =>  {BcMV2.BI=FALSE} | 0.25  2475 | 0.718  31 | 0.351  485 | 1.33  7314 | 10  2 |
| {BcMCV3.BI=FALSE, BcMV2.BI=FALSE} =>  {BcMV11=FALSE} | 0.25  2475 | 0.481  132 | 0.524  752 | 1.25  4047 | 10  2 |
| {BcMV11=FALSE, BcPV4.BI=FALSE} =>  {BcMV2.BI=FALSE} | 0.24  2574 | 0.680  556 | 0.356  436 | 1.26  7025 | 98 |
| {BcMV2.BI=FALSE, BcPV4.BI=FALSE} =>  {BcMV11=FALSE} | 0.24  2574 | 0.480  392 | 0.504  95 | 1.25  2119 | 98 |
| {BcEV6=FALSE, BcMV11=FALSE} =>  {BcMV2.BI=FALSE} | 0.24  505 | 0.682  759 | 0.358  911 | 1.27  1127 | 99 |
| {BcEV6=FALSE, BcMV2.BI=FALSE} =>  {BcMV11=FALSE} | 0.24  505 | 0.487  685 | 0.502  475 | 1.27  1127 | 99 |
| {BcMV11=FALSE, BcOLV17.BI=FALSE} =>  {BcMV2.BI=FALSE} | 0.25  495 | 0.682  119 | 0.373  762 | 1.26  9936 | 10  3 |
| {BcMV2.BI=FALSE, BcOLV17.BI=FALSE} =>  {BcMV11=FALSE} | 0.25  495 | 0.488  152 | 0.522  277 | 1.27  2344 | 10  3 |
| {BcMV11=FALSE, BcOLV15.BI=FALSE} =>  {BcMV2.BI=FALSE} | 0.24  0099 | 0.664  384 | 0.361  386 | 1.23  6917 | 97 |
| {BcMV2.BI=FALSE, BcOLV15.BI=FALSE} =>  {BcMV11=FALSE} | 0.24  0099 | 0.482  587 | 0.497  525 | 1.25  784 | 97 |
| {BcEV5=FALSE, BcMV11=FALSE} => | 0.25 | 0.684 | 0.376 | 1.27 | 10 |

| {BcMV2.BI=FALSE} | 7426 | 211 | 238 | 383 | 4 |
| --- | --- | --- | --- | --- | --- |
| {BcEV5=FALSE, BcMV2.BI=FALSE} =>  {BcMV11=FALSE} | 0.25  7426 | 0.488  263 | 0.527  228 | 1.27  2634 | 10  4 |
| {BcHV1SLR.BI=TRUE, BcMV11=FALSE} =>  {BcMV4.BI=FALSE} | 0.12  6238 | 0.68 | 0.185  644 | 1.22  0978 | 51 |
| {BcHV1SLR.BI=TRUE, BcMV11=FALSE} =>  {BcHV1.BI=TRUE} | 0.17  0792 | 0.92 | 0.185  644 | 1.52  3279 | 69 |
| {BcHV1.BI=TRUE, BcMV11=FALSE} =>  {BcHV1SLR.BI=TRUE} | 0.17  0792 | 0.766  667 | 0.222  772 | 1.41  4307 | 69 |
| {BcHV1SLR.BI=TRUE, BcMV11=FALSE} =>  {BcMV9.BI=TRUE} | 0.14  8515 | 0.8 | 0.185  644 | 1.17  9562 | 60 |
| {BcHV1SLR.BI=TRUE, BcMV11=FALSE} =>  {BcRV1.BI=FALSE} | 0.16  0891 | 0.866  667 | 0.185  644 | 1.19  9087 | 65 |
| {BcHV1SLR.BI=TRUE, BcMV11=FALSE} =>  {BcALV1.BI=FALSE} | 0.16  8317 | 0.906  667 | 0.185  644 | 1.11  3354 | 68 |
| {BcHV1SLR.BI=TRUE, BcMV11=FALSE} =>  {BcDMV1=FALSE} | 0.17  0792 | 0.92 | 0.185  644 | 1.08  9971 | 69 |
| {BcHV1SLR.BI=TRUE, BcMV11=FALSE} =>  {BcOLV4.BI=FALSE} | 0.17  5743 | 0.946  667 | 0.185  644 | 1.08  0377 | 71 |
| {BcHV1SLR.BI=TRUE, BcMV11=FALSE} =>  {BcULV5=FALSE} | 0.17  8218 | 0.96 | 0.185  644 | 1.09  5593 | 72 |
| {BcMV11=FALSE, BcMV3.BI=TRUE} => | 0.13 | 0.828 | 0.158 | 1.14 | 53 |

| {BcRV1.BI=FALSE} | 1188 | 125 | 416 | 5762 |  |
| --- | --- | --- | --- | --- | --- |
| {BcMV11=FALSE, BcMV3.BI=TRUE} => | 0.13 |  | 0.158 | 1.17 |  |
|  |  | 0.875 |  |  | 56 |
| {BcEV2.BI=FALSE} | 8614 |  | 416 | 053 |  |
| {BcMV11=FALSE, BcMV3.BI=TRUE} => | 0.14 | 0.937 | 0.158 | 1.15 |  |
|  |  |  |  |  | 60 |
| {BcALV1.BI=FALSE} | 8515 | 5 | 416 | 1216 |  |
| {BcMV11=FALSE, BcOLV16.BI=FALSE} => | 0.18 | 0.765 | 0.242 | 1.37 |  |
|  |  |  |  |  | 75 |
| {BcMV4.BI=FALSE} | 5644 | 306 | 574 | 415 |  |
| {BcMV4.BI=FALSE, BcOLV16.BI=FALSE} => | 0.18 | 0.506 | 0.366 | 1.32 |  |
|  |  |  |  |  | 75 |
| {BcMV11=FALSE} | 5644 | 757 | 337 | 0837 |  |
| {BcMV11=FALSE, BcULV3=TRUE} => | 0.14 |  | 0.198 | 1.30 |  |
|  |  | 0.725 |  |  | 58 |
| {BcMV4.BI=FALSE} | 3564 |  | 02 | 1778 |  |
| {BcMV4.BI=FALSE, BcULV3=TRUE} => | 0.14 | 0.479 | 0.299 | 1.24 |  |
|  |  |  |  |  | 58 |
| {BcMV11=FALSE} | 3564 | 339 | 505 | 9374 |  |
| {BcHV1.BI=TRUE, BcMV11=FALSE} => | 0.15 | 0.688 | 0.222 | 1.23 |  |
|  |  |  |  |  | 62 |
| {BcMV4.BI=FALSE} | 3465 | 889 | 772 | 6938 |  |
| {BcHV1.BI=TRUE, BcMV4.BI=FALSE} => | 0.15 | 0.504 | 0.304 | 1.31 |  |
|  |  |  |  |  | 62 |
| {BcMV11=FALSE} | 3465 | 065 | 455 | 3821 |  |
| {BcMV11=FALSE, BcMV4.BI=FALSE} => | 0.21 | 0.761 | 0.279 | 1.13 |  |
|  |  |  |  |  | 86 |
| {BcOLV1.BI.MOVA.=FALSE} | 2871 | 062 | 703 | 8774 |  |
| {BcMV11=FALSE, |  |  |  |  |  |
|  | 0.21 | 0.722 | 0.294 | 1.29 |  |
| BcOLV1.BI.MOVA.=FALSE} => |  |  |  |  | 86 |
|  | 2871 | 689 | 554 | 7628 |  |
| {BcMV4.BI=FALSE} |  |  |  |  |  |

| {BcMV4.BI=FALSE, BcOLV1.BI.MOVA.=FALSE} =>  {BcMV11=FALSE} | 0.21  2871 | 0.518  072 | 0.410  891 | 1.35  033 | 86 |
| --- | --- | --- | --- | --- | --- |
| {BcMV11=FALSE, BcMV9.BI=TRUE} =>  {BcMV4.BI=FALSE} | 0.19  0594 | 0.7 | 0.272  277 | 1.25  6889 | 77 |
| {BcMV4.BI=FALSE, BcMV9.BI=TRUE} =>  {BcMV11=FALSE} | 0.19  0594 | 0.557  971 | 0.341  584 | 1.45  4324 | 77 |
| {BcMV11=FALSE, BcULV4=TRUE} =>  {BcMV4.BI=FALSE} | 0.19  802 | 0.727  273 | 0.272  277 | 1.30  5859 | 80 |
| {BcMV4.BI=FALSE, BcULV4=TRUE} =>  {BcMV11=FALSE} | 0.19  802 | 0.540  541 | 0.366  337 | 1.40  8893 | 80 |
| {BcMV11=FALSE, BcMV4.BI=FALSE} =>  {BcMV6.BI=FALSE} | 0.22  2772 | 0.796  46 | 0.279  703 | 1.14  5089 | 90 |
| {BcMV11=FALSE, BcMV6.BI=FALSE} =>  {BcMV4.BI=FALSE} | 0.22  2772 | 0.725  806 | 0.306  931 | 1.30  3226 | 90 |
| {BcMV4.BI=FALSE, BcMV6.BI=FALSE} =>  {BcMV11=FALSE} | 0.22  2772 | 0.520  231 | 0.428  218 | 1.35  5957 | 90 |
| {BcMV11=FALSE, BcMV4.BI=FALSE} =>  {BcPV3.BI=FALSE} | 0.22  5248 | 0.805  31 | 0.279  703 | 1.13  3607 | 91 |
| {BcMV11=FALSE, BcPV3.BI=FALSE} =>  {BcMV4.BI=FALSE} | 0.22  5248 | 0.771  186 | 0.292  079 | 1.38  4708 | 91 |
| {BcMV4.BI=FALSE, BcPV3.BI=FALSE} => | 0.22 | 0.502 | 0.448 | 1.31 | 91 |

| {BcMV11=FALSE} | 5248 | 762 | 02 | 0426 |  |
| --- | --- | --- | --- | --- | --- |
| {BcMV11=FALSE, BcMV4.BI=FALSE} =>  {BcRV1.BI=FALSE} | 0.23  7624 | 0.849  558 | 0.279  703 | 1.17  5415 | 96 |
| {BcMV11=FALSE, BcRV1.BI=FALSE} =>  {BcMV4.BI=FALSE} | 0.23  7624 | 0.755  906 | 0.314  356 | 1.35  727 | 96 |
| {BcMV4.BI=FALSE, BcRV1.BI=FALSE} =>  {BcMV11=FALSE} | 0.23  7624 | 0.533  333 | 0.445  545 | 1.39  0108 | 96 |
| {BcMV11=FALSE, BcMV4.BI=FALSE} =>  {BcEV2.BI=FALSE} | 0.24  2574 | 0.867  257 | 0.279  703 | 1.16  0171 | 98 |
| {BcEV2.BI=FALSE, BcMV11=FALSE} =>  {BcMV4.BI=FALSE} | 0.24  2574 | 0.765  625 | 0.316  832 | 1.37  4722 | 98 |
| {BcEV2.BI=FALSE, BcMV4.BI=FALSE} =>  {BcMV11=FALSE} | 0.24  2574 | 0.56 | 0.433  168 | 1.45  9613 | 98 |
| {BcMV11=FALSE, BcMV4.BI=FALSE} =>  {BcHV3.BI=FALSE} | 0.23  5149 | 0.840  708 | 0.279  703 | 1.10  6339 | 95 |
| {BcHV3.BI=FALSE, BcMV11=FALSE} =>  {BcMV4.BI=FALSE} | 0.23  5149 | 0.742  188 | 0.316  832 | 1.33  2639 | 95 |
| {BcHV3.BI=FALSE, BcMV4.BI=FALSE} =>  {BcMV11=FALSE} | 0.23  5149 | 0.502  646 | 0.467  822 | 1.31  0121 | 95 |
| {BcFV5.BI=FALSE, BcMV11=FALSE} =>  {BcMV4.BI=FALSE} | 0.22  5248 | 0.733  871 | 0.306  931 | 1.31  7706 | 91 |
| {BcFV5.BI=FALSE, BcMV4.BI=FALSE} => | 0.22 | 0.489 | 0.460 | 1.27 | 91 |

| {BcMV11=FALSE} | 5248 | 247 | 396 | 5199 |  |
| --- | --- | --- | --- | --- | --- |
| {BcMV11=FALSE, BcULV1.BI=FALSE} =>  {BcMV4.BI=FALSE} | 0.23  0198 | 0.738  095 | 0.311  881 | 1.32  5291 | 93 |
| {BcMV4.BI=FALSE, BcULV1.BI=FALSE} =>  {BcMV11=FALSE} | 0.23  0198 | 0.510  989 | 0.450  495 | 1.33  1868 | 93 |
| {BcMV1.BI=TRUE, BcMV11=FALSE} =>  {BcMV4.BI=FALSE} | 0.19  5545 | 0.724  771 | 0.269  802 | 1.30  1366 | 79 |
| {BcMV1.BI=TRUE, BcMV4.BI=FALSE} =>  {BcMV11=FALSE} | 0.19  5545 | 0.473  054 | 0.413  366 | 1.23  2992 | 79 |
| {BcMV11=FALSE, BcMV12=FALSE} =>  {BcMV4.BI=FALSE} | 0.21  0396 | 0.720  339 | 0.292  079 | 1.29  3409 | 85 |
| {BcMV12=FALSE, BcMV4.BI=FALSE} =>  {BcMV11=FALSE} | 0.21  0396 | 0.480  226 | 0.438  119 | 1.25  1686 | 85 |
| {BcMV11=FALSE, BcNSRV7.BI=FALSE} =>  {BcMV4.BI=FALSE} | 0.23  7624 | 0.755  906 | 0.314  356 | 1.35  727 | 96 |
| {BcMV4.BI=FALSE, BcNSRV7.BI=FALSE} =>  {BcMV11=FALSE} | 0.23  7624 | 0.513  369 | 0.462  871 | 1.33  8071 | 96 |
| {BcMV11=FALSE, BcVV3.BI=FALSE} =>  {BcMV4.BI=FALSE} | 0.23  7624 | 0.744  186 | 0.319  307 | 1.33  6227 | 96 |
| {BcMV4.BI=FALSE, BcVV3.BI=FALSE} =>  {BcMV11=FALSE} | 0.23  7624 | 0.505  263 | 0.470  297 | 1.31  6944 | 96 |
| {BcMV11=FALSE, BcOLV7.BI=FALSE} => | 0.24 | 0.753 | 0.321 | 1.35 | 98 |

| {BcMV4.BI=FALSE} | 2574 | 846 | 782 | 3573 |  |
| --- | --- | --- | --- | --- | --- |
| {BcMV4.BI=FALSE, BcOLV7.BI=FALSE} =>  {BcMV11=FALSE} | 0.24  2574 | 0.515  789 | 0.470  297 | 1.34  438 | 98 |
| {BcMV11=FALSE, BcMV4.BI=FALSE} =>  {BcALV1.BI=FALSE} | 0.26  4851 | 0.946  903 | 0.279  703 | 1.16  2762 | 10  7 |
| {BcALV1.BI=FALSE, BcMV11=FALSE} =>  {BcMV4.BI=FALSE} | 0.26  4851 | 0.748  252 | 0.353  96 | 1.34  3528 | 10  7 |
| {BcALV1.BI=FALSE, BcMV4.BI=FALSE} =>  {BcMV11=FALSE} | 0.26  4851 | 0.529  703 | 0.5 | 1.38  0645 | 10  7 |
| {BcDMV1=FALSE, BcMV11=FALSE} =>  {BcMV4.BI=FALSE} | 0.23  5149 | 0.703  704 | 0.334  158 | 1.26  3539 | 95 |
| {BcDMV1=FALSE, BcMV4.BI=FALSE} =>  {BcMV11=FALSE} | 0.23  5149 | 0.487  179 | 0.482  673 | 1.26  981 | 95 |
| {BcMV11=FALSE, BcMV4.BI=FALSE} =>  {BcFV9=FALSE} | 0.26  2376 | 0.938  053 | 0.279  703 | 1.07  9696 | 10  6 |
| {BcFV9=FALSE, BcMV11=FALSE} =>  {BcMV4.BI=FALSE} | 0.26  2376 | 0.757  143 | 0.346  535 | 1.35  9492 | 10  6 |
| {BcFV9=FALSE, BcMV4.BI=FALSE} =>  {BcMV11=FALSE} | 0.26  2376 | 0.512  077 | 0.512  376 | 1.33  4705 | 10  6 |
| {BcMV11=FALSE, BcMV4.BI=FALSE} =>  {BcOLV4.BI=FALSE} | 0.26  7327 | 0.955  752 | 0.279  703 | 1.09  0745 | 10  8 |
| {BcMV11=FALSE, BcOLV4.BI=FALSE} => | 0.26 | 0.734 | 0.363 | 1.31 | 10 |

| {BcMV4.BI=FALSE} | 7327 | 694 | 861 | 9184 | 8 |
| --- | --- | --- | --- | --- | --- |
| {BcMV4.BI=FALSE, BcOLV4.BI=FALSE} =>  {BcMV11=FALSE} | 0.26  7327 | 0.529  412 | 0.504  95 | 1.37  9886 | 10  8 |
| {BcMV11=FALSE, BcMV4.BI=FALSE} =>  {BcULV5=FALSE} | 0.26  9802 | 0.964  602 | 0.279  703 | 1.10  0845 | 10  9 |
| {BcMV11=FALSE, BcULV5=FALSE} =>  {BcMV4.BI=FALSE} | 0.26  9802 | 0.726  667 | 0.371  287 | 1.30  477 | 10  9 |
| {BcMV4.BI=FALSE, BcULV5=FALSE} =>  {BcMV11=FALSE} | 0.26  9802 | 0.545 | 0.495  05 | 1.42  0516 | 10  9 |
| {BcBNV5.BI=FALSE, BcMV11=FALSE} =>  {BcMV4.BI=FALSE} | 0.26  9802 | 0.726  667 | 0.371  287 | 1.30  477 | 10  9 |
| {BcBNV5.BI=FALSE, BcMV4.BI=FALSE} =>  {BcMV11=FALSE} | 0.26  9802 | 0.514  151 | 0.524  752 | 1.34  011 | 10  9 |
| {BcMV11=FALSE, BvF.BI=FALSE} =>  {BcMV4.BI=FALSE} | 0.25  495 | 0.746  377 | 0.341  584 | 1.34  0161 | 10  3 |
| {BcMV4.BI=FALSE, BvF.BI=FALSE} =>  {BcMV11=FALSE} | 0.25  495 | 0.502  439 | 0.507  426 | 1.30  9583 | 10  3 |
| {BcMCV5.BI=FALSE, BcMV11=FALSE} =>  {BcMV4.BI=FALSE} | 0.27  7228 | 0.736  842 | 0.376  238 | 1.32  3041 | 11  2 |
| {BcMCV5.BI=FALSE, BcMV4.BI=FALSE} =>  {BcMV11=FALSE} | 0.27  7228 | 0.509  091 | 0.544  554 | 1.32  6921 | 11  2 |
| {BcMV11=FALSE, BcOLV13.BI=FALSE} => | 0.27 | 0.743 | 0.366 | 1.33 | 11 |

| {BcMV4.BI=FALSE} | 2277 | 243 | 337 | 4535 | 0 |
| --- | --- | --- | --- | --- | --- |
| {BcMV4.BI=FALSE, BcOLV13.BI=FALSE} =>  {BcMV11=FALSE} | 0.27  2277 | 0.509  259 | 0.534  653 | 1.32  736 | 11  0 |
| {BcMV11=FALSE, BcOLV2.BI=FALSE} =>  {BcMV4.BI=FALSE} | 0.25  9901 | 0.724  138 | 0.358  911 | 1.30  023 | 10  5 |
| {BcMV4.BI=FALSE, BcOLV2.BI=FALSE} =>  {BcMV11=FALSE} | 0.25  9901 | 0.495  283 | 0.524  752 | 1.29  0931 | 10  5 |
| {BcMCV3.BI=FALSE, BcMV11=FALSE} =>  {BcMV4.BI=FALSE} | 0.26  2376 | 0.746  479 | 0.351  485 | 1.34  0344 | 10  6 |
| {BcMCV3.BI=FALSE, BcMV4.BI=FALSE} =>  {BcMV11=FALSE} | 0.26  2376 | 0.502  37 | 0.522  277 | 1.30  9402 | 10  6 |
| {BcMV11=FALSE, BcPV4.BI=FALSE} =>  {BcMV4.BI=FALSE} | 0.25  7426 | 0.722  222 | 0.356  436 | 1.29  679 | 10  4 |
| {BcMV4.BI=FALSE, BcPV4.BI=FALSE} =>  {BcMV11=FALSE} | 0.25  7426 | 0.5 | 0.514  851 | 1.30  3226 | 10  4 |
| {BcEV6=FALSE, BcMV11=FALSE} =>  {BcMV4.BI=FALSE} | 0.25  7426 | 0.717  241 | 0.358  911 | 1.28  7847 | 10  4 |
| {BcEV6=FALSE, BcMV4.BI=FALSE} =>  {BcMV11=FALSE} | 0.25  7426 | 0.5 | 0.514  851 | 1.30  3226 | 10  4 |
| {BcMV11=FALSE, BcOLV17.BI=FALSE} =>  {BcMV4.BI=FALSE} | 0.27  2277 | 0.728  477 | 0.373  762 | 1.30  8021 | 11  0 |
| {BcMV4.BI=FALSE, BcOLV17.BI=FALSE} => | 0.27 | 0.506 | 0.537 | 1.32 | 11 |

| {BcMV11=FALSE} | 2277 | 912 | 129 | 1243 | 0 |
| --- | --- | --- | --- | --- | --- |
| {BcMV11=FALSE, BcOLV15.BI=FALSE} =>  {BcMV4.BI=FALSE} | 0.26  7327 | 0.739  726 | 0.361  386 | 1.32  8219 | 10  8 |
| {BcMV4.BI=FALSE, BcOLV15.BI=FALSE} =>  {BcMV11=FALSE} | 0.26  7327 | 0.497  696 | 0.537  129 | 1.29  722 | 10  8 |
| {BcEV5=FALSE, BcMV11=FALSE} =>  {BcMV4.BI=FALSE} | 0.27  4752 | 0.730  263 | 0.376  238 | 1.31  1228 | 11  1 |
| {BcEV5=FALSE, BcMV4.BI=FALSE} =>  {BcMV11=FALSE} | 0.27  4752 | 0.497  758 | 0.551  98 | 1.29  7382 | 11  1 |
| {BcMV11=FALSE, BcOLV16.BI=FALSE} =>  {BcOLV1.BI.MOVA.=FALSE} | 0.20  0495 | 0.826  531 | 0.242  574 | 1.23  6735 | 81 |
| {BcMV11=FALSE, BcOLV1.BI.MOVA.=FALSE} =>  {BcOLV16.BI=FALSE} | 0.20  0495 | 0.680  672 | 0.294  554 | 1.16  5219 | 81 |
| {BcOLV16.BI=FALSE, BcULV4=TRUE} =>  {BcMV11=FALSE} | 0.16  3366 | 0.464  789 | 0.351  485 | 1.21  1449 | 66 |
| {BcMV11=FALSE, BcOLV16.BI=FALSE} =>  {BcMV6.BI=FALSE} | 0.20  5446 | 0.846  939 | 0.242  574 | 1.21  7663 | 83 |
| {BcMV6.BI=FALSE, BcOLV16.BI=FALSE} =>  {BcMV11=FALSE} | 0.20  5446 | 0.461  111 | 0.445  545 | 1.20  1864 | 83 |
| {BcMV11=FALSE, BcOLV16.BI=FALSE} =>  {BcRV1.BI=FALSE} | 0.19  802 | 0.816  327 | 0.242  574 | 1.12  9438 | 80 |

| {BcMV11=FALSE, BcOLV16.BI=FALSE} =>  {BcULV1.BI=FALSE} | 0.21  5347 | 0.887  755 | 0.242  574 | 1.13  1398 | 87 |
| --- | --- | --- | --- | --- | --- |
| {BcMV11=FALSE, BcULV1.BI=FALSE} =>  {BcOLV16.BI=FALSE} | 0.21  5347 | 0.690  476 | 0.311  881 | 1.18  2002 | 87 |
| {BcMV11=FALSE, BcOLV16.BI=FALSE} =>  {BcALV1.BI=FALSE} | 0.23  0198 | 0.948  98 | 0.242  574 | 1.16  5312 | 93 |
| {BcALV1.BI=FALSE, BcOLV16.BI=FALSE} =>  {BcMV11=FALSE} | 0.23  0198 | 0.455  882 | 0.504  95 | 1.18  8235 | 93 |
| {BcMV11=FALSE, BcOLV16.BI=FALSE} =>  {BcOLV4.BI=FALSE} | 0.23  5149 | 0.969  388 | 0.242  574 | 1.10  6307 | 95 |
| {BcMV11=FALSE, BcOLV16.BI=FALSE} =>  {BcULV5=FALSE} | 0.23  7624 | 0.979  592 | 0.242  574 | 1.11  7952 | 96 |
| {BcOLV16.BI=FALSE, BcULV5=FALSE} =>  {BcMV11=FALSE} | 0.23  7624 | 0.452  83 | 0.524  752 | 1.18  028 | 96 |
| {BcMCV3.BI=FALSE, BcMV11=FALSE} =>  {BcOLV16.BI=FALSE} | 0.23  7624 | 0.676  056 | 0.351  485 | 1.15  7317 | 96 |
| {BcMV11=FALSE, BcULV3=TRUE} =>  {BcOLV1.BI.MOVA.=FALSE} | 0.15  3465 | 0.775 | 0.198  02 | 1.15  963 | 62 |
| {BcMV11=FALSE, BcULV3=TRUE} =>  {BcRV1.BI=FALSE} | 0.16  3366 | 0.825 | 0.198  02 | 1.14  1438 | 66 |
| {BcMV11=FALSE, BcULV3=TRUE} =>  {BcALV1.BI=FALSE} | 0.19  0594 | 0.962  5 | 0.198  02 | 1.18  1915 | 77 |

| {BcMV11=FALSE, BcULV3=TRUE} =>  {BcOLV4.BI=FALSE} | 0.18  8119 | 0.95 | 0.198  02 | 1.08  4181 | 76 |
| --- | --- | --- | --- | --- | --- |
| {BcMV11=FALSE, BcULV3=TRUE} =>  {BcULV5=FALSE} | 0.19  0594 | 0.962  5 | 0.198  02 | 1.09  8446 | 77 |
| {BcHV1.BI=TRUE, BcMV11=FALSE} =>  {BcOLV1.BI.MOVA.=FALSE} | 0.16  8317 | 0.755  556 | 0.222  772 | 1.13  0535 | 68 |
| {BcHV1.BI=TRUE, BcMV11=FALSE} =>  {BcMV9.BI=TRUE} | 0.18  5644 | 0.833  333 | 0.222  772 | 1.22  871 | 75 |
| {BcHV1.BI=TRUE, BcMV6.BI=FALSE} =>  {BcMV11=FALSE} | 0.17  3267 | 0.460  526 | 0.376  238 | 1.20  034 | 70 |
| {BcHV1.BI=TRUE, BcMV11=FALSE} =>  {BcRV1.BI=FALSE} | 0.18  5644 | 0.833  333 | 0.222  772 | 1.15  2968 | 75 |
| {BcHV1.BI=TRUE, BcMV11=FALSE} =>  {BcALV1.BI=FALSE} | 0.20  297 | 0.911  111 | 0.222  772 | 1.11  8811 | 82 |
| {BcHV1.BI=TRUE, BcMV11=FALSE} =>  {BcDMV1=FALSE} | 0.20  5446 | 0.922  222 | 0.222  772 | 1.09  2603 | 83 |
| {BcHV1.BI=TRUE, BcMV11=FALSE} =>  {BcOLV4.BI=FALSE} | 0.21  2871 | 0.955  556 | 0.222  772 | 1.09  0521 | 86 |
| {BcHV1.BI=TRUE, BcMV11=FALSE} =>  {BcULV5=FALSE} | 0.21  5347 | 0.966  667 | 0.222  772 | 1.10  3202 | 87 |
| {BcMV11=FALSE, BcMV9.BI=TRUE} =>  {BcOLV1.BI.MOVA.=FALSE} | 0.20  7921 | 0.763  636 | 0.272  277 | 1.14  2626 | 84 |

| {BcMV11=FALSE, BcULV4=TRUE} =>  {BcOLV1.BI.MOVA.=FALSE} | 0.20  7921 | 0.763  636 | 0.272  277 | 1.14  2626 | 84 |
| --- | --- | --- | --- | --- | --- |
| {BcOLV1.BI.MOVA.=FALSE, BcULV4=TRUE}  => {BcMV11=FALSE} | 0.20  7921 | 0.466  667 | 0.445  545 | 1.21  6344 | 84 |
| {BcMV11=FALSE, BcOLV1.BI.MOVA.=FALSE} =>  {BcMV6.BI=FALSE} | 0.24  505 | 0.831  933 | 0.294  554 | 1.19  6088 | 99 |
| {BcMV11=FALSE, BcMV6.BI=FALSE} =>  {BcOLV1.BI.MOVA.=FALSE} | 0.24  505 | 0.798  387 | 0.306  931 | 1.19  4624 | 99 |
| {BcMV6.BI=FALSE, BcOLV1.BI.MOVA.=FALSE} =>  {BcMV11=FALSE} | 0.24  505 | 0.464  789 | 0.527  228 | 1.21  1449 | 99 |
| {BcMV11=FALSE, BcOLV1.BI.MOVA.=FALSE} =>  {BcPV3.BI=FALSE} | 0.23  7624 | 0.806  723 | 0.294  554 | 1.13  5596 | 96 |
| {BcMV11=FALSE, BcPV3.BI=FALSE} =>  {BcOLV1.BI.MOVA.=FALSE} | 0.23  7624 | 0.813  559 | 0.292  079 | 1.21  7326 | 96 |
| {BcOLV1.BI.MOVA.=FALSE,  BcPV3.BI=FALSE} => {BcMV11=FALSE} | 0.23  7624 | 0.461  538 | 0.514  851 | 1.20  2978 | 96 |
| {BcMV11=FALSE, BcOLV1.BI.MOVA.=FALSE} =>  {BcRV1.BI=FALSE} | 0.24  7525 | 0.840  336 | 0.294  554 | 1.16  2657 | 10  0 |

| {BcMV11=FALSE, BcRV1.BI=FALSE} =>  {BcOLV1.BI.MOVA.=FALSE} | 0.24  7525 | 0.787  402 | 0.314  356 | 1.17  8186 | 10  0 |
| --- | --- | --- | --- | --- | --- |
| {BcOLV1.BI.MOVA.=FALSE,  BcRV1.BI=FALSE} => {BcMV11=FALSE} | 0.24  7525 | 0.471  698 | 0.524  752 | 1.22  9458 | 10  0 |
| {BcEV2.BI=FALSE, BcMV11=FALSE} =>  {BcOLV1.BI.MOVA.=FALSE} | 0.24  0099 | 0.757  813 | 0.316  832 | 1.13  3912 | 97 |
| {BcEV2.BI=FALSE, BcOLV1.BI.MOVA.=FALSE} =>  {BcMV11=FALSE} | 0.24  0099 | 0.482  587 | 0.497  525 | 1.25  784 | 97 |
| {BcMV11=FALSE, BcOLV1.BI.MOVA.=FALSE} =>  {BcHV3.BI=FALSE} | 0.25  2475 | 0.857  143 | 0.294  554 | 1.12  7966 | 10  2 |
| {BcHV3.BI=FALSE, BcMV11=FALSE} =>  {BcOLV1.BI.MOVA.=FALSE} | 0.25  2475 | 0.796  875 | 0.316  832 | 1.19  2361 | 10  2 |
| {BcHV3.BI=FALSE, BcOLV1.BI.MOVA.=FALSE} =>  {BcMV11=FALSE} | 0.25  2475 | 0.474  419 | 0.532  178 | 1.23  6549 | 10  2 |
| {BcFV5.BI=FALSE, BcMV11=FALSE} =>  {BcOLV1.BI.MOVA.=FALSE} | 0.24  0099 | 0.782  258 | 0.306  931 | 1.17  049 | 97 |
| {BcMV11=FALSE, BcOLV1.BI.MOVA.=FALSE} =>  {BcULV1.BI=FALSE} | 0.25  7426 | 0.873  95 | 0.294  554 | 1.11  3803 | 10  4 |

| {BcMV11=FALSE, BcULV1.BI=FALSE} =>  {BcOLV1.BI.MOVA.=FALSE} | 0.25  7426 | 0.825  397 | 0.311  881 | 1.23  5038 | 10  4 |
| --- | --- | --- | --- | --- | --- |
| {BcOLV1.BI.MOVA.=FALSE,  BcULV1.BI=FALSE} => {BcMV11=FALSE} | 0.25  7426 | 0.460  177 | 0.559  406 | 1.19  9429 | 10  4 |
| {BcMV11=FALSE, BcMV12=FALSE} =>  {BcOLV1.BI.MOVA.=FALSE} | 0.24  0099 | 0.822  034 | 0.292  079 | 1.23  0006 | 97 |
| {BcMV11=FALSE, BcNSRV7.BI=FALSE} =>  {BcOLV1.BI.MOVA.=FALSE} | 0.24  0099 | 0.763  78 | 0.314  356 | 1.14  284 | 97 |
| {BcNSRV7.BI=FALSE, BcOLV1.BI.MOVA.=FALSE} =>  {BcMV11=FALSE} | 0.24  0099 | 0.449  074 | 0.534  653 | 1.17  049 | 97 |
| {BcMV11=FALSE, BcVV3.BI=FALSE} =>  {BcOLV1.BI.MOVA.=FALSE} | 0.24  505 | 0.767  442 | 0.319  307 | 1.14  832 | 99 |
| {BcMV11=FALSE, BcOLV7.BI=FALSE} =>  {BcOLV1.BI.MOVA.=FALSE} | 0.25  495 | 0.792  308 | 0.321  782 | 1.18  5527 | 10  3 |
| {BcOLV1.BI.MOVA.=FALSE,  BcOLV7.BI=FALSE} => {BcMV11=FALSE} | 0.25  495 | 0.447  826 | 0.569  307 | 1.16  7237 | 10  3 |
| {BcMV11=FALSE, BcOLV1.BI.MOVA.=FALSE} =>  {BcALV1.BI=FALSE} | 0.27  2277 | 0.924  37 | 0.294  554 | 1.13  5092 | 11  0 |
| {BcALV1.BI=FALSE, BcMV11=FALSE} =>  {BcOLV1.BI.MOVA.=FALSE} | 0.27  2277 | 0.769  231 | 0.353  96 | 1.15  0997 | 11  0 |

| {BcALV1.BI=FALSE, BcOLV1.BI.MOVA.=FALSE} =>  {BcMV11=FALSE} | 0.27  2277 | 0.462  185 | 0.589  109 | 1.20  4663 | 11  0 |
| --- | --- | --- | --- | --- | --- |
| {BcDMV1=FALSE, BcMV11=FALSE} =>  {BcOLV1.BI.MOVA.=FALSE} | 0.25  9901 | 0.777  778 | 0.334  158 | 1.16  3786 | 10  5 |
| {BcDMV1=FALSE, BcOLV1.BI.MOVA.=FALSE} =>  {BcMV11=FALSE} | 0.25  9901 | 0.456  522 | 0.569  307 | 1.18  9902 | 10  5 |
| {BcFV9=FALSE, BcMV11=FALSE} =>  {BcOLV1.BI.MOVA.=FALSE} | 0.26  7327 | 0.771  429 | 0.346  535 | 1.15  4286 | 10  8 |
| {BcFV9=FALSE, BcOLV1.BI.MOVA.=FALSE}  => {BcMV11=FALSE} | 0.26  7327 | 0.453  782 | 0.589  109 | 1.18  276 | 10  8 |
| {BcMV11=FALSE, BcOLV1.BI.MOVA.=FALSE} =>  {BcOLV4.BI=FALSE} | 0.28  7129 | 0.974  79 | 0.294  554 | 1.11  2472 | 11  6 |
| {BcMV11=FALSE, BcOLV4.BI=FALSE} =>  {BcOLV1.BI.MOVA.=FALSE} | 0.28  7129 | 0.789  116 | 0.363  861 | 1.18  0751 | 11  6 |
| {BcOLV1.BI.MOVA.=FALSE,  BcOLV4.BI=FALSE} => {BcMV11=FALSE} | 0.28  7129 | 0.467  742 | 0.613  861 | 1.21  9147 | 11  6 |
| {BcMV11=FALSE, BcOLV1.BI.MOVA.=FALSE} =>  {BcULV5=FALSE} | 0.28  9604 | 0.983  193 | 0.294  554 | 1.12  2062 | 11  7 |

| {BcMV11=FALSE, BcULV5=FALSE} =>  {BcOLV1.BI.MOVA.=FALSE} | 0.28  9604 | 0.78 | 0.371  287 | 1.16  7111 | 11  7 |
| --- | --- | --- | --- | --- | --- |
| {BcOLV1.BI.MOVA.=FALSE, BcULV5=FALSE}  => {BcMV11=FALSE} | 0.28  9604 | 0.468 | 0.618  812 | 1.21  9819 | 11  7 |
| {BcBNV5.BI=FALSE, BcMV11=FALSE} =>  {BcOLV1.BI.MOVA.=FALSE} | 0.28  4653 | 0.766  667 | 0.371  287 | 1.14  716 | 11  5 |
| {BcBNV5.BI=FALSE, BcOLV1.BI.MOVA.=FALSE} =>  {BcMV11=FALSE} | 0.28  4653 | 0.454  545 | 0.626  238 | 1.18  4751 | 11  5 |
| {BcMV11=FALSE, BvF.BI=FALSE} =>  {BcOLV1.BI.MOVA.=FALSE} | 0.25  9901 | 0.760  87 | 0.341  584 | 1.13  8486 | 10  5 |
| {BcMV11=FALSE, BcOLV1.BI.MOVA.=FALSE} =>  {BcMCV5.BI=FALSE} | 0.29  2079 | 0.991  597 | 0.294  554 | 1.07  4008 | 11  8 |
| {BcMCV5.BI=FALSE, BcMV11=FALSE} =>  {BcOLV1.BI.MOVA.=FALSE} | 0.29  2079 | 0.776  316 | 0.376  238 | 1.16  1598 | 11  8 |
| {BcMCV5.BI=FALSE, BcOLV1.BI.MOVA.=FALSE} =>  {BcMV11=FALSE} | 0.29  2079 | 0.452  107 | 0.646  04 | 1.17  8396 | 11  8 |
| {BcMV11=FALSE, BcOLV13.BI=FALSE} =>  {BcOLV1.BI.MOVA.=FALSE} | 0.28  7129 | 0.783  784 | 0.366  337 | 1.17  2773 | 11  6 |
| {BcOLV1.BI.MOVA.=FALSE, | 0.28 | 0.442 | 0.648 | 1.15 | 11 |

| BcOLV13.BI=FALSE} => {BcMV11=FALSE} | 7129 | 748 | 515 | 4001 | 6 |
| --- | --- | --- | --- | --- | --- |
| {BcMV11=FALSE, BcOLV2.BI=FALSE} =>  {BcOLV1.BI.MOVA.=FALSE} | 0.28  9604 | 0.806  897 | 0.358  911 | 1.20  7356 | 11  7 |
| {BcOLV1.BI.MOVA.=FALSE,  BcOLV2.BI=FALSE} => {BcMV11=FALSE} | 0.28  9604 | 0.444  867 | 0.650  99 | 1.15  9524 | 11  7 |
| {BcMCV3.BI=FALSE, BcMV11=FALSE} =>  {BcOLV1.BI.MOVA.=FALSE} | 0.27  9703 | 0.795  775 | 0.351  485 | 1.19  0715 | 11  3 |
| {BcMV11=FALSE, BcPV4.BI=FALSE} =>  {BcOLV1.BI.MOVA.=FALSE} | 0.27  2277 | 0.763  889 | 0.356  436 | 1.14  3004 | 11  0 |
| {BcOLV1.BI.MOVA.=FALSE,  BcPV4.BI=FALSE} => {BcMV11=FALSE} | 0.27  2277 | 0.441  767 | 0.616  337 | 1.15  1444 | 11  0 |
| {BcEV6=FALSE, BcMV11=FALSE} =>  {BcOLV1.BI.MOVA.=FALSE} | 0.27  9703 | 0.779  31 | 0.358  911 | 1.16  6079 | 11  3 |
| {BcEV6=FALSE, BcOLV1.BI.MOVA.=FALSE}  => {BcMV11=FALSE} | 0.27  9703 | 0.444  882 | 0.628  713 | 1.15  9563 | 11  3 |
| {BcMV11=FALSE, BcOLV17.BI=FALSE} =>  {BcOLV1.BI.MOVA.=FALSE} | 0.28  9604 | 0.774  834 | 0.373  762 | 1.15  9382 | 11  7 |
| {BcOLV1.BI.MOVA.=FALSE,  BcOLV17.BI=FALSE} => {BcMV11=FALSE} | 0.28  9604 | 0.446  565 | 0.648  515 | 1.16  395 | 11  7 |
| {BcMV11=FALSE, BcOLV15.BI=FALSE} =>  {BcOLV1.BI.MOVA.=FALSE} | 0.27  4752 | 0.760  274 | 0.361  386 | 1.13  7595 | 11  1 |
| {BcEV5=FALSE, BcMV11=FALSE} => | 0.28 | 0.769 | 0.376 | 1.15 | 11 |

| {BcOLV1.BI.MOVA.=FALSE} | 9604 | 737 | 238 | 1754 | 7 |
| --- | --- | --- | --- | --- | --- |
| {BcEV5=FALSE, BcOLV1.BI.MOVA.=FALSE}  => {BcMV11=FALSE} | 0.28  9604 | 0.443  182 | 0.653  465 | 1.15  5132 | 11  7 |
| {BcMV11=FALSE, BcULV4=TRUE} =>  {BcMV9.BI=TRUE} | 0.21  0396 | 0.772  727 | 0.272  277 | 1.13  935 | 85 |
| {BcMV11=FALSE, BcMV9.BI=TRUE} =>  {BcMV6.BI=FALSE} | 0.21  7822 | 0.8 | 0.272  277 | 1.15  0178 | 88 |
| {BcMV6.BI=FALSE, BcMV9.BI=TRUE} =>  {BcMV11=FALSE} | 0.21  7822 | 0.491  62 | 0.443  069 | 1.28  1384 | 88 |
| {BcMV9.BI=TRUE, BcRV1.BI=FALSE} =>  {BcMV11=FALSE} | 0.21  5347 | 0.472  826 | 0.455  446 | 1.23  2398 | 87 |
| {BcMV11=FALSE, BcMV9.BI=TRUE} =>  {BcHV3.BI=FALSE} | 0.23  0198 | 0.845  455 | 0.272  277 | 1.11  2585 | 93 |
| {BcHV3.BI=FALSE, BcMV9.BI=TRUE} =>  {BcMV11=FALSE} | 0.23  0198 | 0.455  882 | 0.504  95 | 1.18  8235 | 93 |
| {BcMV1.BI=TRUE, BcMV11=FALSE} =>  {BcMV9.BI=TRUE} | 0.20  7921 | 0.770  642 | 0.269  802 | 1.13  6275 | 84 |
| {BcMV11=FALSE, BcMV9.BI=TRUE} =>  {BcALV1.BI=FALSE} | 0.25  2475 | 0.927  273 | 0.272  277 | 1.13  8657 | 10  2 |
| {BcALV1.BI=FALSE, BcMV9.BI=TRUE} =>  {BcMV11=FALSE} | 0.25  2475 | 0.465  753 | 0.542  079 | 1.21  3964 | 10  2 |
| {BcMV11=FALSE, BcMV9.BI=TRUE} => | 0.26 | 0.981 | 0.272 | 1.12 | 10 |

| {BcOLV4.BI=FALSE} | 7327 | 818 | 277 | 0493 | 8 |
| --- | --- | --- | --- | --- | --- |
| {BcMV11=FALSE, BcMV9.BI=TRUE} =>  {BcULV5=FALSE} | 0.26  7327 | 0.981  818 | 0.272  277 | 1.12  0493 | 10  8 |
| {BcMV11=FALSE, BcULV4=TRUE} =>  {BcMV6.BI=FALSE} | 0.21  7822 | 0.8 | 0.272  277 | 1.15  0178 | 88 |
| {BcMV6.BI=FALSE, BcULV4=TRUE} =>  {BcMV11=FALSE} | 0.21  7822 | 0.486  188 | 0.448  02 | 1.26  7225 | 88 |
| {BcRV1.BI=FALSE, BcULV4=TRUE} =>  {BcMV11=FALSE} | 0.20  7921 | 0.464  088 | 0.448  02 | 1.20  9624 | 84 |
| {BcMV11=FALSE, BcULV4=TRUE} =>  {BcEV2.BI=FALSE} | 0.23  2673 | 0.854  545 | 0.272  277 | 1.14  3167 | 94 |
| {BcMV11=FALSE, BcULV4=TRUE} =>  {BcALV1.BI=FALSE} | 0.24  505 | 0.9 | 0.272  277 | 1.10  5167 | 99 |
| {BcALV1.BI=FALSE, BcULV4=TRUE} =>  {BcMV11=FALSE} | 0.24  505 | 0.462  617 | 0.529  703 | 1.20  5788 | 99 |
| {BcMV11=FALSE, BcULV4=TRUE} =>  {BcOLV4.BI=FALSE} | 0.26  7327 | 0.981  818 | 0.272  277 | 1.12  0493 | 10  8 |
| {BcMV11=FALSE, BcULV4=TRUE} =>  {BcULV5=FALSE} | 0.26  7327 | 0.981  818 | 0.272  277 | 1.12  0493 | 10  8 |
| {BcMV11=FALSE, BcPV3.BI=FALSE} =>  {BcMV6.BI=FALSE} | 0.23  2673 | 0.796  61 | 0.292  079 | 1.14  5304 | 94 |
| {BcMV11=FALSE, BcRV1.BI=FALSE} => | 0.24 | 0.787 | 0.314 | 1.13 | 10 |

| {BcMV6.BI=FALSE} | 7525 | 402 | 356 | 2065 | 0 |
| --- | --- | --- | --- | --- | --- |
| {BcMV6.BI=FALSE, BcRV1.BI=FALSE} =>  {BcMV11=FALSE} | 0.24  7525 | 0.465  116 | 0.532  178 | 1.21  2303 | 10  0 |
| {BcMV11=FALSE, BcMV6.BI=FALSE} =>  {BcEV2.BI=FALSE} | 0.25  7426 | 0.838  71 | 0.306  931 | 1.12  1982 | 10  4 |
| {BcEV2.BI=FALSE, BcMV11=FALSE} =>  {BcMV6.BI=FALSE} | 0.25  7426 | 0.812  5 | 0.316  832 | 1.16  8149 | 10  4 |
| {BcEV2.BI=FALSE, BcMV6.BI=FALSE} =>  {BcMV11=FALSE} | 0.25  7426 | 0.5 | 0.514  851 | 1.30  3226 | 10  4 |
| {BcHV3.BI=FALSE, BcMV11=FALSE} =>  {BcMV6.BI=FALSE} | 0.24  7525 | 0.781  25 | 0.316  832 | 1.12  3221 | 10  0 |
| {BcHV3.BI=FALSE, BcMV6.BI=FALSE} =>  {BcMV11=FALSE} | 0.24  7525 | 0.458  716 | 0.539  604 | 1.19  562 | 10  0 |
| {BcMV11=FALSE, BcULV1.BI=FALSE} =>  {BcMV6.BI=FALSE} | 0.25  2475 | 0.809  524 | 0.311  881 | 1.16  3871 | 10  2 |
| {BcMV1.BI=TRUE, BcMV11=FALSE} =>  {BcMV6.BI=FALSE} | 0.21  5347 | 0.798  165 | 0.269  802 | 1.14  754 | 87 |
| {BcMV11=FALSE, BcMV12=FALSE} =>  {BcMV6.BI=FALSE} | 0.24  505 | 0.838  983 | 0.292  079 | 1.20  6225 | 99 |
| {BcMV11=FALSE, BcNSRV7.BI=FALSE} =>  {BcMV6.BI=FALSE} | 0.24  505 | 0.779  528 | 0.314  356 | 1.12  0744 | 99 |
| {BcMV11=FALSE, BcVV3.BI=FALSE} => | 0.25 | 0.806 | 0.319 | 1.15 | 10 |

| {BcMV6.BI=FALSE} | 7426 | 202 | 307 | 9094 | 4 |
| --- | --- | --- | --- | --- | --- |
| {BcMV6.BI=FALSE, BcVV3.BI=FALSE} =>  {BcMV11=FALSE} | 0.25  7426 | 0.450  216 | 0.571  782 | 1.17  3467 | 10  4 |
| {BcMV11=FALSE, BcOLV7.BI=FALSE} =>  {BcMV6.BI=FALSE} | 0.25  495 | 0.792  308 | 0.321  782 | 1.13  9119 | 10  3 |
| {BcMV11=FALSE, BcMV6.BI=FALSE} =>  {BcALV1.BI=FALSE} | 0.28  4653 | 0.927  419 | 0.306  931 | 1.13  8837 | 11  5 |
| {BcALV1.BI=FALSE, BcMV11=FALSE} =>  {BcMV6.BI=FALSE} | 0.28  4653 | 0.804  196 | 0.353  96 | 1.15  621 | 11  5 |
| {BcALV1.BI=FALSE, BcMV6.BI=FALSE} =>  {BcMV11=FALSE} | 0.28  4653 | 0.477  178 | 0.596  535 | 1.24  3742 | 11  5 |
| {BcFV9=FALSE, BcMV11=FALSE} =>  {BcMV6.BI=FALSE} | 0.27  2277 | 0.785  714 | 0.346  535 | 1.12  9639 | 11  0 |
| {BcFV9=FALSE, BcMV6.BI=FALSE} =>  {BcMV11=FALSE} | 0.27  2277 | 0.452  675 | 0.601  485 | 1.17  9875 | 11  0 |
| {BcMV11=FALSE, BcMV6.BI=FALSE} =>  {BcOLV4.BI=FALSE} | 0.29  703 | 0.967  742 | 0.306  931 | 1.10  4429 | 12  0 |
| {BcMV11=FALSE, BcOLV4.BI=FALSE} =>  {BcMV6.BI=FALSE} | 0.29  703 | 0.816  327 | 0.363  861 | 1.17  3651 | 12  0 |
| {BcMV6.BI=FALSE, BcOLV4.BI=FALSE} =>  {BcMV11=FALSE} | 0.29  703 | 0.481  928 | 0.616  337 | 1.25  6121 | 12  0 |
| {BcMV11=FALSE, BcMV6.BI=FALSE} => | 0.29 | 0.975 | 0.306 | 1.11 | 12 |

| {BcULV5=FALSE} | 9505 | 806 | 931 | 3632 | 1 |
| --- | --- | --- | --- | --- | --- |
| {BcMV11=FALSE, BcULV5=FALSE} =>  {BcMV6.BI=FALSE} | 0.29  9505 | 0.806  667 | 0.371  287 | 1.15  9763 | 12  1 |
| {BcMV6.BI=FALSE, BcULV5=FALSE} =>  {BcMV11=FALSE} | 0.29  9505 | 0.482  072 | 0.621  287 | 1.25  6497 | 12  1 |
| {BcMV11=FALSE, BcMV6.BI=FALSE} =>  {BcBNV5.BI=FALSE} | 0.29  9505 | 0.975  806 | 0.306  931 | 1.07  4185 | 12  1 |
| {BcBNV5.BI=FALSE, BcMV11=FALSE} =>  {BcMV6.BI=FALSE} | 0.29  9505 | 0.806  667 | 0.371  287 | 1.15  9763 | 12  1 |
| {BcBNV5.BI=FALSE, BcMV6.BI=FALSE} =>  {BcMV11=FALSE} | 0.29  9505 | 0.451  493 | 0.663  366 | 1.17  6793 | 12  1 |
| {BcMV11=FALSE, BvF.BI=FALSE} =>  {BcMV6.BI=FALSE} | 0.27  2277 | 0.797  101 | 0.341  584 | 1.14  6011 | 11  0 |
| {BcMCV5.BI=FALSE, BcMV11=FALSE} =>  {BcMV6.BI=FALSE} | 0.30  198 | 0.802  632 | 0.376  238 | 1.15  3961 | 12  2 |
| {BcMCV5.BI=FALSE, BcMV6.BI=FALSE} =>  {BcMV11=FALSE} | 0.30  198 | 0.455  224 | 0.663  366 | 1.18  6519 | 12  2 |
| {BcMV11=FALSE, BcOLV13.BI=FALSE} =>  {BcMV6.BI=FALSE} | 0.29  4554 | 0.804  054 | 0.366  337 | 1.15  6007 | 11  9 |
| {BcMV6.BI=FALSE, BcOLV13.BI=FALSE} =>  {BcMV11=FALSE} | 0.29  4554 | 0.449  057 | 0.655  941 | 1.17  0444 | 11  9 |
| {BcMV11=FALSE, BcOLV2.BI=FALSE} => | 0.29 | 0.820 | 0.358 | 1.17 | 11 |

| {BcMV6.BI=FALSE} | 4554 | 69 | 911 | 9924 | 9 |
| --- | --- | --- | --- | --- | --- |
| {BcMV6.BI=FALSE, BcOLV2.BI=FALSE} =>  {BcMV11=FALSE} | 0.29  4554 | 0.442  379 | 0.665  842 | 1.15  304 | 11  9 |
| {BcMCV3.BI=FALSE, BcMV11=FALSE} =>  {BcMV6.BI=FALSE} | 0.29  2079 | 0.830  986 | 0.351  485 | 1.19  4727 | 11  8 |
| {BcMV11=FALSE, BcPV4.BI=FALSE} =>  {BcMV6.BI=FALSE} | 0.28  7129 | 0.805  556 | 0.356  436 | 1.15  8165 | 11  6 |
| {BcMV6.BI=FALSE, BcPV4.BI=FALSE} =>  {BcMV11=FALSE} | 0.28  7129 | 0.437  736 | 0.655  941 | 1.14  0937 | 11  6 |
| {BcEV6=FALSE, BcMV11=FALSE} =>  {BcMV6.BI=FALSE} | 0.28  7129 | 0.8 | 0.358  911 | 1.15  0178 | 11  6 |
| {BcEV6=FALSE, BcMV6.BI=FALSE} =>  {BcMV11=FALSE} | 0.28  7129 | 0.439  394 | 0.653  465 | 1.14  5259 | 11  6 |
| {BcMV11=FALSE, BcOLV17.BI=FALSE} =>  {BcMV6.BI=FALSE} | 0.29  703 | 0.794  702 | 0.373  762 | 1.14  2561 | 12  0 |
| {BcMV6.BI=FALSE, BcOLV17.BI=FALSE} =>  {BcMV11=FALSE} | 0.29  703 | 0.442  804 | 0.670  792 | 1.15  4148 | 12  0 |
| {BcMV11=FALSE, BcOLV15.BI=FALSE} =>  {BcMV6.BI=FALSE} | 0.28  7129 | 0.794  521 | 0.361  386 | 1.14  23 | 11  6 |
| {BcMV6.BI=FALSE, BcOLV15.BI=FALSE} =>  {BcMV11=FALSE} | 0.28  7129 | 0.441  065 | 0.650  99 | 1.14  9614 | 11  6 |
| {BcEV5=FALSE, BcMV11=FALSE} => | 0.29 | 0.796 | 0.376 | 1.14 | 12 |

| {BcMV6.BI=FALSE} | 9505 | 053 | 238 | 4503 | 1 |
| --- | --- | --- | --- | --- | --- |
| {BcMV11=FALSE, BcPV3.BI=FALSE} =>  {BcRV1.BI=FALSE} | 0.25  2475 | 0.864  407 | 0.292  079 | 1.19  596 | 10  2 |
| {BcMV11=FALSE, BcRV1.BI=FALSE} =>  {BcPV3.BI=FALSE} | 0.25  2475 | 0.803  15 | 0.314  356 | 1.13  0566 | 10  2 |
| {BcPV3.BI=FALSE, BcRV1.BI=FALSE} =>  {BcMV11=FALSE} | 0.25  2475 | 0.451  327 | 0.559  406 | 1.17  6363 | 10  2 |
| {BcMV11=FALSE, BcPV3.BI=FALSE} =>  {BcEV2.BI=FALSE} | 0.24  2574 | 0.830  508 | 0.292  079 | 1.11  1011 | 98 |
| {BcEV2.BI=FALSE, BcPV3.BI=FALSE} =>  {BcMV11=FALSE} | 0.24  2574 | 0.447  489 | 0.542  079 | 1.16  6357 | 98 |
| {BcMV11=FALSE, BcPV3.BI=FALSE} =>  {BcALV1.BI=FALSE} | 0.27  2277 | 0.932  203 | 0.292  079 | 1.14  4712 | 11  0 |
| {BcALV1.BI=FALSE, BcPV3.BI=FALSE} =>  {BcMV11=FALSE} | 0.27  2277 | 0.466  102 | 0.584  158 | 1.21  4872 | 11  0 |
| {BcMV11=FALSE, BcPV3.BI=FALSE} =>  {BcOLV4.BI=FALSE} | 0.28  2178 | 0.966  102 | 0.292  079 | 1.10  2557 | 11  4 |
| {BcOLV4.BI=FALSE, BcPV3.BI=FALSE} =>  {BcMV11=FALSE} | 0.28  2178 | 0.441  86 | 0.638  614 | 1.15  1688 | 11  4 |
| {BcMV11=FALSE, BcPV3.BI=FALSE} =>  {BcULV5=FALSE} | 0.28  7129 | 0.983  051 | 0.292  079 | 1.12  19 | 11  6 |
| {BcPV3.BI=FALSE, BcULV5=FALSE} => | 0.28 | 0.449 | 0.638 | 1.17 | 11 |

| {BcMV11=FALSE} | 7129 | 612 | 614 | 1893 | 6 |
| --- | --- | --- | --- | --- | --- |
| {BcMV11=FALSE, BcPV3.BI=FALSE} =>  {BcBNV5.BI=FALSE} | 0.28  4653 | 0.974  576 | 0.292  079 | 1.07  2831 | 11  5 |
| {BcBNV5.BI=FALSE, BcPV3.BI=FALSE} =>  {BcMV11=FALSE} | 0.28  4653 | 0.442  308 | 0.643  564 | 1.15  2854 | 11  5 |
| {BcMV11=FALSE, BcRV1.BI=FALSE} =>  {BcEV2.BI=FALSE} | 0.25  9901 | 0.826  772 | 0.314  356 | 1.10  6012 | 10  5 |
| {BcEV2.BI=FALSE, BcMV11=FALSE} =>  {BcRV1.BI=FALSE} | 0.25  9901 | 0.820  313 | 0.316  832 | 1.13  4953 | 10  5 |
| {BcEV2.BI=FALSE, BcRV1.BI=FALSE} =>  {BcMV11=FALSE} | 0.25  9901 | 0.486  111 | 0.534  653 | 1.26  7025 | 10  5 |
| {BcHV3.BI=FALSE, BcMV11=FALSE} =>  {BcRV1.BI=FALSE} | 0.25  7426 | 0.812  5 | 0.316  832 | 1.12  4144 | 10  4 |
| {BcHV3.BI=FALSE, BcRV1.BI=FALSE} =>  {BcMV11=FALSE} | 0.25  7426 | 0.470  588 | 0.547  03 | 1.22  6565 | 10  4 |
| {BcMV11=FALSE, BcULV1.BI=FALSE} =>  {BcRV1.BI=FALSE} | 0.25  2475 | 0.809  524 | 0.311  881 | 1.12  0026 | 10  2 |
| {BcMV1.BI=TRUE, BcMV11=FALSE} =>  {BcRV1.BI=FALSE} | 0.22  7723 | 0.844  037 | 0.269  802 | 1.16  7777 | 92 |
| {BcNSRV7.BI=FALSE, BcRV1.BI=FALSE} =>  {BcMV11=FALSE} | 0.25 | 0.446  903 | 0.559  406 | 1.16  483 | 10  1 |
| {BcMV11=FALSE, BcVV3.BI=FALSE} => | 0.26 | 0.837 | 0.319 | 1.15 | 10 |

| {BcRV1.BI=FALSE} | 7327 | 209 | 307 | 8331 | 8 |
| --- | --- | --- | --- | --- | --- |
| {BcRV1.BI=FALSE, BcVV3.BI=FALSE} =>  {BcMV11=FALSE} | 0.26  7327 | 0.469  565 | 0.569  307 | 1.22  3899 | 10  8 |
| {BcMV11=FALSE, BcRV1.BI=FALSE} =>  {BcOLV7.BI=FALSE} | 0.28  2178 | 0.897  638 | 0.314  356 | 1.11  241 | 11  4 |
| {BcMV11=FALSE, BcOLV7.BI=FALSE} =>  {BcRV1.BI=FALSE} | 0.28  2178 | 0.876  923 | 0.321  782 | 1.21  3277 | 11  4 |
| {BcOLV7.BI=FALSE, BcRV1.BI=FALSE} =>  {BcMV11=FALSE} | 0.28  2178 | 0.441  86 | 0.638  614 | 1.15  1688 | 11  4 |
| {BcMV11=FALSE, BcRV1.BI=FALSE} =>  {BcALV1.BI=FALSE} | 0.29  2079 | 0.929  134 | 0.314  356 | 1.14  0942 | 11  8 |
| {BcALV1.BI=FALSE, BcMV11=FALSE} =>  {BcRV1.BI=FALSE} | 0.29  2079 | 0.825  175 | 0.353  96 | 1.14  168 | 11  8 |
| {BcALV1.BI=FALSE, BcRV1.BI=FALSE} =>  {BcMV11=FALSE} | 0.29  2079 | 0.468  254 | 0.623  762 | 1.22  0481 | 11  8 |
| {BcDMV1=FALSE, BcMV11=FALSE} =>  {BcRV1.BI=FALSE} | 0.26  7327 | 0.8 | 0.334  158 | 1.10  6849 | 10  8 |
| {BcDMV1=FALSE, BcRV1.BI=FALSE} =>  {BcMV11=FALSE} | 0.26  7327 | 0.455  696 | 0.586  634 | 1.18  775 | 10  8 |
| {BcFV9=FALSE, BcMV11=FALSE} =>  {BcRV1.BI=FALSE} | 0.28  2178 | 0.814  286 | 0.346  535 | 1.12  6614 | 11  4 |
| {BcFV9=FALSE, BcRV1.BI=FALSE} => | 0.28 | 0.443 | 0.636 | 1.15 | 11 |

| {BcMV11=FALSE} | 2178 | 58 | 139 | 6169 | 4 |
| --- | --- | --- | --- | --- | --- |
| {BcMV11=FALSE, BcRV1.BI=FALSE} =>  {BcOLV4.BI=FALSE} | 0.29  4554 | 0.937  008 | 0.314  356 | 1.06  9354 | 11  9 |
| {BcMV11=FALSE, BcOLV4.BI=FALSE} =>  {BcRV1.BI=FALSE} | 0.29  4554 | 0.809  524 | 0.363  861 | 1.12  0026 | 11  9 |
| {BcOLV4.BI=FALSE, BcRV1.BI=FALSE} =>  {BcMV11=FALSE} | 0.29  4554 | 0.466  667 | 0.631  188 | 1.21  6344 | 11  9 |
| {BcMV11=FALSE, BcRV1.BI=FALSE} =>  {BcULV5=FALSE} | 0.30  198 | 0.960  63 | 0.314  356 | 1.09  6312 | 12  2 |
| {BcMV11=FALSE, BcULV5=FALSE} =>  {BcRV1.BI=FALSE} | 0.30  198 | 0.813  333 | 0.371  287 | 1.12  5297 | 12  2 |
| {BcRV1.BI=FALSE, BcULV5=FALSE} =>  {BcMV11=FALSE} | 0.30  198 | 0.480  315 | 0.628  713 | 1.25  1918 | 12  2 |
| {BcMV11=FALSE, BcRV1.BI=FALSE} =>  {BcBNV5.BI=FALSE} | 0.30  6931 | 0.976  378 | 0.314  356 | 1.07  4814 | 12  4 |
| {BcBNV5.BI=FALSE, BcMV11=FALSE} =>  {BcRV1.BI=FALSE} | 0.30  6931 | 0.826  667 | 0.371  287 | 1.14  3744 | 12  4 |
| {BcBNV5.BI=FALSE, BcRV1.BI=FALSE} =>  {BcMV11=FALSE} | 0.30  6931 | 0.473  282 | 0.648  515 | 1.23  3588 | 12  4 |
| {BcMV11=FALSE, BvF.BI=FALSE} =>  {BcRV1.BI=FALSE} | 0.27  4752 | 0.804  348 | 0.341  584 | 1.11  2865 | 11  1 |
| {BcMCV5.BI=FALSE, BcMV11=FALSE} => | 0.30 | 0.822 | 0.376 | 1.13 | 12 |

| {BcRV1.BI=FALSE} | 9406 | 368 | 238 | 7797 | 5 |
| --- | --- | --- | --- | --- | --- |
| {BcMCV5.BI=FALSE, BcRV1.BI=FALSE} =>  {BcMV11=FALSE} | 0.30  9406 | 0.459  559 | 0.673  267 | 1.19  7818 | 12  5 |
| {BcMV11=FALSE, BcOLV13.BI=FALSE} =>  {BcRV1.BI=FALSE} | 0.30  4455 | 0.831  081 | 0.366  337 | 1.14  9852 | 12  3 |
| {BcOLV13.BI=FALSE, BcRV1.BI=FALSE} =>  {BcMV11=FALSE} | 0.30  4455 | 0.440  86 | 0.690  594 | 1.14  9081 | 12  3 |
| {BcMV11=FALSE, BcOLV2.BI=FALSE} =>  {BcRV1.BI=FALSE} | 0.29  2079 | 0.813  793 | 0.358  911 | 1.12  5933 | 11  8 |
| {BcMCV3.BI=FALSE, BcMV11=FALSE} =>  {BcRV1.BI=FALSE} | 0.28  7129 | 0.816  901 | 0.351  485 | 1.13  0233 | 11  6 |
| {BcMV11=FALSE, BcPV4.BI=FALSE} =>  {BcRV1.BI=FALSE} | 0.28  7129 | 0.805  556 | 0.356  436 | 1.11  4536 | 11  6 |
| {BcEV6=FALSE, BcMV11=FALSE} =>  {BcRV1.BI=FALSE} | 0.29  2079 | 0.813  793 | 0.358  911 | 1.12  5933 | 11  8 |
| {BcEV6=FALSE, BcRV1.BI=FALSE} =>  {BcMV11=FALSE} | 0.29  2079 | 0.437  037 | 0.668  317 | 1.13  9116 | 11  8 |
| {BcMV11=FALSE, BcOLV17.BI=FALSE} =>  {BcRV1.BI=FALSE} | 0.30  9406 | 0.827  815 | 0.373  762 | 1.14  5332 | 12  5 |
| {BcOLV17.BI=FALSE, BcRV1.BI=FALSE} =>  {BcMV11=FALSE} | 0.30  9406 | 0.437  063 | 0.707  921 | 1.13  9183 | 12  5 |
| {BcMV11=FALSE, BcOLV15.BI=FALSE} => | 0.29 | 0.808 | 0.361 | 1.11 | 11 |

| {BcRV1.BI=FALSE} | 2079 | 219 | 386 | 8221 | 8 |
| --- | --- | --- | --- | --- | --- |
| {BcEV5=FALSE, BcMV11=FALSE} =>  {BcRV1.BI=FALSE} | 0.30  6931 | 0.815  789 | 0.376  238 | 1.12  8695 | 12  4 |
| {BcEV5=FALSE, BcRV1.BI=FALSE} =>  {BcMV11=FALSE} | 0.30  6931 | 0.455  882 | 0.673  267 | 1.18  8235 | 12  4 |
| {BcEV2.BI=FALSE, BcMV11=FALSE} =>  {BcHV3.BI=FALSE} | 0.26  7327 | 0.843  75 | 0.316  832 | 1.11  0342 | 10  8 |
| {BcHV3.BI=FALSE, BcMV11=FALSE} =>  {BcEV2.BI=FALSE} | 0.26  7327 | 0.843  75 | 0.316  832 | 1.12  8725 | 10  8 |
| {BcEV2.BI=FALSE, BcHV3.BI=FALSE} =>  {BcMV11=FALSE} | 0.26  7327 | 0.448  133 | 0.596  535 | 1.16  8036 | 10  8 |
| {BcFV5.BI=FALSE, BcMV11=FALSE} =>  {BcEV2.BI=FALSE} | 0.26  2376 | 0.854  839 | 0.306  931 | 1.14  3559 | 10  6 |
| {BcMV11=FALSE, BcNSRV7.BI=FALSE} =>  {BcEV2.BI=FALSE} | 0.26  2376 | 0.834  646 | 0.314  356 | 1.11  6546 | 10  6 |
| {BcMV11=FALSE, BcVV3.BI=FALSE} =>  {BcEV2.BI=FALSE} | 0.27  2277 | 0.852  713 | 0.319  307 | 1.14  0716 | 11  0 |
| {BcEV2.BI=FALSE, BcOLV7.BI=FALSE} =>  {BcMV11=FALSE} | 0.26  2376 | 0.443  515 | 0.591  584 | 1.15  5999 | 10  6 |
| {BcEV2.BI=FALSE, BcMV11=FALSE} =>  {BcALV1.BI=FALSE} | 0.29  2079 | 0.921  875 | 0.316  832 | 1.13  2029 | 11  8 |
| {BcALV1.BI=FALSE, BcMV11=FALSE} => | 0.29 | 0.825 | 0.353 | 1.10 | 11 |

| {BcEV2.BI=FALSE} | 2079 | 175 | 96 | 3876 | 8 |
| --- | --- | --- | --- | --- | --- |
| {BcALV1.BI=FALSE, BcEV2.BI=FALSE} =>  {BcMV11=FALSE} | 0.29  2079 | 0.473  896 | 0.616  337 | 1.23  5186 | 11  8 |
| {BcFV9=FALSE, BcMV11=FALSE} =>  {BcEV2.BI=FALSE} | 0.28  9604 | 0.835  714 | 0.346  535 | 1.11  7975 | 11  7 |
| {BcEV2.BI=FALSE, BcMV11=FALSE} =>  {BcOLV4.BI=FALSE} | 0.29  703 | 0.937  5 | 0.316  832 | 1.06  9915 | 12  0 |
| {BcEV2.BI=FALSE, BcOLV4.BI=FALSE} =>  {BcMV11=FALSE} | 0.29  703 | 0.446  097 | 0.665  842 | 1.16  2729 | 12  0 |
| {BcEV2.BI=FALSE, BcMV11=FALSE} =>  {BcULV5=FALSE} | 0.30  4455 | 0.960  938 | 0.316  832 | 1.09  6663 | 12  3 |
| {BcMV11=FALSE, BcULV5=FALSE} =>  {BcEV2.BI=FALSE} | 0.30  4455 | 0.82 | 0.371  287 | 1.09  6954 | 12  3 |
| {BcEV2.BI=FALSE, BcULV5=FALSE} =>  {BcMV11=FALSE} | 0.30  4455 | 0.464  151 | 0.655  941 | 1.20  9787 | 12  3 |
| {BcBNV5.BI=FALSE, BcMV11=FALSE} =>  {BcEV2.BI=FALSE} | 0.30  6931 | 0.826  667 | 0.371  287 | 1.10  5872 | 12  4 |
| {BcBNV5.BI=FALSE, BcEV2.BI=FALSE} =>  {BcMV11=FALSE} | 0.30  6931 | 0.442  857 | 0.693  069 | 1.15  4286 | 12  4 |
| {BcMV11=FALSE, BvF.BI=FALSE} =>  {BcEV2.BI=FALSE} | 0.29  4554 | 0.862  319 | 0.341  584 | 1.15  3566 | 11  9 |
| {BcMCV5.BI=FALSE, BcMV11=FALSE} => | 0.31 | 0.828 | 0.376 | 1.10 | 12 |

| {BcEV2.BI=FALSE} | 1881 | 947 | 238 | 8923 | 6 |
| --- | --- | --- | --- | --- | --- |
| {BcEV2.BI=FALSE, BcMCV5.BI=FALSE} =>  {BcMV11=FALSE} | 0.31  1881 | 0.440  559 | 0.707  921 | 1.14  8297 | 12  6 |
| {BcMV11=FALSE, BcOLV13.BI=FALSE} =>  {BcEV2.BI=FALSE} | 0.30  4455 | 0.831  081 | 0.366  337 | 1.11  1777 | 12  3 |
| {BcEV2.BI=FALSE, BcOLV13.BI=FALSE} =>  {BcMV11=FALSE} | 0.30  4455 | 0.436  17 | 0.698  02 | 1.13  6857 | 12  3 |
| {BcMV11=FALSE, BcOLV2.BI=FALSE} =>  {BcEV2.BI=FALSE} | 0.29  703 | 0.827  586 | 0.358  911 | 1.10  7102 | 12  0 |
| {BcMCV3.BI=FALSE, BcMV11=FALSE} =>  {BcEV2.BI=FALSE} | 0.29  2079 | 0.830  986 | 0.351  485 | 1.11  165 | 11  8 |
| {BcMV11=FALSE, BcPV4.BI=FALSE} =>  {BcEV2.BI=FALSE} | 0.29  4554 | 0.826  389 | 0.356  436 | 1.10  55 | 11  9 |
| {BcEV6=FALSE, BcMV11=FALSE} =>  {BcEV2.BI=FALSE} | 0.29  703 | 0.827  586 | 0.358  911 | 1.10  7102 | 12  0 |
| {BcMV11=FALSE, BcOLV17.BI=FALSE} =>  {BcEV2.BI=FALSE} | 0.30  9406 | 0.827  815 | 0.373  762 | 1.10  7408 | 12  5 |
| {BcMV11=FALSE, BcOLV15.BI=FALSE} =>  {BcEV2.BI=FALSE} | 0.30  6931 | 0.849  315 | 0.361  386 | 1.13  617 | 12  4 |
| {BcEV5=FALSE, BcMV11=FALSE} =>  {BcEV2.BI=FALSE} | 0.30  9406 | 0.822  368 | 0.376  238 | 1.10  0122 | 12  5 |
| {BcEV2.BI=FALSE, BcEV5=FALSE} => | 0.30 | 0.434 | 0.712 | 1.13 | 12 |

| {BcMV11=FALSE} | 9406 | 028 | 871 | 1272 | 5 |
| --- | --- | --- | --- | --- | --- |
| {BcHV3.BI=FALSE, BcMV11=FALSE} =>  {BcFV5.BI=FALSE} | 0.27  2277 | 0.859  375 | 0.316  832 | 1.09  8695 | 11  0 |
| {BcFV5.BI=FALSE, BcMV11=FALSE} =>  {BcHV3.BI=FALSE} | 0.27  2277 | 0.887  097 | 0.306  931 | 1.16  7385 | 11  0 |
| {BcHV3.BI=FALSE, BcMV11=FALSE} =>  {BcNSRV7.BI=FALSE} | 0.27  7228 | 0.875 | 0.316  832 | 1.09  4427 | 11  2 |
| {BcMV11=FALSE, BcNSRV7.BI=FALSE} =>  {BcHV3.BI=FALSE} | 0.27  7228 | 0.881  89 | 0.314  356 | 1.16  0532 | 11  2 |
| {BcMV11=FALSE, BcVV3.BI=FALSE} =>  {BcHV3.BI=FALSE} | 0.27  4752 | 0.860  465 | 0.319  307 | 1.13  2338 | 11  1 |
| {BcMV11=FALSE, BcOLV7.BI=FALSE} =>  {BcHV3.BI=FALSE} | 0.27  4752 | 0.853  846 | 0.321  782 | 1.12  3628 | 11  1 |
| {BcHV3.BI=FALSE, BcMV11=FALSE} =>  {BcALV1.BI=FALSE} | 0.29  703 | 0.937  5 | 0.316  832 | 1.15  1216 | 12  0 |
| {BcALV1.BI=FALSE, BcMV11=FALSE} =>  {BcHV3.BI=FALSE} | 0.29  703 | 0.839  161 | 0.353  96 | 1.10  4303 | 12  0 |
| {BcALV1.BI=FALSE, BcHV3.BI=FALSE} =>  {BcMV11=FALSE} | 0.29  703 | 0.468  75 | 0.633  663 | 1.22  1774 | 12  0 |
| {BcDMV1=FALSE, BcMV11=FALSE} =>  {BcHV3.BI=FALSE} | 0.28  4653 | 0.851  852 | 0.334  158 | 1.12  1004 | 11  5 |
| {BcFV9=FALSE, BcMV11=FALSE} => | 0.28 | 0.835 | 0.346 | 1.09 | 11 |

| {BcHV3.BI=FALSE} | 9604 | 714 | 535 | 9767 | 7 |
| --- | --- | --- | --- | --- | --- |
| {BcHV3.BI=FALSE, BcMV11=FALSE} =>  {BcOLV4.BI=FALSE} | 0.30  198 | 0.953  125 | 0.316  832 | 1.08  7747 | 12  2 |
| {BcHV3.BI=FALSE, BcOLV4.BI=FALSE} =>  {BcMV11=FALSE} | 0.30  198 | 0.448  529 | 0.673  267 | 1.16  907 | 12  2 |
| {BcHV3.BI=FALSE, BcMV11=FALSE} =>  {BcULV5=FALSE} | 0.30  4455 | 0.960  938 | 0.316  832 | 1.09  6663 | 12  3 |
| {BcHV3.BI=FALSE, BcULV5=FALSE} =>  {BcMV11=FALSE} | 0.30  4455 | 0.455  556 | 0.668  317 | 1.18  7384 | 12  3 |
| {BcHV3.BI=FALSE, BcMV11=FALSE} =>  {BcBNV5.BI=FALSE} | 0.30  9406 | 0.976  563 | 0.316  832 | 1.07  5017 | 12  5 |
| {BcBNV5.BI=FALSE, BcMV11=FALSE} =>  {BcHV3.BI=FALSE} | 0.30  9406 | 0.833  333 | 0.371  287 | 1.09  6634 | 12  5 |
| {BcMV11=FALSE, BcOLV13.BI=FALSE} =>  {BcHV3.BI=FALSE} | 0.30  6931 | 0.837  838 | 0.366  337 | 1.10  2562 | 12  4 |
| {BcMV11=FALSE, BcOLV15.BI=FALSE} =>  {BcHV3.BI=FALSE} | 0.30  4455 | 0.842  466 | 0.361  386 | 1.10  8652 | 12  3 |
| {BcFV5.BI=FALSE, BcMV11=FALSE} =>  {BcNSRV7.BI=FALSE} | 0.26  9802 | 0.879  032 | 0.306  931 | 1.09  9471 | 10  9 |
| {BcMV11=FALSE, BcNSRV7.BI=FALSE} =>  {BcFV5.BI=FALSE} | 0.26  9802 | 0.858  268 | 0.314  356 | 1.09  7279 | 10  9 |
| {BcFV5.BI=FALSE, BcMV11=FALSE} => | 0.28 | 0.927 | 0.306 | 1.13 | 11 |

| {BcALV1.BI=FALSE} | 4653 | 419 | 931 | 8837 | 5 |
| --- | --- | --- | --- | --- | --- |
| {BcFV5.BI=FALSE, BcMV11=FALSE} =>  {BcDMV1=FALSE} | 0.28  2178 | 0.919  355 | 0.306  931 | 1.08  9206 | 11  4 |
| {BcFV5.BI=FALSE, BcMV11=FALSE} =>  {BcFV9=FALSE} | 0.28  7129 | 0.935  484 | 0.306  931 | 1.07  6739 | 11  6 |
| {BcFV5.BI=FALSE, BcMV11=FALSE} =>  {BcOLV4.BI=FALSE} | 0.28  7129 | 0.935  484 | 0.306  931 | 1.06  7614 | 11  6 |
| {BcFV5.BI=FALSE, BcMV11=FALSE} =>  {BcULV5=FALSE} | 0.29  703 | 0.967  742 | 0.306  931 | 1.10  4429 | 12  0 |
| {BcMV11=FALSE, BcULV1.BI=FALSE} =>  {BcALV1.BI=FALSE} | 0.28  7129 | 0.920  635 | 0.311  881 | 1.13  0506 | 11  6 |
| {BcALV1.BI=FALSE, BcULV1.BI=FALSE} =>  {BcMV11=FALSE} | 0.28  7129 | 0.442  748 | 0.648  515 | 1.15  4001 | 11  6 |
| {BcMV11=FALSE, BcULV1.BI=FALSE} =>  {BcOLV4.BI=FALSE} | 0.29  703 | 0.952  381 | 0.311  881 | 1.08  6898 | 12  0 |
| {BcMV11=FALSE, BcULV1.BI=FALSE} =>  {BcULV5=FALSE} | 0.30  9406 | 0.992  063 | 0.311  881 | 1.13  2185 | 12  5 |
| {BcULV1.BI=FALSE, BcULV5=FALSE} =>  {BcMV11=FALSE} | 0.30  9406 | 0.441  696 | 0.700  495 | 1.15  126 | 12  5 |
| {BcMV1.BI=TRUE, BcMV11=FALSE} =>  {BcALV1.BI=FALSE} | 0.25 | 0.926  606 | 0.269  802 | 1.13  7838 | 10  1 |
| {BcMV1.BI=TRUE, BcMV11=FALSE} => | 0.24 | 0.908 | 0.269 | 1.07 | 99 |

| {BcDMV1=FALSE} | 505 | 257 | 802 | 6058 |  |
| --- | --- | --- | --- | --- | --- |
| {BcMV1.BI=TRUE, BcMV11=FALSE} =>  {BcOLV4.BI=FALSE} | 0.25  495 | 0.944  954 | 0.269  802 | 1.07  8422 | 10  3 |
| {BcMV1.BI=TRUE, BcMV11=FALSE} =>  {BcULV5=FALSE} | 0.25  9901 | 0.963  303 | 0.269  802 | 1.09  9362 | 10  5 |
| {BcMV11=FALSE, BcMV12=FALSE} =>  {BcALV1.BI=FALSE} | 0.26  7327 | 0.915  254 | 0.292  079 | 1.12  3899 | 10  8 |
| {BcMV11=FALSE, BcMV12=FALSE} =>  {BcOLV4.BI=FALSE} | 0.28  2178 | 0.966  102 | 0.292  079 | 1.10  2557 | 11  4 |
| {BcMV11=FALSE, BcMV12=FALSE} =>  {BcULV5=FALSE} | 0.28  4653 | 0.974  576 | 0.292  079 | 1.11  2228 | 11  5 |
| {BcMV11=FALSE, BcMV12=FALSE} =>  {BcBNV5.BI=FALSE} | 0.28  4653 | 0.974  576 | 0.292  079 | 1.07  2831 | 11  5 |
| {BcMV11=FALSE, BcNSRV7.BI=FALSE} =>  {BcALV1.BI=FALSE} | 0.28  9604 | 0.921  26 | 0.314  356 | 1.13  1273 | 11  7 |
| {BcALV1.BI=FALSE, BcNSRV7.BI=FALSE} =>  {BcMV11=FALSE} | 0.28  9604 | 0.438  202 | 0.660  891 | 1.14  2153 | 11  7 |
| {BcMV11=FALSE, BcNSRV7.BI=FALSE} =>  {BcDMV1=FALSE} | 0.28  4653 | 0.905  512 | 0.314  356 | 1.07  2806 | 11  5 |
| {BcMV11=FALSE, BcNSRV7.BI=FALSE} =>  {BcFV9=FALSE} | 0.29  2079 | 0.929  134 | 0.314  356 | 1.06  943 | 11  8 |
| {BcMV11=FALSE, BcNSRV7.BI=FALSE} => | 0.29 | 0.937 | 0.314 | 1.06 | 11 |

| {BcOLV4.BI=FALSE} | 4554 | 008 | 356 | 9354 | 9 |
| --- | --- | --- | --- | --- | --- |
| {BcMV11=FALSE, BcNSRV7.BI=FALSE} =>  {BcULV5=FALSE} | 0.30  198 | 0.960  63 | 0.314  356 | 1.09  6312 | 12  2 |
| {BcNSRV7.BI=FALSE, BcULV5=FALSE} =>  {BcMV11=FALSE} | 0.30  198 | 0.437  276 | 0.690  594 | 1.13  9739 | 12  2 |
| {BcMV11=FALSE, BcNSRV7.BI=FALSE} =>  {BcBNV5.BI=FALSE} | 0.30  9406 | 0.984  252 | 0.314  356 | 1.08  3482 | 12  5 |
| {BcMV11=FALSE, BcVV3.BI=FALSE} =>  {BcOLV7.BI=FALSE} | 0.28  2178 | 0.883  721 | 0.319  307 | 1.09  5163 | 11  4 |
| {BcMV11=FALSE, BcOLV7.BI=FALSE} =>  {BcVV3.BI=FALSE} | 0.28  2178 | 0.876  923 | 0.321  782 | 1.09  3447 | 11  4 |
| {BcMV11=FALSE, BcVV3.BI=FALSE} =>  {BcALV1.BI=FALSE} | 0.29  703 | 0.930  233 | 0.319  307 | 1.14  2292 | 12  0 |
| {BcALV1.BI=FALSE, BcVV3.BI=FALSE} =>  {BcMV11=FALSE} | 0.29  703 | 0.451  128 | 0.658  416 | 1.17  5843 | 12  0 |
| {BcMV11=FALSE, BcVV3.BI=FALSE} =>  {BcOLV4.BI=FALSE} | 0.29  9505 | 0.937  984 | 0.319  307 | 1.07  0468 | 12  1 |
| {BcMV11=FALSE, BcVV3.BI=FALSE} =>  {BcULV5=FALSE} | 0.30  6931 | 0.961  24 | 0.319  307 | 1.09  7009 | 12  4 |
| {BcMV11=FALSE, BcOLV7.BI=FALSE} =>  {BcALV1.BI=FALSE} | 0.29  9505 | 0.930  769 | 0.321  782 | 1.14  2951 | 12  1 |
| {BcALV1.BI=FALSE, BcOLV7.BI=FALSE} => | 0.29 | 0.443 | 0.675 | 1.15 | 12 |

| {BcMV11=FALSE} | 9505 | 223 | 743 | 524 | 1 |
| --- | --- | --- | --- | --- | --- |
| {BcMV11=FALSE, BcOLV7.BI=FALSE} =>  {BcOLV4.BI=FALSE} | 0.30  4455 | 0.946  154 | 0.321  782 | 1.07  9791 | 12  3 |
| {BcMV11=FALSE, BcOLV7.BI=FALSE} =>  {BcULV5=FALSE} | 0.31  1881 | 0.969  231 | 0.321  782 | 1.10  6128 | 12  6 |
| {BcOLV7.BI=FALSE, BcULV5=FALSE} =>  {BcMV11=FALSE} | 0.31  1881 | 0.445  23 | 0.700  495 | 1.16  047 | 12  6 |
| {BcMV11=FALSE, BcOLV7.BI=FALSE} =>  {BcBNV5.BI=FALSE} | 0.31  6832 | 0.984  615 | 0.321  782 | 1.08  3882 | 12  8 |
| {BcBNV5.BI=FALSE, BcOLV7.BI=FALSE} =>  {BcMV11=FALSE} | 0.31  6832 | 0.433  898 | 0.730  198 | 1.13  0935 | 12  8 |
| {BcMV11=FALSE, BcOLV7.BI=FALSE} =>  {BcMCV5.BI=FALSE} | 0.31  9307 | 0.992  308 | 0.321  782 | 1.07  4778 | 12  9 |
| {BcDMV1=FALSE, BcMV11=FALSE} =>  {BcALV1.BI=FALSE} | 0.30  9406 | 0.925  926 | 0.334  158 | 1.13  7003 | 12  5 |
| {BcALV1.BI=FALSE, BcDMV1=FALSE} =>  {BcMV11=FALSE} | 0.30  9406 | 0.441  696 | 0.700  495 | 1.15  126 | 12  5 |
| {BcFV9=FALSE, BcMV11=FALSE} =>  {BcALV1.BI=FALSE} | 0.32  4257 | 0.935  714 | 0.346  535 | 1.14  9023 | 13  1 |
| {BcALV1.BI=FALSE, BcFV9=FALSE} =>  {BcMV11=FALSE} | 0.32  4257 | 0.450  172 | 0.720  297 | 1.17  3351 | 13  1 |
| {BcALV1.BI=FALSE, BcMV11=FALSE} => | 0.33 | 0.951 | 0.353 | 1.08 | 13 |

| {BcOLV4.BI=FALSE} | 6634 | 049 | 96 | 5378 | 6 |
| --- | --- | --- | --- | --- | --- |
| {BcMV11=FALSE, BcOLV4.BI=FALSE} =>  {BcALV1.BI=FALSE} | 0.33  6634 | 0.925  17 | 0.363  861 | 1.13  6075 | 13  6 |
| {BcALV1.BI=FALSE, BcOLV4.BI=FALSE} =>  {BcMV11=FALSE} | 0.33  6634 | 0.467  354 | 0.720  297 | 1.21  8135 | 13  6 |
| {BcALV1.BI=FALSE, BcMV11=FALSE} =>  {BcULV5=FALSE} | 0.34  1584 | 0.965  035 | 0.353  96 | 1.10  1339 | 13  8 |
| {BcMV11=FALSE, BcULV5=FALSE} =>  {BcALV1.BI=FALSE} | 0.34  1584 | 0.92 | 0.371  287 | 1.12  9726 | 13  8 |
| {BcALV1.BI=FALSE, BcULV5=FALSE} =>  {BcMV11=FALSE} | 0.34  1584 | 0.484  211 | 0.705  446 | 1.26  2071 | 13  8 |
| {BcBNV5.BI=FALSE, BcMV11=FALSE} =>  {BcALV1.BI=FALSE} | 0.34  4059 | 0.926  667 | 0.371  287 | 1.13  7913 | 13  9 |
| {BcALV1.BI=FALSE, BcBNV5.BI=FALSE} =>  {BcMV11=FALSE} | 0.34  4059 | 0.449  838 | 0.764  851 | 1.17  2481 | 13  9 |
| {BcMV11=FALSE, BvF.BI=FALSE} =>  {BcALV1.BI=FALSE} | 0.31  6832 | 0.927  536 | 0.341  584 | 1.13  8981 | 12  8 |
| {BcMCV5.BI=FALSE, BcMV11=FALSE} =>  {BcALV1.BI=FALSE} | 0.34  6535 | 0.921  053 | 0.376  238 | 1.13  1019 | 14  0 |
| {BcALV1.BI=FALSE, BcMCV5.BI=FALSE} =>  {BcMV11=FALSE} | 0.34  6535 | 0.448  718 | 0.772  277 | 1.16  9562 | 14  0 |
| {BcMV11=FALSE, BcOLV13.BI=FALSE} => | 0.33 | 0.918 | 0.366 | 1.12 | 13 |

| {BcALV1.BI=FALSE} | 6634 | 919 | 337 | 8399 | 6 |
| --- | --- | --- | --- | --- | --- |
| {BcALV1.BI=FALSE, BcOLV13.BI=FALSE} =>  {BcMV11=FALSE} | 0.33  6634 | 0.448  845 | 0.75 | 1.16  9892 | 13  6 |
| {BcMV11=FALSE, BcOLV2.BI=FALSE} =>  {BcALV1.BI=FALSE} | 0.32  9208 | 0.917  241 | 0.358  911 | 1.12  6339 | 13  3 |
| {BcALV1.BI=FALSE, BcOLV2.BI=FALSE} =>  {BcMV11=FALSE} | 0.32  9208 | 0.436  066 | 0.754  95 | 1.13  6584 | 13  3 |
| {BcMCV3.BI=FALSE, BcMV11=FALSE} =>  {BcALV1.BI=FALSE} | 0.32  6733 | 0.929  577 | 0.351  485 | 1.14  1487 | 13  2 |
| {BcALV1.BI=FALSE, BcMCV3.BI=FALSE} =>  {BcMV11=FALSE} | 0.32  6733 | 0.429  967 | 0.759  901 | 1.12  0689 | 13  2 |
| {BcMV11=FALSE, BcPV4.BI=FALSE} =>  {BcALV1.BI=FALSE} | 0.32  9208 | 0.923  611 | 0.356  436 | 1.13  4161 | 13  3 |
| {BcALV1.BI=FALSE, BcPV4.BI=FALSE} =>  {BcMV11=FALSE} | 0.32  9208 | 0.437  5 | 0.752  475 | 1.14  0323 | 13  3 |
| {BcEV6=FALSE, BcMV11=FALSE} =>  {BcALV1.BI=FALSE} | 0.32  9208 | 0.917  241 | 0.358  911 | 1.12  6339 | 13  3 |
| {BcALV1.BI=FALSE, BcEV6=FALSE} =>  {BcMV11=FALSE} | 0.32  9208 | 0.430  421 | 0.764  851 | 1.12  1871 | 13  3 |
| {BcMV11=FALSE, BcOLV17.BI=FALSE} =>  {BcALV1.BI=FALSE} | 0.34  4059 | 0.920  53 | 0.373  762 | 1.13  0377 | 13  9 |
| {BcALV1.BI=FALSE, BcOLV17.BI=FALSE} => | 0.34 | 0.446 | 0.769 | 1.16 | 13 |

| {BcMV11=FALSE} | 4059 | 945 | 802 | 4941 | 9 |
| --- | --- | --- | --- | --- | --- |
| {BcMV11=FALSE, BcOLV15.BI=FALSE} =>  {BcALV1.BI=FALSE} | 0.33  1683 | 0.917  808 | 0.361  386 | 1.12  7035 | 13  4 |
| {BcALV1.BI=FALSE, BcOLV15.BI=FALSE} =>  {BcMV11=FALSE} | 0.33  1683 | 0.432  258 | 0.767  327 | 1.12  666 | 13  4 |
| {BcEV5=FALSE, BcMV11=FALSE} =>  {BcALV1.BI=FALSE} | 0.34  6535 | 0.921  053 | 0.376  238 | 1.13  1019 | 14  0 |
| {BcALV1.BI=FALSE, BcEV5=FALSE} =>  {BcMV11=FALSE} | 0.34  6535 | 0.440  252 | 0.787  129 | 1.14  7494 | 14  0 |
| {BcDMV1=FALSE, BcMV11=FALSE} =>  {BcOLV4.BI=FALSE} | 0.31  4356 | 0.940  741 | 0.334  158 | 1.07  3614 | 12  7 |
| {BcDMV1=FALSE, BcOLV4.BI=FALSE} =>  {BcMV11=FALSE} | 0.31  4356 | 0.434  932 | 0.722  772 | 1.13  3628 | 12  7 |
| {BcDMV1=FALSE, BcMV11=FALSE} =>  {BcULV5=FALSE} | 0.32  4257 | 0.970  37 | 0.334  158 | 1.10  7428 | 13  1 |
| {BcDMV1=FALSE, BcULV5=FALSE} =>  {BcMV11=FALSE} | 0.32  4257 | 0.447  099 | 0.725  248 | 1.16  5342 | 13  1 |
| {BcFV9=FALSE, BcMV11=FALSE} =>  {BcOLV4.BI=FALSE} | 0.32  9208 | 0.95 | 0.346  535 | 1.08  4181 | 13  3 |
| {BcFV9=FALSE, BcMV11=FALSE} =>  {BcULV5=FALSE} | 0.33  6634 | 0.971  429 | 0.346  535 | 1.10  8636 | 13  6 |
| {BcFV9=FALSE, BcULV5=FALSE} => | 0.33 | 0.441 | 0.762 | 1.15 | 13 |

| {BcMV11=FALSE} | 6634 | 558 | 376 | 0901 | 6 |
| --- | --- | --- | --- | --- | --- |
| {BcMV11=FALSE, BcOLV15.BI=FALSE} =>  {BcFV9=FALSE} | 0.33  9109 | 0.938  356 | 0.361  386 | 1.08  0045 | 13  7 |
| {BcMV11=FALSE, BcOLV4.BI=FALSE} =>  {BcULV5=FALSE} | 0.35  6436 | 0.979  592 | 0.363  861 | 1.11  7952 | 14  4 |
| {BcMV11=FALSE, BcULV5=FALSE} =>  {BcOLV4.BI=FALSE} | 0.35  6436 | 0.96 | 0.371  287 | 1.09  5593 | 14  4 |
| {BcOLV4.BI=FALSE, BcULV5=FALSE} =>  {BcMV11=FALSE} | 0.35  6436 | 0.454  259 | 0.784  653 | 1.18  4003 | 14  4 |
| {BcBNV5.BI=FALSE, BcMV11=FALSE} =>  {BcOLV4.BI=FALSE} | 0.35  1485 | 0.946  667 | 0.371  287 | 1.08  0377 | 14  2 |
| {BcBNV5.BI=FALSE, BcOLV4.BI=FALSE} =>  {BcMV11=FALSE} | 0.35  1485 | 0.445  141 | 0.789  604 | 1.16  0239 | 14  2 |
| {BcMV11=FALSE, BvF.BI=FALSE} =>  {BcOLV4.BI=FALSE} | 0.32  6733 | 0.956  522 | 0.341  584 | 1.09  1624 | 13  2 |
| {BcMCV5.BI=FALSE, BcMV11=FALSE} =>  {BcOLV4.BI=FALSE} | 0.35  8911 | 0.953  947 | 0.376  238 | 1.08  8686 | 14  5 |
| {BcMCV5.BI=FALSE, BcOLV4.BI=FALSE} =>  {BcMV11=FALSE} | 0.35  8911 | 0.435  435 | 0.824  257 | 1.13  4941 | 14  5 |
| {BcMV11=FALSE, BcOLV13.BI=FALSE} =>  {BcOLV4.BI=FALSE} | 0.35  1485 | 0.959  459 | 0.366  337 | 1.09  4976 | 14  2 |
| {BcMV11=FALSE, BcOLV2.BI=FALSE} => | 0.34 | 0.958 | 0.358 | 1.09 | 13 |

| {BcOLV4.BI=FALSE} | 4059 | 621 | 911 | 4019 | 9 |
| --- | --- | --- | --- | --- | --- |
| {BcMCV3.BI=FALSE, BcMV11=FALSE} =>  {BcOLV4.BI=FALSE} | 0.33  9109 | 0.964  789 | 0.351  485 | 1.10  1058 | 13  7 |
| {BcMV11=FALSE, BcPV4.BI=FALSE} =>  {BcOLV4.BI=FALSE} | 0.33  9109 | 0.951  389 | 0.356  436 | 1.08  5766 | 13  7 |
| {BcEV6=FALSE, BcMV11=FALSE} =>  {BcOLV4.BI=FALSE} | 0.34  1584 | 0.951  724 | 0.358  911 | 1.08  6148 | 13  8 |
| {BcMV11=FALSE, BcOLV17.BI=FALSE} =>  {BcOLV4.BI=FALSE} | 0.35  396 | 0.947  02 | 0.373  762 | 1.08  078 | 14  3 |
| {BcMV11=FALSE, BcOLV15.BI=FALSE} =>  {BcOLV4.BI=FALSE} | 0.34  1584 | 0.945  205 | 0.361  386 | 1.07  8709 | 13  8 |
| {BcEV5=FALSE, BcMV11=FALSE} =>  {BcOLV4.BI=FALSE} | 0.35  6436 | 0.947  368 | 0.376  238 | 1.08  1178 | 14  4 |
| {BcEV5=FALSE, BcOLV4.BI=FALSE} =>  {BcMV11=FALSE} | 0.35  6436 | 0.429  851 | 0.829  208 | 1.12  0385 | 14  4 |
| {BcBNV5.BI=FALSE, BcMV11=FALSE} =>  {BcULV5=FALSE} | 0.35  8911 | 0.966  667 | 0.371  287 | 1.10  3202 | 14  5 |
| {BcBNV5.BI=FALSE, BcULV5=FALSE} =>  {BcMV11=FALSE} | 0.35  8911 | 0.454  545 | 0.789  604 | 1.18  4751 | 14  5 |
| {BcMV11=FALSE, BvF.BI=FALSE} =>  {BcULV5=FALSE} | 0.33  1683 | 0.971  014 | 0.341  584 | 1.10  8163 | 13  4 |
| {BcMCV5.BI=FALSE, BcMV11=FALSE} => | 0.36 | 0.967 | 0.376 | 1.10 | 14 |

| {BcULV5=FALSE} | 3861 | 105 | 238 | 3702 | 7 |
| --- | --- | --- | --- | --- | --- |
| {BcMCV5.BI=FALSE, BcULV5=FALSE} =>  {BcMV11=FALSE} | 0.36  3861 | 0.446  809 | 0.814  356 | 1.16  4585 | 14  7 |
| {BcMV11=FALSE, BcOLV13.BI=FALSE} =>  {BcULV5=FALSE} | 0.35  8911 | 0.979  73 | 0.366  337 | 1.11  811 | 14  5 |
| {BcOLV13.BI=FALSE, BcULV5=FALSE} =>  {BcMV11=FALSE} | 0.35  8911 | 0.434  132 | 0.826  733 | 1.13  1543 | 14  5 |
| {BcMV11=FALSE, BcOLV2.BI=FALSE} =>  {BcULV5=FALSE} | 0.34  901 | 0.972  414 | 0.358  911 | 1.10  976 | 14  1 |
| {BcMCV3.BI=FALSE, BcMV11=FALSE} =>  {BcULV5=FALSE} | 0.34  4059 | 0.978  873 | 0.351  485 | 1.11  7132 | 13  9 |
| {BcMV11=FALSE, BcPV4.BI=FALSE} =>  {BcULV5=FALSE} | 0.34  6535 | 0.972  222 | 0.356  436 | 1.10  9542 | 14  0 |
| {BcEV6=FALSE, BcMV11=FALSE} =>  {BcULV5=FALSE} | 0.34  901 | 0.972  414 | 0.358  911 | 1.10  976 | 14  1 |
| {BcMV11=FALSE, BcOLV17.BI=FALSE} =>  {BcULV5=FALSE} | 0.36  1386 | 0.966  887 | 0.373  762 | 1.10  3453 | 14  6 |
| {BcOLV17.BI=FALSE, BcULV5=FALSE} =>  {BcMV11=FALSE} | 0.36  1386 | 0.433  234 | 0.834  158 | 1.12  9205 | 14  6 |
| {BcMV11=FALSE, BcOLV15.BI=FALSE} =>  {BcULV5=FALSE} | 0.34  901 | 0.965  753 | 0.361  386 | 1.10  2159 | 14  1 |
| {BcEV5=FALSE, BcMV11=FALSE} => | 0.36 | 0.967 | 0.376 | 1.10 | 14 |

| {BcULV5=FALSE} | 3861 | 105 | 238 | 3702 | 7 |
| --- | --- | --- | --- | --- | --- |
| {BcEV5=FALSE, BcULV5=FALSE} =>  {BcMV11=FALSE} | 0.36  3861 | 0.438  806 | 0.829  208 | 1.14  3727 | 14  7 |
| {BcMCV5.BI=FALSE, BcMV11=FALSE} =>  {BcBNV5.BI=FALSE} | 0.36  6337 | 0.973  684 | 0.376  238 | 1.07  1849 | 14  8 |
| {BcMV11=FALSE, BcOLV15.BI=FALSE} =>  {BcBNV5.BI=FALSE} | 0.35  1485 | 0.972  603 | 0.361  386 | 1.07  0658 | 14  2 |
| {BcHV1.BI=FALSE, BcULV3=FALSE} =>  {BcHV1SLR.BI=FALSE} | 0.20  5446 | 0.912  088 | 0.225  248 | 1.99  1803 | 83 |
| {BcHV1.BI=FALSE, BcHV1SLR.BI=FALSE} =>  {BcULV3=FALSE} | 0.20  5446 | 0.605  839 | 0.339  109 | 1.49  2434 | 83 |
| {BcHV1SLR.BI=FALSE, BcULV3=FALSE} =>  {BcHV1.BI=FALSE} | 0.20  5446 | 0.821  782 | 0.25 | 2.07  5 | 83 |
| {BcHV1.BI=FALSE, BcULV3=FALSE} =>  {BcMV3.BI=FALSE} | 0.12  6238 | 0.560  44 | 0.225  248 | 1.22  3879 | 51 |
| {BcHV1.BI=FALSE, BcMV3.BI=FALSE} =>  {BcULV3=FALSE} | 0.12  6238 | 0.607  143 | 0.207  921 | 1.49  5645 | 51 |
| {BcMV3.BI=FALSE, BcULV3=FALSE} =>  {BcHV1.BI=FALSE} | 0.12  6238 | 0.6 | 0.210  396 | 1.51  5 | 51 |
| {BcHV1.BI=FALSE, BcMV2.BI=TRUE} =>  {BcULV3=FALSE} | 0.09  1584 | 0.536  232 | 0.170  792 | 1.32  0961 | 37 |
| {BcMV2.BI=TRUE, BcULV3=FALSE} => | 0.09 | 0.513 | 0.178 | 1.29 | 37 |

| {BcHV1.BI=FALSE} | 1584 | 889 | 218 | 7569 |  |
| --- | --- | --- | --- | --- | --- |
| {BcHV1.BI=FALSE, BcULV3=FALSE} =>  {BcOLV18=FALSE} | 0.14  3564 | 0.637  363 | 0.225  248 | 1.32  0485 | 58 |
| {BcHV1.BI=FALSE, BcOLV18=FALSE} =>  {BcULV3=FALSE} | 0.14  3564 | 0.585  859 | 0.245  05 | 1.44  3213 | 58 |
| {BcOLV18=FALSE, BcULV3=FALSE} =>  {BcHV1.BI=FALSE} | 0.14  3564 | 0.674  419 | 0.212  871 | 1.70  2907 | 58 |
| {BcHV1.BI=FALSE, BcOLV18=TRUE} =>  {BcULV3=FALSE} | 0.08  1683 | 0.540  984 | 0.150  99 | 1.33  2667 | 33 |
| {BcHV1.BI=FALSE, BcMV2.BI=FALSE} =>  {BcULV3=FALSE} | 0.13  3663 | 0.593  407 | 0.225  248 | 1.46  1806 | 54 |
| {BcMV2.BI=FALSE, BcULV3=FALSE} =>  {BcHV1.BI=FALSE} | 0.13  3663 | 0.586  957 | 0.227  723 | 1.48  2065 | 54 |
| {BcHV1.BI=FALSE, BcMV3.BI=TRUE} =>  {BcULV3=FALSE} | 0.09  901 | 0.526  316 | 0.188  119 | 1.29  6534 | 40 |
| {BcMV3.BI=TRUE, BcULV3=FALSE} =>  {BcHV1.BI=FALSE} | 0.09  901 | 0.506  329 | 0.195  545 | 1.27  8481 | 40 |
| {BcHV1.BI=FALSE, BcULV3=FALSE} =>  {BcMV4.BI=FALSE} | 0.15  3465 | 0.681  319 | 0.225  248 | 1.22  3346 | 62 |
| {BcHV1.BI=FALSE, BcMV4.BI=FALSE} =>  {BcULV3=FALSE} | 0.15  3465 | 0.607  843 | 0.252  475 | 1.49  737 | 62 |
| {BcMV4.BI=FALSE, BcULV3=FALSE} => | 0.15 | 0.596 | 0.257 | 1.50 | 62 |

| {BcHV1.BI=FALSE} | 3465 | 154 | 426 | 5288 |  |
| --- | --- | --- | --- | --- | --- |
| {BcHV1.BI=FALSE, BcULV3=FALSE} => | 0.15 | 0.703 | 0.225 | 1.20 |  |
|  |  |  |  |  | 64 |
| {BcOLV16.BI=FALSE} | 8416 | 297 | 248 | 3949 |  |
| {BcHV1.BI=FALSE, BcOLV16.BI=FALSE} => | 0.15 | 0.633 |  | 1.56 |  |
|  |  |  | 0.25 |  | 64 |
| {BcULV3=FALSE} | 8416 | 663 |  | 0976 |  |
| {BcOLV16.BI=FALSE, BcULV3=FALSE} => | 0.15 | 0.587 | 0.269 | 1.48 |  |
|  |  |  |  |  | 64 |
| {BcHV1.BI=FALSE} | 8416 | 156 | 802 | 2569 |  |
| {BcHV1.BI=FALSE, BcMV11=TRUE} => | 0.12 | 0.526 | 0.235 | 1.29 |  |
|  |  |  |  |  | 50 |
| {BcULV3=FALSE} | 3762 | 316 | 149 | 6534 |  |
| {BcMV11=TRUE, BcULV3=FALSE} => | 0.12 | 0.561 | 0.220 | 1.41 |  |
|  |  |  |  |  | 50 |
| {BcHV1.BI=FALSE} | 3762 | 798 | 297 | 8539 |  |
| {BcHV1.BI=FALSE, |  |  |  |  |  |
|  | 0.15 | 0.571 | 0.277 | 1.40 |  |
| BcOLV1.BI.MOVA.=FALSE} => |  |  |  |  | 64 |
|  | 8416 | 429 | 228 | 7666 |  |
| {BcULV3=FALSE} |  |  |  |  |  |
| {BcOLV1.BI.MOVA.=FALSE, BcULV3=FALSE} | 0.15 | 0.587 | 0.269 | 1.48 |  |
|  |  |  |  |  | 64 |
| => {BcHV1.BI=FALSE} | 8416 | 156 | 802 | 2569 |  |
| {BcHV1.BI=FALSE, BcMV9.BI=TRUE} => | 0.10 | 0.517 | 0.210 | 1.27 |  |
|  |  |  |  |  | 44 |
| {BcULV3=FALSE} | 8911 | 647 | 396 | 5179 |  |
| {BcHV1.BI=FALSE, BcULV4=TRUE} => | 0.12 | 0.547 | 0.235 | 1.34 |  |
|  |  |  |  |  | 52 |
| {BcULV3=FALSE} | 8713 | 368 | 149 | 8395 |  |
| {BcHV1.BI=FALSE, BcULV3=FALSE} => | 0.20 | 0.890 | 0.225 | 1.27 |  |
|  |  |  |  |  | 81 |
| {BcMV6.BI=FALSE} | 0495 | 11 | 248 | 9731 |  |

| {BcHV1.BI=FALSE, BcMV6.BI=FALSE} =>  {BcULV3=FALSE} | 0.20  0495 | 0.627  907 | 0.319  307 | 1.54  6795 | 81 |
| --- | --- | --- | --- | --- | --- |
| {BcMV6.BI=FALSE, BcULV3=FALSE} =>  {BcHV1.BI=FALSE} | 0.20  0495 | 0.632  813 | 0.316  832 | 1.59  7852 | 81 |
| {BcHV1.BI=FALSE, BcULV3=FALSE} =>  {BcPV3.BI=FALSE} | 0.18  5644 | 0.824  176 | 0.225  248 | 1.16  0164 | 75 |
| {BcHV1.BI=FALSE, BcPV3.BI=FALSE} =>  {BcULV3=FALSE} | 0.18  5644 | 0.604  839 | 0.306  931 | 1.48  9969 | 75 |
| {BcPV3.BI=FALSE, BcULV3=FALSE} =>  {BcHV1.BI=FALSE} | 0.18  5644 | 0.641  026 | 0.289  604 | 1.61  859 | 75 |
| {BcHV1.BI=FALSE, BcRV1.BI=FALSE} =>  {BcULV3=FALSE} | 0.17  0792 | 0.556  452 | 0.306  931 | 1.37  0771 | 69 |
| {BcRV1.BI=FALSE, BcULV3=FALSE} =>  {BcHV1.BI=FALSE} | 0.17  0792 | 0.589  744 | 0.289  604 | 1.48  9103 | 69 |
| {BcEV2.BI=FALSE, BcHV1.BI=FALSE} =>  {BcULV3=FALSE} | 0.18  0693 | 0.584 | 0.309  406 | 1.43  8634 | 73 |
| {BcEV2.BI=FALSE, BcULV3=FALSE} =>  {BcHV1.BI=FALSE} | 0.18  0693 | 0.579  365 | 0.311  881 | 1.46  2897 | 73 |
| {BcHV1.BI=FALSE, BcHV3.BI=FALSE} =>  {BcULV3=FALSE} | 0.18  5644 | 0.585  938 | 0.316  832 | 1.44  3407 | 75 |
| {BcHV3.BI=FALSE, BcULV3=FALSE} =>  {BcHV1.BI=FALSE} | 0.18  5644 | 0.551  471 | 0.336  634 | 1.39  2463 | 75 |

| {BcHV1.BI=FALSE, BcULV3=FALSE} =>  {BcFV5.BI=FALSE} | 0.19  5545 | 0.868  132 | 0.225  248 | 1.10  989 | 79 |
| --- | --- | --- | --- | --- | --- |
| {BcFV5.BI=FALSE, BcHV1.BI=FALSE} =>  {BcULV3=FALSE} | 0.19  5545 | 0.593  985 | 0.329  208 | 1.46  3231 | 79 |
| {BcFV5.BI=FALSE, BcULV3=FALSE} =>  {BcHV1.BI=FALSE} | 0.19  5545 | 0.556  338 | 0.351  485 | 1.40  4754 | 79 |
| {BcHV1.BI=FALSE, BcULV3=FALSE} =>  {BcULV1.BI=FALSE} | 0.20  7921 | 0.923  077 | 0.225  248 | 1.17  6413 | 84 |
| {BcHV1.BI=FALSE, BcULV1.BI=FALSE} =>  {BcULV3=FALSE} | 0.20  7921 | 0.583  333 | 0.356  436 | 1.43  6992 | 84 |
| {BcULV1.BI=FALSE, BcULV3=FALSE} =>  {BcHV1.BI=FALSE} | 0.20  7921 | 0.636  364 | 0.326  733 | 1.60  6818 | 84 |
| {BcHV1.BI=FALSE, BcMV1.BI=TRUE} =>  {BcULV3=FALSE} | 0.16  3366 | 0.532  258 | 0.306  931 | 1.31  1172 | 66 |
| {BcMV1.BI=TRUE, BcULV3=FALSE} =>  {BcHV1.BI=FALSE} | 0.16  3366 | 0.536  585 | 0.304  455 | 1.35  4878 | 66 |
| {BcHV1.BI=FALSE, BcMV12=FALSE} =>  {BcULV3=FALSE} | 0.18  8119 | 0.562  963 | 0.334  158 | 1.38  6811 | 76 |
| {BcMV12=FALSE, BcULV3=FALSE} =>  {BcHV1.BI=FALSE} | 0.18  8119 | 0.567  164 | 0.331  683 | 1.43  209 | 76 |
| {BcHV1.BI=FALSE, BcNSRV7.BI=FALSE} =>  {BcULV3=FALSE} | 0.19  5545 | 0.585  185 | 0.334  158 | 1.44  1554 | 79 |

| {BcNSRV7.BI=FALSE, BcULV3=FALSE} =>  {BcHV1.BI=FALSE} | 0.19  5545 | 0.541  096 | 0.361  386 | 1.36  6267 | 79 |
| --- | --- | --- | --- | --- | --- |
| {BcHV1.BI=FALSE, BcVV3.BI=FALSE} =>  {BcULV3=FALSE} | 0.18  3168 | 0.596  774 | 0.306  931 | 1.47  0102 | 74 |
| {BcULV3=FALSE, BcVV3.BI=FALSE} =>  {BcHV1.BI=FALSE} | 0.18  3168 | 0.564  885 | 0.324  257 | 1.42  6336 | 74 |
| {BcHV1.BI=FALSE, BcOLV7.BI=FALSE} =>  {BcULV3=FALSE} | 0.19  0594 | 0.578  947 | 0.329  208 | 1.42  6187 | 77 |
| {BcOLV7.BI=FALSE, BcULV3=FALSE} =>  {BcHV1.BI=FALSE} | 0.19  0594 | 0.574  627 | 0.331  683 | 1.45  0933 | 77 |
| {BcHV1.BI=FALSE, BcULV3=FALSE} =>  {BcALV1.BI=FALSE} | 0.20  0495 | 0.890  11 | 0.225  248 | 1.09  3022 | 81 |
| {BcALV1.BI=FALSE, BcHV1.BI=FALSE} =>  {BcULV3=FALSE} | 0.20  0495 | 0.574  468 | 0.349  01 | 1.41  5153 | 81 |
| {BcALV1.BI=FALSE, BcULV3=FALSE} =>  {BcHV1.BI=FALSE} | 0.20  0495 | 0.604  478 | 0.331  683 | 1.52  6306 | 81 |
| {BcDMV1=FALSE, BcHV1.BI=FALSE} =>  {BcULV3=FALSE} | 0.18  3168 | 0.560  606 | 0.326  733 | 1.38  1005 | 74 |
| {BcDMV1=FALSE, BcULV3=FALSE} =>  {BcHV1.BI=FALSE} | 0.18  3168 | 0.540  146 | 0.339  109 | 1.36  3869 | 74 |
| {BcFV9=FALSE, BcHV1.BI=FALSE} =>  {BcULV3=FALSE} | 0.20  0495 | 0.554  795 | 0.361  386 | 1.36  6689 | 81 |

| {BcFV9=FALSE, BcULV3=FALSE} =>  {BcHV1.BI=FALSE} | 0.20  0495 | 0.558  621 | 0.358  911 | 1.41  0517 | 81 |
| --- | --- | --- | --- | --- | --- |
| {BcHV1.BI=FALSE, BcOLV4.BI=FALSE} =>  {BcULV3=FALSE} | 0.20  0495 | 0.578  571 | 0.346  535 | 1.42  5261 | 81 |
| {BcOLV4.BI=FALSE, BcULV3=FALSE} =>  {BcHV1.BI=FALSE} | 0.20  0495 | 0.543  624 | 0.368  812 | 1.37  2651 | 81 |
| {BcHV1.BI=FALSE, BcULV5=FALSE} =>  {BcULV3=FALSE} | 0.19  802 | 0.575  54 | 0.344  059 | 1.41  7793 | 80 |
| {BcULV3=FALSE, BcULV5=FALSE} =>  {BcHV1.BI=FALSE} | 0.19  802 | 0.551  724 | 0.358  911 | 1.39  3103 | 80 |
| {BcHV1.BI=FALSE, BcULV3=FALSE} =>  {BcBNV5.BI=FALSE} | 0.22  0297 | 0.978  022 | 0.225  248 | 1.07  6624 | 89 |
| {BcBNV5.BI=FALSE, BcHV1.BI=FALSE} =>  {BcULV3=FALSE} | 0.22  0297 | 0.585  526 | 0.376  238 | 1.44  2394 | 89 |
| {BcBNV5.BI=FALSE, BcULV3=FALSE} =>  {BcHV1.BI=FALSE} | 0.22  0297 | 0.563  291 | 0.391  089 | 1.42  231 | 89 |
| {BcHV1.BI=FALSE, BvF.BI=FALSE} =>  {BcULV3=FALSE} | 0.20  5446 | 0.557  047 | 0.368  812 | 1.37  2238 | 83 |
| {BcULV3=FALSE, BvF.BI=FALSE} =>  {BcHV1.BI=FALSE} | 0.20  5446 | 0.568  493 | 0.361  386 | 1.43  5445 | 83 |
| {BcHV1.BI=FALSE, BcMCV5.BI=FALSE} =>  {BcULV3=FALSE} | 0.21  5347 | 0.572  368 | 0.376  238 | 1.40  9981 | 87 |

| {BcMCV5.BI=FALSE, BcULV3=FALSE} =>  {BcHV1.BI=FALSE} | 0.21  5347 | 0.550  633 | 0.391  089 | 1.39  0348 | 87 |
| --- | --- | --- | --- | --- | --- |
| {BcHV1.BI=FALSE, BcOLV13.BI=FALSE} =>  {BcULV3=FALSE} | 0.21  2871 | 0.558  442 | 0.381  188 | 1.37  5673 | 86 |
| {BcOLV13.BI=FALSE, BcULV3=FALSE} =>  {BcHV1.BI=FALSE} | 0.21  2871 | 0.558  442 | 0.381  188 | 1.41  0065 | 86 |
| {BcHV1.BI=FALSE, BcOLV2.BI=FALSE} =>  {BcULV3=FALSE} | 0.22  2772 | 0.592  105 | 0.376  238 | 1.45  8601 | 90 |
| {BcOLV2.BI=FALSE, BcULV3=FALSE} =>  {BcHV1.BI=FALSE} | 0.22  2772 | 0.576  923 | 0.386  139 | 1.45  6731 | 90 |
| {BcHV1.BI=FALSE, BcMCV3.BI=FALSE} =>  {BcULV3=FALSE} | 0.22  5248 | 0.579  618 | 0.388  614 | 1.42  7839 | 91 |
| {BcMCV3.BI=FALSE, BcULV3=FALSE} =>  {BcHV1.BI=FALSE} | 0.22  5248 | 0.575  949 | 0.391  089 | 1.45  4272 | 91 |
| {BcHV1.BI=FALSE, BcPV4.BI=FALSE} =>  {BcULV3=FALSE} | 0.21  0396 | 0.562  914 | 0.373  762 | 1.38  669 | 85 |
| {BcPV4.BI=FALSE, BcULV3=FALSE} =>  {BcHV1.BI=FALSE} | 0.21  0396 | 0.548  387 | 0.383  663 | 1.38  4677 | 85 |
| {BcEV6=FALSE, BcHV1.BI=FALSE} =>  {BcULV3=FALSE} | 0.22  0297 | 0.570  513 | 0.386  139 | 1.40  541 | 89 |
| {BcEV6=FALSE, BcULV3=FALSE} =>  {BcHV1.BI=FALSE} | 0.22  0297 | 0.585  526 | 0.376  238 | 1.47  8454 | 89 |

| {BcHV1.BI=FALSE, BcOLV17.BI=FALSE} =>  {BcULV3=FALSE} | 0.21  7822 | 0.567  742 | 0.383  663 | 1.39  8584 | 88 |
| --- | --- | --- | --- | --- | --- |
| {BcOLV17.BI=FALSE, BcULV3=FALSE} =>  {BcHV1.BI=FALSE} | 0.21  7822 | 0.556  962 | 0.391  089 | 1.40  6329 | 88 |
| {BcHV1.BI=FALSE, BcOLV15.BI=FALSE} =>  {BcULV3=FALSE} | 0.21  7822 | 0.564  103 | 0.386  139 | 1.38  9619 | 88 |
| {BcOLV15.BI=FALSE, BcULV3=FALSE} =>  {BcHV1.BI=FALSE} | 0.21  7822 | 0.553  459 | 0.393  564 | 1.39  7484 | 88 |
| {BcEV5=FALSE, BcHV1.BI=FALSE} =>  {BcULV3=FALSE} | 0.21  7822 | 0.578  947 | 0.376  238 | 1.42  6187 | 88 |
| {BcEV5=FALSE, BcULV3=FALSE} =>  {BcHV1.BI=FALSE} | 0.21  7822 | 0.546  584 | 0.398  515 | 1.38  0124 | 88 |
| {BcHV1.BI=FALSE, BcOLV16.BI=TRUE} =>  {BcHV1SLR.BI=FALSE} | 0.12  8713 | 0.881  356 | 0.146  04 | 1.92  4691 | 52 |
| {BcHV1SLR.BI=FALSE, BcOLV16.BI=TRUE}  => {BcHV1.BI=FALSE} | 0.12  8713 | 0.722  222 | 0.178  218 | 1.82  3611 | 52 |
| {BcHV1.BI=FALSE, BcOLV18=TRUE} =>  {BcOLV16.BI=TRUE} | 0.08  1683 | 0.540  984 | 0.150  99 | 1.30  0937 | 33 |
| {BcHV1.BI=FALSE, BcMV4.BI=TRUE} =>  {BcHV1SLR.BI=FALSE} | 0.12  3762 | 0.862  069 | 0.143  564 | 1.88  2572 | 50 |
| {BcHV1SLR.BI=FALSE, BcMV4.BI=TRUE} =>  {BcHV1.BI=FALSE} | 0.12  3762 | 0.684  932 | 0.180  693 | 1.72  9452 | 50 |

| {BcHV1.BI=FALSE, BcMV4.BI=TRUE} =>  {BcMV2.BI=TRUE} | 0.08  4158 | 0.586  207 | 0.143  564 | 1.26  6458 | 34 |
| --- | --- | --- | --- | --- | --- |
| {BcHV1.BI=FALSE, BcMV4.BI=TRUE} =>  {BcMV11=TRUE} | 0.10  8911 | 0.758  621 | 0.143  564 | 1.23  0854 | 44 |
| {BcHV1.BI=FALSE, BcMV3.BI=FALSE} =>  {BcHV1SLR.BI=FALSE} | 0.18  3168 | 0.880  952 | 0.207  921 | 1.92  381 | 74 |
| {BcHV1SLR.BI=FALSE, BcMV3.BI=FALSE}  => {BcHV1.BI=FALSE} | 0.18  3168 | 0.747  475 | 0.245  05 | 1.88  7374 | 74 |
| {BcHV1.BI=FALSE, BcMV2.BI=TRUE} =>  {BcHV1SLR.BI=FALSE} | 0.14  604 | 0.855  072 | 0.170  792 | 1.86  7293 | 59 |
| {BcHV1SLR.BI=FALSE, BcMV2.BI=TRUE} =>  {BcHV1.BI=FALSE} | 0.14  604 | 0.766  234 | 0.190  594 | 1.93  474 | 59 |
| {BcHV1.BI=FALSE, BcHV1SLR.BI=FALSE} =>  {BcOLV18=FALSE} | 0.21  2871 | 0.627  737 | 0.339  109 | 1.30  0543 | 86 |
| {BcHV1.BI=FALSE, BcOLV18=FALSE} =>  {BcHV1SLR.BI=FALSE} | 0.21  2871 | 0.868  687 | 0.245  05 | 1.89  7024 | 86 |
| {BcHV1SLR.BI=FALSE, BcOLV18=FALSE} =>  {BcHV1.BI=FALSE} | 0.21  2871 | 0.754  386 | 0.282  178 | 1.90  4825 | 86 |
| {BcHV1.BI=FALSE, BcOLV18=TRUE} =>  {BcHV1SLR.BI=FALSE} | 0.12  6238 | 0.836  066 | 0.150  99 | 1.82  5786 | 51 |
| {BcHV1SLR.BI=FALSE, BcOLV18=TRUE} =>  {BcHV1.BI=FALSE} | 0.12  6238 | 0.718  31 | 0.175  743 | 1.81  3732 | 51 |

| {BcHV1.BI=FALSE, BcMV2.BI=FALSE} =>  {BcHV1SLR.BI=FALSE} | 0.19  3069 | 0.857  143 | 0.225  248 | 1.87  1815 | 78 |
| --- | --- | --- | --- | --- | --- |
| {BcHV1SLR.BI=FALSE, BcMV2.BI=FALSE}  => {BcHV1.BI=FALSE} | 0.19  3069 | 0.722  222 | 0.267  327 | 1.82  3611 | 78 |
| {BcHV1.BI=FALSE, BcMV3.BI=TRUE} =>  {BcHV1SLR.BI=FALSE} | 0.15  5941 | 0.828  947 | 0.188  119 | 1.81  0242 | 63 |
| {BcHV1SLR.BI=FALSE, BcMV3.BI=TRUE} =>  {BcHV1.BI=FALSE} | 0.15  5941 | 0.732  558 | 0.212  871 | 1.84  9709 | 63 |
| {BcHV1.BI=FALSE, BcMV4.BI=FALSE} =>  {BcHV1SLR.BI=FALSE} | 0.21  5347 | 0.852  941 | 0.252  475 | 1.86  2639 | 87 |
| {BcHV1SLR.BI=FALSE, BcMV4.BI=FALSE}  => {BcHV1.BI=FALSE} | 0.21  5347 | 0.776  786 | 0.277  228 | 1.96  1384 | 87 |
| {BcHV1.BI=FALSE, BcOLV16.BI=FALSE} =>  {BcHV1SLR.BI=FALSE} | 0.21  0396 | 0.841  584 | 0.25 | 1.83  7838 | 85 |
| {BcHV1SLR.BI=FALSE, BcOLV16.BI=FALSE}  => {BcHV1.BI=FALSE} | 0.21  0396 | 0.752  212 | 0.279  703 | 1.89  9336 | 85 |
| {BcHV1.BI=FALSE, BcULV3=TRUE} =>  {BcHV1SLR.BI=FALSE} | 0.13  3663 | 0.782  609 | 0.170  792 | 1.70  9048 | 54 |
| {BcHV1SLR.BI=FALSE, BcULV3=TRUE} =>  {BcHV1.BI=FALSE} | 0.13  3663 | 0.642  857 | 0.207  921 | 1.62  3214 | 54 |
| {BcHV1.BI=FALSE, BcMV11=TRUE} =>  {BcHV1SLR.BI=FALSE} | 0.19  3069 | 0.821  053 | 0.235  149 | 1.79  3001 | 78 |

| {BcHV1SLR.BI=FALSE, BcMV11=TRUE} =>  {BcHV1.BI=FALSE} | 0.19  3069 | 0.742  857 | 0.259  901 | 1.87  5714 | 78 |
| --- | --- | --- | --- | --- | --- |
| {BcHV1.BI=FALSE, BcOLV1.BI.MOVA.=FALSE} =>  {BcHV1SLR.BI=FALSE} | 0.24  2574 | 0.875 | 0.277  228 | 1.91  0811 | 98 |
| {BcHV1SLR.BI=FALSE, BcOLV1.BI.MOVA.=FALSE} =>  {BcHV1.BI=FALSE} | 0.24  2574 | 0.742  424 | 0.326  733 | 1.87  4621 | 98 |
| {BcHV1.BI=FALSE, BcMV9.BI=TRUE} =>  {BcHV1SLR.BI=FALSE} | 0.17  8218 | 0.847  059 | 0.210  396 | 1.84  9793 | 72 |
| {BcHV1SLR.BI=FALSE, BcMV9.BI=TRUE} =>  {BcHV1.BI=FALSE} | 0.17  8218 | 0.660  55 | 0.269  802 | 1.66  789 | 72 |
| {BcHV1.BI=FALSE, BcULV4=TRUE} =>  {BcHV1SLR.BI=FALSE} | 0.19  802 | 0.842  105 | 0.235  149 | 1.83  8976 | 80 |
| {BcHV1SLR.BI=FALSE, BcULV4=TRUE} =>  {BcHV1.BI=FALSE} | 0.19  802 | 0.683  761 | 0.289  604 | 1.72  6496 | 80 |
| {BcHV1.BI=FALSE, BcHV1SLR.BI=FALSE} =>  {BcMV6.BI=FALSE} | 0.27  7228 | 0.817  518 | 0.339  109 | 1.17  5364 | 11  2 |
| {BcHV1.BI=FALSE, BcMV6.BI=FALSE} =>  {BcHV1SLR.BI=FALSE} | 0.27  7228 | 0.868  217 | 0.319  307 | 1.89  5998 | 11  2 |
| {BcHV1SLR.BI=FALSE, BcMV6.BI=FALSE}  => {BcHV1.BI=FALSE} | 0.27  7228 | 0.772  414 | 0.358  911 | 1.95  0345 | 11  2 |

| {BcHV1.BI=FALSE, BcPV3.BI=FALSE} =>  {BcHV1SLR.BI=FALSE} | 0.25  9901 | 0.846  774 | 0.306  931 | 1.84  9172 | 10  5 |
| --- | --- | --- | --- | --- | --- |
| {BcHV1SLR.BI=FALSE, BcPV3.BI=FALSE} =>  {BcHV1.BI=FALSE} | 0.25  9901 | 0.772  059 | 0.336  634 | 1.94  9449 | 10  5 |
| {BcHV1.BI=FALSE, BcRV1.BI=FALSE} =>  {BcHV1SLR.BI=FALSE} | 0.25  9901 | 0.846  774 | 0.306  931 | 1.84  9172 | 10  5 |
| {BcHV1SLR.BI=FALSE, BcRV1.BI=FALSE} =>  {BcHV1.BI=FALSE} | 0.25  9901 | 0.760  87 | 0.341  584 | 1.92  1196 | 10  5 |
| {BcEV2.BI=FALSE, BcHV1.BI=FALSE} =>  {BcHV1SLR.BI=FALSE} | 0.26  4851 | 0.856 | 0.309  406 | 1.86  9319 | 10  7 |
| {BcEV2.BI=FALSE, BcHV1SLR.BI=FALSE} =>  {BcHV1.BI=FALSE} | 0.26  4851 | 0.732  877 | 0.361  386 | 1.85  0514 | 10  7 |
| {BcHV1.BI=FALSE, BcHV3.BI=FALSE} =>  {BcHV1SLR.BI=FALSE} | 0.26  9802 | 0.851  563 | 0.316  832 | 1.85  9628 | 10  9 |
| {BcHV1SLR.BI=FALSE, BcHV3.BI=FALSE} =>  {BcHV1.BI=FALSE} | 0.26  9802 | 0.736  486 | 0.366  337 | 1.85  9628 | 10  9 |
| {BcFV5.BI=FALSE, BcHV1.BI=FALSE} =>  {BcHV1SLR.BI=FALSE} | 0.28  7129 | 0.872  18 | 0.329  208 | 1.90  4654 | 11  6 |
| {BcFV5.BI=FALSE, BcHV1SLR.BI=FALSE} =>  {BcHV1.BI=FALSE} | 0.28  7129 | 0.734  177 | 0.391  089 | 1.85  3797 | 11  6 |
| {BcHV1.BI=FALSE, BcHV1SLR.BI=FALSE} =>  {BcULV1.BI=FALSE} | 0.30  6931 | 0.905  109 | 0.339  109 | 1.15  3515 | 12  4 |

| {BcHV1.BI=FALSE, BcULV1.BI=FALSE} =>  {BcHV1SLR.BI=FALSE} | 0.30  6931 | 0.861  111 | 0.356  436 | 1.88  048 | 12  4 |
| --- | --- | --- | --- | --- | --- |
| {BcHV1SLR.BI=FALSE, BcULV1.BI=FALSE}  => {BcHV1.BI=FALSE} | 0.30  6931 | 0.765  432 | 0.400  99 | 1.93  2716 | 12  4 |
| {BcHV1.BI=FALSE, BcMV1.BI=TRUE} =>  {BcHV1SLR.BI=FALSE} | 0.25  495 | 0.830  645 | 0.306  931 | 1.81  3949 | 10  3 |
| {BcHV1SLR.BI=FALSE, BcMV1.BI=TRUE} =>  {BcHV1.BI=FALSE} | 0.25  495 | 0.730  496 | 0.349  01 | 1.84  4504 | 10  3 |
| {BcHV1.BI=FALSE, BcMV12=FALSE} =>  {BcHV1SLR.BI=FALSE} | 0.28  9604 | 0.866  667 | 0.334  158 | 1.89  2613 | 11  7 |
| {BcHV1SLR.BI=FALSE, BcMV12=FALSE} =>  {BcHV1.BI=FALSE} | 0.28  9604 | 0.75 | 0.386  139 | 1.89  375 | 11  7 |
| {BcHV1.BI=FALSE, BcNSRV7.BI=FALSE} =>  {BcHV1SLR.BI=FALSE} | 0.28  7129 | 0.859  259 | 0.334  158 | 1.87  6436 | 11  6 |
| {BcHV1SLR.BI=FALSE, BcNSRV7.BI=FALSE}  => {BcHV1.BI=FALSE} | 0.28  7129 | 0.734  177 | 0.391  089 | 1.85  3797 | 11  6 |
| {BcHV1.BI=FALSE, BcVV3.BI=FALSE} =>  {BcHV1SLR.BI=FALSE} | 0.26  2376 | 0.854  839 | 0.306  931 | 1.86  6783 | 10  6 |
| {BcHV1SLR.BI=FALSE, BcVV3.BI=FALSE} =>  {BcHV1.BI=FALSE} | 0.26  2376 | 0.711  409 | 0.368  812 | 1.79  6309 | 10  6 |
| {BcHV1.BI=FALSE, BcOLV7.BI=FALSE} =>  {BcHV1SLR.BI=FALSE} | 0.28  4653 | 0.864  662 | 0.329  208 | 1.88  8234 | 11  5 |

| {BcHV1SLR.BI=FALSE, BcOLV7.BI=FALSE}  => {BcHV1.BI=FALSE} | 0.28  4653 | 0.766  667 | 0.371  287 | 1.93  5833 | 11  5 |
| --- | --- | --- | --- | --- | --- |
| {BcHV1.BI=FALSE, BcHV1SLR.BI=FALSE} =>  {BcALV1.BI=FALSE} | 0.30  4455 | 0.897  81 | 0.339  109 | 1.10  2478 | 12  3 |
| {BcALV1.BI=FALSE, BcHV1.BI=FALSE} =>  {BcHV1SLR.BI=FALSE} | 0.30  4455 | 0.872  34 | 0.349  01 | 1.90  5003 | 12  3 |
| {BcALV1.BI=FALSE, BcHV1SLR.BI=FALSE}  => {BcHV1.BI=FALSE} | 0.30  4455 | 0.763  975 | 0.398  515 | 1.92  9037 | 12  3 |
| {BcDMV1=FALSE, BcHV1.BI=FALSE} =>  {BcHV1SLR.BI=FALSE} | 0.27  4752 | 0.840  909 | 0.326  733 | 1.83  6364 | 11  1 |
| {BcDMV1=FALSE, BcHV1SLR.BI=FALSE} =>  {BcHV1.BI=FALSE} | 0.27  4752 | 0.725  49 | 0.378  713 | 1.83  1863 | 11  1 |
| {BcFV9=FALSE, BcHV1.BI=FALSE} =>  {BcHV1SLR.BI=FALSE} | 0.30  4455 | 0.842  466 | 0.361  386 | 1.83  9763 | 12  3 |
| {BcFV9=FALSE, BcHV1SLR.BI=FALSE} =>  {BcHV1.BI=FALSE} | 0.30  4455 | 0.727  811 | 0.418  317 | 1.83  7722 | 12  3 |
| {BcHV1.BI=FALSE, BcOLV4.BI=FALSE} =>  {BcHV1SLR.BI=FALSE} | 0.30  198 | 0.871  429 | 0.346  535 | 1.90  3012 | 12  2 |
| {BcHV1SLR.BI=FALSE, BcOLV4.BI=FALSE}  => {BcHV1.BI=FALSE} | 0.30  198 | 0.730  539 | 0.413  366 | 1.84  4611 | 12  2 |
| {BcHV1.BI=FALSE, BcULV5=FALSE} =>  {BcHV1SLR.BI=FALSE} | 0.29  9505 | 0.870  504 | 0.344  059 | 1.90  0992 | 12  1 |

| {BcHV1SLR.BI=FALSE, BcULV5=FALSE} =>  {BcHV1.BI=FALSE} | 0.29  9505 | 0.733  333 | 0.408  416 | 1.85  1667 | 12  1 |
| --- | --- | --- | --- | --- | --- |
| {BcBNV5.BI=FALSE, BcHV1.BI=FALSE} =>  {BcHV1SLR.BI=FALSE} | 0.32  1782 | 0.855  263 | 0.376  238 | 1.86  771 | 13  0 |
| {BcBNV5.BI=FALSE, BcHV1SLR.BI=FALSE}  => {BcHV1.BI=FALSE} | 0.32  1782 | 0.747  126 | 0.430  693 | 1.88  6494 | 13  0 |
| {BcHV1.BI=FALSE, BvF.BI=FALSE} =>  {BcHV1SLR.BI=FALSE} | 0.31  9307 | 0.865  772 | 0.368  812 | 1.89  0658 | 12  9 |
| {BcHV1SLR.BI=FALSE, BvF.BI=FALSE} =>  {BcHV1.BI=FALSE} | 0.31  9307 | 0.737  143 | 0.433  168 | 1.86  1286 | 12  9 |
| {BcHV1.BI=FALSE, BcMCV5.BI=FALSE} =>  {BcHV1SLR.BI=FALSE} | 0.32  1782 | 0.855  263 | 0.376  238 | 1.86  771 | 13  0 |
| {BcHV1SLR.BI=FALSE, BcMCV5.BI=FALSE}  => {BcHV1.BI=FALSE} | 0.32  1782 | 0.742  857 | 0.433  168 | 1.87  5714 | 13  0 |
| {BcHV1.BI=FALSE, BcOLV13.BI=FALSE} =>  {BcHV1SLR.BI=FALSE} | 0.32  6733 | 0.857  143 | 0.381  188 | 1.87  1815 | 13  2 |
| {BcHV1SLR.BI=FALSE, BcOLV13.BI=FALSE}  => {BcHV1.BI=FALSE} | 0.32  6733 | 0.75 | 0.435  644 | 1.89  375 | 13  2 |
| {BcHV1.BI=FALSE, BcOLV2.BI=FALSE} =>  {BcHV1SLR.BI=FALSE} | 0.32  6733 | 0.868  421 | 0.376  238 | 1.89  6444 | 13  2 |
| {BcHV1SLR.BI=FALSE, BcOLV2.BI=FALSE}  => {BcHV1.BI=FALSE} | 0.32  6733 | 0.733  333 | 0.445  545 | 1.85  1667 | 13  2 |

| {BcHV1.BI=FALSE, BcMCV3.BI=FALSE} =>  {BcHV1SLR.BI=FALSE} | 0.33  4158 | 0.859  873 | 0.388  614 | 1.87  7776 | 13  5 |
| --- | --- | --- | --- | --- | --- |
| {BcHV1SLR.BI=FALSE, BcMCV3.BI=FALSE}  => {BcHV1.BI=FALSE} | 0.33  4158 | 0.75 | 0.445  545 | 1.89  375 | 13  5 |
| {BcHV1.BI=FALSE, BcPV4.BI=FALSE} =>  {BcHV1SLR.BI=FALSE} | 0.31  9307 | 0.854  305 | 0.373  762 | 1.86  5617 | 12  9 |
| {BcHV1SLR.BI=FALSE, BcPV4.BI=FALSE} =>  {BcHV1.BI=FALSE} | 0.31  9307 | 0.745  665 | 0.428  218 | 1.88  2803 | 12  9 |
| {BcEV6=FALSE, BcHV1.BI=FALSE} =>  {BcHV1SLR.BI=FALSE} | 0.32  9208 | 0.852  564 | 0.386  139 | 1.86  1816 | 13  3 |
| {BcEV6=FALSE, BcHV1SLR.BI=FALSE} =>  {BcHV1.BI=FALSE} | 0.32  9208 | 0.76 | 0.433  168 | 1.91  9 | 13  3 |
| {BcHV1.BI=FALSE, BcOLV17.BI=FALSE} =>  {BcHV1SLR.BI=FALSE} | 0.32  9208 | 0.858  065 | 0.383  663 | 1.87  3827 | 13  3 |
| {BcHV1SLR.BI=FALSE, BcOLV17.BI=FALSE}  => {BcHV1.BI=FALSE} | 0.32  9208 | 0.743  017 | 0.443  069 | 1.87  6117 | 13  3 |
| {BcHV1.BI=FALSE, BcOLV15.BI=FALSE} =>  {BcHV1SLR.BI=FALSE} | 0.33  1683 | 0.858  974 | 0.386  139 | 1.87  5814 | 13  4 |
| {BcHV1SLR.BI=FALSE, BcOLV15.BI=FALSE}  => {BcHV1.BI=FALSE} | 0.33  1683 | 0.736  264 | 0.450  495 | 1.85  9066 | 13  4 |
| {BcEV5=FALSE, BcHV1.BI=FALSE} =>  {BcHV1SLR.BI=FALSE} | 0.32  1782 | 0.855  263 | 0.376  238 | 1.86  771 | 13  0 |

| {BcEV5=FALSE, BcHV1SLR.BI=FALSE} =>  {BcHV1.BI=FALSE} | 0.32  1782 | 0.734  463 | 0.438  119 | 1.85  452 | 13  0 |
| --- | --- | --- | --- | --- | --- |
| {BcHV1.BI=FALSE, BcMV3.BI=FALSE} =>  {BcOLV18=FALSE} | 0.13  8614 | 0.666  667 | 0.207  921 | 1.38  1197 | 56 |
| {BcHV1.BI=FALSE, BcOLV18=FALSE} =>  {BcMV3.BI=FALSE} | 0.13  8614 | 0.565  657 | 0.245  05 | 1.23  5272 | 56 |
| {BcMV3.BI=FALSE, BcOLV18=FALSE} =>  {BcHV1.BI=FALSE} | 0.13  8614 | 0.513  761 | 0.269  802 | 1.29  7248 | 56 |
| {BcHV1.BI=FALSE, BcMV3.BI=FALSE} =>  {BcMV2.BI=FALSE} | 0.13  8614 | 0.666  667 | 0.207  921 | 1.24  1167 | 56 |
| {BcHV1.BI=FALSE, BcMV2.BI=FALSE} =>  {BcMV3.BI=FALSE} | 0.13  8614 | 0.615  385 | 0.225  248 | 1.34  3867 | 56 |
| {BcHV1.BI=FALSE, BcMV3.BI=FALSE} =>  {BcMV4.BI=FALSE} | 0.13  8614 | 0.666  667 | 0.207  921 | 1.19  7037 | 56 |
| {BcMV11=TRUE, BcMV3.BI=FALSE} =>  {BcHV1.BI=FALSE} | 0.11  8812 | 0.510  638 | 0.232  673 | 1.28  9362 | 48 |
| {BcHV1.BI=FALSE, BcMV3.BI=FALSE} =>  {BcOLV1.BI.MOVA.=FALSE} | 0.16  5842 | 0.797  619 | 0.207  921 | 1.19  3474 | 67 |
| {BcHV1.BI=FALSE, BcOLV1.BI.MOVA.=FALSE} =>  {BcMV3.BI=FALSE} | 0.16  5842 | 0.598  214 | 0.277  228 | 1.30  6371 | 67 |
| {BcHV1.BI=FALSE, BcULV4=TRUE} => | 0.13 | 0.568 | 0.235 | 1.24 | 54 |

| {BcMV3.BI=FALSE} | 3663 | 421 | 149 | 1309 |  |
| --- | --- | --- | --- | --- | --- |
| {BcHV1.BI=FALSE, BcMV3.BI=FALSE} =>  {BcMV6.BI=FALSE} | 0.18  3168 | 0.880  952 | 0.207  921 | 1.26  6565 | 74 |
| {BcHV1.BI=FALSE, BcMV6.BI=FALSE} =>  {BcMV3.BI=FALSE} | 0.18  3168 | 0.573  643 | 0.319  307 | 1.25  2713 | 74 |
| {BcMV3.BI=FALSE, BcMV6.BI=FALSE} =>  {BcHV1.BI=FALSE} | 0.18  3168 | 0.486  842 | 0.376  238 | 1.22  9276 | 74 |
| {BcHV1.BI=FALSE, BcMV3.BI=FALSE} =>  {BcFV5.BI=FALSE} | 0.18  8119 | 0.904  762 | 0.207  921 | 1.15  6721 | 76 |
| {BcFV5.BI=FALSE, BcHV1.BI=FALSE} =>  {BcMV3.BI=FALSE} | 0.18  8119 | 0.571  429 | 0.329  208 | 1.24  7876 | 76 |
| {BcFV5.BI=FALSE, BcMV3.BI=FALSE} =>  {BcHV1.BI=FALSE} | 0.18  8119 | 0.477  987 | 0.393  564 | 1.20  6918 | 76 |
| {BcHV1.BI=FALSE, BcMV3.BI=FALSE} =>  {BcULV1.BI=FALSE} | 0.19  0594 | 0.916  667 | 0.207  921 | 1.16  8244 | 77 |
| {BcMV3.BI=FALSE, BcULV1.BI=FALSE} =>  {BcHV1.BI=FALSE} | 0.19  0594 | 0.484  277 | 0.393  564 | 1.22  2799 | 77 |
| {BcMV1.BI=TRUE, BcMV3.BI=FALSE} =>  {BcHV1.BI=FALSE} | 0.15  8416 | 0.477  612 | 0.331  683 | 1.20  597 | 64 |
| {BcHV1.BI=FALSE, BcMV3.BI=FALSE} =>  {BcMV12=FALSE} | 0.19  0594 | 0.916  667 | 0.207  921 | 1.14  6543 | 77 |
| {BcHV1.BI=FALSE, BcMV12=FALSE} => | 0.19 | 0.570 | 0.334 | 1.24 | 77 |

| {BcMV3.BI=FALSE} | 0594 | 37 | 158 | 5566 |  |
| --- | --- | --- | --- | --- | --- |
| {BcHV1.BI=FALSE, BcVV3.BI=FALSE} =>  {BcMV3.BI=FALSE} | 0.17  3267 | 0.564  516 | 0.306  931 | 1.23  2781 | 70 |
| {BcDMV1=FALSE, BcHV1.BI=FALSE} =>  {BcMV3.BI=FALSE} | 0.18  5644 | 0.568  182 | 0.326  733 | 1.24  0786 | 75 |
| {BcHV1.BI=FALSE, BcMV3.BI=FALSE} =>  {BcFV9=FALSE} | 0.19  3069 | 0.928  571 | 0.207  921 | 1.06  8783 | 78 |
| {BcHV1.BI=FALSE, BcOLV4.BI=FALSE} =>  {BcMV3.BI=FALSE} | 0.19  3069 | 0.557  143 | 0.346  535 | 1.21  668 | 78 |
| {BcHV1.BI=FALSE, BcULV5=FALSE} =>  {BcMV3.BI=FALSE} | 0.19  0594 | 0.553  957 | 0.344  059 | 1.20  9722 | 77 |
| {BcHV1.BI=FALSE, BvF.BI=FALSE} =>  {BcMV3.BI=FALSE} | 0.20  0495 | 0.543  624 | 0.368  812 | 1.18  7158 | 81 |
| {BcMV3.BI=FALSE, BvF.BI=FALSE} =>  {BcHV1.BI=FALSE} | 0.20  0495 | 0.479  29 | 0.418  317 | 1.21  0207 | 81 |
| {BcEV6=FALSE, BcMV3.BI=FALSE} =>  {BcHV1.BI=FALSE} | 0.20  0495 | 0.479  29 | 0.418  317 | 1.21  0207 | 81 |
| {BcEV5=FALSE, BcHV1.BI=FALSE} =>  {BcMV3.BI=FALSE} | 0.20  5446 | 0.546  053 | 0.376  238 | 1.19  2461 | 83 |
| {BcMV2.BI=TRUE, BcOLV18=FALSE} =>  {BcHV1.BI=FALSE} | 0.08  6634 | 0.514  706 | 0.168  317 | 1.29  9632 | 35 |
| {BcMV2.BI=TRUE, BcMV6.BI=FALSE} => | 0.12 | 0.495 | 0.254 | 1.25 | 51 |

| {BcHV1.BI=FALSE} | 6238 | 146 | 95 | 0243 |  |
| --- | --- | --- | --- | --- | --- |
| {BcHV1.BI=FALSE, BcMV2.BI=TRUE} =>  {BcMV1.BI=TRUE} | 0.15  099 | 0.884  058 | 0.170  792 | 1.11  9622 | 61 |
| {BcHV1.BI=FALSE, BcOLV18=FALSE} =>  {BcMV2.BI=FALSE} | 0.15  8416 | 0.646  465 | 0.245  05 | 1.20  3556 | 64 |
| {BcHV1.BI=FALSE, BcMV2.BI=FALSE} =>  {BcOLV18=FALSE} | 0.15  8416 | 0.703  297 | 0.225  248 | 1.45  7087 | 64 |
| {BcMV2.BI=FALSE, BcOLV18=FALSE} =>  {BcHV1.BI=FALSE} | 0.15  8416 | 0.503  937 | 0.314  356 | 1.27  2441 | 64 |
| {BcMV3.BI=TRUE, BcOLV18=FALSE} =>  {BcHV1.BI=FALSE} | 0.10  6436 | 0.5 | 0.212  871 | 1.26  25 | 43 |
| {BcHV1.BI=FALSE, BcOLV18=FALSE} =>  {BcMV4.BI=FALSE} | 0.16  8317 | 0.686  869 | 0.245  05 | 1.23  3311 | 68 |
| {BcHV1.BI=FALSE, BcMV4.BI=FALSE} =>  {BcOLV18=FALSE} | 0.16  8317 | 0.666  667 | 0.252  475 | 1.38  1197 | 68 |
| {BcMV4.BI=FALSE, BcOLV18=FALSE} =>  {BcHV1.BI=FALSE} | 0.16  8317 | 0.531  25 | 0.316  832 | 1.34  1406 | 68 |
| {BcHV1.BI=FALSE, BcOLV18=FALSE} =>  {BcOLV16.BI=FALSE} | 0.18  0693 | 0.737  374 | 0.245  05 | 1.26  2284 | 73 |
| {BcHV1.BI=FALSE, BcOLV16.BI=FALSE} =>  {BcOLV18=FALSE} | 0.18  0693 | 0.722  772 | 0.25 | 1.49  7436 | 73 |
| {BcOLV16.BI=FALSE, BcOLV18=FALSE} => | 0.18 | 0.536 | 0.336 | 1.35 | 73 |

| {BcHV1.BI=FALSE} | 0693 | 765 | 634 | 5331 |  |
| --- | --- | --- | --- | --- | --- |
| {BcHV1.BI=FALSE, BcULV3=TRUE} => | 0.10 | 0.594 | 0.170 | 1.23 |  |
|  |  |  |  |  | 41 |
| {BcOLV18=FALSE} | 1485 | 203 | 792 | 1067 |  |
| {BcHV1.BI=FALSE, BcMV11=TRUE} => | 0.14 |  | 0.235 | 1.24 |  |
|  |  | 0.6 |  |  | 57 |
| {BcOLV18=FALSE} | 1089 |  | 149 | 3077 |  |
| {BcMV11=TRUE, BcOLV18=FALSE} => | 0.14 | 0.522 | 0.269 | 1.32 |  |
|  |  |  |  |  | 57 |
| {BcHV1.BI=FALSE} | 1089 | 936 | 802 | 0413 |  |
| {BcHV1.BI=FALSE, BcOLV18=FALSE} => | 0.19 | 0.777 | 0.245 | 1.16 |  |
|  |  |  |  |  | 77 |
| {BcOLV1.BI.MOVA.=FALSE} | 0594 | 778 | 05 | 3786 |  |
| {BcHV1.BI=FALSE, |  |  |  |  |  |
|  | 0.19 | 0.687 | 0.277 | 1.42 |  |
| BcOLV1.BI.MOVA.=FALSE} => |  |  |  |  | 77 |
|  | 0594 | 5 | 228 | 4359 |  |
| {BcOLV18=FALSE} |  |  |  |  |  |
| {BcOLV1.BI.MOVA.=FALSE, | 0.19 |  | 0.381 | 1.26 |  |
|  |  | 0.5 |  |  | 77 |
| BcOLV18=FALSE} => {BcHV1.BI=FALSE} | 0594 |  | 188 | 25 |  |
| {BcHV1.BI=FALSE, BcOLV18=FALSE} => | 0.19 | 0.808 | 0.245 | 1.16 |  |
|  |  |  |  |  | 80 |
| {BcMV6.BI=FALSE} | 802 | 081 | 05 | 1796 |  |
| {BcHV1.BI=FALSE, BcMV6.BI=FALSE} => | 0.19 | 0.620 | 0.319 | 1.28 |  |
|  |  |  |  |  | 80 |
| {BcOLV18=FALSE} | 802 | 155 | 307 | 4834 |  |
| {BcMV6.BI=FALSE, BcOLV18=FALSE} => | 0.19 | 0.563 | 0.351 | 1.42 |  |
|  |  |  |  |  | 80 |
| {BcHV1.BI=FALSE} | 802 | 38 | 485 | 2535 |  |
| {BcHV1.BI=FALSE, BcOLV18=FALSE} => | 0.20 | 0.818 | 0.245 | 1.15 |  |
|  |  |  |  |  | 81 |
| {BcPV3.BI=FALSE} | 0495 | 182 | 05 | 1726 |  |

| {BcHV1.BI=FALSE, BcPV3.BI=FALSE} =>  {BcOLV18=FALSE} | 0.20  0495 | 0.653  226 | 0.306  931 | 1.35  335 | 81 |
| --- | --- | --- | --- | --- | --- |
| {BcOLV18=FALSE, BcPV3.BI=FALSE} =>  {BcHV1.BI=FALSE} | 0.20  0495 | 0.525  974 | 0.381  188 | 1.32  8084 | 81 |
| {BcHV1.BI=FALSE, BcRV1.BI=FALSE} =>  {BcOLV18=FALSE} | 0.18  8119 | 0.612  903 | 0.306  931 | 1.26  981 | 76 |
| {BcOLV18=FALSE, BcRV1.BI=FALSE} =>  {BcHV1.BI=FALSE} | 0.18  8119 | 0.477  987 | 0.393  564 | 1.20  6918 | 76 |
| {BcHV1.BI=FALSE, BcOLV18=FALSE} =>  {BcEV2.BI=FALSE} | 0.20  297 | 0.828  283 | 0.245  05 | 1.10  8034 | 82 |
| {BcEV2.BI=FALSE, BcHV1.BI=FALSE} =>  {BcOLV18=FALSE} | 0.20  297 | 0.656 | 0.309  406 | 1.35  9097 | 82 |
| {BcEV2.BI=FALSE, BcOLV18=FALSE} =>  {BcHV1.BI=FALSE} | 0.20  297 | 0.535  948 | 0.378  713 | 1.35  3268 | 82 |
| {BcHV1.BI=FALSE, BcOLV18=FALSE} =>  {BcHV3.BI=FALSE} | 0.21  5347 | 0.878  788 | 0.245  05 | 1.15  645 | 87 |
| {BcHV1.BI=FALSE, BcHV3.BI=FALSE} =>  {BcOLV18=FALSE} | 0.21  5347 | 0.679  688 | 0.316  832 | 1.40  8173 | 87 |
| {BcHV3.BI=FALSE, BcOLV18=FALSE} =>  {BcHV1.BI=FALSE} | 0.21  5347 | 0.527  273 | 0.408  416 | 1.33  1364 | 87 |
| {BcHV1.BI=FALSE, BcOLV18=FALSE} =>  {BcFV5.BI=FALSE} | 0.21  5347 | 0.878  788 | 0.245  05 | 1.12  3514 | 87 |

| {BcFV5.BI=FALSE, BcHV1.BI=FALSE} =>  {BcOLV18=FALSE} | 0.21  5347 | 0.654  135 | 0.329  208 | 1.35  5234 | 87 |
| --- | --- | --- | --- | --- | --- |
| {BcFV5.BI=FALSE, BcOLV18=FALSE} =>  {BcHV1.BI=FALSE} | 0.21  5347 | 0.520  958 | 0.413  366 | 1.31  5419 | 87 |
| {BcHV1.BI=FALSE, BcOLV18=FALSE} =>  {BcULV1.BI=FALSE} | 0.23  0198 | 0.939  394 | 0.245  05 | 1.19  7209 | 93 |
| {BcHV1.BI=FALSE, BcULV1.BI=FALSE} =>  {BcOLV18=FALSE} | 0.23  0198 | 0.645  833 | 0.356  436 | 1.33  8034 | 93 |
| {BcOLV18=FALSE, BcULV1.BI=FALSE} =>  {BcHV1.BI=FALSE} | 0.23  0198 | 0.537  572 | 0.428  218 | 1.35  737 | 93 |
| {BcHV1.BI=FALSE, BcMV1.BI=TRUE} =>  {BcOLV18=FALSE} | 0.17  8218 | 0.580  645 | 0.306  931 | 1.20  2978 | 72 |
| {BcMV1.BI=TRUE, BcOLV18=FALSE} =>  {BcHV1.BI=FALSE} | 0.17  8218 | 0.510  638 | 0.349  01 | 1.28  9362 | 72 |
| {BcHV1.BI=FALSE, BcMV12=FALSE} =>  {BcOLV18=FALSE} | 0.21  2871 | 0.637  037 | 0.334  158 | 1.31  981 | 86 |
| {BcMV12=FALSE, BcOLV18=FALSE} =>  {BcHV1.BI=FALSE} | 0.21  2871 | 0.527  607 | 0.403  465 | 1.33  2209 | 86 |
| {BcHV1.BI=FALSE, BcOLV18=FALSE} =>  {BcNSRV7.BI=FALSE} | 0.22  5248 | 0.919  192 | 0.245  05 | 1.14  9701 | 91 |
| {BcHV1.BI=FALSE, BcNSRV7.BI=FALSE} =>  {BcOLV18=FALSE} | 0.22  5248 | 0.674  074 | 0.334  158 | 1.39  6543 | 91 |

| {BcNSRV7.BI=FALSE, BcOLV18=FALSE} =>  {BcHV1.BI=FALSE} | 0.22  5248 | 0.548  193 | 0.410  891 | 1.38  4187 | 91 |
| --- | --- | --- | --- | --- | --- |
| {BcHV1.BI=FALSE, BcVV3.BI=FALSE} =>  {BcOLV18=FALSE} | 0.19  802 | 0.645  161 | 0.306  931 | 1.33  6642 | 80 |
| {BcOLV18=FALSE, BcVV3.BI=FALSE} =>  {BcHV1.BI=FALSE} | 0.19  802 | 0.506  329 | 0.391  089 | 1.27  8481 | 80 |
| {BcHV1.BI=FALSE, BcOLV7.BI=FALSE} =>  {BcOLV18=FALSE} | 0.21  5347 | 0.654  135 | 0.329  208 | 1.35  5234 | 87 |
| {BcOLV18=FALSE, BcOLV7.BI=FALSE} =>  {BcHV1.BI=FALSE} | 0.21  5347 | 0.5 | 0.430  693 | 1.26  25 | 87 |
| {BcHV1.BI=FALSE, BcOLV18=FALSE} =>  {BcALV1.BI=FALSE} | 0.22  5248 | 0.919  192 | 0.245  05 | 1.12  8734 | 91 |
| {BcALV1.BI=FALSE, BcHV1.BI=FALSE} =>  {BcOLV18=FALSE} | 0.22  5248 | 0.645  39 | 0.349  01 | 1.33  7116 | 91 |
| {BcALV1.BI=FALSE, BcOLV18=FALSE} =>  {BcHV1.BI=FALSE} | 0.22  5248 | 0.514  124 | 0.438  119 | 1.29  8164 | 91 |
| {BcDMV1=FALSE, BcHV1.BI=FALSE} =>  {BcOLV18=FALSE} | 0.21  0396 | 0.643  939 | 0.326  733 | 1.33  411 | 85 |
| {BcDMV1=FALSE, BcOLV18=FALSE} =>  {BcHV1.BI=FALSE} | 0.21  0396 | 0.505  952 | 0.415  842 | 1.27  753 | 85 |
| {BcHV1.BI=FALSE, BcOLV18=FALSE} =>  {BcFV9=FALSE} | 0.23  5149 | 0.959  596 | 0.245  05 | 1.10  4492 | 95 |

| {BcFV9=FALSE, BcHV1.BI=FALSE} =>  {BcOLV18=FALSE} | 0.23  5149 | 0.650  685 | 0.361  386 | 1.34  8086 | 95 |
| --- | --- | --- | --- | --- | --- |
| {BcFV9=FALSE, BcOLV18=FALSE} =>  {BcHV1.BI=FALSE} | 0.23  5149 | 0.524  862 | 0.448  02 | 1.32  5276 | 95 |
| {BcHV1.BI=FALSE, BcOLV4.BI=FALSE} =>  {BcOLV18=FALSE} | 0.22  0297 | 0.635  714 | 0.346  535 | 1.31  707 | 89 |
| {BcOLV18=FALSE, BcOLV4.BI=FALSE} =>  {BcHV1.BI=FALSE} | 0.22  0297 | 0.514  451 | 0.428  218 | 1.29  8988 | 89 |
| {BcHV1.BI=FALSE, BcULV5=FALSE} =>  {BcOLV18=FALSE} | 0.20  297 | 0.589  928 | 0.344  059 | 1.22  221 | 82 |
| {BcOLV18=FALSE, BcULV5=FALSE} =>  {BcHV1.BI=FALSE} | 0.20  297 | 0.496  97 | 0.408  416 | 1.25  4848 | 82 |
| {BcBNV5.BI=FALSE, BcHV1.BI=FALSE} =>  {BcOLV18=FALSE} | 0.23  7624 | 0.631  579 | 0.376  238 | 1.30  8502 | 96 |
| {BcBNV5.BI=FALSE, BcOLV18=FALSE} =>  {BcHV1.BI=FALSE} | 0.23  7624 | 0.521  739 | 0.455  446 | 1.31  7391 | 96 |
| {BcHV1.BI=FALSE, BvF.BI=FALSE} =>  {BcOLV18=FALSE} | 0.22  5248 | 0.610  738 | 0.368  812 | 1.26  5324 | 91 |
| {BcOLV18=FALSE, BvF.BI=FALSE} =>  {BcHV1.BI=FALSE} | 0.22  5248 | 0.514  124 | 0.438  119 | 1.29  8164 | 91 |
| {BcHV1.BI=FALSE, BcOLV18=FALSE} =>  {BcMCV5.BI=FALSE} | 0.24  505 | 1 | 0.245  05 | 1.08  311 | 99 |

| {BcHV1.BI=FALSE, BcMCV5.BI=FALSE} =>  {BcOLV18=FALSE} | 0.24  505 | 0.651  316 | 0.376  238 | 1.34  9393 | 99 |
| --- | --- | --- | --- | --- | --- |
| {BcMCV5.BI=FALSE, BcOLV18=FALSE} =>  {BcHV1.BI=FALSE} | 0.24  505 | 0.518  325 | 0.472  772 | 1.30  877 | 99 |
| {BcHV1.BI=FALSE, BcOLV13.BI=FALSE} =>  {BcOLV18=FALSE} | 0.23  7624 | 0.623  377 | 0.381  188 | 1.29  1508 | 96 |
| {BcOLV13.BI=FALSE, BcOLV18=FALSE} =>  {BcHV1.BI=FALSE} | 0.23  7624 | 0.527  473 | 0.450  495 | 1.33  1868 | 96 |
| {BcHV1.BI=FALSE, BcOLV2.BI=FALSE} =>  {BcOLV18=FALSE} | 0.23  5149 | 0.625 | 0.376  238 | 1.29  4872 | 95 |
| {BcOLV18=FALSE, BcOLV2.BI=FALSE} =>  {BcHV1.BI=FALSE} | 0.23  5149 | 0.510  753 | 0.460  396 | 1.28  9651 | 95 |
| {BcHV1.BI=FALSE, BcMCV3.BI=FALSE} =>  {BcOLV18=FALSE} | 0.23  7624 | 0.611  465 | 0.388  614 | 1.26  683 | 96 |
| {BcMCV3.BI=FALSE, BcOLV18=FALSE} =>  {BcHV1.BI=FALSE} | 0.23  7624 | 0.518  919 | 0.457  921 | 1.31  027 | 96 |
| {BcHV1.BI=FALSE, BcPV4.BI=FALSE} =>  {BcOLV18=FALSE} | 0.23  2673 | 0.622  517 | 0.373  762 | 1.28  9727 | 94 |
| {BcOLV18=FALSE, BcPV4.BI=FALSE} =>  {BcHV1.BI=FALSE} | 0.23  2673 | 0.510  87 | 0.455  446 | 1.28  9946 | 94 |
| {BcEV6=FALSE, BcHV1.BI=FALSE} =>  {BcOLV18=FALSE} | 0.23  5149 | 0.608  974 | 0.386  139 | 1.26  167 | 95 |

| {BcEV6=FALSE, BcOLV18=FALSE} =>  {BcHV1.BI=FALSE} | 0.23  5149 | 0.519  126 | 0.452  97 | 1.31  0792 | 95 |
| --- | --- | --- | --- | --- | --- |
| {BcHV1.BI=FALSE, BcOLV17.BI=FALSE} =>  {BcOLV18=FALSE} | 0.24  2574 | 0.632  258 | 0.383  663 | 1.30  9909 | 98 |
| {BcOLV17.BI=FALSE, BcOLV18=FALSE} =>  {BcHV1.BI=FALSE} | 0.24  2574 | 0.515  789 | 0.470  297 | 1.30  2368 | 98 |
| {BcHV1.BI=FALSE, BcOLV15.BI=FALSE} =>  {BcOLV18=FALSE} | 0.24  505 | 0.634  615 | 0.386  139 | 1.31  4793 | 99 |
| {BcOLV15.BI=FALSE, BcOLV18=FALSE} =>  {BcHV1.BI=FALSE} | 0.24  505 | 0.518  325 | 0.472  772 | 1.30  877 | 99 |
| {BcEV5=FALSE, BcHV1.BI=FALSE} =>  {BcOLV18=FALSE} | 0.23  7624 | 0.631  579 | 0.376  238 | 1.30  8502 | 96 |
| {BcEV5=FALSE, BcOLV18=FALSE} =>  {BcHV1.BI=FALSE} | 0.23  7624 | 0.513  369 | 0.462  871 | 1.29  6257 | 96 |
| {BcHV1.BI=FALSE, BcOLV18=TRUE} =>  {BcULV4=TRUE} | 0.11  8812 | 0.786  885 | 0.150  99 | 1.13  9432 | 48 |
| {BcHV1.BI=FALSE, BcOLV18=TRUE} =>  {BcMV6.BI=FALSE} | 0.12  1287 | 0.803  279 | 0.150  99 | 1.15  4892 | 49 |
| {BcHV1.BI=FALSE, BcMV2.BI=FALSE} =>  {BcMV4.BI=FALSE} | 0.16  5842 | 0.736  264 | 0.225  248 | 1.32  2002 | 67 |
| {BcHV1.BI=FALSE, BcMV4.BI=FALSE} =>  {BcMV2.BI=FALSE} | 0.16  5842 | 0.656  863 | 0.252  475 | 1.22  2915 | 67 |

| {BcHV1.BI=FALSE, BcMV2.BI=FALSE} =>  {BcOLV16.BI=FALSE} | 0.15  3465 | 0.681  319 | 0.225  248 | 1.16  6325 | 62 |
| --- | --- | --- | --- | --- | --- |
| {BcHV1.BI=FALSE, BcMV2.BI=FALSE} =>  {BcOLV1.BI.MOVA.=FALSE} | 0.19  802 | 0.879  121 | 0.225  248 | 1.31  5425 | 80 |
| {BcHV1.BI=FALSE, BcOLV1.BI.MOVA.=FALSE} =>  {BcMV2.BI=FALSE} | 0.19  802 | 0.714  286 | 0.277  228 | 1.32  9822 | 80 |
| {BcHV1.BI=FALSE, BcMV2.BI=FALSE} =>  {BcMV6.BI=FALSE} | 0.19  3069 | 0.857  143 | 0.225  248 | 1.23  2334 | 78 |
| {BcHV1.BI=FALSE, BcMV2.BI=FALSE} =>  {BcPV3.BI=FALSE} | 0.18  8119 | 0.835  165 | 0.225  248 | 1.17  5633 | 76 |
| {BcHV1.BI=FALSE, BcMV2.BI=FALSE} =>  {BcEV2.BI=FALSE} | 0.19  5545 | 0.868  132 | 0.225  248 | 1.16  1342 | 79 |
| {BcEV2.BI=FALSE, BcHV1.BI=FALSE} =>  {BcMV2.BI=FALSE} | 0.19  5545 | 0.632 | 0.309  406 | 1.17  6627 | 79 |
| {BcHV1.BI=FALSE, BcMV2.BI=FALSE} =>  {BcHV3.BI=FALSE} | 0.19  5545 | 0.868  132 | 0.225  248 | 1.14  2428 | 79 |
| {BcHV1.BI=FALSE, BcMV2.BI=FALSE} =>  {BcFV5.BI=FALSE} | 0.19  5545 | 0.868  132 | 0.225  248 | 1.10  989 | 79 |
| {BcHV1.BI=FALSE, BcMV2.BI=FALSE} =>  {BcULV1.BI=FALSE} | 0.22  0297 | 0.978  022 | 0.225  248 | 1.24  6438 | 89 |
| {BcMV2.BI=FALSE, BcULV1.BI=FALSE} => | 0.22 | 0.478 | 0.460 | 1.20 | 89 |

| {BcHV1.BI=FALSE} | 0297 | 495 | 396 | 8199 |  |
| --- | --- | --- | --- | --- | --- |
| {BcHV1.BI=FALSE, BcMV2.BI=FALSE} =>  {BcMV12=FALSE} | 0.20  5446 | 0.912  088 | 0.225  248 | 1.14  0816 | 83 |
| {BcHV1.BI=FALSE, BcMV2.BI=FALSE} =>  {BcALV1.BI=FALSE} | 0.20  7921 | 0.923  077 | 0.225  248 | 1.13  3505 | 84 |
| {BcHV1.BI=FALSE, BcMV2.BI=FALSE} =>  {BcFV9=FALSE} | 0.21  2871 | 0.945  055 | 0.225  248 | 1.08  7756 | 86 |
| {BcHV1.BI=FALSE, BcMV2.BI=FALSE} =>  {BcBNV5.BI=FALSE} | 0.22  2772 | 0.989  011 | 0.225  248 | 1.08  8721 | 90 |
| {BcHV1.BI=FALSE, BcMV2.BI=FALSE} =>  {BcMCV5.BI=FALSE} | 0.22  5248 | 1 | 0.225  248 | 1.08  311 | 91 |
| {BcHV1.BI=FALSE, BcMV3.BI=TRUE} =>  {BcULV1.BI=FALSE} | 0.16  5842 | 0.881  579 | 0.188  119 | 1.12  3526 | 67 |
| {BcHV1.BI=FALSE, BcMV3.BI=TRUE} =>  {BcALV1.BI=FALSE} | 0.16  8317 | 0.894  737 | 0.188  119 | 1.09  8704 | 68 |
| {BcHV1.BI=FALSE, BcMV4.BI=FALSE} =>  {BcOLV16.BI=FALSE} | 0.17  3267 | 0.686  275 | 0.252  475 | 1.17  4809 | 70 |
| {BcHV1.BI=FALSE, BcOLV16.BI=FALSE} =>  {BcMV4.BI=FALSE} | 0.17  3267 | 0.693  069 | 0.25 | 1.24  4444 | 70 |
| {BcHV1.BI=FALSE, BcMV4.BI=FALSE} =>  {BcOLV1.BI.MOVA.=FALSE} | 0.19  3069 | 0.764  706 | 0.252  475 | 1.14  4227 | 78 |
| {BcHV1.BI=FALSE, | 0.19 | 0.696 | 0.277 | 1.25 | 78 |

| BcOLV1.BI.MOVA.=FALSE} =>  {BcMV4.BI=FALSE} | 3069 | 429 | 228 | 0476 |  |
| --- | --- | --- | --- | --- | --- |
| {BcMV4.BI=FALSE, |  |  |  |  |  |
|  | 0.19 | 0.469 | 0.410 | 1.18 |  |
| BcOLV1.BI.MOVA.=FALSE} => |  |  |  |  | 78 |
|  | 3069 | 88 | 891 | 6446 |  |
| {BcHV1.BI=FALSE} |  |  |  |  |  |
| {BcHV1.BI=FALSE, BcMV4.BI=FALSE} => | 0.21 | 0.852 | 0.252 | 1.22 |  |
|  |  |  |  |  | 87 |
| {BcMV6.BI=FALSE} | 5347 | 941 | 475 | 6293 |  |
| {BcHV1.BI=FALSE, BcMV6.BI=FALSE} => | 0.21 | 0.674 | 0.319 | 1.21 |  |
|  |  |  |  |  | 87 |
| {BcMV4.BI=FALSE} | 5347 | 419 | 307 | 0956 |  |
| {BcMV4.BI=FALSE, BcMV6.BI=FALSE} => | 0.21 | 0.502 | 0.428 | 1.26 |  |
|  |  |  |  |  | 87 |
| {BcHV1.BI=FALSE} | 5347 | 89 | 218 | 9798 |  |
| {BcHV1.BI=FALSE, BcMV4.BI=FALSE} => | 0.21 | 0.843 | 0.252 | 1.18 |  |
|  |  |  |  |  | 86 |
| {BcPV3.BI=FALSE} | 2871 | 137 | 475 | 6855 |  |
| {BcHV1.BI=FALSE, BcPV3.BI=FALSE} => | 0.21 | 0.693 | 0.306 | 1.24 |  |
|  |  |  |  |  | 86 |
| {BcMV4.BI=FALSE} | 2871 | 548 | 931 | 5305 |  |
| {BcMV4.BI=FALSE, BcPV3.BI=FALSE} => | 0.21 | 0.475 | 0.448 | 1.19 |  |
|  |  |  |  |  | 86 |
| {BcHV1.BI=FALSE} | 2871 | 138 | 02 | 9724 |  |
| {BcEV2.BI=FALSE, BcHV1.BI=FALSE} => | 0.20 |  | 0.309 | 1.19 |  |
|  |  | 0.664 |  |  | 83 |
| {BcMV4.BI=FALSE} | 5446 |  | 406 | 2249 |  |
| {BcEV2.BI=FALSE, BcMV4.BI=FALSE} => | 0.20 | 0.474 | 0.433 | 1.19 |  |
|  |  |  |  |  | 83 |
| {BcHV1.BI=FALSE} | 5446 | 286 | 168 | 7571 |  |
| {BcHV1.BI=FALSE, BcMV4.BI=FALSE} => | 0.21 | 0.862 | 0.252 | 1.13 | 88 |

| {BcHV3.BI=FALSE} | 7822 | 745 | 475 | 5339 |  |
| --- | --- | --- | --- | --- | --- |
| {BcHV1.BI=FALSE, BcHV3.BI=FALSE} =>  {BcMV4.BI=FALSE} | 0.21  7822 | 0.687  5 | 0.316  832 | 1.23  4444 | 88 |
| {BcHV3.BI=FALSE, BcMV4.BI=FALSE} =>  {BcHV1.BI=FALSE} | 0.21  7822 | 0.465  608 | 0.467  822 | 1.17  5661 | 88 |
| {BcHV1.BI=FALSE, BcMV4.BI=FALSE} =>  {BcULV1.BI=FALSE} | 0.23  7624 | 0.941  176 | 0.252  475 | 1.19  948 | 96 |
| {BcHV1.BI=FALSE, BcULV1.BI=FALSE} =>  {BcMV4.BI=FALSE} | 0.23  7624 | 0.666  667 | 0.356  436 | 1.19  7037 | 96 |
| {BcMV4.BI=FALSE, BcULV1.BI=FALSE} =>  {BcHV1.BI=FALSE} | 0.23  7624 | 0.527  473 | 0.450  495 | 1.33  1868 | 96 |
| {BcMV12=FALSE, BcMV4.BI=FALSE} =>  {BcHV1.BI=FALSE} | 0.21  2871 | 0.485  876 | 0.438  119 | 1.22  6836 | 86 |
| {BcHV1.BI=FALSE, BcVV3.BI=FALSE} =>  {BcMV4.BI=FALSE} | 0.20  297 | 0.661  29 | 0.306  931 | 1.18  7384 | 82 |
| {BcHV1.BI=FALSE, BcMV4.BI=FALSE} =>  {BcALV1.BI=FALSE} | 0.23  0198 | 0.911  765 | 0.252  475 | 1.11  9614 | 93 |
| {BcALV1.BI=FALSE, BcHV1.BI=FALSE} =>  {BcMV4.BI=FALSE} | 0.23  0198 | 0.659  574 | 0.349  01 | 1.18  4303 | 93 |
| {BcDMV1=FALSE, BcHV1.BI=FALSE} =>  {BcMV4.BI=FALSE} | 0.21  0396 | 0.643  939 | 0.326  733 | 1.15  6229 | 85 |
| {BcHV1.BI=FALSE, BcMV4.BI=FALSE} => | 0.23 | 0.941 | 0.252 | 1.08 | 96 |

| {BcFV9=FALSE} | 7624 | 176 | 475 | 3291 |  |
| --- | --- | --- | --- | --- | --- |
| {BcFV9=FALSE, BcHV1.BI=FALSE} =>  {BcMV4.BI=FALSE} | 0.23  7624 | 0.657  534 | 0.361  386 | 1.18  0639 | 96 |
| {BcHV1.BI=FALSE, BcOLV4.BI=FALSE} =>  {BcMV4.BI=FALSE} | 0.22  7723 | 0.657  143 | 0.346  535 | 1.17  9937 | 92 |
| {BcHV1.BI=FALSE, BcULV5=FALSE} =>  {BcMV4.BI=FALSE} | 0.22  2772 | 0.647  482 | 0.344  059 | 1.16  259 | 90 |
| {BcBNV5.BI=FALSE, BcHV1.BI=FALSE} =>  {BcMV4.BI=FALSE} | 0.24  505 | 0.651  316 | 0.376  238 | 1.16  9474 | 99 |
| {BcBNV5.BI=FALSE, BcMV4.BI=FALSE} =>  {BcHV1.BI=FALSE} | 0.24  505 | 0.466  981 | 0.524  752 | 1.17  9127 | 99 |
| {BcHV1.BI=FALSE, BcMCV5.BI=FALSE} =>  {BcMV4.BI=FALSE} | 0.24  7525 | 0.657  895 | 0.376  238 | 1.18  1287 | 10  0 |
| {BcHV1.BI=FALSE, BcOLV13.BI=FALSE} =>  {BcMV4.BI=FALSE} | 0.24  7525 | 0.649  351 | 0.381  188 | 1.16  5945 | 10  0 |
| {BcMV4.BI=FALSE, BcOLV13.BI=FALSE} =>  {BcHV1.BI=FALSE} | 0.24  7525 | 0.462  963 | 0.534  653 | 1.16  8981 | 10  0 |
| {BcHV1.BI=FALSE, BcOLV2.BI=FALSE} =>  {BcMV4.BI=FALSE} | 0.24  505 | 0.651  316 | 0.376  238 | 1.16  9474 | 99 |
| {BcMV4.BI=FALSE, BcOLV2.BI=FALSE} =>  {BcHV1.BI=FALSE} | 0.24  505 | 0.466  981 | 0.524  752 | 1.17  9127 | 99 |
| {BcHV1.BI=FALSE, BcMCV3.BI=FALSE} => | 0.25 | 0.643 | 0.388 | 1.15 | 10 |

| {BcMV4.BI=FALSE} |  | 312 | 614 | 5103 | 1 |
| --- | --- | --- | --- | --- | --- |
| {BcMCV3.BI=FALSE, BcMV4.BI=FALSE} =>  {BcHV1.BI=FALSE} | 0.25 | 0.478  673 | 0.522  277 | 1.20  8649 | 10  1 |
| {BcHV1.BI=FALSE, BcPV4.BI=FALSE} =>  {BcMV4.BI=FALSE} | 0.24  0099 | 0.642  384 | 0.373  762 | 1.15  3436 | 97 |
| {BcMV4.BI=FALSE, BcPV4.BI=FALSE} =>  {BcHV1.BI=FALSE} | 0.24  0099 | 0.466  346 | 0.514  851 | 1.17  7524 | 97 |
| {BcEV6=FALSE, BcHV1.BI=FALSE} =>  {BcMV4.BI=FALSE} | 0.24  505 | 0.634  615 | 0.386  139 | 1.13  9487 | 99 |
| {BcEV6=FALSE, BcMV4.BI=FALSE} =>  {BcHV1.BI=FALSE} | 0.24  505 | 0.475  962 | 0.514  851 | 1.20  1803 | 99 |
| {BcHV1.BI=FALSE, BcOLV17.BI=FALSE} =>  {BcMV4.BI=FALSE} | 0.24  7525 | 0.645  161 | 0.383  663 | 1.15  8423 | 10  0 |
| {BcMV4.BI=FALSE, BcOLV17.BI=FALSE} =>  {BcHV1.BI=FALSE} | 0.24  7525 | 0.460  829 | 0.537  129 | 1.16  3594 | 10  0 |
| {BcHV1.BI=FALSE, BcOLV15.BI=FALSE} =>  {BcMV4.BI=FALSE} | 0.24  7525 | 0.641  026 | 0.386  139 | 1.15  0997 | 10  0 |
| {BcMV4.BI=FALSE, BcOLV15.BI=FALSE} =>  {BcHV1.BI=FALSE} | 0.24  7525 | 0.460  829 | 0.537  129 | 1.16  3594 | 10  0 |
| {BcEV5=FALSE, BcHV1.BI=FALSE} =>  {BcMV4.BI=FALSE} | 0.24  7525 | 0.657  895 | 0.376  238 | 1.18  1287 | 10  0 |
| {BcHV1.BI=FALSE, BcOLV16.BI=FALSE} => | 0.21 | 0.841 | 0.25 | 1.20 | 85 |

| {BcMV6.BI=FALSE} | 0396 | 584 |  | 9964 |  |
| --- | --- | --- | --- | --- | --- |
| {BcMV6.BI=FALSE, BcOLV16.BI=FALSE} =>  {BcHV1.BI=FALSE} | 0.21  0396 | 0.472  222 | 0.445  545 | 1.19  2361 | 85 |
| {BcHV1.BI=FALSE, BcOLV16.BI=FALSE} =>  {BcULV1.BI=FALSE} | 0.23  5149 | 0.940  594 | 0.25 | 1.19  8738 | 95 |
| {BcHV1.BI=FALSE, BcOLV16.BI=FALSE} =>  {BcMV12=FALSE} | 0.22  5248 | 0.900  99 | 0.25 | 1.12  6935 | 91 |
| {BcHV1.BI=FALSE, BcMV12=FALSE} =>  {BcOLV16.BI=FALSE} | 0.22  5248 | 0.674  074 | 0.334  158 | 1.15  3923 | 91 |
| {BcHV1.BI=FALSE, BcOLV16.BI=FALSE} =>  {BcNSRV7.BI=FALSE} | 0.22  0297 | 0.881  188 | 0.25 | 1.10  2167 | 89 |
| {BcNSRV7.BI=FALSE, BcOLV16.BI=FALSE}  => {BcHV1.BI=FALSE} | 0.22  0297 | 0.473  404 | 0.465  347 | 1.19  5346 | 89 |
| {BcHV1.BI=FALSE, BcOLV16.BI=FALSE} =>  {BcALV1.BI=FALSE} | 0.22  7723 | 0.910  891 | 0.25 | 1.11  8541 | 92 |
| {BcHV1.BI=FALSE, BcOLV16.BI=FALSE} =>  {BcFV9=FALSE} | 0.23  2673 | 0.930  693 | 0.25 | 1.07  1225 | 94 |
| {BcHV1.BI=FALSE, BcULV3=TRUE} =>  {BcFV9=FALSE} | 0.16  0891 | 0.942  029 | 0.170  792 | 1.08  4273 | 65 |
| {BcHV1.BI=FALSE, BcMV11=TRUE} =>  {BcMV6.BI=FALSE} | 0.18  5644 | 0.789  474 | 0.235  149 | 1.13  5044 | 75 |
| {BcMV11=TRUE, BcMV6.BI=FALSE} => | 0.18 | 0.477 | 0.388 | 1.20 | 75 |

| {BcHV1.BI=FALSE} | 5644 | 707 | 614 | 621 |  |
| --- | --- | --- | --- | --- | --- |
| {BcHV1.BI=FALSE, BcMV9.BI=TRUE} =>  {BcOLV1.BI.MOVA.=FALSE} | 0.16  0891 | 0.764  706 | 0.210  396 | 1.14  4227 | 65 |
| {BcHV1.BI=FALSE, BcOLV1.BI.MOVA.=FALSE} =>  {BcMV6.BI=FALSE} | 0.23  2673 | 0.839  286 | 0.277  228 | 1.20  666 | 94 |
| {BcHV1.BI=FALSE, |  |  |  |  |  |
|  | 0.22 | 0.794 | 0.277 | 1.11 |  |
| BcOLV1.BI.MOVA.=FALSE} => |  |  |  |  | 89 |
|  | 0297 | 643 | 228 | 8591 |  |
| {BcPV3.BI=FALSE} |  |  |  |  |  |
| {BcHV1.BI=FALSE, |  |  |  |  |  |
|  | 0.23 | 0.848 | 0.277 | 1.11 |  |
| BcOLV1.BI.MOVA.=FALSE} => |  |  |  |  | 95 |
|  | 5149 | 214 | 228 | 6217 |  |
| {BcHV3.BI=FALSE} |  |  |  |  |  |
| {BcHV1.BI=FALSE, |  |  |  |  |  |
|  |  | 0.901 | 0.277 | 1.15 | 10 |
| BcOLV1.BI.MOVA.=FALSE} => | 0.25 |  |  |  |  |
|  |  | 786 | 228 | 2916 | 1 |
| {BcFV5.BI=FALSE} |  |  |  |  |  |
| {BcFV5.BI=FALSE, BcHV1.BI=FALSE} => |  | 0.759 | 0.329 | 1.13 | 10 |
|  | 0.25 |  |  |  |  |
| {BcOLV1.BI.MOVA.=FALSE} |  | 398 | 208 | 6285 | 1 |
| {BcFV5.BI=FALSE, |  |  |  |  |  |
|  |  | 0.461 | 0.542 | 1.16 | 10 |
| BcOLV1.BI.MOVA.=FALSE} => | 0.25 |  |  |  |  |
|  |  | 187 | 079 | 4498 | 1 |
| {BcHV1.BI=FALSE} |  |  |  |  |  |
| {BcHV1.BI=FALSE, | 0.26 | 0.946 | 0.277 | 1.20 | 10 |
| BcOLV1.BI.MOVA.=FALSE} => | 2376 | 429 | 228 | 6174 | 6 |

| {BcULV1.BI=FALSE} |  |  |  |  |  |
| --- | --- | --- | --- | --- | --- |
| {BcOLV1.BI.MOVA.=FALSE, | 0.26 | 0.469 | 0.559 | 1.18 | 10 |
| BcULV1.BI=FALSE} => {BcHV1.BI=FALSE} | 2376 | 027 | 406 | 4292 | 6 |
| {BcHV1.BI=FALSE, |  |  |  |  |  |
|  | 0.24 |  | 0.277 | 1.09 |  |
| BcOLV1.BI.MOVA.=FALSE} => |  | 0.875 |  |  | 98 |
|  | 2574 |  | 228 | 4427 |  |
| {BcMV12=FALSE} |  |  |  |  |  |
| {BcHV1.BI=FALSE, |  |  |  |  |  |
|  | 0.25 | 0.919 | 0.277 | 1.12 | 10 |
| BcOLV1.BI.MOVA.=FALSE} => |  |  |  |  |  |
|  | 495 | 643 | 228 | 9288 | 3 |
| {BcALV1.BI=FALSE} |  |  |  |  |  |
| {BcHV1.BI=FALSE, |  |  |  |  |  |
|  | 0.25 | 0.937 | 0.277 | 1.07 | 10 |
| BcOLV1.BI.MOVA.=FALSE} => |  |  |  |  |  |
|  | 9901 | 5 | 228 | 906 | 5 |
| {BcFV9=FALSE} |  |  |  |  |  |
| {BcHV1.BI=FALSE, |  |  |  |  |  |
|  | 0.26 | 0.946 | 0.277 | 1.08 | 10 |
| BcOLV1.BI.MOVA.=FALSE} => |  |  |  |  |  |
|  | 2376 | 429 | 228 | 0105 | 6 |
| {BcULV5=FALSE} |  |  |  |  |  |
| {BcHV1.BI=FALSE, BcULV5=FALSE} => | 0.26 | 0.762 | 0.344 | 1.14 | 10 |
| {BcOLV1.BI.MOVA.=FALSE} | 2376 | 59 | 059 | 106 | 6 |
| {BcHV1.BI=FALSE, BcMV9.BI=TRUE} => | 0.16 |  | 0.210 | 1.15 |  |
|  |  | 0.8 |  |  | 68 |
| {BcMV6.BI=FALSE} | 8317 |  | 396 | 0178 |  |
| {BcHV1.BI=FALSE, BcMV9.BI=TRUE} => | 0.20 | 0.988 | 0.210 | 1.12 |  |
|  |  |  |  |  | 84 |
| {BcULV5=FALSE} | 7921 | 235 | 396 | 7817 |  |
| {BcHV1.BI=FALSE, BcULV4=TRUE} => | 0.18 | 0.789 | 0.235 | 1.13 | 75 |

| {BcMV6.BI=FALSE} | 5644 | 474 | 149 | 5044 |  |
| --- | --- | --- | --- | --- | --- |
| {BcHV1.BI=FALSE, BcULV4=TRUE} =>  {BcULV5=FALSE} | 0.22  0297 | 0.936  842 | 0.235  149 | 1.06  9164 | 89 |
| {BcHV1.BI=FALSE, BcPV3.BI=FALSE} =>  {BcMV6.BI=FALSE} | 0.25  2475 | 0.822  581 | 0.306  931 | 1.18  2643 | 10  2 |
| {BcMV6.BI=FALSE, BcPV3.BI=FALSE} =>  {BcHV1.BI=FALSE} | 0.25  2475 | 0.488  038 | 0.517  327 | 1.23  2297 | 10  2 |
| {BcHV1.BI=FALSE, BcRV1.BI=FALSE} =>  {BcMV6.BI=FALSE} | 0.24  2574 | 0.790  323 | 0.306  931 | 1.13  6264 | 98 |
| {BcEV2.BI=FALSE, BcHV1.BI=FALSE} =>  {BcMV6.BI=FALSE} | 0.24  505 | 0.792 | 0.309  406 | 1.13  8676 | 99 |
| {BcEV2.BI=FALSE, BcMV6.BI=FALSE} =>  {BcHV1.BI=FALSE} | 0.24  505 | 0.475  962 | 0.514  851 | 1.20  1803 | 99 |
| {BcHV1.BI=FALSE, BcHV3.BI=FALSE} =>  {BcMV6.BI=FALSE} | 0.25  9901 | 0.820  313 | 0.316  832 | 1.17  9382 | 10  5 |
| {BcHV3.BI=FALSE, BcMV6.BI=FALSE} =>  {BcHV1.BI=FALSE} | 0.25  9901 | 0.481  651 | 0.539  604 | 1.21  617 | 10  5 |
| {BcFV5.BI=FALSE, BcHV1.BI=FALSE} =>  {BcMV6.BI=FALSE} | 0.26  7327 | 0.812  03 | 0.329  208 | 1.16  7474 | 10  8 |
| {BcFV5.BI=FALSE, BcMV6.BI=FALSE} =>  {BcHV1.BI=FALSE} | 0.26  7327 | 0.490  909 | 0.544  554 | 1.23  9545 | 10  8 |
| {BcHV1.BI=FALSE, BcMV6.BI=FALSE} => | 0.29 | 0.914 | 0.319 | 1.16 | 11 |

| {BcULV1.BI=FALSE} | 2079 | 729 | 307 | 5774 | 8 |
| --- | --- | --- | --- | --- | --- |
| {BcHV1.BI=FALSE, BcULV1.BI=FALSE} =>  {BcMV6.BI=FALSE} | 0.29  2079 | 0.819  444 | 0.356  436 | 1.17  8134 | 11  8 |
| {BcMV6.BI=FALSE, BcULV1.BI=FALSE} =>  {BcHV1.BI=FALSE} | 0.29  2079 | 0.510  823 | 0.571  782 | 1.28  9827 | 11  8 |
| {BcHV1.BI=FALSE, BcMV1.BI=TRUE} =>  {BcMV6.BI=FALSE} | 0.24  505 | 0.798  387 | 0.306  931 | 1.14  7859 | 99 |
| {BcHV1.BI=FALSE, BcMV12=FALSE} =>  {BcMV6.BI=FALSE} | 0.27  4752 | 0.822  222 | 0.334  158 | 1.18  2127 | 11  1 |
| {BcMV12=FALSE, BcMV6.BI=FALSE} =>  {BcHV1.BI=FALSE} | 0.27  4752 | 0.472  34 | 0.581  683 | 1.19  266 | 11  1 |
| {BcHV1.BI=FALSE, BcNSRV7.BI=FALSE} =>  {BcMV6.BI=FALSE} | 0.27  4752 | 0.822  222 | 0.334  158 | 1.18  2127 | 11  1 |
| {BcMV6.BI=FALSE, BcNSRV7.BI=FALSE} =>  {BcHV1.BI=FALSE} | 0.27  4752 | 0.484  716 | 0.566  832 | 1.22  3908 | 11  1 |
| {BcHV1.BI=FALSE, BcVV3.BI=FALSE} =>  {BcMV6.BI=FALSE} | 0.25  495 | 0.830  645 | 0.306  931 | 1.19  4237 | 10  3 |
| {BcHV1.BI=FALSE, BcOLV7.BI=FALSE} =>  {BcMV6.BI=FALSE} | 0.26  4851 | 0.804  511 | 0.329  208 | 1.15  6664 | 10  7 |
| {BcMV6.BI=FALSE, BcOLV7.BI=FALSE} =>  {BcHV1.BI=FALSE} | 0.26  4851 | 0.461  207 | 0.574  257 | 1.16  4547 | 10  7 |
| {BcHV1.BI=FALSE, BcMV6.BI=FALSE} => | 0.28 | 0.899 | 0.319 | 1.10 | 11 |

| {BcALV1.BI=FALSE} | 7129 | 225 | 307 | 4215 | 6 |
| --- | --- | --- | --- | --- | --- |
| {BcALV1.BI=FALSE, BcHV1.BI=FALSE} =>  {BcMV6.BI=FALSE} | 0.28  7129 | 0.822  695 | 0.349  01 | 1.18  2807 | 11  6 |
| {BcALV1.BI=FALSE, BcMV6.BI=FALSE} =>  {BcHV1.BI=FALSE} | 0.28  7129 | 0.481  328 | 0.596  535 | 1.21  5353 | 11  6 |
| {BcDMV1=FALSE, BcHV1.BI=FALSE} =>  {BcMV6.BI=FALSE} | 0.26  7327 | 0.818  182 | 0.326  733 | 1.17  6318 | 10  8 |
| {BcFV9=FALSE, BcHV1.BI=FALSE} =>  {BcMV6.BI=FALSE} | 0.28  7129 | 0.794  521 | 0.361  386 | 1.14  23 | 11  6 |
| {BcFV9=FALSE, BcMV6.BI=FALSE} =>  {BcHV1.BI=FALSE} | 0.28  7129 | 0.477  366 | 0.601  485 | 1.20  535 | 11  6 |
| {BcHV1.BI=FALSE, BcOLV4.BI=FALSE} =>  {BcMV6.BI=FALSE} | 0.27  9703 | 0.807  143 | 0.346  535 | 1.16  0447 | 11  3 |
| {BcMV6.BI=FALSE, BcOLV4.BI=FALSE} =>  {BcHV1.BI=FALSE} | 0.27  9703 | 0.453  815 | 0.616  337 | 1.14  5884 | 11  3 |
| {BcHV1.BI=FALSE, BcULV5=FALSE} =>  {BcMV6.BI=FALSE} | 0.28  7129 | 0.834  532 | 0.344  059 | 1.19  9826 | 11  6 |
| {BcMV6.BI=FALSE, BcULV5=FALSE} =>  {BcHV1.BI=FALSE} | 0.28  7129 | 0.462  151 | 0.621  287 | 1.16  6932 | 11  6 |
| {BcHV1.BI=FALSE, BcMV6.BI=FALSE} =>  {BcBNV5.BI=FALSE} | 0.31  1881 | 0.976  744 | 0.319  307 | 1.07  5217 | 12  6 |
| {BcBNV5.BI=FALSE, BcHV1.BI=FALSE} => | 0.31 | 0.828 | 0.376 | 1.19 | 12 |

| {BcMV6.BI=FALSE} | 1881 | 947 | 238 | 1796 | 6 |
| --- | --- | --- | --- | --- | --- |
| {BcBNV5.BI=FALSE, BcMV6.BI=FALSE} =>  {BcHV1.BI=FALSE} | 0.31  1881 | 0.470  149 | 0.663  366 | 1.18  7127 | 12  6 |
| {BcHV1.BI=FALSE, BvF.BI=FALSE} =>  {BcMV6.BI=FALSE} | 0.29  4554 | 0.798  658 | 0.368  812 | 1.14  8248 | 11  9 |
| {BcMV6.BI=FALSE, BvF.BI=FALSE} =>  {BcHV1.BI=FALSE} | 0.29  4554 | 0.472  222 | 0.623  762 | 1.19  2361 | 11  9 |
| {BcHV1.BI=FALSE, BcMCV5.BI=FALSE} =>  {BcMV6.BI=FALSE} | 0.30  6931 | 0.815  789 | 0.376  238 | 1.17  2879 | 12  4 |
| {BcMCV5.BI=FALSE, BcMV6.BI=FALSE} =>  {BcHV1.BI=FALSE} | 0.30  6931 | 0.462  687 | 0.663  366 | 1.16  8284 | 12  4 |
| {BcHV1.BI=FALSE, BcOLV13.BI=FALSE} =>  {BcMV6.BI=FALSE} | 0.30  6931 | 0.805  195 | 0.381  188 | 1.15  7647 | 12  4 |
| {BcMV6.BI=FALSE, BcOLV13.BI=FALSE} =>  {BcHV1.BI=FALSE} | 0.30  6931 | 0.467  925 | 0.655  941 | 1.18  1509 | 12  4 |
| {BcHV1.BI=FALSE, BcOLV2.BI=FALSE} =>  {BcMV6.BI=FALSE} | 0.30  6931 | 0.815  789 | 0.376  238 | 1.17  2879 | 12  4 |
| {BcMV6.BI=FALSE, BcOLV2.BI=FALSE} =>  {BcHV1.BI=FALSE} | 0.30  6931 | 0.460  967 | 0.665  842 | 1.16  3941 | 12  4 |
| {BcHV1.BI=FALSE, BcMCV3.BI=FALSE} =>  {BcMV6.BI=FALSE} | 0.31  9307 | 0.821  656 | 0.388  614 | 1.18  1313 | 12  9 |
| {BcMCV3.BI=FALSE, BcMV6.BI=FALSE} => | 0.31 | 0.474 | 0.673 | 1.19 | 12 |

| {BcHV1.BI=FALSE} | 9307 | 265 | 267 | 7518 | 9 |
| --- | --- | --- | --- | --- | --- |
| {BcHV1.BI=FALSE, BcPV4.BI=FALSE} =>  {BcMV6.BI=FALSE} | 0.30  4455 | 0.814  57 | 0.373  762 | 1.17  1125 | 12  3 |
| {BcMV6.BI=FALSE, BcPV4.BI=FALSE} =>  {BcHV1.BI=FALSE} | 0.30  4455 | 0.464  151 | 0.655  941 | 1.17  1981 | 12  3 |
| {BcEV6=FALSE, BcHV1.BI=FALSE} =>  {BcMV6.BI=FALSE} | 0.31  4356 | 0.814  103 | 0.386  139 | 1.17  0454 | 12  7 |
| {BcEV6=FALSE, BcMV6.BI=FALSE} =>  {BcHV1.BI=FALSE} | 0.31  4356 | 0.481  061 | 0.653  465 | 1.21  4678 | 12  7 |
| {BcHV1.BI=FALSE, BcOLV17.BI=FALSE} =>  {BcMV6.BI=FALSE} | 0.31  1881 | 0.812  903 | 0.383  663 | 1.16  8729 | 12  6 |
| {BcMV6.BI=FALSE, BcOLV17.BI=FALSE} =>  {BcHV1.BI=FALSE} | 0.31  1881 | 0.464  945 | 0.670  792 | 1.17  3985 | 12  6 |
| {BcHV1.BI=FALSE, BcOLV15.BI=FALSE} =>  {BcMV6.BI=FALSE} | 0.31  1881 | 0.807  692 | 0.386  139 | 1.16  1237 | 12  6 |
| {BcMV6.BI=FALSE, BcOLV15.BI=FALSE} =>  {BcHV1.BI=FALSE} | 0.31  1881 | 0.479  087 | 0.650  99 | 1.20  9696 | 12  6 |
| {BcEV5=FALSE, BcHV1.BI=FALSE} =>  {BcMV6.BI=FALSE} | 0.31  1881 | 0.828  947 | 0.376  238 | 1.19  1796 | 12  6 |
| {BcEV5=FALSE, BcMV6.BI=FALSE} =>  {BcHV1.BI=FALSE} | 0.31  1881 | 0.454  874 | 0.685  644 | 1.14  8556 | 12  6 |
| {BcHV1.BI=FALSE, BcHV3.BI=FALSE} => | 0.25 | 0.804 | 0.316 | 1.13 | 10 |

| {BcPV3.BI=FALSE} | 495 | 688 | 832 | 2731 | 3 |
| --- | --- | --- | --- | --- | --- |
| {BcHV3.BI=FALSE, BcPV3.BI=FALSE} =>  {BcHV1.BI=FALSE} | 0.25  495 | 0.461  883 | 0.551  98 | 1.16  6256 | 10  3 |
| {BcFV5.BI=FALSE, BcPV3.BI=FALSE} =>  {BcHV1.BI=FALSE} | 0.25  2475 | 0.461  538 | 0.547  03 | 1.16  5385 | 10  2 |
| {BcHV1.BI=FALSE, BcPV3.BI=FALSE} =>  {BcULV1.BI=FALSE} | 0.27  7228 | 0.903  226 | 0.306  931 | 1.15  1114 | 11  2 |
| {BcPV3.BI=FALSE, BcULV1.BI=FALSE} =>  {BcHV1.BI=FALSE} | 0.27  7228 | 0.484  848 | 0.571  782 | 1.22  4242 | 11  2 |
| {BcNSRV7.BI=FALSE, BcPV3.BI=FALSE} =>  {BcHV1.BI=FALSE} | 0.25  9901 | 0.462  555 | 0.561  881 | 1.16  7952 | 10  5 |
| {BcHV1.BI=FALSE, BcVV3.BI=FALSE} =>  {BcPV3.BI=FALSE} | 0.25 | 0.814  516 | 0.306  931 | 1.14  6566 | 10  1 |
| {BcHV1.BI=FALSE, BcOLV7.BI=FALSE} =>  {BcPV3.BI=FALSE} | 0.26  2376 | 0.796  992 | 0.329  208 | 1.12  1899 | 10  6 |
| {BcALV1.BI=FALSE, BcPV3.BI=FALSE} =>  {BcHV1.BI=FALSE} | 0.26  7327 | 0.457  627 | 0.584  158 | 1.15  5508 | 10  8 |
| {BcHV1.BI=FALSE, BcPV3.BI=FALSE} =>  {BcFV9=FALSE} | 0.28  4653 | 0.927  419 | 0.306  931 | 1.06  7457 | 11  5 |
| {BcFV9=FALSE, BcHV1.BI=FALSE} =>  {BcPV3.BI=FALSE} | 0.28  4653 | 0.787  671 | 0.361  386 | 1.10  8778 | 11  5 |
| {BcFV9=FALSE, BcPV3.BI=FALSE} => | 0.28 | 0.456 | 0.623 | 1.15 | 11 |

| {BcHV1.BI=FALSE} | 4653 | 349 | 762 | 2282 | 5 |
| --- | --- | --- | --- | --- | --- |
| {BcHV1.BI=FALSE, BcOLV4.BI=FALSE} =>  {BcPV3.BI=FALSE} | 0.27  4752 | 0.792  857 | 0.346  535 | 1.11  6078 | 11  1 |
| {BcBNV5.BI=FALSE, BcHV1.BI=FALSE} =>  {BcPV3.BI=FALSE} | 0.29  703 | 0.789  474 | 0.376  238 | 1.11  1315 | 12  0 |
| {BcBNV5.BI=FALSE, BcPV3.BI=FALSE} =>  {BcHV1.BI=FALSE} | 0.29  703 | 0.461  538 | 0.643  564 | 1.16  5385 | 12  0 |
| {BcMCV3.BI=FALSE, BcPV3.BI=FALSE} =>  {BcHV1.BI=FALSE} | 0.30  4455 | 0.447  273 | 0.680  693 | 1.12  9364 | 12  3 |
| {BcEV6=FALSE, BcPV3.BI=FALSE} =>  {BcHV1.BI=FALSE} | 0.29  9505 | 0.453  184 | 0.660  891 | 1.14  4288 | 12  1 |
| {BcHV1.BI=FALSE, BcOLV17.BI=FALSE} =>  {BcPV3.BI=FALSE} | 0.30  198 | 0.787  097 | 0.383  663 | 1.10  7969 | 12  2 |
| {BcFV5.BI=FALSE, BcRV1.BI=FALSE} =>  {BcHV1.BI=FALSE} | 0.25  2475 | 0.463  636 | 0.544  554 | 1.17  0682 | 10  2 |
| {BcHV1.BI=FALSE, BcRV1.BI=FALSE} =>  {BcULV1.BI=FALSE} | 0.27  4752 | 0.895  161 | 0.306  931 | 1.14  0836 | 11  1 |
| {BcRV1.BI=FALSE, BcULV1.BI=FALSE} =>  {BcHV1.BI=FALSE} | 0.27  4752 | 0.470  339 | 0.584  158 | 1.18  7606 | 11  1 |
| {BcHV1.BI=FALSE, BcMV1.BI=TRUE} =>  {BcRV1.BI=FALSE} | 0.25 | 0.814  516 | 0.306  931 | 1.12  6933 | 10  1 |
| {BcHV1.BI=FALSE, BcOLV7.BI=FALSE} => | 0.26 | 0.804 | 0.329 | 1.11 | 10 |

| {BcRV1.BI=FALSE} | 4851 | 511 | 208 | 3091 | 7 |
| --- | --- | --- | --- | --- | --- |
| {BcHV1.BI=FALSE, BcRV1.BI=FALSE} =>  {BcALV1.BI=FALSE} | 0.27  9703 | 0.911  29 | 0.306  931 | 1.11  9031 | 11  3 |
| {BcALV1.BI=FALSE, BcHV1.BI=FALSE} =>  {BcRV1.BI=FALSE} | 0.27  9703 | 0.801  418 | 0.349  01 | 1.10  8812 | 11  3 |
| {BcHV1.BI=FALSE, BcRV1.BI=FALSE} =>  {BcFV9=FALSE} | 0.28  9604 | 0.943  548 | 0.306  931 | 1.08  6022 | 11  7 |
| {BcFV9=FALSE, BcHV1.BI=FALSE} =>  {BcRV1.BI=FALSE} | 0.28  9604 | 0.801  37 | 0.361  386 | 1.10  8745 | 11  7 |
| {BcFV9=FALSE, BcRV1.BI=FALSE} =>  {BcHV1.BI=FALSE} | 0.28  9604 | 0.455  253 | 0.636  139 | 1.14  9514 | 11  7 |
| {BcEV2.BI=FALSE, BcHV1.BI=FALSE} =>  {BcHV3.BI=FALSE} | 0.26  4851 | 0.856 | 0.309  406 | 1.12  6463 | 10  7 |
| {BcHV1.BI=FALSE, BcHV3.BI=FALSE} =>  {BcEV2.BI=FALSE} | 0.26  4851 | 0.835  938 | 0.316  832 | 1.11  8274 | 10  7 |
| {BcEV2.BI=FALSE, BcHV1.BI=FALSE} =>  {BcULV1.BI=FALSE} | 0.28  4653 | 0.92 | 0.309  406 | 1.17  2492 | 11  5 |
| {BcEV2.BI=FALSE, BcULV1.BI=FALSE} =>  {BcHV1.BI=FALSE} | 0.28  4653 | 0.483  193 | 0.589  109 | 1.22  0063 | 11  5 |
| {BcEV2.BI=FALSE, BcHV1.BI=FALSE} =>  {BcFV9=FALSE} | 0.29  2079 | 0.944 | 0.309  406 | 1.08  6541 | 11  8 |
| {BcHV1.BI=FALSE, BcHV3.BI=FALSE} => | 0.28 | 0.890 | 0.316 | 1.13 | 11 |

| {BcFV5.BI=FALSE} | 2178 | 625 | 832 | 8647 | 4 |
| --- | --- | --- | --- | --- | --- |
| {BcFV5.BI=FALSE, BcHV1.BI=FALSE} =>  {BcHV3.BI=FALSE} | 0.28  2178 | 0.857  143 | 0.329  208 | 1.12  7966 | 11  4 |
| {BcHV1.BI=FALSE, BcHV3.BI=FALSE} =>  {BcULV1.BI=FALSE} | 0.29  4554 | 0.929  688 | 0.316  832 | 1.18  4838 | 11  9 |
| {BcHV3.BI=FALSE, BcULV1.BI=FALSE} =>  {BcHV1.BI=FALSE} | 0.29  4554 | 0.487  705 | 0.603  96 | 1.23  1455 | 11  9 |
| {BcHV1.BI=FALSE, BcHV3.BI=FALSE} =>  {BcNSRV7.BI=FALSE} | 0.28  4653 | 0.898  438 | 0.316  832 | 1.12  3742 | 11  5 |
| {BcHV1.BI=FALSE, BcNSRV7.BI=FALSE} =>  {BcHV3.BI=FALSE} | 0.28  4653 | 0.851  852 | 0.334  158 | 1.12  1004 | 11  5 |
| {BcHV1.BI=FALSE, BcVV3.BI=FALSE} =>  {BcHV3.BI=FALSE} | 0.25  9901 | 0.846  774 | 0.306  931 | 1.11  4322 | 10  5 |
| {BcHV1.BI=FALSE, BcHV3.BI=FALSE} =>  {BcALV1.BI=FALSE} | 0.28  7129 | 0.906  25 | 0.316  832 | 1.11  2842 | 11  6 |
| {BcALV1.BI=FALSE, BcHV3.BI=FALSE} =>  {BcHV1.BI=FALSE} | 0.28  7129 | 0.453  125 | 0.633  663 | 1.14  4141 | 11  6 |
| {BcDMV1=FALSE, BcHV1.BI=FALSE} =>  {BcHV3.BI=FALSE} | 0.27  2277 | 0.833  333 | 0.326  733 | 1.09  6634 | 11  0 |
| {BcHV1.BI=FALSE, BcHV3.BI=FALSE} =>  {BcFV9=FALSE} | 0.29  4554 | 0.929  688 | 0.316  832 | 1.07  0068 | 11  9 |
| {BcHV1.BI=FALSE, BcHV3.BI=FALSE} => | 0.30 | 0.976 | 0.316 | 1.07 | 12 |

| {BcBNV5.BI=FALSE} | 9406 | 563 | 832 | 5017 | 5 |
| --- | --- | --- | --- | --- | --- |
| {BcFV5.BI=FALSE, BcHV1.BI=FALSE} =>  {BcULV1.BI=FALSE} | 0.29  9505 | 0.909  774 | 0.329  208 | 1.15  946 | 12  1 |
| {BcFV5.BI=FALSE, BcULV1.BI=FALSE} =>  {BcHV1.BI=FALSE} | 0.29  9505 | 0.489  879 | 0.611  386 | 1.23  6943 | 12  1 |
| {BcFV5.BI=FALSE, BcHV1.BI=FALSE} =>  {BcNSRV7.BI=FALSE} | 0.29  4554 | 0.894  737 | 0.329  208 | 1.11  9114 | 11  9 |
| {BcHV1.BI=FALSE, BcNSRV7.BI=FALSE} =>  {BcFV5.BI=FALSE} | 0.29  4554 | 0.881  481 | 0.334  158 | 1.12  6957 | 11  9 |
| {BcFV5.BI=FALSE, BcHV1.BI=FALSE} =>  {BcALV1.BI=FALSE} | 0.29  703 | 0.902  256 | 0.329  208 | 1.10  7937 | 12  0 |
| {BcDMV1=FALSE, BcHV1.BI=FALSE} =>  {BcFV5.BI=FALSE} | 0.28  7129 | 0.878  788 | 0.326  733 | 1.12  3514 | 11  6 |
| {BcHV1.BI=FALSE, BcMCV5.BI=FALSE} =>  {BcFV5.BI=FALSE} | 0.31  9307 | 0.848  684 | 0.376  238 | 1.08  5027 | 12  9 |
| {BcEV5=FALSE, BcHV1.BI=FALSE} =>  {BcFV5.BI=FALSE} | 0.32  4257 | 0.861  842 | 0.376  238 | 1.10  1849 | 13  1 |
| {BcHV1.BI=FALSE, BcMV1.BI=TRUE} =>  {BcULV1.BI=FALSE} | 0.27  2277 | 0.887  097 | 0.306  931 | 1.13  0559 | 11  0 |
| {BcHV1.BI=FALSE, BcMV12=FALSE} =>  {BcULV1.BI=FALSE} | 0.30  6931 | 0.918  519 | 0.334  158 | 1.17  0604 | 12  4 |
| {BcMV12=FALSE, BcULV1.BI=FALSE} => | 0.30 | 0.469 | 0.653 | 1.18 | 12 |

| {BcHV1.BI=FALSE} | 6931 | 697 | 465 | 5985 | 4 |
| --- | --- | --- | --- | --- | --- |
| {BcHV1.BI=FALSE, BcNSRV7.BI=FALSE} =>  {BcULV1.BI=FALSE} | 0.30  4455 | 0.911  111 | 0.334  158 | 1.16  1164 | 12  3 |
| {BcNSRV7.BI=FALSE, BcULV1.BI=FALSE} =>  {BcHV1.BI=FALSE} | 0.30  4455 | 0.495  968 | 0.613  861 | 1.25  2319 | 12  3 |
| {BcHV1.BI=FALSE, BcVV3.BI=FALSE} =>  {BcULV1.BI=FALSE} | 0.28  4653 | 0.927  419 | 0.306  931 | 1.18  1948 | 11  5 |
| {BcULV1.BI=FALSE, BcVV3.BI=FALSE} =>  {BcHV1.BI=FALSE} | 0.28  4653 | 0.452  756 | 0.628  713 | 1.14  3209 | 11  5 |
| {BcHV1.BI=FALSE, BcOLV7.BI=FALSE} =>  {BcULV1.BI=FALSE} | 0.30  198 | 0.917  293 | 0.329  208 | 1.16  9042 | 12  2 |
| {BcOLV7.BI=FALSE, BcULV1.BI=FALSE} =>  {BcHV1.BI=FALSE} | 0.30  198 | 0.463  878 | 0.650  99 | 1.17  1293 | 12  2 |
| {BcHV1.BI=FALSE, BcULV1.BI=FALSE} =>  {BcALV1.BI=FALSE} | 0.31  6832 | 0.888  889 | 0.356  436 | 1.09  1523 | 12  8 |
| {BcALV1.BI=FALSE, BcHV1.BI=FALSE} =>  {BcULV1.BI=FALSE} | 0.31  6832 | 0.907  801 | 0.349  01 | 1.15  6946 | 12  8 |
| {BcALV1.BI=FALSE, BcULV1.BI=FALSE} =>  {BcHV1.BI=FALSE} | 0.31  6832 | 0.488  55 | 0.648  515 | 1.23  3588 | 12  8 |
| {BcDMV1=FALSE, BcHV1.BI=FALSE} =>  {BcULV1.BI=FALSE} | 0.29  2079 | 0.893  939 | 0.326  733 | 1.13  9279 | 11  8 |
| {BcFV9=FALSE, BcHV1.BI=FALSE} => | 0.32 | 0.897 | 0.361 | 1.14 | 13 |

| {BcULV1.BI=FALSE} | 4257 | 26 | 386 | 3512 | 1 |
| --- | --- | --- | --- | --- | --- |
| {BcFV9=FALSE, BcULV1.BI=FALSE} =>  {BcHV1.BI=FALSE} | 0.32  4257 | 0.478  102 | 0.678  218 | 1.20  7208 | 13  1 |
| {BcHV1.BI=FALSE, BcOLV4.BI=FALSE} =>  {BcULV1.BI=FALSE} | 0.32  1782 | 0.928  571 | 0.346  535 | 1.18  3416 | 13  0 |
| {BcOLV4.BI=FALSE, BcULV1.BI=FALSE} =>  {BcHV1.BI=FALSE} | 0.32  1782 | 0.462  633 | 0.695  545 | 1.16  8149 | 13  0 |
| {BcHV1.BI=FALSE, BcULV5=FALSE} =>  {BcULV1.BI=FALSE} | 0.31  1881 | 0.906  475 | 0.344  059 | 1.15  5255 | 12  6 |
| {BcBNV5.BI=FALSE, BcHV1.BI=FALSE} =>  {BcULV1.BI=FALSE} | 0.34  1584 | 0.907  895 | 0.376  238 | 1.15  7065 | 13  8 |
| {BcBNV5.BI=FALSE, BcULV1.BI=FALSE} =>  {BcHV1.BI=FALSE} | 0.34  1584 | 0.479  167 | 0.712  871 | 1.20  9896 | 13  8 |
| {BcHV1.BI=FALSE, BvF.BI=FALSE} =>  {BcULV1.BI=FALSE} | 0.33  4158 | 0.906  04 | 0.368  812 | 1.15  4701 | 13  5 |
| {BcULV1.BI=FALSE, BvF.BI=FALSE} =>  {BcHV1.BI=FALSE} | 0.33  4158 | 0.468  75 | 0.712  871 | 1.18  3594 | 13  5 |
| {BcHV1.BI=FALSE, BcMCV5.BI=FALSE} =>  {BcULV1.BI=FALSE} | 0.33  9109 | 0.901  316 | 0.376  238 | 1.14  868 | 13  7 |
| {BcMCV5.BI=FALSE, BcULV1.BI=FALSE} =>  {BcHV1.BI=FALSE} | 0.33  9109 | 0.464  407 | 0.730  198 | 1.17  2627 | 13  7 |
| {BcHV1.BI=FALSE, BcOLV13.BI=FALSE} => | 0.34 | 0.909 | 0.381 | 1.15 | 14 |

| {BcULV1.BI=FALSE} | 6535 | 091 | 188 | 8589 | 0 |
| --- | --- | --- | --- | --- | --- |
| {BcOLV13.BI=FALSE, BcULV1.BI=FALSE} =>  {BcHV1.BI=FALSE} | 0.34  6535 | 0.468  227 | 0.740  099 | 1.18  2274 | 14  0 |
| {BcHV1.BI=FALSE, BcOLV2.BI=FALSE} =>  {BcULV1.BI=FALSE} | 0.34  1584 | 0.907  895 | 0.376  238 | 1.15  7065 | 13  8 |
| {BcOLV2.BI=FALSE, BcULV1.BI=FALSE} =>  {BcHV1.BI=FALSE} | 0.34  1584 | 0.46 | 0.742  574 | 1.16  15 | 13  8 |
| {BcHV1.BI=FALSE, BcMCV3.BI=FALSE} =>  {BcULV1.BI=FALSE} | 0.35  1485 | 0.904  459 | 0.388  614 | 1.15  2685 | 14  2 |
| {BcMCV3.BI=FALSE, BcULV1.BI=FALSE} =>  {BcHV1.BI=FALSE} | 0.35  1485 | 0.459  547 | 0.764  851 | 1.16  0356 | 14  2 |
| {BcHV1.BI=FALSE, BcPV4.BI=FALSE} =>  {BcULV1.BI=FALSE} | 0.33  9109 | 0.907  285 | 0.373  762 | 1.15  6287 | 13  7 |
| {BcPV4.BI=FALSE, BcULV1.BI=FALSE} =>  {BcHV1.BI=FALSE} | 0.33  9109 | 0.456  667 | 0.742  574 | 1.15  3083 | 13  7 |
| {BcEV6=FALSE, BcHV1.BI=FALSE} =>  {BcULV1.BI=FALSE} | 0.34  901 | 0.903  846 | 0.386  139 | 1.15  1905 | 14  1 |
| {BcEV6=FALSE, BcULV1.BI=FALSE} =>  {BcHV1.BI=FALSE} | 0.34  901 | 0.473  154 | 0.737  624 | 1.19  4715 | 14  1 |
| {BcHV1.BI=FALSE, BcOLV17.BI=FALSE} =>  {BcULV1.BI=FALSE} | 0.34  6535 | 0.903  226 | 0.383  663 | 1.15  1114 | 14  0 |
| {BcOLV17.BI=FALSE, BcULV1.BI=FALSE} => | 0.34 | 0.462 | 0.75 | 1.16 | 14 |

| {BcHV1.BI=FALSE} | 6535 | 046 |  | 6667 | 0 |
| --- | --- | --- | --- | --- | --- |
| {BcHV1.BI=FALSE, BcOLV15.BI=FALSE} =>  {BcULV1.BI=FALSE} | 0.34  6535 | 0.897  436 | 0.386  139 | 1.14  3735 | 14  0 |
| {BcOLV15.BI=FALSE, BcULV1.BI=FALSE} =>  {BcHV1.BI=FALSE} | 0.34  6535 | 0.468  227 | 0.740  099 | 1.18  2274 | 14  0 |
| {BcEV5=FALSE, BcHV1.BI=FALSE} =>  {BcULV1.BI=FALSE} | 0.33  9109 | 0.901  316 | 0.376  238 | 1.14  868 | 13  7 |
| {BcEV5=FALSE, BcULV1.BI=FALSE} =>  {BcHV1.BI=FALSE} | 0.33  9109 | 0.456  667 | 0.742  574 | 1.15  3083 | 13  7 |
| {BcHV1.BI=FALSE, BcMV12=FALSE} =>  {BcALV1.BI=FALSE} | 0.30  198 | 0.903  704 | 0.334  158 | 1.10  9715 | 12  2 |
| {BcALV1.BI=FALSE, BcMV12=FALSE} =>  {BcHV1.BI=FALSE} | 0.30  198 | 0.462  121 | 0.653  465 | 1.16  6856 | 12  2 |
| {BcHV1.BI=FALSE, BcNSRV7.BI=FALSE} =>  {BcALV1.BI=FALSE} | 0.29  9505 | 0.896  296 | 0.334  158 | 1.10  0619 | 12  1 |
| {BcALV1.BI=FALSE, BcNSRV7.BI=FALSE} =>  {BcHV1.BI=FALSE} | 0.29  9505 | 0.453  184 | 0.660  891 | 1.14  4288 | 12  1 |
| {BcDMV1=FALSE, BcHV1.BI=FALSE} =>  {BcNSRV7.BI=FALSE} | 0.28  9604 | 0.886  364 | 0.326  733 | 1.10  8641 | 11  7 |
| {BcHV1.BI=FALSE, BcNSRV7.BI=FALSE} =>  {BcBNV5.BI=FALSE} | 0.32  6733 | 0.977  778 | 0.334  158 | 1.07  6355 | 13  2 |
| {BcBNV5.BI=FALSE, BcHV1.BI=FALSE} => | 0.32 | 0.868 | 0.376 | 1.08 | 13 |

| {BcNSRV7.BI=FALSE} | 6733 | 421 | 238 | 6198 | 2 |
| --- | --- | --- | --- | --- | --- |
| {BcHV1.BI=FALSE, BcMCV5.BI=FALSE} =>  {BcNSRV7.BI=FALSE} | 0.32  6733 | 0.868  421 | 0.376  238 | 1.08  6198 | 13  2 |
| {BcMCV3.BI=FALSE, BcNSRV7.BI=FALSE}  => {BcHV1.BI=FALSE} | 0.32  6733 | 0.444  444 | 0.735  149 | 1.12  2222 | 13  2 |
| {BcEV5=FALSE, BcHV1.BI=FALSE} =>  {BcNSRV7.BI=FALSE} | 0.32  9208 | 0.875 | 0.376  238 | 1.09  4427 | 13  3 |
| {BcHV1.BI=FALSE, BcVV3.BI=FALSE} =>  {BcALV1.BI=FALSE} | 0.27  2277 | 0.887  097 | 0.306  931 | 1.08  9322 | 11  0 |
| {BcHV1.BI=FALSE, BcVV3.BI=FALSE} =>  {BcFV9=FALSE} | 0.28  4653 | 0.927  419 | 0.306  931 | 1.06  7457 | 11  5 |
| {BcHV1.BI=FALSE, BcVV3.BI=FALSE} =>  {BcBNV5.BI=FALSE} | 0.29  9505 | 0.975  806 | 0.306  931 | 1.07  4185 | 12  1 |
| {BcHV1.BI=FALSE, BcOLV7.BI=FALSE} =>  {BcALV1.BI=FALSE} | 0.29  703 | 0.902  256 | 0.329  208 | 1.10  7937 | 12  0 |
| {BcFV9=FALSE, BcHV1.BI=FALSE} =>  {BcALV1.BI=FALSE} | 0.32  1782 | 0.890  411 | 0.361  386 | 1.09  3392 | 13  0 |
| {BcALV1.BI=FALSE, BcFV9=FALSE} =>  {BcHV1.BI=FALSE} | 0.32  1782 | 0.446  735 | 0.720  297 | 1.12  8007 | 13  0 |
| {BcHV1.BI=FALSE, BcOLV4.BI=FALSE} =>  {BcALV1.BI=FALSE} | 0.30  6931 | 0.885  714 | 0.346  535 | 1.08  7625 | 12  4 |
| {BcBNV5.BI=FALSE, BcHV1.BI=FALSE} => | 0.33 | 0.894 | 0.376 | 1.09 | 13 |

| {BcALV1.BI=FALSE} | 6634 | 737 | 238 | 8704 | 6 |
| --- | --- | --- | --- | --- | --- |
| {BcHV1.BI=FALSE, BcMCV5.BI=FALSE} =>  {BcALV1.BI=FALSE} | 0.33  6634 | 0.894  737 | 0.376  238 | 1.09  8704 | 13  6 |
| {BcHV1.BI=FALSE, BcOLV13.BI=FALSE} =>  {BcALV1.BI=FALSE} | 0.33  4158 | 0.876  623 | 0.381  188 | 1.07  6462 | 13  5 |
| {BcALV1.BI=FALSE, BcOLV13.BI=FALSE} =>  {BcHV1.BI=FALSE} | 0.33  4158 | 0.445  545 | 0.75 | 1.12  5 | 13  5 |
| {BcHV1.BI=FALSE, BcOLV2.BI=FALSE} =>  {BcALV1.BI=FALSE} | 0.33  1683 | 0.881  579 | 0.376  238 | 1.08  2547 | 13  4 |
| {BcHV1.BI=FALSE, BcMCV3.BI=FALSE} =>  {BcALV1.BI=FALSE} | 0.34  1584 | 0.878  981 | 0.388  614 | 1.07  9356 | 13  8 |
| {BcALV1.BI=FALSE, BcMCV3.BI=FALSE} =>  {BcHV1.BI=FALSE} | 0.34  1584 | 0.449  511 | 0.759  901 | 1.13  5016 | 13  8 |
| {BcHV1.BI=FALSE, BcPV4.BI=FALSE} =>  {BcALV1.BI=FALSE} | 0.32  9208 | 0.880  795 | 0.373  762 | 1.08  1584 | 13  3 |
| {BcEV6=FALSE, BcHV1.BI=FALSE} =>  {BcALV1.BI=FALSE} | 0.33  9109 | 0.878  205 | 0.386  139 | 1.07  8404 | 13  7 |
| {BcALV1.BI=FALSE, BcEV6=FALSE} =>  {BcHV1.BI=FALSE} | 0.33  9109 | 0.443  366 | 0.764  851 | 1.11  9498 | 13  7 |
| {BcHV1.BI=FALSE, BcOLV17.BI=FALSE} =>  {BcALV1.BI=FALSE} | 0.33  9109 | 0.883  871 | 0.383  663 | 1.08  5361 | 13  7 |
| {BcHV1.BI=FALSE, BcOLV15.BI=FALSE} => | 0.33 | 0.878 | 0.386 | 1.07 | 13 |

| {BcALV1.BI=FALSE} | 9109 | 205 | 139 | 8404 | 7 |
| --- | --- | --- | --- | --- | --- |
| {BcEV5=FALSE, BcHV1.BI=FALSE} =>  {BcALV1.BI=FALSE} | 0.33  4158 | 0.888  158 | 0.376  238 | 1.09  0626 | 13  5 |
| {BcDMV1=FALSE, BcHV1.BI=FALSE} =>  {BcFV9=FALSE} | 0.30  198 | 0.924  242 | 0.326  733 | 1.06  38 | 12  2 |
| {BcDMV1=FALSE, BcHV1.BI=FALSE} =>  {BcBNV5.BI=FALSE} | 0.31  9307 | 0.977  273 | 0.326  733 | 1.07  5799 | 12  9 |
| {BcHV1.BI=FALSE, BcMCV5.BI=FALSE} =>  {BcFV9=FALSE} | 0.34  901 | 0.927  632 | 0.376  238 | 1.06  7701 | 14  1 |
| {BcHV1.BI=FALSE, BcOLV13.BI=FALSE} =>  {BcFV9=FALSE} | 0.35  396 | 0.928  571 | 0.381  188 | 1.06  8783 | 14  3 |
| {BcHV1.BI=FALSE, BcOLV17.BI=FALSE} =>  {BcFV9=FALSE} | 0.35  396 | 0.922  581 | 0.383  663 | 1.06  1888 | 14  3 |
| {BcHV1.BI=FALSE, BcOLV15.BI=FALSE} =>  {BcFV9=FALSE} | 0.35  6436 | 0.923  077 | 0.386  139 | 1.06  2459 | 14  4 |
| {BcEV5=FALSE, BcHV1.BI=FALSE} =>  {BcBNV5.BI=FALSE} | 0.36  6337 | 0.973  684 | 0.376  238 | 1.07  1849 | 14  8 |
| {BcOLV16.BI=TRUE, BcULV3=FALSE} =>  {BcHV1SLR.BI=FALSE} | 0.07  9208 | 0.581  818 | 0.136  139 | 1.27  0565 | 32 |
| {BcOLV16.BI=TRUE, BcULV3=FALSE} =>  {BcMV2.BI=TRUE} | 0.08  1683 | 0.6 | 0.136  139 | 1.29  6257 | 33 |
| {BcOLV16.BI=TRUE, BcULV3=FALSE} => | 0.08 | 0.654 | 0.136 | 1.26 | 36 |

| {BcOLV18=TRUE} | 9109 | 545 | 139 | 5246 |  |
| --- | --- | --- | --- | --- | --- |
| {BcOLV16.BI=TRUE, BcULV3=FALSE} =>  {BcMV9.BI=TRUE} | 0.11  3861 | 0.836  364 | 0.136  139 | 1.23  3179 | 46 |
| {BcOLV16.BI=TRUE, BcULV3=FALSE} =>  {BcHV3.BI=FALSE} | 0.11  8812 | 0.872  727 | 0.136  139 | 1.14  8475 | 48 |
| {BcOLV16.BI=TRUE, BcULV3=FALSE} =>  {BcBNV5.BI=FALSE} | 0.13  3663 | 0.981  818 | 0.136  139 | 1.08  0803 | 54 |
| {BcMV4.BI=TRUE, BcULV3=FALSE} =>  {BcHV1SLR.BI=FALSE} | 0.08  9109 | 0.6 | 0.148  515 | 1.31  027 | 36 |
| {BcMV4.BI=TRUE, BcULV3=FALSE} =>  {BcMV2.BI=TRUE} | 0.09  1584 | 0.616  667 | 0.148  515 | 1.33  2264 | 37 |
| {BcMV4.BI=TRUE, BcULV3=FALSE} =>  {BcOLV18=TRUE} | 0.09  4059 | 0.633  333 | 0.148  515 | 1.22  4242 | 38 |
| {BcMV4.BI=TRUE, BcULV3=FALSE} =>  {BcNSRV7.BI=FALSE} | 0.13  3663 | 0.9 | 0.148  515 | 1.12  5697 | 54 |
| {BcHV1SLR.BI=FALSE, BcULV3=FALSE} =>  {BcMV3.BI=FALSE} | 0.14  1089 | 0.564  356 | 0.25 | 1.23  2432 | 57 |
| {BcMV3.BI=FALSE, BcULV3=FALSE} =>  {BcHV1SLR.BI=FALSE} | 0.14  1089 | 0.670  588 | 0.210  396 | 1.46  442 | 57 |
| {BcHV1SLR.BI=FALSE, BcMV3.BI=FALSE}  => {BcULV3=FALSE} | 0.14  1089 | 0.575  758 | 0.245  05 | 1.41  833 | 57 |
| {BcHV1SLR.BI=FALSE, BcMV2.BI=TRUE} => | 0.09 | 0.519 | 0.190 | 1.27 | 40 |

| {BcULV3=FALSE} | 901 | 481 | 594 | 9696 |  |
| --- | --- | --- | --- | --- | --- |
| {BcHV1SLR.BI=FALSE, BcULV3=FALSE} =>  {BcOLV18=FALSE} | 0.15  5941 | 0.623  762 | 0.25 | 1.29  2308 | 63 |
| {BcOLV18=FALSE, BcULV3=FALSE} =>  {BcHV1SLR.BI=FALSE} | 0.15  5941 | 0.732  558 | 0.212  871 | 1.59  9749 | 63 |
| {BcHV1SLR.BI=FALSE, BcOLV18=FALSE} =>  {BcULV3=FALSE} | 0.15  5941 | 0.552  632 | 0.282  178 | 1.36  1361 | 63 |
| {BcHV1SLR.BI=FALSE, BcOLV18=TRUE} =>  {BcULV3=FALSE} | 0.09  4059 | 0.535  211 | 0.175  743 | 1.31  8447 | 38 |
| {BcMV2.BI=FALSE, BcULV3=FALSE} =>  {BcHV1SLR.BI=FALSE} | 0.15  099 | 0.663  043 | 0.227  723 | 1.44  7944 | 61 |
| {BcHV1SLR.BI=FALSE, BcMV2.BI=FALSE}  => {BcULV3=FALSE} | 0.15  099 | 0.564  815 | 0.267  327 | 1.39  1373 | 61 |
| {BcHV1SLR.BI=FALSE, BcMV3.BI=TRUE} =>  {BcULV3=FALSE} | 0.10  8911 | 0.511  628 | 0.212  871 | 1.26  0352 | 44 |
| {BcMV4.BI=FALSE, BcULV3=FALSE} =>  {BcHV1SLR.BI=FALSE} | 0.16  0891 | 0.625 | 0.257  426 | 1.36  4865 | 65 |
| {BcHV1SLR.BI=FALSE, BcMV4.BI=FALSE}  => {BcULV3=FALSE} | 0.16  0891 | 0.580  357 | 0.277  228 | 1.42  966 | 65 |
| {BcHV1SLR.BI=FALSE, BcULV3=FALSE} =>  {BcOLV16.BI=FALSE} | 0.17  0792 | 0.683  168 | 0.25 | 1.16  9492 | 69 |
| {BcOLV16.BI=FALSE, BcULV3=FALSE} => | 0.17 | 0.633 | 0.269 | 1.38 | 69 |

| {BcHV1SLR.BI=FALSE} | 0792 | 028 | 802 | 2395 |  |
| --- | --- | --- | --- | --- | --- |
| {BcHV1SLR.BI=FALSE, BcOLV16.BI=FALSE}  => {BcULV3=FALSE} | 0.17  0792 | 0.610  619 | 0.279  703 | 1.50  4209 | 69 |
| {BcMV11=TRUE, BcULV3=FALSE} =>  {BcHV1SLR.BI=FALSE} | 0.13  6139 | 0.617  978 | 0.220  297 | 1.34  9529 | 55 |
| {BcHV1SLR.BI=FALSE, BcMV11=TRUE} =>  {BcULV3=FALSE} | 0.13  6139 | 0.523  81 | 0.259  901 | 1.29  036 | 55 |
| {BcOLV1.BI.MOVA.=FALSE, BcULV3=FALSE}  => {BcHV1SLR.BI=FALSE} | 0.17  5743 | 0.651  376 | 0.269  802 | 1.42  2465 | 71 |
| {BcHV1SLR.BI=FALSE, BcOLV1.BI.MOVA.=FALSE} =>  {BcULV3=FALSE} | 0.17  5743 | 0.537  879 | 0.326  733 | 1.32  5018 | 71 |
| {BcHV1SLR.BI=FALSE, BcMV9.BI=TRUE} =>  {BcULV3=FALSE} | 0.13  6139 | 0.504  587 | 0.269  802 | 1.24  3007 | 55 |
| {BcULV3=FALSE, BcULV4=TRUE} =>  {BcHV1SLR.BI=FALSE} | 0.15  3465 | 0.558  559 | 0.274  752 | 1.21  9771 | 62 |
| {BcHV1SLR.BI=FALSE, BcULV4=TRUE} =>  {BcULV3=FALSE} | 0.15  3465 | 0.529  915 | 0.289  604 | 1.30  5399 | 62 |
| {BcHV1SLR.BI=FALSE, BcULV3=FALSE} =>  {BcMV6.BI=FALSE} | 0.21  2871 | 0.851  485 | 0.25 | 1.22  4199 | 86 |
| {BcMV6.BI=FALSE, BcULV3=FALSE} =>  {BcHV1SLR.BI=FALSE} | 0.21  2871 | 0.671  875 | 0.316  832 | 1.46  723 | 86 |

| {BcHV1SLR.BI=FALSE, BcMV6.BI=FALSE}  => {BcULV3=FALSE} | 0.21  2871 | 0.593  103 | 0.358  911 | 1.46  106 | 86 |
| --- | --- | --- | --- | --- | --- |
| {BcPV3.BI=FALSE, BcULV3=FALSE} =>  {BcHV1SLR.BI=FALSE} | 0.18  8119 | 0.649  573 | 0.289  604 | 1.41  8526 | 76 |
| {BcHV1SLR.BI=FALSE, BcPV3.BI=FALSE} =>  {BcULV3=FALSE} | 0.18  8119 | 0.558  824 | 0.336  634 | 1.37  6614 | 76 |
| {BcRV1.BI=FALSE, BcULV3=FALSE} =>  {BcHV1SLR.BI=FALSE} | 0.18  3168 | 0.632  479 | 0.289  604 | 1.38  1197 | 74 |
| {BcHV1SLR.BI=FALSE, BcRV1.BI=FALSE} =>  {BcULV3=FALSE} | 0.18  3168 | 0.536  232 | 0.341  584 | 1.32  0961 | 74 |
| {BcEV2.BI=FALSE, BcULV3=FALSE} =>  {BcHV1SLR.BI=FALSE} | 0.20  297 | 0.650  794 | 0.311  881 | 1.42  1193 | 82 |
| {BcEV2.BI=FALSE, BcHV1SLR.BI=FALSE} =>  {BcULV3=FALSE} | 0.20  297 | 0.561  644 | 0.361  386 | 1.38  3562 | 82 |
| {BcHV3.BI=FALSE, BcULV3=FALSE} =>  {BcHV1SLR.BI=FALSE} | 0.20  7921 | 0.617  647 | 0.336  634 | 1.34  8808 | 84 |
| {BcHV1SLR.BI=FALSE, BcHV3.BI=FALSE} =>  {BcULV3=FALSE} | 0.20  7921 | 0.567  568 | 0.366  337 | 1.39  8154 | 84 |
| {BcHV1SLR.BI=FALSE, BcULV3=FALSE} =>  {BcFV5.BI=FALSE} | 0.22  2772 | 0.891  089 | 0.25 | 1.13  9241 | 90 |
| {BcFV5.BI=FALSE, BcULV3=FALSE} =>  {BcHV1SLR.BI=FALSE} | 0.22  2772 | 0.633  803 | 0.351  485 | 1.38  4088 | 90 |

| {BcFV5.BI=FALSE, BcHV1SLR.BI=FALSE} =>  {BcULV3=FALSE} | 0.22  2772 | 0.569  62 | 0.391  089 | 1.40  3211 | 90 |
| --- | --- | --- | --- | --- | --- |
| {BcHV1SLR.BI=FALSE, BcULV3=FALSE} =>  {BcULV1.BI=FALSE} | 0.21  7822 | 0.871  287 | 0.25 | 1.11  041 | 88 |
| {BcULV1.BI=FALSE, BcULV3=FALSE} =>  {BcHV1SLR.BI=FALSE} | 0.21  7822 | 0.666  667 | 0.326  733 | 1.45  5856 | 88 |
| {BcHV1SLR.BI=FALSE, BcULV1.BI=FALSE}  => {BcULV3=FALSE} | 0.21  7822 | 0.543  21 | 0.400  99 | 1.33  8151 | 88 |
| {BcMV1.BI=TRUE, BcULV3=FALSE} =>  {BcHV1SLR.BI=FALSE} | 0.17  5743 | 0.577  236 | 0.304  455 | 1.26  0558 | 71 |
| {BcHV1SLR.BI=FALSE, BcMV1.BI=TRUE} =>  {BcULV3=FALSE} | 0.17  5743 | 0.503  546 | 0.349  01 | 1.24  0443 | 71 |
| {BcMV12=FALSE, BcULV3=FALSE} =>  {BcHV1SLR.BI=FALSE} | 0.21  0396 | 0.634  328 | 0.331  683 | 1.38  5236 | 85 |
| {BcHV1SLR.BI=FALSE, BcMV12=FALSE} =>  {BcULV3=FALSE} | 0.21  0396 | 0.544  872 | 0.386  139 | 1.34  2245 | 85 |
| {BcNSRV7.BI=FALSE, BcULV3=FALSE} =>  {BcHV1SLR.BI=FALSE} | 0.21  5347 | 0.595  89 | 0.361  386 | 1.30  1296 | 87 |
| {BcHV1SLR.BI=FALSE, BcNSRV7.BI=FALSE}  => {BcULV3=FALSE} | 0.21  5347 | 0.550  633 | 0.391  089 | 1.35  6437 | 87 |
| {BcULV3=FALSE, BcVV3.BI=FALSE} =>  {BcHV1SLR.BI=FALSE} | 0.20  7921 | 0.641  221 | 0.324  257 | 1.40  0289 | 84 |

| {BcHV1SLR.BI=FALSE, BcVV3.BI=FALSE} =>  {BcULV3=FALSE} | 0.20  7921 | 0.563  758 | 0.368  812 | 1.38  8771 | 84 |
| --- | --- | --- | --- | --- | --- |
| {BcOLV7.BI=FALSE, BcULV3=FALSE} =>  {BcHV1SLR.BI=FALSE} | 0.20  5446 | 0.619  403 | 0.331  683 | 1.35  2642 | 83 |
| {BcHV1SLR.BI=FALSE, BcOLV7.BI=FALSE}  => {BcULV3=FALSE} | 0.20  5446 | 0.553  333 | 0.371  287 | 1.36  3089 | 83 |
| {BcALV1.BI=FALSE, BcULV3=FALSE} =>  {BcHV1SLR.BI=FALSE} | 0.21  5347 | 0.649  254 | 0.331  683 | 1.41  783 | 87 |
| {BcALV1.BI=FALSE, BcHV1SLR.BI=FALSE}  => {BcULV3=FALSE} | 0.21  5347 | 0.540  373 | 0.398  515 | 1.33  1162 | 87 |
| {BcDMV1=FALSE, BcULV3=FALSE} =>  {BcHV1SLR.BI=FALSE} | 0.20  297 | 0.598  54 | 0.339  109 | 1.30  7082 | 82 |
| {BcDMV1=FALSE, BcHV1SLR.BI=FALSE} =>  {BcULV3=FALSE} | 0.20  297 | 0.535  948 | 0.378  713 | 1.32  0261 | 82 |
| {BcFV9=FALSE, BcULV3=FALSE} =>  {BcHV1SLR.BI=FALSE} | 0.22  2772 | 0.620  69 | 0.358  911 | 1.35  5452 | 90 |
| {BcFV9=FALSE, BcHV1SLR.BI=FALSE} =>  {BcULV3=FALSE} | 0.22  2772 | 0.532  544 | 0.418  317 | 1.31  1878 | 90 |
| {BcOLV4.BI=FALSE, BcULV3=FALSE} =>  {BcHV1SLR.BI=FALSE} | 0.22  2772 | 0.604  027 | 0.368  812 | 1.31  9064 | 90 |
| {BcHV1SLR.BI=FALSE, BcOLV4.BI=FALSE}  => {BcULV3=FALSE} | 0.22  2772 | 0.538  922 | 0.413  366 | 1.32  7589 | 90 |

| {BcULV3=FALSE, BcULV5=FALSE} =>  {BcHV1SLR.BI=FALSE} | 0.22  0297 | 0.613  793 | 0.358  911 | 1.34  0391 | 89 |
| --- | --- | --- | --- | --- | --- |
| {BcHV1SLR.BI=FALSE, BcULV5=FALSE} =>  {BcULV3=FALSE} | 0.22  0297 | 0.539  394 | 0.408  416 | 1.32  8751 | 89 |
| {BcHV1SLR.BI=FALSE, BcULV3=FALSE} =>  {BcBNV5.BI=FALSE} | 0.24  505 | 0.980  198 | 0.25 | 1.07  9019 | 99 |
| {BcBNV5.BI=FALSE, BcULV3=FALSE} =>  {BcHV1SLR.BI=FALSE} | 0.24  505 | 0.626  582 | 0.391  089 | 1.36  832 | 99 |
| {BcBNV5.BI=FALSE, BcHV1SLR.BI=FALSE}  => {BcULV3=FALSE} | 0.24  505 | 0.568  966 | 0.430  693 | 1.40  1598 | 99 |
| {BcULV3=FALSE, BvF.BI=FALSE} =>  {BcHV1SLR.BI=FALSE} | 0.23  2673 | 0.643  836 | 0.361  386 | 1.40  5998 | 94 |
| {BcHV1SLR.BI=FALSE, BvF.BI=FALSE} =>  {BcULV3=FALSE} | 0.23  2673 | 0.537  143 | 0.433  168 | 1.32  3206 | 94 |
| {BcMCV5.BI=FALSE, BcULV3=FALSE} =>  {BcHV1SLR.BI=FALSE} | 0.24  0099 | 0.613  924 | 0.391  089 | 1.34  0677 | 97 |
| {BcHV1SLR.BI=FALSE, BcMCV5.BI=FALSE}  => {BcULV3=FALSE} | 0.24  0099 | 0.554  286 | 0.433  168 | 1.36  5436 | 97 |
| {BcOLV13.BI=FALSE, BcULV3=FALSE} =>  {BcHV1SLR.BI=FALSE} | 0.23  7624 | 0.623  377 | 0.381  188 | 1.36  132 | 96 |
| {BcHV1SLR.BI=FALSE, BcOLV13.BI=FALSE}  => {BcULV3=FALSE} | 0.23  7624 | 0.545  455 | 0.435  644 | 1.34  3681 | 96 |

| {BcOLV2.BI=FALSE, BcULV3=FALSE} =>  {BcHV1SLR.BI=FALSE} | 0.24  7525 | 0.641  026 | 0.386  139 | 1.39  9861 | 10  0 |
| --- | --- | --- | --- | --- | --- |
| {BcHV1SLR.BI=FALSE, BcOLV2.BI=FALSE}  => {BcULV3=FALSE} | 0.24  7525 | 0.555  556 | 0.445  545 | 1.36  8564 | 10  0 |
| {BcMCV3.BI=FALSE, BcULV3=FALSE} =>  {BcHV1SLR.BI=FALSE} | 0.25 | 0.639  241 | 0.391  089 | 1.39  5963 | 10  1 |
| {BcHV1SLR.BI=FALSE, BcMCV3.BI=FALSE}  => {BcULV3=FALSE} | 0.25 | 0.561  111 | 0.445  545 | 1.38  2249 | 10  1 |
| {BcPV4.BI=FALSE, BcULV3=FALSE} =>  {BcHV1SLR.BI=FALSE} | 0.23  5149 | 0.612  903 | 0.383  663 | 1.33  8448 | 95 |
| {BcHV1SLR.BI=FALSE, BcPV4.BI=FALSE} =>  {BcULV3=FALSE} | 0.23  5149 | 0.549  133 | 0.428  218 | 1.35  2742 | 95 |
| {BcEV6=FALSE, BcULV3=FALSE} =>  {BcHV1SLR.BI=FALSE} | 0.23  5149 | 0.625 | 0.376  238 | 1.36  4865 | 95 |
| {BcEV6=FALSE, BcHV1SLR.BI=FALSE} =>  {BcULV3=FALSE} | 0.23  5149 | 0.542  857 | 0.433  168 | 1.33  7282 | 95 |
| {BcOLV17.BI=FALSE, BcULV3=FALSE} =>  {BcHV1SLR.BI=FALSE} | 0.24  2574 | 0.620  253 | 0.391  089 | 1.35  4499 | 98 |
| {BcHV1SLR.BI=FALSE, BcOLV17.BI=FALSE}  => {BcULV3=FALSE} | 0.24  2574 | 0.547  486 | 0.443  069 | 1.34  8685 | 98 |
| {BcOLV15.BI=FALSE, BcULV3=FALSE} =>  {BcHV1SLR.BI=FALSE} | 0.24  505 | 0.622  642 | 0.393  564 | 1.35  9714 | 99 |

| {BcHV1SLR.BI=FALSE, BcOLV15.BI=FALSE}  => {BcULV3=FALSE} | 0.24  505 | 0.543  956 | 0.450  495 | 1.33  9989 | 99 |
| --- | --- | --- | --- | --- | --- |
| {BcEV5=FALSE, BcULV3=FALSE} =>  {BcHV1SLR.BI=FALSE} | 0.24  2574 | 0.608  696 | 0.398  515 | 1.32  926 | 98 |
| {BcEV5=FALSE, BcHV1SLR.BI=FALSE} =>  {BcULV3=FALSE} | 0.24  2574 | 0.553  672 | 0.438  119 | 1.36  3924 | 98 |
| {BcMV3.BI=FALSE, BcULV3=FALSE} =>  {BcOLV18=FALSE} | 0.13  1188 | 0.623  529 | 0.210  396 | 1.29  1825 | 53 |
| {BcOLV18=FALSE, BcULV3=FALSE} =>  {BcMV3.BI=FALSE} | 0.13  1188 | 0.616  279 | 0.212  871 | 1.34  582 | 53 |
| {BcMV3.BI=FALSE, BcULV3=FALSE} =>  {BcMV2.BI=FALSE} | 0.14  1089 | 0.670  588 | 0.210  396 | 1.24  8468 | 57 |
| {BcMV2.BI=FALSE, BcULV3=FALSE} =>  {BcMV3.BI=FALSE} | 0.14  1089 | 0.619  565 | 0.227  723 | 1.35  2996 | 57 |
| {BcMV3.BI=FALSE, BcULV3=FALSE} =>  {BcMV4.BI=FALSE} | 0.14  3564 | 0.682  353 | 0.210  396 | 1.22  5203 | 58 |
| {BcMV3.BI=FALSE, BcULV3=FALSE} =>  {BcOLV16.BI=FALSE} | 0.14  604 | 0.694  118 | 0.210  396 | 1.18  8235 | 59 |
| {BcMV3.BI=FALSE, BcULV3=FALSE} =>  {BcOLV1.BI.MOVA.=FALSE} | 0.16  5842 | 0.788  235 | 0.210  396 | 1.17  9434 | 67 |
| {BcOLV1.BI.MOVA.=FALSE, BcULV3=FALSE}  => {BcMV3.BI=FALSE} | 0.16  5842 | 0.614  679 | 0.269  802 | 1.34  2326 | 67 |

| {BcMV3.BI=FALSE, BcULV3=FALSE} =>  {BcMV6.BI=FALSE} | 0.17  5743 | 0.835  294 | 0.210  396 | 1.20  0921 | 71 |
| --- | --- | --- | --- | --- | --- |
| {BcMV6.BI=FALSE, BcULV3=FALSE} =>  {BcMV3.BI=FALSE} | 0.17  5743 | 0.554  688 | 0.316  832 | 1.21  1318 | 71 |
| {BcRV1.BI=FALSE, BcULV3=FALSE} =>  {BcMV3.BI=FALSE} | 0.16  3366 | 0.564  103 | 0.289  604 | 1.23  1878 | 66 |
| {BcMV3.BI=FALSE, BcULV3=FALSE} =>  {BcHV3.BI=FALSE} | 0.17  8218 | 0.847  059 | 0.210  396 | 1.11  4696 | 72 |
| {BcHV3.BI=FALSE, BcMV3.BI=FALSE} =>  {BcULV3=FALSE} | 0.17  8218 | 0.486  486 | 0.366  337 | 1.19  8418 | 72 |
| {BcMV3.BI=FALSE, BcULV3=FALSE} =>  {BcFV5.BI=FALSE} | 0.20  0495 | 0.952  941 | 0.210  396 | 1.21  8317 | 81 |
| {BcFV5.BI=FALSE, BcULV3=FALSE} =>  {BcMV3.BI=FALSE} | 0.20  0495 | 0.570  423 | 0.351  485 | 1.24  5679 | 81 |
| {BcFV5.BI=FALSE, BcMV3.BI=FALSE} =>  {BcULV3=FALSE} | 0.20  0495 | 0.509  434 | 0.393  564 | 1.25  4947 | 81 |
| {BcMV3.BI=FALSE, BcULV3=FALSE} =>  {BcMV12=FALSE} | 0.19  802 | 0.941  176 | 0.210  396 | 1.17  7199 | 80 |
| {BcMV12=FALSE, BcULV3=FALSE} =>  {BcMV3.BI=FALSE} | 0.19  802 | 0.597  015 | 0.331  683 | 1.30  3752 | 80 |
| {BcMV12=FALSE, BcMV3.BI=FALSE} =>  {BcULV3=FALSE} | 0.19  802 | 0.481  928 | 0.410  891 | 1.18  7188 | 80 |

| {BcMV3.BI=FALSE, BcULV3=FALSE} =>  {BcNSRV7.BI=FALSE} | 0.18  5644 | 0.882  353 | 0.210  396 | 1.10  3624 | 75 |
| --- | --- | --- | --- | --- | --- |
| {BcMV3.BI=FALSE, BcNSRV7.BI=FALSE} =>  {BcULV3=FALSE} | 0.18  5644 | 0.493  421 | 0.376  238 | 1.21  5501 | 75 |
| {BcULV3=FALSE, BcVV3.BI=FALSE} =>  {BcMV3.BI=FALSE} | 0.18  3168 | 0.564  885 | 0.324  257 | 1.23  3588 | 74 |
| {BcMV3.BI=FALSE, BcULV3=FALSE} =>  {BcULV5=FALSE} | 0.19  802 | 0.941  176 | 0.210  396 | 1.07  4111 | 80 |
| {BcULV3=FALSE, BcULV5=FALSE} =>  {BcMV3.BI=FALSE} | 0.19  802 | 0.551  724 | 0.358  911 | 1.20  4846 | 80 |
| {BcOLV2.BI=FALSE, BcULV3=FALSE} =>  {BcMV3.BI=FALSE} | 0.20  7921 | 0.538  462 | 0.386  139 | 1.17  5884 | 84 |
| {BcMCV3.BI=FALSE, BcULV3=FALSE} =>  {BcMV3.BI=FALSE} | 0.21  0396 | 0.537  975 | 0.391  089 | 1.17  482 | 85 |
| {BcMV2.BI=TRUE, BcULV3=FALSE} =>  {BcMV1.BI=TRUE} | 0.15  8416 | 0.888  889 | 0.178  218 | 1.12  574 | 64 |
| {BcMV2.BI=TRUE, BcULV3=FALSE} =>  {BcNSRV7.BI=FALSE} | 0.16  0891 | 0.902  778 | 0.178  218 | 1.12  9171 | 65 |
| {BcMV2.BI=TRUE, BcULV3=FALSE} =>  {BcDMV1=FALSE} | 0.16  3366 | 0.916  667 | 0.178  218 | 1.08  6022 | 66 |
| {BcOLV18=FALSE, BcULV3=FALSE} =>  {BcMV2.BI=FALSE} | 0.14  1089 | 0.662  791 | 0.212  871 | 1.23  3951 | 57 |

| {BcMV2.BI=FALSE, BcULV3=FALSE} =>  {BcOLV18=FALSE} | 0.14  1089 | 0.619  565 | 0.227  723 | 1.28  3612 | 57 |
| --- | --- | --- | --- | --- | --- |
| {BcOLV18=FALSE, BcULV3=FALSE} =>  {BcMV4.BI=FALSE} | 0.15  8416 | 0.744  186 | 0.212  871 | 1.33  6227 | 64 |
| {BcMV4.BI=FALSE, BcULV3=FALSE} =>  {BcOLV18=FALSE} | 0.15  8416 | 0.615  385 | 0.257  426 | 1.27  4951 | 64 |
| {BcMV4.BI=FALSE, BcOLV18=FALSE} =>  {BcULV3=FALSE} | 0.15  8416 | 0.5 | 0.316  832 | 1.23  1707 | 64 |
| {BcOLV18=FALSE, BcULV3=FALSE} =>  {BcOLV16.BI=FALSE} | 0.16  5842 | 0.779  07 | 0.212  871 | 1.33  3662 | 67 |
| {BcOLV16.BI=FALSE, BcULV3=FALSE} =>  {BcOLV18=FALSE} | 0.16  5842 | 0.614  679 | 0.269  802 | 1.27  3489 | 67 |
| {BcOLV16.BI=FALSE, BcOLV18=FALSE} =>  {BcULV3=FALSE} | 0.16  5842 | 0.492  647 | 0.336  634 | 1.21  3594 | 67 |
| {BcOLV18=FALSE, BcULV3=FALSE} =>  {BcOLV1.BI.MOVA.=FALSE} | 0.16  8317 | 0.790  698 | 0.212  871 | 1.18  3118 | 68 |
| {BcOLV1.BI.MOVA.=FALSE, BcULV3=FALSE}  => {BcOLV18=FALSE} | 0.16  8317 | 0.623  853 | 0.269  802 | 1.29  2496 | 68 |
| {BcOLV18=FALSE, BcULV3=FALSE} =>  {BcMV6.BI=FALSE} | 0.17  8218 | 0.837  209 | 0.212  871 | 1.20  3675 | 72 |
| {BcMV6.BI=FALSE, BcOLV18=FALSE} =>  {BcULV3=FALSE} | 0.17  8218 | 0.507  042 | 0.351  485 | 1.24  9055 | 72 |

| {BcOLV18=FALSE, BcULV3=FALSE} =>  {BcPV3.BI=FALSE} | 0.18  3168 | 0.860  465 | 0.212  871 | 1.21  1247 | 74 |
| --- | --- | --- | --- | --- | --- |
| {BcPV3.BI=FALSE, BcULV3=FALSE} =>  {BcOLV18=FALSE} | 0.18  3168 | 0.632  479 | 0.289  604 | 1.31  0366 | 74 |
| {BcRV1.BI=FALSE, BcULV3=FALSE} =>  {BcOLV18=FALSE} | 0.17  0792 | 0.589  744 | 0.289  604 | 1.22  1828 | 69 |
| {BcOLV18=FALSE, BcULV3=FALSE} =>  {BcHV3.BI=FALSE} | 0.18  8119 | 0.883  721 | 0.212  871 | 1.16  2942 | 76 |
| {BcOLV18=FALSE, BcULV3=FALSE} =>  {BcFV5.BI=FALSE} | 0.19  0594 | 0.895  349 | 0.212  871 | 1.14  4686 | 77 |
| {BcOLV18=FALSE, BcULV3=FALSE} =>  {BcULV1.BI=FALSE} | 0.19  0594 | 0.895  349 | 0.212  871 | 1.14  1075 | 77 |
| {BcULV1.BI=FALSE, BcULV3=FALSE} =>  {BcOLV18=FALSE} | 0.19  0594 | 0.583  333 | 0.326  733 | 1.20  8547 | 77 |
| {BcOLV18=FALSE, BcULV3=FALSE} =>  {BcMV12=FALSE} | 0.18  8119 | 0.883  721 | 0.212  871 | 1.10  5335 | 76 |
| {BcOLV18=FALSE, BcULV3=FALSE} =>  {BcNSRV7.BI=FALSE} | 0.19  3069 | 0.906  977 | 0.212  871 | 1.13  4423 | 78 |
| {BcOLV18=FALSE, BcULV3=FALSE} =>  {BcALV1.BI=FALSE} | 0.19  5545 | 0.918  605 | 0.212  871 | 1.12  8013 | 79 |
| {BcALV1.BI=FALSE, BcULV3=FALSE} =>  {BcOLV18=FALSE} | 0.19  5545 | 0.589  552 | 0.331  683 | 1.22  1431 | 79 |

| {BcOLV18=FALSE, BcULV3=FALSE} =>  {BcFV9=FALSE} | 0.20  0495 | 0.941  86 | 0.212  871 | 1.08  4079 | 81 |
| --- | --- | --- | --- | --- | --- |
| {BcHV1SLR.BI=TRUE, BcULV3=FALSE} =>  {BcOLV18=TRUE} | 0.09  901 | 0.634  921 | 0.155  941 | 1.22  7311 | 40 |
| {BcOLV18=TRUE, BcULV3=FALSE} =>  {BcMV9.BI=TRUE} | 0.15  099 | 0.782  051 | 0.193  069 | 1.15  3098 | 61 |
| {BcOLV18=TRUE, BcULV3=FALSE} =>  {BcULV4=TRUE} | 0.16  3366 | 0.846  154 | 0.193  069 | 1.22  5255 | 66 |
| {BcMV2.BI=FALSE, BcULV3=FALSE} =>  {BcMV4.BI=FALSE} | 0.17  0792 | 0.75 | 0.227  723 | 1.34  6667 | 69 |
| {BcMV4.BI=FALSE, BcULV3=FALSE} =>  {BcMV2.BI=FALSE} | 0.17  0792 | 0.663  462 | 0.257  426 | 1.23  52 | 69 |
| {BcMV2.BI=FALSE, BcULV3=FALSE} =>  {BcOLV16.BI=FALSE} | 0.17  3267 | 0.760  87 | 0.227  723 | 1.30  2506 | 70 |
| {BcOLV16.BI=FALSE, BcULV3=FALSE} =>  {BcMV2.BI=FALSE} | 0.17  3267 | 0.642  202 | 0.269  802 | 1.19  562 | 70 |
| {BcMV2.BI=FALSE, BcULV3=FALSE} =>  {BcOLV1.BI.MOVA.=FALSE} | 0.19  3069 | 0.847  826 | 0.227  723 | 1.26  8599 | 78 |
| {BcOLV1.BI.MOVA.=FALSE, BcULV3=FALSE}  => {BcMV2.BI=FALSE} | 0.19  3069 | 0.715  596 | 0.269  802 | 1.33  2262 | 78 |
| {BcMV2.BI=FALSE, BcULV3=FALSE} =>  {BcMV6.BI=FALSE} | 0.19  0594 | 0.836  957 | 0.227  723 | 1.20  3311 | 77 |

| {BcMV2.BI=FALSE, BcULV3=FALSE} =>  {BcRV1.BI=FALSE} | 0.18  5644 | 0.815  217 | 0.227  723 | 1.12  7904 | 75 |
| --- | --- | --- | --- | --- | --- |
| {BcRV1.BI=FALSE, BcULV3=FALSE} =>  {BcMV2.BI=FALSE} | 0.18  5644 | 0.641  026 | 0.289  604 | 1.19  343 | 75 |
| {BcMV2.BI=FALSE, BcULV3=FALSE} =>  {BcFV5.BI=FALSE} | 0.20  297 | 0.891  304 | 0.227  723 | 1.13  9516 | 82 |
| {BcMV2.BI=FALSE, BcULV3=FALSE} =>  {BcULV1.BI=FALSE} | 0.20  0495 | 0.880  435 | 0.227  723 | 1.12  2068 | 81 |
| {BcMV2.BI=FALSE, BcULV3=FALSE} =>  {BcNSRV7.BI=FALSE} | 0.20  0495 | 0.880  435 | 0.227  723 | 1.10  1225 | 81 |
| {BcMV2.BI=FALSE, BcULV3=FALSE} =>  {BcALV1.BI=FALSE} | 0.20  5446 | 0.902  174 | 0.227  723 | 1.10  7837 | 83 |
| {BcMV2.BI=FALSE, BcULV3=FALSE} =>  {BcOLV4.BI=FALSE} | 0.21  5347 | 0.945  652 | 0.227  723 | 1.07  9219 | 87 |
| {BcHV1SLR.BI=TRUE, BcULV3=FALSE} =>  {BcHV1.BI=TRUE} | 0.13  6139 | 0.873  016 | 0.155  941 | 1.44  5485 | 55 |
| {BcHV1.BI=TRUE, BcULV3=FALSE} =>  {BcHV1SLR.BI=TRUE} | 0.13  6139 | 0.753  425 | 0.180  693 | 1.38  9879 | 55 |
| {BcHV1SLR.BI=TRUE, BcULV3=FALSE} =>  {BcNSRV7.BI=FALSE} | 0.14  604 | 0.936  508 | 0.155  941 | 1.17  136 | 59 |
| {BcMV3.BI=TRUE, BcULV3=FALSE} =>  {BcNSRV7.BI=FALSE} | 0.17  5743 | 0.898  734 | 0.195  545 | 1.12  4113 | 71 |

| {BcMV4.BI=FALSE, BcULV3=FALSE} =>  {BcOLV16.BI=FALSE} | 0.17  8218 | 0.692  308 | 0.257  426 | 1.18  5137 | 72 |
| --- | --- | --- | --- | --- | --- |
| {BcOLV16.BI=FALSE, BcULV3=FALSE} =>  {BcMV4.BI=FALSE} | 0.17  8218 | 0.660  55 | 0.269  802 | 1.18  6055 | 72 |
| {BcMV4.BI=FALSE, BcOLV16.BI=FALSE} =>  {BcULV3=FALSE} | 0.17  8218 | 0.486  486 | 0.366  337 | 1.19  8418 | 72 |
| {BcOLV1.BI.MOVA.=FALSE, BcULV3=FALSE}  => {BcMV4.BI=FALSE} | 0.18  5644 | 0.688  073 | 0.269  802 | 1.23  5474 | 75 |
| {BcMV4.BI=FALSE, BcULV3=FALSE} =>  {BcMV6.BI=FALSE} | 0.21  2871 | 0.826  923 | 0.257  426 | 1.18  8886 | 86 |
| {BcMV6.BI=FALSE, BcULV3=FALSE} =>  {BcMV4.BI=FALSE} | 0.21  2871 | 0.671  875 | 0.316  832 | 1.20  6389 | 86 |
| {BcMV4.BI=FALSE, BcMV6.BI=FALSE} =>  {BcULV3=FALSE} | 0.21  2871 | 0.497  11 | 0.428  218 | 1.22  4588 | 86 |
| {BcMV4.BI=FALSE, BcULV3=FALSE} =>  {BcPV3.BI=FALSE} | 0.21  2871 | 0.826  923 | 0.257  426 | 1.16  4031 | 86 |
| {BcPV3.BI=FALSE, BcULV3=FALSE} =>  {BcMV4.BI=FALSE} | 0.21  2871 | 0.735  043 | 0.289  604 | 1.31  981 | 86 |
| {BcRV1.BI=FALSE, BcULV3=FALSE} =>  {BcMV4.BI=FALSE} | 0.20  297 | 0.700  855 | 0.289  604 | 1.25  8424 | 82 |
| {BcEV2.BI=FALSE, BcULV3=FALSE} =>  {BcMV4.BI=FALSE} | 0.21  0396 | 0.674  603 | 0.311  881 | 1.21  1287 | 85 |

| {BcEV2.BI=FALSE, BcMV4.BI=FALSE} =>  {BcULV3=FALSE} | 0.21  0396 | 0.485  714 | 0.433  168 | 1.19  6516 | 85 |
| --- | --- | --- | --- | --- | --- |
| {BcMV4.BI=FALSE, BcULV3=FALSE} =>  {BcHV3.BI=FALSE} | 0.22  5248 | 0.875 | 0.257  426 | 1.15  1466 | 91 |
| {BcHV3.BI=FALSE, BcULV3=FALSE} =>  {BcMV4.BI=FALSE} | 0.22  5248 | 0.669  118 | 0.336  634 | 1.20  1438 | 91 |
| {BcHV3.BI=FALSE, BcMV4.BI=FALSE} =>  {BcULV3=FALSE} | 0.22  5248 | 0.481  481 | 0.467  822 | 1.18  6089 | 91 |
| {BcMV4.BI=FALSE, BcULV3=FALSE} =>  {BcFV5.BI=FALSE} | 0.22  7723 | 0.884  615 | 0.257  426 | 1.13  0964 | 92 |
| {BcFV5.BI=FALSE, BcULV3=FALSE} =>  {BcMV4.BI=FALSE} | 0.22  7723 | 0.647  887 | 0.351  485 | 1.16  3318 | 92 |
| {BcFV5.BI=FALSE, BcMV4.BI=FALSE} =>  {BcULV3=FALSE} | 0.22  7723 | 0.494  624 | 0.460  396 | 1.21  8463 | 92 |
| {BcULV1.BI=FALSE, BcULV3=FALSE} =>  {BcMV4.BI=FALSE} | 0.21  5347 | 0.659  091 | 0.326  733 | 1.18  3434 | 87 |
| {BcMV4.BI=FALSE, BcULV1.BI=FALSE} =>  {BcULV3=FALSE} | 0.21  5347 | 0.478  022 | 0.450  495 | 1.17  7566 | 87 |
| {BcMV4.BI=FALSE, BcULV3=FALSE} =>  {BcNSRV7.BI=FALSE} | 0.22  7723 | 0.884  615 | 0.257  426 | 1.10  6454 | 92 |
| {BcMV4.BI=FALSE, BcNSRV7.BI=FALSE} =>  {BcULV3=FALSE} | 0.22  7723 | 0.491  979 | 0.462  871 | 1.21  1947 | 92 |

| {BcULV3=FALSE, BcVV3.BI=FALSE} =>  {BcMV4.BI=FALSE} | 0.21  5347 | 0.664  122 | 0.324  257 | 1.19  2468 | 87 |
| --- | --- | --- | --- | --- | --- |
| {BcOLV7.BI=FALSE, BcULV3=FALSE} =>  {BcMV4.BI=FALSE} | 0.22  2772 | 0.671  642 | 0.331  683 | 1.20  597 | 90 |
| {BcALV1.BI=FALSE, BcULV3=FALSE} =>  {BcMV4.BI=FALSE} | 0.22  5248 | 0.679  104 | 0.331  683 | 1.21  937 | 91 |
| {BcMV4.BI=FALSE, BcULV3=FALSE} =>  {BcFV9=FALSE} | 0.24  2574 | 0.942  308 | 0.257  426 | 1.08  4593 | 98 |
| {BcFV9=FALSE, BcULV3=FALSE} =>  {BcMV4.BI=FALSE} | 0.24  2574 | 0.675  862 | 0.358  911 | 1.21  3548 | 98 |
| {BcFV9=FALSE, BcMV4.BI=FALSE} =>  {BcULV3=FALSE} | 0.24  2574 | 0.473  43 | 0.512  376 | 1.16  6254 | 98 |
| {BcOLV4.BI=FALSE, BcULV3=FALSE} =>  {BcMV4.BI=FALSE} | 0.23  7624 | 0.644  295 | 0.368  812 | 1.15  6868 | 96 |
| {BcULV3=FALSE, BcULV5=FALSE} =>  {BcMV4.BI=FALSE} | 0.23  0198 | 0.641  379 | 0.358  911 | 1.15  1632 | 93 |
| {BcBNV5.BI=FALSE, BcMV4.BI=FALSE} =>  {BcULV3=FALSE} | 0.24  7525 | 0.471  698 | 0.524  752 | 1.16  1988 | 10  0 |
| {BcMCV5.BI=FALSE, BcULV3=FALSE} =>  {BcMV4.BI=FALSE} | 0.25  2475 | 0.645  57 | 0.391  089 | 1.15  9156 | 10  2 |
| {BcOLV13.BI=FALSE, BcULV3=FALSE} =>  {BcMV4.BI=FALSE} | 0.24  7525 | 0.649  351 | 0.381  188 | 1.16  5945 | 10  0 |

| {BcOLV2.BI=FALSE, BcULV3=FALSE} =>  {BcMV4.BI=FALSE} | 0.24  505 | 0.634  615 | 0.386  139 | 1.13  9487 | 99 |
| --- | --- | --- | --- | --- | --- |
| {BcMCV3.BI=FALSE, BcMV4.BI=FALSE} =>  {BcULV3=FALSE} | 0.24  7525 | 0.473  934 | 0.522  277 | 1.16  7495 | 10  0 |
| {BcOLV17.BI=FALSE, BcULV3=FALSE} =>  {BcMV4.BI=FALSE} | 0.25  2475 | 0.645  57 | 0.391  089 | 1.15  9156 | 10  2 |
| {BcOLV15.BI=FALSE, BcULV3=FALSE} =>  {BcMV4.BI=FALSE} | 0.25  2475 | 0.641  509 | 0.393  564 | 1.15  1866 | 10  2 |
| {BcEV5=FALSE, BcULV3=FALSE} =>  {BcMV4.BI=FALSE} | 0.25  495 | 0.639  752 | 0.398  515 | 1.14  8709 | 10  3 |
| {BcMV11=TRUE, BcULV3=FALSE} =>  {BcOLV16.BI=FALSE} | 0.15  099 | 0.685  393 | 0.220  297 | 1.17  33 | 61 |
| {BcOLV1.BI.MOVA.=FALSE, BcULV3=FALSE}  => {BcOLV16.BI=FALSE} | 0.19  0594 | 0.706  422 | 0.269  802 | 1.20  9299 | 77 |
| {BcOLV16.BI=FALSE, BcULV4=TRUE} =>  {BcULV3=FALSE} | 0.17  5743 | 0.5 | 0.351  485 | 1.23  1707 | 71 |
| {BcOLV16.BI=FALSE, BcULV3=FALSE} =>  {BcMV6.BI=FALSE} | 0.21  5347 | 0.798  165 | 0.269  802 | 1.14  754 | 87 |
| {BcMV6.BI=FALSE, BcULV3=FALSE} =>  {BcOLV16.BI=FALSE} | 0.21  5347 | 0.679  688 | 0.316  832 | 1.16  3533 | 87 |
| {BcMV6.BI=FALSE, BcOLV16.BI=FALSE} =>  {BcULV3=FALSE} | 0.21  5347 | 0.483  333 | 0.445  545 | 1.19  065 | 87 |

| {BcPV3.BI=FALSE, BcULV3=FALSE} =>  {BcOLV16.BI=FALSE} | 0.19  5545 | 0.675  214 | 0.289  604 | 1.15  5874 | 79 |
| --- | --- | --- | --- | --- | --- |
| {BcRV1.BI=FALSE, BcULV3=FALSE} =>  {BcOLV16.BI=FALSE} | 0.19  5545 | 0.675  214 | 0.289  604 | 1.15  5874 | 79 |
| {BcEV2.BI=FALSE, BcOLV16.BI=FALSE} =>  {BcULV3=FALSE} | 0.20  297 | 0.482  353 | 0.420  792 | 1.18  8235 | 82 |
| {BcHV3.BI=FALSE, BcOLV16.BI=FALSE} =>  {BcULV3=FALSE} | 0.21  7822 | 0.497  175 | 0.438  119 | 1.22  4749 | 88 |
| {BcOLV16.BI=FALSE, BcULV3=FALSE} =>  {BcFV5.BI=FALSE} | 0.24  0099 | 0.889  908 | 0.269  802 | 1.13  7731 | 97 |
| {BcFV5.BI=FALSE, BcULV3=FALSE} =>  {BcOLV16.BI=FALSE} | 0.24  0099 | 0.683  099 | 0.351  485 | 1.16  9372 | 97 |
| {BcFV5.BI=FALSE, BcOLV16.BI=FALSE} =>  {BcULV3=FALSE} | 0.24  0099 | 0.521  505 | 0.460  396 | 1.28  4684 | 97 |
| {BcULV1.BI=FALSE, BcULV3=FALSE} =>  {BcOLV16.BI=FALSE} | 0.22  2772 | 0.681  818 | 0.326  733 | 1.16  718 | 90 |
| {BcMV1.BI=TRUE, BcULV3=FALSE} =>  {BcOLV16.BI=FALSE} | 0.20  5446 | 0.674  797 | 0.304  455 | 1.15  5161 | 83 |
| {BcMV12=FALSE, BcULV3=FALSE} =>  {BcOLV16.BI=FALSE} | 0.23  5149 | 0.708  955 | 0.331  683 | 1.21  3635 | 95 |
| {BcOLV16.BI=FALSE, BcULV3=FALSE} =>  {BcNSRV7.BI=FALSE} | 0.24  2574 | 0.899  083 | 0.269  802 | 1.12  4549 | 98 |

| {BcNSRV7.BI=FALSE, BcULV3=FALSE} =>  {BcOLV16.BI=FALSE} | 0.24  2574 | 0.671  233 | 0.361  386 | 1.14  906 | 98 |
| --- | --- | --- | --- | --- | --- |
| {BcNSRV7.BI=FALSE, BcOLV16.BI=FALSE}  => {BcULV3=FALSE} | 0.24  2574 | 0.521  277 | 0.465  347 | 1.28  412 | 98 |
| {BcULV3=FALSE, BcVV3.BI=FALSE} =>  {BcOLV16.BI=FALSE} | 0.21  7822 | 0.671  756 | 0.324  257 | 1.14  9955 | 88 |
| {BcOLV7.BI=FALSE, BcULV3=FALSE} =>  {BcOLV16.BI=FALSE} | 0.23  0198 | 0.694  03 | 0.331  683 | 1.18  8085 | 93 |
| {BcALV1.BI=FALSE, BcULV3=FALSE} =>  {BcOLV16.BI=FALSE} | 0.23  0198 | 0.694  03 | 0.331  683 | 1.18  8085 | 93 |
| {BcFV9=FALSE, BcULV3=FALSE} =>  {BcOLV16.BI=FALSE} | 0.24  505 | 0.682  759 | 0.358  911 | 1.16  879 | 99 |
| {BcFV9=FALSE, BcOLV16.BI=FALSE} =>  {BcULV3=FALSE} | 0.24  505 | 0.480  583 | 0.509  901 | 1.18  3874 | 99 |
| {BcOLV4.BI=FALSE, BcULV3=FALSE} =>  {BcOLV16.BI=FALSE} | 0.24  505 | 0.664  43 | 0.368  812 | 1.13  7413 | 99 |
| {BcULV3=FALSE, BcULV5=FALSE} =>  {BcOLV16.BI=FALSE} | 0.24  0099 | 0.668  966 | 0.358  911 | 1.14  5178 | 97 |
| {BcBNV5.BI=FALSE, BcOLV16.BI=FALSE} =>  {BcULV3=FALSE} | 0.25  7426 | 0.481  481 | 0.534  653 | 1.18  6089 | 10  4 |
| {BcMCV5.BI=FALSE, BcULV3=FALSE} =>  {BcOLV16.BI=FALSE} | 0.26  4851 | 0.677  215 | 0.391  089 | 1.15  9301 | 10  7 |

| {BcMCV5.BI=FALSE, BcOLV16.BI=FALSE} =>  {BcULV3=FALSE} | 0.26  4851 | 0.475  556 | 0.556  931 | 1.17  1491 | 10  7 |
| --- | --- | --- | --- | --- | --- |
| {BcOLV13.BI=FALSE, BcULV3=FALSE} =>  {BcOLV16.BI=FALSE} | 0.25  495 | 0.668  831 | 0.381  188 | 1.14  4948 | 10  3 |
| {BcOLV2.BI=FALSE, BcULV3=FALSE} =>  {BcOLV16.BI=FALSE} | 0.25  7426 | 0.666  667 | 0.386  139 | 1.14  1243 | 10  4 |
| {BcMCV3.BI=FALSE, BcULV3=FALSE} =>  {BcOLV16.BI=FALSE} | 0.26  4851 | 0.677  215 | 0.391  089 | 1.15  9301 | 10  7 |
| {BcPV4.BI=FALSE, BcULV3=FALSE} =>  {BcOLV16.BI=FALSE} | 0.25  9901 | 0.677  419 | 0.383  663 | 1.15  965 | 10  5 |
| {BcOLV16.BI=FALSE, BcPV4.BI=FALSE} =>  {BcULV3=FALSE} | 0.25  9901 | 0.468  75 | 0.554  455 | 1.15  4726 | 10  5 |
| {BcEV6=FALSE, BcULV3=FALSE} =>  {BcOLV16.BI=FALSE} | 0.25  2475 | 0.671  053 | 0.376  238 | 1.14  8751 | 10  2 |
| {BcOLV17.BI=FALSE, BcULV3=FALSE} =>  {BcOLV16.BI=FALSE} | 0.25  9901 | 0.664  557 | 0.391  089 | 1.13  7631 | 10  5 |
| {BcOLV15.BI=FALSE, BcULV3=FALSE} =>  {BcOLV16.BI=FALSE} | 0.25  9901 | 0.660  377 | 0.393  564 | 1.13  0476 | 10  5 |
| {BcOLV15.BI=FALSE, BcOLV16.BI=FALSE}  => {BcULV3=FALSE} | 0.25  9901 | 0.475  113 | 0.547  03 | 1.17  0401 | 10  5 |
| {BcEV5=FALSE, BcULV3=FALSE} =>  {BcOLV16.BI=FALSE} | 0.26  4851 | 0.664  596 | 0.398  515 | 1.13  7699 | 10  7 |

| {BcEV5=FALSE, BcOLV16.BI=FALSE} =>  {BcULV3=FALSE} | 0.26  4851 | 0.475  556 | 0.556  931 | 1.17  1491 | 10  7 |
| --- | --- | --- | --- | --- | --- |
| {BcHV1.BI=TRUE, BcULV3=FALSE} =>  {BcMV9.BI=TRUE} | 0.14  604 | 0.808  219 | 0.180  693 | 1.19  1681 | 59 |
| {BcHV1.BI=TRUE, BcULV3=FALSE} =>  {BcULV4=TRUE} | 0.14  604 | 0.808  219 | 0.180  693 | 1.17  0325 | 59 |
| {BcHV1.BI=TRUE, BcULV3=FALSE} =>  {BcNSRV7.BI=FALSE} | 0.16  5842 | 0.917  808 | 0.180  693 | 1.14  7971 | 67 |
| {BcMV11=TRUE, BcULV3=FALSE} =>  {BcFV5.BI=FALSE} | 0.19  5545 | 0.887  64 | 0.220  297 | 1.13  4831 | 79 |
| {BcMV11=TRUE, BcULV3=FALSE} =>  {BcNSRV7.BI=FALSE} | 0.20  297 | 0.921  348 | 0.220  297 | 1.15  2399 | 82 |
| {BcOLV1.BI.MOVA.=FALSE, BcULV3=FALSE}  => {BcMV6.BI=FALSE} | 0.22  2772 | 0.825  688 | 0.269  802 | 1.18  711 | 90 |
| {BcOLV1.BI.MOVA.=FALSE, BcULV3=FALSE}  => {BcHV3.BI=FALSE} | 0.23  0198 | 0.853  211 | 0.269  802 | 1.12  2792 | 93 |
| {BcOLV1.BI.MOVA.=FALSE, BcULV3=FALSE}  => {BcFV5.BI=FALSE} | 0.24  0099 | 0.889  908 | 0.269  802 | 1.13  7731 | 97 |
| {BcOLV1.BI.MOVA.=FALSE, BcULV3=FALSE}  => {BcOLV4.BI=FALSE} | 0.25  495 | 0.944  954 | 0.269  802 | 1.07  8422 | 10  3 |
| {BcOLV1.BI.MOVA.=FALSE, BcULV3=FALSE}  => {BcULV5=FALSE} | 0.25  495 | 0.944  954 | 0.269  802 | 1.07  8422 | 10  3 |

| {BcMV9.BI=TRUE, BcULV3=FALSE} =>  {BcULV4=TRUE} | 0.20  0495 | 0.786  408 | 0.254  95 | 1.13  8741 | 81 |
| --- | --- | --- | --- | --- | --- |
| {BcMV9.BI=TRUE, BcULV3=FALSE} =>  {BcOLV4.BI=FALSE} | 0.24  0099 | 0.941  748 | 0.254  95 | 1.07  4763 | 97 |
| {BcMV9.BI=TRUE, BcULV3=FALSE} =>  {BcULV5=FALSE} | 0.24  505 | 0.961  165 | 0.254  95 | 1.09  6923 | 99 |
| {BcPV3.BI=FALSE, BcULV3=FALSE} =>  {BcMV6.BI=FALSE} | 0.24  0099 | 0.829  06 | 0.289  604 | 1.19  1958 | 97 |
| {BcRV1.BI=FALSE, BcULV3=FALSE} =>  {BcMV6.BI=FALSE} | 0.23  2673 | 0.803  419 | 0.289  604 | 1.15  5093 | 94 |
| {BcEV2.BI=FALSE, BcULV3=FALSE} =>  {BcMV6.BI=FALSE} | 0.24  2574 | 0.777  778 | 0.311  881 | 1.11  8229 | 98 |
| {BcHV3.BI=FALSE, BcULV3=FALSE} =>  {BcMV6.BI=FALSE} | 0.26  4851 | 0.786  765 | 0.336  634 | 1.13  1149 | 10  7 |
| {BcHV3.BI=FALSE, BcMV6.BI=FALSE} =>  {BcULV3=FALSE} | 0.26  4851 | 0.490  826 | 0.539  604 | 1.20  9107 | 10  7 |
| {BcMV6.BI=FALSE, BcULV3=FALSE} =>  {BcFV5.BI=FALSE} | 0.27  2277 | 0.859  375 | 0.316  832 | 1.09  8695 | 11  0 |
| {BcFV5.BI=FALSE, BcMV6.BI=FALSE} =>  {BcULV3=FALSE} | 0.27  2277 | 0.5 | 0.544  554 | 1.23  1707 | 11  0 |
| {BcULV1.BI=FALSE, BcULV3=FALSE} =>  {BcMV6.BI=FALSE} | 0.26  9802 | 0.825  758 | 0.326  733 | 1.18  721 | 10  9 |

| {BcMV6.BI=FALSE, BcULV1.BI=FALSE} =>  {BcULV3=FALSE} | 0.26  9802 | 0.471  861 | 0.571  782 | 1.16  239 | 10  9 |
| --- | --- | --- | --- | --- | --- |
| {BcMV12=FALSE, BcULV3=FALSE} =>  {BcMV6.BI=FALSE} | 0.26  4851 | 0.798  507 | 0.331  683 | 1.14  8032 | 10  7 |
| {BcMV6.BI=FALSE, BcULV3=FALSE} =>  {BcNSRV7.BI=FALSE} | 0.27  7228 | 0.875 | 0.316  832 | 1.09  4427 | 11  2 |
| {BcMV6.BI=FALSE, BcNSRV7.BI=FALSE} =>  {BcULV3=FALSE} | 0.27  7228 | 0.489  083 | 0.566  832 | 1.20  4814 | 11  2 |
| {BcULV3=FALSE, BcVV3.BI=FALSE} =>  {BcMV6.BI=FALSE} | 0.26  4851 | 0.816  794 | 0.324  257 | 1.17  4323 | 10  7 |
| {BcOLV7.BI=FALSE, BcULV3=FALSE} =>  {BcMV6.BI=FALSE} | 0.26  7327 | 0.805  97 | 0.331  683 | 1.15  8761 | 10  8 |
| {BcALV1.BI=FALSE, BcULV3=FALSE} =>  {BcMV6.BI=FALSE} | 0.26  2376 | 0.791  045 | 0.331  683 | 1.13  7303 | 10  6 |
| {BcOLV4.BI=FALSE, BcULV3=FALSE} =>  {BcMV6.BI=FALSE} | 0.28  7129 | 0.778  523 | 0.368  812 | 1.11  9301 | 11  6 |
| {BcMV6.BI=FALSE, BcOLV4.BI=FALSE} =>  {BcULV3=FALSE} | 0.28  7129 | 0.465  863 | 0.616  337 | 1.14  7615 | 11  6 |
| {BcMV6.BI=FALSE, BcULV3=FALSE} =>  {BcBNV5.BI=FALSE} | 0.30  9406 | 0.976  563 | 0.316  832 | 1.07  5017 | 12  5 |
| {BcBNV5.BI=FALSE, BcULV3=FALSE} =>  {BcMV6.BI=FALSE} | 0.30  9406 | 0.791  139 | 0.391  089 | 1.13  7439 | 12  5 |

| {BcBNV5.BI=FALSE, BcMV6.BI=FALSE} =>  {BcULV3=FALSE} | 0.30  9406 | 0.466  418 | 0.663  366 | 1.14  8981 | 12  5 |
| --- | --- | --- | --- | --- | --- |
| {BcULV3=FALSE, BvF.BI=FALSE} =>  {BcMV6.BI=FALSE} | 0.27  9703 | 0.773  973 | 0.361  386 | 1.11  2758 | 11  3 |
| {BcMCV5.BI=FALSE, BcULV3=FALSE} =>  {BcMV6.BI=FALSE} | 0.30  198 | 0.772  152 | 0.391  089 | 1.11  014 | 12  2 |
| {BcOLV13.BI=FALSE, BcULV3=FALSE} =>  {BcMV6.BI=FALSE} | 0.29  9505 | 0.785  714 | 0.381  188 | 1.12  9639 | 12  1 |
| {BcOLV2.BI=FALSE, BcULV3=FALSE} =>  {BcMV6.BI=FALSE} | 0.30  9406 | 0.801  282 | 0.386  139 | 1.15  2021 | 12  5 |
| {BcMV6.BI=FALSE, BcOLV2.BI=FALSE} =>  {BcULV3=FALSE} | 0.30  9406 | 0.464  684 | 0.665  842 | 1.14  4709 | 12  5 |
| {BcMCV3.BI=FALSE, BcULV3=FALSE} =>  {BcMV6.BI=FALSE} | 0.30  6931 | 0.784  81 | 0.391  089 | 1.12  8339 | 12  4 |
| {BcPV4.BI=FALSE, BcULV3=FALSE} =>  {BcMV6.BI=FALSE} | 0.29  9505 | 0.780  645 | 0.383  663 | 1.12  2351 | 12  1 |
| {BcEV6=FALSE, BcULV3=FALSE} =>  {BcMV6.BI=FALSE} | 0.29  4554 | 0.782  895 | 0.376  238 | 1.12  5585 | 11  9 |
| {BcOLV17.BI=FALSE, BcULV3=FALSE} =>  {BcMV6.BI=FALSE} | 0.30  6931 | 0.784  81 | 0.391  089 | 1.12  8339 | 12  4 |
| {BcOLV15.BI=FALSE, BcULV3=FALSE} =>  {BcMV6.BI=FALSE} | 0.30  6931 | 0.779  874 | 0.393  564 | 1.12  1243 | 12  4 |

| {BcMV6.BI=FALSE, BcOLV15.BI=FALSE} =>  {BcULV3=FALSE} | 0.30  6931 | 0.471  483 | 0.650  99 | 1.16  1458 | 12  4 |
| --- | --- | --- | --- | --- | --- |
| {BcEV5=FALSE, BcULV3=FALSE} =>  {BcMV6.BI=FALSE} | 0.31  1881 | 0.782  609 | 0.398  515 | 1.12  5174 | 12  6 |
| {BcRV1.BI=FALSE, BcULV3=FALSE} =>  {BcPV3.BI=FALSE} | 0.23  0198 | 0.794  872 | 0.289  604 | 1.11  8914 | 93 |
| {BcPV3.BI=FALSE, BcULV3=FALSE} =>  {BcHV3.BI=FALSE} | 0.25 | 0.863  248 | 0.289  604 | 1.13  6 | 10  1 |
| {BcRV1.BI=FALSE, BcULV3=FALSE} =>  {BcFV5.BI=FALSE} | 0.25 | 0.863  248 | 0.289  604 | 1.10  3646 | 10  1 |
| {BcRV1.BI=FALSE, BcULV3=FALSE} =>  {BcOLV7.BI=FALSE} | 0.25  495 | 0.880  342 | 0.289  604 | 1.09  0976 | 10  3 |
| {BcRV1.BI=FALSE, BcULV3=FALSE} =>  {BcFV9=FALSE} | 0.26  7327 | 0.923  077 | 0.289  604 | 1.06  2459 | 10  8 |
| {BcEV2.BI=FALSE, BcULV3=FALSE} =>  {BcHV3.BI=FALSE} | 0.26  7327 | 0.857  143 | 0.311  881 | 1.12  7966 | 10  8 |
| {BcEV2.BI=FALSE, BcULV3=FALSE} =>  {BcFV5.BI=FALSE} | 0.27  7228 | 0.888  889 | 0.311  881 | 1.13  6428 | 11  2 |
| {BcEV2.BI=FALSE, BcULV3=FALSE} =>  {BcNSRV7.BI=FALSE} | 0.27  7228 | 0.888  889 | 0.311  881 | 1.11  1799 | 11  2 |
| {BcEV2.BI=FALSE, BcULV3=FALSE} =>  {BcBNV5.BI=FALSE} | 0.30  6931 | 0.984  127 | 0.311  881 | 1.08  3344 | 12  4 |

| {BcHV3.BI=FALSE, BcULV3=FALSE} =>  {BcFV5.BI=FALSE} | 0.30  198 | 0.897  059 | 0.336  634 | 1.14  6873 | 12  2 |
| --- | --- | --- | --- | --- | --- |
| {BcFV5.BI=FALSE, BcULV3=FALSE} =>  {BcHV3.BI=FALSE} | 0.30  198 | 0.859  155 | 0.351  485 | 1.13  0614 | 12  2 |
| {BcFV5.BI=FALSE, BcHV3.BI=FALSE} =>  {BcULV3=FALSE} | 0.30  198 | 0.460  377 | 0.655  941 | 1.13  41 | 12  2 |
| {BcULV1.BI=FALSE, BcULV3=FALSE} =>  {BcHV3.BI=FALSE} | 0.27  7228 | 0.848  485 | 0.326  733 | 1.11  6573 | 11  2 |
| {BcHV3.BI=FALSE, BcULV3=FALSE} =>  {BcNSRV7.BI=FALSE} | 0.30  9406 | 0.919  118 | 0.336  634 | 1.14  9608 | 12  5 |
| {BcNSRV7.BI=FALSE, BcULV3=FALSE} =>  {BcHV3.BI=FALSE} | 0.30  9406 | 0.856  164 | 0.361  386 | 1.12  6679 | 12  5 |
| {BcHV3.BI=FALSE, BcNSRV7.BI=FALSE} =>  {BcULV3=FALSE} | 0.30  9406 | 0.473  485 | 0.653  465 | 1.16  639 | 12  5 |
| {BcULV3=FALSE, BcVV3.BI=FALSE} =>  {BcHV3.BI=FALSE} | 0.27  9703 | 0.862  595 | 0.324  257 | 1.13  5142 | 11  3 |
| {BcOLV7.BI=FALSE, BcULV3=FALSE} =>  {BcHV3.BI=FALSE} | 0.28  7129 | 0.865  672 | 0.331  683 | 1.13  919 | 11  6 |
| {BcDMV1=FALSE, BcULV3=FALSE} =>  {BcHV3.BI=FALSE} | 0.28  7129 | 0.846  715 | 0.339  109 | 1.11  4244 | 11  6 |
| {BcFV9=FALSE, BcULV3=FALSE} =>  {BcHV3.BI=FALSE} | 0.30  198 | 0.841  379 | 0.358  911 | 1.10  7222 | 12  2 |

| {BcOLV4.BI=FALSE, BcULV3=FALSE} =>  {BcHV3.BI=FALSE} | 0.30  9406 | 0.838  926 | 0.368  812 | 1.10  3994 | 12  5 |
| --- | --- | --- | --- | --- | --- |
| {BcHV3.BI=FALSE, BcOLV4.BI=FALSE} =>  {BcULV3=FALSE} | 0.30  9406 | 0.459  559 | 0.673  267 | 1.13  2084 | 12  5 |
| {BcBNV5.BI=FALSE, BcULV3=FALSE} =>  {BcHV3.BI=FALSE} | 0.32  6733 | 0.835  443 | 0.391  089 | 1.09  941 | 13  2 |
| {BcBNV5.BI=FALSE, BcHV3.BI=FALSE} =>  {BcULV3=FALSE} | 0.32  6733 | 0.456  747 | 0.715  347 | 1.12  5158 | 13  2 |
| {BcMCV5.BI=FALSE, BcULV3=FALSE} =>  {BcHV3.BI=FALSE} | 0.32  6733 | 0.835  443 | 0.391  089 | 1.09  941 | 13  2 |
| {BcOLV13.BI=FALSE, BcULV3=FALSE} =>  {BcHV3.BI=FALSE} | 0.32  1782 | 0.844  156 | 0.381  188 | 1.11  0876 | 13  0 |
| {BcOLV2.BI=FALSE, BcULV3=FALSE} =>  {BcHV3.BI=FALSE} | 0.32  4257 | 0.839  744 | 0.386  139 | 1.10  507 | 13  1 |
| {BcHV3.BI=FALSE, BcOLV2.BI=FALSE} =>  {BcULV3=FALSE} | 0.32  4257 | 0.459  649 | 0.705  446 | 1.13  2306 | 13  1 |
| {BcHV3.BI=FALSE, BcMCV3.BI=FALSE} =>  {BcULV3=FALSE} | 0.32  1782 | 0.462  633 | 0.695  545 | 1.13  9658 | 13  0 |
| {BcOLV17.BI=FALSE, BcULV3=FALSE} =>  {BcHV3.BI=FALSE} | 0.32  9208 | 0.841  772 | 0.391  089 | 1.10  7739 | 13  3 |
| {BcHV3.BI=FALSE, BcOLV17.BI=FALSE} =>  {BcULV3=FALSE} | 0.32  9208 | 0.453  925 | 0.725  248 | 1.11  8205 | 13  3 |

| {BcOLV15.BI=FALSE, BcULV3=FALSE} =>  {BcHV3.BI=FALSE} | 0.33  1683 | 0.842  767 | 0.393  564 | 1.10  9049 | 13  4 |
| --- | --- | --- | --- | --- | --- |
| {BcULV1.BI=FALSE, BcULV3=FALSE} =>  {BcFV5.BI=FALSE} | 0.28  2178 | 0.863  636 | 0.326  733 | 1.10  4143 | 11  4 |
| {BcMV1.BI=TRUE, BcULV3=FALSE} =>  {BcFV5.BI=FALSE} | 0.26  2376 | 0.861  789 | 0.304  455 | 1.10  178 | 10  6 |
| {BcMV12=FALSE, BcULV3=FALSE} =>  {BcFV5.BI=FALSE} | 0.28  7129 | 0.865  672 | 0.331  683 | 1.10  6745 | 11  6 |
| {BcFV5.BI=FALSE, BcULV3=FALSE} =>  {BcNSRV7.BI=FALSE} | 0.32  1782 | 0.915  493 | 0.351  485 | 1.14  5075 | 13  0 |
| {BcNSRV7.BI=FALSE, BcULV3=FALSE} =>  {BcFV5.BI=FALSE} | 0.32  1782 | 0.890  411 | 0.361  386 | 1.13  8374 | 13  0 |
| {BcFV5.BI=FALSE, BcNSRV7.BI=FALSE} =>  {BcULV3=FALSE} | 0.32  1782 | 0.474  453 | 0.678  218 | 1.16  8773 | 13  0 |
| {BcULV3=FALSE, BcVV3.BI=FALSE} =>  {BcFV5.BI=FALSE} | 0.28  2178 | 0.870  229 | 0.324  257 | 1.11  2571 | 11  4 |
| {BcOLV7.BI=FALSE, BcULV3=FALSE} =>  {BcFV5.BI=FALSE} | 0.28  4653 | 0.858  209 | 0.331  683 | 1.09  7204 | 11  5 |
| {BcALV1.BI=FALSE, BcULV3=FALSE} =>  {BcFV5.BI=FALSE} | 0.29  703 | 0.895  522 | 0.331  683 | 1.14  4908 | 12  0 |
| {BcDMV1=FALSE, BcULV3=FALSE} =>  {BcFV5.BI=FALSE} | 0.30  198 | 0.890  511 | 0.339  109 | 1.13  8501 | 12  2 |

| {BcFV9=FALSE, BcULV3=FALSE} =>  {BcFV5.BI=FALSE} | 0.31  4356 | 0.875  862 | 0.358  911 | 1.11  9773 | 12  7 |
| --- | --- | --- | --- | --- | --- |
| {BcOLV4.BI=FALSE, BcULV3=FALSE} =>  {BcFV5.BI=FALSE} | 0.31  6832 | 0.859  06 | 0.368  812 | 1.09  8292 | 12  8 |
| {BcFV5.BI=FALSE, BcOLV4.BI=FALSE} =>  {BcULV3=FALSE} | 0.31  6832 | 0.465  455 | 0.680  693 | 1.14  6608 | 12  8 |
| {BcBNV5.BI=FALSE, BcULV3=FALSE} =>  {BcFV5.BI=FALSE} | 0.34  1584 | 0.873  418 | 0.391  089 | 1.11  6648 | 13  8 |
| {BcBNV5.BI=FALSE, BcFV5.BI=FALSE} =>  {BcULV3=FALSE} | 0.34  1584 | 0.458  472 | 0.745  05 | 1.12  9406 | 13  8 |
| {BcULV3=FALSE, BvF.BI=FALSE} =>  {BcFV5.BI=FALSE} | 0.31  9307 | 0.883  562 | 0.361  386 | 1.12  9617 | 12  9 |
| {BcMCV5.BI=FALSE, BcULV3=FALSE} =>  {BcFV5.BI=FALSE} | 0.33  6634 | 0.860  759 | 0.391  089 | 1.10  0465 | 13  6 |
| {BcFV5.BI=FALSE, BcMCV5.BI=FALSE} =>  {BcULV3=FALSE} | 0.33  6634 | 0.453  333 | 0.742  574 | 1.11  6748 | 13  6 |
| {BcOLV13.BI=FALSE, BcULV3=FALSE} =>  {BcFV5.BI=FALSE} | 0.32  6733 | 0.857  143 | 0.381  188 | 1.09  5841 | 13  2 |
| {BcFV5.BI=FALSE, BcOLV13.BI=FALSE} =>  {BcULV3=FALSE} | 0.32  6733 | 0.455  172 | 0.717  822 | 1.12  1278 | 13  2 |
| {BcOLV2.BI=FALSE, BcULV3=FALSE} =>  {BcFV5.BI=FALSE} | 0.33  4158 | 0.865  385 | 0.386  139 | 1.10  6378 | 13  5 |

| {BcFV5.BI=FALSE, BcOLV2.BI=FALSE} =>  {BcULV3=FALSE} | 0.33  4158 | 0.459  184 | 0.727  723 | 1.13  116 | 13  5 |
| --- | --- | --- | --- | --- | --- |
| {BcMCV3.BI=FALSE, BcULV3=FALSE} =>  {BcFV5.BI=FALSE} | 0.34  1584 | 0.873  418 | 0.391  089 | 1.11  6648 | 13  8 |
| {BcFV5.BI=FALSE, BcMCV3.BI=FALSE} =>  {BcULV3=FALSE} | 0.34  1584 | 0.470  99 | 0.725  248 | 1.16  0243 | 13  8 |
| {BcPV4.BI=FALSE, BcULV3=FALSE} =>  {BcFV5.BI=FALSE} | 0.33  4158 | 0.870  968 | 0.383  663 | 1.11  3516 | 13  5 |
| {BcFV5.BI=FALSE, BcPV4.BI=FALSE} =>  {BcULV3=FALSE} | 0.33  4158 | 0.456  081 | 0.732  673 | 1.12  3517 | 13  5 |
| {BcEV6=FALSE, BcULV3=FALSE} =>  {BcFV5.BI=FALSE} | 0.32  1782 | 0.855  263 | 0.376  238 | 1.09  3438 | 13  0 |
| {BcOLV17.BI=FALSE, BcULV3=FALSE} =>  {BcFV5.BI=FALSE} | 0.33  6634 | 0.860  759 | 0.391  089 | 1.10  0465 | 13  6 |
| {BcFV5.BI=FALSE, BcOLV17.BI=FALSE} =>  {BcULV3=FALSE} | 0.33  6634 | 0.457  912 | 0.735  149 | 1.12  8028 | 13  6 |
| {BcOLV15.BI=FALSE, BcULV3=FALSE} =>  {BcFV5.BI=FALSE} | 0.34  6535 | 0.880  503 | 0.393  564 | 1.12  5707 | 14  0 |
| {BcEV5=FALSE, BcULV3=FALSE} =>  {BcFV5.BI=FALSE} | 0.34  901 | 0.875  776 | 0.398  515 | 1.11  9663 | 14  1 |
| {BcULV1.BI=FALSE, BcULV3=FALSE} =>  {BcNSRV7.BI=FALSE} | 0.28  9604 | 0.886  364 | 0.326  733 | 1.10  8641 | 11  7 |

| {BcNSRV7.BI=FALSE, BcULV1.BI=FALSE} =>  {BcULV3=FALSE} | 0.28  9604 | 0.471  774 | 0.613  861 | 1.16  2175 | 11  7 |
| --- | --- | --- | --- | --- | --- |
| {BcMV1.BI=TRUE, BcULV3=FALSE} =>  {BcNSRV7.BI=FALSE} | 0.27  2277 | 0.894  309 | 0.304  455 | 1.11  8578 | 11  0 |
| {BcMV12=FALSE, BcULV3=FALSE} =>  {BcNSRV7.BI=FALSE} | 0.29  703 | 0.895  522 | 0.331  683 | 1.12  0096 | 12  0 |
| {BcULV3=FALSE, BcVV3.BI=FALSE} =>  {BcNSRV7.BI=FALSE} | 0.28  9604 | 0.893  13 | 0.324  257 | 1.11  7103 | 11  7 |
| {BcOLV7.BI=FALSE, BcULV3=FALSE} =>  {BcNSRV7.BI=FALSE} | 0.29  4554 | 0.888  06 | 0.331  683 | 1.11  0762 | 11  9 |
| {BcALV1.BI=FALSE, BcULV3=FALSE} =>  {BcNSRV7.BI=FALSE} | 0.29  703 | 0.895  522 | 0.331  683 | 1.12  0096 | 12  0 |
| {BcDMV1=FALSE, BcULV3=FALSE} =>  {BcNSRV7.BI=FALSE} | 0.30  9406 | 0.912  409 | 0.339  109 | 1.14  1217 | 12  5 |
| {BcFV9=FALSE, BcULV3=FALSE} =>  {BcNSRV7.BI=FALSE} | 0.32  4257 | 0.903  448 | 0.358  911 | 1.13  001 | 13  1 |
| {BcOLV4.BI=FALSE, BcULV3=FALSE} =>  {BcNSRV7.BI=FALSE} | 0.32  4257 | 0.879  195 | 0.368  812 | 1.09  9674 | 13  1 |
| {BcNSRV7.BI=FALSE, BcOLV4.BI=FALSE} =>  {BcULV3=FALSE} | 0.32  4257 | 0.472  924 | 0.685  644 | 1.16  5008 | 13  1 |
| {BcULV3=FALSE, BcULV5=FALSE} =>  {BcNSRV7.BI=FALSE} | 0.31  4356 | 0.875  862 | 0.358  911 | 1.09  5505 | 12  7 |

| {BcNSRV7.BI=FALSE, BcULV3=FALSE} =>  {BcBNV5.BI=FALSE} | 0.35  1485 | 0.972  603 | 0.361  386 | 1.07  0658 | 14  2 |
| --- | --- | --- | --- | --- | --- |
| {BcBNV5.BI=FALSE, BcULV3=FALSE} =>  {BcNSRV7.BI=FALSE} | 0.35  1485 | 0.898  734 | 0.391  089 | 1.12  4113 | 14  2 |
| {BcBNV5.BI=FALSE, BcNSRV7.BI=FALSE}  => {BcULV3=FALSE} | 0.35  1485 | 0.464  052 | 0.757  426 | 1.14  3153 | 14  2 |
| {BcULV3=FALSE, BvF.BI=FALSE} =>  {BcNSRV7.BI=FALSE} | 0.32  1782 | 0.890  411 | 0.361  386 | 1.11  3703 | 13  0 |
| {BcMCV5.BI=FALSE, BcULV3=FALSE} =>  {BcNSRV7.BI=FALSE} | 0.34  901 | 0.892  405 | 0.391  089 | 1.11  6197 | 14  1 |
| {BcMCV5.BI=FALSE, BcNSRV7.BI=FALSE}  => {BcULV3=FALSE} | 0.34  901 | 0.462  295 | 0.754  95 | 1.13  8824 | 14  1 |
| {BcOLV13.BI=FALSE, BcULV3=FALSE} =>  {BcNSRV7.BI=FALSE} | 0.33  6634 | 0.883  117 | 0.381  188 | 1.10  458 | 13  6 |
| {BcNSRV7.BI=FALSE, BcOLV13.BI=FALSE}  => {BcULV3=FALSE} | 0.33  6634 | 0.454  849 | 0.740  099 | 1.12  0483 | 13  6 |
| {BcOLV2.BI=FALSE, BcULV3=FALSE} =>  {BcNSRV7.BI=FALSE} | 0.34  4059 | 0.891  026 | 0.386  139 | 1.11  4472 | 13  9 |
| {BcNSRV7.BI=FALSE, BcOLV2.BI=FALSE} =>  {BcULV3=FALSE} | 0.34  4059 | 0.464  883 | 0.740  099 | 1.14  5199 | 13  9 |
| {BcMCV3.BI=FALSE, BcULV3=FALSE} =>  {BcNSRV7.BI=FALSE} | 0.34  6535 | 0.886  076 | 0.391  089 | 1.10  8281 | 14  0 |

| {BcMCV3.BI=FALSE, BcNSRV7.BI=FALSE}  => {BcULV3=FALSE} | 0.34  6535 | 0.471  38 | 0.735  149 | 1.16  1206 | 14  0 |
| --- | --- | --- | --- | --- | --- |
| {BcPV4.BI=FALSE, BcULV3=FALSE} =>  {BcNSRV7.BI=FALSE} | 0.33  9109 | 0.883  871 | 0.383  663 | 1.10  5523 | 13  7 |
| {BcNSRV7.BI=FALSE, BcPV4.BI=FALSE} =>  {BcULV3=FALSE} | 0.33  9109 | 0.456  667 | 0.742  574 | 1.12  4959 | 13  7 |
| {BcEV6=FALSE, BcULV3=FALSE} =>  {BcNSRV7.BI=FALSE} | 0.33  4158 | 0.888  158 | 0.376  238 | 1.11  0885 | 13  5 |
| {BcOLV17.BI=FALSE, BcULV3=FALSE} =>  {BcNSRV7.BI=FALSE} | 0.34  6535 | 0.886  076 | 0.391  089 | 1.10  8281 | 14  0 |
| {BcNSRV7.BI=FALSE, BcOLV17.BI=FALSE}  => {BcULV3=FALSE} | 0.34  6535 | 0.456  026 | 0.759  901 | 1.12  3381 | 14  0 |
| {BcOLV15.BI=FALSE, BcULV3=FALSE} =>  {BcNSRV7.BI=FALSE} | 0.35  396 | 0.899  371 | 0.393  564 | 1.12  491 | 14  3 |
| {BcNSRV7.BI=FALSE, BcOLV15.BI=FALSE}  => {BcULV3=FALSE} | 0.35  396 | 0.453  968 | 0.779  703 | 1.11  8312 | 14  3 |
| {BcEV5=FALSE, BcULV3=FALSE} =>  {BcNSRV7.BI=FALSE} | 0.35  6436 | 0.894  41 | 0.398  515 | 1.11  8705 | 14  4 |
| {BcEV5=FALSE, BcNSRV7.BI=FALSE} =>  {BcULV3=FALSE} | 0.35  6436 | 0.455  696 | 0.782  178 | 1.12  2569 | 14  4 |
| {BcALV1.BI=FALSE, BcULV3=FALSE} =>  {BcBNV5.BI=FALSE} | 0.32  4257 | 0.977  612 | 0.331  683 | 1.07  6172 | 13  1 |

| {BcDMV1=FALSE, BcULV3=FALSE} =>  {BcBNV5.BI=FALSE} | 0.33  4158 | 0.985  401 | 0.339  109 | 1.08  4747 | 13  5 |
| --- | --- | --- | --- | --- | --- |
| {BcMCV5.BI=FALSE, BcULV3=FALSE} =>  {BcOLV4.BI=FALSE} | 0.36  3861 | 0.930  38 | 0.391  089 | 1.06  1789 | 14  7 |
| {BcOLV13.BI=FALSE, BcULV3=FALSE} =>  {BcOLV4.BI=FALSE} | 0.35  396 | 0.928  571 | 0.381  188 | 1.05  9726 | 14  3 |
| {BcOLV2.BI=FALSE, BcULV3=FALSE} =>  {BcBNV5.BI=FALSE} | 0.37  6238 | 0.974  359 | 0.386  139 | 1.07  2591 | 15  2 |
| {BcMV4.BI=TRUE, BcOLV16.BI=TRUE} =>  {BcMV2.BI=TRUE} | 0.16  0891 | 0.714  286 | 0.225  248 | 1.54  3163 | 65 |
| {BcMV2.BI=TRUE, BcOLV16.BI=TRUE} =>  {BcMV4.BI=TRUE} | 0.16  0891 | 0.637  255 | 0.252  475 | 1.43  8274 | 65 |
| {BcMV2.BI=TRUE, BcMV4.BI=TRUE} =>  {BcOLV16.BI=TRUE} | 0.16  0891 | 0.585  586 | 0.274  752 | 1.40  8194 | 65 |
| {BcMV4.BI=TRUE, BcOLV16.BI=TRUE} =>  {BcOLV18=TRUE} | 0.16  3366 | 0.725  275 | 0.225  248 | 1.40  1966 | 66 |
| {BcOLV16.BI=TRUE, BcOLV18=TRUE} =>  {BcMV4.BI=TRUE} | 0.16  3366 | 0.605  505 | 0.269  802 | 1.36  6614 | 66 |
| {BcMV4.BI=TRUE, BcOLV18=TRUE} =>  {BcOLV16.BI=TRUE} | 0.16  3366 | 0.589  286 | 0.277  228 | 1.41  7092 | 66 |
| {BcHV1SLR.BI=TRUE, BcOLV16.BI=TRUE}  => {BcMV4.BI=TRUE} | 0.13  8614 | 0.583  333 | 0.237  624 | 1.31  6574 | 56 |

| {BcHV1SLR.BI=TRUE, BcMV4.BI=TRUE} =>  {BcOLV16.BI=TRUE} | 0.13  8614 | 0.528  302 | 0.262  376 | 1.27  044 | 56 |
| --- | --- | --- | --- | --- | --- |
| {BcMV4.BI=TRUE, BcOLV16.BI=TRUE} =>  {BcMV3.BI=TRUE} | 0.17  0792 | 0.758  242 | 0.225  248 | 1.39  8766 | 69 |
| {BcMV3.BI=TRUE, BcOLV16.BI=TRUE} =>  {BcMV4.BI=TRUE} | 0.17  0792 | 0.638  889 | 0.267  327 | 1.44  1962 | 69 |
| {BcMV3.BI=TRUE, BcMV4.BI=TRUE} =>  {BcOLV16.BI=TRUE} | 0.17  0792 | 0.579  832 | 0.294  554 | 1.39  4358 | 69 |
| {BcMV4.BI=TRUE, BcOLV16.BI=TRUE} =>  {BcULV3=TRUE} | 0.16  8317 | 0.747  253 | 0.225  248 | 1.25  7875 | 68 |
| {BcOLV16.BI=TRUE, BcULV3=TRUE} =>  {BcMV4.BI=TRUE} | 0.16  8317 | 0.601  77 | 0.279  703 | 1.35  8185 | 68 |
| {BcMV4.BI=TRUE, BcULV3=TRUE} =>  {BcOLV16.BI=TRUE} | 0.16  8317 | 0.571  429 | 0.294  554 | 1.37  415 | 68 |
| {BcMV4.BI=TRUE, BcOLV16.BI=TRUE} =>  {BcHV1.BI=TRUE} | 0.15  8416 | 0.703  297 | 0.225  248 | 1.16  4475 | 64 |
| {BcHV1.BI=TRUE, BcOLV16.BI=TRUE} =>  {BcMV4.BI=TRUE} | 0.15  8416 | 0.587  156 | 0.269  802 | 1.32  5201 | 64 |
| {BcHV1.BI=TRUE, BcMV4.BI=TRUE} =>  {BcOLV16.BI=TRUE} | 0.15  8416 | 0.528  926 | 0.299  505 | 1.27  194 | 64 |
| {BcMV4.BI=TRUE, BcOLV16.BI=TRUE} =>  {BcMV11=TRUE} | 0.17  8218 | 0.791  209 | 0.225  248 | 1.28  3728 | 72 |

| {BcMV11=TRUE, BcOLV16.BI=TRUE} =>  {BcMV4.BI=TRUE} | 0.17  8218 | 0.648  649 | 0.274  752 | 1.46  3989 | 72 |
| --- | --- | --- | --- | --- | --- |
| {BcMV11=TRUE, BcMV4.BI=TRUE} =>  {BcOLV16.BI=TRUE} | 0.17  8218 | 0.525  547 | 0.339  109 | 1.26  3816 | 72 |
| {BcMV4.BI=TRUE, BcOLV16.BI=TRUE} =>  {BcMV9.BI=TRUE} | 0.17  8218 | 0.791  209 | 0.225  248 | 1.16  66 | 72 |
| {BcMV9.BI=TRUE, BcOLV16.BI=TRUE} =>  {BcMV4.BI=TRUE} | 0.17  8218 | 0.537  313 | 0.331  683 | 1.21  2707 | 72 |
| {BcMV4.BI=TRUE, BcMV9.BI=TRUE} =>  {BcOLV16.BI=TRUE} | 0.17  8218 | 0.529  412 | 0.336  634 | 1.27  3109 | 72 |
| {BcMV4.BI=TRUE, BcULV4=TRUE} =>  {BcOLV16.BI=TRUE} | 0.17  3267 | 0.534  351 | 0.324  257 | 1.28  4987 | 70 |
| {BcEV2.BI=FALSE, BcMV4.BI=TRUE} =>  {BcOLV16.BI=TRUE} | 0.16  8317 | 0.535  433 | 0.314  356 | 1.28  7589 | 68 |
| {BcHV3.BI=FALSE, BcMV4.BI=TRUE} =>  {BcOLV16.BI=TRUE} | 0.15  099 | 0.516  949 | 0.292  079 | 1.24  314 | 61 |
| {BcFV5.BI=FALSE, BcMV4.BI=TRUE} =>  {BcOLV16.BI=TRUE} | 0.16  3366 | 0.507  692 | 0.321  782 | 1.22  0879 | 66 |
| {BcOLV16.BI=TRUE, BcULV1.BI=FALSE} =>  {BcMV4.BI=TRUE} | 0.15  099 | 0.559  633 | 0.269  802 | 1.26  3082 | 61 |
| {BcMV1.BI=TRUE, BcOLV16.BI=TRUE} =>  {BcMV4.BI=TRUE} | 0.19  0594 | 0.566  176 | 0.336  634 | 1.27  7851 | 77 |

| {BcMV1.BI=TRUE, BcMV4.BI=TRUE} =>  {BcOLV16.BI=TRUE} | 0.19  0594 | 0.506  579 | 0.376  238 | 1.21  8202 | 77 |
| --- | --- | --- | --- | --- | --- |
| {BcMV12=FALSE, BcOLV16.BI=TRUE} =>  {BcMV4.BI=TRUE} | 0.16  5842 | 0.563  025 | 0.294  554 | 1.27  0738 | 67 |
| {BcNSRV7.BI=FALSE, BcOLV16.BI=TRUE}  => {BcMV4.BI=TRUE} | 0.17  8218 | 0.533  333 | 0.334  158 | 1.20  3724 | 72 |
| {BcMV4.BI=TRUE, BcNSRV7.BI=FALSE} =>  {BcOLV16.BI=TRUE} | 0.17  8218 | 0.529  412 | 0.336  634 | 1.27  3109 | 72 |
| {BcOLV16.BI=TRUE, BcVV3.BI=FALSE} =>  {BcMV4.BI=TRUE} | 0.18  3168 | 0.544  118 | 0.336  634 | 1.22  8064 | 74 |
| {BcMV4.BI=TRUE, BcVV3.BI=FALSE} =>  {BcOLV16.BI=TRUE} | 0.18  3168 | 0.552  239 | 0.331  683 | 1.32  8003 | 74 |
| {BcDMV1=FALSE, BcOLV16.BI=TRUE} =>  {BcMV4.BI=TRUE} | 0.19  0594 | 0.538  462 | 0.353  96 | 1.21  5299 | 77 |
| {BcDMV1=FALSE, BcMV4.BI=TRUE} =>  {BcOLV16.BI=TRUE} | 0.19  0594 | 0.527  397 | 0.361  386 | 1.26  8265 | 77 |
| {BcFV9=FALSE, BcOLV16.BI=TRUE} =>  {BcMV4.BI=TRUE} | 0.19  0594 | 0.531  034 | 0.358  911 | 1.19  8536 | 77 |
| {BcFV9=FALSE, BcMV4.BI=TRUE} =>  {BcOLV16.BI=TRUE} | 0.19  0594 | 0.534  722 | 0.356  436 | 1.28  588 | 77 |
| {BcBNV5.BI=FALSE, BcMV4.BI=TRUE} =>  {BcOLV16.BI=TRUE} | 0.19  5545 | 0.509  677 | 0.383  663 | 1.22  5653 | 79 |

| {BcOLV16.BI=TRUE, BvF.BI=FALSE} =>  {BcMV4.BI=TRUE} | 0.21  2871 | 0.544  304 | 0.391  089 | 1.22  8485 | 86 |
| --- | --- | --- | --- | --- | --- |
| {BcMV4.BI=TRUE, BvF.BI=FALSE} =>  {BcOLV16.BI=TRUE} | 0.21  2871 | 0.524  39 | 0.405  941 | 1.26  1034 | 86 |
| {BcMCV3.BI=FALSE, BcOLV16.BI=TRUE} =>  {BcMV4.BI=TRUE} | 0.19  802 | 0.551  724 | 0.358  911 | 1.24  5232 | 80 |
| {BcOLV16.BI=TRUE, BcPV4.BI=FALSE} =>  {BcMV4.BI=TRUE} | 0.21  0396 | 0.555  556 | 0.378  713 | 1.25  388 | 85 |
| {BcMV4.BI=TRUE, BcPV4.BI=FALSE} =>  {BcOLV16.BI=TRUE} | 0.21  0396 | 0.502  959 | 0.418  317 | 1.20  9496 | 85 |
| {BcEV6=FALSE, BcOLV16.BI=TRUE} =>  {BcMV4.BI=TRUE} | 0.21  5347 | 0.550  633 | 0.391  089 | 1.24  2769 | 87 |
| {BcEV6=FALSE, BcMV4.BI=TRUE} =>  {BcOLV16.BI=TRUE} | 0.21  5347 | 0.511  765 | 0.420  792 | 1.23  0672 | 87 |
| {BcOLV16.BI=TRUE, BcOLV17.BI=FALSE} =>  {BcMV4.BI=TRUE} | 0.20  5446 | 0.532  051 | 0.386  139 | 1.20  0831 | 83 |
| {BcMV4.BI=TRUE, BcOLV17.BI=FALSE} =>  {BcOLV16.BI=TRUE} | 0.20  5446 | 0.5 | 0.410  891 | 1.20  2381 | 83 |
| {BcOLV15.BI=FALSE, BcOLV16.BI=TRUE} =>  {BcMV4.BI=TRUE} | 0.21  2871 | 0.527  607 | 0.403  465 | 1.19  0801 | 86 |
| {BcMV4.BI=TRUE, BcOLV15.BI=FALSE} =>  {BcOLV16.BI=TRUE} | 0.21  2871 | 0.514  97 | 0.413  366 | 1.23  838 | 86 |

| {BcEV5=FALSE, BcMV4.BI=TRUE} =>  {BcOLV16.BI=TRUE} | 0.20  297 | 0.509  317 | 0.398  515 | 1.22  4786 | 82 |
| --- | --- | --- | --- | --- | --- |
| {BcMV3.BI=FALSE, BcOLV16.BI=TRUE} =>  {BcHV1SLR.BI=FALSE} | 0.08  6634 | 0.583  333 | 0.148  515 | 1.27  3874 | 35 |
| {BcOLV16.BI=TRUE, BcOLV18=FALSE} =>  {BcHV1SLR.BI=FALSE} | 0.08  4158 | 0.576  271 | 0.146  04 | 1.25  8452 | 34 |
| {BcHV1SLR.BI=FALSE, BcOLV18=TRUE} =>  {BcOLV16.BI=TRUE} | 0.09  4059 | 0.535  211 | 0.175  743 | 1.28  7056 | 38 |
| {BcMV2.BI=FALSE, BcOLV16.BI=TRUE} =>  {BcHV1SLR.BI=FALSE} | 0.10  1485 | 0.621  212 | 0.163  366 | 1.35  6593 | 41 |
| {BcMV3.BI=FALSE, BcOLV16.BI=TRUE} =>  {BcMV9.BI=TRUE} | 0.11  6337 | 0.783  333 | 0.148  515 | 1.15  4988 | 47 |
| {BcMV3.BI=FALSE, BcOLV16.BI=TRUE} =>  {BcULV4=TRUE} | 0.11  8812 | 0.8 | 0.148  515 | 1.15  8423 | 48 |
| {BcMV3.BI=FALSE, BcOLV16.BI=TRUE} =>  {BcMV6.BI=FALSE} | 0.11  8812 | 0.8 | 0.148  515 | 1.15  0178 | 48 |
| {BcMV3.BI=FALSE, BcOLV16.BI=TRUE} =>  {BcEV2.BI=FALSE} | 0.13  1188 | 0.883  333 | 0.148  515 | 1.18  1678 | 53 |
| {BcMV3.BI=FALSE, BcOLV16.BI=TRUE} =>  {BcMV12=FALSE} | 0.13  6139 | 0.916  667 | 0.148  515 | 1.14  6543 | 55 |
| {BcMV2.BI=TRUE, BcOLV16.BI=TRUE} =>  {BcOLV18=TRUE} | 0.18  3168 | 0.725  49 | 0.252  475 | 1.40  2383 | 74 |

| {BcOLV16.BI=TRUE, BcOLV18=TRUE} =>  {BcMV2.BI=TRUE} | 0.18  3168 | 0.678  899 | 0.269  802 | 1.46  6712 | 74 |
| --- | --- | --- | --- | --- | --- |
| {BcMV2.BI=TRUE, BcOLV18=TRUE} =>  {BcOLV16.BI=TRUE} | 0.18  3168 | 0.621  849 | 0.294  554 | 1.49  5398 | 74 |
| {BcMV2.BI=TRUE, BcOLV16.BI=TRUE} =>  {BcHV1SLR.BI=TRUE} | 0.17  5743 | 0.696  078 | 0.252  475 | 1.28  409 | 71 |
| {BcHV1SLR.BI=TRUE, BcOLV16.BI=TRUE}  => {BcMV2.BI=TRUE} | 0.17  5743 | 0.739  583 | 0.237  624 | 1.59  7816 | 71 |
| {BcHV1SLR.BI=TRUE, BcMV2.BI=TRUE} =>  {BcOLV16.BI=TRUE} | 0.17  5743 | 0.645  455 | 0.272  277 | 1.55  2165 | 71 |
| {BcMV2.BI=TRUE, BcOLV16.BI=TRUE} =>  {BcMV3.BI=TRUE} | 0.19  3069 | 0.764  706 | 0.252  475 | 1.41  069 | 78 |
| {BcMV3.BI=TRUE, BcOLV16.BI=TRUE} =>  {BcMV2.BI=TRUE} | 0.19  3069 | 0.722  222 | 0.267  327 | 1.56  0309 | 78 |
| {BcMV2.BI=TRUE, BcMV3.BI=TRUE} =>  {BcOLV16.BI=TRUE} | 0.19  3069 | 0.614  173 | 0.314  356 | 1.47  694 | 78 |
| {BcOLV16.BI=TRUE, BcULV3=TRUE} =>  {BcMV2.BI=TRUE} | 0.17  0792 | 0.610  619 | 0.279  703 | 1.31  9199 | 69 |
| {BcMV2.BI=TRUE, BcULV3=TRUE} =>  {BcOLV16.BI=TRUE} | 0.17  0792 | 0.6 | 0.284  653 | 1.44  2857 | 69 |
| {BcMV2.BI=TRUE, BcOLV16.BI=TRUE} =>  {BcHV1.BI=TRUE} | 0.17  8218 | 0.705  882 | 0.252  475 | 1.16  8756 | 72 |

| {BcHV1.BI=TRUE, BcOLV16.BI=TRUE} =>  {BcMV2.BI=TRUE} | 0.17  8218 | 0.660  55 | 0.269  802 | 1.42  7072 | 72 |
| --- | --- | --- | --- | --- | --- |
| {BcHV1.BI=TRUE, BcMV2.BI=TRUE} =>  {BcOLV16.BI=TRUE} | 0.17  8218 | 0.610  169 | 0.292  079 | 1.46  7312 | 72 |
| {BcMV2.BI=TRUE, BcOLV16.BI=TRUE} =>  {BcMV11=TRUE} | 0.19  5545 | 0.774  51 | 0.252  475 | 1.25  6634 | 79 |
| {BcMV11=TRUE, BcOLV16.BI=TRUE} =>  {BcMV2.BI=TRUE} | 0.19  5545 | 0.711  712 | 0.274  752 | 1.53  7602 | 79 |
| {BcMV11=TRUE, BcMV2.BI=TRUE} =>  {BcOLV16.BI=TRUE} | 0.19  5545 | 0.572  464 | 0.341  584 | 1.37  6639 | 79 |
| {BcMV2.BI=TRUE, BcOLV16.BI=TRUE} =>  {BcMV9.BI=TRUE} | 0.20  297 | 0.803  922 | 0.252  475 | 1.18  5344 | 82 |
| {BcMV9.BI=TRUE, BcOLV16.BI=TRUE} =>  {BcMV2.BI=TRUE} | 0.20  297 | 0.611  94 | 0.331  683 | 1.32  2053 | 82 |
| {BcMV2.BI=TRUE, BcMV9.BI=TRUE} =>  {BcOLV16.BI=TRUE} | 0.20  297 | 0.630  769 | 0.321  782 | 1.51  685 | 82 |
| {BcMV2.BI=TRUE, BcOLV16.BI=TRUE} =>  {BcULV4=TRUE} | 0.20  7921 | 0.823  529 | 0.252  475 | 1.19  2494 | 84 |
| {BcOLV16.BI=TRUE, BcULV4=TRUE} =>  {BcMV2.BI=TRUE} | 0.20  7921 | 0.613  139 | 0.339  109 | 1.32  4642 | 84 |
| {BcMV2.BI=TRUE, BcULV4=TRUE} =>  {BcOLV16.BI=TRUE} | 0.20  7921 | 0.608  696 | 0.341  584 | 1.46  3768 | 84 |

| {BcMV2.BI=TRUE, BcPV3.BI=FALSE} =>  {BcOLV16.BI=TRUE} | 0.14  8515 | 0.521  739 | 0.284  653 | 1.25  4658 | 60 |
| --- | --- | --- | --- | --- | --- |
| {BcEV2.BI=FALSE, BcOLV16.BI=TRUE} =>  {BcMV2.BI=TRUE} | 0.18  5644 | 0.568  182 | 0.326  733 | 1.22  7516 | 75 |
| {BcEV2.BI=FALSE, BcMV2.BI=TRUE} =>  {BcOLV16.BI=TRUE} | 0.18  5644 | 0.581  395 | 0.319  307 | 1.39  8117 | 75 |
| {BcHV3.BI=FALSE, BcOLV16.BI=TRUE} =>  {BcMV2.BI=TRUE} | 0.18  8119 | 0.584  615 | 0.321  782 | 1.26  3019 | 76 |
| {BcHV3.BI=FALSE, BcMV2.BI=TRUE} =>  {BcOLV16.BI=TRUE} | 0.18  8119 | 0.571  429 | 0.329  208 | 1.37  415 | 76 |
| {BcFV5.BI=FALSE, BcOLV16.BI=TRUE} =>  {BcMV2.BI=TRUE} | 0.18  3168 | 0.569  231 | 0.321  782 | 1.22  9782 | 74 |
| {BcFV5.BI=FALSE, BcMV2.BI=TRUE} =>  {BcOLV16.BI=TRUE} | 0.18  3168 | 0.532  374 | 0.344  059 | 1.28  0233 | 74 |
| {BcMV1.BI=TRUE, BcOLV16.BI=TRUE} =>  {BcMV2.BI=TRUE} | 0.22  0297 | 0.654  412 | 0.336  634 | 1.41  3809 | 89 |
| {BcMV1.BI=TRUE, BcMV2.BI=TRUE} =>  {BcOLV16.BI=TRUE} | 0.22  0297 | 0.539  394 | 0.408  416 | 1.29  7114 | 89 |
| {BcNSRV7.BI=FALSE, BcOLV16.BI=TRUE}  => {BcMV2.BI=TRUE} | 0.20  0495 | 0.6 | 0.334  158 | 1.29  6257 | 81 |
| {BcMV2.BI=TRUE, BcNSRV7.BI=FALSE} =>  {BcOLV16.BI=TRUE} | 0.20  0495 | 0.554  795 | 0.361  386 | 1.33  4149 | 81 |

| {BcOLV16.BI=TRUE, BcVV3.BI=FALSE} =>  {BcMV2.BI=TRUE} | 0.20  5446 | 0.610  294 | 0.336  634 | 1.31  8496 | 83 |
| --- | --- | --- | --- | --- | --- |
| {BcMV2.BI=TRUE, BcVV3.BI=FALSE} =>  {BcOLV16.BI=TRUE} | 0.20  5446 | 0.584  507 | 0.351  485 | 1.40  56 | 83 |
| {BcOLV16.BI=TRUE, BcOLV7.BI=FALSE} =>  {BcMV2.BI=TRUE} | 0.17  0792 | 0.579  832 | 0.294  554 | 1.25  2685 | 69 |
| {BcALV1.BI=FALSE, BcMV2.BI=TRUE} =>  {BcOLV16.BI=TRUE} | 0.16  3366 | 0.503  817 | 0.324  257 | 1.21  1559 | 66 |
| {BcDMV1=FALSE, BcOLV16.BI=TRUE} =>  {BcMV2.BI=TRUE} | 0.21  5347 | 0.608  392 | 0.353  96 | 1.31  4386 | 87 |
| {BcDMV1=FALSE, BcMV2.BI=TRUE} =>  {BcOLV16.BI=TRUE} | 0.21  5347 | 0.543  75 | 0.396  04 | 1.30  7589 | 87 |
| {BcFV9=FALSE, BcOLV16.BI=TRUE} =>  {BcMV2.BI=TRUE} | 0.21  2871 | 0.593  103 | 0.358  911 | 1.28  1357 | 86 |
| {BcFV9=FALSE, BcMV2.BI=TRUE} =>  {BcOLV16.BI=TRUE} | 0.21  2871 | 0.530  864 | 0.400  99 | 1.27  6602 | 86 |
| {BcOLV16.BI=TRUE, BcOLV4.BI=FALSE} =>  {BcMV2.BI=TRUE} | 0.19  0594 | 0.553  957 | 0.344  059 | 1.19  6784 | 77 |
| {BcMV2.BI=TRUE, BcOLV4.BI=FALSE} =>  {BcOLV16.BI=TRUE} | 0.19  0594 | 0.506  579 | 0.376  238 | 1.21  8202 | 77 |
| {BcOLV16.BI=TRUE, BcULV5=FALSE} =>  {BcMV2.BI=TRUE} | 0.20  297 | 0.577  465 | 0.351  485 | 1.24  7571 | 82 |

| {BcMV2.BI=TRUE, BcULV5=FALSE} =>  {BcOLV16.BI=TRUE} | 0.20  297 | 0.539  474 | 0.376  238 | 1.29  7306 | 82 |
| --- | --- | --- | --- | --- | --- |
| {BcBNV5.BI=FALSE, BcOLV16.BI=TRUE} =>  {BcMV2.BI=TRUE} | 0.21  5347 | 0.576  159 | 0.373  762 | 1.24  475 | 87 |
| {BcBNV5.BI=FALSE, BcMV2.BI=TRUE} =>  {BcOLV16.BI=TRUE} | 0.21  5347 | 0.527  273 | 0.408  416 | 1.26  7965 | 87 |
| {BcMV2.BI=TRUE, BcOLV16.BI=TRUE} =>  {BvF.BI=FALSE} | 0.24  7525 | 0.980  392 | 0.252  475 | 1.07  3383 | 10  0 |
| {BcOLV16.BI=TRUE, BvF.BI=FALSE} =>  {BcMV2.BI=TRUE} | 0.24  7525 | 0.632  911 | 0.391  089 | 1.36  7359 | 10  0 |
| {BcMV2.BI=TRUE, BvF.BI=FALSE} =>  {BcOLV16.BI=TRUE} | 0.24  7525 | 0.571  429 | 0.433  168 | 1.37  415 | 10  0 |
| {BcMCV5.BI=FALSE, BcOLV16.BI=TRUE} =>  {BcMV2.BI=TRUE} | 0.20  7921 | 0.567  568 | 0.366  337 | 1.22  6189 | 84 |
| {BcMCV5.BI=FALSE, BcMV2.BI=TRUE} =>  {BcOLV16.BI=TRUE} | 0.20  7921 | 0.518  519 | 0.400  99 | 1.24  6914 | 84 |
| {BcOLV13.BI=FALSE, BcOLV16.BI=TRUE} =>  {BcMV2.BI=TRUE} | 0.21  2871 | 0.581  081 | 0.366  337 | 1.25  5384 | 86 |
| {BcMV2.BI=TRUE, BcOLV13.BI=FALSE} =>  {BcOLV16.BI=TRUE} | 0.21  2871 | 0.521  212 | 0.408  416 | 1.25  3391 | 86 |
| {BcOLV16.BI=TRUE, BcOLV2.BI=FALSE} =>  {BcMV2.BI=TRUE} | 0.21  7822 | 0.590  604 | 0.368  812 | 1.27  5957 | 88 |

| {BcMV2.BI=TRUE, BcOLV2.BI=FALSE} =>  {BcOLV16.BI=TRUE} | 0.21  7822 | 0.517  647 | 0.420  792 | 1.24  4818 | 88 |
| --- | --- | --- | --- | --- | --- |
| {BcMCV3.BI=FALSE, BcOLV16.BI=TRUE} =>  {BcMV2.BI=TRUE} | 0.20  297 | 0.565  517 | 0.358  911 | 1.22  1759 | 82 |
| {BcMCV3.BI=FALSE, BcMV2.BI=TRUE} =>  {BcOLV16.BI=TRUE} | 0.20  297 | 0.496  97 | 0.408  416 | 1.19  5094 | 82 |
| {BcOLV16.BI=TRUE, BcPV4.BI=FALSE} =>  {BcMV2.BI=TRUE} | 0.22  5248 | 0.594  771 | 0.378  713 | 1.28  496 | 91 |
| {BcMV2.BI=TRUE, BcPV4.BI=FALSE} =>  {BcOLV16.BI=TRUE} | 0.22  5248 | 0.526  012 | 0.428  218 | 1.26  4933 | 91 |
| {BcEV6=FALSE, BcOLV16.BI=TRUE} =>  {BcMV2.BI=TRUE} | 0.23  5149 | 0.601  266 | 0.391  089 | 1.29  8991 | 95 |
| {BcEV6=FALSE, BcMV2.BI=TRUE} =>  {BcOLV16.BI=TRUE} | 0.23  5149 | 0.542  857 | 0.433  168 | 1.30  5442 | 95 |
| {BcOLV16.BI=TRUE, BcOLV17.BI=FALSE} =>  {BcMV2.BI=TRUE} | 0.23  0198 | 0.596  154 | 0.386  139 | 1.28  7947 | 93 |
| {BcMV2.BI=TRUE, BcOLV17.BI=FALSE} =>  {BcOLV16.BI=TRUE} | 0.23  0198 | 0.540  698 | 0.425  743 | 1.30  0249 | 93 |
| {BcOLV15.BI=FALSE, BcOLV16.BI=TRUE} =>  {BcMV2.BI=TRUE} | 0.24  505 | 0.607  362 | 0.403  465 | 1.31  2162 | 99 |
| {BcMV2.BI=TRUE, BcOLV15.BI=FALSE} =>  {BcOLV16.BI=TRUE} | 0.24  505 | 0.540  984 | 0.452  97 | 1.30  0937 | 99 |

| {BcEV5=FALSE, BcOLV16.BI=TRUE} =>  {BcMV2.BI=TRUE} | 0.23  0198 | 0.584  906 | 0.393  564 | 1.26  3646 | 93 |
| --- | --- | --- | --- | --- | --- |
| {BcEV5=FALSE, BcMV2.BI=TRUE} =>  {BcOLV16.BI=TRUE} | 0.23  0198 | 0.543  86 | 0.423  267 | 1.30  7853 | 93 |
| {BcOLV16.BI=TRUE, BcOLV18=FALSE} =>  {BcEV2.BI=FALSE} | 0.12  8713 | 0.881  356 | 0.146  04 | 1.17  9032 | 52 |
| {BcOLV16.BI=TRUE, BcOLV18=FALSE} =>  {BcHV3.BI=FALSE} | 0.13  1188 | 0.898  305 | 0.146  04 | 1.18  2134 | 53 |
| {BcOLV16.BI=TRUE, BcOLV18=FALSE} =>  {BcFV5.BI=FALSE} | 0.13  1188 | 0.898  305 | 0.146  04 | 1.14  8466 | 53 |
| {BcOLV16.BI=TRUE, BcOLV18=FALSE} =>  {BcFV9=FALSE} | 0.13  8614 | 0.949  153 | 0.146  04 | 1.09  2472 | 56 |
| {BcOLV16.BI=TRUE, BcOLV18=FALSE} =>  {BcMCV5.BI=FALSE} | 0.14  604 | 1 | 0.146  04 | 1.08  311 | 59 |
| {BcOLV16.BI=TRUE, BcOLV18=TRUE} =>  {BcHV1SLR.BI=TRUE} | 0.17  5743 | 0.651  376 | 0.269  802 | 1.20  1625 | 71 |
| {BcHV1SLR.BI=TRUE, BcOLV16.BI=TRUE}  => {BcOLV18=TRUE} | 0.17  5743 | 0.739  583 | 0.237  624 | 1.42  9625 | 71 |
| {BcHV1SLR.BI=TRUE, BcOLV18=TRUE} =>  {BcOLV16.BI=TRUE} | 0.17  5743 | 0.514  493 | 0.341  584 | 1.23  7233 | 71 |
| {BcOLV16.BI=TRUE, BcOLV18=TRUE} =>  {BcMV3.BI=TRUE} | 0.19  3069 | 0.715  596 | 0.269  802 | 1.32  0096 | 78 |

| {BcMV3.BI=TRUE, BcOLV16.BI=TRUE} =>  {BcOLV18=TRUE} | 0.19  3069 | 0.722  222 | 0.267  327 | 1.39  6066 | 78 |
| --- | --- | --- | --- | --- | --- |
| {BcMV3.BI=TRUE, BcOLV18=TRUE} =>  {BcOLV16.BI=TRUE} | 0.19  3069 | 0.586  466 | 0.329  208 | 1.41  0311 | 78 |
| {BcOLV16.BI=TRUE, BcULV3=TRUE} =>  {BcOLV18=TRUE} | 0.18  0693 | 0.646  018 | 0.279  703 | 1.24  8761 | 73 |
| {BcOLV18=TRUE, BcULV3=TRUE} =>  {BcOLV16.BI=TRUE} | 0.18  0693 | 0.557  252 | 0.324  257 | 1.34  0058 | 73 |
| {BcOLV16.BI=TRUE, BcOLV18=TRUE} =>  {BcHV1.BI=TRUE} | 0.18  8119 | 0.697  248 | 0.269  802 | 1.15  4459 | 76 |
| {BcHV1.BI=TRUE, BcOLV16.BI=TRUE} =>  {BcOLV18=TRUE} | 0.18  8119 | 0.697  248 | 0.269  802 | 1.34  779 | 76 |
| {BcHV1.BI=TRUE, BcOLV18=TRUE} =>  {BcOLV16.BI=TRUE} | 0.18  8119 | 0.513  514 | 0.366  337 | 1.23  4878 | 76 |
| {BcOLV16.BI=TRUE, BcOLV18=TRUE} =>  {BcMV11=TRUE} | 0.19  0594 | 0.706  422 | 0.269  802 | 1.14  6163 | 77 |
| {BcMV11=TRUE, BcOLV16.BI=TRUE} =>  {BcOLV18=TRUE} | 0.19  0594 | 0.693  694 | 0.274  752 | 1.34  092 | 77 |
| {BcMV11=TRUE, BcOLV18=TRUE} =>  {BcOLV16.BI=TRUE} | 0.19  0594 | 0.55 | 0.346  535 | 1.32  2619 | 77 |
| {BcOLV16.BI=TRUE, BcOLV18=TRUE} =>  {BcMV9.BI=TRUE} | 0.24  2574 | 0.899  083 | 0.269  802 | 1.32  5655 | 98 |

| {BcMV9.BI=TRUE, BcOLV16.BI=TRUE} =>  {BcOLV18=TRUE} | 0.24  2574 | 0.731  343 | 0.331  683 | 1.41  3697 | 98 |
| --- | --- | --- | --- | --- | --- |
| {BcMV9.BI=TRUE, BcOLV18=TRUE} =>  {BcOLV16.BI=TRUE} | 0.24  2574 | 0.586  826 | 0.413  366 | 1.41  1178 | 98 |
| {BcOLV16.BI=TRUE, BcOLV18=TRUE} =>  {BcULV4=TRUE} | 0.24  505 | 0.908  257 | 0.269  802 | 1.31  5182 | 99 |
| {BcOLV16.BI=TRUE, BcULV4=TRUE} =>  {BcOLV18=TRUE} | 0.24  505 | 0.722  628 | 0.339  109 | 1.39  685 | 99 |
| {BcOLV18=TRUE, BcULV4=TRUE} =>  {BcOLV16.BI=TRUE} | 0.24  505 | 0.556  18 | 0.440  594 | 1.33  748 | 99 |
| {BcMV6.BI=FALSE, BcOLV16.BI=TRUE} =>  {BcOLV18=TRUE} | 0.16  0891 | 0.643  564 | 0.25 | 1.24  4019 | 65 |
| {BcOLV16.BI=TRUE, BcPV3.BI=FALSE} =>  {BcOLV18=TRUE} | 0.17  0792 | 0.627  273 | 0.272  277 | 1.21  2527 | 69 |
| {BcOLV18=TRUE, BcPV3.BI=FALSE} =>  {BcOLV16.BI=TRUE} | 0.17  0792 | 0.518  797 | 0.329  208 | 1.24  7583 | 69 |
| {BcEV2.BI=FALSE, BcOLV18=TRUE} =>  {BcOLV16.BI=TRUE} | 0.19  802 | 0.536  913 | 0.368  812 | 1.29  1147 | 80 |
| {BcHV3.BI=FALSE, BcOLV18=TRUE} =>  {BcOLV16.BI=TRUE} | 0.19  0594 | 0.542  254 | 0.351  485 | 1.30  3991 | 77 |
| {BcFV5.BI=FALSE, BcOLV18=TRUE} =>  {BcOLV16.BI=TRUE} | 0.19  0594 | 0.516  779 | 0.368  812 | 1.24  2729 | 77 |

| {BcOLV16.BI=TRUE, BcOLV18=TRUE} =>  {BcMV1.BI=TRUE} | 0.24  0099 | 0.889  908 | 0.269  802 | 1.12  7031 | 97 |
| --- | --- | --- | --- | --- | --- |
| {BcMV1.BI=TRUE, BcOLV16.BI=TRUE} =>  {BcOLV18=TRUE} | 0.24  0099 | 0.713  235 | 0.336  634 | 1.37  8694 | 97 |
| {BcMV1.BI=TRUE, BcOLV18=TRUE} =>  {BcOLV16.BI=TRUE} | 0.24  0099 | 0.544  944 | 0.440  594 | 1.31  046 | 97 |
| {BcMV12=FALSE, BcOLV16.BI=TRUE} =>  {BcOLV18=TRUE} | 0.19  0594 | 0.647  059 | 0.294  554 | 1.25  0774 | 77 |
| {BcNSRV7.BI=FALSE, BcOLV16.BI=TRUE}  => {BcOLV18=TRUE} | 0.21  0396 | 0.629  63 | 0.334  158 | 1.21  7083 | 85 |
| {BcNSRV7.BI=FALSE, BcOLV18=TRUE} =>  {BcOLV16.BI=TRUE} | 0.21  0396 | 0.541  401 | 0.388  614 | 1.30  1941 | 85 |
| {BcOLV16.BI=TRUE, BcVV3.BI=FALSE} =>  {BcOLV18=TRUE} | 0.21  7822 | 0.647  059 | 0.336  634 | 1.25  0774 | 88 |
| {BcOLV18=TRUE, BcVV3.BI=FALSE} =>  {BcOLV16.BI=TRUE} | 0.21  7822 | 0.530  12 | 0.410  891 | 1.27  4814 | 88 |
| {BcDMV1=FALSE, BcOLV16.BI=TRUE} =>  {BcOLV18=TRUE} | 0.22  7723 | 0.643  357 | 0.353  96 | 1.24  3618 | 92 |
| {BcDMV1=FALSE, BcOLV18=TRUE} =>  {BcOLV16.BI=TRUE} | 0.22  7723 | 0.531  792 | 0.428  218 | 1.27  8833 | 92 |
| {BcFV9=FALSE, BcOLV16.BI=TRUE} =>  {BcOLV18=TRUE} | 0.22  0297 | 0.613  793 | 0.358  911 | 1.18  6471 | 89 |

| {BcFV9=FALSE, BcOLV18=TRUE} =>  {BcOLV16.BI=TRUE} | 0.22  0297 | 0.523  529 | 0.420  792 | 1.25  8964 | 89 |
| --- | --- | --- | --- | --- | --- |
| {BcOLV16.BI=TRUE, BcOLV4.BI=FALSE} =>  {BcOLV18=TRUE} | 0.22  2772 | 0.647  482 | 0.344  059 | 1.25  1592 | 90 |
| {BcOLV18=TRUE, BcOLV4.BI=FALSE} =>  {BcOLV16.BI=TRUE} | 0.22  2772 | 0.497  238 | 0.448  02 | 1.19  5738 | 90 |
| {BcOLV16.BI=TRUE, BcULV5=FALSE} =>  {BcOLV18=TRUE} | 0.24  0099 | 0.683  099 | 0.351  485 | 1.32  0439 | 97 |
| {BcOLV18=TRUE, BcULV5=FALSE} =>  {BcOLV16.BI=TRUE} | 0.24  0099 | 0.513  228 | 0.467  822 | 1.23  419 | 97 |
| {BcBNV5.BI=FALSE, BcOLV16.BI=TRUE} =>  {BcOLV18=TRUE} | 0.23  5149 | 0.629  139 | 0.373  762 | 1.21  6135 | 95 |
| {BcBNV5.BI=FALSE, BcOLV18=TRUE} =>  {BcOLV16.BI=TRUE} | 0.23  5149 | 0.519  126 | 0.452  97 | 1.24  8374 | 95 |
| {BcOLV16.BI=TRUE, BvF.BI=FALSE} =>  {BcOLV18=TRUE} | 0.25  2475 | 0.645  57 | 0.391  089 | 1.24  7895 | 10  2 |
| {BcOLV18=TRUE, BvF.BI=FALSE} =>  {BcOLV16.BI=TRUE} | 0.25  2475 | 0.531  25 | 0.475  248 | 1.27  753 | 10  2 |
| {BcMCV5.BI=FALSE, BcOLV16.BI=TRUE} =>  {BcOLV18=TRUE} | 0.22  0297 | 0.601  351 | 0.366  337 | 1.16  2421 | 89 |
| {BcMCV5.BI=FALSE, BcOLV18=TRUE} =>  {BcOLV16.BI=TRUE} | 0.22  0297 | 0.489  011 | 0.450  495 | 1.17  5955 | 89 |

| {BcOLV13.BI=FALSE, BcOLV16.BI=TRUE} =>  {BcOLV18=TRUE} | 0.24  505 | 0.668  919 | 0.366  337 | 1.29  303 | 99 |
| --- | --- | --- | --- | --- | --- |
| {BcOLV13.BI=FALSE, BcOLV18=TRUE} =>  {BcOLV16.BI=TRUE} | 0.24  505 | 0.515  625 | 0.475  248 | 1.23  9955 | 99 |
| {BcOLV16.BI=TRUE, BcOLV2.BI=FALSE} =>  {BcOLV18=TRUE} | 0.24  0099 | 0.651  007 | 0.368  812 | 1.25  8405 | 97 |
| {BcOLV18=TRUE, BcOLV2.BI=FALSE} =>  {BcOLV16.BI=TRUE} | 0.24  0099 | 0.513  228 | 0.467  822 | 1.23  419 | 97 |
| {BcMCV3.BI=FALSE, BcOLV16.BI=TRUE} =>  {BcOLV18=TRUE} | 0.23  5149 | 0.655  172 | 0.358  911 | 1.26  6458 | 95 |
| {BcMCV3.BI=FALSE, BcOLV18=TRUE} =>  {BcOLV16.BI=TRUE} | 0.23  5149 | 0.494  792 | 0.475  248 | 1.18  9856 | 95 |
| {BcOLV16.BI=TRUE, BcPV4.BI=FALSE} =>  {BcOLV18=TRUE} | 0.24  2574 | 0.640  523 | 0.378  713 | 1.23  814 | 98 |
| {BcOLV18=TRUE, BcPV4.BI=FALSE} =>  {BcOLV16.BI=TRUE} | 0.24  2574 | 0.507  772 | 0.477  723 | 1.22  1071 | 98 |
| {BcEV6=FALSE, BcOLV16.BI=TRUE} =>  {BcOLV18=TRUE} | 0.25  7426 | 0.658  228 | 0.391  089 | 1.27  2364 | 10  4 |
| {BcEV6=FALSE, BcOLV18=TRUE} =>  {BcOLV16.BI=TRUE} | 0.25  7426 | 0.533  333 | 0.482  673 | 1.28  254 | 10  4 |
| {BcOLV16.BI=TRUE, BcOLV17.BI=FALSE} =>  {BcOLV18=TRUE} | 0.24  2574 | 0.628  205 | 0.386  139 | 1.21  433 | 98 |

| {BcOLV17.BI=FALSE, BcOLV18=TRUE} =>  {BcOLV16.BI=TRUE} | 0.24  2574 | 0.507  772 | 0.477  723 | 1.22  1071 | 98 |
| --- | --- | --- | --- | --- | --- |
| {BcOLV15.BI=FALSE, BcOLV16.BI=TRUE} =>  {BcOLV18=TRUE} | 0.25  7426 | 0.638  037 | 0.403  465 | 1.23  3334 | 10  4 |
| {BcOLV15.BI=FALSE, BcOLV18=TRUE} =>  {BcOLV16.BI=TRUE} | 0.25  7426 | 0.538  86 | 0.477  723 | 1.29  583 | 10  4 |
| {BcEV5=FALSE, BcOLV16.BI=TRUE} =>  {BcOLV18=TRUE} | 0.25 | 0.635  22 | 0.393  564 | 1.22  789 | 10  1 |
| {BcEV5=FALSE, BcOLV18=TRUE} =>  {BcOLV16.BI=TRUE} | 0.25 | 0.512  69 | 0.487  624 | 1.23  2898 | 10  1 |
| {BcMV2.BI=FALSE, BcOLV16.BI=TRUE} =>  {BcOLV1.BI.MOVA.=FALSE} | 0.12  8713 | 0.787  879 | 0.163  366 | 1.17  89 | 52 |
| {BcMV2.BI=FALSE, BcOLV16.BI=TRUE} =>  {BcMV9.BI=TRUE} | 0.12  8713 | 0.787  879 | 0.163  366 | 1.16  169 | 52 |
| {BcMV2.BI=FALSE, BcOLV16.BI=TRUE} =>  {BcULV4=TRUE} | 0.13  1188 | 0.803  03 | 0.163  366 | 1.16  2811 | 53 |
| {BcMV2.BI=FALSE, BcOLV16.BI=TRUE} =>  {BcEV2.BI=FALSE} | 0.14  1089 | 0.863  636 | 0.163  366 | 1.15  5328 | 57 |
| {BcMV2.BI=FALSE, BcOLV16.BI=TRUE} =>  {BcALV1.BI=FALSE} | 0.14  604 | 0.893  939 | 0.163  366 | 1.09  7725 | 59 |
| {BcHV1SLR.BI=TRUE, BcOLV16.BI=TRUE}  => {BcMV3.BI=TRUE} | 0.17  5743 | 0.739  583 | 0.237  624 | 1.36  4346 | 71 |

| {BcMV3.BI=TRUE, BcOLV16.BI=TRUE} =>  {BcHV1SLR.BI=TRUE} | 0.17  5743 | 0.657  407 | 0.267  327 | 1.21  2752 | 71 |
| --- | --- | --- | --- | --- | --- |
| {BcHV1SLR.BI=TRUE, BcMV3.BI=TRUE} =>  {BcOLV16.BI=TRUE} | 0.17  5743 | 0.533  835 | 0.329  208 | 1.28  3745 | 71 |
| {BcHV1SLR.BI=TRUE, BcOLV16.BI=TRUE}  => {BcULV3=TRUE} | 0.18  0693 | 0.760  417 | 0.237  624 | 1.28  0035 | 73 |
| {BcOLV16.BI=TRUE, BcULV3=TRUE} =>  {BcHV1SLR.BI=TRUE} | 0.18  0693 | 0.646  018 | 0.279  703 | 1.19  174 | 73 |
| {BcHV1SLR.BI=TRUE, BcOLV16.BI=TRUE}  => {BcHV1.BI=TRUE} | 0.22  0297 | 0.927  083 | 0.237  624 | 1.53  5007 | 89 |
| {BcHV1.BI=TRUE, BcOLV16.BI=TRUE} =>  {BcHV1SLR.BI=TRUE} | 0.22  0297 | 0.816  514 | 0.269  802 | 1.50  6263 | 89 |
| {BcHV1SLR.BI=TRUE, BcOLV16.BI=TRUE}  => {BcMV9.BI=TRUE} | 0.19  802 | 0.833  333 | 0.237  624 | 1.22  871 | 80 |
| {BcHV1SLR.BI=TRUE, BcOLV16.BI=TRUE}  => {BcULV4=TRUE} | 0.20  0495 | 0.843  75 | 0.237  624 | 1.22  1774 | 81 |
| {BcHV1SLR.BI=TRUE, BcULV4=TRUE} =>  {BcOLV16.BI=TRUE} | 0.20  0495 | 0.5 | 0.400  99 | 1.20  2381 | 81 |
| {BcMV3.BI=TRUE, BcOLV16.BI=TRUE} =>  {BcULV3=TRUE} | 0.19  5545 | 0.731  481 | 0.267  327 | 1.23  1327 | 79 |
| {BcOLV16.BI=TRUE, BcULV3=TRUE} =>  {BcMV3.BI=TRUE} | 0.19  5545 | 0.699  115 | 0.279  703 | 1.28  9692 | 79 |

| {BcMV3.BI=TRUE, BcULV3=TRUE} =>  {BcOLV16.BI=TRUE} | 0.19  5545 | 0.564  286 | 0.346  535 | 1.35  6973 | 79 |
| --- | --- | --- | --- | --- | --- |
| {BcMV3.BI=TRUE, BcOLV16.BI=TRUE} =>  {BcHV1.BI=TRUE} | 0.19  3069 | 0.722  222 | 0.267  327 | 1.19  5811 | 78 |
| {BcHV1.BI=TRUE, BcOLV16.BI=TRUE} =>  {BcMV3.BI=TRUE} | 0.19  3069 | 0.715  596 | 0.269  802 | 1.32  0096 | 78 |
| {BcHV1.BI=TRUE, BcMV3.BI=TRUE} =>  {BcOLV16.BI=TRUE} | 0.19  3069 | 0.545  455 | 0.353  96 | 1.31  1688 | 78 |
| {BcMV3.BI=TRUE, BcOLV16.BI=TRUE} =>  {BcMV11=TRUE} | 0.20  297 | 0.759  259 | 0.267  327 | 1.23  1891 | 82 |
| {BcMV11=TRUE, BcOLV16.BI=TRUE} =>  {BcMV3.BI=TRUE} | 0.20  297 | 0.738  739 | 0.274  752 | 1.36  2787 | 82 |
| {BcMV11=TRUE, BcMV3.BI=TRUE} =>  {BcOLV16.BI=TRUE} | 0.20  297 | 0.529  032 | 0.383  663 | 1.27  2197 | 82 |
| {BcMV3.BI=TRUE, BcOLV16.BI=TRUE} =>  {BcMV9.BI=TRUE} | 0.21  5347 | 0.805  556 | 0.267  327 | 1.18  7753 | 87 |
| {BcMV9.BI=TRUE, BcOLV16.BI=TRUE} =>  {BcMV3.BI=TRUE} | 0.21  5347 | 0.649  254 | 0.331  683 | 1.19  771 | 87 |
| {BcMV3.BI=TRUE, BcMV9.BI=TRUE} =>  {BcOLV16.BI=TRUE} | 0.21  5347 | 0.543  75 | 0.396  04 | 1.30  7589 | 87 |
| {BcMV3.BI=TRUE, BcOLV16.BI=TRUE} =>  {BcULV4=TRUE} | 0.22  0297 | 0.824  074 | 0.267  327 | 1.19  3283 | 89 |

| {BcOLV16.BI=TRUE, BcULV4=TRUE} =>  {BcMV3.BI=TRUE} | 0.22  0297 | 0.649  635 | 0.339  109 | 1.19  8413 | 89 |
| --- | --- | --- | --- | --- | --- |
| {BcMV3.BI=TRUE, BcULV4=TRUE} =>  {BcOLV16.BI=TRUE} | 0.22  0297 | 0.549  383 | 0.400  99 | 1.32  1135 | 89 |
| {BcHV3.BI=FALSE, BcMV3.BI=TRUE} =>  {BcOLV16.BI=TRUE} | 0.19  802 | 0.503  145 | 0.393  564 | 1.20  9943 | 80 |
| {BcFV5.BI=FALSE, BcMV3.BI=TRUE} =>  {BcOLV16.BI=TRUE} | 0.20  0495 | 0.515  924 | 0.388  614 | 1.24  0673 | 81 |
| {BcMV3.BI=TRUE, BcOLV16.BI=TRUE} =>  {BcMV1.BI=TRUE} | 0.23  7624 | 0.888  889 | 0.267  327 | 1.12  574 | 96 |
| {BcMV1.BI=TRUE, BcOLV16.BI=TRUE} =>  {BcMV3.BI=TRUE} | 0.23  7624 | 0.705  882 | 0.336  634 | 1.30  2176 | 96 |
| {BcMV1.BI=TRUE, BcMV3.BI=TRUE} =>  {BcOLV16.BI=TRUE} | 0.23  7624 | 0.518  919 | 0.457  921 | 1.24  7876 | 96 |
| {BcNSRV7.BI=FALSE, BcOLV16.BI=TRUE}  => {BcMV3.BI=TRUE} | 0.22  0297 | 0.659  259 | 0.334  158 | 1.21  6168 | 89 |
| {BcMV3.BI=TRUE, BcNSRV7.BI=FALSE} =>  {BcOLV16.BI=TRUE} | 0.22  0297 | 0.520  468 | 0.423  267 | 1.25  1601 | 89 |
| {BcMV3.BI=TRUE, BcVV3.BI=FALSE} =>  {BcOLV16.BI=TRUE} | 0.20  7921 | 0.5 | 0.415  842 | 1.20  2381 | 84 |
| {BcDMV1=FALSE, BcMV3.BI=TRUE} =>  {BcOLV16.BI=TRUE} | 0.22  0297 | 0.520  468 | 0.423  267 | 1.25  1601 | 89 |

| {BcFV9=FALSE, BcOLV16.BI=TRUE} =>  {BcMV3.BI=TRUE} | 0.23  2673 | 0.648  276 | 0.358  911 | 1.19  5906 | 94 |
| --- | --- | --- | --- | --- | --- |
| {BcFV9=FALSE, BcMV3.BI=TRUE} =>  {BcOLV16.BI=TRUE} | 0.23  2673 | 0.510  87 | 0.455  446 | 1.22  852 | 94 |
| {BcBNV5.BI=FALSE, BcOLV16.BI=TRUE} =>  {BcMV3.BI=TRUE} | 0.23  5149 | 0.629  139 | 0.373  762 | 1.16  0604 | 95 |
| {BcBNV5.BI=FALSE, BcMV3.BI=TRUE} =>  {BcOLV16.BI=TRUE} | 0.23  5149 | 0.5 | 0.470  297 | 1.20  2381 | 95 |
| {BcOLV16.BI=TRUE, BvF.BI=FALSE} =>  {BcMV3.BI=TRUE} | 0.25  9901 | 0.664  557 | 0.391  089 | 1.22  5941 | 10  5 |
| {BcMV3.BI=TRUE, BvF.BI=FALSE} =>  {BcOLV16.BI=TRUE} | 0.25  9901 | 0.525 | 0.495  05 | 1.26  25 | 10  5 |
| {BcEV6=FALSE, BcOLV16.BI=TRUE} =>  {BcMV3.BI=TRUE} | 0.25  7426 | 0.658  228 | 0.391  089 | 1.21  4265 | 10  4 |
| {BcEV6=FALSE, BcMV3.BI=TRUE} =>  {BcOLV16.BI=TRUE} | 0.25  7426 | 0.497  608 | 0.517  327 | 1.19  6628 | 10  4 |
| {BcOLV16.BI=TRUE, BcOLV17.BI=FALSE} =>  {BcMV3.BI=TRUE} | 0.24  2574 | 0.628  205 | 0.386  139 | 1.15  8881 | 98 |
| {BcOLV15.BI=FALSE, BcOLV16.BI=TRUE} =>  {BcMV3.BI=TRUE} | 0.25  7426 | 0.638  037 | 0.403  465 | 1.17  7018 | 10  4 |
| {BcMV3.BI=TRUE, BcOLV15.BI=FALSE} =>  {BcOLV16.BI=TRUE} | 0.25  7426 | 0.507  317 | 0.507  426 | 1.21  9977 | 10  4 |

| {BcEV5=FALSE, BcMV3.BI=TRUE} =>  {BcOLV16.BI=TRUE} | 0.24  505 | 0.495 | 0.495  05 | 1.19  0357 | 99 |
| --- | --- | --- | --- | --- | --- |
| {BcMV4.BI=FALSE, BcOLV16.BI=TRUE} =>  {BcMV9.BI=TRUE} | 0.15  3465 | 0.805  195 | 0.190  594 | 1.18  7222 | 62 |
| {BcMV4.BI=FALSE, BcOLV16.BI=TRUE} =>  {BcULV4=TRUE} | 0.16  5842 | 0.870  13 | 0.190  594 | 1.25  9973 | 67 |
| {BcMV4.BI=FALSE, BcOLV16.BI=TRUE} =>  {BcHV3.BI=FALSE} | 0.17  0792 | 0.896  104 | 0.190  594 | 1.17  9238 | 69 |
| {BcOLV16.BI=TRUE, BcULV3=TRUE} =>  {BcHV1.BI=TRUE} | 0.20  0495 | 0.716  814 | 0.279  703 | 1.18  6856 | 81 |
| {BcHV1.BI=TRUE, BcOLV16.BI=TRUE} =>  {BcULV3=TRUE} | 0.20  0495 | 0.743  119 | 0.269  802 | 1.25  0917 | 81 |
| {BcOLV16.BI=TRUE, BcULV3=TRUE} =>  {BcMV11=TRUE} | 0.20  5446 | 0.734  513 | 0.279  703 | 1.19  174 | 83 |
| {BcMV11=TRUE, BcOLV16.BI=TRUE} =>  {BcULV3=TRUE} | 0.20  5446 | 0.747  748 | 0.274  752 | 1.25  8709 | 83 |
| {BcMV11=TRUE, BcULV3=TRUE} =>  {BcOLV16.BI=TRUE} | 0.20  5446 | 0.518  75 | 0.396  04 | 1.24  747 | 83 |
| {BcOLV16.BI=TRUE, BcULV3=TRUE} =>  {BcMV9.BI=TRUE} | 0.21  7822 | 0.778  761 | 0.279  703 | 1.14  8246 | 88 |
| {BcMV9.BI=TRUE, BcULV3=TRUE} =>  {BcOLV16.BI=TRUE} | 0.21  7822 | 0.514  62 | 0.423  267 | 1.23  7538 | 88 |

| {BcOLV16.BI=TRUE, BcULV3=TRUE} =>  {BcULV4=TRUE} | 0.24  0099 | 0.858  407 | 0.279  703 | 1.24  2998 | 97 |
| --- | --- | --- | --- | --- | --- |
| {BcOLV16.BI=TRUE, BcULV4=TRUE} =>  {BcULV3=TRUE} | 0.24  0099 | 0.708  029 | 0.339  109 | 1.19  1849 | 97 |
| {BcULV3=TRUE, BcULV4=TRUE} =>  {BcOLV16.BI=TRUE} | 0.24  0099 | 0.577  381 | 0.415  842 | 1.38  8464 | 97 |
| {BcEV2.BI=FALSE, BcULV3=TRUE} =>  {BcOLV16.BI=TRUE} | 0.21  7822 | 0.5 | 0.435  644 | 1.20  2381 | 88 |
| {BcMV1.BI=TRUE, BcOLV16.BI=TRUE} =>  {BcULV3=TRUE} | 0.23  7624 | 0.705  882 | 0.336  634 | 1.18  8235 | 96 |
| {BcMV1.BI=TRUE, BcULV3=TRUE} =>  {BcOLV16.BI=TRUE} | 0.23  7624 | 0.489  796 | 0.485  149 | 1.17  7843 | 96 |
| {BcNSRV7.BI=FALSE, BcULV3=TRUE} =>  {BcOLV16.BI=TRUE} | 0.21  5347 | 0.491  525 | 0.438  119 | 1.18  2002 | 87 |
| {BcOLV16.BI=TRUE, BcVV3.BI=FALSE} =>  {BcULV3=TRUE} | 0.23  0198 | 0.683  824 | 0.336  634 | 1.15  1103 | 93 |
| {BcFV9=FALSE, BcOLV16.BI=TRUE} =>  {BcULV3=TRUE} | 0.24  505 | 0.682  759 | 0.358  911 | 1.14  931 | 99 |
| {BcOLV16.BI=TRUE, BvF.BI=FALSE} =>  {BcULV3=TRUE} | 0.26  9802 | 0.689  873 | 0.391  089 | 1.16  1287 | 10  9 |
| {BcULV3=TRUE, BvF.BI=FALSE} =>  {BcOLV16.BI=TRUE} | 0.26  9802 | 0.488  789 | 0.551  98 | 1.17  5422 | 10  9 |

| {BcOLV16.BI=TRUE, BcPV4.BI=FALSE} =>  {BcULV3=TRUE} | 0.25  495 | 0.673  203 | 0.378  713 | 1.13  3224 | 10  3 |
| --- | --- | --- | --- | --- | --- |
| {BcEV6=FALSE, BcOLV16.BI=TRUE} =>  {BcULV3=TRUE} | 0.26  7327 | 0.683  544 | 0.391  089 | 1.15  0633 | 10  8 |
| {BcOLV15.BI=FALSE, BcULV3=TRUE} =>  {BcOLV16.BI=TRUE} | 0.26  9802 | 0.484  444 | 0.556  931 | 1.16  4974 | 10  9 |
| {BcHV1.BI=TRUE, BcOLV16.BI=TRUE} =>  {BcMV9.BI=TRUE} | 0.22  2772 | 0.825  688 | 0.269  802 | 1.21  7438 | 90 |
| {BcHV1.BI=TRUE, BcOLV16.BI=TRUE} =>  {BcULV4=TRUE} | 0.23  0198 | 0.853  211 | 0.269  802 | 1.23  5474 | 93 |
| {BcHV1.BI=TRUE, BcULV4=TRUE} =>  {BcOLV16.BI=TRUE} | 0.23  0198 | 0.505  435 | 0.455  446 | 1.21  545 | 93 |
| {BcDMV1=FALSE, BcOLV16.BI=TRUE} =>  {BcHV1.BI=TRUE} | 0.24  2574 | 0.685  315 | 0.353  96 | 1.13  4701 | 98 |
| {BcMV11=TRUE, BcOLV16.BI=TRUE} =>  {BcMV9.BI=TRUE} | 0.21  2871 | 0.774  775 | 0.274  752 | 1.14  2369 | 86 |
| {BcMV11=TRUE, BcMV9.BI=TRUE} =>  {BcOLV16.BI=TRUE} | 0.21  2871 | 0.524  39 | 0.405  941 | 1.26  1034 | 86 |
| {BcMV11=TRUE, BcOLV16.BI=TRUE} =>  {BcULV4=TRUE} | 0.23  0198 | 0.837  838 | 0.274  752 | 1.21  3213 | 93 |
| {BcMV11=TRUE, BcULV4=TRUE} =>  {BcOLV16.BI=TRUE} | 0.23  0198 | 0.550  296 | 0.418  317 | 1.32  3331 | 93 |

| {BcOLV1.BI.MOVA.=FALSE,  BcOLV16.BI=TRUE} => {BcMV9.BI=TRUE} | 0.19  3069 | 0.857  143 | 0.225  248 | 1.26  3816 | 78 |
| --- | --- | --- | --- | --- | --- |
| {BcOLV1.BI.MOVA.=FALSE,  BcOLV16.BI=TRUE} => {BcULV4=TRUE} | 0.19  3069 | 0.857  143 | 0.225  248 | 1.24  1167 | 78 |
| {BcMV9.BI=TRUE, BcOLV16.BI=TRUE} =>  {BcULV4=TRUE} | 0.29  9505 | 0.902  985 | 0.331  683 | 1.30  7548 | 12  1 |
| {BcOLV16.BI=TRUE, BcULV4=TRUE} =>  {BcMV9.BI=TRUE} | 0.29  9505 | 0.883  212 | 0.339  109 | 1.30  2254 | 12  1 |
| {BcMV9.BI=TRUE, BcULV4=TRUE} =>  {BcOLV16.BI=TRUE} | 0.29  9505 | 0.547  511 | 0.547  03 | 1.31  6634 | 12  1 |
| {BcMV6.BI=FALSE, BcOLV16.BI=TRUE} =>  {BcMV9.BI=TRUE} | 0.19  5545 | 0.782  178 | 0.25 | 1.15  3285 | 79 |
| {BcOLV16.BI=TRUE, BcPV3.BI=FALSE} =>  {BcMV9.BI=TRUE} | 0.22  5248 | 0.827  273 | 0.272  277 | 1.21  9774 | 91 |
| {BcEV2.BI=FALSE, BcOLV16.BI=TRUE} =>  {BcMV9.BI=TRUE} | 0.26  2376 | 0.803  03 | 0.326  733 | 1.18  403 | 10  6 |
| {BcEV2.BI=FALSE, BcMV9.BI=TRUE} =>  {BcOLV16.BI=TRUE} | 0.26  2376 | 0.519  608 | 0.504  95 | 1.24  9533 | 10  6 |
| {BcHV3.BI=FALSE, BcOLV16.BI=TRUE} =>  {BcMV9.BI=TRUE} | 0.25  9901 | 0.807  692 | 0.321  782 | 1.19  0904 | 10  5 |
| {BcHV3.BI=FALSE, BcMV9.BI=TRUE} =>  {BcOLV16.BI=TRUE} | 0.25  9901 | 0.514  706 | 0.504  95 | 1.23  7745 | 10  5 |

| {BcFV5.BI=FALSE, BcOLV16.BI=TRUE} =>  {BcMV9.BI=TRUE} | 0.25  2475 | 0.784  615 | 0.321  782 | 1.15  6878 | 10  2 |
| --- | --- | --- | --- | --- | --- |
| {BcFV5.BI=FALSE, BcMV9.BI=TRUE} =>  {BcOLV16.BI=TRUE} | 0.25  2475 | 0.492  754 | 0.512  376 | 1.18  4955 | 10  2 |
| {BcOLV16.BI=TRUE, BcULV1.BI=FALSE} =>  {BcMV9.BI=TRUE} | 0.21  0396 | 0.779  817 | 0.269  802 | 1.14  9802 | 85 |
| {BcMV1.BI=TRUE, BcOLV16.BI=TRUE} =>  {BcMV9.BI=TRUE} | 0.27  2277 | 0.808  824 | 0.336  634 | 1.19  2572 | 11  0 |
| {BcMV1.BI=TRUE, BcMV9.BI=TRUE} =>  {BcOLV16.BI=TRUE} | 0.27  2277 | 0.495  495 | 0.549  505 | 1.19  1549 | 11  0 |
| {BcMV12=FALSE, BcOLV16.BI=TRUE} =>  {BcMV9.BI=TRUE} | 0.24  505 | 0.831  933 | 0.294  554 | 1.22  6645 | 99 |
| {BcNSRV7.BI=FALSE, BcOLV16.BI=TRUE}  => {BcMV9.BI=TRUE} | 0.25  9901 | 0.777  778 | 0.334  158 | 1.14  6796 | 10  5 |
| {BcMV9.BI=TRUE, BcNSRV7.BI=FALSE} =>  {BcOLV16.BI=TRUE} | 0.25  9901 | 0.502  392 | 0.517  327 | 1.20  8134 | 10  5 |
| {BcOLV16.BI=TRUE, BcVV3.BI=FALSE} =>  {BcMV9.BI=TRUE} | 0.27  9703 | 0.830  882 | 0.336  634 | 1.22  5097 | 11  3 |
| {BcMV9.BI=TRUE, BcVV3.BI=FALSE} =>  {BcOLV16.BI=TRUE} | 0.27  9703 | 0.504  464 | 0.554  455 | 1.21  3116 | 11  3 |
| {BcALV1.BI=FALSE, BcOLV16.BI=TRUE} =>  {BcMV9.BI=TRUE} | 0.23  7624 | 0.768 | 0.309  406 | 1.13  238 | 96 |

| {BcDMV1=FALSE, BcOLV16.BI=TRUE} =>  {BcMV9.BI=TRUE} | 0.27  2277 | 0.769  231 | 0.353  96 | 1.13  4194 | 11  0 |
| --- | --- | --- | --- | --- | --- |
| {BcDMV1=FALSE, BcMV9.BI=TRUE} =>  {BcOLV16.BI=TRUE} | 0.27  2277 | 0.493  274 | 0.551  98 | 1.18  6205 | 11  0 |
| {BcFV9=FALSE, BcOLV16.BI=TRUE} =>  {BcMV9.BI=TRUE} | 0.28  9604 | 0.806  897 | 0.358  911 | 1.18  9731 | 11  7 |
| {BcFV9=FALSE, BcMV9.BI=TRUE} =>  {BcOLV16.BI=TRUE} | 0.28  9604 | 0.495  763 | 0.584  158 | 1.19  2191 | 11  7 |
| {BcOLV16.BI=TRUE, BcOLV4.BI=FALSE} =>  {BcMV9.BI=TRUE} | 0.29  2079 | 0.848  921 | 0.344  059 | 1.25  1694 | 11  8 |
| {BcMV9.BI=TRUE, BcOLV4.BI=FALSE} =>  {BcOLV16.BI=TRUE} | 0.29  2079 | 0.473  896 | 0.616  337 | 1.13  9606 | 11  8 |
| {BcMV9.BI=TRUE, BcOLV16.BI=TRUE} =>  {BcULV5=FALSE} | 0.31  4356 | 0.947  761 | 0.331  683 | 1.08  1626 | 12  7 |
| {BcOLV16.BI=TRUE, BcULV5=FALSE} =>  {BcMV9.BI=TRUE} | 0.31  4356 | 0.894  366 | 0.351  485 | 1.31  8701 | 12  7 |
| {BcMV9.BI=TRUE, BcULV5=FALSE} =>  {BcOLV16.BI=TRUE} | 0.31  4356 | 0.479  245 | 0.655  941 | 1.15  2471 | 12  7 |
| {BcBNV5.BI=FALSE, BcOLV16.BI=TRUE} =>  {BcMV9.BI=TRUE} | 0.29  4554 | 0.788  079 | 0.373  762 | 1.16  1986 | 11  9 |
| {BcBNV5.BI=FALSE, BcMV9.BI=TRUE} =>  {BcOLV16.BI=TRUE} | 0.29  4554 | 0.493  776 | 0.596  535 | 1.18  7414 | 11  9 |

| {BcOLV16.BI=TRUE, BvF.BI=FALSE} =>  {BcMV9.BI=TRUE} | 0.31  1881 | 0.797  468 | 0.391  089 | 1.17  5829 | 12  6 |
| --- | --- | --- | --- | --- | --- |
| {BcMV9.BI=TRUE, BvF.BI=FALSE} =>  {BcOLV16.BI=TRUE} | 0.31  1881 | 0.510  121 | 0.611  386 | 1.22  6721 | 12  6 |
| {BcMCV5.BI=FALSE, BcOLV16.BI=TRUE} =>  {BcMV9.BI=TRUE} | 0.28  9604 | 0.790  541 | 0.366  337 | 1.16  5615 | 11  7 |
| {BcOLV13.BI=FALSE, BcOLV16.BI=TRUE} =>  {BcMV9.BI=TRUE} | 0.30  6931 | 0.837  838 | 0.366  337 | 1.23  5352 | 12  4 |
| {BcMV9.BI=TRUE, BcOLV13.BI=FALSE} =>  {BcOLV16.BI=TRUE} | 0.30  6931 | 0.482  49 | 0.636  139 | 1.16  0274 | 12  4 |
| {BcOLV16.BI=TRUE, BcOLV2.BI=FALSE} =>  {BcMV9.BI=TRUE} | 0.29  9505 | 0.812  081 | 0.368  812 | 1.19  7374 | 12  1 |
| {BcMV9.BI=TRUE, BcOLV2.BI=FALSE} =>  {BcOLV16.BI=TRUE} | 0.29  9505 | 0.482  072 | 0.621  287 | 1.15  9268 | 12  1 |
| {BcMCV3.BI=FALSE, BcOLV16.BI=TRUE} =>  {BcMV9.BI=TRUE} | 0.29  2079 | 0.813  793 | 0.358  911 | 1.19  9899 | 11  8 |
| {BcOLV16.BI=TRUE, BcPV4.BI=FALSE} =>  {BcMV9.BI=TRUE} | 0.30  6931 | 0.810  458 | 0.378  713 | 1.19  4981 | 12  4 |
| {BcMV9.BI=TRUE, BcPV4.BI=FALSE} =>  {BcOLV16.BI=TRUE} | 0.30  6931 | 0.484  375 | 0.633  663 | 1.16  4807 | 12  4 |
| {BcEV6=FALSE, BcOLV16.BI=TRUE} =>  {BcMV9.BI=TRUE} | 0.31  4356 | 0.803  797 | 0.391  089 | 1.18  5161 | 12  7 |

| {BcEV6=FALSE, BcMV9.BI=TRUE} =>  {BcOLV16.BI=TRUE} | 0.31  4356 | 0.498  039 | 0.631  188 | 1.19  7666 | 12  7 |
| --- | --- | --- | --- | --- | --- |
| {BcOLV16.BI=TRUE, BcOLV17.BI=FALSE} =>  {BcMV9.BI=TRUE} | 0.30  6931 | 0.794  872 | 0.386  139 | 1.17  2001 | 12  4 |
| {BcMV9.BI=TRUE, BcOLV17.BI=FALSE} =>  {BcOLV16.BI=TRUE} | 0.30  6931 | 0.480  62 | 0.638  614 | 1.15  5777 | 12  4 |
| {BcOLV15.BI=FALSE, BcOLV16.BI=TRUE} =>  {BcMV9.BI=TRUE} | 0.32  6733 | 0.809  816 | 0.403  465 | 1.19  4035 | 13  2 |
| {BcMV9.BI=TRUE, BcOLV15.BI=FALSE} =>  {BcOLV16.BI=TRUE} | 0.32  6733 | 0.509  653 | 0.641  089 | 1.22  5593 | 13  2 |
| {BcEV5=FALSE, BcOLV16.BI=TRUE} =>  {BcMV9.BI=TRUE} | 0.30  9406 | 0.786  164 | 0.393  564 | 1.15  9161 | 12  5 |
| {BcEV5=FALSE, BcMV9.BI=TRUE} =>  {BcOLV16.BI=TRUE} | 0.30  9406 | 0.484  496 | 0.638  614 | 1.16  5098 | 12  5 |
| {BcOLV16.BI=TRUE, BcPV3.BI=FALSE} =>  {BcULV4=TRUE} | 0.23  2673 | 0.854  545 | 0.272  277 | 1.23  7406 | 94 |
| {BcEV2.BI=FALSE, BcOLV16.BI=TRUE} =>  {BcULV4=TRUE} | 0.26  7327 | 0.818  182 | 0.326  733 | 1.18  4751 | 10  8 |
| {BcEV2.BI=FALSE, BcULV4=TRUE} =>  {BcOLV16.BI=TRUE} | 0.26  7327 | 0.504  673 | 0.529  703 | 1.21  3618 | 10  8 |
| {BcHV3.BI=FALSE, BcOLV16.BI=TRUE} =>  {BcULV4=TRUE} | 0.26  2376 | 0.815  385 | 0.321  782 | 1.18  07 | 10  6 |

| {BcHV3.BI=FALSE, BcULV4=TRUE} =>  {BcOLV16.BI=TRUE} | 0.26  2376 | 0.509  615 | 0.514  851 | 1.22  5504 | 10  6 |
| --- | --- | --- | --- | --- | --- |
| {BcFV5.BI=FALSE, BcOLV16.BI=TRUE} =>  {BcULV4=TRUE} | 0.25  9901 | 0.807  692 | 0.321  782 | 1.16  9562 | 10  5 |
| {BcFV5.BI=FALSE, BcULV4=TRUE} =>  {BcOLV16.BI=TRUE} | 0.25  9901 | 0.490  654 | 0.529  703 | 1.17  9907 | 10  5 |
| {BcMV1.BI=TRUE, BcOLV16.BI=TRUE} =>  {BcULV4=TRUE} | 0.27  2277 | 0.808  824 | 0.336  634 | 1.17  12 | 11  0 |
| {BcMV1.BI=TRUE, BcULV4=TRUE} =>  {BcOLV16.BI=TRUE} | 0.27  2277 | 0.495  495 | 0.549  505 | 1.19  1549 | 11  0 |
| {BcMV12=FALSE, BcOLV16.BI=TRUE} =>  {BcULV4=TRUE} | 0.24  2574 | 0.823  529 | 0.294  554 | 1.19  2494 | 98 |
| {BcNSRV7.BI=FALSE, BcOLV16.BI=TRUE}  => {BcULV4=TRUE} | 0.26  2376 | 0.785  185 | 0.334  158 | 1.13  6971 | 10  6 |
| {BcNSRV7.BI=FALSE, BcULV4=TRUE} =>  {BcOLV16.BI=TRUE} | 0.26  2376 | 0.490  741 | 0.534  653 | 1.18  0115 | 10  6 |
| {BcOLV16.BI=TRUE, BcVV3.BI=FALSE} =>  {BcULV4=TRUE} | 0.28  2178 | 0.838  235 | 0.336  634 | 1.21  3789 | 11  4 |
| {BcULV4=TRUE, BcVV3.BI=FALSE} =>  {BcOLV16.BI=TRUE} | 0.28  2178 | 0.515  837 | 0.547  03 | 1.24  0465 | 11  4 |
| {BcALV1.BI=FALSE, BcOLV16.BI=TRUE} =>  {BcULV4=TRUE} | 0.24  0099 | 0.776 | 0.309  406 | 1.12  367 | 97 |

| {BcDMV1=FALSE, BcOLV16.BI=TRUE} =>  {BcULV4=TRUE} | 0.28  2178 | 0.797  203 | 0.353  96 | 1.15  4373 | 11  4 |
| --- | --- | --- | --- | --- | --- |
| {BcDMV1=FALSE, BcULV4=TRUE} =>  {BcOLV16.BI=TRUE} | 0.28  2178 | 0.493  506 | 0.571  782 | 1.18  6766 | 11  4 |
| {BcFV9=FALSE, BcOLV16.BI=TRUE} =>  {BcULV4=TRUE} | 0.29  2079 | 0.813  793 | 0.358  911 | 1.17  8396 | 11  8 |
| {BcFV9=FALSE, BcULV4=TRUE} =>  {BcOLV16.BI=TRUE} | 0.29  2079 | 0.489  627 | 0.596  535 | 1.17  7435 | 11  8 |
| {BcOLV16.BI=TRUE, BcOLV4.BI=FALSE} =>  {BcULV4=TRUE} | 0.29  9505 | 0.870  504 | 0.344  059 | 1.26  0514 | 12  1 |
| {BcOLV4.BI=FALSE, BcULV4=TRUE} =>  {BcOLV16.BI=TRUE} | 0.29  9505 | 0.482  072 | 0.621  287 | 1.15  9268 | 12  1 |
| {BcOLV16.BI=TRUE, BcULV5=FALSE} =>  {BcULV4=TRUE} | 0.30  198 | 0.859  155 | 0.351  485 | 1.24  4081 | 12  2 |
| {BcULV4=TRUE, BcULV5=FALSE} =>  {BcOLV16.BI=TRUE} | 0.30  198 | 0.478  431 | 0.631  188 | 1.15  0514 | 12  2 |
| {BcBNV5.BI=FALSE, BcOLV16.BI=TRUE} =>  {BcULV4=TRUE} | 0.29  9505 | 0.801  325 | 0.373  762 | 1.16  0341 | 12  1 |
| {BcBNV5.BI=FALSE, BcULV4=TRUE} =>  {BcOLV16.BI=TRUE} | 0.29  9505 | 0.484 | 0.618  812 | 1.16  3905 | 12  1 |
| {BcOLV16.BI=TRUE, BvF.BI=FALSE} =>  {BcULV4=TRUE} | 0.32  6733 | 0.835  443 | 0.391  089 | 1.20  9745 | 13  2 |

| {BcULV4=TRUE, BvF.BI=FALSE} =>  {BcOLV16.BI=TRUE} | 0.32  6733 | 0.505  747 | 0.646  04 | 1.21  6201 | 13  2 |
| --- | --- | --- | --- | --- | --- |
| {BcMCV5.BI=FALSE, BcOLV16.BI=TRUE} =>  {BcULV4=TRUE} | 0.29  703 | 0.810  811 | 0.366  337 | 1.17  4077 | 12  0 |
| {BcOLV13.BI=FALSE, BcOLV16.BI=TRUE} =>  {BcULV4=TRUE} | 0.30  9406 | 0.844  595 | 0.366  337 | 1.22  2997 | 12  5 |
| {BcOLV13.BI=FALSE, BcULV4=TRUE} =>  {BcOLV16.BI=TRUE} | 0.30  9406 | 0.482  625 | 0.641  089 | 1.16  0599 | 12  5 |
| {BcOLV16.BI=TRUE, BcOLV2.BI=FALSE} =>  {BcULV4=TRUE} | 0.30  4455 | 0.825  503 | 0.368  812 | 1.19  5353 | 12  3 |
| {BcOLV2.BI=FALSE, BcULV4=TRUE} =>  {BcOLV16.BI=TRUE} | 0.30  4455 | 0.474  903 | 0.641  089 | 1.14  203 | 12  3 |
| {BcMCV3.BI=FALSE, BcOLV16.BI=TRUE} =>  {BcULV4=TRUE} | 0.29  703 | 0.827  586 | 0.358  911 | 1.19  8369 | 12  0 |
| {BcOLV16.BI=TRUE, BcPV4.BI=FALSE} =>  {BcULV4=TRUE} | 0.31  1881 | 0.823  529 | 0.378  713 | 1.19  2494 | 12  6 |
| {BcPV4.BI=FALSE, BcULV4=TRUE} =>  {BcOLV16.BI=TRUE} | 0.31  1881 | 0.482  759 | 0.646  04 | 1.16  092 | 12  6 |
| {BcEV6=FALSE, BcOLV16.BI=TRUE} =>  {BcULV4=TRUE} | 0.32  1782 | 0.822  785 | 0.391  089 | 1.19  1416 | 13  0 |
| {BcEV6=FALSE, BcULV4=TRUE} =>  {BcOLV16.BI=TRUE} | 0.32  1782 | 0.498  084 | 0.646  04 | 1.19  7774 | 13  0 |

| {BcOLV16.BI=TRUE, BcOLV17.BI=FALSE} =>  {BcULV4=TRUE} | 0.31  1881 | 0.807  692 | 0.386  139 | 1.16  9562 | 12  6 |
| --- | --- | --- | --- | --- | --- |
| {BcOLV17.BI=FALSE, BcULV4=TRUE} =>  {BcOLV16.BI=TRUE} | 0.31  1881 | 0.482  759 | 0.646  04 | 1.16  092 | 12  6 |
| {BcOLV15.BI=FALSE, BcOLV16.BI=TRUE} =>  {BcULV4=TRUE} | 0.33  1683 | 0.822  086 | 0.403  465 | 1.19  0404 | 13  4 |
| {BcOLV15.BI=FALSE, BcULV4=TRUE} =>  {BcOLV16.BI=TRUE} | 0.33  1683 | 0.503  759 | 0.658  416 | 1.21  1421 | 13  4 |
| {BcEV5=FALSE, BcOLV16.BI=TRUE} =>  {BcULV4=TRUE} | 0.31  9307 | 0.811  321 | 0.393  564 | 1.17  4816 | 12  9 |
| {BcEV5=FALSE, BcULV4=TRUE} =>  {BcOLV16.BI=TRUE} | 0.31  9307 | 0.484  962 | 0.658  416 | 1.16  6219 | 12  9 |
| {BcOLV16.BI=TRUE, BcPV3.BI=FALSE} =>  {BcEV2.BI=FALSE} | 0.22  5248 | 0.827  273 | 0.272  277 | 1.10  6683 | 91 |
| {BcHV3.BI=FALSE, BcOLV16.BI=TRUE} =>  {BcEV2.BI=FALSE} | 0.26  7327 | 0.830  769 | 0.321  782 | 1.11  136 | 10  8 |
| {BcNSRV7.BI=FALSE, BcOLV16.BI=TRUE}  => {BcEV2.BI=FALSE} | 0.27  4752 | 0.822  222 | 0.334  158 | 1.09  9926 | 11  1 |
| {BcFV9=FALSE, BcOLV16.BI=TRUE} =>  {BcEV2.BI=FALSE} | 0.29  703 | 0.827  586 | 0.358  911 | 1.10  7102 | 12  0 |
| {BcFV5.BI=FALSE, BcOLV16.BI=TRUE} =>  {BcHV3.BI=FALSE} | 0.27  2277 | 0.846  154 | 0.321  782 | 1.11  3505 | 11  0 |

| {BcHV3.BI=FALSE, BcOLV16.BI=TRUE} =>  {BcVV3.BI=FALSE} | 0.27  9703 | 0.869  231 | 0.321  782 | 1.08  3856 | 11  3 |
| --- | --- | --- | --- | --- | --- |
| {BcMCV5.BI=FALSE, BcOLV16.BI=TRUE} =>  {BcHV3.BI=FALSE} | 0.30  6931 | 0.837  838 | 0.366  337 | 1.10  2562 | 12  4 |
| {BcALV1.BI=FALSE, BcOLV16.BI=TRUE} =>  {BcFV5.BI=FALSE} | 0.26  4851 | 0.856 | 0.309  406 | 1.09  438 | 10  7 |
| {BcFV5.BI=FALSE, BcOLV16.BI=TRUE} =>  {BcDMV1=FALSE} | 0.29  4554 | 0.915  385 | 0.321  782 | 1.08  4503 | 11  9 |
| {BcNSRV7.BI=FALSE, BcOLV16.BI=TRUE}  => {BcDMV1=FALSE} | 0.30  4455 | 0.911  111 | 0.334  158 | 1.07  944 | 12  3 |
| {BcEV5=FALSE, BcOLV16.BI=TRUE} =>  {BcDMV1=FALSE} | 0.35  396 | 0.899  371 | 0.393  564 | 1.06  5531 | 14  3 |
| {BcHV1SLR.BI=FALSE, BcMV4.BI=TRUE} =>  {BcMV2.BI=TRUE} | 0.10  6436 | 0.589  041 | 0.180  693 | 1.27  2581 | 43 |
| {BcHV1SLR.BI=FALSE, BcMV2.BI=TRUE} =>  {BcMV4.BI=TRUE} | 0.10  6436 | 0.558  442 | 0.190  594 | 1.26  0393 | 43 |
| {BcHV1SLR.BI=FALSE, BcMV4.BI=TRUE} =>  {BcMV11=TRUE} | 0.13  6139 | 0.753  425 | 0.180  693 | 1.22  2424 | 55 |
| {BcMV3.BI=FALSE, BcMV4.BI=TRUE} =>  {BcMV6.BI=FALSE} | 0.12  6238 | 0.85 | 0.148  515 | 1.22  2064 | 51 |
| {BcMV3.BI=FALSE, BcMV4.BI=TRUE} =>  {BcDMV1=FALSE} | 0.13  8614 | 0.933  333 | 0.148  515 | 1.10  5767 | 56 |

| {BcMV2.BI=TRUE, BcMV4.BI=TRUE} =>  {BcOLV18=TRUE} | 0.19  3069 | 0.702  703 | 0.274  752 | 1.35  8334 | 78 |
| --- | --- | --- | --- | --- | --- |
| {BcMV4.BI=TRUE, BcOLV18=TRUE} =>  {BcMV2.BI=TRUE} | 0.19  3069 | 0.696  429 | 0.277  228 | 1.50  4584 | 78 |
| {BcMV2.BI=TRUE, BcOLV18=TRUE} =>  {BcMV4.BI=TRUE} | 0.19  3069 | 0.655  462 | 0.294  554 | 1.47  9367 | 78 |
| {BcHV1SLR.BI=TRUE, BcMV4.BI=TRUE} =>  {BcMV2.BI=TRUE} | 0.16  8317 | 0.641  509 | 0.262  376 | 1.38  5935 | 68 |
| {BcHV1SLR.BI=TRUE, BcMV2.BI=TRUE} =>  {BcMV4.BI=TRUE} | 0.16  8317 | 0.618  182 | 0.272  277 | 1.39  5226 | 68 |
| {BcMV2.BI=TRUE, BcMV4.BI=TRUE} =>  {BcMV3.BI=TRUE} | 0.20  5446 | 0.747  748 | 0.274  752 | 1.37  9407 | 83 |
| {BcMV3.BI=TRUE, BcMV4.BI=TRUE} =>  {BcMV2.BI=TRUE} | 0.20  5446 | 0.697  479 | 0.294  554 | 1.50  6853 | 83 |
| {BcMV2.BI=TRUE, BcMV3.BI=TRUE} =>  {BcMV4.BI=TRUE} | 0.20  5446 | 0.653  543 | 0.314  356 | 1.47  5036 | 83 |
| {BcMV4.BI=TRUE, BcULV3=TRUE} =>  {BcMV2.BI=TRUE} | 0.18  3168 | 0.621  849 | 0.294  554 | 1.34  3459 | 74 |
| {BcMV2.BI=TRUE, BcULV3=TRUE} =>  {BcMV4.BI=TRUE} | 0.18  3168 | 0.643  478 | 0.284  653 | 1.45  232 | 74 |
| {BcMV2.BI=TRUE, BcMV4.BI=TRUE} =>  {BcHV1.BI=TRUE} | 0.19  0594 | 0.693  694 | 0.274  752 | 1.14  8575 | 77 |

| {BcHV1.BI=TRUE, BcMV4.BI=TRUE} =>  {BcMV2.BI=TRUE} | 0.19  0594 | 0.636  364 | 0.299  505 | 1.37  4818 | 77 |
| --- | --- | --- | --- | --- | --- |
| {BcHV1.BI=TRUE, BcMV2.BI=TRUE} =>  {BcMV4.BI=TRUE} | 0.19  0594 | 0.652  542 | 0.292  079 | 1.47  2777 | 77 |
| {BcMV2.BI=TRUE, BcMV4.BI=TRUE} =>  {BcMV11=TRUE} | 0.23  5149 | 0.855  856 | 0.274  752 | 1.38  8618 | 95 |
| {BcMV11=TRUE, BcMV4.BI=TRUE} =>  {BcMV2.BI=TRUE} | 0.23  5149 | 0.693  431 | 0.339  109 | 1.49  8107 | 95 |
| {BcMV11=TRUE, BcMV2.BI=TRUE} =>  {BcMV4.BI=TRUE} | 0.23  5149 | 0.688  406 | 0.341  584 | 1.55  372 | 95 |
| {BcMV2.BI=TRUE, BcOLV1.BI.MOVA.=FALSE} =>  {BcMV4.BI=TRUE} | 0.12  1287 | 0.569  767 | 0.212  871 | 1.28  5956 | 49 |
| {BcMV2.BI=TRUE, BcMV4.BI=TRUE} =>  {BcMV9.BI=TRUE} | 0.21  2871 | 0.774  775 | 0.274  752 | 1.14  2369 | 86 |
| {BcMV4.BI=TRUE, BcMV9.BI=TRUE} =>  {BcMV2.BI=TRUE} | 0.21  2871 | 0.632  353 | 0.336  634 | 1.36  6153 | 86 |
| {BcMV2.BI=TRUE, BcMV9.BI=TRUE} =>  {BcMV4.BI=TRUE} | 0.21  2871 | 0.661  538 | 0.321  782 | 1.49  3081 | 86 |
| {BcMV4.BI=TRUE, BcULV4=TRUE} =>  {BcMV2.BI=TRUE} | 0.21  2871 | 0.656  489 | 0.324  257 | 1.41  8296 | 86 |
| {BcMV2.BI=TRUE, BcULV4=TRUE} => | 0.21 | 0.623 | 0.341 | 1.40 | 86 |

| {BcMV4.BI=TRUE} | 2871 | 188 | 584 | 6526 |  |
| --- | --- | --- | --- | --- | --- |
| {BcMV4.BI=TRUE, BcPV3.BI=FALSE} =>  {BcMV2.BI=TRUE} | 0.15  5941 | 0.594  34 | 0.262  376 | 1.28  4028 | 63 |
| {BcMV2.BI=TRUE, BcPV3.BI=FALSE} =>  {BcMV4.BI=TRUE} | 0.15  5941 | 0.547  826 | 0.284  653 | 1.23  6434 | 63 |
| {BcEV2.BI=FALSE, BcMV4.BI=TRUE} =>  {BcMV2.BI=TRUE} | 0.19  0594 | 0.606  299 | 0.314  356 | 1.30  9866 | 77 |
| {BcEV2.BI=FALSE, BcMV2.BI=TRUE} =>  {BcMV4.BI=TRUE} | 0.19  0594 | 0.596  899 | 0.319  307 | 1.34  7192 | 77 |
| {BcHV3.BI=FALSE, BcMV4.BI=TRUE} =>  {BcMV2.BI=TRUE} | 0.17  8218 | 0.610  169 | 0.292  079 | 1.31  8227 | 72 |
| {BcHV3.BI=FALSE, BcMV2.BI=TRUE} =>  {BcMV4.BI=TRUE} | 0.17  8218 | 0.541  353 | 0.329  208 | 1.22  1826 | 72 |
| {BcFV5.BI=FALSE, BcMV4.BI=TRUE} =>  {BcMV2.BI=TRUE} | 0.19  5545 | 0.607  692 | 0.321  782 | 1.31  2875 | 79 |
| {BcFV5.BI=FALSE, BcMV2.BI=TRUE} =>  {BcMV4.BI=TRUE} | 0.19  5545 | 0.568  345 | 0.344  059 | 1.28  2746 | 79 |
| {BcMV4.BI=TRUE, BcULV1.BI=FALSE} =>  {BcMV2.BI=TRUE} | 0.19  5545 | 0.585  185 | 0.334  158 | 1.26  425 | 79 |
| {BcMV2.BI=TRUE, BcULV1.BI=FALSE} =>  {BcMV4.BI=TRUE} | 0.19  5545 | 0.603  053 | 0.324  257 | 1.36  1081 | 79 |
| {BcMV2.BI=TRUE, BcMV4.BI=TRUE} => | 0.24 | 0.873 | 0.274 | 1.10 | 97 |

| {BcMV1.BI=TRUE} | 0099 | 874 | 752 | 6724 |  |
| --- | --- | --- | --- | --- | --- |
| {BcMV1.BI=TRUE, BcMV4.BI=TRUE} =>  {BcMV2.BI=TRUE} | 0.24  0099 | 0.638  158 | 0.376  238 | 1.37  8694 | 97 |
| {BcMV1.BI=TRUE, BcMV2.BI=TRUE} =>  {BcMV4.BI=TRUE} | 0.24  0099 | 0.587  879 | 0.408  416 | 1.32  6833 | 97 |
| {BcMV12=FALSE, BcMV4.BI=TRUE} =>  {BcMV2.BI=TRUE} | 0.21  5347 | 0.595  89 | 0.361  386 | 1.28  7378 | 87 |
| {BcMV12=FALSE, BcMV2.BI=TRUE} =>  {BcMV4.BI=TRUE} | 0.21  5347 | 0.630  435 | 0.341  584 | 1.42  2881 | 87 |
| {BcMV4.BI=TRUE, BcNSRV7.BI=FALSE} =>  {BcMV2.BI=TRUE} | 0.20  5446 | 0.610  294 | 0.336  634 | 1.31  8496 | 83 |
| {BcMV2.BI=TRUE, BcNSRV7.BI=FALSE} =>  {BcMV4.BI=TRUE} | 0.20  5446 | 0.568  493 | 0.361  386 | 1.28  308 | 83 |
| {BcMV4.BI=TRUE, BcVV3.BI=FALSE} =>  {BcMV2.BI=TRUE} | 0.20  0495 | 0.604  478 | 0.331  683 | 1.30  593 | 81 |
| {BcMV2.BI=TRUE, BcVV3.BI=FALSE} =>  {BcMV4.BI=TRUE} | 0.20  0495 | 0.570  423 | 0.351  485 | 1.28  7434 | 81 |
| {BcMV4.BI=TRUE, BcOLV7.BI=FALSE} =>  {BcMV2.BI=TRUE} | 0.21  0396 | 0.625 | 0.336  634 | 1.35  0267 | 85 |
| {BcMV2.BI=TRUE, BcOLV7.BI=FALSE} =>  {BcMV4.BI=TRUE} | 0.21  0396 | 0.586  207 | 0.358  911 | 1.32  3059 | 85 |
| {BcDMV1=FALSE, BcMV4.BI=TRUE} => | 0.21 | 0.602 | 0.361 | 1.30 | 88 |

| {BcMV2.BI=TRUE} | 7822 | 74 | 386 | 2176 |  |
| --- | --- | --- | --- | --- | --- |
| {BcDMV1=FALSE, BcMV2.BI=TRUE} =>  {BcMV4.BI=TRUE} | 0.21  7822 | 0.55 | 0.396  04 | 1.24  1341 | 88 |
| {BcFV9=FALSE, BcMV4.BI=TRUE} =>  {BcMV2.BI=TRUE} | 0.22  7723 | 0.638  889 | 0.356  436 | 1.38  0273 | 92 |
| {BcFV9=FALSE, BcMV2.BI=TRUE} =>  {BcMV4.BI=TRUE} | 0.22  7723 | 0.567  901 | 0.400  99 | 1.28  1744 | 92 |
| {BcMV4.BI=TRUE, BcOLV4.BI=FALSE} =>  {BcMV2.BI=TRUE} | 0.21  5347 | 0.58 | 0.371  287 | 1.25  3048 | 87 |
| {BcMV2.BI=TRUE, BcOLV4.BI=FALSE} =>  {BcMV4.BI=TRUE} | 0.21  5347 | 0.572  368 | 0.376  238 | 1.29  1826 | 87 |
| {BcMV4.BI=TRUE, BcULV5=FALSE} =>  {BcMV2.BI=TRUE} | 0.22  2772 | 0.584  416 | 0.381  188 | 1.26  2588 | 90 |
| {BcMV2.BI=TRUE, BcULV5=FALSE} =>  {BcMV4.BI=TRUE} | 0.22  2772 | 0.592  105 | 0.376  238 | 1.33  6372 | 90 |
| {BcBNV5.BI=FALSE, BcMV4.BI=TRUE} =>  {BcMV2.BI=TRUE} | 0.23  0198 | 0.6 | 0.383  663 | 1.29  6257 | 93 |
| {BcBNV5.BI=FALSE, BcMV2.BI=TRUE} =>  {BcMV4.BI=TRUE} | 0.23  0198 | 0.563  636 | 0.408  416 | 1.27  2118 | 93 |
| {BcMV4.BI=TRUE, BvF.BI=FALSE} =>  {BcMV2.BI=TRUE} | 0.25  7426 | 0.634  146 | 0.405  941 | 1.37  0027 | 10  4 |
| {BcMV2.BI=TRUE, BvF.BI=FALSE} => | 0.25 | 0.594 | 0.433 | 1.34 | 10 |

| {BcMV4.BI=TRUE} | 7426 | 286 | 168 | 1293 | 4 |
| --- | --- | --- | --- | --- | --- |
| {BcMCV5.BI=FALSE, BcMV4.BI=TRUE} =>  {BcMV2.BI=TRUE} | 0.22  5248 | 0.594  771 | 0.378  713 | 1.28  496 | 91 |
| {BcMCV5.BI=FALSE, BcMV2.BI=TRUE} =>  {BcMV4.BI=TRUE} | 0.22  5248 | 0.561  728 | 0.400  99 | 1.26  7812 | 91 |
| {BcMV4.BI=TRUE, BcOLV13.BI=FALSE} =>  {BcMV2.BI=TRUE} | 0.23  5149 | 0.601  266 | 0.391  089 | 1.29  8991 | 95 |
| {BcMV2.BI=TRUE, BcOLV13.BI=FALSE} =>  {BcMV4.BI=TRUE} | 0.23  5149 | 0.575  758 | 0.408  416 | 1.29  9475 | 95 |
| {BcMV4.BI=TRUE, BcOLV2.BI=FALSE} =>  {BcMV2.BI=TRUE} | 0.24  2574 | 0.601  227 | 0.403  465 | 1.29  8908 | 98 |
| {BcMV2.BI=TRUE, BcOLV2.BI=FALSE} =>  {BcMV4.BI=TRUE} | 0.24  2574 | 0.576  471 | 0.420  792 | 1.30  1084 | 98 |
| {BcMCV3.BI=FALSE, BcMV4.BI=TRUE} =>  {BcMV2.BI=TRUE} | 0.24  7525 | 0.602  41 | 0.410  891 | 1.30  1463 | 10  0 |
| {BcMCV3.BI=FALSE, BcMV2.BI=TRUE} =>  {BcMV4.BI=TRUE} | 0.24  7525 | 0.606  061 | 0.408  416 | 1.36  7869 | 10  0 |
| {BcMV4.BI=TRUE, BcPV4.BI=FALSE} =>  {BcMV2.BI=TRUE} | 0.26  2376 | 0.627  219 | 0.418  317 | 1.35  5061 | 10  6 |
| {BcMV2.BI=TRUE, BcPV4.BI=FALSE} =>  {BcMV4.BI=TRUE} | 0.26  2376 | 0.612  717 | 0.428  218 | 1.38  2891 | 10  6 |
| {BcEV6=FALSE, BcMV4.BI=TRUE} => | 0.25 | 0.617 | 0.420 | 1.33 | 10 |

| {BcMV2.BI=TRUE} | 9901 | 647 | 792 | 4382 | 5 |
| --- | --- | --- | --- | --- | --- |
| {BcEV6=FALSE, BcMV2.BI=TRUE} =>  {BcMV4.BI=TRUE} | 0.25  9901 | 0.6 | 0.433  168 | 1.35  419 | 10  5 |
| {BcMV4.BI=TRUE, BcOLV17.BI=FALSE} =>  {BcMV2.BI=TRUE} | 0.24  7525 | 0.602  41 | 0.410  891 | 1.30  1463 | 10  0 |
| {BcMV2.BI=TRUE, BcOLV17.BI=FALSE} =>  {BcMV4.BI=TRUE} | 0.24  7525 | 0.581  395 | 0.425  743 | 1.31  22 | 10  0 |
| {BcMV4.BI=TRUE, BcOLV15.BI=FALSE} =>  {BcMV2.BI=TRUE} | 0.26  4851 | 0.640  719 | 0.413  366 | 1.38  4226 | 10  7 |
| {BcMV2.BI=TRUE, BcOLV15.BI=FALSE} =>  {BcMV4.BI=TRUE} | 0.26  4851 | 0.584  699 | 0.452  97 | 1.31  9657 | 10  7 |
| {BcEV5=FALSE, BcMV4.BI=TRUE} =>  {BcMV2.BI=TRUE} | 0.23  7624 | 0.596  273 | 0.398  515 | 1.28  8205 | 96 |
| {BcEV5=FALSE, BcMV2.BI=TRUE} =>  {BcMV4.BI=TRUE} | 0.23  7624 | 0.561  404 | 0.423  267 | 1.26  7078 | 96 |
| {BcMV4.BI=TRUE, BcOLV18=FALSE} =>  {BcRV1.BI=FALSE} | 0.14  604 | 0.880  597 | 0.165  842 | 1.21  836 | 59 |
| {BcMV4.BI=TRUE, BcOLV18=FALSE} =>  {BcULV1.BI=FALSE} | 0.14  604 | 0.880  597 | 0.165  842 | 1.12  2275 | 59 |
| {BcMV4.BI=TRUE, BcOLV18=FALSE} =>  {BcOLV7.BI=FALSE} | 0.14  8515 | 0.895  522 | 0.165  842 | 1.10  9788 | 60 |
| {BcMV4.BI=TRUE, BcOLV18=TRUE} => | 0.19 | 0.687 | 0.277 | 1.26 | 77 |

| {BcHV1SLR.BI=TRUE} | 0594 | 5 | 228 | 8265 |  |
| --- | --- | --- | --- | --- | --- |
| {BcHV1SLR.BI=TRUE, BcMV4.BI=TRUE} =>  {BcOLV18=TRUE} | 0.19  0594 | 0.726  415 | 0.262  376 | 1.40  4171 | 77 |
| {BcHV1SLR.BI=TRUE, BcOLV18=TRUE} =>  {BcMV4.BI=TRUE} | 0.19  0594 | 0.557  971 | 0.341  584 | 1.25  9331 | 77 |
| {BcMV4.BI=TRUE, BcOLV18=TRUE} =>  {BcMV3.BI=TRUE} | 0.20  7921 | 0.75 | 0.277  228 | 1.38  3562 | 84 |
| {BcMV3.BI=TRUE, BcMV4.BI=TRUE} =>  {BcOLV18=TRUE} | 0.20  7921 | 0.705  882 | 0.294  554 | 1.36  4481 | 84 |
| {BcMV3.BI=TRUE, BcOLV18=TRUE} =>  {BcMV4.BI=TRUE} | 0.20  7921 | 0.631  579 | 0.329  208 | 1.42  5463 | 84 |
| {BcMV4.BI=TRUE, BcULV3=TRUE} =>  {BcOLV18=TRUE} | 0.18  3168 | 0.621  849 | 0.294  554 | 1.20  2043 | 74 |
| {BcOLV18=TRUE, BcULV3=TRUE} =>  {BcMV4.BI=TRUE} | 0.18  3168 | 0.564  885 | 0.324  257 | 1.27  4937 | 74 |
| {BcMV4.BI=TRUE, BcOLV18=TRUE} =>  {BcHV1.BI=TRUE} | 0.21  0396 | 0.758  929 | 0.277  228 | 1.25  6587 | 85 |
| {BcHV1.BI=TRUE, BcMV4.BI=TRUE} =>  {BcOLV18=TRUE} | 0.21  0396 | 0.702  479 | 0.299  505 | 1.35  7903 | 85 |
| {BcHV1.BI=TRUE, BcOLV18=TRUE} =>  {BcMV4.BI=TRUE} | 0.21  0396 | 0.574  324 | 0.366  337 | 1.29  624 | 85 |
| {BcMV4.BI=TRUE, BcOLV18=TRUE} => | 0.22 | 0.803 | 0.277 | 1.30 | 90 |

| {BcMV11=TRUE} | 2772 | 571 | 228 | 3787 |  |
| --- | --- | --- | --- | --- | --- |
| {BcMV11=TRUE, BcMV4.BI=TRUE} =>  {BcOLV18=TRUE} | 0.22  2772 | 0.656  934 | 0.339  109 | 1.26  9863 | 90 |
| {BcMV11=TRUE, BcOLV18=TRUE} =>  {BcMV4.BI=TRUE} | 0.22  2772 | 0.642  857 | 0.346  535 | 1.45  0918 | 90 |
| {BcMV4.BI=TRUE, BcOLV18=TRUE} =>  {BcMV9.BI=TRUE} | 0.24  2574 | 0.875 | 0.277  228 | 1.29  0146 | 98 |
| {BcMV4.BI=TRUE, BcMV9.BI=TRUE} =>  {BcOLV18=TRUE} | 0.24  2574 | 0.720  588 | 0.336  634 | 1.39  2907 | 98 |
| {BcMV9.BI=TRUE, BcOLV18=TRUE} =>  {BcMV4.BI=TRUE} | 0.24  2574 | 0.586  826 | 0.413  366 | 1.32  4457 | 98 |
| {BcMV4.BI=TRUE, BcOLV18=TRUE} =>  {BcULV4=TRUE} | 0.24  2574 | 0.875 | 0.277  228 | 1.26  7025 | 98 |
| {BcMV4.BI=TRUE, BcULV4=TRUE} =>  {BcOLV18=TRUE} | 0.24  2574 | 0.748  092 | 0.324  257 | 1.44  6072 | 98 |
| {BcOLV18=TRUE, BcULV4=TRUE} =>  {BcMV4.BI=TRUE} | 0.24  2574 | 0.550  562 | 0.440  594 | 1.24  2609 | 98 |
| {BcEV2.BI=FALSE, BcMV4.BI=TRUE} =>  {BcOLV18=TRUE} | 0.19  802 | 0.629  921 | 0.314  356 | 1.21  7647 | 80 |
| {BcEV2.BI=FALSE, BcOLV18=TRUE} =>  {BcMV4.BI=TRUE} | 0.19  802 | 0.536  913 | 0.368  812 | 1.21  1803 | 80 |
| {BcOLV18=TRUE, BcULV1.BI=FALSE} => | 0.18 | 0.527 | 0.356 | 1.19 | 76 |

| {BcMV4.BI=TRUE} | 8119 | 778 | 436 | 1186 |  |
| --- | --- | --- | --- | --- | --- |
| {BcMV4.BI=TRUE, BcOLV18=TRUE} =>  {BcMV1.BI=TRUE} | 0.24  2574 | 0.875 | 0.277  228 | 1.10  815 | 98 |
| {BcMV1.BI=TRUE, BcMV4.BI=TRUE} =>  {BcOLV18=TRUE} | 0.24  2574 | 0.644  737 | 0.376  238 | 1.24  6286 | 98 |
| {BcMV1.BI=TRUE, BcOLV18=TRUE} =>  {BcMV4.BI=TRUE} | 0.24  2574 | 0.550  562 | 0.440  594 | 1.24  2609 | 98 |
| {BcMV12=FALSE, BcMV4.BI=TRUE} =>  {BcOLV18=TRUE} | 0.21  7822 | 0.602  74 | 0.361  386 | 1.16  5105 | 88 |
| {BcMV12=FALSE, BcOLV18=TRUE} =>  {BcMV4.BI=TRUE} | 0.21  7822 | 0.55 | 0.396  04 | 1.24  1341 | 88 |
| {BcMV4.BI=TRUE, BcVV3.BI=FALSE} =>  {BcOLV18=TRUE} | 0.20  5446 | 0.619  403 | 0.331  683 | 1.19  7315 | 83 |
| {BcDMV1=FALSE, BcMV4.BI=TRUE} =>  {BcOLV18=TRUE} | 0.22  2772 | 0.616  438 | 0.361  386 | 1.19  1584 | 90 |
| {BcDMV1=FALSE, BcOLV18=TRUE} =>  {BcMV4.BI=TRUE} | 0.22  2772 | 0.520  231 | 0.428  218 | 1.17  4153 | 90 |
| {BcMV4.BI=TRUE, BcOLV4.BI=FALSE} =>  {BcOLV18=TRUE} | 0.23  0198 | 0.62 | 0.371  287 | 1.19  8469 | 93 |
| {BcMV4.BI=TRUE, BcULV5=FALSE} =>  {BcOLV18=TRUE} | 0.25 | 0.655  844 | 0.381  188 | 1.26  7756 | 10  1 |
| {BcOLV18=TRUE, BcULV5=FALSE} => | 0.25 | 0.534 | 0.467 | 1.20 | 10 |

| {BcMV4.BI=TRUE} |  | 392 | 822 | 6113 | 1 |
| --- | --- | --- | --- | --- | --- |
| {BcMV4.BI=TRUE, BvF.BI=FALSE} =>  {BcOLV18=TRUE} | 0.25  7426 | 0.634  146 | 0.405  941 | 1.22  5814 | 10  4 |
| {BcOLV18=TRUE, BvF.BI=FALSE} =>  {BcMV4.BI=TRUE} | 0.25  7426 | 0.541  667 | 0.475  248 | 1.22  2533 | 10  4 |
| {BcMV4.BI=TRUE, BcOLV13.BI=FALSE} =>  {BcOLV18=TRUE} | 0.24  2574 | 0.620  253 | 0.391  089 | 1.19  8958 | 98 |
| {BcMV4.BI=TRUE, BcOLV2.BI=FALSE} =>  {BcOLV18=TRUE} | 0.24  505 | 0.607  362 | 0.403  465 | 1.17  4039 | 99 |
| {BcOLV18=TRUE, BcOLV2.BI=FALSE} =>  {BcMV4.BI=TRUE} | 0.24  505 | 0.523  81 | 0.467  822 | 1.18  2229 | 99 |
| {BcMCV3.BI=FALSE, BcMV4.BI=TRUE} =>  {BcOLV18=TRUE} | 0.25  2475 | 0.614  458 | 0.410  891 | 1.18  7756 | 10  2 |
| {BcMCV3.BI=FALSE, BcOLV18=TRUE} =>  {BcMV4.BI=TRUE} | 0.25  2475 | 0.531  25 | 0.475  248 | 1.19  9022 | 10  2 |
| {BcMV4.BI=TRUE, BcPV4.BI=FALSE} =>  {BcOLV18=TRUE} | 0.25  9901 | 0.621  302 | 0.418  317 | 1.20  0985 | 10  5 |
| {BcOLV18=TRUE, BcPV4.BI=FALSE} =>  {BcMV4.BI=TRUE} | 0.25  9901 | 0.544  041 | 0.477  723 | 1.22  7892 | 10  5 |
| {BcEV6=FALSE, BcMV4.BI=TRUE} =>  {BcOLV18=TRUE} | 0.26  4851 | 0.629  412 | 0.420  792 | 1.21  6662 | 10  7 |
| {BcEV6=FALSE, BcOLV18=TRUE} => | 0.26 | 0.548 | 0.482 | 1.23 | 10 |

| {BcMV4.BI=TRUE} | 4851 | 718 | 673 | 8447 | 7 |
| --- | --- | --- | --- | --- | --- |
| {BcMV4.BI=TRUE, BcOLV17.BI=FALSE} =>  {BcOLV18=TRUE} | 0.24  7525 | 0.602  41 | 0.410  891 | 1.16  4466 | 10  0 |
| {BcOLV17.BI=FALSE, BcOLV18=TRUE} =>  {BcMV4.BI=TRUE} | 0.24  7525 | 0.518  135 | 0.477  723 | 1.16  9421 | 10  0 |
| {BcMV4.BI=TRUE, BcOLV15.BI=FALSE} =>  {BcOLV18=TRUE} | 0.25  2475 | 0.610  778 | 0.413  366 | 1.18  0643 | 10  2 |
| {BcOLV15.BI=FALSE, BcOLV18=TRUE} =>  {BcMV4.BI=TRUE} | 0.25  2475 | 0.528  497 | 0.477  723 | 1.19  281 | 10  2 |
| {BcEV5=FALSE, BcMV4.BI=TRUE} =>  {BcOLV18=TRUE} | 0.25 | 0.627  329 | 0.398  515 | 1.21  2636 | 10  1 |
| {BcMV2.BI=FALSE, BcMV4.BI=TRUE} =>  {BcOLV1.BI.MOVA.=FALSE} | 0.13  6139 | 0.808  824 | 0.168  317 | 1.21  024 | 55 |
| {BcMV2.BI=FALSE, BcMV4.BI=TRUE} =>  {BcMV6.BI=FALSE} | 0.14  3564 | 0.852  941 | 0.168  317 | 1.22  6293 | 58 |
| {BcHV1SLR.BI=TRUE, BcMV4.BI=TRUE} =>  {BcMV3.BI=TRUE} | 0.19  802 | 0.754  717 | 0.262  376 | 1.39  2263 | 80 |
| {BcMV3.BI=TRUE, BcMV4.BI=TRUE} =>  {BcHV1SLR.BI=TRUE} | 0.19  802 | 0.672  269 | 0.294  554 | 1.24  0167 | 80 |
| {BcHV1SLR.BI=TRUE, BcMV3.BI=TRUE} =>  {BcMV4.BI=TRUE} | 0.19  802 | 0.601  504 | 0.329  208 | 1.35  7584 | 80 |
| {BcHV1SLR.BI=TRUE, BcMV4.BI=TRUE} => | 0.20 | 0.773 | 0.262 | 1.30 | 82 |

| {BcULV3=TRUE} | 297 | 585 | 376 | 2201 |  |
| --- | --- | --- | --- | --- | --- |
| {BcMV4.BI=TRUE, BcULV3=TRUE} =>  {BcHV1SLR.BI=TRUE} | 0.20  297 | 0.689  076 | 0.294  554 | 1.27  1171 | 82 |
| {BcHV1SLR.BI=TRUE, BcULV3=TRUE} =>  {BcMV4.BI=TRUE} | 0.20  297 | 0.525  641 | 0.386  139 | 1.18  6363 | 82 |
| {BcHV1SLR.BI=TRUE, BcMV4.BI=TRUE} =>  {BcHV1.BI=TRUE} | 0.24  2574 | 0.924  528 | 0.262  376 | 1.53  0776 | 98 |
| {BcHV1.BI=TRUE, BcMV4.BI=TRUE} =>  {BcHV1SLR.BI=TRUE} | 0.24  2574 | 0.809  917 | 0.299  505 | 1.49  4094 | 98 |
| {BcHV1SLR.BI=TRUE, BcMV4.BI=TRUE} =>  {BcMV11=TRUE} | 0.20  297 | 0.773  585 | 0.262  376 | 1.25  5134 | 82 |
| {BcHV1SLR.BI=TRUE, BcMV11=TRUE} =>  {BcMV4.BI=TRUE} | 0.20  297 | 0.569  444 | 0.356  436 | 1.28  5227 | 82 |
| {BcHV1SLR.BI=TRUE, BcMV4.BI=TRUE} =>  {BcMV9.BI=TRUE} | 0.22  2772 | 0.849  057 | 0.262  376 | 1.25  1894 | 90 |
| {BcMV4.BI=TRUE, BcMV9.BI=TRUE} =>  {BcHV1SLR.BI=TRUE} | 0.22  2772 | 0.661  765 | 0.336  634 | 1.22  079 | 90 |
| {BcHV1SLR.BI=TRUE, BcMV9.BI=TRUE} =>  {BcMV4.BI=TRUE} | 0.22  2772 | 0.545  455 | 0.408  416 | 1.23  1082 | 90 |
| {BcHV1SLR.BI=TRUE, BcMV4.BI=TRUE} =>  {BcULV4=TRUE} | 0.21  2871 | 0.811  321 | 0.262  376 | 1.17  4816 | 86 |
| {BcMV4.BI=TRUE, BcULV4=TRUE} => | 0.21 | 0.656 | 0.324 | 1.21 | 86 |

| {BcHV1SLR.BI=TRUE} | 2871 | 489 | 257 | 1057 |  |
| --- | --- | --- | --- | --- | --- |
| {BcHV1SLR.BI=TRUE, BcULV4=TRUE} =>  {BcMV4.BI=TRUE} | 0.21  2871 | 0.530  864 | 0.400  99 | 1.19  8152 | 86 |
| {BcMV3.BI=TRUE, BcMV4.BI=TRUE} =>  {BcULV3=TRUE} | 0.21  2871 | 0.722  689 | 0.294  554 | 1.21  6527 | 86 |
| {BcMV4.BI=TRUE, BcULV3=TRUE} =>  {BcMV3.BI=TRUE} | 0.21  2871 | 0.722  689 | 0.294  554 | 1.33  318 | 86 |
| {BcMV3.BI=TRUE, BcULV3=TRUE} =>  {BcMV4.BI=TRUE} | 0.21  2871 | 0.614  286 | 0.346  535 | 1.38  6433 | 86 |
| {BcMV3.BI=TRUE, BcMV4.BI=TRUE} =>  {BcHV1.BI=TRUE} | 0.22  0297 | 0.747  899 | 0.294  554 | 1.23  8325 | 89 |
| {BcHV1.BI=TRUE, BcMV4.BI=TRUE} =>  {BcMV3.BI=TRUE} | 0.22  0297 | 0.735  537 | 0.299  505 | 1.35  6881 | 89 |
| {BcHV1.BI=TRUE, BcMV3.BI=TRUE} =>  {BcMV4.BI=TRUE} | 0.22  0297 | 0.622  378 | 0.353  96 | 1.40  4696 | 89 |
| {BcMV3.BI=TRUE, BcMV4.BI=TRUE} =>  {BcMV11=TRUE} | 0.24  0099 | 0.815  126 | 0.294  554 | 1.32  2534 | 97 |
| {BcMV11=TRUE, BcMV4.BI=TRUE} =>  {BcMV3.BI=TRUE} | 0.24  0099 | 0.708  029 | 0.339  109 | 1.30  6136 | 97 |
| {BcMV11=TRUE, BcMV3.BI=TRUE} =>  {BcMV4.BI=TRUE} | 0.24  0099 | 0.625  806 | 0.383  663 | 1.41  2435 | 97 |
| {BcMV3.BI=TRUE, BcMV4.BI=TRUE} => | 0.23 | 0.798 | 0.294 | 1.17 | 95 |

| {BcMV9.BI=TRUE} | 5149 | 319 | 554 | 7084 |  |
| --- | --- | --- | --- | --- | --- |
| {BcMV4.BI=TRUE, BcMV9.BI=TRUE} =>  {BcMV3.BI=TRUE} | 0.23  5149 | 0.698  529 | 0.336  634 | 1.28  8611 | 95 |
| {BcMV3.BI=TRUE, BcMV9.BI=TRUE} =>  {BcMV4.BI=TRUE} | 0.23  5149 | 0.593  75 | 0.396  04 | 1.34  0084 | 95 |
| {BcMV3.BI=TRUE, BcMV4.BI=TRUE} =>  {BcULV4=TRUE} | 0.23  0198 | 0.781  513 | 0.294  554 | 1.13  1653 | 93 |
| {BcMV4.BI=TRUE, BcULV4=TRUE} =>  {BcMV3.BI=TRUE} | 0.23  0198 | 0.709  924 | 0.324  257 | 1.30  9631 | 93 |
| {BcMV3.BI=TRUE, BcULV4=TRUE} =>  {BcMV4.BI=TRUE} | 0.23  0198 | 0.574  074 | 0.400  99 | 1.29  5676 | 93 |
| {BcEV2.BI=FALSE, BcMV4.BI=TRUE} =>  {BcMV3.BI=TRUE} | 0.21  2871 | 0.677  165 | 0.314  356 | 1.24  92 | 86 |
| {BcEV2.BI=FALSE, BcMV3.BI=TRUE} =>  {BcMV4.BI=TRUE} | 0.21  2871 | 0.527  607 | 0.403  465 | 1.19  0801 | 86 |
| {BcHV3.BI=FALSE, BcMV4.BI=TRUE} =>  {BcMV3.BI=TRUE} | 0.18  8119 | 0.644  068 | 0.292  079 | 1.18  8143 | 76 |
| {BcMV4.BI=TRUE, BcULV1.BI=FALSE} =>  {BcMV3.BI=TRUE} | 0.21  2871 | 0.637  037 | 0.334  158 | 1.17  5173 | 86 |
| {BcMV3.BI=TRUE, BcULV1.BI=FALSE} =>  {BcMV4.BI=TRUE} | 0.21  2871 | 0.544  304 | 0.391  089 | 1.22  8485 | 86 |
| {BcMV3.BI=TRUE, BcMV4.BI=TRUE} => | 0.25 | 0.882 | 0.294 | 1.11 | 10 |

| {BcMV1.BI=TRUE} | 9901 | 353 | 554 | 7463 | 5 |
| --- | --- | --- | --- | --- | --- |
| {BcMV1.BI=TRUE, BcMV4.BI=TRUE} =>  {BcMV3.BI=TRUE} | 0.25  9901 | 0.690  789 | 0.376  238 | 1.27  4333 | 10  5 |
| {BcMV1.BI=TRUE, BcMV3.BI=TRUE} =>  {BcMV4.BI=TRUE} | 0.25  9901 | 0.567  568 | 0.457  921 | 1.28  099 | 10  5 |
| {BcMV12=FALSE, BcMV4.BI=TRUE} =>  {BcMV3.BI=TRUE} | 0.23  2673 | 0.643  836 | 0.361  386 | 1.18  7715 | 94 |
| {BcMV12=FALSE, BcMV3.BI=TRUE} =>  {BcMV4.BI=TRUE} | 0.23  2673 | 0.598  726 | 0.388  614 | 1.35  1315 | 94 |
| {BcMV4.BI=TRUE, BcNSRV7.BI=FALSE} =>  {BcMV3.BI=TRUE} | 0.22  0297 | 0.654  412 | 0.336  634 | 1.20  7225 | 89 |
| {BcMV3.BI=TRUE, BcNSRV7.BI=FALSE} =>  {BcMV4.BI=TRUE} | 0.22  0297 | 0.520  468 | 0.423  267 | 1.17  4687 | 89 |
| {BcMV4.BI=TRUE, BcVV3.BI=FALSE} =>  {BcMV3.BI=TRUE} | 0.21  5347 | 0.649  254 | 0.331  683 | 1.19  771 | 87 |
| {BcMV4.BI=TRUE, BcOLV7.BI=FALSE} =>  {BcMV3.BI=TRUE} | 0.21  5347 | 0.639  706 | 0.336  634 | 1.18  0097 | 87 |
| {BcALV1.BI=FALSE, BcMV4.BI=TRUE} =>  {BcMV3.BI=TRUE} | 0.20  297 | 0.645  669 | 0.314  356 | 1.19  1098 | 82 |
| {BcDMV1=FALSE, BcMV3.BI=TRUE} =>  {BcMV4.BI=TRUE} | 0.22  2772 | 0.526  316 | 0.423  267 | 1.18  7886 | 90 |
| {BcFV9=FALSE, BcMV4.BI=TRUE} => | 0.23 | 0.652 | 0.356 | 1.20 | 94 |

| {BcMV3.BI=TRUE} | 2673 | 778 | 436 | 4211 |  |
| --- | --- | --- | --- | --- | --- |
| {BcMV4.BI=TRUE, BcOLV4.BI=FALSE} =>  {BcMV3.BI=TRUE} | 0.24  2574 | 0.653  333 | 0.371  287 | 1.20  5236 | 98 |
| {BcMV3.BI=TRUE, BcOLV4.BI=FALSE} =>  {BcMV4.BI=TRUE} | 0.24  2574 | 0.532  609 | 0.455  446 | 1.20  2089 | 98 |
| {BcMV4.BI=TRUE, BcULV5=FALSE} =>  {BcMV3.BI=TRUE} | 0.24  2574 | 0.636  364 | 0.381  188 | 1.17  3931 | 98 |
| {BcMV3.BI=TRUE, BcULV5=FALSE} =>  {BcMV4.BI=TRUE} | 0.24  2574 | 0.535  519 | 0.452  97 | 1.20  8658 | 98 |
| {BcBNV5.BI=FALSE, BcMV4.BI=TRUE} =>  {BcMV3.BI=TRUE} | 0.24  2574 | 0.632  258 | 0.383  663 | 1.16  6357 | 98 |
| {BcBNV5.BI=FALSE, BcMV3.BI=TRUE} =>  {BcMV4.BI=TRUE} | 0.24  2574 | 0.515  789 | 0.470  297 | 1.16  4128 | 98 |
| {BcMV4.BI=TRUE, BvF.BI=FALSE} =>  {BcMV3.BI=TRUE} | 0.27  2277 | 0.670  732 | 0.405  941 | 1.23  7332 | 11  0 |
| {BcMV3.BI=TRUE, BvF.BI=FALSE} =>  {BcMV4.BI=TRUE} | 0.27  2277 | 0.55 | 0.495  05 | 1.24  1341 | 11  0 |
| {BcMCV5.BI=FALSE, BcMV4.BI=TRUE} =>  {BcMV3.BI=TRUE} | 0.23  7624 | 0.627  451 | 0.378  713 | 1.15  7489 | 96 |
| {BcMV4.BI=TRUE, BcOLV13.BI=FALSE} =>  {BcMV3.BI=TRUE} | 0.24  505 | 0.626  582 | 0.391  089 | 1.15  5887 | 99 |
| {BcMV3.BI=TRUE, BcOLV13.BI=FALSE} => | 0.24 | 0.515 | 0.475 | 1.16 | 99 |

| {BcMV4.BI=TRUE} | 505 | 625 | 248 | 3757 |  |
| --- | --- | --- | --- | --- | --- |
| {BcMV4.BI=TRUE, BcOLV2.BI=FALSE} =>  {BcMV3.BI=TRUE} | 0.25  7426 | 0.638  037 | 0.403  465 | 1.17  7018 | 10  4 |
| {BcMV3.BI=TRUE, BcOLV2.BI=FALSE} =>  {BcMV4.BI=TRUE} | 0.25  7426 | 0.530  612 | 0.485  149 | 1.19  7583 | 10  4 |
| {BcMCV3.BI=FALSE, BcMV4.BI=TRUE} =>  {BcMV3.BI=TRUE} | 0.26  2376 | 0.638  554 | 0.410  891 | 1.17  7972 | 10  6 |
| {BcMCV3.BI=FALSE, BcMV3.BI=TRUE} =>  {BcMV4.BI=TRUE} | 0.26  2376 | 0.552  083 | 0.475  248 | 1.24  6043 | 10  6 |
| {BcMV4.BI=TRUE, BcPV4.BI=FALSE} =>  {BcMV3.BI=TRUE} | 0.27  4752 | 0.656  805 | 0.418  317 | 1.21  164 | 11  1 |
| {BcMV3.BI=TRUE, BcPV4.BI=FALSE} =>  {BcMV4.BI=TRUE} | 0.27  4752 | 0.552  239 | 0.497  525 | 1.24  6394 | 11  1 |
| {BcEV6=FALSE, BcMV4.BI=TRUE} =>  {BcMV3.BI=TRUE} | 0.28  4653 | 0.676  471 | 0.420  792 | 1.24  7918 | 11  5 |
| {BcEV6=FALSE, BcMV3.BI=TRUE} =>  {BcMV4.BI=TRUE} | 0.28  4653 | 0.550  239 | 0.517  327 | 1.24  1881 | 11  5 |
| {BcMV4.BI=TRUE, BcOLV17.BI=FALSE} =>  {BcMV3.BI=TRUE} | 0.26  9802 | 0.656  627 | 0.410  891 | 1.21  1311 | 10  9 |
| {BcMV3.BI=TRUE, BcOLV17.BI=FALSE} =>  {BcMV4.BI=TRUE} | 0.26  9802 | 0.526  57 | 0.512  376 | 1.18  846 | 10  9 |
| {BcMV4.BI=TRUE, BcOLV15.BI=FALSE} => | 0.27 | 0.658 | 0.413 | 1.21 | 11 |

| {BcMV3.BI=TRUE} | 2277 | 683 | 366 | 5104 | 0 |
| --- | --- | --- | --- | --- | --- |
| {BcMV3.BI=TRUE, BcOLV15.BI=FALSE} =>  {BcMV4.BI=TRUE} | 0.27  2277 | 0.536  585 | 0.507  426 | 1.21  1064 | 11  0 |
| {BcEV5=FALSE, BcMV4.BI=TRUE} =>  {BcMV3.BI=TRUE} | 0.25 | 0.627  329 | 0.398  515 | 1.15  7265 | 10  1 |
| {BcMV4.BI=TRUE, BcOLV16.BI=FALSE} =>  {BcMV11=TRUE} | 0.16  0891 | 0.738  636 | 0.217  822 | 1.19  843 | 65 |
| {BcMV4.BI=TRUE, BcOLV16.BI=FALSE} =>  {BcMV12=FALSE} | 0.19  5545 | 0.897  727 | 0.217  822 | 1.12  2854 | 79 |
| {BcMV4.BI=TRUE, BcULV3=TRUE} =>  {BcHV1.BI=TRUE} | 0.22  2772 | 0.756  303 | 0.294  554 | 1.25  2239 | 90 |
| {BcHV1.BI=TRUE, BcMV4.BI=TRUE} =>  {BcULV3=TRUE} | 0.22  2772 | 0.743  802 | 0.299  505 | 1.25  2066 | 90 |
| {BcHV1.BI=TRUE, BcULV3=TRUE} =>  {BcMV4.BI=TRUE} | 0.22  2772 | 0.526  316 | 0.423  267 | 1.18  7886 | 90 |
| {BcMV4.BI=TRUE, BcULV3=TRUE} =>  {BcMV11=TRUE} | 0.24  0099 | 0.815  126 | 0.294  554 | 1.32  2534 | 97 |
| {BcMV11=TRUE, BcMV4.BI=TRUE} =>  {BcULV3=TRUE} | 0.24  0099 | 0.708  029 | 0.339  109 | 1.19  1849 | 97 |
| {BcMV11=TRUE, BcULV3=TRUE} =>  {BcMV4.BI=TRUE} | 0.24  0099 | 0.606  25 | 0.396  04 | 1.36  8296 | 97 |
| {BcMV4.BI=TRUE, BcULV3=TRUE} => | 0.22 | 0.764 | 0.294 | 1.12 | 91 |

| {BcMV9.BI=TRUE} | 5248 | 706 | 554 | 7523 |  |
| --- | --- | --- | --- | --- | --- |
| {BcMV9.BI=TRUE, BcULV3=TRUE} =>  {BcMV4.BI=TRUE} | 0.22  5248 | 0.532  164 | 0.423  267 | 1.20  1085 | 91 |
| {BcULV3=TRUE, BcULV4=TRUE} =>  {BcMV4.BI=TRUE} | 0.21  7822 | 0.523  81 | 0.415  842 | 1.18  2229 | 88 |
| {BcMV4.BI=TRUE, BcPV3.BI=FALSE} =>  {BcULV3=TRUE} | 0.18  5644 | 0.707  547 | 0.262  376 | 1.19  1038 | 75 |
| {BcMV4.BI=TRUE, BcRV1.BI=FALSE} =>  {BcULV3=TRUE} | 0.19  0594 | 0.687  5 | 0.277  228 | 1.15  7292 | 77 |
| {BcEV2.BI=FALSE, BcMV4.BI=TRUE} =>  {BcULV3=TRUE} | 0.21  2871 | 0.677  165 | 0.314  356 | 1.13  9895 | 86 |
| {BcMV1.BI=TRUE, BcMV4.BI=TRUE} =>  {BcULV3=TRUE} | 0.25  2475 | 0.671  053 | 0.376  238 | 1.12  9605 | 10  2 |
| {BcMV1.BI=TRUE, BcULV3=TRUE} =>  {BcMV4.BI=TRUE} | 0.25  2475 | 0.520  408 | 0.485  149 | 1.17  4553 | 10  2 |
| {BcFV9=FALSE, BcMV4.BI=TRUE} =>  {BcULV3=TRUE} | 0.24  0099 | 0.673  611 | 0.356  436 | 1.13  3912 | 97 |
| {BcMV4.BI=TRUE, BvF.BI=FALSE} =>  {BcULV3=TRUE} | 0.27  4752 | 0.676  829 | 0.405  941 | 1.13  9329 | 11  1 |
| {BcHV1.BI=TRUE, BcMV4.BI=TRUE} =>  {BcMV11=TRUE} | 0.23  0198 | 0.768  595 | 0.299  505 | 1.24  7038 | 93 |
| {BcHV1.BI=TRUE, BcMV11=TRUE} => | 0.23 | 0.603 | 0.381 | 1.36 | 93 |

| {BcMV4.BI=TRUE} | 0198 | 896 | 188 | 2983 |  |
| --- | --- | --- | --- | --- | --- |
| {BcHV1.BI=TRUE, BcMV4.BI=TRUE} =>  {BcMV9.BI=TRUE} | 0.25  495 | 0.851  24 | 0.299  505 | 1.25  5113 | 10  3 |
| {BcMV4.BI=TRUE, BcMV9.BI=TRUE} =>  {BcHV1.BI=TRUE} | 0.25  495 | 0.757  353 | 0.336  634 | 1.25  3978 | 10  3 |
| {BcHV1.BI=TRUE, BcMV9.BI=TRUE} =>  {BcMV4.BI=TRUE} | 0.25  495 | 0.544  974 | 0.467  822 | 1.22  9996 | 10  3 |
| {BcHV1.BI=TRUE, BcMV4.BI=TRUE} =>  {BcULV4=TRUE} | 0.24  2574 | 0.809  917 | 0.299  505 | 1.17  2784 | 98 |
| {BcMV4.BI=TRUE, BcULV4=TRUE} =>  {BcHV1.BI=TRUE} | 0.24  2574 | 0.748  092 | 0.324  257 | 1.23  8643 | 98 |
| {BcHV1.BI=TRUE, BcULV4=TRUE} =>  {BcMV4.BI=TRUE} | 0.24  2574 | 0.532  609 | 0.455  446 | 1.20  2089 | 98 |
| {BcMV1.BI=TRUE, BcMV4.BI=TRUE} =>  {BcHV1.BI=TRUE} | 0.25  7426 | 0.684  211 | 0.376  238 | 1.13  2873 | 10  4 |
| {BcHV1.BI=TRUE, BcMV1.BI=TRUE} =>  {BcMV4.BI=TRUE} | 0.25  7426 | 0.533  333 | 0.482  673 | 1.20  3724 | 10  4 |
| {BcHV1.BI=TRUE, BcMV12=FALSE} =>  {BcMV4.BI=TRUE} | 0.24  0099 | 0.515  957 | 0.465  347 | 1.16  4507 | 97 |
| {BcMV4.BI=TRUE, BcVV3.BI=FALSE} =>  {BcHV1.BI=TRUE} | 0.22  7723 | 0.686  567 | 0.331  683 | 1.13  6775 | 92 |
| {BcMV4.BI=TRUE, BcULV5=FALSE} => | 0.25 | 0.681 | 0.381 | 1.12 | 10 |

| {BcHV1.BI=TRUE} | 9901 | 818 | 188 | 8912 | 5 |
| --- | --- | --- | --- | --- | --- |
| {BcMV4.BI=TRUE, BcPV4.BI=FALSE} =>  {BcHV1.BI=TRUE} | 0.28  4653 | 0.680  473 | 0.418  317 | 1.12  6685 | 11  5 |
| {BcHV1.BI=TRUE, BcPV4.BI=FALSE} =>  {BcMV4.BI=TRUE} | 0.28  4653 | 0.508  85 | 0.559  406 | 1.14  8465 | 11  5 |
| {BcEV6=FALSE, BcHV1.BI=TRUE} =>  {BcMV4.BI=TRUE} | 0.27  9703 | 0.509  009 | 0.549  505 | 1.14  8825 | 11  3 |
| {BcMV4.BI=TRUE, BcMV9.BI=TRUE} =>  {BcMV11=TRUE} | 0.25  495 | 0.757  353 | 0.336  634 | 1.22  8798 | 10  3 |
| {BcMV11=TRUE, BcMV9.BI=TRUE} =>  {BcMV4.BI=TRUE} | 0.25  495 | 0.628  049 | 0.405  941 | 1.41  7496 | 10  3 |
| {BcMV4.BI=TRUE, BcULV4=TRUE} =>  {BcMV11=TRUE} | 0.25 | 0.770  992 | 0.324  257 | 1.25  0927 | 10  1 |
| {BcMV11=TRUE, BcULV4=TRUE} =>  {BcMV4.BI=TRUE} | 0.25 | 0.597  633 | 0.418  317 | 1.34  8848 | 10  1 |
| {BcMV4.BI=TRUE, BcPV3.BI=FALSE} =>  {BcMV11=TRUE} | 0.19  5545 | 0.745  283 | 0.262  376 | 1.20  9214 | 79 |
| {BcMV4.BI=TRUE, BcRV1.BI=FALSE} =>  {BcMV11=TRUE} | 0.20  0495 | 0.723  214 | 0.277  228 | 1.17  3408 | 81 |
| {BcEV2.BI=FALSE, BcMV4.BI=TRUE} =>  {BcMV11=TRUE} | 0.24  0099 | 0.763  78 | 0.314  356 | 1.23  9225 | 97 |
| {BcEV2.BI=FALSE, BcMV11=TRUE} => | 0.24 | 0.557 | 0.430 | 1.25 | 97 |

| {BcMV4.BI=TRUE} | 0099 | 471 | 693 | 8203 |  |
| --- | --- | --- | --- | --- | --- |
| {BcHV3.BI=FALSE, BcMV4.BI=TRUE} =>  {BcMV11=TRUE} | 0.21  0396 | 0.720  339 | 0.292  079 | 1.16  8743 | 85 |
| {BcFV5.BI=FALSE, BcMV4.BI=TRUE} =>  {BcMV11=TRUE} | 0.24  0099 | 0.746  154 | 0.321  782 | 1.21  0627 | 97 |
| {BcMV4.BI=TRUE, BcULV1.BI=FALSE} =>  {BcMV11=TRUE} | 0.25  2475 | 0.755  556 | 0.334  158 | 1.22  5881 | 10  2 |
| {BcMV11=TRUE, BcULV1.BI=FALSE} =>  {BcMV4.BI=TRUE} | 0.25  2475 | 0.534  031 | 0.472  772 | 1.20  53 | 10  2 |
| {BcMV11=TRUE, BcMV4.BI=TRUE} =>  {BcMV1.BI=TRUE} | 0.30  198 | 0.890  511 | 0.339  109 | 1.12  7794 | 12  2 |
| {BcMV1.BI=TRUE, BcMV4.BI=TRUE} =>  {BcMV11=TRUE} | 0.30  198 | 0.802  632 | 0.376  238 | 1.30  2262 | 12  2 |
| {BcMV1.BI=TRUE, BcMV11=TRUE} =>  {BcMV4.BI=TRUE} | 0.30  198 | 0.580  952 | 0.519  802 | 1.31  12 | 12  2 |
| {BcMV12=FALSE, BcMV4.BI=TRUE} =>  {BcMV11=TRUE} | 0.27  9703 | 0.773  973 | 0.361  386 | 1.25  5763 | 11  3 |
| {BcMV11=TRUE, BcMV12=FALSE} =>  {BcMV4.BI=TRUE} | 0.27  9703 | 0.551  22 | 0.507  426 | 1.24  4093 | 11  3 |
| {BcMV4.BI=TRUE, BcNSRV7.BI=FALSE} =>  {BcMV11=TRUE} | 0.25  9901 | 0.772  059 | 0.336  634 | 1.25  2658 | 10  5 |
| {BcMV11=TRUE, BcNSRV7.BI=FALSE} => | 0.25 | 0.535 | 0.485 | 1.20 | 10 |

| {BcMV4.BI=TRUE} | 9901 | 714 | 149 | 9098 | 5 |
| --- | --- | --- | --- | --- | --- |
| {BcMV4.BI=TRUE, BcVV3.BI=FALSE} =>  {BcMV11=TRUE} | 0.25 | 0.753  731 | 0.331  683 | 1.22  2922 | 10  1 |
| {BcMV11=TRUE, BcVV3.BI=FALSE} =>  {BcMV4.BI=TRUE} | 0.25 | 0.517  949 | 0.482  673 | 1.16  9002 | 10  1 |
| {BcMV4.BI=TRUE, BcOLV7.BI=FALSE} =>  {BcMV11=TRUE} | 0.25  7426 | 0.764  706 | 0.336  634 | 1.24  0728 | 10  4 |
| {BcMV11=TRUE, BcOLV7.BI=FALSE} =>  {BcMV4.BI=TRUE} | 0.25  7426 | 0.530  612 | 0.485  149 | 1.19  7583 | 10  4 |
| {BcALV1.BI=FALSE, BcMV4.BI=TRUE} =>  {BcMV11=TRUE} | 0.22  5248 | 0.716  535 | 0.314  356 | 1.16  2572 | 91 |
| {BcDMV1=FALSE, BcMV4.BI=TRUE} =>  {BcMV11=TRUE} | 0.26  2376 | 0.726  027 | 0.361  386 | 1.17  7972 | 10  6 |
| {BcDMV1=FALSE, BcMV11=TRUE} =>  {BcMV4.BI=TRUE} | 0.26  2376 | 0.514  563 | 0.509  901 | 1.16  136 | 10  6 |
| {BcFV9=FALSE, BcMV4.BI=TRUE} =>  {BcMV11=TRUE} | 0.27  2277 | 0.763  889 | 0.356  436 | 1.23  9402 | 11  0 |
| {BcFV9=FALSE, BcMV11=TRUE} =>  {BcMV4.BI=TRUE} | 0.27  2277 | 0.521  327 | 0.522  277 | 1.17  6626 | 11  0 |
| {BcMV4.BI=TRUE, BcOLV4.BI=FALSE} =>  {BcMV11=TRUE} | 0.27  4752 | 0.74 | 0.371  287 | 1.20  0643 | 11  1 |
| {BcMV11=TRUE, BcOLV4.BI=FALSE} => | 0.27 | 0.536 | 0.512 | 1.21 | 11 |

| {BcMV4.BI=TRUE} | 4752 | 232 | 376 | 0266 | 1 |
| --- | --- | --- | --- | --- | --- |
| {BcMV4.BI=TRUE, BcULV5=FALSE} =>  {BcMV11=TRUE} | 0.27  9703 | 0.733  766 | 0.381  188 | 1.19  0528 | 11  3 |
| {BcMV11=TRUE, BcULV5=FALSE} =>  {BcMV4.BI=TRUE} | 0.27  9703 | 0.553  922 | 0.504  95 | 1.25  0192 | 11  3 |
| {BcBNV5.BI=FALSE, BcMV4.BI=TRUE} =>  {BcMV11=TRUE} | 0.28  2178 | 0.735  484 | 0.383  663 | 1.19  3315 | 11  4 |
| {BcBNV5.BI=FALSE, BcMV11=TRUE} =>  {BcMV4.BI=TRUE} | 0.28  2178 | 0.525  346 | 0.537  129 | 1.18  5696 | 11  4 |
| {BcMV4.BI=TRUE, BvF.BI=FALSE} =>  {BcMV11=TRUE} | 0.31  9307 | 0.786  585 | 0.405  941 | 1.27  6227 | 12  9 |
| {BcMV11=TRUE, BvF.BI=FALSE} =>  {BcMV4.BI=TRUE} | 0.31  9307 | 0.558  442 | 0.571  782 | 1.26  0393 | 12  9 |
| {BcMCV5.BI=FALSE, BcMV4.BI=TRUE} =>  {BcMV11=TRUE} | 0.27  9703 | 0.738  562 | 0.378  713 | 1.19  831 | 11  3 |
| {BcMCV5.BI=FALSE, BcMV11=TRUE} =>  {BcMV4.BI=TRUE} | 0.27  9703 | 0.511  312 | 0.547  03 | 1.15  4023 | 11  3 |
| {BcMV4.BI=TRUE, BcOLV13.BI=FALSE} =>  {BcMV11=TRUE} | 0.29  703 | 0.759  494 | 0.391  089 | 1.23  2271 | 12  0 |
| {BcMV11=TRUE, BcOLV13.BI=FALSE} =>  {BcMV4.BI=TRUE} | 0.29  703 | 0.530  973 | 0.559  406 | 1.19  8398 | 12  0 |
| {BcMV4.BI=TRUE, BcOLV2.BI=FALSE} => | 0.30 | 0.754 | 0.403 | 1.22 | 12 |

| {BcMV11=TRUE} | 4455 | 601 | 465 | 4333 | 3 |
| --- | --- | --- | --- | --- | --- |
| {BcMV11=TRUE, BcOLV2.BI=FALSE} =>  {BcMV4.BI=TRUE} | 0.30  4455 | 0.534  783 | 0.569  307 | 1.20  6995 | 12  3 |
| {BcMCV3.BI=FALSE, BcMV4.BI=TRUE} =>  {BcMV11=TRUE} | 0.32  1782 | 0.783  133 | 0.410  891 | 1.27  0625 | 13  0 |
| {BcMCV3.BI=FALSE, BcMV11=TRUE} =>  {BcMV4.BI=TRUE} | 0.32  1782 | 0.553  191 | 0.581  683 | 1.24  8544 | 13  0 |
| {BcMV4.BI=TRUE, BcPV4.BI=FALSE} =>  {BcMV11=TRUE} | 0.31  9307 | 0.763  314 | 0.418  317 | 1.23  8469 | 12  9 |
| {BcMV11=TRUE, BcPV4.BI=FALSE} =>  {BcMV4.BI=TRUE} | 0.31  9307 | 0.553  648 | 0.576  733 | 1.24  9574 | 12  9 |
| {BcEV6=FALSE, BcMV4.BI=TRUE} =>  {BcMV11=TRUE} | 0.31  9307 | 0.758  824 | 0.420  792 | 1.23  1184 | 12  9 |
| {BcEV6=FALSE, BcMV11=TRUE} =>  {BcMV4.BI=TRUE} | 0.31  9307 | 0.553  648 | 0.576  733 | 1.24  9574 | 12  9 |
| {BcMV4.BI=TRUE, BcOLV17.BI=FALSE} =>  {BcMV11=TRUE} | 0.30  9406 | 0.753  012 | 0.410  891 | 1.22  1754 | 12  5 |
| {BcMV11=TRUE, BcOLV17.BI=FALSE} =>  {BcMV4.BI=TRUE} | 0.30  9406 | 0.538  793 | 0.574  257 | 1.21  6047 | 12  5 |
| {BcMV4.BI=TRUE, BcOLV15.BI=FALSE} =>  {BcMV11=TRUE} | 0.31  9307 | 0.772  455 | 0.413  366 | 1.25  3301 | 12  9 |
| {BcMV11=TRUE, BcOLV15.BI=FALSE} => | 0.31 | 0.542 | 0.589 | 1.22 | 12 |

| {BcMV4.BI=TRUE} | 9307 | 017 | 109 | 3323 | 9 |
| --- | --- | --- | --- | --- | --- |
| {BcEV5=FALSE, BcMV4.BI=TRUE} =>  {BcMV11=TRUE} | 0.29  703 | 0.745  342 | 0.398  515 | 1.20  9309 | 12  0 |
| {BcEV5=FALSE, BcMV11=TRUE} =>  {BcMV4.BI=TRUE} | 0.29  703 | 0.517  241 | 0.574  257 | 1.16  7405 | 12  0 |
| {BcMV4.BI=TRUE, BcOLV1.BI.MOVA.=FALSE} =>  {BcMV9.BI=TRUE} | 0.20  297 | 0.788  462 | 0.257  426 | 1.16  2549 | 82 |
| {BcMV4.BI=TRUE, BcOLV1.BI.MOVA.=FALSE} =>  {BcULV5=FALSE} | 0.24  2574 | 0.942  308 | 0.257  426 | 1.07  5402 | 98 |
| {BcMV4.BI=TRUE, BcMV9.BI=TRUE} =>  {BcULV4=TRUE} | 0.27  7228 | 0.823  529 | 0.336  634 | 1.19  2494 | 11  2 |
| {BcMV4.BI=TRUE, BcULV4=TRUE} =>  {BcMV9.BI=TRUE} | 0.27  7228 | 0.854  962 | 0.324  257 | 1.26  0601 | 11  2 |
| {BcMV9.BI=TRUE, BcULV4=TRUE} =>  {BcMV4.BI=TRUE} | 0.27  7228 | 0.506  787 | 0.547  03 | 1.14  3811 | 11  2 |
| {BcEV2.BI=FALSE, BcMV4.BI=TRUE} =>  {BcMV9.BI=TRUE} | 0.24  0099 | 0.763  78 | 0.314  356 | 1.12  6157 | 97 |
| {BcMV1.BI=TRUE, BcMV9.BI=TRUE} =>  {BcMV4.BI=TRUE} | 0.28  4653 | 0.518  018 | 0.549  505 | 1.16  9158 | 11  5 |
| {BcMV12=FALSE, BcMV4.BI=TRUE} => | 0.28 | 0.787 | 0.361 | 1.16 | 11 |

| {BcMV9.BI=TRUE} | 4653 | 671 | 386 | 1384 | 5 |
| --- | --- | --- | --- | --- | --- |
| {BcMV12=FALSE, BcMV9.BI=TRUE} =>  {BcMV4.BI=TRUE} | 0.28  4653 | 0.527  523 | 0.539  604 | 1.19  061 | 11  5 |
| {BcMV4.BI=TRUE, BcVV3.BI=FALSE} =>  {BcMV9.BI=TRUE} | 0.26  2376 | 0.791  045 | 0.331  683 | 1.16  6358 | 10  6 |
| {BcMV4.BI=TRUE, BcOLV4.BI=FALSE} =>  {BcMV9.BI=TRUE} | 0.29  9505 | 0.806  667 | 0.371  287 | 1.18  9392 | 12  1 |
| {BcMV4.BI=TRUE, BcMV9.BI=TRUE} =>  {BcULV5=FALSE} | 0.31  9307 | 0.948  529 | 0.336  634 | 1.08  2502 | 12  9 |
| {BcMV4.BI=TRUE, BcULV5=FALSE} =>  {BcMV9.BI=TRUE} | 0.31  9307 | 0.837  662 | 0.381  188 | 1.23  5093 | 12  9 |
| {BcMV4.BI=TRUE, BvF.BI=FALSE} =>  {BcMV9.BI=TRUE} | 0.30  9406 | 0.762  195 | 0.405  941 | 1.12  3821 | 12  5 |
| {BcMV9.BI=TRUE, BvF.BI=FALSE} =>  {BcMV4.BI=TRUE} | 0.30  9406 | 0.506  073 | 0.611  386 | 1.14  2198 | 12  5 |
| {BcMV4.BI=TRUE, BcOLV13.BI=FALSE} =>  {BcMV9.BI=TRUE} | 0.30  4455 | 0.778  481 | 0.391  089 | 1.14  7833 | 12  3 |
| {BcMV4.BI=TRUE, BcOLV2.BI=FALSE} =>  {BcMV9.BI=TRUE} | 0.30  6931 | 0.760  736 | 0.403  465 | 1.12  1669 | 12  4 |
| {BcMCV3.BI=FALSE, BcMV4.BI=TRUE} =>  {BcMV9.BI=TRUE} | 0.31  1881 | 0.759  036 | 0.410  891 | 1.11  9163 | 12  6 |
| {BcMV4.BI=TRUE, BcPV4.BI=FALSE} => | 0.32 | 0.769 | 0.418 | 1.13 | 13 |

| {BcMV9.BI=TRUE} | 1782 | 231 | 317 | 4194 | 0 |
| --- | --- | --- | --- | --- | --- |
| {BcMV9.BI=TRUE, BcPV4.BI=FALSE} =>  {BcMV4.BI=TRUE} | 0.32  1782 | 0.507  813 | 0.633  663 | 1.14  6124 | 13  0 |
| {BcEV6=FALSE, BcMV4.BI=TRUE} =>  {BcMV9.BI=TRUE} | 0.32  1782 | 0.764  706 | 0.420  792 | 1.12  7523 | 13  0 |
| {BcEV6=FALSE, BcMV9.BI=TRUE} =>  {BcMV4.BI=TRUE} | 0.32  1782 | 0.509  804 | 0.631  188 | 1.15  0619 | 13  0 |
| {BcMV4.BI=TRUE, BcOLV17.BI=FALSE} =>  {BcMV9.BI=TRUE} | 0.31  1881 | 0.759  036 | 0.410  891 | 1.11  9163 | 12  6 |
| {BcMV4.BI=TRUE, BcOLV15.BI=FALSE} =>  {BcMV9.BI=TRUE} | 0.31  6832 | 0.766  467 | 0.413  366 | 1.13  0119 | 12  8 |
| {BcMV1.BI=TRUE, BcULV4=TRUE} =>  {BcMV4.BI=TRUE} | 0.27  9703 | 0.509  009 | 0.549  505 | 1.14  8825 | 11  3 |
| {BcMV4.BI=TRUE, BcMV6.BI=FALSE} =>  {BcDMV1=FALSE} | 0.24  505 | 0.916  667 | 0.267  327 | 1.08  6022 | 99 |
| {BcMV4.BI=TRUE, BcRV1.BI=FALSE} =>  {BcMV1.BI=TRUE} | 0.24  7525 | 0.892  857 | 0.277  228 | 1.13  0766 | 10  0 |
| {BcMV4.BI=TRUE, BcRV1.BI=FALSE} =>  {BcOLV7.BI=FALSE} | 0.24  505 | 0.883  929 | 0.277  228 | 1.09  5421 | 99 |
| {BcMV4.BI=TRUE, BvF.BI=FALSE} =>  {BcMV1.BI=TRUE} | 0.34  901 | 0.859  756 | 0.405  941 | 1.08  8845 | 14  1 |
| {BcMCV3.BI=FALSE, BcMV4.BI=TRUE} => | 0.35 | 0.855 | 0.410 | 1.08 | 14 |

| {BcMV1.BI=TRUE} | 1485 | 422 | 891 | 3355 | 2 |
| --- | --- | --- | --- | --- | --- |
| {BcBNV5.BI=FALSE, BcMV4.BI=TRUE} =>  {BcDMV1=FALSE} | 0.34  6535 | 0.903  226 | 0.383  663 | 1.07  0097 | 14  0 |
| {BcEV5=FALSE, BcMV4.BI=TRUE} =>  {BcDMV1=FALSE} | 0.36  1386 | 0.906  832 | 0.398  515 | 1.07  437 | 14  6 |
| {BcHV1SLR.BI=FALSE, BcMV3.BI=FALSE}  => {BcOLV18=FALSE} | 0.16  0891 | 0.656  566 | 0.245  05 | 1.36  0269 | 65 |
| {BcHV1SLR.BI=FALSE, BcOLV18=FALSE} =>  {BcMV3.BI=FALSE} | 0.16  0891 | 0.570  175 | 0.282  178 | 1.24  514 | 65 |
| {BcMV3.BI=FALSE, BcOLV18=FALSE} =>  {BcHV1SLR.BI=FALSE} | 0.16  0891 | 0.596  33 | 0.269  802 | 1.30  2256 | 65 |
| {BcHV1SLR.BI=FALSE, BcMV3.BI=FALSE}  => {BcMV2.BI=FALSE} | 0.16  0891 | 0.656  566 | 0.245  05 | 1.22  2362 | 65 |
| {BcHV1SLR.BI=FALSE, BcMV2.BI=FALSE}  => {BcMV3.BI=FALSE} | 0.16  0891 | 0.601  852 | 0.267  327 | 1.31  4314 | 65 |
| {BcHV1SLR.BI=FALSE, BcMV3.BI=FALSE}  => {BcMV4.BI=FALSE} | 0.16  0891 | 0.656  566 | 0.245  05 | 1.17  89 | 65 |
| {BcHV1SLR.BI=FALSE, BcMV4.BI=FALSE}  => {BcMV3.BI=FALSE} | 0.16  0891 | 0.580  357 | 0.277  228 | 1.26  7375 | 65 |
| {BcHV1SLR.BI=FALSE, BcOLV16.BI=FALSE}  => {BcMV3.BI=FALSE} | 0.15  8416 | 0.566  372 | 0.279  703 | 1.23  6833 | 64 |
| {BcHV1SLR.BI=FALSE, BcMV3.BI=FALSE} | 0.19 | 0.797 | 0.245 | 1.19 | 79 |

| => {BcOLV1.BI.MOVA.=FALSE} | 5545 | 98 | 05 | 4014 |  |
| --- | --- | --- | --- | --- | --- |
| {BcHV1SLR.BI=FALSE, |  |  |  |  |  |
|  | 0.19 | 0.598 | 0.326 | 1.30 |  |
| BcOLV1.BI.MOVA.=FALSE} => |  |  |  |  | 79 |
|  | 5545 | 485 | 733 | 6962 |  |
| {BcMV3.BI=FALSE} |  |  |  |  |  |
| {BcHV1SLR.BI=FALSE, BcULV4=TRUE} => | 0.16 | 0.572 | 0.289 | 1.25 |  |
|  |  |  |  |  | 67 |
| {BcMV3.BI=FALSE} | 5842 | 65 | 604 | 0543 |  |
| {BcMV3.BI=FALSE, BcULV4=TRUE} => | 0.16 | 0.572 | 0.289 | 1.25 |  |
|  |  |  |  |  | 67 |
| {BcHV1SLR.BI=FALSE} | 5842 | 65 | 604 | 0543 |  |
| {BcHV1SLR.BI=FALSE, BcMV3.BI=FALSE} | 0.20 | 0.838 | 0.245 | 1.20 |  |
|  |  |  |  |  | 83 |
| => {BcMV6.BI=FALSE} | 5446 | 384 | 05 | 5363 |  |
| {BcHV1SLR.BI=FALSE, BcMV6.BI=FALSE} | 0.20 | 0.572 | 0.358 | 1.25 |  |
|  |  |  |  |  | 83 |
| => {BcMV3.BI=FALSE} | 5446 | 414 | 911 | 0028 |  |
| {BcMV3.BI=FALSE, BcMV6.BI=FALSE} => | 0.20 | 0.546 | 0.376 | 1.19 |  |
|  |  |  |  |  | 83 |
| {BcHV1SLR.BI=FALSE} | 5446 | 053 | 238 | 2461 |  |
| {BcHV1SLR.BI=FALSE, BcPV3.BI=FALSE} => | 0.18 | 0.544 | 0.336 | 1.18 |  |
|  |  |  |  |  | 74 |
| {BcMV3.BI=FALSE} | 3168 | 118 | 634 | 8235 |  |
| {BcHV1SLR.BI=FALSE, BcRV1.BI=FALSE} => | 0.18 | 0.543 | 0.341 | 1.18 |  |
|  |  |  |  |  | 75 |
| {BcMV3.BI=FALSE} | 5644 | 478 | 584 | 6839 |  |
| {BcEV2.BI=FALSE, BcMV3.BI=FALSE} => | 0.19 | 0.568 | 0.344 | 1.24 |  |
|  |  |  |  |  | 79 |
| {BcHV1SLR.BI=FALSE} | 5545 | 345 | 059 | 1143 |  |
| {BcHV1SLR.BI=FALSE, BcHV3.BI=FALSE} => | 0.20 | 0.547 | 0.366 | 1.19 |  |
|  |  |  |  |  | 81 |
| {BcMV3.BI=FALSE} | 0495 | 297 | 337 | 5179 |  |

| {BcHV3.BI=FALSE, BcMV3.BI=FALSE} =>  {BcHV1SLR.BI=FALSE} | 0.20  0495 | 0.547  297 | 0.366  337 | 1.19  5179 | 81 |
| --- | --- | --- | --- | --- | --- |
| {BcHV1SLR.BI=FALSE, BcMV3.BI=FALSE}  => {BcFV5.BI=FALSE} | 0.22  5248 | 0.919  192 | 0.245  05 | 1.17  5169 | 91 |
| {BcFV5.BI=FALSE, BcHV1SLR.BI=FALSE} =>  {BcMV3.BI=FALSE} | 0.22  5248 | 0.575  949 | 0.391  089 | 1.25  7749 | 91 |
| {BcFV5.BI=FALSE, BcMV3.BI=FALSE} =>  {BcHV1SLR.BI=FALSE} | 0.22  5248 | 0.572  327 | 0.393  564 | 1.24  9839 | 91 |
| {BcHV1SLR.BI=FALSE, BcMV3.BI=FALSE}  => {BcULV1.BI=FALSE} | 0.22  2772 | 0.909  091 | 0.245  05 | 1.15  8589 | 90 |
| {BcHV1SLR.BI=FALSE, BcULV1.BI=FALSE}  => {BcMV3.BI=FALSE} | 0.22  2772 | 0.555  556 | 0.400  99 | 1.21  3213 | 90 |
| {BcMV3.BI=FALSE, BcULV1.BI=FALSE} =>  {BcHV1SLR.BI=FALSE} | 0.22  2772 | 0.566  038 | 0.393  564 | 1.23  6104 | 90 |
| {BcHV1SLR.BI=FALSE, BcMV3.BI=FALSE}  => {BcMV12=FALSE} | 0.22  0297 | 0.898  99 | 0.245  05 | 1.12  4433 | 89 |
| {BcHV1SLR.BI=FALSE, BcMV12=FALSE} =>  {BcMV3.BI=FALSE} | 0.22  0297 | 0.570  513 | 0.386  139 | 1.24  5877 | 89 |
| {BcMV3.BI=FALSE, BcNSRV7.BI=FALSE} =>  {BcHV1SLR.BI=FALSE} | 0.20  7921 | 0.552  632 | 0.376  238 | 1.20  6828 | 84 |
| {BcHV1SLR.BI=FALSE, BcVV3.BI=FALSE} =>  {BcMV3.BI=FALSE} | 0.20  5446 | 0.557  047 | 0.368  812 | 1.21  647 | 83 |

| {BcALV1.BI=FALSE, BcMV3.BI=FALSE} =>  {BcHV1SLR.BI=FALSE} | 0.21  2871 | 0.544  304 | 0.391  089 | 1.18  8642 | 86 |
| --- | --- | --- | --- | --- | --- |
| {BcDMV1=FALSE, BcHV1SLR.BI=FALSE} =>  {BcMV3.BI=FALSE} | 0.22  0297 | 0.581  699 | 0.378  713 | 1.27  0306 | 89 |
| {BcFV9=FALSE, BcHV1SLR.BI=FALSE} =>  {BcMV3.BI=FALSE} | 0.22  5248 | 0.538  462 | 0.418  317 | 1.17  5884 | 91 |
| {BcFV9=FALSE, BcMV3.BI=FALSE} =>  {BcHV1SLR.BI=FALSE} | 0.22  5248 | 0.544  91 | 0.413  366 | 1.18  9966 | 91 |
| {BcHV1SLR.BI=FALSE, BcOLV4.BI=FALSE}  => {BcMV3.BI=FALSE} | 0.22  7723 | 0.550  898 | 0.413  366 | 1.20  3043 | 92 |
| {BcMV3.BI=FALSE, BcOLV4.BI=FALSE} =>  {BcHV1SLR.BI=FALSE} | 0.22  7723 | 0.541  176 | 0.420  792 | 1.18  1812 | 92 |
| {BcHV1SLR.BI=FALSE, BcMV3.BI=FALSE}  => {BcULV5=FALSE} | 0.23  0198 | 0.939  394 | 0.245  05 | 1.07  2077 | 93 |
| {BcHV1SLR.BI=FALSE, BcULV5=FALSE} =>  {BcMV3.BI=FALSE} | 0.23  0198 | 0.563  636 | 0.408  416 | 1.23  086 | 93 |
| {BcMV3.BI=FALSE, BcULV5=FALSE} =>  {BcHV1SLR.BI=FALSE} | 0.23  0198 | 0.543  86 | 0.423  267 | 1.18  7672 | 93 |
| {BcBNV5.BI=FALSE, BcHV1SLR.BI=FALSE}  => {BcMV3.BI=FALSE} | 0.23  5149 | 0.545  977 | 0.430  693 | 1.19  2296 | 95 |
| {BcBNV5.BI=FALSE, BcMV3.BI=FALSE} =>  {BcHV1SLR.BI=FALSE} | 0.23  5149 | 0.536  723 | 0.438  119 | 1.17  2087 | 95 |

| {BcHV1SLR.BI=FALSE, BvF.BI=FALSE} =>  {BcMV3.BI=FALSE} | 0.23  7624 | 0.548  571 | 0.433  168 | 1.19  7961 | 96 |
| --- | --- | --- | --- | --- | --- |
| {BcMV3.BI=FALSE, BvF.BI=FALSE} =>  {BcHV1SLR.BI=FALSE} | 0.23  7624 | 0.568  047 | 0.418  317 | 1.24  0493 | 96 |
| {BcHV1SLR.BI=FALSE, BcMCV5.BI=FALSE}  => {BcMV3.BI=FALSE} | 0.23  7624 | 0.548  571 | 0.433  168 | 1.19  7961 | 96 |
| {BcMCV5.BI=FALSE, BcMV3.BI=FALSE} =>  {BcHV1SLR.BI=FALSE} | 0.23  7624 | 0.539  326 | 0.440  594 | 1.17  7771 | 96 |
| {BcHV1SLR.BI=FALSE, BcOLV13.BI=FALSE}  => {BcMV3.BI=FALSE} | 0.24  2574 | 0.556  818 | 0.435  644 | 1.21  5971 | 98 |
| {BcMV3.BI=FALSE, BcOLV13.BI=FALSE} =>  {BcHV1SLR.BI=FALSE} | 0.24  2574 | 0.538  462 | 0.450  495 | 1.17  5884 | 98 |
| {BcHV1SLR.BI=FALSE, BcOLV2.BI=FALSE}  => {BcMV3.BI=FALSE} | 0.23  7624 | 0.533  333 | 0.445  545 | 1.16  4685 | 96 |
| {BcMV3.BI=FALSE, BcOLV2.BI=FALSE} =>  {BcHV1SLR.BI=FALSE} | 0.23  7624 | 0.536  313 | 0.443  069 | 1.17  1191 | 96 |
| {BcHV1SLR.BI=FALSE, BcMCV3.BI=FALSE}  => {BcMV3.BI=FALSE} | 0.24  505 | 0.55 | 0.445  545 | 1.20  1081 | 99 |
| {BcMCV3.BI=FALSE, BcMV3.BI=FALSE} =>  {BcHV1SLR.BI=FALSE} | 0.24  505 | 0.535  135 | 0.457  921 | 1.16  8619 | 99 |
| {BcHV1SLR.BI=FALSE, BcPV4.BI=FALSE} =>  {BcMV3.BI=FALSE} | 0.23  2673 | 0.543  353 | 0.428  218 | 1.18  6565 | 94 |

| {BcEV6=FALSE, BcMV3.BI=FALSE} =>  {BcHV1SLR.BI=FALSE} | 0.22  7723 | 0.544  379 | 0.418  317 | 1.18  8805 | 92 |
| --- | --- | --- | --- | --- | --- |
| {BcMV3.BI=FALSE, BcOLV17.BI=FALSE} =>  {BcHV1SLR.BI=FALSE} | 0.23  5149 | 0.539  773 | 0.435  644 | 1.17  8747 | 95 |
| {BcHV1SLR.BI=FALSE, BcOLV15.BI=FALSE}  => {BcMV3.BI=FALSE} | 0.24  0099 | 0.532  967 | 0.450  495 | 1.16  3885 | 97 |
| {BcMV3.BI=FALSE, BcOLV15.BI=FALSE} =>  {BcHV1SLR.BI=FALSE} | 0.24  0099 | 0.541  899 | 0.443  069 | 1.18  3391 | 97 |
| {BcEV5=FALSE, BcHV1SLR.BI=FALSE} =>  {BcMV3.BI=FALSE} | 0.24  2574 | 0.553  672 | 0.438  119 | 1.20  9101 | 98 |
| {BcEV5=FALSE, BcMV3.BI=FALSE} =>  {BcHV1SLR.BI=FALSE} | 0.24  2574 | 0.532  609 | 0.455  446 | 1.16  3102 | 98 |
| {BcMV2.BI=TRUE, BcOLV18=FALSE} =>  {BcHV1SLR.BI=FALSE} | 0.09  901 | 0.588  235 | 0.168  317 | 1.28  4579 | 40 |
| {BcMV2.BI=TRUE, BcMV6.BI=FALSE} =>  {BcHV1SLR.BI=FALSE} | 0.14  3564 | 0.563  107 | 0.254  95 | 1.22  9703 | 58 |
| {BcHV1SLR.BI=FALSE, BcMV2.BI=TRUE} =>  {BcMV1.BI=TRUE} | 0.16  8317 | 0.883  117 | 0.190  594 | 1.11  843 | 68 |
| {BcHV1SLR.BI=FALSE, BcOLV18=FALSE} =>  {BcMV2.BI=FALSE} | 0.18  3168 | 0.649  123 | 0.282  178 | 1.20  8505 | 74 |
| {BcHV1SLR.BI=FALSE, BcMV2.BI=FALSE}  => {BcOLV18=FALSE} | 0.18  3168 | 0.685  185 | 0.267  327 | 1.41  9563 | 74 |

| {BcMV2.BI=FALSE, BcOLV18=FALSE} =>  {BcHV1SLR.BI=FALSE} | 0.18  3168 | 0.582  677 | 0.314  356 | 1.27  2441 | 74 |
| --- | --- | --- | --- | --- | --- |
| {BcMV3.BI=TRUE, BcOLV18=FALSE} =>  {BcHV1SLR.BI=FALSE} | 0.12  1287 | 0.569  767 | 0.212  871 | 1.24  4249 | 49 |
| {BcHV1SLR.BI=FALSE, BcOLV18=FALSE} =>  {BcMV4.BI=FALSE} | 0.18  8119 | 0.666  667 | 0.282  178 | 1.19  7037 | 76 |
| {BcHV1SLR.BI=FALSE, BcMV4.BI=FALSE}  => {BcOLV18=FALSE} | 0.18  8119 | 0.678  571 | 0.277  228 | 1.40  5861 | 76 |
| {BcMV4.BI=FALSE, BcOLV18=FALSE} =>  {BcHV1SLR.BI=FALSE} | 0.18  8119 | 0.593  75 | 0.316  832 | 1.29  6622 | 76 |
| {BcHV1SLR.BI=FALSE, BcOLV18=FALSE} =>  {BcOLV16.BI=FALSE} | 0.19  802 | 0.701  754 | 0.282  178 | 1.20  1308 | 80 |
| {BcHV1SLR.BI=FALSE, BcOLV16.BI=FALSE}  => {BcOLV18=FALSE} | 0.19  802 | 0.707  965 | 0.279  703 | 1.46  6757 | 80 |
| {BcOLV16.BI=FALSE, BcOLV18=FALSE} =>  {BcHV1SLR.BI=FALSE} | 0.19  802 | 0.588  235 | 0.336  634 | 1.28  4579 | 80 |
| {BcHV1SLR.BI=FALSE, BcULV3=TRUE} =>  {BcOLV18=FALSE} | 0.12  6238 | 0.607  143 | 0.207  921 | 1.25  7875 | 51 |
| {BcHV1SLR.BI=FALSE, BcMV11=TRUE} =>  {BcOLV18=FALSE} | 0.15  5941 | 0.6 | 0.259  901 | 1.24  3077 | 63 |
| {BcMV11=TRUE, BcOLV18=FALSE} =>  {BcHV1SLR.BI=FALSE} | 0.15  5941 | 0.577  982 | 0.269  802 | 1.26  2187 | 63 |

| {BcHV1SLR.BI=FALSE, BcOLV18=FALSE} =>  {BcOLV1.BI.MOVA.=FALSE} | 0.22  0297 | 0.780  702 | 0.282  178 | 1.16  8161 | 89 |
| --- | --- | --- | --- | --- | --- |
| {BcHV1SLR.BI=FALSE, BcOLV1.BI.MOVA.=FALSE} =>  {BcOLV18=FALSE} | 0.22  0297 | 0.674  242 | 0.326  733 | 1.39  6892 | 89 |
| {BcOLV1.BI.MOVA.=FALSE,  BcOLV18=FALSE} => {BcHV1SLR.BI=FALSE} | 0.22  0297 | 0.577  922 | 0.381  188 | 1.26  2057 | 89 |
| {BcOLV18=FALSE, BcULV4=TRUE} =>  {BcHV1SLR.BI=FALSE} | 0.14  604 | 0.584  158 | 0.25 | 1.27  5676 | 59 |
| {BcHV1SLR.BI=FALSE, BcOLV18=FALSE} =>  {BcMV6.BI=FALSE} | 0.22  2772 | 0.789  474 | 0.282  178 | 1.13  5044 | 90 |
| {BcHV1SLR.BI=FALSE, BcMV6.BI=FALSE}  => {BcOLV18=FALSE} | 0.22  2772 | 0.620  69 | 0.358  911 | 1.28  5942 | 90 |
| {BcMV6.BI=FALSE, BcOLV18=FALSE} =>  {BcHV1SLR.BI=FALSE} | 0.22  2772 | 0.633  803 | 0.351  485 | 1.38  4088 | 90 |
| {BcHV1SLR.BI=FALSE, BcOLV18=FALSE} =>  {BcPV3.BI=FALSE} | 0.22  5248 | 0.798  246 | 0.282  178 | 1.12  3663 | 91 |
| {BcHV1SLR.BI=FALSE, BcPV3.BI=FALSE} =>  {BcOLV18=FALSE} | 0.22  5248 | 0.669  118 | 0.336  634 | 1.38  6275 | 91 |
| {BcOLV18=FALSE, BcPV3.BI=FALSE} =>  {BcHV1SLR.BI=FALSE} | 0.22  5248 | 0.590  909 | 0.381  188 | 1.29  0418 | 91 |
| {BcHV1SLR.BI=FALSE, BcRV1.BI=FALSE} => | 0.21 | 0.630 | 0.341 | 1.30 | 87 |

| {BcOLV18=FALSE} | 5347 | 435 | 584 | 6132 |  |
| --- | --- | --- | --- | --- | --- |
| {BcOLV18=FALSE, BcRV1.BI=FALSE} =>  {BcHV1SLR.BI=FALSE} | 0.21  5347 | 0.547  17 | 0.393  564 | 1.19  4901 | 87 |
| {BcHV1SLR.BI=FALSE, BcOLV18=FALSE} =>  {BcEV2.BI=FALSE} | 0.23  7624 | 0.842  105 | 0.282  178 | 1.12  6525 | 96 |
| {BcEV2.BI=FALSE, BcHV1SLR.BI=FALSE} =>  {BcOLV18=FALSE} | 0.23  7624 | 0.657  534 | 0.361  386 | 1.36  2276 | 96 |
| {BcEV2.BI=FALSE, BcOLV18=FALSE} =>  {BcHV1SLR.BI=FALSE} | 0.23  7624 | 0.627  451 | 0.378  713 | 1.37  0217 | 96 |
| {BcHV1SLR.BI=FALSE, BcOLV18=FALSE} =>  {BcHV3.BI=FALSE} | 0.24  2574 | 0.859  649 | 0.282  178 | 1.13  1265 | 98 |
| {BcHV1SLR.BI=FALSE, BcHV3.BI=FALSE} =>  {BcOLV18=FALSE} | 0.24  2574 | 0.662  162 | 0.366  337 | 1.37  1864 | 98 |
| {BcHV3.BI=FALSE, BcOLV18=FALSE} =>  {BcHV1SLR.BI=FALSE} | 0.24  2574 | 0.593  939 | 0.408  416 | 1.29  7035 | 98 |
| {BcHV1SLR.BI=FALSE, BcOLV18=FALSE} =>  {BcFV5.BI=FALSE} | 0.25  2475 | 0.894  737 | 0.282  178 | 1.14  3904 | 10  2 |
| {BcFV5.BI=FALSE, BcHV1SLR.BI=FALSE} =>  {BcOLV18=FALSE} | 0.25  2475 | 0.645  57 | 0.391  089 | 1.33  7488 | 10  2 |
| {BcFV5.BI=FALSE, BcOLV18=FALSE} =>  {BcHV1SLR.BI=FALSE} | 0.25  2475 | 0.610  778 | 0.413  366 | 1.33  3808 | 10  2 |
| {BcHV1SLR.BI=FALSE, BcOLV18=FALSE} => | 0.26 | 0.947 | 0.282 | 1.20 | 10 |

| {BcULV1.BI=FALSE} | 7327 | 368 | 178 | 7372 | 8 |
| --- | --- | --- | --- | --- | --- |
| {BcHV1SLR.BI=FALSE, BcULV1.BI=FALSE}  => {BcOLV18=FALSE} | 0.26  7327 | 0.666  667 | 0.400  99 | 1.38  1197 | 10  8 |
| {BcOLV18=FALSE, BcULV1.BI=FALSE} =>  {BcHV1SLR.BI=FALSE} | 0.26  7327 | 0.624  277 | 0.428  218 | 1.36  3287 | 10  8 |
| {BcHV1SLR.BI=FALSE, BcMV1.BI=TRUE} =>  {BcOLV18=FALSE} | 0.20  0495 | 0.574  468 | 0.349  01 | 1.19  018 | 81 |
| {BcMV1.BI=TRUE, BcOLV18=FALSE} =>  {BcHV1SLR.BI=FALSE} | 0.20  0495 | 0.574  468 | 0.349  01 | 1.25  4514 | 81 |
| {BcHV1SLR.BI=FALSE, BcOLV18=FALSE} =>  {BcMV12=FALSE} | 0.24  7525 | 0.877  193 | 0.282  178 | 1.09  717 | 10  0 |
| {BcHV1SLR.BI=FALSE, BcMV12=FALSE} =>  {BcOLV18=FALSE} | 0.24  7525 | 0.641  026 | 0.386  139 | 1.32  8074 | 10  0 |
| {BcMV12=FALSE, BcOLV18=FALSE} =>  {BcHV1SLR.BI=FALSE} | 0.24  7525 | 0.613  497 | 0.403  465 | 1.33  9745 | 10  0 |
| {BcHV1SLR.BI=FALSE, BcOLV18=FALSE} =>  {BcNSRV7.BI=FALSE} | 0.25  495 | 0.903  509 | 0.282  178 | 1.13  0085 | 10  3 |
| {BcHV1SLR.BI=FALSE, BcNSRV7.BI=FALSE}  => {BcOLV18=FALSE} | 0.25  495 | 0.651  899 | 0.391  089 | 1.35  06 | 10  3 |
| {BcNSRV7.BI=FALSE, BcOLV18=FALSE} =>  {BcHV1SLR.BI=FALSE} | 0.25  495 | 0.620  482 | 0.410  891 | 1.35  4998 | 10  3 |
| {BcHV1SLR.BI=FALSE, BcVV3.BI=FALSE} => | 0.23 | 0.624 | 0.368 | 1.29 | 93 |

| {BcOLV18=FALSE} | 0198 | 161 | 812 | 3134 |  |
| --- | --- | --- | --- | --- | --- |
| {BcOLV18=FALSE, BcVV3.BI=FALSE} =>  {BcHV1SLR.BI=FALSE} | 0.23  0198 | 0.588  608 | 0.391  089 | 1.28  5392 | 93 |
| {BcHV1SLR.BI=FALSE, BcOLV7.BI=FALSE}  => {BcOLV18=FALSE} | 0.23  2673 | 0.626  667 | 0.371  287 | 1.29  8325 | 94 |
| {BcOLV18=FALSE, BcOLV7.BI=FALSE} =>  {BcHV1SLR.BI=FALSE} | 0.23  2673 | 0.540  23 | 0.430  693 | 1.17  9745 | 94 |
| {BcHV1SLR.BI=FALSE, BcOLV18=FALSE} =>  {BcALV1.BI=FALSE} | 0.25  9901 | 0.921  053 | 0.282  178 | 1.13  1019 | 10  5 |
| {BcALV1.BI=FALSE, BcHV1SLR.BI=FALSE}  => {BcOLV18=FALSE} | 0.25  9901 | 0.652  174 | 0.398  515 | 1.35  1171 | 10  5 |
| {BcALV1.BI=FALSE, BcOLV18=FALSE} =>  {BcHV1SLR.BI=FALSE} | 0.25  9901 | 0.593  22 | 0.438  119 | 1.29  5465 | 10  5 |
| {BcDMV1=FALSE, BcHV1SLR.BI=FALSE} =>  {BcOLV18=FALSE} | 0.23  7624 | 0.627  451 | 0.378  713 | 1.29  995 | 96 |
| {BcDMV1=FALSE, BcOLV18=FALSE} =>  {BcHV1SLR.BI=FALSE} | 0.23  7624 | 0.571  429 | 0.415  842 | 1.24  7876 | 96 |
| {BcHV1SLR.BI=FALSE, BcOLV18=FALSE} =>  {BcFV9=FALSE} | 0.26  7327 | 0.947  368 | 0.282  178 | 1.09  0418 | 10  8 |
| {BcFV9=FALSE, BcHV1SLR.BI=FALSE} =>  {BcOLV18=FALSE} | 0.26  7327 | 0.639  053 | 0.418  317 | 1.32  3987 | 10  8 |
| {BcFV9=FALSE, BcOLV18=FALSE} => | 0.26 | 0.596 | 0.448 | 1.30 | 10 |

| {BcHV1SLR.BI=FALSE} | 7327 | 685 | 02 | 3031 | 8 |
| --- | --- | --- | --- | --- | --- |
| {BcHV1SLR.BI=FALSE, BcOLV4.BI=FALSE}  => {BcOLV18=FALSE} | 0.25  7426 | 0.622  754 | 0.413  366 | 1.29  022 | 10  4 |
| {BcOLV18=FALSE, BcOLV4.BI=FALSE} =>  {BcHV1SLR.BI=FALSE} | 0.25  7426 | 0.601  156 | 0.428  218 | 1.31  2795 | 10  4 |
| {BcHV1SLR.BI=FALSE, BcULV5=FALSE} =>  {BcOLV18=FALSE} | 0.24  0099 | 0.587  879 | 0.408  416 | 1.21  7964 | 97 |
| {BcOLV18=FALSE, BcULV5=FALSE} =>  {BcHV1SLR.BI=FALSE} | 0.24  0099 | 0.587  879 | 0.408  416 | 1.28  38 | 97 |
| {BcHV1SLR.BI=FALSE, BcOLV18=FALSE} =>  {BcBNV5.BI=FALSE} | 0.27  4752 | 0.973  684 | 0.282  178 | 1.07  1849 | 11  1 |
| {BcBNV5.BI=FALSE, BcHV1SLR.BI=FALSE}  => {BcOLV18=FALSE} | 0.27  4752 | 0.637  931 | 0.430  693 | 1.32  1662 | 11  1 |
| {BcBNV5.BI=FALSE, BcOLV18=FALSE} =>  {BcHV1SLR.BI=FALSE} | 0.27  4752 | 0.603  261 | 0.455  446 | 1.31  7391 | 11  1 |
| {BcHV1SLR.BI=FALSE, BvF.BI=FALSE} =>  {BcOLV18=FALSE} | 0.26  7327 | 0.617  143 | 0.433  168 | 1.27  8593 | 10  8 |
| {BcOLV18=FALSE, BvF.BI=FALSE} =>  {BcHV1SLR.BI=FALSE} | 0.26  7327 | 0.610  169 | 0.438  119 | 1.33  2478 | 10  8 |
| {BcHV1SLR.BI=FALSE, BcOLV18=FALSE} =>  {BcMCV5.BI=FALSE} | 0.28  2178 | 1 | 0.282  178 | 1.08  311 | 11  4 |
| {BcHV1SLR.BI=FALSE, BcMCV5.BI=FALSE} | 0.28 | 0.651 | 0.433 | 1.34 | 11 |

| => {BcOLV18=FALSE} | 2178 | 429 | 168 | 9626 | 4 |
| --- | --- | --- | --- | --- | --- |
| {BcMCV5.BI=FALSE, BcOLV18=FALSE} =>  {BcHV1SLR.BI=FALSE} | 0.28  2178 | 0.596  859 | 0.472  772 | 1.30  341 | 11  4 |
| {BcHV1SLR.BI=FALSE, BcOLV13.BI=FALSE}  => {BcOLV18=FALSE} | 0.26  9802 | 0.619  318 | 0.435  644 | 1.28  31 | 10  9 |
| {BcOLV13.BI=FALSE, BcOLV18=FALSE} =>  {BcHV1SLR.BI=FALSE} | 0.26  9802 | 0.598  901 | 0.450  495 | 1.30  7871 | 10  9 |
| {BcHV1SLR.BI=FALSE, BcOLV2.BI=FALSE}  => {BcOLV18=FALSE} | 0.27  4752 | 0.616  667 | 0.445  545 | 1.27  7607 | 11  1 |
| {BcOLV18=FALSE, BcOLV2.BI=FALSE} =>  {BcHV1SLR.BI=FALSE} | 0.27  4752 | 0.596  774 | 0.460  396 | 1.30  3226 | 11  1 |
| {BcHV1SLR.BI=FALSE, BcMCV3.BI=FALSE}  => {BcOLV18=FALSE} | 0.27  7228 | 0.622  222 | 0.445  545 | 1.28  9117 | 11  2 |
| {BcMCV3.BI=FALSE, BcOLV18=FALSE} =>  {BcHV1SLR.BI=FALSE} | 0.27  7228 | 0.605  405 | 0.457  921 | 1.32  2075 | 11  2 |
| {BcHV1SLR.BI=FALSE, BcPV4.BI=FALSE} =>  {BcOLV18=FALSE} | 0.26  7327 | 0.624  277 | 0.428  218 | 1.29  3375 | 10  8 |
| {BcOLV18=FALSE, BcPV4.BI=FALSE} =>  {BcHV1SLR.BI=FALSE} | 0.26  7327 | 0.586  957 | 0.455  446 | 1.28  1786 | 10  8 |
| {BcEV6=FALSE, BcHV1SLR.BI=FALSE} =>  {BcOLV18=FALSE} | 0.26  4851 | 0.611  429 | 0.433  168 | 1.26  6755 | 10  7 |
| {BcEV6=FALSE, BcOLV18=FALSE} => | 0.26 | 0.584 | 0.452 | 1.27 | 10 |

| {BcHV1SLR.BI=FALSE} | 4851 | 699 | 97 | 6857 | 7 |
| --- | --- | --- | --- | --- | --- |
| {BcHV1SLR.BI=FALSE, BcOLV17.BI=FALSE}  => {BcOLV18=FALSE} | 0.27  4752 | 0.620  112 | 0.443  069 | 1.28  4744 | 11  1 |
| {BcOLV17.BI=FALSE, BcOLV18=FALSE} =>  {BcHV1SLR.BI=FALSE} | 0.27  4752 | 0.584  211 | 0.470  297 | 1.27  5789 | 11  1 |
| {BcHV1SLR.BI=FALSE, BcOLV15.BI=FALSE}  => {BcOLV18=FALSE} | 0.28  2178 | 0.626  374 | 0.450  495 | 1.29  7718 | 11  4 |
| {BcOLV15.BI=FALSE, BcOLV18=FALSE} =>  {BcHV1SLR.BI=FALSE} | 0.28  2178 | 0.596  859 | 0.472  772 | 1.30  341 | 11  4 |
| {BcEV5=FALSE, BcHV1SLR.BI=FALSE} =>  {BcOLV18=FALSE} | 0.27  4752 | 0.627  119 | 0.438  119 | 1.29  9261 | 11  1 |
| {BcEV5=FALSE, BcOLV18=FALSE} =>  {BcHV1SLR.BI=FALSE} | 0.27  4752 | 0.593  583 | 0.462  871 | 1.29  6257 | 11  1 |
| {BcHV1SLR.BI=FALSE, BcOLV18=TRUE} =>  {BcULV4=TRUE} | 0.14  3564 | 0.816  901 | 0.175  743 | 1.18  2897 | 58 |
| {BcHV1SLR.BI=FALSE, BcOLV18=TRUE} =>  {BcULV5=FALSE} | 0.16  8317 | 0.957  746 | 0.175  743 | 1.09  3021 | 68 |
| {BcHV1SLR.BI=FALSE, BcMV2.BI=FALSE}  => {BcMV4.BI=FALSE} | 0.19  3069 | 0.722  222 | 0.267  327 | 1.29  679 | 78 |
| {BcHV1SLR.BI=FALSE, BcMV4.BI=FALSE}  => {BcMV2.BI=FALSE} | 0.19  3069 | 0.696  429 | 0.277  228 | 1.29  6577 | 78 |
| {BcHV1SLR.BI=FALSE, BcMV2.BI=FALSE} | 0.22 | 0.851 | 0.267 | 1.27 | 92 |

| => {BcOLV1.BI.MOVA.=FALSE} | 7723 | 852 | 327 | 4623 |  |
| --- | --- | --- | --- | --- | --- |
| {BcHV1SLR.BI=FALSE, BcOLV1.BI.MOVA.=FALSE} =>  {BcMV2.BI=FALSE} | 0.22  7723 | 0.696  97 | 0.326  733 | 1.29  7584 | 92 |
| {BcHV1SLR.BI=FALSE, BcMV2.BI=FALSE} | 0.21 | 0.805 | 0.267 | 1.15 |  |
|  |  |  |  |  | 87 |
| => {BcMV6.BI=FALSE} | 5347 | 556 | 327 | 8165 |  |
| {BcHV1SLR.BI=FALSE, BcMV2.BI=FALSE} | 0.23 | 0.870 | 0.267 | 1.16 |  |
|  |  |  |  |  | 94 |
| => {BcEV2.BI=FALSE} | 2673 | 37 | 327 | 4337 |  |
| {BcEV2.BI=FALSE, BcHV1SLR.BI=FALSE} => | 0.23 | 0.643 | 0.361 | 1.19 |  |
|  |  |  |  |  | 94 |
| {BcMV2.BI=FALSE} | 2673 | 836 | 386 | 8662 |  |
| {BcEV2.BI=FALSE, BcMV2.BI=FALSE} => | 0.23 | 0.543 | 0.428 | 1.18 |  |
|  |  |  |  |  | 94 |
| {BcHV1SLR.BI=FALSE} | 2673 | 353 | 218 | 6565 |  |
| {BcHV1SLR.BI=FALSE, BcMV2.BI=FALSE} | 0.23 | 0.870 | 0.267 | 1.14 |  |
|  |  |  |  |  | 94 |
| => {BcHV3.BI=FALSE} | 2673 | 37 | 327 | 5373 |  |
| {BcHV1SLR.BI=FALSE, BcHV3.BI=FALSE} => | 0.23 | 0.635 | 0.366 | 1.18 |  |
|  |  |  |  |  | 94 |
| {BcMV2.BI=FALSE} | 2673 | 135 | 337 | 2464 |  |
| {BcHV3.BI=FALSE, BcMV2.BI=FALSE} => | 0.23 | 0.540 | 0.430 | 1.17 |  |
|  |  |  |  |  | 94 |
| {BcHV1SLR.BI=FALSE} | 2673 | 23 | 693 | 9745 |  |
| {BcHV1SLR.BI=FALSE, BcMV2.BI=FALSE} | 0.23 | 0.888 | 0.267 | 1.13 |  |
|  |  |  |  |  | 96 |
| => {BcFV5.BI=FALSE} | 7624 | 889 | 327 | 6428 |  |
| {BcFV5.BI=FALSE, BcMV2.BI=FALSE} => | 0.23 | 0.542 | 0.438 | 1.18 |  |
|  |  |  |  |  | 96 |
| {BcHV1SLR.BI=FALSE} | 7624 | 373 | 119 | 4425 |  |

| {BcHV1SLR.BI=FALSE, BcMV2.BI=FALSE}  => {BcULV1.BI=FALSE} | 0.24  7525 | 0.925  926 | 0.267  327 | 1.18  0044 | 10  0 |
| --- | --- | --- | --- | --- | --- |
| {BcMV2.BI=FALSE, BcULV1.BI=FALSE} =>  {BcHV1SLR.BI=FALSE} | 0.24  7525 | 0.537  634 | 0.460  396 | 1.17  4077 | 10  0 |
| {BcHV1SLR.BI=FALSE, BcMV2.BI=FALSE}  => {BcNSRV7.BI=FALSE} | 0.23  5149 | 0.879  63 | 0.267  327 | 1.10  0218 | 95 |
| {BcMV2.BI=FALSE, BcNSRV7.BI=FALSE} =>  {BcHV1SLR.BI=FALSE} | 0.23  5149 | 0.536  723 | 0.438  119 | 1.17  2087 | 95 |
| {BcHV1SLR.BI=FALSE, BcMV2.BI=FALSE}  => {BcALV1.BI=FALSE} | 0.24  505 | 0.916  667 | 0.267  327 | 1.12  5633 | 99 |
| {BcHV1SLR.BI=FALSE, BcMV2.BI=FALSE}  => {BcFV9=FALSE} | 0.25 | 0.935  185 | 0.267  327 | 1.07  6395 | 10  1 |
| {BcFV9=FALSE, BcMV2.BI=FALSE} =>  {BcHV1SLR.BI=FALSE} | 0.25 | 0.534  392 | 0.467  822 | 1.16  6996 | 10  1 |
| {BcHV1SLR.BI=FALSE, BcMV2.BI=FALSE}  => {BcOLV4.BI=FALSE} | 0.25  2475 | 0.944  444 | 0.267  327 | 1.07  7841 | 10  2 |
| {BcMV2.BI=FALSE, BcOLV15.BI=FALSE} =>  {BcHV1SLR.BI=FALSE} | 0.26  4851 | 0.532  338 | 0.497  525 | 1.16  2512 | 10  7 |
| {BcHV1SLR.BI=FALSE, BcOLV16.BI=FALSE}  => {BcMV4.BI=FALSE} | 0.18  5644 | 0.663  717 | 0.279  703 | 1.19  174 | 75 |
| {BcHV1SLR.BI=FALSE, BcMV4.BI=FALSE}  => {BcOLV1.BI.MOVA.=FALSE} | 0.22  0297 | 0.794  643 | 0.277  228 | 1.18  9021 | 89 |

| {BcHV1SLR.BI=FALSE, BcOLV1.BI.MOVA.=FALSE} =>  {BcMV4.BI=FALSE} | 0.22  0297 | 0.674  242 | 0.326  733 | 1.21  064 | 89 |
| --- | --- | --- | --- | --- | --- |
| {BcHV1SLR.BI=FALSE, BcMV4.BI=FALSE}  => {BcMV6.BI=FALSE} | 0.22  2772 | 0.803  571 | 0.277  228 | 1.15  5313 | 90 |
| {BcHV1SLR.BI=FALSE, BcMV4.BI=FALSE}  => {BcPV3.BI=FALSE} | 0.22  5248 | 0.812  5 | 0.277  228 | 1.14  3728 | 91 |
| {BcHV1SLR.BI=FALSE, BcPV3.BI=FALSE} =>  {BcMV4.BI=FALSE} | 0.22  5248 | 0.669  118 | 0.336  634 | 1.20  1438 | 91 |
| {BcHV1SLR.BI=FALSE, BcMV4.BI=FALSE}  => {BcEV2.BI=FALSE} | 0.23  0198 | 0.830  357 | 0.277  228 | 1.11  0809 | 93 |
| {BcHV1SLR.BI=FALSE, BcMV4.BI=FALSE}  => {BcHV3.BI=FALSE} | 0.24  0099 | 0.866  071 | 0.277  228 | 1.13  9716 | 97 |
| {BcHV1SLR.BI=FALSE, BcHV3.BI=FALSE} =>  {BcMV4.BI=FALSE} | 0.24  0099 | 0.655  405 | 0.366  337 | 1.17  6817 | 97 |
| {BcHV1SLR.BI=FALSE, BcMV4.BI=FALSE}  => {BcFV5.BI=FALSE} | 0.24  0099 | 0.866  071 | 0.277  228 | 1.10  7256 | 97 |
| {BcHV1SLR.BI=FALSE, BcMV4.BI=FALSE}  => {BcULV1.BI=FALSE} | 0.25  2475 | 0.910  714 | 0.277  228 | 1.16  0658 | 10  2 |
| {BcMV4.BI=FALSE, BcULV1.BI=FALSE} =>  {BcHV1SLR.BI=FALSE} | 0.25  2475 | 0.560  44 | 0.450  495 | 1.22  3879 | 10  2 |
| {BcMV12=FALSE, BcMV4.BI=FALSE} => | 0.23 | 0.542 | 0.438 | 1.18 | 96 |

| {BcHV1SLR.BI=FALSE} | 7624 | 373 | 119 | 4425 |  |
| --- | --- | --- | --- | --- | --- |
| {BcHV1SLR.BI=FALSE, BcMV4.BI=FALSE}  => {BcALV1.BI=FALSE} | 0.25  2475 | 0.910  714 | 0.277  228 | 1.11  8324 | 10  2 |
| {BcHV1SLR.BI=FALSE, BcMV4.BI=FALSE}  => {BcFV9=FALSE} | 0.25  9901 | 0.937  5 | 0.277  228 | 1.07  906 | 10  5 |
| {BcHV1SLR.BI=FALSE, BcMCV5.BI=FALSE}  => {BcMV4.BI=FALSE} | 0.27  2277 | 0.628  571 | 0.433  168 | 1.12  8635 | 11  0 |
| {BcHV1SLR.BI=FALSE, BcOLV16.BI=FALSE}  => {BcMV6.BI=FALSE} | 0.23  0198 | 0.823  009 | 0.279  703 | 1.18  3258 | 93 |
| {BcHV1SLR.BI=FALSE, BcOLV16.BI=FALSE}  => {BcULV1.BI=FALSE} | 0.25  7426 | 0.920  354 | 0.279  703 | 1.17  2943 | 10  4 |
| {BcHV1SLR.BI=FALSE, BcOLV16.BI=FALSE}  => {BcMV12=FALSE} | 0.24  7525 | 0.884  956 | 0.279  703 | 1.10  688 | 10  0 |
| {BcHV1SLR.BI=FALSE, BcOLV16.BI=FALSE}  => {BcNSRV7.BI=FALSE} | 0.24  7525 | 0.884  956 | 0.279  703 | 1.10  688 | 10  0 |
| {BcHV1SLR.BI=FALSE, BcOLV16.BI=FALSE}  => {BcALV1.BI=FALSE} | 0.25  2475 | 0.902  655 | 0.279  703 | 1.10  8427 | 10  2 |
| {BcHV1SLR.BI=FALSE, BcOLV16.BI=FALSE}  => {BcFV9=FALSE} | 0.25  9901 | 0.929  204 | 0.279  703 | 1.06  9511 | 10  5 |
| {BcHV1SLR.BI=FALSE, BcULV3=TRUE} =>  {BcULV1.BI=FALSE} | 0.18  3168 | 0.880  952 | 0.207  921 | 1.12  2728 | 74 |
| {BcHV1SLR.BI=FALSE, BcULV3=TRUE} => | 0.19 | 0.940 | 0.207 | 1.08 | 79 |

| {BcFV9=FALSE} | 5545 | 476 | 921 | 2485 |  |
| --- | --- | --- | --- | --- | --- |
| {BcHV1.BI=TRUE, BcHV1SLR.BI=FALSE} =>  {BcFV5.BI=FALSE} | 0.10  396 | 0.875 | 0.118  812 | 1.11  8671 | 42 |
| {BcHV1.BI=TRUE, BcHV1SLR.BI=FALSE} => | 0.10 | 0.895 | 0.118 | 1.11 |  |
|  |  |  |  |  | 43 |
| {BcVV3.BI=FALSE} | 6436 | 833 | 812 | 7027 |  |
| {BcHV1.BI=TRUE, BcHV1SLR.BI=FALSE} => | 0.11 | 0.958 | 0.118 | 1.10 |  |
|  |  |  |  |  | 46 |
| {BcFV9=FALSE} | 3861 | 333 | 812 | 3039 |  |
| {BcHV1SLR.BI=FALSE, BcMV9.BI=TRUE} => | 0.20 | 0.761 | 0.269 | 1.13 |  |
|  |  |  |  |  | 83 |
| {BcOLV1.BI.MOVA.=FALSE} | 5446 | 468 | 802 | 9382 |  |
| {BcHV1SLR.BI=FALSE, |  |  |  |  |  |
|  | 0.25 | 0.787 | 0.326 | 1.13 | 10 |
| BcOLV1.BI.MOVA.=FALSE} => |  |  |  |  |  |
|  | 7426 | 879 | 733 | 2751 | 4 |
| {BcMV6.BI=FALSE} |  |  |  |  |  |
| {BcEV2.BI=FALSE, |  |  |  |  |  |
|  | 0.26 | 0.532 | 0.497 | 1.16 | 10 |
| BcOLV1.BI.MOVA.=FALSE} => |  |  |  |  |  |
|  | 4851 | 338 | 525 | 2512 | 7 |
| {BcHV1SLR.BI=FALSE} |  |  |  |  |  |
| {BcHV1SLR.BI=FALSE, |  |  |  |  |  |
|  | 0.27 | 0.840 | 0.326 | 1.10 | 11 |
| BcOLV1.BI.MOVA.=FALSE} => |  |  |  |  |  |
|  | 4752 | 909 | 733 | 6603 | 1 |
| {BcHV3.BI=FALSE} |  |  |  |  |  |
| {BcHV1SLR.BI=FALSE, BcHV3.BI=FALSE} => | 0.27 |  | 0.366 | 1.12 | 11 |
|  |  | 0.75 |  |  |  |
| {BcOLV1.BI.MOVA.=FALSE} | 4752 |  | 337 | 2222 | 1 |
| {BcHV1SLR.BI=FALSE, | 0.29 | 0.909 | 0.326 | 1.16 | 12 |
| BcOLV1.BI.MOVA.=FALSE} => | 703 | 091 | 733 | 2255 | 0 |

| {BcFV5.BI=FALSE} |  |  |  |  |  |
| --- | --- | --- | --- | --- | --- |
| {BcFV5.BI=FALSE, BcHV1SLR.BI=FALSE} => | 0.29 | 0.759 | 0.391 | 1.13 | 12 |
| {BcOLV1.BI.MOVA.=FALSE} | 703 | 494 | 089 | 6428 | 0 |
| {BcFV5.BI=FALSE, |  |  |  |  |  |
|  | 0.29 | 0.547 | 0.542 | 1.19 | 12 |
| BcOLV1.BI.MOVA.=FALSE} => |  |  |  |  |  |
|  | 703 | 945 | 079 | 6594 | 0 |
| {BcHV1SLR.BI=FALSE} |  |  |  |  |  |
| {BcHV1SLR.BI=FALSE, |  |  |  |  |  |
|  | 0.30 | 0.924 | 0.326 | 1.17 | 12 |
| BcOLV1.BI.MOVA.=FALSE} => |  |  |  |  |  |
|  | 198 | 242 | 733 | 7899 | 2 |
| {BcULV1.BI=FALSE} |  |  |  |  |  |
| {BcHV1SLR.BI=FALSE, BcULV1.BI=FALSE} | 0.30 | 0.753 | 0.400 | 1.12 | 12 |
| => {BcOLV1.BI.MOVA.=FALSE} | 198 | 086 | 99 | 684 | 2 |
| {BcOLV1.BI.MOVA.=FALSE, |  |  |  |  |  |
|  | 0.30 | 0.539 | 0.559 | 1.17 | 12 |
| BcULV1.BI=FALSE} => |  |  |  |  |  |
|  | 198 | 823 | 406 | 8857 | 2 |
| {BcHV1SLR.BI=FALSE} |  |  |  |  |  |
| {BcNSRV7.BI=FALSE, |  |  |  |  |  |
|  | 0.28 | 0.527 | 0.534 | 1.15 | 11 |
| BcOLV1.BI.MOVA.=FALSE} => |  |  |  |  |  |
|  | 2178 | 778 | 653 | 2553 | 4 |
| {BcHV1SLR.BI=FALSE} |  |  |  |  |  |
| {BcHV1SLR.BI=FALSE, BcVV3.BI=FALSE} => | 0.27 | 0.751 | 0.368 | 1.12 | 11 |
| {BcOLV1.BI.MOVA.=FALSE} | 7228 | 678 | 812 | 4733 | 2 |
| {BcHV1SLR.BI=FALSE, |  |  |  |  |  |
|  | 0.29 | 0.916 | 0.326 | 1.12 | 12 |
| BcOLV1.BI.MOVA.=FALSE} => |  |  |  |  |  |
|  | 9505 | 667 | 733 | 5633 | 1 |
| {BcALV1.BI=FALSE} |  |  |  |  |  |

| {BcALV1.BI=FALSE, BcHV1SLR.BI=FALSE}  => {BcOLV1.BI.MOVA.=FALSE} | 0.29  9505 | 0.751  553 | 0.398  515 | 1.12  4546 | 12  1 |
| --- | --- | --- | --- | --- | --- |
| {BcHV1SLR.BI=FALSE, BcOLV1.BI.MOVA.=FALSE} =>  {BcFV9=FALSE} | 0.30  4455 | 0.931  818 | 0.326  733 | 1.07  252 | 12  3 |
| {BcFV9=FALSE, BcOLV1.BI.MOVA.=FALSE}  => {BcHV1SLR.BI=FALSE} | 0.30  4455 | 0.516  807 | 0.589  109 | 1.12  8594 | 12  3 |
| {BcHV1SLR.BI=FALSE, BcOLV1.BI.MOVA.=FALSE} =>  {BcOLV4.BI=FALSE} | 0.30  4455 | 0.931  818 | 0.326  733 | 1.06  3431 | 12  3 |
| {BcHV1SLR.BI=FALSE, BcOLV1.BI.MOVA.=FALSE} =>  {BcULV5=FALSE} | 0.31  4356 | 0.962  121 | 0.326  733 | 1.09  8014 | 12  7 |
| {BcHV1SLR.BI=FALSE, BcULV5=FALSE} =>  {BcOLV1.BI.MOVA.=FALSE} | 0.31  4356 | 0.769  697 | 0.408  416 | 1.15  1695 | 12  7 |
| {BcOLV1.BI.MOVA.=FALSE, BvF.BI=FALSE}  => {BcHV1SLR.BI=FALSE} | 0.30  9406 | 0.516  529 | 0.599  01 | 1.12  7987 | 12  5 |
| {BcHV1SLR.BI=FALSE, BcMCV5.BI=FALSE}  => {BcOLV1.BI.MOVA.=FALSE} | 0.32  1782 | 0.742  857 | 0.433  168 | 1.11  1534 | 13  0 |
| {BcHV1SLR.BI=FALSE, BcMV9.BI=TRUE} =>  {BcULV4=TRUE} | 0.21  5347 | 0.798  165 | 0.269  802 | 1.15  5766 | 87 |
| {BcHV1SLR.BI=FALSE, BcMV9.BI=TRUE} => | 0.26 | 0.990 | 0.269 | 1.13 | 10 |

| {BcULV5=FALSE} | 7327 | 826 | 802 | 0773 | 8 |
| --- | --- | --- | --- | --- | --- |
| {BcHV1SLR.BI=FALSE, BcULV4=TRUE} =>  {BcFV9=FALSE} | 0.26  9802 | 0.931  624 | 0.289  604 | 1.07  2296 | 10  9 |
| {BcHV1SLR.BI=FALSE, BcULV4=TRUE} =>  {BcULV5=FALSE} | 0.27  4752 | 0.948  718 | 0.289  604 | 1.08  2718 | 11  1 |
| {BcHV1SLR.BI=FALSE, BcPV3.BI=FALSE} =>  {BcMV6.BI=FALSE} | 0.26  7327 | 0.794  118 | 0.336  634 | 1.14  1721 | 10  8 |
| {BcEV2.BI=FALSE, BcMV6.BI=FALSE} =>  {BcHV1SLR.BI=FALSE} | 0.27  7228 | 0.538  462 | 0.514  851 | 1.17  5884 | 11  2 |
| {BcHV1SLR.BI=FALSE, BcHV3.BI=FALSE} =>  {BcMV6.BI=FALSE} | 0.28  7129 | 0.783  784 | 0.366  337 | 1.12  6864 | 11  6 |
| {BcHV3.BI=FALSE, BcMV6.BI=FALSE} =>  {BcHV1SLR.BI=FALSE} | 0.28  7129 | 0.532  11 | 0.539  604 | 1.16  2013 | 11  6 |
| {BcFV5.BI=FALSE, BcHV1SLR.BI=FALSE} =>  {BcMV6.BI=FALSE} | 0.30  198 | 0.772  152 | 0.391  089 | 1.11  014 | 12  2 |
| {BcFV5.BI=FALSE, BcMV6.BI=FALSE} =>  {BcHV1SLR.BI=FALSE} | 0.30  198 | 0.554  545 | 0.544  554 | 1.21  1007 | 12  2 |
| {BcHV1SLR.BI=FALSE, BcMV6.BI=FALSE}  => {BcULV1.BI=FALSE} | 0.31  9307 | 0.889  655 | 0.358  911 | 1.13  3819 | 12  9 |
| {BcHV1SLR.BI=FALSE, BcULV1.BI=FALSE}  => {BcMV6.BI=FALSE} | 0.31  9307 | 0.796  296 | 0.400  99 | 1.14  4853 | 12  9 |
| {BcMV6.BI=FALSE, BcULV1.BI=FALSE} => | 0.31 | 0.558 | 0.571 | 1.21 | 12 |

| {BcHV1SLR.BI=FALSE} | 9307 | 442 | 782 | 9516 | 9 |
| --- | --- | --- | --- | --- | --- |
| {BcHV1SLR.BI=FALSE, BcMV12=FALSE} =>  {BcMV6.BI=FALSE} | 0.30  9406 | 0.801  282 | 0.386  139 | 1.15  2021 | 12  5 |
| {BcMV12=FALSE, BcMV6.BI=FALSE} =>  {BcHV1SLR.BI=FALSE} | 0.30  9406 | 0.531  915 | 0.581  683 | 1.16  1587 | 12  5 |
| {BcHV1SLR.BI=FALSE, BcMV6.BI=FALSE}  => {BcNSRV7.BI=FALSE} | 0.31  4356 | 0.875  862 | 0.358  911 | 1.09  5505 | 12  7 |
| {BcHV1SLR.BI=FALSE, BcNSRV7.BI=FALSE}  => {BcMV6.BI=FALSE} | 0.31  4356 | 0.803  797 | 0.391  089 | 1.15  5638 | 12  7 |
| {BcMV6.BI=FALSE, BcNSRV7.BI=FALSE} =>  {BcHV1SLR.BI=FALSE} | 0.31  4356 | 0.554  585 | 0.566  832 | 1.21  1094 | 12  7 |
| {BcHV1SLR.BI=FALSE, BcVV3.BI=FALSE} =>  {BcMV6.BI=FALSE} | 0.29  4554 | 0.798  658 | 0.368  812 | 1.14  8248 | 11  9 |
| {BcHV1SLR.BI=FALSE, BcOLV7.BI=FALSE}  => {BcMV6.BI=FALSE} | 0.29  703 | 0.8 | 0.371  287 | 1.15  0178 | 12  0 |
| {BcHV1SLR.BI=FALSE, BcMV6.BI=FALSE}  => {BcALV1.BI=FALSE} | 0.31  6832 | 0.882  759 | 0.358  911 | 1.08  3995 | 12  8 |
| {BcALV1.BI=FALSE, BcHV1SLR.BI=FALSE}  => {BcMV6.BI=FALSE} | 0.31  6832 | 0.795  031 | 0.398  515 | 1.14  3034 | 12  8 |
| {BcALV1.BI=FALSE, BcMV6.BI=FALSE} =>  {BcHV1SLR.BI=FALSE} | 0.31  6832 | 0.531  12 | 0.596  535 | 1.15  9852 | 12  8 |
| {BcDMV1=FALSE, BcHV1SLR.BI=FALSE} => | 0.30 | 0.797 | 0.378 | 1.14 | 12 |

| {BcMV6.BI=FALSE} | 198 | 386 | 713 | 6419 | 2 |
| --- | --- | --- | --- | --- | --- |
| {BcFV9=FALSE, BcHV1SLR.BI=FALSE} =>  {BcMV6.BI=FALSE} | 0.32  4257 | 0.775  148 | 0.418  317 | 1.11  4448 | 13  1 |
| {BcFV9=FALSE, BcMV6.BI=FALSE} =>  {BcHV1SLR.BI=FALSE} | 0.32  4257 | 0.539  095 | 0.601  485 | 1.17  7266 | 13  1 |
| {BcHV1SLR.BI=FALSE, BcOLV4.BI=FALSE}  => {BcMV6.BI=FALSE} | 0.31  9307 | 0.772  455 | 0.413  366 | 1.11  0576 | 12  9 |
| {BcMV6.BI=FALSE, BcOLV4.BI=FALSE} =>  {BcHV1SLR.BI=FALSE} | 0.31  9307 | 0.518  072 | 0.616  337 | 1.13  1358 | 12  9 |
| {BcHV1SLR.BI=FALSE, BcULV5=FALSE} =>  {BcMV6.BI=FALSE} | 0.32  1782 | 0.787  879 | 0.408  416 | 1.13  2751 | 13  0 |
| {BcMV6.BI=FALSE, BcULV5=FALSE} =>  {BcHV1SLR.BI=FALSE} | 0.32  1782 | 0.517  928 | 0.621  287 | 1.13  1043 | 13  0 |
| {BcHV1SLR.BI=FALSE, BcMV6.BI=FALSE}  => {BcBNV5.BI=FALSE} | 0.34  901 | 0.972  414 | 0.358  911 | 1.07  045 | 14  1 |
| {BcBNV5.BI=FALSE, BcHV1SLR.BI=FALSE}  => {BcMV6.BI=FALSE} | 0.34  901 | 0.810  345 | 0.430  693 | 1.16  5051 | 14  1 |
| {BcBNV5.BI=FALSE, BcMV6.BI=FALSE} =>  {BcHV1SLR.BI=FALSE} | 0.34  901 | 0.526  119 | 0.663  366 | 1.14  8931 | 14  1 |
| {BcHV1SLR.BI=FALSE, BvF.BI=FALSE} =>  {BcMV6.BI=FALSE} | 0.33  4158 | 0.771  429 | 0.433  168 | 1.10  91 | 13  5 |
| {BcMV6.BI=FALSE, BvF.BI=FALSE} => | 0.33 | 0.535 | 0.623 | 1.16 | 13 |

| {BcHV1SLR.BI=FALSE} | 4158 | 714 | 762 | 9884 | 5 |
| --- | --- | --- | --- | --- | --- |
| {BcHV1SLR.BI=FALSE, BcMCV5.BI=FALSE}  => {BcMV6.BI=FALSE} | 0.34  4059 | 0.794  286 | 0.433  168 | 1.14  1962 | 13  9 |
| {BcMCV5.BI=FALSE, BcMV6.BI=FALSE} =>  {BcHV1SLR.BI=FALSE} | 0.34  4059 | 0.518  657 | 0.663  366 | 1.13  2634 | 13  9 |
| {BcHV1SLR.BI=FALSE, BcOLV13.BI=FALSE}  => {BcMV6.BI=FALSE} | 0.33  9109 | 0.778  409 | 0.435  644 | 1.11  9136 | 13  7 |
| {BcMV6.BI=FALSE, BcOLV13.BI=FALSE} =>  {BcHV1SLR.BI=FALSE} | 0.33  9109 | 0.516  981 | 0.655  941 | 1.12  8975 | 13  7 |
| {BcHV1SLR.BI=FALSE, BcOLV2.BI=FALSE}  => {BcMV6.BI=FALSE} | 0.34  901 | 0.783  333 | 0.445  545 | 1.12  6216 | 14  1 |
| {BcMV6.BI=FALSE, BcOLV2.BI=FALSE} =>  {BcHV1SLR.BI=FALSE} | 0.34  901 | 0.524  164 | 0.665  842 | 1.14  466 | 14  1 |
| {BcHV1SLR.BI=FALSE, BcMCV3.BI=FALSE}  => {BcMV6.BI=FALSE} | 0.35  396 | 0.794  444 | 0.445  545 | 1.14  2191 | 14  3 |
| {BcMCV3.BI=FALSE, BcMV6.BI=FALSE} =>  {BcHV1SLR.BI=FALSE} | 0.35  396 | 0.525  735 | 0.673  267 | 1.14  8092 | 14  3 |
| {BcHV1SLR.BI=FALSE, BcPV4.BI=FALSE} =>  {BcMV6.BI=FALSE} | 0.33  6634 | 0.786  127 | 0.428  218 | 1.13  0233 | 13  6 |
| {BcMV6.BI=FALSE, BcPV4.BI=FALSE} =>  {BcHV1SLR.BI=FALSE} | 0.33  6634 | 0.513  208 | 0.655  941 | 1.12  0734 | 13  6 |
| {BcEV6=FALSE, BcHV1SLR.BI=FALSE} => | 0.34 | 0.788 | 0.433 | 1.13 | 13 |

| {BcMV6.BI=FALSE} | 1584 | 571 | 168 | 3747 | 8 |
| --- | --- | --- | --- | --- | --- |
| {BcEV6=FALSE, BcMV6.BI=FALSE} =>  {BcHV1SLR.BI=FALSE} | 0.34  1584 | 0.522  727 | 0.653  465 | 1.14  1523 | 13  8 |
| {BcHV1SLR.BI=FALSE, BcOLV17.BI=FALSE}  => {BcMV6.BI=FALSE} | 0.34  901 | 0.787  709 | 0.443  069 | 1.13  2508 | 14  1 |
| {BcMV6.BI=FALSE, BcOLV17.BI=FALSE} =>  {BcHV1SLR.BI=FALSE} | 0.34  901 | 0.520  295 | 0.670  792 | 1.13  6212 | 14  1 |
| {BcHV1SLR.BI=FALSE, BcOLV15.BI=FALSE}  => {BcMV6.BI=FALSE} | 0.35  1485 | 0.780  22 | 0.450  495 | 1.12  1739 | 14  2 |
| {BcMV6.BI=FALSE, BcOLV15.BI=FALSE} =>  {BcHV1SLR.BI=FALSE} | 0.35  1485 | 0.539  924 | 0.650  99 | 1.17  9077 | 14  2 |
| {BcEV5=FALSE, BcHV1SLR.BI=FALSE} =>  {BcMV6.BI=FALSE} | 0.35  1485 | 0.802  26 | 0.438  119 | 1.15  3427 | 14  2 |
| {BcEV5=FALSE, BcMV6.BI=FALSE} =>  {BcHV1SLR.BI=FALSE} | 0.35  1485 | 0.512  635 | 0.685  644 | 1.11  9485 | 14  2 |
| {BcHV1SLR.BI=FALSE, BcPV3.BI=FALSE} =>  {BcULV1.BI=FALSE} | 0.29  703 | 0.882  353 | 0.336  634 | 1.12  4513 | 12  0 |
| {BcFV5.BI=FALSE, BcRV1.BI=FALSE} =>  {BcHV1SLR.BI=FALSE} | 0.28  4653 | 0.522  727 | 0.544  554 | 1.14  1523 | 11  5 |
| {BcHV1SLR.BI=FALSE, BcRV1.BI=FALSE} =>  {BcULV1.BI=FALSE} | 0.29  703 | 0.869  565 | 0.341  584 | 1.10  8216 | 12  0 |
| {BcHV1SLR.BI=FALSE, BcOLV7.BI=FALSE} | 0.29 | 0.8 | 0.371 | 1.10 | 12 |

| => {BcRV1.BI=FALSE} | 703 |  | 287 | 6849 | 0 |
| --- | --- | --- | --- | --- | --- |
| {BcHV1SLR.BI=FALSE, BcRV1.BI=FALSE} =>  {BcALV1.BI=FALSE} | 0.30  6931 | 0.898  551 | 0.341  584 | 1.10  3388 | 12  4 |
| {BcHV1SLR.BI=FALSE, BcRV1.BI=FALSE} =>  {BcFV9=FALSE} | 0.31  9307 | 0.934  783 | 0.341  584 | 1.07  5932 | 12  9 |
| {BcEV2.BI=FALSE, BcHV1SLR.BI=FALSE} =>  {BcHV3.BI=FALSE} | 0.30  9406 | 0.856  164 | 0.361  386 | 1.12  6679 | 12  5 |
| {BcHV1SLR.BI=FALSE, BcHV3.BI=FALSE} =>  {BcEV2.BI=FALSE} | 0.30  9406 | 0.844  595 | 0.366  337 | 1.12  9855 | 12  5 |
| {BcEV2.BI=FALSE, BcHV3.BI=FALSE} =>  {BcHV1SLR.BI=FALSE} | 0.30  9406 | 0.518  672 | 0.596  535 | 1.13  2668 | 12  5 |
| {BcEV2.BI=FALSE, BcHV1SLR.BI=FALSE} =>  {BcFV5.BI=FALSE} | 0.31  9307 | 0.883  562 | 0.361  386 | 1.12  9617 | 12  9 |
| {BcEV2.BI=FALSE, BcHV1SLR.BI=FALSE} =>  {BcULV1.BI=FALSE} | 0.32  4257 | 0.897  26 | 0.361  386 | 1.14  3512 | 13  1 |
| {BcEV2.BI=FALSE, BcULV1.BI=FALSE} =>  {BcHV1SLR.BI=FALSE} | 0.32  4257 | 0.550  42 | 0.589  109 | 1.20  1999 | 13  1 |
| {BcEV2.BI=FALSE, BcHV1SLR.BI=FALSE} =>  {BcFV9=FALSE} | 0.34  1584 | 0.945  205 | 0.361  386 | 1.08  7929 | 13  8 |
| {BcFV9=FALSE, BcHV1SLR.BI=FALSE} =>  {BcEV2.BI=FALSE} | 0.34  1584 | 0.816  568 | 0.418  317 | 1.09  2363 | 13  8 |
| {BcHV1SLR.BI=FALSE, BcMCV5.BI=FALSE} | 0.35 | 0.817 | 0.433 | 1.09 | 14 |

| => {BcEV2.BI=FALSE} | 396 | 143 | 168 | 3132 | 3 |
| --- | --- | --- | --- | --- | --- |
| {BcHV1SLR.BI=FALSE, BcHV3.BI=FALSE} =>  {BcFV5.BI=FALSE} | 0.33  4158 | 0.912  162 | 0.366  337 | 1.16  6182 | 13  5 |
| {BcFV5.BI=FALSE, BcHV1SLR.BI=FALSE} =>  {BcHV3.BI=FALSE} | 0.33  4158 | 0.854  43 | 0.391  089 | 1.12  4397 | 13  5 |
| {BcHV1SLR.BI=FALSE, BcHV3.BI=FALSE} =>  {BcULV1.BI=FALSE} | 0.32  4257 | 0.885  135 | 0.366  337 | 1.12  8059 | 13  1 |
| {BcHV3.BI=FALSE, BcULV1.BI=FALSE} =>  {BcHV1SLR.BI=FALSE} | 0.32  4257 | 0.536  885 | 0.603  96 | 1.17  2441 | 13  1 |
| {BcHV1SLR.BI=FALSE, BcHV3.BI=FALSE} =>  {BcNSRV7.BI=FALSE} | 0.32  6733 | 0.891  892 | 0.366  337 | 1.11  5555 | 13  2 |
| {BcHV1SLR.BI=FALSE, BcNSRV7.BI=FALSE}  => {BcHV3.BI=FALSE} | 0.32  6733 | 0.835  443 | 0.391  089 | 1.09  941 | 13  2 |
| {BcHV1SLR.BI=FALSE, BcVV3.BI=FALSE} =>  {BcHV3.BI=FALSE} | 0.31  4356 | 0.852  349 | 0.368  812 | 1.12  1658 | 12  7 |
| {BcHV1SLR.BI=FALSE, BcHV3.BI=FALSE} =>  {BcALV1.BI=FALSE} | 0.33  1683 | 0.905  405 | 0.366  337 | 1.11  1805 | 13  4 |
| {BcALV1.BI=FALSE, BcHV1SLR.BI=FALSE}  => {BcHV3.BI=FALSE} | 0.33  1683 | 0.832  298 | 0.398  515 | 1.09  5272 | 13  4 |
| {BcALV1.BI=FALSE, BcHV3.BI=FALSE} =>  {BcHV1SLR.BI=FALSE} | 0.33  1683 | 0.523  438 | 0.633  663 | 1.14  3074 | 13  4 |
| {BcHV1SLR.BI=FALSE, BcHV3.BI=FALSE} => | 0.33 | 0.925 | 0.366 | 1.06 | 13 |

| {BcFV9=FALSE} | 9109 | 676 | 337 | 545 | 7 |
| --- | --- | --- | --- | --- | --- |
| {BcHV1SLR.BI=FALSE, BcMCV5.BI=FALSE}  => {BcHV3.BI=FALSE} | 0.35  8911 | 0.828  571 | 0.433  168 | 1.09  0368 | 14  5 |
| {BcFV5.BI=FALSE, BcHV1SLR.BI=FALSE} =>  {BcULV1.BI=FALSE} | 0.34  4059 | 0.879  747 | 0.391  089 | 1.12  1192 | 13  9 |
| {BcHV1SLR.BI=FALSE, BcULV1.BI=FALSE}  => {BcFV5.BI=FALSE} | 0.34  4059 | 0.858  025 | 0.400  99 | 1.09  6968 | 13  9 |
| {BcFV5.BI=FALSE, BcULV1.BI=FALSE} =>  {BcHV1SLR.BI=FALSE} | 0.34  4059 | 0.562  753 | 0.611  386 | 1.22  8931 | 13  9 |
| {BcFV5.BI=FALSE, BcMV12=FALSE} =>  {BcHV1SLR.BI=FALSE} | 0.32  4257 | 0.517  787 | 0.626  238 | 1.13  0734 | 13  1 |
| {BcFV5.BI=FALSE, BcHV1SLR.BI=FALSE} =>  {BcNSRV7.BI=FALSE} | 0.34  6535 | 0.886  076 | 0.391  089 | 1.10  8281 | 14  0 |
| {BcHV1SLR.BI=FALSE, BcNSRV7.BI=FALSE}  => {BcFV5.BI=FALSE} | 0.34  6535 | 0.886  076 | 0.391  089 | 1.13  2831 | 14  0 |
| {BcHV1SLR.BI=FALSE, BcVV3.BI=FALSE} =>  {BcFV5.BI=FALSE} | 0.32  4257 | 0.879  195 | 0.368  812 | 1.12  4034 | 13  1 |
| {BcFV5.BI=FALSE, BcHV1SLR.BI=FALSE} =>  {BcALV1.BI=FALSE} | 0.35  1485 | 0.898  734 | 0.391  089 | 1.10  3613 | 14  2 |
| {BcALV1.BI=FALSE, BcHV1SLR.BI=FALSE}  => {BcFV5.BI=FALSE} | 0.35  1485 | 0.881  988 | 0.398  515 | 1.12  7604 | 14  2 |
| {BcALV1.BI=FALSE, BcFV5.BI=FALSE} => | 0.35 | 0.527 | 0.665 | 1.15 | 14 |

| {BcHV1SLR.BI=FALSE} | 1485 | 881 | 842 | 2778 | 2 |
| --- | --- | --- | --- | --- | --- |
| {BcDMV1=FALSE, BcHV1SLR.BI=FALSE} =>  {BcFV5.BI=FALSE} | 0.33  9109 | 0.895  425 | 0.378  713 | 1.14  4784 | 13  7 |
| {BcHV1SLR.BI=FALSE, BcOLV4.BI=FALSE}  => {BcFV5.BI=FALSE} | 0.35  396 | 0.856  287 | 0.413  366 | 1.09  4747 | 14  3 |
| {BcFV5.BI=FALSE, BcOLV4.BI=FALSE} =>  {BcHV1SLR.BI=FALSE} | 0.35  396 | 0.52 | 0.680  693 | 1.13  5568 | 14  3 |
| {BcBNV5.BI=FALSE, BcHV1SLR.BI=FALSE}  => {BcFV5.BI=FALSE} | 0.37  3762 | 0.867  816 | 0.430  693 | 1.10  9486 | 15  1 |
| {BcHV1SLR.BI=FALSE, BvF.BI=FALSE} =>  {BcFV5.BI=FALSE} | 0.37  1287 | 0.857  143 | 0.433  168 | 1.09  5841 | 15  0 |
| {BcFV5.BI=FALSE, BvF.BI=FALSE} =>  {BcHV1SLR.BI=FALSE} | 0.37  1287 | 0.513  699 | 0.722  772 | 1.12  1807 | 15  0 |
| {BcHV1SLR.BI=FALSE, BcMCV5.BI=FALSE}  => {BcFV5.BI=FALSE} | 0.37  8713 | 0.874  286 | 0.433  168 | 1.11  7758 | 15  3 |
| {BcFV5.BI=FALSE, BcMCV5.BI=FALSE} =>  {BcHV1SLR.BI=FALSE} | 0.37  8713 | 0.51 | 0.742  574 | 1.11  373 | 15  3 |
| {BcHV1SLR.BI=FALSE, BcOLV13.BI=FALSE}  => {BcFV5.BI=FALSE} | 0.37  1287 | 0.852  273 | 0.435  644 | 1.08  9614 | 15  0 |
| {BcFV5.BI=FALSE, BcOLV13.BI=FALSE} =>  {BcHV1SLR.BI=FALSE} | 0.37  1287 | 0.517  241 | 0.717  822 | 1.12  9543 | 15  0 |
| {BcHV1SLR.BI=FALSE, BcOLV2.BI=FALSE} | 0.38 | 0.855 | 0.445 | 1.09 | 15 |

| => {BcFV5.BI=FALSE} | 1188 | 556 | 545 | 3812 | 4 |
| --- | --- | --- | --- | --- | --- |
| {BcFV5.BI=FALSE, BcOLV2.BI=FALSE} =>  {BcHV1SLR.BI=FALSE} | 0.38  1188 | 0.523  81 | 0.727  723 | 1.14  3887 | 15  4 |
| {BcHV1SLR.BI=FALSE, BcMCV3.BI=FALSE}  => {BcFV5.BI=FALSE} | 0.37  8713 | 0.85 | 0.445  545 | 1.08  6709 | 15  3 |
| {BcFV5.BI=FALSE, BcMCV3.BI=FALSE} =>  {BcHV1SLR.BI=FALSE} | 0.37  8713 | 0.522  184 | 0.725  248 | 1.14  0338 | 15  3 |
| {BcHV1SLR.BI=FALSE, BcPV4.BI=FALSE} =>  {BcFV5.BI=FALSE} | 0.36  3861 | 0.849  711 | 0.428  218 | 1.08  6339 | 14  7 |
| {BcHV1SLR.BI=FALSE, BcOLV17.BI=FALSE}  => {BcFV5.BI=FALSE} | 0.37  6238 | 0.849  162 | 0.443  069 | 1.08  5638 | 15  2 |
| {BcFV5.BI=FALSE, BcOLV17.BI=FALSE} =>  {BcHV1SLR.BI=FALSE} | 0.37  6238 | 0.511  785 | 0.735  149 | 1.11  7627 | 15  2 |
| {BcHV1SLR.BI=FALSE, BcOLV15.BI=FALSE}  => {BcFV5.BI=FALSE} | 0.38  6139 | 0.857  143 | 0.450  495 | 1.09  5841 | 15  6 |
| {BcEV5=FALSE, BcHV1SLR.BI=FALSE} =>  {BcFV5.BI=FALSE} | 0.38  6139 | 0.881  356 | 0.438  119 | 1.12  6797 | 15  6 |
| {BcHV1SLR.BI=FALSE, BcMV12=FALSE} =>  {BcULV1.BI=FALSE} | 0.33  9109 | 0.878  205 | 0.386  139 | 1.11  9227 | 13  7 |
| {BcMV12=FALSE, BcULV1.BI=FALSE} =>  {BcHV1SLR.BI=FALSE} | 0.33  9109 | 0.518  939 | 0.653  465 | 1.13  3251 | 13  7 |
| {BcHV1SLR.BI=FALSE, BcULV1.BI=FALSE} | 0.34 | 0.864 | 0.400 | 1.08 | 14 |

| => {BcNSRV7.BI=FALSE} | 6535 | 198 | 99 | 0916 | 0 |
| --- | --- | --- | --- | --- | --- |
| {BcHV1SLR.BI=FALSE, BcNSRV7.BI=FALSE}  => {BcULV1.BI=FALSE} | 0.34  6535 | 0.886  076 | 0.391  089 | 1.12  9258 | 14  0 |
| {BcNSRV7.BI=FALSE, BcULV1.BI=FALSE} =>  {BcHV1SLR.BI=FALSE} | 0.34  6535 | 0.564  516 | 0.613  861 | 1.23  2781 | 14  0 |
| {BcHV1SLR.BI=FALSE, BcVV3.BI=FALSE} =>  {BcULV1.BI=FALSE} | 0.32  9208 | 0.892  617 | 0.368  812 | 1.13  7594 | 13  3 |
| {BcULV1.BI=FALSE, BcVV3.BI=FALSE} =>  {BcHV1SLR.BI=FALSE} | 0.32  9208 | 0.523  622 | 0.628  713 | 1.14  3477 | 13  3 |
| {BcHV1SLR.BI=FALSE, BcOLV7.BI=FALSE}  => {BcULV1.BI=FALSE} | 0.32  6733 | 0.88 | 0.371  287 | 1.12  1514 | 13  2 |
| {BcHV1SLR.BI=FALSE, BcULV1.BI=FALSE}  => {BcALV1.BI=FALSE} | 0.35  396 | 0.882  716 | 0.400  99 | 1.08  3943 | 14  3 |
| {BcALV1.BI=FALSE, BcHV1SLR.BI=FALSE}  => {BcULV1.BI=FALSE} | 0.35  396 | 0.888  199 | 0.398  515 | 1.13  1963 | 14  3 |
| {BcALV1.BI=FALSE, BcULV1.BI=FALSE} =>  {BcHV1SLR.BI=FALSE} | 0.35  396 | 0.545  802 | 0.648  515 | 1.19  1913 | 14  3 |
| {BcDMV1=FALSE, BcHV1SLR.BI=FALSE} =>  {BcULV1.BI=FALSE} | 0.32  9208 | 0.869  281 | 0.378  713 | 1.10  7853 | 13  3 |
| {BcFV9=FALSE, BcHV1SLR.BI=FALSE} =>  {BcULV1.BI=FALSE} | 0.36  3861 | 0.869  822 | 0.418  317 | 1.10  8543 | 14  7 |
| {BcFV9=FALSE, BcULV1.BI=FALSE} => | 0.36 | 0.536 | 0.678 | 1.17 | 14 |

| {BcHV1SLR.BI=FALSE} | 3861 | 496 | 218 | 1592 | 7 |
| --- | --- | --- | --- | --- | --- |
| {BcHV1SLR.BI=FALSE, BcOLV4.BI=FALSE}  => {BcULV1.BI=FALSE} | 0.36  3861 | 0.880  24 | 0.413  366 | 1.12  1819 | 14  7 |
| {BcOLV4.BI=FALSE, BcULV1.BI=FALSE} =>  {BcHV1SLR.BI=FALSE} | 0.36  3861 | 0.523  132 | 0.695  545 | 1.14  2406 | 14  7 |
| {BcHV1SLR.BI=FALSE, BcULV5=FALSE} =>  {BcULV1.BI=FALSE} | 0.35  8911 | 0.878  788 | 0.408  416 | 1.11  9969 | 14  5 |
| {BcULV1.BI=FALSE, BcULV5=FALSE} =>  {BcHV1SLR.BI=FALSE} | 0.35  8911 | 0.512  367 | 0.700  495 | 1.11  89 | 14  5 |
| {BcBNV5.BI=FALSE, BcHV1SLR.BI=FALSE}  => {BcULV1.BI=FALSE} | 0.38  3663 | 0.890  805 | 0.430  693 | 1.13  5284 | 15  5 |
| {BcBNV5.BI=FALSE, BcULV1.BI=FALSE} =>  {BcHV1SLR.BI=FALSE} | 0.38  3663 | 0.538  194 | 0.712  871 | 1.17  53 | 15  5 |
| {BcHV1SLR.BI=FALSE, BvF.BI=FALSE} =>  {BcULV1.BI=FALSE} | 0.38  1188 | 0.88 | 0.433  168 | 1.12  1514 | 15  4 |
| {BcULV1.BI=FALSE, BvF.BI=FALSE} =>  {BcHV1SLR.BI=FALSE} | 0.38  1188 | 0.534  722 | 0.712  871 | 1.16  7718 | 15  4 |
| {BcHV1SLR.BI=FALSE, BcMCV5.BI=FALSE}  => {BcULV1.BI=FALSE} | 0.38  1188 | 0.88 | 0.433  168 | 1.12  1514 | 15  4 |
| {BcMCV5.BI=FALSE, BcULV1.BI=FALSE} =>  {BcHV1SLR.BI=FALSE} | 0.38  1188 | 0.522  034 | 0.730  198 | 1.14  0009 | 15  4 |
| {BcHV1SLR.BI=FALSE, BcOLV13.BI=FALSE} | 0.38 | 0.875 | 0.435 | 1.11 | 15 |

| => {BcULV1.BI=FALSE} | 1188 |  | 644 | 5142 | 4 |
| --- | --- | --- | --- | --- | --- |
| {BcOLV13.BI=FALSE, BcULV1.BI=FALSE} =>  {BcHV1SLR.BI=FALSE} | 0.38  1188 | 0.515  05 | 0.740  099 | 1.12  4758 | 15  4 |
| {BcHV1SLR.BI=FALSE, BcOLV2.BI=FALSE}  => {BcULV1.BI=FALSE} | 0.38  8614 | 0.872  222 | 0.445  545 | 1.11  1602 | 15  7 |
| {BcOLV2.BI=FALSE, BcULV1.BI=FALSE} =>  {BcHV1SLR.BI=FALSE} | 0.38  8614 | 0.523  333 | 0.742  574 | 1.14  2847 | 15  7 |
| {BcHV1SLR.BI=FALSE, BcMCV3.BI=FALSE}  => {BcULV1.BI=FALSE} | 0.39  3564 | 0.883  333 | 0.445  545 | 1.12  5762 | 15  9 |
| {BcMCV3.BI=FALSE, BcULV1.BI=FALSE} =>  {BcHV1SLR.BI=FALSE} | 0.39  3564 | 0.514  563 | 0.764  851 | 1.12  3695 | 15  9 |
| {BcHV1SLR.BI=FALSE, BcPV4.BI=FALSE} =>  {BcULV1.BI=FALSE} | 0.37  8713 | 0.884  393 | 0.428  218 | 1.12  7113 | 15  3 |
| {BcPV4.BI=FALSE, BcULV1.BI=FALSE} =>  {BcHV1SLR.BI=FALSE} | 0.37  8713 | 0.51 | 0.742  574 | 1.11  373 | 15  3 |
| {BcEV6=FALSE, BcHV1SLR.BI=FALSE} =>  {BcULV1.BI=FALSE} | 0.38  3663 | 0.885  714 | 0.433  168 | 1.12  8797 | 15  5 |
| {BcEV6=FALSE, BcULV1.BI=FALSE} =>  {BcHV1SLR.BI=FALSE} | 0.38  3663 | 0.520  134 | 0.737  624 | 1.13  5861 | 15  5 |
| {BcHV1SLR.BI=FALSE, BcOLV17.BI=FALSE}  => {BcULV1.BI=FALSE} | 0.38  6139 | 0.871  508 | 0.443  069 | 1.11  0692 | 15  6 |
| {BcOLV17.BI=FALSE, BcULV1.BI=FALSE} => | 0.38 | 0.514 | 0.75 | 1.12 | 15 |

| {BcHV1SLR.BI=FALSE} | 6139 | 851 |  | 4324 | 6 |
| --- | --- | --- | --- | --- | --- |
| {BcHV1SLR.BI=FALSE, BcOLV15.BI=FALSE}  => {BcULV1.BI=FALSE} | 0.39  3564 | 0.873  626 | 0.450  495 | 1.11  3391 | 15  9 |
| {BcOLV15.BI=FALSE, BcULV1.BI=FALSE} =>  {BcHV1SLR.BI=FALSE} | 0.39  3564 | 0.531  773 | 0.740  099 | 1.16  1276 | 15  9 |
| {BcEV5=FALSE, BcHV1SLR.BI=FALSE} =>  {BcULV1.BI=FALSE} | 0.38  3663 | 0.875  706 | 0.438  119 | 1.11  6042 | 15  5 |
| {BcEV5=FALSE, BcULV1.BI=FALSE} =>  {BcHV1SLR.BI=FALSE} | 0.38  3663 | 0.516  667 | 0.742  574 | 1.12  8288 | 15  5 |
| {BcMV12=FALSE, BcNSRV7.BI=FALSE} =>  {BcHV1SLR.BI=FALSE} | 0.33  4158 | 0.517  241 | 0.646  04 | 1.12  9543 | 13  5 |
| {BcHV1SLR.BI=FALSE, BcMV12=FALSE} =>  {BcALV1.BI=FALSE} | 0.34  4059 | 0.891  026 | 0.386  139 | 1.09  4147 | 13  9 |
| {BcALV1.BI=FALSE, BcHV1SLR.BI=FALSE}  => {BcMV12=FALSE} | 0.34  4059 | 0.863  354 | 0.398  515 | 1.07  9861 | 13  9 |
| {BcALV1.BI=FALSE, BcMV12=FALSE} =>  {BcHV1SLR.BI=FALSE} | 0.34  4059 | 0.526  515 | 0.653  465 | 1.14  9795 | 13  9 |
| {BcHV1SLR.BI=FALSE, BcMV12=FALSE} =>  {BcFV9=FALSE} | 0.35  6436 | 0.923  077 | 0.386  139 | 1.06  2459 | 14  4 |
| {BcFV9=FALSE, BcMV12=FALSE} =>  {BcHV1SLR.BI=FALSE} | 0.35  6436 | 0.512  456 | 0.695  545 | 1.11  9092 | 14  4 |
| {BcBNV5.BI=FALSE, BcMV12=FALSE} => | 0.36 | 0.510 | 0.722 | 1.11 | 14 |

| {BcHV1SLR.BI=FALSE} | 8812 | 274 | 772 | 4328 | 9 |
| --- | --- | --- | --- | --- | --- |
| {BcMV12=FALSE, BvF.BI=FALSE} =>  {BcHV1SLR.BI=FALSE} | 0.36  6337 | 0.510  345 | 0.717  822 | 1.11  4483 | 14  8 |
| {BcHV1SLR.BI=FALSE, BcMCV3.BI=FALSE}  => {BcMV12=FALSE} | 0.38  3663 | 0.861  111 | 0.445  545 | 1.07  7055 | 15  5 |
| {BcHV1SLR.BI=FALSE, BcVV3.BI=FALSE} =>  {BcNSRV7.BI=FALSE} | 0.32  1782 | 0.872  483 | 0.368  812 | 1.09  1279 | 13  0 |
| {BcHV1SLR.BI=FALSE, BcOLV7.BI=FALSE}  => {BcNSRV7.BI=FALSE} | 0.32  1782 | 0.866  667 | 0.371  287 | 1.08  4004 | 13  0 |
| {BcHV1SLR.BI=FALSE, BcNSRV7.BI=FALSE}  => {BcALV1.BI=FALSE} | 0.34  4059 | 0.879  747 | 0.391  089 | 1.08  0297 | 13  9 |
| {BcALV1.BI=FALSE, BcHV1SLR.BI=FALSE}  => {BcNSRV7.BI=FALSE} | 0.34  4059 | 0.863  354 | 0.398  515 | 1.07  9861 | 13  9 |
| {BcALV1.BI=FALSE, BcNSRV7.BI=FALSE} =>  {BcHV1SLR.BI=FALSE} | 0.34  4059 | 0.520  599 | 0.660  891 | 1.13  6876 | 13  9 |
| {BcDMV1=FALSE, BcHV1SLR.BI=FALSE} =>  {BcNSRV7.BI=FALSE} | 0.33  9109 | 0.895  425 | 0.378  713 | 1.11  9974 | 13  7 |
| {BcHV1SLR.BI=FALSE, BcNSRV7.BI=FALSE}  => {BcFV9=FALSE} | 0.36  1386 | 0.924  051 | 0.391  089 | 1.06  358 | 14  6 |
| {BcFV9=FALSE, BcHV1SLR.BI=FALSE} =>  {BcNSRV7.BI=FALSE} | 0.36  1386 | 0.863  905 | 0.418  317 | 1.08  055 | 14  6 |
| {BcBNV5.BI=FALSE, BcHV1SLR.BI=FALSE} | 0.37 | 0.867 | 0.430 | 1.08 | 15 |

| => {BcNSRV7.BI=FALSE} | 3762 | 816 | 693 | 5442 | 1 |
| --- | --- | --- | --- | --- | --- |
| {BcNSRV7.BI=FALSE, BvF.BI=FALSE} =>  {BcHV1SLR.BI=FALSE} | 0.36  8812 | 0.506  803 | 0.727  723 | 1.10  6748 | 14  9 |
| {BcHV1SLR.BI=FALSE, BcMCV5.BI=FALSE}  => {BcNSRV7.BI=FALSE} | 0.37  6238 | 0.868  571 | 0.433  168 | 1.08  6387 | 15  2 |
| {BcNSRV7.BI=FALSE, BcOLV2.BI=FALSE} =>  {BcHV1SLR.BI=FALSE} | 0.38  1188 | 0.515  05 | 0.740  099 | 1.12  4758 | 15  4 |
| {BcMCV3.BI=FALSE, BcNSRV7.BI=FALSE}  => {BcHV1SLR.BI=FALSE} | 0.37  8713 | 0.515  152 | 0.735  149 | 1.12  498 | 15  3 |
| {BcHV1SLR.BI=FALSE, BcOLV17.BI=FALSE}  => {BcNSRV7.BI=FALSE} | 0.38  1188 | 0.860  335 | 0.443  069 | 1.07  6085 | 15  4 |
| {BcEV5=FALSE, BcHV1SLR.BI=FALSE} =>  {BcNSRV7.BI=FALSE} | 0.38  3663 | 0.875  706 | 0.438  119 | 1.09  5311 | 15  5 |
| {BcHV1SLR.BI=FALSE, BcVV3.BI=FALSE} =>  {BcALV1.BI=FALSE} | 0.32  4257 | 0.879  195 | 0.368  812 | 1.07  9619 | 13  1 |
| {BcHV1SLR.BI=FALSE, BcVV3.BI=FALSE} =>  {BcFV9=FALSE} | 0.34  1584 | 0.926  174 | 0.368  812 | 1.06  6024 | 13  8 |
| {BcHV1SLR.BI=FALSE, BcOLV7.BI=FALSE}  => {BcALV1.BI=FALSE} | 0.33  1683 | 0.893  333 | 0.371  287 | 1.09  6981 | 13  4 |
| {BcHV1SLR.BI=FALSE, BcOLV7.BI=FALSE}  => {BcFV9=FALSE} | 0.34  1584 | 0.92 | 0.371  287 | 1.05  8917 | 13  8 |
| {BcALV1.BI=FALSE, BcHV1SLR.BI=FALSE} | 0.36 | 0.925 | 0.398 | 1.06 | 14 |

| => {BcFV9=FALSE} | 8812 | 466 | 515 | 5209 | 9 |
| --- | --- | --- | --- | --- | --- |
| {BcFV9=FALSE, BcHV1SLR.BI=FALSE} =>  {BcALV1.BI=FALSE} | 0.36  8812 | 0.881  657 | 0.418  317 | 1.08  2642 | 14  9 |
| {BcALV1.BI=FALSE, BcFV9=FALSE} =>  {BcHV1SLR.BI=FALSE} | 0.36  8812 | 0.512  027 | 0.720  297 | 1.11  8157 | 14  9 |
| {BcHV1SLR.BI=FALSE, BcOLV4.BI=FALSE}  => {BcALV1.BI=FALSE} | 0.36  3861 | 0.880  24 | 0.413  366 | 1.08  0902 | 14  7 |
| {BcBNV5.BI=FALSE, BcHV1SLR.BI=FALSE}  => {BcALV1.BI=FALSE} | 0.38  1188 | 0.885  057 | 0.430  693 | 1.08  6818 | 15  4 |
| {BcHV1SLR.BI=FALSE, BcMCV5.BI=FALSE}  => {BcALV1.BI=FALSE} | 0.38  3663 | 0.885  714 | 0.433  168 | 1.08  7625 | 15  5 |
| {BcHV1SLR.BI=FALSE, BcOLV13.BI=FALSE}  => {BcALV1.BI=FALSE} | 0.37  8713 | 0.869  318 | 0.435  644 | 1.06  7491 | 15  3 |
| {BcALV1.BI=FALSE, BcOLV2.BI=FALSE} =>  {BcHV1SLR.BI=FALSE} | 0.38  6139 | 0.511  475 | 0.754  95 | 1.11  6952 | 15  6 |
| {BcALV1.BI=FALSE, BcMCV3.BI=FALSE} =>  {BcHV1SLR.BI=FALSE} | 0.38  6139 | 0.508  143 | 0.759  901 | 1.10  9675 | 15  6 |
| {BcEV6=FALSE, BcHV1SLR.BI=FALSE} =>  {BcALV1.BI=FALSE} | 0.38  3663 | 0.885  714 | 0.433  168 | 1.08  7625 | 15  5 |
| {BcALV1.BI=FALSE, BcOLV15.BI=FALSE} =>  {BcHV1SLR.BI=FALSE} | 0.39  1089 | 0.509  677 | 0.767  327 | 1.11  3025 | 15  8 |
| {BcEV5=FALSE, BcHV1SLR.BI=FALSE} => | 0.38 | 0.875 | 0.438 | 1.07 | 15 |

| {BcALV1.BI=FALSE} | 3663 | 706 | 119 | 5335 | 5 |
| --- | --- | --- | --- | --- | --- |
| {BcDMV1=FALSE, BcHV1SLR.BI=FALSE} =>  {BcFV9=FALSE} | 0.34  901 | 0.921  569 | 0.378  713 | 1.06  0723 | 14  1 |
| {BcHV1SLR.BI=FALSE, BcOLV4.BI=FALSE}  => {BcFV9=FALSE} | 0.38  1188 | 0.922  156 | 0.413  366 | 1.06  1399 | 15  4 |
| {BcHV1SLR.BI=FALSE, BcMCV5.BI=FALSE}  => {BcFV9=FALSE} | 0.40  099 | 0.925  714 | 0.433  168 | 1.06  5495 | 16  2 |
| {BcHV1SLR.BI=FALSE, BcOLV13.BI=FALSE}  => {BcFV9=FALSE} | 0.40  3465 | 0.926  136 | 0.435  644 | 1.06  598 | 16  3 |
| {BcFV9=FALSE, BcOLV2.BI=FALSE} =>  {BcHV1SLR.BI=FALSE} | 0.40  8416 | 0.506  135 | 0.806  931 | 1.10  5289 | 16  5 |
| {BcFV9=FALSE, BcMCV3.BI=FALSE} =>  {BcHV1SLR.BI=FALSE} | 0.40  5941 | 0.501  529 | 0.809  406 | 1.09  5231 | 16  4 |
| {BcHV1SLR.BI=FALSE, BcOLV17.BI=FALSE}  => {BcFV9=FALSE} | 0.40  8416 | 0.921  788 | 0.443  069 | 1.06  0975 | 16  5 |
| {BcHV1SLR.BI=FALSE, BcOLV15.BI=FALSE}  => {BcFV9=FALSE} | 0.41  5842 | 0.923  077 | 0.450  495 | 1.06  2459 | 16  8 |
| {BcMCV3.BI=FALSE, BcOLV2.BI=FALSE} =>  {BcHV1SLR.BI=FALSE} | 0.43  5644 | 0.497  175 | 0.876  238 | 1.08  5723 | 17  6 |
| {BcOLV15.BI=FALSE, BcOLV2.BI=FALSE} =>  {BcHV1SLR.BI=FALSE} | 0.44  0594 | 0.494  444 | 0.891  089 | 1.07  976 | 17  8 |
| {BcMCV3.BI=FALSE, BcOLV15.BI=FALSE} => | 0.43 | 0.495 | 0.883 | 1.08 | 17 |

| {BcHV1SLR.BI=FALSE} | 8119 | 798 | 663 | 2716 | 7 |
| --- | --- | --- | --- | --- | --- |
| {BcMV2.BI=TRUE, BcMV3.BI=FALSE} =>  {BcOLV7.BI=FALSE} | 0.13  3663 | 0.9 | 0.148  515 | 1.11  5337 | 54 |
| {BcMV2.BI=TRUE, BcMV3.BI=FALSE} =>  {BcDMV1=FALSE} | 0.13  8614 | 0.933  333 | 0.148  515 | 1.10  5767 | 56 |
| {BcMV3.BI=FALSE, BcOLV18=FALSE} =>  {BcMV2.BI=FALSE} | 0.20  297 | 0.752  294 | 0.269  802 | 1.40  0583 | 82 |
| {BcMV2.BI=FALSE, BcMV3.BI=FALSE} =>  {BcOLV18=FALSE} | 0.20  297 | 0.656 | 0.309  406 | 1.35  9097 | 82 |
| {BcMV2.BI=FALSE, BcOLV18=FALSE} =>  {BcMV3.BI=FALSE} | 0.20  297 | 0.645  669 | 0.314  356 | 1.41  0002 | 82 |
| {BcMV3.BI=FALSE, BcOLV18=FALSE} =>  {BcMV4.BI=FALSE} | 0.19  0594 | 0.706  422 | 0.269  802 | 1.26  842 | 77 |
| {BcMV3.BI=FALSE, BcMV4.BI=FALSE} =>  {BcOLV18=FALSE} | 0.19  0594 | 0.616 | 0.309  406 | 1.27  6226 | 77 |
| {BcMV4.BI=FALSE, BcOLV18=FALSE} =>  {BcMV3.BI=FALSE} | 0.19  0594 | 0.601  563 | 0.316  832 | 1.31  3682 | 77 |
| {BcMV3.BI=FALSE, BcOLV18=FALSE} =>  {BcOLV16.BI=FALSE} | 0.19  802 | 0.733  945 | 0.269  802 | 1.25  6414 | 80 |
| {BcMV3.BI=FALSE, BcOLV16.BI=FALSE} =>  {BcOLV18=FALSE} | 0.19  802 | 0.64 | 0.309  406 | 1.32  5949 | 80 |
| {BcOLV16.BI=FALSE, BcOLV18=FALSE} => | 0.19 | 0.588 | 0.336 | 1.28 | 80 |

| {BcMV3.BI=FALSE} | 802 | 235 | 634 | 4579 |  |
| --- | --- | --- | --- | --- | --- |
| {BcMV3.BI=FALSE, BcOLV18=FALSE} =>  {BcOLV1.BI.MOVA.=FALSE} | 0.24  505 | 0.908  257 | 0.269  802 | 1.35  9021 | 99 |
| {BcMV3.BI=FALSE, BcOLV1.BI.MOVA.=FALSE} =>  {BcOLV18=FALSE} | 0.24  505 | 0.66 | 0.371  287 | 1.36  7385 | 99 |
| {BcOLV1.BI.MOVA.=FALSE, | 0.24 | 0.642 | 0.381 | 1.40 |  |
|  |  |  |  |  | 99 |
| BcOLV18=FALSE} => {BcMV3.BI=FALSE} | 505 | 857 | 188 | 3861 |  |
| {BcMV9.BI=TRUE, BcOLV18=FALSE} => | 0.14 | 0.560 | 0.264 | 1.22 |  |
|  |  |  |  |  | 60 |
| {BcMV3.BI=FALSE} | 8515 | 748 | 851 | 4552 |  |
| {BcOLV18=FALSE, BcULV4=TRUE} => | 0.14 | 0.564 |  | 1.23 |  |
|  |  |  | 0.25 |  | 57 |
| {BcMV3.BI=FALSE} | 1089 | 356 |  | 2432 |  |
| {BcMV3.BI=FALSE, BcOLV18=FALSE} => | 0.22 | 0.816 | 0.269 | 1.17 |  |
|  |  |  |  |  | 89 |
| {BcMV6.BI=FALSE} | 0297 | 514 | 802 | 392 |  |
| {BcMV3.BI=FALSE, BcMV6.BI=FALSE} => | 0.22 | 0.585 | 0.376 | 1.21 |  |
|  |  |  |  |  | 89 |
| {BcOLV18=FALSE} | 0297 | 526 | 238 | 309 |  |
| {BcMV6.BI=FALSE, BcOLV18=FALSE} => | 0.22 | 0.626 | 0.351 | 1.36 |  |
|  |  |  |  |  | 89 |
| {BcMV3.BI=FALSE} | 0297 | 761 | 485 | 871 |  |
| {BcMV3.BI=FALSE, BcOLV18=FALSE} => | 0.21 | 0.807 | 0.269 | 1.13 |  |
|  |  |  |  |  | 88 |
| {BcPV3.BI=FALSE} | 7822 | 339 | 802 | 6464 |  |
| {BcMV3.BI=FALSE, BcPV3.BI=FALSE} => | 0.21 | 0.624 | 0.349 | 1.29 |  |
|  |  |  |  |  | 88 |
| {BcOLV18=FALSE} | 7822 | 113 | 01 | 3035 |  |

| {BcOLV18=FALSE, BcPV3.BI=FALSE} =>  {BcMV3.BI=FALSE} | 0.21  7822 | 0.571  429 | 0.381  188 | 1.24  7876 | 88 |
| --- | --- | --- | --- | --- | --- |
| {BcMV3.BI=FALSE, BcOLV18=FALSE} =>  {BcRV1.BI=FALSE} | 0.22  0297 | 0.816  514 | 0.269  802 | 1.12  9697 | 89 |
| {BcMV3.BI=FALSE, BcRV1.BI=FALSE} =>  {BcOLV18=FALSE} | 0.22  0297 | 0.618  056 | 0.356  436 | 1.28  0484 | 89 |
| {BcOLV18=FALSE, BcRV1.BI=FALSE} =>  {BcMV3.BI=FALSE} | 0.22  0297 | 0.559  748 | 0.393  564 | 1.22  237 | 89 |
| {BcEV2.BI=FALSE, BcMV3.BI=FALSE} =>  {BcOLV18=FALSE} | 0.20  297 | 0.589  928 | 0.344  059 | 1.22  221 | 82 |
| {BcMV3.BI=FALSE, BcOLV18=FALSE} =>  {BcHV3.BI=FALSE} | 0.23  2673 | 0.862  385 | 0.269  802 | 1.13  4865 | 94 |
| {BcHV3.BI=FALSE, BcMV3.BI=FALSE} =>  {BcOLV18=FALSE} | 0.23  2673 | 0.635  135 | 0.366  337 | 1.31  587 | 94 |
| {BcHV3.BI=FALSE, BcOLV18=FALSE} =>  {BcMV3.BI=FALSE} | 0.23  2673 | 0.569  697 | 0.408  416 | 1.24  4095 | 94 |
| {BcMV3.BI=FALSE, BcOLV18=FALSE} =>  {BcFV5.BI=FALSE} | 0.24  505 | 0.908  257 | 0.269  802 | 1.16  1189 | 99 |
| {BcFV5.BI=FALSE, BcMV3.BI=FALSE} =>  {BcOLV18=FALSE} | 0.24  505 | 0.622  642 | 0.393  564 | 1.28  9985 | 99 |
| {BcFV5.BI=FALSE, BcOLV18=FALSE} =>  {BcMV3.BI=FALSE} | 0.24  505 | 0.592  814 | 0.413  366 | 1.29  4578 | 99 |

| {BcMV3.BI=FALSE, BcOLV18=FALSE} =>  {BcULV1.BI=FALSE} | 0.25  2475 | 0.935  78 | 0.269  802 | 1.19  2603 | 10  2 |
| --- | --- | --- | --- | --- | --- |
| {BcMV3.BI=FALSE, BcULV1.BI=FALSE} =>  {BcOLV18=FALSE} | 0.25  2475 | 0.641  509 | 0.393  564 | 1.32  9076 | 10  2 |
| {BcOLV18=FALSE, BcULV1.BI=FALSE} =>  {BcMV3.BI=FALSE} | 0.25  2475 | 0.589  595 | 0.428  218 | 1.28  7549 | 10  2 |
| {BcMV3.BI=FALSE, BcOLV18=FALSE} =>  {BcMV12=FALSE} | 0.24  505 | 0.908  257 | 0.269  802 | 1.13  6024 | 99 |
| {BcMV12=FALSE, BcMV3.BI=FALSE} =>  {BcOLV18=FALSE} | 0.24  505 | 0.596  386 | 0.410  891 | 1.23  5589 | 99 |
| {BcMV12=FALSE, BcOLV18=FALSE} =>  {BcMV3.BI=FALSE} | 0.24  505 | 0.607  362 | 0.403  465 | 1.32  6347 | 99 |
| {BcMV3.BI=FALSE, BcNSRV7.BI=FALSE} =>  {BcOLV18=FALSE} | 0.23  0198 | 0.611  842 | 0.376  238 | 1.26  7611 | 93 |
| {BcNSRV7.BI=FALSE, BcOLV18=FALSE} =>  {BcMV3.BI=FALSE} | 0.23  0198 | 0.560  241 | 0.410  891 | 1.22  3445 | 93 |
| {BcMV3.BI=FALSE, BcVV3.BI=FALSE} =>  {BcOLV18=FALSE} | 0.22  2772 | 0.576  923 | 0.386  139 | 1.19  5266 | 90 |
| {BcOLV18=FALSE, BcVV3.BI=FALSE} =>  {BcMV3.BI=FALSE} | 0.22  2772 | 0.569  62 | 0.391  089 | 1.24  3927 | 90 |
| {BcMV3.BI=FALSE, BcOLV18=FALSE} =>  {BcOLV7.BI=FALSE} | 0.24  0099 | 0.889  908 | 0.269  802 | 1.10  2831 | 97 |

| {BcMV3.BI=FALSE, BcOLV7.BI=FALSE} =>  {BcOLV18=FALSE} | 0.24  0099 | 0.613  924 | 0.391  089 | 1.27  1925 | 97 |
| --- | --- | --- | --- | --- | --- |
| {BcOLV18=FALSE, BcOLV7.BI=FALSE} =>  {BcMV3.BI=FALSE} | 0.24  0099 | 0.557  471 | 0.430  693 | 1.21  7397 | 97 |
| {BcALV1.BI=FALSE, BcMV3.BI=FALSE} =>  {BcOLV18=FALSE} | 0.23  7624 | 0.607  595 | 0.391  089 | 1.25  8812 | 96 |
| {BcALV1.BI=FALSE, BcOLV18=FALSE} =>  {BcMV3.BI=FALSE} | 0.23  7624 | 0.542  373 | 0.438  119 | 1.18  4425 | 96 |
| {BcMV3.BI=FALSE, BcOLV18=FALSE} =>  {BcDMV1=FALSE} | 0.25 | 0.926  606 | 0.269  802 | 1.09  7797 | 10  1 |
| {BcDMV1=FALSE, BcMV3.BI=FALSE} =>  {BcOLV18=FALSE} | 0.25 | 0.594  118 | 0.420  792 | 1.23  089 | 10  1 |
| {BcDMV1=FALSE, BcOLV18=FALSE} =>  {BcMV3.BI=FALSE} | 0.25 | 0.601  19 | 0.415  842 | 1.31  287 | 10  1 |
| {BcMV3.BI=FALSE, BcOLV18=FALSE} =>  {BcFV9=FALSE} | 0.25  9901 | 0.963  303 | 0.269  802 | 1.10  8759 | 10  5 |
| {BcFV9=FALSE, BcMV3.BI=FALSE} =>  {BcOLV18=FALSE} | 0.25  9901 | 0.628  743 | 0.413  366 | 1.30  2626 | 10  5 |
| {BcFV9=FALSE, BcOLV18=FALSE} =>  {BcMV3.BI=FALSE} | 0.25  9901 | 0.580  11 | 0.448  02 | 1.26  6836 | 10  5 |
| {BcMV3.BI=FALSE, BcOLV18=FALSE} =>  {BcOLV4.BI=FALSE} | 0.25  495 | 0.944  954 | 0.269  802 | 1.07  8422 | 10  3 |

| {BcMV3.BI=FALSE, BcOLV4.BI=FALSE} =>  {BcOLV18=FALSE} | 0.25  495 | 0.605  882 | 0.420  792 | 1.25  5264 | 10  3 |
| --- | --- | --- | --- | --- | --- |
| {BcOLV18=FALSE, BcOLV4.BI=FALSE} =>  {BcMV3.BI=FALSE} | 0.25  495 | 0.595  376 | 0.428  218 | 1.30  0172 | 10  3 |
| {BcMV3.BI=FALSE, BcULV5=FALSE} =>  {BcOLV18=FALSE} | 0.25 | 0.590  643 | 0.423  267 | 1.22  3692 | 10  1 |
| {BcOLV18=FALSE, BcULV5=FALSE} =>  {BcMV3.BI=FALSE} | 0.25 | 0.612  121 | 0.408  416 | 1.33  674 | 10  1 |
| {BcBNV5.BI=FALSE, BcMV3.BI=FALSE} =>  {BcOLV18=FALSE} | 0.25  495 | 0.581  921 | 0.438  119 | 1.20  5621 | 10  3 |
| {BcBNV5.BI=FALSE, BcOLV18=FALSE} =>  {BcMV3.BI=FALSE} | 0.25  495 | 0.559  783 | 0.455  446 | 1.22  2444 | 10  3 |
| {BcMV3.BI=FALSE, BvF.BI=FALSE} =>  {BcOLV18=FALSE} | 0.24  2574 | 0.579  882 | 0.418  317 | 1.20  1396 | 98 |
| {BcOLV18=FALSE, BvF.BI=FALSE} =>  {BcMV3.BI=FALSE} | 0.24  2574 | 0.553  672 | 0.438  119 | 1.20  9101 | 98 |
| {BcMCV5.BI=FALSE, BcMV3.BI=FALSE} =>  {BcOLV18=FALSE} | 0.26  7327 | 0.606  742 | 0.440  594 | 1.25  7044 | 10  8 |
| {BcMCV5.BI=FALSE, BcOLV18=FALSE} =>  {BcMV3.BI=FALSE} | 0.26  7327 | 0.565  445 | 0.472  772 | 1.23  481 | 10  8 |
| {BcMV3.BI=FALSE, BcOLV13.BI=FALSE} =>  {BcOLV18=FALSE} | 0.26  9802 | 0.598  901 | 0.450  495 | 1.24  08 | 10  9 |

| {BcOLV13.BI=FALSE, BcOLV18=FALSE} =>  {BcMV3.BI=FALSE} | 0.26  9802 | 0.598  901 | 0.450  495 | 1.30  7871 | 10  9 |
| --- | --- | --- | --- | --- | --- |
| {BcMV3.BI=FALSE, BcOLV2.BI=FALSE} =>  {BcOLV18=FALSE} | 0.26  2376 | 0.592  179 | 0.443  069 | 1.22  6873 | 10  6 |
| {BcOLV18=FALSE, BcOLV2.BI=FALSE} =>  {BcMV3.BI=FALSE} | 0.26  2376 | 0.569  892 | 0.460  396 | 1.24  4522 | 10  6 |
| {BcMCV3.BI=FALSE, BcMV3.BI=FALSE} =>  {BcOLV18=FALSE} | 0.26  9802 | 0.589  189 | 0.457  921 | 1.22  0679 | 10  9 |
| {BcMCV3.BI=FALSE, BcOLV18=FALSE} =>  {BcMV3.BI=FALSE} | 0.26  9802 | 0.589  189 | 0.457  921 | 1.28  6662 | 10  9 |
| {BcMV3.BI=FALSE, BcPV4.BI=FALSE} =>  {BcOLV18=FALSE} | 0.25  9901 | 0.596  591 | 0.435  644 | 1.23  6014 | 10  5 |
| {BcOLV18=FALSE, BcPV4.BI=FALSE} =>  {BcMV3.BI=FALSE} | 0.25  9901 | 0.570  652 | 0.455  446 | 1.24  6181 | 10  5 |
| {BcEV6=FALSE, BcMV3.BI=FALSE} =>  {BcOLV18=FALSE} | 0.24  505 | 0.585  799 | 0.418  317 | 1.21  3655 | 99 |
| {BcEV6=FALSE, BcOLV18=FALSE} =>  {BcMV3.BI=FALSE} | 0.24  505 | 0.540  984 | 0.452  97 | 1.18  1391 | 99 |
| {BcMV3.BI=FALSE, BcOLV17.BI=FALSE} =>  {BcOLV18=FALSE} | 0.25  9901 | 0.596  591 | 0.435  644 | 1.23  6014 | 10  5 |
| {BcOLV17.BI=FALSE, BcOLV18=FALSE} =>  {BcMV3.BI=FALSE} | 0.25  9901 | 0.552  632 | 0.470  297 | 1.20  6828 | 10  5 |

| {BcMV3.BI=FALSE, BcOLV15.BI=FALSE} =>  {BcOLV18=FALSE} | 0.26  4851 | 0.597  765 | 0.443  069 | 1.23  8447 | 10  7 |
| --- | --- | --- | --- | --- | --- |
| {BcOLV15.BI=FALSE, BcOLV18=FALSE} =>  {BcMV3.BI=FALSE} | 0.26  4851 | 0.560  209 | 0.472  772 | 1.22  3376 | 10  7 |
| {BcEV5=FALSE, BcMV3.BI=FALSE} =>  {BcOLV18=FALSE} | 0.26  9802 | 0.592  391 | 0.455  446 | 1.22  7313 | 10  9 |
| {BcEV5=FALSE, BcOLV18=FALSE} =>  {BcMV3.BI=FALSE} | 0.26  9802 | 0.582  888 | 0.462  871 | 1.27  2901 | 10  9 |
| {BcMV3.BI=FALSE, BcOLV18=TRUE} =>  {BcULV4=TRUE} | 0.14  8515 | 0.789  474 | 0.188  119 | 1.14  3181 | 60 |
| {BcMV3.BI=FALSE, BcOLV18=TRUE} =>  {BcMV6.BI=FALSE} | 0.15  5941 | 0.828  947 | 0.188  119 | 1.19  1796 | 63 |
| {BcMV3.BI=FALSE, BcOLV18=TRUE} =>  {BcDMV1=FALSE} | 0.17  0792 | 0.907  895 | 0.188  119 | 1.07  5629 | 69 |
| {BcHV1SLR.BI=TRUE, BcMV3.BI=FALSE} =>  {BcMV2.BI=FALSE} | 0.14  8515 | 0.697  674 | 0.212  871 | 1.29  8896 | 60 |
| {BcHV1SLR.BI=TRUE, BcMV2.BI=FALSE} =>  {BcMV3.BI=FALSE} | 0.14  8515 | 0.550  459 | 0.269  802 | 1.20  2083 | 60 |
| {BcMV2.BI=FALSE, BcMV3.BI=FALSE} =>  {BcMV4.BI=FALSE} | 0.23  0198 | 0.744 | 0.309  406 | 1.33  5893 | 93 |
| {BcMV3.BI=FALSE, BcMV4.BI=FALSE} =>  {BcMV2.BI=FALSE} | 0.23  0198 | 0.744 | 0.309  406 | 1.38  5143 | 93 |

| {BcMV2.BI=FALSE, BcMV4.BI=FALSE} =>  {BcMV3.BI=FALSE} | 0.23  0198 | 0.624  161 | 0.368  812 | 1.36  3033 | 93 |
| --- | --- | --- | --- | --- | --- |
| {BcMV2.BI=FALSE, BcMV3.BI=FALSE} =>  {BcOLV16.BI=FALSE} | 0.22  0297 | 0.712 | 0.309  406 | 1.21  8847 | 89 |
| {BcMV3.BI=FALSE, BcOLV16.BI=FALSE} =>  {BcMV2.BI=FALSE} | 0.22  0297 | 0.712 | 0.309  406 | 1.32  5567 | 89 |
| {BcMV2.BI=FALSE, BcOLV16.BI=FALSE} =>  {BcMV3.BI=FALSE} | 0.22  0297 | 0.589  404 | 0.373  762 | 1.28  7131 | 89 |
| {BcMV3.BI=FALSE, BcULV3=TRUE} =>  {BcMV2.BI=FALSE} | 0.16  8317 | 0.68 | 0.247  525 | 1.26  5991 | 68 |
| {BcHV1.BI=TRUE, BcMV3.BI=FALSE} =>  {BcMV2.BI=FALSE} | 0.17  0792 | 0.683  168 | 0.25 | 1.27  1889 | 69 |
| {BcMV2.BI=FALSE, BcMV3.BI=FALSE} =>  {BcOLV1.BI.MOVA.=FALSE} | 0.26  9802 | 0.872 | 0.309  406 | 1.30  477 | 10  9 |
| {BcMV3.BI=FALSE, BcOLV1.BI.MOVA.=FALSE} =>  {BcMV2.BI=FALSE} | 0.26  9802 | 0.726  667 | 0.371  287 | 1.35  2873 | 10  9 |
| {BcMV2.BI=FALSE, BcOLV1.BI.MOVA.=FALSE} =>  {BcMV3.BI=FALSE} | 0.26  9802 | 0.592  391 | 0.455  446 | 1.29  3655 | 10  9 |
| {BcMV3.BI=FALSE, BcMV9.BI=TRUE} =>  {BcMV2.BI=FALSE} | 0.19  0594 | 0.675  439 | 0.282  178 | 1.25  7499 | 77 |

| {BcMV2.BI=FALSE, BcMV3.BI=FALSE} =>  {BcMV6.BI=FALSE} | 0.25  9901 | 0.84 | 0.309  406 | 1.20  7687 | 10  5 |
| --- | --- | --- | --- | --- | --- |
| {BcMV3.BI=FALSE, BcMV6.BI=FALSE} =>  {BcMV2.BI=FALSE} | 0.25  9901 | 0.690  789 | 0.376  238 | 1.28  6078 | 10  5 |
| {BcMV2.BI=FALSE, BcMV6.BI=FALSE} =>  {BcMV3.BI=FALSE} | 0.25  9901 | 0.589  888 | 0.440  594 | 1.28  8187 | 10  5 |
| {BcMV2.BI=FALSE, BcMV3.BI=FALSE} =>  {BcPV3.BI=FALSE} | 0.24  7525 | 0.8 | 0.309  406 | 1.12  6132 | 10  0 |
| {BcMV3.BI=FALSE, BcPV3.BI=FALSE} =>  {BcMV2.BI=FALSE} | 0.24  7525 | 0.709  22 | 0.349  01 | 1.32  0391 | 10  0 |
| {BcMV2.BI=FALSE, BcPV3.BI=FALSE} =>  {BcMV3.BI=FALSE} | 0.24  7525 | 0.581  395 | 0.425  743 | 1.26  9642 | 10  0 |
| {BcMV2.BI=FALSE, BcMV3.BI=FALSE} =>  {BcRV1.BI=FALSE} | 0.25  495 | 0.824 | 0.309  406 | 1.14  0055 | 10  3 |
| {BcMV3.BI=FALSE, BcRV1.BI=FALSE} =>  {BcMV2.BI=FALSE} | 0.25  495 | 0.715  278 | 0.356  436 | 1.33  1669 | 10  3 |
| {BcMV2.BI=FALSE, BcRV1.BI=FALSE} =>  {BcMV3.BI=FALSE} | 0.25  495 | 0.581  921 | 0.438  119 | 1.27  0789 | 10  3 |
| {BcEV2.BI=FALSE, BcMV3.BI=FALSE} =>  {BcMV2.BI=FALSE} | 0.24  0099 | 0.697  842 | 0.344  059 | 1.29  9208 | 97 |
| {BcEV2.BI=FALSE, BcMV2.BI=FALSE} =>  {BcMV3.BI=FALSE} | 0.24  0099 | 0.560  694 | 0.428  218 | 1.22  4434 | 97 |

| {BcHV3.BI=FALSE, BcMV3.BI=FALSE} =>  {BcMV2.BI=FALSE} | 0.25 | 0.682  432 | 0.366  337 | 1.27  0519 | 10  1 |
| --- | --- | --- | --- | --- | --- |
| {BcHV3.BI=FALSE, BcMV2.BI=FALSE} =>  {BcMV3.BI=FALSE} | 0.25 | 0.580  46 | 0.430  693 | 1.26  7599 | 10  1 |
| {BcMV2.BI=FALSE, BcMV3.BI=FALSE} =>  {BcFV5.BI=FALSE} | 0.27  2277 | 0.88 | 0.309  406 | 1.12  5063 | 11  0 |
| {BcFV5.BI=FALSE, BcMV3.BI=FALSE} =>  {BcMV2.BI=FALSE} | 0.27  2277 | 0.691  824 | 0.393  564 | 1.28  8004 | 11  0 |
| {BcFV5.BI=FALSE, BcMV2.BI=FALSE} =>  {BcMV3.BI=FALSE} | 0.27  2277 | 0.621  469 | 0.438  119 | 1.35  7154 | 11  0 |
| {BcMV2.BI=FALSE, BcMV3.BI=FALSE} =>  {BcULV1.BI=FALSE} | 0.27  7228 | 0.896 | 0.309  406 | 1.14  1905 | 11  2 |
| {BcMV3.BI=FALSE, BcULV1.BI=FALSE} =>  {BcMV2.BI=FALSE} | 0.27  7228 | 0.704  403 | 0.393  564 | 1.31  1422 | 11  2 |
| {BcMV2.BI=FALSE, BcULV1.BI=FALSE} =>  {BcMV3.BI=FALSE} | 0.27  7228 | 0.602  151 | 0.460  396 | 1.31  4967 | 11  2 |
| {BcMV1.BI=TRUE, BcMV3.BI=FALSE} =>  {BcMV2.BI=FALSE} | 0.21  0396 | 0.634  328 | 0.331  683 | 1.18  0962 | 85 |
| {BcMV1.BI=TRUE, BcMV2.BI=FALSE} =>  {BcMV3.BI=FALSE} | 0.21  0396 | 0.551  948 | 0.381  188 | 1.20  5335 | 85 |
| {BcMV2.BI=FALSE, BcMV3.BI=FALSE} =>  {BcMV12=FALSE} | 0.28  2178 | 0.912 | 0.309  406 | 1.14  0706 | 11  4 |

| {BcMV12=FALSE, BcMV3.BI=FALSE} =>  {BcMV2.BI=FALSE} | 0.28  2178 | 0.686  747 | 0.410  891 | 1.27  8552 | 11  4 |
| --- | --- | --- | --- | --- | --- |
| {BcMV12=FALSE, BcMV2.BI=FALSE} =>  {BcMV3.BI=FALSE} | 0.28  2178 | 0.616  216 | 0.457  921 | 1.34  5683 | 11  4 |
| {BcMV3.BI=FALSE, BcNSRV7.BI=FALSE} =>  {BcMV2.BI=FALSE} | 0.25  9901 | 0.690  789 | 0.376  238 | 1.28  6078 | 10  5 |
| {BcMV2.BI=FALSE, BcNSRV7.BI=FALSE} =>  {BcMV3.BI=FALSE} | 0.25  9901 | 0.593  22 | 0.438  119 | 1.29  5465 | 10  5 |
| {BcMV3.BI=FALSE, BcVV3.BI=FALSE} =>  {BcMV2.BI=FALSE} | 0.26  9802 | 0.698  718 | 0.386  139 | 1.30  0839 | 10  9 |
| {BcMV2.BI=FALSE, BcVV3.BI=FALSE} =>  {BcMV3.BI=FALSE} | 0.26  9802 | 0.598  901 | 0.450  495 | 1.30  7871 | 10  9 |
| {BcMV3.BI=FALSE, BcOLV7.BI=FALSE} =>  {BcMV2.BI=FALSE} | 0.25  7426 | 0.658  228 | 0.391  089 | 1.22  5456 | 10  4 |
| {BcMV2.BI=FALSE, BcOLV7.BI=FALSE} =>  {BcMV3.BI=FALSE} | 0.25  7426 | 0.574  586 | 0.448  02 | 1.25  4771 | 10  4 |
| {BcMV2.BI=FALSE, BcMV3.BI=FALSE} =>  {BcALV1.BI=FALSE} | 0.27  7228 | 0.896 | 0.309  406 | 1.10  0255 | 11  2 |
| {BcALV1.BI=FALSE, BcMV3.BI=FALSE} =>  {BcMV2.BI=FALSE} | 0.27  7228 | 0.708  861 | 0.391  089 | 1.31  9722 | 11  2 |
| {BcALV1.BI=FALSE, BcMV2.BI=FALSE} =>  {BcMV3.BI=FALSE} | 0.27  7228 | 0.565  657 | 0.490  099 | 1.23  5272 | 11  2 |

| {BcMV2.BI=FALSE, BcMV3.BI=FALSE} =>  {BcDMV1=FALSE} | 0.28  2178 | 0.912 | 0.309  406 | 1.08  0493 | 11  4 |
| --- | --- | --- | --- | --- | --- |
| {BcDMV1=FALSE, BcMV3.BI=FALSE} =>  {BcMV2.BI=FALSE} | 0.28  2178 | 0.670  588 | 0.420  792 | 1.24  8468 | 11  4 |
| {BcDMV1=FALSE, BcMV2.BI=FALSE} =>  {BcMV3.BI=FALSE} | 0.28  2178 | 0.629  834 | 0.448  02 | 1.37  5422 | 11  4 |
| {BcFV9=FALSE, BcMV3.BI=FALSE} =>  {BcMV2.BI=FALSE} | 0.27  9703 | 0.676  647 | 0.413  366 | 1.25  9748 | 11  3 |
| {BcFV9=FALSE, BcMV2.BI=FALSE} =>  {BcMV3.BI=FALSE} | 0.27  9703 | 0.597  884 | 0.467  822 | 1.30  5649 | 11  3 |
| {BcMV2.BI=FALSE, BcMV3.BI=FALSE} =>  {BcOLV4.BI=FALSE} | 0.29  2079 | 0.944 | 0.309  406 | 1.07  7333 | 11  8 |
| {BcMV3.BI=FALSE, BcOLV4.BI=FALSE} =>  {BcMV2.BI=FALSE} | 0.29  2079 | 0.694  118 | 0.420  792 | 1.29  2274 | 11  8 |
| {BcMV2.BI=FALSE, BcOLV4.BI=FALSE} =>  {BcMV3.BI=FALSE} | 0.29  2079 | 0.584  158 | 0.5 | 1.27  5676 | 11  8 |
| {BcMV2.BI=FALSE, BcMV3.BI=FALSE} =>  {BcULV5=FALSE} | 0.29  2079 | 0.944 | 0.309  406 | 1.07  7333 | 11  8 |
| {BcMV3.BI=FALSE, BcULV5=FALSE} =>  {BcMV2.BI=FALSE} | 0.29  2079 | 0.690  058 | 0.423  267 | 1.28  4717 | 11  8 |
| {BcMV2.BI=FALSE, BcULV5=FALSE} =>  {BcMV3.BI=FALSE} | 0.29  2079 | 0.584  158 | 0.5 | 1.27  5676 | 11  8 |

| {BcBNV5.BI=FALSE, BcMV3.BI=FALSE} =>  {BcMV2.BI=FALSE} | 0.29  9505 | 0.683  616 | 0.438  119 | 1.27  2723 | 12  1 |
| --- | --- | --- | --- | --- | --- |
| {BcBNV5.BI=FALSE, BcMV2.BI=FALSE} =>  {BcMV3.BI=FALSE} | 0.29  9505 | 0.599  01 | 0.5 | 1.30  8108 | 12  1 |
| {BcMV3.BI=FALSE, BvF.BI=FALSE} =>  {BcMV2.BI=FALSE} | 0.27  7228 | 0.662  722 | 0.418  317 | 1.23  3823 | 11  2 |
| {BcMV2.BI=FALSE, BvF.BI=FALSE} =>  {BcMV3.BI=FALSE} | 0.27  7228 | 0.577  32 | 0.480  198 | 1.26  0741 | 11  2 |
| {BcMCV5.BI=FALSE, BcMV3.BI=FALSE} =>  {BcMV2.BI=FALSE} | 0.30  4455 | 0.691  011 | 0.440  594 | 1.28  6491 | 12  3 |
| {BcMCV5.BI=FALSE, BcMV2.BI=FALSE} =>  {BcMV3.BI=FALSE} | 0.30  4455 | 0.582  938 | 0.522  277 | 1.27  3011 | 12  3 |
| {BcMV3.BI=FALSE, BcOLV13.BI=FALSE} =>  {BcMV2.BI=FALSE} | 0.30  4455 | 0.675  824 | 0.450  495 | 1.25  8216 | 12  3 |
| {BcMV2.BI=FALSE, BcOLV13.BI=FALSE} =>  {BcMV3.BI=FALSE} | 0.30  4455 | 0.588  517 | 0.517  327 | 1.28  5193 | 12  3 |
| {BcMV3.BI=FALSE, BcOLV2.BI=FALSE} =>  {BcMV2.BI=FALSE} | 0.29  4554 | 0.664  804 | 0.443  069 | 1.23  77 | 11  9 |
| {BcMV2.BI=FALSE, BcOLV2.BI=FALSE} =>  {BcMV3.BI=FALSE} | 0.29  4554 | 0.580  488 | 0.507  426 | 1.26  766 | 11  9 |
| {BcMCV3.BI=FALSE, BcMV3.BI=FALSE} =>  {BcMV2.BI=FALSE} | 0.30  9406 | 0.675  676 | 0.457  921 | 1.25  794 | 12  5 |

| {BcMCV3.BI=FALSE, BcMV2.BI=FALSE} =>  {BcMV3.BI=FALSE} | 0.30  9406 | 0.589  623 | 0.524  752 | 1.28  7608 | 12  5 |
| --- | --- | --- | --- | --- | --- |
| {BcMV3.BI=FALSE, BcPV4.BI=FALSE} =>  {BcMV2.BI=FALSE} | 0.29  2079 | 0.670  455 | 0.435  644 | 1.24  822 | 11  8 |
| {BcMV2.BI=FALSE, BcPV4.BI=FALSE} =>  {BcMV3.BI=FALSE} | 0.29  2079 | 0.578  431 | 0.504  95 | 1.26  3169 | 11  8 |
| {BcEV6=FALSE, BcMV3.BI=FALSE} =>  {BcMV2.BI=FALSE} | 0.28  4653 | 0.680  473 | 0.418  317 | 1.26  6872 | 11  5 |
| {BcEV6=FALSE, BcMV2.BI=FALSE} =>  {BcMV3.BI=FALSE} | 0.28  4653 | 0.566  502 | 0.502  475 | 1.23  7119 | 11  5 |
| {BcMV3.BI=FALSE, BcOLV17.BI=FALSE} =>  {BcMV2.BI=FALSE} | 0.29  9505 | 0.687  5 | 0.435  644 | 1.27  9954 | 12  1 |
| {BcMV2.BI=FALSE, BcOLV17.BI=FALSE} =>  {BcMV3.BI=FALSE} | 0.29  9505 | 0.573  46 | 0.522  277 | 1.25  2312 | 12  1 |
| {BcMV3.BI=FALSE, BcOLV15.BI=FALSE} =>  {BcMV2.BI=FALSE} | 0.29  703 | 0.670  391 | 0.443  069 | 1.24  8101 | 12  0 |
| {BcMV2.BI=FALSE, BcOLV15.BI=FALSE} =>  {BcMV3.BI=FALSE} | 0.29  703 | 0.597  015 | 0.497  525 | 1.30  3752 | 12  0 |
| {BcEV5=FALSE, BcMV3.BI=FALSE} =>  {BcMV2.BI=FALSE} | 0.30  6931 | 0.673  913 | 0.455  446 | 1.25  4658 | 12  4 |
| {BcEV5=FALSE, BcMV2.BI=FALSE} =>  {BcMV3.BI=FALSE} | 0.30  6931 | 0.582  16 | 0.527  228 | 1.27  1311 | 12  4 |

| {BcHV1SLR.BI=TRUE, BcMV3.BI=FALSE} =>  {BcMV4.BI=FALSE} | 0.14  8515 | 0.697  674 | 0.212  871 | 1.25  2713 | 60 |
| --- | --- | --- | --- | --- | --- |
| {BcHV1SLR.BI=TRUE, BcMV3.BI=FALSE} =>  {BcOLV16.BI=FALSE} | 0.15  099 | 0.709  302 | 0.212  871 | 1.21  4229 | 61 |
| {BcHV1SLR.BI=TRUE, BcMV3.BI=FALSE} =>  {BcHV1.BI=TRUE} | 0.18  8119 | 0.883  721 | 0.212  871 | 1.46  321 | 76 |
| {BcHV1.BI=TRUE, BcMV3.BI=FALSE} =>  {BcHV1SLR.BI=TRUE} | 0.18  8119 | 0.752  475 | 0.25 | 1.38  8128 | 76 |
| {BcHV1SLR.BI=TRUE, BcMV3.BI=FALSE} =>  {BcOLV1.BI.MOVA.=FALSE} | 0.17  5743 | 0.825  581 | 0.212  871 | 1.23  5314 | 71 |
| {BcHV1SLR.BI=TRUE, BcMV3.BI=FALSE} =>  {BcMV6.BI=FALSE} | 0.17  0792 | 0.802  326 | 0.212  871 | 1.15  3521 | 69 |
| {BcHV1SLR.BI=TRUE, BcMV3.BI=FALSE} =>  {BcMV12=FALSE} | 0.19  0594 | 0.895  349 | 0.212  871 | 1.11  9879 | 77 |
| {BcHV1SLR.BI=TRUE, BcMV3.BI=FALSE} =>  {BcOLV7.BI=FALSE} | 0.19  3069 | 0.906  977 | 0.212  871 | 1.12  3983 | 78 |
| {BcHV1SLR.BI=TRUE, BcMV3.BI=FALSE} =>  {BcDMV1=FALSE} | 0.20  0495 | 0.941  86 | 0.212  871 | 1.11  587 | 81 |
| {BcMV3.BI=FALSE, BcMV4.BI=FALSE} =>  {BcOLV16.BI=FALSE} | 0.21  5347 | 0.696 | 0.309  406 | 1.19  1458 | 87 |
| {BcMV3.BI=FALSE, BcOLV16.BI=FALSE} =>  {BcMV4.BI=FALSE} | 0.21  5347 | 0.696 | 0.309  406 | 1.24  9707 | 87 |

| {BcMV4.BI=FALSE, BcOLV16.BI=FALSE} =>  {BcMV3.BI=FALSE} | 0.21  5347 | 0.587  838 | 0.366  337 | 1.28  3711 | 87 |
| --- | --- | --- | --- | --- | --- |
| {BcMV3.BI=FALSE, BcULV3=TRUE} =>  {BcMV4.BI=FALSE} | 0.16  5842 | 0.67 | 0.247  525 | 1.20  3022 | 67 |
| {BcMV4.BI=FALSE, BcULV3=TRUE} =>  {BcMV3.BI=FALSE} | 0.16  5842 | 0.553  719 | 0.299  505 | 1.20  9203 | 67 |
| {BcHV1.BI=TRUE, BcMV3.BI=FALSE} =>  {BcMV4.BI=FALSE} | 0.17  0792 | 0.683  168 | 0.25 | 1.22  6667 | 69 |
| {BcHV1.BI=TRUE, BcMV4.BI=FALSE} =>  {BcMV3.BI=FALSE} | 0.17  0792 | 0.560  976 | 0.304  455 | 1.22  5049 | 69 |
| {BcMV3.BI=FALSE, BcMV4.BI=FALSE} =>  {BcOLV1.BI.MOVA.=FALSE} | 0.25  7426 | 0.832 | 0.309  406 | 1.24  4919 | 10  4 |
| {BcMV3.BI=FALSE, BcOLV1.BI.MOVA.=FALSE} =>  {BcMV4.BI=FALSE} | 0.25  7426 | 0.693  333 | 0.371  287 | 1.24  4919 | 10  4 |
| {BcMV4.BI=FALSE, BcOLV1.BI.MOVA.=FALSE} =>  {BcMV3.BI=FALSE} | 0.25  7426 | 0.626  506 | 0.410  891 | 1.36  8154 | 10  4 |
| {BcMV3.BI=FALSE, BcULV4=TRUE} =>  {BcMV4.BI=FALSE} | 0.19  5545 | 0.675  214 | 0.289  604 | 1.21  2384 | 79 |
| {BcMV3.BI=FALSE, BcMV4.BI=FALSE} =>  {BcMV6.BI=FALSE} | 0.25 | 0.808 | 0.309  406 | 1.16  168 | 10  1 |

| {BcMV3.BI=FALSE, BcMV6.BI=FALSE} =>  {BcMV4.BI=FALSE} | 0.25 | 0.664  474 | 0.376  238 | 1.19  3099 | 10  1 |
| --- | --- | --- | --- | --- | --- |
| {BcMV4.BI=FALSE, BcMV6.BI=FALSE} =>  {BcMV3.BI=FALSE} | 0.25 | 0.583  815 | 0.428  218 | 1.27  4926 | 10  1 |
| {BcMV3.BI=FALSE, BcMV4.BI=FALSE} =>  {BcPV3.BI=FALSE} | 0.25 | 0.808 | 0.309  406 | 1.13  7394 | 10  1 |
| {BcMV3.BI=FALSE, BcPV3.BI=FALSE} =>  {BcMV4.BI=FALSE} | 0.25 | 0.716  312 | 0.349  01 | 1.28  6178 | 10  1 |
| {BcMV4.BI=FALSE, BcPV3.BI=FALSE} =>  {BcMV3.BI=FALSE} | 0.25 | 0.558  011 | 0.448  02 | 1.21  8575 | 10  1 |
| {BcMV3.BI=FALSE, BcMV4.BI=FALSE} =>  {BcRV1.BI=FALSE} | 0.25  2475 | 0.816 | 0.309  406 | 1.12  8986 | 10  2 |
| {BcMV3.BI=FALSE, BcRV1.BI=FALSE} =>  {BcMV4.BI=FALSE} | 0.25  2475 | 0.708  333 | 0.356  436 | 1.27  1852 | 10  2 |
| {BcMV4.BI=FALSE, BcRV1.BI=FALSE} =>  {BcMV3.BI=FALSE} | 0.25  2475 | 0.566  667 | 0.445  545 | 1.23  7477 | 10  2 |
| {BcEV2.BI=FALSE, BcMV3.BI=FALSE} =>  {BcMV4.BI=FALSE} | 0.24  2574 | 0.705  036 | 0.344  059 | 1.26  5931 | 98 |
| {BcEV2.BI=FALSE, BcMV4.BI=FALSE} =>  {BcMV3.BI=FALSE} | 0.24  2574 | 0.56 | 0.433  168 | 1.22  2919 | 98 |
| {BcMV3.BI=FALSE, BcMV4.BI=FALSE} =>  {BcHV3.BI=FALSE} | 0.26  2376 | 0.848 | 0.309  406 | 1.11  5935 | 10  6 |

| {BcHV3.BI=FALSE, BcMV3.BI=FALSE} =>  {BcMV4.BI=FALSE} | 0.26  2376 | 0.716  216 | 0.366  337 | 1.28  6006 | 10  6 |
| --- | --- | --- | --- | --- | --- |
| {BcHV3.BI=FALSE, BcMV4.BI=FALSE} =>  {BcMV3.BI=FALSE} | 0.26  2376 | 0.560  847 | 0.467  822 | 1.22  4768 | 10  6 |
| {BcMV3.BI=FALSE, BcMV4.BI=FALSE} =>  {BcFV5.BI=FALSE} | 0.26  9802 | 0.872 | 0.309  406 | 1.11  4835 | 10  9 |
| {BcFV5.BI=FALSE, BcMV3.BI=FALSE} =>  {BcMV4.BI=FALSE} | 0.26  9802 | 0.685  535 | 0.393  564 | 1.23  0915 | 10  9 |
| {BcFV5.BI=FALSE, BcMV4.BI=FALSE} =>  {BcMV3.BI=FALSE} | 0.26  9802 | 0.586  022 | 0.460  396 | 1.27  9744 | 10  9 |
| {BcMV3.BI=FALSE, BcMV4.BI=FALSE} =>  {BcULV1.BI=FALSE} | 0.27  2277 | 0.88 | 0.309  406 | 1.12  1514 | 11  0 |
| {BcMV3.BI=FALSE, BcULV1.BI=FALSE} =>  {BcMV4.BI=FALSE} | 0.27  2277 | 0.691  824 | 0.393  564 | 1.24  2208 | 11  0 |
| {BcMV4.BI=FALSE, BcULV1.BI=FALSE} =>  {BcMV3.BI=FALSE} | 0.27  2277 | 0.604  396 | 0.450  495 | 1.31  9869 | 11  0 |
| {BcMV1.BI=TRUE, BcMV3.BI=FALSE} =>  {BcMV4.BI=FALSE} | 0.21  5347 | 0.649  254 | 0.331  683 | 1.16  5771 | 87 |
| {BcMV3.BI=FALSE, BcMV4.BI=FALSE} =>  {BcMV12=FALSE} | 0.28  2178 | 0.912 | 0.309  406 | 1.14  0706 | 11  4 |
| {BcMV12=FALSE, BcMV3.BI=FALSE} =>  {BcMV4.BI=FALSE} | 0.28  2178 | 0.686  747 | 0.410  891 | 1.23  3092 | 11  4 |

| {BcMV12=FALSE, BcMV4.BI=FALSE} =>  {BcMV3.BI=FALSE} | 0.28  2178 | 0.644  068 | 0.438  119 | 1.40  6505 | 11  4 |
| --- | --- | --- | --- | --- | --- |
| {BcMV3.BI=FALSE, BcNSRV7.BI=FALSE} =>  {BcMV4.BI=FALSE} | 0.25  9901 | 0.690  789 | 0.376  238 | 1.24  0351 | 10  5 |
| {BcMV4.BI=FALSE, BcNSRV7.BI=FALSE} =>  {BcMV3.BI=FALSE} | 0.25  9901 | 0.561  497 | 0.462  871 | 1.22  6189 | 10  5 |
| {BcMV3.BI=FALSE, BcVV3.BI=FALSE} =>  {BcMV4.BI=FALSE} | 0.26  9802 | 0.698  718 | 0.386  139 | 1.25  4587 | 10  9 |
| {BcMV4.BI=FALSE, BcVV3.BI=FALSE} =>  {BcMV3.BI=FALSE} | 0.26  9802 | 0.573  684 | 0.470  297 | 1.25  2802 | 10  9 |
| {BcMV3.BI=FALSE, BcOLV7.BI=FALSE} =>  {BcMV4.BI=FALSE} | 0.26  9802 | 0.689  873 | 0.391  089 | 1.23  8706 | 10  9 |
| {BcMV4.BI=FALSE, BcOLV7.BI=FALSE} =>  {BcMV3.BI=FALSE} | 0.26  9802 | 0.573  684 | 0.470  297 | 1.25  2802 | 10  9 |
| {BcMV3.BI=FALSE, BcMV4.BI=FALSE} =>  {BcALV1.BI=FALSE} | 0.27  9703 | 0.904 | 0.309  406 | 1.11  0079 | 11  3 |
| {BcALV1.BI=FALSE, BcMV3.BI=FALSE} =>  {BcMV4.BI=FALSE} | 0.27  9703 | 0.715  19 | 0.391  089 | 1.28  4163 | 11  3 |
| {BcALV1.BI=FALSE, BcMV4.BI=FALSE} =>  {BcMV3.BI=FALSE} | 0.27  9703 | 0.559  406 | 0.5 | 1.22  1622 | 11  3 |
| {BcMV3.BI=FALSE, BcMV4.BI=FALSE} =>  {BcDMV1=FALSE} | 0.28  2178 | 0.912 | 0.309  406 | 1.08  0493 | 11  4 |

| {BcDMV1=FALSE, BcMV3.BI=FALSE} =>  {BcMV4.BI=FALSE} | 0.28  2178 | 0.670  588 | 0.420  792 | 1.20  4078 | 11  4 |
| --- | --- | --- | --- | --- | --- |
| {BcDMV1=FALSE, BcMV4.BI=FALSE} =>  {BcMV3.BI=FALSE} | 0.28  2178 | 0.584  615 | 0.482  673 | 1.27  6674 | 11  4 |
| {BcMV3.BI=FALSE, BcMV4.BI=FALSE} =>  {BcFV9=FALSE} | 0.28  9604 | 0.936 | 0.309  406 | 1.07  7333 | 11  7 |
| {BcFV9=FALSE, BcMV3.BI=FALSE} =>  {BcMV4.BI=FALSE} | 0.28  9604 | 0.700  599 | 0.413  366 | 1.25  7964 | 11  7 |
| {BcFV9=FALSE, BcMV4.BI=FALSE} =>  {BcMV3.BI=FALSE} | 0.28  9604 | 0.565  217 | 0.512  376 | 1.23  4313 | 11  7 |
| {BcMV3.BI=FALSE, BcMV4.BI=FALSE} =>  {BcOLV4.BI=FALSE} | 0.29  2079 | 0.944 | 0.309  406 | 1.07  7333 | 11  8 |
| {BcMV3.BI=FALSE, BcOLV4.BI=FALSE} =>  {BcMV4.BI=FALSE} | 0.29  2079 | 0.694  118 | 0.420  792 | 1.24  6327 | 11  8 |
| {BcMV4.BI=FALSE, BcOLV4.BI=FALSE} =>  {BcMV3.BI=FALSE} | 0.29  2079 | 0.578  431 | 0.504  95 | 1.26  3169 | 11  8 |
| {BcMV3.BI=FALSE, BcULV5=FALSE} =>  {BcMV4.BI=FALSE} | 0.28  4653 | 0.672  515 | 0.423  267 | 1.20  7537 | 11  5 |
| {BcMV4.BI=FALSE, BcULV5=FALSE} =>  {BcMV3.BI=FALSE} | 0.28  4653 | 0.575 | 0.495  05 | 1.25  5676 | 11  5 |
| {BcBNV5.BI=FALSE, BcMV3.BI=FALSE} =>  {BcMV4.BI=FALSE} | 0.29  703 | 0.677  966 | 0.438  119 | 1.21  7326 | 12  0 |

| {BcBNV5.BI=FALSE, BcMV4.BI=FALSE} =>  {BcMV3.BI=FALSE} | 0.29  703 | 0.566  038 | 0.524  752 | 1.23  6104 | 12  0 |
| --- | --- | --- | --- | --- | --- |
| {BcMV3.BI=FALSE, BvF.BI=FALSE} =>  {BcMV4.BI=FALSE} | 0.28  4653 | 0.680  473 | 0.418  317 | 1.22  1828 | 11  5 |
| {BcMV4.BI=FALSE, BvF.BI=FALSE} =>  {BcMV3.BI=FALSE} | 0.28  4653 | 0.560  976 | 0.507  426 | 1.22  5049 | 11  5 |
| {BcMCV5.BI=FALSE, BcMV3.BI=FALSE} =>  {BcMV4.BI=FALSE} | 0.29  9505 | 0.679  775 | 0.440  594 | 1.22  0574 | 12  1 |
| {BcMCV5.BI=FALSE, BcMV4.BI=FALSE} =>  {BcMV3.BI=FALSE} | 0.29  9505 | 0.55 | 0.544  554 | 1.20  1081 | 12  1 |
| {BcMV3.BI=FALSE, BcOLV13.BI=FALSE} =>  {BcMV4.BI=FALSE} | 0.30  4455 | 0.675  824 | 0.450  495 | 1.21  348 | 12  3 |
| {BcMV4.BI=FALSE, BcOLV13.BI=FALSE} =>  {BcMV3.BI=FALSE} | 0.30  4455 | 0.569  444 | 0.534  653 | 1.24  3544 | 12  3 |
| {BcMV3.BI=FALSE, BcOLV2.BI=FALSE} =>  {BcMV4.BI=FALSE} | 0.29  703 | 0.670  391 | 0.443  069 | 1.20  3724 | 12  0 |
| {BcMV4.BI=FALSE, BcOLV2.BI=FALSE} =>  {BcMV3.BI=FALSE} | 0.29  703 | 0.566  038 | 0.524  752 | 1.23  6104 | 12  0 |
| {BcMCV3.BI=FALSE, BcMV3.BI=FALSE} =>  {BcMV4.BI=FALSE} | 0.30  9406 | 0.675  676 | 0.457  921 | 1.21  3213 | 12  5 |
| {BcMCV3.BI=FALSE, BcMV4.BI=FALSE} =>  {BcMV3.BI=FALSE} | 0.30  9406 | 0.592  417 | 0.522  277 | 1.29  3711 | 12  5 |

| {BcMV3.BI=FALSE, BcPV4.BI=FALSE} =>  {BcMV4.BI=FALSE} | 0.29  2079 | 0.670  455 | 0.435  644 | 1.20  3838 | 11  8 |
| --- | --- | --- | --- | --- | --- |
| {BcMV4.BI=FALSE, BcPV4.BI=FALSE} =>  {BcMV3.BI=FALSE} | 0.29  2079 | 0.567  308 | 0.514  851 | 1.23  8877 | 11  8 |
| {BcEV6=FALSE, BcMV3.BI=FALSE} =>  {BcMV4.BI=FALSE} | 0.28  2178 | 0.674  556 | 0.418  317 | 1.21  1203 | 11  4 |
| {BcEV6=FALSE, BcMV4.BI=FALSE} =>  {BcMV3.BI=FALSE} | 0.28  2178 | 0.548  077 | 0.514  851 | 1.19  6881 | 11  4 |
| {BcMV3.BI=FALSE, BcOLV17.BI=FALSE} =>  {BcMV4.BI=FALSE} | 0.29  4554 | 0.676  136 | 0.435  644 | 1.21  404 | 11  9 |
| {BcMV4.BI=FALSE, BcOLV17.BI=FALSE} =>  {BcMV3.BI=FALSE} | 0.29  4554 | 0.548  387 | 0.537  129 | 1.19  7559 | 11  9 |
| {BcMV3.BI=FALSE, BcOLV15.BI=FALSE} =>  {BcMV4.BI=FALSE} | 0.30  198 | 0.681  564 | 0.443  069 | 1.22  3786 | 12  2 |
| {BcMV4.BI=FALSE, BcOLV15.BI=FALSE} =>  {BcMV3.BI=FALSE} | 0.30  198 | 0.562  212 | 0.537  129 | 1.22  7749 | 12  2 |
| {BcEV5=FALSE, BcMV3.BI=FALSE} =>  {BcMV4.BI=FALSE} | 0.30  6931 | 0.673  913 | 0.455  446 | 1.21  0048 | 12  4 |
| {BcEV5=FALSE, BcMV4.BI=FALSE} =>  {BcMV3.BI=FALSE} | 0.30  6931 | 0.556  054 | 0.551  98 | 1.21  4301 | 12  4 |
| {BcHV1.BI=TRUE, BcMV3.BI=FALSE} =>  {BcOLV16.BI=FALSE} | 0.17  3267 | 0.693  069 | 0.25 | 1.18  6441 | 70 |

| {BcMV11=TRUE, BcMV3.BI=FALSE} =>  {BcOLV16.BI=FALSE} | 0.16  0891 | 0.691  489 | 0.232  673 | 1.18  3736 | 65 |
| --- | --- | --- | --- | --- | --- |
| {BcMV3.BI=FALSE, BcOLV16.BI=FALSE} =>  {BcOLV1.BI.MOVA.=FALSE} | 0.25  9901 | 0.84 | 0.309  406 | 1.25  6889 | 10  5 |
| {BcMV3.BI=FALSE, BcOLV1.BI.MOVA.=FALSE} =>  {BcOLV16.BI=FALSE} | 0.25  9901 | 0.7 | 0.371  287 | 1.19  8305 | 10  5 |
| {BcOLV1.BI.MOVA.=FALSE,  BcOLV16.BI=FALSE} => {BcMV3.BI=FALSE} | 0.25  9901 | 0.586  592 | 0.443  069 | 1.28  099 | 10  5 |
| {BcMV3.BI=FALSE, BcOLV16.BI=FALSE} =>  {BcMV6.BI=FALSE} | 0.25  7426 | 0.832 | 0.309  406 | 1.19  6185 | 10  4 |
| {BcMV3.BI=FALSE, BcMV6.BI=FALSE} =>  {BcOLV16.BI=FALSE} | 0.25  7426 | 0.684  211 | 0.376  238 | 1.17  1276 | 10  4 |
| {BcMV6.BI=FALSE, BcOLV16.BI=FALSE} =>  {BcMV3.BI=FALSE} | 0.25  7426 | 0.577  778 | 0.445  545 | 1.26  1742 | 10  4 |
| {BcMV3.BI=FALSE, BcPV3.BI=FALSE} =>  {BcOLV16.BI=FALSE} | 0.23  7624 | 0.680  851 | 0.349  01 | 1.16  5525 | 96 |
| {BcOLV16.BI=FALSE, BcPV3.BI=FALSE} =>  {BcMV3.BI=FALSE} | 0.23  7624 | 0.542  373 | 0.438  119 | 1.18  4425 | 96 |
| {BcMV3.BI=FALSE, BcRV1.BI=FALSE} =>  {BcOLV16.BI=FALSE} | 0.24  7525 | 0.694  444 | 0.356  436 | 1.18  8795 | 10  0 |
| {BcOLV16.BI=FALSE, BcRV1.BI=FALSE} => | 0.24 | 0.540 | 0.457 | 1.18 | 10 |

| {BcMV3.BI=FALSE} | 7525 | 541 | 921 | 0424 | 0 |
| --- | --- | --- | --- | --- | --- |
| {BcHV3.BI=FALSE, BcOLV16.BI=FALSE} =>  {BcMV3.BI=FALSE} | 0.24  2574 | 0.553  672 | 0.438  119 | 1.20  9101 | 98 |
| {BcMV3.BI=FALSE, BcOLV16.BI=FALSE} =>  {BcFV5.BI=FALSE} | 0.27  2277 | 0.88 | 0.309  406 | 1.12  5063 | 11  0 |
| {BcFV5.BI=FALSE, BcMV3.BI=FALSE} =>  {BcOLV16.BI=FALSE} | 0.27  2277 | 0.691  824 | 0.393  564 | 1.18  4309 | 11  0 |
| {BcFV5.BI=FALSE, BcOLV16.BI=FALSE} =>  {BcMV3.BI=FALSE} | 0.27  2277 | 0.591  398 | 0.460  396 | 1.29  1485 | 11  0 |
| {BcMV3.BI=FALSE, BcOLV16.BI=FALSE} =>  {BcULV1.BI=FALSE} | 0.27  9703 | 0.904 | 0.309  406 | 1.15  2101 | 11  3 |
| {BcMV3.BI=FALSE, BcULV1.BI=FALSE} =>  {BcOLV16.BI=FALSE} | 0.27  9703 | 0.710  692 | 0.393  564 | 1.21  6608 | 11  3 |
| {BcOLV16.BI=FALSE, BcULV1.BI=FALSE} =>  {BcMV3.BI=FALSE} | 0.27  9703 | 0.543  269 | 0.514  851 | 1.18  6383 | 11  3 |
| {BcMV1.BI=TRUE, BcMV3.BI=FALSE} =>  {BcOLV16.BI=FALSE} | 0.23  2673 | 0.701  493 | 0.331  683 | 1.20  086 | 94 |
| {BcMV3.BI=FALSE, BcOLV16.BI=FALSE} =>  {BcMV12=FALSE} | 0.27  4752 | 0.888 | 0.309  406 | 1.11  0687 | 11  1 |
| {BcMV12=FALSE, BcMV3.BI=FALSE} =>  {BcOLV16.BI=FALSE} | 0.27  4752 | 0.668  675 | 0.410  891 | 1.14  468 | 11  1 |
| {BcMV12=FALSE, BcOLV16.BI=FALSE} => | 0.27 | 0.544 | 0.504 | 1.18 | 11 |

| {BcMV3.BI=FALSE} | 4752 | 118 | 95 | 8235 | 1 |
| --- | --- | --- | --- | --- | --- |
| {BcMV3.BI=FALSE, BcNSRV7.BI=FALSE} =>  {BcOLV16.BI=FALSE} | 0.26  2376 | 0.697  368 | 0.376  238 | 1.19  38 | 10  6 |
| {BcNSRV7.BI=FALSE, BcOLV16.BI=FALSE}  => {BcMV3.BI=FALSE} | 0.26  2376 | 0.563  83 | 0.465  347 | 1.23  1282 | 10  6 |
| {BcMV3.BI=FALSE, BcVV3.BI=FALSE} =>  {BcOLV16.BI=FALSE} | 0.25  7426 | 0.666  667 | 0.386  139 | 1.14  1243 | 10  4 |
| {BcOLV16.BI=FALSE, BcVV3.BI=FALSE} =>  {BcMV3.BI=FALSE} | 0.25  7426 | 0.553  191 | 0.465  347 | 1.20  8051 | 10  4 |
| {BcMV3.BI=FALSE, BcOLV16.BI=FALSE} =>  {BcOLV7.BI=FALSE} | 0.27  7228 | 0.896 | 0.309  406 | 1.11  038 | 11  2 |
| {BcMV3.BI=FALSE, BcOLV7.BI=FALSE} =>  {BcOLV16.BI=FALSE} | 0.27  7228 | 0.708  861 | 0.391  089 | 1.21  3474 | 11  2 |
| {BcOLV16.BI=FALSE, BcOLV7.BI=FALSE} =>  {BcMV3.BI=FALSE} | 0.27  7228 | 0.541  063 | 0.512  376 | 1.18  1564 | 11  2 |
| {BcMV3.BI=FALSE, BcOLV16.BI=FALSE} =>  {BcALV1.BI=FALSE} | 0.27  4752 | 0.888 | 0.309  406 | 1.09  0432 | 11  1 |
| {BcALV1.BI=FALSE, BcMV3.BI=FALSE} =>  {BcOLV16.BI=FALSE} | 0.27  4752 | 0.702  532 | 0.391  089 | 1.20  2639 | 11  1 |
| {BcALV1.BI=FALSE, BcOLV16.BI=FALSE} =>  {BcMV3.BI=FALSE} | 0.27  4752 | 0.544  118 | 0.504  95 | 1.18  8235 | 11  1 |
| {BcMV3.BI=FALSE, BcOLV16.BI=FALSE} => | 0.28 | 0.928 | 0.309 | 1.09 | 11 |

| {BcDMV1=FALSE} | 7129 |  | 406 | 9449 | 6 |
| --- | --- | --- | --- | --- | --- |
| {BcDMV1=FALSE, BcMV3.BI=FALSE} =>  {BcOLV16.BI=FALSE} | 0.28  7129 | 0.682  353 | 0.420  792 | 1.16  8096 | 11  6 |
| {BcDMV1=FALSE, BcOLV16.BI=FALSE} =>  {BcMV3.BI=FALSE} | 0.28  7129 | 0.585  859 | 0.490  099 | 1.27  9388 | 11  6 |
| {BcMV3.BI=FALSE, BcOLV16.BI=FALSE} =>  {BcFV9=FALSE} | 0.28  7129 | 0.928 | 0.309  406 | 1.06  8125 | 11  6 |
| {BcFV9=FALSE, BcMV3.BI=FALSE} =>  {BcOLV16.BI=FALSE} | 0.28  7129 | 0.694  611 | 0.413  366 | 1.18  9079 | 11  6 |
| {BcFV9=FALSE, BcOLV16.BI=FALSE} =>  {BcMV3.BI=FALSE} | 0.28  7129 | 0.563  107 | 0.509  901 | 1.22  9703 | 11  6 |
| {BcMV3.BI=FALSE, BcOLV16.BI=FALSE} =>  {BcOLV4.BI=FALSE} | 0.28  9604 | 0.936 | 0.309  406 | 1.06  8203 | 11  7 |
| {BcMV3.BI=FALSE, BcOLV4.BI=FALSE} =>  {BcOLV16.BI=FALSE} | 0.28  9604 | 0.688  235 | 0.420  792 | 1.17  8166 | 11  7 |
| {BcOLV16.BI=FALSE, BcOLV4.BI=FALSE} =>  {BcMV3.BI=FALSE} | 0.28  9604 | 0.544  186 | 0.532  178 | 1.18  8385 | 11  7 |
| {BcMV3.BI=FALSE, BcULV5=FALSE} =>  {BcOLV16.BI=FALSE} | 0.28  4653 | 0.672  515 | 0.423  267 | 1.15  1254 | 11  5 |
| {BcOLV16.BI=FALSE, BcULV5=FALSE} =>  {BcMV3.BI=FALSE} | 0.28  4653 | 0.542  453 | 0.524  752 | 1.18  46 | 11  5 |
| {BcBNV5.BI=FALSE, BcMV3.BI=FALSE} => | 0.29 | 0.683 | 0.438 | 1.17 | 12 |

| {BcOLV16.BI=FALSE} | 9505 | 616 | 119 | 0258 | 1 |
| --- | --- | --- | --- | --- | --- |
| {BcBNV5.BI=FALSE, BcOLV16.BI=FALSE} =>  {BcMV3.BI=FALSE} | 0.29  9505 | 0.560  185 | 0.534  653 | 1.22  3323 | 12  1 |
| {BcMV3.BI=FALSE, BvF.BI=FALSE} =>  {BcOLV16.BI=FALSE} | 0.28  7129 | 0.686  391 | 0.418  317 | 1.17  5008 | 11  6 |
| {BcOLV16.BI=FALSE, BvF.BI=FALSE} =>  {BcMV3.BI=FALSE} | 0.28  7129 | 0.549  763 | 0.522  277 | 1.20  0564 | 11  6 |
| {BcMCV5.BI=FALSE, BcMV3.BI=FALSE} =>  {BcOLV16.BI=FALSE} | 0.29  703 | 0.674  157 | 0.440  594 | 1.15  4066 | 12  0 |
| {BcMCV5.BI=FALSE, BcOLV16.BI=FALSE} =>  {BcMV3.BI=FALSE} | 0.29  703 | 0.533  333 | 0.556  931 | 1.16  4685 | 12  0 |
| {BcMV3.BI=FALSE, BcOLV13.BI=FALSE} =>  {BcOLV16.BI=FALSE} | 0.30  198 | 0.670  33 | 0.450  495 | 1.14  7514 | 12  2 |
| {BcOLV13.BI=FALSE, BcOLV16.BI=FALSE}  => {BcMV3.BI=FALSE} | 0.30  198 | 0.539  823 | 0.559  406 | 1.17  8857 | 12  2 |
| {BcMV3.BI=FALSE, BcOLV2.BI=FALSE} =>  {BcOLV16.BI=FALSE} | 0.30  198 | 0.681  564 | 0.443  069 | 1.16  6746 | 12  2 |
| {BcOLV16.BI=FALSE, BcOLV2.BI=FALSE} =>  {BcMV3.BI=FALSE} | 0.30  198 | 0.539  823 | 0.559  406 | 1.17  8857 | 12  2 |
| {BcMCV3.BI=FALSE, BcMV3.BI=FALSE} =>  {BcOLV16.BI=FALSE} | 0.30  9406 | 0.675  676 | 0.457  921 | 1.15  6665 | 12  5 |
| {BcMCV3.BI=FALSE, BcOLV16.BI=FALSE} => | 0.30 | 0.538 | 0.574 | 1.17 | 12 |

| {BcMV3.BI=FALSE} | 9406 | 793 | 257 | 6608 | 5 |
| --- | --- | --- | --- | --- | --- |
| {BcMV3.BI=FALSE, BcPV4.BI=FALSE} =>  {BcOLV16.BI=FALSE} | 0.29  2079 | 0.670  455 | 0.435  644 | 1.14  7727 | 11  8 |
| {BcOLV16.BI=FALSE, BcPV4.BI=FALSE} =>  {BcMV3.BI=FALSE} | 0.29  2079 | 0.526  786 | 0.554  455 | 1.15  0386 | 11  8 |
| {BcEV6=FALSE, BcMV3.BI=FALSE} =>  {BcOLV16.BI=FALSE} | 0.28  4653 | 0.680  473 | 0.418  317 | 1.16  4878 | 11  5 |
| {BcEV6=FALSE, BcOLV16.BI=FALSE} =>  {BcMV3.BI=FALSE} | 0.28  4653 | 0.522  727 | 0.544  554 | 1.14  1523 | 11  5 |
| {BcMV3.BI=FALSE, BcOLV17.BI=FALSE} =>  {BcOLV16.BI=FALSE} | 0.29  2079 | 0.670  455 | 0.435  644 | 1.14  7727 | 11  8 |
| {BcMV3.BI=FALSE, BcOLV15.BI=FALSE} =>  {BcOLV16.BI=FALSE} | 0.29  703 | 0.670  391 | 0.443  069 | 1.14  7619 | 12  0 |
| {BcOLV15.BI=FALSE, BcOLV16.BI=FALSE}  => {BcMV3.BI=FALSE} | 0.29  703 | 0.542  986 | 0.547  03 | 1.18  5765 | 12  0 |
| {BcEV5=FALSE, BcMV3.BI=FALSE} =>  {BcOLV16.BI=FALSE} | 0.30  6931 | 0.673  913 | 0.455  446 | 1.15  3648 | 12  4 |
| {BcEV5=FALSE, BcOLV16.BI=FALSE} =>  {BcMV3.BI=FALSE} | 0.30  6931 | 0.551  111 | 0.556  931 | 1.20  3508 | 12  4 |
| {BcMV3.BI=FALSE, BcULV3=TRUE} =>  {BcOLV1.BI.MOVA.=FALSE} | 0.20  5446 | 0.83 | 0.247  525 | 1.24  1926 | 83 |
| {BcMV3.BI=FALSE, BcULV3=TRUE} => | 0.20 | 0.81 | 0.247 | 1.16 | 81 |

| {BcMV6.BI=FALSE} | 0495 |  | 525 | 4555 |  |
| --- | --- | --- | --- | --- | --- |
| {BcMV3.BI=FALSE, BcULV3=TRUE} =>  {BcULV1.BI=FALSE} | 0.21  5347 | 0.87 | 0.247  525 | 1.10  877 | 87 |
| {BcMV3.BI=FALSE, BcULV3=TRUE} =>  {BcDMV1=FALSE} | 0.23  7624 | 0.96 | 0.247  525 | 1.13  7361 | 96 |
| {BcHV1.BI=TRUE, BcMV3.BI=FALSE} =>  {BcOLV1.BI.MOVA.=FALSE} | 0.20  5446 | 0.821  782 | 0.25 | 1.22  963 | 83 |
| {BcHV1.BI=TRUE, BcMV3.BI=FALSE} =>  {BcMV12=FALSE} | 0.22  0297 | 0.881  188 | 0.25 | 1.10  2167 | 89 |
| {BcHV1.BI=TRUE, BcMV3.BI=FALSE} =>  {BcDMV1=FALSE} | 0.23  5149 | 0.940  594 | 0.25 | 1.11  437 | 95 |
| {BcMV11=TRUE, BcMV3.BI=FALSE} =>  {BcOLV1.BI.MOVA.=FALSE} | 0.18  3168 | 0.787  234 | 0.232  673 | 1.17  7935 | 74 |
| {BcMV11=TRUE, BcMV3.BI=FALSE} =>  {BcMV6.BI=FALSE} | 0.18  8119 | 0.808  511 | 0.232  673 | 1.16  2414 | 76 |
| {BcMV11=TRUE, BcMV3.BI=FALSE} =>  {BcMV12=FALSE} | 0.21  2871 | 0.914  894 | 0.232  673 | 1.14  4325 | 86 |
| {BcMV11=TRUE, BcMV3.BI=FALSE} =>  {BcDMV1=FALSE} | 0.21  7822 | 0.936  17 | 0.232  673 | 1.10  9128 | 88 |
| {BcMV3.BI=FALSE, BcMV9.BI=TRUE} =>  {BcOLV1.BI.MOVA.=FALSE} | 0.23  2673 | 0.824  561 | 0.282  178 | 1.23  3788 | 94 |
| {BcMV3.BI=FALSE, BcULV4=TRUE} => | 0.22 | 0.786 | 0.289 | 1.17 | 92 |

| {BcOLV1.BI.MOVA.=FALSE} | 7723 | 325 | 604 | 6575 |  |
| --- | --- | --- | --- | --- | --- |
| {BcMV3.BI=FALSE, |  |  |  |  |  |
|  | 0.30 | 0.833 | 0.371 | 1.19 | 12 |
| BcOLV1.BI.MOVA.=FALSE} => |  |  |  |  |  |
|  | 9406 | 333 | 287 | 8102 | 5 |
| {BcMV6.BI=FALSE} |  |  |  |  |  |
| {BcMV3.BI=FALSE, BcMV6.BI=FALSE} => | 0.30 | 0.822 | 0.376 | 1.23 | 12 |
| {BcOLV1.BI.MOVA.=FALSE} | 9406 | 368 | 238 | 0507 | 5 |
| {BcMV6.BI=FALSE, |  |  |  |  |  |
|  | 0.30 | 0.586 | 0.527 | 1.28 | 12 |
| BcOLV1.BI.MOVA.=FALSE} => |  |  |  |  |  |
|  | 9406 | 854 | 228 | 1563 | 5 |
| {BcMV3.BI=FALSE} |  |  |  |  |  |
| {BcMV3.BI=FALSE, |  |  |  |  |  |
|  | 0.29 | 0.786 | 0.371 | 1.10 | 11 |
| BcOLV1.BI.MOVA.=FALSE} => |  |  |  |  |  |
|  | 2079 | 667 | 287 | 7364 | 8 |
| {BcPV3.BI=FALSE} |  |  |  |  |  |
| {BcMV3.BI=FALSE, BcPV3.BI=FALSE} => | 0.29 | 0.836 | 0.349 | 1.25 | 11 |
| {BcOLV1.BI.MOVA.=FALSE} | 2079 | 879 | 01 | 222 | 8 |
| {BcOLV1.BI.MOVA.=FALSE, | 0.29 | 0.567 | 0.514 | 1.23 | 11 |
| BcPV3.BI=FALSE} => {BcMV3.BI=FALSE} | 2079 | 308 | 851 | 8877 | 8 |
| {BcMV3.BI=FALSE, |  |  |  |  |  |
|  | 0.29 |  | 0.371 | 1.10 | 12 |
| BcOLV1.BI.MOVA.=FALSE} => |  | 0.8 |  |  |  |
|  | 703 |  | 287 | 6849 | 0 |
| {BcRV1.BI=FALSE} |  |  |  |  |  |
| {BcMV3.BI=FALSE, BcRV1.BI=FALSE} => | 0.29 | 0.833 | 0.356 | 1.24 | 12 |
| {BcOLV1.BI.MOVA.=FALSE} | 703 | 333 | 436 | 6914 | 0 |
| {BcOLV1.BI.MOVA.=FALSE, | 0.29 | 0.566 | 0.524 | 1.23 | 12 |

| BcRV1.BI=FALSE} => {BcMV3.BI=FALSE} | 703 | 038 | 752 | 6104 | 0 |
| --- | --- | --- | --- | --- | --- |
| {BcEV2.BI=FALSE, BcMV3.BI=FALSE} =>  {BcOLV1.BI.MOVA.=FALSE} | 0.27  2277 | 0.791  367 | 0.344  059 | 1.18  4119 | 11  0 |
| {BcEV2.BI=FALSE, BcOLV1.BI.MOVA.=FALSE} =>  {BcMV3.BI=FALSE} | 0.27  2277 | 0.547  264 | 0.497  525 | 1.19  5106 | 11  0 |
| {BcHV3.BI=FALSE, BcMV3.BI=FALSE} =>  {BcOLV1.BI.MOVA.=FALSE} | 0.29  9505 | 0.817  568 | 0.366  337 | 1.22  3323 | 12  1 |
| {BcHV3.BI=FALSE, BcOLV1.BI.MOVA.=FALSE} =>  {BcMV3.BI=FALSE} | 0.29  9505 | 0.562  791 | 0.532  178 | 1.22  9013 | 12  1 |
| {BcMV3.BI=FALSE, BcOLV1.BI.MOVA.=FALSE} =>  {BcFV5.BI=FALSE} | 0.32  6733 | 0.88 | 0.371  287 | 1.12  5063 | 13  2 |
| {BcFV5.BI=FALSE, BcMV3.BI=FALSE} =>  {BcOLV1.BI.MOVA.=FALSE} | 0.32  6733 | 0.830  189 | 0.393  564 | 1.24  2208 | 13  2 |
| {BcFV5.BI=FALSE, BcOLV1.BI.MOVA.=FALSE} =>  {BcMV3.BI=FALSE} | 0.32  6733 | 0.602  74 | 0.542  079 | 1.31  6253 | 13  2 |
| {BcMV3.BI=FALSE, BcOLV1.BI.MOVA.=FALSE} =>  {BcULV1.BI=FALSE} | 0.31  9307 | 0.86 | 0.371  287 | 1.09  6025 | 12  9 |

| {BcMV3.BI=FALSE, BcULV1.BI=FALSE} =>  {BcOLV1.BI.MOVA.=FALSE} | 0.31  9307 | 0.811  321 | 0.393  564 | 1.21  3976 | 12  9 |
| --- | --- | --- | --- | --- | --- |
| {BcOLV1.BI.MOVA.=FALSE,  BcULV1.BI=FALSE} => {BcMV3.BI=FALSE} | 0.31  9307 | 0.570  796 | 0.559  406 | 1.24  6496 | 12  9 |
| {BcMV1.BI=TRUE, BcMV3.BI=FALSE} =>  {BcOLV1.BI.MOVA.=FALSE} | 0.25  7426 | 0.776  119 | 0.331  683 | 1.16  1305 | 10  4 |
| {BcMV3.BI=FALSE, BcOLV1.BI.MOVA.=FALSE} =>  {BcMV12=FALSE} | 0.33  4158 | 0.9 | 0.371  287 | 1.12  5697 | 13  5 |
| {BcMV12=FALSE, BcMV3.BI=FALSE} =>  {BcOLV1.BI.MOVA.=FALSE} | 0.33  4158 | 0.813  253 | 0.410  891 | 1.21  6867 | 13  5 |
| {BcMV12=FALSE, BcOLV1.BI.MOVA.=FALSE} =>  {BcMV3.BI=FALSE} | 0.33  4158 | 0.586  957 | 0.569  307 | 1.28  1786 | 13  5 |
| {BcMV3.BI=FALSE, BcNSRV7.BI=FALSE} =>  {BcOLV1.BI.MOVA.=FALSE} | 0.30  198 | 0.802  632 | 0.376  238 | 1.20  0975 | 12  2 |
| {BcNSRV7.BI=FALSE, BcOLV1.BI.MOVA.=FALSE} =>  {BcMV3.BI=FALSE} | 0.30  198 | 0.564  815 | 0.534  653 | 1.23  3433 | 12  2 |
| {BcMV3.BI=FALSE, BcVV3.BI=FALSE} =>  {BcOLV1.BI.MOVA.=FALSE} | 0.31  4356 | 0.814  103 | 0.386  139 | 1.21  8139 | 12  7 |
| {BcOLV1.BI.MOVA.=FALSE, | 0.31 | 0.561 | 0.559 | 1.22 | 12 |

| BcVV3.BI=FALSE} => {BcMV3.BI=FALSE} | 4356 | 947 | 406 | 7171 | 7 |
| --- | --- | --- | --- | --- | --- |
| {BcMV3.BI=FALSE, BcOLV1.BI.MOVA.=FALSE} =>  {BcOLV7.BI=FALSE} | 0.32  4257 | 0.873  333 | 0.371  287 | 1.08  229 | 13  1 |
| {BcMV3.BI=FALSE, BcOLV7.BI=FALSE} =>  {BcOLV1.BI.MOVA.=FALSE} | 0.32  4257 | 0.829  114 | 0.391  089 | 1.24  06 | 13  1 |
| {BcOLV1.BI.MOVA.=FALSE,  BcOLV7.BI=FALSE} => {BcMV3.BI=FALSE} | 0.32  4257 | 0.569  565 | 0.569  307 | 1.24  3807 | 13  1 |
| {BcMV3.BI=FALSE, BcOLV1.BI.MOVA.=FALSE} =>  {BcALV1.BI=FALSE} | 0.32  9208 | 0.886  667 | 0.371  287 | 1.08  8794 | 13  3 |
| {BcALV1.BI=FALSE, BcMV3.BI=FALSE} =>  {BcOLV1.BI.MOVA.=FALSE} | 0.32  9208 | 0.841  772 | 0.391  089 | 1.25  9541 | 13  3 |
| {BcALV1.BI=FALSE, BcOLV1.BI.MOVA.=FALSE} =>  {BcMV3.BI=FALSE} | 0.32  9208 | 0.558  824 | 0.589  109 | 1.22  035 | 13  3 |
| {BcMV3.BI=FALSE, BcOLV1.BI.MOVA.=FALSE} =>  {BcDMV1=FALSE} | 0.34  1584 | 0.92 | 0.371  287 | 1.08  9971 | 13  8 |
| {BcDMV1=FALSE, BcMV3.BI=FALSE} =>  {BcOLV1.BI.MOVA.=FALSE} | 0.34  1584 | 0.811  765 | 0.420  792 | 1.21  4641 | 13  8 |
| {BcDMV1=FALSE, | 0.34 | 0.6 | 0.569 | 1.31 | 13 |

| BcOLV1.BI.MOVA.=FALSE} =>  {BcMV3.BI=FALSE} | 1584 |  | 307 | 027 | 8 |
| --- | --- | --- | --- | --- | --- |
| {BcFV9=FALSE, BcMV3.BI=FALSE} => | 0.33 | 0.820 | 0.413 | 1.22 | 13 |
| {BcOLV1.BI.MOVA.=FALSE} | 9109 | 359 | 366 | 7501 | 7 |
| {BcFV9=FALSE, BcOLV1.BI.MOVA.=FALSE} | 0.33 | 0.575 | 0.589 | 1.25 | 13 |
| => {BcMV3.BI=FALSE} | 9109 | 63 | 109 | 7052 | 7 |
| {BcMV3.BI=FALSE, |  |  |  |  |  |
|  | 0.34 | 0.933 | 0.371 | 1.06 | 14 |
| BcOLV1.BI.MOVA.=FALSE} => |  |  |  |  |  |
|  | 6535 | 333 | 287 | 516 | 0 |
| {BcOLV4.BI=FALSE} |  |  |  |  |  |
| {BcMV3.BI=FALSE, BcOLV4.BI=FALSE} => | 0.34 | 0.823 | 0.420 | 1.23 | 14 |
| {BcOLV1.BI.MOVA.=FALSE} | 6535 | 529 | 792 | 2244 | 0 |
| {BcOLV1.BI.MOVA.=FALSE, | 0.34 | 0.564 | 0.613 | 1.23 | 14 |
| BcOLV4.BI=FALSE} => {BcMV3.BI=FALSE} | 6535 | 516 | 861 | 2781 | 0 |
| {BcMV3.BI=FALSE, |  |  |  |  |  |
|  | 0.35 | 0.953 | 0.371 | 1.08 | 14 |
| BcOLV1.BI.MOVA.=FALSE} => |  |  |  |  |  |
|  | 396 | 333 | 287 | 7985 | 3 |
| {BcULV5=FALSE} |  |  |  |  |  |
| {BcMV3.BI=FALSE, BcULV5=FALSE} => | 0.35 | 0.836 | 0.423 | 1.25 | 14 |
| {BcOLV1.BI.MOVA.=FALSE} | 396 | 257 | 267 | 1289 | 3 |
| {BcOLV1.BI.MOVA.=FALSE, BcULV5=FALSE} | 0.35 |  | 0.618 | 1.24 | 14 |
|  |  | 0.572 |  |  |  |
| => {BcMV3.BI=FALSE} | 396 |  | 812 | 9124 | 3 |
| {BcBNV5.BI=FALSE, BcMV3.BI=FALSE} => | 0.35 | 0.807 | 0.438 | 1.20 | 14 |
| {BcOLV1.BI.MOVA.=FALSE} | 396 | 91 | 119 | 8872 | 3 |

| {BcBNV5.BI=FALSE, BcOLV1.BI.MOVA.=FALSE} =>  {BcMV3.BI=FALSE} | 0.35  396 | 0.565  217 | 0.626  238 | 1.23  4313 | 14  3 |
| --- | --- | --- | --- | --- | --- |
| {BcMV3.BI=FALSE, BvF.BI=FALSE} =>  {BcOLV1.BI.MOVA.=FALSE} | 0.33  4158 | 0.798  817 | 0.418  317 | 1.19  5266 | 13  5 |
| {BcOLV1.BI.MOVA.=FALSE, BvF.BI=FALSE}  => {BcMV3.BI=FALSE} | 0.33  4158 | 0.557  851 | 0.599  01 | 1.21  8226 | 13  5 |
| {BcMCV5.BI=FALSE, BcMV3.BI=FALSE} =>  {BcOLV1.BI.MOVA.=FALSE} | 0.36  1386 | 0.820  225 | 0.440  594 | 1.22  7299 | 14  6 |
| {BcMCV5.BI=FALSE, BcOLV1.BI.MOVA.=FALSE} =>  {BcMV3.BI=FALSE} | 0.36  1386 | 0.559  387 | 0.646  04 | 1.22  158 | 14  6 |
| {BcMV3.BI=FALSE, BcOLV13.BI=FALSE} =>  {BcOLV1.BI.MOVA.=FALSE} | 0.36  8812 | 0.818  681 | 0.450  495 | 1.22  499 | 14  9 |
| {BcOLV1.BI.MOVA.=FALSE,  BcOLV13.BI=FALSE} => {BcMV3.BI=FALSE} | 0.36  8812 | 0.568  702 | 0.648  515 | 1.24  1923 | 14  9 |
| {BcMV3.BI=FALSE, BcOLV2.BI=FALSE} =>  {BcOLV1.BI.MOVA.=FALSE} | 0.36  6337 | 0.826  816 | 0.443  069 | 1.23  7161 | 14  8 |
| {BcOLV1.BI.MOVA.=FALSE,  BcOLV2.BI=FALSE} => {BcMV3.BI=FALSE} | 0.36  6337 | 0.562  738 | 0.650  99 | 1.22  8897 | 14  8 |
| {BcMCV3.BI=FALSE, BcMV3.BI=FALSE} =>  {BcOLV1.BI.MOVA.=FALSE} | 0.37  1287 | 0.810  811 | 0.457  921 | 1.21  3213 | 15  0 |

| {BcMCV3.BI=FALSE, BcOLV1.BI.MOVA.=FALSE} =>  {BcMV3.BI=FALSE} | 0.37  1287 | 0.574  713 | 0.646  04 | 1.25  5048 | 15  0 |
| --- | --- | --- | --- | --- | --- |
| {BcMV3.BI=FALSE, BcPV4.BI=FALSE} =>  {BcOLV1.BI.MOVA.=FALSE} | 0.34  901 | 0.801  136 | 0.435  644 | 1.19  8737 | 14  1 |
| {BcOLV1.BI.MOVA.=FALSE,  BcPV4.BI=FALSE} => {BcMV3.BI=FALSE} | 0.34  901 | 0.566  265 | 0.616  337 | 1.23  66 | 14  1 |
| {BcEV6=FALSE, BcMV3.BI=FALSE} =>  {BcOLV1.BI.MOVA.=FALSE} | 0.34  4059 | 0.822  485 | 0.418  317 | 1.23  0682 | 13  9 |
| {BcEV6=FALSE, BcOLV1.BI.MOVA.=FALSE}  => {BcMV3.BI=FALSE} | 0.34  4059 | 0.547  244 | 0.628  713 | 1.19  5063 | 13  9 |
| {BcMV3.BI=FALSE, BcOLV17.BI=FALSE} =>  {BcOLV1.BI.MOVA.=FALSE} | 0.35  396 | 0.812  5 | 0.435  644 | 1.21  5741 | 14  3 |
| {BcOLV1.BI.MOVA.=FALSE,  BcOLV17.BI=FALSE} => {BcMV3.BI=FALSE} | 0.35  396 | 0.545  802 | 0.648  515 | 1.19  1913 | 14  3 |
| {BcMV3.BI=FALSE, BcOLV15.BI=FALSE} =>  {BcOLV1.BI.MOVA.=FALSE} | 0.35  8911 | 0.810  056 | 0.443  069 | 1.21  2084 | 14  5 |
| {BcOLV1.BI.MOVA.=FALSE,  BcOLV15.BI=FALSE} => {BcMV3.BI=FALSE} | 0.35  8911 | 0.570  866 | 0.628  713 | 1.24  6648 | 14  5 |
| {BcEV5=FALSE, BcMV3.BI=FALSE} =>  {BcOLV1.BI.MOVA.=FALSE} | 0.36  8812 | 0.809  783 | 0.455  446 | 1.21  1675 | 14  9 |
| {BcEV5=FALSE, BcOLV1.BI.MOVA.=FALSE} | 0.36 | 0.564 | 0.653 | 1.23 | 14 |

| => {BcMV3.BI=FALSE} | 8812 | 394 | 465 | 2514 | 9 |
| --- | --- | --- | --- | --- | --- |
| {BcMV3.BI=FALSE, BcMV9.BI=TRUE} =>  {BcULV4=TRUE} | 0.22  0297 | 0.780  702 | 0.282  178 | 1.13  0479 | 89 |
| {BcMV3.BI=FALSE, BcMV9.BI=TRUE} =>  {BcMV6.BI=FALSE} | 0.22  2772 | 0.789  474 | 0.282  178 | 1.13  5044 | 90 |
| {BcMV3.BI=FALSE, BcMV9.BI=TRUE} =>  {BcMV12=FALSE} | 0.25  2475 | 0.894  737 | 0.282  178 | 1.11  9114 | 10  2 |
| {BcMV3.BI=FALSE, BcMV9.BI=TRUE} =>  {BcDMV1=FALSE} | 0.25  495 | 0.903  509 | 0.282  178 | 1.07  0433 | 10  3 |
| {BcMV3.BI=FALSE, BcMV9.BI=TRUE} =>  {BcOLV4.BI=FALSE} | 0.26  9802 | 0.956  14 | 0.282  178 | 1.09  1188 | 10  9 |
| {BcMV3.BI=FALSE, BcMV9.BI=TRUE} =>  {BcULV5=FALSE} | 0.27  9703 | 0.991  228 | 0.282  178 | 1.13  1232 | 11  3 |
| {BcMV3.BI=FALSE, BcULV4=TRUE} =>  {BcMV6.BI=FALSE} | 0.22  5248 | 0.777  778 | 0.289  604 | 1.11  8229 | 91 |
| {BcMV3.BI=FALSE, BcULV4=TRUE} =>  {BcPV3.BI=FALSE} | 0.23  0198 | 0.794  872 | 0.289  604 | 1.11  8914 | 93 |
| {BcMV3.BI=FALSE, BcULV4=TRUE} =>  {BcEV2.BI=FALSE} | 0.24  2574 | 0.837  607 | 0.289  604 | 1.12  0507 | 98 |
| {BcMV3.BI=FALSE, BcULV4=TRUE} =>  {BcFV5.BI=FALSE} | 0.25 | 0.863  248 | 0.289  604 | 1.10  3646 | 10  1 |
| {BcMV3.BI=FALSE, BcULV4=TRUE} => | 0.26 | 0.905 | 0.289 | 1.13 | 10 |

| {BcMV12=FALSE} | 2376 | 983 | 604 | 318 | 6 |
| --- | --- | --- | --- | --- | --- |
| {BcMV3.BI=FALSE, BcULV4=TRUE} =>  {BcDMV1=FALSE} | 0.26  2376 | 0.905  983 | 0.289  604 | 1.07  3364 | 10  6 |
| {BcMV3.BI=FALSE, BcULV4=TRUE} =>  {BcOLV4.BI=FALSE} | 0.27  2277 | 0.940  171 | 0.289  604 | 1.07  2963 | 11  0 |
| {BcMV3.BI=FALSE, BcULV4=TRUE} =>  {BcULV5=FALSE} | 0.27  9703 | 0.965  812 | 0.289  604 | 1.10  2226 | 11  3 |
| {BcMV3.BI=FALSE, BcPV3.BI=FALSE} =>  {BcMV6.BI=FALSE} | 0.28  4653 | 0.815  603 | 0.349  01 | 1.17  261 | 11  5 |
| {BcMV6.BI=FALSE, BcPV3.BI=FALSE} =>  {BcMV3.BI=FALSE} | 0.28  4653 | 0.550  239 | 0.517  327 | 1.20  1604 | 11  5 |
| {BcMV3.BI=FALSE, BcRV1.BI=FALSE} =>  {BcMV6.BI=FALSE} | 0.29  2079 | 0.819  444 | 0.356  436 | 1.17  8134 | 11  8 |
| {BcMV6.BI=FALSE, BcRV1.BI=FALSE} =>  {BcMV3.BI=FALSE} | 0.29  2079 | 0.548  837 | 0.532  178 | 1.19  8542 | 11  8 |
| {BcEV2.BI=FALSE, BcMV3.BI=FALSE} =>  {BcMV6.BI=FALSE} | 0.27  9703 | 0.812  95 | 0.344  059 | 1.16  8796 | 11  3 |
| {BcEV2.BI=FALSE, BcMV6.BI=FALSE} =>  {BcMV3.BI=FALSE} | 0.27  9703 | 0.543  269 | 0.514  851 | 1.18  6383 | 11  3 |
| {BcHV3.BI=FALSE, BcMV3.BI=FALSE} =>  {BcMV6.BI=FALSE} | 0.29  9505 | 0.817  568 | 0.366  337 | 1.17  5435 | 12  1 |
| {BcHV3.BI=FALSE, BcMV6.BI=FALSE} => | 0.29 | 0.555 | 0.539 | 1.21 | 12 |

| {BcMV3.BI=FALSE} | 9505 | 046 | 604 | 21 | 1 |
| --- | --- | --- | --- | --- | --- |
| {BcMV3.BI=FALSE, BcMV6.BI=FALSE} =>  {BcFV5.BI=FALSE} | 0.32  1782 | 0.855  263 | 0.376  238 | 1.09  3438 | 13  0 |
| {BcFV5.BI=FALSE, BcMV3.BI=FALSE} =>  {BcMV6.BI=FALSE} | 0.32  1782 | 0.817  61 | 0.393  564 | 1.17  5496 | 13  0 |
| {BcFV5.BI=FALSE, BcMV6.BI=FALSE} =>  {BcMV3.BI=FALSE} | 0.32  1782 | 0.590  909 | 0.544  554 | 1.29  0418 | 13  0 |
| {BcMV3.BI=FALSE, BcMV6.BI=FALSE} =>  {BcULV1.BI=FALSE} | 0.32  4257 | 0.861  842 | 0.376  238 | 1.09  8373 | 13  1 |
| {BcMV3.BI=FALSE, BcULV1.BI=FALSE} =>  {BcMV6.BI=FALSE} | 0.32  4257 | 0.823  899 | 0.393  564 | 1.18  4539 | 13  1 |
| {BcMV6.BI=FALSE, BcULV1.BI=FALSE} =>  {BcMV3.BI=FALSE} | 0.32  4257 | 0.567  1 | 0.571  782 | 1.23  8423 | 13  1 |
| {BcMV1.BI=TRUE, BcMV3.BI=FALSE} =>  {BcMV6.BI=FALSE} | 0.27  7228 | 0.835  821 | 0.331  683 | 1.20  1678 | 11  2 |
| {BcMV3.BI=FALSE, BcMV6.BI=FALSE} =>  {BcMV12=FALSE} | 0.33  1683 | 0.881  579 | 0.376  238 | 1.10  2656 | 13  4 |
| {BcMV12=FALSE, BcMV3.BI=FALSE} =>  {BcMV6.BI=FALSE} | 0.33  1683 | 0.807  229 | 0.410  891 | 1.16  0571 | 13  4 |
| {BcMV12=FALSE, BcMV6.BI=FALSE} =>  {BcMV3.BI=FALSE} | 0.33  1683 | 0.570  213 | 0.581  683 | 1.24  5221 | 13  4 |
| {BcMV3.BI=FALSE, BcNSRV7.BI=FALSE} => | 0.30 | 0.815 | 0.376 | 1.17 | 12 |

| {BcMV6.BI=FALSE} | 6931 | 789 | 238 | 2879 | 4 |
| --- | --- | --- | --- | --- | --- |
| {BcMV6.BI=FALSE, BcNSRV7.BI=FALSE} =>  {BcMV3.BI=FALSE} | 0.30  6931 | 0.541  485 | 0.566  832 | 1.18  2486 | 12  4 |
| {BcMV3.BI=FALSE, BcVV3.BI=FALSE} =>  {BcMV6.BI=FALSE} | 0.32  1782 | 0.833  333 | 0.386  139 | 1.19  8102 | 13  0 |
| {BcMV6.BI=FALSE, BcVV3.BI=FALSE} =>  {BcMV3.BI=FALSE} | 0.32  1782 | 0.562  771 | 0.571  782 | 1.22  8969 | 13  0 |
| {BcMV3.BI=FALSE, BcMV6.BI=FALSE} =>  {BcOLV7.BI=FALSE} | 0.32  9208 | 0.875 | 0.376  238 | 1.08  4356 | 13  3 |
| {BcMV3.BI=FALSE, BcOLV7.BI=FALSE} =>  {BcMV6.BI=FALSE} | 0.32  9208 | 0.841  772 | 0.391  089 | 1.21  0235 | 13  3 |
| {BcMV6.BI=FALSE, BcOLV7.BI=FALSE} =>  {BcMV3.BI=FALSE} | 0.32  9208 | 0.573  276 | 0.574  257 | 1.25  1911 | 13  3 |
| {BcALV1.BI=FALSE, BcMV3.BI=FALSE} =>  {BcMV6.BI=FALSE} | 0.32  9208 | 0.841  772 | 0.391  089 | 1.21  0235 | 13  3 |
| {BcALV1.BI=FALSE, BcMV6.BI=FALSE} =>  {BcMV3.BI=FALSE} | 0.32  9208 | 0.551  867 | 0.596  535 | 1.20  5159 | 13  3 |
| {BcMV3.BI=FALSE, BcMV6.BI=FALSE} =>  {BcDMV1=FALSE} | 0.34  1584 | 0.907  895 | 0.376  238 | 1.07  5629 | 13  8 |
| {BcDMV1=FALSE, BcMV3.BI=FALSE} =>  {BcMV6.BI=FALSE} | 0.34  1584 | 0.811  765 | 0.420  792 | 1.16  7092 | 13  8 |
| {BcDMV1=FALSE, BcMV6.BI=FALSE} => | 0.34 | 0.563 | 0.606 | 1.23 | 13 |

| {BcMV3.BI=FALSE} | 1584 | 265 | 436 | 005 | 8 |
| --- | --- | --- | --- | --- | --- |
| {BcFV9=FALSE, BcMV3.BI=FALSE} =>  {BcMV6.BI=FALSE} | 0.33  4158 | 0.808  383 | 0.413  366 | 1.16  2231 | 13  5 |
| {BcFV9=FALSE, BcMV6.BI=FALSE} =>  {BcMV3.BI=FALSE} | 0.33  4158 | 0.555  556 | 0.601  485 | 1.21  3213 | 13  5 |
| {BcMV3.BI=FALSE, BcOLV4.BI=FALSE} =>  {BcMV6.BI=FALSE} | 0.34  901 | 0.829  412 | 0.420  792 | 1.19  2464 | 14  1 |
| {BcMV6.BI=FALSE, BcOLV4.BI=FALSE} =>  {BcMV3.BI=FALSE} | 0.34  901 | 0.566  265 | 0.616  337 | 1.23  66 | 14  1 |
| {BcMV3.BI=FALSE, BcULV5=FALSE} =>  {BcMV6.BI=FALSE} | 0.34  6535 | 0.818  713 | 0.423  267 | 1.17  7083 | 14  0 |
| {BcMV6.BI=FALSE, BcULV5=FALSE} =>  {BcMV3.BI=FALSE} | 0.34  6535 | 0.557  769 | 0.621  287 | 1.21  8047 | 14  0 |
| {BcMV3.BI=FALSE, BcMV6.BI=FALSE} =>  {BcBNV5.BI=FALSE} | 0.36  6337 | 0.973  684 | 0.376  238 | 1.07  1849 | 14  8 |
| {BcBNV5.BI=FALSE, BcMV3.BI=FALSE} =>  {BcMV6.BI=FALSE} | 0.36  6337 | 0.836  158 | 0.438  119 | 1.20  2163 | 14  8 |
| {BcBNV5.BI=FALSE, BcMV6.BI=FALSE} =>  {BcMV3.BI=FALSE} | 0.36  6337 | 0.552  239 | 0.663  366 | 1.20  597 | 14  8 |
| {BcMV3.BI=FALSE, BvF.BI=FALSE} =>  {BcMV6.BI=FALSE} | 0.34  1584 | 0.816  568 | 0.418  317 | 1.17  3998 | 13  8 |
| {BcMV6.BI=FALSE, BvF.BI=FALSE} => | 0.34 | 0.547 | 0.623 | 1.19 | 13 |

| {BcMV3.BI=FALSE} | 1584 | 619 | 762 | 5882 | 8 |
| --- | --- | --- | --- | --- | --- |
| {BcMCV5.BI=FALSE, BcMV3.BI=FALSE} =>  {BcMV6.BI=FALSE} | 0.35  8911 | 0.814  607 | 0.440  594 | 1.17  1178 | 14  5 |
| {BcMCV5.BI=FALSE, BcMV6.BI=FALSE} =>  {BcMV3.BI=FALSE} | 0.35  8911 | 0.541  045 | 0.663  366 | 1.18  1525 | 14  5 |
| {BcMV3.BI=FALSE, BcOLV13.BI=FALSE} =>  {BcMV6.BI=FALSE} | 0.36  8812 | 0.818  681 | 0.450  495 | 1.17  7036 | 14  9 |
| {BcMV6.BI=FALSE, BcOLV13.BI=FALSE} =>  {BcMV3.BI=FALSE} | 0.36  8812 | 0.562  264 | 0.655  941 | 1.22  7863 | 14  9 |
| {BcMV3.BI=FALSE, BcOLV2.BI=FALSE} =>  {BcMV6.BI=FALSE} | 0.36  3861 | 0.821  229 | 0.443  069 | 1.18  0699 | 14  7 |
| {BcMV6.BI=FALSE, BcOLV2.BI=FALSE} =>  {BcMV3.BI=FALSE} | 0.36  3861 | 0.546  468 | 0.665  842 | 1.19  3369 | 14  7 |
| {BcMCV3.BI=FALSE, BcMV3.BI=FALSE} =>  {BcMV6.BI=FALSE} | 0.37  6238 | 0.821  622 | 0.457  921 | 1.18  1264 | 15  2 |
| {BcMCV3.BI=FALSE, BcMV6.BI=FALSE} =>  {BcMV3.BI=FALSE} | 0.37  6238 | 0.558  824 | 0.673  267 | 1.22  035 | 15  2 |
| {BcMV3.BI=FALSE, BcPV4.BI=FALSE} =>  {BcMV6.BI=FALSE} | 0.36  1386 | 0.829  545 | 0.435  644 | 1.19  2656 | 14  6 |
| {BcMV6.BI=FALSE, BcPV4.BI=FALSE} =>  {BcMV3.BI=FALSE} | 0.36  1386 | 0.550  943 | 0.655  941 | 1.20  3141 | 14  6 |
| {BcEV6=FALSE, BcMV3.BI=FALSE} => | 0.34 | 0.822 | 0.418 | 1.18 | 13 |

| {BcMV6.BI=FALSE} | 4059 | 485 | 317 | 2505 | 9 |
| --- | --- | --- | --- | --- | --- |
| {BcEV6=FALSE, BcMV6.BI=FALSE} =>  {BcMV3.BI=FALSE} | 0.34  4059 | 0.526  515 | 0.653  465 | 1.14  9795 | 13  9 |
| {BcMV3.BI=FALSE, BcOLV17.BI=FALSE} =>  {BcMV6.BI=FALSE} | 0.35  6436 | 0.818  182 | 0.435  644 | 1.17  6318 | 14  4 |
| {BcMV6.BI=FALSE, BcOLV17.BI=FALSE} =>  {BcMV3.BI=FALSE} | 0.35  6436 | 0.531  365 | 0.670  792 | 1.16  0387 | 14  4 |
| {BcMV3.BI=FALSE, BcOLV15.BI=FALSE} =>  {BcMV6.BI=FALSE} | 0.36  1386 | 0.815  642 | 0.443  069 | 1.17  2667 | 14  6 |
| {BcMV6.BI=FALSE, BcOLV15.BI=FALSE} =>  {BcMV3.BI=FALSE} | 0.36  1386 | 0.555  133 | 0.650  99 | 1.21  2291 | 14  6 |
| {BcEV5=FALSE, BcMV3.BI=FALSE} =>  {BcMV6.BI=FALSE} | 0.37  3762 | 0.820  652 | 0.455  446 | 1.17  987 | 15  1 |
| {BcEV5=FALSE, BcMV6.BI=FALSE} =>  {BcMV3.BI=FALSE} | 0.37  3762 | 0.545  126 | 0.685  644 | 1.19  0438 | 15  1 |
| {BcMV3.BI=FALSE, BcPV3.BI=FALSE} =>  {BcFV5.BI=FALSE} | 0.30  198 | 0.865  248 | 0.349  01 | 1.10  6203 | 12  2 |
| {BcFV5.BI=FALSE, BcPV3.BI=FALSE} =>  {BcMV3.BI=FALSE} | 0.30  198 | 0.552  036 | 0.547  03 | 1.20  5528 | 12  2 |
| {BcPV3.BI=FALSE, BcULV1.BI=FALSE} =>  {BcMV3.BI=FALSE} | 0.29  9505 | 0.523  81 | 0.571  782 | 1.14  3887 | 12  1 |
| {BcMV3.BI=FALSE, BcPV3.BI=FALSE} => | 0.30 | 0.886 | 0.349 | 1.10 | 12 |

| {BcMV12=FALSE} | 9406 | 525 | 01 | 8842 | 5 |
| --- | --- | --- | --- | --- | --- |
| {BcMV12=FALSE, BcPV3.BI=FALSE} =>  {BcMV3.BI=FALSE} | 0.30  9406 | 0.531  915 | 0.581  683 | 1.16  1587 | 12  5 |
| {BcMV3.BI=FALSE, BcPV3.BI=FALSE} =>  {BcDMV1=FALSE} | 0.31  9307 | 0.914  894 | 0.349  01 | 1.08  3921 | 12  9 |
| {BcDMV1=FALSE, BcPV3.BI=FALSE} =>  {BcMV3.BI=FALSE} | 0.31  9307 | 0.535  27 | 0.596  535 | 1.16  8913 | 12  9 |
| {BcBNV5.BI=FALSE, BcPV3.BI=FALSE} =>  {BcMV3.BI=FALSE} | 0.33  4158 | 0.519  231 | 0.643  564 | 1.13  3888 | 13  5 |
| {BcMCV3.BI=FALSE, BcPV3.BI=FALSE} =>  {BcMV3.BI=FALSE} | 0.34  901 | 0.512  727 | 0.680  693 | 1.11  9686 | 14  1 |
| {BcEV5=FALSE, BcPV3.BI=FALSE} =>  {BcMV3.BI=FALSE} | 0.34  6535 | 0.514  706 | 0.673  267 | 1.12  4006 | 14  0 |
| {BcFV5.BI=FALSE, BcRV1.BI=FALSE} =>  {BcMV3.BI=FALSE} | 0.30  198 | 0.554  545 | 0.544  554 | 1.21  1007 | 12  2 |
| {BcRV1.BI=FALSE, BcULV1.BI=FALSE} =>  {BcMV3.BI=FALSE} | 0.30  4455 | 0.521  186 | 0.584  158 | 1.13  8158 | 12  3 |
| {BcMV1.BI=TRUE, BcMV3.BI=FALSE} =>  {BcRV1.BI=FALSE} | 0.26  7327 | 0.805  97 | 0.331  683 | 1.11  5109 | 10  8 |
| {BcMV3.BI=FALSE, BcRV1.BI=FALSE} =>  {BcMV12=FALSE} | 0.31  9307 | 0.895  833 | 0.356  436 | 1.12  0485 | 12  9 |
| {BcMV12=FALSE, BcRV1.BI=FALSE} => | 0.31 | 0.551 | 0.579 | 1.20 | 12 |

| {BcMV3.BI=FALSE} | 9307 | 282 | 208 | 3881 | 9 |
| --- | --- | --- | --- | --- | --- |
| {BcRV1.BI=FALSE, BcVV3.BI=FALSE} =>  {BcMV3.BI=FALSE} | 0.30  198 | 0.530  435 | 0.569  307 | 1.15  8355 | 12  2 |
| {BcMV3.BI=FALSE, BcRV1.BI=FALSE} =>  {BcOLV7.BI=FALSE} | 0.32  1782 | 0.902  778 | 0.356  436 | 1.11  878 | 13  0 |
| {BcMV3.BI=FALSE, BcOLV7.BI=FALSE} =>  {BcRV1.BI=FALSE} | 0.32  1782 | 0.822  785 | 0.391  089 | 1.13  8374 | 13  0 |
| {BcMV3.BI=FALSE, BcRV1.BI=FALSE} =>  {BcALV1.BI=FALSE} | 0.31  6832 | 0.888  889 | 0.356  436 | 1.09  1523 | 12  8 |
| {BcALV1.BI=FALSE, BcMV3.BI=FALSE} =>  {BcRV1.BI=FALSE} | 0.31  6832 | 0.810  127 | 0.391  089 | 1.12  086 | 12  8 |
| {BcMV3.BI=FALSE, BcRV1.BI=FALSE} =>  {BcDMV1=FALSE} | 0.32  1782 | 0.902  778 | 0.356  436 | 1.06  9567 | 13  0 |
| {BcDMV1=FALSE, BcRV1.BI=FALSE} =>  {BcMV3.BI=FALSE} | 0.32  1782 | 0.548  523 | 0.586  634 | 1.19  7856 | 13  0 |
| {BcRV1.BI=FALSE, BcULV5=FALSE} =>  {BcMV3.BI=FALSE} | 0.32  6733 | 0.519  685 | 0.628  713 | 1.13  488 | 13  2 |
| {BcBNV5.BI=FALSE, BcRV1.BI=FALSE} =>  {BcMV3.BI=FALSE} | 0.34  4059 | 0.530  534 | 0.648  515 | 1.15  8572 | 13  9 |
| {BcOLV13.BI=FALSE, BcRV1.BI=FALSE} =>  {BcMV3.BI=FALSE} | 0.35  396 | 0.512  545 | 0.690  594 | 1.11  9287 | 14  3 |
| {BcMCV3.BI=FALSE, BcRV1.BI=FALSE} => | 0.35 | 0.523 | 0.680 | 1.14 | 14 |

| {BcMV3.BI=FALSE} | 6436 | 636 | 693 | 3509 | 4 |
| --- | --- | --- | --- | --- | --- |
| {BcEV5=FALSE, BcRV1.BI=FALSE} =>  {BcMV3.BI=FALSE} | 0.35  396 | 0.525  735 | 0.673  267 | 1.14  8092 | 14  3 |
| {BcEV2.BI=FALSE, BcMV3.BI=FALSE} =>  {BcHV3.BI=FALSE} | 0.28  9604 | 0.841  727 | 0.344  059 | 1.10  7679 | 11  7 |
| {BcEV2.BI=FALSE, BcMV3.BI=FALSE} =>  {BcFV5.BI=FALSE} | 0.30  9406 | 0.899  281 | 0.344  059 | 1.14  9713 | 12  5 |
| {BcEV2.BI=FALSE, BcMV3.BI=FALSE} =>  {BcULV1.BI=FALSE} | 0.29  703 | 0.863  309 | 0.344  059 | 1.10  0243 | 12  0 |
| {BcEV2.BI=FALSE, BcMV3.BI=FALSE} =>  {BcMV12=FALSE} | 0.30  9406 | 0.899  281 | 0.344  059 | 1.12  4797 | 12  5 |
| {BcEV2.BI=FALSE, BcMV12=FALSE} =>  {BcMV3.BI=FALSE} | 0.30  9406 | 0.531  915 | 0.581  683 | 1.16  1587 | 12  5 |
| {BcEV2.BI=FALSE, BcMV3.BI=FALSE} =>  {BcVV3.BI=FALSE} | 0.30  6931 | 0.892  086 | 0.344  059 | 1.11  2355 | 12  4 |
| {BcEV2.BI=FALSE, BcMV3.BI=FALSE} =>  {BcDMV1=FALSE} | 0.31  1881 | 0.906  475 | 0.344  059 | 1.07  3947 | 12  6 |
| {BcEV2.BI=FALSE, BcMV3.BI=FALSE} =>  {BcFV9=FALSE} | 0.32  1782 | 0.935  252 | 0.344  059 | 1.07  6472 | 13  0 |
| {BcEV2.BI=FALSE, BcMV3.BI=FALSE} =>  {BcULV5=FALSE} | 0.32  1782 | 0.935  252 | 0.344  059 | 1.06  735 | 13  0 |
| {BcHV3.BI=FALSE, BcMV3.BI=FALSE} => | 0.32 | 0.885 | 0.366 | 1.13 | 13 |

| {BcFV5.BI=FALSE} | 4257 | 135 | 337 | 1628 | 1 |
| --- | --- | --- | --- | --- | --- |
| {BcHV3.BI=FALSE, BcMV3.BI=FALSE} =>  {BcULV1.BI=FALSE} | 0.31  6832 | 0.864  865 | 0.366  337 | 1.10  2225 | 12  8 |
| {BcHV3.BI=FALSE, BcULV1.BI=FALSE} =>  {BcMV3.BI=FALSE} | 0.31  6832 | 0.524  59 | 0.603  96 | 1.14  5591 | 12  8 |
| {BcHV3.BI=FALSE, BcMV3.BI=FALSE} =>  {BcMV12=FALSE} | 0.33  1683 | 0.905  405 | 0.366  337 | 1.13  2458 | 13  4 |
| {BcHV3.BI=FALSE, BcMV12=FALSE} =>  {BcMV3.BI=FALSE} | 0.33  1683 | 0.551  44 | 0.601  485 | 1.20  4226 | 13  4 |
| {BcHV3.BI=FALSE, BcMV3.BI=FALSE} =>  {BcVV3.BI=FALSE} | 0.32  4257 | 0.885  135 | 0.366  337 | 1.10  3687 | 13  1 |
| {BcMV3.BI=FALSE, BcVV3.BI=FALSE} =>  {BcHV3.BI=FALSE} | 0.32  4257 | 0.839  744 | 0.386  139 | 1.10  507 | 13  1 |
| {BcHV3.BI=FALSE, BcMV3.BI=FALSE} =>  {BcDMV1=FALSE} | 0.34  1584 | 0.932  432 | 0.366  337 | 1.10  47 | 13  8 |
| {BcHV3.BI=FALSE, BcMV3.BI=FALSE} =>  {BcOLV4.BI=FALSE} | 0.34  1584 | 0.932  432 | 0.366  337 | 1.06  4132 | 13  8 |
| {BcHV3.BI=FALSE, BcMV3.BI=FALSE} =>  {BcULV5=FALSE} | 0.34  6535 | 0.945  946 | 0.366  337 | 1.07  9554 | 14  0 |
| {BcHV3.BI=FALSE, BcULV5=FALSE} =>  {BcMV3.BI=FALSE} | 0.34  6535 | 0.518  519 | 0.668  317 | 1.13  2332 | 14  0 |
| {BcHV3.BI=FALSE, BcMCV3.BI=FALSE} => | 0.36 | 0.526 | 0.695 | 1.15 | 14 |

| {BcMV3.BI=FALSE} | 6337 | 69 | 545 | 0178 | 8 |
| --- | --- | --- | --- | --- | --- |
| {BcFV5.BI=FALSE, BcMV3.BI=FALSE} =>  {BcULV1.BI=FALSE} | 0.33  6634 | 0.855  346 | 0.393  564 | 1.09  0094 | 13  6 |
| {BcMV3.BI=FALSE, BcULV1.BI=FALSE} =>  {BcFV5.BI=FALSE} | 0.33  6634 | 0.855  346 | 0.393  564 | 1.09  3544 | 13  6 |
| {BcFV5.BI=FALSE, BcULV1.BI=FALSE} =>  {BcMV3.BI=FALSE} | 0.33  6634 | 0.550  607 | 0.611  386 | 1.20  2407 | 13  6 |
| {BcMV1.BI=TRUE, BcMV3.BI=FALSE} =>  {BcFV5.BI=FALSE} | 0.28  4653 | 0.858  209 | 0.331  683 | 1.09  7204 | 11  5 |
| {BcFV5.BI=FALSE, BcMV3.BI=FALSE} =>  {BcMV12=FALSE} | 0.36  3861 | 0.924  528 | 0.393  564 | 1.15  6376 | 14  7 |
| {BcMV12=FALSE, BcMV3.BI=FALSE} =>  {BcFV5.BI=FALSE} | 0.36  3861 | 0.885  542 | 0.410  891 | 1.13  2149 | 14  7 |
| {BcFV5.BI=FALSE, BcMV12=FALSE} =>  {BcMV3.BI=FALSE} | 0.36  3861 | 0.581  028 | 0.626  238 | 1.26  8839 | 14  7 |
| {BcMV3.BI=FALSE, BcNSRV7.BI=FALSE} =>  {BcFV5.BI=FALSE} | 0.33  4158 | 0.888  158 | 0.376  238 | 1.13  5493 | 13  5 |
| {BcMV3.BI=FALSE, BcOLV7.BI=FALSE} =>  {BcFV5.BI=FALSE} | 0.33  4158 | 0.854  43 | 0.391  089 | 1.09  2373 | 13  5 |
| {BcFV5.BI=FALSE, BcOLV7.BI=FALSE} =>  {BcMV3.BI=FALSE} | 0.33  4158 | 0.535  714 | 0.623  762 | 1.16  9884 | 13  5 |
| {BcALV1.BI=FALSE, BcMV3.BI=FALSE} => | 0.33 | 0.860 | 0.391 | 1.10 | 13 |

| {BcFV5.BI=FALSE} | 6634 | 759 | 089 | 0465 | 6 |
| --- | --- | --- | --- | --- | --- |
| {BcFV5.BI=FALSE, BcMV3.BI=FALSE} =>  {BcDMV1=FALSE} | 0.36  6337 | 0.930  818 | 0.393  564 | 1.10  2787 | 14  8 |
| {BcDMV1=FALSE, BcMV3.BI=FALSE} =>  {BcFV5.BI=FALSE} | 0.36  6337 | 0.870  588 | 0.420  792 | 1.11  3031 | 14  8 |
| {BcDMV1=FALSE, BcFV5.BI=FALSE} =>  {BcMV3.BI=FALSE} | 0.36  6337 | 0.521  127 | 0.702  97 | 1.13  8028 | 14  8 |
| {BcFV9=FALSE, BcMV3.BI=FALSE} =>  {BcFV5.BI=FALSE} | 0.35  8911 | 0.868  263 | 0.413  366 | 1.11  0058 | 14  5 |
| {BcFV5.BI=FALSE, BcFV9=FALSE} =>  {BcMV3.BI=FALSE} | 0.35  8911 | 0.514  184 | 0.698  02 | 1.12  2868 | 14  5 |
| {BcMV3.BI=FALSE, BcOLV4.BI=FALSE} =>  {BcFV5.BI=FALSE} | 0.36  1386 | 0.858  824 | 0.420  792 | 1.09  799 | 14  6 |
| {BcFV5.BI=FALSE, BcOLV4.BI=FALSE} =>  {BcMV3.BI=FALSE} | 0.36  1386 | 0.530  909 | 0.680  693 | 1.15  9391 | 14  6 |
| {BcMV3.BI=FALSE, BcULV5=FALSE} =>  {BcFV5.BI=FALSE} | 0.36  3861 | 0.859  649 | 0.423  267 | 1.09  9045 | 14  7 |
| {BcFV5.BI=FALSE, BcULV5=FALSE} =>  {BcMV3.BI=FALSE} | 0.36  3861 | 0.534  545 | 0.680  693 | 1.16  7332 | 14  7 |
| {BcBNV5.BI=FALSE, BcMV3.BI=FALSE} =>  {BcFV5.BI=FALSE} | 0.37  8713 | 0.864  407 | 0.438  119 | 1.10  5128 | 15  3 |
| {BcBNV5.BI=FALSE, BcFV5.BI=FALSE} => | 0.37 | 0.508 | 0.745 | 1.11 | 15 |

| {BcMV3.BI=FALSE} | 8713 | 306 | 05 | 003 | 3 |
| --- | --- | --- | --- | --- | --- |
| {BcMV3.BI=FALSE, BvF.BI=FALSE} =>  {BcFV5.BI=FALSE} | 0.36  3861 | 0.869  822 | 0.418  317 | 1.11  2052 | 14  7 |
| {BcMCV5.BI=FALSE, BcMV3.BI=FALSE} =>  {BcFV5.BI=FALSE} | 0.37  8713 | 0.859  551 | 0.440  594 | 1.09  8919 | 15  3 |
| {BcFV5.BI=FALSE, BcMCV5.BI=FALSE} =>  {BcMV3.BI=FALSE} | 0.37  8713 | 0.51 | 0.742  574 | 1.11  373 | 15  3 |
| {BcMV3.BI=FALSE, BcOLV13.BI=FALSE} =>  {BcFV5.BI=FALSE} | 0.38  8614 | 0.862  637 | 0.450  495 | 1.10  2865 | 15  7 |
| {BcFV5.BI=FALSE, BcOLV13.BI=FALSE} =>  {BcMV3.BI=FALSE} | 0.38  8614 | 0.541  379 | 0.717  822 | 1.18  2255 | 15  7 |
| {BcMV3.BI=FALSE, BcOLV2.BI=FALSE} =>  {BcFV5.BI=FALSE} | 0.38  3663 | 0.865  922 | 0.443  069 | 1.10  7065 | 15  5 |
| {BcFV5.BI=FALSE, BcOLV2.BI=FALSE} =>  {BcMV3.BI=FALSE} | 0.38  3663 | 0.527  211 | 0.727  723 | 1.15  1315 | 15  5 |
| {BcMCV3.BI=FALSE, BcMV3.BI=FALSE} =>  {BcFV5.BI=FALSE} | 0.39  3564 | 0.859  459 | 0.457  921 | 1.09  8803 | 15  9 |
| {BcFV5.BI=FALSE, BcMCV3.BI=FALSE} =>  {BcMV3.BI=FALSE} | 0.39  3564 | 0.542  662 | 0.725  248 | 1.18  5057 | 15  9 |
| {BcMV3.BI=FALSE, BcPV4.BI=FALSE} =>  {BcFV5.BI=FALSE} | 0.37  3762 | 0.857  955 | 0.435  644 | 1.09  6879 | 15  1 |
| {BcFV5.BI=FALSE, BcPV4.BI=FALSE} => | 0.37 | 0.510 | 0.732 | 1.11 | 15 |

| {BcMV3.BI=FALSE} | 3762 | 135 | 673 | 4025 | 1 |
| --- | --- | --- | --- | --- | --- |
| {BcEV6=FALSE, BcMV3.BI=FALSE} =>  {BcFV5.BI=FALSE} | 0.35  6436 | 0.852  071 | 0.418  317 | 1.08  9357 | 14  4 |
| {BcMV3.BI=FALSE, BcOLV17.BI=FALSE} =>  {BcFV5.BI=FALSE} | 0.37  3762 | 0.857  955 | 0.435  644 | 1.09  6879 | 15  1 |
| {BcFV5.BI=FALSE, BcOLV17.BI=FALSE} =>  {BcMV3.BI=FALSE} | 0.37  3762 | 0.508  418 | 0.735  149 | 1.11  0274 | 15  1 |
| {BcMV3.BI=FALSE, BcOLV15.BI=FALSE} =>  {BcFV5.BI=FALSE} | 0.38  8614 | 0.877  095 | 0.443  069 | 1.12  1349 | 15  7 |
| {BcFV5.BI=FALSE, BcOLV15.BI=FALSE} =>  {BcMV3.BI=FALSE} | 0.38  8614 | 0.504  823 | 0.769  802 | 1.10  2425 | 15  7 |
| {BcEV5=FALSE, BcMV3.BI=FALSE} =>  {BcFV5.BI=FALSE} | 0.39  1089 | 0.858  696 | 0.455  446 | 1.09  7826 | 15  8 |
| {BcEV5=FALSE, BcFV5.BI=FALSE} =>  {BcMV3.BI=FALSE} | 0.39  1089 | 0.506  41 | 0.772  277 | 1.10  5891 | 15  8 |
| {BcMV3.BI=FALSE, BcULV1.BI=FALSE} =>  {BcMV12=FALSE} | 0.35  6436 | 0.905  66 | 0.393  564 | 1.13  2776 | 14  4 |
| {BcMV12=FALSE, BcMV3.BI=FALSE} =>  {BcULV1.BI=FALSE} | 0.35  6436 | 0.867  47 | 0.410  891 | 1.10  5545 | 14  4 |
| {BcMV12=FALSE, BcULV1.BI=FALSE} =>  {BcMV3.BI=FALSE} | 0.35  6436 | 0.545  455 | 0.653  465 | 1.19  1155 | 14  4 |
| {BcNSRV7.BI=FALSE, BcULV1.BI=FALSE} => | 0.31 | 0.520 | 0.613 | 1.13 | 12 |

| {BcMV3.BI=FALSE} | 9307 | 161 | 861 | 592 | 9 |
| --- | --- | --- | --- | --- | --- |
| {BcMV3.BI=FALSE, BcVV3.BI=FALSE} =>  {BcULV1.BI=FALSE} | 0.33  4158 | 0.865  385 | 0.386  139 | 1.10  2888 | 13  5 |
| {BcULV1.BI=FALSE, BcVV3.BI=FALSE} =>  {BcMV3.BI=FALSE} | 0.33  4158 | 0.531  496 | 0.628  713 | 1.16  0672 | 13  5 |
| {BcMV3.BI=FALSE, BcOLV7.BI=FALSE} =>  {BcULV1.BI=FALSE} | 0.33  4158 | 0.854  43 | 0.391  089 | 1.08  8927 | 13  5 |
| {BcOLV7.BI=FALSE, BcULV1.BI=FALSE} =>  {BcMV3.BI=FALSE} | 0.33  4158 | 0.513  308 | 0.650  99 | 1.12  0954 | 13  5 |
| {BcALV1.BI=FALSE, BcMV3.BI=FALSE} =>  {BcULV1.BI=FALSE} | 0.33  4158 | 0.854  43 | 0.391  089 | 1.08  8927 | 13  5 |
| {BcALV1.BI=FALSE, BcULV1.BI=FALSE} =>  {BcMV3.BI=FALSE} | 0.33  4158 | 0.515  267 | 0.648  515 | 1.12  5232 | 13  5 |
| {BcMV3.BI=FALSE, BcULV1.BI=FALSE} =>  {BcDMV1=FALSE} | 0.36  1386 | 0.918  239 | 0.393  564 | 1.08  7884 | 14  6 |
| {BcDMV1=FALSE, BcMV3.BI=FALSE} =>  {BcULV1.BI=FALSE} | 0.36  1386 | 0.858  824 | 0.420  792 | 1.09  4526 | 14  6 |
| {BcDMV1=FALSE, BcULV1.BI=FALSE} =>  {BcMV3.BI=FALSE} | 0.36  1386 | 0.557  252 | 0.648  515 | 1.21  6918 | 14  6 |
| {BcFV9=FALSE, BcMV3.BI=FALSE} =>  {BcULV1.BI=FALSE} | 0.35  8911 | 0.868  263 | 0.413  366 | 1.10  6557 | 14  5 |
| {BcFV9=FALSE, BcULV1.BI=FALSE} => | 0.35 | 0.529 | 0.678 | 1.15 | 14 |

| {BcMV3.BI=FALSE} | 8911 | 197 | 218 | 5652 | 5 |
| --- | --- | --- | --- | --- | --- |
| {BcMV3.BI=FALSE, BcOLV4.BI=FALSE} =>  {BcULV1.BI=FALSE} | 0.36  3861 | 0.864  706 | 0.420  792 | 1.10  2023 | 14  7 |
| {BcOLV4.BI=FALSE, BcULV1.BI=FALSE} =>  {BcMV3.BI=FALSE} | 0.36  3861 | 0.523  132 | 0.695  545 | 1.14  2406 | 14  7 |
| {BcMV3.BI=FALSE, BcULV1.BI=FALSE} =>  {BcULV5=FALSE} | 0.36  6337 | 0.930  818 | 0.393  564 | 1.06  2289 | 14  8 |
| {BcMV3.BI=FALSE, BcULV5=FALSE} =>  {BcULV1.BI=FALSE} | 0.36  6337 | 0.865  497 | 0.423  267 | 1.10  3031 | 14  8 |
| {BcULV1.BI=FALSE, BcULV5=FALSE} =>  {BcMV3.BI=FALSE} | 0.36  6337 | 0.522  968 | 0.700  495 | 1.14  2049 | 14  8 |
| {BcBNV5.BI=FALSE, BcMV3.BI=FALSE} =>  {BcULV1.BI=FALSE} | 0.37  8713 | 0.864  407 | 0.438  119 | 1.10  1641 | 15  3 |
| {BcBNV5.BI=FALSE, BcULV1.BI=FALSE} =>  {BcMV3.BI=FALSE} | 0.37  8713 | 0.531  25 | 0.712  871 | 1.16  0135 | 15  3 |
| {BcMV3.BI=FALSE, BvF.BI=FALSE} =>  {BcULV1.BI=FALSE} | 0.35  8911 | 0.857  988 | 0.418  317 | 1.09  3461 | 14  5 |
| {BcMCV5.BI=FALSE, BcMV3.BI=FALSE} =>  {BcULV1.BI=FALSE} | 0.38  1188 | 0.865  169 | 0.440  594 | 1.10  2612 | 15  4 |
| {BcMCV5.BI=FALSE, BcULV1.BI=FALSE} =>  {BcMV3.BI=FALSE} | 0.38  1188 | 0.522  034 | 0.730  198 | 1.14  0009 | 15  4 |
| {BcMV3.BI=FALSE, BcOLV13.BI=FALSE} => | 0.38 | 0.862 | 0.450 | 1.09 | 15 |

| {BcULV1.BI=FALSE} | 8614 | 637 | 495 | 9386 | 7 |
| --- | --- | --- | --- | --- | --- |
| {BcOLV13.BI=FALSE, BcULV1.BI=FALSE} =>  {BcMV3.BI=FALSE} | 0.38  8614 | 0.525  084 | 0.740  099 | 1.14  6669 | 15  7 |
| {BcMV3.BI=FALSE, BcOLV2.BI=FALSE} =>  {BcULV1.BI=FALSE} | 0.38  1188 | 0.860  335 | 0.443  069 | 1.09  6452 | 15  4 |
| {BcOLV2.BI=FALSE, BcULV1.BI=FALSE} =>  {BcMV3.BI=FALSE} | 0.38  1188 | 0.513  333 | 0.742  574 | 1.12  1009 | 15  4 |
| {BcMCV3.BI=FALSE, BcMV3.BI=FALSE} =>  {BcULV1.BI=FALSE} | 0.39  3564 | 0.859  459 | 0.457  921 | 1.09  5336 | 15  9 |
| {BcMCV3.BI=FALSE, BcULV1.BI=FALSE} =>  {BcMV3.BI=FALSE} | 0.39  3564 | 0.514  563 | 0.764  851 | 1.12  3695 | 15  9 |
| {BcMV3.BI=FALSE, BcPV4.BI=FALSE} =>  {BcULV1.BI=FALSE} | 0.37  6238 | 0.863  636 | 0.435  644 | 1.10  066 | 15  2 |
| {BcPV4.BI=FALSE, BcULV1.BI=FALSE} =>  {BcMV3.BI=FALSE} | 0.37  6238 | 0.506  667 | 0.742  574 | 1.10  645 | 15  2 |
| {BcEV6=FALSE, BcMV3.BI=FALSE} =>  {BcULV1.BI=FALSE} | 0.36  6337 | 0.875  74 | 0.418  317 | 1.11  6085 | 14  8 |
| {BcMV3.BI=FALSE, BcOLV17.BI=FALSE} =>  {BcULV1.BI=FALSE} | 0.37  6238 | 0.863  636 | 0.435  644 | 1.10  066 | 15  2 |
| {BcMV3.BI=FALSE, BcOLV15.BI=FALSE} =>  {BcULV1.BI=FALSE} | 0.38  1188 | 0.860  335 | 0.443  069 | 1.09  6452 | 15  4 |
| {BcOLV15.BI=FALSE, BcULV1.BI=FALSE} => | 0.38 | 0.515 | 0.740 | 1.12 | 15 |

| {BcMV3.BI=FALSE} | 1188 | 05 | 099 | 4758 | 4 |
| --- | --- | --- | --- | --- | --- |
| {BcEV5=FALSE, BcMV3.BI=FALSE} =>  {BcULV1.BI=FALSE} | 0.39  1089 | 0.858  696 | 0.455  446 | 1.09  4363 | 15  8 |
| {BcEV5=FALSE, BcULV1.BI=FALSE} =>  {BcMV3.BI=FALSE} | 0.39  1089 | 0.526  667 | 0.742  574 | 1.15  0126 | 15  8 |
| {BcMV1.BI=TRUE, BcMV3.BI=FALSE} =>  {BcMV12=FALSE} | 0.29  9505 | 0.902  985 | 0.331  683 | 1.12  943 | 12  1 |
| {BcMV1.BI=TRUE, BcMV3.BI=FALSE} =>  {BcDMV1=FALSE} | 0.30  4455 | 0.917  91 | 0.331  683 | 1.08  7495 | 12  3 |
| {BcMV3.BI=FALSE, BcNSRV7.BI=FALSE} =>  {BcMV12=FALSE} | 0.34  1584 | 0.907  895 | 0.376  238 | 1.13  5571 | 13  8 |
| {BcMV12=FALSE, BcNSRV7.BI=FALSE} =>  {BcMV3.BI=FALSE} | 0.34  1584 | 0.528  736 | 0.646  04 | 1.15  4644 | 13  8 |
| {BcMV3.BI=FALSE, BcVV3.BI=FALSE} =>  {BcMV12=FALSE} | 0.34  6535 | 0.897  436 | 0.386  139 | 1.12  2489 | 14  0 |
| {BcMV12=FALSE, BcVV3.BI=FALSE} =>  {BcMV3.BI=FALSE} | 0.34  6535 | 0.542  636 | 0.638  614 | 1.18  4999 | 14  0 |
| {BcMV3.BI=FALSE, BcOLV7.BI=FALSE} =>  {BcMV12=FALSE} | 0.35  1485 | 0.898  734 | 0.391  089 | 1.12  4113 | 14  2 |
| {BcMV12=FALSE, BcOLV7.BI=FALSE} =>  {BcMV3.BI=FALSE} | 0.35  1485 | 0.535  849 | 0.655  941 | 1.17  0178 | 14  2 |
| {BcMV12=FALSE, BcMV3.BI=FALSE} => | 0.35 | 0.873 | 0.410 | 1.07 | 14 |

| {BcALV1.BI=FALSE} | 8911 | 494 | 891 | 2619 | 5 |
| --- | --- | --- | --- | --- | --- |
| {BcALV1.BI=FALSE, BcMV3.BI=FALSE} =>  {BcMV12=FALSE} | 0.35  8911 | 0.917  722 | 0.391  089 | 1.14  7862 | 14  5 |
| {BcALV1.BI=FALSE, BcMV12=FALSE} =>  {BcMV3.BI=FALSE} | 0.35  8911 | 0.549  242 | 0.653  465 | 1.19  9427 | 14  5 |
| {BcMV12=FALSE, BcMV3.BI=FALSE} =>  {BcDMV1=FALSE} | 0.38  1188 | 0.927  711 | 0.410  891 | 1.09  9106 | 15  4 |
| {BcDMV1=FALSE, BcMV3.BI=FALSE} =>  {BcMV12=FALSE} | 0.38  1188 | 0.905  882 | 0.420  792 | 1.13  3054 | 15  4 |
| {BcDMV1=FALSE, BcMV12=FALSE} =>  {BcMV3.BI=FALSE} | 0.38  1188 | 0.570  37 | 0.668  317 | 1.24  5566 | 15  4 |
| {BcFV9=FALSE, BcMV3.BI=FALSE} =>  {BcMV12=FALSE} | 0.37  1287 | 0.898  204 | 0.413  366 | 1.12  345 | 15  0 |
| {BcFV9=FALSE, BcMV12=FALSE} =>  {BcMV3.BI=FALSE} | 0.37  1287 | 0.533  808 | 0.695  545 | 1.16  5721 | 15  0 |
| {BcMV3.BI=FALSE, BcOLV4.BI=FALSE} =>  {BcMV12=FALSE} | 0.37  6238 | 0.894  118 | 0.420  792 | 1.11  8339 | 15  2 |
| {BcMV12=FALSE, BcOLV4.BI=FALSE} =>  {BcMV3.BI=FALSE} | 0.37  6238 | 0.527  778 | 0.712  871 | 1.15  2553 | 15  2 |
| {BcMV3.BI=FALSE, BcULV5=FALSE} =>  {BcMV12=FALSE} | 0.37  8713 | 0.894  737 | 0.423  267 | 1.11  9114 | 15  3 |
| {BcMV12=FALSE, BcULV5=FALSE} => | 0.37 | 0.529 | 0.715 | 1.15 | 15 |

| {BcMV3.BI=FALSE} | 8713 | 412 | 347 | 6121 | 3 |
| --- | --- | --- | --- | --- | --- |
| {BcBNV5.BI=FALSE, BcMV3.BI=FALSE} =>  {BcMV12=FALSE} | 0.39  604 | 0.903  955 | 0.438  119 | 1.13  0643 | 16  0 |
| {BcBNV5.BI=FALSE, BcMV12=FALSE} =>  {BcMV3.BI=FALSE} | 0.39  604 | 0.547  945 | 0.722  772 | 1.19  6594 | 16  0 |
| {BcMV3.BI=FALSE, BvF.BI=FALSE} =>  {BcMV12=FALSE} | 0.37  3762 | 0.893  491 | 0.418  317 | 1.11  7555 | 15  1 |
| {BcMV12=FALSE, BvF.BI=FALSE} =>  {BcMV3.BI=FALSE} | 0.37  3762 | 0.520  69 | 0.717  822 | 1.13  7074 | 15  1 |
| {BcMCV5.BI=FALSE, BcMV3.BI=FALSE} =>  {BcMV12=FALSE} | 0.39  3564 | 0.893  258 | 0.440  594 | 1.11  7264 | 15  9 |
| {BcMCV5.BI=FALSE, BcMV12=FALSE} =>  {BcMV3.BI=FALSE} | 0.39  3564 | 0.533  557 | 0.737  624 | 1.16  5173 | 15  9 |
| {BcMV3.BI=FALSE, BcOLV13.BI=FALSE} =>  {BcMV12=FALSE} | 0.40  3465 | 0.895  604 | 0.450  495 | 1.12  0199 | 16  3 |
| {BcMV12=FALSE, BcOLV13.BI=FALSE} =>  {BcMV3.BI=FALSE} | 0.40  3465 | 0.534  426 | 0.754  95 | 1.16  7071 | 16  3 |
| {BcMV3.BI=FALSE, BcOLV2.BI=FALSE} =>  {BcMV12=FALSE} | 0.39  8515 | 0.899  441 | 0.443  069 | 1.12  4998 | 16  1 |
| {BcMV12=FALSE, BcOLV2.BI=FALSE} =>  {BcMV3.BI=FALSE} | 0.39  8515 | 0.526  144 | 0.757  426 | 1.14  8984 | 16  1 |
| {BcMCV3.BI=FALSE, BcMV3.BI=FALSE} => | 0.41 | 0.897 | 0.457 | 1.12 | 16 |

| {BcMV12=FALSE} | 0891 | 297 | 921 | 2316 | 6 |
| --- | --- | --- | --- | --- | --- |
| {BcMCV3.BI=FALSE, BcMV12=FALSE} =>  {BcMV3.BI=FALSE} | 0.41  0891 | 0.526  984 | 0.779  703 | 1.15  0819 | 16  6 |
| {BcMV3.BI=FALSE, BcPV4.BI=FALSE} =>  {BcMV12=FALSE} | 0.39  604 | 0.909  091 | 0.435  644 | 1.13  7067 | 16  0 |
| {BcMV12=FALSE, BcPV4.BI=FALSE} =>  {BcMV3.BI=FALSE} | 0.39  604 | 0.522  876 | 0.757  426 | 1.14  1848 | 16  0 |
| {BcEV6=FALSE, BcMV3.BI=FALSE} =>  {BcMV12=FALSE} | 0.37  8713 | 0.905  325 | 0.418  317 | 1.13  2358 | 15  3 |
| {BcEV6=FALSE, BcMV12=FALSE} =>  {BcMV3.BI=FALSE} | 0.37  8713 | 0.51 | 0.742  574 | 1.11  373 | 15  3 |
| {BcMV3.BI=FALSE, BcOLV17.BI=FALSE} =>  {BcMV12=FALSE} | 0.38  8614 | 0.892  045 | 0.435  644 | 1.11  5747 | 15  7 |
| {BcMV12=FALSE, BcOLV17.BI=FALSE} =>  {BcMV3.BI=FALSE} | 0.38  8614 | 0.511  401 | 0.759  901 | 1.11  6788 | 15  7 |
| {BcMV3.BI=FALSE, BcOLV15.BI=FALSE} =>  {BcMV12=FALSE} | 0.39  8515 | 0.899  441 | 0.443  069 | 1.12  4998 | 16  1 |
| {BcMV12=FALSE, BcOLV15.BI=FALSE} =>  {BcMV3.BI=FALSE} | 0.39  8515 | 0.526  144 | 0.757  426 | 1.14  8984 | 16  1 |
| {BcEV5=FALSE, BcMV3.BI=FALSE} =>  {BcMV12=FALSE} | 0.40  8416 | 0.896  739 | 0.455  446 | 1.12  1618 | 16  5 |
| {BcEV5=FALSE, BcMV12=FALSE} => | 0.40 | 0.540 | 0.754 | 1.18 | 16 |

| {BcMV3.BI=FALSE} | 8416 | 984 | 95 | 1391 | 5 |
| --- | --- | --- | --- | --- | --- |
| {BcMV3.BI=FALSE, BcNSRV7.BI=FALSE} =>  {BcDMV1=FALSE} | 0.35  1485 | 0.934  211 | 0.376  238 | 1.10  6807 | 14  2 |
| {BcMV3.BI=FALSE, BcNSRV7.BI=FALSE} =>  {BcFV9=FALSE} | 0.34  901 | 0.927  632 | 0.376  238 | 1.06  7701 | 14  1 |
| {BcMCV3.BI=FALSE, BcNSRV7.BI=FALSE}  => {BcMV3.BI=FALSE} | 0.37  6238 | 0.511  785 | 0.735  149 | 1.11  7627 | 15  2 |
| {BcMV3.BI=FALSE, BcVV3.BI=FALSE} =>  {BcDMV1=FALSE} | 0.35  1485 | 0.910  256 | 0.386  139 | 1.07  8427 | 14  2 |
| {BcMV3.BI=FALSE, BcVV3.BI=FALSE} =>  {BcULV5=FALSE} | 0.37  1287 | 0.961  538 | 0.386  139 | 1.09  7349 | 15  0 |
| {BcMV3.BI=FALSE, BcULV5=FALSE} =>  {BcVV3.BI=FALSE} | 0.37  1287 | 0.877  193 | 0.423  267 | 1.09  3784 | 15  0 |
| {BcULV5=FALSE, BcVV3.BI=FALSE} =>  {BcMV3.BI=FALSE} | 0.37  1287 | 0.517  241 | 0.717  822 | 1.12  9543 | 15  0 |
| {BcMCV3.BI=FALSE, BcVV3.BI=FALSE} =>  {BcMV3.BI=FALSE} | 0.38  6139 | 0.521  739 | 0.740  099 | 1.13  9365 | 15  6 |
| {BcMV3.BI=FALSE, BcOLV7.BI=FALSE} =>  {BcALV1.BI=FALSE} | 0.34  4059 | 0.879  747 | 0.391  089 | 1.08  0297 | 13  9 |
| {BcALV1.BI=FALSE, BcMV3.BI=FALSE} =>  {BcOLV7.BI=FALSE} | 0.34  4059 | 0.879  747 | 0.391  089 | 1.09  0238 | 13  9 |
| {BcMV3.BI=FALSE, BcOLV7.BI=FALSE} => | 0.35 | 0.917 | 0.391 | 1.08 | 14 |

| {BcDMV1=FALSE} | 8911 | 722 | 089 | 7271 | 5 |
| --- | --- | --- | --- | --- | --- |
| {BcDMV1=FALSE, BcOLV7.BI=FALSE} =>  {BcMV3.BI=FALSE} | 0.35  8911 | 0.539  033 | 0.665  842 | 1.17  7133 | 14  5 |
| {BcFV9=FALSE, BcMV3.BI=FALSE} =>  {BcOLV7.BI=FALSE} | 0.35  8911 | 0.868  263 | 0.413  366 | 1.07  6007 | 14  5 |
| {BcOLV7.BI=FALSE, BcULV5=FALSE} =>  {BcMV3.BI=FALSE} | 0.35  8911 | 0.512  367 | 0.700  495 | 1.11  89 | 14  5 |
| {BcBNV5.BI=FALSE, BcOLV7.BI=FALSE} =>  {BcMV3.BI=FALSE} | 0.37  6238 | 0.515  254 | 0.730  198 | 1.12  5204 | 15  2 |
| {BcOLV13.BI=FALSE, BcOLV7.BI=FALSE} =>  {BcMV3.BI=FALSE} | 0.38  3663 | 0.509  868 | 0.752  475 | 1.11  3442 | 15  5 |
| {BcMCV3.BI=FALSE, BcOLV7.BI=FALSE} =>  {BcMV3.BI=FALSE} | 0.39  1089 | 0.514  658 | 0.759  901 | 1.12  3902 | 15  8 |
| {BcEV5=FALSE, BcOLV7.BI=FALSE} =>  {BcMV3.BI=FALSE} | 0.38  8614 | 0.511  401 | 0.759  901 | 1.11  6788 | 15  7 |
| {BcALV1.BI=FALSE, BcMV3.BI=FALSE} =>  {BcDMV1=FALSE} | 0.35  8911 | 0.917  722 | 0.391  089 | 1.08  7271 | 14  5 |
| {BcALV1.BI=FALSE, BcDMV1=FALSE} =>  {BcMV3.BI=FALSE} | 0.35  8911 | 0.512  367 | 0.700  495 | 1.11  89 | 14  5 |
| {BcALV1.BI=FALSE, BcMV3.BI=FALSE} =>  {BcOLV4.BI=FALSE} | 0.36  3861 | 0.930  38 | 0.391  089 | 1.06  1789 | 14  7 |
| {BcALV1.BI=FALSE, BcOLV13.BI=FALSE} => | 0.38 | 0.511 | 0.75 | 1.11 | 15 |

| {BcMV3.BI=FALSE} | 3663 | 551 |  | 7117 | 5 |
| --- | --- | --- | --- | --- | --- |
| {BcALV1.BI=FALSE, BcMCV3.BI=FALSE} =>  {BcMV3.BI=FALSE} | 0.39  1089 | 0.514  658 | 0.759  901 | 1.12  3902 | 15  8 |
| {BcFV9=FALSE, BcMV3.BI=FALSE} =>  {BcDMV1=FALSE} | 0.38  1188 | 0.922  156 | 0.413  366 | 1.09  2525 | 15  4 |
| {BcDMV1=FALSE, BcFV9=FALSE} =>  {BcMV3.BI=FALSE} | 0.38  1188 | 0.511  628 | 0.745  05 | 1.11  7285 | 15  4 |
| {BcMV3.BI=FALSE, BcOLV4.BI=FALSE} =>  {BcDMV1=FALSE} | 0.38  3663 | 0.911  765 | 0.420  792 | 1.08  0214 | 15  5 |
| {BcDMV1=FALSE, BcOLV4.BI=FALSE} =>  {BcMV3.BI=FALSE} | 0.38  3663 | 0.530  822 | 0.722  772 | 1.15  92 | 15  5 |
| {BcMV3.BI=FALSE, BcULV5=FALSE} =>  {BcDMV1=FALSE} | 0.38  6139 | 0.912  281 | 0.423  267 | 1.08  0825 | 15  6 |
| {BcDMV1=FALSE, BcULV5=FALSE} =>  {BcMV3.BI=FALSE} | 0.38  6139 | 0.532  423 | 0.725  248 | 1.16  2697 | 15  6 |
| {BcBNV5.BI=FALSE, BcMV3.BI=FALSE} =>  {BcDMV1=FALSE} | 0.40  5941 | 0.926  554 | 0.438  119 | 1.09  7735 | 16  4 |
| {BcBNV5.BI=FALSE, BcDMV1=FALSE} =>  {BcMV3.BI=FALSE} | 0.40  5941 | 0.504  615 | 0.804  455 | 1.10  1971 | 16  4 |
| {BcMV3.BI=FALSE, BvF.BI=FALSE} =>  {BcDMV1=FALSE} | 0.38  6139 | 0.923  077 | 0.418  317 | 1.09  3616 | 15  6 |
| {BcMCV5.BI=FALSE, BcMV3.BI=FALSE} => | 0.40 | 0.921 | 0.440 | 1.09 | 16 |

| {BcDMV1=FALSE} | 5941 | 348 | 594 | 1568 | 4 |
| --- | --- | --- | --- | --- | --- |
| {BcDMV1=FALSE, BcMCV5.BI=FALSE} =>  {BcMV3.BI=FALSE} | 0.40  5941 | 0.506  173 | 0.801  98 | 1.10  5372 | 16  4 |
| {BcMV3.BI=FALSE, BcOLV13.BI=FALSE} =>  {BcDMV1=FALSE} | 0.41  3366 | 0.917  582 | 0.450  495 | 1.08  7106 | 16  7 |
| {BcDMV1=FALSE, BcOLV13.BI=FALSE} =>  {BcMV3.BI=FALSE} | 0.41  3366 | 0.533  546 | 0.774  752 | 1.16  515 | 16  7 |
| {BcMV3.BI=FALSE, BcOLV2.BI=FALSE} =>  {BcDMV1=FALSE} | 0.40  8416 | 0.921  788 | 0.443  069 | 1.09  2089 | 16  5 |
| {BcDMV1=FALSE, BcOLV2.BI=FALSE} =>  {BcMV3.BI=FALSE} | 0.40  8416 | 0.523  81 | 0.779  703 | 1.14  3887 | 16  5 |
| {BcDMV1=FALSE, BcMV3.BI=FALSE} =>  {BcMCV3.BI=FALSE} | 0.42  0792 | 1 | 0.420  792 | 1.07  1618 | 17  0 |
| {BcMCV3.BI=FALSE, BcMV3.BI=FALSE} =>  {BcDMV1=FALSE} | 0.42  0792 | 0.918  919 | 0.457  921 | 1.08  869 | 17  0 |
| {BcDMV1=FALSE, BcMCV3.BI=FALSE} =>  {BcMV3.BI=FALSE} | 0.42  0792 | 0.541  401 | 0.777  228 | 1.18  2303 | 17  0 |
| {BcMV3.BI=FALSE, BcPV4.BI=FALSE} =>  {BcDMV1=FALSE} | 0.39  8515 | 0.914  773 | 0.435  644 | 1.08  3778 | 16  1 |
| {BcDMV1=FALSE, BcPV4.BI=FALSE} =>  {BcMV3.BI=FALSE} | 0.39  8515 | 0.509  494 | 0.782  178 | 1.11  2624 | 16  1 |
| {BcEV6=FALSE, BcMV3.BI=FALSE} => | 0.38 | 0.923 | 0.418 | 1.09 | 15 |

| {BcDMV1=FALSE} | 6139 | 077 | 317 | 3616 | 6 |
| --- | --- | --- | --- | --- | --- |
| {BcMV3.BI=FALSE, BcOLV17.BI=FALSE} =>  {BcDMV1=FALSE} | 0.39  8515 | 0.914  773 | 0.435  644 | 1.08  3778 | 16  1 |
| {BcMV3.BI=FALSE, BcOLV15.BI=FALSE} =>  {BcDMV1=FALSE} | 0.40  8416 | 0.921  788 | 0.443  069 | 1.09  2089 | 16  5 |
| {BcDMV1=FALSE, BcOLV15.BI=FALSE} =>  {BcMV3.BI=FALSE} | 0.40  8416 | 0.507  692 | 0.804  455 | 1.10  869 | 16  5 |
| {BcEV5=FALSE, BcMV3.BI=FALSE} =>  {BcDMV1=FALSE} | 0.41  8317 | 0.918  478 | 0.455  446 | 1.08  8168 | 16  9 |
| {BcFV9=FALSE, BcMV3.BI=FALSE} =>  {BcOLV4.BI=FALSE} | 0.38  8614 | 0.940  12 | 0.413  366 | 1.07  2905 | 15  7 |
| {BcMV3.BI=FALSE, BcOLV4.BI=FALSE} =>  {BcFV9=FALSE} | 0.38  8614 | 0.923  529 | 0.420  792 | 1.06  298 | 15  7 |
| {BcFV9=FALSE, BcOLV4.BI=FALSE} =>  {BcMV3.BI=FALSE} | 0.38  8614 | 0.504  823 | 0.769  802 | 1.10  2425 | 15  7 |
| {BcFV9=FALSE, BcMV3.BI=FALSE} =>  {BcULV5=FALSE} | 0.38  6139 | 0.934  132 | 0.413  366 | 1.06  6071 | 15  6 |
| {BcFV9=FALSE, BcULV5=FALSE} =>  {BcMV3.BI=FALSE} | 0.38  6139 | 0.506  494 | 0.762  376 | 1.10  6072 | 15  6 |
| {BcFV9=FALSE, BcMV3.BI=FALSE} =>  {BcOLV13.BI=FALSE} | 0.41  0891 | 0.994  012 | 0.413  366 | 1.07  3746 | 16  6 |
| {BcFV9=FALSE, BcOLV13.BI=FALSE} => | 0.41 | 0.504 | 0.814 | 1.10 | 16 |

| {BcMV3.BI=FALSE} | 0891 | 559 | 356 | 1848 | 6 |
| --- | --- | --- | --- | --- | --- |
| {BcFV9=FALSE, BcMCV3.BI=FALSE} =>  {BcMV3.BI=FALSE} | 0.41  3366 | 0.510  703 | 0.809  406 | 1.11  5266 | 16  7 |
| {BcMV3.BI=FALSE, BcOLV15.BI=FALSE} =>  {BcFV9=FALSE} | 0.40  8416 | 0.921  788 | 0.443  069 | 1.06  0975 | 16  5 |
| {BcMV3.BI=FALSE, BcOLV4.BI=FALSE} =>  {BcULV5=FALSE} | 0.39  604 | 0.941  176 | 0.420  792 | 1.07  4111 | 16  0 |
| {BcMV3.BI=FALSE, BcULV5=FALSE} =>  {BcOLV4.BI=FALSE} | 0.39  604 | 0.935  673 | 0.423  267 | 1.06  783 | 16  0 |
| {BcOLV4.BI=FALSE, BcULV5=FALSE} =>  {BcMV3.BI=FALSE} | 0.39  604 | 0.504  732 | 0.784  653 | 1.10  2225 | 16  0 |
| {BcBNV5.BI=FALSE, BcOLV4.BI=FALSE} =>  {BcMV3.BI=FALSE} | 0.40  099 | 0.507  837 | 0.789  604 | 1.10  9006 | 16  2 |
| {BcMV3.BI=FALSE, BvF.BI=FALSE} =>  {BcOLV4.BI=FALSE} | 0.38  8614 | 0.928  994 | 0.418  317 | 1.06  0208 | 15  7 |
| {BcMCV5.BI=FALSE, BcMV3.BI=FALSE} =>  {BcOLV4.BI=FALSE} | 0.40  8416 | 0.926  966 | 0.440  594 | 1.05  7894 | 16  5 |
| {BcMV3.BI=FALSE, BcOLV4.BI=FALSE} =>  {BcMCV3.BI=FALSE} | 0.42  0792 | 1 | 0.420  792 | 1.07  1618 | 17  0 |
| {BcMCV3.BI=FALSE, BcOLV4.BI=FALSE} =>  {BcMV3.BI=FALSE} | 0.42  0792 | 0.508  982 | 0.826  733 | 1.11  1507 | 17  0 |
| {BcMV3.BI=FALSE, BcOLV15.BI=FALSE} => | 0.41 | 0.927 | 0.443 | 1.05 | 16 |

| {BcOLV4.BI=FALSE} | 0891 | 374 | 069 | 8359 | 6 |
| --- | --- | --- | --- | --- | --- |
| {BcEV5=FALSE, BcOLV4.BI=FALSE} =>  {BcMV3.BI=FALSE} | 0.41  8317 | 0.504  478 | 0.829  208 | 1.10  167 | 16  9 |
| {BcBNV5.BI=FALSE, BcULV5=FALSE} =>  {BcMV3.BI=FALSE} | 0.40  5941 | 0.514  107 | 0.789  604 | 1.12  2698 | 16  4 |
| {BcMCV5.BI=FALSE, BcMV3.BI=FALSE} =>  {BcULV5=FALSE} | 0.41  0891 | 0.932  584 | 0.440  594 | 1.06  4305 | 16  6 |
| {BcMCV5.BI=FALSE, BcULV5=FALSE} =>  {BcMV3.BI=FALSE} | 0.41  0891 | 0.504  559 | 0.814  356 | 1.10  1848 | 16  6 |
| {BcMV3.BI=FALSE, BcOLV13.BI=FALSE} =>  {BcULV5=FALSE} | 0.41  8317 | 0.928  571 | 0.450  495 | 1.05  9726 | 16  9 |
| {BcOLV13.BI=FALSE, BcULV5=FALSE} =>  {BcMV3.BI=FALSE} | 0.41  8317 | 0.505  988 | 0.826  733 | 1.10  4968 | 16  9 |
| {BcMCV3.BI=FALSE, BcULV5=FALSE} =>  {BcMV3.BI=FALSE} | 0.42  3267 | 0.515  06 | 0.821  782 | 1.12  478 | 17  1 |
| {BcEV6=FALSE, BcMV3.BI=FALSE} =>  {BcULV5=FALSE} | 0.38  8614 | 0.928  994 | 0.418  317 | 1.06  0208 | 15  7 |
| {BcMV3.BI=FALSE, BcOLV17.BI=FALSE} =>  {BcULV5=FALSE} | 0.40  5941 | 0.931  818 | 0.435  644 | 1.06  3431 | 16  4 |
| {BcMV3.BI=FALSE, BcOLV15.BI=FALSE} =>  {BcULV5=FALSE} | 0.41  0891 | 0.927  374 | 0.443  069 | 1.05  8359 | 16  6 |
| {BcEV5=FALSE, BcULV5=FALSE} => | 0.42 | 0.507 | 0.829 | 1.10 | 17 |

| {BcMV3.BI=FALSE} | 0792 | 463 | 208 | 8189 | 0 |
| --- | --- | --- | --- | --- | --- |
| {BcBNV5.BI=FALSE, BcOLV13.BI=FALSE} =>  {BcMV3.BI=FALSE} | 0.43  0693 | 0.514  793 | 0.836  634 | 1.12  4196 | 17  4 |
| {BcBNV5.BI=FALSE, BcOLV2.BI=FALSE} =>  {BcMV3.BI=FALSE} | 0.42  3267 | 0.5 | 0.846  535 | 1.09  1892 | 17  1 |
| {BcBNV5.BI=FALSE, BcMCV3.BI=FALSE} =>  {BcMV3.BI=FALSE} | 0.43  8119 | 0.519  062 | 0.844  059 | 1.13  3518 | 17  7 |
| {BcMV3.BI=FALSE, BvF.BI=FALSE} =>  {BcMCV3.BI=FALSE} | 0.41  8317 | 1 | 0.418  317 | 1.07  1618 | 16  9 |
| {BcMCV5.BI=FALSE, BcOLV13.BI=FALSE} =>  {BcMV3.BI=FALSE} | 0.43  5644 | 0.505  747 | 0.861  386 | 1.10  4442 | 17  6 |
| {BcMCV5.BI=FALSE, BcOLV2.BI=FALSE} =>  {BcMV3.BI=FALSE} | 0.42  5743 | 0.498  551 | 0.853  96 | 1.08  8727 | 17  2 |
| {BcMCV3.BI=FALSE, BcMCV5.BI=FALSE} =>  {BcMV3.BI=FALSE} | 0.44  0594 | 0.510  029 | 0.863  861 | 1.11  3792 | 17  8 |
| {BcOLV13.BI=FALSE, BcOLV2.BI=FALSE} =>  {BcMV3.BI=FALSE} | 0.43  8119 | 0.502  841 | 0.871  287 | 1.09  8096 | 17  7 |
| {BcMCV3.BI=FALSE, BcOLV13.BI=FALSE} =>  {BcMV3.BI=FALSE} | 0.45  0495 | 0.515  581 | 0.873  762 | 1.12  5917 | 18  2 |
| {BcOLV13.BI=FALSE, BcOLV15.BI=FALSE}  => {BcMV3.BI=FALSE} | 0.44  0594 | 0.495  822 | 0.888  614 | 1.08  2767 | 17  8 |
| {BcEV5=FALSE, BcOLV13.BI=FALSE} => | 0.44 | 0.511 | 0.876 | 1.11 | 18 |

| {BcMV3.BI=FALSE} | 802 | 299 | 238 | 6567 | 1 |
| --- | --- | --- | --- | --- | --- |
| {BcMCV3.BI=FALSE, BcOLV2.BI=FALSE} =>  {BcMV3.BI=FALSE} | 0.44  3069 | 0.505  65 | 0.876  238 | 1.10  423 | 17  9 |
| {BcEV5=FALSE, BcOLV2.BI=FALSE} =>  {BcMV3.BI=FALSE} | 0.44  0594 | 0.501  408 | 0.878  713 | 1.09  4968 | 17  8 |
| {BcEV6=FALSE, BcMV3.BI=FALSE} =>  {BcMCV3.BI=FALSE} | 0.41  8317 | 1 | 0.418  317 | 1.07  1618 | 16  9 |
| {BcMCV3.BI=FALSE, BcOLV15.BI=FALSE} =>  {BcMV3.BI=FALSE} | 0.44  3069 | 0.501  401 | 0.883  663 | 1.09  495 | 17  9 |
| {BcEV5=FALSE, BcMCV3.BI=FALSE} =>  {BcMV3.BI=FALSE} | 0.45  5446 | 0.515  406 | 0.883  663 | 1.12  5536 | 18  4 |
| {BcMV2.BI=TRUE, BcOLV18=FALSE} =>  {BcRV1.BI=FALSE} | 0.13  8614 | 0.823  529 | 0.168  317 | 1.13  9404 | 56 |
| {BcMV2.BI=TRUE, BcOLV18=FALSE} =>  {BcOLV7.BI=FALSE} | 0.15  5941 | 0.926  471 | 0.168  317 | 1.14  8141 | 63 |
| {BcMV2.BI=TRUE, BcOLV18=FALSE} =>  {BcALV1.BI=FALSE} | 0.15  099 | 0.897  059 | 0.168  317 | 1.10  1556 | 61 |
| {BcMV2.BI=TRUE, BcOLV18=FALSE} =>  {BcDMV1=FALSE} | 0.15  3465 | 0.911  765 | 0.168  317 | 1.08  0214 | 62 |
| {BcMV2.BI=TRUE, BcOLV18=FALSE} =>  {BcFV9=FALSE} | 0.16  0891 | 0.955  882 | 0.168  317 | 1.10  0218 | 65 |
| {BcMV2.BI=TRUE, BcOLV18=TRUE} => | 0.20 | 0.689 | 0.294 | 1.27 | 82 |

| {BcHV1SLR.BI=TRUE} | 297 | 076 | 554 | 1171 |  |
| --- | --- | --- | --- | --- | --- |
| {BcHV1SLR.BI=TRUE, BcMV2.BI=TRUE} =>  {BcOLV18=TRUE} | 0.20  297 | 0.745  455 | 0.272  277 | 1.44  0974 | 82 |
| {BcHV1SLR.BI=TRUE, BcOLV18=TRUE} =>  {BcMV2.BI=TRUE} | 0.20  297 | 0.594  203 | 0.341  584 | 1.28  3732 | 82 |
| {BcMV2.BI=TRUE, BcOLV18=TRUE} =>  {BcMV3.BI=TRUE} | 0.21  2871 | 0.722  689 | 0.294  554 | 1.33  318 | 86 |
| {BcMV2.BI=TRUE, BcMV3.BI=TRUE} =>  {BcOLV18=TRUE} | 0.21  2871 | 0.677  165 | 0.314  356 | 1.30  897 | 86 |
| {BcMV3.BI=TRUE, BcOLV18=TRUE} =>  {BcMV2.BI=TRUE} | 0.21  2871 | 0.646  617 | 0.329  208 | 1.39  6968 | 86 |
| {BcMV2.BI=TRUE, BcULV3=TRUE} =>  {BcOLV18=TRUE} | 0.18  8119 | 0.660  87 | 0.284  653 | 1.27  747 | 76 |
| {BcOLV18=TRUE, BcULV3=TRUE} =>  {BcMV2.BI=TRUE} | 0.18  8119 | 0.580  153 | 0.324  257 | 1.25  3378 | 76 |
| {BcMV2.BI=TRUE, BcOLV18=TRUE} =>  {BcHV1.BI=TRUE} | 0.21  0396 | 0.714  286 | 0.294  554 | 1.18  267 | 85 |
| {BcHV1.BI=TRUE, BcMV2.BI=TRUE} =>  {BcOLV18=TRUE} | 0.21  0396 | 0.720  339 | 0.292  079 | 1.39  2426 | 85 |
| {BcHV1.BI=TRUE, BcOLV18=TRUE} =>  {BcMV2.BI=TRUE} | 0.21  0396 | 0.574  324 | 0.366  337 | 1.24  0786 | 85 |
| {BcMV2.BI=TRUE, BcOLV18=TRUE} => | 0.23 | 0.789 | 0.294 | 1.28 | 94 |

| {BcMV11=TRUE} | 2673 | 916 | 554 | 1631 |  |
| --- | --- | --- | --- | --- | --- |
| {BcMV11=TRUE, BcMV2.BI=TRUE} =>  {BcOLV18=TRUE} | 0.23  2673 | 0.681  159 | 0.341  584 | 1.31  6691 | 94 |
| {BcMV11=TRUE, BcOLV18=TRUE} =>  {BcMV2.BI=TRUE} | 0.23  2673 | 0.671  429 | 0.346  535 | 1.45  0573 | 94 |
| {BcMV2.BI=TRUE, BcOLV18=TRUE} =>  {BcMV9.BI=TRUE} | 0.24  505 | 0.831  933 | 0.294  554 | 1.22  6645 | 99 |
| {BcMV2.BI=TRUE, BcMV9.BI=TRUE} =>  {BcOLV18=TRUE} | 0.24  505 | 0.761  538 | 0.321  782 | 1.47  2065 | 99 |
| {BcMV9.BI=TRUE, BcOLV18=TRUE} =>  {BcMV2.BI=TRUE} | 0.24  505 | 0.592  814 | 0.413  366 | 1.28  0733 | 99 |
| {BcMV2.BI=TRUE, BcOLV18=TRUE} =>  {BcULV4=TRUE} | 0.25  9901 | 0.882  353 | 0.294  554 | 1.27  7672 | 10  5 |
| {BcMV2.BI=TRUE, BcULV4=TRUE} =>  {BcOLV18=TRUE} | 0.25  9901 | 0.760  87 | 0.341  584 | 1.47  0772 | 10  5 |
| {BcOLV18=TRUE, BcULV4=TRUE} =>  {BcMV2.BI=TRUE} | 0.25  9901 | 0.589  888 | 0.440  594 | 1.27  441 | 10  5 |
| {BcFV5.BI=FALSE, BcMV2.BI=TRUE} =>  {BcOLV18=TRUE} | 0.20  7921 | 0.604  317 | 0.344  059 | 1.16  8153 | 84 |
| {BcFV5.BI=FALSE, BcOLV18=TRUE} =>  {BcMV2.BI=TRUE} | 0.20  7921 | 0.563  758 | 0.368  812 | 1.21  7959 | 84 |
| {BcMV2.BI=TRUE, BcOLV18=TRUE} => | 0.26 | 0.899 | 0.294 | 1.13 | 10 |

| {BcMV1.BI=TRUE} | 4851 | 16 | 554 | 8748 | 7 |
| --- | --- | --- | --- | --- | --- |
| {BcMV1.BI=TRUE, BcMV2.BI=TRUE} =>  {BcOLV18=TRUE} | 0.26  4851 | 0.648  485 | 0.408  416 | 1.25  3531 | 10  7 |
| {BcMV1.BI=TRUE, BcOLV18=TRUE} =>  {BcMV2.BI=TRUE} | 0.26  4851 | 0.601  124 | 0.440  594 | 1.29  8684 | 10  7 |
| {BcMV12=FALSE, BcMV2.BI=TRUE} =>  {BcOLV18=TRUE} | 0.22  2772 | 0.652  174 | 0.341  584 | 1.26  0662 | 90 |
| {BcMV12=FALSE, BcOLV18=TRUE} =>  {BcMV2.BI=TRUE} | 0.22  2772 | 0.562  5 | 0.396  04 | 1.21  5241 | 90 |
| {BcMV2.BI=TRUE, BcNSRV7.BI=FALSE} =>  {BcOLV18=TRUE} | 0.21  7822 | 0.602  74 | 0.361  386 | 1.16  5105 | 88 |
| {BcNSRV7.BI=FALSE, BcOLV18=TRUE} =>  {BcMV2.BI=TRUE} | 0.21  7822 | 0.560  51 | 0.388  614 | 1.21  094 | 88 |
| {BcMV2.BI=TRUE, BcVV3.BI=FALSE} =>  {BcOLV18=TRUE} | 0.22  0297 | 0.626  761 | 0.351  485 | 1.21  1537 | 89 |
| {BcDMV1=FALSE, BcMV2.BI=TRUE} =>  {BcOLV18=TRUE} | 0.24  2574 | 0.612  5 | 0.396  04 | 1.18  3971 | 98 |
| {BcDMV1=FALSE, BcOLV18=TRUE} =>  {BcMV2.BI=TRUE} | 0.24  2574 | 0.566  474 | 0.428  218 | 1.22  3826 | 98 |
| {BcFV9=FALSE, BcMV2.BI=TRUE} =>  {BcOLV18=TRUE} | 0.24  0099 | 0.598  765 | 0.400  99 | 1.15  7422 | 97 |
| {BcFV9=FALSE, BcOLV18=TRUE} => | 0.24 | 0.570 | 0.420 | 1.23 | 97 |

| {BcMV2.BI=TRUE} | 0099 | 588 | 792 | 2715 |  |
| --- | --- | --- | --- | --- | --- |
| {BcMV2.BI=TRUE, BcOLV4.BI=FALSE} =>  {BcOLV18=TRUE} | 0.24  2574 | 0.644  737 | 0.376  238 | 1.24  6286 | 98 |
| {BcOLV18=TRUE, BcOLV4.BI=FALSE} =>  {BcMV2.BI=TRUE} | 0.24  2574 | 0.541  436 | 0.448  02 | 1.16  9734 | 98 |
| {BcMV2.BI=TRUE, BcULV5=FALSE} =>  {BcOLV18=TRUE} | 0.25  7426 | 0.684  211 | 0.376  238 | 1.32  2589 | 10  4 |
| {BcOLV18=TRUE, BcULV5=FALSE} =>  {BcMV2.BI=TRUE} | 0.25  7426 | 0.550  265 | 0.467  822 | 1.18  8807 | 10  4 |
| {BcBNV5.BI=FALSE, BcMV2.BI=TRUE} =>  {BcOLV18=TRUE} | 0.25  2475 | 0.618  182 | 0.408  416 | 1.19  4954 | 10  2 |
| {BcBNV5.BI=FALSE, BcOLV18=TRUE} =>  {BcMV2.BI=TRUE} | 0.25  2475 | 0.557  377 | 0.452  97 | 1.20  4173 | 10  2 |
| {BcMV2.BI=TRUE, BvF.BI=FALSE} =>  {BcOLV18=TRUE} | 0.27  7228 | 0.64 | 0.433  168 | 1.23  7129 | 11  2 |
| {BcOLV18=TRUE, BvF.BI=FALSE} =>  {BcMV2.BI=TRUE} | 0.27  7228 | 0.583  333 | 0.475  248 | 1.26  025 | 11  2 |
| {BcMV2.BI=TRUE, BcOLV13.BI=FALSE} =>  {BcOLV18=TRUE} | 0.26  2376 | 0.642  424 | 0.408  416 | 1.24  1815 | 10  6 |
| {BcOLV13.BI=FALSE, BcOLV18=TRUE} =>  {BcMV2.BI=TRUE} | 0.26  2376 | 0.552  083 | 0.475  248 | 1.19  2736 | 10  6 |
| {BcMV2.BI=TRUE, BcOLV2.BI=FALSE} => | 0.26 | 0.623 | 0.420 | 1.20 | 10 |

| {BcOLV18=TRUE} | 2376 | 529 | 792 | 5291 | 6 |
| --- | --- | --- | --- | --- | --- |
| {BcOLV18=TRUE, BcOLV2.BI=FALSE} =>  {BcMV2.BI=TRUE} | 0.26  2376 | 0.560  847 | 0.467  822 | 1.21  1669 | 10  6 |
| {BcMCV3.BI=FALSE, BcMV2.BI=TRUE} =>  {BcOLV18=TRUE} | 0.26  2376 | 0.642  424 | 0.408  416 | 1.24  1815 | 10  6 |
| {BcMCV3.BI=FALSE, BcOLV18=TRUE} =>  {BcMV2.BI=TRUE} | 0.26  2376 | 0.552  083 | 0.475  248 | 1.19  2736 | 10  6 |
| {BcMV2.BI=TRUE, BcPV4.BI=FALSE} =>  {BcOLV18=TRUE} | 0.26  9802 | 0.630  058 | 0.428  218 | 1.21  7911 | 10  9 |
| {BcOLV18=TRUE, BcPV4.BI=FALSE} =>  {BcMV2.BI=TRUE} | 0.26  9802 | 0.564  767 | 0.477  723 | 1.22  0138 | 10  9 |
| {BcEV6=FALSE, BcMV2.BI=TRUE} =>  {BcOLV18=TRUE} | 0.27  2277 | 0.628  571 | 0.433  168 | 1.21  5038 | 11  0 |
| {BcEV6=FALSE, BcOLV18=TRUE} =>  {BcMV2.BI=TRUE} | 0.27  2277 | 0.564  103 | 0.482  673 | 1.21  8703 | 11  0 |
| {BcMV2.BI=TRUE, BcOLV17.BI=FALSE} =>  {BcOLV18=TRUE} | 0.26  4851 | 0.622  093 | 0.425  743 | 1.20  2515 | 10  7 |
| {BcOLV17.BI=FALSE, BcOLV18=TRUE} =>  {BcMV2.BI=TRUE} | 0.26  4851 | 0.554  404 | 0.477  723 | 1.19  775 | 10  7 |
| {BcMV2.BI=TRUE, BcOLV15.BI=FALSE} =>  {BcOLV18=TRUE} | 0.28  4653 | 0.628  415 | 0.452  97 | 1.21  4736 | 11  5 |
| {BcOLV15.BI=FALSE, BcOLV18=TRUE} => | 0.28 | 0.595 | 0.477 | 1.28 | 11 |

| {BcMV2.BI=TRUE} | 4653 | 855 | 723 | 7302 | 5 |
| --- | --- | --- | --- | --- | --- |
| {BcEV5=FALSE, BcMV2.BI=TRUE} =>  {BcOLV18=TRUE} | 0.26  7327 | 0.631  579 | 0.423  267 | 1.22  0851 | 10  8 |
| {BcEV5=FALSE, BcOLV18=TRUE} =>  {BcMV2.BI=TRUE} | 0.26  7327 | 0.548  223 | 0.487  624 | 1.18  4397 | 10  8 |
| {BcHV1SLR.BI=TRUE, BcMV2.BI=TRUE} =>  {BcMV3.BI=TRUE} | 0.20  7921 | 0.763  636 | 0.272  277 | 1.40  8717 | 84 |
| {BcMV2.BI=TRUE, BcMV3.BI=TRUE} =>  {BcHV1SLR.BI=TRUE} | 0.20  7921 | 0.661  417 | 0.314  356 | 1.22  0149 | 84 |
| {BcHV1SLR.BI=TRUE, BcMV3.BI=TRUE} =>  {BcMV2.BI=TRUE} | 0.20  7921 | 0.631  579 | 0.329  208 | 1.36  4481 | 84 |
| {BcHV1SLR.BI=TRUE, BcMV2.BI=TRUE} =>  {BcULV3=TRUE} | 0.19  3069 | 0.709  091 | 0.272  277 | 1.19  3636 | 78 |
| {BcMV2.BI=TRUE, BcULV3=TRUE} =>  {BcHV1SLR.BI=TRUE} | 0.19  3069 | 0.678  261 | 0.284  653 | 1.25  1221 | 78 |
| {BcHV1SLR.BI=TRUE, BcMV2.BI=TRUE} =>  {BcHV1.BI=TRUE} | 0.24  7525 | 0.909  091 | 0.272  277 | 1.50  5216 | 10  0 |
| {BcHV1.BI=TRUE, BcMV2.BI=TRUE} =>  {BcHV1SLR.BI=TRUE} | 0.24  7525 | 0.847  458 | 0.292  079 | 1.56  3346 | 10  0 |
| {BcHV1SLR.BI=TRUE, BcMV2.BI=TRUE} =>  {BcMV11=TRUE} | 0.21  2871 | 0.781  818 | 0.272  277 | 1.26  8492 | 86 |
| {BcHV1SLR.BI=TRUE, BcMV11=TRUE} => | 0.21 | 0.597 | 0.356 | 1.29 | 86 |

| {BcMV2.BI=TRUE} | 2871 | 222 | 436 | 0255 |  |
| --- | --- | --- | --- | --- | --- |
| {BcHV1SLR.BI=TRUE, BcMV2.BI=TRUE} =>  {BcMV9.BI=TRUE} | 0.21  0396 | 0.772  727 | 0.272  277 | 1.13  935 | 85 |
| {BcMV2.BI=TRUE, BcMV9.BI=TRUE} =>  {BcHV1SLR.BI=TRUE} | 0.21  0396 | 0.653  846 | 0.321  782 | 1.20  6182 | 85 |
| {BcHV1SLR.BI=TRUE, BcMV2.BI=TRUE} =>  {BcULV4=TRUE} | 0.22  5248 | 0.827  273 | 0.272  277 | 1.19  7915 | 91 |
| {BcMV2.BI=TRUE, BcULV4=TRUE} =>  {BcHV1SLR.BI=TRUE} | 0.22  5248 | 0.659  42 | 0.341  584 | 1.21  6465 | 91 |
| {BcHV1SLR.BI=TRUE, BcULV4=TRUE} =>  {BcMV2.BI=TRUE} | 0.22  5248 | 0.561  728 | 0.400  99 | 1.21  3574 | 91 |
| {BcHV1SLR.BI=TRUE, BcMV2.BI=TRUE} =>  {BcMV1.BI=TRUE} | 0.24  0099 | 0.881  818 | 0.272  277 | 1.11  6785 | 97 |
| {BcHV1SLR.BI=TRUE, BcMV1.BI=TRUE} =>  {BcMV2.BI=TRUE} | 0.24  0099 | 0.544  944 | 0.440  594 | 1.17  7312 | 97 |
| {BcHV1SLR.BI=TRUE, BcOLV15.BI=FALSE}  => {BcMV2.BI=TRUE} | 0.26  7327 | 0.534  653 | 0.5 | 1.15  508 | 10  8 |
| {BcMV2.BI=TRUE, BcULV3=TRUE} =>  {BcMV3.BI=TRUE} | 0.20  5446 | 0.721  739 | 0.284  653 | 1.33  1427 | 83 |
| {BcMV3.BI=TRUE, BcULV3=TRUE} =>  {BcMV2.BI=TRUE} | 0.20  5446 | 0.592  857 | 0.346  535 | 1.28  0825 | 83 |
| {BcHV1.BI=TRUE, BcMV2.BI=TRUE} => | 0.21 | 0.728 | 0.292 | 1.34 | 86 |

| {BcMV3.BI=TRUE} | 2871 | 814 | 079 | 4478 |  |
| --- | --- | --- | --- | --- | --- |
| {BcHV1.BI=TRUE, BcMV3.BI=TRUE} =>  {BcMV2.BI=TRUE} | 0.21  2871 | 0.601  399 | 0.353  96 | 1.29  9278 | 86 |
| {BcMV2.BI=TRUE, BcMV3.BI=TRUE} =>  {BcMV11=TRUE} | 0.25 | 0.795  276 | 0.314  356 | 1.29  0327 | 10  1 |
| {BcMV11=TRUE, BcMV2.BI=TRUE} =>  {BcMV3.BI=TRUE} | 0.25 | 0.731  884 | 0.341  584 | 1.35  0142 | 10  1 |
| {BcMV11=TRUE, BcMV3.BI=TRUE} =>  {BcMV2.BI=TRUE} | 0.25 | 0.651  613 | 0.383  663 | 1.40  7763 | 10  1 |
| {BcMV2.BI=TRUE, BcMV9.BI=TRUE} =>  {BcMV3.BI=TRUE} | 0.23  0198 | 0.715  385 | 0.321  782 | 1.31  9705 | 93 |
| {BcMV3.BI=TRUE, BcMV9.BI=TRUE} =>  {BcMV2.BI=TRUE} | 0.23  0198 | 0.581  25 | 0.396  04 | 1.25  5749 | 93 |
| {BcMV2.BI=TRUE, BcULV4=TRUE} =>  {BcMV3.BI=TRUE} | 0.23  2673 | 0.681  159 | 0.341  584 | 1.25  6568 | 94 |
| {BcMV3.BI=TRUE, BcULV4=TRUE} =>  {BcMV2.BI=TRUE} | 0.23  2673 | 0.580  247 | 0.400  99 | 1.25  3582 | 94 |
| {BcMV2.BI=TRUE, BcPV3.BI=FALSE} =>  {BcMV3.BI=TRUE} | 0.18  3168 | 0.643  478 | 0.284  653 | 1.18  7056 | 74 |
| {BcMV2.BI=TRUE, BcRV1.BI=FALSE} =>  {BcMV3.BI=TRUE} | 0.18  3168 | 0.643  478 | 0.284  653 | 1.18  7056 | 74 |
| {BcEV2.BI=FALSE, BcMV2.BI=TRUE} => | 0.21 | 0.674 | 0.319 | 1.24 | 87 |

| {BcMV3.BI=TRUE} | 5347 | 419 | 307 | 4133 |  |
| --- | --- | --- | --- | --- | --- |
| {BcHV3.BI=FALSE, BcMV2.BI=TRUE} =>  {BcMV3.BI=TRUE} | 0.21  2871 | 0.646  617 | 0.329  208 | 1.19  2845 | 86 |
| {BcFV5.BI=FALSE, BcMV2.BI=TRUE} =>  {BcMV3.BI=TRUE} | 0.22  2772 | 0.647  482 | 0.344  059 | 1.19  4442 | 90 |
| {BcFV5.BI=FALSE, BcMV3.BI=TRUE} =>  {BcMV2.BI=TRUE} | 0.22  2772 | 0.573  248 | 0.388  614 | 1.23  8462 | 90 |
| {BcMV2.BI=TRUE, BcULV1.BI=FALSE} =>  {BcMV3.BI=TRUE} | 0.20  7921 | 0.641  221 | 0.324  257 | 1.18  2892 | 84 |
| {BcMV2.BI=TRUE, BcMV3.BI=TRUE} =>  {BcMV1.BI=TRUE} | 0.28  7129 | 0.913  386 | 0.314  356 | 1.15  6764 | 11  6 |
| {BcMV1.BI=TRUE, BcMV2.BI=TRUE} =>  {BcMV3.BI=TRUE} | 0.28  7129 | 0.703  03 | 0.408  416 | 1.29  6914 | 11  6 |
| {BcMV1.BI=TRUE, BcMV3.BI=TRUE} =>  {BcMV2.BI=TRUE} | 0.28  7129 | 0.627  027 | 0.457  921 | 1.35  4647 | 11  6 |
| {BcMV12=FALSE, BcMV3.BI=TRUE} =>  {BcMV2.BI=TRUE} | 0.21  2871 | 0.547  771 | 0.388  614 | 1.18  3419 | 86 |
| {BcMV2.BI=TRUE, BcNSRV7.BI=FALSE} =>  {BcMV3.BI=TRUE} | 0.24  505 | 0.678  082 | 0.361  386 | 1.25  0891 | 99 |
| {BcMV3.BI=TRUE, BcNSRV7.BI=FALSE} =>  {BcMV2.BI=TRUE} | 0.24  505 | 0.578  947 | 0.423  267 | 1.25  0774 | 99 |
| {BcMV2.BI=TRUE, BcVV3.BI=FALSE} => | 0.23 | 0.669 | 0.351 | 1.23 | 95 |

| {BcMV3.BI=TRUE} | 5149 | 014 | 485 | 4163 |  |
| --- | --- | --- | --- | --- | --- |
| {BcMV3.BI=TRUE, BcVV3.BI=FALSE} =>  {BcMV2.BI=TRUE} | 0.23  5149 | 0.565  476 | 0.415  842 | 1.22  167 | 95 |
| {BcMV2.BI=TRUE, BcOLV7.BI=FALSE} =>  {BcMV3.BI=TRUE} | 0.22  5248 | 0.627  586 | 0.358  911 | 1.15  7739 | 91 |
| {BcALV1.BI=FALSE, BcMV2.BI=TRUE} =>  {BcMV3.BI=TRUE} | 0.21  0396 | 0.648  855 | 0.324  257 | 1.19  6974 | 85 |
| {BcDMV1=FALSE, BcMV2.BI=TRUE} =>  {BcMV3.BI=TRUE} | 0.25  7426 | 0.65 | 0.396  04 | 1.19  9087 | 10  4 |
| {BcDMV1=FALSE, BcMV3.BI=TRUE} =>  {BcMV2.BI=TRUE} | 0.25  7426 | 0.608  187 | 0.423  267 | 1.31  3944 | 10  4 |
| {BcFV9=FALSE, BcMV2.BI=TRUE} =>  {BcMV3.BI=TRUE} | 0.26  7327 | 0.666  667 | 0.400  99 | 1.22  9833 | 10  8 |
| {BcFV9=FALSE, BcMV3.BI=TRUE} =>  {BcMV2.BI=TRUE} | 0.26  7327 | 0.586  957 | 0.455  446 | 1.26  8077 | 10  8 |
| {BcMV2.BI=TRUE, BcOLV4.BI=FALSE} =>  {BcMV3.BI=TRUE} | 0.24  7525 | 0.657  895 | 0.376  238 | 1.21  3651 | 10  0 |
| {BcMV3.BI=TRUE, BcOLV4.BI=FALSE} =>  {BcMV2.BI=TRUE} | 0.24  7525 | 0.543  478 | 0.455  446 | 1.17  4146 | 10  0 |
| {BcMV2.BI=TRUE, BcULV5=FALSE} =>  {BcMV3.BI=TRUE} | 0.24  505 | 0.651  316 | 0.376  238 | 1.20  1514 | 99 |
| {BcMV3.BI=TRUE, BcULV5=FALSE} => | 0.24 | 0.540 | 0.452 | 1.16 | 99 |

| {BcMV2.BI=TRUE} | 505 | 984 | 97 | 8756 |  |
| --- | --- | --- | --- | --- | --- |
| {BcBNV5.BI=FALSE, BcMV2.BI=TRUE} =>  {BcMV3.BI=TRUE} | 0.26  9802 | 0.660  606 | 0.408  416 | 1.21  8652 | 10  9 |
| {BcBNV5.BI=FALSE, BcMV3.BI=TRUE} =>  {BcMV2.BI=TRUE} | 0.26  9802 | 0.573  684 | 0.470  297 | 1.23  9403 | 10  9 |
| {BcMV2.BI=TRUE, BvF.BI=FALSE} =>  {BcMV3.BI=TRUE} | 0.29  2079 | 0.674  286 | 0.433  168 | 1.24  3888 | 11  8 |
| {BcMV3.BI=TRUE, BvF.BI=FALSE} =>  {BcMV2.BI=TRUE} | 0.29  2079 | 0.59 | 0.495  05 | 1.27  4652 | 11  8 |
| {BcMCV5.BI=FALSE, BcMV2.BI=TRUE} =>  {BcMV3.BI=TRUE} | 0.26  4851 | 0.660  494 | 0.400  99 | 1.21  8445 | 10  7 |
| {BcMCV5.BI=FALSE, BcMV3.BI=TRUE} =>  {BcMV2.BI=TRUE} | 0.26  4851 | 0.548  718 | 0.482  673 | 1.18  5466 | 10  7 |
| {BcMV2.BI=TRUE, BcOLV13.BI=FALSE} =>  {BcMV3.BI=TRUE} | 0.26  2376 | 0.642  424 | 0.408  416 | 1.18  5111 | 10  6 |
| {BcMV3.BI=TRUE, BcOLV13.BI=FALSE} =>  {BcMV2.BI=TRUE} | 0.26  2376 | 0.552  083 | 0.475  248 | 1.19  2736 | 10  6 |
| {BcMV2.BI=TRUE, BcOLV2.BI=FALSE} =>  {BcMV3.BI=TRUE} | 0.27  2277 | 0.647  059 | 0.420  792 | 1.19  3661 | 11  0 |
| {BcMV3.BI=TRUE, BcOLV2.BI=FALSE} =>  {BcMV2.BI=TRUE} | 0.27  2277 | 0.561  224 | 0.485  149 | 1.21  2485 | 11  0 |
| {BcMCV3.BI=FALSE, BcMV2.BI=TRUE} => | 0.25 | 0.636 | 0.408 | 1.17 | 10 |

| {BcMV3.BI=TRUE} | 9901 | 364 | 416 | 3931 | 5 |
| --- | --- | --- | --- | --- | --- |
| {BcMCV3.BI=FALSE, BcMV3.BI=TRUE} =>  {BcMV2.BI=TRUE} | 0.25  9901 | 0.546  875 | 0.475  248 | 1.18  1484 | 10  5 |
| {BcMV2.BI=TRUE, BcPV4.BI=FALSE} =>  {BcMV3.BI=TRUE} | 0.28  4653 | 0.664  74 | 0.428  218 | 1.22  6278 | 11  5 |
| {BcMV3.BI=TRUE, BcPV4.BI=FALSE} =>  {BcMV2.BI=TRUE} | 0.28  4653 | 0.572  139 | 0.497  525 | 1.23  6066 | 11  5 |
| {BcEV6=FALSE, BcMV2.BI=TRUE} =>  {BcMV3.BI=TRUE} | 0.29  9505 | 0.691  429 | 0.433  168 | 1.27  5512 | 12  1 |
| {BcEV6=FALSE, BcMV3.BI=TRUE} =>  {BcMV2.BI=TRUE} | 0.29  9505 | 0.578  947 | 0.517  327 | 1.25  0774 | 12  1 |
| {BcMV2.BI=TRUE, BcOLV17.BI=FALSE} =>  {BcMV3.BI=TRUE} | 0.28  9604 | 0.680  233 | 0.425  743 | 1.25  4858 | 11  7 |
| {BcMV3.BI=TRUE, BcOLV17.BI=FALSE} =>  {BcMV2.BI=TRUE} | 0.28  9604 | 0.565  217 | 0.512  376 | 1.22  1111 | 11  7 |
| {BcMV2.BI=TRUE, BcOLV15.BI=FALSE} =>  {BcMV3.BI=TRUE} | 0.30  6931 | 0.677  596 | 0.452  97 | 1.24  9994 | 12  4 |
| {BcMV3.BI=TRUE, BcOLV15.BI=FALSE} =>  {BcMV2.BI=TRUE} | 0.30  6931 | 0.604  878 | 0.507  426 | 1.30  6795 | 12  4 |
| {BcEV5=FALSE, BcMV2.BI=TRUE} =>  {BcMV3.BI=TRUE} | 0.27  4752 | 0.649  123 | 0.423  267 | 1.19  7469 | 11  1 |
| {BcEV5=FALSE, BcMV3.BI=TRUE} => | 0.27 | 0.555 | 0.495 | 1.19 | 11 |

| {BcMV2.BI=TRUE} | 4752 |  | 05 | 9037 | 1 |
| --- | --- | --- | --- | --- | --- |
| {BcMV2.BI=TRUE, BcMV4.BI=FALSE} =>  {BcMV1.BI=TRUE} | 0.16  8317 | 0.894  737 | 0.188  119 | 1.13  3146 | 68 |
| {BcMV2.BI=TRUE, BcMV4.BI=FALSE} =>  {BcDMV1=FALSE} | 0.17  8218 | 0.947  368 | 0.188  119 | 1.12  2395 | 72 |
| {BcMV2.BI=TRUE, BcOLV16.BI=FALSE} =>  {BcMV1.BI=TRUE} | 0.18  8119 | 0.894  118 | 0.210  396 | 1.13  2362 | 76 |
| {BcMV2.BI=TRUE, BcOLV16.BI=FALSE} =>  {BcOLV7.BI=FALSE} | 0.18  8119 | 0.894  118 | 0.210  396 | 1.10  8048 | 76 |
| {BcMV2.BI=TRUE, BcULV3=TRUE} =>  {BcHV1.BI=TRUE} | 0.20  5446 | 0.721  739 | 0.284  653 | 1.19  5011 | 83 |
| {BcHV1.BI=TRUE, BcMV2.BI=TRUE} =>  {BcULV3=TRUE} | 0.20  5446 | 0.703  39 | 0.292  079 | 1.18  404 | 83 |
| {BcMV2.BI=TRUE, BcULV3=TRUE} =>  {BcMV11=TRUE} | 0.23  7624 | 0.834  783 | 0.284  653 | 1.35  4426 | 96 |
| {BcMV11=TRUE, BcMV2.BI=TRUE} =>  {BcULV3=TRUE} | 0.23  7624 | 0.695  652 | 0.341  584 | 1.17  1014 | 96 |
| {BcMV11=TRUE, BcULV3=TRUE} =>  {BcMV2.BI=TRUE} | 0.23  7624 | 0.6 | 0.396  04 | 1.29  6257 | 96 |
| {BcMV2.BI=TRUE, BcULV3=TRUE} =>  {BcMV1.BI=TRUE} | 0.25 | 0.878  261 | 0.284  653 | 1.11  228 | 10  1 |
| {BcHV1.BI=TRUE, BcMV2.BI=TRUE} => | 0.23 | 0.788 | 0.292 | 1.27 | 93 |

| {BcMV11=TRUE} | 0198 | 136 | 079 | 8742 |  |
| --- | --- | --- | --- | --- | --- |
| {BcHV1.BI=TRUE, BcMV11=TRUE} =>  {BcMV2.BI=TRUE} | 0.23  0198 | 0.603  896 | 0.381  188 | 1.30  4674 | 93 |
| {BcHV1.BI=TRUE, BcMV2.BI=TRUE} =>  {BcMV9.BI=TRUE} | 0.23  0198 | 0.788  136 | 0.292  079 | 1.16  2069 | 93 |
| {BcMV2.BI=TRUE, BcMV9.BI=TRUE} =>  {BcHV1.BI=TRUE} | 0.23  0198 | 0.715  385 | 0.321  782 | 1.18  4489 | 93 |
| {BcHV1.BI=TRUE, BcMV2.BI=TRUE} =>  {BcULV4=TRUE} | 0.23  5149 | 0.805  085 | 0.292  079 | 1.16  5786 | 95 |
| {BcMV2.BI=TRUE, BcULV4=TRUE} =>  {BcHV1.BI=TRUE} | 0.23  5149 | 0.688  406 | 0.341  584 | 1.13  9819 | 95 |
| {BcHV1.BI=TRUE, BcMV2.BI=TRUE} =>  {BcMV1.BI=TRUE} | 0.25  7426 | 0.881  356 | 0.292  079 | 1.11  62 | 10  4 |
| {BcMV2.BI=TRUE, BcMV9.BI=TRUE} =>  {BcMV11=TRUE} | 0.23  0198 | 0.715  385 | 0.321  782 | 1.16  0704 | 93 |
| {BcMV11=TRUE, BcMV9.BI=TRUE} =>  {BcMV2.BI=TRUE} | 0.23  0198 | 0.567  073 | 0.405  941 | 1.22  5121 | 93 |
| {BcMV2.BI=TRUE, BcULV4=TRUE} =>  {BcMV11=TRUE} | 0.25  495 | 0.746  377 | 0.341  584 | 1.21  0989 | 10  3 |
| {BcMV11=TRUE, BcULV4=TRUE} =>  {BcMV2.BI=TRUE} | 0.25  495 | 0.609  467 | 0.418  317 | 1.31  671 | 10  3 |
| {BcMV2.BI=TRUE, BcPV3.BI=FALSE} => | 0.20 | 0.704 | 0.284 | 1.14 | 81 |

| {BcMV11=TRUE} | 0495 | 348 | 653 | 2797 |  |
| --- | --- | --- | --- | --- | --- |
| {BcEV2.BI=FALSE, BcMV2.BI=TRUE} =>  {BcMV11=TRUE} | 0.22  2772 | 0.697  674 | 0.319  307 | 1.13  197 | 90 |
| {BcHV3.BI=FALSE, BcMV2.BI=TRUE} =>  {BcMV11=TRUE} | 0.23  0198 | 0.699  248 | 0.329  208 | 1.13  4523 | 93 |
| {BcFV5.BI=FALSE, BcMV2.BI=TRUE} =>  {BcMV11=TRUE} | 0.24  0099 | 0.697  842 | 0.344  059 | 1.13  2241 | 97 |
| {BcMV2.BI=TRUE, BcULV1.BI=FALSE} =>  {BcMV11=TRUE} | 0.23  5149 | 0.725  191 | 0.324  257 | 1.17  6615 | 95 |
| {BcMV11=TRUE, BcMV2.BI=TRUE} =>  {BcMV1.BI=TRUE} | 0.31  4356 | 0.920  29 | 0.341  584 | 1.16  5508 | 12  7 |
| {BcMV1.BI=TRUE, BcMV2.BI=TRUE} =>  {BcMV11=TRUE} | 0.31  4356 | 0.769  697 | 0.408  416 | 1.24  8826 | 12  7 |
| {BcMV1.BI=TRUE, BcMV11=TRUE} =>  {BcMV2.BI=TRUE} | 0.31  4356 | 0.604  762 | 0.519  802 | 1.30  6544 | 12  7 |
| {BcMV12=FALSE, BcMV2.BI=TRUE} =>  {BcMV11=TRUE} | 0.25  9901 | 0.760  87 | 0.341  584 | 1.23  4503 | 10  5 |
| {BcMV2.BI=TRUE, BcNSRV7.BI=FALSE} =>  {BcMV11=TRUE} | 0.25  9901 | 0.719  178 | 0.361  386 | 1.16  6859 | 10  5 |
| {BcMV11=TRUE, BcNSRV7.BI=FALSE} =>  {BcMV2.BI=TRUE} | 0.25  9901 | 0.535  714 | 0.485  149 | 1.15  7372 | 10  5 |
| {BcMV2.BI=TRUE, BcVV3.BI=FALSE} => | 0.25 | 0.725 | 0.351 | 1.17 | 10 |

| {BcMV11=TRUE} | 495 | 352 | 485 | 6877 | 3 |
| --- | --- | --- | --- | --- | --- |
| {BcMV2.BI=TRUE, BcOLV7.BI=FALSE} =>  {BcMV11=TRUE} | 0.25  9901 | 0.724  138 | 0.358  911 | 1.17  4907 | 10  5 |
| {BcMV11=TRUE, BcOLV7.BI=FALSE} =>  {BcMV2.BI=TRUE} | 0.25  9901 | 0.535  714 | 0.485  149 | 1.15  7372 | 10  5 |
| {BcDMV1=FALSE, BcMV2.BI=TRUE} =>  {BcMV11=TRUE} | 0.28  2178 | 0.712  5 | 0.396  04 | 1.15  6024 | 11  4 |
| {BcDMV1=FALSE, BcMV11=TRUE} =>  {BcMV2.BI=TRUE} | 0.28  2178 | 0.553  398 | 0.509  901 | 1.19  5577 | 11  4 |
| {BcFV9=FALSE, BcMV2.BI=TRUE} =>  {BcMV11=TRUE} | 0.28  9604 | 0.722  222 | 0.400  99 | 1.17  1798 | 11  7 |
| {BcFV9=FALSE, BcMV11=TRUE} =>  {BcMV2.BI=TRUE} | 0.28  9604 | 0.554  502 | 0.522  277 | 1.19  7962 | 11  7 |
| {BcMV2.BI=TRUE, BcOLV4.BI=FALSE} =>  {BcMV11=TRUE} | 0.26  7327 | 0.710  526 | 0.376  238 | 1.15  2822 | 10  8 |
| {BcMV2.BI=TRUE, BcULV5=FALSE} =>  {BcMV11=TRUE} | 0.26  2376 | 0.697  368 | 0.376  238 | 1.13  1473 | 10  6 |
| {BcBNV5.BI=FALSE, BcMV2.BI=TRUE} =>  {BcMV11=TRUE} | 0.28  9604 | 0.709  091 | 0.408  416 | 1.15  0493 | 11  7 |
| {BcBNV5.BI=FALSE, BcMV11=TRUE} =>  {BcMV2.BI=TRUE} | 0.28  9604 | 0.539  171 | 0.537  129 | 1.16  4839 | 11  7 |
| {BcMV2.BI=TRUE, BvF.BI=FALSE} => | 0.32 | 0.748 | 0.433 | 1.21 | 13 |

| {BcMV11=TRUE} | 4257 | 571 | 168 | 455 | 1 |
| --- | --- | --- | --- | --- | --- |
| {BcMV11=TRUE, BvF.BI=FALSE} =>  {BcMV2.BI=TRUE} | 0.32  4257 | 0.567  1 | 0.571  782 | 1.22  5178 | 13  1 |
| {BcMCV5.BI=FALSE, BcMV2.BI=TRUE} =>  {BcMV11=TRUE} | 0.28  2178 | 0.703  704 | 0.400  99 | 1.14  1752 | 11  4 |
| {BcMV2.BI=TRUE, BcOLV13.BI=FALSE} =>  {BcMV11=TRUE} | 0.29  4554 | 0.721  212 | 0.408  416 | 1.17  0159 | 11  9 |
| {BcMV2.BI=TRUE, BcOLV2.BI=FALSE} =>  {BcMV11=TRUE} | 0.30  9406 | 0.735  294 | 0.420  792 | 1.19  3007 | 12  5 |
| {BcMV11=TRUE, BcOLV2.BI=FALSE} =>  {BcMV2.BI=TRUE} | 0.30  9406 | 0.543  478 | 0.569  307 | 1.17  4146 | 12  5 |
| {BcMCV3.BI=FALSE, BcMV2.BI=TRUE} =>  {BcMV11=TRUE} | 0.30  9406 | 0.757  576 | 0.408  416 | 1.22  9159 | 12  5 |
| {BcMCV3.BI=FALSE, BcMV11=TRUE} =>  {BcMV2.BI=TRUE} | 0.30  9406 | 0.531  915 | 0.581  683 | 1.14  9164 | 12  5 |
| {BcMV2.BI=TRUE, BcPV4.BI=FALSE} =>  {BcMV11=TRUE} | 0.31  4356 | 0.734  104 | 0.428  218 | 1.19  1076 | 12  7 |
| {BcMV11=TRUE, BcPV4.BI=FALSE} =>  {BcMV2.BI=TRUE} | 0.31  4356 | 0.545  064 | 0.576  733 | 1.17  7572 | 12  7 |
| {BcEV6=FALSE, BcMV2.BI=TRUE} =>  {BcMV11=TRUE} | 0.31  9307 | 0.737  143 | 0.433  168 | 1.19  6007 | 12  9 |
| {BcEV6=FALSE, BcMV11=TRUE} => | 0.31 | 0.553 | 0.576 | 1.19 | 12 |

| {BcMV2.BI=TRUE} | 9307 | 648 | 733 | 6117 | 9 |
| --- | --- | --- | --- | --- | --- |
| {BcMV2.BI=TRUE, BcOLV17.BI=FALSE} =>  {BcMV11=TRUE} | 0.30  6931 | 0.720  93 | 0.425  743 | 1.16  9702 | 12  4 |
| {BcMV11=TRUE, BcOLV17.BI=FALSE} =>  {BcMV2.BI=TRUE} | 0.30  6931 | 0.534  483 | 0.574  257 | 1.15  4711 | 12  4 |
| {BcMV2.BI=TRUE, BcOLV15.BI=FALSE} =>  {BcMV11=TRUE} | 0.33  1683 | 0.732  24 | 0.452  97 | 1.18  8053 | 13  4 |
| {BcMV11=TRUE, BcOLV15.BI=FALSE} =>  {BcMV2.BI=TRUE} | 0.33  1683 | 0.563  025 | 0.589  109 | 1.21  6375 | 13  4 |
| {BcEV5=FALSE, BcMV2.BI=TRUE} =>  {BcMV11=TRUE} | 0.30  4455 | 0.719  298 | 0.423  267 | 1.16  7054 | 12  3 |
| {BcEV5=FALSE, BcMV11=TRUE} =>  {BcMV2.BI=TRUE} | 0.30  4455 | 0.530  172 | 0.574  257 | 1.14  5399 | 12  3 |
| {BcMV2.BI=TRUE, BcMV9.BI=TRUE} =>  {BcULV4=TRUE} | 0.27  9703 | 0.869  231 | 0.321  782 | 1.25  8671 | 11  3 |
| {BcMV2.BI=TRUE, BcULV4=TRUE} =>  {BcMV9.BI=TRUE} | 0.27  9703 | 0.818  841 | 0.341  584 | 1.20  7342 | 11  3 |
| {BcMV2.BI=TRUE, BcMV9.BI=TRUE} =>  {BcMV1.BI=TRUE} | 0.28  2178 | 0.876  923 | 0.321  782 | 1.11  0586 | 11  4 |
| {BcMV2.BI=TRUE, BcMV9.BI=TRUE} =>  {BcULV5=FALSE} | 0.30  4455 | 0.946  154 | 0.321  782 | 1.07  9791 | 12  3 |
| {BcMV2.BI=TRUE, BcULV5=FALSE} => | 0.30 | 0.809 | 0.376 | 1.19 | 12 |

| {BcMV9.BI=TRUE} | 4455 | 211 | 238 | 3143 | 3 |
| --- | --- | --- | --- | --- | --- |
| {BcMV2.BI=TRUE, BcULV4=TRUE} =>  {BcMV1.BI=TRUE} | 0.29  703 | 0.869  565 | 0.341  584 | 1.10  1268 | 12  0 |
| {BcMV1.BI=TRUE, BcULV4=TRUE} =>  {BcMV2.BI=TRUE} | 0.29  703 | 0.540  541 | 0.549  505 | 1.16  7799 | 12  0 |
| {BcMV2.BI=TRUE, BcOLV4.BI=FALSE} =>  {BcULV4=TRUE} | 0.29  2079 | 0.776  316 | 0.376  238 | 1.12  4128 | 11  8 |
| {BcMV2.BI=TRUE, BcULV5=FALSE} =>  {BcULV4=TRUE} | 0.29  9505 | 0.796  053 | 0.376  238 | 1.15  2707 | 12  1 |
| {BcMV2.BI=TRUE, BcMV6.BI=FALSE} =>  {BcMV1.BI=TRUE} | 0.22  2772 | 0.873  786 | 0.254  95 | 1.10  6614 | 90 |
| {BcMV2.BI=TRUE, BcMV6.BI=FALSE} =>  {BcDMV1=FALSE} | 0.24  2574 | 0.951  456 | 0.254  95 | 1.12  7239 | 98 |
| {BcMV2.BI=TRUE, BcMV6.BI=FALSE} =>  {BcBNV5.BI=FALSE} | 0.25 | 0.980  583 | 0.254  95 | 1.07  9442 | 10  1 |
| {BcMV2.BI=TRUE, BcRV1.BI=FALSE} =>  {BcMV1.BI=TRUE} | 0.25  9901 | 0.913  043 | 0.284  653 | 1.15  6331 | 10  5 |
| {BcMV2.BI=TRUE, BcRV1.BI=FALSE} =>  {BcOLV7.BI=FALSE} | 0.25  495 | 0.895  652 | 0.284  653 | 1.10  9949 | 10  3 |
| {BcHV3.BI=FALSE, BcMV2.BI=TRUE} =>  {BcFV5.BI=FALSE} | 0.28  4653 | 0.864  662 | 0.329  208 | 1.10  5454 | 11  5 |
| {BcHV3.BI=FALSE, BcMV2.BI=TRUE} => | 0.30 | 0.939 | 0.329 | 1.11 | 12 |

| {BcDMV1=FALSE} | 9406 | 85 | 208 | 3488 | 5 |
| --- | --- | --- | --- | --- | --- |
| {BcFV5.BI=FALSE, BcMV2.BI=TRUE} =>  {BcMV1.BI=TRUE} | 0.29  9505 | 0.870  504 | 0.344  059 | 1.10  2456 | 12  1 |
| {BcFV5.BI=FALSE, BcMV2.BI=TRUE} =>  {BcDMV1=FALSE} | 0.32  1782 | 0.935  252 | 0.344  059 | 1.10  804 | 13  0 |
| {BcMV2.BI=TRUE, BcULV1.BI=FALSE} =>  {BcMV1.BI=TRUE} | 0.28  4653 | 0.877  863 | 0.324  257 | 1.11  1776 | 11  5 |
| {BcMV12=FALSE, BcMV2.BI=TRUE} =>  {BcMV1.BI=TRUE} | 0.30  198 | 0.884  058 | 0.341  584 | 1.11  9622 | 12  2 |
| {BcMV2.BI=TRUE, BcNSRV7.BI=FALSE} =>  {BcMV1.BI=TRUE} | 0.31  4356 | 0.869  863 | 0.361  386 | 1.10  1645 | 12  7 |
| {BcMV2.BI=TRUE, BcVV3.BI=FALSE} =>  {BcMV1.BI=TRUE} | 0.30  198 | 0.859  155 | 0.351  485 | 1.08  8083 | 12  2 |
| {BcMV2.BI=TRUE, BcOLV7.BI=FALSE} =>  {BcMV1.BI=TRUE} | 0.31  1881 | 0.868  966 | 0.358  911 | 1.10  0508 | 12  6 |
| {BcALV1.BI=FALSE, BcMV2.BI=TRUE} =>  {BcMV1.BI=TRUE} | 0.28  4653 | 0.877  863 | 0.324  257 | 1.11  1776 | 11  5 |
| {BcDMV1=FALSE, BcMV2.BI=TRUE} =>  {BcMV1.BI=TRUE} | 0.34  6535 | 0.875 | 0.396  04 | 1.10  815 | 14  0 |
| {BcDMV1=FALSE, BcMV1.BI=TRUE} =>  {BcMV2.BI=TRUE} | 0.34  6535 | 0.520  446 | 0.665  842 | 1.12  4386 | 14  0 |
| {BcFV9=FALSE, BcMV2.BI=TRUE} => | 0.35 | 0.888 | 0.400 | 1.12 | 14 |

| {BcMV1.BI=TRUE} | 6436 | 889 | 99 | 574 | 4 |
| --- | --- | --- | --- | --- | --- |
| {BcFV9=FALSE, BcMV1.BI=TRUE} =>  {BcMV2.BI=TRUE} | 0.35  6436 | 0.525  547 | 0.678  218 | 1.13  5407 | 14  4 |
| {BcMV2.BI=TRUE, BcOLV4.BI=FALSE} =>  {BcMV1.BI=TRUE} | 0.32  4257 | 0.861  842 | 0.376  238 | 1.09  1487 | 13  1 |
| {BcMV2.BI=TRUE, BcULV5=FALSE} =>  {BcMV1.BI=TRUE} | 0.32  4257 | 0.861  842 | 0.376  238 | 1.09  1487 | 13  1 |
| {BcBNV5.BI=FALSE, BcMV2.BI=TRUE} =>  {BcMV1.BI=TRUE} | 0.35  6436 | 0.872  727 | 0.408  416 | 1.10  5272 | 14  4 |
| {BcMV2.BI=TRUE, BvF.BI=FALSE} =>  {BcMV1.BI=TRUE} | 0.38  1188 | 0.88 | 0.433  168 | 1.11  4483 | 15  4 |
| {BcMV1.BI=TRUE, BvF.BI=FALSE} =>  {BcMV2.BI=TRUE} | 0.38  1188 | 0.522  034 | 0.730  198 | 1.12  7817 | 15  4 |
| {BcMCV5.BI=FALSE, BcMV2.BI=TRUE} =>  {BcMV1.BI=TRUE} | 0.34  6535 | 0.864  198 | 0.400  99 | 1.09  447 | 14  0 |
| {BcMV2.BI=TRUE, BcOLV13.BI=FALSE} =>  {BcMV1.BI=TRUE} | 0.36  1386 | 0.884  848 | 0.408  416 | 1.12  0623 | 14  6 |
| {BcMV2.BI=TRUE, BcOLV2.BI=FALSE} =>  {BcMV1.BI=TRUE} | 0.37  3762 | 0.888  235 | 0.420  792 | 1.12  4912 | 15  1 |
| {BcMCV3.BI=FALSE, BcMV2.BI=TRUE} =>  {BcMV1.BI=TRUE} | 0.36  1386 | 0.884  848 | 0.408  416 | 1.12  0623 | 14  6 |
| {BcMV2.BI=TRUE, BcPV4.BI=FALSE} => | 0.37 | 0.872 | 0.428 | 1.10 | 15 |

| {BcMV1.BI=TRUE} | 3762 | 832 | 218 | 5405 | 1 |
| --- | --- | --- | --- | --- | --- |
| {BcMV1.BI=TRUE, BcPV4.BI=FALSE} =>  {BcMV2.BI=TRUE} | 0.37  3762 | 0.513  605 | 0.727  723 | 1.10  9607 | 15  1 |
| {BcEV6=FALSE, BcMV2.BI=TRUE} =>  {BcMV1.BI=TRUE} | 0.38  1188 | 0.88 | 0.433  168 | 1.11  4483 | 15  4 |
| {BcEV6=FALSE, BcMV1.BI=TRUE} =>  {BcMV2.BI=TRUE} | 0.38  1188 | 0.515  05 | 0.740  099 | 1.11  2729 | 15  4 |
| {BcMV2.BI=TRUE, BcOLV17.BI=FALSE} =>  {BcMV1.BI=TRUE} | 0.37  3762 | 0.877  907 | 0.425  743 | 1.11  1832 | 15  1 |
| {BcMV2.BI=TRUE, BcOLV15.BI=FALSE} =>  {BcMV1.BI=TRUE} | 0.40  099 | 0.885  246 | 0.452  97 | 1.12  1126 | 16  2 |
| {BcMV1.BI=TRUE, BcOLV15.BI=FALSE} =>  {BcMV2.BI=TRUE} | 0.40  099 | 0.538  206 | 0.745  05 | 1.16  2755 | 16  2 |
| {BcEV5=FALSE, BcMV2.BI=TRUE} =>  {BcMV1.BI=TRUE} | 0.36  8812 | 0.871  345 | 0.423  267 | 1.10  3522 | 14  9 |
| {BcMV2.BI=TRUE, BcNSRV7.BI=FALSE} =>  {BcDMV1=FALSE} | 0.33  9109 | 0.938  356 | 0.361  386 | 1.11  1718 | 13  7 |
| {BcALV1.BI=FALSE, BcMV2.BI=TRUE} =>  {BcDMV1=FALSE} | 0.29  2079 | 0.900  763 | 0.324  257 | 1.06  718 | 11  8 |
| {BcBNV5.BI=FALSE, BcMV2.BI=TRUE} =>  {BcDMV1=FALSE} | 0.37  8713 | 0.927  273 | 0.408  416 | 1.09  8587 | 15  3 |
| {BcMCV5.BI=FALSE, BcMV2.BI=TRUE} => | 0.36 | 0.907 | 0.400 | 1.07 | 14 |

| {BcDMV1=FALSE} | 3861 | 407 | 99 | 5052 | 7 |
| --- | --- | --- | --- | --- | --- |
| {BcEV5=FALSE, BcMV2.BI=TRUE} =>  {BcDMV1=FALSE} | 0.39  3564 | 0.929  825 | 0.423  267 | 1.10  161 | 15  9 |
| {BcHV1SLR.BI=TRUE, BcOLV18=FALSE} =>  {BcMV2.BI=FALSE} | 0.13  1188 | 0.654  321 | 0.200  495 | 1.21  8183 | 53 |
| {BcMV2.BI=FALSE, BcOLV18=FALSE} =>  {BcMV4.BI=FALSE} | 0.23  0198 | 0.732  283 | 0.314  356 | 1.31  4856 | 93 |
| {BcMV4.BI=FALSE, BcOLV18=FALSE} =>  {BcMV2.BI=FALSE} | 0.23  0198 | 0.726  563 | 0.316  832 | 1.35  2679 | 93 |
| {BcMV2.BI=FALSE, BcMV4.BI=FALSE} =>  {BcOLV18=FALSE} | 0.23  0198 | 0.624  161 | 0.368  812 | 1.29  3134 | 93 |
| {BcMV2.BI=FALSE, BcOLV18=FALSE} =>  {BcOLV16.BI=FALSE} | 0.23  7624 | 0.755  906 | 0.314  356 | 1.29  4008 | 96 |
| {BcOLV16.BI=FALSE, BcOLV18=FALSE} =>  {BcMV2.BI=FALSE} | 0.23  7624 | 0.705  882 | 0.336  634 | 1.31  4177 | 96 |
| {BcMV2.BI=FALSE, BcOLV16.BI=FALSE} =>  {BcOLV18=FALSE} | 0.23  7624 | 0.635  762 | 0.373  762 | 1.31  7168 | 96 |
| {BcOLV18=FALSE, BcULV3=TRUE} =>  {BcMV2.BI=FALSE} | 0.17  3267 | 0.642  202 | 0.269  802 | 1.19  562 | 70 |
| {BcHV1.BI=TRUE, BcOLV18=FALSE} =>  {BcMV2.BI=FALSE} | 0.15  5941 | 0.656  25 | 0.237  624 | 1.22  1774 | 63 |
| {BcMV11=TRUE, BcMV2.BI=FALSE} => | 0.16 | 0.585 | 0.274 | 1.21 | 65 |

| {BcOLV18=FALSE} | 0891 | 586 | 752 | 3213 |  |
| --- | --- | --- | --- | --- | --- |
| {BcMV2.BI=FALSE, BcOLV18=FALSE} => | 0.28 | 0.905 | 0.314 | 1.35 | 11 |
| {BcOLV1.BI.MOVA.=FALSE} | 4653 | 512 | 356 | 4914 | 5 |
| {BcOLV1.BI.MOVA.=FALSE, | 0.28 | 0.746 | 0.381 | 1.39 | 11 |
| BcOLV18=FALSE} => {BcMV2.BI=FALSE} | 4653 | 753 | 188 | 0269 | 5 |
| {BcMV2.BI=FALSE, |  |  |  |  |  |
|  | 0.28 |  | 0.455 | 1.29 | 11 |
| BcOLV1.BI.MOVA.=FALSE} => |  | 0.625 |  |  |  |
|  | 4653 |  | 446 | 4872 | 5 |
| {BcOLV18=FALSE} |  |  |  |  |  |
| {BcMV9.BI=TRUE, BcOLV18=FALSE} => | 0.18 | 0.710 | 0.264 | 1.32 |  |
|  |  |  |  |  | 76 |
| {BcMV2.BI=FALSE} | 8119 | 28 | 851 | 2365 |  |
| {BcOLV18=FALSE, BcULV4=TRUE} => | 0.16 | 0.673 |  | 1.25 |  |
|  |  |  | 0.25 |  | 68 |
| {BcMV2.BI=FALSE} | 8317 | 267 |  | 3456 |  |
| {BcMV2.BI=FALSE, BcOLV18=FALSE} => | 0.24 | 0.779 | 0.314 | 1.12 |  |
|  |  |  |  |  | 99 |
| {BcMV6.BI=FALSE} | 505 | 528 | 356 | 0744 |  |
| {BcMV6.BI=FALSE, BcOLV18=FALSE} => | 0.24 | 0.697 | 0.351 | 1.29 |  |
|  |  |  |  |  | 99 |
| {BcMV2.BI=FALSE} | 505 | 183 | 485 | 7981 |  |
| {BcMV2.BI=FALSE, BcOLV18=FALSE} => | 0.26 | 0.842 | 0.314 | 1.18 | 10 |
| {BcPV3.BI=FALSE} | 4851 | 52 | 356 | 5986 | 7 |
| {BcOLV18=FALSE, BcPV3.BI=FALSE} => | 0.26 | 0.694 | 0.381 | 1.29 | 10 |
| {BcMV2.BI=FALSE} | 4851 | 805 | 188 | 3554 | 7 |
| {BcMV2.BI=FALSE, BcPV3.BI=FALSE} => | 0.26 | 0.622 | 0.425 | 1.28 | 10 |
| {BcOLV18=FALSE} | 4851 | 093 | 743 | 8849 | 7 |

| {BcMV2.BI=FALSE, BcOLV18=FALSE} =>  {BcRV1.BI=FALSE} | 0.25  495 | 0.811  024 | 0.314  356 | 1.12  2101 | 10  3 |
| --- | --- | --- | --- | --- | --- |
| {BcOLV18=FALSE, BcRV1.BI=FALSE} =>  {BcMV2.BI=FALSE} | 0.25  495 | 0.647  799 | 0.393  564 | 1.20  604 | 10  3 |
| {BcMV2.BI=FALSE, BcRV1.BI=FALSE} =>  {BcOLV18=FALSE} | 0.25  495 | 0.581  921 | 0.438  119 | 1.20  5621 | 10  3 |
| {BcEV2.BI=FALSE, BcOLV18=FALSE} =>  {BcMV2.BI=FALSE} | 0.24  2574 | 0.640  523 | 0.378  713 | 1.19  2494 | 98 |
| {BcEV2.BI=FALSE, BcMV2.BI=FALSE} =>  {BcOLV18=FALSE} | 0.24  2574 | 0.566  474 | 0.428  218 | 1.17  3618 | 98 |
| {BcMV2.BI=FALSE, BcOLV18=FALSE} =>  {BcHV3.BI=FALSE} | 0.26  9802 | 0.858  268 | 0.314  356 | 1.12  9447 | 10  9 |
| {BcHV3.BI=FALSE, BcOLV18=FALSE} =>  {BcMV2.BI=FALSE} | 0.26  9802 | 0.660  606 | 0.408  416 | 1.22  9884 | 10  9 |
| {BcHV3.BI=FALSE, BcMV2.BI=FALSE} =>  {BcOLV18=FALSE} | 0.26  9802 | 0.626  437 | 0.430  693 | 1.29  7849 | 10  9 |
| {BcMV2.BI=FALSE, BcOLV18=FALSE} =>  {BcFV5.BI=FALSE} | 0.27  7228 | 0.881  89 | 0.314  356 | 1.12  7479 | 11  2 |
| {BcFV5.BI=FALSE, BcOLV18=FALSE} =>  {BcMV2.BI=FALSE} | 0.27  7228 | 0.670  659 | 0.413  366 | 1.24  86 | 11  2 |
| {BcFV5.BI=FALSE, BcMV2.BI=FALSE} =>  {BcOLV18=FALSE} | 0.27  7228 | 0.632  768 | 0.438  119 | 1.31  0966 | 11  2 |

| {BcMV2.BI=FALSE, BcOLV18=FALSE} =>  {BcULV1.BI=FALSE} | 0.28  7129 | 0.913  386 | 0.314  356 | 1.16  4063 | 11  6 |
| --- | --- | --- | --- | --- | --- |
| {BcOLV18=FALSE, BcULV1.BI=FALSE} =>  {BcMV2.BI=FALSE} | 0.28  7129 | 0.670  52 | 0.428  218 | 1.24  8342 | 11  6 |
| {BcMV2.BI=FALSE, BcULV1.BI=FALSE} =>  {BcOLV18=FALSE} | 0.28  7129 | 0.623  656 | 0.460  396 | 1.29  2087 | 11  6 |
| {BcMV2.BI=FALSE, BcOLV18=FALSE} =>  {BcMV12=FALSE} | 0.28  4653 | 0.905  512 | 0.314  356 | 1.13  2591 | 11  5 |
| {BcMV12=FALSE, BcOLV18=FALSE} =>  {BcMV2.BI=FALSE} | 0.28  4653 | 0.705  521 | 0.403  465 | 1.31  3505 | 11  5 |
| {BcMV12=FALSE, BcMV2.BI=FALSE} =>  {BcOLV18=FALSE} | 0.28  4653 | 0.621  622 | 0.457  921 | 1.28  7872 | 11  5 |
| {BcNSRV7.BI=FALSE, BcOLV18=FALSE} =>  {BcMV2.BI=FALSE} | 0.26  7327 | 0.650  602 | 0.410  891 | 1.21  126 | 10  8 |
| {BcMV2.BI=FALSE, BcNSRV7.BI=FALSE} =>  {BcOLV18=FALSE} | 0.26  7327 | 0.610  169 | 0.438  119 | 1.26  4146 | 10  8 |
| {BcOLV18=FALSE, BcVV3.BI=FALSE} =>  {BcMV2.BI=FALSE} | 0.25  9901 | 0.664  557 | 0.391  089 | 1.23  724 | 10  5 |
| {BcMV2.BI=FALSE, BcVV3.BI=FALSE} =>  {BcOLV18=FALSE} | 0.25  9901 | 0.576  923 | 0.450  495 | 1.19  5266 | 10  5 |
| {BcOLV18=FALSE, BcOLV7.BI=FALSE} =>  {BcMV2.BI=FALSE} | 0.27  4752 | 0.637  931 | 0.430  693 | 1.18  7669 | 11  1 |

| {BcMV2.BI=FALSE, BcOLV7.BI=FALSE} =>  {BcOLV18=FALSE} | 0.27  4752 | 0.613  26 | 0.448  02 | 1.27  0548 | 11  1 |
| --- | --- | --- | --- | --- | --- |
| {BcMV2.BI=FALSE, BcOLV18=FALSE} =>  {BcALV1.BI=FALSE} | 0.28  7129 | 0.913  386 | 0.314  356 | 1.12  1604 | 11  6 |
| {BcALV1.BI=FALSE, BcOLV18=FALSE} =>  {BcMV2.BI=FALSE} | 0.28  7129 | 0.655  367 | 0.438  119 | 1.22  0131 | 11  6 |
| {BcALV1.BI=FALSE, BcMV2.BI=FALSE} =>  {BcOLV18=FALSE} | 0.28  7129 | 0.585  859 | 0.490  099 | 1.21  3779 | 11  6 |
| {BcDMV1=FALSE, BcOLV18=FALSE} =>  {BcMV2.BI=FALSE} | 0.26  2376 | 0.630  952 | 0.415  842 | 1.17  4676 | 10  6 |
| {BcDMV1=FALSE, BcMV2.BI=FALSE} =>  {BcOLV18=FALSE} | 0.26  2376 | 0.585  635 | 0.448  02 | 1.21  3316 | 10  6 |
| {BcFV9=FALSE, BcOLV18=FALSE} =>  {BcMV2.BI=FALSE} | 0.28  7129 | 0.640  884 | 0.448  02 | 1.19  3166 | 11  6 |
| {BcFV9=FALSE, BcMV2.BI=FALSE} =>  {BcOLV18=FALSE} | 0.28  7129 | 0.613  757 | 0.467  822 | 1.27  1578 | 11  6 |
| {BcMV2.BI=FALSE, BcOLV18=FALSE} =>  {BcOLV4.BI=FALSE} | 0.29  4554 | 0.937  008 | 0.314  356 | 1.06  9354 | 11  9 |
| {BcOLV18=FALSE, BcOLV4.BI=FALSE} =>  {BcMV2.BI=FALSE} | 0.29  4554 | 0.687  861 | 0.428  218 | 1.28  0627 | 11  9 |
| {BcMV2.BI=FALSE, BcOLV4.BI=FALSE} =>  {BcOLV18=FALSE} | 0.29  4554 | 0.589  109 | 0.5 | 1.22  0513 | 11  9 |

| {BcOLV18=FALSE, BcULV5=FALSE} =>  {BcMV2.BI=FALSE} | 0.28  9604 | 0.709  091 | 0.408  416 | 1.32  0151 | 11  7 |
| --- | --- | --- | --- | --- | --- |
| {BcMV2.BI=FALSE, BcULV5=FALSE} =>  {BcOLV18=FALSE} | 0.28  9604 | 0.579  208 | 0.5 | 1.2 | 11  7 |
| {BcBNV5.BI=FALSE, BcOLV18=FALSE} =>  {BcMV2.BI=FALSE} | 0.29  9505 | 0.657  609 | 0.455  446 | 1.22  4304 | 12  1 |
| {BcBNV5.BI=FALSE, BcMV2.BI=FALSE} =>  {BcOLV18=FALSE} | 0.29  9505 | 0.599  01 | 0.5 | 1.24  1026 | 12  1 |
| {BcOLV18=FALSE, BvF.BI=FALSE} =>  {BcMV2.BI=FALSE} | 0.28  2178 | 0.644  068 | 0.438  119 | 1.19  9094 | 11  4 |
| {BcMV2.BI=FALSE, BvF.BI=FALSE} =>  {BcOLV18=FALSE} | 0.28  2178 | 0.587  629 | 0.480  198 | 1.21  7446 | 11  4 |
| {BcMCV5.BI=FALSE, BcOLV18=FALSE} =>  {BcMV2.BI=FALSE} | 0.30  9406 | 0.654  45 | 0.472  772 | 1.21  8424 | 12  5 |
| {BcMCV5.BI=FALSE, BcMV2.BI=FALSE} =>  {BcOLV18=FALSE} | 0.30  9406 | 0.592  417 | 0.522  277 | 1.22  7367 | 12  5 |
| {BcOLV13.BI=FALSE, BcOLV18=FALSE} =>  {BcMV2.BI=FALSE} | 0.30  4455 | 0.675  824 | 0.450  495 | 1.25  8216 | 12  3 |
| {BcMV2.BI=FALSE, BcOLV13.BI=FALSE} =>  {BcOLV18=FALSE} | 0.30  4455 | 0.588  517 | 0.517  327 | 1.21  9286 | 12  3 |
| {BcOLV18=FALSE, BcOLV2.BI=FALSE} =>  {BcMV2.BI=FALSE} | 0.30  198 | 0.655  914 | 0.460  396 | 1.22  1149 | 12  2 |

| {BcMV2.BI=FALSE, BcOLV2.BI=FALSE} =>  {BcOLV18=FALSE} | 0.30  198 | 0.595  122 | 0.507  426 | 1.23  2971 | 12  2 |
| --- | --- | --- | --- | --- | --- |
| {BcMCV3.BI=FALSE, BcOLV18=FALSE} =>  {BcMV2.BI=FALSE} | 0.31  1881 | 0.681  081 | 0.457  921 | 1.26  8003 | 12  6 |
| {BcMCV3.BI=FALSE, BcMV2.BI=FALSE} =>  {BcOLV18=FALSE} | 0.31  1881 | 0.594  34 | 0.524  752 | 1.23  135 | 12  6 |
| {BcOLV18=FALSE, BcPV4.BI=FALSE} =>  {BcMV2.BI=FALSE} | 0.29  703 | 0.652  174 | 0.455  446 | 1.21  4186 | 12  0 |
| {BcMV2.BI=FALSE, BcPV4.BI=FALSE} =>  {BcOLV18=FALSE} | 0.29  703 | 0.588  235 | 0.504  95 | 1.21  8703 | 12  0 |
| {BcEV6=FALSE, BcOLV18=FALSE} =>  {BcMV2.BI=FALSE} | 0.29  2079 | 0.644  809 | 0.452  97 | 1.20  0473 | 11  8 |
| {BcEV6=FALSE, BcMV2.BI=FALSE} =>  {BcOLV18=FALSE} | 0.29  2079 | 0.581  281 | 0.502  475 | 1.20  4295 | 11  8 |
| {BcOLV17.BI=FALSE, BcOLV18=FALSE} =>  {BcMV2.BI=FALSE} | 0.30  9406 | 0.657  895 | 0.470  297 | 1.22  4836 | 12  5 |
| {BcMV2.BI=FALSE, BcOLV17.BI=FALSE} =>  {BcOLV18=FALSE} | 0.30  9406 | 0.592  417 | 0.522  277 | 1.22  7367 | 12  5 |
| {BcOLV15.BI=FALSE, BcOLV18=FALSE} =>  {BcMV2.BI=FALSE} | 0.30  4455 | 0.643  979 | 0.472  772 | 1.19  8929 | 12  3 |
| {BcMV2.BI=FALSE, BcOLV15.BI=FALSE} =>  {BcOLV18=FALSE} | 0.30  4455 | 0.611  94 | 0.497  525 | 1.26  7815 | 12  3 |

| {BcEV5=FALSE, BcOLV18=FALSE} =>  {BcMV2.BI=FALSE} | 0.30  6931 | 0.663  102 | 0.462  871 | 1.23  453 | 12  4 |
| --- | --- | --- | --- | --- | --- |
| {BcEV5=FALSE, BcMV2.BI=FALSE} =>  {BcOLV18=FALSE} | 0.30  6931 | 0.582  16 | 0.527  228 | 1.20  6115 | 12  4 |
| {BcHV1SLR.BI=TRUE, BcOLV18=FALSE} =>  {BcOLV16.BI=FALSE} | 0.13  8614 | 0.691  358 | 0.200  495 | 1.18  3511 | 56 |
| {BcHV1SLR.BI=TRUE, BcOLV18=FALSE} =>  {BcULV3=TRUE} | 0.14  3564 | 0.716  049 | 0.200  495 | 1.20  535 | 58 |
| {BcHV1SLR.BI=TRUE, BcOLV18=FALSE} =>  {BcHV1.BI=TRUE} | 0.16  8317 | 0.839  506 | 0.200  495 | 1.39  0002 | 68 |
| {BcHV1.BI=TRUE, BcOLV18=FALSE} =>  {BcHV1SLR.BI=TRUE} | 0.16  8317 | 0.708  333 | 0.237  624 | 1.30  6697 | 68 |
| {BcHV1SLR.BI=TRUE, BcOLV18=FALSE} =>  {BcOLV1.BI.MOVA.=FALSE} | 0.16  0891 | 0.802  469 | 0.200  495 | 1.20  0732 | 65 |
| {BcHV1SLR.BI=TRUE, BcOLV18=FALSE} =>  {BcRV1.BI=FALSE} | 0.17  8218 | 0.888  889 | 0.200  495 | 1.22  9833 | 72 |
| {BcHV1SLR.BI=TRUE, BcOLV18=FALSE} =>  {BcOLV7.BI=FALSE} | 0.19  802 | 0.987  654 | 0.200  495 | 1.22  3964 | 80 |
| {BcMV3.BI=TRUE, BcOLV18=FALSE} =>  {BcRV1.BI=FALSE} | 0.17  3267 | 0.813  953 | 0.212  871 | 1.12  6155 | 70 |
| {BcMV3.BI=TRUE, BcOLV18=FALSE} =>  {BcOLV7.BI=FALSE} | 0.19  0594 | 0.895  349 | 0.212  871 | 1.10  9573 | 77 |

| {BcMV3.BI=TRUE, BcOLV18=FALSE} =>  {BcALV1.BI=FALSE} | 0.20  0495 | 0.941  86 | 0.212  871 | 1.15  657 | 81 |
| --- | --- | --- | --- | --- | --- |
| {BcMV4.BI=FALSE, BcOLV18=FALSE} =>  {BcOLV16.BI=FALSE} | 0.23  2673 | 0.734  375 | 0.316  832 | 1.25  715 | 94 |
| {BcOLV16.BI=FALSE, BcOLV18=FALSE} =>  {BcMV4.BI=FALSE} | 0.23  2673 | 0.691  176 | 0.336  634 | 1.24  1046 | 94 |
| {BcMV4.BI=FALSE, BcOLV16.BI=FALSE} =>  {BcOLV18=FALSE} | 0.23  2673 | 0.635  135 | 0.366  337 | 1.31  587 | 94 |
| {BcMV4.BI=FALSE, BcOLV18=FALSE} =>  {BcOLV1.BI.MOVA.=FALSE} | 0.26  4851 | 0.835  938 | 0.316  832 | 1.25  081 | 10  7 |
| {BcOLV1.BI.MOVA.=FALSE,  BcOLV18=FALSE} => {BcMV4.BI=FALSE} | 0.26  4851 | 0.694  805 | 0.381  188 | 1.24  7561 | 10  7 |
| {BcMV4.BI=FALSE, BcOLV1.BI.MOVA.=FALSE} =>  {BcOLV18=FALSE} | 0.26  4851 | 0.644  578 | 0.410  891 | 1.33  5434 | 10  7 |
| {BcOLV18=FALSE, BcULV4=TRUE} =>  {BcMV4.BI=FALSE} | 0.16  8317 | 0.673  267 | 0.25 | 1.20  8889 | 68 |
| {BcMV6.BI=FALSE, BcOLV18=FALSE} =>  {BcMV4.BI=FALSE} | 0.24  0099 | 0.683  099 | 0.351  485 | 1.22  6541 | 97 |
| {BcMV4.BI=FALSE, BcOLV18=FALSE} =>  {BcPV3.BI=FALSE} | 0.26  9802 | 0.851  563 | 0.316  832 | 1.19  8715 | 10  9 |
| {BcOLV18=FALSE, BcPV3.BI=FALSE} => | 0.26 | 0.707 | 0.381 | 1.27 | 10 |

| {BcMV4.BI=FALSE} | 9802 | 792 | 188 | 088 | 9 |
| --- | --- | --- | --- | --- | --- |
| {BcMV4.BI=FALSE, BcPV3.BI=FALSE} =>  {BcOLV18=FALSE} | 0.26  9802 | 0.602  21 | 0.448  02 | 1.24  7655 | 10  9 |
| {BcMV4.BI=FALSE, BcRV1.BI=FALSE} =>  {BcOLV18=FALSE} | 0.24  7525 | 0.555  556 | 0.445  545 | 1.15  0997 | 10  0 |
| {BcMV4.BI=FALSE, BcOLV18=FALSE} =>  {BcEV2.BI=FALSE} | 0.26  2376 | 0.828  125 | 0.316  832 | 1.10  7823 | 10  6 |
| {BcEV2.BI=FALSE, BcOLV18=FALSE} =>  {BcMV4.BI=FALSE} | 0.26  2376 | 0.692  81 | 0.378  713 | 1.24  398 | 10  6 |
| {BcEV2.BI=FALSE, BcMV4.BI=FALSE} =>  {BcOLV18=FALSE} | 0.26  2376 | 0.605  714 | 0.433  168 | 1.25  4916 | 10  6 |
| {BcMV4.BI=FALSE, BcOLV18=FALSE} =>  {BcHV3.BI=FALSE} | 0.28  4653 | 0.898  438 | 0.316  832 | 1.18  2309 | 11  5 |
| {BcHV3.BI=FALSE, BcOLV18=FALSE} =>  {BcMV4.BI=FALSE} | 0.28  4653 | 0.696  97 | 0.408  416 | 1.25  1448 | 11  5 |
| {BcHV3.BI=FALSE, BcMV4.BI=FALSE} =>  {BcOLV18=FALSE} | 0.28  4653 | 0.608  466 | 0.467  822 | 1.26  0616 | 11  5 |
| {BcMV4.BI=FALSE, BcOLV18=FALSE} =>  {BcFV5.BI=FALSE} | 0.28  2178 | 0.890  625 | 0.316  832 | 1.13  8647 | 11  4 |
| {BcFV5.BI=FALSE, BcOLV18=FALSE} =>  {BcMV4.BI=FALSE} | 0.28  2178 | 0.682  635 | 0.413  366 | 1.22  5709 | 11  4 |
| {BcFV5.BI=FALSE, BcMV4.BI=FALSE} => | 0.28 | 0.612 | 0.460 | 1.26 | 11 |

| {BcOLV18=FALSE} | 2178 | 903 | 396 | 981 | 4 |
| --- | --- | --- | --- | --- | --- |
| {BcMV4.BI=FALSE, BcOLV18=FALSE} =>  {BcULV1.BI=FALSE} | 0.28  2178 | 0.890  625 | 0.316  832 | 1.13  5055 | 11  4 |
| {BcOLV18=FALSE, BcULV1.BI=FALSE} =>  {BcMV4.BI=FALSE} | 0.28  2178 | 0.658  96 | 0.428  218 | 1.18  3198 | 11  4 |
| {BcMV4.BI=FALSE, BcULV1.BI=FALSE} =>  {BcOLV18=FALSE} | 0.28  2178 | 0.626  374 | 0.450  495 | 1.29  7718 | 11  4 |
| {BcMV12=FALSE, BcOLV18=FALSE} =>  {BcMV4.BI=FALSE} | 0.25  9901 | 0.644  172 | 0.403  465 | 1.15  6646 | 10  5 |
| {BcMV12=FALSE, BcMV4.BI=FALSE} =>  {BcOLV18=FALSE} | 0.25  9901 | 0.593  22 | 0.438  119 | 1.22  9031 | 10  5 |
| {BcNSRV7.BI=FALSE, BcOLV18=FALSE} =>  {BcMV4.BI=FALSE} | 0.27  4752 | 0.668  675 | 0.410  891 | 1.20  0643 | 11  1 |
| {BcMV4.BI=FALSE, BcNSRV7.BI=FALSE} =>  {BcOLV18=FALSE} | 0.27  4752 | 0.593  583 | 0.462  871 | 1.22  9782 | 11  1 |
| {BcOLV18=FALSE, BcVV3.BI=FALSE} =>  {BcMV4.BI=FALSE} | 0.26  4851 | 0.677  215 | 0.391  089 | 1.21  5977 | 10  7 |
| {BcMV4.BI=FALSE, BcVV3.BI=FALSE} =>  {BcOLV18=FALSE} | 0.26  4851 | 0.563  158 | 0.470  297 | 1.16  6748 | 10  7 |
| {BcMV4.BI=FALSE, BcOLV18=FALSE} =>  {BcOLV7.BI=FALSE} | 0.28  2178 | 0.890  625 | 0.316  832 | 1.10  3719 | 11  4 |
| {BcOLV18=FALSE, BcOLV7.BI=FALSE} => | 0.28 | 0.655 | 0.430 | 1.17 | 11 |

| {BcMV4.BI=FALSE} | 2178 | 172 | 693 | 6398 | 4 |
| --- | --- | --- | --- | --- | --- |
| {BcMV4.BI=FALSE, BcOLV7.BI=FALSE} =>  {BcOLV18=FALSE} | 0.28  2178 | 0.6 | 0.470  297 | 1.24  3077 | 11  4 |
| {BcMV4.BI=FALSE, BcOLV18=FALSE} =>  {BcALV1.BI=FALSE} | 0.29  703 | 0.937  5 | 0.316  832 | 1.15  1216 | 12  0 |
| {BcALV1.BI=FALSE, BcOLV18=FALSE} =>  {BcMV4.BI=FALSE} | 0.29  703 | 0.677  966 | 0.438  119 | 1.21  7326 | 12  0 |
| {BcALV1.BI=FALSE, BcMV4.BI=FALSE} =>  {BcOLV18=FALSE} | 0.29  703 | 0.594  059 | 0.5 | 1.23  0769 | 12  0 |
| {BcDMV1=FALSE, BcOLV18=FALSE} =>  {BcMV4.BI=FALSE} | 0.27  7228 | 0.666  667 | 0.415  842 | 1.19  7037 | 11  2 |
| {BcDMV1=FALSE, BcMV4.BI=FALSE} =>  {BcOLV18=FALSE} | 0.27  7228 | 0.574  359 | 0.482  673 | 1.18  9954 | 11  2 |
| {BcMV4.BI=FALSE, BcOLV18=FALSE} =>  {BcFV9=FALSE} | 0.29  703 | 0.937  5 | 0.316  832 | 1.07  906 | 12  0 |
| {BcFV9=FALSE, BcOLV18=FALSE} =>  {BcMV4.BI=FALSE} | 0.29  703 | 0.662  983 | 0.448  02 | 1.19  0424 | 12  0 |
| {BcFV9=FALSE, BcMV4.BI=FALSE} =>  {BcOLV18=FALSE} | 0.29  703 | 0.579  71 | 0.512  376 | 1.20  1041 | 12  0 |
| {BcOLV18=FALSE, BcOLV4.BI=FALSE} =>  {BcMV4.BI=FALSE} | 0.28  7129 | 0.670  52 | 0.428  218 | 1.20  3956 | 11  6 |
| {BcMV4.BI=FALSE, BcOLV4.BI=FALSE} => | 0.28 | 0.568 | 0.504 | 1.17 | 11 |

| {BcOLV18=FALSE} | 7129 | 627 | 95 | 8079 | 6 |
| --- | --- | --- | --- | --- | --- |
| {BcOLV18=FALSE, BcULV5=FALSE} =>  {BcMV4.BI=FALSE} | 0.27  7228 | 0.678  788 | 0.408  416 | 1.21  8801 | 11  2 |
| {BcMV4.BI=FALSE, BcULV5=FALSE} =>  {BcOLV18=FALSE} | 0.27  7228 | 0.56 | 0.495  05 | 1.16  0205 | 11  2 |
| {BcBNV5.BI=FALSE, BcOLV18=FALSE} =>  {BcMV4.BI=FALSE} | 0.30  198 | 0.663  043 | 0.455  446 | 1.19  0531 | 12  2 |
| {BcBNV5.BI=FALSE, BcMV4.BI=FALSE} =>  {BcOLV18=FALSE} | 0.30  198 | 0.575  472 | 0.524  752 | 1.19  2259 | 12  2 |
| {BcOLV18=FALSE, BvF.BI=FALSE} =>  {BcMV4.BI=FALSE} | 0.28  9604 | 0.661  017 | 0.438  119 | 1.18  6893 | 11  7 |
| {BcMV4.BI=FALSE, BvF.BI=FALSE} =>  {BcOLV18=FALSE} | 0.28  9604 | 0.570  732 | 0.507  426 | 1.18  2439 | 11  7 |
| {BcMV4.BI=FALSE, BcOLV18=FALSE} =>  {BcMCV5.BI=FALSE} | 0.31  6832 | 1 | 0.316  832 | 1.08  311 | 12  8 |
| {BcMCV5.BI=FALSE, BcOLV18=FALSE} =>  {BcMV4.BI=FALSE} | 0.31  6832 | 0.670  157 | 0.472  772 | 1.20  3304 | 12  8 |
| {BcMCV5.BI=FALSE, BcMV4.BI=FALSE} =>  {BcOLV18=FALSE} | 0.31  6832 | 0.581  818 | 0.544  554 | 1.20  5408 | 12  8 |
| {BcOLV13.BI=FALSE, BcOLV18=FALSE} =>  {BcMV4.BI=FALSE} | 0.30  198 | 0.670  33 | 0.450  495 | 1.20  3614 | 12  2 |
| {BcMV4.BI=FALSE, BcOLV13.BI=FALSE} => | 0.30 | 0.564 | 0.534 | 1.17 | 12 |

| {BcOLV18=FALSE} | 198 | 815 | 653 | 018 | 2 |
| --- | --- | --- | --- | --- | --- |
| {BcOLV18=FALSE, BcOLV2.BI=FALSE} =>  {BcMV4.BI=FALSE} | 0.30  198 | 0.655  914 | 0.460  396 | 1.17  773 | 12  2 |
| {BcMV4.BI=FALSE, BcOLV2.BI=FALSE} =>  {BcOLV18=FALSE} | 0.30  198 | 0.575  472 | 0.524  752 | 1.19  2259 | 12  2 |
| {BcMCV3.BI=FALSE, BcOLV18=FALSE} =>  {BcMV4.BI=FALSE} | 0.29  9505 | 0.654  054 | 0.457  921 | 1.17  439 | 12  1 |
| {BcMCV3.BI=FALSE, BcMV4.BI=FALSE} =>  {BcOLV18=FALSE} | 0.29  9505 | 0.573  46 | 0.522  277 | 1.18  8091 | 12  1 |
| {BcOLV18=FALSE, BcPV4.BI=FALSE} =>  {BcMV4.BI=FALSE} | 0.29  703 | 0.652  174 | 0.455  446 | 1.17  1014 | 12  0 |
| {BcMV4.BI=FALSE, BcPV4.BI=FALSE} =>  {BcOLV18=FALSE} | 0.29  703 | 0.576  923 | 0.514  851 | 1.19  5266 | 12  0 |
| {BcEV6=FALSE, BcOLV18=FALSE} =>  {BcMV4.BI=FALSE} | 0.29  703 | 0.655  738 | 0.452  97 | 1.17  7413 | 12  0 |
| {BcEV6=FALSE, BcMV4.BI=FALSE} =>  {BcOLV18=FALSE} | 0.29  703 | 0.576  923 | 0.514  851 | 1.19  5266 | 12  0 |
| {BcOLV17.BI=FALSE, BcOLV18=FALSE} =>  {BcMV4.BI=FALSE} | 0.30  6931 | 0.652  632 | 0.470  297 | 1.17  1836 | 12  4 |
| {BcMV4.BI=FALSE, BcOLV17.BI=FALSE} =>  {BcOLV18=FALSE} | 0.30  6931 | 0.571  429 | 0.537  129 | 1.18  3883 | 12  4 |
| {BcOLV15.BI=FALSE, BcOLV18=FALSE} => | 0.31 | 0.659 | 0.472 | 1.18 | 12 |

| {BcMV4.BI=FALSE} | 1881 | 686 | 772 | 4503 | 6 |
| --- | --- | --- | --- | --- | --- |
| {BcMV4.BI=FALSE, BcOLV15.BI=FALSE} =>  {BcOLV18=FALSE} | 0.31  1881 | 0.580  645 | 0.537  129 | 1.20  2978 | 12  6 |
| {BcEV5=FALSE, BcOLV18=FALSE} =>  {BcMV4.BI=FALSE} | 0.31  4356 | 0.679  144 | 0.462  871 | 1.21  9441 | 12  7 |
| {BcEV5=FALSE, BcMV4.BI=FALSE} =>  {BcOLV18=FALSE} | 0.31  4356 | 0.569  507 | 0.551  98 | 1.17  9901 | 12  7 |
| {BcMV11=TRUE, BcOLV18=FALSE} =>  {BcOLV16.BI=FALSE} | 0.18  5644 | 0.688  073 | 0.269  802 | 1.17  7888 | 75 |
| {BcOLV16.BI=FALSE, BcOLV18=FALSE} =>  {BcOLV1.BI.MOVA.=FALSE} | 0.28  9604 | 0.860  294 | 0.336  634 | 1.28  7255 | 11  7 |
| {BcOLV1.BI.MOVA.=FALSE,  BcOLV18=FALSE} => {BcOLV16.BI=FALSE} | 0.28  9604 | 0.759  74 | 0.381  188 | 1.30  0572 | 11  7 |
| {BcOLV1.BI.MOVA.=FALSE,  BcOLV16.BI=FALSE} => {BcOLV18=FALSE} | 0.28  9604 | 0.653  631 | 0.443  069 | 1.35  419 | 11  7 |
| {BcOLV16.BI=FALSE, BcOLV18=FALSE} =>  {BcMV6.BI=FALSE} | 0.26  2376 | 0.779  412 | 0.336  634 | 1.12  0578 | 10  6 |
| {BcMV6.BI=FALSE, BcOLV18=FALSE} =>  {BcOLV16.BI=FALSE} | 0.26  2376 | 0.746  479 | 0.351  485 | 1.27  7871 | 10  6 |
| {BcMV6.BI=FALSE, BcOLV16.BI=FALSE} =>  {BcOLV18=FALSE} | 0.26  2376 | 0.588  889 | 0.445  545 | 1.22  0057 | 10  6 |
| {BcOLV16.BI=FALSE, BcOLV18=FALSE} => | 0.27 | 0.830 | 0.336 | 1.16 | 11 |

| {BcPV3.BI=FALSE} | 9703 | 882 | 634 | 9604 | 3 |
| --- | --- | --- | --- | --- | --- |
| {BcOLV18=FALSE, BcPV3.BI=FALSE} =>  {BcOLV16.BI=FALSE} | 0.27  9703 | 0.733  766 | 0.381  188 | 1.25  6108 | 11  3 |
| {BcOLV16.BI=FALSE, BcPV3.BI=FALSE} =>  {BcOLV18=FALSE} | 0.27  9703 | 0.638  418 | 0.438  119 | 1.32  2671 | 11  3 |
| {BcOLV16.BI=FALSE, BcOLV18=FALSE} =>  {BcRV1.BI=FALSE} | 0.28  2178 | 0.838  235 | 0.336  634 | 1.15  975 | 11  4 |
| {BcOLV18=FALSE, BcRV1.BI=FALSE} =>  {BcOLV16.BI=FALSE} | 0.28  2178 | 0.716  981 | 0.393  564 | 1.22  7374 | 11  4 |
| {BcOLV16.BI=FALSE, BcRV1.BI=FALSE} =>  {BcOLV18=FALSE} | 0.28  2178 | 0.616  216 | 0.457  921 | 1.27  6674 | 11  4 |
| {BcEV2.BI=FALSE, BcOLV18=FALSE} =>  {BcOLV16.BI=FALSE} | 0.25 | 0.660  131 | 0.378  713 | 1.13  0054 | 10  1 |
| {BcEV2.BI=FALSE, BcOLV16.BI=FALSE} =>  {BcOLV18=FALSE} | 0.25 | 0.594  118 | 0.420  792 | 1.23  089 | 10  1 |
| {BcHV3.BI=FALSE, BcOLV18=FALSE} =>  {BcOLV16.BI=FALSE} | 0.27  7228 | 0.678  788 | 0.408  416 | 1.16  1993 | 11  2 |
| {BcHV3.BI=FALSE, BcOLV16.BI=FALSE} =>  {BcOLV18=FALSE} | 0.27  7228 | 0.632  768 | 0.438  119 | 1.31  0966 | 11  2 |
| {BcFV5.BI=FALSE, BcOLV18=FALSE} =>  {BcOLV16.BI=FALSE} | 0.28  2178 | 0.682  635 | 0.413  366 | 1.16  8578 | 11  4 |
| {BcFV5.BI=FALSE, BcOLV16.BI=FALSE} => | 0.28 | 0.612 | 0.460 | 1.26 | 11 |

| {BcOLV18=FALSE} | 2178 | 903 | 396 | 981 | 4 |
| --- | --- | --- | --- | --- | --- |
| {BcOLV16.BI=FALSE, BcOLV18=FALSE} =>  {BcULV1.BI=FALSE} | 0.31  1881 | 0.926  471 | 0.336  634 | 1.18  0739 | 12  6 |
| {BcOLV18=FALSE, BcULV1.BI=FALSE} =>  {BcOLV16.BI=FALSE} | 0.31  1881 | 0.728  324 | 0.428  218 | 1.24  6791 | 12  6 |
| {BcOLV16.BI=FALSE, BcULV1.BI=FALSE} =>  {BcOLV18=FALSE} | 0.31  1881 | 0.605  769 | 0.514  851 | 1.25  503 | 12  6 |
| {BcMV1.BI=TRUE, BcOLV18=FALSE} =>  {BcOLV16.BI=FALSE} | 0.25  2475 | 0.723  404 | 0.349  01 | 1.23  837 | 10  2 |
| {BcMV1.BI=TRUE, BcOLV16.BI=FALSE} =>  {BcOLV18=FALSE} | 0.25  2475 | 0.557  377 | 0.452  97 | 1.15  4771 | 10  2 |
| {BcOLV16.BI=FALSE, BcOLV18=FALSE} =>  {BcMV12=FALSE} | 0.29  9505 | 0.889  706 | 0.336  634 | 1.11  2821 | 12  1 |
| {BcMV12=FALSE, BcOLV18=FALSE} =>  {BcOLV16.BI=FALSE} | 0.29  9505 | 0.742  331 | 0.403  465 | 1.27  0771 | 12  1 |
| {BcMV12=FALSE, BcOLV16.BI=FALSE} =>  {BcOLV18=FALSE} | 0.29  9505 | 0.593  137 | 0.504  95 | 1.22  8859 | 12  1 |
| {BcNSRV7.BI=FALSE, BcOLV18=FALSE} =>  {BcOLV16.BI=FALSE} | 0.28  7129 | 0.698  795 | 0.410  891 | 1.19  6243 | 11  6 |
| {BcNSRV7.BI=FALSE, BcOLV16.BI=FALSE}  => {BcOLV18=FALSE} | 0.28  7129 | 0.617  021 | 0.465  347 | 1.27  8342 | 11  6 |
| {BcOLV18=FALSE, BcVV3.BI=FALSE} => | 0.27 | 0.696 | 0.391 | 1.19 | 11 |

| {BcOLV16.BI=FALSE} | 2277 | 203 | 089 | 1804 | 0 |
| --- | --- | --- | --- | --- | --- |
| {BcOLV16.BI=FALSE, BcVV3.BI=FALSE} =>  {BcOLV18=FALSE} | 0.27  2277 | 0.585  106 | 0.465  347 | 1.21  222 | 11  0 |
| {BcOLV16.BI=FALSE, BcOLV18=FALSE} =>  {BcOLV7.BI=FALSE} | 0.30  198 | 0.897  059 | 0.336  634 | 1.11  1693 | 12  2 |
| {BcOLV18=FALSE, BcOLV7.BI=FALSE} =>  {BcOLV16.BI=FALSE} | 0.30  198 | 0.701  149 | 0.430  693 | 1.20  0273 | 12  2 |
| {BcOLV16.BI=FALSE, BcOLV7.BI=FALSE} =>  {BcOLV18=FALSE} | 0.30  198 | 0.589  372 | 0.512  376 | 1.22  1058 | 12  2 |
| {BcOLV16.BI=FALSE, BcOLV18=FALSE} =>  {BcALV1.BI=FALSE} | 0.30  9406 | 0.919  118 | 0.336  634 | 1.12  8643 | 12  5 |
| {BcALV1.BI=FALSE, BcOLV18=FALSE} =>  {BcOLV16.BI=FALSE} | 0.30  9406 | 0.706  215 | 0.438  119 | 1.20  8944 | 12  5 |
| {BcALV1.BI=FALSE, BcOLV16.BI=FALSE} =>  {BcOLV18=FALSE} | 0.30  9406 | 0.612  745 | 0.504  95 | 1.26  9482 | 12  5 |
| {BcDMV1=FALSE, BcOLV18=FALSE} =>  {BcOLV16.BI=FALSE} | 0.28  9604 | 0.696  429 | 0.415  842 | 1.19  2191 | 11  7 |
| {BcDMV1=FALSE, BcOLV16.BI=FALSE} =>  {BcOLV18=FALSE} | 0.28  9604 | 0.590  909 | 0.490  099 | 1.22  4242 | 11  7 |
| {BcFV9=FALSE, BcOLV18=FALSE} =>  {BcOLV16.BI=FALSE} | 0.30  9406 | 0.690  608 | 0.448  02 | 1.18  2227 | 12  5 |
| {BcFV9=FALSE, BcOLV16.BI=FALSE} => | 0.30 | 0.606 | 0.509 | 1.25 | 12 |

| {BcOLV18=FALSE} | 9406 | 796 | 901 | 7157 | 5 |
| --- | --- | --- | --- | --- | --- |
| {BcOLV18=FALSE, BcOLV4.BI=FALSE} =>  {BcOLV16.BI=FALSE} | 0.30  6931 | 0.716  763 | 0.428  218 | 1.22  7001 | 12  4 |
| {BcOLV16.BI=FALSE, BcOLV4.BI=FALSE} =>  {BcOLV18=FALSE} | 0.30  6931 | 0.576  744 | 0.532  178 | 1.19  4896 | 12  4 |
| {BcOLV18=FALSE, BcULV5=FALSE} =>  {BcOLV16.BI=FALSE} | 0.29  703 | 0.727  273 | 0.408  416 | 1.24  4992 | 12  0 |
| {BcOLV16.BI=FALSE, BcULV5=FALSE} =>  {BcOLV18=FALSE} | 0.29  703 | 0.566  038 | 0.524  752 | 1.17  2714 | 12  0 |
| {BcBNV5.BI=FALSE, BcOLV18=FALSE} =>  {BcOLV16.BI=FALSE} | 0.31  6832 | 0.695  652 | 0.455  446 | 1.19  0862 | 12  8 |
| {BcBNV5.BI=FALSE, BcOLV16.BI=FALSE} =>  {BcOLV18=FALSE} | 0.31  6832 | 0.592  593 | 0.534  653 | 1.22  773 | 12  8 |
| {BcOLV18=FALSE, BvF.BI=FALSE} =>  {BcOLV16.BI=FALSE} | 0.29  9505 | 0.683  616 | 0.438  119 | 1.17  0258 | 12  1 |
| {BcOLV16.BI=FALSE, BvF.BI=FALSE} =>  {BcOLV18=FALSE} | 0.29  9505 | 0.573  46 | 0.522  277 | 1.18  8091 | 12  1 |
| {BcMCV5.BI=FALSE, BcOLV18=FALSE} =>  {BcOLV16.BI=FALSE} | 0.32  6733 | 0.691  099 | 0.472  772 | 1.18  3069 | 13  2 |
| {BcMCV5.BI=FALSE, BcOLV16.BI=FALSE} =>  {BcOLV18=FALSE} | 0.32  6733 | 0.586  667 | 0.556  931 | 1.21  5453 | 13  2 |
| {BcOLV13.BI=FALSE, BcOLV18=FALSE} => | 0.32 | 0.730 | 0.450 | 1.25 | 13 |

| {BcOLV16.BI=FALSE} | 9208 | 769 | 495 | 0978 | 3 |
| --- | --- | --- | --- | --- | --- |
| {BcOLV13.BI=FALSE, BcOLV16.BI=FALSE}  => {BcOLV18=FALSE} | 0.32  9208 | 0.588  496 | 0.559  406 | 1.21  9242 | 13  3 |
| {BcOLV18=FALSE, BcOLV2.BI=FALSE} =>  {BcOLV16.BI=FALSE} | 0.33  1683 | 0.720  43 | 0.460  396 | 1.23  3279 | 13  4 |
| {BcOLV16.BI=FALSE, BcOLV2.BI=FALSE} =>  {BcOLV18=FALSE} | 0.33  1683 | 0.592  92 | 0.559  406 | 1.22  8409 | 13  4 |
| {BcMCV3.BI=FALSE, BcOLV18=FALSE} =>  {BcOLV16.BI=FALSE} | 0.33  4158 | 0.729  73 | 0.457  921 | 1.24  9198 | 13  5 |
| {BcMCV3.BI=FALSE, BcOLV16.BI=FALSE} =>  {BcOLV18=FALSE} | 0.33  4158 | 0.581  897 | 0.574  257 | 1.20  557 | 13  5 |
| {BcOLV18=FALSE, BcPV4.BI=FALSE} =>  {BcOLV16.BI=FALSE} | 0.31  9307 | 0.701  087 | 0.455  446 | 1.20  0166 | 12  9 |
| {BcOLV16.BI=FALSE, BcPV4.BI=FALSE} =>  {BcOLV18=FALSE} | 0.31  9307 | 0.575  893 | 0.554  455 | 1.19  3132 | 12  9 |
| {BcEV6=FALSE, BcOLV18=FALSE} =>  {BcOLV16.BI=FALSE} | 0.31  9307 | 0.704  918 | 0.452  97 | 1.20  6724 | 12  9 |
| {BcEV6=FALSE, BcOLV16.BI=FALSE} =>  {BcOLV18=FALSE} | 0.31  9307 | 0.586  364 | 0.544  554 | 1.21  4825 | 12  9 |
| {BcOLV17.BI=FALSE, BcOLV18=FALSE} =>  {BcOLV16.BI=FALSE} | 0.32  6733 | 0.694  737 | 0.470  297 | 1.18  9295 | 13  2 |
| {BcOLV16.BI=FALSE, BcOLV17.BI=FALSE} | 0.32 | 0.581 | 0.561 | 1.20 | 13 |

| => {BcOLV18=FALSE} | 6733 | 498 | 881 | 4744 | 2 |
| --- | --- | --- | --- | --- | --- |
| {BcOLV15.BI=FALSE, BcOLV18=FALSE} =>  {BcOLV16.BI=FALSE} | 0.32  6733 | 0.691  099 | 0.472  772 | 1.18  3069 | 13  2 |
| {BcOLV15.BI=FALSE, BcOLV16.BI=FALSE}  => {BcOLV18=FALSE} | 0.32  6733 | 0.597  285 | 0.547  03 | 1.23  7452 | 13  2 |
| {BcEV5=FALSE, BcOLV18=FALSE} =>  {BcOLV16.BI=FALSE} | 0.31  9307 | 0.689  84 | 0.462  871 | 1.18  0912 | 12  9 |
| {BcEV5=FALSE, BcOLV16.BI=FALSE} =>  {BcOLV18=FALSE} | 0.31  9307 | 0.573  333 | 0.556  931 | 1.18  7829 | 12  9 |
| {BcHV1.BI=TRUE, BcOLV18=FALSE} =>  {BcULV3=TRUE} | 0.16  8317 | 0.708  333 | 0.237  624 | 1.19  2361 | 68 |
| {BcOLV18=FALSE, BcULV3=TRUE} =>  {BcOLV1.BI.MOVA.=FALSE} | 0.21  2871 | 0.788  991 | 0.269  802 | 1.18  0564 | 86 |
| {BcOLV18=FALSE, BcULV3=TRUE} =>  {BcRV1.BI=FALSE} | 0.22  2772 | 0.825  688 | 0.269  802 | 1.14  239 | 90 |
| {BcOLV18=FALSE, BcULV3=TRUE} =>  {BcEV2.BI=FALSE} | 0.22  5248 | 0.834  862 | 0.269  802 | 1.11  6836 | 91 |
| {BcOLV18=FALSE, BcULV3=TRUE} =>  {BcULV1.BI=FALSE} | 0.23  7624 | 0.880  734 | 0.269  802 | 1.12  245 | 96 |
| {BcOLV18=FALSE, BcULV3=TRUE} =>  {BcOLV7.BI=FALSE} | 0.24  7525 | 0.917  431 | 0.269  802 | 1.13  6939 | 10  0 |
| {BcOLV18=FALSE, BcULV3=TRUE} => | 0.24 | 0.899 | 0.269 | 1.10 | 98 |

| {BcALV1.BI=FALSE} | 2574 | 083 | 802 | 4041 |  |
| --- | --- | --- | --- | --- | --- |
| {BcHV1.BI=TRUE, BcOLV18=FALSE} =>  {BcOLV1.BI.MOVA.=FALSE} | 0.19  0594 | 0.802  083 | 0.237  624 | 1.20  0154 | 77 |
| {BcHV1.BI=TRUE, BcOLV18=FALSE} =>  {BcRV1.BI=FALSE} | 0.20  5446 | 0.864  583 | 0.237  624 | 1.19  6204 | 83 |
| {BcHV1.BI=TRUE, BcOLV18=FALSE} =>  {BcOLV7.BI=FALSE} | 0.21  5347 | 0.906  25 | 0.237  624 | 1.12  3083 | 87 |
| {BcHV1.BI=TRUE, BcOLV18=FALSE} =>  {BcALV1.BI=FALSE} | 0.21  2871 | 0.895  833 | 0.237  624 | 1.10  0051 | 86 |
| {BcMV11=TRUE, BcOLV18=FALSE} =>  {BcOLV7.BI=FALSE} | 0.24  505 | 0.908  257 | 0.269  802 | 1.12  557 | 99 |
| {BcMV9.BI=TRUE, BcOLV18=FALSE} =>  {BcOLV1.BI.MOVA.=FALSE} | 0.23  7624 | 0.897  196 | 0.264  851 | 1.34  2471 | 96 |
| {BcOLV18=FALSE, BcULV4=TRUE} =>  {BcOLV1.BI.MOVA.=FALSE} | 0.21  7822 | 0.871  287 | 0.25 | 1.30  3704 | 88 |
| {BcMV6.BI=FALSE, BcOLV18=FALSE} =>  {BcOLV1.BI.MOVA.=FALSE} | 0.28  9604 | 0.823  944 | 0.351  485 | 1.23  2864 | 11  7 |
| {BcOLV1.BI.MOVA.=FALSE,  BcOLV18=FALSE} => {BcPV3.BI=FALSE} | 0.30  9406 | 0.811  688 | 0.381  188 | 1.14  2586 | 12  5 |
| {BcOLV18=FALSE, BcPV3.BI=FALSE} =>  {BcOLV1.BI.MOVA.=FALSE} | 0.30  9406 | 0.811  688 | 0.381  188 | 1.21  4526 | 12  5 |
| {BcOLV1.BI.MOVA.=FALSE, | 0.30 | 0.600 | 0.514 | 1.24 | 12 |

| BcPV3.BI=FALSE} => {BcOLV18=FALSE} | 9406 | 962 | 851 | 5069 | 5 |
| --- | --- | --- | --- | --- | --- |
| {BcOLV1.BI.MOVA.=FALSE,  BcOLV18=FALSE} => {BcRV1.BI=FALSE} | 0.30  4455 | 0.798  701 | 0.381  188 | 1.10  5052 | 12  3 |
| {BcOLV18=FALSE, BcRV1.BI=FALSE} =>  {BcOLV1.BI.MOVA.=FALSE} | 0.30  4455 | 0.773  585 | 0.393  564 | 1.15  7512 | 12  3 |
| {BcOLV1.BI.MOVA.=FALSE,  BcRV1.BI=FALSE} => {BcOLV18=FALSE} | 0.30  4455 | 0.580  189 | 0.524  752 | 1.20  2032 | 12  3 |
| {BcEV2.BI=FALSE, BcOLV18=FALSE} =>  {BcOLV1.BI.MOVA.=FALSE} | 0.29  4554 | 0.777  778 | 0.378  713 | 1.16  3786 | 11  9 |
| {BcEV2.BI=FALSE, BcOLV1.BI.MOVA.=FALSE} =>  {BcOLV18=FALSE} | 0.29  4554 | 0.592  04 | 0.497  525 | 1.22  6585 | 11  9 |
| {BcOLV1.BI.MOVA.=FALSE,  BcOLV18=FALSE} => {BcHV3.BI=FALSE} | 0.32  6733 | 0.857  143 | 0.381  188 | 1.12  7966 | 13  2 |
| {BcHV3.BI=FALSE, BcOLV18=FALSE} =>  {BcOLV1.BI.MOVA.=FALSE} | 0.32  6733 | 0.8 | 0.408  416 | 1.19  7037 | 13  2 |
| {BcHV3.BI=FALSE, BcOLV1.BI.MOVA.=FALSE} =>  {BcOLV18=FALSE} | 0.32  6733 | 0.613  953 | 0.532  178 | 1.27  1986 | 13  2 |
| {BcOLV1.BI.MOVA.=FALSE,  BcOLV18=FALSE} => {BcFV5.BI=FALSE} | 0.32  6733 | 0.857  143 | 0.381  188 | 1.09  5841 | 13  2 |
| {BcFV5.BI=FALSE, BcOLV18=FALSE} => | 0.32 | 0.790 | 0.413 | 1.18 | 13 |

| {BcOLV1.BI.MOVA.=FALSE} | 6733 | 419 | 366 | 2701 | 2 |
| --- | --- | --- | --- | --- | --- |
| {BcFV5.BI=FALSE, BcOLV1.BI.MOVA.=FALSE} =>  {BcOLV18=FALSE} | 0.32  6733 | 0.602  74 | 0.542  079 | 1.24  8753 | 13  2 |
| {BcOLV1.BI.MOVA.=FALSE,  BcOLV18=FALSE} => {BcULV1.BI=FALSE} | 0.35  1485 | 0.922  078 | 0.381  188 | 1.17  514 | 14  2 |
| {BcOLV18=FALSE, BcULV1.BI=FALSE} =>  {BcOLV1.BI.MOVA.=FALSE} | 0.35  1485 | 0.820  809 | 0.428  218 | 1.22  8174 | 14  2 |
| {BcOLV1.BI.MOVA.=FALSE,  BcULV1.BI=FALSE} => {BcOLV18=FALSE} | 0.35  1485 | 0.628  319 | 0.559  406 | 1.30  1747 | 14  2 |
| {BcOLV1.BI.MOVA.=FALSE,  BcOLV18=FALSE} => {BcMV12=FALSE} | 0.34  1584 | 0.896  104 | 0.381  188 | 1.12  0823 | 13  8 |
| {BcMV12=FALSE, BcOLV18=FALSE} =>  {BcOLV1.BI.MOVA.=FALSE} | 0.34  1584 | 0.846  626 | 0.403  465 | 1.26  6803 | 13  8 |
| {BcMV12=FALSE, BcOLV1.BI.MOVA.=FALSE} =>  {BcOLV18=FALSE} | 0.34  1584 | 0.6 | 0.569  307 | 1.24  3077 | 13  8 |
| {BcNSRV7.BI=FALSE, BcOLV18=FALSE} =>  {BcOLV1.BI.MOVA.=FALSE} | 0.31  4356 | 0.765  06 | 0.410  891 | 1.14  4757 | 12  7 |
| {BcNSRV7.BI=FALSE, BcOLV1.BI.MOVA.=FALSE} =>  {BcOLV18=FALSE} | 0.31  4356 | 0.587  963 | 0.534  653 | 1.21  8139 | 12  7 |

| {BcOLV18=FALSE, BcVV3.BI=FALSE} =>  {BcOLV1.BI.MOVA.=FALSE} | 0.31  9307 | 0.816  456 | 0.391  089 | 1.22  166 | 12  9 |
| --- | --- | --- | --- | --- | --- |
| {BcOLV1.BI.MOVA.=FALSE,  BcVV3.BI=FALSE} => {BcOLV18=FALSE} | 0.31  9307 | 0.570  796 | 0.559  406 | 1.18  2573 | 12  9 |
| {BcOLV1.BI.MOVA.=FALSE,  BcOLV18=FALSE} => {BcOLV7.BI=FALSE} | 0.33  9109 | 0.889  61 | 0.381  188 | 1.10  2462 | 13  7 |
| {BcOLV18=FALSE, BcOLV7.BI=FALSE} =>  {BcOLV1.BI.MOVA.=FALSE} | 0.33  9109 | 0.787  356 | 0.430  693 | 1.17  8118 | 13  7 |
| {BcOLV1.BI.MOVA.=FALSE,  BcOLV7.BI=FALSE} => {BcOLV18=FALSE} | 0.33  9109 | 0.595  652 | 0.569  307 | 1.23  4069 | 13  7 |
| {BcOLV1.BI.MOVA.=FALSE,  BcOLV18=FALSE} => {BcALV1.BI=FALSE} | 0.34  1584 | 0.896  104 | 0.381  188 | 1.10  0383 | 13  8 |
| {BcALV1.BI=FALSE, BcOLV18=FALSE} =>  {BcOLV1.BI.MOVA.=FALSE} | 0.34  1584 | 0.779  661 | 0.438  119 | 1.16  6604 | 13  8 |
| {BcALV1.BI=FALSE, BcOLV1.BI.MOVA.=FALSE} =>  {BcOLV18=FALSE} | 0.34  1584 | 0.579  832 | 0.589  109 | 1.20  1293 | 13  8 |
| {BcDMV1=FALSE, BcOLV18=FALSE} =>  {BcOLV1.BI.MOVA.=FALSE} | 0.32  1782 | 0.773  81 | 0.415  842 | 1.15  7848 | 13  0 |
| {BcDMV1=FALSE, BcOLV1.BI.MOVA.=FALSE} =>  {BcOLV18=FALSE} | 0.32  1782 | 0.565  217 | 0.569  307 | 1.17  1014 | 13  0 |

| {BcOLV1.BI.MOVA.=FALSE,  BcOLV18=FALSE} => {BcFV9=FALSE} | 0.35  396 | 0.928  571 | 0.381  188 | 1.06  8783 | 14  3 |
| --- | --- | --- | --- | --- | --- |
| {BcFV9=FALSE, BcOLV18=FALSE} =>  {BcOLV1.BI.MOVA.=FALSE} | 0.35  396 | 0.790  055 | 0.448  02 | 1.18  2157 | 14  3 |
| {BcFV9=FALSE, BcOLV1.BI.MOVA.=FALSE}  => {BcOLV18=FALSE} | 0.35  396 | 0.600  84 | 0.589  109 | 1.24  4818 | 14  3 |
| {BcOLV1.BI.MOVA.=FALSE,  BcOLV18=FALSE} => {BcOLV4.BI=FALSE} | 0.35  6436 | 0.935  065 | 0.381  188 | 1.06  7136 | 14  4 |
| {BcOLV18=FALSE, BcOLV4.BI=FALSE} =>  {BcOLV1.BI.MOVA.=FALSE} | 0.35  6436 | 0.832  37 | 0.428  218 | 1.24  5472 | 14  4 |
| {BcOLV1.BI.MOVA.=FALSE,  BcOLV4.BI=FALSE} => {BcOLV18=FALSE} | 0.35  6436 | 0.580  645 | 0.613  861 | 1.20  2978 | 14  4 |
| {BcOLV1.BI.MOVA.=FALSE,  BcOLV18=FALSE} => {BcULV5=FALSE} | 0.35  8911 | 0.941  558 | 0.381  188 | 1.07  4547 | 14  5 |
| {BcOLV18=FALSE, BcULV5=FALSE} =>  {BcOLV1.BI.MOVA.=FALSE} | 0.35  8911 | 0.878  788 | 0.408  416 | 1.31  4927 | 14  5 |
| {BcOLV1.BI.MOVA.=FALSE, BcULV5=FALSE}  => {BcOLV18=FALSE} | 0.35  8911 | 0.58 | 0.618  812 | 1.20  1641 | 14  5 |
| {BcBNV5.BI=FALSE, BcOLV18=FALSE} =>  {BcOLV1.BI.MOVA.=FALSE} | 0.36  1386 | 0.793  478 | 0.455  446 | 1.18  7279 | 14  6 |
| {BcBNV5.BI=FALSE,  BcOLV1.BI.MOVA.=FALSE} => | 0.36  1386 | 0.577  075 | 0.626  238 | 1.19  5581 | 14  6 |

| {BcOLV18=FALSE} |  |  |  |  |  |
| --- | --- | --- | --- | --- | --- |
| {BcOLV18=FALSE, BvF.BI=FALSE} => | 0.34 | 0.779 | 0.438 | 1.16 | 13 |
| {BcOLV1.BI.MOVA.=FALSE} | 1584 | 661 | 119 | 6604 | 8 |
| {BcOLV1.BI.MOVA.=FALSE, BvF.BI=FALSE} | 0.34 | 0.570 | 0.599 | 1.18 | 13 |
| => {BcOLV18=FALSE} | 1584 | 248 | 01 | 1437 | 8 |
| {BcMCV5.BI=FALSE, BcOLV18=FALSE} => | 0.37 | 0.795 | 0.472 | 1.19 | 15 |
| {BcOLV1.BI.MOVA.=FALSE} | 6238 | 812 | 772 | 077 | 2 |
| {BcMCV5.BI=FALSE, |  |  |  |  |  |
|  | 0.37 | 0.582 | 0.646 | 1.20 | 15 |
| BcOLV1.BI.MOVA.=FALSE} => |  |  |  |  |  |
|  | 6238 | 375 | 04 | 6563 | 2 |
| {BcOLV18=FALSE} |  |  |  |  |  |
| {BcOLV13.BI=FALSE, BcOLV18=FALSE} => | 0.37 | 0.835 | 0.450 | 1.24 | 15 |
| {BcOLV1.BI.MOVA.=FALSE} | 6238 | 165 | 495 | 9654 | 2 |
| {BcOLV1.BI.MOVA.=FALSE, | 0.37 | 0.580 | 0.648 | 1.20 | 15 |
| BcOLV13.BI=FALSE} => {BcOLV18=FALSE} | 6238 | 153 | 515 | 1957 | 2 |
| {BcOLV18=FALSE, BcOLV2.BI=FALSE} => | 0.37 | 0.817 | 0.460 | 1.22 | 15 |
| {BcOLV1.BI.MOVA.=FALSE} | 6238 | 204 | 396 | 278 | 2 |
| {BcOLV1.BI.MOVA.=FALSE, | 0.37 | 0.577 | 0.650 | 1.19 | 15 |
| BcOLV2.BI=FALSE} => {BcOLV18=FALSE} | 6238 | 947 | 99 | 7387 | 2 |
| {BcMCV3.BI=FALSE, BcOLV18=FALSE} => | 0.37 | 0.827 | 0.457 | 1.23 | 15 |
| {BcOLV1.BI.MOVA.=FALSE} | 8713 | 027 | 921 | 7477 | 3 |
| {BcMCV3.BI=FALSE, | 0.37 | 0.586 | 0.646 | 1.21 | 15 |
| BcOLV1.BI.MOVA.=FALSE} => | 8713 | 207 | 04 | 45 | 3 |

| {BcOLV18=FALSE} |  |  |  |  |  |
| --- | --- | --- | --- | --- | --- |
| {BcOLV18=FALSE, BcPV4.BI=FALSE} =>  {BcOLV1.BI.MOVA.=FALSE} | 0.36  1386 | 0.793  478 | 0.455  446 | 1.18  7279 | 14  6 |
| {BcOLV1.BI.MOVA.=FALSE,  BcPV4.BI=FALSE} => {BcOLV18=FALSE} | 0.36  1386 | 0.586  345 | 0.616  337 | 1.21  4787 | 14  6 |
| {BcEV6=FALSE, BcOLV18=FALSE} =>  {BcOLV1.BI.MOVA.=FALSE} | 0.35  6436 | 0.786  885 | 0.452  97 | 1.17  7413 | 14  4 |
| {BcEV6=FALSE, BcOLV1.BI.MOVA.=FALSE}  => {BcOLV18=FALSE} | 0.35  6436 | 0.566  929 | 0.628  713 | 1.17  4561 | 14  4 |
| {BcOLV17.BI=FALSE, BcOLV18=FALSE} =>  {BcOLV1.BI.MOVA.=FALSE} | 0.36  8812 | 0.784  211 | 0.470  297 | 1.17  3411 | 14  9 |
| {BcOLV1.BI.MOVA.=FALSE,  BcOLV17.BI=FALSE} => {BcOLV18=FALSE} | 0.36  8812 | 0.568  702 | 0.648  515 | 1.17  8234 | 14  9 |
| {BcOLV15.BI=FALSE, BcOLV18=FALSE} =>  {BcOLV1.BI.MOVA.=FALSE} | 0.37  1287 | 0.785  34 | 0.472  772 | 1.17  5102 | 15  0 |
| {BcOLV1.BI.MOVA.=FALSE,  BcOLV15.BI=FALSE} => {BcOLV18=FALSE} | 0.37  1287 | 0.590  551 | 0.628  713 | 1.22  3501 | 15  0 |
| {BcEV5=FALSE, BcOLV18=FALSE} =>  {BcOLV1.BI.MOVA.=FALSE} | 0.37  1287 | 0.802  139 | 0.462  871 | 1.20  0238 | 15  0 |
| {BcEV5=FALSE, BcOLV1.BI.MOVA.=FALSE}  => {BcOLV18=FALSE} | 0.37  1287 | 0.568  182 | 0.653  465 | 1.17  7156 | 15  0 |
| {BcMV9.BI=TRUE, BcOLV18=FALSE} => | 0.22 | 0.831 | 0.264 | 1.17 | 89 |

| {BcPV3.BI=FALSE} | 0297 | 776 | 851 | 0862 |  |
| --- | --- | --- | --- | --- | --- |
| {BcMV9.BI=TRUE, BcOLV18=FALSE} =>  {BcHV3.BI=FALSE} | 0.22  5248 | 0.850  467 | 0.264  851 | 1.11  9182 | 91 |
| {BcMV9.BI=TRUE, BcOLV18=FALSE} =>  {BcULV1.BI=FALSE} | 0.23  7624 | 0.897  196 | 0.264  851 | 1.14  343 | 96 |
| {BcMV9.BI=TRUE, BcOLV18=FALSE} =>  {BcALV1.BI=FALSE} | 0.24  7525 | 0.934  579 | 0.264  851 | 1.14  7629 | 10  0 |
| {BcMV9.BI=TRUE, BcOLV18=FALSE} =>  {BcOLV4.BI=FALSE} | 0.25  2475 | 0.953  271 | 0.264  851 | 1.08  7914 | 10  2 |
| {BcMV9.BI=TRUE, BcOLV18=FALSE} =>  {BcULV5=FALSE} | 0.26  4851 | 1 | 0.264  851 | 1.14  1243 | 10  7 |
| {BcOLV18=FALSE, BcULV4=TRUE} =>  {BcEV2.BI=FALSE} | 0.21  7822 | 0.871  287 | 0.25 | 1.16  5563 | 88 |
| {BcOLV18=FALSE, BcULV4=TRUE} =>  {BcHV3.BI=FALSE} | 0.21  5347 | 0.861  386 | 0.25 | 1.13  355 | 87 |
| {BcOLV18=FALSE, BcULV4=TRUE} =>  {BcFV5.BI=FALSE} | 0.21  7822 | 0.871  287 | 0.25 | 1.11  3924 | 88 |
| {BcOLV18=FALSE, BcULV4=TRUE} =>  {BcALV1.BI=FALSE} | 0.22  2772 | 0.891  089 | 0.25 | 1.09  4225 | 90 |
| {BcOLV18=FALSE, BcULV4=TRUE} =>  {BcFV9=FALSE} | 0.23  7624 | 0.950  495 | 0.25 | 1.09  4017 | 96 |
| {BcMV6.BI=FALSE, BcOLV18=FALSE} => | 0.29 | 0.845 | 0.351 | 1.11 | 12 |

| {BcHV3.BI=FALSE} | 703 | 07 | 485 | 208 | 0 |
| --- | --- | --- | --- | --- | --- |
| {BcHV3.BI=FALSE, BcMV6.BI=FALSE} =>  {BcOLV18=FALSE} | 0.29  703 | 0.550  459 | 0.539  604 | 1.14  0438 | 12  0 |
| {BcMV6.BI=FALSE, BcOLV18=FALSE} =>  {BcULV1.BI=FALSE} | 0.32  6733 | 0.929  577 | 0.351  485 | 1.18  4698 | 13  2 |
| {BcMV6.BI=FALSE, BcULV1.BI=FALSE} =>  {BcOLV18=FALSE} | 0.32  6733 | 0.571  429 | 0.571  782 | 1.18  3883 | 13  2 |
| {BcMV6.BI=FALSE, BcOLV18=FALSE} =>  {BcOLV7.BI=FALSE} | 0.31  4356 | 0.894  366 | 0.351  485 | 1.10  8356 | 12  7 |
| {BcMV6.BI=FALSE, BcOLV7.BI=FALSE} =>  {BcOLV18=FALSE} | 0.31  4356 | 0.547  414 | 0.574  257 | 1.13  4129 | 12  7 |
| {BcMV6.BI=FALSE, BcOLV18=FALSE} =>  {BcALV1.BI=FALSE} | 0.31  9307 | 0.908  451 | 0.351  485 | 1.11  5544 | 12  9 |
| {BcMV6.BI=FALSE, BcOLV18=FALSE} =>  {BcFV9=FALSE} | 0.32  6733 | 0.929  577 | 0.351  485 | 1.06  9941 | 13  2 |
| {BcFV9=FALSE, BcMV6.BI=FALSE} =>  {BcOLV18=FALSE} | 0.32  6733 | 0.543  21 | 0.601  485 | 1.12  5419 | 13  2 |
| {BcMV6.BI=FALSE, BcOLV18=FALSE} =>  {BcMCV5.BI=FALSE} | 0.34  901 | 0.992  958 | 0.351  485 | 1.07  5482 | 14  1 |
| {BcOLV18=FALSE, BcPV3.BI=FALSE} =>  {BcRV1.BI=FALSE} | 0.31  6832 | 0.831  169 | 0.381  188 | 1.14  9973 | 12  8 |
| {BcOLV18=FALSE, BcRV1.BI=FALSE} => | 0.31 | 0.805 | 0.393 | 1.13 | 12 |

| {BcPV3.BI=FALSE} | 6832 | 031 | 564 | 3215 | 8 |
| --- | --- | --- | --- | --- | --- |
| {BcPV3.BI=FALSE, BcRV1.BI=FALSE} =>  {BcOLV18=FALSE} | 0.31  6832 | 0.566  372 | 0.559  406 | 1.17  3406 | 12  8 |
| {BcOLV18=FALSE, BcPV3.BI=FALSE} =>  {BcHV3.BI=FALSE} | 0.32  4257 | 0.850  649 | 0.381  188 | 1.11  9421 | 13  1 |
| {BcHV3.BI=FALSE, BcOLV18=FALSE} =>  {BcPV3.BI=FALSE} | 0.32  4257 | 0.793  939 | 0.408  416 | 1.11  7601 | 13  1 |
| {BcHV3.BI=FALSE, BcPV3.BI=FALSE} =>  {BcOLV18=FALSE} | 0.32  4257 | 0.587  444 | 0.551  98 | 1.21  7063 | 13  1 |
| {BcFV5.BI=FALSE, BcPV3.BI=FALSE} =>  {BcOLV18=FALSE} | 0.32  1782 | 0.588  235 | 0.547  03 | 1.21  8703 | 13  0 |
| {BcOLV18=FALSE, BcPV3.BI=FALSE} =>  {BcULV1.BI=FALSE} | 0.33  6634 | 0.883  117 | 0.381  188 | 1.12  5487 | 13  6 |
| {BcOLV18=FALSE, BcULV1.BI=FALSE} =>  {BcPV3.BI=FALSE} | 0.33  6634 | 0.786  127 | 0.428  218 | 1.10  6604 | 13  6 |
| {BcPV3.BI=FALSE, BcULV1.BI=FALSE} =>  {BcOLV18=FALSE} | 0.33  6634 | 0.588  745 | 0.571  782 | 1.21  9758 | 13  6 |
| {BcMV12=FALSE, BcOLV18=FALSE} =>  {BcPV3.BI=FALSE} | 0.32  9208 | 0.815  951 | 0.403  465 | 1.14  8586 | 13  3 |
| {BcMV12=FALSE, BcPV3.BI=FALSE} =>  {BcOLV18=FALSE} | 0.32  9208 | 0.565  957 | 0.581  683 | 1.17  2548 | 13  3 |
| {BcNSRV7.BI=FALSE, BcPV3.BI=FALSE} => | 0.32 | 0.572 | 0.561 | 1.18 | 13 |

| {BcOLV18=FALSE} | 1782 | 687 | 881 | 649 | 0 |
| --- | --- | --- | --- | --- | --- |
| {BcOLV18=FALSE, BcVV3.BI=FALSE} =>  {BcPV3.BI=FALSE} | 0.30  9406 | 0.791  139 | 0.391  089 | 1.11  3659 | 12  5 |
| {BcOLV18=FALSE, BcPV3.BI=FALSE} =>  {BcOLV7.BI=FALSE} | 0.34  4059 | 0.902  597 | 0.381  188 | 1.11  8556 | 13  9 |
| {BcOLV18=FALSE, BcOLV7.BI=FALSE} =>  {BcPV3.BI=FALSE} | 0.34  4059 | 0.798  851 | 0.430  693 | 1.12  4514 | 13  9 |
| {BcOLV7.BI=FALSE, BcPV3.BI=FALSE} =>  {BcOLV18=FALSE} | 0.34  4059 | 0.574  38 | 0.599  01 | 1.18  9998 | 13  9 |
| {BcOLV18=FALSE, BcPV3.BI=FALSE} =>  {BcALV1.BI=FALSE} | 0.34  4059 | 0.902  597 | 0.381  188 | 1.10  8357 | 13  9 |
| {BcALV1.BI=FALSE, BcOLV18=FALSE} =>  {BcPV3.BI=FALSE} | 0.34  4059 | 0.785  311 | 0.438  119 | 1.10  5455 | 13  9 |
| {BcALV1.BI=FALSE, BcPV3.BI=FALSE} =>  {BcOLV18=FALSE} | 0.34  4059 | 0.588  983 | 0.584  158 | 1.22  0252 | 13  9 |
| {BcDMV1=FALSE, BcPV3.BI=FALSE} =>  {BcOLV18=FALSE} | 0.32  4257 | 0.543  568 | 0.596  535 | 1.12  6162 | 13  1 |
| {BcFV9=FALSE, BcPV3.BI=FALSE} =>  {BcOLV18=FALSE} | 0.34  901 | 0.559  524 | 0.623  762 | 1.15  9219 | 14  1 |
| {BcOLV18=FALSE, BcOLV4.BI=FALSE} =>  {BcPV3.BI=FALSE} | 0.34  901 | 0.815  029 | 0.428  218 | 1.14  7288 | 14  1 |
| {BcOLV4.BI=FALSE, BcPV3.BI=FALSE} => | 0.34 | 0.546 | 0.638 | 1.13 | 14 |

| {BcOLV18=FALSE} | 901 | 512 | 614 | 226 | 1 |
| --- | --- | --- | --- | --- | --- |
| {BcOLV18=FALSE, BcULV5=FALSE} =>  {BcPV3.BI=FALSE} | 0.32  9208 | 0.806  061 | 0.408  416 | 1.13  4664 | 13  3 |
| {BcBNV5.BI=FALSE, BcOLV18=FALSE} =>  {BcPV3.BI=FALSE} | 0.35  8911 | 0.788  043 | 0.455  446 | 1.10  9302 | 14  5 |
| {BcBNV5.BI=FALSE, BcPV3.BI=FALSE} =>  {BcOLV18=FALSE} | 0.35  8911 | 0.557  692 | 0.643  564 | 1.15  5424 | 14  5 |
| {BcOLV18=FALSE, BvF.BI=FALSE} =>  {BcPV3.BI=FALSE} | 0.34  4059 | 0.785  311 | 0.438  119 | 1.10  5455 | 13  9 |
| {BcMCV5.BI=FALSE, BcOLV18=FALSE} =>  {BcPV3.BI=FALSE} | 0.37  1287 | 0.785  34 | 0.472  772 | 1.10  5496 | 15  0 |
| {BcMCV5.BI=FALSE, BcPV3.BI=FALSE} =>  {BcOLV18=FALSE} | 0.37  1287 | 0.563  91 | 0.658  416 | 1.16  8305 | 15  0 |
| {BcOLV13.BI=FALSE, BcOLV18=FALSE} =>  {BcPV3.BI=FALSE} | 0.36  3861 | 0.807  692 | 0.450  495 | 1.13  6961 | 14  7 |
| {BcOLV13.BI=FALSE, BcPV3.BI=FALSE} =>  {BcOLV18=FALSE} | 0.36  3861 | 0.536  496 | 0.678  218 | 1.11  151 | 14  7 |
| {BcOLV18=FALSE, BcOLV2.BI=FALSE} =>  {BcPV3.BI=FALSE} | 0.36  3861 | 0.790  323 | 0.460  396 | 1.11  251 | 14  7 |
| {BcOLV2.BI=FALSE, BcPV3.BI=FALSE} =>  {BcOLV18=FALSE} | 0.36  3861 | 0.546  468 | 0.665  842 | 1.13  217 | 14  7 |
| {BcMCV3.BI=FALSE, BcOLV18=FALSE} => | 0.36 | 0.805 | 0.457 | 1.13 | 14 |

| {BcPV3.BI=FALSE} | 8812 | 405 | 921 | 3741 | 9 |
| --- | --- | --- | --- | --- | --- |
| {BcMCV3.BI=FALSE, BcPV3.BI=FALSE} =>  {BcOLV18=FALSE} | 0.36  8812 | 0.541  818 | 0.680  693 | 1.12  2536 | 14  9 |
| {BcOLV18=FALSE, BcPV4.BI=FALSE} =>  {BcPV3.BI=FALSE} | 0.36  1386 | 0.793  478 | 0.455  446 | 1.11  6952 | 14  6 |
| {BcPV3.BI=FALSE, BcPV4.BI=FALSE} =>  {BcOLV18=FALSE} | 0.36  1386 | 0.546  816 | 0.660  891 | 1.13  2892 | 14  6 |
| {BcEV6=FALSE, BcOLV18=FALSE} =>  {BcPV3.BI=FALSE} | 0.35  6436 | 0.786  885 | 0.452  97 | 1.10  7671 | 14  4 |
| {BcEV6=FALSE, BcPV3.BI=FALSE} =>  {BcOLV18=FALSE} | 0.35  6436 | 0.539  326 | 0.660  891 | 1.11  7373 | 14  4 |
| {BcOLV17.BI=FALSE, BcOLV18=FALSE} =>  {BcPV3.BI=FALSE} | 0.37  8713 | 0.805  263 | 0.470  297 | 1.13  3541 | 15  3 |
| {BcOLV17.BI=FALSE, BcPV3.BI=FALSE} =>  {BcOLV18=FALSE} | 0.37  8713 | 0.548  387 | 0.690  594 | 1.13  6146 | 15  3 |
| {BcOLV15.BI=FALSE, BcOLV18=FALSE} =>  {BcPV3.BI=FALSE} | 0.37  1287 | 0.785  34 | 0.472  772 | 1.10  5496 | 15  0 |
| {BcOLV15.BI=FALSE, BcPV3.BI=FALSE} =>  {BcOLV18=FALSE} | 0.37  1287 | 0.557  621 | 0.665  842 | 1.15  5276 | 15  0 |
| {BcEV5=FALSE, BcOLV18=FALSE} =>  {BcPV3.BI=FALSE} | 0.36  3861 | 0.786  096 | 0.462  871 | 1.10  6561 | 14  7 |
| {BcEV5=FALSE, BcPV3.BI=FALSE} => | 0.36 | 0.540 | 0.673 | 1.11 | 14 |

| {BcOLV18=FALSE} | 3861 | 441 | 267 | 9683 | 7 |
| --- | --- | --- | --- | --- | --- |
| {BcEV2.BI=FALSE, BcOLV18=FALSE} =>  {BcRV1.BI=FALSE} | 0.30  4455 | 0.803  922 | 0.378  713 | 1.11  2275 | 12  3 |
| {BcEV2.BI=FALSE, BcRV1.BI=FALSE} =>  {BcOLV18=FALSE} | 0.30  4455 | 0.569  444 | 0.534  653 | 1.17  9772 | 12  3 |
| {BcHV3.BI=FALSE, BcOLV18=FALSE} =>  {BcRV1.BI=FALSE} | 0.32  6733 | 0.8 | 0.408  416 | 1.10  6849 | 13  2 |
| {BcHV3.BI=FALSE, BcRV1.BI=FALSE} =>  {BcOLV18=FALSE} | 0.32  6733 | 0.597  285 | 0.547  03 | 1.23  7452 | 13  2 |
| {BcFV5.BI=FALSE, BcOLV18=FALSE} =>  {BcRV1.BI=FALSE} | 0.32  9208 | 0.796  407 | 0.413  366 | 1.10  1878 | 13  3 |
| {BcFV5.BI=FALSE, BcRV1.BI=FALSE} =>  {BcOLV18=FALSE} | 0.32  9208 | 0.604  545 | 0.544  554 | 1.25  2494 | 13  3 |
| {BcOLV18=FALSE, BcRV1.BI=FALSE} =>  {BcULV1.BI=FALSE} | 0.34  6535 | 0.880  503 | 0.393  564 | 1.12  2155 | 14  0 |
| {BcOLV18=FALSE, BcULV1.BI=FALSE} =>  {BcRV1.BI=FALSE} | 0.34  6535 | 0.809  249 | 0.428  218 | 1.11  9645 | 14  0 |
| {BcRV1.BI=FALSE, BcULV1.BI=FALSE} =>  {BcOLV18=FALSE} | 0.34  6535 | 0.593  22 | 0.584  158 | 1.22  9031 | 14  0 |
| {BcMV1.BI=TRUE, BcOLV18=FALSE} =>  {BcRV1.BI=FALSE} | 0.29  2079 | 0.836  879 | 0.349  01 | 1.15  7874 | 11  8 |
| {BcMV12=FALSE, BcOLV18=FALSE} => | 0.32 | 0.815 | 0.403 | 1.12 | 13 |

| {BcRV1.BI=FALSE} | 9208 | 951 | 465 | 8918 | 3 |
| --- | --- | --- | --- | --- | --- |
| {BcMV12=FALSE, BcRV1.BI=FALSE} =>  {BcOLV18=FALSE} | 0.32  9208 | 0.568  376 | 0.579  208 | 1.17  7559 | 13  3 |
| {BcNSRV7.BI=FALSE, BcOLV18=FALSE} =>  {BcRV1.BI=FALSE} | 0.33  1683 | 0.807  229 | 0.410  891 | 1.11  6851 | 13  4 |
| {BcNSRV7.BI=FALSE, BcRV1.BI=FALSE} =>  {BcOLV18=FALSE} | 0.33  1683 | 0.592  92 | 0.559  406 | 1.22  8409 | 13  4 |
| {BcOLV18=FALSE, BcVV3.BI=FALSE} =>  {BcRV1.BI=FALSE} | 0.31  9307 | 0.816  456 | 0.391  089 | 1.12  9617 | 12  9 |
| {BcRV1.BI=FALSE, BcVV3.BI=FALSE} =>  {BcOLV18=FALSE} | 0.31  9307 | 0.560  87 | 0.569  307 | 1.16  2007 | 12  9 |
| {BcOLV18=FALSE, BcRV1.BI=FALSE} =>  {BcOLV7.BI=FALSE} | 0.36  1386 | 0.918  239 | 0.393  564 | 1.13  794 | 14  6 |
| {BcOLV18=FALSE, BcOLV7.BI=FALSE} =>  {BcRV1.BI=FALSE} | 0.36  1386 | 0.839  08 | 0.430  693 | 1.16  092 | 14  6 |
| {BcOLV7.BI=FALSE, BcRV1.BI=FALSE} =>  {BcOLV18=FALSE} | 0.36  1386 | 0.565  891 | 0.638  614 | 1.17  2411 | 14  6 |
| {BcOLV18=FALSE, BcRV1.BI=FALSE} =>  {BcALV1.BI=FALSE} | 0.35  6436 | 0.905  66 | 0.393  564 | 1.11  2118 | 14  4 |
| {BcALV1.BI=FALSE, BcOLV18=FALSE} =>  {BcRV1.BI=FALSE} | 0.35  6436 | 0.813  559 | 0.438  119 | 1.12  5609 | 14  4 |
| {BcALV1.BI=FALSE, BcRV1.BI=FALSE} => | 0.35 | 0.571 | 0.623 | 1.18 | 14 |

| {BcOLV18=FALSE} | 6436 | 429 | 762 | 3883 | 4 |
| --- | --- | --- | --- | --- | --- |
| {BcDMV1=FALSE, BcOLV18=FALSE} =>  {BcRV1.BI=FALSE} | 0.33  6634 | 0.809  524 | 0.415  842 | 1.12  0026 | 13  6 |
| {BcDMV1=FALSE, BcRV1.BI=FALSE} =>  {BcOLV18=FALSE} | 0.33  6634 | 0.573  84 | 0.586  634 | 1.18  8878 | 13  6 |
| {BcOLV18=FALSE, BcRV1.BI=FALSE} =>  {BcFV9=FALSE} | 0.36  3861 | 0.924  528 | 0.393  564 | 1.06  4129 | 14  7 |
| {BcFV9=FALSE, BcOLV18=FALSE} =>  {BcRV1.BI=FALSE} | 0.36  3861 | 0.812  155 | 0.448  02 | 1.12  3666 | 14  7 |
| {BcFV9=FALSE, BcRV1.BI=FALSE} =>  {BcOLV18=FALSE} | 0.36  3861 | 0.571  984 | 0.636  139 | 1.18  5034 | 14  7 |
| {BcOLV18=FALSE, BcOLV4.BI=FALSE} =>  {BcRV1.BI=FALSE} | 0.34  1584 | 0.797  688 | 0.428  218 | 1.10  365 | 13  8 |
| {BcOLV4.BI=FALSE, BcRV1.BI=FALSE} =>  {BcOLV18=FALSE} | 0.34  1584 | 0.541  176 | 0.631  188 | 1.12  1207 | 13  8 |
| {BcOLV18=FALSE, BcULV5=FALSE} =>  {BcRV1.BI=FALSE} | 0.32  6733 | 0.8 | 0.408  416 | 1.10  6849 | 13  2 |
| {BcBNV5.BI=FALSE, BcOLV18=FALSE} =>  {BcRV1.BI=FALSE} | 0.36  8812 | 0.809  783 | 0.455  446 | 1.12  0384 | 14  9 |
| {BcBNV5.BI=FALSE, BcRV1.BI=FALSE} =>  {BcOLV18=FALSE} | 0.36  8812 | 0.568  702 | 0.648  515 | 1.17  8234 | 14  9 |
| {BcOLV18=FALSE, BvF.BI=FALSE} => | 0.35 | 0.802 | 0.438 | 1.10 | 14 |

| {BcRV1.BI=FALSE} | 1485 | 26 | 119 | 9976 | 2 |
| --- | --- | --- | --- | --- | --- |
| {BcRV1.BI=FALSE, BvF.BI=FALSE} =>  {BcOLV18=FALSE} | 0.35  1485 | 0.544  061 | 0.646  04 | 1.12  7183 | 14  2 |
| {BcMCV5.BI=FALSE, BcOLV18=FALSE} =>  {BcRV1.BI=FALSE} | 0.38  3663 | 0.811  518 | 0.472  772 | 1.12  2786 | 15  5 |
| {BcMCV5.BI=FALSE, BcRV1.BI=FALSE} =>  {BcOLV18=FALSE} | 0.38  3663 | 0.569  853 | 0.673  267 | 1.18  0618 | 15  5 |
| {BcOLV13.BI=FALSE, BcOLV18=FALSE} =>  {BcRV1.BI=FALSE} | 0.36  8812 | 0.818  681 | 0.450  495 | 1.13  2696 | 14  9 |
| {BcOLV13.BI=FALSE, BcRV1.BI=FALSE} =>  {BcOLV18=FALSE} | 0.36  8812 | 0.534  05 | 0.690  594 | 1.10  6442 | 14  9 |
| {BcOLV18=FALSE, BcOLV2.BI=FALSE} =>  {BcRV1.BI=FALSE} | 0.37  3762 | 0.811  828 | 0.460  396 | 1.12  3214 | 15  1 |
| {BcOLV2.BI=FALSE, BcRV1.BI=FALSE} =>  {BcOLV18=FALSE} | 0.37  3762 | 0.547  101 | 0.683  168 | 1.13  3482 | 15  1 |
| {BcMCV3.BI=FALSE, BcOLV18=FALSE} =>  {BcRV1.BI=FALSE} | 0.37  1287 | 0.810  811 | 0.457  921 | 1.12  1807 | 15  0 |
| {BcMCV3.BI=FALSE, BcRV1.BI=FALSE} =>  {BcOLV18=FALSE} | 0.37  1287 | 0.545  455 | 0.680  693 | 1.13  007 | 15  0 |
| {BcOLV18=FALSE, BcPV4.BI=FALSE} =>  {BcRV1.BI=FALSE} | 0.37  3762 | 0.820  652 | 0.455  446 | 1.13  5423 | 15  1 |
| {BcPV4.BI=FALSE, BcRV1.BI=FALSE} => | 0.37 | 0.557 | 0.670 | 1.15 | 15 |

| {BcOLV18=FALSE} | 3762 | 196 | 792 | 4395 | 1 |
| --- | --- | --- | --- | --- | --- |
| {BcEV6=FALSE, BcOLV18=FALSE} =>  {BcRV1.BI=FALSE} | 0.36  3861 | 0.803  279 | 0.452  97 | 1.11  1386 | 14  7 |
| {BcEV6=FALSE, BcRV1.BI=FALSE} =>  {BcOLV18=FALSE} | 0.36  3861 | 0.544  444 | 0.668  317 | 1.12  7977 | 14  7 |
| {BcOLV17.BI=FALSE, BcOLV18=FALSE} =>  {BcRV1.BI=FALSE} | 0.38  6139 | 0.821  053 | 0.470  297 | 1.13  5977 | 15  6 |
| {BcOLV17.BI=FALSE, BcRV1.BI=FALSE} =>  {BcOLV18=FALSE} | 0.38  6139 | 0.545  455 | 0.707  921 | 1.13  007 | 15  6 |
| {BcOLV15.BI=FALSE, BcOLV18=FALSE} =>  {BcRV1.BI=FALSE} | 0.38  3663 | 0.811  518 | 0.472  772 | 1.12  2786 | 15  5 |
| {BcOLV15.BI=FALSE, BcRV1.BI=FALSE} =>  {BcOLV18=FALSE} | 0.38  3663 | 0.563  636 | 0.680  693 | 1.16  7739 | 15  5 |
| {BcEV5=FALSE, BcOLV18=FALSE} =>  {BcRV1.BI=FALSE} | 0.37  3762 | 0.807  487 | 0.462  871 | 1.11  7208 | 15  1 |
| {BcEV5=FALSE, BcRV1.BI=FALSE} =>  {BcOLV18=FALSE} | 0.37  3762 | 0.555  147 | 0.673  267 | 1.15  0151 | 15  1 |
| {BcEV2.BI=FALSE, BcOLV18=FALSE} =>  {BcHV3.BI=FALSE} | 0.32  9208 | 0.869  281 | 0.378  713 | 1.14  394 | 13  3 |
| {BcEV2.BI=FALSE, BcHV3.BI=FALSE} =>  {BcOLV18=FALSE} | 0.32  9208 | 0.551  867 | 0.596  535 | 1.14  3356 | 13  3 |
| {BcEV2.BI=FALSE, BcOLV18=FALSE} => | 0.33 | 0.882 | 0.378 | 1.12 | 13 |

| {BcFV5.BI=FALSE} | 4158 | 353 | 713 | 8071 | 5 |
| --- | --- | --- | --- | --- | --- |
| {BcEV2.BI=FALSE, BcOLV18=FALSE} =>  {BcULV1.BI=FALSE} | 0.34  1584 | 0.901  961 | 0.378  713 | 1.14  9502 | 13  8 |
| {BcEV2.BI=FALSE, BcULV1.BI=FALSE} =>  {BcOLV18=FALSE} | 0.34  1584 | 0.579  832 | 0.589  109 | 1.20  1293 | 13  8 |
| {BcOLV18=FALSE, BcVV3.BI=FALSE} =>  {BcEV2.BI=FALSE} | 0.32  1782 | 0.822  785 | 0.391  089 | 1.10  0679 | 13  0 |
| {BcEV2.BI=FALSE, BcOLV18=FALSE} =>  {BcOLV7.BI=FALSE} | 0.33  1683 | 0.875  817 | 0.378  713 | 1.08  5368 | 13  4 |
| {BcEV2.BI=FALSE, BcOLV7.BI=FALSE} =>  {BcOLV18=FALSE} | 0.33  1683 | 0.560  669 | 0.591  584 | 1.16  1592 | 13  4 |
| {BcEV2.BI=FALSE, BcOLV18=FALSE} =>  {BcALV1.BI=FALSE} | 0.34  901 | 0.921  569 | 0.378  713 | 1.13  1653 | 14  1 |
| {BcALV1.BI=FALSE, BcEV2.BI=FALSE} =>  {BcOLV18=FALSE} | 0.34  901 | 0.566  265 | 0.616  337 | 1.17  3185 | 14  1 |
| {BcEV2.BI=FALSE, BcOLV18=FALSE} =>  {BcFV9=FALSE} | 0.36  1386 | 0.954  248 | 0.378  713 | 1.09  8337 | 14  6 |
| {BcEV2.BI=FALSE, BcFV9=FALSE} =>  {BcOLV18=FALSE} | 0.36  1386 | 0.536  765 | 0.673  267 | 1.11  2066 | 14  6 |
| {BcOLV18=FALSE, BvF.BI=FALSE} =>  {BcEV2.BI=FALSE} | 0.36  1386 | 0.824  859 | 0.438  119 | 1.10  3453 | 14  6 |
| {BcHV3.BI=FALSE, BcOLV18=FALSE} => | 0.36 | 0.896 | 0.408 | 1.14 | 14 |

| {BcFV5.BI=FALSE} | 6337 | 97 | 416 | 6759 | 8 |
| --- | --- | --- | --- | --- | --- |
| {BcFV5.BI=FALSE, BcOLV18=FALSE} =>  {BcHV3.BI=FALSE} | 0.36  6337 | 0.886  228 | 0.413  366 | 1.16  6241 | 14  8 |
| {BcFV5.BI=FALSE, BcHV3.BI=FALSE} =>  {BcOLV18=FALSE} | 0.36  6337 | 0.558  491 | 0.655  941 | 1.15  7078 | 14  8 |
| {BcHV3.BI=FALSE, BcOLV18=FALSE} =>  {BcULV1.BI=FALSE} | 0.36  1386 | 0.884  848 | 0.408  416 | 1.12  7693 | 14  6 |
| {BcOLV18=FALSE, BcULV1.BI=FALSE} =>  {BcHV3.BI=FALSE} | 0.36  1386 | 0.843  931 | 0.428  218 | 1.11  058 | 14  6 |
| {BcHV3.BI=FALSE, BcULV1.BI=FALSE} =>  {BcOLV18=FALSE} | 0.36  1386 | 0.598  361 | 0.603  96 | 1.23  9681 | 14  6 |
| {BcMV12=FALSE, BcOLV18=FALSE} =>  {BcHV3.BI=FALSE} | 0.33  4158 | 0.828  221 | 0.403  465 | 1.08  9906 | 13  5 |
| {BcHV3.BI=FALSE, BcMV12=FALSE} =>  {BcOLV18=FALSE} | 0.33  4158 | 0.555  556 | 0.601  485 | 1.15  0997 | 13  5 |
| {BcHV3.BI=FALSE, BcOLV18=FALSE} =>  {BcNSRV7.BI=FALSE} | 0.35  396 | 0.866  667 | 0.408  416 | 1.08  4004 | 14  3 |
| {BcNSRV7.BI=FALSE, BcOLV18=FALSE} =>  {BcHV3.BI=FALSE} | 0.35  396 | 0.861  446 | 0.410  891 | 1.13  3629 | 14  3 |
| {BcHV3.BI=FALSE, BcNSRV7.BI=FALSE} =>  {BcOLV18=FALSE} | 0.35  396 | 0.541  667 | 0.653  465 | 1.12  2222 | 14  3 |
| {BcOLV18=FALSE, BcVV3.BI=FALSE} => | 0.34 | 0.879 | 0.391 | 1.15 | 13 |

| {BcHV3.BI=FALSE} | 4059 | 747 | 089 | 7712 | 9 |
| --- | --- | --- | --- | --- | --- |
| {BcHV3.BI=FALSE, BcOLV18=FALSE} =>  {BcOLV7.BI=FALSE} | 0.36  8812 | 0.903  03 | 0.408  416 | 1.11  9093 | 14  9 |
| {BcOLV18=FALSE, BcOLV7.BI=FALSE} =>  {BcHV3.BI=FALSE} | 0.36  8812 | 0.856  322 | 0.430  693 | 1.12  6886 | 14  9 |
| {BcHV3.BI=FALSE, BcOLV7.BI=FALSE} =>  {BcOLV18=FALSE} | 0.36  8812 | 0.584  314 | 0.631  188 | 1.21  0578 | 14  9 |
| {BcHV3.BI=FALSE, BcOLV18=FALSE} =>  {BcALV1.BI=FALSE} | 0.38  1188 | 0.933  333 | 0.408  416 | 1.14  6099 | 15  4 |
| {BcALV1.BI=FALSE, BcOLV18=FALSE} =>  {BcHV3.BI=FALSE} | 0.38  1188 | 0.870  056 | 0.438  119 | 1.14  496 | 15  4 |
| {BcALV1.BI=FALSE, BcHV3.BI=FALSE} =>  {BcOLV18=FALSE} | 0.38  1188 | 0.601  563 | 0.633  663 | 1.24  6314 | 15  4 |
| {BcDMV1=FALSE, BcOLV18=FALSE} =>  {BcHV3.BI=FALSE} | 0.36  1386 | 0.869  048 | 0.415  842 | 1.14  3633 | 14  6 |
| {BcHV3.BI=FALSE, BcOLV18=FALSE} =>  {BcFV9=FALSE} | 0.38  3663 | 0.939  394 | 0.408  416 | 1.08  124 | 15  5 |
| {BcFV9=FALSE, BcOLV18=FALSE} =>  {BcHV3.BI=FALSE} | 0.38  3663 | 0.856  354 | 0.448  02 | 1.12  6928 | 15  5 |
| {BcFV9=FALSE, BcHV3.BI=FALSE} =>  {BcOLV18=FALSE} | 0.38  3663 | 0.571  956 | 0.670  792 | 1.18  4975 | 15  5 |
| {BcOLV18=FALSE, BcOLV4.BI=FALSE} => | 0.36 | 0.855 | 0.428 | 1.12 | 14 |

| {BcHV3.BI=FALSE} | 6337 | 491 | 218 | 5793 | 8 |
| --- | --- | --- | --- | --- | --- |
| {BcHV3.BI=FALSE, BcOLV4.BI=FALSE} =>  {BcOLV18=FALSE} | 0.36  6337 | 0.544  118 | 0.673  267 | 1.12  73 | 14  8 |
| {BcOLV18=FALSE, BcULV5=FALSE} =>  {BcHV3.BI=FALSE} | 0.34  4059 | 0.842  424 | 0.408  416 | 1.10  8597 | 13  9 |
| {BcBNV5.BI=FALSE, BcOLV18=FALSE} =>  {BcHV3.BI=FALSE} | 0.39  3564 | 0.864  13 | 0.455  446 | 1.13  7162 | 15  9 |
| {BcBNV5.BI=FALSE, BcHV3.BI=FALSE} =>  {BcOLV18=FALSE} | 0.39  3564 | 0.550  173 | 0.715  347 | 1.13  9846 | 15  9 |
| {BcOLV18=FALSE, BvF.BI=FALSE} =>  {BcHV3.BI=FALSE} | 0.37  3762 | 0.853  107 | 0.438  119 | 1.12  2656 | 15  1 |
| {BcHV3.BI=FALSE, BvF.BI=FALSE} =>  {BcOLV18=FALSE} | 0.37  3762 | 0.535  461 | 0.698  02 | 1.10  9365 | 15  1 |
| {BcHV3.BI=FALSE, BcOLV18=FALSE} =>  {BcMCV5.BI=FALSE} | 0.40  5941 | 0.993  939 | 0.408  416 | 1.07  6546 | 16  4 |
| {BcMCV5.BI=FALSE, BcOLV18=FALSE} =>  {BcHV3.BI=FALSE} | 0.40  5941 | 0.858  639 | 0.472  772 | 1.12  9935 | 16  4 |
| {BcHV3.BI=FALSE, BcMCV5.BI=FALSE} =>  {BcOLV18=FALSE} | 0.40  5941 | 0.550  336 | 0.737  624 | 1.14  0182 | 16  4 |
| {BcOLV13.BI=FALSE, BcOLV18=FALSE} =>  {BcHV3.BI=FALSE} | 0.38  3663 | 0.851  648 | 0.450  495 | 1.12  0736 | 15  5 |
| {BcHV3.BI=FALSE, BcOLV13.BI=FALSE} => | 0.38 | 0.540 | 0.710 | 1.11 | 15 |

| {BcOLV18=FALSE} | 3663 | 07 | 396 | 8914 | 5 |
| --- | --- | --- | --- | --- | --- |
| {BcOLV18=FALSE, BcOLV2.BI=FALSE} =>  {BcHV3.BI=FALSE} | 0.38  6139 | 0.838  71 | 0.460  396 | 1.10  3709 | 15  6 |
| {BcHV3.BI=FALSE, BcOLV2.BI=FALSE} =>  {BcOLV18=FALSE} | 0.38  6139 | 0.547  368 | 0.705  446 | 1.13  4035 | 15  6 |
| {BcMCV3.BI=FALSE, BcOLV18=FALSE} =>  {BcHV3.BI=FALSE} | 0.38  3663 | 0.837  838 | 0.457  921 | 1.10  2562 | 15  5 |
| {BcHV3.BI=FALSE, BcMCV3.BI=FALSE} =>  {BcOLV18=FALSE} | 0.38  3663 | 0.551  601 | 0.695  545 | 1.14  2805 | 15  5 |
| {BcOLV18=FALSE, BcPV4.BI=FALSE} =>  {BcHV3.BI=FALSE} | 0.38  3663 | 0.842  391 | 0.455  446 | 1.10  8554 | 15  5 |
| {BcHV3.BI=FALSE, BcPV4.BI=FALSE} =>  {BcOLV18=FALSE} | 0.38  3663 | 0.543  86 | 0.705  446 | 1.12  6766 | 15  5 |
| {BcEV6=FALSE, BcOLV18=FALSE} =>  {BcHV3.BI=FALSE} | 0.38  3663 | 0.846  995 | 0.452  97 | 1.11  4612 | 15  5 |
| {BcEV6=FALSE, BcHV3.BI=FALSE} =>  {BcOLV18=FALSE} | 0.38  3663 | 0.541  958 | 0.707  921 | 1.12  2826 | 15  5 |
| {BcOLV17.BI=FALSE, BcOLV18=FALSE} =>  {BcHV3.BI=FALSE} | 0.39  8515 | 0.847  368 | 0.470  297 | 1.11  5104 | 16  1 |
| {BcHV3.BI=FALSE, BcOLV17.BI=FALSE} =>  {BcOLV18=FALSE} | 0.39  8515 | 0.549  488 | 0.725  248 | 1.13  8427 | 16  1 |
| {BcOLV15.BI=FALSE, BcOLV18=FALSE} => | 0.40 | 0.848 | 0.472 | 1.11 | 16 |

| {BcHV3.BI=FALSE} | 099 | 168 | 772 | 6155 | 2 |
| --- | --- | --- | --- | --- | --- |
| {BcHV3.BI=FALSE, BcOLV15.BI=FALSE} =>  {BcOLV18=FALSE} | 0.40  099 | 0.547  297 | 0.732  673 | 1.13  3888 | 16  2 |
| {BcEV5=FALSE, BcOLV18=FALSE} =>  {BcHV3.BI=FALSE} | 0.40  3465 | 0.871  658 | 0.462  871 | 1.14  7068 | 16  3 |
| {BcEV5=FALSE, BcHV3.BI=FALSE} =>  {BcOLV18=FALSE} | 0.40  3465 | 0.536  184 | 0.752  475 | 1.11  0864 | 16  3 |
| {BcFV5.BI=FALSE, BcOLV18=FALSE} =>  {BcULV1.BI=FALSE} | 0.36  6337 | 0.886  228 | 0.413  366 | 1.12  9451 | 14  8 |
| {BcOLV18=FALSE, BcULV1.BI=FALSE} =>  {BcFV5.BI=FALSE} | 0.36  6337 | 0.855  491 | 0.428  218 | 1.09  3729 | 14  8 |
| {BcFV5.BI=FALSE, BcULV1.BI=FALSE} =>  {BcOLV18=FALSE} | 0.36  6337 | 0.599  19 | 0.611  386 | 1.24  1399 | 14  8 |
| {BcMV1.BI=TRUE, BcOLV18=FALSE} =>  {BcFV5.BI=FALSE} | 0.29  9505 | 0.858  156 | 0.349  01 | 1.09  7136 | 12  1 |
| {BcMV12=FALSE, BcOLV18=FALSE} =>  {BcFV5.BI=FALSE} | 0.34  4059 | 0.852  761 | 0.403  465 | 1.09  0238 | 13  9 |
| {BcFV5.BI=FALSE, BcMV12=FALSE} =>  {BcOLV18=FALSE} | 0.34  4059 | 0.549  407 | 0.626  238 | 1.13  8259 | 13  9 |
| {BcFV5.BI=FALSE, BcOLV18=FALSE} =>  {BcNSRV7.BI=FALSE} | 0.36  6337 | 0.886  228 | 0.413  366 | 1.10  847 | 14  8 |
| {BcNSRV7.BI=FALSE, BcOLV18=FALSE} => | 0.36 | 0.891 | 0.410 | 1.13 | 14 |

| {BcFV5.BI=FALSE} | 6337 | 566 | 891 | 9851 | 8 |
| --- | --- | --- | --- | --- | --- |
| {BcFV5.BI=FALSE, BcNSRV7.BI=FALSE} =>  {BcOLV18=FALSE} | 0.36  6337 | 0.540  146 | 0.678  218 | 1.11  9072 | 14  8 |
| {BcOLV18=FALSE, BcVV3.BI=FALSE} =>  {BcFV5.BI=FALSE} | 0.33  6634 | 0.860  759 | 0.391  089 | 1.10  0465 | 13  6 |
| {BcFV5.BI=FALSE, BcOLV18=FALSE} =>  {BcOLV7.BI=FALSE} | 0.36  6337 | 0.886  228 | 0.413  366 | 1.09  827 | 14  8 |
| {BcOLV18=FALSE, BcOLV7.BI=FALSE} =>  {BcFV5.BI=FALSE} | 0.36  6337 | 0.850  575 | 0.430  693 | 1.08  7444 | 14  8 |
| {BcFV5.BI=FALSE, BcOLV7.BI=FALSE} =>  {BcOLV18=FALSE} | 0.36  6337 | 0.587  302 | 0.623  762 | 1.21  6768 | 14  8 |
| {BcFV5.BI=FALSE, BcOLV18=FALSE} =>  {BcALV1.BI=FALSE} | 0.37  8713 | 0.916  168 | 0.413  366 | 1.12  502 | 15  3 |
| {BcALV1.BI=FALSE, BcOLV18=FALSE} =>  {BcFV5.BI=FALSE} | 0.37  8713 | 0.864  407 | 0.438  119 | 1.10  5128 | 15  3 |
| {BcALV1.BI=FALSE, BcFV5.BI=FALSE} =>  {BcOLV18=FALSE} | 0.37  8713 | 0.568  773 | 0.665  842 | 1.17  8381 | 15  3 |
| {BcDMV1=FALSE, BcOLV18=FALSE} =>  {BcFV5.BI=FALSE} | 0.36  3861 | 0.875 | 0.415  842 | 1.11  8671 | 14  7 |
| {BcFV5.BI=FALSE, BcOLV18=FALSE} =>  {BcFV9=FALSE} | 0.38  6139 | 0.934  132 | 0.413  366 | 1.07  5183 | 15  6 |
| {BcFV9=FALSE, BcOLV18=FALSE} => | 0.38 | 0.861 | 0.448 | 1.10 | 15 |

| {BcFV5.BI=FALSE} | 6139 | 878 | 02 | 1895 | 6 |
| --- | --- | --- | --- | --- | --- |
| {BcFV5.BI=FALSE, BcFV9=FALSE} =>  {BcOLV18=FALSE} | 0.38  6139 | 0.553  191 | 0.698  02 | 1.14  6099 | 15  6 |
| {BcOLV18=FALSE, BcOLV4.BI=FALSE} =>  {BcFV5.BI=FALSE} | 0.36  3861 | 0.849  711 | 0.428  218 | 1.08  6339 | 14  7 |
| {BcFV5.BI=FALSE, BcOLV4.BI=FALSE} =>  {BcOLV18=FALSE} | 0.36  3861 | 0.534  545 | 0.680  693 | 1.10  7469 | 14  7 |
| {BcOLV18=FALSE, BcULV5=FALSE} =>  {BcFV5.BI=FALSE} | 0.34  901 | 0.854  545 | 0.408  416 | 1.09  252 | 14  1 |
| {BcBNV5.BI=FALSE, BcOLV18=FALSE} =>  {BcFV5.BI=FALSE} | 0.39  3564 | 0.864  13 | 0.455  446 | 1.10  4774 | 15  9 |
| {BcOLV18=FALSE, BvF.BI=FALSE} =>  {BcFV5.BI=FALSE} | 0.38  3663 | 0.875  706 | 0.438  119 | 1.11  9574 | 15  5 |
| {BcMCV5.BI=FALSE, BcOLV18=FALSE} =>  {BcFV5.BI=FALSE} | 0.40  5941 | 0.858  639 | 0.472  772 | 1.09  7753 | 16  4 |
| {BcFV5.BI=FALSE, BcMCV5.BI=FALSE} =>  {BcOLV18=FALSE} | 0.40  5941 | 0.546  667 | 0.742  574 | 1.13  2581 | 16  4 |
| {BcOLV13.BI=FALSE, BcOLV18=FALSE} =>  {BcFV5.BI=FALSE} | 0.38  3663 | 0.851  648 | 0.450  495 | 1.08  8816 | 15  5 |
| {BcFV5.BI=FALSE, BcOLV13.BI=FALSE} =>  {BcOLV18=FALSE} | 0.38  3663 | 0.534  483 | 0.717  822 | 1.10  7339 | 15  5 |
| {BcOLV18=FALSE, BcOLV2.BI=FALSE} => | 0.39 | 0.854 | 0.460 | 1.09 | 15 |

| {BcFV5.BI=FALSE} | 3564 | 839 | 396 | 2895 | 9 |
| --- | --- | --- | --- | --- | --- |
| {BcFV5.BI=FALSE, BcOLV2.BI=FALSE} =>  {BcOLV18=FALSE} | 0.39  3564 | 0.540  816 | 0.727  723 | 1.12  046 | 15  9 |
| {BcMCV3.BI=FALSE, BcOLV18=FALSE} =>  {BcFV5.BI=FALSE} | 0.38  8614 | 0.848  649 | 0.457  921 | 1.08  4981 | 15  7 |
| {BcFV5.BI=FALSE, BcMCV3.BI=FALSE} =>  {BcOLV18=FALSE} | 0.38  8614 | 0.535  836 | 0.725  248 | 1.11  0143 | 15  7 |
| {BcOLV18=FALSE, BcPV4.BI=FALSE} =>  {BcFV5.BI=FALSE} | 0.39  1089 | 0.858  696 | 0.455  446 | 1.09  7826 | 15  8 |
| {BcFV5.BI=FALSE, BcPV4.BI=FALSE} =>  {BcOLV18=FALSE} | 0.39  1089 | 0.533  784 | 0.732  673 | 1.10  5891 | 15  8 |
| {BcEV6=FALSE, BcOLV18=FALSE} =>  {BcFV5.BI=FALSE} | 0.38  3663 | 0.846  995 | 0.452  97 | 1.08  2866 | 15  5 |
| {BcOLV17.BI=FALSE, BcOLV18=FALSE} =>  {BcFV5.BI=FALSE} | 0.40  3465 | 0.857  895 | 0.470  297 | 1.09  6802 | 16  3 |
| {BcFV5.BI=FALSE, BcOLV17.BI=FALSE} =>  {BcOLV18=FALSE} | 0.40  3465 | 0.548  822 | 0.735  149 | 1.13  7046 | 16  3 |
| {BcOLV15.BI=FALSE, BcOLV18=FALSE} =>  {BcFV5.BI=FALSE} | 0.41  0891 | 0.869  11 | 0.472  772 | 1.11  1141 | 16  6 |
| {BcFV5.BI=FALSE, BcOLV15.BI=FALSE} =>  {BcOLV18=FALSE} | 0.41  0891 | 0.533  762 | 0.769  802 | 1.10  5845 | 16  6 |
| {BcEV5=FALSE, BcOLV18=FALSE} => | 0.40 | 0.882 | 0.462 | 1.12 | 16 |

| {BcFV5.BI=FALSE} | 8416 | 353 | 871 | 8071 | 5 |
| --- | --- | --- | --- | --- | --- |
| {BcEV5=FALSE, BcFV5.BI=FALSE} =>  {BcOLV18=FALSE} | 0.40  8416 | 0.528  846 | 0.772  277 | 1.09  5661 | 16  5 |
| {BcMV1.BI=TRUE, BcOLV18=FALSE} =>  {BcULV1.BI=FALSE} | 0.30  4455 | 0.872  34 | 0.349  01 | 1.11  1752 | 12  3 |
| {BcMV12=FALSE, BcOLV18=FALSE} =>  {BcULV1.BI=FALSE} | 0.36  8812 | 0.914  11 | 0.403  465 | 1.16  4986 | 14  9 |
| {BcMV12=FALSE, BcULV1.BI=FALSE} =>  {BcOLV18=FALSE} | 0.36  8812 | 0.564  394 | 0.653  465 | 1.16  9308 | 14  9 |
| {BcNSRV7.BI=FALSE, BcOLV18=FALSE} =>  {BcULV1.BI=FALSE} | 0.36  1386 | 0.879  518 | 0.410  891 | 1.12  09 | 14  6 |
| {BcNSRV7.BI=FALSE, BcULV1.BI=FALSE} =>  {BcOLV18=FALSE} | 0.36  1386 | 0.588  71 | 0.613  861 | 1.21  9686 | 14  6 |
| {BcOLV18=FALSE, BcVV3.BI=FALSE} =>  {BcULV1.BI=FALSE} | 0.34  901 | 0.892  405 | 0.391  089 | 1.13  7324 | 14  1 |
| {BcULV1.BI=FALSE, BcVV3.BI=FALSE} =>  {BcOLV18=FALSE} | 0.34  901 | 0.555  118 | 0.628  713 | 1.15  0091 | 14  1 |
| {BcOLV18=FALSE, BcULV1.BI=FALSE} =>  {BcOLV7.BI=FALSE} | 0.37  8713 | 0.884  393 | 0.428  218 | 1.09  5996 | 15  3 |
| {BcOLV18=FALSE, BcOLV7.BI=FALSE} =>  {BcULV1.BI=FALSE} | 0.37  8713 | 0.879  31 | 0.430  693 | 1.12  0635 | 15  3 |
| {BcOLV7.BI=FALSE, BcULV1.BI=FALSE} => | 0.37 | 0.581 | 0.650 | 1.20 | 15 |

| {BcOLV18=FALSE} | 8713 | 749 | 99 | 5265 | 3 |
| --- | --- | --- | --- | --- | --- |
| {BcOLV18=FALSE, BcULV1.BI=FALSE} =>  {BcALV1.BI=FALSE} | 0.39  1089 | 0.913  295 | 0.428  218 | 1.12  1493 | 15  8 |
| {BcALV1.BI=FALSE, BcOLV18=FALSE} =>  {BcULV1.BI=FALSE} | 0.39  1089 | 0.892  655 | 0.438  119 | 1.13  7643 | 15  8 |
| {BcALV1.BI=FALSE, BcULV1.BI=FALSE} =>  {BcOLV18=FALSE} | 0.39  1089 | 0.603  053 | 0.648  515 | 1.24  9403 | 15  8 |
| {BcDMV1=FALSE, BcOLV18=FALSE} =>  {BcULV1.BI=FALSE} | 0.36  3861 | 0.875 | 0.415  842 | 1.11  5142 | 14  7 |
| {BcDMV1=FALSE, BcULV1.BI=FALSE} =>  {BcOLV18=FALSE} | 0.36  3861 | 0.561  069 | 0.648  515 | 1.16  2419 | 14  7 |
| {BcOLV18=FALSE, BcULV1.BI=FALSE} =>  {BcFV9=FALSE} | 0.39  8515 | 0.930  636 | 0.428  218 | 1.07  1159 | 16  1 |
| {BcFV9=FALSE, BcOLV18=FALSE} =>  {BcULV1.BI=FALSE} | 0.39  8515 | 0.889  503 | 0.448  02 | 1.13  3625 | 16  1 |
| {BcFV9=FALSE, BcULV1.BI=FALSE} =>  {BcOLV18=FALSE} | 0.39  8515 | 0.587  591 | 0.678  218 | 1.21  7369 | 16  1 |
| {BcOLV18=FALSE, BcOLV4.BI=FALSE} =>  {BcULV1.BI=FALSE} | 0.38  8614 | 0.907  514 | 0.428  218 | 1.15  658 | 15  7 |
| {BcOLV4.BI=FALSE, BcULV1.BI=FALSE} =>  {BcOLV18=FALSE} | 0.38  8614 | 0.558  719 | 0.695  545 | 1.15  7551 | 15  7 |
| {BcOLV18=FALSE, BcULV5=FALSE} => | 0.37 | 0.909 | 0.408 | 1.15 | 15 |

| {BcULV1.BI=FALSE} | 1287 | 091 | 416 | 8589 | 0 |
| --- | --- | --- | --- | --- | --- |
| {BcBNV5.BI=FALSE, BcOLV18=FALSE} =>  {BcULV1.BI=FALSE} | 0.40  8416 | 0.896  739 | 0.455  446 | 1.14  2847 | 16  5 |
| {BcBNV5.BI=FALSE, BcULV1.BI=FALSE} =>  {BcOLV18=FALSE} | 0.40  8416 | 0.572  917 | 0.712  871 | 1.18  6966 | 16  5 |
| {BcOLV18=FALSE, BvF.BI=FALSE} =>  {BcULV1.BI=FALSE} | 0.39  1089 | 0.892  655 | 0.438  119 | 1.13  7643 | 15  8 |
| {BcULV1.BI=FALSE, BvF.BI=FALSE} =>  {BcOLV18=FALSE} | 0.39  1089 | 0.548  611 | 0.712  871 | 1.13  661 | 15  8 |
| {BcMCV5.BI=FALSE, BcOLV18=FALSE} =>  {BcULV1.BI=FALSE} | 0.42  0792 | 0.890  052 | 0.472  772 | 1.13  4325 | 17  0 |
| {BcMCV5.BI=FALSE, BcULV1.BI=FALSE} =>  {BcOLV18=FALSE} | 0.42  0792 | 0.576  271 | 0.730  198 | 1.19  3916 | 17  0 |
| {BcOLV13.BI=FALSE, BcOLV18=FALSE} =>  {BcULV1.BI=FALSE} | 0.41  0891 | 0.912  088 | 0.450  495 | 1.16  2409 | 16  6 |
| {BcOLV13.BI=FALSE, BcULV1.BI=FALSE} =>  {BcOLV18=FALSE} | 0.41  0891 | 0.555  184 | 0.740  099 | 1.15  0227 | 16  6 |
| {BcOLV18=FALSE, BcOLV2.BI=FALSE} =>  {BcULV1.BI=FALSE} | 0.41  5842 | 0.903  226 | 0.460  396 | 1.15  1114 | 16  8 |
| {BcOLV2.BI=FALSE, BcULV1.BI=FALSE} =>  {BcOLV18=FALSE} | 0.41  5842 | 0.56 | 0.742  574 | 1.16  0205 | 16  8 |
| {BcMCV3.BI=FALSE, BcOLV18=FALSE} => | 0.41 | 0.913 | 0.457 | 1.16 | 16 |

| {BcULV1.BI=FALSE} | 8317 | 514 | 921 | 4225 | 9 |
| --- | --- | --- | --- | --- | --- |
| {BcMCV3.BI=FALSE, BcULV1.BI=FALSE} =>  {BcOLV18=FALSE} | 0.41  8317 | 0.546  926 | 0.764  851 | 1.13  3118 | 16  9 |
| {BcOLV18=FALSE, BcPV4.BI=FALSE} =>  {BcULV1.BI=FALSE} | 0.41  0891 | 0.902  174 | 0.455  446 | 1.14  9774 | 16  6 |
| {BcPV4.BI=FALSE, BcULV1.BI=FALSE} =>  {BcOLV18=FALSE} | 0.41  0891 | 0.553  333 | 0.742  574 | 1.14  6393 | 16  6 |
| {BcEV6=FALSE, BcOLV18=FALSE} =>  {BcULV1.BI=FALSE} | 0.40  3465 | 0.890  71 | 0.452  97 | 1.13  5164 | 16  3 |
| {BcEV6=FALSE, BcULV1.BI=FALSE} =>  {BcOLV18=FALSE} | 0.40  3465 | 0.546  98 | 0.737  624 | 1.13  323 | 16  3 |
| {BcOLV17.BI=FALSE, BcOLV18=FALSE} =>  {BcULV1.BI=FALSE} | 0.41  5842 | 0.884  211 | 0.470  297 | 1.12  688 | 16  8 |
| {BcOLV17.BI=FALSE, BcULV1.BI=FALSE} =>  {BcOLV18=FALSE} | 0.41  5842 | 0.554  455 | 0.75 | 1.14  8718 | 16  8 |
| {BcOLV15.BI=FALSE, BcOLV18=FALSE} =>  {BcULV1.BI=FALSE} | 0.42  0792 | 0.890  052 | 0.472  772 | 1.13  4325 | 17  0 |
| {BcOLV15.BI=FALSE, BcULV1.BI=FALSE} =>  {BcOLV18=FALSE} | 0.42  0792 | 0.568  562 | 0.740  099 | 1.17  7944 | 17  0 |
| {BcEV5=FALSE, BcOLV18=FALSE} =>  {BcULV1.BI=FALSE} | 0.40  8416 | 0.882  353 | 0.462  871 | 1.12  4513 | 16  5 |
| {BcEV5=FALSE, BcULV1.BI=FALSE} => | 0.40 | 0.55 | 0.742 | 1.13 | 16 |

| {BcOLV18=FALSE} | 8416 |  | 574 | 9487 | 5 |
| --- | --- | --- | --- | --- | --- |
| {BcMV1.BI=TRUE, BcOLV18=FALSE} =>  {BcOLV7.BI=FALSE} | 0.30  9406 | 0.886  525 | 0.349  01 | 1.09  8638 | 12  5 |
| {BcMV1.BI=TRUE, BcOLV18=FALSE} =>  {BcALV1.BI=FALSE} | 0.31  9307 | 0.914  894 | 0.349  01 | 1.12  3456 | 12  9 |
| {BcMV12=FALSE, BcOLV18=FALSE} =>  {BcOLV7.BI=FALSE} | 0.36  3861 | 0.901  84 | 0.403  465 | 1.11  7618 | 14  7 |
| {BcMV12=FALSE, BcOLV7.BI=FALSE} =>  {BcOLV18=FALSE} | 0.36  3861 | 0.554  717 | 0.655  941 | 1.14  926 | 14  7 |
| {BcMV12=FALSE, BcOLV18=FALSE} =>  {BcALV1.BI=FALSE} | 0.37  1287 | 0.920  245 | 0.403  465 | 1.13  0028 | 15  0 |
| {BcALV1.BI=FALSE, BcMV12=FALSE} =>  {BcOLV18=FALSE} | 0.37  1287 | 0.568  182 | 0.653  465 | 1.17  7156 | 15  0 |
| {BcMV12=FALSE, BcOLV18=FALSE} =>  {BcFV9=FALSE} | 0.37  6238 | 0.932  515 | 0.403  465 | 1.07  3322 | 15  2 |
| {BcFV9=FALSE, BcMV12=FALSE} =>  {BcOLV18=FALSE} | 0.37  6238 | 0.540  925 | 0.695  545 | 1.12  0686 | 15  2 |
| {BcOLV18=FALSE, BcOLV4.BI=FALSE} =>  {BcMV12=FALSE} | 0.37  1287 | 0.867  052 | 0.428  218 | 1.08  4486 | 15  0 |
| {BcOLV18=FALSE, BcULV5=FALSE} =>  {BcMV12=FALSE} | 0.35  6436 | 0.872  727 | 0.408  416 | 1.09  1585 | 14  4 |
| {BcMCV5.BI=FALSE, BcMV12=FALSE} => | 0.39 | 0.533 | 0.737 | 1.10 | 15 |

| {BcOLV18=FALSE} | 3564 | 557 | 624 | 5421 | 9 |
| --- | --- | --- | --- | --- | --- |
| {BcOLV13.BI=FALSE, BcOLV18=FALSE} =>  {BcMV12=FALSE} | 0.39  1089 | 0.868  132 | 0.450  495 | 1.08  5837 | 15  8 |
| {BcMCV3.BI=FALSE, BcOLV18=FALSE} =>  {BcMV12=FALSE} | 0.40  3465 | 0.881  081 | 0.457  921 | 1.10  2033 | 16  3 |
| {BcNSRV7.BI=FALSE, BcOLV18=FALSE} =>  {BcOLV7.BI=FALSE} | 0.37  1287 | 0.903  614 | 0.410  891 | 1.11  9817 | 15  0 |
| {BcOLV18=FALSE, BcOLV7.BI=FALSE} =>  {BcNSRV7.BI=FALSE} | 0.37  1287 | 0.862  069 | 0.430  693 | 1.07  8253 | 15  0 |
| {BcNSRV7.BI=FALSE, BcOLV7.BI=FALSE} =>  {BcOLV18=FALSE} | 0.37  1287 | 0.581  395 | 0.638  614 | 1.20  4532 | 15  0 |
| {BcNSRV7.BI=FALSE, BcOLV18=FALSE} =>  {BcALV1.BI=FALSE} | 0.37  8713 | 0.921  687 | 0.410  891 | 1.13  1798 | 15  3 |
| {BcALV1.BI=FALSE, BcOLV18=FALSE} =>  {BcNSRV7.BI=FALSE} | 0.37  8713 | 0.864  407 | 0.438  119 | 1.08  1178 | 15  3 |
| {BcALV1.BI=FALSE, BcNSRV7.BI=FALSE} =>  {BcOLV18=FALSE} | 0.37  8713 | 0.573  034 | 0.660  891 | 1.18  7208 | 15  3 |
| {BcDMV1=FALSE, BcOLV18=FALSE} =>  {BcNSRV7.BI=FALSE} | 0.36  3861 | 0.875 | 0.415  842 | 1.09  4427 | 14  7 |
| {BcNSRV7.BI=FALSE, BcOLV18=FALSE} =>  {BcFV9=FALSE} | 0.39  1089 | 0.951  807 | 0.410  891 | 1.09  5527 | 15  8 |
| {BcFV9=FALSE, BcOLV18=FALSE} => | 0.39 | 0.872 | 0.448 | 1.09 | 15 |

| {BcNSRV7.BI=FALSE} | 1089 | 928 | 02 | 1836 | 8 |
| --- | --- | --- | --- | --- | --- |
| {BcFV9=FALSE, BcNSRV7.BI=FALSE} =>  {BcOLV18=FALSE} | 0.39  1089 | 0.542  955 | 0.720  297 | 1.12  4892 | 15  8 |
| {BcBNV5.BI=FALSE, BcOLV18=FALSE} =>  {BcNSRV7.BI=FALSE} | 0.39  604 | 0.869  565 | 0.455  446 | 1.08  763 | 16  0 |
| {BcMCV5.BI=FALSE, BcOLV18=FALSE} =>  {BcNSRV7.BI=FALSE} | 0.40  5941 | 0.858  639 | 0.472  772 | 1.07  3963 | 16  4 |
| {BcMCV5.BI=FALSE, BcNSRV7.BI=FALSE}  => {BcOLV18=FALSE} | 0.40  5941 | 0.537  705 | 0.754  95 | 1.11  4014 | 16  4 |
| {BcOLV15.BI=FALSE, BcOLV18=FALSE} =>  {BcNSRV7.BI=FALSE} | 0.40  8416 | 0.863  874 | 0.472  772 | 1.08  0512 | 16  5 |
| {BcEV5=FALSE, BcOLV18=FALSE} =>  {BcNSRV7.BI=FALSE} | 0.40  099 | 0.866  31 | 0.462  871 | 1.08  3558 | 16  2 |
| {BcOLV18=FALSE, BcVV3.BI=FALSE} =>  {BcOLV7.BI=FALSE} | 0.35  1485 | 0.898  734 | 0.391  089 | 1.11  3769 | 14  2 |
| {BcOLV18=FALSE, BcVV3.BI=FALSE} =>  {BcALV1.BI=FALSE} | 0.35  8911 | 0.917  722 | 0.391  089 | 1.12  6929 | 14  5 |
| {BcALV1.BI=FALSE, BcVV3.BI=FALSE} =>  {BcOLV18=FALSE} | 0.35  8911 | 0.545  113 | 0.658  416 | 1.12  9362 | 14  5 |
| {BcOLV18=FALSE, BcVV3.BI=FALSE} =>  {BcFV9=FALSE} | 0.36  6337 | 0.936  709 | 0.391  089 | 1.07  8149 | 14  8 |
| {BcOLV18=FALSE, BcOLV7.BI=FALSE} => | 0.39 | 0.913 | 0.430 | 1.12 | 15 |

| {BcALV1.BI=FALSE} | 3564 | 793 | 693 | 2105 | 9 |
| --- | --- | --- | --- | --- | --- |
| {BcALV1.BI=FALSE, BcOLV18=FALSE} =>  {BcOLV7.BI=FALSE} | 0.39  3564 | 0.898  305 | 0.438  119 | 1.11  3237 | 15  9 |
| {BcALV1.BI=FALSE, BcOLV7.BI=FALSE} =>  {BcOLV18=FALSE} | 0.39  3564 | 0.582  418 | 0.675  743 | 1.20  665 | 15  9 |
| {BcDMV1=FALSE, BcOLV18=FALSE} =>  {BcOLV7.BI=FALSE} | 0.37  6238 | 0.904  762 | 0.415  842 | 1.12  1239 | 15  2 |
| {BcDMV1=FALSE, BcOLV7.BI=FALSE} =>  {BcOLV18=FALSE} | 0.37  6238 | 0.565  056 | 0.665  842 | 1.17  068 | 15  2 |
| {BcOLV18=FALSE, BcOLV7.BI=FALSE} =>  {BcFV9=FALSE} | 0.40  099 | 0.931  034 | 0.430  693 | 1.07  1618 | 16  2 |
| {BcFV9=FALSE, BcOLV18=FALSE} =>  {BcOLV7.BI=FALSE} | 0.40  099 | 0.895  028 | 0.448  02 | 1.10  9175 | 16  2 |
| {BcFV9=FALSE, BcOLV7.BI=FALSE} =>  {BcOLV18=FALSE} | 0.40  099 | 0.568  421 | 0.705  446 | 1.17  7652 | 16  2 |
| {BcOLV18=FALSE, BcOLV4.BI=FALSE} =>  {BcOLV7.BI=FALSE} | 0.37  8713 | 0.884  393 | 0.428  218 | 1.09  5996 | 15  3 |
| {BcOLV18=FALSE, BcULV5=FALSE} =>  {BcOLV7.BI=FALSE} | 0.35  6436 | 0.872  727 | 0.408  416 | 1.08  1539 | 14  4 |
| {BcBNV5.BI=FALSE, BcOLV18=FALSE} =>  {BcOLV7.BI=FALSE} | 0.40  8416 | 0.896  739 | 0.455  446 | 1.11  1296 | 16  5 |
| {BcBNV5.BI=FALSE, BcOLV7.BI=FALSE} => | 0.40 | 0.559 | 0.730 | 1.15 | 16 |

| {BcOLV18=FALSE} | 8416 | 322 | 198 | 8801 | 5 |
| --- | --- | --- | --- | --- | --- |
| {BcOLV18=FALSE, BvF.BI=FALSE} =>  {BcOLV7.BI=FALSE} | 0.38  6139 | 0.881  356 | 0.438  119 | 1.09  2233 | 15  6 |
| {BcMCV5.BI=FALSE, BcOLV18=FALSE} =>  {BcOLV7.BI=FALSE} | 0.42  0792 | 0.890  052 | 0.472  772 | 1.10  301 | 17  0 |
| {BcMCV5.BI=FALSE, BcOLV7.BI=FALSE} =>  {BcOLV18=FALSE} | 0.42  0792 | 0.561  056 | 0.75 | 1.16  2393 | 17  0 |
| {BcOLV13.BI=FALSE, BcOLV18=FALSE} =>  {BcOLV7.BI=FALSE} | 0.40  3465 | 0.895  604 | 0.450  495 | 1.10  989 | 16  3 |
| {BcOLV13.BI=FALSE, BcOLV7.BI=FALSE} =>  {BcOLV18=FALSE} | 0.40  3465 | 0.536  184 | 0.752  475 | 1.11  0864 | 16  3 |
| {BcOLV18=FALSE, BcOLV2.BI=FALSE} =>  {BcOLV7.BI=FALSE} | 0.40  8416 | 0.887  097 | 0.460  396 | 1.09  9347 | 16  5 |
| {BcOLV2.BI=FALSE, BcOLV7.BI=FALSE} =>  {BcOLV18=FALSE} | 0.40  8416 | 0.542  763 | 0.752  475 | 1.12  4494 | 16  5 |
| {BcMCV3.BI=FALSE, BcOLV18=FALSE} =>  {BcOLV7.BI=FALSE} | 0.40  8416 | 0.891  892 | 0.457  921 | 1.10  5289 | 16  5 |
| {BcMCV3.BI=FALSE, BcOLV7.BI=FALSE} =>  {BcOLV18=FALSE} | 0.40  8416 | 0.537  459 | 0.759  901 | 1.11  3505 | 16  5 |
| {BcOLV18=FALSE, BcPV4.BI=FALSE} =>  {BcOLV7.BI=FALSE} | 0.41  3366 | 0.907  609 | 0.455  446 | 1.12  4767 | 16  7 |
| {BcOLV7.BI=FALSE, BcPV4.BI=FALSE} => | 0.41 | 0.545 | 0.757 | 1.13 | 16 |

| {BcOLV18=FALSE} | 3366 | 752 | 426 | 0685 | 7 |
| --- | --- | --- | --- | --- | --- |
| {BcEV6=FALSE, BcOLV18=FALSE} =>  {BcOLV7.BI=FALSE} | 0.40  5941 | 0.896  175 | 0.452  97 | 1.11  0597 | 16  4 |
| {BcEV6=FALSE, BcOLV7.BI=FALSE} =>  {BcOLV18=FALSE} | 0.40  5941 | 0.539  474 | 0.752  475 | 1.11  7679 | 16  4 |
| {BcOLV17.BI=FALSE, BcOLV18=FALSE} =>  {BcOLV7.BI=FALSE} | 0.42  0792 | 0.894  737 | 0.470  297 | 1.10  8815 | 17  0 |
| {BcOLV17.BI=FALSE, BcOLV7.BI=FALSE} =>  {BcOLV18=FALSE} | 0.42  0792 | 0.541  401 | 0.777  228 | 1.12  1672 | 17  0 |
| {BcOLV15.BI=FALSE, BcOLV18=FALSE} =>  {BcOLV7.BI=FALSE} | 0.42  0792 | 0.890  052 | 0.472  772 | 1.10  301 | 17  0 |
| {BcOLV15.BI=FALSE, BcOLV7.BI=FALSE} =>  {BcOLV18=FALSE} | 0.42  0792 | 0.551  948 | 0.762  376 | 1.14  3523 | 17  0 |
| {BcEV5=FALSE, BcOLV18=FALSE} =>  {BcOLV7.BI=FALSE} | 0.41  3366 | 0.893  048 | 0.462  871 | 1.10  6722 | 16  7 |
| {BcEV5=FALSE, BcOLV7.BI=FALSE} =>  {BcOLV18=FALSE} | 0.41  3366 | 0.543  974 | 0.759  901 | 1.12  7002 | 16  7 |
| {BcDMV1=FALSE, BcOLV18=FALSE} =>  {BcALV1.BI=FALSE} | 0.38  1188 | 0.916  667 | 0.415  842 | 1.12  5633 | 15  4 |
| {BcALV1.BI=FALSE, BcDMV1=FALSE} =>  {BcOLV18=FALSE} | 0.38  1188 | 0.544  17 | 0.700  495 | 1.12  7408 | 15  4 |
| {BcALV1.BI=FALSE, BcOLV18=FALSE} => | 0.40 | 0.932 | 0.438 | 1.07 | 16 |

| {BcFV9=FALSE} | 8416 | 203 | 119 | 2963 | 5 |
| --- | --- | --- | --- | --- | --- |
| {BcFV9=FALSE, BcOLV18=FALSE} =>  {BcALV1.BI=FALSE} | 0.40  8416 | 0.911  602 | 0.448  02 | 1.11  9414 | 16  5 |
| {BcALV1.BI=FALSE, BcFV9=FALSE} =>  {BcOLV18=FALSE} | 0.40  8416 | 0.567  01 | 0.720  297 | 1.17  4729 | 16  5 |
| {BcOLV18=FALSE, BcOLV4.BI=FALSE} =>  {BcALV1.BI=FALSE} | 0.39  3564 | 0.919  075 | 0.428  218 | 1.12  8591 | 15  9 |
| {BcALV1.BI=FALSE, BcOLV4.BI=FALSE} =>  {BcOLV18=FALSE} | 0.39  3564 | 0.546  392 | 0.720  297 | 1.13  2012 | 15  9 |
| {BcOLV18=FALSE, BcULV5=FALSE} =>  {BcALV1.BI=FALSE} | 0.37  1287 | 0.909  091 | 0.408  416 | 1.11  633 | 15  0 |
| {BcBNV5.BI=FALSE, BcOLV18=FALSE} =>  {BcALV1.BI=FALSE} | 0.41  8317 | 0.918  478 | 0.455  446 | 1.12  7858 | 16  9 |
| {BcALV1.BI=FALSE, BcBNV5.BI=FALSE} =>  {BcOLV18=FALSE} | 0.41  8317 | 0.546  926 | 0.764  851 | 1.13  3118 | 16  9 |
| {BcOLV18=FALSE, BvF.BI=FALSE} =>  {BcALV1.BI=FALSE} | 0.40  3465 | 0.920  904 | 0.438  119 | 1.13  0836 | 16  3 |
| {BcALV1.BI=FALSE, BvF.BI=FALSE} =>  {BcOLV18=FALSE} | 0.40  3465 | 0.539  735 | 0.747  525 | 1.11  822 | 16  3 |
| {BcMCV5.BI=FALSE, BcOLV18=FALSE} =>  {BcALV1.BI=FALSE} | 0.43  3168 | 0.916  23 | 0.472  772 | 1.12  5097 | 17  5 |
| {BcALV1.BI=FALSE, BcMCV5.BI=FALSE} => | 0.43 | 0.560 | 0.772 | 1.16 | 17 |

| {BcOLV18=FALSE} | 3168 | 897 | 277 | 2064 | 5 |
| --- | --- | --- | --- | --- | --- |
| {BcOLV13.BI=FALSE, BcOLV18=FALSE} =>  {BcALV1.BI=FALSE} | 0.40  8416 | 0.906  593 | 0.450  495 | 1.11  3264 | 16  5 |
| {BcALV1.BI=FALSE, BcOLV13.BI=FALSE} =>  {BcOLV18=FALSE} | 0.40  8416 | 0.544  554 | 0.75 | 1.12  8205 | 16  5 |
| {BcOLV18=FALSE, BcOLV2.BI=FALSE} =>  {BcALV1.BI=FALSE} | 0.41  5842 | 0.903  226 | 0.460  396 | 1.10  9128 | 16  8 |
| {BcALV1.BI=FALSE, BcOLV2.BI=FALSE} =>  {BcOLV18=FALSE} | 0.41  5842 | 0.550  82 | 0.754  95 | 1.14  1185 | 16  8 |
| {BcMCV3.BI=FALSE, BcOLV18=FALSE} =>  {BcALV1.BI=FALSE} | 0.41  3366 | 0.902  703 | 0.457  921 | 1.10  8486 | 16  7 |
| {BcALV1.BI=FALSE, BcMCV3.BI=FALSE} =>  {BcOLV18=FALSE} | 0.41  3366 | 0.543  974 | 0.759  901 | 1.12  7002 | 16  7 |
| {BcOLV18=FALSE, BcPV4.BI=FALSE} =>  {BcALV1.BI=FALSE} | 0.41  3366 | 0.907  609 | 0.455  446 | 1.11  451 | 16  7 |
| {BcALV1.BI=FALSE, BcPV4.BI=FALSE} =>  {BcOLV18=FALSE} | 0.41  3366 | 0.549  342 | 0.752  475 | 1.13  8124 | 16  7 |
| {BcEV6=FALSE, BcOLV18=FALSE} =>  {BcALV1.BI=FALSE} | 0.41  3366 | 0.912  568 | 0.452  97 | 1.12  0601 | 16  7 |
| {BcALV1.BI=FALSE, BcEV6=FALSE} =>  {BcOLV18=FALSE} | 0.41  3366 | 0.540  453 | 0.764  851 | 1.11  9708 | 16  7 |
| {BcOLV17.BI=FALSE, BcOLV18=FALSE} => | 0.42 | 0.905 | 0.470 | 1.11 | 17 |

| {BcALV1.BI=FALSE} | 5743 | 263 | 297 | 163 | 2 |
| --- | --- | --- | --- | --- | --- |
| {BcALV1.BI=FALSE, BcOLV17.BI=FALSE} =>  {BcOLV18=FALSE} | 0.42  5743 | 0.553  055 | 0.769  802 | 1.14  5816 | 17  2 |
| {BcOLV15.BI=FALSE, BcOLV18=FALSE} =>  {BcALV1.BI=FALSE} | 0.42  8218 | 0.905  759 | 0.472  772 | 1.11  2239 | 17  3 |
| {BcALV1.BI=FALSE, BcOLV15.BI=FALSE} =>  {BcOLV18=FALSE} | 0.42  8218 | 0.558  065 | 0.767  327 | 1.15  6195 | 17  3 |
| {BcEV5=FALSE, BcOLV18=FALSE} =>  {BcALV1.BI=FALSE} | 0.42  0792 | 0.909  091 | 0.462  871 | 1.11  633 | 17  0 |
| {BcALV1.BI=FALSE, BcEV5=FALSE} =>  {BcOLV18=FALSE} | 0.42  0792 | 0.534  591 | 0.787  129 | 1.10  7563 | 17  0 |
| {BcDMV1=FALSE, BcOLV18=FALSE} =>  {BcFV9=FALSE} | 0.39  1089 | 0.940  476 | 0.415  842 | 1.08  2485 | 15  8 |
| {BcDMV1=FALSE, BcOLV18=FALSE} =>  {BcMCV5.BI=FALSE} | 0.41  5842 | 1 | 0.415  842 | 1.08  311 | 16  8 |
| {BcEV5=FALSE, BcOLV18=FALSE} =>  {BcDMV1=FALSE} | 0.41  3366 | 0.893  048 | 0.462  871 | 1.05  8039 | 16  7 |
| {BcOLV18=FALSE, BcOLV4.BI=FALSE} =>  {BcFV9=FALSE} | 0.39  3564 | 0.919  075 | 0.428  218 | 1.05  7853 | 15  9 |
| {BcOLV18=FALSE, BcULV5=FALSE} =>  {BcFV9=FALSE} | 0.37  6238 | 0.921  212 | 0.408  416 | 1.06  0313 | 15  2 |
| {BcBNV5.BI=FALSE, BcOLV18=FALSE} => | 0.42 | 0.940 | 0.455 | 1.08 | 17 |

| {BcFV9=FALSE} | 8218 | 217 | 446 | 2188 | 3 |
| --- | --- | --- | --- | --- | --- |
| {BcBNV5.BI=FALSE, BcFV9=FALSE} =>  {BcOLV18=FALSE} | 0.42  8218 | 0.530  675 | 0.806  931 | 1.09  9449 | 17  3 |
| {BcOLV18=FALSE, BvF.BI=FALSE} =>  {BcFV9=FALSE} | 0.40  3465 | 0.920  904 | 0.438  119 | 1.05  9958 | 16  3 |
| {BcMCV5.BI=FALSE, BcOLV18=FALSE} =>  {BcFV9=FALSE} | 0.44  3069 | 0.937  173 | 0.472  772 | 1.07  8683 | 17  9 |
| {BcFV9=FALSE, BcMCV5.BI=FALSE} =>  {BcOLV18=FALSE} | 0.44  3069 | 0.544  073 | 0.814  356 | 1.12  7208 | 17  9 |
| {BcOLV13.BI=FALSE, BcOLV18=FALSE} =>  {BcFV9=FALSE} | 0.42  0792 | 0.934  066 | 0.450  495 | 1.07  5107 | 17  0 |
| {BcOLV18=FALSE, BcOLV2.BI=FALSE} =>  {BcFV9=FALSE} | 0.43  0693 | 0.935  484 | 0.460  396 | 1.07  6739 | 17  4 |
| {BcFV9=FALSE, BcOLV2.BI=FALSE} =>  {BcOLV18=FALSE} | 0.43  0693 | 0.533  742 | 0.806  931 | 1.10  5805 | 17  4 |
| {BcMCV3.BI=FALSE, BcOLV18=FALSE} =>  {BcFV9=FALSE} | 0.42  3267 | 0.924  324 | 0.457  921 | 1.06  3895 | 17  1 |
| {BcFV9=FALSE, BcMCV3.BI=FALSE} =>  {BcOLV18=FALSE} | 0.42  3267 | 0.522  936 | 0.809  406 | 1.08  3416 | 17  1 |
| {BcOLV18=FALSE, BcPV4.BI=FALSE} =>  {BcFV9=FALSE} | 0.42  0792 | 0.923  913 | 0.455  446 | 1.06  3421 | 17  0 |
| {BcEV6=FALSE, BcOLV18=FALSE} => | 0.42 | 0.928 | 0.452 | 1.06 | 17 |

| {BcFV9=FALSE} | 0792 | 962 | 97 | 9232 | 0 |
| --- | --- | --- | --- | --- | --- |
| {BcOLV17.BI=FALSE, BcOLV18=FALSE} =>  {BcFV9=FALSE} | 0.43  5644 | 0.926  316 | 0.470  297 | 1.06  6187 | 17  6 |
| {BcOLV15.BI=FALSE, BcOLV18=FALSE} =>  {BcFV9=FALSE} | 0.44  5545 | 0.942  408 | 0.472  772 | 1.08  4709 | 18  0 |
| {BcFV9=FALSE, BcOLV15.BI=FALSE} =>  {BcOLV18=FALSE} | 0.44  5545 | 0.524  781 | 0.849  01 | 1.08  7239 | 18  0 |
| {BcEV5=FALSE, BcOLV18=FALSE} =>  {BcFV9=FALSE} | 0.43  5644 | 0.941  176 | 0.462  871 | 1.08  3291 | 17  6 |
| {BcEV5=FALSE, BcFV9=FALSE} =>  {BcOLV18=FALSE} | 0.43  5644 | 0.525  373 | 0.829  208 | 1.08  8465 | 17  6 |
| {BcBNV5.BI=FALSE, BcOLV18=FALSE} =>  {BcMCV5.BI=FALSE} | 0.45  5446 | 1 | 0.455  446 | 1.08  311 | 18  4 |
| {BcBNV5.BI=FALSE, BcMCV5.BI=FALSE} =>  {BcOLV18=FALSE} | 0.45  5446 | 0.522  727 | 0.871  287 | 1.08  2984 | 18  4 |
| {BcMCV5.BI=FALSE, BcOLV2.BI=FALSE} =>  {BcOLV18=FALSE} | 0.45  0495 | 0.527  536 | 0.853  96 | 1.09  2947 | 18  2 |
| {BcMCV5.BI=FALSE, BcOLV17.BI=FALSE} =>  {BcOLV18=FALSE} | 0.46  0396 | 0.522  472 | 0.881  188 | 1.08  2455 | 18  6 |
| {BcMCV5.BI=FALSE, BcOLV15.BI=FALSE} =>  {BcOLV18=FALSE} | 0.46  2871 | 0.526  761 | 0.878  713 | 1.09  134 | 18  7 |
| {BcEV5=FALSE, BcOLV18=FALSE} => | 0.46 | 0.994 | 0.462 | 1.07 | 18 |

| {BcMCV5.BI=FALSE} | 0396 | 652 | 871 | 7318 | 6 |
| --- | --- | --- | --- | --- | --- |
| {BcMV2.BI=FALSE, BcOLV18=TRUE} =>  {BcHV1.BI=TRUE} | 0.15  5941 | 0.7 | 0.222  772 | 1.15  9016 | 63 |
| {BcMV2.BI=FALSE, BcOLV18=TRUE} =>  {BcOLV1.BI.MOVA.=FALSE} | 0.17  0792 | 0.766  667 | 0.222  772 | 1.14  716 | 69 |
| {BcMV2.BI=FALSE, BcOLV18=TRUE} =>  {BcULV4=TRUE} | 0.18  0693 | 0.811  111 | 0.222  772 | 1.17  4512 | 73 |
| {BcMV2.BI=FALSE, BcOLV18=TRUE} =>  {BcMV6.BI=FALSE} | 0.19  5545 | 0.877  778 | 0.222  772 | 1.26  2001 | 79 |
| {BcMV2.BI=FALSE, BcOLV18=TRUE} =>  {BcRV1.BI=FALSE} | 0.18  3168 | 0.822  222 | 0.222  772 | 1.13  7595 | 74 |
| {BcMV2.BI=FALSE, BcOLV18=TRUE} =>  {BcEV2.BI=FALSE} | 0.18  5644 | 0.833  333 | 0.222  772 | 1.11  479 | 75 |
| {BcMV2.BI=FALSE, BcOLV18=TRUE} =>  {BcALV1.BI=FALSE} | 0.20  297 | 0.911  111 | 0.222  772 | 1.11  8811 | 82 |
| {BcMV2.BI=FALSE, BcOLV18=TRUE} =>  {BcULV5=FALSE} | 0.21  0396 | 0.944  444 | 0.222  772 | 1.07  7841 | 85 |
| {BcHV1SLR.BI=TRUE, BcOLV18=TRUE} =>  {BcMV3.BI=TRUE} | 0.23  7624 | 0.695  652 | 0.341  584 | 1.28  3304 | 96 |
| {BcMV3.BI=TRUE, BcOLV18=TRUE} =>  {BcHV1SLR.BI=TRUE} | 0.23  7624 | 0.721  805 | 0.329  208 | 1.33  1548 | 96 |
| {BcHV1SLR.BI=TRUE, BcMV3.BI=TRUE} => | 0.23 | 0.721 | 0.329 | 1.39 | 96 |

| {BcOLV18=TRUE} | 7624 | 805 | 208 | 5258 |  |
| --- | --- | --- | --- | --- | --- |
| {BcOLV16.BI=FALSE, BcOLV18=TRUE} =>  {BcHV1SLR.BI=TRUE} | 0.16  5842 | 0.67 | 0.247  525 | 1.23  5982 | 67 |
| {BcHV1SLR.BI=TRUE, BcOLV18=TRUE} =>  {BcULV3=TRUE} | 0.24  2574 | 0.710  145 | 0.341  584 | 1.19  5411 | 98 |
| {BcOLV18=TRUE, BcULV3=TRUE} =>  {BcHV1SLR.BI=TRUE} | 0.24  2574 | 0.748  092 | 0.324  257 | 1.38  0041 | 98 |
| {BcHV1SLR.BI=TRUE, BcULV3=TRUE} =>  {BcOLV18=TRUE} | 0.24  2574 | 0.628  205 | 0.386  139 | 1.21  433 | 98 |
| {BcHV1SLR.BI=TRUE, BcOLV18=TRUE} =>  {BcHV1.BI=TRUE} | 0.31  6832 | 0.927  536 | 0.341  584 | 1.53  5757 | 12  8 |
| {BcHV1.BI=TRUE, BcOLV18=TRUE} =>  {BcHV1SLR.BI=TRUE} | 0.31  6832 | 0.864  865 | 0.366  337 | 1.59  5458 | 12  8 |
| {BcHV1.BI=TRUE, BcHV1SLR.BI=TRUE} =>  {BcOLV18=TRUE} | 0.31  6832 | 0.653  061 | 0.485  149 | 1.26  2377 | 12  8 |
| {BcHV1SLR.BI=TRUE, BcOLV18=TRUE} =>  {BcMV11=TRUE} | 0.24  2574 | 0.710  145 | 0.341  584 | 1.15  2203 | 98 |
| {BcMV11=TRUE, BcOLV18=TRUE} =>  {BcHV1SLR.BI=TRUE} | 0.24  2574 | 0.7 | 0.346  535 | 1.29  1324 | 98 |
| {BcHV1SLR.BI=TRUE, BcMV11=TRUE} =>  {BcOLV18=TRUE} | 0.24  2574 | 0.680  556 | 0.356  436 | 1.31  5524 | 98 |
| {BcHV1SLR.BI=TRUE, BcOLV18=TRUE} => | 0.27 | 0.818 | 0.341 | 1.20 | 11 |

| {BcMV9.BI=TRUE} | 9703 | 841 | 584 | 7342 | 3 |
| --- | --- | --- | --- | --- | --- |
| {BcMV9.BI=TRUE, BcOLV18=TRUE} =>  {BcHV1SLR.BI=TRUE} | 0.27  9703 | 0.676  647 | 0.413  366 | 1.24  8243 | 11  3 |
| {BcHV1SLR.BI=TRUE, BcMV9.BI=TRUE} =>  {BcOLV18=TRUE} | 0.27  9703 | 0.684  848 | 0.408  416 | 1.32  3822 | 11  3 |
| {BcHV1SLR.BI=TRUE, BcOLV18=TRUE} =>  {BcULV4=TRUE} | 0.29  703 | 0.869  565 | 0.341  584 | 1.25  9155 | 12  0 |
| {BcOLV18=TRUE, BcULV4=TRUE} =>  {BcHV1SLR.BI=TRUE} | 0.29  703 | 0.674  157 | 0.440  594 | 1.24  3651 | 12  0 |
| {BcHV1SLR.BI=TRUE, BcULV4=TRUE} =>  {BcOLV18=TRUE} | 0.29  703 | 0.740  741 | 0.400  99 | 1.43  1862 | 12  0 |
| {BcHV1SLR.BI=TRUE, BcMV6.BI=FALSE} =>  {BcOLV18=TRUE} | 0.20  7921 | 0.617  647 | 0.336  634 | 1.19  3921 | 84 |
| {BcOLV18=TRUE, BcPV3.BI=FALSE} =>  {BcHV1SLR.BI=TRUE} | 0.21  7822 | 0.661  654 | 0.329  208 | 1.22  0586 | 88 |
| {BcEV2.BI=FALSE, BcOLV18=TRUE} =>  {BcHV1SLR.BI=TRUE} | 0.24  505 | 0.664  43 | 0.368  812 | 1.22  5706 | 99 |
| {BcEV2.BI=FALSE, BcHV1SLR.BI=TRUE} =>  {BcOLV18=TRUE} | 0.24  505 | 0.634  615 | 0.386  139 | 1.22  6721 | 99 |
| {BcHV3.BI=FALSE, BcOLV18=TRUE} =>  {BcHV1SLR.BI=TRUE} | 0.22  7723 | 0.647  887 | 0.351  485 | 1.19  5189 | 92 |
| {BcFV5.BI=FALSE, BcOLV18=TRUE} => | 0.23 | 0.624 | 0.368 | 1.15 | 93 |

| {BcHV1SLR.BI=TRUE} | 0198 | 161 | 812 | 142 |  |
| --- | --- | --- | --- | --- | --- |
| {BcOLV18=TRUE, BcULV1.BI=FALSE} =>  {BcHV1SLR.BI=TRUE} | 0.22  2772 | 0.625 | 0.356  436 | 1.15  2968 | 90 |
| {BcMV1.BI=TRUE, BcOLV18=TRUE} =>  {BcHV1SLR.BI=TRUE} | 0.29  2079 | 0.662  921 | 0.440  594 | 1.22  2923 | 11  8 |
| {BcHV1SLR.BI=TRUE, BcMV1.BI=TRUE} =>  {BcOLV18=TRUE} | 0.29  2079 | 0.662  921 | 0.440  594 | 1.28  1436 | 11  8 |
| {BcMV12=FALSE, BcOLV18=TRUE} =>  {BcHV1SLR.BI=TRUE} | 0.25  7426 | 0.65 | 0.396  04 | 1.19  9087 | 10  4 |
| {BcHV1SLR.BI=TRUE, BcMV12=FALSE} =>  {BcOLV18=TRUE} | 0.25  7426 | 0.622  754 | 0.413  366 | 1.20  3793 | 10  4 |
| {BcNSRV7.BI=FALSE, BcOLV18=TRUE} =>  {BcHV1SLR.BI=TRUE} | 0.25  2475 | 0.649  682 | 0.388  614 | 1.19  8499 | 10  2 |
| {BcHV1SLR.BI=TRUE, BcNSRV7.BI=FALSE}  => {BcOLV18=TRUE} | 0.25  2475 | 0.618  182 | 0.408  416 | 1.19  4954 | 10  2 |
| {BcOLV18=TRUE, BcVV3.BI=FALSE} =>  {BcHV1SLR.BI=TRUE} | 0.27  2277 | 0.662  651 | 0.410  891 | 1.22  2424 | 11  0 |
| {BcHV1SLR.BI=TRUE, BcVV3.BI=FALSE} =>  {BcOLV18=TRUE} | 0.27  2277 | 0.628  571 | 0.433  168 | 1.21  5038 | 11  0 |
| {BcOLV18=TRUE, BcOLV7.BI=FALSE} =>  {BcHV1SLR.BI=TRUE} | 0.23  7624 | 0.631  579 | 0.376  238 | 1.16  5105 | 96 |
| {BcALV1.BI=FALSE, BcOLV18=TRUE} => | 0.23 | 0.631 | 0.376 | 1.16 | 96 |

| {BcHV1SLR.BI=TRUE} | 7624 | 579 | 238 | 5105 |  |
| --- | --- | --- | --- | --- | --- |
| {BcDMV1=FALSE, BcOLV18=TRUE} =>  {BcHV1SLR.BI=TRUE} | 0.28  7129 | 0.670  52 | 0.428  218 | 1.23  6941 | 11  6 |
| {BcDMV1=FALSE, BcHV1SLR.BI=TRUE} =>  {BcOLV18=TRUE} | 0.28  7129 | 0.617  021 | 0.465  347 | 1.19  2711 | 11  6 |
| {BcFV9=FALSE, BcOLV18=TRUE} =>  {BcHV1SLR.BI=TRUE} | 0.26  9802 | 0.641  176 | 0.420  792 | 1.18  281 | 10  9 |
| {BcFV9=FALSE, BcHV1SLR.BI=TRUE} =>  {BcOLV18=TRUE} | 0.26  9802 | 0.598  901 | 0.450  495 | 1.15  7684 | 10  9 |
| {BcOLV18=TRUE, BcOLV4.BI=FALSE} =>  {BcHV1SLR.BI=TRUE} | 0.29  2079 | 0.651  934 | 0.448  02 | 1.20  2654 | 11  8 |
| {BcHV1SLR.BI=TRUE, BcOLV4.BI=FALSE}  => {BcOLV18=TRUE} | 0.29  2079 | 0.631  016 | 0.462  871 | 1.21  9763 | 11  8 |
| {BcOLV18=TRUE, BcULV5=FALSE} =>  {BcHV1SLR.BI=TRUE} | 0.29  9505 | 0.640  212 | 0.467  822 | 1.18  103 | 12  1 |
| {BcHV1SLR.BI=TRUE, BcULV5=FALSE} =>  {BcOLV18=TRUE} | 0.29  9505 | 0.640  212 | 0.467  822 | 1.23  7538 | 12  1 |
| {BcBNV5.BI=FALSE, BcOLV18=TRUE} =>  {BcHV1SLR.BI=TRUE} | 0.29  703 | 0.655  738 | 0.452  97 | 1.20  9671 | 12  0 |
| {BcBNV5.BI=FALSE, BcHV1SLR.BI=TRUE}  => {BcOLV18=TRUE} | 0.29  703 | 0.621  762 | 0.477  723 | 1.20  1874 | 12  0 |
| {BcOLV18=TRUE, BvF.BI=FALSE} => | 0.30 | 0.651 | 0.475 | 1.20 | 12 |

| {BcHV1SLR.BI=TRUE} | 9406 | 042 | 248 | 1008 | 5 |
| --- | --- | --- | --- | --- | --- |
| {BcHV1SLR.BI=TRUE, BvF.BI=FALSE} =>  {BcOLV18=TRUE} | 0.30  9406 | 0.644  33 | 0.480  198 | 1.24  5499 | 12  5 |
| {BcMCV5.BI=FALSE, BcOLV18=TRUE} =>  {BcHV1SLR.BI=TRUE} | 0.29  9505 | 0.664  835 | 0.450  495 | 1.22  6454 | 12  1 |
| {BcHV1SLR.BI=TRUE, BcMCV5.BI=FALSE}  => {BcOLV18=TRUE} | 0.29  9505 | 0.611  111 | 0.490  099 | 1.18  1287 | 12  1 |
| {BcOLV13.BI=FALSE, BcOLV18=TRUE} =>  {BcHV1SLR.BI=TRUE} | 0.30  9406 | 0.651  042 | 0.475  248 | 1.20  1008 | 12  5 |
| {BcHV1SLR.BI=TRUE, BcOLV13.BI=FALSE}  => {BcOLV18=TRUE} | 0.30  9406 | 0.631  313 | 0.490  099 | 1.22  0337 | 12  5 |
| {BcOLV18=TRUE, BcOLV2.BI=FALSE} =>  {BcHV1SLR.BI=TRUE} | 0.29  703 | 0.634  921 | 0.467  822 | 1.17  1269 | 12  0 |
| {BcHV1SLR.BI=TRUE, BcOLV2.BI=FALSE}  => {BcOLV18=TRUE} | 0.29  703 | 0.615  385 | 0.482  673 | 1.18  9547 | 12  0 |
| {BcMCV3.BI=FALSE, BcOLV18=TRUE} =>  {BcHV1SLR.BI=TRUE} | 0.30  6931 | 0.645  833 | 0.475  248 | 1.19  14 | 12  4 |
| {BcHV1SLR.BI=TRUE, BcMCV3.BI=FALSE}  => {BcOLV18=TRUE} | 0.30  6931 | 0.629  442 | 0.487  624 | 1.21  672 | 12  4 |
| {BcOLV18=TRUE, BcPV4.BI=FALSE} =>  {BcHV1SLR.BI=TRUE} | 0.31  6832 | 0.663  212 | 0.477  723 | 1.22  346 | 12  8 |
| {BcHV1SLR.BI=TRUE, BcPV4.BI=FALSE} => | 0.31 | 0.627 | 0.504 | 1.21 | 12 |

| {BcOLV18=TRUE} | 6832 | 451 | 95 | 2872 | 8 |
| --- | --- | --- | --- | --- | --- |
| {BcEV6=FALSE, BcOLV18=TRUE} =>  {BcHV1SLR.BI=TRUE} | 0.31  4356 | 0.651  282 | 0.482  673 | 1.20  1452 | 12  7 |
| {BcEV6=FALSE, BcHV1SLR.BI=TRUE} =>  {BcOLV18=TRUE} | 0.31  4356 | 0.625  616 | 0.502  475 | 1.20  9324 | 12  7 |
| {BcOLV17.BI=FALSE, BcOLV18=TRUE} =>  {BcHV1SLR.BI=TRUE} | 0.30  9406 | 0.647  668 | 0.477  723 | 1.19  4786 | 12  5 |
| {BcHV1SLR.BI=TRUE, BcOLV17.BI=FALSE}  => {BcOLV18=TRUE} | 0.30  9406 | 0.612  745 | 0.504  95 | 1.18  4445 | 12  5 |
| {BcOLV15.BI=FALSE, BcOLV18=TRUE} =>  {BcHV1SLR.BI=TRUE} | 0.30  9406 | 0.647  668 | 0.477  723 | 1.19  4786 | 12  5 |
| {BcHV1SLR.BI=TRUE, BcOLV15.BI=FALSE}  => {BcOLV18=TRUE} | 0.30  9406 | 0.618  812 | 0.5 | 1.19  6172 | 12  5 |
| {BcEV5=FALSE, BcOLV18=TRUE} =>  {BcHV1SLR.BI=TRUE} | 0.32  4257 | 0.664  975 | 0.487  624 | 1.22  6711 | 13  1 |
| {BcEV5=FALSE, BcHV1SLR.BI=TRUE} =>  {BcOLV18=TRUE} | 0.32  4257 | 0.632  85 | 0.512  376 | 1.22  3309 | 13  1 |
| {BcOLV18=TRUE, BcULV3=TRUE} =>  {BcMV3.BI=TRUE} | 0.21  5347 | 0.664  122 | 0.324  257 | 1.22  5139 | 87 |
| {BcMV3.BI=TRUE, BcULV3=TRUE} =>  {BcOLV18=TRUE} | 0.21  5347 | 0.621  429 | 0.346  535 | 1.20  123 | 87 |
| {BcMV3.BI=TRUE, BcOLV18=TRUE} => | 0.24 | 0.751 | 0.329 | 1.24 | 10 |

| {BcHV1.BI=TRUE} | 7525 | 88 | 208 | 4916 | 0 |
| --- | --- | --- | --- | --- | --- |
| {BcHV1.BI=TRUE, BcOLV18=TRUE} =>  {BcMV3.BI=TRUE} | 0.24  7525 | 0.675  676 | 0.366  337 | 1.24  6452 | 10  0 |
| {BcHV1.BI=TRUE, BcMV3.BI=TRUE} =>  {BcOLV18=TRUE} | 0.24  7525 | 0.699  301 | 0.353  96 | 1.35  1758 | 10  0 |
| {BcMV3.BI=TRUE, BcOLV18=TRUE} =>  {BcMV11=TRUE} | 0.24  0099 | 0.729  323 | 0.329  208 | 1.18  332 | 97 |
| {BcMV11=TRUE, BcOLV18=TRUE} =>  {BcMV3.BI=TRUE} | 0.24  0099 | 0.692  857 | 0.346  535 | 1.27  8147 | 97 |
| {BcMV11=TRUE, BcMV3.BI=TRUE} =>  {BcOLV18=TRUE} | 0.24  0099 | 0.625  806 | 0.383  663 | 1.20  9693 | 97 |
| {BcMV3.BI=TRUE, BcOLV18=TRUE} =>  {BcMV9.BI=TRUE} | 0.27  9703 | 0.849  624 | 0.329  208 | 1.25  273 | 11  3 |
| {BcMV9.BI=TRUE, BcOLV18=TRUE} =>  {BcMV3.BI=TRUE} | 0.27  9703 | 0.676  647 | 0.413  366 | 1.24  8243 | 11  3 |
| {BcMV3.BI=TRUE, BcMV9.BI=TRUE} =>  {BcOLV18=TRUE} | 0.27  9703 | 0.706  25 | 0.396  04 | 1.36  5191 | 11  3 |
| {BcMV3.BI=TRUE, BcOLV18=TRUE} =>  {BcULV4=TRUE} | 0.29  2079 | 0.887  218 | 0.329  208 | 1.28  4717 | 11  8 |
| {BcOLV18=TRUE, BcULV4=TRUE} =>  {BcMV3.BI=TRUE} | 0.29  2079 | 0.662  921 | 0.440  594 | 1.22  2923 | 11  8 |
| {BcMV3.BI=TRUE, BcULV4=TRUE} => | 0.29 | 0.728 | 0.400 | 1.40 | 11 |

| {BcOLV18=TRUE} | 2079 | 395 | 99 | 7998 | 8 |
| --- | --- | --- | --- | --- | --- |
| {BcMV3.BI=TRUE, BcOLV18=TRUE} =>  {BcMV1.BI=TRUE} | 0.28  7129 | 0.872  18 | 0.329  208 | 1.10  458 | 11  6 |
| {BcMV1.BI=TRUE, BcOLV18=TRUE} =>  {BcMV3.BI=TRUE} | 0.28  7129 | 0.651  685 | 0.440  594 | 1.20  2196 | 11  6 |
| {BcMV1.BI=TRUE, BcMV3.BI=TRUE} =>  {BcOLV18=TRUE} | 0.28  7129 | 0.627  027 | 0.457  921 | 1.21  2052 | 11  6 |
| {BcNSRV7.BI=FALSE, BcOLV18=TRUE} =>  {BcMV3.BI=TRUE} | 0.24  2574 | 0.624  204 | 0.388  614 | 1.15  1499 | 98 |
| {BcMV3.BI=TRUE, BcVV3.BI=FALSE} =>  {BcOLV18=TRUE} | 0.24  7525 | 0.595  238 | 0.415  842 | 1.15  0604 | 10  0 |
| {BcDMV1=FALSE, BcMV3.BI=TRUE} =>  {BcOLV18=TRUE} | 0.25  7426 | 0.608  187 | 0.423  267 | 1.17  5634 | 10  4 |
| {BcFV9=FALSE, BcOLV18=TRUE} =>  {BcMV3.BI=TRUE} | 0.26  7327 | 0.635  294 | 0.420  792 | 1.17  1958 | 10  8 |
| {BcOLV18=TRUE, BcOLV4.BI=FALSE} =>  {BcMV3.BI=TRUE} | 0.28  2178 | 0.629  834 | 0.448  02 | 1.16  1886 | 11  4 |
| {BcMV3.BI=TRUE, BcOLV4.BI=FALSE} =>  {BcOLV18=TRUE} | 0.28  2178 | 0.619  565 | 0.455  446 | 1.19  7628 | 11  4 |
| {BcOLV18=TRUE, BcULV5=FALSE} =>  {BcMV3.BI=TRUE} | 0.29  4554 | 0.629  63 | 0.467  822 | 1.16  1509 | 11  9 |
| {BcMV3.BI=TRUE, BcULV5=FALSE} => | 0.29 | 0.650 | 0.452 | 1.25 | 11 |

| {BcOLV18=TRUE} | 4554 | 273 | 97 | 6987 | 9 |
| --- | --- | --- | --- | --- | --- |
| {BcOLV18=TRUE, BvF.BI=FALSE} =>  {BcMV3.BI=TRUE} | 0.29  9505 | 0.630  208 | 0.475  248 | 1.16  2576 | 12  1 |
| {BcMV3.BI=TRUE, BvF.BI=FALSE} =>  {BcOLV18=TRUE} | 0.29  9505 | 0.605 | 0.495  05 | 1.16  9474 | 12  1 |
| {BcOLV13.BI=FALSE, BcOLV18=TRUE} =>  {BcMV3.BI=TRUE} | 0.29  4554 | 0.619  792 | 0.475  248 | 1.14  336 | 11  9 |
| {BcMV3.BI=TRUE, BcOLV13.BI=FALSE} =>  {BcOLV18=TRUE} | 0.29  4554 | 0.619  792 | 0.475  248 | 1.19  8066 | 11  9 |
| {BcOLV18=TRUE, BcOLV2.BI=FALSE} =>  {BcMV3.BI=TRUE} | 0.28  7129 | 0.613  757 | 0.467  822 | 1.13  2227 | 11  6 |
| {BcMV3.BI=TRUE, BcOLV2.BI=FALSE} =>  {BcOLV18=TRUE} | 0.28  7129 | 0.591  837 | 0.485  149 | 1.14  4029 | 11  6 |
| {BcMCV3.BI=FALSE, BcMV3.BI=TRUE} =>  {BcOLV18=TRUE} | 0.28  7129 | 0.604  167 | 0.475  248 | 1.16  7863 | 11  6 |
| {BcOLV18=TRUE, BcPV4.BI=FALSE} =>  {BcMV3.BI=TRUE} | 0.30  198 | 0.632  124 | 0.477  723 | 1.16  6111 | 12  2 |
| {BcMV3.BI=TRUE, BcPV4.BI=FALSE} =>  {BcOLV18=TRUE} | 0.30  198 | 0.606  965 | 0.497  525 | 1.17  3272 | 12  2 |
| {BcEV6=FALSE, BcOLV18=TRUE} =>  {BcMV3.BI=TRUE} | 0.30  9406 | 0.641  026 | 0.482  673 | 1.18  2531 | 12  5 |
| {BcEV6=FALSE, BcMV3.BI=TRUE} => | 0.30 | 0.598 | 0.517 | 1.15 | 12 |

| {BcOLV18=TRUE} | 9406 | 086 | 327 | 6109 | 5 |
| --- | --- | --- | --- | --- | --- |
| {BcOLV17.BI=FALSE, BcOLV18=TRUE} =>  {BcMV3.BI=TRUE} | 0.30  198 | 0.632  124 | 0.477  723 | 1.16  6111 | 12  2 |
| {BcMV3.BI=TRUE, BcOLV17.BI=FALSE} =>  {BcOLV18=TRUE} | 0.30  198 | 0.589  372 | 0.512  376 | 1.13  9264 | 12  2 |
| {BcOLV15.BI=FALSE, BcOLV18=TRUE} =>  {BcMV3.BI=TRUE} | 0.29  9505 | 0.626  943 | 0.477  723 | 1.15  6552 | 12  1 |
| {BcMV3.BI=TRUE, BcOLV15.BI=FALSE} =>  {BcOLV18=TRUE} | 0.29  9505 | 0.590  244 | 0.507  426 | 1.14  095 | 12  1 |
| {BcEV5=FALSE, BcOLV18=TRUE} =>  {BcMV3.BI=TRUE} | 0.30  198 | 0.619  289 | 0.487  624 | 1.14  2433 | 12  2 |
| {BcEV5=FALSE, BcMV3.BI=TRUE} =>  {BcOLV18=TRUE} | 0.30  198 | 0.61 | 0.495  05 | 1.17  9139 | 12  2 |
| {BcMV4.BI=FALSE, BcOLV18=TRUE} =>  {BcULV4=TRUE} | 0.19  802 | 0.824  742 | 0.240  099 | 1.19  425 | 80 |
| {BcMV4.BI=FALSE, BcOLV18=TRUE} =>  {BcRV1.BI=FALSE} | 0.19  802 | 0.824  742 | 0.240  099 | 1.14  1082 | 80 |
| {BcOLV16.BI=FALSE, BcOLV18=TRUE} =>  {BcHV1.BI=TRUE} | 0.17  8218 | 0.72 | 0.247  525 | 1.19  2131 | 72 |
| {BcOLV16.BI=FALSE, BcOLV18=TRUE} =>  {BcULV4=TRUE} | 0.19  5545 | 0.79 | 0.247  525 | 1.14  3943 | 79 |
| {BcOLV18=TRUE, BcULV3=TRUE} => | 0.25 | 0.786 | 0.324 | 1.30 | 10 |

| {BcHV1.BI=TRUE} | 495 | 26 | 257 | 184 | 3 |
| --- | --- | --- | --- | --- | --- |
| {BcHV1.BI=TRUE, BcOLV18=TRUE} =>  {BcULV3=TRUE} | 0.25  495 | 0.695  946 | 0.366  337 | 1.17  1509 | 10  3 |
| {BcHV1.BI=TRUE, BcULV3=TRUE} =>  {BcOLV18=TRUE} | 0.25  495 | 0.602  339 | 0.423  267 | 1.16  433 | 10  3 |
| {BcOLV18=TRUE, BcULV3=TRUE} =>  {BcMV11=TRUE} | 0.24  2574 | 0.748  092 | 0.324  257 | 1.21  3771 | 98 |
| {BcMV11=TRUE, BcOLV18=TRUE} =>  {BcULV3=TRUE} | 0.24  2574 | 0.7 | 0.346  535 | 1.17  8333 | 98 |
| {BcMV11=TRUE, BcULV3=TRUE} =>  {BcOLV18=TRUE} | 0.24  2574 | 0.612  5 | 0.396  04 | 1.18  3971 | 98 |
| {BcOLV18=TRUE, BcULV3=TRUE} =>  {BcMV9.BI=TRUE} | 0.26  2376 | 0.809  16 | 0.324  257 | 1.19  3068 | 10  6 |
| {BcMV9.BI=TRUE, BcULV3=TRUE} =>  {BcOLV18=TRUE} | 0.26  2376 | 0.619  883 | 0.423  267 | 1.19  8243 | 10  6 |
| {BcOLV18=TRUE, BcULV3=TRUE} =>  {BcULV4=TRUE} | 0.27  7228 | 0.854  962 | 0.324  257 | 1.23  8009 | 11  2 |
| {BcULV3=TRUE, BcULV4=TRUE} =>  {BcOLV18=TRUE} | 0.27  7228 | 0.666  667 | 0.415  842 | 1.28  8676 | 11  2 |
| {BcOLV18=TRUE, BcPV3.BI=FALSE} =>  {BcULV3=TRUE} | 0.22  2772 | 0.676  692 | 0.329  208 | 1.13  9098 | 90 |
| {BcMV11=TRUE, BcOLV18=TRUE} => | 0.25 | 0.728 | 0.346 | 1.20 | 10 |

| {BcHV1.BI=TRUE} | 2475 | 571 | 535 | 6323 | 2 |
| --- | --- | --- | --- | --- | --- |
| {BcHV1.BI=TRUE, BcMV11=TRUE} =>  {BcOLV18=TRUE} | 0.25  2475 | 0.662  338 | 0.381  188 | 1.28  0308 | 10  2 |
| {BcOLV1.BI.MOVA.=FALSE,  BcOLV18=TRUE} => {BcHV1.BI=TRUE} | 0.20  0495 | 0.698  276 | 0.287  129 | 1.15  6162 | 81 |
| {BcHV1.BI=TRUE, BcOLV18=TRUE} =>  {BcMV9.BI=TRUE} | 0.30  9406 | 0.844  595 | 0.366  337 | 1.24  5315 | 12  5 |
| {BcMV9.BI=TRUE, BcOLV18=TRUE} =>  {BcHV1.BI=TRUE} | 0.30  9406 | 0.748  503 | 0.413  366 | 1.23  9325 | 12  5 |
| {BcHV1.BI=TRUE, BcMV9.BI=TRUE} =>  {BcOLV18=TRUE} | 0.30  9406 | 0.661  376 | 0.467  822 | 1.27  8449 | 12  5 |
| {BcHV1.BI=TRUE, BcOLV18=TRUE} =>  {BcULV4=TRUE} | 0.32  1782 | 0.878  378 | 0.366  337 | 1.27  1917 | 13  0 |
| {BcOLV18=TRUE, BcULV4=TRUE} =>  {BcHV1.BI=TRUE} | 0.32  1782 | 0.730  337 | 0.440  594 | 1.20  9247 | 13  0 |
| {BcHV1.BI=TRUE, BcULV4=TRUE} =>  {BcOLV18=TRUE} | 0.32  1782 | 0.706  522 | 0.455  446 | 1.36  5717 | 13  0 |
| {BcEV2.BI=FALSE, BcOLV18=TRUE} =>  {BcHV1.BI=TRUE} | 0.26  2376 | 0.711  409 | 0.368  812 | 1.17  7907 | 10  6 |
| {BcEV2.BI=FALSE, BcHV1.BI=TRUE} =>  {BcOLV18=TRUE} | 0.26  2376 | 0.598  87 | 0.438  119 | 1.15  7624 | 10  6 |
| {BcHV3.BI=FALSE, BcOLV18=TRUE} => | 0.25 | 0.711 | 0.351 | 1.17 | 10 |

| {BcHV1.BI=TRUE} |  | 268 | 485 | 7673 | 1 |
| --- | --- | --- | --- | --- | --- |
| {BcFV5.BI=FALSE, BcOLV18=TRUE} =>  {BcHV1.BI=TRUE} | 0.25  495 | 0.691  275 | 0.368  812 | 1.14  457 | 10  3 |
| {BcMV1.BI=TRUE, BcOLV18=TRUE} =>  {BcHV1.BI=TRUE} | 0.31  1881 | 0.707  865 | 0.440  594 | 1.17  2039 | 12  6 |
| {BcHV1.BI=TRUE, BcMV1.BI=TRUE} =>  {BcOLV18=TRUE} | 0.31  1881 | 0.646  154 | 0.482  673 | 1.24  9025 | 12  6 |
| {BcMV12=FALSE, BcOLV18=TRUE} =>  {BcHV1.BI=TRUE} | 0.27  4752 | 0.693  75 | 0.396  04 | 1.14  8668 | 11  1 |
| {BcHV1.BI=TRUE, BcMV12=FALSE} =>  {BcOLV18=TRUE} | 0.27  4752 | 0.590  426 | 0.465  347 | 1.14  1301 | 11  1 |
| {BcNSRV7.BI=FALSE, BcOLV18=TRUE} =>  {BcHV1.BI=TRUE} | 0.27  9703 | 0.719  745 | 0.388  614 | 1.19  1709 | 11  3 |
| {BcHV1.BI=TRUE, BcNSRV7.BI=FALSE} =>  {BcOLV18=TRUE} | 0.27  9703 | 0.601  064 | 0.465  347 | 1.16  1865 | 11  3 |
| {BcOLV18=TRUE, BcVV3.BI=FALSE} =>  {BcHV1.BI=TRUE} | 0.30  198 | 0.734  94 | 0.410  891 | 1.21  6867 | 12  2 |
| {BcHV1.BI=TRUE, BcVV3.BI=FALSE} =>  {BcOLV18=TRUE} | 0.30  198 | 0.61 | 0.495  05 | 1.17  9139 | 12  2 |
| {BcOLV18=TRUE, BcOLV7.BI=FALSE} =>  {BcHV1.BI=TRUE} | 0.26  2376 | 0.697  368 | 0.376  238 | 1.15  4659 | 10  6 |
| {BcDMV1=FALSE, BcOLV18=TRUE} => | 0.31 | 0.728 | 0.428 | 1.20 | 12 |

| {BcHV1.BI=TRUE} | 1881 | 324 | 218 | 5913 | 6 |
| --- | --- | --- | --- | --- | --- |
| {BcDMV1=FALSE, BcHV1.BI=TRUE} =>  {BcOLV18=TRUE} | 0.31  1881 | 0.602  871 | 0.517  327 | 1.16  5358 | 12  6 |
| {BcFV9=FALSE, BcOLV18=TRUE} =>  {BcHV1.BI=TRUE} | 0.29  4554 | 0.7 | 0.420  792 | 1.15  9016 | 11  9 |
| {BcOLV18=TRUE, BcOLV4.BI=FALSE} =>  {BcHV1.BI=TRUE} | 0.32  1782 | 0.718  232 | 0.448  02 | 1.18  9204 | 13  0 |
| {BcHV1.BI=TRUE, BcOLV4.BI=FALSE} =>  {BcOLV18=TRUE} | 0.32  1782 | 0.607  477 | 0.529  703 | 1.17  4261 | 13  0 |
| {BcOLV18=TRUE, BcULV5=FALSE} =>  {BcHV1.BI=TRUE} | 0.32  6733 | 0.698  413 | 0.467  822 | 1.15  6388 | 13  2 |
| {BcHV1.BI=TRUE, BcULV5=FALSE} =>  {BcOLV18=TRUE} | 0.32  6733 | 0.613  953 | 0.532  178 | 1.18  6781 | 13  2 |
| {BcBNV5.BI=FALSE, BcOLV18=TRUE} =>  {BcHV1.BI=TRUE} | 0.31  4356 | 0.693  989 | 0.452  97 | 1.14  9064 | 12  7 |
| {BcBNV5.BI=FALSE, BcHV1.BI=TRUE} =>  {BcOLV18=TRUE} | 0.31  4356 | 0.590  698 | 0.532  178 | 1.14  1827 | 12  7 |
| {BcOLV18=TRUE, BvF.BI=FALSE} =>  {BcHV1.BI=TRUE} | 0.33  1683 | 0.697  917 | 0.475  248 | 1.15  5567 | 13  4 |
| {BcHV1.BI=TRUE, BvF.BI=FALSE} =>  {BcOLV18=TRUE} | 0.33  1683 | 0.609  091 | 0.544  554 | 1.17  7381 | 13  4 |
| {BcMCV5.BI=FALSE, BcOLV18=TRUE} => | 0.31 | 0.708 | 0.450 | 1.17 | 12 |

| {BcHV1.BI=TRUE} | 9307 | 791 | 495 | 3572 | 9 |
| --- | --- | --- | --- | --- | --- |
| {BcHV1.BI=TRUE, BcMCV5.BI=FALSE} =>  {BcOLV18=TRUE} | 0.31  9307 | 0.583  71 | 0.547  03 | 1.12  8321 | 12  9 |
| {BcOLV13.BI=FALSE, BcOLV18=TRUE} =>  {BcHV1.BI=TRUE} | 0.33  1683 | 0.697  917 | 0.475  248 | 1.15  5567 | 13  4 |
| {BcHV1.BI=TRUE, BcOLV13.BI=FALSE} =>  {BcOLV18=TRUE} | 0.33  1683 | 0.609  091 | 0.544  554 | 1.17  7381 | 13  4 |
| {BcOLV18=TRUE, BcOLV2.BI=FALSE} =>  {BcHV1.BI=TRUE} | 0.32  6733 | 0.698  413 | 0.467  822 | 1.15  6388 | 13  2 |
| {BcHV1.BI=TRUE, BcOLV2.BI=FALSE} =>  {BcOLV18=TRUE} | 0.32  6733 | 0.591  928 | 0.551  98 | 1.14  4206 | 13  2 |
| {BcMCV3.BI=FALSE, BcOLV18=TRUE} =>  {BcHV1.BI=TRUE} | 0.32  4257 | 0.682  292 | 0.475  248 | 1.12  9696 | 13  1 |
| {BcHV1.BI=TRUE, BcMCV3.BI=FALSE} =>  {BcOLV18=TRUE} | 0.32  4257 | 0.595  455 | 0.544  554 | 1.15  1022 | 13  1 |
| {BcOLV18=TRUE, BcPV4.BI=FALSE} =>  {BcHV1.BI=TRUE} | 0.33  6634 | 0.704  663 | 0.477  723 | 1.16  6737 | 13  6 |
| {BcHV1.BI=TRUE, BcPV4.BI=FALSE} =>  {BcOLV18=TRUE} | 0.33  6634 | 0.601  77 | 0.559  406 | 1.16  323 | 13  6 |
| {BcEV6=FALSE, BcOLV18=TRUE} =>  {BcHV1.BI=TRUE} | 0.33  1683 | 0.687  179 | 0.482  673 | 1.13  7789 | 13  4 |
| {BcEV6=FALSE, BcHV1.BI=TRUE} => | 0.33 | 0.603 | 0.549 | 1.16 | 13 |

| {BcOLV18=TRUE} | 1683 | 604 | 505 | 6774 | 4 |
| --- | --- | --- | --- | --- | --- |
| {BcOLV17.BI=FALSE, BcOLV18=TRUE} =>  {BcHV1.BI=TRUE} | 0.33  6634 | 0.704  663 | 0.477  723 | 1.16  6737 | 13  6 |
| {BcHV1.BI=TRUE, BcOLV17.BI=FALSE} =>  {BcOLV18=TRUE} | 0.33  6634 | 0.596  491 | 0.564  356 | 1.15  3026 | 13  6 |
| {BcOLV15.BI=FALSE, BcOLV18=TRUE} =>  {BcHV1.BI=TRUE} | 0.33  6634 | 0.704  663 | 0.477  723 | 1.16  6737 | 13  6 |
| {BcHV1.BI=TRUE, BcOLV15.BI=FALSE} =>  {BcOLV18=TRUE} | 0.33  6634 | 0.596  491 | 0.564  356 | 1.15  3026 | 13  6 |
| {BcEV5=FALSE, BcOLV18=TRUE} =>  {BcHV1.BI=TRUE} | 0.34  901 | 0.715  736 | 0.487  624 | 1.18  5071 | 14  1 |
| {BcEV5=FALSE, BcHV1.BI=TRUE} =>  {BcOLV18=TRUE} | 0.34  901 | 0.607  759 | 0.574  257 | 1.17  4806 | 14  1 |
| {BcMV11=TRUE, BcOLV18=TRUE} =>  {BcMV9.BI=TRUE} | 0.27  4752 | 0.792  857 | 0.346  535 | 1.16  903 | 11  1 |
| {BcMV11=TRUE, BcMV9.BI=TRUE} =>  {BcOLV18=TRUE} | 0.27  4752 | 0.676  829 | 0.405  941 | 1.30  8321 | 11  1 |
| {BcMV11=TRUE, BcOLV18=TRUE} =>  {BcULV4=TRUE} | 0.29  703 | 0.857  143 | 0.346  535 | 1.24  1167 | 12  0 |
| {BcMV11=TRUE, BcULV4=TRUE} =>  {BcOLV18=TRUE} | 0.29  703 | 0.710  059 | 0.418  317 | 1.37  2555 | 12  0 |
| {BcMV11=TRUE, BcOLV18=TRUE} => | 0.30 | 0.871 | 0.346 | 1.10 | 12 |

| {BcMV1.BI=TRUE} | 198 | 429 | 535 | 3627 | 2 |
| --- | --- | --- | --- | --- | --- |
| {BcMV12=FALSE, BcOLV18=TRUE} =>  {BcMV11=TRUE} | 0.27  7228 | 0.7 | 0.396  04 | 1.13  5743 | 11  2 |
| {BcMV11=TRUE, BcULV5=FALSE} =>  {BcOLV18=TRUE} | 0.30  4455 | 0.602  941 | 0.504  95 | 1.16  5494 | 12  3 |
| {BcMCV3.BI=FALSE, BcOLV18=TRUE} =>  {BcMV11=TRUE} | 0.32  4257 | 0.682  292 | 0.475  248 | 1.10  7011 | 13  1 |
| {BcOLV1.BI.MOVA.=FALSE,  BcOLV18=TRUE} => {BcMV9.BI=TRUE} | 0.22  7723 | 0.793  103 | 0.287  129 | 1.16  9393 | 92 |
| {BcOLV1.BI.MOVA.=FALSE,  BcOLV18=TRUE} => {BcULV4=TRUE} | 0.22  7723 | 0.793  103 | 0.287  129 | 1.14  8437 | 92 |
| {BcOLV1.BI.MOVA.=FALSE,  BcOLV18=TRUE} => {BcMV6.BI=FALSE} | 0.23  7624 | 0.827  586 | 0.287  129 | 1.18  9839 | 96 |
| {BcMV9.BI=TRUE, BcOLV18=TRUE} =>  {BcULV4=TRUE} | 0.37  6238 | 0.910  18 | 0.413  366 | 1.31  7966 | 15  2 |
| {BcOLV18=TRUE, BcULV4=TRUE} =>  {BcMV9.BI=TRUE} | 0.37  6238 | 0.853  933 | 0.440  594 | 1.25  9083 | 15  2 |
| {BcMV9.BI=TRUE, BcULV4=TRUE} =>  {BcOLV18=TRUE} | 0.37  6238 | 0.687  783 | 0.547  03 | 1.32  9494 | 15  2 |
| {BcMV6.BI=FALSE, BcOLV18=TRUE} =>  {BcMV9.BI=TRUE} | 0.26  2376 | 0.762  59 | 0.344  059 | 1.12  4403 | 10  6 |
| {BcMV6.BI=FALSE, BcMV9.BI=TRUE} => | 0.26 | 0.592 | 0.443 | 1.14 | 10 |

| {BcOLV18=TRUE} | 2376 | 179 | 069 | 469 | 6 |
| --- | --- | --- | --- | --- | --- |
| {BcOLV18=TRUE, BcPV3.BI=FALSE} =>  {BcMV9.BI=TRUE} | 0.25  2475 | 0.766  917 | 0.329  208 | 1.13  0783 | 10  2 |
| {BcEV2.BI=FALSE, BcOLV18=TRUE} =>  {BcMV9.BI=TRUE} | 0.30  198 | 0.818  792 | 0.368  812 | 1.20  727 | 12  2 |
| {BcEV2.BI=FALSE, BcMV9.BI=TRUE} =>  {BcOLV18=TRUE} | 0.30  198 | 0.598  039 | 0.504  95 | 1.15  6018 | 12  2 |
| {BcHV3.BI=FALSE, BcOLV18=TRUE} =>  {BcMV9.BI=TRUE} | 0.27  9703 | 0.795  775 | 0.351  485 | 1.17  3332 | 11  3 |
| {BcFV5.BI=FALSE, BcOLV18=TRUE} =>  {BcMV9.BI=TRUE} | 0.28  9604 | 0.785  235 | 0.368  812 | 1.15  7792 | 11  7 |
| {BcMV9.BI=TRUE, BcOLV18=TRUE} =>  {BcMV1.BI=TRUE} | 0.35  8911 | 0.868  263 | 0.413  366 | 1.09  9619 | 14  5 |
| {BcMV1.BI=TRUE, BcOLV18=TRUE} =>  {BcMV9.BI=TRUE} | 0.35  8911 | 0.814  607 | 0.440  594 | 1.20  1099 | 14  5 |
| {BcMV1.BI=TRUE, BcMV9.BI=TRUE} =>  {BcOLV18=TRUE} | 0.35  8911 | 0.653  153 | 0.549  505 | 1.26  2554 | 14  5 |
| {BcMV12=FALSE, BcOLV18=TRUE} =>  {BcMV9.BI=TRUE} | 0.31  1881 | 0.787  5 | 0.396  04 | 1.16  1131 | 12  6 |
| {BcMV12=FALSE, BcMV9.BI=TRUE} =>  {BcOLV18=TRUE} | 0.31  1881 | 0.577  982 | 0.539  604 | 1.11  7247 | 12  6 |
| {BcNSRV7.BI=FALSE, BcOLV18=TRUE} => | 0.30 | 0.777 | 0.388 | 1.14 | 12 |

| {BcMV9.BI=TRUE} | 198 | 07 | 614 | 5753 | 2 |
| --- | --- | --- | --- | --- | --- |
| {BcMV9.BI=TRUE, BcNSRV7.BI=FALSE} =>  {BcOLV18=TRUE} | 0.30  198 | 0.583  732 | 0.517  327 | 1.12  8362 | 12  2 |
| {BcOLV18=TRUE, BcVV3.BI=FALSE} =>  {BcMV9.BI=TRUE} | 0.33  1683 | 0.807  229 | 0.410  891 | 1.19  0221 | 13  4 |
| {BcMV9.BI=TRUE, BcVV3.BI=FALSE} =>  {BcOLV18=TRUE} | 0.33  1683 | 0.598  214 | 0.554  455 | 1.15  6357 | 13  4 |
| {BcALV1.BI=FALSE, BcOLV18=TRUE} =>  {BcMV9.BI=TRUE} | 0.29  4554 | 0.782  895 | 0.376  238 | 1.15  4341 | 11  9 |
| {BcDMV1=FALSE, BcOLV18=TRUE} =>  {BcMV9.BI=TRUE} | 0.33  9109 | 0.791  908 | 0.428  218 | 1.16  763 | 13  7 |
| {BcDMV1=FALSE, BcMV9.BI=TRUE} =>  {BcOLV18=TRUE} | 0.33  9109 | 0.614  35 | 0.551  98 | 1.18  7547 | 13  7 |
| {BcFV9=FALSE, BcOLV18=TRUE} =>  {BcMV9.BI=TRUE} | 0.34  1584 | 0.811  765 | 0.420  792 | 1.19  6909 | 13  8 |
| {BcFV9=FALSE, BcMV9.BI=TRUE} =>  {BcOLV18=TRUE} | 0.34  1584 | 0.584  746 | 0.584  158 | 1.13  0322 | 13  8 |
| {BcOLV18=TRUE, BcOLV4.BI=FALSE} =>  {BcMV9.BI=TRUE} | 0.36  3861 | 0.812  155 | 0.448  02 | 1.19  7484 | 14  7 |
| {BcMV9.BI=TRUE, BcOLV4.BI=FALSE} =>  {BcOLV18=TRUE} | 0.36  3861 | 0.590  361 | 0.616  337 | 1.14  1177 | 14  7 |
| {BcMV9.BI=TRUE, BcOLV18=TRUE} => | 0.39 | 0.946 | 0.413 | 1.07 | 15 |

| {BcULV5=FALSE} | 1089 | 108 | 366 | 9739 | 8 |
| --- | --- | --- | --- | --- | --- |
| {BcOLV18=TRUE, BcULV5=FALSE} =>  {BcMV9.BI=TRUE} | 0.39  1089 | 0.835  979 | 0.467  822 | 1.23  2611 | 15  8 |
| {BcMV9.BI=TRUE, BcULV5=FALSE} =>  {BcOLV18=TRUE} | 0.39  1089 | 0.596  226 | 0.655  941 | 1.15  2514 | 15  8 |
| {BcBNV5.BI=FALSE, BcOLV18=TRUE} =>  {BcMV9.BI=TRUE} | 0.35  396 | 0.781  421 | 0.452  97 | 1.15  2168 | 14  3 |
| {BcBNV5.BI=FALSE, BcMV9.BI=TRUE} =>  {BcOLV18=TRUE} | 0.35  396 | 0.593  361 | 0.596  535 | 1.14  6975 | 14  3 |
| {BcOLV18=TRUE, BvF.BI=FALSE} =>  {BcMV9.BI=TRUE} | 0.37  8713 | 0.796  875 | 0.475  248 | 1.17  4954 | 15  3 |
| {BcMV9.BI=TRUE, BvF.BI=FALSE} =>  {BcOLV18=TRUE} | 0.37  8713 | 0.619  433 | 0.611  386 | 1.19  7373 | 15  3 |
| {BcMCV5.BI=FALSE, BcOLV18=TRUE} =>  {BcMV9.BI=TRUE} | 0.36  1386 | 0.802  198 | 0.450  495 | 1.18  2803 | 14  6 |
| {BcMCV5.BI=FALSE, BcMV9.BI=TRUE} =>  {BcOLV18=TRUE} | 0.36  1386 | 0.586  345 | 0.616  337 | 1.13  3414 | 14  6 |
| {BcOLV13.BI=FALSE, BcOLV18=TRUE} =>  {BcMV9.BI=TRUE} | 0.37  8713 | 0.796  875 | 0.475  248 | 1.17  4954 | 15  3 |
| {BcMV9.BI=TRUE, BcOLV13.BI=FALSE} =>  {BcOLV18=TRUE} | 0.37  8713 | 0.595  331 | 0.636  139 | 1.15  0783 | 15  3 |
| {BcOLV18=TRUE, BcOLV2.BI=FALSE} => | 0.36 | 0.783 | 0.467 | 1.15 | 14 |

| {BcMV9.BI=TRUE} | 6337 | 069 | 822 | 4598 | 8 |
| --- | --- | --- | --- | --- | --- |
| {BcMV9.BI=TRUE, BcOLV2.BI=FALSE} =>  {BcOLV18=TRUE} | 0.36  6337 | 0.589  641 | 0.621  287 | 1.13  9785 | 14  8 |
| {BcMCV3.BI=FALSE, BcOLV18=TRUE} =>  {BcMV9.BI=TRUE} | 0.37  1287 | 0.781  25 | 0.475  248 | 1.15  1916 | 15  0 |
| {BcMCV3.BI=FALSE, BcMV9.BI=TRUE} =>  {BcOLV18=TRUE} | 0.37  1287 | 0.590  551 | 0.628  713 | 1.14  1544 | 15  0 |
| {BcOLV18=TRUE, BcPV4.BI=FALSE} =>  {BcMV9.BI=TRUE} | 0.38  8614 | 0.813  472 | 0.477  723 | 1.19  9425 | 15  7 |
| {BcMV9.BI=TRUE, BcPV4.BI=FALSE} =>  {BcOLV18=TRUE} | 0.38  8614 | 0.613  281 | 0.633  663 | 1.18  5481 | 15  7 |
| {BcEV6=FALSE, BcOLV18=TRUE} =>  {BcMV9.BI=TRUE} | 0.38  3663 | 0.794  872 | 0.482  673 | 1.17  2001 | 15  5 |
| {BcEV6=FALSE, BcMV9.BI=TRUE} =>  {BcOLV18=TRUE} | 0.38  3663 | 0.607  843 | 0.631  188 | 1.17  497 | 15  5 |
| {BcOLV17.BI=FALSE, BcOLV18=TRUE} =>  {BcMV9.BI=TRUE} | 0.38  1188 | 0.797  927 | 0.477  723 | 1.17  6506 | 15  4 |
| {BcMV9.BI=TRUE, BcOLV17.BI=FALSE} =>  {BcOLV18=TRUE} | 0.38  1188 | 0.596  899 | 0.638  614 | 1.15  3815 | 15  4 |
| {BcOLV15.BI=FALSE, BcOLV18=TRUE} =>  {BcMV9.BI=TRUE} | 0.38  6139 | 0.808  29 | 0.477  723 | 1.19  1785 | 15  6 |
| {BcMV9.BI=TRUE, BcOLV15.BI=FALSE} => | 0.38 | 0.602 | 0.641 | 1.16 | 15 |

| {BcOLV18=TRUE} | 6139 | 317 | 089 | 4287 | 6 |
| --- | --- | --- | --- | --- | --- |
| {BcEV5=FALSE, BcOLV18=TRUE} =>  {BcMV9.BI=TRUE} | 0.38  6139 | 0.791  878 | 0.487  624 | 1.16  7587 | 15  6 |
| {BcEV5=FALSE, BcMV9.BI=TRUE} =>  {BcOLV18=TRUE} | 0.38  6139 | 0.604  651 | 0.638  614 | 1.16  8799 | 15  6 |
| {BcMV6.BI=FALSE, BcOLV18=TRUE} =>  {BcULV4=TRUE} | 0.28  2178 | 0.820  144 | 0.344  059 | 1.18  7592 | 11  4 |
| {BcMV6.BI=FALSE, BcULV4=TRUE} =>  {BcOLV18=TRUE} | 0.28  2178 | 0.629  834 | 0.448  02 | 1.21  7479 | 11  4 |
| {BcOLV18=TRUE, BcPV3.BI=FALSE} =>  {BcULV4=TRUE} | 0.28  7129 | 0.872  18 | 0.329  208 | 1.26  2942 | 11  6 |
| {BcPV3.BI=FALSE, BcULV4=TRUE} =>  {BcOLV18=TRUE} | 0.28  7129 | 0.591  837 | 0.485  149 | 1.14  4029 | 11  6 |
| {BcOLV18=TRUE, BcRV1.BI=FALSE} =>  {BcULV4=TRUE} | 0.26  2376 | 0.796  992 | 0.329  208 | 1.15  4068 | 10  6 |
| {BcEV2.BI=FALSE, BcOLV18=TRUE} =>  {BcULV4=TRUE} | 0.31  1881 | 0.845  638 | 0.368  812 | 1.22  4507 | 12  6 |
| {BcEV2.BI=FALSE, BcULV4=TRUE} =>  {BcOLV18=TRUE} | 0.31  1881 | 0.588  785 | 0.529  703 | 1.13  813 | 12  6 |
| {BcHV3.BI=FALSE, BcOLV18=TRUE} =>  {BcULV4=TRUE} | 0.29  9505 | 0.852  113 | 0.351  485 | 1.23  3884 | 12  1 |
| {BcFV5.BI=FALSE, BcOLV18=TRUE} => | 0.31 | 0.845 | 0.368 | 1.22 | 12 |

| {BcULV4=TRUE} | 1881 | 638 | 812 | 4507 | 6 |
| --- | --- | --- | --- | --- | --- |
| {BcFV5.BI=FALSE, BcULV4=TRUE} =>  {BcOLV18=TRUE} | 0.31  1881 | 0.588  785 | 0.529  703 | 1.13  813 | 12  6 |
| {BcOLV18=TRUE, BcULV1.BI=FALSE} =>  {BcULV4=TRUE} | 0.28  4653 | 0.798  611 | 0.356  436 | 1.15  6412 | 11  5 |
| {BcOLV18=TRUE, BcULV4=TRUE} =>  {BcMV1.BI=TRUE} | 0.37  8713 | 0.859  551 | 0.440  594 | 1.08  8584 | 15  3 |
| {BcMV1.BI=TRUE, BcOLV18=TRUE} =>  {BcULV4=TRUE} | 0.37  8713 | 0.859  551 | 0.440  594 | 1.24  4654 | 15  3 |
| {BcMV1.BI=TRUE, BcULV4=TRUE} =>  {BcOLV18=TRUE} | 0.37  8713 | 0.689  189 | 0.549  505 | 1.33  2213 | 15  3 |
| {BcMV12=FALSE, BcOLV18=TRUE} =>  {BcULV4=TRUE} | 0.34  1584 | 0.862  5 | 0.396  04 | 1.24  8925 | 13  8 |
| {BcMV12=FALSE, BcULV4=TRUE} =>  {BcOLV18=TRUE} | 0.34  1584 | 0.621  622 | 0.549  505 | 1.20  1604 | 13  8 |
| {BcNSRV7.BI=FALSE, BcOLV18=TRUE} =>  {BcULV4=TRUE} | 0.32  6733 | 0.840  764 | 0.388  614 | 1.21  7451 | 13  2 |
| {BcNSRV7.BI=FALSE, BcULV4=TRUE} =>  {BcOLV18=TRUE} | 0.32  6733 | 0.611  111 | 0.534  653 | 1.18  1287 | 13  2 |
| {BcOLV18=TRUE, BcVV3.BI=FALSE} =>  {BcULV4=TRUE} | 0.34  6535 | 0.843  373 | 0.410  891 | 1.22  1229 | 14  0 |
| {BcULV4=TRUE, BcVV3.BI=FALSE} => | 0.34 | 0.633 | 0.547 | 1.22 | 14 |

| {BcOLV18=TRUE} | 6535 | 484 | 03 | 4534 | 0 |
| --- | --- | --- | --- | --- | --- |
| {BcOLV18=TRUE, BcOLV7.BI=FALSE} =>  {BcULV4=TRUE} | 0.30  198 | 0.802  632 | 0.376  238 | 1.16  2234 | 12  2 |
| {BcOLV7.BI=FALSE, BcULV4=TRUE} =>  {BcOLV18=TRUE} | 0.30  198 | 0.589  372 | 0.512  376 | 1.13  9264 | 12  2 |
| {BcALV1.BI=FALSE, BcOLV18=TRUE} =>  {BcULV4=TRUE} | 0.30  6931 | 0.815  789 | 0.376  238 | 1.18  1287 | 12  4 |
| {BcDMV1=FALSE, BcOLV18=TRUE} =>  {BcULV4=TRUE} | 0.36  3861 | 0.849  711 | 0.428  218 | 1.23  0406 | 14  7 |
| {BcDMV1=FALSE, BcULV4=TRUE} =>  {BcOLV18=TRUE} | 0.36  3861 | 0.636  364 | 0.571  782 | 1.23  01 | 14  7 |
| {BcFV9=FALSE, BcOLV18=TRUE} =>  {BcULV4=TRUE} | 0.35  8911 | 0.852  941 | 0.420  792 | 1.23  5083 | 14  5 |
| {BcFV9=FALSE, BcULV4=TRUE} =>  {BcOLV18=TRUE} | 0.35  8911 | 0.601  66 | 0.596  535 | 1.16  3017 | 14  5 |
| {BcOLV18=TRUE, BcOLV4.BI=FALSE} =>  {BcULV4=TRUE} | 0.38  8614 | 0.867  403 | 0.448  02 | 1.25  6025 | 15  7 |
| {BcOLV4.BI=FALSE, BcULV4=TRUE} =>  {BcOLV18=TRUE} | 0.38  8614 | 0.625  498 | 0.621  287 | 1.20  9097 | 15  7 |
| {BcOLV18=TRUE, BcULV5=FALSE} =>  {BcULV4=TRUE} | 0.40  3465 | 0.862  434 | 0.467  822 | 1.24  8829 | 16  3 |
| {BcULV4=TRUE, BcULV5=FALSE} => | 0.40 | 0.639 | 0.631 | 1.23 | 16 |

| {BcOLV18=TRUE} | 3465 | 216 | 188 | 5613 | 3 |
| --- | --- | --- | --- | --- | --- |
| {BcBNV5.BI=FALSE, BcOLV18=TRUE} =>  {BcULV4=TRUE} | 0.38  1188 | 0.841  53 | 0.452  97 | 1.21  856 | 15  4 |
| {BcBNV5.BI=FALSE, BcULV4=TRUE} =>  {BcOLV18=TRUE} | 0.38  1188 | 0.616 | 0.618  812 | 1.19  0737 | 15  4 |
| {BcOLV18=TRUE, BvF.BI=FALSE} =>  {BcULV4=TRUE} | 0.41  3366 | 0.869  792 | 0.475  248 | 1.25  9483 | 16  7 |
| {BcULV4=TRUE, BvF.BI=FALSE} =>  {BcOLV18=TRUE} | 0.41  3366 | 0.639  847 | 0.646  04 | 1.23  6833 | 16  7 |
| {BcMCV5.BI=FALSE, BcOLV18=TRUE} =>  {BcULV4=TRUE} | 0.38  3663 | 0.851  648 | 0.450  495 | 1.23  3211 | 15  5 |
| {BcMCV5.BI=FALSE, BcULV4=TRUE} =>  {BcOLV18=TRUE} | 0.38  3663 | 0.607  843 | 0.631  188 | 1.17  497 | 15  5 |
| {BcOLV13.BI=FALSE, BcOLV18=TRUE} =>  {BcULV4=TRUE} | 0.40  5941 | 0.854  167 | 0.475  248 | 1.23  6858 | 16  4 |
| {BcOLV13.BI=FALSE, BcULV4=TRUE} =>  {BcOLV18=TRUE} | 0.40  5941 | 0.633  205 | 0.641  089 | 1.22  3994 | 16  4 |
| {BcOLV18=TRUE, BcOLV2.BI=FALSE} =>  {BcULV4=TRUE} | 0.39  8515 | 0.851  852 | 0.467  822 | 1.23  3506 | 16  1 |
| {BcOLV2.BI=FALSE, BcULV4=TRUE} =>  {BcOLV18=TRUE} | 0.39  8515 | 0.621  622 | 0.641  089 | 1.20  1604 | 16  1 |
| {BcMCV3.BI=FALSE, BcOLV18=TRUE} => | 0.39 | 0.838 | 0.475 | 1.21 | 16 |

| {BcULV4=TRUE} | 8515 | 542 | 248 | 4232 | 1 |
| --- | --- | --- | --- | --- | --- |
| {BcMCV3.BI=FALSE, BcULV4=TRUE} =>  {BcOLV18=TRUE} | 0.39  8515 | 0.621  622 | 0.641  089 | 1.20  1604 | 16  1 |
| {BcOLV18=TRUE, BcPV4.BI=FALSE} =>  {BcULV4=TRUE} | 0.41  0891 | 0.860  104 | 0.477  723 | 1.24  5455 | 16  6 |
| {BcPV4.BI=FALSE, BcULV4=TRUE} =>  {BcOLV18=TRUE} | 0.41  0891 | 0.636  015 | 0.646  04 | 1.22  9427 | 16  6 |
| {BcEV6=FALSE, BcOLV18=TRUE} =>  {BcULV4=TRUE} | 0.41  3366 | 0.856  41 | 0.482  673 | 1.24  0107 | 16  7 |
| {BcEV6=FALSE, BcULV4=TRUE} =>  {BcOLV18=TRUE} | 0.41  3366 | 0.639  847 | 0.646  04 | 1.23  6833 | 16  7 |
| {BcOLV17.BI=FALSE, BcOLV18=TRUE} =>  {BcULV4=TRUE} | 0.40  3465 | 0.844  56 | 0.477  723 | 1.22  2946 | 16  3 |
| {BcOLV17.BI=FALSE, BcULV4=TRUE} =>  {BcOLV18=TRUE} | 0.40  3465 | 0.624  521 | 0.646  04 | 1.20  7208 | 16  3 |
| {BcOLV15.BI=FALSE, BcOLV18=TRUE} =>  {BcULV4=TRUE} | 0.41  3366 | 0.865  285 | 0.477  723 | 1.25  2957 | 16  7 |
| {BcOLV15.BI=FALSE, BcULV4=TRUE} =>  {BcOLV18=TRUE} | 0.41  3366 | 0.627  82 | 0.658  416 | 1.21  3584 | 16  7 |
| {BcEV5=FALSE, BcOLV18=TRUE} =>  {BcULV4=TRUE} | 0.41  3366 | 0.847  716 | 0.487  624 | 1.22  7517 | 16  7 |
| {BcEV5=FALSE, BcULV4=TRUE} => | 0.41 | 0.627 | 0.658 | 1.21 | 16 |

| {BcOLV18=TRUE} | 3366 | 82 | 416 | 3584 | 7 |
| --- | --- | --- | --- | --- | --- |
| {BcOLV18=TRUE, BcPV3.BI=FALSE} =>  {BcULV5=FALSE} | 0.30  9406 | 0.939  85 | 0.329  208 | 1.07  2597 | 12  5 |
| {BcOLV18=TRUE, BcRV1.BI=FALSE} =>  {BcMV1.BI=TRUE} | 0.29  4554 | 0.894  737 | 0.329  208 | 1.13  3146 | 11  9 |
| {BcHV3.BI=FALSE, BcOLV18=TRUE} =>  {BcDMV1=FALSE} | 0.31  9307 | 0.908  451 | 0.351  485 | 1.07  6288 | 12  9 |
| {BcFV5.BI=FALSE, BcOLV18=TRUE} =>  {BcDMV1=FALSE} | 0.33  9109 | 0.919  463 | 0.368  812 | 1.08  9335 | 13  7 |
| {BcMV1.BI=TRUE, BcULV5=FALSE} =>  {BcOLV18=TRUE} | 0.39  3564 | 0.584  559 | 0.673  267 | 1.12  9961 | 15  9 |
| {BcOLV18=TRUE, BvF.BI=FALSE} =>  {BcMV1.BI=TRUE} | 0.40  8416 | 0.859  375 | 0.475  248 | 1.08  8362 | 16  5 |
| {BcOLV13.BI=FALSE, BcOLV18=TRUE} =>  {BcMV1.BI=TRUE} | 0.40  5941 | 0.854  167 | 0.475  248 | 1.08  1766 | 16  4 |
| {BcOLV18=TRUE, BcOLV2.BI=FALSE} =>  {BcMV1.BI=TRUE} | 0.39  8515 | 0.851  852 | 0.467  822 | 1.07  8834 | 16  1 |
| {BcMCV3.BI=FALSE, BcOLV18=TRUE} =>  {BcMV1.BI=TRUE} | 0.40  3465 | 0.848  958 | 0.475  248 | 1.07  517 | 16  3 |
| {BcOLV17.BI=FALSE, BcOLV18=TRUE} =>  {BcMV1.BI=TRUE} | 0.40  5941 | 0.849  741 | 0.477  723 | 1.07  6161 | 16  4 |
| {BcOLV15.BI=FALSE, BcOLV18=TRUE} => | 0.40 | 0.849 | 0.477 | 1.07 | 16 |

| {BcMV1.BI=TRUE} | 5941 | 741 | 723 | 6161 | 4 |
| --- | --- | --- | --- | --- | --- |
| {BcEV5=FALSE, BcOLV18=TRUE} =>  {BcMV1.BI=TRUE} | 0.41  3366 | 0.847  716 | 0.487  624 | 1.07  3596 | 16  7 |
| {BcNSRV7.BI=FALSE, BcOLV18=TRUE} =>  {BcDMV1=FALSE} | 0.35  1485 | 0.904  459 | 0.388  614 | 1.07  1558 | 14  2 |
| {BcHV1SLR.BI=TRUE, BcMV2.BI=FALSE} =>  {BcMV4.BI=FALSE} | 0.17  5743 | 0.651  376 | 0.269  802 | 1.16  9582 | 71 |
| {BcHV1SLR.BI=TRUE, BcMV2.BI=FALSE} =>  {BcOLV16.BI=FALSE} | 0.20  7921 | 0.770  642 | 0.269  802 | 1.31  9235 | 84 |
| {BcHV1SLR.BI=TRUE, BcOLV16.BI=FALSE}  => {BcMV2.BI=FALSE} | 0.20  7921 | 0.682  927 | 0.304  455 | 1.27  144 | 84 |
| {BcHV1SLR.BI=TRUE, BcMV2.BI=FALSE} =>  {BcULV3=TRUE} | 0.19  3069 | 0.715  596 | 0.269  802 | 1.20  4587 | 78 |
| {BcHV1SLR.BI=TRUE, BcMV2.BI=FALSE} =>  {BcHV1.BI=TRUE} | 0.23  7624 | 0.880  734 | 0.269  802 | 1.45  8264 | 96 |
| {BcHV1.BI=TRUE, BcMV2.BI=FALSE} =>  {BcHV1SLR.BI=TRUE} | 0.23  7624 | 0.761  905 | 0.311  881 | 1.40  5523 | 96 |
| {BcHV1SLR.BI=TRUE, BcMV2.BI=FALSE} =>  {BcOLV1.BI.MOVA.=FALSE} | 0.22  7723 | 0.844  037 | 0.269  802 | 1.26  2929 | 92 |
| {BcHV1SLR.BI=TRUE, BcOLV1.BI.MOVA.=FALSE} =>  {BcMV2.BI=FALSE} | 0.22  7723 | 0.666  667 | 0.341  584 | 1.24  1167 | 92 |

| {BcHV1SLR.BI=TRUE, BcMV2.BI=FALSE} =>  {BcMV6.BI=FALSE} | 0.22  5248 | 0.834  862 | 0.269  802 | 1.20  03 | 91 |
| --- | --- | --- | --- | --- | --- |
| {BcHV1SLR.BI=TRUE, BcMV6.BI=FALSE} =>  {BcMV2.BI=FALSE} | 0.22  5248 | 0.669  118 | 0.336  634 | 1.24  5731 | 91 |
| {BcHV1SLR.BI=TRUE, BcMV2.BI=FALSE} =>  {BcPV3.BI=FALSE} | 0.21  7822 | 0.807  339 | 0.269  802 | 1.13  6464 | 88 |
| {BcHV1SLR.BI=TRUE, BcMV2.BI=FALSE} =>  {BcRV1.BI=FALSE} | 0.23  7624 | 0.880  734 | 0.269  802 | 1.21  855 | 96 |
| {BcHV1SLR.BI=TRUE, BcRV1.BI=FALSE} =>  {BcMV2.BI=FALSE} | 0.23  7624 | 0.623  377 | 0.381  188 | 1.16  0572 | 96 |
| {BcHV1SLR.BI=TRUE, BcMV2.BI=FALSE} =>  {BcALV1.BI=FALSE} | 0.24  505 | 0.908  257 | 0.269  802 | 1.11  5306 | 99 |
| {BcMV2.BI=FALSE, BcMV3.BI=TRUE} =>  {BcOLV1.BI.MOVA.=FALSE} | 0.18  5644 | 0.815  217 | 0.227  723 | 1.21  9807 | 75 |
| {BcMV2.BI=FALSE, BcMV3.BI=TRUE} =>  {BcMV6.BI=FALSE} | 0.18  0693 | 0.793  478 | 0.227  723 | 1.14  0801 | 73 |
| {BcMV2.BI=FALSE, BcMV3.BI=TRUE} =>  {BcALV1.BI=FALSE} | 0.21  2871 | 0.934  783 | 0.227  723 | 1.14  7879 | 86 |
| {BcMV2.BI=FALSE, BcMV4.BI=FALSE} =>  {BcOLV16.BI=FALSE} | 0.26  9802 | 0.731  544 | 0.368  812 | 1.25  2303 | 10  9 |
| {BcMV2.BI=FALSE, BcOLV16.BI=FALSE} =>  {BcMV4.BI=FALSE} | 0.26  9802 | 0.721  854 | 0.373  762 | 1.29  613 | 10  9 |

| {BcMV4.BI=FALSE, BcOLV16.BI=FALSE} =>  {BcMV2.BI=FALSE} | 0.26  9802 | 0.736  486 | 0.366  337 | 1.37  1155 | 10  9 |
| --- | --- | --- | --- | --- | --- |
| {BcMV4.BI=FALSE, BcULV3=TRUE} =>  {BcMV2.BI=FALSE} | 0.19  802 | 0.661  157 | 0.299  505 | 1.23  091 | 80 |
| {BcHV1.BI=TRUE, BcMV2.BI=FALSE} =>  {BcMV4.BI=FALSE} | 0.20  297 | 0.650  794 | 0.311  881 | 1.16  8536 | 82 |
| {BcHV1.BI=TRUE, BcMV4.BI=FALSE} =>  {BcMV2.BI=FALSE} | 0.20  297 | 0.666  667 | 0.304  455 | 1.24  1167 | 82 |
| {BcMV2.BI=FALSE, BcMV4.BI=FALSE} =>  {BcOLV1.BI.MOVA.=FALSE} | 0.31  9307 | 0.865  772 | 0.368  812 | 1.29  5451 | 12  9 |
| {BcMV2.BI=FALSE, BcOLV1.BI.MOVA.=FALSE} =>  {BcMV4.BI=FALSE} | 0.31  9307 | 0.701  087 | 0.455  446 | 1.25  8841 | 12  9 |
| {BcMV4.BI=FALSE, BcOLV1.BI.MOVA.=FALSE} =>  {BcMV2.BI=FALSE} | 0.31  9307 | 0.777  108 | 0.410  891 | 1.44  6783 | 12  9 |
| {BcMV2.BI=FALSE, BcMV9.BI=TRUE} =>  {BcMV4.BI=FALSE} | 0.23  2673 | 0.652  778 | 0.356  436 | 1.17  2099 | 94 |
| {BcMV4.BI=FALSE, BcMV9.BI=TRUE} =>  {BcMV2.BI=FALSE} | 0.23  2673 | 0.681  159 | 0.341  584 | 1.26  8149 | 94 |
| {BcMV2.BI=FALSE, BcULV4=TRUE} =>  {BcMV4.BI=FALSE} | 0.23  7624 | 0.680  851 | 0.349  01 | 1.22  2506 | 96 |

| {BcMV4.BI=FALSE, BcULV4=TRUE} =>  {BcMV2.BI=FALSE} | 0.23  7624 | 0.648  649 | 0.366  337 | 1.20  7622 | 96 |
| --- | --- | --- | --- | --- | --- |
| {BcMV2.BI=FALSE, BcMV4.BI=FALSE} =>  {BcMV6.BI=FALSE} | 0.29  703 | 0.805  369 | 0.368  812 | 1.15  7897 | 12  0 |
| {BcMV2.BI=FALSE, BcMV6.BI=FALSE} =>  {BcMV4.BI=FALSE} | 0.29  703 | 0.674  157 | 0.440  594 | 1.21  0487 | 12  0 |
| {BcMV4.BI=FALSE, BcMV6.BI=FALSE} =>  {BcMV2.BI=FALSE} | 0.29  703 | 0.693  642 | 0.428  218 | 1.29  1388 | 12  0 |
| {BcMV2.BI=FALSE, BcMV4.BI=FALSE} =>  {BcPV3.BI=FALSE} | 0.31  9307 | 0.865  772 | 0.368  812 | 1.21  8717 | 12  9 |
| {BcMV2.BI=FALSE, BcPV3.BI=FALSE} =>  {BcMV4.BI=FALSE} | 0.31  9307 | 0.75 | 0.425  743 | 1.34  6667 | 12  9 |
| {BcMV4.BI=FALSE, BcPV3.BI=FALSE} =>  {BcMV2.BI=FALSE} | 0.31  9307 | 0.712  707 | 0.448  02 | 1.32  6883 | 12  9 |
| {BcMV2.BI=FALSE, BcMV4.BI=FALSE} =>  {BcRV1.BI=FALSE} | 0.30  6931 | 0.832  215 | 0.368  812 | 1.15  142 | 12  4 |
| {BcMV2.BI=FALSE, BcRV1.BI=FALSE} =>  {BcMV4.BI=FALSE} | 0.30  6931 | 0.700  565 | 0.438  119 | 1.25  7903 | 12  4 |
| {BcMV4.BI=FALSE, BcRV1.BI=FALSE} =>  {BcMV2.BI=FALSE} | 0.30  6931 | 0.688  889 | 0.445  545 | 1.28  254 | 12  4 |
| {BcMV2.BI=FALSE, BcMV4.BI=FALSE} =>  {BcEV2.BI=FALSE} | 0.30  4455 | 0.825  503 | 0.368  812 | 1.10  4316 | 12  3 |

| {BcEV2.BI=FALSE, BcMV2.BI=FALSE} =>  {BcMV4.BI=FALSE} | 0.30  4455 | 0.710  983 | 0.428  218 | 1.27  6609 | 12  3 |
| --- | --- | --- | --- | --- | --- |
| {BcEV2.BI=FALSE, BcMV4.BI=FALSE} =>  {BcMV2.BI=FALSE} | 0.30  4455 | 0.702  857 | 0.433  168 | 1.30  8545 | 12  3 |
| {BcMV2.BI=FALSE, BcMV4.BI=FALSE} =>  {BcHV3.BI=FALSE} | 0.31  6832 | 0.859  06 | 0.368  812 | 1.13  049 | 12  8 |
| {BcHV3.BI=FALSE, BcMV2.BI=FALSE} =>  {BcMV4.BI=FALSE} | 0.31  6832 | 0.735  632 | 0.430  693 | 1.32  0868 | 12  8 |
| {BcHV3.BI=FALSE, BcMV4.BI=FALSE} =>  {BcMV2.BI=FALSE} | 0.31  6832 | 0.677  249 | 0.467  822 | 1.26  0869 | 12  8 |
| {BcFV5.BI=FALSE, BcMV2.BI=FALSE} =>  {BcMV4.BI=FALSE} | 0.31  1881 | 0.711  864 | 0.438  119 | 1.27  8192 | 12  6 |
| {BcFV5.BI=FALSE, BcMV4.BI=FALSE} =>  {BcMV2.BI=FALSE} | 0.31  1881 | 0.677  419 | 0.460  396 | 1.26  1186 | 12  6 |
| {BcMV2.BI=FALSE, BcMV4.BI=FALSE} =>  {BcULV1.BI=FALSE} | 0.32  1782 | 0.872  483 | 0.368  812 | 1.11  1934 | 13  0 |
| {BcMV2.BI=FALSE, BcULV1.BI=FALSE} =>  {BcMV4.BI=FALSE} | 0.32  1782 | 0.698  925 | 0.460  396 | 1.25  4958 | 13  0 |
| {BcMV4.BI=FALSE, BcULV1.BI=FALSE} =>  {BcMV2.BI=FALSE} | 0.32  1782 | 0.714  286 | 0.450  495 | 1.32  9822 | 13  0 |
| {BcMV1.BI=TRUE, BcMV2.BI=FALSE} =>  {BcMV4.BI=FALSE} | 0.24  505 | 0.642  857 | 0.381  188 | 1.15  4286 | 99 |

| {BcMV12=FALSE, BcMV2.BI=FALSE} =>  {BcMV4.BI=FALSE} | 0.31  1881 | 0.681  081 | 0.457  921 | 1.22  2919 | 12  6 |
| --- | --- | --- | --- | --- | --- |
| {BcMV12=FALSE, BcMV4.BI=FALSE} =>  {BcMV2.BI=FALSE} | 0.31  1881 | 0.711  864 | 0.438  119 | 1.32  5314 | 12  6 |
| {BcMV2.BI=FALSE, BcNSRV7.BI=FALSE} =>  {BcMV4.BI=FALSE} | 0.30  6931 | 0.700  565 | 0.438  119 | 1.25  7903 | 12  4 |
| {BcMV4.BI=FALSE, BcNSRV7.BI=FALSE} =>  {BcMV2.BI=FALSE} | 0.30  6931 | 0.663  102 | 0.462  871 | 1.23  453 | 12  4 |
| {BcMV2.BI=FALSE, BcVV3.BI=FALSE} =>  {BcMV4.BI=FALSE} | 0.31  9307 | 0.708  791 | 0.450  495 | 1.27  2674 | 12  9 |
| {BcMV4.BI=FALSE, BcVV3.BI=FALSE} =>  {BcMV2.BI=FALSE} | 0.31  9307 | 0.678  947 | 0.470  297 | 1.26  4031 | 12  9 |
| {BcMV2.BI=FALSE, BcMV4.BI=FALSE} =>  {BcOLV7.BI=FALSE} | 0.32  1782 | 0.872  483 | 0.368  812 | 1.08  1237 | 13  0 |
| {BcMV2.BI=FALSE, BcOLV7.BI=FALSE} =>  {BcMV4.BI=FALSE} | 0.32  1782 | 0.718  232 | 0.448  02 | 1.28  9626 | 13  0 |
| {BcMV4.BI=FALSE, BcOLV7.BI=FALSE} =>  {BcMV2.BI=FALSE} | 0.32  1782 | 0.684  211 | 0.470  297 | 1.27  383 | 13  0 |
| {BcMV2.BI=FALSE, BcMV4.BI=FALSE} =>  {BcALV1.BI=FALSE} | 0.34  4059 | 0.932  886 | 0.368  812 | 1.14  555 | 13  9 |
| {BcALV1.BI=FALSE, BcMV2.BI=FALSE} =>  {BcMV4.BI=FALSE} | 0.34  4059 | 0.702  02 | 0.490  099 | 1.26  0516 | 13  9 |

| {BcALV1.BI=FALSE, BcMV4.BI=FALSE} =>  {BcMV2.BI=FALSE} | 0.34  4059 | 0.688  119 | 0.5 | 1.28  1106 | 13  9 |
| --- | --- | --- | --- | --- | --- |
| {BcDMV1=FALSE, BcMV2.BI=FALSE} =>  {BcMV4.BI=FALSE} | 0.30  4455 | 0.679  558 | 0.448  02 | 1.22  0184 | 12  3 |
| {BcDMV1=FALSE, BcMV4.BI=FALSE} =>  {BcMV2.BI=FALSE} | 0.30  4455 | 0.630  769 | 0.482  673 | 1.17  4335 | 12  3 |
| {BcMV2.BI=FALSE, BcMV4.BI=FALSE} =>  {BcFV9=FALSE} | 0.33  9109 | 0.919  463 | 0.368  812 | 1.05  8299 | 13  7 |
| {BcFV9=FALSE, BcMV2.BI=FALSE} =>  {BcMV4.BI=FALSE} | 0.33  9109 | 0.724  868 | 0.467  822 | 1.30  154 | 13  7 |
| {BcFV9=FALSE, BcMV4.BI=FALSE} =>  {BcMV2.BI=FALSE} | 0.33  9109 | 0.661  836 | 0.512  376 | 1.23  2173 | 13  7 |
| {BcMV2.BI=FALSE, BcMV4.BI=FALSE} =>  {BcOLV4.BI=FALSE} | 0.34  4059 | 0.932  886 | 0.368  812 | 1.06  4649 | 13  9 |
| {BcMV2.BI=FALSE, BcOLV4.BI=FALSE} =>  {BcMV4.BI=FALSE} | 0.34  4059 | 0.688  119 | 0.5 | 1.23  5556 | 13  9 |
| {BcMV4.BI=FALSE, BcOLV4.BI=FALSE} =>  {BcMV2.BI=FALSE} | 0.34  4059 | 0.681  373 | 0.504  95 | 1.26  8546 | 13  9 |
| {BcMV2.BI=FALSE, BcULV5=FALSE} =>  {BcMV4.BI=FALSE} | 0.34  1584 | 0.683  168 | 0.5 | 1.22  6667 | 13  8 |
| {BcMV4.BI=FALSE, BcULV5=FALSE} =>  {BcMV2.BI=FALSE} | 0.34  1584 | 0.69 | 0.495  05 | 1.28  4608 | 13  8 |

| {BcBNV5.BI=FALSE, BcMV2.BI=FALSE} =>  {BcMV4.BI=FALSE} | 0.34  6535 | 0.693  069 | 0.5 | 1.24  4444 | 14  0 |
| --- | --- | --- | --- | --- | --- |
| {BcBNV5.BI=FALSE, BcMV4.BI=FALSE} =>  {BcMV2.BI=FALSE} | 0.34  6535 | 0.660  377 | 0.524  752 | 1.22  9458 | 14  0 |
| {BcMV2.BI=FALSE, BvF.BI=FALSE} =>  {BcMV4.BI=FALSE} | 0.33  1683 | 0.690  722 | 0.480  198 | 1.24  0229 | 13  4 |
| {BcMV4.BI=FALSE, BvF.BI=FALSE} =>  {BcMV2.BI=FALSE} | 0.33  1683 | 0.653  659 | 0.507  426 | 1.21  695 | 13  4 |
| {BcMV2.BI=FALSE, BcMV4.BI=FALSE} =>  {BcMCV5.BI=FALSE} | 0.36  8812 | 1 | 0.368  812 | 1.08  311 | 14  9 |
| {BcMCV5.BI=FALSE, BcMV2.BI=FALSE} =>  {BcMV4.BI=FALSE} | 0.36  8812 | 0.706  161 | 0.522  277 | 1.26  7952 | 14  9 |
| {BcMCV5.BI=FALSE, BcMV4.BI=FALSE} =>  {BcMV2.BI=FALSE} | 0.36  8812 | 0.677  273 | 0.544  554 | 1.26  0913 | 14  9 |
| {BcMV2.BI=FALSE, BcOLV13.BI=FALSE} =>  {BcMV4.BI=FALSE} | 0.36  1386 | 0.698  565 | 0.517  327 | 1.25  4312 | 14  6 |
| {BcMV4.BI=FALSE, BcOLV13.BI=FALSE} =>  {BcMV2.BI=FALSE} | 0.36  1386 | 0.675  926 | 0.534  653 | 1.25  8406 | 14  6 |
| {BcMV2.BI=FALSE, BcOLV2.BI=FALSE} =>  {BcMV4.BI=FALSE} | 0.34  6535 | 0.682  927 | 0.507  426 | 1.22  6233 | 14  0 |
| {BcMV4.BI=FALSE, BcOLV2.BI=FALSE} =>  {BcMV2.BI=FALSE} | 0.34  6535 | 0.660  377 | 0.524  752 | 1.22  9458 | 14  0 |

| {BcMCV3.BI=FALSE, BcMV2.BI=FALSE} =>  {BcMV4.BI=FALSE} | 0.36  1386 | 0.688  679 | 0.524  752 | 1.23  6562 | 14  6 |
| --- | --- | --- | --- | --- | --- |
| {BcMCV3.BI=FALSE, BcMV4.BI=FALSE} =>  {BcMV2.BI=FALSE} | 0.36  1386 | 0.691  943 | 0.522  277 | 1.28  8226 | 14  6 |
| {BcMV2.BI=FALSE, BcPV4.BI=FALSE} =>  {BcMV4.BI=FALSE} | 0.34  901 | 0.691  176 | 0.504  95 | 1.24  1046 | 14  1 |
| {BcMV4.BI=FALSE, BcPV4.BI=FALSE} =>  {BcMV2.BI=FALSE} | 0.34  901 | 0.677  885 | 0.514  851 | 1.26  2052 | 14  1 |
| {BcEV6=FALSE, BcMV2.BI=FALSE} =>  {BcMV4.BI=FALSE} | 0.34  1584 | 0.679  803 | 0.502  475 | 1.22  0624 | 13  8 |
| {BcEV6=FALSE, BcMV4.BI=FALSE} =>  {BcMV2.BI=FALSE} | 0.34  1584 | 0.663  462 | 0.514  851 | 1.23  52 | 13  8 |
| {BcMV2.BI=FALSE, BcOLV17.BI=FALSE} =>  {BcMV4.BI=FALSE} | 0.35  8911 | 0.687  204 | 0.522  277 | 1.23  3913 | 14  5 |
| {BcMV4.BI=FALSE, BcOLV17.BI=FALSE} =>  {BcMV2.BI=FALSE} | 0.35  8911 | 0.668  203 | 0.537  129 | 1.24  4027 | 14  5 |
| {BcMV2.BI=FALSE, BcOLV15.BI=FALSE} =>  {BcMV4.BI=FALSE} | 0.34  901 | 0.701  493 | 0.497  525 | 1.25  9569 | 14  1 |
| {BcMV4.BI=FALSE, BcOLV15.BI=FALSE} =>  {BcMV2.BI=FALSE} | 0.34  901 | 0.649  77 | 0.537  129 | 1.20  9709 | 14  1 |
| {BcEV5=FALSE, BcMV2.BI=FALSE} =>  {BcMV4.BI=FALSE} | 0.36  6337 | 0.694  836 | 0.527  228 | 1.24  7616 | 14  8 |

| {BcEV5=FALSE, BcMV4.BI=FALSE} =>  {BcMV2.BI=FALSE} | 0.36  6337 | 0.663  677 | 0.551  98 | 1.23  5602 | 14  8 |
| --- | --- | --- | --- | --- | --- |
| {BcOLV16.BI=FALSE, BcULV3=TRUE} =>  {BcMV2.BI=FALSE} | 0.20  0495 | 0.637  795 | 0.314  356 | 1.18  7416 | 81 |
| {BcHV1.BI=TRUE, BcMV2.BI=FALSE} =>  {BcOLV16.BI=FALSE} | 0.22  0297 | 0.706  349 | 0.311  881 | 1.20  9174 | 89 |
| {BcHV1.BI=TRUE, BcOLV16.BI=FALSE} =>  {BcMV2.BI=FALSE} | 0.22  0297 | 0.659  259 | 0.334  158 | 1.22  7377 | 89 |
| {BcMV11=TRUE, BcMV2.BI=FALSE} =>  {BcOLV16.BI=FALSE} | 0.19  5545 | 0.711  712 | 0.274  752 | 1.21  8354 | 79 |
| {BcMV2.BI=FALSE, BcOLV16.BI=FALSE} =>  {BcOLV1.BI.MOVA.=FALSE} | 0.32  6733 | 0.874  172 | 0.373  762 | 1.30  8021 | 13  2 |
| {BcMV2.BI=FALSE, BcOLV1.BI.MOVA.=FALSE} =>  {BcOLV16.BI=FALSE} | 0.32  6733 | 0.717  391 | 0.455  446 | 1.22  8077 | 13  2 |
| {BcOLV1.BI.MOVA.=FALSE,  BcOLV16.BI=FALSE} => {BcMV2.BI=FALSE} | 0.32  6733 | 0.737  43 | 0.443  069 | 1.37  2911 | 13  2 |
| {BcMV9.BI=TRUE, BcOLV16.BI=FALSE} =>  {BcMV2.BI=FALSE} | 0.22  7723 | 0.657  143 | 0.346  535 | 1.22  3436 | 92 |
| {BcMV2.BI=FALSE, BcOLV16.BI=FALSE} =>  {BcMV6.BI=FALSE} | 0.31  1881 | 0.834  437 | 0.373  762 | 1.19  9689 | 12  6 |
| {BcMV2.BI=FALSE, BcMV6.BI=FALSE} => | 0.31 | 0.707 | 0.440 | 1.21 | 12 |

| {BcOLV16.BI=FALSE} | 1881 | 865 | 594 | 1769 | 6 |
| --- | --- | --- | --- | --- | --- |
| {BcMV6.BI=FALSE, BcOLV16.BI=FALSE} =>  {BcMV2.BI=FALSE} | 0.31  1881 | 0.7 | 0.445  545 | 1.30  3226 | 12  6 |
| {BcMV2.BI=FALSE, BcOLV16.BI=FALSE} =>  {BcPV3.BI=FALSE} | 0.30  198 | 0.807  947 | 0.373  762 | 1.13  7319 | 12  2 |
| {BcMV2.BI=FALSE, BcPV3.BI=FALSE} =>  {BcOLV16.BI=FALSE} | 0.30  198 | 0.709  302 | 0.425  743 | 1.21  4229 | 12  2 |
| {BcOLV16.BI=FALSE, BcPV3.BI=FALSE} =>  {BcMV2.BI=FALSE} | 0.30  198 | 0.689  266 | 0.438  119 | 1.28  3241 | 12  2 |
| {BcMV2.BI=FALSE, BcOLV16.BI=FALSE} =>  {BcRV1.BI=FALSE} | 0.30  9406 | 0.827  815 | 0.373  762 | 1.14  5332 | 12  5 |
| {BcMV2.BI=FALSE, BcRV1.BI=FALSE} =>  {BcOLV16.BI=FALSE} | 0.30  9406 | 0.706  215 | 0.438  119 | 1.20  8944 | 12  5 |
| {BcOLV16.BI=FALSE, BcRV1.BI=FALSE} =>  {BcMV2.BI=FALSE} | 0.30  9406 | 0.675  676 | 0.457  921 | 1.25  794 | 12  5 |
| {BcEV2.BI=FALSE, BcMV2.BI=FALSE} =>  {BcOLV16.BI=FALSE} | 0.28  7129 | 0.670  52 | 0.428  218 | 1.14  784 | 11  6 |
| {BcEV2.BI=FALSE, BcOLV16.BI=FALSE} =>  {BcMV2.BI=FALSE} | 0.28  7129 | 0.682  353 | 0.420  792 | 1.27  0371 | 11  6 |
| {BcHV3.BI=FALSE, BcMV2.BI=FALSE} =>  {BcOLV16.BI=FALSE} | 0.29  703 | 0.689  655 | 0.430  693 | 1.18  0596 | 12  0 |
| {BcHV3.BI=FALSE, BcOLV16.BI=FALSE} => | 0.29 | 0.677 | 0.438 | 1.26 | 12 |

| {BcMV2.BI=FALSE} | 703 | 966 | 119 | 2204 | 0 |
| --- | --- | --- | --- | --- | --- |
| {BcFV5.BI=FALSE, BcMV2.BI=FALSE} =>  {BcOLV16.BI=FALSE} | 0.29  9505 | 0.683  616 | 0.438  119 | 1.17  0258 | 12  1 |
| {BcFV5.BI=FALSE, BcOLV16.BI=FALSE} =>  {BcMV2.BI=FALSE} | 0.29  9505 | 0.650  538 | 0.460  396 | 1.21  1139 | 12  1 |
| {BcMV2.BI=FALSE, BcOLV16.BI=FALSE} =>  {BcULV1.BI=FALSE} | 0.33  9109 | 0.907  285 | 0.373  762 | 1.15  6287 | 13  7 |
| {BcMV2.BI=FALSE, BcULV1.BI=FALSE} =>  {BcOLV16.BI=FALSE} | 0.33  9109 | 0.736  559 | 0.460  396 | 1.26  0889 | 13  7 |
| {BcOLV16.BI=FALSE, BcULV1.BI=FALSE} =>  {BcMV2.BI=FALSE} | 0.33  9109 | 0.658  654 | 0.514  851 | 1.22  625 | 13  7 |
| {BcMV1.BI=TRUE, BcMV2.BI=FALSE} =>  {BcOLV16.BI=FALSE} | 0.26  4851 | 0.694  805 | 0.381  188 | 1.18  9412 | 10  7 |
| {BcMV2.BI=FALSE, BcOLV16.BI=FALSE} =>  {BcMV12=FALSE} | 0.32  4257 | 0.867  55 | 0.373  762 | 1.08  5109 | 13  1 |
| {BcMV12=FALSE, BcMV2.BI=FALSE} =>  {BcOLV16.BI=FALSE} | 0.32  4257 | 0.708  108 | 0.457  921 | 1.21  2185 | 13  1 |
| {BcMV12=FALSE, BcOLV16.BI=FALSE} =>  {BcMV2.BI=FALSE} | 0.32  4257 | 0.642  157 | 0.504  95 | 1.19  5536 | 13  1 |
| {BcMV2.BI=FALSE, BcNSRV7.BI=FALSE} =>  {BcOLV16.BI=FALSE} | 0.30  4455 | 0.694  915 | 0.438  119 | 1.18  9601 | 12  3 |
| {BcNSRV7.BI=FALSE, BcOLV16.BI=FALSE} | 0.30 | 0.654 | 0.465 | 1.21 | 12 |

| => {BcMV2.BI=FALSE} | 4455 | 255 | 347 | 8061 | 3 |
| --- | --- | --- | --- | --- | --- |
| {BcMV2.BI=FALSE, BcVV3.BI=FALSE} =>  {BcOLV16.BI=FALSE} | 0.31  9307 | 0.708  791 | 0.450  495 | 1.21  3354 | 12  9 |
| {BcOLV16.BI=FALSE, BcVV3.BI=FALSE} =>  {BcMV2.BI=FALSE} | 0.31  9307 | 0.686  17 | 0.465  347 | 1.27  7478 | 12  9 |
| {BcMV2.BI=FALSE, BcOLV7.BI=FALSE} =>  {BcOLV16.BI=FALSE} | 0.32  4257 | 0.723  757 | 0.448  02 | 1.23  8974 | 13  1 |
| {BcOLV16.BI=FALSE, BcOLV7.BI=FALSE} =>  {BcMV2.BI=FALSE} | 0.32  4257 | 0.632  85 | 0.512  376 | 1.17  821 | 13  1 |
| {BcMV2.BI=FALSE, BcOLV16.BI=FALSE} =>  {BcALV1.BI=FALSE} | 0.34  4059 | 0.920  53 | 0.373  762 | 1.13  0377 | 13  9 |
| {BcALV1.BI=FALSE, BcMV2.BI=FALSE} =>  {BcOLV16.BI=FALSE} | 0.34  4059 | 0.702  02 | 0.490  099 | 1.20  1763 | 13  9 |
| {BcALV1.BI=FALSE, BcOLV16.BI=FALSE} =>  {BcMV2.BI=FALSE} | 0.34  4059 | 0.681  373 | 0.504  95 | 1.26  8546 | 13  9 |
| {BcDMV1=FALSE, BcMV2.BI=FALSE} =>  {BcOLV16.BI=FALSE} | 0.30  9406 | 0.690  608 | 0.448  02 | 1.18  2227 | 12  5 |
| {BcDMV1=FALSE, BcOLV16.BI=FALSE} =>  {BcMV2.BI=FALSE} | 0.30  9406 | 0.631  313 | 0.490  099 | 1.17  5348 | 12  5 |
| {BcFV9=FALSE, BcMV2.BI=FALSE} =>  {BcOLV16.BI=FALSE} | 0.32  1782 | 0.687  831 | 0.467  822 | 1.17  7473 | 13  0 |
| {BcFV9=FALSE, BcOLV16.BI=FALSE} => | 0.32 | 0.631 | 0.509 | 1.17 | 13 |

| {BcMV2.BI=FALSE} | 1782 | 068 | 901 | 4892 | 0 |
| --- | --- | --- | --- | --- | --- |
| {BcMV2.BI=FALSE, BcOLV4.BI=FALSE} =>  {BcOLV16.BI=FALSE} | 0.34  6535 | 0.693  069 | 0.5 | 1.18  6441 | 14  0 |
| {BcOLV16.BI=FALSE, BcOLV4.BI=FALSE} =>  {BcMV2.BI=FALSE} | 0.34  6535 | 0.651  163 | 0.532  178 | 1.21  2303 | 14  0 |
| {BcMV2.BI=FALSE, BcOLV16.BI=FALSE} =>  {BcULV5=FALSE} | 0.35  1485 | 0.940  397 | 0.373  762 | 1.07  3222 | 14  2 |
| {BcMV2.BI=FALSE, BcULV5=FALSE} =>  {BcOLV16.BI=FALSE} | 0.35  1485 | 0.702  97 | 0.5 | 1.20  339 | 14  2 |
| {BcOLV16.BI=FALSE, BcULV5=FALSE} =>  {BcMV2.BI=FALSE} | 0.35  1485 | 0.669  811 | 0.524  752 | 1.24  7022 | 14  2 |
| {BcBNV5.BI=FALSE, BcMV2.BI=FALSE} =>  {BcOLV16.BI=FALSE} | 0.34  1584 | 0.683  168 | 0.5 | 1.16  9492 | 13  8 |
| {BcBNV5.BI=FALSE, BcOLV16.BI=FALSE} =>  {BcMV2.BI=FALSE} | 0.34  1584 | 0.638  889 | 0.534  653 | 1.18  9452 | 13  8 |
| {BcMV2.BI=FALSE, BvF.BI=FALSE} =>  {BcOLV16.BI=FALSE} | 0.33  6634 | 0.701  031 | 0.480  198 | 1.20  007 | 13  6 |
| {BcOLV16.BI=FALSE, BvF.BI=FALSE} =>  {BcMV2.BI=FALSE} | 0.33  6634 | 0.644  55 | 0.522  277 | 1.19  9991 | 13  6 |
| {BcMCV5.BI=FALSE, BcMV2.BI=FALSE} =>  {BcOLV16.BI=FALSE} | 0.36  3861 | 0.696  682 | 0.522  277 | 1.19  2626 | 14  7 |
| {BcMCV5.BI=FALSE, BcOLV16.BI=FALSE} => | 0.36 | 0.653 | 0.556 | 1.21 | 14 |

| {BcMV2.BI=FALSE} | 3861 | 333 | 931 | 6344 | 7 |
| --- | --- | --- | --- | --- | --- |
| {BcMV2.BI=FALSE, BcOLV13.BI=FALSE} =>  {BcOLV16.BI=FALSE} | 0.36  3861 | 0.703  349 | 0.517  327 | 1.20  4039 | 14  7 |
| {BcOLV13.BI=FALSE, BcOLV16.BI=FALSE}  => {BcMV2.BI=FALSE} | 0.36  3861 | 0.650  442 | 0.559  406 | 1.21  0962 | 14  7 |
| {BcMV2.BI=FALSE, BcOLV2.BI=FALSE} =>  {BcOLV16.BI=FALSE} | 0.35  6436 | 0.702  439 | 0.507  426 | 1.20  248 | 14  4 |
| {BcOLV16.BI=FALSE, BcOLV2.BI=FALSE} =>  {BcMV2.BI=FALSE} | 0.35  6436 | 0.637  168 | 0.559  406 | 1.18  6249 | 14  4 |
| {BcMCV3.BI=FALSE, BcMV2.BI=FALSE} =>  {BcOLV16.BI=FALSE} | 0.36  8812 | 0.702  83 | 0.524  752 | 1.20  315 | 14  9 |
| {BcMCV3.BI=FALSE, BcOLV16.BI=FALSE} =>  {BcMV2.BI=FALSE} | 0.36  8812 | 0.642  241 | 0.574  257 | 1.19  5694 | 14  9 |
| {BcMV2.BI=FALSE, BcPV4.BI=FALSE} =>  {BcOLV16.BI=FALSE} | 0.35  1485 | 0.696  078 | 0.504  95 | 1.19  1592 | 14  2 |
| {BcOLV16.BI=FALSE, BcPV4.BI=FALSE} =>  {BcMV2.BI=FALSE} | 0.35  1485 | 0.633  929 | 0.554  455 | 1.18  0217 | 14  2 |
| {BcEV6=FALSE, BcMV2.BI=FALSE} =>  {BcOLV16.BI=FALSE} | 0.34  6535 | 0.689  655 | 0.502  475 | 1.18  0596 | 14  0 |
| {BcEV6=FALSE, BcOLV16.BI=FALSE} =>  {BcMV2.BI=FALSE} | 0.34  6535 | 0.636  364 | 0.544  554 | 1.18  4751 | 14  0 |
| {BcMV2.BI=FALSE, BcOLV17.BI=FALSE} => | 0.36 | 0.701 | 0.522 | 1.20 | 14 |

| {BcOLV16.BI=FALSE} | 6337 | 422 | 277 | 0739 | 8 |
| --- | --- | --- | --- | --- | --- |
| {BcOLV16.BI=FALSE, BcOLV17.BI=FALSE}  => {BcMV2.BI=FALSE} | 0.36  6337 | 0.651  982 | 0.561  881 | 1.21  3829 | 14  8 |
| {BcMV2.BI=FALSE, BcOLV15.BI=FALSE} =>  {BcOLV16.BI=FALSE} | 0.33  9109 | 0.681  592 | 0.497  525 | 1.16  6793 | 13  7 |
| {BcOLV15.BI=FALSE, BcOLV16.BI=FALSE}  => {BcMV2.BI=FALSE} | 0.33  9109 | 0.619  91 | 0.547  03 | 1.15  4117 | 13  7 |
| {BcEV5=FALSE, BcMV2.BI=FALSE} =>  {BcOLV16.BI=FALSE} | 0.36  3861 | 0.690  141 | 0.527  228 | 1.18  1428 | 14  7 |
| {BcEV5=FALSE, BcOLV16.BI=FALSE} =>  {BcMV2.BI=FALSE} | 0.36  3861 | 0.653  333 | 0.556  931 | 1.21  6344 | 14  7 |
| {BcMV2.BI=FALSE, BcULV3=TRUE} =>  {BcHV1.BI=TRUE} | 0.21  7822 | 0.704 | 0.309  406 | 1.16  5639 | 88 |
| {BcHV1.BI=TRUE, BcMV2.BI=FALSE} =>  {BcULV3=TRUE} | 0.21  7822 | 0.698  413 | 0.311  881 | 1.17  5661 | 88 |
| {BcMV2.BI=FALSE, BcULV3=TRUE} =>  {BcOLV1.BI.MOVA.=FALSE} | 0.26  2376 | 0.848 | 0.309  406 | 1.26  8859 | 10  6 |
| {BcOLV1.BI.MOVA.=FALSE, BcULV3=TRUE}  => {BcMV2.BI=FALSE} | 0.26  2376 | 0.658  385 | 0.398  515 | 1.22  5749 | 10  6 |
| {BcMV2.BI=FALSE, BcULV3=TRUE} =>  {BcMV6.BI=FALSE} | 0.25 | 0.808 | 0.309  406 | 1.16  168 | 10  1 |
| {BcMV6.BI=FALSE, BcULV3=TRUE} => | 0.25 | 0.660 | 0.378 | 1.22 | 10 |

| {BcMV2.BI=FALSE} |  | 131 | 713 | 8999 | 1 |
| --- | --- | --- | --- | --- | --- |
| {BcMV2.BI=FALSE, BcULV3=TRUE} => |  |  | 0.309 | 1.13 | 10 |
|  | 0.25 | 0.808 |  |  |  |
| {BcPV3.BI=FALSE} |  |  | 406 | 7394 | 1 |
| {BcMV2.BI=FALSE, BcULV3=TRUE} => | 0.25 |  | 0.309 | 1.12 | 10 |
|  |  | 0.816 |  |  |  |
| {BcRV1.BI=FALSE} | 2475 |  | 406 | 8986 | 2 |
| {BcMV2.BI=FALSE, BcULV3=TRUE} => | 0.28 |  | 0.309 | 1.12 | 11 |
|  |  | 0.92 |  |  |  |
| {BcALV1.BI=FALSE} | 4653 |  | 406 | 9726 | 5 |
| {BcHV1.BI=TRUE, BcMV2.BI=FALSE} => | 0.25 | 0.825 | 0.311 | 1.23 | 10 |
| {BcOLV1.BI.MOVA.=FALSE} | 7426 | 397 | 881 | 5038 | 4 |
| {BcHV1.BI=TRUE, |  |  |  |  |  |
|  | 0.25 | 0.658 | 0.391 | 1.22 | 10 |
| BcOLV1.BI.MOVA.=FALSE} => |  |  |  |  |  |
|  | 7426 | 228 | 089 | 5456 | 4 |
| {BcMV2.BI=FALSE} |  |  |  |  |  |
| {BcHV1.BI=TRUE, BcMV2.BI=FALSE} => | 0.23 | 0.761 | 0.311 | 1.12 |  |
|  |  |  |  |  | 96 |
| {BcMV9.BI=TRUE} | 7624 | 905 | 881 | 3392 |  |
| {BcHV1.BI=TRUE, BcMV2.BI=FALSE} => | 0.24 | 0.793 | 0.311 | 1.14 | 10 |
| {BcMV6.BI=FALSE} | 7525 | 651 | 881 | 105 | 0 |
| {BcHV1.BI=TRUE, BcMV6.BI=FALSE} => | 0.24 | 0.657 | 0.376 | 1.22 | 10 |
| {BcMV2.BI=FALSE} | 7525 | 895 | 238 | 4836 | 0 |
| {BcHV1.BI=TRUE, BcMV2.BI=FALSE} => | 0.25 | 0.825 | 0.311 | 1.14 | 10 |
| {BcRV1.BI=FALSE} | 7426 | 397 | 881 | 1987 | 4 |
| {BcHV1.BI=TRUE, BcRV1.BI=FALSE} => | 0.25 | 0.619 | 0.415 | 1.15 | 10 |
| {BcMV2.BI=FALSE} | 7426 | 048 | 842 | 2513 | 4 |

| {BcHV1.BI=TRUE, BcMV2.BI=FALSE} =>  {BcALV1.BI=FALSE} | 0.28  2178 | 0.904  762 | 0.311  881 | 1.11  1015 | 11  4 |
| --- | --- | --- | --- | --- | --- |
| {BcALV1.BI=FALSE, BcHV1.BI=TRUE} =>  {BcMV2.BI=FALSE} | 0.28  2178 | 0.606  383 | 0.465  347 | 1.12  8934 | 11  4 |
| {BcMV11=TRUE, BcMV2.BI=FALSE} =>  {BcOLV1.BI.MOVA.=FALSE} | 0.23  5149 | 0.855  856 | 0.274  752 | 1.28  0614 | 95 |
| {BcMV11=TRUE, BcOLV1.BI.MOVA.=FALSE}  => {BcMV2.BI=FALSE} | 0.23  5149 | 0.629  139 | 0.373  762 | 1.17  13 | 95 |
| {BcMV11=TRUE, BcMV2.BI=FALSE} =>  {BcMV6.BI=FALSE} | 0.22  2772 | 0.810  811 | 0.274  752 | 1.16  5721 | 90 |
| {BcMV11=TRUE, BcMV2.BI=FALSE} =>  {BcMV12=FALSE} | 0.24  7525 | 0.900  901 | 0.274  752 | 1.12  6823 | 10  0 |
| {BcMV11=TRUE, BcMV2.BI=FALSE} =>  {BcALV1.BI=FALSE} | 0.24  7525 | 0.900  901 | 0.274  752 | 1.10  6273 | 10  0 |
| {BcMV2.BI=FALSE, BcMV9.BI=TRUE} =>  {BcOLV1.BI.MOVA.=FALSE} | 0.30  9406 | 0.868  056 | 0.356  436 | 1.29  8868 | 12  5 |
| {BcMV9.BI=TRUE, BcOLV1.BI.MOVA.=FALSE} =>  {BcMV2.BI=FALSE} | 0.30  9406 | 0.664  894 | 0.465  347 | 1.23  7866 | 12  5 |
| {BcMV2.BI=FALSE, BcULV4=TRUE} =>  {BcOLV1.BI.MOVA.=FALSE} | 0.28  9604 | 0.829  787 | 0.349  01 | 1.24  1608 | 11  7 |
| {BcOLV1.BI.MOVA.=FALSE, BcULV4=TRUE} | 0.28 | 0.65 | 0.445 | 1.21 | 11 |

| => {BcMV2.BI=FALSE} | 9604 |  | 545 | 0138 | 7 |
| --- | --- | --- | --- | --- | --- |
| {BcMV2.BI=FALSE, |  |  |  |  |  |
|  | 0.37 | 0.831 | 0.455 | 1.19 | 15 |
| BcOLV1.BI.MOVA.=FALSE} => |  |  |  |  |  |
|  | 8713 | 522 | 446 | 5497 | 3 |
| {BcMV6.BI=FALSE} |  |  |  |  |  |
| {BcMV2.BI=FALSE, BcMV6.BI=FALSE} => | 0.37 | 0.859 | 0.440 | 1.28 | 15 |
| {BcOLV1.BI.MOVA.=FALSE} | 8713 | 551 | 594 | 6142 | 3 |
| {BcMV6.BI=FALSE, |  |  |  |  |  |
|  | 0.37 | 0.718 | 0.527 | 1.33 | 15 |
| BcOLV1.BI.MOVA.=FALSE} => |  |  |  |  |  |
|  | 8713 | 31 | 228 | 7314 | 3 |
| {BcMV2.BI=FALSE} |  |  |  |  |  |
| {BcMV2.BI=FALSE, |  |  |  |  |  |
|  | 0.37 | 0.820 | 0.455 | 1.15 | 15 |
| BcOLV1.BI.MOVA.=FALSE} => |  |  |  |  |  |
|  | 3762 | 652 | 446 | 5204 | 1 |
| {BcPV3.BI=FALSE} |  |  |  |  |  |
| {BcMV2.BI=FALSE, BcPV3.BI=FALSE} => | 0.37 | 0.877 | 0.425 | 1.31 | 15 |
| {BcOLV1.BI.MOVA.=FALSE} | 3762 | 907 | 743 | 3609 | 1 |
| {BcOLV1.BI.MOVA.=FALSE, | 0.37 | 0.725 | 0.514 | 1.35 | 15 |
| BcPV3.BI=FALSE} => {BcMV2.BI=FALSE} | 3762 | 962 | 851 | 156 | 1 |
| {BcMV2.BI=FALSE, |  |  |  |  |  |
|  | 0.38 | 0.842 | 0.455 | 1.16 | 15 |
| BcOLV1.BI.MOVA.=FALSE} => |  |  |  |  |  |
|  | 3663 | 391 | 446 | 55 | 5 |
| {BcRV1.BI=FALSE} |  |  |  |  |  |
| {BcMV2.BI=FALSE, BcRV1.BI=FALSE} => | 0.38 | 0.875 | 0.438 | 1.31 | 15 |
| {BcOLV1.BI.MOVA.=FALSE} | 3663 | 706 | 119 | 0316 | 5 |
| {BcOLV1.BI.MOVA.=FALSE, | 0.38 | 0.731 | 0.524 | 1.36 | 15 |

| BcRV1.BI=FALSE} => {BcMV2.BI=FALSE} | 3663 | 132 | 752 | 1186 | 5 |
| --- | --- | --- | --- | --- | --- |
| {BcEV2.BI=FALSE, BcMV2.BI=FALSE} =>  {BcOLV1.BI.MOVA.=FALSE} | 0.35  396 | 0.826  59 | 0.428  218 | 1.23  6823 | 14  3 |
| {BcEV2.BI=FALSE, BcOLV1.BI.MOVA.=FALSE} =>  {BcMV2.BI=FALSE} | 0.35  396 | 0.711  443 | 0.497  525 | 1.32  4529 | 14  3 |
| {BcHV3.BI=FALSE, BcMV2.BI=FALSE} =>  {BcOLV1.BI.MOVA.=FALSE} | 0.36  8812 | 0.856  322 | 0.430  693 | 1.28  1311 | 14  9 |
| {BcHV3.BI=FALSE, BcOLV1.BI.MOVA.=FALSE} =>  {BcMV2.BI=FALSE} | 0.36  8812 | 0.693  023 | 0.532  178 | 1.29  0237 | 14  9 |
| {BcFV5.BI=FALSE, BcMV2.BI=FALSE} =>  {BcOLV1.BI.MOVA.=FALSE} | 0.37  3762 | 0.853  107 | 0.438  119 | 1.27  6501 | 15  1 |
| {BcFV5.BI=FALSE, BcOLV1.BI.MOVA.=FALSE} =>  {BcMV2.BI=FALSE} | 0.37  3762 | 0.689  498 | 0.542  079 | 1.28  3673 | 15  1 |
| {BcMV2.BI=FALSE, BcOLV1.BI.MOVA.=FALSE} =>  {BcULV1.BI=FALSE} | 0.39  3564 | 0.864  13 | 0.455  446 | 1.10  1289 | 15  9 |
| {BcMV2.BI=FALSE, BcULV1.BI=FALSE} =>  {BcOLV1.BI.MOVA.=FALSE} | 0.39  3564 | 0.854  839 | 0.460  396 | 1.27  9092 | 15  9 |
| {BcOLV1.BI.MOVA.=FALSE, | 0.39 | 0.703 | 0.559 | 1.30 | 15 |

| BcULV1.BI=FALSE} => {BcMV2.BI=FALSE} | 3564 | 54 | 406 | 9816 | 9 |
| --- | --- | --- | --- | --- | --- |
| {BcMV1.BI=TRUE, BcMV2.BI=FALSE} =>  {BcOLV1.BI.MOVA.=FALSE} | 0.31  6832 | 0.831  169 | 0.381  188 | 1.24  3675 | 12  8 |
| {BcMV1.BI=TRUE, BcOLV1.BI.MOVA.=FALSE} =>  {BcMV2.BI=FALSE} | 0.31  6832 | 0.64 | 0.495  05 | 1.19  1521 | 12  8 |
| {BcMV12=FALSE, BcMV2.BI=FALSE} =>  {BcOLV1.BI.MOVA.=FALSE} | 0.38  8614 | 0.848  649 | 0.457  921 | 1.26  983 | 15  7 |
| {BcMV12=FALSE, BcOLV1.BI.MOVA.=FALSE} =>  {BcMV2.BI=FALSE} | 0.38  8614 | 0.682  609 | 0.569  307 | 1.27  0848 | 15  7 |
| {BcMV2.BI=FALSE, BcNSRV7.BI=FALSE} =>  {BcOLV1.BI.MOVA.=FALSE} | 0.36  6337 | 0.836  158 | 0.438  119 | 1.25  114 | 14  8 |
| {BcNSRV7.BI=FALSE, BcOLV1.BI.MOVA.=FALSE} =>  {BcMV2.BI=FALSE} | 0.36  6337 | 0.685  185 | 0.534  653 | 1.27  5644 | 14  8 |
| {BcMV2.BI=FALSE, BcVV3.BI=FALSE} =>  {BcOLV1.BI.MOVA.=FALSE} | 0.38  8614 | 0.862  637 | 0.450  495 | 1.29  0761 | 15  7 |
| {BcOLV1.BI.MOVA.=FALSE,  BcVV3.BI=FALSE} => {BcMV2.BI=FALSE} | 0.38  8614 | 0.694  69 | 0.559  406 | 1.29  334 | 15  7 |
| {BcMV2.BI=FALSE, BcOLV7.BI=FALSE} =>  {BcOLV1.BI.MOVA.=FALSE} | 0.39  1089 | 0.872  928 | 0.448  02 | 1.30  6159 | 15  8 |

| {BcOLV1.BI.MOVA.=FALSE,  BcOLV7.BI=FALSE} => {BcMV2.BI=FALSE} | 0.39  1089 | 0.686  957 | 0.569  307 | 1.27  8942 | 15  8 |
| --- | --- | --- | --- | --- | --- |
| {BcMV2.BI=FALSE, BcOLV1.BI.MOVA.=FALSE} =>  {BcALV1.BI=FALSE} | 0.41  8317 | 0.918  478 | 0.455  446 | 1.12  7858 | 16  9 |
| {BcALV1.BI=FALSE, BcMV2.BI=FALSE} =>  {BcOLV1.BI.MOVA.=FALSE} | 0.41  8317 | 0.853  535 | 0.490  099 | 1.27  7142 | 16  9 |
| {BcALV1.BI=FALSE, BcOLV1.BI.MOVA.=FALSE} =>  {BcMV2.BI=FALSE} | 0.41  8317 | 0.710  084 | 0.589  109 | 1.32  2 | 16  9 |
| {BcDMV1=FALSE, BcMV2.BI=FALSE} =>  {BcOLV1.BI.MOVA.=FALSE} | 0.37  8713 | 0.845  304 | 0.448  02 | 1.26  4825 | 15  3 |
| {BcDMV1=FALSE, BcOLV1.BI.MOVA.=FALSE} =>  {BcMV2.BI=FALSE} | 0.37  8713 | 0.665  217 | 0.569  307 | 1.23  8469 | 15  3 |
| {BcFV9=FALSE, BcMV2.BI=FALSE} =>  {BcOLV1.BI.MOVA.=FALSE} | 0.39  604 | 0.846  561 | 0.467  822 | 1.26  6706 | 16  0 |
| {BcFV9=FALSE, BcOLV1.BI.MOVA.=FALSE}  => {BcMV2.BI=FALSE} | 0.39  604 | 0.672  269 | 0.589  109 | 1.25  1597 | 16  0 |
| {BcMV2.BI=FALSE, BcOLV1.BI.MOVA.=FALSE} =>  {BcOLV4.BI=FALSE} | 0.42  3267 | 0.929  348 | 0.455  446 | 1.06  0612 | 17  1 |

| {BcMV2.BI=FALSE, BcOLV4.BI=FALSE} =>  {BcOLV1.BI.MOVA.=FALSE} | 0.42  3267 | 0.846  535 | 0.5 | 1.26  6667 | 17  1 |
| --- | --- | --- | --- | --- | --- |
| {BcOLV1.BI.MOVA.=FALSE,  BcOLV4.BI=FALSE} => {BcMV2.BI=FALSE} | 0.42  3267 | 0.689  516 | 0.613  861 | 1.28  3707 | 17  1 |
| {BcMV2.BI=FALSE, BcOLV1.BI.MOVA.=FALSE} =>  {BcULV5=FALSE} | 0.43  0693 | 0.945  652 | 0.455  446 | 1.07  9219 | 17  4 |
| {BcMV2.BI=FALSE, BcULV5=FALSE} =>  {BcOLV1.BI.MOVA.=FALSE} | 0.43  0693 | 0.861  386 | 0.5 | 1.28  8889 | 17  4 |
| {BcOLV1.BI.MOVA.=FALSE, BcULV5=FALSE}  => {BcMV2.BI=FALSE} | 0.43  0693 | 0.696 | 0.618  812 | 1.29  5779 | 17  4 |
| {BcBNV5.BI=FALSE, BcMV2.BI=FALSE} =>  {BcOLV1.BI.MOVA.=FALSE} | 0.42  8218 | 0.856  436 | 0.5 | 1.28  1481 | 17  3 |
| {BcBNV5.BI=FALSE, BcOLV1.BI.MOVA.=FALSE} =>  {BcMV2.BI=FALSE} | 0.42  8218 | 0.683  794 | 0.626  238 | 1.27  3055 | 17  3 |
| {BcMV2.BI=FALSE, BvF.BI=FALSE} =>  {BcOLV1.BI.MOVA.=FALSE} | 0.39  8515 | 0.829  897 | 0.480  198 | 1.24  1772 | 16  1 |
| {BcOLV1.BI.MOVA.=FALSE, BvF.BI=FALSE}  => {BcMV2.BI=FALSE} | 0.39  8515 | 0.665  289 | 0.599  01 | 1.23  8603 | 16  1 |
| {BcMCV5.BI=FALSE, BcMV2.BI=FALSE} =>  {BcOLV1.BI.MOVA.=FALSE} | 0.44  802 | 0.857  82 | 0.522  277 | 1.28  3553 | 18  1 |

| {BcMCV5.BI=FALSE, BcOLV1.BI.MOVA.=FALSE} =>  {BcMV2.BI=FALSE} | 0.44  802 | 0.693  487 | 0.646  04 | 1.29  1099 | 18  1 |
| --- | --- | --- | --- | --- | --- |
| {BcMV2.BI=FALSE, BcOLV13.BI=FALSE} =>  {BcOLV1.BI.MOVA.=FALSE} | 0.44  5545 | 0.861  244 | 0.517  327 | 1.28  8676 | 18  0 |
| {BcOLV1.BI.MOVA.=FALSE,  BcOLV13.BI=FALSE} => {BcMV2.BI=FALSE} | 0.44  5545 | 0.687  023 | 0.648  515 | 1.27  9066 | 18  0 |
| {BcMV2.BI=FALSE, BcOLV2.BI=FALSE} =>  {BcOLV1.BI.MOVA.=FALSE} | 0.44  0594 | 0.868  293 | 0.507  426 | 1.29  9223 | 17  8 |
| {BcOLV1.BI.MOVA.=FALSE,  BcOLV2.BI=FALSE} => {BcMV2.BI=FALSE} | 0.44  0594 | 0.676  806 | 0.650  99 | 1.26  0045 | 17  8 |
| {BcMCV3.BI=FALSE, BcMV2.BI=FALSE} =>  {BcOLV1.BI.MOVA.=FALSE} | 0.44  5545 | 0.849  057 | 0.524  752 | 1.27  044 | 18  0 |
| {BcMCV3.BI=FALSE, BcOLV1.BI.MOVA.=FALSE} =>  {BcMV2.BI=FALSE} | 0.44  5545 | 0.689  655 | 0.646  04 | 1.28  3966 | 18  0 |
| {BcMV2.BI=FALSE, BcPV4.BI=FALSE} =>  {BcOLV1.BI.MOVA.=FALSE} | 0.42  5743 | 0.843  137 | 0.504  95 | 1.26  1583 | 17  2 |
| {BcOLV1.BI.MOVA.=FALSE,  BcPV4.BI=FALSE} => {BcMV2.BI=FALSE} | 0.42  5743 | 0.690  763 | 0.616  337 | 1.28  6029 | 17  2 |
| {BcEV6=FALSE, BcMV2.BI=FALSE} =>  {BcOLV1.BI.MOVA.=FALSE} | 0.42  5743 | 0.847  291 | 0.502  475 | 1.26  7798 | 17  2 |

| {BcEV6=FALSE, BcOLV1.BI.MOVA.=FALSE}  => {BcMV2.BI=FALSE} | 0.42  5743 | 0.677  165 | 0.628  713 | 1.26  0713 | 17  2 |
| --- | --- | --- | --- | --- | --- |
| {BcMV2.BI=FALSE, BcOLV17.BI=FALSE} =>  {BcOLV1.BI.MOVA.=FALSE} | 0.44  5545 | 0.853  081 | 0.522  277 | 1.27  6461 | 18  0 |
| {BcOLV1.BI.MOVA.=FALSE,  BcOLV17.BI=FALSE} => {BcMV2.BI=FALSE} | 0.44  5545 | 0.687  023 | 0.648  515 | 1.27  9066 | 18  0 |
| {BcMV2.BI=FALSE, BcOLV15.BI=FALSE} =>  {BcOLV1.BI.MOVA.=FALSE} | 0.41  8317 | 0.840  796 | 0.497  525 | 1.25  808 | 16  9 |
| {BcOLV1.BI.MOVA.=FALSE,  BcOLV15.BI=FALSE} => {BcMV2.BI=FALSE} | 0.41  8317 | 0.665  354 | 0.628  713 | 1.23  8724 | 16  9 |
| {BcEV5=FALSE, BcMV2.BI=FALSE} =>  {BcOLV1.BI.MOVA.=FALSE} | 0.44  802 | 0.849  765 | 0.527  228 | 1.27  1501 | 18  1 |
| {BcEV5=FALSE, BcOLV1.BI.MOVA.=FALSE}  => {BcMV2.BI=FALSE} | 0.44  802 | 0.685  606 | 0.653  465 | 1.27  6428 | 18  1 |
| {BcMV2.BI=FALSE, BcULV4=TRUE} =>  {BcMV9.BI=TRUE} | 0.26  7327 | 0.765  957 | 0.349  01 | 1.12  9368 | 10  8 |
| {BcMV2.BI=FALSE, BcMV9.BI=TRUE} =>  {BcMV6.BI=FALSE} | 0.28  9604 | 0.812  5 | 0.356  436 | 1.16  8149 | 11  7 |
| {BcMV6.BI=FALSE, BcMV9.BI=TRUE} =>  {BcMV2.BI=FALSE} | 0.28  9604 | 0.653  631 | 0.443  069 | 1.21  6899 | 11  7 |
| {BcMV2.BI=FALSE, BcMV9.BI=TRUE} =>  {BcPV3.BI=FALSE} | 0.28  7129 | 0.805  556 | 0.356  436 | 1.13  3953 | 11  6 |

| {BcMV9.BI=TRUE, BcPV3.BI=FALSE} =>  {BcMV2.BI=FALSE} | 0.28  7129 | 0.607  33 | 0.472  772 | 1.13  0697 | 11  6 |
| --- | --- | --- | --- | --- | --- |
| {BcMV2.BI=FALSE, BcMV9.BI=TRUE} =>  {BcHV3.BI=FALSE} | 0.29  9505 | 0.840  278 | 0.356  436 | 1.10  5773 | 12  1 |
| {BcMV2.BI=FALSE, BcMV9.BI=TRUE} =>  {BcALV1.BI=FALSE} | 0.33  4158 | 0.937  5 | 0.356  436 | 1.15  1216 | 13  5 |
| {BcALV1.BI=FALSE, BcMV9.BI=TRUE} =>  {BcMV2.BI=FALSE} | 0.33  4158 | 0.616  438 | 0.542  079 | 1.14  7655 | 13  5 |
| {BcMV2.BI=FALSE, BcMV9.BI=TRUE} =>  {BcOLV4.BI=FALSE} | 0.34  1584 | 0.958  333 | 0.356  436 | 1.09  3691 | 13  8 |
| {BcMV2.BI=FALSE, BcMV9.BI=TRUE} =>  {BcULV5=FALSE} | 0.35  1485 | 0.986  111 | 0.356  436 | 1.12  5392 | 14  2 |
| {BcMV2.BI=FALSE, BcULV4=TRUE} =>  {BcMV6.BI=FALSE} | 0.27  9703 | 0.801  418 | 0.349  01 | 1.15  2217 | 11  3 |
| {BcMV6.BI=FALSE, BcULV4=TRUE} =>  {BcMV2.BI=FALSE} | 0.27  9703 | 0.624  309 | 0.448  02 | 1.16  2309 | 11  3 |
| {BcMV2.BI=FALSE, BcULV4=TRUE} =>  {BcPV3.BI=FALSE} | 0.27  9703 | 0.801  418 | 0.349  01 | 1.12  8129 | 11  3 |
| {BcMV2.BI=FALSE, BcULV4=TRUE} =>  {BcEV2.BI=FALSE} | 0.29  9505 | 0.858  156 | 0.349  01 | 1.14  7997 | 12  1 |
| {BcMV2.BI=FALSE, BcULV4=TRUE} =>  {BcALV1.BI=FALSE} | 0.31  1881 | 0.893  617 | 0.349  01 | 1.09  7329 | 12  6 |

| {BcMV2.BI=FALSE, BcULV4=TRUE} =>  {BcOLV4.BI=FALSE} | 0.32  9208 | 0.943  262 | 0.349  01 | 1.07  6492 | 13  3 |
| --- | --- | --- | --- | --- | --- |
| {BcMV2.BI=FALSE, BcULV4=TRUE} =>  {BcULV5=FALSE} | 0.33  1683 | 0.950  355 | 0.349  01 | 1.08  4585 | 13  4 |
| {BcMV2.BI=FALSE, BcMV6.BI=FALSE} =>  {BcPV3.BI=FALSE} | 0.34  901 | 0.792  135 | 0.440  594 | 1.11  5061 | 14  1 |
| {BcMV2.BI=FALSE, BcPV3.BI=FALSE} =>  {BcMV6.BI=FALSE} | 0.34  901 | 0.819  767 | 0.425  743 | 1.17  8598 | 14  1 |
| {BcMV6.BI=FALSE, BcPV3.BI=FALSE} =>  {BcMV2.BI=FALSE} | 0.34  901 | 0.674  641 | 0.517  327 | 1.25  6014 | 14  1 |
| {BcMV2.BI=FALSE, BcMV6.BI=FALSE} =>  {BcRV1.BI=FALSE} | 0.36  1386 | 0.820  225 | 0.440  594 | 1.13  4831 | 14  6 |
| {BcMV2.BI=FALSE, BcRV1.BI=FALSE} =>  {BcMV6.BI=FALSE} | 0.36  1386 | 0.824  859 | 0.438  119 | 1.18  5918 | 14  6 |
| {BcMV6.BI=FALSE, BcRV1.BI=FALSE} =>  {BcMV2.BI=FALSE} | 0.36  1386 | 0.679  07 | 0.532  178 | 1.26  4259 | 14  6 |
| {BcEV2.BI=FALSE, BcMV2.BI=FALSE} =>  {BcMV6.BI=FALSE} | 0.34  901 | 0.815  029 | 0.428  218 | 1.17  1785 | 14  1 |
| {BcEV2.BI=FALSE, BcMV6.BI=FALSE} =>  {BcMV2.BI=FALSE} | 0.34  901 | 0.677  885 | 0.514  851 | 1.26  2052 | 14  1 |
| {BcHV3.BI=FALSE, BcMV2.BI=FALSE} =>  {BcMV6.BI=FALSE} | 0.34  6535 | 0.804  598 | 0.430  693 | 1.15  6788 | 14  0 |

| {BcHV3.BI=FALSE, BcMV6.BI=FALSE} =>  {BcMV2.BI=FALSE} | 0.34  6535 | 0.642  202 | 0.539  604 | 1.19  562 | 14  0 |
| --- | --- | --- | --- | --- | --- |
| {BcFV5.BI=FALSE, BcMV2.BI=FALSE} =>  {BcMV6.BI=FALSE} | 0.34  6535 | 0.790  96 | 0.438  119 | 1.13  7182 | 14  0 |
| {BcFV5.BI=FALSE, BcMV6.BI=FALSE} =>  {BcMV2.BI=FALSE} | 0.34  6535 | 0.636  364 | 0.544  554 | 1.18  4751 | 14  0 |
| {BcMV2.BI=FALSE, BcMV6.BI=FALSE} =>  {BcULV1.BI=FALSE} | 0.38  3663 | 0.870  787 | 0.440  594 | 1.10  9772 | 15  5 |
| {BcMV2.BI=FALSE, BcULV1.BI=FALSE} =>  {BcMV6.BI=FALSE} | 0.38  3663 | 0.833  333 | 0.460  396 | 1.19  8102 | 15  5 |
| {BcMV6.BI=FALSE, BcULV1.BI=FALSE} =>  {BcMV2.BI=FALSE} | 0.38  3663 | 0.670  996 | 0.571  782 | 1.24  9227 | 15  5 |
| {BcMV1.BI=TRUE, BcMV2.BI=FALSE} =>  {BcMV6.BI=FALSE} | 0.31  1881 | 0.818  182 | 0.381  188 | 1.17  6318 | 12  6 |
| {BcMV12=FALSE, BcMV2.BI=FALSE} =>  {BcMV6.BI=FALSE} | 0.37  6238 | 0.821  622 | 0.457  921 | 1.18  1264 | 15  2 |
| {BcMV12=FALSE, BcMV6.BI=FALSE} =>  {BcMV2.BI=FALSE} | 0.37  6238 | 0.646  809 | 0.581  683 | 1.20  4196 | 15  2 |
| {BcMV2.BI=FALSE, BcNSRV7.BI=FALSE} =>  {BcMV6.BI=FALSE} | 0.35  6436 | 0.813  559 | 0.438  119 | 1.16  9672 | 14  4 |
| {BcMV6.BI=FALSE, BcNSRV7.BI=FALSE} =>  {BcMV2.BI=FALSE} | 0.35  6436 | 0.628  821 | 0.566  832 | 1.17  0708 | 14  4 |

| {BcMV2.BI=FALSE, BcVV3.BI=FALSE} =>  {BcMV6.BI=FALSE} | 0.37  3762 | 0.829  67 | 0.450  495 | 1.19  2836 | 15  1 |
| --- | --- | --- | --- | --- | --- |
| {BcMV6.BI=FALSE, BcVV3.BI=FALSE} =>  {BcMV2.BI=FALSE} | 0.37  3762 | 0.653  68 | 0.571  782 | 1.21  6989 | 15  1 |
| {BcMV2.BI=FALSE, BcOLV7.BI=FALSE} =>  {BcMV6.BI=FALSE} | 0.37  3762 | 0.834  254 | 0.448  02 | 1.19  9426 | 15  1 |
| {BcMV6.BI=FALSE, BcOLV7.BI=FALSE} =>  {BcMV2.BI=FALSE} | 0.37  3762 | 0.650  862 | 0.574  257 | 1.21  1743 | 15  1 |
| {BcMV2.BI=FALSE, BcMV6.BI=FALSE} =>  {BcALV1.BI=FALSE} | 0.40  3465 | 0.915  73 | 0.440  594 | 1.12  4483 | 16  3 |
| {BcALV1.BI=FALSE, BcMV2.BI=FALSE} =>  {BcMV6.BI=FALSE} | 0.40  3465 | 0.823  232 | 0.490  099 | 1.18  358 | 16  3 |
| {BcALV1.BI=FALSE, BcMV6.BI=FALSE} =>  {BcMV2.BI=FALSE} | 0.40  3465 | 0.676  349 | 0.596  535 | 1.25  9193 | 16  3 |
| {BcDMV1=FALSE, BcMV2.BI=FALSE} =>  {BcMV6.BI=FALSE} | 0.36  3861 | 0.812  155 | 0.448  02 | 1.16  7653 | 14  7 |
| {BcDMV1=FALSE, BcMV6.BI=FALSE} =>  {BcMV2.BI=FALSE} | 0.36  3861 | 0.6 | 0.606  436 | 1.11  7051 | 14  7 |
| {BcFV9=FALSE, BcMV2.BI=FALSE} =>  {BcMV6.BI=FALSE} | 0.37  8713 | 0.809  524 | 0.467  822 | 1.16  3871 | 15  3 |
| {BcFV9=FALSE, BcMV6.BI=FALSE} =>  {BcMV2.BI=FALSE} | 0.37  8713 | 0.629  63 | 0.601  485 | 1.17  2214 | 15  3 |

| {BcMV2.BI=FALSE, BcMV6.BI=FALSE} =>  {BcOLV4.BI=FALSE} | 0.40  8416 | 0.926  966 | 0.440  594 | 1.05  7894 | 16  5 |
| --- | --- | --- | --- | --- | --- |
| {BcMV2.BI=FALSE, BcOLV4.BI=FALSE} =>  {BcMV6.BI=FALSE} | 0.40  8416 | 0.816  832 | 0.5 | 1.17  4377 | 16  5 |
| {BcMV6.BI=FALSE, BcOLV4.BI=FALSE} =>  {BcMV2.BI=FALSE} | 0.40  8416 | 0.662  651 | 0.616  337 | 1.23  3691 | 16  5 |
| {BcMV2.BI=FALSE, BcMV6.BI=FALSE} =>  {BcULV5=FALSE} | 0.41  5842 | 0.943  82 | 0.440  594 | 1.07  7128 | 16  8 |
| {BcMV2.BI=FALSE, BcULV5=FALSE} =>  {BcMV6.BI=FALSE} | 0.41  5842 | 0.831  683 | 0.5 | 1.19  573 | 16  8 |
| {BcMV6.BI=FALSE, BcULV5=FALSE} =>  {BcMV2.BI=FALSE} | 0.41  5842 | 0.669  323 | 0.621  287 | 1.24  6112 | 16  8 |
| {BcBNV5.BI=FALSE, BcMV2.BI=FALSE} =>  {BcMV6.BI=FALSE} | 0.41  3366 | 0.826  733 | 0.5 | 1.18  8612 | 16  7 |
| {BcBNV5.BI=FALSE, BcMV6.BI=FALSE} =>  {BcMV2.BI=FALSE} | 0.41  3366 | 0.623  134 | 0.663  366 | 1.16  0121 | 16  7 |
| {BcMV2.BI=FALSE, BvF.BI=FALSE} =>  {BcMV6.BI=FALSE} | 0.38  8614 | 0.809  278 | 0.480  198 | 1.16  3518 | 15  7 |
| {BcMV6.BI=FALSE, BvF.BI=FALSE} =>  {BcMV2.BI=FALSE} | 0.38  8614 | 0.623  016 | 0.623  762 | 1.15  9901 | 15  7 |
| {BcMCV5.BI=FALSE, BcMV2.BI=FALSE} =>  {BcMV6.BI=FALSE} | 0.43  0693 | 0.824  645 | 0.522  277 | 1.18  561 | 17  4 |

| {BcMCV5.BI=FALSE, BcMV6.BI=FALSE} =>  {BcMV2.BI=FALSE} | 0.43  0693 | 0.649  254 | 0.663  366 | 1.20  8749 | 17  4 |
| --- | --- | --- | --- | --- | --- |
| {BcMV2.BI=FALSE, BcOLV13.BI=FALSE} =>  {BcMV6.BI=FALSE} | 0.42  3267 | 0.818  182 | 0.517  327 | 1.17  6318 | 17  1 |
| {BcMV6.BI=FALSE, BcOLV13.BI=FALSE} =>  {BcMV2.BI=FALSE} | 0.42  3267 | 0.645  283 | 0.655  941 | 1.20  1356 | 17  1 |
| {BcMV2.BI=FALSE, BcOLV2.BI=FALSE} =>  {BcMV6.BI=FALSE} | 0.41  8317 | 0.824  39 | 0.507  426 | 1.18  5244 | 16  9 |
| {BcMV6.BI=FALSE, BcOLV2.BI=FALSE} =>  {BcMV2.BI=FALSE} | 0.41  8317 | 0.628  253 | 0.665  842 | 1.16  965 | 16  9 |
| {BcMCV3.BI=FALSE, BcMV2.BI=FALSE} =>  {BcMV6.BI=FALSE} | 0.43  3168 | 0.825  472 | 0.524  752 | 1.18  6799 | 17  5 |
| {BcMCV3.BI=FALSE, BcMV6.BI=FALSE} =>  {BcMV2.BI=FALSE} | 0.43  3168 | 0.643  382 | 0.673  267 | 1.19  7818 | 17  5 |
| {BcMV2.BI=FALSE, BcPV4.BI=FALSE} =>  {BcMV6.BI=FALSE} | 0.41  8317 | 0.828  431 | 0.504  95 | 1.19  1054 | 16  9 |
| {BcMV6.BI=FALSE, BcPV4.BI=FALSE} =>  {BcMV2.BI=FALSE} | 0.41  8317 | 0.637  736 | 0.655  941 | 1.18  7305 | 16  9 |
| {BcEV6=FALSE, BcMV2.BI=FALSE} =>  {BcMV6.BI=FALSE} | 0.41  3366 | 0.822  66 | 0.502  475 | 1.18  2757 | 16  7 |
| {BcEV6=FALSE, BcMV6.BI=FALSE} =>  {BcMV2.BI=FALSE} | 0.41  3366 | 0.632  576 | 0.653  465 | 1.17  7699 | 16  7 |

| {BcMV2.BI=FALSE, BcOLV17.BI=FALSE} =>  {BcMV6.BI=FALSE} | 0.43  0693 | 0.824  645 | 0.522  277 | 1.18  561 | 17  4 |
| --- | --- | --- | --- | --- | --- |
| {BcMV6.BI=FALSE, BcOLV17.BI=FALSE} =>  {BcMV2.BI=FALSE} | 0.43  0693 | 0.642  066 | 0.670  792 | 1.19  5368 | 17  4 |
| {BcMV2.BI=FALSE, BcOLV15.BI=FALSE} =>  {BcMV6.BI=FALSE} | 0.40  3465 | 0.810  945 | 0.497  525 | 1.16  5914 | 16  3 |
| {BcMV6.BI=FALSE, BcOLV15.BI=FALSE} =>  {BcMV2.BI=FALSE} | 0.40  3465 | 0.619  772 | 0.650  99 | 1.15  3861 | 16  3 |
| {BcEV5=FALSE, BcMV2.BI=FALSE} =>  {BcMV6.BI=FALSE} | 0.43  3168 | 0.821  596 | 0.527  228 | 1.18  1227 | 17  5 |
| {BcEV5=FALSE, BcMV6.BI=FALSE} =>  {BcMV2.BI=FALSE} | 0.43  3168 | 0.631  769 | 0.685  644 | 1.17  6197 | 17  5 |
| {BcMV2.BI=FALSE, BcPV3.BI=FALSE} =>  {BcRV1.BI=FALSE} | 0.35  6436 | 0.837  209 | 0.425  743 | 1.15  8331 | 14  4 |
| {BcMV2.BI=FALSE, BcRV1.BI=FALSE} =>  {BcPV3.BI=FALSE} | 0.35  6436 | 0.813  559 | 0.438  119 | 1.14  5219 | 14  4 |
| {BcPV3.BI=FALSE, BcRV1.BI=FALSE} =>  {BcMV2.BI=FALSE} | 0.35  6436 | 0.637  168 | 0.559  406 | 1.18  6249 | 14  4 |
| {BcEV2.BI=FALSE, BcMV2.BI=FALSE} =>  {BcPV3.BI=FALSE} | 0.33  6634 | 0.786  127 | 0.428  218 | 1.10  6604 | 13  6 |
| {BcEV2.BI=FALSE, BcPV3.BI=FALSE} =>  {BcMV2.BI=FALSE} | 0.33  6634 | 0.621  005 | 0.542  079 | 1.15  6156 | 13  6 |

| {BcHV3.BI=FALSE, BcMV2.BI=FALSE} =>  {BcPV3.BI=FALSE} | 0.35  1485 | 0.816  092 | 0.430  693 | 1.14  8784 | 14  2 |
| --- | --- | --- | --- | --- | --- |
| {BcHV3.BI=FALSE, BcPV3.BI=FALSE} =>  {BcMV2.BI=FALSE} | 0.35  1485 | 0.636  771 | 0.551  98 | 1.18  551 | 14  2 |
| {BcFV5.BI=FALSE, BcPV3.BI=FALSE} =>  {BcMV2.BI=FALSE} | 0.33  9109 | 0.619  91 | 0.547  03 | 1.15  4117 | 13  7 |
| {BcMV2.BI=FALSE, BcPV3.BI=FALSE} =>  {BcULV1.BI=FALSE} | 0.36  6337 | 0.860  465 | 0.425  743 | 1.09  6618 | 14  8 |
| {BcMV2.BI=FALSE, BcULV1.BI=FALSE} =>  {BcPV3.BI=FALSE} | 0.36  6337 | 0.795  699 | 0.460  396 | 1.12  0078 | 14  8 |
| {BcPV3.BI=FALSE, BcULV1.BI=FALSE} =>  {BcMV2.BI=FALSE} | 0.36  6337 | 0.640  693 | 0.571  782 | 1.19  281 | 14  8 |
| {BcMV12=FALSE, BcMV2.BI=FALSE} =>  {BcPV3.BI=FALSE} | 0.36  6337 | 0.8 | 0.457  921 | 1.12  6132 | 14  8 |
| {BcMV12=FALSE, BcPV3.BI=FALSE} =>  {BcMV2.BI=FALSE} | 0.36  6337 | 0.629  787 | 0.581  683 | 1.17  2507 | 14  8 |
| {BcNSRV7.BI=FALSE, BcPV3.BI=FALSE} =>  {BcMV2.BI=FALSE} | 0.34  1584 | 0.607  93 | 0.561  881 | 1.13  1813 | 13  8 |
| {BcMV2.BI=FALSE, BcVV3.BI=FALSE} =>  {BcPV3.BI=FALSE} | 0.36  3861 | 0.807  692 | 0.450  495 | 1.13  6961 | 14  7 |
| {BcPV3.BI=FALSE, BcVV3.BI=FALSE} =>  {BcMV2.BI=FALSE} | 0.36  3861 | 0.622  881 | 0.584  158 | 1.15  965 | 14  7 |

| {BcMV2.BI=FALSE, BcOLV7.BI=FALSE} =>  {BcPV3.BI=FALSE} | 0.36  3861 | 0.812  155 | 0.448  02 | 1.14  3242 | 14  7 |
| --- | --- | --- | --- | --- | --- |
| {BcOLV7.BI=FALSE, BcPV3.BI=FALSE} =>  {BcMV2.BI=FALSE} | 0.36  3861 | 0.607  438 | 0.599  01 | 1.13  0898 | 14  7 |
| {BcMV2.BI=FALSE, BcPV3.BI=FALSE} =>  {BcALV1.BI=FALSE} | 0.38  6139 | 0.906  977 | 0.425  743 | 1.11  3734 | 15  6 |
| {BcALV1.BI=FALSE, BcMV2.BI=FALSE} =>  {BcPV3.BI=FALSE} | 0.38  6139 | 0.787  879 | 0.490  099 | 1.10  907 | 15  6 |
| {BcALV1.BI=FALSE, BcPV3.BI=FALSE} =>  {BcMV2.BI=FALSE} | 0.38  6139 | 0.661  017 | 0.584  158 | 1.23  0649 | 15  6 |
| {BcDMV1=FALSE, BcMV2.BI=FALSE} =>  {BcPV3.BI=FALSE} | 0.35  1485 | 0.784  53 | 0.448  02 | 1.10  4356 | 14  2 |
| {BcFV9=FALSE, BcMV2.BI=FALSE} =>  {BcPV3.BI=FALSE} | 0.37  3762 | 0.798  942 | 0.467  822 | 1.12  4643 | 15  1 |
| {BcFV9=FALSE, BcPV3.BI=FALSE} =>  {BcMV2.BI=FALSE} | 0.37  3762 | 0.599  206 | 0.623  762 | 1.11  5573 | 15  1 |
| {BcMV2.BI=FALSE, BcPV3.BI=FALSE} =>  {BcOLV4.BI=FALSE} | 0.39  8515 | 0.936  047 | 0.425  743 | 1.06  8256 | 16  1 |
| {BcMV2.BI=FALSE, BcOLV4.BI=FALSE} =>  {BcPV3.BI=FALSE} | 0.39  8515 | 0.797  03 | 0.5 | 1.12  1951 | 16  1 |
| {BcOLV4.BI=FALSE, BcPV3.BI=FALSE} =>  {BcMV2.BI=FALSE} | 0.39  8515 | 0.624  031 | 0.638  614 | 1.16  179 | 16  1 |

| {BcMV2.BI=FALSE, BcPV3.BI=FALSE} =>  {BcULV5=FALSE} | 0.39  8515 | 0.936  047 | 0.425  743 | 1.06  8256 | 16  1 |
| --- | --- | --- | --- | --- | --- |
| {BcMV2.BI=FALSE, BcULV5=FALSE} =>  {BcPV3.BI=FALSE} | 0.39  8515 | 0.797  03 | 0.5 | 1.12  1951 | 16  1 |
| {BcPV3.BI=FALSE, BcULV5=FALSE} =>  {BcMV2.BI=FALSE} | 0.39  8515 | 0.624  031 | 0.638  614 | 1.16  179 | 16  1 |
| {BcBNV5.BI=FALSE, BcMV2.BI=FALSE} =>  {BcPV3.BI=FALSE} | 0.39  3564 | 0.787  129 | 0.5 | 1.10  8014 | 15  9 |
| {BcBNV5.BI=FALSE, BcPV3.BI=FALSE} =>  {BcMV2.BI=FALSE} | 0.39  3564 | 0.611  538 | 0.643  564 | 1.13  8532 | 15  9 |
| {BcMV2.BI=FALSE, BvF.BI=FALSE} =>  {BcPV3.BI=FALSE} | 0.37  6238 | 0.783  505 | 0.480  198 | 1.10  2913 | 15  2 |
| {BcMCV5.BI=FALSE, BcMV2.BI=FALSE} =>  {BcPV3.BI=FALSE} | 0.41  3366 | 0.791  469 | 0.522  277 | 1.11  4124 | 16  7 |
| {BcMCV5.BI=FALSE, BcPV3.BI=FALSE} =>  {BcMV2.BI=FALSE} | 0.41  3366 | 0.627  82 | 0.658  416 | 1.16  8844 | 16  7 |
| {BcMV2.BI=FALSE, BcOLV13.BI=FALSE} =>  {BcPV3.BI=FALSE} | 0.41  0891 | 0.794  258 | 0.517  327 | 1.11  805 | 16  6 |
| {BcOLV13.BI=FALSE, BcPV3.BI=FALSE} =>  {BcMV2.BI=FALSE} | 0.41  0891 | 0.605  839 | 0.678  218 | 1.12  7922 | 16  6 |
| {BcMV2.BI=FALSE, BcOLV2.BI=FALSE} =>  {BcPV3.BI=FALSE} | 0.39  8515 | 0.785  366 | 0.507  426 | 1.10  5532 | 16  1 |

| {BcOLV2.BI=FALSE, BcPV3.BI=FALSE} =>  {BcMV2.BI=FALSE} | 0.39  8515 | 0.598  513 | 0.665  842 | 1.11  4282 | 16  1 |
| --- | --- | --- | --- | --- | --- |
| {BcMCV3.BI=FALSE, BcMV2.BI=FALSE} =>  {BcPV3.BI=FALSE} | 0.41  8317 | 0.797  17 | 0.524  752 | 1.12  2148 | 16  9 |
| {BcMCV3.BI=FALSE, BcPV3.BI=FALSE} =>  {BcMV2.BI=FALSE} | 0.41  8317 | 0.614  545 | 0.680  693 | 1.14  4131 | 16  9 |
| {BcMV2.BI=FALSE, BcPV4.BI=FALSE} =>  {BcPV3.BI=FALSE} | 0.39  604 | 0.784  314 | 0.504  95 | 1.10  4051 | 16  0 |
| {BcPV3.BI=FALSE, BcPV4.BI=FALSE} =>  {BcMV2.BI=FALSE} | 0.39  604 | 0.599  251 | 0.660  891 | 1.11  5656 | 16  0 |
| {BcEV6=FALSE, BcMV2.BI=FALSE} =>  {BcPV3.BI=FALSE} | 0.39  604 | 0.788  177 | 0.502  475 | 1.10  949 | 16  0 |
| {BcEV6=FALSE, BcPV3.BI=FALSE} =>  {BcMV2.BI=FALSE} | 0.39  604 | 0.599  251 | 0.660  891 | 1.11  5656 | 16  0 |
| {BcMV2.BI=FALSE, BcOLV17.BI=FALSE} =>  {BcPV3.BI=FALSE} | 0.41  8317 | 0.800  948 | 0.522  277 | 1.12  7467 | 16  9 |
| {BcOLV17.BI=FALSE, BcPV3.BI=FALSE} =>  {BcMV2.BI=FALSE} | 0.41  8317 | 0.605  735 | 0.690  594 | 1.12  7727 | 16  9 |
| {BcMV2.BI=FALSE, BcOLV15.BI=FALSE} =>  {BcPV3.BI=FALSE} | 0.39  1089 | 0.786  07 | 0.497  525 | 1.10  6523 | 15  8 |
| {BcEV5=FALSE, BcMV2.BI=FALSE} =>  {BcPV3.BI=FALSE} | 0.41  8317 | 0.793  427 | 0.527  228 | 1.11  688 | 16  9 |

| {BcEV5=FALSE, BcPV3.BI=FALSE} =>  {BcMV2.BI=FALSE} | 0.41  8317 | 0.621  324 | 0.673  267 | 1.15  675 | 16  9 |
| --- | --- | --- | --- | --- | --- |
| {BcEV2.BI=FALSE, BcMV2.BI=FALSE} =>  {BcRV1.BI=FALSE} | 0.34  1584 | 0.797  688 | 0.428  218 | 1.10  365 | 13  8 |
| {BcEV2.BI=FALSE, BcRV1.BI=FALSE} =>  {BcMV2.BI=FALSE} | 0.34  1584 | 0.638  889 | 0.534  653 | 1.18  9452 | 13  8 |
| {BcHV3.BI=FALSE, BcMV2.BI=FALSE} =>  {BcRV1.BI=FALSE} | 0.35  396 | 0.821  839 | 0.430  693 | 1.13  7065 | 14  3 |
| {BcHV3.BI=FALSE, BcRV1.BI=FALSE} =>  {BcMV2.BI=FALSE} | 0.35  396 | 0.647  059 | 0.547  03 | 1.20  4663 | 14  3 |
| {BcFV5.BI=FALSE, BcRV1.BI=FALSE} =>  {BcMV2.BI=FALSE} | 0.34  1584 | 0.627  273 | 0.544  554 | 1.16  7826 | 13  8 |
| {BcMV2.BI=FALSE, BcRV1.BI=FALSE} =>  {BcULV1.BI=FALSE} | 0.37  8713 | 0.864  407 | 0.438  119 | 1.10  1641 | 15  3 |
| {BcMV2.BI=FALSE, BcULV1.BI=FALSE} =>  {BcRV1.BI=FALSE} | 0.37  8713 | 0.822  581 | 0.460  396 | 1.13  8091 | 15  3 |
| {BcRV1.BI=FALSE, BcULV1.BI=FALSE} =>  {BcMV2.BI=FALSE} | 0.37  8713 | 0.648  305 | 0.584  158 | 1.20  6983 | 15  3 |
| {BcMV1.BI=TRUE, BcMV2.BI=FALSE} =>  {BcRV1.BI=FALSE} | 0.32  6733 | 0.857  143 | 0.381  188 | 1.18  591 | 13  2 |
| {BcMV12=FALSE, BcMV2.BI=FALSE} =>  {BcRV1.BI=FALSE} | 0.37  3762 | 0.816  216 | 0.457  921 | 1.12  9285 | 15  1 |

| {BcMV12=FALSE, BcRV1.BI=FALSE} =>  {BcMV2.BI=FALSE} | 0.37  3762 | 0.645  299 | 0.579  208 | 1.20  1386 | 15  1 |
| --- | --- | --- | --- | --- | --- |
| {BcMV2.BI=FALSE, BcNSRV7.BI=FALSE} =>  {BcRV1.BI=FALSE} | 0.34  6535 | 0.790  96 | 0.438  119 | 1.09  4343 | 14  0 |
| {BcNSRV7.BI=FALSE, BcRV1.BI=FALSE} =>  {BcMV2.BI=FALSE} | 0.34  6535 | 0.619  469 | 0.559  406 | 1.15  3297 | 14  0 |
| {BcMV2.BI=FALSE, BcVV3.BI=FALSE} =>  {BcRV1.BI=FALSE} | 0.37  1287 | 0.824  176 | 0.450  495 | 1.14  0298 | 15  0 |
| {BcRV1.BI=FALSE, BcVV3.BI=FALSE} =>  {BcMV2.BI=FALSE} | 0.37  1287 | 0.652  174 | 0.569  307 | 1.21  4186 | 15  0 |
| {BcMV2.BI=FALSE, BcRV1.BI=FALSE} =>  {BcOLV7.BI=FALSE} | 0.38  3663 | 0.875  706 | 0.438  119 | 1.08  5231 | 15  5 |
| {BcMV2.BI=FALSE, BcOLV7.BI=FALSE} =>  {BcRV1.BI=FALSE} | 0.38  3663 | 0.856  354 | 0.448  02 | 1.18  4818 | 15  5 |
| {BcOLV7.BI=FALSE, BcRV1.BI=FALSE} =>  {BcMV2.BI=FALSE} | 0.38  3663 | 0.600  775 | 0.638  614 | 1.11  8494 | 15  5 |
| {BcMV2.BI=FALSE, BcRV1.BI=FALSE} =>  {BcALV1.BI=FALSE} | 0.39  8515 | 0.909  605 | 0.438  119 | 1.11  6961 | 16  1 |
| {BcALV1.BI=FALSE, BcMV2.BI=FALSE} =>  {BcRV1.BI=FALSE} | 0.39  8515 | 0.813  131 | 0.490  099 | 1.12  5017 | 16  1 |
| {BcALV1.BI=FALSE, BcRV1.BI=FALSE} =>  {BcMV2.BI=FALSE} | 0.39  8515 | 0.638  889 | 0.623  762 | 1.18  9452 | 16  1 |

| {BcDMV1=FALSE, BcMV2.BI=FALSE} =>  {BcRV1.BI=FALSE} | 0.35  8911 | 0.801  105 | 0.448  02 | 1.10  8378 | 14  5 |
| --- | --- | --- | --- | --- | --- |
| {BcDMV1=FALSE, BcRV1.BI=FALSE} =>  {BcMV2.BI=FALSE} | 0.35  8911 | 0.611  814 | 0.586  634 | 1.13  9046 | 14  5 |
| {BcFV9=FALSE, BcMV2.BI=FALSE} =>  {BcRV1.BI=FALSE} | 0.37  3762 | 0.798  942 | 0.467  822 | 1.10  5385 | 15  1 |
| {BcMV2.BI=FALSE, BcOLV4.BI=FALSE} =>  {BcRV1.BI=FALSE} | 0.40  3465 | 0.806  931 | 0.5 | 1.11  6438 | 16  3 |
| {BcOLV4.BI=FALSE, BcRV1.BI=FALSE} =>  {BcMV2.BI=FALSE} | 0.40  3465 | 0.639  216 | 0.631  188 | 1.19  0061 | 16  3 |
| {BcMV2.BI=FALSE, BcULV5=FALSE} =>  {BcRV1.BI=FALSE} | 0.40  3465 | 0.806  931 | 0.5 | 1.11  6438 | 16  3 |
| {BcRV1.BI=FALSE, BcULV5=FALSE} =>  {BcMV2.BI=FALSE} | 0.40  3465 | 0.641  732 | 0.628  713 | 1.19  4746 | 16  3 |
| {BcBNV5.BI=FALSE, BcMV2.BI=FALSE} =>  {BcRV1.BI=FALSE} | 0.40  5941 | 0.811  881 | 0.5 | 1.12  3288 | 16  4 |
| {BcBNV5.BI=FALSE, BcRV1.BI=FALSE} =>  {BcMV2.BI=FALSE} | 0.40  5941 | 0.625  954 | 0.648  515 | 1.16  5371 | 16  4 |
| {BcMV2.BI=FALSE, BvF.BI=FALSE} =>  {BcRV1.BI=FALSE} | 0.38  3663 | 0.798  969 | 0.480  198 | 1.10  5423 | 15  5 |
| {BcRV1.BI=FALSE, BvF.BI=FALSE} =>  {BcMV2.BI=FALSE} | 0.38  3663 | 0.593  87 | 0.646  04 | 1.10  5638 | 15  5 |

| {BcMCV5.BI=FALSE, BcMV2.BI=FALSE} =>  {BcRV1.BI=FALSE} | 0.42  8218 | 0.819  905 | 0.522  277 | 1.13  4389 | 17  3 |
| --- | --- | --- | --- | --- | --- |
| {BcMCV5.BI=FALSE, BcRV1.BI=FALSE} =>  {BcMV2.BI=FALSE} | 0.42  8218 | 0.636  029 | 0.673  267 | 1.18  4128 | 17  3 |
| {BcMV2.BI=FALSE, BcOLV13.BI=FALSE} =>  {BcRV1.BI=FALSE} | 0.42  8218 | 0.827  751 | 0.517  327 | 1.14  5245 | 17  3 |
| {BcOLV13.BI=FALSE, BcRV1.BI=FALSE} =>  {BcMV2.BI=FALSE} | 0.42  8218 | 0.620  072 | 0.690  594 | 1.15  4419 | 17  3 |
| {BcMV2.BI=FALSE, BcOLV2.BI=FALSE} =>  {BcRV1.BI=FALSE} | 0.41  3366 | 0.814  634 | 0.507  426 | 1.12  7097 | 16  7 |
| {BcOLV2.BI=FALSE, BcRV1.BI=FALSE} =>  {BcMV2.BI=FALSE} | 0.41  3366 | 0.605  072 | 0.683  168 | 1.12  6494 | 16  7 |
| {BcMCV3.BI=FALSE, BcMV2.BI=FALSE} =>  {BcRV1.BI=FALSE} | 0.42  8218 | 0.816  038 | 0.524  752 | 1.12  9039 | 17  3 |
| {BcMCV3.BI=FALSE, BcRV1.BI=FALSE} =>  {BcMV2.BI=FALSE} | 0.42  8218 | 0.629  091 | 0.680  693 | 1.17  1211 | 17  3 |
| {BcMV2.BI=FALSE, BcPV4.BI=FALSE} =>  {BcRV1.BI=FALSE} | 0.40  8416 | 0.808  824 | 0.504  95 | 1.11  9057 | 16  5 |
| {BcPV4.BI=FALSE, BcRV1.BI=FALSE} =>  {BcMV2.BI=FALSE} | 0.40  8416 | 0.608  856 | 0.670  792 | 1.13  3539 | 16  5 |
| {BcEV6=FALSE, BcMV2.BI=FALSE} =>  {BcRV1.BI=FALSE} | 0.40  5941 | 0.807  882 | 0.502  475 | 1.11  7754 | 16  4 |

| {BcEV6=FALSE, BcRV1.BI=FALSE} =>  {BcMV2.BI=FALSE} | 0.40  5941 | 0.607  407 | 0.668  317 | 1.13  0841 | 16  4 |
| --- | --- | --- | --- | --- | --- |
| {BcMV2.BI=FALSE, BcOLV17.BI=FALSE} =>  {BcRV1.BI=FALSE} | 0.43  0693 | 0.824  645 | 0.522  277 | 1.14  0947 | 17  4 |
| {BcOLV17.BI=FALSE, BcRV1.BI=FALSE} =>  {BcMV2.BI=FALSE} | 0.43  0693 | 0.608  392 | 0.707  921 | 1.13  2674 | 17  4 |
| {BcMV2.BI=FALSE, BcOLV15.BI=FALSE} =>  {BcRV1.BI=FALSE} | 0.39  8515 | 0.800  995 | 0.497  525 | 1.10  8226 | 16  1 |
| {BcEV5=FALSE, BcMV2.BI=FALSE} =>  {BcRV1.BI=FALSE} | 0.42  8218 | 0.812  207 | 0.527  228 | 1.12  3738 | 17  3 |
| {BcEV5=FALSE, BcRV1.BI=FALSE} =>  {BcMV2.BI=FALSE} | 0.42  8218 | 0.636  029 | 0.673  267 | 1.18  4128 | 17  3 |
| {BcEV2.BI=FALSE, BcMV2.BI=FALSE} =>  {BcFV5.BI=FALSE} | 0.36  8812 | 0.861  272 | 0.428  218 | 1.10  1119 | 14  9 |
| {BcFV5.BI=FALSE, BcMV2.BI=FALSE} =>  {BcEV2.BI=FALSE} | 0.36  8812 | 0.841  808 | 0.438  119 | 1.12  6127 | 14  9 |
| {BcEV2.BI=FALSE, BcFV5.BI=FALSE} =>  {BcMV2.BI=FALSE} | 0.36  8812 | 0.593  625 | 0.621  287 | 1.10  5183 | 14  9 |
| {BcEV2.BI=FALSE, BcMV2.BI=FALSE} =>  {BcULV1.BI=FALSE} | 0.37  1287 | 0.867  052 | 0.428  218 | 1.10  5013 | 15  0 |
| {BcEV2.BI=FALSE, BcULV1.BI=FALSE} =>  {BcMV2.BI=FALSE} | 0.37  1287 | 0.630  252 | 0.589  109 | 1.17  3373 | 15  0 |

| {BcEV2.BI=FALSE, BcMV12=FALSE} =>  {BcMV2.BI=FALSE} | 0.36  1386 | 0.621  277 | 0.581  683 | 1.15  6662 | 14  6 |
| --- | --- | --- | --- | --- | --- |
| {BcMV2.BI=FALSE, BcNSRV7.BI=FALSE} =>  {BcEV2.BI=FALSE} | 0.36  8812 | 0.841  808 | 0.438  119 | 1.12  6127 | 14  9 |
| {BcEV2.BI=FALSE, BcNSRV7.BI=FALSE} =>  {BcMV2.BI=FALSE} | 0.36  8812 | 0.598  394 | 0.616  337 | 1.11  406 | 14  9 |
| {BcEV2.BI=FALSE, BcMV2.BI=FALSE} =>  {BcALV1.BI=FALSE} | 0.38  8614 | 0.907  514 | 0.428  218 | 1.11  4395 | 15  7 |
| {BcALV1.BI=FALSE, BcEV2.BI=FALSE} =>  {BcMV2.BI=FALSE} | 0.38  8614 | 0.630  522 | 0.616  337 | 1.17  3875 | 15  7 |
| {BcFV9=FALSE, BcMV2.BI=FALSE} =>  {BcEV2.BI=FALSE} | 0.39  1089 | 0.835  979 | 0.467  822 | 1.11  8329 | 15  8 |
| {BcEV2.BI=FALSE, BcMV2.BI=FALSE} =>  {BcOLV4.BI=FALSE} | 0.39  8515 | 0.930  636 | 0.428  218 | 1.06  2082 | 16  1 |
| {BcEV2.BI=FALSE, BcOLV4.BI=FALSE} =>  {BcMV2.BI=FALSE} | 0.39  8515 | 0.598  513 | 0.665  842 | 1.11  4282 | 16  1 |
| {BcEV2.BI=FALSE, BcMV2.BI=FALSE} =>  {BcULV5=FALSE} | 0.40  3465 | 0.942  197 | 0.428  218 | 1.07  5275 | 16  3 |
| {BcMV2.BI=FALSE, BcULV5=FALSE} =>  {BcEV2.BI=FALSE} | 0.40  3465 | 0.806  931 | 0.5 | 1.07  947 | 16  3 |
| {BcEV2.BI=FALSE, BcULV5=FALSE} =>  {BcMV2.BI=FALSE} | 0.40  3465 | 0.615  094 | 0.655  941 | 1.14  5153 | 16  3 |

| {BcBNV5.BI=FALSE, BcMV2.BI=FALSE} =>  {BcEV2.BI=FALSE} | 0.40  8416 | 0.816  832 | 0.5 | 1.09  2715 | 16  5 |
| --- | --- | --- | --- | --- | --- |
| {BcBNV5.BI=FALSE, BcEV2.BI=FALSE} =>  {BcMV2.BI=FALSE} | 0.40  8416 | 0.589  286 | 0.693  069 | 1.09  7103 | 16  5 |
| {BcMV2.BI=FALSE, BvF.BI=FALSE} =>  {BcEV2.BI=FALSE} | 0.39  1089 | 0.814  433 | 0.480  198 | 1.08  9506 | 15  8 |
| {BcEV2.BI=FALSE, BcMCV5.BI=FALSE} =>  {BcMV2.BI=FALSE} | 0.41  8317 | 0.590  909 | 0.707  921 | 1.10  0126 | 16  9 |
| {BcMV2.BI=FALSE, BcOLV13.BI=FALSE} =>  {BcEV2.BI=FALSE} | 0.41  5842 | 0.803  828 | 0.517  327 | 1.07  5319 | 16  8 |
| {BcEV2.BI=FALSE, BcOLV13.BI=FALSE} =>  {BcMV2.BI=FALSE} | 0.41  5842 | 0.595  745 | 0.698  02 | 1.10  9128 | 16  8 |
| {BcMV2.BI=FALSE, BcOLV2.BI=FALSE} =>  {BcEV2.BI=FALSE} | 0.40  8416 | 0.804  878 | 0.507  426 | 1.07  6724 | 16  5 |
| {BcEV2.BI=FALSE, BcOLV2.BI=FALSE} =>  {BcMV2.BI=FALSE} | 0.40  8416 | 0.589  286 | 0.693  069 | 1.09  7103 | 16  5 |
| {BcEV2.BI=FALSE, BcMCV3.BI=FALSE} =>  {BcMV2.BI=FALSE} | 0.41  8317 | 0.599  291 | 0.698  02 | 1.11  573 | 16  9 |
| {BcMV2.BI=FALSE, BcOLV15.BI=FALSE} =>  {BcEV2.BI=FALSE} | 0.41  5842 | 0.835  821 | 0.497  525 | 1.11  8118 | 16  8 |
| {BcEV2.BI=FALSE, BcEV5=FALSE} =>  {BcMV2.BI=FALSE} | 0.41  8317 | 0.586  806 | 0.712  871 | 1.09  2486 | 16  9 |

| {BcHV3.BI=FALSE, BcMV2.BI=FALSE} =>  {BcFV5.BI=FALSE} | 0.37  1287 | 0.862  069 | 0.430  693 | 1.10  2139 | 15  0 |
| --- | --- | --- | --- | --- | --- |
| {BcFV5.BI=FALSE, BcMV2.BI=FALSE} =>  {BcHV3.BI=FALSE} | 0.37  1287 | 0.847  458 | 0.438  119 | 1.11  5221 | 15  0 |
| {BcHV3.BI=FALSE, BcMV2.BI=FALSE} =>  {BcULV1.BI=FALSE} | 0.36  6337 | 0.850  575 | 0.430  693 | 1.08  4013 | 14  8 |
| {BcHV3.BI=FALSE, BcULV1.BI=FALSE} =>  {BcMV2.BI=FALSE} | 0.36  6337 | 0.606  557 | 0.603  96 | 1.12  9259 | 14  8 |
| {BcHV3.BI=FALSE, BcMV12=FALSE} =>  {BcMV2.BI=FALSE} | 0.36  1386 | 0.600  823 | 0.601  485 | 1.11  8583 | 14  6 |
| {BcMV2.BI=FALSE, BcNSRV7.BI=FALSE} =>  {BcHV3.BI=FALSE} | 0.36  8812 | 0.841  808 | 0.438  119 | 1.10  7786 | 14  9 |
| {BcMV2.BI=FALSE, BcOLV7.BI=FALSE} =>  {BcHV3.BI=FALSE} | 0.36  8812 | 0.823  204 | 0.448  02 | 1.08  3305 | 14  9 |
| {BcHV3.BI=FALSE, BcMV2.BI=FALSE} =>  {BcALV1.BI=FALSE} | 0.39  3564 | 0.913  793 | 0.430  693 | 1.12  2105 | 15  9 |
| {BcALV1.BI=FALSE, BcHV3.BI=FALSE} =>  {BcMV2.BI=FALSE} | 0.39  3564 | 0.621  094 | 0.633  663 | 1.15  6322 | 15  9 |
| {BcDMV1=FALSE, BcMV2.BI=FALSE} =>  {BcHV3.BI=FALSE} | 0.37  1287 | 0.828  729 | 0.448  02 | 1.09  0575 | 15  0 |
| {BcHV3.BI=FALSE, BcMV2.BI=FALSE} =>  {BcOLV4.BI=FALSE} | 0.40  3465 | 0.936  782 | 0.430  693 | 1.06  9095 | 16  3 |

| {BcHV3.BI=FALSE, BcOLV4.BI=FALSE} =>  {BcMV2.BI=FALSE} | 0.40  3465 | 0.599  265 | 0.673  267 | 1.11  5682 | 16  3 |
| --- | --- | --- | --- | --- | --- |
| {BcHV3.BI=FALSE, BcMV2.BI=FALSE} =>  {BcULV5=FALSE} | 0.40  5941 | 0.942  529 | 0.430  693 | 1.07  5654 | 16  4 |
| {BcHV3.BI=FALSE, BcULV5=FALSE} =>  {BcMV2.BI=FALSE} | 0.40  5941 | 0.607  407 | 0.668  317 | 1.13  0841 | 16  4 |
| {BcHV3.BI=FALSE, BcOLV13.BI=FALSE} =>  {BcMV2.BI=FALSE} | 0.42  0792 | 0.592  334 | 0.710  396 | 1.10  2779 | 17  0 |
| {BcHV3.BI=FALSE, BcMCV3.BI=FALSE} =>  {BcMV2.BI=FALSE} | 0.41  8317 | 0.601  423 | 0.695  545 | 1.11  9701 | 16  9 |
| {BcMV2.BI=FALSE, BcOLV15.BI=FALSE} =>  {BcHV3.BI=FALSE} | 0.41  0891 | 0.825  871 | 0.497  525 | 1.08  6813 | 16  6 |
| {BcFV5.BI=FALSE, BcMV2.BI=FALSE} =>  {BcULV1.BI=FALSE} | 0.37  3762 | 0.853  107 | 0.438  119 | 1.08  7241 | 15  1 |
| {BcFV5.BI=FALSE, BcULV1.BI=FALSE} =>  {BcMV2.BI=FALSE} | 0.37  3762 | 0.611  336 | 0.611  386 | 1.13  8156 | 15  1 |
| {BcFV5.BI=FALSE, BcMV12=FALSE} =>  {BcMV2.BI=FALSE} | 0.37  3762 | 0.596  838 | 0.626  238 | 1.11  1164 | 15  1 |
| {BcFV5.BI=FALSE, BcMV2.BI=FALSE} =>  {BcNSRV7.BI=FALSE} | 0.39  1089 | 0.892  655 | 0.438  119 | 1.11  651 | 15  8 |
| {BcMV2.BI=FALSE, BcNSRV7.BI=FALSE} =>  {BcFV5.BI=FALSE} | 0.39  1089 | 0.892  655 | 0.438  119 | 1.14  1243 | 15  8 |

| {BcFV5.BI=FALSE, BcMV2.BI=FALSE} =>  {BcALV1.BI=FALSE} | 0.40  099 | 0.915  254 | 0.438  119 | 1.12  3899 | 16  2 |
| --- | --- | --- | --- | --- | --- |
| {BcALV1.BI=FALSE, BcFV5.BI=FALSE} =>  {BcMV2.BI=FALSE} | 0.40  099 | 0.602  23 | 0.665  842 | 1.12  1203 | 16  2 |
| {BcDMV1=FALSE, BcMV2.BI=FALSE} =>  {BcFV5.BI=FALSE} | 0.38  1188 | 0.850  829 | 0.448  02 | 1.08  7768 | 15  4 |
| {BcFV9=FALSE, BcMV2.BI=FALSE} =>  {BcFV5.BI=FALSE} | 0.39  8515 | 0.851  852 | 0.467  822 | 1.08  9076 | 16  1 |
| {BcFV5.BI=FALSE, BcOLV4.BI=FALSE} =>  {BcMV2.BI=FALSE} | 0.40  3465 | 0.592  727 | 0.680  693 | 1.10  3511 | 16  3 |
| {BcFV5.BI=FALSE, BcMV2.BI=FALSE} =>  {BcULV5=FALSE} | 0.40  8416 | 0.932  203 | 0.438  119 | 1.06  3871 | 16  5 |
| {BcFV5.BI=FALSE, BcULV5=FALSE} =>  {BcMV2.BI=FALSE} | 0.40  8416 | 0.6 | 0.680  693 | 1.11  7051 | 16  5 |
| {BcBNV5.BI=FALSE, BcMV2.BI=FALSE} =>  {BcFV5.BI=FALSE} | 0.41  8317 | 0.836  634 | 0.5 | 1.06  962 | 16  9 |
| {BcFV5.BI=FALSE, BcOLV13.BI=FALSE} =>  {BcMV2.BI=FALSE} | 0.42  5743 | 0.593  103 | 0.717  822 | 1.10  4211 | 17  2 |
| {BcFV5.BI=FALSE, BcMCV3.BI=FALSE} =>  {BcMV2.BI=FALSE} | 0.42  5743 | 0.587  031 | 0.725  248 | 1.09  2905 | 17  2 |
| {BcMV2.BI=FALSE, BcOLV15.BI=FALSE} =>  {BcFV5.BI=FALSE} | 0.43  5644 | 0.875  622 | 0.497  525 | 1.11  9466 | 17  6 |

| {BcMV12=FALSE, BcMV2.BI=FALSE} =>  {BcULV1.BI=FALSE} | 0.39  3564 | 0.859  459 | 0.457  921 | 1.09  5336 | 15  9 |
| --- | --- | --- | --- | --- | --- |
| {BcMV12=FALSE, BcULV1.BI=FALSE} =>  {BcMV2.BI=FALSE} | 0.39  3564 | 0.602  273 | 0.653  465 | 1.12  1282 | 15  9 |
| {BcNSRV7.BI=FALSE, BcULV1.BI=FALSE} =>  {BcMV2.BI=FALSE} | 0.36  8812 | 0.600  806 | 0.613  861 | 1.11  8552 | 14  9 |
| {BcMV2.BI=FALSE, BcVV3.BI=FALSE} =>  {BcULV1.BI=FALSE} | 0.38  3663 | 0.851  648 | 0.450  495 | 1.08  5381 | 15  5 |
| {BcULV1.BI=FALSE, BcVV3.BI=FALSE} =>  {BcMV2.BI=FALSE} | 0.38  3663 | 0.610  236 | 0.628  713 | 1.13  6108 | 15  5 |
| {BcMV2.BI=FALSE, BcOLV7.BI=FALSE} =>  {BcULV1.BI=FALSE} | 0.38  6139 | 0.861  878 | 0.448  02 | 1.09  8419 | 15  6 |
| {BcOLV7.BI=FALSE, BcULV1.BI=FALSE} =>  {BcMV2.BI=FALSE} | 0.38  6139 | 0.593  156 | 0.650  99 | 1.10  4309 | 15  6 |
| {BcMV2.BI=FALSE, BcULV1.BI=FALSE} =>  {BcALV1.BI=FALSE} | 0.42  5743 | 0.924  731 | 0.460  396 | 1.13  5536 | 17  2 |
| {BcALV1.BI=FALSE, BcMV2.BI=FALSE} =>  {BcULV1.BI=FALSE} | 0.42  5743 | 0.868  687 | 0.490  099 | 1.10  7096 | 17  2 |
| {BcALV1.BI=FALSE, BcULV1.BI=FALSE} =>  {BcMV2.BI=FALSE} | 0.42  5743 | 0.656  489 | 0.648  515 | 1.22  2218 | 17  2 |
| {BcFV9=FALSE, BcMV2.BI=FALSE} =>  {BcULV1.BI=FALSE} | 0.40  3465 | 0.862  434 | 0.467  822 | 1.09  9127 | 16  3 |

| {BcFV9=FALSE, BcULV1.BI=FALSE} =>  {BcMV2.BI=FALSE} | 0.40  3465 | 0.594  891 | 0.678  218 | 1.10  7538 | 16  3 |
| --- | --- | --- | --- | --- | --- |
| {BcMV2.BI=FALSE, BcOLV4.BI=FALSE} =>  {BcULV1.BI=FALSE} | 0.42  5743 | 0.851  485 | 0.5 | 1.08  5174 | 17  2 |
| {BcOLV4.BI=FALSE, BcULV1.BI=FALSE} =>  {BcMV2.BI=FALSE} | 0.42  5743 | 0.612  1 | 0.695  545 | 1.13  9577 | 17  2 |
| {BcMV2.BI=FALSE, BcULV1.BI=FALSE} =>  {BcULV5=FALSE} | 0.43  3168 | 0.940  86 | 0.460  396 | 1.07  375 | 17  5 |
| {BcMV2.BI=FALSE, BcULV5=FALSE} =>  {BcULV1.BI=FALSE} | 0.43  3168 | 0.866  337 | 0.5 | 1.10  4101 | 17  5 |
| {BcULV1.BI=FALSE, BcULV5=FALSE} =>  {BcMV2.BI=FALSE} | 0.43  3168 | 0.618  375 | 0.700  495 | 1.15  126 | 17  5 |
| {BcBNV5.BI=FALSE, BcMV2.BI=FALSE} =>  {BcULV1.BI=FALSE} | 0.43  3168 | 0.866  337 | 0.5 | 1.10  4101 | 17  5 |
| {BcBNV5.BI=FALSE, BcULV1.BI=FALSE} =>  {BcMV2.BI=FALSE} | 0.43  3168 | 0.607  639 | 0.712  871 | 1.13  1272 | 17  5 |
| {BcMV2.BI=FALSE, BvF.BI=FALSE} =>  {BcULV1.BI=FALSE} | 0.41  0891 | 0.855  67 | 0.480  198 | 1.09  0507 | 16  6 |
| {BcMV2.BI=FALSE, BcULV1.BI=FALSE} =>  {BcMCV5.BI=FALSE} | 0.45  297 | 0.983  871 | 0.460  396 | 1.06  564 | 18  3 |
| {BcMCV5.BI=FALSE, BcMV2.BI=FALSE} =>  {BcULV1.BI=FALSE} | 0.45  297 | 0.867  299 | 0.522  277 | 1.10  5327 | 18  3 |

| {BcMCV5.BI=FALSE, BcULV1.BI=FALSE} =>  {BcMV2.BI=FALSE} | 0.45  297 | 0.620  339 | 0.730  198 | 1.15  4917 | 18  3 |
| --- | --- | --- | --- | --- | --- |
| {BcMV2.BI=FALSE, BcOLV13.BI=FALSE} =>  {BcULV1.BI=FALSE} | 0.44  802 | 0.866  029 | 0.517  327 | 1.10  3709 | 18  1 |
| {BcOLV13.BI=FALSE, BcULV1.BI=FALSE} =>  {BcMV2.BI=FALSE} | 0.44  802 | 0.605  351 | 0.740  099 | 1.12  7013 | 18  1 |
| {BcMV2.BI=FALSE, BcOLV2.BI=FALSE} =>  {BcULV1.BI=FALSE} | 0.43  8119 | 0.863  415 | 0.507  426 | 1.10  0377 | 17  7 |
| {BcOLV2.BI=FALSE, BcULV1.BI=FALSE} =>  {BcMV2.BI=FALSE} | 0.43  8119 | 0.59 | 0.742  574 | 1.09  8433 | 17  7 |
| {BcMCV3.BI=FALSE, BcMV2.BI=FALSE} =>  {BcULV1.BI=FALSE} | 0.45  5446 | 0.867  925 | 0.524  752 | 1.10  6125 | 18  4 |
| {BcMCV3.BI=FALSE, BcULV1.BI=FALSE} =>  {BcMV2.BI=FALSE} | 0.45  5446 | 0.595  469 | 0.764  851 | 1.10  8616 | 18  4 |
| {BcMV2.BI=FALSE, BcPV4.BI=FALSE} =>  {BcULV1.BI=FALSE} | 0.43  5644 | 0.862  745 | 0.504  95 | 1.09  9524 | 17  6 |
| {BcPV4.BI=FALSE, BcULV1.BI=FALSE} =>  {BcMV2.BI=FALSE} | 0.43  5644 | 0.586  667 | 0.742  574 | 1.09  2227 | 17  6 |
| {BcEV6=FALSE, BcMV2.BI=FALSE} =>  {BcULV1.BI=FALSE} | 0.43  5644 | 0.866  995 | 0.502  475 | 1.10  494 | 17  6 |
| {BcEV6=FALSE, BcULV1.BI=FALSE} =>  {BcMV2.BI=FALSE} | 0.43  5644 | 0.590  604 | 0.737  624 | 1.09  9558 | 17  6 |

| {BcMV2.BI=FALSE, BcOLV17.BI=FALSE} =>  {BcULV1.BI=FALSE} | 0.45  0495 | 0.862  559 | 0.522  277 | 1.09  9287 | 18  2 |
| --- | --- | --- | --- | --- | --- |
| {BcOLV17.BI=FALSE, BcULV1.BI=FALSE} =>  {BcMV2.BI=FALSE} | 0.45  0495 | 0.600  66 | 0.75 | 1.11  828 | 18  2 |
| {BcMV2.BI=FALSE, BcOLV15.BI=FALSE} =>  {BcULV1.BI=FALSE} | 0.42  5743 | 0.855  721 | 0.497  525 | 1.09  0572 | 17  2 |
| {BcEV5=FALSE, BcMV2.BI=FALSE} =>  {BcULV1.BI=FALSE} | 0.45  0495 | 0.854  46 | 0.527  228 | 1.08  8965 | 18  2 |
| {BcEV5=FALSE, BcULV1.BI=FALSE} =>  {BcMV2.BI=FALSE} | 0.45  0495 | 0.606  667 | 0.742  574 | 1.12  9462 | 18  2 |
| {BcMV1.BI=TRUE, BcMV2.BI=FALSE} =>  {BcALV1.BI=FALSE} | 0.34  6535 | 0.909  091 | 0.381  188 | 1.11  633 | 14  0 |
| {BcMV12=FALSE, BcVV3.BI=FALSE} =>  {BcMV2.BI=FALSE} | 0.38  1188 | 0.596  899 | 0.638  614 | 1.11  1278 | 15  4 |
| {BcMV2.BI=FALSE, BcOLV7.BI=FALSE} =>  {BcMV12=FALSE} | 0.39  1089 | 0.872  928 | 0.448  02 | 1.09  1836 | 15  8 |
| {BcMV12=FALSE, BcOLV7.BI=FALSE} =>  {BcMV2.BI=FALSE} | 0.39  1089 | 0.596  226 | 0.655  941 | 1.11  0025 | 15  8 |
| {BcMV12=FALSE, BcMV2.BI=FALSE} =>  {BcALV1.BI=FALSE} | 0.42  0792 | 0.918  919 | 0.457  921 | 1.12  8399 | 17  0 |
| {BcALV1.BI=FALSE, BcMV2.BI=FALSE} =>  {BcMV12=FALSE} | 0.42  0792 | 0.858  586 | 0.490  099 | 1.07  3897 | 17  0 |

| {BcALV1.BI=FALSE, BcMV12=FALSE} =>  {BcMV2.BI=FALSE} | 0.42  0792 | 0.643  939 | 0.653  465 | 1.19  8855 | 17  0 |
| --- | --- | --- | --- | --- | --- |
| {BcFV9=FALSE, BcMV2.BI=FALSE} =>  {BcMV12=FALSE} | 0.40  3465 | 0.862  434 | 0.467  822 | 1.07  871 | 16  3 |
| {BcMV12=FALSE, BcOLV4.BI=FALSE} =>  {BcMV2.BI=FALSE} | 0.42  3267 | 0.593  75 | 0.712  871 | 1.10  5415 | 17  1 |
| {BcMV12=FALSE, BcULV5=FALSE} =>  {BcMV2.BI=FALSE} | 0.42  3267 | 0.591  696 | 0.715  347 | 1.10  159 | 17  1 |
| {BcBNV5.BI=FALSE, BcMV12=FALSE} =>  {BcMV2.BI=FALSE} | 0.42  5743 | 0.589  041 | 0.722  772 | 1.09  6648 | 17  2 |
| {BcMCV5.BI=FALSE, BcMV2.BI=FALSE} =>  {BcMV12=FALSE} | 0.44  5545 | 0.853  081 | 0.522  277 | 1.06  7011 | 18  0 |
| {BcMCV5.BI=FALSE, BcMV12=FALSE} =>  {BcMV2.BI=FALSE} | 0.44  5545 | 0.604  027 | 0.737  624 | 1.12  4548 | 18  0 |
| {BcMV2.BI=FALSE, BcOLV13.BI=FALSE} =>  {BcMV12=FALSE} | 0.44  0594 | 0.851  675 | 0.517  327 | 1.06  5252 | 17  8 |
| {BcMV12=FALSE, BcOLV13.BI=FALSE} =>  {BcMV2.BI=FALSE} | 0.44  0594 | 0.583  607 | 0.754  95 | 1.08  653 | 17  8 |
| {BcMV2.BI=FALSE, BcOLV2.BI=FALSE} =>  {BcMV12=FALSE} | 0.43  3168 | 0.853  659 | 0.507  426 | 1.06  7734 | 17  5 |
| {BcMV12=FALSE, BcMV2.BI=FALSE} =>  {BcMCV3.BI=FALSE} | 0.45  7921 | 1 | 0.457  921 | 1.07  1618 | 18  5 |

| {BcMCV3.BI=FALSE, BcMV2.BI=FALSE} =>  {BcMV12=FALSE} | 0.45  7921 | 0.872  642 | 0.524  752 | 1.09  1477 | 18  5 |
| --- | --- | --- | --- | --- | --- |
| {BcMCV3.BI=FALSE, BcMV12=FALSE} =>  {BcMV2.BI=FALSE} | 0.45  7921 | 0.587  302 | 0.779  703 | 1.09  3409 | 18  5 |
| {BcMV2.BI=FALSE, BcOLV17.BI=FALSE} =>  {BcMV12=FALSE} | 0.44  5545 | 0.853  081 | 0.522  277 | 1.06  7011 | 18  0 |
| {BcMV12=FALSE, BcOLV17.BI=FALSE} =>  {BcMV2.BI=FALSE} | 0.44  5545 | 0.586  319 | 0.759  901 | 1.09  158 | 18  0 |
| {BcMV2.BI=FALSE, BcOLV15.BI=FALSE} =>  {BcMV12=FALSE} | 0.42  5743 | 0.855  721 | 0.497  525 | 1.07  0314 | 17  2 |
| {BcEV5=FALSE, BcMV2.BI=FALSE} =>  {BcMV12=FALSE} | 0.44  802 | 0.849  765 | 0.527  228 | 1.06  2864 | 18  1 |
| {BcEV5=FALSE, BcMV12=FALSE} =>  {BcMV2.BI=FALSE} | 0.44  802 | 0.593  443 | 0.754  95 | 1.10  4842 | 18  1 |
| {BcMV2.BI=FALSE, BcNSRV7.BI=FALSE} =>  {BcALV1.BI=FALSE} | 0.40  3465 | 0.920  904 | 0.438  119 | 1.13  0836 | 16  3 |
| {BcALV1.BI=FALSE, BcNSRV7.BI=FALSE} =>  {BcMV2.BI=FALSE} | 0.40  3465 | 0.610  487 | 0.660  891 | 1.13  6575 | 16  3 |
| {BcFV9=FALSE, BcMV2.BI=FALSE} =>  {BcNSRV7.BI=FALSE} | 0.40  099 | 0.857  143 | 0.467  822 | 1.07  2092 | 16  2 |
| {BcNSRV7.BI=FALSE, BcOLV4.BI=FALSE} =>  {BcMV2.BI=FALSE} | 0.40  3465 | 0.588  448 | 0.685  644 | 1.09  5543 | 16  3 |

| {BcMV2.BI=FALSE, BcNSRV7.BI=FALSE} =>  {BcULV5=FALSE} | 0.41  0891 | 0.937  853 | 0.438  119 | 1.07  0318 | 16  6 |
| --- | --- | --- | --- | --- | --- |
| {BcNSRV7.BI=FALSE, BcULV5=FALSE} =>  {BcMV2.BI=FALSE} | 0.41  0891 | 0.594  982 | 0.690  594 | 1.10  7709 | 16  6 |
| {BcMV2.BI=FALSE, BcOLV15.BI=FALSE} =>  {BcNSRV7.BI=FALSE} | 0.42  8218 | 0.860  697 | 0.497  525 | 1.07  6537 | 17  3 |
| {BcMV2.BI=FALSE, BcVV3.BI=FALSE} =>  {BcOLV7.BI=FALSE} | 0.38  8614 | 0.862  637 | 0.450  495 | 1.06  9035 | 15  7 |
| {BcMV2.BI=FALSE, BcOLV7.BI=FALSE} =>  {BcVV3.BI=FALSE} | 0.38  8614 | 0.867  403 | 0.448  02 | 1.08  1577 | 15  7 |
| {BcOLV7.BI=FALSE, BcVV3.BI=FALSE} =>  {BcMV2.BI=FALSE} | 0.38  8614 | 0.594  697 | 0.653  465 | 1.10  7178 | 15  7 |
| {BcMV2.BI=FALSE, BcVV3.BI=FALSE} =>  {BcALV1.BI=FALSE} | 0.40  8416 | 0.906  593 | 0.450  495 | 1.11  3264 | 16  5 |
| {BcALV1.BI=FALSE, BcVV3.BI=FALSE} =>  {BcMV2.BI=FALSE} | 0.40  8416 | 0.620  301 | 0.658  416 | 1.15  4846 | 16  5 |
| {BcOLV4.BI=FALSE, BcVV3.BI=FALSE} =>  {BcMV2.BI=FALSE} | 0.41  5842 | 0.591  549 | 0.702  97 | 1.10  1318 | 16  8 |
| {BcMV2.BI=FALSE, BcVV3.BI=FALSE} =>  {BcULV5=FALSE} | 0.42  8218 | 0.950  549 | 0.450  495 | 1.08  4808 | 17  3 |
| {BcMV2.BI=FALSE, BcULV5=FALSE} =>  {BcVV3.BI=FALSE} | 0.42  8218 | 0.856  436 | 0.5 | 1.06  7901 | 17  3 |

| {BcULV5=FALSE, BcVV3.BI=FALSE} =>  {BcMV2.BI=FALSE} | 0.42  8218 | 0.596  552 | 0.717  822 | 1.11  0631 | 17  3 |
| --- | --- | --- | --- | --- | --- |
| {BcMCV5.BI=FALSE, BcVV3.BI=FALSE} =>  {BcMV2.BI=FALSE} | 0.44  3069 | 0.581  169 | 0.762  376 | 1.08  1992 | 17  9 |
| {BcMCV3.BI=FALSE, BcVV3.BI=FALSE} =>  {BcMV2.BI=FALSE} | 0.43  8119 | 0.591  973 | 0.740  099 | 1.10  2107 | 17  7 |
| {BcMV2.BI=FALSE, BcOLV7.BI=FALSE} =>  {BcALV1.BI=FALSE} | 0.41  5842 | 0.928  177 | 0.448  02 | 1.13  9767 | 16  8 |
| {BcALV1.BI=FALSE, BcOLV7.BI=FALSE} =>  {BcMV2.BI=FALSE} | 0.41  5842 | 0.615  385 | 0.675  743 | 1.14  5693 | 16  8 |
| {BcMV2.BI=FALSE, BcOLV7.BI=FALSE} =>  {BcOLV4.BI=FALSE} | 0.41  8317 | 0.933  702 | 0.448  02 | 1.06  558 | 16  9 |
| {BcOLV4.BI=FALSE, BcOLV7.BI=FALSE} =>  {BcMV2.BI=FALSE} | 0.41  8317 | 0.586  806 | 0.712  871 | 1.09  2486 | 16  9 |
| {BcMV2.BI=FALSE, BcOLV7.BI=FALSE} =>  {BcULV5=FALSE} | 0.41  5842 | 0.928  177 | 0.448  02 | 1.05  9275 | 16  8 |
| {BcOLV7.BI=FALSE, BcULV5=FALSE} =>  {BcMV2.BI=FALSE} | 0.41  5842 | 0.593  64 | 0.700  495 | 1.10  5209 | 16  8 |
| {BcMCV5.BI=FALSE, BcOLV7.BI=FALSE} =>  {BcMV2.BI=FALSE} | 0.44  3069 | 0.590  759 | 0.75 | 1.09  9846 | 17  9 |
| {BcMCV3.BI=FALSE, BcOLV7.BI=FALSE} =>  {BcMV2.BI=FALSE} | 0.44  0594 | 0.579  805 | 0.759  901 | 1.07  9452 | 17  8 |

| {BcDMV1=FALSE, BcMV2.BI=FALSE} =>  {BcALV1.BI=FALSE} | 0.40  8416 | 0.911  602 | 0.448  02 | 1.11  9414 | 16  5 |
| --- | --- | --- | --- | --- | --- |
| {BcFV9=FALSE, BcMV2.BI=FALSE} =>  {BcALV1.BI=FALSE} | 0.42  8218 | 0.915  344 | 0.467  822 | 1.12  4009 | 17  3 |
| {BcALV1.BI=FALSE, BcFV9=FALSE} =>  {BcMV2.BI=FALSE} | 0.42  8218 | 0.594  502 | 0.720  297 | 1.10  6814 | 17  3 |
| {BcALV1.BI=FALSE, BcMV2.BI=FALSE} =>  {BcOLV4.BI=FALSE} | 0.46  0396 | 0.939  394 | 0.490  099 | 1.07  2077 | 18  6 |
| {BcMV2.BI=FALSE, BcOLV4.BI=FALSE} =>  {BcALV1.BI=FALSE} | 0.46  0396 | 0.920  792 | 0.5 | 1.13  0699 | 18  6 |
| {BcALV1.BI=FALSE, BcOLV4.BI=FALSE} =>  {BcMV2.BI=FALSE} | 0.46  0396 | 0.639  175 | 0.720  297 | 1.18  9985 | 18  6 |
| {BcALV1.BI=FALSE, BcMV2.BI=FALSE} =>  {BcULV5=FALSE} | 0.45  5446 | 0.929  293 | 0.490  099 | 1.06  0549 | 18  4 |
| {BcMV2.BI=FALSE, BcULV5=FALSE} =>  {BcALV1.BI=FALSE} | 0.45  5446 | 0.910  891 | 0.5 | 1.11  8541 | 18  4 |
| {BcALV1.BI=FALSE, BcULV5=FALSE} =>  {BcMV2.BI=FALSE} | 0.45  5446 | 0.645  614 | 0.705  446 | 1.20  1973 | 18  4 |
| {BcBNV5.BI=FALSE, BcMV2.BI=FALSE} =>  {BcALV1.BI=FALSE} | 0.46  2871 | 0.925  743 | 0.5 | 1.13  6778 | 18  7 |
| {BcALV1.BI=FALSE, BcBNV5.BI=FALSE} =>  {BcMV2.BI=FALSE} | 0.46  2871 | 0.605  178 | 0.764  851 | 1.12  6691 | 18  7 |

| {BcMV2.BI=FALSE, BvF.BI=FALSE} =>  {BcALV1.BI=FALSE} | 0.43  8119 | 0.912  371 | 0.480  198 | 1.12  0358 | 17  7 |
| --- | --- | --- | --- | --- | --- |
| {BcALV1.BI=FALSE, BvF.BI=FALSE} =>  {BcMV2.BI=FALSE} | 0.43  8119 | 0.586  093 | 0.747  525 | 1.09  1159 | 17  7 |
| {BcMCV5.BI=FALSE, BcMV2.BI=FALSE} =>  {BcALV1.BI=FALSE} | 0.48  0198 | 0.919  431 | 0.522  277 | 1.12  9028 | 19  4 |
| {BcALV1.BI=FALSE, BcMCV5.BI=FALSE} =>  {BcMV2.BI=FALSE} | 0.48  0198 | 0.621  795 | 0.772  277 | 1.15  7627 | 19  4 |
| {BcMV2.BI=FALSE, BcOLV13.BI=FALSE} =>  {BcALV1.BI=FALSE} | 0.47  0297 | 0.909  091 | 0.517  327 | 1.11  633 | 19  0 |
| {BcALV1.BI=FALSE, BcOLV13.BI=FALSE} =>  {BcMV2.BI=FALSE} | 0.47  0297 | 0.627  063 | 0.75 | 1.16  7435 | 19  0 |
| {BcMV2.BI=FALSE, BcOLV2.BI=FALSE} =>  {BcALV1.BI=FALSE} | 0.46  0396 | 0.907  317 | 0.507  426 | 1.11  4152 | 18  6 |
| {BcALV1.BI=FALSE, BcOLV2.BI=FALSE} =>  {BcMV2.BI=FALSE} | 0.46  0396 | 0.609  836 | 0.754  95 | 1.13  5363 | 18  6 |
| {BcMCV3.BI=FALSE, BcMV2.BI=FALSE} =>  {BcALV1.BI=FALSE} | 0.47  7723 | 0.910  377 | 0.524  752 | 1.11  791 | 19  3 |
| {BcALV1.BI=FALSE, BcMCV3.BI=FALSE} =>  {BcMV2.BI=FALSE} | 0.47  7723 | 0.628  664 | 0.759  901 | 1.17  0417 | 19  3 |
| {BcMV2.BI=FALSE, BcPV4.BI=FALSE} =>  {BcALV1.BI=FALSE} | 0.46  0396 | 0.911  765 | 0.504  95 | 1.11  9614 | 18  6 |

| {BcALV1.BI=FALSE, BcPV4.BI=FALSE} =>  {BcMV2.BI=FALSE} | 0.46  0396 | 0.611  842 | 0.752  475 | 1.13  9098 | 18  6 |
| --- | --- | --- | --- | --- | --- |
| {BcEV6=FALSE, BcMV2.BI=FALSE} =>  {BcALV1.BI=FALSE} | 0.45  7921 | 0.911  33 | 0.502  475 | 1.11  908 | 18  5 |
| {BcALV1.BI=FALSE, BcEV6=FALSE} =>  {BcMV2.BI=FALSE} | 0.45  7921 | 0.598  706 | 0.764  851 | 1.11  4641 | 18  5 |
| {BcMV2.BI=FALSE, BcOLV17.BI=FALSE} =>  {BcALV1.BI=FALSE} | 0.47  5248 | 0.909  953 | 0.522  277 | 1.11  7389 | 19  2 |
| {BcALV1.BI=FALSE, BcOLV17.BI=FALSE} =>  {BcMV2.BI=FALSE} | 0.47  5248 | 0.617  363 | 0.769  802 | 1.14  9377 | 19  2 |
| {BcMV2.BI=FALSE, BcOLV15.BI=FALSE} =>  {BcALV1.BI=FALSE} | 0.45  0495 | 0.905  473 | 0.497  525 | 1.11  1887 | 18  2 |
| {BcALV1.BI=FALSE, BcOLV15.BI=FALSE} =>  {BcMV2.BI=FALSE} | 0.45  0495 | 0.587  097 | 0.767  327 | 1.09  3028 | 18  2 |
| {BcEV5=FALSE, BcMV2.BI=FALSE} =>  {BcALV1.BI=FALSE} | 0.48  0198 | 0.910  798 | 0.527  228 | 1.11  8427 | 19  4 |
| {BcALV1.BI=FALSE, BcEV5=FALSE} =>  {BcMV2.BI=FALSE} | 0.48  0198 | 0.610  063 | 0.787  129 | 1.13  5785 | 19  4 |
| {BcFV9=FALSE, BcMV2.BI=FALSE} =>  {BcOLV4.BI=FALSE} | 0.43  8119 | 0.936  508 | 0.467  822 | 1.06  8783 | 17  7 |
| {BcFV9=FALSE, BcMV2.BI=FALSE} =>  {BcULV5=FALSE} | 0.43  8119 | 0.936  508 | 0.467  822 | 1.06  8783 | 17  7 |

| {BcMV2.BI=FALSE, BcOLV4.BI=FALSE} =>  {BcULV5=FALSE} | 0.46  5347 | 0.930  693 | 0.5 | 1.06  2147 | 18  8 |
| --- | --- | --- | --- | --- | --- |
| {BcMV2.BI=FALSE, BcULV5=FALSE} =>  {BcOLV4.BI=FALSE} | 0.46  5347 | 0.930  693 | 0.5 | 1.06  2147 | 18  8 |
| {BcOLV4.BI=FALSE, BcULV5=FALSE} =>  {BcMV2.BI=FALSE} | 0.46  5347 | 0.593  06 | 0.784  653 | 1.10  413 | 18  8 |
| {BcBNV5.BI=FALSE, BcMV2.BI=FALSE} =>  {BcOLV4.BI=FALSE} | 0.46  5347 | 0.930  693 | 0.5 | 1.06  2147 | 18  8 |
| {BcBNV5.BI=FALSE, BcOLV4.BI=FALSE} =>  {BcMV2.BI=FALSE} | 0.46  5347 | 0.589  342 | 0.789  604 | 1.09  7208 | 18  8 |
| {BcMV2.BI=FALSE, BvF.BI=FALSE} =>  {BcOLV4.BI=FALSE} | 0.45  0495 | 0.938  144 | 0.480  198 | 1.07  0651 | 18  2 |
| {BcMCV5.BI=FALSE, BcMV2.BI=FALSE} =>  {BcOLV4.BI=FALSE} | 0.48  7624 | 0.933  649 | 0.522  277 | 1.06  5521 | 19  7 |
| {BcMCV5.BI=FALSE, BcOLV4.BI=FALSE} =>  {BcMV2.BI=FALSE} | 0.48  7624 | 0.591  592 | 0.824  257 | 1.10  1396 | 19  7 |
| {BcMV2.BI=FALSE, BcOLV13.BI=FALSE} =>  {BcOLV4.BI=FALSE} | 0.48  0198 | 0.928  23 | 0.517  327 | 1.05  9336 | 19  4 |
| {BcMV2.BI=FALSE, BcOLV2.BI=FALSE} =>  {BcOLV4.BI=FALSE} | 0.47  2772 | 0.931  707 | 0.507  426 | 1.06  3304 | 19  1 |
| {BcOLV2.BI=FALSE, BcOLV4.BI=FALSE} =>  {BcMV2.BI=FALSE} | 0.47  2772 | 0.577  039 | 0.819  307 | 1.07  4304 | 19  1 |

| {BcMCV3.BI=FALSE, BcMV2.BI=FALSE} =>  {BcOLV4.BI=FALSE} | 0.48  7624 | 0.929  245 | 0.524  752 | 1.06  0495 | 19  7 |
| --- | --- | --- | --- | --- | --- |
| {BcMCV3.BI=FALSE, BcOLV4.BI=FALSE} =>  {BcMV2.BI=FALSE} | 0.48  7624 | 0.589  82 | 0.826  733 | 1.09  8099 | 19  7 |
| {BcMV2.BI=FALSE, BcPV4.BI=FALSE} =>  {BcOLV4.BI=FALSE} | 0.47  0297 | 0.931  373 | 0.504  95 | 1.06  2922 | 19  0 |
| {BcEV6=FALSE, BcMV2.BI=FALSE} =>  {BcOLV4.BI=FALSE} | 0.46  5347 | 0.926  108 | 0.502  475 | 1.05  6915 | 18  8 |
| {BcMV2.BI=FALSE, BcOLV17.BI=FALSE} =>  {BcOLV4.BI=FALSE} | 0.48  5149 | 0.928  91 | 0.522  277 | 1.06  0112 | 19  6 |
| {BcMV2.BI=FALSE, BcOLV15.BI=FALSE} =>  {BcOLV4.BI=FALSE} | 0.46  2871 | 0.930  348 | 0.497  525 | 1.06  1753 | 18  7 |
| {BcEV5=FALSE, BcMV2.BI=FALSE} =>  {BcOLV4.BI=FALSE} | 0.49  0099 | 0.929  577 | 0.527  228 | 1.06  0874 | 19  8 |
| {BcEV5=FALSE, BcOLV4.BI=FALSE} =>  {BcMV2.BI=FALSE} | 0.49  0099 | 0.591  045 | 0.829  208 | 1.10  0378 | 19  8 |
| {BcBNV5.BI=FALSE, BcMV2.BI=FALSE} =>  {BcULV5=FALSE} | 0.46  2871 | 0.925  743 | 0.5 | 1.05  6497 | 18  7 |
| {BcBNV5.BI=FALSE, BcULV5=FALSE} =>  {BcMV2.BI=FALSE} | 0.46  2871 | 0.586  207 | 0.789  604 | 1.09  1371 | 18  7 |
| {BcMV2.BI=FALSE, BvF.BI=FALSE} =>  {BcULV5=FALSE} | 0.44  802 | 0.932  99 | 0.480  198 | 1.06  4768 | 18  1 |

| {BcMCV5.BI=FALSE, BcMV2.BI=FALSE} =>  {BcULV5=FALSE} | 0.48  5149 | 0.928  91 | 0.522  277 | 1.06  0112 | 19  6 |
| --- | --- | --- | --- | --- | --- |
| {BcMCV5.BI=FALSE, BcULV5=FALSE} =>  {BcMV2.BI=FALSE} | 0.48  5149 | 0.595  745 | 0.814  356 | 1.10  9128 | 19  6 |
| {BcMV2.BI=FALSE, BcOLV13.BI=FALSE} =>  {BcULV5=FALSE} | 0.48  2673 | 0.933  014 | 0.517  327 | 1.06  4796 | 19  5 |
| {BcOLV13.BI=FALSE, BcULV5=FALSE} =>  {BcMV2.BI=FALSE} | 0.48  2673 | 0.583  832 | 0.826  733 | 1.08  6951 | 19  5 |
| {BcMV2.BI=FALSE, BcOLV2.BI=FALSE} =>  {BcULV5=FALSE} | 0.47  0297 | 0.926  829 | 0.507  426 | 1.05  7737 | 19  0 |
| {BcMCV3.BI=FALSE, BcMV2.BI=FALSE} =>  {BcULV5=FALSE} | 0.48  7624 | 0.929  245 | 0.524  752 | 1.06  0495 | 19  7 |
| {BcMCV3.BI=FALSE, BcULV5=FALSE} =>  {BcMV2.BI=FALSE} | 0.48  7624 | 0.593  373 | 0.821  782 | 1.10  4714 | 19  7 |
| {BcMV2.BI=FALSE, BcPV4.BI=FALSE} =>  {BcULV5=FALSE} | 0.47  2772 | 0.936  275 | 0.504  95 | 1.06  8517 | 19  1 |
| {BcEV6=FALSE, BcMV2.BI=FALSE} =>  {BcULV5=FALSE} | 0.47  0297 | 0.935  961 | 0.502  475 | 1.06  8158 | 19  0 |
| {BcMV2.BI=FALSE, BcOLV17.BI=FALSE} =>  {BcULV5=FALSE} | 0.48  5149 | 0.928  91 | 0.522  277 | 1.06  0112 | 19  6 |
| {BcOLV17.BI=FALSE, BcULV5=FALSE} =>  {BcMV2.BI=FALSE} | 0.48  5149 | 0.581  602 | 0.834  158 | 1.08  2799 | 19  6 |

| {BcMV2.BI=FALSE, BcOLV15.BI=FALSE} =>  {BcULV5=FALSE} | 0.46  2871 | 0.930  348 | 0.497  525 | 1.06  1753 | 18  7 |
| --- | --- | --- | --- | --- | --- |
| {BcEV5=FALSE, BcMV2.BI=FALSE} =>  {BcULV5=FALSE} | 0.49  0099 | 0.929  577 | 0.527  228 | 1.06  0874 | 19  8 |
| {BcEV5=FALSE, BcULV5=FALSE} =>  {BcMV2.BI=FALSE} | 0.49  0099 | 0.591  045 | 0.829  208 | 1.10  0378 | 19  8 |
| {BcBNV5.BI=FALSE, BcMV2.BI=FALSE} =>  {BcMCV5.BI=FALSE} | 0.49  505 | 0.990  099 | 0.5 | 1.07  2386 | 20  0 |
| {BcBNV5.BI=FALSE, BcOLV13.BI=FALSE} =>  {BcMV2.BI=FALSE} | 0.48  2673 | 0.576  923 | 0.836  634 | 1.07  4087 | 19  5 |
| {BcBNV5.BI=FALSE, BcMCV3.BI=FALSE} =>  {BcMV2.BI=FALSE} | 0.49  0099 | 0.580  645 | 0.844  059 | 1.08  1017 | 19  8 |
| {BcMCV5.BI=FALSE, BcOLV13.BI=FALSE} =>  {BcMV2.BI=FALSE} | 0.50  2475 | 0.583  333 | 0.861  386 | 1.08  6022 | 20  3 |
| {BcMCV5.BI=FALSE, BcOLV2.BI=FALSE} =>  {BcMV2.BI=FALSE} | 0.49  2574 | 0.576  812 | 0.853  96 | 1.07  388 | 19  9 |
| {BcMCV3.BI=FALSE, BcMCV5.BI=FALSE} =>  {BcMV2.BI=FALSE} | 0.51  2376 | 0.593  123 | 0.863  861 | 1.10  4248 | 20  7 |
| {BcMCV5.BI=FALSE, BcOLV17.BI=FALSE} =>  {BcMV2.BI=FALSE} | 0.50  7426 | 0.575  843 | 0.881  188 | 1.07  2076 | 20  5 |
| {BcMCV3.BI=FALSE, BcOLV13.BI=FALSE} =>  {BcMV2.BI=FALSE} | 0.50  495 | 0.577  904 | 0.873  762 | 1.07  5913 | 20  4 |

| {BcEV5=FALSE, BcOLV13.BI=FALSE} =>  {BcMV2.BI=FALSE} | 0.50  7426 | 0.579  096 | 0.876  238 | 1.07  8133 | 20  5 |
| --- | --- | --- | --- | --- | --- |
| {BcMCV3.BI=FALSE, BcOLV17.BI=FALSE} =>  {BcMV2.BI=FALSE} | 0.50  9901 | 0.575  419 | 0.886  139 | 1.07  1287 | 20  6 |
| {BcEV5=FALSE, BcMCV3.BI=FALSE} =>  {BcMV2.BI=FALSE} | 0.51  4851 | 0.582  633 | 0.883  663 | 1.08  4718 | 20  8 |
| {BcHV1SLR.BI=TRUE, BcMV3.BI=TRUE} =>  {BcULV3=TRUE} | 0.24  2574 | 0.736  842 | 0.329  208 | 1.24  0351 | 98 |
| {BcHV1SLR.BI=TRUE, BcULV3=TRUE} =>  {BcMV3.BI=TRUE} | 0.24  2574 | 0.628  205 | 0.386  139 | 1.15  8881 | 98 |
| {BcMV3.BI=TRUE, BcULV3=TRUE} =>  {BcHV1SLR.BI=TRUE} | 0.24  2574 | 0.7 | 0.346  535 | 1.29  1324 | 98 |
| {BcHV1SLR.BI=TRUE, BcMV3.BI=TRUE} =>  {BcHV1.BI=TRUE} | 0.29  703 | 0.902  256 | 0.329  208 | 1.49  3899 | 12  0 |
| {BcHV1.BI=TRUE, BcHV1SLR.BI=TRUE} =>  {BcMV3.BI=TRUE} | 0.29  703 | 0.612  245 | 0.485  149 | 1.12  9438 | 12  0 |
| {BcHV1.BI=TRUE, BcMV3.BI=TRUE} =>  {BcHV1SLR.BI=TRUE} | 0.29  703 | 0.839  161 | 0.353  96 | 1.54  8041 | 12  0 |
| {BcHV1SLR.BI=TRUE, BcMV3.BI=TRUE} =>  {BcMV11=TRUE} | 0.24  505 | 0.744  361 | 0.329  208 | 1.20  7718 | 99 |
| {BcHV1SLR.BI=TRUE, BcMV11=TRUE} =>  {BcMV3.BI=TRUE} | 0.24  505 | 0.687  5 | 0.356  436 | 1.26  8265 | 99 |

| {BcMV11=TRUE, BcMV3.BI=TRUE} =>  {BcHV1SLR.BI=TRUE} | 0.24  505 | 0.638  71 | 0.383  663 | 1.17  8259 | 99 |
| --- | --- | --- | --- | --- | --- |
| {BcHV1SLR.BI=TRUE, BcMV3.BI=TRUE} =>  {BcMV9.BI=TRUE} | 0.26  4851 | 0.804  511 | 0.329  208 | 1.18  6214 | 10  7 |
| {BcHV1SLR.BI=TRUE, BcMV9.BI=TRUE} =>  {BcMV3.BI=TRUE} | 0.26  4851 | 0.648  485 | 0.408  416 | 1.19  6292 | 10  7 |
| {BcMV3.BI=TRUE, BcMV9.BI=TRUE} =>  {BcHV1SLR.BI=TRUE} | 0.26  4851 | 0.668  75 | 0.396  04 | 1.23  3676 | 10  7 |
| {BcHV1SLR.BI=TRUE, BcMV3.BI=TRUE} =>  {BcULV4=TRUE} | 0.27  7228 | 0.842  105 | 0.329  208 | 1.21  9393 | 11  2 |
| {BcHV1SLR.BI=TRUE, BcULV4=TRUE} =>  {BcMV3.BI=TRUE} | 0.27  7228 | 0.691  358 | 0.400  99 | 1.27  5382 | 11  2 |
| {BcMV3.BI=TRUE, BcULV4=TRUE} =>  {BcHV1SLR.BI=TRUE} | 0.27  7228 | 0.691  358 | 0.400  99 | 1.27  5382 | 11  2 |
| {BcHV1SLR.BI=TRUE, BcMV3.BI=TRUE} =>  {BcMV1.BI=TRUE} | 0.28  9604 | 0.879  699 | 0.329  208 | 1.11  4102 | 11  7 |
| {BcHV1SLR.BI=TRUE, BcMV1.BI=TRUE} =>  {BcMV3.BI=TRUE} | 0.28  9604 | 0.657  303 | 0.440  594 | 1.21  256 | 11  7 |
| {BcMV1.BI=TRUE, BcMV3.BI=TRUE} =>  {BcHV1SLR.BI=TRUE} | 0.28  9604 | 0.632  432 | 0.457  921 | 1.16  6679 | 11  7 |
| {BcDMV1=FALSE, BcMV3.BI=TRUE} =>  {BcHV1SLR.BI=TRUE} | 0.26  4851 | 0.625  731 | 0.423  267 | 1.15  4317 | 10  7 |

| {BcHV1SLR.BI=TRUE, BvF.BI=FALSE} =>  {BcMV3.BI=TRUE} | 0.29  9505 | 0.623  711 | 0.480  198 | 1.15  0591 | 12  1 |
| --- | --- | --- | --- | --- | --- |
| {BcEV6=FALSE, BcHV1SLR.BI=TRUE} =>  {BcMV3.BI=TRUE} | 0.31  1881 | 0.620  69 | 0.502  475 | 1.14  5017 | 12  6 |
| {BcHV1SLR.BI=TRUE, BcMV4.BI=FALSE} =>  {BcHV1.BI=TRUE} | 0.24  2574 | 0.867  257 | 0.279  703 | 1.43  595 | 98 |
| {BcHV1.BI=TRUE, BcMV4.BI=FALSE} =>  {BcHV1SLR.BI=TRUE} | 0.24  2574 | 0.796  748 | 0.304  455 | 1.46  98 | 98 |
| {BcHV1SLR.BI=TRUE, BcMV4.BI=FALSE} =>  {BcPV3.BI=FALSE} | 0.22  2772 | 0.796  46 | 0.279  703 | 1.12  115 | 90 |
| {BcHV1SLR.BI=TRUE, BcMV4.BI=FALSE} =>  {BcRV1.BI=FALSE} | 0.23  7624 | 0.849  558 | 0.279  703 | 1.17  5415 | 96 |
| {BcHV1SLR.BI=TRUE, BcMV4.BI=FALSE} =>  {BcDMV1=FALSE} | 0.25  495 | 0.911  504 | 0.279  703 | 1.07  9906 | 10  3 |
| {BcOLV16.BI=FALSE, BcULV3=TRUE} =>  {BcHV1SLR.BI=TRUE} | 0.20  5446 | 0.653  543 | 0.314  356 | 1.20  5623 | 83 |
| {BcHV1SLR.BI=TRUE, BcOLV16.BI=FALSE}  => {BcHV1.BI=TRUE} | 0.26  4851 | 0.869  919 | 0.304  455 | 1.44  0357 | 10  7 |
| {BcHV1.BI=TRUE, BcOLV16.BI=FALSE} =>  {BcHV1SLR.BI=TRUE} | 0.26  4851 | 0.792  593 | 0.334  158 | 1.46  2134 | 10  7 |
| {BcHV1SLR.BI=TRUE, BcOLV16.BI=FALSE}  => {BcOLV1.BI.MOVA.=FALSE} | 0.23  5149 | 0.772  358 | 0.304  455 | 1.15  5676 | 95 |

| {BcHV1SLR.BI=TRUE, BcOLV1.BI.MOVA.=FALSE} =>  {BcOLV16.BI=FALSE} | 0.23  5149 | 0.688  406 | 0.341  584 | 1.17  8457 | 95 |
| --- | --- | --- | --- | --- | --- |
| {BcHV1SLR.BI=TRUE, BcULV1.BI=FALSE}  => {BcOLV16.BI=FALSE} | 0.25  7426 | 0.670  968 | 0.383  663 | 1.14  8606 | 10  4 |
| {BcHV1SLR.BI=TRUE, BcOLV16.BI=FALSE}  => {BcOLV7.BI=FALSE} | 0.27  9703 | 0.918  699 | 0.304  455 | 1.13  8511 | 11  3 |
| {BcHV1SLR.BI=TRUE, BcULV3=TRUE} =>  {BcHV1.BI=TRUE} | 0.34  901 | 0.903  846 | 0.386  139 | 1.49  6532 | 14  1 |
| {BcHV1.BI=TRUE, BcHV1SLR.BI=TRUE} =>  {BcULV3=TRUE} | 0.34  901 | 0.719  388 | 0.485  149 | 1.21  0969 | 14  1 |
| {BcHV1.BI=TRUE, BcULV3=TRUE} =>  {BcHV1SLR.BI=TRUE} | 0.34  901 | 0.824  561 | 0.423  267 | 1.52  1109 | 14  1 |
| {BcHV1SLR.BI=TRUE, BcULV3=TRUE} =>  {BcMV11=TRUE} | 0.27  2277 | 0.705  128 | 0.386  139 | 1.14  4063 | 11  0 |
| {BcHV1SLR.BI=TRUE, BcMV11=TRUE} =>  {BcULV3=TRUE} | 0.27  2277 | 0.763  889 | 0.356  436 | 1.28  588 | 11  0 |
| {BcMV11=TRUE, BcULV3=TRUE} =>  {BcHV1SLR.BI=TRUE} | 0.27  2277 | 0.687  5 | 0.396  04 | 1.26  8265 | 11  0 |
| {BcHV1SLR.BI=TRUE, BcOLV1.BI.MOVA.=FALSE} =>  {BcULV3=TRUE} | 0.24  7525 | 0.724  638 | 0.341  584 | 1.21  9807 | 10  0 |

| {BcHV1SLR.BI=TRUE, BcMV9.BI=TRUE} =>  {BcULV3=TRUE} | 0.28  9604 | 0.709  091 | 0.408  416 | 1.19  3636 | 11  7 |
| --- | --- | --- | --- | --- | --- |
| {BcMV9.BI=TRUE, BcULV3=TRUE} =>  {BcHV1SLR.BI=TRUE} | 0.28  9604 | 0.684  211 | 0.423  267 | 1.26  2197 | 11  7 |
| {BcHV1SLR.BI=TRUE, BcULV4=TRUE} =>  {BcULV3=TRUE} | 0.27  9703 | 0.697  531 | 0.400  99 | 1.17  4177 | 11  3 |
| {BcULV3=TRUE, BcULV4=TRUE} =>  {BcHV1SLR.BI=TRUE} | 0.27  9703 | 0.672  619 | 0.415  842 | 1.24  0813 | 11  3 |
| {BcHV1SLR.BI=TRUE, BcMV6.BI=FALSE} =>  {BcULV3=TRUE} | 0.23  2673 | 0.691  176 | 0.336  634 | 1.16  348 | 94 |
| {BcHV1SLR.BI=TRUE, BcPV3.BI=FALSE} =>  {BcULV3=TRUE} | 0.27  2277 | 0.728  477 | 0.373  762 | 1.22  6269 | 11  0 |
| {BcPV3.BI=FALSE, BcULV3=TRUE} =>  {BcHV1SLR.BI=TRUE} | 0.27  2277 | 0.647  059 | 0.420  792 | 1.19  3661 | 11  0 |
| {BcHV1SLR.BI=TRUE, BcRV1.BI=FALSE} =>  {BcULV3=TRUE} | 0.27  4752 | 0.720  779 | 0.381  188 | 1.21  3312 | 11  1 |
| {BcRV1.BI=FALSE, BcULV3=TRUE} =>  {BcHV1SLR.BI=TRUE} | 0.27  4752 | 0.634  286 | 0.433  168 | 1.17  0098 | 11  1 |
| {BcEV2.BI=FALSE, BcHV1SLR.BI=TRUE} =>  {BcULV3=TRUE} | 0.27  7228 | 0.717  949 | 0.386  139 | 1.20  8547 | 11  2 |
| {BcEV2.BI=FALSE, BcULV3=TRUE} =>  {BcHV1SLR.BI=TRUE} | 0.27  7228 | 0.636  364 | 0.435  644 | 1.17  3931 | 11  2 |

| {BcHV1SLR.BI=TRUE, BcHV3.BI=FALSE} =>  {BcULV3=TRUE} | 0.26  4851 | 0.672  956 | 0.393  564 | 1.13  2809 | 10  7 |
| --- | --- | --- | --- | --- | --- |
| {BcHV3.BI=FALSE, BcULV3=TRUE} =>  {BcHV1SLR.BI=TRUE} | 0.26  4851 | 0.625  731 | 0.423  267 | 1.15  4317 | 10  7 |
| {BcFV5.BI=FALSE, BcHV1SLR.BI=TRUE} =>  {BcULV3=TRUE} | 0.26  2376 | 0.670  886 | 0.391  089 | 1.12  9325 | 10  6 |
| {BcHV1SLR.BI=TRUE, BcULV1.BI=FALSE}  => {BcULV3=TRUE} | 0.27  4752 | 0.716  129 | 0.383  663 | 1.20  5484 | 11  1 |
| {BcHV1SLR.BI=TRUE, BcMV1.BI=TRUE} =>  {BcULV3=TRUE} | 0.31  1881 | 0.707  865 | 0.440  594 | 1.19  1573 | 12  6 |
| {BcMV1.BI=TRUE, BcULV3=TRUE} =>  {BcHV1SLR.BI=TRUE} | 0.31  1881 | 0.642  857 | 0.485  149 | 1.18  591 | 12  6 |
| {BcHV1SLR.BI=TRUE, BcMV12=FALSE} =>  {BcULV3=TRUE} | 0.29  2079 | 0.706  587 | 0.413  366 | 1.18  9421 | 11  8 |
| {BcMV12=FALSE, BcULV3=TRUE} =>  {BcHV1SLR.BI=TRUE} | 0.29  2079 | 0.624  339 | 0.467  822 | 1.15  1748 | 11  8 |
| {BcHV1SLR.BI=TRUE, BcVV3.BI=FALSE} =>  {BcULV3=TRUE} | 0.31  6832 | 0.731  429 | 0.433  168 | 1.23  1238 | 12  8 |
| {BcULV3=TRUE, BcVV3.BI=FALSE} =>  {BcHV1SLR.BI=TRUE} | 0.31  6832 | 0.663  212 | 0.477  723 | 1.22  346 | 12  8 |
| {BcHV1SLR.BI=TRUE, BcOLV7.BI=FALSE}  => {BcULV3=TRUE} | 0.30  9406 | 0.710  227 | 0.435  644 | 1.19  5549 | 12  5 |

| {BcOLV7.BI=FALSE, BcULV3=TRUE} =>  {BcHV1SLR.BI=TRUE} | 0.30  9406 | 0.651  042 | 0.475  248 | 1.20  1008 | 12  5 |
| --- | --- | --- | --- | --- | --- |
| {BcALV1.BI=FALSE, BcHV1SLR.BI=TRUE} =>  {BcULV3=TRUE} | 0.29  9505 | 0.720  238 | 0.415  842 | 1.21  2401 | 12  1 |
| {BcALV1.BI=FALSE, BcULV3=TRUE} =>  {BcHV1SLR.BI=TRUE} | 0.29  9505 | 0.620  513 | 0.482  673 | 1.14  469 | 12  1 |
| {BcDMV1=FALSE, BcHV1SLR.BI=TRUE} =>  {BcULV3=TRUE} | 0.32  9208 | 0.707  447 | 0.465  347 | 1.19  0869 | 13  3 |
| {BcDMV1=FALSE, BcULV3=TRUE} =>  {BcHV1SLR.BI=TRUE} | 0.32  9208 | 0.651  961 | 0.504  95 | 1.20  2704 | 13  3 |
| {BcFV9=FALSE, BcHV1SLR.BI=TRUE} =>  {BcULV3=TRUE} | 0.31  4356 | 0.697  802 | 0.450  495 | 1.17  4634 | 12  7 |
| {BcFV9=FALSE, BcULV3=TRUE} =>  {BcHV1SLR.BI=TRUE} | 0.31  4356 | 0.616  505 | 0.509  901 | 1.13  7297 | 12  7 |
| {BcHV1SLR.BI=TRUE, BcOLV4.BI=FALSE}  => {BcULV3=TRUE} | 0.31  6832 | 0.684  492 | 0.462  871 | 1.15  2228 | 12  8 |
| {BcOLV4.BI=FALSE, BcULV3=TRUE} =>  {BcHV1SLR.BI=TRUE} | 0.31  6832 | 0.624  39 | 0.507  426 | 1.15  1843 | 12  8 |
| {BcHV1SLR.BI=TRUE, BcULV5=FALSE} =>  {BcULV3=TRUE} | 0.32  9208 | 0.703  704 | 0.467  822 | 1.18  4568 | 13  3 |
| {BcULV3=TRUE, BcULV5=FALSE} =>  {BcHV1SLR.BI=TRUE} | 0.32  9208 | 0.636  364 | 0.517  327 | 1.17  3931 | 13  3 |

| {BcBNV5.BI=FALSE, BcHV1SLR.BI=TRUE}  => {BcULV3=TRUE} | 0.33  1683 | 0.694  301 | 0.477  723 | 1.16  8739 | 13  4 |
| --- | --- | --- | --- | --- | --- |
| {BcBNV5.BI=FALSE, BcULV3=TRUE} =>  {BcHV1SLR.BI=TRUE} | 0.33  1683 | 0.641  148 | 0.517  327 | 1.18  2758 | 13  4 |
| {BcHV1SLR.BI=TRUE, BvF.BI=FALSE} =>  {BcULV3=TRUE} | 0.35  1485 | 0.731  959 | 0.480  198 | 1.23  2131 | 14  2 |
| {BcULV3=TRUE, BvF.BI=FALSE} =>  {BcHV1SLR.BI=TRUE} | 0.35  1485 | 0.636  771 | 0.551  98 | 1.17  4683 | 14  2 |
| {BcHV1SLR.BI=TRUE, BcMCV5.BI=FALSE}  => {BcULV3=TRUE} | 0.33  9109 | 0.691  919 | 0.490  099 | 1.16  4731 | 13  7 |
| {BcMCV5.BI=FALSE, BcULV3=TRUE} =>  {BcHV1SLR.BI=TRUE} | 0.33  9109 | 0.637  209 | 0.532  178 | 1.17  5491 | 13  7 |
| {BcHV1SLR.BI=TRUE, BcOLV13.BI=FALSE}  => {BcULV3=TRUE} | 0.34  6535 | 0.707  071 | 0.490  099 | 1.19  0236 | 14  0 |
| {BcOLV13.BI=FALSE, BcULV3=TRUE} =>  {BcHV1SLR.BI=TRUE} | 0.34  6535 | 0.636  364 | 0.544  554 | 1.17  3931 | 14  0 |
| {BcHV1SLR.BI=TRUE, BcOLV2.BI=FALSE}  => {BcULV3=TRUE} | 0.34  4059 | 0.712  821 | 0.482  673 | 1.19  9915 | 13  9 |
| {BcOLV2.BI=FALSE, BcULV3=TRUE} =>  {BcHV1SLR.BI=TRUE} | 0.34  4059 | 0.634  703 | 0.542  079 | 1.17  0868 | 13  9 |
| {BcHV1SLR.BI=TRUE, BcMCV3.BI=FALSE}  => {BcULV3=TRUE} | 0.34  6535 | 0.710  66 | 0.487  624 | 1.19  6277 | 14  0 |

| {BcMCV3.BI=FALSE, BcULV3=TRUE} =>  {BcHV1SLR.BI=TRUE} | 0.34  6535 | 0.639  269 | 0.542  079 | 1.17  9292 | 14  0 |
| --- | --- | --- | --- | --- | --- |
| {BcHV1SLR.BI=TRUE, BcPV4.BI=FALSE} =>  {BcULV3=TRUE} | 0.35  6436 | 0.705  882 | 0.504  95 | 1.18  8235 | 14  4 |
| {BcPV4.BI=FALSE, BcULV3=TRUE} =>  {BcHV1SLR.BI=TRUE} | 0.35  6436 | 0.648  649 | 0.549  505 | 1.19  6594 | 14  4 |
| {BcEV6=FALSE, BcHV1SLR.BI=TRUE} =>  {BcULV3=TRUE} | 0.36  1386 | 0.719  212 | 0.502  475 | 1.21  0673 | 14  6 |
| {BcEV6=FALSE, BcULV3=TRUE} =>  {BcHV1SLR.BI=TRUE} | 0.36  1386 | 0.646  018 | 0.559  406 | 1.19  174 | 14  6 |
| {BcHV1SLR.BI=TRUE, BcOLV17.BI=FALSE}  => {BcULV3=TRUE} | 0.35  6436 | 0.705  882 | 0.504  95 | 1.18  8235 | 14  4 |
| {BcOLV17.BI=FALSE, BcULV3=TRUE} =>  {BcHV1SLR.BI=TRUE} | 0.35  6436 | 0.64 | 0.556  931 | 1.18  0639 | 14  4 |
| {BcHV1SLR.BI=TRUE, BcOLV15.BI=FALSE}  => {BcULV3=TRUE} | 0.35  1485 | 0.702  97 | 0.5 | 1.18  3333 | 14  2 |
| {BcOLV15.BI=FALSE, BcULV3=TRUE} =>  {BcHV1SLR.BI=TRUE} | 0.35  1485 | 0.631  111 | 0.556  931 | 1.16  4242 | 14  2 |
| {BcEV5=FALSE, BcHV1SLR.BI=TRUE} =>  {BcULV3=TRUE} | 0.35  6436 | 0.695  652 | 0.512  376 | 1.17  1014 | 14  4 |
| {BcEV5=FALSE, BcULV3=TRUE} =>  {BcHV1SLR.BI=TRUE} | 0.35  6436 | 0.645  74 | 0.551  98 | 1.19  1228 | 14  4 |

| {BcHV1SLR.BI=TRUE, BcMV11=TRUE} =>  {BcHV1.BI=TRUE} | 0.31  4356 | 0.881  944 | 0.356  436 | 1.46  0269 | 12  7 |
| --- | --- | --- | --- | --- | --- |
| {BcHV1.BI=TRUE, BcMV11=TRUE} =>  {BcHV1SLR.BI=TRUE} | 0.31  4356 | 0.824  675 | 0.381  188 | 1.52  1319 | 12  7 |
| {BcHV1SLR.BI=TRUE, BcOLV1.BI.MOVA.=FALSE} =>  {BcHV1.BI=TRUE} | 0.30  6931 | 0.898  551 | 0.341  584 | 1.48  7764 | 12  4 |
| {BcHV1.BI=TRUE, BcOLV1.BI.MOVA.=FALSE} =>  {BcHV1SLR.BI=TRUE} | 0.30  6931 | 0.784  81 | 0.391  089 | 1.44  7778 | 12  4 |
| {BcHV1.BI=TRUE, BcHV1SLR.BI=TRUE} =>  {BcMV9.BI=TRUE} | 0.37  6238 | 0.775  51 | 0.485  149 | 1.14  3453 | 15  2 |
| {BcHV1SLR.BI=TRUE, BcMV9.BI=TRUE} =>  {BcHV1.BI=TRUE} | 0.37  6238 | 0.921  212 | 0.408  416 | 1.52  5286 | 15  2 |
| {BcHV1.BI=TRUE, BcMV9.BI=TRUE} =>  {BcHV1SLR.BI=TRUE} | 0.37  6238 | 0.804  233 | 0.467  822 | 1.48  3608 | 15  2 |
| {BcHV1SLR.BI=TRUE, BcULV4=TRUE} =>  {BcHV1.BI=TRUE} | 0.36  3861 | 0.907  407 | 0.400  99 | 1.50  2429 | 14  7 |
| {BcHV1.BI=TRUE, BcULV4=TRUE} =>  {BcHV1SLR.BI=TRUE} | 0.36  3861 | 0.798  913 | 0.455  446 | 1.47  3794 | 14  7 |
| {BcHV1SLR.BI=TRUE, BcMV6.BI=FALSE} =>  {BcHV1.BI=TRUE} | 0.29  4554 | 0.875 | 0.336  634 | 1.44  877 | 11  9 |

| {BcHV1.BI=TRUE, BcMV6.BI=FALSE} =>  {BcHV1SLR.BI=TRUE} | 0.29  4554 | 0.782  895 | 0.376  238 | 1.44  4244 | 11  9 |
| --- | --- | --- | --- | --- | --- |
| {BcHV1SLR.BI=TRUE, BcPV3.BI=FALSE} =>  {BcHV1.BI=TRUE} | 0.32  6733 | 0.874  172 | 0.373  762 | 1.44  74 | 13  2 |
| {BcHV1.BI=TRUE, BcPV3.BI=FALSE} =>  {BcHV1SLR.BI=TRUE} | 0.32  6733 | 0.809  816 | 0.403  465 | 1.49  3907 | 13  2 |
| {BcHV1SLR.BI=TRUE, BcRV1.BI=FALSE} =>  {BcHV1.BI=TRUE} | 0.33  4158 | 0.876  623 | 0.381  188 | 1.45  1458 | 13  5 |
| {BcHV1.BI=TRUE, BcRV1.BI=FALSE} =>  {BcHV1SLR.BI=TRUE} | 0.33  4158 | 0.803  571 | 0.415  842 | 1.48  2387 | 13  5 |
| {BcEV2.BI=FALSE, BcHV1SLR.BI=TRUE} =>  {BcHV1.BI=TRUE} | 0.34  1584 | 0.884  615 | 0.386  139 | 1.46  4691 | 13  8 |
| {BcEV2.BI=FALSE, BcHV1.BI=TRUE} =>  {BcHV1SLR.BI=TRUE} | 0.34  1584 | 0.779  661 | 0.438  119 | 1.43  8279 | 13  8 |
| {BcHV1SLR.BI=TRUE, BcHV3.BI=FALSE} =>  {BcHV1.BI=TRUE} | 0.34  6535 | 0.880  503 | 0.393  564 | 1.45  7882 | 14  0 |
| {BcHV1.BI=TRUE, BcHV3.BI=FALSE} =>  {BcHV1SLR.BI=TRUE} | 0.34  6535 | 0.782  123 | 0.443  069 | 1.44  282 | 14  0 |
| {BcFV5.BI=FALSE, BcHV1SLR.BI=TRUE} =>  {BcHV1.BI=TRUE} | 0.34  901 | 0.892  405 | 0.391  089 | 1.47  7589 | 14  1 |
| {BcFV5.BI=FALSE, BcHV1.BI=TRUE} =>  {BcHV1SLR.BI=TRUE} | 0.34  901 | 0.770  492 | 0.452  97 | 1.42  1364 | 14  1 |

| {BcHV1SLR.BI=TRUE, BcULV1.BI=FALSE}  => {BcHV1.BI=TRUE} | 0.33  4158 | 0.870  968 | 0.383  663 | 1.44  2094 | 13  5 |
| --- | --- | --- | --- | --- | --- |
| {BcHV1.BI=TRUE, BcULV1.BI=FALSE} =>  {BcHV1SLR.BI=TRUE} | 0.33  4158 | 0.780  347 | 0.428  218 | 1.43  9544 | 13  5 |
| {BcHV1SLR.BI=TRUE, BcMV1.BI=TRUE} =>  {BcHV1.BI=TRUE} | 0.38  8614 | 0.882  022 | 0.440  594 | 1.46  0398 | 15  7 |
| {BcHV1.BI=TRUE, BcMV1.BI=TRUE} =>  {BcHV1SLR.BI=TRUE} | 0.38  8614 | 0.805  128 | 0.482  673 | 1.48  5259 | 15  7 |
| {BcHV1SLR.BI=TRUE, BcMV12=FALSE} =>  {BcHV1.BI=TRUE} | 0.36  8812 | 0.892  216 | 0.413  366 | 1.47  7275 | 14  9 |
| {BcHV1.BI=TRUE, BcMV12=FALSE} =>  {BcHV1SLR.BI=TRUE} | 0.36  8812 | 0.792  553 | 0.465  347 | 1.46  2062 | 14  9 |
| {BcHV1SLR.BI=TRUE, BcNSRV7.BI=FALSE}  => {BcHV1.BI=TRUE} | 0.36  1386 | 0.884  848 | 0.408  416 | 1.46  5077 | 14  6 |
| {BcHV1.BI=TRUE, BcNSRV7.BI=FALSE} =>  {BcHV1SLR.BI=TRUE} | 0.36  1386 | 0.776  596 | 0.465  347 | 1.43  2624 | 14  6 |
| {BcHV1SLR.BI=TRUE, BcVV3.BI=FALSE} =>  {BcHV1.BI=TRUE} | 0.38  8614 | 0.897  143 | 0.433  168 | 1.48  5433 | 15  7 |
| {BcHV1.BI=TRUE, BcVV3.BI=FALSE} =>  {BcHV1SLR.BI=TRUE} | 0.38  8614 | 0.785 | 0.495  05 | 1.44  8128 | 15  7 |
| {BcHV1SLR.BI=TRUE, BcOLV7.BI=FALSE}  => {BcHV1.BI=TRUE} | 0.39  1089 | 0.897  727 | 0.435  644 | 1.48  6401 | 15  8 |

| {BcHV1.BI=TRUE, BcOLV7.BI=FALSE} =>  {BcHV1SLR.BI=TRUE} | 0.39  1089 | 0.818  653 | 0.477  723 | 1.51  0209 | 15  8 |
| --- | --- | --- | --- | --- | --- |
| {BcALV1.BI=FALSE, BcHV1SLR.BI=TRUE} =>  {BcHV1.BI=TRUE} | 0.37  1287 | 0.892  857 | 0.415  842 | 1.47  8337 | 15  0 |
| {BcALV1.BI=FALSE, BcHV1.BI=TRUE} =>  {BcHV1SLR.BI=TRUE} | 0.37  1287 | 0.797  872 | 0.465  347 | 1.47  1874 | 15  0 |
| {BcDMV1=FALSE, BcHV1SLR.BI=TRUE} =>  {BcHV1.BI=TRUE} | 0.41  3366 | 0.888  298 | 0.465  347 | 1.47  0788 | 16  7 |
| {BcDMV1=FALSE, BcHV1.BI=TRUE} =>  {BcHV1SLR.BI=TRUE} | 0.41  3366 | 0.799  043 | 0.517  327 | 1.47  4034 | 16  7 |
| {BcFV9=FALSE, BcHV1SLR.BI=TRUE} =>  {BcHV1.BI=TRUE} | 0.39  3564 | 0.873  626 | 0.450  495 | 1.44  6496 | 15  9 |
| {BcFV9=FALSE, BcHV1.BI=TRUE} =>  {BcHV1SLR.BI=TRUE} | 0.39  3564 | 0.775  61 | 0.507  426 | 1.43  0805 | 15  9 |
| {BcHV1SLR.BI=TRUE, BcOLV4.BI=FALSE}  => {BcHV1.BI=TRUE} | 0.41  8317 | 0.903  743 | 0.462  871 | 1.49  6362 | 16  9 |
| {BcHV1.BI=TRUE, BcOLV4.BI=FALSE} =>  {BcHV1SLR.BI=TRUE} | 0.41  8317 | 0.789  72 | 0.529  703 | 1.45  6834 | 16  9 |
| {BcHV1SLR.BI=TRUE, BcULV5=FALSE} =>  {BcHV1.BI=TRUE} | 0.42  3267 | 0.904  762 | 0.467  822 | 1.49  8048 | 17  1 |
| {BcHV1.BI=TRUE, BcULV5=FALSE} =>  {BcHV1SLR.BI=TRUE} | 0.42  3267 | 0.795  349 | 0.532  178 | 1.46  7219 | 17  1 |

| {BcBNV5.BI=FALSE, BcHV1SLR.BI=TRUE}  => {BcHV1.BI=TRUE} | 0.42  3267 | 0.886  01 | 0.477  723 | 1.46  7001 | 17  1 |
| --- | --- | --- | --- | --- | --- |
| {BcBNV5.BI=FALSE, BcHV1.BI=TRUE} =>  {BcHV1SLR.BI=TRUE} | 0.42  3267 | 0.795  349 | 0.532  178 | 1.46  7219 | 17  1 |
| {BcHV1SLR.BI=TRUE, BvF.BI=FALSE} =>  {BcHV1.BI=TRUE} | 0.43  0693 | 0.896  907 | 0.480  198 | 1.48  5043 | 17  4 |
| {BcHV1.BI=TRUE, BvF.BI=FALSE} =>  {BcHV1SLR.BI=TRUE} | 0.43  0693 | 0.790  909 | 0.544  554 | 1.45  9029 | 17  4 |
| {BcHV1SLR.BI=TRUE, BcMCV5.BI=FALSE}  => {BcHV1.BI=TRUE} | 0.43  5644 | 0.888  889 | 0.490  099 | 1.47  1767 | 17  6 |
| {BcHV1.BI=TRUE, BcMCV5.BI=FALSE} =>  {BcHV1SLR.BI=TRUE} | 0.43  5644 | 0.796  38 | 0.547  03 | 1.46  9121 | 17  6 |
| {BcHV1SLR.BI=TRUE, BcOLV13.BI=FALSE}  => {BcHV1.BI=TRUE} | 0.43  5644 | 0.888  889 | 0.490  099 | 1.47  1767 | 17  6 |
| {BcHV1.BI=TRUE, BcOLV13.BI=FALSE} =>  {BcHV1SLR.BI=TRUE} | 0.43  5644 | 0.8 | 0.544  554 | 1.47  5799 | 17  6 |
| {BcHV1SLR.BI=TRUE, BcOLV2.BI=FALSE}  => {BcHV1.BI=TRUE} | 0.43  3168 | 0.897  436 | 0.482  673 | 1.48  5918 | 17  5 |
| {BcHV1.BI=TRUE, BcOLV2.BI=FALSE} =>  {BcHV1SLR.BI=TRUE} | 0.43  3168 | 0.784  753 | 0.551  98 | 1.44  7673 | 17  5 |
| {BcHV1SLR.BI=TRUE, BcMCV3.BI=FALSE}  => {BcHV1.BI=TRUE} | 0.43  3168 | 0.888  325 | 0.487  624 | 1.47  0833 | 17  5 |

| {BcHV1.BI=TRUE, BcMCV3.BI=FALSE} =>  {BcHV1SLR.BI=TRUE} | 0.43  3168 | 0.795  455 | 0.544  554 | 1.46  7414 | 17  5 |
| --- | --- | --- | --- | --- | --- |
| {BcHV1SLR.BI=TRUE, BcPV4.BI=FALSE} =>  {BcHV1.BI=TRUE} | 0.45  0495 | 0.892  157 | 0.504  95 | 1.47  7178 | 18  2 |
| {BcHV1.BI=TRUE, BcPV4.BI=FALSE} =>  {BcHV1SLR.BI=TRUE} | 0.45  0495 | 0.805  31 | 0.559  406 | 1.48  5594 | 18  2 |
| {BcEV6=FALSE, BcHV1SLR.BI=TRUE} =>  {BcHV1.BI=TRUE} | 0.44  5545 | 0.886  7 | 0.502  475 | 1.46  8142 | 18  0 |
| {BcEV6=FALSE, BcHV1.BI=TRUE} =>  {BcHV1SLR.BI=TRUE} | 0.44  5545 | 0.810  811 | 0.549  505 | 1.49  5742 | 18  0 |
| {BcHV1SLR.BI=TRUE, BcOLV17.BI=FALSE}  => {BcHV1.BI=TRUE} | 0.45  0495 | 0.892  157 | 0.504  95 | 1.47  7178 | 18  2 |
| {BcHV1.BI=TRUE, BcOLV17.BI=FALSE} =>  {BcHV1SLR.BI=TRUE} | 0.45  0495 | 0.798  246 | 0.564  356 | 1.47  2563 | 18  2 |
| {BcHV1SLR.BI=TRUE, BcOLV15.BI=FALSE}  => {BcHV1.BI=TRUE} | 0.44  5545 | 0.891  089 | 0.5 | 1.47  541 | 18  0 |
| {BcHV1.BI=TRUE, BcOLV15.BI=FALSE} =>  {BcHV1SLR.BI=TRUE} | 0.44  5545 | 0.789  474 | 0.564  356 | 1.45  6381 | 18  0 |
| {BcEV5=FALSE, BcHV1SLR.BI=TRUE} =>  {BcHV1.BI=TRUE} | 0.45  7921 | 0.893  72 | 0.512  376 | 1.47  9766 | 18  5 |
| {BcEV5=FALSE, BcHV1.BI=TRUE} =>  {BcHV1SLR.BI=TRUE} | 0.45  7921 | 0.797  414 | 0.574  257 | 1.47  1028 | 18  5 |

| {BcMV11=TRUE, BcMV9.BI=TRUE} =>  {BcHV1SLR.BI=TRUE} | 0.25  9901 | 0.640  244 | 0.405  941 | 1.18  1089 | 10  5 |
| --- | --- | --- | --- | --- | --- |
| {BcMV11=TRUE, BcULV4=TRUE} =>  {BcHV1SLR.BI=TRUE} | 0.27  4752 | 0.656  805 | 0.418  317 | 1.21  164 | 11  1 |
| {BcHV1SLR.BI=TRUE, BcOLV1.BI.MOVA.=FALSE} =>  {BcMV9.BI=TRUE} | 0.25  9901 | 0.760  87 | 0.341  584 | 1.12  1866 | 10  5 |
| {BcHV1SLR.BI=TRUE, BcOLV1.BI.MOVA.=FALSE} =>  {BcMV6.BI=FALSE} | 0.26  9802 | 0.789  855 | 0.341  584 | 1.13  5592 | 10  9 |
| {BcHV1SLR.BI=TRUE, BcMV6.BI=FALSE} =>  {BcOLV1.BI.MOVA.=FALSE} | 0.26  9802 | 0.801  471 | 0.336  634 | 1.19  9237 | 10  9 |
| {BcHV1SLR.BI=TRUE, BcOLV1.BI.MOVA.=FALSE} =>  {BcRV1.BI=FALSE} | 0.27  4752 | 0.804  348 | 0.341  584 | 1.11  2865 | 11  1 |
| {BcHV1SLR.BI=TRUE, BcOLV1.BI.MOVA.=FALSE} =>  {BcOLV7.BI=FALSE} | 0.29  9505 | 0.876  812 | 0.341  584 | 1.08  6601 | 12  1 |
| {BcHV1SLR.BI=TRUE, BcMV9.BI=TRUE} =>  {BcULV4=TRUE} | 0.33  1683 | 0.812  121 | 0.408  416 | 1.17  5975 | 13  4 |
| {BcHV1SLR.BI=TRUE, BcULV4=TRUE} =>  {BcMV9.BI=TRUE} | 0.33  1683 | 0.827  16 | 0.400  99 | 1.21  9609 | 13  4 |

| {BcMV9.BI=TRUE, BcULV4=TRUE} =>  {BcHV1SLR.BI=TRUE} | 0.33  1683 | 0.606  335 | 0.547  03 | 1.11  8536 | 13  4 |
| --- | --- | --- | --- | --- | --- |
| {BcHV1SLR.BI=TRUE, BcPV3.BI=FALSE} =>  {BcMV9.BI=TRUE} | 0.28  4653 | 0.761  589 | 0.373  762 | 1.12  2927 | 11  5 |
| {BcEV2.BI=FALSE, BcHV1SLR.BI=TRUE} =>  {BcMV9.BI=TRUE} | 0.29  2079 | 0.756  41 | 0.386  139 | 1.11  5291 | 11  8 |
| {BcHV1SLR.BI=TRUE, BcMV1.BI=TRUE} =>  {BcMV9.BI=TRUE} | 0.34  1584 | 0.775  281 | 0.440  594 | 1.14  3115 | 13  8 |
| {BcMV1.BI=TRUE, BcMV9.BI=TRUE} =>  {BcHV1SLR.BI=TRUE} | 0.34  1584 | 0.621  622 | 0.549  505 | 1.14  6736 | 13  8 |
| {BcHV1SLR.BI=TRUE, BcMV12=FALSE} =>  {BcMV9.BI=TRUE} | 0.31  6832 | 0.766  467 | 0.413  366 | 1.13  0119 | 12  8 |
| {BcHV1SLR.BI=TRUE, BcVV3.BI=FALSE} =>  {BcMV9.BI=TRUE} | 0.33  4158 | 0.771  429 | 0.433  168 | 1.13  7435 | 13  5 |
| {BcMV9.BI=TRUE, BcOLV7.BI=FALSE} =>  {BcHV1SLR.BI=TRUE} | 0.31  4356 | 0.619  512 | 0.507  426 | 1.14  2844 | 12  7 |
| {BcDMV1=FALSE, BcMV9.BI=TRUE} =>  {BcHV1SLR.BI=TRUE} | 0.34  1584 | 0.618  834 | 0.551  98 | 1.14  1593 | 13  8 |
| {BcHV1SLR.BI=TRUE, BcOLV4.BI=FALSE}  => {BcMV9.BI=TRUE} | 0.36  3861 | 0.786  096 | 0.462  871 | 1.15  9062 | 14  7 |
| {BcHV1SLR.BI=TRUE, BcMV9.BI=TRUE} =>  {BcULV5=FALSE} | 0.38  8614 | 0.951  515 | 0.408  416 | 1.08  591 | 15  7 |

| {BcHV1SLR.BI=TRUE, BcULV5=FALSE} =>  {BcMV9.BI=TRUE} | 0.38  8614 | 0.830  688 | 0.467  822 | 1.22  481 | 15  7 |
| --- | --- | --- | --- | --- | --- |
| {BcHV1SLR.BI=TRUE, BvF.BI=FALSE} =>  {BcMV9.BI=TRUE} | 0.35  8911 | 0.747  423 | 0.480  198 | 1.10  2039 | 14  5 |
| {BcHV1SLR.BI=TRUE, BcMCV5.BI=FALSE}  => {BcMV9.BI=TRUE} | 0.36  6337 | 0.747  475 | 0.490  099 | 1.10  2116 | 14  8 |
| {BcHV1SLR.BI=TRUE, BcOLV13.BI=FALSE}  => {BcMV9.BI=TRUE} | 0.37  6238 | 0.767  677 | 0.490  099 | 1.13  1903 | 15  2 |
| {BcHV1SLR.BI=TRUE, BcMCV3.BI=FALSE}  => {BcMV9.BI=TRUE} | 0.36  6337 | 0.751  269 | 0.487  624 | 1.10  7711 | 14  8 |
| {BcHV1SLR.BI=TRUE, BcPV4.BI=FALSE} =>  {BcMV9.BI=TRUE} | 0.38  6139 | 0.764  706 | 0.504  95 | 1.12  7523 | 15  6 |
| {BcMV9.BI=TRUE, BcPV4.BI=FALSE} =>  {BcHV1SLR.BI=TRUE} | 0.38  6139 | 0.609  375 | 0.633  663 | 1.12  4144 | 15  6 |
| {BcEV6=FALSE, BcHV1SLR.BI=TRUE} =>  {BcMV9.BI=TRUE} | 0.37  6238 | 0.748  768 | 0.502  475 | 1.10  4024 | 15  2 |
| {BcHV1SLR.BI=TRUE, BcOLV17.BI=FALSE}  => {BcMV9.BI=TRUE} | 0.37  8713 | 0.75 | 0.504  95 | 1.10  5839 | 15  3 |
| {BcHV1SLR.BI=TRUE, BcOLV15.BI=FALSE}  => {BcMV9.BI=TRUE} | 0.37  3762 | 0.747  525 | 0.5 | 1.10  219 | 15  1 |
| {BcEV5=FALSE, BcHV1SLR.BI=TRUE} =>  {BcMV9.BI=TRUE} | 0.38  1188 | 0.743  961 | 0.512  376 | 1.09  6936 | 15  4 |

| {BcHV1SLR.BI=TRUE, BcULV5=FALSE} =>  {BcULV4=TRUE} | 0.35  6436 | 0.761  905 | 0.467  822 | 1.10  326 | 14  4 |
| --- | --- | --- | --- | --- | --- |
| {BcHV1SLR.BI=TRUE, BvF.BI=FALSE} =>  {BcULV4=TRUE} | 0.36  8812 | 0.768  041 | 0.480  198 | 1.11  2146 | 14  9 |
[truncated: 747,630 more chars]
